# Global, regional, and national prevalence and mortality burden of sickle cell disease, 2000–2021: a systematic analysis from the Global Burden of Disease Study 2021

**DOI:** 10.1016/S2352-3026(23)00118-7

**Published:** 2023-06-15

**Authors:** GBD 2021 Sickle Cell Disease Collaborators, Azalea M Thomson, Theresa A McHugh, Assaf P Oron, Corey Teply, Nikhil Lonberg, Victor Vilchis Tella, Lauren B Wilner, Kia Fuller, Hailey Hagins, Richard Gyan Aboagye, Melka Biratu Aboye, Eman Abu-Gharbieh, Ahmed Abu-Zaid, Isaac Yeboah Addo, Bright Opoku Ahinkorah, Aqeel Ahmad, Saif Aldeen S AlRyalat, Hubert Amu, Aleksandr Y Aravkin, Judie Arulappan, Maha Moh'd Wahbi Atout, Ashish D Badiye, Sara Bagherieh, Maciej Banach, Morteza Banakar, Mainak Bardhan, Amadou Barrow, Deriba Abera Bedane, Isabela M Bensenor, Akshaya Srikanth Bhagavathula, Pankaj Bhardwaj, Prarthna V Bhardwaj, Ajay Nagesh Bhat, Zulfiqar A Bhutta, Mariah Malak Bilalaga, Jessica Devin Bishai, Saeid Bitaraf, Archith Boloor, Muhammad Hammad Butt, Vijay Kumar Chattu, Dinh-Toi Chu, Omid Dadras, Xiaochen Dai, Bardia Danaei, Anh Kim Dang, Fitsum Wolde Demisse, Meghnath Dhimal, Daniel Diaz, Shirin Djalalinia, Deepa Dongarwar, Muhammed Elhadi, Mohamed A Elmonem, Christopher Imokhuede Esezobor, Farshid Etaee, Oghenowede Eyawo, Adeniyi Francis Fagbamigbe, Ali Fatehizadeh, Lisa M Force, William M Gardner, Kazem Ghaffari, Paramjit Singh Gill, Mahaveer Golechha, Pouya Goleij, Vivek Kumar Gupta, Hamidreza Hasani, Treska S Hassan, Mohammed Bheser Hassen, Segun Emmanuel Ibitoye, Adalia I Ikiroma, Chidozie C D Iwu, Peter Bai James, Shubha Jayaram, Rime Jebai, Ravi Prakash Jha, Nitin Joseph, Farnaz Kalantar, Himal Kandel, Ibraheem M Karaye, Woldeteklehaymanot Dagne Kassahun, Imteyaz A Khan, Shaghayegh Khanmohammadi, Adnan Kisa, Farzad Kompani, Kewal Krishan, Iván Landires, Stephen S Lim, Preetam Bhalchandra Mahajan, Soleiman Mahjoub, Azeem Majeed, Bishnu P Marasini, Haftu Asmerom Meresa, Tomislav Mestrovic, Sonica Minhas, Awoke Misganaw, Ali H Mokdad, Lorenzo Monasta, Ghulam Mustafa, Tapas Sadasivan Nair, Sreenivas Narasimha Swamy, Hasan Nassereldine, Zuhair S Natto, Muhammad Naveed, Biswa Prakash Nayak, Jean Jacques Noubiap, Taylor Noyes, Chisom Adaobi Nri-ezedi, Vincent Ebuka Nwatah, Chimezie Igwegbe Nzoputam, Ogochukwu Janet Nzoputam, Osaretin Christabel Okonji, Adeyinka Omoniyi Onikan, Mayowa O Owolabi, Jay Patel, Siddhartha Pati, Shrikant Pawar, Ionela-Roxana Petcu, Frédéric B Piel, Ibrahim Qattea, Mehran Rahimi, Mosiur Rahman, Salman Rawaf, Elrashdy Moustafa Mohamed Redwan, Nazila Rezaei, Basema Saddik, Umar Saeed, Fatemeh Saheb Sharif-Askari, Abdallah M Samy, Austin E Schumacher, Elaheh Shaker, Adithi Shetty, Migbar Mekonnen Sibhat, Jasvinder A Singh, Muhammad Suleman, Dev Ram Sunuwar, Mindy D Szeto, Jacques JL Lukenze Tamuzi, Nathan Y Tat, Birhan Tsegaw Taye, Mohamad-Hani Temsah, Muhammad Umair, Sahel Valadan Tahbaz, Cong Wang, Nuwan Darshana Wickramasinghe, Arzu Yigit, Vahit Yiğit, Ismaeel Yunusa, Burhan Abdullah Zaman, Moein Zangiabadian, Peng Zheng, Simon I Hay, Mohsen Naghavi, Christopher J L Murray, Nicholas J Kassebaum

## Abstract

**Background:**

Previous global analyses, with known underdiagnosis and single cause per death attribution systems, provide only a small insight into the suspected high population health effect of sickle cell disease. Completed as part of the Global Burden of Diseases, Injuries, and Risk Factors Study (GBD) 2021, this study delivers a comprehensive global assessment of prevalence of sickle cell disease and mortality burden by age and sex for 204 countries and territories from 2000 to 2021.

**Methods:**

We estimated cause-specific sickle cell disease mortality using standardised GBD approaches, in which each death is assigned to a single underlying cause, to estimate mortality rates from the International Classification of Diseases (ICD)-coded vital registration, surveillance, and verbal autopsy data. In parallel, our goal was to estimate a more accurate account of sickle cell disease health burden using four types of epidemiological data on sickle cell disease: birth incidence, age-specific prevalence, with-condition mortality (total deaths), and excess mortality (excess deaths). Systematic reviews, supplemented with ICD-coded hospital discharge and insurance claims data, informed this modelling approach. We employed DisMod-MR 2.1 to triangulate between these measures—borrowing strength from predictive covariates and across age, time, and geography—and generated internally consistent estimates of incidence, prevalence, and mortality for three distinct genotypes of sickle cell disease: homozygous sickle cell disease and severe sickle cell β-thalassaemia, sickle-haemoglobin C disease, and mild sickle cell β-thalassaemia. Summing the three models yielded final estimates of incidence at birth, prevalence by age and sex, and total sickle cell disease mortality, the latter of which was compared directly against cause-specific mortality estimates to evaluate differences in mortality burden assessment and implications for the Sustainable Development Goals (SDGs).

**Findings:**

Between 2000 and 2021, national incidence rates of sickle cell disease were relatively stable, but total births of babies with sickle cell disease increased globally by 13·7% (95% uncertainty interval 11·1–16·5), to 515 000 (425 000–614 000), primarily due to population growth in the Caribbean and western and central sub-Saharan Africa. The number of people living with sickle cell disease globally increased by 41·4% (38·3–44·9), from 5·46 million (4·62–6·45) in 2000 to 7·74 million (6·51–9·2) in 2021. We estimated 34 400 (25 000–45 200) cause-specific all-age deaths globally in 2021, but total sickle cell disease mortality burden was nearly 11-times higher at 376 000 (303 000–467 000). In children younger than 5 years, there were 81 100 (58 800–108 000) deaths, ranking total sickle cell disease mortality as 12th (compared to 40th for cause-specific sickle cell disease mortality) across all causes estimated by the GBD in 2021.

**Interpretation:**

Our findings show a strikingly high contribution of sickle cell disease to all-cause mortality that is not apparent when each death is assigned to only a single cause. Sickle cell disease mortality burden is highest in children, especially in countries with the greatest under-5 mortality rates. Without comprehensive strategies to address morbidity and mortality associated with sickle cell disease, attainment of SDG 3.1, 3.2, and 3.4 is uncertain. Widespread data gaps and correspondingly high uncertainty in the estimates highlight the urgent need for routine and sustained surveillance efforts, further research to assess the contribution of conditions associated with sickle cell disease, and widespread deployment of evidence-based prevention and treatment for those with sickle cell disease.

**Funding:**

Bill & Melinda Gates Foundation.

## Introduction

Sickle cell arises from a missense mutation in the *HBB* gene encoding the β-globin subunit of haemoglobin.^1^ An individual with one sickle mutation (usually sickle haemoglobin [HbS]) will have sickle cell trait, whereas someone with a mutation on both *HBB* genes (at least one of which is HbS) will have sickle cell disease. The differences between sickle cell trait and sickle cell disease are stark. Although sickle cell trait confers protection against severe malaria and is otherwise largely a benign condition,^2^, ^3^ those with sickle cell disease have a lifelong, severely-disabling disease with lower quality of life, high use of medical resources, increased economic burden, and nearly guaranteed early death.^4^, ^5^, ^6^ Sickle cell disease causes malformed, sickle-shaped red blood cells that occlude capillaries and prevent tissue oxygen delivery, leading to acute and chronic pain, severe anaemia, kidney dysfunction, acute chest syndrome, stroke and other cardiovascular diseases, increased susceptibility to infectious diseases (including malaria), pregnancy complications, and maternal mortality.^7^, ^8^, ^9^, ^10^

**Research in context**

**Evidence before this study**

Previous efforts to quantify the burden of sickle cell disease have included systematic reviews and meta-analyses, along with scarce modelled estimates of incidence at birth and under-5 child mortality. However, up to this point, the Global Burden of Diseases, Injuries, and Risk Factors Study (GBD), is the only attempt at comprehensive assessment of global morbidity and mortality from sickle cell disease. Building upon a gene frequency dataset compiled by an expert group in 2010, a systematic literature review in PubMed was done for GBD 2013, with updates in 2016. Additions of clinical administrative data were added for GBD 2017, 2019, and 2021. Yet, even within GBD, previous mortality estimates have only focused on single, underlying causes of death in accordance with international death certification guidelines; thus, they have the potential to underestimate the full mortality effect of conditions such as sickle cell disease, especially in locations where the frequency of sickle cell disease is high, comorbid conditions are common, and diagnosis of sickle cell disease is rare.

**Added value of this study**

This study is the first report on sickle cell disease burden using GBD 2021 results and, to our knowledge, is the first to estimate the full global mortality burden of sickle cell disease. We produced modelled estimates for prevalence, cause-specific mortality, and total sickle cell disease mortality for each of the 204 countries and territories, 23 age groups, and two sexes from 2000 to 2021. Total sickle cell disease mortality was estimated from a combination of cohort survival data and age-specific prevalence data using meta-regression on the basis of a compartmental model of disease. The mismatch between cause-specific and total sickle cell disease mortality shows the enormous and underappreciated mortality burden of sickle cell disease, suggests potential shortcomings of a one death to one cause heuristic, and highlights substantial data gaps in both disease frequency of sickle cell disease and its consequences.

**Implications of all the available evidence**

Incidence at birth and all-age sickle cell disease prevalence increased most notably in sub-Saharan Africa, likely to be largely due to population growth and increased survival in early ages. We found that, globally, among children younger than 5 years, sickle cell disease cause-specific mortality ranked 40th among all estimated GBD causes of death. In contrast, using the total sickle cell disease mortality metric resulted in a cause ranking of 12th among children younger than 5 years. Given this vast burden, as previous research has called for, sickle cell disease should be integrated into pre-existing health surveillance systems, with resources dedicated to newborn screening, improved treatment access (eg, hydroxyurea and blood transfusion) and prevention (eg, transcranial Doppler screening, point-of-care tests, and genetic counselling). Particular attention toward implementation of these supportive measures should be employed in sub-Saharan Africa and south Asia, where the double burden of communicable and non-communicable diseases exposes sickle cell disease as both a cause and risk factor for premature mortality.

Comprehensive global estimates on disease burden of sickle cell disease are scarce. Piel and colleagues^11^ generated granular maps of birth incidence of sickle cell trait for 2010 and back-calculated sickle cell disease at birth (HbS–HbS only) of 312 000 (95% CI 294 000–330 000) assuming Hardy-Weinberg equilibrium.^11^, ^12^ Under-5 sickle cell disease mortality has been suggested by cross-sectional studies to be as high as 90%,^13^ and a 2018 meta-analysis estimated that sickle cell disease explains 7·3% (4·03–10·57%) of under-5 mortality in Africa.^14^ To our knowledge, the Global Burden of Diseases, Injuries, and Risk Factors Study (GBD) is the only effort to have approached sickle cell disease estimation comprehensively by simultaneously assessing disease frequency of sickle cell disease and cause-specific mortality (in which deaths are assigned to a single underlying cause according to the International Classification of Diseases [ICD]). GBD 2019 estimated a higher number of annual births of babies with sickle cell disease (homozygous sickle cell disease and severe sickle cell β-thalassaemia, sickle-haemoglobin C disease, and mild sickle cell β-thalassaemia) globally in 2010 at 586 000 (453 000–752 000), but only 41 900 (95% CI 29 500–57 900) cause-specific deaths of individuals with sickle cell disease globally in 2019, including 0·66% (0·41–0·94) of all under-5 deaths in sub-Saharan Africa.^15^

High sickle cell disease burden in historically malaria-endemic regions of Africa, the Middle East, the Caribbean, and south Asia dictate that sickle cell disease monitoring is relevant to at least three Sustainable Development Goal (SDG) reduction targets: maternal mortality (SDG 3.1), neonatal and under-5 mortality (SDG 3.2), and premature mortality due to non-communicable disease (SDG 3.4).^16^ The substantial gap in estimated sickle cell disease mortality burden between GBD and cross-sectional studies is most likely multifactorial, but a crucial first step is quantifying the gap globally. To that end, this analysis aims to simultaneously provide an updated assessment of births and cases of babies with sickle cell disease throughout the world and quantify the degree of under-ascertainment of sickle cell disease mortality burden implied by the disconnect between cause-specific deaths of individuals with sickle cell disease (ICD rules=one death per one cause) and total sickle cell disease mortality. We did this by developing internally consistent models informed by available data on birth incidence, age-specific prevalence, and mortality in those with sickle cell disease. This manuscript was produced as part of the GBD Collaborator Network and in accordance with the GBD Protocol.

## Methods

### Overview, definitions, and input data

**Figure 1:**
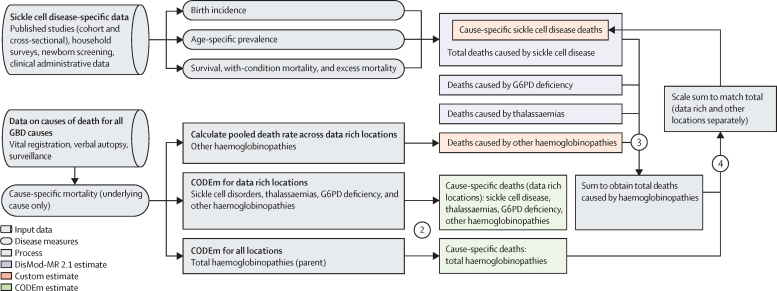
Flowchart showing prevalence, cause-specific mortality, and total sickle cell disease mortality estimation process Overview of the estimation process for sickle cell disorders for both cause-specific mortality on the basis of the International Classification of Diseases definition of one cause per death and total sickle cell disease mortality. Shapes differentiate input data, disease measures, processes, and estimates, whereas colours distinguish different modelling tools. The four steps are highlighted: (1) sickle cell disease-specific data on disease epidemiology were inputted into DisMod-MR 2.1 models to generate internally consistent incidence, prevalence, and overall mortality; (2) causes of death data processed across GBD were run through a series of CODEm to generate mortality estimates for total haemoglobinopathies (parent GBD cause) for all locations and for data rich locations only, for each of the child causes, including sickle cell disease, thalassaemias, G6PD deficiency, and other haemoglobinopathies; (3) the mortality results from each of the four subcauses were scaled to 100%; and (4) these mortality results were used to divide total haemoglobinopathies CODEm results into cause-specific mortality results for sickle cell disease and other subcauses. CODEm=Causes of Death Ensemble model. G6PD=glucose-6-phosphate dehydrogenase.

Overall methods and approaches used in GBD have been extensively described previously.^15^ We present a summary of the most salient details related to estimation of the disease burden of sickle cell disease, including a description of the distinction between cause-specific mortality and total sickle cell disease mortality—a key focus of this analysis. Further details are available in the appendix (pp 5–59), and the overall estimation process is shown in figure 1 and the appendix (p 9). This analysis complies with the Guidelines for Accurate and Transparent Health Estimates Reporting (appendix pp 4–5). All input data sources are in the appendix (pp 15–49); inputs and results can also be downloaded from the Global Health Data Exchange.

GBD 2021 produced estimates for 369 diseases and injuries and 87 risk factors for each of the 204 countries and territories (appendix p 6), 22 of which were estimated subnationally, for 23 age groups and two sexes from 2000 to 2021. Sickle cell disease is a Level 4 cause within the broader Level 3 cause category of haemoglobinopathies and haemolytic anaemias, a subclassification of Level 2 other non-communicable diseases, and Level 1 non-communicable diseases. Sickle cell disease includes International Classification of Disease Version 10 (ICD-10) codes D57-D57·8 (excluding sickle cell trait D57·3) and ICD-9 code 282·6 (appendix p 8).

Following ICD rules for death certification, GBD assigns each death to a single underlying cause and processes all resulting cause-specific mortality data for all GBD causes in parallel. Sickle cell disease inputs included vital registration, verbal autopsy, and mortality surveillance sources that were processed using standardised methods to account for under-reporting, misclassification, and stochastic variability, according to the methodology previously described.^17^ Of note, all verbal autopsy data were considered as being implausibly low and designated as outliers for sickle cell disease datasets, given that sickle cell disease is not even included as a potential cause of death in many such studies.

**Figure 2:**
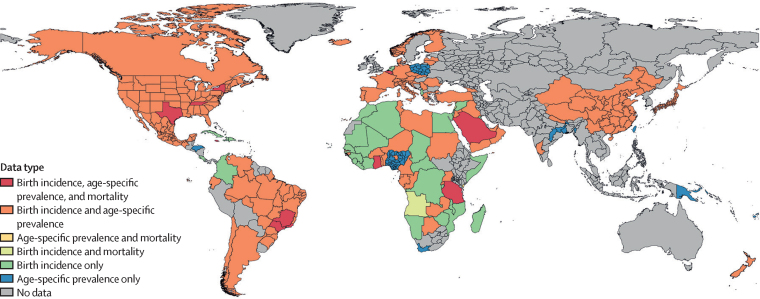
Data availability map showing types of input data source measures present in each country for sickle cell disease DisMod-MR 2.1 models Each colour represents a different combination of birth incidence, age-specific prevalence, and survival or mortality data that were used as inputs for DisMod-MR 2.1 models for each of the three estimated genotypes of sickle cell disease. The outputs of these models include estimated birth incidence, age-specific prevalence, and total sickle cell disease mortality for each GBD location, age group, sex, and year (see appendix for genotype specific counts [pp 95–178] and for genotype specific rates [pp 207–90]). The specific sources are in the appendix (pp 15–22) and online at the Global Health Data Exchange; contributing counts of prevalence and mortality measures from each genotype are also in the appendix (p 50).

Systematic reviews for sickle cell disease birth incidence, age-specific prevalence, and total sickle cell disease mortality were completed in 2013 and 2016. Search terms are in the appendix (p 10) along with a PRISMA diagram (appendix p 10) and case definitions (appendix p 7). Prevalence data were extracted for each of three mutually exclusive groupings based on phenotypic severity and geographical distribution: homozygous sickle cell disease (SS) and severe sickle cell β-thalassaemia (Sβ°), sickle-haemoglobin C disease (SC), and mild sickle cell β-thalassaemia (Sβ+). Excess mortality data (all deaths in those with sickle cell disease) were derived from published population-level longitudinal studies and published clinical cohorts. Data sources not ICD-coded were only included if diagnosis was on the basis of blood testing or physician diagnosis. Data additions since 2016 have included ICD-coded administrative sources (ie, hospital discharges and insurance claims) and a small number of household surveys (eg, 2018 Nigeria Demographic and Health Survey), newborn screening sources, published studies, and reports referred by GBD collaborators. We processed all data to be age-specific and sex-specific before modelling. We evaluated but did not find any systematic differences between ICD-coded data and those based on blood testing, so both were considered equally. Geographic input data coverage for the types of data informing the total sickle cell disease model is seen in figure 2, and further data coverage maps are in the appendix (pp 50–51).

### Incidence, prevalence, and total mortality

In the absence of ideal data, as is the case with sickle cell disease, predictions are strengthened by triangulating between epidemiologically related quantities. We accomplished this triangulation using DisMod-MR 2.1, a Bayesian meta-regression tool with a compartmental model of disease developed for GBD,^15^ to generate internally consistent epidemiological estimates. The compartmental model relies on input data of each of the aforementioned measures and country-level predictive covariates to produce estimates of four transition hazards—incidence, remission, case fatality, and all other mortality—for each age, sex, location, and year combination. Since sickle cell disease is a genetic disorder with no widespread cure, we assumed both remission and incidence after birth to be zero. Total sickle cell disease mortality was calculated as the sum of prevalence times excess mortality rate results from each of the three separate genotype-specific sickle cell disease models: SS and Sβ°; SC; and Sβ+. Predictive covariates of prevalence included haemoglobin S trait (all models)^18^ and haemoglobin C trait (SC model only).^19^ Universal health coverage index, a composite metric assessing coverage of essential health services within a location,^17^ was used as an additional covariate for excess mortality rate. More detailed information on the DisMod-MR 2.1 modelling process are in the appendix (pp 53–55).

### Cause-specific sickle cell disease mortality

We estimated cause-specific mortality for sickle cell disease (not separately by genotype) using a hybrid approach (appendix p 56). For data rich locations, we used the Cause of Death Ensemble model (CODEm) tool, scaling the CODEm results of sickle cell disease along with those of glucose-6 phosphate dehydrogenase (G6PD) deficiency, thalassaemias, and other haemoglobinopathies, to match deaths for total haemoglobinopathies.^15^ For non-data rich locations, the four subcauses were again scaled to match the CODEm result for total haemoglobinopathies, but in this case the proportional split was based on the summed total of sickle cell disease mortality estimates from DisMod-MR 2.1 models, along with total mortality DisMod-MR 2.1 results for G6PD deficiency, thalassaemias and other haemoglobinopathies. Further details of CODEm, including model specification and included covariates, are in the appendix (pp 55–57) and in the GBD 2019 appendix (pp 48–50).^15^

### Secondary analyses: decomposition and comparative mortality rankings

We completed two secondary analyses. First, to evaluate time trends and the variable contribution of population growth, ageing, and changes in disease frequency and survival, we completed a decomposition analysis of change over time using counterfactual scenarios in which demographic and epidemiological factors were held constant from 2000 or changed to reflect 2021. Further detail on the decomposition analysis are in the appendix (p 58). Second, we re-ranked the GBD causes of death and evaluated the cause fraction of total sickle cell disease mortality as compared with cause-specific sickle cell disease mortality to show the gap between the two assessments.

### Uncertainty

Uncertainty was propagated through data processing (including sampling variance in input data and variance from data processing steps such as age-sex splitting and causes of death redistribution algorithms), and model fits. Final 95% uncertainty intervals (UIs) of modelled point estimates were generated for every metric using the 25th and 975th ordered draws from 1000 values of the posterior distribution.

### Role of the funding source

The funder of the study had no role in study design, data collection, data analysis, data interpretation, or writing of the report.

## Results

Nearly all countries had stable sickle cell disease birth rates over time, but due to demographic changes, the number of births of babies with sickle cell disease increased from 453 000 (95% UI 370 000–542 000) to 515 000 (425 000–614 000) between 2000 and 2021—a 13·7% (11·1–16·5) rise in global sickle cell disease birth rate to 382 (95% UI 316–456) per 100 000 livebirths (appendix pp 67–94, 179–206). Of these births, 394 000 (76·5%) of 515 000 were of the SS and Sβ° genotypes, 101 000 (19·6%) of 515 000 were of genotype SC, and 19 800 (3·9%) of 515 000 were of genotype Sβ+ (appendix pp 95–178). Increased sickle cell disease birth rates were most notable in Latin America and the Caribbean, and decreased sickle cell disease birth rates were highest in central Europe, eastern Europe, and central Asia, as well as north Africa and the Middle East. All-age sickle cell disease prevalent cases increased by 41·4% (95% UI 38·3–44·9) from 5·46 million (4·62–6·45) cases in 2000 to 7·74 million cases (6·51–9·20) in 2021 (appendix pp 67–94), which our decomposition analysis identified as being due to population growth (31·1% [95% UI 30·7–31·5]), changes in disease frequency associated with increased survival (29·3% [26·4–32·8]), and partially offset by ageing of the general population (–18·4% [–18·8 to –18·1]).

There were 28 400 (95% UI 22 100–34 500) cause-specific deaths of individuals with sickle cell disease globally in 2000, which increased by 20·8% (–5·3 to 57·6) to 34 400 (25 000–45 200) in 2021. In comparison, there was a 43·4% (95% UI 39·8–47·3) increase in total deaths of individuals with sickle cell disease globally, from 262 000 (211 000–327 000) in 2000 to 376 000 (303 000–467 000) in 2021. Although the 2021 global all-age and age-standardised cause-specific sickle cell disease mortality rates of 0·4 deaths (95% UI 0·3–0·6) and 0·5 deaths (0·4–0·7) per 100 000 population (appendix pp 459–486) changed only slightly from 2000, total sickle cell disease mortality rates for the same age groups were markedly higher with 4·8 deaths (3·8–5·9) per 100 000 population and 5·1 deaths (4·2–6·3) per 100 000 population, respectively.

Across super-regions, sickle cell disease incidence and prevalence increased noticeably in sub-Saharan Africa, where births in 2021 were 405 000 (95% UI 343 000–478 000), rising 27·2% (23·4–30·2) since 2000; all-age prevalence in 2021 was 5·68 million cases (4·78–6·62), a 67·4% (62·9–71·5) increase since 2000 (appendix pp 67–94). In contrast to observed trends at the global level, a greater proportion of the increased number of births of babies with sickle cell disease in sub-Saharan Africa was due to population growth (74·0% [95% UI 73·0–74·9]), rather than changes in age structure (–7·1% [–7·7 to –6·5) or disease frequency (0·9% [–2·4 to 4·0]; see appendix pp 487–514 for location specific changes decomposed). Apart from this 27·2% (95% UI 23·4–30·2) upsurge in births of babies with sickle cell disease in sub-Saharan Africa and a slight increase (5·6% [2·1–10·5]) in Latin America and the Caribbean, incident cases decreased in all other super-regions. In the high-income super-region, an 11·5% (95% UI 9·8–12·8) reduction occurred: 2860 births (95% UI 2550–3190) in 2000 and 2530 (95% UI 2250–2830) in 2021. Across super regions, examining all-age sickle cell disease prevalence, central Europe, eastern Europe, and central Asia had the largest percentage decline: down 33·3% (95% UI 28·9–37·9); 425 (95% UI 345–507) cases in 2000 to 285 cases (95% UI 232–340) in 2021.

**Figure 3:**
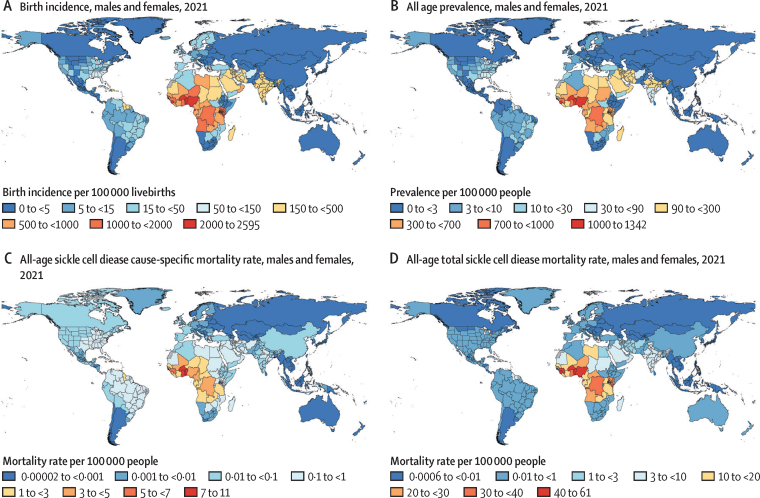
Maps of total sickle cell disease rates per 100 000 population (A) Birth incidence. (B) All-age prevalence. (C) All-age cause-specific mortality. (D) All-age total sickle cell disease mortality among males and females combined in 2021. See the appendix (p 63) for all measures in 2000.

The highest sickle cell disease disability burden was concentrated in western and central sub-Saharan Africa and India (figure 3A, B). In 2021, countries with a sickle cell disease incidence at birth between 1000 and 2000 per 100 000 livebirths included Bahrain, Angola, Democratic Republic of the Congo, Kenya, Ghana, Guinea, Niger, and Sao Tome and Principe. Countries that have consistently exceeded an incidence at birth of 2000 per 100 000 livebirths since 2000 were Equatorial Guinea, Benin, Burkina Faso, Nigeria, Sierra Leone, and Togo. These six countries made up 44% (227 000/515 000) of the global incidence at birth in 2021, similar to 2000, when they accounted for 41% (184 000/453 000) of the global incidence at birth. At the super-region level, the global burden incurred in sub-Saharan Africa increased from 70% (318 000/453 000) in 2000 to 79% (405 000/515 000) in 2021. In contrast, incidence at birth in India accounted for 21% (96 700/453 000) of the global burden in 2000 but dropped to 16% (82 500/515 000) in 2021.

The highest mortality burden from sickle cell disease was concentrated in sub-Saharan Africa (figure 3C, D), where our cause-specific analysis indicated 29 400 (95% UI 20 300–40 000) people died of sickle cell disease in 2021, an increase of 30·1% (–2·3 to 77·6) since 2000. Total deaths of individuals with sickle cell disease were 9-times greater in sub-Saharan Africa: 265 000 (95% UI 219 000–322 000) deaths in 2021, up 65·1% (60·7–69·2) from 160 000 (133 000–194 000) deaths in 2000.

**Figure 4:**
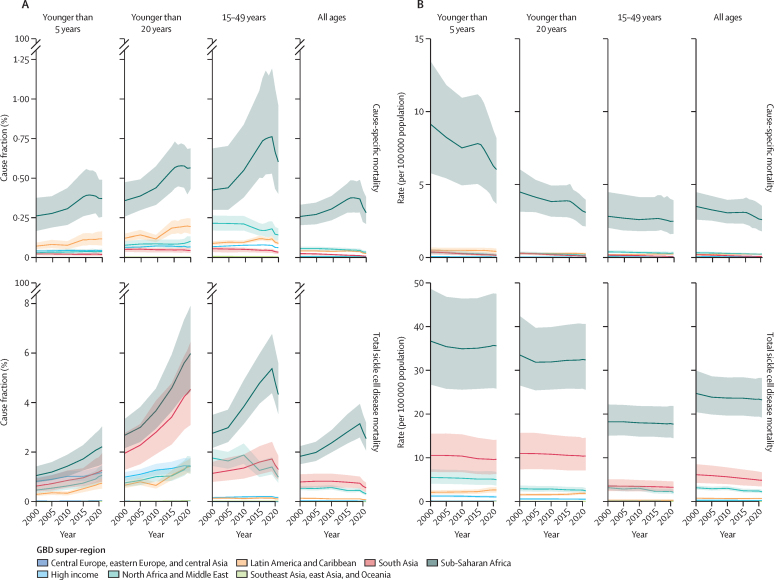
Time trends of sickle cell disease cause-specific mortality (top) and total sickle cell disease mortality (bottom) as a cause fraction of total mortality within the super-region (A) and rate within the super-region (B), by age group Solid lines are coloured by super-region and display the trends of sickle cell disease mortality measures between 2000 and 2021. The shaded area represents the 95% uncertainty interval.

Across age groups (younger than 5 years, younger than 20 years, and 15–49 years), both cause-specific and total mortality metrics showed increasing sickle cell disease mortality cause fractions over time (figure 4A; appendix pp 291–486). Particularly in sub-Saharan Africa, sickle cell disease mortality as a fraction of all-cause mortality increased with age. In 2021, cause-specific deaths of individuals with sickle cell disease accounted for 0·4% (95% UI 0·3–0·5) of all deaths in children younger than 5 years and 0·6% (0·4–1·0) of all deaths in individuals aged 15–49 years. In comparison, total sickle cell disease deaths accounted for 2·2% (1·5–3·0) of all deaths in children younger than 5 years and 4·3% (3·5–5·6) of all deaths in individuals aged 15–49 years. Cause-specific sickle cell disease mortality rates declined between 2000 and 2021, whereas total sickle cell disease mortality rates remained fairly constant or declined less noticeably. Sickle cell disease mortality rates were highest in the under-5 age group (figure 4B); in 2021 in Sub-Saharan Africa, there were 6·0 (95% UI 3·7–8·2) cause-specific deaths per 100 000 and 35·6 (25·8–47·5) total deaths of individuals with sickle cell disease per 100 000.

Females and males had comparable disease patterns. Globally, all-age prevalence of sickle cell disease among females in 2021 was 3·90 million (95% UI 3·28–4·61), similar to the all-age prevalence of sickle cell disease among males, which was 3·84 million (3·22–4·58; appendix p 66).

Global all-age total deaths of individuals with sickle cell disease were nearly 11-times higher than cause-specific deaths of individuals with sickle cell disease (appendix pp 67–94). This difference was especially pronounced in south Asia and sub-Saharan Africa in 2021, where total deaths of individuals with sickle cell disease were nearly 67-times and 9-times higher, respectively, than cause-specific deaths of individuals with sickle cell disease.

**Figure 5:**
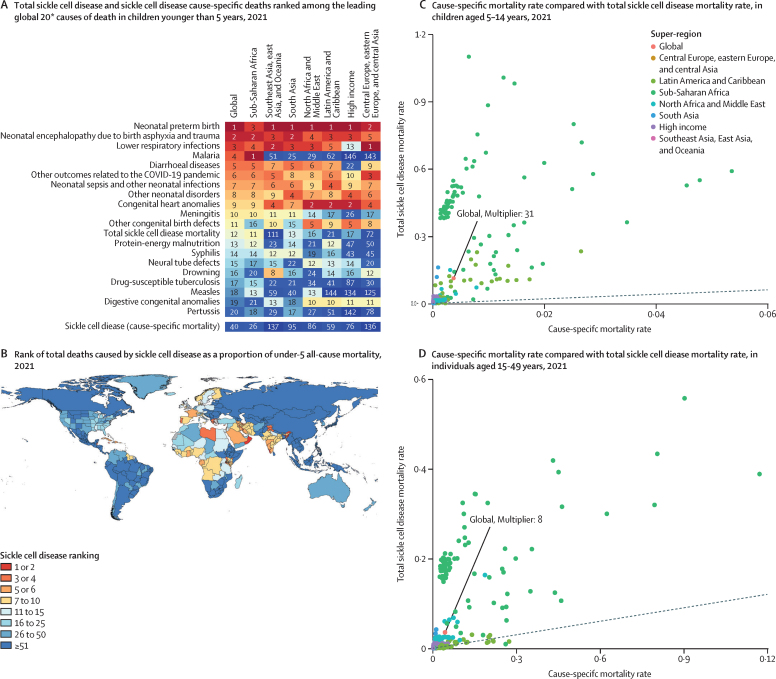
Ranking of total and cause-specific sickle cell disease mortality in children younger than 5 years compared with other GBD causes in 2021, and comparison of cause-specific mortality rates to total sickle cell disease mortality rates in individuals aged 5–14 years and 15–49 years Total under-5 mortality deaths of children with sickle cell disease ranked together with sickle cell disease cause-specific deaths and the leading 20 causes of death globally for each super-region (A) and ranked as a proportion of all-cause under-5 mortality by country (B). The 2021 cause-specific mortality rates are plotted along the x-axis compared with total sickle cell disease mortality rates plotted along the y-axis for the 5–14 years age group (C) and 15–49 years age group (D). *Sickle cell disease cause-specific deaths are not included in the 20 leading causes but are included for comparison to total sickle cell disease. See the appendix (p 65) for rankings by country in 2000.

We gauged the public health effect of sickle cell disease and implications for SDGs 3.2 and 3.4 in three ways. First, examining the mortality burden in children younger than 5 years, there were 81 100 (58 800–108 000) deaths, ranking total sickle cell disease mortality 12th among all GBD causes globally, whereas cause-specific sickle cell disease was ranked 40th (figure 5A). In Portugal, Jamaica, Libya, Oman, and San Marino, total sickle cell disease mortality was in the top three causes of death for children younger than 5 years (figure 5B). Total sickle cell disease mortality rates exceeded cause-specific mortality rates by a factor of 50 or greater in 70 countries in children younger than 5 years, in 51 countries among those aged 5–14 years (figure 5C), and in nine countries in those aged 15–49 years (figure 5D). Second, in countries with high sickle cell disease incidence at birth (more than 500 per 100 000 births), total sickle cell disease mortality comprised, on average, 3·8% (SD 3·6) of total under-5 deaths (appendix p 64). Assessing the total sickle cell disease mortality cause fractions across locations currently not meeting SDG target 3.2, sickle cell disease contributed, on average, 1·9% (SD 2·9) of the under-5 mortality rate (appendix p 64). After multiplying the total sickle cell disease cause fraction by the observed under-5 mortality rate in this subset of locations not meeting SDG target 3.2, total deaths of individuals with sickle cell disease accounted for at least three under-5 deaths per 1000 livebirths in Equatorial Guinea, Benin, Burkina Faso, and Sierra Leone (appendix p 64). Finally, in countries where malaria incidence was above 10 000 per 100 000 population, the contribution of total sickle cell disease mortality to all-cause mortality in the under-5 age group averaged 2·3% (SD 1·9) and was as high as 8·4% (95% UI 4·8–12·0) in Equatorial Guinea (appendix p 64).

## Discussion

Sickle cell disease has a large and growing global public health significance. Over half a million babies were born with sickle cell disease in 2021—with more than three quarters in countries of sub-Saharan Africa—and almost 8 million people were living with sickle cell disease globally. The increase in global births of babies with sickle cell disease was primarily due to a larger proportion of global births occurring in locations with higher sickle cell disease rates, with potential additional effects from migration,^11^ which are difficult to measure due to data lags and absence of universal newborn screening. Total sickle cell disease mortality was nearly 11-times higher than cause-specific sickle cell disease mortality and increased as a proportion of all deaths across all super-regions (except high-income super region) since 2000. Total sickle cell disease mortality consistently ranked in the top 20 causes of death in children younger than 5 years, children aged 5–14 years, and individuals aged 15–49 years in more than half of the super-regions, whereas cause-specific sickle cell disease mortality ranked no higher than 26th—showing the under-recognition of sickle cell disease mortality burden in conventional cause-of-death attribution systems.

Achievement of the SDGs for child mortality will unequivocally require sustained local and global efforts on combating sickle cell disease. Global organisational efforts to fight diseases, such as measles^20^ and tuberculosis,^21^ have contributed to cause-specific mortality declines in children younger than 5 years, yet sickle cell disease has had no such global push. In the face of decreasing all-cause under-5 mortality,^22^ failure to reduce mortality among those with sickle cell disease is exacerbating health inequities and hindering progress towards SDG targets 3.1, 3.2, and 3.4, especially in high-burden regions. Although sickle cell disease can occur in people of all races and ethnicities, it is most commonly found in individuals of African descent. Underlying structural racism has been linked to improper pain management,^23^ differences in research funding,^24^ and stigma^25^, ^26^ that perpetuate inequities. As noted in the WHO report on the African Region sickle-cell strategy from 2010–2020, while all 23 countries defined as high-burden (sickle cell trait prevalence between 20% and 30%) had established a sickle cell disease unit in their ministries of health, only eight countries had allocated annual budget funds for health promotion of sickle cell disease, and only five had allocated funding for newborn screening.^27^

Scarcity of proper diagnostics, data collection, and linkage for sickle cell disease monitoring makes mortality burden estimation challenging and hinders accurate sickle cell disease incidence assessment, even in high-income countries and locations with well resourced vital registration systems.^28^ Relatedly, even in the presence of a known diagnosis of sickle cell disease, physicians or coroners might be reluctant to assign sickle cell disease as the underlying cause of death—a situation that has similarities to mortality certification challenges in conditions such as atrial fibrillation,^29^ HIV,^30^ dementia,^31^ preterm birth,^32^ and congenital heart disease.^33^ The first step in improving outcomes and data quality is early diagnosis.

Universal newborn screening combined with preventive treatment is feasible and effective even in low-resource locations.^34^ Early diagnosis of sickle cell disease allows for risk mitigation and early treatment intervention, which most likely explains some improvement in survival among women with sickle cell disease.^35^, ^36^ This effect is self-evident when considering the other diseases for which sickle cell disease is closely linked. Those with sickle cell disease exposed to malaria are at greater risk of sickle cell crisis and death,^3^, ^37^ have higher risk of pneumococcal disease (particularly children younger than 5 years),^38^, ^39^ diarrhoeal disease, and bone infections linked to vaso-occlusive crisis.^40^ If pregnant, those with sickle cell disease are at increased risk for gestational hypertension, pre-eclampsia and eclampsia, and other diseases contributing to maternal mortality, along with increased likelihood of stillbirth, neonatal mortality, and low birthweight.^9^, ^10^

For newborn screening to be most effective, funding must be designated both for scientific research and building health system capacity. Even in the USA, where newborn screening is universal, there is not a yet a population-based national registry; and in Brazil, a national newborn screening programme exists,^41^ but implementation of a strong system of sickle cell disease care has been very challenging.^42^ In low-income and middle-income countries, newborn screening is rare,^34^ and at present is not universal in any African country—although efforts on this front have accelerated recently in Ghana, Uganda, and Tanzania,^43^ alongside the Consortium on Newborn Screening in Africa.^44^ Importantly, new diagnostic point-of-care test kits^45^, ^46^ can facilitate more widespread adoption of newborn and catch-up screenings; these kits have enabled Nigeria to do the first nationally representative Demographic and Health Survey with sickle cell disease testing and an unprecedented case-control multisite African study of sickle cell disease child mortality.^47^, ^48^ In addition, the SickleInAfrica consortium has established one of the largest registries of individuals with sickle cell disease that spans Ghana, Nigeria, and Tanzania.^49^ Such efforts have the potential to uncover the true sickle cell disease burden in resource-constrained settings. As new methods of screening are implemented and registry systems improve, the number of reported sickle cell disease cases will most likely continue to rise, underscoring the importance of efforts that build better data coverage and widen access to health care.

Newborn screening for sickle cell provides an opportunity for integration with other screening programmes,^50^ genetic counselling and education,^51^ outpatient clinic care,^52^ and intersectoral interventions.^52^, ^53^ While insufficient alone, prevention techniques, such as those in Greece^54^ and Bahrain,^55^ including systematic carrier screening and counselling, knowledge of genotype in partner selection, and option of prenatal diagnosis, can be successful in the reduction of births of babies with sickle cell disease. Integrating sickle cell disease testing into HIV screening,^56^, ^57^ provision of prophylactic antibiotics,^58^, ^59^ malaria chemoprophylaxis,^60^ and routine vaccinations^61^ can help prevent potentially life-threatening infections, and transcranial Doppler screening should be implemented for stroke prevention beginning at the age of 2 years.^62^ In Jamaica, since 2000, a decline in the proportion of all-cause deaths that are deaths of individuals with sickle cell disease might be attributed to implementation of newborn screening, pneumococcal conjugate vaccine use in young children, and tracking neonates in sickle cell clinics.^63^

Alongside prevention, affordable, comprehensive, and timely health care should be made more accessible.^64^, ^65^ Pain management for patients with sickle cell disease must be prioritised,^66^ with improved education for physicians^67^ and policy action to correct the documented disparities in treatment for Black people.^68^ Blood transfusion therapy, parenteral analgesics, and disease-modifying drugs are effective at managing pain, reducing the incidence of acute chest syndrome, infections, malaria, and ultimately decreasing mortality.^69^, ^70^, ^71^, ^72^ Particular attention should be focused on scaling the use of hydroxyurea (also known as hydroxycarbamide), which in a 2020 review, was missing from the list of national drug formularies in India and many countries of southeast Asia.^66^ Pioneering efforts, such as the government of Ghana's 2019 commitment to ensure free access to hydroxyurea for all patients with sickle cell disease through the National Health Insurance Scheme, might serve as a model for other countries.^73^

Although haematopoietic stem-cell transplantation and gene therapy can result in complete disease cure,^74^, ^75^ both require high levels of health-care resources and both can have their own potentially serious health consequences. As of 2021, haematopoietic stem-cell transplantation is only available in six centres in all of Africa,^76^ whereas in the USA, there are 215 centres that report transplantations to the Center for International Blood and Marrow Transplant Research.^77^ Although high-resource settings might be better equipped to provide care to those with sickle cell disease, there is much room for improvement in access to quality treatment, and further research is needed on the multiplicity of factors contributing to negative outcomes.

Our analysis estimated a 2021 global sickle cell disease incidence at birth for the SS and Sβ° genotypes of 386 000 (95% UI 307 000–485 000), within the uncertainty interval of previous estimates produced by Piel and colleagues^12^ of 305 773 (238 400–398 775) in 2010. Our 2021 estimate of 515 000 births (425 000–614 000) already exceeds Piel's forecasted estimate of 404 190 (242 530–657 634) by 2050. In addition to including more genotypes of sickle cell disease (Piel and colleagues only estimated the SS genotype) and estimating disease frequency of sickle cell disease directly (Piel and colleagues back-calculated approximate birth prevalence from estimated gene frequency at birth), our incorporation of data sources not available to Piel and colleagues, inclusion of age-specific prevalence data and mortality data to inform birth incidence in the compartmental model of disease, and spatial-temporal covariates, lend strength to our estimates. Finally, although mortality data are particularly sparse, our results support the findings of Ranque and colleagues' case-control study^47^ in six centres across Africa showing considerably higher mortality risk in children younger than 5 years with sickle cell disease, and even higher risk in children aged 5–9 years. Additionally, our estimates of under-5 total sickle cell disease mortality align with recent studies from Nigeria^78^ and the Kilifi district of Kenya,^79^ which found excess deaths of individuals with sickle cell disease comprised 4·2% and 15·0% of total under-5 mortality (per livebirths), respectively (we estimated 3·1% [95% UI 2·0–4·6%] in Nigeria and 5·6% [95% UI 3·7–8·2%] in Kilifi).

As with all estimation efforts, this study has limitations. First, we were limited by data sparsity in locations with likely high burden. Combined with the strong geographic trends, this creates the potential for location-based modelling to underestimate geographic gradients (such as between Nepal and India). Second, although the GBD quantifies net migration in estimation of population and mortality, specific migration patterns are not captured, such that recent time trends in some countries might not yet be reflected here. Third, our ability to quantify time trends in ICD-coded data for specific genotypes of sickle cell disease might be incomplete since ICD-9 required 5-digit codes for sickle cell disease subtype classification (a granularity unavailable in some data systems), whereas ICD-10 required only 4-digit codes. Fourth, the absence of newborn screening, in combination with deaths assigned to only a single underlying cause, leads to a virtual guarantee of under-reporting of sickle cell disease, since patients are at increased risk for many common causes of death and health-care staff might not have awareness of sickle cell disease.^80^ Explicit guidance for sickle cell disease assessment and consideration as cause of death or contributing factor in death is crucial for addressing these shortcomings.^81^ Fifth, scarce evidence suggests that patients with sickle cell disease infected with SARS-CoV-2 might have more severe clinical outcomes, including increased susceptibility to additional infection, COVID-19-related vaso-occlusive crisis, and amplification of acute chest syndrome or other comorbidities.^82^

The findings of this study highlight the need for a coalescence of efforts to address the large and under-recognised burden of sickle cell disease. As countries strive to reduce child mortality and mortality due to non-communicable diseases, policy makers must consider the growing number of individuals living with sickle cell disease, and the increasing contribution of this disease to all-cause mortality. Progress in improving sickle cell disease health outcomes requires global action, including efficient diagnostic screening, effective case monitoring through population registries, and implementation of high-quality prevention and treatment.

## Data sharing

This study follows the Guidelines for Accurate and Transparent Health Estimates Reporting. To download the data used in these analyses and corresponding results, please visit the Global Health Data Exchange at https://ghdx.healthdata.org/record/ihme-data/gbd-2021-scd-birth-incidence-age-specific-prevalence-total-mortality.

**This online publication has been corrected. The corrected version first appeared at thelancet.com on July 31, 2023**

## Declaration of interests

L M Force reports support for this Article from the Bill & Melinda Gates Foundation; grants or contracts from Conquer Cancer Foundation, St Jude Children's Research Hospital, St Baldrick's Foundation, and National Institutes of Health (NIH) Loan Repayment Program; leadership or fiduciary roles in board, society, committee or advocacy groups, unpaid with Lancet Oncology International Advisory Board, outside the submitted work. K Fuller reports support for this Article from the Institute for Health Metrics and Evaluation and the Gates Foundation. P Gill reports other support as a National Institute for Health and Care Research (NIHR) Senior Investigator; the views expressed in this publication are those of the author and not necessarily those of the NIHR or the UK Department of Health and Social Care; all outside the submitted work. N J Kassebaum reports grant funding support for this Article from the Gates Foundation. K Kewal reports other non-financial support from UGC Centre of Advanced Study, CAS II, Department of Anthropology, Panjab University, Chandigarh, India; all outside the submitted work. F B Piel reports grants or contracts from the National Health Service Race and Health Observatory; consulting fees from Bluebird Bio; payment or honoraria for lectures, presentations, speakers bureaus, manuscript writing or educational events from King's Sickle Cell Preceptorship 2023; support for attending meetings or travel from 4th Global Sickle Cell Disease Congress; participation on a data safety monitoring board or advisory board with Fondation Pierre Fabre; all outside the submitted work. J A Singh reports consulting fees from Crealta/Horizon, Medisys, Fidia, PK Med, Two labs, Adept Field Solutions, Clinical Care Options, ClearView Healthcare Partners, Putnam Associates, Focus Forward, Navigant Consulting, Spherix, MedIQ, Jupiter Life Science, UBM, Trio Health, Medscape, WebMD, and Practice Point Communications; and the NIH and the American College of Rheumatology; payment or honoraria for lectures, presentations, speakers bureaus, manuscript writing or educational events from the speaker's bureau of Simply Speaking; support for attending meetings or travel from the steering committee of OMERACT; participation on a data safety monitoring board or advisory board as a member of the US Food and Drug Administration Arthritis Advisory Committee; leadership or fiduciary roles in board, society, committee or advocacy groups, paid or unpaid with OMERACT as a steering committee member; leadership or fiduciary roles in board, society, committee or advocacy groups, paid or unpaid with the Veterans Affairs of Rheumatology Field Advisory Committee as a chair; leadership or fiduciary roles in board, society, committee or advocacy groups, paid or unpaid with UAB Cochrane Musculoskeletal Group Satellite Center on Network Meta-analysis as the editor and director; stock or stock options in TPT Global Tech, Vaxart Pharmaceuticals, Atyu Biopharma, Adaptimmune Therapeutics, GeoVax Labs, Pieris Pharmaceuticals, Enzolytics, Seres Therapeutics, Tonix Pharmaceuticals, and Charlotte's Web Holdings, with previously owned stock options in Amarin, Viking, and Moderna Pharmaceuticals; all outside the submitted work.
