# Correction to *Lancet Haematol* 2023; 10: e585–99

**DOI:** 10.1016/S2352-3026(23)00215-6

**Published:** 2023-07-31

*GBD 2021 Sickle Cell Disease Collaborators. Global, regional, and national prevalence and mortality burden of sickle cell disease, 2000–2021: a systematic analysis from the Global Burden of Disease Study 2021.* Lancet Haematol 2023; **10:** e585–99—In this Article, the colour fill of Ukraine and India in the maps in figures 3 and 5 have been corrected, and the appendix has also been updated. These corrections have been made as of July 31, 2023.

