# Supplementary material for: Global, regional, and national prevalence and mortality burden of sickle cell disease, 2000–2021: a systematic analysis from the Global Burden of Disease Study 2021
Source: Lancet Haematol. 2023 Jun 15;10(8):e585–99. doi: 10.1016/S2352-3026(23)00118-7 (PMC10390339; doi:10.1016/S2352-3026(23)00118-7)
Supplement: Supplementary appendix [file mmc1.pdf]

THE LANCET
Haematology

Supplementary appendix

This appendix formed part of the original submission and has been peer reviewed. We post it as supplied by the authors.

Supplement to: GBD 2021 Sickle Cell Disease Collaborators. Global, regional, and national prevalence and mortality burden of sickle cell disease, 2000–2021: a systematic analysis from the Global Burden of Disease Study 2021. *Lancet Haematol* 2023; published online June 15. https://doi.org/10.1016/S2352-3026(23)00118-7.

Appendix to "Global, regional, and national prevalence and mortality burden of sickle cell disease among children and adults, 2000–2021: a systematic analysis from the Global Burden of Disease Study 2021"

Outline

Contents

Outline 1

GATHER Checklist 4

Supplementary methods 5

Dimensions of GBD 5

Figure S1. Map of locations in each GBD region and super region 6

Overview and definitions 7

Table S1. International Classification of Diseases versions 9 and 10 (ICD-9 and ICD-10) codes used for estimation of three subtypes of sickle cell disorders 8

Figure S2. Analytical flowchart for the estimation of sickle cell disorders 9

Data identification and processing 9

Scientific literature 10

Figure S3A. Prisma diagram for sickle cell disorders systematic reviews, 2013 & 2016 10

Clinical administrative data 11

Household surveys 11

Age and sex splitting 12

Systematic evaluation of input data and identification of outliers 12

Cause-specific mortality data 12

Table S2A. Mortality input data source counts of nationals with subnationals 14

Table S2B. Prevalence input data source counts of nationals with subnationals 14

Table S2C. Cause of death mortality input data source counts of nationals with subnationals 14

Data citations 15

Table S3. Clinical data citations for SCD genotypes 15

Table S4. Scientific literature data citations for SCD genotypes. 17

Table S5. Cause of death cause specific mortality data citations for sickle cell disorders and haemoglobinopathies and haemolytic anaemias. 23

Figure S3B. Data availability maps showing number of sources of prevalence and mortality data for homozygous sickle cell and severe sickle cell/beta-thalassaemia (A, B), haemoglobin SC disease (C, D), and mild sickle cell disease (E, F). 50

Figure S3C. Data availability maps showing types of cause-specific mortality data input sources including demographic surveillance, scientific literature, survey, vital registration, verbal autopsy, and estimates, for haemoglobinopathies and hemolytic anaemias (F) and sickle cell disorders (I). 51

Figure S4. Sankey diagram showing cause-specific mortality due to sickle cell disease before and after garbage code redistribution of International Classification of Diseases (ICD) version 9 and version 10 codes for all years, all age groups, and both sexes combined for selected examples including global ICD-10 (A), USA ICD-9 (B), USA ICD-10 (C), Ghana ICD-10 (D), South Africa ICD-10 (E), and India ICD-10 (F) 52

Modelling 53

DisMod-MR 2.1 53

Figure S5. Compartmental model of disease that describes how a population moves from health to a diseased state, to death (or back to health) 53

Table S6. DisMod-MR 2.1 covariate betas 55

Causes of Death Ensemble modelling (CODEm) 55

Hybridization approach for final cause-specific mortality estimates 56

Covariates used in modelling 56

Table S7. Covariates, including level and direction, used in Cause of Death Ensemble modelling of haemoglobinopathies and haemolytic anaemia 57

Table S8. Covariates, including level and direction, used in Cause of Death Ensemble modelling of sickle cell disorders 57

Anaemia causal attribution 58

Decomposition Analysis 58

CoDCorrect 58

References 60

Author Contributions 61

Supplemental Results: Figures 63

Figure S6. Geographical distribution of total sickle cell disease death and disability burden (per 100,000) in males and females combined in 2000 by four measures: birth incidence (A), all-ages prevalence (B), all-ages cause-specific mortality (C) and total SCD mortality (D) 63

Figure S7. Among males and females combined in 2021, in locations with birth incidence rate above 500 per 100,000 livebirths, total SCD deaths as a percentage of under-5 deaths (A), in locations not currently meeting Sustainable Development Goal 3.2 for U5MR (under-5 mortality rate), total SCD deaths as a percentage of under-5 deaths (B), U5MR attributed

to total SCD deaths alone (C), and in locations with over 10,000 incident malaria cases per 100,000, total SCD deaths as a percentage of under-5 deaths 64

Figure S8. Total sickle cell disease deaths ranked by super-region and country as a proportion of all-cause mortality in children under 5 years (A and B) and cause-specific mortality rate compared to total SCD mortality rate in 5-14 years age group and 15-49 years age group (C and D). 65

Figure S9. Geographical distribution of total sickle cell disease all-age prevalence (per 100,000) in males (A) and females (B) 66

Table S9. Number of births, cases, total deaths, and under-5 deaths for sickle cell disease, 2000 - 2021 67

Table S10. Number of births, cases, total deaths, and under-5 deaths for Homozygous sickle cell and severe sickle cell/beta-thalassemia, 2000 - 2021 95

Table S11. Number of births, cases, total deaths, and under-5 deaths for Hemoglobin SC disease, 2000 - 2021 123

Table S12. Number of births, cases, total deaths, and under-5 deaths for Mild sickle cell/beta-thalassemia, 2000 - 2021 151

Table S13. Total sickle cell disease frequency: Birth rate, all-age prevalence and mortality rates, under-5 mortality rates, and mortality ranks, 2000 - 2021....................................... 179

Table S14. Homozygous sickle cell and severe sickle cell/beta-thalassemia disease frequency: Birth rate, age-specific and all-age prevalence rates, 2000 - 2021.................... 207

Table S15. Hemoglobin SC disease frequency: Birth rate, age-specific and all-age prevalence rates, 2000 - 2021 235

Table S16. Mild sickle cell/beta-thalassemia disease frequency: Birth rate, age-specific and all-age prevalence rates, 2000 - 2021 263

Table S17. Under-5 sickle cell disease mortality metrics in 2000, 2010, and 2021 291

Table S18. 5-14 sickle cell disease mortality metrics in 2000, 2010, and 2021 319

Table S19. Under-20 sickle cell disease mortality metrics in 2000, 2010, and 2021 347

Table S20. 15-49 sickle cell disease mortality metrics in 2000, 2010, and 2021 375

Table S21. 50 plus sickle cell disease mortality metrics in 2000, 2010, and 2021 403

Table S22. All age sickle cell disease mortality metrics in 2000, 2010, and 2021................ 431

Table S23. Age standardized sickle cell disease mortality metrics in 2000 and 2021 459

Table S24. Decomposition of change, sickle cell disease cases, 2000-2021........................ 487

GATHER Checklist

Item #	Checklist item	Reported on page #
**Objectives and funding**		
**1**	Define the indicator(s), populations (including age, sex, and geographic entities), and time period(s) for which estimates were made.	Main manuscript methods dimensions and definitions section and appendix supplementary methods dimensions of GBD section
**2**	List the funding sources for the work.	Main manuscript funding statement
**Data Inputs**		
For all data inputs from multiple sources that are synthesized as part of the study:		
**3**	Describe how the data were identified and how the data were accessed.	Main manuscript methods data sources and processing section and appendix supplementary methods data identification and processing section
**4**	Specify the inclusion and exclusion criteria. Identify all ad-hoc exclusions.	Main manuscript methods data sources and processing section and appendix supplementary methods data identification and processing section
**5**	Provide information on all included data sources and their main characteristics. For each data source used, report reference information or contact name/institution, population represented, data collection method, year(s) of data collection, sex and age range, diagnostic criteria or measurement method, and sample size, as relevant.	Main manuscript methods data sources and processing section and appendix supplementary methods data identification and processing section. Detailed data sources for each component available online (https://ghdx.healthdata.org/record/ihme-data/gbd-2021-scd-birth-incidence-age-specific-prevalence-total-mortality)
**6**	Identify and describe any categories of input data that have potentially important biases (e.g., based on characteristics listed in item 5).	Main manuscript methods data sources and processing section and appendix supplementary methods data identification and processing section
For data inputs that contribute to the analysis but were not synthesized as part of the study:		
**7**	Describe and give sources for any other data inputs.	Main manuscript methods data sources and processing section and appendix supplementary methods data identification and processing section
For all data inputs:		
**8**	Provide all data inputs in a file format from which data can be efficiently extracted (e.g., a spreadsheet rather than a PDF), including all relevant meta-data listed in item 5. For any data inputs that cannot be shared because of ethical or legal reasons, such as third-party ownership, provide a contact name or the name of the institution that retains the right to the data.	Detailed data sources for each component available online (https://ghdx.healthdata.org/record/ihme-data/gbd-2021-scd-birth-incidence-age-specific-prevalence-total-mortality)
**Data analysis**		
**9**	Provide a conceptual overview of the data analysis method. A diagram may be helpful.	Main manuscript methods modelling strategy section and appendix supplementary methods modelling section
**10**	Provide a detailed description of all steps of the analysis, including mathematical formulae. This description should cover, as relevant, data cleaning, data pre-processing, data adjustments and weighting of data sources, and mathematical or statistical model(s).	Main manuscript methods data sources and processing, and modelling strategy sections and appendix supplementary methods data

		identification and processing, and modelling sections
**11**	Describe how candidate models were evaluated and how the final model(s) were selected.	
**12**	Provide the results of an evaluation of model performance, if done, as well as the results of any relevant sensitivity analysis.	
**13**	Describe methods for calculating uncertainty of the estimates. State which sources of uncertainty were, and were not, accounted for in the uncertainty analysis.	Main manuscript methods uncertainty section and appendix supplementary methods modelling section
**14**	State how analytic or statistical source code used to generate estimates can be accessed.	All statistical code for SCD-specific methods available online through our GitHub project: sickle_cell_disease_gbd_2021 (https://ghdx.healthdata.org/record/ihme-data/gbd-2021-scd-birth-incidence-age-specific-prevalence-total-mortality). Code pages for shared GBD processes will be available at https://github.com/ihmeuw/ihme-modeling/tree/master/gbd_2021/shared_code.)
**Results and Discussion**		
**15**	Provide published estimates in a file format from which data can be efficiently extracted.	Results are publicly available from the GHDx (https://ghdx.healthdata.org/record/ihme-data/gbd-2021-scd-birth-incidence-age-specific-prevalence-total-mortality)
**16**	Report a quantitative measure of the uncertainty of the estimates (e.g., uncertainty intervals).	Main manuscript results text includes 95% uncertainty intervals. Uncertainty intervals are also present in the online Results Tool.
**17**	Interpret results in light of existing evidence. If updating a previous set of estimates, describe the reasons for changes in estimates.	Main manuscript discussion section
**18**	Discuss limitations of the estimates. Include a discussion of any modelling assumptions or data limitations that affect interpretation of the estimates.	Main manuscript discussion section

This checklist should be used in conjunction with the GATHER statement and Explanation and Elaboration document, found on gather-statement.org

Supplementary methods

Dimensions of GBD

This analysis uses estimates from the 2021 Global Burden of Diseases, Injuries, and Risk Factors (GBD) Study which evaluated age, geographical, and temporal patterns for 369 diseases and injuries and 87 risks. The GBD methods and input data comply with the Guidelines for Accurate and Transparent Health Estimates Reporting (GATHER), as reported in Table S1.

The GBD estimates health loss for 204 countries and territories grouped across 21 regions and seven super-regions, with additional subnational estimates produced for 21 countries (Italy, Nigeria, Pakistan, the Philippines, Poland, Brazil, China, Ethiopia, India, Indonesia, Iran, Japan, Kenya, Mexico, New

Zealand, Norway, Russia, South Africa, Sweden, the United Kingdom, and the United States of America) at a more granular level. The GBD produces prevalence and disability estimates between the years 2000 and 2021, and cause-specific mortality estimates for years 1980 to 2021, for 23 age groups and two sexes. We note that sex and gender are not mutually exclusive and that while sex is a biological variable most often assigned at birth, gender is a social construct. Due to limitations in data collection, data presented here refer only to males and females.

Within the GBD, diseases and injuries are organised in a tiered cause hierarchy from Level 1 being most broad to Level 4 being most specific. Sickle cell disorders are classified as "Other non-communicable diseases" and are a fourth-level cause of death and disability. All input data for all data measures used to generate estimates within the GBD are publicly available in the Global Health Data Exchange (GHDx) interactive tool, and further visualizations outside the scope of this analysis using GBD 2019 results can be viewed in the GBD Compare tool at https://vizhub.healthdata.org/gbd-compare/.

Figure S1. Map of locations in each GBD region and super region

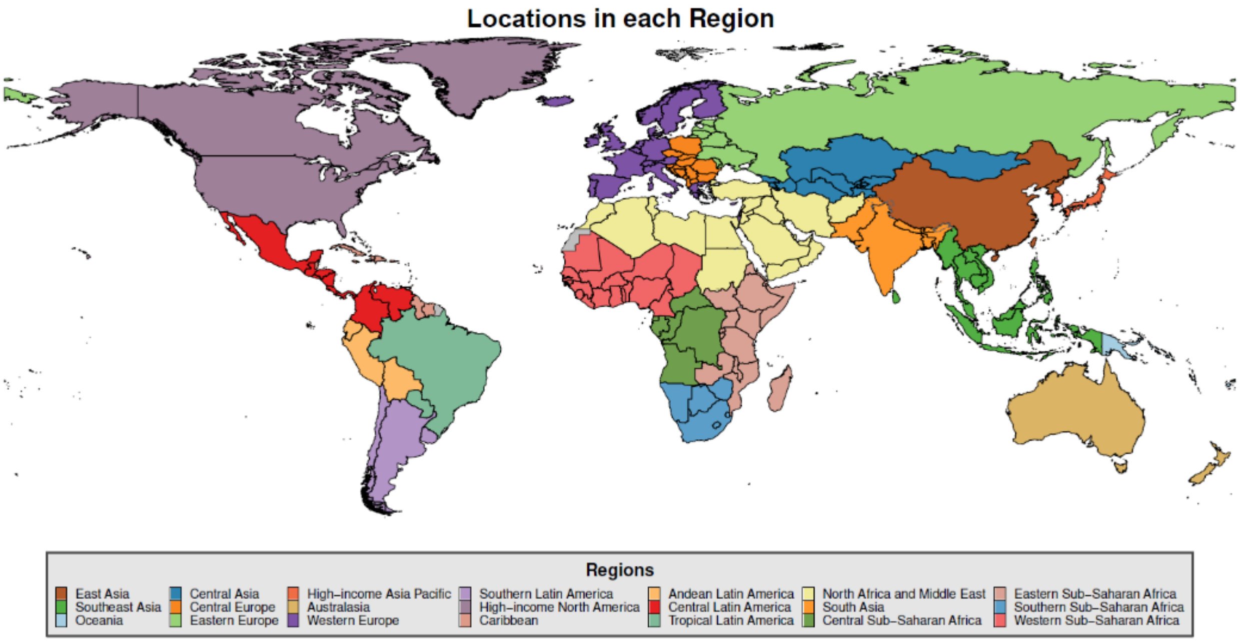

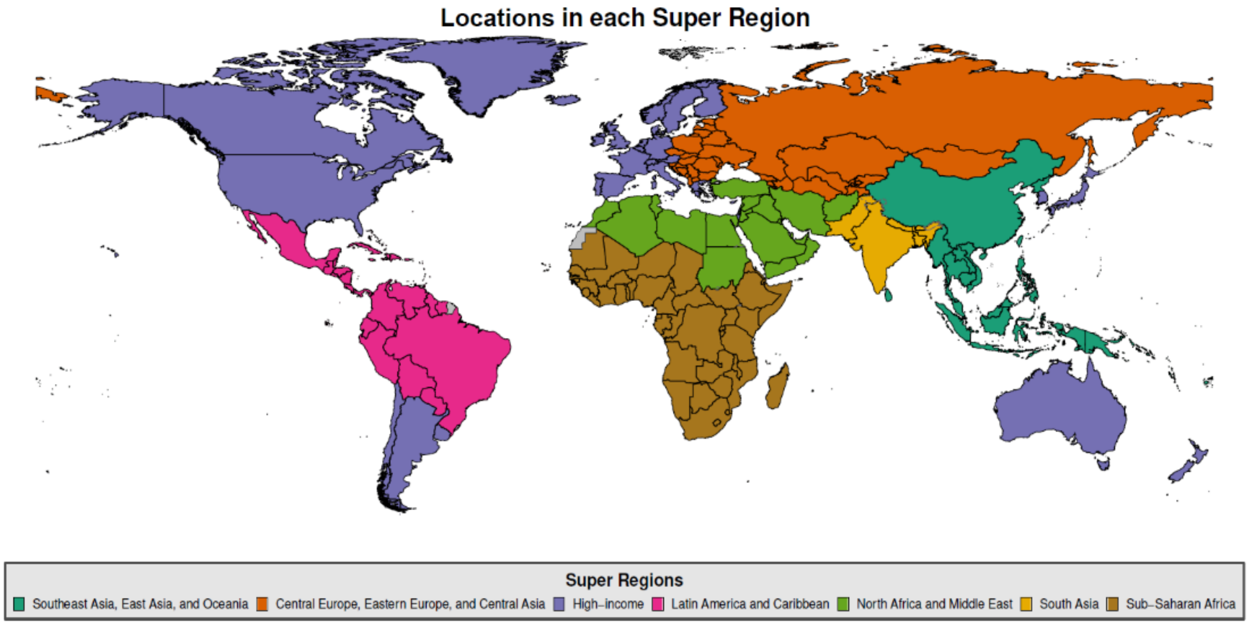

Overview and definitions

Sickle cell disorders as modelled within the GBD include ICD-10 codes D57-D57.8 (excluding sickle cell trait D57.3) and ICD-9 code 282.6 as shown in Table S2. Three distinct sickle cell genotypes, each of differing severity, are together classified as sickle cell disorders in the GBD modelling framework and are referred to throughout this analysis as sickle cell genotypes: 1) homozygous sickle cell and severe sickle cell/beta thalassaemia (SS and Sβ°), 2) sickle cell-haemoglobin C disease (SC), and 3) mild sickle cell/beta-thalassaemia ($S\beta^{+}$). Sickle cell trait is excluded from classification as a sickle cell disorder and is not covered in this paper but is included in GBD overall. Clinical data with ICD codes classified as "Other sickle-cell disorders" were mapped to Sβ+.

As illustrated in the estimation flowchart in Figure S2, the estimation processes for cause-specific mortality and non-fatal health outcomes from sickle cell disorders were closely linked both to each other and to estimation of burden due to the overall group of haemoglobinopathies and haemolytic anaemias. In brief, our non-fatal and mortality estimates were produced in tandem methods using two primary tools, the Cause of Death Ensemble model (CODEm), and DisMod-MR 2.1, an age-integrating mixed effects Bayesian meta-regression tool that is built on a compartmental model of disease to leverage all available input data on prevalence and survival to generate age-specific prevalence models that were internally consistent with respect to prevalence, incidence, remission, and excess mortality. We modelled non-fatal disease burden for the three sickle cell sub-causes using DisMod-MR 2.1, summing the three sub-cause estimates to create a total. CODEm was used to estimate cause-specific mortality for haemoglobinopathies overall for all GBD locations. We then developed a second set of CODEm models for each of the sub-causes of haemoglobinopathies (sickle cell disorders, thalassaemias, glucose-6-phosphate dehydrogenase (G6PD) deficiency, and other) only for "data-rich" (DR) locations with long time series of high-quality vital registration (VR) data. Final estimates for non-DR locations were generated by summing and scaling the DisMod-MR 2.1 cause-specific mortality rate (CSMR) results for three subtypes of sickle cell disorders, three subtypes of thalassaemias, and G6PD deficiency. What

follows is a more complete description of each of these steps including data identification, data processing, modelling, and processing of model results.

Table S1. International Classification of Diseases versions 9 and 10 (ICD-9 and ICD-10) codes used for estimation of three subtypes of sickle cell disorders

ICD-10	ICD-10 name	Causes of death (COD)	Non-fatal		
			HbSS and HbSβ°	HbSC	HbSβ+
**D57**	Sickle-cell disorders	x		x	
**D57.0**	Hb-SS disease with crisis	x	x		
**D57.00**	Hb-SS disease with crisis, unspecified	x	x		
**D57.01**	Hb-SS disease with acute chest syndrome	x	x		
**D57.02**	Hb-SS disease with splenic sequestration	x	x		
**D57.1**	Sickle-cell disease without crisis	x	x		
**D57.2**	Sickle-cell/Hb-C disease	x		x	
**D57.20**	Sickle-cell/Hb-C disease without crisis	x		x	
**D57.21**	Sickle-cell/Hb-C disease with crisis	x		x	
**D57.211**	Sickle-cell/Hb-C disease with acute chest syndrome	x		x	
**D57.212**	Sickle-cell/Hb-C disease with splenic sequestration	x		x	
**D57.219**	Sickle-cell/Hb-C disease with crisis, unspecified	x		x	
**D57.3**	Sickle-cell trait	x			
**D57.4**	Sickle-cell thalassaemia	x	x		
**D57.40**	Sickle-cell thalassaemia without crisis	x	x		
**D57.41**	Sickle-cell thalassaemia with crisis	x	x		
**D57.411**	Sickle-cell thalassaemia with acute chest syndrome	x	x		
**D57.412**	Sickle-cell thalassaemia with splenic sequestration	x	x		
**D57.419**	Sickle-cell thalassaemia with crisis, unspecified	x	x		
**D57.8**	Other sickle-cell disorders	x			x
**D57.80**	Other sickle-cell disorders without crisis	x			x
**D57.81**	Other sickle-cell disorders with crisis	x			x
**D57.811**	Other sickle-cell disorders with acute chest syndrome	x			x
**D57.812**	Other sickle-cell disorders with splenic sequestration	x			x
**D57.819**	Other sickle-cell disorders with crisis, unspecified	x			x
**ICD-9**	**ICD-9 name**	**Causes of death (COD)**	**Non-fatal**		
			HbSS and HbSβ°	HbSC	HbSβ+
**282.41**	Sickle-cell thalassaemia without crisis		x		
**282.42**	Sickle-cell thalassaemia with crisis		x		
**282.44**	Beta thalassaemia				
**282.6**	Sickle-cell disease	x	x		
**282.61**	Hb-SS disease without crisis	x	x		
**282.62**	Hb-SS disease with crisis	x	x		
**282.63**	Sickle-cell/Hb-C disease without crisis	x		x	
**282.64**	Sickle-cell/Hb-C disease with crisis	x		x	
**282.68**	Other sickle-cell disease without crisis	x			x
**282.69**	Other sickle cell disease with crisis	x			x

Figure S2. Analytical flowchart for the estimation of sickle cell disorders

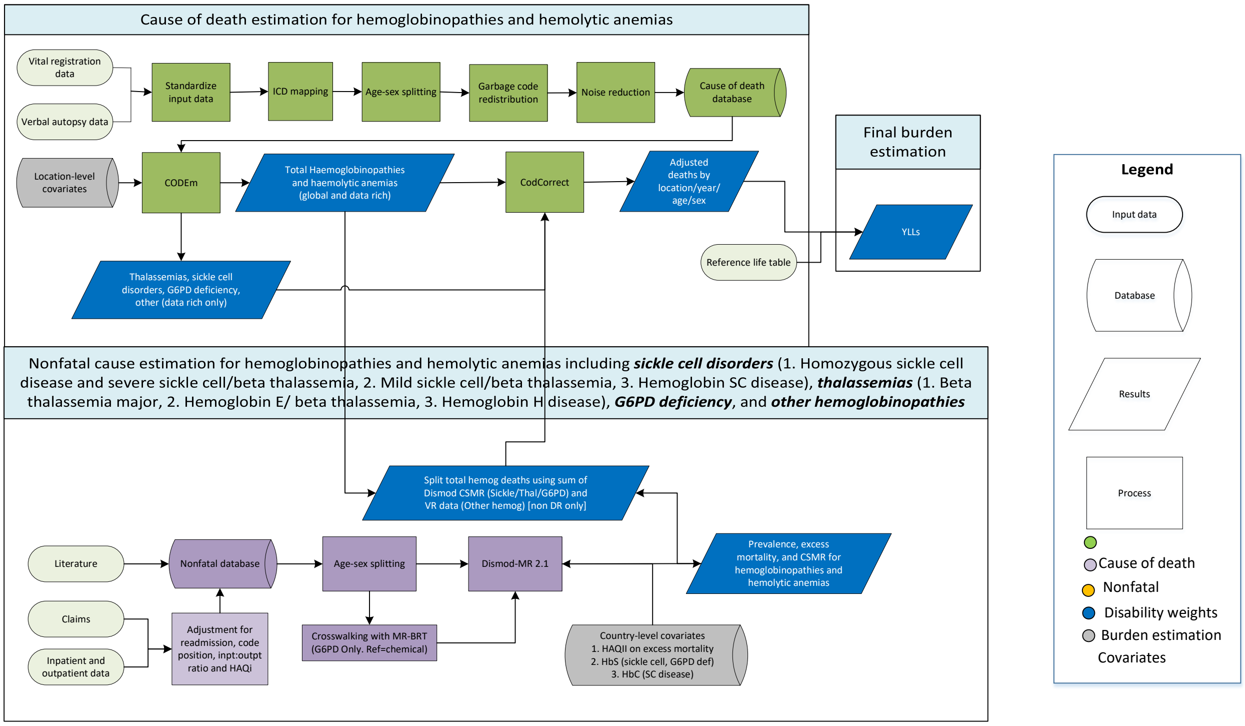

<u>Hemoglobinopathies and hemolytic anemias:</u> Sickle cell disorders, Thalassemias, Glucose-6-phosphate dehydrogenase (G6PD) deficiency, Sickle cell trait, Thalassemia trait, Hemizygous G6PD deficiency, Other hemoglobinopathies and hemolytic anemias

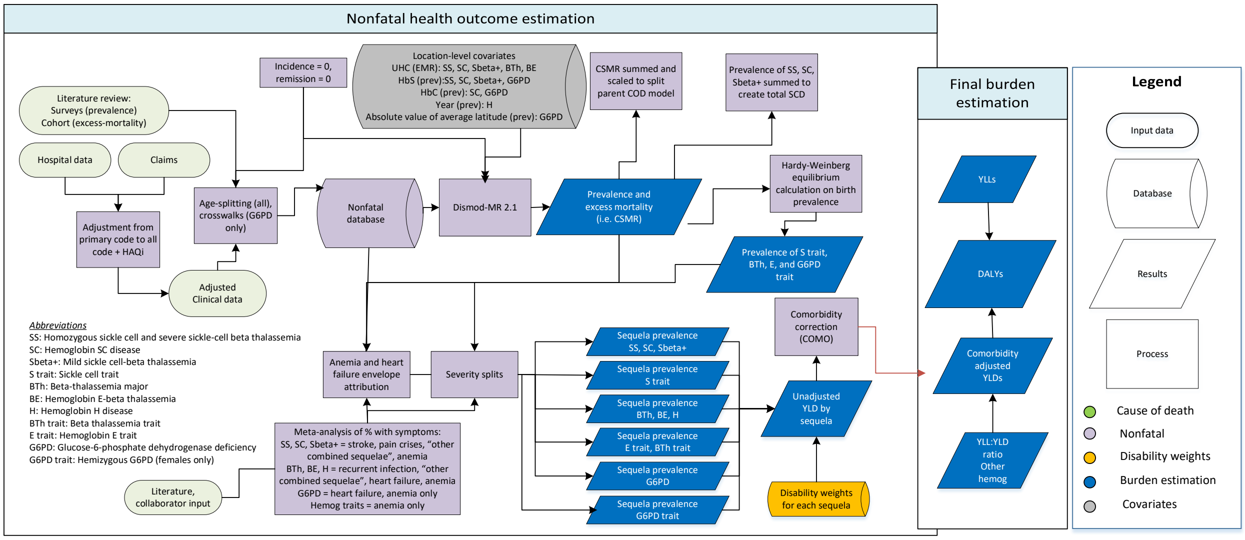

Data identification and processing

Data inputs for cause-specific mortality models came primarily from vital registration (VR), verbal autopsy (VA), and mortality surveillance sources that were processed for all GBD causes in parallel and stored in a central Causes of Death (COD) database. Prevalence data were extracted primarily from clinical administrative data sources, published scientific literature, and a small number of household surveys and newborn screening sources. Excess mortality rate (EMR) data that were input into DisMod-

MR 2.1 models came from mortality and survival data identified and extracted from population-based registries and cohort studies for specific subtypes of sickle cell disorders. Each of the steps of data identification and processing are described in more detail below.

Scientific literature

We conducted a systematic review for GBD 2013 and GBD 2016 using the following search strings for sickle cell disorders:

> (sickle cell[Title/Abstract] AND (mortality[Title/Abstract] OR survival[Title/Abstract] OR prevalence[Title/Abstract] OR incidence[Title/Abstract]) AND (2013/04/01[PDat] : 2016/12/31[PDat])) AND "humans"[MeSH Terms]

As shown in the PRISMA diagram in Figure S3A, for GBD 2013, a total of 639 sources were screened, 151 were selected for full text review, and 85 were extracted. For GBD 2016, a total of 488 titles and abstracts were screened in the sickle cell search; 49 were reviewed, and 22 were extracted. We extracted any data which fit our case definitions for sickle cell disorders (homozygous sickle cell, compound heterozygous HbS-thal, hemoglobin SC disease, compound heterozygous HbS-beta+), excluding any data defined as sickle cell trait, and excluding any data where the results were themselves a product of modelling. When sources reported multiple levels of granularity, data were extracted at the most granular level possible. Data measures extracted included prevalence, with-condition mortality, and excess mortality. Of note, presence of disease at birth is extracted as birth prevalence. However, we report this measure as "incidence" within the GBD outputs.

Figure S3A. Prisma diagram for sickle cell disorders systematic reviews, 2013 & 2016

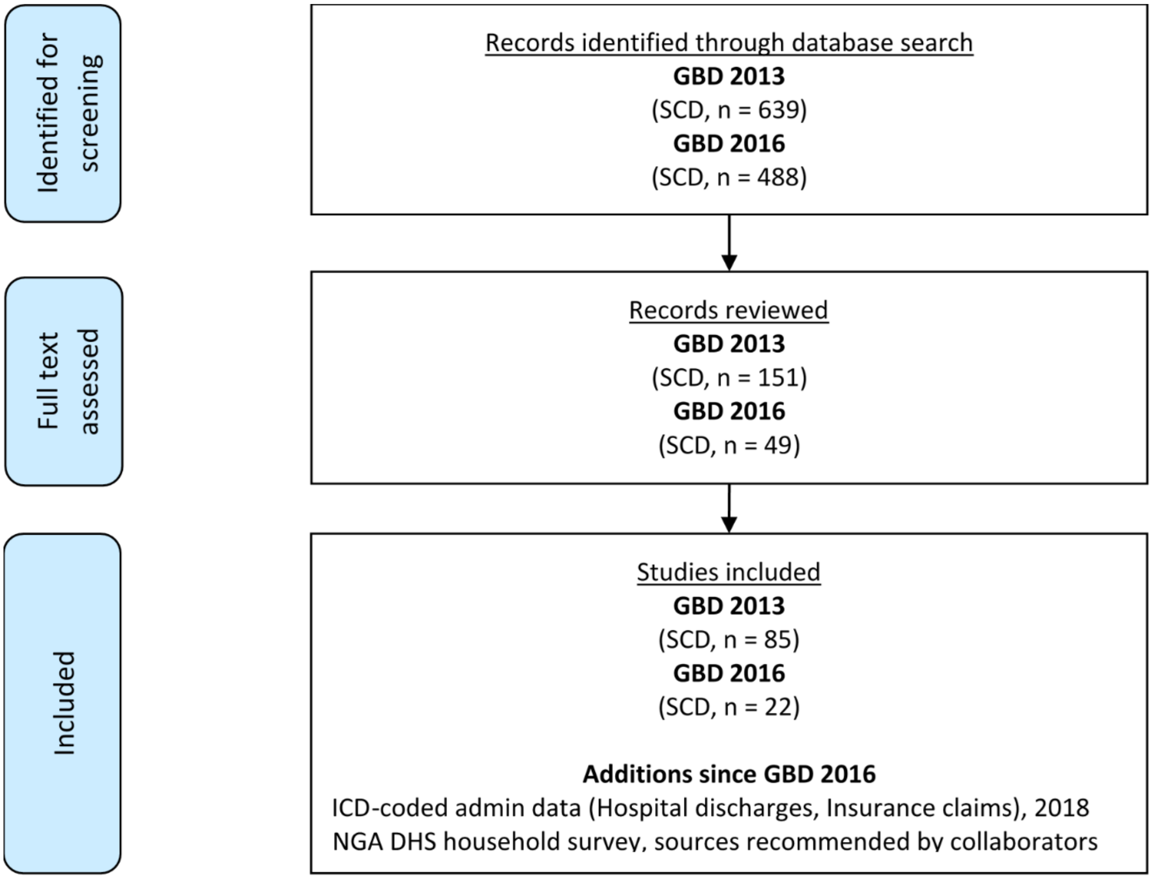

Clinical administrative data

We utilised clinical administrative data extracted from hospital inpatient discharges (admission for at least one overnight stay), outpatient visits, and health insurance claims data. This process is described in more detail in the most recent GBD publication on diseases and injuries.[1] Briefly, clinical data were processed to account for five main potential sources of bias: 1) sources that do not report on population denominators, 2) multiple admissions for the same patient, 3) multiple diagnoses within a single inpatient admission, 4) ratio between outpatient cases observed for any inpatient case (accounting for cases that did not go to the hospital), and 5) population access to, and quality of, health care.

To convert discharge data without population denominators into usable fractions, we multiplied source-specific admission fractions (cause-specific admissions / total admissions) from administrative data sources by matching inpatient utilisation rates (total admissions / population) by location, year, age group, and sex. Input data were modelled using spatiotemporal Gaussian process regression (ST-GPR) to smooth over age, time, and location to complete estimates for the annual inpatient utilisation envelope. Subsequent adjustments ratios (2–4) were calculated using all clinical inpatient sources including MarketScan and Taiwan (province of China) claims data, claims and inpatient data from Singapore, the Philippines, Ecuador, New Zealand, and the Healthcare Cost Utilization Project State Inpatient Database (HCUP SID). Estimates for the log of ratios were created through a mixed-effects model using the MR-BRT (meta-regression—Bayesian, regularised, trimmed) tool to estimate the log of the ratios between non-primary to primary diagnosis, as well as outpatient to inpatient care. Finally, to account for inequitable access to health care, a scalar derived from the Healthcare Access and Quality (HAQ) Index was applied to the estimates.[1] This scalar approximates the proportion of the population that would be represented in hospital admissions based on access. A scalar of less than 1 would increase the number of admissions to account for individuals in the population who would have been represented in hospital admissions, if they had access to a facility.

The HAQ Index is estimated from causes for which disease burden is reduced with access to quality health care. These include vaccine-preventable diseases, maternal and child infectious diseases, and cancers.[2] With an understanding that differences in mortality exist across regions, underlying geographical variation in risk exposure is captured by standardising mortality rates by location and age. The standardised mortality rates are then log transformed and scaled from zero to 100 across the GBD estimation framework.

Household surveys

This analysis used data from the 2018 Nigeria Demographic and Health Survey (DHS) Biomarker Questionnaire, which conducted genotype testing for sickle cell disease and sickle cell trait among children ages 6–59 months and measured haemoglobin levels among women and children. The BioMedomics™ Sickle SCAN® was used as a diagnostic test, which provides rapid results within five minutes. Results were validated against a sample of confirmatory high-performance liquid chromatography testing. Microdata were extracted at the most detailed subnational level and aggregated to the national level for a total of three age groups: 6–11 months, 12–23 months, and 2–4 years. Data were age pooled in subnational locations for a single age group of 6 months to 4 years.

Age and sex splitting

All data from clinical administrative sources were age- and sex-specific. A portion of the scientific literature data were available only in broader age ranges or for both sexes combined. Based on multiple rounds of experience modelling for GBD, we have found that models perform more consistently when data are sex-specific and divided into specific age groups prior to modelling, rather than relying on DisMod-MR 2.1 to infer the relationships. When input prevalence data existed in broader age groups or aggregated over sexes rather than in GBD specific categorisations, we pulled GBD 2019 model estimates to generate age and sex weights, which we then used to split input data. We used previous round model results rather than clinical informatics data because the latter come preferentially from higher-resource settings, so is thought to not reflect as accurately the age pattern of sickle cell disorders as did previous model results.

Systematic evaluation of input data and identification of outliers

Our data processing methods for clinical administrative data are robust and were applied across all GBD causes in an equivalent manner. One notable issue identified for sickle cell disorders is that when location-specific sample sizes of uncorrected data were quite small and stochastic, the application of sequential correction factors compounded implausible age patterns, leading to apparently increasing prevalence with increasing age (which is known to be impossible). We additionally outliered data from sources with small sample sizes that reported implausibly high or low prevalence values. Examples include prevalence >2% in any location, >1% in high-income super-regions, and prevalence of 0% at birth in western and central sub-Saharan Africa. Outliered data remained in the dataset but were excluded from the model itself. Final input datasets for each of the sickle cell disorders models (3 genotypes) are illustrated in Figure S3A for prevalence and 3B for excess mortality.

Cause-specific mortality data

Sickle cell disorders in GBD are one of four sub-causes of the overall cause of "haemoglobinopathies and haemolytic anaemias." The remaining three sub-causes are thalassaemias, G6PD deficiency, and other haemoglobinopathies and haemolytic anaemias. Data processing and modelling for each of the four sub-causes and parent cause were completed in parallel. A complete description of Cause of Death (CoD) data processing and modelling is available in the most recent GBD publication on Diseases and injuries.[1] Briefly, VR, verbal autopsy, surveillance, survey, and minimally invasive tissue sample (MITS) data were assigned to International Classification of Diseases and Injuries (ICD) codes and systematically processed and stored in the CoD database as cause-specific mortality data. Viable data from 1980 to 2019 are updated biannually or more frequently when new sources become available. Deaths from causes that cannot ultimately cause death or lack specificity are reassigned to underlying causes of death via a process of redistribution. Redistribution algorithms consist of proportionate redistribution, fixed proportion redistribution, and statistical algorithms. Causes redistributed to sickle cell disorders can be seen in Figure S4.

When data exhibit high spatial and temporal stochasticity, there is risk of inappropriate overfitting and underfitting with many modelling approaches. Additionally, observations of zero deaths are eminently possible in countries with small populations and/or for comparatively uncommon conditions, but because many modelling approaches use log- or logit-transformation, all of these observations would be ignored and lead to upward bias. To solve this, we apply an empirical Bayesian noise reduction algorithm to the CoD data such that locations with stochastic trends and smaller populations give higher weight to

the prior, and less weight to the observed value, and vice versa in larger populations. This prior is calculated via predictions from cause- and sex-specific Poisson regressions on data grouped by criteria such as source type (eg, VR), population size, and endemicity of malaria.

Additionally, if a datum was originally zero, and after noise reduction, it was adjusted to an extraordinarily small non-zero number, there is a risk of extreme downward bias in modelling approaches with log- and logit-transformed input data. To solve this, we applied a "non-zero floor" to each dataset. This floor is specific by age and sex and is the minimum cause fraction a given datapoint can be across all locations and years, determined by national-level VR data that were originally non-zero prior to noise reduction. Next, after the non-zero floor has been applied, we calculate a linear floor—or an overall minimum value for the model—by converting the non-zero cause fractions to rates per 100,000, taking the lowest non-zero floor rate across ages and calculating $e^{\ln(non-zero\ floor)-2}$. Outliers were identified in an iterative modelling process during which model fit was evaluated for consistency with epidemiological evidence relative to age, sex, and geographical and temporal patterns. This resulted in near total outliering of data from many verbal autopsy sources in locations with sparse data coverage and low data quality. Input datasets for each of haemoglobinopathies and haemolytic anaemias and sickle cell disorders are illustrated in Figure S3 with national input data counts in Tables S2A–S2C.

Table S2A. Mortality input data source counts of nationals with subnationals

Country	Homozygous sickle cell and severe sickle cell/beta-thalassaemia	Haemoglobin SC disease	Mild sickle cell/ beta-thalassaemia	Total SCD
Brazil	2	2	2	0
India	0	0	1	0
Iran	2	3	1	0
Italy	0	0	2	0
Kenya	0	0	1	0
Mexico	1	1	1	0
New Zealand Maori	18	18	18	0
New Zealand non-Maori	18	18	18	0
Nigeria	3	3	1	0
Pakistan	0	0	1	0
Philippines	3	3	3	0
Sweden	19	19	19	0
USA	25	24	24	9

Table S2B. Prevalence input data source counts of nationals with subnationals

Country	Homozygous sickle cell and severe sickle cell/beta-thalassaemia	Haemoglobin SC disease	Mild sickle cell/ beta-thalasseamia	Total SCD
Brazil	2	2	2	6
Ethiopia	0	0	0	1
India	0	0	1	2
Iran	2	3	1	6
Italy	0	0	2	4
Japan	0	0	0	1
Kenya	0	0	1	2
Mexico	1	1	1	3
New Zealand Maori	18	18	18	54
New Zealand non-Maori	18	18	18	54
Nigeria	3	3	1	7
Pakistan	0	0	1	2
Philippines	3	3	3	9
Poland	0	0	0	2
South Africa	0	0	0	1
Sweden	19	19	19	57
USA	25	24	24	73

Table S2C. Cause of death mortality input data source counts of nationals with subnationals

Country	Haemoglobinopathies	Sickle cell disorders
New Zealand Maori population	36	36
New Zealand non-Maori population	36	36
Ukraine	38	7

Data citations

Table S3. Clinical data citations for SCD genotypes

Citation	NID	Type of clinical data	Locations covered	Genotype HbSS/SS and Sβ°	Hemoglobin SC disease	Mild sickle cell/ beta thalassaemia (Sβ+)
Center for Health Statistics and Information, National Health and Family Planning Commission (China), Shanghai Municipal Center for Disease Control and Prevention (Shanghai CDC), Shanghai Health Information Center. China Hospital Inpatient Data 2013-2016.	337619	inpatient	China (some subnationals)	0	1	0
Ministry of Health (Italy). Italy Hospital Inpatient Discharges 2005-2007.	334464	inpatient	Italy (all subnationals)	1	1	1
Ministry of Health (Italy). Italy Hospital Inpatient Discharges 2008-2012.	334465	inpatient	Italy (all subnationals)	1	1	1
Ministry of Health (Mexico). Mexico Ministry of Health Hospital Discharges 2013-2015	404395	inpatient	Mexico (all subnationals)	1	1	1
Nazareth Hospital, Shillong, JSS Hospital, Mysore, King George's Medical University (India). India Hospital Inpatient Data 2014-2017.	337129	inpatient	Karnataka	1	1	0
St. John's Medical College Hospital (India). India - Bangalore St. John's Medical College Hospital Inpatient Data 2017.	354896	inpatient	Karnataka, Urban	1	1	0
Department of Health Services, Ministry of Health and Population (Nepal). Nepal Hospital Inpatient Discharges 2010-2012.	292575	inpatient	Nepal	1	1	0
Department of Health Services, Ministry of Health and Population (Nepal). Nepal Hospital Inpatient Discharges 2013-2014.	292437	inpatient	Nepal	1	1	0
Directorate of Health Statistics and Information, Ministry of Health (Argentina). Argentina Public Hospital Injury Discharges, Hospital Discharges by Diagnosis and Public Hospital Discharge Statistics 2010-2012. Buenos Aires, Argentina: Directorate of Health Statistics and Information, Ministry of Health (Argentina).	433061	inpatient	Argentina	1	0	0
Federal Ministry of Health (Austria), Statistics Austria. Austria Hospital Inpatient Discharges 1989-1992.	234670	inpatient	Austria	1	1	1
Federal Ministry of Health (Austria), Statistics Austria. Austria Hospital Inpatient Discharges 1993-1997.	234671	inpatient	Austria	1	1	1
Federal Ministry of Health (Austria), Statistics Austria. Austria Hospital Inpatient Discharges 1998-2002.	234672	inpatient	Austria	1	1	1
Federal Ministry of Health (Austria), Statistics Austria. Austria Hospital Inpatient Discharges 2003-2007.	234673	inpatient	Austria	1	1	1
Federal Ministry of Health (Austria), Statistics Austria. Austria Hospital Inpatient Discharges 2008-2012.	234674	inpatient	Austria	1	1	1
Federal Ministry of Health (Austria), Statistics Austria. Austria Hospital Inpatient Discharges 2013-2014.	284419	inpatient	Austria	1	1	1
Federal Statistical Office (Germany). Germany Hospital Discharges by Diagnosis 2009. Wiesbaden, Germany: Federal Statistical Office (Germany), 2011.	67132	inpatient	Germany	1	1	1
Greece - Regional General University Hospital of Larissa Inpatient Data 2013-2017.	433059	inpatient	Greece	1	1	1
Greece - Regional General University Hospital of Larissa Inpatient Data 2018-2019.	433060	inpatient	Greece	1	1	1
Healthcare Cost and Utilization Project (HCUP), Agency for Healthcare Research and Quality (AHRQ). United States State Inpatient Databases 2003-2007.	284421	inpatient	United States of America (some subnationals)	1	1	1
Healthcare Cost and Utilization Project (HCUP), Agency for Healthcare Research and Quality (AHRQ). United States State Inpatient Databases 2008-2009.	284422	inpatient	United States of America (some subnationals)	1	1	1
Institute for Maternal and Child Health - IRCCS "Burlo Garofolo", Ministry of Health (Italy). Italy Hospital Inpatient Discharges 2018.	421047	inpatient	Italy (all subnationals)	1	1	1
Ministry of Health (Botswana). Botswana Health Management Data System 2008-2009.	411100	inpatient	Botswana	1	1	0
Ministry of Health (Brazil). Brazil Hospital Information System 1992.	104241	inpatient	Brazil (all subnationals)	1	1	1
Ministry of Health (Brazil). Brazil Hospital Information System 1993-1997.	439739	inpatient	Brazil (all subnationals)	1	1	1
Ministry of Health (Brazil). Brazil Hospital Information System 1998-2002.	234692	inpatient	Brazil (all subnationals)	1	1	1
Ministry of Health (Brazil). Brazil Hospital Information System 2003-2007.	234693	inpatient	Brazil (all subnationals)	1	1	1
Ministry of Health (Brazil). Brazil Hospital Information System 2008-2012.	234703	inpatient	Brazil (all subnationals)	1	1	1
Ministry of Health (Brazil). Brazil Hospital Information System 2013-2014.	234704	inpatient	Brazil (all subnationals)	1	1	1
Ministry of Health (Chile). Chile Hospital Discharges 2001-2002.	287201	inpatient	Chile	1	1	1
Ministry of Health (Chile). Chile Hospital Discharges 2003-2007.	287202	inpatient	Chile	1	1	1
Ministry of Health (Chile). Chile Hospital Discharges 2008-2012.	287203	inpatient	Chile	1	1	1
Ministry of Health (Chile). Chile Hospital Discharges 2013-2017. Santiago, Chile: Ministry of Health (Chile).	430710	inpatient	Chile	1	1	1
Ministry of Health (Chile). Chile Hospital Discharges 2018. Santiago, Chile: Ministry of Health (Chile).	422874	inpatient	Chile	1	1	1
Ministry of Health (Italy). Italy Hospital Inpatient Discharges 2013-2017.	439738	inpatient	Italy (all subnationals)	1	1	1
Ministry of Health (Jordan). Jordan Al-Bashir Hospital Discharges 2016.	317423	inpatient	Jordan	0	1	0
Ministry of Health (Kenya). Kenya National Inpatient Morbidity and Mortality Statistics 1999.	133665	inpatient	Kenya	0	0	1
Ministry of Health (Mexico). Mexico Ministry of Health Hospital Discharges 2000-2002.	234745	inpatient	Mexico (all subnationals)	1	1	1
Ministry of Health (Mexico). Mexico Ministry of Health Hospital Discharges 2003-2007.	234746	inpatient	Mexico (all subnationals)	1	1	1
Ministry of Health (Mexico). Mexico Ministry of Health Hospital Discharges 2008-2012.	234747	inpatient	Mexico (all subnationals)	1	1	1
Ministry of Health (New Zealand). New Zealand National Minimum Dataset 2000-2002.	284439	inpatient	New Zealand (all subnationals)	1	1	1
Ministry of Health (New Zealand). New Zealand National Minimum Dataset 2003-2007.	284440	inpatient	New Zealand (all subnationals)	1	1	1
Ministry of Health (New Zealand). New Zealand National Minimum Dataset 2008-2012.	284442	inpatient	New Zealand (all subnationals)	1	1	1
Ministry of Health (New Zealand). New Zealand National Minimum Dataset 2013-2017.	439737	inpatient	New Zealand (all subnationals)	1	1	1
Ministry of Health (Portugal). Portugal Hospital Inpatient Discharges 2015.	285520	inpatient	Portugal	1	0	0
Ministry of Health (Turkey). Turkey Diagnosis-Related Group Hospital Inpatient Database 2011-2012.	287207	inpatient	Türkiye	1	1	1
Ministry of Health and Medical Education (Iran). Iran Hospital Data 2001-2010.	331084	inpatient	Iran (Islamic Republic of)	0	1	0
Ministry of Health and Welfare (Taiwan). Taiwan National Health Insurance Claims Data 2016.	336203	claims	Taiwan (Province of China)	1	0	1
Ministry of Health, Labour and Welfare (Japan). Japan Diagnosis Procedure Combination Database 2010-2012.	336851	inpatient	Japan (all subnationals)	1	1	0
Ministry of Health, Labour and Welfare (Japan). Japan Diagnosis Procedure Combination Database 2013-2015.	336852	inpatient	Japan (all subnationals)	1	1	0
NHS England. United Kingdom - England Hospital Episode Statistics 2001-2002.	234764	inpatient	England (subnationals)	0	1	0

NHS England. United Kingdom - England Hospital Episode Statistics 2003-2007.	234762	inpatient	England (subnationals)	0	1	0
NHS England. United Kingdom - England Hospital Episode Statistics 2008-2012.	234765	inpatient	England (subnationals)	0	1	0
NHS England. United Kingdom - England Hospital Episode Statistics 2013-2014.	292577	inpatient	England (subnationals)	0	1	0
National Board of Health and Welfare (Sweden). Sweden National Patient Register 1998-2002.	234758	inpatient	Sweden	1	1	1
National Board of Health and Welfare (Sweden). Sweden National Patient Register 2003-2007.	234760	inpatient	Sweden	1	1	1
National Board of Health and Welfare (Sweden). Sweden National Patient Register 2008-2012.	234761	inpatient	Sweden	1	1	1
National Board of Health and Welfare (Sweden). Sweden National Patient Register 2013-2016.	408336	inpatient	Sweden	1	1	1
National Center for Disease Control and Public Health (Georgia). Georgia Hospital Data 2013-2014.	321359	inpatient	Georgia	1	1	0
National Center for Disease Control and Public Health (Georgia). Georgia Hospital Discharges 2016-2017.	407536	inpatient	Georgia	1	1	0
National Center for Health Statistics (NCHS), Centers for Disease Control and Prevention (CDC). United States National Hospital Discharge Survey 1988-1992.	234766	inpatient	United States of America	1	1	1
National Center for Health Statistics (NCHS), Centers for Disease Control and Prevention (CDC). United States National Hospital Discharge Survey 1993-1997.	234769	inpatient	United States of America	1	1	1
National Center for Health Statistics (NCHS), Centers for Disease Control and Prevention (CDC). United States National Hospital Discharge Survey 1998-2002.	234771	inpatient	United States of America	1	1	1
National Center for Health Statistics (NCHS), Centers for Disease Control and Prevention (CDC). United States National Hospital Discharge Survey 2003-2007.	234772	inpatient	United States of America	1	1	1
National Center for Health Statistics (NCHS), Centers for Disease Control and Prevention (CDC). United States National Hospital Discharge Survey 2008-2010.	234774	inpatient	United States of America	1	1	1
National Health Fund (Poland). Poland National Health Fund Patient Claims 2015.	397812	claims	Poland (all subnationals)	1	1	1
National Health Fund (Poland). Poland National Health Fund Patient Claims 2016.	397813	claims	Poland (all subnationals)	1	1	1
National Health Fund (Poland). Poland National Health Fund Patient Claims 2017.	397814	claims	Poland (all subnationals)	1	1	1
National Health Fund (Poland). Poland National Health Fund Patient Claims 2018.	431674	claims	Poland (all subnationals)	1	1	1
National Institute of Statistics and Censuses (Ecuador). Ecuador Hospital Inpatient Discharges 1993-1997.	234738	inpatient	Ecuador	1	1	1
National Institute of Statistics and Censuses (Ecuador). Ecuador Hospital Inpatient Discharges 1998-2002.	234740	inpatient	Ecuador	1	1	1
National Institute of Statistics and Censuses (Ecuador). Ecuador Hospital Inpatient Discharges 2003-2007.	234741	inpatient	Ecuador	1	1	1
National Institute of Statistics and Censuses (Ecuador). Ecuador Hospital Inpatient Discharges 2008-2012.	234742	inpatient	Ecuador	1	1	1
National Institute of Statistics and Censuses (Ecuador). Ecuador Statistical Registry of Hospital Beds and Discharges 2018. Quito, Ecuador: National Institute of Statistics and Censuses (Ecuador).	422318	inpatient	Ecuador	1	1	1
National Institute of Statistics and Censuses (Ecuador). Ecuador Statistical Registry of Hospital Beds and Expenses 2013-2017.	439735	inpatient	Ecuador	1	1	1
National Statistics Institute (Spain). Spain Hospital Morbidity Survey 2015-2017. Madrid, Spain: National Statistics Institute (Spain).	433115	inpatient	Spain	1	1	1
Norwegian Directorate of Health. Norway Patient Register 2008-2012.	234750	inpatient	Norway (all subnationals)	1	1	1
Philippine Health Insurance Corporation. Philippine Health Insurance Corporation Claims 2013-2016.	292574	inpatient	Philippines	1	1	1
Regional General University Hospital of Larissa (Greece). Greece - Regional General University Hospital of Larissa Inpatient Data 2012.	432298	inpatient	Greece	1	1	1
Truven Health Analytics. United States MarketScan Claims and Medicare Data - 2000. Ann Arbor, United States: Truven Health Analytics.	244369	claims	United States of America (all subnationals)	1	1	1
Truven Health Analytics. United States MarketScan Claims and Medicare Data - 2010. Ann Arbor, United States: Truven Health Analytics.	244370	claims	United States of America (all subnationals)	1	1	1
Truven Health Analytics. United States MarketScan Claims and Medicare Data - 2012. Ann Arbor, United States: Truven Health Analytics.	244371	claims	United States of America (all subnationals)	1	1	1
Truven Health Analytics. United States MarketScan Claims and Medicare Data 2011. Ann Arbor, United States: Truven Health Analytics.	336850	claims	United States of America (all subnationals)	1	1	1
Truven Health Analytics. United States MarketScan Claims and Medicare Data 2013. Ann Arbor, United States: Truven Health Analytics.	336849	claims	United States of America (all subnationals)	1	1	1
Truven Health Analytics. United States MarketScan Claims and Medicare Data 2014. Ann Arbor, United States: Truven Health Analytics.	336848	claims	United States of America (all subnationals)	1	1	1
Truven Health Analytics. United States MarketScan Claims and Medicare Data 2015. Ann Arbor, United States: Truven Health Analytics.	336847	claims	United States of America (all subnationals)	1	1	1
Truven Health Analytics. United States MarketScan Claims and Medicare Data 2016. Ann Arbor, United States: Truven Health Analytics.	408680	claims	United States of America (all subnationals)	1	1	1
Truven Health Analytics. United States MarketScan Claims and Medicare Data 2017. Ann Arbor, United States of America: Truven Health Analytics.	433114	claims	United States of America (all subnationals)	1	1	1
World Health Organization Regional Office for Europe (WHO/Europe). European Hospital Morbidity Database, 2008. Copenhagen, Denmark: World Health Organization Regional Office for Europe (WHO/Europe).	3822	inpatient	Belgium, Switzerland, Cyprus, Czechia, Denmark, Finland, Croatia, Iceland, Lithuania, Luxembourg, Latvia, Malta, Poland, Romania, Serbia, Slovakia, Slovenia	0	1	0

* NID (node identifier) corresponds to a unique identifier assigned upon extraction and upload into the Global Health Data Exchange (GHDx). The NID can be used to search for the source on ghdx.healthdata.org. The 0 and 1 represent whether the citation applies to the genotype (1= yes, 0 = no)

Table S4. Scientific literature data citations for SCD genotypes.

Citation	NID	Locations covered	Genotype HbSS/SS and Sβ°	Hemoglobin SC disease	Mild sickle cell/ beta thalassaemia (Sβ+)
Abarca G, Navarrete M, Trejos R, de Cspedes C, Saboro M. Abnormal haemoglobins in the newborn human population of Costa Rica. Rev Biol Trop. 2008; 56(3): 995-1001.	116039	Costa Rica	1	1	0
Adewuyi JO, Gwanzura C. Frequency of the sickle haemoglobin gene in Zimbabwe. East Afr Med J. 1994; 71(3): 204-6.	142070	Zimbabwe	1	0	0
Agasa B, Bosunga K, Opara A, Tshilumba K, Dupont E, Vertongen F, Cotton F, Gulbis B. Prevalence of sickle cell disease in a northeastern region of the Democratic Republic of Congo: what impact on transfusion policy. Transfus Med. 2010; 20(1): 62-5.	116026	Democratic Republic of the Congo	1	0	0
Aidoo M, Terlouw DJ, Kolczak MS, McElroy PD, ter Kuile FO, Kariuki S, Nahlen BL, Lal AA, Udhayakumar V. Protective effects of the sickle cell gene against malaria morbidity and mortality. Lancet. 2002; 359(9314): 1311-2.	110042	Kisumu	1	0	0
Akinkugbe FM. Anaemia in a rural population in Nigeria (Ilora). Ann Trop Med Parasitol. 1980; 74(6): 625-33.	141384	Oyo	1	1	0
Akinyanju OO. A profile of sickle cell disease in Nigeria. Ann N Y Acad Sci. 1989; 565: 126-36.	96445	Lagos	1	1	0
Al Hosani H, Salah M, Osman HM, Farag HM, Anvery SM. Incidence of haemoglobinopathies detected through neonatal screening in the United Arab Emirates. East Mediterr Health J. 2005; 11(3): 300-7.	96446	United Arab Emirates	1	0	0
Al Hosani H, Salah M, Osman HM, Farag HM, El-Assiouty L, Saade D, Hertecant J. Expanding the comprehensive national neonatal screening programme in the United Arab Emirates from 1995 to 2011. East Mediterr Health J. 2014; 20(1): 17-23.	292589	United Arab Emirates	1	1	1
Al Kahtani MA, AlQahtani M, Alshebaily MM, Abd Elzaher M, Moawad A, Aljohani N. Morbidity and pregnancy outcomes associated with sickle cell anemia among Saudi women. Int J Gynaecol Obstet. 2012; 119(3): 224-6.	116024	Saudi Arabia	1	0	0
Al-Allawi 0, Al-Dousky AA. Frequency of haemoglobinopathies at premarital health screening in Dohuk, Iraq: implications for a regional prevention programme. East Mediterr Health J. 2010; 16(4): 381-5.	116044	Iraq	0	0	1
Al-Awamy BH, Al-Muzan M, Al-Turki M, Serjeant GR. Neonatal screening for sickle cell disease in the Eastern Province of Saudi Arabia. Trans R Soc Trop Med Hyg. 1984; 78(6): 79294.	142076	Saudi Arabia	1	0	0
Al-Riyami A, Ebrahim GJ. Genetic Blood Disorders Survey in the Sultanate of Oman. J Trop Pediatr. 2003; 49(Suppl 1): 1-20.	110030	Oman	1	0	0
Allen SJ, Bennett S, Riley EM, Rowe PA, Jakobsen PH, O'Donnell A, Greenwood BM. Morbidity from malaria and immune responses to defined Plasmodium falciparum antigens in children with sickle cell trait in The Gambia. Trans R Soc Trop Med Hyg. 1992; 86(5): 494-8.	142051	Gambia	1	1	0
Almeida AM, Henthorn JS, Davies SC. Neonatal screening for haemoglobinopathies: the results of a 10-year programme in an English Health Region. Br J Haematol. 2001; 112(1): 32-5.	96447	United Kingdom	1	1	1
Amoako N, Asante KP, Adjei G, Awandare GA, Bimi L, Owusu-Agyei S. Associations between red cell polymorphisms and Plasmodium falciparum infection in the middle belt of Ghana. PLoS One. 2014; 9(12): e112868.	287701	Ghana	1	1	0
Angastiniotis M, Modell B, Englezos P, Boulyjenkov V, World Health Organization (WHO). Prevention and control of haemoglobinopathies. Bull World Health Organ. 1995; 73(3): 375-86.	96448	Bahrain, France, Nigeria	1	1	0
Arjunan A. Prevalence of the beta-s Gene and Sickle Cell Disease in India [masters thesis]. Pittsburgh, Pennsylvania: University of Pittsburgh; 2013.	227982	Maharashtra, Urban	1	0	0
Atkinson SH, Rockett K, Sirugo G, Bejon PA, Fulford A, O'Connell MA, Bailey R, Kwiatkowski DP, Prentice AM. Seasonal childhood anaemia in West Africa is associated with the haptoglobin 2-2 genotype. PLoS Med. 2006; 3(5): e172.	142050	Gambia	1	0	0
Azev<ea>do ES, Alves AF, Da Silva MC, Souza MG, Muniz Dias Lima AM, Azevedo WC. Distribution of abnormal hemoglobins and glucose-6-phosphate dehydrogenase variants in 1200 school children of Bahia, Brazil. Am J Phys Anthropol. 1980; 53(4): 509-12.	142040	Bahia	1	1	0
Ballardini E, Tarocco A, Marsella M, Bernardoni R, Carandina G, Melandri C, Guerra G, Patella A, Zucchelli M, Ferlini A, Bigoni S, Ravani A, Garani G, Borgna-Pignatti C. Universal neonatal screening for sickle cell disease and other haemoglobinopathies in Ferrara, Italy. Blood Transfus. 2013; 11(2): 245-9.	292593	Emilia-Romagna	1	1	1
Ballas SK, Park D, Wapner RJ. Neonatal screening for sickle cell disease in a metropolitan university hospital: efficacy and problems. J Med Screen. 1994; 1(4): 229-32.	142064	United States of America	1	0	0
Bardakdjian-Michau J, Bahuau M, Hurtrel D, Godart C, Riou J, Mathis M, Goossens M, Badens C, Ducrocq R, Elion J, Perini JM. Neonatal screening for sickle cell disease in France. J Clin Pathol. 2009; 62(1): 31-3.	96449	France	1	1	1
Bernstein SC, Bowman JE, Kaptue Noche L. Population studies in Cameroon: hemoglobin S, glucose-6-phosphate dehydrogenase deficiency and falciparum malaria. Hum Hered. 1980; 30(4): 251-8.	116020	Cameroon	1	0	0
Berthet S, Monpoux F, Soummer A-M, Brard E, Sarles J, Badens C. Neonatal screening for sickle cell disease at the Nice University Hospital: the last 8 years. Arch Pediatr. 2010; 17(12): 1652-6.	116040	France	1	0	1
Biondi G, Calandra PL, Coppa A, Falcone G, Rickards O, Vecchi F. Distribution of the S and C hemoglobins in Atakora District (Benin). Hum Biol. 1980; 52(2): 205-13.	142035	Benin	1	1	0
Bird AR, Ellis P, Wood K, Mathew C, Karabus C. Inherited haemoglobin variants in a South African population. J Med Genet. 1987; 24(4): 215-9.	110035	Western Cape	1	1	0
Boemer F, Vanbellinghen J-F, Bours V, Schoos R. Screening for sickle cell disease on dried blood: a new approach evaluated on 27,000 Belgian newborns. J Med Screen. 2006; 13(3): 132-6.	142033	Belgium	1	0	0
Bouva MJ, Mohrmann K, Brinkman HBJM, Kemper-Proper EA, Elvers B, Loeber JG, Verheul FEAM, Giordano PC. Implementing neonatal screening for haemoglobinopathies in the Netherlands. J Med Screen. 2010; 17(2): 58-65.	116043	Netherlands	1	1	0
Brandelise S, Pinheiro V, Gabetta CS, Hambleton I, Serjeant B, Serjeant G. Newborn screening for sickle cell disease in Brazil: the Campinas experience. Clin Lab Haematol. 2004; 26(1): 15-9.	110040	São Paulo	1	1	1
Buchanan JG, Jha BK, Matthews JR, Nixon AD. The prevalence and nature of anemia among apparently normal subjects in Fiji. Pathology. 1979; 11(3): 36976.	227962	Fiji	1	1	1

Cardoso MA, Scopel KKG, Muniz PT, Villamor E, Ferreira MU. Underlying factors associated with anemia in Amazonian children: a population-based, cross-sectional study. PLoS One. 2012; 7(5): e36341.	116013	Acre	1	0	0
Carlos AM, Souza RA, Souza BM, Pereira Gde A, Tostes Junior S, Martins PR, Moraes-Souza H. Hemoglobinopathies in newborns in the southern region of the Triangulo Mineiro, Brazil. Cross-sectional study. Sao Paulo Med J. 2015; 133(5): 439-44.	285961	Minas Gerais	1	1	1
Cholnoky P. Is breast-feeding in Hungary satisfactory. Acta Paediatr Hung. 1984; 25(1-2): 33-8.	145045	France	1	1	0
Cresta M, Avoundgoba N. Risultati dello studio longitudinale dalla nascita a 5 anni in un gruppo di bambini di Porto Novo (Repubblica Popolare del Benin). Riv Antropol. 1980; 61: 43-132.	142037	Benin	1	1	0
Danquah I, Ziniel P, Eggelte TA, Ehrhardt S, Mockenhaupt FP. Influence of haemoglobins S and C on predominantly asymptomatic Plasmodium infections in northern Ghana. Trans R Soc Trop Med Hyg. 2010; 104(11): 713-9.	116042	Ghana	1	1	0
Dash S, Chhanhimi L, Chhakchhuak L, Zomawaia E. Screening for haemoglobinopathies and G6PD deficiency among the Mizos of Mizoram: a preliminary study. Indian J Pathol Microbiol. 2005; 48(1): 178.	291106	Mizoram	1	1	1
Day TG, Thein SL, Drasar E, Dick MC, Height SE, O'Driscoll S, Rees DC. Changing pattern of hospital admissions of children with sickle cell disease over the last 50 years. J Pediatr Hematol Oncol. 2011; 33(7): 491-5.	116037	Greater London	1	0	0
De Ara<fa>jo MCPE, Serafim ESS, de Castro Jr WAP, de Medeiros TMD. Prevalence of abnormal hemoglobins in newborns in Natal, Rio Grande do Norte, Brazil . Cad Saude Publica. 2004; 20(1): 123-8.	110020	Rio Grande do Norte	1	0	0
Delicat-Loembet LM, Elguero E, Arnathau C, Durand P, Ollomo B, Ossari S, Mezui-me-ndong J, Mbang Mboro T, Becquart P, Nkoghe D, Leroy E, Sica L, Gonzalez JP, Prugnolle F, Renaud F. Prevalence of the sickle cell trait in Gabon: a nationwide study. Infect Genet Evol. 2014; 25: 52-6.	292591	Gabon	1	1	1
Devoucoux R, Hurpin C, Baudon D, Molez JF, Roux JF, Guilloud-Bataille M, Carnevale P, Feingold J. Population genetics of abnormal haemoglobins in Burkina Faso, West Africa. Ann Hum Biol. 1991; 18(4): 295-302.	142036	Burkina Faso, Niger	1	1	0
Diniz D, Guedes C, Barbosa L, Tauil PL, Magalh<e3>es I. Prevalence of sickle cell trait and sickle cell anemia among newborns in the Federal District, Brazil, 2004 to 2006. Cad Saude Publica. 2009; 25(1): 188-94.	110021	Distrito Federal	1	0	0
El-Hazmi MAF, Warsy AS. Appraisal of sickle-cell and thalassaemia genes in Saudi Arabia. East Mediterr Health J. 1999; 5(6): 1147-53.	96451	Saudi Arabia	1	0	0
Elderdery AY, Mohamed BA, Cooper AJ, Knight G, Mills J. Tribal distribution of haemoglobinopathies in a Sudanese patient population. J Med Lab Diagn. 2011; 2(4): 317.	292787	Sudan	1	1	1
Erazo BM, Ramirez GA, Cerrato LE, Pinto LJ, Castro EJ, Yanez NJ, Montoya B, Fontecha GA. Prevalence of Hb S (HHB: c.20A > T) in a Honduran population of African descent. Hemoglobin. 2015; 39(2): 134-7.	292583	Honduras	1	1	1
Ewing N, Powars D, Hilburn J, Schroeder WA. Newborn diagnosis of abnormal hemoglobins from a large municipal hospital in Los Angeles. Am J Public Health. 1981; 71(6): 629-31.	142065	California	1	1	0
Fattoum S, Abbes S. Some data on the epidemiology of hemoglobinopathies in Tunisia. Hemoglobin. 1985; 9(4): 423-9.	110047	Tunisia	1	0	0
Federal Ministry of Health (Nigeria), ICF International, National Population Commission (NPC). Nigeria Demographic and Health Survey 2018. Fairfax, United States of America: ICF International, 2020.	408484	Nigeria (all subnationals)	1	1	0
Fernandes APPC, Janurio JN, Cangussu CB, Macedo DL de, Viana MB. Mortality of children with sickle cell disease: a population study. J Pediatr (Rio J). 2010; 86(4): 279-84.	96452	Minas Gerais	1	1	1
Fleming AF, Storey J, Molineaux L, Iroko EA, Attai ED. Abnormal haemoglobins in the Sudan savanna of Nigeria. I. Prevalence of haemoglobins and relationships between sickle cell trait, malaria and survival. Ann Trop Med Parasitol. 1979; 73(2): 16172.	138582	Kano	1	1	0
Fonseca GHH, Souza R, Salemi VMC, Jardim CVP, Gualandro SFM. Pulmonary hypertension diagnosed by right heart catheterisation in sickle cell disease. Eur Respir J. 2012; 39(1): 112-8.	116038	São Paulo	1	0	0
Foote EM, Sullivan KM, Ruth LJ, Oremo J, Sadumah I, Williams TN, Suchdev PS. Determinants of anemia among preschool children in rural, western Kenya. Am J Trop Med Hyg. 2013; 88(4): 757-64.	116022	Kisumu	1	0	0
Gahutu J-B, Musemakweri A, Harms G, Mockenhaupt FP. Prevalence of classic erythrocyte polymorphisms among 749 children in southern highland Rwanda. Trans R Soc Trop Med Hyg. 2012; 106(1): 63-5.	116018	Rwanda	1	0	0
Gali AE, Polat G, Dogdu O, Akgol M, Parlar HR, Arpact A, Aksoy K, Yuregir G. A premarital screening programme of hemoglobinopathies in Hatay. Ann Med Sci. 1999; 8(Pt 2): 88-92.	142062	Türkiye	1	0	0
Gill FM, Sleeper LA, Weiner SJ, Brown AK, Bellevue R, Grover R, Pegelow CH, Vichinsky E. Clinical events in the first decade in a cohort of infants with sickle cell disease. Cooperative Study of Sickle Cell Disease. Blood. 1995; 86(2): 77683.	227960	United States of America	1	1	1
Gimnez OG, Torrealba MC, Urquiola MB, Ortiz GG, Fonseca SM, Merzn R, de Donato M, Castillo O, Arends de Prez A. Diagnosis of hemoglobinopathies in newborns in Venezuela hospitals. An Pediatr (Barc). 2009; 71(4): 314-8.	116041	Venezuela (Bolivarian Republic of)	1	1	0
Giordano PC. Starting neonatal screening for haemoglobinopathies in The Netherlands. J Clin Pathol. 2009; 62(1): 18-21.	110048	Netherlands	1	0	0
Gouagna LC, Bancone G, Yao F, Yameogo B, Dabir KR, Costantini C, Simpor J, Ouedraogo JB, Modiano D. Genetic variation in human HBB is associated with Plasmodium falciparum transmission. Nat Genet. 2010; 42(4): 328-31.	142038	Burkina Faso	1	1	0
Granda H, Gispert S, Dortics A, Martn M, Cuadras Y, Calvo M, Martnez G, Zayas MA, Oliva JA, Heredero L. Cuban programme for prevention of sickle cell disease. Lancet. 1991; 337(8734): 1523.	227954	Cuba	1	1	0
Grosse R, Lukacs Z, Cobos PN, Oyen F, Ehmen C, Muntau B, Timmann C, Noack B. The Prevalence of Sickle Cell Disease and Its Implication for Newborn Screening in Germany (Hamburg Metropolitan Area). Pediatr Blood Cancer. 2016; 63(1): 168-70.	285963	Germany	1	1	1
Gulbis B, Cotton F, Ferster A, Ketelslegers O, Dresse MF, Rong-Collard E, Minon JM, L PQ, Vertongen F. Neonatal haemoglobinopathy screening in Belgium. J Clin Pathol. 2009; 62(1): 49-52.	96453	Belgium	1	1	1
Guler E, Garipardic M, Dalkiran T, Davutoglu M. Premarital screening test results for -thalassemia and sickle cell anemia trait in east Mediterranean region of Turkey. Pediatr Hematol Oncol. 2010; 27(8): 60813.	293156	Türkiye	1	1	1
Hajer S, Neila T, Sondess HF, Fekria O, Nabila A, Mahbouba K, Melika D, Faida O, Amina B, Raja B, Hedi R, Sadok M, Naima K, Slaheddine F, Taieb M. A lower-cost protocol for sickle cell disease neonatal screening in Tunisia. Ann Saudi Med. 2012; 32(1): 49-52.	116025	Tunisia	1	1	0

Henthorn J, Anionwu E, Brozovic M. Screening cord blood for sickle haemoglobinopathies in Brent. Br Med J (Clin Res Ed). 1984; 289(6443): 479-80.	142048	Greater London	1	1	0
Heredero-Baute L. Community-based program for the diagnosis and prevention of genetic disorders in Cuba Twenty years of experience. Community Genet. 2004; 7(2-3): 130-6.	110025	Cuba	1	1	0
Inati A, Jradi O, Tarabay H, Moallem H, Rachkidi Y, El Accaoui R, Isma'eel H, Wehbe R, Mfarrej BG, Dabbous I, Taher A. Sickle cell disease: the Lebanese experience. Int J Lab Hematol. 2007; 29(6): 399-408.	110046	Lebanon	1	0	0
Jain DL, Sarathi V, Upadhye D, Gulhane R, Nadkarni AH, Ghosh K, Colah RB. Newborn screening shows a high incidence of sickle cell anemia in Central India. Hemoglobin. 2012; 36(4): 31622.	293235	Maharashtra, Urban	1	1	1
Jain RC. Abnormal haemoglobin, thalassaemia and G-6-PD deficiency in Libya. Br J Haematol. 1983; 54(1): 154-5.	110023	Libya	1	0	1
Kafando E, Sawadogo M, Cotton F, Vertongen F, Gulbis B. Neonatal screening for sickle cell disorders in Ouagadougou, Burkina Faso: a pilot study. J Med Screen. 2005; 12(3): 112-4.	96456	Burkina Faso	1	1	0
Kamble M, Chatruvedi P. Epidemiology of sickle cell disease in a rural hospital of central India. Indian Pediatr. 2000; 37(4): 3916.	227970	Maharashtra, Rural	1	0	0
Kaptue-Noche L, Mbantenkhu J, Nsangou JFI. Geographic Distribution of Human Haemoglobins and Thalassemia in Cameroon, Middle and East Africa. In: Bowman JE, ed. Distribution and Evolution of the Haemoglobin and Globin Loci. New York City, United States: Elsevier, 1983. 159-66.	142043	Cameroon	1	0	0
Kaur M, Das GP, Verma IC. Sickle cell trait & disease among tribal communities in Orissa, Madhya Pradesh & Kerala. Indian J Med Res. 1997; 1116.	227972	Kerala, Rural, Madhya Pradesh, Rural, Odisha, Rural	1	0	0
Khoriaty E, Halaby R, Berro M, Sweid A, Abbas HA, Inati A. Incidence of sickle cell disease and other hemoglobin variants in 10,095 Lebanese neonates. PLoS One. 2014; 9(9): e105109.	292585	Lebanon	1	1	1
King L, Fraser R, Forbes M, Grindley M, Ali S, Reid M. Newborn sickle cell disease screening: the Jamaican experience (1995-2006). J Med Screen. 2007; 14(3): 117-22.	96457	Jamaica	1	1	0
King LG, Bortolusso-Ali S, Cunningham-Myrie CA, Reid ME. Impact of a Comprehensive Sickle Cell Center on Early Childhood Mortality in a Developing Country: The Jamaican Experience. J Pediatr. 2015; 167(3): 702-5e1.	285967	Jamaica	1	0	0
Kofi Ekue JM, Wurapa FK, Rwabwogo-Atenyi J. Haemoglobin genotypes, glucose - 6 - phosphate dehydrogenase deficiency and bronchial asthma. East Afr Med J. 1983; 60(10): 676-8.	142069	Zambia	1	1	0
Koren A, Zalman L, Palmor H, Zamir RB, Levin C, Openheim A, Daniel-Spiegel E, Shalev S, Filon D. Sickle cell anemia in northern Israel: screening and prevention. Isr Med Assoc J. 2009; 11(4): 229-34.	110044	Israel	1	0	0
Kreuels B, Kreuzberg C, Kobbe R, Ayim-Akonor M, Apiah-Thompson P, Thompson B, Ehmen C, Adjei S, Langefeld I, Adjei O, May J. Differing effects of HbS and HbC traits on uncomplicated falciparum malaria, anemia, and child growth. Blood. 2010; 115(22): 4551-8.	116048	Ghana	1	1	0
Kulkarni AG, Jekeme SD. Cord blood screening for haemoglobinopathies in northern Nigeria. Ann Trop Med Parasitol. 1986; 80(5): 549-51.	142056	Kaduna	1	0	0
Kunz JB, Awad S, Happich M, Muckenthaler L, Lindner M, Gramer G, Okun JG, Hoffmann GF, Bruckner T, Muckenthaler MU, Kulozik AE. Significant prevalence of sickle cell disease in Southwest Germany: results from a birth cohort study indicate the necessity for newborn screening. Ann Hematol. 2016; 95(3): 397-402.	292581	Germany	1	1	1
Labie D, Richin C, Pagnier J, Gentilini M, Nagel RL. Hemoglobins S and C in Upper Volta. Hum Genet. 1984; 65(3): 300-2.	116047	Burkina Faso	1	1	0
Lallemant M, Galacteros F, Lallemant-Lecoeur S, Feingold J, Carnevale P, Boukila V, Mouchet J, Rosa J. Hemoglobin abnormalities. An evaluation on new-born infants and their mothers in a maternity unit close to Brazzaville (P.R. Congo). Hum Genet. 1986; 74(1): 54-8.	142044	Congo	1	0	0
Lavu EK, Oswyn G, Vince JD. Sickle-cell/beta+-thalassaemia in a Papua New Guinean: the first reported case of the sickle gene in Papua New Guinea. Med J Aust. 2002; 176(2): 70-1.	96458	Papua New Guinea	0	0	1
Le PQ, Gulbis B, Dedeken L, Dupont S, Vanderfaeillie A, Heijmans C, Huybrechts S, Devalck C, Efira A, Dresse MF, Rozen L, Benghiat FS, Ferster A. Survival among children and adults with sickle cell disease in Belgium: Benefit from hydroxyurea treatment. Pediatr Blood Cancer. 2015; 62(11): 1956-61.	285965	Belgium	1	1	1
Leikin SL, Gallagher D, Kinney TR, Sloane D, Klug P, Rida W. Mortality in children and adolescents with sickle cell disease. Pediatrics. 1989; 84(3): 500-8.	110026	United States of America	1	1	1
Lerner NB, Platania BL, LaBella S. Newborn sickle cell screening in a region of Western New York State. J Pediatr. 2009; 154(1): 121-5.	116031	New York	1	1	1
Lin M, Yang LY, Xie DD, Chen JT, Nguba SM, Ehapo CS, Zhan XF, Eyi JU, Matesa RA, Obono MM, Yang H, Yang HT, Cheng JD. G6PD Deficiency and Hemoglobinopathies: Molecular Epidemiological Characteristics and Healthy Effects on Malaria Endemic Bioko Island, Equatorial Guinea. PLoS One. 2015; 10(4): e0123991.	283173	Equatorial Guinea	0	1	0
Lobitz S, Frommel C, Brose A, Klein J, Blankenstein O. Incidence of sickle cell disease in an unselected cohort of neonates born in Berlin, Germany. Eur J Hum Genet. 2014; 22(8): 1051-3.	292587	Germany	1	1	1
Lobo CL de C, Bueno LM, Moura P, Ogeda LL, Castilho S, de Carvalho SMF. Neonatal screening for hemoglobinopathies in Rio de Janeiro, Brazil. Rev Panam Salud Publica. 2003; 13(2-3): 154-9.	96459	Rio de Janeiro	1	1	0
Lobo CL de C, Pinto JFC, Nascimento EM, Moura PG, Cardoso GP, Hankins JS. The effect of hydroxcarbamide therapy on survival of children with sickle cell disease. Br J Haematol. 2013; 161(6): 852-60.	116021	Rio de Janeiro	1	1	1
Lobo CL, Ballas SK, Domingos AC, Moura PG, do Nascimento EM, Cardoso GP, de Carvalho SM. Newborn screening program for hemoglobinopathies in Rio de Janeiro, Brazil. Pediatr Blood Cancer. 2014; 61(1): 34-9.	292597	Rio de Janeiro	1	1	1
Lwanira CN, Mukasa MK, Swedberg G, Kironde F. Frequency of RANTES gene polymorphisms and their association with incidence of malaria: a longitudinal study on children in Iganga district, Uganda. Malar J. 2015; 14: 341.	285957	Uganda	1	1	1
Makani J, Cox SE, Soka D, Komba AN, Oruo J, Mwamtemi H, Magesa P, Rwezaula S, Meda E, Mgaya J, Lowe B, Muturi D, Roberts DJ, Williams TN, Pallangyo K, Kitundu J, Fegan G, Kirkham FJ, Marsh K, Newton CR. Mortality in sickle cell anemia in Africa: a prospective cohort study in Tanzania. PLoS One. 2011; 6(2): e14699.	110038	United Republic of Tanzania	1	0	0
Mann JR. Sickle cell haemoglobinopathies in England. Arch Dis Child. 1981; 56(9): 676-83.	96461	West Midlands	1	1	1

Masmas TN, Garly M-L, Lisse IM, Rodriques A, Petersen PT, Birgens H. Inherited hemoglobin disorders in Guinea-Bissau, West Africa: a population study. Hemoglobin. 2006; 30(3): 355-64.	110036	Guinea-Bissau	1	1	0
McGann PT, Ferris MG, Ramamurthy U, Santos B, de Oliveira V, Bernardino L, Ware RE. A prospective newborn screening and treatment program for sickle cell anemia in Luanda, Angola. Am J Hematol. 2013; 88(12): 984-9.	292599	Angola	1	1	1
Melo-Reis PR de, Naoum PC, Diniz-Filho JAF, Dias-Penna KGB, Mesquita MM de, Balestra FA, Ternes YMF, Mascarenhas C do C, Chen LC. Prevalence of thalassemias and variant hemoglobins in the state of Gois, Brazil. J Bras Patologia Med Laborat. 2006; 42(6): 42530.	228000	Goiás	1	1	1
Miall WE, Milner PF, Lovell HG, Standard KL. Haematological investigations of population samples in Jamaica. Br J Prev Soc Med. 1967; 21(2): 45-55.	96462	Jamaica	1	1	0
Michlitsch J, Azimi M, Hoppe C, Walters MC, Lubin B, Lorey F, Vichinsky E. Newborn screening for hemoglobinopathies in California. Pediatr Blood Cancer. 2009; 52(4): 486-90.	116035	California	1	1	1
Mockenhaupt FP, Rong B, G<fc>nther M, Beck S, Till H, Kohne E, Thompson WN, Bienzle U. Anaemia in pregnant Ghanaian women: importance of malaria, iron deficiency, and haemoglobinopathies. Trans R Soc Trop Med Hyg. 2000; 94(5): 477-83.	142049	Ghana	1	1	0
Modell B, Razzak A, Hindley N. Thalassaemia in the Maldives. Lancet. 1990; 335(8698): 1169-70.	110275	Maldives	0	0	1
Mohanty D, Colah R B, Gorakshakar A C, Patel R Z, Matsre D C, Mahanta J, Sharma S K, Chaudhari U, Ghosh M, Das S, Britt R P, Singh S, Ross C, Jagannathan L, Kaul R, Shukla D K, Muthuswamy V. Prevalence of -thalassemia and other haemoglobinopathies in six cities in India: a multicentre study. J Community Genet. 2013; 2013: 4:3342.	281461	Assam, Urban, Gujarat, Urban, Karnataka, Urban, Maharashtra, Urban, Punjab, Urban, West Bengal	1	1	1
Monplaisir N, Casius de Linval JC, Sellaye M, Galacteros F, Braconnier F, Beuzard Y, Hilbert J, Mezin R, Quist C, Duville S, Rosa J. Depistage des hemolgobinopathies a la naissance par isoelectrofocalisation. Etude de la population de la Martinique. Nouv Presse Med. 1981; 10(38): 3127-30.	142046	France	1	1	0
Monteiro C, Rueff J, Falcao AB, Portugal S, Weatherall DJ, Kulozik AE. The frequency and origin of the sickle cell mutation in the district of Coruche/Portugal. Hum Genet. 1989; 82(3): 255-8.	142057	Portugal	1	0	0
Moreno JL, Baribwira C. The epidemiology of neonatal sickle cell anemia in Bujumbura (Burundi). Ann Pediatr (Paris). 1994; 41(4): 215.	142032	Burundi	1	0	0
Munyanganizi R, Cotton F, Vertongen F, Gulbis B. Red blood cell disorders in Rwandese neonates: screening for sickle cell disease and glucose-6-phosphate dehydrogenase deficiency. J Med Screen. 2006; 13(3): 129-31.	128461	Rwanda	1	1	0
Mutesa L, Boemer F, Ngendahayo L, Rulisa S, Rusingiza EK, Cwinya-Ay N, Mazina D, Kariyo PC, Bours V, Schoos R. Neonatal screening for sickle cell disease in Central Africa: a study of 1825 newborns with a new enzyme-linked immunosorbent assay test. J Med Screen. 2007; 14(3): 113-6.	142031	Burundi, Democratic Republic of the Congo, Rwanda	1	0	0
Nagar R, Raman R. Diversity of sickle cell trait in Jharkhand state in India: Is it the zone of contact between two geographically and ethnically distinct populations in India. J Biosci. 2015; 40(3): 539-47.	285955	India (some subnationals)	1	1	1
Naoum PC, Angulo I de L, Brandao AC, Graciano RA, Spir M, Nomura E, Anjos ID. [Detection and awareness of patients with hemoglobinopathies in the regions of Sao Jose do Rio Preto and Presidente Prudente, SP (Brazil)]. Rev Saude Publica. 1985; 19(4): 36473.	227958	São Paulo	1	1	0
Nasserullah Z, Al Jame A, Abu Srair H, Al Qatari G, Al Naim S, Al Aqib A, Mokhtar M. Neonatal screening for sickle cell disease, glucose-6-phosphate dehydrogenase deficiency and a-thalassemia in Qatif and Al Hasa. Ann Saudi Med. 1998; 18(4): 289-92.	141404	Saudi Arabia	1	0	0
Nasserullah Z, Alshammari A, Abbas MA, Abu-Khamseen Y, Qadri M, Jafer SA, Wabel MA. Regional experience with newborn screening for sickle cell disease, other hemoglobinopathies and G6PD deficiency. Ann Saudi Med. 2003; 23(6): 354-7.	142059	Saudi Arabia	1	0	0
Nnaji GA, Ezeagwuna DA, Nnaji I, Osakwe JO, Nwigwe AC, Onwurah OW. Prevalence and pattern of sickle cell disease in premarital couples in Southeastern Nigeria. Niger J Clin Pract. 2013; 16(3): 309-14.	292595	Anambra	1	1	1
North ML, Piffaut MC, Duwig I, Locoh-Donou AG, Locoh-Donou AM. Detection of haemoglobinopathies at birth in Togo. Nouv Rev Fr Hematol. 1988; 30(4): 237-41.	142061	Togo	1	1	0
O'Leary JD, Odame I, Pehora C, Chakraborty P, Crawford MW. Effectiveness of preoperative screening for sickle cell disease in a population with a newborn screening program: a cohort study. Can J Anaesth. 2013; 60(1): 54-9.	116023	Canada	1	0	0
Odunvbun ME, Okolo AA, Rahimy CM. Newborn screening for sickle cell disease in a Nigerian hospital. Public Health. 2008; 122(10): 1111-6.	96465	Benin	1	1	0
Ohene-Frempong K, Oduro J, Tetteh H, Nkrumah F. Screening newborns for sickle cell disease in Ghana. Pediatrics. 2008; 121(Suppl): S120-21.	116045	Ghana	1	1	0
Okpara RA, Nkan EU, Essien EM. Haemoglobin patterns in the Cross River State of Nigeria with particular reference to haemoglobin C. East Afr Med J. 1986; 63(6): 41721.	227991	Rivers	1	1	0
Orsini A, Vovan L, Lena-Russo D, Maarouf I, Merono F, Jaujou M, Nebbia D, Fruitet B. School screening of hemoglobinopathies in the Marseilles area An analytic study of 35,289 tests. Arch Fr Pediatr. 1987; 44(6): 455-9.	96466	France	1	1	0
Paixao MC, Cunha Ferraz MH, Januario JN, Viana MB, Lima JM. Reliability of isoelectrofocusing for the detection of Hb S, Hb C, and HB D in a pioneering population-based program of newborn screening in Brazil. Hemoglobin. 2001; 25(3): 297303.	227974	Minas Gerais	1	1	1
Panigrahi S, Patra PK, Khodiar PK. Neonatal screening of sickle cell anemia: a preliminary report. Indian J Pediatr. 2012; 79(6): 74750.	295194	Chhattisgarh, Urban	1	1	1
Patel J, Patel B, Gamit N, Serjeant GR. Screening for the sickle cell gene in Gujarat, India: a village-based model. J Community Genet. 2013; 4(1): 437.	227966	Gujarat, Rural	1	0	0
Patra PK, Chauhan VS, Khodiar PK, Dalla AR, Serjeant GR. Screening for the sickle cell gene in Chhattisgarh state, India: an approach to a major public health problem. J Community Genet. 2011; 2(3): 14751.	227968	Chhattisgarh, Rural	1	0	0
Peres MJ, Carreiro MH, Machado MC, Seixas T, Pican<e7>o I, Batalha L, Lavinha J, Martins MC. [Neonatal screening of hemoglobinopathies in a population residing in Portugal]. Acta Med Port. 1996; 9(4-6): 135-9.	142058	Portugal	1	0	0
Perrine RP, Pembrey ME, John P, Perrine S, Shoup F. Natural history of sickle cell anemia in Saudi Arabs A study of 270 subjects. Ann Intern Med. 1978; 88(1): 1-6.	96467	Saudi Arabia	1	0	0

Pinheiro LS, Gon<e7>alves RP, Tom CAS, Alc<e2>ntara AEE, Marques ARC, Silva MM da. Preval<ea>ncia de hemoglobina S em recm-nascidos de Fortaleza: import<e2>ncia da investiga<e7><e3>o neonatal. Rev Bras Ginecol Obstet. 2006; 28(2): 122-5.	142041	Ceará	1	0	0
Platt OS, Brambilla DJ, Rosse WF, Milner PF, Castro O, Steinberg MH, Klug PP. Mortality in sickle cell disease Life expectancy and risk factors for early death. N Engl J Med. 1994; 330(23): 1639-44.	96468	United States of America	1	1	1
Quinn CT, Rogers ZR, Buchanan GR. Survival of children with sickle cell disease. Blood. 2004; 103(11): 4023-7.	96469	Texas	1	1	1
Quinn CT, Rogers ZR, McCavit TL, Buchanan GR. Improved survival of children and adolescents with sickle cell disease. Blood. 2010; 115(17): 3447-52.	116032	Texas	1	1	0
Rahgozar S, Poorfathollah AA, Moafi AR, Old JM. Beta s gene in Central Iran is in linkage disequilibrium with the Indian-Arab haplotype. Am J Hematol. 2000; 65(3): 192-5.	142053	Iran (Islamic Republic of)	1	0	0
Rahimy MC, Gangbo A, Ahouignan G, Alihonou E. Newborn screening for sickle cell disease in the Republic of Benin. J Clin Pathol. 2009; 62(1): 46-8.	96470	Benin	1	1	0
Robitaille N, Delvin EE, Hume HA. Newborn screening for sickle cell disease: A 1988-2003 Quebec experience. Paediatr Child Health. 2006; 11(4): 223-7.	96471	Canada	1	1	1
Ropero P, Molina L, Gonzlez FA, Villegas A. [Incidence of sickle cell disease in Equatorial Guinea]. Med Clin (Barc). 2011; 136(7): 309-10.	142052	Equatorial Guinea	1	0	0
Rotz S, Arty G, Dall'Amico R, De Zen L, Zanolli F, Bodas P. Prevalence of sickle cell disease, hemoglobin S, and hemoglobin C among Haitian newborns. Am J Hematol. 2013; 88(9): 827-8.	292647	Haiti	1	1	1
Russo-Mancuso G, La Spina M, Schilir<f2> G. The changing profile of sickle cell disease in Italy. Eur J Epidemiol. 2003; 18(9): 923-4.	110050	Italy	1	0	1
Sadarangani M, Makani J, Komba AN, Ajala-Agbo T, Newton CR, Marsh K, Williams TN. An observational study of children with sickle cell disease in Kilifi, Kenya. Br J Haematol. 2009; 146(6): 675-82.	116033	Kilifi	1	0	0
Salzano FM, Tondo CV. Hemoglobin types in Brazilian populations. Hemoglobin. 1982; 6(1): 8597.	227964	Brazil	1	1	1
Schedlbauer LM, Pass KA. Cellulose acetate/citrate agar electrophoresis of filter paper hemolysates from heel stick. Pediatrics. 1989; 83(5 Pt 2): 839-42.	142067	New York	1	1	0
Scott JAG, Berkley JA, Mwangi I, Ochola L, Uyoga S, Macharia A, Ndila C, Lowe BS, Mwarumba S, Bauni E, Marsh K, Williams TN. Relation between falciparum malaria and bacteraemia in Kenyan children: a population-based, case-control study and a longitudinal study. Lancet. 2011; 378(9799): 1316-23.	116034	Kilifi	1	0	0
Segbena AY, Prehu C, Wajcman H, Bardakdjian-Michau J, Messie K, Feteke L, Vovor A, David M, Feingold J, Galacteros F. Hemoglobins in Togolese newborns: Hb S, Hb C, Hb Bart's, and alpha-globin gene status. Am J Hematol. 1998; 59(3): 208-13.	142060	Togo	1	1	0
Serjeant GR. Observations on the epidemiology of sickle cell disease. Trans R Soc Trop Med Hyg. 1981; 75(2): 228-33.	96472	Jamaica	1	1	1
Shankar SM, Arbogast PG, Mitchel E, Cooper WO, Wang WC, Griffin MR. Medical care utilization and mortality in sickle cell disease: a population-based study. Am J Hematol. 2005; 80(4): 262-70.	110037	Tennessee	1	0	0
Shibata S. Hemoglobinopathies in Japan. Hemoglobin. 1981; 5(5): 509-15.	96473	Japan	0	1	0
Simpore J, Ilboudo D, Damintoti K, Sawadogo L, Maria E, Binet S, Nitiema H, Ouedraogo P, Pignatelli S, Nikiema J-B. Glucose-6-phosphate dehydrogenase deficiency and sickle cell disease in Burkina Faso . Pak J Biol Sci. 2007; 10(3): 409-14.	96474	Burkina Faso	1	1	0
Simpore J, Pignatelli S, Barlati S, Musumeci S. Modification in the frequency of Hb C and Hb S in Burkina Faso: an influence of migratory fluxes and improvement of patient health care. Hemoglobin. 2002; 26(2): 113-20.	116046	Burkina Faso	1	1	0
Stirnadel HA, St<f6>ckle M, Felger I, Smith T, Tanner M, Beck HP. Malaria infection and morbidity in infants in relation to genetic polymorphisms in Tanzania. Trop Med Int Health. 1999; 4(3): 187-93.	142063	United Republic of Tanzania	1	0	0
Streetly A, Clarke M, Downing M, Farrar L, Foo Y, Hall K, Kemp H, Newbold J, Walsh P, Yates J, Henthorn J. Implementation of the newborn screening programme for sickle cell disease in England: results for 2003-2005. J Med Screen. 2008; 15(1): 9-13.	96475	England (some subnationals)	1	1	0
Streetly A, Latinovic R, Hall K, Henthorn J. Implementation of universal newborn bloodspot screening for sickle cell disease and other clinically significant haemoglobinopathies in England: screening results for 2005-7. J Clin Pathol. 2009; 62(1): 26-30.	96476	England (all subnationals)	1	1	0
Suchdev PS, Ruth LJ, Earley M, Macharia A, Williams TN. The burden and consequences of inherited blood disorders among young children in western Kenya. Matern Child Nutr. 2014; 10(1): 135-44.	282368	Kisumu	1	0	0
Telfer P, Coen P, Chakravorty S, Wilkey O, Evans J, Newell H, Smalling B, Amos R, Stephens A, Rogers D, Kirkham F. Clinical outcomes in children with sickle cell disease living in England: a neonatal cohort in East London. Haematologica. 2007; 92(7): 905-12.	110024	Greater London	1	1	1
Therrell BL, Lloyd-Puryear MA, Eckman JR, Mann MY. Newborn screening for sickle cell diseases in the United States: A review of data spanning 2 decades. Semin Perinatol. 2015; 39(3): 238-51.	292579	United States of America (all subnationals)	1	1	1
Topal Y, Topal H, Ceyhan MN, Azik F, Capanoglu M, Kocabas CN. The Prevalence of Hemoglobinopathies in Young Adolescents in the Province of Mugla in Turkey: Results of a Screening Program. Hemoglobin. 2015; 39(4): 247-50.	285953	Türkiye	1	1	1
Torres-Guerra E, Torres-Guerra T, Valbuena G, Arteaga Vizcano M, Soto L. [Frequency of sickle-cell anemia in the population of ""Cuatro Bocas,"" Parroquia Ricaurte, Mara municipality, Zulia state, Venezuela]. Invest Clin. 1993; 34(2): 99-105.	142068	Venezuela (Bolivarian Republic of)	1	1	0
Tshilolo L, Aissi LM, Lukusa D, Kinsiama C, Wembonyama S, Gulbis B, Vertongen F. Neonatal screening for sickle cell anaemia in the Democratic Republic of the Congo: experience from a pioneer project on 31,204 newborns. J Clin Pathol. 2009; 62(1): 35-8.	96478	Democratic Republic of the Congo	1	0	0
Tshilolo L, Gulbis B, Barlow P, Damis E, Denis-Song R, Ferster A, Haumont D, Vanderpas JB, Pardou A, Schwers J, Vokaer A, Vertongen F. [Neonatal screening for hemoglobinopathies in the Brussels region]. Rev Med Brux. 1997; 18(2): 70-3.	142034	Belgium	1	0	0
Umoh AV, Abah GM, Ekanem TI, Essien EM. Haemoglobin genotypes: a prevalence study and implications for reproductive health in Uyo, Nigeria. Niger J Med. 2010; 19(1): 36-41.	96479	Akwa Ibom	1	1	0
Uyoga S, Macharia AW, Mochamah G, Ndila CM, Nyutu G, Makale J, Tendwa M, Nyatichi E, Ojal J, Otiende M, Shebe M, Awuondo KO, Mturi N, Peshu N, Tsofa B, Maitland K, Scott JAG, Williams TN. The epidemiology of sickle cell disease in children recruited in infancy in Kilifi, Kenya: a prospective cohort study. Lancet Glob Health. 2019; 7(10): e1458-e1466.	448123	Kilifi	1	1	0
Vierin Nzame Y, Badinga BB, Koko J, Blot P, Moussavou A. [Neonatal screening of the sickle cell disease in Gabon]. Med Afr Noire. 2012; 59(2): 959.	292652	Gabon	1	1	0

Vovor A, Feteke L, Kueviakoe IM, Kpatarou L, Mawussi K, Magnang H, Segbena AY. Blood typing profile of a school-aged population of a North Togo township. Hemoglobin. 2014; 38(5): 316-8.	285969	Togo	1	1	0
Wagner SC, de Castro SM, Gonzalez TP, Santin AP, Zaleski CF, Azevedo LA, Dreau H, Henderson S, Old J, Hutz MH. Neonatal screening for hemoglobinopathies: results of a public health system in South Brazil. Genet Test Mol Biomarkers. 2010; 14(4): 565-9.	116036	Rio Grande do Sul	1	1	1
Wang Y, Liu G, Caggana M, Kennedy J, Zimmerman R, Oyeku SO, Werner EM, Grant AM, Green NS, Grosse SD. Mortality of New York children with sickle cell disease identified through newborn screening. Genet Med. 2015; 17(6): 4529.	227988	New York	1	1	0
Watanabe AM, Pianovski MAD, Zanis Neto J, Lichtvan LCL, Chautard-Freire-Maia EA, Domingos MT, Wittig EO. [Prevalence of hemoglobin S in the State of Parana, Brazil, based on neonatal screening]. Cad Saude Publica. 2008; 24(5): 9931000.	227976	Paraná	1	1	1
Weatherall D. Sickle Cell and Thalassemias Prevalence Data, Personal Correspondence with David Weatherall. [Unpublished].	96480	Angola, Albania, United Arab Emirates, Burundi, Benin, Burkina Faso, Bahrain, Bahamas, Brazil, Botswana, Central African Republic, Canada, Côte d'Ivoire, Cameroon, Democratic Republic of the Congo, Congo, Colombia, Comoros, Cabo Verde, Cuba, Germany, Dominican Republic, Algeria, Egypt, Ethiopia, France, Gabon, United Kingdom, Ghana, Guinea, Gambia, Guinea-Bissau, Equatorial Guinea, Greece, Haiti, India, Iran (Islamic Republic of), Iraq, Italy, Jamaica, Kenya, Kuwait, Lebanon, Liberia, Libya, Morocco, Madagascar, Mexico, Mali, Mozambique, Mauritania, Malawi, Namibia, Niger, Nigeria, Oman, Pakistan, Rwanda, Saudi Arabia, Sudan, Senegal, Sierra Leone, Somalia, Sao Tome and Principe, Syrian Arab Republic, Chad, Togo, Tunisia, Türkiye, United Republic of Tanzania, Uganda, United States of America, Venezuela (Bolivarian Republic of), Yemen, South Africa, Zambia, Zimbabwe	1	1	1
White JM, Byrne M, Richards R, Buchanan T, Katsoulis E, Weerasingh K. Red cell genetic abnormalities in Peninsular Arabs: sickle haemoglobin, G6PD deficiency, and alpha and beta thalassaemia. J Med Genet. 1986; 23(3): 245-51.	110043	United Arab Emirates, Oman, Yemen	1	0	0
Williams TN, Mwangi TW, Wambua S, Alexander ND, Kortok M, Snow RW, Marsh K. Sickle cell trait and the risk of Plasmodium falciparum malaria and other childhood diseases. J Infect Dis. 2005; 192(1): 178-86.	116049	Kilifi	1	0	0
Yorke D, Mitchell J, Clow C, Nuguid E, Cadogan R, Sinclair D, Luscombe S, Rozen R, Meredith P, Esseltine D. Newborn screening for sickle cell and other hemoglobinopathies: a Canadian pilot study. Clin Invest Med. 1992; 15(4): 376-83.	96482	Canada	1	0	0
Zago MA, Costa FF, Tone LG, Bottura C. Hereditary hemoglobin disorders in a Brazilian population. Hum Hered. 1983; 33(2): 125-9.	142042	São Paulo	1	0	0
el-Hazmi MA, Warsy AS, Bahakim HH, al-Swailem A. Glucose-6-phosphate dehydrogenase deficiency and the sickle cell gene in Makkah, Saudi Arabia. J Trop Pediatr. 1994; 40(1): 12-6.	128621	Saudi Arabia	1	0	0
el-Hazmi MA, Warsy AS. Interaction between glucose-6-phosphate dehydrogenase deficiency and sickle cell gene in Saudi Arabia. Trop Geogr Med. 1987; 39(1): 32-5.	141403	Saudi Arabia	1	0	0

*NID (node identifier) corresponds to a unique identifier assigned upon extraction and upload into the Global Health Data Exchange (GHDx). The NID can be used to search for the source on ghdx.healthdata.org. The 0 and 1 represent whether the citation applies to the genotype (1= yes, 0 = no)

Table S5. Cause of death cause specific mortality data citations for sickle cell disorders and haemoglobinopathies and haemolytic anaemias.

Citation	Locations covered	Data type	Haemoglobinopathies and haemolytic anaemias	Sickle cell disorders
Center for Demographic Research, New Economic School (Russia). Russia Mortality Rates by Region, 1-Year Age Groups, and Sex, 2015-2017. Moscow, Russia: Center for Demographic Research, New Economic School (Russia). http://demogr.nes.ru/index.php/ru/demogr_indicat/data	Russia (subnationals) and Ukraine	VR	1	1
Center for Demographic Research, New Economic School (Russia). Russia Mortality Rates by Region, Age, Sex, and Cause of Death 1989-1998. Moscow, Russia: Center for Demographic Research, New Economic School (Russia). http://demogr.nes.ru/index.php/ru/demogr_indicat/data	Russian Federation (some subnationals)	VR	1	1
Center for Demographic Research, New Economic School (Russia). Russia Mortality Rates by Region, Age, Sex, and Cause of Death 1999-2005. Moscow, Russia: Center for Demographic Research, New Economic School (Russia). http://demogr.nes.ru/index.php/ru/demogr_indicat/data	Russian Federation (all subnationals)	VR	1	1
Center for Demographic Research, New Economic School (Russia). Russia Mortality Rates by Region, Age, Sex, and Cause of Death 2006-2012. Moscow, Russia: Center for Demographic Research, New Economic School (Russia). http://demogr.nes.ru/index.php/ru/demogr_indicat/data	Russian Federation (all subnationals)	VR	1	1
Center for Demographic Research, New Economic School (Russia). Russia Mortality Rates by Region, Cause of Death, 5-Year Age Groups, and Sex, 2011-2014. Moscow, Russia: Center for Demographic Research, New Economic School (Russia). http://demogr.nes.ru/index.php/ru/demogr_indicat/data	Russian Federation (all subnationals)	VR	1	1
Center for Demographic Research, New Economic School (Russia). USSR - Russia Mortality Rates by Region, Age, Sex, and Cause of Death 1969-1989. Moscow, Russia: Center for Demographic Research, New Economic School (Russia). http://demogr.nes.ru/index.php/ru/demogr_indicat/data	Russian Federation (some subnationals)	VR	1	1
Centers for Disease Control and Prevention (CDC), Child Health and Mortality Prevention Surveillance (CHAMPS) Network, Deloitte Consulting LLP, Emory Global Health Institute (EGHI), International Association of National Public Health Institutes (IANPHI), Task Force for Global Health, Emory University. Child Health and Mortality Prevention Surveillance (CHAMPS) Network Program.	Ethiopia, Kenya, Mali, Kisumu, Harari	MITS	1	1
Guyana Vital Registration - Deaths 2000 ICD10 - PAHO.	Guyana	VR	1	1
Hoa NP, Rao C, Hoy DG, Hinh ND, Chuc NT, Ngo DA. Mortality measures from sample-based surveillance: evidence of the epidemiological transition in Viet Nam. Bull World Health Organ. 2012; 90(10): 764-72. [Unpublished data].	Viet Nam	VA	0	1
Hospital Mortality Data 2002-2007.	Oman	VR	1	1
INDEPTH. Tanzania - Ifakara Health and Demographic Surveillance System.	United Republic of Tanzania	VA	1	1
India SRS Verbal Autopsy 2004-2006. [Unpublished].	India (some subnationals)	VA	1	1
India SRS Verbal Autopsy 2007-2009. [Unpublished].	India (some subnationals)	VA	1	1
India SRS Verbal Autopsy 2010-2013. [Unpublished].	India (some subnationals)	VA	1	1
Iraq Causes of Death 2012.	Iraq	VR	1	1
Iraq Causes of Death 2013.	Iraq	VR	1	1
Iraq Causes of Death 2014.	Iraq	VR	1	1
Iraq Causes of Death 2015.	Iraq	VR	1	1
Iraq Causes of Death 2016.	Iraq	VR	1	1
MRC/Wits Rural Public Health and Health Transitions Research Unit (Agincourt), INDEPTH. South Africa - Agincourt Health and Socio-Demographic Surveillance System.	South Africa (some subnationals)	VA	1	1
Muhwava W. Contributions of the Africa Centre Demographic Surveillance to the Community. Umbiko. 2011; 12. 3-4.	South Africa (some subnationals)	VA	1	1
Munthali CV, Kangoma S, Nasasara K, Zaina LM, Lupafya C, Mziya J, Harries AD, Takarinda KC, Kwataine M, Dambula I. Can a village headman use an electronic village register and a simplified community-based verbal autopsy tool to record numbers and causes of death in rural Malawi? [Unpublished data]. Front Public Health. 2018; 6(246): 1-11.	Malawi	VA	0	1
Office of Civil Service (Monaco), Government of Monaco. Monaco Vital Registration - Causes of Death 2011.	Monaco	VR	1	1
Office of Civil Service (Monaco), Government of Monaco. Monaco Vital Registration - Causes of Death 2012.	Monaco	VR	1	1
Office of Civil Service (Monaco), Government of Monaco. Monaco Vital Registration - Causes of Death 2013.	Monaco	VR	1	1
Peru Vital Statistics - Deaths 2001-2006.	Peru	VR	1	1
Porapakkham Y, Rao C, Pattaraarchachai J, Polprasert W, Vos T, Adair T, Lopez AD. Estimated causes of death in Thailand, 2005: implications for health policy. Popul Health Metr. 2010; 8:14.	Thailand	VA	1	1
Russian Fertility and Mortality database (RusFMD). Center for Demographic Research, New Economic School (Russia). Russia and regions, one-year age groups, 2015-2019. Moscow, Russia: Center for Demographic Research, New Economic School (Russia). http://demogr.nes.ru/index.php/ru/demogr_indicat/data	Russia (subnationals) and Ukraine	VR	1	1

Zimbabwe Vital Statistics - Deaths 1995.	Zimbabwe	VR	1	1
Addis Ababa University. Ethiopia - Addis Ababa Mortality Surveillance Program 2006.	Addis Ababa	VA	1	0
African Population and Health Research Center, INDEPTH. Kenya - Nairobi Urban Health and Demographic Surveillance System.	Kenya (some subnationals)	VA	1	1
Agency of Health Research and Development (Indonesia). Indonesia Basic Health Research 2007-2008.	Indonesia (some subnationals)	VA	1	1
Agency of Health Research and Development (Indonesia). Indonesia Mortality Registration System Strengthening Project.	Indonesia (some subnationals)	VA	1	1
Agency of Health Research and Development (Indonesia). Indonesia Sample Registration System - Deaths 2015.	Indonesia (some subnationals)	VA	1	1
Antigua and Barbuda Vital Statistics - Deaths 1996-1999.	Antigua and Barbuda	VR	1	1
Associates for Community and Population Research (ACPR), International Centre for Diarrhoeal Disease Research, Bangladesh (ICDDR,B), Johns Hopkins University, Mitra and Associates, National Institute of Population Research and Training (NIPORT), ORC Macro. Bangladesh Special Demographic and Health Survey 2001. Fairfax, United States of America: ICF International.	Bangladesh	VA	1	1
Australian Bureau of Statistics. Australia Vital Registration - Deaths 2005.	Australia	VR	1	1
BRAC. Bangladesh Verbal Autopsy Study 2015.	Bangladesh	VA	0	1
Baskent University, Ministry of Health (Turkey), State Institute of Statistics (Turkey). Turkey Verbal Autopsy Survey 2003.	Türkiye	VA	1	1
Births and Deaths Registry (Ghana). Ghana - Accra Births and Deaths Registry - Deaths 2000-2007.	Ghana	VR	1	1
Centers for Disease Control and Prevention (CDC), INDEPTH, International Vaccine Institute. Bangladesh - Abhoynagar, Mirsarai, and Kamalapur Health and Demographic Surveillance System.	Bangladesh	VA	1	1
Centers for Disease Control and Prevention (CDC), Kenya Medical Research Institute (KEMRI). Kenya KEMRI/CDC Health and Demographic Surveillance System.	Kenya (some subnationals)	VA	0	1
Centers for Disease Control and Prevention (CDC), MEASURE Evaluation Project, Carolina Population Center, University of North Carolina, Ministry of Health (Mozambique), National Institute of Statistics (INE) (Mozambique), United States Census Bureau (USCB). Mozambique National Survey on the Causes of Death 2007-2008.	Mozambique	VA	0	1
Central Statistical Office (Poland), National Institute of Public Health-National Institute of Hygiene (NIPH-NIH) (Poland). Poland Vital Registration - Causes of Death Data 1999.	Poland (all subnationals)	VR	1	1
Central Statistical Office (Poland), National Institute of Public Health-National Institute of Hygiene (NIPH-NIH) (Poland). Poland Vital Registration - Causes of Death Data 2000.	Poland (all subnationals)	VR	1	1
Central Statistical Office (Poland), National Institute of Public Health-National Institute of Hygiene (NIPH-NIH) (Poland). Poland Vital Registration - Causes of Death Data 2001.	Poland (all subnationals)	VR	1	1
Central Statistical Office (Poland), National Institute of Public Health-National Institute of Hygiene (NIPH-NIH) (Poland). Poland Vital Registration - Causes of Death Data 2002.	Poland (all subnationals)	VR	1	1
Central Statistical Office (Poland), National Institute of Public Health-National Institute of Hygiene (NIPH-NIH) (Poland). Poland Vital Registration - Causes of Death Data 2003.	Poland (all subnationals)	VR	1	1
Central Statistical Office (Poland), National Institute of Public Health-National Institute of Hygiene (NIPH-NIH) (Poland). Poland Vital Registration - Causes of Death Data 2004.	Poland (all subnationals)	VR	1	1
Central Statistical Office (Poland), National Institute of Public Health-National Institute of Hygiene (NIPH-NIH) (Poland). Poland Vital Registration - Causes of Death Data 2005.	Poland (all subnationals)	VR	1	1
Central Statistical Office (Poland), National Institute of Public Health-National Institute of Hygiene (NIPH-NIH) (Poland). Poland Vital Registration - Causes of Death Data 2006.	Poland (all subnationals)	VR	1	1
Central Statistical Office (Poland), National Institute of Public Health-National Institute of Hygiene (NIPH-NIH) (Poland). Poland Vital Registration - Causes of Death Data 2007.	Poland (all subnationals)	VR	1	1
Central Statistical Office (Poland), National Institute of Public Health-National Institute of Hygiene (NIPH-NIH) (Poland). Poland Vital Registration - Causes of Death Data 2008.	Poland (all subnationals)	VR	1	1
Central Statistical Office (Poland), National Institute of Public Health-National Institute of Hygiene (NIPH-NIH) (Poland). Poland Vital Registration - Causes of Death Data 2009.	Poland (all subnationals)	VR	1	1
Central Statistical Office (Poland), National Institute of Public Health-National Institute of Hygiene (NIPH-NIH) (Poland). Poland Vital Registration - Causes of Death Data 2010.	Poland (all subnationals)	VR	1	1
Central Statistical Office (Poland), National Institute of Public Health-National Institute of Hygiene (NIPH-NIH) (Poland). Poland Vital Registration - Causes of Death Data 2011.	Poland (all subnationals)	VR	1	1
Central Statistical Office (Poland), National Institute of Public Health-National Institute of Hygiene (NIPH-NIH) (Poland). Poland Vital Registration - Causes of Death Data 2012.	Poland (all subnationals)	VR	1	1
Central Statistical Office (Poland), National Institute of Public Health-National Institute of Hygiene (NIPH-NIH) (Poland). Poland Vital Registration - Causes of Death Data 2013.	Poland (all subnationals)	VR	1	1
Central Statistical Office (Poland), National Institute of Public Health-National Institute of Hygiene (NIPH-NIH) (Poland). Poland Vital Registration - Causes of Death Data 2014.	Poland (all subnationals)	VR	1	1

Central Statistical Office (Poland), National Institute of Public Health-National Institute of Hygiene (NIPH-NIH) (Poland). Poland Vital Registration - Causes of Death Data 2015.	Poland (all subnationals)	VR	1	1
Central Statistical Office (Poland), National Institute of Public Health-National Institute of Hygiene (NIPH-NIH) (Poland). Poland Vital Registration - Causes of Death Data 2016.	Poland (all subnationals)	VR	1	1
Central Statistical Office (Poland), National Institute of Public Health-National Institute of Hygiene (NIPH-NIH) (Poland). Poland Vital Registration - Causes of Death Data 2017.	Poland (all subnationals)	VR	1	1
Central Statistical Office (Poland), National Institute of Public Health-National Institute of Hygiene (NIPH-NIH) (Poland). Poland Vital Registration - Causes of Death Data 2018.	Poland (all subnationals)	VR	1	1
Central Statistical Office (Poland), National Institute of Public Health-National Institute of Hygiene (NIPH-NIH) (Poland). Poland Vital Registration - Causes of Death Data 2019.	Poland (all subnationals)	VR	1	1
Central Statistical Office (Poland), National Institute of Public Health-National Institute of Hygiene (NIPH-NIH) (Poland). Poland Vital Registration - Death Data 1980.	Poland (all subnationals)	VR	1	1
Central Statistical Office (Poland), National Institute of Public Health-National Institute of Hygiene (NIPH-NIH) (Poland). Poland Vital Registration - Death Data 1981.	Poland (all subnationals)	VR	1	1
Central Statistical Office (Poland), National Institute of Public Health-National Institute of Hygiene (NIPH-NIH) (Poland). Poland Vital Registration - Death Data 1982.	Poland (all subnationals)	VR	1	1
Central Statistical Office (Poland), National Institute of Public Health-National Institute of Hygiene (NIPH-NIH) (Poland). Poland Vital Registration - Death Data 1983.	Poland (all subnationals)	VR	1	1
Central Statistical Office (Poland), National Institute of Public Health-National Institute of Hygiene (NIPH-NIH) (Poland). Poland Vital Registration - Death Data 1984.	Poland (all subnationals)	VR	1	1
Central Statistical Office (Poland), National Institute of Public Health-National Institute of Hygiene (NIPH-NIH) (Poland). Poland Vital Registration - Death Data 1985.	Poland (all subnationals)	VR	1	1
Central Statistical Office (Poland), National Institute of Public Health-National Institute of Hygiene (NIPH-NIH) (Poland). Poland Vital Registration - Death Data 1986.	Poland (all subnationals)	VR	1	1
Central Statistical Office (Poland), National Institute of Public Health-National Institute of Hygiene (NIPH-NIH) (Poland). Poland Vital Registration - Death Data 1987.	Poland (all subnationals)	VR	1	1
Central Statistical Office (Poland), National Institute of Public Health-National Institute of Hygiene (NIPH-NIH) (Poland). Poland Vital Registration - Death Data 1988.	Poland (all subnationals)	VR	1	1
Central Statistical Office (Poland), National Institute of Public Health-National Institute of Hygiene (NIPH-NIH) (Poland). Poland Vital Registration - Death Data 1989.	Poland (all subnationals)	VR	1	1
Central Statistical Office (Poland), National Institute of Public Health-National Institute of Hygiene (NIPH-NIH) (Poland). Poland Vital Registration - Death Data 1990.	Poland (all subnationals)	VR	1	1
Central Statistical Office (Poland), National Institute of Public Health-National Institute of Hygiene (NIPH-NIH) (Poland). Poland Vital Registration - Death Data 1991.	Poland (all subnationals)	VR	1	1
Central Statistical Office (Poland), National Institute of Public Health-National Institute of Hygiene (NIPH-NIH) (Poland). Poland Vital Registration - Death Data 1992.	Poland (all subnationals)	VR	1	1
Central Statistical Office (Poland), National Institute of Public Health-National Institute of Hygiene (NIPH-NIH) (Poland). Poland Vital Registration - Death Data 1993.	Poland (all subnationals)	VR	1	1
Central Statistical Office (Poland), National Institute of Public Health-National Institute of Hygiene (NIPH-NIH) (Poland). Poland Vital Registration - Death Data 1994.	Poland (all subnationals)	VR	1	1
Central Statistical Office (Poland), National Institute of Public Health-National Institute of Hygiene (NIPH-NIH) (Poland). Poland Vital Registration - Death Data 1995.	Poland (all subnationals)	VR	1	1
Central Statistical Office (Poland), National Institute of Public Health-National Institute of Hygiene (NIPH-NIH) (Poland). Poland Vital Registration - Death Data 1996.	Poland (all subnationals)	VR	1	1
Chief Medical Office of Greenland. Greenland Vital Registration - Deaths 1995.	Greenland	VR	1	1
Chief Medical Office of Greenland. Greenland Vital Registration - Deaths 1996.	Greenland	VR	1	1
Chief Medical Office of Greenland. Greenland Vital Registration - Deaths 1997.	Greenland	VR	1	1
Chief Medical Office of Greenland. Greenland Vital Registration - Deaths 1998.	Greenland	VR	1	1
Chief Medical Office of Greenland. Greenland Vital Registration - Deaths 1999.	Greenland	VR	1	1
Chief Medical Office of Greenland. Greenland Vital Registration - Deaths 2000.	Greenland	VR	1	1
Chief Medical Office of Greenland. Greenland Vital Registration - Deaths 2001.	Greenland	VR	1	1
Chief Medical Office of Greenland. Greenland Vital Registration - Deaths 2002.	Greenland	VR	1	1
Chief Medical Office of Greenland. Greenland Vital Registration - Deaths 2003.	Greenland	VR	1	1

Chief Medical Office of Greenland. Greenland Vital Registration - Deaths 2004.	Greenland	VR	1	1
Chief Medical Office of Greenland. Greenland Vital Registration - Deaths 2005.	Greenland	VR	1	1
Chief Medical Office of Greenland. Greenland Vital Registration - Deaths 2006.	Greenland	VR	1	1
Chief Medical Office of Greenland. Greenland Vital Registration - Deaths 2007.	Greenland	VR	1	1
Chief Medical Office of Greenland. Greenland Vital Registration - Deaths 2008.	Greenland	VR	1	1
Chief Medical Office of Greenland. Greenland Vital Registration - Deaths 2009.	Greenland	VR	1	1
Chief Medical Office of Greenland. Greenland Vital Registration - Deaths 2010.	Greenland	VR	1	1
Chief Medical Office of Greenland. Greenland Vital Registration - Deaths 2011.	Greenland	VR	1	1
Chief Medical Office of Greenland. Greenland Vital Registration - Deaths 2012.	Greenland	VR	1	1
Chief Medical Office of Greenland. Greenland Vital Registration - Deaths 2013.	Greenland	VR	1	1
Chief Medical Office of Greenland. Greenland Vital Registration - Deaths 2014.	Greenland	VR	1	1
Chief Medical Office of Greenland. Greenland Vital Registration - Deaths 2015.	Greenland	VR	1	1
Chinese Center for Disease Control and Prevention (CCDC). China Disease Surveillance Points 2004 - China CDC.	China (some subnationals)	VR	1	1
Chinese Center for Disease Control and Prevention (CCDC). China Disease Surveillance Points 2004 - China CDC.	China (some subnationals)	VR-S	1	1
Chinese Center for Disease Control and Prevention (CCDC). China Disease Surveillance Points 2005 - China CDC.	China (some subnationals)	VR	1	1
Chinese Center for Disease Control and Prevention (CCDC). China Disease Surveillance Points 2005 - China CDC.	China (some subnationals)	VR-S	1	1
Chinese Center for Disease Control and Prevention (CCDC). China Disease Surveillance Points 2006 - China CDC.	China (some subnationals)	VR	1	1
Chinese Center for Disease Control and Prevention (CCDC). China Disease Surveillance Points 2006 - China CDC.	China (some subnationals)	VR-S	1	1
Chinese Center for Disease Control and Prevention (CCDC). China Disease Surveillance Points 2007 - China CDC.	China (some subnationals)	VR	1	1
Chinese Center for Disease Control and Prevention (CCDC). China Disease Surveillance Points 2007 - China CDC.	China (some subnationals)	VR-S	1	1
Chinese Center for Disease Control and Prevention (CCDC). China Disease Surveillance Points 2013 - China CDC.	China (some subnationals)	VR	1	1
Chinese Center for Disease Control and Prevention (CCDC). China Disease Surveillance Points 2013 - China CDC.	China (some subnationals)	VR-S	1	1
Chinese Center for Disease Control and Prevention (CCDC). China Disease Surveillance Points 2014 - China CDC.	China (some subnationals)	VR	1	1
Chinese Center for Disease Control and Prevention (CCDC). China Disease Surveillance Points 2014 - China CDC.	China (some subnationals)	VR-S	1	1
Chinese Center for Disease Control and Prevention (CCDC). China Disease Surveillance Points 2015 - China CDC.	China (some subnationals)	VR	1	1
Chinese Center for Disease Control and Prevention (CCDC). China Disease Surveillance Points 2015 - China CDC.	China (some subnationals)	VR-S	1	1
Chinese Center for Disease Control and Prevention (CCDC). China Disease Surveillance Points 2016 - China CDC.	China (some subnationals)	VR	1	1
Chinese Center for Disease Control and Prevention (CCDC). China Disease Surveillance Points 2016 - China CDC.	China (some subnationals)	VR-S	1	1
Chinese Center for Disease Control and Prevention (CCDC). China Disease Surveillance Points 2017 - China CDC.	China (some subnationals)	VR	1	1
Chinese Center for Disease Control and Prevention (CCDC). China Disease Surveillance Points 2017 - China CDC.	China (some subnationals)	VR-S	1	1
Chinese Center for Disease Control and Prevention (CCDC). China Disease Surveillance Points and Death Registration 2008 - China CDC.	China (some subnationals)	VR	1	1
Chinese Center for Disease Control and Prevention (CCDC). China Disease Surveillance Points and Death Registration 2008 - China CDC.	China (some subnationals)	VR-S	1	1
Chinese Center for Disease Control and Prevention (CCDC). China Disease Surveillance Points and Death Registration 2009 - China CDC.	China (some subnationals)	VR	1	1
Chinese Center for Disease Control and Prevention (CCDC). China Disease Surveillance Points and Death Registration 2009 - China CDC.	China (some subnationals)	VR-S	1	1
Chinese Center for Disease Control and Prevention (CCDC). China Disease Surveillance Points and Death Registration 2010 - China CDC.	China (some subnationals)	VR	1	1
Chinese Center for Disease Control and Prevention (CCDC). China Disease Surveillance Points and Death Registration 2010 - China CDC.	China (some subnationals)	VR-S	1	1
Chinese Center for Disease Control and Prevention (CCDC). China Disease Surveillance Points and Death Registration 2011 - China CDC.	China (some subnationals)	VR	1	1
Chinese Center for Disease Control and Prevention (CCDC). China Disease Surveillance Points and Death Registration 2011 - China CDC.	China (some subnationals)	VR-S	1	1
Chinese Center for Disease Control and Prevention (CCDC). China Disease Surveillance Points and Death Registration 2012 - China CDC.	China (some subnationals)	VR	1	1
Chinese Center for Disease Control and Prevention (CCDC). China Disease Surveillance Points and Death Registration 2012 - China CDC.	China (some subnationals)	VR-S	1	1
Chisumpa VH, Odimegwu CO, Saikia N. Adult mortality in sub-Saharan Africa: cross-sectional study of causes of death in Zambia. <i>Trop Med Int Health</i>. 2019; 24(10): 1208-20.	Zambia	VA	1	1
Cook Islands Statistics Office, Ministry of Health (Cook Islands). Cook Islands Vital Registration - Causes of Death 2000.	Cook Islands	VR	1	0
Cook Islands Statistics Office, Ministry of Health (Cook Islands). Cook Islands Vital Registration - Causes of Death 2001.	Cook Islands	VR	1	0

Cook Islands Statistics Office, Ministry of Health (Cook Islands). Cook Islands Vital Registration - Causes of Death 2002.	Cook Islands	VR	1	0
Cook Islands Statistics Office, Ministry of Health (Cook Islands). Cook Islands Vital Registration - Causes of Death 2003.	Cook Islands	VR	1	0
Cook Islands Statistics Office, Ministry of Health (Cook Islands). Cook Islands Vital Registration - Causes of Death 2004.	Cook Islands	VR	1	0
Cook Islands Statistics Office, Ministry of Health (Cook Islands). Cook Islands Vital Registration - Causes of Death 2005.	Cook Islands	VR	1	0
Cook Islands Statistics Office, Ministry of Health (Cook Islands). Cook Islands Vital Registration - Causes of Death 2006.	Cook Islands	VR	1	0
Cook Islands Statistics Office, Ministry of Health (Cook Islands). Cook Islands Vital Registration - Causes of Death 2007.	Cook Islands	VR	1	0
Cook Islands Statistics Office, Ministry of Health (Cook Islands). Cook Islands Vital Registration - Causes of Death 2009.	Cook Islands	VR	1	0
Cook Islands Statistics Office, Ministry of Health (Cook Islands). Cook Islands Vital Registration - Causes of Death 2010.	Cook Islands	VR	1	0
Cook Islands Statistics Office, Ministry of Health (Cook Islands). Cook Islands Vital Registration - Causes of Death 2011.	Cook Islands	VR	1	0
Cook Islands Statistics Office, Ministry of Health (Cook Islands). Cook Islands Vital Registration - Causes of Death 2012.	Cook Islands	VR	1	0
Cook Islands Statistics Office, Ministry of Health (Cook Islands). Cook Islands Vital Registration - Causes of Death 2013.	Cook Islands	VR	1	0
Cook Islands Statistics Office, Ministry of Health (Cook Islands). Cook Islands Vital Registration - Causes of Death 2014.	Cook Islands	VR	1	0
Cook Islands Statistics Office, Ministry of Health (Cook Islands). Cook Islands Vital Registration - Causes of Death 2015.	Cook Islands	VR	1	0
Cook Islands Statistics Office, Ministry of Health (Cook Islands). Cook Islands Vital Registration - Causes of Death 2016.	Cook Islands	VR	1	0
Department of Census and Statistics (Sri Lanka). Sri Lanka Vital Statistics - Deaths 2009. Colombo, Sri Lanka: Department of Census and Statistics (Sri Lanka).	Sri Lanka	VR	1	1
Department of Census and Statistics (Sri Lanka). Sri Lanka Vital Statistics - Deaths 2010. Colombo, Sri Lanka: Department of Census and Statistics (Sri Lanka).	Sri Lanka	VR	1	1
Department of Economics and Statistics, Government of Kerala. India - Kerala Medical Certification of Cause of Death Report 2011. Thiruvananthapuram, India: Department of Economics and Statistics, Government of Kerala.	Kerala (some subnationals)	VR	1	0
Department of Economics and Statistics, Government of Kerala. India - Kerala Medical Certification of Cause of Death Report 2012. Thiruvananthapuram, India: Department of Economics and Statistics, Government of Kerala.	Kerala (some subnationals)	VR	1	0
Department of Economics and Statistics, Government of Kerala. India - Kerala Medical Certification of Cause of Death Report 2016. Thiruvananthapuram, India: Department of Economics and Statistics, Government of Kerala.	Kerala (some subnationals)	VR	1	0
Department of Economics and Statistics, Government of Kerala. India - Kerala Medical Certification of Cause of Death Report 2017. Thiruvananthapuram, India: Department of Economics and Statistics, Government of Kerala.	Kerala (some subnationals)	VR	1	0
Department of Health (Taiwan). Taiwan Vital Registration - Deaths 1980.	Taiwan (Province of China)	VR	1	1
Department of Health (Taiwan). Taiwan Vital Registration - Deaths 1981.	Taiwan (Province of China)	VR	1	1
Department of Health (Taiwan). Taiwan Vital Registration - Deaths 1982.	Taiwan (Province of China)	VR	1	1
Department of Health (Taiwan). Taiwan Vital Registration - Deaths 1983.	Taiwan (Province of China)	VR	1	1
Department of Health (Taiwan). Taiwan Vital Registration - Deaths 1984.	Taiwan (Province of China)	VR	1	1
Department of Health (Taiwan). Taiwan Vital Registration - Deaths 1985.	Taiwan (Province of China)	VR	1	1
Department of Health (Taiwan). Taiwan Vital Registration - Deaths 1986.	Taiwan (Province of China)	VR	1	1
Department of Health (Taiwan). Taiwan Vital Registration - Deaths 1987.	Taiwan (Province of China)	VR	1	1
Department of Health (Taiwan). Taiwan Vital Registration - Deaths 1988.	Taiwan (Province of China)	VR	1	1
Department of Health (Taiwan). Taiwan Vital Registration - Deaths 1989.	Taiwan (Province of China)	VR	1	1
Department of Health (Taiwan). Taiwan Vital Registration - Deaths 1990.	Taiwan (Province of China)	VR	1	1
Department of Health (Taiwan). Taiwan Vital Registration - Deaths 1991.	Taiwan (Province of China)	VR	1	1
Department of Health (Taiwan). Taiwan Vital Registration - Deaths 1992. Taipei City, Taiwan: Ministry of Health and Welfare (Taiwan).	Taiwan (Province of China)	VR	1	1
Department of Health (Taiwan). Taiwan Vital Registration - Deaths 1993. Taipei City, Taiwan: Ministry of Health and Welfare (Taiwan).	Taiwan (Province of China)	VR	1	1
Department of Health (Taiwan). Taiwan Vital Registration - Deaths 1994. Taipei City, Taiwan: Ministry of Health and Welfare (Taiwan).	Taiwan (Province of China)	VR	1	1
Department of Health (Taiwan). Taiwan Vital Registration - Deaths 1995. Taipei City, Taiwan: Ministry of Health and Welfare (Taiwan).	Taiwan (Province of China)	VR	1	1
Department of Health (Taiwan). Taiwan Vital Registration - Deaths 1996. Taipei City, Taiwan: Ministry of Health and Welfare (Taiwan).	Taiwan (Province of China)	VR	1	1
Department of Health (Taiwan). Taiwan Vital Registration - Deaths 1997. Taipei City, Taiwan: Ministry of Health and Welfare (Taiwan).	Taiwan (Province of China)	VR	1	1
Department of Health (Taiwan). Taiwan Vital Registration - Deaths 1998. Taipei City, Taiwan: Ministry of Health and Welfare (Taiwan).	Taiwan (Province of China)	VR	1	1
Department of Health (Taiwan). Taiwan Vital Registration - Deaths 1999. Taipei City, Taiwan: Ministry of Health and Welfare (Taiwan).	Taiwan (Province of China)	VR	1	1

Department of Health (Taiwan). Taiwan Vital Registration - Deaths 2000. Taipei City, Taiwan (Province of China): Ministry of Health and Welfare (Taiwan).	Taiwan (Province of China)	VR	1	1
Department of Health (Taiwan). Taiwan Vital Registration - Deaths 2001. Taipei City, Taiwan (Province of China): Ministry of Health and Welfare (Taiwan).	Taiwan (Province of China)	VR	1	1
Department of Health (Taiwan). Taiwan Vital Registration - Deaths 2002. Taipei City, Taiwan (Province of China): Ministry of Health and Welfare (Taiwan).	Taiwan (Province of China)	VR	1	1
Department of Health (Taiwan). Taiwan Vital Registration - Deaths 2003. Taipei City, Taiwan (Province of China): Ministry of Health and Welfare (Taiwan).	Taiwan (Province of China)	VR	1	1
Department of Health (Taiwan). Taiwan Vital Registration - Deaths 2004. Taipei City, Taiwan (Province of China): Ministry of Health and Welfare (Taiwan).	Taiwan (Province of China)	VR	1	1
Department of Health (Taiwan). Taiwan Vital Registration - Deaths 2005. Taipei City, Taiwan (Province of China): Ministry of Health and Welfare (Taiwan).	Taiwan (Province of China)	VR	1	1
Department of Health (Taiwan). Taiwan Vital Registration - Deaths 2006. Taipei City, Taiwan (Province of China): Ministry of Health and Welfare (Taiwan).	Taiwan (Province of China)	VR	1	1
Department of Health (Taiwan). Taiwan Vital Registration - Deaths 2007. Taipei City, Taiwan (Province of China): Ministry of Health and Welfare (Taiwan).	Taiwan (Province of China)	VR	1	1
Department of Health (Taiwan). Taiwan Vital Registration - Deaths 2008. Taipei City, Taiwan (Province of China): Ministry of Health and Welfare (Taiwan).	Taiwan (Province of China)	VR	1	1
Department of Health (Taiwan). Taiwan Vital Registration - Deaths 2009. Taipei City, Taiwan (Province of China): Ministry of Health and Welfare (Taiwan).	Taiwan (Province of China)	VR	1	1
Department of Health (Taiwan). Taiwan Vital Registration - Deaths 2010. Taipei City, Taiwan (Province of China): Ministry of Health and Welfare (Taiwan).	Taiwan (Province of China)	VR	1	1
Department of Health (Taiwan). Taiwan Vital Registration - Deaths 2011. Taipei City, Taiwan (Province of China): Ministry of Health and Welfare (Taiwan).	Taiwan (Province of China)	VR	1	1
Department of Health (Taiwan). Taiwan Vital Registration - Deaths 2012. Taipei City, Taiwan (Province of China): Ministry of Health and Welfare (Taiwan).	Taiwan (Province of China)	VR	1	1
Department of Home Affairs (South Africa), Statistics South Africa. South Africa Vital Registration - Causes of Death 1997-2005. Pretoria, South Africa: Statistics South Africa.	South Africa (all subnationals)	VR	1	1
Department of Home Affairs (South Africa), Statistics South Africa. South Africa Vital Registration - Causes of Death 2006. Pretoria, South Africa: Statistics South Africa.	South Africa (all subnationals)	VR	1	1
Department of Home Affairs (South Africa), Statistics South Africa. South Africa Vital Registration - Causes of Death 2007. Pretoria, South Africa: Statistics South Africa.	South Africa (all subnationals)	VR	1	1
Department of Home Affairs (South Africa), Statistics South Africa. South Africa Vital Registration - Causes of Death 2008. Pretoria, South Africa: Statistics South Africa.	South Africa (all subnationals)	VR	1	1
Department of Home Affairs (South Africa), Statistics South Africa. South Africa Vital Registration - Causes of Death 2009. Pretoria, South Africa: Statistics South Africa.	South Africa (all subnationals)	VR	1	1
Department of Home Affairs (South Africa), Statistics South Africa. South Africa Vital Registration - Causes of Death 2010. Pretoria, South Africa: Statistics South Africa.	South Africa (all subnationals)	VR	1	1
Department of Home Affairs (South Africa), Statistics South Africa. South Africa Vital Registration - Causes of Death 2011. Pretoria, South Africa: Statistics South Africa.	South Africa (all subnationals)	VR	1	1
Department of Home Affairs (South Africa), Statistics South Africa. South Africa Vital Registration - Causes of Death 2012. Pretoria, South Africa: Statistics South Africa.	South Africa (all subnationals)	VR	1	1
Department of Home Affairs (South Africa), Statistics South Africa. South Africa Vital Registration - Causes of Death 2013. Pretoria, South Africa: Statistics South Africa.	South Africa (all subnationals)	VR	1	1
Department of Home Affairs (South Africa), Statistics South Africa. South Africa Vital Registration - Causes of Death 2014. Pretoria, South Africa: Statistics South Africa.	South Africa (all subnationals)	VR	1	1
Department of Home Affairs (South Africa), Statistics South Africa. South Africa Vital Registration - Causes of Death 2015. Pretoria, South Africa: Statistics South Africa.	South Africa (all subnationals)	VR	1	1
Department of Home Affairs (South Africa), Statistics South Africa. South Africa Vital Registration - Causes of Death 2016. Pretoria, South Africa: Statistics South Africa, 2019.	South Africa (all subnationals)	VR	1	1
Department of Home Affairs (South Africa), Statistics South Africa. South Africa Vital Registration - Causes of Death 2017. Pretoria, South Africa: Statistics South Africa, 2020.	South Africa (all subnationals)	VR	1	1
Department of Public Health (Palau), Ministry of Health (Palau), Office of Planning and Statistics (Palau). Palau Vital Statistics - Causes of Death 2013.	Palau	VR	1	1
Department of Statistics and Health Information, Ministry of Health (Chile), National Institute of Statistics (Chile). Chile Vital Statistics - Deaths 1990. Santiago, Chile: Department of Statistics and Health Information, Ministry of Health (Chile).	Chile	VR	1	1

Department of Statistics and Health Information, Ministry of Health (Chile), National Institute of Statistics (Chile). Chile Vital Statistics - Deaths 1991. Santiago, Chile: Department of Statistics and Health Information, Ministry of Health (Chile).	Chile	VR	1	1
Department of Statistics and Health Information, Ministry of Health (Chile), National Institute of Statistics (Chile). Chile Vital Statistics - Deaths 1992. Santiago, Chile: Department of Statistics and Health Information, Ministry of Health (Chile).	Chile	VR	1	1
Department of Statistics and Health Information, Ministry of Health (Chile), National Institute of Statistics (Chile). Chile Vital Statistics - Deaths 1993. Santiago, Chile: Department of Statistics and Health Information, Ministry of Health (Chile).	Chile	VR	1	1
Department of Statistics and Health Information, Ministry of Health (Chile), National Institute of Statistics (Chile). Chile Vital Statistics - Deaths 1994. Santiago, Chile: Department of Statistics and Health Information, Ministry of Health (Chile).	Chile	VR	1	1
Department of Statistics and Health Information, Ministry of Health (Chile), National Institute of Statistics (Chile). Chile Vital Statistics - Deaths 1995. Santiago, Chile: Department of Statistics and Health Information, Ministry of Health (Chile).	Chile	VR	1	1
Department of Statistics and Health Information, Ministry of Health (Chile), National Institute of Statistics (Chile). Chile Vital Statistics - Deaths 1996. Santiago, Chile: Department of Statistics and Health Information, Ministry of Health (Chile).	Chile	VR	1	1
Directorate of Economics and Statistics, Government of Karnataka, Office of the Chief Registrar of Births and Deaths, Government of Karnataka. India - Karnataka Medical Certification of Cause of Death Report 2016. Bangalore, India: Directorate of Economics and Statistics, Government of Karnataka, 2018.	Karnataka (some subnationals)	VR	1	0
Directorate of Economics and Statistics, Government of Karnataka, Office of the Chief Registrar of Births and Deaths, Government of Karnataka. India - Karnataka Medical Certification of Cause of Death Report 2017. Bangalore, India: Directorate of Economics and Statistics, Government of Karnataka, 2018.	Karnataka (some subnationals)	VR	1	0
Directorate of Health (Iceland). Iceland Causes of Death Register 2017.	Iceland	VR	1	1
Directorate of Health (Iceland). Iceland Causes of Death Register 2018.	Iceland	VR	1	1
Directorate of Health (Iceland). Iceland Causes of Death Register 2019.	Iceland	VR	1	1
Directorate of Health (Iceland). Iceland Causes of Death Register 2020.	Iceland	VR	1	1
Ekanem EE, Asindi AA, Okoi OU. Community-based surveillance of paediatric deaths in Cross River State, Nigeria. <i>Trop Geogr Med</i>. 1994; 46(5): 305-8.	Nigeria (some subnationals)	VA	1	1
Ethiopian Public Health Association. Ethiopia Demographic Surveillance Verbal Autopsy Data 2009-2016.	Ethiopia (some subnationals)	VA	1	1
Federal Competitiveness and Statistics Authority (United Arab Emirates), Ministry of Health and Prevention (United Arab Emirates). United Arab Emirates - Abu Dhabi Causes of Death 2014.	United Arab Emirates	VR	1	1
Federal Competitiveness and Statistics Authority (United Arab Emirates), Ministry of Health and Prevention (United Arab Emirates). United Arab Emirates - Abu Dhabi Causes of Death 2015.	United Arab Emirates	VR	1	1
Federal Competitiveness and Statistics Authority (United Arab Emirates), Ministry of Health and Prevention (United Arab Emirates). United Arab Emirates - Abu Dhabi Causes of Death 2016.	United Arab Emirates	VR	1	1
Federal Competitiveness and Statistics Authority (United Arab Emirates), Ministry of Health and Prevention (United Arab Emirates). United Arab Emirates - Abu Dhabi Causes of Death 2017.	United Arab Emirates	VR	1	1
Federal Competitiveness and Statistics Authority (United Arab Emirates), Ministry of Health and Prevention (United Arab Emirates). United Arab Emirates - Abu Dhabi Causes of Death 2018.	United Arab Emirates	VR	1	1
General Directorate of Strategic Health Information (DIGIES) (Paraguay), Ministry of Health (Paraguay). Paraguay Vital Registration - Deaths 2004.	Paraguay	VR	1	1
General Directorate of Strategic Health Information (DIGIES) (Paraguay), Ministry of Health (Paraguay). Paraguay Vital Registration - Deaths 2005.	Paraguay	VR	1	1
General Directorate of Strategic Health Information (DIGIES) (Paraguay), Ministry of Health (Paraguay). Paraguay Vital Registration - Deaths 2006.	Paraguay	VR	1	1
General Directorate of Strategic Health Information (DIGIES) (Paraguay), Ministry of Health (Paraguay). Paraguay Vital Registration - Deaths 2007.	Paraguay	VR	1	1
General Directorate of Strategic Health Information (DIGIES) (Paraguay), Ministry of Health (Paraguay). Paraguay Vital Registration - Deaths 2008.	Paraguay	VR	1	1
General Directorate of Strategic Health Information (DIGIES) (Paraguay), Ministry of Health (Paraguay). Paraguay Vital Registration - Deaths 2009.	Paraguay	VR	1	1
General Directorate of Strategic Health Information (DIGIES) (Paraguay), Ministry of Health (Paraguay). Paraguay Vital Registration - Deaths 2010.	Paraguay	VR	1	1
General Directorate of Strategic Health Information (DIGIES) (Paraguay), Ministry of Health (Paraguay). Paraguay Vital Registration - Deaths 2011.	Paraguay	VR	1	1
General Directorate of Strategic Health Information (DIGIES) (Paraguay), Ministry of Health (Paraguay). Paraguay Vital Registration - Deaths 2012.	Paraguay	VR	1	1
General Directorate of Strategic Health Information (DIGIES) (Paraguay), Ministry of Health (Paraguay). Paraguay Vital Registration - Deaths 2013.	Paraguay	VR	1	1

General Directorate of Strategic Health Information (DIGIES) (Paraguay), Ministry of Health (Paraguay). Paraguay Vital Registration - Deaths 2014.	Paraguay	VR	1	1
General Directorate of Strategic Health Information (DIGIES) (Paraguay), Ministry of Health (Paraguay). Paraguay Vital Registration - Deaths 2015.	Paraguay	VR	1	1
General Directorate of Strategic Health Information (DIGIES) (Paraguay), Ministry of Health (Paraguay). Paraguay Vital Registration - Deaths 2016.	Paraguay	VR	1	1
General Directorate of Strategic Health Information (DIGIES) (Paraguay), Ministry of Health (Paraguay). Paraguay Vital Registration - Deaths 2017.	Paraguay	VR	1	1
Ghana Health Service, Ghana Statistical Service, ICF International. Ghana Special Demographic and Health Survey 2017. Fairfax, United States of America: ICF International, 2018.	Ghana	VA	1	1
Ghana Health Service, Ghana Statistical Service, Macro International, Inc. Ghana Special Demographic and Health Survey 2007-2008. Fairfax, United States of America: ICF International.	Ghana	VA	0	1
ICF International, Ministry of Health (Nepal), New ERA. Nepal Demographic and Health Survey 2016-2017. Fairfax, United States of America: ICF International, 2017.	Nepal	VA	1	1
INDEPTH, International Centre for Diarrhoeal Disease Research, Bangladesh (ICDDR,B). Bangladesh - Matlab Health and Demographic Surveillance System.	Bangladesh	VA	1	1
INDEPTH, Manhica Health Research Center (CISM). Mozambique - Manhiça Health and Demographic Surveillance System.	Mozambique	VA	0	1
INDEPTH, Nouna Health Research Center (CRSN) (Burkina Faso). Burkina Faso - Nouna Health and Demographic Surveillance System.	Burkina Faso	VA	1	1
Indian Council of Medical Research (ICMR). India Study on Causes of Death by Verbal Autopsy 2003.	India, Assam, Urban, Maharashtra, Urban, Rajasthan, Urban, Tamil Nadu, Urban, Bihar, Rural, Maharashtra, Rural, Rajasthan, Rural, Tamil Nadu, Rural	VA	1	1
Institute for Health Metrics and Evaluation (IHME). IHME GBD India - Andhra Pradesh Cause-Specific Mortality Estimates.	Andhra Pradesh	VA	1	0
Institute for Health Metrics and Evaluation (IHME). IHME GBD India - Assam Cause-Specific Mortality Estimates.	Assam	VA	1	0
Institute for Health Metrics and Evaluation (IHME). IHME GBD India - Bihar Cause-Specific Mortality Estimates.	Bihar	VA	1	0
Institute for Health Metrics and Evaluation (IHME). IHME GBD India - Gujarat Cause-Specific Mortality Estimates.	Gujarat	VA	1	0
Institute for Health Metrics and Evaluation (IHME). IHME GBD India - Haryana Cause-Specific Mortality Estimates.	Haryana	VA	1	0
Institute for Health Metrics and Evaluation (IHME). IHME GBD India - Himachal Pradesh Cause-Specific Mortality Estimates.	Himachal Pradesh	VA	1	0
Institute for Health Metrics and Evaluation (IHME). IHME GBD India - Jammu and Kashmir Cause-Specific Mortality Estimates.	Jammu & Kashmir and Ladakh	VA	1	0
Institute for Health Metrics and Evaluation (IHME). IHME GBD India - Karnataka Cause-Specific Mortality Estimates.	Karnataka	VA	1	0
Institute for Health Metrics and Evaluation (IHME). IHME GBD India - Kerala Cause-Specific Mortality Estimates.	Kerala	VA	1	0
Institute for Health Metrics and Evaluation (IHME). IHME GBD India - Madhya Pradesh Cause-Specific Mortality Estimates.	Madhya Pradesh	VA	1	0
Institute for Health Metrics and Evaluation (IHME). IHME GBD India - Maharashtra Cause-Specific Mortality Estimates.	Maharashtra	VA	1	0
Institute for Health Metrics and Evaluation (IHME). IHME GBD India - Meghalaya Cause-Specific Mortality Estimates.	Meghalaya	VA	1	0
Institute for Health Metrics and Evaluation (IHME). IHME GBD India - Odisha Cause-Specific Mortality Estimates.	Odisha	VA	1	0
Institute for Health Metrics and Evaluation (IHME). IHME GBD India - Punjab Cause-Specific Mortality Estimates.	Punjab	VA	1	0
Institute for Health Metrics and Evaluation (IHME). IHME GBD India - Rajasthan Cause-Specific Mortality Estimates.	Rajasthan	VA	1	0
Institute for Health Metrics and Evaluation (IHME). IHME GBD India - Tamil Nadu Cause-Specific Mortality Estimates.	Tamil Nadu	VA	1	0
Institute for Health Metrics and Evaluation (IHME). IHME GBD India - Telangana Cause-Specific Mortality Estimates.	Telangana	VA	1	0
Institute for Health Metrics and Evaluation (IHME). IHME GBD India - Uttar Pradesh Cause-Specific Mortality Estimates.	Uttar Pradesh	VA	1	0
Institute for Health Metrics and Evaluation (IHME). IHME GBD India - West Bengal Cause-Specific Mortality Estimates.	West Bengal	VA	1	0
Institute for Health Metrics and Evaluation (IHME). IHME GBD India Cause-Specific Mortality Estimates.	India	VA	1	0
Institute of Health Systems (India). India Cause of Death Dataset Version 1.3 1980-1998. Hyperabad, India: Institute of Health Systems (India), 2002.	India (some subnationals)	VA	1	0
International Centre for Diarrhoeal Disease Research, Bangladesh (ICDDR,B). Bangladesh - Chandpur and Comilla District Verbal Autopsy Study 2011-2014.	Bangladesh	VA	1	1
Italian National Institute of Statistics. Italy Vital Registration - Deaths 1980. Rome, Italy: Italian National Institute of Statistics.	Italy (all subnationals)	VR	1	1
Italian National Institute of Statistics. Italy Vital Registration - Deaths 1981. Rome, Italy: Italian National Institute of Statistics.	Italy (all subnationals)	VR	1	1
Italian National Institute of Statistics. Italy Vital Registration - Deaths 1982. Rome, Italy: Italian National Institute of Statistics.	Italy (all subnationals)	VR	1	1

Italian National Institute of Statistics. Italy Vital Registration - Deaths 1983. Rome, Italy: Italian National Institute of Statistics.	Italy (all subnationals)	VR	1	1
Italian National Institute of Statistics. Italy Vital Registration - Deaths 1984. Rome, Italy: Italian National Institute of Statistics.	Italy (all subnationals)	VR	1	1
Italian National Institute of Statistics. Italy Vital Registration - Deaths 1985. Rome, Italy: Italian National Institute of Statistics.	Italy (all subnationals)	VR	1	1
Italian National Institute of Statistics. Italy Vital Registration - Deaths 1986. Rome, Italy: Italian National Institute of Statistics.	Italy (all subnationals)	VR	1	1
Italian National Institute of Statistics. Italy Vital Registration - Deaths 1987. Rome, Italy: Italian National Institute of Statistics.	Italy (all subnationals)	VR	1	1
Italian National Institute of Statistics. Italy Vital Registration - Deaths 1988. Rome, Italy: Italian National Institute of Statistics.	Italy (all subnationals)	VR	1	1
Italian National Institute of Statistics. Italy Vital Registration - Deaths 1989. Rome, Italy: Italian National Institute of Statistics.	Italy (all subnationals)	VR	1	1
Italian National Institute of Statistics. Italy Vital Registration - Deaths 1990. Rome, Italy: Italian National Institute of Statistics.	Italy (all subnationals)	VR	1	1
Italian National Institute of Statistics. Italy Vital Registration - Deaths 1991. Rome, Italy: Italian National Institute of Statistics.	Italy (all subnationals)	VR	1	1
Italian National Institute of Statistics. Italy Vital Registration - Deaths 1992. Rome, Italy: Italian National Institute of Statistics.	Italy (all subnationals)	VR	1	1
Italian National Institute of Statistics. Italy Vital Registration - Deaths 1993. Rome, Italy: Italian National Institute of Statistics.	Italy (all subnationals)	VR	1	1
Italian National Institute of Statistics. Italy Vital Registration - Deaths 1994. Rome, Italy: Italian National Institute of Statistics.	Italy (all subnationals)	VR	1	1
Italian National Institute of Statistics. Italy Vital Registration - Deaths 1995. Rome, Italy: Italian National Institute of Statistics.	Italy (all subnationals)	VR	1	1
Italian National Institute of Statistics. Italy Vital Registration - Deaths 1996. Rome, Italy: Italian National Institute of Statistics.	Italy (all subnationals)	VR	1	1
Italian National Institute of Statistics. Italy Vital Registration - Deaths 1997. Rome, Italy: Italian National Institute of Statistics.	Italy (all subnationals)	VR	1	1
Italian National Institute of Statistics. Italy Vital Registration - Deaths 1998. Rome, Italy: Italian National Institute of Statistics.	Italy (all subnationals)	VR	1	1
Italian National Institute of Statistics. Italy Vital Registration - Deaths 1999. Rome, Italy: Italian National Institute of Statistics.	Italy (all subnationals)	VR	1	1
Italian National Institute of Statistics. Italy Vital Registration - Deaths 2000. Rome, Italy: Italian National Institute of Statistics.	Italy (all subnationals)	VR	1	1
Italian National Institute of Statistics. Italy Vital Registration - Deaths 2001. Rome, Italy: Italian National Institute of Statistics.	Italy (all subnationals)	VR	1	1
Italian National Institute of Statistics. Italy Vital Registration - Deaths 2002. Rome, Italy: Italian National Institute of Statistics.	Italy (all subnationals)	VR	1	1
Italian National Institute of Statistics. Italy Vital Registration - Deaths 2003. Rome, Italy: Italian National Institute of Statistics.	Italy (all subnationals)	VR	1	1
Italian National Institute of Statistics. Italy Vital Registration - Deaths 2004. Rome, Italy: Italian National Institute of Statistics.	Italy (all subnationals)	VR	1	1
Italian National Institute of Statistics. Italy Vital Registration - Deaths 2005. Rome, Italy: Italian National Institute of Statistics.	Italy (all subnationals)	VR	1	1
Italian National Institute of Statistics. Italy Vital Registration - Deaths 2006. Rome, Italy: Italian National Institute of Statistics.	Italy (all subnationals)	VR	1	1
Italian National Institute of Statistics. Italy Vital Registration - Deaths 2007. Rome, Italy: Italian National Institute of Statistics.	Italy (all subnationals)	VR	1	1
Italian National Institute of Statistics. Italy Vital Registration - Deaths 2008. Rome, Italy: Italian National Institute of Statistics.	Italy (all subnationals)	VR	1	1
Italian National Institute of Statistics. Italy Vital Registration - Deaths 2009. Rome, Italy: Italian National Institute of Statistics.	Italy (all subnationals)	VR	1	1
Italian National Institute of Statistics. Italy Vital Registration - Deaths 2010. Rome, Italy: Italian National Institute of Statistics.	Italy (all subnationals)	VR	1	1
Italian National Institute of Statistics. Italy Vital Registration - Deaths 2011. Rome, Italy: Italian National Institute of Statistics.	Italy (all subnationals)	VR	1	1
Italian National Institute of Statistics. Italy Vital Registration - Deaths 2012. Rome, Italy: Italian National Institute of Statistics.	Italy (all subnationals)	VR	1	1
Italian National Institute of Statistics. Italy Vital Registration - Deaths 2014. Rome, Italy: Italian National Institute of Statistics.	Italy (all subnationals)	VR	1	1
Italian National Institute of Statistics. Italy Vital Registration - Deaths 2015. Rome, Italy: Italian National Institute of Statistics.	Italy (all subnationals)	VR	1	1
Italian National Institute of Statistics. Italy Vital Registration - Deaths 2016. Rome, Italy: Italian National Institute of Statistics.	Italy (all subnationals)	VR	1	1
Italian National Institute of Statistics. Italy Vital Registration - Deaths 2017. Rome, Italy: Italian National Institute of Statistics.	Italy (all subnationals)	VR	1	1
Italian National Institute of Statistics. Italy Vital Registration - Deaths 2018. Rome, Italy: Italian National Institute of Statistics.	Italy (all subnationals)	VR	1	1
Italy Vital Registration - Deaths 2013. Rome, Italy: Italian National Institute of Statistics.	Italy (all subnationals)	VR	1	1
Kaatano GM, Mashauri FM, Kinung'hi SM, Mwanga JR, Malima RC, Kishamawe C, Nnko SE, Magesa SM, Mboera LE. Patterns of malaria related mortality based on verbal autopsy in Muleba District, north-western Tanzania. <i>Tanzan J Health Res</i>. 2009; 11(4): 210-8.	United Republic of Tanzania	VA	1	1
Macro International, Inc, National Institute of Population Studies (Pakistan). Pakistan Demographic and Health Survey 2006-2007. Fairfax, United States of America: ICF International.	Pakistan (some subnationals)	VA	1	0
Ministry of Health (Brazil). Brazil Mortality Information System - Deaths 1980. Rio de Janeiro, Brazil: Ministry of Health (Brazil).	Brazil (some subnationals)	VR	1	1
Ministry of Health (Brazil). Brazil Mortality Information System - Deaths 1981. Rio de Janeiro, Brazil: Ministry of Health (Brazil).	Brazil (some subnationals)	VR	1	1
Ministry of Health (Brazil). Brazil Mortality Information System - Deaths 1982. Rio de Janeiro, Brazil: Ministry of Health (Brazil).	Brazil (some subnationals)	VR	1	1
Ministry of Health (Brazil). Brazil Mortality Information System - Deaths 1983. Rio de Janeiro, Brazil: Ministry of Health (Brazil).	Brazil (some subnationals)	VR	1	1
Ministry of Health (Brazil). Brazil Mortality Information System - Deaths 1984. Rio de Janeiro, Brazil: Ministry of Health (Brazil).	Brazil (some subnationals)	VR	1	1

Ministry of Health (Brazil). Brazil Mortality Information System - Deaths 1985. Rio de Janeiro, Brazil: Ministry of Health (Brazil).	Brazil (some subnationals)	VR	1	1
Ministry of Health (Brazil). Brazil Mortality Information System - Deaths 1986. Rio de Janeiro, Brazil: Ministry of Health (Brazil).	Brazil (some subnationals)	VR	1	1
Ministry of Health (Brazil). Brazil Mortality Information System - Deaths 1987. Rio de Janeiro, Brazil: Ministry of Health (Brazil).	Brazil (some subnationals)	VR	1	1
Ministry of Health (Brazil). Brazil Mortality Information System - Deaths 1988. Rio de Janeiro, Brazil: Ministry of Health (Brazil).	Brazil (some subnationals)	VR	1	1
Ministry of Health (Brazil). Brazil Mortality Information System - Deaths 1989. Rio de Janeiro, Brazil: Ministry of Health (Brazil).	Brazil (some subnationals)	VR	1	1
Ministry of Health (Brazil). Brazil Mortality Information System - Deaths 1990. Rio de Janeiro, Brazil: Ministry of Health (Brazil).	Brazil (some subnationals)	VR	1	1
Ministry of Health (Brazil). Brazil Mortality Information System - Deaths 1991. Rio de Janeiro, Brazil: Ministry of Health (Brazil).	Brazil (some subnationals)	VR	1	1
Ministry of Health (Brazil). Brazil Mortality Information System - Deaths 1992. Rio de Janeiro, Brazil: Ministry of Health (Brazil).	Brazil (some subnationals)	VR	1	1
Ministry of Health (Brazil). Brazil Mortality Information System - Deaths 1993. Rio de Janeiro, Brazil: Ministry of Health (Brazil).	Brazil (some subnationals)	VR	1	1
Ministry of Health (Brazil). Brazil Mortality Information System - Deaths 1994. Rio de Janeiro, Brazil: Ministry of Health (Brazil).	Brazil (some subnationals)	VR	1	1
Ministry of Health (Brazil). Brazil Mortality Information System - Deaths 1995. Rio de Janeiro, Brazil: Ministry of Health (Brazil).	Brazil (some subnationals)	VR	1	1
Ministry of Health (Brazil). Brazil Mortality Information System - Deaths 1996. Rio de Janeiro, Brazil: Ministry of Health (Brazil).	Brazil (some subnationals)	VR	1	1
Ministry of Health (Brazil). Brazil Mortality Information System - Deaths 1997. Rio de Janeiro, Brazil: Ministry of Health (Brazil).	Brazil (some subnationals)	VR	1	1
Ministry of Health (Brazil). Brazil Mortality Information System - Deaths 1998. Rio de Janeiro, Brazil: Ministry of Health (Brazil).	Brazil (some subnationals)	VR	1	1
Ministry of Health (Brazil). Brazil Mortality Information System - Deaths 1999. Rio de Janeiro, Brazil: Ministry of Health (Brazil).	Brazil (some subnationals)	VR	1	1
Ministry of Health (Brazil). Brazil Mortality Information System - Deaths 2000. Rio de Janeiro, Brazil: Ministry of Health (Brazil).	Brazil (some subnationals)	VR	1	1
Ministry of Health (Brazil). Brazil Mortality Information System - Deaths 2001. Rio de Janeiro, Brazil: Ministry of Health (Brazil).	Brazil (some subnationals)	VR	1	1
Ministry of Health (Brazil). Brazil Mortality Information System - Deaths 2002. Rio de Janeiro, Brazil: Ministry of Health (Brazil).	Brazil (some subnationals)	VR	1	1
Ministry of Health (Brazil). Brazil Mortality Information System - Deaths 2003. Rio de Janeiro, Brazil: Ministry of Health (Brazil).	Brazil (some subnationals)	VR	1	1
Ministry of Health (Brazil). Brazil Mortality Information System - Deaths 2004. Rio de Janeiro, Brazil: Ministry of Health (Brazil).	Brazil (some subnationals)	VR	1	1
Ministry of Health (Brazil). Brazil Mortality Information System - Deaths 2005. Rio de Janeiro, Brazil: Ministry of Health (Brazil).	Brazil (all subnationals)	VR	1	1
Ministry of Health (Brazil). Brazil Mortality Information System - Deaths 2006. Rio de Janeiro, Brazil: Ministry of Health (Brazil).	Brazil (all subnationals)	VR	1	1
Ministry of Health (Brazil). Brazil Mortality Information System - Deaths 2007. Rio de Janeiro, Brazil: Ministry of Health (Brazil).	Brazil (all subnationals)	VR	1	1
Ministry of Health (Brazil). Brazil Mortality Information System - Deaths 2008. Rio de Janeiro, Brazil: Ministry of Health (Brazil).	Brazil (all subnationals)	VR	1	1
Ministry of Health (Brazil). Brazil Mortality Information System - Deaths 2009. Rio de Janeiro, Brazil: Ministry of Health (Brazil).	Brazil (all subnationals)	VR	1	1
Ministry of Health (Brazil). Brazil Mortality Information System - Deaths 2010. Rio de Janeiro, Brazil: Ministry of Health (Brazil).	Brazil (all subnationals)	VR	1	1
Ministry of Health (Brazil). Brazil Mortality Information System - Deaths 2011. Rio de Janeiro, Brazil: Ministry of Health (Brazil).	Brazil (all subnationals)	VR	1	1
Ministry of Health (Brazil). Brazil Mortality Information System - Deaths 2012. Rio de Janeiro, Brazil: Ministry of Health (Brazil).	Brazil (all subnationals)	VR	1	1
Ministry of Health (Brazil). Brazil Mortality Information System - Deaths 2013. Rio de Janeiro, Brazil: Ministry of Health (Brazil).	Brazil (all subnationals)	VR	1	1
Ministry of Health (Brazil). Brazil Mortality Information System - Deaths 2014. Rio de Janeiro, Brazil: Ministry of Health (Brazil).	Brazil (all subnationals)	VR	1	1
Ministry of Health (Brazil). Brazil Mortality Information System - Deaths 2015. Rio de Janeiro, Brazil: Ministry of Health (Brazil).	Brazil (all subnationals)	VR	1	1
Ministry of Health (Brazil). Brazil Mortality Information System - Deaths 2016. Rio de Janeiro, Brazil: Ministry of Health (Brazil).	Brazil (all subnationals)	VR	1	1
Ministry of Health (Brazil). Brazil Mortality Information System - Deaths 2017. Rio de Janeiro, Brazil: Ministry of Health (Brazil).	Brazil (all subnationals)	VR	1	1
Ministry of Health (Brazil). Brazil Mortality Information System - Deaths 2018. Rio de Janeiro, Brazil: Ministry of Health (Brazil).	Brazil (all subnationals)	VR	1	1
Ministry of Health (Brazil). Brazil Mortality Information System - Deaths 2019. Rio de Janeiro, Brazil: Ministry of Health (Brazil).	Brazil (all subnationals)	VR	1	1
Ministry of Health (Indonesia). Indonesia Cause of Death Survey 2010-2011.	Indonesia (some subnationals)	VA	1	1
Ministry of Health (Indonesia). Indonesia Sample Registration System - Deaths 2012.	Indonesia (some subnationals)	VA	1	0
Ministry of Health (Indonesia). Indonesia Sample Registration System - Deaths 2013.	Indonesia (some subnationals)	VA	1	1
Ministry of Health (Indonesia). Indonesia Sample Registration System - Deaths 2014.	Indonesia (some subnationals)	VA	1	1
Ministry of Health (Jordan). Jordan Causes of Death and Population 2013.	Jordan	VR	1	1
Ministry of Health (Jordan). Jordan Vital Registration - Deaths 2004-2006.	Jordan	VR	1	1
Ministry of Health (Kyrgyzstan). Kyrgyzstan Vital Registration - Deaths 2017.	Kyrgyzstan	VR	1	1
Ministry of Health (Mongolia). Mongolia Vital Registration - Deaths 2010.	Mongolia	VR	1	1
Ministry of Health (Mongolia). Mongolia Vital Registration - Deaths 2018.	Mongolia	VR	1	1

Ministry of Health (Mongolia). Mongolia Vital Registration - Deaths 2019.	Mongolia	VR	1	1
Ministry of Health (Mongolia). Mongolia Vital Registration - Deaths 2020.	Mongolia	VR	1	1
Ministry of Health (New Zealand). New Zealand Mortality Collection 1988. Wellington, New Zealand: Ministry of Health (New Zealand).	New Zealand (all subnationals)	VR	1	1
Ministry of Health (New Zealand). New Zealand Mortality Collection 1989. Wellington, New Zealand: Ministry of Health (New Zealand).	New Zealand (all subnationals)	VR	1	1
Ministry of Health (New Zealand). New Zealand Mortality Collection 1990. Wellington, New Zealand: Ministry of Health (New Zealand).	New Zealand (all subnationals)	VR	1	1
Ministry of Health (New Zealand). New Zealand Mortality Collection 1991. Wellington, New Zealand: Ministry of Health (New Zealand).	New Zealand (all subnationals)	VR	1	1
Ministry of Health (New Zealand). New Zealand Mortality Collection 1992. Wellington, New Zealand: Ministry of Health (New Zealand).	New Zealand (all subnationals)	VR	1	1
Ministry of Health (New Zealand). New Zealand Mortality Collection 1993. Wellington, New Zealand: Ministry of Health (New Zealand).	New Zealand (all subnationals)	VR	1	1
Ministry of Health (New Zealand). New Zealand Mortality Collection 1994. Wellington, New Zealand: Ministry of Health (New Zealand).	New Zealand (all subnationals)	VR	1	1
Ministry of Health (New Zealand). New Zealand Mortality Collection 1996. Wellington, New Zealand: Ministry of Health (New Zealand).	New Zealand (all subnationals)	VR	1	1
Ministry of Health (New Zealand). New Zealand Mortality Collection 1997. Wellington, New Zealand: Ministry of Health (New Zealand).	New Zealand (all subnationals)	VR	1	1
Ministry of Health (New Zealand). New Zealand Mortality Collection 1998. Wellington, New Zealand: Ministry of Health (New Zealand).	New Zealand (all subnationals)	VR	1	1
Ministry of Health (New Zealand). New Zealand Mortality Collection 1999. Wellington, New Zealand: Ministry of Health (New Zealand).	New Zealand (all subnationals)	VR	1	1
Ministry of Health (New Zealand). New Zealand Mortality Collection 2000. Wellington, New Zealand: Ministry of Health (New Zealand).	New Zealand (all subnationals)	VR	1	1
Ministry of Health (New Zealand). New Zealand Mortality Collection 2001. Wellington, New Zealand: Ministry of Health (New Zealand).	New Zealand (all subnationals)	VR	1	1
Ministry of Health (New Zealand). New Zealand Mortality Collection 2002. Wellington, New Zealand: Ministry of Health (New Zealand).	New Zealand (all subnationals)	VR	1	1
Ministry of Health (New Zealand). New Zealand Mortality Collection 2003. Wellington, New Zealand: Ministry of Health (New Zealand).	New Zealand (all subnationals)	VR	1	1
Ministry of Health (New Zealand). New Zealand Mortality Collection 2004. Wellington, New Zealand: Ministry of Health (New Zealand).	New Zealand (all subnationals)	VR	1	1
Ministry of Health (New Zealand). New Zealand Mortality Collection 2005. Wellington, New Zealand: Ministry of Health (New Zealand).	New Zealand (all subnationals)	VR	1	1
Ministry of Health (New Zealand). New Zealand Mortality Collection 2006. Wellington, New Zealand: Ministry of Health (New Zealand).	New Zealand (all subnationals)	VR	1	1
Ministry of Health (New Zealand). New Zealand Mortality Collection 2007. Wellington, New Zealand: Ministry of Health (New Zealand).	New Zealand (all subnationals)	VR	1	1
Ministry of Health (New Zealand). New Zealand Mortality Collection 2008. Wellington, New Zealand: Ministry of Health (New Zealand).	New Zealand (all subnationals)	VR	1	1
Ministry of Health (New Zealand). New Zealand Mortality Collection 2009. Wellington, New Zealand: Ministry of Health (New Zealand).	New Zealand (all subnationals)	VR	1	1
Ministry of Health (New Zealand). New Zealand Mortality Collection 2010. Wellington, New Zealand: Ministry of Health (New Zealand).	New Zealand (all subnationals)	VR	1	1
Ministry of Health (New Zealand). New Zealand Mortality Collection 2011. Wellington, New Zealand: Ministry of Health (New Zealand).	New Zealand (all subnationals)	VR	1	1
Ministry of Health (New Zealand). New Zealand Mortality Collection 2012. Wellington, New Zealand: Ministry of Health (New Zealand).	New Zealand (all subnationals)	VR	1	1
Ministry of Health (New Zealand). New Zealand Mortality Collection 2013. Wellington, New Zealand: Ministry of Health (New Zealand).	New Zealand (all subnationals)	VR	1	1
Ministry of Health (New Zealand). New Zealand Mortality Collection 2014. Wellington, New Zealand: Ministry of Health (New Zealand).	New Zealand (all subnationals)	VR	1	1
Ministry of Health (New Zealand). New Zealand Mortality Collection 2015. Wellington, New Zealand: Ministry of Health (New Zealand).	New Zealand (all subnationals)	VR	1	1
Ministry of Health (New Zealand). New Zealand Mortality Collection 2016. Wellington, New Zealand: Ministry of Health (New Zealand).	New Zealand (all subnationals)	VR	1	1
Ministry of Health (New Zealand). New Zealand Mortality Collection 2017. Wellington, New Zealand: Ministry of Health (New Zealand).	New Zealand (all subnationals)	VR	1	1
Ministry of Health (New Zealand). New Zealand Mortality Data 1980. Wellington, New Zealand: Ministry of Health (New Zealand).	New Zealand (all subnationals)	VR	1	1
Ministry of Health (New Zealand). New Zealand Mortality Data 1981. Wellington, New Zealand: Ministry of Health (New Zealand).	New Zealand (all subnationals)	VR	1	1
Ministry of Health (New Zealand). New Zealand Mortality Data 1982. Wellington, New Zealand: Ministry of Health (New Zealand).	New Zealand (all subnationals)	VR	1	1
Ministry of Health (New Zealand). New Zealand Mortality Data 1983. Wellington, New Zealand: Ministry of Health (New Zealand).	New Zealand (all subnationals)	VR	1	1
Ministry of Health (New Zealand). New Zealand Mortality Data 1984. Wellington, New Zealand: Ministry of Health (New Zealand).	New Zealand (all subnationals)	VR	1	1
Ministry of Health (New Zealand). New Zealand Mortality Data 1985. Wellington, New Zealand: Ministry of Health (New Zealand).	New Zealand (all subnationals)	VR	1	1
Ministry of Health (New Zealand). New Zealand Mortality Data 1986. Wellington, New Zealand: Ministry of Health (New Zealand).	New Zealand (all subnationals)	VR	1	1
Ministry of Health (New Zealand). New Zealand Mortality Data 1987. Wellington, New Zealand: Ministry of Health (New Zealand).	New Zealand (all subnationals)	VR	1	1
Ministry of Health (Palestine). Palestine - Gaza Strip and West Bank Mortality by Sex, Age, and Cause 2012.	Palestine	VR	1	1
Ministry of Health (Palestine). Palestine - Gaza Strip and West Bank Mortality by Sex, Age, and Cause 2013.	Palestine	VR	1	1
Ministry of Health (Palestine). Palestine - Gaza Strip and West Bank Mortality by Sex, Age, and Cause 2014.	Palestine	VR	1	1
Ministry of Health (Palestine). Palestine - Gaza Strip and West Bank Mortality by Sex, Age, and Cause 2015.	Palestine	VR	1	1
Ministry of Health (Palestine). Palestine - Gaza Strip and West Bank Mortality by Sex, Age, and Cause 2016.	Palestine	VR	1	1

Ministry of Health (Palestine). Palestine - Gaza Strip and West Bank Mortality by Sex, Age, and Cause 2017.	Palestine	VR	1	1
Ministry of Health (Palestine). Palestine - Gaza Strip and West Bank Mortality by Sex, Age, and Cause 2018.	Palestine	VR	1	1
Ministry of Health (Saudi Arabia). Saudi Arabia Vital Registration - Deaths 1996-2011.	Saudi Arabia	VR	1	1
Ministry of Health (Saudi Arabia). Saudi Arabia Vital Registration - Deaths 2009-2013.	Saudi Arabia	VR	1	1
Ministry of Health (Singapore). Singapore Causes of Death 1980.	Singapore	VR	1	1
Ministry of Health (Singapore). Singapore Causes of Death 1981.	Singapore	VR	1	1
Ministry of Health (Singapore). Singapore Causes of Death 1982.	Singapore	VR	1	1
Ministry of Health (Singapore). Singapore Causes of Death 1983.	Singapore	VR	1	1
Ministry of Health (Singapore). Singapore Causes of Death 1984.	Singapore	VR	1	1
Ministry of Health (Singapore). Singapore Causes of Death 1985.	Singapore	VR	1	1
Ministry of Health (Singapore). Singapore Causes of Death 1986.	Singapore	VR	1	1
Ministry of Health (Singapore). Singapore Causes of Death 1987.	Singapore	VR	1	1
Ministry of Health (Singapore). Singapore Causes of Death 1988.	Singapore	VR	1	1
Ministry of Health (Singapore). Singapore Causes of Death 1989.	Singapore	VR	1	1
Ministry of Health (Singapore). Singapore Causes of Death 1990.	Singapore	VR	1	1
Ministry of Health (Singapore). Singapore Causes of Death 1991.	Singapore	VR	1	1
Ministry of Health (Singapore). Singapore Causes of Death 1992.	Singapore	VR	1	1
Ministry of Health (Singapore). Singapore Causes of Death 1993.	Singapore	VR	1	1
Ministry of Health (Singapore). Singapore Causes of Death 1994.	Singapore	VR	1	1
Ministry of Health (Singapore). Singapore Causes of Death 1995.	Singapore	VR	1	1
Ministry of Health (Singapore). Singapore Causes of Death 1996.	Singapore	VR	1	1
Ministry of Health (Singapore). Singapore Causes of Death 1997.	Singapore	VR	1	1
Ministry of Health (Singapore). Singapore Causes of Death 1998.	Singapore	VR	1	1
Ministry of Health (Singapore). Singapore Causes of Death 1999.	Singapore	VR	1	1
Ministry of Health (Singapore). Singapore Causes of Death 2000.	Singapore	VR	1	1
Ministry of Health (Singapore). Singapore Causes of Death 2001.	Singapore	VR	1	1
Ministry of Health (Singapore). Singapore Causes of Death 2002.	Singapore	VR	1	1
Ministry of Health (Singapore). Singapore Causes of Death 2003.	Singapore	VR	1	1
Ministry of Health (Singapore). Singapore Causes of Death 2004.	Singapore	VR	1	1
Ministry of Health (Singapore). Singapore Causes of Death 2005.	Singapore	VR	1	1
Ministry of Health (Singapore). Singapore Causes of Death 2006.	Singapore	VR	1	1
Ministry of Health (Singapore). Singapore Causes of Death 2007.	Singapore	VR	1	1
Ministry of Health (Singapore). Singapore Causes of Death 2008.	Singapore	VR	1	1
Ministry of Health (Singapore). Singapore Causes of Death 2009.	Singapore	VR	1	1
Ministry of Health (Singapore). Singapore Causes of Death 2010.	Singapore	VR	1	1
Ministry of Health (Singapore). Singapore Causes of Death 2011.	Singapore	VR	1	1
Ministry of Health (Singapore). Singapore Causes of Death 2012.	Singapore	VR	1	1
Ministry of Health (Singapore). Singapore Causes of Death 2013.	Singapore	VR	1	1
Ministry of Health (Singapore). Singapore Causes of Death 2014.	Singapore	VR	1	1
Ministry of Health (Singapore). Singapore Causes of Death 2015.	Singapore	VR	1	1
Ministry of Health (Singapore). Singapore Causes of Death 2016.	Singapore	VR	1	1
Ministry of Health (Singapore). Singapore Causes of Death 2017.	Singapore	VR	1	1
Ministry of Health and Medical Education (Iran). Iran Death Registration System 2001.	Iran (Islamic Republic of) (some subnationals)	VR	1	1
Ministry of Health and Medical Education (Iran). Iran Death Registration System 2002.	Iran (Islamic Republic of) (some subnationals)	VR	1	1

Ministry of Health and Medical Education (Iran). Iran Death Registration System 2003.	Iran (Islamic Republic of) (some subnationals)	VR	1	1
Ministry of Health and Medical Education (Iran). Iran Death Registration System 2004.	Iran (Islamic Republic of) (some subnationals)	VR	1	1
Ministry of Health and Medical Education (Iran). Iran Death Registration System 2005.	Iran (Islamic Republic of) (some subnationals)	VR	1	1
Ministry of Health and Medical Education (Iran). Iran Death Registration System 2006.	Iran (Islamic Republic of) (some subnationals)	VR	1	1
Ministry of Health and Medical Education (Iran). Iran Death Registration System 2007.	Iran (Islamic Republic of) (some subnationals)	VR	1	1
Ministry of Health and Medical Education (Iran). Iran Death Registration System 2008.	Iran (Islamic Republic of) (some subnationals)	VR	1	1
Ministry of Health and Medical Education (Iran). Iran Death Registration System 2009.	Iran (Islamic Republic of) (some subnationals)	VR	1	1
Ministry of Health and Medical Education (Iran). Iran Death Registration System 2010.	Iran (Islamic Republic of) (some subnationals)	VR	1	1
Ministry of Health and Medical Education (Iran). Iran Death Registration System 2011.	Iran (Islamic Republic of) (some subnationals)	VR	1	1
Ministry of Health and Medical Education (Iran). Iran Death Registration System 2012.	Iran (Islamic Republic of) (some subnationals)	VR	1	1
Ministry of Health and Medical Education (Iran). Iran Death Registration System 2013.	Iran (Islamic Republic of) (some subnationals)	VR	1	1
Ministry of Health and Medical Education (Iran). Iran Death Registration System 2014.	Iran (Islamic Republic of) (some subnationals)	VR	1	1
Ministry of Health and Medical Education (Iran). Iran Death Registration System 2015.	Iran (Islamic Republic of) (all subnationals)	VR	1	1
Ministry of Health and Medical Education (Iran). Iran Death Registration System 2016.	Iran (Islamic Republic of) (all subnationals)	VR	1	1
Ministry of Health and Welfare (Taiwan). Taiwan Statistics of Causes of Death 2017. Taipei City, Taiwan (Province of China): Ministry of Health and Welfare (Taiwan), 2018.	Taiwan (Province of China)	VR	1	1
Ministry of Health and Welfare (Taiwan). Taiwan Vital Registration - Deaths 2013. Taipei City, Taiwan (Province of China): Ministry of Health and Welfare (Taiwan).	Taiwan (Province of China)	VR	1	1
Ministry of Health and Welfare (Taiwan). Taiwan Vital Registration - Deaths 2014. Taipei City, Taiwan (Province of China): Ministry of Health and Welfare (Taiwan).	Taiwan (Province of China)	VR	1	1
Ministry of Health and Welfare (Taiwan). Taiwan Vital Registration - Deaths 2015. Taipei City, Taiwan (Province of China): Ministry of Health and Welfare (Taiwan).	Taiwan (Province of China)	VR	1	1
Ministry of Health and Welfare (Taiwan). Taiwan Vital Registration - Deaths 2016. Taipei City, Taiwan (Province of China): Ministry of Health and Welfare (Taiwan).	Taiwan (Province of China)	VR	1	1
Ministry of Health, Labour and Welfare (Japan). Japan Vital Registration - Deaths 1980.	Japan (all subnationals)	VR	1	1
Ministry of Health, Labour and Welfare (Japan). Japan Vital Registration - Deaths 1981.	Japan (all subnationals)	VR	1	1
Ministry of Health, Labour and Welfare (Japan). Japan Vital Registration - Deaths 1982.	Japan (all subnationals)	VR	1	1
Ministry of Health, Labour and Welfare (Japan). Japan Vital Registration - Deaths 1983.	Japan (all subnationals)	VR	1	1
Ministry of Health, Labour and Welfare (Japan). Japan Vital Registration - Deaths 1984.	Japan (all subnationals)	VR	1	1
Ministry of Health, Labour and Welfare (Japan). Japan Vital Registration - Deaths 1985.	Japan (all subnationals)	VR	1	1
Ministry of Health, Labour and Welfare (Japan). Japan Vital Registration - Deaths 1986.	Japan (all subnationals)	VR	1	1
Ministry of Health, Labour and Welfare (Japan). Japan Vital Registration - Deaths 1987.	Japan (all subnationals)	VR	1	1
Ministry of Health, Labour and Welfare (Japan). Japan Vital Registration - Deaths 1988.	Japan (all subnationals)	VR	1	1
Ministry of Health, Labour and Welfare (Japan). Japan Vital Registration - Deaths 1989.	Japan (all subnationals)	VR	1	1
Ministry of Health, Labour and Welfare (Japan). Japan Vital Registration - Deaths 1990.	Japan (all subnationals)	VR	1	1
Ministry of Health, Labour and Welfare (Japan). Japan Vital Registration - Deaths 1991.	Japan (all subnationals)	VR	1	1
Ministry of Health, Labour and Welfare (Japan). Japan Vital Registration - Deaths 1992.	Japan (all subnationals)	VR	1	1
Ministry of Health, Labour and Welfare (Japan). Japan Vital Registration - Deaths 1993.	Japan (all subnationals)	VR	1	1
Ministry of Health, Labour and Welfare (Japan). Japan Vital Registration - Deaths 1994.	Japan (all subnationals)	VR	1	1
Ministry of Health, Labour and Welfare (Japan). Japan Vital Registration - Deaths 1995.	Japan (all subnationals)	VR	1	1
Ministry of Health, Labour and Welfare (Japan). Japan Vital Registration - Deaths 1996.	Japan (all subnationals)	VR	1	1
Ministry of Health, Labour and Welfare (Japan). Japan Vital Registration - Deaths 1997.	Japan (all subnationals)	VR	1	1
Ministry of Health, Labour and Welfare (Japan). Japan Vital Registration - Deaths 1998.	Japan (all subnationals)	VR	1	1
Ministry of Health, Labour and Welfare (Japan). Japan Vital Registration - Deaths 1999.	Japan (all subnationals)	VR	1	1
Ministry of Health, Labour and Welfare (Japan). Japan Vital Registration - Deaths 2000.	Japan (all subnationals)	VR	1	1
Ministry of Health, Labour and Welfare (Japan). Japan Vital Registration - Deaths 2001.	Japan (all subnationals)	VR	1	1

Ministry of Health, Labour and Welfare (Japan). Japan Vital Registration - Deaths 2002.	Japan (all subnationals)	VR	1	1
Ministry of Health, Labour and Welfare (Japan). Japan Vital Registration - Deaths 2003.	Japan (all subnationals)	VR	1	1
Ministry of Health, Labour and Welfare (Japan). Japan Vital Registration - Deaths 2004.	Japan (all subnationals)	VR	1	1
Ministry of Health, Labour and Welfare (Japan). Japan Vital Registration - Deaths 2005.	Japan (all subnationals)	VR	1	1
Ministry of Health, Labour and Welfare (Japan). Japan Vital Registration - Deaths 2006.	Japan (all subnationals)	VR	1	1
Ministry of Health, Labour and Welfare (Japan). Japan Vital Registration - Deaths 2007.	Japan (all subnationals)	VR	1	1
Ministry of Health, Labour and Welfare (Japan). Japan Vital Registration - Deaths 2008.	Japan (all subnationals)	VR	1	1
Ministry of Health, Labour and Welfare (Japan). Japan Vital Registration - Deaths 2009.	Japan (all subnationals)	VR	1	1
Ministry of Health, Labour and Welfare (Japan). Japan Vital Registration - Deaths 2010.	Japan (all subnationals)	VR	1	1
Ministry of Health, Labour and Welfare (Japan). Japan Vital Registration - Deaths 2011.	Japan (all subnationals)	VR	1	1
Ministry of Health, Labour and Welfare (Japan). Japan Vital Registration - Deaths 2012.	Japan (all subnationals)	VR	1	1
Ministry of Health, Labour and Welfare (Japan). Japan Vital Registration - Deaths 2013.	Japan (all subnationals)	VR	1	1
Ministry of Health, Labour and Welfare (Japan). Japan Vital Registration - Deaths 2014.	Japan (all subnationals)	VR	1	1
Ministry of Health, Labour and Welfare (Japan). Japan Vital Registration - Deaths 2015.	Japan (all subnationals)	VR	1	1
Ministry of Health, Labour and Welfare (Japan). Japan Vital Registration - Deaths 2016.	Japan (all subnationals)	VR	1	1
Ministry of Health, Labour and Welfare (Japan). Japan Vital Registration - Deaths 2017.	Japan (all subnationals)	VR	1	1
Ministry of Health, Labour and Welfare (Japan). Japan Vital Registration - Deaths 2018.	Japan (all subnationals)	VR	1	1
Ministry of Health, Labour and Welfare (Japan). Japan Vital Registration - Deaths 2019.	Japan (all subnationals)	VR	1	1
Ministry of Public Health (Thailand). Thailand Burden of Disease and Injuries 1998-1999.	Thailand	VA	1	1
National Administrative Department of Statistics (DANE) (Colombia). Colombia Vital Statistics - Deaths 2008. Bogotá, Colombia: National Administrative Department of Statistics (DANE) (Colombia).	Colombia	VR	1	1
National Administrative Department of Statistics (DANE) (Colombia). Colombia Vital Statistics - Deaths 2016. Bogotá, Colombia: National Administrative Department of Statistics (DANE) (Colombia).	Colombia	VR	1	1
National Administrative Department of Statistics (DANE) (Colombia). Colombia Vital Statistics - Deaths 2017. Bogotá, Colombia: National Administrative Department of Statistics (DANE) (Colombia).	Colombia	VR	1	1
National Administrative Department of Statistics (DANE) (Colombia). Colombia Vital Statistics - Deaths 2018. Bogotá, Colombia: National Administrative Department of Statistics (DANE) (Colombia).	Colombia	VR	1	1
National Administrative Department of Statistics (DANE) (Colombia). Colombia Vital Statistics - Deaths 2019. Bogotá, Colombia: National Administrative Department of Statistics (DANE) (Colombia).	Colombia	VR	1	1
National Board of Health and Welfare (Sweden). Sweden - Stockholm County Vital Registration - Deaths 1980.	Sweden (some subnationals)	VR	1	1
National Board of Health and Welfare (Sweden). Sweden - Stockholm County Vital Registration - Deaths 1981.	Sweden (some subnationals)	VR	1	1
National Board of Health and Welfare (Sweden). Sweden - Stockholm County Vital Registration - Deaths 1982.	Sweden (some subnationals)	VR	1	1
National Board of Health and Welfare (Sweden). Sweden - Stockholm County Vital Registration - Deaths 1983.	Sweden (some subnationals)	VR	1	1
National Board of Health and Welfare (Sweden). Sweden - Stockholm County Vital Registration - Deaths 1984.	Sweden (some subnationals)	VR	1	1
National Board of Health and Welfare (Sweden). Sweden - Stockholm County Vital Registration - Deaths 1985.	Sweden (some subnationals)	VR	1	1
National Board of Health and Welfare (Sweden). Sweden - Stockholm County Vital Registration - Deaths 1986.	Sweden (some subnationals)	VR	1	1
National Board of Health and Welfare (Sweden). Sweden Cause of Death Register 1987.	Sweden (all subnationals)	VR	1	1
National Board of Health and Welfare (Sweden). Sweden Cause of Death Register 1988.	Sweden (all subnationals)	VR	1	1
National Board of Health and Welfare (Sweden). Sweden Cause of Death Register 1989.	Sweden (all subnationals)	VR	1	1
National Board of Health and Welfare (Sweden). Sweden Cause of Death Register 1990.	Sweden (all subnationals)	VR	1	1
National Board of Health and Welfare (Sweden). Sweden Cause of Death Register 1991.	Sweden (all subnationals)	VR	1	1
National Board of Health and Welfare (Sweden). Sweden Cause of Death Register 1992.	Sweden (all subnationals)	VR	1	1
National Board of Health and Welfare (Sweden). Sweden Cause of Death Register 1993.	Sweden (all subnationals)	VR	1	1
National Board of Health and Welfare (Sweden). Sweden Cause of Death Register 1994.	Sweden (all subnationals)	VR	1	1
National Board of Health and Welfare (Sweden). Sweden Cause of Death Register 1995.	Sweden (all subnationals)	VR	1	1
National Board of Health and Welfare (Sweden). Sweden Cause of Death Register 1996.	Sweden (all subnationals)	VR	1	1

National Board of Health and Welfare (Sweden). Sweden Cause of Death Register 1997.	Sweden (all subnationals)	VR	1	1
National Board of Health and Welfare (Sweden). Sweden Cause of Death Register 1998.	Sweden (all subnationals)	VR	1	1
National Board of Health and Welfare (Sweden). Sweden Cause of Death Register 1999.	Sweden (all subnationals)	VR	1	1
National Board of Health and Welfare (Sweden). Sweden Cause of Death Register 2000.	Sweden (all subnationals)	VR	1	1
National Board of Health and Welfare (Sweden). Sweden Cause of Death Register 2001.	Sweden (all subnationals)	VR	1	1
National Board of Health and Welfare (Sweden). Sweden Cause of Death Register 2002.	Sweden (all subnationals)	VR	1	1
National Board of Health and Welfare (Sweden). Sweden Cause of Death Register 2003.	Sweden (all subnationals)	VR	1	1
National Board of Health and Welfare (Sweden). Sweden Cause of Death Register 2004.	Sweden (all subnationals)	VR	1	1
National Board of Health and Welfare (Sweden). Sweden Cause of Death Register 2005.	Sweden (all subnationals)	VR	1	1
National Board of Health and Welfare (Sweden). Sweden Cause of Death Register 2006.	Sweden (all subnationals)	VR	1	1
National Board of Health and Welfare (Sweden). Sweden Cause of Death Register 2007.	Sweden (all subnationals)	VR	1	1
National Board of Health and Welfare (Sweden). Sweden Cause of Death Register 2008.	Sweden (all subnationals)	VR	1	1
National Board of Health and Welfare (Sweden). Sweden Cause of Death Register 2009.	Sweden (all subnationals)	VR	1	1
National Board of Health and Welfare (Sweden). Sweden Cause of Death Register 2010.	Sweden (all subnationals)	VR	1	1
National Board of Health and Welfare (Sweden). Sweden Cause of Death Register 2011.	Sweden (all subnationals)	VR	1	1
National Board of Health and Welfare (Sweden). Sweden Cause of Death Register 2012. Stockholm, Sweden: National Board of Health and Welfare (Sweden).	Sweden (all subnationals)	VR	1	1
National Board of Health and Welfare (Sweden). Sweden Cause of Death Register 2013. Stockholm, Sweden: National Board of Health and Welfare (Sweden).	Sweden (all subnationals)	VR	1	1
National Board of Health and Welfare (Sweden). Sweden Cause of Death Register 2014. Stockholm, Sweden: National Board of Health and Welfare (Sweden).	Sweden (all subnationals)	VR	1	1
National Board of Health and Welfare (Sweden). Sweden Cause of Death Register 2015. Stockholm, Sweden: National Board of Health and Welfare (Sweden).	Sweden (all subnationals)	VR	1	1
National Board of Health and Welfare (Sweden). Sweden Cause of Death Register 2016. Stockholm, Sweden: National Board of Health and Welfare (Sweden).	Sweden (all subnationals)	VR	1	1
National Board of Health and Welfare (Sweden). Sweden Cause of Death Register 2017. Stockholm, Sweden: National Board of Health and Welfare (Sweden).	Sweden (all subnationals)	VR	1	1
National Board of Health and Welfare (Sweden). Sweden Cause of Death Register 2018. Stockholm, Sweden: National Board of Health and Welfare (Sweden).	Sweden (all subnationals)	VR	1	1
National Board of Health and Welfare (Sweden). Sweden Cause of Death Register 2019. Stockholm, Sweden: National Board of Health and Welfare (Sweden).	Sweden (all subnationals)	VR	1	1
National Board of Health and Welfare (Sweden). Sweden Vital Registration - Deaths 1980.	Sweden (some subnationals)	VR	1	1
National Board of Health and Welfare (Sweden). Sweden Vital Registration - Deaths 1981.	Sweden (some subnationals)	VR	1	1
National Board of Health and Welfare (Sweden). Sweden Vital Registration - Deaths 1982.	Sweden (some subnationals)	VR	1	1
National Board of Health and Welfare (Sweden). Sweden Vital Registration - Deaths 1983.	Sweden (some subnationals)	VR	1	1
National Board of Health and Welfare (Sweden). Sweden Vital Registration - Deaths 1984.	Sweden (some subnationals)	VR	1	1
National Board of Health and Welfare (Sweden). Sweden Vital Registration - Deaths 1985.	Sweden (some subnationals)	VR	1	1
National Board of Health and Welfare (Sweden). Sweden Vital Registration - Deaths 1986.	Sweden (some subnationals)	VR	1	1
National Center for Health Statistics, Centers for Disease Control and Prevention, U.S. Department of Defense. United States NVSS Custom Mortality Data 2005 and United States Military Deaths 1980-2014.	United States of America (all subnationals)	VR	1	1
National Center for Health Statistics, Centers for Disease Control and Prevention, U.S. Department of Defense. United States NVSS Custom Mortality Data 2006 and United States Military Deaths 1980-2014.	United States of America (all subnationals)	VR	1	1
National Center for Health Statistics, Centers for Disease Control and Prevention, U.S. Department of Defense. United States NVSS Custom Mortality Data 2007 and United States Military Deaths 1980-2014.	United States of America (all subnationals)	VR	1	1
National Center for Health Statistics, Centers for Disease Control and Prevention, U.S. Department of Defense. United States NVSS Custom Mortality Data 2008 and United States Military Deaths 1980-2014.	United States of America (all subnationals)	VR	1	1
National Center for Health Statistics, Centers for Disease Control and Prevention, U.S. Department of Defense. United States NVSS Custom Mortality Data 2009 and United States Military Deaths 1980-2014.	United States of America (all subnationals)	VR	1	1
National Center for Health Statistics, Centers for Disease Control and Prevention, U.S. Department of Defense. United States NVSS Custom Mortality Data 2010 and United States Military Deaths 1980-2014.	United States of America (all subnationals)	VR	1	1

National Center for Health Statistics, Centers for Disease Control and Prevention, U.S. Department of Defense. United States NVSS Custom Mortality Data 2011 and United States Military Deaths 1980-2014.	United States of America (all subnationals)	VR	1	1
National Center for Health Statistics, Centers for Disease Control and Prevention, U.S. Department of Defense. United States NVSS Custom Mortality Data 2012 and United States Military Deaths 1980-2014.	United States of America (all subnationals)	VR	1	1
National Center for Health Statistics, Centers for Disease Control and Prevention, U.S. Department of Defense. United States NVSS Custom Mortality Data 2013 and United States Military Deaths 1980-2014.	United States of America (all subnationals)	VR	1	1
National Center for Health Statistics, Centers for Disease Control and Prevention, U.S. Department of Defense. United States NVSS Custom Mortality Data 2014 and United States Military Deaths 1980-2014.	United States of America (all subnationals)	VR	1	1
National Center for Health Statistics, Centers for Disease Control and Prevention, U.S. Department of Defense. United States NVSS Custom Mortality Data 2015 and United States Military Deaths 2000-2018.	United States of America (all subnationals)	VR	1	1
National Center for Health Statistics, Centers for Disease Control and Prevention, U.S. Department of Defense. United States NVSS Custom Mortality Data 2016 and United States Military Deaths 2000-2018.	United States of America (all subnationals)	VR	1	1
National Center for Health Statistics, Centers for Disease Control and Prevention, U.S. Department of Defense. United States NVSS Custom Mortality Data 2017 and United States Military Deaths 2000-2018.	United States of America (all subnationals)	VR	1	1
National Center for Health Statistics, Centers for Disease Control and Prevention, U.S. Department of Defense. United States NVSS Custom Mortality Data 2018 and United States Military Deaths 2000-2018.	United States of America (all subnationals)	VR	1	1
National Center for Health Statistics, Centers for Disease Control and Prevention, U.S. Department of Defense. United States NVSS Mortality Data 1980 - NBER and United States Military Deaths 1980-2014.	United States of America (all subnationals)	VR	1	1
National Center for Health Statistics, Centers for Disease Control and Prevention, U.S. Department of Defense. United States NVSS Mortality Data 1981 - NBER and United States Military Deaths 1980-2014.	United States of America (all subnationals)	VR	1	1
National Center for Health Statistics, Centers for Disease Control and Prevention, U.S. Department of Defense. United States NVSS Mortality Data 1982 - NBER and United States Military Deaths 1980-2014.	United States of America (all subnationals)	VR	1	1
National Center for Health Statistics, Centers for Disease Control and Prevention, U.S. Department of Defense. United States NVSS Mortality Data 1983 - NBER and United States Military Deaths 1980-2014.	United States of America (all subnationals)	VR	1	1
National Center for Health Statistics, Centers for Disease Control and Prevention, U.S. Department of Defense. United States NVSS Mortality Data 1984 - NBER and United States Military Deaths 1980-2014.	United States of America (all subnationals)	VR	1	1
National Center for Health Statistics, Centers for Disease Control and Prevention, U.S. Department of Defense. United States NVSS Mortality Data 1985 - NBER and United States Military Deaths 1980-2014.	United States of America (all subnationals)	VR	1	1
National Center for Health Statistics, Centers for Disease Control and Prevention, U.S. Department of Defense. United States NVSS Mortality Data 1986 - NBER and United States Military Deaths 1980-2014.	United States of America (all subnationals)	VR	1	1
National Center for Health Statistics, Centers for Disease Control and Prevention, U.S. Department of Defense. United States NVSS Mortality Data 1987 - NBER and United States Military Deaths 1980-2014.	United States of America (all subnationals)	VR	1	1
National Center for Health Statistics, Centers for Disease Control and Prevention, U.S. Department of Defense. United States NVSS Mortality Data 1988 - NBER and United States Military Deaths 1980-2014.	United States of America (all subnationals)	VR	1	1
National Center for Health Statistics, Centers for Disease Control and Prevention, U.S. Department of Defense. United States NVSS Mortality Data 1989 - NBER and United States Military Deaths 1980-2014.	United States of America (all subnationals)	VR	1	1
National Center for Health Statistics, Centers for Disease Control and Prevention, U.S. Department of Defense. United States NVSS Mortality Data 1990 - NBER and United States Military Deaths 1980-2014.	United States of America (all subnationals)	VR	1	1
National Center for Health Statistics, Centers for Disease Control and Prevention, U.S. Department of Defense. United States NVSS Mortality Data 1991 - NBER and United States Military Deaths 1980-2014.	United States of America (all subnationals)	VR	1	1
National Center for Health Statistics, Centers for Disease Control and Prevention, U.S. Department of Defense. United States NVSS Mortality Data 1992 - NBER and United States Military Deaths 1980-2014.	United States of America (all subnationals)	VR	1	1
National Center for Health Statistics, Centers for Disease Control and Prevention, U.S. Department of Defense. United States NVSS Mortality Data 1993 - NBER and United States Military Deaths 1980-2014.	United States of America (all subnationals)	VR	1	1
National Center for Health Statistics, Centers for Disease Control and Prevention, U.S. Department of Defense. United States NVSS Mortality Data 1994 - NBER and United States Military Deaths 1980-2014.	United States of America (all subnationals)	VR	1	1
National Center for Health Statistics, Centers for Disease Control and Prevention, U.S. Department of Defense. United States NVSS Mortality Data 1995 - NBER and United States Military Deaths 1980-2014.	United States of America (all subnationals)	VR	1	1
National Center for Health Statistics, Centers for Disease Control and Prevention, U.S. Department of Defense. United States NVSS Mortality Data 1996 - NBER and United States Military Deaths 1980-2014.	United States of America (all subnationals)	VR	1	1
National Center for Health Statistics, Centers for Disease Control and Prevention, U.S. Department of Defense. United States NVSS Mortality Data 1997 - NBER and United States Military Deaths 1980-2014.	United States of America (all subnationals)	VR	1	1
National Center for Health Statistics, Centers for Disease Control and Prevention, U.S. Department of Defense. United States NVSS Mortality Data 1998 - NBER and United States Military Deaths 1980-2014.	United States of America (some subnationals)	VR	1	1
National Center for Health Statistics, Centers for Disease Control and Prevention, U.S. Department of Defense. United States NVSS Mortality Data 1999 - NBER and United States Military Deaths 1980-2014.	United States of America (some subnationals)	VR	1	1

National Center for Health Statistics, Centers for Disease Control and Prevention, U.S. Department of Defense. United States NVSS Mortality Data 2000 - NBER and United States Military Deaths 1980-2014.	United States of America (some subnationals)	VR	1	1
National Center for Health Statistics, Centers for Disease Control and Prevention, U.S. Department of Defense. United States NVSS Mortality Data 2001 - NBER and United States Military Deaths 1980-2014.	United States of America (all subnationals)	VR	1	1
National Center for Health Statistics, Centers for Disease Control and Prevention, U.S. Department of Defense. United States NVSS Mortality Data 2002 - NBER and United States Military Deaths 1980-2014.	United States of America (all subnationals)	VR	1	1
National Center for Health Statistics, Centers for Disease Control and Prevention, U.S. Department of Defense. United States NVSS Mortality Data 2003 - NBER and United States Military Deaths 1980-2014.	United States of America (all subnationals)	VR	1	1
National Center for Health Statistics, Centers for Disease Control and Prevention, U.S. Department of Defense. United States NVSS Mortality Data 2004 - NBER and United States Military Deaths 1980-2014.	United States of America (all subnationals)	VR	1	1
National Center for Health Statistics, Centers for Disease Control and Prevention. American Samoa NVSS Custom Mortality Data 2005. Hyattsville, United States of America: National Center for Health Statistics, Centers for Disease Control and Prevention.	American Samoa	VR	1	1
National Center for Health Statistics, Centers for Disease Control and Prevention. American Samoa NVSS Custom Mortality Data 2006. Hyattsville, United States of America: National Center for Health Statistics, Centers for Disease Control and Prevention.	American Samoa	VR	1	1
National Center for Health Statistics, Centers for Disease Control and Prevention. American Samoa NVSS Custom Mortality Data 2007. Hyattsville, United States of America: National Center for Health Statistics, Centers for Disease Control and Prevention.	American Samoa	VR	1	1
National Center for Health Statistics, Centers for Disease Control and Prevention. American Samoa NVSS Custom Mortality Data 2008. Hyattsville, United States of America: National Center for Health Statistics, Centers for Disease Control and Prevention.	American Samoa	VR	1	1
National Center for Health Statistics, Centers for Disease Control and Prevention. American Samoa NVSS Custom Mortality Data 2009. Hyattsville, United States of America: National Center for Health Statistics, Centers for Disease Control and Prevention.	American Samoa	VR	1	1
National Center for Health Statistics, Centers for Disease Control and Prevention. American Samoa NVSS Custom Mortality Data 2010. Hyattsville, United States of America: National Center for Health Statistics, Centers for Disease Control and Prevention.	American Samoa	VR	1	1
National Center for Health Statistics, Centers for Disease Control and Prevention. American Samoa NVSS Custom Mortality Data 2011. Hyattsville, United States of America: National Center for Health Statistics, Centers for Disease Control and Prevention.	American Samoa	VR	1	1
National Center for Health Statistics, Centers for Disease Control and Prevention. American Samoa NVSS Custom Mortality Data 2012. Hyattsville, United States of America: National Center for Health Statistics, Centers for Disease Control and Prevention.	American Samoa	VR	1	1
National Center for Health Statistics, Centers for Disease Control and Prevention. American Samoa NVSS Custom Mortality Data 2013. Hyattsville, United States of America: National Center for Health Statistics, Centers for Disease Control and Prevention.	American Samoa	VR	1	1
National Center for Health Statistics, Centers for Disease Control and Prevention. American Samoa NVSS Custom Mortality Data 2014. Hyattsville, United States of America: National Center for Health Statistics, Centers for Disease Control and Prevention.	American Samoa	VR	1	1
National Center for Health Statistics, Centers for Disease Control and Prevention. American Samoa NVSS Custom Mortality Data 2015. Hyattsville, United States of America: National Center for Health Statistics, Centers for Disease Control and Prevention.	American Samoa	VR	1	1
National Center for Health Statistics, Centers for Disease Control and Prevention. American Samoa NVSS Mortality Data 1997 - NBER. Hyattsville, United States: National Center for Health Statistics, Centers for Disease Control and Prevention.	American Samoa	VR	1	1
National Center for Health Statistics, Centers for Disease Control and Prevention. American Samoa NVSS Mortality Data 1998 - NBER. Hyattsville, United States: National Center for Health Statistics, Centers for Disease Control and Prevention.	American Samoa	VR	1	1
National Center for Health Statistics, Centers for Disease Control and Prevention. American Samoa NVSS Mortality Data 1999 - NBER. Hyattsville, United States: National Center for Health Statistics, Centers for Disease Control and Prevention.	American Samoa	VR	1	1
National Center for Health Statistics, Centers for Disease Control and Prevention. American Samoa NVSS Mortality Data 2000 - NBER. Hyattsville, United States of America: National Center for Health Statistics, Centers for Disease Control and Prevention.	American Samoa	VR	1	1
National Center for Health Statistics, Centers for Disease Control and Prevention. American Samoa NVSS Mortality Data 2001 - NBER. Hyattsville, United States of America: National Center for Health Statistics, Centers for Disease Control and Prevention.	American Samoa	VR	1	1
National Center for Health Statistics, Centers for Disease Control and Prevention. American Samoa NVSS Mortality Data 2002 - NBER. Hyattsville, United States of America: National Center for Health Statistics, Centers for Disease Control and Prevention.	American Samoa	VR	1	1
National Center for Health Statistics, Centers for Disease Control and Prevention. American Samoa NVSS Mortality Data 2003 - NBER. Hyattsville, United States of America: National Center for Health Statistics, Centers for Disease Control and Prevention.	American Samoa	VR	1	1
National Center for Health Statistics, Centers for Disease Control and Prevention. American Samoa NVSS Mortality Data 2004 - NBER. Hyattsville, United States of America: National Center for Health Statistics, Centers for Disease Control and Prevention.	American Samoa	VR	1	1
National Center for Health Statistics, Centers for Disease Control and Prevention. Guam NVSS Custom Mortality Data 2005. Hyattsville, United States of America: National Center for Health Statistics, Centers for Disease Control and Prevention.	Guam	VR	1	1
National Center for Health Statistics, Centers for Disease Control and Prevention. Guam NVSS Custom Mortality Data 2006. Hyattsville, United States of America: National Center for Health Statistics, Centers for Disease Control and Prevention.	Guam	VR	1	1
National Center for Health Statistics, Centers for Disease Control and Prevention. Guam NVSS Custom Mortality Data 2007. Hyattsville, United States of America: National Center for Health Statistics, Centers for Disease Control and Prevention.	Guam	VR	1	1
National Center for Health Statistics, Centers for Disease Control and Prevention. Guam NVSS Custom Mortality Data 2008. Hyattsville, United States of America: National Center for Health Statistics, Centers for Disease Control and Prevention.	Guam	VR	1	1

National Center for Health Statistics, Centers for Disease Control and Prevention. Guam NVSS Custom Mortality Data 2009. Hyattsville, United States of America: National Center for Health Statistics, Centers for Disease Control and Prevention.	Guam	VR	1	1
National Center for Health Statistics, Centers for Disease Control and Prevention. Guam NVSS Custom Mortality Data 2010. Hyattsville, United States of America: National Center for Health Statistics, Centers for Disease Control and Prevention.	Guam	VR	1	1
National Center for Health Statistics, Centers for Disease Control and Prevention. Guam NVSS Custom Mortality Data 2011. Hyattsville, United States of America: National Center for Health Statistics, Centers for Disease Control and Prevention.	Guam	VR	1	1
National Center for Health Statistics, Centers for Disease Control and Prevention. Guam NVSS Custom Mortality Data 2012. Hyattsville, United States of America: National Center for Health Statistics, Centers for Disease Control and Prevention.	Guam	VR	1	1
National Center for Health Statistics, Centers for Disease Control and Prevention. Guam NVSS Custom Mortality Data 2013. Hyattsville, United States of America: National Center for Health Statistics, Centers for Disease Control and Prevention.	Guam	VR	1	1
National Center for Health Statistics, Centers for Disease Control and Prevention. Guam NVSS Custom Mortality Data 2014. Hyattsville, United States of America: National Center for Health Statistics, Centers for Disease Control and Prevention.	Guam	VR	1	1
National Center for Health Statistics, Centers for Disease Control and Prevention. Guam NVSS Custom Mortality Data 2015. Hyattsville, United States of America: National Center for Health Statistics, Centers for Disease Control and Prevention.	Guam	VR	1	1
National Center for Health Statistics, Centers for Disease Control and Prevention. Guam NVSS Mortality Data 1994 - NBER. Hyattsville, United States: National Center for Health Statistics, Centers for Disease Control and Prevention.	Guam	VR	1	1
National Center for Health Statistics, Centers for Disease Control and Prevention. Guam NVSS Mortality Data 1995 - NBER. Hyattsville, United States: National Center for Health Statistics, Centers for Disease Control and Prevention.	Guam	VR	1	1
National Center for Health Statistics, Centers for Disease Control and Prevention. Guam NVSS Mortality Data 1996 - NBER. Hyattsville, United States: National Center for Health Statistics, Centers for Disease Control and Prevention.	Guam	VR	1	1
National Center for Health Statistics, Centers for Disease Control and Prevention. Guam NVSS Mortality Data 1997 - NBER. Hyattsville, United States: National Center for Health Statistics, Centers for Disease Control and Prevention.	Guam	VR	1	1
National Center for Health Statistics, Centers for Disease Control and Prevention. Guam NVSS Mortality Data 1998 - NBER. Hyattsville, United States: National Center for Health Statistics, Centers for Disease Control and Prevention.	Guam	VR	1	1
National Center for Health Statistics, Centers for Disease Control and Prevention. Guam NVSS Mortality Data 1999 - NBER. Hyattsville, United States: National Center for Health Statistics, Centers for Disease Control and Prevention.	Guam	VR	1	1
National Center for Health Statistics, Centers for Disease Control and Prevention. Guam NVSS Mortality Data 2000 - NBER. Hyattsville, United States of America: National Center for Health Statistics, Centers for Disease Control and Prevention.	Guam	VR	1	1
National Center for Health Statistics, Centers for Disease Control and Prevention. Guam NVSS Mortality Data 2001 - NBER. Hyattsville, United States of America: National Center for Health Statistics, Centers for Disease Control and Prevention.	Guam	VR	1	1
National Center for Health Statistics, Centers for Disease Control and Prevention. Guam NVSS Mortality Data 2002 - NBER. Hyattsville, United States of America: National Center for Health Statistics, Centers for Disease Control and Prevention.	Guam	VR	1	1
National Center for Health Statistics, Centers for Disease Control and Prevention. Guam NVSS Mortality Data 2003 - NBER. Hyattsville, United States of America: National Center for Health Statistics, Centers for Disease Control and Prevention.	Guam	VR	1	1
National Center for Health Statistics, Centers for Disease Control and Prevention. Guam NVSS Mortality Data 2004 - NBER. Hyattsville, United States of America: National Center for Health Statistics, Centers for Disease Control and Prevention.	Guam	VR	1	1
National Center for Health Statistics, Centers for Disease Control and Prevention. Northern Mariana Islands NVSS Custom Mortality Data 2005. Hyattsville, United States of America: National Center for Health Statistics, Centers for Disease Control and Prevention.	Northern Mariana Islands	VR	1	1
National Center for Health Statistics, Centers for Disease Control and Prevention. Northern Mariana Islands NVSS Custom Mortality Data 2006. Hyattsville, United States of America: National Center for Health Statistics, Centers for Disease Control and Prevention.	Northern Mariana Islands	VR	1	1
National Center for Health Statistics, Centers for Disease Control and Prevention. Northern Mariana Islands NVSS Custom Mortality Data 2007. Hyattsville, United States of America: National Center for Health Statistics, Centers for Disease Control and Prevention.	Northern Mariana Islands	VR	1	1
National Center for Health Statistics, Centers for Disease Control and Prevention. Northern Mariana Islands NVSS Custom Mortality Data 2008. Hyattsville, United States of America: National Center for Health Statistics, Centers for Disease Control and Prevention.	Northern Mariana Islands	VR	1	1
National Center for Health Statistics, Centers for Disease Control and Prevention. Northern Mariana Islands NVSS Custom Mortality Data 2009. Hyattsville, United States of America: National Center for Health Statistics, Centers for Disease Control and Prevention.	Northern Mariana Islands	VR	1	1
National Center for Health Statistics, Centers for Disease Control and Prevention. Northern Mariana Islands NVSS Custom Mortality Data 2010. Hyattsville, United States of America: National Center for Health Statistics, Centers for Disease Control and Prevention.	Northern Mariana Islands	VR	1	1
National Center for Health Statistics, Centers for Disease Control and Prevention. Northern Mariana Islands NVSS Custom Mortality Data 2011. Hyattsville, United States of America: National Center for Health Statistics, Centers for Disease Control and Prevention.	Northern Mariana Islands	VR	1	1
National Center for Health Statistics, Centers for Disease Control and Prevention. Northern Mariana Islands NVSS Custom Mortality Data 2012. Hyattsville, United States of America: National Center for Health Statistics, Centers for Disease Control and Prevention.	Northern Mariana Islands	VR	1	1
National Center for Health Statistics, Centers for Disease Control and Prevention. Northern Mariana Islands NVSS Custom Mortality Data 2013. Hyattsville, United States of America: National Center for Health Statistics, Centers for Disease Control and Prevention.	Northern Mariana Islands	VR	1	1
National Center for Health Statistics, Centers for Disease Control and Prevention. Northern Mariana Islands NVSS Custom Mortality Data 2014. Hyattsville, United States of America: National Center for Health Statistics, Centers for Disease Control and Prevention.	Northern Mariana Islands	VR	1	1

National Center for Health Statistics, Centers for Disease Control and Prevention. Northern Mariana Islands NVSS Custom Mortality Data 2015. Hyattsville, United States of America: National Center for Health Statistics, Centers for Disease Control and Prevention.	Northern Mariana Islands	VR	1	1
National Center for Health Statistics, Centers for Disease Control and Prevention. Northern Mariana Islands NVSS Mortality Data 1998 - NBER. Hyattsville, United States: National Center for Health Statistics, Centers for Disease Control and Prevention.	Northern Mariana Islands	VR	1	1
National Center for Health Statistics, Centers for Disease Control and Prevention. Northern Mariana Islands NVSS Mortality Data 1999 - NBER. Hyattsville, United States: National Center for Health Statistics, Centers for Disease Control and Prevention.	Northern Mariana Islands	VR	1	1
National Center for Health Statistics, Centers for Disease Control and Prevention. Northern Mariana Islands NVSS Mortality Data 2000 - NBER. Hyattsville, United States of America: National Center for Health Statistics, Centers for Disease Control and Prevention.	Northern Mariana Islands	VR	1	1
National Center for Health Statistics, Centers for Disease Control and Prevention. Northern Mariana Islands NVSS Mortality Data 2001 - NBER. Hyattsville, United States of America: National Center for Health Statistics, Centers for Disease Control and Prevention.	Northern Mariana Islands	VR	1	1
National Center for Health Statistics, Centers for Disease Control and Prevention. Northern Mariana Islands NVSS Mortality Data 2002 - NBER. Hyattsville, United States of America: National Center for Health Statistics, Centers for Disease Control and Prevention.	Northern Mariana Islands	VR	1	1
National Center for Health Statistics, Centers for Disease Control and Prevention. Northern Mariana Islands NVSS Mortality Data 2003 - NBER. Hyattsville, United States of America: National Center for Health Statistics, Centers for Disease Control and Prevention.	Northern Mariana Islands	VR	1	1
National Center for Health Statistics, Centers for Disease Control and Prevention. Northern Mariana Islands NVSS Mortality Data 2004 - NBER. Hyattsville, United States of America: National Center for Health Statistics, Centers for Disease Control and Prevention.	Northern Mariana Islands	VR	1	1
National Center for Health Statistics, Centers for Disease Control and Prevention. Puerto Rico NVSS Custom Mortality Data 2005. Hyattsville, United States of America: National Center for Health Statistics, Centers for Disease Control and Prevention.	Puerto Rico	VR	1	1
National Center for Health Statistics, Centers for Disease Control and Prevention. Puerto Rico NVSS Custom Mortality Data 2006. Hyattsville, United States of America: National Center for Health Statistics, Centers for Disease Control and Prevention.	Puerto Rico	VR	1	1
National Center for Health Statistics, Centers for Disease Control and Prevention. Puerto Rico NVSS Custom Mortality Data 2007. Hyattsville, United States of America: National Center for Health Statistics, Centers for Disease Control and Prevention.	Puerto Rico	VR	1	1
National Center for Health Statistics, Centers for Disease Control and Prevention. Puerto Rico NVSS Custom Mortality Data 2008. Hyattsville, United States of America: National Center for Health Statistics, Centers for Disease Control and Prevention.	Puerto Rico	VR	1	1
National Center for Health Statistics, Centers for Disease Control and Prevention. Puerto Rico NVSS Custom Mortality Data 2009. Hyattsville, United States of America: National Center for Health Statistics, Centers for Disease Control and Prevention.	Puerto Rico	VR	1	1
National Center for Health Statistics, Centers for Disease Control and Prevention. Puerto Rico NVSS Custom Mortality Data 2010. Hyattsville, United States of America: National Center for Health Statistics, Centers for Disease Control and Prevention.	Puerto Rico	VR	1	1
National Center for Health Statistics, Centers for Disease Control and Prevention. Puerto Rico NVSS Custom Mortality Data 2011. Hyattsville, United States of America: National Center for Health Statistics, Centers for Disease Control and Prevention.	Puerto Rico	VR	1	1
National Center for Health Statistics, Centers for Disease Control and Prevention. Puerto Rico NVSS Custom Mortality Data 2012. Hyattsville, United States of America: National Center for Health Statistics, Centers for Disease Control and Prevention.	Puerto Rico	VR	1	1
National Center for Health Statistics, Centers for Disease Control and Prevention. Puerto Rico NVSS Custom Mortality Data 2013. Hyattsville, United States of America: National Center for Health Statistics, Centers for Disease Control and Prevention.	Puerto Rico	VR	1	1
National Center for Health Statistics, Centers for Disease Control and Prevention. Puerto Rico NVSS Custom Mortality Data 2014. Hyattsville, United States of America: National Center for Health Statistics, Centers for Disease Control and Prevention.	Puerto Rico	VR	1	1
National Center for Health Statistics, Centers for Disease Control and Prevention. Puerto Rico NVSS Custom Mortality Data 2015. Hyattsville, United States of America: National Center for Health Statistics, Centers for Disease Control and Prevention.	Puerto Rico	VR	1	1
National Center for Health Statistics, Centers for Disease Control and Prevention. Puerto Rico NVSS Mortality Data 1994 - NBER. Hyattsville, United States: National Center for Health Statistics, Centers for Disease Control and Prevention.	Puerto Rico	VR	1	1
National Center for Health Statistics, Centers for Disease Control and Prevention. Puerto Rico NVSS Mortality Data 1995 - NBER. Hyattsville, United States: National Center for Health Statistics, Centers for Disease Control and Prevention.	Puerto Rico	VR	1	1
National Center for Health Statistics, Centers for Disease Control and Prevention. Puerto Rico NVSS Mortality Data 1996 - NBER. Hyattsville, United States: National Center for Health Statistics, Centers for Disease Control and Prevention.	Puerto Rico	VR	1	1
National Center for Health Statistics, Centers for Disease Control and Prevention. Puerto Rico NVSS Mortality Data 1997 - NBER. Hyattsville, United States: National Center for Health Statistics, Centers for Disease Control and Prevention.	Puerto Rico	VR	1	1
National Center for Health Statistics, Centers for Disease Control and Prevention. Puerto Rico NVSS Mortality Data 1998 - NBER. Hyattsville, United States: National Center for Health Statistics, Centers for Disease Control and Prevention.	Puerto Rico	VR	1	1
National Center for Health Statistics, Centers for Disease Control and Prevention. Puerto Rico NVSS Mortality Data 1999 - NBER. Hyattsville, United States: National Center for Health Statistics, Centers for Disease Control and Prevention.	Puerto Rico	VR	1	1
National Center for Health Statistics, Centers for Disease Control and Prevention. Puerto Rico NVSS Mortality Data 2000 - NBER. Hyattsville, United States of America: National Center for Health Statistics, Centers for Disease Control and Prevention.	Puerto Rico	VR	1	1
National Center for Health Statistics, Centers for Disease Control and Prevention. Puerto Rico NVSS Mortality Data 2001 - NBER. Hyattsville, United States of America: National Center for Health Statistics, Centers for Disease Control and Prevention.	Puerto Rico	VR	1	1
National Center for Health Statistics, Centers for Disease Control and Prevention. Puerto Rico NVSS Mortality Data 2002 - NBER. Hyattsville, United States of America: National Center for Health Statistics, Centers for Disease Control and Prevention.	Puerto Rico	VR	1	1

National Center for Health Statistics, Centers for Disease Control and Prevention. Puerto Rico NVSS Mortality Data 2003 - NBER. Hyattsville, United States of America: National Center for Health Statistics, Centers for Disease Control and Prevention.	Puerto Rico	VR	1	1
National Center for Health Statistics, Centers for Disease Control and Prevention. Puerto Rico NVSS Mortality Data 2004 - NBER. Hyattsville, United States of America: National Center for Health Statistics, Centers for Disease Control and Prevention.	Puerto Rico	VR	1	1
National Center for Health Statistics, Centers for Disease Control and Prevention. United States NVSS Custom Mortality Data 2016. Hyattsville, United States of America: National Center for Health Statistics, Centers for Disease Control and Prevention.	American Samoa, Guam, Northern Mariana Islands, Puerto Rico, United States Virgin Islands	VR	1	1
National Center for Health Statistics, Centers for Disease Control and Prevention. United States NVSS Custom Mortality Data 2017. Hyattsville, United States of America: National Center for Health Statistics, Centers for Disease Control and Prevention.	American Samoa, Guam, Northern Mariana Islands, Puerto Rico, United States Virgin Islands	VR	1	1
National Center for Health Statistics, Centers for Disease Control and Prevention. United States NVSS Custom Mortality Data 2018. Hyattsville, United States of America: National Center for Health Statistics, Centers for Disease Control and Prevention.	Guam, Northern Mariana Islands, Puerto Rico	VR	1	1
National Center for Health Statistics, Centers for Disease Control and Prevention. United States NVSS Custom Mortality Data 2019. Hyattsville, United States of America: National Center for Health Statistics, Centers for Disease Control and Prevention.	United States of America (all subnationals)	VR	1	1
National Center for Health Statistics, Centers for Disease Control and Prevention. United States Virgin Islands NVSS Custom Mortality Data 2005. Hyattsville, United States of America: National Center for Health Statistics, Centers for Disease Control and Prevention.	United States Virgin Islands	VR	1	1
National Center for Health Statistics, Centers for Disease Control and Prevention. United States Virgin Islands NVSS Custom Mortality Data 2006. Hyattsville, United States of America: National Center for Health Statistics, Centers for Disease Control and Prevention.	United States Virgin Islands	VR	1	1
National Center for Health Statistics, Centers for Disease Control and Prevention. United States Virgin Islands NVSS Custom Mortality Data 2007. Hyattsville, United States of America: National Center for Health Statistics, Centers for Disease Control and Prevention.	United States Virgin Islands	VR	1	1
National Center for Health Statistics, Centers for Disease Control and Prevention. United States Virgin Islands NVSS Custom Mortality Data 2008. Hyattsville, United States of America: National Center for Health Statistics, Centers for Disease Control and Prevention.	United States Virgin Islands	VR	1	1
National Center for Health Statistics, Centers for Disease Control and Prevention. United States Virgin Islands NVSS Custom Mortality Data 2009. Hyattsville, United States of America: National Center for Health Statistics, Centers for Disease Control and Prevention.	United States Virgin Islands	VR	1	1
National Center for Health Statistics, Centers for Disease Control and Prevention. United States Virgin Islands NVSS Custom Mortality Data 2010. Hyattsville, United States of America: National Center for Health Statistics, Centers for Disease Control and Prevention.	United States Virgin Islands	VR	1	1
National Center for Health Statistics, Centers for Disease Control and Prevention. United States Virgin Islands NVSS Custom Mortality Data 2011. Hyattsville, United States of America: National Center for Health Statistics, Centers for Disease Control and Prevention.	United States Virgin Islands	VR	1	1
National Center for Health Statistics, Centers for Disease Control and Prevention. United States Virgin Islands NVSS Custom Mortality Data 2012. Hyattsville, United States of America: National Center for Health Statistics, Centers for Disease Control and Prevention.	United States Virgin Islands	VR	1	1
National Center for Health Statistics, Centers for Disease Control and Prevention. United States Virgin Islands NVSS Custom Mortality Data 2015. Hyattsville, United States of America: National Center for Health Statistics, Centers for Disease Control and Prevention.	United States Virgin Islands	VR	1	1
National Center for Health Statistics, Centers for Disease Control and Prevention. United States Virgin Islands NVSS Mortality Data 1994 - NBER. Hyattsville, United States: National Center for Health Statistics, Centers for Disease Control and Prevention.	United States Virgin Islands	VR	1	1
National Center for Health Statistics, Centers for Disease Control and Prevention. United States Virgin Islands NVSS Mortality Data 1995 - NBER. Hyattsville, United States: National Center for Health Statistics, Centers for Disease Control and Prevention.	United States Virgin Islands	VR	1	1
National Center for Health Statistics, Centers for Disease Control and Prevention. United States Virgin Islands NVSS Mortality Data 1996 - NBER. Hyattsville, United States: National Center for Health Statistics, Centers for Disease Control and Prevention.	United States Virgin Islands	VR	1	1
National Center for Health Statistics, Centers for Disease Control and Prevention. United States Virgin Islands NVSS Mortality Data 1997 - NBER. Hyattsville, United States: National Center for Health Statistics, Centers for Disease Control and Prevention.	United States Virgin Islands	VR	1	1
National Center for Health Statistics, Centers for Disease Control and Prevention. United States Virgin Islands NVSS Mortality Data 1998 - NBER. Hyattsville, United States: National Center for Health Statistics, Centers for Disease Control and Prevention.	United States Virgin Islands	VR	1	1
National Center for Health Statistics, Centers for Disease Control and Prevention. United States Virgin Islands NVSS Mortality Data 1999 - NBER. Hyattsville, United States: National Center for Health Statistics, Centers for Disease Control and Prevention.	United States Virgin Islands	VR	1	1
National Center for Health Statistics, Centers for Disease Control and Prevention. United States Virgin Islands NVSS Mortality Data 2000 - NBER. Hyattsville, United States of America: National Center for Health Statistics, Centers for Disease Control and Prevention.	United States Virgin Islands	VR	1	1
National Center for Health Statistics, Centers for Disease Control and Prevention. United States Virgin Islands NVSS Mortality Data 2001 - NBER. Hyattsville, United States of America: National Center for Health Statistics, Centers for Disease Control and Prevention.	United States Virgin Islands	VR	1	1
National Center for Health Statistics, Centers for Disease Control and Prevention. United States Virgin Islands NVSS Mortality Data 2002 - NBER. Hyattsville, United States of America: National Center for Health Statistics, Centers for Disease Control and Prevention.	United States Virgin Islands	VR	1	1
National Center for Health Statistics, Centers for Disease Control and Prevention. United States Virgin Islands NVSS Mortality Data 2003 - NBER. Hyattsville, United States of America: National Center for Health Statistics, Centers for Disease Control and Prevention.	United States Virgin Islands	VR	1	1
National Center for Health Statistics, Centers for Disease Control and Prevention. United States Virgin Islands NVSS Mortality Data 2004 - NBER. Hyattsville, United States of America: National Center for Health Statistics, Centers for Disease Control and Prevention.	United States Virgin Islands	VR	1	1
National Institute of Statistics and Censuses (Costa Rica). Costa Rica Registered Deaths 2015. San José, Costa Rica: National Institute of Statistics and Censuses (Costa Rica).	Costa Rica	VR	1	1
National Institute of Statistics and Censuses (Costa Rica). Costa Rica Registered Deaths 2016. San José, Costa Rica: National Institute of Statistics and Censuses (Costa Rica).	Costa Rica	VR	1	1

National Institute of Statistics and Censuses (Costa Rica). Costa Rica Registered Deaths 2017. San José, Costa Rica: National Institute of Statistics and Censuses (Costa Rica).	Costa Rica	VR	1	1
National Institute of Statistics and Censuses (Costa Rica). Costa Rica Registered Deaths 2018. San José, Costa Rica: National Institute of Statistics and Censuses (Costa Rica).	Costa Rica	VR	1	1
National Institute of Statistics and Censuses (Costa Rica). Costa Rica Registered Deaths 2019. San José, Costa Rica: National Institute of Statistics and Censuses (Costa Rica).	Costa Rica	VR	1	1
National Institute of Statistics and Censuses (Ecuador). Ecuador General Deaths 2017. Quito, Ecuador: National Institute of Statistics and Censuses (Ecuador).	Ecuador	VR	1	1
National Institute of Statistics and Censuses (Ecuador). Ecuador General Deaths 2018. Quito, Ecuador: National Institute of Statistics and Censuses (Ecuador).	Ecuador	VR	1	1
National Institute of Statistics and Censuses (Ecuador). Ecuador General Deaths 2019. Quito, Ecuador: National Institute of Statistics and Censuses (Ecuador).	Ecuador	VR	1	1
National Institute of Statistics and Geography (INEGI) (Mexico), Secretariat of Health (Mexico). Mexico Vital Registration - Deaths 1980.	Mexico (all subnationals)	VR	1	1
National Institute of Statistics and Geography (INEGI) (Mexico), Secretariat of Health (Mexico). Mexico Vital Registration - Deaths 1981.	Mexico (all subnationals)	VR	1	1
National Institute of Statistics and Geography (INEGI) (Mexico), Secretariat of Health (Mexico). Mexico Vital Registration - Deaths 1982.	Mexico (all subnationals)	VR	1	1
National Institute of Statistics and Geography (INEGI) (Mexico), Secretariat of Health (Mexico). Mexico Vital Registration - Deaths 1983.	Mexico (some subnationals)	VR	1	1
National Institute of Statistics and Geography (INEGI) (Mexico), Secretariat of Health (Mexico). Mexico Vital Registration - Deaths 1984.	Mexico (some subnationals)	VR	1	1
National Institute of Statistics and Geography (INEGI) (Mexico), Secretariat of Health (Mexico). Mexico Vital Registration - Deaths 1985.	Mexico (all subnationals)	VR	1	1
National Institute of Statistics and Geography (INEGI) (Mexico), Secretariat of Health (Mexico). Mexico Vital Registration - Deaths 1986.	Mexico (all subnationals)	VR	1	1
National Institute of Statistics and Geography (INEGI) (Mexico), Secretariat of Health (Mexico). Mexico Vital Registration - Deaths 1987.	Mexico (all subnationals)	VR	1	1
National Institute of Statistics and Geography (INEGI) (Mexico), Secretariat of Health (Mexico). Mexico Vital Registration - Deaths 1988.	Mexico (some subnationals)	VR	1	1
National Institute of Statistics and Geography (INEGI) (Mexico), Secretariat of Health (Mexico). Mexico Vital Registration - Deaths 1989.	Mexico (all subnationals)	VR	1	1
National Institute of Statistics and Geography (INEGI) (Mexico), Secretariat of Health (Mexico). Mexico Vital Registration - Deaths 1990.	Mexico (all subnationals)	VR	1	1
National Institute of Statistics and Geography (INEGI) (Mexico), Secretariat of Health (Mexico). Mexico Vital Registration - Deaths 1991.	Mexico (some subnationals)	VR	1	1
National Institute of Statistics and Geography (INEGI) (Mexico), Secretariat of Health (Mexico). Mexico Vital Registration - Deaths 1992.	Mexico (all subnationals)	VR	1	1
National Institute of Statistics and Geography (INEGI) (Mexico), Secretariat of Health (Mexico). Mexico Vital Registration - Deaths 1993.	Mexico (all subnationals)	VR	1	1
National Institute of Statistics and Geography (INEGI) (Mexico), Secretariat of Health (Mexico). Mexico Vital Registration - Deaths 1994.	Mexico (all subnationals)	VR	1	1
National Institute of Statistics and Geography (INEGI) (Mexico), Secretariat of Health (Mexico). Mexico Vital Registration - Deaths 1995.	Mexico (all subnationals)	VR	1	1
National Institute of Statistics and Geography (INEGI) (Mexico), Secretariat of Health (Mexico). Mexico Vital Registration - Deaths 1996.	Mexico (all subnationals)	VR	1	1
National Institute of Statistics and Geography (INEGI) (Mexico), Secretariat of Health (Mexico). Mexico Vital Registration - Deaths 1997.	Mexico (all subnationals)	VR	1	1
National Institute of Statistics and Geography (INEGI) (Mexico), Secretariat of Health (Mexico). Mexico Vital Registration - Deaths 1998.	Mexico (all subnationals)	VR	1	1
National Institute of Statistics and Geography (INEGI) (Mexico), Secretariat of Health (Mexico). Mexico Vital Registration - Deaths 1999.	Mexico (all subnationals)	VR	1	1
National Institute of Statistics and Geography (INEGI) (Mexico), Secretariat of Health (Mexico). Mexico Vital Registration - Deaths 2000.	Mexico (all subnationals)	VR	1	1
National Institute of Statistics and Geography (INEGI) (Mexico), Secretariat of Health (Mexico). Mexico Vital Registration - Deaths 2001.	Mexico (all subnationals)	VR	1	1
National Institute of Statistics and Geography (INEGI) (Mexico), Secretariat of Health (Mexico). Mexico Vital Registration - Deaths 2002.	Mexico (all subnationals)	VR	1	1
National Institute of Statistics and Geography (INEGI) (Mexico), Secretariat of Health (Mexico). Mexico Vital Registration - Deaths 2003.	Mexico (all subnationals)	VR	1	1
National Institute of Statistics and Geography (INEGI) (Mexico), Secretariat of Health (Mexico). Mexico Vital Registration - Deaths 2004.	Mexico (all subnationals)	VR	1	1
National Institute of Statistics and Geography (INEGI) (Mexico), Secretariat of Health (Mexico). Mexico Vital Registration - Deaths 2005.	Mexico (all subnationals)	VR	1	1
National Institute of Statistics and Geography (INEGI) (Mexico), Secretariat of Health (Mexico). Mexico Vital Registration - Deaths 2006.	Mexico (all subnationals)	VR	1	1
National Institute of Statistics and Geography (INEGI) (Mexico), Secretariat of Health (Mexico). Mexico Vital Registration - Deaths 2007.	Mexico (all subnationals)	VR	1	1
National Institute of Statistics and Geography (INEGI) (Mexico), Secretariat of Health (Mexico). Mexico Vital Registration - Deaths 2008.	Mexico (all subnationals)	VR	1	1
National Institute of Statistics and Geography (INEGI) (Mexico), Secretariat of Health (Mexico). Mexico Vital Registration - Deaths 2010.	Mexico (all subnationals)	VR	1	1
National Institute of Statistics and Geography (INEGI) (Mexico), Secretariat of Health (Mexico). Mexico Vital Registration - Deaths 2011.	Mexico (all subnationals)	VR	1	1
National Institute of Statistics and Geography (INEGI) (Mexico). Mexico Vital Registration - Deaths 2012.	Mexico (all subnationals)	VR	1	1
National Institute of Statistics and Geography (INEGI) (Mexico). Mexico Vital Registration - Deaths 2013.	Mexico (all subnationals)	VR	1	1
National Institute of Statistics and Geography (INEGI) (Mexico). Mexico Vital Registration - Deaths 2014. Mexico City, Mexico: National Institute of Statistics and Geography (INEGI) (Mexico).	Mexico (all subnationals)	VR	1	1

National Institute of Statistics and Geography (INEGI) (Mexico). Mexico Vital Registration - Deaths 2015. Mexico City, Mexico: National Institute of Statistics and Geography (INEGI) (Mexico).	Mexico (all subnationals)	VR	1	1
National Institute of Statistics and Geography (INEGI) (Mexico). Mexico Vital Registration - Deaths 2016. Mexico City, Mexico: National Institute of Statistics and Geography (INEGI) (Mexico).	Mexico (all subnationals)	VR	1	1
National Institute of Statistics and Geography (INEGI) (Mexico). Mexico Vital Registration - Deaths 2017. Mexico City, Mexico: National Institute of Statistics and Geography (INEGI) (Mexico).	Mexico (all subnationals)	VR	1	1
National Institute of Statistics and Geography (INEGI) (Mexico). Mexico Vital Registration - Deaths 2018. Mexico City, Mexico: National Institute of Statistics and Geography (INEGI) (Mexico).	Mexico (all subnationals)	VR	1	1
National Institute of Statistics and Geography (INEGI) (Mexico). Mexico Vital Registration - Deaths 2019. Mexico City, Mexico: National Institute of Statistics and Geography (INEGI) (Mexico).	Mexico (all subnationals)	VR	1	1
National Institute of Statistics and Geography (INEGI) (Mexico). Mexico Vital Statistics - Deaths 2009.	Mexico (all subnationals)	VR	1	1
National Records of Scotland. United Kingdom - Scotland Vital Events Reference Tables 2015. Edinburgh, Scotland: National Records of Scotland, 2016.	United Kingdom (some subnationals)	VR	1	1
National Records of Scotland. United Kingdom - Scotland Vital Events Reference Tables 2016. Edinburgh, Scotland: National Records of Scotland, 2017.	United Kingdom (some subnationals)	VR	1	1
National Statistics Institute (Guatemala). Guatemala Vital Statistics 2010. Guatemala City, Guatemala: National Statistics Institute (Guatemala), 2011.	Guatemala	VR	1	1
National Statistics Institute (Guatemala). Guatemala Vital Statistics 2011. Guatemala City, Guatemala: National Statistics Institute (Guatemala), 2012.	Guatemala	VR	1	1
National Statistics Institute (Guatemala). Guatemala Vital Statistics 2012. Guatemala City, Guatemala: National Statistics Institute (Guatemala), 2013.	Guatemala	VR	1	1
National Statistics Institute (Guatemala). Guatemala Vital Statistics 2013. Guatemala City, Guatemala: National Statistics Institute (Guatemala), 2014.	Guatemala	VR	1	1
National Statistics Institute (Guatemala). Guatemala Vital Statistics 2014. Guatemala City, Guatemala: National Statistics Institute (Guatemala), 2015.	Guatemala	VR	1	1
National Statistics Institute (Guatemala). Guatemala Vital Statistics 2015. Guatemala City, Guatemala: National Statistics Institute (Guatemala), 2016.	Guatemala	VR	1	1
National Statistics Institute (Guatemala). Guatemala Vital Statistics 2016. Guatemala City, Guatemala: National Statistics Institute (Guatemala), 2017.	Guatemala	VR	1	1
National Statistics Institute (Guatemala). Guatemala Vital Statistics 2017. Guatemala City, Guatemala: National Statistics Institute (Guatemala), 2018.	Guatemala	VR	1	1
National Statistics Institute (Guatemala). Guatemala Vital Statistics 2018. Guatemala City, Guatemala: National Statistics Institute (Guatemala), 2019.	Guatemala	VR	1	1
National Statistics Institute (Guatemala). Guatemala Vital Statistics 2019. Guatemala City, Guatemala: National Statistics Institute (Guatemala), 2020.	Guatemala	VR	1	1
National Statistics Office (Philippines). Philippines Vital Registration - Deaths 2006.	Philippines (some subnationals)	VR	1	1
National Statistics Office (Philippines). Philippines Vital Registration - Deaths 2007.	Philippines (some subnationals)	VR	1	1
National Statistics Office (Philippines). Philippines Vital Registration - Deaths 2008.	Philippines (some subnationals)	VR	1	1
National Statistics Office (Philippines). Philippines Vital Registration - Deaths 2009.	Philippines (some subnationals)	VR	1	1
National Statistics Office (Philippines). Philippines Vital Registration - Deaths 2010.	Philippines (some subnationals)	VR	1	1
National Statistics Office (Philippines). Philippines Vital Registration - Deaths 2011.	Philippines (some subnationals)	VR	1	1
National Statistics Office (Philippines). Philippines Vital Registration - Deaths 2012.	Philippines (some subnationals)	VR	1	1
Navrongo Health Research Centre. Ghana - Navrongo Health and Demographic Surveillance System.	Ghana	VA	0	1
Norwegian Institute of Public Health. Norway Cause of Death Registry 1980. Oslo, Norway: Norwegian Institute of Public Health.	Norway (all subnationals)	VR	1	1
Norwegian Institute of Public Health. Norway Cause of Death Registry 1981. Oslo, Norway: Norwegian Institute of Public Health.	Norway (all subnationals)	VR	1	1
Norwegian Institute of Public Health. Norway Cause of Death Registry 1982. Oslo, Norway: Norwegian Institute of Public Health.	Norway (all subnationals)	VR	1	1
Norwegian Institute of Public Health. Norway Cause of Death Registry 1983. Oslo, Norway: Norwegian Institute of Public Health.	Norway (all subnationals)	VR	1	1
Norwegian Institute of Public Health. Norway Cause of Death Registry 1984. Oslo, Norway: Norwegian Institute of Public Health.	Norway (all subnationals)	VR	1	1
Norwegian Institute of Public Health. Norway Cause of Death Registry 1985. Oslo, Norway: Norwegian Institute of Public Health.	Norway (all subnationals)	VR	1	1
Norwegian Institute of Public Health. Norway Cause of Death Registry 1986. Oslo, Norway: Norwegian Institute of Public Health.	Norway (all subnationals)	VR	1	1
Norwegian Institute of Public Health. Norway Cause of Death Registry 1987. Oslo, Norway: Norwegian Institute of Public Health.	Norway (all subnationals)	VR	1	1

Norwegian Institute of Public Health. Norway Cause of Death Registry 1988. Oslo, Norway: Norwegian Institute of Public Health.	Norway (all subnationals)	VR	1	1
Norwegian Institute of Public Health. Norway Cause of Death Registry 1989. Oslo, Norway: Norwegian Institute of Public Health.	Norway (all subnationals)	VR	1	1
Norwegian Institute of Public Health. Norway Cause of Death Registry 1990. Oslo, Norway: Norwegian Institute of Public Health.	Norway (all subnationals)	VR	1	1
Norwegian Institute of Public Health. Norway Cause of Death Registry 1991. Oslo, Norway: Norwegian Institute of Public Health.	Norway (all subnationals)	VR	1	1
Norwegian Institute of Public Health. Norway Cause of Death Registry 1992. Oslo, Norway: Norwegian Institute of Public Health.	Norway (all subnationals)	VR	1	1
Norwegian Institute of Public Health. Norway Cause of Death Registry 1993. Oslo, Norway: Norwegian Institute of Public Health.	Norway (all subnationals)	VR	1	1
Norwegian Institute of Public Health. Norway Cause of Death Registry 1994. Oslo, Norway: Norwegian Institute of Public Health.	Norway (all subnationals)	VR	1	1
Norwegian Institute of Public Health. Norway Cause of Death Registry 1995. Oslo, Norway: Norwegian Institute of Public Health.	Norway (all subnationals)	VR	1	1
Norwegian Institute of Public Health. Norway Cause of Death Registry 1996. Oslo, Norway: Norwegian Institute of Public Health.	Norway (all subnationals)	VR	1	1
Norwegian Institute of Public Health. Norway Cause of Death Registry 1997. Oslo, Norway: Norwegian Institute of Public Health.	Norway (all subnationals)	VR	1	1
Norwegian Institute of Public Health. Norway Cause of Death Registry 1998. Oslo, Norway: Norwegian Institute of Public Health.	Norway (all subnationals)	VR	1	1
Norwegian Institute of Public Health. Norway Cause of Death Registry 1999. Oslo, Norway: Norwegian Institute of Public Health.	Norway (all subnationals)	VR	1	1
Norwegian Institute of Public Health. Norway Cause of Death Registry 2000. Oslo, Norway: Norwegian Institute of Public Health.	Norway (all subnationals)	VR	1	1
Norwegian Institute of Public Health. Norway Cause of Death Registry 2001. Oslo, Norway: Norwegian Institute of Public Health.	Norway (all subnationals)	VR	1	1
Norwegian Institute of Public Health. Norway Cause of Death Registry 2002. Oslo, Norway: Norwegian Institute of Public Health.	Norway (all subnationals)	VR	1	1
Norwegian Institute of Public Health. Norway Cause of Death Registry 2003. Oslo, Norway: Norwegian Institute of Public Health.	Norway (all subnationals)	VR	1	1
Norwegian Institute of Public Health. Norway Cause of Death Registry 2004. Oslo, Norway: Norwegian Institute of Public Health.	Norway (all subnationals)	VR	1	1
Norwegian Institute of Public Health. Norway Cause of Death Registry 2005. Oslo, Norway: Norwegian Institute of Public Health.	Norway (all subnationals)	VR	1	1
Norwegian Institute of Public Health. Norway Cause of Death Registry 2006. Oslo, Norway: Norwegian Institute of Public Health.	Norway (all subnationals)	VR	1	1
Norwegian Institute of Public Health. Norway Cause of Death Registry 2007. Oslo, Norway: Norwegian Institute of Public Health.	Norway (all subnationals)	VR	1	1
Norwegian Institute of Public Health. Norway Cause of Death Registry 2008. Oslo, Norway: Norwegian Institute of Public Health.	Norway (all subnationals)	VR	1	1
Norwegian Institute of Public Health. Norway Cause of Death Registry 2009. Oslo, Norway: Norwegian Institute of Public Health.	Norway (all subnationals)	VR	1	1
Norwegian Institute of Public Health. Norway Cause of Death Registry 2010. Oslo, Norway: Norwegian Institute of Public Health.	Norway (all subnationals)	VR	1	1
Norwegian Institute of Public Health. Norway Cause of Death Registry 2011. Oslo, Norway: Norwegian Institute of Public Health.	Norway (all subnationals)	VR	1	1
Norwegian Institute of Public Health. Norway Cause of Death Registry 2012. Oslo, Norway: Norwegian Institute of Public Health.	Norway (all subnationals)	VR	1	1
Norwegian Institute of Public Health. Norway Cause of Death Registry 2013. Oslo, Norway: Norwegian Institute of Public Health.	Norway (all subnationals)	VR	1	1
Norwegian Institute of Public Health. Norway Cause of Death Registry 2014. Oslo, Norway: Norwegian Institute of Public Health.	Norway (all subnationals)	VR	1	1
Norwegian Institute of Public Health. Norway Cause of Death Registry 2015. Oslo, Norway: Norwegian Institute of Public Health.	Norway (all subnationals)	VR	1	1
Norwegian Institute of Public Health. Norway Cause of Death Registry 2016. Oslo, Norway: Norwegian Institute of Public Health.	Norway (all subnationals)	VR	1	1
Norwegian Institute of Public Health. Norway Cause of Death Registry 2017. Oslo, Norway: Norwegian Institute of Public Health.	Norway (all subnationals)	VR	1	1
Norwegian Institute of Public Health. Norway Cause of Death Registry 2018. Oslo, Norway: Norwegian Institute of Public Health.	Norway (all subnationals)	VR	1	1
Norwegian Institute of Public Health. Norway Cause of Death Registry 2019. Oslo, Norway: Norwegian Institute of Public Health.	Norway (all subnationals)	VR	1	1
Office for National Statistics (ONS) (United Kingdom). United Kingdom - England Mortality Statistics 1981.	England (subnationals)	VR	1	1
Office for National Statistics (ONS) (United Kingdom). United Kingdom - England Mortality Statistics 1982.	England (subnationals)	VR	1	1
Office for National Statistics (ONS) (United Kingdom). United Kingdom - England Mortality Statistics 1983.	England (subnationals)	VR	1	1
Office for National Statistics (ONS) (United Kingdom). United Kingdom - England Mortality Statistics 1984.	England (subnationals)	VR	1	1
Office for National Statistics (ONS) (United Kingdom). United Kingdom - England Mortality Statistics 1985.	England (subnationals)	VR	1	1
Office for National Statistics (ONS) (United Kingdom). United Kingdom - England Mortality Statistics 1986.	England (subnationals)	VR	1	1
Office for National Statistics (ONS) (United Kingdom). United Kingdom - England Mortality Statistics 1987.	England (subnationals)	VR	1	1
Office for National Statistics (ONS) (United Kingdom). United Kingdom - England Mortality Statistics 1988.	England (subnationals)	VR	1	1
Office for National Statistics (ONS) (United Kingdom). United Kingdom - England Mortality Statistics 1989.	England (subnationals)	VR	1	1
Office for National Statistics (ONS) (United Kingdom). United Kingdom - England Mortality Statistics 1990.	England (subnationals)	VR	1	1
Office for National Statistics (ONS) (United Kingdom). United Kingdom - England Mortality Statistics 1991.	England (subnationals)	VR	1	1
Office for National Statistics (ONS) (United Kingdom). United Kingdom - England Mortality Statistics 1992.	England (subnationals)	VR	1	1

Office for National Statistics (ONS) (United Kingdom). United Kingdom - England Mortality Statistics 1993.	England (subnationals)	VR	1	1
Office for National Statistics (ONS) (United Kingdom). United Kingdom - England Mortality Statistics 1994.	England (subnationals)	VR	1	1
Office for National Statistics (ONS) (United Kingdom). United Kingdom - England Mortality Statistics 1995.	England (subnationals)	VR	1	1
Office for National Statistics (ONS) (United Kingdom). United Kingdom - England Mortality Statistics 1996.	England (subnationals)	VR	1	1
Office for National Statistics (ONS) (United Kingdom). United Kingdom - England Mortality Statistics 1997.	England (subnationals)	VR	1	1
Office for National Statistics (ONS) (United Kingdom). United Kingdom - England Mortality Statistics 1998.	England (subnationals)	VR	1	1
Office for National Statistics (ONS) (United Kingdom). United Kingdom - England Mortality Statistics 1999.	England (subnationals)	VR	1	1
Office for National Statistics (ONS) (United Kingdom). United Kingdom - England Mortality Statistics 2000.	England (subnationals)	VR	1	1
Office for National Statistics (ONS) (United Kingdom). United Kingdom - England Mortality Statistics 2001.	England (subnationals)	VR	1	1
Office for National Statistics (ONS) (United Kingdom). United Kingdom - England Mortality Statistics 2002.	England (subnationals)	VR	1	1
Office for National Statistics (ONS) (United Kingdom). United Kingdom - England Mortality Statistics 2003.	England (subnationals)	VR	1	1
Office for National Statistics (ONS) (United Kingdom). United Kingdom - England Mortality Statistics 2004.	England (subnationals)	VR	1	1
Office for National Statistics (ONS) (United Kingdom). United Kingdom - England Mortality Statistics 2005.	England (subnationals)	VR	1	1
Office for National Statistics (ONS) (United Kingdom). United Kingdom - England Mortality Statistics 2006.	England (subnationals)	VR	1	1
Office for National Statistics (ONS) (United Kingdom). United Kingdom - England Mortality Statistics 2007.	England (subnationals)	VR	1	1
Office for National Statistics (ONS) (United Kingdom). United Kingdom - England Mortality Statistics 2008.	England (subnationals)	VR	1	1
Office for National Statistics (ONS) (United Kingdom). United Kingdom - England Mortality Statistics 2009.	England (subnationals)	VR	1	1
Office for National Statistics (ONS) (United Kingdom). United Kingdom - England Mortality Statistics 2010.	England (subnationals)	VR	1	1
Office for National Statistics (ONS) (United Kingdom). United Kingdom - England Mortality Statistics 2011.	England (subnationals)	VR	1	1
Office for National Statistics (ONS) (United Kingdom). United Kingdom - England Mortality Statistics 2012.	England (subnationals)	VR	1	1
Office for National Statistics (ONS) (United Kingdom). United Kingdom - England and Wales Mortality Data 1981-1994.	United Kingdom (some subnationals)	VR	1	1
Office for National Statistics (ONS) (United Kingdom). United Kingdom - England and Wales Mortality Data 1995-2000.	United Kingdom (some subnationals)	VR	1	1
Office for National Statistics (ONS) (United Kingdom). United Kingdom - England and Wales Mortality Data 2001-2012.	United Kingdom (some subnationals)	VR	1	1
Office for National Statistics (ONS) (United Kingdom). United Kingdom - England and Wales Mortality Statistics 2013.	England (subnationals)	VR	1	1
Office for National Statistics (ONS) (United Kingdom). United Kingdom - England and Wales Mortality Statistics 2014.	England (subnationals)	VR	1	1
Office for National Statistics (ONS) (United Kingdom). United Kingdom - England and Wales Mortality Statistics 2015.	England (subnationals)	VR	1	1
Office for National Statistics (ONS) (United Kingdom). United Kingdom - England and Wales Mortality Statistics 2016.	England (subnationals)	VR	1	1
Office for National Statistics (ONS) (United Kingdom). United Kingdom - England and Wales Mortality Statistics 2017.	England (subnationals)	VR	1	1
Office for National Statistics (ONS) (United Kingdom). United Kingdom - England and Wales Mortality Statistics 2018.	England (subnationals)	VR	1	1
Office for National Statistics (ONS) (United Kingdom). United Kingdom - England and Wales Mortality Statistics 2019.	England (subnationals)	VR	1	1
Office of the Registrar General and Census Commissioner (India). India Medical Certification of Cause of Death Report 2008. New Delhi, India: Office of the Registrar General and Census Commissioner (India).	India	VR	1	0
Office of the Registrar General and Census Commissioner (India). India Medical Certification of Cause of Death Report 2012. New Delhi, India: Office of the Registrar General and Census Commissioner (India), 2015.	Karnataka (some subnationals)	VR	1	0
Office of the Registrar General and Census Commissioner (India). India Survey of Causes of Death (Rural) 1980.	India (some subnationals)	VA	1	0
Office of the Registrar General and Census Commissioner (India). India Survey of Causes of Death (Rural) 1981.	India (some subnationals)	VA	1	0
Office of the Registrar General and Census Commissioner (India). India Survey of Causes of Death (Rural) 1982.	India (some subnationals)	VA	1	0
Office of the Registrar General and Census Commissioner (India). India Survey of Causes of Death (Rural) 1983.	India (some subnationals)	VA	1	0
Office of the Registrar General and Census Commissioner (India). India Survey of Causes of Death (Rural) 1984.	India (some subnationals)	VA	1	0
Office of the Registrar General and Census Commissioner (India). India Survey of Causes of Death (Rural) 1985.	India (some subnationals)	VA	1	0
Office of the Registrar General and Census Commissioner (India). India Survey of Causes of Death (Rural) 1986.	India (some subnationals)	VA	1	0
Office of the Registrar General and Census Commissioner (India). India Vital Statistics 1980. New Delhi, India: Office of the Registrar General and Census Commissioner (India).	Telangana, Urban	VR	1	0
Palestinian Central Bureau of Statistics. Palestine - West Bank Vital Registration - Deaths 1997.	Palestine	VR	1	1
Palestinian Central Bureau of Statistics. Palestine - West Bank Vital Registration - Deaths 1998.	Palestine	VR	1	1

Palestinian Central Bureau of Statistics. Palestine - West Bank Vital Registration - Deaths 1999.	Palestine	VR	1	1
Palestinian Central Bureau of Statistics. Palestine - West Bank Vital Registration - Deaths 2000.	Palestine	VR	1	1
Palestinian Central Bureau of Statistics. Palestine - West Bank Vital Registration - Deaths 2001.	Palestine	VR	1	1
Palestinian Central Bureau of Statistics. Palestine - West Bank Vital Registration - Deaths 2002.	Palestine	VR	1	1
Palestinian Central Bureau of Statistics. Palestine - West Bank Vital Registration - Deaths 2003.	Palestine	VR	1	1
Palestinian Central Bureau of Statistics. Palestine - West Bank Vital Registration - Deaths 2004.	Palestine	VR	1	1
Palestinian Central Bureau of Statistics. Palestine - West Bank Vital Registration - Deaths 2005.	Palestine	VR	1	1
Palestinian Central Bureau of Statistics. Palestine - West Bank Vital Registration - Deaths 2007.	Palestine	VR	1	1
Philippines Statistics Authority. Philippines Vital Registration - Deaths 2013.	Philippines (some subnationals)	VR	1	1
Philippines Statistics Authority. Philippines Vital Registration - Deaths 2014.	Philippines (some subnationals)	VR	1	1
Philippines Statistics Authority. Philippines Vital Registration - Deaths 2015.	Philippines (some subnationals)	VR	1	1
Philippines Statistics Authority. Philippines Vital Registration - Deaths 2016.	Philippines (some subnationals)	VR	1	1
Philippines Statistics Authority. Philippines Vital Registration - Deaths 2017.	Philippines (some subnationals)	VR	1	1
Philippines Statistics Authority. Philippines Vital Registration - Deaths 2018.	Philippines (some subnationals)	VR	1	1
Portugal Vital Statistics - Deaths 2005-2006.	Portugal	VR	1	1
Registrar General's Department (Zimbabwe), Zimbabwe National Statistics Agency. Zimbabwe Mortality Report 2007.	Zimbabwe	VR	1	1
State Statistics Service (Ukraine). Ukraine Vital Registration - Deaths 2013.	Ukraine (all subnationals)	VR	1	1
State Statistics Service (Ukraine). Ukraine Vital Registration - Deaths 2015.	Ukraine (some subnationals)	VR	1	1
State Statistics Service (Ukraine). Ukraine Vital Registration - Deaths 2016.	Ukraine (some subnationals)	VR	1	1
Statistics Belgium. Belgium Vital Registration - Causes of Deaths 2017.	Belgium	VR	1	1
Statistics Belgium. Belgium Vital Registration - Causes of Deaths 2018.	Belgium	VR	1	1
Statistics Portugal. Portugal Vital Registration - Deaths 1980.	Portugal	VR	1	1
Statistics Portugal. Portugal Vital Registration - Deaths 1981.	Portugal	VR	1	1
Statistics Portugal. Portugal Vital Registration - Deaths 1982.	Portugal	VR	1	1
Statistics Portugal. Portugal Vital Registration - Deaths 1983.	Portugal	VR	1	1
Statistics Portugal. Portugal Vital Registration - Deaths 2004.	Portugal	VR	1	1
Suriname Vital Statistics - Deaths 2007.	Suriname	VR	1	1
World Health Organization (WHO). WHO Mortality Database Version April 2018. Geneva, Switzerland: World Health Organization (WHO).	China, Thailand, Armenia, Mongolia, Tajikistan, Croatia, Czechia, Hungary, Montenegro, Romania, Lithuania, Republic of Moldova, Belgium, Cyprus, Greece, Israel, Malta, Netherlands, Switzerland, United Kingdom, Argentina, Dominican Republic, Colombia, Bahrain, Qatar, Türkiye, Hong Kong Special Administrative Region of China, Saint Kitts and Nevis, Northern Ireland, Kazakhstan, Turkmenistan, Seychelles, San Marino	VR	1	1
World Health Organization (WHO). WHO Mortality Database Version December 2018. Geneva, Switzerland: World Health Organization (WHO).	Maldives, Armenia, Kyrgyzstan, Uzbekistan, Estonia, Lithuania, Brunei Darussalam, Republic of Korea, Australia, Austria, France, Ireland, Portugal, Spain, Argentina, Chile, Uruguay, Canada, Antigua and Barbuda, Bahamas, Belize, Cuba, Grenada, Guyana, Jamaica, Saint Vincent and the Grenadines, Trinidad and Tobago, Ecuador, Nicaragua, Panama, Türkiye, Mauritius, Bermuda, Saint Kitts and Nevis	VR	1	1
World Health Organization (WHO). WHO Mortality Database Version December 2019. Geneva, Switzerland: World Health Organization (WHO).	China (some subnationals)	VR	1	1
World Health Organization (WHO). WHO Mortality Database Version February 2014. Geneva, Switzerland: World Health Organization (WHO).	Panama	VR	1	1

World Health Organization (WHO). WHO Mortality Database Version July 2012. Geneva, Switzerland: World Health Organization (WHO).	Finland, Iceland, Cabo Verde	VR	1	0
World Health Organization (WHO). WHO Mortality Database Version June 2021. Geneva, Switzerland: World Health Organization (WHO).	United Kingdom (some subnationals)	VR	1	1
World Health Organization (WHO). WHO Mortality Database Version March 2017. Geneva, Switzerland: World Health Organization (WHO).	Armenia, Kyrgyzstan, Uzbekistan, Albania, Czechia, Hungary, North Macedonia, Romania, Serbia, Slovenia, Lithuania, Republic of Moldova, Belgium, Cyprus, Denmark, Greece, Iceland, Israel, Netherlands, Canada, Jordan, Qatar, Monaco, Saint Kitts and Nevis, San Marino	VR	1	1
World Health Organization (WHO). WHO Mortality Database Version March 2021. Geneva, Switzerland: World Health Organization (WHO).	United Kingdom (some subnationals)	VR	1	1
World Health Organization (WHO). WHO Mortality Database Version May 2019. Geneva, Switzerland: World Health Organization (WHO).	China, Kazakhstan, Bosnia and Herzegovina, Bulgaria, Czechia, Hungary, Romania, Serbia, Republic of Moldova, Australia, Belgium, Finland, Germany, Greece, Israel, Luxembourg, Switzerland, United Kingdom, Jordan, Qatar, Hong Kong Special Administrative Region of China, Northern Ireland	VR	1	1
World Health Organization (WHO). WHO Mortality Database Version November 2015. Geneva, Switzerland: World Health Organization (WHO).	China, Kiribati, Albania, Bulgaria, Croatia, Czechia, Hungary, North Macedonia, Slovakia, Slovenia, Estonia, Lithuania, Australia, Austria, Belgium, France, Germany, Greece, Iceland, Ireland, Israel, Luxembourg, Malta, Netherlands, Portugal, Spain, United Kingdom, Canada, Cuba, Dominica, Dominican Republic, Guyana, Saint Vincent and the Grenadines, Ecuador, Guatemala, Mauritius, Macao Special Administrative Region of China, San Marino, Northern Ireland, Scotland, Sri Lanka, Armenia, Azerbaijan, Kazakhstan, Turkmenistan, Belarus, Ukraine, Brunei Darussalam, Oman, Syrian Arab Republic, Seychelles, Republic of Crimea, Sevastopol, Ukraine (without Crimea & Sevastopol)	VR	1	1
World Health Organization (WHO). WHO Mortality Database Version October 2017. Geneva, Switzerland: World Health Organization (WHO).	China, Malaysia, Maldives, Sri Lanka, Thailand, Fiji, Kiribati, Armenia, Azerbaijan, Georgia, Kazakhstan, Kyrgyzstan, Uzbekistan, Bosnia and Herzegovina, Bulgaria, Croatia, Czechia, Hungary, North Macedonia, Montenegro, Romania, Serbia, Slovakia, Slovenia, Estonia, Latvia, Lithuania, Republic of Moldova, Brunei Darussalam, Republic of Korea, Australia, Austria, Belgium, Cyprus, Denmark, Finland, France, Germany, Iceland, Ireland, Israel, Luxembourg, Malta, Netherlands, Portugal, Spain, Switzerland, United Kingdom, Argentina, Chile, Uruguay, Canada, Antigua and Barbuda, Bahamas, Barbados, Belize, Cuba, Dominica, Dominican Republic, Grenada, Guyana, Jamaica, Saint Lucia, Saint Vincent and the Grenadines, Suriname, Trinidad and Tobago, Ecuador, Peru, Colombia, Costa Rica, El Salvador, Guatemala, Nicaragua, Panama, Venezuela (Bolivarian Republic of), Paraguay, Bahrain, Iraq, Jordan, Kuwait, Palestine, Qatar, Syrian Arab Republic, Türkiye, Mauritius, Cabo Verde, Bermuda, Hong Kong Special Administrative Region of China, Northern Ireland, Scotland, Andorra	VR	1	1
World Health Organization (WHO). WHO Mortality Database Version September 2016. Geneva, Switzerland: World Health Organization (WHO).	China, Albania, Bosnia and Herzegovina, Bulgaria, North Macedonia, Romania, Lithuania, Republic	VR	1	0

	of Moldova, Republic of Korea, Austria, Belgium, Finland, Germany, Greece, Iceland, Israel, Luxembourg, Argentina, Chile, Uruguay, Canada, Grenada, Saint Vincent and the Grenadines, Ecuador, Peru, Costa Rica, El Salvador, Guatemala, Honduras, Nicaragua, Paraguay, Bahrain, Egypt, Kuwait, Mauritius, Zimbabwe, Puerto Rico, United States Virgin Islands			

*The 0 and 1 represent whether the citation applies to the cause (1= yes, 0 = no)

Figure S3B. Data availability maps showing number of sources of prevalence and mortality data for homozygous sickle cell and severe sickle cell/beta-thalassaemia (A, B), haemoglobin SC disease (C, D), and mild sickle cell disease (E, F).

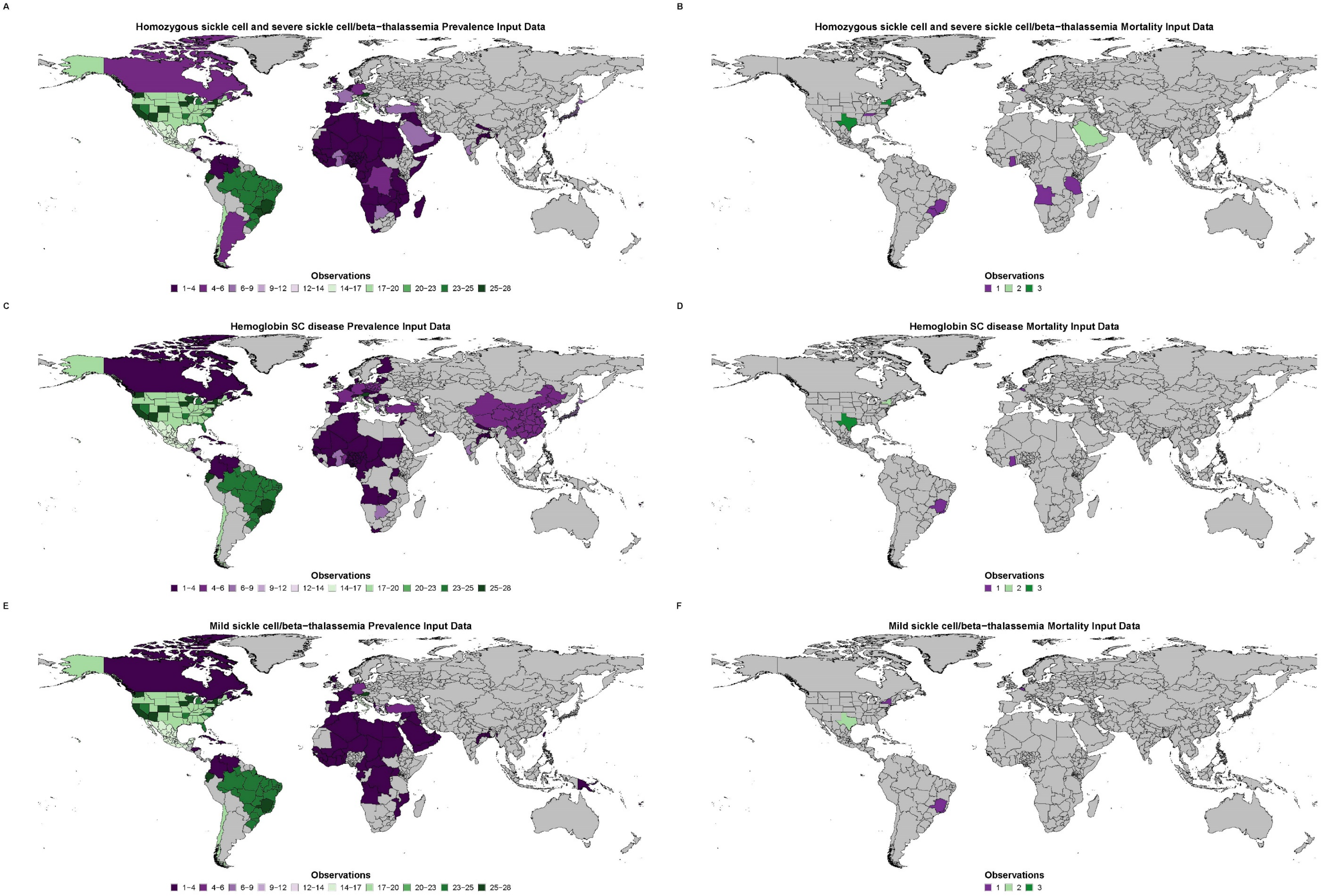

Figure S3C. Data availability maps showing types of cause-specific mortality data input sources including demographic surveillance, scientific literature, survey, vital registration, verbal autopsy, and estimates, for haemoglobinopathies and hemolytic anaemias (F) and sickle cell disorders (I).

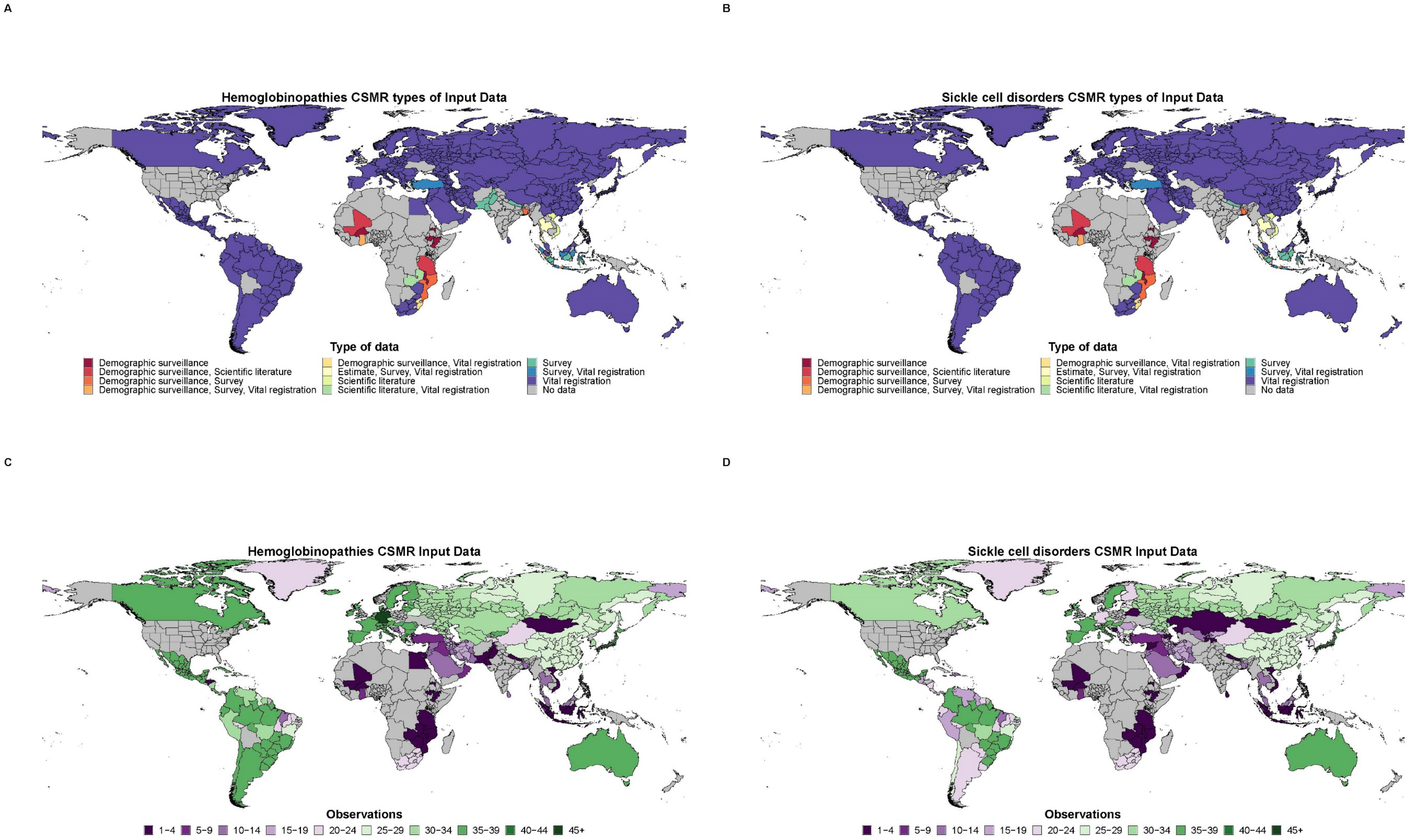

Figure S4. Sankey diagram showing cause-specific mortality due to sickle cell disease before and after garbage code redistribution of International Classification of Diseases (ICD) version 9 and version 10 codes for all years, all age groups, and both sexes combined for selected examples including global ICD-10 (A), USA ICD-9 (B), USA ICD-10 (C), Ghana ICD-10 (D), South Africa ICD-10 (E), and India ICD-10 (F).

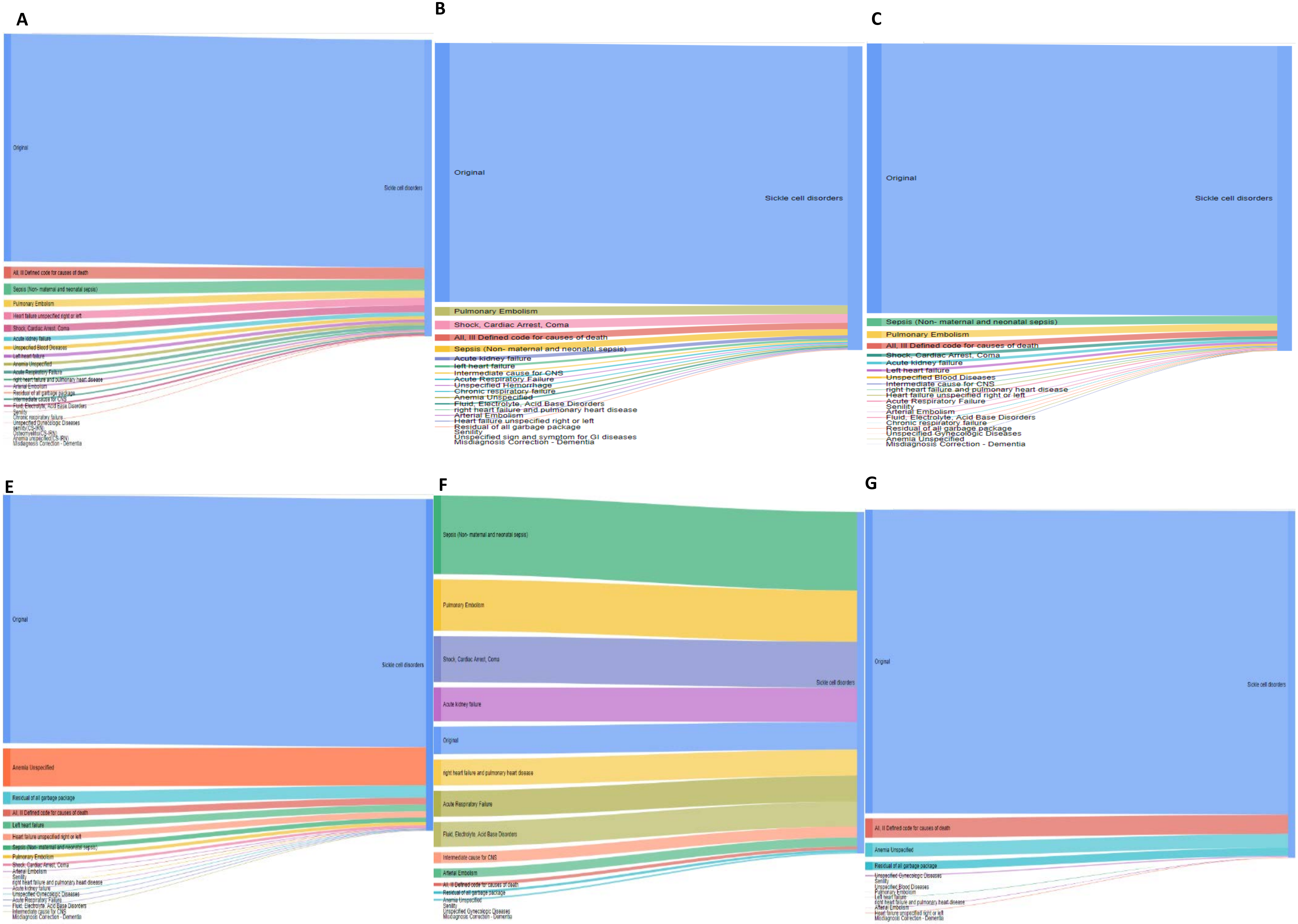

Modelling

DisMod-MR 2.1

DisMod-MR 2.1 is a compartmental model that describes how a population is affected by a single disease, as well as mortality from other causes. Using specified priors, the model considers input data to balance linked equations, which calculate the value of four log rates as a function of age: incidence, prevalence, remission, and mortality (Figure S5). These four rates define how for a given disease, people move between three states: a disease susceptible state, to the prevalent diseased population, to death (or remit back to the susceptible population).

Figure S5. Compartmental model of disease that describes how a population moves from health to a diseased state, to death (or back to health).

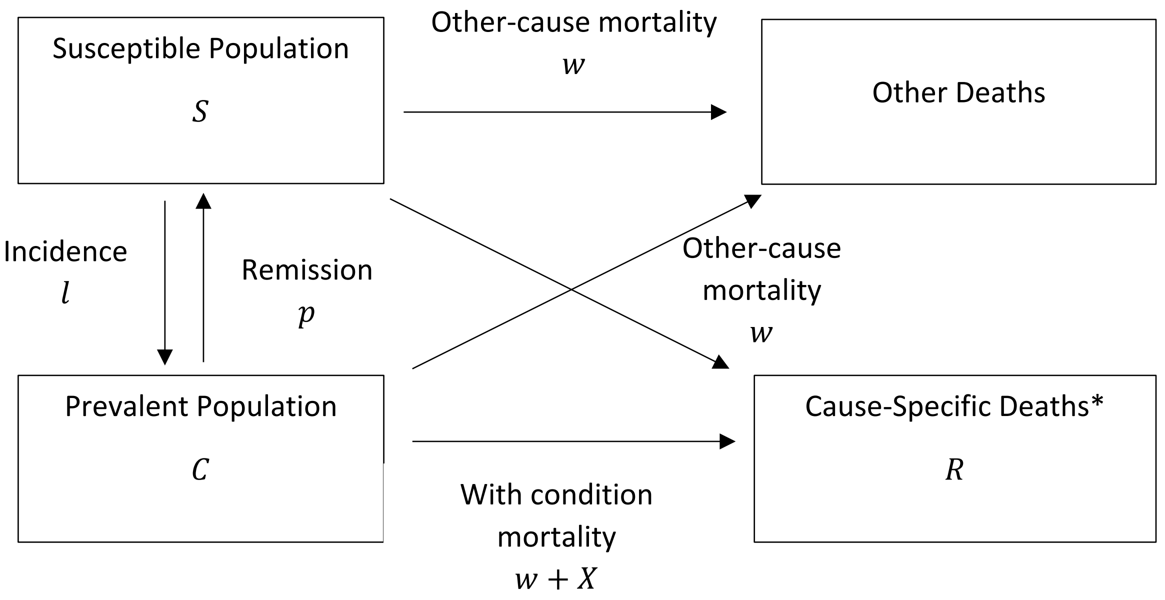

*Mortality from all other causes is assumed to be independent from the disease of interest which means that in the model, cause-specific deaths actually represent all excess mortality caused by the disease, which may differ from statistical accounting of cause-specific mortality from national and multinational offices.

The Dismod-MR 2.1 process model is described by the ordinary differential equation as follows:

$$S'(a) = -\big(l(a) + w(a)\big)S(a) + p(a)C(a)$$
$$C'(a) = l(a)S(a) - \big(p(a) + X(a) + w(a)\big)C(a)$$

Where,
$S(a)$= the population susceptible to the condition
$C(a)$= those with the condition
$l(a)$ - Incidence rate as a function of age
$p(a)$ - Remission as a function of age
$X(a)$ - Excess mortality as a function of age
$w(a)$ - Other-cause mortality as a function of age

Assuming candidate rates of $l(a)$, $p(a)$, $X(a)$, and $w(a)$, the data model assigns a likelihood to each input data value given area effects and the x-covariate effect. The total effect u_i can be defined as:

$$u_i = t_i + r_i + s_i + \sum_j x_{ij}\beta_{ji}$$

t_i=the sub-region random effect
r_i=the region random effect
s_i=the super-region random effect

The input data value and its standard deviation are scaled by the total effect and the residual is then computed against the expected mean using the following equation (written below in the linear case for ease of interpretation):

$$residual = \frac{y_i e^{-u_i} - v_i}{\sigma_i e^{-u_i} + \sum_k z_{ik}\zeta_{ki}}$$

Where,
$z\zeta$= z covariates and multiplier zeta

Ultimately, for each measure, DisMod-MR 2.1 produces an internally consistent estimation for each GBD location, age, sex, and year.[1] Estimation occurs in a geographical cascade at five levels: global, super-region, region, country, and subnational location (where available). Beginning at the highest tier, priors are generated for the next tier down in a mixed effect, non-linear regression, using all non-excluded input data. The global fit informs the super-region fit, which informs the global fit, and so on. Random effects are included at the super-region-, region-, and country-level estimates, and due to computational limitations, are imitated for the subnational level by adjusting based on an average of the residuals between the subnational-level data and its prior. Country covariates are estimated in the same geographical hierarchy where the coefficient is calculated using both data and priors in a given location, and when data is absent, the coefficient of the parent location is used. Further details on the DisMod-MR 2.1 computational process can be found in the 2003 open access paper by Barendregt and colleagues.[3] Further details on the use of DisMod in the GBD modeling framework, including the log-Gaussian equation for the likelihood function, can be found in Appendix 1, Section 4.5 of the GBD 2019 diseases and injuries capstone.[1]

We ran three models in DisMod-MR 2.1 to estimate three mutually exclusive sickle cell disorders: homozygous sickle cell disease and severe sickle cell/beta thalassaemia (SS and Sβ°), sickle cell-haemoglobin C disease (SC), and mild sickle cell/beta thalassaemia ($S\beta^+$). These models contained prevalence data and genotype-specific EMR data. We then summed these three genotype modelled estimates across location, age, sex, and year, to create a fourth model estimate for total sickle cell disorders.

We established priors for all sickle cell models for zero incidence as sickle cell is a genetic condition, with zero remission for the same logic. A prior on excess mortality was set for the neonatal period (up to 27 days) with a maximum of 1% mortality per person-year. All models except haemoglobin SC disease also had an additional excess mortality prior from 28 days to 100 years of 40% mortality per person-year. A coefficient of variation (which determines the uncertainty around priors) of 0.8 was also supplied across all three genotype models such that in data sparse areas the model could pull strength from the priors.

Further information on simulation testing for the coefficient of variation can be in Appendix 1, Section 4.5 of the GBD 2019 diseases and injuries capstone.[1]

Prevalence of haemoglobin S trait and haemoglobin C trait are strong predictive covariates for sickle cell disorders and were included from invariant age and temporal estimates by the Malaria Atlas Project. Universal health coverage index (UHC) was also included as a predictive covariate. UHC is a performance metric for the number of people covered by health services within a country and is estimated within the GBD. Effective coverage indicators were based on the level of implementation of a health-improving intervention, or outcome measures such as mortality-to-incidence ratio. The UHC index weighted each effective coverage input indicator based on the number of disability-adjusted life-years (DALYs). Further information on UHC can be found elsewhere.[4] Covariate betas for each of the included factors for each of the models can be seen in Table S3.

Table S6. DisMod-MR 2.1 covariate betas

Covariate name	Measure	Beta value	Exponentiated value
Homozygous sickle cell and severe sickle cell/beta-thalassaemia (HbSS and HbSβ°)			
Haemoglobin S (sickle type S) trait	Prevalence	49.97 (49.90 to 50.00)	5.02e+21 (4.69e+21 to 5.18e+21)
Universal health coverage index	Excess mortality rate	–0.028 (–0.048 to –0.0036)	0.97 (0.95 to 1.00)
Haemoglobin SC Disease (HbSC)			
Haemoglobin C (sickle type C) trait	Prevalence	10.00 (9.99 to 10.00)	2.19e+4 (2.18e+4 to 2.20e+4)
Haemoglobin S (sickle type S) trait	Prevalence	19.99 (19.98 to 20.00)	4.82e+8 (4.76e+8 to 4.85e+8)
Universal health coverage	Excess mortality rate	–0.024 (–0.046 to –0.0038)	0.98 (0.96 to 1.00)
Mild sickle cell/beta-thalassaemia (HbSβ+)			
Haemoglobin S (sickle type S) trait	Prevalence	19.99 (19.97 to 20.00)	4.80e+8 (4.71e+8 to 4.85e+8)
Universal health coverage	Excess mortality rate	–0.025 (–0.048 to –0.0029)	0.98 (0.95 to 1.00)

Causes of Death Ensemble modelling (CODEm)

Mortality estimates due to sickle cell disorders estimates were generated using Cause of Death Ensemble modelling (CODEm). CODEm uses input data, ranked covariates, and combinations of two model types and two outcome measures, to generate death estimates in every location, age, sex, and year within the GBD estimation framework.[1] The ensemble model ultimately selected by CODEm is determined by the out-of-sample predictive validity, or how well the model developed on a training dataset fits a hold-out test dataset.

The two model families used in the ensemble are linear mixed effects models and spatiotemporal models. The linear mixed effects models facilitate the selection of covariates and drive differences in geographical location, while spatiotemporal models add an additional component that allows us to increase "smoothing" of estimates when data are sparse and adjust "smoothing" down when data are expected to vary more across time, space, and age. Additionally, spatiotemporal models incorporate Gaussian process regression in the final step, which allows for the estimation of uncertainty over time, increasing the accuracy of temporal trend estimates. The two outcome measures are cause fractions, modelled using a logit transformation, and death rates, modelled using the natural logarithm. Both outcome estimates are assessed using out-of-sample predictive validity in natural log rate space.

Covariates are pre-selected and are used to inform estimates where data are lacking. Then CODEm software selects a combination of covariates based on statistical significance and epidemiological plausibility. Generating different hold-out datasets, CODEm iterates through this process multiple times,

creating a set of submodels consisting of both linear mixed effects and spatiotemporal submodels. Each submodel is then weighted with respect to the out-of-sample predictive validity, and ensemble models are created from a set of submodels. After two more iterations of weighting both submodels and ensemble models, the final model is selected based on highest performance over the domains of root mean square error, correct prediction of temporal trend, and accuracy of prediction uncertainty (test data coverage in the 95% prediction interval).

Typically, for each modelled cause, we run a pair of data-rich and global models for each sex. Running a data-rich model allows us to avoid unnecessarily inflating uncertainty in locations with higher-quality VR data, and running a global model with all available data allows us to pull strength from those high-quality VR locations to inform more data-sparse locations. The data-rich and global models are then hybridised so that data-rich estimates replace the global estimates where applicable. However, a global model for sickle cell disease produces implausible patterns of mortality; therefore, in addition to the data-rich CODEm model for sickle cell disease, we used a custom hybridization approach.

Hybridization approach for final cause-specific mortality estimates

In order to generate mortality estimates for sickle cell disease in data-sparse locations, we assembled three inputs: 1) VR CSMR data from the CoD database coded as "Other haemoglobinopathies," 2) CSMR estimates from the seven non-fatal DisMod-MR 2.1 haemoglobinopathy models (beta-thalassaemia major, haemoglobin E/beta-thalassaemia, haemoglobin H disease, homozygous sickle cell and severe sickle cell/beta-thalassaemia, haemoglobin SC disease, mild sickle cell/beta-thalassaemia, and G6PD deficiency), and 3) CSMR estimates from the single parent haemoglobinopathies CODEm model. Details for estimation of all non-sickle cell models can be found elsewhere.[1] First, we pooled "other haemoglobinopathies" CSMR data across ten-year spans to obtain mortality rates specific by age and sex, averaged across data-rich locations. Next, we interpolated DisMod model results from all causes classified as haemoglobinopathies to obtain a set of complete estimation years (1980–2022). We then summed all interpolated haemoglobinopathies model estimates, plus the "other haemoglobinopathies" year-pooled, data-rich average mortality rate, to obtain a total haemoglobinopathies estimate. We next summed the sickle cell model estimates and calculated the proportion of total haemoglobinopathies attributable to sickle cell disease. Finally, we then applied this proportion to the CODEm parent haemoglobinopathies estimates to create a set of scaled results attributable to sickle cell disorders in data-sparse locations.

Covariates used in modelling

Covariates used in the parent haemoglobinopathies and haemolytic anaemias and the sickle cell disorders CODEm models, along with their level and direction, are shown in Tables S4 and S5. Level indicates the strength of correlation, where 1 is most highly correlated and 3 is less correlated, and direction indicates the positive or negative correlation with mortality due to sickle cell disorders. Prevalence of haemoglobin S trait[5] and haemoglobin C trait[6] have a strong positive correlation with mortality due to sickle cell disorders and were included from estimates by the Malaria Atlas Project. Malaria lysenko holoendemicity[7] has long been shown to follow a similar global geographical distribution as prevalence of sickle cell disorders and thalassaemias. This trend is a result of the protective interaction of the heterozygous genotype (HbAS) associated with sickle cell trait and malaria. Healthcare Access and Quality (HAQ) Index and maternal care and immunization (MCI) were included as level 2 covariates and have a negative correlation with mortality due to sickle cell disorders. This trend is

associated with whether a patient can access high-quality medical care, including both preventive care such as immunisations and adequate maternal care, as well as life-saving drugs and surgical interventions. A weak (level 3) negative correlation was specified for education, log-transformed lag-distributed income[8] (LDI), and Socio-demographic Index (SDI),[8] as there is some evidence to show improved levels across these categories would reduce mortality due to sickle cell disorders. Finally, we specified a level 3 negative correlation with the proportion of population living in the latitudinal ranges of under 15 and 15–30 for the decreased likelihood of malaria holoendemicity at these latitudes, and a level 3 positive correlation with the proportion of population living in the latitudinal ranges of 30–45, and over 45 for the increased likelihood of malaria holoendemicity. Covariates were retained in the model when the relationship with observed data was statistically significant at the p<0.05 level and in the prior specified direction.

Table S7. Covariates, including level and direction, used in Cause of Death Ensemble modelling of haemoglobinopathies and haemolytic anaemia

Covariate	Transformation	Level*	Direction*
Sickle C trait from Malaria Atlas Project	Squared	1	1
Sickle S trait from Malaria Atlas Project	Squared	1	1
Malaria Lysenko PFPR 1 (Holoendemic)	None	1	1
Healthcare Access and Quality (HAQ) Index	None	2	-1
Maternal care and immunization (MCI)	None	2	-1
Education (years per capita)	None	3	-1
LDI (lag distributed income per capita)	Log	3	-1
Latitude under 15 (proportion)	None	3	1
Latitude 15 to 30 (proportion)	None	3	1
Latitude 30 to 45 (proportion)	None	3	-1
Latitude over 45 (proportion)	None	3	-1
Socio-demographic Index	None	3	-1

*Level refers to the likelihood of strength in correlation between the covariate and mortality (1 = more likely; 3 = less likely); direction refers to the direction of the relationship (1 = positive correlation; -1 = negative correlation).

Table S8. Covariates, including level and direction, used in Cause of Death Ensemble modelling of sickle cell disorders

Covariate	Transformation	Level*	Direction*
Sickle C trait from Malaria Atlas Project	None	1	1
Sickle S trait from Malaria Atlas Project	None	1	1
Haemoglobinopathies (sum of prevalence * excess-mortality from DisMod)	None	1	1
Healthcare Access and Quality (HAQ) Index	None	2	-1
Maternal care and immunization	None	2	-1
Education (years per capita)	None	3	-1
LDI (lag distributed income per capita)	Log	3	-1
Latitude under 15 (proportion)	None	3	1
Latitude 15 to 30 (proportion)	None	3	1
Latitude over 45 (proportion)	None	3	-1
Socio-demographic Index	None	3	-1
Malaria Lysenko PFPR 1 (Holoendemic)	None	3	1
Proportion of the population with at least 6 years of education	None	3	-1
Proportion of the population with at least 12 years of education	None	3	-1

*Level refers to the likelihood of strength in correlation between the covariate and mortality (1 = more likely; 3 = less likely); direction refers to the direction of the relationship (1 = positive correlation; -1 = negative correlation).

Anaemia causal attribution

Prevalence estimates of sickle cell disease and other diseases that cause anaemia were used to estimate the prevalence of anaemia attributable to each cause. To calculate the total prevalence of anaemia, mean haemoglobin concentration and prevalence of severe, moderate and severe combined, and total anaemia were modeled in ST-GPR.[11] These estimates were then used to generate distributions of haemoglobin concentration by location, year, age, and sex, from which estimates of mild, moderate, severe, and total anaemia were calculated using the World Health Organization (WHO) anaemia thresholds.[12] To calculate the proportion of anaemia attributable to a given disease, we used haemoglobin shifts associated with each disease from published literature and expert knowledge. For each cause shift, we adjusted our estimated haemoglobin distribution by the cause prevalence-weighted haemoglobin shift, resulting in a counterfactual distribution representing the anaemia prevalence in the absence of the given cause. The difference between the anaemia prevalence estimates from our original distribution and those from this counterfactual distribution represent the prevalence of anaemia attributed to this disease. After this process was completed for each cause of anaemia, estimates of attributable mild, moderate, severe, and total anaemia were squeezed to the original prevalence totals so that the attributable prevalence across all causes was equal to the total estimated anaemia prevalence.

Decomposition Analysis

Decomposition of change from 2000 to 2021 was analyzed by demographic and epidemiological factors including total population size, change in population age and sex structure, and change in rates of disease frequency by age and sex. The counterfactual mortality estimates were generated using two scenarios developed in GBD 2010[13]: (1) A population growth scenario in which population age-sex structure and age-sex disease frequency rates remained the same as 2000 but population size increased to that of 2021 and (2) a population growth and population aging scenario in which age-sex disease frequency rates remained the same as 2000 but population age-sex structure and population size changed to that of 2021. The difference in 2000 mortality results and scenario 1 is the change in deaths due to only population growth. The difference in mortality from scenario 1 and scenario 2 is the change due to aging of the population and population growth. Finally, the difference between 2010 mortality results and scenario 2 is the change due to epidemiological changes in rates of disease frequency by age and sex. The differences are reported as a percent change relative to the 2000 number.

CoDCorrect

To ensure internal consistency across mortality estimation of all GBD causes, that is, to ensure that summed cause-specific modelled estimates did not exceed all-cause mortality estimates, a core algorithm was used in the process known as CoDCorrect. Level 1 causes were rescaled to match all-cause mortality estimates; Level 2 causes were rescaled to match the rescaled Level 1 causes, and so on.

$$CD_{lyasjd} = D_{lyasjd}\left(\frac{PD_{lyasjd}}{\sum_{j=i}^{j=k} D_{lyasjd}}\right.$$

Where:

CD_{lyasjd} is the corrected number of deaths for a location l, year y, age a, sex s, cause j, and draw d

PD_{lyasjd} is the parent CoD for a location l, year y, age a, sex s, cause j, and draw d

D_{lyasjd} is the uncorrected number of deaths estimated from a cause-specific model for a location l, year y, age a, sex s, cause j, and draw d

References

1 Vos T, Lim SS, Abbafati C, *et al.* Global burden of 369 diseases and injuries in 204 countries and territories, 1990–2019: a systematic analysis for the Global Burden of Disease Study 2019. *The Lancet* 2020; **396**: 1204–22.

2 Fullman N, Yearwood J, Abay SM, *et al.* Measuring performance on the Healthcare Access and Quality Index for 195 countries and territories and selected subnational locations: a systematic analysis from the Global Burden of Disease Study 2016. *The Lancet* 2018; **391**: 2236–71.

3 Barendregt JJ, van Oortmarssen GJ, Vos T, Murray CJ. A generic model for the assessment of disease epidemiology: the computational basis of DisMod II. *Popul Health Metr* 2003; **1**: 4.

4 Lozano R, Fullman N, Mumford JE, *et al.* Measuring universal health coverage based on an index of effective coverage of health services in 204 countries and territories, 1990–2019: a systematic analysis for the Global Burden of Disease Study 2019. *The Lancet* 2020; **396**: 1250–84.

5 Malaria Atlas Project. Sickle Haemoglobin (Hbs) Allele Frequency Layer. 2013.

6 Malaria Atlas Project. Haemoglobin C (HbC) Allele Frequency Layer. 2013.

7 Lysenko A, Semashko I. Geography of malaria: A medico-geographic profile of an ancient disease. *Itogi Nauk Med Geogr* 1968; **25**: 146.

8 Wang H, Abbas KM, Abbasifard M, *et al.* Global age-sex-specific fertility, mortality, healthy life expectancy (HALE), and population estimates in 204 countries and territories, 1950–2019: a comprehensive demographic analysis for the Global Burden of Disease Study 2019. *The Lancet* 2020; **396**: 1160–203.

9 Global Burden of Disease Collaborative Network. Global Burden of Disease Study 2010 (GBD 2010) Results by Cause 1990-2010. 2012. DOI:10.6069/J86D-DZ43.

10 AlRyalat SA, Nawaiseh M, Aladwan B, Roto A, Alessa Z, Al-Omar A. Ocular Manifestations of Sickle Cell Disease: Signs, Symptoms and Complications. *Ophthalmic Epidemiol* 2020; **27**: 259–64.

11 Gardner W. Levels and trends in anaemia burden by severity and cause from 1990–2020: findings from the Global Burden of Disease Study 2020. 2022.

12 World Health Organization. Haemoglobin concentrations for the diagnosis of anaemia and assessment of severity. 2011. https://www.who.int/vmnis/indicators/haemoglobin.pdf (accessed July 29, 2021).

13 Lozano R, Naghavi M, Foreman K, *et al.* Global and regional mortality from 235 causes of death for 20 age groups in 1990 and 2010: a systematic analysis for the Global Burden of Disease Study 2010. *The Lancet* 2012; **380**: 2095–128.

Author Contributions

Managing the overall research enterprise: Nicholas J Kassebaum and Christopher J L Murray.

Writing the first draft of the manuscript: Azalea M Thomson and Theresa A McHugh.

Primary responsibility for applying analytical methods to produce estimates: Azalea M Thomson, Corey Teply, Nikhil Lonberg, Victor M Vilchis Tella, and Nicholas J Kassebaum.

Primary responsibility for seeking, cataloguing, extracting, or cleaning data; designing or coding figures and tables: Azalea M Thomson, Corey Teply, Nikhil Lonberg, Victor M Vilchis Tella, and Nicholas J Kassebaum.

Providing data or critical feedback on data sources:

Richard Gyan Aboagye, Ahmed Abu-Zaid, Bright Opoku Ahinkorah, Saif Aldeen S AlRyalat, Hubert Amu, Judie Arulappan, Maha Moh'd Wahbi Atout, Ashish D Badiye, Sara Bagherieh, Maciej Banach, Mainak Bardhan, Amadou Barrow, Ajay Nagesh Bhat, Jessica Devin Bishai, Archith Boloor, Muhammad Hammad Butt, Vijay Kumar Chattu, Dinh-Toi Chu, Xiaochen Dai, Anh Kim Dang, Fitsum Wolde Demisse, Meghnath Dhimal, Adeniyi Francis Fagbamigbe, Ali Fatehizadeh, Kazem Ghaffari, Mahaveer Golechha, Pouya Goleij, Vivek Kumar Gupta, Treska S Hassan, Segun Emmanuel Ibitoye, Shubha Jayaram, Himal Kandel, Nicholas J Kassebaum, Imteyaz A Khan, Shaghayegh Khanmohammadi, Adnan Kisa, Kewal Krishan, Stephen S Lim, Soleiman Mahjoub, Azeem Majeed, Haftu Asmerom Meresa, Awoke Misganaw, Ali H Mokdad, Lorenzo Monasta, Christopher J L Murray, Ghulam Mustafa, Mohsen Naghavi, Sreenivas Narasimha Swamy, Zuhair S Natto, Biswa Prakash Nayak, Jean Jacques Noubiap, Vincent Ebuka Nwatah, Osaretin Christabel Okonji, Adeyinka Omoniyi Onikan, Mayowa O Owolabi, Jay Patel, Shrikant Pawar, Frédéric B Piel, Mehran Rahimi, Salman Rawaf, Elrashdy Moustafa Mohamed Redwan, Basema Saddik, Umar Saeed, Abdallah M Samy, Adithi Shetty, Jasvinder A Singh, Muhammad Suleman, Mindy D Szeto, Azalea M Thomson, Muhammad Umair, Sahel Valadan Tahbaz, and Ismaeel Yunusa.

Developing methods or computational machinery:

Hubert Amu, Aleksandr Y Aravkin, Judie Arulappan, Muhammad Hammad Butt, Xiaochen Dai, Fitsum Wolde Demisse, Ali Fatehizadeh, Kazem Ghaffari, Nicholas J Kassebaum, Adnan Kisa, Nikhil Lonberg, Ali H Mokdad, Christopher J L Murray, Mohsen Naghavi, Ogochukwu Igwegbe Nzoputam, Chimezie Janet Nzoputam, Umar Saeed, Abdallah M Samy, Austin E Schumacher, Muhammad Suleman, Corey Teply, Azalea M Thomson, Burhan Abdullah Zaman, and Peng Zheng.

Providing critical feedback on methods or results:

Richard Gyan Aboagye, Melka Biratu Aboye, Ahmed Abu-Zaid, Isaac Yeboah Addo, Bright Opoku Ahinkorah, Aqeel Ahmad, Saif Aldeen S AlRyalat, Hubert Amu, Judie Arulappan, Maha Moh'd Wahbi Atout, Ashish D Badiye, Sara Bagherieh, Maciej Banach, Morteza Banakar, Mainak Bardhan, Amadou Barrow, Isabela M Bensenor, Akshaya Srikanth Bhagavathula, Pankaj Bhardwaj, Prarthna V Bhardwaj, Ajay Nagesh Bhat, Zulfiqar A Bhutta, Mariah Malak Bilalaga, Jessica Devin Bishai, Archith Boloor, Muhammad Hammad Butt, Vijay Kumar Chattu, Dinh-Toi Chu, Omid Dadras, Xiaochen Dai, Bardia Danaei, Anh Kim Dang, Fitsum Wolde Demisse, Meghnath Dhimal, Daniel Diaz, Shirin Djalalinia, Deepa Dongarwar, Muhammed Elhadi, Mohamed A Elmonem, Farshid Etaee, Oghenowede Eyawo, Adeniyi Francis Fagbamigbe, Ali Fatehizadeh, Lisa M Force, William M Gardner, Kazem Ghaffari, Paramjit Singh

Gill, Mahaveer Golechha, Vivek Kumar Gupta, Hamidreza Hasani, Treska S Hassan, Mohammed Bheser Hassen, Simon I Hay, Segun Emmanuel Ibitoye, Chidozie C D Iwu, Shubha Jayaram, Rime Jebai, Ravi Prakash Prakash Jha, Farnaz Kalantar, Himal Kandel, Ibraheem M Karaye, Nicholas J Kassebaum, Imteyaz A Khan, Shaghayegh Khanmohammadi, Adnan Kisa, Farzad Kompani, Kewal Krishan, Stephen S Lim, Preetam Bhalchandra Mahajan, Soleiman Mahjoub, Azeem Majeed, Bishnu P Marasini, Haftu Asmerom Meresa, Tomislav Mestrovic, Awoke Misganaw, Ali H Mokdad, Christopher J L Murray, Ghulam Mustafa, Mohsen Naghavi, Tapas Sadasivan Nair, Sreenivas Narasimha Swamy, Hasan Nassereldine, Zuhair S Natto, Muhammad Naveed, Biswa Prakash Nayak, Jean Jacques Noubiap, Taylor Noyes, Chisom Adaobi Nri-ezedi, Vincent Ebuka Nwatah, Ogochukwu Igwegbe Nzoputam, Chimezie Janet Nzoputam, Osaretin Christabel Okonji, Adeyinka Omoniyi Onikan, Assaf P Oron, Mayowa O Owolabi, Jay Patel, Siddhartha Pati, Shrikant Pawar, Ionela-Roxana Petcu, Frédéric B Piel, Ibrahim Qattea, Mehran Rahimi, Mosiur Rahman, Salman Rawaf, Elrashdy Moustafa Mohamed Redwan, Nazila Rezaei, Basema Saddik, Umar Saeed, Fatemeh Saheb Sharif-Askari, Abdallah M Samy, Elaheh Shaker, Migbar Mekonnen Sibhat, Jasvinder A Singh, Muhammad Suleman, Dev Ram Sunuwar, Mindy D Szeto, Jacques JL Lukenze Tamuzi, Birhan Tsegaw Taye, Mohamad-Hani Temsah, Azalea M Thomson, Muhammad Umair, Sahel Valadan Tahbaz, Cong Wang, Nuwan Darshana Wickramasinghe, Lauren B Wilner, Vahit Yiğit, Arzu Yigit, Ismaeel Yunusa, Burhan Abdullah Zaman, and Moein Zangiabadian.

Drafting the work or revising it critically for important intellectual content:

Eman Abu-Gharbieh, Ahmed Abu-Zaid, Isaac Yeboah Addo, Bright Opoku Ahinkorah, Saif Aldeen S AlRyalat, Hubert Amu, Judie Arulappan, Maha Moh'd Wahbi Atout, Ashish D Badiye, Sara Bagherieh, Maciej Banach, Morteza Banakar, Mainak Bardhan, Amadou Barrow, Deriba Abera Bedane, Isabela M Bensenor, Akshaya Srikanth Bhagavathula, Prarthna V Bhardwaj, Mariah Malak Bilalaga, Saeid Bitaraf, Vijay Kumar Chattu, Dinh-Toi Chu, Bardia Danaei, Anh Kim Dang, Meghnath Dhimal, Daniel Diaz, Deepa Dongarwar, Muhammed Elhadi, Mohamed A Elmonem, Christopher Imokhuede Esezobor, Farshid Etaee, Oghenowede Eyawo, Adeniyi Francis Fagbamigbe, Ali Fatehizadeh, Kia Fuller, Kazem Ghaffari, Paramjit Singh Gill, Vivek Kumar Gupta, Hailey Hagins, Hamidreza Hasani, Treska S Hassan, Simon I Hay, Segun Emmanuel Ibitoye, Adalia I Ikiroma, Chidozie C D Iwu, Peter Bai James, Shubha Jayaram, Rime Jebai, Ravi Prakash Prakash Jha, Nitin Joseph, Farnaz Kalantar, Himal Kandel, Woldeteklehaymanot Dagne Kassahun, Nicholas J Kassebaum, Imteyaz A Khan, Adnan Kisa, Farzad Kompani, Kewal Krishan, Iván Landires, Preetam Bhalchandra Mahajan, Soleiman Mahjoub, Bishnu P Marasini, Theresa A McHugh, Haftu Asmerom Meresa, Tomislav Mestrovic, Sonica Minhas, Awoke Misganaw, Ali H Mokdad, Lorenzo Monasta, Christopher J L Murray, Ghulam Mustafa, Mohsen Naghavi, Sreenivas Narasimha Swamy, Hasan Nassereldine, Zuhair S Natto, Biswa Prakash Nayak, Chisom Adaobi Nri-ezedi, Vincent Ebuka Nwatah, Ogochukwu Igwegbe Nzoputam, Chimezie Janet Nzoputam, Assaf P Oron, Mayowa O Owolabi, Jay Patel, Shrikant Pawar, Ionela-Roxana Petcu, Frédéric B Piel, Mehran Rahimi, Salman Rawaf, Elrashdy Moustafa Mohamed Redwan, Nazila Rezaei, Basema Saddik, Umar Saeed, Fatemeh Saheb Sharif-Askari, Abdallah M Samy, Elaheh Shaker, Migbar Mekonnen Sibhat, Jasvinder A Singh, Muhammad Suleman, Dev Ram Sunuwar, Mindy D Szeto, Jacques JL Lukenze Tamuzi, Nathan Y Tat, Mohamad-Hani Temsah, Azalea M Thomson, Sahel Valadan Tahbaz, Cong Wang, Nuwan Darshana Wickramasinghe, Vahit Yiğit, Arzu Yigit, and Burhan Abdullah Zaman.

Managing the estimation or publications process:

Ali Fatehizadeh, Kia Fuller, Kazem Ghaffari, Hailey Hagins, Simon I Hay, Nicholas J Kassebaum, Soleiman Mahjoub, Theresa A McHugh, Ali H Mokdad, Christopher J L Murray, Mohsen Naghavi, Abdallah M Samy, Azalea M Thomson, and Lauren B Wilner.

Supplemental Results: Figures

Figure S6. Geographical distribution of total sickle cell disease death and disability burden (per 100,000) in males and females combined in 2000 by four measures: birth incidence (A), all-ages prevalence (B), all-ages cause-specific mortality (C) and total SCD mortality (D)

This figure demonstrates four different lenses on geographical distribution of sickle cell disease. These include incidence rate at birth in panel A, prevalence across all age groups in the population in panel B, all-ages cause-specific mortality in panel C, and all-ages SCD-associated mortality rate in panel D. Panels A and B demonstrate highest birth incidence and all-age prevalence rates in 2000 were concentrated in sub-Saharan Africa and south Asia (India). Panels C and D show all age cause-specific and total SCD mortality rates followed a similar geographical distribution but are particularly high in western and central sub-Saharan Africa. These patterns align with the highest death and disability burden in 2021.

A

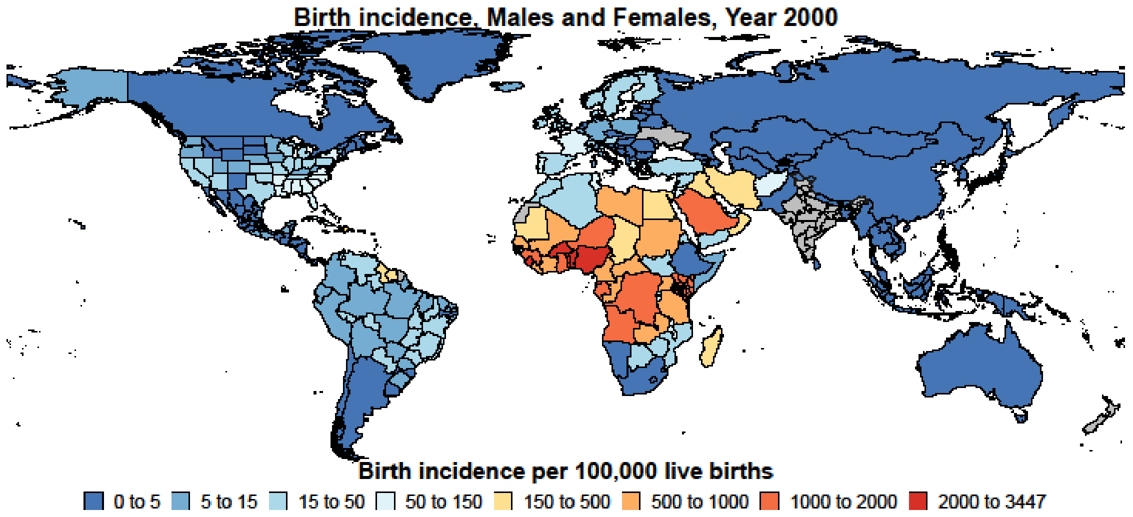

B

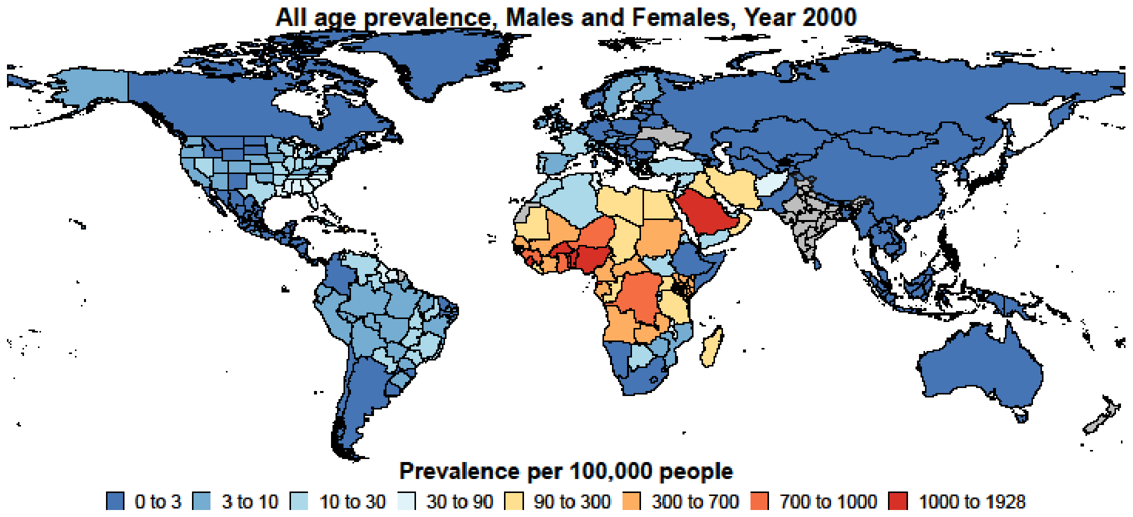

C

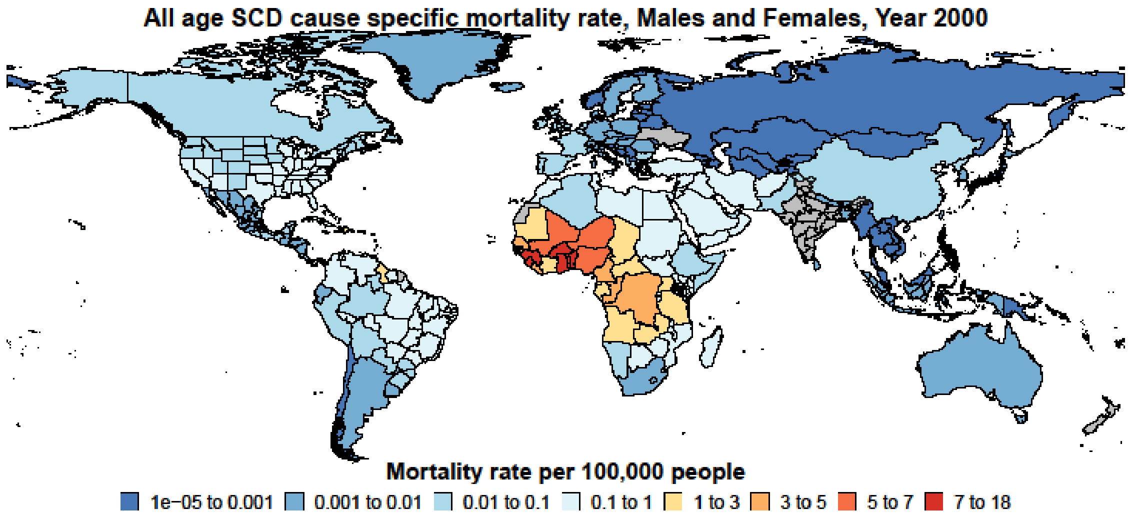

D

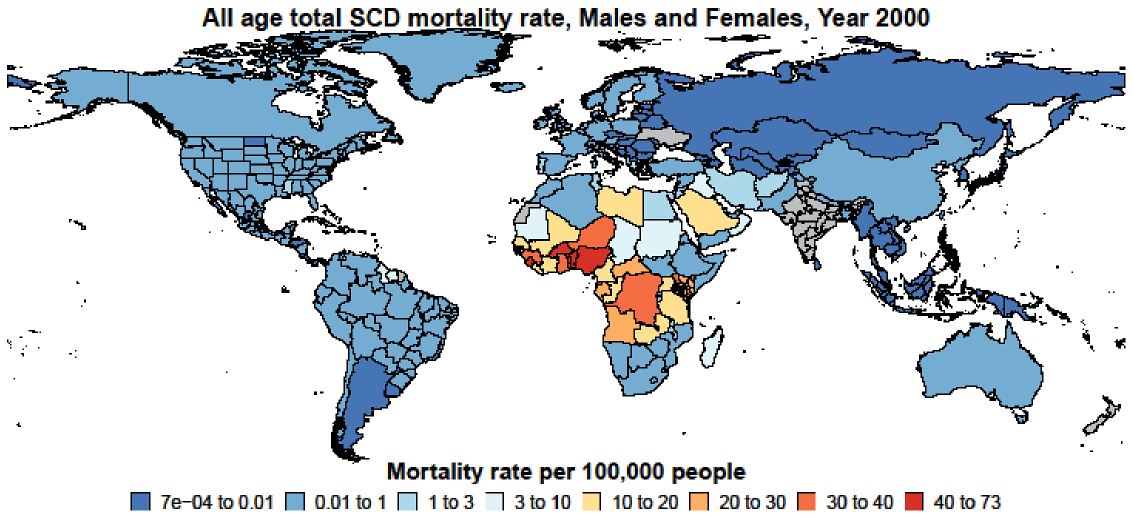

Figure S7. Among males and females combined in 2021, in locations with birth incidence rate above 500 per 100,000 livebirths, total SCD deaths as a percentage of under-5 deaths (A), in locations not currently meeting Sustainable Development Goal 3.2 for U5MR (under-5 mortality rate), total SCD deaths as a percentage of under-5 deaths (B), U5MR attributed to total SCD deaths alone (C), and in locations with over 10,000 incident malaria cases per 100,000, total SCD deaths as a percentage of under-5 deaths .

This figure shows the percentage of under-5 total SCD deaths in locations with SCD birth incidence above 500 per 100,000 livebirths in panel A, the under-5 total SCD deaths in locations not currently meeting SDG 3.2 (U5MR below 25 under-5 deaths per 1000 livebirths) in panel B, the U5MR from total SCD deaths alone in panel C, and the percentage of under-5 total SCD deaths in locations with malaria all-age incidence rate above 10,000 per 100,000 livebirths. Examining only locations with high sickle cell disease birth incidence and locations suffering from high numbers of deaths in children under 5 per 1000 livebirths, these maps indicate that sickle cell disease has the greatest impact on mortality in countries in western and central sub-Saharan Africa.

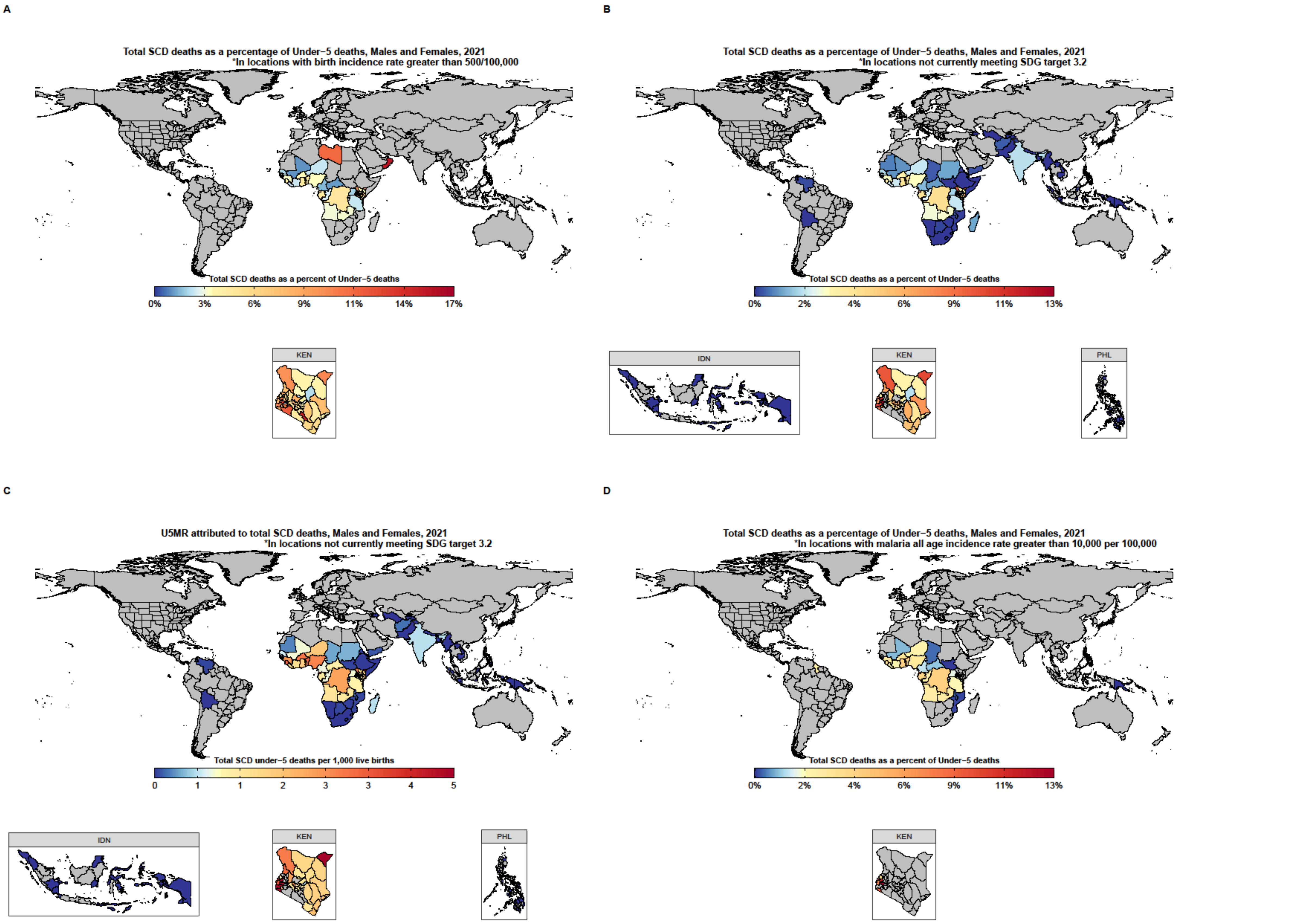

Figure S8. Total sickle cell disease deaths ranked by super-region and country as a proportion of all-cause mortality in children under 5 years (A and B) and cause-specific mortality rate compared to total SCD mortality rate in 5-14 years age group and 15-49 years age group (C and D).

This figure shows the 2000 ranking of total SCD deaths as a proportion of all-cause mortality, with warmer colors indicating countries with a higher rank. In 2000 total SCD was ranked higher than cause-specific deaths. Compared to 2021, these rankings have increased, demonstrating the increasing burden of sickle cell disease to childhood mortality. Similarly, in the 5-14 years age group and 15-49 years age group in 2000, panels C and D show that total SCD mortality rates exceeds cause-specific mortality rates across all locations.

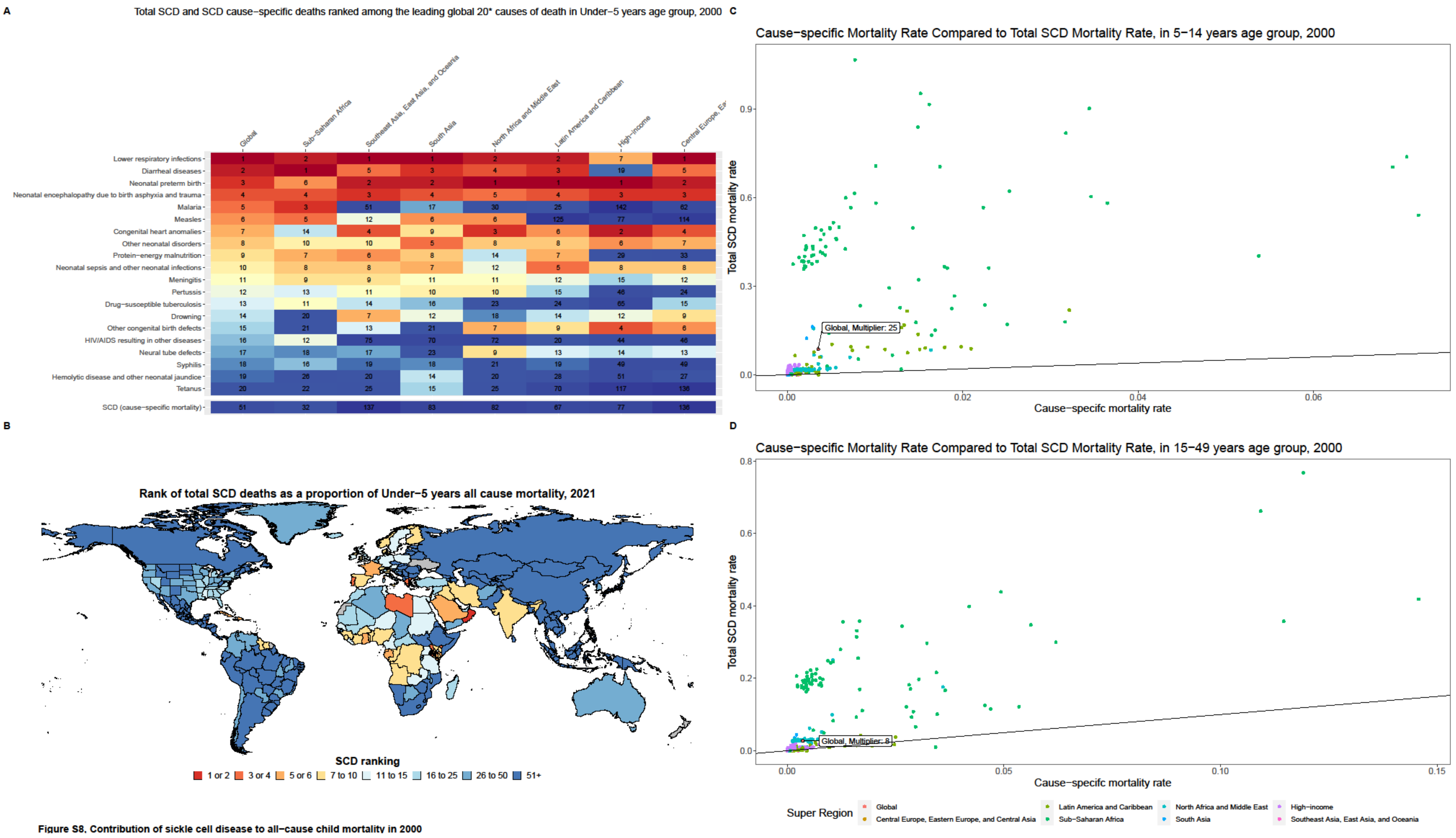

Figure S9. Geographical distribution of total sickle cell disease all-age prevalence (per 100,000) in males (A) and females (B)

This figure shows the 2021 all age prevalence for males and females.

A

B

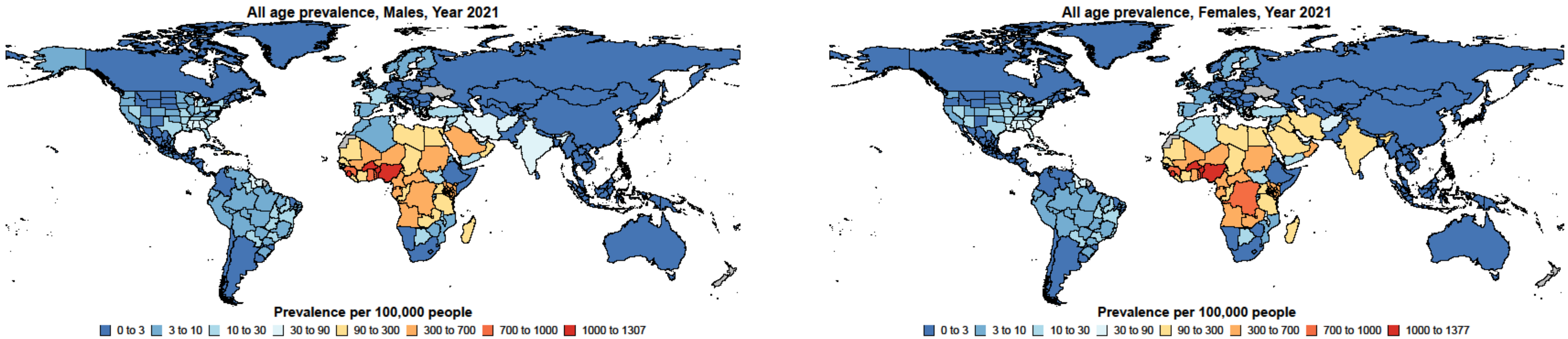

Table S9: Number of births, cases, total deaths, and under-5 deaths for sickle cell disease, 2000 - 2021

Location	Births, 2000	Births, 2021	Births percent change (2000-2021)	All age cases, 2000	All age cases, 2021	All age cases percent change (2000-2021)	All age deaths (cause-specific), 2000	All age deaths (cause-specific), 2021	All age deaths (cause-specific) percent change (2000-2021)	All age deaths (total), 2000	All age deaths (total), 2021	All age deaths (total) percent change (2000-2021)	Under-5 deaths (cause-specific), 2000	Under-5 deaths (cause-specific), 2021	Under-5 deaths (cause-specific) percent change (2000-2021)	Under-5 deaths (total), 2000	Under-5 deaths (total), 2021	Under-5 deaths (total) percent change (2000-2021)	Under-5 cause-specific mortality cause fraction, 2000	Under-5 total SCD mortality cause fraction, 2000	Under-5 cause-specific mortality cause fraction, 2021	Under-5 total SCD mortality cause fraction, 2021	Under-5 cause-specific mortality rank 2000	Under-5 cause-specific mortality rank 2021	Under-5 total SCD mortality rank 2000	Under-5 total SCD mortality rank 2021
Global	453,000 (370,000-542,000)	515,000 (425,000-614,000)	13.7 (11.1-16.5)	5,460,000 (4,620,000-6,450,000)	7,740,000 (6,510,000-9,200,000)	41.4 (38.3-44.9)	28,400 (22,100-34,500)	34,400 (25,000-45,200)	20.8 (-5.3-57.6)	262,000 (211,000-327,000)	376,000 (303,000-467,000)	43.4 (39.8-47.3)	11,600 (7,590-16,900)	10,800 (6,720-14,600)	-7.1 (-33.8-34.6)	65,200 (47,200-87,600)	81,100 (58,800-108,000)	24.4 (19.7-29.3)	0.1 (0.1-0.2)	0.7 (0.5-0.9)	0.2 (0.2-0.3)	1.7 (1.1-2.3)	51	40	22	12
Central Europe, Eastern Europe, and Central Asia	45 (32-62)	31 (23-40)	-31 (-38.4--24.3)	425 (345-507)	285 (232-340)	-33.3 (-37.9--28.9)	5 (4-5)	3 (3-3)	-32.2 (-38.1--26.8)	55 (39-76)	35 (26-46)	-36.5 (-43.3--28.7)	0 (0-0)	0 (0-0)	-57.3 (-71.4--36)	29 (19-43)	20 (13-29)	-31.2 (-40.4--19.7)	0 (0-0)	0 (0-0)	0 (0-0)	0 (0-0)	136	136	81	72
**Central Asia**	1 (1-2)	1 (1-3)	17.9 (4.2-21.7)	8 (4-12)	9 (4-14)	15.7 (-0.2-23.6)	0 (0-0)	0 (0-0)	36.1 (-19.5-134)	1 (0-2)	1 (1-3)	18.5 (-9.6-22.8)	0 (0-0)	0 (0-0)	7.2 (-43.8-98)	1 (0-2)	1 (0-2)	22.4 (-7.4-27.5)	0 (0-0)	0 (0-0)	0 (0-0)	0 (0-0)	136	135	126	118
Armenia	0 (0-0)	0 (0-0)	-20.3 (-29.4--11.3)	0 (0-1)	0 (0-0)	-20.9 (-27--11.8)	0 (0-0)	0 (0-0)	47.1 (-17.1-136)	0 (0-0)	0 (0-0)	-17.7 (-36.9--7.6)	0 (0-0)	0 (0-0)	-43.1 (-72.8-26.9)	0 (0-0)	0 (0-0)	-11.1 (-32.3--1.1)	0 (0-0)	0 (0-0)	0 (0-0)	0 (0-0)	132	132	106	100
Azerbaijan	0 (0-0)	0 (0-0)	-5.6 (-25.9--2.5)	1 (0-1)	1 (0-1)	0.8 (-10.9-8.7)	0 (0-0)	0 (0-0)	-46.5 (-72.9-12.7)	0 (0-0)	0 (0-0)	-0.9 (-29.9-5.4)	0 (0-0)	0 (0-0)	-49.3 (-79.2-19.1)	0 (0-0)	0 (0-0)	0.6 (-28-5.7)	0 (0-0)	0 (0-0)	0 (0-0)	0 (0-0)	128	127	119	107
Georgia	0 (0-0)	0 (0-0)	-14.2 (-23.8--9.9)	0 (0-1)	0 (0-1)	-27.4 (-44.2--19.7)	0 (0-0)	0 (0-0)	-29.9 (-50.6--8.1)	0 (0-0)	0 (0-0)	-21.3 (-38.3--17.9)	0 (0-0)	0 (0-0)	-68.8 (-81.1--51.7)	0 (0-0)	0 (0-0)	-11.6 (-31.7--6.9)	0 (0-0)	0 (0-0)	0 (0-0)	0 (0-0)	136	135	108	100
Kazakhstan	0 (0-0)	0 (0-1)	45.5 (20.8-100)	1 (0-2)	2 (1-3)	33.1 (8.6-97.1)	0 (0-0)	0 (0-0)	190 (129-256)	0 (0-0)	0 (0-1)	43.3 (12.1-82.8)	0 (0-0)	0 (0-0)	212 (46.5-586)	0 (0-0)	0 (0-0)	57.8 (24.7-94.5)	0 (0-0)	0 (0-0)	0 (0-0)	0 (0-0)	134	132	115	104
Kyrgyzstan	0 (0-0)	0 (0-0)	40.4 (8.2-44.9)	1 (0-1)	1 (0-1)	34.6 (9.5-47.8)	0 (0-0)	0 (0-0)	61 (25.4-105)	0 (0-0)	0 (0-0)	39.9 (6.3-44.7)	0 (0-0)	0 (0-0)	-44.8 (-58.4--30.2)	0 (0-0)	0 (0-0)	46.9 (12.5-54.2)	0 (0-0)	0 (0-0)	0 (0-0)	0 (0-0)	134	134	114	111
Mongolia	0 (0-0)	0 (0-0)	33.9 (4.8-42.3)	0 (0-0)	0 (0-1)	30.8 (1-46.3)	0 (0-0)	0 (0-0)	-37.8 (-71.8-61)	0 (0-0)	0 (0-0)	40.6 (6.1-47.1)	0 (0-0)	0 (0-0)	-45.7 (-82.1-64.3)	0 (0-0)	0 (0-0)	49.9 (9-58.7)	0 (0-0)	0 (0-0)	0 (0-0)	0 (0-0)	126	128	124	110
Tajikistan	0 (0-0)	0 (0-0)	34.8 (8.6-40.3)	1 (0-1)	1 (0-2)	35.3 (18.1-43)	0 (0-0)	0 (0-0)	7.1 (-47.2-132)	0 (0-0)	0 (0-0)	33.3 (2.8-38.5)	0 (0-0)	0 (0-0)	15 (-48.6-172)	0 (0-0)	0 (0-0)	34.6 (3.9-40.6)	0 (0-0)	0 (0-0)	0 (0-0)	0 (0-0)	127	129	116	114
Turkmenistan	0 (0-0)	0 (0-0)	7.2 (-17.8-11.3)	0 (0-1)	0 (0-1)	5.5 (-16.5-14.5)	0 (0-0)	0 (0-0)	166 (56.9-370)	0 (0-0)	0 (0-0)	8 (-19.7-11.5)	0 (0-0)	0 (0-0)	79 (-0.6-254)	0 (0-0)	0 (0-0)	10.9 (-17-16.3)	0 (0-0)	0 (0-0)	0 (0-0)	0 (0-0)	135	129	121	113
Uzbekistan	0 (0-1)	1 (0-1)	10.2 (-13.7-15.8)	3 (1-4)	3 (2-5)	16.4 (-3.5-27.3)	0 (0-0)	0 (0-0)	427 (276-655)	0 (0-1)	1 (0-1)	19 (-10.3-24.3)	0 (0-0)	0 (0-0)	314 (183-472)	0 (0-1)	0 (0-1)	24.4 (-7.3-32)	0 (0-0)	0 (0-0)	0 (0-0)	0 (0-0)	132	125	113	111
**Central Europe**	41 (29-58)	26 (19-36)	-35.1 (-41.3--28.1)	361 (284-454)	226 (180-277)	-37.6 (-42.5--32.8)	4 (3-4)	2 (2-3)	-39.1 (-45--32.4)	50 (35-70)	30 (22-41)	-40.3 (-47.2--33.4)	0 (0-0)	0 (0-0)	-78.4 (-86.4--66.6)	27 (17-41)	17 (11-26)	-35.4 (-44.1--25.1)	0 (0-0)	0.2 (0.1-0.3)	0 (0-0)	0.3 (0.2-0.5)	122	126	45	32
Albania	0 (0-1)	0 (0-0)	-39.5 (-45.1--37.6)	3 (2-4)	2 (1-2)	-41.9 (-51.7--37.9)	0 (0-0)	0 (0-0)	-88.4 (-94.1--74.1)	0 (0-1)	0 (0-0)	-44.7 (-49.7--42.3)	0 (0-0)	0 (0-0)	-92 (-97.5--76.1)	0 (0-0)	0 (0-0)	-45.3 (-50.2--43.3)	0 (0-0)	0 (0-0)	0 (0-0)	0 (0-0.1)	102	110	88	71
Bosnia and Herzegovina	0 (0-0)	0 (0-0)	-40.3 (-45.1--34.1)	2 (1-3)	1 (1-2)	-38.3 (-44.6--32.2)	0 (0-0)	0 (0-0)	-57.2 (-79--14.3)	0 (0-0)	0 (0-0)	-35.8 (-45.8--30.5)	0 (0-0)	0 (0-0)	-84.5 (-96.5--50.6)	0 (0-0)	0 (0-0)	-43.4 (-51--39.3)	0 (0-0)	0 (0-0)	0 (0-0)	0 (0-0)	123	127	97	89
Bulgaria	0 (0-0)	0 (0-0)	-20.7 (-31.7--18.6)	3 (2-4)	2 (2-3)	-25 (-35.9--19.2)	1 (1-1)	1 (1-1)	-22.1 (-36.3--0.5)	0 (0-0)	0 (0-0)	-22.1 (-39.6--16.8)	0 (0-0)	0 (0-0)	-58.5 (-71.9--37)	0 (0-0)	0 (0-0)	-9.4 (-28.5--1.1)	0 (0-0)	0 (0-0)	0 (0-0)	0 (0-0)	109	108	101	80
Croatia	1 (0-1)	0 (0-1)	-25.6 (-33.6--16)	18 (14-22)	13 (11-17)	-24.5 (-32.1--15.6)	0 (0-0)	0 (0-0)	-6.1 (-25-15.2)	1 (1-1)	1 (0-1)	-24.7 (-31.9--17.2)	0 (0-0)	0 (0-0)	-40.9 (-61.4--16.3)	0 (0-0)	0 (0-0)	-37.2 (-45.4--29.5)	0 (0-0)	0.1 (0-0.1)	0 (0-0)	0.1 (0-0.1)	118	113	56	50
Czechia	0 (0-0)	0 (0-0)	17.4 (3.5-23.3)	1 (1-2)	1 (1-2)	-5.6 (-12.6-4.3)	0 (0-0)	0 (0-0)	-37.5 (-50.2--19.9)	0 (0-0)	0 (0-0)	2.3 (-14.5-13.2)	0 (0-0)	0 (0-0)	-38.2 (-56.1--17)	0 (0-0)	0 (0-0)	26.5 (7.1-39.6)	0 (0-0)	0 (0-0)	0 (0-0)	0 (0-0)	122	119	105	83
Hungary	0 (0-0)	0 (0-0)	-21.1 (-36--17.2)	4 (3-6)	3 (2-5)	-21.4 (-42.7--16.2)	0 (0-0)	0 (0-0)	-26.2 (-48.2--7.2)	0 (0-0)	0 (0-0)	-20.1 (-38.4--16.2)	0 (0-0)	0 (0-0)	-48.2 (-68.4--24.6)	0 (0-0)	0 (0-0)	-15.3 (-31.3--9.2)	0 (0-0)	0 (0-0)	0 (0-0)	0 (0-0)	124	115	101	85
Montenegro	0 (0-0)	0 (0-0)	-24.5 (-31.9--17.4)	0 (0-0)	0 (0-0)	-18.6 (-28.4--14.2)	0 (0-0)	0 (0-0)	-55 (-75.1--15.8)	0 (0-0)	0 (0-0)	-17.4 (-34.3--8.2)	0 (0-0)	0 (0-0)	-75.5 (-95.2--12.6)	0 (0-0)	0 (0-0)	-24.7 (-39.4--9.5)	0 (0-0)	0 (0-0)	0 (0-0)	0 (0-0)	119	117	95	71
North Macedonia	0 (0-0)	0 (0-0)	-13.9 (-27.6--1.3)	1 (1-2)	1 (1-1)	-11.8 (-29-4.1)	0 (0-0)	0 (0-0)	-55.3 (-74.2--19.7)	0 (0-0)	0 (0-0)	-9.9 (-30.5--2.5)	0 (0-0)	0 (0-0)	-92.8 (-98.4--78.8)	0 (0-0)	0 (0-0)	-15.3 (-34.2--6)	0 (0-0)	0 (0-0)	0 (0-0)	0 (0-0)	121	123	99	64
Poland	38 (26-55)	24 (17-34)	-36.1 (-42.7--28.5)	292 (217-381)	174 (130-226)	-40.9 (-46.3--35.5)	1 (1-1)	1 (1-1)	-52.1 (-56.3--47.1)	46 (32-66)	26 (19-38)	-42.3 (-49.6--35)	0 (0-0)	0 (0-0)	-63.6 (-71.4--55.7)	25 (16-40)	16 (10-25)	-36.8 (-45.9--26.2)	0 (0-0)	0.7 (0.5-1.1)	0 (0-0)	1 (0.6-1.6)	117	115	19	13
Romania	1 (1-2)	1 (1-1)	-26 (-31.4--9.3)	27 (20-34)	19 (14-26)	-29.3 (-36.9--11.9)	0 (0-1)	0 (0-0)	-29.4 (-45.1--11.2)	2 (1-2)	1 (1-2)	-25.2 (-30.1--8.4)	0 (0-0)	0 (0-0)	-59.5 (-72.6--44.2)	1 (0-1)	0 (0-1)	-19.2 (-28.2-2.8)	0 (0-0)	0 (0-0)	0 (0-0)	0 (0-0.1)	123	124	92	70
Serbia	0 (0-0)	0 (0-0)	-25.4 (-28.9--4.4)	1 (0-2)	1 (0-1)	-23.1 (-32.1-17.5)	0 (0-0)	0 (0-0)	-68.1 (-83.2--46.7)	0 (0-0)	0 (0-0)	-27.3 (-31.1--15.2)	0 (0-0)	0 (0-0)	-87.5 (-95.9--62.3)	0 (0-0)	0 (0-0)	-33.4 (-36.1--27)	0 (0-0)	0 (0-0)	0 (0-0)	0 (0-0)	130	131	115	92
Slovakia	0 (0-0)	0 (0-0)	-0.8 (-21.1-21.7)	7 (5-8)	5 (4-7)	-19.5 (-36.1--2.8)	0 (0-0)	0 (0-0)	-58.7 (-77.2--22.1)	0 (0-0)	0 (0-0)	-16.2 (-33-5.7)	0 (0-0)	0 (0-0)	-73.5 (-92.3--15.4)	0 (0-0)	0 (0-0)	-5.3 (-24.9-18.2)	0 (0-0)	0 (0-0)	0 (0-0)	0 (0-0)	120	124	82	70
Slovenia	0 (0-0)	0 (0-0)	-1.3 (-24.8-5.2)	2 (1-2)	2 (1-2)	-13.2 (-32.8--5)	0 (0-0)	0 (0-0)	-60 (-70--48.2)	0 (0-0)	0 (0-0)	-8.3 (-35--1.6)	0 (0-0)	0 (0-0)	-64 (-75.6--49.3)	0 (0-0)	0 (0-0)	1.8 (-24.9-13.3)	0 (0-0)	0 (0-0)	0 (0-0)	0 (0-0)	125	125	82	63
**Eastern Europe**	3 (2-4)	3 (2-5)	9.1 (4.3-11)	56 (41-74)	50 (36-66)	-11.6 (-17.7--7.1)	1 (1-1)	1 (1-1)	-7.3 (-15.9-2.1)	4 (2-5)	4 (2-6)	-0.3 (-9.5-6.5)	0 (0-0)	0 (0-0)	-13 (-41.2-23.8)	1 (1-2)	2 (1-3)	22.9 (17.9-25.9)	0 (0-0)	0 (0-0)	0 (0-0)	0 (0-0)	132	130	115	91
Belarus	0 (0-0)	0 (0-0)	2.6 (-18.4-6.1)	3 (2-4)	2 (1-3)	-16.9 (-45.1--9.5)	0 (0-0)	0 (0-0)	73.5 (12.5-187)	0 (0-0)	0 (0-0)	-4.3 (-22.3-1.8)	0 (0-0)	0 (0-0)	-16.8 (-64.2-107)	0 (0-0)	0 (0-0)	15.3 (3-20.8)	0 (0-0)	0 (0-0)	0 (0-0)	0 (0-0)	134	135	106	84
Estonia	0 (0-0)	0 (0-0)	0.6 (-25.3-4.6)	0 (0-0)	0 (0-0)	-16.6 (-48.7--9.2)	0 (0-0)	0 (0-0)	-19.1 (-39.2-7.5)	0 (0-0)	0 (0-0)	-9.3 (-27.6--4.5)	0 (0-0)	0 (0-0)	-48.3 (-66.2--24.5)	0 (0-0)	0 (0-0)	7.2 (-7.3-12.2)	0 (0-0)	0 (0-0)	0 (0-0)	0 (0-0)	135	132	104	82

Location	Births, 2000	Births, 2021	Births percent change (2000-2021)	All age cases, 2000	All age cases, 2021	All age cases percent change (2000-2021)	All age deaths (cause-specific), 2000	All age deaths (cause-specific), 2021	All age deaths (cause-specific) percent change (2000-2021)	All age deaths (total), 2000	All age deaths (total), 2021	All age deaths (total) percent change (2000-2021)	Under-5 deaths (cause-specific), 2000	Under-5 deaths (cause-specific), 2021	Under-5 deaths (cause-specific) percent change (2000-2021)	Under-5 deaths (total), 2000	Under-5 deaths (total), 2021	Under-5 deaths (total) percent change (2000-2021)	Under-5 cause-specific mortality cause fraction, 2000	Under-5 total SCD mortality cause fraction, 2000	Under-5 cause-specific mortality cause fraction, 2021	Under-5 total SCD mortality cause fraction, 2021	Under-5 cause-specific mortality rank 2000	Under-5 cause-specific mortality rank 2021	Under-5 total SCD mortality rank 2000	Under-5 total SCD mortality rank 2021
Latvia	0 (0-0)	0 (0-0)	-12.1 (-45.8--2.5)	1 (1-1)	1 (0-1)	-32.6 (-60.2--26.3)	0 (0-0)	0 (0-0)	-46.2 (-65.1--18.5)	0 (0-0)	0 (0-0)	-23.3 (-47.5--16.5)	0 (0-0)	0 (0-0)	-77.8 (-84.8--69.5)	0 (0-0)	0 (0-0)	2.9 (-19.7-9.6)	0 (0-0)	0 (0-0)	0 (0-0)	0 (0-0)	134	135	109	81
Lithuania	0 (0-0)	0 (0-0)	-19.3 (-25.6-12.7)	2 (1-2)	1 (1-2)	-34.5 (-39.5--3)	0 (0-0)	0 (0-0)	-23.1 (-43.9-4.8)	0 (0-0)	0 (0-0)	-28.6 (-32.4--10)	0 (0-0)	0 (0-0)	-70.6 (-80.4--60.5)	0 (0-0)	0 (0-0)	-24.4 (-30.1--10.4)	0 (0-0)	0 (0-0)	0 (0-0)	0 (0-0)	133	129	101	75
Republic of Moldova	0 (0-0)	0 (0-0)	-35.1 (-46.2--32.8)	1 (1-2)	1 (1-1)	-34.3 (-49.7--26)	0 (0-0)	0 (0-0)	-29.1 (-40.2--11.9)	0 (0-0)	0 (0-0)	-31.4 (-41.6--26.7)	0 (0-0)	0 (0-0)	-62.9 (-76.3--43.4)	0 (0-0)	0 (0-0)	-33.9 (-41.5--30.6)	0 (0-0)	0 (0-0)	0 (0-0)	0 (0-0)	134	135	110	101
Russian Federation	2 (1-3)	2 (1-4)	19.6 (13.6-21.8)	37 (26-50)	35 (25-46)	-5.4 (-11.6-0.4)	1 (1-1)	1 (1-1)	-9.1 (-16.8--0.5)	2 (2-4)	3 (2-4)	7.6 (-4.2-16.6)	0 (0-0)	0 (0-0)	55.2 (28.4-87.3)	1 (0-2)	1 (0-2)	37.4 (31.2-39.5)	0 (0-0)	0 (0-0)	0 (0-0)	0 (0-0)	133	133	117	90
Ukraine	1 (0-1)	1 (0-1)	-13.8 (-20.1--11.4)	12 (9-17)	10 (7-13)	-21.7 (-31.3--18.3)	0 (0-0)	0 (0-0)	2.3 (-28.3-39.4)	1 (0-1)	1 (0-1)	-16.3 (-27.2--13)	0 (0-0)	0 (0-0)	-29 (-58.3-16.6)	0 (0-1)	0 (0-1)	-8.4 (-15.9--4.8)	0 (0-0)	0 (0-0)	0 (0-0)	0 (0-0)	122	119	98	89
High-income	2,860 (2,550-3,190)	2,530 (2,250-2,830)	-11.5 (-12.8--9.8)	79,800 (73,200-86,500)	84,100 (77,800-90,600)	5.2 (3.6-6.6)	622 (609-633)	596 (578-614)	-4.1 (-7.6--1.1)	2,720 (2,440-3,060)	2,720 (2,450-3,040)	-0.1 (-1.9-1.8)	37 (36-38)	22 (19-23)	-41.3 (-47.9--36.1)	729 (587-886)	578 (461-718)	-20.6 (-24--17)	0 (0-0)	0.8 (0.7-1)	0 (0-0)	1.2 (0.9-1.5)	77	76	21	17
**Australasia**	6 (5-8)	8 (6-10)	31.5 (23-42.9)	123 (100-155)	148 (120-188)	20.2 (14-28)	1 (1-1)	0 (0-0)	-66.1 (-71.4--58.1)	6 (5-8)	8 (6-10)	23.4 (15.2-34)	0 (0-0)	0 (0-0)	-85.7 (-89.8--81.1)	3 (2-4)	3 (2-5)	21 (10.1-35.5)	0 (0-0)	0.1 (0.1-0.2)	0 (0-0)	0.3 (0.2-0.4)	102	113	44	39
Australia	4 (3-5)	5 (4-7)	29.5 (17.6-42.1)	81 (64-105)	98 (78-128)	20.9 (11.7-32)	1 (1-1)	0 (0-0)	-69.5 (-74.8--61.9)	4 (3-6)	5 (4-7)	22 (11.9-35.1)	0 (0-0)	0 (0-0)	-90.6 (-93.7--87.1)	2 (1-3)	2 (2-3)	19.8 (6.4-37.2)	0 (0-0)	0.1 (0.1-0.2)	0 (0-0)	0.3 (0.2-0.4)	102	114	46	37
New Zealand	2 (2-2)	3 (2-3)	35.5 (24.6-49.2)	43 (35-50)	51 (42-62)	18.9 (9.8-28.8)	0 (0-0)	0 (0-0)	40.8 (16.8-74.1)	2 (2-2)	3 (2-3)	26.5 (11.9-41.5)	0 (0-0)	0 (0-0)	17.8 (-23.3-64.8)	1 (1-1)	1 (1-1)	24.3 (4.4-44)	0 (0-0)	0.2 (0.1-0.2)	0 (0-0)	0.3 (0.2-0.4)	102	99	40	35
**High-income Asia Pacific**	6 (4-7)	4 (3-5)	-26.7 (-31.2--23.4)	109 (81-147)	93 (69-123)	-15.8 (-20.2--11.9)	6 (5-7)	2 (2-2)	-67.5 (-71.7--62.8)	7 (5-9)	5 (4-7)	-16.2 (-22.4--9.9)	1 (0-1)	0 (0-0)	-86.5 (-92.8--71)	3 (2-4)	2 (2-3)	-27 (-33.4--21.2)	0 (0-0)	0 (0-0)	0 (0-0)	0.1 (0.1-0.1)	109	110	87	66
Brunei Darussalam	0 (0-0)	0 (0-0)	-11.3 (-19.7--2)	0 (0-1)	0 (0-1)	10.1 (0.4-26)	0 (0-0)	0 (0-0)	46.7 (-18.1-145)	0 (0-0)	0 (0-0)	-2.4 (-14.2-8.2)	0 (0-0)	0 (0-0)	-5.8 (-75.3-172)	0 (0-0)	0 (0-0)	-14 (-27--4.4)	0 (0-0)	0 (0-0)	0 (0-0)	0 (0-0.1)	101	94	84	77
Japan	3 (2-4)	2 (1-3)	-24.5 (-29.3--18.5)	58 (37-90)	49 (33-73)	-15.6 (-20.4--9.3)	5 (4-5)	1 (1-1)	-69.2 (-70.7--67.4)	3 (2-5)	3 (2-4)	-14.1 (-20.1--7.4)	0 (0-0)	0 (0-0)	-79.9 (-82.3--77.3)	1 (1-2)	1 (1-1)	-21.3 (-27.2--14.6)	0 (0-0)	0 (0-0)	0 (0-0)	0.1 (0-0.1)	107	109	82	72
Aichi	1 (1-1)	1 (0-1)	-22.4 (-32--11.5)	18 (14-24)	16 (12-21)	-10.3 (-21.5-1.6)	0 (0-0)	0 (0-0)	-69 (-73.6--64.2)	1 (1-1)	1 (1-1)	-9.2 (-20.7-3.2)	0 (0-0)	0 (0-0)	-77.4 (-83.7--71.1)	0 (0-0)	0 (0-0)	-18.9 (-31.8--5.4)	0 (0-0)	0.1 (0.1-0.2)	0 (0-0)	0.2 (0.2-0.3)	106	108	58	44
Akita	0 (0-0)	0 (0-0)	-49.2 (-50.1--48.3)	0 (0-1)	0 (0-0)	-42.9 (-47.7--37.8)	0 (0-0)	0 (0-0)	-68.5 (-73.6--60.3)	0 (0-0)	0 (0-0)	-42.8 (-48.8--40.4)	0 (0-0)	0 (0-0)	-84.7 (-89--79)	0 (0-0)	0 (0-0)	-49 (-53.1--46)	0 (0-0)	0 (0-0)	0 (0-0)	0 (0-0.1)	107	112	92	78
Aomori	0 (0-0)	0 (0-0)	-44.4 (-44.6--43.7)	0 (0-1)	0 (0-0)	-38.6 (-43.2--34.5)	0 (0-0)	0 (0-0)	-70.6 (-75.4--63.5)	0 (0-0)	0 (0-0)	-37.9 (-40.6--36.4)	0 (0-0)	0 (0-0)	-83.4 (-87.8--77.6)	0 (0-0)	0 (0-0)	-43.8 (-46.7--40.6)	0 (0-0)	0 (0-0)	0 (0-0)	0 (0-0.1)	105	106	92	78
Chiba	0 (0-0)	0 (0-0)	-27.8 (-32.8--15.3)	5 (3-7)	4 (3-6)	-17.8 (-25.4--5.4)	0 (0-0)	0 (0-0)	-68.7 (-72.5--63.7)	0 (0-0)	0 (0-0)	-15.1 (-18.9-4.6)	0 (0-0)	0 (0-0)	-77.4 (-81.6--72.4)	0 (0-0)	0 (0-0)	-23.6 (-31.4--6.7)	0 (0-0)	0 (0-0.1)	0 (0-0)	0.1 (0-0.1)	106	107	74	64
Ehime	0 (0-0)	0 (0-0)	-38.4 (-46--36.7)	0 (0-1)	0 (0-1)	-29.2 (-41--24.8)	0 (0-0)	0 (0-0)	-71.2 (-74.7--66.5)	0 (0-0)	0 (0-0)	-29.2 (-39.1--26.9)	0 (0-0)	0 (0-0)	-82.8 (-87.3--77.3)	0 (0-0)	0 (0-0)	-35.2 (-40.2--31.9)	0 (0-0)	0 (0-0)	0 (0-0)	0 (0-0.1)	106	108	94	81
Fukui	0 (0-0)	0 (0-0)	-34.1 (-48.7--29)	0 (0-0)	0 (0-0)	-24.8 (-44.5--16.5)	0 (0-0)	0 (0-0)	-67.6 (-73.1--61.3)	0 (0-0)	0 (0-0)	-25.9 (-29.6--23.6)	0 (0-0)	0 (0-0)	-79.6 (-86--72.2)	0 (0-0)	0 (0-0)	-34.1 (-39--30.3)	0 (0-0)	0 (0-0)	0 (0-0)	0 (0-0.1)	105	109	87	84
Fukuoka	0 (0-0)	0 (0-0)	-17.1 (-19.3--12.6)	1 (0-2)	1 (0-2)	-13.8 (-20.1--5.6)	0 (0-0)	0 (0-0)	-70.2 (-74.9--64.9)	0 (0-0)	0 (0-0)	-11 (-15.3--10)	0 (0-0)	0 (0-0)	-74.2 (-80.7--65.7)	0 (0-0)	0 (0-0)	-12.4 (-18.4--8.2)	0 (0-0)	0 (0-0)	0 (0-0)	0 (0-0)	108	109	93	84
Fukushima	0 (0-0)	0 (0-0)	-44.3 (-46.2--41.1)	0 (0-1)	0 (0-1)	-37.1 (-42.1--31.3)	0 (0-0)	0 (0-0)	-69 (-73.9--61.7)	0 (0-0)	0 (0-0)	-36.3 (-40.2--33.5)	0 (0-0)	0 (0-0)	-82.1 (-87--76.5)	0 (0-0)	0 (0-0)	-42.3 (-45.3--39)	0 (0-0)	0 (0-0)	0 (0-0)	0 (0-0.1)	108	110	92	84
Gifu	0 (0-0)	0 (0-0)	-37.5 (-42.4--33.1)	0 (0-1)	0 (0-1)	-25.4 (-33.3--17.5)	0 (0-0)	0 (0-0)	-71.2 (-76.6--66.1)	0 (0-0)	0 (0-0)	-26.8 (-32.7--23.5)	0 (0-0)	0 (0-0)	-82.3 (-86.4--76.3)	0 (0-0)	0 (0-0)	-34.6 (-38.6--30.1)	0 (0-0)	0 (0-0)	0 (0-0)	0 (0-0.1)	106	107	90	80
Gunma	0 (0-0)	0 (0-0)	-39.3 (-39.2--34.9)	0 (0-1)	0 (0-1)	-25.4 (-31.1--18.1)	0 (0-0)	0 (0-0)	-68.4 (-73.4--62.8)	0 (0-0)	0 (0-0)	-26.9 (-31.5--23.5)	0 (0-0)	0 (0-0)	-84 (-88.7--79.3)	0 (0-0)	0 (0-0)	-36.5 (-40.2--32.5)	0 (0-0)	0 (0-0)	0 (0-0)	0 (0-0.1)	108	109	94	81
Hiroshima	0 (0-0)	0 (0-0)	-28.2 (-29.2--26.1)	1 (0-1)	1 (0-1)	-17.7 (-22.7--12.2)	0 (0-0)	0 (0-0)	-70.7 (-74.9--64.9)	0 (0-0)	0 (0-0)	-17.9 (-21.5--15.7)	0 (0-0)	0 (0-0)	-77.7 (-84.6--69.3)	0 (0-0)	0 (0-0)	-24.2 (-28.8--19.9)	0 (0-0)	0 (0-0)	0 (0-0)	0 (0-0.1)	105	105	88	79
Hokkaidō	0 (0-0)	0 (0-0)	-39.9 (-44.2--35)	1 (0-3)	1 (0-2)	-32.4 (-37.7--25.1)	0 (0-0)	0 (0-0)	-69.6 (-76--62.4)	0 (0-0)	0 (0-0)	-31.1 (-61--22.9)	0 (0-0)	0 (0-0)	-78 (-84.1--71.2)	0 (0-0)	0 (0-0)	-36.2 (-60.9--28.8)	0 (0-0)	0 (0-0)	0 (0-0)	0 (0-0.1)	105	109	92	87
Hyōgo	0 (0-0)	0 (0-0)	-30.6 (-41.8--28.4)	2 (1-4)	2 (1-3)	-22.1 (-36.5--10.1)	0 (0-0)	0 (0-0)	-69.6 (-74.5--63.4)	0 (0-0)	0 (0-0)	-21.3 (-43.7--17.5)	0 (0-0)	0 (0-0)	-81 (-86.2--74.6)	0 (0-0)	0 (0-0)	-28.9 (-50.5--23)	0 (0-0)	0 (0-0)	0 (0-0)	0.1 (0-0.1)	106	107	82	73
Ibaraki	0 (0-0)	0 (0-0)	-38.4 (-55.5--36)	2 (2-4)	2 (1-3)	-26.8 (-41.2--17)	0 (0-0)	0 (0-0)	-68 (-72.8--61.4)	0 (0-0)	0 (0-0)	-26.9 (-48.8--22.1)	0 (0-0)	0 (0-0)	-79.3 (-84.7--71.4)	0 (0-0)	0 (0-0)	-35.5 (-57.9--27.9)	0 (0-0)	0 (0-0.1)	0 (0-0)	0.1 (0-0.1)	106	107	72	64
Ishikawa	0 (0-0)	0 (0-0)	-31.7 (-33.3--29)	0 (0-1)	0 (0-0)	-22.1 (-28.1--14.5)	0 (0-0)	0 (0-0)	-71.5 (-75.9--66.9)	0 (0-0)	0 (0-0)	-22.5 (-25.3--16.5)	0 (0-0)	0 (0-0)	-83.8 (-88.3--74.7)	0 (0-0)	0 (0-0)	-29.5 (-34--23.4)	0 (0-0)	0 (0-0)	0 (0-0)	0 (0-0.1)	104	108	88	79
Iwate	0 (0-0)	0 (0-0)	-43.2 (-45.4--41.1)	0 (0-1)	0 (0-0)	-33.3 (-38.1--29.2)	0 (0-0)	0 (0-0)	-73 (-76.8--67)	0 (0-0)	0 (0-0)	-33.2 (-35.9--31.2)	0 (0-0)	0 (0-0)	-82.7 (-87.2--77.6)	0 (0-0)	0 (0-0)	-40 (-43.6--36)	0 (0-0)	0 (0-0)	0 (0-0)	0 (0-0.1)	107	108	90	77
Kagawa	0 (0-0)	0 (0-0)	-36.7 (-64.9--24.6)	0 (0-1)	0 (0-0)	-25.2 (-58.1--5.7)	0 (0-0)	0 (0-0)	-70.4 (-74.4--65.5)	0 (0-0)	0 (0-0)	-26.3 (-30--22.3)	0 (0-0)	0 (0-0)	-78.1 (-84.2--71.1)	0 (0-0)	0 (0-0)	-33.8 (-40.4--28.6)	0 (0-0)	0 (0-0)	0 (0-0)	0 (0-0.1)	105	105	90	80

Location	Births, 2000	Births, 2021	Births percent change (2000-2021)	All age cases, 2000	All age cases, 2021	All age cases percent change (2000-2021)	All age deaths (cause-specific), 2000	All age deaths (cause-specific), 2021	All age deaths (cause-specific) percent change (2000-2021)
Kagoshima	0 (0-0)	0 (0-0)	-29.5 (-31.4--27.4)	0 (0-1)	0 (0-1)	-29.1 (-33.4--23.6)	0 (0-0)	0 (0-0)	-67.9 (-72.2--64)
Kanagawa	0 (0-0)	0 (0-0)	-27.6 (-36.5--23.2)	3 (1-7)	3 (1-6)	-15.2 (-23.2--6.8)	0 (0-0)	0 (0-0)	-67.6 (-72.5--61.4)
Kōchi	0 (0-0)	0 (0-0)	-39.2 (-44--13.5)	0 (0-0)	0 (0-0)	-32.4 (-39.6-1.1)	0 (0-0)	0 (0-0)	-70.4 (-74.5--65.4)
Kumamoto	0 (0-0)	0 (0-0)	-27.7 (-39.1--23.6)	0 (0-1)	0 (0-1)	-24.7 (-38.5--16.9)	0 (0-0)	0 (0-0)	-69.5 (-74.7--64)
Kyōto	0 (0-0)	0 (0-0)	-25.9 (-28.4--17.3)	1 (0-1)	0 (0-1)	-19 (-26.3--4.1)	0 (0-0)	0 (0-0)	-70.2 (-75--62.8)
Mie	0 (0-0)	0 (0-0)	-35.8 (-41.6--24.1)	0 (0-1)	0 (0-1)	-24.1 (-32.3--10.6)	0 (0-0)	0 (0-0)	-71 (-77.2--64.8)
Miyagi	0 (0-0)	0 (0-0)	-33 (-50--23.8)	1 (0-1)	0 (0-1)	-23.8 (-48.6--14.4)	0 (0-0)	0 (0-0)	-68.5 (-74.1--62.2)
Miyazaki	0 (0-0)	0 (0-0)	-34.5 (-37.2--29.4)	0 (0-1)	0 (0-0)	-27.3 (-33.4--20.8)	0 (0-0)	0 (0-0)	-67.4 (-72.2--62.1)
Nagano	0 (0-0)	0 (0-0)	-39.8 (-46.3--37.8)	0 (0-1)	0 (0-1)	-27.3 (-36.9--20)	0 (0-0)	0 (0-0)	-70.8 (-75.2--65.8)
Nagasaki	0 (0-0)	0 (0-0)	-35.5 (-49.3--32.3)	0 (0-1)	0 (0-1)	-32.7 (-50.9--23.6)	0 (0-0)	0 (0-0)	-71.2 (-76.6--65.1)
Nara	0 (0-0)	0 (0-0)	-38.4 (-39.4--35.7)	0 (0-1)	0 (0-1)	-28.9 (-34.2--23.5)	0 (0-0)	0 (0-0)	-69 (-74.3--63.3)
Niigata	0 (0-0)	0 (0-0)	-37.1 (-38.9--35)	1 (0-1)	0 (0-1)	-28.2 (-33.1--20.2)	0 (0-0)	0 (0-0)	-70.2 (-75.2--63.5)
Ōita	0 (0-0)	0 (0-0)	-31.4 (-49.9--23.1)	0 (0-1)	0 (0-0)	-25.7 (-48.9--14.6)	0 (0-0)	0 (0-0)	-70.3 (-75.2--65.2)
Okayama	0 (0-0)	0 (0-0)	-28.2 (-30.4--26.7)	0 (0-1)	0 (0-1)	-19.5 (-23.8--13.5)	0 (0-0)	0 (0-0)	-69.8 (-75--63.5)
Okinawa	0 (0-0)	0 (0-0)	-12.5 (-17.3--9.6)	1 (0-1)	1 (0-1)	-9.1 (-19.9--0.2)	0 (0-0)	0 (0-0)	-64.7 (-70.4--57.5)
Ōsaka	0 (0-0)	0 (0-0)	-30.6 (-31.8--28.9)	3 (1-6)	2 (1-4)	-20.3 (-26.8--10.7)	0 (0-0)	0 (0-0)	-69.7 (-73.5--64.5)
Saga	0 (0-0)	0 (0-0)	-31.9 (-32.3--31.1)	0 (0-0)	0 (0-0)	-25.1 (-30.9--20.1)	0 (0-0)	0 (0-0)	-69.5 (-74.4--61.6)
Saitama	0 (0-0)	0 (0-0)	-28.3 (-28.6--24.5)	2 (0-4)	1 (0-3)	-18.6 (-24.4--11.2)	0 (0-0)	0 (0-0)	-68.5 (-73.3--64)
Shiga	0 (0-0)	0 (0-0)	-28.3 (-39.7--23.4)	0 (0-1)	0 (0-1)	-15.6 (-28.9--5)	0 (0-0)	0 (0-0)	-66.9 (-72.2--61)
Shimane	0 (0-0)	0 (0-0)	-29.3 (-34.6--7)	0 (0-0)	0 (0-0)	-24.4 (-31.9-4.1)	0 (0-0)	0 (0-0)	-73.8 (-76.8--68.8)
Shizuoka	0 (0-0)	0 (0-0)	-35 (-41.2--32.3)	1 (0-2)	1 (0-1)	-25.3 (-33.6--19.7)	0 (0-0)	0 (0-0)	-68.3 (-73.5--63.1)
Tochigi	0 (0-0)	0 (0-0)	-33.9 (-34.5--33.5)	0 (0-1)	0 (0-1)	-23 (-27.5--18.2)	0 (0-0)	0 (0-0)	-67.8 (-74.2--60.4)
Tokushima	0 (0-0)	0 (0-0)	-36.7 (-57.5--29.5)	0 (0-0)	0 (0-0)	-31.7 (-53.8--20)	0 (0-0)	0 (0-0)	-68.5 (-74--62.3)
Tōkyō	0 (0-0)	0 (0-0)	5.7 (-18.5-10.3)	8 (4-13)	8 (4-13)	-0.1 (-21.6-10.2)	0 (0-0)	0 (0-0)	-67.3 (-72.6--60.2)
Tottori	0 (0-0)	0 (0-0)	-30.7 (-31.9--29.9)	0 (0-0)	0 (0-0)	-25.3 (-30.1--20.9)	0 (0-0)	0 (0-0)	-70.9 (-76.6--64.2)
Toyama	0 (0-0)	0 (0-0)	-38.4 (-39.3--34.4)	0 (0-1)	0 (0-0)	-25.3 (-33.2--17.6)	0 (0-0)	0 (0-0)	-68.9 (-73.8--63.3)
Wakayama	0 (0-0)	0 (0-0)	-41.1 (-43--40.5)	0 (0-1)	0 (0-0)	-32.4 (-36.5--27.4)	0 (0-0)	0 (0-0)	-72.7 (-76.2--68.2)
Yamagata	0 (0-0)	0 (0-0)	-41 (-42.1--37.1)	0 (0-1)	0 (0-0)	-32.3 (-40--25.1)	0 (0-0)	0 (0-0)	-66.9 (-72.1--62.5)
Yamaguchi	0 (0-0)	0 (0-0)	-37.3 (-59.2--9.4)	0 (0-1)	0 (0-1)	-29.1 (-57.2-5.8)	0 (0-0)	0 (0-0)	-71.7 (-77.6--65.4)

Location	All age deaths (total), 2000	All age deaths (total), 2021	All age deaths (total) percent change (2000-2021)	Under-5 deaths (cause-specific), 2000	Under-5 deaths (cause-specific), 2021	Under-5 deaths (cause-specific) percent change (2000-2021)	Under-5 deaths (total), 2000	Under-5 deaths (total), 2021	Under-5 deaths (total) percent change (2000-2021)
Kagoshima	0 (0-0)	0 (0-0)	-26.2 (-27.4--24.2)	0 (0-0)	0 (0-0)	-81 (-85.4--75.1)	0 (0-0)	0 (0-0)	-26.5 (-33.2--21.7)
Kanagawa	0 (0-0)	0 (0-0)	-12.7 (-31.3--8.2)	0 (0-0)	0 (0-0)	-81.7 (-85.3--76.7)	0 (0-0)	0 (0-0)	-21.1 (-41.4--12.5)
Kōchi	0 (0-0)	0 (0-0)	-31.9 (-34.8--29.6)	0 (0-0)	0 (0-0)	-79.8 (-84.6--70.9)	0 (0-0)	0 (0-0)	-36.1 (-39.4--31.7)
Kumamoto	0 (0-0)	0 (0-0)	-21.9 (-50.5--18)	0 (0-0)	0 (0-0)	-76.9 (-83.4--67.2)	0 (0-0)	0 (0-0)	-22.6 (-51.8--15.8)
Kyōto	0 (0-0)	0 (0-0)	-18.2 (-32--3.9)	0 (0-0)	0 (0-0)	-80.9 (-86.8--74.6)	0 (0-0)	0 (0-0)	-24.1 (-37.9--12.1)
Mie	0 (0-0)	0 (0-0)	-24.7 (-28.4--21.1)	0 (0-0)	0 (0-0)	-82.3 (-87.6--76.7)	0 (0-0)	0 (0-0)	-33.1 (-37--25.7)
Miyagi	0 (0-0)	0 (0-0)	-22 (-24.8--18.9)	0 (0-0)	0 (0-0)	-78.5 (-84.1--72.2)	0 (0-0)	0 (0-0)	-28.3 (-31.7--24.8)
Miyazaki	0 (0-0)	0 (0-0)	-27 (-28.9--25.2)	0 (0-0)	0 (0-0)	-77.3 (-83.5--68.6)	0 (0-0)	0 (0-0)	-31.2 (-36--24.1)
Nagano	0 (0-0)	0 (0-0)	-28.6 (-33.5--25.9)	0 (0-0)	0 (0-0)	-80.2 (-84.5--73.1)	0 (0-0)	0 (0-0)	-36.9 (-45--32.6)
Nagasaki	0 (0-0)	0 (0-0)	-31.1 (-45.6--28.4)	0 (0-0)	0 (0-0)	-79.7 (-85.7--72.6)	0 (0-0)	0 (0-0)	-34.4 (-46.1--29.6)
Nara	0 (0-0)	0 (0-0)	-28.7 (-32.1--26.8)	0 (0-0)	0 (0-0)	-77.8 (-84--69)	0 (0-0)	0 (0-0)	-34.8 (-39.3--31.5)
Niigata	0 (0-0)	0 (0-0)	-28.4 (-32.2--13.3)	0 (0-0)	0 (0-0)	-84.8 (-88.2--80.9)	0 (0-0)	0 (0-0)	-35.3 (-40.6--15.8)
Ōita	0 (0-0)	0 (0-0)	-24.8 (-32.8--21.7)	0 (0-0)	0 (0-0)	-80.2 (-85.7--72.9)	0 (0-0)	0 (0-0)	-28.2 (-37.7--23)
Okayama	0 (0-0)	0 (0-0)	-20.1 (-22.2--17.5)	0 (0-0)	0 (0-0)	-81.1 (-85.6--74.5)	0 (0-0)	0 (0-0)	-25.4 (-29.4--20.4)
Okinawa	0 (0-0)	0 (0-0)	-5.6 (-19.5--2)	0 (0-0)	0 (0-0)	-75.9 (-82.3--67.5)	0 (0-0)	0 (0-0)	-7.5 (-24.1-3.1)
Ōsaka	0 (0-0)	0 (0-0)	-20 (-23--16.8)	0 (0-0)	0 (0-0)	-79.9 (-85.3--72.8)	0 (0-0)	0 (0-0)	-27.8 (-31.7--22.6)
Saga	0 (0-0)	0 (0-0)	-24.4 (-26.2--23)	0 (0-0)	0 (0-0)	-82.8 (-88.1--76.7)	0 (0-0)	0 (0-0)	-29.5 (-33.6--26)
Saitama	0 (0-0)	0 (0-0)	-17.4 (-21.2--13.9)	0 (0-0)	0 (0-0)	-83.2 (-87.2--77.3)	0 (0-0)	0 (0-0)	-23.9 (-28.6--17.8)
Shiga	0 (0-0)	0 (0-0)	-16.2 (-50.4--11.4)	0 (0-0)	0 (0-0)	-81.3 (-86.9--73.3)	0 (0-0)	0 (0-0)	-24.2 (-53.5--17.9)
Shimane	0 (0-0)	0 (0-0)	-23.5 (-27.2--5.4)	0 (0-0)	0 (0-0)	-81.7 (-87.2--74.1)	0 (0-0)	0 (0-0)	-26 (-31.1-0.3)
Shizuoka	0 (0-0)	0 (0-0)	-25 (-27.4--21.9)	0 (0-0)	0 (0-0)	-78.1 (-82.7--70.7)	0 (0-0)	0 (0-0)	-31.4 (-36.5--27.1)
Tochigi	0 (0-0)	0 (0-0)	-22.6 (-25.5--20.2)	0 (0-0)	0 (0-0)	-81.7 (-86.5--75.4)	0 (0-0)	0 (0-0)	-28.7 (-31.9--24.8)
Tokushima	0 (0-0)	0 (0-0)	-30.4 (-45--27)	0 (0-0)	0 (0-0)	-81.4 (-87.2--73.3)	0 (0-0)	0 (0-0)	-33.4 (-42.5--28)
Tōkyō	0 (0-1)	0 (0-1)	8.5 (-16.4-22.1)	0 (0-0)	0 (0-0)	-77.4 (-83.7--69.5)	0 (0-0)	0 (0-0)	11 (-19.8-23.5)
Tottori	0 (0-0)	0 (0-0)	-24.1 (-26.2--22.7)	0 (0-0)	0 (0-0)	-75.8 (-82.4--65.2)	0 (0-0)	0 (0-0)	-26.9 (-31.2--22.5)
Toyama	0 (0-0)	0 (0-0)	-26 (-29.6--23.5)	0 (0-0)	0 (0-0)	-83.2 (-88.9--78.4)	0 (0-0)	0 (0-0)	-32.8 (-36.4--28.8)
Wakayama	0 (0-0)	0 (0-0)	-32.4 (-34.5--30.1)	0 (0-0)	0 (0-0)	-80.1 (-86.6--74)	0 (0-0)	0 (0-0)	-38.9 (-41.9--34.6)
Yamagata	0 (0-0)	0 (0-0)	-32.8 (-48.3--17.2)	0 (0-0)	0 (0-0)	-80.9 (-85.8--75.5)	0 (0-0)	0 (0-0)	-39.2 (-56.8--18.4)
Yamaguchi	0 (0-0)	0 (0-0)	-29 (-42.1--26.3)	0 (0-0)	0 (0-0)	-78.9 (-85.2--71.6)	0 (0-0)	0 (0-0)	-33.4 (-41.3--27.2)

Location	Under-5 cause-specific mortality cause fraction, 2000	Under-5 total SCD mortality cause fraction, 2000	Under-5 cause-specific mortality cause fraction, 2021	Under-5 total SCD mortality cause fraction, 2021	Under-5 cause-specific mortality rank 2000	Under-5 cause-specific mortality rank 2021	Under-5 total SCD mortality rank 2000	Under-5 total SCD mortality rank 2021
Kagoshima	0 (0-0)	0 (0-0)	0 (0-0)	0 (0-0.1)	109	109	95	83
Kanagawa	0 (0-0)	0 (0-0)	0 (0-0)	0 (0-0.1)	108	112	87	74
Kōchi	0 (0-0)	0 (0-0)	0 (0-0)	0 (0-0.1)	106	109	88	81
Kumamoto	0 (0-0)	0 (0-0)	0 (0-0)	0 (0-0.1)	104	108	95	84
Kyōto	0 (0-0)	0 (0-0)	0 (0-0)	0 (0-0.1)	105	110	92	79
Mie	0 (0-0)	0 (0-0)	0 (0-0)	0 (0-0.1)	108	109	94	80
Miyagi	0 (0-0)	0 (0-0)	0 (0-0)	0 (0-0.1)	107	106	89	79
Miyazaki	0 (0-0)	0 (0-0)	0 (0-0)	0 (0-0.1)	109	112	92	85
Nagano	0 (0-0)	0 (0-0)	0 (0-0)	0 (0-0.1)	105	108	92	78
Nagasaki	0 (0-0)	0 (0-0)	0 (0-0)	0 (0-0.1)	110	114	94	83
Nara	0 (0-0)	0 (0-0)	0 (0-0)	0 (0-0.1)	102	104	90	83
Niigata	0 (0-0)	0 (0-0)	0 (0-0)	0 (0-0.1)	107	110	91	76
Ōita	0 (0-0)	0 (0-0)	0 (0-0)	0 (0-0.1)	108	109	93	81
Okayama	0 (0-0)	0 (0-0)	0 (0-0)	0 (0-0.1)	107	106	92	78
Okinawa	0 (0-0)	0 (0-0)	0 (0-0)	0.1 (0-0.1)	110	114	84	75
Ōsaka	0 (0-0)	0 (0-0)	0 (0-0)	0 (0-0.1)	106	107	87	80
Saga	0 (0-0)	0 (0-0)	0 (0-0)	0 (0-0.1)	110	108	95	81
Saitama	0 (0-0)	0 (0-0)	0 (0-0)	0 (0-0.1)	105	107	92	79
Shiga	0 (0-0)	0 (0-0)	0 (0-0)	0 (0-0.1)	105	107	91	78
Shimane	0 (0-0)	0 (0-0)	0 (0-0)	0 (0-0.1)	104	106	90	78
Shizuoka	0 (0-0)	0 (0-0)	0 (0-0)	0 (0-0)	105	108	90	84
Tochigi	0 (0-0)	0 (0-0)	0 (0-0)	0 (0-0.1)	107	108	92	80
Tokushima	0 (0-0)	0 (0-0)	0 (0-0)	0 (0-0.1)	107	110	93	83
Tōkyō	0 (0-0)	0 (0-0)	0 (0-0)	0.1 (0-0.1)	110	112	76	65
Tottori	0 (0-0)	0 (0-0)	0 (0-0)	0 (0-0.1)	107	109	89	81
Toyama	0 (0-0)	0 (0-0)	0 (0-0)	0 (0-0.1)	105	107	87	79
Wakayama	0 (0-0)	0 (0-0)	0 (0-0)	0 (0-0.1)	106	106	91	81
Yamagata	0 (0-0)	0 (0-0)	0 (0-0)	0 (0-0.1)	107	108	90	76
Yamaguchi	0 (0-0)	0 (0-0)	0 (0-0)	0 (0-0.1)	107	111	94	84

Location	Births, 2000	Births, 2021	Births percent change (2000-2021)	All age cases, 2000	All age cases, 2021	All age cases percent change (2000-2021)	All age deaths (cause-specific), 2000	All age deaths (cause-specific), 2021	All age deaths (cause-specific) percent change (2000-2021)	All age deaths (total), 2000	All age deaths (total), 2021	All age deaths (total) percent change (2000-2021)	Under-5 deaths (cause-specific), 2000	Under-5 deaths (cause-specific), 2021	Under-5 deaths (cause-specific) percent change (2000-2021)	Under-5 deaths (total), 2000	Under-5 deaths (total), 2021	Under-5 deaths (total) percent change (2000-2021)	Under-5 cause-specific mortality cause fraction, 2000	Under-5 total SCD mortality cause fraction, 2000	Under-5 cause-specific mortality cause fraction, 2021	Under-5 total SCD mortality cause fraction, 2021	Under-5 cause-specific mortality rank 2000	Under-5 cause-specific mortality rank 2021	Under-5 total SCD mortality rank 2000	Under-5 total SCD mortality rank 2021
Yamanashi	0 (0-0)	0 (0-0)	-40 (-42.3--38.3)	0 (0-0)	0 (0-0)	-29.9 (-34.4--24.4)	0 (0-0)	0 (0-0)	-67.4 (-72.7--59.3)	0 (0-0)	0 (0-0)	-31.2 (-40.6--28.3)	0 (0-0)	0 (0-0)	-85.4 (-89.5--80.5)	0 (0-0)	0 (0-0)	-40.9 (-46.3--37)	0 (0-0)	0 (0-0)	0 (0-0)	0 (0-0.1)	104	104	90	77
Republic of Korea	3 (2-4)	2 (1-3)	-30.6 (-38.8--28.6)	46 (33-60)	38 (26-50)	-18.8 (-29.9--11.6)	1 (1-2)	0 (0-1)	-63 (-78.2--36.6)	3 (2-4)	3 (2-3)	-21 (-31.5--9.6)	0 (0-1)	0 (0-0)	-91.7 (-98.6--58.9)	2 (1-3)	1 (1-2)	-33.8 (-43.3--24.3)	0 (0-0)	0 (0-0.1)	0 (0-0)	0.3 (0.2-0.4)	103	99	81	39
Singapore	0 (0-0)	0 (0-0)	-7.6 (-14.7--0.9)	5 (3-6)	5 (4-7)	10.2 (-2.1-20.1)	0 (0-0)	0 (0-0)	-65.7 (-73.2--55.9)	0 (0-0)	0 (0-0)	21.8 (8.2-36.4)	0 (0-0)	0 (0-0)	-74.5 (-85.4--60.8)	0 (0-0)	0 (0-0)	19.8 (6.7-38.2)	0 (0-0)	0.1 (0-0.1)	0 (0-0)	0.1 (0.1-0.2)	116	116	74	50
**High-income North America**	1,480 (1,380-1,580)	1,260 (1,170-1,340)	-14.9 (-16--13.3)	54,500 (50,800-57,900)	59,400 (56,200-63,400)	8.9 (6.9-10.6)	546 (535-558)	517 (500-535)	-5.3 (-9.2--1.7)	1,290 (1,190-1,390)	1,300 (1,210-1,400)	1.2 (-1.4-4)	29 (28-30)	16 (14-17)	-45.4 (-51.8--40.5)	277 (230-334)	152 (124-184)	-45 (-48.6--41.7)	0.1 (0.1-0.1)	0.8 (0.7-0.9)	0.1 (0.1-0.1)	0.6 (0.5-0.8)	57	63	22	23
Canada	6 (4-7)	3 (2-4)	-45.6 (-59.9--26.4)	146 (119-176)	130 (96-166)	-10.9 (-37.9-13.5)	14 (13-15)	13 (12-14)	-7.5 (-16.2-5.3)	6 (5-8)	4 (3-5)	-42.2 (-58.8--20.6)	1 (1-1)	0 (0-0)	-46.4 (-60--32.1)	3 (2-5)	1 (1-1)	-75.3 (-83.6--60.6)	0 (0-0)	0.2 (0.1-0.2)	0 (0-0)	0.1 (0-0.1)	73	77	45	62
Greenland	0 (0-0)	0 (0-0)	-16.9 (-25.5--6.3)	0 (0-0)	0 (0-0)	-13.6 (-22.1-8)	0 (0-0)	0 (0-0)	-30.5 (-60.3-23.8)	0 (0-0)	0 (0-0)	-12.1 (-21.4-1)	0 (0-0)	0 (0-0)	-58.1 (-88.6-21.8)	0 (0-0)	0 (0-0)	-17.4 (-28.7--1.9)	0 (0-0)	0.1 (0-0.1)	0 (0-0)	0.1 (0.1-0.2)	103	104	63	48
United States of America	1,470 (1,370-1,560)	1,220 (1,130-1,290)	-17.3 (-18.4--15.9)	54,300 (50,600-57,600)	59,800 (56,600-63,900)	10.3 (8.2-12)	533 (521-544)	505 (486-522)	-5.3 (-9.2--1.7)	1,280 (1,180-1,380)	1,310 (1,220-1,410)	2.6 (0.1-5.4)	28 (27-30)	16 (14-17)	-45.4 (-51.6--40.7)	273 (226-328)	153 (125-186)	-43.8 (-47.5--40.4)	0.1 (0.1-0.1)	0.8 (0.7-1)	0.1 (0.1-0.1)	0.7 (0.5-0.9)	57	63	22	23
Alabama	48 (44-52)	40 (37-43)	-16.7 (-20.5--12.9)	1,650 (1,530-1,780)	1,760 (1,640-1,930)	6.6 (0-13.1)	18 (17-19)	17 (14-20)	-8 (-24.6-8.1)	43 (40-47)	42 (38-45)	-4.2 (-9.2-1.2)	1 (1-1)	1 (1-1)	-35.1 (-45.4--25.3)	11 (9-13)	6 (5-8)	-44 (-51.4--35.9)	0.1 (0.1-0.1)	1.6 (1.3-1.9)	0.1 (0.1-0.1)	1.3 (0.9-1.7)	51	54	14	18
Alaska	1 (1-1)	1 (1-1)	-22.8 (-28.9--16.8)	40 (29-53)	40 (29-53)	2.1 (-4.6-9.3)	0 (0-0)	0 (0-0)	4.8 (-13.8-24.1)	1 (1-1)	1 (1-1)	-1.8 (-10.6-6.9)	0 (0-0)	0 (0-0)	-40.6 (-55.2--9.4)	0 (0-0)	0 (0-0)	-32.1 (-47.7--19.6)	0 (0-0)	0.1 (0.1-0.2)	0 (0-0)	0.1 (0.1-0.2)	85	87	55	56
Arizona	15 (14-16)	14 (13-15)	-6.2 (-11.4--0.2)	496 (453-536)	700 (636-751)	41.1 (33.3-49.3)	5 (5-6)	6 (5-7)	16.7 (-2.9-39.5)	11 (10-12)	16 (14-17)	39.6 (32.6-47.4)	0 (0-0)	0 (0-0)	-43.7 (-56.8--31.5)	2 (2-3)	2 (2-2)	-19.3 (-27.9--9.8)	0 (0-0.1)	0.3 (0.3-0.4)	0 (0-0)	0.4 (0.3-0.5)	71	75	35	35
Arkansas	19 (17-20)	14 (12-15)	-27.4 (-31.2--23.2)	633 (573-683)	641 (581-703)	1.2 (-5.2-6.9)	8 (7-8)	8 (7-10)	4.7 (-13.3-24.4)	17 (15-19)	14 (13-16)	-16 (-21.2--11.2)	0 (0-0)	0 (0-0)	-39.1 (-52.3--23.1)	5 (4-6)	2 (1-2)	-60 (-65.3--54.2)	0.1 (0.1-0.1)	1.2 (0.9-1.5)	0.1 (0.1-0.1)	0.7 (0.4-0.9)	55	58	18	25
California	84 (78-90)	62 (58-66)	-26.5 (-29.6--21.7)	3,140 (2,920-3,370)	3,350 (3,130-3,580)	6.8 (2.6-11.6)	40 (38-42)	35 (29-39)	-12.7 (-29.4-1)	64 (59-70)	68 (63-73)	5.4 (0.5-11.5)	2 (2-3)	1 (1-1)	-63.4 (-70.3--56)	13 (11-16)	7 (6-9)	-45.6 (-52--38.1)	0.1 (0.1-0.1)	0.4 (0.3-0.4)	0 (0-0.1)	0.4 (0.3-0.5)	60	71	33	30
Colorado	7 (7-8)	6 (5-7)	-16.1 (-20.7--11.9)	256 (226-286)	287 (245-330)	12 (6-18.2)	3 (3-4)	4 (3-4)	9.1 (-10.4-30.6)	6 (5-7)	7 (6-8)	5.4 (-1.4-12.5)	0 (0-0)	0 (0-0)	-41.4 (-55.7--27.2)	1 (1-2)	1 (1-1)	-42.1 (-49.5--34)	0 (0-0)	0.3 (0.2-0.4)	0 (0-0)	0.2 (0.2-0.3)	73	76	37	40
Connecticut	16 (15-17)	11 (10-12)	-33 (-36.3--28.9)	645 (590-685)	615 (565-666)	-4.7 (-9-0.9)	5 (5-5)	4 (3-5)	-20 (-35.3--7.9)	16 (14-17)	14 (13-16)	-9.2 (-13.6--3.8)	0 (0-0)	0 (0-0)	-58.5 (-67.8--46.9)	3 (2-4)	1 (1-2)	-54.1 (-60.3--47.4)	0.1 (0.1-0.1)	1 (0.8-1.2)	0.1 (0-0.1)	1 (0.7-1.3)	56	66	17	17
Delaware	9 (9-10)	9 (8-10)	-5.4 (-10.4--1.2)	333 (304-357)	402 (370-431)	20.5 (15.1-26.9)	2 (2-2)	2 (1-2)	1.1 (-13.3-18.5)	8 (8-9)	10 (9-11)	16.6 (10.7-23.2)	0 (0-0)	0 (0-0)	-21.6 (-39.8-4)	2 (2-2)	1 (1-2)	-25.7 (-35.7--15.8)	0.1 (0.1-0.1)	1.7 (1.3-2.1)	0.1 (0.1-0.1)	1.7 (1.2-2.2)	52	56	11	13
District of Columbia	12 (10-13)	11 (9-12)	-7.6 (-14--0.1)	346 (302-390)	362 (310-411)	4.7 (-1.8-12.8)	4 (3-4)	2 (2-3)	-36.8 (-44.4--28.3)	11 (9-12)	10 (8-11)	-8.7 (-16.1--1.6)	0 (0-0)	0 (0-0)	-49 (-63--30.6)	3 (2-4)	2 (1-2)	-43.6 (-52.9--33.8)	0.2 (0.1-0.2)	2.5 (1.9-3.1)	0.1 (0.1-0.2)	2.4 (1.5-3.3)	45	49	11	11
Florida	142 (133-150)	134 (126-143)	-5.8 (-9.2--1)	5,540 (5,180-5,930)	7,070 (6,650-7,540)	27.6 (22.2-34.4)	38 (37-41)	47 (41-54)	21.3 (1.3-42.8)	133 (123-144)	160 (149-171)	20.8 (15.1-26.6)	2 (2-2)	1 (1-1)	-44.5 (-53.3--35.8)	23 (20-28)	16 (14-20)	-29.1 (-37--20.4)	0.1 (0.1-0.1)	1.3 (1.1-1.5)	0.1 (0.1-0.1)	1.2 (1-1.5)	51	56	16	16
Georgia	96 (90-103)	87 (81-93)	-10 (-13.3--6.3)	3,270 (3,030-3,510)	4,130 (3,890-4,440)	26.3 (21-31.6)	30 (28-31)	31 (27-36)	3.8 (-9.7-23.5)	76 (70-83)	90 (84-97)	18.8 (12.6-24.9)	2 (2-2)	1 (1-1)	-38 (-49.4--26.7)	17 (14-21)	12 (10-15)	-30 (-39.4--19.3)	0.1 (0.1-0.1)	1.3 (1.1-1.6)	0.1 (0.1-0.1)	1.3 (1-1.6)	50	53	16	16
Hawaii	1 (1-1)	0 (0-1)	-49.9 (-72.5--34.4)	29 (16-46)	21 (10-38)	-26.3 (-64--3.2)	1 (1-1)	0 (0-1)	-11.8 (-28.8-11.8)	1 (0-1)	0 (0-1)	-34.3 (-63.1--13.3)	0 (0-0)	0 (0-0)	-45.3 (-60--25.6)	0 (0-0)	0 (0-0)	-64.9 (-82.2--47.5)	0 (0-0)	0.1 (0.1-0.2)	0 (0-0)	0.1 (0-0.1)	84	89	48	56
Idaho	1 (0-1)	1 (1-1)	11.8 (3.1-21.3)	19 (15-25)	27 (22-34)	39.7 (28.1-50.4)	0 (0-0)	0 (0-1)	14.4 (-7.8-40.5)	1 (0-1)	1 (1-1)	29.3 (17.7-41.8)	0 (0-0)	0 (0-0)	-42.7 (-58.7--22.1)	0 (0-0)	0 (0-0)	-17.5 (-30.1--3.4)	0 (0-0)	0.1 (0-0.1)	0 (0-0)	0.1 (0.1-0.1)	86	86	65	64
Illinois	53 (49-58)	28 (26-30)	-47.5 (-50.1--44.5)	1,750 (1,600-1,920)	1,510 (1,400-1,640)	-13.7 (-18.4--8.8)	27 (26-29)	22 (18-25)	-21.7 (-34--8)	45 (41-50)	33 (30-36)	-27.2 (-31.3--22.3)	2 (1-2)	1 (0-1)	-65.6 (-71.5--57.7)	13 (10-16)	4 (3-5)	-70 (-74.9--64.1)	0.1 (0.1-0.1)	0.7 (0.6-0.9)	0.1 (0.1-0.1)	0.5 (0.4-0.7)	54	60	22	24
Indiana	23 (21-24)	19 (17-20)	-18.5 (-22.7--15.4)	807 (745-874)	839 (778-903)	4 (-1.6-8.8)	10 (9-11)	10 (8-12)	-1.1 (-17.6-18.1)	20 (18-22)	19 (18-21)	-5.2 (-10.1-0.2)	1 (1-1)	0 (0-0)	-40.1 (-51.2--24)	5 (4-6)	3 (2-3)	-43.2 (-50.3--35)	0.1 (0.1-0.1)	0.6 (0.5-0.7)	0.1 (0.1-0.1)	0.5 (0.3-0.6)	59	64	25	31
Iowa	6 (5-6)	5 (4-6)	-9.6 (-14.3--4.4)	230 (201-254)	244 (212-276)	6 (0.3-11.9)	2 (2-2)	2 (2-2)	-9.4 (-25.6-8.9)	5 (4-6)	5 (4-6)	-2.1 (-7.5-4.1)	0 (0-0)	0 (0-0)	-38.5 (-53.1--15.4)	1 (1-1)	0 (0-1)	-42.7 (-49.2--35.5)	0 (0-0)	0.3 (0.2-0.4)	0 (0-0)	0.3 (0.2-0.4)	70	75	36	43
Kansas	8 (7-9)	5 (4-6)	-34.1 (-38.9--30.5)	266 (233-303)	241 (208-280)	-9.4 (-16.6--2.9)	4 (4-4)	4 (3-5)	-8.2 (-26-11.9)	7 (6-8)	5 (5-6)	-20.2 (-25.3--14.1)	0 (0-0)	0 (0-0)	-55.2 (-65.3--41.5)	2 (1-2)	1 (1-1)	-52.9 (-59.4--45.9)	0.1 (0.1-0.1)	0.5 (0.4-0.7)	0.1 (0.1-0.1)	0.4 (0.3-0.5)	57	67	26	32
Kentucky	8 (7-8)	6 (6-7)	-15.3 (-18.3--11.7)	266 (234-297)	284 (245-316)	6.7 (2.3-12.2)	6 (6-7)	7 (6-8)	6.3 (-11.7-23.4)	7 (6-8)	7 (6-8)	-3.3 (-8.7-2)	0 (0-0)	0 (0-0)	-40.4 (-52.2--23.6)	2 (1-2)	1 (1-1)	-41.9 (-48.1--34.4)	0.1 (0.1-0.1)	0.4 (0.3-0.5)	0.1 (0-0.1)	0.3 (0.2-0.4)	65	70	32	36
Louisiana	44 (40-47)	30 (28-33)	-30.3 (-34--26.2)	1,390 (1,290-1,500)	1,260 (1,170-1,360)	-9.1 (-13.4--4.2)	18 (18-19)	16 (14-19)	-12.8 (-25.5-1.9)	37 (34-41)	29 (27-32)	-21.8 (-26.3--17)	1 (1-1)	1 (1-1)	-46.6 (-56--35.8)	11 (9-14)	5 (4-6)	-55.5 (-62.1--47.6)	0.1 (0.1-0.2)	1.5 (1.2-1.8)	0.1 (0.1-0.1)	1 (0.8-1.2)	49	54	14	18
Maine	0 (0-0)	0 (0-0)	-50.4 (-66.6--29.7)	12 (8-19)	7 (4-10)	-43.7 (-64.8--17.1)	0 (0-1)	0 (0-1)	-1.9 (-20.3-19.6)	0 (0-0)	0 (0-0)	-40.3 (-61.5--16.5)	0 (0-0)	0 (0-0)	-45.2 (-61.9--21.1)	0 (0-0)	0 (0-0)	-55.2 (-79.4--41)	0 (0-0)	0 (0-0.1)	0 (0-0)	0 (0-0)	82	84	69	77
Maryland	67 (62-72)	57 (53-61)	-15.1 (-19.1--11.4)	2,600 (2,430-2,800)	2,850 (2,660-3,060)	9.6 (4.8-15)	16 (15-16)	13 (11-16)	-15 (-28.9--0.5)	59 (54-65)	62 (58-67)	4.5 (-0.9-10.3)	1 (1-1)	0 (0-0)	-48.3 (-57.2--37.2)	12 (10-15)	7 (6-9)	-41 (-48.8--32.4)	0.1 (0.1-0.1)	1.9 (1.5-2.3)	0.1 (0.1-0.1)	1.9 (1.4-2.4)	50	55	11	12
Massachusetts	25 (22-26)	19 (18-21)	-21.9 (-25--17.4)	986 (908-1,050)	993 (904-1,070)	0.7 (-4.1-6)	7 (6-7)	5 (4-6)	-22.3 (-36.5--4)	24 (22-26)	23 (21-25)	-3.2 (-8.4-2)	0 (0-0)	0 (0-0)	-50.2 (-62.9--33.8)	4 (4-6)	2 (2-3)	-48.1 (-54.9--40.7)	0.1 (0-0.1)	1 (0.8-1.2)	0 (0-0.1)	0.8 (0.6-1.1)	63	70	17	18

Location	Births, 2000	Births, 2021	Births percent change (2000-2021)	All age cases, 2000	All age cases, 2021	All age cases percent change (2000-2021)	All age deaths (cause-specific), 2000	All age deaths (cause-specific), 2021	All age deaths (cause-specific) percent change (2000-2021)	All age deaths (total), 2000	All age deaths (total), 2021	All age deaths (total) percent change (2000-2021)	Under-5 deaths (cause-specific), 2000	Under-5 deaths (cause-specific), 2021	Under-5 deaths (cause-specific) percent change (2000-2021)	Under-5 deaths (total), 2000	Under-5 deaths (total), 2021	Under-5 deaths (total) percent change (2000-2021)	Under-5 cause-specific mortality cause fraction, 2000	Under-5 total SCD mortality cause fraction, 2000	Under-5 cause-specific mortality cause fraction, 2021	Under-5 total SCD mortality cause fraction, 2021	Under-5 cause-specific mortality rank 2000	Under-5 cause-specific mortality rank 2021	Under-5 total SCD mortality rank 2000	Under-5 total SCD mortality rank 2021
Michigan	53 (49-57)	38 (36-40)	-28.5 (-31.7--25.4)	1,980 (1,830-2,110)	1,790 (1,700-1,920)	-9.4 (-14.5--4.9)	20 (18-21)	16 (14-19)	-17.9 (-29.6--3.6)	48 (44-52)	41 (38-44)	-15.7 (-20--11.1)	1 (1-1)	1 (0-1)	-48.1 (-57.9--39)	11 (9-14)	5 (4-6)	-55.7 (-62.5--49.1)	0.1 (0.1-0.1)	0.9 (0.7-1.1)	0.1 (0.1-0.1)	0.7 (0.5-0.8)	54	59	19	22
Minnesota	9 (8-10)	8 (7-9)	-15.2 (-19.8--9.7)	354 (304-398)	374 (322-428)	5.5 (-0.3-13.1)	4 (4-4)	4 (3-5)	-4.3 (-23-15.4)	8 (7-9)	8 (7-9)	-0.2 (-7.2-8)	0 (0-0)	0 (0-0)	-36.4 (-52.7--18)	2 (1-2)	1 (1-1)	-42.6 (-50.2--34.9)	0.1 (0-0.1)	0.4 (0.3-0.5)	0 (0-0.1)	0.3 (0.2-0.4)	67	74	33	38
Mississippi	41 (38-44)	27 (25-29)	-33.9 (-36.4--30.7)	1,220 (1,120-1,290)	1,130 (1,040-1,220)	-7.1 (-11--2.5)	13 (12-13)	12 (10-13)	-7.4 (-19.4-8.4)	35 (32-38)	28 (25-30)	-20.7 (-24.9--16.1)	1 (1-1)	0 (0-0)	-54.1 (-63--44.7)	11 (9-13)	5 (4-6)	-55.9 (-62.2--49.3)	0.1 (0.1-0.2)	2 (1.6-2.4)	0.1 (0.1-0.1)	1.6 (1.1-2.1)	48	53	13	15
Missouri	28 (26-31)	22 (20-23)	-23.9 (-27.4--19.9)	1,000 (921-1,090)	1,000 (932-1,080)	0.4 (-3.7-6.3)	11 (10-12)	11 (9-13)	-2.4 (-17.8-15.8)	26 (24-28)	23 (21-25)	-9.7 (-14.2--4.8)	1 (0-1)	0 (0-0)	-31.8 (-45.1--19.5)	6 (5-8)	3 (3-4)	-48.5 (-55.1--41.5)	0.1 (0.1-0.1)	1 (0.8-1.1)	0.1 (0.1-0.1)	0.7 (0.5-0.8)	54	62	20	25
Montana	0 (0-0)	0 (0-0)	-4.1 (-34.6-31.8)	4 (2-7)	5 (3-8)	9.7 (-23.9-52)	0 (0-0)	0 (0-0)	7.5 (-9.9-27.9)	0 (0-0)	0 (0-0)	6.9 (-23.4-42.5)	0 (0-0)	0 (0-0)	-34.6 (-52.3--13.2)	0 (0-0)	0 (0-0)	-27.1 (-65.7-1.5)	0 (0-0)	0 (0-0)	0 (0-0)	0 (0-0)	87	87	94	92
Nebraska	3 (3-4)	2 (2-3)	-25.4 (-32.1--18.5)	109 (89-134)	100 (78-126)	-8.2 (-16.4--0.9)	2 (2-2)	2 (1-2)	-8.1 (-21.8-6.7)	3 (2-3)	2 (2-3)	-17.7 (-25--9.8)	0 (0-0)	0 (0-0)	-26.3 (-46.6-3.1)	1 (0-1)	0 (0-1)	-47.5 (-54.9--39.6)	0 (0-0.1)	0.3 (0.2-0.5)	0 (0-0.1)	0.2 (0.2-0.4)	68	73	34	40
Nevada	10 (9-11)	9 (8-10)	-3.6 (-9-2.7)	340 (309-376)	512 (460-572)	50.5 (41.2-61.4)	3 (3-3)	3 (3-4)	10.3 (-6.2-26.2)	8 (7-9)	11 (10-12)	39.1 (31.6-46.8)	0 (0-0)	0 (0-0)	-37.9 (-52.8--22.3)	2 (1-2)	1 (1-2)	-26 (-36.2--15.4)	0.1 (0.1-0.1)	0.7 (0.6-1)	0.1 (0-0.1)	0.7 (0.5-1)	66	71	23	24
New Hampshire	0 (0-1)	0 (0-0)	-22 (-32.6--12.3)	19 (14-25)	19 (14-25)	-0.6 (-10.7-12.9)	0 (0-0)	0 (0-1)	-0.5 (-17.7-20.3)	1 (0-1)	1 (0-1)	-1.3 (-15.9-11.6)	0 (0-0)	0 (0-0)	-44.1 (-58.5--20.1)	0 (0-0)	0 (0-0)	-50.1 (-64.3--38.1)	0 (0-0)	0.1 (0.1-0.2)	0 (0-0)	0.1 (0.1-0.1)	80	85	49	57
New Jersey	57 (53-62)	44 (41-47)	-22.7 (-26.1--19.1)	2,200 (2,050-2,380)	2,230 (2,080-2,400)	1.3 (-2.9-5.9)	18 (17-19)	13 (10-15)	-28.1 (-41--15.2)	51 (47-55)	48 (45-52)	-4.4 (-9-0.3)	1 (1-1)	0 (0-0)	-64.4 (-72.2--56.9)	10 (8-12)	5 (4-6)	-48.1 (-54.4--41.1)	0.1 (0.1-0.1)	1.1 (0.9-1.3)	0.1 (0.1-0.1)	1.3 (1-1.7)	52	57	15	14
New Mexico	1 (1-1)	1 (1-1)	-26.1 (-32.1--18.2)	36 (26-47)	38 (27-50)	5 (-2.5-16.5)	1 (1-1)	1 (1-2)	16.6 (-4.5-40.5)	1 (1-1)	1 (1-1)	5.1 (-4-18.1)	0 (0-0)	0 (0-0)	-54.4 (-65.7--38.9)	0 (0-0)	0 (0-0)	-27.3 (-40.4--12.5)	0 (0-0)	0 (0-0)	0 (0-0)	0 (0-0.1)	80	83	79	77
New York	144 (135-155)	116 (108-123)	-19.8 (-23.8--16.4)	6,390 (6,030-6,840)	6,440 (6,060-6,910)	0.8 (-3.2-5.4)	43 (40-45)	33 (28-39)	-22.5 (-34.8--8.7)	124 (113-136)	124 (115-135)	0.4 (-5.5-5.8)	2 (2-2)	1 (1-1)	-62.6 (-68.3--54.2)	17 (13-21)	9 (7-11)	-48.1 (-55.8--39.9)	0.1 (0.1-0.1)	0.9 (0.7-1.1)	0.1 (0.1-0.1)	0.9 (0.7-1.2)	54	59	22	18
North Carolina	69 (64-73)	61 (57-65)	-11.3 (-15.1--7.5)	2,480 (2,300-2,660)	2,990 (2,820-3,190)	20.3 (15.1-25.8)	22 (20-23)	23 (20-27)	5 (-9.1-24.5)	59 (54-64)	66 (61-71)	12.2 (6.2-18.1)	1 (1-1)	1 (1-1)	-33.1 (-43.8--22.4)	12 (10-15)	8 (6-10)	-34.7 (-43.5--25.7)	0.1 (0.1-0.1)	1 (0.9-1.3)	0.1 (0.1-0.1)	0.9 (0.7-1.2)	52	58	17	19
North Dakota	0 (0-0)	0 (0-0)	21.7 (-36.9-40.6)	3 (1-5)	3 (1-6)	12 (-36.6-33.4)	0 (0-0)	0 (0-0)	18.1 (-3.8-37.3)	0 (0-0)	0 (0-0)	6.2 (-44.6-24.1)	0 (0-0)	0 (0-0)	-0.8 (-26.1-33.5)	0 (0-0)	0 (0-0)	-12.8 (-62.9-18.6)	0 (0-0)	0 (0-0)	0 (0-0)	0 (0-0)	76	82	89	94
Ohio	44 (41-48)	34 (32-36)	-23.3 (-26.2--19.5)	1,640 (1,510-1,770)	1,590 (1,490-1,710)	-2.8 (-6.9-1.3)	21 (19-22)	20 (17-23)	-0.9 (-16.1-15.4)	41 (37-45)	36 (33-39)	-12.2 (-17.4--7.2)	1 (1-1)	1 (1-1)	-36.3 (-46.9--21.8)	10 (8-12)	5 (4-6)	-53.4 (-60.3--45.5)	0.1 (0.1-0.1)	0.7 (0.6-0.9)	0.1 (0.1-0.1)	0.4 (0.3-0.6)	54	59	21	30
Oklahoma	10 (9-11)	7 (6-7)	-34.4 (-38.3--29)	311 (274-349)	279 (243-317)	-10.3 (-15.4--2.4)	6 (6-6)	6 (5-7)	-3.2 (-19.6-9.9)	9 (8-10)	7 (6-8)	-26.1 (-31.7--20.5)	0 (0-0)	0 (0-0)	-35.6 (-46.2--20.6)	3 (2-3)	1 (1-2)	-57.2 (-63.3--50.6)	0.1 (0.1-0.1)	0.5 (0.4-0.7)	0.1 (0.1-0.1)	0.3 (0.2-0.4)	62	67	24	38
Oregon	3 (3-4)	3 (3-4)	-12.6 (-16.7--6.5)	125 (104-146)	146 (122-173)	16.8 (11-25.7)	2 (1-2)	2 (1-2)	-0.2 (-17.9-24.5)	3 (3-4)	4 (3-4)	11.5 (4-19.8)	0 (0-0)	0 (0-0)	-48.8 (-62.6--28)	1 (0-1)	0 (0-1)	-39 (-45.3--31)	0 (0-0)	0.2 (0.2-0.3)	0 (0-0)	0.2 (0.1-0.3)	80	84	39	42
Pennsylvania	38 (36-41)	30 (28-33)	-20.1 (-23.5--15.8)	1,520 (1,410-1,650)	1,500 (1,390-1,600)	-1.2 (-6.2-3.2)	21 (20-22)	18 (15-20)	-17.5 (-32.3--3.1)	39 (36-43)	36 (33-38)	-9.4 (-14.1--4.3)	1 (1-1)	1 (0-1)	-37.8 (-49.7--25)	8 (7-10)	4 (3-5)	-48.8 (-56.9--40.5)	0.1 (0.1-0.1)	0.7 (0.6-0.9)	0.1 (0.1-0.1)	0.5 (0.4-0.6)	55	60	21	27
Rhode Island	4 (4-5)	3 (2-3)	-36.6 (-42--30.4)	179 (157-207)	152 (130-176)	-15 (-20.5--7.5)	1 (1-1)	1 (1-1)	-33.1 (-44.4--17.9)	4 (3-5)	3 (3-4)	-23.7 (-31--14.5)	0 (0-0)	0 (0-0)	-44.9 (-60.2--24.4)	1 (1-1)	0 (0-0)	-73.7 (-78.1--67.7)	0 (0-0)	0.8 (0.5-1.2)	0 (0-0)	0.3 (0.2-0.4)	68	72	19	33
South Carolina	39 (36-42)	32 (30-34)	-17.5 (-21.3--13.1)	1,420 (1,300-1,540)	1,550 (1,450-1,670)	9.4 (5.9-13.3)	18 (17-19)	19 (16-22)	6.3 (-11-22.4)	35 (32-38)	34 (32-37)	-2.2 (-7.3-3.5)	1 (1-1)	0 (0-1)	-35.2 (-46.1--23.5)	8 (7-10)	4 (3-5)	-48.2 (-55.2--39.9)	0.1 (0.1-0.1)	1.4 (1.2-1.7)	0.1 (0.1-0.1)	1.1 (0.8-1.4)	51	52	14	17
South Dakota	0 (0-0)	0 (0-0)	-5.3 (-25.9-11.4)	9 (5-15)	10 (6-16)	12.6 (-0.8-28.8)	0 (0-0)	0 (0-1)	-2.7 (-17.8-16.6)	0 (0-0)	0 (0-0)	-0.1 (-25.3-19.9)	0 (0-0)	0 (0-0)	-22.6 (-42.9-1.9)	0 (0-0)	0 (0-0)	-38.8 (-68--9.2)	0 (0-0)	0.1 (0-0.1)	0 (0-0)	0 (0-0.1)	77	81	69	77
Tennessee	36 (33-39)	33 (31-35)	-8.6 (-13.8--3.3)	1,220 (1,130-1,320)	1,380 (1,280-1,470)	12.8 (7.8-18)	14 (13-15)	15 (13-18)	8.7 (-10.5-26.2)	33 (30-36)	34 (31-37)	2.5 (-2.6-7.6)	1 (1-1)	0 (0-1)	-32.4 (-43.2--18.1)	8 (7-10)	5 (4-7)	-36.6 (-46.4--27.3)	0.1 (0.1-0.1)	1 (0.8-1.2)	0.1 (0.1-0.1)	0.8 (0.6-1.1)	54	63	19	20
Texas	118 (109-126)	122 (113-132)	3.9 (-1.6-9.2)	3,980 (3,690-4,250)	5,630 (5,280-6,040)	41.4 (34.3-47.4)	38 (36-40)	42 (36-47)	9.1 (-7.6-26.7)	83 (76-92)	113 (104-123)	35.7 (28.8-43.1)	2 (2-3)	2 (1-2)	-30.4 (-41.2--18.4)	16 (13-20)	13 (10-16)	-21.5 (-33.2--8.9)	0.1 (0.1-0.1)	0.6 (0.5-0.8)	0.1 (0.1-0.1)	0.5 (0.4-0.7)	56	64	25	27
Utah	2 (2-3)	2 (2-3)	-11.5 (-17.2--4)	67 (54-83)	91 (74-112)	35.2 (26.5-46.9)	1 (1-1)	1 (1-1)	8.8 (-9.3-26.3)	1 (1-2)	2 (1-2)	22.3 (9.8-33)	0 (0-0)	0 (0-0)	-36.6 (-54.4--9.1)	0 (0-1)	0 (0-0)	-36.1 (-49.1--24.6)	0 (0-0)	0.1 (0.1-0.2)	0 (0-0)	0.1 (0.1-0.1)	77	81	54	57
Vermont	0 (0-1)	0 (0-0)	-21.9 (-35.8--6)	20 (13-30)	20 (13-30)	-2.1 (-13.3-15.5)	0 (0-0)	0 (0-0)	-10 (-24.8-8.3)	0 (0-1)	0 (0-1)	-1.5 (-23.2-19.1)	0 (0-0)	0 (0-0)	-47.4 (-61.4--20.9)	0 (0-0)	0 (0-0)	-61.6 (-83.3--25)	0 (0-0)	0.1 (0-0.2)	0 (0-0)	0.1 (0-0.1)	85	87	58	68
Virginia	50 (47-54)	43 (40-46)	-14.5 (-18.6--10.1)	1,860 (1,740-1,990)	2,130 (1,990-2,280)	14.8 (9.8-20.2)	16 (16-17)	15 (13-17)	-6.1 (-21.5-9.5)	45 (41-49)	47 (43-51)	4.3 (-1.1-9.9)	1 (1-1)	0 (0-1)	-37.3 (-45.9--26.5)	11 (9-13)	6 (5-8)	-43.6 (-52.3--33.9)	0.1 (0.1-0.1)	1.3 (1.1-1.6)	0.1 (0.1-0.1)	1 (0.8-1.3)	52	58	15	17
Washington	9 (8-10)	9 (8-10)	0.7 (-4.3-5.4)	381 (337-418)	456 (405-498)	19.5 (14.4-26.4)	4 (4-4)	4 (3-5)	6.7 (-11-26.2)	9 (8-10)	10 (9-11)	17.4 (11.1-24.4)	0 (0-0)	0 (0-0)	-36.1 (-52.4--17.8)	1 (1-2)	1 (1-1)	-28.1 (-34.7--21)	0 (0-0)	0.3 (0.2-0.4)	0 (0-0)	0.3 (0.2-0.3)	73	78	35	40
West Virginia	1 (1-2)	1 (1-1)	-31.7 (-36.6--27.2)	64 (50-80)	55 (44-68)	-13.3 (-19.8--6.8)	2 (2-2)	2 (2-3)	6.2 (-13.3-28.1)	2 (1-2)	1 (1-2)	-19.8 (-25.5--12.6)	0 (0-0)	0 (0-0)	-36.9 (-53.9--13.6)	0 (0-0)	0 (0-0)	-53.3 (-60.3--45)	0 (0-0)	0.1 (0.1-0.2)	0 (0-0)	0.1 (0.1-0.1)	70	77	45	56
Wisconsin	15 (14-17)	11 (10-12)	-28.7 (-32.4--24.7)	609 (549-667)	551 (498-606)	-9.6 (-15.1--4.7)	7 (6-7)	6 (5-7)	-8.2 (-23.9-8.2)	15 (13-16)	13 (11-14)	-14.9 (-19.4--9.8)	0 (0-0)	0 (0-0)	-45 (-58.9--29.1)	3 (2-4)	1 (1-2)	-51.7 (-57.4--45.1)	0.1 (0.1-0.1)	0.6 (0.4-0.7)	0.1 (0-0.1)	0.4 (0.3-0.5)	61	70	25	32
Wyoming	0 (0-0)	0 (0-0)	-51.7 (-66.1--38.6)	13 (8-22)	8 (4-16)	-36.6 (-53.2--22.1)	0 (0-0)	0 (0-0)	-4.6 (-20.9-13.5)	0 (0-0)	0 (0-0)	-42.2 (-61.7--25.8)	0 (0-0)	0 (0-0)	-43.2 (-57.9--20.9)	0 (0-0)	0 (0-0)	-70.7 (-88.9--37.9)	0 (0-0)	0.1 (0-0.1)	0 (0-0)	0 (0-0.1)	80	84	67	78

Location	Births, 2000	Births, 2021	Births percent change (2000-2021)	All age cases, 2000	All age cases, 2021	All age cases percent change (2000-2021)	All age deaths (cause-specific), 2000	All age deaths (cause-specific), 2021	All age deaths (cause-specific) percent change (2000-2021)	All age deaths (total), 2000	All age deaths (total), 2021	All age deaths (total) percent change (2000-2021)	Under-5 deaths (cause-specific), 2000	Under-5 deaths (cause-specific), 2021	Under-5 deaths (cause-specific) percent change (2000-2021)	Under-5 deaths (total), 2000	Under-5 deaths (total), 2021	Under-5 deaths (total) percent change (2000-2021)	Under-5 cause-specific mortality cause fraction, 2000	Under-5 total SCD mortality cause fraction, 2000	Under-5 cause-specific mortality cause fraction, 2021	Under-5 total SCD mortality cause fraction, 2021	Under-5 cause-specific mortality rank 2000	Under-5 cause-specific mortality rank 2021	Under-5 total SCD mortality rank 2000	Under-5 total SCD mortality rank 2021
**Southern Latin America**	6 (5-8)	7 (6-9)	17.9 (8.1-36.7)	68 (50-87)	91 (70-114)	34.3 (22-50.7)	1 (1-1)	1 (0-1)	-2.4 (-17.8-12.8)	6 (4-8)	8 (6-9)	27.6 (14.5-54)	0 (0-0)	0 (0-0)	-63.8 (-77.2--47.2)	4 (3-5)	5 (4-6)	19.9 (5.5-48.5)	0 (0-0)	0 (0-0)	0 (0-0)	0.1 (0-0.1)	127	130	81	57
Argentina	3 (2-4)	3 (2-4)	-1.2 (-10.6-32)	26 (18-36)	29 (20-41)	12.2 (-1.4-35.5)	0 (0-0)	0 (0-0)	-12.2 (-29-6.8)	2 (2-3)	3 (2-4)	3.6 (-9.8-37.4)	0 (0-0)	0 (0-0)	-69.2 (-80.9--50.8)	2 (1-2)	2 (1-2)	-1.5 (-14.6-33)	0 (0-0)	0 (0-0)	0 (0-0)	0 (0-0)	126	129	93	74
Chile	3 (2-4)	4 (4-6)	34.5 (16.7-63)	40 (29-52)	60 (47-75)	50.7 (32.1-78.6)	0 (0-0)	0 (0-0)	-41.8 (-53.3--27.8)	3 (2-4)	5 (4-6)	47.9 (27.2-96)	0 (0-0)	0 (0-0)	-73.1 (-82.4--58.7)	2 (1-3)	3 (2-4)	39.9 (18-89)	0 (0-0)	0.1 (0-0.1)	0 (0-0)	0.3 (0.2-0.4)	132	132	63	33
Uruguay	0 (0-0)	0 (0-0)	-13.8 (-16.3--1)	2 (1-3)	2 (1-3)	-6.8 (-19.1-9)	0 (0-0)	0 (0-0)	52.3 (20.2-89.4)	0 (0-0)	0 (0-0)	-12 (-21.1--4.7)	0 (0-0)	0 (0-0)	-36.5 (-67.1-16.1)	0 (0-0)	0 (0-0)	-15.5 (-23--8.3)	0 (0-0)	0 (0-0)	0 (0-0)	0.1 (0-0.1)	108	102	88	67
**Western Europe**	1,360 (1,130-1,630)	1,250 (1,030-1,480)	-8.1 (-10.5--5.4)	24,900 (21,700-28,400)	24,400 (21,100-27,900)	-2.3 (-4.4--0.5)	68 (66-71)	76 (73-80)	11.5 (6.7-15.6)	1,420 (1,210-1,680)	1,400 (1,190-1,650)	-1.2 (-3.6-1.5)	7 (7-8)	6 (5-6)	-19.9 (-28.9--8.1)	442 (337-568)	416 (319-534)	-5.9 (-9.7--1.7)	0 (0-0)	1.8 (1.4-2.2)	0 (0-0)	2.8 (2.1-3.6)	80	72	14	10
Andorra	0 (0-0)	0 (0-0)	-16.2 (-22--9.6)	3 (2-3)	3 (2-3)	8.8 (1.7-14.9)	0 (0-0)	0 (0-0)	-28.4 (-56.4-13.1)	0 (0-0)	0 (0-0)	6.4 (-0.9-14.6)	0 (0-0)	0 (0-0)	-78.2 (-93.3--46.6)	0 (0-0)	0 (0-0)	-27.4 (-34.4--19.7)	0.1 (0.1-0.3)	1.9 (1.2-2.7)	0.1 (0.1-0.3)	5.6 (3.3-8.4)	55	56	16	5
Austria	12 (11-14)	21 (18-23)	64.1 (52.6-76.5)	303 (273-334)	376 (337-421)	23.9 (19.1-30.5)	1 (1-1)	1 (1-1)	22.7 (9.7-39.5)	15 (13-17)	22 (19-24)	44.7 (34.1-55.7)	0 (0-0)	0 (0-0)	-10.8 (-35.8-14.8)	3 (3-4)	6 (4-7)	62.7 (43.7-83.4)	0 (0-0)	0.8 (0.6-1)	0 (0-0)	2.4 (1.8-3)	85	80	21	11
Belgium	15 (12-19)	15 (12-20)	-0.2 (-8.3-8.3)	222 (183-266)	231 (192-275)	3.9 (-3-11.3)	2 (2-2)	2 (2-3)	22.8 (8-38.5)	16 (13-20)	16 (13-21)	3.1 (-4.5-13.1)	0 (0-0)	0 (0-0)	-23.5 (-42.3-2)	6 (4-8)	6 (4-8)	-0.8 (-12.9-11.7)	0 (0-0)	0.8 (0.6-1.2)	0 (0-0)	1.4 (0.9-2.1)	74	72	23	17
Cyprus	6 (5-7)	9 (7-10)	41 (31.1-52.5)	137 (120-156)	166 (145-189)	21.3 (14.9-29)	1 (1-1)	1 (0-1)	-46.2 (-61.1--28.6)	7 (6-8)	9 (8-11)	27.9 (18.5-38.4)	0 (0-0)	0 (0-0)	-57.6 (-75.1--16.4)	2 (2-3)	3 (2-4)	34.1 (18.8-52.8)	0 (0-0)	3 (2.2-3.8)	0 (0-0)	8.2 (5.3-11.2)	77	77	10	4
Denmark	16 (13-20)	16 (13-19)	-1.3 (-9.3-5.5)	310 (264-363)	312 (266-358)	0.3 (-5.8-6.9)	0 (0-0)	0 (0-0)	15.6 (-0.2-37.7)	16 (13-19)	16 (14-20)	1.2 (-5.8-7.8)	0 (0-0)	0 (0-0)	-16.9 (-45.2-14)	5 (4-7)	5 (4-6)	-7.7 (-16.9-1.9)	0 (0-0)	1.4 (1-1.8)	0 (0-0)	2 (1.4-2.7)	104	93	14	9
Finland	13 (10-15)	10 (8-13)	-17.5 (-25.3--11.6)	266 (227-304)	243 (203-284)	-8.8 (-14.6--4)	0 (0-0)	0 (0-0)	26 (9.7-39.1)	14 (12-17)	13 (11-16)	-7.7 (-14.3--1)	0 (0-0)	0 (0-0)	-17.8 (-41.6-4)	4 (3-5)	3 (2-4)	-17.4 (-26.3--7.9)	0 (0-0)	1.6 (1.1-2)	0 (0-0)	3 (2.1-4.1)	104	95	14	10
France	450 (384-531)	374 (312-442)	-17 (-22.6--10.5)	6,650 (5,690-7,700)	6,570 (5,540-7,720)	-1.3 (-6-4.4)	19 (18-20)	23 (21-25)	19.6 (7.6-33.1)	437 (371-518)	412 (347-488)	-5.8 (-11.6-0.3)	2 (2-2)	2 (2-2)	-5.8 (-25.7-18)	157 (120-202)	134 (104-172)	-14.8 (-22.4--6.8)	0.1 (0-0.1)	3.8 (3-4.9)	0.1 (0.1-0.1)	4.9 (3.6-6.3)	68	61	7	6
Germany	98 (83-115)	98 (83-113)	0.2 (-5.7-6.4)	2,340 (2,020-2,760)	2,240 (1,910-2,600)	-4.8 (-8.8-0.3)	6 (6-7)	8 (8-9)	35.5 (21.4-49.5)	116 (99-135)	114 (97-132)	-1.6 (-8.4-5)	1 (1-1)	1 (1-1)	-2.4 (-21.4-18.5)	30 (23-38)	31 (24-40)	3.8 (-5.9-14.9)	0 (0-0)	0.7 (0.6-0.9)	0 (0-0)	1.2 (0.9-1.5)	86	76	23	15
Greece	97 (79-120)	74 (61-88)	-23.9 (-32.9--13.2)	2,550 (2,200-2,950)	1,920 (1,680-2,160)	-24.8 (-28.9--19)	4 (4-4)	6 (5-7)	54.2 (33.8-79.8)	114 (94-141)	91 (76-108)	-20.8 (-29.8--11.4)	0 (0-0)	0 (0-0)	-12.2 (-31.2-10)	30 (21-42)	25 (18-34)	-18.8 (-31.3--4.7)	0 (0-0)	4.8 (3.3-6.6)	0.1 (0-0.1)	6.8 (4.7-9.7)	63	57	4	4
Iceland	1 (0-1)	1 (0-1)	3.4 (-5.1-13.1)	9 (7-11)	10 (8-13)	10.5 (2.6-18.4)	0 (0-0)	0 (0-0)	74.3 (51.1-105)	1 (0-1)	1 (0-1)	8.7 (0-19.7)	0 (0-0)	0 (0-0)	-20 (-40.2-7.9)	0 (0-0)	0 (0-0)	-4.6 (-14.2-8.7)	0 (0-0)	1 (0.6-1.5)	0 (0-0)	1.2 (0.7-2)	82	78	21	16
Ireland	10 (8-13)	11 (8-14)	4.1 (-5.4-12.8)	176 (142-211)	204 (162-242)	15.6 (9.3-23.4)	1 (0-1)	1 (0-1)	4 (-12-20.4)	10 (8-12)	12 (9-15)	17.6 (8-26.9)	0 (0-0)	0 (0-0)	-29.3 (-46.7--9.8)	3 (2-4)	3 (2-5)	11.4 (-0.9-25.2)	0 (0-0)	0.8 (0.6-1.1)	0 (0-0)	2 (1.4-2.9)	82	66	21	14
Israel	62 (49-78)	70 (55-87)	12.9 (-1.5-23.8)	667 (558-799)	835 (675-1,010)	25 (14.1-32.7)	1 (1-2)	2 (2-2)	28.9 (13.4-50.9)	52 (41-68)	58 (45-75)	10.5 (-3.4-27)	0 (0-0)	0 (0-0)	-35.2 (-53.5--16.4)	24 (16-33)	25 (18-35)	7.2 (-9.7-28.5)	0 (0-0)	2.6 (1.8-3.5)	0.1 (0-0.1)	6.7 (4.5-9.7)	71	66	9	5
Italy	109 (93-126)	90 (77-102)	-17.4 (-19.4--14.7)	2,620 (2,270-2,970)	2,630 (2,310-3,000)	0.2 (-3.1-2.9)	5 (5-6)	3 (3-3)	-39.7 (-45.9--33.3)	117 (101-136)	124 (108-144)	6 (3-9.6)	1 (1-1)	0 (0-0)	-59.5 (-68.4--51.5)	23 (18-29)	21 (17-26)	-8.3 (-12.1--4.3)	0 (0-0)	0.8 (0.6-1)	0 (0-0)	1.8 (1.4-2.2)	83	81	21	11
Abruzzo	1 (1-1)	1 (1-1)	-11.9 (-22.8--2.3)	19 (14-25)	18 (14-24)	-3.6 (-14.6-5.9)	0 (0-0)	0 (0-0)	-42.4 (-50.3--32.3)	1 (1-1)	1 (1-1)	0 (-9.8-11.3)	0 (0-0)	0 (0-0)	-56.9 (-69.8--43.1)	0 (0-0)	0 (0-0)	-6.1 (-18.8-8.1)	0 (0-0)	0.3 (0.2-0.4)	0 (0-0)	0.6 (0.4-0.9)	89	85	36	26
Basilicata	0 (0-0)	0 (0-0)	-13.3 (-23-0.8)	9 (6-12)	9 (6-12)	-1 (-11.7-10.5)	0 (0-0)	0 (0-0)	-46.2 (-55.2--37.6)	0 (0-1)	0 (0-1)	-0.7 (-12.9-14.8)	0 (0-0)	0 (0-0)	-61.4 (-75.1--38)	0 (0-0)	0 (0-0)	-11.6 (-26.6-7)	0 (0-0)	0.2 (0.1-0.3)	0 (0-0)	0.5 (0.3-0.8)	86	85	47	29
Calabria	5 (4-5)	3 (3-4)	-25.5 (-31.7--17.8)	142 (123-162)	118 (103-136)	-16.7 (-22--10)	0 (0-0)	0 (0-0)	-34.8 (-45.1--18.8)	6 (5-7)	5 (4-5)	-18 (-25.4--10.3)	0 (0-0)	0 (0-0)	-59.1 (-69.2--41.5)	1 (1-1)	1 (1-1)	-24.8 (-34.5--14.5)	0 (0-0)	0.7 (0.5-1)	0 (0-0)	1.2 (0.9-1.5)	76	79	16	12
Campania	7 (6-8)	5 (5-6)	-19.6 (-25.9--13.5)	168 (144-193)	167 (144-191)	-0.5 (-7.5-5.5)	0 (0-0)	0 (0-0)	-40.7 (-50--24.4)	7 (6-9)	7 (6-9)	0.2 (-6.6-8.1)	0 (0-0)	0 (0-0)	-64.6 (-73--52.9)	2 (1-2)	1 (1-2)	-14.8 (-22.5--5.4)	0 (0-0)	0.4 (0.3-0.5)	0 (0-0)	1 (0.7-1.4)	84	84	27	14
Emilia-Romagna	17 (14-20)	16 (14-18)	-7.7 (-15.5--1.4)	319 (283-359)	378 (336-419)	18.1 (11.9-24.8)	0 (0-0)	0 (0-0)	-39.3 (-51.6--29)	17 (14-19)	22 (19-24)	29.6 (21.1-38.9)	0 (0-0)	0 (0-0)	-54.2 (-67.3--38.4)	3 (3-4)	4 (3-5)	15.5 (4.8-28.7)	0 (0-0)	2 (1.6-2.5)	0 (0-0)	4.8 (3.7-6.1)	84	81	11	7
Friuli-Venezia Giulia	3 (3-4)	3 (2-3)	-21.6 (-27.5--10.4)	66 (56-76)	66 (56-75)	-0.1 (-7-9.1)	0 (0-0)	0 (0-0)	-48.8 (-56.9--39.2)	4 (3-4)	4 (3-4)	5.9 (-2.4-15.4)	0 (0-0)	0 (0-0)	-45.8 (-63.5--19.1)	1 (0-1)	1 (0-1)	-10.4 (-19.7--0.2)	0 (0-0)	1.9 (1.4-2.7)	0 (0-0)	3.3 (2.4-4.5)	90	82	13	9
Lazio	7 (6-8)	7 (6-8)	-1.3 (-7.3-6.9)	169 (146-196)	195 (169-224)	14.8 (9.4-23)	0 (0-0)	0 (0-0)	-39.5 (-50--27.1)	7 (6-9)	9 (8-10)	21.7 (14-30.9)	0 (0-0)	0 (0-0)	-63.5 (-72.8--53.1)	1 (1-2)	1 (1-2)	8.7 (-1.8-20.4)	0 (0-0)	0.5 (0.4-0.6)	0 (0-0)	1.5 (1.1-2)	81	83	27	13
Liguria	4 (3-5)	3 (3-4)	-17.7 (-23.3--12.4)	102 (89-115)	100 (89-112)	-3 (-7.9-2.3)	0 (0-0)	0 (0-0)	-46.3 (-56.5--36.4)	5 (4-5)	5 (4-6)	2.9 (-4-9.7)	0 (0-0)	0 (0-0)	-56.5 (-68.8--36.1)	1 (1-1)	1 (1-1)	-2.9 (-14.2-9.6)	0 (0-0)	1.5 (1.1-1.9)	0 (0-0)	3.5 (2.6-4.5)	80	79	11	9
Lombardia	11 (9-14)	10 (9-12)	-11.1 (-18.4--4.6)	281 (237-329)	304 (266-351)	7.7 (-0.1-15.2)	1 (1-1)	0 (0-0)	-43.1 (-53.5--30)	12 (10-14)	14 (12-16)	15.7 (5.9-25.7)	0 (0-0)	0 (0-0)	-53 (-68.6--37.9)	2 (2-3)	2 (2-3)	4 (-6.4-16.3)	0 (0-0)	0.6 (0.4-0.7)	0 (0-0)	1.2 (0.9-1.5)	86	85	23	16
Marche	4 (3-4)	3 (2-3)	-18 (-23.9--11.6)	77 (65-90)	80 (70-95)	3.3 (-2.1-9)	0 (0-0)	0 (0-0)	-41.3 (-52.5--28)	4 (3-5)	4 (3-5)	8.5 (0.6-17.1)	0 (0-0)	0 (0-0)	-61.6 (-71.6--52.2)	1 (1-1)	1 (1-1)	-6.5 (-16.1-3.9)	0 (0-0)	1.2 (0.9-1.5)	0 (0-0)	2.9 (2.1-3.7)	85	80	19	9
Molise	0 (0-1)	0 (0-0)	-34.3 (-40.6--25.8)	9 (6-13)	7 (5-9)	-25.2 (-31--17.2)	0 (0-0)	0 (0-0)	-48.5 (-57.8--39.8)	0 (0-1)	0 (0-1)	-25.3 (-32.6--16.5)	0 (0-0)	0 (0-0)	-61.9 (-76.8--41.9)	0 (0-0)	0 (0-0)	-32.8 (-42.2--23.3)	0 (0-0)	0.5 (0.3-0.9)	0 (0-0)	1 (0.6-1.7)	97	94	25	17

Location	Births, 2000	Births, 2021	Births percent change (2000-2021)	All age cases, 2000	All age cases, 2021	All age cases percent change (2000-2021)	All age deaths (cause-specific), 2000	All age deaths (cause-specific), 2021	All age deaths (cause-specific) percent change (2000-2021)	All age deaths (total), 2000	All age deaths (total), 2021	All age deaths (total) percent change (2000-2021)	Under-5 deaths (cause-specific), 2000	Under-5 deaths (cause-specific), 2021	Under-5 deaths (cause-specific) percent change (2000-2021)	Under-5 deaths (total), 2000	Under-5 deaths (total), 2021	Under-5 deaths (total) percent change (2000-2021)	Under-5 cause-specific mortality cause fraction, 2000	Under-5 total SCD mortality cause fraction, 2000	Under-5 cause-specific mortality cause fraction, 2021	Under-5 total SCD mortality cause fraction, 2021	Under-5 cause-specific mortality rank 2000	Under-5 cause-specific mortality rank 2021	Under-5 total SCD mortality rank 2000	Under-5 total SCD mortality rank 2021
Piemonte	7 (6-8)	6 (5-7)	-10.3 (-16.3--4.7)	133 (112-156)	140 (120-161)	5.7 (-0.8-11.1)	0 (0-0)	0 (0-0)	-46.3 (-54.5--36.3)	7 (6-9)	9 (7-10)	15.4 (7.5-24.4)	0 (0-0)	0 (0-0)	-54.6 (-71.1--34.6)	1 (1-2)	1 (1-2)	4.4 (-6.9-17.1)	0 (0-0)	0.8 (0.6-1.1)	0 (0-0)	2.1 (1.5-2.6)	92	89	22	11
Provincia autonoma di Bolzano	1 (1-1)	1 (1-1)	-8.8 (-17.2-5.2)	16 (12-20)	17 (13-21)	5.9 (-0.6-15.1)	0 (0-0)	0 (0-0)	-48.1 (-56.8--34.1)	1 (1-1)	1 (1-1)	8.7 (-1.5-21.3)	0 (0-0)	0 (0-0)	-47.7 (-66.2--19.9)	0 (0-0)	0 (0-0)	-6.3 (-17.1-7.9)	0 (0-0)	0.7 (0.4-1)	0 (0-0)	1.2 (0.7-1.7)	91	91	24	16
Provincia autonoma di Trento	1 (1-1)	1 (0-1)	-10.9 (-20.9--1.8)	14 (11-18)	15 (12-19)	8.2 (-1.2-17.7)	0 (0-0)	0 (0-0)	-39.8 (-49.6--23.8)	1 (0-1)	1 (1-1)	13.5 (2.2-26)	0 (0-0)	0 (0-0)	-48.3 (-66.6--27.3)	0 (0-0)	0 (0-0)	-4.8 (-17.9-10)	0 (0-0)	0.7 (0.5-1)	0 (0-0)	1.2 (0.8-1.7)	86	84	24	19
Puglia	4 (3-4)	2 (2-3)	-36.5 (-43.3--30)	94 (75-115)	77 (63-89)	-18.7 (-26--11.3)	0 (0-0)	0 (0-0)	-39.5 (-49.2--26.2)	4 (3-5)	3 (3-4)	-18.5 (-26.8--9.4)	0 (0-0)	0 (0-0)	-69.7 (-77.6--57.7)	1 (0-1)	0 (0-1)	-34 (-42.9--24.2)	0 (0-0)	0.3 (0.2-0.3)	0 (0-0)	0.6 (0.4-0.7)	88	85	35	25
Sardegna	1 (1-1)	1 (1-1)	-28 (-35.1--16.3)	27 (20-34)	22 (17-28)	-16.2 (-21.9--8.8)	0 (0-0)	0 (0-0)	-28 (-44.3--9.9)	1 (1-2)	1 (1-1)	-12.1 (-20.6--1.6)	0 (0-0)	0 (0-0)	-64.1 (-75.6--47)	0 (0-0)	0 (0-0)	-21.1 (-32.9--7.8)	0 (0-0)	0.3 (0.2-0.4)	0 (0-0)	0.7 (0.4-1)	88	89	35	21
Sicilia	21 (18-24)	14 (12-16)	-32.7 (-36.8--28.6)	601 (529-693)	522 (466-584)	-13.3 (-17.8--8.8)	1 (1-1)	1 (1-1)	-34.3 (-45.2--19.9)	23 (20-26)	19 (17-22)	-16.2 (-21.5--10.6)	0 (0-0)	0 (0-0)	-59.6 (-71--45.5)	5 (4-7)	4 (3-4)	-31.5 (-38.8--23.9)	0 (0-0.1)	1.3 (1-1.7)	0 (0-0.1)	2.3 (1.8-3.2)	64	68	11	10
Toscana	4 (3-5)	4 (3-5)	-11.5 (-18.8--1.8)	94 (78-110)	98 (84-116)	3.2 (-4.6-11.3)	0 (0-0)	0 (0-0)	-40.7 (-51.9--29.5)	5 (4-6)	5 (4-6)	14.1 (5.2-24.8)	0 (0-0)	0 (0-0)	-62.9 (-75.2--50.6)	1 (1-1)	1 (1-1)	3.8 (-7.7-17.5)	0 (0-0)	0.6 (0.4-0.8)	0 (0-0)	1.6 (1.1-2.1)	87	88	23	11
Umbria	1 (1-2)	1 (1-2)	-9.7 (-17.3--0.9)	31 (26-36)	34 (29-40)	9.8 (2.3-16.4)	0 (0-0)	0 (0-0)	-44 (-53.4--30.4)	2 (1-2)	2 (1-2)	15.3 (6.3-25.6)	0 (0-0)	0 (0-0)	-64.7 (-75.7--48.2)	0 (0-0)	0 (0-0)	6.3 (-5.9-20.7)	0 (0-0)	0.9 (0.7-1.3)	0 (0-0)	2.3 (1.5-3)	68	73	20	11
Valle d'Aosta	0 (0-0)	0 (0-0)	-31.6 (-43.7--18.8)	2 (1-2)	1 (1-2)	-11.1 (-22.2-2.9)	0 (0-0)	0 (0-0)	-57.5 (-65.2--50.1)	0 (0-0)	0 (0-0)	-3.9 (-17-8.5)	0 (0-0)	0 (0-0)	-57.7 (-70.8--37.2)	0 (0-0)	0 (0-0)	-21.2 (-33.1--7.8)	0 (0-0)	0.4 (0.2-0.7)	0 (0-0)	1.1 (0.5-1.8)	95	95	32	25
Veneto	11 (9-12)	9 (8-10)	-14.9 (-21.5--10.7)	248 (219-277)	265 (236-294)	6.7 (-0.5-11.5)	0 (0-0)	0 (0-0)	-44.3 (-54--32.2)	11 (10-13)	13 (11-15)	13.3 (6.7-20.5)	0 (0-0)	0 (0-0)	-52.9 (-67.1--37.6)	2 (2-3)	2 (2-3)	-5.3 (-13.8-4.1)	0 (0-0)	1.3 (1.1-1.7)	0 (0-0)	2.6 (2-3.3)	86	85	13	10
Luxembourg	5 (4-5)	5 (4-6)	10.7 (1.7-20.2)	103 (90-118)	130 (114-152)	26.2 (18.9-34.3)	0 (0-0)	0 (0-0)	28.3 (13-49.5)	5 (4-5)	6 (5-7)	31.4 (22.3-42.9)	0 (0-0)	0 (0-0)	8.4 (-19.8-59.8)	1 (1-2)	2 (1-2)	11.3 (-1.6-27.2)	0 (0-0)	5.7 (4.1-7.3)	0 (0-0)	6.7 (4.3-9.8)	76	68	6	5
Malta	1 (1-1)	1 (1-1)	2.9 (-3.2-10.5)	28 (23-33)	24 (20-28)	-12.8 (-17.2--8)	0 (0-0)	0 (0-0)	56.2 (30.2-91.7)	1 (1-2)	1 (1-2)	-10.3 (-15.8--3.4)	0 (0-0)	0 (0-0)	16.8 (-19-83)	0 (0-1)	0 (0-1)	-5.8 (-14.1-4.4)	0 (0-0)	1.3 (1-1.7)	0 (0-0)	1.7 (1.1-2.4)	84	72	12	12
Monaco	0 (0-0)	0 (0-0)	-4.2 (-11.1-5.8)	1 (1-1)	1 (1-1)	13.2 (7.8-20.6)	0 (0-0)	0 (0-0)	-11.4 (-36.7-35.6)	0 (0-0)	0 (0-0)	17.1 (8.7-26.8)	0 (0-0)	0 (0-0)	-69.3 (-88.8--13)	0 (0-0)	0 (0-0)	19.9 (7.4-35.3)	0 (0-0.1)	1.2 (0.8-1.8)	0 (0-0.1)	3.5 (2.4-5)	67	70	16	7
Netherlands	5 (4-6)	4 (4-5)	-11.7 (-18.2--3.3)	78 (64-98)	74 (60-93)	-5 (-10.4-1.8)	4 (3-4)	4 (4-4)	10.5 (-0.5-22.6)	5 (4-6)	5 (4-6)	-3.3 (-9.9-7.2)	0 (0-1)	0 (0-0)	-38.7 (-53.1--21.1)	2 (1-2)	2 (1-2)	-10.1 (-18.6-2.7)	0 (0-0)	0.1 (0.1-0.2)	0 (0-0.1)	0.2 (0.2-0.3)	70	69	45	33
Norway	10 (7-14)	9 (6-14)	-4.3 (-8.4-0.1)	109 (78-153)	116 (83-162)	6.5 (2.8-9.9)	0 (0-0)	0 (0-0)	-43.3 (-49.6--37.2)	10 (6-15)	10 (6-15)	4.7 (0.1-10.4)	0 (0-0)	0 (0-0)	-59.5 (-68.5--47.7)	4 (2-7)	4 (2-6)	-5.6 (-11.9-1.3)	0 (0-0)	1.4 (0.7-2.3)	0 (0-0)	3.5 (1.8-5.8)	101	100	13	9
Agder	1 (0-1)	1 (0-1)	-7.5 (-21.9-5)	7 (5-10)	7 (5-10)	2.4 (-6.6-11.8)	0 (0-0)	0 (0-0)	-50.8 (-59.1--36.3)	1 (0-1)	1 (0-1)	1.3 (-11.8-16.2)	0 (0-0)	0 (0-0)	-64.5 (-74.6--52.2)	0 (0-0)	0 (0-0)	-11.1 (-27.9-6.8)	0 (0-0)	1.4 (0.7-2.3)	0 (0-0)	3.2 (1.5-5.5)	107	110	15	9
Innlandet	1 (0-1)	1 (0-1)	-20.7 (-31.5--7.9)	8 (6-12)	7 (5-10)	-11 (-19.9--1.9)	0 (0-0)	0 (0-0)	-51 (-61.9--36.3)	1 (0-1)	1 (0-1)	-12.2 (-24.5-1.1)	0 (0-0)	0 (0-0)	-64.6 (-72.7--51.4)	0 (0-1)	0 (0-0)	-22 (-38.2--4.2)	0 (0-0)	1.5 (0.7-2.4)	0 (0-0)	3.6 (1.9-6.1)	106	109	14	8
Møre og Romsdal	1 (0-1)	0 (0-1)	-17.9 (-28.2--4.1)	6 (4-8)	6 (4-8)	-5.2 (-14.8-3.2)	0 (0-0)	0 (0-0)	-43.8 (-55.1--26.9)	1 (0-1)	1 (0-1)	-6.6 (-17.5-6.2)	0 (0-0)	0 (0-0)	-58.1 (-69.8--42)	0 (0-0)	0 (0-0)	-16.5 (-32.5-2.7)	0 (0-0)	1.6 (0.8-2.7)	0 (0-0)	3.5 (1.6-5.7)	103	104	14	9
Nordland	0 (0-0)	0 (0-0)	-18.2 (-27.6--7.6)	6 (4-8)	5 (3-7)	-16 (-23.5--7.9)	0 (0-0)	0 (0-0)	-36.1 (-50.2--17.6)	1 (0-1)	0 (0-1)	-18.5 (-28.2--8.4)	0 (0-0)	0 (0-0)	-53.7 (-68.3--36.2)	0 (0-0)	0 (0-0)	-27.9 (-39.9--13.1)	0 (0-0)	1.6 (0.8-2.7)	0 (0-0)	3 (1.6-4.8)	103	102	12	9
Oslo	1 (1-2)	2 (1-3)	24.9 (12.1-38.7)	12 (8-16)	16 (11-22)	38.3 (28.3-46.7)	0 (0-0)	0 (0-0)	-51.7 (-63.3--34.7)	1 (1-1)	1 (1-2)	38.2 (22.4-57.2)	0 (0-0)	0 (0-0)	-51.9 (-67.5--31.5)	0 (0-1)	1 (0-1)	31.1 (9.1-57.5)	0 (0-0)	1.1 (0.6-1.7)	0 (0-0)	4.1 (2-7.1)	108	107	16	7
Rogaland	1 (1-1)	1 (1-1)	-3.2 (-14.4-9.8)	10 (7-14)	11 (8-16)	12.3 (3.1-23.5)	0 (0-0)	0 (0-0)	-36.4 (-49.6--21.8)	1 (1-1)	1 (1-2)	10.9 (-3.6-28)	0 (0-0)	0 (0-0)	-59 (-70.4--44.6)	0 (0-1)	0 (0-1)	-1.3 (-19.2-22.6)	0 (0-0)	1.3 (0.7-2.2)	0 (0-0)	3.5 (1.7-6)	91	87	14	8
Troms og Finnmark	1 (1-1)	1 (0-1)	-20.9 (-32.9--7.1)	6 (4-8)	5 (4-7)	-11 (-21.8--0.8)	0 (0-0)	0 (0-0)	-48.1 (-59.6--30.9)	1 (0-1)	0 (0-1)	-14.5 (-25.7-0.1)	0 (0-0)	0 (0-0)	-66.1 (-76.7--51.5)	0 (0-0)	0 (0-0)	-24.1 (-39.1--2.8)	0 (0-0)	1.5 (0.7-2.4)	0 (0-0)	3.4 (1.7-5.8)	101	99	15	9
Trøndelag	1 (0-1)	1 (0-1)	4.5 (-10.8-23.4)	10 (7-13)	10 (7-14)	6.2 (-4.5-16.9)	0 (0-0)	0 (0-0)	-37.2 (-52.8--15.1)	1 (0-1)	1 (1-1)	3.6 (-10.6-19.7)	0 (0-0)	0 (0-0)	-54.5 (-66.2--34.7)	0 (0-1)	0 (0-1)	-6.1 (-24.3-17.1)	0 (0-0)	1.6 (0.8-2.7)	0 (0-0)	3.7 (1.8-6.5)	104	104	13	8
Vestfold og Telemark	1 (0-1)	1 (0-1)	-14.1 (-23.8--1.8)	9 (6-13)	9 (6-12)	-3.5 (-10.5-5.4)	0 (0-0)	0 (0-0)	-59.2 (-66.8--48.3)	1 (0-1)	1 (0-1)	-4.8 (-16.3-8.4)	0 (0-0)	0 (0-0)	-65 (-74.3--48.7)	0 (0-1)	0 (0-0)	-16.1 (-30.8-5)	0 (0-0)	1.4 (0.7-2.3)	0 (0-0)	3.5 (1.8-5.9)	89	90	14	9
Vestland	1 (1-2)	1 (1-2)	-4 (-15.1-9.4)	14 (10-19)	14 (10-20)	4.9 (-3.3-16.2)	0 (0-0)	0 (0-0)	-36.8 (-48.3--18.5)	1 (1-2)	1 (1-2)	3 (-10.6-17.2)	0 (0-0)	0 (0-0)	-44.9 (-59.8--24.6)	0 (0-1)	0 (0-1)	-5.7 (-23.5-16)	0 (0-0)	1.5 (0.8-2.5)	0 (0-0)	3.2 (1.6-5)	107	100	14	9
Viken	2 (1-3)	2 (1-3)	-4.1 (-20.3-9.4)	23 (16-32)	26 (19-36)	14 (3.7-25.4)	0 (0-0)	0 (0-0)	-42.7 (-53--29)	2 (1-3)	2 (1-4)	13.4 (-3.7-29.5)	0 (0-0)	0 (0-0)	-46.9 (-61.4--22.2)	1 (0-1)	1 (0-1)	0 (-22.4-22.3)	0 (0-0)	1.4 (0.7-2.3)	0 (0-0)	3.5 (1.7-6.1)	106	105	13	9
Portugal	115 (90-147)	69 (54-89)	-39.6 (-46.9--30.4)	2,170 (1,860-2,500)	1,520 (1,260-1,820)	-30 (-38.6--24.6)	4 (4-4)	3 (3-3)	-17.3 (-23.8--9.1)	119 (94-150)	87 (69-111)	-26.7 (-35--16.6)	0 (0-0)	0 (0-0)	-54.1 (-67.6--41.4)	40 (27-56)	26 (18-38)	-33.2 (-44.7--19.7)	0 (0-0.1)	4.7 (3.2-6.6)	0.1 (0.1-0.1)	10.2 (6.4-14.6)	76	68	6	3
San Marino	0 (0-0)	0 (0-0)	-4.1 (-11-4.4)	1 (1-1)	1 (1-1)	8.9 (2.2-15.9)	0 (0-0)	0 (0-0)	-30.1 (-55.7-23.9)	0 (0-0)	0 (0-0)	7.7 (-1.1-16.8)	0 (0-0)	0 (0-0)	-78.6 (-94.5--43.4)	0 (0-0)	0 (0-0)	-6.1 (-15.7-5.6)	0.1 (0-0.2)	1.2 (0.8-1.8)	0.1 (0-0.2)	6.5 (4.1-9.9)	63	62	22	3
Spain	96 (80-115)	84 (68-101)	-12.9 (-21.1--5.3)	1,840 (1,540-2,140)	1,870 (1,590-2,170)	1.5 (-5.5-8.5)	5 (4-5)	5 (5-6)	17.2 (3-31.2)	105 (88-124)	111 (92-134)	5.5 (-3.3-16.3)	0 (0-1)	0 (0-0)	-15.3 (-32.3-6.2)	26 (19-35)	26 (19-35)	0.5 (-12.1-15.8)	0 (0-0)	1.2 (0.9-1.6)	0 (0-0)	2.8 (2.1-3.9)	84	74	16	10

Location	Births, 2000	Births, 2021	Births percent change (2000-2021)	All age cases, 2000	All age cases, 2021	All age cases percent change (2000-2021)	All age deaths (cause-specific), 2000	All age deaths (cause-specific), 2021	All age deaths (cause-specific) percent change (2000-2021)	All age deaths (total), 2000	All age deaths (total), 2021	All age deaths (total) percent change (2000-2021)	Under-5 deaths (cause-specific), 2000	Under-5 deaths (cause-specific), 2021	Under-5 deaths (cause-specific) percent change (2000-2021)	Under-5 deaths (total), 2000	Under-5 deaths (total), 2021	Under-5 deaths (total) percent change (2000-2021)	Under-5 cause-specific mortality cause fraction, 2000	Under-5 total SCD mortality cause fraction, 2000	Under-5 cause-specific mortality cause fraction, 2021	Under-5 total SCD mortality cause fraction, 2021	Under-5 cause-specific mortality rank 2000	Under-5 cause-specific mortality rank 2021	Under-5 total SCD mortality rank 2000	Under-5 total SCD mortality rank 2021
Sweden	16 (12-21)	22 (16-29)	41.8 (28.9-55.8)	365 (296-449)	455 (353-565)	24.1 (16.6-32.5)	0 (0-0)	0 (0-0)	140 (101-182)	18 (14-24)	23 (17-30)	26.4 (15-38.1)	0 (0-0)	0 (0-0)	49.8 (19-95.5)	4 (2-5)	5 (4-8)	44.3 (29.7-60.2)	0 (0-0)	1 (0.7-1.5)	0 (0-0)	2.1 (1.4-3.1)	90	81	16	13
Stockholm	4 (3-5)	5 (4-7)	41.4 (29.7-55.6)	76 (60-94)	109 (85-134)	43.8 (34.8-53.6)	0 (0-0)	0 (0-0)	74.6 (40.1-117)	4 (3-5)	5 (4-7)	48.3 (33.6-63.8)	0 (0-0)	0 (0-0)	24.6 (-6.5-68.5)	1 (1-1)	1 (1-2)	55.3 (35.5-77.8)	0 (0-0)	1 (0.7-1.5)	0 (0-0)	2.4 (1.4-3.5)	89	82	17	12
Sweden except Stockholm	12 (9-16)	17 (13-22)	41.9 (27.7-61.8)	290 (233-357)	345 (268-430)	18.9 (10.6-29.5)	0 (0-0)	0 (0-0)	165 (115-208)	14 (11-19)	18 (13-23)	20.9 (7.8-35.6)	0 (0-0)	0 (0-0)	59.1 (19.4-105)	3 (2-4)	4 (3-6)	41.1 (23.3-59.7)	0 (0-0)	1.1 (0.7-1.5)	0 (0-0)	2.1 (1.4-3)	91	82	17	14
Switzerland	36 (33-42)	44 (40-49)	20.6 (14.9-27.5)	919 (848-998)	1,030 (942-1,120)	11.6 (6-17.4)	0 (0-1)	1 (1-1)	17.5 (2.5-36)	43 (38-48)	50 (44-56)	17.2 (10.9-23.7)	0 (0-0)	0 (0-0)	-8.9 (-40.3-23.2)	10 (8-13)	12 (10-16)	21.7 (8-37)	0 (0-0)	2.2 (1.7-2.7)	0 (0-0)	4 (3-5.1)	87	77	12	8
United Kingdom	182 (148-224)	219 (179-271)	20.5 (19.1-21.9)	3,020 (2,410-3,630)	3,400 (2,700-4,120)	12.2 (10.8-14)	16 (15-16)	16 (16-17)	3.5 (0.4-6.9)	193 (152-238)	216 (169-266)	12 (10.1-14.1)	1 (1-1)	1 (1-1)	-13.6 (-19.9--7.1)	66 (49-87)	75 (55-100)	14.3 (11.3-17.2)	0 (0-0)	1.5 (1.1-2)	0 (0-0)	2 (1.4-2.6)	76	70	13	11
England	162 (131-198)	198 (160-243)	22.4 (21-23.8)	2,620 (2,070-3,180)	3,010 (2,370-3,660)	14.4 (12.8-16.3)	12 (12-12)	12 (12-13)	1.5 (-2.3-5.3)	170 (133-209)	194 (150-238)	14.1 (12.2-16.2)	1 (1-1)	1 (1-1)	-8.1 (-14.8--1.4)	59 (43-78)	69 (50-91)	17 (14.2-19.9)	0 (0-0)	1.6 (1.1-2.1)	0 (0-0)	2 (1.4-2.7)	77	69	13	11
East Midlands	9 (7-12)	12 (9-15)	25.5 (21.1-30.8)	151 (118-185)	174 (137-211)	14.8 (11.2-20.6)	1 (0-1)	1 (1-1)	12.5 (3-24)	10 (8-13)	12 (9-15)	14.5 (9.8-19.8)	0 (0-0)	0 (0-0)	11.7 (-1.2-28.4)	3 (2-5)	4 (3-5)	16.9 (11.1-24)	0 (0-0)	1.2 (0.8-1.6)	0 (0-0)	1.5 (1.1-2)	90	80	14	12
Derby	1 (0-1)	1 (1-1)	18.6 (7.1-31.5)	9 (7-12)	11 (8-14)	17.1 (8.4-26.1)	0 (0-0)	0 (0-0)	8.6 (-3.6-23.2)	1 (0-1)	1 (1-1)	17.3 (5.9-28.4)	0 (0-0)	0 (0-0)	81.4 (43.7-132)	0 (0-0)	0 (0-0)	14.7 (-0.5-29.4)	0 (0-0)	1 (0.7-1.5)	0 (0-0)	1 (0.7-1.5)	79	68	14	14
Derbyshire	1 (1-1)	1 (1-1)	6.6 (-5.2-18.5)	12 (9-16)	12 (9-16)	-3.4 (-11.1-10.8)	0 (0-0)	0 (0-0)	32.1 (10.8-58.1)	1 (1-1)	1 (1-1)	-5.6 (-16.2-6.2)	0 (0-0)	0 (0-0)	23.5 (-2.6-58.5)	0 (0-1)	0 (0-0)	-7.5 (-21.5-8.8)	0 (0-0)	0.8 (0.5-1.2)	0 (0-0)	0.8 (0.5-1.3)	95	85	17	19
Leicester	1 (1-2)	2 (1-2)	36.5 (27.9-47.4)	20 (15-26)	27 (21-35)	32.7 (23.6-42.2)	0 (0-0)	0 (0-0)	6.7 (-6.4-23.4)	1 (1-2)	2 (1-2)	33 (22.1-44.8)	0 (0-0)	0 (0-0)	30.4 (-3.8-79.3)	0 (0-1)	1 (0-1)	31.8 (18.3-46.8)	0 (0-0)	1.2 (0.8-1.7)	0 (0-0)	1.4 (1-1.9)	77	72	13	14
Leicestershire	1 (0-1)	1 (1-1)	14.4 (0.9-32.4)	10 (7-13)	10 (8-14)	3.6 (-5.8-20)	0 (0-0)	0 (0-0)	39.2 (16.8-65.6)	1 (1-1)	1 (1-1)	1.7 (-9.8-18.6)	0 (0-0)	0 (0-0)	37.2 (15.3-71.9)	0 (0-0)	0 (0-0)	1.6 (-13.7-22.1)	0 (0-0)	0.8 (0.5-1.2)	0 (0-0)	0.8 (0.4-1.2)	103	97	17	20
Lincolnshire	1 (0-1)	1 (1-1)	33.1 (15.8-54.6)	10 (8-14)	12 (9-15)	11.1 (0.7-36.2)	0 (0-0)	0 (0-0)	47 (21.9-75.1)	1 (1-1)	1 (1-1)	8 (-5.6-27)	0 (0-0)	0 (0-0)	18.9 (-7.3-45.1)	0 (0-0)	0 (0-1)	11.8 (-4-33.6)	0 (0-0)	0.7 (0.4-1.1)	0 (0-0)	1 (0.6-1.5)	104	98	18	17
Northamptonshire	3 (2-3)	3 (2-4)	23 (13.6-32.5)	47 (36-62)	53 (41-68)	11.7 (2.9-20.1)	0 (0-0)	0 (0-0)	1.9 (-13.2-18.5)	3 (2-4)	3 (2-4)	13.4 (5.6-21.6)	0 (0-0)	0 (0-0)	-47.8 (-59.5--35.2)	1 (1-1)	1 (1-1)	17.1 (6.2-28.9)	0 (0-0)	1.9 (1.3-2.5)	0 (0-0)	5 (3.5-7.1)	79	71	9	5
Nottingham	2 (1-2)	2 (2-3)	39.7 (31.3-49.7)	29 (22-37)	36 (28-47)	25.3 (17.1-33.2)	0 (0-0)	0 (0-0)	8.6 (-5.3-25.7)	2 (1-2)	2 (2-3)	29.6 (19.1-40.4)	0 (0-0)	0 (0-0)	25.7 (-1.6-49.7)	1 (0-1)	1 (1-1)	38.9 (21.7-56.3)	0 (0-0)	2.1 (1.4-2.8)	0 (0-0)	3 (2-4.2)	89	80	9	5
Nottinghamshire	1 (1-1)	1 (1-1)	13.3 (3.3-29.6)	12 (9-16)	13 (10-16)	1.9 (-4.6-11.3)	0 (0-0)	0 (0-0)	14.3 (-2.3-33.8)	1 (1-1)	1 (1-1)	0.7 (-9.3-15)	0 (0-0)	0 (0-0)	25.6 (-2.8-59.2)	0 (0-1)	0 (0-1)	1.7 (-11.3-20.6)	0 (0-0)	0.8 (0.5-1.3)	0 (0-0)	0.8 (0.5-1.3)	95	89	17	19
Rutland	0 (0-0)	0 (0-0)	19.4 (3.4-41.5)	1 (0-1)	1 (0-1)	5.2 (-7.5-33.9)	0 (0-0)	0 (0-0)	-0.8 (-20.5-21.4)	0 (0-0)	0 (0-0)	1.6 (-10.9-22)	0 (0-0)	0 (0-0)	-55 (-70.4--32.2)	0 (0-0)	0 (0-0)	0.9 (-14.3-21.4)	0.1 (0-0.1)	1.1 (0.6-1.7)	0 (0-0.1)	2.4 (1.3-3.6)	68	65	19	11
East of England	14 (11-18)	16 (12-20)	12.5 (8.3-15.8)	242 (184-304)	252 (188-318)	3.8 (0.4-6.9)	1 (1-1)	1 (1-1)	15.8 (6.8-25.9)	15 (12-20)	16 (12-21)	4.2 (0.6-7.7)	0 (0-0)	0 (0-0)	22.1 (8.8-35.8)	5 (3-7)	5 (4-7)	3.5 (-1.1-8.1)	0 (0-0)	1.5 (1-2.1)	0 (0-0)	1.6 (1.1-2.1)	84	74	13	12
Bedford	1 (1-1)	1 (1-1)	-3.5 (-12.3-5.4)	14 (10-19)	12 (8-16)	-12.9 (-21--4.3)	0 (0-0)	0 (0-0)	10.4 (-6.9-27.8)	1 (1-1)	1 (1-1)	-13.3 (-20.5--5.2)	0 (0-0)	0 (0-0)	20.5 (-13.3-54)	0 (0-1)	0 (0-0)	-13.5 (-23.7--1.7)	0 (0-0)	2.9 (1.8-4.1)	0 (0-0)	2.3 (1.4-3.1)	78	75	7	11
Cambridgeshire	1 (1-1)	1 (1-1)	26.6 (16.2-39.3)	14 (11-19)	17 (12-22)	17.1 (8.3-27)	0 (0-0)	0 (0-0)	27.9 (8.7-47.8)	1 (1-1)	1 (1-2)	16.1 (5.7-27.2)	0 (0-0)	0 (0-0)	19.2 (-9.3-55.1)	0 (0-0)	0 (0-1)	14.4 (0.8-30.1)	0 (0-0)	1.1 (0.7-1.6)	0 (0-0)	1.3 (0.8-1.9)	95	86	17	14
Central Bedfordshire	0 (0-0)	0 (0-0)	16.6 (5.7-35.3)	4 (3-6)	4 (3-6)	7.7 (-1.1-28.6)	0 (0-0)	0 (0-0)	6.7 (-8.6-26.1)	0 (0-0)	0 (0-0)	6.1 (-3.5-20.4)	0 (0-0)	0 (0-0)	62.3 (24.7-107)	0 (0-0)	0 (0-0)	4.8 (-9.7-22.6)	0 (0-0)	0.9 (0.5-1.3)	0 (0-0)	0.8 (0.5-1.2)	87	75	18	22
Essex	3 (2-4)	4 (3-5)	18.8 (10.3-25.8)	57 (41-74)	60 (43-77)	5.8 (-0.8-11.1)	0 (0-0)	0 (0-0)	24.2 (6.6-41.4)	4 (3-5)	4 (3-5)	6.1 (-1.7-13.2)	0 (0-0)	0 (0-0)	17.9 (-7.2-47.4)	1 (1-2)	1 (1-2)	6.4 (-5.6-17.6)	0 (0-0)	1.5 (1-2.1)	0 (0-0)	1.6 (1.1-2.4)	84	71	13	11
Hertfordshire	3 (2-3)	3 (2-4)	18.4 (10.8-25.6)	46 (34-59)	52 (39-68)	13.1 (6.3-19.3)	0 (0-0)	0 (0-0)	7.4 (-6.3-22.4)	3 (2-4)	3 (2-4)	13.8 (6.4-22.9)	0 (0-0)	0 (0-0)	19.4 (-5.5-59)	1 (1-1)	1 (1-1)	13 (2-26.1)	0 (0-0)	1.5 (1-2)	0 (0-0)	1.6 (1.1-2.3)	83	70	14	12
Luton	1 (1-1)	1 (1-1)	21 (14.1-28)	12 (8-16)	14 (10-19)	20.5 (14.8-27.2)	0 (0-0)	0 (0-0)	3.7 (-9-18.7)	1 (1-1)	1 (1-1)	21.7 (13-30.5)	0 (0-0)	0 (0-0)	15.4 (-8.2-43.2)	0 (0-0)	0 (0-1)	24.7 (12.4-36.2)	0 (0-0.1)	1.3 (0.8-1.8)	0.1 (0-0.1)	1.6 (1-2.2)	65	64	14	12
Norfolk	1 (1-2)	1 (1-2)	19.8 (11-30.7)	21 (16-27)	23 (18-30)	8.8 (2.4-15.7)	0 (0-0)	0 (0-0)	25.8 (7-50)	1 (1-2)	2 (1-2)	8.3 (0-18)	0 (0-0)	0 (0-0)	10.6 (-19.2-42)	0 (0-1)	0 (0-1)	7.9 (-4.7-21.8)	0 (0-0)	1 (0.7-1.5)	0 (0-0)	1.2 (0.9-1.9)	104	97	17	14
Peterborough	0 (0-0)	1 (0-1)	52.2 (40-67.9)	4 (3-6)	6 (4-9)	42.5 (30.6-53.7)	0 (0-0)	0 (0-0)	64.6 (42-94.1)	0 (0-0)	0 (0-1)	41.5 (29.1-55.7)	0 (0-0)	0 (0-0)	37.3 (2.7-80.1)	0 (0-0)	0 (0-0)	44 (26.7-64.4)	0 (0-0)	0.9 (0.5-1.3)	0 (0-0)	1.2 (0.8-1.8)	102	92	18	13
Southend-on-Sea	0 (0-0)	0 (0-0)	18.5 (8-34.1)	3 (2-4)	3 (2-4)	14.7 (4.5-29.5)	0 (0-0)	0 (0-0)	4 (-12.1-20.7)	0 (0-0)	0 (0-0)	12.8 (1.8-27.2)	0 (0-0)	0 (0-0)	5.1 (-19.9-53.4)	0 (0-0)	0 (0-0)	9.9 (-5.1-27.4)	0 (0-0)	0.9 (0.5-1.3)	0 (0-0)	1.1 (0.6-1.7)	84	77	18	18
Suffolk	3 (2-4)	2 (2-3)	-14.9 (-25--4.7)	53 (39-70)	43 (30-56)	-19.2 (-28.4--10.8)	0 (0-0)	0 (0-0)	26 (7.3-45.4)	3 (2-4)	3 (2-3)	-20.2 (-28.6--11.7)	0 (0-0)	0 (0-0)	19.2 (-6.1-48.7)	1 (1-1)	1 (1-1)	-25.3 (-35.8--13.7)	0 (0-0)	2.9 (2-4.1)	0 (0-0)	2.3 (1.5-3.1)	84	76	7	9
Thurrock	1 (1-1)	1 (1-1)	23.1 (14.5-30.2)	14 (10-18)	16 (11-22)	18.8 (9.8-28.6)	0 (0-0)	0 (0-0)	10.9 (-4.9-28.4)	1 (1-1)	1 (1-1)	19.3 (10.4-27.6)	0 (0-0)	0 (0-0)	43.3 (13.6-77.5)	0 (0-0)	0 (0-1)	20.4 (8.8-31.2)	0.1 (0.1-0.1)	2.7 (1.8-3.8)	0.1 (0.1-0.1)	2.6 (1.7-3.5)	65	60	8	9
Greater London	69 (56-85)	88 (71-108)	26.7 (24-29.1)	1,100 (895-1,370)	1,340 (1,080-1,670)	21.7 (19.4-24.4)	6 (5-6)	5 (4-5)	-14.4 (-20.7--8.3)	66 (52-80)	80 (63-98)	22.2 (19.2-25.3)	1 (1-1)	0 (0-0)	-27 (-32.3--20.6)	23 (17-30)	29 (22-39)	27.5 (23.7-31.3)	0.1 (0.1-0.1)	3.1 (2.3-4.2)	0.1 (0.1-0.1)	5.2 (3.9-6.8)	58	58	8	5

Location	Births, 2000	Births, 2021	Births percent change (2000-2021)	All age cases, 2000	All age cases, 2021	All age cases percent change (2000-2021)	All age deaths (cause-specific), 2000	All age deaths (cause-specific), 2021	All age deaths (cause-specific) percent change (2000-2021)	All age deaths (total), 2000	All age deaths (total), 2021	All age deaths (total) percent change (2000-2021)	Under-5 deaths (cause-specific), 2000	Under-5 deaths (cause-specific), 2021	Under-5 deaths (cause-specific) percent change (2000-2021)	Under-5 deaths (total), 2000	Under-5 deaths (total), 2021	Under-5 deaths (total) percent change (2000-2021)	Under-5 cause-specific mortality cause fraction, 2000	Under-5 total SCD mortality cause fraction, 2000	Under-5 cause-specific mortality cause fraction, 2021	Under-5 total SCD mortality cause fraction, 2021	Under-5 cause-specific mortality rank 2000	Under-5 cause-specific mortality rank 2021	Under-5 total SCD mortality rank 2000	Under-5 total SCD mortality rank 2021
Barking and Dagenham	2 (2-2)	3 (2-4)	55.1 (43.3-67.2)	33 (27-40)	48 (39-58)	43.4 (32.9-52.9)	0 (0-0)	0 (0-0)	1.8 (-7.2-13.9)	2 (2-2)	3 (2-3)	41.6 (31-53.2)	0 (0-0)	0 (0-0)	14.3 (-4.4-32)	1 (1-1)	1 (1-1)	51.6 (35.4-69.7)	0.1 (0.1-0.1)	4.5 (3.2-6)	0.1 (0.1-0.1)	5.5 (3.9-7.3)	55	55	7	5
Barnet	2 (2-3)	3 (2-4)	32.1 (21.8-41.1)	44 (35-55)	54 (42-68)	22.9 (15.8-33.1)	0 (0-0)	0 (0-0)	-7.9 (-18.6-2)	2 (2-3)	3 (2-4)	23.4 (15.2-32.9)	0 (0-0)	0 (0-0)	1.1 (-15.2-22.5)	1 (1-1)	1 (1-1)	31.8 (18.2-47.8)	0.1 (0-0.1)	3.3 (2.4-4.4)	0.1 (0.1-0.1)	4.7 (3.3-6.4)	58	55	7	5
Bexley	1 (1-1)	1 (1-2)	15.3 (6.1-26)	20 (14-26)	20 (14-26)	0.3 (-8.2-10.2)	0 (0-0)	0 (0-0)	-10 (-22.4-3.6)	1 (1-1)	1 (1-1)	1.9 (-5.8-10.6)	0 (0-0)	0 (0-0)	20.3 (-2.4-47.4)	0 (0-0)	0 (0-0)	4.3 (-6.3-17)	0.1 (0-0.1)	2.5 (1.7-3.5)	0.1 (0-0.1)	2.2 (1.4-3.1)	66	61	9	10
Brent	2 (2-3)	3 (2-4)	40.3 (29.2-52.9)	33 (27-42)	45 (36-57)	34.1 (25.5-47.3)	0 (0-0)	0 (0-0)	0 (-11.9-9.7)	2 (2-3)	3 (2-3)	37 (26.6-48.9)	0 (0-0)	0 (0-0)	-11.3 (-26-1.3)	1 (1-1)	1 (1-2)	52.6 (36-73.2)	0.1 (0.1-0.1)	2.1 (1.5-2.9)	0.1 (0.1-0.1)	4.2 (3-5.8)	55	51	10	6
Bromley	1 (1-1)	1 (1-2)	25.1 (16.9-34.7)	20 (13-27)	22 (14-29)	10.4 (2.3-19.9)	0 (0-0)	0 (0-0)	-14.7 (-26.4--4)	1 (1-1)	1 (1-2)	11.9 (4.6-20.3)	0 (0-0)	0 (0-0)	2.6 (-14.9-21.2)	0 (0-0)	0 (0-1)	14.1 (2.6-26.7)	0 (0-0)	1.9 (1.3-2.7)	0 (0-0)	2.5 (1.6-3.6)	72	68	11	8
Camden	2 (1-2)	1 (1-2)	-11.3 (-17.3--3.2)	26 (20-34)	22 (18-29)	-12.7 (-19.3--5.5)	0 (0-0)	0 (0-0)	-4.1 (-40.4-21.8)	2 (1-2)	1 (1-2)	-14 (-20.9--7)	0 (0-0)	0 (0-0)	-35.7 (-49.5--20.7)	0 (0-1)	0 (0-1)	-13.6 (-22.5--3.2)	0.1 (0-0.1)	2.9 (2.1-4)	0.1 (0.1-0.1)	4.7 (3.3-6.5)	68	62	8	5
Croydon	3 (2-3)	4 (3-5)	40 (29.9-49.8)	50 (40-63)	63 (51-78)	24.7 (15.1-34.3)	0 (0-0)	0 (0-0)	-1.9 (-14.5-8.8)	3 (2-4)	4 (3-5)	27.5 (18.9-38.3)	0 (0-0)	0 (0-0)	-10.6 (-24.8-3.4)	1 (1-1)	1 (1-2)	39.4 (25-54.8)	0.1 (0.1-0.1)	2.9 (2.2-4)	0.1 (0.1-0.1)	5 (3.5-6.8)	59	55	8	5
Ealing	2 (1-2)	2 (2-2)	12.9 (5.5-22.4)	28 (21-37)	31 (24-39)	9.3 (4-17.8)	0 (0-0)	0 (0-0)	-7.2 (-16.1-2.6)	2 (1-2)	2 (1-2)	11.1 (3.8-19.5)	0 (0-0)	0 (0-0)	-11.2 (-24.1-9.9)	1 (0-1)	1 (0-1)	19.2 (7.9-31.8)	0.1 (0.1-0.1)	2 (1.5-2.8)	0.1 (0.1-0.1)	3.3 (2.3-4.6)	59	52	10	7
Enfield	5 (4-6)	6 (5-8)	28.4 (17.9-39)	92 (77-112)	117 (96-144)	26.6 (17.1-36)	0 (0-0)	0 (0-0)	-3.3 (-17.8-11)	5 (4-6)	6 (5-8)	26.1 (16.3-35.4)	0 (0-0)	0 (0-0)	-9 (-22-10.2)	2 (1-2)	2 (1-3)	32.8 (18.6-50.5)	0.1 (0.1-0.1)	5.9 (4.1-7.8)	0.1 (0.1-0.1)	8.7 (6.3-11.6)	53	52	5	2
Greenwich	2 (2-3)	3 (3-4)	50.9 (39.5-61)	36 (29-45)	51 (42-64)	40.7 (31.8-51.3)	0 (0-0)	0 (0-0)	-2.2 (-14.2-8.2)	2 (2-3)	3 (2-4)	41.4 (30.8-51.9)	0 (0-0)	0 (0-0)	19.3 (6.3-37.9)	1 (1-1)	1 (1-1)	47.5 (33.1-61.5)	0.1 (0.1-0.1)	3.3 (2.4-4.5)	0.1 (0.1-0.1)	4.3 (3.1-5.7)	63	57	8	5
Hackney	7 (6-9)	9 (7-11)	22.1 (11.3-33.3)	101 (83-123)	126 (102-153)	24.2 (15.1-36)	0 (0-0)	0 (0-0)	-27.8 (-38.9--16.6)	6 (5-7)	7 (6-9)	24.2 (14.2-35.6)	0 (0-0)	0 (0-0)	-66.7 (-71.9--60.4)	2 (2-3)	3 (2-4)	20.2 (7.2-34.9)	0.1 (0.1-0.1)	6 (4.4-8)	0.1 (0.1-0.2)	21.7 (15.2-30.8)	45	48	5	2
Hammersmith and Fulham	1 (1-2)	1 (1-2)	-5.5 (-13-3.4)	20 (15-28)	19 (14-26)	-7.2 (-15.4-0.8)	0 (0-0)	0 (0-0)	-32.6 (-42.8--25.4)	1 (1-2)	1 (1-2)	-7.4 (-14.6-1.3)	0 (0-0)	0 (0-0)	-28.4 (-39.5--12.9)	0 (0-1)	0 (0-1)	-0.5 (-10.2-10.7)	0.1 (0.1-0.1)	2.6 (1.8-3.7)	0.1 (0.1-0.1)	4.2 (2.8-5.9)	55	51	9	6
Haringey	7 (5-8)	7 (6-9)	9.2 (1.5-17.7)	101 (83-123)	119 (99-148)	18.5 (10.2-30.4)	0 (0-0)	0 (0-0)	-11.4 (-20.8--2.2)	6 (5-7)	7 (6-9)	16.2 (7.7-26.5)	0 (0-0)	0 (0-0)	-48.8 (-56.6--39.3)	2 (2-3)	2 (2-3)	17.5 (5.3-31.3)	0.1 (0.1-0.1)	6.6 (4.5-9.3)	0.1 (0.1-0.1)	14.7 (10.3-20.5)	48	52	3	2
Harrow	0 (0-1)	1 (0-1)	59 (45.8-73.5)	6 (4-8)	8 (6-12)	41.7 (31-54.7)	0 (0-0)	0 (0-0)	16.2 (1.2-33)	0 (0-1)	1 (0-1)	44.8 (32.6-56.7)	0 (0-0)	0 (0-0)	41.7 (18.1-72.8)	0 (0-0)	0 (0-0)	56.1 (38.2-75.7)	0 (0-0)	0.7 (0.5-1.1)	0 (0-0.1)	1 (0.6-1.5)	65	61	21	18
Havering	0 (0-1)	1 (1-1)	57.9 (44.8-72.2)	9 (6-14)	11 (7-17)	19.9 (11.6-27.9)	0 (0-0)	0 (0-0)	24.4 (6.6-44.6)	1 (0-1)	1 (0-1)	21.6 (12.8-32)	0 (0-0)	0 (0-0)	14.4 (-16.1-56)	0 (0-0)	0 (0-0)	38.5 (23.9-54.9)	0 (0-0)	1.4 (0.9-2.1)	0 (0-0)	2 (1.2-3)	82	75	12	11
Hillingdon	1 (0-1)	1 (1-2)	54 (41-66.1)	11 (7-16)	15 (10-22)	36.5 (24-47.6)	0 (0-0)	0 (0-0)	17.5 (1-35.5)	1 (0-1)	1 (1-1)	37.7 (27.9-47.6)	0 (0-0)	0 (0-0)	34.1 (1.6-64.6)	0 (0-0)	0 (0-1)	44.6 (29.5-60.4)	0 (0-0)	1.3 (0.8-2)	0 (0-0)	1.8 (1.1-2.7)	94	83	13	11
Hounslow	1 (1-2)	2 (1-2)	41.8 (29.3-54.7)	19 (13-26)	26 (19-34)	36 (23.1-49.5)	0 (0-0)	0 (0-0)	-4.3 (-19.3-13.5)	1 (1-2)	2 (1-2)	37.2 (26.3-48.4)	0 (0-0)	0 (0-0)	26.9 (3.2-57)	0 (0-1)	1 (0-1)	45.5 (32.3-61.5)	0 (0-0)	1.9 (1.3-2.8)	0 (0-0)	2.5 (1.7-3.6)	76	66	10	8
Islington	1 (1-1)	1 (1-2)	40.2 (30.7-51.7)	12 (8-16)	15 (10-20)	22.9 (12.8-30.3)	0 (0-0)	0 (0-0)	-29.8 (-38.8--19.9)	1 (1-1)	1 (1-1)	26.1 (16.5-36)	0 (0-0)	0 (0-0)	-39.9 (-47.3--26.5)	0 (0-0)	0 (0-1)	32.5 (19.8-46.7)	0.1 (0.1-0.1)	1.6 (1.1-2.2)	0.1 (0.1-0.1)	3.9 (2.5-5.3)	65	59	13	6
Kensington and Chelsea	0 (0-0)	0 (0-0)	-8.3 (-19.1-3.2)	3 (2-4)	2 (2-3)	-9.1 (-16.5-8)	0 (0-0)	0 (0-0)	-35.9 (-44.9--28.3)	0 (0-0)	0 (0-0)	-10.1 (-20.4-4.2)	0 (0-0)	0 (0-0)	-29.8 (-43.9--14.7)	0 (0-0)	0 (0-0)	-17.2 (-29.3--1.5)	0.1 (0.1-0.1)	0.8 (0.5-1.3)	0.1 (0.1-0.1)	1.3 (0.8-2)	67	60	24	16
Kingston upon Thames	0 (0-1)	1 (0-1)	36.7 (25.4-49.1)	8 (6-10)	9 (7-12)	14.4 (4.4-25.8)	0 (0-0)	0 (0-0)	22.4 (6.8-41.8)	0 (0-1)	1 (0-1)	16 (7-26.7)	0 (0-0)	0 (0-0)	74.2 (15.6-137)	0 (0-0)	0 (0-0)	26.5 (13.1-42.1)	0 (0-0)	1.8 (1.2-2.8)	0 (0-0)	1.9 (1.4-2.6)	93	83	12	12
Lambeth	6 (5-7)	6 (5-8)	7 (-1.4-15.8)	91 (75-110)	96 (78-119)	6 (-1-14)	0 (0-0)	0 (0-0)	-31.2 (-41.8--21.6)	5 (4-7)	6 (5-7)	5.5 (-3-14.1)	0 (0-0)	0 (0-0)	-48.4 (-56.5--38.4)	2 (1-2)	2 (1-3)	8.1 (-3.5-20.6)	0.1 (0.1-0.1)	4.8 (3.5-6.5)	0.1 (0.1-0.1)	9.4 (6.7-12.4)	47	51	7	2
Lewisham	4 (3-5)	5 (4-6)	24.6 (16.9-33.5)	62 (53-76)	71 (59-86)	13.4 (6.5-23.6)	0 (0-0)	0 (0-0)	-22.7 (-33.4--11.8)	4 (3-4)	4 (3-5)	13.9 (5-22.4)	0 (0-0)	0 (0-0)	-22.5 (-35--6.1)	1 (1-2)	2 (1-2)	22.8 (9.3-36.9)	0.1 (0.1-0.1)	4 (2.9-5.4)	0.1 (0.1-0.1)	6.5 (4.8-8.7)	53	52	7	3
Merton	1 (1-2)	2 (1-2)	28 (20.3-37.4)	21 (16-29)	24 (18-32)	12.1 (4.5-21.7)	0 (0-0)	0 (0-0)	-7.4 (-20.3-8.3)	1 (1-2)	1 (1-2)	12.8 (4.7-23)	0 (0-0)	0 (0-0)	8.7 (-13.6-42.5)	0 (0-1)	1 (0-1)	23.1 (11.6-35.8)	0 (0-0)	2.9 (2-4)	0 (0-0)	4.3 (3-6)	74	66	8	6
Newham	4 (3-4)	5 (4-7)	44.4 (35-54.1)	51 (43-63)	73 (60-89)	41.7 (33-52.1)	0 (0-0)	0 (0-0)	-3.2 (-14.4-7.4)	3 (3-4)	5 (4-6)	43.7 (32.9-55.7)	0 (0-0)	0 (0-0)	-26.8 (-36.9--13.7)	1 (1-2)	2 (1-3)	41.2 (26.1-57.4)	0.1 (0.1-0.1)	3.1 (2.3-4.1)	0.1 (0.1-0.2)	6.4 (4.8-8.4)	48	49	9	3
Redbridge	1 (1-1)	2 (1-2)	66 (55.4-78.1)	18 (13-24)	27 (19-36)	51.1 (41.1-61)	0 (0-0)	0 (0-0)	5.2 (-11.6-17.3)	1 (1-1)	2 (1-2)	52.3 (42.1-65.1)	0 (0-0)	0 (0-0)	13.3 (-5.2-41.2)	0 (0-1)	1 (0-1)	62.8 (49.3-79.3)	0 (0-0.1)	1.8 (1.1-2.4)	0.1 (0-0.1)	2.9 (1.9-4)	66	62	12	7
Richmond upon Thames	1 (0-1)	1 (0-1)	9.4 (0.2-14.8)	9 (6-13)	11 (7-15)	15.6 (6.3-24.1)	0 (0-0)	0 (0-0)	1.4 (-15.7-17.4)	1 (0-1)	1 (0-1)	15.5 (7-23.3)	0 (0-0)	0 (0-0)	-4 (-25.3-19.2)	0 (0-0)	0 (0-0)	11.6 (0.9-22.6)	0 (0-0)	1.7 (1.1-2.5)	0 (0-0)	2.5 (1.6-3.8)	93	85	13	8
Southwark	5 (4-6)	6 (5-8)	28.7 (20-37.8)	74 (60-92)	90 (74-109)	19.8 (12.6-27.6)	0 (0-0)	0 (0-0)	-26.4 (-34.3--17.5)	5 (4-6)	6 (4-7)	22.4 (13.9-32.6)	0 (0-0)	0 (0-0)	-56.3 (-62.9--48.6)	2 (1-2)	2 (2-3)	27.6 (14.7-42.6)	0.1 (0.1-0.1)	4.2 (3-5.7)	0.1 (0.1-0.1)	11.2 (7.9-15.5)	45	48	7	2
Sutton	0 (0-1)	1 (0-1)	25.9 (16.9-36.5)	7 (5-9)	8 (6-10)	7.6 (-0.6-17.8)	0 (0-0)	0 (0-0)	-14.7 (-25.9-0.8)	0 (0-1)	0 (0-1)	9.6 (1.3-19.1)	0 (0-0)	0 (0-0)	24.2 (-3.1-52.1)	0 (0-0)	0 (0-0)	12.2 (0.2-25.3)	0 (0-0)	1.3 (0.8-1.9)	0 (0-0)	1.4 (1-2)	81	73	15	14
Tower Hamlets	1 (1-2)	2 (1-2)	32.9 (19.1-45.8)	16 (13-21)	20 (16-27)	24.1 (11.7-39.4)	0 (0-0)	0 (0-0)	-10.9 (-20.6-1.2)	1 (1-1)	1 (1-2)	26.5 (13.3-41.9)	0 (0-0)	0 (0-0)	-13.5 (-29.8-4.8)	0 (0-1)	1 (0-1)	28.5 (12.6-46.8)	0 (0-0.1)	1.8 (1.2-2.4)	0.1 (0-0.1)	2.8 (2-3.9)	72	63	12	6

Location	Births, 2000	Births, 2021	Births percent change (2000-2021)	All age cases, 2000	All age cases, 2021	All age cases percent change (2000-2021)	All age deaths (cause-specific), 2000	All age deaths (cause-specific), 2021	All age deaths (cause-specific) percent change (2000-2021)	All age deaths (total), 2000	All age deaths (total), 2021	All age deaths (total) percent change (2000-2021)	Under-5 deaths (cause-specific), 2000	Under-5 deaths (cause-specific), 2021	Under-5 deaths (cause-specific) percent change (2000-2021)	Under-5 deaths (total), 2000	Under-5 deaths (total), 2021	Under-5 deaths (total) percent change (2000-2021)	Under-5 cause-specific mortality cause fraction, 2000	Under-5 total SCD mortality cause fraction, 2000	Under-5 cause-specific mortality cause fraction, 2021	Under-5 total SCD mortality cause fraction, 2021	Under-5 cause-specific mortality rank 2000	Under-5 cause-specific mortality rank 2021	Under-5 total SCD mortality rank 2000	Under-5 total SCD mortality rank 2021
Waltham Forest	3 (2-3)	4 (3-5)	41.6 (32.4-51)	40 (31-51)	55 (43-70)	36 (25.8-46.3)	0 (0-0)	0 (0-0)	-11 (-23.8-0.7)	3 (2-3)	3 (3-4)	36.6 (26.2-48.4)	0 (0-0)	0 (0-0)	-24 (-34.5--11.8)	1 (1-1)	1 (1-2)	43.8 (27.2-60.1)	0.1 (0.1-0.1)	3.2 (2.3-4.4)	0.1 (0.1-0.1)	6.1 (4.3-8.4)	46	51	8	5
Wandsworth	2 (1-2)	2 (2-3)	37.4 (28.4-45.6)	22 (16-30)	29 (22-38)	30.5 (20.5-41.3)	0 (0-0)	0 (0-0)	-29.4 (-41.9--17.8)	1 (1-2)	2 (1-2)	31.2 (21.9-42.5)	0 (0-0)	0 (0-0)	-34.2 (-45.2--19.3)	0 (0-1)	1 (0-1)	45.5 (33.3-59.4)	0.1 (0.1-0.1)	1.8 (1.3-2.4)	0.1 (0.1-0.1)	4.2 (2.9-5.7)	57	53	11	5
Westminster	1 (0-1)	1 (0-1)	-9.2 (-21.4-2.6)	10 (7-13)	9 (6-13)	-9.7 (-22-2.5)	0 (0-0)	0 (0-0)	-26.5 (-40.4--17.1)	1 (0-1)	1 (0-1)	-8.8 (-20.5-2.1)	0 (0-0)	0 (0-0)	-39.7 (-52.4--24)	0 (0-0)	0 (0-0)	-5.6 (-19.8-8.8)	0.1 (0.1-0.1)	1.3 (0.8-1.9)	0.1 (0.1-0.1)	2.7 (1.8-4)	65	57	15	9
North East England	4 (3-6)	5 (4-6)	11.9 (7.7-15.8)	72 (55-91)	72 (55-93)	0 (-3.7-4.1)	0 (0-0)	0 (0-0)	37.2 (26.5-48.6)	5 (4-7)	5 (4-7)	-0.2 (-3.7-3.9)	0 (0-0)	0 (0-0)	8.4 (-14.4-32.1)	2 (1-3)	2 (1-3)	2.2 (-2.9-7.1)	0 (0-0)	1.1 (0.7-1.5)	0 (0-0)	1.5 (1-2.1)	107	101	15	14
County Durham	1 (0-1)	1 (0-1)	9 (-1.7-27.2)	8 (6-11)	8 (6-11)	-0.7 (-9.4-14.2)	0 (0-0)	0 (0-0)	39.6 (16.9-63.9)	1 (0-1)	1 (0-1)	-2.3 (-11.7-15.7)	0 (0-0)	0 (0-0)	15.6 (-14.2-58.3)	0 (0-0)	0 (0-0)	-2 (-16-17)	0 (0-0)	0.7 (0.4-1.1)	0 (0-0)	0.9 (0.5-1.4)	103	101	19	19
Darlington	0 (0-0)	0 (0-0)	9.8 (-3.8-26)	2 (1-2)	2 (1-3)	9.1 (-1.2-27.6)	0 (0-0)	0 (0-0)	60.2 (36.6-89.1)	0 (0-0)	0 (0-0)	6.6 (-6.3-23)	0 (0-0)	0 (0-0)	-11.5 (-36.9-32.2)	0 (0-0)	0 (0-0)	2.9 (-13.3-21.6)	0 (0-0)	0.8 (0.4-1.3)	0 (0-0)	1 (0.6-1.5)	103	96	21	16
Gateshead	0 (0-1)	0 (0-1)	11.9 (2.1-20.7)	7 (5-9)	7 (5-10)	4.3 (-3.5-14.5)	0 (0-0)	0 (0-0)	37.6 (17.3-59.7)	0 (0-1)	0 (0-1)	4.2 (-4.7-13.2)	0 (0-0)	0 (0-0)	28.4 (-7.1-87.3)	0 (0-0)	0 (0-0)	1.9 (-10.7-14.5)	0 (0-0)	1.1 (0.7-1.6)	0 (0-0)	1.4 (0.9-2.1)	104	96	14	13
Hartlepool	0 (0-0)	0 (0-0)	2.3 (-8.6-26.4)	2 (1-2)	2 (1-2)	-4.6 (-13-30.7)	0 (0-0)	0 (0-0)	56.4 (19.5-99)	0 (0-0)	0 (0-0)	-8.6 (-20.7-11.2)	0 (0-0)	0 (0-0)	14.1 (-13.9-54.8)	0 (0-0)	0 (0-0)	-10 (-24.5-14)	0 (0-0)	0.8 (0.5-1.2)	0 (0-0)	1.3 (0.8-1.9)	104	98	18	17
Middlesbrough	1 (0-1)	1 (0-1)	9.3 (0.3-18.8)	8 (6-10)	7 (5-10)	-6.8 (-15.6-1.1)	0 (0-0)	0 (0-0)	31.5 (5.6-53.4)	1 (0-1)	1 (0-1)	-2.3 (-9.9-6.8)	0 (0-0)	0 (0-0)	7.8 (-24.6-46.9)	0 (0-0)	0 (0-0)	6.5 (-4.2-19.5)	0 (0-0)	1.7 (1.1-2.4)	0 (0-0)	2.4 (1.5-3.3)	99	95	12	11
Newcastle upon Tyne	1 (1-1)	1 (1-2)	29.9 (21.5-38.9)	16 (12-22)	18 (13-25)	11.1 (4.2-18)	0 (0-0)	0 (0-0)	23.1 (5.4-44.5)	1 (1-1)	1 (1-2)	13.9 (6-21.8)	0 (0-0)	0 (0-0)	37 (5.2-81)	0 (0-0)	0 (0-1)	20.5 (9.9-31.8)	0 (0-0)	1.9 (1.2-2.8)	0 (0-0)	2.5 (1.6-3.5)	103	101	12	10
North Tyneside	0 (0-1)	1 (0-1)	14.6 (5-24.3)	9 (6-11)	9 (6-12)	1.8 (-7.6-10.4)	0 (0-0)	0 (0-0)	30.2 (9.1-55.2)	1 (0-1)	1 (0-1)	3.9 (-3.8-12.6)	0 (0-0)	0 (0-0)	-4.6 (-37.8-27.3)	0 (0-0)	0 (0-0)	7.7 (-2.7-20.1)	0 (0-0)	1.8 (1.1-2.5)	0 (0-0)	2.6 (1.7-3.6)	98	99	10	9
Northumberland	0 (0-0)	0 (0-0)	0.8 (-10.2-16.9)	5 (4-7)	5 (3-6)	-7.6 (-16.4-9.2)	0 (0-0)	0 (0-0)	30.8 (12.6-53.5)	0 (0-1)	0 (0-0)	-8.9 (-18.6-4.1)	0 (0-0)	0 (0-0)	-8.5 (-36.9-32.9)	0 (0-0)	0 (0-0)	-11.3 (-24.8-4.3)	0 (0-0)	0.8 (0.4-1.2)	0 (0-0)	1 (0.6-1.6)	109	104	17	18
Redcar and Cleveland	0 (0-0)	0 (0-0)	-1.6 (-13.7-12.5)	2 (2-3)	2 (2-3)	-14 (-22--2)	0 (0-0)	0 (0-0)	73.5 (43.3-118)	0 (0-0)	0 (0-0)	-16.1 (-25.9--3)	0 (0-0)	0 (0-0)	-15.7 (-41-8.2)	0 (0-0)	0 (0-0)	-12.7 (-26.4-4.6)	0 (0-0)	0.9 (0.6-1.3)	0 (0-0)	1.2 (0.7-1.9)	101	94	18	16
South Tyneside	0 (0-0)	0 (0-0)	3.8 (-7.8-18.9)	3 (2-4)	2 (2-3)	-12.2 (-20.1-4.2)	0 (0-0)	0 (0-0)	13.3 (-5.3-36.4)	0 (0-0)	0 (0-0)	-14.6 (-24-0.3)	0 (0-0)	0 (0-0)	12 (-20.4-64.2)	0 (0-0)	0 (0-0)	-11.2 (-24.5-7.7)	0 (0-0)	0.9 (0.5-1.3)	0 (0-0)	1 (0.6-1.5)	101	100	18	17
Stockton-on-Tees	0 (0-0)	0 (0-1)	13.1 (2.1-23)	6 (4-8)	6 (4-8)	1.1 (-8.4-10.3)	0 (0-0)	0 (0-0)	53.2 (25.1-80.3)	0 (0-1)	0 (0-1)	1.2 (-7.7-9.9)	0 (0-0)	0 (0-0)	8.5 (-23.9-43.7)	0 (0-0)	0 (0-0)	0.1 (-12.2-12.1)	0 (0-0)	1 (0.7-1.5)	0 (0-0)	1.2 (0.8-1.7)	104	100	15	14
Sunderland	0 (0-0)	0 (0-0)	-6.6 (-16.1-6.1)	5 (4-7)	4 (3-6)	-16.5 (-24.6--3.6)	0 (0-0)	0 (0-0)	31 (14-51.5)	0 (0-1)	0 (0-0)	-17.5 (-26--6.7)	0 (0-0)	0 (0-0)	-1.5 (-25.2-32)	0 (0-0)	0 (0-0)	-15.2 (-27.1--2.7)	0 (0-0)	0.7 (0.4-1.1)	0 (0-0)	1.1 (0.6-1.6)	103	100	18	16
North West England	17 (14-22)	22 (17-27)	24.1 (20.5-28.1)	281 (222-340)	314 (248-381)	11.5 (7.7-15.1)	1 (1-1)	1 (1-1)	10.3 (3.3-17.6)	20 (15-25)	22 (17-27)	11.3 (7.6-15.6)	0 (0-0)	0 (0-0)	26.9 (11.5-41.4)	7 (5-9)	8 (6-11)	15.1 (9.5-21.5)	0 (0-0)	1.3 (0.9-1.7)	0 (0-0)	1.5 (1.1-2)	88	73	13	12
Blackburn with Darwen	1 (0-1)	1 (0-1)	1.3 (-6.2-11.1)	8 (5-10)	8 (6-10)	2.6 (-3.7-10)	0 (0-0)	0 (0-0)	11.1 (-1.6-29.9)	1 (0-1)	1 (0-1)	1.2 (-6.5-9.4)	0 (0-0)	0 (0-0)	-5.1 (-25.5-23.2)	0 (0-0)	0 (0-0)	-5.9 (-15.4-5)	0 (0-0)	1.1 (0.8-1.6)	0 (0-0.1)	1.4 (1-2)	73	67	16	13
Blackpool	0 (0-0)	0 (0-1)	12.9 (4.7-20.2)	5 (3-6)	5 (3-6)	-0.5 (-7.8-5.3)	0 (0-0)	0 (0-0)	36.8 (18.3-64.8)	0 (0-1)	0 (0-1)	0.1 (-8-8.1)	0 (0-0)	0 (0-0)	28.3 (3.5-73.6)	0 (0-0)	0 (0-0)	2.3 (-8.1-13.4)	0 (0-0)	1.3 (0.9-1.9)	0 (0-0)	1.3 (0.9-1.8)	88	77	14	14
Bolton	1 (1-1)	1 (1-2)	19.4 (10.3-29.7)	15 (11-20)	17 (12-22)	7.7 (-0.7-17.7)	0 (0-0)	0 (0-0)	11.5 (-4.7-32.9)	1 (1-1)	1 (1-2)	8.4 (0.1-17.6)	0 (0-0)	0 (0-0)	37.7 (1.6-77.1)	0 (0-1)	0 (0-1)	9.8 (-1.5-22.2)	0 (0-0)	1.7 (1.2-2.4)	0 (0-0)	1.8 (1.2-2.5)	100	91	12	11
Bury	0 (0-0)	0 (0-1)	12.8 (0.4-26.3)	5 (3-6)	5 (3-7)	2.5 (-7.5-10.8)	0 (0-0)	0 (0-0)	15 (1.5-32.1)	0 (0-1)	0 (0-1)	2.3 (-7.1-12.1)	0 (0-0)	0 (0-0)	58.8 (18.9-97)	0 (0-0)	0 (0-0)	3.8 (-9.1-18.7)	0 (0-0)	1.2 (0.8-1.7)	0 (0-0)	1 (0.7-1.5)	77	68	14	19
Cheshire East	0 (0-1)	1 (0-1)	9.9 (0.5-22.5)	8 (6-10)	8 (6-10)	-0.7 (-6.3-6.8)	0 (0-0)	0 (0-0)	20.1 (1.6-38)	1 (0-1)	1 (0-1)	-2.4 (-11.5-7.1)	0 (0-0)	0 (0-0)	47.1 (18.9-89)	0 (0-0)	0 (0-0)	-5.3 (-16.8-8.2)	0 (0-0)	1 (0.6-1.5)	0 (0-0)	1 (0.6-1.5)	96	86	16	18
Cheshire West and Chester	0 (0-1)	0 (0-1)	7.4 (-6.3-20.5)	5 (4-7)	5 (4-7)	-6.2 (-14.8-10.4)	0 (0-0)	0 (0-0)	19.2 (1.4-40.7)	0 (0-1)	0 (0-1)	-9.6 (-21.1-3.2)	0 (0-0)	0 (0-0)	59 (20-120)	0 (0-0)	0 (0-0)	-8.6 (-23.8-10.7)	0 (0-0)	0.9 (0.5-1.4)	0 (0-0)	0.9 (0.5-1.4)	102	95	18	20
Cumbria	1 (0-1)	1 (0-1)	1.7 (-6.5-13.1)	10 (8-14)	10 (7-13)	-7.5 (-15.3-0.8)	0 (0-0)	0 (0-0)	36.6 (18.7-55.5)	1 (1-1)	1 (1-1)	-8.9 (-16.7-0.6)	0 (0-0)	0 (0-0)	10.8 (-17-51.5)	0 (0-0)	0 (0-0)	-9.4 (-19.3-5)	0 (0-0)	1 (0.7-1.5)	0 (0-0)	1.1 (0.8-1.7)	106	96	16	16
Halton	0 (0-0)	0 (0-0)	3.9 (-5.4-19.7)	2 (2-3)	2 (2-3)	1.5 (-6.3-15.3)	0 (0-0)	0 (0-0)	23 (2.8-43.7)	0 (0-0)	0 (0-0)	0.6 (-10.8-15.3)	0 (0-0)	0 (0-0)	12.5 (-18.5-56.5)	0 (0-0)	0 (0-0)	-1.4 (-15.7-17.1)	0 (0-0)	0.7 (0.4-1.1)	0 (0-0)	1 (0.6-1.5)	104	98	22	20
Knowsley	0 (0-0)	0 (0-0)	6.4 (-6.7-27.2)	3 (2-4)	3 (2-3)	-11.3 (-19.5-6.3)	0 (0-0)	0 (0-0)	7 (-12.1-21.5)	0 (0-0)	0 (0-0)	-13 (-22.6-0.7)	0 (0-0)	0 (0-0)	-0.9 (-28.7-33.1)	0 (0-0)	0 (0-0)	-5.7 (-20-11.4)	0 (0-0)	0.8 (0.5-1.1)	0 (0-0)	0.8 (0.5-1.3)	100	94	20	22
Lancashire	2 (1-2)	2 (1-2)	2.6 (-7-15.9)	24 (19-32)	22 (16-28)	-11.8 (-20.1--3.9)	0 (0-0)	0 (0-0)	35.1 (20.5-53.7)	2 (1-3)	2 (1-2)	-10.3 (-19.1--1.1)	0 (0-0)	0 (0-0)	24.9 (-9.3-67.9)	1 (0-1)	1 (0-1)	-9 (-20.2-4.2)	0 (0-0)	0.8 (0.5-1.2)	0 (0-0)	0.8 (0.5-1.4)	96	83	18	20
Liverpool	2 (1-2)	2 (2-3)	25.1 (16.1-35.1)	31 (23-41)	32 (24-43)	3.1 (-3.1-11.1)	0 (0-0)	0 (0-0)	-8.6 (-19-3.5)	2 (1-3)	2 (2-3)	6.7 (-0.6-14.7)	0 (0-0)	0 (0-0)	41.3 (8.3-82.3)	1 (0-1)	1 (1-1)	15.2 (4.9-26.6)	0 (0-0)	1.8 (1.2-2.4)	0 (0-0)	1.8 (1.2-2.5)	73	68	11	12
Manchester	4 (3-5)	6 (5-8)	58.4 (47.7-69.5)	66 (52-82)	95 (76-118)	44.1 (34.8-53.6)	0 (0-0)	0 (0-0)	1.6 (-9.9-15)	4 (3-5)	6 (5-8)	48 (37.5-58.1)	0 (0-0)	0 (0-0)	14.6 (-10.6-42.8)	1 (1-2)	2 (2-3)	57.3 (40.9-73.8)	0 (0-0.1)	2.6 (1.9-3.6)	0 (0-0.1)	3.6 (2.6-5.1)	66	62	8	6

Location	Births, 2000	Births, 2021	Births percent change (2000-2021)	All age cases, 2000	All age cases, 2021	All age cases percent change (2000-2021)	All age deaths (cause-specific), 2000	All age deaths (cause-specific), 2021	All age deaths (cause-specific) percent change (2000-2021)	All age deaths (total), 2000	All age deaths (total), 2021	All age deaths (total) percent change (2000-2021)	Under-5 deaths (cause-specific), 2000	Under-5 deaths (cause-specific), 2021	Under-5 deaths (cause-specific) percent change (2000-2021)	Under-5 deaths (total), 2000	Under-5 deaths (total), 2021	Under-5 deaths (total) percent change (2000-2021)	Under-5 cause-specific mortality cause fraction, 2000	Under-5 total SCD mortality cause fraction, 2000	Under-5 cause-specific mortality cause fraction, 2021	Under-5 total SCD mortality cause fraction, 2021	Under-5 cause-specific mortality rank 2000	Under-5 cause-specific mortality rank 2021	Under-5 total SCD mortality rank 2000	Under-5 total SCD mortality rank 2021
Oldham	0 (0-1)	1 (0-1)	14.2 (5.7-22.3)	6 (4-8)	6 (5-8)	7.4 (0.9-15.5)	0 (0-0)	0 (0-0)	25 (11.2-39.7)	0 (0-1)	0 (0-1)	7 (-3.6-16.9)	0 (0-0)	0 (0-0)	32.9 (7.3-68.3)	0 (0-0)	0 (0-0)	5.4 (-9.7-19.5)	0 (0-0)	0.7 (0.5-1.1)	0 (0-0)	0.7 (0.4-1)	74	70	19	22
Rochdale	0 (0-0)	0 (0-1)	13.3 (5-25)	4 (3-5)	4 (3-5)	1.1 (-6.1-10.7)	0 (0-0)	0 (0-0)	38.7 (17.4-57.3)	0 (0-1)	0 (0-0)	-0.3 (-10.2-10.9)	0 (0-0)	0 (0-0)	62.8 (25.5-111)	0 (0-0)	0 (0-0)	0.3 (-11.7-16.1)	0 (0-0)	0.7 (0.4-1)	0 (0-0)	0.7 (0.4-1.1)	101	93	20	23
Salford	2 (1-2)	2 (2-3)	42.4 (27.4-52.6)	28 (22-35)	34 (27-43)	22.3 (11-31.3)	0 (0-0)	0 (0-0)	7.9 (-6.5-27)	2 (1-2)	2 (2-3)	24.9 (15.3-36.8)	0 (0-0)	0 (0-0)	127 (80.3-187)	1 (0-1)	1 (1-1)	40.1 (26.6-57)	0 (0-0)	2.9 (2-4)	0 (0-0)	3 (2.1-4.1)	85	69	8	7
Sefton	0 (0-0)	0 (0-0)	2.5 (-6.5-18)	5 (4-7)	4 (3-6)	-14.6 (-23.4-2.9)	0 (0-0)	0 (0-0)	11.1 (-4.8-30.2)	0 (0-1)	0 (0-0)	-16.7 (-25.6--3.9)	0 (0-0)	0 (0-0)	7.5 (-20.9-38.6)	0 (0-0)	0 (0-0)	-10.5 (-24.8-5.6)	0 (0-0)	0.8 (0.5-1.3)	0 (0-0)	0.8 (0.5-1.2)	85	77	18	22
St Helens	0 (0-0)	0 (0-0)	3.9 (-8.6-15.7)	3 (2-4)	3 (2-4)	-10.8 (-19--0.4)	0 (0-0)	0 (0-0)	35.5 (13.2-62.8)	0 (0-0)	0 (0-0)	-12.3 (-22.1--1.1)	0 (0-0)	0 (0-0)	2.7 (-28.9-44.7)	0 (0-0)	0 (0-0)	-8.3 (-22.9-7.6)	0 (0-0)	0.7 (0.4-1.1)	0 (0-0)	1 (0.6-1.5)	99	93	20	19
Stockport	0 (0-1)	1 (0-1)	9.6 (0.4-20.7)	8 (6-11)	8 (6-10)	-4.6 (-10.7-4.2)	0 (0-0)	0 (0-0)	3.2 (-13.7-21)	1 (0-1)	1 (0-1)	-3.8 (-10.9-5)	0 (0-0)	0 (0-0)	40.8 (14.1-76)	0 (0-0)	0 (0-0)	0.2 (-10.9-12.9)	0 (0-0)	1.1 (0.8-1.7)	0 (0-0)	1.1 (0.7-1.6)	88	76	13	17
Tameside	1 (1-1)	1 (1-1)	15.6 (7.2-25.2)	17 (12-22)	17 (12-22)	0.8 (-5.5-8.1)	0 (0-0)	0 (0-0)	43.1 (20.3-71.2)	1 (1-1)	1 (1-1)	3 (-3.8-10.2)	0 (0-0)	0 (0-0)	22.4 (-7.3-65)	0 (0-1)	0 (0-1)	7.8 (-1.3-18.1)	0 (0-0)	2.2 (1.5-3.1)	0 (0-0)	2.7 (1.8-3.7)	99	95	8	9
Trafford	0 (0-0)	0 (0-0)	13.2 (0.8-24.8)	4 (3-5)	4 (3-6)	16.1 (4.4-36.6)	0 (0-0)	0 (0-0)	-14.3 (-29.2-1.4)	0 (0-0)	0 (0-0)	17.3 (2.6-33)	0 (0-0)	0 (0-0)	8.7 (-14.3-42.2)	0 (0-0)	0 (0-0)	13.7 (-4.2-33.4)	0 (0-0)	0.8 (0.5-1.2)	0 (0-0)	1.1 (0.7-1.7)	86	77	19	15
Warrington	0 (0-0)	0 (0-0)	3 (-6.4-15.7)	3 (3-5)	3 (3-5)	0.9 (-6.4-10.7)	0 (0-0)	0 (0-0)	3.7 (-10.9-21.5)	0 (0-0)	0 (0-0)	-1.6 (-11.5-10.8)	0 (0-0)	0 (0-0)	25.7 (-6.3-60.4)	0 (0-0)	0 (0-0)	-6.1 (-19.3-8.2)	0 (0-0)	0.8 (0.5-1.2)	0 (0-0)	1 (0.6-1.5)	86	75	17	18
Wigan	1 (1-1)	1 (1-1)	7.9 (-1.7-17.3)	12 (9-16)	13 (9-17)	1.9 (-5.8-11.3)	0 (0-0)	0 (0-0)	41.2 (19.8-65.3)	1 (1-1)	1 (1-1)	2.3 (-5.5-11.1)	0 (0-0)	0 (0-0)	27.2 (-8.2-68.5)	0 (0-0)	0 (0-0)	0.2 (-11.1-14.4)	0 (0-0)	1.1 (0.8-1.6)	0 (0-0)	1.3 (0.8-1.8)	99	91	15	16
Wirral	1 (0-1)	1 (0-1)	-4.3 (-12.6-7.7)	9 (7-11)	7 (6-10)	-15.1 (-22.6--5.3)	0 (0-0)	0 (0-0)	12.3 (-1.7-25.6)	1 (0-1)	1 (0-1)	-13 (-21--4.4)	0 (0-0)	0 (0-0)	14 (-13.3-47)	0 (0-0)	0 (0-0)	-12.2 (-23.3--0.3)	0 (0-0)	0.9 (0.6-1.4)	0 (0-0)	0.9 (0.6-1.4)	82	70	17	20
South East England	16 (13-22)	19 (15-25)	16.9 (13.6-20.1)	278 (214-353)	306 (237-385)	9.9 (7.3-13)	1 (1-1)	1 (1-1)	16.5 (6.2-26.4)	18 (14-24)	20 (15-26)	10.2 (6.7-13.4)	0 (0-0)	0 (0-0)	16.5 (6.2-29.1)	6 (4-8)	7 (5-9)	9.9 (5.2-14.3)	0 (0-0)	1.3 (0.9-1.8)	0 (0-0)	1.5 (1-2)	86	79	14	12
Bracknell Forest	0 (0-0)	0 (0-0)	-1.7 (-15.4-12.2)	2 (2-3)	2 (2-3)	-1.7 (-12-20.2)	0 (0-0)	0 (0-0)	-14.3 (-29.9-1)	0 (0-0)	0 (0-0)	-2.2 (-15.4-14.7)	0 (0-0)	0 (0-0)	-54.2 (-66.5--40.8)	0 (0-0)	0 (0-0)	-8.3 (-23.4-10.1)	0 (0-0)	1 (0.6-1.5)	0 (0-0)	1.8 (1-2.8)	76	74	19	12
Brighton and Hove	0 (0-1)	1 (0-1)	12.7 (4.5-23.8)	7 (5-10)	8 (6-12)	18 (11.2-25.3)	0 (0-0)	0 (0-0)	-2.4 (-18.7-15.3)	0 (0-1)	1 (0-1)	15.5 (6.2-25.8)	0 (0-0)	0 (0-0)	-26.9 (-45.8--1.6)	0 (0-0)	0 (0-0)	5.7 (-6.7-20.4)	0 (0-0)	0.9 (0.6-1.3)	0 (0-0)	1.7 (1.1-2.5)	90	79	18	11
Buckinghamshire	1 (1-2)	1 (1-2)	9.4 (0.5-17.6)	23 (16-31)	24 (16-32)	3.5 (-3.9-10.2)	0 (0-0)	0 (0-0)	4.8 (-14.2-24.3)	1 (1-2)	1 (1-2)	4.4 (-2.6-12.3)	0 (0-0)	0 (0-0)	28.4 (1-55.2)	0 (0-1)	0 (0-1)	0.4 (-9.7-11.2)	0 (0-0)	1.5 (1-2.2)	0 (0-0)	1.4 (0.9-2)	94	84	13	13
East Sussex	1 (0-1)	1 (0-1)	14.4 (0.3-30.8)	8 (6-10)	8 (6-11)	3.5 (-8.6-18.7)	0 (0-0)	0 (0-0)	17.2 (-0.7-35.6)	1 (0-1)	1 (0-1)	-1 (-13-13.4)	0 (0-0)	0 (0-0)	-0.7 (-26.3-32.3)	0 (0-0)	0 (0-0)	-4.7 (-19.9-12.8)	0 (0-0)	0.9 (0.5-1.4)	0 (0-0)	1 (0.6-1.6)	91	78	18	17
Hampshire	2 (2-3)	3 (2-3)	9.7 (2.1-19.5)	43 (33-54)	44 (34-56)	1.5 (-5-9.4)	0 (0-0)	0 (0-0)	25.7 (10.4-45.8)	3 (2-4)	3 (2-4)	1.8 (-6.5-10.3)	0 (0-0)	0 (0-0)	34.3 (3.2-80.8)	1 (1-1)	1 (1-1)	-2.6 (-15.2-9.4)	0 (0-0)	1.3 (0.9-1.9)	0 (0-0)	1.1 (0.8-1.7)	98	88	14	14
Isle of Wight	0 (0-0)	0 (0-0)	19.4 (6.1-38.9)	2 (1-3)	2 (2-3)	-1.1 (-10.5-15.5)	0 (0-0)	0 (0-0)	39.7 (15.4-63.1)	0 (0-0)	0 (0-0)	-4.6 (-14.8-9.7)	0 (0-0)	0 (0-0)	-16 (-39.8-18.7)	0 (0-0)	0 (0-0)	-2.7 (-17.6-16.7)	0 (0-0)	1.1 (0.6-1.6)	0 (0-0)	1.4 (0.9-2.3)	102	97	17	12
Kent	3 (2-4)	4 (3-5)	16.8 (6.8-28.8)	55 (40-72)	58 (44-74)	4.5 (-4.3-16.1)	0 (0-0)	0 (0-0)	39.6 (18.9-56.9)	4 (3-5)	4 (3-5)	5.7 (-4.2-15.9)	0 (0-0)	0 (0-0)	40.6 (8.3-85.8)	1 (1-2)	1 (1-2)	5.9 (-7.2-19.4)	0 (0-0)	1.6 (1-2.2)	0 (0-0)	1.4 (1-2)	86	77	13	13
Medway	1 (1-1)	1 (1-1)	22.4 (11.8-33.9)	12 (9-16)	12 (9-16)	5.6 (-4.4-13.1)	0 (0-0)	0 (0-0)	14.1 (-2.5-31.3)	1 (1-1)	1 (1-1)	7.1 (-1.3-15.6)	0 (0-0)	0 (0-0)	5 (-22.6-37.4)	0 (0-0)	0 (0-0)	9.3 (-2.2-21.9)	0 (0-0)	1.3 (0.9-1.7)	0 (0-0)	1.7 (1.1-2.4)	91	77	15	14
Milton Keynes	1 (1-2)	2 (2-2)	27.6 (19.1-37.7)	27 (21-34)	34 (26-45)	26.9 (17.9-38.1)	0 (0-0)	0 (0-0)	17.9 (2.1-36.5)	2 (1-2)	2 (2-3)	27.4 (18.3-35.8)	0 (0-0)	0 (0-0)	1.8 (-27.1-31.9)	1 (0-1)	1 (1-1)	33.7 (21.1-48.8)	0 (0-0)	2.5 (1.9-3.5)	0 (0-0)	3.6 (2.4-4.8)	80	75	8	6
Oxfordshire	1 (1-2)	2 (1-2)	13.6 (6.8-22.1)	24 (17-31)	26 (19-35)	8.8 (2.2-15.9)	0 (0-0)	0 (0-0)	3.3 (-11-21.9)	2 (1-2)	2 (1-2)	8.7 (1.7-16)	0 (0-0)	0 (0-0)	-0.2 (-18.8-32.2)	0 (0-1)	1 (0-1)	7.3 (-3.5-19.2)	0 (0-0)	1.4 (1-2.1)	0 (0-0)	1.9 (1.3-2.7)	80	76	14	12
Portsmouth	0 (0-0)	0 (0-0)	27.4 (16.2-41.5)	3 (2-5)	4 (3-5)	20.9 (10.9-36.5)	0 (0-0)	0 (0-0)	7.3 (-9.8-24.7)	0 (0-0)	0 (0-0)	20.9 (9.1-36)	0 (0-0)	0 (0-0)	-6.4 (-25.3-24.3)	0 (0-0)	0 (0-0)	21.9 (5.7-42.7)	0 (0-0)	0.7 (0.4-1.1)	0 (0-0)	1.4 (0.8-2.1)	103	91	19	14
Reading	0 (0-0)	0 (0-0)	30 (15.2-50.8)	3 (2-4)	3 (2-5)	26.8 (15.6-43.9)	0 (0-0)	0 (0-0)	9.4 (-6-25)	0 (0-0)	0 (0-0)	32 (16.6-58.1)	0 (0-0)	0 (0-0)	9.6 (-17-39.1)	0 (0-0)	0 (0-0)	36.5 (15.6-69.6)	0 (0-0)	0.6 (0.4-0.9)	0 (0-0)	0.9 (0.5-1.4)	74	72	25	18
Slough	1 (0-1)	1 (1-1)	40 (31.3-50)	9 (6-12)	14 (9-18)	48.3 (38.1-64.8)	0 (0-0)	0 (0-0)	-7 (-17.6-3.4)	1 (0-1)	1 (1-1)	48.8 (39.2-59.8)	0 (0-0)	0 (0-0)	49.2 (19.9-93.3)	0 (0-0)	0 (0-1)	57 (44.7-71.7)	0.1 (0.1-0.1)	1.9 (1.2-2.8)	0.1 (0.1-0.1)	2 (1.4-2.9)	64	61	11	10
Southampton	1 (0-1)	1 (0-1)	37.2 (26.6-48.5)	8 (6-11)	9 (7-13)	16.8 (6.7-26.3)	0 (0-0)	0 (0-0)	4.7 (-8.8-19.9)	1 (0-1)	1 (0-1)	22 (12.6-33)	0 (0-0)	0 (0-0)	8.8 (-20.4-58)	0 (0-0)	0 (0-0)	34.8 (19.6-51.8)	0 (0-0)	1.3 (0.8-1.9)	0 (0-0)	1.8 (1.1-2.6)	88	78	14	12
Surrey	2 (1-2)	2 (2-3)	13.8 (4.3-24.2)	30 (23-38)	33 (26-41)	11.3 (3.1-19.1)	0 (0-0)	0 (0-0)	9.9 (-4.1-25.7)	2 (1-3)	2 (2-3)	11.4 (2-21.2)	0 (0-0)	0 (0-0)	22.3 (-5.2-48.9)	1 (0-1)	1 (0-1)	8.4 (-5.2-21.9)	0 (0-0)	1.3 (0.8-1.8)	0 (0-0)	1.4 (0.9-2)	91	78	14	14
West Berkshire	0 (0-0)	0 (0-0)	1.6 (-8.9-18.7)	3 (2-3)	3 (2-4)	2.9 (-6-16.9)	0 (0-0)	0 (0-0)	18.4 (0.6-38.6)	0 (0-0)	0 (0-0)	3.9 (-7.1-17.8)	0 (0-0)	0 (0-0)	23.2 (-10-67)	0 (0-0)	0 (0-0)	-2 (-17.5-14.4)	0 (0-0)	0.9 (0.5-1.4)	0 (0-0)	1.1 (0.6-1.6)	102	98	18	18
West Sussex	1 (1-1)	1 (1-2)	17.2 (4.8-29.2)	15 (11-20)	17 (12-22)	9.4 (2.3-20.1)	0 (0-0)	0 (0-0)	29 (14.7-49.9)	1 (1-2)	1 (1-2)	7.4 (-4.6-20.7)	0 (0-0)	0 (0-0)	13.5 (-10.7-38.7)	0 (0-1)	0 (0-1)	5.2 (-10-22.7)	0 (0-0)	1 (0.7-1.6)	0 (0-0)	1 (0.7-1.5)	77	71	17	17

Location	Births, 2000	Births, 2021	Births percent change (2000-2021)	All age cases, 2000	All age cases, 2021	All age cases percent change (2000-2021)	All age deaths (cause-specific), 2000	All age deaths (cause-specific), 2021	All age deaths (cause-specific) percent change (2000-2021)	All age deaths (total), 2000	All age deaths (total), 2021	All age deaths (total) percent change (2000-2021)	Under-5 deaths (cause-specific), 2000	Under-5 deaths (cause-specific), 2021	Under-5 deaths (cause-specific) percent change (2000-2021)	Under-5 deaths (total), 2000	Under-5 deaths (total), 2021	Under-5 deaths (total) percent change (2000-2021)	Under-5 cause-specific mortality cause fraction, 2000	Under-5 total SCD mortality cause fraction, 2000	Under-5 cause-specific mortality cause fraction, 2021	Under-5 total SCD mortality cause fraction, 2021	Under-5 cause-specific mortality rank 2000	Under-5 cause-specific mortality rank 2021	Under-5 total SCD mortality rank 2000	Under-5 total SCD mortality rank 2021
Windsor and Maidenhead	0 (0-0)	0 (0-0)	7.4 (-4.8-23.3)	2 (2-3)	3 (2-4)	11.8 (1.6-28.4)	0 (0-0)	0 (0-0)	0.2 (-14-15.1)	0 (0-0)	0 (0-0)	11.2 (-1.6-28.7)	0 (0-0)	0 (0-0)	-1.5 (-26.3-27.7)	0 (0-0)	0 (0-0)	2.5 (-13.3-19.7)	0 (0-0)	0.8 (0.5-1.3)	0 (0-0)	1.2 (0.7-1.8)	78	72	21	17
Wokingham	0 (0-0)	0 (0-0)	-5.9 (-18.1-7.2)	3 (2-4)	3 (2-4)	-4.3 (-12.8-7.2)	0 (0-0)	0 (0-0)	33.7 (13.8-54.1)	0 (0-0)	0 (0-0)	-2.5 (-12.9-10.3)	0 (0-0)	0 (0-0)	19.1 (-10-61)	0 (0-0)	0 (0-0)	-9.2 (-22.7-4.3)	0 (0-0)	1 (0.6-1.5)	0 (0-0)	1 (0.6-1.5)	81	73	16	17
South West England	8 (6-10)	9 (7-12)	20.2 (16.2-26.8)	131 (104-165)	141 (111-177)	7.1 (3.5-14.7)	0 (0-0)	1 (0-1)	21.2 (11.1-31.9)	9 (7-12)	10 (7-13)	5.8 (1.8-11.7)	0 (0-0)	0 (0-0)	-4.4 (-20.6-12.3)	3 (2-4)	3 (2-5)	8 (2.6-14.2)	0 (0-0)	1.1 (0.7-1.5)	0 (0-0)	1.5 (1-2)	93	88	17	12
Bath and North East Somerset	0 (0-0)	0 (0-1)	17.2 (5.9-24.3)	7 (5-9)	7 (5-9)	2.3 (-5.5-10.2)	0 (0-0)	0 (0-0)	-15.2 (-28.6--0.3)	0 (0-1)	0 (0-1)	2.3 (-6.7-10.1)	0 (0-0)	0 (0-0)	-12.7 (-34.4-6.9)	0 (0-0)	0 (0-0)	3 (-9.8-14.1)	0 (0-0)	1.8 (1.1-2.5)	0 (0-0)	2.4 (1.5-3.3)	84	77	12	12
Bournemouth	1 (0-1)	1 (0-1)	19.3 (5.3-34.9)	11 (8-14)	11 (8-14)	-1.8 (-13.1-8.9)	0 (0-0)	0 (0-0)	4.7 (-14-29.6)	1 (0-1)	1 (0-1)	-1.8 (-14.4-12.1)	0 (0-0)	0 (0-0)	-56.6 (-70--36.8)	0 (0-0)	0 (0-0)	9.6 (-7.4-28.6)	0 (0-0)	1.8 (1.2-2.5)	0 (0-0)	5.7 (3.5-8.3)	102	92	11	5
Bristol, City of	2 (2-2)	3 (2-3)	28.9 (19.7-39.6)	36 (28-46)	41 (32-52)	13.6 (5.1-24.2)	0 (0-0)	0 (0-0)	16.7 (2.3-31.4)	2 (2-3)	2 (2-3)	14.3 (5.6-22.7)	0 (0-0)	0 (0-0)	3.8 (-19.9-34.2)	1 (0-1)	1 (1-1)	21.5 (8.5-34.8)	0 (0-0)	2.3 (1.6-3)	0 (0-0)	3.6 (2.4-4.7)	78	68	8	7
Cornwall	1 (0-1)	1 (0-1)	21.2 (3.5-45.3)	8 (6-10)	8 (6-11)	5.3 (-5.8-23.8)	0 (0-0)	0 (0-0)	49.9 (26.3-78.1)	1 (0-1)	1 (0-1)	1.6 (-10.9-18.4)	0 (0-0)	0 (0-0)	16.3 (-10.9-46.6)	0 (0-0)	0 (0-0)	0.9 (-16.2-22.3)	0 (0-0)	0.8 (0.5-1.4)	0 (0-0)	0.9 (0.6-1.4)	106	103	17	17
Devon	1 (0-1)	1 (1-1)	16 (1.4-37.9)	11 (8-14)	11 (8-15)	2.9 (-8.7-23.2)	0 (0-0)	0 (0-0)	34.3 (9.9-54.9)	1 (1-1)	1 (1-1)	0.4 (-10.8-18)	0 (0-0)	0 (0-0)	5.7 (-21.6-49.6)	0 (0-0)	0 (0-0)	2 (-14.2-21.6)	0 (0-0)	0.8 (0.5-1.2)	0 (0-0)	1 (0.6-1.4)	93	88	17	16
Dorset	0 (0-0)	0 (0-1)	9.6 (-5.2-23.6)	6 (4-8)	6 (4-8)	-1.3 (-11.2-17.2)	0 (0-0)	0 (0-0)	16 (-3.1-42.7)	0 (0-1)	0 (0-1)	-3.8 (-15.3-11.3)	0 (0-0)	0 (0-0)	-0.2 (-22.8-35.1)	0 (0-0)	0 (0-0)	-4 (-18.1-16.3)	0 (0-0)	0.9 (0.5-1.4)	0 (0-0)	1.2 (0.8-1.9)	104	99	18	13
Gloucestershire	1 (1-1)	1 (1-1)	16.5 (4-29.6)	15 (11-20)	16 (12-21)	3.9 (-5.1-12.3)	0 (0-0)	0 (0-0)	29.9 (9.4-51.5)	1 (1-1)	1 (1-1)	3.1 (-6.8-13.3)	0 (0-0)	0 (0-0)	-2.9 (-24.9-29.5)	0 (0-0)	0 (0-1)	1.9 (-12.5-16)	0 (0-0)	1 (0.7-1.4)	0 (0-0)	1.2 (0.8-1.7)	81	75	17	15
North Somerset	0 (0-0)	0 (0-0)	21.2 (8.7-33.9)	3 (2-4)	3 (2-5)	8.6 (0.6-20.5)	0 (0-0)	0 (0-0)	18.5 (1.3-44.4)	0 (0-0)	0 (0-0)	8.1 (-3.9-20.5)	0 (0-0)	0 (0-0)	-3.6 (-26.8-29.1)	0 (0-0)	0 (0-0)	6.5 (-8.9-23.3)	0 (0-0)	0.8 (0.5-1.2)	0 (0-0)	1.2 (0.7-1.9)	102	93	19	15
Plymouth	0 (0-0)	0 (0-1)	22.9 (4.6-45.4)	4 (3-6)	5 (3-6)	9.8 (-1.1-28.5)	0 (0-0)	0 (0-0)	33.6 (15.3-62.3)	0 (0-0)	0 (0-0)	7.1 (-4.8-22.8)	0 (0-0)	0 (0-0)	1.1 (-24.1-37.3)	0 (0-0)	0 (0-0)	13.3 (-2.9-33.8)	0 (0-0)	0.7 (0.4-1.1)	0 (0-0)	1.2 (0.7-1.7)	103	96	20	15
Poole	0 (0-0)	0 (0-0)	13.9 (3.4-29.5)	2 (2-3)	2 (2-3)	10.5 (2.5-28.3)	0 (0-0)	0 (0-0)	-5 (-23.8-19.7)	0 (0-0)	0 (0-0)	8.9 (-2-24.7)	0 (0-0)	0 (0-0)	-76.9 (-83.3--67.3)	0 (0-0)	0 (0-0)	8 (-7.5-29.1)	0 (0-0)	0.8 (0.5-1.3)	0 (0-0)	3.4 (1.8-5)	79	77	21	9
Somerset	1 (0-1)	1 (0-1)	17.8 (1.8-39.5)	8 (6-11)	8 (6-11)	4.1 (-6.2-24.8)	0 (0-0)	0 (0-0)	23.3 (5.8-44.6)	1 (0-1)	1 (0-1)	1.6 (-10.4-19.6)	0 (0-0)	0 (0-0)	6.2 (-30.2-45.5)	0 (0-0)	0 (0-0)	1.9 (-14.4-24.3)	0 (0-0)	0.9 (0.5-1.3)	0 (0-0)	0.9 (0.6-1.4)	93	88	17	18
South Gloucestershire	1 (0-1)	1 (0-1)	15.9 (5.9-26.2)	8 (6-11)	9 (6-12)	6.6 (-1.5-15.2)	0 (0-0)	0 (0-0)	1.7 (-12.5-18.5)	1 (0-1)	1 (0-1)	5.7 (-3.5-14.5)	0 (0-0)	0 (0-0)	28.6 (1.9-59.2)	0 (0-0)	0 (0-0)	7.8 (-5.3-20.8)	0 (0-0)	1.4 (0.9-1.9)	0 (0-0)	1.7 (1.1-2.4)	101	92	13	12
Swindon	0 (0-0)	0 (0-0)	33.2 (22.3-52.2)	3 (2-5)	4 (3-6)	29 (17.9-41.3)	0 (0-0)	0 (0-0)	25.9 (8.3-51.8)	0 (0-0)	0 (0-0)	27.3 (15.3-44.4)	0 (0-0)	0 (0-0)	9.3 (-18.8-49.6)	0 (0-0)	0 (0-0)	24.8 (9.2-48.7)	0 (0-0)	0.8 (0.5-1.1)	0 (0-0)	1.3 (0.7-1.9)	106	97	18	14
Torbay	0 (0-0)	0 (0-0)	17.5 (4.2-33.4)	2 (1-3)	2 (2-3)	-0.7 (-11.2-16.5)	0 (0-0)	0 (0-0)	30 (11.2-51.4)	0 (0-0)	0 (0-0)	-1.2 (-12.8-14.4)	0 (0-0)	0 (0-0)	-0.2 (-25.9-47.2)	0 (0-0)	0 (0-0)	5.1 (-11.7-24.8)	0 (0-0)	0.9 (0.5-1.4)	0 (0-0)	1.4 (0.8-2)	103	99	20	15
Wiltshire	1 (0-1)	1 (0-1)	9 (-2.9-29)	7 (5-10)	8 (6-11)	7.1 (-2.8-30)	0 (0-0)	0 (0-0)	28 (6.9-46.2)	1 (0-1)	1 (0-1)	4.7 (-7.5-22.7)	0 (0-0)	0 (0-0)	49.2 (9.8-97.7)	0 (0-0)	0 (0-0)	-3.3 (-16.7-15)	0 (0-0)	0.9 (0.5-1.4)	0 (0-0)	0.8 (0.5-1.3)	108	99	18	21
West Midlands	15 (12-18)	18 (14-22)	19.8 (15.8-24.7)	234 (185-287)	261 (209-319)	11.3 (7.9-16.5)	2 (2-2)	2 (2-2)	11.4 (4.1-20.3)	16 (12-20)	18 (14-22)	11.2 (7.2-15.4)	0 (0-0)	0 (0-0)	10.8 (-0.6-24.6)	6 (4-8)	6 (5-9)	11.6 (6.1-17.4)	0 (0-0)	1.2 (0.9-1.6)	0 (0-0)	1.2 (0.9-1.7)	75	68	14	14
Birmingham	6 (5-7)	8 (6-10)	30.5 (22.1-42.5)	96 (76-120)	121 (97-154)	25.8 (19.2-39.8)	1 (1-1)	1 (1-1)	11.5 (1-25.3)	6 (5-7)	8 (6-9)	26.3 (18.1-36.2)	0 (0-0)	0 (0-0)	4.8 (-13-25)	2 (2-3)	3 (2-4)	24.5 (12.8-38)	0 (0-0.1)	1.5 (1.1-2)	0 (0-0)	1.8 (1.3-2.4)	66	57	13	10
Coventry	1 (1-2)	2 (1-2)	31.1 (23.4-41.4)	20 (14-27)	22 (16-30)	11.4 (2.3-18.7)	0 (0-0)	0 (0-0)	12.8 (-0.3-26.8)	1 (1-2)	2 (1-2)	14.2 (6.6-22)	0 (0-0)	0 (0-0)	20.7 (0.4-46.8)	0 (0-1)	1 (0-1)	22.2 (11.3-34.3)	0 (0-0)	1.5 (1.1-2.2)	0 (0-0)	2.1 (1.3-3)	73	71	13	10
Dudley	1 (1-1)	1 (1-1)	-7.3 (-16.5-5)	16 (12-22)	14 (10-18)	-16.5 (-24.6--6.2)	0 (0-0)	0 (0-0)	15.3 (-1.3-35.1)	1 (1-1)	1 (1-1)	-13.5 (-21.8--4.8)	0 (0-0)	0 (0-0)	25.6 (-1.3-53)	0 (0-0)	0 (0-0)	-11.4 (-21.8-1)	0 (0-0)	1.4 (1-1.9)	0 (0-0)	1.4 (0.9-1.9)	82	72	14	14
Herefordshire, County of	0 (0-0)	0 (0-0)	11.2 (1.2-27.1)	3 (2-4)	3 (2-4)	-2 (-10.8-12.6)	0 (0-0)	0 (0-0)	72.3 (47.9-113)	0 (0-0)	0 (0-0)	-6.1 (-17.3-8.1)	0 (0-0)	0 (0-0)	47.6 (3.3-88.5)	0 (0-0)	0 (0-0)	-8 (-21.8-10.2)	0 (0-0)	1.1 (0.6-1.8)	0 (0-0)	0.9 (0.5-1.4)	102	95	16	22
Sandwell	2 (1-2)	2 (1-2)	11.2 (4.1-21.1)	24 (18-32)	26 (19-34)	7.1 (0.7-14.9)	0 (0-0)	0 (0-0)	5.3 (-5.5-16.3)	2 (1-2)	2 (1-2)	7.1 (0.1-15.5)	0 (0-0)	0 (0-0)	9.3 (-6-29.5)	1 (0-1)	1 (0-1)	6 (-2.8-16.5)	0 (0-0.1)	1.8 (1.2-2.4)	0 (0-0.1)	1.6 (1.1-2.3)	64	58	11	12
Shropshire	0 (0-0)	0 (0-0)	8.9 (-2-22.2)	5 (3-6)	5 (3-6)	1.8 (-7-19.4)	0 (0-0)	0 (0-0)	35.5 (16.8-57.7)	0 (0-1)	0 (0-1)	-1.6 (-11.1-11)	0 (0-0)	0 (0-0)	28.2 (-1-64.9)	0 (0-0)	0 (0-0)	-5.7 (-19.3-11.5)	0 (0-0)	0.9 (0.5-1.4)	0 (0-0)	0.9 (0.6-1.5)	104	96	20	19
Solihull	0 (0-0)	0 (0-1)	20.6 (11.4-35.7)	7 (5-9)	7 (5-9)	1.4 (-7.4-10.9)	0 (0-0)	0 (0-0)	11.3 (-3.7-29.9)	0 (0-1)	0 (0-1)	1.5 (-7.6-11.9)	0 (0-0)	0 (0-0)	21.3 (-6.9-68.9)	0 (0-0)	0 (0-0)	3.8 (-8.3-19)	0 (0-0)	1.2 (0.8-1.8)	0 (0-0)	1 (0.6-1.5)	86	79	14	17
Staffordshire	1 (1-1)	1 (1-1)	1.9 (-6.4-16.1)	17 (13-23)	16 (12-20)	-10.4 (-18.1-4.9)	0 (0-0)	0 (0-0)	13.4 (-3.2-32.5)	1 (1-2)	1 (1-2)	-9.1 (-18.9-4.4)	0 (0-0)	0 (0-0)	35.6 (12.6-59)	0 (0-1)	0 (0-1)	-9.7 (-23.9-8)	0 (0-0)	0.8 (0.5-1.2)	0 (0-0)	0.6 (0.4-1)	85	75	17	23
Stoke-on-Trent	0 (0-0)	0 (0-1)	15.7 (2.1-34.4)	4 (3-6)	5 (3-6)	9.1 (-0.4-25.7)	0 (0-0)	0 (0-0)	53.6 (27.2-78.4)	0 (0-1)	0 (0-1)	10.8 (-1.3-27.9)	0 (0-0)	0 (0-0)	71.3 (25-132)	0 (0-0)	0 (0-0)	12 (-3.4-35.2)	0 (0-0)	0.5 (0.3-0.8)	0 (0-0)	0.5 (0.3-0.7)	104	102	21	26
Telford and Wrekin	1 (1-1)	1 (1-1)	9 (-2-19.2)	11 (9-15)	11 (9-15)	-1.3 (-11.4-9.4)	0 (0-0)	0 (0-0)	32.6 (12.4-55.1)	1 (1-1)	1 (1-1)	-0.9 (-9.4-8.4)	0 (0-0)	0 (0-0)	20.5 (-9.2-56)	0 (0-0)	0 (0-0)	-0.2 (-11.9-13.6)	0 (0-0)	2.2 (1.5-3)	0 (0-0)	2.5 (1.7-3.6)	97	91	9	8

Location	Births, 2000	Births, 2021	Births percent change (2000-2021)	All age cases, 2000	All age cases, 2021	All age cases percent change (2000-2021)	All age deaths (cause-specific), 2000	All age deaths (cause-specific), 2021	All age deaths (cause-specific) percent change (2000-2021)	All age deaths (total), 2000	All age deaths (total), 2021	All age deaths (total) percent change (2000-2021)	Under-5 deaths (cause-specific), 2000	Under-5 deaths (cause-specific), 2021	Under-5 deaths (cause-specific) percent change (2000-2021)	Under-5 deaths (total), 2000	Under-5 deaths (total), 2021	Under-5 deaths (total) percent change (2000-2021)	Under-5 cause-specific mortality cause fraction, 2000	Under-5 total SCD mortality cause fraction, 2000	Under-5 cause-specific mortality cause fraction, 2021	Under-5 total SCD mortality cause fraction, 2021	Under-5 cause-specific mortality rank 2000	Under-5 cause-specific mortality rank 2021	Under-5 total SCD mortality rank 2000	Under-5 total SCD mortality rank 2021
Walsall	0 (0-1)	0 (0-1)	21.5 (10.6-37.4)	5 (4-6)	5 (4-7)	12.2 (2.8-30.7)	0 (0-0)	0 (0-0)	-0.7 (-14.3-14)	0 (0-1)	0 (0-1)	11.4 (-1.5-27.2)	0 (0-0)	0 (0-0)	27.2 (3.4-49.2)	0 (0-0)	0 (0-0)	11.8 (-6-31.8)	0 (0-0)	0.7 (0.4-1)	0 (0-0)	0.6 (0.4-1)	81	74	20	25
Warwickshire	1 (0-1)	1 (0-1)	17.6 (6.8-34.4)	8 (6-11)	9 (7-12)	7.3 (-3.3-26)	0 (0-0)	0 (0-0)	43.9 (22.1-70.3)	1 (0-1)	1 (1-1)	5.9 (-4.7-23.1)	0 (0-0)	0 (0-0)	61.5 (24.6-109)	0 (0-0)	0 (0-0)	5.1 (-8.7-25.7)	0 (0-0)	0.8 (0.4-1.3)	0 (0-0)	0.7 (0.4-1)	93	82	18	23
Wolverhampton	1 (0-1)	1 (1-1)	20.8 (11.8-29)	8 (6-11)	9 (7-12)	11.6 (4.7-19.7)	0 (0-0)	0 (0-0)	-6.4 (-15.2-6.4)	1 (0-1)	1 (0-1)	12.5 (3.5-22.5)	0 (0-0)	0 (0-0)	-11.5 (-28.5-6)	0 (0-0)	0 (0-0)	16.9 (4-31.6)	0.1 (0-0.1)	0.8 (0.6-1.1)	0.1 (0-0.1)	1 (0.7-1.5)	63	58	16	17
Worcestershire	1 (0-1)	1 (0-1)	6.6 (-3.5-25.4)	9 (7-12)	9 (6-12)	-2.5 (-11.6-13.7)	0 (0-0)	0 (0-0)	49.2 (29.6-73.4)	1 (1-1)	1 (0-1)	-4 (-14.6-12.6)	0 (0-0)	0 (0-0)	20 (-4.6-54.2)	0 (0-0)	0 (0-0)	-5.6 (-19.9-16)	0 (0-0)	0.7 (0.5-1.2)	0 (0-0)	0.7 (0.5-1.2)	96	84	20	22
Yorkshire and the Humber	9 (7-12)	11 (8-14)	19.8 (14.9-24.7)	136 (106-174)	150 (115-193)	9.4 (5.8-14.3)	1 (1-1)	1 (1-1)	24.4 (14.4-33.2)	10 (8-13)	11 (8-14)	8.1 (4.1-12.3)	0 (0-0)	0 (0-0)	3.7 (-7.6-15.8)	4 (2-5)	4 (3-6)	9.5 (4.2-15)	0 (0-0)	0.8 (0.5-1.2)	0 (0-0)	1.1 (0.8-1.6)	90	77	17	17
Barnsley	0 (0-0)	0 (0-0)	19.1 (8.2-34.3)	4 (3-5)	4 (3-6)	5.6 (-2.1-16.5)	0 (0-0)	0 (0-0)	55.4 (31.2-81.6)	0 (0-0)	0 (0-0)	3.7 (-6.8-16)	0 (0-0)	0 (0-0)	23 (-7.5-59.2)	0 (0-0)	0 (0-0)	3.7 (-11.6-19.8)	0 (0-0)	0.7 (0.5-1.1)	0 (0-0)	0.9 (0.5-1.3)	102	95	20	20
Bradford	1 (1-2)	2 (1-2)	14.3 (3.9-27.2)	18 (13-22)	22 (16-28)	22.7 (11.1-36.1)	0 (0-0)	0 (0-0)	49.1 (28.4-72.2)	1 (1-2)	2 (1-2)	20.5 (10-32.1)	0 (0-0)	0 (0-0)	6.3 (-18.3-41.4)	1 (0-1)	1 (0-1)	14.2 (0.8-29.8)	0 (0-0)	0.7 (0.5-1)	0 (0-0)	1 (0.7-1.5)	84	71	20	18
Calderdale	0 (0-0)	0 (0-0)	10.9 (-2.2-23.8)	3 (3-5)	4 (3-5)	3.3 (-6.3-17.4)	0 (0-0)	0 (0-0)	37.1 (16.2-65.6)	0 (0-0)	0 (0-0)	0.9 (-11.2-13.6)	0 (0-0)	0 (0-0)	5.1 (-23.9-38)	0 (0-0)	0 (0-0)	-2.8 (-17.7-13.3)	0 (0-0)	0.6 (0.4-1)	0 (0-0)	1 (0.6-1.5)	101	93	21	17
Doncaster	0 (0-1)	0 (0-1)	10.7 (-1.6-24.2)	5 (4-7)	5 (4-7)	3.6 (-5.8-23.5)	0 (0-0)	0 (0-0)	49.5 (27.8-74.8)	0 (0-1)	0 (0-1)	1.6 (-9-15.9)	0 (0-0)	0 (0-0)	29 (-3-70.1)	0 (0-0)	0 (0-0)	0.8 (-12.9-17.9)	0 (0-0)	0.7 (0.4-1.1)	0 (0-0)	0.8 (0.5-1.1)	103	98	20	21
East Riding of Yorkshire	0 (0-0)	0 (0-0)	5.9 (-8.2-18.9)	5 (4-7)	5 (4-7)	-4.3 (-13.1-12.5)	0 (0-0)	0 (0-0)	14.5 (-5.4-42.2)	0 (0-1)	0 (0-1)	-6.7 (-17.9-5.3)	0 (0-0)	0 (0-0)	-6.8 (-30.6-23.2)	0 (0-0)	0 (0-0)	-8.6 (-23.4-5.8)	0 (0-0)	0.8 (0.5-1.3)	0 (0-0)	1.1 (0.6-1.7)	107	102	19	18
Kingston upon Hull, City of	1 (0-1)	1 (1-1)	16.7 (7.7-24.6)	12 (8-16)	12 (8-18)	4.4 (-4.4-11.2)	0 (0-0)	0 (0-0)	12.6 (-8.8-30.9)	1 (1-1)	1 (1-1)	6.1 (-1.1-13.1)	0 (0-0)	0 (0-0)	1.4 (-24.2-29)	0 (0-0)	0 (0-0)	13.5 (3.4-23.2)	0 (0-0)	1.3 (0.9-1.9)	0 (0-0)	1.8 (1.1-2.6)	86	76	15	12
Kirklees	1 (0-1)	1 (0-1)	8.8 (-1.5-25.7)	7 (5-9)	8 (6-10)	8.4 (-1.6-25.9)	0 (0-0)	0 (0-0)	23.2 (5.5-44.3)	1 (0-1)	1 (0-1)	5.7 (-6.8-22.5)	0 (0-0)	0 (0-0)	16.6 (-5.8-47)	0 (0-0)	0 (0-0)	-0.3 (-15.7-18.1)	0 (0-0)	0.6 (0.4-1)	0 (0-0)	0.8 (0.5-1.1)	84	71	20	20
Leeds	2 (2-3)	3 (2-4)	36.1 (27.4-46)	38 (27-49)	42 (30-54)	10.7 (2.1-20.4)	0 (0-0)	0 (0-0)	20.4 (4.4-34.6)	2 (2-3)	3 (2-3)	12.8 (5.5-20.4)	0 (0-0)	0 (0-0)	2.9 (-17.1-25.9)	1 (1-1)	1 (1-1)	25 (14-37.5)	0 (0-0)	1.3 (0.9-1.7)	0 (0-0)	1.7 (1.2-2.5)	78	69	13	11
North East Lincolnshire	0 (0-0)	0 (0-0)	9.4 (-3.5-26.9)	3 (2-4)	3 (2-4)	-5.4 (-14.4-10.1)	0 (0-0)	0 (0-0)	20.5 (3.1-38.6)	0 (0-0)	0 (0-0)	-8.3 (-18.8-6.3)	0 (0-0)	0 (0-0)	8.8 (-16.2-43.9)	0 (0-0)	0 (0-0)	-6.2 (-21-11)	0 (0-0)	0.8 (0.5-1.2)	0 (0-0)	1 (0.6-1.5)	100	95	19	19
North Lincolnshire	0 (0-0)	0 (0-0)	19.7 (8.7-33.6)	3 (2-4)	3 (2-4)	10.7 (3-23.1)	0 (0-0)	0 (0-0)	6.9 (-11.5-24)	0 (0-0)	0 (0-0)	9.3 (-1.2-21.9)	0 (0-0)	0 (0-0)	0.5 (-25.5-34.4)	0 (0-0)	0 (0-0)	8.2 (-5.8-25.3)	0 (0-0)	0.7 (0.4-1.1)	0 (0-0)	1.2 (0.7-1.9)	105	97	21	16
North Yorkshire	1 (0-1)	1 (0-1)	6.3 (-8.9-25.8)	9 (7-12)	9 (7-12)	-1.6 (-10.9-20.6)	0 (0-0)	0 (0-0)	18.2 (-1.9-41.3)	1 (1-1)	1 (0-1)	-3.5 (-15.5-11.5)	0 (0-0)	0 (0-0)	-9.4 (-30.1-27.5)	0 (0-0)	0 (0-0)	-5 (-21.2-13)	0 (0-0)	0.9 (0.5-1.4)	0 (0-0)	1.1 (0.7-1.8)	94	85	18	15
Rotherham	0 (0-0)	0 (0-1)	12.2 (0.6-26.9)	4 (3-6)	4 (3-6)	-0.1 (-9.5-17)	0 (0-0)	0 (0-0)	24.4 (4.5-47)	0 (0-1)	0 (0-1)	-2.4 (-12.6-10.6)	0 (0-0)	0 (0-0)	-25.6 (-43.1--1.2)	0 (0-0)	0 (0-0)	-2.9 (-17.7-14.3)	0 (0-0)	0.7 (0.4-1)	0 (0-0)	1 (0.6-1.4)	88	80	21	18
Sheffield	1 (1-1)	1 (1-2)	27.7 (17.2-38.5)	18 (13-24)	21 (15-29)	17.5 (7.4-30.1)	0 (0-0)	0 (0-0)	12.4 (-1.2-28.7)	1 (1-2)	1 (1-2)	15.9 (6.3-26.1)	0 (0-0)	0 (0-0)	-5.7 (-24.8-19.2)	0 (0-1)	0 (0-1)	16.2 (1.9-31.4)	0 (0-0)	0.8 (0.5-1.2)	0 (0-0)	1.4 (0.9-2)	77	69	17	16
Wakefield	0 (0-1)	0 (0-1)	13.9 (1.1-29.7)	6 (4-7)	6 (4-8)	-0.2 (-8.7-19.2)	0 (0-0)	0 (0-0)	31.1 (14.4-49.6)	0 (0-1)	0 (0-1)	-1.4 (-13.4-10.9)	0 (0-0)	0 (0-0)	27.7 (-3.8-58.5)	0 (0-0)	0 (0-0)	0.6 (-14.6-18.6)	0 (0-0)	0.7 (0.4-1)	0 (0-0)	0.8 (0.5-1.3)	90	79	19	22
York	0 (0-0)	0 (0-0)	23.3 (8.9-36.3)	3 (2-4)	3 (2-4)	11.6 (0.1-27.9)	0 (0-0)	0 (0-0)	44.3 (23.7-73.1)	0 (0-0)	0 (0-0)	8 (-4.9-20.3)	0 (0-0)	0 (0-0)	-14.7 (-36.4-14.9)	0 (0-0)	0 (0-0)	8.1 (-8.1-25.5)	0 (0-0)	0.7 (0.4-1.1)	0 (0-0)	1.3 (0.8-2.1)	105	97	22	15
Northern Ireland	4 (3-5)	5 (4-6)	14.9 (4.6-22.9)	76 (60-91)	78 (63-93)	2.6 (-5.3-8.4)	1 (1-1)	1 (1-1)	4.7 (-5.5-17)	4 (3-6)	5 (4-6)	1.8 (-5.7-10.2)	0 (0-0)	0 (0-0)	-49.3 (-60.4--33.9)	1 (1-2)	1 (1-2)	-2 (-11.4-9.7)	0 (0-0)	1 (0.7-1.5)	0 (0-0)	1.9 (1.3-2.6)	71	63	14	10
Scotland	10 (8-13)	10 (8-13)	0.1 (-7.9-8)	204 (168-246)	196 (155-234)	-4.3 (-10.5-3)	2 (2-2)	2 (2-2)	14.1 (-0.8-26.1)	12 (9-15)	11 (9-14)	-5.2 (-12.2-1.9)	0 (0-0)	0 (0-0)	-39.5 (-56.3--21.5)	4 (3-5)	3 (2-4)	-12.2 (-21.4--2.7)	0 (0-0)	1 (0.7-1.4)	0 (0-0)	1.7 (1.2-2.4)	70	64	15	12
Wales	6 (5-7)	6 (5-8)	7.7 (-2.5-17.1)	124 (100-148)	123 (101-149)	-0.8 (-7-5.1)	1 (1-1)	1 (1-1)	6.2 (-2.6-16.9)	7 (6-9)	7 (5-9)	-2 (-11.1-7.6)	0 (0-0)	0 (0-0)	-35.5 (-46.6--22.6)	2 (2-3)	2 (1-3)	-4.9 (-16.5-6.9)	0 (0-0)	1.1 (0.8-1.4)	0 (0-0)	1.5 (1.1-2.1)	71	64	13	10
Latin America and Caribbean	4,110 (3,580-4,630)	4,340 (3,800-4,960)	5.6 (2.1-10.5)	51,800 (44,400-58,800)	56,700 (48,400-64,900)	9.2 (6.4-12.1)	1,050 (926-1,200)	1,330 (1,160-1,550)	26.6 (14-44.4)	3,460 (2,960-3,990)	4,080 (3,480-4,710)	17.9 (13.8-22.6)	262 (196-346)	200 (118-288)	-23.7 (-45.2-0)	1,070 (854-1,290)	1,250 (983-1,550)	16.9 (10-25.4)	0.1 (0.1-0.1)	0.3 (0.2-0.4)	0.1 (0.1-0.2)	0.7 (0.5-0.9)	67	59	35	21
**Andean Latin America**	133 (105-163)	145 (113-179)	9.4 (3.5-15.9)	2,080 (1,700-2,550)	2,570 (2,090-3,120)	23.5 (17.6-28.9)	22 (14-32)	17 (12-26)	-22.1 (-48.2-22.9)	93 (72-121)	116 (90-149)	24.3 (15.8-32.7)	1 (0-2)	0 (0-1)	-59.3 (-80.6--21.9)	8 (5-11)	9 (6-13)	18.3 (9.3-29.2)	0 (0-0)	0 (0-0)	0 (0-0)	0 (0-0.1)	124	128	97	74
Bolivia (Plurinational State of)	30 (22-39)	38 (28-51)	24.3 (12-38.1)	429 (328-553)	559 (423-715)	30.2 (19.9-41.3)	8 (5-12)	6 (4-11)	-26.4 (-51.2-28.6)	20 (14-28)	25 (18-36)	25.1 (10.7-39.2)	0 (0-1)	0 (0-0)	-63.8 (-85.4--24.8)	2 (1-3)	3 (2-4)	32.1 (14.2-54.2)	0 (0-0)	0 (0-0)	0 (0-0)	0 (0-0.1)	124	123	99	77
Ecuador	34 (30-36)	40 (36-44)	20 (12.3-27.4)	566 (513-613)	723 (655-797)	27.9 (18.5-35.2)	1 (1-1)	4 (3-5)	415 (306-554)	24 (22-28)	34 (30-38)	39.4 (29.5-52.5)	0 (0-0)	0 (0-0)	151 (40.7-313)	2 (1-2)	2 (2-3)	17.4 (4.1-32.4)	0 (0-0)	0 (0-0)	0 (0-0)	0 (0-0.1)	129	114	93	67
Peru	69 (51-91)	67 (49-89)	-2.4 (-12.8-5.2)	1,110 (865-1,430)	1,280 (981-1,630)	15.2 (5.1-23)	13 (9-20)	7 (4-12)	-45.4 (-68.6--8.2)	50 (35-69)	56 (40-77)	13.5 (1.7-25.5)	1 (0-1)	0 (0-0)	-78.1 (-92.7--55.4)	4 (3-6)	4 (3-6)	6.9 (-6.4-22.9)	0 (0-0)	0 (0-0)	0 (0-0)	0 (0-0.1)	122	129	95	74
**Caribbean**	2,950 (2,550-3,360)	3,210 (2,750-3,690)	8.6 (3.5-15.1)	29,800 (25,300-34,400)	31,700 (26,400-37,300)	6.1 (2-9.8)	556 (424-711)	586 (434-780)	5.3 (-13.8-31.5)	2,460 (2,080-2,840)	2,910 (2,480-3,370)	18.1 (12.9-24.1)	148 (74-228)	122 (51-203)	-17.9 (-47.2-23.7)	922 (736-1,110)	1,110 (867-1,380)	20.5 (12.6-30.2)	0.3 (0.2-0.5)	2.1 (1.7-2.6)	0.4 (0.2-0.6)	3.2 (2.3-4)	33	31	12	9

Location	Births, 2000	Births, 2021	Births percent change (2000-2021)	All age cases, 2000	All age cases, 2021	All age cases percent change (2000-2021)	All age deaths (cause-specific), 2000
Antigua and Barbuda	2 (2-3)	2 (1-2)	-25.9 (-34.2--16.8)	37 (28-47)	31 (23-39)	-15.9 (-24.2--8.4)	2 (1-2)
Bahamas	15 (11-20)	14 (11-18)	-7.8 (-19.2-5.6)	266 (196-338)	249 (191-306)	-6.7 (-17.9-4.9)	6 (5-6)
Barbados	7 (5-8)	5 (4-7)	-17.3 (-25.9--8.4)	107 (82-133)	93 (69-116)	-13.2 (-21.5--5)	3 (3-3)
Belize	16 (13-21)	23 (17-29)	41.7 (25.6-61)	189 (146-238)	266 (200-342)	40 (24.6-55)	2 (1-2)
Bermuda	1 (1-1)	1 (0-1)	-34.4 (-43.2--23.4)	20 (15-24)	14 (11-18)	-29.7 (-35.4--21.2)	1 (1-1)
Cuba	91 (76-107)	97 (78-119)	6.2 (-5.9-21)	1,520 (1,260-1,860)	1,400 (1,130-1,740)	-8.4 (-17.2-3.6)	43 (42-45)
Dominica	2 (2-3)	2 (1-2)	-30.4 (-38.1--23)	35 (27-45)	25 (19-32)	-29.9 (-35.3--22.4)	1 (1-1)
Dominican Republic	892 (730-1,040)	927 (771-1,100)	3.9 (-6-12.8)	8,000 (6,290-9,530)	8,600 (6,810-10,400)	7.4 (-3-16.4)	98 (76-119)
Grenada	4 (3-5)	3 (2-4)	-21.7 (-31--13.1)	57 (44-72)	43 (32-55)	-25.2 (-32.8--17.2)	3 (2-3)
Guyana	37 (28-48)	34 (25-44)	-8.7 (-19.2-2.6)	512 (394-668)	411 (310-529)	-20 (-28.8--10.5)	17 (14-19)
Haiti	1,360 (1,180-1,540)	1,690 (1,440-1,900)	24.4 (15.6-36.8)	11,400 (9,690-13,300)	15,100 (12,500-17,500)	31.7 (22.5-45.2)	287 (164-424)
Jamaica	277 (263-291)	200 (174-228)	-27.9 (-36.3--17)	3,980 (3,600-4,390)	2,590 (2,220-3,080)	-35.1 (-42.5--26.8)	44 (42-48)
Puerto Rico	77 (56-102)	33 (24-45)	-56.6 (-61.5--51.6)	1,490 (1,110-1,870)	834 (623-1,070)	-44.1 (-50.5--38.8)	6 (6-6)
Saint Kitts and Nevis	1 (1-2)	1 (1-2)	-9.6 (-22.1-1)	24 (18-30)	22 (17-28)	-9.4 (-19.3-0.4)	1 (1-1)
Saint Lucia	6 (4-8)	4 (3-5)	-34.4 (-41.4--26.8)	94 (74-120)	65 (50-82)	-31.6 (-38.6--25.2)	3 (3-3)
Saint Vincent and the Grenadines	4 (3-6)	3 (3-4)	-24.7 (-32.6--14.9)	65 (51-85)	48 (37-62)	-26.9 (-34--19.3)	2 (2-2)
Suriname	21 (16-26)	21 (16-26)	-0.9 (-10.7-9.3)	256 (196-324)	276 (212-358)	7.7 (-2-18.1)	3 (2-4)
Trinidad and Tobago	35 (27-44)	32 (24-41)	-8.5 (-23.4-4.6)	650 (496-826)	532 (410-682)	-18.5 (-27.4--8.2)	17 (16-17)
United States Virgin Islands	3 (2-4)	2 (2-3)	-31.5 (-37.9--23.9)	49 (38-62)	34 (25-44)	-31.7 (-38.3--24.1)	0 (0-1)
**Central Latin America**	280 (232-332)	180 (146-218)	-35.9 (-40.2--31)	3,720 (2,990-4,470)	2,000 (1,580-2,600)	-46.4 (-51.6--40.8)	102 (97-106)
Colombia	56 (41-71)	48 (37-64)	-13.9 (-24.7--1.1)	205 (170-244)	179 (145-223)	-12.9 (-19.3--1.4)	42 (39-46)
Costa Rica	0 (0-0)	0 (0-0)	-9.3 (-15.9-11)	2 (1-3)	2 (1-3)	-3.1 (-12.7-12.8)	3 (3-3)
El Salvador	1 (1-1)	1 (0-1)	-17.7 (-24.3-10)	4 (2-5)	3 (2-5)	-15.7 (-28.1-6.7)	0 (0-0)
Guatemala	2 (2-3)	3 (2-4)	22.3 (7.7-87.4)	7 (4-10)	7 (4-10)	0.3 (-18.3-35.9)	0 (0-0)
Honduras	0 (0-1)	0 (0-1)	33.3 (8.8-154)	1 (1-2)	2 (1-3)	37.7 (14.8-127)	0 (0-0)
Mexico	65 (49-86)	54 (40-74)	-16.5 (-20.1--12.1)	1,100 (838-1,500)	1,080 (808-1,500)	-1.6 (-5.5-2.5)	11 (10-12)
Aguascalientes	0 (0-0)	0 (0-0)	13.5 (-7.6-225)	1 (0-3)	1 (0-3)	29.6 (2.8-489)	0 (0-0)
Baja California	1 (1-2)	2 (1-2)	14.3 (3.5-41.4)	24 (13-38)	33 (18-52)	37.9 (24.8-71.6)	0 (0-0)
Baja California Sur	0 (0-1)	1 (0-1)	32.4 (16.7-47.5)	9 (5-13)	14 (9-21)	61.6 (47.1-80.2)	0 (0-0)

Location	All age deaths (cause-specific), 2021	All age deaths (cause-specific) percent change (2000-2021)	All age deaths (total), 2000	All age deaths (total), 2021	All age deaths (total) percent change (2000-2021)	Under-5 deaths (cause-specific), 2000	Under-5 deaths (cause-specific), 2021	Under-5 deaths (cause-specific) percent change (2000-2021)
Antigua and Barbuda	2 (2-2)	19.7 (6.2-32.3)	2 (2-3)	2 (2-3)	2.8 (-8.8-16.1)	0 (0-0)	0 (0-0)	-47.5 (-64.3--23.7)
Bahamas	7 (5-8)	20 (-6.2-55.1)	17 (12-23)	19 (14-25)	13.2 (-2.1-30)	0 (0-0)	0 (0-0)	-35.7 (-55.4-5.9)
Barbados	2 (2-3)	-27.6 (-43.7--5.5)	6 (5-8)	7 (5-9)	6 (-7.1-19.2)	0 (0-0)	0 (0-0)	-63 (-74.3--47.7)
Belize	2 (2-3)	50.1 (32.9-74.4)	15 (11-19)	24 (18-32)	65.2 (47.5-86.7)	0 (0-0)	0 (0-0)	-36.7 (-50.9--17.9)
Bermuda	1 (1-1)	4.1 (-10-29.5)	1 (1-1)	1 (1-1)	-10.2 (-20.8-2.5)	0 (0-0)	0 (0-0)	-54.1 (-69.9--27.3)
Cuba	43 (36-49)	0 (-17.2-13)	100 (83-121)	116 (93-142)	15.4 (1.7-30.8)	2 (2-3)	1 (1-1)	-52.9 (-66.2--37.7)
Dominica	1 (1-1)	-7.5 (-31.1-21.3)	2 (2-3)	2 (1-3)	-18 (-28.2--7.8)	0 (0-0)	0 (0-0)	-23 (-55.4-26.3)
Dominican Republic	75 (60-96)	-23.6 (-42.3--3.8)	714 (568-859)	814 (656-971)	14 (4.5-25.6)	32 (24-43)	13 (8-21)	-59.2 (-73.7--36.4)
Grenada	3 (3-3)	18 (2.1-33.9)	4 (3-5)	3 (2-4)	-13.6 (-24.3--2.2)	0 (0-0)	0 (0-0)	-50.6 (-62.4--35.2)
Guyana	14 (11-18)	-14.5 (-34.6-15.6)	34 (25-44)	32 (24-42)	-6.6 (-16.5-4.9)	2 (2-3)	1 (1-2)	-49.7 (-66.7--28.2)
Haiti	346 (216-515)	20.5 (-10.5-74.4)	1,030 (875-1,180)	1,440 (1,230-1,630)	39.4 (30.1-51.1)	98 (29-170)	99 (31-176)	1.2 (-35.7-91.2)
Jamaica	40 (31-51)	-9.2 (-29-13.8)	298 (276-321)	229 (197-263)	-23.2 (-32.6--13.9)	5 (4-6)	2 (1-2)	-67.7 (-76.1--53.3)
Puerto Rico	5 (4-6)	-11.6 (-28.8-4.9)	79 (55-107)	56 (39-76)	-29.2 (-37.3--19.2)	0 (0-0)	0 (0-0)	-77.6 (-84.8--68.4)
Saint Kitts and Nevis	1 (1-1)	13 (-7.8-36)	2 (1-2)	2 (1-2)	3.6 (-8.2-16.5)	0 (0-0)	0 (0-0)	-53.6 (-67.9--31.7)
Saint Lucia	3 (3-4)	6 (-11.4-27.2)	6 (5-8)	5 (4-6)	-20.2 (-28.4--9.2)	0 (0-0)	0 (0-0)	-64.9 (-75.8--45.7)
Saint Vincent and the Grenadines	2 (2-3)	2.3 (-10.5-20.4)	4 (3-6)	4 (3-5)	-16.3 (-25.2--5.6)	0 (0-0)	0 (0-0)	-61.5 (-73.3--44.7)
Suriname	4 (3-4)	2.6 (-19.8-28.9)	18 (14-24)	22 (17-29)	20.6 (8.4-34.6)	1 (0-1)	1 (0-1)	-23.3 (-50.8-29.6)
Trinidad and Tobago	14 (11-18)	-13.2 (-32.5-8.5)	45 (33-60)	39 (29-52)	-12.6 (-24.9--1.5)	1 (1-2)	1 (0-1)	-58.5 (-70.6--40.5)
United States Virgin Islands	0 (0-0)	-32.3 (-51.1-0.2)	3 (2-4)	2 (2-3)	-21.1 (-30.2--11.3)	0 (0-0)	0 (0-0)	-81.6 (-90.6--63.7)
**Central Latin America**	133 (112-153)	30.1 (11.2-50.7)	239 (195-291)	179 (142-220)	-25 (-30.4--19)	17 (15-19)	13 (9-17)	-27.1 (-44.6--0.1)
Colombia	50 (42-59)	18.6 (-0.1-45)	54 (39-69)	47 (35-62)	-12.3 (-20.6--0.4)	7 (6-8)	4 (2-5)	-44.6 (-66.1--9.7)
Costa Rica	5 (4-5)	50.7 (29.3-69.6)	0 (0-0)	0 (0-0)	-3.3 (-11.5-26.4)	0 (0-0)	0 (0-0)	-50.5 (-62--35.8)
El Salvador	0 (0-0)	-64.1 (-80--30.1)	1 (1-1)	1 (1-1)	-17.8 (-28.9-21.8)	0 (0-0)	0 (0-0)	-77.3 (-90.5--41.3)
Guatemala	0 (0-1)	18.9 (-2.5-42.2)	2 (2-4)	2 (1-3)	-7.3 (-20.1-35.7)	0 (0-0)	0 (0-0)	-41.5 (-64.4--16.1)
Honduras	0 (0-0)	-49.2 (-76.4-26.8)	0 (0-1)	0 (0-1)	36.4 (1.1-156)	0 (0-0)	0 (0-0)	-62.9 (-91.2-4.7)
Mexico	15 (14-17)	42.4 (28.5-57.4)	50 (36-71)	53 (38-76)	5.3 (0.3-11.5)	4 (3-4)	2 (2-3)	-44.8 (-57.1--27)
Aguascalientes	0 (0-0)	82.3 (47.5-118)	0 (0-0)	0 (0-0)	35.9 (4.9-229)	0 (0-0)	0 (0-0)	-24.8 (-48.9-10.7)
Baja California	0 (0-1)	124 (85.6-162)	1 (1-2)	2 (1-3)	53.7 (41.3-83.9)	0 (0-0)	0 (0-0)	-28.8 (-49.6--1.5)
Baja California Sur	0 (0-0)	112 (81.6-148)	0 (0-1)	1 (0-1)	79.2 (59.5-99.6)	0 (0-0)	0 (0-0)	-17 (-45-19)

Location	Under-5 deaths (total), 2000	Under-5 deaths (total), 2021	Under-5 deaths (total) percent change (2000-2021)	Under-5 cause-specific mortality cause fraction, 2000	Under-5 total SCD mortality cause fraction, 2000	Under-5 cause-specific mortality cause fraction, 2021	Under-5 total SCD mortality cause fraction, 2021	Under-5 cause-specific mortality rank 2000	Under-5 cause-specific mortality rank 2021	Under-5 total SCD mortality rank 2000	Under-5 total SCD mortality rank 2021
Antigua and Barbuda	0 (0-1)	0 (0-1)	7.1 (-13.6-30.6)	0.8 (0.7-0.8)	2.1 (1.3-3.1)	1 (0.9-1.1)	5.7 (3.1-8.4)	18	16	11	5
Bahamas	2 (1-3)	2 (2-4)	28 (8-55.5)	0.4 (0.4-0.5)	3 (2-3.9)	0.5 (0.4-0.5)	6.3 (3.6-8.9)	24	28	8	8
Barbados	1 (1-2)	1 (1-2)	9.1 (-11.6-32.8)	0.3 (0.3-0.3)	2.1 (1.3-2.9)	0.2 (0.2-0.2)	4.4 (2.4-6.5)	32	38	10	7
Belize	5 (4-8)	9 (6-14)	63 (33-99.2)	0.2 (0.1-0.2)	2.8 (1.7-4)	0.2 (0.2-0.2)	8.1 (5-12.1)	47	43	8	4
Bermuda	0 (0-0)	0 (0-0)	-10.6 (-28.8-9.6)	0.6 (0.5-0.7)	2 (1.3-2.8)	0.8 (0.7-1.1)	5 (3-7.5)	24	21	12	5
Cuba	27 (20-34)	34 (23-47)	26.3 (6.5-53.7)	0.2 (0.2-0.2)	2.1 (1.6-2.7)	0.2 (0.2-0.3)	6.8 (4.3-9.8)	42	40	10	5
Dominica	1 (0-1)	0 (0-1)	-19.2 (-34.1--3)	0.4 (0.3-0.6)	2.4 (1.5-3.3)	0.4 (0.3-0.6)	2.6 (1.4-3.8)	28	27	10	8
Dominican Republic	292 (215-381)	340 (257-436)	16.4 (3.1-35)	0.4 (0.3-0.4)	3.3 (2.2-4.3)	0.2 (0.1-0.3)	6.2 (4.5-8.1)	30	29	7	5
Grenada	1 (1-1)	1 (1-1)	-6.4 (-21.3-12.9)	0.6 (0.6-0.7)	2.4 (1.4-3.2)	0.7 (0.6-0.8)	5.1 (3-7.3)	19	19	7	7
Guyana	9 (6-13)	9 (6-13)	0.9 (-16.7-21.1)	0.3 (0.2-0.3)	1.2 (0.7-1.6)	0.3 (0.3-0.4)	2.9 (1.7-4.1)	26	27	12	8
Haiti	417 (327-503)	570 (458-698)	36.7 (24.1-53)	0.3 (0.1-0.6)	1.5 (1.1-1.8)	0.4 (0.1-0.7)	2.2 (1.6-2.8)	33	30	14	11
Jamaica	112 (97-126)	83 (64-104)	-25.9 (-39.4--11.2)	0.4 (0.4-0.5)	10.2 (7.7-13.1)	0.3 (0.3-0.4)	17.1 (11.7-23.7)	20	25	3	2
Puerto Rico	7 (5-10)	4 (3-6)	-44.2 (-53.9--34.8)	0.1 (0-0.1)	1.1 (0.8-1.6)	0.1 (0.1-0.1)	4.1 (2.5-5.7)	65	56	13	6
Saint Kitts and Nevis	0 (0-0)	0 (0-1)	10.4 (-6.9-34)	0.4 (0.4-0.5)	1.7 (1.1-2.4)	0.5 (0.4-0.5)	4.5 (2.5-6.6)	23	26	11	6
Saint Lucia	1 (1-2)	1 (1-2)	-20.4 (-33.2--3.8)	0.5 (0.5-0.6)	2.9 (1.7-4.4)	0.4 (0.3-0.4)	4.8 (2.7-7.5)	18	24	8	6
Saint Vincent and the Grenadines	1 (1-2)	1 (1-1)	-10.9 (-24.7-6.4)	0.4 (0.4-0.4)	2 (1.4-2.9)	0.4 (0.4-0.5)	5.4 (3.1-8.1)	24	26	11	6
Suriname	5 (3-7)	7 (4-9)	27.6 (7.5-48.4)	0.2 (0.1-0.2)	1.4 (0.9-2)	0.2 (0.1-0.3)	2.7 (1.7-3.9)	38	39	13	9
Trinidad and Tobago	8 (5-11)	9 (6-14)	17.5 (-2.1-39.2)	0.3 (0.2-0.3)	1.7 (1.1-2.4)	0.3 (0.2-0.3)	4.6 (2.6-6.5)	31	32	12	7
United States Virgin Islands	0 (0-1)	0 (0-0)	-11.9 (-26.3-4.4)	0.1 (0.1-0.2)	1.6 (1-2.1)	0.2 (0.1-0.2)	7.7 (4.3-11)	46	53	14	4
**Central Latin America**	96 (71-124)	83 (61-109)	-13.8 (-21.3--4.2)	0 (0-0)	0.1 (0-0.1)	0 (0-0)	0.1 (0.1-0.2)	102	95	71	52
Colombia	45 (32-61)	40 (28-54)	-12.6 (-22.5-0.8)	0 (0-0)	0.2 (0.1-0.2)	0 (0-0.1)	0.5 (0.3-0.8)	87	75	38	27
Costa Rica	0 (0-0)	0 (0-0)	-8.4 (-17.8-37)	0 (0-0)	0 (0-0)	0 (0-0)	0 (0-0)	77	75	105	90
El Salvador	1 (1-1)	1 (0-1)	-19.2 (-31.4-23.9)	0 (0-0)	0 (0-0)	0 (0-0)	0.1 (0-0.1)	121	121	84	67
Guatemala	2 (1-3)	2 (1-3)	-9.8 (-22.7-36.2)	0 (0-0)	0 (0-0)	0 (0-0)	0 (0-0)	125	123	88	75
Honduras	0 (0-0)	0 (0-1)	34.7 (-2-170)	0 (0-0)	0 (0-0)	0 (0-0)	0 (0-0)	124	126	116	95
Mexico	5 (3-7)	5 (3-7)	-6.6 (-16-12.7)	0 (0-0)	0 (0-0)	0 (0-0)	0 (0-0)	112	103	109	91
Aguascalientes	0 (0-0)	0 (0-0)	22.8 (-5.7-239)	0 (0-0)	0 (0-0)	0 (0-0)	0 (0-0)	120	113	116	102
Baja California	0 (0-0)	0 (0-0)	25 (5.4-85.5)	0 (0-0)	0 (0-0)	0 (0-0)	0 (0-0)	110	104	105	93
Baja California Sur	0 (0-0)	0 (0-0)	41.6 (20.3-88.3)	0 (0-0)	0 (0-0)	0 (0-0)	0 (0-0)	97	94	97	85

Location	Births, 2000	Births, 2021	Births percent change (2000-2021)	All age cases, 2000	All age cases, 2021	All age cases percent change (2000-2021)	All age deaths (cause-specific), 2000	All age deaths (cause-specific), 2021	All age deaths (cause-specific) percent change (2000-2021)	All age deaths (total), 2000	All age deaths (total), 2021	All age deaths (total) percent change (2000-2021)	Under-5 deaths (cause-specific), 2000	Under-5 deaths (cause-specific), 2021	Under-5 deaths (cause-specific) percent change (2000-2021)	Under-5 deaths (total), 2000	Under-5 deaths (total), 2021	Under-5 deaths (total) percent change (2000-2021)	Under-5 cause-specific mortality cause fraction, 2000	Under-5 total SCD mortality cause fraction, 2000	Under-5 cause-specific mortality cause fraction, 2021	Under-5 total SCD mortality cause fraction, 2021	Under-5 cause-specific mortality rank 2000	Under-5 cause-specific mortality rank 2021	Under-5 total SCD mortality rank 2000	Under-5 total SCD mortality rank 2021
Campeche	1 (1-1)	1 (1-1)	-0.2 (-7.9-12.5)	16 (11-23)	18 (12-25)	7.3 (-2.1-18)	0 (0-0)	0 (0-0)	60.4 (30.5-102)	1 (0-1)	1 (1-1)	11.9 (-1.1-26.7)	0 (0-0)	0 (0-0)	-38.1 (-58.4--10.6)	0 (0-0)	0 (0-0)	5.3 (-11.4-39.9)	0 (0-0)	0 (0-0)	0 (0-0)	0 (0-0)	103	94	100	84
Chiapas	4 (3-5)	4 (3-6)	7.7 (-4.3-21)	59 (42-81)	69 (50-96)	16.7 (4.7-25.8)	1 (1-1)	1 (1-2)	32.5 (5.8-59.9)	3 (2-4)	3 (2-5)	18.6 (4.7-33.1)	0 (0-0)	0 (0-0)	-24.6 (-46.1-11.6)	0 (0-0)	0 (0-1)	20.3 (-1.3-77.2)	0 (0-0)	0 (0-0)	0 (0-0)	0 (0-0)	109	100	112	95
Chihuahua	1 (0-1)	1 (0-1)	-13.7 (-29.1-26.3)	14 (7-26)	15 (7-27)	3.4 (-12.9-56.4)	0 (0-0)	0 (0-1)	63.9 (36.2-101)	1 (0-1)	1 (0-1)	12.8 (-4-74.9)	0 (0-0)	0 (0-0)	-28.3 (-55.2--0.2)	0 (0-0)	0 (0-0)	-5 (-25.9-85.8)	0 (0-0)	0 (0-0)	0 (0-0)	0 (0-0)	115	111	116	107
Coahuila	1 (1-1)	1 (1-1)	9.1 (-0.4-34.7)	16 (10-23)	19 (12-28)	20.4 (11.1-37.7)	0 (0-0)	0 (0-0)	59.2 (38.9-83.6)	1 (0-1)	1 (1-1)	29.6 (13.7-64.1)	0 (0-0)	0 (0-0)	-28.7 (-54.1-10.2)	0 (0-0)	0 (0-0)	21.6 (-4-122)	0 (0-0)	0 (0-0)	0 (0-0)	0 (0-0)	114	108	106	91
Colima	0 (0-0)	0 (0-0)	-3.4 (-35.1-13.9)	4 (2-9)	4 (2-10)	10.2 (-31.1-26.2)	0 (0-0)	0 (0-0)	89.5 (53.9-129)	0 (0-0)	0 (0-0)	17.2 (-18.4-37.6)	0 (0-0)	0 (0-0)	-51 (-66.8--26.6)	0 (0-0)	0 (0-0)	7.6 (-17.2-66.9)	0 (0-0)	0 (0-0)	0 (0-0)	0 (0-0)	104	101	116	94
Durango	1 (0-1)	1 (0-1)	-9 (-25.6-16.1)	10 (4-19)	11 (4-22)	11.3 (-9.2-50.2)	0 (0-0)	0 (0-0)	17.2 (-0.4-43.4)	0 (0-1)	0 (0-1)	18.9 (0.3-50.8)	0 (0-0)	0 (0-0)	-51.6 (-69.8--31.8)	0 (0-0)	0 (0-0)	-1.9 (-20.4-57.9)	0 (0-0)	0 (0-0)	0 (0-0)	0 (0-0)	118	113	114	99
Guanajuato	2 (1-3)	1 (1-2)	-19.4 (-46.5-21.1)	25 (15-37)	24 (15-38)	-3.5 (-36.8-39.2)	0 (0-0)	0 (0-1)	94.1 (57.5-141)	1 (1-2)	1 (1-2)	7.9 (-24-56.8)	0 (0-0)	0 (0-0)	-43.6 (-59.9--25.1)	0 (0-0)	0 (0-0)	-1.7 (-26.9-80)	0 (0-0)	0 (0-0)	0 (0-0)	0 (0-0)	115	110	104	91
Guerrero	7 (5-9)	5 (4-6)	-30.3 (-36.7--24.7)	100 (79-121)	86 (68-109)	-13.8 (-21.5--7.4)	1 (1-1)	1 (1-1)	-3.1 (-27.8-24.8)	4 (3-6)	4 (3-5)	-9.7 (-18.2-0)	1 (0-1)	0 (0-0)	-52.3 (-73--12.3)	1 (0-1)	0 (0-1)	-21.6 (-34.7-4)	0 (0-0)	0 (0-0)	0 (0-0)	0 (0-0)	105	95	108	89
Hidalgo	1 (0-1)	1 (0-1)	-10.6 (-22.8-16.7)	12 (6-22)	13 (6-24)	10.5 (-4-35.1)	0 (0-0)	0 (0-0)	42.4 (17.7-71.4)	1 (0-1)	1 (0-1)	18.3 (-0.1-52.9)	0 (0-0)	0 (0-0)	-51.3 (-66.2--29.9)	0 (0-0)	0 (0-0)	-0.4 (-21.1-104)	0 (0-0)	0 (0-0)	0 (0-0)	0 (0-0)	119	109	113	95
Jalisco	3 (2-4)	2 (1-3)	-26.8 (-51--11.9)	52 (32-78)	42 (23-68)	-19.5 (-44.2--3.5)	0 (0-1)	1 (1-1)	68.3 (40.5-106)	2 (1-4)	2 (1-4)	-8.2 (-33.5-6.8)	0 (0-0)	0 (0-0)	-33.7 (-53.9--10.2)	0 (0-0)	0 (0-0)	-11.7 (-34.1-42.5)	0 (0-0)	0 (0-0)	0 (0-0)	0 (0-0)	110	106	109	98
México	2 (1-4)	2 (1-4)	4 (-19.4-142)	29 (10-53)	35 (13-74)	22.9 (-3.7-215)	1 (1-1)	1 (1-1)	42.6 (22.7-65.6)	1 (1-2)	2 (1-4)	30.5 (7.6-202)	0 (0-0)	0 (0-0)	-61.3 (-73.7--42.5)	0 (0-0)	0 (0-1)	16.4 (-7.7-151)	0 (0-0)	0 (0-0)	0 (0-0)	0 (0-0)	114	105	110	93
Mexico City	3 (2-4)	2 (1-2)	-36.2 (-42--24.3)	50 (35-67)	40 (29-52)	-20.1 (-26.7--8.8)	0 (0-0)	1 (0-1)	50.1 (24.9-85.1)	2 (2-3)	2 (1-3)	-14.1 (-22.8-2.7)	0 (0-0)	0 (0-0)	-35.6 (-56.8--4.8)	0 (0-0)	0 (0-0)	-25.8 (-39-6)	0 (0-0)	0 (0-0)	0 (0-0)	0 (0-0)	116	106	94	87
Michoacán de Ocampo	2 (1-3)	1 (1-2)	-19.2 (-36.8--1)	29 (17-50)	27 (15-47)	-7.3 (-27.6-17.2)	0 (0-0)	0 (0-0)	41.8 (20.2-72.1)	1 (1-3)	1 (1-2)	-4 (-23.1-18.6)	0 (0-0)	0 (0-0)	-34.9 (-56.7--9.3)	0 (0-0)	0 (0-0)	-10 (-28.6-47.6)	0 (0-0)	0 (0-0)	0 (0-0)	0 (0-0)	117	112	114	99
Morelos	1 (0-1)	1 (0-1)	-20.1 (-45.6--5)	12 (5-25)	11 (5-24)	-14.2 (-46.7-8.8)	0 (0-0)	0 (0-0)	88.5 (45.6-140)	1 (0-1)	1 (0-1)	-4.9 (-35.6-11.8)	0 (0-0)	0 (0-0)	-46.6 (-62--23.2)	0 (0-0)	0 (0-0)	-4.8 (-29.8-65.6)	0 (0-0)	0 (0-0)	0 (0-0)	0 (0-0)	111	106	106	91
Nayarit	0 (0-1)	0 (0-1)	4.5 (-10.5-21.9)	9 (5-16)	11 (5-20)	17.2 (-0.4-33.7)	0 (0-0)	0 (0-0)	44.9 (20.1-75.7)	0 (0-1)	0 (0-1)	24.1 (7.6-48.6)	0 (0-0)	0 (0-0)	-43.5 (-61.9--14.2)	0 (0-0)	0 (0-0)	11.2 (-10.8-80.1)	0 (0-0)	0 (0-0)	0 (0-0)	0 (0-0)	114	109	111	95
Nuevo León	3 (2-4)	2 (2-3)	-26.3 (-33.8--19.1)	58 (40-81)	53 (36-75)	-8.4 (-17-1.7)	0 (0-0)	1 (0-1)	89.8 (60.4-126)	2 (2-3)	2 (2-3)	-0.8 (-13.2-10.6)	0 (0-0)	0 (0-0)	-21.3 (-48.9-25.7)	0 (0-0)	0 (0-0)	-13.2 (-29.3-21.8)	0 (0-0)	0 (0-0)	0 (0-0)	0 (0-0)	114	107	109	95
Oaxaca	6 (4-7)	4 (3-6)	-22.9 (-28.2--15.9)	90 (71-116)	80 (62-102)	-11.6 (-18.2--5.4)	1 (1-1)	1 (1-1)	1.4 (-16.1-24.2)	4 (3-6)	4 (3-5)	-6.6 (-14.4-2.3)	0 (0-0)	0 (0-0)	-54 (-69.8--26.9)	0 (0-1)	0 (0-0)	-16.7 (-29.2-10.2)	0 (0-0)	0 (0-0)	0 (0-0)	0 (0-0)	105	98	100	85
Puebla	1 (1-3)	1 (1-2)	-6.4 (-22.4-73.8)	19 (10-37)	21 (11-40)	10 (-8.7-96.2)	0 (0-1)	1 (1-1)	24.3 (4.5-50.4)	1 (1-2)	1 (1-2)	16.7 (-3.5-98.7)	0 (0-0)	0 (0-0)	-47.2 (-64--19)	0 (0-0)	0 (0-0)	2.3 (-18.5-102)	0 (0-0)	0 (0-0)	0 (0-0)	0 (0-0)	116	109	117	98
Querétaro	0 (0-0)	0 (0-0)	18.4 (-2.6-151)	1 (0-4)	2 (0-5)	32.5 (5.8-339)	0 (0-0)	0 (0-0)	99.2 (64.6-139)	0 (0-0)	0 (0-0)	39.1 (8.9-242)	0 (0-0)	0 (0-0)	-10.6 (-36.2-34.9)	0 (0-0)	0 (0-0)	27.9 (-1.9-242)	0 (0-0)	0 (0-0)	0 (0-0)	0 (0-0)	114	105	112	102
Quintana Roo	1 (1-2)	1 (1-2)	27 (14.2-43.3)	19 (12-28)	28 (17-42)	44.4 (32-61.7)	0 (0-0)	0 (0-0)	135 (81.1-205)	1 (1-1)	1 (1-2)	55.7 (42.9-73.5)	0 (0-0)	0 (0-0)	-9.5 (-37.6-42.1)	0 (0-0)	0 (0-0)	40.6 (19.1-99.5)	0 (0-0)	0 (0-0)	0 (0-0)	0 (0-0)	106	99	100	90
San Luis Potosí	1 (0-2)	1 (0-2)	-18.1 (-32.7-19.9)	18 (8-32)	18 (8-32)	-2.9 (-18.5-43.7)	0 (0-0)	0 (0-0)	39.7 (18-71.1)	1 (0-2)	1 (0-2)	3.7 (-12-33.5)	0 (0-0)	0 (0-0)	-56.6 (-69--39.3)	0 (0-0)	0 (0-0)	-10.5 (-26.1-57.3)	0 (0-0)	0 (0-0)	0 (0-0)	0 (0-0)	113	104	111	93
Sinaloa	2 (1-3)	1 (1-2)	-23 (-30.3--15.3)	34 (22-49)	30 (20-44)	-9.8 (-20.5--1.8)	0 (0-0)	0 (0-0)	31.6 (12.6-55.3)	1 (1-2)	1 (1-2)	-3.2 (-13.7-9.8)	0 (0-0)	0 (0-0)	-54.3 (-68--35)	0 (0-0)	0 (0-0)	-18.9 (-32.8-12.2)	0 (0-0)	0 (0-0)	0 (0-0)	0 (0-0)	119	113	109	91
Sonora	1 (1-2)	1 (1-1)	-30.5 (-45.6--18.1)	21 (13-33)	18 (11-30)	-15.4 (-28.2--2.3)	0 (0-0)	0 (0-0)	88.6 (60.4-129)	1 (1-2)	1 (1-2)	-7.7 (-31.3-10.6)	0 (0-0)	0 (0-0)	-50.8 (-65.4--33)	0 (0-0)	0 (0-0)	-16.6 (-35.9-30)	0 (0-0)	0 (0-0)	0 (0-0)	0 (0-0)	115	111	109	94
Tabasco	6 (5-7)	5 (4-7)	-9.1 (-15.9--0.4)	108 (91-128)	110 (92-130)	1.9 (-5.4-9.5)	1 (1-1)	1 (1-1)	74 (47.3-101)	5 (4-6)	6 (4-7)	8 (-3-20.2)	0 (0-0)	0 (0-0)	-39.2 (-54.9--16.1)	0 (0-1)	0 (0-1)	-2.1 (-16.9-19.9)	0 (0-0)	0 (0-0)	0 (0-0)	0.1 (0-0.1)	91	90	86	66
Tamaulipas	5 (4-6)	4 (3-5)	-22.5 (-28.8--14.2)	77 (62-97)	78 (61-100)	1 (-6.4-10.6)	0 (0-0)	0 (0-1)	57.5 (33.1-87.8)	3 (2-4)	4 (3-5)	11.2 (-0.7-23)	0 (0-0)	0 (0-0)	-30.4 (-51.7--6.2)	0 (0-0)	0 (0-0)	-14.3 (-28.8-10.6)	0 (0-0)	0 (0-0)	0 (0-0)	0 (0-0)	111	101	90	84
Tlaxcala	0 (0-0)	0 (0-0)	-2.4 (-19.2-28.9)	5 (2-9)	5 (3-10)	15.9 (-8.5-43)	0 (0-0)	0 (0-0)	61.4 (30-94.4)	0 (0-0)	0 (0-0)	25.5 (5.9-67.9)	0 (0-0)	0 (0-0)	-34.6 (-57.5--4.7)	0 (0-0)	0 (0-0)	10.6 (-13.1-125)	0 (0-0)	0 (0-0)	0 (0-0)	0 (0-0)	117	105	109	97
Veracruz de Ignacio de la Llave	8 (6-10)	6 (5-8)	-24.2 (-30.2--17.1)	139 (109-173)	127 (100-164)	-8.8 (-17.3--1)	2 (2-2)	2 (2-2)	21 (3.2-40.5)	6 (5-8)	6 (4-8)	-2.8 (-12.1-8.3)	1 (0-1)	0 (0-0)	-64.7 (-73.9--52.8)	1 (0-1)	0 (0-1)	-20.5 (-33.5-8)	0 (0-0)	0 (0-0)	0 (0-0)	0 (0-0)	101	91	103	83
Yucatán	2 (1-3)	2 (1-2)	-9.6 (-18.9--1.3)	36 (25-52)	37 (25-52)	2.8 (-8.2-12.2)	0 (0-0)	0 (0-0)	68.9 (43.2-95.8)	2 (1-2)	2 (1-3)	8.3 (-4.1-21.3)	0 (0-0)	0 (0-0)	-20.7 (-44.6-9.9)	0 (0-0)	0 (0-0)	-4.4 (-19.3-25.6)	0 (0-0)	0 (0-0)	0 (0-0)	0 (0-0)	105	100	97	84
Zacatecas	0 (0-0)	0 (0-0)	-6.8 (-25.8-92.6)	1 (0-4)	1 (0-4)	2.3 (-20.3-157)	0 (0-0)	0 (0-0)	87.9 (58.5-117)	0 (0-0)	0 (0-0)	6.2 (-15.3-180)	0 (0-0)	0 (0-0)	-41.3 (-57.4--21)	0 (0-0)	0 (0-0)	1.5 (-21.1-184)	0 (0-0)	0 (0-0)	0 (0-0)	0 (0-0)	117	112	115	106

Location	Births, 2000	Births, 2021	Births percent change (2000-2021)	All age cases, 2000	All age cases, 2021	All age cases percent change (2000-2021)	All age deaths (cause-specific), 2000	All age deaths (cause-specific), 2021	All age deaths (cause-specific) percent change (2000-2021)	All age deaths (total), 2000	All age deaths (total), 2021	All age deaths (total) percent change (2000-2021)	Under-5 deaths (cause-specific), 2000	Under-5 deaths (cause-specific), 2021	Under-5 deaths (cause-specific) percent change (2000-2021)	Under-5 deaths (total), 2000	Under-5 deaths (total), 2021	Under-5 deaths (total) percent change (2000-2021)	Under-5 cause-specific mortality cause fraction, 2000	Under-5 total SCD mortality cause fraction, 2000	Under-5 cause-specific mortality cause fraction, 2021	Under-5 total SCD mortality cause fraction, 2021	Under-5 cause-specific mortality rank 2000	Under-5 cause-specific mortality rank 2021	Under-5 total SCD mortality rank 2000	Under-5 total SCD mortality rank 2021
Nicaragua	1 (1-2)	1 (1-2)	9 (-3.9-38.2)	4 (3-6)	5 (3-6)	13.6 (-7.8-47.7)	0 (0-1)	0 (0-0)	-60.7 (-79.5--15.3)	1 (1-2)	1 (1-2)	11.3 (-4.3-54.9)	0 (0-1)	0 (0-0)	-69 (-86--14.6)	1 (1-2)	1 (1-2)	10.1 (-6.7-55.2)	0 (0-0)	0 (0-0)	0 (0-0)	0.1 (0-0.1)	105	109	88	60
Panama	0 (0-1)	1 (0-1)	31.2 (-0.9-75.5)	2 (2-3)	3 (2-4)	31.4 (10.4-61.1)	13 (12-14)	15 (12-18)	18.9 (0.2-39.7)	0 (0-1)	1 (0-1)	33 (8.4-86.3)	2 (2-2)	1 (1-1)	-41 (-56.3--17.8)	0 (0-1)	1 (0-1)	32 (5.8-91.6)	0.1 (0.1-0.1)	0 (0-0)	0.1 (0.1-0.1)	0.1 (0-0.1)	49	57	85	68
Venezuela (Bolivarian Republic of)	154 (127-182)	71 (58-86)	-53.8 (-60.1--47.9)	2,410 (1,950-2,850)	690 (549-865)	-71.5 (-74.3--67.8)	32 (30-34)	46 (36-58)	46.1 (15.8-81.4)	130 (106-157)	70 (55-85)	-46 (-52.5--38.1)	4 (3-4)	5 (4-7)	36.5 (0.8-81.1)	41 (30-52)	32 (22-43)	-22.7 (-35.6--5.4)	0 (0-0)	0.3 (0.2-0.4)	0 (0-0)	0.2 (0.1-0.3)	85	76	22	34
**Tropical Latin America**	748 (656-833)	812 (683-925)	8.6 (3.5-13.1)	16,200 (14,500-17,600)	20,400 (17,900-22,500)	26 (21.5-30.6)	374 (351-393)	598 (569-630)	60.1 (49.5-73.4)	665 (575-769)	875 (737-1,030)	31.5 (24.5-38.8)	95 (81-111)	65 (50-82)	-31.8 (-49.6--9.4)	44 (34-56)	49 (37-62)	10 (3.9-16.8)	0.1 (0.1-0.1)	0 (0-0)	0.1 (0.1-0.2)	0.1 (0.1-0.1)	60	54	77	60
Brazil	707 (624-786)	771 (651-878)	9.1 (3.6-13.9)	15,400 (13,800-16,700)	19,500 (17,100-21,400)	26.4 (21.5-31.3)	369 (347-388)	592 (562-624)	60.4 (49.8-73.7)	633 (550-727)	837 (706-982)	32.3 (24.8-39.9)	94 (80-110)	64 (49-81)	-31.9 (-49.8--9.6)	41 (32-52)	45 (34-57)	10.9 (4.2-18.3)	0.1 (0.1-0.1)	0 (0-0)	0.1 (0.1-0.2)	0.1 (0.1-0.1)	59	52	79	59
Acre	2 (2-3)	4 (3-5)	58.4 (41.1-76.6)	43 (35-51)	75 (62-90)	74.1 (62.7-88.7)	1 (1-1)	2 (2-2)	89.2 (52-136)	2 (2-3)	4 (3-5)	82.5 (63.5-104)	0 (0-1)	1 (0-1)	2.8 (-34.2-70.2)	0 (0-0)	0 (0-0)	48.1 (29.3-69.9)	0.1 (0-0.1)	0 (0-0)	0.1 (0.1-0.2)	0.1 (0-0.1)	69	58	90	77
Alagoas	5 (4-6)	4 (3-5)	-16.8 (-27.5--2.5)	86 (72-100)	91 (73-111)	6.1 (-8.5-26.6)	6 (5-7)	7 (7-8)	26.2 (4.1-54.8)	3 (3-4)	3 (3-4)	3.8 (-10.5-20.9)	3 (2-4)	1 (1-2)	-52.5 (-70--28.7)	0 (0-0)	0 (0-0)	-5.4 (-23.1-19.8)	0.1 (0-0.1)	0 (0-0)	0.1 (0.1-0.2)	0 (0-0)	72	56	109	84
Amapá	1 (1-1)	1 (1-2)	55.5 (32.3-77)	14 (10-19)	25 (17-35)	73.3 (54.3-93.4)	1 (0-1)	2 (2-2)	225 (165-328)	1 (1-1)	1 (1-2)	60.6 (40.7-85.2)	0 (0-0)	0 (0-0)	128 (42-232)	0 (0-0)	0 (0-0)	40.5 (13.8-70.5)	0 (0-0.1)	0 (0-0)	0.1 (0.1-0.1)	0 (0-0)	69	53	96	90
Amazonas	6 (5-6)	9 (7-10)	52.5 (41.6-61.9)	159 (135-184)	278 (232-320)	74.7 (64.3-85.3)	2 (2-2)	6 (6-7)	219 (160-308)	4 (3-5)	8 (6-9)	97.1 (82-113)	1 (0-1)	1 (1-1)	73.9 (18.8-154)	0 (0-0)	1 (0-1)	56.4 (40.3-76.9)	0 (0-0)	0 (0-0)	0.1 (0.1-0.1)	0 (0-0)	80	64	93	85
Bahia	63 (54-71)	95 (79-112)	52 (36-68.2)	1,260 (1,100-1,410)	2,110 (1,830-2,370)	67.6 (55-77.8)	57 (52-63)	86 (76-96)	51.1 (29.7-77.3)	44 (37-52)	84 (69-101)	88.9 (68.7-111)	20 (16-24)	11 (7-15)	-44.4 (-61.7--18.6)	3 (2-4)	5 (4-7)	58.4 (37.9-85.4)	0.1 (0.1-0.2)	0 (0-0)	0.3 (0.2-0.3)	0.1 (0.1-0.2)	46	40	84	55
Ceará	10 (8-12)	13 (11-16)	32.3 (21.6-46.1)	178 (151-207)	263 (221-308)	47.2 (34.2-64.8)	10 (8-11)	15 (13-17)	57.2 (34.8-85.5)	7 (5-9)	10 (8-13)	46.6 (32.5-62.7)	3 (2-4)	2 (1-3)	-39.1 (-60.3--5)	0 (0-1)	1 (0-1)	37 (19.8-58.2)	0 (0-0)	0 (0-0)	0.1 (0.1-0.1)	0 (0-0)	72	62	105	78
Distrito Federal	32 (28-36)	27 (24-30)	-16.9 (-26.7--9.1)	711 (642-782)	819 (714-904)	14.9 (7.8-22.5)	7 (7-8)	9 (8-10)	23.4 (11.6-38.3)	26 (22-30)	30 (26-36)	16 (4.1-27.4)	1 (1-2)	1 (1-1)	-35.7 (-48.1--18.2)	1 (1-2)	1 (1-1)	-12.1 (-22.8-1)	0.1 (0.1-0.2)	0.1 (0.1-0.2)	0.2 (0.2-0.2)	0.2 (0.2-0.3)	43	39	45	32
Espírito Santo	22 (18-25)	24 (20-29)	9.9 (-2.2-22.6)	388 (340-430)	501 (429-572)	29 (17.2-39.3)	8 (7-8)	12 (11-13)	57.7 (39.4-76.7)	20 (17-24)	26 (21-31)	27.9 (13-44.3)	1 (1-2)	1 (1-2)	-4.2 (-31.7-33.3)	1 (1-1)	1 (1-1)	11.3 (-6.9-32.8)	0.1 (0.1-0.1)	0.1 (0-0.1)	0.2 (0.2-0.2)	0.1 (0.1-0.2)	54	45	64	53
Goiás	24 (20-27)	27 (22-31)	13.4 (2.9-26)	528 (470-589)	712 (618-798)	34.6 (24.5-44.7)	13 (12-14)	24 (22-26)	83.1 (58.7-114)	22 (18-25)	32 (26-37)	46.3 (31.1-63.4)	3 (2-4)	2 (2-3)	-28 (-50.2-4.7)	1 (1-1)	1 (1-2)	12 (-3.8-35.4)	0.1 (0.1-0.1)	0 (0-0.1)	0.2 (0.1-0.2)	0.1 (0.1-0.1)	54	45	76	58
Maranhão	26 (22-31)	32 (26-39)	22.2 (8-36.9)	334 (284-388)	526 (438-619)	57.5 (42.9-71)	30 (25-40)	32 (28-35)	4.5 (-24-29.1)	16 (13-20)	24 (19-31)	47.1 (29.8-68.2)	13 (8-22)	6 (4-8)	-54.9 (-75.7--23.2)	1 (1-2)	2 (1-3)	37 (17.4-58.8)	0.1 (0.1-0.2)	0 (0-0)	0.2 (0.2-0.3)	0.1 (0-0.1)	51	43	100	75
Mato Grosso	6 (5-6)	7 (6-9)	30.2 (19.1-40.1)	135 (114-152)	195 (162-224)	44.2 (34-54.5)	5 (4-6)	10 (10-11)	105 (81.8-130)	5 (4-6)	8 (7-10)	58.5 (43.9-74.3)	1 (1-1)	2 (1-2)	32.5 (-1.4-74.1)	0 (0-0)	0 (0-1)	26.9 (13-45.3)	0.1 (0.1-0.1)	0 (0-0)	0.2 (0.1-0.2)	0 (0-0.1)	57	51	91	78
Mato Grosso do Sul	5 (4-6)	6 (5-8)	22.2 (12-32.3)	133 (114-152)	181 (151-206)	35.2 (26.1-43.1)	2 (2-3)	5 (4-5)	99.6 (72.7-131)	5 (4-6)	7 (6-9)	53.6 (38.9-70.7)	0 (0-1)	1 (0-1)	5.7 (-20.7-46.5)	0 (0-0)	0 (0-0)	23.3 (6.4-45.5)	0 (0-0)	0 (0-0)	0.1 (0.1-0.1)	0.1 (0-0.1)	74	64	86	75
Minas Gerais	95 (82-109)	92 (78-108)	-3.1 (-12.1-8.9)	1,900 (1,680-2,090)	2,130 (1,830-2,390)	12 (3.3-24)	48 (43-52)	78 (71-84)	63.3 (48.2-83.2)	91 (76-106)	104 (87-122)	14.1 (3-27.8)	10 (8-13)	7 (5-9)	-34.4 (-51.3-0.1)	5 (4-7)	5 (4-7)	-7.5 (-20.8-10.9)	0.1 (0.1-0.1)	0.1 (0-0.1)	0.2 (0.2-0.2)	0.1 (0.1-0.2)	48	44	65	51
Pará	14 (12-16)	17 (14-20)	22 (9.1-33.7)	295 (254-332)	438 (371-497)	48 (34.2-58)	10 (8-11)	20 (17-22)	99.8 (66.3-149)	12 (10-15)	17 (13-21)	35.3 (20.7-52.2)	4 (3-5)	3 (2-4)	-17.1 (-45.7-26.5)	1 (1-1)	1 (1-1)	23.8 (7-43.1)	0.1 (0-0.1)	0 (0-0)	0.1 (0.1-0.2)	0 (0-0.1)	71	58	96	80
Paraíba	8 (6-9)	10 (8-12)	35.5 (27.6-42.8)	130 (108-149)	197 (165-233)	51.7 (42.6-61.1)	5 (5-6)	7 (7-8)	40.2 (23.8-66.6)	6 (5-7)	8 (7-10)	52.1 (40.3-65.1)	2 (1-2)	1 (1-1)	-57.1 (-74.6--31.8)	1 (1-1)	1 (1-2)	53.4 (37.5-71.9)	0.1 (0-0.1)	0 (0-0)	0.1 (0.1-0.1)	0.1 (0.1-0.2)	69	61	80	52
Paraná	12 (10-13)	14 (11-16)	18.6 (8.4-28.6)	317 (279-356)	412 (354-475)	29.7 (21.3-38.6)	8 (7-8)	14 (13-16)	86.5 (65.1-112)	10 (9-12)	16 (13-19)	53.8 (40.4-69.9)	1 (1-2)	1 (1-2)	-9 (-35.9-28.8)	1 (0-1)	1 (0-1)	14.9 (-0.1-33)	0 (0-0)	0 (0-0)	0.1 (0.1-0.1)	0 (0-0)	72	64	90	76
Pernambuco	10 (8-12)	12 (10-14)	25.4 (13.2-38.8)	190 (159-218)	286 (243-347)	50.4 (35.6-65.9)	16 (14-17)	21 (19-23)	37.1 (19.1-58.1)	6 (5-8)	11 (9-13)	65.3 (48.8-84.1)	5 (4-6)	2 (2-3)	-54.1 (-68.8--27.5)	0 (0-1)	1 (1-1)	45 (20.5-77)	0.1 (0-0.1)	0 (0-0)	0.1 (0.1-0.1)	0 (0-0)	66	58	107	78
Piaui	23 (20-27)	24 (21-29)	4.9 (-4.8-17)	386 (335-430)	472 (406-533)	22.1 (14.8-31)	12 (9-14)	17 (15-19)	46.1 (25-71.3)	16 (13-18)	19 (16-23)	22.2 (9.1-37)	4 (3-5)	2 (1-3)	-51 (-68.6--24.2)	1 (1-1)	1 (1-2)	18.6 (0.5-40.6)	0.1 (0.1-0.2)	0 (0-0.1)	0.2 (0.2-0.3)	0.1 (0.1-0.2)	46	39	79	48
Rio de Janeiro	99 (88-111)	89 (75-101)	-10.6 (-18.6--3.6)	2,200 (2,000-2,400)	2,250 (1,970-2,490)	2.3 (-4.8-8.5)	44 (41-46)	75 (70-79)	72.3 (55.1-87.6)	101 (89-115)	109 (94-127)	8.2 (-2.1-18.8)	6 (5-7)	6 (4-7)	-4.7 (-26.6-23.6)	7 (5-9)	7 (5-9)	-7.1 (-20-8.1)	0.1 (0.1-0.1)	0.1 (0.1-0.2)	0.2 (0.2-0.2)	0.2 (0.2-0.3)	52	47	46	40
Rio Grande do Norte	5 (4-6)	6 (5-8)	22.5 (11-42.5)	85 (69-102)	119 (96-147)	39.9 (26.9-58.7)	4 (3-4)	6 (6-7)	64.4 (39.6-96.9)	3 (3-4)	5 (4-7)	47.8 (32.1-70.2)	1 (1-2)	1 (1-1)	-48.2 (-67--18.9)	0 (0-0)	0 (0-0)	33.1 (12.6-60.2)	0 (0-0.1)	0 (0-0)	0.1 (0.1-0.1)	0 (0-0.1)	72	61	107	77
Rio Grande do Sul	13 (11-14)	14 (12-16)	10.7 (1.1-20.7)	398 (345-450)	501 (430-582)	25.5 (16.6-33.5)	9 (8-9)	15 (14-16)	70.2 (51.9-88.5)	13 (11-15)	18 (15-22)	40.6 (28.4-54)	1 (1-1)	1 (1-1)	-8.6 (-28.5-23.4)	1 (0-1)	1 (0-1)	9.4 (-4.5-25.3)	0 (0-0)	0 (0-0)	0.1 (0.1-0.1)	0 (0-0.1)	71	60	84	73
Rondônia	6 (5-7)	6 (5-7)	8.4 (-2.5-20.7)	109 (92-124)	133 (113-152)	21.3 (11.6-32.1)	2 (1-2)	2 (2-2)	34.6 (16.6-58.9)	6 (5-7)	7 (5-9)	21.4 (7.5-35.6)	0 (0-1)	0 (0-0)	-21 (-41.1-18.8)	0 (0-0)	0 (0-0)	0.9 (-15.2-18.3)	0 (0-0.1)	0 (0-0)	0.1 (0.1-0.1)	0.1 (0.1-0.1)	71	59	82	66
Roraima	3 (2-4)	4 (3-4)	19.5 (8.4-31.6)	42 (34-49)	63 (52-76)	51.8 (36.5-64.9)	0 (0-0)	1 (1-1)	191 (116-295)	2 (2-3)	3 (2-4)	43.1 (23.7-63.7)	0 (0-0)	0 (0-0)	271 (125-553)	0 (0-0)	0 (0-0)	17.2 (-3.1-42.1)	0 (0-0)	0.1 (0-0.1)	0.1 (0.1-0.1)	0.1 (0-0.1)	76	66	64	74
Santa Catarina	4 (3-5)	5 (4-6)	21.2 (13.4-30)	126 (103-145)	173 (143-208)	37.4 (28.3-49.6)	3 (3-3)	6 (5-6)	103 (75.8-133)	4 (3-5)	6 (5-7)	53.6 (39.9-69.6)	1 (0-1)	1 (0-1)	2.7 (-29.8-51.6)	0 (0-0)	0 (0-0)	28.2 (9.7-53.7)	0 (0-0)	0 (0-0)	0.1 (0-0.1)	0 (0-0)	78	68	93	82

Location	Births, 2000	Births, 2021	Births percent change (2000-2021)	All age cases, 2000	All age cases, 2021	All age cases percent change (2000-2021)	All age deaths (cause-specific), 2000	All age deaths (cause-specific), 2021	All age deaths (cause-specific) percent change (2000-2021)	All age deaths (total), 2000	All age deaths (total), 2021	All age deaths (total) percent change (2000-2021)	Under-5 deaths (cause-specific), 2000	Under-5 deaths (cause-specific), 2021	Under-5 deaths (cause-specific) percent change (2000-2021)	Under-5 deaths (total), 2000	Under-5 deaths (total), 2021	Under-5 deaths (total) percent change (2000-2021)	Under-5 cause-specific mortality cause fraction, 2000	Under-5 total SCD mortality cause fraction, 2000	Under-5 cause-specific mortality cause fraction, 2021	Under-5 total SCD mortality cause fraction, 2021	Under-5 cause-specific mortality rank 2000	Under-5 cause-specific mortality rank 2021	Under-5 total SCD mortality rank 2000	Under-5 total SCD mortality rank 2021
São Paulo	197 (175-220)	207 (175-239)	4.8 (-4.7-13.7)	4,940 (4,410-5,330)	6,090 (5,380-6,790)	22.9 (16-29.9)	60 (56-64)	105 (97-114)	75.6 (58.6-93.9)	193 (168-220)	256 (219-299)	32.7 (21.5-45.3)	9 (7-10)	9 (7-11)	4.7 (-21.1-31.3)	12 (9-16)	13 (10-17)	5.9 (-4.2-17.2)	0.1 (0.1-0.1)	0.1 (0.1-0.1)	0.1 (0.1-0.1)	0.2 (0.1-0.2)	65	54	55	44
Sergipe	7 (6-8)	8 (6-9)	11 (0.8-23.1)	139 (119-157)	181 (152-216)	29.8 (18.4-40.5)	5 (5-6)	8 (7-8)	50 (31.3-79.8)	6 (5-7)	8 (6-10)	32.6 (16-48.9)	2 (1-2)	1 (1-2)	-29.1 (-52.4-4.8)	0 (0-1)	0 (0-1)	17.4 (0.4-37.6)	0.1 (0.1-0.1)	0 (0-0)	0.2 (0.1-0.2)	0.1 (0-0.1)	55	47	80	69
Tocantins	11 (9-13)	14 (11-16)	25.3 (16.4-39.4)	184 (159-207)	287 (245-321)	55.2 (43.9-68.5)	4 (3-4)	6 (5-7)	62 (38.6-91.9)	8 (7-10)	13 (11-16)	57.1 (42.9-75.8)	1 (1-2)	1 (1-1)	-24.1 (-51.6-11.9)	1 (0-1)	1 (1-1)	28 (14.1-44.9)	0.1 (0.1-0.1)	0 (0-0.1)	0.2 (0.2-0.3)	0.2 (0.1-0.3)	51	42	76	49
Paraguay	40 (32-51)	40 (32-50)	-0.1 (-7.8-7.4)	767 (619-910)	900 (734-1,090)	17.1 (11.3-23.7)	5 (4-7)	6 (4-9)	32.4 (-3.4-86.2)	32 (24-44)	38 (28-49)	15.8 (5.6-27.3)	1 (0-1)	1 (0-1)	-16.8 (-55.9-66)	4 (3-5)	4 (3-5)	0.4 (-8.6-10.1)	0 (0-0)	0.1 (0.1-0.1)	0 (0-0)	0.1 (0.1-0.2)	90	93	51	48
North Africa and Middle East	29,900 (27,000-33,600)	19,700 (17,000-22,800)	-34.2 (-38--30)	723,000 (643,000-807,000)	618,000 (514,000-719,000)	-14.8 (-20.4--9.2)	1,400 (1,110-1,740)	1,510 (1,180-1,910)	8 (-9.8-29.4)	13,500 (11,800-15,500)	14,000 (11,800-16,700)	3.6 (-3.2-10.6)	199 (120-318)	108 (71-161)	-45.6 (-62.9--6.9)	3,060 (2,260-4,110)	2,970 (2,170-4,020)	-3 (-7.2-1.9)	0 (0-0)	0.5 (0.3-0.6)	0 (0-0.1)	1.1 (0.8-1.4)	82	86	29	16
**North Africa and Middle East**	29,900 (27,000-33,600)	19,700 (17,000-22,800)	-34.2 (-38--30)	723,000 (643,000-807,000)	618,000 (514,000-719,000)	-14.8 (-20.4--9.2)	1,400 (1,110-1,740)	1,510 (1,180-1,910)	8 (-9.8-29.4)	13,500 (11,800-15,500)	14,000 (11,800-16,700)	3.6 (-3.2-10.6)	199 (120-318)	108 (71-161)	-45.6 (-62.9--6.9)	3,060 (2,260-4,110)	2,970 (2,170-4,020)	-3 (-7.2-1.9)	0 (0-0)	0.5 (0.3-0.6)	0 (0-0.1)	1.1 (0.8-1.4)	82	86	29	16
Afghanistan	1,200 (965-1,470)	2,050 (1,650-2,500)	70.5 (60.6-80.5)	16,400 (13,000-20,600)	33,700 (26,400-43,300)	104 (91.1-118)	165 (100-260)	257 (171-416)	55.7 (4-122)	445 (344-577)	916 (715-1,170)	106 (88.3-123)	31 (12-64)	30 (18-50)	-2.4 (-39.6-129)	201 (136-298)	360 (240-542)	79.3 (58.6-100)	0 (0-0)	0.1 (0.1-0.2)	0 (0-0.1)	0.4 (0.3-0.6)	78	79	44	31
Algeria	163 (139-188)	199 (172-233)	22 (15.9-27.8)	3,490 (2,900-4,020)	4,270 (3,610-4,980)	22 (15.6-30.2)	29 (19-44)	23 (14-38)	-20.1 (-47.4-16.7)	113 (92-137)	155 (129-187)	37.4 (28.8-47.3)	6 (3-9)	3 (2-5)	-46.8 (-70.3--4.2)	49 (35-67)	65 (45-90)	31 (17.3-47.8)	0 (0-0)	0.2 (0.1-0.3)	0 (0-0)	0.4 (0.3-0.5)	83	85	39	28
Bahrain	166 (137-198)	126 (101-156)	-24.3 (-35.2--16.4)	6,160 (5,020-7,500)	9,330 (7,450-11,500)	47.4 (26.9-62.6)	21 (17-24)	28 (23-34)	35.1 (13.7-66.6)	103 (81-130)	227 (173-292)	120 (82.2-151)	1 (1-1)	1 (1-2)	11.6 (-18.1-55.7)	10 (7-15)	10 (7-14)	-6.7 (-18.1-5.5)	0.6 (0.4-0.9)	7.1 (5-10.2)	0.7 (0.5-0.9)	6.2 (4.3-8.7)	26	27	4	4
Egypt	2,900 (2,520-3,300)	3,100 (2,690-3,470)	6.9 (-1.6-13.2)	74,800 (63,200-85,500)	107,000 (91,800-120,000)	42.6 (28.3-51.2)	100 (77-133)	157 (120-204)	57.5 (17.7-121)	1,350 (1,150-1,560)	2,050 (1,750-2,370)	51.5 (41.6-62.1)	12 (7-19)	11 (6-17)	-6.5 (-49.5-84.9)	272 (209-350)	337 (266-432)	23.9 (12.3-35.7)	0 (0-0)	0.3 (0.2-0.4)	0 (0-0)	0.9 (0.7-1.2)	80	79	33	14
Iran (Islamic Republic of)	3,270 (2,600-4,000)	1,960 (1,570-2,420)	-40 (-41.1--38.3)	114,000 (89,400-142,000)	82,200 (64,200-103,000)	-28.6 (-30.4--26.3)	220 (154-273)	157 (119-186)	-28.6 (-41.4--8.8)	1,780 (1,360-2,300)	1,770 (1,360-2,270)	-0.6 (-6.7-5.4)	11 (5-18)	1 (0-2)	-90.5 (-94.7--83.7)	189 (138-262)	166 (118-235)	-12.4 (-18.2--7)	0 (0-0)	0.4 (0.3-0.6)	0 (0-0)	3.8 (2.7-5.2)	80	85	27	7
Alborz	120 (93-147)	76 (60-96)	-36.8 (-41--31.9)	3,400 (2,640-4,250)	2,860 (2,230-3,600)	-16.6 (-22.1--9.5)	3 (2-4)	3 (2-4)	-9.7 (-30.2-21.5)	50 (37-65)	61 (46-80)	22 (8.7-35.6)	0 (0-0)	0 (0-0)	-98 (-99.6--93.3)	5 (3-6)	4 (3-6)	-6 (-17.4-8.1)	0 (0-0)	0.6 (0.4-0.9)	0 (0-0)	28.5 (16.2-46.4)	88	94	22	2
Ardebil	61 (48-76)	28 (22-34)	-54.8 (-57.4--51.5)	2,100 (1,630-2,610)	1,210 (932-1,510)	-42.8 (-47.4--38.4)	2 (1-3)	1 (1-2)	-36.3 (-53.1--11.5)	31 (24-40)	26 (20-34)	-13.9 (-23.2--3.7)	0 (0-0)	0 (0-0)	-94.5 (-97.4--88.8)	4 (3-5)	2 (2-3)	-35.5 (-43.2--27.7)	0 (0-0)	0.3 (0.2-0.4)	0 (0-0)	2.9 (2-3.9)	84	92	34	7
Bushehr	47 (37-57)	35 (28-43)	-26.2 (-31.9--20.9)	1,510 (1,160-1,890)	1,390 (1,070-1,740)	-8 (-14.5--1.6)	3 (2-4)	3 (2-4)	-14.4 (-37.4-14.7)	23 (18-29)	29 (22-37)	23.9 (13.6-34.3)	0 (0-0)	0 (0-0)	-88.1 (-94.1--78.8)	3 (2-5)	4 (2-5)	9.2 (-3.8-24.8)	0 (0-0.1)	0.6 (0.4-0.9)	0 (0-0.1)	4.7 (3.1-7)	64	73	22	6
Chahar Mahaal and Bakhtiari	47 (37-58)	27 (21-33)	-42.8 (-46.9--38.7)	1,540 (1,190-1,910)	1,060 (813-1,340)	-32 (-36.3--27.1)	2 (2-3)	1 (1-2)	-42.1 (-60.2--19.3)	23 (18-29)	22 (17-29)	-0.7 (-9.8-8.9)	0 (0-0)	0 (0-0)	-93.1 (-96--86.9)	4 (3-5)	3 (2-4)	-14.9 (-25.7--3.4)	0 (0-0.1)	0.7 (0.5-1)	0 (0-0.1)	7.5 (4.8-11)	68	76	21	4
East Azarbayejan	164 (126-202)	85 (67-105)	-48.3 (-52.1--43.2)	5,830 (4,400-7,370)	3,800 (2,950-4,770)	-35.2 (-40.6--28.8)	6 (4-10)	3 (2-6)	-46.6 (-61.8--26)	96 (73-126)	86 (66-111)	-10 (-19.1-1.1)	0 (0-1)	0 (0-0)	-94.7 (-97.5--89.8)	9 (7-13)	7 (5-10)	-18.8 (-27.3--9)	0 (0-0)	0.3 (0.2-0.5)	0 (0-0)	5 (3.4-7.3)	86	95	30	6
Fars	200 (158-249)	115 (92-142)	-42.3 (-46.8--38.3)	7,560 (5,830-9,460)	5,090 (3,860-6,440)	-33.4 (-39--28.5)	25 (15-32)	16 (9-19)	-37 (-53.3--12.7)	126 (96-165)	119 (92-152)	-5.9 (-16.4-3.7)	1 (0-2)	0 (0-0)	-91.1 (-94.9--84)	14 (10-20)	12 (8-17)	-16.4 (-25.7--6.4)	0 (0-0.1)	0.6 (0.4-0.8)	0 (0-0.1)	4.3 (3-6.1)	64	78	21	5
Gilan	88 (68-110)	39 (30-50)	-55.2 (-58.6--52.2)	3,830 (3,020-4,860)	2,230 (1,680-2,830)	-42.2 (-46.1--37.9)	7 (4-10)	5 (3-6)	-31.4 (-47.4--9.2)	61 (46-80)	53 (40-70)	-13.1 (-22--3.7)	0 (0-0)	0 (0-0)	-97.9 (-99.1--95.5)	4 (3-6)	2 (2-3)	-38.6 (-45.7--30.5)	0 (0-0)	0.5 (0.4-0.7)	0 (0-0)	14.1 (7.9-24.1)	74	78	22	3
Golestan	85 (66-106)	50 (40-63)	-40.4 (-44.8--35.9)	2,750 (2,110-3,460)	2,010 (1,540-2,450)	-27.7 (-32.4--20.8)	6 (4-9)	5 (3-7)	-17.2 (-40.3-13.3)	43 (32-56)	43 (33-55)	-0.1 (-9.7-12.8)	0 (0-1)	0 (0-0)	-91 (-94.9--81.8)	5 (3-7)	4 (3-6)	-12.3 (-22.4--1.3)	0 (0-0.1)	0.4 (0.2-0.5)	0 (0-0.1)	3 (2.1-4.1)	73	83	31	7
Hamadan	82 (65-102)	36 (28-44)	-56.3 (-59.1--52.8)	2,950 (2,310-3,720)	1,590 (1,210-2,020)	-46.6 (-50.1--42.3)	3 (2-5)	2 (2-3)	-42 (-58.5--22.5)	49 (37-63)	37 (29-48)	-23.4 (-31.3--15.5)	0 (0-0)	0 (0-0)	-93.9 (-96.7--87.1)	5 (4-7)	3 (2-5)	-35.7 (-42.7--28)	0 (0-0)	0.4 (0.2-0.5)	0 (0-0)	3 (2.2-4.1)	81	90	26	8
Hormozgan	74 (58-92)	58 (46-72)	-21.2 (-26.3--15.6)	2,210 (1,710-2,830)	2,100 (1,620-2,620)	-5.8 (-12.9-0.6)	8 (3-11)	8 (3-10)	-0.1 (-28-45.6)	33 (25-43)	42 (32-54)	26.2 (14.4-38.8)	1 (0-1)	0 (0-0)	-87.7 (-93.5--75.9)	4 (3-6)	5 (4-7)	14 (1.1-28.2)	0 (0-0.1)	0.4 (0.3-0.5)	0 (0-0.1)	3.2 (2.4-4.6)	66	74	31	7
Ilam	28 (22-35)	12 (10-15)	-55.5 (-57.8--52)	951 (732-1,200)	591 (453-745)	-38.6 (-42.5--33.8)	2 (1-2)	1 (1-1)	-21.4 (-40.9-10.2)	14 (11-18)	13 (10-17)	-8.9 (-18.9-1.3)	0 (0-0)	0 (0-0)	-92.4 (-96.6--81.7)	2 (1-2)	1 (1-2)	-35 (-42.3--26.9)	0 (0-0.1)	0.4 (0.3-0.6)	0 (0-0)	3 (1.8-4.6)	72	81	25	7
Isfahan	165 (130-204)	88 (70-107)	-46.4 (-50--42.5)	7,190 (5,620-9,070)	5,030 (3,820-6,260)	-30.9 (-35.5--24.8)	9 (6-12)	6 (5-8)	-28.7 (-42.6--6.5)	115 (87-148)	112 (85-144)	-2 (-11-8.4)	0 (0-1)	0 (0-0)	-88.7 (-93.6--78.5)	11 (8-16)	9 (6-13)	-21.6 (-31.1--9)	0 (0-0)	0.6 (0.4-0.8)	0 (0-0)	3.6 (2.5-5.1)	76	87	23	7
Kerman	116 (91-146)	87 (68-109)	-25.1 (-30--19.4)	4,080 (3,160-5,160)	3,470 (2,690-4,420)	-15.7 (-21--8.9)	10 (6-13)	7 (5-9)	-23.7 (-46.1-6.9)	63 (47-82)	72 (55-92)	14.5 (3.1-27.1)	0 (0-1)	0 (0-0)	-82.7 (-89.9--70.4)	7 (5-9)	7 (5-10)	7.1 (-5.2-20)	0 (0-0)	0.4 (0.3-0.6)	0 (0-0)	2.4 (1.7-3.4)	73	83	25	8
Kermanshah	92 (71-115)	46 (36-57)	-50 (-53.4--47)	3,360 (2,640-4,250)	1,900 (1,420-2,390)	-44 (-47.6--39.8)	6 (4-7)	3 (3-4)	-44.3 (-57.6--26.1)	55 (41-72)	45 (34-58)	-18.7 (-27.4--9.6)	0 (0-0)	0 (0-0)	-94.3 (-97.3--86.8)	5 (4-8)	4 (3-6)	-28.6 (-36.3--19)	0 (0-0)	0.3 (0.2-0.5)	0 (0-0)	3.4 (2.4-4.7)	82	89	28	6
Khorasan-e-Razavi	294 (225-363)	186 (148-231)	-36.8 (-41.6--31.4)	9,110 (6,960-11,500)	6,850 (5,290-8,580)	-25.4 (-31.7--18.6)	12 (9-16)	8 (6-10)	-36.9 (-52.3--13.3)	144 (109-188)	146 (111-187)	1.4 (-8.5-12.5)	1 (0-1)	0 (0-0)	-92.4 (-96.5--84.1)	17 (12-24)	16 (11-22)	-7.4 (-17.2-7.1)	0 (0-0)	0.3 (0.2-0.5)	0 (0-0)	3.7 (2.4-5.3)	85	90	31	7
Khuzestan	251 (197-312)	152 (121-190)	-39.3 (-43.3--35.9)	7,970 (6,160-9,910)	5,510 (4,300-6,900)	-31.6 (-36.1--27.6)	22 (12-29)	16 (10-20)	-27.1 (-43.4--2.6)	125 (95-161)	118 (91-150)	-6.1 (-15-2.8)	2 (1-3)	0 (0-0)	-88.7 (-94--83)	18 (13-26)	16 (11-24)	-9.6 (-21-2.7)	0 (0-0.1)	0.6 (0.4-0.8)	0 (0-0.1)	4.3 (3-6)	64	72	23	5
Kohgiluyeh and Boyer-Ahmad	44 (35-55)	26 (21-32)	-41 (-45.2--35.7)	1,160 (896-1,450)	840 (650-1,070)	-27.8 (-33.3--20.6)	4 (3-6)	3 (2-4)	-28 (-47.9-1.6)	20 (15-25)	19 (15-25)	-2.7 (-13-9.1)	0 (0-1)	0 (0-0)	-93.5 (-96.9--85.1)	3 (2-5)	3 (2-5)	-11.9 (-23.9-0.5)	0.1 (0-0.1)	0.6 (0.4-0.9)	0 (0-0.1)	7.4 (4.8-11.1)	66	71	22	4
Kurdistan	69 (55-86)	39 (30-49)	-43.8 (-47.8--39.9)	2,470 (1,890-3,160)	1,630 (1,250-2,050)	-34.3 (-38.6--28.4)	2 (1-3)	1 (1-2)	-41.5 (-58.8--21.4)	38 (29-50)	36 (28-47)	-5.3 (-15.2-4.8)	0 (0-0)	0 (0-0)	-94.7 (-97.4--88.2)	5 (3-7)	4 (3-5)	-21.6 (-30.3--11.5)	0 (0-0)	0.3 (0.2-0.4)	0 (0-0)	3.3 (2.3-4.6)	85	94	30	6

Location	Births, 2000	Births, 2021	Births percent change (2000-2021)	All age cases, 2000	All age cases, 2021	All age cases percent change (2000-2021)	All age deaths (cause-specific), 2000	All age deaths (cause-specific), 2021	All age deaths (cause-specific) percent change (2000-2021)	All age deaths (total), 2000	All age deaths (total), 2021	All age deaths (total) percent change (2000-2021)	Under-5 deaths (cause-specific), 2000	Under-5 deaths (cause-specific), 2021	Under-5 deaths (cause-specific) percent change (2000-2021)	Under-5 deaths (total), 2000	Under-5 deaths (total), 2021	Under-5 deaths (total) percent change (2000-2021)	Under-5 cause-specific mortality cause fraction, 2000	Under-5 total SCD mortality cause fraction, 2000	Under-5 cause-specific mortality cause fraction, 2021	Under-5 total SCD mortality cause fraction, 2021	Under-5 cause-specific mortality rank 2000	Under-5 cause-specific mortality rank 2021	Under-5 total SCD mortality rank 2000	Under-5 total SCD mortality rank 2021
Lorestan	93 (72-117)	43 (34-53)	-54 (-57.2--50.2)	3,080 (2,350-3,880)	1,760 (1,340-2,250)	-43.4 (-48.2--39)	5 (3-7)	3 (2-4)	-36.3 (-51.9--5.5)	45 (35-59)	38 (30-50)	-15.4 (-23.6--6.9)	0 (0-1)	0 (0-0)	-95.9 (-97.8--91)	6 (4-8)	4 (3-6)	-33.8 (-41.1--25.6)	0 (0-0.1)	0.5 (0.3-0.7)	0 (0-0.1)	7.6 (5.3-10.3)	76	79	26	4
Markazi	53 (41-65)	27 (21-33)	-49.9 (-53.9--46)	2,190 (1,680-2,710)	1,320 (1,020-1,630)	-40.2 (-45.3--33.5)	2 (2-4)	1 (1-2)	-49.2 (-62.8--31.6)	35 (26-46)	30 (23-39)	-13.3 (-21.8--3.4)	0 (0-0)	0 (0-0)	-93.5 (-96.9--87.1)	3 (2-4)	2 (2-3)	-28.7 (-37.1--20.2)	0 (0-0)	0.4 (0.3-0.6)	0 (0-0)	3.8 (2.6-5.5)	81	92	23	8
Mazandaran	111 (87-139)	56 (44-70)	-49.4 (-52.7--46.4)	4,560 (3,500-5,750)	3,090 (2,390-3,850)	-33 (-36.9--28.6)	11 (5-15)	9 (4-11)	-18.2 (-40.3-13.5)	71 (53-92)	70 (53-89)	-1.5 (-11-8.2)	0 (0-0)	0 (0-0)	-94 (-97.4--88.2)	5 (4-7)	4 (3-5)	-28.8 (-37.4--20.1)	0 (0-0)	0.5 (0.3-0.7)	0 (0-0.1)	6.2 (4.1-9.4)	74	78	25	6
North Khorasan	51 (40-64)	22 (18-28)	-56.2 (-58.9--53.1)	1,320 (1,010-1,660)	852 (648-1,080)	-35.9 (-39.4--31.4)	1 (1-2)	1 (0-1)	-28.8 (-43--6.4)	21 (16-28)	18 (14-24)	-14.6 (-22.3--6.1)	0 (0-0)	0 (0-0)	-92.7 (-95.8--80.8)	3 (2-4)	2 (1-3)	-35 (-43.3--27.3)	0 (0-0)	0.4 (0.3-0.5)	0 (0-0)	3.3 (2.4-4.7)	89	97	32	8
Qazvin	47 (36-58)	28 (22-34)	-41 (-44.3--37)	1,880 (1,440-2,340)	1,270 (966-1,590)	-32.9 (-37.2--27.1)	1 (1-2)	1 (1-2)	-32.7 (-44.9--8.8)	28 (21-36)	28 (21-36)	0.3 (-11.3-11.3)	0 (0-0)	0 (0-0)	-94.6 (-97.6--87.5)	3 (2-4)	2 (2-3)	-17.4 (-25.9--6.2)	0 (0-0)	0.4 (0.3-0.5)	0 (0-0)	5 (3.4-7.3)	82	92	29	6
Qom	43 (34-53)	32 (25-40)	-24.3 (-29.6--17.9)	1,630 (1,270-2,040)	1,410 (1,090-1,750)	-14.3 (-19.9--7.8)	2 (1-2)	1 (1-2)	-28.9 (-44--1.1)	24 (18-31)	28 (21-36)	19.1 (7-31.1)	0 (0-0)	0 (0-0)	-89.6 (-94.9--79.8)	2 (2-3)	3 (2-4)	7.6 (-4.8-22.5)	0 (0-0)	0.5 (0.3-0.7)	0 (0-0)	5 (3.3-7.2)	87	95	24	6
Semnan	21 (16-26)	16 (12-20)	-23.8 (-31.3--17)	896 (721-1,150)	740 (569-928)	-17.8 (-25.7--10.1)	1 (1-2)	1 (1-1)	-30.8 (-52.9--2.4)	14 (10-18)	15 (12-20)	12 (-0.4-24.5)	0 (0-0)	0 (0-0)	-91.2 (-96.3--78.3)	1 (1-1)	1 (1-2)	0.8 (-11.1-15.5)	0 (0-0)	0.4 (0.3-0.6)	0 (0-0)	4 (2.4-6.4)	77	84	25	8
Sistan and Baluchistan	183 (143-232)	139 (109-174)	-24.2 (-30.5--17.6)	3,950 (3,050-5,040)	3,610 (2,790-4,560)	-9 (-16.2--2.2)	13 (6-18)	14 (6-19)	7.3 (-20.6-51.5)	60 (46-79)	67 (52-88)	11.8 (1.6-23.1)	2 (1-3)	0 (0-0)	-84.3 (-91.5--72.7)	11 (8-15)	12 (9-17)	12.2 (-0.1-26)	0 (0-0.1)	0.3 (0.2-0.4)	0 (0-0.1)	1.9 (1.4-2.6)	71	82	31	10
South Khorasan	36 (28-45)	23 (18-28)	-37.5 (-41.2--32.9)	1,060 (805-1,360)	848 (649-1,080)	-20.4 (-25.4--14.9)	1 (0-1)	1 (0-1)	-16 (-35.1-17.4)	16 (12-20)	17 (13-22)	5.9 (-6.4-18.5)	0 (0-0)	0 (0-0)	-90.7 (-95--78.3)	2 (2-3)	2 (1-3)	-9.9 (-20.3-2.6)	0 (0-0)	0.3 (0.2-0.4)	0 (0-0)	2.8 (2-3.9)	90	96	34	8
Tehran	390 (309-482)	250 (194-308)	-35.8 (-40.4--31.6)	16,000 (12,500-19,800)	12,500 (9,660-15,800)	-22.6 (-28.4--17.1)	43 (18-58)	28 (13-35)	-34.7 (-51.5--8.4)	239 (183-309)	260 (196-337)	8.6 (-2.2-20.2)	1 (0-1)	0 (0-0)	-94.4 (-98.3--85.9)	15 (10-20)	14 (10-21)	-3.9 (-15.3-8.5)	0 (0-0)	0.5 (0.3-0.7)	0 (0-0)	6.5 (3.1-13.1)	78	82	24	5
West Azarbayejan	137 (108-172)	92 (72-114)	-32.9 (-36.4--27.6)	4,600 (3,640-5,840)	3,440 (2,640-4,340)	-25.8 (-29.7--20.3)	5 (4-9)	3 (2-5)	-40.4 (-56--17.8)	74 (55-96)	74 (56-94)	-0.1 (-8.9-10.9)	0 (0-1)	0 (0-0)	-92.2 (-95.9--82.6)	8 (6-11)	8 (6-11)	0 (-11.5-14.4)	0 (0-0)	0.3 (0.2-0.4)	0 (0-0)	3.4 (2.4-4.7)	83	92	31	6
Yazd	41 (32-50)	28 (22-35)	-31.3 (-38.6--25.6)	1,460 (1,130-1,820)	1,180 (918-1,490)	-19.5 (-28.2--12.7)	1 (1-2)	1 (1-1)	-32.7 (-51.2--7.3)	22 (17-28)	24 (18-31)	7.5 (-2.7-18)	0 (0-0)	0 (0-0)	-86.6 (-91.9--75.2)	2 (2-3)	2 (2-3)	4.2 (-8.8-19.3)	0 (0-0)	0.4 (0.3-0.6)	0 (0-0)	2.7 (1.9-3.8)	80	88	23	8
Zanjan	44 (34-54)	23 (18-28)	-48 (-51.4--43.8)	1,610 (1,240-2,040)	1,010 (776-1,270)	-37.8 (-42--34.4)	1 (1-2)	1 (0-1)	-48.9 (-63.3--29.8)	24 (18-30)	22 (17-28)	-6.8 (-16.1-2)	0 (0-0)	0 (0-0)	-94.4 (-97.4--87.9)	3 (2-4)	2 (1-3)	-24.6 (-33--14.9)	0 (0-0)	0.3 (0.2-0.4)	0 (0-0)	3.1 (2.2-4.6)	83	96	31	8
Iraq	2,060 (1,780-2,460)	2,050 (1,750-2,380)	-0.7 (-8.1-6.4)	30,000 (26,400-34,200)	42,800 (37,800-48,600)	42.4 (34.2-51)	124 (90-161)	102 (76-142)	-17.3 (-36.6-14.1)	1,180 (936-1,510)	1,560 (1,280-1,910)	32.7 (20.9-46)	18 (12-27)	9 (6-14)	-51 (-69.4--12.3)	594 (414-854)	607 (416-872)	2.3 (-10.9-18.4)	0.1 (0-0.1)	1.8 (1.3-2.6)	0.1 (0-0.1)	5.1 (3.2-7.1)	64	62	9	7
Jordan	145 (117-175)	260 (211-312)	79 (69.1-94)	3,400 (2,740-4,240)	8,380 (6,640-10,600)	145 (132-161)	13 (10-17)	17 (12-22)	27.1 (-8.4-74.5)	66 (51-84)	180 (142-225)	171 (145-198)	1 (1-2)	1 (0-2)	-25 (-53.7-16.2)	21 (13-34)	39 (24-62)	84.9 (61-115)	0 (0-0)	0.6 (0.3-0.9)	0 (0-0.1)	1.5 (0.9-2.4)	71	75	25	11
Kuwait	29 (24-35)	44 (36-54)	49.8 (40.9-60.8)	750 (592-898)	1,620 (1,290-1,920)	115 (101-126)	4 (4-4)	4 (4-5)	11.4 (-5.3-33.6)	21 (16-27)	48 (39-61)	131 (107-156)	0 (0-0)	0 (0-0)	-30.8 (-51.7--9)	7 (4-11)	10 (6-17)	53.9 (28.8-80.7)	0 (0-0)	1.5 (0.9-2.4)	0 (0-0)	2.8 (1.6-4.7)	67	74	11	8
Lebanon	78 (65-92)	81 (67-95)	3.9 (-2.4-11)	1,830 (1,480-2,190)	2,420 (1,940-2,890)	31.3 (22-40.7)	9 (7-12)	9 (7-11)	-6.3 (-26.3-26.6)	38 (31-46)	56 (45-68)	49.2 (39.1-60.3)	0 (0-0)	0 (0-0)	-77.5 (-88.3--48.5)	6 (4-9)	8 (5-11)	19.3 (5.7-34.9)	0 (0-0)	0.4 (0.2-0.5)	0 (0-0)	1.7 (1-2.9)	86	89	31	13
Libya	1,080 (833-1,440)	676 (519-881)	-37.1 (-43.1--32.3)	15,200 (12,800-17,900)	15,600 (13,300-18,200)	2.2 (-6.2-9.5)	24 (19-34)	37 (27-56)	55.3 (11-129)	883 (644-1,220)	794 (611-1,030)	-10.1 (-20.4-3.4)	5 (3-7)	5 (3-7)	6.4 (-27.1-73.1)	505 (322-777)	335 (213-504)	-33.7 (-42.7--22.4)	0.1 (0.1-0.2)	15.5 (10.2-23.2)	0.2 (0.1-0.2)	11.4 (7-17.6)	53	49	2	3
Morocco	243 (202-283)	167 (136-195)	-31.3 (-34.9--26.9)	3,890 (3,190-4,510)	4,090 (3,380-4,800)	4.8 (-0.7-11.3)	38 (24-57)	28 (16-46)	-25.3 (-53.9-30.7)	154 (123-190)	156 (127-188)	1.4 (-6.7-10)	6 (3-11)	2 (1-4)	-70.2 (-85.2--42)	80 (55-111)	63 (44-86)	-20.9 (-30.1--10.5)	0 (0-0)	0.2 (0.1-0.3)	0 (0-0)	0.6 (0.5-0.9)	82	88	38	19
Oman	298 (254-337)	391 (325-463)	31.4 (19.6-43.4)	6,720 (5,770-7,610)	12,800 (10,500-15,000)	90.9 (73.1-109)	16 (13-20)	17 (13-22)	8 (-16.4-44)	175 (143-210)	355 (291-427)	103 (81.3-127)	2 (1-3)	1 (1-2)	-33.3 (-53.3--9.1)	64 (42-91)	97 (64-137)	53.2 (31.2-74.1)	0.2 (0.2-0.3)	7.4 (4.7-10.7)	0.2 (0.1-0.3)	15.7 (10.4-23.1)	34	40	5	2
Palestine	126 (100-154)	122 (99-147)	-3.3 (-8.9-2.8)	2,230 (1,760-2,840)	3,470 (2,720-4,370)	55.3 (43.5-66.1)	4 (3-6)	5 (4-6)	9.7 (-18.2-48.7)	43 (33-56)	68 (54-88)	58.2 (42-75)	1 (0-1)	0 (0-1)	-48.4 (-70.9-2)	17 (11-26)	19 (12-30)	10 (-4.1-28.7)	0 (0-0)	0.5 (0.4-0.8)	0 (0-0)	1.5 (0.9-2.4)	76	84	22	11
Qatar	13 (11-16)	32 (27-39)	143 (128-160)	430 (340-542)	2,070 (1,610-2,590)	382 (342-418)	1 (1-1)	2 (1-2)	114 (55.3-201)	10 (8-13)	51 (39-65)	392 (346-443)	0 (0-0)	0 (0-0)	-3.1 (-46.6-68.4)	2 (1-3)	6 (4-10)	194 (152-249)	0 (0-0.1)	1.5 (0.9-2.5)	0 (0-0.1)	4.8 (2.8-8.3)	73	74	15	5
Saudi Arabia	7,790 (7,230-8,300)	1,700 (1,420-2,010)	-78.2 (-81.5--74.5)	263,000 (246,000-281,000)	109,000 (89,300-130,000)	-58.7 (-66.1--52)	172 (133-219)	279 (205-378)	62.2 (3.5-138)	3,950 (3,530-4,420)	2,070 (1,640-2,570)	-47.6 (-58--36.2)	10 (6-14)	2 (1-4)	-80.2 (-89.9--65.2)	195 (148-250)	93 (68-122)	-52.2 (-61.3--42)	0.1 (0.1-0.1)	1.7 (1.2-2.2)	0.1 (0.1-0.2)	4.4 (2.9-6.1)	59	61	9	6
Sudan	9,070 (7,620-10,700)	5,870 (4,780-7,100)	-35.2 (-41.6--27.1)	153,000 (128,000-182,000)	154,000 (123,000-188,000)	0.3 (-10.7-13.2)	260 (164-359)	254 (157-404)	-2.1 (-32.3-46.9)	2,410 (1,990-2,940)	2,720 (2,200-3,340)	13 (0.7-25.6)	62 (24-107)	29 (16-46)	-52.9 (-71.8-23.3)	524 (409-676)	506 (399-654)	-3.5 (-13-7)	0.1 (0-0.1)	0.5 (0.4-0.6)	0.1 (0-0.1)	1.2 (0.9-1.6)	68	70	26	15
Syrian Arab Republic	169 (139-207)	68 (56-82)	-60 (-63--56.8)	2,120 (1,760-2,420)	1,370 (1,140-1,590)	-36.4 (-41.4--32.5)	30 (22-42)	18 (12-27)	-41.9 (-63.1--9.1)	120 (93-155)	65 (53-80)	-45.9 (-51.4--39.4)	4 (2-6)	2 (1-3)	-56.7 (-76.8--24.6)	72 (50-102)	29 (20-41)	-59.3 (-64--53.6)	0 (0-0.1)	0.8 (0.6-1.2)	0 (0-0.1)	0.7 (0.4-0.9)	73	77	23	25
Tunisia	222 (177-268)	167 (139-200)	-24.6 (-29.3--19.5)	6,690 (5,290-8,440)	6,670 (5,230-8,310)	-0.6 (-9.1-7.7)	16 (11-24)	15 (9-25)	-9.7 (-38.3-30.1)	135 (108-167)	164 (132-203)	21.6 (11.4-32.4)	1 (1-2)	0 (0-1)	-74.2 (-85.8--47.1)	31 (22-45)	27 (18-38)	-13.7 (-24.5--1.2)	0 (0-0)	0.6 (0.4-0.8)	0 (0-0)	1.5 (1-2.2)	78	82	26	13
Türkiye	574 (512-668)	306 (267-353)	-46.7 (-51--42)	14,600 (13,100-16,400)	10,900 (9,730-12,400)	-25.5 (-32--17.4)	126 (90-166)	67 (50-84)	-46.8 (-62.5--26.8)	380 (327-447)	335 (290-391)	-12 (-19.1--4.9)	18 (10-34)	4 (2-6)	-80 (-88--55)	133 (98-184)	86 (63-122)	-35.2 (-43.6--25.8)	0 (0-0.1)	0.2 (0.2-0.3)	0 (0-0)	0.7 (0.5-1)	79	90	40	23
United Arab Emirates	15 (12-17)	14 (12-16)	-4 (-8.2-1.6)	680 (563-806)	1,580 (1,290-1,830)	132 (119-151)	3 (2-4)	5 (3-7)	53.6 (10.8-119)	12 (10-15)	44 (35-53)	253 (213-292)	0 (0-0)	0 (0-0)	-60.7 (-76.2--34.8)	1 (1-2)	2 (1-3)	67.5 (50.1-89.3)	0 (0-0)	0.2 (0.1-0.3)	0 (0-0)	0.7 (0.5-1)	105	112	40	22
Yemen	253 (216-302)	273 (231-322)	7.6 (2.7-12.5)	2,580 (2,150-3,030)	4,270 (3,530-4,900)	64.7 (53.2-73.8)	25 (16-37)	30 (18-47)	20 (-26.3-113)	129 (102-162)	187 (153-234)	45.6 (34.9-57.8)	9 (4-17)	6 (3-11)	-33.9 (-62.4-57)	82 (59-111)	101 (73-140)	23.5 (11.3-37.2)	0 (0-0)	0.1 (0.1-0.1)	0 (0-0)	0.3 (0.2-0.4)	84	88	47	38

Location	Births, 2000	Births, 2021	Births percent change (2000-2021)	All age cases, 2000	All age cases, 2021	All age cases percent change (2000-2021)	All age deaths (cause-specific), 2000	All age deaths (cause-specific), 2021	All age deaths (cause-specific) percent change (2000-2021)	All age deaths (total), 2000	All age deaths (total), 2021	All age deaths (total) percent change (2000-2021)
South Asia	96,900 (68,700-129,000)	82,600 (58,800-110,000)	-14.7 (-16.8--12.3)	1,190,000 (881,000-1,560,000)	1,280,000 (949,000-1,680,000)	7.1 (4.3-9.9)	2,530 (1,770-3,040)	1,360 (932-1,730)	-46.4 (-58.4--32.3)	81,300 (56,800-113,000)	89,900 (64,200-123,000)	10.6 (6.4-15.5)
**South Asia**	96,900 (68,700-129,000)	82,600 (58,800-110,000)	-14.7 (-16.8--12.3)	1,190,000 (881,000-1,560,000)	1,280,000 (949,000-1,680,000)	7.1 (4.3-9.9)	2,530 (1,770-3,040)	1,360 (932-1,730)	-46.4 (-58.4--32.3)	81,300 (56,800-113,000)	89,900 (64,200-123,000)	10.6 (6.4-15.5)
Bangladesh	59 (44-77)	44 (32-58)	-25.4 (-34.8--18.3)	443 (327-578)	378 (263-499)	-14.7 (-29.8--6.5)	2 (1-3)	1 (1-1)	-56.9 (-76.8--6.3)	56 (42-75)	47 (35-62)	-16 (-27.1--8.4)
Bhutan	0 (0-0)	0 (0-0)	-32.3 (-37.9--26.8)	2 (2-3)	2 (1-2)	-18.7 (-28.7--12.8)	0 (0-0)	0 (0-0)	-55.9 (-77.4--8.7)	0 (0-0)	0 (0-0)	-20.4 (-28--13.7)
India	96,700 (68,600-129,000)	82,500 (58,700-110,000)	-14.7 (-16.8--12.3)	1,190,000 (880,000-1,560,000)	1,280,000 (948,000-1,680,000)	7 (4.3-9.9)	2,510 (1,760-3,010)	1,340 (921-1,710)	-46.6 (-58.4--32.7)	81,200 (56,700-113,000)	89,800 (64,100-123,000)	10.6 (6.4-15.5)
Nepal	27 (23-32)	20 (17-24)	-24.4 (-30.7--18.8)	248 (203-295)	242 (200-296)	-2.2 (-9.5-7.8)	2 (1-4)	2 (1-3)	-24.6 (-62.7-51.3)	20 (16-25)	20 (16-24)	-1.5 (-10.1-7.9)
Pakistan	48 (31-72)	53 (36-79)	10.8 (3-18.4)	178 (118-248)	231 (158-320)	29.3 (19.4-39.1)	16 (7-30)	13 (5-25)	-18.1 (-65.5-94.1)	38 (24-59)	48 (32-72)	25.9 (13.2-41)
Azad Jammu & Kashmir	1 (1-1)	1 (0-1)	-12.6 (-30.3--3.2)	3 (2-5)	3 (2-5)	1.3 (-9.5-9.6)	0 (0-1)	0 (0-0)	-45.6 (-76.8-44.3)	1 (0-1)	1 (0-1)	-2.1 (-19.6-9.1)
Balochistan	2 (2-4)	4 (3-7)	84.8 (60.7-116)	9 (6-12)	17 (11-24)	97.9 (77-128)	1 (0-1)	1 (0-2)	26.7 (-37.2-182)	2 (1-3)	4 (2-6)	96.5 (65-136)
Gilgit-Baltistan	0 (0-1)	1 (0-1)	84.4 (60.9-108)	1 (1-2)	3 (2-4)	98.3 (77.1-120)	0 (0-0)	0 (0-0)	-14.5 (-67.3-202)	0 (0-0)	1 (0-1)	97 (64.8-129)
Islamabad Capital Territory	0 (0-0)	0 (0-0)	91 (54.2-164)	1 (1-1)	2 (1-3)	121 (93.2-167)	0 (0-0)	0 (0-0)	-25.5 (-72.9-135)	0 (0-0)	0 (0-1)	118 (72.6-193)
Khyber Pakhtunkhwa	7 (4-10)	10 (6-15)	58.6 (42.5-78.4)	28 (19-40)	48 (32-67)	69.2 (50.5-86.3)	2 (1-3)	2 (1-4)	11.4 (-63.8-218)	5 (3-8)	9 (6-14)	67.1 (30.2-96.2)
Punjab	26 (17-40)	24 (16-36)	-7.2 (-16.9-5.8)	95 (61-131)	106 (70-148)	11.1 (-0.8-22.3)	10 (4-19)	8 (3-15)	-21.5 (-68.2-113)	21 (13-32)	22 (15-33)	8.2 (-7.7-28.6)
Sindh	11 (7-17)	12 (8-18)	6.9 (-6.9-19)	41 (26-57)	52 (34-72)	27.2 (14.4-41.2)	3 (1-7)	2 (1-6)	-29 (-70.8-65.1)	9 (5-14)	11 (7-17)	25.4 (7.8-42.5)
Southeast Asia, East Asia, and Oceania	687 (539-864)	610 (482-767)	-11.2 (-12.5--9.9)	25,300 (19,400-32,000)	22,300 (17,000-28,000)	-11.7 (-13.6--9.7)	237 (147-354)	176 (119-249)	-26 (-45.7-0.6)	754 (564-985)	744 (567-952)	-1.3 (-6.3-3.6)
**East Asia**	653 (510-828)	580 (456-735)	-11.2 (-12.6--9.8)	25,000 (19,200-31,800)	22,100 (16,800-27,800)	-11.7 (-13.6--9.7)	226 (136-343)	170 (114-243)	-25.1 (-45.5-3)	724 (532-954)	715 (540-926)	-1.2 (-6.3-4)
China	623 (486-788)	562 (441-712)	-9.8 (-11.3--8.4)	24,200 (18,500-30,600)	21,200 (16,100-26,700)	-12.1 (-14.1--10.2)	221 (132-333)	163 (110-237)	-26 (-46.4-2.2)	702 (516-922)	691 (520-895)	-1.5 (-6.6-3.6)
Democratic People's Republic of Korea	22 (17-29)	12 (9-16)	-44.4 (-48.6--40.3)	499 (377-644)	442 (330-563)	-12 (-18.6--6)	5 (3-9)	6 (4-10)	16.3 (-21.8-67.1)	12 (9-17)	13 (10-17)	4.6 (-5.8-14.8)
Taiwan (Province of China)	9 (7-11)	6 (5-8)	-28.5 (-36.3--18.4)	386 (296-485)	438 (362-519)	12.6 (-0.1-29.6)	0 (0-0)	0 (0-0)	-88.8 (-90.4--86.9)	10 (7-13)	11 (9-14)	16 (0.3-35.4)
**Oceania**	0 (0-0)	0 (0-0)	50.8 (43.9-58.9)	2 (0-4)	3 (0-7)	54.2 (45.5-68.5)	0 (0-0)	0 (0-0)	27.7 (-14.3-80.6)	0 (0-0)	0 (0-0)	58.9 (55.1-65.6)
American Samoa	0 (0-0)	0 (0-0)	-38.3 (-53.3--35.3)	0 (0-0)	0 (0-0)	-19.9 (-36.6--14.6)	0 (0-0)	0 (0-0)	41.2 (-24.9-118)	0 (0-0)	0 (0-0)	-19.3 (-34.5--12.3)
Cook Islands	0 (0-0)	0 (0-0)	-36.8 (-53.4--32.6)	0 (0-0)	0 (0-0)	-25.4 (-55.4--19.6)	0 (0-0)	0 (0-0)	-39.6 (-62.3--5.8)	0 (0-0)	0 (0-0)	-22.5 (-42.6--17)
Fiji	0 (0-0)	0 (0-0)	-6.7 (-6.7--6.7)	0 (0-0)	0 (0-0)	-0.1 (-4.2-7.5)	0 (0-0)	0 (0-0)	1.1 (-29.4-46.2)	0 (0-0)	0 (0-0)	1.6 (0.1-3.1)
Guam	0 (0-0)	0 (0-0)	-9.4 (-9.9--9.3)	0 (0-0)	0 (0-0)	-4.4 (-8.2--0.2)	0 (0-0)	0 (0-0)	-44.1 (-63.5-16.6)	0 (0-0)	0 (0-0)	-3 (-6.2--0.6)
Kiribati	0 (0-0)	0 (0-0)	20.3 (9.9-131)	0 (0-0)	0 (0-0)	31 (15.9-292)	0 (0-0)	0 (0-0)	14.6 (-35.2-88.3)	0 (0-0)	0 (0-0)	30.3 (24.3-71.6)
Marshall Islands	0 (0-0)	0 (0-0)	-20.4 (-36.3--2)	0 (0-0)	0 (0-0)	-10.4 (-26.5-22.1)	0 (0-0)	0 (0-0)	-18.7 (-46.9-33)	0 (0-0)	0 (0-0)	-8.2 (-29.5--1.4)
Micronesia (Federated States of)	0 (0-0)	0 (0-0)	-36.2 (-36.3--36.2)	0 (0-0)	0 (0-0)	-25 (-30.2--21.6)	0 (0-0)	0 (0-0)	-35.7 (-59.6-5)	0 (0-0)	0 (0-0)	-22.8 (-30.2--16.5)
Nauru	0 (0-0)	0 (0-0)	-28.6 (-61.7--23.1)	0 (0-0)	0 (0-0)	-8.8 (-68.5-2.7)	0 (0-0)	0 (0-0)	-38.8 (-61-13.4)	0 (0-0)	0 (0-0)	-9.6 (-33.7--3.2)
Niue	0 (0-0)	0 (0-0)	-35.2 (-46.8--28.3)	0 (0-0)	0 (0-0)	-25.3 (-31.8--20.5)	0 (0-0)	0 (0-0)	-44.3 (-62.5--13.8)	0 (0-0)	0 (0-0)	-24.4 (-46.9--19)

Location	Under-5 deaths (cause-specific), 2000	Under-5 deaths (cause-specific), 2021	Under-5 deaths (cause-specific) percent change (2000-2021)	Under-5 deaths (total), 2000	Under-5 deaths (total), 2021	Under-5 deaths (total) percent change (2000-2021)	Under-5 cause-specific mortality cause fraction, 2000	Under-5 total SCD mortality cause fraction, 2000	Under-5 cause-specific mortality cause fraction, 2021	Under-5 total SCD mortality cause fraction, 2021	Under-5 cause-specific mortality rank 2000	Under-5 cause-specific mortality rank 2021	Under-5 total SCD mortality rank 2000	Under-5 total SCD mortality rank 2021
South Asia	658 (410-920)	231 (111-372)	-64.9 (-81.9--31.4)	18,100 (11,600-26,600)	15,600 (10,200-22,800)	-13.8 (-17.8--9.7)	0 (0-0)	0.6 (0.4-0.9)	0 (0-0)	1.3 (0.8-2)	83	95	22	13
**South Asia**	658 (410-920)	231 (111-372)	-64.9 (-81.9--31.4)	18,100 (11,600-26,600)	15,600 (10,200-22,800)	-13.8 (-17.8--9.7)	0 (0-0)	0.6 (0.4-0.9)	0 (0-0)	1.3 (0.8-2)	83	95	22	13
Bangladesh	1 (0-2)	0 (0-1)	-72.6 (-88.7--1.8)	32 (22-47)	25 (17-36)	-23.1 (-31.1--14.2)	0 (0-0)	0 (0-0)	0 (0-0)	0 (0-0)	130	130	87	67
Bhutan	0 (0-0)	0 (0-0)	-61.6 (-88-32.6)	0 (0-0)	0 (0-0)	-26 (-35.2--16.5)	0 (0-0)	0 (0-0)	0 (0-0)	0 (0-0)	120	119	100	85
India	652 (406-910)	227 (108-366)	-65.3 (-82.2--31.5)	18,100 (11,600-26,500)	15,600 (10,100-22,700)	-13.8 (-17.8--9.7)	0 (0-0)	0.9 (0.6-1.3)	0 (0-0)	2 (1.3-3.2)	75	80	19	10
Nepal	1 (0-1)	0 (0-0)	-68 (-88.9-22.3)	8 (6-12)	7 (5-10)	-19.3 (-28--9.6)	0 (0-0)	0 (0-0)	0 (0-0)	0 (0-0.1)	126	124	90	72
Pakistan	4 (2-9)	4 (2-8)	-12.8 (-58.7-140)	29 (17-47)	34 (22-54)	17.8 (6.2-33.5)	0 (0-0)	0 (0-0)	0 (0-0)	0 (0-0)	114	126	97	102
Azad Jammu & Kashmir	0 (0-0)	0 (0-0)	-49.7 (-80.5-28.2)	1 (0-1)	1 (0-1)	-7.5 (-27.5-3.7)	0 (0-0)	0 (0-0)	0 (0-0)	0 (0-0)	114	128	99	100
Balochistan	0 (0-0)	0 (0-1)	41.6 (-33.3-291)	1 (1-2)	3 (2-4)	91.1 (57.5-134)	0 (0-0)	0 (0-0)	0 (0-0)	0 (0-0)	116	127	97	107
Gilgit-Baltistan	0 (0-0)	0 (0-0)	-35.7 (-73.2-83.1)	0 (0-0)	0 (0-1)	91.8 (55.5-124)	0 (0-0)	0 (0-0)	0 (0-0)	0 (0-0)	117	128	100	97
Islamabad Capital Territory	0 (0-0)	0 (0-0)	-39.9 (-81.1-86.1)	0 (0-0)	0 (0-0)	105 (53-195)	0 (0-0)	0 (0-0)	0 (0-0)	0 (0-0.1)	118	130	89	80
Khyber Pakhtunkhwa	0 (0-1)	0 (0-1)	25.4 (-53.7-240)	4 (2-7)	6 (4-10)	61.9 (21.9-96.2)	0 (0-0)	0 (0-0)	0 (0-0)	0 (0-0)	118	128	95	98
Punjab	3 (1-5)	2 (1-5)	-11.6 (-60.9-205)	16 (9-26)	16 (10-25)	-0.1 (-15.8-22.2)	0 (0-0)	0 (0-0)	0 (0-0)	0 (0-0)	113	125	95	103
Sindh	1 (0-2)	1 (0-2)	-31.4 (-74.5-108)	7 (4-11)	8 (5-13)	17 (-0.5-34.7)	0 (0-0)	0 (0-0)	0 (0-0)	0 (0-0)	111	126	98	97
Southeast Asia, East Asia, and Oceania	10 (6-17)	2 (2-4)	-74.6 (-85.8--53.8)	61 (47-77)	56 (43-70)	-9.4 (-12.2--6.3)	0 (0-0)	0 (0-0)	0 (0-0)	0 (0-0)	137	137	126	111
**East Asia**	6 (3-12)	1 (0-2)	-85.5 (-91.9--71.6)	43 (31-57)	38 (28-52)	-10.3 (-13.8--6.8)	0 (0-0)	0 (0-0)	0 (0-0)	0 (0-0.1)	134	136	121	87
China	6 (3-12)	1 (0-2)	-85.5 (-91.9--71.4)	42 (30-56)	38 (27-51)	-9.6 (-13.1--6)	0 (0-0)	0 (0-0)	0 (0-0)	0 (0-0.1)	134	134	121	87
Democratic People's Republic of Korea	0 (0-0)	0 (0-0)	-67.1 (-84.1--30.2)	1 (1-1)	1 (0-1)	-38.6 (-46.3--27.5)	0 (0-0)	0 (0-0)	0 (0-0)	0 (0-0)	132	132	115	106
Taiwan (Province of China)	0 (0-0)	0 (0-0)	-97.3 (-98.2--96.2)	0 (0-0)	0 (0-0)	-21.3 (-32.5--7.8)	0 (0-0)	0 (0-0)	0 (0-0)	0 (0-0)	112	127	92	80
**Oceania**	0 (0-0)	0 (0-0)	-12.6 (-47.9-41.9)	0 (0-0)	0 (0-0)	58.6 (53.1-66.3)	0 (0-0)	0 (0-0)	0 (0-0)	0 (0-0)	139	141	127	124
American Samoa	0 (0-0)	0 (0-0)	-61.3 (-83.8--7.8)	0 (0-0)	0 (0-0)	-39.6 (-56.2--35.6)	0 (0-0)	0 (0-0)	0 (0-0)	0 (0-0)	131	132	121	106
Cook Islands	0 (0-0)	0 (0-0)	-91.9 (-98.2--73.2)	0 (0-0)	0 (0-0)	-37.6 (-42--34)	0 (0-0)	0 (0-0)	0 (0-0)	0 (0-0)	132	131	110	93
Fiji	0 (0-0)	0 (0-0)	-7.3 (-61-136)	0 (0-0)	0 (0-0)	-0.6 (-5.8-4.5)	0 (0-0)	0 (0-0)	0 (0-0)	0 (0-0)	128	133	116	114
Guam	0 (0-0)	0 (0-0)	-64.3 (-84.9--19.4)	0 (0-0)	0 (0-0)	-12.7 (-17.5--8.2)	0 (0-0)	0 (0-0)	0 (0-0)	0 (0-0)	133	137	114	111
Kiribati	0 (0-0)	0 (0-0)	-35.9 (-71-33)	0 (0-0)	0 (0-0)	22.1 (16.7-30)	0 (0-0)	0 (0-0)	0 (0-0)	0 (0-0)	131	131	121	119
Marshall Islands	0 (0-0)	0 (0-0)	-62.5 (-83.7-49.7)	0 (0-0)	0 (0-0)	-17.5 (-40.8--11.1)	0 (0-0)	0 (0-0)	0 (0-0)	0 (0-0)	134	135	117	112
Micronesia (Federated States of)	0 (0-0)	0 (0-0)	-74.9 (-87.1--9)	0 (0-0)	0 (0-0)	-36.4 (-39.2--33.9)	0 (0-0)	0 (0-0)	0 (0-0)	0 (0-0)	135	135	113	107
Nauru	0 (0-0)	0 (0-0)	-60.8 (-81.5-12.2)	0 (0-0)	0 (0-0)	-21 (-27.7--16.5)	0 (0-0)	0 (0-0)	0 (0-0)	0 (0-0)	135	135	119	114
Niue	0 (0-0)	0 (0-0)	-58.7 (-85.2-2.1)	0 (0-0)	0 (0-0)	-35 (-59.3--29.3)	0 (0-0)	0 (0-0)	0 (0-0)	0 (0-0)	135	136	114	111

Location	Births, 2000	Births, 2021	Births percent change (2000-2021)	All age cases, 2000	All age cases, 2021	All age cases percent change (2000-2021)	All age deaths (cause-specific), 2000	All age deaths (cause-specific), 2021	All age deaths (cause-specific) percent change (2000-2021)	All age deaths (total), 2000	All age deaths (total), 2021	All age deaths (total) percent change (2000-2021)	Under-5 deaths (cause-specific), 2000	Under-5 deaths (cause-specific), 2021	Under-5 deaths (cause-specific) percent change (2000-2021)	Under-5 deaths (total), 2000	Under-5 deaths (total), 2021	Under-5 deaths (total) percent change (2000-2021)	Under-5 cause-specific mortality cause fraction, 2000	Under-5 total SCD mortality cause fraction, 2000	Under-5 cause-specific mortality cause fraction, 2021	Under-5 total SCD mortality cause fraction, 2021	Under-5 cause-specific mortality rank 2000	Under-5 cause-specific mortality rank 2021	Under-5 total SCD mortality rank 2000	Under-5 total SCD mortality rank 2021
Northern Mariana Islands	0 (0-0)	0 (0-0)	-81 (-81.8--77.3)	0 (0-0)	0 (0-0)	-61.5 (-63.7--51.4)	0 (0-0)	0 (0-0)	-65.2 (-78.6--35.4)	0 (0-0)	0 (0-0)	-59.2 (-64.1--52.8)	0 (0-0)	0 (0-0)	-89.1 (-95.9--71.3)	0 (0-0)	0 (0-0)	-73.9 (-75.7--70.5)	0 (0-0)	0 (0-0)	0 (0-0)	0 (0-0)	134	137	117	116
Palau	0 (0-0)	0 (0-0)	-54.5 (-56.7--43.3)	0 (0-0)	0 (0-0)	-33.4 (-38.3--13.5)	0 (0-0)	0 (0-0)	-34.5 (-56.3--7)	0 (0-0)	0 (0-0)	-32.5 (-39.4--27.6)	0 (0-0)	0 (0-0)	-66.5 (-82.1--27.3)	0 (0-0)	0 (0-0)	-49.9 (-52.4--47.2)	0 (0-0)	0 (0-0)	0 (0-0)	0 (0-0)	129	131	110	109
Papua New Guinea	0 (0-0)	0 (0-0)	68.7 (60.7-74.7)	1 (0-3)	2 (0-5)	75.8 (64.7-95.9)	0 (0-0)	0 (0-0)	44.8 (-13.7-112)	0 (0-0)	0 (0-0)	76.7 (72.8-80.8)	0 (0-0)	0 (0-0)	-2.7 (-43.9-56.7)	0 (0-0)	0 (0-0)	73.1 (66.9-80)	0 (0-0)	0 (0-0)	0 (0-0)	0 (0-0)	137	139	124	121
Samoa	0 (0-0)	0 (0-0)	30.3 (-4.3-34.4)	0 (0-0)	0 (0-0)	17.9 (-8.5-28.8)	0 (0-0)	0 (0-0)	-0.1 (-47.2-65)	0 (0-0)	0 (0-0)	18.2 (-9.3-23)	0 (0-0)	0 (0-0)	-50 (-80.3-12)	0 (0-0)	0 (0-0)	13.9 (-17.4-20.9)	0 (0-0)	0 (0-0)	0 (0-0)	0 (0-0)	134	136	115	108
Solomon Islands	0 (0-0)	0 (0-0)	9.3 (2.8-12.2)	0 (0-0)	0 (0-0)	37 (27.8-44.7)	0 (0-0)	0 (0-0)	18.7 (-28.8-97.6)	0 (0-0)	0 (0-0)	35.1 (6.7-43.7)	0 (0-0)	0 (0-0)	-47.5 (-73.7-9.9)	0 (0-0)	0 (0-0)	19.6 (-6.5-26)	0 (0-0)	0 (0-0)	0 (0-0)	0 (0-0)	136	137	118	116
Tokelau	0 (0-0)	0 (0-0)	-30.7 (-37--20.1)	0 (0-0)	0 (0-0)	-20.7 (-27.7--9.4)	0 (0-0)	0 (0-0)	-43.3 (-64-2.5)	0 (0-0)	0 (0-0)	-17.4 (-25.6--12.6)	0 (0-0)	0 (0-0)	-77 (-92--48.1)	0 (0-0)	0 (0-0)	-22.5 (-32.4--16.1)	0 (0-0)	0 (0-0)	0 (0-0)	0 (0-0)	135	135	115	103
Tonga	0 (0-0)	0 (0-0)	-14.9 (-20.4--13.6)	0 (0-0)	0 (0-0)	-8.1 (-15.8--4.3)	0 (0-0)	0 (0-0)	-24.7 (-47.7-10.1)	0 (0-0)	0 (0-0)	-7.6 (-16.5--3.9)	0 (0-0)	0 (0-0)	-53.5 (-78.4--12)	0 (0-0)	0 (0-0)	-13.4 (-22.3--8.9)	0 (0-0)	0 (0-0)	0 (0-0)	0 (0-0)	134	135	118	115
Tuvalu	0 (0-0)	0 (0-0)	-4.8 (-6.6-3.5)	0 (0-0)	0 (0-0)	13.7 (5.6-26.1)	0 (0-0)	0 (0-0)	-25.2 (-52.7-6.7)	0 (0-0)	0 (0-0)	12.6 (1.2-24.2)	0 (0-0)	0 (0-0)	-64.2 (-83.5--20.2)	0 (0-0)	0 (0-0)	-6.8 (-11.8--0.3)	0 (0-0)	0 (0-0)	0 (0-0)	0 (0-0)	135	135	118	111
Vanuatu	0 (0-0)	0 (0-0)	15.8 (-16-19.5)	0 (0-0)	0 (0-0)	43 (7-54.9)	0 (0-0)	0 (0-0)	17.8 (-26.7-83.2)	0 (0-0)	0 (0-0)	39.6 (4.8-49.2)	0 (0-0)	0 (0-0)	-47.3 (-74.4-24.1)	0 (0-0)	0 (0-0)	21.2 (-14-29)	0 (0-0)	0 (0-0)	0 (0-0)	0 (0-0)	134	137	117	113
**Southeast Asia**	33 (24-42)	29 (22-36)	-12.4 (-14.6--7.8)	237 (152-385)	233 (146-384)	-2.2 (-6.4-2.7)	11 (8-15)	6 (4-8)	-45.9 (-63.5--27)	30 (22-40)	30 (22-39)	-3.5 (-7.6-3.5)	4 (2-6)	2 (1-2)	-55.6 (-74.9--25.7)	19 (13-26)	17 (12-24)	-7.6 (-11.1--0.1)	0 (0-0)	0 (0-0)	0 (0-0)	0 (0-0)	132	136	123	113
Cambodia	1 (1-1)	1 (1-1)	-0.7 (-7.4-21.2)	6 (3-9)	6 (3-10)	6.8 (-4.6-39.9)	0 (0-0)	0 (0-0)	-44.9 (-69.9-4.7)	1 (1-1)	1 (1-1)	5.3 (-5.5-61.9)	0 (0-0)	0 (0-0)	-61.4 (-80.7--0.8)	1 (0-1)	1 (0-1)	5 (-8.7-65.3)	0 (0-0)	0 (0-0)	0 (0-0)	0 (0-0)	138	138	123	107
Indonesia	14 (10-17)	11 (8-14)	-20.8 (-23.6--11.1)	96 (61-153)	91 (55-150)	-5.8 (-11-0.2)	4 (2-5)	2 (1-3)	-44.8 (-67.4--15.3)	12 (9-17)	12 (8-16)	-6.9 (-12.3-5.6)	2 (1-3)	1 (0-1)	-52.5 (-72--18.7)	8 (5-11)	7 (5-9)	-13.9 (-18.1-0.9)	0 (0-0)	0 (0-0)	0 (0-0)	0 (0-0)	129	130	122	109
Aceh	0 (0-0)	0 (0-0)	-19.4 (-25.5--6)	2 (1-3)	2 (1-3)	-2.6 (-13.2-8.1)	0 (0-0)	0 (0-0)	-45 (-74.8-12.3)	0 (0-0)	0 (0-0)	-4.8 (-11.4-20.4)	0 (0-0)	0 (0-0)	-59.2 (-83.7-16.7)	0 (0-0)	0 (0-0)	-11.7 (-18.8-17.3)	0 (0-0)	0 (0-0)	0 (0-0)	0 (0-0)	128	130	121	106
Bali	0 (0-0)	0 (0-0)	-28.4 (-31--1.3)	1 (1-2)	1 (1-2)	-6.8 (-13.9-8.8)	0 (0-0)	0 (0-0)	-55.3 (-76.1--23)	0 (0-0)	0 (0-0)	-7.7 (-13.8-16.9)	0 (0-0)	0 (0-0)	-66.2 (-83.8--28.3)	0 (0-0)	0 (0-0)	-20.6 (-26.6-7)	0 (0-0)	0 (0-0)	0 (0-0)	0 (0-0)	127	133	106	103
Bangka-Belitung Islands	0 (0-0)	0 (0-0)	-16.9 (-32.2-1.5)	0 (0-1)	0 (0-1)	0.5 (-8.1-14.2)	0 (0-0)	0 (0-0)	-31.2 (-63-10.2)	0 (0-0)	0 (0-0)	-1.5 (-15.1-27.2)	0 (0-0)	0 (0-0)	-29.2 (-61.4-49.9)	0 (0-0)	0 (0-0)	-9.6 (-25.6-21.6)	0 (0-0)	0 (0-0)	0 (0-0)	0 (0-0)	128	129	112	110
Banten	1 (0-1)	1 (0-1)	-28.9 (-33.5--3)	5 (3-8)	4 (2-7)	-18.4 (-25.3--3.6)	0 (0-0)	0 (0-0)	-62.4 (-80.4--35.2)	1 (0-1)	1 (0-1)	-19.1 (-23.6-10.8)	0 (0-0)	0 (0-0)	-68.8 (-85.3--29.8)	0 (0-1)	0 (0-1)	-22.9 (-29.3-10.5)	0 (0-0)	0 (0-0)	0 (0-0)	0 (0-0)	126	129	118	106
Bengkulu	0 (0-0)	0 (0-0)	-21 (-26.6-24.3)	1 (0-1)	1 (0-1)	-6.3 (-15.6-21.9)	0 (0-0)	0 (0-0)	-54.7 (-78.3--10.7)	0 (0-0)	0 (0-0)	-7.8 (-12.8-26.6)	0 (0-0)	0 (0-0)	-61.7 (-82.9--7.6)	0 (0-0)	0 (0-0)	-13.7 (-19.9-24.5)	0 (0-0)	0 (0-0)	0 (0-0)	0 (0-0)	124	129	120	109
Gorontalo	0 (0-0)	0 (0-0)	10.4 (5.4-30.4)	0 (0-1)	0 (0-1)	33.5 (21.3-50.3)	0 (0-0)	0 (0-0)	3.9 (-50-95.3)	0 (0-0)	0 (0-0)	24.5 (15.7-64.8)	0 (0-0)	0 (0-0)	-30.7 (-67.6-60.4)	0 (0-0)	0 (0-0)	11.9 (3.3-55.2)	0 (0-0)	0 (0-0)	0 (0-0)	0 (0-0)	123	125	117	109
Jakarta	1 (0-1)	0 (0-1)	-38.5 (-46--21.9)	4 (3-7)	3 (2-6)	-21.4 (-28.6--13.8)	0 (0-0)	0 (0-0)	-61.7 (-77--36.3)	1 (0-1)	0 (0-1)	-21.8 (-32.6-8.1)	0 (0-0)	0 (0-0)	-67.8 (-82.1--37.4)	0 (0-0)	0 (0-0)	-28.1 (-40.4-7.5)	0 (0-0)	0 (0-0)	0 (0-0)	0 (0-0)	127	129	117	108
Jambi	0 (0-0)	0 (0-0)	-13.4 (-19.4-15.7)	1 (1-2)	1 (1-2)	2.7 (-7.9-22.2)	0 (0-0)	0 (0-0)	-43.6 (-68.3--4.3)	0 (0-0)	0 (0-0)	1.4 (-4.6-38)	0 (0-0)	0 (0-0)	-48.2 (-76.1-16.1)	0 (0-0)	0 (0-0)	-6.5 (-13.1-36.1)	0 (0-0)	0 (0-0)	0 (0-0)	0 (0-0)	130	132	119	107
West Java	2 (2-3)	2 (1-3)	-8.5 (-15.6-36.3)	16 (10-26)	18 (11-30)	10.4 (1.7-35.9)	1 (0-1)	0 (0-1)	-36.3 (-63.5-10.4)	2 (1-3)	2 (2-3)	7.1 (-1-54.1)	0 (0-1)	0 (0-0)	-48.7 (-72.3-15.5)	1 (1-2)	1 (1-2)	-1.6 (-10.8-53.3)	0 (0-0)	0 (0-0)	0 (0-0)	0 (0-0)	127	128	116	103
Central Java	2 (1-2)	1 (1-1)	-35.2 (-39.8--12.7)	14 (8-22)	11 (7-18)	-21.9 (-29.8--9.5)	0 (0-1)	0 (0-0)	-56.8 (-77.2--25.9)	2 (1-2)	1 (1-2)	-24.1 (-34.4-3.5)	0 (0-0)	0 (0-0)	-63.9 (-82.2--11)	1 (1-1)	1 (0-1)	-31.5 (-42.4-1.1)	0 (0-0)	0 (0-0)	0 (0-0)	0 (0-0)	127	129	115	102
East Java	2 (1-2)	1 (1-1)	-35.2 (-38.6--28.4)	14 (9-23)	11 (6-19)	-20.3 (-27--12)	0 (0-1)	0 (0-0)	-51.2 (-75.8--27.9)	2 (1-2)	1 (1-2)	-22 (-27--0.4)	0 (0-0)	0 (0-0)	-64.1 (-83.2--27.8)	1 (1-1)	1 (0-1)	-30.8 (-36.7--7.4)	0 (0-0)	0 (0-0)	0 (0-0)	0 (0-0)	125	129	113	102
West Kalimantan	0 (0-0)	0 (0-0)	-18.1 (-22.1-13.3)	2 (1-3)	2 (1-3)	-2.1 (-10.5-19.7)	0 (0-0)	0 (0-0)	-48.6 (-71.3-11.4)	0 (0-0)	0 (0-0)	-3.6 (-10.2-48.9)	0 (0-0)	0 (0-0)	-54.7 (-79.2-24.2)	0 (0-0)	0 (0-0)	-10.1 (-18.2-48.2)	0 (0-0)	0 (0-0)	0 (0-0)	0 (0-0)	128	131	122	114
South Kalimantan	0 (0-0)	0 (0-0)	-22.1 (-26.2-3.3)	1 (1-2)	1 (1-2)	-4.3 (-13.1-12.8)	0 (0-0)	0 (0-0)	-48.4 (-71.1--12.9)	0 (0-0)	0 (0-0)	-6.8 (-12.7-24.2)	0 (0-0)	0 (0-0)	-59.4 (-77.8-9.2)	0 (0-0)	0 (0-0)	-14.4 (-20.7-21)	0 (0-0)	0 (0-0)	0 (0-0)	0 (0-0)	127	131	120	107
Central Kalimantan	0 (0-0)	0 (0-0)	-15.1 (-18.9-16.2)	1 (1-1)	1 (1-2)	2.6 (-5.9-19.2)	0 (0-0)	0 (0-0)	-30.3 (-59-18.7)	0 (0-0)	0 (0-0)	0.3 (-5.7-34.9)	0 (0-0)	0 (0-0)	-32.7 (-66.1-40.7)	0 (0-0)	0 (0-0)	-7.7 (-14.4-30.4)	0 (0-0)	0 (0-0)	0 (0-0)	0 (0-0)	125	129	116	111
East Kalimantan	0 (0-0)	0 (0-0)	0.2 (-4.7-28)	1 (1-2)	1 (1-2)	24.7 (16.9-42.7)	0 (0-0)	0 (0-0)	-35.5 (-61-3.7)	0 (0-0)	0 (0-0)	24.8 (18.2-68.8)	0 (0-0)	0 (0-0)	-47.8 (-74.7-9.1)	0 (0-0)	0 (0-0)	14.3 (4.9-64.6)	0 (0-0)	0 (0-0)	0 (0-0)	0 (0-0)	130	131	115	108
North Kalimantan	0 (0-0)	0 (0-0)	11.7 (3.9-31.1)	0 (0-0)	0 (0-0)	47.1 (30.9-63.4)	0 (0-0)	0 (0-0)	-31.3 (-56.7-28)	0 (0-0)	0 (0-0)	35.9 (27.1-74)	0 (0-0)	0 (0-0)	-36.7 (-64.9-48.7)	0 (0-0)	0 (0-0)	20.7 (11.6-63.9)	0 (0-0)	0 (0-0)	0 (0-0)	0 (0-0)	128	128	118	111
Riau Islands	0 (0-0)	0 (0-0)	-14.4 (-20.2-10.5)	1 (0-1)	1 (0-1)	6.3 (-2.5-23.1)	0 (0-0)	0 (0-0)	-54.5 (-73.7--3.2)	0 (0-0)	0 (0-0)	10.5 (4.7-47.8)	0 (0-0)	0 (0-0)	-59.2 (-77.7-1.7)	0 (0-0)	0 (0-0)	6.3 (-3-48.9)	0 (0-0)	0 (0-0)	0 (0-0)	0 (0-0)	128	128	116	108

Location	Births, 2000	Births, 2021	Births percent change (2000-2021)	All age cases, 2000	All age cases, 2021	All age cases percent change (2000-2021)	All age deaths (cause-specific), 2000	All age deaths (cause-specific), 2021	All age deaths (cause-specific) percent change (2000-2021)	All age deaths (total), 2000	All age deaths (total), 2021	All age deaths (total) percent change (2000-2021)	Under-5 deaths (cause-specific), 2000	Under-5 deaths (cause-specific), 2021	Under-5 deaths (cause-specific) percent change (2000-2021)	Under-5 deaths (total), 2000	Under-5 deaths (total), 2021	Under-5 deaths (total) percent change (2000-2021)	Under-5 cause-specific mortality cause fraction, 2000	Under-5 total SCD mortality cause fraction, 2000	Under-5 cause-specific mortality cause fraction, 2021	Under-5 total SCD mortality cause fraction, 2021	Under-5 cause-specific mortality rank 2000	Under-5 cause-specific mortality rank 2021	Under-5 total SCD mortality rank 2000	Under-5 total SCD mortality rank 2021
Lampung	0 (0-1)	0 (0-0)	-30.8 (-38.4--11.2)	3 (2-6)	3 (2-5)	-18.5 (-26.7--5.2)	0 (0-0)	0 (0-0)	-40.8 (-63.8-6.9)	0 (0-1)	0 (0-0)	-19.6 (-25.2-11.1)	0 (0-0)	0 (0-0)	-42.1 (-68.2-43.9)	0 (0-0)	0 (0-0)	-26.4 (-32.6-7.3)	0 (0-0)	0 (0-0)	0 (0-0)	0 (0-0)	128	129	113	110
Maluku	0 (0-0)	0 (0-0)	-20.1 (-28.9-5.3)	1 (0-1)	1 (0-1)	0.1 (-10.7-19.1)	0 (0-0)	0 (0-0)	-53.5 (-75.7-11.8)	0 (0-0)	0 (0-0)	-3.5 (-9.4-39.7)	0 (0-0)	0 (0-0)	-60 (-79.6-7.5)	0 (0-0)	0 (0-0)	-11 (-18.1-35.2)	0 (0-0)	0 (0-0)	0 (0-0)	0 (0-0)	129	130	124	110
North Maluku	0 (0-0)	0 (0-0)	-19.8 (-25.1-23.9)	0 (0-1)	0 (0-1)	-1.7 (-12.7-27.6)	0 (0-0)	0 (0-0)	-37.9 (-62.5-23.1)	0 (0-0)	0 (0-0)	-5.7 (-11.2-23)	0 (0-0)	0 (0-0)	-41 (-71.1-48)	0 (0-0)	0 (0-0)	-13.2 (-18.8-18.7)	0 (0-0)	0 (0-0)	0 (0-0)	0 (0-0)	128	129	121	115
West Nusa Tenggara	0 (0-0)	0 (0-0)	-15.2 (-19.3-13.4)	2 (1-3)	2 (1-3)	-0.9 (-9.4-22.8)	0 (0-0)	0 (0-0)	-47.2 (-81.8--2.1)	0 (0-0)	0 (0-0)	-2.1 (-7.7-37.5)	0 (0-0)	0 (0-0)	-60.4 (-83.4-15.1)	0 (0-0)	0 (0-0)	-7.7 (-15-37.4)	0 (0-0)	0 (0-0)	0 (0-0)	0 (0-0)	131	131	117	101
East Nusa Tenggara	0 (0-0)	0 (0-0)	-9.8 (-18.5-33.8)	2 (1-3)	2 (1-4)	10.1 (-1-39.6)	0 (0-0)	0 (0-0)	-37.2 (-67.9-20.7)	0 (0-0)	0 (0-0)	5.3 (-1.1-40.8)	0 (0-0)	0 (0-0)	-49.6 (-75.8-30.1)	0 (0-0)	0 (0-0)	-3.4 (-10-37.3)	0 (0-0)	0 (0-0)	0 (0-0)	0 (0-0)	126	128	116	111
Papua	0 (0-0)	0 (0-0)	63.4 (52.9-142)	1 (1-1)	2 (1-3)	92.5 (72.3-148)	0 (0-0)	0 (0-0)	42.2 (-15.3-156)	0 (0-0)	0 (0-0)	87.4 (78-150)	0 (0-0)	0 (0-0)	44.9 (-20.3-201)	0 (0-0)	0 (0-0)	75.2 (63.5-142)	0 (0-0)	0 (0-0)	0 (0-0)	0 (0-0)	128	131	122	122
West Papua	0 (0-0)	0 (0-0)	-16.7 (-24.3-48.8)	0 (0-1)	0 (0-1)	-3.5 (-15-39.7)	0 (0-0)	0 (0-0)	-40.9 (-68.4-19.3)	0 (0-0)	0 (0-0)	-3.8 (-9.2-36.2)	0 (0-0)	0 (0-0)	-34.2 (-69.3-54.8)	0 (0-0)	0 (0-0)	-8.1 (-15.3-36.3)	0 (0-0)	0 (0-0)	0 (0-0)	0 (0-0)	129	131	117	115
Riau	0 (0-0)	0 (0-1)	51.6 (45.7-92.2)	2 (1-3)	3 (2-6)	83.6 (71.1-114)	0 (0-0)	0 (0-0)	-27.2 (-58.2-18)	0 (0-0)	0 (0-1)	81.4 (71.4-146)	0 (0-0)	0 (0-0)	-41.1 (-69.9-7.2)	0 (0-0)	0 (0-0)	67.4 (55.1-146)	0 (0-0)	0 (0-0)	0 (0-0)	0 (0-0)	129	132	122	106
West Sulawesi	0 (0-0)	0 (0-0)	-5 (-7.7-14.6)	0 (0-1)	1 (0-1)	18.9 (7-35.7)	0 (0-0)	0 (0-0)	-28.4 (-56.1-34.6)	0 (0-0)	0 (0-0)	12.5 (6.2-38)	0 (0-0)	0 (0-0)	-33.5 (-66.3-97.5)	0 (0-0)	0 (0-0)	2.3 (-3.3-28.8)	0 (0-0)	0 (0-0)	0 (0-0)	0 (0-0)	128	129	119	114
South Sulawesi	1 (0-1)	0 (0-1)	-27.8 (-33.1-5.8)	4 (2-5)	3 (2-5)	-11.5 (-20.8-9.2)	0 (0-0)	0 (0-0)	-42.9 (-68.4-4.6)	0 (0-1)	0 (0-1)	-14.2 (-20.2-13.6)	0 (0-0)	0 (0-0)	-55 (-77-11.6)	0 (0-0)	0 (0-0)	-22.2 (-28.6-7.5)	0 (0-0)	0 (0-0)	0 (0-0)	0 (0-0)	131	133	114	101
Central Sulawesi	0 (0-0)	0 (0-0)	-18.5 (-23.1-13.9)	1 (1-2)	1 (1-2)	3.2 (-8.6-26.5)	0 (0-0)	0 (0-0)	-37.2 (-63.6-4.8)	0 (0-0)	0 (0-0)	-0.8 (-7.9-35.3)	0 (0-0)	0 (0-0)	-45.4 (-69.9-33.7)	0 (0-0)	0 (0-0)	-11 (-18.5-27)	0 (0-0)	0 (0-0)	0 (0-0)	0 (0-0)	128	131	119	115
Southeast Sulawesi	0 (0-0)	0 (0-0)	-3.1 (-7.7-27.3)	1 (1-1)	1 (1-2)	15.9 (6.3-34.3)	0 (0-0)	0 (0-0)	-16.7 (-41.7-28.7)	0 (0-0)	0 (0-0)	12.8 (6.8-47.2)	0 (0-0)	0 (0-0)	-13.4 (-50.9-62.5)	0 (0-0)	0 (0-0)	4.5 (-3.7-43.5)	0 (0-0)	0 (0-0)	0 (0-0)	0 (0-0)	126	128	117	116
North Sulawesi	0 (0-0)	0 (0-0)	-42.2 (-47--33.9)	1 (1-2)	1 (0-1)	-26.2 (-33--20.1)	0 (0-0)	0 (0-0)	-45 (-65.5--10.2)	0 (0-0)	0 (0-0)	-29.4 (-34.6--13.6)	0 (0-0)	0 (0-0)	-32.4 (-60.4-12.8)	0 (0-0)	0 (0-0)	-38.9 (-43.9--21.7)	0 (0-0)	0 (0-0)	0 (0-0)	0 (0-0)	129	128	108	114
West Sumatra	0 (0-0)	0 (0-0)	-30.8 (-34.6--15.7)	2 (1-3)	2 (1-3)	-14.1 (-23.7--4)	0 (0-0)	0 (0-0)	-61.2 (-80.9--27.8)	0 (0-0)	0 (0-0)	-18 (-34.4-7.9)	0 (0-0)	0 (0-0)	-69.8 (-86.6--22.4)	0 (0-0)	0 (0-0)	-25.2 (-42.3-4.7)	0 (0-0)	0 (0-0)	0 (0-0)	0 (0-0)	123	128	117	105
South Sumatra	0 (0-1)	0 (0-0)	-22.9 (-30--7.4)	3 (2-6)	3 (2-5)	-8.5 (-23-3.2)	0 (0-0)	0 (0-0)	-50.1 (-71.4--4)	0 (0-1)	0 (0-1)	-9.7 (-20.1-19.6)	0 (0-0)	0 (0-0)	-59.9 (-77.9--8.6)	0 (0-0)	0 (0-0)	-16.1 (-27-19.2)	0 (0-0)	0 (0-0)	0 (0-0)	0 (0-0)	125	127	117	106
North Sumatra	1 (1-1)	1 (0-1)	-22.1 (-26.1--5.7)	6 (4-10)	5 (3-9)	-8.2 (-17.2-5.3)	0 (0-0)	0 (0-0)	-31.7 (-59.4-3.6)	1 (1-1)	1 (1-1)	-10.3 (-16-19.4)	0 (0-0)	0 (0-0)	-32.2 (-60.2-24.1)	1 (0-1)	0 (0-1)	-16.6 (-24.6-17.4)	0 (0-0)	0 (0-0)	0 (0-0)	0 (0-0)	123	126	113	112
Yogyakarta	0 (0-0)	0 (0-0)	-26.7 (-30.6--13.9)	1 (1-2)	1 (1-2)	-13.8 (-20.1-11.9)	0 (0-0)	0 (0-0)	-34.8 (-60.4-3.8)	0 (0-0)	0 (0-0)	-13.9 (-18.6-7.1)	0 (0-0)	0 (0-0)	-50.6 (-73.5-0)	0 (0-0)	0 (0-0)	-22.1 (-28.5-4.8)	0 (0-0)	0 (0-0)	0 (0-0)	0 (0-0)	128	130	103	99
Lao People's Democratic Republic	1 (0-1)	1 (0-1)	-4.4 (-8.3-28.1)	2 (1-4)	3 (1-4)	6.5 (-1.9-33.9)	0 (0-0)	0 (0-0)	-51 (-70.9-5.5)	0 (0-1)	0 (0-1)	3.1 (-0.5-39)	0 (0-0)	0 (0-0)	-64 (-80.4--8.9)	0 (0-0)	0 (0-0)	1.4 (-5.8-41.2)	0 (0-0)	0 (0-0)	0 (0-0)	0 (0-0)	136	136	121	110
Malaysia	1 (1-2)	1 (1-2)	6.5 (-2-19)	11 (6-19)	12 (6-21)	6 (-3.2-15.4)	0 (0-0)	0 (0-0)	-15.1 (-48.6-47.7)	1 (1-2)	1 (1-2)	2.1 (-10.5-46.6)	0 (0-0)	0 (0-0)	-62.2 (-81.3--31.1)	1 (0-1)	1 (0-1)	-2.3 (-18-50)	0 (0-0)	0 (0-0)	0 (0-0)	0 (0-0)	133	135	98	93
Maldives	0 (0-0)	0 (0-0)	14.3 (10.8-38.2)	0 (0-0)	0 (0-0)	25.2 (9.3-49.3)	0 (0-0)	0 (0-0)	-26.6 (-61.5-11.7)	0 (0-0)	0 (0-0)	16.4 (6.2-52.2)	0 (0-0)	0 (0-0)	-61.9 (-79.1--8.4)	0 (0-0)	0 (0-0)	14 (3-55.4)	0 (0-0)	0 (0-0)	0 (0-0)	0 (0-0)	132	132	102	84
Mauritius	0 (0-0)	0 (0-0)	-35.6 (-40.1--14.3)	0 (0-1)	0 (0-1)	-27.7 (-34.6--13.3)	4 (3-4)	1 (1-1)	-67.9 (-72.3--63.4)	0 (0-0)	0 (0-0)	-30.1 (-37.6--10.8)	0 (0-0)	0 (0-0)	-86.5 (-90.6--82.2)	0 (0-0)	0 (0-0)	-36.8 (-45.8--12.7)	0.1 (0.1-0.1)	0 (0-0)	0 (0-0)	0 (0-0)	49	73	89	87
Myanmar	4 (2-5)	3 (2-5)	-15.4 (-17.6--4.9)	18 (9-30)	17 (9-30)	-3.8 (-12.2-25.2)	0 (0-1)	0 (0-0)	-60.5 (-75.3--23.1)	3 (2-5)	3 (2-4)	-6.6 (-11.5-19.4)	0 (0-0)	0 (0-0)	-68.2 (-81.8--18.9)	2 (1-3)	2 (1-3)	-9.3 (-14-19)	0 (0-0)	0 (0-0)	0 (0-0)	0 (0-0)	133	137	123	115
Philippines	6 (5-8)	7 (5-9)	15.1 (13.3-23.8)	41 (25-63)	50 (30-79)	23.1 (19.9-29.2)	2 (1-3)	2 (1-3)	-16.2 (-53.7-33.3)	5 (4-7)	6 (4-9)	21.9 (18.6-30.3)	1 (0-2)	0 (0-1)	-47.3 (-78.3-3)	3 (2-5)	4 (3-5)	18 (15.3-27.2)	0 (0-0)	0 (0-0)	0 (0-0)	0 (0-0)	129	131	115	112
Abra	0 (0-0)	0 (0-0)	-17.6 (-25.8-8.4)	0 (0-0)	0 (0-0)	-3.2 (-15.6-14.9)	0 (0-0)	0 (0-0)	11.5 (-46-89.6)	0 (0-0)	0 (0-0)	-4.1 (-13.8-26.2)	0 (0-0)	0 (0-0)	-60.6 (-84.4--7.4)	0 (0-0)	0 (0-0)	-11.4 (-21.4-24.9)	0 (0-0)	0 (0-0)	0 (0-0)	0 (0-0)	130	132	105	96
Agusan Del Norte	0 (0-0)	0 (0-0)	10.4 (6.7-26.4)	0 (0-0)	0 (0-1)	15.3 (7.1-26.9)	0 (0-0)	0 (0-0)	-50.1 (-76.3--18)	0 (0-0)	0 (0-0)	14.5 (10.6-52.1)	0 (0-0)	0 (0-0)	-59 (-81.6--23.5)	0 (0-0)	0 (0-0)	11.3 (2.4-56.7)	0 (0-0)	0 (0-0)	0 (0-0)	0 (0-0)	125	127	118	107
Agusan Del Sur	0 (0-0)	0 (0-0)	-7 (-10.2-20.3)	0 (0-0)	0 (0-1)	8.7 (0.6-25.3)	0 (0-0)	0 (0-0)	22.5 (-33-103)	0 (0-0)	0 (0-0)	6.8 (-8.3-45.8)	0 (0-0)	0 (0-0)	-7.4 (-63.3-115)	0 (0-0)	0 (0-0)	1.3 (-15.3-43.1)	0 (0-0)	0 (0-0)	0 (0-0)	0 (0-0)	130	133	118	118
Aklan	0 (0-0)	0 (0-0)	21.7 (13.6-61.6)	0 (0-0)	0 (0-0)	21.6 (13.9-47.5)	0 (0-0)	0 (0-0)	8.1 (-47.1-79.8)	0 (0-0)	0 (0-0)	22.8 (19.1-61.8)	0 (0-0)	0 (0-0)	-25.7 (-70.2-59.7)	0 (0-0)	0 (0-0)	21 (12-66.2)	0 (0-0)	0 (0-0)	0 (0-0)	0 (0-0)	125	128	111	111
Albay	0 (0-0)	0 (0-0)	5.7 (4.5-15.6)	1 (0-1)	1 (0-1)	8.8 (3.3-16.6)	0 (0-0)	0 (0-0)	-35.2 (-64.7-3.5)	0 (0-0)	0 (0-0)	7.3 (4.6-22.8)	0 (0-0)	0 (0-0)	-57.5 (-83.1--11)	0 (0-0)	0 (0-0)	4.2 (-2-22.2)	0 (0-0)	0 (0-0)	0 (0-0)	0 (0-0)	126	128	108	100
Antique	0 (0-0)	0 (0-0)	6 (2.2-36.3)	0 (0-0)	0 (0-0)	13.6 (5.3-35.4)	0 (0-0)	0 (0-0)	-19.4 (-61-40.3)	0 (0-0)	0 (0-0)	12.9 (8.8-51.2)	0 (0-0)	0 (0-0)	-58.6 (-86--5.2)	0 (0-0)	0 (0-0)	7 (-0.7-48.3)	0 (0-0)	0 (0-0)	0 (0-0)	0 (0-0)	124	128	114	103

Location	Births, 2000	Births, 2021	Births percent change (2000-2021)	All age cases, 2000	All age cases, 2021	All age cases percent change (2000-2021)	All age deaths (cause-specific), 2000	All age deaths (cause-specific), 2021	All age deaths (cause-specific) percent change (2000-2021)	All age deaths (total), 2000	All age deaths (total), 2021	All age deaths (total) percent change (2000-2021)	Under-5 deaths (cause-specific), 2000	Under-5 deaths (cause-specific), 2021	Under-5 deaths (cause-specific) percent change (2000-2021)	Under-5 deaths (total), 2000	Under-5 deaths (total), 2021	Under-5 deaths (total) percent change (2000-2021)	Under-5 cause-specific mortality cause fraction, 2000	Under-5 total SCD mortality cause fraction, 2000	Under-5 cause-specific mortality cause fraction, 2021	Under-5 total SCD mortality cause fraction, 2021	Under-5 cause-specific mortality rank 2000	Under-5 cause-specific mortality rank 2021	Under-5 total SCD mortality rank 2000	Under-5 total SCD mortality rank 2021
Apayao	0 (0-0)	0 (0-0)	-22.5 (-29--10.8)	0 (0-0)	0 (0-0)	-1.3 (-9.5-9.1)	0 (0-0)	0 (0-0)	-35.8 (-70.1-14.4)	0 (0-0)	0 (0-0)	-4.2 (-16.5-29.9)	0 (0-0)	0 (0-0)	-67.2 (-87.5-6.5)	0 (0-0)	0 (0-0)	-10.3 (-23.6-28.9)	0 (0-0)	0 (0-0)	0 (0-0)	0 (0-0)	124	127	117	109
Aurora	0 (0-0)	0 (0-0)	-2.3 (-6.2-14.3)	0 (0-0)	0 (0-0)	8.9 (1.1-23.1)	0 (0-0)	0 (0-0)	-35.2 (-70.6-18.8)	0 (0-0)	0 (0-0)	8.4 (3.6-46.4)	0 (0-0)	0 (0-0)	-74 (-90.6--36.9)	0 (0-0)	0 (0-0)	4.1 (-2.5-48.6)	0 (0-0)	0 (0-0)	0 (0-0)	0 (0-0)	124	127	119	100
Basilan	0 (0-0)	0 (0-0)	30.3 (25.1-66.2)	0 (0-0)	0 (0-0)	43.5 (35.5-65.7)	0 (0-0)	0 (0-0)	-20.3 (-65.6-53.8)	0 (0-0)	0 (0-0)	41.9 (37.6-72.2)	0 (0-0)	0 (0-0)	-48.9 (-81.6-48.1)	0 (0-0)	0 (0-0)	37.1 (26.6-73.4)	0 (0-0)	0 (0-0)	0 (0-0)	0 (0-0)	127	130	105	93
Bataan	0 (0-0)	0 (0-0)	15.3 (-0.3-61.9)	0 (0-0)	0 (0-1)	23.3 (10.6-49.9)	0 (0-0)	0 (0-0)	13.2 (-43.4-106)	0 (0-0)	0 (0-0)	23 (18.7-61.4)	0 (0-0)	0 (0-0)	-16.9 (-69.4-110)	0 (0-0)	0 (0-0)	18.3 (10.6-63.6)	0 (0-0)	0 (0-0)	0 (0-0)	0 (0-0)	129	129	111	111
Batanes	0 (0-0)	0 (0-0)	-27.8 (-30.9--12.2)	0 (0-0)	0 (0-0)	-14.4 (-19.9--3.5)	0 (0-0)	0 (0-0)	-39.5 (-70.8-11.6)	0 (0-0)	0 (0-0)	-15.9 (-20.2--8.8)	0 (0-0)	0 (0-0)	-68.4 (-87.4--19.4)	0 (0-0)	0 (0-0)	-22.8 (-28.8--15.3)	0 (0-0)	0 (0-0)	0 (0-0)	0 (0-0)	125	127	106	97
Batangas	0 (0-0)	0 (0-0)	43.5 (26.2-62.6)	1 (1-2)	1 (1-2)	39.6 (28.7-52.9)	0 (0-0)	0 (0-0)	-4.2 (-51.5-59)	0 (0-0)	0 (0-0)	39.7 (22-75)	0 (0-0)	0 (0-0)	-48.8 (-77-36.4)	0 (0-0)	0 (0-0)	40.4 (18.6-82.1)	0 (0-0)	0 (0-0)	0 (0-0)	0 (0-0)	127	129	109	98
Benguet	0 (0-0)	0 (0-0)	30.2 (18.5-60.4)	0 (0-0)	0 (0-1)	28.1 (17-46.3)	0 (0-0)	0 (0-0)	-52.3 (-75--15.6)	0 (0-0)	0 (0-0)	28.4 (15.2-62.9)	0 (0-0)	0 (0-0)	-75 (-89.9--42.1)	0 (0-0)	0 (0-0)	29.9 (14.4-73.6)	0 (0-0)	0 (0-0)	0 (0-0)	0 (0-0)	127	130	113	94
Biliran	0 (0-0)	0 (0-0)	1.7 (-2.4-35.4)	0 (0-0)	0 (0-0)	10.2 (2.2-33.8)	0 (0-0)	0 (0-0)	3.1 (-45-71.6)	0 (0-0)	0 (0-0)	10.8 (7.3-22.2)	0 (0-0)	0 (0-0)	-57.4 (-82.7--8.8)	0 (0-0)	0 (0-0)	5.6 (-2.9-24.4)	0 (0-0)	0 (0-0)	0 (0-0)	0 (0-0)	123	130	105	95
Bohol	0 (0-0)	0 (0-0)	-22.3 (-26.2-7.3)	1 (0-1)	1 (0-1)	-4.8 (-15.4-16)	0 (0-0)	0 (0-0)	-16.7 (-57.3-48.1)	0 (0-0)	0 (0-0)	-7.5 (-11.8-24.8)	0 (0-0)	0 (0-0)	-52.5 (-81.8--3.4)	0 (0-0)	0 (0-0)	-15.6 (-21.2-20)	0 (0-0)	0 (0-0)	0 (0-0)	0 (0-0)	130	131	111	111
Bukidnon	0 (0-0)	0 (0-0)	-4.9 (-16.8-15.1)	1 (0-1)	1 (0-1)	13.7 (-1.2-28.9)	0 (0-0)	0 (0-0)	6.8 (-50.1-96.8)	0 (0-0)	0 (0-0)	11.6 (-1.1-56.8)	0 (0-0)	0 (0-0)	-25.3 (-72.7-77.3)	0 (0-0)	0 (0-0)	4.8 (-10.5-53.8)	0 (0-0)	0 (0-0)	0 (0-0)	0 (0-0)	127	130	115	118
Bulacan	0 (0-0)	0 (0-0)	31.8 (26-82.5)	1 (1-2)	2 (1-3)	37.8 (28.1-61.9)	0 (0-0)	0 (0-0)	30.2 (-35.3-112)	0 (0-0)	0 (0-0)	34.9 (29.9-64.4)	0 (0-0)	0 (0-0)	-31 (-69.4-68.3)	0 (0-0)	0 (0-0)	28.6 (20.1-66.5)	0 (0-0)	0 (0-0)	0 (0-0)	0 (0-0)	128	132	107	103
Cagayan	0 (0-0)	0 (0-0)	-0.5 (-7.6-6.8)	0 (0-1)	0 (0-1)	5.6 (-2-16.4)	0 (0-0)	0 (0-0)	-26.4 (-65.3-24.9)	0 (0-0)	0 (0-0)	4.4 (-7.8-36.8)	0 (0-0)	0 (0-0)	-56.7 (-84.6--7)	0 (0-0)	0 (0-0)	1.9 (-12.5-37.8)	0 (0-0)	0 (0-0)	0 (0-0)	0 (0-0)	125	127	117	112
Camarines Norte	0 (0-0)	0 (0-0)	5 (2-28.4)	0 (0-0)	0 (0-0)	13.4 (7.7-24.7)	0 (0-0)	0 (0-0)	25.7 (-32-123)	0 (0-0)	0 (0-0)	12.5 (9.2-44.1)	0 (0-0)	0 (0-0)	0.4 (-60.9-110)	0 (0-0)	0 (0-0)	7.7 (-0.7-45.2)	0 (0-0)	0 (0-0)	0 (0-0)	0 (0-0)	127	127	105	110
Camarines Sur	0 (0-0)	0 (0-0)	9.7 (6.7-19.5)	1 (1-1)	1 (1-2)	17.9 (10.4-29.7)	0 (0-0)	0 (0-0)	-13.8 (-61.2-48.6)	0 (0-0)	0 (0-0)	16.1 (12.7-42.6)	0 (0-0)	0 (0-0)	-44.6 (-78.1-28.4)	0 (0-0)	0 (0-0)	11.8 (5.4-43.2)	0 (0-0)	0 (0-0)	0 (0-0)	0 (0-0)	129	128	116	114
Camiguin	0 (0-0)	0 (0-0)	5.2 (-0.9-53.2)	0 (0-0)	0 (0-0)	9.5 (3.2-30.1)	0 (0-0)	0 (0-0)	-25.1 (-67.8-37.9)	0 (0-0)	0 (0-0)	10.7 (-3.2-52.6)	0 (0-0)	0 (0-0)	-73.7 (-92.2--11.1)	0 (0-0)	0 (0-0)	8.3 (-5.6-56.6)	0 (0-0)	0 (0-0)	0 (0-0)	0 (0-0)	124	127	107	91
Capiz	0 (0-0)	0 (0-0)	1.3 (-1.6-23.3)	0 (0-1)	0 (0-1)	3 (-2.2-16.8)	0 (0-0)	0 (0-0)	-26.9 (-60.8-26.7)	0 (0-0)	0 (0-0)	3 (-1.7-37.5)	0 (0-0)	0 (0-0)	-47.6 (-81.8-9.2)	0 (0-0)	0 (0-0)	0.1 (-9.1-42.1)	0 (0-0)	0 (0-0)	0 (0-0)	0 (0-0)	126	130	113	112
Catanduanes	0 (0-0)	0 (0-0)	-6.8 (-12.8-12.6)	0 (0-0)	0 (0-0)	7.2 (-1.6-19.9)	0 (0-0)	0 (0-0)	-11.2 (-50.8-43.7)	0 (0-0)	0 (0-0)	5.8 (-5.3-41.7)	0 (0-0)	0 (0-0)	-31.8 (-70.3-38.1)	0 (0-0)	0 (0-0)	0.5 (-9.8-38.3)	0 (0-0)	0 (0-0)	0 (0-0)	0 (0-0)	124	125	117	117
Cavite	0 (0-0)	0 (0-0)	83.5 (72.4-158)	1 (1-2)	2 (1-3)	82 (69.8-127)	0 (0-0)	0 (0-0)	72.6 (-14-204)	0 (0-0)	0 (0-0)	80.7 (72.6-151)	0 (0-0)	0 (0-0)	-2.8 (-64.4-120)	0 (0-0)	0 (0-0)	78 (61.7-165)	0 (0-0)	0 (0-0)	0 (0-0)	0 (0-0)	127	130	107	99
Cebu	0 (0-0)	0 (0-0)	36.3 (29.5-89.3)	2 (1-3)	2 (1-4)	38.2 (28.4-72.9)	0 (0-0)	0 (0-0)	-24 (-65.4-34.2)	0 (0-0)	0 (0-0)	39.7 (36.7-83.2)	0 (0-0)	0 (0-0)	-62.1 (-86.8-3)	0 (0-0)	0 (0-0)	39.2 (29.2-97.9)	0 (0-0)	0 (0-0)	0 (0-0)	0 (0-0)	129	129	116	101
Cotabato (North Cotabato)	0 (0-0)	0 (0-0)	35.9 (20.5-45.1)	1 (0-1)	1 (0-1)	43.4 (32.9-50.4)	0 (0-0)	0 (0-0)	8.2 (-45.9-94.8)	0 (0-0)	0 (0-0)	44.2 (34.7-92.5)	0 (0-0)	0 (0-0)	-41 (-78.5-54.5)	0 (0-0)	0 (0-0)	43.5 (27.3-101)	0 (0-0)	0 (0-0)	0 (0-0)	0 (0-0)	126	129	114	112
Davao de Oro	0 (0-0)	0 (0-0)	-7.9 (-13.2-12.5)	0 (0-1)	0 (0-1)	9.2 (1.2-18.9)	0 (0-0)	0 (0-0)	23.6 (-39.3-105)	0 (0-0)	0 (0-0)	8.7 (1.4-27)	0 (0-0)	0 (0-0)	-28.8 (-71.4-75.2)	0 (0-0)	0 (0-0)	3.6 (-4.7-26.3)	0 (0-0)	0 (0-0)	0 (0-0)	0 (0-0)	129	131	112	112
Davao Del Norte	0 (0-0)	0 (0-0)	-10.8 (-14.3-15.8)	0 (0-1)	0 (0-1)	10.6 (3.7-29.6)	0 (0-0)	0 (0-0)	9.5 (-45-78.7)	0 (0-0)	0 (0-0)	10.5 (4.9-44.5)	0 (0-0)	0 (0-0)	-31.4 (-74.5-68)	0 (0-0)	0 (0-0)	-0.1 (-7.4-39.4)	0 (0-0)	0 (0-0)	0 (0-0)	0 (0-0)	128	132	112	108
Davao Del Sur	0 (0-0)	0 (0-0)	26.5 (23-50.1)	1 (0-1)	1 (1-2)	30.8 (23.2-45.4)	0 (0-0)	0 (0-0)	-76.3 (-87.7--61.3)	0 (0-0)	0 (0-0)	31.4 (27.8-72.1)	0 (0-0)	0 (0-0)	-88 (-95.5--73.1)	0 (0-0)	0 (0-0)	30 (21.5-78.3)	0 (0-0)	0 (0-0)	0 (0-0)	0 (0-0.1)	129	129	118	85
Davao Occidental	0 (0-0)	0 (0-0)	-11.4 (-13.5-12.3)	0 (0-0)	0 (0-0)	18.7 (9.5-39.1)	0 (0-0)	0 (0-0)	29.5 (-39.9-115)	0 (0-0)	0 (0-0)	13.1 (5.7-51.2)	0 (0-0)	0 (0-0)	-31.1 (-69.5-43.4)	0 (0-0)	0 (0-0)	-0.9 (-8.9-37.8)	0 (0-0)	0 (0-0)	0 (0-0)	0 (0-0)	124	128	116	111
Davao Oriental	0 (0-0)	0 (0-0)	-13.3 (-16.3--3.7)	0 (0-0)	0 (0-0)	3.2 (-3.7-10.5)	0 (0-0)	0 (0-0)	25.2 (-35.6-120)	0 (0-0)	0 (0-0)	-0.9 (-5.5-23.9)	0 (0-0)	0 (0-0)	-13.8 (-61.6-118)	0 (0-0)	0 (0-0)	-8.7 (-15.5-19.8)	0 (0-0)	0 (0-0)	0 (0-0)	0 (0-0)	129	130	113	117
Dinagat Islands	0 (0-0)	0 (0-0)	-29.1 (-34.8--17.2)	0 (0-0)	0 (0-0)	-10.3 (-16.8-2.3)	0 (0-0)	0 (0-0)	28.1 (-34.6-137)	0 (0-0)	0 (0-0)	-12.2 (-20.3-15.4)	0 (0-0)	0 (0-0)	-52.6 (-81.5-13.2)	0 (0-0)	0 (0-0)	-17.6 (-25.7-13.5)	0 (0-0)	0 (0-0)	0 (0-0)	0 (0-0)	127	131	111	105
Eastern Samar	0 (0-0)	0 (0-0)	7.2 (-1.1-25.8)	0 (0-0)	0 (0-0)	15.3 (3.4-29.8)	0 (0-0)	0 (0-0)	2.3 (-48.5-81.9)	0 (0-0)	0 (0-0)	12.7 (5-47.4)	0 (0-0)	0 (0-0)	-22.5 (-67.9-72.3)	0 (0-0)	0 (0-0)	9.8 (2.4-48.8)	0 (0-0)	0 (0-0)	0 (0-0)	0 (0-0)	126	132	113	114
Guimaras	0 (0-0)	0 (0-0)	0.2 (-30.9-30.1)	0 (0-0)	0 (0-0)	8.4 (-12.4-24.3)	0 (0-0)	0 (0-0)	-3 (-52.2-70.6)	0 (0-0)	0 (0-0)	9.3 (4.6-42.2)	0 (0-0)	0 (0-0)	-54 (-84.2-13.8)	0 (0-0)	0 (0-0)	7.3 (-0.5-47.3)	0 (0-0)	0 (0-0)	0 (0-0)	0 (0-0)	127	133	105	93
Ifugao	0 (0-0)	0 (0-0)	17.6 (11.9-51.3)	0 (0-0)	0 (0-0)	19.4 (12-48.1)	0 (0-0)	0 (0-0)	-23.3 (-60.3-35.8)	0 (0-0)	0 (0-0)	21.9 (17.9-59.4)	0 (0-0)	0 (0-0)	-44.5 (-81-36.1)	0 (0-0)	0 (0-0)	24.5 (15.6-70.9)	0 (0-0)	0 (0-0)	0 (0-0)	0 (0-0)	126	131	114	108

Location	Births, 2000	Births, 2021	Births percent change (2000-2021)	All age cases, 2000	All age cases, 2021	All age cases percent change (2000-2021)	All age deaths (cause-specific), 2000	All age deaths (cause-specific), 2021	All age deaths (cause-specific) percent change (2000-2021)	All age deaths (total), 2000	All age deaths (total), 2021	All age deaths (total) percent change (2000-2021)	Under-5 deaths (cause-specific), 2000	Under-5 deaths (cause-specific), 2021	Under-5 deaths (cause-specific) percent change (2000-2021)	Under-5 deaths (total), 2000	Under-5 deaths (total), 2021	Under-5 deaths (total) percent change (2000-2021)	Under-5 cause-specific mortality cause fraction, 2000	Under-5 total SCD mortality cause fraction, 2000	Under-5 cause-specific mortality cause fraction, 2021	Under-5 total SCD mortality cause fraction, 2021	Under-5 cause-specific mortality rank 2000	Under-5 cause-specific mortality rank 2021	Under-5 total SCD mortality rank 2000	Under-5 total SCD mortality rank 2021
Ilocos Norte	0 (0-0)	0 (0-0)	4.4 (-6.9-43.9)	0 (0-0)	0 (0-0)	5.6 (-3.5-29.8)	0 (0-0)	0 (0-0)	-31 (-69.1-20.5)	0 (0-0)	0 (0-0)	7.3 (4.8-41)	0 (0-0)	0 (0-0)	-68.7 (-87.7--24)	0 (0-0)	0 (0-0)	7.4 (-0.7-49.5)	0 (0-0)	0 (0-0)	0 (0-0)	0 (0-0)	127	128	113	100
Ilocos Sur	0 (0-0)	0 (0-0)	-2.1 (-31.1-27)	0 (0-0)	0 (0-0)	4.2 (-14.9-21.8)	0 (0-0)	0 (0-0)	-26.6 (-65.6-30.2)	0 (0-0)	0 (0-0)	5.3 (2.8-34.9)	0 (0-0)	0 (0-0)	-63.6 (-84.8--1.6)	0 (0-0)	0 (0-0)	2.6 (-5.8-38.8)	0 (0-0)	0 (0-0)	0 (0-0)	0 (0-0)	127	129	109	101
Iloilo	0 (0-0)	0 (0-0)	22 (15.4-62.6)	1 (1-2)	1 (1-2)	20.1 (12.8-55.1)	0 (0-0)	0 (0-0)	-20.4 (-58.5-29.6)	0 (0-0)	0 (0-0)	21.7 (18-58.2)	0 (0-0)	0 (0-0)	-53.8 (-79.3-4.5)	0 (0-0)	0 (0-0)	23.1 (12.6-71.6)	0 (0-0)	0 (0-0)	0 (0-0)	0 (0-0)	127	131	109	99
Isabela	0 (0-0)	0 (0-0)	15.3 (12.9-21)	1 (0-1)	1 (0-1)	14.5 (6.2-22.8)	0 (0-0)	0 (0-0)	-6.2 (-50.1-54.7)	0 (0-0)	0 (0-0)	15.5 (12.9-32.4)	0 (0-0)	0 (0-0)	-51.8 (-79.1-4.1)	0 (0-0)	0 (0-0)	16.7 (9.5-40.1)	0 (0-0)	0 (0-0)	0 (0-0)	0 (0-0)	124	125	113	98
Kalinga	0 (0-0)	0 (0-0)	-13.6 (-16.5-8.4)	0 (0-0)	0 (0-0)	5.4 (-0.7-18.5)	0 (0-0)	0 (0-0)	32.9 (-40.9-133)	0 (0-0)	0 (0-0)	6 (2.1-35.6)	0 (0-0)	0 (0-0)	-57.8 (-87.1-9.5)	0 (0-0)	0 (0-0)	-0.7 (-9-32.4)	0 (0-0)	0 (0-0)	0 (0-0)	0 (0-0)	122	123	104	94
La Union	0 (0-0)	0 (0-0)	7.4 (3.8-25.8)	0 (0-1)	0 (0-1)	8.2 (2.3-30)	0 (0-0)	0 (0-0)	-26.8 (-67.4-23.7)	0 (0-0)	0 (0-0)	8.6 (6.1-25)	0 (0-0)	0 (0-0)	-59.5 (-86.3-2.6)	0 (0-0)	0 (0-0)	9.5 (3.4-30.4)	0 (0-0)	0 (0-0)	0 (0-0)	0 (0-0)	122	126	114	103
Laguna	0 (0-0)	0 (0-0)	59.1 (51-106)	1 (1-2)	2 (1-3)	56.6 (48-91.3)	0 (0-0)	0 (0-0)	18.6 (-39.7-103)	0 (0-0)	0 (0-0)	59.2 (54-114)	0 (0-0)	0 (0-0)	-31.3 (-68.2-38.3)	0 (0-0)	0 (0-0)	60.9 (47.8-126)	0 (0-0)	0 (0-0)	0 (0-0)	0 (0-0)	127	130	109	101
Lanao Del Norte	0 (0-0)	0 (0-0)	16.8 (11.3-59.5)	0 (0-1)	1 (0-1)	29.9 (20.8-58)	0 (0-0)	0 (0-0)	-56.4 (-76.7--29.5)	0 (0-0)	0 (0-0)	30.3 (26.4-68.1)	0 (0-0)	0 (0-0)	-69 (-88.5--30.9)	0 (0-0)	0 (0-0)	27.1 (17.5-70.1)	0 (0-0)	0 (0-0)	0 (0-0)	0 (0-0)	126	126	114	95
Lanao Del Sur	0 (0-0)	0 (0-0)	16.3 (11.4-18.9)	1 (0-1)	1 (0-1)	33.4 (24.2-38.7)	0 (0-0)	0 (0-0)	-36.5 (-73.5-26.7)	0 (0-0)	0 (0-0)	33.9 (26.5-61.5)	0 (0-0)	0 (0-0)	-51.9 (-82.3-113)	0 (0-0)	0 (0-0)	30.5 (21.3-61.5)	0 (0-0)	0 (0-0)	0 (0-0)	0 (0-0)	126	130	120	113
Leyte	0 (0-0)	0 (0-0)	12.4 (6.4-17.7)	1 (1-1)	1 (1-2)	16.2 (7.2-22.4)	0 (0-0)	0 (0-0)	-24.9 (-61.5-35.8)	0 (0-0)	0 (0-0)	14.6 (10.9-51)	0 (0-0)	0 (0-0)	-51.5 (-81-0.5)	0 (0-0)	0 (0-0)	12.9 (3.7-51.7)	0 (0-0)	0 (0-0)	0 (0-0)	0 (0-0)	126	128	113	107
Maguindanao	0 (0-0)	0 (0-0)	33.6 (30.3-53.7)	1 (0-1)	1 (1-2)	60 (50.5-83.1)	0 (0-0)	0 (0-0)	16.6 (-39.1-129)	0 (0-0)	0 (0-0)	59.1 (53.2-98.5)	0 (0-0)	0 (0-0)	13.5 (-55.7-213)	0 (0-0)	0 (0-0)	51.8 (42.5-95)	0 (0-0)	0 (0-0)	0 (0-0)	0 (0-0)	128	133	119	118
Marinduque	0 (0-0)	0 (0-0)	-28.9 (-32.1--12.4)	0 (0-0)	0 (0-0)	-15.6 (-22.5--5.2)	0 (0-0)	0 (0-0)	-34.5 (-69.3-20)	0 (0-0)	0 (0-0)	-18.3 (-21.9-1.1)	0 (0-0)	0 (0-0)	-57.4 (-81.6--15.7)	0 (0-0)	0 (0-0)	-26.3 (-31.5--6.9)	0 (0-0)	0 (0-0)	0 (0-0)	0 (0-0)	125	129	110	112
Masbate	0 (0-0)	0 (0-0)	10.7 (8.4-14)	0 (0-1)	0 (0-1)	21.1 (14.6-26.5)	0 (0-0)	0 (0-0)	22.3 (-34.7-111)	0 (0-0)	0 (0-0)	19.7 (12.6-60.8)	0 (0-0)	0 (0-0)	-2.1 (-60.6-108)	0 (0-0)	0 (0-0)	14.3 (5.5-58.3)	0 (0-0)	0 (0-0)	0 (0-0)	0 (0-0)	125	127	114	117
Misamis Occidental	0 (0-0)	0 (0-0)	11.9 (2.2-24.1)	0 (0-0)	0 (0-0)	16.1 (5.6-26)	0 (0-0)	0 (0-0)	-29.9 (-63.7-26.2)	0 (0-0)	0 (0-0)	17.3 (6.8-60.8)	0 (0-0)	0 (0-0)	-57.6 (-86.5-2.8)	0 (0-0)	0 (0-0)	15.7 (5.9-67.6)	0 (0-0)	0 (0-0)	0 (0-0)	0 (0-0)	126	130	118	113
Misamis Oriental	0 (0-0)	0 (0-0)	37.2 (32.7-67.4)	1 (0-1)	1 (0-1)	37.7 (30.2-58)	0 (0-0)	0 (0-0)	-45.5 (-74.5--8.2)	0 (0-0)	0 (0-0)	39.4 (31.8-84.3)	0 (0-0)	0 (0-0)	-65.6 (-86.7--20.5)	0 (0-0)	0 (0-0)	39 (29.2-95.4)	0 (0-0)	0 (0-0)	0 (0-0)	0 (0-0)	129	129	119	98
Mountain Province	0 (0-0)	0 (0-0)	-10.7 (-24.9-10.3)	0 (0-0)	0 (0-0)	-3.2 (-11.6-12.2)	0 (0-0)	0 (0-0)	-23.7 (-66.3-29.6)	0 (0-0)	0 (0-0)	-2.9 (-8.8-26.6)	0 (0-0)	0 (0-0)	-58.1 (-85--9.2)	0 (0-0)	0 (0-0)	-4.9 (-14.3-25.3)	0 (0-0)	0 (0-0)	0 (0-0)	0 (0-0)	128	130	108	102
National Capital Region	1 (1-1)	1 (1-1)	2.5 (-19.5-55.4)	5 (3-8)	6 (3-10)	14.7 (-3.5-43.4)	0 (0-0)	0 (0-0)	-33.4 (-68-14.3)	1 (0-1)	1 (0-1)	12.5 (6.9-49)	0 (0-0)	0 (0-0)	-62.7 (-83.4--22.9)	0 (0-1)	0 (0-1)	5.4 (-3.1-50.1)	0 (0-0)	0 (0-0)	0 (0-0)	0 (0-0)	129	132	112	99
Negros Occidental	0 (0-0)	0 (0-0)	3 (-0.4-31.2)	1 (1-2)	1 (1-2)	5.8 (-1.2-20.4)	0 (0-0)	0 (0-0)	-12.3 (-50.9-50.9)	0 (0-0)	0 (0-0)	4.9 (2.2-30.3)	0 (0-0)	0 (0-0)	-25.5 (-73-60.9)	0 (0-0)	0 (0-0)	2.3 (-4.7-30.2)	0 (0-0)	0 (0-0)	0 (0-0)	0 (0-0)	124	125	111	111
Negros Oriental	0 (0-0)	0 (0-0)	-2.1 (-4.1-3.7)	1 (0-1)	1 (0-1)	4.7 (-0.4-11.5)	0 (0-0)	0 (0-0)	-4.2 (-53.7-58.7)	0 (0-0)	0 (0-0)	3.5 (-0.1-39.1)	0 (0-0)	0 (0-0)	-28.4 (-72.6-59.6)	0 (0-0)	0 (0-0)	0.6 (-6.5-46.3)	0 (0-0)	0 (0-0)	0 (0-0)	0 (0-0)	124	128	113	116
Northern Samar	0 (0-0)	0 (0-0)	14.4 (8.8-30.5)	0 (0-0)	0 (0-1)	19.7 (8.3-35.1)	0 (0-0)	0 (0-0)	-41.2 (-77.2-20.8)	0 (0-0)	0 (0-0)	18.1 (9.2-58.7)	0 (0-0)	0 (0-0)	-64.4 (-86.9--16.3)	0 (0-0)	0 (0-0)	15.5 (5.1-61.9)	0 (0-0)	0 (0-0)	0 (0-0)	0 (0-0)	125	128	117	104
Nueva Ecija	0 (0-0)	0 (0-0)	22.9 (16.1-68.4)	1 (1-1)	1 (1-2)	25.6 (18.6-50.3)	0 (0-0)	0 (0-0)	2.4 (-49.5-79.4)	0 (0-0)	0 (0-0)	26.2 (22.2-66.8)	0 (0-0)	0 (0-0)	-37.3 (-70.8-39)	0 (0-0)	0 (0-0)	26.8 (16.4-80.2)	0 (0-0)	0 (0-0)	0 (0-0)	0 (0-0)	127	130	107	99
Nueva Vizcaya	0 (0-0)	0 (0-0)	5.5 (2.9-19)	0 (0-0)	0 (0-0)	11.7 (6.4-18.5)	0 (0-0)	0 (0-0)	-20.1 (-64.1-40.7)	0 (0-0)	0 (0-0)	12.2 (9.5-29.5)	0 (0-0)	0 (0-0)	-60.7 (-83.7--6)	0 (0-0)	0 (0-0)	10.6 (2.6-34.6)	0 (0-0)	0 (0-0)	0 (0-0)	0 (0-0)	125	127	112	101
Occidental Mindoro	0 (0-0)	0 (0-0)	-3.5 (-7.5-4.6)	0 (0-0)	0 (0-0)	14 (7.1-26.9)	0 (0-0)	0 (0-0)	-31 (-66.6-49.6)	0 (0-0)	0 (0-0)	12.6 (-0.4-53.3)	0 (0-0)	0 (0-0)	-63.7 (-87.3--0.8)	0 (0-0)	0 (0-0)	6.6 (-8.6-55.2)	0 (0-0)	0 (0-0)	0 (0-0)	0 (0-0)	129	129	116	109
Oriental Mindoro	0 (0-0)	0 (0-0)	-6.4 (-12.5--0.9)	0 (0-1)	0 (0-1)	6.4 (-2-11.5)	0 (0-0)	0 (0-0)	-7.5 (-58.6-60.4)	0 (0-0)	0 (0-0)	3.5 (-1.6-37.7)	0 (0-0)	0 (0-0)	-40.7 (-77.4-39.6)	0 (0-0)	0 (0-0)	-3.5 (-10.3-35.2)	0 (0-0)	0 (0-0)	0 (0-0)	0 (0-0)	129	132	118	110
Palawan	0 (0-0)	0 (0-0)	27.2 (23.8-44.8)	0 (0-1)	1 (0-1)	40.1 (32.8-60.9)	0 (0-0)	0 (0-0)	-21.9 (-59.5-28.2)	0 (0-0)	0 (0-0)	37.7 (31.9-81.2)	0 (0-0)	0 (0-0)	-50.5 (-81.1-13.5)	0 (0-0)	0 (0-0)	31.8 (21.3-81)	0 (0-0)	0 (0-0)	0 (0-0)	0 (0-0)	127	128	116	109
Pampanga	0 (0-0)	0 (0-0)	33.7 (25.7-85.2)	1 (1-2)	1 (1-2)	33.5 (24.6-60)	0 (0-0)	0 (0-0)	10.4 (-42.8-103)	0 (0-0)	0 (0-0)	32.7 (28.1-83)	0 (0-0)	0 (0-0)	-9 (-60.9-64.6)	0 (0-0)	0 (0-0)	31.4 (22.2-90.2)	0 (0-0)	0 (0-0)	0 (0-0)	0 (0-0)	127	129	105	105
Pangasinan	0 (0-0)	0 (0-0)	8.9 (5.6-12.3)	1 (1-2)	1 (1-2)	12.5 (7.3-18.6)	0 (0-0)	0 (0-0)	-24.1 (-65.5-38.5)	0 (0-0)	0 (0-0)	13.1 (10.4-40.9)	0 (0-0)	0 (0-0)	-59.2 (-83.1-3.5)	0 (0-0)	0 (0-0)	11.9 (3.2-47.9)	0 (0-0)	0 (0-0)	0 (0-0)	0 (0-0)	126	127	109	103
Quezon	0 (0-0)	0 (0-0)	10.2 (6.3-42.5)	1 (1-1)	1 (1-2)	16.5 (9.8-35.2)	0 (0-0)	0 (0-0)	-34.8 (-68-19.8)	0 (0-0)	0 (0-0)	15.2 (11.5-48.5)	0 (0-0)	0 (0-0)	-70.5 (-88.5--26.7)	0 (0-0)	0 (0-0)	11.2 (4.2-46.8)	0 (0-0)	0 (0-0)	0 (0-0)	0 (0-0)	127	127	116	99
Quirino	0 (0-0)	0 (0-0)	-12.6 (-15.9-11)	0 (0-0)	0 (0-0)	4.4 (-1.7-17.8)	0 (0-0)	0 (0-0)	-34.3 (-66.2-24.7)	0 (0-0)	0 (0-0)	3.2 (-1.4-37.8)	0 (0-0)	0 (0-0)	-66.3 (-83.6--26.1)	0 (0-0)	0 (0-0)	-2.4 (-8.8-37.2)	0 (0-0)	0 (0-0)	0 (0-0)	0 (0-0)	124	126	112	103

Location	Births, 2000	Births, 2021	Births percent change (2000-2021)	All age cases, 2000	All age cases, 2021	All age cases percent change (2000-2021)	All age deaths (cause-specific), 2000	All age deaths (cause-specific), 2021	All age deaths (cause-specific) percent change (2000-2021)	All age deaths (total), 2000	All age deaths (total), 2021	All age deaths (total) percent change (2000-2021)	Under-5 deaths (cause-specific), 2000	Under-5 deaths (cause-specific), 2021	Under-5 deaths (cause-specific) percent change (2000-2021)	Under-5 deaths (total), 2000	Under-5 deaths (total), 2021	Under-5 deaths (total) percent change (2000-2021)	Under-5 cause-specific mortality cause fraction, 2000	Under-5 total SCD mortality cause fraction, 2000	Under-5 cause-specific mortality cause fraction, 2021	Under-5 total SCD mortality cause fraction, 2021	Under-5 cause-specific mortality rank 2000	Under-5 cause-specific mortality rank 2021	Under-5 total SCD mortality rank 2000	Under-5 total SCD mortality rank 2021
Rizal	0 (0-0)	0 (0-0)	78.2 (53.5-93.8)	1 (1-1)	2 (1-2)	75.3 (57.1-83.5)	0 (0-0)	0 (0-0)	46.5 (-31.2-133)	0 (0-0)	0 (0-0)	73.7 (68.8-122)	0 (0-0)	0 (0-0)	-15.8 (-67.9-66)	0 (0-0)	0 (0-0)	71.9 (59.5-128)	0 (0-0)	0 (0-0)	0 (0-0)	0 (0-0)	130	131	110	107
Romblon	0 (0-0)	0 (0-0)	-17.3 (-26.3--12)	0 (0-0)	0 (0-0)	-6.2 (-13.4--0.8)	0 (0-0)	0 (0-0)	-35.1 (-69-27.3)	0 (0-0)	0 (0-0)	-7.1 (-14.2-24.6)	0 (0-0)	0 (0-0)	-69.3 (-87.8--26.9)	0 (0-0)	0 (0-0)	-11.7 (-20-23.6)	0 (0-0)	0 (0-0)	0 (0-0)	0 (0-0)	125	127	116	107
Samar (Western Samar)	0 (0-0)	0 (0-0)	5.1 (-1.8-68.7)	0 (0-1)	0 (0-1)	9.7 (1.1-43.5)	0 (0-0)	0 (0-0)	-8 (-53.1-71.8)	0 (0-0)	0 (0-0)	7.6 (2.4-51.3)	0 (0-0)	0 (0-0)	-23.7 (-70.6-93)	0 (0-0)	0 (0-0)	5.6 (-1.9-54.9)	0 (0-0)	0 (0-0)	0 (0-0)	0 (0-0)	127	129	122	121
Sarangani	0 (0-0)	0 (0-0)	18.4 (13.7-63.2)	0 (0-0)	0 (0-0)	21.2 (13.6-40.1)	0 (0-0)	0 (0-0)	24.7 (-34.4-109)	0 (0-0)	0 (0-0)	19.2 (14.8-62)	0 (0-0)	0 (0-0)	-25.4 (-73.9-42.9)	0 (0-0)	0 (0-0)	15.8 (8.1-63.6)	0 (0-0)	0 (0-0)	0 (0-0)	0 (0-0)	131	133	114	113
Siquijor	0 (0-0)	0 (0-0)	-11.7 (-16.3-27.5)	0 (0-0)	0 (0-0)	1.5 (-4.1-20.4)	0 (0-0)	0 (0-0)	-21.3 (-61.6-39)	0 (0-0)	0 (0-0)	1.2 (-5-37.3)	0 (0-0)	0 (0-0)	-70.6 (-88.7--15.3)	0 (0-0)	0 (0-0)	-4.5 (-12.8-37.9)	0 (0-0)	0 (0-0)	0 (0-0)	0 (0-0)	124	129	111	96
Sorsogon	0 (0-0)	0 (0-0)	11.7 (7.4-44.3)	0 (0-1)	0 (0-1)	11.9 (3.7-29.4)	0 (0-0)	0 (0-0)	-20.1 (-64.8-37.4)	0 (0-0)	0 (0-0)	10.2 (6.6-41)	0 (0-0)	0 (0-0)	-60.4 (-86.1-9.9)	0 (0-0)	0 (0-0)	7.5 (0-42.3)	0 (0-0)	0 (0-0)	0 (0-0)	0 (0-0)	127	129	111	99
South Cotabato	0 (0-0)	0 (0-0)	21.4 (18.3-35)	1 (0-1)	1 (0-1)	25.9 (18.1-34.9)	0 (0-0)	0 (0-0)	-33.9 (-64.4-5.2)	0 (0-0)	0 (0-0)	25.7 (19.6-61.5)	0 (0-0)	0 (0-0)	-54.2 (-82.7-1.6)	0 (0-0)	0 (0-0)	23.6 (15.4-64.2)	0 (0-0)	0 (0-0)	0 (0-0)	0 (0-0)	128	127	116	108
Southern Leyte	0 (0-0)	0 (0-0)	-2.3 (-9.2-4.1)	0 (0-0)	0 (0-0)	4.3 (-5-9.7)	0 (0-0)	0 (0-0)	-45.2 (-76.5--0.8)	0 (0-0)	0 (0-0)	2.6 (-15.8-36)	0 (0-0)	0 (0-0)	-67.7 (-87.1--28.8)	0 (0-0)	0 (0-0)	-0.9 (-21.4-37)	0 (0-0)	0 (0-0)	0 (0-0)	0 (0-0)	125	130	116	107
Sultan Kudarat	0 (0-0)	0 (0-0)	0.9 (-1.1-18.5)	0 (0-1)	0 (0-1)	21.5 (15.5-35.5)	0 (0-0)	0 (0-0)	25.8 (-33.9-89.7)	0 (0-0)	0 (0-0)	20 (15.3-54)	0 (0-0)	0 (0-0)	-10.9 (-64.6-82.9)	0 (0-0)	0 (0-0)	12.4 (4.8-48.2)	0 (0-0)	0 (0-0)	0 (0-0)	0 (0-0)	129	133	112	114
Sulu	0 (0-0)	0 (0-0)	-9.2 (-11.3-1.4)	0 (0-1)	0 (0-1)	23.7 (13.5-35)	0 (0-0)	0 (0-0)	-17.9 (-53.5-63.6)	0 (0-0)	0 (0-0)	17.4 (11.5-48.9)	0 (0-0)	0 (0-0)	-37.4 (-69.9-96.6)	0 (0-0)	0 (0-0)	5.8 (-1.2-38.7)	0 (0-0)	0 (0-0)	0 (0-0)	0 (0-0)	127	130	118	116
Surigao Del Norte	0 (0-0)	0 (0-0)	20.8 (14.6-51.8)	0 (0-0)	0 (0-0)	22.9 (15.9-36.1)	0 (0-0)	0 (0-0)	-21.8 (-63.9-34.1)	0 (0-0)	0 (0-0)	22.8 (14.3-63.4)	0 (0-0)	0 (0-0)	-57.8 (-82.3--10.9)	0 (0-0)	0 (0-0)	20.5 (10.3-68.8)	0 (0-0)	0 (0-0)	0 (0-0)	0 (0-0)	127	128	117	107
Surigao Del Sur	0 (0-0)	0 (0-0)	-1.7 (-11-19.3)	0 (0-0)	0 (0-0)	2.5 (-6.2-13.2)	0 (0-0)	0 (0-0)	-19.1 (-62.4-44.2)	0 (0-0)	0 (0-0)	2.1 (-9.3-41.5)	0 (0-0)	0 (0-0)	-48.1 (-78.6-43.9)	0 (0-0)	0 (0-0)	-0.3 (-11.4-44.2)	0 (0-0)	0 (0-0)	0 (0-0)	0 (0-0)	128	130	119	114
Tarlac	0 (0-0)	0 (0-0)	4.5 (-2.2-45.2)	1 (0-1)	1 (0-1)	12.2 (4-37.2)	0 (0-0)	0 (0-0)	10.9 (-45.3-91.7)	0 (0-0)	0 (0-0)	11.6 (8.2-38.6)	0 (0-0)	0 (0-0)	-27.3 (-68.9-69.8)	0 (0-0)	0 (0-0)	6.8 (-1-35.3)	0 (0-0)	0 (0-0)	0 (0-0)	0 (0-0)	127	128	109	111
Tawi-Tawi	0 (0-0)	0 (0-0)	-2.3 (-7.8-28.8)	0 (0-0)	0 (0-0)	12.4 (5.6-33.9)	0 (0-0)	0 (0-0)	-12.4 (-55.4-58.8)	0 (0-0)	0 (0-0)	12.3 (7.2-46.9)	0 (0-0)	0 (0-0)	-40.5 (-80.7-58.2)	0 (0-0)	0 (0-0)	7.5 (-4.7-47.2)	0 (0-0)	0 (0-0)	0 (0-0)	0 (0-0)	128	130	107	103
Zambales	0 (0-0)	0 (0-0)	21.4 (12.3-30.2)	0 (0-0)	0 (0-1)	23.2 (12.2-31.5)	0 (0-0)	0 (0-0)	-33.6 (-65.7-8.6)	0 (0-0)	0 (0-0)	23 (18.1-47.6)	0 (0-0)	0 (0-0)	-58.1 (-82.5--6.2)	0 (0-0)	0 (0-0)	20 (10.1-52.9)	0 (0-0)	0 (0-0)	0 (0-0)	0 (0-0)	128	129	112	96
Zamboanga Del Norte	0 (0-0)	0 (0-0)	-5.4 (-12.4-0.6)	0 (0-1)	0 (0-1)	7.6 (-2.9-16.4)	0 (0-0)	0 (0-0)	-17.9 (-58.9-48.2)	0 (0-0)	0 (0-0)	6.8 (2.9-38.3)	0 (0-0)	0 (0-0)	-56.4 (-82.9-21.7)	0 (0-0)	0 (0-0)	1.9 (-5.4-38.2)	0 (0-0)	0 (0-0)	0 (0-0)	0 (0-0)	126	128	113	108
Zamboanga Del Sur	0 (0-0)	0 (0-0)	9.8 (5.7-19.7)	1 (0-1)	1 (1-2)	15.5 (8.5-25.9)	0 (0-0)	0 (0-0)	-43.1 (-67.6--2.8)	0 (0-0)	0 (0-0)	14.1 (10.7-33.4)	0 (0-0)	0 (0-0)	-68.3 (-88.8--40.8)	0 (0-0)	0 (0-0)	11.9 (4.7-34.3)	0 (0-0)	0 (0-0)	0 (0-0)	0 (0-0)	128	131	114	97
Zamboanga Sibugay	0 (0-0)	0 (0-0)	9.6 (6.9-40.9)	0 (0-0)	0 (0-1)	24.4 (15.9-46.4)	0 (0-0)	0 (0-0)	-24.2 (-61.9-46.3)	0 (0-0)	0 (0-0)	22.4 (18.3-61.3)	0 (0-0)	0 (0-0)	-62 (-86.5-5.4)	0 (0-0)	0 (0-0)	16.1 (8.3-56.4)	0 (0-0)	0 (0-0)	0 (0-0)	0 (0-0)	127	131	115	110
Seychelles	0 (0-0)	0 (0-0)	-6.3 (-35.3-18.8)	0 (0-0)	0 (0-0)	0.5 (-18-18)	0 (0-0)	0 (0-0)	-36.2 (-58.8-0.6)	0 (0-0)	0 (0-0)	0.3 (-17.9-20.5)	0 (0-0)	0 (0-0)	-38.6 (-64.4-10.1)	0 (0-0)	0 (0-0)	-1.3 (-22.5-24.3)	0 (0-0)	0 (0-0)	0 (0-0)	0 (0-0)	118	121	93	93
Sri Lanka	1 (1-1)	1 (0-1)	-16.8 (-23.3-0.9)	8 (4-14)	7 (4-13)	-4.6 (-11.6-6.2)	0 (0-0)	0 (0-0)	-66.2 (-83--26.4)	1 (1-1)	1 (1-1)	-3 (-15-32.1)	0 (0-0)	0 (0-0)	-84.6 (-92.3--71.8)	0 (0-1)	0 (0-0)	-8.9 (-23.3-37.1)	0 (0-0)	0 (0-0)	0 (0-0)	0 (0-0)	129	131	106	86
Thailand	2 (1-3)	1 (1-2)	-38.4 (-42.2--10.2)	21 (11-37)	15 (8-28)	-28.7 (-36.3--14.5)	0 (0-0)	0 (0-0)	-68.5 (-80.7--45.6)	2 (2-3)	2 (1-2)	-30.6 (-35.3--8.2)	0 (0-0)	0 (0-0)	-92 (-96.3--76.3)	1 (1-2)	1 (0-1)	-37.2 (-42.6--8.7)	0 (0-0)	0 (0-0)	0 (0-0)	0 (0-0)	136	141	106	93
Timor-Leste	0 (0-0)	0 (0-0)	5 (-15.2-55.6)	0 (0-1)	1 (0-1)	28.8 (5.4-71.8)	0 (0-0)	0 (0-0)	-17.2 (-52.7-63.4)	0 (0-0)	0 (0-0)	20.7 (5.8-62.4)	0 (0-0)	0 (0-0)	-38 (-66-54.2)	0 (0-0)	0 (0-0)	14.3 (0.7-59.5)	0 (0-0)	0 (0-0)	0 (0-0)	0 (0-0)	133	133	120	109
Viet Nam	4 (3-5)	3 (2-4)	-18.9 (-21.1-6.9)	33 (18-56)	30 (15-52)	-10.3 (-16.6-12.2)	0 (0-1)	0 (0-1)	-4.5 (-42.2-35.7)	4 (3-6)	3 (2-5)	-11.5 (-16.2-18.1)	0 (0-0)	0 (0-0)	-48.7 (-73.5--4.5)	2 (1-3)	2 (1-3)	-12.3 (-19.2-24.9)	0 (0-0)	0 (0-0)	0 (0-0)	0 (0-0)	133	136	106	93
Sub-Saharan Africa	318,000 (269,000-377,000)	405,000 (343,000-478,000)	27.2 (23.4-30.2)	3,390,000 (2,900,000-3,950,000)	5,680,000 (4,780,000-6,620,000)	67.4 (62.9-71.5)	22,600 (16,800-28,900)	29,400 (20,300-40,000)	30.1 (-2.3-77.6)	160,000 (133,000-194,000)	265,000 (219,000-322,000)	65.1 (60.7-69.2)	10,500 (6,610-15,400)	10,200 (6,240-13,900)	-2.2 (-31.7-45)	42,100 (30,700-55,800)	60,600 (43,900-80,800)	43.9 (38.3-48.8)	0.3 (0.2-0.4)	1 (0.8-1.4)	0.4 (0.3-0.5)	2.2 (1.5-3)	32	26	17	11
**Central Sub-Saharan Africa**	49,100 (42,800-56,500)	64,900 (56,100-74,600)	32.2 (22.5-38.6)	508,000 (430,000-594,000)	891,000 (747,000-1,030,000)	75.4 (63.6-85.8)	2,460 (611-6,520)	3,620 (1,020-11,400)	47 (-4.1-115)	25,300 (21,000-29,700)	45,800 (37,900-53,200)	81 (69.9-91.5)	844 (162-1,700)	731 (221-1,910)	-13.3 (-51.7-87.3)	6,890 (4,780-9,400)	10,800 (7,310-15,100)	56.9 (41.3-72.6)	0.2 (0-0.3)	1.4 (1-1.9)	0.3 (0.1-0.6)	3.7 (2.3-5.1)	35	31	15	8
Angola	8,930 (7,440-10,700)	13,700 (11,500-16,300)	53.2 (41.9-64.6)	87,600 (73,400-105,000)	182,000 (149,000-218,000)	107 (92.3-125)	307 (84-580)	451 (151-835)	47.1 (2.2-132)	4,330 (3,510-5,370)	9,190 (7,310-11,500)	112 (91.9-133)	124 (25-286)	132 (33-240)	6 (-30.5-202)	1,170 (787-1,700)	2,190 (1,430-3,250)	86.8 (59.2-115)	0.1 (0-0.3)	1 (0.7-1.4)	0.2 (0-0.3)	2.9 (1.9-4.2)	44	39	19	10
Central African Republic	1,420 (1,190-1,680)	1,790 (1,530-2,110)	26.3 (16.1-38)	14,700 (12,200-17,300)	21,200 (17,700-25,600)	44.4 (36.1-56.7)	96 (23-435)	139 (35-636)	45 (-5.6-134)	756 (622-938)	1,110 (920-1,350)	47.1 (35.3-59.3)	27 (4-78)	36 (8-140)	33.8 (-25.7-191)	192 (128-276)	266 (176-379)	38.7 (18.9-60.7)	0.1 (0-0.3)	0.7 (0.5-1.1)	0.1 (0-0.6)	1.1 (0.7-1.5)	44	40	24	19
Congo	782 (671-900)	851 (725-985)	8.8 (2.3-17)	9,420 (7,960-10,900)	13,800 (11,700-16,500)	46.5 (35.4-57.4)	97 (31-178)	102 (32-199)	5.7 (-41.5-84.2)	442 (367-526)	672 (563-796)	52.3 (40.4-63.8)	23 (6-52)	11 (4-21)	-51.5 (-78.2-36.8)	100 (71-133)	131 (93-176)	31.4 (17.4-47.3)	0.2 (0-0.4)	0.9 (0.6-1.2)	0.2 (0.1-0.4)	2.5 (1.6-3.3)	33	36	22	10
Democratic Republic of the Congo	36,900 (32,000-42,500)	47,400 (40,500-54,200)	28.5 (15.5-37.4)	384,000 (321,000-447,000)	655,000 (550,000-753,000)	70.6 (56-84.9)	1,920 (385-5,710)	2,880 (654-10,200)	50.1 (-6.1-129)	19,100 (15,900-22,400)	33,800 (27,800-38,900)	76.4 (62.7-89.4)	659 (103-1,420)	545 (127-1,680)	-17.3 (-60.4-88.4)	5,250 (3,620-7,220)	8,010 (5,440-11,200)	52.4 (34.4-73.7)	0.2 (0-0.4)	1.6 (1.1-2.1)	0.3 (0.1-0.9)	4.5 (2.8-6.2)	35	27	14	7

Location	Births, 2000	Births, 2021	Births percent change (2000-2021)	All age cases, 2000	All age cases, 2021	All age cases percent change (2000-2021)	All age deaths (cause-specific), 2000	All age deaths (cause-specific), 2021	All age deaths (cause-specific) percent change (2000-2021)	All age deaths (total), 2000	All age deaths (total), 2021	All age deaths (total) percent change (2000-2021)	Under-5 deaths (cause-specific), 2000	Under-5 deaths (cause-specific), 2021	Under-5 deaths (cause-specific) percent change (2000-2021)	Under-5 deaths (total), 2000	Under-5 deaths (total), 2021	Under-5 deaths (total) percent change (2000-2021)	Under-5 cause-specific mortality cause fraction, 2000	Under-5 total SCD mortality cause fraction, 2000	Under-5 cause-specific mortality cause fraction, 2021	Under-5 total SCD mortality cause fraction, 2021	Under-5 cause-specific mortality rank 2000	Under-5 cause-specific mortality rank 2021	Under-5 total SCD mortality rank 2000	Under-5 total SCD mortality rank 2021
Equatorial Guinea	663 (558-794)	801 (662-962)	20.7 (9.8-30.9)	6,770 (5,680-7,920)	12,900 (10,700-15,200)	90.3 (74.8-104)	17 (2-32)	21 (2-49)	23.9 (-36.3-120)	348 (281-430)	701 (568-846)	101 (86-119)	5 (0-10)	3 (0-6)	-34.4 (-62-74.3)	111 (81-149)	156 (113-210)	40.2 (25.3-56)	0.2 (0-0.3)	3.4 (2.3-4.4)	0.2 (0-0.3)	8.4 (4.8-12)	34	35	6	5
Gabon	439 (351-527)	412 (337-493)	-6.2 (-12.6-0.6)	5,810 (4,660-7,000)	6,930 (5,760-8,420)	19.1 (10.7-26.8)	28 (1-55)	29 (2-71)	3 (-44.8-282)	265 (214-328)	329 (267-405)	24.3 (15.1-34.9)	5 (0-10)	4 (0-9)	-27.5 (-60.7-93.4)	61 (43-81)	61 (44-81)	0.5 (-10.1-12.8)	0.2 (0-0.3)	2.1 (1.5-2.9)	0.3 (0-0.5)	4.2 (2.5-6.3)	32	31	12	6
**Eastern Sub-Saharan Africa**	39,500 (33,500-47,000)	50,100 (42,000-59,100)	26.6 (22.6-31.5)	416,000 (351,000-493,000)	652,000 (542,000-767,000)	56.7 (52-61.6)	1,580 (901-2,900)	1,990 (1,130-4,110)	26 (-14.3-110)	22,300 (17,600-27,700)	36,900 (29,400-45,900)	65.7 (60.8-71.5)	548 (200-899)	461 (223-734)	-15.9 (-45.7-68.3)	5,970 (4,050-8,230)	8,700 (5,880-11,900)	45.6 (38.3-55.2)	0 (0-0.1)	0.4 (0.3-0.6)	0.1 (0-0.1)	1 (0.7-1.4)	77	71	25	18
Burundi	188 (154-234)	316 (257-391)	68.4 (44-91.5)	1,860 (1,510-2,290)	3,340 (2,740-4,100)	79.8 (60.3-99.7)	32 (19-50)	42 (27-63)	33.7 (-30.9-154)	115 (90-146)	205 (164-265)	78.8 (53.1-100)	10 (3-19)	7 (4-12)	-26.3 (-63.8-160)	38 (26-52)	68 (48-94)	81.1 (54.1-111)	0 (0-0)	0.1 (0.1-0.1)	0 (0-0)	0.2 (0.2-0.3)	83	77	47	28
Comoros	1 (1-2)	1 (1-1)	-19.6 (-26.9--11.8)	12 (9-15)	12 (9-16)	3.2 (-4.1-12.2)	0 (0-1)	0 (0-1)	-1 (-47.6-83.3)	1 (1-1)	1 (1-1)	4.1 (-5.1-15.9)	0 (0-0)	0 (0-0)	-39.3 (-72.9-45)	0 (0-1)	0 (0-1)	-12.6 (-24--0.2)	0 (0-0)	0 (0-0)	0 (0-0)	0 (0-0.1)	104	103	83	74
Djibouti	6 (5-8)	8 (6-10)	22.3 (11.5-31)	60 (46-76)	98 (75-124)	63 (50.6-73.8)	1 (1-2)	1 (1-2)	13.4 (-37.9-126)	3 (2-4)	6 (4-7)	71.6 (54.7-86.8)	0 (0-1)	0 (0-0)	-20.9 (-64.7-113)	1 (1-1)	1 (1-2)	51.2 (30.2-72.3)	0 (0-0)	0 (0-0.1)	0 (0-0)	0.1 (0.1-0.2)	101	102	74	56
Eritrea	42 (31-53)	49 (37-63)	19.1 (9.7-30.7)	430 (323-556)	635 (488-817)	47.7 (37.1-58.7)	8 (5-13)	11 (7-20)	45.7 (-18.2-153)	26 (19-35)	39 (29-52)	51.3 (38.4-68.5)	2 (1-6)	3 (1-5)	6.1 (-46.2-250)	9 (6-12)	11 (7-15)	28.7 (12.8-48.8)	0 (0-0)	0.1 (0-0.1)	0 (0-0)	0.1 (0.1-0.2)	100	98	66	60
Ethiopia	147 (110-205)	184 (138-254)	25.4 (20.8-30.2)	1,100 (824-1,430)	1,630 (1,230-2,090)	47.4 (40.4-54.7)	32 (17-55)	30 (17-50)	-6.4 (-55.4-92.6)	96 (68-138)	144 (103-203)	49.1 (40.8-56.9)	10 (3-25)	6 (3-12)	-42.2 (-72.6-32)	47 (30-74)	64 (41-103)	37.3 (28.2-46.5)	0 (0-0)	0 (0-0)	0 (0-0)	0 (0-0.1)	122	123	99	80
Addis Ababa	3 (2-4)	2 (2-3)	-25.4 (-31.2--19)	34 (26-43)	33 (25-43)	-1.3 (-13.3-7.1)	1 (1-2)	1 (0-1)	-51.3 (-80.7-9.6)	2 (2-3)	3 (2-3)	2.9 (-6.7-13.3)	0 (0-0)	0 (0-0)	-56 (-81.4--7.7)	1 (0-1)	1 (0-1)	-3.7 (-17.3-10.1)	0 (0-0)	0 (0-0)	0 (0-0)	0.1 (0.1-0.1)	121	122	85	68
Afar	3 (3-5)	3 (2-4)	-4.6 (-12.5-7)	25 (19-33)	27 (20-36)	6.6 (-3.1-16.6)	1 (0-2)	1 (0-1)	-26.9 (-62.9-71.9)	2 (2-3)	3 (2-4)	7.2 (-3.7-19)	0 (0-1)	0 (0-0)	-43.2 (-71-52.2)	1 (1-2)	1 (1-2)	6.3 (-8-22.4)	0 (0-0)	0 (0-0)	0 (0-0)	0 (0-0)	120	122	98	85
Amhara	33 (24-47)	35 (25-48)	4.9 (-3.5-12.9)	257 (194-338)	315 (234-407)	22.5 (12.5-31.8)	7 (4-13)	5 (3-10)	-28.8 (-68.7-75.1)	22 (16-32)	28 (20-39)	25.1 (13.1-38.8)	2 (1-5)	1 (0-2)	-61.6 (-85.3-27.3)	11 (7-17)	12 (8-19)	12.5 (-2.5-29)	0 (0-0)	0 (0-0)	0 (0-0)	0 (0-0.1)	122	124	100	77
Benishangul-Gumuz	2 (1-2)	2 (2-3)	43.4 (28.9-56)	10 (8-13)	18 (14-23)	75.7 (60.6-90.3)	0 (0-1)	1 (0-1)	29.1 (-37.8-185)	1 (1-1)	2 (1-2)	77.4 (60.6-94.5)	0 (0-0)	0 (0-0)	-14.7 (-58.8-167)	1 (0-1)	1 (1-1)	61.6 (41.2-81.2)	0 (0-0)	0 (0-0)	0 (0-0)	0 (0-0.1)	122	121	101	83
Dire Dawa	1 (0-1)	1 (0-1)	5.6 (-2.3-14.6)	4 (3-6)	6 (5-8)	42.9 (32.8-55.2)	0 (0-0)	0 (0-0)	-32.2 (-69.9-44.1)	0 (0-1)	1 (0-1)	43.5 (30.7-57.1)	0 (0-0)	0 (0-0)	-62.1 (-84.6-13.2)	0 (0-0)	0 (0-0)	23.7 (8.4-43)	0 (0-0)	0 (0-0)	0 (0-0)	0.1 (0-0.1)	118	123	104	74
Gambella	1 (0-1)	1 (1-1)	31.7 (20.5-42.8)	4 (3-5)	7 (5-9)	86.4 (73.6-102)	0 (0-0)	0 (0-0)	5 (-53.2-118)	0 (0-1)	1 (0-1)	86.7 (63.4-106)	0 (0-0)	0 (0-0)	-49.4 (-80.9-59.7)	0 (0-0)	0 (0-0)	50.9 (27.2-72)	0 (0-0)	0 (0-0)	0 (0-0)	0.1 (0-0.1)	120	124	97	76
Harari	0 (0-0)	0 (0-0)	-7.3 (-14.1-0.7)	2 (2-3)	3 (2-4)	35.8 (25.2-48.5)	0 (0-0)	0 (0-0)	-23.8 (-67.2-78.2)	0 (0-0)	0 (0-0)	36.3 (25.2-50.6)	0 (0-0)	0 (0-0)	-54.1 (-82.7-47)	0 (0-0)	0 (0-0)	11.8 (-2.1-27)	0 (0-0)	0 (0-0)	0 (0-0)	0 (0-0.1)	119	123	93	74
Oromia	55 (41-76)	75 (55-103)	36.9 (26.2-47.6)	401 (300-528)	654 (490-830)	62.8 (49.8-77.7)	11 (6-19)	11 (6-19)	0 (-53.4-103)	35 (24-50)	57 (40-80)	63.7 (48.1-80.4)	4 (1-9)	2 (1-5)	-37.1 (-67.5-58.5)	17 (11-28)	26 (16-41)	50.4 (31.3-71.4)	0 (0-0)	0 (0-0)	0 (0-0)	0 (0-0.1)	121	122	97	78
Somali	10 (7-14)	14 (10-20)	48.7 (35.8-62)	74 (56-95)	113 (83-148)	51.8 (38.8-63.4)	2 (1-4)	2 (1-4)	24.8 (-39.8-187)	6 (5-9)	10 (7-14)	54 (38.7-69.6)	1 (0-2)	1 (0-1)	9.5 (-49.1-224)	3 (2-5)	5 (3-8)	62.3 (42.5-85.9)	0 (0-0)	0 (0-0)	0 (0-0)	0 (0-0)	120	122	98	87
Southern Nations, Nationalities, and Peoples	32 (24-44)	42 (31-58)	32 (20.9-42.7)	230 (169-301)	366 (270-481)	59 (47.2-71)	7 (3-14)	8 (4-13)	8.1 (-48.7-129)	21 (15-30)	33 (24-47)	59 (42.8-75.7)	3 (1-7)	2 (1-3)	-42.3 (-75.4-68)	11 (7-17)	15 (10-24)	41.9 (22.2-61.6)	0 (0-0)	0 (0-0)	0 (0-0)	0 (0-0.1)	122	122	103	79
Tigray	8 (6-11)	8 (6-12)	8.2 (0.7-16.8)	61 (45-81)	85 (64-110)	39.4 (27.2-52.9)	2 (1-3)	1 (1-2)	-16.7 (-68.6-66.7)	5 (4-7)	7 (5-10)	43 (29.6-57.7)	0 (0-1)	0 (0-0)	-57 (-82.8-45.2)	2 (1-4)	3 (2-4)	21.7 (6.8-38.4)	0 (0-0)	0 (0-0)	0 (0-0)	0 (0-0.1)	122	125	98	75
Kenya	15,600 (11,500-20,900)	18,100 (13,300-24,100)	16.3 (13.4-19)	181,000 (139,000-237,000)	264,000 (204,000-341,000)	45 (42.3-48.6)	208 (131-317)	262 (172-421)	25.5 (-6.2-67.3)	9,600 (6,890-13,400)	15,300 (11,000-21,200)	59.7 (54.8-64.5)	66 (30-113)	42 (23-67)	-36.6 (-54.8-7.9)	2,180 (1,370-3,240)	2,980 (1,910-4,360)	36.5 (30.3-43.5)	0.1 (0-0.1)	2.1 (1.3-3.3)	0.1 (0.1-0.1)	7.1 (4.4-10.7)	47	51	13	7
Baringo	220 (160-300)	226 (163-310)	3.1 (-8.8-15.6)	2,360 (1,760-3,140)	3,100 (2,280-4,180)	30.9 (18.1-45.5)	3 (3-4)	3 (2-5)	-1.7 (-36-50.8)	132 (92-185)	189 (131-268)	43.3 (26.2-63.3)	1 (0-2)	1 (0-1)	-49.6 (-68.2--0.6)	33 (21-51)	38 (24-60)	15.3 (-2.9-40.6)	0.1 (0-0.1)	1.8 (1.2-3)	0.1 (0-0.1)	5 (2.9-8.3)	56	59	14	7
Bomet	229 (162-306)	470 (337-636)	105 (85.9-127)	2,840 (2,090-3,730)	7,690 (5,710-10,000)	170 (146-192)	1 (0-2)	4 (2-7)	367 (223-642)	126 (87-176)	390 (276-553)	209 (175-246)	0 (0-0)	0 (0-0)	12.1 (-29.5-92)	25 (15-36)	55 (35-82)	124 (86.6-165)	0 (0-0)	2.6 (1.5-4)	0 (0-0.1)	10.5 (6.3-16.3)	58	56	11	5
Bungoma	958 (710-1,290)	454 (331-621)	-52.6 (-57.3--47.6)	10,400 (7,760-13,800)	6,520 (4,810-8,590)	-37.3 (-42.7--31.4)	12 (7-17)	6 (3-10)	-50.9 (-65.6--23.1)	542 (381-771)	374 (265-529)	-31 (-38.9--22.2)	6 (2-11)	2 (1-4)	-62.6 (-80--11.5)	137 (87-205)	83 (52-124)	-39.3 (-48.5--28.1)	0.1 (0-0.2)	2 (1.3-3)	0.1 (0.1-0.2)	4.2 (2.5-6.7)	46	46	13	7
Busia	445 (323-590)	527 (379-705)	18.4 (2.1-33.6)	5,050 (3,910-6,520)	7,140 (5,430-9,310)	41.1 (23.7-54.6)	9 (3-14)	9 (3-17)	3.8 (-38.2-63.5)	274 (193-387)	422 (292-590)	54.1 (34.5-72.5)	4 (1-7)	2 (1-3)	-55.5 (-69.6--12.5)	73 (45-110)	100 (64-152)	37.5 (15.5-64.8)	0.1 (0-0.2)	2.2 (1.4-3.3)	0.1 (0-0.2)	8.4 (5.1-13.2)	41	41	14	4
Elgeyo-Marakwet	214 (154-297)	62 (45-84)	-71.1 (-73.9--68.1)	2,600 (1,940-3,420)	1,090 (835-1,430)	-58.2 (-61.4--53.7)	2 (2-4)	1 (1-2)	-55.4 (-71.2--29.6)	130 (88-188)	61 (42-88)	-53 (-58.1--46.6)	0 (0-1)	0 (0-0)	-61.7 (-78.3--26.2)	25 (15-37)	9 (6-13)	-63.7 (-69.5--56.3)	0 (0-0)	2.2 (1.3-3.5)	0.1 (0-0.1)	4.5 (2.6-7.3)	58	55	11	9
Embu	158 (114-216)	97 (69-135)	-38.5 (-45.9--29.6)	2,120 (1,580-2,830)	1,860 (1,380-2,440)	-13 (-20.7--4.1)	1 (1-3)	2 (1-3)	9.3 (-30.8-73.7)	108 (75-153)	104 (73-150)	-3.2 (-16.2-10.9)	0 (0-0)	0 (0-0)	-28 (-53.7-16.8)	20 (12-30)	14 (9-21)	-28.8 (-42.6--13.8)	0 (0-0.1)	2.2 (1.3-3.5)	0.1 (0-0.1)	4.2 (2.5-7)	59	57	11	8
Garissa	139 (101-188)	519 (379-717)	273 (242-304)	1,690 (1,300-2,220)	6,170 (4,810-8,310)	265 (237-295)	2 (1-3)	4 (2-7)	142 (40.9-283)	88 (61-126)	331 (230-474)	275 (235-322)	0 (0-1)	0 (0-1)	0.4 (-45.9-115)	20 (12-30)	73 (44-114)	274 (213-338)	0 (0-0.1)	1.9 (1.1-3.3)	0 (0-0.1)	7.4 (4.4-11.7)	56	60	15	6
Homa Bay	933 (675-1,230)	1,000 (756-1,290)	7.6 (-5.2-19)	8,560 (6,480-11,000)	12,300 (9,380-15,600)	43.2 (27.4-57.2)	17 (4-31)	19 (5-36)	11.9 (-30.5-116)	494 (348-687)	776 (555-1,050)	57.1 (37.3-75)	7 (2-13)	4 (1-8)	-35.6 (-59.5-40.5)	135 (85-203)	193 (124-283)	42.6 (18.4-67.5)	0.1 (0-0.2)	1.8 (1.1-2.7)	0.2 (0.1-0.4)	10.1 (6.2-15.2)	39	37	13	2
Isiolo	54 (38-71)	67 (48-91)	25.1 (12-40)	566 (413-732)	861 (646-1,140)	51.8 (38.8-68.7)	1 (0-1)	1 (1-1)	37.9 (-13.9-143)	33 (22-47)	54 (38-75)	63.1 (43.3-89.4)	0 (0-1)	0 (0-1)	0.5 (-39.5-155)	9 (5-13)	12 (7-19)	43 (16.8-73.4)	0 (0-0.1)	1.2 (0.7-1.9)	0.1 (0-0.1)	2 (1.2-3.4)	57	63	18	13

Location	Births, 2000	Births, 2021	Births percent change (2000-2021)	All age cases, 2000	All age cases, 2021	All age cases percent change (2000-2021)	All age deaths (cause-specific), 2000	All age deaths (cause-specific), 2021	All age deaths (cause-specific) percent change (2000-2021)	All age deaths (total), 2000	All age deaths (total), 2021	All age deaths (total) percent change (2000-2021)	Under-5 deaths (cause-specific), 2000	Under-5 deaths (cause-specific), 2021	Under-5 deaths (cause-specific) percent change (2000-2021)	Under-5 deaths (total), 2000	Under-5 deaths (total), 2021	Under-5 deaths (total) percent change (2000-2021)	Under-5 cause-specific mortality cause fraction, 2000	Under-5 total SCD mortality cause fraction, 2000	Under-5 cause-specific mortality cause fraction, 2021	Under-5 total SCD mortality cause fraction, 2021	Under-5 cause-specific mortality rank 2000	Under-5 cause-specific mortality rank 2021	Under-5 total SCD mortality rank 2000	Under-5 total SCD mortality rank 2021
Kajiado	170 (124-235)	238 (172-329)	40.2 (26.6-57.7)	2,200 (1,680-2,870)	4,170 (3,150-5,470)	88.7 (70.9-108)	2 (1-3)	3 (1-6)	63.3 (-22.6-197)	109 (75-156)	232 (165-329)	113 (88.7-142)	1 (0-1)	1 (0-1)	11.9 (-58.4-138)	21 (13-33)	34 (21-51)	56.3 (31.6-86.4)	0 (0-0.1)	2 (1.2-3.2)	0.1 (0-0.1)	4.1 (2.3-7)	52	52	12	8
Kakamega	641 (470-865)	1,170 (855-1,530)	81.9 (60-102)	7,250 (5,490-9,530)	15,700 (12,100-20,400)	117 (94.8-138)	11 (6-15)	16 (10-26)	50.8 (-8.4-169)	394 (273-561)	938 (677-1,280)	138 (105-172)	5 (2-10)	3 (1-5)	-44.6 (-64.2-18.3)	108 (65-167)	218 (138-318)	101 (65.5-147)	0.1 (0-0.2)	2 (1.2-2.9)	0.1 (0.1-0.2)	10.8 (6.5-17.3)	45	46	14	2
Kericho	370 (263-507)	118 (86-153)	-68.3 (-71.3--64.7)	4,110 (3,050-5,560)	1,920 (1,470-2,500)	-53.4 (-57.4--48.6)	2 (1-4)	2 (1-3)	-39 (-57.7--7.5)	199 (137-288)	105 (75-147)	-47.1 (-53.1--40.5)	0 (0-1)	1 (0-1)	29.5 (-17-97.8)	43 (26-63)	17 (11-26)	-59.6 (-66.6--52.1)	0 (0-0)	2.5 (1.6-3.9)	0.1 (0-0.1)	2.1 (1.2-3.4)	56	54	10	11
Kiambu	538 (387-722)	376 (268-507)	-30 (-37.5--20.7)	6,680 (4,990-8,700)	6,560 (4,950-8,580)	-2 (-10.7-7.5)	7 (4-11)	7 (3-11)	-6.3 (-35.1-32.2)	347 (239-482)	378 (269-529)	9 (-3.3-24.8)	1 (1-2)	1 (0-2)	-15.7 (-73-69)	60 (37-89)	51 (31-78)	-15.3 (-30.4-1.2)	0 (0-0.1)	2.3 (1.5-3.5)	0.1 (0-0.1)	4.2 (2.4-6.9)	55	55	12	7
Kilifi	393 (306-488)	494 (392-624)	25.8 (17.9-35.2)	5,050 (4,080-6,220)	7,720 (6,180-9,570)	52.6 (42.1-63.2)	7 (3-14)	11 (5-22)	50.3 (-2.2-165)	253 (193-325)	437 (332-580)	72.9 (56.4-89.2)	3 (1-5)	2 (1-4)	-11 (-45.7-74.7)	62 (42-88)	94 (64-132)	51.4 (29.8-74.7)	0.1 (0-0.1)	1.5 (1-2.1)	0.1 (0.1-0.2)	5.6 (3.7-8.2)	46	44	14	7
Kirinyaga	163 (119-223)	104 (74-144)	-36.5 (-42.5--30)	2,030 (1,540-2,710)	1,830 (1,370-2,460)	-10.4 (-19--2.3)	2 (1-3)	2 (1-3)	7 (-38-61.4)	108 (76-151)	108 (77-155)	0.3 (-10.1-14.5)	0 (0-1)	0 (0-0)	-40.7 (-85.9-11.6)	20 (12-30)	15 (9-23)	-25.7 (-38.2--9.2)	0 (0-0.1)	2.3 (1.4-3.3)	0.1 (0-0.1)	4.3 (2.5-7)	56	60	12	8
Kisii	673 (497-903)	370 (274-481)	-45.1 (-49.7--40.4)	8,360 (6,360-10,800)	6,810 (5,310-8,700)	-18.9 (-25.9--12.4)	17 (8-26)	12 (6-20)	-27.3 (-52.3-13.9)	456 (325-652)	420 (298-583)	-8.1 (-18.1-3)	4 (2-7)	1 (1-2)	-68.6 (-80.8--32.3)	100 (63-147)	68 (43-99)	-31.9 (-42.9--19.8)	0.1 (0-0.2)	2.2 (1.5-3.3)	0.1 (0.1-0.2)	7.7 (4.5-11.7)	45	43	13	6
Kisumu	836 (665-1,040)	663 (539-836)	-20.7 (-28.7--14.3)	8,560 (6,890-10,700)	9,620 (7,700-11,600)	12 (1.8-22.5)	10 (6-17)	11 (6-18)	7.2 (-29.9-68)	559 (433-717)	682 (539-878)	22 (10.1-35.8)	3 (1-5)	2 (1-3)	-37.5 (-59.5-24.2)	164 (104-246)	157 (105-228)	-4.4 (-19.5-14.6)	0.1 (0-0.1)	3.7 (2.4-5.8)	0.1 (0.1-0.2)	12.9 (8.2-19.4)	45	41	9	2
Kitui	393 (282-540)	336 (243-459)	-14.5 (-23.4--5.5)	4,560 (3,430-5,970)	5,060 (3,820-6,670)	10.8 (0.9-23.5)	3 (2-5)	3 (2-5)	-11.4 (-46.4-35)	230 (158-329)	283 (199-409)	23 (8.8-38.5)	1 (0-1)	0 (0-1)	-46.9 (-70.3-10.2)	52 (32-78)	50 (31-76)	-3.2 (-20.8-16.7)	0 (0-0.1)	2.1 (1.3-3.3)	0.1 (0-0.1)	6.5 (3.7-10.8)	58	59	12	6
Kwale	297 (215-396)	342 (250-451)	15.2 (1.3-27.8)	3,850 (2,910-4,950)	5,330 (4,130-6,980)	38.2 (24.4-51.4)	5 (2-9)	7 (3-12)	29.1 (-21-123)	205 (143-284)	306 (211-423)	49.6 (32.7-71)	2 (1-3)	1 (1-2)	-17 (-48.7-60.4)	48 (30-75)	63 (39-95)	30.5 (9.3-57.7)	0.1 (0-0.1)	2.4 (1.6-3.5)	0.1 (0.1-0.2)	5.7 (3.4-8.9)	43	43	12	6
Laikipia	145 (105-193)	94 (67-126)	-35 (-42.6--27.1)	1,790 (1,350-2,360)	1,690 (1,280-2,230)	-5.8 (-14.7-3.1)	1 (1-2)	1 (0-2)	-24.9 (-57.1-20)	83 (56-120)	89 (61-124)	7.4 (-5.6-23.3)	0 (0-0)	0 (0-0)	-47.1 (-76.4-17.8)	15 (9-22)	12 (7-17)	-22.2 (-36.5--5)	0 (0-0)	2.4 (1.4-3.7)	0.1 (0-0.1)	7.9 (4.4-12.7)	59	59	11	5
Lamu	29 (22-39)	32 (24-44)	11 (-1.9-22.5)	376 (288-484)	525 (404-687)	39.2 (27.1-50.5)	0 (0-1)	1 (0-1)	28.2 (-29.9-133)	20 (14-29)	31 (22-43)	51.1 (33.4-69.9)	0 (0-0)	0 (0-0)	-29 (-60.9-57.1)	4 (3-7)	5 (3-9)	24.4 (3.6-48.3)	0 (0-0.1)	1.5 (0.9-2.5)	0.1 (0-0.1)	3.7 (2.2-5.9)	55	61	14	10
Machakos	398 (286-535)	183 (130-246)	-54 (-57.9--48.9)	5,600 (4,190-7,400)	3,630 (2,770-4,700)	-35.4 (-41.4--28.5)	3 (2-5)	2 (1-4)	-19.4 (-44.7-19.3)	275 (187-394)	202 (140-281)	-26.7 (-35.4--16.4)	1 (0-1)	0 (0-1)	-41.6 (-72.6-8.8)	50 (30-76)	27 (16-41)	-45.7 (-54.9--34.4)	0 (0-0)	2.2 (1.3-3.5)	0.1 (0-0.1)	4 (2.2-6.7)	58	56	10	8
Makueni	271 (198-378)	370 (263-505)	36.3 (24.6-51.8)	3,200 (2,390-4,290)	5,720 (4,250-7,560)	78.4 (65.5-93.3)	1 (1-2)	2 (1-4)	67.5 (-8.5-152)	156 (108-229)	314 (216-440)	101 (79.7-128)	0 (0-0)	0 (0-0)	-49.8 (-74.7--1.4)	33 (21-50)	50 (31-76)	49.9 (28.2-78.4)	0 (0-0)	2.5 (1.6-3.8)	0 (0-0.1)	14.9 (8.8-23.8)	61	58	10	1
Mandera	121 (88-163)	1,390 (975-1,890)	1050 (940-1200)	1,720 (1,280-2,240)	17,700 (13,300-22,900)	921 (813-1060)	2 (1-2)	13 (8-22)	721 (442-1190)	95 (65-138)	1,020 (706-1,440)	967 (844-1110)	1 (0-1)	1 (0-2)	122 (33.9-311)	24 (14-37)	224 (135-338)	844 (679-1040)	0 (0-0.1)	1.9 (1.1-3.2)	0.1 (0-0.1)	10 (6-16)	58	59	15	4
Marsabit	88 (65-119)	175 (127-239)	98.9 (75.4-119)	1,000 (742-1,340)	2,190 (1,630-2,810)	119 (99.5-138)	1 (0-1)	2 (1-3)	161 (69-331)	53 (37-77)	123 (87-176)	134 (108-164)	0 (0-0)	0 (0-1)	85.4 (21.5-208)	13 (8-20)	26 (16-39)	102 (68.4-145)	0 (0-0.1)	2 (1.2-3.1)	0 (0-0.1)	3.3 (2-5.4)	59	64	12	7
Meru	486 (355-665)	337 (238-449)	-30.6 (-36.3--21.9)	6,440 (4,830-8,520)	6,260 (4,670-8,090)	-3.2 (-10.9-7)	3 (2-6)	6 (3-11)	76.8 (5.9-192)	334 (228-465)	362 (249-502)	8.5 (-3.1-23.2)	1 (1-1)	1 (1-2)	36.6 (-35.3-112)	67 (41-102)	53 (32-81)	-21.7 (-33.1--5.2)	0 (0-0)	2.3 (1.5-3.5)	0.1 (0-0.1)	3.6 (2.1-5.7)	59	57	11	9
Migori	816 (614-1,080)	1,130 (817-1,480)	39 (28.1-57.6)	7,310 (5,610-9,360)	12,700 (9,610-16,500)	73 (60-90.1)	16 (4-32)	24 (6-44)	43.1 (-18.1-167)	426 (302-592)	810 (574-1,120)	90 (69.8-113)	8 (2-15)	5 (1-10)	-31.3 (-58.7-89.1)	126 (80-189)	225 (146-338)	79.2 (52.5-110)	0.1 (0-0.2)	1.8 (1.1-2.7)	0.2 (0-0.4)	8.6 (5.7-13.6)	38	36	14	5
Mombasa	288 (205-387)	355 (258-470)	23.2 (11.2-35.2)	3,910 (2,970-5,000)	6,440 (4,930-8,350)	63.9 (48.8-75)	3 (2-7)	6 (3-11)	75.1 (-17.9-217)	202 (145-280)	360 (262-489)	78.6 (60.2-98.1)	0 (0-1)	0 (0-1)	-22.4 (-58.4-44)	36 (23-53)	55 (35-80)	55 (30.8-85.9)	0 (0-0.1)	2.4 (1.5-3.5)	0.1 (0-0.1)	11.1 (6.8-17.4)	51	49	10	3
Murang'a	250 (175-339)	169 (122-225)	-32.6 (-38.7--26.3)	3,550 (2,690-4,660)	3,310 (2,530-4,300)	-7.1 (-14.7-1.1)	2 (1-4)	3 (2-5)	26.7 (-18.8-114)	176 (121-252)	188 (133-265)	6.7 (-5.3-20.8)	0 (0-1)	0 (0-1)	-32.5 (-78.4-15.9)	31 (18-48)	22 (14-33)	-28.3 (-40.1--15.5)	0 (0-0.1)	2.6 (1.6-4.2)	0.1 (0-0.1)	5.2 (3-8)	57	56	12	7
Nairobi	830 (598-1,120)	950 (678-1,290)	14.4 (3.6-27.9)	9,710 (7,330-12,500)	16,000 (12,100-21,400)	64.3 (50.2-79.8)	12 (8-19)	16 (9-23)	31.7 (-19.5-117)	529 (372-734)	941 (670-1,300)	77.9 (58.4-99.2)	2 (1-3)	1 (0-1)	-71.4 (-93.9--21.4)	86 (52-128)	130 (80-194)	52.1 (28.1-82)	0 (0-0.1)	2.1 (1.2-3.2)	0.1 (0-0.1)	14.5 (7.6-24.7)	56	61	11	1
Nakuru	495 (357-676)	379 (271-507)	-23.5 (-32.6--14.2)	6,420 (4,890-8,380)	7,260 (5,540-9,380)	12.6 (1.6-22.2)	4 (2-7)	5 (3-8)	25.3 (-10.7-86.9)	311 (211-440)	399 (283-551)	28.2 (13.6-43.2)	1 (0-1)	1 (0-1)	7.6 (-36.8-76.4)	57 (35-86)	50 (31-74)	-12.7 (-26.1-4.9)	0 (0-0.1)	2.7 (1.7-4.2)	0.1 (0-0.1)	4.8 (2.7-7.9)	55	56	10	8
Nandi	268 (192-365)	220 (161-292)	-18 (-26.1--8.4)	3,590 (2,720-4,660)	4,090 (3,130-5,420)	13.6 (4-26.2)	2 (1-3)	3 (2-4)	24 (-14.5-73.8)	165 (114-237)	212 (150-302)	28.8 (14.2-43)	0 (0-1)	0 (0-1)	5.3 (-31.9-72.1)	32 (20-50)	31 (19-45)	-5.9 (-20.8-10.6)	0 (0-0)	2.8 (1.7-4.6)	0.1 (0-0.1)	6.1 (3.8-9.5)	54	54	10	7
Narok	211 (153-282)	800 (558-1,100)	280 (236-321)	2,430 (1,800-3,160)	10,300 (7,600-13,700)	324 (281-370)	1 (1-2)	6 (3-11)	331 (158-550)	116 (81-167)	541 (379-765)	365 (314-429)	0 (0-0)	0 (0-1)	86.6 (11.5-225)	28 (17-43)	108 (66-163)	282 (231-347)	0 (0-0)	3 (1.8-4.9)	0.1 (0-0.1)	12.1 (7.3-19.5)	60	59	12	3
Nyamira	182 (129-240)	203 (142-271)	11.8 (2.1-24.1)	2,210 (1,660-2,880)	3,510 (2,560-4,580)	58.2 (47.5-72.5)	2 (2-4)	5 (3-8)	127 (47.7-252)	111 (77-158)	199 (137-290)	79.6 (59.1-102)	0 (0-0)	0 (0-0)	7.1 (-34.5-96.5)	20 (12-30)	26 (16-38)	29.9 (10.8-57.3)	0 (0-0.1)	3 (1.9-4.5)	0.1 (0-0.1)	9.3 (5.7-13.5)	50	50	10	5
Nyandarua	213 (155-292)	162 (118-220)	-23.9 (-34--17.2)	2,630 (2,000-3,490)	2,720 (2,040-3,530)	3 (-6.6-10.5)	2 (1-3)	3 (2-5)	44.7 (-5.3-126)	130 (90-185)	152 (107-217)	17.5 (6.5-30.4)	0 (0-0)	0 (0-0)	-34.2 (-79.3-39.3)	23 (14-35)	19 (11-29)	-18.2 (-31.6--3.1)	0 (0-0)	3 (1.8-4.9)	0.1 (0-0.1)	7.1 (4.1-11.6)	58	62	11	6
Nyeri	221 (162-295)	117 (83-160)	-47.2 (-52.4--41.3)	3,050 (2,300-4,010)	2,260 (1,720-2,940)	-26.3 (-31.6--19.5)	3 (1-4)	2 (1-4)	-5.6 (-41.3-76.9)	156 (111-222)	131 (92-183)	-15.9 (-25.5--4.7)	0 (0-0)	0 (0-0)	-31.8 (-77.8-23.1)	24 (15-37)	14 (9-22)	-41.4 (-51.4--27.8)	0 (0-0)	2.9 (1.7-4.6)	0.1 (0-0.1)	6.4 (3.7-10.4)	61	59	10	7
Samburu	85 (60-116)	136 (99-189)	59.4 (46.2-76.3)	903 (669-1,200)	1,680 (1,250-2,260)	85.8 (75.1-101)	1 (0-1)	1 (1-2)	122 (45.9-281)	44 (30-64)	90 (62-132)	104 (78.7-131)	0 (0-0)	0 (0-0)	60.3 (4.2-169)	10 (6-15)	17 (10-26)	68.5 (42.3-102)	0 (0-0)	1.8 (1.1-2.7)	0 (0-0.1)	3.3 (1.9-5.4)	65	65	16	8
Siaya	680 (504-859)	605 (439-791)	-11 (-19-2.4)	6,900 (5,330-8,790)	8,070 (6,130-10,500)	16.8 (7-30.6)	16 (4-32)	12 (3-24)	-28.2 (-53.4-25)	400 (290-543)	515 (366-702)	28.7 (14.5-46)	8 (2-16)	3 (1-6)	-55.6 (-69.8-3.8)	110 (70-165)	127 (82-187)	15 (-1.5-37.9)	0.1 (0-0.3)	1.8 (1.1-2.7)	0.2 (0-0.4)	7.5 (4.6-11.4)	37	40	14	6

Location	Births, 2000	Births, 2021	Births percent change (2000-2021)	All age cases, 2000	All age cases, 2021	All age cases percent change (2000-2021)	All age deaths (cause-specific), 2000	All age deaths (cause-specific), 2021	All age deaths (cause-specific) percent change (2000-2021)	All age deaths (total), 2000	All age deaths (total), 2021	All age deaths (total) percent change (2000-2021)	Under-5 deaths (cause-specific), 2000	Under-5 deaths (cause-specific), 2021	Under-5 deaths (cause-specific) percent change (2000-2021)	Under-5 deaths (total), 2000	Under-5 deaths (total), 2021	Under-5 deaths (total) percent change (2000-2021)	Under-5 cause-specific mortality cause fraction, 2000	Under-5 total SCD mortality cause fraction, 2000	Under-5 cause-specific mortality cause fraction, 2021	Under-5 total SCD mortality cause fraction, 2021	Under-5 cause-specific mortality rank 2000	Under-5 cause-specific mortality rank 2021	Under-5 total SCD mortality rank 2000	Under-5 total SCD mortality rank 2021
Taita Taveta	83 (60-111)	56 (41-76)	-31.9 (-39.7--24.4)	1,250 (954-1,620)	1,140 (870-1,510)	-9.7 (-17.9-0)	1 (1-2)	1 (1-2)	14.3 (-41.5-95.6)	68 (48-96)	68 (48-95)	0.8 (-11.3-13.8)	0 (0-0)	0 (0-0)	-37.1 (-69.7-14.3)	12 (7-19)	9 (6-14)	-23.3 (-37.2--7.2)	0 (0-0.1)	1.9 (1.3-3)	0.1 (0-0.1)	5.7 (3.4-9.7)	52	56	11	6
Tana River	94 (69-130)	134 (96-180)	41.9 (25.6-58.4)	1,130 (859-1,500)	1,750 (1,290-2,290)	55.4 (41.6-68.5)	1 (1-2)	2 (1-3)	34.6 (-17.8-126)	61 (43-86)	100 (70-141)	64.3 (45.3-84.8)	0 (0-1)	0 (0-1)	-3.9 (-47.1-85.4)	15 (9-22)	22 (14-35)	52.5 (26.2-79.8)	0 (0-0.1)	1.8 (1.1-2.8)	0.1 (0-0.1)	4.1 (2.3-6.3)	55	55	15	8
Tharaka Nithi	41 (29-56)	35 (26-47)	-16 (-22.9--8.5)	522 (385-686)	599 (463-791)	14.4 (5-25)	1 (0-1)	1 (0-1)	-7 (-42.9-34.7)	28 (19-40)	35 (25-50)	26.6 (12.1-44.5)	0 (0-0)	0 (0-0)	7.6 (-52.6-154)	6 (4-9)	6 (3-9)	-6.4 (-20.5-12.8)	0.1 (0-0.1)	2 (1.2-3.3)	0.1 (0-0.1)	2.5 (1.5-4.1)	56	57	15	11
Trans Nzoia	297 (219-405)	286 (207-388)	-3.6 (-12.8-7.5)	3,680 (2,780-4,820)	4,830 (3,580-6,230)	30.9 (18.7-41.8)	3 (2-4)	3 (2-6)	29.2 (-18.3-115)	183 (126-256)	271 (191-376)	47.7 (31.8-68.2)	1 (0-1)	0 (0-1)	-6.2 (-44.2-61.7)	40 (25-62)	44 (28-67)	9.5 (-7.5-33.5)	0 (0-0.1)	2.9 (1.7-4.3)	0.1 (0.1-0.1)	8.7 (5.2-13.2)	47	52	10	3
Turkana	277 (198-382)	763 (554-1,020)	175 (150-207)	2,880 (2,190-3,800)	9,050 (7,040-11,700)	214 (189-246)	2 (1-3)	5 (2-11)	166 (43-404)	149 (104-213)	503 (356-693)	237 (193-284)	0 (0-1)	0 (0-1)	-11.5 (-49.7-71.5)	36 (22-55)	110 (70-164)	208 (160-257)	0 (0-0)	2 (1.2-3.1)	0 (0-0.1)	9.4 (5.7-14.6)	61	62	13	4
Uasin Gishu	282 (204-388)	260 (188-352)	-7.8 (-16.6-3.5)	3,570 (2,660-4,710)	4,850 (3,790-6,240)	35.2 (23.9-49)	2 (1-4)	3 (2-6)	41.1 (-1.8-105)	172 (118-251)	264 (187-371)	53.2 (37.2-72.2)	0 (0-1)	1 (0-1)	66.8 (1.5-152)	35 (22-52)	38 (24-56)	9.6 (-8.1-29.7)	0 (0-0)	2.6 (1.7-4)	0.1 (0.1-0.1)	4.7 (2.8-7.3)	54	54	10	7
Vihiga	212 (156-280)	264 (193-355)	24.4 (8.3-46)	2,660 (2,050-3,480)	4,270 (3,280-5,580)	60.1 (43-80.1)	4 (2-6)	6 (3-12)	76.1 (4.7-188)	142 (97-201)	253 (181-357)	78.2 (57.4-106)	1 (0-2)	0 (0-1)	-53.8 (-68.7--17.7)	34 (21-52)	46 (29-68)	34.2 (12.4-63.9)	0.1 (0-0.1)	2.5 (1.5-3.7)	0.1 (0.1-0.2)	12.1 (7.7-19.6)	41	40	11	2
Wajir	185 (132-247)	514 (369-685)	178 (141-211)	2,110 (1,590-2,770)	5,740 (4,280-7,420)	171 (144-202)	2 (1-3)	4 (2-6)	74 (14-169)	117 (82-164)	327 (228-463)	179 (141-220)	1 (0-2)	1 (0-1)	-10.4 (-44.5-77.5)	28 (17-44)	77 (48-121)	175 (124-226)	0 (0-0.1)	1.3 (0.8-2.3)	0 (0-0)	3.4 (2.1-5.7)	61	66	15	8
West Pokot	200 (145-273)	354 (259-478)	76.5 (58.3-93.3)	2,020 (1,520-2,730)	4,180 (3,170-5,460)	106 (86.8-124)	2 (2-3)	4 (2-5)	63.7 (-0.8-180)	108 (76-158)	242 (167-344)	123 (94.5-150)	1 (0-1)	0 (0-1)	-29.5 (-54.5-40.7)	30 (18-47)	57 (35-88)	90.8 (57.7-128)	0 (0-0.1)	2.2 (1.3-3.2)	0.1 (0-0.1)	7 (4.2-11.3)	53	56	14	6
Madagascar	2,530 (2,140-2,980)	3,240 (2,720-3,860)	28 (17.5-39.8)	25,600 (21,600-30,300)	40,600 (34,000-48,000)	58.4 (46.8-73.4)	142 (72-203)	196 (108-302)	38.2 (-2.6-116)	1,460 (1,200-1,780)	2,370 (1,970-2,870)	62.5 (50.2-75.9)	47 (15-92)	38 (16-68)	-19.3 (-49.2-88.2)	432 (295-602)	600 (399-820)	38.9 (21.8-58.8)	0.1 (0-0.1)	0.6 (0.4-0.8)	0.1 (0-0.1)	1.2 (0.7-1.6)	52	57	20	17
Malawi	404 (351-488)	422 (364-519)	4.5 (-4.8-13)	3,590 (3,020-4,380)	5,780 (4,800-6,940)	60.8 (47-76.2)	58 (35-94)	61 (39-101)	5.6 (-44.1-107)	214 (179-265)	354 (292-436)	65.5 (51.9-78.6)	21 (6-44)	11 (5-19)	-49 (-73.1-52.9)	74 (53-100)	94 (65-126)	26.1 (9.8-42.9)	0 (0-0.1)	0.1 (0.1-0.1)	0 (0-0.1)	0.3 (0.2-0.4)	81	80	50	32
Mozambique	155 (128-184)	220 (183-264)	41.7 (34.4-49.4)	1,140 (943-1,300)	2,010 (1,690-2,320)	75.9 (67.5-83.9)	31 (17-52)	36 (22-56)	15.8 (-37.2-141)	92 (74-112)	161 (130-198)	75.9 (64.2-87.6)	14 (4-28)	9 (5-16)	-31.2 (-65.8-117)	44 (33-60)	71 (52-96)	59.2 (43.5-75.6)	0 (0-0)	0 (0-0)	0 (0-0)	0.1 (0.1-0.1)	96	96	75	54
Rwanda	76 (61-92)	84 (67-103)	10.4 (2.6-19.1)	747 (608-906)	1,030 (829-1,230)	37.5 (28.4-52)	23 (13-38)	17 (10-27)	-28 (-61.6-55.8)	49 (38-62)	69 (54-87)	41.8 (29.7-53.7)	7 (1-14)	3 (1-6)	-52.9 (-77.4-46.8)	18 (12-24)	21 (15-30)	20.2 (6.9-34.9)	0 (0-0)	0 (0-0.1)	0 (0-0)	0.1 (0.1-0.2)	100	92	77	47
Somalia	27 (19-37)	48 (34-66)	80.8 (63.1-99.9)	168 (121-224)	342 (251-450)	103 (86.9-121)	6 (3-10)	9 (5-15)	62.4 (-9.9-197)	17 (12-24)	34 (23-47)	100 (78.2-123)	3 (1-7)	4 (1-7)	30.9 (-35.4-224)	10 (6-15)	19 (12-30)	94.5 (67.9-122)	0 (0-0)	0 (0-0)	0 (0-0)	0 (0-0)	116	121	101	94
South Sudan	97 (72-120)	105 (81-131)	9 (-1.1-18.5)	831 (633-1,040)	1,010 (789-1,270)	20.5 (11.1-28.4)	12 (7-23)	21 (12-38)	73.4 (9.7-189)	57 (42-74)	68 (51-90)	20.7 (9.6-32.2)	5 (1-11)	9 (2-18)	84.6 (4.9-393)	22 (15-32)	25 (17-35)	11.7 (-1.4-26.9)	0 (0-0)	0 (0-0.1)	0 (0-0)	0 (0-0.1)	101	103	64	77
Uganda	7,250 (6,200-8,310)	9,410 (7,840-10,700)	29.7 (20.3-41)	65,800 (56,100-76,200)	107,000 (90,300-124,000)	63 (50.2-79.1)	262 (133-395)	322 (170-525)	23.1 (-20.6-96.8)	3,710 (3,040-4,320)	6,240 (4,950-7,350)	68.4 (54.3-81.3)	90 (27-163)	79 (36-140)	-12.4 (-45.2-125)	1,150 (768-1,620)	1,700 (1,050-2,410)	47.5 (27.3-70.5)	0.1 (0-0.1)	0.7 (0.5-1)	0.1 (0-0.1)	1.8 (1.1-2.7)	52	52	19	13
United Republic of Tanzania	9,600 (8,130-11,100)	13,100 (10,900-15,000)	37 (25.4-52.5)	99,100 (82,600-114,000)	164,000 (136,000-190,000)	65.6 (51.7-82.4)	582 (239-1,940)	762 (300-2,820)	30.8 (-24.9-116)	4,960 (4,020-5,800)	8,450 (6,720-10,100)	70.3 (54.4-90.5)	205 (51-442)	197 (64-516)	-3.5 (-47.3-93.3)	1,390 (885-1,910)	2,190 (1,500-3,040)	57.2 (36.9-90.2)	0.1 (0-0.2)	0.8 (0.5-1)	0.2 (0.1-0.6)	2.2 (1.4-3.2)	46	45	19	12
Zambia	3,420 (2,950-4,180)	4,700 (4,090-5,700)	37.6 (29.3-52.4)	33,500 (28,700-40,500)	60,100 (50,900-73,600)	78.9 (66-96.7)	179 (73-291)	216 (78-363)	20.3 (-18.1-96.5)	1,870 (1,540-2,310)	3,400 (2,810-4,230)	82.2 (66.6-100)	68 (20-134)	53 (18-91)	-22.3 (-48.8-67.2)	549 (368-758)	853 (594-1,180)	55.4 (34.7-76.2)	0.1 (0-0.2)	0.9 (0.6-1.2)	0.2 (0.1-0.3)	3 (1.8-4)	45	45	19	11
**Southern Sub-Saharan Africa**	114 (95-133)	111 (95-135)	-2.9 (-8.8-7.2)	1,370 (1,150-1,600)	1,490 (1,270-1,750)	9 (3-18.8)	26 (18-35)	34 (23-55)	33.2 (-13.7-89.8)	84 (68-103)	92 (75-114)	10.4 (3.5-21.3)	2 (1-4)	4 (3-8)	88.1 (2.1-220)	27 (20-37)	28 (21-39)	5 (-3.4-15)	0 (0-0)	0 (0-0)	0 (0-0)	0 (0-0)	109	109	63	73
Botswana	20 (16-23)	21 (17-25)	6 (-1.6-14.2)	357 (304-422)	422 (357-490)	17.6 (8.2-26.8)	3 (2-5)	3 (2-4)	-5.3 (-40.1-50.1)	15 (12-18)	19 (16-23)	28.6 (17.1-39)	0 (0-0)	0 (0-1)	43.9 (-39-173)	3 (2-4)	3 (2-4)	12.7 (0.1-26.6)	0 (0-0)	0.1 (0.1-0.1)	0 (0-0)	0.2 (0.1-0.2)	93	98	38	42
Eswatini	1 (1-1)	1 (1-1)	-17.3 (-22.8--11.9)	10 (7-12)	9 (7-11)	-6.7 (-13.9-2.4)	0 (0-0)	0 (0-0)	-9.9 (-48.1-39.7)	1 (1-1)	1 (1-1)	-4.7 (-11.3-2.2)	0 (0-0)	0 (0-0)	-4.1 (-44.2-51.5)	0 (0-1)	0 (0-1)	-11 (-19.4--1)	0 (0-0)	0 (0-0)	0 (0-0)	0 (0-0)	117	120	75	77
Lesotho	2 (1-2)	1 (1-2)	-23.2 (-30.6--15.8)	15 (12-18)	13 (10-16)	-11.6 (-20.2--2.8)	0 (0-0)	0 (0-0)	23.4 (-27.9-114)	1 (1-2)	1 (1-2)	-9.9 (-17.4--1.9)	0 (0-0)	0 (0-0)	18.7 (-35.1-108)	1 (0-1)	1 (0-1)	-16.2 (-25.5--6.2)	0 (0-0)	0 (0-0)	0 (0-0)	0 (0-0)	114	118	67	76
Namibia	2 (1-2)	2 (2-3)	12.6 (4.8-19.6)	17 (14-21)	20 (15-24)	12.8 (5.3-21.5)	0 (0-0)	0 (0-0)	-8.7 (-52.6-55.5)	1 (1-2)	2 (1-2)	16.3 (6.2-26.3)	0 (0-0)	0 (0-0)	30 (-30.3-138)	1 (0-1)	1 (1-1)	14.2 (0.1-28.2)	0 (0-0)	0 (0-0)	0 (0-0)	0 (0-0.1)	117	120	66	69
South Africa	19 (13-27)	18 (13-25)	-6.2 (-9.9--2.5)	155 (112-195)	155 (113-193)	0.2 (-4-3.6)	2 (1-3)	1 (1-2)	-21.2 (-48.4-21.7)	17 (12-24)	17 (13-24)	0.4 (-3.6-4.6)	0 (0-1)	1 (0-1)	18.7 (-28.1-88.1)	8 (6-13)	8 (5-13)	-0.7 (-6-5.1)	0 (0-0)	0 (0-0)	0 (0-0)	0 (0-0)	122	121	79	81
Eastern Cape	3 (2-4)	2 (2-3)	-27.5 (-33--19.6)	24 (17-31)	18 (13-23)	-24 (-29.5--18)	0 (0-0)	0 (0-0)	-35.3 (-55.7-2.4)	3 (2-4)	2 (2-3)	-23 (-30.8--13.3)	0 (0-0)	0 (0-0)	-35.4 (-63-18.1)	1 (1-2)	1 (1-2)	-24 (-34.1--11.2)	0 (0-0)	0 (0-0)	0 (0-0)	0 (0-0)	120	128	85	80
Free State	1 (1-1)	1 (1-1)	-24 (-29.8--14.9)	9 (6-11)	7 (5-9)	-19.3 (-25.4--11)	0 (0-0)	0 (0-0)	-35.7 (-60.4-7)	1 (1-1)	1 (1-1)	-18.4 (-27.3--6.5)	0 (0-0)	0 (0-0)	10.1 (-34.3-104)	0 (0-1)	0 (0-1)	-17.2 (-28.7--0.7)	0 (0-0)	0 (0-0)	0 (0-0)	0 (0-0)	113	117	77	83
Gauteng	3 (2-4)	4 (3-6)	42.2 (29.7-66.9)	24 (18-30)	38 (28-47)	59.6 (46-74)	0 (0-1)	0 (0-1)	-1.8 (-38.9-60.2)	3 (2-4)	4 (3-6)	59.6 (43.7-86.8)	0 (0-0)	0 (0-0)	75.5 (-18.4-248)	1 (1-2)	2 (1-3)	65 (42.4-105)	0 (0-0)	0 (0-0)	0 (0-0)	0 (0-0)	114	118	78	80
KwaZulu-Natal	4 (3-6)	4 (3-5)	-18.3 (-25.9--11.1)	36 (25-45)	32 (23-40)	-10.5 (-17.3--0.2)	0 (0-1)	0 (0-0)	-41.9 (-62.8--5.4)	4 (3-6)	4 (3-5)	-9.9 (-18.2-0.3)	0 (0-0)	0 (0-0)	27.1 (-34.2-134)	2 (1-3)	2 (1-3)	-12.7 (-23.5-0.4)	0 (0-0)	0 (0-0)	0 (0-0)	0 (0-0)	120	123	71	78
Limpopo	3 (2-4)	2 (2-3)	-23.8 (-29.2--16)	25 (18-32)	20 (14-25)	-22.7 (-28.1--16.5)	0 (0-0)	0 (0-0)	22.8 (-25.7-94.3)	3 (2-4)	2 (1-3)	-20.7 (-27.3--10.8)	0 (0-0)	0 (0-0)	42.7 (-25.6-167)	1 (1-2)	1 (1-2)	-20.6 (-30.3--7)	0 (0-0)	0 (0-0)	0 (0-0)	0 (0-0)	116	119	72	87

Location	Births, 2000	Births, 2021	Births percent change (2000-2021)	All age cases, 2000	All age cases, 2021	All age cases percent change (2000-2021)	All age deaths (cause-specific), 2000	All age deaths (cause-specific), 2021	All age deaths (cause-specific) percent change (2000-2021)	All age deaths (total), 2000	All age deaths (total), 2021	All age deaths (total) percent change (2000-2021)	Under-5 deaths (cause-specific), 2000	Under-5 deaths (cause-specific), 2021	Under-5 deaths (cause-specific) percent change (2000-2021)	Under-5 deaths (total), 2000	Under-5 deaths (total), 2021	Under-5 deaths (total) percent change (2000-2021)	Under-5 cause-specific mortality cause fraction, 2000	Under-5 total SCD mortality cause fraction, 2000	Under-5 cause-specific mortality cause fraction, 2021	Under-5 total SCD mortality cause fraction, 2021	Under-5 cause-specific mortality rank 2000	Under-5 cause-specific mortality rank 2021	Under-5 total SCD mortality rank 2000	Under-5 total SCD mortality rank 2021
Mpumalanga	1 (1-2)	2 (1-3)	21.9 (9-32.8)	13 (9-16)	16 (11-20)	24.4 (11.3-37.1)	0 (0-0)	0 (0-0)	-8.8 (-44.7-62.7)	1 (1-2)	2 (1-3)	26.1 (8.8-40.3)	0 (0-0)	0 (0-0)	98.4 (11.3-243)	1 (0-1)	1 (1-1)	26.7 (3.5-47.6)	0 (0-0)	0 (0-0)	0 (0-0)	0 (0-0)	115	117	74	79
North-West	1 (1-2)	1 (1-1)	-29.4 (-36--22.4)	12 (9-15)	9 (6-12)	-24.3 (-31.2--15.9)	0 (0-0)	0 (0-0)	-32.3 (-57.4-11)	1 (1-2)	1 (1-2)	-24.2 (-32.6--13.3)	0 (0-0)	0 (0-0)	-23.1 (-56.7-26.6)	1 (0-1)	1 (0-1)	-22.9 (-34--6.4)	0 (0-0)	0 (0-0)	0 (0-0)	0 (0-0)	114	118	79	77
Northern Cape	0 (0-0)	0 (0-1)	54.3 (32.1-76.8)	3 (2-3)	4 (3-5)	55.6 (40.5-74)	0 (0-0)	0 (0-0)	-2.5 (-37.9-61.3)	0 (0-0)	0 (0-1)	57.4 (38.6-83.6)	0 (0-0)	0 (0-0)	28.2 (-33.7-122)	0 (0-0)	0 (0-0)	51.5 (26.8-85.9)	0 (0-0)	0 (0-0)	0 (0-0)	0 (0-0)	117	118	83	80
Western Cape	1 (1-1)	1 (1-2)	4.8 (-14.9-25.2)	10 (7-13)	11 (8-16)	14.4 (-5.4-35.1)	0 (0-0)	0 (0-0)	-38.4 (-61.5--4.8)	1 (1-1)	1 (1-2)	15.6 (-4.6-36.8)	0 (0-0)	0 (0-0)	-38.8 (-76.4-7.2)	0 (0-1)	0 (0-1)	13.6 (-11.1-38.6)	0 (0-0)	0 (0-0)	0 (0-0)	0 (0-0)	120	122	87	78
Zimbabwe	71 (58-87)	68 (56-88)	-4.1 (-12.9-11.4)	813 (663-993)	873 (694-1,080)	7.4 (-1.7-24)	20 (14-29)	30 (19-49)	44.2 (-14.8-122)	48 (38-61)	53 (41-69)	9.1 (-2.2-28.5)	2 (1-3)	4 (2-6)	116 (6.8-281)	14 (10-19)	15 (11-21)	7.8 (-6.7-27.3)	0 (0-0)	0 (0-0.1)	0 (0-0)	0.1 (0-0.1)	87	90	48	62
**Western Sub-Saharan Africa**	229,000 (190,000-275,000)	290,000 (239,000-347,000)	26.3 (23-29.9)	2,460,000 (2,110,000-2,890,000)	4,130,000 (3,460,000-4,860,000)	67.6 (62.1-73)	18,500 (12,800-24,200)	23,700 (15,200-33,700)	28.1 (-3-74.1)	113,000 (91,900-138,000)	182,000 (149,000-225,000)	61.4 (56.3-66.4)	9,070 (5,380-13,400)	9,040 (5,100-12,900)	-0.3 (-28.9-43.1)	29,200 (21,600-39,200)	41,000 (30,000-54,300)	40.5 (33.4-46.6)	0.5 (0.3-0.7)	1.5 (1.1-2)	0.6 (0.4-0.8)	2.7 (1.9-3.6)	22	21	14	8
Benin	10,600 (8,770-12,300)	13,400 (11,100-16,100)	27 (14.3-39)	129,000 (110,000-147,000)	182,000 (148,000-215,000)	40.3 (26.4-53.1)	1,170 (540-1,890)	1,490 (772-2,370)	27.4 (-7.7-96.4)	4,900 (4,060-5,860)	7,450 (5,990-9,130)	52.3 (34.9-69.1)	660 (213-1,190)	686 (293-1,240)	3.9 (-32.9-87.1)	1,210 (879-1,640)	1,730 (1,240-2,350)	43.5 (24.3-64.2)	1.6 (0.5-2.8)	2.9 (2.1-3.9)	1.7 (0.8-2.8)	4.3 (2.9-6.2)	13	11	9	7
Burkina Faso	18,100 (16,800-19,400)	22,000 (18,100-25,700)	21 (-0.1-42.9)	221,000 (207,000-236,000)	281,000 (231,000-329,000)	27.2 (5.6-48.3)	2,180 (173-9,350)	2,650 (199-13,200)	21.6 (-21.3-89)	8,070 (7,120-9,210)	11,400 (9,380-13,800)	41.4 (19.3-66.8)	1,320 (74-5,820)	1,370 (70-7,560)	4.2 (-46.6-74.9)	2,070 (1,580-2,730)	2,910 (2,170-3,830)	40.7 (15.7-73.3)	1.3 (0.1-5.6)	2 (1.5-2.7)	1.5 (0.1-8.5)	3.3 (2.3-4.4)	14	13	12	9
Cabo Verde	15 (12-18)	12 (10-15)	-16.6 (-22--10.6)	291 (240-357)	291 (238-359)	-0.4 (-6.8-6.7)	3 (2-4)	2 (1-3)	-33.2 (-54.3--3.5)	10 (8-12)	11 (9-14)	17.9 (9.7-28.1)	1 (0-1)	0 (0-0)	-79.8 (-92.5--38.5)	2 (1-2)	1 (1-2)	-12.8 (-20.2--3.8)	0.1 (0.1-0.2)	0.3 (0.2-0.3)	0.1 (0.1-0.2)	1.2 (0.8-1.6)	46	52	35	16
Cameroon	5,050 (4,300-5,660)	7,310 (6,390-8,270)	44.7 (35.7-54.2)	64,300 (53,800-73,300)	126,000 (107,000-143,000)	95.6 (83.1-111)	686 (177-1,100)	970 (309-1,790)	41.4 (1.5-121)	2,470 (2,030-2,870)	4,980 (4,160-5,800)	101 (84.2-118)	265 (49-518)	281 (67-520)	5.9 (-27.5-76.3)	621 (419-852)	1,040 (715-1,440)	67.5 (45.7-90.5)	0.3 (0.1-0.7)	0.8 (0.6-1.1)	0.4 (0.1-0.7)	1.4 (1-2.1)	28	26	21	12
Chad	940 (825-1,060)	1,790 (1,560-2,010)	90.9 (78.1-104)	9,090 (7,670-10,500)	19,400 (16,600-22,400)	114 (96.9-129)	199 (90-337)	322 (155-579)	62.5 (15.7-143)	428 (353-502)	909 (748-1,050)	112 (98.2-128)	100 (32-199)	134 (50-237)	33.9 (-7.6-163)	167 (126-214)	341 (252-437)	104 (85.7-122)	0.1 (0-0.3)	0.2 (0.2-0.3)	0.1 (0-0.2)	0.3 (0.2-0.5)	37	36	34	26
Côte d'Ivoire	5,080 (4,550-5,470)	6,050 (5,400-6,640)	18.9 (12.5-26.2)	51,500 (45,400-56,300)	74,900 (66,800-83,600)	45.4 (36-56.2)	494 (316-748)	531 (337-737)	7.4 (-23.6-60.5)	2,790 (2,450-3,160)	4,080 (3,600-4,580)	46.4 (38.3-55)	180 (103-294)	153 (96-224)	-14.7 (-44.8-41.6)	1,090 (876-1,340)	1,470 (1,180-1,810)	34.8 (23.8-47.4)	0.2 (0.1-0.3)	1.2 (0.9-1.5)	0.2 (0.2-0.3)	2.3 (1.8-3.1)	34	34	15	10
Gambia	414 (365-470)	443 (380-513)	7 (-1.2-14.9)	4,830 (4,220-5,470)	7,480 (6,240-8,560)	54.6 (41.2-65.8)	74 (18-320)	94 (24-392)	26.9 (-19.1-111)	184 (155-214)	288 (242-336)	57 (46-70.2)	34 (6-149)	26 (6-129)	-24.5 (-73-83)	48 (36-63)	62 (46-81)	29.4 (16.1-45.2)	0.6 (0.1-2.6)	0.8 (0.6-1)	0.8 (0.2-4.1)	1.8 (1.2-2.4)	21	22	19	14
Ghana	11,900 (11,100-12,600)	13,700 (12,100-15,600)	15.1 (2.5-30.2)	171,000 (157,000-183,000)	239,000 (212,000-273,000)	39.4 (26.9-56.8)	2,110 (1,430-2,580)	2,390 (1,520-3,370)	13.2 (-17.3-58.5)	6,650 (6,110-7,210)	10,200 (8,940-11,600)	52.9 (34.1-73.8)	736 (454-1,040)	641 (265-1,120)	-12.9 (-47.9-46.3)	1,330 (1,050-1,660)	1,780 (1,380-2,280)	33.5 (15.4-54)	1.1 (0.7-1.6)	2 (1.6-2.5)	1.5 (0.7-2.5)	4.3 (3-6.4)	16	14	11	6
Guinea	6,560 (5,800-7,530)	9,200 (7,990-10,500)	40.2 (30-49.9)	76,700 (66,000-87,000)	129,000 (112,000-151,000)	68.2 (54.9-83.1)	653 (106-1,110)	803 (160-1,400)	22.9 (-13.4-127)	3,190 (2,630-3,740)	5,360 (4,430-6,280)	68.1 (56.1-83)	346 (39-658)	290 (57-524)	-16 (-46.2-98.6)	865 (588-1,190)	1,340 (933-1,830)	54.8 (36.3-77.1)	0.5 (0.1-1)	1.4 (0.9-1.9)	0.7 (0.1-1.2)	3 (2-4.2)	20	21	13	9
Guinea-Bissau	37 (30-44)	41 (33-50)	11.5 (4.6-21.3)	375 (304-449)	523 (429-633)	39.3 (28.9-51.5)	40 (9-179)	38 (8-172)	-5.2 (-49.9-70.6)	21 (17-26)	30 (24-37)	39.3 (29.8-50.9)	16 (2-71)	8 (1-44)	-49 (-80.5-26.4)	9 (6-12)	11 (8-16)	26.9 (14.9-41.2)	0.2 (0-0.9)	0.1 (0.1-0.1)	0.2 (0-1)	0.2 (0.2-0.3)	37	41	48	38
Liberia	669 (580-760)	682 (586-776)	2 (-1.9-6.3)	5,790 (4,920-6,570)	8,690 (7,360-9,790)	49.7 (42.6-56.7)	104 (36-293)	109 (34-401)	4.6 (-44.5-87.1)	387 (331-459)	554 (478-644)	43.1 (34.3-52.4)	50 (13-116)	24 (7-91)	-51 (-80-5.7)	194 (152-248)	229 (181-295)	17.7 (7.7-28.3)	0.2 (0.1-0.5)	0.8 (0.6-1)	0.2 (0.1-0.9)	2.3 (1.6-3.1)	34	32	19	12
Mali	3,620 (3,200-4,040)	6,720 (5,980-7,470)	86 (77.4-98.5)	36,900 (32,700-41,600)	78,300 (68,700-88,500)	112 (101-127)	769 (333-1,340)	995 (499-1,860)	29.3 (-11.9-90)	1,650 (1,420-1,890)	3,530 (3,030-4,050)	114 (101-127)	410 (135-807)	389 (166-754)	-5.1 (-42.1-94.8)	526 (404-673)	1,040 (799-1,350)	98.2 (82.6-116)	0.4 (0.1-0.7)	0.5 (0.4-0.6)	0.4 (0.2-0.7)	1 (0.7-1.2)	23	25	20	17
Mauritania	208 (174-243)	221 (183-251)	6.2 (-1.9-12.6)	2,880 (2,400-3,430)	4,310 (3,590-5,110)	49.5 (37.4-60.1)	38 (20-58)	34 (19-52)	-11.6 (-37.8-33.1)	108 (87-128)	162 (130-191)	50.4 (38.6-62.9)	15 (7-26)	8 (4-13)	-46.3 (-64.8--3)	29 (21-38)	34 (25-44)	17.3 (5.2-30)	0.2 (0.1-0.3)	0.4 (0.2-0.5)	0.2 (0.1-0.3)	0.8 (0.5-1.1)	36	40	28	21
Niger	9,640 (8,280-11,300)	14,500 (12,500-16,600)	50 (34.5-63.2)	89,400 (78,200-104,000)	157,000 (134,000-183,000)	75.3 (55-91.9)	599 (225-1,020)	728 (349-1,070)	21.7 (-15.5-111)	3,940 (3,370-4,650)	7,160 (6,120-8,490)	81.5 (66.2-97.4)	363 (105-725)	345 (143-531)	-5.1 (-35-106)	1,380 (1,080-1,770)	2,510 (1,980-3,180)	81.7 (63.6-103)	0.3 (0.1-0.5)	1 (0.8-1.4)	0.3 (0.1-0.5)	2.2 (1.6-2.9)	27	25	15	13
Nigeria	145,000 (110,000-185,000)	179,000 (138,000-224,000)	23.3 (19.2-27.8)	1,460,000 (1,160,000-1,830,000)	2,590,000 (2,090,000-3,220,000)	77.4 (72-84.6)	7,970 (1,870-13,000)	11,000 (3,540-18,800)	37.7 (-5.9-116)	72,000 (54,500-94,300)	116,000 (89,300-150,000)	60.9 (53.9-67.5)	3,890 (675-7,320)	4,200 (1,030-7,350)	8.1 (-26.6-84.9)	18,200 (12,500-25,900)	24,400 (17,000-33,900)	34 (25-42)	0.4 (0.1-0.7)	1.9 (1.3-2.6)	0.5 (0.2-0.9)	3.1 (2-4.6)	23	21	11	8
Sao Tome and Principe	54 (44-67)	46 (37-56)	-14.9 (-21.1--7.4)	835 (670-1,030)	1,090 (871-1,340)	30.1 (18-39.1)	5 (1-8)	4 (1-8)	-11.5 (-41.2-53.9)	28 (22-36)	39 (30-49)	39 (25.6-53.7)	1 (0-2)	1 (0-1)	-56.9 (-77.4--10.4)	7 (4-9)	6 (4-9)	-3.2 (-15.7-10.5)	0.3 (0.1-0.6)	1.6 (1-2.2)	0.7 (0.2-1.6)	7.3 (4.5-10.7)	28	24	11	5
Senegal	2,160 (1,860-2,440)	2,620 (2,300-2,940)	21.6 (13-28.5)	29,800 (25,800-34,500)	45,000 (39,000-52,500)	51.3 (39.9-63.8)	331 (124-779)	347 (146-846)	4.8 (-32.1-65.4)	1,120 (936-1,300)	1,700 (1,430-1,970)	52.3 (42.1-63.4)	149 (50-297)	90 (34-238)	-39.4 (-66-10.7)	281 (207-362)	374 (278-495)	33.1 (18.9-48.3)	0.3 (0.1-0.6)	0.6 (0.5-0.8)	0.5 (0.2-1.2)	1.9 (1.4-2.5)	28	29	19	11
Sierra Leone	5,040 (4,440-5,640)	7,250 (6,360-8,170)	43.9 (33.8-53.4)	56,900 (48,700-65,900)	108,000 (92,600-125,000)	89.3 (75.5-102)	341 (44-604)	425 (69-798)	24.7 (-12.6-136)	2,390 (1,970-2,770)	4,580 (3,770-5,310)	91.3 (76.2-107)	214 (22-432)	156 (24-272)	-27.1 (-48.3-69.8)	635 (433-871)	1,060 (702-1,440)	66.2 (46.7-90.6)	0.5 (0-0.9)	1.4 (0.9-1.8)	0.5 (0.1-0.9)	3.7 (2.3-5.5)	21	21	15	8
Togo	4,200 (3,720-4,710)	4,870 (4,290-5,510)	16 (8.2-23.8)	54,000 (47,900-62,000)	80,600 (71,100-93,100)	49.1 (37.7-60.4)	772 (415-1,230)	849 (399-1,570)	10 (-25.3-74.3)	2,230 (1,950-2,580)	3,440 (2,990-3,960)	53.9 (44.9-64.2)	323 (139-534)	231 (112-440)	-28.6 (-57-38.8)	543 (414-696)	707 (535-918)	30.2 (20.1-42.8)	1.4 (0.7-2.3)	2.3 (1.7-3)	1.6 (0.8-3.1)	5.1 (3.5-6.8)	14	10	9	7

Table S10: Number of births, cases, total deaths, and under-5 deaths for Homozygous sickle cell and severe sickle cell/beta-thalassemia, 2000 - 2021

Location	Birth , 2000	Births percent change (2000-2021)	All age cases, 2000	All age cases percent change (2000-2021)	All age deaths, 2000	All age deaths percent change (2000-2021)	Under-5 deaths, 2000	Under-5 deaths percent change (2000-2021)	Births, 2021	All age cases, 2021	All age deaths, 2021	Under-5 deaths, 2021
Global	347,000 (273,000-443,000)	13.5 (10.9-16.4)	4,180,000 (3,400,000-5,160,000)	40.5 (36.8-44.4)	203,000 (154,000-269,000)	42.9 (38.9-47.4)	44,500 (27,600-67,000)	22.3 (16.6-28.4)	394,000 (312,000-498,000)	5,880,000 (4,720,000-7,280,000)	290,000 (221,000-378,000)	54,400 (34,000-81,500)
Central Europe, Eastern Europe, and Central Asia	3 (1-7)	1.9 (-26.6-15.7)	19 (7-39)	-5.4 (-35.7-9.5)	4 (1-8)	-0.3 (-31.4-14.1)	2 (1-5)	6.7 (-26-21.2)	3 (1-7)	18 (7-38)	4 (1-8)	3 (1-6)
**Central Asia**	1 (0-2)	17 (-18.5-21.5)	3 (0-8)	16.2 (-19.1-23.7)	1 (0-2)	17.7 (-18-22.6)	1 (0-1)	21.8 (-15.4-27.9)	1 (0-2)	3 (0-10)	1 (0-2)	1 (0-2)
Armenia	0 (0-0)	-21.2 (-45.3--19.3)	0 (0-0)	-19.8 (-45.1--9.4)	0 (0-0)	-18.6 (-44--14.4)	0 (0-0)	-11.7 (-39--4.2)	0 (0-0)	0 (0-0)	0 (0-0)	0 (0-0)
Azerbaijan	0 (0-0)	-6 (-34.1--2.9)	0 (0-1)	-2.1 (-33.5-8.2)	0 (0-0)	-3.2 (-32.7-1)	0 (0-0)	0.2 (-30.8-6.3)	0 (0-0)	0 (0-1)	0 (0-0)	0 (0-0)
Georgia	0 (0-0)	-14.7 (-41.4--12.6)	0 (0-0)	-22.9 (-48--13.1)	0 (0-0)	-20.2 (-46.5--16.2)	0 (0-0)	-12 (-40.3--5.7)	0 (0-0)	0 (0-0)	0 (0-0)	0 (0-0)
Kazakhstan	0 (0-0)	43 (-2.5-46.5)	1 (0-1)	39.9 (-1.6-55.6)	0 (0-0)	44.8 (-0.2-51.5)	0 (0-0)	56 (13.5-66.5)	0 (0-0)	1 (0-2)	0 (0-0)	0 (0-0)
Kyrgyzstan	0 (0-0)	40.4 (-4.4-44)	0 (0-1)	40.1 (0.7-55.4)	0 (0-0)	40.8 (-3.5-45.8)	0 (0-0)	46.7 (2.8-55.4)	0 (0-0)	0 (0-1)	0 (0-0)	0 (0-0)
Mongolia	0 (0-0)	32.6 (-8.7-37.6)	0 (0-0)	39.1 (-3.9-53)	0 (0-0)	41.8 (-3-48.5)	0 (0-0)	48.7 (-1.1-59.2)	0 (0-0)	0 (0-0)	0 (0-0)	0 (0-0)
Tajikistan	0 (0-0)	34.4 (-8.2-37.9)	0 (0-1)	32.7 (-5.6-43.4)	0 (0-0)	31.9 (-9.9-36.1)	0 (0-0)	34.3 (-6.3-41.2)	0 (0-0)	0 (0-1)	0 (0-0)	0 (0-0)
Turkmenistan	0 (0-0)	7.5 (-26.9-10)	0 (0-0)	7.8 (-24.7-17)	0 (0-0)	8.3 (-26.1-11.4)	0 (0-0)	11.4 (-23.7-17.4)	0 (0-0)	0 (0-1)	0 (0-0)	0 (0-0)
Uzbekistan	0 (0-1)	9.5 (-24-14.2)	1 (0-3)	18.6 (-19-31.4)	0 (0-1)	17.9 (-18.7-24.1)	0 (0-1)	23.6 (-15.7-33.1)	0 (0-1)	1 (0-3)	0 (0-1)	0 (0-1)
**Central Europe**	2 (1-3)	-10 (-41-16.7)	10 (5-20)	-19.3 (-48-5.5)	2 (1-4)	-16.1 (-44.8-8)	1 (1-2)	-10.5 (-41.2-14.9)	1 (1-2)	8 (5-14)	2 (1-3)	1 (1-2)
Albania	0 (0-0)	-38.9 (-39--38.9)	1 (0-1)	-44.2 (-47.5--40.9)	0 (0-0)	-46.6 (-47.3--46.2)	0 (0-0)	-44.7 (-46.1--43.6)	0 (0-0)	0 (0-0)	0 (0-0)	0 (0-0)
Bosnia and Herzegovina	0 (0-0)	-39.8 (-40--40)	0 (0-0)	-41.2 (-46.7--35.1)	0 (0-0)	-41.8 (-42.5--41.1)	0 (0-0)	-42.5 (-45--40)	0 (0-0)	0 (0-0)	0 (0-0)	0 (0-0)
Bulgaria	0 (0-0)	-19.5 (-19.6--19.6)	0 (0-0)	-15.7 (-22.2--8.7)	0 (0-0)	-11.2 (-13.1--10.4)	0 (0-0)	-7.5 (-10.9--4.5)	0 (0-0)	0 (0-0)	0 (0-0)	0 (0-0)
Croatia	0 (0-0)	-20.5 (-20.2--20.2)	0 (0-0)	-25.4 (-31.6--18.4)	0 (0-0)	-26.6 (-27.2--25)	0 (0-0)	-29.6 (-32.1--26.3)	0 (0-0)	0 (0-0)	0 (0-0)	0 (0-0)
Czechia	0 (0-0)	18 (17.9-17.9)	0 (0-1)	5 (-4.9-16.2)	0 (0-0)	13.7 (9.3-17.4)	0 (0-0)	27.6 (22.4-33)	0 (0-0)	0 (0-1)	0 (0-0)	0 (0-0)
Hungary	0 (0-0)	-20.6 (-56.6--19.3)	0 (0-1)	-19 (-56.8--10)	0 (0-0)	-17.9 (-55.2--15.7)	0 (0-0)	-14.2 (-52.8--9.1)	0 (0-0)	0 (0-0)	0 (0-0)	0 (0-0)
Montenegro	0 (0-0)	-22.1 (-23.8-52.1)	0 (0-0)	-19.7 (-28.2-56.8)	0 (0-0)	-21.4 (-23.5-52.6)	0 (0-0)	-22.3 (-26.4-45.6)	0 (0-0)	0 (0-0)	0 (0-0)	0 (0-0)
North Macedonia	0 (0-0)	-15.1 (-12.8--12.8)	0 (0-0)	-16.3 (-22.8--5.6)	0 (0-0)	-17.6 (-16.6--14.6)	0 (0-0)	-15.5 (-16.9--10.2)	0 (0-0)	0 (0-0)	0 (0-0)	0 (0-0)
Poland	1 (1-3)	-5.1 (-42-31.8)	8 (4-17)	-18.6 (-50-13.2)	2 (1-3)	-14 (-47.5-18.9)	1 (0-2)	-6.8 (-43.2-28.7)	1 (1-2)	7 (4-12)	1 (1-2)	1 (1-2)
Romania	0 (0-0)	-27.6 (-27.6--27.6)	0 (0-1)	-25.7 (-31.5--20.2)	0 (0-0)	-23.1 (-24--22.6)	0 (0-0)	-20.7 (-22.9--18.6)	0 (0-0)	0 (0-1)	0 (0-0)	0 (0-0)
Serbia	0 (0-0)	-25.8 (-25.8--25.8)	0 (0-1)	-29.8 (-36.4--22.9)	0 (0-0)	-32 (-32.9--30.9)	0 (0-0)	-33.4 (-35.9--30.9)	0 (0-0)	0 (0-0)	0 (0-0)	0 (0-0)
Slovakia	0 (0-0)	3 (-38.9-4.4)	0 (0-0)	-10.4 (-44--0.4)	0 (0-0)	-7.9 (-44.8--4.5)	0 (0-0)	-1.2 (-41.5-4.1)	0 (0-0)	0 (0-0)	0 (0-0)	0 (0-0)
Slovenia	0 (0-0)	1.6 (1.6-2)	0 (0-0)	-3.2 (-12.5-6.6)	0 (0-0)	3.7 (1.2-5.4)	0 (0-0)	6.4 (1.8-11.2)	0 (0-0)	0 (0-0)	0 (0-0)	0 (0-0)
**Eastern Europe**	1 (0-2)	10.6 (9.2-12.5)	6 (0-15)	8.1 (1.6-13.9)	1 (0-3)	14.7 (9.1-18.6)	1 (0-2)	24.9 (22.8-27.7)	1 (0-3)	6 (0-16)	1 (0-3)	1 (0-2)
Belarus	0 (0-0)	4.2 (4.2-4.2)	0 (0-1)	-1.4 (-10.1-8.3)	0 (0-0)	5.8 (0.9-8.8)	0 (0-0)	17.3 (12.4-22)	0 (0-0)	0 (0-1)	0 (0-0)	0 (0-0)
Estonia	0 (0-0)	1.7 (1.8-1.8)	0 (0-0)	-4.9 (-13.4-5.8)	0 (0-0)	-0.9 (-4.3-1.9)	0 (0-0)	8.4 (4-13.3)	0 (0-0)	0 (0-0)	0 (0-0)	0 (0-0)

Location	Birth , 2000	Births percent change (2000-2021)	All age cases, 2000	All age cases percent change (2000-2021)	All age deaths, 2000	All age deaths percent change (2000-2021)	Under-5 deaths, 2000	Under-5 deaths percent change (2000-2021)	Births, 2021	All age cases, 2021	All age deaths, 2021	Under-5 deaths, 2021
Latvia	0 (0-0)	-9 (-9.6--9.6)	0 (0-0)	-15.1 (-23.9--5.2)	0 (0-0)	-9.9 (-15.8--6.6)	0 (0-0)	6.1 (0.8-11.1)	0 (0-0)	0 (0-0)	0 (0-0)	0 (0-0)
Lithuania	0 (0-0)	-21.1 (-20.9--20.9)	0 (0-0)	-32.9 (-38.7--26.2)	0 (0-0)	-31.5 (-33.8--29.5)	0 (0-0)	-24.6 (-27.3--21)	0 (0-0)	0 (0-0)	0 (0-0)	0 (0-0)
Republic of Moldova	0 (0-0)	-34.3 (-34.3--34.3)	0 (0-0)	-34.7 (-40--29.5)	0 (0-0)	-35 (-35.9--34.1)	0 (0-0)	-32.4 (-34.4--30.4)	0 (0-0)	0 (0-0)	0 (0-0)	0 (0-0)
Russian Federation	1 (0-2)	20.9 (20.2-20.9)	4 (0-10)	19.4 (12-26.5)	1 (0-2)	27.1 (20.7-31.2)	0 (0-1)	39.4 (37.7-40.6)	1 (0-2)	5 (0-11)	1 (0-2)	1 (0-2)
Ukraine	0 (0-1)	-13.3 (-13.2--13.2)	1 (0-4)	-14.9 (-21.6--7.1)	0 (0-1)	-11.8 (-14.2--10)	0 (0-0)	-7.2 (-10.6--2.9)	0 (0-0)	1 (0-3)	0 (0-1)	0 (0-0)
High-income	1,880 (1,650-2,190)	-10.6 (-12.3--8.9)	57,400 (52,100-63,300)	4.4 (3-5.9)	1,760 (1,520-2,070)	2.3 (-0.2-5.1)	354 (245-483)	-10.2 (-14.2--5.9)	1,680 (1,470-1,950)	60,000 (54,700-66,000)	1,800 (1,560-2,100)	318 (220-438)
**Australasia**	6 (4-7)	32.3 (23.3-43.9)	111 (88-139)	21 (13.3-30.5)	6 (4-7)	24.1 (15.2-35.6)	3 (2-4)	21.5 (9.6-37.1)	7 (6-9)	135 (106-172)	7 (5-9)	3 (2-4)
Australia	4 (3-5)	29.3 (17.8-44.3)	74 (56-95)	21 (10.1-35)	4 (3-5)	21.8 (10.7-35.8)	2 (1-3)	19.6 (5.2-38)	5 (4-6)	89 (67-117)	5 (4-6)	2 (2-3)
New Zealand	2 (1-2)	38.7 (22.8-58.3)	38 (30-46)	21.1 (8.8-34.3)	2 (1-2)	29.1 (13.6-47.3)	1 (0-1)	26.8 (5.7-50.4)	2 (2-3)	46 (37-56)	2 (2-3)	1 (1-1)
**High-income Asia Pacific**	4 (3-6)	-26.3 (-33.3--21.6)	83 (56-120)	-14.8 (-22.3--7.3)	5 (3-7)	-15.6 (-23.4--9.2)	2 (2-4)	-26.7 (-35.1--20.6)	3 (2-4)	70 (48-101)	4 (3-6)	2 (1-3)
Brunei Darussalam	0 (0-0)	-11.6 (-21.8--10.7)	0 (0-0)	11.9 (-4-28.1)	0 (0-0)	-3.6 (-17.4-2.5)	0 (0-0)	-14.2 (-29.2--7)	0 (0-0)	0 (0-0)	0 (0-0)	0 (0-0)
Japan	2 (1-3)	-23.9 (-29.6--16.3)	50 (32-84)	-14.8 (-20.5--7)	3 (2-4)	-12.7 (-19.7--4.2)	1 (1-2)	-20.7 (-27.6--11.9)	2 (1-3)	43 (28-69)	2 (1-3)	1 (0-1)
Aichi	1 (1-1)	-22.4 (-32.5--10.7)	17 (13-23)	-10.3 (-20.7-2.9)	1 (1-1)	-9 (-20.7-3.8)	0 (0-0)	-19 (-32.2--4.9)	1 (0-1)	16 (12-20)	1 (1-1)	0 (0-0)
Akita	0 (0-0)	-49.1 (-48.9--48.8)	0 (0-1)	-42.1 (-47.1--36.6)	0 (0-0)	-41.7 (-43.9--39)	0 (0-0)	-49.2 (-52.5--45.1)	0 (0-0)	0 (0-0)	0 (0-0)	0 (0-0)
Aomori	0 (0-0)	-44.2 (-44.7--44.6)	0 (0-1)	-37.9 (-43.7--32.3)	0 (0-0)	-36.9 (-39.2--35)	0 (0-0)	-43.8 (-47.6--39.3)	0 (0-0)	0 (0-0)	0 (0-0)	0 (0-0)
Chiba	0 (0-0)	-27.7 (-28.2--8.5)	5 (3-7)	-17.9 (-27-2.8)	0 (0-0)	-14.6 (-18.7-7.4)	0 (0-0)	-23.5 (-31.9--4.4)	0 (0-0)	4 (2-6)	0 (0-0)	0 (0-0)
Ehime	0 (0-0)	-38 (-38.2--38.1)	0 (0-1)	-27.9 (-33.5--22.3)	0 (0-0)	-27.6 (-29.8--25.7)	0 (0-0)	-34.9 (-39.3--31)	0 (0-0)	0 (0-0)	0 (0-0)	0 (0-0)
Fukui	0 (0-0)	-34.2 (-34.1--33.9)	0 (0-0)	-24 (-33.1--16.3)	0 (0-0)	-24.9 (-27.7--22.1)	0 (0-0)	-34.2 (-41.4--28.5)	0 (0-0)	0 (0-0)	0 (0-0)	0 (0-0)
Fukuoka	0 (0-0)	-16.5 (-18.3--18.1)	1 (0-2)	-12.6 (-21.8--5.4)	0 (0-0)	-9.1 (-13.4--8.6)	0 (0-0)	-12 (-19.9--6)	0 (0-0)	1 (0-2)	0 (0-0)	0 (0-0)
Fukushima	0 (0-0)	-44.4 (-44.3--44.1)	0 (0-1)	-36.2 (-42.3--30.6)	0 (0-0)	-35.2 (-38--32.2)	0 (0-0)	-42.4 (-46.7--38)	0 (0-0)	0 (0-1)	0 (0-0)	0 (0-0)
Gifu	0 (0-0)	-37.6 (-36.9--36.8)	0 (0-1)	-24.7 (-30.1--17)	0 (0-0)	-25.5 (-27--21.7)	0 (0-0)	-34.9 (-39--29.3)	0 (0-0)	0 (0-1)	0 (0-0)	0 (0-0)
Gunma	0 (0-0)	-39.5 (-42.6--38.7)	0 (0-1)	-24.8 (-33--16.9)	0 (0-0)	-25.8 (-29.9--21.9)	0 (0-0)	-36.9 (-42.4--31.1)	0 (0-0)	0 (0-1)	0 (0-0)	0 (0-0)
Hiroshima	0 (0-0)	-28.2 (-28.6--28.5)	0 (0-1)	-16.8 (-24.7--9.2)	0 (0-0)	-16.3 (-19--14.1)	0 (0-0)	-24.4 (-31.2--18.7)	0 (0-0)	0 (0-1)	0 (0-0)	0 (0-0)
Hokkaidō	0 (0-0)	-41 (-72--37.9)	1 (0-2)	-32.9 (-69.7--22.1)	0 (0-0)	-31 (-68--24.9)	0 (0-0)	-37.2 (-67.4--29.4)	0 (0-0)	1 (0-2)	0 (0-0)	0 (0-0)
Hyōgo	0 (0-0)	-30.6 (-54.4--29.3)	2 (1-4)	-21.3 (-46.1--11.4)	0 (0-0)	-20.4 (-47.1--15.9)	0 (0-0)	-29.2 (-56--21.9)	0 (0-0)	2 (1-3)	0 (0-0)	0 (0-0)
Ibaraki	0 (0-0)	-38.6 (-58.9--36.9)	2 (1-3)	-26.2 (-47.5--11.8)	0 (0-0)	-26.7 (-49.8--21.3)	0 (0-0)	-35.7 (-59--27.2)	0 (0-0)	2 (1-2)	0 (0-0)	0 (0-0)
Ishikawa	0 (0-0)	-32 (-31.5--31.3)	0 (0-1)	-21.9 (-29.2--13.5)	0 (0-0)	-21.6 (-23.6--18)	0 (0-0)	-29.9 (-36.1--23.2)	0 (0-0)	0 (0-0)	0 (0-0)	0 (0-0)
Iwate	0 (0-0)	-43.3 (-43.2--43.1)	0 (0-1)	-32.5 (-38.1--25.8)	0 (0-0)	-32.2 (-34.3--29.9)	0 (0-0)	-40.2 (-44.7--35.2)	0 (0-0)	0 (0-0)	0 (0-0)	0 (0-0)
Kagawa	0 (0-0)	-37.2 (-35.6--35.3)	0 (0-0)	-24.9 (-33--15.9)	0 (0-0)	-25.5 (-27.7--20.8)	0 (0-0)	-34.3 (-41.3--27.4)	0 (0-0)	0 (0-0)	0 (0-0)	0 (0-0)

Location	Birth , 2000	Births percent change (2000-2021)	All age cases, 2000	All age cases percent change (2000-2021)	All age deaths, 2000	All age deaths percent change (2000-2021)	Under-5 deaths, 2000	Under-5 deaths percent change (2000-2021)	Births, 2021	All age cases, 2021	All age deaths, 2021	Under-5 deaths, 2021
Kagoshima	0 (0-0)	-29.8 (-29--28.8)	0 (0-1)	-28.5 (-36.3--19.9)	0 (0-0)	-25.9 (-27.4--23.3)	0 (0-0)	-26.8 (-35.7--20.1)	0 (0-0)	0 (0-1)	0 (0-0)	0 (0-0)
Kanagawa	0 (0-0)	-27.8 (-48.5--26.8)	2 (0-6)	-15.1 (-26.2--4.3)	0 (0-0)	-11.5 (-33.6--6.2)	0 (0-0)	-21.4 (-45.2--10.5)	0 (0-0)	2 (0-5)	0 (0-0)	0 (0-0)
Kōchi	0 (0-0)	-39.1 (-39.8--39.7)	0 (0-0)	-31.6 (-37.6--24.8)	0 (0-0)	-30.7 (-33.3--28.9)	0 (0-0)	-35.9 (-40.6--29.7)	0 (0-0)	0 (0-0)	0 (0-0)	0 (0-0)
Kumamoto	0 (0-0)	-28.4 (-58.9--25.2)	0 (0-1)	-24.9 (-56.1--12.5)	0 (0-0)	-22.2 (-54.8--16.7)	0 (0-0)	-23.6 (-55.7--12.9)	0 (0-0)	0 (0-1)	0 (0-0)	0 (0-0)
Kyōto	0 (0-0)	-25.8 (-43.6--18.3)	0 (0-1)	-18.4 (-31.3--6.7)	0 (0-0)	-16.5 (-36.3--7)	0 (0-0)	-24.2 (-43.5--13.4)	0 (0-0)	0 (0-1)	0 (0-0)	0 (0-0)
Mie	0 (0-0)	-36 (-36--35.9)	0 (0-1)	-23.4 (-30.4--12.5)	0 (0-0)	-23.4 (-26.3--19.5)	0 (0-0)	-33.3 (-37.9--24.9)	0 (0-0)	0 (0-1)	0 (0-0)	0 (0-0)
Miyagi	0 (0-0)	-33.4 (-32.4--32.2)	0 (0-1)	-23.3 (-28.5--15.2)	0 (0-0)	-21 (-22.1--17.2)	0 (0-0)	-28.8 (-33.4--23.6)	0 (0-0)	0 (0-1)	0 (0-0)	0 (0-0)
Miyazaki	0 (0-0)	-34.4 (-34.6--34.5)	0 (0-1)	-26.6 (-34.4--17.2)	0 (0-0)	-26.4 (-28.5--24)	0 (0-0)	-31.3 (-37.9--22.6)	0 (0-0)	0 (0-0)	0 (0-0)	0 (0-0)
Nagano	0 (0-0)	-39.9 (-45.4--39.6)	0 (0-1)	-26.8 (-35--19.2)	0 (0-0)	-27.3 (-33.2--24)	0 (0-0)	-37.2 (-46.9--31.4)	0 (0-0)	0 (0-1)	0 (0-0)	0 (0-0)
Nagasaki	0 (0-0)	-35.4 (-42--34.2)	0 (0-1)	-31.7 (-42.7--23.8)	0 (0-0)	-30.4 (-37.6--27.4)	0 (0-0)	-34.7 (-44.8--28.1)	0 (0-0)	0 (0-0)	0 (0-0)	0 (0-0)
Nara	0 (0-0)	-38.3 (-39--38.9)	0 (0-1)	-28.3 (-35--20.3)	0 (0-0)	-27.2 (-30.6--25)	0 (0-0)	-34.7 (-40.5--30.6)	0 (0-0)	0 (0-0)	0 (0-0)	0 (0-0)
Niigata	0 (0-0)	-36.8 (-37.9--17.4)	0 (0-1)	-27.4 (-33.7--14.3)	0 (0-0)	-26.9 (-30.4--7)	0 (0-0)	-35.1 (-42--8.6)	0 (0-0)	0 (0-1)	0 (0-0)	0 (0-0)
Ōita	0 (0-0)	-31.7 (-41.4--30.1)	0 (0-1)	-25.3 (-35.3--16.6)	0 (0-0)	-24 (-34.3--20.4)	0 (0-0)	-28.6 (-39.9--21.7)	0 (0-0)	0 (0-0)	0 (0-0)	0 (0-0)
Okayama	0 (0-0)	-28.6 (-27.7--27.6)	0 (0-1)	-18.9 (-26.9--10.1)	0 (0-0)	-19.2 (-20.3--16.2)	0 (0-0)	-25.8 (-31.2--19.4)	0 (0-0)	0 (0-1)	0 (0-0)	0 (0-0)
Okinawa	0 (0-0)	-12.5 (-25.4--10.9)	1 (0-1)	-8.9 (-22.3-3.1)	0 (0-0)	-5.3 (-18--1.3)	0 (0-0)	-7.7 (-28.9-4.5)	0 (0-0)	1 (0-1)	0 (0-0)	0 (0-0)
Ōsaka	0 (0-0)	-30.7 (-30.3--30.2)	2 (0-5)	-20.2 (-27.2--11.8)	0 (0-0)	-18.8 (-21.1--14.5)	0 (0-0)	-28.1 (-33.7--21.5)	0 (0-0)	2 (0-4)	0 (0-0)	0 (0-0)
Saga	0 (0-0)	-31.9 (-32--31.9)	0 (0-0)	-24.4 (-30.4--18.2)	0 (0-0)	-23.7 (-25.6--21.7)	0 (0-0)	-29.8 (-34.7--24.5)	0 (0-0)	0 (0-0)	0 (0-0)	0 (0-0)
Saitama	0 (0-0)	-28.6 (-28.1--27.9)	1 (0-3)	-18.2 (-25.5--9.6)	0 (0-0)	-15.7 (-18--11)	0 (0-0)	-24.2 (-30.9--15.7)	0 (0-0)	1 (0-3)	0 (0-0)	0 (0-0)
Shiga	0 (0-0)	-29 (-64.7--26.2)	0 (0-1)	-15.4 (-59.1--5.5)	0 (0-0)	-15.5 (-58.7--9.4)	0 (0-0)	-25 (-61--16.4)	0 (0-0)	0 (0-1)	0 (0-0)	0 (0-0)
Shimane	0 (0-0)	-28.9 (-30.8--5.8)	0 (0-0)	-23 (-31.5--8.3)	0 (0-0)	-22 (-25.6-0.5)	0 (0-0)	-25.5 (-32.4-11.2)	0 (0-0)	0 (0-0)	0 (0-0)	0 (0-0)
Shizuoka	0 (0-0)	-35.4 (-34.2--34)	1 (0-2)	-25 (-32.9--16.3)	0 (0-0)	-24.1 (-25.4--19.9)	0 (0-0)	-31.8 (-38.1--25.5)	0 (0-0)	0 (0-1)	0 (0-0)	0 (0-0)
Tochigi	0 (0-0)	-33.9 (-33.9--33.7)	0 (0-1)	-22.2 (-29--15.8)	0 (0-0)	-21.3 (-23.6--18.6)	0 (0-0)	-28.8 (-33.6--23.4)	0 (0-0)	0 (0-1)	0 (0-0)	0 (0-0)
Tokushima	0 (0-0)	-36.7 (-35.6--35.5)	0 (0-0)	-31 (-37.3--23.4)	0 (0-0)	-29.5 (-30--26.1)	0 (0-0)	-33.4 (-37.9--26.9)	0 (0-0)	0 (0-0)	0 (0-0)	0 (0-0)
Tōkyō	0 (0-0)	5.3 (-23.4-7.4)	7 (3-12)	0 (-24.4-11.4)	0 (0-1)	8.8 (-19.5-14.8)	0 (0-0)	10.5 (-23.2-23.3)	0 (0-0)	7 (3-12)	0 (0-1)	0 (0-0)
Tottori	0 (0-0)	-30.7 (-30.9--30.8)	0 (0-0)	-24.8 (-30.4--17.9)	0 (0-0)	-23.2 (-24.9--21.9)	0 (0-0)	-26.8 (-32.5--21)	0 (0-0)	0 (0-0)	0 (0-0)	0 (0-0)
Toyama	0 (0-0)	-38.3 (-38.7--38.5)	0 (0-1)	-24.4 (-31.6--17.9)	0 (0-0)	-24.4 (-27.3--21.8)	0 (0-0)	-32.9 (-38--26.8)	0 (0-0)	0 (0-0)	0 (0-0)	0 (0-0)
Wakayama	0 (0-0)	-41.3 (-40.7--40.5)	0 (0-0)	-32 (-37.7--25.4)	0 (0-0)	-31.7 (-33.1--28.6)	0 (0-0)	-39.1 (-43.7--33.2)	0 (0-0)	0 (0-0)	0 (0-0)	0 (0-0)
Yamagata	0 (0-0)	-41.1 (-56.6--12)	0 (0-1)	-31.9 (-53.4--7)	0 (0-0)	-32.3 (-50.8--1.4)	0 (0-0)	-39.6 (-61.8--0.3)	0 (0-0)	0 (0-0)	0 (0-0)	0 (0-0)
Yamaguchi	0 (0-0)	-37 (-36.9--36.8)	0 (0-1)	-27.9 (-35.6--20.1)	0 (0-0)	-27.4 (-29.5--24.8)	0 (0-0)	-33.1 (-38.6--25.7)	0 (0-0)	0 (0-0)	0 (0-0)	0 (0-0)

Location	Birth , 2000	Births percent change (2000-2021)	All age cases, 2000	All age cases percent change (2000-2021)	All age deaths, 2000	All age deaths percent change (2000-2021)	Under-5 deaths, 2000	Under-5 deaths percent change (2000-2021)	Births, 2021	All age cases, 2021	All age deaths, 2021	Under-5 deaths, 2021
Yamanashi	0 (0-0)	-39.8 (-40.1--40)	0 (0-0)	-28.8 (-34.7--22.1)	0 (0-0)	-29.8 (-32.6--26.9)	0 (0-0)	-40.9 (-45.9--36)	0 (0-0)	0 (0-0)	0 (0-0)	0 (0-0)
Republic of Korea	2 (1-3)	-30.9 (-43.2--30.5)	29 (18-43)	-17.7 (-32.7--3.4)	2 (1-3)	-22 (-35.6--15.5)	1 (1-2)	-34.3 (-46.9--25.9)	1 (1-2)	24 (15-36)	2 (1-2)	1 (1-1)
Singapore	0 (0-0)	-7.5 (-18-3)	3 (2-4)	11.3 (-4.9-28.6)	0 (0-0)	23.5 (11.2-38.6)	0 (0-0)	20.7 (5.3-41.3)	0 (0-0)	3 (2-5)	0 (0-0)	0 (0-0)
**High-income North America**	921 (846-1,010)	-9.2 (-11--7.8)	40,500 (37,400-44,000)	8.6 (6.6-10.4)	755 (675-841)	10.7 (6.8-15.1)	46 (34-59)	-13.7 (-16.6--10.6)	836 (767-911)	44,000 (40,800-47,700)	836 (760-919)	39 (30-51)
Canada	2 (1-2)	23.3 (-28.5-78.6)	80 (58-110)	18.9 (-32.1-66.8)	2 (1-2)	32.3 (-23.1-94)	0 (0-0)	18.3 (-32.9-80.6)	2 (1-3)	94 (60-134)	2 (1-3)	0 (0-0)
Greenland	0 (0-0)	-8.9 (-16.8-252)	0 (0-0)	-5.5 (-15.9-239)	0 (0-0)	-0.6 (-13.6-302)	0 (0-0)	-10.8 (-24.7-261)	0 (0-0)	0 (0-0)	0 (0-0)	0 (0-0)
United States of America	915 (841-1,000)	-12 (-13.7--10.6)	40,400 (37,300-43,900)	9.8 (7.9-11.7)	751 (672-837)	11.8 (7.9-16.3)	45 (34-58)	-12.5 (-15.6--9.4)	805 (739-877)	44,300 (41,100-48,100)	840 (764-924)	40 (30-51)
Alabama	29 (26-32)	-10.3 (-16--4.4)	1,240 (1,130-1,350)	5.8 (-0.1-12.1)	25 (22-28)	5.5 (-1.7-12.9)	2 (1-2)	-13.7 (-23.6--3.4)	26 (24-29)	1,310 (1,210-1,430)	26 (24-29)	1 (1-2)
Alaska	1 (1-1)	-21.1 (-28.7--14.1)	31 (21-46)	2.5 (-7.3-11)	1 (0-1)	2.3 (-7.9-13)	0 (0-0)	-17.8 (-28.8--6.6)	1 (0-1)	32 (21-46)	1 (0-1)	0 (0-0)
Arizona	9 (8-10)	-4.2 (-10.8-2.6)	359 (322-399)	40.3 (31.1-49.6)	6 (6-7)	49.2 (38.4-60.9)	0 (0-1)	1.9 (-9.3-13.4)	9 (8-10)	504 (451-559)	10 (8-11)	0 (0-1)
Arkansas	12 (10-13)	-17.6 (-23.4--12)	502 (445-560)	0.4 (-6.5-6.5)	10 (9-12)	-1.8 (-9.1-5.4)	1 (1-1)	-19.1 (-28.1--9.5)	10 (9-11)	504 (446-563)	10 (9-11)	1 (0-1)
California	53 (49-59)	-22.7 (-27.2--17.5)	2,310 (2,120-2,540)	7.7 (1.5-14.3)	38 (33-42)	16 (8.5-25.6)	3 (2-3)	-21.6 (-30.3--12.4)	41 (38-45)	2,490 (2,270-2,720)	44 (39-48)	2 (2-3)
Colorado	4 (3-4)	-9.4 (-17.4--2.5)	162 (136-188)	14.7 (5.3-23.9)	3 (2-4)	18.4 (7.8-29.7)	0 (0-0)	-15.2 (-25.3--4.3)	3 (3-4)	186 (155-221)	4 (3-4)	0 (0-0)
Connecticut	9 (9-10)	-28.3 (-32.5--23.1)	460 (415-507)	-3.7 (-9.2-2.5)	9 (8-10)	-1.1 (-8.3-7.9)	0 (0-1)	-29.5 (-36.5--21.2)	7 (6-8)	443 (401-489)	9 (8-10)	0 (0-0)
Delaware	5 (5-6)	-3.3 (-9.3-3.9)	225 (200-250)	19 (11.6-27.6)	4 (4-5)	22.6 (13.8-34.2)	0 (0-0)	-5.7 (-15.1-5.4)	5 (4-6)	267 (241-296)	5 (5-6)	0 (0-0)
District of Columbia	7 (6-8)	1 (-8.5-11.6)	268 (229-310)	-0.3 (-8.6-8.8)	6 (5-7)	-2.4 (-13.1-8.7)	0 (0-1)	-8.5 (-21.9-7.2)	7 (5-8)	267 (221-313)	6 (5-7)	0 (0-1)
Florida	93 (86-102)	-1.5 (-7.4-4.5)	4,220 (3,910-4,580)	26.4 (19.9-33.4)	86 (77-95)	27 (18.7-35.2)	5 (4-7)	0.1 (-11.7-12.8)	92 (84-100)	5,330 (4,930-5,780)	109 (98-119)	5 (4-7)
Georgia	61 (56-67)	-6.4 (-11.4--1.1)	2,450 (2,260-2,670)	25.5 (18.8-32.4)	45 (40-51)	27.7 (18.9-37.6)	3 (2-4)	-5.7 (-15.8-5.3)	57 (52-63)	3,080 (2,830-3,340)	58 (52-64)	3 (2-4)
Hawaii	1 (0-1)	-44.1 (-76.6--27)	22 (10-40)	-26.3 (-68.8--2.7)	0 (0-1)	-24.8 (-67.8-0.1)	0 (0-0)	-40.5 (-74.5--19.2)	0 (0-1)	16 (6-35)	0 (0-1)	0 (0-0)
Idaho	0 (0-0)	15 (0.9-31.7)	11 (7-15)	39.3 (21.5-59.7)	0 (0-0)	36.5 (20.1-56.1)	0 (0-0)	9.7 (-7.1-33.1)	0 (0-0)	15 (11-21)	0 (0-0)	0 (0-0)
Illinois	32 (29-35)	-39 (-42.7--35)	1,330 (1,210-1,460)	-12.3 (-16.9--6.8)	25 (22-28)	-11.3 (-17.3--3.9)	2 (1-2)	-36.9 (-43.8--29)	19 (17-21)	1,170 (1,060-1,280)	22 (20-25)	1 (1-1)
Indiana	14 (12-15)	-14.3 (-20.1--8.5)	580 (528-640)	3.3 (-2.9-9.5)	11 (10-13)	1.7 (-5.9-9.4)	1 (1-1)	-18.2 (-27--7.9)	12 (11-13)	599 (545-658)	12 (10-13)	1 (0-1)
Iowa	3 (3-4)	2.6 (-4.2-10.5)	164 (141-189)	12.5 (5.3-20.9)	3 (3-4)	13.1 (5.5-23.4)	0 (0-0)	0.2 (-10.3-12.4)	4 (3-4)	185 (158-213)	3 (3-4)	0 (0-0)
Kansas	4 (3-5)	-24 (-30--18.1)	178 (151-209)	-3.5 (-11.1-4)	3 (3-4)	-5 (-13-3.5)	0 (0-0)	-23.8 (-32.5--14.1)	3 (3-4)	172 (144-205)	3 (3-4)	0 (0-0)
Kentucky	5 (4-5)	-10.1 (-16--4.2)	208 (183-235)	4.4 (-2.4-11.3)	4 (4-5)	4.6 (-3.1-12.5)	0 (0-0)	-11.4 (-19.9--0.8)	4 (4-5)	217 (191-246)	4 (4-5)	0 (0-0)
Louisiana	25 (22-27)	-24.3 (-29.5--18.9)	1,020 (927-1,120)	-10.6 (-16.4--4.9)	20 (17-22)	-12.4 (-19.6--5.4)	2 (1-2)	-30.3 (-38.6--21.1)	19 (17-21)	909 (829-989)	17 (15-19)	1 (1-1)
Maine	0 (0-0)	-55.7 (-75.6--34.9)	10 (6-17)	-49.1 (-71.5--23.7)	0 (0-0)	-46.8 (-70.7--20.8)	0 (0-0)	-58.5 (-77.7--37.2)	0 (0-0)	5 (3-9)	0 (0-0)	0 (0-0)
Maryland	43 (39-47)	-9.9 (-15.6--4.2)	1,980 (1,820-2,150)	9 (2.9-15.5)	36 (32-40)	13 (5-21.9)	2 (2-3)	-8.7 (-18.2-1.9)	39 (36-42)	2,160 (2,000-2,330)	41 (37-45)	2 (1-2)
Massachusetts	14 (12-15)	-20.4 (-25.7--15.1)	662 (600-729)	-2.2 (-8.2-4)	13 (11-14)	-1 (-8.6-7.4)	1 (0-1)	-25.5 (-32.8--16.3)	11 (10-12)	648 (586-719)	13 (11-14)	0 (0-1)

Location	Birth , 2000	Births percent change (2000-2021)	All age cases, 2000	All age cases percent change (2000-2021)	All age deaths, 2000	All age deaths percent change (2000-2021)	Under-5 deaths, 2000	Under-5 deaths percent change (2000-2021)	Births, 2021	All age cases, 2021	All age deaths, 2021	Under-5 deaths, 2021
Michigan	31 (28-34)	-23.1 (-27.4--18.4)	1,430 (1,320-1,560)	-9.4 (-14.2--4.4)	27 (24-30)	-6.5 (-13.2-0.7)	2 (1-2)	-29.8 (-36.9--22.1)	24 (22-26)	1,300 (1,210-1,410)	25 (23-28)	1 (1-1)
Minnesota	6 (5-7)	-11 (-17.9--2.2)	277 (234-322)	4 (-4.1-13.5)	5 (4-6)	6.4 (-3-18.6)	0 (0-0)	-13.6 (-22.7--1.4)	5 (4-6)	288 (243-340)	5 (4-6)	0 (0-0)
Mississippi	22 (20-25)	-26 (-30.8--20.8)	880 (798-968)	-6.8 (-12.1--1)	18 (16-20)	-7.1 (-13.7--0.3)	1 (1-2)	-28.5 (-37.1--19.3)	17 (15-18)	821 (751-892)	16 (15-18)	1 (1-1)
Missouri	17 (15-18)	-18.3 (-23.8--12.4)	733 (663-810)	-0.3 (-6-6)	14 (13-16)	-1.2 (-8.2-6.8)	1 (1-1)	-20.9 (-29--12.2)	14 (12-15)	731 (667-805)	14 (13-16)	1 (1-1)
Montana	0 (0-0)	-2 (-32.8-36.8)	4 (2-8)	9.3 (-25.7-52.9)	0 (0-0)	9.8 (-23.8-50.8)	0 (0-0)	-8.6 (-37.5-30.8)	0 (0-0)	4 (2-8)	0 (0-0)	0 (0-0)
Nebraska	2 (1-2)	-25.2 (-34.1--15.7)	82 (63-104)	-14 (-23.8--3)	1 (1-2)	-16.6 (-26.4--3.9)	0 (0-0)	-27.5 (-38.2--16.7)	1 (1-2)	70 (51-93)	1 (1-2)	0 (0-0)
Nevada	6 (5-7)	1.7 (-5.9-9.1)	245 (218-274)	49.3 (37.7-59.9)	5 (4-5)	52 (40.2-64.9)	0 (0-0)	12.2 (0.2-26.3)	6 (5-7)	367 (325-412)	7 (6-8)	0 (0-0)
New Hampshire	0 (0-0)	-17.3 (-26.5--3.3)	12 (8-18)	1.1 (-9.6-19.3)	0 (0-0)	6.4 (-7.7-26)	0 (0-0)	-26.3 (-37.3--11.9)	0 (0-0)	12 (8-18)	0 (0-0)	0 (0-0)
New Jersey	37 (34-40)	-18.3 (-23.6--13.6)	1,660 (1,530-1,810)	0.9 (-4.5-6.3)	31 (28-35)	2.2 (-5.3-9.6)	2 (1-2)	-19.8 (-27.1--11.3)	30 (27-33)	1,670 (1,540-1,810)	32 (29-35)	1 (1-2)
New Mexico	1 (1-1)	-26.8 (-32.8--18.2)	33 (24-44)	3.4 (-4.7-14.2)	1 (0-1)	4.9 (-4.6-17.6)	0 (0-0)	-24.3 (-33.7--12.7)	1 (0-1)	34 (24-46)	1 (1-1)	0 (0-0)
New York	98 (89-107)	-17 (-22--12.4)	4,880 (4,480-5,330)	0 (-5-4.9)	79 (69-89)	5.4 (-2.7-13.8)	3 (2-4)	-18 (-26.2--8.1)	81 (74-89)	4,880 (4,510-5,300)	84 (75-93)	3 (2-4)
North Carolina	44 (40-48)	-7.3 (-12.9--2)	1,870 (1,720-2,040)	19.1 (12.2-25.4)	36 (32-40)	19.8 (11.3-28.4)	2 (2-3)	-6.7 (-16.5-3.6)	41 (38-44)	2,230 (2,060-2,410)	43 (39-47)	2 (2-3)
North Dakota	0 (0-0)	23.4 (-40.8-36.8)	2 (1-5)	10.4 (-45.5-24.6)	0 (0-0)	6.9 (-50.1-24.6)	0 (0-0)	8.5 (-50.2-31.6)	0 (0-0)	2 (1-6)	0 (0-0)	0 (0-0)
Ohio	27 (25-30)	-18.5 (-23.9--13.9)	1,240 (1,130-1,360)	-4.9 (-11-0.2)	24 (21-27)	-4.9 (-12.2-1.9)	1 (1-2)	-24.8 (-33.1--16.6)	22 (20-25)	1,180 (1,080-1,280)	23 (20-25)	1 (1-1)
Oklahoma	5 (4-6)	-20.9 (-26.8--14.8)	213 (184-243)	-3.8 (-10.2-3.4)	4 (4-5)	-8.2 (-16.1--0.8)	0 (0-0)	-24.7 (-33.8--15.3)	4 (4-5)	205 (177-238)	4 (3-5)	0 (0-0)
Oregon	2 (2-3)	-12 (-19--3.7)	95 (77-116)	12.5 (4-22.3)	2 (2-2)	15.6 (5.7-28.1)	0 (0-0)	-18.1 (-27.8--6.8)	2 (1-2)	107 (86-132)	2 (2-3)	0 (0-0)
Pennsylvania	22 (19-24)	-15.3 (-20.7--8.9)	1,070 (970-1,170)	-2.7 (-8.3-4)	22 (19-24)	-4.1 (-11.4-4)	1 (1-1)	-21.8 (-29.2--12.5)	18 (17-20)	1,040 (945-1,140)	21 (19-23)	1 (1-1)
Rhode Island	3 (2-3)	-22.4 (-29--16.1)	138 (115-164)	-8.9 (-16.4--1.3)	3 (2-3)	-5.4 (-13.9-5.1)	0 (0-0)	-30.4 (-38.2--21.7)	2 (2-2)	126 (104-151)	2 (2-3)	0 (0-0)
South Carolina	24 (22-27)	-9.8 (-15.6--3.9)	1,080 (989-1,180)	9.9 (2.9-16.3)	21 (19-24)	9.6 (1.2-17.9)	1 (1-2)	-14.3 (-24.1--4.2)	22 (20-24)	1,190 (1,090-1,290)	23 (21-26)	1 (1-2)
South Dakota	0 (0-0)	4.2 (-6.2-17.8)	8 (4-14)	14.6 (4-28.8)	0 (0-0)	11 (-1.6-26.3)	0 (0-0)	-1.1 (-16.6-19.6)	0 (0-0)	9 (5-15)	0 (0-0)	0 (0-0)
Tennessee	19 (17-21)	-2 (-8.1-5.1)	812 (741-891)	11.7 (5.4-18.3)	16 (15-18)	12.2 (4.8-21.3)	1 (1-1)	3.5 (-8.2-16.2)	18 (17-20)	907 (830-994)	18 (17-20)	1 (1-1)
Texas	78 (71-86)	8.3 (2.1-14.5)	3,030 (2,780-3,290)	40.8 (33.1-48)	52 (45-58)	46.1 (36.4-57.3)	3 (2-4)	18.1 (5.3-31.9)	85 (77-94)	4,260 (3,940-4,660)	75 (67-84)	4 (3-5)
Utah	2 (1-2)	-8.1 (-16--0.2)	52 (40-67)	33.7 (22.4-45.9)	1 (1-1)	37.9 (26-49.5)	0 (0-0)	-6.1 (-17-5.1)	1 (1-2)	70 (54-89)	1 (1-1)	0 (0-0)
Vermont	0 (0-1)	-12.1 (-24.1-0.3)	18 (11-30)	1.9 (-9.6-14.9)	0 (0-1)	11.4 (-4-29)	0 (0-0)	-14.1 (-27.8-2)	0 (0-0)	19 (11-29)	0 (0-1)	0 (0-0)
Virginia	30 (27-33)	-7.7 (-13.2--2.3)	1,370 (1,260-1,510)	13.6 (7.2-20.1)	26 (23-29)	14.7 (6.7-22.1)	2 (1-2)	-7.4 (-17-3.1)	28 (25-30)	1,560 (1,430-1,700)	30 (27-33)	1 (1-2)
Washington	5 (4-6)	10.5 (2.8-19.2)	240 (211-269)	26.8 (18.3-35.9)	4 (4-5)	32.7 (21.9-44.4)	0 (0-0)	2.2 (-9.5-18.4)	6 (5-6)	304 (265-345)	6 (5-7)	0 (0-0)
West Virginia	1 (1-1)	-28.1 (-33.4--23.2)	55 (41-70)	-15.1 (-21.7--8.1)	1 (1-2)	-15.5 (-22.3--7.1)	0 (0-0)	-28.5 (-36.5--18.4)	1 (1-1)	46 (36-59)	1 (1-1)	0 (0-0)
Wisconsin	9 (8-10)	-22.5 (-27.9--17)	431 (375-488)	-8 (-13.9--1.4)	8 (7-10)	-5.8 (-12.2-2.5)	0 (0-1)	-24.6 (-32.5--15.3)	7 (6-8)	396 (344-447)	8 (7-9)	0 (0-0)
Wyoming	0 (0-0)	-46.8 (-61.1--34.6)	12 (6-21)	-34.9 (-52.7--20.3)	0 (0-0)	-36 (-53.5--21.8)	0 (0-0)	-43.6 (-58.7--30.2)	0 (0-0)	8 (4-16)	0 (0-0)	0 (0-0)

Location	Birth , 2000	Births percent change (2000-2021)	All age cases, 2000	All age cases percent change (2000-2021)	All age deaths, 2000	All age deaths percent change (2000-2021)	Under-5 deaths, 2000	Under-5 deaths percent change (2000-2021)	Births, 2021	All age cases, 2021	All age deaths, 2021	Under-5 deaths, 2021
**Southern Latin America**	6 (4-7)	19.4 (6.8-48.4)	60 (45-79)	38 (24.2-63.4)	5 (4-7)	29.6 (14.8-60.4)	4 (3-5)	21.6 (5.8-54.3)	7 (5-8)	83 (64-105)	7 (5-9)	4 (3-6)
Argentina	2 (1-3)	-1.1 (-9.6-36.9)	21 (14-30)	12.8 (-8.5-44.9)	2 (1-3)	3.9 (-4.5-43.3)	1 (1-2)	-1.4 (-11.3-42)	2 (1-3)	24 (15-34)	2 (1-3)	1 (1-2)
Chile	3 (2-4)	34.8 (15.7-77)	38 (27-50)	53.8 (34.2-91.2)	3 (2-4)	48.3 (26.7-96.4)	2 (1-3)	40.1 (17.5-91.4)	4 (3-5)	58 (45-73)	5 (3-6)	3 (2-4)
Uruguay	0 (0-0)	-14 (-24.5--13.9)	2 (1-3)	-6.2 (-19.6-11.7)	0 (0-0)	-12.2 (-22.7--9.8)	0 (0-0)	-15.8 (-24--8.1)	0 (0-0)	2 (1-2)	0 (0-0)	0 (0-0)
**Western Europe**	949 (760-1,190)	-12.3 (-15.3--8.9)	16,700 (14,100-19,600)	-5.2 (-8--2.6)	985 (791-1,230)	-4.1 (-7.5--0.8)	300 (202-417)	-10.2 (-15--5.1)	832 (657-1,060)	15,800 (13,300-18,800)	944 (751-1,190)	269 (180-376)
Andorra	0 (0-0)	-15.9 (-24.3--8)	2 (1-2)	10.6 (2.5-19.6)	0 (0-0)	7.9 (-2.3-19.7)	0 (0-0)	-27.2 (-36.7--16.6)	0 (0-0)	2 (1-2)	0 (0-0)	0 (0-0)
Austria	10 (9-11)	69.7 (53.8-87.1)	240 (215-267)	25.2 (16.7-33.5)	12 (10-13)	48.9 (34.5-62.9)	2 (2-3)	72.9 (47.3-100)	17 (14-20)	301 (266-339)	17 (15-20)	4 (3-6)
Belgium	12 (9-16)	-0.9 (-10-11.1)	163 (130-205)	3.9 (-4.5-13)	13 (10-17)	2.7 (-6.6-14.9)	5 (3-7)	-2 (-16.5-13.7)	12 (9-16)	169 (135-213)	13 (10-17)	4 (3-7)
Cyprus	2 (1-2)	40.9 (24-55.9)	28 (21-36)	27.9 (17.5-39.2)	2 (1-2)	18 (4.1-30.8)	1 (0-1)	31.1 (10.8-51.4)	2 (2-3)	35 (27-45)	2 (2-3)	1 (0-1)
Denmark	8 (6-11)	-0.8 (-10.9-8.9)	146 (113-186)	1.2 (-6.4-9.9)	8 (6-11)	1.3 (-8.7-11.6)	3 (2-4)	-8.5 (-21.3-3.4)	8 (6-11)	147 (113-189)	8 (6-11)	2 (1-3)
Finland	7 (5-10)	-15.5 (-24.8--4.8)	140 (108-182)	-5.1 (-12.5-3.5)	8 (6-11)	-4.9 (-15-7.1)	2 (1-3)	-15.5 (-27--1.9)	6 (4-8)	133 (103-170)	8 (5-10)	2 (1-3)
France	301 (243-376)	-19 (-25.8--11.3)	3,960 (3,230-4,810)	-0.9 (-8.6-6.7)	288 (232-361)	-6.8 (-15-1.8)	104 (71-146)	-17.7 (-27.4--6.5)	244 (192-307)	3,930 (3,180-4,830)	269 (212-336)	85 (58-120)
Germany	77 (64-94)	-0.1 (-8.6-8.1)	1,780 (1,500-2,100)	-3.8 (-10-3)	90 (74-109)	-2.6 (-11.2-5.7)	22 (16-30)	3 (-8.5-17.4)	77 (64-92)	1,720 (1,450-2,010)	88 (73-104)	23 (16-31)
Greece	89 (71-113)	-24.7 (-33.8--14.7)	2,400 (2,060-2,800)	-25.1 (-30.4--19.4)	105 (85-132)	-21.4 (-31.1--11.6)	26 (17-37)	-20.1 (-34.5--3.8)	67 (54-82)	1,800 (1,560-2,040)	83 (67-100)	21 (14-30)
Iceland	0 (0-1)	3.5 (-4.7-14)	9 (7-11)	11 (3.4-19.3)	0 (0-1)	8.6 (-0.4-20.2)	0 (0-0)	-4.6 (-14.9-8.9)	1 (0-1)	10 (7-12)	1 (0-1)	0 (0-0)
Ireland	7 (5-10)	4.6 (-8-15.6)	118 (91-150)	19 (10.1-28.7)	7 (5-9)	20.8 (6.6-33.6)	2 (1-3)	11.8 (-3.3-30.2)	8 (5-10)	140 (108-179)	8 (6-11)	2 (1-4)
Israel	51 (38-68)	6 (-9.3-24.2)	489 (394-612)	21 (7.9-34.9)	43 (32-58)	3.9 (-12.5-23)	19 (12-28)	-1.4 (-20.4-20.7)	54 (39-72)	592 (467-745)	45 (32-62)	19 (12-29)
Italy	72 (58-89)	-17.4 (-20.6--13.6)	1,780 (1,500-2,090)	-1 (-4.4-2.6)	74 (61-91)	9.1 (4.8-14.1)	12 (8-17)	-4.6 (-9.8-1.2)	60 (49-72)	1,760 (1,490-2,040)	81 (67-98)	11 (8-16)
Abruzzo	1 (0-1)	-7.1 (-16.2-4.3)	15 (11-20)	2.1 (-6.4-14.2)	1 (1-1)	6.8 (-2.6-19.4)	0 (0-0)	2.4 (-11.9-19.8)	1 (0-1)	15 (11-20)	1 (1-1)	0 (0-0)
Basilicata	0 (0-0)	-10.4 (-22.2-4.1)	8 (5-12)	2.1 (-9.1-18.5)	0 (0-1)	2.7 (-9-19.8)	0 (0-0)	-5.5 (-19.9-15.2)	0 (0-0)	8 (6-11)	0 (0-1)	0 (0-0)
Calabria	4 (3-5)	-24.3 (-32--16.8)	124 (107-143)	-14.9 (-20.3--8.7)	5 (4-6)	-16.4 (-24.6--7.8)	1 (0-1)	-22 (-34.1--8.9)	3 (2-4)	106 (91-121)	4 (3-5)	1 (0-1)
Campania	4 (3-4)	-24.4 (-31.6--15.6)	96 (79-116)	-4.8 (-12.5-3.8)	4 (3-5)	-4 (-13.2-7.7)	1 (0-1)	-19.1 (-29.8--5.3)	3 (2-3)	92 (76-108)	3 (3-4)	0 (0-1)
Emilia-Romagna	12 (9-14)	-7.7 (-17.3-2.9)	210 (179-244)	15 (7.3-23.5)	10 (9-13)	36.1 (23.1-50.9)	2 (1-3)	18.8 (1.3-43.2)	11 (9-13)	241 (209-275)	14 (12-17)	2 (1-3)
Friuli-Venezia Giulia	2 (2-3)	-20.1 (-28.7--9.1)	45 (37-54)	1.6 (-6.3-11.2)	2 (2-3)	13.3 (1.4-28.2)	0 (0-1)	-6.4 (-20.4-9.8)	2 (1-2)	45 (38-54)	3 (2-3)	0 (0-1)
Lazio	4 (3-5)	1.6 (-7.5-12.7)	114 (96-136)	16.9 (8.7-25.8)	5 (4-6)	29 (17.8-42.1)	1 (0-1)	18.6 (3.1-36.6)	4 (4-5)	134 (112-157)	6 (5-7)	1 (1-1)
Liguria	2 (2-3)	-20.2 (-28.8--11.3)	68 (56-79)	-5.8 (-12.2-1.1)	3 (2-3)	3.5 (-6.7-13.2)	0 (0-1)	-3.5 (-19.4-17.6)	2 (1-2)	64 (54-73)	3 (2-3)	0 (0-1)
Lombardia	8 (6-10)	-15.2 (-25.5--4.8)	206 (167-251)	1.9 (-7.8-11.9)	8 (6-10)	14.6 (1.1-28.4)	1 (1-2)	-0.5 (-15.4-16.2)	7 (6-9)	210 (175-250)	9 (8-12)	1 (1-2)
Marche	3 (2-3)	-17.2 (-25.9--8.2)	55 (45-67)	2.7 (-5.1-10.5)	3 (2-3)	11.9 (0.8-23.9)	0 (0-1)	-1.8 (-16.3-14)	2 (2-3)	56 (46-68)	3 (2-4)	0 (0-1)
Molise	0 (0-1)	-33.8 (-39.7--25.7)	8 (6-12)	-24.1 (-30.4--16.6)	0 (0-1)	-24.7 (-32--15.3)	0 (0-0)	-32.3 (-42.1--22.1)	0 (0-0)	6 (4-9)	0 (0-1)	0 (0-0)

Location	Birth , 2000	Births percent change (2000-2021)	All age cases, 2000	All age cases percent change (2000-2021)	All age deaths, 2000	All age deaths percent change (2000-2021)	Under-5 deaths, 2000	Under-5 deaths percent change (2000-2021)	Births, 2021	All age cases, 2021	All age deaths, 2021	Under-5 deaths, 2021
Piemonte	5 (4-6)	-14.2 (-22.3--4)	89 (74-108)	0 (-8.1-8.5)	5 (4-6)	14.6 (4.7-27.7)	1 (1-1)	0.6 (-14.1-19.1)	4 (3-5)	89 (74-105)	6 (5-7)	1 (1-1)
Provincia autonoma di Bolzano	1 (0-1)	-8.4 (-19-5.4)	13 (9-18)	6.2 (-2.6-18.7)	1 (0-1)	10.5 (-1.7-26.2)	0 (0-0)	-4.4 (-17.7-12.9)	1 (0-1)	14 (10-18)	1 (0-1)	0 (0-0)
Provincia autonoma di Trento	1 (0-1)	-8.2 (-17.9-3.5)	12 (9-16)	11.9 (2.3-22.8)	1 (0-1)	18.3 (6.2-32.7)	0 (0-0)	0.6 (-13.1-17.7)	1 (0-1)	13 (10-17)	1 (0-1)	0 (0-0)
Puglia	3 (2-4)	-36.9 (-42.7--29.5)	77 (60-99)	-18.7 (-26--10.6)	3 (2-4)	-18.4 (-26.9--8.7)	1 (0-1)	-33.6 (-42.7--23.1)	2 (1-2)	63 (50-78)	3 (2-3)	0 (0-1)
Sardegna	1 (1-1)	-24.5 (-32.8--14.8)	22 (15-29)	-11.5 (-19.1--2.4)	1 (1-1)	-6.5 (-15.9-5.5)	0 (0-0)	-13.3 (-25.1-1.9)	1 (0-1)	19 (14-25)	1 (1-1)	0 (0-0)
Sicilia	12 (10-14)	-31.2 (-36.8--24.5)	379 (322-438)	-11.1 (-16.9--4.2)	12 (10-15)	-13 (-20.2--4.6)	2 (1-3)	-27.2 (-36.3--16.1)	8 (7-10)	337 (293-384)	11 (9-13)	1 (1-2)
Toscana	3 (2-4)	-9.7 (-18.9-1.3)	71 (57-88)	5.4 (-1.9-14.7)	3 (3-4)	20.2 (8.8-34)	0 (0-1)	8 (-7.4-25.8)	3 (2-4)	75 (61-91)	4 (3-5)	1 (0-1)
Umbria	1 (1-1)	-5.6 (-16.7-5.1)	21 (17-26)	13.9 (4.9-23.8)	1 (1-1)	24.1 (11.2-38.5)	0 (0-0)	14.1 (-3.3-36.8)	1 (1-1)	24 (19-30)	1 (1-2)	0 (0-0)
Valle d'Aosta	0 (0-0)	-31.3 (-42--19.1)	1 (1-2)	-11.6 (-24.3-2.6)	0 (0-0)	-0.8 (-16.2-15.1)	0 (0-0)	-20 (-36.2--3.1)	0 (0-0)	1 (1-2)	0 (0-0)	0 (0-0)
Veneto	6 (5-7)	-15.6 (-23.6--6.9)	141 (118-168)	3.1 (-4-11.7)	6 (5-8)	17.9 (6.9-29.3)	1 (1-1)	-1.9 (-14.6-15.5)	5 (4-6)	145 (122-169)	7 (6-9)	1 (1-1)
Luxembourg	1 (0-1)	19 (8.1-31.1)	12 (9-16)	37.4 (27.5-48.4)	1 (0-1)	33 (21-48.2)	0 (0-0)	17.1 (1.4-35.6)	1 (1-1)	17 (13-21)	1 (1-1)	0 (0-0)
Malta	1 (0-1)	4.3 (-4.7-18.8)	12 (9-15)	-7.7 (-14.7-0.6)	1 (0-1)	-11.5 (-19.7-0.7)	0 (0-0)	-5.8 (-17.5-10.9)	1 (0-1)	11 (8-14)	1 (0-1)	0 (0-0)
Monaco	0 (0-0)	-3.9 (-14-7.5)	1 (1-1)	14.1 (6-22.5)	0 (0-0)	18.1 (6.5-31.6)	0 (0-0)	20.1 (4.7-41.3)	0 (0-0)	1 (1-1)	0 (0-0)	0 (0-0)
Netherlands	3 (2-4)	-10.8 (-19.3-4.1)	36 (26-49)	-3.4 (-12.2-11.4)	3 (2-4)	-3.5 (-12.9-13.4)	1 (1-2)	-9.9 (-20.9-8.9)	3 (2-4)	35 (25-47)	3 (2-4)	1 (1-1)
Norway	9 (5-14)	-4.4 (-8.6-1)	96 (64-136)	6.3 (2.5-10.5)	9 (5-14)	4.3 (-0.6-10.3)	4 (2-6)	-5.7 (-12.5-1.7)	9 (5-13)	103 (68-145)	10 (6-15)	4 (2-6)
Agder	1 (0-1)	-7.6 (-21.1-6.3)	6 (4-9)	2.3 (-9.2-14.4)	1 (0-1)	1.1 (-12.7-17.1)	0 (0-0)	-11.2 (-29-8.2)	1 (0-1)	6 (4-9)	1 (0-1)	0 (0-0)
Innlandet	1 (0-1)	-20.7 (-32.4--7.5)	7 (5-11)	-11.1 (-21.4-0.9)	1 (0-1)	-12.6 (-25.4-1.9)	0 (0-0)	-22 (-38.8--3.3)	1 (0-1)	7 (4-9)	1 (0-1)	0 (0-0)
Møre og Romsdal	1 (0-1)	-17.8 (-28.5--6)	5 (3-8)	-5.1 (-15.6-5.9)	1 (0-1)	-6.8 (-18.3-6.8)	0 (0-0)	-16.4 (-33.4-4.3)	0 (0-1)	5 (3-7)	0 (0-1)	0 (0-0)
Nordland	0 (0-0)	-18.3 (-28.4--8.2)	5 (3-8)	-16.4 (-24.8--6.4)	1 (0-1)	-19.1 (-29.3--8.4)	0 (0-0)	-28 (-40.6--12.3)	0 (0-0)	4 (3-6)	0 (0-1)	0 (0-0)
Oslo	1 (1-2)	25 (10-42.8)	10 (7-14)	38.5 (25.1-53.1)	1 (1-1)	38.3 (20.9-59.2)	0 (0-1)	31.3 (7.9-59.9)	2 (1-2)	14 (9-20)	1 (1-2)	1 (0-1)
Rogaland	1 (0-1)	-3.2 (-16.6-12.5)	9 (6-12)	12.2 (0.6-25.6)	1 (0-1)	10.5 (-4.7-29.2)	0 (0-1)	-1.4 (-20.2-25.3)	1 (0-1)	10 (7-14)	1 (1-1)	0 (0-1)
Troms og Finnmark	1 (0-1)	-20.9 (-31.7--6.4)	5 (3-7)	-11 (-21.6-1.4)	1 (0-1)	-15.1 (-26.8-0.4)	0 (0-0)	-24.3 (-40.3--1.1)	1 (0-1)	5 (3-7)	0 (0-1)	0 (0-0)
Trøndelag	1 (0-1)	4.5 (-10-22.5)	8 (6-12)	5.7 (-4.7-17.9)	1 (0-1)	3 (-12.1-20.6)	0 (0-1)	-6.2 (-25.1-18.1)	1 (0-1)	9 (6-13)	1 (0-1)	0 (0-1)
Vestfold og Telemark	1 (0-1)	-14.1 (-24.6--0.4)	8 (5-11)	-3.7 (-12.9-8)	1 (0-1)	-5.1 (-17.4-9.8)	0 (0-0)	-16.1 (-31.1-6.6)	1 (0-1)	8 (5-11)	1 (0-1)	0 (0-0)
Vestland	1 (1-2)	-3.9 (-17.7-10.8)	12 (8-17)	4.7 (-6.4-16.1)	1 (1-2)	2.6 (-11.9-18.1)	0 (0-1)	-5.7 (-24.2-17.8)	1 (1-2)	13 (8-18)	1 (1-2)	0 (0-1)
Viken	2 (1-3)	-4.2 (-19.1-9.7)	20 (13-28)	13.6 (0.2-26.4)	2 (1-3)	13.1 (-4.9-29.5)	1 (0-1)	0 (-23.3-23.8)	2 (1-3)	23 (15-32)	2 (1-3)	1 (0-1)
Portugal	106 (81-138)	-40.1 (-48.2--30.4)	1,940 (1,620-2,240)	-29.4 (-37.8--21.4)	109 (84-140)	-27.1 (-36.1--16)	36 (23-53)	-33.8 (-46.2--19)	64 (47-84)	1,370 (1,110-1,670)	80 (61-103)	24 (15-35)
San Marino	0 (0-0)	-3.9 (-13.4-6.5)	1 (1-1)	10.3 (1.4-19)	0 (0-0)	8.2 (-3.4-20.9)	0 (0-0)	-6.3 (-18.8-10)	0 (0-0)	1 (1-1)	0 (0-0)	0 (0-0)
Spain	91 (75-110)	-12.9 (-20.3--3.5)	1,710 (1,450-2,020)	2.6 (-4.6-10.8)	98 (82-118)	6.2 (-3-17.8)	24 (17-33)	0.5 (-13-16.6)	79 (65-98)	1,750 (1,480-2,050)	105 (86-128)	24 (17-33)

Location	Birth , 2000	Births percent change (2000-2021)	All age cases, 2000	All age cases percent change (2000-2021)	All age deaths, 2000	All age deaths percent change (2000-2021)	Under-5 deaths, 2000	Under-5 deaths percent change (2000-2021)	Births, 2021	All age cases, 2021	All age deaths, 2021	Under-5 deaths, 2021
Sweden	14 (10-19)	42.3 (29.1-55.8)	321 (251-409)	25.1 (15.8-34.9)	16 (12-22)	26.7 (14.1-40)	3 (2-5)	45 (28.2-63.2)	20 (14-27)	402 (315-515)	20 (15-28)	5 (3-7)
Stockholm	3 (2-5)	41.9 (26.4-58)	66 (51-85)	45.4 (33.1-57.7)	3 (2-4)	49.3 (33-66.8)	1 (0-1)	56.2 (33.3-81.7)	5 (4-6)	96 (75-122)	5 (4-6)	1 (1-2)
Sweden except Stockholm	11 (8-14)	42.5 (26.5-61.5)	255 (199-325)	19.8 (9.4-31.3)	13 (9-17)	21 (6.8-37.5)	2 (2-4)	41.7 (21-63.9)	15 (11-21)	305 (238-390)	16 (11-21)	3 (2-5)
Switzerland	10 (7-13)	18.5 (6.6-30.7)	187 (144-241)	14.4 (5.3-23.9)	10 (7-14)	13.5 (1.7-25.6)	3 (2-4)	17.6 (1-35.8)	11 (8-16)	214 (167-274)	12 (8-16)	3 (2-5)
United Kingdom	77 (54-107)	16.8 (15-18.5)	1,080 (832-1,390)	6.5 (5-8.1)	85 (60-119)	6.2 (4.6-8)	31 (18-46)	6.5 (4.3-8.4)	90 (63-124)	1,150 (881-1,480)	91 (64-125)	33 (20-49)
England	63 (44-88)	19.4 (18-20.8)	802 (613-1,040)	9.3 (8.2-10.5)	69 (48-97)	8.6 (7.2-10.1)	26 (15-39)	9.4 (7.5-11.3)	75 (52-104)	877 (672-1,140)	75 (52-104)	29 (17-43)
East Midlands	5 (4-7)	20.3 (15.2-25.3)	68 (51-88)	8.3 (4.6-11.8)	6 (4-8)	7.2 (2.7-11.7)	2 (1-3)	8.7 (2.5-15)	6 (4-8)	73 (56-96)	6 (4-9)	2 (1-3)
Derby	0 (0-0)	17.3 (2-32.1)	4 (3-5)	14.9 (4.3-25.1)	0 (0-0)	15.6 (0.5-30.3)	0 (0-0)	12.8 (-6.1-31.9)	0 (0-1)	4 (3-6)	0 (0-1)	0 (0-0)
Derbyshire	1 (1-1)	5.8 (-5.9-16.5)	12 (9-15)	-4.3 (-12.4-4.6)	1 (1-1)	-6.4 (-17-3.2)	0 (0-1)	-8.1 (-23-7.2)	1 (1-1)	11 (8-14)	1 (1-1)	0 (0-0)
Leicester	0 (0-1)	36.3 (22.6-52.9)	5 (4-7)	31.8 (20.7-44.1)	0 (0-1)	29.3 (16.5-45.5)	0 (0-0)	29.8 (11.8-53.3)	1 (0-1)	7 (5-9)	1 (0-1)	0 (0-0)
Leicestershire	1 (0-1)	13.2 (1-28.4)	10 (7-12)	1.9 (-6.7-13)	1 (1-1)	0.5 (-10.3-13.8)	0 (0-0)	0.7 (-14-20.8)	1 (1-1)	10 (7-13)	1 (1-1)	0 (0-0)
Lincolnshire	1 (0-1)	31.7 (15.8-48.2)	10 (8-13)	9.6 (-0.4-21)	1 (1-1)	6.8 (-6.3-21.4)	0 (0-0)	10.8 (-5.1-30.5)	1 (1-1)	11 (8-14)	1 (1-1)	0 (0-0)
Northamptonshire	1 (1-1)	22.3 (10.6-35.1)	10 (8-14)	13 (4.4-22.8)	1 (1-1)	13.1 (2.1-25)	0 (0-1)	13.7 (-2.8-32.4)	1 (1-1)	12 (9-16)	1 (1-1)	0 (0-1)
Nottingham	0 (0-1)	38.2 (24-54.9)	5 (3-6)	28.2 (17.5-40.4)	0 (0-1)	26.1 (13.2-41.6)	0 (0-0)	32.9 (15.3-57.6)	1 (0-1)	6 (4-8)	1 (0-1)	0 (0-0)
Nottinghamshire	1 (1-1)	12.5 (2-26.9)	12 (9-16)	0.9 (-6.7-10)	1 (1-1)	0 (-9.5-13.1)	0 (0-1)	1 (-11.9-19.5)	1 (1-1)	12 (9-16)	1 (1-1)	0 (0-1)
Rutland	0 (0-0)	16 (2.6-30.5)	1 (0-1)	0.1 (-8.4-9)	0 (0-0)	-1.9 (-13.1-10.6)	0 (0-0)	-1.5 (-16.5-17.5)	0 (0-0)	1 (0-1)	0 (0-0)	0 (0-0)
East of England	7 (5-9)	18.3 (13.5-22.6)	87 (67-113)	9.4 (6-12.8)	7 (5-10)	8.6 (3.9-12.7)	3 (2-4)	7.8 (1.9-14.3)	8 (5-11)	95 (73-124)	8 (6-11)	3 (2-4)
Bedford	0 (0-0)	18.4 (5.7-33.5)	2 (2-3)	7 (-1.9-15.9)	0 (0-0)	7.1 (-4.2-20.9)	0 (0-0)	7.3 (-7.9-24)	0 (0-0)	3 (2-3)	0 (0-0)	0 (0-0)
Cambridgeshire	1 (0-1)	23.4 (10.1-36.8)	9 (7-12)	14.3 (5.1-24.6)	1 (0-1)	12.8 (0.8-24.8)	0 (0-0)	10.3 (-5.6-29)	1 (1-1)	10 (8-13)	1 (1-1)	0 (0-0)
Central Bedfordshire	0 (0-0)	15.6 (4.8-29.7)	4 (3-5)	6.3 (-1.9-15.9)	0 (0-0)	5.1 (-4.5-18.4)	0 (0-0)	3.9 (-10.1-21.4)	0 (0-0)	4 (3-5)	0 (0-0)	0 (0-0)
Essex	2 (1-2)	15.7 (2.3-29.5)	21 (16-27)	3.4 (-5.5-11.8)	2 (1-2)	1.8 (-9.7-13.7)	1 (0-1)	1.1 (-14.6-19.8)	2 (1-2)	22 (17-29)	2 (1-2)	1 (0-1)
Hertfordshire	1 (1-2)	16.6 (3.6-32.5)	17 (13-22)	11.4 (2.2-23)	1 (1-2)	11.4 (-0.9-26.6)	0 (0-1)	9.6 (-6.8-27.8)	1 (1-2)	19 (14-25)	2 (1-2)	1 (0-1)
Luton	0 (0-0)	19.4 (4.3-33.2)	3 (3-4)	20.8 (8.2-32.7)	0 (0-0)	21.1 (5.1-35.8)	0 (0-0)	20.6 (2-39.4)	0 (0-1)	4 (3-5)	0 (0-1)	0 (0-0)
Norfolk	1 (1-1)	18.5 (5.8-31.8)	12 (9-16)	6.6 (-2.3-16.7)	1 (1-1)	5.5 (-5.4-17.2)	0 (0-1)	5.8 (-9.3-22.2)	1 (1-1)	13 (10-17)	1 (1-1)	0 (0-1)
Peterborough	0 (0-0)	48.9 (33-66.9)	3 (2-4)	38.5 (26.2-51)	0 (0-0)	38.5 (23.8-55.7)	0 (0-0)	39.9 (20.5-63)	0 (0-1)	4 (3-5)	0 (0-0)	0 (0-0)
Southend-on-Sea	0 (0-0)	17.7 (6.6-30.8)	3 (2-3)	13.5 (4.3-23.7)	0 (0-0)	11.9 (1.1-24.6)	0 (0-0)	9.2 (-5.5-25.7)	0 (0-0)	3 (2-4)	0 (0-0)	0 (0-0)
Suffolk	1 (1-1)	11.7 (0.4-24.6)	11 (8-14)	4.8 (-4-14.4)	1 (1-1)	2.5 (-8.2-14)	0 (0-0)	-0.9 (-15.3-14.7)	1 (1-1)	11 (8-15)	1 (1-1)	0 (0-0)
Thurrock	0 (0-0)	22.1 (9.5-35.9)	2 (2-3)	18.7 (9.1-29.1)	0 (0-0)	20.2 (7.9-34)	0 (0-0)	16.7 (-0.1-36.7)	0 (0-0)	3 (2-4)	0 (0-0)	0 (0-0)
Greater London	11 (8-16)	34.4 (31.4-37.2)	123 (94-159)	28.6 (26.2-30.9)	10 (7-14)	30.5 (27.5-33.2)	4 (3-7)	30.5 (26.6-34.2)	15 (11-21)	159 (121-205)	14 (9-19)	6 (3-9)

Location	Birth , 2000	Births percent change (2000-2021)	All age cases, 2000	All age cases percent change (2000-2021)	All age deaths, 2000	All age deaths percent change (2000-2021)	Under-5 deaths, 2000	Under-5 deaths percent change (2000-2021)	Births, 2021	All age cases, 2021	All age deaths, 2021	Under-5 deaths, 2021
Barking and Dagenham	0 (0-0)	63.3 (44.9-81.5)	3 (2-4)	52.5 (39.1-67.1)	0 (0-0)	57.8 (39.6-76.4)	0 (0-0)	56.9 (32.8-82.8)	0 (0-1)	5 (4-6)	0 (0-1)	0 (0-0)
Barnet	0 (0-1)	36.7 (21.3-54.9)	5 (4-7)	29.8 (19.8-41.6)	0 (0-1)	33.4 (18.3-52.2)	0 (0-0)	31.3 (11.9-56)	1 (0-1)	7 (5-9)	1 (0-1)	0 (0-0)
Bexley	0 (0-0)	31.6 (19-49.2)	4 (3-5)	15.5 (5.3-26)	0 (0-0)	15 (4-30.7)	0 (0-0)	18.8 (-0.4-40.7)	0 (0-0)	4 (3-5)	0 (0-0)	0 (0-0)
Brent	0 (0-1)	39.5 (24.9-56)	4 (3-6)	40.5 (27.3-52.7)	0 (0-1)	46.7 (30.7-64.5)	0 (0-0)	48.6 (26.7-74.4)	1 (0-1)	6 (5-8)	1 (0-1)	0 (0-0)
Bromley	0 (0-0)	22.7 (8.7-39.2)	5 (4-6)	9.1 (0.3-19.4)	0 (0-1)	10 (-3.1-25.6)	0 (0-0)	9.1 (-8-31.3)	0 (0-1)	5 (4-7)	0 (0-1)	0 (0-0)
Camden	0 (0-0)	9.3 (-1-21.6)	3 (2-4)	9.3 (0.2-19.9)	0 (0-0)	8.8 (-2.5-20.8)	0 (0-0)	3.8 (-10.2-19.7)	0 (0-0)	4 (3-5)	0 (0-0)	0 (0-0)
Croydon	0 (0-1)	37.1 (22.5-51.9)	6 (4-7)	26 (15.7-37.2)	0 (0-1)	28.7 (14.5-43.3)	0 (0-0)	31 (15.5-50.8)	1 (0-1)	7 (6-10)	1 (0-1)	0 (0-0)
Ealing	0 (0-1)	24.9 (10.7-39.2)	5 (4-7)	26 (15.2-37.8)	0 (0-1)	31.4 (16.7-46.9)	0 (0-0)	27.5 (9.1-49)	1 (0-1)	6 (5-8)	1 (0-1)	0 (0-0)
Enfield	0 (0-1)	40.5 (24.3-59.5)	5 (4-6)	37.6 (25.6-52)	0 (0-1)	42.8 (26.3-63)	0 (0-0)	40.4 (19.2-65.1)	1 (0-1)	6 (5-8)	1 (0-1)	0 (0-0)
Greenwich	0 (0-1)	53.6 (37-68.5)	4 (3-5)	44.6 (32.7-57.3)	0 (0-0)	46 (30.1-60.8)	0 (0-0)	46.3 (27.8-67.1)	1 (0-1)	6 (4-7)	1 (0-1)	0 (0-0)
Hackney	0 (0-1)	29.8 (17-44.1)	4 (3-5)	30.7 (19.9-42.3)	0 (0-1)	24.7 (11.6-39.3)	0 (0-0)	25.4 (9.2-46.3)	1 (0-1)	5 (4-7)	0 (0-1)	0 (0-0)
Hammersmith and Fulham	0 (0-0)	11.4 (0.6-26.6)	3 (2-4)	13.3 (4-24.1)	0 (0-0)	14.3 (2.8-30.3)	0 (0-0)	11.1 (-4.5-32.4)	0 (0-0)	3 (2-4)	0 (0-0)	0 (0-0)
Haringey	0 (0-1)	14.7 (1.4-30.6)	4 (3-5)	25.8 (14.5-38.1)	0 (0-0)	27.2 (11.8-45)	0 (0-0)	21.7 (2.8-43.1)	0 (0-1)	5 (4-6)	0 (0-1)	0 (0-0)
Harrow	0 (0-0)	56.5 (40.6-72.7)	3 (3-4)	40.9 (29.5-51.6)	0 (0-0)	45.6 (29.7-61.5)	0 (0-0)	54.1 (32.6-77.5)	0 (0-1)	5 (4-6)	0 (0-1)	0 (0-0)
Havering	0 (0-0)	52.7 (36.8-71)	4 (3-5)	17.7 (8.4-28)	0 (0-0)	15.9 (3.9-31.6)	0 (0-0)	30.7 (11.5-54)	0 (0-1)	4 (3-5)	0 (0-0)	0 (0-0)
Hillingdon	0 (0-0)	49.3 (34.6-66.5)	4 (3-5)	33.4 (23.1-45.3)	0 (0-0)	34.1 (20.4-49.7)	0 (0-0)	37 (16.7-61.1)	1 (0-1)	6 (4-7)	0 (0-1)	0 (0-0)
Hounslow	0 (0-0)	46 (27.7-61.2)	4 (3-5)	44.2 (31.5-57.2)	0 (0-0)	49.1 (30.2-65)	0 (0-0)	48.3 (25.4-71.3)	1 (0-1)	5 (4-7)	0 (0-1)	0 (0-0)
Islington	0 (0-0)	39.7 (25.9-57.6)	3 (2-4)	26.8 (15.4-38.1)	0 (0-0)	21.6 (8.7-37.6)	0 (0-0)	27.4 (10.5-48.3)	0 (0-1)	4 (3-5)	0 (0-0)	0 (0-0)
Kensington and Chelsea	0 (0-0)	-9 (-18.5-3.5)	2 (2-3)	-10.3 (-18.2--1.5)	0 (0-0)	-11 (-20.7-2.1)	0 (0-0)	-17.8 (-29.5--1.3)	0 (0-0)	2 (2-3)	0 (0-0)	0 (0-0)
Kingston upon Thames	0 (0-0)	35.8 (22.3-50.1)	2 (2-3)	19.7 (8.9-31.7)	0 (0-0)	22.9 (10.2-36.6)	0 (0-0)	23.7 (5.3-43.1)	0 (0-0)	3 (2-4)	0 (0-0)	0 (0-0)
Lambeth	1 (0-1)	17.1 (3.6-30.9)	5 (4-6)	18.2 (7.5-28.7)	0 (0-1)	18.1 (4.2-31.5)	0 (0-0)	16.7 (1-34.9)	1 (0-1)	6 (4-7)	0 (0-1)	0 (0-0)
Lewisham	0 (0-1)	32.2 (17.5-48)	5 (3-6)	23.9 (13.2-35.3)	0 (0-1)	27.1 (12.3-42.9)	0 (0-0)	26.9 (6.5-48.5)	1 (0-1)	6 (4-7)	0 (0-1)	0 (0-0)
Merton	0 (0-0)	28.8 (16-42.3)	3 (2-4)	16.4 (7-27)	0 (0-0)	21.2 (8.9-34)	0 (0-0)	21.2 (5.2-39.8)	0 (0-1)	4 (3-5)	0 (0-0)	0 (0-0)
Newham	1 (0-1)	43.2 (29.7-59.7)	5 (4-7)	42 (30.1-55.1)	0 (0-1)	35 (21.2-51.4)	0 (0-0)	35.8 (17.9-58.4)	1 (1-1)	7 (5-9)	1 (0-1)	0 (0-0)
Redbridge	0 (0-0)	64.9 (46.8-83.6)	4 (3-5)	51.5 (38-65.5)	0 (0-0)	56.6 (38.8-75.5)	0 (0-0)	59.4 (35.5-84.5)	1 (0-1)	6 (5-8)	1 (0-1)	0 (0-0)
Richmond upon Thames	0 (0-0)	7.3 (-4.4-18.6)	3 (2-4)	14.2 (5.1-23.6)	0 (0-0)	19.8 (6.7-33)	0 (0-0)	8.2 (-7.7-26)	0 (0-0)	3 (2-4)	0 (0-0)	0 (0-0)
Southwark	0 (0-1)	28.4 (15.2-46.2)	5 (3-6)	23.7 (12.9-35.5)	0 (0-1)	21.3 (8.7-38)	0 (0-0)	23.3 (5.8-44.7)	1 (0-1)	6 (4-7)	0 (0-1)	0 (0-0)
Sutton	0 (0-0)	24.9 (13.1-38.7)	3 (2-4)	9.6 (1-19.3)	0 (0-0)	10 (-0.4-22.6)	0 (0-0)	10 (-4.8-26.7)	0 (0-0)	3 (2-4)	0 (0-0)	0 (0-0)
Tower Hamlets	0 (0-1)	55.2 (33.8-76.7)	4 (3-5)	51.7 (37.7-66.2)	0 (0-0)	44 (24.5-65.4)	0 (0-0)	49.8 (25.7-76.2)	1 (0-1)	6 (4-7)	0 (0-1)	0 (0-0)

Location	Birth , 2000	Births percent change (2000-2021)	All age cases, 2000	All age cases percent change (2000-2021)	All age deaths, 2000	All age deaths percent change (2000-2021)	Under-5 deaths, 2000	Under-5 deaths percent change (2000-2021)	Births, 2021	All age cases, 2021	All age deaths, 2021	Under-5 deaths, 2021
Waltham Forest	0 (0-1)	45.7 (30.7-65.6)	4 (3-5)	43.7 (30.5-58.3)	0 (0-0)	45.7 (30.5-65.4)	0 (0-0)	44.4 (24.3-70.2)	1 (0-1)	6 (4-7)	0 (0-1)	0 (0-0)
Wandsworth	0 (0-1)	36.4 (21.9-51.3)	4 (3-6)	36.2 (24.7-48.7)	0 (0-0)	45.3 (30.2-61.4)	0 (0-0)	42.2 (22.4-65)	1 (0-1)	6 (4-8)	0 (0-1)	0 (0-0)
Westminster	0 (0-0)	12.6 (-1.6-25.9)	3 (2-4)	18.6 (8.5-30.2)	0 (0-0)	25.2 (9.7-40.1)	0 (0-0)	18.2 (-0.9-38.6)	0 (0-0)	3 (3-4)	0 (0-0)	0 (0-0)
North East England	3 (2-4)	7.2 (3.6-11.6)	41 (31-52)	-4 (-6.5--1)	4 (2-5)	-5.2 (-8.3--1.3)	1 (1-2)	-3.5 (-8.2-1.3)	3 (2-4)	39 (30-51)	3 (2-5)	1 (1-2)
County Durham	1 (0-1)	7.6 (-1.8-21.7)	8 (6-10)	-2.5 (-10.1-5.8)	1 (0-1)	-3.6 (-12.1-8.9)	0 (0-0)	-3 (-16.4-14.3)	1 (0-1)	8 (6-10)	1 (0-1)	0 (0-0)
Darlington	0 (0-0)	8.1 (-4.8-20.7)	2 (1-2)	6.8 (-2.3-16.1)	0 (0-0)	4.8 (-7.9-17.2)	0 (0-0)	1.3 (-14.5-17.1)	0 (0-0)	2 (1-2)	0 (0-0)	0 (0-0)
Gateshead	0 (0-0)	9.9 (-2-22.2)	3 (2-4)	2.6 (-5.4-12)	0 (0-0)	0.5 (-10.6-12)	0 (0-0)	-0.6 (-16.3-16.7)	0 (0-0)	3 (2-4)	0 (0-0)	0 (0-0)
Hartlepool	0 (0-0)	-0.5 (-12.6-11.2)	2 (1-2)	-8.5 (-16.4-0.4)	0 (0-0)	-11.5 (-21.6--0.9)	0 (0-0)	-12.3 (-25.7-2.6)	0 (0-0)	1 (1-2)	0 (0-0)	0 (0-0)
Middlesbrough	0 (0-0)	7.7 (-3.4-20.2)	2 (2-3)	-4.5 (-12.9-3.9)	0 (0-0)	-3.8 (-13.9-8.3)	0 (0-0)	1.6 (-11.9-19.9)	0 (0-0)	2 (2-3)	0 (0-0)	0 (0-0)
Newcastle upon Tyne	0 (0-0)	28.7 (14.9-43.2)	4 (3-6)	11.1 (1.6-21.2)	0 (0-1)	9.3 (-2.7-21.9)	0 (0-0)	16.1 (-0.5-32.6)	0 (0-1)	5 (4-6)	0 (0-1)	0 (0-0)
North Tyneside	0 (0-0)	13.1 (1-26.4)	3 (2-4)	3.6 (-5-13.3)	0 (0-0)	2.9 (-7.9-15.2)	0 (0-0)	4.6 (-11.2-24.1)	0 (0-0)	3 (2-4)	0 (0-0)	0 (0-0)
Northumberland	0 (0-0)	-0.1 (-10.7-11.4)	5 (4-6)	-8.8 (-16.4--0.1)	0 (0-1)	-9.8 (-19.1-0.2)	0 (0-0)	-12 (-25.2-2.6)	0 (0-0)	4 (3-6)	0 (0-0)	0 (0-0)
Redcar and Cleveland	0 (0-0)	-3 (-14.8-9.7)	2 (2-3)	-15.6 (-23.2--7.9)	0 (0-0)	-17.5 (-27.2--7.2)	0 (0-0)	-13.9 (-27.4-1.4)	0 (0-0)	2 (1-2)	0 (0-0)	0 (0-0)
South Tyneside	0 (0-0)	2.3 (-8.7-15.7)	2 (2-3)	-13.9 (-21.5--5)	0 (0-0)	-16 (-25.4--4.2)	0 (0-0)	-12.5 (-25.4-5)	0 (0-0)	2 (2-3)	0 (0-0)	0 (0-0)
Stockton-on-Tees	0 (0-0)	11.3 (0.8-24.9)	3 (2-4)	-0.8 (-8.9-8.6)	0 (0-0)	-1.7 (-11.2-10.3)	0 (0-0)	-1.4 (-16.3-14.1)	0 (0-0)	3 (2-4)	0 (0-0)	0 (0-0)
Sunderland	0 (0-0)	-7.4 (-16.5-3.1)	5 (3-6)	-17.5 (-24.2--9.8)	0 (0-1)	-18.3 (-26.4--8.4)	0 (0-0)	-16 (-27.5--4.4)	0 (0-0)	4 (3-5)	0 (0-0)	0 (0-0)
North West England	9 (6-12)	13.4 (10.1-16.5)	111 (85-144)	1.3 (-1-3.6)	10 (7-14)	0 (-3.1-2.8)	4 (2-6)	2.1 (-2-6)	10 (7-14)	113 (86-146)	10 (7-14)	4 (2-6)
Blackburn with Darwen	0 (0-0)	-0.4 (-11.7-12.8)	3 (2-3)	0.3 (-9.6-9.8)	0 (0-0)	-3.2 (-14.7-9.4)	0 (0-0)	-8.9 (-23.3-6.9)	0 (0-0)	3 (2-3)	0 (0-0)	0 (0-0)
Blackpool	0 (0-0)	11.5 (0.3-25.1)	2 (2-3)	-1.6 (-9.7-7.5)	0 (0-0)	-2.4 (-12.3-9.7)	0 (0-0)	-1.3 (-14.1-14.4)	0 (0-0)	2 (2-3)	0 (0-0)	0 (0-0)
Bolton	0 (0-1)	16.4 (2.1-33.2)	4 (3-6)	6.3 (-4-16.2)	0 (0-1)	4.9 (-8.4-20.1)	0 (0-0)	3.9 (-13.1-23.5)	0 (0-1)	5 (4-6)	0 (0-1)	0 (0-0)
Bury	0 (0-0)	10 (-2.4-24.6)	3 (2-4)	0.4 (-7.6-10.4)	0 (0-0)	-0.2 (-11.5-13.4)	0 (0-0)	-0.1 (-16.5-19.3)	0 (0-0)	3 (2-4)	0 (0-0)	0 (0-0)
Cheshire East	0 (0-1)	8.4 (-2.3-21.2)	5 (4-7)	-3.2 (-11-5.6)	0 (0-1)	-5.2 (-14.7-6.2)	0 (0-0)	-7.1 (-19.2-8.2)	0 (0-1)	5 (4-7)	0 (0-1)	0 (0-0)
Cheshire West and Chester	0 (0-1)	6.1 (-6.6-18.2)	5 (4-7)	-8.3 (-15.6--0.1)	0 (0-1)	-10.9 (-21.8--0.7)	0 (0-0)	-9.5 (-23.9-7.3)	0 (0-1)	5 (4-6)	0 (0-1)	0 (0-0)
Cumbria	1 (0-1)	-0.4 (-9.6-12)	7 (6-10)	-10 (-16.6--1.8)	1 (0-1)	-11.4 (-19.4--0.3)	0 (0-0)	-12 (-23.4-5.6)	1 (0-1)	7 (5-9)	1 (0-1)	0 (0-0)
Halton	0 (0-0)	2.6 (-8.1-16.1)	2 (2-3)	-0.1 (-9.5-9.9)	0 (0-0)	-0.8 (-11.3-11.7)	0 (0-0)	-2.7 (-16.6-13.3)	0 (0-0)	2 (2-3)	0 (0-0)	0 (0-0)
Knowsley	0 (0-0)	4.7 (-5.4-17.1)	3 (2-4)	-12.9 (-19.7--4.4)	0 (0-0)	-14.5 (-23.3--3.6)	0 (0-0)	-7.1 (-20.7-8.9)	0 (0-0)	2 (2-3)	0 (0-0)	0 (0-0)
Lancashire	1 (1-2)	7.7 (-3.2-20.6)	18 (14-24)	-5 (-12.6-4)	2 (1-2)	-6.6 (-16.3-4)	1 (0-1)	-6.2 (-19.1-9.4)	1 (1-2)	17 (13-23)	2 (1-2)	1 (0-1)
Liverpool	1 (0-1)	23.5 (9.1-37.5)	7 (6-10)	5.2 (-4.1-15.1)	1 (0-1)	0.4 (-11.4-11.8)	0 (0-0)	10.2 (-7-29.3)	1 (1-1)	8 (6-10)	1 (0-1)	0 (0-0)
Manchester	1 (0-1)	56.3 (40.8-77.1)	7 (5-9)	46.2 (32.9-60.2)	1 (0-1)	43.4 (28.6-62.2)	0 (0-0)	51 (29.8-78.6)	1 (1-2)	11 (8-14)	1 (1-1)	0 (0-1)

Location	Birth , 2000	Births percent change (2000-2021)	All age cases, 2000	All age cases percent change (2000-2021)	All age deaths, 2000	All age deaths percent change (2000-2021)	Under-5 deaths, 2000	Under-5 deaths percent change (2000-2021)	Births, 2021	All age cases, 2021	All age deaths, 2021	Under-5 deaths, 2021
Oldham	0 (0-0)	12.3 (1-25.2)	4 (3-5)	4.8 (-3.7-13.5)	0 (0-1)	4.6 (-6.2-16.6)	0 (0-0)	3.7 (-13.1-20.2)	0 (0-1)	4 (3-5)	0 (0-1)	0 (0-0)
Rochdale	0 (0-0)	12.6 (2-25.4)	4 (3-5)	0.2 (-7.4-8.4)	0 (0-0)	-1 (-10.5-9.9)	0 (0-0)	-0.3 (-12.6-15.4)	0 (0-0)	4 (3-5)	0 (0-0)	0 (0-0)
Salford	0 (0-0)	40.2 (24.2-57)	4 (3-5)	25.8 (14.4-37)	0 (0-0)	25.2 (11-40.7)	0 (0-0)	34.5 (12.6-58.6)	0 (0-1)	5 (3-6)	0 (0-1)	0 (0-0)
Sefton	0 (0-0)	1.6 (-9.1-15.2)	4 (3-6)	-15.8 (-22.9--7.8)	0 (0-1)	-17.5 (-26--6.7)	0 (0-0)	-11.2 (-25.2-3.6)	0 (0-0)	4 (3-5)	0 (0-0)	0 (0-0)
St Helens	0 (0-0)	3.3 (-8.3-15.9)	3 (2-4)	-11.5 (-19--3.2)	0 (0-0)	-13 (-22.8--2.1)	0 (0-0)	-8.8 (-23.7-6.7)	0 (0-0)	3 (2-3)	0 (0-0)	0 (0-0)
Stockport	0 (0-0)	7 (-3.4-19.6)	5 (3-6)	-5.7 (-13.1-2.9)	0 (0-1)	-5.3 (-14.6-5.9)	0 (0-0)	-3.5 (-17.9-14.3)	0 (0-0)	4 (3-6)	0 (0-1)	0 (0-0)
Tameside	0 (0-0)	14.4 (1.9-30.3)	4 (3-5)	2 (-6.9-11.6)	0 (0-0)	1.4 (-9.7-15.5)	0 (0-0)	3.7 (-10.6-23.6)	0 (0-0)	4 (3-5)	0 (0-0)	0 (0-0)
Trafford	0 (0-0)	12.2 (-1.5-25.2)	3 (3-4)	14.7 (4.2-24.6)	0 (0-0)	16.4 (1.9-29.9)	0 (0-0)	12.9 (-5-31.8)	0 (0-0)	4 (3-5)	0 (0-0)	0 (0-0)
Warrington	0 (0-0)	2.2 (-7.8-15)	3 (2-4)	-0.5 (-8.7-8.8)	0 (0-0)	-2.4 (-11.8-9.7)	0 (0-0)	-6.7 (-19.7-7.8)	0 (0-0)	3 (2-4)	0 (0-0)	0 (0-0)
Wigan	0 (0-1)	7 (-4.4-21.5)	5 (4-7)	0.6 (-7.8-9.9)	0 (0-1)	-0.4 (-11.3-13.8)	0 (0-0)	-2.4 (-16.5-16.8)	0 (0-1)	5 (4-7)	0 (0-1)	0 (0-0)
Wirral	0 (0-1)	2.6 (-8.3-13.7)	5 (4-7)	-6.6 (-14-1.7)	0 (0-1)	-8.9 (-18-1.3)	0 (0-0)	-8.6 (-21.6-5.9)	0 (0-1)	5 (4-6)	0 (0-1)	0 (0-0)
South East England	10 (7-14)	15.6 (11.5-19)	129 (99-166)	8.3 (5.4-11.1)	11 (7-15)	8.1 (4.3-11.5)	4 (2-6)	6.3 (1.5-10.9)	11 (8-16)	139 (106-180)	12 (8-16)	4 (2-6)
Bracknell Forest	0 (0-0)	-3.2 (-16.2-8.6)	2 (1-3)	-3.9 (-12.8-4.9)	0 (0-0)	-3.7 (-16.8-8.4)	0 (0-0)	-9.7 (-24.2-7.2)	0 (0-0)	2 (1-2)	0 (0-0)	0 (0-0)
Brighton and Hove	0 (0-0)	10 (-2.1-25.1)	4 (3-5)	13.1 (3.3-23.6)	0 (0-0)	9.3 (-2.4-23.3)	0 (0-0)	2.3 (-12.1-21.2)	0 (0-0)	4 (3-6)	0 (0-0)	0 (0-0)
Buckinghamshire	1 (0-1)	7.2 (-5.5-22)	8 (6-10)	1 (-8-10.4)	1 (0-1)	1.3 (-10.2-15.5)	0 (0-0)	-2.7 (-15.9-16.5)	1 (0-1)	8 (6-10)	1 (0-1)	0 (0-0)
East Sussex	1 (0-1)	13.1 (-1.1-28.5)	7 (6-10)	1.6 (-7.9-12.7)	1 (0-1)	-2.3 (-14.4-10.5)	0 (0-0)	-5.7 (-20.4-11.2)	1 (0-1)	8 (6-10)	1 (0-1)	0 (0-0)
Hampshire	1 (1-2)	8.1 (-5.8-20.2)	20 (15-26)	0.2 (-8.2-8.5)	2 (1-2)	-1 (-13.3-9.9)	1 (0-1)	-4.7 (-21.1-13.7)	1 (1-2)	20 (15-26)	2 (1-2)	1 (0-1)
Isle of Wight	0 (0-0)	17.4 (4.8-31.7)	2 (1-2)	-3.5 (-11.4-5.5)	0 (0-0)	-6.5 (-16.4-4.6)	0 (0-0)	-4.3 (-18.2-12.9)	0 (0-0)	2 (1-2)	0 (0-0)	0 (0-0)
Kent	2 (1-2)	25.3 (11.5-40.2)	22 (16-28)	13 (3.1-23)	2 (1-3)	11.7 (-0.6-25.2)	1 (0-1)	11.6 (-5.6-28.4)	2 (1-3)	24 (19-32)	2 (1-3)	1 (0-1)
Medway	0 (0-0)	21.9 (7.4-37.2)	4 (3-6)	6.2 (-3.2-15.1)	0 (0-1)	4.3 (-8.7-18.1)	0 (0-0)	8.1 (-9.6-27.4)	0 (0-1)	5 (4-6)	0 (0-1)	0 (0-0)
Milton Keynes	0 (0-0)	27.9 (14.1-44)	4 (3-5)	31 (19.9-43.3)	0 (0-0)	35 (20.1-52.5)	0 (0-0)	31 (11.9-52.3)	0 (0-1)	5 (4-6)	0 (0-1)	0 (0-0)
Oxfordshire	1 (1-1)	11.2 (-1.3-24.8)	10 (7-13)	6.8 (-1.6-16.5)	1 (1-1)	6 (-6.4-19.2)	0 (0-0)	3.6 (-12.2-21.1)	1 (1-1)	10 (8-14)	1 (1-1)	0 (0-0)
Portsmouth	0 (0-0)	26.6 (13.2-42.9)	3 (2-4)	20.1 (10.8-30.8)	0 (0-0)	20 (7.6-35.7)	0 (0-0)	21.2 (4-42.9)	0 (0-0)	4 (3-5)	0 (0-0)	0 (0-0)
Reading	0 (0-0)	27.8 (13.6-51.1)	2 (2-3)	24.4 (13.8-38.3)	0 (0-0)	30.3 (15.9-54.9)	0 (0-0)	34.4 (13.9-64.8)	0 (0-0)	3 (2-4)	0 (0-0)	0 (0-0)
Slough	0 (0-0)	38.7 (24.3-56.1)	2 (2-3)	50.2 (36-66.2)	0 (0-0)	58.3 (40.6-79)	0 (0-0)	53.6 (32.5-82.9)	0 (0-0)	3 (2-4)	0 (0-0)	0 (0-0)
Southampton	0 (0-0)	34 (20-48.5)	4 (3-5)	18.4 (9.1-28.7)	0 (0-0)	19.8 (7.3-33.5)	0 (0-0)	28.8 (10.2-46.7)	0 (0-1)	4 (3-6)	0 (0-1)	0 (0-0)
Surrey	1 (1-2)	12.4 (0.3-26.4)	17 (13-22)	8.9 (-0.9-18.2)	1 (1-2)	10.6 (-1.6-24.5)	0 (0-1)	5.8 (-11.2-25.5)	1 (1-2)	18 (14-24)	1 (1-2)	0 (0-1)
West Berkshire	0 (0-0)	1 (-10.4-14.6)	2 (2-3)	1.9 (-6.2-11.8)	0 (0-0)	3.3 (-7.4-17.3)	0 (0-0)	-2.5 (-18-14.5)	0 (0-0)	2 (2-3)	0 (0-0)	0 (0-0)
West Sussex	1 (1-1)	14.7 (1.5-31.3)	12 (9-15)	5.7 (-3.2-15.9)	1 (1-1)	4.5 (-7.4-20.1)	0 (0-1)	3.3 (-12.5-23.3)	1 (1-1)	12 (9-16)	1 (1-1)	0 (0-1)

Location	Birth , 2000	Births percent change (2000-2021)	All age cases, 2000	All age cases percent change (2000-2021)	All age deaths, 2000	All age deaths percent change (2000-2021)	Under-5 deaths, 2000	Under-5 deaths percent change (2000-2021)	Births, 2021	All age cases, 2021	All age deaths, 2021	Under-5 deaths, 2021
Windsor and Maidenhead	0 (0-0)	5.6 (-6.4-16.6)	2 (2-3)	8.6 (0.2-18.1)	0 (0-0)	9.4 (-2.2-20.4)	0 (0-0)	1 (-13.9-15.1)	0 (0-0)	2 (2-3)	0 (0-0)	0 (0-0)
Wokingham	0 (0-0)	-6.6 (-16.6-4.7)	2 (2-3)	-5.3 (-13.9-3.2)	0 (0-0)	-3 (-13.1-9.2)	0 (0-0)	-9.8 (-23.6-4)	0 (0-0)	2 (2-3)	0 (0-0)	0 (0-0)
South West England	5 (4-7)	19 (15.2-23.5)	76 (58-98)	6.1 (3.3-9)	6 (4-9)	4.5 (0.8-8.4)	2 (1-3)	5.5 (0.3-10.7)	6 (4-9)	80 (62-104)	7 (5-9)	2 (1-4)
Bath and North East Somerset	0 (0-0)	13.7 (0.9-27.3)	3 (2-3)	-0.9 (-9.2-7.7)	0 (0-0)	-3.9 (-14.7-7.9)	0 (0-0)	-3.4 (-17.8-13.3)	0 (0-0)	3 (2-3)	0 (0-0)	0 (0-0)
Bournemouth	0 (0-0)	55.2 (35-75.2)	2 (2-3)	30.3 (18.3-42.8)	0 (0-0)	27.6 (12.2-44.6)	0 (0-0)	38.5 (13.5-62.5)	0 (0-0)	3 (2-4)	0 (0-0)	0 (0-0)
Bristol, City of	1 (0-1)	39.2 (24.4-58.4)	6 (5-8)	25.3 (14.5-36.6)	1 (0-1)	25.7 (12.7-42.7)	0 (0-0)	29.4 (9.3-53)	1 (0-1)	8 (6-10)	1 (0-1)	0 (0-0)
Cornwall	1 (0-1)	19.6 (6-36.7)	7 (6-10)	3 (-6.3-13.3)	1 (0-1)	0.1 (-11.2-15)	0 (0-0)	-0.3 (-16.7-19.2)	1 (0-1)	8 (6-10)	1 (0-1)	0 (0-0)
Devon	1 (0-1)	14.6 (2.5-28.3)	10 (8-13)	0.8 (-8-9.9)	1 (1-1)	-0.8 (-11.3-10.9)	0 (0-0)	1 (-14.4-18.2)	1 (1-1)	10 (8-14)	1 (1-1)	0 (0-0)
Dorset	0 (0-0)	8.7 (-4.8-24.2)	5 (4-7)	-2.9 (-11.7-5.8)	0 (0-1)	-4.9 (-15.7-9.5)	0 (0-0)	-4.7 (-18.9-15.2)	0 (0-1)	5 (4-7)	0 (0-1)	0 (0-0)
Gloucestershire	1 (0-1)	14.3 (1-27.2)	9 (7-12)	1.3 (-6.4-10.5)	1 (1-1)	-0.6 (-12.6-10.7)	0 (0-0)	-0.2 (-17-16.6)	1 (1-1)	9 (7-12)	1 (1-1)	0 (0-0)
North Somerset	0 (0-0)	20.5 (7.6-34.7)	3 (2-4)	7.4 (-0.8-16.7)	0 (0-0)	7.3 (-4.1-19.6)	0 (0-0)	5.8 (-9.5-23.4)	0 (0-0)	3 (2-4)	0 (0-0)	0 (0-0)
Plymouth	0 (0-0)	21.8 (8.9-37.4)	4 (3-5)	8.6 (-0.5-19.5)	0 (0-0)	6 (-5.7-19.6)	0 (0-0)	12.3 (-3.7-32.9)	0 (0-0)	4 (3-5)	0 (0-0)	0 (0-0)
Poole	0 (0-0)	12.2 (1-27.3)	2 (2-3)	8.1 (-0.6-17.4)	0 (0-0)	7.2 (-3.2-20.9)	0 (0-0)	6.5 (-8.2-25.4)	0 (0-0)	2 (2-3)	0 (0-0)	0 (0-0)
Somerset	1 (0-1)	16.1 (3.9-33)	8 (6-10)	1.9 (-6.5-11.6)	1 (0-1)	0.1 (-10.6-14.8)	0 (0-0)	0.7 (-15.1-20.1)	1 (0-1)	8 (6-10)	1 (0-1)	0 (0-0)
South Gloucestershire	0 (0-0)	13.1 (0-26.6)	4 (3-5)	3.2 (-5.5-12.1)	0 (0-0)	2 (-9.9-14.7)	0 (0-0)	2.5 (-11.7-19.6)	0 (0-0)	4 (3-5)	0 (0-0)	0 (0-0)
Swindon	0 (0-0)	32.1 (19.9-48.9)	3 (2-4)	27.5 (18-39.3)	0 (0-0)	26.2 (14.2-42.3)	0 (0-0)	23.9 (7.8-47)	0 (0-0)	4 (3-5)	0 (0-0)	0 (0-0)
Torbay	0 (0-0)	15.5 (3-28.8)	2 (1-2)	-2.7 (-10.5-5.8)	0 (0-0)	-2.9 (-13.4-7.7)	0 (0-0)	3.4 (-12.8-20.2)	0 (0-0)	2 (1-2)	0 (0-0)	0 (0-0)
Wiltshire	1 (0-1)	7.5 (-2.9-19.8)	7 (5-9)	4.5 (-4.8-14.2)	1 (0-1)	3.1 (-7.7-16.8)	0 (0-0)	-4.5 (-17.2-12.5)	1 (0-1)	7 (6-10)	1 (0-1)	0 (0-0)
West Midlands	7 (5-10)	16.7 (12.2-22.1)	87 (66-113)	6.8 (3.4-10.4)	8 (5-11)	6.1 (2-11)	3 (2-5)	6.3 (0.8-12.4)	8 (6-11)	93 (71-120)	8 (6-11)	3 (2-5)
Birmingham	2 (1-2)	23.6 (12-42.2)	18 (14-23)	18.9 (8.6-32.3)	2 (1-2)	17 (5.8-35.3)	1 (0-1)	15.2 (-0.5-36.8)	2 (1-3)	21 (16-28)	2 (1-3)	1 (0-1)
Coventry	0 (0-1)	29.2 (11.6-44.7)	5 (4-7)	12.8 (2.4-23.3)	0 (0-1)	12.5 (-2.8-26.6)	0 (0-0)	16.9 (-0.4-35.6)	1 (0-1)	6 (4-8)	1 (0-1)	0 (0-0)
Dudley	0 (0-1)	9.5 (-4.3-24)	5 (4-6)	1.8 (-8.2-11.3)	0 (0-1)	1.7 (-11.1-15.1)	0 (0-0)	1.9 (-14.3-21)	0 (0-1)	5 (4-6)	0 (0-1)	0 (0-0)
Herefordshire, County of	0 (0-0)	9.6 (-2.5-23.3)	3 (2-3)	-4.3 (-12.5-4.1)	0 (0-0)	-7.8 (-17.9-3.2)	0 (0-0)	-9.3 (-22.5-6.1)	0 (0-0)	3 (2-3)	0 (0-0)	0 (0-0)
Sandwell	0 (0-1)	27.2 (13.5-41.9)	5 (4-6)	22.8 (12.1-33.2)	0 (0-1)	23 (9.8-37.6)	0 (0-0)	21.7 (5.9-40.5)	1 (0-1)	6 (5-8)	1 (0-1)	0 (0-0)
Shropshire	0 (0-0)	7.9 (-3-21.3)	4 (3-6)	0.1 (-8.6-8.5)	0 (0-1)	-2.8 (-11.9-9.7)	0 (0-0)	-6.5 (-20.1-11.3)	0 (0-0)	4 (3-6)	0 (0-0)	0 (0-0)
Solihull	0 (0-0)	18.6 (6.4-32.8)	3 (2-4)	-0.6 (-8.5-8.2)	0 (0-0)	-2.5 (-12.8-9.3)	0 (0-0)	1.5 (-12.8-20.4)	0 (0-0)	3 (2-4)	0 (0-0)	0 (0-0)
Staffordshire	1 (1-1)	6.8 (-5.7-20.8)	13 (9-16)	-4 (-13-4.7)	1 (1-1)	-5.7 (-16.9-6.1)	0 (0-1)	-7.3 (-22.8-10.5)	1 (1-1)	12 (9-16)	1 (1-1)	0 (0-1)
Stoke-on-Trent	0 (0-0)	14.8 (2.7-32.2)	4 (3-5)	8.2 (-1-19.5)	0 (0-1)	10.1 (-1.6-25.7)	0 (0-0)	11.2 (-3.6-33.7)	0 (0-1)	4 (3-6)	0 (0-1)	0 (0-0)
Telford and Wrekin	0 (0-0)	9.1 (-3.1-22.9)	3 (2-4)	-0.7 (-8.5-8.7)	0 (0-0)	-1.1 (-12.3-11.8)	0 (0-0)	-3.1 (-17.8-13.5)	0 (0-0)	3 (2-4)	0 (0-0)	0 (0-0)

Location	Birth , 2000	Births percent change (2000-2021)	All age cases, 2000	All age cases percent change (2000-2021)	All age deaths, 2000	All age deaths percent change (2000-2021)	Under-5 deaths, 2000	Under-5 deaths percent change (2000-2021)	Births, 2021	All age cases, 2021	All age deaths, 2021	Under-5 deaths, 2021
Walsall	0 (0-1)	20.4 (7.2-34.3)	4 (3-6)	11 (1.8-21.3)	0 (0-1)	10.4 (-1.8-23.5)	0 (0-0)	11 (-7.2-29.9)	0 (0-1)	5 (4-6)	0 (0-1)	0 (0-0)
Warwickshire	1 (0-1)	16.3 (5.7-30.2)	8 (6-10)	5.6 (-3-15.2)	1 (0-1)	4.6 (-5.1-17.4)	0 (0-0)	4.1 (-9.4-22.7)	1 (0-1)	8 (6-11)	1 (0-1)	0 (0-0)
Wolverhampton	0 (0-0)	19.2 (6.9-33.5)	4 (3-5)	11.5 (2.6-22.2)	0 (0-1)	11.3 (-0.5-25.7)	0 (0-0)	14.9 (-0.4-33)	0 (0-1)	4 (3-6)	0 (0-1)	0 (0-0)
Worcestershire	1 (0-1)	5.3 (-5.9-21.2)	8 (6-11)	-4.5 (-12.2-4.5)	1 (0-1)	-5.3 (-15-8.6)	0 (0-0)	-6.6 (-20.7-14.7)	1 (0-1)	8 (6-11)	1 (0-1)	0 (0-0)
Yorkshire and the Humber	6 (4-9)	15.7 (11.1-19.6)	81 (62-105)	5.7 (2.8-8.5)	7 (5-10)	4.4 (0.4-8.2)	3 (2-4)	4.9 (-0.6-9.9)	7 (5-10)	86 (66-111)	8 (5-10)	3 (2-4)
Barnsley	0 (0-0)	18.1 (6.5-30.6)	4 (3-5)	4.4 (-3.5-13.2)	0 (0-0)	2.8 (-7.6-14.3)	0 (0-0)	2.9 (-12.7-19.5)	0 (0-0)	4 (3-5)	0 (0-0)	0 (0-0)
Bradford	1 (1-1)	11.9 (-0.7-26.8)	8 (6-11)	17.7 (6.3-29)	1 (1-1)	16.7 (3.5-31.9)	0 (0-1)	11.2 (-5.7-30.8)	1 (1-1)	10 (8-13)	1 (1-1)	0 (0-1)
Calderdale	0 (0-0)	10.1 (-2.9-22.9)	3 (2-4)	2.3 (-7.2-13.2)	0 (0-0)	0.1 (-12.2-12)	0 (0-0)	-3.5 (-18.8-13.2)	0 (0-0)	3 (2-4)	0 (0-0)	0 (0-0)
Doncaster	0 (0-1)	9.8 (-1.4-22.9)	5 (4-6)	2.2 (-6.4-12.7)	0 (0-1)	0.6 (-9.7-13.3)	0 (0-0)	0 (-14.4-15.9)	0 (0-1)	5 (4-6)	0 (0-1)	0 (0-0)
East Riding of Yorkshire	0 (0-0)	5 (-7.3-16.5)	5 (4-6)	-5.8 (-14.4-2.9)	0 (0-1)	-7.7 (-18.6-2.7)	0 (0-0)	-9.4 (-24.3-4.6)	0 (0-0)	4 (3-6)	0 (0-1)	0 (0-0)
Kingston upon Hull, City of	0 (0-1)	13.8 (2.4-27.2)	4 (3-6)	3.8 (-5.5-13.1)	0 (0-1)	3 (-7.9-15.3)	0 (0-0)	8.9 (-5.5-25)	0 (0-1)	4 (3-6)	0 (0-1)	0 (0-0)
Kirklees	1 (0-1)	7.5 (-4-20.8)	7 (5-9)	6.4 (-2.7-15.5)	1 (0-1)	4.3 (-7.2-17.8)	0 (0-0)	-1.4 (-16.6-16.3)	1 (0-1)	7 (5-9)	1 (0-1)	0 (0-0)
Leeds	1 (1-1)	35.1 (20.2-50.8)	12 (9-15)	12.6 (3-22.9)	1 (1-1)	12.1 (-0.5-25.3)	0 (0-1)	21.2 (3.1-43.6)	1 (1-2)	13 (10-17)	1 (1-2)	0 (0-1)
North East Lincolnshire	0 (0-0)	7.9 (-3.6-20)	3 (2-4)	-7.3 (-15.4-1.4)	0 (0-0)	-9.8 (-19.5-0.3)	0 (0-0)	-7.6 (-21.6-8.4)	0 (0-0)	2 (2-3)	0 (0-0)	0 (0-0)
North Lincolnshire	0 (0-0)	19.2 (8.2-32.6)	2 (2-3)	10.1 (1.7-19.6)	0 (0-0)	8.7 (-1.6-21.2)	0 (0-0)	7.7 (-6.3-24.2)	0 (0-0)	3 (2-3)	0 (0-0)	0 (0-0)
North Yorkshire	1 (0-1)	5.2 (-7.7-17.8)	9 (7-11)	-3.4 (-11.7-5.5)	1 (0-1)	-4.6 (-15.7-6.4)	0 (0-0)	-5.8 (-21.6-10.2)	1 (0-1)	8 (6-11)	1 (0-1)	0 (0-0)
Rotherham	0 (0-0)	11.3 (0.3-23.9)	4 (3-5)	-1.6 (-9.5-6.9)	0 (0-1)	-3.3 (-13.5-7.7)	0 (0-0)	-3.8 (-18.2-12.5)	0 (0-0)	4 (3-5)	0 (0-0)	0 (0-0)
Sheffield	1 (0-1)	25.2 (10.8-39.2)	8 (6-10)	14.2 (3.8-24.9)	1 (0-1)	11.9 (-0.7-24.3)	0 (0-0)	13.2 (-4-31.2)	1 (1-1)	9 (7-12)	1 (1-1)	0 (0-0)
Wakefield	0 (0-1)	12.9 (-0.3-26.1)	5 (4-7)	-1.4 (-10.5-7.3)	0 (0-1)	-2.3 (-13.6-9.6)	0 (0-0)	-0.3 (-15.7-17)	0 (0-1)	5 (4-7)	0 (0-1)	0 (0-0)
York	0 (0-0)	22.4 (7.4-34.6)	3 (2-3)	9.9 (0.3-19.5)	0 (0-0)	6.8 (-5.9-17.2)	0 (0-0)	7.3 (-8.9-23.9)	0 (0-0)	3 (2-4)	0 (0-0)	0 (0-0)
Northern Ireland	3 (2-4)	15.2 (5.6-27)	52 (40-66)	3.5 (-4.8-11.8)	3 (2-4)	1.4 (-7.9-13)	1 (1-2)	-2.8 (-15.4-13.3)	3 (2-5)	53 (41-68)	3 (2-4)	1 (1-1)
Scotland	7 (5-10)	0 (-9.6-9.9)	142 (110-183)	-4.1 (-11.1-4.1)	8 (6-12)	-6.1 (-15-3.8)	2 (2-4)	-13.1 (-24.8-0.7)	7 (5-10)	136 (105-173)	8 (6-11)	2 (1-3)
Wales	4 (3-6)	7.4 (-5.1-21.2)	81 (62-104)	-0.8 (-9.2-7.4)	5 (3-7)	-3.5 (-15.4-9.1)	1 (1-2)	-6.4 (-21.2-10.3)	4 (3-6)	80 (62-103)	5 (3-6)	1 (1-2)
Latin America and Caribbean	2,960 (2,450-3,500)	3.5 (-0.6-7.9)	44,000 (37,500-51,200)	9 (5.6-12.6)	2,380 (1,950-2,860)	18 (13-23.5)	129 (82-184)	12.4 (3.4-21.3)	3,060 (2,500-3,630)	48,000 (40,500-56,400)	2,810 (2,290-3,370)	145 (93-210)
**Andean Latin America**	131 (103-168)	9.3 (3.1-15.5)	1,990 (1,620-2,450)	24.7 (18.6-31)	92 (70-119)	24.3 (15.7-32.7)	7 (4-10)	17.5 (7.5-28.7)	143 (111-182)	2,480 (2,020-3,030)	114 (88-147)	8 (5-12)
Bolivia (Plurinational State of)	30 (22-40)	24.3 (11.8-36.9)	407 (309-530)	31.4 (21.2-41.9)	20 (14-28)	24.9 (10.4-39.2)	2 (1-3)	31 (11-54.6)	37 (27-52)	534 (397-705)	25 (17-35)	2 (1-4)
Ecuador	33 (30-36)	20 (12.2-29.6)	551 (503-602)	30 (22.6-38.3)	24 (21-27)	39.8 (29.4-53)	1 (1-2)	17.8 (2.3-36.1)	40 (36-44)	716 (652-793)	33 (29-37)	2 (1-2)
Peru	68 (50-93)	-2.5 (-12.1-7.2)	1,060 (807-1,380)	15.7 (6-25.6)	49 (34-68)	13.5 (1.6-25.6)	3 (2-5)	5.7 (-8.9-22.4)	66 (48-89)	1,220 (932-1,560)	56 (39-76)	4 (2-6)
**Caribbean**	2,030 (1,650-2,420)	5.8 (0.4-12.4)	26,700 (22,300-31,200)	7.4 (2.5-13.4)	1,580 (1,280-1,900)	18 (11.3-25.7)	86 (52-124)	18.9 (6.1-34)	2,140 (1,720-2,540)	28,700 (23,900-33,800)	1,870 (1,480-2,230)	102 (64-149)

Location	Birth , 2000	Births percent change (2000-2021)	All age cases, 2000	All age cases percent change (2000-2021)	All age deaths, 2000	All age deaths percent change (2000-2021)	Under-5 deaths, 2000	Under-5 deaths percent change (2000-2021)	Births, 2021	All age cases, 2021	All age deaths, 2021	Under-5 deaths, 2021
Antigua and Barbuda	2 (1-3)	-29.9 (-38.6--19.4)	34 (25-44)	-14.4 (-22.8--4.8)	2 (1-2)	2 (-11.3-18.8)	0 (0-0)	-22.9 (-34.4--8.2)	1 (1-2)	29 (22-38)	2 (1-2)	0 (0-0)
Bahamas	14 (10-19)	-14 (-25.6--0.6)	252 (190-324)	-6.4 (-17.4-6.4)	15 (11-22)	10.4 (-5.8-28.1)	0 (0-1)	-24.9 (-38.8--6.6)	12 (8-16)	236 (179-304)	17 (12-23)	0 (0-1)
Barbados	5 (4-8)	-21.7 (-31.7--8.5)	97 (74-127)	-11.5 (-20.4--1.7)	5 (4-7)	6.1 (-8.1-23)	0 (0-0)	-19.5 (-31.7--2)	4 (3-6)	86 (65-112)	5 (4-8)	0 (0-0)
Belize	11 (8-15)	32.8 (14.9-53.6)	168 (125-223)	42.9 (26.9-60.7)	9 (6-13)	67.2 (42.9-96.6)	0 (0-1)	30.5 (7.7-62)	15 (10-20)	240 (180-314)	16 (11-22)	1 (0-1)
Bermuda	1 (1-1)	-36.2 (-44.4--26)	18 (14-24)	-28.2 (-35.2--19.9)	1 (1-1)	-10 (-21.5-3.7)	0 (0-0)	-35.6 (-45.1--21)	1 (0-1)	13 (10-17)	1 (1-1)	0 (0-0)
Cuba	64 (51-81)	1.6 (-13.7-17.4)	1,350 (1,110-1,660)	-5.9 (-17.7-8.9)	72 (56-91)	13.9 (-3.8-31.8)	2 (1-2)	7.7 (-11.9-27.5)	65 (50-83)	1,270 (1,010-1,600)	82 (63-104)	2 (1-3)
Dominica	2 (1-3)	-33.8 (-43.4--23.9)	33 (25-43)	-29.1 (-36.5--20.8)	2 (1-3)	-17.6 (-30.3--4.7)	0 (0-0)	-37 (-48.4--24.7)	1 (1-2)	23 (18-30)	2 (1-2)	0 (0-0)
Dominican Republic	594 (449-745)	0.4 (-10.7-13.6)	7,230 (5,630-8,770)	8.9 (-2.1-20.4)	431 (318-551)	12.8 (-1-28.5)	24 (14-36)	3.6 (-12.4-26.2)	597 (443-751)	7,870 (6,140-9,600)	486 (359-616)	24 (15-37)
Grenada	3 (2-4)	-26 (-36.8--15.3)	52 (40-67)	-24 (-32.3--14.2)	3 (2-4)	-15.5 (-27.9--2)	0 (0-0)	-27 (-40.5--9.9)	2 (2-3)	39 (30-52)	2 (2-3)	0 (0-0)
Guyana	29 (20-40)	-12 (-22.1--0.4)	461 (347-612)	-18.4 (-26.5--10)	25 (17-35)	-9 (-20.2-3.5)	1 (1-2)	-18.7 (-30.7--4)	26 (18-35)	377 (280-496)	23 (16-32)	1 (1-2)
Haiti	940 (764-1,090)	22.6 (13.3-36.2)	10,200 (8,480-12,000)	32.3 (22.8-44.9)	649 (520-766)	41.4 (29.4-58)	50 (29-73)	33.9 (12.5-56.5)	1,150 (940-1,340)	13,600 (11,400-15,900)	918 (741-1,070)	67 (41-99)
Jamaica	168 (156-183)	-29.7 (-39.4--19.4)	3,450 (3,090-3,870)	-32.8 (-40.8--24)	180 (164-199)	-20.1 (-31.4--8.5)	3 (2-4)	-35.6 (-46.6--24.1)	118 (99-141)	2,320 (1,970-2,770)	144 (120-173)	2 (1-2)
Puerto Rico	70 (48-96)	-57.8 (-62.6--51.4)	1,390 (1,050-1,790)	-43.1 (-48.5--36.6)	72 (48-100)	-27.8 (-36.5--17)	1 (1-2)	-59.2 (-66.3--50.4)	30 (20-42)	792 (600-1,030)	52 (35-73)	1 (0-1)
Saint Kitts and Nevis	1 (1-2)	-14 (-24.9--2.6)	22 (16-28)	-7.9 (-17.2-1.9)	1 (1-2)	2.3 (-11.4-17.9)	0 (0-0)	-14.8 (-30.4-0.5)	1 (1-1)	20 (15-26)	1 (1-2)	0 (0-0)
Saint Lucia	5 (3-6)	-38.3 (-45.7--28.6)	84 (63-112)	-30.2 (-36.9--22.1)	5 (3-7)	-19.7 (-29.9--5.3)	0 (0-0)	-40.4 (-48.3--28.3)	3 (2-4)	59 (45-77)	4 (3-5)	0 (0-0)
Saint Vincent and the Grenadines	3 (2-5)	-28.3 (-36.9--17.9)	59 (44-78)	-25.8 (-33.4--16.9)	3 (2-5)	-17.5 (-28.5--4.9)	0 (0-0)	-30.1 (-41.7--14.5)	2 (2-3)	44 (33-57)	3 (2-4)	0 (0-0)
Suriname	16 (11-21)	-6.2 (-17.6-7.3)	231 (175-300)	9.4 (-1.9-22)	13 (9-18)	18.5 (4.2-37.2)	1 (0-1)	6.2 (-10.7-29.1)	15 (10-20)	253 (191-334)	16 (11-22)	1 (0-1)
Trinidad and Tobago	27 (19-38)	-13.6 (-28--1.2)	588 (449-768)	-17 (-27.6--7.8)	37 (26-52)	-17.9 (-31.4--5.8)	1 (0-1)	-9 (-26.4-6.4)	24 (16-32)	488 (370-639)	30 (21-41)	1 (0-1)
United States Virgin Islands	3 (2-4)	-33.3 (-41.6--24)	46 (34-59)	-30.6 (-37.5--22.4)	2 (2-3)	-22.2 (-32--11.6)	0 (0-0)	-32 (-42.8--19.8)	2 (1-2)	32 (24-42)	2 (1-3)	0 (0-0)
**Central Latin America**	182 (142-232)	-47.6 (-53--41)	3,400 (2,720-4,230)	-48.3 (-54--42.1)	142 (108-184)	-33.8 (-40.9--26.3)	9 (6-13)	-42.8 (-50.7--34)	95 (70-128)	1,760 (1,330-2,320)	94 (68-128)	5 (3-8)
Colombia	6 (4-8)	-17.2 (-30.9--0.3)	82 (62-105)	-11 (-23.9-5.1)	5 (4-6)	-10.2 (-24.1-8.3)	0 (0-0)	-17.1 (-34.6-5.5)	5 (4-6)	73 (54-95)	4 (3-6)	0 (0-0)
Costa Rica	0 (0-0)	-12.1 (-28.1--12.2)	1 (0-2)	-2.4 (-21.3-2.4)	0 (0-0)	-2.5 (-21.2-4.1)	0 (0-0)	-13.8 (-34.5--3.2)	0 (0-0)	1 (0-2)	0 (0-0)	0 (0-0)
El Salvador	0 (0-0)	-22.8 (-22.8--22.8)	0 (0-2)	-15 (-19.6--11.2)	0 (0-0)	-12.2 (-16--7.8)	0 (0-0)	-25.8 (-35--17.6)	0 (0-0)	0 (0-2)	0 (0-0)	0 (0-0)
Guatemala	0 (0-0)	11.7 (12.8-12.8)	1 (0-4)	6.7 (1.6-12.9)	0 (0-0)	16.2 (11.7-22.8)	0 (0-0)	-14.8 (-26--1.6)	0 (0-0)	1 (0-4)	0 (0-0)	0 (0-0)
Honduras	0 (0-0)	25.3 (24.3-24.4)	0 (0-1)	39.1 (31.9-45.3)	0 (0-0)	41.9 (36.6-48.8)	0 (0-0)	24.5 (10.7-52.7)	0 (0-0)	1 (0-2)	0 (0-0)	0 (0-0)
Mexico	61 (44-86)	-17.3 (-20.8--13)	1,060 (765-1,440)	-1.8 (-5.6-2.8)	47 (33-68)	5 (0-11.1)	3 (2-5)	-14 (-18.9--9.3)	51 (36-74)	1,050 (736-1,430)	50 (34-72)	3 (2-4)
Aguascalientes	0 (0-0)	7.6 (-4.1-601)	1 (0-3)	28.9 (10.3-732)	0 (0-0)	38 (18.6-799)	0 (0-0)	10.5 (-12.6-574)	0 (0-0)	1 (0-3)	0 (0-0)	0 (0-0)
Baja California	1 (1-2)	13.4 (4.2-36.6)	23 (12-38)	38 (27-72.7)	1 (1-2)	53.7 (41.2-83.6)	0 (0-0)	16.4 (2.1-45)	1 (1-3)	32 (17-53)	2 (1-3)	0 (0-0)
Baja California Sur	0 (0-1)	31.9 (16.6-45)	9 (5-13)	63.4 (47.7-79.9)	0 (0-1)	79.2 (58.8-99.9)	0 (0-0)	35.3 (14.4-64)	1 (0-1)	14 (8-21)	1 (0-1)	0 (0-0)

Location	Birth , 2000	Births percent change (2000-2021)	All age cases, 2000	All age cases percent change (2000-2021)	All age deaths, 2000	All age deaths percent change (2000-2021)	Under-5 deaths, 2000	Under-5 deaths percent change (2000-2021)	Births, 2021	All age cases, 2021	All age deaths, 2021	Under-5 deaths, 2021
Campeche	1 (1-1)	-0.7 (-11.6-10.4)	16 (11-23)	6.4 (-3.3-17.4)	1 (0-1)	11.6 (-1.4-26.6)	0 (0-0)	0.8 (-16.3-18.5)	1 (1-1)	17 (11-24)	1 (1-1)	0 (0-0)
Chiapas	4 (3-5)	6.4 (-5.6-16.5)	58 (41-80)	16.3 (5.5-27)	3 (2-4)	17.2 (4-29.4)	0 (0-0)	9 (-8.3-26.9)	4 (3-6)	68 (47-92)	3 (2-5)	0 (0-0)
Chihuahua	1 (0-1)	-14.9 (-25.7-30.7)	14 (6-26)	3.2 (-10.8-60.8)	1 (0-1)	13.7 (-2-81.6)	0 (0-0)	-10.6 (-30.2-45.2)	1 (0-1)	14 (6-27)	1 (0-1)	0 (0-0)
Coahuila	1 (0-1)	5.3 (-6.5-21.5)	14 (8-22)	19.6 (6.5-37.3)	1 (0-1)	28 (13.9-50.1)	0 (0-0)	5.5 (-13.5-34.6)	1 (0-1)	17 (10-26)	1 (0-1)	0 (0-0)
Colima	0 (0-0)	-4.6 (-36.7-11.2)	4 (1-8)	10.5 (-21.9-28.6)	0 (0-0)	16.6 (-23.5-35.4)	0 (0-0)	-1.6 (-34.6-19.2)	0 (0-0)	4 (1-10)	0 (0-0)	0 (0-0)
Durango	1 (0-1)	-9.6 (-22.2-14.6)	10 (4-19)	11.3 (-4.4-38.3)	0 (0-1)	20.1 (1.9-53.4)	0 (0-0)	-4.7 (-23.3-23.2)	1 (0-1)	11 (4-22)	0 (0-1)	0 (0-0)
Guanajuato	1 (1-2)	-24.1 (-49.7-14.4)	22 (13-37)	-5.2 (-36.3-37.1)	1 (1-2)	5.4 (-29.2-67.1)	0 (0-0)	-19.9 (-49.1-21.4)	1 (1-2)	21 (12-36)	1 (1-2)	0 (0-0)
Guerrero	7 (5-9)	-30.8 (-37.3--24.3)	98 (78-122)	-14.3 (-21.4--6.3)	4 (3-6)	-10 (-18.6-0)	0 (0-1)	-26.2 (-37.4--14.1)	5 (4-6)	84 (66-107)	4 (3-5)	0 (0-0)
Hidalgo	1 (0-1)	-11.8 (-24.1-15.1)	11 (5-21)	10.2 (-4.9-38.6)	0 (0-1)	19.3 (1.8-54.5)	0 (0-0)	-8.1 (-26.9-22.5)	1 (0-1)	12 (5-24)	1 (0-1)	0 (0-0)
Jalisco	3 (2-4)	-28.2 (-51.4--16.7)	51 (28-78)	-20.1 (-46.1--7.1)	2 (1-4)	-9.4 (-37-5.5)	0 (0-0)	-25.4 (-48.4--8.2)	2 (1-3)	41 (20-67)	2 (1-3)	0 (0-0)
México	2 (0-3)	3.5 (-10.7-187)	26 (8-50)	23.3 (2.8-226)	1 (0-2)	32.6 (10.8-279)	0 (0-0)	11.6 (-14.9-214)	2 (0-4)	33 (10-74)	2 (0-3)	0 (0-0)
Mexico City	2 (1-3)	-38.1 (-42.5--27.5)	43 (29-61)	-20.2 (-27.4--8.3)	2 (1-3)	-13.2 (-20.5-2.7)	0 (0-0)	-32 (-41.3--17.6)	1 (1-2)	34 (23-49)	1 (1-2)	0 (0-0)
Michoacán de Ocampo	2 (1-3)	-20.1 (-36.5--4.4)	29 (15-49)	-7.6 (-25.5-7.9)	1 (1-2)	-4.5 (-25.1-16.5)	0 (0-0)	-16.8 (-34.6-2.3)	1 (1-2)	26 (14-46)	1 (1-2)	0 (0-0)
Morelos	1 (0-1)	-22 (-52.3--10.9)	12 (5-24)	-14.8 (-47.9-2)	1 (0-1)	-6.5 (-40.9-8.6)	0 (0-0)	-19.4 (-50--0.7)	0 (0-1)	10 (4-22)	1 (0-1)	0 (0-0)
Nayarit	0 (0-1)	3.7 (-7.8-21.9)	9 (4-16)	17.1 (1.7-35.4)	0 (0-1)	24.1 (6.9-50.2)	0 (0-0)	4.9 (-15.2-32.2)	0 (0-1)	11 (5-19)	0 (0-1)	0 (0-0)
Nuevo León	3 (2-4)	-27.3 (-36.6--19.6)	57 (39-79)	-8.8 (-19.3-1.3)	2 (1-3)	-1.7 (-14.8-10.1)	0 (0-0)	-23.1 (-35.5--10.9)	2 (1-3)	52 (35-74)	2 (1-3)	0 (0-0)
Oaxaca	6 (4-7)	-23.2 (-28.5--17.2)	89 (68-116)	-11.7 (-17.5--5.1)	4 (3-6)	-6.8 (-14.5-2)	0 (0-0)	-20.3 (-31.5--7.7)	4 (3-6)	79 (61-101)	4 (3-5)	0 (0-0)
Puebla	1 (1-2)	-7.3 (-21.8-65.6)	18 (8-36)	9.9 (-8.7-86.4)	1 (0-2)	18 (-1.3-119)	0 (0-0)	-2 (-23.8-92.6)	1 (0-2)	20 (8-38)	1 (0-2)	0 (0-0)
Querétaro	0 (0-0)	11.8 (0.4-632)	1 (0-4)	31.1 (12.4-739)	0 (0-0)	39.4 (20.2-790)	0 (0-0)	13.9 (-11.4-709)	0 (0-0)	2 (0-5)	0 (0-0)	0 (0-0)
Quintana Roo	1 (1-2)	25.8 (14.3-37.6)	19 (12-28)	43.2 (30-59.9)	1 (0-1)	54.9 (42-72.8)	0 (0-0)	29.7 (12-49.5)	1 (1-2)	27 (16-42)	1 (1-2)	0 (0-0)
San Luis Potosí	1 (0-2)	-18.8 (-33.4-5.7)	18 (8-33)	-2.9 (-20.7-28.3)	1 (0-2)	3.8 (-13.9-37.2)	0 (0-0)	-16.1 (-33.6-13.9)	1 (0-2)	17 (8-32)	1 (0-2)	0 (0-0)
Sinaloa	2 (1-3)	-23.5 (-31.8--13.9)	33 (21-48)	-9.6 (-19.1-1.1)	1 (1-2)	-3.2 (-13.8-10.3)	0 (0-0)	-23 (-36.8--6.6)	1 (1-2)	30 (19-44)	1 (1-2)	0 (0-0)
Sonora	1 (1-2)	-32 (-50.3--19.3)	20 (11-33)	-15.2 (-33.2--1)	1 (1-2)	-9 (-33.3-9.3)	0 (0-0)	-29.4 (-48.3--13.5)	1 (0-1)	17 (9-28)	1 (0-1)	0 (0-0)
Tabasco	6 (5-7)	-9.8 (-18.3-0.2)	104 (88-123)	1.4 (-6.3-10.2)	5 (4-6)	7.5 (-3.3-20.2)	0 (0-0)	-7.2 (-22-9.3)	5 (4-6)	106 (88-125)	5 (4-7)	0 (0-0)
Tamaulipas	4 (3-6)	-22.7 (-30.2--15.7)	76 (60-96)	1 (-7.9-10.1)	3 (2-4)	11.2 (-0.7-22.3)	0 (0-0)	-17.1 (-30.7-0.9)	3 (3-5)	77 (60-98)	4 (3-5)	0 (0-0)
Tlaxcala	0 (0-0)	-4.4 (-18.3-28.9)	4 (2-9)	15.6 (-2.5-53.5)	0 (0-0)	25.9 (6.7-72.3)	0 (0-0)	-1.3 (-24.8-41.3)	0 (0-0)	5 (2-10)	0 (0-0)	0 (0-0)
Veracruz de Ignacio de la Llave	8 (6-10)	-24.5 (-31.7--17.1)	137 (106-174)	-8.7 (-16.3--0.1)	6 (4-8)	-2.7 (-12.2-8.6)	0 (0-1)	-23.6 (-35.7--8.8)	6 (4-8)	125 (96-161)	6 (4-8)	0 (0-0)
Yucatán	2 (1-3)	-9.9 (-20.2--0.8)	36 (24-50)	2.1 (-7.5-11.7)	2 (1-2)	8.2 (-4.4-20.9)	0 (0-0)	-7.7 (-21.8-7.5)	2 (1-2)	36 (24-52)	2 (1-3)	0 (0-0)
Zacatecas	0 (0-0)	-10 (-21-304)	1 (0-4)	2.4 (-13.9-368)	0 (0-0)	6.8 (-9.1-381)	0 (0-0)	-6.9 (-24.8-322)	0 (0-0)	1 (0-4)	0 (0-0)	0 (0-0)

Location	Birth , 2000	Births percent change (2000-2021)	All age cases, 2000	All age cases percent change (2000-2021)	All age deaths, 2000	All age deaths percent change (2000-2021)	Under-5 deaths, 2000	Under-5 deaths percent change (2000-2021)	Births, 2021	All age cases, 2021	All age deaths, 2021	Under-5 deaths, 2021
Nicaragua	0 (0-0)	11.9 (0.9-1570)	0 (0-1)	23.9 (5.8-1580)	0 (0-0)	23.9 (6.4-1730)	0 (0-0)	13.6 (-12.6-1580)	0 (0-0)	0 (0-1)	0 (0-0)	0 (0-0)
Panama	0 (0-0)	18.8 (22.8-22.9)	0 (0-1)	28.6 (24.8-37.2)	0 (0-0)	29.7 (30-37.8)	0 (0-0)	19.8 (-8.3-35.1)	0 (0-0)	0 (0-1)	0 (0-0)	0 (0-0)
Venezuela (Bolivarian Republic of)	114 (90-144)	-65.5 (-70.3--59.1)	2,260 (1,820-2,710)	-73 (-76.6--68.6)	90 (68-117)	-57.2 (-63.4--49.4)	5 (4-8)	-63.5 (-71.2--53.7)	40 (30-52)	609 (468-783)	39 (28-51)	2 (1-3)
**Tropical Latin America**	620 (533-725)	9.9 (3-16.4)	11,900 (10,500-13,400)	26.1 (19.8-32.5)	567 (480-672)	30 (21.9-38.3)	28 (19-40)	8.4 (-0.4-18.3)	682 (554-818)	15,100 (12,800-17,400)	737 (602-890)	30 (20-43)
Brazil	588 (508-686)	10.4 (3.3-17.6)	11,400 (10,000-12,800)	26.6 (20-33)	540 (460-636)	30.8 (22-39.2)	26 (18-37)	9.1 (-0.2-20)	649 (531-782)	14,400 (12,300-16,600)	707 (581-853)	29 (19-41)
Acre	2 (2-3)	62 (45.7-80.1)	34 (27-41)	77 (62.5-95)	2 (2-3)	81.9 (61.9-104)	0 (0-0)	49 (27.9-73.7)	4 (3-5)	60 (48-74)	4 (3-5)	0 (0-0)
Alagoas	4 (4-6)	-24.1 (-34.3--12.6)	68 (58-82)	-8.7 (-20.4-5)	3 (2-4)	-6 (-19.1-8.7)	0 (0-0)	-20.8 (-34.9--3.2)	3 (3-4)	62 (49-79)	3 (2-4)	0 (0-0)
Amapá	1 (1-1)	55 (34-79.7)	14 (10-18)	72.5 (54-92.5)	1 (1-1)	60.1 (38.7-84.8)	0 (0-0)	37.7 (12.1-65.8)	1 (1-2)	24 (16-33)	1 (1-2)	0 (0-0)
Amazonas	3 (2-3)	61.1 (45.2-78.5)	54 (43-67)	78.7 (62.1-97)	2 (2-3)	83.2 (63.8-105)	0 (0-0)	48.2 (20.9-73.7)	4 (3-6)	96 (75-125)	4 (3-6)	0 (0-0)
Bahia	51 (43-60)	62.9 (44.4-82.5)	896 (775-1,030)	82.4 (66-100)	38 (31-46)	94.5 (71.8-121)	2 (1-3)	71.6 (44.8-105)	83 (67-101)	1,630 (1,380-1,920)	74 (59-91)	4 (2-5)
Ceará	8 (6-10)	33.5 (21.4-44.3)	121 (97-150)	46.8 (34.6-58.8)	6 (5-8)	40.6 (26.5-54.9)	0 (0-1)	31.3 (13.2-52.8)	11 (8-14)	177 (142-220)	9 (7-11)	0 (0-1)
Distrito Federal	28 (24-32)	-18.8 (-27.2--9.6)	554 (493-620)	10.5 (1.6-20)	24 (20-28)	9.5 (-2.6-22.1)	1 (1-1)	-20.2 (-32.4--4.6)	23 (19-28)	613 (528-706)	26 (22-32)	1 (1-1)
Espírito Santo	21 (18-25)	10.3 (-3-25)	345 (299-391)	29.9 (19.1-40.9)	20 (16-23)	27 (11.2-44.2)	1 (1-1)	9 (-10.5-33)	23 (19-28)	448 (384-519)	25 (20-30)	1 (1-1)
Goiás	21 (18-24)	15.6 (2.7-29.3)	407 (360-458)	36 (24-48.4)	20 (16-23)	44.2 (27.7-62.2)	1 (1-1)	11.1 (-8.2-38.2)	24 (19-29)	553 (472-643)	28 (23-34)	1 (1-1)
Maranhão	24 (20-29)	21.6 (8.1-39.6)	271 (229-320)	57 (41.8-73.9)	15 (12-19)	44.5 (26.7-66.5)	1 (1-2)	32.4 (11.1-57.4)	29 (23-38)	425 (349-522)	22 (17-29)	2 (1-2)
Mato Grosso	5 (4-6)	30.6 (19.1-43)	102 (85-121)	41.2 (29.5-53.5)	5 (4-6)	53.4 (37.9-70.1)	0 (0-0)	23.1 (7.5-45.5)	6 (5-8)	145 (118-176)	7 (6-9)	0 (0-0)
Mato Grosso do Sul	4 (3-5)	27 (13.2-41.9)	88 (74-105)	40.1 (27.4-52.5)	4 (3-5)	51.2 (34.1-71)	0 (0-0)	20.1 (0.4-44.9)	5 (4-7)	123 (100-150)	6 (4-7)	0 (0-0)
Minas Gerais	84 (71-99)	-0.9 (-11.2-12)	1,480 (1,310-1,670)	13.6 (4.2-24.2)	83 (69-98)	13.6 (1.6-28.1)	5 (3-6)	-5.1 (-20.1-16.8)	83 (68-101)	1,680 (1,430-1,950)	94 (77-113)	4 (3-6)
Pará	12 (10-14)	18.5 (4.3-34.3)	212 (184-244)	41.8 (26.4-56.6)	11 (9-14)	27.5 (11.5-46.1)	1 (0-1)	12.3 (-6.4-34.6)	14 (11-18)	301 (244-361)	14 (11-18)	1 (0-1)
Paraíba	4 (3-5)	35.9 (20.3-50.1)	65 (52-80)	50 (34.4-64.2)	3 (2-4)	48.1 (30.4-64.6)	0 (0-0)	38.5 (13.6-64.1)	6 (4-7)	97 (76-124)	5 (3-6)	0 (0-0)
Paraná	9 (7-10)	26.1 (13.4-40.9)	193 (163-227)	35.8 (24-49)	8 (7-10)	52.2 (36.6-72.4)	0 (0-0)	17.4 (-0.7-40)	11 (8-13)	263 (214-316)	12 (10-15)	0 (0-1)
Pernambuco	7 (6-9)	24 (11-39.8)	123 (100-150)	44.1 (32.8-57.3)	5 (4-7)	56.7 (40.6-76.9)	0 (0-0)	30.8 (8.4-55.6)	9 (7-12)	177 (141-220)	8 (6-11)	0 (0-1)
Piaui	21 (18-24)	3.3 (-8.4-15.8)	324 (281-369)	19 (8.5-29.6)	14 (12-17)	18.3 (3.5-33.8)	1 (1-1)	10.4 (-8.7-33.4)	22 (18-26)	385 (326-449)	17 (14-21)	1 (1-1)
Rio de Janeiro	86 (75-98)	-11 (-21--0.3)	1,730 (1,560-1,940)	0.7 (-8-9)	88 (76-102)	6.3 (-5.7-18.3)	5 (4-8)	-9.1 (-25.3-10.7)	76 (64-90)	1,750 (1,510-2,010)	93 (78-111)	5 (3-7)
Rio Grande do Norte	4 (3-5)	21.6 (7.8-38.8)	62 (50-77)	34.2 (19.9-51.1)	3 (2-4)	41 (24-63)	0 (0-0)	24.1 (3.6-46.2)	5 (4-7)	83 (64-109)	4 (3-6)	0 (0-0)
Rio Grande do Sul	9 (7-11)	13.7 (2.2-27.5)	221 (187-258)	25.8 (15.3-38.4)	10 (8-12)	34.7 (21.4-51.3)	0 (0-0)	5.3 (-12.1-25.3)	10 (8-13)	278 (228-337)	13 (10-16)	0 (0-1)
Rondônia	5 (4-6)	9.6 (-3.2-23.9)	87 (72-104)	20.9 (10.3-32.7)	5 (4-7)	19.1 (4.7-33.8)	0 (0-0)	1.5 (-16.8-21.7)	6 (4-7)	105 (86-127)	6 (5-8)	0 (0-0)
Roraima	3 (2-4)	19.1 (4.7-34.2)	40 (34-48)	50.9 (37-65.1)	2 (2-3)	42.6 (23-63.2)	0 (0-0)	15.7 (-5.5-40.6)	4 (3-4)	60 (49-74)	3 (2-4)	0 (0-0)
Santa Catarina	3 (2-3)	16.6 (5.3-29.6)	66 (53-82)	24.8 (13-37.3)	3 (2-3)	34.3 (20.3-52.3)	0 (0-0)	9.1 (-8-30.6)	3 (2-4)	83 (64-105)	4 (3-5)	0 (0-0)

Location	Birth , 2000	Births percent change (2000-2021)	All age cases, 2000	All age cases percent change (2000-2021)	All age deaths, 2000	All age deaths percent change (2000-2021)	Under-5 deaths, 2000	Under-5 deaths percent change (2000-2021)	Births, 2021	All age cases, 2021	All age deaths, 2021	Under-5 deaths, 2021
São Paulo	154 (132-178)	7.5 (-3.2-19.6)	3,550 (3,120-3,990)	24.2 (14.4-33.9)	152 (128-179)	34.3 (20.2-49.8)	5 (3-7)	6.5 (-10.3-27.5)	166 (136-200)	4,410 (3,790-5,100)	204 (169-245)	5 (3-8)
Sergipe	6 (5-7)	8.4 (-5.5-22)	107 (89-126)	23.3 (10.2-36.3)	5 (4-6)	25.9 (8.5-43.3)	0 (0-0)	5.7 (-11.9-25.3)	7 (5-8)	132 (106-162)	7 (5-8)	0 (0-0)
Tocantins	10 (8-12)	26.3 (14.1-40.6)	151 (129-176)	55.6 (44.5-69.2)	8 (6-9)	55.7 (40-75.9)	0 (0-1)	25.1 (6.2-46.8)	12 (10-15)	235 (199-272)	12 (9-14)	1 (0-1)
Paraguay	32 (23-44)	-0.2 (-9.4-9)	569 (434-729)	15.7 (6.2-25.4)	27 (18-38)	13.7 (2.1-27.4)	2 (1-3)	-0.9 (-14.8-16.8)	32 (23-44)	658 (501-853)	30 (21-42)	2 (1-3)
North Africa and Middle East	25,700 (22,600-29,500)	-38.7 (-43.4--34.4)	663,000 (586,000-747,000)	-16.3 (-22.9--10.1)	9,940 (8,540-11,700)	2.1 (-7.4-11.3)	593 (407-830)	-21.2 (-28.4--14)	15,800 (12,900-18,800)	555,000 (457,000-660,000)	10,100 (8,220-12,500)	467 (314-679)
**North Africa and Middle East**	25,700 (22,600-29,500)	-38.7 (-43.4--34.4)	663,000 (586,000-747,000)	-16.3 (-22.9--10.1)	9,940 (8,540-11,700)	2.1 (-7.4-11.3)	593 (407-830)	-21.2 (-28.4--14)	15,800 (12,900-18,800)	555,000 (457,000-660,000)	10,100 (8,220-12,500)	467 (314-679)
Afghanistan	938 (701-1,210)	70.4 (57.5-83.3)	14,900 (11,200-19,200)	106 (91.1-122)	234 (173-319)	129 (110-151)	29 (17-46)	79.7 (55.7-108)	1,600 (1,210-2,060)	30,700 (23,100-39,700)	536 (395-715)	53 (32-83)
Algeria	86 (68-105)	21.5 (13.7-31.5)	2,780 (2,210-3,340)	23.2 (15.3-32.2)	42 (32-52)	58.3 (45.6-72.6)	2 (1-3)	31.2 (16.5-48.3)	105 (83-128)	3,430 (2,750-4,160)	67 (52-83)	3 (2-4)
Bahrain	155 (124-189)	-25.5 (-37--16.7)	5,650 (4,590-6,920)	48.1 (26.8-65.3)	92 (71-118)	132 (89.7-168)	3 (2-5)	-20 (-35.4--5.5)	115 (90-146)	8,370 (6,620-10,500)	214 (161-278)	3 (2-4)
Egypt	2,580 (2,180-2,980)	6.9 (-0.9-15.2)	66,400 (56,200-76,500)	44.6 (34.2-55.4)	1,080 (896-1,290)	57 (44.9-70.1)	71 (48-101)	27 (12.6-44.3)	2,760 (2,340-3,160)	96,000 (81,600-110,000)	1,700 (1,420-1,990)	90 (61-132)
Iran (Islamic Republic of)	3,100 (2,360-3,920)	-41.7 (-42.9--40.3)	107,000 (82,200-136,000)	-28.5 (-30.3--26.4)	1,610 (1,190-2,120)	-0.4 (-7.3-6.3)	83 (54-122)	-30.8 (-33.5--27.6)	1,810 (1,380-2,310)	76,800 (59,400-96,700)	1,600 (1,190-2,090)	57 (38-84)
Alborz	114 (86-146)	-38.2 (-42.9--32.2)	3,210 (2,440-4,070)	-16.7 (-23.7--9.3)	46 (33-61)	23.1 (7.9-37.8)	2 (1-3)	-25.4 (-33.7--14.4)	71 (54-93)	2,670 (2,040-3,430)	56 (42-75)	2 (1-2)
Ardebil	58 (44-74)	-55.9 (-59.3--52)	1,980 (1,510-2,520)	-42.8 (-47.1--37.5)	28 (21-37)	-12.7 (-23.2--1)	2 (1-3)	-48.5 (-56.4--41.2)	26 (19-33)	1,140 (857-1,440)	24 (18-32)	1 (1-1)
Bushehr	43 (33-55)	-28.9 (-34.4--22.8)	1,410 (1,080-1,780)	-7.5 (-14.5-0.4)	20 (15-26)	24 (12-35.7)	1 (1-2)	-15.7 (-26.2--5.4)	31 (23-40)	1,300 (994-1,660)	24 (18-32)	1 (1-1)
Chahar Mahaal and Bakhtiari	43 (33-55)	-45.1 (-49.2--40.3)	1,440 (1,100-1,830)	-31.9 (-37.1--26.2)	19 (14-25)	0.6 (-9.9-11.9)	1 (1-2)	-36.6 (-45--27)	24 (18-30)	983 (748-1,250)	19 (14-25)	1 (0-1)
East Azarbayejan	156 (119-200)	-49.6 (-53.5--44.8)	5,470 (4,220-6,900)	-34.8 (-39.6--28.6)	89 (66-119)	-10.4 (-19.9-1.6)	4 (3-6)	-34.6 (-43.1--26.2)	79 (59-100)	3,570 (2,750-4,500)	80 (59-104)	3 (2-4)
Fars	185 (140-241)	-44.3 (-49.3--39.6)	7,070 (5,360-9,090)	-33.4 (-39.1--27.7)	111 (82-150)	-5.2 (-17.2-5.9)	5 (3-8)	-35.6 (-44.2--26.4)	103 (77-133)	4,710 (3,580-6,000)	105 (77-139)	3 (2-5)
Gilan	84 (64-108)	-56.1 (-59.4--52.5)	3,600 (2,750-4,640)	-41.9 (-46.4--37.4)	58 (42-76)	-12.2 (-21.6--2.2)	2 (1-3)	-50.6 (-57.5--43.1)	37 (28-48)	2,090 (1,590-2,710)	50 (38-67)	1 (1-1)
Golestan	81 (61-104)	-41.8 (-46.7--36.5)	2,590 (1,980-3,300)	-27.7 (-33.7--21.2)	39 (28-52)	-0.3 (-10.5-13.7)	2 (2-3)	-28.9 (-38.1--17.9)	47 (36-60)	1,870 (1,430-2,370)	39 (29-51)	2 (1-2)
Hamadan	77 (59-99)	-57.6 (-61.3--54.1)	2,780 (2,140-3,530)	-46.6 (-50.9--42.4)	44 (33-59)	-22.8 (-31.6--14.2)	2 (1-3)	-49.3 (-55.1--41.9)	33 (25-42)	1,480 (1,130-1,900)	34 (25-44)	1 (1-2)
Hormozgan	70 (53-90)	-23.4 (-29.6--16.5)	2,070 (1,580-2,650)	-5.6 (-13.3-3.1)	30 (22-39)	25.3 (12.6-39.9)	2 (1-3)	-11.8 (-21.3-0.5)	54 (41-68)	1,960 (1,500-2,460)	37 (27-49)	2 (1-3)
Ilam	26 (20-33)	-56.8 (-60.3--52.9)	893 (675-1,140)	-38.6 (-43.5--32.6)	13 (9-17)	-6.8 (-17.8-4.8)	1 (0-1)	-49.4 (-55.7--42)	11 (9-15)	548 (417-701)	12 (9-16)	0 (0-1)
Isfahan	153 (116-196)	-48.2 (-52--43.9)	6,750 (5,090-8,620)	-31.1 (-36.4--24.9)	102 (75-136)	-0.9 (-10.8-10.3)	4 (3-6)	-39.8 (-47.9--31.5)	79 (60-101)	4,660 (3,510-5,880)	101 (74-132)	2 (2-4)
Kerman	110 (84-144)	-26.9 (-33.1--21.6)	3,840 (2,910-4,930)	-15.2 (-22.2--8.6)	57 (41-76)	13.8 (1.1-27.5)	3 (2-5)	-14.9 (-24.8--1.3)	81 (61-104)	3,250 (2,500-4,110)	65 (48-85)	3 (2-4)
Kermanshah	86 (66-112)	-51.4 (-54.7--47.7)	3,150 (2,410-4,040)	-44 (-48.2--39.4)	50 (37-67)	-18.2 (-27.8--7.6)	2 (2-3)	-43.6 (-50--35.9)	42 (32-54)	1,770 (1,360-2,270)	41 (30-54)	1 (1-2)
Khorasan-e-Razavi	280 (212-360)	-38.3 (-42.7--32.4)	8,560 (6,540-10,900)	-25 (-30.5--18.4)	131 (96-175)	0.9 (-9.8-12.8)	8 (5-12)	-25.4 (-34.6--14)	173 (131-222)	6,420 (4,970-8,080)	132 (98-174)	6 (4-9)
Khuzestan	231 (174-295)	-41.6 (-46.5--36.4)	7,450 (5,650-9,520)	-31.6 (-37.3--25.8)	106 (77-142)	-6.7 (-16.8-3.8)	6 (4-9)	-31.4 (-40.4--20.7)	135 (102-174)	5,100 (3,900-6,500)	99 (73-131)	4 (3-6)
Kohgiluyeh and Boyer-Ahmad	40 (30-51)	-43.7 (-48.3--37.7)	1,080 (818-1,380)	-27.9 (-33.7--20.6)	16 (12-21)	-2.1 (-14.4-11.8)	1 (1-2)	-34 (-41.7--25.2)	23 (17-29)	779 (597-999)	16 (11-21)	1 (0-1)
Kurdistan	66 (50-84)	-45.4 (-49.5--40.8)	2,320 (1,770-2,960)	-34.2 (-38.9--28.9)	34 (26-46)	-4.3 (-15.4-6.7)	2 (1-3)	-37.5 (-45.8--26.7)	36 (27-46)	1,530 (1,170-1,930)	33 (24-43)	1 (1-2)

Location	Birth , 2000	Births percent change (2000-2021)	All age cases, 2000	All age cases percent change (2000-2021)	All age deaths, 2000	All age deaths percent change (2000-2021)	Under-5 deaths, 2000	Under-5 deaths percent change (2000-2021)	Births, 2021	All age cases, 2021	All age deaths, 2021	Under-5 deaths, 2021
Lorestan	87 (66-113)	-55.4 (-58.3--52.1)	2,890 (2,200-3,700)	-43.3 (-47.4--39)	40 (29-54)	-13.9 (-23.3--4.7)	2 (2-4)	-48.3 (-54.8--39.7)	39 (30-50)	1,640 (1,280-2,090)	34 (26-45)	1 (1-2)
Markazi	50 (38-65)	-51.3 (-55.1--46.6)	2,050 (1,570-2,630)	-40 (-44.6--34.7)	32 (23-43)	-12.5 (-22--1.6)	1 (1-2)	-43.9 (-51.3--35.5)	24 (18-31)	1,230 (941-1,560)	28 (20-36)	1 (0-1)
Mazandaran	106 (81-137)	-50.4 (-54.4--46.9)	4,310 (3,310-5,470)	-32.9 (-38.4--28.3)	66 (48-88)	-0.5 (-10.7-10.3)	3 (2-4)	-42.9 (-50.9--35.2)	53 (40-67)	2,890 (2,210-3,630)	66 (49-85)	1 (1-2)
North Khorasan	49 (37-62)	-57.2 (-60.7--53.4)	1,240 (943-1,570)	-35.6 (-40.6--29.8)	19 (14-26)	-13.9 (-22.3--4.2)	1 (1-2)	-47.1 (-54.3--40.6)	21 (16-27)	799 (604-1,020)	17 (12-22)	1 (0-1)
Qazvin	45 (33-57)	-42.4 (-46.6--37.6)	1,770 (1,330-2,280)	-32.8 (-38.2--27.1)	25 (18-34)	1 (-11.7-13.1)	1 (1-2)	-34.6 (-42.6--27.2)	26 (20-33)	1,190 (910-1,510)	26 (19-34)	1 (1-1)
Qom	40 (31-52)	-26.2 (-33.1--20.1)	1,530 (1,180-1,940)	-13.9 (-21.3--7.4)	21 (16-29)	18.9 (6.1-32.1)	1 (1-2)	-15.7 (-25.6--5.2)	30 (22-38)	1,310 (1,010-1,650)	25 (18-34)	1 (1-1)
Semnan	20 (15-25)	-25.5 (-32.8--18.6)	843 (650-1,070)	-17.7 (-25.4--9.8)	13 (9-17)	12 (-1.3-25.5)	1 (0-1)	-19.4 (-30--6.4)	15 (11-19)	694 (533-891)	14 (11-19)	0 (0-1)
Sistan and Baluchistan	174 (131-221)	-26.2 (-32--19.2)	3,710 (2,830-4,710)	-8.8 (-15.7--0.2)	53 (38-71)	9 (-1.7-21.8)	5 (3-8)	-10 (-22.9-4.2)	128 (97-168)	3,380 (2,560-4,370)	57 (42-77)	5 (3-7)
South Khorasan	34 (26-44)	-39.1 (-45.3--33.4)	998 (767-1,280)	-20.1 (-27.5--12.8)	14 (11-19)	5.9 (-7.9-20.2)	1 (1-2)	-28.2 (-40.1--13.3)	21 (16-27)	798 (615-1,010)	15 (11-20)	1 (0-1)
Tehran	374 (288-476)	-37.2 (-42.6--32)	15,100 (11,700-19,000)	-22.3 (-28.7--15.8)	224 (166-296)	8.5 (-2.7-21.3)	7 (5-10)	-23.9 (-33.8--12.3)	235 (177-299)	11,800 (9,050-14,900)	243 (181-319)	5 (4-8)
West Azarbayejan	131 (100-166)	-34.6 (-40--28.7)	4,330 (3,310-5,500)	-25.4 (-31.2--18.7)	67 (50-90)	-1.4 (-10.9-10.9)	4 (2-6)	-20.6 (-31.8--8.4)	85 (65-109)	3,230 (2,450-4,050)	66 (49-86)	3 (2-4)
Yazd	38 (29-49)	-33.2 (-38.6--26.6)	1,370 (1,050-1,740)	-19.3 (-25.5--11.2)	20 (15-26)	6.4 (-5.7-18)	1 (1-1)	-19.3 (-30.3--7.3)	26 (20-33)	1,110 (847-1,410)	21 (16-28)	1 (0-1)
Zanjan	42 (32-54)	-49.4 (-53.1--45.7)	1,520 (1,160-1,960)	-37.5 (-41.7--32.9)	21 (16-28)	-5.9 (-15.9-4.1)	1 (1-2)	-40.2 (-47.8--30.8)	21 (16-27)	948 (722-1,200)	20 (15-26)	1 (0-1)
Iraq	986 (825-1,160)	-0.7 (-7.4-6.9)	21,400 (18,200-25,000)	48.4 (38.8-59.7)	303 (250-367)	84.8 (70.7-101)	26 (18-36)	3.2 (-8.1-16.9)	979 (834-1,150)	31,800 (27,200-36,800)	559 (470-665)	27 (18-37)
Jordan	112 (86-146)	79.3 (66.1-94.2)	2,980 (2,270-3,820)	149 (130-169)	37 (28-50)	225 (195-255)	2 (2-4)	87.4 (64.7-111)	201 (153-258)	7,410 (5,700-9,570)	122 (91-161)	5 (3-7)
Kuwait	17 (13-22)	49.5 (36.6-61.6)	622 (481-775)	119 (100-136)	10 (7-13)	195 (164-225)	0 (0-0)	53.8 (35.3-71.8)	26 (20-32)	1,360 (1,060-1,700)	29 (22-38)	1 (0-1)
Lebanon	67 (54-83)	3.7 (-3.5-11.5)	1,670 (1,330-2,060)	34.3 (25.3-44.7)	29 (23-37)	56.5 (44.1-69.6)	2 (1-2)	17.6 (5.1-33.5)	69 (55-85)	2,240 (1,800-2,750)	46 (36-56)	2 (1-3)
Libya	285 (223-361)	-37.4 (-42.6--31.6)	8,860 (6,970-11,300)	13.7 (4.2-23.6)	128 (98-167)	73.6 (56.1-94.2)	8 (5-12)	-33.5 (-40.9--23.5)	178 (138-225)	10,100 (7,870-12,600)	222 (170-289)	6 (4-8)
Morocco	118 (92-145)	-31.4 (-35.9--26.2)	3,030 (2,370-3,700)	8.1 (1-16.4)	50 (38-62)	36.8 (26.7-48.6)	3 (2-4)	-19.9 (-29.2--9.7)	81 (63-99)	3,280 (2,580-3,960)	68 (52-84)	2 (2-3)
Oman	194 (165-221)	34.9 (17.8-51.4)	5,620 (4,790-6,440)	97.6 (73.2-122)	82 (68-97)	160 (122-200)	4 (3-6)	64.7 (35.8-91.9)	261 (208-313)	11,100 (8,750-13,300)	213 (165-266)	7 (5-10)
Palestine	99 (75-129)	-3.5 (-12.7-4.3)	1,960 (1,490-2,530)	57.3 (43.8-69.9)	23 (17-30)	92.6 (72.8-111)	2 (2-3)	9.6 (-4.5-24.5)	96 (73-124)	3,080 (2,340-3,960)	44 (32-58)	3 (2-4)
Qatar	10 (7-12)	142 (123-161)	374 (285-485)	401 (363-445)	7 (5-9)	461 (406-519)	0 (0-0)	188 (151-227)	23 (18-29)	1,880 (1,430-2,360)	38 (28-51)	1 (0-1)
Saudi Arabia	7,660 (7,120-8,200)	-79.3 (-83--75.3)	255,000 (239,000-273,000)	-58.8 (-66--51.1)	3,830 (3,400-4,300)	-49.1 (-59.7--37.3)	117 (78-165)	-79 (-83.5--74.2)	1,590 (1,290-1,920)	105,000 (85,500-127,000)	1,950 (1,520-2,450)	25 (16-36)
Sudan	8,540 (7,080-10,200)	-37 (-44.8--29.6)	141,000 (118,000-167,000)	-0.3 (-12.4-11.6)	2,030 (1,630-2,540)	12.3 (-2.1-27.2)	220 (143-320)	-22.1 (-35.1--8)	5,380 (4,340-6,620)	141,000 (113,000-173,000)	2,280 (1,780-2,890)	171 (109-261)
Syrian Arab Republic	56 (43-69)	-59.7 (-62.2--56.8)	1,370 (1,080-1,680)	-30.7 (-35.2--25.5)	19 (14-24)	2.6 (-6.1-13.7)	2 (1-2)	-58.4 (-62.9--53.4)	22 (18-28)	951 (749-1,160)	20 (15-24)	1 (0-1)
Tunisia	177 (137-228)	-24.7 (-31.3--18.3)	6,070 (4,710-7,800)	0.9 (-7.4-9.9)	94 (71-124)	36.3 (23.6-51.2)	4 (3-6)	-13.4 (-24.7--2.6)	133 (103-169)	6,130 (4,770-7,650)	128 (97-165)	4 (2-5)
Türkiye	373 (324-427)	-52.7 (-56.7--48.4)	12,300 (10,700-13,900)	-25.7 (-32--19)	196 (165-232)	0.9 (-8.6-12.1)	10 (7-13)	-50 (-56.9--41.6)	176 (150-206)	9,120 (7,900-10,600)	198 (165-237)	5 (3-7)
United Arab Emirates	13 (11-15)	-4.2 (-10.3-2.5)	619 (510-721)	136 (118-156)	10 (8-13)	284 (240-333)	0 (0-0)	66.1 (50.1-88)	12 (10-14)	1,460 (1,190-1,710)	40 (32-49)	0 (0-1)
Yemen	122 (98-147)	7.5 (0.3-14.8)	2,040 (1,650-2,460)	67.3 (57.1-77.6)	30 (24-38)	92.1 (77.9-109)	3 (2-5)	26.7 (8.1-45.5)	132 (106-158)	3,420 (2,790-4,090)	58 (46-72)	4 (3-6)

Location	Birth , 2000	Births percent change (2000-2021)	All age cases, 2000	All age cases percent change (2000-2021)	All age deaths, 2000	All age deaths percent change (2000-2021)	Under-5 deaths, 2000	Under-5 deaths percent change (2000-2021)	Births, 2021	All age cases, 2021	All age deaths, 2021	Under-5 deaths, 2021
South Asia	92,200 (63,900-128,000)	-14.9 (-17.2--12.2)	1,110,000 (825,000-1,500,000)	6.4 (3.2-9.8)	76,800 (52,900-109,000)	11.4 (7-16.7)	14,800 (8,620-23,400)	-15.4 (-19.8--10.7)	78,400 (54,600-109,000)	1,190,000 (881,000-1,580,000)	85,600 (59,600-119,000)	12,500 (7,410-19,900)
**South Asia**	92,200 (63,900-128,000)	-14.9 (-17.2--12.2)	1,110,000 (825,000-1,500,000)	6.4 (3.2-9.8)	76,800 (52,900-109,000)	11.4 (7-16.7)	14,800 (8,620-23,400)	-15.4 (-19.8--10.7)	78,400 (54,600-109,000)	1,190,000 (881,000-1,580,000)	85,600 (59,600-119,000)	12,500 (7,410-19,900)
Bangladesh	24 (17-34)	-26.2 (-45.1--17.4)	308 (213-433)	-12.6 (-34--1.7)	21 (15-30)	-8.2 (-31.7-1.7)	4 (2-7)	-24.5 (-47.3--8.7)	18 (12-25)	269 (184-379)	20 (14-27)	3 (2-5)
Bhutan	0 (0-0)	-32.6 (-45.5--27.9)	1 (1-2)	-17.3 (-32.4--10.2)	0 (0-0)	-12.8 (-28.3--7.1)	0 (0-0)	-25.6 (-39.5--12.4)	0 (0-0)	1 (1-2)	0 (0-0)	0 (0-0)
India	92,100 (63,900-128,000)	-14.9 (-17.2--12.2)	1,110,000 (825,000-1,500,000)	6.4 (3.2-9.8)	76,700 (52,800-109,000)	11.4 (7-16.7)	14,800 (8,620-23,400)	-15.4 (-19.8--10.7)	78,400 (54,500-109,000)	1,180,000 (880,000-1,580,000)	85,500 (59,500-119,000)	12,500 (7,400-19,800)
Nepal	19 (15-23)	-24.3 (-32.5--15.1)	212 (171-256)	-1.2 (-11.5-10.2)	12 (10-16)	7.5 (-5.2-22.5)	3 (2-4)	-17.8 (-34.1-1.3)	14 (12-18)	209 (170-254)	14 (11-17)	2 (1-3)
Pakistan	4 (1-8)	12.7 (10.4-13.1)	41 (10-82)	43.1 (37.5-48)	3 (1-5)	54.8 (51-59.1)	0 (0-1)	22.1 (12.8-32.1)	5 (1-10)	59 (15-119)	4 (1-8)	1 (0-1)
Azad Jammu & Kashmir	0 (0-0)	-10.9 (-10.8--10.8)	1 (0-2)	13 (6.4-19.3)	0 (0-0)	22.7 (17.9-28.3)	0 (0-0)	-4.2 (-17.9-11.5)	0 (0-0)	1 (0-2)	0 (0-0)	0 (0-0)
Balochistan	0 (0-0)	85.1 (85.4-85.4)	2 (1-4)	107 (95.1-115)	0 (0-0)	117 (109-123)	0 (0-0)	91.7 (67-127)	0 (0-1)	4 (1-9)	0 (0-1)	0 (0-0)
Gilgit-Baltistan	0 (0-0)	87.4 (87.6-87.6)	0 (0-1)	107 (98.9-116)	0 (0-0)	114 (109-119)	0 (0-0)	97.2 (72.5-134)	0 (0-0)	1 (0-1)	0 (0-0)	0 (0-0)
Islamabad Capital Territory	0 (0-0)	91.4 (92.1-92.1)	0 (0-1)	132 (123-142)	0 (0-0)	148 (141-159)	0 (0-0)	105 (78.9-135)	0 (0-0)	1 (0-1)	0 (0-0)	0 (0-0)
Khyber Pakhtunkhwa	1 (0-1)	61.1 (61.5-61.5)	7 (2-13)	80.6 (72.6-87.1)	0 (0-1)	88.8 (85-93.2)	0 (0-0)	72.8 (50.9-95.5)	1 (0-2)	12 (3-24)	1 (0-1)	0 (0-0)
Punjab	2 (1-4)	-6.7 (-6.5--6.5)	22 (5-43)	25.4 (17.9-31.9)	1 (0-3)	39.2 (34.9-44.4)	0 (0-1)	2.3 (-9.1-17.5)	2 (1-4)	27 (7-55)	2 (0-4)	0 (0-1)
Sindh	1 (0-2)	7 (7.2-7.2)	9 (2-18)	41.2 (32.7-48.9)	1 (0-1)	54.2 (48.9-59.2)	0 (0-0)	20.2 (4.3-39.6)	1 (0-2)	13 (3-26)	1 (0-2)	0 (0-0)
Southeast Asia, East Asia, and Oceania	630 (467-821)	-11.2 (-12.7--9.6)	23,700 (17,700-30,400)	-9.9 (-12.2--7.4)	696 (504-926)	-0.8 (-6.2-4.7)	31 (21-45)	-8.3 (-12--4.2)	559 (419-726)	21,400 (16,200-27,200)	691 (519-901)	29 (19-41)
**East Asia**	626 (464-819)	-11.2 (-12.7--9.6)	23,600 (17,600-30,300)	-9.9 (-12.2--7.3)	693 (503-923)	-0.8 (-6.2-4.7)	31 (20-45)	-8.3 (-12.1--4.1)	556 (414-724)	21,300 (16,100-27,200)	688 (514-899)	29 (19-41)
China	596 (442-779)	-9.7 (-11.4--8)	22,800 (17,000-29,300)	-10.3 (-12.5--7.8)	672 (487-893)	-1.1 (-6.4-4.3)	30 (20-43)	-7.3 (-11.2--3)	538 (400-701)	20,500 (15,500-26,100)	664 (495-868)	28 (18-40)
Democratic People's Republic of Korea	22 (16-29)	-44.4 (-49.7--39.8)	457 (343-595)	-11.5 (-19.5--4.1)	12 (9-17)	4.9 (-5.6-15)	1 (1-1)	-38.6 (-46.9--26.5)	12 (9-16)	404 (304-528)	13 (10-17)	1 (0-1)
Taiwan (Province of China)	9 (6-11)	-28.4 (-36.8--17.8)	368 (279-473)	12.9 (-2.3-31.3)	10 (7-13)	16.3 (0.4-35.9)	0 (0-0)	-20.1 (-32.2--4.8)	6 (5-8)	416 (342-500)	11 (9-14)	0 (0-0)
**Oceania**	0 (0-0)	50.4 (49.8-60.8)	1 (0-4)	55.4 (49.7-67.9)	0 (0-0)	62.1 (57.1-77.9)	0 (0-0)	59.5 (36.8-86.1)	0 (0-0)	2 (0-6)	0 (0-0)	0 (0-0)
American Samoa	0 (0-0)	-37.8 (-37.8--37.6)	0 (0-0)	-18.6 (-20.7--17.1)	0 (0-0)	-9.7 (-13.4--6)	0 (0-0)	-38.3 (-44--29)	0 (0-0)	0 (0-0)	0 (0-0)	0 (0-0)
Cook Islands	0 (0-0)	-37.7 (-65.9--35.9)	0 (0-0)	-25.9 (-62.7--21.5)	0 (0-0)	-18.9 (-57--12.3)	0 (0-0)	-38.9 (-67.3--29.4)	0 (0-0)	0 (0-0)	0 (0-0)	0 (0-0)
Fiji	0 (0-0)	-6.7 (-6.7--6.7)	0 (0-0)	0.2 (-2.5-2.9)	0 (0-0)	2.8 (-0.1-5.2)	0 (0-0)	-0.6 (-13.2-12.5)	0 (0-0)	0 (0-0)	0 (0-0)	0 (0-0)
Guam	0 (0-0)	-9.4 (-9.4--9.2)	0 (0-0)	-4.5 (-6.3--2.3)	0 (0-0)	-0.8 (-3.3-2.2)	0 (0-0)	-12.1 (-20.7--1.5)	0 (0-0)	0 (0-0)	0 (0-0)	0 (0-0)
Kiribati	0 (0-0)	22.4 (18.4-1500)	0 (0-0)	32.8 (24.6-1490)	0 (0-0)	39.8 (31-1650)	0 (0-0)	26.4 (6.4-1270)	0 (0-0)	0 (0-0)	0 (0-0)	0 (0-0)
Marshall Islands	0 (0-0)	-18.9 (-19.9--19.9)	0 (0-0)	-8.2 (-12--6.5)	0 (0-0)	1.3 (-4.1-5.2)	0 (0-0)	-14.5 (-25.7-1.8)	0 (0-0)	0 (0-0)	0 (0-0)	0 (0-0)
Micronesia (Federated States of)	0 (0-0)	-36.2 (-36.3--36.2)	0 (0-0)	-24 (-26.4--22)	0 (0-0)	-12.9 (-16.4--9.6)	0 (0-0)	-36.2 (-43.8--27)	0 (0-0)	0 (0-0)	0 (0-0)	0 (0-0)
Nauru	0 (0-0)	-29.1 (-94.6--27.4)	0 (0-0)	-7.3 (-92.5--2.5)	0 (0-0)	-2.2 (-92.6-2.1)	0 (0-0)	-20.8 (-93.6--4.9)	0 (0-0)	0 (0-0)	0 (0-0)	0 (0-0)
Niue	0 (0-0)	-33.7 (-33.7--33.7)	0 (0-0)	-23.6 (-25.8--21.8)	0 (0-0)	-17.1 (-18.9--15.2)	0 (0-0)	-31.4 (-40.7--20)	0 (0-0)	0 (0-0)	0 (0-0)	0 (0-0)

Location	Birth , 2000	Births percent change (2000-2021)	All age cases, 2000	All age cases percent change (2000-2021)	All age deaths, 2000	All age deaths percent change (2000-2021)	Under-5 deaths, 2000	Under-5 deaths percent change (2000-2021)	Births, 2021	All age cases, 2021	All age deaths, 2021	Under-5 deaths, 2021
Northern Mariana Islands	0 (0-0)	-81.1 (-81.1--81)	0 (0-0)	-61.1 (-62.6--55.7)	0 (0-0)	-56.5 (-61.7--49.7)	0 (0-0)	-73.8 (-77.3--70.2)	0 (0-0)	0 (0-0)	0 (0-0)	0 (0-0)
Palau	0 (0-0)	-54.2 (-54.8--54.7)	0 (0-0)	-32.3 (-35.2--30.2)	0 (0-0)	-25.8 (-29.6--22.3)	0 (0-0)	-48.9 (-55.8--36.5)	0 (0-0)	0 (0-0)	0 (0-0)	0 (0-0)
Papua New Guinea	0 (0-0)	68.6 (68.7-68.8)	1 (0-3)	75.8 (68-82.2)	0 (0-0)	81.7 (75.4-86.8)	0 (0-0)	74.8 (44.1-107)	0 (0-0)	1 (0-4)	0 (0-0)	0 (0-0)
Samoa	0 (0-0)	32.2 (32.2-32.3)	0 (0-0)	19.4 (15.5-22.1)	0 (0-0)	23 (19-26)	0 (0-0)	15.1 (1.4-29.8)	0 (0-0)	0 (0-0)	0 (0-0)	0 (0-0)
Solomon Islands	0 (0-0)	10.5 (10.5-10.5)	0 (0-0)	39.7 (35.8-42.9)	0 (0-0)	48 (44.1-52.1)	0 (0-0)	22.9 (9.4-41.1)	0 (0-0)	0 (0-0)	0 (0-0)	0 (0-0)
Tokelau	0 (0-0)	-30.8 (-30.6--30.6)	0 (0-0)	-20.1 (-21.6--17.8)	0 (0-0)	-13.6 (-16--10.6)	0 (0-0)	-21.6 (-32.9--11.3)	0 (0-0)	0 (0-0)	0 (0-0)	0 (0-0)
Tonga	0 (0-0)	-14.5 (-14.5--14.4)	0 (0-0)	-7.5 (-10.2--5.9)	0 (0-0)	-2.7 (-6.2-0)	0 (0-0)	-12.6 (-24.3--0.7)	0 (0-0)	0 (0-0)	0 (0-0)	0 (0-0)
Tuvalu	0 (0-0)	-5.5 (-5.5--5.5)	0 (0-0)	15.5 (11-20.1)	0 (0-0)	27.2 (23.4-33.4)	0 (0-0)	-8 (-20.3-9.2)	0 (0-0)	0 (0-0)	0 (0-0)	0 (0-0)
Vanuatu	0 (0-0)	17.6 (17.4-17.6)	0 (0-0)	46.3 (42.3-49.6)	0 (0-0)	54.6 (50.2-58.1)	0 (0-0)	24.3 (9.6-42.5)	0 (0-0)	0 (0-0)	0 (0-0)	0 (0-0)
**Southeast Asia**	4 (0-11)	-13.2 (-14.3--3.1)	84 (4-231)	4.6 (1.7-16.2)	3 (0-8)	15.3 (11.2-25.1)	0 (0-1)	-8.3 (-12-1.6)	3 (0-9)	87 (4-237)	3 (0-9)	0 (0-1)
Cambodia	0 (0-0)	-1.4 (-3.4-82.1)	2 (0-6)	12.3 (5.2-112)	0 (0-0)	24.9 (16.2-126)	0 (0-0)	3.7 (-9.3-64.8)	0 (0-0)	2 (0-6)	0 (0-0)	0 (0-0)
Indonesia	2 (0-4)	-23 (-23.1--21.9)	36 (1-97)	2.1 (0.2-4)	1 (0-3)	12.7 (9.8-16.2)	0 (0-0)	-16.3 (-19.8--12.8)	1 (0-3)	36 (1-100)	1 (0-4)	0 (0-0)
Aceh	0 (0-0)	-20.7 (-20.7--20.7)	1 (0-2)	5 (1.8-8.8)	0 (0-0)	14.8 (11.5-18.5)	0 (0-0)	-12.7 (-23.8--3.1)	0 (0-0)	1 (0-2)	0 (0-0)	0 (0-0)
Bali	0 (0-0)	-29.3 (-29.3--29.3)	1 (0-1)	3.4 (-0.2-7.3)	0 (0-0)	13.8 (10.3-17.7)	0 (0-0)	-22.3 (-33.3--12)	0 (0-0)	1 (0-2)	0 (0-0)	0 (0-0)
Bangka-Belitung Islands	0 (0-0)	-18.4 (-18.4--18.4)	0 (0-0)	9.1 (4.7-13)	0 (0-0)	20.8 (15.4-27.1)	0 (0-0)	-11.3 (-22.6--0.1)	0 (0-0)	0 (0-1)	0 (0-0)	0 (0-0)
Banten	0 (0-0)	-31 (-31--31)	2 (0-5)	-12.9 (-16.6--10)	0 (0-0)	-5.1 (-8--1.7)	0 (0-0)	-25.3 (-35--10.2)	0 (0-0)	2 (0-4)	0 (0-0)	0 (0-0)
Bengkulu	0 (0-0)	-22.5 (-23.1--23.1)	0 (0-1)	1 (-3.4-6.5)	0 (0-0)	10.5 (6.3-14.3)	0 (0-0)	-14.9 (-27.5-1.8)	0 (0-0)	0 (0-1)	0 (0-0)	0 (0-0)
Gorontalo	0 (0-0)	8.1 (8.1-8.1)	0 (0-0)	51.6 (45.3-58.7)	0 (0-0)	66.7 (61.8-71.8)	0 (0-0)	8.9 (-6.7-26.1)	0 (0-0)	0 (0-1)	0 (0-0)	0 (0-0)
Jakarta	0 (0-0)	-39.5 (-39.6--39.6)	2 (0-5)	-17.3 (-20.5--14.9)	0 (0-0)	-8.9 (-13.7--4.4)	0 (0-0)	-28.2 (-36--17.4)	0 (0-0)	1 (0-4)	0 (0-0)	0 (0-0)
Jambi	0 (0-0)	-15.7 (-15.7--15.7)	0 (0-1)	10.5 (7.2-14.1)	0 (0-0)	21.6 (17.8-25.7)	0 (0-0)	-9.7 (-20-0.7)	0 (0-0)	0 (0-1)	0 (0-0)	0 (0-0)
West Java	0 (0-1)	-12 (-12--12)	6 (0-16)	19.7 (15.3-23.5)	0 (0-1)	32.8 (28.8-37.5)	0 (0-0)	-5.5 (-15.7-9.2)	0 (0-1)	7 (0-20)	0 (0-1)	0 (0-0)
Central Java	0 (0-1)	-36.1 (-36.1--36.1)	5 (0-14)	-14.5 (-17.3--11.7)	0 (0-0)	-3.9 (-6.9--0.7)	0 (0-0)	-33.1 (-41.4--24.5)	0 (0-0)	4 (0-12)	0 (0-0)	0 (0-0)
East Java	0 (0-1)	-36.1 (-36.1--36.1)	5 (0-15)	-13.4 (-15.9--10.8)	0 (0-1)	-4.8 (-7.2--1.7)	0 (0-0)	-32.4 (-40.3--23.3)	0 (0-0)	5 (0-13)	0 (0-1)	0 (0-0)
West Kalimantan	0 (0-0)	-21.1 (-21.1--21.1)	1 (0-2)	5.2 (0.8-8.9)	0 (0-0)	14.8 (11.5-18.4)	0 (0-0)	-14.4 (-25--2.3)	0 (0-0)	1 (0-2)	0 (0-0)	0 (0-0)
South Kalimantan	0 (0-0)	-24.1 (-24.1--24.1)	1 (0-1)	4.3 (-0.5-8.3)	0 (0-0)	14.1 (11.4-17.7)	0 (0-0)	-16.7 (-26.7--5.5)	0 (0-0)	1 (0-1)	0 (0-0)	0 (0-0)
Central Kalimantan	0 (0-0)	-17 (-17--17)	0 (0-1)	11.1 (6.6-14.7)	0 (0-0)	22.7 (18.8-27.9)	0 (0-0)	-9.1 (-19.3-2.5)	0 (0-0)	0 (0-1)	0 (0-0)	0 (0-0)
East Kalimantan	0 (0-0)	-2.1 (-2.1--2.1)	0 (0-1)	32 (27.5-35.8)	0 (0-0)	40.1 (35.9-44.4)	0 (0-0)	11.8 (-1.2-32)	0 (0-0)	1 (0-2)	0 (0-0)	0 (0-0)
North Kalimantan	0 (0-0)	11.9 (9.8-9.8)	0 (0-0)	67.3 (58.2-71.4)	0 (0-0)	87.8 (78-93.9)	0 (0-0)	20.9 (4.1-37.6)	0 (0-0)	0 (0-0)	0 (0-0)	0 (0-0)
Riau Islands	0 (0-0)	-16.8 (-16.8--16.8)	0 (0-1)	7.6 (3.5-12.1)	0 (0-0)	14.7 (6.5-23.3)	0 (0-0)	4.2 (-12.1-22.1)	0 (0-0)	0 (0-1)	0 (0-0)	0 (0-0)

Location	Birth , 2000	Births percent change (2000-2021)	All age cases, 2000	All age cases percent change (2000-2021)	All age deaths, 2000	All age deaths percent change (2000-2021)	Under-5 deaths, 2000	Under-5 deaths percent change (2000-2021)	Births, 2021	All age cases, 2021	All age deaths, 2021	Under-5 deaths, 2021
Lampung	0 (0-0)	-32.8 (-32.8--32.8)	1 (0-3)	-9.7 (-13--6.9)	0 (0-0)	1.8 (-2-5.9)	0 (0-0)	-28.7 (-36.8--19.7)	0 (0-0)	1 (0-3)	0 (0-0)	0 (0-0)
Maluku	0 (0-0)	-22.9 (-22.3--22.3)	0 (0-1)	9.4 (3.7-14.1)	0 (0-0)	18 (15.2-21.6)	0 (0-0)	-12.9 (-27.6-1)	0 (0-0)	0 (0-1)	0 (0-0)	0 (0-0)
North Maluku	0 (0-0)	-22 (-21.5--21.5)	0 (0-0)	8.6 (3.9-13.3)	0 (0-0)	19.6 (16.5-24.4)	0 (0-0)	-15 (-27.9-2.1)	0 (0-0)	0 (0-0)	0 (0-0)	0 (0-0)
West Nusa Tenggara	0 (0-0)	-17.2 (-17.8--17.8)	1 (0-2)	6.3 (1.1-11.4)	0 (0-0)	16 (11.6-20.4)	0 (0-0)	-10 (-22.3-6.3)	0 (0-0)	1 (0-2)	0 (0-0)	0 (0-0)
East Nusa Tenggara	0 (0-0)	-12.3 (-12.2--12.2)	1 (0-2)	21.8 (17.1-25.5)	0 (0-0)	32.1 (28.7-35.9)	0 (0-0)	-5.4 (-17.1-11.3)	0 (0-0)	1 (0-2)	0 (0-0)	0 (0-0)
Papua	0 (0-0)	59.6 (59.6-59.6)	0 (0-1)	109 (99.5-117)	0 (0-0)	123 (119-129)	0 (0-0)	71.9 (45.4-100)	0 (0-0)	1 (0-2)	0 (0-0)	0 (0-0)
West Papua	0 (0-0)	-19 (-19.1--19.1)	0 (0-0)	2.8 (-1.1-6.8)	0 (0-0)	12.4 (8.4-16.6)	0 (0-0)	-10.8 (-23.5-3.2)	0 (0-0)	0 (0-0)	0 (0-0)	0 (0-0)
Riau	0 (0-0)	47.8 (47.8-47.8)	1 (0-2)	96.6 (89.9-103)	0 (0-0)	114 (107-121)	0 (0-0)	62.9 (44.6-88.9)	0 (0-0)	1 (0-4)	0 (0-0)	0 (0-0)
West Sulawesi	0 (0-0)	-6.8 (-6.8--6.8)	0 (0-0)	33.7 (27.8-39.2)	0 (0-0)	48.1 (44.2-52.1)	0 (0-0)	1 (-13.5-18.1)	0 (0-0)	0 (0-1)	0 (0-0)	0 (0-0)
South Sulawesi	0 (0-0)	-29.8 (-29.8--29.8)	1 (0-3)	-2.1 (-6.2-1.8)	0 (0-0)	9.4 (6.4-12.4)	0 (0-0)	-24.5 (-38.1--13.6)	0 (0-0)	1 (0-3)	0 (0-0)	0 (0-0)
Central Sulawesi	0 (0-0)	-20.5 (-20.6--20.6)	0 (0-1)	14.7 (10.4-18.8)	0 (0-0)	25.9 (22.8-29.3)	0 (0-0)	-12.6 (-23.9-3.1)	0 (0-0)	0 (0-1)	0 (0-0)	0 (0-0)
Southeast Sulawesi	0 (0-0)	-5.2 (-5.2--5.2)	0 (0-1)	26.4 (21.8-30.4)	0 (0-0)	38.1 (34.7-42.1)	0 (0-0)	3.4 (-8.1-20.1)	0 (0-0)	0 (0-1)	0 (0-0)	0 (0-0)
North Sulawesi	0 (0-0)	-42.9 (-42.9--42.9)	0 (0-1)	-17.9 (-21--15)	0 (0-0)	-8.7 (-11.1--5.7)	0 (0-0)	-39.6 (-47.4--29.9)	0 (0-0)	0 (0-1)	0 (0-0)	0 (0-0)
West Sumatra	0 (0-0)	-31.4 (-31.4--31.4)	1 (0-2)	-5.5 (-8.8--1.9)	0 (0-0)	3.8 (1.4-6.6)	0 (0-0)	-25.8 (-35.4--14.8)	0 (0-0)	1 (0-2)	0 (0-0)	0 (0-0)
South Sumatra	0 (0-0)	-24.6 (-24.6--24.6)	1 (0-3)	0 (-3.5-3.6)	0 (0-0)	11 (6.8-15.7)	0 (0-0)	-18.1 (-28.1--8.2)	0 (0-0)	1 (0-3)	0 (0-0)	0 (0-0)
North Sumatra	0 (0-0)	-23.9 (-23.9--23.9)	2 (0-6)	-1 (-4.5-2.6)	0 (0-0)	8.7 (5.8-12)	0 (0-0)	-18.2 (-28--5.8)	0 (0-0)	2 (0-6)	0 (0-0)	0 (0-0)
Yogyakarta	0 (0-0)	-27.2 (-28--28)	0 (0-1)	-8.6 (-12.3--6.2)	0 (0-0)	1 (-3.3-4.1)	0 (0-0)	-23.3 (-33.8--11.6)	0 (0-0)	0 (0-1)	0 (0-0)	0 (0-0)
Lao People's Democratic Republic	0 (0-0)	-4.6 (-5.7--5.7)	1 (0-2)	14.8 (8.4-19.4)	0 (0-0)	28.2 (20.6-35)	0 (0-0)	1.6 (-12.2-21.7)	0 (0-0)	1 (0-3)	0 (0-0)	0 (0-0)
Malaysia	0 (0-0)	5.4 (5.4-5.6)	3 (0-12)	13.8 (10.9-17)	0 (0-0)	29.7 (25.5-34)	0 (0-0)	-4.1 (-13.9-5.8)	0 (0-0)	4 (0-13)	0 (0-0)	0 (0-0)
Maldives	0 (0-0)	16.4 (12.7-118)	0 (0-0)	45.6 (32.7-168)	0 (0-0)	93 (65.1-266)	0 (0-0)	15.9 (0.2-114)	0 (0-0)	0 (0-0)	0 (0-0)	0 (0-0)
Mauritius	0 (0-0)	-35.7 (-36.5--36)	0 (0-1)	-19.8 (-23--13.8)	0 (0-0)	-7.2 (-11.4--1.8)	0 (0-0)	-37.7 (-45.3--23.9)	0 (0-0)	0 (0-0)	0 (0-0)	0 (0-0)
Myanmar	0 (0-1)	-15.4 (-16.7-56.6)	6 (0-18)	2.2 (-3.9-97.8)	0 (0-1)	9.7 (4.7-103)	0 (0-0)	-9.2 (-27.4-58)	0 (0-1)	6 (0-18)	0 (0-1)	0 (0-0)
Philippines	1 (0-2)	13.8 (13.8-14.3)	15 (1-40)	28.6 (27-30.3)	0 (0-1)	37.7 (34.8-40.8)	0 (0-0)	16.2 (14-18.7)	1 (0-2)	19 (1-51)	1 (0-2)	0 (0-0)
Abra	0 (0-0)	-18.2 (-18.8--18.8)	0 (0-0)	2.6 (-1.5-4.5)	0 (0-0)	11.1 (7.6-13.9)	0 (0-0)	-11.6 (-22.5-6.6)	0 (0-0)	0 (0-0)	0 (0-0)	0 (0-0)
Agusan Del Norte	0 (0-0)	8.6 (8.6-8.6)	0 (0-0)	19.5 (15.3-23.3)	0 (0-0)	26.9 (22.9-30.4)	0 (0-0)	9.8 (-4.2-26.5)	0 (0-0)	0 (0-0)	0 (0-0)	0 (0-0)
Agusan Del Sur	0 (0-0)	-7.8 (-7.8--7.8)	0 (0-0)	15.5 (12-19)	0 (0-0)	22.9 (19.2-26.8)	0 (0-0)	0.6 (-11.1-15.8)	0 (0-0)	0 (0-0)	0 (0-0)	0 (0-0)
Aklan	0 (0-0)	19.7 (19.7-19.7)	0 (0-0)	24.3 (21.2-27)	0 (0-0)	32.5 (28.2-36.5)	0 (0-0)	18.4 (5.4-34)	0 (0-0)	0 (0-0)	0 (0-0)	0 (0-0)
Albay	0 (0-0)	5.1 (5.1-5.1)	0 (0-1)	14.6 (11.1-17.6)	0 (0-0)	23.7 (19.9-27.5)	0 (0-0)	3.8 (-9.4-15.3)	0 (0-0)	0 (0-1)	0 (0-0)	0 (0-0)
Antique	0 (0-0)	4.4 (4.4-4.4)	0 (0-0)	18.9 (15.2-22.2)	0 (0-0)	25.6 (22.4-28.9)	0 (0-0)	4.8 (-6.4-21.2)	0 (0-0)	0 (0-0)	0 (0-0)	0 (0-0)

Location	Birth , 2000	Births percent change (2000-2021)	All age cases, 2000	All age cases percent change (2000-2021)	All age deaths, 2000	All age deaths percent change (2000-2021)	Under-5 deaths, 2000	Under-5 deaths percent change (2000-2021)	Births, 2021	All age cases, 2021	All age deaths, 2021	Under-5 deaths, 2021
Apayao	0 (0-0)	-23.2 (-23.1--23.1)	0 (0-0)	7.9 (4-12)	0 (0-0)	17.9 (13.9-21.8)	0 (0-0)	-9.9 (-24.2-2.9)	0 (0-0)	0 (0-0)	0 (0-0)	0 (0-0)
Aurora	0 (0-0)	-4.4 (-3.7--3.7)	0 (0-0)	13.2 (9.6-17.6)	0 (0-0)	20.3 (17.6-24.9)	0 (0-0)	1.8 (-15.1-17.5)	0 (0-0)	0 (0-0)	0 (0-0)	0 (0-0)
Basilan	0 (0-0)	28.9 (28.9-28.9)	0 (0-0)	50.5 (47-54.7)	0 (0-0)	60.3 (56.2-64.6)	0 (0-0)	35.6 (18.8-52.6)	0 (0-0)	0 (0-0)	0 (0-0)	0 (0-0)
Bataan	0 (0-0)	14 (14-14)	0 (0-0)	29.4 (24.9-32.4)	0 (0-0)	40.2 (36.8-43.8)	0 (0-0)	16.6 (5-32)	0 (0-0)	0 (0-0)	0 (0-0)	0 (0-0)
Batanes	0 (0-0)	-28.1 (-28.3--28.3)	0 (0-0)	-8.1 (-11--5.8)	0 (0-0)	1 (-2.5-4.7)	0 (0-0)	-23.4 (-32.8--12.1)	0 (0-0)	0 (0-0)	0 (0-0)	0 (0-0)
Batangas	0 (0-0)	42.4 (42.4-42.4)	0 (0-1)	44 (40.3-47.7)	0 (0-0)	55.3 (50.8-60.1)	0 (0-0)	37.6 (21.3-55.2)	0 (0-0)	1 (0-1)	0 (0-0)	0 (0-0)
Benguet	0 (0-0)	29.7 (29.3-29.3)	0 (0-0)	33.2 (29.4-35.7)	0 (0-0)	42.3 (37.3-47)	0 (0-0)	29.2 (16.1-48.8)	0 (0-0)	0 (0-0)	0 (0-0)	0 (0-0)
Biliran	0 (0-0)	0.9 (0.9-0.9)	0 (0-0)	14.8 (12.7-17.1)	0 (0-0)	22.5 (18.9-26.1)	0 (0-0)	3.8 (-8.3-14.5)	0 (0-0)	0 (0-0)	0 (0-0)	0 (0-0)
Bohol	0 (0-0)	-22.8 (-23.5--23.5)	0 (0-1)	3.9 (-0.2-7)	0 (0-0)	13.8 (9.6-16.2)	0 (0-0)	-15.3 (-26.6-1.7)	0 (0-0)	0 (0-1)	0 (0-0)	0 (0-0)
Bukidnon	0 (0-0)	-7.6 (-6.9--6.9)	0 (0-1)	21.9 (17.4-26.6)	0 (0-0)	31.7 (27.6-37.2)	0 (0-0)	2.5 (-14.1-21.4)	0 (0-0)	0 (0-1)	0 (0-0)	0 (0-0)
Bulacan	0 (0-0)	30.6 (30.6-30.6)	0 (0-1)	46.5 (41.3-50.5)	0 (0-0)	60.3 (56.3-64.4)	0 (0-0)	26.4 (9.4-45.7)	0 (0-0)	1 (0-2)	0 (0-0)	0 (0-0)
Cagayan	0 (0-0)	-1.6 (-1.6--1.6)	0 (0-0)	10.7 (7.1-14.2)	0 (0-0)	19.8 (15.9-24)	0 (0-0)	1.5 (-12.7-16.3)	0 (0-0)	0 (0-1)	0 (0-0)	0 (0-0)
Camarines Norte	0 (0-0)	3.8 (3.8-3.8)	0 (0-0)	19.1 (15.9-21.9)	0 (0-0)	25.6 (22.8-28.5)	0 (0-0)	6 (-5.1-18.5)	0 (0-0)	0 (0-0)	0 (0-0)	0 (0-0)
Camarines Sur	0 (0-0)	8.5 (8.4-8.4)	0 (0-1)	24 (19.7-28)	0 (0-0)	30.8 (27.2-34.5)	0 (0-0)	10.2 (-6.6-23.9)	0 (0-0)	0 (0-1)	0 (0-0)	0 (0-0)
Camiguin	0 (0-0)	4 (4-4)	0 (0-0)	12.5 (9.7-15.1)	0 (0-0)	18.6 (15.8-21.7)	0 (0-0)	5.9 (-6.9-18.9)	0 (0-0)	0 (0-0)	0 (0-0)	0 (0-0)
Capiz	0 (0-0)	-1 (-1--1)	0 (0-0)	6.4 (3.3-9.4)	0 (0-0)	14.6 (10.4-18.6)	0 (0-0)	-3.5 (-16.2-7.6)	0 (0-0)	0 (0-0)	0 (0-0)	0 (0-0)
Catanduanes	0 (0-0)	-8.6 (-8.6--8.6)	0 (0-0)	14.4 (10.6-18.7)	0 (0-0)	22.2 (18.2-26)	0 (0-0)	-0.8 (-12.8-11.7)	0 (0-0)	0 (0-0)	0 (0-0)	0 (0-0)
Cavite	0 (0-0)	79.9 (79.9-79.9)	0 (0-1)	89.5 (84.2-94.5)	0 (0-0)	107 (102-113)	0 (0-0)	73.3 (56.4-92.3)	0 (0-0)	1 (0-2)	0 (0-0)	0 (0-0)
Cebu	0 (0-0)	34.8 (34.8-34.8)	1 (0-2)	41.6 (37.2-45.4)	0 (0-0)	48.3 (44.6-52)	0 (0-0)	36.6 (19.9-56.1)	0 (0-0)	1 (0-2)	0 (0-0)	0 (0-0)
Cotabato (North Cotabato)	0 (0-0)	34.2 (34.2-34.2)	0 (0-1)	46.8 (43-51)	0 (0-0)	54.8 (50-59.8)	0 (0-0)	40.7 (23.6-63.3)	0 (0-0)	0 (0-1)	0 (0-0)	0 (0-0)
Davao de Oro	0 (0-0)	-8.2 (-8.2--8.2)	0 (0-0)	17.2 (14.3-19.8)	0 (0-0)	26 (23.1-29.6)	0 (0-0)	3.6 (-8.5-17.2)	0 (0-0)	0 (0-0)	0 (0-0)	0 (0-0)
Davao Del Norte	0 (0-0)	-12.9 (-12.2--12.2)	0 (0-0)	19 (15.7-22.8)	0 (0-0)	30 (26.8-34.8)	0 (0-0)	-2.9 (-17.3-11.4)	0 (0-0)	0 (0-0)	0 (0-0)	0 (0-0)
Davao Del Sur	0 (0-0)	25.4 (25.4-25.4)	0 (0-1)	34.6 (30.1-38.5)	0 (0-0)	43.8 (39.4-48.2)	0 (0-0)	28.4 (12.3-45.8)	0 (0-0)	0 (0-1)	0 (0-0)	0 (0-0)
Davao Occidental	0 (0-0)	-12.7 (-12.7--12.7)	0 (0-0)	34.8 (31.2-37.7)	0 (0-0)	45.3 (41.3-50.1)	0 (0-0)	0 (-11.5-15.2)	0 (0-0)	0 (0-0)	0 (0-0)	0 (0-0)
Davao Oriental	0 (0-0)	-14.3 (-14.3--14.3)	0 (0-0)	13.1 (9.7-16.5)	0 (0-0)	23.2 (19.9-26.9)	0 (0-0)	-8.7 (-19.6-3.7)	0 (0-0)	0 (0-0)	0 (0-0)	0 (0-0)
Dinagat Islands	0 (0-0)	-29.4 (-30.1--30.1)	0 (0-0)	-2.5 (-6.5--0.8)	0 (0-0)	5.6 (1.6-8)	0 (0-0)	-17 (-25.7--1.9)	0 (0-0)	0 (0-0)	0 (0-0)	0 (0-0)
Eastern Samar	0 (0-0)	5.6 (5.6-5.6)	0 (0-0)	22.4 (17.6-26.2)	0 (0-0)	31.3 (26.5-37.1)	0 (0-0)	8.2 (-4.5-25.7)	0 (0-0)	0 (0-0)	0 (0-0)	0 (0-0)
Guimaras	0 (0-0)	-0.9 (-0.9--0.9)	0 (0-0)	12.5 (9.9-15.2)	0 (0-0)	22.7 (18.1-27.6)	0 (0-0)	5.5 (-6.9-18.6)	0 (0-0)	0 (0-0)	0 (0-0)	0 (0-0)
Ifugao	0 (0-0)	16.9 (15.7-15.7)	0 (0-0)	21.2 (16.4-23.8)	0 (0-0)	29.6 (23.8-34)	0 (0-0)	24.4 (10.4-47.1)	0 (0-0)	0 (0-0)	0 (0-0)	0 (0-0)

Location	Birth , 2000	Births percent change (2000-2021)	All age cases, 2000	All age cases percent change (2000-2021)	All age deaths, 2000	All age deaths percent change (2000-2021)	Under-5 deaths, 2000	Under-5 deaths percent change (2000-2021)	Births, 2021	All age cases, 2021	All age deaths, 2021	Under-5 deaths, 2021
Ilocos Norte	0 (0-0)	3.1 (3.1-3.1)	0 (0-0)	7.4 (4.5-9.9)	0 (0-0)	13.3 (10.4-16.5)	0 (0-0)	5.9 (-5.8-18.7)	0 (0-0)	0 (0-0)	0 (0-0)	0 (0-0)
Ilocos Sur	0 (0-0)	-3.1 (-3.1--3.1)	0 (0-0)	7.2 (3.6-10)	0 (0-0)	13.9 (11.2-17)	0 (0-0)	2.1 (-8-19)	0 (0-0)	0 (0-0)	0 (0-0)	0 (0-0)
Iloilo	0 (0-0)	21.2 (19.9-19.9)	0 (0-1)	21.7 (17.2-23.9)	0 (0-0)	28.5 (23.7-32.1)	0 (0-0)	21.3 (7.4-45.8)	0 (0-0)	0 (0-1)	0 (0-0)	0 (0-0)
Isabela	0 (0-0)	14.3 (14.3-14.3)	0 (0-1)	16.2 (13.4-19.8)	0 (0-0)	23.6 (19.7-27.4)	0 (0-0)	15.3 (0.2-30.6)	0 (0-0)	0 (0-1)	0 (0-0)	0 (0-0)
Kalinga	0 (0-0)	-14.4 (-14.7--14.7)	0 (0-0)	12.2 (8.8-14.7)	0 (0-0)	22 (18-25.6)	0 (0-0)	-2.3 (-13.7-9.6)	0 (0-0)	0 (0-0)	0 (0-0)	0 (0-0)
La Union	0 (0-0)	7.4 (6.3-6.3)	0 (0-0)	11.3 (6.8-14.8)	0 (0-0)	19.1 (14.1-22.3)	0 (0-0)	8.4 (-7.8-24.1)	0 (0-0)	0 (0-0)	0 (0-0)	0 (0-0)
Laguna	0 (0-0)	56.3 (56.3-56.3)	0 (0-1)	58.9 (54.1-62.8)	0 (0-0)	69.5 (64.8-74.3)	0 (0-0)	56.8 (39.5-78.1)	0 (0-0)	1 (0-2)	0 (0-0)	0 (0-0)
Lanao Del Norte	0 (0-0)	15.2 (15.2-15.2)	0 (0-0)	34.5 (31.4-38.4)	0 (0-0)	40.8 (37.6-44.6)	0 (0-0)	24.5 (10-41.9)	0 (0-0)	0 (0-1)	0 (0-0)	0 (0-0)
Lanao Del Sur	0 (0-0)	15.6 (15.6-15.6)	0 (0-0)	38.3 (34.2-41.8)	0 (0-0)	45 (39.9-50.2)	0 (0-0)	29.5 (11.5-49.6)	0 (0-0)	0 (0-1)	0 (0-0)	0 (0-0)
Leyte	0 (0-0)	11.1 (11.1-11.1)	0 (0-1)	21.1 (17.2-25.2)	0 (0-0)	30.2 (25.5-35.6)	0 (0-0)	10.9 (-5.2-26.8)	0 (0-0)	0 (0-1)	0 (0-0)	0 (0-0)
Maguindanao	0 (0-0)	31.7 (31.7-31.7)	0 (0-1)	68.5 (63.6-73.7)	0 (0-0)	77.2 (72.1-81.9)	0 (0-0)	51.4 (29.7-70.3)	0 (0-0)	0 (0-1)	0 (0-0)	0 (0-0)
Marinduque	0 (0-0)	-29.3 (-29.3--29.3)	0 (0-0)	-7.2 (-9.8--4.7)	0 (0-0)	1.8 (-1-4.5)	0 (0-0)	-26.3 (-33.4--16.6)	0 (0-0)	0 (0-0)	0 (0-0)	0 (0-0)
Masbate	0 (0-0)	8.7 (8.6-8.6)	0 (0-0)	27 (22.6-30.2)	0 (0-0)	33.1 (29.3-37.6)	0 (0-0)	12.6 (0.1-32.4)	0 (0-0)	0 (0-0)	0 (0-0)	0 (0-0)
Misamis Occidental	0 (0-0)	9.7 (9.8-9.8)	0 (0-0)	18.7 (14.9-22.8)	0 (0-0)	24.3 (21-27.6)	0 (0-0)	13.2 (-0.8-30.5)	0 (0-0)	0 (0-0)	0 (0-0)	0 (0-0)
Misamis Oriental	0 (0-0)	34.6 (34.6-34.6)	0 (0-1)	39.4 (35.5-43.6)	0 (0-0)	45.8 (42.2-49.9)	0 (0-0)	35.7 (19-54.5)	0 (0-0)	0 (0-1)	0 (0-0)	0 (0-0)
Mountain Province	0 (0-0)	-11.6 (-11.6--11.6)	0 (0-0)	0.2 (-2.5-2.8)	0 (0-0)	5.6 (2.3-8.8)	0 (0-0)	-6.1 (-18.6-4)	0 (0-0)	0 (0-0)	0 (0-0)	0 (0-0)
National Capital Region	0 (0-0)	0.9 (0.9-0.9)	2 (0-5)	22.2 (18.3-25.7)	0 (0-0)	33.9 (31-37)	0 (0-0)	2.6 (-8.6-14.6)	0 (0-0)	2 (0-6)	0 (0-0)	0 (0-0)
Negros Occidental	0 (0-0)	2 (2-2)	0 (0-1)	9.5 (6.5-12.5)	0 (0-0)	17.4 (13.8-21.1)	0 (0-0)	1.4 (-10.3-16.6)	0 (0-0)	1 (0-2)	0 (0-0)	0 (0-0)
Negros Oriental	0 (0-0)	-3.5 (-3.5--3.5)	0 (0-1)	9.6 (6.3-12.7)	0 (0-0)	17.8 (13.8-21.6)	0 (0-0)	-1.8 (-12.7-14.2)	0 (0-0)	0 (0-1)	0 (0-0)	0 (0-0)
Northern Samar	0 (0-0)	12.1 (12.1-12.1)	0 (0-0)	26 (21.8-29.9)	0 (0-0)	35.6 (30.6-41.2)	0 (0-0)	12.9 (-1.7-29.2)	0 (0-0)	0 (0-0)	0 (0-0)	0 (0-0)
Nueva Ecija	0 (0-0)	20.7 (20.7-20.7)	0 (0-1)	28.4 (24.5-31.5)	0 (0-0)	36.6 (33.3-40)	0 (0-0)	23.5 (10.6-39.1)	0 (0-0)	0 (0-1)	0 (0-0)	0 (0-0)
Nueva Vizcaya	0 (0-0)	4.5 (4.5-4.5)	0 (0-0)	14.6 (10.8-18.1)	0 (0-0)	21.9 (18.7-25.1)	0 (0-0)	10.6 (-2.7-24.7)	0 (0-0)	0 (0-0)	0 (0-0)	0 (0-0)
Occidental Mindoro	0 (0-0)	-4.9 (-5--5)	0 (0-0)	20.8 (17.7-23.9)	0 (0-0)	29.5 (26.1-32.7)	0 (0-0)	6.8 (-4.4-21.7)	0 (0-0)	0 (0-0)	0 (0-0)	0 (0-0)
Oriental Mindoro	0 (0-0)	-8.1 (-8.2--8.2)	0 (0-0)	14.2 (10.9-17.5)	0 (0-0)	23.1 (19-26.9)	0 (0-0)	-5.3 (-16.8-9.4)	0 (0-0)	0 (0-0)	0 (0-0)	0 (0-0)
Palawan	0 (0-0)	26 (24.7-24.7)	0 (0-0)	48.9 (42.8-52.9)	0 (0-0)	59.8 (54-64.4)	0 (0-0)	31.4 (15.3-50.8)	0 (0-0)	0 (0-1)	0 (0-0)	0 (0-0)
Pampanga	0 (0-0)	32 (31.3-31.3)	0 (0-1)	38.4 (33.4-42.4)	0 (0-0)	50.1 (44.9-54.6)	0 (0-0)	28.5 (11-47.7)	0 (0-0)	1 (0-1)	0 (0-0)	0 (0-0)
Pangasinan	0 (0-0)	7.6 (7.6-7.6)	0 (0-1)	15.3 (11.8-18.4)	0 (0-0)	22.4 (18.9-26)	0 (0-0)	11 (-1.6-27.2)	0 (0-0)	1 (0-1)	0 (0-0)	0 (0-0)
Quezon	0 (0-0)	8.9 (8.9-8.9)	0 (0-1)	21.3 (18.1-24.4)	0 (0-0)	29.1 (26-32.3)	0 (0-0)	8.6 (-4.8-23.5)	0 (0-0)	0 (0-1)	0 (0-0)	0 (0-0)
Quirino	0 (0-0)	-13.8 (-13.8--13.8)	0 (0-0)	11.3 (7.7-15.3)	0 (0-0)	20.6 (16.9-24.4)	0 (0-0)	-3.3 (-17-8.7)	0 (0-0)	0 (0-0)	0 (0-0)	0 (0-0)

Location	Birth , 2000	Births percent change (2000-2021)	All age cases, 2000	All age cases percent change (2000-2021)	All age deaths, 2000	All age deaths percent change (2000-2021)	Under-5 deaths, 2000	Under-5 deaths percent change (2000-2021)	Births, 2021	All age cases, 2021	All age deaths, 2021	Under-5 deaths, 2021
Rizal	0 (0-0)	76.3 (76.3-76.3)	0 (0-1)	82.4 (76.6-87.2)	0 (0-0)	92.8 (88.4-96.9)	0 (0-0)	69.5 (47.7-90.5)	0 (0-0)	1 (0-2)	0 (0-0)	0 (0-0)
Romblon	0 (0-0)	-18.6 (-18.6--18.6)	0 (0-0)	-0.2 (-3.1-2.1)	0 (0-0)	6.5 (3.6-10)	0 (0-0)	-13 (-22.4--0.6)	0 (0-0)	0 (0-0)	0 (0-0)	0 (0-0)
Samar (Western Samar)	0 (0-0)	1.5 (2.2-2.2)	0 (0-0)	15.7 (10.5-20.5)	0 (0-0)	23.8 (19.1-29.6)	0 (0-0)	1.3 (-14.3-15.8)	0 (0-0)	0 (0-0)	0 (0-0)	0 (0-0)
Sarangani	0 (0-0)	16.5 (16.5-16.5)	0 (0-0)	27.5 (23.4-30.9)	0 (0-0)	38.9 (34.7-43.9)	0 (0-0)	13.2 (-0.1-25.6)	0 (0-0)	0 (0-0)	0 (0-0)	0 (0-0)
Siquijor	0 (0-0)	-12.6 (-12.6--12.6)	0 (0-0)	7.6 (5-10.3)	0 (0-0)	17.4 (13.7-21)	0 (0-0)	-5.6 (-16.5-5.5)	0 (0-0)	0 (0-0)	0 (0-0)	0 (0-0)
Sorsogon	0 (0-0)	10.2 (10.2-10.2)	0 (0-0)	18 (14.6-21)	0 (0-0)	28.3 (24.6-32.3)	0 (0-0)	5.9 (-5.2-18.9)	0 (0-0)	0 (0-0)	0 (0-0)	0 (0-0)
South Cotabato	0 (0-0)	20.1 (20.1-20.1)	0 (0-1)	31.1 (27.4-35)	0 (0-0)	39.9 (35.7-44.1)	0 (0-0)	20.9 (6.1-35.6)	0 (0-0)	0 (0-1)	0 (0-0)	0 (0-0)
Southern Leyte	0 (0-0)	-3.5 (-2.8--2.8)	0 (0-0)	9.3 (5.2-13.2)	0 (0-0)	16.9 (13.7-21.1)	0 (0-0)	-2.5 (-19.8-10.5)	0 (0-0)	0 (0-0)	0 (0-0)	0 (0-0)
Sultan Kudarat	0 (0-0)	-0.1 (-0.3--0.3)	0 (0-0)	30.5 (27-33.4)	0 (0-0)	42.1 (37.6-46)	0 (0-0)	11.9 (0.5-24.1)	0 (0-0)	0 (0-0)	0 (0-0)	0 (0-0)
Sulu	0 (0-0)	-9.6 (-10.4--10.4)	0 (0-0)	37.9 (32.6-45.6)	0 (0-0)	44.1 (38.2-49.8)	0 (0-0)	8.3 (-7-26.5)	0 (0-0)	0 (0-0)	0 (0-0)	0 (0-0)
Surigao Del Norte	0 (0-0)	19.4 (19.4-19.4)	0 (0-0)	27.1 (22.3-31.1)	0 (0-0)	35.8 (31.3-41)	0 (0-0)	17.9 (3.4-39.2)	0 (0-0)	0 (0-0)	0 (0-0)	0 (0-0)
Surigao Del Sur	0 (0-0)	-3.5 (-3.5--3.5)	0 (0-0)	6.6 (3.7-9.5)	0 (0-0)	14.7 (11-18.9)	0 (0-0)	-2.6 (-13.4-10)	0 (0-0)	0 (0-0)	0 (0-0)	0 (0-0)
Tarlac	0 (0-0)	3.4 (3.4-3.4)	0 (0-1)	18.4 (15-21.6)	0 (0-0)	27.6 (24.8-30.9)	0 (0-0)	5.6 (-4.9-17.9)	0 (0-0)	0 (0-1)	0 (0-0)	0 (0-0)
Tawi-Tawi	0 (0-0)	-3.8 (-4--4)	0 (0-0)	17.1 (14.1-19.4)	0 (0-0)	24.1 (20.4-27.3)	0 (0-0)	5 (-7.6-20.1)	0 (0-0)	0 (0-0)	0 (0-0)	0 (0-0)
Zambales	0 (0-0)	20.5 (20.5-20.5)	0 (0-0)	27.7 (23.9-31)	0 (0-0)	34.9 (31.9-38.2)	0 (0-0)	18.8 (3.4-35.9)	0 (0-0)	0 (0-0)	0 (0-0)	0 (0-0)
Zamboanga Del Norte	0 (0-0)	-7.2 (-6.3--6.3)	0 (0-0)	12.5 (9.6-17)	0 (0-0)	18.8 (16.5-23)	0 (0-0)	0 (-16.3-16.4)	0 (0-0)	0 (0-1)	0 (0-0)	0 (0-0)
Zamboanga Del Sur	0 (0-0)	8.9 (8.9-8.9)	0 (0-1)	19.7 (17-22.4)	0 (0-0)	28.1 (24.8-31.8)	0 (0-0)	11.1 (-1-24.6)	0 (0-0)	0 (0-1)	0 (0-0)	0 (0-0)
Zamboanga Sibugay	0 (0-0)	8.2 (8.2-8.2)	0 (0-0)	31.1 (27.8-34.5)	0 (0-0)	39.3 (35.7-43.2)	0 (0-0)	14.2 (1.5-27.5)	0 (0-0)	0 (0-0)	0 (0-0)	0 (0-0)
Seychelles	0 (0-0)	-3.6 (-6.6-73.2)	0 (0-0)	5.7 (-0.7-98.3)	0 (0-0)	18.8 (10.2-104)	0 (0-0)	1.2 (-11.6-71.4)	0 (0-0)	0 (0-0)	0 (0-0)	0 (0-0)
Sri Lanka	0 (0-0)	-17.6 (-18--18)	2 (0-8)	0.4 (-2.6-2.2)	0 (0-0)	6.3 (3.2-8.5)	0 (0-0)	-10.8 (-20.2--0.6)	0 (0-0)	2 (0-8)	0 (0-0)	0 (0-0)
Thailand	0 (0-1)	-39.5 (-39.5--39.5)	7 (0-24)	-22.1 (-24.8--19.3)	0 (0-1)	-9.9 (-13.8--5.4)	0 (0-0)	-38.2 (-45.7--31)	0 (0-0)	6 (0-18)	0 (0-1)	0 (0-0)
Timor-Leste	0 (0-0)	6 (3.7-1290)	0 (0-0)	45.8 (38-1740)	0 (0-0)	54.9 (45.7-1860)	0 (0-0)	15.8 (-1.9-1310)	0 (0-0)	0 (0-1)	0 (0-0)	0 (0-0)
Viet Nam	0 (0-1)	-19.7 (-20.4--20.4)	11 (1-37)	-4.5 (-8.8--1.3)	0 (0-1)	11.7 (4.3-18.4)	0 (0-0)	-14.5 (-24.6--2.3)	0 (0-1)	11 (1-35)	0 (0-1)	0 (0-0)
Sub-Saharan Africa	224,000 (180,000-281,000)	31.5 (27.6-35.2)	2,280,000 (1,870,000-2,810,000)	75.9 (71.2-80.6)	111,000 (87,100-143,000)	69.7 (63.6-75.6)	28,600 (18,100-42,400)	43.2 (35.8-50.5)	294,000 (238,000-367,000)	4,010,000 (3,270,000-4,960,000)	189,000 (148,000-242,000)	40,900 (25,900-60,300)
**Central Sub-Saharan Africa**	46,300 (39,500-53,800)	32.4 (25.3-39.7)	458,000 (389,000-544,000)	75.8 (65.3-86.3)	23,700 (19,300-27,900)	81.6 (69.8-92.8)	6,130 (4,000-8,650)	57.8 (40.2-74.7)	61,300 (51,600-71,100)	805,000 (675,000-959,000)	43,000 (35,100-50,600)	9,670 (6,320-13,900)
Angola	8,700 (7,140-10,600)	53.4 (40.8-67.1)	82,400 (67,800-101,000)	106 (88.6-123)	4,200 (3,360-5,230)	113 (91.9-134)	1,110 (715-1,640)	87.6 (57.7-119)	13,400 (11,000-16,300)	169,000 (139,000-209,000)	8,940 (7,080-11,300)	2,070 (1,300-3,130)
Central African Republic	1,400 (1,160-1,730)	26.3 (16.9-35.3)	13,700 (11,400-16,700)	44.3 (35.5-56)	747 (613-929)	47.2 (35.3-59.5)	187 (124-271)	38.7 (18.4-61.4)	1,770 (1,490-2,140)	19,800 (16,500-24,100)	1,100 (904-1,340)	260 (170-374)
Congo	679 (559-810)	9.6 (1-18.2)	7,860 (6,570-9,540)	47.6 (36.2-59)	377 (304-457)	55.3 (41.3-70.1)	77 (50-109)	33.7 (16.5-54.3)	744 (616-880)	11,600 (9,660-14,000)	586 (479-700)	103 (68-147)
Democratic Republic of the Congo	34,800 (29,600-40,200)	28.3 (18.8-37.5)	345,000 (293,000-408,000)	71.1 (58.9-84.8)	17,900 (14,700-21,100)	76.6 (61.9-90.7)	4,670 (3,000-6,650)	52.5 (31.7-75.6)	44,700 (37,300-51,600)	591,000 (496,000-704,000)	31,700 (25,600-36,800)	7,120 (4,620-10,300)

Location	Birth , 2000	Births percent change (2000-2021)	All age cases, 2000	All age cases percent change (2000-2021)	All age deaths, 2000	All age deaths percent change (2000-2021)	Under-5 deaths, 2000	Under-5 deaths percent change (2000-2021)	Births, 2021	All age cases, 2021	All age deaths, 2021	Under-5 deaths, 2021
Equatorial Guinea	413 (334-519)	21.2 (10.1-33.3)	4,170 (3,380-5,190)	91.7 (74.2-110)	209 (163-272)	101 (81.7-127)	53 (35-79)	43.4 (20.8-68.4)	500 (400-619)	8,000 (6,450-10,000)	421 (329-536)	76 (49-114)
Gabon	320 (253-408)	-5.6 (-13.9-5.1)	4,150 (3,310-5,250)	19.8 (9.8-31.6)	189 (145-246)	25.6 (14-39.9)	36 (24-54)	1.9 (-12.5-21.1)	302 (236-383)	4,980 (3,940-6,240)	237 (182-308)	37 (24-55)
**Eastern Sub-Saharan Africa**	37,800 (30,500-46,300)	26.3 (22.5-30.2)	381,000 (311,000-466,000)	56.6 (52.4-61.4)	21,200 (16,600-26,600)	65.2 (60.2-71.4)	5,480 (3,600-7,690)	43.1 (34.9-53.2)	47,700 (38,600-58,100)	596,000 (489,000-735,000)	35,000 (27,500-43,800)	7,840 (5,110-11,200)
Burundi	157 (122-203)	64.7 (39.2-87.6)	1,550 (1,210-2,050)	76.9 (50.8-98.9)	94 (70-125)	74 (45-99.1)	26 (16-40)	73.3 (39.9-110)	258 (204-342)	2,740 (2,160-3,530)	163 (126-220)	46 (29-71)
Comoros	0 (0-0)	-18.4 (-19.5-0.6)	0 (0-0)	8.2 (-0.5-37.2)	0 (0-0)	14 (7.6-41.9)	0 (0-0)	-10.5 (-22.5-15.6)	0 (0-0)	0 (0-0)	0 (0-0)	0 (0-0)
Djibouti	5 (4-7)	22 (8.6-33.5)	48 (36-64)	64 (48.1-78.9)	3 (2-4)	73.9 (53.7-93)	1 (0-1)	52.7 (24.3-84.1)	6 (4-9)	79 (58-106)	4 (3-6)	1 (1-2)
Eritrea	33 (24-47)	19.2 (8.6-33.4)	343 (253-465)	47.4 (34.9-62.1)	20 (14-29)	53.4 (37.6-75.7)	6 (3-9)	29.8 (7.3-57.1)	40 (28-55)	505 (373-679)	31 (21-44)	7 (4-12)
Ethiopia	5 (3-7)	18.3 (-34.8-80.9)	46 (25-70)	40.5 (-22.7-117)	3 (2-4)	46.5 (-18.8-123)	1 (0-1)	33.2 (-25.2-103)	6 (3-9)	64 (29-106)	4 (2-7)	1 (0-2)
Addis Ababa	0 (0-0)	-30.9 (-65.5-10.8)	2 (1-2)	-4.9 (-53-52.7)	0 (0-0)	3.6 (-49-70.6)	0 (0-0)	-7.5 (-52.2-51.3)	0 (0-0)	1 (1-2)	0 (0-0)	0 (0-0)
Afar	0 (0-0)	-10.6 (-53-47.6)	1 (1-2)	0.5 (-46.4-68.3)	0 (0-0)	2.1 (-45.4-73.4)	0 (0-0)	3 (-46-76.3)	0 (0-0)	1 (0-2)	0 (0-0)	0 (0-0)
Amhara	1 (1-2)	-1.3 (-47.9-54.7)	11 (6-17)	17.5 (-39.7-85.1)	1 (0-1)	22.4 (-35.8-94.2)	0 (0-0)	8.3 (-42.3-74)	1 (1-2)	13 (6-21)	1 (0-1)	0 (0-0)
Benishangul-Gumuz	0 (0-0)	35.4 (-32.3-122)	0 (0-1)	68.1 (-15.5-174)	0 (0-0)	76 (-11.3-186)	0 (0-0)	58.1 (-16.5-172)	0 (0-0)	1 (0-1)	0 (0-0)	0 (0-0)
Dire Dawa	0 (0-0)	-0.4 (-50-57.6)	0 (0-0)	37.5 (-31.5-117)	0 (0-0)	46.4 (-27.1-130)	0 (0-0)	20.5 (-39.1-95.5)	0 (0-0)	0 (0-0)	0 (0-0)	0 (0-0)
Gambella	0 (0-0)	23.9 (-31.6-90.5)	0 (0-0)	79.3 (-2.4-175)	0 (0-0)	94 (10.1-208)	0 (0-0)	45.8 (-16.6-129)	0 (0-0)	0 (0-0)	0 (0-0)	0 (0-0)
Harari	0 (0-0)	-12.6 (-56.2-37)	0 (0-0)	30 (-33.3-102)	0 (0-0)	40.7 (-29.1-124)	0 (0-0)	10.1 (-42.3-82.6)	0 (0-0)	0 (0-0)	0 (0-0)	0 (0-0)
Oromia	2 (1-3)	29.1 (-35.5-104)	17 (9-26)	54.3 (-21.2-147)	1 (1-2)	60.9 (-18.8-151)	0 (0-0)	45.5 (-23.9-136)	2 (1-4)	26 (12-43)	2 (1-3)	0 (0-1)
Somali	0 (0-0)	41.7 (-29.7-136)	3 (2-5)	45.8 (-24.2-151)	0 (0-0)	45.9 (-26.2-152)	0 (0-0)	58.4 (-21.3-180)	0 (0-1)	4 (2-8)	0 (0-0)	0 (0-0)
Southern Nations, Nationalities, and Peoples	1 (1-1)	26.2 (-32.9-98.8)	9 (5-14)	52.6 (-18.4-138)	1 (0-1)	60.3 (-15.2-153)	0 (0-0)	40.7 (-26.7-126)	1 (1-2)	14 (6-24)	1 (0-2)	0 (0-0)
Tigray	0 (0-0)	1.9 (-46.1-61.2)	3 (1-4)	34.9 (-29.5-110)	0 (0-0)	43.4 (-24.7-129)	0 (0-0)	16.6 (-37.9-96.8)	0 (0-0)	3 (2-6)	0 (0-0)	0 (0-0)
Kenya	14,700 (10,700-20,200)	15.3 (12.8-17.9)	167,000 (123,000-221,000)	45.8 (42.6-49)	9,060 (6,340-12,800)	57.9 (53.1-63)	1,990 (1,190-3,040)	29.2 (23.7-34.2)	17,000 (12,200-23,200)	243,000 (180,000-322,000)	14,300 (10,100-20,000)	2,570 (1,520-4,000)
Baringo	212 (150-291)	2.3 (-10-15.7)	2,240 (1,640-3,020)	32 (18.6-46.7)	127 (87-181)	42.1 (24.1-62.8)	32 (19-50)	11.2 (-7.6-37.4)	217 (152-306)	2,950 (2,170-4,000)	180 (123-261)	35 (21-57)
Bomet	221 (155-303)	104 (83.8-128)	2,670 (1,930-3,570)	171 (146-199)	121 (82-172)	206 (171-245)	23 (14-34)	116 (77.9-161)	450 (320-632)	7,250 (5,280-9,780)	372 (259-535)	50 (30-78)
Bungoma	890 (629-1,250)	-53.2 (-58.5--47.8)	9,210 (6,830-12,500)	-36.4 (-42.5--30)	503 (346-732)	-32.3 (-40.4--23)	122 (72-192)	-43.5 (-53.3--31.5)	416 (296-586)	5,860 (4,320-7,940)	340 (233-497)	69 (40-111)
Busia	405 (286-564)	16.3 (1.6-29)	4,330 (3,160-5,840)	41.7 (25.3-55.6)	249 (169-363)	50.3 (29.4-69.4)	63 (36-100)	25.7 (3-55.4)	471 (322-657)	6,140 (4,460-8,350)	374 (250-544)	79 (45-130)
Elgeyo-Marakwet	206 (145-292)	-71.2 (-74--67.6)	2,460 (1,770-3,330)	-57.6 (-61.5--52.7)	125 (83-183)	-53.3 (-58.6--46.5)	23 (14-36)	-65 (-70.7--57.1)	59 (41-84)	1,040 (757-1,430)	58 (39-85)	8 (5-13)
Embu	154 (109-211)	-38.8 (-46--30.8)	2,050 (1,490-2,730)	-12.8 (-22.4--3)	105 (72-151)	-3.6 (-16.8-10.9)	19 (11-29)	-30.8 (-44.6--15.5)	94 (66-134)	1,790 (1,320-2,390)	101 (70-147)	13 (8-20)
Garissa	135 (97-188)	271 (235-312)	1,590 (1,170-2,130)	261 (231-295)	85 (58-123)	272 (231-319)	19 (11-29)	263 (199-329)	501 (354-702)	5,740 (4,210-7,870)	317 (217-461)	67 (39-109)
Homa Bay	839 (593-1,170)	5.1 (-8.2-17.1)	7,390 (5,390-9,880)	43.3 (28.4-57.2)	442 (299-634)	52.7 (31.3-71.9)	114 (65-182)	28 (3.7-53.8)	882 (629-1,210)	10,600 (7,800-14,300)	675 (463-948)	146 (83-234)
Isiolo	52 (36-72)	24.2 (10.3-39.3)	525 (382-698)	52.7 (38.7-66.6)	32 (21-46)	61.6 (41.2-88.4)	8 (5-13)	38.3 (11.7-69.2)	65 (46-90)	802 (591-1,080)	52 (36-74)	12 (6-18)

Location	Birth , 2000	Births percent change (2000-2021)	All age cases, 2000	All age cases percent change (2000-2021)	All age deaths, 2000	All age deaths percent change (2000-2021)	Under-5 deaths, 2000	Under-5 deaths percent change (2000-2021)	Births, 2021	All age cases, 2021	All age deaths, 2021	Under-5 deaths, 2021
Kajiado	165 (116-229)	39.7 (25.3-56.9)	2,110 (1,550-2,800)	89.9 (72-110)	106 (72-153)	112 (87-143)	20 (12-32)	52 (25.3-82.7)	231 (163-323)	4,000 (2,920-5,390)	225 (157-321)	31 (18-48)
Kakamega	598 (422-846)	79.4 (56.3-101)	6,450 (4,700-8,760)	118 (94.8-145)	367 (248-538)	134 (99.4-170)	98 (55-156)	87.9 (50.2-134)	1,070 (773-1,480)	14,100 (10,400-18,800)	858 (600-1,200)	183 (108-283)
Kericho	355 (248-503)	-68.5 (-72.1--65)	3,890 (2,800-5,330)	-52.9 (-57.7--48.2)	191 (129-279)	-47.5 (-53.6--40.9)	40 (24-60)	-61.2 (-68.6--53.4)	112 (80-154)	1,830 (1,350-2,440)	100 (70-142)	16 (9-24)
Kiambu	523 (366-721)	-30.4 (-37.9--21.2)	6,480 (4,770-8,750)	-1.7 (-10.8-9.5)	337 (230-469)	8.6 (-3.8-24.8)	57 (34-87)	-17.6 (-32.7--0.5)	364 (259-510)	6,370 (4,640-8,400)	366 (257-518)	47 (28-75)
Kilifi	351 (270-456)	23.9 (12.6-34.8)	4,280 (3,340-5,500)	54.8 (41.1-68.8)	225 (165-296)	69.6 (52-86.7)	52 (33-77)	37.9 (15.4-63.4)	436 (328-581)	6,620 (5,150-8,580)	382 (280-525)	72 (44-108)
Kirinyaga	159 (112-218)	-36.8 (-42.9--27.8)	1,980 (1,460-2,670)	-10.2 (-18.6-0)	105 (72-148)	0 (-10.9-14.5)	19 (11-30)	-27.6 (-40.7--10.9)	100 (71-144)	1,770 (1,300-2,410)	105 (74-151)	14 (8-22)
Kisii	624 (447-879)	-45.9 (-51.7--40.2)	7,500 (5,550-10,100)	-17.7 (-27.4--9.2)	423 (292-620)	-9.3 (-19.7-2.4)	89 (53-137)	-36.6 (-47.9--24.1)	338 (239-470)	6,170 (4,550-8,200)	384 (266-547)	56 (33-89)
Kisumu	794 (620-1,010)	-21.6 (-28.7--14.5)	7,690 (6,080-9,820)	12 (2.5-22.3)	532 (405-694)	20.5 (7.9-35.1)	154 (94-236)	-8.5 (-23.1-10.8)	622 (489-804)	8,610 (6,800-11,100)	641 (498-839)	141 (89-211)
Kitui	381 (271-535)	-15 (-23.9--5.5)	4,350 (3,180-5,820)	12.2 (1.8-24.4)	223 (153-321)	22 (7.5-38.2)	50 (30-76)	-6.2 (-24.3-13.9)	324 (233-458)	4,880 (3,570-6,580)	272 (188-398)	47 (28-72)
Kwale	274 (194-384)	13.2 (-0.4-28.1)	3,320 (2,450-4,480)	38.3 (24-53.6)	188 (129-267)	46.6 (27.7-70)	43 (25-69)	20.2 (-2.7-50.1)	310 (215-434)	4,590 (3,330-6,190)	276 (185-397)	51 (29-83)
Laikipia	141 (100-200)	-35.3 (-42.2--26.8)	1,720 (1,270-2,320)	-4.9 (-14.3-6.2)	81 (54-118)	6.9 (-6.6-23.3)	14 (8-22)	-24.1 (-38.3--6.5)	92 (64-126)	1,630 (1,190-2,190)	86 (59-122)	11 (6-17)
Lamu	28 (20-39)	10.3 (-2.7-22.2)	346 (256-464)	40.4 (26.1-54)	20 (14-28)	50.1 (32-69.4)	4 (2-7)	20.2 (-2.2-45)	31 (22-44)	486 (357-654)	30 (21-42)	5 (3-8)
Machakos	387 (270-538)	-54.2 (-59--48.6)	5,290 (3,840-7,110)	-34.8 (-40.9--27.8)	267 (179-387)	-27 (-35.9--16.3)	48 (27-74)	-47.2 (-56.4--35.8)	177 (124-246)	3,450 (2,560-4,620)	195 (135-274)	25 (15-40)
Makueni	263 (187-373)	35.7 (22.8-51.6)	3,090 (2,240-4,250)	79.7 (64.7-97.5)	152 (103-225)	99.7 (78-127)	32 (20-49)	45.6 (22.3-75.2)	357 (248-495)	5,550 (4,030-7,440)	303 (206-430)	46 (28-73)
Mandera	118 (83-164)	1040 (917-1190)	1,610 (1,160-2,180)	921 (827-1040)	93 (62-135)	957 (833-1110)	23 (13-36)	816 (642-1010)	1,340 (948-1,870)	16,400 (12,100-22,000)	980 (667-1,400)	208 (120-322)
Marsabit	86 (61-120)	97.6 (78.3-120)	946 (691-1,280)	120 (99.7-142)	51 (36-76)	132 (105-163)	12 (7-19)	95.7 (61.7-140)	169 (120-235)	2,080 (1,530-2,780)	119 (82-171)	24 (14-37)
Meru	473 (327-653)	-31 (-37.4--21.6)	6,190 (4,480-8,310)	-3 (-11.8-8.2)	324 (219-456)	8 (-3.9-23.2)	64 (37-99)	-24 (-35.7--6.3)	326 (226-448)	6,000 (4,420-8,030)	350 (237-490)	49 (28-78)
Migori	732 (522-1,020)	36.1 (21.4-52.8)	6,280 (4,620-8,360)	74.4 (57.9-92.6)	381 (263-548)	84.5 (63.2-110)	106 (61-171)	61.5 (34.4-92.9)	997 (696-1,390)	11,000 (8,030-14,800)	702 (475-1,020)	171 (97-282)
Mombasa	271 (192-379)	21.8 (8.6-35.8)	3,380 (2,490-4,500)	66.3 (50.9-82.7)	189 (132-268)	77.7 (58.2-99)	32 (20-50)	46.3 (22.4-79.6)	330 (235-452)	5,620 (4,200-7,610)	335 (235-463)	48 (28-72)
Murang'a	244 (173-340)	-32.9 (-39.3--25.3)	3,450 (2,540-4,610)	-6.7 (-15.2-2.9)	171 (115-247)	6.2 (-6.2-20.7)	30 (17-47)	-30.3 (-42.4--17)	163 (116-227)	3,220 (2,370-4,300)	182 (127-259)	21 (12-32)
Nairobi	807 (565-1,120)	13.9 (1.1-27.5)	9,320 (6,860-12,400)	65.1 (48.4-81)	512 (355-721)	77.6 (57.8-99.8)	82 (49-125)	48 (23-78.1)	919 (651-1,280)	15,400 (11,200-20,800)	909 (643-1,270)	121 (71-187)
Nakuru	481 (335-670)	-23.8 (-32.4--15.5)	6,150 (4,470-8,240)	13.5 (2.5-24.6)	302 (202-430)	27.6 (12.9-42.9)	55 (33-84)	-15.1 (-29.1-3.3)	366 (260-505)	6,970 (5,180-9,350)	385 (267-537)	46 (28-71)
Nandi	256 (182-357)	-18.5 (-26.3--10.8)	3,220 (2,380-4,360)	15.1 (3.9-25.8)	157 (106-231)	27.7 (13-42.6)	30 (18-48)	-9.9 (-25.6-7.5)	208 (148-290)	3,710 (2,720-5,020)	201 (137-291)	27 (16-42)
Narok	203 (144-283)	277 (239-327)	2,290 (1,660-3,110)	326 (287-372)	112 (76-162)	361 (307-429)	27 (16-42)	268 (217-336)	766 (539-1,060)	9,760 (7,140-13,100)	516 (354-742)	98 (58-154)
Nyamira	171 (121-239)	10.8 (-0.8-23.8)	2,080 (1,510-2,810)	59 (44.8-75.1)	105 (71-151)	77.7 (56.1-101)	19 (11-29)	23 (0.6-51.4)	190 (131-268)	3,310 (2,380-4,440)	186 (124-272)	23 (13-35)
Nyandarua	207 (147-286)	-24.2 (-30.9--18.3)	2,560 (1,870-3,440)	2.9 (-5.1-12.1)	126 (86-182)	17 (5.3-30)	22 (13-34)	-20.1 (-33.9--4.7)	157 (110-222)	2,630 (1,930-3,510)	148 (102-213)	18 (10-28)
Nyeri	216 (156-301)	-47.4 (-53.4--41.5)	2,960 (2,170-4,000)	-26 (-33.2--18.7)	152 (107-218)	-16.2 (-25.9--4.7)	23 (14-36)	-42.8 (-53--28.8)	113 (80-158)	2,190 (1,600-2,940)	127 (89-177)	13 (8-21)
Samburu	83 (59-116)	58.7 (41.7-79.3)	870 (634-1,180)	86.1 (67.5-108)	43 (29-62)	102 (76.2-130)	10 (6-14)	63.1 (35.7-98.9)	131 (92-187)	1,620 (1,170-2,210)	87 (59-128)	16 (9-24)
Siaya	613 (442-826)	-13.2 (-22.7--2.6)	5,980 (4,440-7,950)	18 (6.3-30.6)	360 (248-504)	25.1 (9.7-43.3)	93 (54-146)	3.4 (-13.8-24.9)	532 (376-737)	7,060 (5,190-9,430)	450 (306-640)	97 (56-157)

Location	Birth , 2000	Births percent change (2000-2021)	All age cases, 2000	All age cases percent change (2000-2021)	All age deaths, 2000	All age deaths percent change (2000-2021)	Under-5 deaths, 2000	Under-5 deaths percent change (2000-2021)	Births, 2021	All age cases, 2021	All age deaths, 2021	Under-5 deaths, 2021
Taita Taveta	80 (57-111)	-32.4 (-40.5--24.1)	1,190 (877-1,610)	-9 (-19.3-0.7)	65 (45-94)	0.2 (-12.4-13.7)	12 (7-18)	-26.2 (-40.8--10)	54 (38-75)	1,080 (805-1,460)	65 (45-92)	9 (5-14)
Tana River	91 (64-126)	40.8 (25.7-56.8)	1,020 (758-1,380)	56.2 (41.1-71.2)	59 (41-84)	62.5 (42.9-84.5)	14 (8-22)	46.6 (19.6-75.6)	128 (90-176)	1,590 (1,160-2,150)	95 (65-137)	20 (12-33)
Tharaka Nithi	40 (28-56)	-16.5 (-25.1--5.8)	502 (365-672)	15 (4.8-27.9)	27 (18-39)	25.9 (11.2-44.3)	6 (3-9)	-9.3 (-23.6-10.9)	33 (24-47)	578 (426-769)	34 (24-48)	5 (3-8)
Trans Nzoia	282 (201-387)	-4.4 (-14-6.4)	3,440 (2,510-4,650)	32.1 (19.8-47)	174 (119-248)	46.2 (29.2-67.5)	38 (22-60)	4.3 (-13.1-29.1)	269 (191-374)	4,550 (3,310-6,090)	254 (173-365)	39 (22-62)
Turkana	267 (188-372)	173 (140-205)	2,670 (1,970-3,590)	216 (183-251)	143 (98-206)	233 (186-282)	34 (20-53)	196 (143-247)	728 (516-1,010)	8,450 (6,240-11,200)	478 (332-666)	99 (60-154)
Uasin Gishu	271 (191-384)	-8.4 (-17.5-3.4)	3,330 (2,440-4,550)	36.5 (24.2-50.9)	165 (112-243)	52.2 (35.2-72.2)	33 (20-51)	5.4 (-13-25.3)	248 (178-347)	4,540 (3,330-6,120)	251 (175-358)	34 (21-53)
Vihiga	196 (137-277)	22.6 (10.3-40.2)	2,360 (1,690-3,190)	60.9 (45.4-80.2)	131 (88-193)	75 (52.5-105)	31 (18-48)	24.2 (3.2-54.6)	240 (169-338)	3,790 (2,790-5,180)	230 (157-334)	38 (22-60)
Wajir	179 (129-246)	177 (141-211)	1,950 (1,430-2,630)	171 (140-204)	114 (79-162)	176 (138-218)	27 (16-43)	166 (115-219)	496 (352-699)	5,300 (3,900-7,130)	314 (216-453)	72 (43-117)
West Pokot	191 (137-268)	74.8 (53.6-94.5)	1,890 (1,390-2,600)	108 (86.8-128)	103 (71-153)	120 (90.5-149)	28 (16-45)	81.9 (47.9-121)	334 (236-468)	3,920 (2,850-5,270)	227 (153-329)	51 (28-81)
Madagascar	2,440 (2,030-2,960)	28 (18.6-37.7)	23,600 (19,600-28,500)	58 (46.5-69.9)	1,400 (1,140-1,720)	62.9 (50.2-77)	401 (268-569)	39.2 (20.9-60.8)	3,130 (2,630-3,810)	37,300 (31,100-45,500)	2,280 (1,890-2,800)	559 (365-773)
Malawi	351 (297-448)	4.4 (-4.5-12.7)	3,050 (2,530-3,900)	59.7 (47.3-72.9)	181 (148-233)	68.6 (52.8-83.8)	55 (35-78)	27.5 (6.2-50.1)	366 (305-459)	4,870 (4,020-6,210)	305 (249-385)	70 (45-101)
Mozambique	67 (51-85)	40.4 (29.5-52.4)	570 (448-721)	75.7 (61.7-90.8)	36 (26-46)	80.7 (63.4-99.1)	11 (7-16)	60.4 (35.9-84)	94 (72-118)	1,000 (786-1,260)	64 (49-82)	18 (11-26)
Rwanda	53 (40-71)	10 (-0.2-20.5)	546 (416-721)	39.6 (27-52.4)	32 (23-44)	44.6 (28.9-61.6)	9 (6-14)	19.9 (0.2-42.2)	58 (44-78)	762 (585-990)	47 (35-64)	11 (7-16)
Somalia	1 (0-2)	89.9 (-19.5-213)	5 (1-14)	113 (-10.4-248)	0 (0-1)	120 (-3.6-279)	0 (0-0)	112 (-0.7-277)	1 (0-4)	12 (1-31)	1 (0-2)	0 (0-1)
South Sudan	70 (49-97)	8.3 (-2.9-20.8)	617 (449-828)	19.6 (8.1-31.7)	39 (27-57)	21.5 (7-36.6)	12 (7-20)	10.3 (-8-35.1)	76 (54-105)	738 (547-1,010)	48 (33-67)	14 (8-22)
Uganda	7,170 (6,070-8,250)	29.7 (19.9-39.2)	61,800 (52,200-72,600)	62.7 (50.4-75.4)	3,660 (2,980-4,280)	68.5 (54.2-81.6)	1,130 (743-1,600)	47.5 (27-71.2)	9,300 (7,600-10,800)	101,000 (83,600-119,000)	6,160 (4,870-7,280)	1,670 (1,010-2,380)
United Republic of Tanzania	9,380 (7,750-10,800)	36.9 (25.2-52)	90,700 (75,700-106,000)	65.3 (51.1-81.4)	4,820 (3,900-5,630)	70.4 (54.2-91.1)	1,320 (823-1,830)	57.3 (36-92.2)	12,800 (10,400-15,200)	150,000 (124,000-180,000)	8,220 (6,490-9,850)	2,070 (1,380-2,930)
Zambia	3,300 (2,820-4,080)	37.6 (26.2-50.1)	31,100 (26,200-38,800)	75.1 (61.4-90.4)	1,790 (1,480-2,240)	82.6 (66.1-102)	510 (332-715)	55.5 (33.5-78.6)	4,550 (3,850-5,710)	54,400 (45,400-68,700)	3,280 (2,710-4,140)	793 (534-1,110)
**Southern Sub-Saharan Africa**	72 (56-92)	-4.8 (-13.4-10.3)	962 (767-1,210)	9.5 (0.9-23.3)	48 (36-62)	11.9 (0.2-30)	10 (6-14)	2.5 (-14.8-29.5)	68 (52-90)	1,050 (829-1,350)	54 (40-72)	10 (6-15)
Botswana	17 (14-21)	5.9 (-3.5-15)	304 (254-369)	19.4 (10.2-28.3)	12 (10-16)	30.8 (17.4-43.4)	2 (1-3)	12.9 (-3.7-32.3)	18 (15-22)	363 (302-434)	16 (13-20)	2 (1-3)
Eswatini	0 (0-0)	-18.6 (-40.3--17.3)	1 (0-1)	-1.5 (-26.8-7.8)	0 (0-0)	4.4 (-23.3-10.8)	0 (0-0)	-8.4 (-35.1-10.4)	0 (0-0)	1 (0-1)	0 (0-0)	0 (0-0)
Lesotho	0 (0-0)	-24.9 (-46.2--23.4)	1 (1-2)	-9.2 (-32.9-3.3)	0 (0-0)	-3.8 (-30.9-3.9)	0 (0-0)	-16.9 (-40.6-5)	0 (0-0)	1 (1-2)	0 (0-0)	0 (0-0)
Namibia	0 (0-0)	12.9 (-18-14.8)	1 (1-1)	17.5 (-13.1-26.1)	0 (0-0)	21.8 (-11.5-28.5)	0 (0-0)	16.1 (-22.2-40.4)	0 (0-0)	1 (1-2)	0 (0-0)	0 (0-0)
South Africa	0 (0-1)	-5.4 (-7.9--4.6)	5 (0-14)	4.3 (-0.4-8.1)	0 (0-1)	8.6 (3.4-14.4)	0 (0-0)	2 (-11.6-9.3)	0 (0-1)	5 (0-14)	0 (0-1)	0 (0-0)
Eastern Cape	0 (0-0)	-28 (-27.4--27.4)	1 (0-2)	-21.7 (-25.1--17.5)	0 (0-0)	-18 (-20.7--14.2)	0 (0-0)	-23.3 (-32.6--13.3)	0 (0-0)	1 (0-2)	0 (0-0)	0 (0-0)
Free State	0 (0-0)	-24.3 (-24.2--24.2)	0 (0-1)	-18.6 (-22.4--14.2)	0 (0-0)	-14.9 (-17.3--12.4)	0 (0-0)	-15.1 (-24--3.2)	0 (0-0)	0 (0-1)	0 (0-0)	0 (0-0)
Gauteng	0 (0-0)	42.7 (41.1-41.1)	1 (0-3)	60.1 (51.2-69.3)	0 (0-0)	63.1 (57-66.9)	0 (0-0)	71.3 (45.7-105)	0 (0-0)	1 (0-4)	0 (0-0)	0 (0-0)
KwaZulu-Natal	0 (0-0)	-18 (-17.7--17.7)	1 (0-3)	-6.9 (-10.9--1.1)	0 (0-0)	-1.2 (-4.5-3)	0 (0-0)	-9.5 (-23.1-3.3)	0 (0-0)	1 (0-3)	0 (0-0)	0 (0-0)
Limpopo	0 (0-0)	-24.4 (-24.1--24.1)	1 (0-2)	-19.7 (-23.4--15.5)	0 (0-0)	-14.3 (-17.7--10.2)	0 (0-0)	-20 (-30.1--7.2)	0 (0-0)	1 (0-2)	0 (0-0)	0 (0-0)

Location	Birth , 2000	Births percent change (2000-2021)	All age cases, 2000	All age cases percent change (2000-2021)	All age deaths, 2000	All age deaths percent change (2000-2021)	Under-5 deaths, 2000	Under-5 deaths percent change (2000-2021)	Births, 2021	All age cases, 2021	All age deaths, 2021	Under-5 deaths, 2021
Mpumalanga	0 (0-0)	23.9 (23.9-23.9)	0 (0-1)	30.2 (23.6-35.8)	0 (0-0)	37.3 (32.7-42.4)	0 (0-0)	31.3 (16.9-49.5)	0 (0-0)	0 (0-1)	0 (0-0)	0 (0-0)
North-West	0 (0-0)	-31 (-29.1--29.1)	0 (0-1)	-25.9 (-28.7--20.1)	0 (0-0)	-24.1 (-24.7--20.1)	0 (0-0)	-21.4 (-33.8--8.5)	0 (0-0)	0 (0-1)	0 (0-0)	0 (0-0)
Northern Cape	0 (0-0)	53.8 (54-54)	0 (0-0)	60.7 (51.8-68.7)	0 (0-0)	68.9 (64.4-73.2)	0 (0-0)	53.9 (31.3-78.7)	0 (0-0)	0 (0-0)	0 (0-0)	0 (0-0)
Western Cape	0 (0-0)	4.7 (4.7-4.7)	0 (0-1)	19.7 (14.2-25.4)	0 (0-0)	27.4 (23-31.9)	0 (0-0)	16.7 (2.8-33.4)	0 (0-0)	0 (0-1)	0 (0-0)	0 (0-0)
Zimbabwe	54 (41-71)	-8.1 (-19.4-13.2)	650 (501-837)	5 (-7.2-25.4)	35 (25-47)	5.3 (-9.1-31.8)	8 (4-11)	-0.2 (-22.5-33.9)	50 (36-68)	682 (513-910)	37 (26-52)	7 (4-12)
**Western Sub-Saharan Africa**	140,000 (108,000-183,000)	32.7 (27.4-38.2)	1,440,000 (1,160,000-1,820,000)	81 (74.8-87.9)	66,600 (50,000-89,900)	67 (58.3-75)	16,900 (10,100-25,900)	38 (27.6-47.1)	185,000 (143,000-239,000)	2,610,000 (2,060,000-3,290,000)	111,000 (84,000-149,000)	23,400 (14,600-35,300)
Benin	3,890 (3,210-4,730)	23.7 (2.7-42.3)	48,500 (40,400-57,900)	37 (15.2-55.8)	1,700 (1,320-2,170)	46.4 (19.5-70.5)	421 (262-614)	37.2 (5-72)	4,820 (3,730-6,210)	66,400 (51,500-85,000)	2,490 (1,830-3,330)	578 (360-904)
Burkina Faso	8,300 (7,340-9,380)	32.8 (6.9-61.7)	110,000 (100,000-122,000)	32 (6.8-59.4)	3,130 (2,590-3,790)	71.1 (35.1-116)	746 (503-1,100)	66.3 (23-125)	11,000 (8,540-14,000)	146,000 (114,000-184,000)	5,360 (4,050-7,080)	1,240 (777-1,850)
Cabo Verde	8 (6-10)	-14.9 (-22.4--7.1)	158 (120-202)	7.7 (-2-17.8)	4 (3-6)	26.2 (12.4-41.8)	1 (0-1)	-10.7 (-23.7-5.7)	6 (5-8)	170 (129-219)	6 (4-7)	1 (0-1)
Cameroon	4,810 (4,050-5,530)	44.6 (34.7-55.6)	58,900 (49,600-69,600)	94.8 (81.1-110)	2,320 (1,880-2,710)	102 (84-120)	550 (351-792)	67.8 (44.4-94)	6,950 (5,890-7,980)	115,000 (96,600-135,000)	4,690 (3,860-5,510)	923 (595-1,320)
Chad	765 (627-892)	91.7 (78.7-107)	7,700 (6,390-9,240)	113 (98.5-131)	317 (249-384)	115 (98.1-135)	91 (58-131)	109 (81.2-141)	1,470 (1,180-1,710)	16,400 (13,600-19,900)	683 (531-824)	191 (115-279)
Côte d'Ivoire	2,270 (1,880-2,610)	19.5 (10-29)	27,000 (22,200-31,800)	48.1 (37.2-59.8)	1,060 (850-1,230)	55 (40.4-68.9)	245 (155-350)	38.3 (18.8-59.4)	2,720 (2,240-3,160)	40,000 (33,400-48,000)	1,640 (1,330-1,920)	339 (218-491)
Gambia	311 (258-368)	11.4 (2.5-22.1)	3,600 (3,030-4,290)	59.7 (46.6-73.3)	131 (105-159)	65.7 (51.1-85)	31 (20-45)	35.7 (17.5-61.3)	346 (283-411)	5,740 (4,730-6,860)	217 (174-261)	42 (27-60)
Ghana	5,970 (5,270-6,760)	7.2 (-6.4-28.2)	86,500 (74,800-99,600)	34.8 (18.7-59.2)	3,180 (2,690-3,680)	39.4 (20.2-70.4)	628 (430-882)	18.6 (-3.7-52.8)	6,400 (5,680-7,960)	117,000 (99,500-146,000)	4,440 (3,870-5,610)	745 (513-1,060)
Guinea	5,440 (4,490-6,410)	39.9 (29.1-53.4)	61,300 (51,100-74,000)	66.5 (52.9-81.2)	2,620 (2,060-3,110)	67.1 (52.3-85.3)	699 (445-1,030)	54.7 (33-82.6)	7,610 (6,260-8,850)	102,000 (85,600-122,000)	4,380 (3,480-5,200)	1,080 (708-1,570)
Guinea-Bissau	19 (15-25)	8 (-3.6-21.8)	233 (181-303)	38 (24.1-52.5)	10 (7-13)	39.9 (23.7-62.6)	2 (1-3)	22.7 (2.1-53.2)	21 (16-27)	322 (249-415)	13 (10-18)	3 (2-4)
Liberia	275 (226-322)	1.7 (-5-10.1)	3,150 (2,600-3,780)	52.5 (42.3-63.9)	126 (99-152)	61.4 (49.3-77.6)	32 (20-48)	21.6 (5.8-41.3)	279 (228-328)	4,800 (3,910-5,800)	203 (162-244)	39 (25-57)
Mali	1,760 (1,490-2,100)	87.3 (73.1-101)	18,300 (15,400-22,300)	113 (97.6-127)	756 (615-924)	113 (94-132)	215 (137-316)	101 (72.9-131)	3,300 (2,750-3,930)	39,000 (32,500-47,400)	1,610 (1,280-1,970)	433 (272-638)
Mauritania	164 (128-198)	6.6 (-2.5-15.7)	2,290 (1,820-2,780)	49.5 (36.7-63.1)	80 (61-100)	53.1 (37.5-70.8)	17 (11-25)	19.1 (-0.1-38.7)	175 (137-209)	3,420 (2,740-4,120)	123 (93-150)	20 (13-30)
Niger	3,380 (3,020-4,050)	89.1 (72-115)	33,000 (28,600-39,700)	118 (98.6-147)	1,260 (1,070-1,580)	128 (103-163)	364 (243-532)	125 (84.8-165)	6,400 (5,530-8,060)	72,000 (60,800-93,100)	2,870 (2,390-3,760)	818 (532-1,200)
Nigeria	93,900 (66,800-132,000)	30.6 (23.3-38.6)	878,000 (635,000-1,200,000)	94.5 (85.5-104)	45,800 (31,700-66,500)	64.5 (53.4-75.2)	11,900 (6,720-19,700)	29.5 (17.6-41)	123,000 (89,900-168,000)	1,710,000 (1,270,000-2,270,000)	75,400 (53,500-108,000)	15,400 (9,000-24,600)
Sao Tome and Principe	48 (37-62)	-14.7 (-22.6--5.9)	715 (559-898)	30.4 (19.2-42)	24 (18-32)	41.7 (25.7-58.7)	5 (3-8)	-2.6 (-18.6-14.9)	41 (32-52)	932 (725-1,170)	34 (26-45)	5 (3-7)
Senegal	1,770 (1,440-2,070)	21.5 (12.3-30.1)	23,900 (20,100-28,800)	49.7 (39.1-60.6)	883 (708-1,050)	52.9 (40.4-66.7)	200 (129-277)	33.7 (15.5-53.9)	2,150 (1,780-2,530)	35,800 (29,900-42,600)	1,350 (1,090-1,600)	267 (174-383)
Sierra Leone	4,290 (3,550-4,930)	44 (32-55.5)	46,300 (39,200-54,800)	88.4 (73.5-103)	2,020 (1,590-2,380)	89.9 (72-109)	523 (321-754)	66.9 (42.8-96.6)	6,170 (5,080-7,230)	87,300 (72,800-104,000)	3,840 (3,030-4,560)	874 (544-1,250)
Togo	2,230 (1,870-2,610)	19.1 (10.2-27.6)	28,700 (24,200-34,300)	54.1 (42.7-66.2)	1,120 (913-1,320)	61 (48.5-74.7)	258 (166-369)	35.6 (19.6-55.1)	2,650 (2,220-3,120)	44,300 (37,400-52,900)	1,800 (1,490-2,140)	349 (227-506)

Table S11: Number of births, cases, total deaths, and under-5 deaths for Hemoglobin SC disease, 2000 - 2021

Location	Birth , 2000	Births percent change (2000-2021)	All age cases, 2000	All age cases percent change (2000-2021)	All age deaths, 2000	All age deaths percent change (2000-2021)	Under-5 deaths, 2000	Under-5 deaths percent change (2000-2021)	Births, 2021	All age cases, 2021	All age deaths, 2021	Under-5 deaths, 2021
Global	88,100 (68,900-112,000)	14.8 (9.5-19.6)	932,000 (748,000-1,160,000)	41.8 (34.3-48.1)	44,800 (34,100-58,700)	51.1 (42.7-58)	10,800 (7,140-16,100)	35.7 (27.3-43.2)	101,000 (76,200-132,000)	1,320,000 (1,010,000-1,690,000)	67,700 (50,100-90,900)	14,700 (9,360-22,200)
Central Europe, Eastern Europe, and Central Asia	6 (5-7)	-5.5 (-11.1--0.2)	155 (128-195)	-20.1 (-24.6--16.1)	8 (6-9)	-14.1 (-20.1--8.2)	2 (1-3)	-1 (-7.7-6.3)	5 (4-6)	124 (102-153)	7 (5-8)	2 (1-2)
**Central Asia**	0 (0-1)	18.7 (2.4-21.4)	4 (1-8)	14.6 (0.3-22.6)	0 (0-1)	19.7 (5.2-24.5)	0 (0-0)	22.1 (2.3-28.4)	0 (0-1)	4 (1-9)	0 (0-1)	0 (0-0)
Armenia	0 (0-0)	-18.2 (-19.6-16.7)	0 (0-0)	-22.4 (-27.2-3.9)	0 (0-0)	-13.9 (-19.3-21.4)	0 (0-0)	-8.8 (-15.8-28.3)	0 (0-0)	0 (0-0)	0 (0-0)	0 (0-0)
Azerbaijan	0 (0-0)	-5.9 (-3.1--3)	0 (0-1)	2.5 (-4.2-11.5)	0 (0-0)	4.6 (3.1-11.2)	0 (0-0)	-0.7 (-7.9-8.8)	0 (0-0)	0 (0-1)	0 (0-0)	0 (0-0)
Georgia	0 (0-0)	-14 (-14.2--12.6)	0 (0-0)	-31.5 (-37.7--27.2)	0 (0-0)	-25.6 (-28.9--21.9)	0 (0-0)	-11.9 (-20.6--3.5)	0 (0-0)	0 (0-0)	0 (0-0)	0 (0-0)
Kazakhstan	0 (0-0)	51.9 (14.1-604)	1 (0-1)	27.2 (-6.4-409)	0 (0-0)	37.9 (5.4-514)	0 (0-0)	63.9 (25.2-616)	0 (0-0)	1 (0-2)	0 (0-0)	0 (0-0)
Kyrgyzstan	0 (0-0)	38.9 (-21.8-43.5)	0 (0-1)	28.7 (-17.9-40.3)	0 (0-0)	35.4 (-23.1-42.9)	0 (0-0)	44.2 (-17.9-58.9)	0 (0-0)	0 (0-1)	0 (0-0)	0 (0-0)
Mongolia	0 (0-0)	34.6 (36.7-37.6)	0 (0-0)	22.3 (9.8-35.6)	0 (0-0)	35 (26.8-43.3)	0 (0-0)	50.1 (34.8-63.4)	0 (0-0)	0 (0-0)	0 (0-0)	0 (0-0)
Tajikistan	0 (0-0)	34.2 (37.9-37.9)	0 (0-1)	35.7 (24.9-48.1)	0 (0-0)	35.8 (36-42.5)	0 (0-0)	33 (20.5-44)	0 (0-0)	0 (0-1)	0 (0-0)	0 (0-0)
Turkmenistan	0 (0-0)	4.8 (-25.2-9.8)	0 (0-1)	2.9 (-27.2-15.9)	0 (0-0)	5.8 (-23.5-13.3)	0 (0-0)	7.1 (-21.1-19.5)	0 (0-0)	0 (0-1)	0 (0-0)	0 (0-0)
Uzbekistan	0 (0-0)	10.4 (-34.5-14)	1 (0-3)	14.4 (-33.6-26.9)	0 (0-0)	20.5 (-26.4-27.2)	0 (0-0)	24.1 (-22.3-35.5)	0 (0-0)	2 (0-3)	0 (0-0)	0 (0-0)
**Central Europe**	4 (3-5)	-12 (-17.6--5.4)	104 (87-132)	-23.9 (-28.9--18.6)	5 (4-7)	-17.5 (-23.8--10.9)	1 (1-2)	-8.8 (-15.8-0.4)	4 (3-4)	79 (66-99)	4 (4-5)	1 (1-2)
Albania	0 (0-0)	-40 (-52.7--39)	2 (1-3)	-39.8 (-52.1--35.5)	0 (0-0)	-39.6 (-52.4--35.3)	0 (0-0)	-46.6 (-60--41.5)	0 (0-0)	1 (1-2)	0 (0-0)	0 (0-0)
Bosnia and Herzegovina	0 (0-0)	-40.3 (-50.8--40)	2 (1-3)	-38.5 (-50--35.1)	0 (0-0)	-34.2 (-46.1--29.6)	0 (0-0)	-43.8 (-54--37.3)	0 (0-0)	1 (1-2)	0 (0-0)	0 (0-0)
Bulgaria	0 (0-0)	-20.9 (-42.6--19.6)	3 (2-4)	-25.5 (-45--16)	0 (0-0)	-23.8 (-44--18.7)	0 (0-0)	-10.4 (-37.8-2.1)	0 (0-0)	2 (1-3)	0 (0-0)	0 (0-0)
Croatia	1 (0-1)	-25.7 (-33.5--18.1)	17 (14-22)	-24.8 (-31.8--17.8)	1 (1-1)	-24.6 (-32--17)	0 (0-0)	-37.6 (-46.2--29.4)	0 (0-1)	13 (10-16)	1 (0-1)	0 (0-0)
Czechia	0 (0-0)	17.2 (-3.7-17.9)	1 (0-2)	-8.7 (-25.4--3)	0 (0-0)	-8.1 (-24.1--1.2)	0 (0-0)	24.7 (2.3-39.4)	0 (0-0)	1 (0-1)	0 (0-0)	0 (0-0)
Hungary	0 (0-0)	-21 (-41.4--19.2)	4 (2-6)	-21.8 (-41.8--17)	0 (0-0)	-20.3 (-41.3--16)	0 (0-0)	-15.8 (-40.5--7.5)	0 (0-0)	3 (2-4)	0 (0-0)	0 (0-0)
Montenegro	0 (0-0)	-24.9 (-42.9--23.8)	0 (0-0)	-18.7 (-38.8--12.9)	0 (0-0)	-16.4 (-36.8--12)	0 (0-0)	-25.9 (-44.8--17.1)	0 (0-0)	0 (0-0)	0 (0-0)	0 (0-0)
North Macedonia	0 (0-0)	-13.8 (-35.2--4.7)	1 (1-2)	-11.6 (-31.8--3.2)	0 (0-0)	-8.4 (-30.8--0.6)	0 (0-0)	-15.4 (-38.3--4.4)	0 (0-0)	1 (1-1)	0 (0-0)	0 (0-0)
Poland	1 (1-2)	9.4 (-7.9-28)	39 (30-54)	-21.1 (-32.4--8)	2 (1-3)	-7.1 (-22.3-8.7)	0 (0-1)	19.6 (-1-42.8)	2 (1-2)	31 (25-41)	2 (1-2)	0 (0-1)
Romania	1 (1-2)	-25.9 (-30.1--7.4)	26 (19-33)	-28.8 (-36--14.4)	1 (1-2)	-25.3 (-30.4--7)	0 (0-1)	-19 (-29.1-6.7)	1 (1-1)	18 (14-24)	1 (1-1)	0 (0-1)
Serbia	0 (0-0)	-24.6 (-25.8--1.1)	1 (0-2)	-20.6 (-25.4-0.1)	0 (0-0)	-20.7 (-24.4-1.4)	0 (0-0)	-33.5 (-39.3--8.6)	0 (0-0)	1 (0-1)	0 (0-0)	0 (0-0)
Slovakia	0 (0-0)	-1.1 (-22.5-27)	7 (5-8)	-20.2 (-36.2-2.5)	0 (0-0)	-16.8 (-35-7.1)	0 (0-0)	-6.3 (-29.2-22)	0 (0-0)	5 (4-7)	0 (0-0)	0 (0-0)
Slovenia	0 (0-0)	-1.7 (-31.7-2)	2 (1-2)	-13.7 (-38.1--6.6)	0 (0-0)	-9.4 (-38.1--2.3)	0 (0-0)	0 (-33.1-14.8)	0 (0-0)	2 (1-2)	0 (0-0)	0 (0-0)
**Eastern Europe**	1 (1-2)	7.8 (-0.1-10.3)	48 (32-69)	-14.5 (-20.6--11.6)	2 (2-3)	-9.8 (-18.4--4.2)	0 (0-1)	17.9 (7.4-22)	2 (1-2)	41 (28-58)	2 (1-3)	0 (0-1)
Belarus	0 (0-0)	1.3 (-35.1-4.2)	2 (1-3)	-19 (-46.4--13)	0 (0-0)	-10.5 (-40.4--3.3)	0 (0-0)	10.2 (-25-26)	0 (0-0)	2 (1-3)	0 (0-0)	0 (0-0)
Estonia	0 (0-0)	-0.3 (-41.1-1.8)	0 (0-0)	-18.2 (-53.6--12.6)	0 (0-0)	-14.5 (-49.5--9.8)	0 (0-0)	3.9 (-35-14.8)	0 (0-0)	0 (0-0)	0 (0-0)	0 (0-0)

Location	Birth , 2000	Births percent change (2000-2021)	All age cases, 2000	All age cases percent change (2000-2021)	All age deaths, 2000	All age deaths percent change (2000-2021)	Under-5 deaths, 2000	Under-5 deaths percent change (2000-2021)	Births, 2021	All age cases, 2021	All age deaths, 2021	Under-5 deaths, 2021
Latvia	0 (0-0)	-13.8 (-47.4--10.1)	1 (1-1)	-34.2 (-59.9--28.2)	0 (0-0)	-28.6 (-56.4--22.8)	0 (0-0)	-2.8 (-43-13.3)	0 (0-0)	1 (0-1)	0 (0-0)	0 (0-0)
Lithuania	0 (0-0)	-18.7 (-20.9-7.8)	2 (1-2)	-34.8 (-39.2--10.6)	0 (0-0)	-27.5 (-32.3--2.8)	0 (0-0)	-24.2 (-32.8-5.1)	0 (0-0)	1 (1-2)	0 (0-0)	0 (0-0)
Republic of Moldova	0 (0-0)	-35.7 (-48.9--34.2)	1 (1-1)	-33.4 (-47.8--27.6)	0 (0-0)	-28.6 (-44--23.2)	0 (0-0)	-36.2 (-52.1--28)	0 (0-0)	1 (0-1)	0 (0-0)	0 (0-0)
Russian Federation	1 (1-1)	18.6 (7-21)	31 (20-46)	-9.1 (-16.7--5.9)	1 (1-2)	-4.9 (-15.8-1.6)	0 (0-0)	32.7 (19.5-37.2)	1 (1-2)	28 (19-40)	1 (1-2)	0 (0-1)
Ukraine	0 (0-0)	-14.4 (-31.5--13.2)	11 (7-16)	-22.8 (-38.3--18.3)	0 (0-1)	-19 (-36.5--14.5)	0 (0-0)	-12.1 (-27.8--4.2)	0 (0-0)	8 (5-12)	0 (0-1)	0 (0-0)
High-income	799 (672-943)	-12.8 (-15.4--10.3)	15,100 (13,000-17,800)	8.7 (6.5-11.1)	797 (667-942)	-5.4 (-8--3)	342 (262-435)	-31.7 (-37.4--26.9)	697 (579-834)	16,400 (14,200-19,400)	754 (629-895)	234 (169-313)
**Australasia**	1 (0-1)	22.8 (5-43.4)	11 (9-14)	11.5 (-4.3-26.1)	1 (0-1)	17.3 (0.3-37.5)	0 (0-0)	16 (-0.8-40.3)	1 (1-1)	13 (10-16)	1 (1-1)	0 (0-0)
Australia	0 (0-0)	31 (15.4-63.6)	7 (5-9)	19.8 (8.7-42.3)	0 (0-0)	24.7 (10.9-57.6)	0 (0-0)	23 (6.6-66.8)	0 (0-1)	8 (6-11)	0 (0-1)	0 (0-0)
New Zealand	0 (0-0)	11.2 (-17.4-22.8)	5 (4-6)	-0.2 (-25-14.3)	0 (0-0)	6.4 (-21.6-19.1)	0 (0-0)	3.5 (-25.1-22.6)	0 (0-0)	5 (4-6)	0 (0-0)	0 (0-0)
**High-income Asia Pacific**	1 (1-2)	-27.9 (-38.4--23.5)	24 (16-36)	-18.2 (-29.5--11)	1 (1-2)	-16.6 (-29.2--10)	1 (0-1)	-28.6 (-41.3--19.9)	1 (1-1)	20 (13-30)	1 (1-2)	0 (0-1)
Brunei Darussalam	0 (0-0)	-10.8 (-11.5-18.5)	0 (0-0)	6.8 (-6.1-33.7)	0 (0-0)	1.2 (-4.2-34)	0 (0-0)	-13.7 (-22-21.4)	0 (0-0)	0 (0-0)	0 (0-0)	0 (0-0)
Japan	0 (0-1)	-28 (-31.9--27.5)	6 (0-17)	-20.9 (-24.3--18.9)	0 (0-1)	-22.1 (-25.7--19.6)	0 (0-0)	-24.4 (-26.8--22.7)	0 (0-1)	5 (0-14)	0 (0-1)	0 (0-0)
Aichi	0 (0-0)	-23.2 (-40.4--22.1)	0 (0-1)	-13.1 (-31.8--8)	0 (0-0)	-15.9 (-34.7--12.9)	0 (0-0)	-19.2 (-34.5--12)	0 (0-0)	0 (0-1)	0 (0-0)	0 (0-0)
Akita	0 (0-0)	-50.3 (-71.9--48.8)	0 (0-0)	-45.6 (-66.5--40.5)	0 (0-0)	-44.2 (-67--40.7)	0 (0-0)	-49.1 (-64.1--43.4)	0 (0-0)	0 (0-0)	0 (0-0)	0 (0-0)
Aomori	0 (0-0)	-44.6 (-44.6--44.5)	0 (0-0)	-40.2 (-43.5--36.8)	0 (0-0)	-38.9 (-40.5--37.2)	0 (0-0)	-43.7 (-47.7--39.7)	0 (0-0)	0 (0-0)	0 (0-0)	0 (0-0)
Chiba	0 (0-0)	-28 (-28--27.9)	0 (0-1)	-18.7 (-24.1--13.8)	0 (0-0)	-21 (-23.1--18.9)	0 (0-0)	-24.2 (-30.6--17.5)	0 (0-0)	0 (0-1)	0 (0-0)	0 (0-0)
Ehime	0 (0-0)	-39.9 (-66--38)	0 (0-0)	-32.9 (-61.3--26.9)	0 (0-0)	-33.4 (-61.8--29.8)	0 (0-0)	-36.8 (-56.2--30.9)	0 (0-0)	0 (0-0)	0 (0-0)	0 (0-0)
Fukui	0 (0-0)	-33.9 (-33.9--33.8)	0 (0-0)	-26.4 (-29.8--22.4)	0 (0-0)	-26.7 (-28.1--25)	0 (0-0)	-33.7 (-38.4--29)	0 (0-0)	0 (0-0)	0 (0-0)	0 (0-0)
Fukuoka	0 (0-0)	-18.1 (-18.1--18)	0 (0-1)	-16.7 (-21.3--12.7)	0 (0-0)	-16.5 (-18.4--14.7)	0 (0-0)	-13.2 (-19.3--7.3)	0 (0-0)	0 (0-1)	0 (0-0)	0 (0-0)
Fukushima	0 (0-0)	-44 (-44.1--44.1)	0 (0-0)	-39.5 (-42.9--35.9)	0 (0-0)	-36.7 (-38.7--34.7)	0 (0-0)	-42.1 (-46.4--38)	0 (0-0)	0 (0-0)	0 (0-0)	0 (0-0)
Gifu	0 (0-0)	-37.5 (-51.5--36.6)	0 (0-0)	-27.1 (-43.1--22)	0 (0-0)	-29.3 (-44.7--26.6)	0 (0-0)	-34.3 (-45.4--28.4)	0 (0-0)	0 (0-0)	0 (0-0)	0 (0-0)
Gunma	0 (0-0)	-38.7 (-38.7--38.6)	0 (0-0)	-26.2 (-30.5--22.6)	0 (0-0)	-28 (-29.9--26.2)	0 (0-0)	-35.8 (-40.1--30.9)	0 (0-0)	0 (0-0)	0 (0-0)	0 (0-0)
Hiroshima	0 (0-0)	-28.5 (-28.5--28.3)	0 (0-0)	-19.8 (-24.5--15.4)	0 (0-0)	-21.8 (-23.4--20.2)	0 (0-0)	-23.8 (-29.1--17.6)	0 (0-0)	0 (0-0)	0 (0-0)	0 (0-0)
Hokkaidō	0 (0-0)	-37.1 (-37.9--18.9)	0 (0-1)	-30.7 (-35.1--12.5)	0 (0-0)	-30.6 (-33.3--10.9)	0 (0-0)	-33.5 (-39.5--18.8)	0 (0-0)	0 (0-1)	0 (0-0)	0 (0-0)
Hyōgo	0 (0-0)	-30.3 (-46--29.4)	0 (0-1)	-23.7 (-40.1--18.8)	0 (0-0)	-25.3 (-41.5--22.4)	0 (0-0)	-28.2 (-40.3--21.4)	0 (0-0)	0 (0-1)	0 (0-0)	0 (0-0)
Ibaraki	0 (0-0)	-37 (-37--36.9)	0 (0-0)	-28.3 (-32.5--24.1)	0 (0-0)	-28.7 (-30.4--27)	0 (0-0)	-33.6 (-37.8--28.2)	0 (0-0)	0 (0-0)	0 (0-0)	0 (0-0)
Ishikawa	0 (0-0)	-30.4 (-31.3--10.3)	0 (0-0)	-22 (-26.7--0.9)	0 (0-0)	-23.6 (-26--1.5)	0 (0-0)	-28.1 (-32.9--12.9)	0 (0-0)	0 (0-0)	0 (0-0)	0 (0-0)
Iwate	0 (0-0)	-43.2 (-43.1--43.1)	0 (0-0)	-35 (-38.1--31.4)	0 (0-0)	-33.9 (-35.7--32)	0 (0-0)	-39.8 (-44.2--36)	0 (0-0)	0 (0-0)	0 (0-0)	0 (0-0)
Kagawa	0 (0-0)	-35.2 (-35.3--35.1)	0 (0-0)	-25.5 (-29.5--21.5)	0 (0-0)	-27.3 (-29--25.5)	0 (0-0)	-32.6 (-37.3--27.4)	0 (0-0)	0 (0-0)	0 (0-0)	0 (0-0)

Location	Birth , 2000	Births percent change (2000-2021)	All age cases, 2000	All age cases percent change (2000-2021)	All age deaths, 2000	All age deaths percent change (2000-2021)	Under-5 deaths, 2000	Under-5 deaths percent change (2000-2021)	Births, 2021	All age cases, 2021	All age deaths, 2021	Under-5 deaths, 2021
Kagoshima	0 (0-0)	-28.8 (-28.8--28.8)	0 (0-0)	-30.2 (-33.4--25.8)	0 (0-0)	-27.1 (-28.6--25.5)	0 (0-0)	-25.5 (-30.9--20.7)	0 (0-0)	0 (0-0)	0 (0-0)	0 (0-0)
Kanagawa	0 (0-0)	-27.5 (-26.8--26.6)	0 (0-1)	-14.9 (-20.1--9.8)	0 (0-0)	-17.5 (-19.5--14.1)	0 (0-0)	-20.8 (-27.6--13.5)	0 (0-0)	0 (0-1)	0 (0-0)	0 (0-0)
Kōchi	0 (0-0)	-39.5 (-39.7--39.7)	0 (0-0)	-34.1 (-37.7--30.7)	0 (0-0)	-34.4 (-35.8--32.8)	0 (0-0)	-36.6 (-41.2--31.5)	0 (0-0)	0 (0-0)	0 (0-0)	0 (0-0)
Kumamoto	0 (0-0)	-25.2 (-25.2--25.1)	0 (0-0)	-23.7 (-27.4--18.1)	0 (0-0)	-21.8 (-23.1--20.1)	0 (0-0)	-20.1 (-27.2--14.8)	0 (0-0)	0 (0-0)	0 (0-0)	0 (0-0)
Kyōto	0 (0-0)	-25.4 (-26.1--26)	0 (0-0)	-20.1 (-25--15.5)	0 (0-0)	-21.6 (-24.2--20.5)	0 (0-0)	-23.2 (-29.5--15.6)	0 (0-0)	0 (0-0)	0 (0-0)	0 (0-0)
Mie	0 (0-0)	-35.9 (-35.9--35.8)	0 (0-0)	-25.3 (-29.4--21.4)	0 (0-0)	-26.7 (-28.4--25)	0 (0-0)	-32.6 (-37.4--26.8)	0 (0-0)	0 (0-0)	0 (0-0)	0 (0-0)
Miyagi	0 (0-0)	-32.2 (-32.2--32.1)	0 (0-0)	-24.8 (-28.2--20.7)	0 (0-0)	-23.4 (-25.3--21.3)	0 (0-0)	-27.4 (-32.5--22.1)	0 (0-0)	0 (0-0)	0 (0-0)	0 (0-0)
Miyazaki	0 (0-0)	-34.5 (-34.5--34.4)	0 (0-0)	-28.8 (-32.7--25)	0 (0-0)	-28.5 (-29.7--27)	0 (0-0)	-31 (-35.3--25.7)	0 (0-0)	0 (0-0)	0 (0-0)	0 (0-0)
Nagano	0 (0-0)	-39.6 (-39.6--39.5)	0 (0-0)	-28.6 (-32.4--25.5)	0 (0-0)	-30.2 (-32--28.2)	0 (0-0)	-36.3 (-39.8--31.6)	0 (0-0)	0 (0-0)	0 (0-0)	0 (0-0)
Nagasaki	0 (0-0)	-36.2 (-63.9--34.2)	0 (0-0)	-35 (-61.1--29.5)	0 (0-0)	-32.7 (-60.4--29.4)	0 (0-0)	-34.6 (-55.4--28.3)	0 (0-0)	0 (0-0)	0 (0-0)	0 (0-0)
Nara	0 (0-0)	-38.9 (-38.9--38.8)	0 (0-0)	-30.6 (-33.9--27.3)	0 (0-0)	-31.8 (-33.5--30.2)	0 (0-0)	-35 (-38.8--30.3)	0 (0-0)	0 (0-0)	0 (0-0)	0 (0-0)
Niigata	0 (0-0)	-37.7 (-37.8--37.6)	0 (0-0)	-30.5 (-33.7--26.8)	0 (0-0)	-30.5 (-32.1--28.9)	0 (0-0)	-35.7 (-40--30.9)	0 (0-0)	0 (0-0)	0 (0-0)	0 (0-0)
Ōita	0 (0-0)	-30.1 (-30.1--30)	0 (0-0)	-26.6 (-30.3--23.2)	0 (0-0)	-26.5 (-27.8--24.9)	0 (0-0)	-27.6 (-33--22.9)	0 (0-0)	0 (0-0)	0 (0-0)	0 (0-0)
Okayama	0 (0-0)	-27.5 (-27.6--27.5)	0 (0-0)	-20.3 (-25--14.7)	0 (0-0)	-21.7 (-23--20.2)	0 (0-0)	-24.3 (-30.1--16.9)	0 (0-0)	0 (0-0)	0 (0-0)	0 (0-0)
Okinawa	0 (0-0)	-13.6 (-51.1--10.9)	0 (0-0)	-10.2 (-46.4--2.2)	0 (0-0)	-8.2 (-46.6--3.5)	0 (0-0)	-7.7 (-38.3-1.7)	0 (0-0)	0 (0-0)	0 (0-0)	0 (0-0)
Ōsaka	0 (0-0)	-30.2 (-30.2--30.1)	0 (0-1)	-20.3 (-24.5--15.1)	0 (0-0)	-23.7 (-25.6--21.7)	0 (0-0)	-27 (-33.4--21.9)	0 (0-0)	0 (0-1)	0 (0-0)	0 (0-0)
Saga	0 (0-0)	-32 (-31.9--31.9)	0 (0-0)	-26.2 (-30.4--22)	0 (0-0)	-25.5 (-26.7--24)	0 (0-0)	-28.7 (-33.6--22.8)	0 (0-0)	0 (0-0)	0 (0-0)	0 (0-0)
Saitama	0 (0-0)	-27.9 (-27.9--27.8)	0 (0-1)	-19 (-23.5--14.6)	0 (0-0)	-21.1 (-23.1--19)	0 (0-0)	-23.4 (-29.3--17.3)	0 (0-0)	0 (0-1)	0 (0-0)	0 (0-0)
Shiga	0 (0-0)	-27.1 (-43.4--26.1)	0 (0-0)	-15.8 (-34.5--9.6)	0 (0-0)	-17.4 (-35.8--14.1)	0 (0-0)	-22.8 (-36.8--15.9)	0 (0-0)	0 (0-0)	0 (0-0)	0 (0-0)
Shimane	0 (0-0)	-30.6 (-30.6--30.5)	0 (0-0)	-28.3 (-31.5--24.8)	0 (0-0)	-27.5 (-28.7--26.2)	0 (0-0)	-27.3 (-32.2--22.4)	0 (0-0)	0 (0-0)	0 (0-0)	0 (0-0)
Shizuoka	0 (0-0)	-34 (-34--33.9)	0 (0-1)	-25.3 (-29--20.8)	0 (0-0)	-26.6 (-28.2--24.7)	0 (0-0)	-30.4 (-37.1--25.6)	0 (0-0)	0 (0-0)	0 (0-0)	0 (0-0)
Tochigi	0 (0-0)	-33.7 (-33.7--33.7)	0 (0-0)	-24.7 (-28.5--20.5)	0 (0-0)	-25 (-26.7--23.1)	0 (0-0)	-28.5 (-33.3--23.2)	0 (0-0)	0 (0-0)	0 (0-0)	0 (0-0)
Tokushima	0 (0-0)	-37.2 (-64.6--35.4)	0 (0-0)	-33.8 (-59.1--28)	0 (0-0)	-32.6 (-60.4--29.2)	0 (0-0)	-34.2 (-57.7--26.9)	0 (0-0)	0 (0-0)	0 (0-0)	0 (0-0)
Tōkyō	0 (0-0)	10.6 (7.4-7.6)	1 (0-1)	3.9 (-4.5-9.9)	0 (0-0)	4 (-2.1-4.7)	0 (0-0)	16.3 (5.4-28.8)	0 (0-0)	1 (0-2)	0 (0-0)	0 (0-0)
Tottori	0 (0-0)	-30.8 (-30.8--30.6)	0 (0-0)	-26.8 (-30.3--23.1)	0 (0-0)	-26.2 (-27.4--24.9)	0 (0-0)	-27.3 (-32.8--21)	0 (0-0)	0 (0-0)	0 (0-0)	0 (0-0)
Toyama	0 (0-0)	-38.5 (-38.5--38.4)	0 (0-0)	-27.5 (-31.7--23.9)	0 (0-0)	-29.5 (-31--27.9)	0 (0-0)	-32.7 (-36.8--26.5)	0 (0-0)	0 (0-0)	0 (0-0)	0 (0-0)
Wakayama	0 (0-0)	-40.5 (-40.5--40.4)	0 (0-0)	-33 (-36.3--29.7)	0 (0-0)	-33.4 (-34.8--31.9)	0 (0-0)	-38.2 (-42--33.6)	0 (0-0)	0 (0-0)	0 (0-0)	0 (0-0)
Yamagata	0 (0-0)	-40.5 (-40.6--40.4)	0 (0-0)	-33.1 (-36.8--29)	0 (0-0)	-32.4 (-34--30.5)	0 (0-0)	-38.1 (-42.7--32.6)	0 (0-0)	0 (0-0)	0 (0-0)	0 (0-0)
Yamaguchi	0 (0-0)	-38.7 (-65.3--36.7)	0 (0-0)	-32.2 (-60.5--25.8)	0 (0-0)	-33.5 (-62--30)	0 (0-0)	-35 (-55.4--28.9)	0 (0-0)	0 (0-0)	0 (0-0)	0 (0-0)

Location	Birth , 2000	Births percent change (2000-2021)	All age cases, 2000	All age cases percent change (2000-2021)	All age deaths, 2000	All age deaths percent change (2000-2021)	Under-5 deaths, 2000	Under-5 deaths percent change (2000-2021)	Births, 2021	All age cases, 2021	All age deaths, 2021	Under-5 deaths, 2021
Yamanashi	0 (0-0)	-41.8 (-67.1--40)	0 (0-0)	-33 (-60.5--26.6)	0 (0-0)	-33 (-61.7--28.6)	0 (0-0)	-41 (-59--35.1)	0 (0-0)	0 (0-0)	0 (0-0)	0 (0-0)
Republic of Korea	1 (1-1)	-30 (-44.4--24.5)	17 (11-24)	-20.2 (-36--9.7)	1 (1-1)	-18 (-35.5--9.1)	0 (0-1)	-32.5 (-50--21.7)	1 (0-1)	13 (9-19)	1 (1-1)	0 (0-0)
Singapore	0 (0-0)	-7.7 (-26.6--6.8)	2 (1-2)	9.9 (-10.9-22.7)	0 (0-0)	19 (-2.9-28.7)	0 (0-0)	17.2 (-0.9-35.5)	0 (0-0)	2 (1-2)	0 (0-0)	0 (0-0)
**High-income North America**	421 (367-477)	-27.4 (-30--25.1)	7,730 (6,910-8,690)	11.6 (8.1-15.1)	399 (347-455)	-16.9 (-20.3--13.7)	216 (171-268)	-53.3 (-58.1--49.2)	306 (265-346)	8,630 (7,760-9,580)	332 (291-373)	101 (75-130)
Canada	4 (3-5)	-71.2 (-79.8--57.6)	56 (44-71)	-49.4 (-61.5--30.8)	4 (3-6)	-69.5 (-78.3--54.9)	3 (2-5)	-80.8 (-87.9--67.9)	1 (1-2)	28 (22-36)	1 (1-2)	1 (0-1)
Greenland	0 (0-0)	-17.6 (-25.9--7.1)	0 (0-0)	-14.5 (-22.9--4.6)	0 (0-0)	-13.1 (-21.9--0.9)	0 (0-0)	-17.6 (-28.9--1.8)	0 (0-0)	0 (0-0)	0 (0-0)	0 (0-0)
United States of America	415 (362-470)	-29.1 (-31.7--26.9)	7,660 (6,840-8,600)	13.5 (9.9-16.9)	394 (342-448)	-15.2 (-18.6--11.9)	212 (168-263)	-52.2 (-57.2--47.9)	294 (255-333)	8,690 (7,810-9,650)	334 (292-376)	101 (76-131)
Alabama	15 (12-17)	-30.4 (-37.5--22.7)	227 (198-261)	11.2 (1.4-21.5)	14 (12-17)	-22 (-29.9--13.7)	9 (7-11)	-51.7 (-60.8--42)	10 (8-12)	253 (218-287)	11 (9-13)	4 (3-6)
Alaska	0 (0-0)	-37.9 (-76.7--16.8)	1 (0-3)	5.9 (-58.8-51.6)	0 (0-0)	-25.1 (-72.2-2.7)	0 (0-0)	-51.9 (-84.9--30.1)	0 (0-0)	1 (0-3)	0 (0-0)	0 (0-0)
Arizona	3 (3-4)	-10.6 (-18.1--1.2)	60 (49-72)	51.8 (38.9-66.2)	3 (2-4)	20.8 (10.1-32.5)	2 (1-2)	-27.6 (-39.3--14.9)	3 (2-4)	91 (75-110)	4 (3-4)	1 (1-2)
Arkansas	5 (4-7)	-54.6 (-60.4--47.8)	61 (48-76)	-7.7 (-20.3-6.9)	5 (4-7)	-49.5 (-56--42.1)	4 (3-5)	-70.3 (-76--63.8)	2 (2-3)	56 (43-72)	3 (2-4)	1 (1-2)
California	20 (17-23)	-36.9 (-42.2--30.2)	377 (331-423)	6.8 (-0.6-15)	18 (15-21)	-16.9 (-23.7--8.4)	9 (7-12)	-54.2 (-62.4--44.7)	12 (11-14)	402 (357-451)	15 (13-17)	4 (3-6)
Colorado	2 (2-3)	-23.4 (-32.1--14.5)	49 (37-63)	15.7 (1-30.8)	2 (2-3)	-7.8 (-18.3-4.4)	1 (1-2)	-47.8 (-56.7--37.9)	2 (1-3)	56 (41-75)	2 (1-3)	1 (0-1)
Connecticut	5 (4-6)	-40.4 (-45.9--34.6)	108 (93-125)	-2.4 (-10.7-6.1)	5 (4-6)	-20.5 (-28--13)	2 (2-3)	-59.5 (-67--51.7)	3 (2-4)	105 (90-124)	4 (3-5)	1 (1-1)
Delaware	3 (3-4)	-7.5 (-16.9-0.7)	58 (50-68)	31.9 (21.8-44)	3 (2-3)	10.2 (-1-20)	1 (1-2)	-30.3 (-42.5--18)	3 (2-3)	77 (66-90)	3 (3-4)	1 (1-1)
District of Columbia	4 (3-5)	-34.2 (-44.8--23.2)	32 (25-41)	24.9 (4.8-49.1)	3 (3-4)	-31.2 (-41.5--19.8)	3 (2-3)	-53.7 (-65--41.8)	2 (2-3)	40 (30-52)	2 (2-3)	1 (1-2)
Florida	33 (29-37)	-17.1 (-23.7--9.5)	631 (572-700)	34.8 (26.1-44.6)	32 (28-36)	5 (-3.2-14.2)	16 (13-20)	-40.4 (-51--29.9)	28 (24-31)	850 (779-930)	34 (30-37)	10 (7-12)
Georgia	28 (25-33)	-17.3 (-24.7--9.5)	520 (470-576)	30.8 (22.7-39.6)	25 (22-29)	3.5 (-6.2-13.4)	13 (10-16)	-37 (-47.9--24.3)	23 (21-26)	681 (622-746)	26 (23-29)	8 (6-11)
Hawaii	0 (0-1)	-59 (-83.2--37.1)	6 (3-11)	-30.3 (-71.4-11.8)	0 (0-1)	-47.7 (-78.5--19.2)	0 (0-0)	-68.6 (-87.5--50.4)	0 (0-0)	4 (2-9)	0 (0-0)	0 (0-0)
Idaho	0 (0-0)	8.2 (-6.7-26.5)	4 (3-6)	47.9 (27.9-71.7)	0 (0-0)	22.9 (5.3-43)	0 (0-0)	-25.7 (-41--8)	0 (0-0)	6 (4-9)	0 (0-0)	0 (0-0)
Illinois	17 (14-20)	-62.7 (-67.2--58)	215 (184-250)	-11.5 (-19.7--2.7)	16 (13-19)	-51.4 (-57.4--45)	11 (8-14)	-75.9 (-81.5--69.4)	6 (5-7)	191 (163-220)	8 (6-9)	3 (2-4)
Indiana	7 (6-8)	-26.3 (-33.3--19)	127 (111-145)	9.9 (0.9-18.5)	7 (6-8)	-16.2 (-24.2--8)	4 (3-5)	-49.6 (-58.1--40)	5 (4-6)	140 (122-161)	6 (5-6)	2 (1-2)
Iowa	1 (1-2)	-38 (-48.2--27.4)	24 (18-34)	-14.1 (-29.2-0.7)	1 (1-2)	-32.2 (-43.3--20.7)	1 (0-1)	-58.7 (-66.8--49.3)	1 (0-1)	21 (13-30)	1 (0-1)	0 (0-0)
Kansas	2 (2-3)	-37.2 (-44.2--29.4)	42 (32-53)	6.1 (-6.6-19)	2 (2-3)	-25.6 (-33.7--16.4)	1 (1-2)	-56.1 (-64.4--47)	2 (1-2)	44 (34-56)	2 (1-2)	1 (0-1)
Kentucky	2 (2-3)	-26.3 (-33.3--18.6)	40 (30-52)	12.1 (1.1-25)	2 (2-3)	-18.1 (-25.4--9.9)	1 (1-2)	-48.3 (-55.8--40)	2 (1-2)	45 (34-59)	2 (2-3)	1 (0-1)
Louisiana	15 (13-18)	-40.7 (-46.6--33.5)	215 (190-244)	-1.9 (-10.4-6.8)	14 (12-17)	-35.6 (-42.1--27.8)	9 (7-12)	-60.7 (-68.3--51.6)	9 (8-10)	211 (188-236)	9 (8-11)	4 (3-5)
Maine	0 (0-0)	-31 (-76.5--16.8)	1 (0-2)	-3.8 (-64.1-23.6)	0 (0-0)	-16.4 (-71.7-4.1)	0 (0-0)	-54.4 (-86.8--38.8)	0 (0-0)	1 (0-2)	0 (0-0)	0 (0-0)
Maryland	18 (16-21)	-27.5 (-34.5--20.9)	341 (304-381)	14.3 (5.8-23.1)	18 (15-21)	-12.8 (-21.4--4.6)	10 (7-12)	-49.4 (-58.9--39.4)	13 (12-15)	390 (347-434)	15 (13-18)	5 (3-6)
Massachusetts	9 (7-10)	-29.3 (-35.2--23.1)	207 (178-239)	1.9 (-6.2-10.2)	8 (7-10)	-12.4 (-19.3--4.7)	4 (3-5)	-54.2 (-61.8--45.6)	6 (5-7)	211 (180-244)	7 (6-9)	2 (1-2)

Location	Birth , 2000	Births percent change (2000-2021)	All age cases, 2000	All age cases percent change (2000-2021)	All age deaths, 2000	All age deaths percent change (2000-2021)	Under-5 deaths, 2000	Under-5 deaths percent change (2000-2021)	Births, 2021	All age cases, 2021	All age deaths, 2021	Under-5 deaths, 2021
Michigan	17 (14-19)	-38.7 (-44.7--33.4)	315 (282-354)	-7.6 (-14.1--0.5)	17 (14-19)	-30 (-36.7--23.6)	9 (7-11)	-61.5 (-69.5--53.5)	10 (9-12)	291 (260-324)	12 (10-13)	3 (3-5)
Minnesota	2 (2-3)	-28.2 (-38.5--18.4)	49 (36-65)	6.8 (-9-25.3)	2 (2-3)	-17.3 (-29--5.9)	1 (1-2)	-50.6 (-60.3--41.5)	2 (1-2)	52 (36-71)	2 (1-3)	1 (0-1)
Mississippi	14 (12-17)	-44.4 (-49.9--38.5)	187 (162-215)	-1.2 (-9.3-7.4)	14 (11-16)	-36.6 (-43--29.5)	9 (7-11)	-60.9 (-68.6--53.9)	8 (7-9)	185 (160-211)	9 (7-10)	4 (3-5)
Missouri	9 (8-11)	-34.1 (-39.8--27.7)	169 (146-196)	3.2 (-4.9-11.8)	9 (8-11)	-23.2 (-29.9--15.8)	5 (4-6)	-54.6 (-62.1--46.3)	6 (5-7)	174 (151-202)	7 (6-8)	2 (2-3)
Montana	0 (0-0)	-17.6 (-88.1-1.6)	0 (0-1)	14.5 (-81.4-52.5)	0 (0-0)	-9.6 (-86.8-14.2)	0 (0-0)	-44.2 (-92.8--24.5)	0 (0-0)	0 (0-1)	0 (0-0)	0 (0-0)
Nebraska	1 (1-1)	-32.7 (-43.3--23)	16 (11-23)	4 (-14-22.1)	1 (1-1)	-26.8 (-38.7--16.8)	1 (0-1)	-52.9 (-61.5--42.7)	1 (0-1)	17 (11-25)	1 (0-1)	0 (0-0)
Nevada	2 (2-3)	-22.6 (-32.5--13.6)	34 (28-42)	54.8 (37.7-74.6)	2 (2-3)	3.5 (-9.7-16.5)	1 (1-2)	-39.9 (-52.1--26.7)	2 (1-2)	53 (43-65)	2 (2-3)	1 (1-1)
New Hampshire	0 (0-0)	-28 (-45.8--12.5)	5 (3-8)	-1.9 (-25.8-20.4)	0 (0-0)	-9.6 (-32.2-9.4)	0 (0-0)	-55 (-70.5--40.7)	0 (0-0)	5 (3-7)	0 (0-0)	0 (0-0)
New Jersey	15 (13-18)	-34.8 (-39.9--29.1)	313 (276-354)	1.2 (-6.4-8.7)	15 (13-17)	-20.7 (-27--13.9)	7 (6-9)	-56.7 (-64.3--48.1)	10 (9-11)	317 (281-354)	12 (10-13)	3 (2-4)
New Mexico	0 (0-0)	-30.5 (-78.6--23.8)	0 (0-1)	21.7 (-58.9-44.3)	0 (0-0)	-9.9 (-72.8-1.7)	0 (0-0)	-47.8 (-86--38.2)	0 (0-0)	0 (0-1)	0 (0-0)	0 (0-0)
New York	34 (30-38)	-30.3 (-36.2--24.5)	858 (785-946)	0.8 (-5-6.8)	33 (29-37)	-14.2 (-21.3--6.5)	12 (9-17)	-58.9 (-68.1--49.4)	24 (21-26)	866 (794-942)	28 (25-31)	5 (4-7)
North Carolina	19 (16-21)	-20.8 (-27.6--13.3)	346 (314-383)	27.5 (19.4-36.3)	17 (15-20)	-3.3 (-11.7-6)	9 (7-12)	-42.9 (-53.3--32.1)	15 (13-17)	442 (400-486)	17 (15-19)	5 (4-7)
North Dakota	0 (0-0)	17.1 (-68.6-36.8)	0 (0-1)	33.8 (-61.5-68.6)	0 (0-0)	7.3 (-71.1-27.7)	0 (0-0)	-25 (-83--1.6)	0 (0-0)	0 (0-1)	0 (0-0)	0 (0-0)
Ohio	14 (12-16)	-35.9 (-42--29.4)	249 (218-281)	0.2 (-7.8-7.9)	14 (12-16)	-28.4 (-35.5--20.6)	8 (6-10)	-60.3 (-68.2--51.2)	9 (8-10)	249 (221-279)	10 (8-11)	3 (2-4)
Oklahoma	3 (3-4)	-44 (-51.1--36.9)	47 (37-60)	-2.8 (-15.8-11.6)	3 (3-4)	-38.2 (-45.9--30.5)	2 (2-3)	-61.2 (-68.3--53.5)	2 (1-3)	46 (35-59)	2 (2-3)	1 (1-1)
Oregon	1 (1-2)	-15.4 (-22.4--4.4)	24 (17-33)	28.9 (17.2-44.1)	1 (1-2)	2.8 (-6.3-15.2)	1 (0-1)	-43.9 (-51.3--35.4)	1 (1-1)	31 (22-43)	1 (1-2)	0 (0-0)
Pennsylvania	12 (11-14)	-30.6 (-37.5--23.8)	251 (222-285)	3.5 (-4.4-11.5)	13 (11-15)	-20.3 (-27.9--12.7)	7 (5-9)	-55.2 (-64.4--45.8)	9 (7-10)	259 (230-293)	10 (9-12)	3 (2-4)
Rhode Island	1 (1-2)	-76.1 (-81.6--70)	21 (14-31)	-61.6 (-70.6--51.5)	1 (1-2)	-71.6 (-78--64.6)	1 (0-1)	-85.3 (-88.9--81.5)	0 (0-0)	8 (5-13)	0 (0-0)	0 (0-0)
South Carolina	11 (9-12)	-33 (-39--25.3)	166 (148-188)	15.8 (7-24.7)	10 (9-12)	-23.2 (-30.3--14.7)	6 (5-8)	-56.9 (-64.8--47.1)	7 (6-8)	193 (173-214)	8 (7-9)	3 (2-4)
South Dakota	0 (0-0)	-30.6 (-76.4-5)	1 (0-3)	0 (-63.1-58.9)	0 (0-0)	-26.7 (-74.9-12.4)	0 (0-0)	-50.3 (-85.3--20.3)	0 (0-0)	1 (0-3)	0 (0-0)	0 (0-0)
Tennessee	13 (11-15)	-16 (-23.4--8.2)	229 (204-258)	23.1 (14.2-32.5)	12 (11-14)	-7.4 (-15.7-0.9)	7 (5-9)	-42.9 (-53.9--31.4)	11 (9-12)	282 (250-316)	12 (10-13)	4 (3-5)
Texas	30 (26-34)	-8 (-15.3-1.3)	580 (526-645)	41.9 (33.1-51)	25 (21-29)	12.8 (3.2-25.9)	12 (9-16)	-34.3 (-48.4--18.5)	27 (24-31)	823 (751-903)	28 (24-32)	8 (6-11)
Utah	1 (1-1)	-17.9 (-31.4--4.8)	12 (8-17)	38.9 (16.7-63.1)	1 (0-1)	2.2 (-15.6-18.6)	0 (0-1)	-43 (-56.5--30.1)	1 (0-1)	17 (12-23)	1 (0-1)	0 (0-0)
Vermont	0 (0-0)	-86.9 (-99.2--17.2)	1 (0-3)	-81.4 (-98.8-15.4)	0 (0-0)	-84 (-99-0.8)	0 (0-0)	-91.4 (-99.5--43.4)	0 (0-0)	0 (0-1)	0 (0-0)	0 (0-0)
Virginia	16 (13-18)	-28 (-35.1--19.9)	273 (238-311)	21.5 (11.5-32.4)	15 (13-18)	-13.9 (-22.6--4)	9 (7-11)	-51.3 (-60.9--40.1)	11 (10-13)	331 (293-376)	13 (11-15)	4 (3-6)
Washington	2 (2-3)	-3.4 (-12.5-6.1)	53 (41-68)	27.7 (15.7-41.4)	2 (2-3)	9.3 (-1.5-20.7)	1 (1-1)	-35.4 (-44.2--26.5)	2 (2-3)	67 (52-87)	2 (2-3)	1 (0-1)
West Virginia	0 (0-1)	-46.3 (-54.9--35.7)	5 (3-8)	-8.1 (-27.4-13.8)	0 (0-1)	-37.7 (-47.8--25)	0 (0-0)	-61.2 (-68.6--53.2)	0 (0-0)	5 (3-8)	0 (0-0)	0 (0-0)
Wisconsin	5 (4-6)	-36.4 (-43--29.8)	104 (85-126)	-7.2 (-16.3-2.7)	5 (4-6)	-25.2 (-32.5--17.9)	2 (2-3)	-57.2 (-64.2--49.1)	3 (2-4)	96 (78-116)	4 (3-4)	1 (1-1)
Wyoming	0 (0-0)	-85 (-99.1--8.7)	1 (0-2)	-76.2 (-98.6-41)	0 (0-0)	-82.1 (-99-9)	0 (0-0)	-88.9 (-99.3--29.9)	0 (0-0)	0 (0-1)	0 (0-0)	0 (0-0)

Location	Birth , 2000	Births percent change (2000-2021)	All age cases, 2000	All age cases percent change (2000-2021)	All age deaths, 2000	All age deaths percent change (2000-2021)	Under-5 deaths, 2000	Under-5 deaths percent change (2000-2021)	Births, 2021	All age cases, 2021	All age deaths, 2021	Under-5 deaths, 2021
**Southern Latin America**	0 (0-1)	-4.2 (-9.4-19.6)	4 (1-7)	4.9 (-6.1-31.1)	0 (0-1)	2.3 (-5.2-26.7)	0 (0-0)	-4 (-12.8-15.8)	0 (0-1)	4 (1-8)	0 (0-1)	0 (0-0)
Argentina	0 (0-0)	-2.2 (-6.6-29.8)	3 (1-5)	7.3 (-6.1-42.8)	0 (0-0)	3.2 (0.4-36.2)	0 (0-0)	-2.9 (-11.6-23.2)	0 (0-0)	3 (1-5)	0 (0-0)	0 (0-0)
Chile	0 (0-0)	-9.1 (-11.7-66.7)	1 (0-2)	1.5 (-18-75.7)	0 (0-0)	2.1 (-4.2-87.2)	0 (0-0)	-5.9 (-17-83.1)	0 (0-0)	1 (0-2)	0 (0-0)	0 (0-0)
Uruguay	0 (0-0)	-11.5 (-13.9-18.5)	0 (0-0)	-7.2 (-17.8-32.8)	0 (0-0)	-8.2 (-12-22.6)	0 (0-0)	-12.8 (-20.9-12.5)	0 (0-0)	0 (0-0)	0 (0-0)	0 (0-0)
**Western Europe**	376 (296-468)	3.5 (0.3-6.8)	7,340 (5,950-9,150)	5.9 (2.7-9.1)	395 (311-489)	6.4 (3.3-9.6)	126 (88-174)	5.2 (0-10.1)	389 (305-488)	7,780 (6,260-9,750)	420 (332-524)	132 (93-184)
Andorra	0 (0-0)	-16.6 (-23.3--8.8)	1 (1-1)	6.4 (-2.7-15.8)	0 (0-0)	3.8 (-5.3-15.2)	0 (0-0)	-27.7 (-37.1--16.2)	0 (0-0)	1 (1-1)	0 (0-0)	0 (0-0)
Austria	2 (2-3)	45.1 (30.7-60)	55 (44-68)	23.2 (12.8-33.7)	3 (2-4)	32.2 (19.3-45.6)	1 (1-1)	43.1 (21.3-67.7)	3 (3-4)	68 (54-85)	4 (3-5)	1 (1-2)
Belgium	3 (2-4)	2.6 (-5.1-12.2)	59 (45-76)	3.4 (-4.3-11)	3 (2-4)	4.9 (-2.7-14.2)	1 (1-2)	4 (-8.1-20.3)	3 (2-4)	61 (46-79)	3 (3-5)	1 (1-2)
Cyprus	5 (4-6)	41.1 (29.4-55.5)	107 (91-125)	20.8 (12.5-30.6)	5 (4-7)	31.2 (19.3-45.8)	2 (1-2)	35.1 (16.8-58.2)	6 (5-8)	129 (109-151)	7 (6-8)	2 (2-3)
Denmark	8 (6-10)	-1.8 (-9.7-6.7)	158 (131-190)	0.9 (-6-8.5)	8 (6-10)	1.2 (-7.3-10.3)	3 (2-4)	-7 (-20.7-8.7)	8 (6-9)	160 (133-192)	8 (7-10)	2 (2-3)
Finland	6 (5-7)	-20.2 (-27.6--11.9)	124 (106-145)	-12.5 (-19.9--5.1)	6 (5-8)	-11.2 (-19.2--1.7)	2 (1-2)	-19.6 (-31.5--4.8)	4 (4-5)	108 (90-130)	6 (5-7)	1 (1-2)
France	146 (116-178)	-13.2 (-19.5--5.9)	2,660 (2,150-3,280)	-1.8 (-9.4-6)	145 (115-177)	-3.8 (-10.7-4.4)	51 (35-69)	-9.6 (-19.7-2.6)	126 (102-155)	2,620 (2,100-3,260)	140 (113-172)	46 (32-62)
Germany	21 (15-28)	1.7 (-6.6-11)	466 (355-608)	-4 (-10.8-4.4)	26 (19-33)	1.8 (-6.4-10.9)	8 (5-12)	5.9 (-7.3-20.3)	21 (16-28)	447 (341-580)	26 (20-34)	8 (6-12)
Greece	1 (1-2)	-21.8 (-28.5--15.1)	35 (26-47)	-25.6 (-31.7--19.1)	2 (1-3)	-24.7 (-31.4--17.1)	1 (0-1)	-16.2 (-26--4.6)	1 (1-2)	26 (19-35)	1 (1-2)	0 (0-1)
Iceland	0 (0-0)	2 (-17.3-20.7)	1 (0-1)	7.2 (-9-26.2)	0 (0-0)	10.1 (-9.4-32)	0 (0-0)	-3.8 (-23.7-16.5)	0 (0-0)	1 (0-1)	0 (0-0)	0 (0-0)
Ireland	3 (2-4)	3.1 (-6-12.6)	56 (41-72)	11.7 (3.5-20.6)	3 (2-4)	10.1 (0.7-20.8)	1 (1-1)	10.5 (-3.3-25.3)	3 (2-4)	62 (47-81)	3 (2-4)	1 (1-2)
Israel	11 (8-15)	44.3 (32-56.8)	164 (124-214)	40.4 (29.8-51.6)	9 (6-12)	42.3 (29-56)	4 (3-6)	48.5 (29.1-70.1)	16 (12-21)	230 (172-298)	13 (9-17)	6 (4-9)
Italy	18 (13-23)	-10 (-13.5--6)	406 (306-531)	11.6 (6.6-17)	21 (16-27)	8.3 (2.4-14.3)	4 (3-6)	0.2 (-5-5.8)	16 (12-20)	454 (351-577)	23 (17-29)	4 (3-6)
Abruzzo	0 (0-0)	-31.2 (-63.6--17.2)	3 (2-5)	-23.9 (-59--7.5)	0 (0-0)	-24.9 (-59.9--8.5)	0 (0-0)	-27.9 (-61.8--10.7)	0 (0-0)	2 (1-4)	0 (0-0)	0 (0-0)
Basilicata	0 (0-0)	-43 (-88.5--34.4)	0 (0-0)	-37.3 (-87.1--24.7)	0 (0-0)	-36 (-86.8--23.2)	0 (0-0)	-44 (-89.2--30.1)	0 (0-0)	0 (0-0)	0 (0-0)	0 (0-0)
Calabria	0 (0-0)	-37.4 (-59--22.8)	5 (3-7)	-36 (-56.5--21.3)	0 (0-0)	-34.3 (-56.2--18.2)	0 (0-0)	-38.9 (-60.9--21.8)	0 (0-0)	3 (2-5)	0 (0-0)	0 (0-0)
Campania	2 (1-2)	-19.2 (-28.4--8.3)	36 (24-52)	-2.2 (-12.6-10)	2 (1-3)	-1.6 (-12.9-11.1)	0 (0-1)	-18 (-29.4--4.1)	1 (1-2)	36 (25-50)	2 (1-3)	0 (0-1)
Emilia-Romagna	4 (3-5)	-4.8 (-12.5-2.7)	76 (63-93)	27.8 (18.3-37.8)	4 (3-5)	20.5 (11.1-31.2)	1 (1-1)	18.4 (4.5-36.7)	4 (3-4)	98 (81-117)	5 (4-6)	1 (1-1)
Friuli-Venezia Giulia	1 (0-1)	-22.6 (-29.4--14)	13 (9-17)	-0.3 (-8.5-10.1)	1 (1-1)	-7.6 (-16.2-3.9)	0 (0-0)	-11.9 (-23-2.1)	0 (0-1)	13 (9-17)	1 (0-1)	0 (0-0)
Lazio	1 (1-2)	11.6 (-0.1-24.4)	30 (21-42)	28.6 (15.9-42.3)	2 (1-2)	27.9 (13.8-42.5)	0 (0-0)	23 (7.1-41)	1 (1-2)	39 (28-52)	2 (1-3)	0 (0-1)
Liguria	1 (0-1)	-16.9 (-24.7--10.1)	13 (9-18)	-0.3 (-9.2-8.2)	1 (1-1)	-3.8 (-12.8-5.3)	0 (0-0)	-6 (-17.1-6.3)	0 (0-1)	13 (9-18)	1 (0-1)	0 (0-0)
Lombardia	2 (1-3)	-1.8 (-10.4-7.3)	46 (34-62)	23.7 (12.9-36.2)	2 (2-3)	15.2 (4.5-27.2)	0 (0-1)	9.3 (-3.6-23.1)	2 (1-2)	58 (44-74)	3 (2-4)	0 (0-1)
Marche	1 (0-1)	-9.6 (-18.2-0.5)	13 (9-17)	19.1 (8.1-31.5)	1 (0-1)	12.1 (1-24.7)	0 (0-0)	0.1 (-14.4-15.3)	0 (0-1)	15 (11-20)	1 (1-1)	0 (0-0)
Molise	0 (0-0)	-42.6 (-73.9--31.5)	0 (0-1)	-35.4 (-70.9--19.8)	0 (0-0)	-34.4 (-70.3--19.3)	0 (0-0)	-39.9 (-72--22.8)	0 (0-0)	0 (0-1)	0 (0-0)	0 (0-0)

Location	Birth , 2000	Births percent change (2000-2021)	All age cases, 2000	All age cases percent change (2000-2021)	All age deaths, 2000	All age deaths percent change (2000-2021)	Under-5 deaths, 2000	Under-5 deaths percent change (2000-2021)	Births, 2021	All age cases, 2021	All age deaths, 2021	Under-5 deaths, 2021
Piemonte	1 (1-2)	0 (-10.1-9.4)	33 (24-44)	17.9 (8.2-29.1)	2 (1-2)	15.1 (3.2-27.2)	0 (0-1)	9.7 (-6.2-26)	1 (1-2)	38 (29-51)	2 (1-3)	0 (0-1)
Provincia autonoma di Bolzano	0 (0-0)	0.3 (-14.2-15.9)	1 (1-2)	18.6 (1.3-35.7)	0 (0-0)	12.4 (-4.1-29.5)	0 (0-0)	-1 (-17.7-21.6)	0 (0-0)	2 (1-3)	0 (0-0)	0 (0-0)
Provincia autonoma di Trento	0 (0-0)	-25.5 (-59.6--13.4)	1 (0-2)	-8.8 (-49.5-9.1)	0 (0-0)	-10.6 (-51.1-6.3)	0 (0-0)	-21.9 (-55.6--2.7)	0 (0-0)	1 (0-2)	0 (0-0)	0 (0-0)
Puglia	0 (0-1)	-29.3 (-46.3--12.4)	12 (7-18)	-8.9 (-30.7-11.4)	1 (0-1)	-12.9 (-33.7-7.5)	0 (0-0)	-27.4 (-47.9--6.8)	0 (0-0)	10 (7-15)	1 (0-1)	0 (0-0)
Sardegna	0 (0-0)	-43.5 (-65.3--29.8)	3 (1-5)	-37 (-59.8--22.1)	0 (0-0)	-35.8 (-60.8--19.9)	0 (0-0)	-40.6 (-64.8--24.3)	0 (0-0)	2 (1-3)	0 (0-0)	0 (0-0)
Sicilia	2 (1-3)	-21.4 (-28--13.8)	48 (36-64)	-7.6 (-14.9-1.5)	2 (2-3)	-4.9 (-13-5.7)	0 (0-1)	-16.5 (-27.8--4.7)	2 (1-2)	45 (34-57)	2 (2-3)	0 (0-1)
Toscana	1 (0-1)	-20.4 (-27.4--12.4)	14 (9-21)	-4 (-11.4-5.3)	1 (0-1)	-8.3 (-17.3-1.3)	0 (0-0)	-10.4 (-21.1-2.6)	0 (0-1)	14 (9-20)	1 (0-1)	0 (0-0)
Umbria	0 (0-0)	-22.4 (-32.7--14.2)	5 (3-8)	-3.4 (-17.6-6.3)	0 (0-0)	-7.9 (-20.6-2.1)	0 (0-0)	-8.9 (-24.7-3.8)	0 (0-0)	5 (3-7)	0 (0-0)	0 (0-0)
Valle d'Aosta	0 (0-0)	-32.1 (-83.1--23.8)	0 (0-0)	-11.6 (-77.9-3.8)	0 (0-0)	-16 (-79.1--2)	0 (0-0)	-22.3 (-80.8--7.2)	0 (0-0)	0 (0-0)	0 (0-0)	0 (0-0)
Veneto	2 (2-3)	-9.3 (-16.3--0.9)	53 (42-67)	15 (6.7-24.5)	3 (2-3)	11.6 (1.5-22.7)	1 (0-1)	-1.5 (-12.6-13.1)	2 (2-3)	61 (48-76)	3 (2-4)	0 (0-1)
Luxembourg	4 (3-5)	9.3 (0-21.7)	90 (78-104)	25.1 (16.6-35.2)	4 (3-5)	31.1 (20.5-43.9)	1 (1-2)	10.4 (-4.8-28.7)	4 (4-5)	113 (96-132)	5 (4-6)	1 (1-2)
Malta	1 (0-1)	1.6 (-6.4-10.4)	16 (12-20)	-15.8 (-22.5--9.6)	1 (1-1)	-9.2 (-16.4--0.4)	0 (0-0)	-5.9 (-17.4-10.1)	1 (0-1)	13 (10-17)	1 (1-1)	0 (0-0)
Monaco	0 (0-0)	-5.1 (-13.4-4.9)	0 (0-0)	12.6 (3.1-22.2)	0 (0-0)	14.5 (4-25.5)	0 (0-0)	19.2 (4.1-36.3)	0 (0-0)	0 (0-0)	0 (0-0)	0 (0-0)
Netherlands	2 (2-3)	-12.7 (-21.4-0.1)	41 (31-54)	-5.3 (-12.2-6.1)	2 (2-3)	-3 (-11.8-10.6)	1 (0-1)	-10.2 (-22.1-7.4)	2 (1-3)	39 (29-52)	2 (2-3)	1 (0-1)
Norway	1 (0-1)	-4 (-10.4-1.4)	11 (7-17)	10.3 (3.2-15.8)	1 (0-1)	11.9 (4-18)	0 (0-0)	-4.6 (-11.6-2.3)	1 (0-1)	12 (8-18)	1 (0-1)	0 (0-0)
Agder	0 (0-0)	-6.6 (-18.1-6.4)	1 (0-1)	6.1 (-6.2-19.4)	0 (0-0)	6.4 (-5.8-21.2)	0 (0-0)	-9.4 (-27.5-5.2)	0 (0-0)	1 (0-1)	0 (0-0)	0 (0-0)
Innlandet	0 (0-0)	-21.6 (-43.9--8.9)	1 (1-1)	-8.2 (-32.3-6.9)	0 (0-0)	-5.6 (-32.7-10.2)	0 (0-0)	-22.9 (-44.9--8.5)	0 (0-0)	1 (0-1)	0 (0-0)	0 (0-0)
Møre og Romsdal	0 (0-0)	-19.3 (-40.7--12.2)	1 (0-1)	-3.6 (-28.9-5.9)	0 (0-0)	-2.4 (-29.1-6.9)	0 (0-0)	-18.5 (-40.6--6.7)	0 (0-0)	1 (0-1)	0 (0-0)	0 (0-0)
Nordland	0 (0-0)	-17.3 (-27.7--9)	1 (0-1)	-12.5 (-23.2--2.2)	0 (0-0)	-9.2 (-21.2-0.4)	0 (0-0)	-26.4 (-37.2--14.2)	0 (0-0)	1 (0-1)	0 (0-0)	0 (0-0)
Oslo	0 (0-0)	23.3 (6.6-44.8)	1 (1-2)	39.4 (20.7-60.1)	0 (0-0)	37.9 (20.5-65.2)	0 (0-0)	28.6 (4.1-58)	0 (0-0)	2 (1-3)	0 (0-0)	0 (0-0)
Rogaland	0 (0-0)	-1.8 (-14.6-10.4)	1 (1-2)	15.9 (0.9-28.5)	0 (0-0)	17.3 (1.9-31.8)	0 (0-0)	0.6 (-15.3-17.3)	0 (0-0)	1 (1-2)	0 (0-0)	0 (0-0)
Troms og Finnmark	0 (0-0)	-21.1 (-30.4--12)	1 (0-1)	-6.1 (-17.6-4.3)	0 (0-0)	-2 (-13.3-9.3)	0 (0-0)	-21.8 (-32.6--9.4)	0 (0-0)	1 (0-1)	0 (0-0)	0 (0-0)
Trøndelag	0 (0-0)	4.8 (-14.6-19.2)	1 (1-1)	10.4 (-9.8-28.3)	0 (0-0)	14.6 (-5.8-31)	0 (0-0)	-5.5 (-25.5-7.6)	0 (0-0)	1 (1-2)	0 (0-0)	0 (0-0)
Vestfold og Telemark	0 (0-0)	-14.2 (-26.1--0.1)	1 (1-1)	-0.2 (-13.3-15.4)	0 (0-0)	1.7 (-11.2-18.6)	0 (0-0)	-15.7 (-28.9-1.9)	0 (0-0)	1 (1-1)	0 (0-0)	0 (0-0)
Vestland	0 (0-0)	-3.8 (-13.9-9.2)	1 (1-2)	7.9 (-3.3-23.2)	0 (0-0)	10.8 (-1.4-26.8)	0 (0-0)	-4.4 (-17.7-11)	0 (0-0)	2 (1-2)	0 (0-0)	0 (0-0)
Viken	0 (0-0)	-3.5 (-16.5-18)	3 (2-4)	18.8 (4.2-42.7)	0 (0-0)	19.3 (3.7-44.4)	0 (0-0)	-0.3 (-14.9-23.8)	0 (0-0)	3 (2-4)	0 (0-0)	0 (0-0)
Portugal	9 (6-12)	-33.8 (-38.6--26.7)	161 (121-210)	-22.1 (-28--15.2)	10 (7-13)	-22.2 (-28.3--14.2)	3 (2-5)	-27.7 (-36.2--15.8)	6 (4-8)	125 (94-164)	8 (6-10)	2 (2-4)
San Marino	0 (0-0)	-4.7 (-11.6-3.1)	0 (0-0)	6.6 (-1.5-15.2)	0 (0-0)	6.6 (-1.6-16.1)	0 (0-0)	-5.5 (-16.2-7.1)	0 (0-0)	0 (0-0)	0 (0-0)	0 (0-0)
Spain	3 (2-5)	-13.7 (-27.4--4.5)	77 (55-105)	-5.1 (-18-3.3)	4 (3-6)	-6.3 (-20.9-4.4)	1 (1-2)	-0.4 (-17.7-17)	3 (2-4)	74 (52-99)	4 (3-5)	1 (1-2)

Location	Birth , 2000	Births percent change (2000-2021)	All age cases, 2000	All age cases percent change (2000-2021)	All age deaths, 2000	All age deaths percent change (2000-2021)	Under-5 deaths, 2000	Under-5 deaths percent change (2000-2021)	Births, 2021	All age cases, 2021	All age deaths, 2021	Under-5 deaths, 2021
Sweden	2 (1-2)	37.4 (27.5-47.2)	38 (29-50)	19.9 (12.4-28.4)	2 (1-2)	23.9 (15.3-33.1)	0 (0-1)	41.4 (26.1-58.5)	2 (2-3)	45 (35-60)	2 (2-3)	1 (0-1)
Stockholm	0 (0-1)	37.3 (25.7-51.1)	8 (6-10)	39.7 (29.3-53.5)	0 (0-1)	40.9 (28.9-55.4)	0 (0-0)	51.2 (32-72.6)	1 (0-1)	11 (8-14)	1 (0-1)	0 (0-0)
Sweden except Stockholm	1 (1-2)	37.4 (26.2-49.5)	30 (23-39)	14.9 (7-24.2)	1 (1-2)	19.4 (9.7-30.3)	0 (0-1)	38.5 (21.6-58)	2 (1-2)	34 (26-46)	2 (1-2)	0 (0-1)
Switzerland	27 (24-30)	21.4 (13.3-30.7)	685 (628-752)	13.1 (6.4-20)	32 (29-36)	18.4 (10.6-27)	7 (5-9)	23.4 (5.4-45.7)	32 (28-37)	774 (698-859)	38 (34-43)	9 (7-12)
United Kingdom	105 (76-139)	23.3 (21.1-25.5)	1,920 (1,410-2,580)	15.3 (13.5-17.3)	108 (78-144)	16.6 (14.3-19)	35 (23-51)	21.4 (18.4-24.3)	129 (93-173)	2,210 (1,620-2,970)	126 (90-169)	42 (28-62)
England	99 (72-131)	24.3 (22.1-26.6)	1,790 (1,310-2,420)	16.7 (14.7-18.7)	101 (73-135)	17.8 (15.4-20.3)	32 (22-48)	23.2 (20.2-26.3)	123 (88-165)	2,090 (1,520-2,820)	119 (86-160)	40 (27-59)
East Midlands	4 (3-6)	31.7 (24.9-40.2)	82 (57-115)	20.5 (14.6-29.8)	5 (3-6)	23.8 (17.4-32.5)	1 (1-2)	29.8 (19.9-41.5)	6 (4-8)	99 (67-141)	6 (4-8)	2 (1-3)
Derby	0 (0-0)	20.2 (4.7-35.9)	5 (3-9)	18.8 (4.9-36.1)	0 (0-0)	19.5 (4.9-35.2)	0 (0-0)	18.4 (-2-42)	0 (0-1)	6 (3-10)	0 (0-1)	0 (0-0)
Derbyshire	0 (0-0)	43.9 (5.1-2590)	0 (0-2)	30.2 (-8.3-2320)	0 (0-0)	35.1 (-3.5-2430)	0 (0-0)	33 (-11-2470)	0 (0-0)	0 (0-2)	0 (0-0)	0 (0-0)
Leicester	1 (1-1)	36.8 (20.6-52.5)	15 (10-21)	33.2 (17.9-48.6)	1 (1-1)	35 (19-50.6)	0 (0-0)	33.4 (13.5-54.4)	1 (1-2)	20 (14-28)	1 (1-2)	0 (0-0)
Leicestershire	0 (0-0)	71.8 (12-2920)	0 (0-2)	53.5 (-4.3-2540)	0 (0-0)	58.2 (1.4-2640)	0 (0-0)	60.1 (-2.7-2610)	0 (0-0)	1 (0-3)	0 (0-0)	0 (0-0)
Lincolnshire	0 (0-0)	99.4 (30.7-3420)	0 (0-2)	64 (3.2-2640)	0 (0-0)	70.2 (9.3-2850)	0 (0-0)	76.1 (6.4-3050)	0 (0-0)	1 (0-3)	0 (0-0)	0 (0-0)
Northamptonshire	2 (1-2)	23.4 (11.7-35.4)	36 (26-51)	11.3 (1-22.5)	2 (1-3)	13.5 (3-24.6)	1 (0-1)	19.2 (4.3-33.8)	2 (2-3)	41 (29-56)	2 (2-3)	1 (0-1)
Nottingham	1 (1-2)	40.2 (26-55.6)	24 (16-33)	25.3 (13.4-35.8)	1 (1-2)	30.5 (17.2-45.2)	0 (0-1)	40.9 (18.9-66.5)	2 (1-3)	30 (21-43)	2 (1-3)	1 (0-1)
Nottinghamshire	0 (0-0)	53.9 (12.7-2440)	0 (0-2)	35.4 (-4-2060)	0 (0-0)	39.8 (0.8-2200)	0 (0-0)	47.1 (-3-2700)	0 (0-0)	1 (0-2)	0 (0-0)	0 (0-0)
Rutland	0 (0-0)	76.9 (15.5-1120)	0 (0-0)	54.3 (-4.9-875)	0 (0-0)	58.2 (-0.2-949)	0 (0-0)	56.1 (-2.7-1050)	0 (0-0)	0 (0-0)	0 (0-0)	0 (0-0)
East of England	7 (5-11)	7.4 (0.6-13.1)	154 (94-230)	0.5 (-5.7-5.5)	8 (5-12)	0.3 (-6.2-5.2)	2 (1-4)	-1.1 (-9.3-5.6)	8 (5-12)	155 (92-237)	8 (5-12)	2 (1-4)
Bedford	1 (0-1)	-10.7 (-20.6--0.7)	11 (7-17)	-17.2 (-25.8--7.8)	1 (0-1)	-19.8 (-29.2--10.6)	0 (0-0)	-21.4 (-32.1--7.6)	1 (0-1)	9 (6-14)	1 (0-1)	0 (0-0)
Cambridgeshire	0 (0-0)	34.8 (19.4-55.3)	5 (2-10)	21.4 (6.7-39.9)	0 (0-1)	24 (10.2-42.2)	0 (0-0)	26.7 (8.5-49.6)	0 (0-1)	7 (3-13)	0 (0-1)	0 (0-0)
Central Bedfordshire	0 (0-0)	48.4 (14.8-1730)	0 (0-1)	34.5 (0.2-1560)	0 (0-0)	39.2 (6-1630)	0 (0-0)	40.3 (-1.1-1550)	0 (0-0)	0 (0-1)	0 (0-0)	0 (0-0)
Essex	2 (1-2)	21.9 (12.6-31.4)	36 (20-55)	7.1 (-0.9-15)	2 (1-3)	10.4 (1.8-18.9)	0 (0-1)	13.1 (2.1-25.5)	2 (1-3)	38 (21-60)	2 (1-3)	1 (0-1)
Hertfordshire	1 (1-2)	20.2 (13.1-28.4)	29 (18-43)	14 (5.4-22.6)	1 (1-2)	16.1 (8.3-24)	0 (0-1)	17.2 (5.8-30.5)	2 (1-2)	33 (20-50)	2 (1-2)	0 (0-1)
Luton	1 (0-1)	22.2 (13.7-31.4)	8 (5-13)	20.5 (12-30.3)	0 (0-1)	22.1 (13.6-31.2)	0 (0-0)	28 (15.9-43.8)	1 (0-1)	10 (6-15)	1 (0-1)	0 (0-0)
Norfolk	0 (0-1)	22.9 (10.3-38.7)	9 (5-15)	11.3 (-0.9-25.5)	0 (0-1)	14.3 (2.8-29.1)	0 (0-0)	15.2 (-1.2-36.9)	0 (0-1)	10 (6-17)	1 (0-1)	0 (0-0)
Peterborough	0 (0-0)	61.3 (43.3-91.5)	2 (1-3)	49.3 (32.1-74.5)	0 (0-0)	50.2 (31.7-78)	0 (0-0)	60.1 (35.5-92)	0 (0-0)	2 (1-5)	0 (0-0)	0 (0-0)
Southend-on-Sea	0 (0-0)	41.6 (16.9-1220)	0 (0-1)	36.8 (7.8-1130)	0 (0-0)	38.3 (13.2-1190)	0 (0-0)	35.4 (4.8-1170)	0 (0-0)	0 (0-1)	0 (0-0)	0 (0-0)
Suffolk	2 (1-3)	-24.9 (-36.2--13.4)	42 (28-59)	-25.5 (-36.5--15.4)	2 (2-3)	-28.8 (-39.5--17.9)	1 (0-1)	-35.5 (-47.2--21.7)	1 (1-2)	31 (19-47)	2 (1-2)	0 (0-1)
Thurrock	1 (0-1)	23.6 (12.8-33.5)	11 (7-16)	18.6 (7.8-28.4)	1 (0-1)	19 (8.7-29.1)	0 (0-0)	22.1 (7.8-34.9)	1 (0-1)	13 (8-18)	1 (0-1)	0 (0-0)
Greater London	58 (44-73)	25.2 (21.8-28.4)	961 (753-1,220)	20.9 (18-23.5)	55 (43-70)	20.7 (16.9-24.1)	18 (13-26)	26.8 (22.3-31.4)	72 (56-92)	1,160 (902-1,490)	67 (52-84)	24 (16-34)

Location	Birth , 2000	Births percent change (2000-2021)	All age cases, 2000	All age cases percent change (2000-2021)	All age deaths, 2000	All age deaths percent change (2000-2021)	Under-5 deaths, 2000	Under-5 deaths percent change (2000-2021)	Births, 2021	All age cases, 2021	All age deaths, 2021	Under-5 deaths, 2021
Barking and Dagenham	2 (1-2)	53.7 (41-69.4)	30 (24-37)	42.6 (31.9-54.7)	2 (1-2)	38.8 (26.7-52)	1 (0-1)	50.6 (32.8-72.2)	3 (2-3)	42 (34-53)	2 (2-3)	1 (1-1)
Barnet	2 (1-2)	31.1 (21-41.6)	38 (29-49)	21.9 (12.8-31.4)	2 (2-3)	21.4 (11.8-31.6)	1 (0-1)	31.9 (16.1-49.4)	2 (2-3)	47 (35-60)	2 (2-3)	1 (0-1)
Bexley	1 (0-1)	9.2 (-1.8-19.8)	16 (10-23)	-3.2 (-12.7-5.8)	1 (1-1)	-3 (-13.2-7)	0 (0-0)	-2.7 (-15.2-10.4)	1 (1-1)	15 (10-22)	1 (0-1)	0 (0-0)
Brent	2 (1-2)	40.5 (28.7-53.1)	29 (22-37)	33.3 (22.6-44.1)	2 (1-2)	34.8 (22.6-48)	1 (0-1)	53.7 (32.9-79.9)	2 (2-3)	38 (30-50)	2 (2-3)	1 (1-1)
Bromley	1 (0-1)	26.4 (15.7-37)	15 (8-23)	10.5 (2.4-21)	1 (0-1)	12.8 (3.2-22.3)	0 (0-0)	17.7 (6.2-32.6)	1 (0-1)	16 (9-25)	1 (0-1)	0 (0-0)
Camden	1 (1-2)	-16.3 (-24.5--8.3)	22 (16-30)	-15.7 (-23.1--7.8)	1 (1-2)	-18.4 (-26.2--10.4)	0 (0-1)	-18.7 (-28.8--6.3)	1 (1-1)	19 (13-26)	1 (1-1)	0 (0-0)
Croydon	2 (2-3)	40.6 (29.7-54.7)	44 (34-57)	24.4 (15.3-35.6)	2 (2-3)	27.2 (17.4-40.1)	1 (1-1)	41.4 (25.2-61.3)	3 (2-4)	55 (42-70)	3 (2-4)	1 (1-2)
Ealing	1 (1-2)	8.3 (-0.4-17.3)	23 (16-31)	5.6 (-2.8-14.4)	1 (1-2)	4.3 (-3.7-13.2)	0 (0-1)	15.3 (1.8-31.3)	1 (1-2)	24 (17-33)	1 (1-2)	0 (0-1)
Enfield	5 (4-6)	27.3 (17.2-37.8)	87 (71-109)	25.9 (16.3-35.3)	5 (4-6)	24.7 (14.7-34.6)	1 (1-2)	32 (17-51.9)	6 (5-7)	110 (88-136)	6 (4-7)	2 (1-3)
Greenwich	2 (1-2)	50.3 (38-62.7)	32 (25-41)	40.2 (29.2-51.9)	2 (1-2)	40.6 (28.9-52.6)	1 (0-1)	47.8 (30.9-65.2)	3 (2-4)	45 (35-58)	3 (2-3)	1 (1-1)
Hackney	7 (5-8)	21.6 (11.4-33.6)	96 (78-118)	23.9 (14.8-35.4)	6 (4-7)	24.2 (13.6-36.5)	2 (1-3)	19.7 (5.7-36.1)	8 (6-10)	119 (94-148)	7 (6-9)	3 (2-4)
Hammersmith and Fulham	1 (1-2)	-9.8 (-18.1-0.5)	18 (12-25)	-10.3 (-18.6--0.8)	1 (1-2)	-11.7 (-19.8--1.5)	0 (0-0)	-4 (-15.4-10.2)	1 (1-1)	16 (10-22)	1 (1-1)	0 (0-0)
Haringey	6 (5-8)	8.8 (0.5-18.5)	96 (78-118)	18.2 (9.9-27.8)	6 (5-7)	15.5 (6.3-26.3)	2 (1-3)	17.2 (4.6-31.8)	7 (5-9)	113 (91-142)	7 (5-8)	2 (2-3)
Harrow	0 (0-0)	64.6 (45.4-89.2)	3 (1-5)	42.8 (24.7-63.4)	0 (0-0)	43.1 (27.1-65.6)	0 (0-0)	63 (39.5-98.7)	0 (0-0)	4 (2-7)	0 (0-0)	0 (0-0)
Havering	0 (0-0)	63 (49.6-84.5)	6 (2-11)	21 (10.8-37.1)	0 (0-1)	27.3 (16.9-44.1)	0 (0-0)	48.5 (34.7-68.8)	0 (0-1)	7 (3-13)	0 (0-1)	0 (0-0)
Hillingdon	0 (0-1)	59 (42.4-75.1)	7 (3-12)	38.8 (25.2-50.1)	0 (0-1)	41.5 (26-55.6)	0 (0-0)	54.4 (34.2-75.2)	1 (0-1)	9 (4-16)	0 (0-1)	0 (0-0)
Hounslow	1 (1-1)	40.3 (26.8-54.1)	16 (9-23)	34.3 (21.4-46.4)	1 (1-1)	33 (19.2-45.9)	0 (0-0)	44.2 (27.3-65.2)	1 (1-2)	21 (13-30)	1 (1-2)	0 (0-1)
Islington	1 (0-1)	40.5 (26.7-54.6)	9 (5-13)	22.1 (10.1-32.9)	1 (0-1)	28 (14.4-40.8)	0 (0-0)	35.3 (18.9-55.3)	1 (0-1)	11 (7-16)	1 (0-1)	0 (0-0)
Kensington and Chelsea	0 (0-0)	15.8 (-9.8-1310)	0 (0-1)	13.4 (-15.6-1230)	0 (0-0)	11.2 (-15.7-1250)	0 (0-0)	8.3 (-19.9-1250)	0 (0-0)	0 (0-1)	0 (0-0)	0 (0-0)
Kingston upon Thames	0 (0-0)	37.5 (22.5-60.3)	6 (4-8)	12 (1-30.5)	0 (0-0)	11.4 (-0.8-30)	0 (0-0)	29.6 (11.6-53.8)	0 (0-0)	6 (4-9)	0 (0-0)	0 (0-0)
Lambeth	5 (4-7)	6 (-2.2-14.5)	85 (68-107)	5.4 (-2-13.8)	5 (4-6)	4.5 (-4.3-14)	2 (1-2)	7.1 (-5.6-20.7)	6 (4-7)	90 (71-112)	5 (4-7)	2 (1-2)
Lewisham	3 (3-4)	23.6 (13.1-33.7)	57 (46-71)	12.7 (4.4-21.3)	3 (3-4)	12.4 (2.7-22)	1 (1-2)	22.1 (7.7-38.7)	4 (3-5)	64 (52-80)	4 (3-5)	1 (1-2)
Merton	1 (1-1)	27.8 (17.1-38.5)	18 (12-25)	11.2 (2.3-21.2)	1 (1-1)	10.6 (1.6-21.3)	0 (0-0)	23.8 (9.3-39.3)	1 (1-2)	20 (14-28)	1 (1-2)	0 (0-1)
Newham	3 (3-4)	44.6 (32.8-57.8)	46 (36-58)	41.8 (31.8-54.3)	3 (2-4)	45.3 (33.6-58.6)	1 (1-2)	42.3 (24.3-61.2)	5 (4-6)	65 (51-81)	4 (3-5)	2 (1-2)
Redbridge	1 (0-1)	66.5 (52.4-82.1)	14 (8-21)	51.1 (39-66.7)	1 (0-1)	50.2 (37.9-65.3)	0 (0-0)	65 (46.9-87.7)	1 (1-2)	21 (13-31)	1 (1-2)	0 (0-1)
Richmond upon Thames	0 (0-1)	10.9 (1.3-19.2)	6 (3-10)	16.1 (5.5-25.5)	0 (0-1)	12.7 (3.3-22)	0 (0-0)	14.8 (1.6-27.5)	0 (0-1)	7 (4-12)	0 (0-1)	0 (0-0)
Southwark	5 (3-6)	28.7 (18.7-40.4)	69 (56-87)	19.8 (11.7-29.3)	4 (3-5)	22.5 (13.2-34.2)	1 (1-2)	28.2 (13.6-44.2)	6 (5-7)	83 (65-103)	5 (4-6)	2 (1-3)
Sutton	0 (0-0)	27 (13.3-43.5)	4 (2-7)	6.1 (-5.2-19.5)	0 (0-0)	9.1 (-2.9-24.6)	0 (0-0)	16.6 (0.1-36.9)	0 (0-0)	4 (3-7)	0 (0-0)	0 (0-0)
Tower Hamlets	1 (1-1)	22.4 (4.8-41)	12 (9-17)	16 (1.7-33.5)	1 (1-1)	19.3 (2.6-39.4)	0 (0-0)	18.6 (-2.7-43)	1 (1-2)	14 (10-20)	1 (1-1)	0 (0-1)

Location	Birth , 2000	Births percent change (2000-2021)	All age cases, 2000	All age cases percent change (2000-2021)	All age deaths, 2000	All age deaths percent change (2000-2021)	Under-5 deaths, 2000	Under-5 deaths percent change (2000-2021)	Births, 2021	All age cases, 2021	All age deaths, 2021	Under-5 deaths, 2021
Waltham Forest	2 (2-3)	40.9 (28.5-53.7)	36 (28-46)	35.6 (24.9-46)	2 (2-3)	35.1 (22.6-47.8)	1 (1-1)	43.7 (24.5-62.9)	3 (2-4)	48 (37-63)	3 (2-4)	1 (1-2)
Wandsworth	1 (1-1)	37.8 (29.2-49.8)	17 (12-25)	29.4 (18.8-41.4)	1 (1-1)	26.8 (16.5-40.8)	0 (0-0)	47.2 (32.2-66)	1 (1-2)	22 (16-32)	1 (1-2)	0 (0-1)
Westminster	0 (0-1)	-25.6 (-43--10.6)	7 (3-11)	-21.6 (-40--6.8)	0 (0-1)	-27 (-45.3--13)	0 (0-0)	-25 (-43.7--9)	0 (0-1)	5 (2-9)	0 (0-1)	0 (0-0)
North East England	1 (1-2)	21.3 (14.8-28.4)	30 (16-50)	5.4 (-0.1-13.2)	2 (1-3)	9.9 (3.3-17.6)	1 (0-1)	15.2 (7.8-23.5)	2 (1-3)	32 (17-54)	2 (1-3)	1 (0-1)
County Durham	0 (0-0)	73.5 (6.6-4440)	0 (0-1)	56.3 (-8.2-3860)	0 (0-0)	60.8 (-3.3-4070)	0 (0-0)	64.2 (-6.1-4260)	0 (0-0)	0 (0-2)	0 (0-0)	0 (0-0)
Darlington	0 (0-0)	53.5 (8.8-3910)	0 (0-0)	49.6 (3.5-3640)	0 (0-0)	54.3 (7.8-3980)	0 (0-0)	54.1 (-1.9-4360)	0 (0-0)	0 (0-1)	0 (0-0)	0 (0-0)
Gateshead	0 (0-0)	14.6 (-1.9-28.7)	4 (2-6)	5.8 (-7.9-20.1)	0 (0-0)	9.2 (-5.9-23.3)	0 (0-0)	7.2 (-9.6-22.3)	0 (0-0)	4 (2-7)	0 (0-0)	0 (0-0)
Hartlepool	0 (0-0)	76.2 (-0.9-5660)	0 (0-0)	60.1 (-13.9-5140)	0 (0-0)	68.1 (-7.7-5450)	0 (0-0)	64.5 (-13.6-5640)	0 (0-0)	0 (0-1)	0 (0-0)	0 (0-0)
Middlesbrough	0 (0-0)	10.4 (-0.6-26.4)	5 (3-8)	-7.7 (-18.1-4.7)	0 (0-1)	-1.4 (-11.4-12.6)	0 (0-0)	9.8 (-5.3-27.1)	0 (0-1)	5 (3-8)	0 (0-1)	0 (0-0)
Newcastle upon Tyne	1 (0-1)	30.6 (20.6-41.4)	12 (7-19)	11.1 (2.8-19.2)	1 (0-1)	16.4 (7.1-26.3)	0 (0-0)	23.6 (10.8-41.4)	1 (0-1)	13 (7-21)	1 (0-1)	0 (0-0)
North Tyneside	0 (0-0)	15.8 (3-30.8)	6 (3-9)	0.8 (-9.9-14.5)	0 (0-1)	4.7 (-6.8-18.5)	0 (0-0)	10.2 (-5.4-29.7)	0 (0-0)	6 (3-9)	0 (0-1)	0 (0-0)
Northumberland	0 (0-0)	29.3 (-1.1-1470)	0 (0-1)	17 (-13.7-1320)	0 (0-0)	21.2 (-8.8-1380)	0 (0-0)	19.5 (-15.6-1310)	0 (0-0)	0 (0-1)	0 (0-0)	0 (0-0)
Redcar and Cleveland	0 (0-0)	34.5 (-2.7-3520)	0 (0-1)	13 (-20-2790)	0 (0-0)	20.4 (-14.7-3160)	0 (0-0)	27.7 (-16.6-4020)	0 (0-0)	0 (0-1)	0 (0-0)	0 (0-0)
South Tyneside	0 (0-0)	41.2 (1.5-3670)	0 (0-1)	13.7 (-20.1-2890)	0 (0-0)	23.1 (-14-3250)	0 (0-0)	31.9 (-15-3930)	0 (0-0)	0 (0-1)	0 (0-0)	0 (0-0)
Stockton-on-Tees	0 (0-0)	16.5 (2.1-33.7)	3 (1-5)	3.4 (-9.4-21.7)	0 (0-0)	7.1 (-6.5-23.6)	0 (0-0)	4.5 (-11.3-27.4)	0 (0-0)	3 (1-5)	0 (0-0)	0 (0-0)
Sunderland	0 (0-0)	20.1 (-8.4-1390)	0 (0-1)	3.6 (-23.5-1190)	0 (0-0)	8.5 (-18.9-1260)	0 (0-0)	15.6 (-19.3-1270)	0 (0-0)	0 (0-1)	0 (0-0)	0 (0-0)
North West England	9 (6-12)	34.9 (28.9-41.7)	167 (116-236)	18.2 (12.9-24.3)	10 (7-13)	23.1 (17.5-29.3)	3 (2-5)	31.3 (23.7-40.6)	12 (8-16)	197 (140-273)	12 (8-16)	4 (3-6)
Blackburn with Darwen	0 (0-0)	2.8 (-6.9-13.2)	5 (3-8)	3.6 (-5.5-14.7)	0 (0-0)	5.2 (-4.3-16.1)	0 (0-0)	-2.5 (-13.5-10.8)	0 (0-0)	5 (3-8)	0 (0-0)	0 (0-0)
Blackpool	0 (0-0)	14.6 (2.3-28.5)	2 (1-4)	0.8 (-10.6-14.1)	0 (0-0)	3 (-8.1-14.8)	0 (0-0)	6.6 (-8.4-23.5)	0 (0-0)	2 (1-4)	0 (0-0)	0 (0-0)
Bolton	1 (0-1)	21.4 (11.4-33.9)	11 (7-16)	8.1 (-1.8-19.1)	1 (0-1)	10.6 (0.6-22.1)	0 (0-0)	13.8 (-0.5-30.9)	1 (0-1)	12 (7-18)	1 (0-1)	0 (0-0)
Bury	0 (0-0)	20 (8.2-35)	2 (1-4)	5.8 (-8.5-23.6)	0 (0-0)	8.4 (-2.3-22)	0 (0-0)	13.7 (-3.6-28.3)	0 (0-0)	2 (1-4)	0 (0-0)	0 (0-0)
Cheshire East	0 (0-0)	16.1 (1.3-37)	2 (1-5)	4.7 (-6.8-24.3)	0 (0-0)	9.4 (-3.8-30.8)	0 (0-0)	5.8 (-10-34.1)	0 (0-0)	2 (1-5)	0 (0-0)	0 (0-0)
Cheshire West and Chester	0 (0-0)	56.2 (5.7-1560)	0 (0-1)	34.2 (-13.2-1340)	0 (0-0)	38.5 (-7.6-1370)	0 (0-0)	37.7 (-12.1-1300)	0 (0-0)	0 (0-1)	0 (0-0)	0 (0-0)
Cumbria	0 (0-0)	9.9 (-5.7-28.2)	3 (1-6)	-1.8 (-14.6-18)	0 (0-0)	0.5 (-13.5-16.1)	0 (0-0)	1.6 (-13.8-16.5)	0 (0-0)	3 (1-6)	0 (0-0)	0 (0-0)
Halton	0 (0-0)	39.7 (2.1-3690)	0 (0-1)	31.7 (-6.7-3480)	0 (0-0)	36.9 (-1.6-3680)	0 (0-0)	41.5 (-5.5-4260)	0 (0-0)	0 (0-1)	0 (0-0)	0 (0-0)
Knowsley	0 (0-0)	52.9 (4.1-4110)	0 (0-1)	21.2 (-19.9-2970)	0 (0-0)	30.9 (-13.5-3640)	0 (0-0)	45.6 (-9.7-4400)	0 (0-0)	0 (0-1)	0 (0-0)	0 (0-0)
Lancashire	0 (0-0)	-25.1 (-47.3--4.2)	6 (3-10)	-33.9 (-53.4--14.5)	0 (0-1)	-31.1 (-51.7--11.4)	0 (0-0)	-31.6 (-53.1--7.1)	0 (0-0)	4 (2-8)	0 (0-0)	0 (0-0)
Liverpool	1 (1-2)	25.9 (15.1-37.6)	23 (15-34)	2.3 (-7.4-11.3)	1 (1-2)	9.8 (0.7-20.4)	0 (0-1)	18.3 (6.9-32.3)	1 (1-2)	24 (16-35)	1 (1-2)	0 (0-1)
Manchester	3 (2-4)	58.9 (47-71)	58 (45-74)	43.7 (32.9-55)	3 (3-4)	49 (37.4-60.2)	1 (1-2)	58.9 (40.1-79.6)	5 (4-7)	83 (64-107)	5 (4-7)	2 (1-3)

Location	Birth , 2000	Births percent change (2000-2021)	All age cases, 2000	All age cases percent change (2000-2021)	All age deaths, 2000	All age deaths percent change (2000-2021)	Under-5 deaths, 2000	Under-5 deaths percent change (2000-2021)	Births, 2021	All age cases, 2021	All age deaths, 2021	Under-5 deaths, 2021
Oldham	0 (0-0)	21.3 (10.4-52.6)	2 (1-4)	13.7 (2.2-40.4)	0 (0-0)	16.2 (4.9-47.6)	0 (0-0)	14.7 (-3-54.2)	0 (0-0)	2 (1-4)	0 (0-0)	0 (0-0)
Rochdale	0 (0-0)	36.3 (11.4-1250)	0 (0-1)	21.1 (-5.3-1100)	0 (0-0)	23.8 (0.3-1140)	0 (0-0)	24.7 (-4.3-1150)	0 (0-0)	0 (0-1)	0 (0-0)	0 (0-0)
Salford	1 (1-2)	42.9 (30.5-59.4)	24 (18-31)	21.8 (11.8-34)	1 (1-2)	24.8 (13.2-39)	0 (0-1)	42 (26-63.2)	2 (1-2)	30 (22-38)	2 (1-2)	1 (0-1)
Sefton	0 (0-0)	29.8 (0.8-1500)	0 (0-1)	5.1 (-21.5-1150)	0 (0-0)	11.5 (-15.3-1280)	0 (0-0)	19.6 (-14.3-1460)	0 (0-0)	0 (0-1)	0 (0-0)	0 (0-0)
St Helens	0 (0-0)	22.7 (2.8-786)	0 (0-1)	2.8 (-16.8-628)	0 (0-0)	6.8 (-11.9-670)	0 (0-0)	13 (-11.4-811)	0 (0-0)	0 (0-1)	0 (0-0)	0 (0-0)
Stockport	0 (0-0)	14.6 (4.2-32.2)	4 (2-7)	-3.5 (-13.4-12.1)	0 (0-0)	-1 (-10.4-13.8)	0 (0-0)	7.4 (-4.9-26.9)	0 (0-0)	3 (2-6)	0 (0-0)	0 (0-0)
Tameside	1 (0-1)	16.1 (7.7-26.9)	13 (8-19)	0.4 (-7.2-8)	1 (0-1)	3.8 (-4.4-13.5)	0 (0-0)	10 (-1.7-24.6)	1 (0-1)	13 (8-19)	1 (0-1)	0 (0-0)
Trafford	0 (0-0)	41.7 (11.5-1650)	0 (0-1)	43.2 (7.2-1670)	0 (0-0)	43.9 (12-1680)	0 (0-0)	48.8 (7.5-1700)	0 (0-0)	0 (0-1)	0 (0-0)	0 (0-0)
Warrington	0 (0-0)	24.4 (2.5-1080)	0 (0-1)	21.4 (-4.4-1050)	0 (0-0)	23.1 (0.4-1070)	0 (0-0)	16.8 (-9-1020)	0 (0-0)	0 (0-1)	0 (0-0)	0 (0-0)
Wigan	0 (0-1)	8.9 (1-20.4)	7 (4-12)	2.6 (-6.9-14.4)	0 (0-1)	5.4 (-2.6-16.3)	0 (0-0)	4.7 (-8.8-24.8)	0 (0-1)	7 (4-12)	0 (0-1)	0 (0-0)
Wirral	0 (0-0)	-20.9 (-36.3--4.7)	4 (2-6)	-27.3 (-41.4--11.8)	0 (0-0)	-23.7 (-38.8--7.1)	0 (0-0)	-25.9 (-42.2--3.6)	0 (0-0)	3 (1-5)	0 (0-0)	0 (0-0)
South East England	7 (4-10)	18.7 (13.6-23.6)	147 (87-225)	11.1 (6.4-15.6)	8 (5-12)	13.1 (8-18)	2 (1-4)	16.2 (9-22.8)	8 (5-12)	163 (96-251)	9 (5-13)	3 (1-4)
Bracknell Forest	0 (0-0)	35 (-3.4-3820)	0 (0-1)	30.2 (-9-3600)	0 (0-0)	33.8 (-6.2-3810)	0 (0-0)	34.6 (-12.2-4210)	0 (0-0)	0 (0-1)	0 (0-0)	0 (0-0)
Brighton and Hove	0 (0-0)	18.5 (6.8-29.9)	3 (1-6)	24.6 (12-39)	0 (0-0)	27 (14.5-40.2)	0 (0-0)	15.1 (1.6-27)	0 (0-0)	4 (1-8)	0 (0-0)	0 (0-0)
Buckinghamshire	1 (0-1)	11.3 (4.1-21)	15 (8-24)	4.7 (-3.7-13.3)	1 (0-1)	7.2 (-0.2-16)	0 (0-0)	4.2 (-6.2-15.7)	1 (0-1)	16 (9-25)	1 (0-1)	0 (0-0)
East Sussex	0 (0-0)	63.9 (13.2-1000)	0 (0-1)	50.1 (-1.4-902)	0 (0-0)	53.4 (4.4-938)	0 (0-0)	40.1 (-8.3-966)	0 (0-0)	0 (0-2)	0 (0-0)	0 (0-0)
Hampshire	1 (1-1)	11.9 (2-21.3)	23 (14-35)	2.4 (-6.5-12)	1 (1-2)	6 (-3.4-15.5)	0 (0-0)	2.1 (-10.2-16)	1 (1-2)	24 (14-36)	1 (1-2)	0 (0-0)
Isle of Wight	0 (0-0)	66.3 (16.4-4220)	0 (0-1)	34.3 (-7.2-3180)	0 (0-0)	43 (-1.5-3580)	0 (0-0)	50.2 (-5.6-4400)	0 (0-0)	0 (0-1)	0 (0-0)	0 (0-0)
Kent	2 (1-2)	8.2 (-9.1-24.8)	33 (18-50)	-1.3 (-17.2-13.5)	2 (1-3)	-0.4 (-17.1-15.3)	1 (0-1)	-0.7 (-18.9-17.2)	2 (1-3)	32 (17-52)	2 (1-3)	1 (0-1)
Medway	0 (0-1)	22.9 (9.5-35.9)	7 (4-11)	5.3 (-6.8-16.3)	0 (0-1)	10.2 (-1.2-22.2)	0 (0-0)	11.2 (-4.5-27.1)	0 (0-1)	8 (4-12)	0 (0-1)	0 (0-0)
Milton Keynes	1 (1-2)	27.5 (16.2-38)	23 (17-30)	26.2 (16-36.7)	1 (1-2)	25.5 (14.6-35.7)	0 (0-1)	34.5 (19.4-53.3)	2 (1-2)	29 (22-38)	2 (1-2)	1 (0-1)
Oxfordshire	1 (0-1)	16.3 (8.3-23.6)	14 (8-23)	9.8 (0.8-17.1)	1 (0-1)	11.7 (4.4-19)	0 (0-0)	12.7 (2.7-24.3)	1 (0-1)	16 (8-26)	1 (0-1)	0 (0-0)
Portsmouth	0 (0-0)	50.5 (25.4-1320)	0 (0-1)	42.8 (14.3-1260)	0 (0-0)	44.3 (18-1250)	0 (0-0)	47.4 (17.3-1300)	0 (0-0)	0 (0-1)	0 (0-0)	0 (0-0)
Reading	0 (0-0)	90.5 (26.6-4600)	0 (0-1)	72.5 (10.8-4070)	0 (0-0)	74.2 (11-4320)	0 (0-0)	110 (28.7-5650)	0 (0-0)	0 (0-1)	0 (0-0)	0 (0-0)
Slough	0 (0-1)	40.7 (30.6-57.3)	7 (4-11)	47.8 (37.3-64.1)	0 (0-1)	44.2 (33.4-59.9)	0 (0-0)	59.1 (45.6-80.1)	1 (0-1)	10 (6-16)	1 (0-1)	0 (0-0)
Southampton	0 (0-0)	41.5 (24.1-64.3)	4 (2-7)	15.7 (0.7-33.7)	0 (0-0)	24.7 (8.3-43.6)	0 (0-0)	43.5 (21.8-69)	0 (0-1)	5 (2-8)	0 (0-1)	0 (0-0)
Surrey	1 (0-1)	17.1 (3.3-29)	13 (7-21)	14 (1.9-26.2)	1 (0-1)	13 (-0.7-24.5)	0 (0-0)	14.5 (-1.7-33.7)	1 (0-1)	14 (8-24)	1 (0-1)	0 (0-0)
West Berkshire	0 (0-0)	16.7 (0-1030)	0 (0-1)	17.7 (-3.8-1030)	0 (0-0)	17.9 (-0.6-1030)	0 (0-0)	15.1 (-7.8-990)	0 (0-0)	0 (0-1)	0 (0-0)	0 (0-0)
West Sussex	0 (0-0)	31 (13.1-62)	3 (1-7)	21.6 (4.8-48.1)	0 (0-0)	25.6 (7-55.4)	0 (0-0)	23.1 (2.3-64)	0 (0-0)	4 (2-9)	0 (0-0)	0 (0-0)

Location	Birth , 2000	Births percent change (2000-2021)	All age cases, 2000	All age cases percent change (2000-2021)	All age deaths, 2000	All age deaths percent change (2000-2021)	Under-5 deaths, 2000	Under-5 deaths percent change (2000-2021)	Births, 2021	All age cases, 2021	All age deaths, 2021	Under-5 deaths, 2021
Windsor and Maidenhead	0 (0-0)	54.9 (5.2-3800)	0 (0-1)	58.9 (5.4-3760)	0 (0-0)	58.6 (6.5-3880)	0 (0-0)	55.7 (-4.1-4520)	0 (0-0)	0 (0-1)	0 (0-0)	0 (0-0)
Wokingham	0 (0-0)	11.2 (-6.9-689)	0 (0-1)	10.7 (-11.5-670)	0 (0-0)	10.1 (-9.6-675)	0 (0-0)	9.6 (-12.2-742)	0 (0-0)	0 (0-1)	0 (0-0)	0 (0-0)
South West England	3 (2-4)	22.6 (13.5-36.7)	54 (36-82)	7.8 (-0.7-21.6)	3 (2-4)	8.7 (-0.4-22.3)	1 (1-1)	14.7 (2.5-29.6)	3 (2-5)	58 (37-92)	3 (2-5)	1 (1-2)
Bath and North East Somerset	0 (0-0)	20.5 (7.7-33.6)	4 (2-7)	3.6 (-8.8-16.2)	0 (0-0)	8 (-4.2-19.4)	0 (0-0)	9.3 (-7.6-24.7)	0 (0-0)	4 (2-7)	0 (0-0)	0 (0-0)
Bournemouth	0 (0-1)	1.9 (-14.9-18.5)	8 (6-12)	-11.3 (-24.9-3.6)	0 (0-1)	-14.8 (-29.5-0)	0 (0-0)	-12 (-29.6-11.3)	0 (0-1)	7 (5-11)	0 (0-1)	0 (0-0)
Bristol, City of	1 (1-2)	25.3 (12.6-36.8)	29 (21-40)	10.4 (-0.1-19.1)	2 (1-2)	10.7 (-0.6-21.2)	0 (0-1)	18.4 (1.8-33.9)	2 (1-3)	32 (23-45)	2 (1-2)	1 (0-1)
Cornwall	0 (0-0)	94 (19.5-4920)	0 (0-1)	66.3 (-1.4-3990)	0 (0-0)	72.1 (4-4350)	0 (0-0)	72.9 (-1.8-4480)	0 (0-0)	0 (0-2)	0 (0-0)	0 (0-0)
Devon	0 (0-0)	75.5 (14.2-3050)	0 (0-2)	54 (-3.5-2750)	0 (0-0)	58.6 (1.4-2760)	0 (0-0)	61.6 (-0.5-2750)	0 (0-0)	1 (0-3)	0 (0-0)	0 (0-0)
Dorset	0 (0-0)	40.8 (7.9-1620)	0 (0-2)	26.6 (-6.3-1440)	0 (0-0)	31.2 (-1.3-1520)	0 (0-0)	29.3 (-7.7-1480)	0 (0-0)	0 (0-2)	0 (0-0)	0 (0-0)
Gloucestershire	0 (0-0)	22.2 (4.8-39)	6 (2-11)	7.6 (-7.1-22.5)	0 (0-1)	13.1 (-2.8-28.9)	0 (0-0)	10.6 (-7.7-32)	0 (0-1)	6 (3-12)	0 (0-1)	0 (0-0)
North Somerset	0 (0-0)	43.1 (18.8-1260)	0 (0-1)	28.1 (1.8-1110)	0 (0-0)	29.6 (6.5-1140)	0 (0-0)	30.1 (1.1-1220)	0 (0-0)	0 (0-1)	0 (0-0)	0 (0-0)
Plymouth	0 (0-0)	56.5 (21.4-1800)	0 (0-1)	36.1 (1.3-1460)	0 (0-0)	42.3 (7.5-1630)	0 (0-0)	51.9 (9.5-1770)	0 (0-0)	0 (0-1)	0 (0-0)	0 (0-0)
Poole	0 (0-0)	54.2 (11.4-4040)	0 (0-1)	46.3 (3.6-3730)	0 (0-0)	51.4 (8.5-3930)	0 (0-0)	55.4 (1.8-4740)	0 (0-0)	0 (0-1)	0 (0-0)	0 (0-0)
Somerset	0 (0-0)	91.3 (15.9-4840)	0 (0-1)	66.1 (-2.8-4070)	0 (0-0)	73.2 (3-4370)	0 (0-0)	78.1 (-2.5-4410)	0 (0-0)	0 (0-2)	0 (0-0)	0 (0-0)
South Gloucestershire	0 (0-0)	20 (4.5-38.2)	4 (2-7)	9.5 (-4.1-25.2)	0 (0-0)	11.1 (-4.8-28.6)	0 (0-0)	16.4 (-1.6-36.4)	0 (0-0)	5 (2-8)	0 (0-0)	0 (0-0)
Swindon	0 (0-0)	64.2 (31.7-1970)	0 (0-1)	56.3 (20.6-1820)	0 (0-0)	58.6 (25.8-1930)	0 (0-0)	59.7 (19.7-1970)	0 (0-0)	0 (0-1)	0 (0-0)	0 (0-0)
Torbay	0 (0-0)	64.2 (14.9-4170)	0 (0-1)	32.9 (-9-3180)	0 (0-0)	41 (-3.2-3640)	0 (0-0)	60.2 (-0.8-4830)	0 (0-0)	0 (0-1)	0 (0-0)	0 (0-0)
Wiltshire	0 (0-0)	80.4 (7.3-4470)	0 (0-1)	78.5 (2.4-4320)	0 (0-0)	81 (5.6-4460)	0 (0-0)	69 (-6.7-4150)	0 (0-0)	0 (0-2)	0 (0-0)	0 (0-0)
West Midlands	8 (5-11)	22.5 (16.3-29.4)	145 (101-201)	14.1 (8.8-20.7)	8 (6-11)	16 (10.5-22.3)	3 (2-4)	17.5 (8.6-26.9)	9 (7-13)	166 (117-234)	9 (7-13)	3 (2-5)
Birmingham	4 (3-6)	33.1 (23.1-46)	78 (58-103)	27.5 (18.5-39.5)	4 (3-6)	29.9 (20.2-42.5)	2 (1-2)	29 (14.6-45.7)	6 (4-8)	99 (74-133)	6 (4-7)	2 (1-3)
Coventry	1 (0-1)	32.2 (21.7-43.2)	15 (8-23)	10.9 (1.3-19.6)	1 (1-1)	15.2 (5.8-24.4)	0 (0-0)	25.9 (12.6-41.4)	1 (1-1)	16 (9-25)	1 (1-1)	0 (0-1)
Dudley	1 (0-1)	-19.7 (-30.8--7.5)	11 (8-17)	-24.5 (-35.1--13.7)	1 (0-1)	-24.8 (-35.4--12.8)	0 (0-0)	-25.6 (-39.4--11.8)	0 (0-1)	9 (5-13)	0 (0-1)	0 (0-0)
Herefordshire, County of	0 (0-0)	53.8 (9.7-4340)	0 (0-1)	31.7 (-8.2-3400)	0 (0-0)	39.1 (-2.6-3900)	0 (0-0)	38.4 (-11.2-4500)	0 (0-0)	0 (0-1)	0 (0-0)	0 (0-0)
Sandwell	1 (1-2)	4.7 (-4.8-15.2)	19 (13-27)	3 (-5.9-12.6)	1 (1-2)	0.7 (-8.9-11.3)	0 (0-1)	-1.4 (-12.6-11.4)	1 (1-2)	20 (13-28)	1 (1-2)	0 (0-1)
Shropshire	0 (0-0)	41.5 (8.1-1620)	0 (0-1)	30.7 (-4.5-1460)	0 (0-0)	34.7 (0.9-1550)	0 (0-0)	28.8 (-9.3-1460)	0 (0-0)	0 (0-1)	0 (0-0)	0 (0-0)
Solihull	0 (0-0)	23.8 (12.2-42.1)	3 (2-6)	3.1 (-7-18.9)	0 (0-0)	8.3 (-1.8-24.4)	0 (0-0)	9.1 (-5.3-26.9)	0 (0-0)	3 (2-6)	0 (0-0)	0 (0-0)
Staffordshire	0 (0-0)	-21 (-42.2-17.5)	5 (2-9)	-28.1 (-47.5-7.7)	0 (0-0)	-25.1 (-45.2-12.7)	0 (0-0)	-27.4 (-48-12.1)	0 (0-0)	3 (1-8)	0 (0-0)	0 (0-0)
Stoke-on-Trent	0 (0-0)	46.2 (13.4-1680)	0 (0-1)	34.5 (0.5-1490)	0 (0-0)	36.7 (4.7-1560)	0 (0-0)	47.4 (4.3-1730)	0 (0-0)	0 (0-1)	0 (0-0)	0 (0-0)
Telford and Wrekin	0 (0-1)	9 (-2.7-23)	9 (6-12)	-1.6 (-12.2-11.4)	1 (0-1)	-0.8 (-11.6-11.9)	0 (0-0)	1.2 (-14.7-19.3)	1 (0-1)	8 (6-12)	1 (0-1)	0 (0-0)

Location	Birth , 2000	Births percent change (2000-2021)	All age cases, 2000	All age cases percent change (2000-2021)	All age deaths, 2000	All age deaths percent change (2000-2021)	Under-5 deaths, 2000	Under-5 deaths percent change (2000-2021)	Births, 2021	All age cases, 2021	All age deaths, 2021	Under-5 deaths, 2021
Walsall	0 (0-0)	56.2 (19.1-1800)	0 (0-1)	44.2 (3.9-1660)	0 (0-0)	47 (11.1-1700)	0 (0-0)	50.1 (6.3-1690)	0 (0-0)	0 (0-1)	0 (0-0)	0 (0-0)
Warwickshire	0 (0-0)	77.4 (15-2840)	0 (0-1)	61.6 (0.9-2600)	0 (0-0)	65.2 (5.7-2640)	0 (0-0)	65.9 (-0.1-2680)	0 (0-0)	0 (0-2)	0 (0-0)	0 (0-0)
Wolverhampton	0 (0-0)	23.1 (11.4-39.2)	4 (3-7)	11.8 (0.6-25.6)	0 (0-0)	14.3 (3.5-30.1)	0 (0-0)	21.2 (5.2-44.3)	0 (0-0)	5 (3-8)	0 (0-0)	0 (0-0)
Worcestershire	0 (0-0)	69.7 (4.8-3130)	0 (0-1)	53.4 (-8.6-2610)	0 (0-0)	57.3 (-4.3-2890)	0 (0-0)	59.2 (-9.6-3040)	0 (0-0)	0 (0-2)	0 (0-0)	0 (0-0)
Yorkshire and the Humber	3 (1-4)	29.9 (22.5-39.3)	54 (31-88)	14.7 (7.8-23.2)	3 (2-5)	17.5 (10.8-26.5)	1 (0-1)	25.1 (16.2-35.3)	3 (2-5)	62 (34-103)	3 (2-6)	1 (1-2)
Barnsley	0 (0-0)	48.3 (17.7-1750)	0 (0-1)	29.4 (-0.9-1480)	0 (0-0)	32.6 (3.8-1590)	0 (0-0)	34.3 (0.3-1670)	0 (0-0)	0 (0-1)	0 (0-0)	0 (0-0)
Bradford	1 (0-1)	18 (1.5-35.4)	9 (5-15)	27.2 (9.4-47.2)	1 (0-1)	27 (8.9-46.3)	0 (0-0)	21 (-0.6-42.8)	1 (0-1)	12 (6-19)	1 (0-1)	0 (0-0)
Calderdale	0 (0-0)	33.2 (8.7-1140)	0 (0-1)	24.6 (-2.8-1060)	0 (0-0)	26.7 (2.1-1080)	0 (0-0)	21.2 (-8.6-1020)	0 (0-0)	0 (0-1)	0 (0-0)	0 (0-0)
Doncaster	0 (0-0)	42.6 (8.6-1630)	0 (0-1)	33.2 (-2.6-1540)	0 (0-0)	36.6 (2-1560)	0 (0-0)	34.5 (-5.6-1410)	0 (0-0)	0 (0-1)	0 (0-0)	0 (0-0)
East Riding of Yorkshire	0 (0-0)	36.3 (4.2-1560)	0 (0-1)	20.8 (-11.3-1380)	0 (0-0)	25.1 (-6.1-1440)	0 (0-0)	23.3 (-12.6-1350)	0 (0-0)	0 (0-1)	0 (0-0)	0 (0-0)
Kingston upon Hull, City of	0 (0-1)	19.6 (9.8-29.1)	7 (3-12)	4.1 (-4.8-13.8)	0 (0-1)	9 (0.6-17.4)	0 (0-0)	19 (8.6-30.2)	0 (0-1)	7 (3-13)	0 (0-1)	0 (0-0)
Kirklees	0 (0-0)	73.5 (7.6-4410)	0 (0-1)	71.2 (1.9-4010)	0 (0-0)	73.1 (6-4400)	0 (0-0)	67.6 (-4.3-4170)	0 (0-0)	0 (0-2)	0 (0-0)	0 (0-0)
Leeds	1 (1-2)	36.7 (28.7-47.5)	26 (16-38)	9.6 (2.3-18.9)	1 (1-2)	13.3 (5.8-23.4)	0 (0-1)	28.6 (17.2-42.5)	2 (1-2)	28 (18-41)	2 (1-2)	0 (0-1)
North East Lincolnshire	0 (0-0)	50 (7.1-4230)	0 (0-1)	25.1 (-13.1-3280)	0 (0-0)	33.9 (-7.3-3810)	0 (0-0)	38.7 (-11.7-4250)	0 (0-0)	0 (0-1)	0 (0-0)	0 (0-0)
North Lincolnshire	0 (0-0)	33.3 (18.4-1240)	0 (0-1)	22.6 (5.2-1130)	0 (0-0)	26 (10.2-1160)	0 (0-0)	25.1 (3.9-1170)	0 (0-0)	0 (0-1)	0 (0-0)	0 (0-0)
North Yorkshire	0 (0-0)	58.8 (4.3-2780)	0 (0-1)	46.5 (-7.8-2600)	0 (0-0)	51.3 (-2.4-2650)	0 (0-0)	47.9 (-9.9-2550)	0 (0-0)	0 (0-2)	0 (0-0)	0 (0-0)
Rotherham	0 (0-0)	45.1 (11-1640)	0 (0-1)	26.7 (-6.3-1400)	0 (0-0)	30.3 (-1.7-1460)	0 (0-0)	32.5 (-6.6-1500)	0 (0-0)	0 (0-1)	0 (0-0)	0 (0-0)
Sheffield	0 (0-1)	31.1 (16.7-48.3)	10 (5-16)	19.9 (6-36.9)	0 (0-1)	21.7 (7.9-38.3)	0 (0-0)	22.9 (5.7-45.6)	1 (0-1)	12 (6-20)	1 (0-1)	0 (0-0)
Wakefield	0 (0-0)	47.3 (12.8-1700)	0 (0-1)	24.8 (-8-1410)	0 (0-0)	29.6 (-2.2-1490)	0 (0-0)	37.5 (-4.2-1460)	0 (0-0)	0 (0-1)	0 (0-0)	0 (0-0)
York	0 (0-0)	50.6 (23.4-1310)	0 (0-1)	34.1 (6.1-1140)	0 (0-0)	37.8 (10.4-1200)	0 (0-0)	37.5 (6.9-1210)	0 (0-0)	0 (0-1)	0 (0-0)	0 (0-0)
Northern Ireland	1 (1-2)	13.9 (5.1-23.8)	24 (17-31)	-0.8 (-7.7-6.4)	1 (1-2)	2.9 (-5.1-12.4)	0 (0-1)	-0.1 (-10.7-13)	1 (1-2)	23 (17-31)	1 (1-2)	0 (0-1)
Scotland	3 (2-4)	0.4 (-8.2-10.1)	62 (47-82)	-5.9 (-13.1-2.1)	3 (2-5)	-3.1 (-11.7-6.6)	1 (1-2)	-10.3 (-22.9-2.2)	3 (2-4)	59 (43-77)	3 (2-4)	1 (1-1)
Wales	2 (1-2)	8.3 (0.1-17.2)	41 (31-54)	-2.6 (-9.4-4.7)	2 (2-3)	1.2 (-6.7-10.1)	1 (0-1)	-1.8 (-13.4-11)	2 (1-3)	40 (30-52)	2 (2-3)	1 (0-1)
Latin America and Caribbean	951 (729-1,190)	12.3 (4.6-21.2)	2,740 (2,270-3,260)	-5.8 (-10.7--0.3)	912 (697-1,140)	16.4 (8.4-25.7)	843 (633-1,060)	18.5 (9.9-29.2)	1,070 (805-1,370)	2,580 (2,080-3,140)	1,060 (800-1,360)	998 (740-1,290)
**Andean Latin America**	1 (1-2)	13.4 (6-51.2)	7 (4-11)	16.1 (2.4-46.7)	1 (1-2)	22.7 (13.2-64.8)	1 (1-2)	22.6 (11.8-67.6)	2 (1-3)	8 (4-13)	1 (1-3)	1 (1-2)
Bolivia (Plurinational State of)	0 (0-1)	31.3 (24.9-123)	1 (0-2)	26.2 (7.2-89.1)	0 (0-0)	38.8 (29.8-137)	0 (0-0)	40.3 (27.4-142)	0 (0-1)	1 (1-2)	0 (0-1)	0 (0-1)
Ecuador	0 (0-1)	14.4 (8.4-60.6)	2 (1-4)	14.5 (-1.1-60.7)	0 (0-1)	14.7 (6.3-61)	0 (0-1)	11.5 (0.4-58.9)	0 (0-1)	3 (1-4)	0 (0-1)	0 (0-1)
Peru	1 (0-1)	5 (-2.3-75.1)	3 (2-6)	10.1 (-8.2-78.9)	1 (0-1)	15.1 (5.4-94.1)	1 (0-1)	14.9 (2.7-104)	1 (0-1)	4 (2-6)	1 (0-1)	1 (0-1)
**Caribbean**	805 (610-1,010)	16.2 (7.8-26.9)	1,440 (1,180-1,740)	-18.6 (-25.4--10.9)	769 (581-963)	19.5 (10.6-30.2)	746 (559-937)	21.9 (12.6-33.1)	936 (702-1,200)	1,170 (908-1,450)	919 (688-1,180)	910 (678-1,170)

Location	Birth , 2000	Births percent change (2000-2021)	All age cases, 2000	All age cases percent change (2000-2021)	All age deaths, 2000	All age deaths percent change (2000-2021)	Under-5 deaths, 2000	Under-5 deaths percent change (2000-2021)	Births, 2021	All age cases, 2021	All age deaths, 2021	Under-5 deaths, 2021
Antigua and Barbuda	0 (0-1)	-5.9 (-25.4-16)	1 (1-2)	-36.8 (-47.2--25.8)	0 (0-1)	8.2 (-13.9-33.5)	0 (0-1)	12.8 (-10.8-41)	0 (0-1)	1 (1-1)	0 (0-1)	0 (0-1)
Bahamas	1 (1-2)	53.6 (28.7-90.8)	4 (3-5)	-3.5 (-18.4-15.8)	2 (1-2)	40.4 (17.9-73.4)	1 (1-2)	44 (19.5-78.2)	2 (1-3)	4 (3-5)	2 (1-3)	2 (1-3)
Barbados	1 (1-2)	3.7 (-16.7-29.4)	4 (3-6)	-38.1 (-48.5--27.3)	1 (1-2)	7.7 (-13.3-33.9)	1 (1-2)	14 (-10.9-42.5)	1 (1-2)	3 (2-4)	1 (1-2)	1 (1-2)
Belize	5 (3-8)	61.3 (30.8-99.3)	12 (9-16)	10.6 (-6.4-26.2)	5 (3-8)	63.4 (32.1-103)	5 (3-8)	67.1 (33.9-111)	8 (5-13)	13 (9-18)	8 (5-13)	8 (5-13)
Bermuda	0 (0-0)	-13 (-31.7-8)	0 (0-1)	-51.2 (-58.3--42.9)	0 (0-0)	-9.4 (-28.7-12.1)	0 (0-0)	-1.6 (-24.3-27.2)	0 (0-0)	0 (0-0)	0 (0-0)	0 (0-0)
Cuba	22 (16-29)	29.3 (6.4-61.2)	62 (48-78)	-33.1 (-46.3--18.3)	23 (16-31)	29.9 (7.1-60.9)	21 (15-29)	36.7 (12.7-69.1)	28 (18-40)	42 (31-54)	30 (19-42)	29 (19-42)
Dominica	0 (0-1)	-16.8 (-34.2-1.8)	1 (1-2)	-47.7 (-56.2--38.2)	0 (0-1)	-18.4 (-34.9--0.4)	0 (0-1)	-15.9 (-34-4.4)	0 (0-1)	1 (1-1)	0 (0-1)	0 (0-1)
Dominican Republic	247 (167-338)	14.3 (-0.4-36.2)	407 (305-516)	-12 (-23.1-0.4)	236 (159-324)	19.2 (3.5-42.3)	231 (154-319)	20.6 (4.2-44.6)	283 (198-380)	358 (269-455)	282 (197-379)	278 (195-376)
Grenada	1 (1-1)	-7.8 (-23.4-12.2)	3 (2-3)	-42.3 (-51.7--31.5)	1 (1-1)	-6.7 (-22.5-13.7)	1 (1-1)	-3.2 (-20.1-18.8)	1 (1-1)	2 (1-2)	1 (1-1)	1 (1-1)
Guyana	8 (5-12)	3.8 (-15.8-27.4)	19 (14-25)	-34.1 (-45.1--22.2)	8 (5-12)	1.7 (-17.6-24.8)	8 (5-11)	5 (-15.9-29.5)	8 (5-12)	13 (9-17)	8 (5-12)	8 (5-12)
Haiti	367 (276-461)	29.4 (15.1-48.7)	500 (394-610)	6.4 (-4.1-19)	333 (250-420)	36.6 (21.3-58)	328 (245-413)	37.8 (22.3-60.2)	474 (366-605)	532 (416-662)	455 (351-581)	452 (348-577)
Jamaica	103 (90-116)	-25.1 (-39.4--10)	300 (252-362)	-57.8 (-66--49.2)	111 (97-125)	-27.9 (-41.7--13.5)	105 (90-119)	-25.5 (-39.8--9.5)	77 (59-98)	126 (102-156)	80 (61-101)	78 (59-100)
Puerto Rico	6 (4-9)	-43.5 (-54--33.7)	33 (24-42)	-64 (-69.5--57.7)	7 (4-10)	-42.7 (-53.2--33.2)	6 (3-9)	-39.5 (-52.3--28.2)	4 (2-6)	12 (9-16)	4 (2-6)	3 (2-5)
Saint Kitts and Nevis	0 (0-0)	9.6 (-8.6-36.3)	1 (1-1)	-32.1 (-42.8--20.6)	0 (0-0)	10.7 (-7.7-37.4)	0 (0-0)	15.3 (-5.1-44.2)	0 (0-0)	1 (0-1)	0 (0-0)	0 (0-0)
Saint Lucia	1 (1-2)	-20.7 (-34.5--2.9)	5 (3-6)	-50.5 (-58--41.4)	1 (1-2)	-20.5 (-34--2.4)	1 (1-2)	-17.2 (-32.4-3.5)	1 (1-2)	2 (2-3)	1 (1-2)	1 (1-2)
Saint Vincent and the Grenadines	1 (1-1)	-11.3 (-26.9-8.1)	3 (2-4)	-45.4 (-54.8--35.4)	1 (1-1)	-11.2 (-26.5-8.2)	1 (1-1)	-7.6 (-23.7-13.8)	1 (0-1)	2 (1-2)	1 (1-1)	1 (0-1)
Suriname	5 (3-7)	16.4 (-3.4-36.2)	11 (8-14)	-17.8 (-29.6--4.6)	5 (3-7)	27.5 (6.4-50.2)	4 (3-7)	31.3 (8.8-57.4)	6 (4-9)	9 (6-12)	6 (4-9)	6 (4-9)
Trinidad and Tobago	7 (5-10)	11.1 (-9-33.2)	26 (20-34)	-38.9 (-49.5--27.1)	8 (5-11)	13.6 (-6.9-35.2)	7 (4-10)	22.1 (-1.3-48.5)	8 (5-12)	16 (12-22)	9 (5-13)	8 (5-13)
United States Virgin Islands	0 (0-0)	-16.2 (-31.1-0.5)	1 (1-2)	-50.3 (-58.3--42)	0 (0-0)	-12 (-27.5-5.7)	0 (0-0)	-6.2 (-24-14.8)	0 (0-0)	1 (0-1)	0 (0-0)	0 (0-0)
**Central Latin America**	92 (66-118)	-14.4 (-21.9--5)	251 (194-322)	-25.3 (-32.9--18.3)	90 (64-117)	-12.4 (-20.3--2.5)	84 (59-111)	-10.9 (-19.7--0.1)	78 (56-104)	187 (142-243)	79 (57-104)	75 (52-100)
Colombia	46 (32-62)	-13.2 (-22.3-0.2)	102 (75-134)	-12.2 (-21.9--1.9)	45 (31-61)	-12 (-21.7-1.7)	43 (29-59)	-12.3 (-22.7-2)	40 (28-54)	89 (64-118)	40 (28-54)	38 (26-52)
Costa Rica	0 (0-0)	-7.5 (-12.3-43.2)	1 (0-1)	-2.6 (-18.1-54.5)	0 (0-0)	-3.2 (-10.6-51.3)	0 (0-0)	-8.1 (-18.1-39.1)	0 (0-0)	1 (0-1)	0 (0-0)	0 (0-0)
El Salvador	1 (1-1)	-17.5 (-29.2-23.3)	3 (2-4)	-15.7 (-31.6-14.6)	1 (1-1)	-17.9 (-29.5-22.6)	1 (1-1)	-19.2 (-31.4-24.1)	1 (0-1)	2 (2-4)	1 (0-1)	1 (0-1)
Guatemala	2 (2-3)	22.8 (5-83)	5 (3-8)	-1.1 (-21.8-39)	2 (2-3)	-7.8 (-21.4-37.5)	2 (1-3)	-9.8 (-22.9-36.7)	3 (2-4)	5 (3-8)	2 (1-3)	2 (1-3)
Honduras	0 (0-1)	34.4 (-3-161)	1 (1-1)	38.1 (-6.9-132)	0 (0-0)	36.3 (-1.5-169)	0 (0-0)	34.9 (-2.2-175)	0 (0-1)	1 (1-2)	0 (0-1)	0 (0-1)
Mexico	1 (0-3)	3.8 (-17.3-81)	7 (1-19)	14.5 (-10.5-95.5)	1 (0-3)	11.2 (-11.8-92.5)	1 (0-3)	7.6 (-14.6-93.7)	1 (0-4)	8 (1-22)	1 (0-4)	1 (0-3)
Aguascalientes	0 (0-0)	18.8 (-4.8-302)	0 (0-0)	29.5 (-7.2-271)	0 (0-0)	25.2 (-1.8-333)	0 (0-0)	20.8 (-6.6-349)	0 (0-0)	0 (0-0)	0 (0-0)	0 (0-0)
Baja California	0 (0-0)	39.6 (4.4-513)	0 (0-0)	59.7 (11.2-511)	0 (0-0)	52 (11.6-564)	0 (0-0)	42.5 (2.5-589)	0 (0-0)	0 (0-1)	0 (0-0)	0 (0-0)
Baja California Sur	0 (0-0)	60.7 (23.6-628)	0 (0-0)	82.8 (32.2-570)	0 (0-0)	76.1 (32.9-687)	0 (0-0)	65.5 (21.9-705)	0 (0-0)	0 (0-0)	0 (0-0)	0 (0-0)

Location	Birth , 2000	Births percent change (2000-2021)	All age cases, 2000	All age cases percent change (2000-2021)	All age deaths, 2000	All age deaths percent change (2000-2021)	Under-5 deaths, 2000	Under-5 deaths percent change (2000-2021)	Births, 2021	All age cases, 2021	All age deaths, 2021	Under-5 deaths, 2021
Campeche	0 (0-0)	17 (-6.2-294)	0 (0-0)	21.2 (-14.2-247)	0 (0-0)	20.5 (-4.7-313)	0 (0-0)	18.3 (-8-330)	0 (0-0)	0 (0-0)	0 (0-0)	0 (0-0)
Chiapas	0 (0-0)	44.1 (14.6-551)	0 (0-1)	44.7 (2.5-392)	0 (0-0)	47.1 (15.8-580)	0 (0-0)	46.5 (13.1-616)	0 (0-0)	0 (0-1)	0 (0-0)	0 (0-0)
Chihuahua	0 (0-0)	-5.3 (-26.2-319)	0 (0-1)	4.8 (-25.8-265)	0 (0-0)	0.6 (-23.2-352)	0 (0-0)	-2.5 (-26.5-387)	0 (0-0)	0 (0-1)	0 (0-0)	0 (0-0)
Coahuila	0 (0-0)	23.3 (-2.6-436)	0 (0-0)	28.4 (-7.4-332)	0 (0-0)	27.7 (-0.1-449)	0 (0-0)	24.7 (-5.8-474)	0 (0-0)	0 (0-1)	0 (0-0)	0 (0-0)
Colima	0 (0-0)	17.3 (-5.5-460)	0 (0-0)	25.8 (-7.4-413)	0 (0-0)	24.6 (-1-478)	0 (0-0)	20.2 (-7.3-499)	0 (0-0)	0 (0-0)	0 (0-0)	0 (0-0)
Durango	0 (0-0)	-3.7 (-23.1-226)	0 (0-0)	8.1 (-22.9-215)	0 (0-0)	3.4 (-18.7-257)	0 (0-0)	-0.1 (-22.8-267)	0 (0-0)	0 (0-0)	0 (0-0)	0 (0-0)
Guanajuato	0 (0-0)	-1.5 (-23.6-358)	0 (0-1)	13 (-18.8-392)	0 (0-0)	7.9 (-18.1-410)	0 (0-0)	2.9 (-23.6-420)	0 (0-0)	0 (0-1)	0 (0-0)	0 (0-0)
Guerrero	0 (0-0)	-9.2 (-27-154)	0 (0-1)	1 (-27.5-150)	0 (0-0)	-3.2 (-23.1-177)	0 (0-0)	-5.2 (-25.5-180)	0 (0-0)	0 (0-1)	0 (0-0)	0 (0-0)
Hidalgo	0 (0-0)	-1.8 (-20.4-269)	0 (0-1)	11.5 (-18.1-264)	0 (0-0)	6.9 (-15.6-303)	0 (0-0)	2.5 (-19.9-311)	0 (0-0)	0 (0-1)	0 (0-0)	0 (0-0)
Jalisco	0 (0-0)	10.1 (-15-411)	0 (0-1)	18.9 (-16.5-355)	0 (0-0)	16.7 (-11.1-444)	0 (0-0)	13 (-15.3-471)	0 (0-0)	0 (0-1)	0 (0-0)	0 (0-0)
México	0 (0-0)	14.3 (-11.4-395)	1 (0-2)	28.5 (-7-345)	0 (0-0)	26.3 (-3.5-451)	0 (0-0)	22 (-9.7-479)	0 (0-1)	1 (0-3)	0 (0-0)	0 (0-0)
Mexico City	0 (0-0)	-29.1 (-41.6-480)	1 (0-2)	-14 (-33.1-522)	0 (0-0)	-15.9 (-32.3-573)	0 (0-0)	-22.6 (-39.2-562)	0 (0-0)	1 (0-1)	0 (0-0)	0 (0-0)
Michoacán de Ocampo	0 (0-0)	-4.6 (-24.8-346)	0 (0-1)	0.6 (-27.5-327)	0 (0-0)	0.7 (-21.6-363)	0 (0-0)	-1.6 (-25.9-376)	0 (0-0)	0 (0-1)	0 (0-0)	0 (0-0)
Morelos	0 (0-0)	9.5 (-11.6-240)	0 (0-0)	16.1 (-14.1-177)	0 (0-0)	15.3 (-8.3-254)	0 (0-0)	12 (-12.8-278)	0 (0-0)	0 (0-0)	0 (0-0)	0 (0-0)
Nayarit	0 (0-0)	18.7 (-7.3-321)	0 (0-0)	23.4 (-13-288)	0 (0-0)	22.1 (-5.8-335)	0 (0-0)	19.8 (-9.6-347)	0 (0-0)	0 (0-0)	0 (0-0)	0 (0-0)
Nuevo León	0 (0-0)	11.3 (-11.7-424)	0 (0-1)	29.9 (-4.6-425)	0 (0-0)	23 (-4.4-472)	0 (0-0)	15.5 (-12-488)	0 (0-0)	0 (0-1)	0 (0-0)	0 (0-0)
Oaxaca	0 (0-0)	-7.5 (-25.1-325)	0 (0-1)	-1.2 (-27.1-244)	0 (0-0)	-3.1 (-22.9-364)	0 (0-0)	-4.9 (-24.9-419)	0 (0-0)	0 (0-1)	0 (0-0)	0 (0-0)
Puebla	0 (0-0)	-2.5 (-19.2-256)	0 (0-1)	8.7 (-21.8-218)	0 (0-0)	3.6 (-15.4-285)	0 (0-0)	1.3 (-18.2-300)	0 (0-0)	0 (0-1)	0 (0-0)	0 (0-0)
Querétaro	0 (0-0)	23.1 (-0.4-261)	0 (0-0)	34.5 (-2.4-222)	0 (0-0)	29.5 (2.8-287)	0 (0-0)	24.6 (-3.1-323)	0 (0-0)	0 (0-0)	0 (0-0)	0 (0-0)
Quintana Roo	0 (0-0)	67.5 (34-462)	0 (0-0)	80.2 (30.7-416)	0 (0-0)	75.5 (38.3-497)	0 (0-0)	70.2 (31.9-522)	0 (0-0)	0 (0-0)	0 (0-0)	0 (0-0)
San Luis Potosí	0 (0-0)	-9.2 (-25.7-212)	0 (0-1)	0.2 (-25.5-169)	0 (0-0)	-3.2 (-22.7-241)	0 (0-0)	-6.8 (-26.3-247)	0 (0-0)	0 (0-1)	0 (0-0)	0 (0-0)
Sinaloa	0 (0-0)	-10.8 (-29-292)	0 (0-0)	-2.9 (-29.7-279)	0 (0-0)	-6.2 (-26.4-308)	0 (0-0)	-9.7 (-30.2-328)	0 (0-0)	0 (0-0)	0 (0-0)	0 (0-0)
Sonora	0 (0-0)	5.4 (-17.7-384)	0 (0-0)	20 (-12.9-386)	0 (0-0)	14.3 (-12.4-423)	0 (0-0)	8.4 (-18.8-435)	0 (0-0)	0 (0-0)	0 (0-0)	0 (0-0)
Tabasco	0 (0-0)	6.5 (-14.3-199)	0 (0-0)	13 (-21.4-187)	0 (0-0)	10.3 (-12.3-216)	0 (0-0)	8 (-15.2-220)	0 (0-0)	0 (0-0)	0 (0-0)	0 (0-0)
Tamaulipas	0 (0-0)	-6.3 (-26-254)	0 (0-1)	12.3 (-17.8-283)	0 (0-0)	5.7 (-18.2-298)	0 (0-0)	-0.6 (-24.8-298)	0 (0-0)	0 (0-1)	0 (0-0)	0 (0-0)
Tlaxcala	0 (0-0)	12 (-11.5-387)	0 (0-0)	24.5 (-12.3-250)	0 (0-0)	18.4 (-8.3-411)	0 (0-0)	14.3 (-12.2-440)	0 (0-0)	0 (0-0)	0 (0-0)	0 (0-0)
Veracruz de Ignacio de la Llave	0 (0-0)	-12.7 (-30.2-194)	0 (0-1)	-4.3 (-30.4-162)	0 (0-0)	-7.6 (-27.4-217)	0 (0-0)	-10.8 (-31.3-231)	0 (0-0)	0 (0-1)	0 (0-0)	0 (0-0)
Yucatán	0 (0-0)	4.8 (-14.3-265)	0 (0-0)	11.7 (-16.8-228)	0 (0-0)	9.8 (-11.7-290)	0 (0-0)	6.5 (-16.5-297)	0 (0-0)	0 (0-0)	0 (0-0)	0 (0-0)
Zacatecas	0 (0-0)	-2.6 (-21.6-331)	0 (0-0)	1.5 (-26.2-244)	0 (0-0)	2.4 (-19-351)	0 (0-0)	0.7 (-21.6-380)	0 (0-0)	0 (0-0)	0 (0-0)	0 (0-0)

Location	Birth , 2000	Births percent change (2000-2021)	All age cases, 2000	All age cases percent change (2000-2021)	All age deaths, 2000	All age deaths percent change (2000-2021)	Under-5 deaths, 2000	Under-5 deaths percent change (2000-2021)	Births, 2021	All age cases, 2021	All age deaths, 2021	Under-5 deaths, 2021
Nicaragua	1 (1-2)	9.1 (-6.9-51.3)	4 (3-5)	12.9 (-7.5-47.6)	1 (1-2)	11.2 (-5.4-55.3)	1 (1-1)	10.2 (-7-55.9)	1 (1-2)	4 (3-6)	1 (1-2)	1 (1-2)
Panama	0 (0-1)	31.7 (7.2-84.9)	2 (1-3)	33 (11.8-73.5)	0 (0-1)	33.2 (7.8-88.1)	0 (0-1)	32.1 (5.7-92.2)	1 (0-1)	3 (2-4)	1 (0-1)	1 (0-1)
Venezuela (Bolivarian Republic of)	39 (28-50)	-20.4 (-33.8--2.6)	127 (98-161)	-45.3 (-53--35.8)	39 (28-50)	-20.7 (-34.2--2.6)	36 (24-46)	-16.6 (-31.6-3.1)	31 (22-42)	70 (53-90)	31 (22-42)	30 (20-40)
**Tropical Latin America**	53 (43-66)	-1.5 (-7.1-4.6)	1,040 (861-1,260)	16.3 (9.5-23.3)	52 (42-66)	18.6 (10.7-26.9)	11 (8-16)	10.1 (0.6-20.8)	52 (42-65)	1,210 (984-1,500)	62 (50-77)	13 (9-18)
Brazil	50 (41-62)	-1.6 (-7.5-4.7)	1,000 (828-1,200)	16.4 (9.4-23.7)	50 (40-63)	18.6 (10.4-27.2)	10 (7-15)	10.9 (0.4-22.4)	50 (40-62)	1,170 (948-1,440)	59 (48-73)	11 (8-17)
Acre	0 (0-0)	104 (22.6-3180)	0 (0-0)	139 (35.8-3650)	0 (0-0)	128 (34.7-3650)	0 (0-0)	113 (21.1-3930)	0 (0-0)	0 (0-1)	0 (0-0)	0 (0-0)
Alagoas	0 (0-0)	54.3 (-12.7-2230)	0 (0-1)	86.5 (0.1-2630)	0 (0-0)	80.4 (0.1-2680)	0 (0-0)	76.6 (-5.6-3040)	0 (0-0)	1 (0-2)	0 (0-0)	0 (0-0)
Amapá	0 (0-0)	120 (39.7-3630)	0 (0-0)	142 (47.6-3970)	0 (0-0)	138 (47.4-4000)	0 (0-0)	126 (34.3-4070)	0 (0-0)	0 (0-1)	0 (0-0)	0 (0-0)
Amazonas	0 (0-0)	106 (14.5-605)	1 (0-2)	131 (24.5-670)	0 (0-0)	124 (22.2-668)	0 (0-0)	119 (14.3-716)	0 (0-0)	2 (1-5)	0 (0-0)	0 (0-0)
Bahia	2 (1-3)	22 (-17.6-103)	30 (19-43)	40.8 (-5.4-132)	2 (1-3)	38.7 (-7.2-132)	1 (0-1)	44.2 (-6.7-158)	3 (2-4)	42 (26-60)	2 (2-4)	1 (0-1)
Ceará	0 (0-0)	81.6 (-0.8-1070)	1 (0-2)	102 (5.3-1230)	0 (0-0)	99.5 (6.3-1220)	0 (0-0)	106 (5.2-1310)	0 (0-0)	2 (0-4)	0 (0-0)	0 (0-0)
Distrito Federal	0 (0-1)	42.2 (-5.5-141)	7 (4-10)	83.9 (23.4-206)	0 (0-1)	82.6 (20.7-211)	0 (0-0)	58.3 (5.5-181)	1 (0-1)	12 (8-18)	1 (0-1)	0 (0-0)
Espírito Santo	0 (0-0)	62.6 (-15.8-263)	3 (1-4)	82.8 (-5.9-308)	0 (0-0)	91.3 (-2-327)	0 (0-0)	93 (-4.5-321)	0 (0-0)	5 (3-9)	0 (0-0)	0 (0-0)
Goiás	0 (0-0)	21.1 (-13.9-129)	5 (3-8)	51 (3-179)	0 (0-0)	47 (1.9-178)	0 (0-0)	31.8 (-12.7-160)	0 (0-1)	8 (5-14)	0 (0-1)	0 (0-0)
Maranhão	0 (0-0)	73.2 (3-1450)	1 (0-2)	113 (20.9-1810)	0 (0-0)	107 (20.1-1720)	0 (0-0)	110 (18.3-1690)	0 (0-0)	1 (0-3)	0 (0-0)	0 (0-0)
Mato Grosso	0 (0-0)	67.1 (-3-1750)	0 (0-1)	91.9 (6-2010)	0 (0-0)	88.5 (6.2-2010)	0 (0-0)	76.5 (-2.4-1960)	0 (0-0)	0 (0-1)	0 (0-0)	0 (0-0)
Mato Grosso do Sul	0 (0-0)	75 (-7.2-326)	2 (1-3)	111 (9.9-406)	0 (0-0)	101 (4.8-395)	0 (0-0)	84.7 (-7.3-364)	0 (0-0)	3 (2-6)	0 (0-0)	0 (0-0)
Minas Gerais	3 (2-4)	-54 (-68.1--34.9)	54 (42-73)	-44.9 (-61.2--23.5)	3 (2-4)	-45.2 (-62.6--22.1)	0 (0-1)	-39 (-60--9.8)	1 (1-2)	30 (20-44)	2 (1-2)	0 (0-0)
Pará	0 (0-0)	108 (12.1-844)	1 (0-3)	136 (21.7-898)	0 (0-0)	126 (19.5-921)	0 (0-0)	121 (13.1-1060)	0 (0-0)	3 (1-7)	0 (0-0)	0 (0-0)
Paraíba	2 (2-3)	35.4 (23.6-48.7)	31 (24-42)	49.7 (35-64.5)	2 (1-3)	45.6 (31.6-62.1)	1 (0-1)	56.9 (36.3-83.4)	3 (2-4)	47 (34-63)	3 (2-4)	1 (1-1)
Paraná	0 (0-0)	45.9 (-22.6-268)	2 (1-5)	75.3 (-10.5-342)	0 (0-0)	71 (-11.5-323)	0 (0-0)	53.1 (-23.2-295)	0 (0-0)	4 (2-8)	0 (0-0)	0 (0-0)
Pernambuco	0 (0-1)	64 (-15.6-330)	4 (2-7)	92 (-4.2-400)	0 (0-0)	96.6 (-0.2-416)	0 (0-0)	98.6 (-2.6-407)	0 (0-1)	8 (4-15)	0 (0-1)	0 (0-0)
Piaui	0 (0-0)	73.1 (-9.2-291)	3 (1-5)	99.4 (1.9-338)	0 (0-0)	94.2 (1.3-348)	0 (0-0)	103 (4.9-409)	0 (0-1)	5 (3-9)	0 (0-1)	0 (0-0)
Rio de Janeiro	9 (7-11)	-8.9 (-16.1--1.3)	187 (158-222)	7.1 (-0.7-15.1)	9 (7-11)	11.3 (1.6-20.9)	2 (1-2)	-2.2 (-14.2-11.7)	8 (6-10)	200 (165-243)	10 (8-12)	1 (1-2)
Rio Grande do Norte	0 (0-0)	74.1 (-10-605)	1 (0-2)	111 (4.4-731)	0 (0-0)	101 (0.9-720)	0 (0-0)	98.4 (-5.3-731)	0 (0-0)	2 (1-3)	0 (0-0)	0 (0-0)
Rio Grande do Sul	0 (0-0)	24.1 (-27.1-227)	6 (3-10)	44.3 (-16.7-271)	0 (0-1)	49.6 (-14.1-292)	0 (0-0)	31 (-26-242)	0 (0-1)	9 (4-15)	0 (0-1)	0 (0-0)
Rondônia	0 (0-0)	-25.5 (-79.2-17.4)	1 (0-2)	-9.3 (-73.6-40)	0 (0-0)	-16.9 (-76.8-32.2)	0 (0-0)	-24.4 (-78-22.3)	0 (0-0)	1 (0-2)	0 (0-0)	0 (0-0)
Roraima	0 (0-0)	106 (26.6-2450)	0 (0-0)	150 (47.3-2830)	0 (0-0)	144 (47-2940)	0 (0-0)	129 (31.8-3400)	0 (0-0)	0 (0-0)	0 (0-0)	0 (0-0)
Santa Catarina	0 (0-0)	73.2 (-8.6-355)	3 (1-5)	88.8 (-3.1-398)	0 (0-0)	103 (4.8-438)	0 (0-0)	89.1 (-4.8-374)	0 (0-0)	5 (2-8)	0 (0-0)	0 (0-0)

Location	Birth , 2000	Births percent change (2000-2021)	All age cases, 2000	All age cases percent change (2000-2021)	All age deaths, 2000	All age deaths percent change (2000-2021)	Under-5 deaths, 2000	Under-5 deaths percent change (2000-2021)	Births, 2021	All age cases, 2021	All age deaths, 2021	Under-5 deaths, 2021
São Paulo	31 (25-39)	-4.1 (-11.7-3.5)	650 (527-789)	17.1 (8.9-26.3)	32 (25-40)	18.7 (8.7-28.9)	7 (4-9)	5.4 (-8.5-20.5)	30 (24-37)	761 (612-936)	38 (30-47)	7 (5-10)
Sergipe	0 (0-0)	44.6 (-8.6-172)	3 (2-4)	60.1 (-0.1-204)	0 (0-0)	67.3 (4.2-216)	0 (0-0)	67.5 (-0.5-211)	0 (0-0)	4 (2-7)	0 (0-0)	0 (0-0)
Tocantins	0 (0-1)	23.4 (-1-49.4)	7 (5-9)	55.8 (26-90.8)	0 (0-1)	51.2 (21.5-83.2)	0 (0-0)	35.3 (6.3-65.4)	1 (0-1)	10 (7-14)	1 (0-1)	0 (0-0)
Paraguay	3 (2-4)	0.4 (-8.5-11.7)	41 (30-54)	13.8 (4.2-24.2)	2 (2-3)	18.2 (6.1-32.3)	1 (1-2)	2.5 (-9.2-19.1)	3 (2-4)	47 (35-61)	3 (2-4)	1 (1-2)
North Africa and Middle East	44 (38-53)	-7.9 (-14.2--2.6)	436 (365-529)	6.8 (0.3-13.4)	37 (31-45)	6.8 (-0.6-14)	22 (16-29)	1.5 (-6.9-9.6)	41 (34-49)	466 (385-575)	40 (33-48)	22 (17-30)
**North Africa and Middle East**	44 (38-53)	-7.9 (-14.2--2.6)	436 (365-529)	6.8 (0.3-13.4)	37 (31-45)	6.8 (-0.6-14)	22 (16-29)	1.5 (-6.9-9.6)	41 (34-49)	466 (385-575)	40 (33-48)	22 (17-30)
Afghanistan	2 (1-2)	66.4 (14.9-70.4)	7 (4-10)	84.3 (29.7-111)	1 (1-2)	78.1 (23.3-88.5)	1 (1-2)	74.7 (21.3-90.7)	3 (2-4)	12 (7-18)	2 (1-3)	2 (1-3)
Algeria	8 (6-10)	22.1 (10.1-37.6)	78 (59-102)	16.3 (4.6-29.4)	7 (5-9)	23.3 (11-38.2)	4 (3-6)	31.1 (13.7-54.2)	9 (7-13)	91 (69-121)	8 (6-11)	5 (3-8)
Bahrain	0 (0-0)	-11.4 (-35.2--8.1)	1 (0-1)	48.6 (11.2-66.1)	0 (0-0)	37.3 (-0.5-54.6)	0 (0-0)	-5.3 (-32.6-6.6)	0 (0-0)	1 (1-1)	0 (0-0)	0 (0-0)
Egypt	4 (3-6)	5.2 (-26.7-29.6)	34 (24-49)	35.9 (-3.2-74.8)	3 (2-5)	29 (-10.6-62.2)	2 (1-3)	20.5 (-18-51.4)	4 (3-6)	46 (32-65)	4 (3-6)	3 (2-4)
Iran (Islamic Republic of)	7 (6-8)	-12.9 (-18.3--6.7)	87 (73-107)	-4.7 (-10.6-1.5)	6 (5-8)	-2.4 (-9.6-5.5)	3 (2-4)	0.6 (-6.3-8.3)	6 (5-7)	83 (68-102)	6 (5-8)	3 (2-4)
Alborz	0 (0-0)	-8.9 (-35.7-16.8)	2 (2-3)	8 (-19.8-42.9)	0 (0-0)	8.3 (-24.3-41)	0 (0-0)	7.6 (-30.9-39.1)	0 (0-0)	3 (2-3)	0 (0-0)	0 (0-0)
Ardebil	0 (0-0)	-34.2 (-53.6--14.4)	1 (1-2)	-26.8 (-45.6--3.4)	0 (0-0)	-24.8 (-46.6-0.7)	0 (0-0)	-25.1 (-50.2-0.9)	0 (0-0)	1 (1-1)	0 (0-0)	0 (0-0)
Bushehr	0 (0-0)	3.3 (-22.1-26.6)	2 (1-2)	23.6 (-3.2-51.1)	0 (0-0)	24 (-6.4-53)	0 (0-0)	19 (-13.2-51.5)	0 (0-0)	2 (2-3)	0 (0-0)	0 (0-0)
Chahar Mahaal and Bakhtiari	0 (0-0)	-17 (-35.9-0.3)	2 (1-2)	-8.7 (-25.8-11.4)	0 (0-0)	-6.2 (-27.6-13.8)	0 (0-0)	-6 (-30.9-15.3)	0 (0-0)	2 (1-2)	0 (0-0)	0 (0-0)
East Azarbayejan	0 (0-0)	-24.3 (-48.5--0.4)	3 (2-4)	-11.1 (-35-18.8)	0 (0-0)	-8.7 (-37.7-21.6)	0 (0-0)	-5.8 (-38.2-24.2)	0 (0-0)	3 (2-4)	0 (0-0)	0 (0-0)
Fars	1 (0-1)	-16.6 (-37.9-1.3)	8 (6-10)	-14.5 (-32.1-2.8)	1 (0-1)	-11.2 (-34.8-8.3)	0 (0-0)	-5.1 (-32.9-17.7)	0 (0-1)	7 (5-9)	1 (0-1)	0 (0-0)
Gilan	0 (0-0)	-33.9 (-52.1--14.5)	2 (2-3)	-24.4 (-41.9-0.4)	0 (0-0)	-22.9 (-44.2--0.8)	0 (0-0)	-27.3 (-49.6--5.7)	0 (0-0)	2 (1-2)	0 (0-0)	0 (0-0)
Golestan	0 (0-0)	-12.5 (-39.2-11.9)	2 (1-2)	-1.8 (-27.7-28.8)	0 (0-0)	1.1 (-30.5-31.9)	0 (0-0)	3.2 (-30.3-34)	0 (0-0)	2 (1-2)	0 (0-0)	0 (0-0)
Hamadan	0 (0-0)	-37.2 (-57.7--18.8)	2 (2-3)	-31 (-50.2--11)	0 (0-0)	-29.3 (-52.4--8.6)	0 (0-0)	-27.4 (-54.2--3.4)	0 (0-0)	1 (1-2)	0 (0-0)	0 (0-0)
Hormozgan	0 (0-0)	20.5 (-4.3-55.1)	1 (1-2)	32.4 (7.2-72.7)	0 (0-0)	34.7 (6.2-74.1)	0 (0-0)	36.1 (3.5-81)	0 (0-0)	2 (1-3)	0 (0-0)	0 (0-0)
Ilam	0 (0-0)	-35.1 (-53.6--17.8)	1 (1-1)	-22.8 (-42.1--1.2)	0 (0-0)	-22.5 (-44.6--1.2)	0 (0-0)	-25.7 (-50--3.5)	0 (0-0)	1 (0-1)	0 (0-0)	0 (0-0)
Isfahan	0 (0-1)	-22.3 (-43.8--3.4)	7 (5-9)	-9.2 (-29.9-16.2)	0 (0-1)	-9.6 (-34.7-12.7)	0 (0-0)	-12.2 (-39.4-12.4)	0 (0-0)	7 (5-9)	0 (0-1)	0 (0-0)
Kerman	0 (0-0)	10.8 (-22.4-39.3)	3 (2-4)	15.1 (-16.6-48.3)	0 (0-0)	18.8 (-17.3-52.3)	0 (0-0)	25.5 (-15.6-65.7)	0 (0-0)	3 (2-4)	0 (0-0)	0 (0-0)
Kermanshah	0 (0-0)	-27.3 (-50.7--4.5)	2 (2-3)	-27.9 (-49.1--4.4)	0 (0-0)	-23.8 (-48.7--0.6)	0 (0-0)	-17.7 (-46.9-8.4)	0 (0-0)	2 (1-2)	0 (0-0)	0 (0-0)
Khorasan-e-Razavi	1 (0-1)	-7.3 (-35-21.8)	5 (4-7)	1.7 (-23.9-34.1)	0 (0-1)	4.6 (-26.2-38.4)	0 (0-0)	8.3 (-25.4-43.6)	0 (0-1)	5 (4-7)	0 (0-1)	0 (0-0)
Khuzestan	1 (1-1)	-12.3 (-33.3-8)	9 (7-12)	-7.8 (-26.8-12.8)	1 (1-1)	-4.4 (-28.9-18.9)	0 (0-0)	1.1 (-26.4-27.9)	1 (1-1)	8 (7-11)	1 (0-1)	0 (0-0)
Kohgiluyeh and Boyer-Ahmad	0 (0-0)	-16.2 (-36.3-5.7)	2 (1-2)	-7.8 (-26.8-15.3)	0 (0-0)	-5.8 (-27.4-19.6)	0 (0-0)	-4.5 (-30.4-22.9)	0 (0-0)	1 (1-2)	0 (0-0)	0 (0-0)
Kurdistan	0 (0-0)	-18.7 (-44.7-4.4)	2 (1-2)	-15.5 (-38.9-9.1)	0 (0-0)	-12.7 (-40.8-13.7)	0 (0-0)	-10.1 (-41.3-20.8)	0 (0-0)	1 (1-2)	0 (0-0)	0 (0-0)

Location	Birth , 2000	Births percent change (2000-2021)	All age cases, 2000	All age cases percent change (2000-2021)	All age deaths, 2000	All age deaths percent change (2000-2021)	Under-5 deaths, 2000	Under-5 deaths percent change (2000-2021)	Births, 2021	All age cases, 2021	All age deaths, 2021	Under-5 deaths, 2021
Lorestan	0 (0-0)	-34.1 (-54.1--15.6)	3 (2-4)	-26.7 (-45.7--2.1)	0 (0-0)	-25.3 (-48.4--4.2)	0 (0-0)	-26.1 (-50.9--4.3)	0 (0-0)	2 (1-3)	0 (0-0)	0 (0-0)
Markazi	0 (0-0)	-27.1 (-50--4.1)	2 (1-2)	-21.4 (-43.1-3.4)	0 (0-0)	-20.1 (-45.6-5.1)	0 (0-0)	-17.8 (-45.5-9.2)	0 (0-0)	1 (1-2)	0 (0-0)	0 (0-0)
Mazandaran	0 (0-0)	-25.9 (-48.8--4.7)	3 (2-4)	-13.2 (-36.2-14.2)	0 (0-0)	-12.6 (-39.8-12.4)	0 (0-0)	-16.6 (-44.1-5.3)	0 (0-0)	3 (2-3)	0 (0-0)	0 (0-0)
North Khorasan	0 (0-0)	-36.1 (-55.3--12.5)	1 (1-1)	-16.1 (-38.4-13.9)	0 (0-0)	-18.2 (-42.8-11.4)	0 (0-0)	-23.5 (-48.4-5.7)	0 (0-0)	1 (0-1)	0 (0-0)	0 (0-0)
Qazvin	0 (0-0)	-12.9 (-36.2-16)	1 (1-2)	-10.3 (-30.9-18.3)	0 (0-0)	-6.6 (-31.6-22.8)	0 (0-0)	-2.4 (-31.5-32.3)	0 (0-0)	1 (1-1)	0 (0-0)	0 (0-0)
Qom	0 (0-0)	12.1 (-16.2-44.9)	1 (1-2)	18.6 (-8.4-52.5)	0 (0-0)	21.2 (-10.4-56.6)	0 (0-0)	26 (-10.2-66.9)	0 (0-0)	1 (1-2)	0 (0-0)	0 (0-0)
Semnan	0 (0-0)	12 (-20.9-44.8)	1 (0-1)	10.3 (-17.4-45.1)	0 (0-0)	14.8 (-18.6-53.5)	0 (0-0)	19.5 (-21.6-59.3)	0 (0-0)	1 (0-1)	0 (0-0)	0 (0-0)
Sistan and Baluchistan	0 (0-0)	12 (-22.8-49.5)	2 (2-3)	29.9 (-7.3-75.1)	0 (0-0)	31.7 (-9.7-75.1)	0 (0-0)	32.8 (-11.5-77.2)	0 (0-0)	3 (2-4)	0 (0-0)	0 (0-0)
South Khorasan	0 (0-0)	-7.8 (-35-20.1)	1 (0-1)	7.7 (-17.6-41.9)	0 (0-0)	8 (-24.3-41.6)	0 (0-0)	6.3 (-28.4-39.1)	0 (0-0)	1 (1-1)	0 (0-0)	0 (0-0)
Tehran	1 (1-1)	-5.3 (-28.7-18.2)	12 (9-15)	6 (-17.3-33.5)	1 (0-1)	8.8 (-18.6-36.4)	0 (0-0)	13.3 (-19.3-43.6)	1 (0-1)	12 (9-16)	1 (1-1)	0 (0-0)
West Azarbayejan	0 (0-0)	-1 (-27.7-28.9)	3 (2-4)	4.7 (-21.1-38.2)	0 (0-0)	9.7 (-22-43.4)	0 (0-0)	17.2 (-20.7-56.2)	0 (0-0)	3 (2-4)	0 (0-0)	0 (0-0)
Yazd	0 (0-0)	1.1 (-27.5-26.5)	1 (1-2)	11.8 (-16.9-41.5)	0 (0-0)	14 (-18.6-43.5)	0 (0-0)	20.4 (-15.9-56.4)	0 (0-0)	1 (1-2)	0 (0-0)	0 (0-0)
Zanjan	0 (0-0)	-23.8 (-44.9-1.4)	1 (1-1)	-17.5 (-37-10)	0 (0-0)	-14.3 (-38.7-14.9)	0 (0-0)	-12.2 (-39.7-18)	0 (0-0)	1 (1-1)	0 (0-0)	0 (0-0)
Iraq	2 (1-3)	-2.3 (-28.5--0.5)	16 (12-23)	27.9 (-3.4-39.7)	1 (1-2)	18.7 (-12.2-29.1)	1 (1-1)	0.2 (-29-11.6)	2 (1-3)	21 (14-29)	2 (1-2)	1 (1-1)
Jordan	0 (0-0)	77.4 (55.2-78.7)	1 (0-1)	130 (108-145)	0 (0-0)	118 (92.8-130)	0 (0-0)	83.1 (59.3-106)	0 (0-0)	2 (1-3)	0 (0-0)	0 (0-0)
Kuwait	0 (0-0)	47.8 (15.3-50.2)	2 (1-2)	99.8 (61.2-117)	0 (0-0)	88.5 (43.8-105)	0 (0-0)	49.3 (6.5-66.6)	0 (0-0)	3 (2-4)	0 (0-0)	0 (0-0)
Lebanon	0 (0-0)	2 (-16.1-25.8)	4 (3-5)	24.9 (3-50.3)	0 (0-0)	24 (0.9-53)	0 (0-0)	14.2 (-6.7-39.8)	0 (0-0)	4 (3-6)	0 (0-0)	0 (0-0)
Libya	0 (0-0)	-39.1 (-58.7--37.5)	3 (2-4)	-9.6 (-34.4-3.2)	0 (0-0)	-19 (-44.7--9.4)	0 (0-0)	-36.3 (-57.3--28.3)	0 (0-0)	3 (2-4)	0 (0-0)	0 (0-0)
Morocco	1 (1-2)	-33.1 (-52.5--31.5)	13 (9-18)	-5 (-30.7-5.7)	1 (1-2)	-11.9 (-38.1--5.6)	1 (0-1)	-24 (-47.6--15.4)	1 (1-1)	12 (8-17)	1 (1-1)	1 (0-1)
Oman	0 (0-0)	23.6 (-9.1-25.5)	1 (1-1)	59.1 (20.7-74.6)	0 (0-0)	60.9 (15.9-73.2)	0 (0-0)	49.8 (7.7-67.1)	0 (0-0)	2 (1-2)	0 (0-0)	0 (0-0)
Palestine	0 (0-0)	-5.3 (-30.6-12.7)	2 (1-2)	40.1 (3-71.9)	0 (0-0)	26.2 (-10.2-45.9)	0 (0-0)	5.2 (-27.6-19.8)	0 (0-0)	3 (2-4)	0 (0-0)	0 (0-0)
Qatar	0 (0-0)	137 (83.2-142)	1 (0-1)	336 (240-383)	0 (0-0)	350 (233-406)	0 (0-0)	178 (103-210)	0 (0-0)	2 (2-3)	0 (0-0)	0 (0-0)
Saudi Arabia	1 (1-1)	-14.2 (-34.3--13.3)	12 (8-17)	24.2 (-1.6-37)	1 (1-1)	20 (-7.4-32.5)	0 (0-1)	-13.9 (-36.5--4.6)	1 (1-1)	15 (10-21)	1 (1-1)	0 (0-1)
Sudan	4 (3-6)	-3.4 (-25.5-39.8)	18 (12-25)	34.5 (4.2-87.8)	3 (2-4)	21.2 (-6.9-76.5)	2 (1-3)	12.2 (-14.1-68.7)	4 (3-5)	24 (17-34)	3 (2-5)	2 (2-4)
Syrian Arab Republic	1 (1-2)	-60.8 (-70.7--55.6)	14 (10-19)	-41.5 (-54.4--32.4)	1 (1-1)	-48.7 (-61.3--39.8)	1 (0-1)	-60.4 (-71.6--54.2)	1 (0-1)	8 (6-11)	1 (0-1)	0 (0-0)
Tunisia	0 (0-0)	-29 (-53.6--24.2)	4 (3-6)	-12.4 (-40.2--1.5)	0 (0-0)	-12.7 (-43.9--3.3)	0 (0-0)	-20.3 (-49.9--8)	0 (0-0)	4 (2-5)	0 (0-0)	0 (0-0)
Türkiye	11 (8-15)	-35.5 (-46.4--23.4)	132 (103-171)	-10.6 (-22.7-4.8)	10 (7-13)	-18.6 (-32.6--1.9)	6 (4-8)	-34.4 (-48--19.7)	7 (6-9)	118 (93-155)	8 (6-10)	4 (3-5)
United Arab Emirates	0 (0-0)	-3.5 (-34.7--1.4)	0 (0-1)	103 (33.9-123)	0 (0-0)	85.9 (23.8-111)	0 (0-0)	63.2 (3-81.2)	0 (0-0)	1 (0-1)	0 (0-0)	0 (0-0)
Yemen	2 (1-2)	4.8 (-22.8-7.2)	8 (5-12)	42.7 (7.4-59.7)	1 (1-2)	29 (-5.5-38)	1 (1-1)	18.5 (-14.2-30.6)	2 (1-2)	11 (7-16)	1 (1-2)	1 (1-2)

Location	Birth , 2000	Births percent change (2000-2021)	All age cases, 2000	All age cases percent change (2000-2021)	All age deaths, 2000	All age deaths percent change (2000-2021)	Under-5 deaths, 2000	Under-5 deaths percent change (2000-2021)	Births, 2021	All age cases, 2021	All age deaths, 2021	Under-5 deaths, 2021
South Asia	31 (23-40)	-15.6 (-21.4--11.8)	274 (198-372)	6.4 (-1.1-12.5)	26 (20-34)	1.1 (-6.2-7.6)	15 (11-21)	-11.5 (-18.7--7)	26 (20-34)	292 (208-403)	27 (20-35)	14 (9-19)
**South Asia**	31 (23-40)	-15.6 (-21.4--11.8)	274 (198-372)	6.4 (-1.1-12.5)	26 (20-34)	1.1 (-6.2-7.6)	15 (11-21)	-11.5 (-18.7--7)	26 (20-34)	292 (208-403)	27 (20-35)	14 (9-19)
Bangladesh	1 (0-2)	-27.1 (-58.8--25)	6 (1-15)	-8.6 (-45.1-1.7)	1 (0-1)	-16.6 (-50.2--10.2)	0 (0-1)	-25.5 (-54.9--19)	1 (0-1)	5 (1-13)	1 (0-1)	0 (0-1)
Bhutan	0 (0-0)	-30.4 (-32.1-23.8)	0 (0-0)	-9 (-18.7-56.4)	0 (0-0)	-15 (-20.4-49.8)	0 (0-0)	-24.7 (-31.1-26.3)	0 (0-0)	0 (0-0)	0 (0-0)	0 (0-0)
India	28 (21-38)	-16.5 (-22.7--12.5)	258 (187-355)	5.6 (-1.7-12)	25 (18-33)	0.4 (-7-7.3)	14 (10-20)	-12.3 (-19.1--7.7)	24 (18-32)	273 (194-379)	25 (19-32)	12 (9-18)
Nepal	0 (0-0)	-24.3 (-24.2--24.2)	1 (0-2)	2.1 (-5.4-10.9)	0 (0-0)	-7.4 (-11.2--3)	0 (0-0)	-19.6 (-25--13.9)	0 (0-0)	1 (0-2)	0 (0-0)	0 (0-0)
Pakistan	2 (0-4)	7.2 (-44.2-13.1)	9 (3-22)	37.4 (-26.4-50.9)	1 (0-2)	27.1 (-29.7-38.4)	1 (0-2)	13.5 (-36.5-24.4)	2 (1-4)	13 (4-31)	1 (0-3)	1 (0-2)
Azad Jammu & Kashmir	0 (0-0)	-13.1 (-57.2--10.8)	0 (0-0)	13.3 (-39.8-25.3)	0 (0-0)	4.7 (-46.3-12.5)	0 (0-0)	-8.2 (-52.1-0.6)	0 (0-0)	0 (0-1)	0 (0-0)	0 (0-0)
Balochistan	0 (0-0)	74.5 (-59.4-85.4)	0 (0-1)	95 (-53.2-119)	0 (0-0)	87 (-56.1-104)	0 (0-0)	78.2 (-56.1-99.8)	0 (0-0)	1 (0-2)	0 (0-0)	0 (0-0)
Gilgit-Baltistan	0 (0-0)	83.4 (50.8-87.6)	0 (0-0)	103 (65.8-121)	0 (0-0)	97.3 (59.7-106)	0 (0-0)	90.1 (53.4-105)	0 (0-0)	0 (0-0)	0 (0-0)	0 (0-0)
Islamabad Capital Territory	0 (0-0)	86.9 (37.9-92.1)	0 (0-0)	135 (74.8-152)	0 (0-0)	126 (64.6-139)	0 (0-0)	96.2 (35.9-117)	0 (0-0)	0 (0-0)	0 (0-0)	0 (0-0)
Khyber Pakhtunkhwa	0 (0-1)	58.4 (22.9-61.5)	1 (0-4)	79.2 (38-91.4)	0 (0-0)	73.3 (34.6-81)	0 (0-0)	64.8 (25.6-80)	0 (0-1)	3 (1-7)	0 (0-1)	0 (0-0)
Punjab	1 (0-2)	-11 (-49--6.5)	5 (2-12)	20 (-30.2-36.5)	1 (0-1)	10.1 (-34.3-21)	0 (0-1)	-4.4 (-40.9-6.2)	1 (0-2)	6 (2-14)	1 (0-2)	0 (0-1)
Sindh	0 (0-1)	2.4 (-41.5-7.2)	2 (1-5)	34.3 (-25.8-51.1)	0 (0-1)	24.6 (-26.2-35.6)	0 (0-0)	11 (-30.4-23)	0 (0-1)	3 (1-7)	0 (0-1)	0 (0-0)
Southeast Asia, East Asia, and Oceania	28 (24-34)	-10.8 (-15.9--6.6)	517 (423-631)	-11.8 (-16.8--7.4)	30 (26-36)	-9.4 (-15.1--4.8)	11 (8-15)	-15.5 (-21.1--10.8)	25 (21-31)	456 (368-567)	27 (23-33)	10 (7-13)
**East Asia**	26 (22-30)	-10.7 (-16.7--6.3)	485 (405-582)	-12.7 (-17.7--8)	28 (24-33)	-10.4 (-16.3--5.4)	10 (8-13)	-16.5 (-22.8--11.1)	23 (20-27)	424 (350-515)	25 (22-29)	9 (6-11)
China	26 (22-30)	-10.5 (-16.4--6.1)	482 (402-580)	-12.6 (-17.7--7.9)	28 (24-33)	-10.4 (-16.2--5.3)	10 (7-13)	-16.4 (-22.7--11)	23 (20-27)	421 (348-513)	25 (22-29)	8 (6-11)
Democratic People's Republic of Korea	0 (0-0)	-44.9 (-44.2--44.2)	2 (0-4)	-16.8 (-21.5--11.7)	0 (0-0)	-17.7 (-21.9--11.9)	0 (0-0)	-40 (-43.9--35.2)	0 (0-0)	1 (0-4)	0 (0-0)	0 (0-0)
Taiwan (Province of China)	0 (0-0)	-43.1 (-42.9--42.9)	2 (0-4)	-24 (-27.2--20.6)	0 (0-0)	-14.6 (-20.3--8.9)	0 (0-0)	-36.3 (-42.2--30.3)	0 (0-0)	1 (0-3)	0 (0-0)	0 (0-0)
**Oceania**	0 (0-0)	51.6 (49.3-62.1)	0 (0-1)	51.5 (42.7-64.4)	0 (0-0)	58.4 (54.9-67.1)	0 (0-0)	59.5 (51.1-69.6)	0 (0-0)	1 (0-2)	0 (0-0)	0 (0-0)
American Samoa	0 (0-0)	-39.5 (-73.7--37.8)	0 (0-0)	-21.5 (-64.6--16.8)	0 (0-0)	-22.8 (-66.9--16.8)	0 (0-0)	-40.6 (-73.4--34)	0 (0-0)	0 (0-0)	0 (0-0)	0 (0-0)
Cook Islands	0 (0-0)	-36 (-35.9--35.9)	0 (0-0)	-23 (-25.4--20.4)	0 (0-0)	-21.6 (-24.1--18.3)	0 (0-0)	-37.6 (-42.4--32.6)	0 (0-0)	0 (0-0)	0 (0-0)	0 (0-0)
Fiji	0 (0-0)	-6.7 (-6.7--6.7)	0 (0-0)	0.3 (-4.6-5.8)	0 (0-0)	0.9 (-0.8-2.6)	0 (0-0)	-0.9 (-7.7-7.2)	0 (0-0)	0 (0-0)	0 (0-0)	0 (0-0)
Guam	0 (0-0)	-9.5 (-9.4--9.4)	0 (0-0)	-4.3 (-7.3--0.2)	0 (0-0)	-2.7 (-4.5--0.3)	0 (0-0)	-12.8 (-20.1--5.7)	0 (0-0)	0 (0-0)	0 (0-0)	0 (0-0)
Kiribati	0 (0-0)	18.4 (18.4-18.4)	0 (0-0)	26.6 (18-35.4)	0 (0-0)	26.1 (23.7-28.6)	0 (0-0)	21.8 (14.5-29.9)	0 (0-0)	0 (0-0)	0 (0-0)	0 (0-0)
Marshall Islands	0 (0-0)	-22.9 (-56.4--19.9)	0 (0-0)	-14.4 (-48.3--6.8)	0 (0-0)	-13.6 (-50.1--8.2)	0 (0-0)	-19.1 (-55.3--10.2)	0 (0-0)	0 (0-0)	0 (0-0)	0 (0-0)
Micronesia (Federated States of)	0 (0-0)	-36.2 (-36.2--36.2)	0 (0-0)	-25.9 (-29.7--21.9)	0 (0-0)	-25.7 (-28.3--22.8)	0 (0-0)	-36.5 (-40.7--32.3)	0 (0-0)	0 (0-0)	0 (0-0)	0 (0-0)
Nauru	0 (0-0)	-28.2 (-27.5--27.5)	0 (0-0)	-8.8 (-13.9--2.8)	0 (0-0)	-12.7 (-14.7--9)	0 (0-0)	-21.8 (-26.4--14.7)	0 (0-0)	0 (0-0)	0 (0-0)	0 (0-0)
Niue	0 (0-0)	-36 (-86.3--33.7)	0 (0-0)	-26.9 (-81.9--21.1)	0 (0-0)	-26.5 (-84.1--21.9)	0 (0-0)	-35 (-86.7--28.7)	0 (0-0)	0 (0-0)	0 (0-0)	0 (0-0)

Location	Birth , 2000	Births percent change (2000-2021)	All age cases, 2000	All age cases percent change (2000-2021)	All age deaths, 2000	All age deaths percent change (2000-2021)	Under-5 deaths, 2000	Under-5 deaths percent change (2000-2021)	Births, 2021	All age cases, 2021	All age deaths, 2021	Under-5 deaths, 2021
Northern Mariana Islands	0 (0-0)	-80.7 (-81--80.6)	0 (0-0)	-60.1 (-62.2--56.6)	0 (0-0)	-59 (-62.7--52)	0 (0-0)	-74.1 (-76.8--69.9)	0 (0-0)	0 (0-0)	0 (0-0)	0 (0-0)
Palau	0 (0-0)	-54.8 (-54.8--54.8)	0 (0-0)	-33.7 (-37.3--30.3)	0 (0-0)	-35.7 (-38.1--32.5)	0 (0-0)	-50.4 (-53.9--46.9)	0 (0-0)	0 (0-0)	0 (0-0)	0 (0-0)
Papua New Guinea	0 (0-0)	68.7 (68.7-68.7)	0 (0-1)	74.9 (63.8-86.2)	0 (0-0)	74.7 (72-77.7)	0 (0-0)	72.8 (63.6-82.5)	0 (0-0)	0 (0-1)	0 (0-0)	0 (0-0)
Samoa	0 (0-0)	27 (-23.5-32.2)	0 (0-0)	14.6 (-33.8-26.5)	0 (0-0)	15.1 (-30-22)	0 (0-0)	11.8 (-31.3-23)	0 (0-0)	0 (0-0)	0 (0-0)	0 (0-0)
Solomon Islands	0 (0-0)	7.8 (-36.3-10.5)	0 (0-0)	33.4 (-20.8-43.8)	0 (0-0)	28.5 (-20.5-34.8)	0 (0-0)	17.7 (-23.8-26.7)	0 (0-0)	0 (0-0)	0 (0-0)	0 (0-0)
Tokelau	0 (0-0)	-30.4 (-46.9--30.6)	0 (0-0)	-20.2 (-39.3--16.4)	0 (0-0)	-18.1 (-38.1--15)	0 (0-0)	-22.5 (-42.4--9.9)	0 (0-0)	0 (0-0)	0 (0-0)	0 (0-0)
Tonga	0 (0-0)	-15.6 (-33.3--14.5)	0 (0-0)	-8.6 (-28.1--3.7)	0 (0-0)	-9.8 (-28.3--6.8)	0 (0-0)	-14.4 (-30.2--6.4)	0 (0-0)	0 (0-0)	0 (0-0)	0 (0-0)
Tuvalu	0 (0-0)	-3.6 (-5.5--5.5)	0 (0-0)	13.2 (4.1-18.9)	0 (0-0)	8.2 (2.4-11.5)	0 (0-0)	-5.9 (-13.5-4.3)	0 (0-0)	0 (0-0)	0 (0-0)	0 (0-0)
Vanuatu	0 (0-0)	12.4 (-32.6-17.6)	0 (0-0)	35.9 (-22.1-49.2)	0 (0-0)	31.4 (-20.4-40.8)	0 (0-0)	18.5 (-29-31.6)	0 (0-0)	0 (0-0)	0 (0-0)	0 (0-0)
**Southeast Asia**	3 (1-6)	-12.8 (-14.2-14.4)	31 (10-64)	2.3 (-1.4-28)	2 (1-5)	1.6 (-2.3-32.4)	1 (0-3)	-8.2 (-10.8-24.4)	2 (1-5)	32 (12-67)	2 (1-5)	1 (0-2)
Cambodia	0 (0-0)	-2.4 (-3.4--3.4)	1 (0-2)	7.7 (-0.6-17.3)	0 (0-0)	7.9 (4.2-10.7)	0 (0-0)	2.9 (-3.7-10.1)	0 (0-0)	1 (0-2)	0 (0-0)	0 (0-0)
Indonesia	1 (0-3)	-21.2 (-23.1-30.8)	12 (4-31)	0.2 (-4.4-59.1)	1 (0-3)	-2.6 (-7.6-61.3)	1 (0-1)	-14.6 (-18-39.5)	1 (0-2)	12 (4-30)	1 (0-2)	0 (0-1)
Aceh	0 (0-0)	-21.9 (-61.7--5.2)	0 (0-1)	0.4 (-49.1-19.2)	0 (0-0)	-3.6 (-52.7-15.9)	0 (0-0)	-14.8 (-58.1-4.4)	0 (0-0)	0 (0-1)	0 (0-0)	0 (0-0)
Bali	0 (0-0)	-29.2 (-29.3--29.3)	0 (0-1)	1.3 (-4.1-6.6)	0 (0-0)	-3 (-6.8-1.2)	0 (0-0)	-22.4 (-27--16.3)	0 (0-0)	0 (0-1)	0 (0-0)	0 (0-0)
Bangka-Belitung Islands	0 (0-0)	-15.4 (-18.4-152)	0 (0-0)	7.9 (-2.9-207)	0 (0-0)	4.4 (-2.8-202)	0 (0-0)	-8.3 (-17.1-159)	0 (0-0)	0 (0-0)	0 (0-0)	0 (0-0)
Banten	0 (0-0)	-28 (-31-19.8)	1 (0-1)	-11.9 (-21.7-50.5)	0 (0-0)	-15.1 (-20.8-40.3)	0 (0-0)	-22.4 (-29.9-26)	0 (0-0)	1 (0-1)	0 (0-0)	0 (0-0)
Bengkulu	0 (0-0)	-20.8 (-23.1-14.5)	0 (0-0)	-0.6 (-9.2-45.7)	0 (0-0)	-4 (-9.4-39.8)	0 (0-0)	-13.6 (-20.8-23.5)	0 (0-0)	0 (0-0)	0 (0-0)	0 (0-0)
Gorontalo	0 (0-0)	9.9 (8.1-96.5)	0 (0-0)	44.4 (30.1-149)	0 (0-0)	32 (23.4-149)	0 (0-0)	11.5 (3.9-109)	0 (0-0)	0 (0-0)	0 (0-0)	0 (0-0)
Jakarta	0 (0-0)	-38.7 (-39.6--20.9)	1 (0-2)	-17.3 (-24.3-7.4)	0 (0-0)	-19.7 (-23.4-4)	0 (0-0)	-28.8 (-34.5--7.6)	0 (0-0)	0 (0-1)	0 (0-0)	0 (0-0)
Jambi	0 (0-0)	-12.9 (-15.7-35.1)	0 (0-0)	10.3 (0.8-69.6)	0 (0-0)	6.9 (0.1-67.3)	0 (0-0)	-6.8 (-14.7-51.1)	0 (0-0)	0 (0-0)	0 (0-0)	0 (0-0)
West Java	0 (0-0)	-10.1 (-12-60)	2 (1-5)	17.7 (8.2-128)	0 (0-0)	11.6 (5.4-102)	0 (0-0)	-4.2 (-11.7-60)	0 (0-0)	2 (1-6)	0 (0-0)	0 (0-0)
Central Java	0 (0-0)	-35.6 (-36.1--36.1)	2 (1-5)	-16 (-21.8--9.8)	0 (0-0)	-18.3 (-22--14.3)	0 (0-0)	-32.4 (-37--25.7)	0 (0-0)	2 (0-4)	0 (0-0)	0 (0-0)
East Java	0 (0-0)	-35.6 (-36.1--36.1)	2 (1-5)	-15 (-20.7--5.6)	0 (0-0)	-17 (-20.6--13.4)	0 (0-0)	-31.3 (-37.4--24)	0 (0-0)	2 (0-4)	0 (0-0)	0 (0-0)
West Kalimantan	0 (0-0)	-18.3 (-21.1-18)	0 (0-1)	4.4 (-6.8-53.4)	0 (0-0)	-0.2 (-6.4-45.9)	0 (0-0)	-11.2 (-19.2-28)	0 (0-0)	0 (0-1)	0 (0-0)	0 (0-0)
South Kalimantan	0 (0-0)	-21.5 (-24.1-13.6)	0 (0-0)	3.4 (-7.8-56.5)	0 (0-0)	-1.8 (-8.2-43.8)	0 (0-0)	-14.3 (-22.1-24.9)	0 (0-0)	0 (0-0)	0 (0-0)	0 (0-0)
Central Kalimantan	0 (0-0)	-14.2 (-17-71.9)	0 (0-0)	10.8 (0-115)	0 (0-0)	6.1 (-0.6-114)	0 (0-0)	-7.3 (-15.1-89.3)	0 (0-0)	0 (0-0)	0 (0-0)	0 (0-0)
East Kalimantan	0 (0-0)	-1.4 (-2.1--2)	0 (0-0)	30.2 (22.9-41.6)	0 (0-0)	26 (22.1-30.4)	0 (0-0)	11.5 (1.9-20.8)	0 (0-0)	0 (0-1)	0 (0-0)	0 (0-0)
North Kalimantan	0 (0-0)	12.4 (9.8-88.3)	0 (0-0)	60.8 (47.2-169)	0 (0-0)	45.4 (36.3-137)	0 (0-0)	21.5 (11.5-100)	0 (0-0)	0 (0-0)	0 (0-0)	0 (0-0)
Riau Islands	0 (0-0)	-15.4 (-16.8-9)	0 (0-0)	8.3 (-0.2-37.7)	0 (0-0)	6.7 (0.7-38.6)	0 (0-0)	3.8 (-4-39.2)	0 (0-0)	0 (0-0)	0 (0-0)	0 (0-0)

Location	Birth , 2000	Births percent change (2000-2021)	All age cases, 2000	All age cases percent change (2000-2021)	All age deaths, 2000	All age deaths percent change (2000-2021)	Under-5 deaths, 2000	Under-5 deaths percent change (2000-2021)	Births, 2021	All age cases, 2021	All age deaths, 2021	Under-5 deaths, 2021
Lampung	0 (0-0)	-31.1 (-32.8--17.6)	0 (0-1)	-10.5 (-19.3-13.4)	0 (0-0)	-14.6 (-19.9-6)	0 (0-0)	-27.1 (-33.3--8.8)	0 (0-0)	0 (0-1)	0 (0-0)	0 (0-0)
Maluku	0 (0-0)	-20.1 (-22.3-35.1)	0 (0-0)	7.4 (-2.7-81.6)	0 (0-0)	-0.1 (-5.9-64.2)	0 (0-0)	-11.6 (-18.3-39.7)	0 (0-0)	0 (0-0)	0 (0-0)	0 (0-0)
North Maluku	0 (0-0)	-19.4 (-21.5-36.5)	0 (0-0)	5.7 (-4.2-75.5)	0 (0-0)	-1 (-6.8-62.7)	0 (0-0)	-13 (-20.3-34.6)	0 (0-0)	0 (0-0)	0 (0-0)	0 (0-0)
West Nusa Tenggara	0 (0-0)	-14.1 (-17.8-49.4)	0 (0-1)	6.2 (-6.4-80.6)	0 (0-0)	2 (-5-85.5)	0 (0-0)	-7.4 (-17-71.5)	0 (0-0)	0 (0-1)	0 (0-0)	0 (0-0)
East Nusa Tenggara	0 (0-0)	-8.4 (-12.2-59.6)	0 (0-1)	20.1 (7.1-123)	0 (0-0)	11.9 (3.2-98.4)	0 (0-0)	-1.9 (-11.4-65.8)	0 (0-0)	0 (0-1)	0 (0-0)	0 (0-0)
Papua	0 (0-0)	63.2 (59.6-177)	0 (0-0)	103 (84.6-208)	0 (0-0)	92.6 (83.5-212)	0 (0-0)	75.1 (62.2-176)	0 (0-0)	0 (0-0)	0 (0-0)	0 (0-0)
West Papua	0 (0-0)	-16.6 (-19.1-63)	0 (0-0)	2.5 (-7.1-92.9)	0 (0-0)	-0.3 (-5.7-96)	0 (0-0)	-8.9 (-17-79.5)	0 (0-0)	0 (0-0)	0 (0-0)	0 (0-0)
Riau	0 (0-0)	51.7 (47.8-206)	0 (0-1)	95.4 (79.4-271)	0 (0-0)	88.6 (79-282)	0 (0-0)	66.6 (52.2-251)	0 (0-0)	0 (0-1)	0 (0-0)	0 (0-0)
West Sulawesi	0 (0-0)	-4.3 (-6.8-62)	0 (0-0)	28.1 (16-111)	0 (0-0)	18 (10.3-96.8)	0 (0-0)	2.3 (-5.5-72)	0 (0-0)	0 (0-0)	0 (0-0)	0 (0-0)
South Sulawesi	0 (0-0)	-28.2 (-29.8-12.6)	0 (0-1)	-4.2 (-12.5-45.6)	0 (0-0)	-9.1 (-14.6-42.6)	0 (0-0)	-23 (-28.6-22)	0 (0-0)	0 (0-1)	0 (0-0)	0 (0-0)
Central Sulawesi	0 (0-0)	-17.5 (-20.6-121)	0 (0-0)	13.3 (1.6-217)	0 (0-0)	5.6 (-2.2-183)	0 (0-0)	-10.5 (-19.9-144)	0 (0-0)	0 (0-0)	0 (0-0)	0 (0-0)
Southeast Sulawesi	0 (0-0)	-3.5 (-5.2-16)	0 (0-0)	23.1 (12.5-61.9)	0 (0-0)	17 (11.2-44.7)	0 (0-0)	4 (-4.2-25.3)	0 (0-0)	0 (0-0)	0 (0-0)	0 (0-0)
North Sulawesi	0 (0-0)	-42.7 (-42.9--42.9)	0 (0-0)	-19.9 (-25.6--15)	0 (0-0)	-24.4 (-27.9--20.9)	0 (0-0)	-39.8 (-44--35.1)	0 (0-0)	0 (0-0)	0 (0-0)	0 (0-0)
West Sumatra	0 (0-0)	-30.5 (-32.2--10.3)	0 (0-1)	-8.1 (-21.1-23)	0 (0-0)	-13 (-19.4-11.5)	0 (0-0)	-25.1 (-36.3--0.5)	0 (0-0)	0 (0-1)	0 (0-0)	0 (0-0)
South Sumatra	0 (0-0)	-24.4 (-24.6-12.4)	0 (0-1)	-2.9 (-12.3-44.3)	0 (0-0)	-7.2 (-11-37.2)	0 (0-0)	-18.1 (-24.3-20.4)	0 (0-0)	0 (0-1)	0 (0-0)	0 (0-0)
North Sumatra	0 (0-0)	-22.2 (-23.9-3.4)	1 (0-2)	-2.5 (-9.9-32.2)	0 (0-0)	-6.6 (-11.2-28.3)	0 (0-0)	-17.1 (-23.3-11.7)	0 (0-0)	1 (0-2)	0 (0-0)	0 (0-0)
Yogyakarta	0 (0-0)	-25.2 (-28-21.8)	0 (0-0)	-6.9 (-14.2-55.4)	0 (0-0)	-6 (-12.2-52.7)	0 (0-0)	-20.8 (-28.7-27.7)	0 (0-0)	0 (0-0)	0 (0-0)	0 (0-0)
Lao People's Democratic Republic	0 (0-0)	-5.4 (-5.7--5.7)	0 (0-1)	9 (0.6-16.3)	0 (0-0)	5.9 (2.7-9.3)	0 (0-0)	-0.2 (-6-6)	0 (0-0)	0 (0-1)	0 (0-0)	0 (0-0)
Malaysia	0 (0-0)	5.8 (5.4-5.5)	2 (0-3)	12.3 (7.2-17.5)	0 (0-0)	16.3 (11.5-20.5)	0 (0-0)	-2.9 (-12-7)	0 (0-0)	2 (0-4)	0 (0-0)	0 (0-0)
Maldives	0 (0-0)	16.7 (12.7-441)	0 (0-0)	38.4 (22-535)	0 (0-0)	39 (23.5-559)	0 (0-0)	17 (6-428)	0 (0-0)	0 (0-0)	0 (0-0)	0 (0-0)
Mauritius	0 (0-0)	-36.4 (-36.5--36.4)	0 (0-0)	-21.6 (-25.4--18.3)	0 (0-0)	-16.7 (-20.5--12.5)	0 (0-0)	-37.9 (-42.5--31.7)	0 (0-0)	0 (0-0)	0 (0-0)	0 (0-0)
Myanmar	0 (0-1)	-16.7 (-16.7--16.7)	2 (0-5)	-2.6 (-10.1-5.7)	0 (0-1)	-5.6 (-7.4--3.4)	0 (0-0)	-11.1 (-16.7--6.5)	0 (0-1)	2 (0-5)	0 (0-1)	0 (0-0)
Philippines	1 (0-1)	13.8 (8.6-17)	6 (2-14)	26.9 (22.4-30.4)	0 (0-1)	24.6 (19.7-28.2)	0 (0-1)	15.6 (10.7-19.2)	1 (0-2)	7 (3-18)	1 (0-1)	0 (0-1)
Abra	0 (0-0)	-16.4 (-18.8-32.1)	0 (0-0)	2.8 (-4.8-55.9)	0 (0-0)	1.8 (-3.5-59.9)	0 (0-0)	-10.4 (-19.2-37.9)	0 (0-0)	0 (0-0)	0 (0-0)	0 (0-0)
Agusan Del Norte	0 (0-0)	8.4 (8.6-8.6)	0 (0-0)	17.9 (9.5-25.5)	0 (0-0)	15 (12.8-17.6)	0 (0-0)	8.3 (0.8-16.4)	0 (0-0)	0 (0-0)	0 (0-0)	0 (0-0)
Agusan Del Sur	0 (0-0)	-7.7 (-7.8-5.3)	0 (0-0)	12.5 (3.3-27.5)	0 (0-0)	8 (4.7-22.5)	0 (0-0)	-0.4 (-7.8-16.8)	0 (0-0)	0 (0-0)	0 (0-0)	0 (0-0)
Aklan	0 (0-0)	19.7 (19.7-19.7)	0 (0-0)	22.9 (17.5-29.1)	0 (0-0)	24.2 (21.8-26.7)	0 (0-0)	18.4 (10.7-25.5)	0 (0-0)	0 (0-0)	0 (0-0)	0 (0-0)
Albay	0 (0-0)	4.8 (5.1-5.1)	0 (0-0)	12.6 (6.7-18.4)	0 (0-0)	10.6 (8.2-14)	0 (0-0)	2.7 (-3.8-8.7)	0 (0-0)	0 (0-0)	0 (0-0)	0 (0-0)
Antique	0 (0-0)	4.4 (4.4-4.4)	0 (0-0)	16.5 (10.6-22.6)	0 (0-0)	13.8 (11.4-16.2)	0 (0-0)	4.2 (-1.9-10.1)	0 (0-0)	0 (0-0)	0 (0-0)	0 (0-0)

Location	Birth , 2000	Births percent change (2000-2021)	All age cases, 2000	All age cases percent change (2000-2021)	All age deaths, 2000	All age deaths percent change (2000-2021)	Under-5 deaths, 2000	Under-5 deaths percent change (2000-2021)	Births, 2021	All age cases, 2021	All age deaths, 2021	Under-5 deaths, 2021
Apayao	0 (0-0)	-23 (-23.1--23.1)	0 (0-0)	3.4 (-4.8-13.9)	0 (0-0)	-2.2 (-6.4-2.5)	0 (0-0)	-11.9 (-18.7--3)	0 (0-0)	0 (0-0)	0 (0-0)	0 (0-0)
Aurora	0 (0-0)	-2.3 (-23.4--0.9)	0 (0-0)	13.7 (-5.2-33.5)	0 (0-0)	10.8 (-13.3-16.9)	0 (0-0)	3.1 (-21.6-13.4)	0 (0-0)	0 (0-0)	0 (0-0)	0 (0-0)
Basilan	0 (0-0)	28.9 (28.9-28.9)	0 (0-0)	48.3 (41.2-55.5)	0 (0-0)	44.6 (41.3-48.4)	0 (0-0)	34.6 (26.7-44.3)	0 (0-0)	0 (0-0)	0 (0-0)	0 (0-0)
Bataan	0 (0-0)	14 (14-14)	0 (0-0)	27.4 (21.6-32.8)	0 (0-0)	26.8 (24.1-30.1)	0 (0-0)	16.2 (10-22.7)	0 (0-0)	0 (0-0)	0 (0-0)	0 (0-0)
Batanes	0 (0-0)	-28.7 (-28.3--28.3)	0 (0-0)	-10.2 (-14.9--4.9)	0 (0-0)	-13.6 (-16--10.5)	0 (0-0)	-24.4 (-29.8--19.1)	0 (0-0)	0 (0-0)	0 (0-0)	0 (0-0)
Batangas	0 (0-0)	42.9 (42.4-42.4)	0 (0-0)	43.7 (37.1-51.2)	0 (0-0)	44.9 (41.4-47.9)	0 (0-0)	38.9 (28.5-47.2)	0 (0-0)	0 (0-0)	0 (0-0)	0 (0-0)
Benguet	0 (0-0)	26.3 (29.3-29.3)	0 (0-0)	28.6 (22.5-38.1)	0 (0-0)	31.2 (30.5-38.1)	0 (0-0)	25.1 (17.3-36.1)	0 (0-0)	0 (0-0)	0 (0-0)	0 (0-0)
Biliran	0 (0-0)	0.3 (0.9-0.9)	0 (0-0)	12.8 (7.3-18.3)	0 (0-0)	12.8 (10.6-16)	0 (0-0)	2.9 (-3.7-10.4)	0 (0-0)	0 (0-0)	0 (0-0)	0 (0-0)
Bohol	0 (0-0)	-23.1 (-23.5--23.5)	0 (0-0)	0.2 (-5.4-5.1)	0 (0-0)	-4.1 (-7.4--1.2)	0 (0-0)	-17.1 (-22.7--10.7)	0 (0-0)	0 (0-0)	0 (0-0)	0 (0-0)
Bukidnon	0 (0-0)	-8.1 (-19.3--6.9)	0 (0-0)	16.4 (0.2-26.5)	0 (0-0)	10.6 (-3.3-15.9)	0 (0-0)	0.3 (-12.2-7.9)	0 (0-0)	0 (0-0)	0 (0-0)	0 (0-0)
Bulacan	0 (0-0)	30.6 (30.6-30.6)	0 (0-0)	44.2 (37.2-51)	0 (0-0)	41.6 (37.4-46.4)	0 (0-0)	26.8 (18.5-37)	0 (0-0)	0 (0-1)	0 (0-0)	0 (0-0)
Cagayan	0 (0-0)	0.7 (-1.6-33.9)	0 (0-0)	10.8 (2.4-46.6)	0 (0-0)	9.7 (4.4-48.8)	0 (0-0)	2.8 (-5.3-37.9)	0 (0-0)	0 (0-0)	0 (0-0)	0 (0-0)
Camarines Norte	0 (0-0)	3.8 (3.8-3.8)	0 (0-0)	16.5 (10.6-22)	0 (0-0)	14.5 (12.1-17.4)	0 (0-0)	6.3 (0-12.9)	0 (0-0)	0 (0-0)	0 (0-0)	0 (0-0)
Camarines Sur	0 (0-0)	11.1 (8.4-43.7)	0 (0-0)	23.8 (12.4-62.8)	0 (0-0)	20.6 (15.2-59.1)	0 (0-0)	12.8 (3.4-51.7)	0 (0-0)	0 (0-0)	0 (0-0)	0 (0-0)
Camiguin	0 (0-0)	3.2 (4-4)	0 (0-0)	11.4 (6.6-16.3)	0 (0-0)	11.4 (9.8-14.3)	0 (0-0)	5 (-3.1-15.1)	0 (0-0)	0 (0-0)	0 (0-0)	0 (0-0)
Capiz	0 (0-0)	-0.4 (-1--1)	0 (0-0)	5.2 (-1.2-11.6)	0 (0-0)	4 (1.2-6.3)	0 (0-0)	-2.6 (-8.9-2.2)	0 (0-0)	0 (0-0)	0 (0-0)	0 (0-0)
Catanduanes	0 (0-0)	-8.6 (-8.6--8.6)	0 (0-0)	10.4 (3-16.6)	0 (0-0)	6 (3.3-9.1)	0 (0-0)	-2.3 (-7.1-3.8)	0 (0-0)	0 (0-0)	0 (0-0)	0 (0-0)
Cavite	0 (0-0)	84.2 (79.9-79.9)	0 (0-0)	92.2 (78.8-99.6)	0 (0-0)	91.9 (82.5-94.1)	0 (0-0)	77.5 (62.7-92.6)	0 (0-0)	0 (0-1)	0 (0-0)	0 (0-0)
Cebu	0 (0-0)	34.9 (34.8-34.8)	0 (0-1)	40.9 (33.7-49.5)	0 (0-0)	40.9 (38.6-43.5)	0 (0-0)	36.9 (28.9-45.8)	0 (0-0)	0 (0-1)	0 (0-0)	0 (0-0)
Cotabato (North Cotabato)	0 (0-0)	32.8 (34.2-34.3)	0 (0-0)	43.3 (30.8-51.7)	0 (0-0)	43.5 (41.1-48.1)	0 (0-0)	39.6 (29.6-50.2)	0 (0-0)	0 (0-0)	0 (0-0)	0 (0-0)
Davao de Oro	0 (0-0)	-10.2 (-38.2--8.2)	0 (0-0)	12.2 (-21.1-20.6)	0 (0-0)	9.7 (-24.5-15.2)	0 (0-0)	0.1 (-25.8-7.7)	0 (0-0)	0 (0-0)	0 (0-0)	0 (0-0)
Davao Del Norte	0 (0-0)	-12.2 (-12.2--12.2)	0 (0-0)	16.1 (10.4-21.5)	0 (0-0)	14.3 (10.9-18)	0 (0-0)	-2.8 (-10.3-3.3)	0 (0-0)	0 (0-0)	0 (0-0)	0 (0-0)
Davao Del Sur	0 (0-0)	24.1 (25.4-25.4)	0 (0-0)	32.2 (24.8-40.5)	0 (0-0)	32.3 (30.7-36.5)	0 (0-0)	26.8 (20-36.1)	0 (0-0)	0 (0-0)	0 (0-0)	0 (0-0)
Davao Occidental	0 (0-0)	-12.7 (-12.7--12.7)	0 (0-0)	28.1 (19.8-35.8)	0 (0-0)	16.9 (12.2-22.9)	0 (0-0)	-3.2 (-10.3-3.1)	0 (0-0)	0 (0-0)	0 (0-0)	0 (0-0)
Davao Oriental	0 (0-0)	-14.3 (-14.3--14.3)	0 (0-0)	8.8 (1.9-15.3)	0 (0-0)	1.5 (-1.9-5.2)	0 (0-0)	-10.8 (-15.4--5.8)	0 (0-0)	0 (0-0)	0 (0-0)	0 (0-0)
Dinagat Islands	0 (0-0)	-30.3 (-30.1--30.1)	0 (0-0)	-7 (-13.2-0.4)	0 (0-0)	-10.1 (-13--6.6)	0 (0-0)	-19.7 (-25.5--12.4)	0 (0-0)	0 (0-0)	0 (0-0)	0 (0-0)
Eastern Samar	0 (0-0)	4.4 (5.6-5.6)	0 (0-0)	16.9 (9.3-26.2)	0 (0-0)	12.6 (10.6-17)	0 (0-0)	6.7 (0-14.7)	0 (0-0)	0 (0-0)	0 (0-0)	0 (0-0)
Guimaras	0 (0-0)	-0.1 (-0.9--0.9)	0 (0-0)	12.4 (6.5-18.8)	0 (0-0)	13.4 (9.3-16.1)	0 (0-0)	6.1 (-2.6-15.5)	0 (0-0)	0 (0-0)	0 (0-0)	0 (0-0)
Ifugao	0 (0-0)	16.1 (15.7-15.7)	0 (0-0)	19.6 (13-25.4)	0 (0-0)	22.7 (19.5-25.6)	0 (0-0)	22.4 (14.1-31.1)	0 (0-0)	0 (0-0)	0 (0-0)	0 (0-0)

Location	Birth , 2000	Births percent change (2000-2021)	All age cases, 2000	All age cases percent change (2000-2021)	All age deaths, 2000	All age deaths percent change (2000-2021)	Under-5 deaths, 2000	Under-5 deaths percent change (2000-2021)	Births, 2021	All age cases, 2021	All age deaths, 2021	Under-5 deaths, 2021
Ilocos Norte	0 (0-0)	3.1 (3.1-3.1)	0 (0-0)	7.2 (2.2-12.3)	0 (0-0)	8 (6.5-9.9)	0 (0-0)	5.4 (-1.3-12.5)	0 (0-0)	0 (0-0)	0 (0-0)	0 (0-0)
Ilocos Sur	0 (0-0)	-3.1 (-3.1--3.1)	0 (0-0)	6.5 (1.8-12.2)	0 (0-0)	6.8 (4.7-8.9)	0 (0-0)	1.1 (-6.1-8)	0 (0-0)	0 (0-0)	0 (0-0)	0 (0-0)
Iloilo	0 (0-0)	19.9 (19.9-19.9)	0 (0-0)	20.3 (15.1-25.9)	0 (0-0)	22.1 (20-24.4)	0 (0-0)	19.9 (11.6-29.6)	0 (0-0)	0 (0-0)	0 (0-0)	0 (0-0)
Isabela	0 (0-0)	13.7 (14.3-14.3)	0 (0-0)	15.6 (7.7-22.4)	0 (0-0)	16.9 (15.1-20.2)	0 (0-0)	14.4 (6.4-24.1)	0 (0-0)	0 (0-0)	0 (0-0)	0 (0-0)
Kalinga	0 (0-0)	-14.7 (-14.7--14.7)	0 (0-0)	9.5 (4.6-14)	0 (0-0)	8 (5-11.4)	0 (0-0)	-3.9 (-9.3-3.3)	0 (0-0)	0 (0-0)	0 (0-0)	0 (0-0)
La Union	0 (0-0)	6.3 (6.3-6.3)	0 (0-0)	9.3 (4.3-15.1)	0 (0-0)	10.7 (8.7-13.2)	0 (0-0)	8.1 (1.4-14.8)	0 (0-0)	0 (0-0)	0 (0-0)	0 (0-0)
Laguna	0 (0-0)	56.8 (56.3-56.3)	0 (0-0)	58.6 (52.3-64.9)	0 (0-0)	61.1 (57.6-64)	0 (0-0)	57.8 (47.9-67.6)	0 (0-0)	0 (0-1)	0 (0-0)	0 (0-0)
Lanao Del Norte	0 (0-0)	15.9 (15.2-15.2)	0 (0-0)	33.4 (26.9-40.9)	0 (0-0)	32.1 (28.6-34.6)	0 (0-0)	24.8 (16.6-36)	0 (0-0)	0 (0-0)	0 (0-0)	0 (0-0)
Lanao Del Sur	0 (0-0)	14.4 (-22-15.6)	0 (0-0)	33.8 (-6.6-44.1)	0 (0-0)	31.9 (-9.1-36.9)	0 (0-0)	26.7 (-11.5-36.4)	0 (0-0)	0 (0-0)	0 (0-0)	0 (0-0)
Leyte	0 (0-0)	11.1 (11.1-11.1)	0 (0-0)	18.7 (12.5-25.7)	0 (0-0)	17.1 (14.3-20.3)	0 (0-0)	11.1 (4.4-18.4)	0 (0-0)	0 (0-0)	0 (0-0)	0 (0-0)
Maguindanao	0 (0-0)	34.7 (31.7-95.3)	0 (0-0)	68 (54.6-155)	0 (0-0)	61.9 (54.3-137)	0 (0-0)	51.1 (38.7-125)	0 (0-0)	0 (0-0)	0 (0-0)	0 (0-0)
Marinduque	0 (0-0)	-30 (-29.3--29.3)	0 (0-0)	-11.1 (-16.3--5.5)	0 (0-0)	-15.8 (-18.3--11.7)	0 (0-0)	-27.8 (-33.7--21.9)	0 (0-0)	0 (0-0)	0 (0-0)	0 (0-0)
Masbate	0 (0-0)	7.1 (-26.8-8.7)	0 (0-0)	22.5 (-16-33.3)	0 (0-0)	17.8 (-18.7-23)	0 (0-0)	9.7 (-20.7-21.6)	0 (0-0)	0 (0-0)	0 (0-0)	0 (0-0)
Misamis Occidental	0 (0-0)	7.5 (-42.9-9.8)	0 (0-0)	15.6 (-37.3-24.4)	0 (0-0)	14.4 (-39.9-19)	0 (0-0)	10 (-42-20.4)	0 (0-0)	0 (0-0)	0 (0-0)	0 (0-0)
Misamis Oriental	0 (0-0)	34.5 (34.6-64.4)	0 (0-0)	38.9 (23.5-64.7)	0 (0-0)	38.6 (35.6-69.3)	0 (0-0)	34.7 (22.9-62.7)	0 (0-0)	0 (0-0)	0 (0-0)	0 (0-0)
Mountain Province	0 (0-0)	-11.1 (-11.6-16.1)	0 (0-0)	-0.4 (-6.8-27.1)	0 (0-0)	-1 (-3.4-27.4)	0 (0-0)	-6.2 (-12.8-19.2)	0 (0-0)	0 (0-0)	0 (0-0)	0 (0-0)
National Capital Region	0 (0-0)	1.4 (0.9-0.9)	1 (0-2)	19.6 (13-27.3)	0 (0-0)	18.2 (14-21.7)	0 (0-0)	3.7 (-3.1-11.4)	0 (0-0)	1 (0-2)	0 (0-0)	0 (0-0)
Negros Occidental	0 (0-0)	2.3 (2-2)	0 (0-0)	8.5 (3.5-14.2)	0 (0-0)	7.6 (4.9-10.1)	0 (0-0)	1.6 (-5.4-7.3)	0 (0-0)	0 (0-1)	0 (0-0)	0 (0-0)
Negros Oriental	0 (0-0)	-2.4 (-3.5-17.9)	0 (0-0)	8.5 (1.4-37.8)	0 (0-0)	6.5 (2.9-29.4)	0 (0-0)	-0.3 (-8.1-20)	0 (0-0)	0 (0-0)	0 (0-0)	0 (0-0)
Northern Samar	0 (0-0)	11.9 (-24.4-12.5)	0 (0-0)	22.2 (-19.5-36.2)	0 (0-0)	18.9 (-18.6-24.7)	0 (0-0)	12.2 (-22.3-25.1)	0 (0-0)	0 (0-0)	0 (0-0)	0 (0-0)
Nueva Ecija	0 (0-0)	21.3 (20.7-20.7)	0 (0-0)	28.9 (22-38)	0 (0-0)	29.1 (26-31.4)	0 (0-0)	24.4 (14.3-34.2)	0 (0-0)	0 (0-0)	0 (0-0)	0 (0-0)
Nueva Vizcaya	0 (0-0)	4.5 (4.5-4.5)	0 (0-0)	13.8 (8.9-19.7)	0 (0-0)	14.3 (12.1-16.6)	0 (0-0)	9.3 (2-16.4)	0 (0-0)	0 (0-0)	0 (0-0)	0 (0-0)
Occidental Mindoro	0 (0-0)	-2.7 (-5-29.3)	0 (0-0)	19.9 (10.2-58.5)	0 (0-0)	16.2 (10.5-59.8)	0 (0-0)	7 (-1.9-48.6)	0 (0-0)	0 (0-0)	0 (0-0)	0 (0-0)
Oriental Mindoro	0 (0-0)	-7.9 (-8.2--8.2)	0 (0-0)	10.8 (2.3-18.6)	0 (0-0)	5.2 (2-8.4)	0 (0-0)	-6.2 (-13.5-1.6)	0 (0-0)	0 (0-0)	0 (0-0)	0 (0-0)
Palawan	0 (0-0)	25.3 (24.7-24.8)	0 (0-0)	44.3 (36-51.8)	0 (0-0)	39.7 (35.8-43.3)	0 (0-0)	28.8 (19.8-39.3)	0 (0-0)	0 (0-0)	0 (0-0)	0 (0-0)
Pampanga	0 (0-0)	33.9 (31.3-32.6)	0 (0-0)	39.4 (29.8-49.9)	0 (0-0)	39 (33.2-42.6)	0 (0-0)	30.7 (19.1-48.1)	0 (0-0)	0 (0-1)	0 (0-0)	0 (0-0)
Pangasinan	0 (0-0)	7.6 (7.6-7.6)	0 (0-0)	14.7 (10-19.9)	0 (0-0)	14.4 (12.5-16.6)	0 (0-0)	9.5 (3.1-15.2)	0 (0-0)	0 (0-1)	0 (0-0)	0 (0-0)
Quezon	0 (0-0)	8.5 (8.9-8.9)	0 (0-0)	18.9 (12.9-25.3)	0 (0-0)	17.1 (15-20.4)	0 (0-0)	9.3 (2.2-17.1)	0 (0-0)	0 (0-0)	0 (0-0)	0 (0-0)
Quirino	0 (0-0)	-14.7 (-25.3--13.8)	0 (0-0)	7 (-6.4-16)	0 (0-0)	3.3 (-9.3-7.3)	0 (0-0)	-5.4 (-19.4-2.7)	0 (0-0)	0 (0-0)	0 (0-0)	0 (0-0)

Location	Birth , 2000	Births percent change (2000-2021)	All age cases, 2000	All age cases percent change (2000-2021)	All age deaths, 2000	All age deaths percent change (2000-2021)	Under-5 deaths, 2000	Under-5 deaths percent change (2000-2021)	Births, 2021	All age cases, 2021	All age deaths, 2021	Under-5 deaths, 2021
Rizal	0 (0-0)	75.8 (76.3-76.3)	0 (0-0)	80.2 (72.7-87.9)	0 (0-0)	77.5 (73.8-82.4)	0 (0-0)	69.1 (60.6-80.2)	0 (0-0)	0 (0-1)	0 (0-0)	0 (0-0)
Romblon	0 (0-0)	-18.8 (-18.6--18.5)	0 (0-0)	-3 (-9.7-4)	0 (0-0)	-6.4 (-8.9--3.2)	0 (0-0)	-14.4 (-21--7.6)	0 (0-0)	0 (0-0)	0 (0-0)	0 (0-0)
Samar (Western Samar)	0 (0-0)	2.2 (2.2-2.2)	0 (0-0)	12.6 (4.6-21.1)	0 (0-0)	6.8 (4.2-10.1)	0 (0-0)	1.5 (-3.6-8.1)	0 (0-0)	0 (0-0)	0 (0-0)	0 (0-0)
Sarangani	0 (0-0)	15.9 (16.5-16.5)	0 (0-0)	24.6 (17.8-30.8)	0 (0-0)	22.3 (19.1-26.8)	0 (0-0)	13.1 (6-20.7)	0 (0-0)	0 (0-0)	0 (0-0)	0 (0-0)
Siquijor	0 (0-0)	-12.6 (-12.6--12.6)	0 (0-0)	5.6 (0.5-11.4)	0 (0-0)	4.3 (1.7-7.3)	0 (0-0)	-6.4 (-12.7--0.1)	0 (0-0)	0 (0-0)	0 (0-0)	0 (0-0)
Sorsogon	0 (0-0)	11.1 (10.2-44.6)	0 (0-0)	16.7 (9.8-44.2)	0 (0-0)	15.1 (11.3-48.7)	0 (0-0)	7.1 (0.5-36.2)	0 (0-0)	0 (0-0)	0 (0-0)	0 (0-0)
South Cotabato	0 (0-0)	18.8 (20.1-20.1)	0 (0-0)	27.8 (18.2-37.7)	0 (0-0)	26.4 (24.6-30.4)	0 (0-0)	20 (6.3-28.8)	0 (0-0)	0 (0-0)	0 (0-0)	0 (0-0)
Southern Leyte	0 (0-0)	-4.4 (-2.8--2.7)	0 (0-0)	5.9 (0.5-13.1)	0 (0-0)	3.3 (2.4-7.8)	0 (0-0)	-3 (-8.2-4.3)	0 (0-0)	0 (0-0)	0 (0-0)	0 (0-0)
Sultan Kudarat	0 (0-0)	-0.3 (-0.3--0.3)	0 (0-0)	26.6 (20-32.6)	0 (0-0)	23.3 (19.8-26.9)	0 (0-0)	10.2 (4-17.9)	0 (0-0)	0 (0-0)	0 (0-0)	0 (0-0)
Sulu	0 (0-0)	-10.4 (-10.4--10.4)	0 (0-0)	28.1 (17.1-36.9)	0 (0-0)	17.1 (12.6-22.3)	0 (0-0)	3.9 (-1.3-10.5)	0 (0-0)	0 (0-0)	0 (0-0)	0 (0-0)
Surigao Del Norte	0 (0-0)	18 (-42.6-19.5)	0 (0-0)	23.9 (-35.6-36)	0 (0-0)	22.8 (-39.3-28)	0 (0-0)	16.3 (-42.7-30)	0 (0-0)	0 (0-0)	0 (0-0)	0 (0-0)
Surigao Del Sur	0 (0-0)	-1.8 (-3.5-43.2)	0 (0-0)	6.4 (-2.5-57.2)	0 (0-0)	4.5 (0.3-51.7)	0 (0-0)	-1.4 (-11-41.8)	0 (0-0)	0 (0-0)	0 (0-0)	0 (0-0)
Tarlac	0 (0-0)	3.4 (3.4-3.4)	0 (0-0)	16.7 (10.9-22.3)	0 (0-0)	15.1 (12.3-17.8)	0 (0-0)	4.8 (-2.2-12)	0 (0-0)	0 (0-0)	0 (0-0)	0 (0-0)
Tawi-Tawi	0 (0-0)	-1.7 (-4--4)	0 (0-0)	17.7 (9.3-24.2)	0 (0-0)	16 (10.5-17.2)	0 (0-0)	6.7 (-2.6-18)	0 (0-0)	0 (0-0)	0 (0-0)	0 (0-0)
Zambales	0 (0-0)	19.6 (-8.2-20.5)	0 (0-0)	25.1 (0.2-34.1)	0 (0-0)	24.9 (-2.8-28.5)	0 (0-0)	18.1 (-4.7-26)	0 (0-0)	0 (0-0)	0 (0-0)	0 (0-0)
Zamboanga Del Norte	0 (0-0)	-5.3 (-6.3--6.2)	0 (0-0)	12.4 (5.6-18.9)	0 (0-0)	9.3 (5.7-11)	0 (0-0)	1 (-6-7.3)	0 (0-0)	0 (0-0)	0 (0-0)	0 (0-0)
Zamboanga Del Sur	0 (0-0)	8.3 (8.9-8.9)	0 (0-0)	17.5 (11.1-24)	0 (0-0)	16.7 (14.5-20.2)	0 (0-0)	9.7 (1.5-17.3)	0 (0-0)	0 (0-0)	0 (0-0)	0 (0-0)
Zamboanga Sibugay	0 (0-0)	8.8 (8.2-8.7)	0 (0-0)	28.9 (21.1-40.3)	0 (0-0)	24.7 (21.1-30)	0 (0-0)	14 (6.7-27.2)	0 (0-0)	0 (0-0)	0 (0-0)	0 (0-0)
Seychelles	0 (0-0)	-6.7 (-6.6--6.6)	0 (0-0)	2.4 (-2-6.2)	0 (0-0)	7.5 (3.9-11.7)	0 (0-0)	-1.5 (-8.2-5.9)	0 (0-0)	0 (0-0)	0 (0-0)	0 (0-0)
Sri Lanka	0 (0-0)	-17.6 (-18--17.8)	1 (0-2)	-0.3 (-5.4-4.1)	0 (0-0)	0 (-2.8-2.2)	0 (0-0)	-11.6 (-18.4--4.8)	0 (0-0)	1 (0-2)	0 (0-0)	0 (0-0)
Thailand	0 (0-0)	-39.5 (-39.5--39.5)	3 (1-6)	-23.1 (-27.5--19.3)	0 (0-0)	-21.4 (-25--17.2)	0 (0-0)	-38.7 (-42.2--34.1)	0 (0-0)	2 (1-5)	0 (0-0)	0 (0-0)
Timor-Leste	0 (0-0)	4.1 (3.7-3.7)	0 (0-0)	35.8 (26.6-46)	0 (0-0)	23.9 (19.1-29.1)	0 (0-0)	12.7 (6.1-19.5)	0 (0-0)	0 (0-0)	0 (0-0)	0 (0-0)
Viet Nam	0 (0-1)	-20.4 (-20.4--19.9)	5 (1-9)	-6.6 (-11--1.3)	0 (0-1)	-3.8 (-8.2-0.9)	0 (0-0)	-14.9 (-21.6--9.5)	0 (0-0)	4 (1-9)	0 (0-1)	0 (0-0)
Sub-Saharan Africa	86,200 (67,300-110,000)	15.1 (9.8-20)	912,000 (730,000-1,140,000)	42.6 (35-49)	43,000 (32,700-56,500)	53.1 (44.6-60.1)	9,580 (6,120-14,700)	39.9 (30.7-48)	99,200 (74,700-130,000)	1,300,000 (994,000-1,670,000)	65,900 (48,600-88,500)	13,400 (8,320-20,800)
**Central Sub-Saharan Africa**	2,520 (1,770-3,420)	28.4 (17.8-39.8)	22,200 (15,900-29,600)	69.6 (56.9-84.4)	1,420 (966-2,000)	74.8 (61.1-91)	597 (373-911)	49.2 (34.9-67.3)	3,240 (2,290-4,510)	37,700 (26,900-50,300)	2,480 (1,700-3,530)	890 (548-1,380)
Angola	201 (144-272)	47.2 (34.9-60.2)	1,610 (1,160-2,160)	96 (80.1-114)	108 (76-152)	92.7 (74.4-112)	48 (30-76)	74.3 (50.2-99.3)	295 (208-402)	3,160 (2,270-4,190)	208 (144-294)	84 (51-132)
Central African Republic	13 (9-18)	25.9 (15.9-35.9)	106 (76-142)	42.8 (31.7-53.8)	7 (5-10)	45.5 (33.5-58.2)	3 (2-5)	37 (21.7-58.1)	16 (11-22)	151 (108-203)	11 (8-15)	4 (3-7)
Congo	99 (68-137)	3.7 (-6.4-13.5)	991 (697-1,340)	37.1 (24.6-50.9)	61 (42-86)	34.7 (20.9-48)	20 (12-31)	23.5 (8.6-41.6)	103 (71-143)	1,360 (946-1,820)	82 (56-117)	25 (15-39)
Democratic Republic of the Congo	1,840 (1,270-2,520)	31.2 (18.1-46.2)	15,900 (11,200-21,300)	71.8 (55.2-91.8)	1,030 (696-1,460)	75.7 (57.3-97.1)	445 (273-687)	51.8 (33.4-75.8)	2,420 (1,690-3,380)	27,300 (19,300-36,600)	1,810 (1,240-2,610)	676 (411-1,050)

Location	Birth , 2000	Births percent change (2000-2021)	All age cases, 2000	All age cases percent change (2000-2021)	All age deaths, 2000	All age deaths percent change (2000-2021)	Under-5 deaths, 2000	Under-5 deaths percent change (2000-2021)	Births, 2021	All age cases, 2021	All age deaths, 2021	Under-5 deaths, 2021
Equatorial Guinea	249 (175-344)	19.7 (6.7-35.3)	2,260 (1,630-3,020)	84.3 (63.8-109)	138 (95-197)	102 (76.6-133)	57 (36-90)	37.3 (18.8-60.1)	298 (212-406)	4,170 (3,000-5,580)	278 (196-382)	78 (48-122)
Gabon	116 (80-160)	-7.6 (-17.1-3.2)	1,310 (924-1,770)	12.6 (1.8-25.5)	74 (50-104)	21.7 (9.2-36.4)	23 (14-35)	-1.5 (-14.4-13.6)	107 (76-149)	1,480 (1,070-1,980)	90 (64-126)	22 (14-35)
**Eastern Sub-Saharan Africa**	231 (167-318)	29.8 (26.1-34.2)	1,990 (1,470-2,660)	59.5 (54.8-64.5)	133 (92-188)	63.1 (57-70.1)	52 (32-81)	45 (38.2-52.4)	300 (216-413)	3,180 (2,330-4,300)	217 (152-305)	75 (46-117)
Burundi	5 (4-8)	83.7 (65.4-99.1)	49 (33-67)	92.4 (74.4-107)	3 (2-5)	97 (76.7-116)	1 (1-2)	96.4 (71.7-124)	10 (7-14)	94 (64-130)	7 (4-9)	3 (2-4)
Comoros	0 (0-0)	-20.2 (-30.1--14.2)	3 (2-3)	3.1 (-9.4-12.4)	0 (0-0)	9.8 (-3.5-20.5)	0 (0-0)	-12.9 (-25.8--3.4)	0 (0-0)	3 (2-4)	0 (0-0)	0 (0-0)
Djibouti	0 (0-0)	22.4 (-0.2-23.6)	2 (1-3)	59.6 (30.2-66.6)	0 (0-0)	60.9 (32.2-66.8)	0 (0-0)	49.2 (22.3-65.2)	0 (0-0)	3 (2-4)	0 (0-0)	0 (0-0)
Eritrea	1 (1-2)	18.2 (-0.6-26)	14 (9-19)	44.6 (23.1-53.5)	1 (1-1)	51.4 (26.6-59.7)	0 (0-1)	27.2 (2.2-42.1)	2 (1-2)	20 (14-28)	1 (1-2)	0 (0-1)
Ethiopia	26 (17-36)	24.6 (12.9-27.3)	233 (158-319)	47.7 (36-52.6)	16 (10-22)	53.1 (38.5-58.6)	6 (3-9)	37.5 (22.6-44.6)	32 (22-46)	344 (236-473)	24 (16-34)	8 (4-12)
Addis Ababa	1 (0-1)	-26 (-42.2--24.6)	7 (5-10)	-2.8 (-23.5-2.1)	0 (0-1)	-3 (-25.2-3.6)	0 (0-0)	-3.7 (-26.6-8.1)	0 (0-1)	7 (5-10)	0 (0-1)	0 (0-0)
Afar	1 (0-1)	-5.3 (-24.6--0.8)	5 (3-7)	4.8 (-15.6-11.8)	0 (0-1)	6.8 (-14.5-12.2)	0 (0-0)	6.3 (-15.4-19.7)	1 (0-1)	5 (4-8)	0 (0-1)	0 (0-0)
Amhara	6 (4-8)	4.5 (-11.9-8.6)	54 (36-75)	23.3 (5.2-29.3)	4 (2-5)	30.3 (9.4-36.5)	1 (1-2)	13.4 (-5.9-24.7)	6 (4-9)	66 (44-92)	5 (3-7)	1 (1-2)
Benishangul-Gumuz	0 (0-0)	42.6 (15-50.2)	2 (1-3)	76.6 (43.8-89.8)	0 (0-0)	82.1 (46.1-92.3)	0 (0-0)	62.5 (27.1-79.1)	0 (0-1)	4 (2-5)	0 (0-0)	0 (0-0)
Dire Dawa	0 (0-0)	5.9 (-11-7)	1 (1-1)	44.3 (23.1-51.1)	0 (0-0)	48.1 (24.7-53.6)	0 (0-0)	26 (3.9-40.3)	0 (0-0)	1 (1-2)	0 (0-0)	0 (0-0)
Gambella	0 (0-0)	30.8 (10-35.9)	1 (1-1)	88.4 (58.6-99.3)	0 (0-0)	99.7 (66.5-112)	0 (0-0)	51.5 (24-64.9)	0 (0-0)	2 (1-2)	0 (0-0)	0 (0-0)
Harari	0 (0-0)	-7.2 (-24.1--6)	0 (0-1)	37.2 (12.6-45.7)	0 (0-0)	42.5 (18-49.5)	0 (0-0)	13.5 (-8.1-23.6)	0 (0-0)	1 (0-1)	0 (0-0)	0 (0-0)
Oromia	10 (6-14)	35 (9.3-37.6)	85 (57-118)	62.1 (34.3-71.2)	6 (4-8)	66.6 (33.9-74.2)	2 (1-3)	48.3 (15.7-63.2)	13 (9-19)	138 (93-191)	9 (6-14)	3 (2-5)
Somali	2 (1-2)	48.3 (25.5-58.2)	16 (11-22)	48.9 (25.6-59.2)	1 (1-2)	50.3 (26.7-61.7)	0 (0-1)	62.9 (34.1-80.6)	2 (2-4)	24 (16-33)	2 (1-2)	1 (0-1)
Southern Nations, Nationalities, and Peoples	6 (4-8)	32.6 (11.4-37.4)	49 (32-68)	61.3 (36.1-70.1)	3 (2-5)	68.1 (41.3-76.9)	1 (1-2)	44.8 (20.2-58.6)	7 (5-11)	79 (52-110)	6 (4-8)	2 (1-3)
Tigray	1 (1-2)	8 (-7-12.2)	13 (8-18)	41.6 (23.9-48.6)	1 (1-1)	51.2 (31-60.1)	0 (0-0)	22.1 (3.8-35.9)	2 (1-2)	18 (12-25)	1 (1-2)	0 (0-1)
Kenya	24 (18-33)	6.9 (4.4-9.3)	248 (187-336)	36.5 (33.3-39.4)	15 (11-21)	45.4 (40.9-49.9)	4 (3-7)	19.8 (15.5-23.9)	26 (19-35)	339 (257-450)	22 (16-30)	5 (3-8)
Baringo	0 (0-0)	-0.4 (-10.1-9.3)	2 (2-3)	28.1 (15.1-40.4)	0 (0-0)	38.4 (21.8-52.8)	0 (0-0)	7.4 (-7.4-24.4)	0 (0-0)	3 (2-4)	0 (0-0)	0 (0-0)
Bomet	0 (0-0)	96.8 (74.1-126)	3 (2-4)	160 (131-197)	0 (0-0)	195 (161-240)	0 (0-0)	106 (77.7-142)	1 (0-1)	8 (6-11)	0 (0-1)	0 (0-0)
Bungoma	2 (1-3)	-54.9 (-58.1--50)	19 (13-26)	-38.2 (-42.1--32.4)	1 (1-2)	-36.1 (-41.4--28.4)	0 (0-1)	-47.2 (-53.3--39.1)	1 (1-1)	12 (8-16)	1 (0-1)	0 (0-0)
Busia	1 (1-2)	12.1 (3.2-22.6)	11 (8-16)	36.2 (26.7-48.7)	1 (0-1)	44 (30.6-59.4)	0 (0-0)	20.5 (6.9-36)	1 (1-2)	15 (11-21)	1 (1-1)	0 (0-0)
Elgeyo-Marakwet	0 (0-0)	-72.3 (-74.5--69.6)	3 (2-4)	-59.4 (-62.8--55.7)	0 (0-0)	-55.3 (-59.2--50.5)	0 (0-0)	-66.8 (-71.2--62.4)	0 (0-0)	1 (1-2)	0 (0-0)	0 (0-0)
Embu	0 (0-0)	-40.3 (-50.7--34.7)	2 (1-2)	-16.3 (-30.1--8.6)	0 (0-0)	-9.3 (-25.3--0.4)	0 (0-0)	-33.6 (-46.4--25.6)	0 (0-0)	1 (1-2)	0 (0-0)	0 (0-0)
Garissa	0 (0-0)	257 (195-268)	1 (1-2)	250 (187-268)	0 (0-0)	264 (197-279)	0 (0-0)	247 (176-289)	0 (0-1)	5 (3-7)	0 (0-0)	0 (0-0)
Homa Bay	2 (2-3)	2.9 (-4.2-10.6)	20 (14-28)	39.3 (28.8-48.8)	1 (1-2)	45.6 (35.2-57)	0 (0-1)	24.1 (10.3-40.1)	3 (2-3)	28 (20-38)	2 (1-3)	1 (0-1)
Isiolo	0 (0-0)	20.1 (-2.2-24)	0 (0-1)	46.4 (21.4-53.8)	0 (0-0)	55.8 (28.3-64)	0 (0-0)	31.5 (8.1-44.4)	0 (0-0)	1 (0-1)	0 (0-0)	0 (0-0)

Location	Birth , 2000	Births percent change (2000-2021)	All age cases, 2000	All age cases percent change (2000-2021)	All age deaths, 2000	All age deaths percent change (2000-2021)	Under-5 deaths, 2000	Under-5 deaths percent change (2000-2021)	Births, 2021	All age cases, 2021	All age deaths, 2021	Under-5 deaths, 2021
Kajiado	0 (0-0)	36.1 (12-50.7)	2 (1-2)	82.5 (52.1-99.7)	0 (0-0)	97.4 (62.7-118)	0 (0-0)	45.3 (16.4-62.1)	0 (0-0)	3 (2-4)	0 (0-0)	0 (0-0)
Kakamega	1 (1-2)	74.2 (61-90.7)	12 (8-17)	110 (93.5-128)	1 (0-1)	123 (104-144)	0 (0-0)	80.2 (59.5-105)	2 (1-3)	26 (18-35)	2 (1-2)	0 (0-1)
Kericho	0 (0-1)	-69.5 (-72.5--67)	5 (3-7)	-54.8 (-59.1--51)	0 (0-0)	-51.2 (-56.2--46.7)	0 (0-0)	-63.9 (-68.4--59.4)	0 (0-0)	2 (2-3)	0 (0-0)	0 (0-0)
Kiambu	0 (0-1)	-32.2 (-43.6--25)	5 (4-7)	-6 (-21.1-2.2)	0 (0-0)	-1.6 (-19-9.2)	0 (0-0)	-21.5 (-35.6--9.8)	0 (0-0)	5 (3-7)	0 (0-0)	0 (0-0)
Kilifi	1 (1-2)	13.4 (3-23.1)	12 (9-17)	44.3 (33.3-56.9)	1 (0-1)	52.7 (36.7-68)	0 (0-0)	23 (5.4-39.5)	1 (1-2)	18 (13-25)	1 (1-2)	0 (0-0)
Kirinyaga	0 (0-0)	-39.5 (-49.9--35.2)	2 (1-2)	-14.8 (-29.1--10.1)	0 (0-0)	-11 (-26.6--4.6)	0 (0-0)	-32.4 (-46.5--24.7)	0 (0-0)	1 (1-2)	0 (0-0)	0 (0-0)
Kisii	1 (1-2)	-47.1 (-51.1--43.2)	16 (11-22)	-20 (-26.7--13.6)	1 (1-1)	-12.6 (-19.3--5.3)	0 (0-0)	-39.5 (-45.5--32.2)	1 (0-1)	13 (9-17)	1 (1-1)	0 (0-0)
Kisumu	1 (1-2)	-22.7 (-28.7--17.1)	11 (8-16)	11.1 (2.9-20.1)	1 (0-1)	19 (9.6-29.5)	0 (0-0)	-10.2 (-21.3-0.7)	1 (1-1)	12 (9-17)	1 (1-1)	0 (0-0)
Kitui	0 (0-1)	-17.5 (-32.3--7.7)	4 (3-6)	8.6 (-10.4-20.5)	0 (0-0)	19.3 (-2.7-34.4)	0 (0-0)	-10.4 (-28.9-5)	0 (0-0)	4 (3-6)	0 (0-0)	0 (0-0)
Kwale	1 (0-1)	10.3 (2.1-18.6)	7 (5-10)	34.9 (24.8-46.1)	0 (0-1)	41.7 (30-53.8)	0 (0-0)	15.7 (2.7-31.5)	1 (0-1)	10 (7-14)	1 (0-1)	0 (0-0)
Laikipia	0 (0-0)	-37.6 (-48.1--36.9)	1 (1-2)	-9 (-23.2--5.8)	0 (0-0)	1.5 (-15.7-6)	0 (0-0)	-27.8 (-41--19.5)	0 (0-0)	1 (1-2)	0 (0-0)	0 (0-0)
Lamu	0 (0-0)	7.2 (-6.7-11.3)	0 (0-0)	36.3 (18.2-43.1)	0 (0-0)	42.9 (23.7-49.8)	0 (0-0)	15.6 (0.2-28.9)	0 (0-0)	0 (0-1)	0 (0-0)	0 (0-0)
Machakos	0 (0-0)	-55.6 (-62.9--52)	5 (3-6)	-38.3 (-48--33.8)	0 (0-0)	-31.6 (-43.1--25.7)	0 (0-0)	-49.9 (-58.5--41.5)	0 (0-0)	3 (2-4)	0 (0-0)	0 (0-0)
Makueni	0 (0-0)	32.6 (20.5-33.5)	3 (2-4)	73.6 (57.5-79.4)	0 (0-0)	98.3 (81.6-109)	0 (0-0)	40.4 (26.1-52.2)	0 (0-0)	5 (3-6)	0 (0-0)	0 (0-0)
Mandera	0 (0-0)	1010 (860-1130)	1 (1-2)	903 (760-986)	0 (0-0)	1000 (833-1120)	0 (0-0)	802 (655-942)	1 (1-1)	12 (8-17)	1 (1-1)	0 (0-0)
Marsabit	0 (0-0)	93 (66.7-95.9)	1 (1-1)	114 (85.3-122)	0 (0-0)	134 (103-145)	0 (0-0)	92.7 (65.1-109)	0 (0-0)	2 (1-2)	0 (0-0)	0 (0-0)
Meru	0 (0-1)	-32.8 (-42.6--25.5)	5 (3-7)	-6.9 (-19.3-2.6)	0 (0-0)	2.4 (-11.6-13.1)	0 (0-0)	-26.9 (-37.3--17.1)	0 (0-0)	5 (3-7)	0 (0-0)	0 (0-0)
Migori	2 (2-3)	31.2 (21.4-42.9)	17 (12-24)	69 (56.9-82.4)	1 (1-2)	73.3 (60.1-89.6)	0 (0-1)	52.7 (37.3-71.5)	3 (2-4)	29 (20-40)	2 (1-3)	1 (0-1)
Mombasa	0 (0-1)	19.1 (10.2-30.5)	5 (4-8)	62.9 (50.4-76.6)	0 (0-1)	57.6 (45.7-71)	0 (0-0)	39.3 (24.6-54.9)	1 (0-1)	9 (6-12)	1 (0-1)	0 (0-0)
Murang'a	0 (0-0)	-34.5 (-45--30.6)	3 (2-4)	-11.2 (-24.1--7.1)	0 (0-0)	3.1 (-13.8-9.3)	0 (0-0)	-32 (-44.2--24.3)	0 (0-0)	3 (2-4)	0 (0-0)	0 (0-0)
Nairobi	1 (0-1)	10.6 (-9.8-23.1)	7 (5-10)	60.1 (32.6-78.7)	0 (0-1)	53 (27.2-70.9)	0 (0-0)	40 (13.5-59.7)	1 (0-1)	12 (8-16)	1 (0-1)	0 (0-0)
Nakuru	0 (0-1)	-25.6 (-39.2--19.3)	5 (4-7)	9.7 (-8.6-20.4)	0 (0-0)	21 (-0.5-32.2)	0 (0-0)	-18.1 (-33.7--5.9)	0 (0-0)	6 (4-8)	0 (0-0)	0 (0-0)
Nandi	0 (0-0)	-21 (-26.7--14.7)	4 (3-6)	10.6 (1.4-19.5)	0 (0-0)	22.4 (12.1-32.7)	0 (0-0)	-13.8 (-24.1--3.2)	0 (0-0)	5 (3-7)	0 (0-0)	0 (0-0)
Narok	0 (0-0)	266 (225-299)	2 (2-3)	311 (269-349)	0 (0-0)	346 (300-392)	0 (0-0)	255 (209-302)	1 (1-1)	10 (7-14)	1 (0-1)	0 (0-0)
Nyamira	0 (0-0)	8.2 (0.5-15.4)	3 (2-5)	53.8 (41.9-64.8)	0 (0-0)	72.6 (59.3-85.6)	0 (0-0)	19.6 (7.3-32.8)	0 (0-0)	5 (4-7)	0 (0-0)	0 (0-0)
Nyandarua	0 (0-0)	-27.1 (-39.8--24.6)	2 (1-3)	-1.5 (-18.3-2.9)	0 (0-0)	14.5 (-5.9-21.5)	0 (0-0)	-24 (-38.2--16.4)	0 (0-0)	2 (1-3)	0 (0-0)	0 (0-0)
Nyeri	0 (0-0)	-48.8 (-55.4--48.5)	2 (1-3)	-29.3 (-37.6--26.5)	0 (0-0)	-20.4 (-31.4--17.2)	0 (0-0)	-44.8 (-53.5--39.2)	0 (0-0)	2 (1-2)	0 (0-0)	0 (0-0)
Samburu	0 (0-0)	52.6 (25.3-65.8)	1 (1-1)	79.1 (51.5-94.1)	0 (0-0)	97.6 (63.6-118)	0 (0-0)	55.7 (22.7-76.6)	0 (0-0)	1 (1-2)	0 (0-0)	0 (0-0)
Siaya	2 (1-3)	-15.3 (-24.8--9)	16 (12-22)	13.7 (3.5-22.6)	1 (1-2)	19.4 (6.2-29)	0 (0-1)	-1 (-13.5-10.5)	2 (1-2)	19 (13-26)	1 (1-2)	0 (0-1)

Location	Birth , 2000	Births percent change (2000-2021)	All age cases, 2000	All age cases percent change (2000-2021)	All age deaths, 2000	All age deaths percent change (2000-2021)	Under-5 deaths, 2000	Under-5 deaths percent change (2000-2021)	Births, 2021	All age cases, 2021	All age deaths, 2021	Under-5 deaths, 2021
Taita Taveta	0 (0-0)	-34.7 (-43.3--28)	1 (1-2)	-13.8 (-25.3--4.3)	0 (0-0)	-6.3 (-19.2-4.9)	0 (0-0)	-29.3 (-40.6--19.7)	0 (0-0)	1 (1-1)	0 (0-0)	0 (0-0)
Tana River	0 (0-0)	34.1 (13.3-45.3)	1 (1-1)	50.1 (27.9-62.3)	0 (0-0)	53.3 (29.8-67)	0 (0-0)	37.8 (15.9-52.9)	0 (0-0)	2 (1-2)	0 (0-0)	0 (0-0)
Tharaka Nithi	0 (0-0)	-18.2 (-30.4--7.2)	0 (0-1)	12.4 (-3.2-24.6)	0 (0-0)	22.6 (4-39.2)	0 (0-0)	-11.9 (-27-2.9)	0 (0-0)	0 (0-1)	0 (0-0)	0 (0-0)
Trans Nzoia	0 (0-1)	-7 (-13.4-1.6)	5 (4-7)	27.8 (18.2-39.9)	0 (0-0)	42.7 (29.1-57.3)	0 (0-0)	0.3 (-11.1-12.7)	0 (0-1)	6 (4-9)	0 (0-1)	0 (0-0)
Turkana	0 (0-0)	166 (147-191)	3 (2-4)	209 (186-238)	0 (0-0)	229 (203-262)	0 (0-0)	182 (151-216)	1 (1-1)	9 (6-12)	1 (0-1)	0 (0-0)
Uasin Gishu	0 (0-0)	-10.5 (-17.8--3.7)	4 (3-5)	30.9 (20.9-40.4)	0 (0-0)	43.6 (31.2-55.5)	0 (0-0)	1.3 (-9.1-13.8)	0 (0-0)	5 (4-7)	0 (0-0)	0 (0-0)
Vihiga	0 (0-1)	18.4 (9.4-29.3)	5 (4-7)	55.9 (42.6-70)	0 (0-0)	73.6 (56.6-93.9)	0 (0-0)	19.4 (6.7-35.6)	1 (0-1)	8 (6-11)	1 (0-1)	0 (0-0)
Wajir	0 (0-0)	168 (126-170)	2 (1-2)	164 (121-173)	0 (0-0)	177 (130-188)	0 (0-0)	160 (122-181)	0 (0-1)	4 (3-6)	0 (0-0)	0 (0-0)
West Pokot	0 (0-0)	69.7 (56-83.7)	2 (2-3)	102 (86.4-120)	0 (0-0)	115 (95.9-138)	0 (0-0)	76 (58.2-101)	0 (0-1)	5 (3-7)	0 (0-0)	0 (0-0)
Madagascar	16 (10-22)	25.6 (13.5-38.7)	139 (97-191)	54.4 (42-68.8)	9 (6-13)	58.1 (41.7-78.4)	4 (2-6)	35.4 (16.6-55.3)	20 (13-27)	215 (148-299)	15 (10-21)	5 (3-8)
Malawi	9 (6-13)	4.4 (-5.1-12.8)	70 (47-99)	60.2 (47.3-73.1)	5 (3-8)	58.5 (43.5-73.9)	2 (1-4)	23.3 (10-38.5)	9 (6-14)	112 (75-156)	8 (5-12)	3 (2-4)
Mozambique	12 (8-16)	42 (31-59)	92 (64-126)	78.8 (64.3-98.7)	6 (4-9)	78.4 (63.9-102)	3 (2-4)	61.2 (43.8-90.3)	16 (12-23)	164 (115-225)	12 (8-16)	4 (3-7)
Rwanda	2 (1-3)	10.4 (-7.9-37.6)	19 (12-28)	36 (13.1-70)	1 (1-2)	41.8 (17.2-77.9)	0 (0-1)	21.4 (1-52.4)	2 (1-3)	26 (17-38)	2 (1-3)	1 (0-1)
Somalia	4 (3-6)	80.4 (45-108)	34 (22-47)	103 (64.8-129)	3 (2-4)	101 (59.6-133)	1 (1-2)	96.9 (55.4-133)	8 (5-11)	69 (46-96)	5 (3-7)	2 (1-3)
South Sudan	5 (3-6)	10 (0.4-19.7)	37 (26-51)	24.4 (14.1-34.9)	3 (2-4)	20.4 (9.6-31.5)	1 (1-2)	13.4 (1-28.1)	5 (3-7)	46 (32-64)	3 (2-5)	1 (1-2)
Uganda	58 (40-81)	29.2 (19.4-42.8)	470 (332-643)	61.8 (50.9-76.5)	31 (21-45)	65.3 (51.8-83.7)	13 (8-21)	44.8 (27.9-65.8)	75 (51-107)	761 (532-1,060)	52 (34-73)	19 (11-31)
United Republic of Tanzania	40 (27-55)	38 (26.8-52.2)	340 (237-463)	66.3 (54.2-81.4)	22 (15-32)	66.4 (52-83.5)	9 (5-14)	54 (36.5-75.3)	55 (38-76)	566 (394-778)	37 (25-53)	13 (8-21)
Zambia	28 (19-39)	36.7 (26.7-49.2)	241 (170-328)	71.8 (58.4-84.7)	17 (11-24)	76 (62.5-93.6)	7 (4-11)	52.3 (36.5-72.3)	39 (26-55)	415 (290-576)	29 (19-42)	10 (6-16)
**Southern Sub-Saharan Africa**	14 (11-19)	3.5 (-1.4-9.1)	175 (131-230)	11.5 (6.4-17.1)	11 (8-15)	13.1 (7.9-19.5)	3 (2-5)	12.2 (4.9-20.1)	15 (11-20)	195 (146-256)	13 (9-17)	4 (2-5)
Botswana	2 (1-3)	7.2 (0.1-18.4)	25 (18-35)	12.1 (4.6-22.1)	2 (1-2)	17.2 (8.4-29.3)	0 (0-1)	13.2 (-0.4-27.3)	2 (1-3)	28 (20-39)	2 (1-3)	0 (0-1)
Eswatini	0 (0-1)	-17 (-24.9--10.1)	5 (4-7)	-6 (-15-2)	0 (0-0)	-0.5 (-9.6-8.8)	0 (0-0)	-10.4 (-20.9-2.3)	0 (0-1)	5 (3-7)	0 (0-0)	0 (0-0)
Lesotho	1 (1-1)	-23 (-32.4--12.7)	8 (6-11)	-11.2 (-22.6-0.6)	1 (0-1)	-6.4 (-17.9-6.5)	0 (0-0)	-15.4 (-29.6--0.6)	1 (0-1)	7 (5-10)	1 (0-1)	0 (0-0)
Namibia	1 (1-1)	13.4 (2-21.5)	10 (7-14)	14.4 (1.3-23.1)	1 (0-1)	19.3 (6.4-28.5)	0 (0-0)	16.5 (1.7-30.4)	1 (1-1)	11 (8-16)	1 (0-1)	0 (0-0)
South Africa	3 (2-4)	-5.5 (-12.8-1)	44 (31-62)	-0.9 (-8.8-5.6)	3 (2-4)	1.6 (-5.9-8.9)	1 (0-1)	1.5 (-6.3-10.1)	3 (2-4)	43 (31-61)	3 (2-4)	1 (0-1)
Eastern Cape	1 (0-1)	-26.6 (-38--1.4)	7 (5-11)	-23.2 (-35.5-1.7)	0 (0-1)	-19.3 (-31.2-8.5)	0 (0-0)	-22.4 (-36.6-6.9)	0 (0-1)	5 (4-8)	0 (0-1)	0 (0-0)
Free State	0 (0-0)	-23.7 (-37.3--0.3)	3 (2-4)	-20.5 (-35.3-2.2)	0 (0-0)	-19.8 (-34.4-5)	0 (0-0)	-15.8 (-31.3-15.8)	0 (0-0)	2 (1-3)	0 (0-0)	0 (0-0)
Gauteng	1 (0-1)	42.3 (24.2-84.3)	7 (4-11)	56.3 (37.3-104)	0 (0-1)	48.2 (28.2-94)	0 (0-0)	68.2 (45.5-123)	1 (1-1)	11 (7-16)	1 (0-1)	0 (0-0)
KwaZulu-Natal	1 (0-1)	-16.7 (-26.7-9.2)	10 (7-16)	-9.5 (-19.5-18.6)	1 (0-1)	-5.3 (-16.2-26.1)	0 (0-0)	-9 (-20.7-21.8)	1 (0-1)	9 (6-14)	1 (0-1)	0 (0-0)
Limpopo	1 (0-1)	-23.8 (-36--6.7)	8 (5-11)	-23.1 (-34.5--6.2)	0 (0-1)	-18.2 (-31.7--1.4)	0 (0-0)	-19 (-30.3-0.5)	0 (0-1)	6 (4-8)	0 (0-1)	0 (0-0)

Location	Birth , 2000	Births percent change (2000-2021)	All age cases, 2000	All age cases percent change (2000-2021)	All age deaths, 2000	All age deaths percent change (2000-2021)	Under-5 deaths, 2000	Under-5 deaths percent change (2000-2021)	Births, 2021	All age cases, 2021	All age deaths, 2021	Under-5 deaths, 2021
Mpumalanga	0 (0-0)	23.1 (-2.2-44.4)	4 (2-6)	23.6 (-1.8-44.7)	0 (0-0)	29.2 (4-52.6)	0 (0-0)	29.3 (2.2-61.3)	0 (0-0)	5 (3-7)	0 (0-0)	0 (0-0)
North-West	0 (0-0)	-28.3 (-41.3--2.2)	4 (2-5)	-24.8 (-38.5-1.9)	0 (0-0)	-25.8 (-39.3-0.4)	0 (0-0)	-19.8 (-36.2-9.7)	0 (0-0)	3 (2-4)	0 (0-0)	0 (0-0)
Northern Cape	0 (0-0)	55.4 (30.5-101)	1 (0-1)	56.5 (30.2-102)	0 (0-0)	65.9 (38.6-115)	0 (0-0)	55.1 (29.5-102)	0 (0-0)	1 (1-2)	0 (0-0)	0 (0-0)
Western Cape	0 (0-0)	4.8 (4.7-4.7)	1 (0-3)	13.1 (10.7-15.7)	0 (0-0)	16.6 (13.4-20.1)	0 (0-0)	15.3 (7.1-24.1)	0 (0-0)	1 (0-3)	0 (0-0)	0 (0-0)
Zimbabwe	7 (5-11)	9.2 (1.1-18.6)	83 (57-114)	21 (12-32)	5 (4-8)	20.2 (10.7-30.7)	2 (1-3)	19.6 (6.5-33.8)	8 (5-12)	100 (69-139)	6 (4-9)	2 (1-3)
**Western Sub-Saharan Africa**	83,400 (65,300-106,000)	14.6 (9.2-19.6)	888,000 (712,000-1,110,000)	41.9 (34.3-48.5)	41,500 (31,600-54,300)	52.3 (43.8-59.5)	8,930 (5,670-13,700)	39.2 (29.4-47.9)	95,700 (72,000-125,000)	1,260,000 (963,000-1,610,000)	63,100 (46,600-84,700)	12,400 (7,700-19,400)
Benin	6,650 (5,210-8,220)	28.8 (10.5-48.6)	76,000 (60,700-92,800)	39.5 (19.6-60.7)	3,170 (2,400-4,000)	55.1 (31.8-80.1)	766 (481-1,140)	45.9 (20.2-74.7)	8,570 (6,440-10,900)	106,000 (79,400-135,000)	4,920 (3,660-6,420)	1,120 (704-1,700)
Burkina Faso	9,620 (8,400-10,900)	9.6 (-13.1-37.2)	100,000 (89,600-111,000)	20.8 (-5.6-51.7)	4,760 (3,990-5,560)	20.4 (-5.6-52.1)	1,190 (779-1,730)	20.1 (-9.1-60.5)	10,500 (8,080-13,600)	121,000 (92,300-156,000)	5,730 (4,290-7,510)	1,430 (905-2,160)
Cabo Verde	6 (4-8)	-18.3 (-26.1--10.2)	111 (80-149)	-11.3 (-19.7--1.8)	4 (3-6)	15.2 (3.5-29)	1 (0-1)	-14.1 (-24.3--0.6)	5 (4-7)	98 (69-133)	5 (3-7)	0 (0-1)
Cameroon	157 (125-203)	46.8 (35.9-58.4)	1,770 (1,400-2,270)	93.5 (79.2-109)	83 (64-108)	103 (87.9-120)	20 (12-30)	66.1 (46.5-90.1)	231 (184-301)	3,430 (2,670-4,410)	169 (133-222)	32 (20-50)
Chad	53 (39-71)	87.4 (73.8-106)	497 (369-653)	111 (95.5-131)	23 (17-32)	111 (95.1-133)	7 (4-11)	99.9 (76.5-130)	99 (73-132)	1,050 (770-1,390)	49 (35-65)	14 (8-21)
Côte d'Ivoire	1,560 (1,200-1,910)	17.9 (6.8-28.2)	16,500 (12,900-20,200)	43.8 (30.6-55.7)	820 (634-1,020)	43.6 (29.5-56.6)	186 (118-276)	33.8 (15.4-52)	1,830 (1,430-2,220)	23,800 (18,300-28,600)	1,180 (909-1,430)	249 (161-357)
Gambia	86 (64-111)	-9.6 (-20.4-0.4)	900 (681-1,160)	28 (13.2-42.6)	41 (31-54)	34.2 (18.7-50.1)	9 (6-14)	8.1 (-8.1-24.2)	78 (59-103)	1,150 (866-1,500)	55 (42-73)	10 (6-15)
Ghana	5,770 (5,550-6,060)	22.9 (1.1-45.1)	75,000 (69,700-80,700)	43 (18.1-68.4)	3,340 (3,160-3,550)	66.1 (36.5-97.6)	620 (430-834)	48.9 (16.7-78.9)	7,100 (5,770-8,450)	107,000 (87,100-128,000)	5,560 (4,520-6,700)	923 (613-1,300)
Guinea	1,110 (774-1,550)	41.4 (28.1-57.4)	11,500 (8,080-15,800)	66.2 (50.6-85.8)	549 (372-791)	73.2 (55.8-96.1)	153 (86-248)	55.5 (33.2-82.5)	1,560 (1,080-2,140)	19,200 (13,600-26,300)	951 (645-1,330)	238 (135-382)
Guinea-Bissau	8 (5-11)	15.8 (6.4-25.7)	84 (59-115)	41.4 (30-52.8)	4 (3-6)	49.6 (35.9-63.3)	1 (1-2)	30.3 (14.9-47.7)	9 (6-12)	119 (83-165)	6 (4-9)	1 (1-2)
Liberia	143 (100-197)	2.4 (-6.3-12.2)	1,480 (1,060-2,000)	49.5 (36.7-64.2)	75 (52-106)	53.7 (39.6-70.1)	19 (11-30)	19.3 (3.7-36.4)	147 (103-202)	2,210 (1,570-3,030)	115 (80-161)	22 (13-36)
Mali	1,730 (1,360-2,100)	84.3 (68.8-103)	16,400 (12,800-19,800)	110 (93.1-131)	800 (624-1,000)	115 (95.6-138)	237 (151-353)	94.9 (72.1-123)	3,180 (2,480-3,840)	34,500 (26,500-41,800)	1,720 (1,320-2,120)	463 (293-685)
Mauritania	28 (20-36)	5.5 (-3.5-16.1)	339 (253-434)	45.8 (32.8-59.7)	15 (11-20)	55.8 (41.5-73)	3 (2-5)	16.5 (-0.3-33.9)	29 (22-38)	494 (373-644)	23 (17-31)	4 (2-6)
Niger	5,580 (4,380-6,920)	22.2 (6.6-39.2)	50,500 (39,900-61,900)	43.9 (24.9-63.3)	2,210 (1,710-2,830)	49.6 (29.3-71.7)	636 (404-956)	45.9 (22.2-73.6)	6,820 (5,100-8,780)	72,600 (55,200-93,100)	3,310 (2,420-4,340)	928 (568-1,420)
Nigeria	48,000 (34,100-65,800)	8.1 (4.3-12.1)	504,000 (365,000-671,000)	42.4 (37.8-47.5)	24,000 (16,600-33,700)	53.6 (48.3-59.9)	4,710 (2,730-7,650)	38 (30.9-45.6)	51,900 (37,000-71,500)	717,000 (524,000-958,000)	36,800 (25,700-51,900)	6,510 (3,770-10,700)
Sao Tome and Principe	4 (3-6)	-16.3 (-24--7.9)	58 (42-76)	19.3 (8.6-30.9)	3 (2-3)	31.5 (18.2-45.4)	0 (0-1)	-5.7 (-18.7-7.7)	4 (3-5)	70 (50-91)	3 (2-4)	0 (0-1)
Senegal	308 (235-377)	21.7 (12.3-31.4)	3,700 (2,880-4,560)	47.7 (35.4-59.2)	174 (130-216)	53.8 (41-66.7)	38 (25-57)	30.9 (16.7-48.6)	375 (284-465)	5,470 (4,200-6,760)	267 (199-335)	50 (33-73)
Sierra Leone	721 (564-891)	43.3 (30.2-56.3)	7,150 (5,510-8,790)	83 (66.8-99.6)	353 (271-442)	101 (81.3-121)	95 (60-141)	63.1 (43.9-86.3)	1,030 (804-1,270)	13,100 (10,200-16,200)	708 (551-872)	155 (98-234)
Togo	1,880 (1,510-2,370)	12.3 (2.9-22.2)	21,900 (17,300-27,500)	42 (30-54.9)	1,050 (820-1,330)	47.3 (34.7-60.8)	237 (154-349)	24 (8.1-42.3)	2,110 (1,670-2,690)	31,000 (24,400-39,000)	1,540 (1,200-1,960)	294 (188-448)

Table S12: Number of births, cases, total deaths, and under-5 deaths for Mild sickle cell/beta-thalassemia , 2000 - 2021

Location	Birth , 2000	Births percent change (2000-2021)	All age cases, 2000	All age cases percent change (2000-2021)	All age deaths, 2000	All age deaths percent change (2000-2021)	Under-5 deaths, 2000	Under-5 deaths percent change (2000-2021)	Births, 2021	All age cases, 2021	All age deaths, 2021	Under-5 deaths, 2021
Global	17,600 (12,600-24,900)	12.7 (10.7-15)	91,300 (68,400-122,000)	27.3 (24.7-30.2)	14,200 (10,000-20,400)	27.5 (24.8-30.5)	9,900 (6,670-14,800)	21.5 (18.4-24.9)	19,800 (14,300-28,200)	116,000 (87,300-154,000)	18,100 (12,900-25,900)	12,000 (8,150-17,900)
Central Europe, Eastern Europe, and Central Asia	36 (24-53)	-38.2 (-46.4--30)	243 (174-330)	-43.7 (-49.7--37.7)	43 (29-64)	-43.6 (-51--35.6)	24 (15-39)	-37.4 (-47.1--26.1)	22 (15-32)	137 (99-187)	24 (16-35)	15 (9-24)
**Central Asia**	0 (0-0)	20.4 (20.2-20.7)	1 (0-2)	18.8 (12.6-23.3)	0 (0-0)	20.2 (17.4-21.8)	0 (0-0)	25.4 (22.5-28.2)	0 (0-0)	1 (0-2)	0 (0-1)	0 (0-0)
Armenia	0 (0-0)	-20.4 (-19.3--19.3)	0 (0-0)	-21.3 (-29.2--12.6)	0 (0-0)	-19.5 (-22.3--15.7)	0 (0-0)	-10.4 (-16--5.2)	0 (0-0)	0 (0-0)	0 (0-0)	0 (0-0)
Azerbaijan	0 (0-0)	-3.1 (-3.1--3.1)	0 (0-0)	-1.1 (-8.6-6)	0 (0-0)	-0.9 (-2.5-0.3)	0 (0-0)	3.2 (0-7.5)	0 (0-0)	0 (0-0)	0 (0-0)	0 (0-0)
Georgia	0 (0-0)	-12.6 (-12.6--12.6)	0 (0-0)	-21.5 (-27.8--13.7)	0 (0-0)	-20.1 (-23.6--17)	0 (0-0)	-9.9 (-14.3--4.6)	0 (0-0)	0 (0-0)	0 (0-0)	0 (0-0)
Kazakhstan	0 (0-0)	46.1 (46.5-46.5)	0 (0-0)	43.1 (32.7-55.4)	0 (0-0)	45.4 (39.5-50.3)	0 (0-0)	59.6 (52.6-67.6)	0 (0-0)	0 (0-0)	0 (0-0)	0 (0-0)
Kyrgyzstan	0 (0-0)	43.4 (43.5-43.5)	0 (0-0)	41.3 (30-52)	0 (0-0)	42.7 (39.7-45.2)	0 (0-0)	50.5 (43.9-58.2)	0 (0-0)	0 (0-0)	0 (0-0)	0 (0-0)
Mongolia	0 (0-0)	36.9 (37.6-37.6)	0 (0-0)	42.2 (30.5-53.3)	0 (0-0)	45.1 (40.7-47.7)	0 (0-0)	54 (47.8-59.9)	0 (0-0)	0 (0-0)	0 (0-0)	0 (0-0)
Tajikistan	0 (0-0)	37.9 (37.9-37.9)	0 (0-0)	35 (26.8-43.2)	0 (0-0)	35 (34-35.7)	0 (0-0)	37.8 (33.5-42.1)	0 (0-0)	0 (0-0)	0 (0-0)	0 (0-0)
Turkmenistan	0 (0-0)	9.8 (9.8-9.8)	0 (0-0)	9.1 (2.2-17.2)	0 (0-0)	9.9 (8.4-11)	0 (0-0)	13.7 (9.7-17.9)	0 (0-0)	0 (0-0)	0 (0-0)	0 (0-0)
Uzbekistan	0 (0-0)	13.2 (13.6-13.6)	0 (0-1)	21.1 (9.9-29.8)	0 (0-0)	20.9 (18.2-23.3)	0 (0-0)	27.7 (22.5-33.3)	0 (0-0)	0 (0-1)	0 (0-0)	0 (0-0)
**Central Europe**	35 (23-53)	-38.9 (-47--30.6)	240 (171-328)	-44.2 (-50.2--38.2)	43 (28-63)	-44.2 (-51.8--36.4)	24 (15-39)	-38.1 (-47.8--27.3)	22 (14-32)	134 (96-184)	24 (16-35)	15 (9-24)
Albania	0 (0-0)	-39.6 (-52.7--39)	1 (0-1)	-45.9 (-56.2--41.4)	0 (0-0)	-47.2 (-58.6--46)	0 (0-0)	-45.3 (-57.2--42.5)	0 (0-0)	0 (0-0)	0 (0-0)	0 (0-0)
Bosnia and Herzegovina	0 (0-0)	-41.3 (-98--39.9)	0 (0-0)	-44.1 (-97.8--37.7)	0 (0-0)	-43.9 (-98.1--42.1)	0 (0-0)	-43.7 (-98.2--39.3)	0 (0-0)	0 (0-0)	0 (0-0)	0 (0-0)
Bulgaria	0 (0-0)	-19.6 (-19.7--19.6)	0 (0-0)	-15.2 (-22.7--8.3)	0 (0-0)	-12.7 (-16--10.9)	0 (0-0)	-7 (-11--2.5)	0 (0-0)	0 (0-0)	0 (0-0)	0 (0-0)
Croatia	0 (0-0)	-24.2 (-56.3--20.2)	0 (0-0)	-30.1 (-62.3--19.6)	0 (0-0)	-30.3 (-60--25.4)	0 (0-0)	-33.1 (-59.1--26.1)	0 (0-0)	0 (0-0)	0 (0-0)	0 (0-0)
Czechia	0 (0-0)	16 (-74.4-101)	0 (0-0)	4.2 (-78.5-87.4)	0 (0-0)	6.6 (-76.8-85.7)	0 (0-0)	25.2 (-70.2-108)	0 (0-0)	0 (0-0)	0 (0-0)	0 (0-0)
Hungary	0 (0-0)	-23.2 (-55.8--19.3)	0 (0-0)	-24.2 (-61--12)	0 (0-0)	-22.7 (-55.8--17.3)	0 (0-0)	-15.8 (-48.7--7.1)	0 (0-0)	0 (0-0)	0 (0-0)	0 (0-0)
Montenegro	0 (0-0)	-24 (-23.8--23.8)	0 (0-0)	-23.3 (-29--16.9)	0 (0-0)	-23.6 (-23.8--22.8)	0 (0-0)	-23.6 (-27.5--18.5)	0 (0-0)	0 (0-0)	0 (0-0)	0 (0-0)
North Macedonia	0 (0-0)	-12.8 (-12.9--12.8)	0 (0-0)	-16.7 (-23.6--9.9)	0 (0-0)	-16.7 (-18--15.5)	0 (0-0)	-13.3 (-16.9--9.1)	0 (0-0)	0 (0-0)	0 (0-0)	0 (0-0)
Poland	35 (23-53)	-39 (-47--30.6)	239 (170-327)	-44.8 (-50.7--38.8)	42 (28-63)	-44.9 (-52.3--37.1)	24 (14-38)	-39 (-48.6--28.1)	21 (14-31)	132 (95-181)	23 (15-34)	15 (9-23)
Romania	0 (0-0)	-27.6 (-27.7--27.6)	0 (0-0)	-25.5 (-31--19.1)	0 (0-0)	-24 (-25.8--23)	0 (0-0)	-20.6 (-23.6--17.5)	0 (0-0)	0 (0-0)	0 (0-0)	0 (0-0)
Serbia	0 (0-0)	-25.8 (-25.8--25.8)	0 (0-0)	-31.3 (-37.7--24.3)	0 (0-0)	-31.7 (-32.8--27.7)	0 (0-0)	-33 (-36.5--28.9)	0 (0-0)	0 (0-0)	0 (0-0)	0 (0-0)
Slovakia	0 (0-0)	4.4 (4.4-4.4)	0 (0-0)	-11 (-18.3--3.4)	0 (0-0)	-10.3 (-13.7--7.6)	0 (0-0)	-0.4 (-5.4-4.3)	0 (0-0)	0 (0-0)	0 (0-0)	0 (0-0)
Slovenia	0 (0-0)	3.3 (-28.2-110)	0 (0-0)	-1.2 (-31.4-77.7)	0 (0-0)	2 (-27.6-99.4)	0 (0-0)	9.1 (-23.2-125)	0 (0-0)	0 (0-0)	0 (0-0)	0 (0-0)
**Eastern Europe**	0 (0-1)	10 (2.3-12.2)	2 (1-4)	6.9 (-0.2-13.6)	0 (0-1)	9.1 (1.6-15.3)	0 (0-0)	25.6 (18.6-29)	0 (0-1)	2 (1-4)	0 (0-1)	0 (0-0)
Belarus	0 (0-0)	4.2 (4.2-4.2)	0 (0-0)	-2.6 (-11.3-7.4)	0 (0-0)	0.5 (-6.1-5.7)	0 (0-0)	18.1 (11.4-24.9)	0 (0-0)	0 (0-0)	0 (0-0)	0 (0-0)
Estonia	0 (0-0)	1.8 (1.8-1.8)	0 (0-0)	-6.2 (-14.7-2.3)	0 (0-0)	-5.3 (-10--1.5)	0 (0-0)	8.9 (2.7-15.2)	0 (0-0)	0 (0-0)	0 (0-0)	0 (0-0)

Location	Birth , 2000	Births percent change (2000-2021)	All age cases, 2000	All age cases percent change (2000-2021)	All age deaths, 2000	All age deaths percent change (2000-2021)	Under-5 deaths, 2000	Under-5 deaths percent change (2000-2021)	Births, 2021	All age cases, 2021	All age deaths, 2021	Under-5 deaths, 2021
Latvia	0 (0-0)	-9.5 (-9.6--9.6)	0 (0-0)	-17.4 (-25--7.7)	0 (0-0)	-15.9 (-22--10.7)	0 (0-0)	6.6 (1.1-12.3)	0 (0-0)	0 (0-0)	0 (0-0)	0 (0-0)
Lithuania	0 (0-0)	-20.9 (-20.9--20.9)	0 (0-0)	-34.1 (-39.7--27.8)	0 (0-0)	-34.1 (-36.9--31.8)	0 (0-0)	-24.6 (-29.2--20.5)	0 (0-0)	0 (0-0)	0 (0-0)	0 (0-0)
Republic of Moldova	0 (0-0)	-34.7 (-34.3--34.3)	0 (0-0)	-37.5 (-43.3--32.2)	0 (0-0)	-37.1 (-38.7--35.4)	0 (0-0)	-32.8 (-36.3--29.7)	0 (0-0)	0 (0-0)	0 (0-0)	0 (0-0)
Russian Federation	0 (0-0)	19.8 (7.2-20.9)	1 (0-3)	18.1 (7.4-26.2)	0 (0-0)	20.3 (8.4-27.9)	0 (0-0)	39.7 (26.3-42.2)	0 (0-0)	1 (0-3)	0 (0-0)	0 (0-0)
Ukraine	0 (0-0)	-13.2 (-13.5--13.2)	0 (0-1)	-15.9 (-22.3--8.7)	0 (0-0)	-14.8 (-18.6--11.8)	0 (0-0)	-6.2 (-11--1.1)	0 (0-0)	0 (0-1)	0 (0-0)	0 (0-0)
High-income	176 (148-211)	-14.9 (-16.4--13.3)	5,860 (4,990-6,890)	-0.5 (-2.4-1.4)	168 (140-202)	0 (-2.6-2.8)	32 (24-44)	-16.9 (-20--13.6)	150 (126-179)	5,830 (5,000-6,850)	168 (142-201)	27 (20-36)
**Australasia**	0 (0-0)	29 (27.9-88.7)	0 (0-0)	17.9 (10.4-70.8)	0 (0-0)	17.6 (16-81.5)	0 (0-0)	18.8 (13.9-84.6)	0 (0-0)	0 (0-0)	0 (0-0)	0 (0-0)
Australia	0 (0-0)	30.2 (30.2-30.2)	0 (0-0)	19.3 (11.1-27.3)	0 (0-0)	18.9 (18.2-19.5)	0 (0-0)	20.3 (15.8-25.4)	0 (0-0)	0 (0-0)	0 (0-0)	0 (0-0)
New Zealand	0 (0-0)	23.3 (17.8-340)	0 (0-0)	11.2 (0-302)	0 (0-0)	11.8 (5.2-365)	0 (0-0)	11.4 (1.9-387)	0 (0-0)	0 (0-0)	0 (0-0)	0 (0-0)
**High-income Asia Pacific**	0 (0-0)	-27.7 (-27.8--27.7)	1 (0-2)	-24.5 (-26.5--22.1)	0 (0-1)	-24.9 (-25.6--22.9)	0 (0-0)	-25.9 (-27--24.2)	0 (0-0)	1 (0-2)	0 (0-0)	0 (0-0)
Brunei Darussalam	0 (0-0)	-11.2 (-11.2--11.2)	0 (0-0)	-12.3 (-17.5--6.2)	0 (0-0)	-13 (-13.4--12)	0 (0-0)	-14.2 (-16.5--11.5)	0 (0-0)	0 (0-0)	0 (0-0)	0 (0-0)
Japan	0 (0-0)	-27.8 (-27.8--27.8)	1 (0-2)	-23 (-24.4--21.5)	0 (0-0)	-22.8 (-23.5--22.2)	0 (0-0)	-24.1 (-24.9--23.5)	0 (0-0)	1 (0-1)	0 (0-0)	0 (0-0)
Aichi	0 (0-0)	-22.2 (-22.2--22.2)	0 (0-0)	-15.7 (-21.8--8.9)	0 (0-0)	-15.7 (-16.8--14.7)	0 (0-0)	-18.2 (-21.7--15.1)	0 (0-0)	0 (0-0)	0 (0-0)	0 (0-0)
Akita	0 (0-0)	-48.8 (-48.8--48.8)	0 (0-0)	-48.2 (-52.2--43.9)	0 (0-0)	-48 (-48.4--47.6)	0 (0-0)	-48.2 (-50.1--46)	0 (0-0)	0 (0-0)	0 (0-0)	0 (0-0)
Aomori	0 (0-0)	-44.6 (-44.6--44.6)	0 (0-0)	-43.4 (-47.3--38.5)	0 (0-0)	-43.6 (-43.9--43.2)	0 (0-0)	-43.8 (-46--41.8)	0 (0-0)	0 (0-0)	0 (0-0)	0 (0-0)
Chiba	0 (0-0)	-28 (-28--28)	0 (0-0)	-22 (-28--15.3)	0 (0-0)	-22 (-23--20.9)	0 (0-0)	-24.2 (-27.1--20.9)	0 (0-0)	0 (0-0)	0 (0-0)	0 (0-0)
Ehime	0 (0-0)	-38.1 (-38.1--38.1)	0 (0-0)	-33.3 (-38.7--27.8)	0 (0-0)	-33.1 (-34--32.4)	0 (0-0)	-35 (-37.5--32.4)	0 (0-0)	0 (0-0)	0 (0-0)	0 (0-0)
Fukui	0 (0-0)	-33.9 (-33.9--33.9)	0 (0-0)	-31.6 (-37--25.8)	0 (0-0)	-31.8 (-32.6--30.9)	0 (0-0)	-34 (-36.6--31.1)	0 (0-0)	0 (0-0)	0 (0-0)	0 (0-0)
Fukuoka	0 (0-0)	-18.1 (-18.1--18.1)	0 (0-0)	-14.1 (-21.2--7)	0 (0-0)	-12.9 (-14.4--11.9)	0 (0-0)	-13.1 (-16.7--9)	0 (0-0)	0 (0-0)	0 (0-0)	0 (0-0)
Fukushima	0 (0-0)	-44.2 (-44.1--44.1)	0 (0-0)	-43.9 (-47.7--39.6)	0 (0-0)	-43.4 (-43.8--43.1)	0 (0-0)	-42.2 (-44.3--40)	0 (0-0)	0 (0-0)	0 (0-0)	0 (0-0)
Gifu	0 (0-0)	-36.8 (-36.8--36.8)	0 (0-0)	-30.4 (-36.5--24.2)	0 (0-0)	-30.7 (-31.7--29.5)	0 (0-0)	-33.3 (-36.1--30)	0 (0-0)	0 (0-0)	0 (0-0)	0 (0-0)
Gunma	0 (0-0)	-38.7 (-38.7--38.7)	0 (0-0)	-32.8 (-38.9--27.2)	0 (0-0)	-33 (-34--31.9)	0 (0-0)	-35.7 (-38.3--32.5)	0 (0-0)	0 (0-0)	0 (0-0)	0 (0-0)
Hiroshima	0 (0-0)	-28.5 (-28.5--28.5)	0 (0-0)	-21.5 (-28.1--14.8)	0 (0-0)	-21.3 (-22.4--20.3)	0 (0-0)	-23.8 (-26.8--20.5)	0 (0-0)	0 (0-0)	0 (0-0)	0 (0-0)
Hokkaidō	0 (0-0)	-37.9 (-37.9--37.9)	0 (0-0)	-33.4 (-39--27.6)	0 (0-0)	-33 (-33.9--32.3)	0 (0-0)	-33.9 (-36.3--31.1)	0 (0-0)	0 (0-0)	0 (0-0)	0 (0-0)
Hyōgo	0 (0-0)	-29.5 (-29.5--29.5)	0 (0-0)	-25.3 (-31--18.7)	0 (0-0)	-25.6 (-26.4--24.7)	0 (0-0)	-27.4 (-30.4--24.5)	0 (0-0)	0 (0-0)	0 (0-0)	0 (0-0)
Ibaraki	0 (0-0)	-37 (-37--37)	0 (0-0)	-32.3 (-37.3--26.3)	0 (0-0)	-32.2 (-32.9--31.4)	0 (0-0)	-33.7 (-36.6--31)	0 (0-0)	0 (0-0)	0 (0-0)	0 (0-0)
Ishikawa	0 (0-0)	-31.3 (-31.3--31.3)	0 (0-0)	-26.8 (-32.7--20.1)	0 (0-0)	-27.1 (-27.9--26.2)	0 (0-0)	-29 (-32.3--25.6)	0 (0-0)	0 (0-0)	0 (0-0)	0 (0-0)
Iwate	0 (0-0)	-43 (-43.1--43.1)	0 (0-0)	-38.5 (-44.1--33.1)	0 (0-0)	-38.6 (-39.2--38)	0 (0-0)	-39.5 (-42.2--37)	0 (0-0)	0 (0-0)	0 (0-0)	0 (0-0)
Kagawa	0 (0-0)	-35.3 (-35.3--35.3)	0 (0-0)	-29.4 (-35.4--23.4)	0 (0-0)	-29.9 (-30.8--28.9)	0 (0-0)	-32.6 (-35.5--29.6)	0 (0-0)	0 (0-0)	0 (0-0)	0 (0-0)

Location	Birth , 2000	Births percent change (2000-2021)	All age cases, 2000	All age cases percent change (2000-2021)	All age deaths, 2000	All age deaths percent change (2000-2021)	Under-5 deaths, 2000	Under-5 deaths percent change (2000-2021)	Births, 2021	All age cases, 2021	All age deaths, 2021	Under-5 deaths, 2021
Kagoshima	0 (0-0)	-28.8 (-28.8--28.8)	0 (0-0)	-27.7 (-33.5--22.2)	0 (0-0)	-26.5 (-27.4--25.8)	0 (0-0)	-25.9 (-28.7--22.9)	0 (0-0)	0 (0-0)	0 (0-0)	0 (0-0)
Kanagawa	0 (0-0)	-26.7 (-26.8--26.8)	0 (0-0)	-16.8 (-24.6--9)	0 (0-0)	-16.8 (-18.2--15.3)	0 (0-0)	-19.7 (-23.8--15.5)	0 (0-0)	0 (0-0)	0 (0-0)	0 (0-0)
Kōchi	0 (0-0)	-39.6 (-39.7--39.7)	0 (0-0)	-36 (-40.9--30.9)	0 (0-0)	-35.9 (-36.5--35.4)	0 (0-0)	-36.8 (-39.2--34.4)	0 (0-0)	0 (0-0)	0 (0-0)	0 (0-0)
Kumamoto	0 (0-0)	-25.2 (-25.2--25.2)	0 (0-0)	-21.4 (-27.5--15.7)	0 (0-0)	-20 (-21.3--19.2)	0 (0-0)	-20.1 (-22.9--16.7)	0 (0-0)	0 (0-0)	0 (0-0)	0 (0-0)
Kyōto	0 (0-0)	-26.1 (-26.1--26.1)	0 (0-0)	-22.9 (-30--16.2)	0 (0-0)	-22.9 (-23.8--22)	0 (0-0)	-24.1 (-27.3--20.1)	0 (0-0)	0 (0-0)	0 (0-0)	0 (0-0)
Mie	0 (0-0)	-35.7 (-35.9--35.9)	0 (0-0)	-29.6 (-35.6--23.8)	0 (0-0)	-30 (-31.2--29.1)	0 (0-0)	-32.7 (-35.6--29.7)	0 (0-0)	0 (0-0)	0 (0-0)	0 (0-0)
Miyagi	0 (0-0)	-32.2 (-32.2--32.2)	0 (0-0)	-27.5 (-33.4--21.1)	0 (0-0)	-26.6 (-27.6--25.8)	0 (0-0)	-27.2 (-30.2--24)	0 (0-0)	0 (0-0)	0 (0-0)	0 (0-0)
Miyazaki	0 (0-0)	-34.5 (-34.5--34.5)	0 (0-0)	-29.3 (-34.9--23.6)	0 (0-0)	-29.1 (-29.9--28.3)	0 (0-0)	-30.9 (-33.6--28.2)	0 (0-0)	0 (0-0)	0 (0-0)	0 (0-0)
Nagano	0 (0-0)	-39.6 (-39.6--39.6)	0 (0-0)	-33.7 (-39--28.3)	0 (0-0)	-33.8 (-34.7--32.9)	0 (0-0)	-36.2 (-38.6--33.3)	0 (0-0)	0 (0-0)	0 (0-0)	0 (0-0)
Nagasaki	0 (0-0)	-34.2 (-34.2--34.2)	0 (0-0)	-33.4 (-38.8--27.8)	0 (0-0)	-33.2 (-33.7--32.8)	0 (0-0)	-33.2 (-35.9--30.5)	0 (0-0)	0 (0-0)	0 (0-0)	0 (0-0)
Nara	0 (0-0)	-38.9 (-38.9--38.9)	0 (0-0)	-32.7 (-38.7--26.9)	0 (0-0)	-32.8 (-33.8--31.8)	0 (0-0)	-35 (-37.9--32.1)	0 (0-0)	0 (0-0)	0 (0-0)	0 (0-0)
Niigata	0 (0-0)	-37.8 (-37.8--37.8)	0 (0-0)	-34.2 (-39.4--28.3)	0 (0-0)	-34.2 (-34.8--33.6)	0 (0-0)	-35.8 (-38.4--33.1)	0 (0-0)	0 (0-0)	0 (0-0)	0 (0-0)
Ōita	0 (0-0)	-30.1 (-30.1--30.1)	0 (0-0)	-27.5 (-33.5--20.9)	0 (0-0)	-26.6 (-27.3--26)	0 (0-0)	-26.9 (-30.5--23.5)	0 (0-0)	0 (0-0)	0 (0-0)	0 (0-0)
Okayama	0 (0-0)	-27.5 (-27.6--27.6)	0 (0-0)	-23.2 (-29.2--16.6)	0 (0-0)	-22.9 (-23.7--22.4)	0 (0-0)	-24.3 (-27.5--21.1)	0 (0-0)	0 (0-0)	0 (0-0)	0 (0-0)
Okinawa	0 (0-0)	-10.9 (-10.9--10.9)	0 (0-0)	-6.8 (-12.9-1)	0 (0-0)	-5.3 (-6.4--4.5)	0 (0-0)	-4.9 (-8.7--1.4)	0 (0-0)	0 (0-0)	0 (0-0)	0 (0-0)
Ōsaka	0 (0-0)	-30.2 (-30.2--30.2)	0 (0-0)	-24.2 (-30.8--16.7)	0 (0-0)	-24.6 (-25.6--23.4)	0 (0-0)	-27.1 (-30.4--24)	0 (0-0)	0 (0-0)	0 (0-0)	0 (0-0)
Saga	0 (0-0)	-31.9 (-31.9--31.9)	0 (0-0)	-27.6 (-33.3--21.9)	0 (0-0)	-27.2 (-27.9--26.5)	0 (0-0)	-28.9 (-31.8--26.1)	0 (0-0)	0 (0-0)	0 (0-0)	0 (0-0)
Saitama	0 (0-0)	-27.9 (-27.9--27.9)	0 (0-0)	-21.9 (-27.9--15.1)	0 (0-0)	-21.7 (-22.5--20.8)	0 (0-0)	-23.3 (-26.5--20.1)	0 (0-0)	0 (0-0)	0 (0-0)	0 (0-0)
Shiga	0 (0-0)	-26.2 (-26.2--26.2)	0 (0-0)	-19.1 (-25.6--11.9)	0 (0-0)	-19 (-20.1--17.8)	0 (0-0)	-21.9 (-25.4--18.5)	0 (0-0)	0 (0-0)	0 (0-0)	0 (0-0)
Shimane	0 (0-0)	-30.6 (-30.6--30.6)	0 (0-0)	-28 (-33--21.7)	0 (0-0)	-27.5 (-28.1--26.9)	0 (0-0)	-27.2 (-30.4--24.3)	0 (0-0)	0 (0-0)	0 (0-0)	0 (0-0)
Shizuoka	0 (0-0)	-34 (-34--34)	0 (0-0)	-28.3 (-33.6--22.2)	0 (0-0)	-28.2 (-29.1--27.2)	0 (0-0)	-30.6 (-33.4--27.7)	0 (0-0)	0 (0-0)	0 (0-0)	0 (0-0)
Tochigi	0 (0-0)	-33.7 (-33.7--33.7)	0 (0-0)	-27.7 (-33.3--22)	0 (0-0)	-27.3 (-28.1--26.6)	0 (0-0)	-28.2 (-31--25.4)	0 (0-0)	0 (0-0)	0 (0-0)	0 (0-0)
Tokushima	0 (0-0)	-35.6 (-35.5--35.5)	0 (0-0)	-33.7 (-38.9--28.5)	0 (0-0)	-32.7 (-33.2--32.1)	0 (0-0)	-32.5 (-35--29.5)	0 (0-0)	0 (0-0)	0 (0-0)	0 (0-0)
Tōkyō	0 (0-0)	7.4 (7.4-7.4)	0 (0-0)	7.3 (-2-17.3)	0 (0-0)	10.4 (7.3-12.4)	0 (0-0)	13.8 (8.8-18.8)	0 (0-0)	0 (0-0)	0 (0-0)	0 (0-0)
Tottori	0 (0-0)	-30.8 (-30.8--30.8)	0 (0-0)	-26.8 (-31.8--20.6)	0 (0-0)	-26.4 (-27.2--25.7)	0 (0-0)	-26.9 (-30--24.3)	0 (0-0)	0 (0-0)	0 (0-0)	0 (0-0)
Toyama	0 (0-0)	-38.5 (-38.5--38.5)	0 (0-0)	-30.9 (-36.3--25.3)	0 (0-0)	-30.7 (-31.6--29.8)	0 (0-0)	-32.6 (-35.5--29.8)	0 (0-0)	0 (0-0)	0 (0-0)	0 (0-0)
Wakayama	0 (0-0)	-40.5 (-40.5--40.5)	0 (0-0)	-35.9 (-40.5--30.5)	0 (0-0)	-36.1 (-36.9--35.4)	0 (0-0)	-38.3 (-41.1--35.4)	0 (0-0)	0 (0-0)	0 (0-0)	0 (0-0)
Yamagata	0 (0-0)	-40.6 (-40.6--40.6)	0 (0-0)	-37.2 (-41.8--31.3)	0 (0-0)	-37.3 (-37.9--36.8)	0 (0-0)	-38.4 (-40.8--36.1)	0 (0-0)	0 (0-0)	0 (0-0)	0 (0-0)
Yamaguchi	0 (0-0)	-36.8 (-36.8--36.8)	0 (0-0)	-31.7 (-37.7--25.6)	0 (0-0)	-31.5 (-32.3--30.7)	0 (0-0)	-33.2 (-36.2--30.3)	0 (0-0)	0 (0-0)	0 (0-0)	0 (0-0)

Location	Birth , 2000	Births percent change (2000-2021)	All age cases, 2000	All age cases percent change (2000-2021)	All age deaths, 2000	All age deaths percent change (2000-2021)	Under-5 deaths, 2000	Under-5 deaths percent change (2000-2021)	Births, 2021	All age cases, 2021	All age deaths, 2021	Under-5 deaths, 2021
Yamanashi	0 (0-0)	-40 (-40--40)	0 (0-0)	-37.1 (-42.7--31.8)	0 (0-0)	-37.9 (-38.8--36.7)	0 (0-0)	-40.4 (-42.9--37.5)	0 (0-0)	0 (0-0)	0 (0-0)	0 (0-0)
Republic of Korea	0 (0-0)	-29.9 (-29.9--29.9)	0 (0-1)	-32.3 (-36.9--26.9)	0 (0-0)	-32.9 (-33.3--32.4)	0 (0-0)	-33.1 (-35.4--31.1)	0 (0-0)	0 (0-0)	0 (0-0)	0 (0-0)
Singapore	0 (0-0)	-7.3 (-7.3--7.3)	0 (0-0)	8.6 (-0.2-17.2)	0 (0-0)	12.7 (9.8-14.3)	0 (0-0)	20 (15-25.8)	0 (0-0)	0 (0-0)	0 (0-0)	0 (0-0)
**High-income North America**	143 (121-167)	-14.1 (-16--12.3)	5,360 (4,590-6,320)	-0.1 (-2.2-2)	131 (110-155)	1.9 (-1.2-5.4)	16 (11-22)	-22.5 (-25.7--19.7)	123 (104-144)	5,360 (4,610-6,290)	134 (113-158)	12 (9-17)
Canada	0 (0-0)	-27.9 (-58.9-11.5)	2 (1-3)	-32.2 (-61.4-7.6)	0 (0-0)	-30.3 (-61.2-9.9)	0 (0-0)	-30 (-63-10.8)	0 (0-0)	1 (1-2)	0 (0-0)	0 (0-0)
Greenland	0 (0-0)	-16.7 (-16.8--16.8)	0 (0-0)	-16.7 (-22.7--11)	0 (0-0)	-17.4 (-19.8--14.6)	0 (0-0)	-18.3 (-24.1--11.8)	0 (0-0)	0 (0-0)	0 (0-0)	0 (0-0)
United States of America	142 (120-166)	-16.7 (-18.5--15)	5,350 (4,580-6,300)	1.2 (-1-3.2)	131 (110-155)	3.2 (0.1-6.7)	16 (11-22)	-21.4 (-24.6--18.5)	118 (100-139)	5,410 (4,660-6,350)	135 (114-159)	13 (9-18)
Alabama	5 (4-5)	-13.8 (-20.4--7)	163 (137-193)	-0.8 (-8.1-7)	4 (4-5)	-1.1 (-8.6-7.1)	1 (0-1)	-19.7 (-27.9--8)	4 (3-5)	162 (134-192)	4 (4-5)	0 (0-1)
Alaska	0 (0-0)	-23.9 (-33.9--12.7)	7 (5-10)	-2.4 (-15-11.5)	0 (0-0)	-7.2 (-18.6-7.6)	0 (0-0)	-27.8 (-39.6--14)	0 (0-0)	7 (5-9)	0 (0-0)	0 (0-0)
Arizona	2 (2-3)	-7.9 (-15.1--0.3)	73 (58-93)	31.3 (21-42.6)	2 (1-2)	36.4 (24.5-49.4)	0 (0-0)	-5.8 (-17.9-6)	2 (2-3)	96 (77-121)	2 (2-3)	0 (0-0)
Arkansas	2 (1-2)	-8.6 (-16.8-0.7)	57 (46-72)	7.9 (-0.8-18.7)	2 (1-2)	7 (-2.8-19.4)	0 (0-0)	-12 (-24.3-3.5)	1 (1-2)	62 (50-79)	2 (1-2)	0 (0-0)
California	11 (9-13)	-26.4 (-32.8--20.2)	408 (347-489)	-1.9 (-10.2-5.5)	9 (8-11)	5.7 (-3.9-15.4)	1 (1-2)	-28.4 (-38.6--18)	8 (7-10)	401 (334-489)	10 (8-12)	1 (1-1)
Colorado	1 (1-2)	-22.7 (-38.6--10.8)	42 (30-58)	-6 (-25.1-8.1)	1 (1-1)	-3.2 (-24.3-13.6)	0 (0-0)	-29.6 (-45.3--16.9)	1 (1-1)	40 (27-58)	1 (1-1)	0 (0-0)
Connecticut	2 (1-2)	-38.2 (-44.2--32.3)	68 (53-85)	-20.4 (-27.8--13.2)	2 (1-2)	-17.8 (-26.1--9.5)	0 (0-0)	-40 (-48--31.6)	1 (1-1)	54 (42-70)	1 (1-2)	0 (0-0)
Delaware	1 (1-1)	-9 (-16.5--2.1)	44 (35-55)	8.1 (-0.5-16.2)	1 (1-1)	9.8 (-0.1-19.1)	0 (0-0)	-15.6 (-26.4--5.3)	1 (1-1)	48 (39-59)	1 (1-2)	0 (0-0)
District of Columbia	1 (1-2)	26.7 (10.3-43.6)	39 (31-48)	20.4 (5.5-35.5)	1 (1-1)	23.6 (7.6-41.3)	0 (0-0)	11.6 (-11.4-37.2)	2 (1-2)	47 (36-60)	1 (1-2)	0 (0-0)
Florida	16 (14-18)	-7 (-13.8--0.1)	605 (533-681)	17 (8.9-25.2)	15 (13-18)	18.8 (9.3-28.3)	2 (1-3)	-11.3 (-22.4-3.3)	15 (13-16)	708 (631-786)	18 (16-20)	2 (1-2)
Georgia	7 (6-8)	-12.5 (-19.1--5.8)	231 (198-270)	13.8 (4.9-21.7)	6 (5-7)	16.4 (6.9-26.5)	1 (1-1)	-14.7 (-24.9--2.5)	6 (5-7)	263 (230-302)	7 (6-8)	1 (0-1)
Hawaii	0 (0-0)	-16 (-14.2--14.2)	0 (0-1)	6.6 (5-10.9)	0 (0-0)	9.3 (6.6-16.3)	0 (0-0)	-13.2 (-19.5--1.8)	0 (0-0)	0 (0-1)	0 (0-0)	0 (0-0)
Idaho	0 (0-0)	10.3 (-9.9-59)	4 (2-7)	31 (7.2-96.9)	0 (0-0)	26.1 (3.2-85.5)	0 (0-0)	-2.7 (-24.9-45.5)	0 (0-0)	5 (3-9)	0 (0-0)	0 (0-0)
Illinois	5 (4-6)	-51.4 (-55.9--46.4)	181 (150-221)	-32.4 (-38.8--26.2)	4 (4-5)	-31 (-37.7--23.7)	1 (0-1)	-51 (-57.9--43.5)	2 (2-3)	122 (99-152)	3 (2-4)	0 (0-0)
Indiana	2 (2-3)	-20.2 (-27.5--13.7)	88 (72-108)	-7.4 (-15.7-0.2)	2 (2-3)	-7.5 (-16.2-0.5)	0 (0-0)	-25.6 (-34.8--14.8)	2 (1-2)	81 (65-102)	2 (2-3)	0 (0-0)
Iowa	1 (1-1)	-19.4 (-28.1--10.5)	39 (28-54)	-13.5 (-22.8--3.8)	1 (1-1)	-14.7 (-24.5--5)	0 (0-0)	-25.7 (-36.2--13.8)	1 (1-1)	34 (23-48)	1 (1-1)	0 (0-0)
Kansas	1 (1-2)	-63.3 (-70.8--56)	42 (28-64)	-54.2 (-63.2--45.3)	1 (1-2)	-55.7 (-64.7--46.2)	0 (0-0)	-65.3 (-73.1--56.9)	0 (0-1)	19 (13-30)	0 (0-1)	0 (0-0)
Kentucky	0 (0-1)	-6.4 (-22.2-29.2)	13 (9-19)	4.9 (-12.1-48.7)	0 (0-1)	4.6 (-12.6-46.3)	0 (0-0)	-11.8 (-29.6-16.2)	0 (0-0)	14 (10-19)	0 (0-1)	0 (0-0)
Louisiana	4 (3-5)	-28.2 (-33.8--23)	126 (105-151)	-17.6 (-23--11.4)	3 (3-4)	-19.2 (-25.8--12.5)	1 (0-1)	-36.6 (-44.9--26.3)	3 (2-3)	104 (87-124)	3 (2-3)	0 (0-0)
Maine	0 (0-0)	-24.1 (-83.4--16.8)	0 (0-1)	-16.5 (-80.2--6.4)	0 (0-0)	-11.2 (-80.1-3)	0 (0-0)	-30.4 (-86.4--16.1)	0 (0-0)	0 (0-1)	0 (0-0)	0 (0-0)
Maryland	6 (5-7)	-14.5 (-21.4--7.3)	229 (196-263)	1.5 (-5.8-9.1)	6 (5-6)	4.1 (-4.5-13.5)	1 (0-1)	-17.3 (-27.9--6.1)	5 (4-6)	233 (199-272)	6 (5-7)	1 (0-1)
Massachusetts	2 (2-3)	-4.2 (-11.7-3.2)	103 (82-130)	10.3 (2.4-19.1)	2 (2-3)	17.4 (7.8-28.2)	0 (0-0)	-7.8 (-17.5-3.6)	2 (2-3)	114 (92-146)	3 (2-4)	0 (0-0)

Location	Birth , 2000	Births percent change (2000-2021)	All age cases, 2000	All age cases percent change (2000-2021)	All age deaths, 2000	All age deaths percent change (2000-2021)	Under-5 deaths, 2000	Under-5 deaths percent change (2000-2021)	Births, 2021	All age cases, 2021	All age deaths, 2021	Under-5 deaths, 2021
Michigan	5 (4-6)	-28.5 (-33.9--22)	200 (169-235)	-19.5 (-25.1--13.2)	5 (4-6)	-17.2 (-24.4--8.4)	1 (0-1)	-37.3 (-44.4--28.4)	4 (3-4)	161 (135-191)	4 (3-5)	0 (0-1)
Minnesota	1 (0-1)	-2.8 (-26.4-34.1)	24 (16-38)	8.4 (-17.8-50.9)	1 (0-1)	15 (-14.2-55.5)	0 (0-0)	-2.8 (-28.6-33.8)	1 (0-1)	27 (18-40)	1 (0-1)	0 (0-0)
Mississippi	4 (3-5)	-40 (-45.8--33.8)	124 (102-150)	-25.6 (-32.8--17.6)	3 (3-4)	-27.6 (-35.3--18.6)	1 (0-1)	-46.1 (-53.5--35.9)	2 (2-3)	92 (74-115)	3 (2-3)	0 (0-0)
Missouri	2 (2-3)	-22.7 (-30.8--13.5)	84 (68-105)	-8.9 (-17.9-0.1)	2 (2-3)	-9.3 (-18.9-0.9)	0 (0-0)	-27.7 (-37.5--15.7)	2 (1-2)	76 (59-98)	2 (2-3)	0 (0-0)
Montana	0 (0-0)	-9.3 (-83.9-1.6)	0 (0-1)	-2.6 (-81.8-12)	0 (0-0)	-3.4 (-83.1-13.3)	0 (0-0)	-17.6 (-86.9-0.4)	0 (0-0)	0 (0-1)	0 (0-0)	0 (0-0)
Nebraska	0 (0-0)	2.3 (-24.7-48.7)	9 (5-16)	11.9 (-17.4-63.7)	0 (0-0)	13 (-17.1-63.5)	0 (0-0)	1.7 (-28.4-42.7)	0 (0-0)	11 (6-17)	0 (0-0)	0 (0-0)
Nevada	2 (1-2)	3.2 (-3.9-11.9)	54 (43-67)	45.8 (34.8-57.6)	1 (1-2)	49.4 (38.5-61.8)	0 (0-0)	12.9 (0.4-28.5)	2 (1-2)	78 (64-98)	2 (2-2)	0 (0-0)
New Hampshire	0 (0-0)	-21.4 (-53--18.1)	2 (1-4)	-9.4 (-46.8--2.1)	0 (0-0)	-5.1 (-43.8-5.9)	0 (0-0)	-34.2 (-60.8--21.3)	0 (0-0)	2 (1-4)	0 (0-0)	0 (0-0)
New Jersey	5 (4-6)	-17.1 (-23.4--10.2)	198 (167-236)	-1.5 (-8.5-6)	5 (4-6)	2.1 (-5.7-11.1)	1 (0-1)	-20.1 (-29.7--10.3)	4 (4-5)	195 (165-233)	5 (4-6)	0 (0-1)
New Mexico	0 (0-0)	-16.9 (-23.8-134)	2 (1-5)	14.1 (1.4-204)	0 (0-0)	11.8 (-0.5-212)	0 (0-0)	-20.2 (-35.9-127)	0 (0-0)	3 (1-5)	0 (0-0)	0 (0-0)
New York	13 (11-14)	-13.3 (-18.4--7.4)	547 (490-605)	0.5 (-5.4-6.6)	12 (10-14)	7.3 (0.1-15.9)	1 (1-2)	-15.1 (-25.2--4.5)	11 (10-12)	549 (495-607)	13 (11-14)	1 (1-1)
North Carolina	6 (5-7)	-11.7 (-17.7--4.9)	222 (192-255)	10.2 (3.3-18)	5 (5-6)	12 (3.9-21.8)	1 (0-1)	-13.9 (-23.7--1)	5 (5-6)	245 (211-281)	6 (5-7)	1 (0-1)
North Dakota	0 (0-0)	12.9 (-82.2-36.8)	0 (0-1)	-1.1 (-83.4-22.2)	0 (0-0)	-3.3 (-85.1-22)	0 (0-0)	-4.7 (-84.6-28)	0 (0-0)	0 (0-1)	0 (0-0)	0 (0-0)
Ohio	3 (3-4)	-8.7 (-14.7--0.8)	123 (102-151)	0.8 (-6.2-9.7)	3 (2-4)	3.5 (-4-11.9)	0 (0-0)	-15.5 (-25.6--3.5)	3 (2-4)	124 (103-152)	3 (3-4)	0 (0-0)
Oklahoma	1 (1-2)	-62.1 (-76.7--51.2)	45 (29-68)	-55.8 (-72.8--42.5)	1 (1-2)	-57.2 (-73.7--44.5)	0 (0-0)	-64.4 (-78.7--52.8)	0 (0-1)	20 (12-34)	1 (0-1)	0 (0-0)
Oregon	0 (0-0)	4.2 (-14.8-168)	5 (2-9)	27.9 (2.1-226)	0 (0-0)	27.7 (3.4-232)	0 (0-0)	-9.8 (-32.6-143)	0 (0-0)	6 (3-10)	0 (0-0)	0 (0-0)
Pennsylvania	4 (3-5)	-13.3 (-20.4--6.3)	177 (148-216)	-4.7 (-12.1-3.6)	5 (4-6)	-3.9 (-12.2-5.4)	0 (0-1)	-19.9 (-30.4--7.2)	4 (3-4)	169 (137-208)	4 (4-5)	0 (0-1)
Rhode Island	0 (0-0)	-23.4 (-33.9--13.6)	16 (13-21)	-14.6 (-25.9--3.9)	0 (0-0)	-7.1 (-19.3-6.2)	0 (0-0)	-27 (-40--12.7)	0 (0-0)	14 (11-18)	0 (0-0)	0 (0-0)
South Carolina	4 (3-5)	-23.3 (-29.3--16.3)	141 (119-163)	-8.5 (-15.4--1.3)	4 (3-4)	-10.9 (-17.7--2.5)	1 (0-1)	-32.2 (-41.7--21.4)	3 (3-3)	129 (111-148)	3 (3-4)	0 (0-0)
South Dakota	0 (0-0)	-16.6 (-86.3-5)	0 (0-1)	-11.1 (-84.9-13.9)	0 (0-0)	-12.2 (-86.2-15)	0 (0-0)	-22.6 (-88.3-8.1)	0 (0-0)	0 (0-1)	0 (0-0)	0 (0-0)
Tennessee	4 (4-5)	-15.2 (-22.2--8.6)	158 (133-189)	-3.1 (-10.5-4.1)	4 (4-5)	-5.7 (-13.4-2.2)	1 (0-1)	-28 (-38.1--16.7)	4 (3-4)	153 (128-181)	4 (3-5)	0 (0-1)
Texas	9 (8-11)	5.6 (-1.4-12.2)	323 (277-375)	34.5 (25.6-42.6)	7 (6-8)	40.1 (30.1-50.1)	1 (1-1)	5.7 (-6.7-20.4)	10 (8-12)	435 (377-503)	10 (8-12)	1 (1-1)
Utah	0 (0-0)	-18.6 (-69.8--1)	2 (0-4)	15 (-56.3-42.4)	0 (0-0)	18.6 (-56-48.4)	0 (0-0)	-19.9 (-73-6.7)	0 (0-0)	2 (1-5)	0 (0-0)	0 (0-0)
Vermont	0 (0-0)	-23.9 (-78.7--17.2)	0 (0-1)	-16.4 (-74.6--7.3)	0 (0-0)	-11.5 (-74.8-1.9)	0 (0-0)	-28.6 (-82.5--15.1)	0 (0-0)	0 (0-0)	0 (0-0)	0 (0-0)
Virginia	4 (4-5)	-12.8 (-20.2--5.5)	175 (146-211)	4.4 (-4.4-13.6)	4 (4-5)	5.9 (-3-15.7)	0 (0-1)	-15.8 (-26.7--4.8)	4 (3-5)	183 (150-224)	5 (4-6)	0 (0-1)
Washington	2 (2-3)	-18.6 (-27.7--7.7)	85 (66-110)	-8.7 (-18.7-2.9)	2 (2-3)	-6.5 (-17.7-7.1)	0 (0-0)	-28.9 (-40.2--16.9)	2 (1-2)	78 (56-105)	2 (1-3)	0 (0-0)
West Virginia	0 (0-0)	-15.4 (-30-139)	2 (1-5)	-6.3 (-21.8-171)	0 (0-0)	-4.1 (-21.6-177)	0 (0-0)	-10.4 (-29.3-146)	0 (0-0)	2 (1-4)	0 (0-0)	0 (0-0)
Wisconsin	2 (1-2)	-39.1 (-48.9--27.2)	68 (53-88)	-30.1 (-41.2--16.6)	2 (1-2)	-28.9 (-40.3--14.8)	0 (0-0)	-43.4 (-53.3--31.9)	1 (1-1)	47 (34-65)	1 (1-2)	0 (0-0)
Wyoming	0 (0-0)	-10.5 (-8.7--8.7)	0 (0-0)	5.3 (2.8-10.4)	0 (0-0)	3.8 (0.3-10.3)	0 (0-0)	-6 (-16.1-4.6)	0 (0-0)	0 (0-0)	0 (0-0)	0 (0-0)

Location	Birth , 2000	Births percent change (2000-2021)	All age cases, 2000	All age cases percent change (2000-2021)	All age deaths, 2000	All age deaths percent change (2000-2021)	Under-5 deaths, 2000	Under-5 deaths percent change (2000-2021)	Births, 2021	All age cases, 2021	All age deaths, 2021	Under-5 deaths, 2021
**Southern Latin America**	0 (0-0)	17.3 (-4.9-71.6)	1 (0-1)	23.5 (-4.9-88.4)	0 (0-0)	19.8 (-3.6-76.1)	0 (0-0)	17.1 (-5.6-71.6)	0 (0-0)	1 (1-2)	0 (0-0)	0 (0-0)
Argentina	0 (0-0)	-1.6 (-1.9--1.7)	1 (0-1)	-0.2 (-7.8-7.5)	0 (0-0)	-0.4 (-1-0.1)	0 (0-0)	-1.9 (-5.6-1.7)	0 (0-0)	1 (0-1)	0 (0-0)	0 (0-0)
Chile	0 (0-0)	86.1 (-11.5-429)	0 (0-0)	99.3 (-8.6-448)	0 (0-0)	91.8 (-8.9-445)	0 (0-0)	89.5 (-12.1-453)	0 (0-0)	0 (0-1)	0 (0-0)	0 (0-0)
Uruguay	0 (0-0)	-13.9 (-14--13.7)	0 (0-0)	-15.1 (-21.7--7.8)	0 (0-0)	-15.3 (-15.6--14.8)	0 (0-0)	-15.6 (-19.1--11.8)	0 (0-0)	0 (0-0)	0 (0-0)	0 (0-0)
**Western Europe**	33 (26-44)	-18.5 (-22.2--14.7)	490 (383-625)	-3 (-6.5-0.7)	36 (29-48)	-6.2 (-10.5--2.2)	16 (12-23)	-11.5 (-17.3--5.8)	27 (21-36)	476 (374-605)	34 (27-45)	14 (10-20)
Andorra	0 (0-0)	-16.8 (-16.9--16.8)	0 (0-0)	-15.5 (-23.3--7)	0 (0-0)	-18.3 (-21.2--14.4)	0 (0-0)	-28.2 (-30.6--25.3)	0 (0-0)	0 (0-0)	0 (0-0)	0 (0-0)
Austria	0 (0-1)	32.5 (10.4-94.2)	2 (2-3)	15.6 (-5.3-61.2)	0 (0-1)	16.8 (-2.7-71)	0 (0-0)	28.6 (0.1-98.3)	0 (0-1)	3 (2-4)	0 (0-1)	0 (0-0)
Belgium	0 (0-0)	4.8 (-12.8-37.6)	0 (0-0)	6.8 (-11.7-41.5)	0 (0-0)	6.8 (-11.2-40.1)	0 (0-0)	6.2 (-11.2-39.4)	0 (0-0)	0 (0-0)	0 (0-0)	0 (0-0)
Cyprus	0 (0-0)	39.9 (39.8-40)	0 (0-0)	23.2 (14.1-32.5)	0 (0-0)	23.6 (20.4-25.7)	0 (0-0)	32.5 (27.3-37.4)	0 (0-0)	0 (0-0)	0 (0-0)	0 (0-0)
Denmark	0 (0-0)	-5.2 (-52.6--2)	0 (0-0)	-8.6 (-53.1-2.3)	0 (0-0)	-10.2 (-54.6--5.3)	0 (0-0)	-11.4 (-53.8--3.9)	0 (0-0)	0 (0-0)	0 (0-0)	0 (0-0)
Finland	0 (0-0)	-15.9 (-16--15.9)	0 (0-0)	-12.8 (-19.7--5.5)	0 (0-0)	-12.5 (-13.6--11.3)	0 (0-0)	-15.2 (-19--11)	0 (0-0)	0 (0-0)	0 (0-0)	0 (0-0)
France	4 (3-5)	-10.1 (-13.3-6)	12 (9-16)	-3.2 (-11-8.9)	4 (3-5)	-2.7 (-6.2-14.8)	3 (2-4)	-4.8 (-9.6-13.6)	3 (2-5)	12 (9-16)	3 (3-5)	3 (2-4)
Germany	0 (0-1)	-1.3 (-26.8--0.2)	2 (1-3)	-3 (-29.8-6.3)	0 (0-1)	-1.3 (-26.7-0.9)	0 (0-0)	2.6 (-23.8-8.7)	0 (0-1)	2 (1-3)	0 (0-1)	0 (0-0)
Greece	7 (5-9)	-13.9 (-24.4--2.6)	71 (56-90)	-12.7 (-22--3.4)	7 (6-10)	-11.6 (-22.3--0.1)	4 (3-6)	-10.7 (-25-5.2)	6 (4-8)	62 (48-78)	7 (5-9)	4 (2-5)
Iceland	0 (0-0)	2.3 (2.2-2.3)	0 (0-0)	-1.4 (-8.8-6.9)	0 (0-0)	-1.4 (-2.5-0.2)	0 (0-0)	-3.8 (-8.6-1.1)	0 (0-0)	0 (0-0)	0 (0-0)	0 (0-0)
Ireland	0 (0-0)	-4.2 (-43.6-3.2)	0 (0-0)	4.3 (-40-22.4)	0 (0-0)	5.4 (-37.8-15.2)	0 (0-0)	2.2 (-39.1-14.3)	0 (0-0)	0 (0-0)	0 (0-0)	0 (0-0)
Israel	0 (0-0)	46.3 (46.3-46.4)	0 (0-0)	47.8 (37.4-57.3)	0 (0-0)	48.6 (47.6-49.4)	0 (0-0)	50 (44.8-56)	0 (0-0)	0 (0-0)	0 (0-0)	0 (0-0)
Italy	19 (14-26)	-24.5 (-28--20.7)	383 (293-500)	-1.5 (-5.3-2.9)	22 (16-29)	-6.6 (-10.9--1.7)	7 (5-11)	-19.5 (-25.1--13.6)	15 (11-20)	377 (293-488)	20 (15-28)	6 (4-9)
Abruzzo	0 (0-0)	-36.8 (-79.2--19.8)	1 (0-1)	-24.7 (-75.2-4.3)	0 (0-0)	-27.7 (-76.1--5.9)	0 (0-0)	-35.2 (-79--9.8)	0 (0-0)	0 (0-1)	0 (0-0)	0 (0-0)
Basilicata	0 (0-0)	-43.5 (-80.4--3)	0 (0-1)	-33.2 (-75.6-15.8)	0 (0-0)	-35.5 (-77.4-9.1)	0 (0-0)	-45.1 (-81.9--4.7)	0 (0-0)	0 (0-1)	0 (0-0)	0 (0-0)
Calabria	0 (0-1)	-31.5 (-38.6--22.2)	9 (5-15)	-21.9 (-30.3--10.4)	1 (0-1)	-25.1 (-33.3--14.8)	0 (0-0)	-32.3 (-41.1--20.6)	0 (0-0)	7 (4-12)	0 (0-1)	0 (0-0)
Campania	2 (1-2)	-9.7 (-21.1-6.7)	31 (20-44)	18.5 (3.8-37.5)	2 (1-3)	10.9 (-3.2-31.8)	1 (0-1)	-8.6 (-24.4-15.6)	1 (1-2)	37 (26-50)	2 (1-3)	1 (0-1)
Emilia-Romagna	2 (1-2)	-14.6 (-23.1--5.7)	28 (21-36)	22.6 (10.9-34.7)	2 (1-2)	13.8 (3.2-25.4)	1 (0-1)	1.6 (-13.3-17.9)	2 (1-2)	34 (26-45)	2 (2-3)	1 (0-1)
Friuli-Venezia Giulia	0 (0-1)	-28.7 (-37.1--18.3)	7 (5-11)	-5.6 (-15.5-7.6)	0 (0-1)	-11.2 (-21.4-1.5)	0 (0-0)	-19.7 (-32.5--3.5)	0 (0-0)	7 (4-10)	0 (0-1)	0 (0-0)
Lazio	1 (1-2)	-27.1 (-36.4--18)	21 (14-31)	-8.6 (-20.4-1.9)	1 (1-2)	-12.4 (-24.2--1.9)	0 (0-1)	-19.2 (-32--4.7)	1 (1-1)	20 (13-28)	1 (1-2)	0 (0-1)
Liguria	1 (1-1)	-12.5 (-22.4--0.5)	20 (15-25)	7.8 (-1.9-19)	1 (1-2)	5.4 (-5.9-18.4)	0 (0-1)	-1.3 (-17.7-20)	1 (1-1)	21 (16-27)	1 (1-2)	0 (0-1)
Lombardia	1 (1-2)	0.4 (-11.4-28.3)	24 (16-36)	32.6 (17.2-62.8)	1 (1-2)	23.3 (8.8-57)	0 (0-1)	11.4 (-6.4-49.5)	1 (1-2)	32 (22-46)	2 (1-3)	0 (0-1)
Marche	0 (0-1)	-33.8 (-41.7--27.3)	8 (5-12)	-10.8 (-20.7--0.2)	0 (0-1)	-15.8 (-25--6.7)	0 (0-0)	-26.3 (-37.3--15.9)	0 (0-0)	7 (4-11)	0 (0-1)	0 (0-0)
Molise	0 (0-0)	-40.3 (-93.2--31.5)	0 (0-0)	-27.8 (-91.7--9.7)	0 (0-0)	-32.5 (-92.3--20)	0 (0-0)	-40.7 (-93.1--26.6)	0 (0-0)	0 (0-0)	0 (0-0)	0 (0-0)

Location	Birth , 2000	Births percent change (2000-2021)	All age cases, 2000	All age cases percent change (2000-2021)	All age deaths, 2000	All age deaths percent change (2000-2021)	Under-5 deaths, 2000	Under-5 deaths percent change (2000-2021)	Births, 2021	All age cases, 2021	All age deaths, 2021	Under-5 deaths, 2021
Piemonte	0 (0-1)	2.8 (-12.7-34.7)	9 (6-14)	29.9 (10.9-65)	1 (0-1)	24.1 (5.1-61.9)	0 (0-0)	13.8 (-7.8-54.9)	0 (0-1)	11 (8-17)	1 (0-1)	0 (0-0)
Provincia autonoma di Bolzano	0 (0-0)	-21.4 (-35.4--5.4)	1 (1-2)	-4.5 (-21.9-17)	0 (0-0)	-10 (-26.6-8.2)	0 (0-0)	-18.2 (-34.6-3.1)	0 (0-0)	1 (1-2)	0 (0-0)	0 (0-0)
Provincia autonoma di Trento	0 (0-0)	-31.8 (-62.5--13.4)	1 (0-2)	-11.6 (-50.6-18.4)	0 (0-0)	-18.2 (-54.9-6.1)	0 (0-0)	-29.2 (-61.6--4.5)	0 (0-0)	1 (0-1)	0 (0-0)	0 (0-0)
Puglia	0 (0-0)	-51.6 (-82.4--35.3)	3 (1-6)	-37.5 (-77.7--9.3)	0 (0-0)	-39.9 (-78.3--17)	0 (0-0)	-50.9 (-82.5--27.3)	0 (0-0)	2 (1-4)	0 (0-0)	0 (0-0)
Sardegna	0 (0-0)	-48 (-76.8--34)	1 (1-3)	-36 (-70--10.2)	0 (0-0)	-38.7 (-73.1--19.1)	0 (0-0)	-44.9 (-76.4--25.1)	0 (0-0)	1 (0-2)	0 (0-0)	0 (0-0)
Sicilia	7 (6-10)	-38 (-45.1--30.5)	158 (129-192)	-18.2 (-24.7--11.1)	8 (7-11)	-23.9 (-32.8--14.9)	3 (2-4)	-36.9 (-46.1--25.7)	5 (4-6)	129 (107-153)	6 (5-8)	2 (1-3)
Toscana	0 (0-1)	-13.8 (-33.7-21)	7 (4-12)	5.7 (-19-42.1)	0 (0-1)	3.5 (-20.3-44)	0 (0-0)	0.6 (-24.2-41.1)	0 (0-0)	7 (5-12)	0 (0-1)	0 (0-0)
Umbria	0 (0-0)	-14.1 (-28.1-1.7)	4 (3-6)	7.8 (-10.1-26)	0 (0-0)	5.5 (-11.5-23.5)	0 (0-0)	-0.3 (-20.4-25.1)	0 (0-0)	4 (3-6)	0 (0-0)	0 (0-0)
Valle d'Aosta	0 (0-0)	-32.9 (-43.1--21.2)	0 (0-1)	-8.7 (-24.9-12.2)	0 (0-0)	-14.9 (-27.7-0.1)	0 (0-0)	-23.5 (-38.3--7.2)	0 (0-0)	0 (0-0)	0 (0-0)	0 (0-0)
Veneto	2 (2-3)	-18.4 (-25.7--10.4)	49 (39-60)	11.3 (2.4-20.8)	3 (2-3)	4.7 (-3.6-15.5)	1 (1-1)	-11.8 (-25.1-2.5)	2 (1-3)	54 (44-67)	3 (2-4)	1 (0-1)
Luxembourg	0 (0-0)	17.9 (17.6-17.9)	0 (0-0)	20.7 (11.3-30.3)	0 (0-0)	19.7 (18.5-21.6)	0 (0-0)	16.2 (11-21.5)	0 (0-0)	0 (0-0)	0 (0-0)	0 (0-0)
Malta	0 (0-0)	2.9 (2.9-3.4)	0 (0-0)	-12 (-19.1--4.2)	0 (0-0)	-11.5 (-13.4--10)	0 (0-0)	-6 (-9.8--1.7)	0 (0-0)	0 (0-0)	0 (0-0)	0 (0-0)
Monaco	0 (0-0)	-12 (-48.1--5.1)	0 (0-0)	7.7 (-32.2-24.3)	0 (0-0)	7.7 (-37.6-17)	0 (0-0)	8.3 (-40.1-22.4)	0 (0-0)	0 (0-0)	0 (0-0)	0 (0-0)
Netherlands	0 (0-0)	-13.4 (-13.4--13.4)	0 (0-0)	-10.3 (-17.2--3.8)	0 (0-0)	-11 (-11.6--10)	0 (0-0)	-12.1 (-15.3--7.9)	0 (0-0)	0 (0-0)	0 (0-0)	0 (0-0)
Norway	0 (0-0)	-4.1 (-18.2-8.5)	0 (0-1)	-0.3 (-14-14.5)	0 (0-0)	-1.1 (-14.6-13.3)	0 (0-0)	-4.9 (-18.7-9.6)	0 (0-0)	0 (0-1)	0 (0-0)	0 (0-0)
Agder	0 (0-0)	-4.9 (-5.3--5.3)	0 (0-0)	-3.8 (-14.2-8.1)	0 (0-0)	-4.6 (-6.4--2.3)	0 (0-0)	-9.3 (-15.1--4.3)	0 (0-0)	0 (0-0)	0 (0-0)	0 (0-0)
Innlandet	0 (0-0)	-21 (-20.9--20.9)	0 (0-0)	-17.8 (-26.8--6.3)	0 (0-0)	-19 (-20.1--16.8)	0 (0-0)	-22.2 (-26.8--16.7)	0 (0-0)	0 (0-0)	0 (0-0)	0 (0-0)
Møre og Romsdal	0 (0-0)	-18.2 (-17.9--17.9)	0 (0-0)	-12.3 (-22.9--0.6)	0 (0-0)	-12.7 (-14--10.1)	0 (0-0)	-16.5 (-21.8--10.3)	0 (0-0)	0 (0-0)	0 (0-0)	0 (0-0)
Nordland	0 (0-0)	-15.5 (-17--17)	0 (0-0)	-21.7 (-29.9--12.7)	0 (0-0)	-22.5 (-24.7--21.7)	0 (0-0)	-25.4 (-31.2--22.3)	0 (0-0)	0 (0-0)	0 (0-0)	0 (0-0)
Oslo	0 (0-0)	24.8 (24.7-24.7)	0 (0-0)	32.4 (19.3-48.2)	0 (0-0)	31.6 (29.5-33.7)	0 (0-0)	27.6 (17.9-36.4)	0 (0-0)	0 (0-0)	0 (0-0)	0 (0-0)
Rogaland	0 (0-0)	-3 (-1.5--1.5)	0 (0-0)	5 (-5.7-18)	0 (0-0)	4.7 (4.1-8.5)	0 (0-0)	-0.6 (-7.1-7.3)	0 (0-0)	0 (0-0)	0 (0-0)	0 (0-0)
Troms og Finnmark	0 (0-0)	-21 (-21--21)	0 (0-0)	-16.6 (-24.7--8)	0 (0-0)	-17.6 (-18.6--15.9)	0 (0-0)	-19.7 (-23.8--15.3)	0 (0-0)	0 (0-0)	0 (0-0)	0 (0-0)
Trøndelag	0 (0-0)	3.5 (-48.1-4.8)	0 (0-0)	-3.5 (-49.5-11.9)	0 (0-0)	-4.3 (-52.2--0.6)	0 (0-0)	-6.9 (-51.1-5)	0 (0-0)	0 (0-0)	0 (0-0)	0 (0-0)
Vestfold og Telemark	0 (0-0)	-15 (-13.9--13.9)	0 (0-0)	-12.9 (-22.5--0.3)	0 (0-0)	-12.9 (-13.1--9.7)	0 (0-0)	-15.8 (-22.2--9.1)	0 (0-0)	0 (0-0)	0 (0-0)	0 (0-0)
Vestland	0 (0-0)	-5.5 (-4.1--4.1)	0 (0-0)	-3.3 (-14.8-11.6)	0 (0-0)	-4.5 (-4.3--0.6)	0 (0-0)	-7.8 (-13.7-1.6)	0 (0-0)	0 (0-0)	0 (0-0)	0 (0-0)
Viken	0 (0-0)	-5.2 (-4.5--4.5)	0 (0-0)	4.6 (-8.7-18.8)	0 (0-0)	4.3 (2.6-8.2)	0 (0-0)	-1 (-12.4-7.7)	0 (0-0)	0 (0-0)	0 (0-0)	0 (0-0)
Portugal	0 (0-0)	-33.5 (-33.7--33.4)	0 (0-0)	-26.7 (-31.7--21.8)	0 (0-0)	-26.5 (-26.9--26)	0 (0-0)	-27.7 (-29.9--25.2)	0 (0-0)	0 (0-0)	0 (0-0)	0 (0-0)
San Marino	0 (0-0)	-4.4 (-4.5--4.4)	0 (0-0)	-2 (-9-5.7)	0 (0-0)	-3 (-3.9--1.4)	0 (0-0)	-6 (-10--2.1)	0 (0-0)	0 (0-0)	0 (0-0)	0 (0-0)
Spain	2 (2-3)	-13.2 (-30.3-15.8)	16 (12-21)	-0.4 (-17.3-26)	2 (2-3)	-0.9 (-20.4-32)	1 (1-2)	0.3 (-23-39.9)	2 (1-3)	16 (12-20)	2 (2-3)	1 (1-2)

Location	Birth , 2000	Births percent change (2000-2021)	All age cases, 2000	All age cases percent change (2000-2021)	All age deaths, 2000	All age deaths percent change (2000-2021)	Under-5 deaths, 2000	Under-5 deaths percent change (2000-2021)	Births, 2021	All age cases, 2021	All age deaths, 2021	Under-5 deaths, 2021
Sweden	0 (0-0)	33.9 (21.8-121)	2 (1-3)	22.4 (3.6-81.7)	0 (0-0)	24.5 (15.8-104)	0 (0-0)	36.4 (18.6-131)	0 (0-0)	2 (2-4)	0 (0-0)	0 (0-0)
Stockholm	0 (0-0)	33.5 (28.3-127)	0 (0-1)	40.8 (14.8-138)	0 (0-0)	41 (33.9-139)	0 (0-0)	46.3 (21.8-151)	0 (0-0)	1 (0-1)	0 (0-0)	0 (0-0)
Sweden except Stockholm	0 (0-0)	34 (29.1-121)	2 (1-2)	17.7 (-2.5-88)	0 (0-0)	20.2 (12.7-97.4)	0 (0-0)	33.7 (13-131)	0 (0-0)	2 (1-3)	0 (0-0)	0 (0-0)
Switzerland	0 (0-0)	15.9 (-16.7-17.9)	0 (0-0)	11.9 (-20.1-22)	0 (0-0)	12.2 (-19.1-15)	0 (0-0)	15.7 (-16.4-22.2)	0 (0-0)	0 (0-0)	0 (0-0)	0 (0-0)
United Kingdom	0 (0-0)	17.3 (16.5-30.3)	0 (0-1)	7.2 (5-17.3)	0 (0-0)	6.9 (5.4-17.7)	0 (0-0)	8.3 (6.5-19.4)	0 (0-0)	0 (0-1)	0 (0-0)	0 (0-0)
England	0 (0-0)	18.9 (17.8-34.3)	0 (0-1)	9 (7.5-21.2)	0 (0-0)	8.9 (8-22.1)	0 (0-0)	10.5 (9.3-24.4)	0 (0-0)	0 (0-1)	0 (0-0)	0 (0-0)
East Midlands	0 (0-0)	19.4 (19.2-21)	0 (0-0)	7.3 (4.1-10.8)	0 (0-0)	7.1 (6.2-8.5)	0 (0-0)	9.4 (7.4-12)	0 (0-0)	0 (0-0)	0 (0-0)	0 (0-0)
Derby	0 (0-0)	15.8 (15.9-15.9)	0 (0-0)	13.4 (5.4-21.5)	0 (0-0)	13.2 (13-13.8)	0 (0-0)	12.8 (9-18.1)	0 (0-0)	0 (0-0)	0 (0-0)	0 (0-0)
Derbyshire	0 (0-0)	5.4 (5.1-5.1)	0 (0-0)	-7.1 (-13.5--0.1)	0 (0-0)	-8 (-9.1--7.1)	0 (0-0)	-6.7 (-10.9--2.4)	0 (0-0)	0 (0-0)	0 (0-0)	0 (0-0)
Leicester	0 (0-0)	36.5 (35.7-35.7)	0 (0-0)	30.5 (20.8-40.8)	0 (0-0)	30.2 (28.8-30.2)	0 (0-0)	31.6 (25.3-39.4)	0 (0-0)	0 (0-0)	0 (0-0)	0 (0-0)
Leicestershire	0 (0-0)	12 (12-12)	0 (0-0)	0.6 (-6.4-7.9)	0 (0-0)	-0.5 (-1.3-0.5)	0 (0-0)	1.2 (-3-5.4)	0 (0-0)	0 (0-0)	0 (0-0)	0 (0-0)
Lincolnshire	0 (0-0)	30.9 (30.7-30.7)	0 (0-0)	10 (1.6-18.1)	0 (0-0)	8.5 (7-9.8)	0 (0-0)	11.9 (6.4-18)	0 (0-0)	0 (0-0)	0 (0-0)	0 (0-0)
Northamptonshire	0 (0-0)	21.2 (21.2-21.2)	0 (0-0)	13.1 (4.4-23)	0 (0-0)	12.8 (12.1-13.4)	0 (0-0)	14.3 (8.7-19.9)	0 (0-0)	0 (0-0)	0 (0-0)	0 (0-0)
Nottingham	0 (0-0)	37.8 (37.8-37.8)	0 (0-0)	30.6 (20.6-40.5)	0 (0-0)	30.5 (29-31.8)	0 (0-0)	34.9 (29.7-41.1)	0 (0-0)	0 (0-0)	0 (0-0)	0 (0-0)
Nottinghamshire	0 (0-0)	12.3 (12.7-12.7)	0 (0-0)	1.3 (-5.2-9.9)	0 (0-0)	0.6 (0.1-1.8)	0 (0-0)	2.8 (-1.4-7.8)	0 (0-0)	0 (0-0)	0 (0-0)	0 (0-0)
Rutland	0 (0-0)	16.1 (15.5-15.5)	0 (0-0)	-0.8 (-9.1-8.8)	0 (0-0)	-1.6 (-3.2--0.6)	0 (0-0)	0.4 (-4-5.7)	0 (0-0)	0 (0-0)	0 (0-0)	0 (0-0)
East of England	0 (0-0)	17.7 (13.4-20.7)	0 (0-0)	8 (3.3-11.7)	0 (0-0)	7.6 (4.1-9.7)	0 (0-0)	8.5 (4.6-11.1)	0 (0-0)	0 (0-0)	0 (0-0)	0 (0-0)
Bedford	0 (0-0)	17.8 (17.4-17.5)	0 (0-0)	7.6 (0.4-17.7)	0 (0-0)	7.2 (6.2-7.2)	0 (0-0)	8.1 (3.6-12.3)	0 (0-0)	0 (0-0)	0 (0-0)	0 (0-0)
Cambridgeshire	0 (0-0)	22.3 (22.8-22.8)	0 (0-0)	11.1 (0.7-20)	0 (0-0)	10.6 (10.2-11.8)	0 (0-0)	11.5 (6.5-17.8)	0 (0-0)	0 (0-0)	0 (0-0)	0 (0-0)
Central Bedfordshire	0 (0-0)	14.8 (14.8-14.8)	0 (0-0)	4.4 (-3.3-12.6)	0 (0-0)	3.9 (3.2-4.6)	0 (0-0)	4.9 (0.3-10)	0 (0-0)	0 (0-0)	0 (0-0)	0 (0-0)
Essex	0 (0-0)	14.6 (15.1-15.1)	0 (0-0)	1.9 (-7-10.8)	0 (0-0)	0.8 (0.2-2.1)	0 (0-0)	2.1 (-3.7-8)	0 (0-0)	0 (0-0)	0 (0-0)	0 (0-0)
Hertfordshire	0 (0-0)	16.1 (16.1-16.1)	0 (0-0)	10.8 (2.7-19.7)	0 (0-0)	10 (9.5-10.7)	0 (0-0)	9.8 (4.6-15.5)	0 (0-0)	0 (0-0)	0 (0-0)	0 (0-0)
Luton	0 (0-0)	20.3 (18.7-18.7)	0 (0-0)	22.3 (13-31.8)	0 (0-0)	22.3 (20.4-21)	0 (0-0)	22.6 (16.8-28.5)	0 (0-0)	0 (0-0)	0 (0-0)	0 (0-0)
Norfolk	0 (0-0)	17.5 (17.5-17.5)	0 (0-0)	6 (-1.6-14)	0 (0-0)	5.4 (4.6-6.3)	0 (0-0)	7.1 (2.3-12)	0 (0-0)	0 (0-0)	0 (0-0)	0 (0-0)
Peterborough	0 (0-0)	49.6 (49.5-49.6)	0 (0-0)	41.5 (30-54.5)	0 (0-0)	41.6 (40.6-42.5)	0 (0-0)	43.3 (36.4-50.2)	0 (0-0)	0 (0-0)	0 (0-0)	0 (0-0)
Southend-on-Sea	0 (0-0)	16.9 (16.9-16.9)	0 (0-0)	10.7 (3.7-19.7)	0 (0-0)	10.1 (9.5-10.9)	0 (0-0)	10.1 (5.1-14.4)	0 (0-0)	0 (0-0)	0 (0-0)	0 (0-0)
Suffolk	0 (0-0)	13.1 (12.3-38.6)	0 (0-0)	3 (-5.9-23.3)	0 (0-0)	2 (0.5-24.4)	0 (0-0)	1.9 (-5.2-21.7)	0 (0-0)	0 (0-0)	0 (0-0)	0 (0-0)
Thurrock	0 (0-0)	20 (21.8-21.8)	0 (0-0)	16.8 (7.9-27.7)	0 (0-0)	16.3 (17.4-18.8)	0 (0-0)	14.9 (5.9-21.3)	0 (0-0)	0 (0-0)	0 (0-0)	0 (0-0)
Greater London	0 (0-0)	34.3 (31.8-133)	0 (0-0)	30.5 (26.6-128)	0 (0-0)	30.8 (27.4-132)	0 (0-0)	31.7 (28.1-133)	0 (0-0)	0 (0-0)	0 (0-0)	0 (0-0)

Location	Birth , 2000	Births percent change (2000-2021)	All age cases, 2000	All age cases percent change (2000-2021)	All age deaths, 2000	All age deaths percent change (2000-2021)	Under-5 deaths, 2000	Under-5 deaths percent change (2000-2021)	Births, 2021	All age cases, 2021	All age deaths, 2021	Under-5 deaths, 2021
Barking and Dagenham	0 (0-0)	64.9 (63.5-63.5)	0 (0-0)	61.2 (44.7-78.8)	0 (0-0)	60.7 (58.2-60.7)	0 (0-0)	59.8 (49.3-70.8)	0 (0-0)	0 (0-0)	0 (0-0)	0 (0-0)
Barnet	0 (0-0)	35.7 (36.3-36.3)	0 (0-0)	32.2 (22.7-43.4)	0 (0-0)	32.1 (31.6-33.4)	0 (0-0)	31.6 (24.2-37.8)	0 (0-0)	0 (0-0)	0 (0-0)	0 (0-0)
Bexley	0 (0-0)	31.6 (31.3-41.5)	0 (0-0)	18.6 (10.2-29.2)	0 (0-0)	18.1 (16.8-26.4)	0 (0-0)	21.1 (15.2-30.8)	0 (0-0)	0 (0-0)	0 (0-0)	0 (0-0)
Brent	0 (0-0)	39.6 (39.6-39.6)	0 (0-0)	47.3 (37.7-58.2)	0 (0-0)	48.6 (47.1-49.6)	0 (0-0)	49.8 (44.3-55.4)	0 (0-0)	0 (0-0)	0 (0-0)	0 (0-0)
Bromley	0 (0-0)	22 (22.5-24)	0 (0-0)	10.3 (1.4-19.3)	0 (0-0)	9.4 (9.1-10.8)	0 (0-0)	9.8 (4.4-15.7)	0 (0-0)	0 (0-0)	0 (0-0)	0 (0-0)
Camden	0 (0-0)	7.9 (9-17.5)	0 (0-0)	5.3 (-3.2-14.9)	0 (0-0)	5.1 (5.3-14.4)	0 (0-0)	4.6 (0.1-13.3)	0 (0-0)	0 (0-0)	0 (0-0)	0 (0-0)
Croydon	0 (0-0)	44.3 (27.8-2180)	0 (0-0)	37.3 (19.4-1820)	0 (0-0)	38 (22.4-2040)	0 (0-0)	40.1 (24.8-2050)	0 (0-0)	0 (0-0)	0 (0-0)	0 (0-0)
Ealing	0 (0-0)	25.5 (24.8-24.8)	0 (0-0)	30.1 (17.8-44.7)	0 (0-0)	30.4 (28.4-31.2)	0 (0-0)	28.8 (20.2-36.1)	0 (0-0)	0 (0-0)	0 (0-0)	0 (0-0)
Enfield	0 (0-0)	38.8 (38.7-38.7)	0 (0-0)	40.3 (30.1-50.6)	0 (0-0)	40.6 (39.8-41.4)	0 (0-0)	40.1 (33.7-46.4)	0 (0-0)	0 (0-0)	0 (0-0)	0 (0-0)
Greenwich	0 (0-0)	52.4 (41.4-52.4)	0 (0-0)	45.7 (30.5-59.1)	0 (0-0)	45.9 (34.6-46.8)	0 (0-0)	46.3 (30.1-57.6)	0 (0-0)	0 (0-0)	0 (0-0)	0 (0-0)
Hackney	0 (0-0)	28.9 (28.9-28.9)	0 (0-0)	23.7 (15.8-31.8)	0 (0-0)	24 (23-25)	0 (0-0)	25.9 (21.1-31.3)	0 (0-0)	0 (0-0)	0 (0-0)	0 (0-0)
Hammersmith and Fulham	0 (0-0)	11.3 (11.3-11.3)	0 (0-0)	12 (4.7-19.7)	0 (0-0)	12.3 (11.2-13.4)	0 (0-0)	12.2 (7-17.2)	0 (0-0)	0 (0-0)	0 (0-0)	0 (0-0)
Haringey	0 (0-0)	14.4 (14.4-14.4)	0 (0-0)	22.5 (15-31)	0 (0-0)	22.9 (22.2-23.7)	0 (0-0)	21.5 (16.7-25.9)	0 (0-0)	0 (0-0)	0 (0-0)	0 (0-0)
Harrow	0 (0-0)	55.2 (55.1-55.1)	0 (0-0)	48.6 (37.1-62)	0 (0-0)	49.6 (47.6-51)	0 (0-0)	53.8 (46.7-60.4)	0 (0-0)	0 (0-0)	0 (0-0)	0 (0-0)
Havering	0 (0-0)	54.4 (53.9-53.9)	0 (0-0)	26.4 (17.3-38.4)	0 (0-0)	26.5 (23-29.8)	0 (0-0)	35.9 (28.9-44.7)	0 (0-0)	0 (0-0)	0 (0-0)	0 (0-0)
Hillingdon	0 (0-0)	48.8 (38.4-49.2)	0 (0-0)	37.1 (26.3-48.3)	0 (0-0)	37 (27.4-38.3)	0 (0-0)	39.3 (31.2-45.7)	0 (0-0)	0 (0-0)	0 (0-0)	0 (0-0)
Hounslow	0 (0-0)	44.2 (44.2-44.2)	0 (0-0)	45.9 (36.4-55.7)	0 (0-0)	47.4 (46-48.4)	0 (0-0)	48 (42.8-53.8)	0 (0-0)	0 (0-0)	0 (0-0)	0 (0-0)
Islington	0 (0-0)	38.5 (38.5-38.5)	0 (0-0)	24.3 (16.7-32.8)	0 (0-0)	24.5 (22.8-26)	0 (0-0)	29.2 (24-34.2)	0 (0-0)	0 (0-0)	0 (0-0)	0 (0-0)
Kensington and Chelsea	0 (0-0)	-9.8 (-9.8--9.8)	0 (0-0)	-14.4 (-19.9--7.7)	0 (0-0)	-15.6 (-16.3--14.3)	0 (0-0)	-17.4 (-20.6--13.7)	0 (0-0)	0 (0-0)	0 (0-0)	0 (0-0)
Kingston upon Thames	0 (0-0)	34.9 (35.4-35.5)	0 (0-0)	23.4 (13.9-34.1)	0 (0-0)	23.6 (22.6-25)	0 (0-0)	24.7 (18.9-30.3)	0 (0-0)	0 (0-0)	0 (0-0)	0 (0-0)
Lambeth	0 (0-0)	15.5 (15.5-15.5)	0 (0-0)	15.9 (8.6-23.6)	0 (0-0)	15.4 (14.5-16.2)	0 (0-0)	16 (11-20.7)	0 (0-0)	0 (0-0)	0 (0-0)	0 (0-0)
Lewisham	0 (0-0)	30.7 (30.7-30.7)	0 (0-0)	26.5 (17.9-35.3)	0 (0-0)	26.6 (25.7-27.4)	0 (0-0)	27.4 (22.2-33.2)	0 (0-0)	0 (0-0)	0 (0-0)	0 (0-0)
Merton	0 (0-0)	27.7 (27.1-321)	0 (0-0)	19.9 (9-291)	0 (0-0)	20.7 (18.3-298)	0 (0-0)	21.2 (13.7-301)	0 (0-0)	0 (0-0)	0 (0-0)	0 (0-0)
Newham	0 (0-0)	42 (42-42)	0 (0-0)	34.9 (26.2-44.3)	0 (0-0)	34.8 (34-35.5)	0 (0-0)	36.7 (31.4-42)	0 (0-0)	0 (0-0)	0 (0-0)	0 (0-0)
Redbridge	0 (0-0)	61.9 (63.8-64.4)	0 (0-0)	56.2 (43-72)	0 (0-0)	56.4 (57-59.2)	0 (0-0)	57.2 (48.8-66.5)	0 (0-0)	0 (0-0)	0 (0-0)	0 (0-0)
Richmond upon Thames	0 (0-0)	5.7 (-15.2-6.5)	0 (0-0)	12.3 (-10.7-21.6)	0 (0-0)	11.7 (-10.3-14.7)	0 (0-0)	6.7 (-12.7-12.3)	0 (0-0)	0 (0-0)	0 (0-0)	0 (0-0)
Southwark	0 (0-0)	28 (27.6-27.6)	0 (0-0)	22.7 (12.5-38.7)	0 (0-0)	22.6 (20.8-23.6)	0 (0-0)	24.9 (15.7-32.9)	0 (0-0)	0 (0-0)	0 (0-0)	0 (0-0)
Sutton	0 (0-0)	24.7 (24.7-24.7)	0 (0-0)	10.9 (3.5-19.6)	0 (0-0)	10.9 (10.1-11.7)	0 (0-0)	12.8 (8.1-17.6)	0 (0-0)	0 (0-0)	0 (0-0)	0 (0-0)
Tower Hamlets	0 (0-0)	56.2 (56-56)	0 (0-0)	48.3 (39-58.8)	0 (0-0)	49.1 (47-50.4)	0 (0-0)	53.8 (48-59.5)	0 (0-0)	0 (0-0)	0 (0-0)	0 (0-0)

Location	Birth , 2000	Births percent change (2000-2021)	All age cases, 2000	All age cases percent change (2000-2021)	All age deaths, 2000	All age deaths percent change (2000-2021)	Under-5 deaths, 2000	Under-5 deaths percent change (2000-2021)	Births, 2021	All age cases, 2021	All age deaths, 2021	Under-5 deaths, 2021
Waltham Forest	0 (0-0)	44.8 (45.4-45.4)	0 (0-0)	45.4 (35.4-55.4)	0 (0-0)	44.8 (44.4-46.1)	0 (0-0)	44.6 (38.9-50.8)	0 (0-0)	0 (0-0)	0 (0-0)	0 (0-0)
Wandsworth	0 (0-0)	35.7 (35.4-44.7)	0 (0-0)	42.1 (33.2-52.3)	0 (0-0)	42.8 (40.3-52.1)	0 (0-0)	41.7 (36-50.1)	0 (0-0)	0 (0-0)	0 (0-0)	0 (0-0)
Westminster	0 (0-0)	11.7 (11.7-11.7)	0 (0-0)	19.6 (12.3-29.4)	0 (0-0)	20 (18.6-21.7)	0 (0-0)	17.4 (12.5-21.7)	0 (0-0)	0 (0-0)	0 (0-0)	0 (0-0)
North East England	0 (0-0)	6.3 (6.3-8.2)	0 (0-0)	-4.4 (-8-0.3)	0 (0-0)	-4.7 (-5.7--2.9)	0 (0-0)	-2.5 (-4.2-0.9)	0 (0-0)	0 (0-0)	0 (0-0)	0 (0-0)
County Durham	0 (0-0)	6.6 (6.6-6.6)	0 (0-0)	-3.3 (-10.5-3.4)	0 (0-0)	-3.9 (-4.7--3.1)	0 (0-0)	-2.1 (-6-2.8)	0 (0-0)	0 (0-0)	0 (0-0)	0 (0-0)
Darlington	0 (0-0)	7.6 (8.8-8.8)	0 (0-0)	3.6 (-5-13)	0 (0-0)	2.8 (3.2-5)	0 (0-0)	2.5 (-1.3-7.6)	0 (0-0)	0 (0-0)	0 (0-0)	0 (0-0)
Gateshead	0 (0-0)	9.7 (9.7-9.7)	0 (0-0)	0.6 (-6.5-8.1)	0 (0-0)	0.3 (-0.3-0.9)	0 (0-0)	1.1 (-3-5.2)	0 (0-0)	0 (0-0)	0 (0-0)	0 (0-0)
Hartlepool	0 (0-0)	-1.9 (-0.9--0.9)	0 (0-0)	-11.7 (-18.6--4.3)	0 (0-0)	-12.5 (-12.6--10.8)	0 (0-0)	-11.1 (-14.6--6.8)	0 (0-0)	0 (0-0)	0 (0-0)	0 (0-0)
Middlesbrough	0 (0-0)	8.2 (7-7)	0 (0-0)	0.2 (-7.2-9.4)	0 (0-0)	0.5 (-1.8-0.5)	0 (0-0)	3.5 (-1.4-8)	0 (0-0)	0 (0-0)	0 (0-0)	0 (0-0)
Newcastle upon Tyne	0 (0-0)	25.8 (27.3-27.3)	0 (0-0)	12.5 (2.8-22)	0 (0-0)	11.7 (10.9-14.3)	0 (0-0)	15.5 (9.6-21.2)	0 (0-0)	0 (0-0)	0 (0-0)	0 (0-0)
North Tyneside	0 (0-0)	12.2 (12.2-12.2)	0 (0-0)	3.6 (-3.7-11.8)	0 (0-0)	3.2 (2.4-4)	0 (0-0)	5.2 (0.8-10)	0 (0-0)	0 (0-0)	0 (0-0)	0 (0-0)
Northumberland	0 (0-0)	-0.9 (-1.1--1.1)	0 (0-0)	-11.7 (-18.3--2.2)	0 (0-0)	-11.9 (-12.8--11)	0 (0-0)	-10.9 (-15.2--6)	0 (0-0)	0 (0-0)	0 (0-0)	0 (0-0)
Redcar and Cleveland	0 (0-0)	-1.1 (-2.7--2.6)	0 (0-0)	-13.1 (-20.9--4.8)	0 (0-0)	-13.7 (-16.2--13.8)	0 (0-0)	-10.8 (-15.6--7)	0 (0-0)	0 (0-0)	0 (0-0)	0 (0-0)
South Tyneside	0 (0-0)	1.5 (1.5-1.5)	0 (0-0)	-14.8 (-20.2--8.7)	0 (0-0)	-14.8 (-16--13.6)	0 (0-0)	-11.4 (-14.9--7.5)	0 (0-0)	0 (0-0)	0 (0-0)	0 (0-0)
Stockton-on-Tees	0 (0-0)	11.6 (10.7-15)	0 (0-0)	0.2 (-11-20.6)	0 (0-0)	-0.7 (-2.5-3.2)	0 (0-0)	0.5 (-6.1-11.4)	0 (0-0)	0 (0-0)	0 (0-0)	0 (0-0)
Sunderland	0 (0-0)	-8.4 (-8.4--8.4)	0 (0-0)	-18.1 (-23.7--12)	0 (0-0)	-18 (-19--17.1)	0 (0-0)	-15.1 (-18.3--11.7)	0 (0-0)	0 (0-0)	0 (0-0)	0 (0-0)
North West England	0 (0-0)	12.5 (12.5-13.7)	0 (0-0)	0.7 (-1.6-3.3)	0 (0-0)	0.8 (-0.2-2.7)	0 (0-0)	3.2 (1.8-6)	0 (0-0)	0 (0-0)	0 (0-0)	0 (0-0)
Blackburn with Darwen	0 (0-0)	0.5 (-0.7--0.7)	0 (0-0)	-5.2 (-12-1.5)	0 (0-0)	-5.5 (-7--5.4)	0 (0-0)	-6.6 (-10.1--2.6)	0 (0-0)	0 (0-0)	0 (0-0)	0 (0-0)
Blackpool	0 (0-0)	10.7 (10.7-10.7)	0 (0-0)	-1.1 (-7.5-5.3)	0 (0-0)	-1.8 (-2.5--1.4)	0 (0-0)	-0.7 (-3.8-2.7)	0 (0-0)	0 (0-0)	0 (0-0)	0 (0-0)
Bolton	0 (0-0)	15.3 (17.1-17.1)	0 (0-0)	4.7 (-2.6-13.9)	0 (0-0)	4.4 (5.2-6.5)	0 (0-0)	5.3 (1.6-10.7)	0 (0-0)	0 (0-0)	0 (0-0)	0 (0-0)
Bury	0 (0-0)	9.2 (9.2-9.2)	0 (0-0)	0.5 (-6.7-8.2)	0 (0-0)	-0.1 (-0.7-0.4)	0 (0-0)	1 (-2.9-4.9)	0 (0-0)	0 (0-0)	0 (0-0)	0 (0-0)
Cheshire East	0 (0-0)	8.4 (8.4-8.4)	0 (0-0)	-4.7 (-12-2.6)	0 (0-0)	-5.8 (-6.7--4.6)	0 (0-0)	-4.7 (-8.8-0.2)	0 (0-0)	0 (0-0)	0 (0-0)	0 (0-0)
Cheshire West and Chester	0 (0-0)	3.6 (4-5.7)	0 (0-0)	-11.9 (-22--3.2)	0 (0-0)	-12.1 (-13.4--9.3)	0 (0-0)	-9.5 (-16.3--4.1)	0 (0-0)	0 (0-0)	0 (0-0)	0 (0-0)
Cumbria	0 (0-0)	-1.8 (-1.7--1.7)	0 (0-0)	-13 (-20.2--5.8)	0 (0-0)	-13.4 (-14.1--12.4)	0 (0-0)	-12 (-16.3--7.5)	0 (0-0)	0 (0-0)	0 (0-0)	0 (0-0)
Halton	0 (0-0)	2.9 (2.1-3.7)	0 (0-0)	-0.8 (-10.7-13.5)	0 (0-0)	-1.5 (-2.8--0.3)	0 (0-0)	-1.1 (-7.3-7.3)	0 (0-0)	0 (0-0)	0 (0-0)	0 (0-0)
Knowsley	0 (0-0)	3.3 (4.1-4.1)	0 (0-0)	-10.7 (-18.1--2.8)	0 (0-0)	-11.1 (-12.5--8.9)	0 (0-0)	-6.4 (-10.5--1.8)	0 (0-0)	0 (0-0)	0 (0-0)	0 (0-0)
Lancashire	0 (0-0)	7.2 (7.2-7.2)	0 (0-0)	-5.6 (-12.1-1.6)	0 (0-0)	-6 (-6.9--5.3)	0 (0-0)	-4.1 (-7.9--0.3)	0 (0-0)	0 (0-0)	0 (0-0)	0 (0-0)
Liverpool	0 (0-0)	23 (23-23)	0 (0-0)	6.7 (-1.3-14.7)	0 (0-0)	6.3 (3.9-8.2)	0 (0-0)	12.3 (7.9-16.9)	0 (0-0)	0 (0-0)	0 (0-0)	0 (0-0)
Manchester	0 (0-0)	55.8 (55.8-55.8)	0 (0-0)	48 (37.9-58.7)	0 (0-0)	47.9 (46.3-49.1)	0 (0-0)	52 (47.1-57.8)	0 (0-0)	0 (0-0)	0 (0-0)	0 (0-0)

Location	Birth , 2000	Births percent change (2000-2021)	All age cases, 2000	All age cases percent change (2000-2021)	All age deaths, 2000	All age deaths percent change (2000-2021)	Under-5 deaths, 2000	Under-5 deaths percent change (2000-2021)	Births, 2021	All age cases, 2021	All age deaths, 2021	Under-5 deaths, 2021
Oldham	0 (0-0)	10.7 (10.7-10.7)	0 (0-0)	3.6 (-3.1-10.9)	0 (0-0)	3.3 (2.8-3.9)	0 (0-0)	3.9 (0.1-7.7)	0 (0-0)	0 (0-0)	0 (0-0)	0 (0-0)
Rochdale	0 (0-0)	11.4 (11.4-11.4)	0 (0-0)	-0.1 (-6.5-7.1)	0 (0-0)	-0.8 (-1.5--0.3)	0 (0-0)	0.7 (-2.8-4.7)	0 (0-0)	0 (0-0)	0 (0-0)	0 (0-0)
Salford	0 (0-0)	42.2 (41.1-43.3)	0 (0-0)	33.6 (19.3-47.9)	0 (0-0)	33.5 (30.1-36)	0 (0-0)	38.2 (30.8-51.2)	0 (0-0)	0 (0-0)	0 (0-0)	0 (0-0)
Sefton	0 (0-0)	0.8 (0.8-0.8)	0 (0-0)	-14.4 (-21--7.4)	0 (0-0)	-14.7 (-16.4--13.1)	0 (0-0)	-10 (-13.8--5.8)	0 (0-0)	0 (0-0)	0 (0-0)	0 (0-0)
St Helens	0 (0-0)	4.4 (2.8-2.9)	0 (0-0)	-9.1 (-16.6--1.4)	0 (0-0)	-9.5 (-12.2--9.5)	0 (0-0)	-5.8 (-10.8--0.3)	0 (0-0)	0 (0-0)	0 (0-0)	0 (0-0)
Stockport	0 (0-0)	6.7 (6.7-6.7)	0 (0-0)	-4.1 (-12.1-3.6)	0 (0-0)	-4 (-4.8--3.2)	0 (0-0)	-1.7 (-5.8-3.4)	0 (0-0)	0 (0-0)	0 (0-0)	0 (0-0)
Tameside	0 (0-0)	13.5 (12.7-14.4)	0 (0-0)	2.3 (-9.4-14)	0 (0-0)	2.6 (0.5-4.2)	0 (0-0)	5 (-2.8-11.7)	0 (0-0)	0 (0-0)	0 (0-0)	0 (0-0)
Trafford	0 (0-0)	11.5 (11.5-11.5)	0 (0-0)	13.8 (5.4-22.8)	0 (0-0)	14 (13.5-14.7)	0 (0-0)	13.5 (9-18.6)	0 (0-0)	0 (0-0)	0 (0-0)	0 (0-0)
Warrington	0 (0-0)	2.5 (2.5-2.5)	0 (0-0)	-3.6 (-10.4-3.1)	0 (0-0)	-4.1 (-4.7--3.3)	0 (0-0)	-4.6 (-8.7--0.4)	0 (0-0)	0 (0-0)	0 (0-0)	0 (0-0)
Wigan	0 (0-0)	7.5 (6.3-6.3)	0 (0-0)	-0.3 (-9.1-10.3)	0 (0-0)	-0.7 (-2.5--1)	0 (0-0)	0.4 (-5.2-6.3)	0 (0-0)	0 (0-0)	0 (0-0)	0 (0-0)
Wirral	0 (0-0)	2.6 (2.6-2.6)	0 (0-0)	-7.6 (-14.4--0.5)	0 (0-0)	-8.2 (-9--7.4)	0 (0-0)	-6.5 (-10.4--2.3)	0 (0-0)	0 (0-0)	0 (0-0)	0 (0-0)
South East England	0 (0-0)	15.3 (14.5-17.6)	0 (0-0)	7 (4.1-10.3)	0 (0-0)	6.9 (5.4-9.5)	0 (0-0)	7.4 (4.8-10.3)	0 (0-0)	0 (0-0)	0 (0-0)	0 (0-0)
Bracknell Forest	0 (0-0)	-1.5 (-3.4-4.1)	0 (0-0)	-5.2 (-13.2-7.9)	0 (0-0)	-5.2 (-7.4-0.8)	0 (0-0)	-6.3 (-12.2-4.3)	0 (0-0)	0 (0-0)	0 (0-0)	0 (0-0)
Brighton and Hove	0 (0-0)	10.4 (10.4-10.4)	0 (0-0)	6.7 (-1-14.5)	0 (0-0)	6 (4.9-7.7)	0 (0-0)	4.6 (0-9.6)	0 (0-0)	0 (0-0)	0 (0-0)	0 (0-0)
Buckinghamshire	0 (0-0)	6.2 (5.4-6.7)	0 (0-0)	-1.4 (-10-7.1)	0 (0-0)	-2 (-3.2--0.4)	0 (0-0)	-2.8 (-7.3-2.6)	0 (0-0)	0 (0-0)	0 (0-0)	0 (0-0)
East Sussex	0 (0-0)	14.5 (13.2-13.3)	0 (0-0)	-1.1 (-9.4-11.6)	0 (0-0)	-2.1 (-4.3--1)	0 (0-0)	-1.5 (-6.8-5.9)	0 (0-0)	0 (0-0)	0 (0-0)	0 (0-0)
Hampshire	0 (0-0)	7.9 (8-8)	0 (0-0)	-2.2 (-9.2-5.3)	0 (0-0)	-2.9 (-3.6--1.8)	0 (0-0)	-2.7 (-7.1-2.2)	0 (0-0)	0 (0-0)	0 (0-0)	0 (0-0)
Isle of Wight	0 (0-0)	16.4 (16.4-16.4)	0 (0-0)	-4.8 (-11.7-2.1)	0 (0-0)	-5.7 (-7.1--4.1)	0 (0-0)	-2.2 (-6.4-2.2)	0 (0-0)	0 (0-0)	0 (0-0)	0 (0-0)
Kent	0 (0-0)	26.3 (24.4-24.5)	0 (0-0)	13.4 (3.4-24.3)	0 (0-0)	13.1 (10.3-13)	0 (0-0)	14.7 (7-20.7)	0 (0-0)	0 (0-0)	0 (0-0)	0 (0-0)
Medway	0 (0-0)	19.9 (19.9-19.9)	0 (0-0)	5.7 (-1.5-12.8)	0 (0-0)	5 (3.8-6.1)	0 (0-0)	8.2 (4.2-13.2)	0 (0-0)	0 (0-0)	0 (0-0)	0 (0-0)
Milton Keynes	0 (0-0)	26.6 (26.6-26.6)	0 (0-0)	30.7 (20.8-40.6)	0 (0-0)	31.2 (30.3-32)	0 (0-0)	30.2 (24-36)	0 (0-0)	0 (0-0)	0 (0-0)	0 (0-0)
Oxfordshire	0 (0-0)	11.1 (10.8-10.8)	0 (0-0)	5.5 (-1.8-12.7)	0 (0-0)	4.7 (3.9-5.1)	0 (0-0)	4.7 (0.3-9.5)	0 (0-0)	0 (0-0)	0 (0-0)	0 (0-0)
Portsmouth	0 (0-0)	26.5 (25.4-57.5)	0 (0-0)	21.8 (12.2-52.4)	0 (0-0)	21.5 (19.7-51.2)	0 (0-0)	23.3 (16.9-52.8)	0 (0-0)	0 (0-0)	0 (0-0)	0 (0-0)
Reading	0 (0-0)	27.1 (26.6-26.7)	0 (0-0)	30.6 (20.9-43.2)	0 (0-0)	32.3 (29.7-33.2)	0 (0-0)	34.5 (28.9-40.9)	0 (0-0)	0 (0-0)	0 (0-0)	0 (0-0)
Slough	0 (0-0)	37.4 (37.1-37.1)	0 (0-0)	53.1 (42.8-63.4)	0 (0-0)	54.3 (52.7-55.3)	0 (0-0)	51.8 (46-57)	0 (0-0)	0 (0-0)	0 (0-0)	0 (0-0)
Southampton	0 (0-0)	33.6 (33.2-42.4)	0 (0-0)	24.4 (15.4-36.1)	0 (0-0)	25.1 (22.6-32.1)	0 (0-0)	30.3 (24.8-38.1)	0 (0-0)	0 (0-0)	0 (0-0)	0 (0-0)
Surrey	0 (0-0)	11.1 (11.1-11.1)	0 (0-0)	7.4 (-0.2-16)	0 (0-0)	7 (6.2-8.1)	0 (0-0)	5.5 (0.1-11.2)	0 (0-0)	0 (0-0)	0 (0-0)	0 (0-0)
West Berkshire	0 (0-0)	0 (0-0)	0 (0-0)	-0.8 (-7.3-6.8)	0 (0-0)	-1.2 (-1.9--0.1)	0 (0-0)	-2.7 (-6.8-1.5)	0 (0-0)	0 (0-0)	0 (0-0)	0 (0-0)
West Sussex	0 (0-0)	16.3 (14.3-22.1)	0 (0-0)	5.7 (-2.2-17.2)	0 (0-0)	5.4 (2.9-11.1)	0 (0-0)	6.1 (-0.8-15.6)	0 (0-0)	0 (0-0)	0 (0-0)	0 (0-0)

Location	Birth , 2000	Births percent change (2000-2021)	All age cases, 2000	All age cases percent change (2000-2021)	All age deaths, 2000	All age deaths percent change (2000-2021)	Under-5 deaths, 2000	Under-5 deaths percent change (2000-2021)	Births, 2021	All age cases, 2021	All age deaths, 2021	Under-5 deaths, 2021
Windsor and Maidenhead	0 (0-0)	4.8 (5.2-5.2)	0 (0-0)	4.8 (-4-13.2)	0 (0-0)	4.4 (3.7-6.2)	0 (0-0)	1.6 (-2.9-7.2)	0 (0-0)	0 (0-0)	0 (0-0)	0 (0-0)
Wokingham	0 (0-0)	-7 (-6.9--6.9)	0 (0-0)	-7.3 (-13.5-1.4)	0 (0-0)	-7.3 (-8--6.2)	0 (0-0)	-9.1 (-13.6--5)	0 (0-0)	0 (0-0)	0 (0-0)	0 (0-0)
South West England	0 (0-0)	18.4 (16.7-22.9)	0 (0-0)	5.2 (2.7-9.1)	0 (0-0)	4.8 (3.4-9.7)	0 (0-0)	7 (5.1-11.7)	0 (0-0)	0 (0-0)	0 (0-0)	0 (0-0)
Bath and North East Somerset	0 (0-0)	14.1 (13.9-13.9)	0 (0-0)	-1.6 (-9.5-6.2)	0 (0-0)	-2.6 (-3.8--1.3)	0 (0-0)	-1 (-5.3-4.3)	0 (0-0)	0 (0-0)	0 (0-0)	0 (0-0)
Bournemouth	0 (0-0)	55.4 (45-55.8)	0 (0-0)	34 (21.8-46.5)	0 (0-0)	34.7 (26-37.3)	0 (0-0)	41.7 (34.1-49)	0 (0-0)	0 (0-0)	0 (0-0)	0 (0-0)
Bristol, City of	0 (0-0)	37.8 (38.4-38.4)	0 (0-0)	28.1 (17.4-38)	0 (0-0)	27.9 (26.4-29.7)	0 (0-0)	29.9 (23.6-37.2)	0 (0-0)	0 (0-0)	0 (0-0)	0 (0-0)
Cornwall	0 (0-0)	19.5 (19.5-19.5)	0 (0-0)	1.2 (-6.2-9.2)	0 (0-0)	0.5 (-0.5-1.7)	0 (0-0)	2.5 (-2-8)	0 (0-0)	0 (0-0)	0 (0-0)	0 (0-0)
Devon	0 (0-0)	14 (14.2-14.2)	0 (0-0)	0.6 (-6.7-8.4)	0 (0-0)	-0.3 (-1.2-1.1)	0 (0-0)	2.5 (-2.5-7.8)	0 (0-0)	0 (0-0)	0 (0-0)	0 (0-0)
Dorset	0 (0-0)	7.4 (-12.5-7.9)	0 (0-0)	-5.1 (-18.5-3.6)	0 (0-0)	-6.2 (-23.4--4.1)	0 (0-0)	-4.1 (-22.2-2.1)	0 (0-0)	0 (0-0)	0 (0-0)	0 (0-0)
Gloucestershire	0 (0-0)	13.2 (13.3-13.3)	0 (0-0)	-0.8 (-8.3-6.8)	0 (0-0)	-1.3 (-2--0.2)	0 (0-0)	0.4 (-4.1-6)	0 (0-0)	0 (0-0)	0 (0-0)	0 (0-0)
North Somerset	0 (0-0)	18.6 (18.8-18.8)	0 (0-0)	6.2 (-0.7-14.1)	0 (0-0)	5.5 (4.9-6.4)	0 (0-0)	6.2 (1.5-10.9)	0 (0-0)	0 (0-0)	0 (0-0)	0 (0-0)
Plymouth	0 (0-0)	22 (21.4-21.4)	0 (0-0)	10 (1.7-20.1)	0 (0-0)	10.1 (8.2-11.1)	0 (0-0)	14.5 (9.1-20.5)	0 (0-0)	0 (0-0)	0 (0-0)	0 (0-0)
Poole	0 (0-0)	10.9 (11.4-11.4)	0 (0-0)	5.8 (-4.4-15.4)	0 (0-0)	6.2 (5.9-7.1)	0 (0-0)	7.2 (1.3-12.4)	0 (0-0)	0 (0-0)	0 (0-0)	0 (0-0)
Somerset	0 (0-0)	15.4 (15.9-15.9)	0 (0-0)	1 (-7-8.2)	0 (0-0)	0 (-0.7-1.5)	0 (0-0)	1.9 (-2.6-7)	0 (0-0)	0 (0-0)	0 (0-0)	0 (0-0)
South Gloucestershire	0 (0-0)	11.9 (12.1-12.1)	0 (0-0)	1.5 (-5.2-9.1)	0 (0-0)	1 (0.2-2.3)	0 (0-0)	3 (-1.4-7.4)	0 (0-0)	0 (0-0)	0 (0-0)	0 (0-0)
Swindon	0 (0-0)	31.7 (31.7-31.7)	0 (0-0)	26 (17.6-35.8)	0 (0-0)	25.3 (24.8-25.8)	0 (0-0)	25.8 (20.2-31.4)	0 (0-0)	0 (0-0)	0 (0-0)	0 (0-0)
Torbay	0 (0-0)	15.8 (14.9-14.9)	0 (0-0)	1.3 (-7.3-9.4)	0 (0-0)	1.3 (-1.1-2.1)	0 (0-0)	5.6 (0.8-11)	0 (0-0)	0 (0-0)	0 (0-0)	0 (0-0)
Wiltshire	0 (0-0)	8.8 (7.3-61.3)	0 (0-0)	1.1 (-7.2-48.3)	0 (0-0)	0.6 (-1.9-50.5)	0 (0-0)	-1 (-6.4-47.4)	0 (0-0)	0 (0-0)	0 (0-0)	0 (0-0)
West Midlands	0 (0-0)	15.6 (15.6-15.6)	0 (0-0)	5.5 (3-8)	0 (0-0)	5.4 (4.8-6.1)	0 (0-0)	6.6 (5.2-8.3)	0 (0-0)	0 (0-0)	0 (0-0)	0 (0-0)
Birmingham	0 (0-0)	22.8 (22.8-22.8)	0 (0-0)	16.9 (9.5-24.5)	0 (0-0)	16.1 (15.6-16.6)	0 (0-0)	15.9 (11.9-20.2)	0 (0-0)	0 (0-0)	0 (0-0)	0 (0-0)
Coventry	0 (0-0)	27.9 (27.7-27.7)	0 (0-0)	15 (7-23.4)	0 (0-0)	15.4 (13.9-16.3)	0 (0-0)	18.7 (14.3-23.5)	0 (0-0)	0 (0-0)	0 (0-0)	0 (0-0)
Dudley	0 (0-0)	8.8 (8.7-8.7)	0 (0-0)	2.6 (-4.5-9.4)	0 (0-0)	1.7 (1-2.1)	0 (0-0)	2.3 (-1.5-6.2)	0 (0-0)	0 (0-0)	0 (0-0)	0 (0-0)
Herefordshire, County of	0 (0-0)	9.8 (9.7-9.7)	0 (0-0)	-6.3 (-12.8-1.5)	0 (0-0)	-7.6 (-8.6--6.5)	0 (0-0)	-6.5 (-10.3--3.2)	0 (0-0)	0 (0-0)	0 (0-0)	0 (0-0)
Sandwell	0 (0-0)	26.6 (26.7-26.7)	0 (0-0)	23.3 (15.5-32)	0 (0-0)	22.7 (22.5-23.3)	0 (0-0)	22.5 (18.4-27.6)	0 (0-0)	0 (0-0)	0 (0-0)	0 (0-0)
Shropshire	0 (0-0)	8.1 (8.1-8.1)	0 (0-0)	-3.2 (-9.4-3.9)	0 (0-0)	-4.4 (-5.3--3.3)	0 (0-0)	-4.4 (-8.3--0.7)	0 (0-0)	0 (0-0)	0 (0-0)	0 (0-0)
Solihull	0 (0-0)	17.9 (18.2-18.2)	0 (0-0)	0.1 (-7-7.8)	0 (0-0)	-0.4 (-1.5-1.3)	0 (0-0)	3.5 (-0.6-8.3)	0 (0-0)	0 (0-0)	0 (0-0)	0 (0-0)
Staffordshire	0 (0-0)	6.1 (6.1-6.1)	0 (0-0)	-6.4 (-13.4-1)	0 (0-0)	-7.2 (-7.9--6.3)	0 (0-0)	-6.2 (-10.3--2.1)	0 (0-0)	0 (0-0)	0 (0-0)	0 (0-0)
Stoke-on-Trent	0 (0-0)	13.4 (13.4-13.4)	0 (0-0)	9.6 (2.4-18)	0 (0-0)	9.8 (9.2-10.2)	0 (0-0)	10.6 (6.7-14.6)	0 (0-0)	0 (0-0)	0 (0-0)	0 (0-0)
Telford and Wrekin	0 (0-0)	7.6 (7.6-7.6)	0 (0-0)	-2 (-9.3-5.1)	0 (0-0)	-3.2 (-3.8--2.5)	0 (0-0)	-3.5 (-7.2-0.3)	0 (0-0)	0 (0-0)	0 (0-0)	0 (0-0)

Location	Birth , 2000	Births percent change (2000-2021)	All age cases, 2000	All age cases percent change (2000-2021)	All age deaths, 2000	All age deaths percent change (2000-2021)	Under-5 deaths, 2000	Under-5 deaths percent change (2000-2021)	Births, 2021	All age cases, 2021	All age deaths, 2021	Under-5 deaths, 2021
Walsall	0 (0-0)	19.1 (19.1-19.1)	0 (0-0)	10.9 (3.8-17.8)	0 (0-0)	10.3 (9.7-10.8)	0 (0-0)	11.4 (7.7-16.2)	0 (0-0)	0 (0-0)	0 (0-0)	0 (0-0)
Warwickshire	0 (0-0)	15 (15-15)	0 (0-0)	3.6 (-3.5-11)	0 (0-0)	3.1 (2.4-3.9)	0 (0-0)	4.2 (-0.4-9.5)	0 (0-0)	0 (0-0)	0 (0-0)	0 (0-0)
Wolverhampton	0 (0-0)	18.7 (18.8-18.8)	0 (0-0)	12.7 (5.5-20.5)	0 (0-0)	13.4 (12.6-14.2)	0 (0-0)	16.1 (12.4-20.4)	0 (0-0)	0 (0-0)	0 (0-0)	0 (0-0)
Worcestershire	0 (0-0)	4.8 (4.8-4.8)	0 (0-0)	-6 (-13-0.8)	0 (0-0)	-6.4 (-7.1--5.7)	0 (0-0)	-5.4 (-9.4--0.2)	0 (0-0)	0 (0-0)	0 (0-0)	0 (0-0)
Yorkshire and the Humber	0 (0-0)	15 (15.3-17.6)	0 (0-0)	4.9 (1.5-8.2)	0 (0-0)	4.5 (3.8-6.9)	0 (0-0)	6.2 (4.6-8.6)	0 (0-0)	0 (0-0)	0 (0-0)	0 (0-0)
Barnsley	0 (0-0)	17.7 (17.7-17.7)	0 (0-0)	3.9 (-2.7-12)	0 (0-0)	3.4 (2.4-4.3)	0 (0-0)	5.3 (1.1-9.7)	0 (0-0)	0 (0-0)	0 (0-0)	0 (0-0)
Bradford	0 (0-0)	10.9 (11.7-11.7)	0 (0-0)	13.7 (6-22.7)	0 (0-0)	12.7 (13.1-14.4)	0 (0-0)	11 (6.6-15.2)	0 (0-0)	0 (0-0)	0 (0-0)	0 (0-0)
Calderdale	0 (0-0)	10.6 (8.7-10.2)	0 (0-0)	0.6 (-8.4-9.3)	0 (0-0)	-0.9 (-3.3--0.1)	0 (0-0)	-1.1 (-6.3-5.7)	0 (0-0)	0 (0-0)	0 (0-0)	0 (0-0)
Doncaster	0 (0-0)	8.6 (8.6-8.6)	0 (0-0)	-0.5 (-7.9-7.5)	0 (0-0)	-0.8 (-1.4--0.2)	0 (0-0)	-0.2 (-4.4-4.9)	0 (0-0)	0 (0-0)	0 (0-0)	0 (0-0)
East Riding of Yorkshire	0 (0-0)	4.3 (4.2-4.2)	0 (0-0)	-8.8 (-15.3--1.4)	0 (0-0)	-9.4 (-10.2--8.4)	0 (0-0)	-8 (-12.2--3.9)	0 (0-0)	0 (0-0)	0 (0-0)	0 (0-0)
Kingston upon Hull, City of	0 (0-0)	13.5 (13.5-13.5)	0 (0-0)	5.2 (-2.3-13)	0 (0-0)	6.2 (4.9-7.2)	0 (0-0)	10 (6.2-14.2)	0 (0-0)	0 (0-0)	0 (0-0)	0 (0-0)
Kirklees	0 (0-0)	4.6 (-31.2-7.6)	0 (0-0)	-1 (-35-9)	0 (0-0)	-1 (-35.2-2.6)	0 (0-0)	-1.8 (-35.5-4.2)	0 (0-0)	0 (0-0)	0 (0-0)	0 (0-0)
Leeds	0 (0-0)	33.2 (33.6-33.7)	0 (0-0)	16.4 (7.7-25.9)	0 (0-0)	16.8 (14.8-18.9)	0 (0-0)	22.3 (17.7-28.3)	0 (0-0)	0 (0-0)	0 (0-0)	0 (0-0)
North East Lincolnshire	0 (0-0)	7.2 (7.1-7.1)	0 (0-0)	-7.9 (-14.5--0.6)	0 (0-0)	-8.5 (-9.6--7.8)	0 (0-0)	-6.2 (-10.2--2.3)	0 (0-0)	0 (0-0)	0 (0-0)	0 (0-0)
North Lincolnshire	0 (0-0)	18.8 (18.4-18.4)	0 (0-0)	9.2 (0.5-19.8)	0 (0-0)	8.5 (7.2-9.2)	0 (0-0)	9.6 (4.7-15.6)	0 (0-0)	0 (0-0)	0 (0-0)	0 (0-0)
North Yorkshire	0 (0-0)	4.9 (4.3-4.3)	0 (0-0)	-4.1 (-15.3-6.6)	0 (0-0)	-5.4 (-7--4.8)	0 (0-0)	-4.5 (-9.9-2.8)	0 (0-0)	0 (0-0)	0 (0-0)	0 (0-0)
Rotherham	0 (0-0)	11 (11-11)	0 (0-0)	-1.9 (-9.1-4.8)	0 (0-0)	-2.7 (-3.6--1.9)	0 (0-0)	-1.3 (-5.4-3.2)	0 (0-0)	0 (0-0)	0 (0-0)	0 (0-0)
Sheffield	0 (0-0)	26.2 (25.8-25.8)	0 (0-0)	14.7 (5.7-22.8)	0 (0-0)	14.7 (13.5-15.2)	0 (0-0)	16.9 (11.9-21.8)	0 (0-0)	0 (0-0)	0 (0-0)	0 (0-0)
Wakefield	0 (0-0)	13.1 (12.8-12.8)	0 (0-0)	0.7 (-6.6-7.9)	0 (0-0)	-0.3 (-1.5-0.3)	0 (0-0)	2 (-2.1-6.5)	0 (0-0)	0 (0-0)	0 (0-0)	0 (0-0)
York	0 (0-0)	23.4 (23.4-23.4)	0 (0-0)	9.8 (2-18.5)	0 (0-0)	9.2 (8.3-10.2)	0 (0-0)	10.9 (6.3-15.5)	0 (0-0)	0 (0-0)	0 (0-0)	0 (0-0)
Northern Ireland	0 (0-0)	13.7 (13.6-13.8)	0 (0-0)	-1.6 (-8.9-6.5)	0 (0-0)	-2.6 (-3.4--1.8)	0 (0-0)	-2.2 (-6-2.5)	0 (0-0)	0 (0-0)	0 (0-0)	0 (0-0)
Scotland	0 (0-0)	0.4 (0.3-0.5)	0 (0-0)	-10.7 (-18.4--1.6)	0 (0-0)	-11.2 (-12--9.9)	0 (0-0)	-11.3 (-15.6--6.2)	0 (0-0)	0 (0-0)	0 (0-0)	0 (0-0)
Wales	0 (0-0)	7.9 (7.9-8)	0 (0-0)	-4.4 (-11.8-2.8)	0 (0-0)	-5 (-5.8--4.2)	0 (0-0)	-3.7 (-7.2-0.4)	0 (0-0)	0 (0-0)	0 (0-0)	0 (0-0)
Latin America and Caribbean	206 (160-274)	3.9 (-0.5-8)	2,950 (2,400-3,680)	27.1 (20.6-32.7)	164 (125-220)	24.6 (19.3-29.8)	99 (71-139)	10 (4-16.5)	214 (162-287)	3,750 (2,960-4,830)	204 (155-275)	109 (78-150)
**Andean Latin America**	0 (0-1)	22.5 (6.2-66.3)	2 (1-3)	38 (11.2-102)	0 (0-0)	31.2 (12.5-78.3)	0 (0-0)	25.5 (4.5-66.1)	0 (0-1)	2 (1-4)	0 (0-1)	0 (0-0)
Bolivia (Plurinational State of)	0 (0-0)	26.8 (24.8-32.5)	0 (0-0)	31.3 (21.3-41.7)	0 (0-0)	31.9 (28.3-37.1)	0 (0-0)	34.3 (25.4-42.6)	0 (0-0)	0 (0-0)	0 (0-0)	0 (0-0)
Ecuador	0 (0-0)	46.5 (8.2-151)	1 (0-1)	60.3 (8.1-195)	0 (0-0)	48.4 (7.8-153)	0 (0-0)	34.8 (-7.7-129)	0 (0-0)	1 (1-2)	0 (0-0)	0 (0-0)
Peru	0 (0-0)	-1.7 (-2.4-20.5)	1 (0-1)	6.3 (-2-27.1)	0 (0-0)	7.4 (6-32.3)	0 (0-0)	6.9 (0.1-27.9)	0 (0-0)	1 (0-1)	0 (0-0)	0 (0-0)
**Caribbean**	125 (90-176)	4.3 (-1.6-10.4)	383 (278-529)	7.4 (1.7-13.9)	112 (80-157)	9.8 (3.6-16.3)	90 (64-129)	10.3 (3.6-17.5)	130 (93-179)	411 (298-561)	123 (88-169)	99 (70-139)

Location	Birth , 2000	Births percent change (2000-2021)	All age cases, 2000	All age cases percent change (2000-2021)	All age deaths, 2000	All age deaths percent change (2000-2021)	Under-5 deaths, 2000	Under-5 deaths percent change (2000-2021)	Births, 2021	All age cases, 2021	All age deaths, 2021	Under-5 deaths, 2021
Antigua and Barbuda	0 (0-0)	-33.8 (-40.7--25.7)	0 (0-0)	-23.5 (-30.7--14.2)	0 (0-0)	-23.9 (-31.6--14.6)	0 (0-0)	-26.5 (-36.1--16.5)	0 (0-0)	0 (0-0)	0 (0-0)	0 (0-0)
Bahamas	0 (0-0)	-21.1 (-43.1-6.9)	0 (0-0)	-24.1 (-41--2.5)	0 (0-0)	-25.6 (-44.4--0.5)	0 (0-0)	-31 (-51.3--6.1)	0 (0-0)	0 (0-0)	0 (0-0)	0 (0-0)
Barbados	0 (0-0)	-27.2 (-34.6--18.8)	0 (0-1)	-21 (-28.6--12.9)	0 (0-0)	-20.3 (-28.2--11)	0 (0-0)	-24.2 (-35.1--12.7)	0 (0-0)	0 (0-0)	0 (0-0)	0 (0-0)
Belize	0 (0-0)	26.9 (14.4-41.8)	1 (1-2)	29.5 (16.3-43.6)	0 (0-0)	28.9 (16.5-43.6)	0 (0-0)	26.8 (12.4-43.8)	0 (0-1)	1 (1-2)	0 (0-1)	0 (0-0)
Bermuda	0 (0-0)	-41.7 (-54--31.9)	0 (0-0)	-35.9 (-47.3--27.4)	0 (0-0)	-35.9 (-48.7--25.4)	0 (0-0)	-40.5 (-54.1--28.3)	0 (0-0)	0 (0-0)	0 (0-0)	0 (0-0)
Cuba	5 (4-8)	-33.2 (-41.3--26.2)	24 (17-34)	-26.9 (-33.6--19.7)	5 (4-8)	-25.2 (-34.1--17.5)	4 (2-5)	-27.5 (-37.7--18.2)	4 (2-5)	18 (13-25)	4 (3-6)	3 (2-4)
Dominica	0 (0-0)	-37.8 (-45--31.1)	0 (0-0)	-37.9 (-45.2--31.2)	0 (0-0)	-38.5 (-45.5--31.9)	0 (0-0)	-40.3 (-47.8--32.8)	0 (0-0)	0 (0-0)	0 (0-0)	0 (0-0)
Dominican Republic	51 (37-72)	-6.1 (-16-2.6)	156 (113-218)	-1.1 (-10.9-8.4)	46 (33-65)	-1 (-11.5-8.6)	38 (26-54)	-1.5 (-12.3-8.3)	48 (34-65)	155 (112-210)	46 (32-62)	37 (26-51)
Grenada	0 (0-0)	-30.7 (-39--23.8)	0 (0-0)	-30.2 (-37.3--23.3)	0 (0-0)	-30.6 (-38.5--23.5)	0 (0-0)	-30.8 (-39.8--22.4)	0 (0-0)	0 (0-0)	0 (0-0)	0 (0-0)
Guyana	0 (0-1)	-17.7 (-25--8.9)	2 (1-2)	-22.8 (-29.7--15.4)	0 (0-1)	-23.8 (-30.8--15.6)	0 (0-1)	-22.2 (-30.2--13.4)	0 (0-1)	1 (1-2)	0 (0-1)	0 (0-0)
Haiti	57 (41-80)	20.9 (11.2-31.4)	154 (111-213)	30 (18.5-41)	48 (34-67)	31.3 (20.8-42.8)	40 (28-57)	30.4 (19.3-42.2)	68 (49-95)	200 (144-272)	63 (45-87)	52 (37-73)
Jamaica	6 (4-8)	-25 (-32.5--15.8)	25 (18-36)	-28.6 (-34.8--21.1)	6 (4-9)	-29.7 (-36.9--21.4)	4 (3-6)	-30 (-39--19.9)	4 (3-6)	18 (13-25)	4 (3-6)	3 (2-4)
Puerto Rico	0 (0-1)	-61.3 (-68.7--52.7)	3 (2-4)	-54.6 (-62.2--45.7)	1 (0-1)	-54 (-63.3--42.6)	0 (0-0)	-62.2 (-70.7--52)	0 (0-0)	1 (1-2)	0 (0-0)	0 (0-0)
Saint Kitts and Nevis	0 (0-0)	-18.3 (-26.8--8.6)	0 (0-0)	-16.5 (-24.8--7.6)	0 (0-0)	-16.8 (-25.5--7.4)	0 (0-0)	-17.8 (-27.5--7)	0 (0-0)	0 (0-0)	0 (0-0)	0 (0-0)
Saint Lucia	0 (0-0)	-42.3 (-47.7--36.5)	0 (0-1)	-41.4 (-47--35.4)	0 (0-0)	-42 (-47.4--36.1)	0 (0-0)	-43.5 (-49.8--36.1)	0 (0-0)	0 (0-0)	0 (0-0)	0 (0-0)
Saint Vincent and the Grenadines	0 (0-0)	-33 (-38.9--25.3)	0 (0-0)	-32.8 (-39.2--24.9)	0 (0-0)	-33 (-39--25.4)	0 (0-0)	-33.8 (-41.3--25)	0 (0-0)	0 (0-0)	0 (0-0)	0 (0-0)
Suriname	0 (0-0)	-10.7 (-19.5--0.5)	1 (1-1)	2.5 (-8.1-15.1)	0 (0-0)	3.6 (-6.6-15.9)	0 (0-0)	0.9 (-10.1-14.4)	0 (0-0)	1 (1-1)	0 (0-0)	0 (0-0)
Trinidad and Tobago	0 (0-1)	-18.6 (-25.9--11.2)	2 (1-3)	-15.1 (-22--7.4)	0 (0-1)	-14.6 (-22.2--7.7)	0 (0-0)	-13.4 (-22.4--3.8)	0 (0-1)	2 (1-3)	0 (0-1)	0 (0-0)
United States Virgin Islands	0 (0-0)	-38.1 (-49.9--25.4)	0 (0-0)	-35.4 (-46.3--23.5)	0 (0-0)	-35 (-47.4--21.6)	0 (0-0)	-35.9 (-49.3--19.2)	0 (0-0)	0 (0-0)	0 (0-0)	0 (0-0)
**Central Latin America**	7 (5-10)	-13.2 (-25.7-8.8)	43 (28-64)	-6.9 (-20.3-20.8)	6 (4-9)	-8.2 (-21.6-15.3)	4 (2-5)	-10.8 (-25.7-10.7)	6 (4-9)	40 (26-65)	6 (4-9)	3 (2-5)
Colombia	4 (3-6)	-17.7 (-37.7--4.2)	19 (13-27)	-17.8 (-34.7--7.6)	4 (3-5)	-18.9 (-38.9--5.6)	2 (1-3)	-17.8 (-38.3--2.2)	3 (2-5)	16 (11-22)	3 (2-4)	2 (1-3)
Costa Rica	0 (0-0)	-9 (-81.7-55.6)	0 (0-0)	-9.3 (-80.5-43.7)	0 (0-0)	-8.9 (-81.8-53.6)	0 (0-0)	-10.8 (-82.3-51.6)	0 (0-0)	0 (0-0)	0 (0-0)	0 (0-0)
El Salvador	0 (0-0)	-18.3 (-22.8-48.4)	0 (0-0)	-18.9 (-28.8-41.1)	0 (0-0)	-19.1 (-24.8-45.3)	0 (0-0)	-21.1 (-29.5-41.7)	0 (0-0)	0 (0-0)	0 (0-0)	0 (0-0)
Guatemala	0 (0-0)	9.7 (-44.2-12.8)	0 (0-0)	-11.5 (-53.6--1.5)	0 (0-0)	-10.5 (-54--4.9)	0 (0-0)	-16.8 (-57.7--9.8)	0 (0-0)	0 (0-0)	0 (0-0)	0 (0-0)
Honduras	0 (0-0)	24.3 (24.3-24.3)	0 (0-0)	27.1 (18.9-35.7)	0 (0-0)	26.3 (25.1-28.5)	0 (0-0)	23.6 (16.6-30.6)	0 (0-0)	0 (0-0)	0 (0-0)	0 (0-0)
Mexico	2 (1-4)	-4.6 (-29-62)	19 (9-36)	2.1 (-22.6-72.6)	2 (1-4)	9 (-17-85.1)	1 (0-2)	5.1 (-21.8-88.9)	2 (1-4)	20 (8-40)	2 (1-5)	1 (0-2)
Aguascalientes	0 (0-0)	29.1 (-4.1-773)	0 (0-0)	34.8 (-7.6-857)	0 (0-0)	42.3 (2.7-840)	0 (0-0)	33.4 (-7.2-774)	0 (0-0)	0 (0-0)	0 (0-0)	0 (0-0)
Baja California	0 (0-0)	35.5 (5-701)	0 (0-1)	47.5 (7.6-848)	0 (0-0)	56.7 (17.7-827)	0 (0-0)	41.2 (-0.2-567)	0 (0-0)	0 (0-1)	0 (0-0)	0 (0-0)
Baja California Sur	0 (0-0)	58.9 (24.7-1030)	0 (0-0)	73.2 (26.9-1030)	0 (0-0)	83.5 (40-1160)	0 (0-0)	65.1 (18.3-1020)	0 (0-0)	0 (0-0)	0 (0-0)	0 (0-0)

Location	Birth , 2000	Births percent change (2000-2021)	All age cases, 2000	All age cases percent change (2000-2021)	All age deaths, 2000	All age deaths percent change (2000-2021)	Under-5 deaths, 2000	Under-5 deaths percent change (2000-2021)	Births, 2021	All age cases, 2021	All age deaths, 2021	Under-5 deaths, 2021
Campeche	0 (0-0)	22.2 (-5.5-1130)	0 (0-0)	20.1 (-12.8-1210)	0 (0-0)	26.1 (-3.8-1160)	0 (0-0)	26.1 (-9.1-1050)	0 (0-0)	0 (0-0)	0 (0-0)	0 (0-0)
Chiapas	0 (0-0)	53.4 (15.5-781)	0 (0-1)	51.7 (7-786)	0 (0-0)	58.4 (18-806)	0 (0-0)	57.9 (12.2-776)	0 (0-0)	1 (0-2)	0 (0-0)	0 (0-0)
Chihuahua	0 (0-0)	-1.2 (-25.7-467)	0 (0-1)	5.6 (-26.1-485)	0 (0-0)	11.2 (-18.6-532)	0 (0-0)	4.3 (-26.9-405)	0 (0-0)	0 (0-1)	0 (0-0)	0 (0-0)
Coahuila	0 (0-0)	27.6 (-1.5-276)	1 (0-3)	29 (-7.5-288)	0 (0-0)	37 (4.6-305)	0 (0-0)	32.3 (-9.5-314)	0 (0-0)	2 (1-3)	0 (0-0)	0 (0-0)
Colima	0 (0-0)	20.6 (-4.9-1140)	0 (0-0)	22.3 (-9.3-1240)	0 (0-0)	29.7 (0.6-1230)	0 (0-0)	27.2 (-7.2-1090)	0 (0-0)	0 (0-0)	0 (0-0)	0 (0-0)
Durango	0 (0-0)	-1 (-22.6-408)	0 (0-0)	6.3 (-22.7-469)	0 (0-0)	11.1 (-15-472)	0 (0-0)	4.7 (-22.5-402)	0 (0-0)	0 (0-0)	0 (0-0)	0 (0-0)
Guanajuato	0 (0-1)	0.5 (-23.2-256)	2 (1-5)	10.4 (-22-291)	0 (0-1)	18.7 (-11-320)	0 (0-0)	8.7 (-26.5-278)	0 (0-1)	2 (1-5)	0 (0-1)	0 (0-0)
Guerrero	0 (0-0)	0.1 (-26.5-1030)	0 (0-1)	7.1 (-27.9-1180)	0 (0-0)	12.1 (-19.2-1160)	0 (0-0)	7.9 (-25.2-1030)	0 (0-0)	0 (0-2)	0 (0-0)	0 (0-0)
Hidalgo	0 (0-0)	3.5 (-19.8-948)	0 (0-1)	10.6 (-18.8-1070)	0 (0-0)	17.7 (-11-1070)	0 (0-0)	11.7 (-20.9-912)	0 (0-0)	0 (0-1)	0 (0-0)	0 (0-0)
Jalisco	0 (0-0)	-2.7 (-14.3-340)	1 (0-2)	2.2 (-16.9-396)	0 (0-0)	6.6 (-7.9-372)	0 (0-0)	2.1 (-16.8-309)	0 (0-0)	1 (0-2)	0 (0-0)	0 (0-0)
México	0 (0-0)	0.5 (-10.7-343)	1 (0-4)	11.1 (-8.2-396)	0 (0-0)	16 (1.8-407)	0 (0-0)	10 (-9.4-321)	0 (0-0)	1 (0-4)	0 (0-0)	0 (0-0)
Mexico City	1 (0-1)	-31.3 (-41.5-18.1)	7 (4-11)	-20.4 (-37.1-39.8)	1 (0-1)	-16.4 (-30.2-44)	0 (0-0)	-24.3 (-41-26.3)	1 (0-1)	6 (3-9)	1 (0-1)	0 (0-0)
Michoacán de Ocampo	0 (0-0)	-0.6 (-24.4-588)	0 (0-1)	2.2 (-27.6-580)	0 (0-0)	6.1 (-20.5-623)	0 (0-0)	3.2 (-26.4-600)	0 (0-0)	0 (0-1)	0 (0-0)	0 (0-0)
Morelos	0 (0-0)	21.2 (-10.9-711)	0 (0-0)	22.8 (-14.8-760)	0 (0-0)	29.3 (-6.5-758)	0 (0-0)	26.5 (-13.7-749)	0 (0-0)	0 (0-1)	0 (0-0)	0 (0-0)
Nayarit	0 (0-0)	21.9 (-6.6-916)	0 (0-0)	20.5 (-12.7-980)	0 (0-0)	27 (-4-949)	0 (0-0)	26.1 (-10.1-884)	0 (0-0)	0 (0-0)	0 (0-0)	0 (0-0)
Nuevo León	0 (0-0)	10.4 (-11.1-432)	0 (0-1)	21.3 (-9.3-549)	0 (0-0)	27.9 (0.6-534)	0 (0-0)	18.6 (-11-463)	0 (0-0)	0 (0-1)	0 (0-0)	0 (0-0)
Oaxaca	0 (0-0)	1.1 (-24.5-702)	0 (0-1)	2.8 (-27.3-713)	0 (0-0)	8.4 (-20.6-764)	0 (0-0)	5.9 (-25.9-661)	0 (0-0)	0 (0-1)	0 (0-0)	0 (0-0)
Puebla	0 (0-0)	4.8 (-18.5-920)	1 (0-2)	10.8 (-18.9-1050)	0 (0-0)	17.7 (-10.5-1030)	0 (0-0)	13.4 (-18-858)	0 (0-0)	1 (0-2)	0 (0-0)	0 (0-0)
Querétaro	0 (0-0)	38 (0.4-874)	0 (0-0)	42 (-3.4-968)	0 (0-0)	49.8 (6.5-926)	0 (0-0)	42.5 (-4-843)	0 (0-0)	0 (0-1)	0 (0-0)	0 (0-0)
Quintana Roo	0 (0-0)	79.6 (34.9-1690)	0 (0-0)	83.2 (28.4-1880)	0 (0-0)	92.1 (41.6-1820)	0 (0-0)	86.7 (31.3-1620)	0 (0-0)	0 (0-1)	0 (0-0)	0 (0-0)
San Luis Potosí	0 (0-0)	-1.2 (-25.1-581)	0 (0-1)	2.8 (-27.2-629)	0 (0-0)	9.4 (-19.3-643)	0 (0-0)	3.8 (-27.4-615)	0 (0-0)	0 (0-1)	0 (0-0)	0 (0-0)
Sinaloa	0 (0-0)	-7.8 (-28.4-323)	0 (0-1)	-4.5 (-31.3-366)	0 (0-0)	0 (-24.1-366)	0 (0-0)	-5.4 (-30.8-289)	0 (0-0)	0 (0-1)	0 (0-0)	0 (0-0)
Sonora	0 (0-0)	7.4 (-17.2-653)	0 (0-1)	17 (-15.7-675)	0 (0-0)	23 (-8.2-733)	0 (0-0)	12.6 (-18.5-676)	0 (0-0)	0 (0-1)	0 (0-0)	0 (0-0)
Tabasco	0 (0-0)	15.7 (-13.2-228)	1 (0-3)	17.4 (-18.2-230)	0 (0-0)	23.9 (-8-253)	0 (0-0)	21.5 (-16.4-227)	0 (0-0)	2 (1-3)	0 (0-0)	0 (0-0)
Tamaulipas	0 (0-0)	-3 (-25.6-389)	0 (0-1)	10.9 (-20.7-470)	0 (0-0)	18 (-12.3-498)	0 (0-0)	6.6 (-23.5-418)	0 (0-0)	0 (0-1)	0 (0-0)	0 (0-0)
Tlaxcala	0 (0-0)	16.8 (-10.8-1070)	0 (0-0)	22.1 (-11.6-1320)	0 (0-0)	29.9 (-2.8-1180)	0 (0-0)	23.7 (-14.2-957)	0 (0-0)	0 (0-0)	0 (0-0)	0 (0-0)
Veracruz de Ignacio de la Llave	0 (0-0)	-9.9 (-29.7-438)	1 (0-2)	-5.8 (-31.9-474)	0 (0-0)	-1.4 (-25-496)	0 (0-0)	-7.2 (-31.7-421)	0 (0-0)	1 (0-2)	0 (0-0)	0 (0-0)
Yucatán	0 (0-0)	9.3 (-13.6-686)	0 (0-0)	11.3 (-18.2-648)	0 (0-0)	17 (-9.5-715)	0 (0-0)	12 (-17.8-677)	0 (0-0)	0 (0-1)	0 (0-0)	0 (0-0)
Zacatecas	0 (0-0)	1.5 (-21-421)	0 (0-0)	3 (-25-521)	0 (0-0)	8.8 (-16.6-463)	0 (0-0)	7.4 (-23.3-368)	0 (0-0)	0 (0-0)	0 (0-0)	0 (0-0)

Location	Birth , 2000	Births percent change (2000-2021)	All age cases, 2000	All age cases percent change (2000-2021)	All age deaths, 2000	All age deaths percent change (2000-2021)	Under-5 deaths, 2000	Under-5 deaths percent change (2000-2021)	Births, 2021	All age cases, 2021	All age deaths, 2021	Under-5 deaths, 2021
Nicaragua	0 (0-0)	1 (0.9-1.5)	0 (0-0)	2.6 (-4.9-10.4)	0 (0-0)	2.2 (1.3-3.9)	0 (0-0)	1.5 (-4.5-7)	0 (0-0)	0 (0-0)	0 (0-0)	0 (0-0)
Panama	0 (0-0)	22.9 (22.9-22.9)	0 (0-0)	27.3 (19.7-34.9)	0 (0-0)	26.9 (25.9-28)	0 (0-0)	23.6 (16-32.1)	0 (0-0)	0 (0-0)	0 (0-0)	0 (0-0)
Venezuela (Bolivarian Republic of)	1 (0-1)	-17 (-38.5-3.9)	4 (3-6)	-17.5 (-36.4-3.7)	1 (0-1)	-17.9 (-38.7-2.4)	0 (0-1)	-18.8 (-39.6-0.3)	1 (0-1)	3 (2-5)	1 (0-1)	0 (0-0)
**Tropical Latin America**	74 (61-92)	4.7 (-1.3-10.4)	2,520 (2,080-3,100)	30.6 (23.2-37.7)	46 (37-56)	64.9 (55.1-74.9)	5 (3-7)	18.9 (11.5-26)	78 (62-100)	3,290 (2,620-4,220)	75 (59-96)	6 (4-9)
Brazil	69 (57-85)	5 (-1.3-11.2)	2,390 (1,980-2,930)	30.9 (23-38.6)	42 (35-52)	67.4 (56.6-78.2)	4 (3-6)	22.1 (13.5-31.1)	72 (58-92)	3,130 (2,500-4,000)	71 (56-90)	5 (3-8)
Acre	0 (0-0)	27 (6.5-45.1)	8 (5-12)	62 (36.4-83.6)	0 (0-0)	90.1 (62.1-119)	0 (0-0)	37.7 (14.6-60.8)	0 (0-1)	13 (8-20)	0 (0-0)	0 (0-0)
Alagoas	1 (0-1)	38.1 (-12.7-145)	14 (9-20)	78.5 (11.7-211)	0 (0-0)	119 (35.3-289)	0 (0-0)	66 (2.4-196)	1 (0-1)	25 (16-39)	1 (0-1)	0 (0-0)
Amapá	0 (0-0)	95.8 (39.7-2740)	0 (0-0)	133 (63.4-3050)	0 (0-0)	194 (99.3-4280)	0 (0-0)	106 (34-3260)	0 (0-0)	0 (0-1)	0 (0-0)	0 (0-0)
Amazonas	3 (2-4)	43.7 (31.4-59.2)	98 (81-122)	72.3 (58.5-89.7)	1 (1-2)	118 (96.4-144)	0 (0-0)	56.1 (37.2-79.4)	4 (3-5)	169 (136-212)	3 (3-4)	0 (0-0)
Bahia	10 (8-11)	0.8 (-7.5-10.7)	270 (225-323)	25.7 (15.3-37.8)	5 (4-6)	61.9 (47.3-78.5)	1 (0-1)	23 (7.8-39.8)	10 (8-12)	339 (279-415)	7 (6-9)	1 (0-1)
Ceará	2 (1-3)	25.5 (-0.8-70.5)	50 (37-68)	49 (17.8-102)	1 (1-1)	85 (45.3-152)	0 (0-0)	45 (10.1-106)	2 (2-3)	75 (53-102)	2 (1-2)	0 (0-0)
Distrito Federal	4 (3-4)	-8.3 (-15.9--1.4)	130 (109-153)	30 (19.5-40.2)	2 (2-3)	78.9 (62.2-96.4)	0 (0-0)	4.4 (-8.2-19.1)	3 (3-4)	169 (141-200)	4 (3-5)	0 (0-0)
Espírito Santo	1 (1-1)	-11 (-31.5-8.8)	26 (19-36)	13 (-12.3-38.2)	0 (0-1)	46.5 (12.2-80.6)	0 (0-0)	2.3 (-24.2-31.2)	1 (0-1)	29 (20-44)	1 (0-1)	0 (0-0)
Goiás	3 (2-3)	-3.5 (-12-5.8)	97 (80-118)	28.8 (17.4-40.4)	2 (1-2)	69.6 (53.2-88.8)	0 (0-0)	8.1 (-6.2-23.7)	3 (2-3)	125 (100-157)	3 (2-4)	0 (0-0)
Maranhão	2 (2-3)	26.9 (9.5-50.8)	51 (39-68)	62.3 (40.9-91.5)	1 (1-1)	89.8 (63.6-128)	0 (0-0)	62.3 (37.4-92.9)	3 (2-4)	83 (61-112)	2 (1-2)	0 (0-0)
Mato Grosso	1 (1-1)	27 (12.4-45.5)	27 (21-37)	58 (39.8-81.6)	0 (0-1)	108 (82.4-137)	0 (0-0)	40.4 (20.3-63.2)	1 (1-1)	43 (31-60)	1 (1-1)	0 (0-0)
Mato Grosso do Sul	1 (1-1)	-0.8 (-13.1-9.3)	38 (29-51)	23 (8.2-35.1)	1 (0-1)	60.9 (41.3-79)	0 (0-0)	10.3 (-5.6-26)	1 (1-1)	47 (34-66)	1 (1-1)	0 (0-0)
Minas Gerais	8 (6-9)	-9.2 (-19.3-0.8)	294 (244-352)	15.9 (3.5-27.7)	5 (4-6)	54.5 (37.6-73.3)	0 (0-1)	1.4 (-13.1-16.8)	7 (6-9)	341 (276-427)	8 (6-10)	0 (0-1)
Pará	2 (2-3)	37.3 (26.7-48.8)	72 (56-93)	65.4 (51.4-80.3)	1 (1-2)	102 (83.3-122)	0 (0-0)	52.7 (34.4-74.3)	3 (2-4)	119 (91-153)	2 (2-3)	0 (0-0)
Paraíba	1 (1-2)	34.3 (9.7-64.7)	30 (22-42)	58.3 (29.1-95.1)	1 (0-1)	100 (64.8-146)	0 (0-0)	60.7 (29.4-102)	1 (1-2)	47 (32-69)	1 (1-1)	0 (0-0)
Paraná	3 (2-4)	-5.1 (-13.1-2.1)	109 (85-139)	19.7 (10.6-29.2)	2 (2-2)	59.4 (44.7-73.7)	0 (0-0)	3.1 (-11.4-16.9)	3 (2-3)	130 (102-169)	3 (2-4)	0 (0-0)
Pernambuco	2 (2-3)	25 (-1.7-71.1)	56 (41-80)	62.3 (27.1-123)	1 (1-1)	102 (58.4-176)	0 (0-0)	52.2 (19.7-112)	3 (2-4)	90 (64-134)	2 (1-3)	0 (0-0)
Piaui	2 (2-3)	13.5 (3-22.4)	53 (42-70)	38.3 (24.6-49.4)	1 (1-1)	72.5 (55.3-89.2)	0 (0-0)	38.9 (21.4-57.1)	2 (2-3)	74 (57-96)	2 (1-2)	0 (0-0)
Rio de Janeiro	5 (4-6)	-5.6 (-14.3-5.1)	197 (160-240)	17.3 (7-29.1)	4 (3-5)	43.5 (29.7-59)	0 (0-0)	4.4 (-10-23.3)	5 (4-6)	231 (184-295)	6 (4-7)	0 (0-0)
Rio Grande do Norte	1 (0-1)	23.8 (-10-90.5)	19 (13-28)	57.5 (14.6-141)	0 (0-0)	105 (47.6-213)	0 (0-0)	47.6 (7.7-131)	1 (1-1)	30 (20-45)	1 (0-1)	0 (0-0)
Rio Grande do Sul	4 (3-4)	2 (-8.9-14.9)	155 (122-200)	25.3 (12.2-41.2)	3 (2-4)	58.6 (40.1-78.5)	0 (0-0)	10.5 (-4.7-29.4)	4 (3-5)	195 (146-259)	5 (4-6)	0 (0-0)
Rondônia	0 (0-1)	0.3 (-9.8-12.2)	18 (12-25)	27.4 (14.5-44.1)	0 (0-0)	71.9 (52.5-99.1)	0 (0-0)	10 (-4.7-27.8)	0 (0-1)	23 (15-32)	1 (0-1)	0 (0-0)
Roraima	0 (0-0)	58.8 (-32.9-493)	0 (0-1)	101 (-15.4-651)	0 (0-0)	158 (7.5-857)	0 (0-0)	92.5 (-21.3-590)	0 (0-0)	1 (0-2)	0 (0-0)	0 (0-0)
Santa Catarina	1 (1-2)	26.1 (11-41.9)	52 (40-69)	53.1 (35.7-73.4)	1 (1-1)	104 (78.3-136)	0 (0-0)	37.4 (17-63.9)	2 (1-2)	79 (56-108)	2 (1-3)	0 (0-0)

Location	Birth , 2000	Births percent change (2000-2021)	All age cases, 2000	All age cases percent change (2000-2021)	All age deaths, 2000	All age deaths percent change (2000-2021)	Under-5 deaths, 2000	Under-5 deaths percent change (2000-2021)	Births, 2021	All age cases, 2021	All age deaths, 2021	Under-5 deaths, 2021
São Paulo	12 (10-14)	-6.5 (-15.7-3.3)	484 (403-580)	21.2 (9.3-32.9)	9 (7-11)	56 (40.1-73.5)	1 (0-1)	5.7 (-7.7-22.7)	11 (9-14)	587 (481-732)	14 (11-17)	1 (0-1)
Sergipe	1 (1-1)	22.9 (10.8-37)	25 (18-35)	55.5 (40.2-71.4)	0 (0-1)	101 (79.9-125)	0 (0-0)	40 (21.8-61)	1 (1-1)	39 (27-54)	1 (1-1)	0 (0-0)
Tocantins	1 (1-1)	14.2 (2.6-25.3)	21 (15-28)	54.9 (40-71)	0 (0-0)	95.5 (73.8-118)	0 (0-0)	35.5 (19.1-53.2)	1 (1-1)	32 (23-43)	1 (0-1)	0 (0-0)
Paraguay	5 (4-7)	-0.1 (-9.1-8.7)	130 (96-172)	24.5 (14.6-35.6)	3 (2-4)	31.7 (18.5-45.3)	1 (0-1)	0.4 (-13-15.5)	5 (4-7)	162 (119-218)	4 (3-6)	1 (0-1)
North Africa and Middle East	4,140 (3,060-5,570)	-6.2 (-9.6--2.1)	24,100 (18,500-31,000)	11.8 (6.9-17.2)	3,530 (2,580-4,850)	7.9 (3.6-13.2)	2,440 (1,680-3,500)	1.4 (-3-6.7)	3,880 (2,870-5,260)	26,900 (20,800-34,600)	3,810 (2,790-5,170)	2,480 (1,700-3,550)
**North Africa and Middle East**	4,140 (3,060-5,570)	-6.2 (-9.6--2.1)	24,100 (18,500-31,000)	11.8 (6.9-17.2)	3,530 (2,580-4,850)	7.9 (3.6-13.2)	2,440 (1,680-3,500)	1.4 (-3-6.7)	3,880 (2,870-5,260)	26,900 (20,800-34,600)	3,810 (2,790-5,170)	2,480 (1,700-3,550)
Afghanistan	264 (170-405)	70.9 (51.1-91.4)	937 (653-1,320)	84.9 (66.9-105)	209 (133-325)	80.5 (58-104)	171 (106-269)	79.2 (55.2-103)	451 (288-674)	1,730 (1,220-2,420)	378 (239-578)	306 (187-484)
Algeria	69 (49-92)	22.5 (11.8-34.5)	397 (298-510)	19.7 (8.1-31.4)	64 (46-86)	25 (12.5-38)	43 (29-61)	31 (15.5-50.2)	84 (61-116)	475 (360-609)	80 (57-110)	57 (38-81)
Bahrain	11 (8-16)	-8.1 (-19.3-3.2)	83 (60-109)	25.1 (12.8-38.5)	11 (7-16)	17 (2.7-31.8)	7 (4-11)	-0.1 (-15.4-16.9)	10 (7-15)	104 (77-136)	13 (9-18)	7 (4-11)
Egypt	313 (240-407)	6.8 (-3.6-17.9)	1,500 (1,170-1,890)	32.9 (20.8-46.3)	267 (203-349)	29.7 (16.7-43.2)	199 (143-267)	22.9 (8.9-38)	334 (258-427)	2,000 (1,570-2,500)	346 (267-443)	244 (179-325)
Iran (Islamic Republic of)	172 (114-259)	-12.2 (-14.8--9.3)	1,120 (806-1,530)	-5.7 (-8.3--3.2)	167 (110-251)	-2 (-5.6-1.7)	104 (63-171)	1.7 (-2.2-6.2)	151 (99-229)	1,050 (759-1,450)	164 (107-250)	106 (64-174)
Alborz	5 (3-8)	-6.3 (-16.1-6.3)	30 (22-42)	5.6 (-4.7-17.9)	4 (3-6)	9.6 (-2.8-25.6)	2 (1-4)	13 (-3.5-34)	5 (3-7)	32 (23-44)	4 (3-7)	3 (1-4)
Ardebil	3 (2-4)	-32.8 (-41.7--23.2)	16 (11-22)	-28.2 (-36.4--19.9)	3 (2-4)	-25.7 (-35.6--15)	2 (1-3)	-23.5 (-35.2--10.4)	2 (1-3)	12 (8-16)	2 (1-3)	1 (1-2)
Bushehr	4 (2-6)	4.6 (-7.9-20)	23 (17-32)	20.8 (8.4-34.5)	3 (2-5)	23.3 (8-41.5)	2 (1-4)	21.3 (3.2-43.8)	4 (2-6)	28 (20-38)	4 (3-6)	3 (2-4)
Chahar Mahaal and Bakhtiari	4 (2-6)	-15.7 (-26.7--3.1)	23 (17-31)	-10.3 (-19.9-0.7)	4 (2-5)	-6.9 (-19.2-6.5)	2 (1-4)	-3.9 (-18.5-13.2)	3 (2-4)	21 (15-28)	3 (2-5)	2 (1-3)
East Azarbayejan	8 (5-12)	-23.6 (-31.1--12.7)	43 (30-59)	-10.8 (-19.9--0.2)	7 (5-11)	-5.8 (-15.3-6.7)	5 (3-8)	-4 (-16-11.7)	6 (4-9)	38 (27-53)	7 (4-10)	4 (3-7)
Fars	14 (9-21)	-16.8 (-25.8--6.9)	101 (71-139)	-15.1 (-24.2--6.2)	15 (10-22)	-11.6 (-21.6--0.3)	9 (5-14)	-5.4 (-17.7-8.9)	12 (8-18)	86 (60-120)	13 (9-20)	8 (5-14)
Gilan	3 (2-5)	-33.1 (-40.8--24.3)	28 (20-39)	-28.6 (-36.3--20.9)	4 (2-5)	-27 (-35.3--17.3)	2 (1-3)	-25.9 (-36.5--13.3)	2 (1-3)	20 (14-28)	3 (2-4)	1 (1-2)
Golestan	4 (2-6)	-12.1 (-24-0.3)	22 (16-30)	-2.1 (-12.8-9.9)	4 (2-5)	1.9 (-12.7-15)	2 (1-4)	3.9 (-14.2-22.1)	3 (2-5)	21 (15-30)	4 (2-6)	2 (1-4)
Hamadan	4 (3-7)	-35.6 (-42.8--27.8)	28 (20-39)	-30.9 (-38.2--23.5)	5 (3-7)	-28.3 (-36.5--19.1)	3 (2-5)	-25.5 (-36.6--13.3)	3 (2-4)	20 (14-27)	3 (2-5)	2 (1-4)
Hormozgan	4 (2-6)	20.2 (4.9-37.2)	21 (15-29)	29.7 (17-44.3)	3 (2-5)	33.3 (17-53.3)	2 (1-4)	36.2 (16.4-62.1)	4 (3-6)	27 (19-37)	5 (3-7)	3 (2-5)
Ilam	2 (1-2)	-34.8 (-41.6--26.2)	10 (7-14)	-27.3 (-34.3--18.8)	2 (1-2)	-26.1 (-34.2--15.9)	1 (1-2)	-24.8 (-34.6--12.6)	1 (1-2)	7 (5-10)	1 (1-2)	1 (0-1)
Isfahan	11 (7-17)	-22 (-32.1--9.5)	87 (62-118)	-12.6 (-21.5--2.7)	12 (8-18)	-11.1 (-22-3.3)	7 (4-12)	-11.2 (-24.7-6.6)	9 (6-13)	76 (54-102)	11 (7-16)	6 (4-10)
Kerman	5 (3-8)	11.5 (-1.6-25.8)	36 (26-51)	15.7 (4.2-29.1)	5 (4-8)	20.9 (7-37.5)	3 (2-6)	26.9 (9.8-47.3)	6 (4-9)	42 (30-58)	7 (4-10)	4 (3-7)
Kermanshah	5 (3-8)	-26.8 (-35.5--17.3)	33 (23-45)	-27.3 (-35.3--19)	5 (3-8)	-23.7 (-34.1--13.5)	3 (2-5)	-17.3 (-29.5--4.2)	4 (2-5)	24 (17-33)	4 (2-6)	2 (1-4)
Khorasan-e-Razavi	14 (9-21)	-6.4 (-17.3-8.1)	73 (51-102)	2.2 (-7.9-14.3)	13 (8-19)	6.1 (-5.7-24)	9 (5-14)	9.4 (-5.3-29.6)	13 (8-19)	74 (53-104)	13 (9-21)	9 (6-15)
Khuzestan	19 (12-29)	-11.9 (-23.4-1.9)	121 (86-167)	-6.7 (-17.3-4.5)	18 (12-28)	-2.9 (-15.5-12.4)	12 (7-19)	2 (-14.2-20.7)	17 (11-25)	113 (81-155)	18 (11-27)	12 (7-19)
Kohgiluyeh and Boyer-Ahmad	4 (3-6)	-14.3 (-26.3--2.5)	22 (15-29)	-8.2 (-19.2-2.8)	3 (2-5)	-5.4 (-18.3-8.2)	2 (1-4)	-1.8 (-18.5-15.4)	3 (2-5)	20 (14-28)	3 (2-5)	2 (1-4)
Kurdistan	4 (2-6)	-18 (-27.2--5.7)	22 (15-30)	-16.7 (-24.9--6.3)	4 (2-6)	-13.7 (-23.5--0.7)	3 (2-4)	-9.2 (-21.4-7)	3 (2-5)	18 (13-25)	3 (2-5)	2 (1-4)

Location	Birth , 2000	Births percent change (2000-2021)	All age cases, 2000	All age cases percent change (2000-2021)	All age deaths, 2000	All age deaths percent change (2000-2021)	Under-5 deaths, 2000	Under-5 deaths percent change (2000-2021)	Births, 2021	All age cases, 2021	All age deaths, 2021	Under-5 deaths, 2021
Lorestan	5 (4-8)	-32.6 (-40.1--24.3)	35 (25-48)	-27.7 (-35.6--19.7)	5 (3-8)	-25.6 (-34.2--16.9)	3 (2-6)	-23.7 (-35--12)	4 (2-5)	25 (18-35)	4 (3-6)	3 (2-4)
Markazi	3 (2-4)	-27.1 (-36.6--16)	21 (15-29)	-23.7 (-31.5--15.1)	3 (2-5)	-21.4 (-31.6--9.8)	2 (1-3)	-17.4 (-30.2--1.6)	2 (1-3)	16 (12-22)	2 (2-4)	1 (1-2)
Mazandaran	4 (3-7)	-25.1 (-33.5--14.7)	36 (26-48)	-17.3 (-25.1--8.2)	5 (3-7)	-15.6 (-25.5--4.7)	3 (1-4)	-14.9 (-27.7--1.5)	3 (2-5)	30 (21-41)	4 (3-6)	2 (1-4)
North Khorasan	2 (2-4)	-36.1 (-45.2--26.8)	12 (8-16)	-20.7 (-29.9--10.2)	2 (1-3)	-21 (-32.3--9.2)	2 (1-3)	-23.6 (-35.3--10.4)	2 (1-2)	9 (6-13)	2 (1-3)	1 (1-2)
Qazvin	2 (1-3)	-12.9 (-23.4--0.7)	15 (11-20)	-11.8 (-21--0.8)	2 (1-3)	-7.5 (-19.3-5.6)	1 (1-2)	-2.2 (-15.7-13.7)	2 (1-3)	13 (9-18)	2 (1-3)	1 (1-2)
Qom	2 (1-3)	11.8 (-2.3-28.1)	15 (11-21)	16.4 (4.4-29)	2 (1-3)	21 (5.9-38.9)	1 (1-2)	26.2 (5.8-48.4)	2 (1-4)	18 (12-25)	3 (2-4)	2 (1-3)
Semnan	1 (1-1)	11.6 (-2.5-27.6)	7 (5-10)	8.3 (-4.2-21.3)	1 (1-1)	12.3 (-1.7-28.3)	1 (0-1)	19.4 (0.7-40.8)	1 (1-1)	8 (5-10)	1 (1-2)	1 (0-1)
Sistan and Baluchistan	9 (6-14)	11.6 (-0.7-23.7)	39 (27-55)	28.5 (15-42.6)	7 (5-12)	31.2 (16.7-45.8)	6 (3-9)	32.3 (15.7-50.5)	10 (6-15)	50 (35-70)	10 (6-15)	7 (5-12)
South Khorasan	2 (1-3)	-8 (-17.9-5.6)	9 (6-12)	4.7 (-5.8-16.8)	2 (1-3)	5.8 (-5.4-21.6)	1 (1-2)	6.1 (-7.4-24.9)	2 (1-2)	9 (7-13)	2 (1-3)	1 (1-2)
Tehran	16 (10-23)	-5 (-15.8-6.3)	126 (90-172)	3.9 (-6.4-14.2)	15 (10-22)	9.5 (-2.5-22.4)	7 (4-12)	15.2 (-2.7-39.7)	15 (10-23)	131 (93-177)	16 (10-24)	8 (5-15)
West Azarbayejan	6 (4-10)	0.5 (-10.7-15.2)	36 (26-50)	8 (-2.9-20.4)	6 (4-9)	13.5 (0.3-30.1)	4 (2-7)	18.8 (3-39.8)	6 (4-10)	39 (27-54)	7 (4-11)	5 (3-8)
Yazd	2 (1-3)	2 (-11.8-15.4)	15 (11-20)	12.8 (0.9-26.2)	2 (1-3)	17.9 (2.3-32.7)	1 (1-2)	21.7 (2.3-43.7)	2 (1-3)	17 (12-23)	2 (2-4)	1 (1-2)
Zanjan	2 (1-3)	-23.2 (-34.4--12.3)	13 (9-18)	-19.1 (-28.4--8.9)	2 (1-3)	-15.2 (-27.2--3.5)	1 (1-2)	-11.4 (-25.1-3.1)	2 (1-3)	10 (7-14)	2 (1-3)	1 (1-2)
Iraq	1,080 (802-1,450)	-0.7 (-11.7-12.4)	7,240 (5,530-9,230)	23 (11.2-36.5)	872 (633-1,210)	14.7 (0.8-30.7)	567 (387-830)	2.3 (-11.6-19.6)	1,070 (783-1,430)	8,900 (6,840-11,400)	1,000 (732-1,350)	580 (385-844)
Jordan	33 (22-50)	78.2 (57-105)	217 (157-298)	111 (90.9-136)	29 (19-45)	101 (75.6-132)	19 (11-32)	84.6 (57.5-119)	59 (38-90)	459 (332-621)	58 (37-88)	34 (20-58)
Kuwait	12 (8-18)	50.3 (30-71.4)	97 (70-132)	80 (60.7-100)	11 (7-17)	73.6 (50.2-99.1)	6 (3-10)	53.9 (27.3-82.6)	18 (12-27)	174 (126-236)	19 (13-28)	10 (5-16)
Lebanon	11 (7-16)	5.6 (-5.4-19.7)	71 (53-95)	24.5 (12.1-37.3)	8 (5-12)	24.1 (10.5-41.7)	5 (3-8)	19.9 (3.2-41)	11 (8-16)	89 (64-118)	10 (7-15)	6 (3-9)
Libya	791 (544-1,130)	-37 (-43.8--28.2)	5,540 (4,070-7,340)	-17.4 (-26.1--6.9)	755 (512-1,090)	-24.3 (-32.9--13.1)	497 (313-767)	-33.8 (-42.9--22.2)	498 (343-705)	4,580 (3,380-6,070)	572 (398-795)	329 (208-498)
Morocco	123 (90-163)	-31.1 (-38.4--23.5)	591 (444-755)	-11.4 (-20.5--2.5)	103 (74-138)	-15.4 (-24.5--5.8)	76 (52-107)	-20.9 (-30.6--9.9)	85 (61-113)	523 (395-666)	87 (63-117)	60 (41-83)
Oman	104 (74-140)	25 (9.1-39.4)	627 (477-798)	53.2 (38.7-70.6)	93 (65-126)	53.2 (33.9-72.6)	59 (38-87)	52.3 (29.1-74.6)	130 (91-176)	960 (725-1,220)	142 (99-193)	90 (57-130)
Palestine	26 (17-40)	-2.2 (-14.3-12)	134 (95-184)	28 (14-47.1)	20 (13-32)	20.6 (4.8-40.3)	15 (9-24)	10.1 (-6.2-31.3)	26 (17-39)	171 (123-232)	25 (16-38)	16 (10-27)
Qatar	4 (2-5)	145 (115-182)	32 (23-44)	281 (240-323)	3 (2-5)	257 (211-314)	2 (1-3)	194 (149-257)	9 (6-13)	123 (90-166)	12 (8-19)	6 (3-10)
Saudi Arabia	126 (93-167)	-12.3 (-20.5--3.9)	864 (659-1,100)	8.5 (-2.9-19.9)	120 (88-161)	0.2 (-10.1-11.3)	78 (52-112)	-12.1 (-22.6--0.5)	110 (81-144)	937 (716-1,200)	121 (89-156)	68 (45-95)
Sudan	524 (413-700)	-6.6 (-15.4-3)	1,850 (1,480-2,360)	23.1 (10-35.8)	375 (291-504)	16.3 (5.1-29.2)	302 (224-417)	9.9 (-0.7-23.7)	489 (381-641)	2,270 (1,790-2,860)	436 (337-576)	332 (249-449)
Syrian Arab Republic	112 (83-150)	-60.1 (-63.8--54.9)	636 (485-819)	-50 (-54.9--44.6)	100 (73-136)	-55 (-59.7--49)	70 (48-100)	-59.4 (-64.2--53.4)	45 (33-59)	318 (241-406)	45 (34-59)	28 (19-40)
Tunisia	45 (31-63)	-24.2 (-33.3--14.6)	282 (212-367)	-12.4 (-20.9--3)	41 (29-58)	-11.7 (-22.1--0.8)	27 (17-41)	-13.7 (-26.2-0.7)	34 (24-47)	247 (183-318)	36 (26-50)	23 (15-34)
Türkiye	189 (144-253)	-35.3 (-42.4--27.8)	1,340 (1,070-1,640)	-18.1 (-25.1--10.1)	174 (131-236)	-26.2 (-34.3--17.5)	118 (83-167)	-34.1 (-43.4--23.5)	122 (92-166)	1,100 (875-1,360)	129 (97-173)	78 (54-114)
United Arab Emirates	2 (1-3)	-3.1 (-12.3-9.1)	17 (13-23)	78.8 (62-97.2)	2 (1-3)	82 (64.8-104)	1 (1-1)	67.9 (46.7-95.4)	2 (1-2)	31 (23-41)	3 (2-5)	2 (1-2)
Yemen	130 (97-172)	7.8 (-1.1-19.2)	479 (369-615)	37.4 (23.8-50.8)	97 (72-130)	31.4 (19.5-45.5)	78 (55-106)	23.4 (10.6-38.1)	140 (104-187)	659 (506-858)	128 (95-172)	96 (68-135)

Location	Birth , 2000	Births percent change (2000-2021)	All age cases, 2000	All age cases percent change (2000-2021)	All age deaths, 2000	All age deaths percent change (2000-2021)	Under-5 deaths, 2000	Under-5 deaths percent change (2000-2021)	Births, 2021	All age cases, 2021	All age deaths, 2021	Under-5 deaths, 2021
South Asia	4,720 (3,040-7,100)	-11.7 (-14.3--9.3)	17,700 (11,700-26,000)	-4 (-6.9--1.3)	4,480 (2,890-6,760)	-3.5 (-6.3--0.9)	3,300 (2,040-5,170)	-6.4 (-9.7--3.4)	4,160 (2,680-6,250)	17,000 (11,300-24,800)	4,320 (2,780-6,460)	3,090 (1,920-4,850)
**South Asia**	4,720 (3,040-7,100)	-11.7 (-14.3--9.3)	17,700 (11,700-26,000)	-4 (-6.9--1.3)	4,480 (2,890-6,760)	-3.5 (-6.3--0.9)	3,300 (2,040-5,170)	-6.4 (-9.7--3.4)	4,160 (2,680-6,250)	17,000 (11,300-24,800)	4,320 (2,780-6,460)	3,090 (1,920-4,850)
Bangladesh	34 (22-52)	-24.9 (-33.2--16.2)	115 (75-176)	-21.1 (-30.3--10.7)	34 (22-52)	-20.8 (-29.7--11.3)	27 (17-42)	-22.8 (-31.6--13.1)	26 (16-39)	91 (58-138)	27 (17-41)	21 (13-32)
Bhutan	0 (0-0)	-32.1 (-40.7--23.7)	1 (0-1)	-25.4 (-35.2--15.5)	0 (0-0)	-25 (-34.4--15.7)	0 (0-0)	-26.1 (-36.1--15.4)	0 (0-0)	0 (0-1)	0 (0-0)	0 (0-0)
India	4,630 (2,980-6,970)	-11.8 (-14.5--9.4)	17,500 (11,500-25,600)	-4.1 (-6.9--1.4)	4,410 (2,840-6,640)	-3.6 (-6.5--0.9)	3,240 (2,000-5,070)	-6.4 (-9.8--3.4)	4,090 (2,620-6,130)	16,800 (11,100-24,400)	4,250 (2,720-6,340)	3,030 (1,880-4,750)
Nepal	8 (5-12)	-24.7 (-33.8--14.5)	25 (16-38)	-17.3 (-27--4.4)	7 (5-11)	-17.2 (-27.1--5.5)	6 (4-9)	-19.9 (-29.9--8.3)	6 (4-9)	20 (13-31)	6 (4-9)	5 (3-7)
Pakistan	42 (25-67)	10.8 (-2.1-24.5)	125 (79-191)	23.5 (10.1-38.7)	34 (20-55)	23.7 (9.9-40.1)	28 (16-45)	17.8 (5.1-34.4)	47 (30-73)	155 (100-238)	42 (27-66)	33 (20-52)
Azad Jammu & Kashmir	1 (0-1)	-12.8 (-31.4--2.9)	2 (1-4)	-4.9 (-22.1-9)	1 (0-1)	-4.4 (-25-7.3)	1 (0-1)	-7.6 (-28.9-4.5)	1 (0-1)	2 (1-3)	1 (0-1)	0 (0-1)
Balochistan	2 (1-3)	85.1 (55.1-126)	6 (4-10)	94.3 (59.2-135)	2 (1-3)	95.3 (61.9-139)	1 (1-2)	91.4 (56.9-135)	4 (2-6)	12 (7-19)	3 (2-5)	3 (1-4)
Gilgit-Baltistan	0 (0-1)	84.1 (50.1-118)	1 (1-2)	95.1 (61.6-129)	0 (0-0)	95.7 (58.8-130)	0 (0-0)	91.8 (53.7-126)	1 (0-1)	2 (1-3)	1 (0-1)	0 (0-1)
Islamabad Capital Territory	0 (0-0)	91.2 (45.7-167)	1 (0-1)	112 (67.5-179)	0 (0-0)	114 (64-199)	0 (0-0)	106 (51-198)	0 (0-0)	1 (1-2)	0 (0-0)	0 (0-0)
Khyber Pakhtunkhwa	6 (3-9)	58.3 (19.7-89.4)	19 (12-30)	64.6 (27.1-100)	5 (3-8)	65.3 (25.2-97.5)	4 (2-6)	61.7 (19.7-98)	9 (5-14)	32 (19-49)	8 (5-13)	6 (3-10)
Punjab	23 (13-38)	-7 (-20.8-11.6)	67 (41-102)	5 (-11.1-27.9)	19 (11-30)	5.9 (-10.7-28.4)	16 (9-25)	0 (-16.4-22.9)	22 (14-33)	71 (45-109)	20 (13-30)	16 (10-24)
Sindh	10 (6-16)	7.1 (-8.6-23.6)	29 (17-45)	21.6 (2.5-42.3)	8 (4-13)	23.4 (4.7-42.8)	7 (3-11)	17.1 (-1.1-36.1)	11 (6-17)	35 (20-54)	10 (6-15)	8 (4-12)
Southeast Asia, East Asia, and Oceania	29 (21-38)	-12.1 (-15.4--5.9)	127 (94-170)	-7.5 (-11--0.4)	28 (20-36)	-6.1 (-9.5-1.1)	19 (13-25)	-7.6 (-11.2--0.3)	25 (19-33)	118 (87-158)	26 (19-33)	17 (12-23)
**East Asia**	2 (0-5)	-11.3 (-15.1--9.9)	11 (1-24)	-12.1 (-16.4--9.7)	2 (0-5)	-9.6 (-14.1--6.8)	1 (0-3)	-10.4 (-15.5--7.5)	2 (0-4)	10 (1-22)	2 (0-4)	1 (0-2)
China	2 (0-4)	-10.1 (-14.3--9.7)	11 (1-24)	-11.1 (-15.8--8.9)	2 (0-4)	-8.7 (-13.7--5.7)	1 (0-3)	-9.5 (-15--6.4)	2 (0-4)	9 (1-21)	2 (0-4)	1 (0-2)
Democratic People's Republic of Korea	0 (0-0)	-44.2 (-44.2--44.2)	0 (0-1)	-34.8 (-39.3--30)	0 (0-0)	-34.8 (-36.5--32.1)	0 (0-0)	-38.8 (-42--35.3)	0 (0-0)	0 (0-0)	0 (0-0)	0 (0-0)
Taiwan (Province of China)	0 (0-0)	-43 (-43.3--42.9)	0 (0-0)	-35.3 (-40.9--29.2)	0 (0-0)	-34.9 (-36.1--33.3)	0 (0-0)	-35.3 (-40.8--29.4)	0 (0-0)	0 (0-0)	0 (0-0)	0 (0-0)
**Oceania**	0 (0-0)	50.3 (34.8-52.5)	0 (0-0)	49.2 (29.2-56.5)	0 (0-0)	54.6 (42-56.5)	0 (0-0)	56.9 (49.1-61.6)	0 (0-0)	0 (0-0)	0 (0-0)	0 (0-0)
American Samoa	0 (0-0)	-37.8 (-38--37.6)	0 (0-0)	-31.4 (-36.5--26)	0 (0-0)	-31.6 (-34.4--28.3)	0 (0-0)	-38.8 (-42.4--34)	0 (0-0)	0 (0-0)	0 (0-0)	0 (0-0)
Cook Islands	0 (0-0)	-35.9 (-35.9--35.9)	0 (0-0)	-31.5 (-36.7--26)	0 (0-0)	-31.2 (-33.4--29.1)	0 (0-0)	-37.5 (-42.3--32.9)	0 (0-0)	0 (0-0)	0 (0-0)	0 (0-0)
Fiji	0 (0-0)	-6.7 (-6.7--6.7)	0 (0-0)	-0.5 (-7.5-5.8)	0 (0-0)	0.5 (-0.3-1.1)	0 (0-0)	-0.3 (-5.2-5.4)	0 (0-0)	0 (0-0)	0 (0-0)	0 (0-0)
Guam	0 (0-0)	-9.4 (-9.4--9.4)	0 (0-0)	-8.4 (-14.1--2.5)	0 (0-0)	-8.7 (-10--7.1)	0 (0-0)	-12.6 (-18.8--6.8)	0 (0-0)	0 (0-0)	0 (0-0)	0 (0-0)
Kiribati	0 (0-0)	18.4 (18.4-18.4)	0 (0-0)	22.1 (14.5-29.7)	0 (0-0)	22.4 (21.9-22.9)	0 (0-0)	21.8 (17.6-26.6)	0 (0-0)	0 (0-0)	0 (0-0)	0 (0-0)
Marshall Islands	0 (0-0)	-19.9 (-19.9--19.9)	0 (0-0)	-15.8 (-21.8--9.4)	0 (0-0)	-15.6 (-16--15.2)	0 (0-0)	-16 (-20.5--11.2)	0 (0-0)	0 (0-0)	0 (0-0)	0 (0-0)
Micronesia (Federated States of)	0 (0-0)	-36.2 (-36.2--36.2)	0 (0-0)	-34.6 (-39--29.4)	0 (0-0)	-34.7 (-35.5--33.5)	0 (0-0)	-36.4 (-39.5--33.3)	0 (0-0)	0 (0-0)	0 (0-0)	0 (0-0)
Nauru	0 (0-0)	-27.5 (-27.5--27.5)	0 (0-0)	-17.7 (-22.7--12.6)	0 (0-0)	-16.3 (-17.9--14.6)	0 (0-0)	-20 (-23.7--16.7)	0 (0-0)	0 (0-0)	0 (0-0)	0 (0-0)
Niue	0 (0-0)	-37.2 (-63.5--33.7)	0 (0-0)	-35.6 (-65.9--26.3)	0 (0-0)	-35 (-62.5--30.4)	0 (0-0)	-35.8 (-59.6--28.8)	0 (0-0)	0 (0-0)	0 (0-0)	0 (0-0)

Location	Birth , 2000	Births percent change (2000-2021)	All age cases, 2000	All age cases percent change (2000-2021)	All age deaths, 2000	All age deaths percent change (2000-2021)	Under-5 deaths, 2000	Under-5 deaths percent change (2000-2021)	Births, 2021	All age cases, 2021	All age deaths, 2021	Under-5 deaths, 2021
Northern Mariana Islands	0 (0-0)	-81 (-81--81)	0 (0-0)	-68.5 (-71.8--64.8)	0 (0-0)	-67.5 (-69.7--64.4)	0 (0-0)	-73.6 (-75.9--71.6)	0 (0-0)	0 (0-0)	0 (0-0)	0 (0-0)
Palau	0 (0-0)	-54.8 (-54.8--54.8)	0 (0-0)	-44.1 (-48.4--39.4)	0 (0-0)	-43.2 (-45.5--40.7)	0 (0-0)	-49.6 (-52.3--46.5)	0 (0-0)	0 (0-0)	0 (0-0)	0 (0-0)
Papua New Guinea	0 (0-0)	68.7 (68.7-68.7)	0 (0-0)	72.4 (62.2-82.3)	0 (0-0)	72.9 (71.7-74.3)	0 (0-0)	73.4 (67.5-80)	0 (0-0)	0 (0-0)	0 (0-0)	0 (0-0)
Samoa	0 (0-0)	32.2 (32.2-32.2)	0 (0-0)	15 (6.9-24.2)	0 (0-0)	14.4 (12.9-16.5)	0 (0-0)	16.3 (11-22)	0 (0-0)	0 (0-0)	0 (0-0)	0 (0-0)
Solomon Islands	0 (0-0)	10.4 (10.5-10.5)	0 (0-0)	25.9 (18.4-36.2)	0 (0-0)	27.2 (24.9-30)	0 (0-0)	21.4 (14.9-27.8)	0 (0-0)	0 (0-0)	0 (0-0)	0 (0-0)
Tokelau	0 (0-0)	-30.6 (-30.6--30.6)	0 (0-0)	-23.3 (-27.7--18.1)	0 (0-0)	-22.8 (-23.5--22)	0 (0-0)	-22.6 (-27.4--16.8)	0 (0-0)	0 (0-0)	0 (0-0)	0 (0-0)
Tonga	0 (0-0)	-14.5 (-14.5--14.5)	0 (0-0)	-11.9 (-18--6.3)	0 (0-0)	-11.4 (-11.9--10.7)	0 (0-0)	-12.4 (-16.6--7.6)	0 (0-0)	0 (0-0)	0 (0-0)	0 (0-0)
Tuvalu	0 (0-0)	-5.5 (-5.5--5.5)	0 (0-0)	-3.9 (-10.1-3.1)	0 (0-0)	-4.7 (-5.8--2.3)	0 (0-0)	-7.8 (-11.6--4.1)	0 (0-0)	0 (0-0)	0 (0-0)	0 (0-0)
Vanuatu	0 (0-0)	17.6 (17.6-17.6)	0 (0-0)	30.5 (20.9-40.4)	0 (0-0)	31 (28.6-33.7)	0 (0-0)	23.9 (17.6-30.1)	0 (0-0)	0 (0-0)	0 (0-0)	0 (0-0)
**Southeast Asia**	27 (19-36)	-12.3 (-15.7--5.5)	116 (84-155)	-7.3 (-11.1-0.6)	25 (18-34)	-6 (-9.8-1.7)	17 (12-24)	-7.5 (-11.3-0.6)	23 (17-31)	108 (78-144)	24 (17-31)	16 (11-22)
Cambodia	1 (1-1)	-0.4 (-11.9-57.5)	3 (2-4)	3 (-10.7-67.5)	1 (1-1)	3.6 (-8.5-63.5)	1 (0-1)	5.2 (-8.9-69.2)	1 (1-1)	3 (2-5)	1 (1-1)	1 (0-1)
Indonesia	11 (7-15)	-20.4 (-24--6.7)	45 (31-62)	-11.5 (-16.7-1.7)	10 (7-14)	-9.8 (-14.4-5.5)	7 (5-10)	-13.8 (-18.2-1.9)	9 (6-12)	40 (28-56)	9 (6-13)	6 (4-9)
Aceh	0 (0-0)	-19 (-20.7-7.7)	1 (1-1)	-8 (-17.7-17.6)	0 (0-0)	-7.1 (-10.8-23.2)	0 (0-0)	-11.5 (-18.4-20.9)	0 (0-0)	1 (1-1)	0 (0-0)	0 (0-0)
Bali	0 (0-0)	-28.2 (-29.3--3.8)	1 (0-1)	-13.3 (-22.5-15.3)	0 (0-0)	-11.4 (-15.7-19.4)	0 (0-0)	-20.5 (-27-10.7)	0 (0-0)	1 (0-1)	0 (0-0)	0 (0-0)
Bangka-Belitung Islands	0 (0-0)	-16.8 (-31.5-13.4)	0 (0-0)	-6.2 (-20.5-23.7)	0 (0-0)	-4.8 (-22.2-31)	0 (0-0)	-9.7 (-26.7-25.6)	0 (0-0)	0 (0-0)	0 (0-0)	0 (0-0)
Banten	1 (0-1)	-28.8 (-31-5.6)	2 (1-3)	-22.2 (-30.6-10.3)	1 (0-1)	-21.1 (-24.4-16.9)	0 (0-1)	-22.9 (-29.6-17.3)	0 (0-1)	2 (1-3)	0 (0-1)	0 (0-0)
Bengkulu	0 (0-0)	-20.9 (-23.1-18.8)	0 (0-1)	-11.4 (-20.6-30)	0 (0-0)	-10.3 (-14-33.6)	0 (0-0)	-13.7 (-20.3-30.1)	0 (0-0)	0 (0-0)	0 (0-0)	0 (0-0)
Gorontalo	0 (0-0)	10.8 (8.1-56.3)	0 (0-0)	19.5 (4.8-64.2)	0 (0-0)	19.1 (13.7-68.4)	0 (0-0)	11.9 (2.6-59.4)	0 (0-0)	0 (0-0)	0 (0-0)	0 (0-0)
Jakarta	0 (0-1)	-38.3 (-48.6--7.6)	2 (1-3)	-25.1 (-36.7-9.3)	0 (0-1)	-23.8 (-37.5-14.7)	0 (0-0)	-28 (-41.2-10.2)	0 (0-0)	1 (1-2)	0 (0-0)	0 (0-0)
Jambi	0 (0-0)	-13.1 (-15.7-27.6)	1 (0-1)	-3.9 (-12.6-38.2)	0 (0-0)	-1.8 (-6.1-43.8)	0 (0-0)	-6.4 (-13.5-40.9)	0 (0-0)	1 (0-1)	0 (0-0)	0 (0-0)
West Java	2 (1-3)	-7.8 (-12-44.1)	7 (5-11)	2.6 (-9.4-54.9)	2 (1-3)	3.6 (-2.8-61.5)	1 (1-2)	-1.3 (-11.4-57.8)	2 (1-3)	8 (5-11)	2 (1-3)	1 (1-2)
Central Java	1 (1-2)	-35 (-45.4--1.9)	6 (4-9)	-28 (-39.9-2.3)	1 (1-2)	-27.3 (-39.3-8.4)	1 (1-1)	-31.4 (-42.7-4.1)	1 (1-1)	4 (3-6)	1 (1-1)	1 (0-1)
East Java	1 (1-2)	-35 (-36.1--10.5)	6 (4-9)	-26.1 (-34.5--3)	1 (1-2)	-25.2 (-28.8-2.8)	1 (1-1)	-30.8 (-37.2--5)	1 (1-1)	5 (3-7)	1 (1-1)	1 (0-1)
West Kalimantan	0 (0-0)	-17.6 (-21.1-37.3)	1 (1-1)	-7.4 (-18.2-50.7)	0 (0-0)	-6 (-11.6-55.7)	0 (0-0)	-10 (-18.8-51.9)	0 (0-0)	1 (1-1)	0 (0-0)	0 (0-0)
South Kalimantan	0 (0-0)	-21.8 (-24.1-11.8)	1 (0-1)	-11.6 (-21.1-20.7)	0 (0-0)	-10 (-14.3-27.3)	0 (0-0)	-14.4 (-21.1-23.8)	0 (0-0)	1 (0-1)	0 (0-0)	0 (0-0)
Central Kalimantan	0 (0-0)	-15 (-17-22.7)	0 (0-1)	-4.2 (-14.5-36.8)	0 (0-0)	-3.1 (-7.2-42.2)	0 (0-0)	-7.8 (-14.9-35)	0 (0-0)	0 (0-1)	0 (0-0)	0 (0-0)
East Kalimantan	0 (0-0)	0.7 (-2.1-42.6)	1 (0-1)	19.7 (8.3-67.7)	0 (0-0)	22.7 (16.7-74.7)	0 (0-0)	14.6 (4.1-69.5)	0 (0-0)	1 (0-1)	0 (0-0)	0 (0-0)
North Kalimantan	0 (0-0)	11.5 (9.8-50.8)	0 (0-0)	29.7 (15.5-65.8)	0 (0-0)	30 (24.4-75)	0 (0-0)	20.6 (11-67.4)	0 (0-0)	0 (0-0)	0 (0-0)	0 (0-0)
Riau Islands	0 (0-0)	-14.1 (-16.8-21.7)	0 (0-0)	7.3 (-5.2-46)	0 (0-0)	10.4 (4.1-55)	0 (0-0)	6.5 (-3.7-54.4)	0 (0-0)	0 (0-1)	0 (0-0)	0 (0-0)

Location	Birth , 2000	Births percent change (2000-2021)	All age cases, 2000	All age cases percent change (2000-2021)	All age deaths, 2000	All age deaths percent change (2000-2021)	Under-5 deaths, 2000	Under-5 deaths percent change (2000-2021)	Births, 2021	All age cases, 2021	All age deaths, 2021	Under-5 deaths, 2021
Lampung	0 (0-1)	-30.5 (-32.8-4.5)	2 (1-2)	-23.1 (-31.4-11.6)	0 (0-1)	-22.6 (-26.6-17.6)	0 (0-0)	-26.3 (-32.8-13.3)	0 (0-0)	1 (1-2)	0 (0-0)	0 (0-0)
Maluku	0 (0-0)	-19.8 (-22.3-25.2)	0 (0-1)	-6.5 (-17.4-43.1)	0 (0-0)	-5.9 (-10.4-46.2)	0 (0-0)	-10.9 (-18.8-39.5)	0 (0-0)	0 (0-0)	0 (0-0)	0 (0-0)
North Maluku	0 (0-0)	-19.6 (-21.5-11.4)	0 (0-0)	-9.6 (-20-24.9)	0 (0-0)	-8.8 (-12.3-26.5)	0 (0-0)	-13.2 (-19.2-22.3)	0 (0-0)	0 (0-0)	0 (0-0)	0 (0-0)
West Nusa Tenggara	0 (0-0)	-15 (-17.8-30.7)	1 (1-1)	-5.6 (-15.8-45)	0 (0-0)	-4.7 (-8.9-46.5)	0 (0-0)	-7.7 (-15.3-47)	0 (0-0)	1 (1-1)	0 (0-0)	0 (0-0)
East Nusa Tenggara	0 (0-0)	-9.7 (-12.2-28.4)	1 (1-1)	1.6 (-9.6-40.1)	0 (0-0)	2.2 (-2.5-44.9)	0 (0-0)	-3.5 (-10.2-40.9)	0 (0-0)	1 (1-2)	0 (0-0)	0 (0-0)
Papua	0 (0-0)	63.9 (59.6-132)	0 (0-1)	81.6 (63.2-149)	0 (0-0)	83.3 (76.1-159)	0 (0-0)	75.2 (62.7-152)	0 (0-0)	1 (1-1)	0 (0-0)	0 (0-0)
West Papua	0 (0-0)	-16.4 (-19.1-25.9)	0 (0-0)	-7.4 (-17.5-38)	0 (0-0)	-5.8 (-10-42.5)	0 (0-0)	-8 (-15.6-40)	0 (0-0)	0 (0-0)	0 (0-0)	0 (0-0)
Riau	0 (0-0)	52.1 (47.8-121)	1 (1-1)	74.3 (58.2-139)	0 (0-0)	77 (68.7-155)	0 (0-0)	67.5 (54.4-142)	0 (0-0)	2 (1-2)	0 (0-1)	0 (0-0)
West Sulawesi	0 (0-0)	-4.8 (-6.8-22.5)	0 (0-0)	7.5 (-2-38.7)	0 (0-0)	8.4 (4.4-39.4)	0 (0-0)	2.3 (-3.5-31.6)	0 (0-0)	0 (0-0)	0 (0-0)	0 (0-0)
South Sulawesi	0 (0-1)	-27.4 (-29.8-3.6)	2 (1-3)	-18.3 (-27.4-12.2)	0 (0-1)	-17.4 (-21.9-17.5)	0 (0-0)	-22.2 (-28.8-13.4)	0 (0-0)	1 (1-2)	0 (0-0)	0 (0-0)
Central Sulawesi	0 (0-0)	-18.3 (-20.6-17.3)	1 (0-1)	-5.8 (-16.3-35.5)	0 (0-0)	-4.4 (-9.7-38.6)	0 (0-0)	-11 (-19.1-30.5)	0 (0-0)	0 (0-1)	0 (0-0)	0 (0-0)
Southeast Sulawesi	0 (0-0)	-2.8 (-5.2-34.5)	0 (0-1)	8.9 (-3.4-47.1)	0 (0-0)	9.8 (5.3-52.2)	0 (0-0)	4.5 (-4.3-47.2)	0 (0-0)	0 (0-1)	0 (0-0)	0 (0-0)
North Sulawesi	0 (0-0)	-42 (-47.2--25)	0 (0-1)	-33.3 (-40.9--15.7)	0 (0-0)	-32.8 (-37.9--13.3)	0 (0-0)	-38.8 (-44.2--20.2)	0 (0-0)	0 (0-0)	0 (0-0)	0 (0-0)
West Sumatra	0 (0-0)	-30.8 (-46.8--3.2)	1 (1-2)	-21.2 (-40.5-7.5)	0 (0-0)	-20.8 (-39.8-10.5)	0 (0-0)	-25.2 (-43.9-6.6)	0 (0-0)	1 (1-1)	0 (0-0)	0 (0-0)
South Sumatra	0 (0-1)	-22.5 (-32.3-10.3)	2 (1-2)	-13.1 (-23.8-18.3)	0 (0-1)	-12.3 (-23.5-24.5)	0 (0-0)	-16 (-27.5-24)	0 (0-0)	1 (1-2)	0 (0-0)	0 (0-0)
North Sumatra	1 (1-1)	-21.8 (-28.2-11.5)	3 (2-4)	-13.3 (-24.7-21.1)	1 (0-1)	-12.7 (-20-24)	1 (0-1)	-16.5 (-25.3-21)	1 (0-1)	3 (2-4)	1 (0-1)	0 (0-1)
Yogyakarta	0 (0-0)	-26.7 (-28--2.8)	1 (0-1)	-18.5 (-26.8-2.9)	0 (0-0)	-17.3 (-20.3-10.1)	0 (0-0)	-22.1 (-29-7.5)	0 (0-0)	0 (0-1)	0 (0-0)	0 (0-0)
Lao People's Democratic Republic	0 (0-1)	-4.2 (-5.8-36.4)	1 (1-2)	0.6 (-8.5-39.8)	0 (0-1)	1 (-1.7-44.1)	0 (0-0)	1.5 (-6.3-44.6)	0 (0-1)	1 (1-2)	0 (0-1)	0 (0-0)
Malaysia	1 (1-1)	6.8 (-10.7-64.2)	6 (4-9)	-1.2 (-15.1-44.9)	1 (1-1)	-1.6 (-17.6-50.7)	0 (0-1)	-2.2 (-19-53.8)	1 (1-1)	6 (4-8)	1 (1-1)	0 (0-1)
Maldives	0 (0-0)	13.9 (4.1-57)	0 (0-0)	9.7 (-2.2-43.6)	0 (0-0)	9 (1.7-48.2)	0 (0-0)	13.8 (2.6-57)	0 (0-0)	0 (0-0)	0 (0-0)	0 (0-0)
Mauritius	0 (0-0)	-35.5 (-45.5--11.3)	0 (0-0)	-34.4 (-43.6--14.1)	0 (0-0)	-34 (-44.2--9.8)	0 (0-0)	-36.8 (-46--11)	0 (0-0)	0 (0-0)	0 (0-0)	0 (0-0)
Myanmar	3 (2-5)	-15.3 (-16.7-16.8)	9 (6-15)	-8.6 (-16.1-22.1)	3 (2-4)	-8.1 (-10-26.9)	2 (1-3)	-9.2 (-14.4-23)	3 (2-4)	9 (6-13)	2 (2-4)	2 (1-3)
Philippines	5 (3-7)	15.5 (13.3-25.1)	20 (14-27)	19.7 (16.5-29.9)	4 (3-6)	19.9 (17.3-29.6)	3 (2-4)	18.2 (15.3-28.4)	6 (4-8)	24 (17-33)	5 (4-7)	4 (2-5)
Abra	0 (0-0)	-17.7 (-22.3-15.3)	0 (0-0)	-7.6 (-17.2-23.8)	0 (0-0)	-6.4 (-12.3-30.5)	0 (0-0)	-11.4 (-22-28.1)	0 (0-0)	0 (0-0)	0 (0-0)	0 (0-0)
Agusan Del Norte	0 (0-0)	11 (8.6-58.1)	0 (0-0)	12.6 (0.8-54.2)	0 (0-0)	13.2 (9.9-61.4)	0 (0-0)	11.5 (2-65.2)	0 (0-0)	0 (0-0)	0 (0-0)	0 (0-0)
Agusan Del Sur	0 (0-0)	-6.7 (-21.8-34.2)	0 (0-0)	4.2 (-8.7-48.7)	0 (0-0)	5.1 (-12.1-51.2)	0 (0-0)	1.5 (-16.7-47.2)	0 (0-0)	0 (0-0)	0 (0-0)	0 (0-0)
Aklan	0 (0-0)	22.3 (19.7-68.4)	0 (0-0)	20.9 (8.6-65.2)	0 (0-0)	21.5 (18.2-67.3)	0 (0-0)	21.2 (11.5-69.6)	0 (0-0)	0 (0-0)	0 (0-0)	0 (0-0)
Albay	0 (0-0)	5.9 (5.1-24.5)	0 (0-0)	5.3 (-3-21.8)	0 (0-0)	5.2 (3.8-23.5)	0 (0-0)	4.4 (-2.3-24.2)	0 (0-0)	0 (0-0)	0 (0-0)	0 (0-0)
Antique	0 (0-0)	6.4 (4.4-52.1)	0 (0-0)	10.3 (-0.5-56.7)	0 (0-0)	11.5 (7.8-60.6)	0 (0-0)	7.3 (-1.4-54.4)	0 (0-0)	0 (0-0)	0 (0-0)	0 (0-0)

Location	Birth , 2000	Births percent change (2000-2021)	All age cases, 2000	All age cases percent change (2000-2021)	All age deaths, 2000	All age deaths percent change (2000-2021)	Under-5 deaths, 2000	Under-5 deaths percent change (2000-2021)	Births, 2021	All age cases, 2021	All age deaths, 2021	Under-5 deaths, 2021
Apayao	0 (0-0)	-22.3 (-34-12)	0 (0-0)	-8.1 (-22.1-29.1)	0 (0-0)	-6.5 (-21.7-34.8)	0 (0-0)	-10.2 (-24.4-32.5)	0 (0-0)	0 (0-0)	0 (0-0)	0 (0-0)
Aurora	0 (0-0)	-1.9 (-3.7-41)	0 (0-0)	6.1 (-3.2-47.5)	0 (0-0)	6.9 (3.7-55.1)	0 (0-0)	4.2 (-3-54.6)	0 (0-0)	0 (0-0)	0 (0-0)	0 (0-0)
Basilan	0 (0-0)	30.6 (28.9-62.9)	0 (0-0)	39.6 (26.4-73.4)	0 (0-0)	40 (36-75.6)	0 (0-0)	37.3 (25.9-78.5)	0 (0-0)	0 (0-0)	0 (0-0)	0 (0-0)
Bataan	0 (0-0)	15.6 (14-64)	0 (0-0)	20 (8.6-64.9)	0 (0-0)	20.6 (17.5-71.5)	0 (0-0)	18.5 (9.9-73.6)	0 (0-0)	0 (0-0)	0 (0-0)	0 (0-0)
Batanes	0 (0-0)	-27.6 (-28.3--21.6)	0 (0-0)	-18.9 (-25.4--9.7)	0 (0-0)	-18.1 (-21--10.2)	0 (0-0)	-22.7 (-29.1--14.8)	0 (0-0)	0 (0-0)	0 (0-0)	0 (0-0)
Batangas	0 (0-0)	43.8 (23.2-92.5)	0 (0-1)	37.9 (18.6-78.2)	0 (0-0)	37.3 (17.2-83.6)	0 (0-0)	40.6 (16.8-86)	0 (0-0)	1 (0-1)	0 (0-0)	0 (0-0)
Benguet	0 (0-0)	30.8 (17-77.7)	0 (0-0)	26.5 (11.6-63.7)	0 (0-0)	26.3 (12.8-72.2)	0 (0-0)	30.3 (13.7-79.8)	0 (0-0)	0 (0-0)	0 (0-0)	0 (0-0)
Biliran	0 (0-0)	2 (0.9-14.8)	0 (0-0)	8.3 (-0.2-21.6)	0 (0-0)	9.3 (6.9-22.8)	0 (0-0)	5.8 (-3.4-26)	0 (0-0)	0 (0-0)	0 (0-0)	0 (0-0)
Bohol	0 (0-0)	-22.2 (-23.5-10.7)	0 (0-0)	-11.2 (-19.2-22.5)	0 (0-0)	-10 (-13.8-28.7)	0 (0-0)	-15.5 (-21.7-22.9)	0 (0-0)	0 (0-0)	0 (0-0)	0 (0-0)
Bukidnon	0 (0-0)	-4.2 (-18.1-41.8)	0 (0-0)	8.4 (-5.1-55.1)	0 (0-0)	9.8 (-6.7-62.6)	0 (0-0)	5.2 (-11.3-61.4)	0 (0-0)	0 (0-0)	0 (0-0)	0 (0-0)
Bulacan	0 (0-0)	32.2 (30.6-70.5)	1 (0-1)	31.6 (20.9-61.7)	0 (0-0)	31.2 (28.3-68.3)	0 (0-0)	28.8 (19.8-71.2)	0 (0-0)	1 (1-1)	0 (0-0)	0 (0-0)
Cagayan	0 (0-0)	-0.4 (-16-39.4)	0 (0-0)	1.7 (-11.4-36.5)	0 (0-0)	2.1 (-13.7-42.9)	0 (0-0)	1.8 (-13.6-43.1)	0 (0-0)	0 (0-0)	0 (0-0)	0 (0-0)
Camarines Norte	0 (0-0)	5.3 (3.8-40.4)	0 (0-0)	10.2 (-1.1-41.8)	0 (0-0)	11 (8.1-48.9)	0 (0-0)	7.8 (-1.2-48.4)	0 (0-0)	0 (0-0)	0 (0-0)	0 (0-0)
Camarines Sur	0 (0-0)	9.7 (8.4-42.2)	0 (0-1)	13.8 (4.4-43)	0 (0-0)	14.2 (11.8-48.2)	0 (0-0)	11.7 (4.8-46.1)	0 (0-0)	0 (0-1)	0 (0-0)	0 (0-0)
Camiguin	0 (0-0)	5.6 (-9-55.8)	0 (0-0)	8.7 (-7-56.1)	0 (0-0)	9.8 (-5.4-61.7)	0 (0-0)	8.6 (-6-62.3)	0 (0-0)	0 (0-0)	0 (0-0)	0 (0-0)
Capiz	0 (0-0)	1.9 (-1.1-44.2)	0 (0-0)	1.1 (-11.9-38.4)	0 (0-0)	1.6 (-2.4-44.3)	0 (0-0)	0.4 (-9.6-48.1)	0 (0-0)	0 (0-0)	0 (0-0)	0 (0-0)
Catanduanes	0 (0-0)	-6.3 (-18.1-31.6)	0 (0-0)	2.9 (-9.3-44.3)	0 (0-0)	4.3 (-8.8-46.7)	0 (0-0)	0.8 (-10.4-41.8)	0 (0-0)	0 (0-0)	0 (0-0)	0 (0-0)
Cavite	0 (0-0)	84 (73.7-173)	1 (0-1)	76.6 (60.1-154)	0 (0-0)	76.3 (66.7-161)	0 (0-0)	78.1 (60.6-174)	0 (0-0)	1 (1-1)	0 (0-0)	0 (0-0)
Cebu	0 (0-0)	36.7 (34.8-95.4)	1 (1-1)	38 (24.1-89.3)	0 (0-0)	38.5 (35.9-97.9)	0 (0-0)	39.4 (28.5-102)	0 (0-0)	1 (1-2)	0 (0-0)	0 (0-0)
Cotabato (North Cotabato)	0 (0-0)	36.4 (33-96.5)	0 (0-0)	42.9 (26.5-96.3)	0 (0-0)	43.2 (38.4-107)	0 (0-0)	43.9 (30.6-111)	0 (0-0)	0 (0-1)	0 (0-0)	0 (0-0)
Davao de Oro	0 (0-0)	-7.6 (-12.7-11)	0 (0-0)	4.9 (-5.2-23.6)	0 (0-0)	6.8 (0.6-29)	0 (0-0)	3.9 (-5.2-29.4)	0 (0-0)	0 (0-0)	0 (0-0)	0 (0-0)
Davao Del Norte	0 (0-0)	-10.3 (-12.2-23.7)	0 (0-0)	4.5 (-3.8-38.6)	0 (0-0)	7.8 (3.3-49)	0 (0-0)	0.1 (-7.8-43.9)	0 (0-0)	0 (0-0)	0 (0-0)	0 (0-0)
Davao Del Sur	0 (0-0)	26.9 (25.4-76.9)	0 (0-1)	29.5 (18.7-78.1)	0 (0-0)	29.8 (27.4-81)	0 (0-0)	30.3 (21.6-85.3)	0 (0-0)	1 (0-1)	0 (0-0)	0 (0-0)
Davao Occidental	0 (0-0)	-11 (-12.7-24.1)	0 (0-0)	7.5 (-3-49.7)	0 (0-0)	9.6 (3-55.1)	0 (0-0)	-0.7 (-9.7-42.6)	0 (0-0)	0 (0-0)	0 (0-0)	0 (0-0)
Davao Oriental	0 (0-0)	-12.9 (-14.3-13.7)	0 (0-0)	-4 (-13-24.7)	0 (0-0)	-3.5 (-6.8-25.6)	0 (0-0)	-8.5 (-15.6-22.3)	0 (0-0)	0 (0-0)	0 (0-0)	0 (0-0)
Dinagat Islands	0 (0-0)	-29 (-30.3--1.2)	0 (0-0)	-15.7 (-24.1-13.2)	0 (0-0)	-14.1 (-17.6-19.5)	0 (0-0)	-17.5 (-25.6-17.8)	0 (0-0)	0 (0-0)	0 (0-0)	0 (0-0)
Eastern Samar	0 (0-0)	7.8 (5.6-49.9)	0 (0-0)	11.1 (0.6-54.9)	0 (0-0)	11 (7.7-54.3)	0 (0-0)	10.2 (2.7-54.3)	0 (0-0)	0 (0-0)	0 (0-0)	0 (0-0)
Guimaras	0 (0-0)	0.4 (-4.6-37.8)	0 (0-0)	6.5 (-4.3-38.3)	0 (0-0)	7.5 (2.2-47.4)	0 (0-0)	7.4 (-0.9-50.2)	0 (0-0)	0 (0-0)	0 (0-0)	0 (0-0)
Ifugao	0 (0-0)	18 (15.7-64.4)	0 (0-0)	20.5 (9.1-64.6)	0 (0-0)	21.1 (16.8-69.4)	0 (0-0)	24.6 (14.4-76.7)	0 (0-0)	0 (0-0)	0 (0-0)	0 (0-0)

Location	Birth , 2000	Births percent change (2000-2021)	All age cases, 2000	All age cases percent change (2000-2021)	All age deaths, 2000	All age deaths percent change (2000-2021)	Under-5 deaths, 2000	Under-5 deaths percent change (2000-2021)	Births, 2021	All age cases, 2021	All age deaths, 2021	Under-5 deaths, 2021
Ilocos Norte	0 (0-0)	4.9 (3.1-45.1)	0 (0-0)	6.1 (-4.3-42.2)	0 (0-0)	6.5 (4.1-47.2)	0 (0-0)	7.5 (-1.2-52.7)	0 (0-0)	0 (0-0)	0 (0-0)	0 (0-0)
Ilocos Sur	0 (0-0)	-1.8 (-3.1-31.9)	0 (0-0)	3.4 (-5.7-33.8)	0 (0-0)	4.1 (2-40.3)	0 (0-0)	2.8 (-6.4-42.6)	0 (0-0)	0 (0-0)	0 (0-0)	0 (0-0)
Iloilo	0 (0-0)	22.4 (20-67.3)	0 (0-1)	20.6 (10.2-60.8)	0 (0-0)	20.8 (17.1-64.9)	0 (0-0)	23.3 (12.3-74.9)	0 (0-0)	1 (0-1)	0 (0-0)	0 (0-0)
Isabela	0 (0-0)	15.5 (14.3-35.4)	0 (0-0)	14.7 (5-29.2)	0 (0-0)	14.5 (12.3-34.5)	0 (0-0)	16.9 (9.4-42.9)	0 (0-0)	0 (0-1)	0 (0-0)	0 (0-0)
Kalinga	0 (0-0)	-13.4 (-14.7-17.3)	0 (0-0)	1.4 (-6.3-36.5)	0 (0-0)	4.1 (0.7-41.1)	0 (0-0)	-0.5 (-9.2-35)	0 (0-0)	0 (0-0)	0 (0-0)	0 (0-0)
La Union	0 (0-0)	7.5 (6.3-27.2)	0 (0-0)	6.9 (-1.2-26.2)	0 (0-0)	7.2 (5-27)	0 (0-0)	9.6 (3.1-32.4)	0 (0-0)	0 (0-0)	0 (0-0)	0 (0-0)
Laguna	0 (0-0)	59.9 (56.3-131)	1 (0-1)	57.5 (44.1-129)	0 (0-0)	57.7 (52.3-128)	0 (0-0)	61.2 (46.8-132)	0 (0-0)	1 (1-1)	0 (0-0)	0 (0-0)
Lanao Del Norte	0 (0-0)	17.1 (15.2-56.3)	0 (0-0)	27.5 (15.6-65.9)	0 (0-0)	29 (25.4-72.7)	0 (0-0)	27.3 (17.3-74.5)	0 (0-0)	0 (0-0)	0 (0-0)	0 (0-0)
Lanao Del Sur	0 (0-0)	16.6 (8.4-45.7)	0 (0-0)	31.2 (19.9-65.9)	0 (0-0)	33 (23.8-64.2)	0 (0-0)	30.8 (21.2-63.4)	0 (0-0)	0 (0-1)	0 (0-0)	0 (0-0)
Leyte	0 (0-0)	12.7 (11.1-55.6)	0 (0-1)	13.1 (2.9-53.3)	0 (0-0)	12.8 (10.3-55.6)	0 (0-0)	13.1 (2.8-55)	0 (0-0)	0 (0-1)	0 (0-0)	0 (0-0)
Maguindanao	0 (0-0)	33.8 (31.7-74.7)	0 (0-0)	54.5 (42.1-97.9)	0 (0-0)	57.1 (51.8-106)	0 (0-0)	51.9 (42.2-103)	0 (0-0)	0 (0-1)	0 (0-0)	0 (0-0)
Marinduque	0 (0-0)	-28.6 (-29.3--7.2)	0 (0-0)	-21.3 (-28.5-0.9)	0 (0-0)	-20.5 (-23-3.9)	0 (0-0)	-26.1 (-31.3--2.3)	0 (0-0)	0 (0-0)	0 (0-0)	0 (0-0)
Masbate	0 (0-0)	11.4 (8.6-64.5)	0 (0-0)	18.1 (6.6-67.3)	0 (0-0)	18.7 (14-72.2)	0 (0-0)	14.8 (5.7-61.4)	0 (0-0)	0 (0-0)	0 (0-0)	0 (0-0)
Misamis Occidental	0 (0-0)	12.8 (9.8-62.1)	0 (0-0)	16.4 (5.9-68.1)	0 (0-0)	16.8 (12.7-68.9)	0 (0-0)	16.2 (6.8-73.5)	0 (0-0)	0 (0-0)	0 (0-0)	0 (0-0)
Misamis Oriental	0 (0-0)	37.9 (34.6-97)	0 (0-0)	38.5 (26.8-91.6)	0 (0-0)	38.8 (34.8-97.3)	0 (0-0)	39.4 (29.2-102)	0 (0-0)	0 (0-1)	0 (0-0)	0 (0-0)
Mountain Province	0 (0-0)	-10.6 (-11.6-20)	0 (0-0)	-4.9 (-14.6-23.2)	0 (0-0)	-3.9 (-6.6-29.9)	0 (0-0)	-4.8 (-15.3-31.6)	0 (0-0)	0 (0-0)	0 (0-0)	0 (0-0)
National Capital Region	1 (0-1)	2.9 (0.9-49.1)	2 (2-3)	8 (-2.1-46.4)	0 (0-1)	9.1 (5.3-58.4)	0 (0-0)	5.6 (-3.8-55.8)	1 (0-1)	3 (2-4)	1 (0-1)	0 (0-1)
Negros Occidental	0 (0-0)	3.2 (2-31.3)	1 (0-1)	3.6 (-5.7-33.8)	0 (0-0)	3.2 (1.5-31.2)	0 (0-0)	2.4 (-4.9-32.9)	0 (0-0)	1 (0-1)	0 (0-0)	0 (0-0)
Negros Oriental	0 (0-0)	-1.8 (-3.5-37.3)	0 (0-0)	1.3 (-8.9-39.6)	0 (0-0)	1.7 (-0.7-43.2)	0 (0-0)	0.7 (-7-51.8)	0 (0-0)	0 (0-0)	0 (0-0)	0 (0-0)
Northern Samar	0 (0-0)	15.1 (5.9-64.1)	0 (0-0)	16 (3-66.3)	0 (0-0)	16.3 (6.6-65.6)	0 (0-0)	15.9 (5.3-66.2)	0 (0-0)	0 (0-0)	0 (0-0)	0 (0-0)
Nueva Ecija	0 (0-0)	23.6 (20.7-74.4)	0 (0-1)	24.6 (14.2-69.8)	0 (0-0)	24.6 (20.9-75.8)	0 (0-0)	27 (15.9-83.6)	0 (0-0)	0 (0-1)	0 (0-0)	0 (0-0)
Nueva Vizcaya	0 (0-0)	5.7 (4.5-25.3)	0 (0-0)	10.7 (2.6-28.3)	0 (0-0)	10.9 (8.7-31.7)	0 (0-0)	10.7 (2-36.3)	0 (0-0)	0 (0-0)	0 (0-0)	0 (0-0)
Occidental Mindoro	0 (0-0)	-3.4 (-16.7-39.1)	0 (0-0)	9.2 (-4.8-54.9)	0 (0-0)	10.6 (-5.4-61.6)	0 (0-0)	6.6 (-9.8-59.2)	0 (0-0)	0 (0-0)	0 (0-0)	0 (0-0)
Oriental Mindoro	0 (0-0)	-6 (-8.2-33.1)	0 (0-0)	0.4 (-11-39.8)	0 (0-0)	1.4 (-2.5-43.4)	0 (0-0)	-3.2 (-10.9-39.7)	0 (0-0)	0 (0-0)	0 (0-0)	0 (0-0)
Palawan	0 (0-0)	27.5 (24-77.1)	0 (0-0)	34.5 (21.3-82.4)	0 (0-0)	35.4 (30.3-88.4)	0 (0-0)	32.1 (20.9-87)	0 (0-0)	0 (0-0)	0 (0-0)	0 (0-0)
Pampanga	0 (0-0)	34 (31.3-96.8)	0 (0-1)	30.3 (18.9-82.6)	0 (0-0)	30.1 (26.5-90.7)	0 (0-0)	31.5 (21.7-95.7)	0 (0-0)	1 (0-1)	0 (0-0)	0 (0-0)
Pangasinan	0 (0-0)	9.2 (7.7-46)	1 (0-1)	11.7 (2-41.5)	0 (0-0)	12 (9.6-50.3)	0 (0-0)	12 (2.8-52.1)	0 (0-0)	1 (0-1)	0 (0-0)	0 (0-0)
Quezon	0 (0-0)	10.7 (8.9-51.5)	0 (0-1)	13.6 (3.7-50.8)	0 (0-0)	13.5 (10.9-55.4)	0 (0-0)	11.3 (3.6-49.4)	0 (0-0)	1 (0-1)	0 (0-0)	0 (0-0)
Quirino	0 (0-0)	-12.2 (-13.8-26.1)	0 (0-0)	0.3 (-9.3-38.5)	0 (0-0)	1.4 (-2-46.8)	0 (0-0)	-2.1 (-9-46.1)	0 (0-0)	0 (0-0)	0 (0-0)	0 (0-0)

Location	Birth , 2000	Births percent change (2000-2021)	All age cases, 2000	All age cases percent change (2000-2021)	All age deaths, 2000	All age deaths percent change (2000-2021)	Under-5 deaths, 2000	Under-5 deaths percent change (2000-2021)	Births, 2021	All age cases, 2021	All age deaths, 2021	Under-5 deaths, 2021
Rizal	0 (0-0)	78.8 (76.3-138)	0 (0-1)	71.7 (55.6-119)	0 (0-0)	71.2 (67.2-129)	0 (0-0)	72.2 (59.4-134)	0 (0-0)	1 (1-1)	0 (0-0)	0 (0-0)
Romblon	0 (0-0)	-16.9 (-25.8-19.2)	0 (0-0)	-9.7 (-21.4-26.1)	0 (0-0)	-8.4 (-17.4-32.1)	0 (0-0)	-11.4 (-20.6-30.3)	0 (0-0)	0 (0-0)	0 (0-0)	0 (0-0)
Samar (Western Samar)	0 (0-0)	5.9 (2.2-59)	0 (0-0)	5.8 (-4.3-58.3)	0 (0-0)	6.2 (1.8-59.2)	0 (0-0)	6.1 (-2.1-62.1)	0 (0-0)	0 (0-0)	0 (0-0)	0 (0-0)
Sarangani	0 (0-0)	18.9 (16.5-72.2)	0 (0-0)	17.2 (6.4-64.8)	0 (0-0)	16.8 (13.8-68.8)	0 (0-0)	16.1 (7.7-70.7)	0 (0-0)	0 (0-0)	0 (0-0)	0 (0-0)
Siquijor	0 (0-0)	-11.4 (-18.6-25.7)	0 (0-0)	-2.5 (-11.9-36.1)	0 (0-0)	-0.9 (-8.7-41.6)	0 (0-0)	-4.3 (-13-40.1)	0 (0-0)	0 (0-0)	0 (0-0)	0 (0-0)
Sorsogon	0 (0-0)	12 (10.2-48.8)	0 (0-0)	8.2 (-1.2-37.8)	0 (0-0)	7.9 (5.5-43.7)	0 (0-0)	7.5 (-0.6-46.5)	0 (0-0)	0 (0-0)	0 (0-0)	0 (0-0)
South Cotabato	0 (0-0)	21.8 (20.1-65.3)	0 (0-0)	23.3 (11.9-61.7)	0 (0-0)	24.1 (21.7-68.8)	0 (0-0)	23.9 (15.9-71.4)	0 (0-0)	0 (0-0)	0 (0-0)	0 (0-0)
Southern Leyte	0 (0-0)	-1.8 (-22.2-39.8)	0 (0-0)	1.3 (-18.2-38.4)	0 (0-0)	1.1 (-20.3-43.3)	0 (0-0)	-0.7 (-23-40.8)	0 (0-0)	0 (0-0)	0 (0-0)	0 (0-0)
Sultan Kudarat	0 (0-0)	1.2 (-0.3-36.9)	0 (0-0)	15.3 (5.4-53.8)	0 (0-0)	17.6 (13.5-57.2)	0 (0-0)	12.6 (4.3-52.7)	0 (0-0)	0 (0-0)	0 (0-0)	0 (0-0)
Sulu	0 (0-0)	-8.9 (-10.4-24.1)	0 (0-0)	13.3 (3.1-52.8)	0 (0-0)	14.7 (10.3-53.7)	0 (0-0)	5.9 (-2-43.4)	0 (0-0)	0 (0-0)	0 (0-0)	0 (0-0)
Surigao Del Norte	0 (0-0)	21.4 (19.4-69.6)	0 (0-0)	21.2 (9.1-65.7)	0 (0-0)	21.4 (18.6-70.4)	0 (0-0)	20.9 (11.4-73.5)	0 (0-0)	0 (0-0)	0 (0-0)	0 (0-0)
Surigao Del Sur	0 (0-0)	-1.3 (-13.4-47.9)	0 (0-0)	0.1 (-12.7-44.8)	0 (0-0)	0.5 (-11.9-50.8)	0 (0-0)	-0.2 (-11.9-49.9)	0 (0-0)	0 (0-0)	0 (0-0)	0 (0-0)
Tarlac	0 (0-0)	4.8 (3.4-35.3)	0 (0-0)	8.6 (-0.6-39.6)	0 (0-0)	9.4 (7-40.6)	0 (0-0)	6.9 (-1.8-36.9)	0 (0-0)	0 (0-0)	0 (0-0)	0 (0-0)
Tawi-Tawi	0 (0-0)	-2 (-8.1-35.1)	0 (0-0)	9.1 (-2.4-43.4)	0 (0-0)	10.8 (4.4-51.7)	0 (0-0)	7.6 (-5.3-49.2)	0 (0-0)	0 (0-0)	0 (0-0)	0 (0-0)
Zambales	0 (0-0)	21.7 (20.5-52.6)	0 (0-0)	21.5 (11-45.9)	0 (0-0)	21.4 (19.5-52.4)	0 (0-0)	20.2 (8.9-56.3)	0 (0-0)	0 (0-0)	0 (0-0)	0 (0-0)
Zamboanga Del Norte	0 (0-0)	-5.1 (-6.2-29)	0 (0-0)	4.3 (-5-40)	0 (0-0)	5.3 (2.4-44.1)	0 (0-0)	2 (-5.7-42.6)	0 (0-0)	0 (0-0)	0 (0-0)	0 (0-0)
Zamboanga Del Sur	0 (0-0)	10.1 (8.9-32)	0 (0-1)	12.3 (2.5-34.5)	0 (0-0)	12.4 (10.4-35.2)	0 (0-0)	12 (4.4-37.5)	0 (0-0)	0 (0-1)	0 (0-0)	0 (0-0)
Zamboanga Sibugay	0 (0-0)	9.9 (8.2-53.2)	0 (0-0)	19.8 (8.6-64.6)	0 (0-0)	20.5 (16.9-67.7)	0 (0-0)	16.2 (8.1-61.7)	0 (0-0)	0 (0-0)	0 (0-0)	0 (0-0)
Seychelles	0 (0-0)	-6.7 (-27.2-16.2)	0 (0-0)	-2.8 (-23.6-15.8)	0 (0-0)	-2.3 (-24.1-21.8)	0 (0-0)	-1.3 (-23.8-25.8)	0 (0-0)	0 (0-0)	0 (0-0)	0 (0-0)
Sri Lanka	1 (0-1)	-16.5 (-29.7-21.2)	4 (3-5)	-6.2 (-21.7-30)	1 (0-1)	-4.3 (-19.1-38.5)	0 (0-1)	-8.8 (-23.5-38.9)	1 (0-1)	4 (2-5)	1 (0-1)	0 (0-0)
Thailand	2 (1-2)	-38.2 (-39.5--12)	10 (7-15)	-35.1 (-41.8--11.9)	2 (1-3)	-34.3 (-36.8--6.1)	1 (1-2)	-37.1 (-42.9--6.5)	1 (1-2)	7 (5-10)	1 (1-2)	1 (0-1)
Timor-Leste	0 (0-0)	4.9 (-10.1-47.1)	0 (0-0)	18.4 (-1.8-61.3)	0 (0-0)	18.5 (1.4-65.8)	0 (0-0)	14.4 (-0.8-62.6)	0 (0-0)	0 (0-0)	0 (0-0)	0 (0-0)
Viet Nam	3 (2-5)	-18.6 (-20.4-15.6)	16 (11-24)	-15.1 (-23.9-13)	3 (2-5)	-14.5 (-17.1-21.3)	2 (1-3)	-12.1 (-19.7-28.2)	3 (2-4)	14 (9-20)	3 (2-4)	2 (1-3)
Sub-Saharan Africa	8,300 (5,980-11,800)	37.1 (34-40.7)	40,300 (29,800-54,400)	54.8 (50.5-59.1)	5,800 (4,190-8,390)	65 (60.2-70)	3,970 (2,660-5,890)	58.2 (53.1-63.9)	11,400 (8,240-16,000)	62,400 (46,000-83,400)	9,570 (6,870-13,900)	6,290 (4,210-9,300)
**Central Sub-Saharan Africa**	266 (188-363)	32.7 (21.9-44.6)	870 (647-1,150)	57.8 (43.9-71.9)	204 (144-282)	57.9 (43.6-72.7)	163 (112-232)	51.2 (36-67)	353 (251-473)	1,370 (1,000-1,810)	322 (229-434)	247 (169-347)
Angola	28 (19-40)	47 (33.2-62.2)	87 (61-121)	79.7 (62.3-98.8)	21 (14-30)	78.9 (60.6-97.7)	17 (11-25)	70.4 (51.8-90.6)	42 (28-58)	157 (110-215)	38 (26-54)	30 (19-43)
Central African Republic	3 (2-4)	27 (14.9-44.6)	8 (5-12)	38.1 (25.5-57.1)	2 (1-3)	37.3 (23.7-55.9)	2 (1-3)	34.9 (20.7-54.2)	3 (2-5)	11 (7-16)	3 (2-4)	2 (1-3)
Congo	5 (3-7)	5.5 (-5.8-15.7)	18 (12-26)	31.2 (18.1-45.5)	4 (2-6)	31.2 (16.3-44.9)	3 (2-4)	23.5 (6.7-38.9)	5 (3-8)	24 (16-35)	5 (3-7)	3 (2-5)
Democratic Republic of the Congo	225 (162-306)	32.2 (19.5-47.2)	738 (552-972)	57.1 (41.5-73.8)	174 (123-238)	56.9 (40.2-75.3)	139 (96-197)	50.2 (32.2-69)	298 (213-400)	1,160 (848-1,520)	273 (195-368)	209 (144-293)

Location	Birth , 2000	Births percent change (2000-2021)	All age cases, 2000	All age cases percent change (2000-2021)	All age deaths, 2000	All age deaths percent change (2000-2021)	Under-5 deaths, 2000	Under-5 deaths percent change (2000-2021)	Births, 2021	All age cases, 2021	All age deaths, 2021	Under-5 deaths, 2021
Equatorial Guinea	2 (1-3)	20.4 (7-35.6)	6 (4-9)	53.2 (35.6-72)	1 (1-2)	49.1 (31.7-68.5)	1 (1-2)	35.6 (17.8-55.6)	2 (1-3)	10 (6-14)	2 (1-3)	1 (1-2)
Gabon	3 (2-4)	-7.5 (-17.8-3.5)	12 (8-18)	4.6 (-5.9-16.2)	2 (1-4)	3.1 (-8.4-15.7)	2 (1-3)	-2.5 (-15.8-12.1)	3 (2-4)	13 (9-18)	2 (2-4)	2 (1-3)
**Eastern Sub-Saharan Africa**	1,550 (1,070-2,200)	33.9 (29-38.8)	13,400 (9,410-18,600)	42 (35.6-48.6)	980 (672-1,440)	76.4 (68.4-83.8)	446 (270-709)	76.2 (65.5-86.6)	2,080 (1,410-2,990)	19,000 (13,400-26,500)	1,730 (1,170-2,520)	785 (477-1,270)
Burundi	25 (17-37)	88.6 (69-111)	174 (119-246)	98.5 (78.5-119)	18 (12-27)	101 (76.9-129)	10 (6-16)	99.6 (70.3-137)	48 (32-70)	345 (237-488)	35 (24-54)	20 (12-33)
Comoros	1 (1-2)	-19.5 (-27.3--9.6)	9 (6-12)	2.6 (-7.3-14.6)	1 (0-1)	2.9 (-8-17)	0 (0-1)	-12.6 (-25.2-1)	1 (1-1)	9 (6-12)	1 (1-1)	0 (0-1)
Djibouti	1 (1-1)	23.6 (9.9-36.3)	6 (4-9)	61.3 (43.4-78.3)	1 (0-1)	62.7 (43.3-80.5)	0 (0-0)	47.9 (26-71.3)	1 (1-2)	10 (7-14)	1 (1-1)	0 (0-1)
Eritrea	7 (5-10)	18.9 (7.5-31.6)	49 (34-69)	43.8 (31.7-57.7)	5 (3-7)	42.6 (28-59.8)	3 (2-4)	26.2 (9.4-45.2)	8 (5-12)	70 (48-100)	7 (4-10)	3 (2-5)
Ethiopia	116 (78-172)	25.9 (19.2-32.1)	807 (557-1,150)	47.8 (40.5-55.3)	78 (51-120)	48.4 (38.6-57.6)	41 (24-68)	37.3 (27.1-47.6)	146 (98-214)	1,190 (829-1,670)	116 (77-172)	56 (33-94)
Addis Ababa	2 (2-4)	-25 (-32.6--15.5)	24 (17-35)	-0.2 (-10.7-11.9)	2 (1-3)	4.4 (-6.8-18.6)	1 (0-1)	-3.6 (-19-12.9)	2 (1-3)	24 (17-35)	2 (1-3)	1 (0-1)
Afar	3 (2-4)	-4.1 (-14.3-8.6)	19 (13-27)	6.7 (-4.1-19.8)	2 (1-3)	7.4 (-5.3-22.1)	1 (1-2)	6.4 (-9.4-26.2)	3 (2-4)	20 (14-29)	2 (1-3)	1 (1-2)
Amhara	26 (18-39)	5.3 (-6-17.4)	187 (131-263)	23.5 (10.7-37.7)	18 (12-28)	24.2 (9.5-40.9)	9 (5-16)	12.4 (-4.7-32.8)	28 (19-41)	231 (161-327)	22 (15-34)	10 (6-17)
Benishangul-Gumuz	1 (1-2)	43.9 (29.9-60.6)	8 (5-11)	76.6 (59.4-97.2)	1 (1-1)	76.6 (57.7-96.7)	0 (0-1)	61.5 (37.7-84.3)	2 (1-3)	14 (9-19)	1 (1-2)	1 (0-1)
Dire Dawa	0 (0-1)	5.8 (-5.5-18.4)	3 (2-4)	42.3 (28.8-56.1)	0 (0-0)	42.4 (27.7-59.4)	0 (0-0)	23.4 (6.7-45.4)	0 (0-1)	4 (3-6)	0 (0-1)	0 (0-0)
Gambella	0 (0-1)	32.2 (15.3-46)	3 (2-4)	85.6 (65.1-106)	0 (0-0)	83.8 (57.1-108)	0 (0-0)	50.9 (24.6-74.6)	1 (0-1)	5 (4-8)	1 (0-1)	0 (0-0)
Harari	0 (0-0)	-7 (-16.2-4.2)	2 (1-2)	34.7 (22.6-49.4)	0 (0-0)	34.8 (22-52.4)	0 (0-0)	11.5 (-4.8-29.6)	0 (0-0)	2 (2-3)	0 (0-0)	0 (0-0)
Oromia	43 (28-64)	37.7 (24.5-51.8)	293 (201-417)	62.7 (46.9-80.6)	28 (18-43)	63.2 (45.2-83.8)	15 (9-25)	50.8 (29.8-74.6)	60 (40-86)	477 (330-672)	46 (30-68)	23 (13-38)
Somali	8 (5-12)	49.2 (34.5-66.1)	54 (37-77)	52.5 (38-68.3)	5 (3-8)	55 (36.6-74.3)	3 (2-4)	62.3 (38.9-89.3)	11 (8-17)	83 (56-118)	8 (5-12)	4 (2-7)
Southern Nations, Nationalities, and Peoples	25 (17-38)	32.1 (16.9-47.1)	169 (114-244)	59.4 (41.4-75.7)	17 (11-26)	57.2 (38.2-77.7)	9 (5-16)	41.5 (19.3-64.5)	33 (22-49)	269 (183-379)	27 (18-40)	13 (8-22)
Tigray	6 (4-9)	8.5 (-1.9-20.3)	44 (30-64)	39.8 (26.4-54.1)	4 (3-6)	41.3 (26-58.2)	2 (1-3)	21.7 (4.7-42)	7 (4-10)	62 (42-89)	6 (4-8)	2 (1-4)
Kenya	848 (585-1,200)	33.1 (25.2-41.3)	8,760 (6,220-12,100)	31.3 (23.1-39.5)	520 (353-760)	90.4 (78.7-102)	183 (108-294)	116 (93.6-139)	1,130 (762-1,650)	11,500 (8,110-16,000)	990 (667-1,450)	397 (235-648)
Baringo	7 (5-10)	24.3 (10.1-39.2)	75 (53-105)	23.1 (8.9-38.1)	4 (3-6)	79.7 (56-106)	2 (1-3)	93 (59.6-135)	9 (6-14)	92 (64-131)	8 (5-12)	3 (2-5)
Bomet	9 (6-12)	132 (105-161)	104 (74-143)	152 (124-183)	5 (3-7)	267 (219-322)	1 (1-2)	260 (194-347)	20 (14-28)	261 (178-365)	17 (12-25)	5 (3-8)
Bungoma	66 (46-95)	-43.7 (-49.6--35.3)	649 (455-913)	-41.2 (-47.6--33.3)	38 (25-57)	-14 (-25.3-0.1)	14 (8-23)	-4 (-21.5-17.9)	37 (25-55)	382 (266-537)	33 (21-49)	14 (8-22)
Busia	39 (27-56)	40.8 (23.9-61.8)	392 (273-541)	32.3 (17.6-50.1)	24 (16-36)	93.8 (68.8-125)	9 (6-15)	117 (81.5-167)	55 (36-81)	519 (355-720)	47 (30-69)	20 (12-33)
Elgeyo-Marakwet	8 (6-11)	-66.7 (-70.5--62.1)	93 (66-130)	-61.3 (-65.7--56.2)	5 (3-7)	-43.9 (-51.2--35.3)	1 (1-2)	-41.2 (-53--27.2)	3 (2-4)	36 (25-50)	3 (2-4)	1 (0-1)
Embu	4 (3-6)	-28.7 (-36.9--20.3)	57 (40-79)	-21.6 (-31.2--12.5)	3 (2-4)	11.1 (-3.7-25.3)	1 (0-1)	17 (-4.7-43.2)	3 (2-5)	45 (31-62)	3 (2-5)	1 (1-2)
Garissa	4 (3-6)	344 (286-411)	48 (34-66)	244 (203-291)	3 (2-4)	396 (324-479)	1 (0-1)	533 (407-694)	18 (12-27)	164 (115-229)	13 (8-19)	5 (3-9)
Homa Bay	92 (64-135)	29.9 (14-46.5)	752 (525-1,050)	34.5 (19.5-51.2)	50 (33-76)	96 (69.9-124)	21 (12-34)	123 (78.8-175)	120 (81-178)	1,010 (707-1,410)	99 (65-148)	46 (27-76)
Isiolo	2 (1-2)	54.5 (35.7-77.5)	14 (10-20)	42.6 (24.3-62.5)	1 (1-1)	112 (83.1-146)	0 (0-1)	144 (102-195)	2 (2-4)	21 (14-29)	2 (1-3)	1 (1-2)

Location	Birth , 2000	Births percent change (2000-2021)	All age cases, 2000	All age cases percent change (2000-2021)	All age deaths, 2000	All age deaths percent change (2000-2021)	Under-5 deaths, 2000	Under-5 deaths percent change (2000-2021)	Births, 2021	All age cases, 2021	All age deaths, 2021	Under-5 deaths, 2021
Kajiado	5 (3-7)	63.1 (43.3-82.8)	57 (40-80)	73.6 (56.2-94.6)	3 (2-4)	144 (114-179)	1 (1-1)	154 (108-213)	8 (5-11)	99 (69-138)	7 (5-11)	2 (1-4)
Kakamega	42 (29-61)	118 (92.9-147)	428 (301-591)	102 (78.8-131)	26 (17-40)	199 (162-246)	11 (6-18)	226 (172-298)	92 (62-134)	866 (613-1,210)	78 (52-116)	34 (21-56)
Kericho	15 (11-21)	-63.3 (-68.5--58.1)	164 (116-227)	-56.9 (-62.4--51.1)	8 (6-12)	-37.8 (-47.5--28)	3 (2-4)	-35.7 (-49--22.1)	6 (4-8)	70 (49-98)	5 (3-8)	2 (1-3)
Kiambu	15 (10-21)	-18.3 (-27.9--8.3)	181 (128-253)	-10.8 (-20.7-0.1)	10 (7-14)	22.3 (7.8-38.7)	2 (1-4)	40.6 (10.8-72.4)	12 (8-18)	161 (111-221)	12 (8-18)	3 (2-6)
Kilifi	40 (28-57)	42.7 (25.3-64.3)	445 (316-611)	37.8 (20.3-56.8)	27 (18-39)	101 (72.8-133)	10 (6-16)	124 (84.9-175)	58 (40-85)	613 (429-857)	53 (36-79)	22 (13-36)
Kirinyaga	4 (3-6)	-26.3 (-34.4--16.9)	54 (38-76)	-20 (-28.5--9.7)	3 (2-4)	9.8 (-2.5-23.9)	1 (0-1)	20.5 (-2.7-48)	3 (2-5)	44 (30-62)	3 (2-5)	1 (1-2)
Kisii	48 (33-69)	-35 (-43--24.8)	560 (393-775)	-25.3 (-34.5--14.7)	33 (22-47)	8.1 (-7.2-26.4)	10 (6-17)	8.3 (-12.6-33.4)	32 (21-47)	418 (298-587)	35 (24-52)	11 (7-19)
Kisumu	41 (28-58)	-2.9 (-15.6-15)	415 (289-574)	5.9 (-6-19.8)	27 (18-39)	51.9 (33.7-80.2)	10 (6-15)	61.4 (30.8-102)	40 (26-58)	440 (303-606)	40 (26-59)	16 (9-26)
Kitui	12 (8-17)	1.6 (-12.2-16.8)	134 (94-181)	5.4 (-7.1-18.8)	7 (5-10)	54.7 (33.7-79.2)	2 (1-4)	61.8 (31.9-95.8)	12 (8-18)	141 (100-193)	11 (7-16)	4 (2-6)
Kwale	23 (16-33)	37.3 (21.2-57.4)	264 (184-370)	28.6 (12.8-45.2)	16 (10-24)	85.1 (62.3-115)	6 (3-9)	110 (75-156)	32 (21-46)	340 (237-475)	29 (19-44)	12 (7-19)
Laikipia	4 (3-6)	-26.1 (-35.5--17.8)	47 (33-65)	-11.9 (-22.1--1.9)	2 (2-3)	25.4 (9.4-39.7)	1 (0-1)	26.5 (3-56.2)	3 (2-4)	42 (29-58)	3 (2-4)	1 (0-1)
Lamu	1 (1-1)	33.6 (18.2-52.8)	11 (8-15)	28 (13.2-45.5)	1 (0-1)	82.2 (58.8-110)	0 (0-0)	110 (73.4-159)	1 (1-2)	14 (10-20)	1 (1-2)	0 (0-1)
Machakos	11 (8-16)	-47.1 (-53.3--40.1)	154 (109-211)	-41.9 (-48.4--34.1)	8 (5-11)	-14.7 (-25.7--1.9)	2 (1-3)	-11.5 (-27.7-8.7)	6 (4-9)	90 (63-124)	7 (4-10)	2 (1-3)
Makueni	8 (5-11)	58 (39.4-79.5)	91 (63-126)	66.8 (48.6-87.7)	4 (3-7)	147 (113-186)	1 (1-2)	148 (101-204)	12 (8-18)	152 (106-211)	11 (7-16)	3 (2-6)
Mandera	3 (2-5)	1280 (1110-1440)	43 (30-62)	870 (761-990)	3 (2-4)	1320 (1130-1540)	1 (1-2)	1530 (1250-1870)	45 (30-66)	417 (293-587)	36 (24-54)	15 (9-25)
Marsabit	3 (2-4)	140 (111-171)	27 (19-38)	110 (83.7-137)	2 (1-2)	208 (164-257)	1 (0-1)	248 (183-323)	6 (4-9)	57 (39-81)	5 (3-7)	2 (1-3)
Meru	14 (9-19)	-18.5 (-27.9--6.7)	172 (122-239)	-12.7 (-22.7--1.5)	9 (6-14)	26 (10.7-46.3)	3 (2-4)	30.3 (8.3-60.7)	11 (7-17)	150 (105-213)	12 (8-18)	4 (2-6)
Migori	82 (55-118)	66 (47.5-92.5)	640 (446-888)	62.6 (45.7-86.7)	45 (29-66)	137 (108-177)	20 (11-32)	176 (128-251)	136 (93-200)	1,040 (724-1,450)	105 (71-160)	54 (32-89)
Mombasa	17 (12-24)	44.1 (25.4-64.9)	204 (143-280)	49.9 (29.3-71.9)	13 (9-18)	93.3 (64.2-123)	3 (2-5)	148 (96.3-202)	24 (17-36)	306 (214-433)	25 (17-36)	8 (5-13)
Murang'a	7 (5-10)	-22.1 (-30.4--12.5)	96 (66-136)	-15.7 (-25.1--4.6)	5 (3-7)	24.4 (8.8-41.7)	1 (1-2)	20.5 (-1.1-49.8)	5 (4-8)	81 (56-112)	6 (4-8)	1 (1-2)
Nairobi	23 (16-32)	33 (17.8-55.1)	261 (183-358)	48.8 (31.8-67.7)	16 (11-23)	87.3 (65.8-118)	3 (2-5)	149 (101-220)	31 (21-44)	389 (274-541)	30 (21-43)	9 (5-14)
Nakuru	14 (10-20)	-12.2 (-22.1-0.7)	178 (126-244)	3.7 (-8.4-16.9)	9 (6-13)	45.8 (28.3-68.9)	2 (1-4)	43.3 (17.3-75.5)	12 (8-18)	185 (129-260)	13 (9-19)	3 (2-6)
Nandi	12 (8-18)	-5.7 (-16.7-7.9)	151 (106-210)	5.9 (-5.8-19.3)	7 (5-11)	51.9 (33.5-75.9)	2 (1-3)	52.6 (24.5-90.7)	12 (8-16)	160 (113-224)	11 (8-17)	3 (2-5)
Narok	8 (5-11)	339 (289-402)	84 (60-118)	303 (253-358)	4 (3-6)	483 (398-582)	1 (1-2)	538 (414-692)	34 (23-50)	340 (239-493)	25 (16-38)	9 (5-15)
Nyamira	10 (7-14)	28.5 (11.1-46.3)	122 (87-170)	48.4 (30.4-67.9)	6 (4-9)	113 (82.8-146)	2 (1-2)	112 (68.8-164)	13 (9-19)	181 (126-257)	13 (9-19)	3 (2-6)
Nyandarua	6 (4-8)	-13.3 (-23.3--1.1)	70 (49-98)	-4.3 (-15-7.3)	3 (2-5)	37.5 (19.7-57.5)	1 (0-1)	32.6 (5.5-66)	5 (3-7)	67 (48-93)	5 (3-7)	1 (1-2)
Nyeri	6 (4-8)	-39.4 (-46.6--32.1)	77 (54-108)	-32.9 (-40.2--24.3)	4 (3-6)	-5.3 (-16.7-7)	1 (1-1)	-2.4 (-20.6-18.8)	3 (2-5)	52 (35-71)	4 (2-5)	1 (0-1)
Samburu	3 (2-4)	85.4 (59-113)	26 (18-36)	77.2 (54.2-100)	1 (1-2)	160 (123-202)	0 (0-1)	185 (130-254)	5 (3-7)	45 (32-64)	3 (2-5)	1 (1-2)
Siaya	66 (45-95)	8.5 (-4.8-27.9)	594 (409-828)	9.4 (-2.9-25)	39 (26-59)	62 (40.3-88.9)	16 (9-27)	81.8 (44.3-125)	71 (47-106)	650 (461-906)	64 (42-95)	30 (17-48)

Location	Birth , 2000	Births percent change (2000-2021)	All age cases, 2000	All age cases percent change (2000-2021)	All age deaths, 2000	All age deaths percent change (2000-2021)	Under-5 deaths, 2000	Under-5 deaths percent change (2000-2021)	Births, 2021	All age cases, 2021	All age deaths, 2021	Under-5 deaths, 2021
Taita Taveta	3 (2-4)	-19.8 (-29.1--9.6)	44 (31-62)	-17.9 (-28--8.6)	2 (2-4)	17.5 (3.2-32.6)	1 (0-1)	27.5 (4.3-54.1)	2 (2-4)	36 (26-51)	3 (2-4)	1 (0-1)
Tana River	4 (2-5)	68.4 (49.6-91.5)	39 (27-54)	45.6 (28.2-66.4)	2 (2-3)	108 (81.7-140)	1 (0-1)	150 (106-199)	6 (4-9)	57 (39-80)	5 (3-8)	2 (1-4)
Tharaka Nithi	1 (1-2)	0.1 (-12.8-14.6)	14 (10-20)	5.6 (-7.4-19.8)	1 (1-1)	51.1 (31.2-76.1)	0 (0-0)	58.1 (27.8-93.9)	1 (1-2)	15 (10-21)	1 (1-2)	0 (0-1)
Trans Nzoia	15 (10-21)	11.7 (0.9-26.7)	176 (124-248)	21.7 (7-37.8)	9 (6-14)	76.7 (53.7-104)	3 (2-5)	76.7 (44.7-118)	17 (11-23)	214 (149-301)	16 (11-23)	5 (3-8)
Turkana	10 (7-15)	227 (186-275)	99 (68-137)	202 (166-240)	6 (4-8)	337 (273-408)	2 (1-3)	407 (307-523)	34 (23-50)	299 (209-419)	25 (16-37)	10 (6-17)
Uasin Gishu	11 (8-15)	7.5 (-5.2-23.1)	134 (94-186)	24.5 (10.6-42.2)	7 (5-10)	76.8 (54.7-106)	2 (1-3)	80.2 (48.2-122)	12 (8-17)	167 (117-233)	12 (8-18)	4 (2-6)
Vihiga	16 (11-22)	47.8 (29.9-68.6)	182 (128-252)	49.2 (32.5-68.4)	10 (7-15)	119 (87.2-152)	4 (2-6)	119 (75.4-169)	23 (16-34)	272 (191-384)	23 (15-34)	8 (5-14)
Wajir	5 (4-7)	236 (188-288)	55 (38-76)	157 (122-193)	3 (2-5)	276 (216-342)	1 (1-2)	372 (279-481)	18 (12-25)	142 (99-196)	12 (8-18)	6 (3-9)
West Pokot	9 (6-13)	112 (86.1-144)	85 (59-119)	97.4 (74.8-124)	5 (3-7)	187 (145-239)	2 (1-3)	216 (153-292)	19 (13-28)	168 (119-233)	14 (10-22)	6 (4-10)
Madagascar	72 (49-106)	26.4 (13.8-40.6)	497 (347-712)	52.6 (37.7-69.6)	48 (32-73)	50.6 (33.9-70)	27 (16-45)	35.2 (16.4-56.8)	91 (62-133)	758 (526-1,070)	73 (49-109)	36 (22-59)
Malawi	44 (29-65)	5 (-6.7-16.6)	262 (180-370)	55.3 (37.5-71.4)	28 (18-42)	46.8 (28-66.4)	17 (10-28)	22.1 (4-42.6)	46 (31-67)	406 (278-584)	41 (27-60)	21 (12-34)
Mozambique	77 (55-102)	42.8 (29.5-58.2)	446 (320-586)	76.2 (60-94.2)	50 (35-68)	72.1 (54.7-91.4)	31 (20-45)	58.6 (37.4-80.5)	110 (78-148)	786 (568-1,050)	86 (60-118)	49 (31-73)
Rwanda	21 (14-31)	11.3 (0.5-24.7)	151 (105-217)	36.7 (24.4-52.4)	15 (10-22)	35.5 (21.1-54.1)	8 (5-14)	20.5 (3.6-41.5)	23 (16-34)	207 (142-295)	20 (13-30)	10 (6-16)
Somalia	22 (14-31)	80.5 (60.3-102)	123 (85-177)	102 (82.6-124)	14 (9-21)	99.5 (75.1-125)	9 (5-14)	94 (64.2-126)	39 (26-57)	248 (171-348)	28 (18-42)	17 (10-27)
South Sudan	22 (15-34)	10.9 (-0.3-22)	133 (92-186)	22.8 (11.8-36.2)	15 (10-23)	18.5 (5.8-31.8)	9 (5-15)	13.5 (-1.1-32.1)	24 (16-36)	163 (113-229)	18 (12-26)	10 (6-17)
Uganda	24 (17-35)	29.9 (15.5-43.4)	145 (103-200)	59.6 (43.3-73.7)	16 (11-23)	56.2 (37.1-75.8)	10 (6-15)	42.7 (21.6-66.3)	32 (22-45)	231 (163-322)	24 (17-35)	14 (8-21)
United Republic of Tanzania	185 (123-273)	39.5 (23.2-55.4)	1,230 (842-1,770)	66.4 (50.3-83.2)	117 (77-176)	64.4 (42-84.8)	66 (40-106)	55 (27.5-84.9)	258 (171-374)	2,040 (1,420-2,870)	192 (127-288)	102 (61-165)
Zambia	85 (57-123)	39.9 (24.3-59.8)	562 (394-789)	72.9 (56.6-93.8)	56 (37-83)	71.5 (53.3-98)	32 (19-50)	54.5 (32.5-80.8)	119 (80-175)	972 (675-1,350)	96 (64-142)	50 (30-80)
**Southern Sub-Saharan Africa**	28 (19-43)	-1.5 (-6-3.6)	175 (120-251)	6.1 (1.4-11)	25 (17-38)	6.2 (1-11.5)	14 (9-24)	5.1 (-1.1-11.5)	28 (19-42)	186 (129-269)	26 (18-39)	15 (9-25)
Botswana	1 (0-1)	6.2 (-6.6-27.1)	4 (3-6)	11.4 (-2.1-29.3)	1 (0-1)	12.6 (-1.1-34.9)	0 (0-1)	11.4 (-6.3-36.7)	1 (0-1)	5 (3-7)	1 (0-1)	0 (0-1)
Eswatini	1 (0-1)	-17.4 (-24.5--8.2)	3 (2-5)	-9.2 (-18.2-1.9)	1 (0-1)	-8.3 (-16.8-2.5)	0 (0-1)	-11.2 (-22.3-2)	1 (0-1)	3 (2-4)	0 (0-1)	0 (0-0)
Lesotho	1 (1-1)	-23.1 (-32.7--13.8)	5 (3-7)	-13.5 (-24.4--3.8)	1 (0-1)	-13 (-24.4--2.3)	0 (0-1)	-16.4 (-29--3.7)	1 (0-1)	4 (3-6)	1 (0-1)	0 (0-1)
Namibia	1 (1-1)	12 (-4.1-26.3)	6 (4-9)	13 (0.1-26.7)	1 (1-1)	13.9 (-2.6-29.4)	0 (0-1)	13.3 (-5.4-32.1)	1 (1-2)	7 (5-10)	1 (1-1)	1 (0-1)
South Africa	15 (11-23)	-6.4 (-10.8--2)	104 (71-148)	0.6 (-3.6-5.2)	14 (10-21)	0.1 (-4.7-5)	8 (5-13)	-0.8 (-6.8-5.3)	14 (10-22)	104 (73-150)	14 (10-21)	8 (5-12)
Eastern Cape	2 (2-4)	-27.6 (-36.3--17)	16 (10-23)	-24.4 (-32.4--13.5)	2 (2-4)	-23.8 (-32.8--12.6)	1 (1-2)	-24.2 (-35.1--10.6)	2 (1-3)	12 (8-18)	2 (1-3)	1 (1-2)
Free State	1 (1-1)	-24 (-33.5--12)	6 (4-8)	-19.1 (-28.1--6.2)	1 (1-1)	-18.2 (-28.5--5.7)	0 (0-1)	-17.3 (-30.1-0.9)	1 (0-1)	5 (3-7)	1 (0-1)	0 (0-1)
Gauteng	2 (2-4)	42.2 (24.3-75.9)	16 (11-22)	61.5 (43.3-92.9)	2 (1-3)	62.2 (42.1-99.6)	1 (1-2)	64.7 (39.3-109)	3 (2-5)	25 (18-37)	3 (2-5)	2 (1-3)
KwaZulu-Natal	4 (2-5)	-18.7 (-27.4--8.6)	24 (16-35)	-11.2 (-20.5-2.1)	3 (2-5)	-11 (-20.8-0.3)	2 (1-3)	-13 (-24.9-1.5)	3 (2-4)	21 (14-30)	3 (2-5)	2 (1-3)
Limpopo	2 (1-4)	-23.8 (-31.1--12.3)	16 (11-24)	-22.2 (-29.4--12.9)	2 (1-3)	-21.3 (-29.3--9.2)	1 (1-2)	-20.7 (-31.2--6)	2 (1-3)	13 (9-18)	2 (1-3)	1 (1-2)

Location	Birth , 2000	Births percent change (2000-2021)	All age cases, 2000	All age cases percent change (2000-2021)	All age deaths, 2000	All age deaths percent change (2000-2021)	Under-5 deaths, 2000	Under-5 deaths percent change (2000-2021)	Births, 2021	All age cases, 2021	All age deaths, 2021	Under-5 deaths, 2021
Mpumalanga	1 (1-2)	21.6 (2.2-38.5)	8 (6-12)	24 (7.8-38.5)	1 (1-2)	25.3 (5.3-42.8)	1 (0-1)	26.4 (0.9-49.9)	1 (1-2)	10 (7-15)	1 (1-2)	1 (0-1)
North-West	1 (1-2)	-29.7 (-38.6--15.8)	8 (5-12)	-24.3 (-34.4--12.8)	1 (1-2)	-23.9 (-34.1--8.9)	1 (0-1)	-23.1 (-34.9--4.4)	1 (1-1)	6 (4-9)	1 (1-1)	0 (0-1)
Northern Cape	0 (0-0)	54.1 (30-85.3)	2 (1-3)	54.6 (32.6-83.2)	0 (0-0)	55.4 (31.4-87.1)	0 (0-0)	51.1 (24.5-88.5)	0 (0-1)	3 (2-4)	0 (0-1)	0 (0-0)
Western Cape	1 (1-1)	4.8 (-14.5-25.2)	9 (6-12)	14.3 (-6.2-35.9)	1 (1-1)	15.4 (-6-37.6)	0 (0-1)	13.6 (-11.2-39.3)	1 (1-2)	10 (7-14)	1 (1-2)	0 (0-1)
Zimbabwe	10 (6-15)	7.5 (-5.1-21)	53 (35-77)	18.5 (5.6-31.7)	8 (5-12)	18.5 (4-33.2)	5 (3-8)	15.8 (-0.6-32.5)	10 (7-16)	63 (42-91)	9 (6-14)	6 (4-10)
**Western Sub-Saharan Africa**	6,450 (4,710-9,120)	38.2 (34.5-42.2)	25,900 (19,400-34,400)	61.6 (57.3-66.4)	4,590 (3,330-6,550)	63.2 (58.2-68.9)	3,350 (2,260-4,950)	56.4 (51.2-62.4)	8,920 (6,480-12,400)	41,900 (31,200-55,300)	7,490 (5,400-10,600)	5,240 (3,540-7,680)
Benin	35 (25-50)	71.4 (54.6-88.5)	142 (105-191)	83.8 (67-102)	27 (19-39)	84.1 (64.8-105)	20 (14-30)	81.4 (58.7-108)	60 (43-84)	262 (193-350)	50 (35-70)	36 (24-53)
Burkina Faso	228 (175-302)	73.7 (57.9-92.3)	878 (678-1,130)	82.3 (64.6-102)	174 (130-232)	83.1 (65.3-103)	133 (95-183)	81.4 (60.7-105)	396 (308-516)	1,600 (1,240-2,060)	318 (245-418)	241 (176-327)
Cabo Verde	1 (1-2)	-18.5 (-27.5--7.1)	8 (5-11)	-12.9 (-22.5--2.9)	1 (1-1)	-10.9 (-21.8-2.5)	0 (0-1)	-14.2 (-27.2-2)	1 (1-1)	7 (5-10)	1 (1-1)	0 (0-1)
Cameroon	93 (70-123)	46.9 (34.4-61.7)	394 (296-514)	76.5 (59.4-92.6)	70 (53-95)	75 (59.2-93.1)	51 (36-72)	64.9 (47.6-87.3)	136 (102-181)	696 (523-912)	123 (92-165)	84 (59-120)
Chad	123 (91-165)	86.7 (69.6-105)	442 (341-575)	102 (83.3-121)	88 (65-121)	101 (82.9-122)	69 (48-97)	98 (77.2-121)	229 (174-301)	891 (688-1,150)	178 (133-236)	136 (98-188)
Côte d'Ivoire	1,250 (992-1,570)	19.3 (8.2-31)	5,220 (4,120-6,610)	39.3 (27.4-54.1)	911 (708-1,150)	39.1 (26.2-54.3)	657 (477-866)	33.7 (18.4-52.3)	1,490 (1,170-1,930)	7,270 (5,720-9,240)	1,270 (987-1,650)	878 (635-1,160)
Gambia	17 (10-26)	11.5 (-0.6-28.5)	70 (47-102)	43 (28.5-59.6)	12 (7-18)	40.7 (24.7-63)	8 (5-14)	29.2 (12.8-52.5)	18 (12-29)	101 (70-142)	16 (10-25)	10 (6-18)
Ghana	148 (111-193)	25.9 (14.8-38.5)	722 (547-927)	41.4 (29.3-54.5)	120 (88-158)	39.4 (26.7-52.9)	82 (56-114)	31.9 (16.9-50)	187 (139-245)	1,020 (777-1,320)	167 (124-221)	108 (74-154)
Guinea	24 (16-34)	39.3 (24-55.6)	91 (65-126)	55.1 (39.5-73)	18 (12-27)	55.3 (36.2-74.5)	14 (9-21)	52.2 (32-73.4)	33 (23-47)	141 (102-193)	28 (19-41)	21 (14-31)
Guinea-Bissau	10 (6-15)	15 (3-28.8)	41 (28-59)	33.3 (20.7-48.4)	8 (5-12)	32.9 (18.1-49.4)	6 (3-9)	27.9 (11.6-49.2)	11 (7-18)	55 (37-79)	10 (6-16)	7 (4-12)
Liberia	252 (194-332)	2 (-7.5-12.1)	951 (749-1,210)	30.7 (18.4-43.7)	187 (143-248)	26.5 (14-41.1)	143 (105-197)	16.5 (3.5-30.5)	257 (196-331)	1,240 (958-1,590)	236 (181-306)	167 (121-228)
Mali	128 (98-169)	90.6 (70.1-109)	465 (359-603)	106 (86.6-126)	94 (69-126)	105 (82.6-126)	73 (52-100)	101 (74.5-124)	245 (185-330)	960 (742-1,250)	192 (145-264)	146 (104-207)
Mauritania	16 (10-25)	4.1 (-7.3-17.6)	83 (57-119)	29.9 (17.2-44.7)	13 (8-20)	27.1 (11.9-44.3)	8 (5-14)	14 (-2.4-33)	17 (11-25)	108 (74-153)	16 (10-24)	10 (5-16)
Niger	684 (542-901)	83.2 (67.3-102)	2,300 (1,820-2,930)	107 (89.6-129)	474 (370-632)	106 (84.5-127)	380 (285-517)	100 (76.9-126)	1,250 (994-1,640)	4,760 (3,760-6,060)	976 (763-1,280)	761 (578-1,030)
Nigeria	3,250 (2,080-4,990)	33.6 (30.1-37.1)	13,200 (9,150-18,800)	62.5 (57.9-67.5)	2,250 (1,430-3,530)	64.7 (59.4-70.9)	1,600 (973-2,730)	55.6 (50.7-62)	4,340 (2,780-6,670)	21,500 (15,000-30,200)	3,700 (2,350-5,740)	2,490 (1,500-4,220)
Sao Tome and Principe	2 (1-3)	-15.3 (-25.6--3.4)	9 (6-13)	8.7 (-3.8-21.3)	1 (1-2)	6.7 (-7.5-22.6)	1 (1-2)	-5.1 (-20.6-12.8)	1 (1-2)	10 (7-14)	1 (1-2)	1 (0-1)
Senegal	77 (57-105)	23.1 (14-34.6)	357 (270-466)	39.1 (27.2-52.4)	61 (44-84)	38.7 (27.1-52.9)	42 (28-60)	32.5 (18.3-50.1)	95 (72-129)	497 (373-653)	84 (63-115)	56 (39-81)
Sierra Leone	30 (22-43)	43.3 (28.2-59.9)	109 (80-147)	67.2 (50.8-83.2)	22 (16-32)	66.2 (48-86.9)	17 (11-26)	60 (40.4-84.2)	43 (30-62)	182 (134-246)	36 (25-53)	27 (18-41)
Togo	86 (65-117)	19.4 (9.4-31)	386 (293-507)	40.7 (28-55)	67 (50-92)	39.3 (27-53.7)	48 (33-68)	31.8 (16-48.5)	103 (76-137)	543 (409-704)	94 (69-126)	63 (43-88)

Table S13: Total sickle cell disease frequency: Birth rate, all-age prevalence and mortality rates, under-5 mortality rates, and mortality ranks , 2000 - 2021

Location	Birth Incidence rate (per 100,000 live births), 2000	Birth Incidence rate (per 100,000 live births), 2021	Birth Incidence rate (per 100,000 live births) percent change (2000-2021)	All age rate (per 100,000 population), 2000	All age rate (per 100,000 population), 2021	All age rate (per 100,000 population) percent change (2000-2021)	All age cause-specific SCD mortality rate (per 100,000 population), 2000	All age cause-specific SCD mortality rate (per 100,000 population), 2021	All age cause-specific SCD mortality rate (per 100,000 population) percent change (2000-2021)	All age total SCD mortality rate (per 100,000 population), 2000	All age total SCD mortality rate (per 100,000 population), 2021	All age total SCD mortality rate (per 100,000 population) percent change (2000-2021)	Under-5 cause-specific SCD mortality rate (per 100,000 population), 2000	Under-5 cause-specific SCD mortality rate (per 100,000 population), 2021	Under-5 cause-specific SCD mortality rate (per 100,000 population) percent change (2000-2021)	Under-5 total SCD mortality rate (per 100,000 population), 2000	Under-5 total SCD mortality rate (per 100,000 population), 2021	Under-5 total SCD mortality rate (per 100,000 population) percent change (2000-2021)	Under-5 cause-specific mortality cause fraction, 2000	Under-5 total SCD mortality cause fraction, 2000	Under-5 cause-specific mortality cause fraction, 2021	Under-5 total SCD mortality cause fraction, 2021	Under-5 cause-specific mortality rank 2000	Under-5 cause-specific mortality rank 2021	Under-5 total SCD mortality rank 2000	Under-5 total SCD mortality rank 2021
Global	336 (275-403)	382 (316-456)	13.6 (11-16.4)	89.3 (75.5-105)	97.8 (82.2-116)	9.5 (7.1-12.2)	0.5 (0.4-0.6)	0.4 (0.3-0.6)	-6.5 (-26.7-22)	4.3 (3.5-5.3)	4.8 (3.8-5.9)	11 (8.3-14)	1.9 (1.2-2.7)	1.7 (1-2.2)	-11.8 (-37.2-27.8)	10.6 (7.7-14.2)	12.5 (9-16.6)	18.1 (13.6-22.8)	0.1 (0.1-0.2)	0.7 (0.5-0.9)	0.2 (0.2-0.3)	1.7 (1.1-2.3)	51	40	22	12
Central Europe, Eastern Europe, and Central Asia	1 (0.7-1.3)	0.6 (0.5-0.8)	-35.8 (-42.7--29.5)	0.1 (0.1-0.1)	0.1 (0.1-0.1)	-33.9 (-38.4--29.5)	0 (0-0)	0 (0-0)	-32.8 (-38.7--27.5)	0 (0-0)	0 (0-0)	-37 (-43.8--29.4)	0 (0-0)	0 (0-0)	-63 (-75.2--44.5)	0.1 (0.1-0.2)	0.1 (0-0.1)	-40.3 (-48.3--30.3)	0 (0-0)	0 (0-0)	0 (0-0)	0 (0-0)	136	136	81	72
**Central Asia**	0.1 (0-0.2)	0.1 (0-0.1)	-2.3 (-13.7-0.8)	0 (0-0)	0 (0-0)	-10.2 (-22.6--4.1)	0 (0-0)	0 (0-0)	5.6 (-37.5-81.4)	0 (0-0)	0 (0-0)	-8 (-29.9--4.7)	0 (0-0)	0 (0-0)	-14.5 (-55.2-57.9)	0 (0-0)	0 (0-0)	-2.4 (-26.2-1.6)	0 (0-0)	0 (0-0)	0 (0-0)	0 (0-0)	136	135	126	118
Armenia	0.1 (0-0.1)	0.1 (0-0.1)	-1.3 (-12.6-9.9)	0 (0-0)	0 (0-0)	-12.5 (-19.3--2.5)	0 (0-0)	0 (0-0)	62.7 (-8.4-161)	0 (0-0)	0 (0-0)	-9 (-30.3-2.1)	0 (0-0)	0 (0-0)	-37.2 (-70-40.1)	0 (0-0)	0 (0-0)	-1.8 (-25.2-9.2)	0 (0-0)	0 (0-0)	0 (0-0)	0 (0-0)	132	132	106	100
Azerbaijan	0.1 (0-0.2)	0.1 (0-0.1)	-2.6 (-23.6-0.6)	0 (0-0)	0 (0-0)	-20.5 (-29.7--14.3)	0 (0-0)	0 (0-0)	-57.8 (-78.6--11.1)	0 (0-0)	0 (0-0)	-21.9 (-44.7--16.9)	0 (0-0)	0 (0-0)	-50.5 (-79.7-16.3)	0 (0-0)	0 (0-0)	-1.8 (-29.7-3.2)	0 (0-0)	0 (0-0)	0 (0-0)	0 (0-0)	128	127	119	107
Georgia	0.1 (0-0.2)	0.1 (0-0.2)	-1.9 (-12.9-3.1)	0 (0-0)	0 (0-0)	-4.7 (-26.8-5.3)	0 (0-0)	0 (0-0)	-8.1 (-35.2-20.5)	0 (0-0)	0 (0-0)	3.2 (-19-7.6)	0 (0-0)	0 (0-0)	-65.3 (-79--46.2)	0 (0-0)	0 (0-0)	-1.6 (-24-3.7)	0 (0-0)	0 (0-0)	0 (0-0)	0 (0-0)	136	135	108	100
Kazakhstan	0.1 (0-0.1)	0.1 (0-0.1)	-0.7 (-17.5-36.6)	0 (0-0)	0 (0-0)	5.6 (-13.9-56.4)	0 (0-0)	0 (0-0)	130 (81.4-183)	0 (0-0)	0 (0-0)	13.7 (-11.1-45)	0 (0-0)	0 (0-0)	95.6 (-8.3-330)	0 (0-0)	0 (0-0)	-1.2 (-21.9-21.8)	0 (0-0)	0 (0-0)	0 (0-0)	0 (0-0)	134	132	115	104
Kyrgyzstan	0.1 (0-0.1)	0.1 (0-0.1)	-2.1 (-24.5-1)	0 (0-0)	0 (0-0)	-1.9 (-20.2-7.7)	0 (0-0)	0 (0-0)	17.4 (-8.6-49.7)	0 (0-0)	0 (0-0)	2 (-22.5-5.5)	0 (0-0)	0 (0-0)	-63 (-72.2--53.3)	0 (0-0)	0 (0-0)	-1.6 (-24.7-3.2)	0 (0-0)	0 (0-0)	0 (0-0)	0 (0-0)	134	134	114	111
Mongolia	0.1 (0-0.2)	0.1 (0-0.1)	-2.7 (-23.8-3.4)	0 (0-0)	0 (0-0)	-8.9 (-29.7-1.9)	0 (0-0)	0 (0-0)	-56.7 (-80.4-12.1)	0 (0-0)	0 (0-0)	-2.1 (-26.2-2.4)	0 (0-0)	0 (0-0)	-65 (-88.4-6)	0 (0-0)	0 (0-0)	-3.3 (-29.7-2.4)	0 (0-0)	0 (0-0)	0 (0-0)	0 (0-0)	126	128	124	110
Tajikistan	0.1 (0-0.2)	0.1 (0-0.2)	-2.2 (-21.3-1.8)	0 (0-0)	0 (0-0)	-13.2 (-24.3--8.3)	0 (0-0)	0 (0-0)	-31.3 (-66.1-48.5)	0 (0-0)	0 (0-0)	-14.5 (-34.1--11.2)	0 (0-0)	0 (0-0)	-16.4 (-62.6-98)	0 (0-0)	0 (0-0)	-2.1 (-24.5-2.2)	0 (0-0)	0 (0-0)	0 (0-0)	0 (0-0)	127	129	116	114
Turkmenistan	0.1 (0-0.2)	0.1 (0-0.1)	-2.4 (-25.1-1.4)	0 (0-0)	0 (0-0)	-14.4 (-32.3--7.1)	0 (0-0)	0 (0-0)	116 (27.4-282)	0 (0-0)	0 (0-0)	-12.3 (-34.8--9.5)	0 (0-0)	0 (0-0)	57.6 (-12.5-212)	0 (0-0)	0 (0-0)	-2.3 (-26.9-2.4)	0 (0-0)	0 (0-0)	0 (0-0)	0 (0-0)	135	129	121	113
Uzbekistan	0.1 (0-0.2)	0.1 (0-0.1)	-3 (-24-2)	0 (0-0)	0 (0-0)	-14.2 (-28.9--6.1)	0 (0-0)	0 (0-0)	289 (178-457)	0 (0-0)	0 (0-0)	-12.3 (-33.9--8.4)	0 (0-0)	0 (0-0)	225 (122-350)	0 (0-0)	0 (0-0)	-2.2 (-27.1-3.8)	0 (0-0)	0 (0-0)	0 (0-0)	0 (0-0)	132	125	113	111
**Central Europe**	3.4 (2.4-4.9)	2.6 (1.9-3.5)	-24.8 (-32--16.7)	0.3 (0.2-0.4)	0.2 (0.2-0.2)	-33.8 (-38.9--28.7)	0 (0-0)	0 (0-0)	-35.3 (-41.6--28.2)	0 (0-0.1)	0 (0-0)	-36.7 (-44--29.3)	0 (0-0)	0 (0-0)	-75.5 (-84.6--62.2)	0.4 (0.3-0.7)	0.3 (0.2-0.5)	-26.9 (-36.8--15.3)	0 (0-0)	0.2 (0.1-0.3)	0 (0-0)	0.3 (0.2-0.5)	122	126	45	32
Albania	0.7 (0.6-0.9)	0.7 (0.6-0.9)	-0.8 (-10-2.4)	0.1 (0.1-0.1)	0.1 (0.1-0.1)	-31.1 (-42.7--26.4)	0 (0-0)	0 (0-0)	-86.3 (-93.1--69.3)	0 (0-0)	0 (0-0)	-34.5 (-40.3--31.6)	0 (0-0.1)	0 (0-0)	-85.3 (-95.4--56.1)	0.1 (0.1-0.1)	0.1 (0.1-0.1)	0.2 (-8.7-3.9)	0 (0-0)	0 (0-0)	0 (0-0)	0 (0-0.1)	102	110	88	71
Bosnia and Herzegovina	0.2 (0.1-0.3)	0.2 (0.1-0.3)	-0.6 (-8.5-9.8)	0 (0-0.1)	0 (0-0.1)	-21.2 (-29.2--13.3)	0 (0-0)	0 (0-0)	-45.3 (-73.2-9.5)	0 (0-0)	0 (0-0)	-17.9 (-30.7--11.2)	0 (0-0)	0 (0-0)	-72.9 (-93.8--13.9)	0 (0-0)	0 (0-0)	-1.3 (-14.5-5.9)	0 (0-0)	0 (0-0)	0 (0-0)	0 (0-0)	123	127	97	89
Bulgaria	0.2 (0.1-0.3)	0.2 (0.1-0.3)	-1.3 (-15-1.3)	0 (0-0.1)	0 (0-0)	-12.8 (-25.5--6.1)	0 (0-0)	0 (0-0)	-9.4 (-25.9-15.7)	0 (0-0)	0 (0-0)	-9.4 (-29.8--3.3)	0 (0-0)	0 (0-0)	-55.6 (-69.9--32.5)	0 (0-0)	0 (0-0)	-3 (-23.4-6)	0 (0-0)	0 (0-0)	0 (0-0)	0 (0-0)	109	108	101	80
Croatia	1.4 (1.1-1.9)	1.4 (1.1-1.7)	-6.8 (-16.8-5.2)	0.4 (0.3-0.5)	0.3 (0.3-0.4)	-18.2 (-26.5--8.6)	0 (0-0)	0 (0-0)	1.8 (-18.7-24.8)	0 (0-0)	0 (0-0)	-18.4 (-26.2--10.3)	0 (0-0)	0 (0-0)	-16.5 (-45.5-18.3)	0.1 (0.1-0.1)	0.1 (0-0.1)	-11.2 (-22.8--0.4)	0 (0-0)	0.1 (0-0.1)	0 (0-0)	0.1 (0-0.1)	118	113	56	50
Czechia	0.1 (0-0.1)	0.1 (0-0.1)	-0.5 (-12.3-4.5)	0 (0-0)	0 (0-0)	-9.4 (-16.1-0)	0 (0-0)	0 (0-0)	-40 (-52.2--23.2)	0 (0-0)	0 (0-0)	-1.9 (-18-8.6)	0 (0-0)	0 (0-0)	-51.3 (-65.4--34.7)	0 (0-0)	0 (0-0)	-0.4 (-15.7-9.9)	0 (0-0)	0 (0-0)	0 (0-0)	0 (0-0)	122	119	105	83
Hungary	0.2 (0.1-0.3)	0.2 (0.1-0.3)	-2.2 (-20.7-2.5)	0 (0-0.1)	0 (0-0)	-16.5 (-39.1--11)	0 (0-0)	0 (0-0)	-21.6 (-45--1.4)	0 (0-0)	0 (0-0)	-15.2 (-34.6--11)	0 (0-0)	0 (0-0)	-40.7 (-63.8--13.8)	0 (0-0)	0 (0-0)	-3.1 (-21.4-3.9)	0 (0-0)	0 (0-0)	0 (0-0)	0 (0-0)	124	115	101	85
Montenegro	0.2 (0.1-0.3)	0.2 (0.1-0.3)	-0.9 (-10.6-8.4)	0.1 (0-0.1)	0 (0-0.1)	-16.2 (-26.3--11.8)	0 (0-0)	0 (0-0)	-53.7 (-74.3--13.4)	0 (0-0)	0 (0-0)	-15.1 (-32.5--5.5)	0 (0-0)	0 (0-0)	-67.9 (-93.7-14.3)	0 (0-0)	0 (0-0)	-1.5 (-20.7-18.4)	0 (0-0)	0 (0-0)	0 (0-0)	0 (0-0)	119	117	95	71
North Macedonia	0.2 (0.2-0.3)	0.2 (0.2-0.3)	-1.3 (-16.9-13.3)	0.1 (0-0.1)	0 (0-0.1)	-18.2 (-34.2--3.4)	0 (0-0)	0 (0-0)	-58.6 (-76--25.5)	0 (0-0)	0 (0-0)	-16.4 (-35.5--9.6)	0 (0-0)	0 (0-0)	-91.7 (-98.2--75.4)	0 (0-0)	0 (0-0)	-1.9 (-23.8-8.9)	0 (0-0)	0 (0-0)	0 (0-0)	0 (0-0)	121	123	99	64
Poland	10.4 (7-15.2)	6.8 (4.7-9.5)	-34.7 (-41.4--27)	0.8 (0.6-1)	0.4 (0.3-0.6)	-41.1 (-46.5--35.8)	0 (0-0)	0 (0-0)	-52.3 (-56.5--47.2)	0.1 (0.1-0.2)	0.1 (0-0.1)	-42.5 (-49.7--35.2)	0 (0-0)	0 (0-0)	-61.9 (-70.1--53.7)	1.3 (0.8-2)	0.8 (0.5-1.3)	-33.9 (-43.4--22.8)	0 (0-0)	0.7 (0.5-1.1)	0 (0-0)	1 (0.6-1.6)	117	115	19	13
Romania	0.6 (0.4-0.7)	0.6 (0.4-0.8)	2.2 (-5.2-25.3)	0.1 (0.1-0.2)	0.1 (0.1-0.1)	-16.3 (-25.3-4.4)	0 (0-0)	0 (0-0)	-16.4 (-35-5.1)	0 (0-0)	0 (0-0)	-11.4 (-17.2-8.5)	0 (0-0)	0 (0-0)	-49.1 (-65.6--29.9)	0 (0-0.1)	0 (0-0.1)	1.4 (-9.8-29)	0 (0-0)	0 (0-0)	0 (0-0)	0 (0-0.1)	123	124	92	70
Serbia	0.1 (0-0.1)	0.1 (0-0.1)	0.6 (-4.1-28.9)	0 (0-0)	0 (0-0)	-16.6 (-26.3-27.4)	0 (0-0)	0 (0-0)	-65.5 (-81.8--42.2)	0 (0-0)	0 (0-0)	-21.1 (-25.3--8)	0 (0-0)	0 (0-0)	-81.1 (-93.9--43.2)	0 (0-0)	0 (0-0)	0.5 (-3.6-10.1)	0 (0-0)	0 (0-0)	0 (0-0)	0 (0-0)	130	131	115	92
Slovakia	0.5 (0.4-0.6)	0.4 (0.3-0.5)	-5 (-24.4-16.5)	0.1 (0.1-0.2)	0.1 (0.1-0.1)	-20.3 (-36.7--3.7)	0 (0-0)	0 (0-0)	-59.1 (-77.5--22.9)	0 (0-0)	0 (0-0)	-17.1 (-33.7-4.7)	0 (0-0)	0 (0-0)	-73.4 (-92.3--15)	0 (0-0)	0 (0-0)	-4.9 (-24.6-18.7)	0 (0-0)	0 (0-0)	0 (0-0)	0 (0-0)	120	124	82	70
Slovenia	0.3 (0.2-0.4)	0.3 (0.2-0.4)	-2.9 (-26-3.6)	0.1 (0.1-0.1)	0.1 (0.1-0.1)	-16.8 (-35.6--8.9)	0 (0-0)	0 (0-0)	-61.6 (-71.3--50.3)	0 (0-0)	0 (0-0)	-12.1 (-37.7--5.7)	0 (0-0)	0 (0-0)	-66.2 (-77.1--52.4)	0 (0-0)	0 (0-0)	-4.4 (-29.5-6.4)	0 (0-0)	0 (0-0)	0 (0-0)	0 (0-0)	125	125	82	63
**Eastern Europe**	0.1 (0.1-0.2)	0.1 (0.1-0.2)	-1 (-5.4-0.7)	0 (0-0)	0 (0-0)	-7 (-13.4--2.2)	0 (0-0)	0 (0-0)	-2.5 (-11.6-7.5)	0 (0-0)	0 (0-0)	4.9 (-4.8-12.1)	0 (0-0)	0 (0-0)	-30.2 (-52.8--0.7)	0 (0-0)	0 (0-0)	-1.4 (-5.4-1)	0 (0-0)	0 (0-0)	0 (0-0)	0 (0-0)	132	130	115	91
Belarus	0.1 (0.1-0.2)	0.1 (0.1-0.2)	-1.6 (-21.7-1.8)	0 (0-0)	0 (0-0)	-10.2 (-40.7--2.2)	0 (0-0)	0 (0-0)	87.6 (21.6-211)	0 (0-0)	0 (0-0)	3.5 (-16-10.1)	0 (0-0)	0 (0-0)	-29.4 (-69.6-75.7)	0 (0-0)	0 (0-0)	-2.1 (-12.6-2.5)	0 (0-0)	0 (0-0)	0 (0-0)	0 (0-0)	134	135	106	84
Estonia	0.1 (0.1-0.2)	0.1 (0.1-0.2)	-1.1 (-26.6-2.8)	0 (0-0)	0 (0-0)	-11.3 (-45.5--3.4)	0 (0-0)	0 (0-0)	-14 (-35.4-14.4)	0 (0-0)	0 (0-0)	-3.5 (-23-1.6)	0 (0-0)	0 (0-0)	-52.2 (-68.8--30.3)	0 (0-0)	0 (0-0)	-1 (-14.4-3.6)	0 (0-0)	0 (0-0)	0 (0-0)	0 (0-0)	135	132	104	82

Location	Birth Incidence rate (per 100,000 live births), 2000	Birth Incidence rate (per 100,000 live births), 2021	Birth Incidence rate (per 100,000 live births) percent change (2000-2021)	All age rate (per 100,000 population), 2000	All age rate (per 100,000 population), 2021	All age rate (per 100,000 population) percent change (2000-2021)	All age cause-specific SCD mortality rate (per 100,000 population), 2000	All age cause-specific SCD mortality rate (per 100,000 population), 2021	All age cause-specific SCD mortality rate (per 100,000 population) percent change (2000-2021)	All age total SCD mortality rate (per 100,000 population), 2000	All age total SCD mortality rate (per 100,000 population), 2021	All age total SCD mortality rate (per 100,000 population) percent change (2000-2021)	Under-5 cause-specific SCD mortality rate (per 100,000 population), 2000	Under-5 cause-specific SCD mortality rate (per 100,000 population), 2021	Under-5 cause-specific SCD mortality rate (per 100,000 population) percent change (2000-2021)	Under-5 total SCD mortality rate (per 100,000 population), 2000	Under-5 total SCD mortality rate (per 100,000 population), 2021	Under-5 total SCD mortality rate (per 100,000 population) percent change (2000-2021)	Under-5 cause-specific mortality cause fraction, 2000	Under-5 total SCD mortality cause fraction, 2000	Under-5 cause-specific mortality cause fraction, 2021	Under-5 total SCD mortality cause fraction, 2021	Under-5 mortality rank 2000	Under-5 mortality rank 2021	Under-5 total SCD mortality rank 2000	Under-5 total SCD mortality rank 2021
Latvia	0.2 (0.1-0.2)	0.1 (0.1-0.2)	-2.8 (-40-7.8)	0 (0-0.1)	0 (0-0)	-14.5 (-49.5--6.5)	0 (0-0)	0 (0-0)	-31.8 (-55.8-3.3)	0 (0-0)	0 (0-0)	-2.7 (-33.5-6)	0 (0-0)	0 (0-0)	-79 (-85.6--71.2)	0 (0-0)	0 (0-0)	-2.6 (-23.9-3.8)	0 (0-0)	0 (0-0)	0 (0-0)	0 (0-0)	134	135	109	81
Lithuania	0.2 (0.2-0.3)	0.2 (0.2-0.3)	2 (-6-42.5)	0.1 (0-0.1)	0 (0-0.1)	-15.7 (-22.2-24.9)	0 (0-0)	0 (0-0)	-1 (-27.9-34.9)	0 (0-0)	0 (0-0)	-8.1 (-13-15.8)	0 (0-0)	0 (0-0)	-61.1 (-74.1--47.7)	0 (0-0)	0 (0-0)	0 (-7.5-18.6)	0 (0-0)	0 (0-0)	0 (0-0)	0 (0-0)	133	129	101	75
Republic of Moldova	0.1 (0.1-0.2)	0.1 (0.1-0.2)	-1.2 (-18.2-2.2)	0 (0-0)	0 (0-0)	-22.5 (-40.6--12.7)	0 (0-0)	0 (0-0)	-16.3 (-29.5-3.9)	0 (0-0)	0 (0-0)	-19 (-31.1--13.5)	0 (0-0)	0 (0-0)	-44.9 (-64.8--15.9)	0 (0-0)	0 (0-0)	-1.7 (-13.1-3.2)	0 (0-0)	0 (0-0)	0 (0-0)	0 (0-0)	134	135	110	101
Russian Federation	0.1 (0.1-0.2)	0.1 (0.1-0.2)	-1 (-6-0.8)	0 (0-0)	0 (0-0)	-4.1 (-10.4-1.8)	0 (0-0)	0 (0-0)	-7.8 (-15.7-0.9)	0 (0-0)	0 (0-0)	9.1 (-2.8-18.2)	0 (0-0)	0 (0-0)	11 (-8.2-34)	0 (0-0)	0 (0-0)	-1.7 (-6.2--0.2)	0 (0-0)	0 (0-0)	0 (0-0)	0 (0-0)	133	133	117	90
Ukraine	0.1 (0.1-0.2)	0.1 (0.1-0.2)	-0.7 (-8-2.1)	0 (0-0)	0 (0-0)	-10.5 (-21.5--6.6)	0 (0-0)	0 (0-0)	16.9 (-18-59.4)	0 (0-0)	0 (0-0)	-4.3 (-16.8--0.6)	0 (0-0)	0 (0-0)	-23.7 (-55.2-25.3)	0 (0-0)	0 (0-0)	-1.6 (-9.7-2.3)	0 (0-0)	0 (0-0)	0 (0-0)	0 (0-0)	122	119	98	89
High-income	24.1 (21.5-26.9)	23 (20.4-25.7)	-4.7 (-6.1--2.9)	8.2 (7.6-8.9)	7.7 (7.1-8.3)	-6.7 (-8.1--5.4)	0.1 (0.1-0.1)	0.1 (0.1-0.1)	-15 (-18.1--12.3)	0.3 (0.3-0.3)	0.2 (0.2-0.3)	-11.4 (-13--9.7)	0.1 (0.1-0.1)	0 (0-0)	-37.7 (-44.6--32.1)	1.2 (1-1.5)	1 (0.8-1.3)	-15.7 (-19.3--11.9)	0 (0-0)	0.8 (0.7-1)	0 (0-0)	1.2 (0.9-1.5)	77	76	21	17
**Australasia**	2 (1.6-2.5)	2.1 (1.7-2.6)	2.8 (-3.9-11.7)	0.5 (0.4-0.7)	0.5 (0.4-0.6)	-9.7 (-14.4--3.9)	0 (0-0)	0 (0-0)	-74.5 (-78.5--68.6)	0 (0-0)	0 (0-0)	-7.3 (-13.5-0.6)	0 (0-0)	0 (0-0)	-87.9 (-91.3--83.9)	0.2 (0.1-0.2)	0.2 (0.1-0.2)	2.9 (-6.4-15.2)	0 (0-0)	0.1 (0.1-0.2)	0 (0-0)	0.3 (0.2-0.4)	102	113	44	39
Australia	1.7 (1.3-2.1)	1.7 (1.3-2.1)	-0.6 (-9.7-9.1)	0.4 (0.3-0.6)	0.4 (0.3-0.5)	-9.6 (-16.5--1.3)	0 (0-0)	0 (0-0)	-77.2 (-81.2--71.5)	0 (0-0)	0 (0-0)	-8.7 (-16.3-1.1)	0 (0-0)	0 (0-0)	-92.2 (-94.8--89.3)	0.2 (0.1-0.2)	0.2 (0.1-0.2)	-0.4 (-11.6-14)	0 (0-0)	0.1 (0.1-0.2)	0 (0-0)	0.3 (0.2-0.4)	102	114	46	37
New Zealand	3.5 (2.8-4.3)	4.1 (3.3-5.1)	15.1 (5.8-26.7)	1.1 (0.9-1.3)	1 (0.8-1.2)	-8.9 (-15.9--1.4)	0 (0-0)	0 (0-0)	7.8 (-10.6-33.3)	0.1 (0-0.1)	0 (0-0.1)	-3.2 (-14.3-8.3)	0 (0-0)	0 (0-0)	11.8 (-27.2-56.5)	0.3 (0.2-0.4)	0.3 (0.2-0.4)	18 (-0.9-36.7)	0 (0-0)	0.2 (0.1-0.2)	0 (0-0)	0.3 (0.2-0.4)	102	99	40	35
**High-income Asia Pacific**	0.3 (0.2-0.4)	0.3 (0.2-0.4)	1.4 (-4.8-6)	0.1 (0-0.1)	0 (0-0.1)	-19.1 (-23.4--15.4)	0 (0-0)	0 (0-0)	-68.8 (-72.8--64.3)	0 (0-0)	0 (0-0)	-19.5 (-25.5--13.5)	0 (0-0)	0 (0-0)	-81.9 (-90.4--61)	0 (0-0)	0 (0-0)	-1.7 (-10.3-6.1)	0 (0-0)	0 (0-0)	0 (0-0)	0.1 (0.1-0.1)	109	110	87	66
Brunei Darussalam	0.5 (0.4-0.7)	0.5 (0.4-0.7)	-0.1 (-9.6-10.3)	0.1 (0.1-0.2)	0.1 (0.1-0.1)	-18.1 (-25.3--6.2)	0 (0-0)	0 (0-0)	9.2 (-39-82.6)	0 (0-0)	0 (0-0)	-27.3 (-36.2--19.4)	0 (0-0)	0 (0-0)	9.7 (-71.3-217)	0.1 (0-0.1)	0.1 (0-0.1)	0.1 (-15.1-11.2)	0 (0-0)	0 (0-0)	0 (0-0)	0 (0-0.1)	101	94	84	77
Japan	0.2 (0.1-0.3)	0.2 (0.2-0.3)	4.5 (-2-12.8)	0 (0-0.1)	0 (0-0.1)	-14.4 (-19.3--7.9)	0 (0-0)	0 (0-0)	-68.8 (-70.3--66.9)	0 (0-0)	0 (0-0)	-12.9 (-18.9--6)	0 (0-0)	0 (0-0)	-73.6 (-76.8--70.2)	0 (0-0)	0 (0-0)	3.3 (-4.4-12.1)	0 (0-0)	0 (0-0)	0 (0-0)	0.1 (0-0.1)	107	109	82	72
Aichi	1 (0.8-1.4)	1 (0.8-1.4)	-0.2 (-12.5-13.8)	0.2 (0.2-0.3)	0.2 (0.2-0.3)	-16.1 (-26.5--4.9)	0 (0-0)	0 (0-0)	-71 (-75.3--66.5)	0 (0-0)	0 (0-0)	-15 (-25.7--3.4)	0 (0-0)	0 (0-0)	-72.5 (-80.2--64.8)	0.1 (0.1-0.1)	0.1 (0.1-0.1)	-1.3 (-16.9-15.2)	0 (0-0)	0.1 (0.1-0.2)	0 (0-0)	0.2 (0.2-0.3)	106	108	58	44
Akita	0.1 (0-0.3)	0.1 (0-0.3)	-0.8 (-2.4-0.9)	0 (0-0)	0 (0-0)	-27.4 (-33.5--21)	0 (0-0)	0 (0-0)	-60 (-66.4--49.5)	0 (0-0)	0 (0-0)	-27.2 (-34.9--24.3)	0 (0-0)	0 (0-0)	-70.4 (-78.8--59.4)	0 (0-0)	0 (0-0)	-1.4 (-9.3-4.3)	0 (0-0)	0 (0-0)	0 (0-0)	0 (0-0.1)	107	112	92	78
Aomori	0.1 (0-0.3)	0.1 (0-0.3)	0.4 (-0.1-1.5)	0 (0-0.1)	0 (0-0)	-26.3 (-31.8--21.3)	0 (0-0)	0 (0-0)	-64.7 (-70.5--56.2)	0 (0-0)	0 (0-0)	-25.5 (-28.7--23.7)	0 (0-0)	0 (0-0)	-70.6 (-78.4--60.2)	0 (0-0)	0 (0-0)	-0.3 (-5.4-5.4)	0 (0-0)	0 (0-0)	0 (0-0)	0 (0-0.1)	105	106	92	78
Chiba	0.4 (0.2-0.6)	0.4 (0.2-0.6)	0.4 (-6.6-17.8)	0.1 (0.1-0.1)	0.1 (0-0.1)	-21.5 (-28.7--9.7)	0 (0-0)	0 (0-0)	-70.1 (-73.7--65.4)	0 (0-0)	0 (0-0)	-18.9 (-22.5--0.1)	0 (0-0)	0 (0-0)	-70.2 (-75.7--63.7)	0 (0-0.1)	0 (0-0.1)	0.6 (-9.6-22.8)	0 (0-0)	0 (0-0.1)	0 (0-0)	0.1 (0-0.1)	106	107	74	64
Ehime	0.1 (0-0.3)	0.1 (0-0.3)	-0.6 (-12.8-2.2)	0 (0-0.1)	0 (0-0)	-19.9 (-33.2--14.9)	0 (0-0)	0 (0-0)	-67.4 (-71.4--62)	0 (0-0)	0 (0-0)	-19.9 (-31--17.2)	0 (0-0)	0 (0-0)	-73.7 (-80.6--65.2)	0 (0-0)	0 (0-0)	-0.7 (-8.4-4.3)	0 (0-0)	0 (0-0)	0 (0-0)	0 (0-0.1)	106	108	94	81
Fukui	0.1 (0-0.3)	0.1 (0-0.3)	-0.3 (-22.3-7.5)	0 (0-0.1)	0 (0-0)	-19 (-40.3--10.1)	0 (0-0)	0 (0-0)	-65.2 (-71.1--58.3)	0 (0-0)	0 (0-0)	-20.2 (-24.1--17.7)	0 (0-0)	0 (0-0)	-69.2 (-78.8--57.8)	0 (0-0)	0 (0-0)	-0.3 (-7.7-5.5)	0 (0-0)	0 (0-0)	0 (0-0)	0 (0-0.1)	105	109	87	84
Fukuoka	0.1 (0-0.2)	0.1 (0-0.2)	1.3 (-1.5-6.8)	0 (0-0)	0 (0-0)	-15 (-21.1--6.8)	0 (0-0)	0 (0-0)	-70.6 (-75.2--65.4)	0 (0-0)	0 (0-0)	-12.2 (-16.4--11.2)	0 (0-0)	0 (0-0)	-70.5 (-77.9--60.7)	0 (0-0)	0 (0-0)	0.4 (-6.5-5.2)	0 (0-0)	0 (0-0)	0 (0-0)	0 (0-0)	108	109	93	84
Fukushima	0.1 (0-0.3)	0.1 (0-0.2)	-0.3 (-3.6-5.4)	0 (0-0.1)	0 (0-0)	-25.4 (-31.3--18.5)	0 (0-0)	0 (0-0)	-63.2 (-69.1--54.5)	0 (0-0)	0 (0-0)	-24.5 (-29.1--21.2)	0 (0-0)	0 (0-0)	-69 (-77.6--59.3)	0 (0-0)	0 (0-0)	-0.2 (-5.4-5.5)	0 (0-0)	0 (0-0)	0 (0-0)	0 (0-0.1)	108	110	92	84
Gifu	0.1 (0-0.2)	0.1 (0-0.2)	-1.1 (-9-5.8)	0 (0-0.1)	0 (0-0)	-19.5 (-28.1--11)	0 (0-0)	0 (0-0)	-68.9 (-74.7--63.4)	0 (0-0)	0 (0-0)	-21 (-27.4--17.4)	0 (0-0)	0 (0-0)	-73.6 (-79.6--64.5)	0 (0-0)	0 (0-0)	-2.2 (-8.2-4.5)	0 (0-0)	0 (0-0)	0 (0-0)	0 (0-0.1)	106	107	90	80
Gunma	0.1 (0-0.2)	0.1 (0-0.2)	-0.9 (-0.8-6.2)	0 (0-0.1)	0 (0-0)	-20.8 (-26.8--13.1)	0 (0-0)	0 (0-0)	-66.5 (-71.7--60.5)	0 (0-0)	0 (0-0)	-22.5 (-27.3--18.8)	0 (0-0)	0 (0-0)	-75.2 (-82.5--67.9)	0 (0-0)	0 (0-0)	-1.5 (-7.2-4.7)	0 (0-0)	0 (0-0)	0 (0-0)	0 (0-0.1)	108	109	94	81
Hiroshima	0.1 (0-0.2)	0.1 (0-0.2)	0.4 (-1.1-3.2)	0 (0-0)	0 (0-0)	-15.7 (-20.9--10.1)	0 (0-0)	0 (0-0)	-70 (-74.4--64.1)	0 (0-0)	0 (0-0)	-16 (-19.6--13.7)	0 (0-0)	0 (0-0)	-70.8 (-79.8--59.8)	0 (0-0)	0 (0-0)	-0.9 (-7-4.7)	0 (0-0)	0 (0-0)	0 (0-0)	0 (0-0.1)	105	105	88	79
Hokkaidō	0.1 (0-0.2)	0.1 (0-0.2)	-3.2 (-10.2-4.7)	0 (0-0)	0 (0-0)	-25.8 (-31.6--17.7)	0 (0-0)	0 (0-0)	-66.6 (-73.6--58.6)	0 (0-0)	0 (0-0)	-24.3 (-57.2--15.3)	0 (0-0)	0 (0-0)	-66.8 (-76.1--56.6)	0 (0-0)	0 (0-0)	-3.8 (-41.1-7.4)	0 (0-0)	0 (0-0)	0 (0-0)	0 (0-0.1)	105	109	92	87
Hyōgo	0.2 (0.1-0.3)	0.2 (0.1-0.3)	-1.5 (-17.5-1.6)	0 (0-0.1)	0 (0-0.1)	-20.1 (-34.9--7.8)	0 (0-0)	0 (0-0)	-68.8 (-73.8--62.4)	0 (0-0)	0 (0-0)	-19.3 (-42.3--15.4)	0 (0-0)	0 (0-0)	-73.9 (-81.1--65.2)	0 (0-0)	0 (0-0)	-2.5 (-32-5.6)	0 (0-0)	0 (0-0)	0 (0-0)	0.1 (0-0.1)	106	107	82	73
Ibaraki	0.4 (0.2-0.6)	0.4 (0.2-0.6)	-2.2 (-29.3-1.6)	0.1 (0.1-0.1)	0.1 (0-0.1)	-22.4 (-37.7--12)	0 (0-0)	0 (0-0)	-66 (-71.2--59.1)	0 (0-0)	0 (0-0)	-22.5 (-45.7--17.3)	0 (0-0)	0 (0-0)	-68.9 (-77.1--57.1)	0 (0-0.1)	0 (0-0.1)	-3.2 (-36.7-8.3)	0 (0-0)	0 (0-0.1)	0 (0-0)	0.1 (0-0.1)	106	107	72	64
Ishikawa	0.1 (0-0.2)	0.1 (0-0.2)	-0.5 (-3-3.3)	0 (0-0)	0 (0-0)	-18.4 (-24.7--10.4)	0 (0-0)	0 (0-0)	-70.1 (-74.7--65.3)	0 (0-0)	0 (0-0)	-18.8 (-21.7--12.5)	0 (0-0)	0 (0-0)	-77.2 (-83.6--64.4)	0 (0-0)	0 (0-0)	-1 (-7.3-7.6)	0 (0-0)	0 (0-0)	0 (0-0)	0 (0-0.1)	104	108	88	79
Iwate	0.1 (0-0.3)	0.1 (0-0.3)	-0.2 (-4-3.5)	0 (0-0)	0 (0-0)	-22.3 (-27.9--17.5)	0 (0-0)	0 (0-0)	-68.6 (-73--61.6)	0 (0-0)	0 (0-0)	-22.2 (-25.4--19.8)	0 (0-0)	0 (0-0)	-71.6 (-79--63.2)	0 (0-0)	0 (0-0)	-1.4 (-7.3-5.2)	0 (0-0)	0 (0-0)	0 (0-0)	0 (0-0.1)	107	108	90	77
Kagawa	0.1 (0-0.3)	0.1 (0-0.2)	-2.3 (-45.7-16.5)	0 (0-0.1)	0 (0-0)	-20.2 (-55.3-0.6)	0 (0-0)	0 (0-0)	-68.4 (-72.7--63.2)	0 (0-0)	0 (0-0)	-21.4 (-25.3--17.1)	0 (0-0)	0 (0-0)	-67.7 (-76.7--57.4)	0 (0-0)	0 (0-0)	-2.5 (-12.2-5)	0 (0-0)	0 (0-0)	0 (0-0)	0 (0-0.1)	105	105	90	80

Location	Birth Incidence rate (per 100,000 live births), 2000	Birth Incidence rate (per 100,000 live births), 2021	Birth Incidence rate (per 100,000 live births) percent change (2000-2021)	All age rate (per 100,000 population), 2000	All age rate (per 100,000 population), 2021	All age rate (per 100,000 population) percent change (2000-2021)	All age cause-specific SCD mortality rate (per 100,000 population), 2000	All age cause-specific SCD mortality rate (per 100,000 population), 2021	All age cause-specific SCD mortality rate (per 100,000 population) percent change (2000-2021)	All age total SCD mortality rate (per 100,000 population), 2000	All age total SCD mortality rate (per 100,000 population), 2021	All age total SCD mortality rate (per 100,000 population) percent change (2000-2021)	Under-5 cause-specific SCD mortality rate (per 100,000 population), 2000	Under-5 cause-specific SCD mortality rate (per 100,000 population), 2021	Under-5 cause-specific SCD mortality rate (per 100,000 population) percent change (2000-2021)	Under-5 total SCD mortality rate (per 100,000 population), 2000	Under-5 total SCD mortality rate (per 100,000 population), 2021	Under-5 total SCD mortality rate (per 100,000 population) percent change (2000-2021)	Under-5 cause-specific mortality cause fraction, 2000	Under-5 total SCD mortality cause fraction, 2000	Under-5 cause-specific mortality cause fraction, 2021	Under-5 total SCD mortality cause fraction, 2021	Under-5 cause-specific mortality rank 2000	Under-5 cause-specific mortality rank 2021	Under-5 total SCD mortality rank 2000	Under-5 total SCD mortality rank 2021
Kagoshima	0.1 (0-0.3)	0.1 (0-0.3)	-0.9 (-3.6-2)	0 (0-0)	0 (0-0)	-19.3 (-24.2--13.1)	0 (0-0)	0 (0-0)	-63.4 (-68.3--59.1)	0 (0-0)	0 (0-0)	-16 (-17.4--13.7)	0 (0-0)	0 (0-0)	-74.5 (-80.4--66.5)	0 (0-0)	0 (0-0)	-1.1 (-10.1-5.4)	0 (0-0)	0 (0-0)	0 (0-0)	0 (0-0.1)	109	109	95	83
Kanagawa	0.1 (0-0.3)	0.1 (0-0.3)	-1.2 (-13.4-4.9)	0 (0-0.1)	0 (0-0.1)	-20.9 (-28.4--13.1)	0 (0-0)	0 (0-0)	-69.8 (-74.4--64)	0 (0-0)	0 (0-0)	-18.6 (-36--14.4)	0 (0-0)	0 (0-0)	-77.4 (-81.8--71.2)	0 (0-0)	0 (0-0)	-2.4 (-27.5-8.4)	0 (0-0)	0 (0-0)	0 (0-0)	0 (0-0.1)	108	112	87	74
Kōchi	0.1 (0-0.3)	0.1 (0-0.3)	0.7 (-7.2-43.5)	0 (0-0)	0 (0-0)	-20 (-28.5-19.6)	0 (0-0)	0 (0-0)	-65 (-69.9--59)	0 (0-0)	0 (0-0)	-19.4 (-22.8--16.7)	0 (0-0)	0 (0-0)	-68.2 (-75.8--54.1)	0 (0-0)	0 (0-0)	0.8 (-4.4-7.7)	0 (0-0)	0 (0-0)	0 (0-0)	0 (0-0.1)	106	109	88	81
Kumamoto	0.1 (0-0.2)	0.1 (0-0.2)	-3.3 (-18.6-2.2)	0 (0-0.1)	0 (0-0)	-19.2 (-33.9--10.8)	0 (0-0)	0 (0-0)	-67.3 (-72.8--61.4)	0 (0-0)	0 (0-0)	-16.2 (-46.9--11.9)	0 (0-0)	0 (0-0)	-71.2 (-79.3--59.2)	0 (0-0)	0 (0-0)	-3.5 (-39.9-4.8)	0 (0-0)	0 (0-0)	0 (0-0)	0 (0-0.1)	104	108	95	84
Kyōto	0.1 (0-0.2)	0.1 (0-0.2)	0.3 (-3.1-11.9)	0 (0-0)	0 (0-0)	-17.8 (-25.2--2.7)	0 (0-0)	0 (0-0)	-69.8 (-74.6--62.2)	0 (0-0)	0 (0-0)	-17 (-31--2.4)	0 (0-0)	0 (0-0)	-74.9 (-82.6--66.6)	0 (0-0)	0 (0-0)	-0.1 (-18.4-15.6)	0 (0-0)	0 (0-0)	0 (0-0)	0 (0-0.1)	105	110	92	79
Mie	0.1 (0-0.2)	0.1 (0-0.2)	0.1 (-9-18.4)	0 (0-0.1)	0 (0-0)	-19.4 (-28.1--5.1)	0 (0-0)	0 (0-0)	-69.2 (-75.8--62.6)	0 (0-0)	0 (0-0)	-20.1 (-24--16.2)	0 (0-0)	0 (0-0)	-73.7 (-81.7--65.4)	0 (0-0)	0 (0-0)	-1 (-6.6-10.1)	0 (0-0)	0 (0-0)	0 (0-0)	0 (0-0.1)	108	109	94	80
Miyagi	0.1 (0-0.2)	0.1 (0-0.2)	-1.2 (-26.3-12.5)	0 (0-0.1)	0 (0-0)	-22 (-47.4--12.4)	0 (0-0)	0 (0-0)	-67.8 (-73.4--61.3)	0 (0-0)	0 (0-0)	-20.1 (-23--16.9)	0 (0-0)	0 (0-0)	-70.5 (-78.3--62)	0 (0-0)	0 (0-0)	-2 (-6.7-2.8)	0 (0-0)	0 (0-0)	0 (0-0)	0 (0-0.1)	107	106	89	79
Miyazaki	0.1 (0-0.3)	0.1 (0-0.3)	-0.1 (-4.2-7.8)	0 (0-0.1)	0 (0-0)	-19.2 (-26--12)	0 (0-0)	0 (0-0)	-63.7 (-69.1--57.9)	0 (0-0)	0 (0-0)	-18.9 (-20.9--16.8)	0 (0-0)	0 (0-0)	-67.4 (-76.2--54.9)	0 (0-0)	0 (0-0)	-1 (-8-9.1)	0 (0-0)	0 (0-0)	0 (0-0)	0 (0-0.1)	109	112	92	85
Nagano	0.1 (0-0.2)	0.1 (0-0.2)	-0.3 (-11.1-3)	0 (0-0)	0 (0-0)	-19.6 (-30.2--11.4)	0 (0-0)	0 (0-0)	-67.6 (-72.6--62.2)	0 (0-0)	0 (0-0)	-20.9 (-26.4--18)	0 (0-0)	0 (0-0)	-69 (-75.7--57.9)	0 (0-0)	0 (0-0)	-1.3 (-14-5.5)	0 (0-0)	0 (0-0)	0 (0-0)	0 (0-0.1)	105	108	92	78
Nagasaki	0.1 (0-0.3)	0.1 (0-0.2)	-2 (-23-2.9)	0 (0-0)	0 (0-0)	-22.2 (-43.2--11.7)	0 (0-0)	0 (0-0)	-66.7 (-72.9--59.7)	0 (0-0)	0 (0-0)	-20.3 (-37.1--17.2)	0 (0-0)	0 (0-0)	-69.8 (-78.7--59.1)	0 (0-0)	0 (0-0)	-2.1 (-19.6-5)	0 (0-0)	0 (0-0)	0 (0-0)	0 (0-0.1)	110	114	94	83
Nara	0.1 (0-0.2)	0.1 (0-0.2)	0.8 (-0.8-5.2)	0 (0-0)	0 (0-0)	-22.1 (-27.8--16.1)	0 (0-0)	0 (0-0)	-66.1 (-71.8--59.7)	0 (0-0)	0 (0-0)	-21.8 (-25.6--19.8)	0 (0-0)	0 (0-0)	-66.1 (-75.5--52.6)	0 (0-0)	0 (0-0)	-0.3 (-7.1-4.8)	0 (0-0)	0 (0-0)	0 (0-0)	0 (0-0.1)	102	104	90	83
Niigata	0.1 (0-0.2)	0.1 (0-0.2)	1 (-1.8-4.4)	0 (0-0)	0 (0-0)	-19 (-24.5--9.9)	0 (0-0)	0 (0-0)	-66.3 (-72--58.8)	0 (0-0)	0 (0-0)	-19.1 (-23.5--2.1)	0 (0-0)	0 (0-0)	-76.4 (-81.8--70.4)	0 (0-0)	0 (0-0)	0.4 (-7.8-30.7)	0 (0-0)	0 (0-0)	0 (0-0)	0 (0-0.1)	107	110	91	76
Ōita	0.1 (0-0.3)	0.1 (0-0.2)	-1.9 (-28.3-10)	0 (0-0.1)	0 (0-0)	-18.9 (-44.2--6.8)	0 (0-0)	0 (0-0)	-67.6 (-73--62)	0 (0-0)	0 (0-0)	-17.8 (-26.6--14.5)	0 (0-0)	0 (0-0)	-72.9 (-80.5--62.9)	0 (0-0)	0 (0-0)	-1.6 (-14.7-5.5)	0 (0-0)	0 (0-0)	0 (0-0)	0 (0-0.1)	108	109	93	81
Okayama	0.1 (0-0.3)	0.1 (0-0.3)	-0.9 (-3.8-1.1)	0 (0-0.1)	0 (0-0)	-16.7 (-21.2--10.5)	0 (0-0)	0 (0-0)	-68.8 (-74.2--62.2)	0 (0-0)	0 (0-0)	-17.3 (-19.5--14.6)	0 (0-0)	0 (0-0)	-75 (-81--66.3)	0 (0-0)	0 (0-0)	-1.5 (-6.8-5.1)	0 (0-0)	0 (0-0)	0 (0-0)	0 (0-0.1)	107	106	92	78
Okinawa	0.2 (0.1-0.4)	0.2 (0.1-0.4)	-1.7 (-7.2-1.5)	0.1 (0-0.1)	0 (0-0.1)	-19 (-28.6--11.1)	0 (0-0)	0 (0-0)	-68.6 (-73.6--62.1)	0 (0-0)	0 (0-0)	-15.8 (-28.3--12.7)	0 (0-0)	0 (0-0)	-74.7 (-81.4--65.8)	0 (0-0)	0 (0-0)	-2.8 (-20.2-8.4)	0 (0-0)	0 (0-0)	0 (0-0)	0.1 (0-0.1)	110	114	84	75
Ōsaka	0.1 (0-0.3)	0.1 (0-0.3)	-0.6 (-2.4-1.9)	0 (0-0.1)	0 (0-0)	-20 (-26.5--10.3)	0 (0-0)	0 (0-0)	-69.6 (-73.4--64.4)	0 (0-0)	0 (0-0)	-19.7 (-22.7--16.4)	0 (0-0)	0 (0-0)	-72.6 (-79.9--63)	0 (0-0)	0 (0-0)	-1.6 (-6.9-5.6)	0 (0-0)	0 (0-0)	0 (0-0)	0 (0-0.1)	106	107	87	80
Saga	0.1 (0-0.3)	0.1 (0-0.3)	0 (-0.7-1.1)	0 (0-0.1)	0 (0-0)	-18.5 (-24.8--13)	0 (0-0)	0 (0-0)	-66.8 (-72.2--58.3)	0 (0-0)	0 (0-0)	-17.7 (-19.7--16.2)	0 (0-0)	0 (0-0)	-76 (-83.4--67.4)	0 (0-0)	0 (0-0)	-1.4 (-7.1-3.5)	0 (0-0)	0 (0-0)	0 (0-0)	0 (0-0.1)	110	108	95	81
Saitama	0.1 (0-0.2)	0.1 (0-0.2)	-0.6 (-1-4.8)	0 (0-0.1)	0 (0-0)	-21.8 (-27.5--14.8)	0 (0-0)	0 (0-0)	-69.8 (-74.4--65.4)	0 (0-0)	0 (0-0)	-20.7 (-24.3--17.3)	0 (0-0)	0 (0-0)	-78.2 (-83.3--70.5)	0 (0-0)	0 (0-0)	-1.1 (-7.2-6.9)	0 (0-0)	0 (0-0)	0 (0-0)	0 (0-0.1)	105	107	92	79
Shiga	0.1 (0-0.2)	0.1 (0-0.2)	-2.9 (-18.2-3.8)	0 (0-0.1)	0 (0-0)	-19.4 (-32.1--9.3)	0 (0-0)	0 (0-0)	-68.4 (-73.4--62.7)	0 (0-0)	0 (0-0)	-20 (-52.6--15.4)	0 (0-0)	0 (0-0)	-76.1 (-83.3--65.9)	0 (0-0)	0 (0-0)	-3.4 (-40.8-4.5)	0 (0-0)	0 (0-0)	0 (0-0)	0 (0-0.1)	105	107	91	78
Shimane	0.1 (0-0.2)	0.1 (0-0.3)	1.8 (-5.8-34)	0 (0-0)	0 (0-0)	-13.8 (-22.4-18.7)	0 (0-0)	0 (0-0)	-70.1 (-73.6--64.4)	0 (0-0)	0 (0-0)	-12.8 (-17-7.8)	0 (0-0)	0 (0-0)	-75 (-82.4--64.5)	0 (0-0)	0 (0-0)	1.3 (-5.6-37.4)	0 (0-0)	0 (0-0)	0 (0-0)	0 (0-0.1)	104	106	90	78
Shizuoka	0.1 (0-0.2)	0.1 (0-0.2)	-1.5 (-10.9-2.7)	0 (0-0)	0 (0-0)	-21.4 (-30.2--15.5)	0 (0-0)	0 (0-0)	-66.6 (-72.1--61.2)	0 (0-0)	0 (0-0)	-21.1 (-23.7--17.8)	0 (0-0)	0 (0-0)	-68.6 (-75.1--57.9)	0 (0-0)	0 (0-0)	-1.6 (-8.9-4.6)	0 (0-0)	0 (0-0)	0 (0-0)	0 (0-0)	105	108	90	84
Tochigi	0.1 (0-0.3)	0.1 (0-0.3)	-0.2 (-1.2-0.3)	0 (0-0.1)	0 (0-0)	-19.9 (-24.6--14.9)	0 (0-0)	0 (0-0)	-66.5 (-73.1--58.8)	0 (0-0)	0 (0-0)	-19.5 (-22.5--17)	0 (0-0)	0 (0-0)	-74.7 (-81.3--65.8)	0 (0-0)	0 (0-0)	-1.1 (-5.6-4.3)	0 (0-0)	0 (0-0)	0 (0-0)	0 (0-0.1)	107	108	92	80
Tokushima	0.1 (0-0.3)	0.1 (0-0.3)	-2 (-34.2-9.3)	0 (0-0)	0 (0-0)	-21.4 (-46.8--8)	0 (0-0)	0 (0-0)	-63.7 (-70.1--56.6)	0 (0-0)	0 (0-0)	-19.9 (-36.8--16)	0 (0-0)	0 (0-0)	-72.5 (-81--60.5)	0 (0-0)	0 (0-0)	-1.4 (-14.9-6.6)	0 (0-0)	0 (0-0)	0 (0-0)	0 (0-0.1)	107	110	93	83
Tōkyō	0.3 (0.1-0.4)	0.3 (0.1-0.4)	-1.6 (-24.1-2.7)	0.1 (0-0.1)	0.1 (0-0.1)	-13.6 (-32.1--4.7)	0 (0-0)	0 (0-0)	-71.7 (-76.3--65.6)	0 (0-0)	0 (0-0)	-6.1 (-27.7-5.6)	0 (0-0)	0 (0-0)	-80.2 (-85.7--73.2)	0 (0-0)	0 (0-0)	-2.6 (-29.6-8.3)	0 (0-0)	0 (0-0)	0 (0-0)	0.1 (0-0.1)	110	112	76	65
Tottori	0.1 (0-0.3)	0.1 (0-0.3)	0 (-1.7-1.3)	0 (0-0.1)	0 (0-0)	-16.5 (-21.9--11.6)	0 (0-0)	0 (0-0)	-67.4 (-73.9--59.9)	0 (0-0)	0 (0-0)	-15.1 (-17.5--13.6)	0 (0-0)	0 (0-0)	-67.1 (-76--52.6)	0 (0-0)	0 (0-0)	-0.5 (-6.3-5.4)	0 (0-0)	0 (0-0)	0 (0-0)	0 (0-0.1)	107	109	89	81
Toyama	0.1 (0-0.3)	0.1 (0-0.2)	0.2 (-1.3-6.8)	0 (0-0.1)	0 (0-0)	-18.4 (-27.1--10)	0 (0-0)	0 (0-0)	-66.1 (-71.3--60)	0 (0-0)	0 (0-0)	-19.2 (-23.1--16.4)	0 (0-0)	0 (0-0)	-75.2 (-83.6--68.2)	0 (0-0)	0 (0-0)	-0.8 (-6-5)	0 (0-0)	0 (0-0)	0 (0-0)	0 (0-0.1)	105	107	87	79
Wakayama	0.1 (0-0.3)	0.1 (0-0.2)	-0.9 (-4.2-0)	0 (0-0.1)	0 (0-0)	-20.5 (-25.4--14.7)	0 (0-0)	0 (0-0)	-67.9 (-72--62.6)	0 (0-0)	0 (0-0)	-20.5 (-22.9--17.7)	0 (0-0)	0 (0-0)	-68 (-78.3--58)	0 (0-0)	0 (0-0)	-1.4 (-6.3-5.4)	0 (0-0)	0 (0-0)	0 (0-0)	0 (0-0.1)	106	106	91	81
Yamagata	0.1 (0-0.2)	0.1 (0-0.2)	-0.7 (-2.6-5.9)	0 (0-0)	0 (0-0)	-21.6 (-30.5--13.3)	0 (0-0)	0 (0-0)	-61.7 (-67.7--56.5)	0 (0-0)	0 (0-0)	-22.2 (-40.1--4.1)	0 (0-0)	0 (0-0)	-69.1 (-77.1--60.3)	0 (0-0)	0 (0-0)	-1.6 (-30.1-32)	0 (0-0)	0 (0-0)	0 (0-0)	0 (0-0.1)	107	108	90	76
Yamaguchi	0.1 (0-0.2)	0.1 (0-0.2)	-0.9 (-35.5-43.3)	0 (0-0)	0 (0-0)	-18.8 (-51-21.1)	0 (0-0)	0 (0-0)	-67.6 (-74.3--60.4)	0 (0-0)	0 (0-0)	-18.7 (-33.8--15.7)	0 (0-0)	0 (0-0)	-68.6 (-78--57.8)	0 (0-0)	0 (0-0)	-0.9 (-12.8-8.2)	0 (0-0)	0 (0-0)	0 (0-0)	0 (0-0.1)	107	111	94	84

Location	Birth Incidence rate (per 100,000 live births), 2000	Birth Incidence rate (per 100,000 live births), 2021	Birth Incidence rate (per 100,000 live births) percent change (2000-2021)	All age rate (per 100,000 population), 2000	All age rate (per 100,000 population), 2021	All age rate (per 100,000 population) percent change (2000-2021)	All age cause-specific SCD mortality rate (per 100,000 population), 2000	All age cause-specific SCD mortality rate (per 100,000 population), 2021	All age cause-specific SCD mortality rate (per 100,000 population) percent change (2000-2021)	All age total SCD mortality rate (per 100,000 population), 2000	All age total SCD mortality rate (per 100,000 population), 2021	All age total SCD mortality rate (per 100,000 population) percent change (2000-2021)	Under-5 cause-specific SCD mortality rate (per 100,000 population), 2000	Under-5 cause-specific SCD mortality rate (per 100,000 population), 2021	Under-5 cause-specific SCD mortality rate (per 100,000 population) percent change (2000-2021)	Under-5 total SCD mortality rate (per 100,000 population), 2000	Under-5 total SCD mortality rate (per 100,000 population), 2021	Under-5 total SCD mortality rate (per 100,000 population) percent change (2000-2021)	Under-5 cause-specific mortality cause fraction, 2000	Under-5 total SCD mortality cause fraction, 2000	Under-5 cause-specific mortality cause fraction, 2021	Under-5 total SCD mortality cause fraction, 2021	Under-5 mortality rank 2000	Under-5 mortality rank 2021	Under-5 total SCD mortality rank 2000	Under-5 total SCD mortality rank 2021
Yamanashi	0.1 (0-0.3)	0.1 (0-0.3)	-0.1 (-3.9-2.8)	0 (0-0.1)	0 (0-0)	-22.5 (-27.4--16.4)	0 (0-0)	0 (0-0)	-64 (-69.9--55)	0 (0-0)	0 (0-0)	-23.9 (-34.3--20.7)	0 (0-0)	0 (0-0)	-75.6 (-82.5--67.3)	0 (0-0)	0 (0-0)	-1 (-10-5.4)	0 (0-0)	0 (0-0)	0 (0-0)	0 (0-0.1)	104	104	90	77
Republic of Korea	0.5 (0.4-0.7)	0.5 (0.4-0.7)	-1 (-12.7-1.9)	0.1 (0.1-0.1)	0.1 (0-0.1)	-30 (-39.5--23.8)	0 (0-0)	0 (0-0)	-68.1 (-81.2--45.4)	0 (0-0)	0 (0-0)	-31.9 (-41--22.1)	0 (0-0)	0 (0-0)	-87.5 (-97.9--38.6)	0.1 (0-0.1)	0.1 (0-0.1)	-1.1 (-15.3-13.2)	0 (0-0)	0 (0-0.1)	0 (0-0)	0.3 (0.2-0.4)	103	99	81	39
Singapore	0.4 (0.3-0.6)	0.4 (0.3-0.6)	-0.2 (-7.9-7)	0.1 (0.1-0.2)	0.1 (0.1-0.1)	-22.7 (-31.4--15.8)	0 (0-0)	0 (0-0)	-76 (-81.3--69.1)	0 (0-0)	0 (0-0)	-14.6 (-24.2--4.4)	0 (0-0)	0 (0-0)	-78.9 (-87.9--67.6)	0 (0-0.1)	0 (0-0.1)	-0.9 (-11.7-14.4)	0 (0-0)	0.1 (0-0.1)	0 (0-0)	0.1 (0.1-0.2)	116	116	74	50
**High-income North America**	33.9 (31.5-36)	30.5 (28.4-32.5)	-9.8 (-11--8.2)	17.5 (16.3-18.6)	16.2 (15.3-17.3)	-7.6 (-9.3--6.2)	0.2 (0.2-0.2)	0.1 (0.1-0.1)	-19.7 (-23--16.6)	0.4 (0.4-0.4)	0.4 (0.3-0.4)	-14.2 (-16.3--11.8)	0.1 (0.1-0.1)	0.1 (0.1-0.1)	-42.3 (-49--37.2)	1.3 (1.1-1.6)	0.8 (0.6-0.9)	-41.9 (-45.7--38.4)	0.1 (0.1-0.1)	0.8 (0.7-0.9)	0.1 (0.1-0.1)	0.6 (0.5-0.8)	57	63	22	23
Canada	1.7 (1.4-2.1)	0.8 (0.6-1.1)	-51.2 (-64.1--34)	0.5 (0.4-0.6)	0.3 (0.3-0.4)	-27.3 (-49.3--7.4)	0 (0-0)	0 (0-0)	-24.5 (-31.6--14)	0 (0-0)	0 (0-0)	-52.8 (-66.4--35.2)	0 (0-0)	0 (0-0)	-50.3 (-62.9--37)	0.2 (0.1-0.3)	0 (0-0.1)	-77.1 (-84.8--63.4)	0 (0-0)	0.2 (0.1-0.2)	0 (0-0)	0.1 (0-0.1)	73	77	45	62
Greenland	2.3 (1.7-3.2)	2.3 (1.6-3.1)	-0.2 (-10.6-12.6)	0.5 (0.4-0.8)	0.5 (0.3-0.7)	-13.3 (-21.9-8.3)	0 (0-0)	0 (0-0)	-30.3 (-60.2-24.1)	0 (0-0.1)	0 (0-0)	-11.9 (-21.1-1.3)	0 (0-0)	0 (0-0)	-48.6 (-86-49.6)	0.3 (0.2-0.4)	0.3 (0.2-0.4)	1.4 (-12.4-20.6)	0 (0-0)	0.1 (0-0.1)	0 (0-0)	0.1 (0.1-0.2)	103	104	63	48
United States of America	36.5 (34-38.8)	33.6 (31.2-35.7)	-8 (-9.2--6.4)	19.4 (18.1-20.5)	18 (17-19.2)	-7.2 (-8.9--5.7)	0.2 (0.2-0.2)	0.2 (0.1-0.2)	-20.2 (-23.5--17.2)	0.5 (0.4-0.5)	0.4 (0.4-0.4)	-13.6 (-15.7--11.2)	0.1 (0.1-0.2)	0.1 (0.1-0.1)	-42.5 (-49.1--37.5)	1.4 (1.2-1.7)	0.8 (0.7-1)	-40.9 (-44.7--37.3)	0.1 (0.1-0.1)	0.8 (0.7-1)	0.1 (0.1-0.1)	0.7 (0.5-0.9)	57	63	22	23
Alabama	77.7 (71.9-83.4)	69.7 (64.3-75)	-10.2 (-14.3--6)	37.3 (34.4-40.1)	35 (32.5-38.2)	-6.2 (-12.1--0.6)	0.4 (0.4-0.4)	0.3 (0.3-0.4)	-19.1 (-33.7--4.9)	1 (0.9-1.1)	0.8 (0.8-0.9)	-15.7 (-20.2--11)	0.3 (0.3-0.3)	0.2 (0.2-0.2)	-33.2 (-43.8--23)	3.6 (2.9-4.4)	2.1 (1.6-2.6)	-42.3 (-50--34)	0.1 (0.1-0.1)	1.6 (1.3-1.9)	0.1 (0.1-0.1)	1.3 (0.9-1.7)	51	54	14	18
Alaska	11 (8.2-14.3)	10.2 (7.6-13)	-7.2 (-14.5-0.1)	6.3 (4.6-8.6)	5.5 (4-7.2)	-12.9 (-18.6--6.8)	0 (0-0.1)	0 (0-0)	-10.6 (-26.5-5.8)	0.1 (0.1-0.2)	0.1 (0.1-0.2)	-16.3 (-23.7--8.9)	0 (0-0)	0 (0-0)	-36.3 (-51.9--2.9)	0.3 (0.2-0.4)	0.2 (0.1-0.3)	-27.1 (-43.9--13.8)	0 (0-0)	0.1 (0.1-0.2)	0 (0-0)	0.1 (0.1-0.2)	85	87	55	56
Arizona	17.8 (16.1-19.2)	18.7 (17.1-20.2)	5 (-0.8-11.7)	9.7 (8.9-10.5)	9.7 (8.8-10.4)	0.2 (-5.4-6)	0.1 (0.1-0.1)	0.1 (0.1-0.1)	-17.2 (-31.1--1)	0.2 (0.2-0.2)	0.2 (0.2-0.2)	-0.9 (-5.9-4.6)	0.1 (0.1-0.1)	0.1 (0-0.1)	-43.2 (-56.4--30.9)	0.6 (0.5-0.7)	0.5 (0.4-0.6)	-18.6 (-27.3--9.1)	0 (0-0.1)	0.3 (0.3-0.4)	0 (0-0)	0.4 (0.3-0.5)	71	75	35	35
Arkansas	50.7 (45.7-55)	40.6 (36.3-44.8)	-20 (-24.2--15.4)	23.8 (21.6-25.7)	21.3 (19.3-23.4)	-10.6 (-16.2--5.5)	0.3 (0.3-0.3)	0.3 (0.2-0.3)	-7.5 (-23.4-9.9)	0.6 (0.6-0.7)	0.5 (0.4-0.5)	-25.8 (-30.3--21.5)	0.2 (0.2-0.3)	0.1 (0.1-0.2)	-37.2 (-50.8--20.6)	2.5 (2-3.2)	1 (0.8-1.4)	-58.8 (-64.3--52.7)	0.1 (0.1-0.1)	1.2 (0.9-1.5)	0.1 (0.1-0.1)	0.7 (0.4-0.9)	55	58	18	25
California	15.9 (14.8-17)	14.8 (13.8-15.9)	-7 (-10.9--0.8)	9.3 (8.6-10)	8.4 (7.9-9)	-9.1 (-12.6--5)	0.1 (0.1-0.1)	0.1 (0.1-0.1)	-25.7 (-39.9--14)	0.2 (0.2-0.2)	0.2 (0.2-0.2)	-10.2 (-14.5--5.1)	0.1 (0.1-0.1)	0 (0-0)	-57.1 (-65.1--48.4)	0.5 (0.4-0.6)	0.3 (0.3-0.4)	-36.2 (-43.7--27.4)	0.1 (0.1-0.1)	0.4 (0.3-0.4)	0 (0-0.1)	0.4 (0.3-0.5)	60	71	33	30
Colorado	11.4 (9.9-12.8)	9.5 (8-10.8)	-16.6 (-21.2--12.4)	6 (5.3-6.7)	4.9 (4.2-5.7)	-17.8 (-22.2--13.3)	0.1 (0.1-0.1)	0.1 (0.1-0.1)	-19.9 (-34.2--4.2)	0.1 (0.1-0.2)	0.1 (0.1-0.1)	-22.6 (-27.6--17.5)	0.1 (0.1-0.1)	0 (0-0)	-42.1 (-56.2--28.1)	0.5 (0.3-0.6)	0.3 (0.2-0.4)	-42.8 (-50.1--34.9)	0 (0-0)	0.3 (0.2-0.4)	0 (0-0)	0.2 (0.2-0.3)	73	76	37	40
Connecticut	37 (34.3-39.2)	33.5 (30.6-36.3)	-9.5 (-13.9--3.9)	19 (17.4-20.2)	17.1 (15.7-18.5)	-10.1 (-14.2--4.8)	0.1 (0.1-0.2)	0.1 (0.1-0.1)	-24.6 (-39--13.2)	0.5 (0.4-0.5)	0.4 (0.4-0.4)	-14.3 (-18.5--9.3)	0.1 (0.1-0.1)	0 (0-0.1)	-46.9 (-58.7--32)	1.3 (1.1-1.7)	0.8 (0.6-1)	-41.2 (-49.1--32.6)	0.1 (0.1-0.1)	1 (0.8-1.2)	0.1 (0-0.1)	1 (0.7-1.3)	56	66	17	17
Delaware	85.5 (77.8-91.1)	83.8 (76.2-89.8)	-2 (-7.1-2.4)	42.7 (39-45.7)	40.4 (37.2-43.3)	-5.5 (-9.8--0.6)	0.2 (0.2-0.2)	0.2 (0.1-0.2)	-20.8 (-32--7.1)	1.1 (1-1.2)	1 (0.9-1.1)	-8.6 (-13.2--3.5)	0.2 (0.2-0.2)	0.1 (0.1-0.2)	-21.8 (-39.9-3.8)	3.6 (2.9-4.4)	2.7 (2.1-3.4)	-25.9 (-35.8--16)	0.1 (0.1-0.1)	1.7 (1.3-2.1)	0.1 (0.1-0.1)	1.7 (1.2-2.2)	52	56	11	13
District of Columbia	150 (132-168)	110 (94.6-125)	-26.8 (-31.9--20.8)	60.7 (53-68.5)	51.9 (44.5-59)	-14.4 (-19.7--7.8)	0.6 (0.6-0.7)	0.3 (0.3-0.4)	-48.4 (-54.5--41.4)	1.9 (1.6-2.1)	1.4 (1.2-1.6)	-25.4 (-31.4--19.6)	0.6 (0.6-0.7)	0.3 (0.2-0.3)	-59.8 (-70.8--45.3)	9.7 (7.5-12.1)	4.3 (3.2-5.6)	-55.6 (-62.9--47.8)	0.2 (0.1-0.2)	2.5 (1.9-3.1)	0.1 (0.1-0.2)	2.4 (1.5-3.3)	45	49	11	11
Florida	69.7 (65-73.6)	63.5 (59.7-67.8)	-8.9 (-12.2--4.2)	34.8 (32.6-37.3)	32.5 (30.6-34.6)	-6.7 (-10.6--1.7)	0.2 (0.2-0.3)	0.2 (0.2-0.2)	-11.3 (-25.9-4.4)	0.8 (0.8-0.9)	0.7 (0.7-0.8)	-11.7 (-15.8--7.4)	0.2 (0.2-0.2)	0.1 (0.1-0.1)	-51.2 (-58.9--43.5)	2.4 (2-2.9)	1.5 (1.2-1.8)	-37.6 (-44.6--30)	0.1 (0.1-0.1)	1.3 (1.1-1.5)	0.1 (0.1-0.1)	1.2 (1-1.5)	51	56	16	16
Georgia	73.2 (68.8-78.4)	70.7 (65.8-75.6)	-3.5 (-6.9-0.6)	40.1 (37.1-43)	38.3 (36.1-41.2)	-4.4 (-8.4--0.4)	0.4 (0.3-0.4)	0.3 (0.2-0.3)	-21.5 (-31.7--6.6)	0.9 (0.9-1)	0.8 (0.8-0.9)	-10.1 (-14.8--5.5)	0.3 (0.3-0.3)	0.2 (0.1-0.2)	-38.8 (-50--27.7)	2.8 (2.3-3.4)	1.9 (1.6-2.4)	-30.9 (-40.2--20.4)	0.1 (0.1-0.1)	1.3 (1.1-1.6)	0.1 (0.1-0.1)	1.3 (1-1.6)	50	53	16	16
Hawaii	5 (3.2-7.6)	2.9 (1.4-5)	-41.6 (-67.9--23.5)	2.4 (1.4-3.8)	1.4 (0.7-2.6)	-39.1 (-70.3--19.9)	0 (0-0.1)	0 (0-0)	-27 (-41.1--7.5)	0.1 (0-0.1)	0 (0-0.1)	-45.6 (-69.5--28.3)	0 (0-0)	0 (0-0)	-44.8 (-59.6--24.9)	0.3 (0.1-0.4)	0.1 (0-0.2)	-64.5 (-82--47)	0 (0-0)	0.1 (0.1-0.2)	0 (0-0)	0.1 (0-0.1)	84	89	48	56
Idaho	3 (2.4-3.8)	3 (2.4-3.8)	-1.4 (-9.1-7)	1.5 (1.2-1.9)	1.4 (1.1-1.9)	-3.3 (-11.3-4.1)	0 (0-0)	0 (0-0)	-20.8 (-36.2--2.8)	0 (0-0.1)	0 (0-0)	-10.5 (-18.5--1.9)	0 (0-0)	0 (0-0)	-51.1 (-64.8--33.5)	0.1 (0.1-0.2)	0.1 (0.1-0.1)	-29.6 (-40.3--17.5)	0 (0-0)	0.1 (0-0.1)	0 (0-0)	0.1 (0.1-0.1)	86	86	65	64
Illinois	28.8 (26.3-31.1)	21.9 (20-23.5)	-23.8 (-27.5--19.4)	14.2 (12.9-15.5)	11.8 (11-12.8)	-16.5 (-21.1--11.8)	0.2 (0.2-0.2)	0.2 (0.1-0.2)	-24.3 (-36.2--11)	0.4 (0.3-0.4)	0.3 (0.2-0.3)	-29.6 (-33.6--24.8)	0.2 (0.2-0.2)	0.1 (0.1-0.1)	-56.3 (-63.8--46.3)	1.5 (1.2-1.8)	0.6 (0.4-0.7)	-61.9 (-68.1--54.4)	0.1 (0.1-0.1)	0.7 (0.6-0.9)	0.1 (0.1-0.1)	0.5 (0.4-0.7)	54	60	22	24
Indiana	26.4 (24.1-28.1)	23.9 (21.8-25.6)	-9.5 (-14.1--6)	13.3 (12.3-14.4)	12.3 (11.4-13.3)	-7.4 (-12.4--3.2)	0.2 (0.2-0.2)	0.1 (0.1-0.2)	-11.9 (-26.7-5.2)	0.3 (0.3-0.4)	0.3 (0.3-0.3)	-15.6 (-20--10.8)	0.1 (0.1-0.2)	0.1 (0.1-0.1)	-35.7 (-47.6--18.4)	1.1 (0.9-1.3)	0.7 (0.5-0.8)	-39 (-46.6--30.2)	0.1 (0.1-0.1)	0.6 (0.5-0.7)	0.1 (0.1-0.1)	0.5 (0.3-0.6)	59	64	25	31
Iowa	14.7 (12.8-16.3)	13.8 (11.9-15.6)	-6.1 (-11--0.7)	7.9 (6.9-8.7)	7.6 (6.6-8.6)	-3.5 (-8.6-2)	0.1 (0.1-0.1)	0.1 (0-0.1)	-17.5 (-32.2--0.8)	0.2 (0.2-0.2)	0.2 (0.1-0.2)	-10.8 (-15.7--5.2)	0.1 (0.1-0.1)	0 (0-0)	-38.1 (-52.7--14.7)	0.4 (0.3-0.6)	0.3 (0.2-0.3)	-42.3 (-48.8--35)	0 (0-0)	0.3 (0.2-0.4)	0 (0-0)	0.3 (0.2-0.4)	70	75	36	43
Kansas	19.8 (17.4-22.2)	15.4 (13.1-17.5)	-21.9 (-27.5--17.5)	9.9 (8.7-11.3)	8.2 (7.1-9.5)	-17.4 (-24--11.5)	0.2 (0.1-0.2)	0.1 (0.1-0.2)	-16.4 (-32.5-2)	0.2 (0.2-0.3)	0.2 (0.2-0.2)	-27.3 (-31.9--21.7)	0.1 (0.1-0.2)	0.1 (0.1-0.1)	-50.8 (-62--35.8)	0.9 (0.7-1.1)	0.5 (0.3-0.6)	-48.3 (-55.4--40.7)	0.1 (0.1-0.1)	0.5 (0.4-0.7)	0.1 (0.1-0.1)	0.4 (0.3-0.5)	57	67	26	32
Kentucky	13.5 (11.9-15)	12.5 (10.9-13.8)	-7.8 (-11.1--3.8)	6.6 (5.8-7.4)	6.3 (5.4-7)	-4.7 (-8.7-0.1)	0.2 (0.1-0.2)	0.1 (0.1-0.2)	-5.1 (-21.2-10.1)	0.2 (0.2-0.2)	0.1 (0.1-0.2)	-13.7 (-18.5--8.9)	0.1 (0.1-0.1)	0.1 (0.1-0.1)	-38.5 (-50.7--21.2)	0.6 (0.4-0.8)	0.4 (0.3-0.5)	-40.1 (-46.5--32.3)	0.1 (0.1-0.1)	0.4 (0.3-0.5)	0.1 (0-0.1)	0.3 (0.2-0.4)	65	70	32	36
Louisiana	65.3 (59.9-70.3)	52.8 (48.6-57.1)	-19.1 (-23.4--14.3)	31.3 (29-33.6)	27.1 (25.2-29.1)	-13.3 (-17.4--8.6)	0.4 (0.4-0.4)	0.3 (0.3-0.4)	-16.7 (-28.9--2.7)	0.8 (0.8-0.9)	0.6 (0.6-0.7)	-25.4 (-29.6--20.8)	0.3 (0.3-0.4)	0.2 (0.2-0.2)	-39.7 (-50.3--27.4)	3.5 (2.9-4.2)	1.8 (1.4-2.2)	-49.7 (-57.2--40.8)	0.1 (0.1-0.2)	1.5 (1.2-1.8)	0.1 (0.1-0.1)	1 (0.8-1.2)	49	54	14	18
Maine	1.8 (1.2-2.9)	1.1 (0.6-1.7)	-40.4 (-59.9--15.5)	0.9 (0.6-1.5)	0.5 (0.3-0.8)	-47.4 (-67.1--22.7)	0 (0-0)	0 (0-0)	-8.5 (-25.6-11.6)	0 (0-0)	0 (0-0)	-44.3 (-64--22.1)	0 (0-0)	0 (0-0)	-34.9 (-54.7--6.1)	0 (0-0.1)	0 (0-0)	-46.7 (-75.5--29.8)	0 (0-0)	0 (0-0.1)	0 (0-0)	0 (0-0)	82	84	69	77
Maryland	90.8 (84.2-97.5)	84.4 (79-90.1)	-7 (-11.4--2.9)	49.3 (46-53)	46 (42.9-49.3)	-6.7 (-10.8--2)	0.3 (0.3-0.3)	0.2 (0.2-0.3)	-27.6 (-39.4--15.3)	1.1 (1-1.2)	1 (0.9-1.1)	-11 (-15.7--6.1)	0.2 (0.2-0.2)	0.1 (0.1-0.1)	-47.8 (-56.8--36.6)	3.4 (2.8-4.2)	2 (1.6-2.5)	-40.4 (-48.3--31.8)	0.1 (0.1-0.1)	1.9 (1.5-2.3)	0.1 (0.1-0.1)	1.9 (1.4-2.4)	50	55	11	12
Massachusetts	30 (27.4-32.1)	27.1 (24.6-28.9)	-10 (-13.6--4.9)	15.6 (14.4-16.6)	14 (12.8-15.1)	-9.9 (-14.2--5.2)	0.1 (0.1-0.1)	0.1 (0.1-0.1)	-30.5 (-43.2--14.1)	0.4 (0.3-0.4)	0.3 (0.3-0.4)	-13.4 (-18.1--8.8)	0.1 (0.1-0.1)	0 (0-0)	-43.5 (-57.9--24.8)	1.1 (0.9-1.4)	0.6 (0.5-0.8)	-41.1 (-48.8--32.7)	0.1 (0-0.1)	1 (0.8-1.2)	0 (0-0.1)	0.8 (0.6-1.1)	63	70	17	18

Location	Birth Incidence rate (per 100,000 live births), 2000	Birth Incidence rate (per 100,000 live births), 2021	Birth Incidence rate (per 100,000 live births) percent change (2000-2021)	All age rate (per 100,000 population), 2000	All age rate (per 100,000 population), 2021	All age rate (per 100,000 population) percent change (2000-2021)	All age cause-specific SCD mortality rate (per 100,000 population), 2000	All age cause-specific SCD mortality rate (per 100,000 population), 2021	All age cause-specific SCD mortality rate (per 100,000 population) percent change (2000-2021)	All age total SCD mortality rate (per 100,000 population), 2000	All age total SCD mortality rate (per 100,000 population), 2021	All age total SCD mortality rate (per 100,000 population) percent change (2000-2021)	Under-5 cause-specific SCD mortality rate (per 100,000 population), 2000	Under-5 cause-specific SCD mortality rate (per 100,000 population), 2021	Under-5 cause-specific SCD mortality rate (per 100,000 population) percent change (2000-2021)	Under-5 total SCD mortality rate (per 100,000 population), 2000	Under-5 total SCD mortality rate (per 100,000 population), 2021	Under-5 total SCD mortality rate (per 100,000 population) percent change (2000-2021)	Under-5 cause-specific mortality cause fraction, 2000	Under-5 total SCD mortality cause fraction, 2000	Under-5 cause-specific mortality cause fraction, 2021	Under-5 total SCD mortality cause fraction, 2021	Under-5 cause-specific mortality rank 2000	Under-5 cause-specific mortality rank 2021	Under-5 total SCD mortality rank 2000	Under-5 total SCD mortality rank 2021
Michigan	39.2 (36.4-42)	34.8 (32.6-37.2)	-11.2 (-15--7.3)	20 (18.5-21.3)	17.8 (16.8-19)	-11.2 (-16.2--6.8)	0.2 (0.2-0.2)	0.2 (0.1-0.2)	-19.5 (-30.9--5.4)	0.5 (0.4-0.5)	0.4 (0.4-0.4)	-17.3 (-21.5--12.8)	0.1 (0.1-0.2)	0.1 (0.1-0.1)	-34.9 (-47.2--23.4)	1.6 (1.3-2)	0.9 (0.7-1.1)	-44.5 (-53--36.2)	0.1 (0.1-0.1)	0.9 (0.7-1.1)	0.1 (0.1-0.1)	0.7 (0.5-0.8)	54	59	19	22
Minnesota	13.3 (11.5-14.9)	11.9 (10.2-13.7)	-10.6 (-15.5--4.8)	7.2 (6.2-8.1)	6.5 (5.6-7.5)	-9.9 (-14.9--3.4)	0.1 (0.1-0.1)	0.1 (0.1-0.1)	-18.3 (-34.3--1.5)	0.2 (0.1-0.2)	0.1 (0.1-0.2)	-14.7 (-20.7--7.8)	0.1 (0.1-0.1)	0 (0-0.1)	-36.2 (-52.5--17.7)	0.5 (0.4-0.6)	0.3 (0.2-0.4)	-42.4 (-50--34.7)	0.1 (0-0.1)	0.4 (0.3-0.5)	0 (0-0.1)	0.3 (0.2-0.4)	67	74	33	38
Mississippi	94.8 (87.4-102)	79.1 (72.8-85.6)	-16.6 (-19.7--12.5)	43 (39.7-45.6)	38.3 (35.2-41.2)	-10.9 (-14.6--6.5)	0.4 (0.4-0.5)	0.4 (0.3-0.5)	-11.1 (-22.7-4)	1.2 (1.1-1.4)	0.9 (0.9-1)	-23.9 (-27.9--19.5)	0.4 (0.3-0.4)	0.2 (0.2-0.2)	-45.7 (-56.3--34.6)	5.3 (4.4-6.5)	2.8 (2.2-3.5)	-47.9 (-55.3--40.1)	0.1 (0.1-0.2)	2 (1.6-2.4)	0.1 (0.1-0.1)	1.6 (1.1-2.1)	48	53	13	15
Missouri	37.2 (34.2-40.4)	32.5 (29.7-34.8)	-12.7 (-16.8--8.2)	18 (16.5-19.5)	16.3 (15.1-17.6)	-9.1 (-12.7--3.7)	0.2 (0.2-0.2)	0.2 (0.1-0.2)	-11.6 (-25.5-4.9)	0.5 (0.4-0.5)	0.4 (0.3-0.4)	-18.2 (-22.3--13.7)	0.1 (0.1-0.2)	0.1 (0.1-0.1)	-26.1 (-40.4--12.8)	1.6 (1.3-2)	0.9 (0.7-1.1)	-44.2 (-51.3--36.5)	0.1 (0.1-0.1)	1 (0.8-1.1)	0.1 (0.1-0.1)	0.7 (0.5-0.8)	54	62	20	25
Montana	0.9 (0.5-1.5)	0.9 (0.5-1.3)	-5.6 (-35.6-29.8)	0.5 (0.2-0.8)	0.4 (0.2-0.7)	-9.6 (-37.3-25.3)	0 (0-0)	0 (0-0)	-11.4 (-25.8-5.4)	0 (0-0)	0 (0-0)	-11.8 (-36.9-17.5)	0 (0-0)	0 (0-0)	-35.7 (-53.1--14.7)	0 (0-0)	0 (0-0)	-28.3 (-66.2--0.1)	0 (0-0)	0 (0-0)	0 (0-0)	0 (0-0)	87	87	94	92
Nebraska	12.5 (10.2-15.2)	9.4 (7.6-11.7)	-24.9 (-31.7--18)	6.4 (5.2-7.9)	5.1 (4-6.4)	-20.6 (-27.8--14.3)	0.1 (0.1-0.1)	0.1 (0.1-0.1)	-20.6 (-32.4--7.8)	0.2 (0.1-0.2)	0.1 (0.1-0.1)	-28.9 (-35.2--22)	0.1 (0.1-0.1)	0.1 (0-0.1)	-29.6 (-49--1.6)	0.6 (0.4-0.8)	0.3 (0.2-0.4)	-49.9 (-57--42.3)	0 (0-0.1)	0.3 (0.2-0.5)	0 (0-0.1)	0.2 (0.2-0.4)	68	73	34	40
Nevada	31.7 (28.1-34.9)	29.8 (27-33.1)	-5.8 (-11-0.4)	17.1 (15.5-18.9)	16.3 (14.7-18.3)	-4.4 (-10.3-2.5)	0.1 (0.1-0.1)	0.1 (0.1-0.1)	-29.9 (-40.4--19.9)	0.4 (0.4-0.4)	0.4 (0.3-0.4)	-11.7 (-16.5--6.8)	0.1 (0.1-0.1)	0.1 (0-0.1)	-48.1 (-60.5--35)	1.2 (0.9-1.5)	0.7 (0.6-0.9)	-38.1 (-46.6--29.3)	0.1 (0.1-0.1)	0.7 (0.6-1)	0.1 (0-0.1)	0.7 (0.5-1)	66	71	23	24
New Hampshire	3.3 (2.3-4.4)	3.1 (2.3-4)	-4.8 (-17.7-7)	1.5 (1.1-2)	1.4 (1-1.8)	-11.3 (-20.3-0.7)	0 (0-0)	0 (0-0)	-11.2 (-26.6-7.3)	0 (0-0.1)	0 (0-0)	-11.9 (-24.9--0.4)	0 (0-0)	0 (0-0)	-30 (-48-0.2)	0.1 (0.1-0.2)	0.1 (0.1-0.1)	-37.4 (-55.2--22.4)	0 (0-0)	0.1 (0.1-0.2)	0 (0-0)	0.1 (0.1-0.1)	80	85	49	57
New Jersey	49 (45.3-52.9)	44 (41-46.9)	-10.2 (-14.2--6.1)	26.3 (24.4-28.3)	23.9 (22.3-25.7)	-9 (-12.7--4.9)	0.2 (0.2-0.2)	0.1 (0.1-0.2)	-35.4 (-47--23.8)	0.6 (0.6-0.7)	0.5 (0.5-0.6)	-14.1 (-18.2--9.8)	0.1 (0.1-0.2)	0.1 (0-0.1)	-61 (-69.5--52.8)	1.7 (1.4-2)	0.9 (0.8-1.2)	-43.2 (-50.1--35.6)	0.1 (0.1-0.1)	1.1 (0.9-1.3)	0.1 (0.1-0.1)	1.3 (1-1.7)	52	57	15	14
New Mexico	3.4 (2.6-4.3)	3.3 (2.5-4.3)	-3 (-10.9-7.4)	2 (1.5-2.6)	1.8 (1.3-2.4)	-10.1 (-16.5--0.2)	0.1 (0.1-0.1)	0.1 (0.1-0.1)	-0.1 (-18.2-20.4)	0 (0-0.1)	0 (0-0.1)	-9.9 (-17.7-1.2)	0 (0-0.1)	0 (0-0)	-46.2 (-59.5--28)	0.1 (0-0.1)	0 (0-0.1)	-14.3 (-29.7-3.1)	0 (0-0)	0 (0-0)	0 (0-0)	0 (0-0.1)	80	83	79	77
New York	56.2 (52.5-60.4)	53.4 (49.9-56.9)	-5 (-9.8--1)	33.8 (31.9-36.2)	31.8 (29.9-34.1)	-6 (-9.6--1.6)	0.2 (0.2-0.2)	0.2 (0.1-0.2)	-27.7 (-39.2--14.8)	0.7 (0.6-0.7)	0.6 (0.6-0.7)	-6.3 (-11.8--1.3)	0.2 (0.1-0.2)	0.1 (0.1-0.1)	-58 (-64.4--48.6)	1.3 (1-1.7)	0.8 (0.6-1)	-41.8 (-50.4--32.5)	0.1 (0.1-0.1)	0.9 (0.7-1.1)	0.1 (0.1-0.1)	0.9 (0.7-1.2)	54	59	22	18
North Carolina	58.5 (54.6-62.2)	53.7 (50.2-57.7)	-8.1 (-12--4.2)	31 (28.7-33.1)	28.5 (26.9-30.4)	-8 (-12--3.8)	0.3 (0.3-0.3)	0.2 (0.2-0.3)	-19.7 (-30.5--4.8)	0.7 (0.7-0.8)	0.6 (0.6-0.7)	-14.2 (-18.8--9.7)	0.2 (0.2-0.2)	0.1 (0.1-0.1)	-36 (-46.2--25.7)	2.2 (1.8-2.7)	1.4 (1.1-1.7)	-37.5 (-45.9--28.9)	0.1 (0.1-0.1)	1 (0.9-1.3)	0.1 (0.1-0.1)	0.9 (0.7-1.2)	52	58	17	19
North Dakota	0.7 (0.3-1.3)	0.6 (0.2-1.2)	-11 (-53.9-2.8)	0.4 (0.2-0.8)	0.4 (0.1-0.8)	-9.2 (-48.6-8.2)	0 (0-0.1)	0 (0-0.1)	-4.2 (-22-11.3)	0 (0-0)	0 (0-0)	-13.9 (-55.1-0.6)	0 (0-0)	0 (0-0)	-23.6 (-43.1-2.8)	0 (0-0)	0 (0-0)	-32.8 (-71.4--8.7)	0 (0-0)	0 (0-0)	0 (0-0)	0 (0-0)	76	82	89	94
Ohio	29.1 (26.9-31.4)	26.1 (24.2-27.7)	-10.3 (-13.7--5.9)	14.5 (13.3-15.6)	13.5 (12.6-14.5)	-6.9 (-10.8--2.9)	0.2 (0.2-0.2)	0.2 (0.1-0.2)	-5 (-19.6-10.5)	0.4 (0.3-0.4)	0.3 (0.3-0.3)	-15.9 (-20.9--11.1)	0.1 (0.1-0.2)	0.1 (0.1-0.1)	-25.6 (-38--8.6)	1.3 (1-1.5)	0.7 (0.6-0.8)	-45.5 (-53.6--36.3)	0.1 (0.1-0.1)	0.7 (0.6-0.9)	0.1 (0.1-0.1)	0.4 (0.3-0.6)	54	59	21	30
Oklahoma	20.1 (17.9-22.5)	14 (12.2-16)	-30.3 (-34.5--24.6)	9 (8-10.2)	7 (6.1-8)	-22.3 (-26.7--15.5)	0.2 (0.2-0.2)	0.1 (0.1-0.2)	-16.1 (-30.4--4.8)	0.3 (0.2-0.3)	0.2 (0.1-0.2)	-36 (-40.8--31.1)	0.1 (0.1-0.2)	0.1 (0.1-0.1)	-34.8 (-45.6--19.7)	1.1 (0.9-1.4)	0.5 (0.3-0.6)	-56.7 (-62.9--50)	0.1 (0.1-0.1)	0.5 (0.4-0.7)	0.1 (0.1-0.1)	0.3 (0.2-0.4)	62	67	24	38
Oregon	7.4 (6.2-8.9)	7.2 (6.1-8.5)	-3.9 (-8.4-2.8)	3.7 (3-4.3)	3.4 (2.9-4.1)	-6.7 (-11.4-0.3)	0 (0-0)	0 (0-0)	-20.3 (-34.5--0.6)	0.1 (0.1-0.1)	0.1 (0.1-0.1)	-11 (-17--4.4)	0 (0-0)	0 (0-0)	-44.2 (-59.3--21.6)	0.3 (0.2-0.4)	0.2 (0.1-0.3)	-33.6 (-40.5--24.8)	0 (0-0)	0.2 (0.2-0.3)	0 (0-0)	0.2 (0.1-0.3)	80	84	39	42
Pennsylvania	26.2 (24.4-28)	23.3 (21.6-25)	-10.9 (-14.7--6.2)	12.4 (11.6-13.5)	11.5 (10.7-12.3)	-7 (-11.7--2.9)	0.2 (0.2-0.2)	0.1 (0.1-0.2)	-22.3 (-36.3--8.8)	0.3 (0.3-0.3)	0.3 (0.3-0.3)	-14.8 (-19.2--9.9)	0.1 (0.1-0.1)	0.1 (0.1-0.1)	-31.8 (-44.8--17.8)	1.1 (0.9-1.4)	0.6 (0.5-0.8)	-43.9 (-52.7--34.8)	0.1 (0.1-0.1)	0.7 (0.6-0.9)	0.1 (0.1-0.1)	0.5 (0.4-0.6)	55	60	21	27
Rhode Island	32 (27.6-37.5)	23.8 (20.3-27.7)	-25.6 (-32--18.4)	17.1 (15-19.9)	13.8 (11.8-15.9)	-19.5 (-24.6--12.3)	0.1 (0.1-0.1)	0.1 (0-0.1)	-36.5 (-47.3--22.2)	0.4 (0.3-0.5)	0.3 (0.2-0.3)	-27.7 (-34.6--19)	0.1 (0-0.1)	0 (0-0)	-33.1 (-51.7--8.2)	1.2 (0.8-1.6)	0.4 (0.3-0.5)	-68 (-73.3--60.7)	0 (0-0)	0.8 (0.5-1.2)	0 (0-0)	0.3 (0.2-0.4)	68	72	19	33
South Carolina	70.4 (65-75.7)	57.4 (52.9-61.6)	-18.5 (-22.2--14.1)	35.5 (32.6-38.5)	30.2 (28.2-32.4)	-15.1 (-17.8--12.1)	0.4 (0.4-0.5)	0.4 (0.3-0.4)	-17.5 (-30.9--5)	0.9 (0.8-1)	0.7 (0.6-0.7)	-24.1 (-28.1--19.7)	0.3 (0.3-0.3)	0.2 (0.1-0.2)	-39.1 (-49.3--28.1)	3 (2.5-3.7)	1.5 (1.2-1.8)	-51.3 (-57.8--43.5)	0.1 (0.1-0.1)	1.4 (1.2-1.7)	0.1 (0.1-0.1)	1.1 (0.8-1.4)	51	52	14	17
South Dakota	2.5 (1.5-4.4)	2.3 (1.4-3.5)	-9.7 (-29.4-6.1)	1.2 (0.7-2)	1.1 (0.7-1.9)	-5.1 (-16.3-8.6)	0.1 (0.1-0.1)	0.1 (0-0.1)	-17.9 (-30.7--1.6)	0 (0-0.1)	0 (0-0)	-15.8 (-37-1.1)	0.1 (0-0.1)	0 (0-0.1)	-29.2 (-47.7--6.7)	0.1 (0-0.2)	0.1 (0-0.1)	-44 (-70.8--16.8)	0 (0-0)	0.1 (0-0.1)	0 (0-0)	0 (0-0.1)	77	81	69	77
Tennessee	45.7 (42.3-49.1)	41.1 (38.3-43.8)	-10 (-15.1--4.8)	21.6 (19.9-23.3)	19.8 (18.5-21.2)	-8 (-12.1--3.8)	0.2 (0.2-0.3)	0.2 (0.2-0.3)	-11.4 (-27-2.9)	0.6 (0.5-0.6)	0.5 (0.5-0.5)	-16.4 (-20.6--12.3)	0.2 (0.2-0.2)	0.1 (0.1-0.1)	-33 (-43.8--18.9)	2.2 (1.8-2.6)	1.4 (1.1-1.7)	-37.3 (-46.9--28)	0.1 (0.1-0.1)	1 (0.8-1.2)	0.1 (0.1-0.1)	0.8 (0.6-1.1)	54	63	19	20
Texas	32.6 (30.3-34.9)	32.8 (30.4-35.5)	0.7 (-4.7-5.8)	19.2 (17.8-20.5)	19.1 (17.9-20.5)	-0.3 (-5.3-4)	0.2 (0.2-0.2)	0.1 (0.1-0.2)	-23 (-34.8--10.6)	0.4 (0.4-0.4)	0.4 (0.4-0.4)	-4.3 (-9.1-1)	0.1 (0.1-0.2)	0.1 (0.1-0.1)	-36.5 (-46.4--25.6)	1 (0.8-1.2)	0.7 (0.5-0.9)	-28.4 (-39--16.9)	0.1 (0.1-0.1)	0.6 (0.5-0.8)	0.1 (0.1-0.1)	0.5 (0.4-0.7)	56	64	25	27
Utah	5 (4.1-6.2)	4.5 (3.7-5.5)	-10.6 (-16.4--3)	3 (2.4-3.7)	2.7 (2.2-3.4)	-9.3 (-15.1--1.4)	0.1 (0-0.1)	0 (0-0)	-27 (-39.2--15.3)	0.1 (0.1-0.1)	0.1 (0-0.1)	-18 (-26.4--10.8)	0 (0-0.1)	0 (0-0)	-41.3 (-57.8--15.9)	0.2 (0.1-0.3)	0.1 (0.1-0.1)	-40.9 (-53--30.3)	0 (0-0)	0.1 (0.1-0.2)	0 (0-0)	0.1 (0.1-0.1)	77	81	54	57
Vermont	5.4 (3.5-8.3)	5 (3.2-7.6)	-5.7 (-22.5-13.5)	3.3 (2.1-5)	3.1 (2-4.6)	-7.8 (-18.3-8.8)	0 (0-0)	0 (0-0)	-15.2 (-29.2-2)	0.1 (0-0.1)	0.1 (0-0.1)	-7.3 (-27.7-12.2)	0 (0-0)	0 (0-0)	-38.5 (-54.9--7.4)	0.1 (0.1-0.2)	0.1 (0-0.1)	-55.1 (-80.4--12.2)	0 (0-0)	0.1 (0-0.2)	0 (0-0)	0.1 (0-0.1)	85	87	58	68
Virginia	51 (47.5-54.5)	45.9 (42.4-49)	-10 (-14.3--5.4)	26.3 (24.7-28.1)	24.6 (22.9-26.3)	-6.5 (-10.6--2.1)	0.2 (0.2-0.2)	0.2 (0.1-0.2)	-23.5 (-36.1--10.8)	0.6 (0.6-0.7)	0.5 (0.5-0.6)	-15.1 (-19.4--10.5)	0.2 (0.1-0.2)	0.1 (0.1-0.1)	-39.2 (-47.6--28.8)	2.3 (1.8-2.8)	1.2 (1-1.6)	-45.4 (-53.8--36)	0.1 (0.1-0.1)	1.3 (1.1-1.6)	0.1 (0.1-0.1)	1 (0.8-1.3)	52	58	15	17
Washington	11.7 (10.4-12.8)	11 (9.8-12.2)	-6.4 (-11--2)	6.5 (5.8-7.1)	5.8 (5.2-6.4)	-9.9 (-13.8--4.7)	0.1 (0.1-0.1)	0.1 (0-0.1)	-19.6 (-32.9--4.9)	0.1 (0.1-0.2)	0.1 (0.1-0.1)	-11.5 (-16.3--6.2)	0 (0-0.1)	0 (0-0)	-39.6 (-55--22.2)	0.4 (0.3-0.5)	0.2 (0.2-0.3)	-31.9 (-38.2--25.2)	0 (0-0)	0.3 (0.2-0.4)	0 (0-0)	0.3 (0.2-0.3)	73	78	35	40
West Virginia	7.1 (5.7-8.9)	6.2 (5-7.7)	-13.7 (-19.9--8)	3.5 (2.8-4.5)	3.1 (2.4-3.8)	-12.4 (-18.9--5.8)	0.1 (0.1-0.1)	0.1 (0.1-0.2)	7.3 (-12.3-29.5)	0.1 (0.1-0.1)	0.1 (0.1-0.1)	-19 (-24.7--11.7)	0.1 (0.1-0.1)	0.1 (0-0.1)	-25 (-45.2-2.7)	0.3 (0.2-0.4)	0.2 (0.1-0.2)	-44.5 (-52.8--34.6)	0 (0-0)	0.1 (0.1-0.2)	0 (0-0)	0.1 (0.1-0.1)	70	77	45	56
Wisconsin	22.4 (20.3-24.4)	18.2 (16.3-19.9)	-18.6 (-22.8--14.1)	11.4 (10.3-12.5)	9.3 (8.4-10.3)	-18.2 (-23.2--13.7)	0.1 (0.1-0.1)	0.1 (0.1-0.1)	-16.9 (-31.1--2)	0.3 (0.2-0.3)	0.2 (0.2-0.2)	-23 (-27.1--18.3)	0.1 (0.1-0.1)	0.1 (0.1-0.1)	-40.5 (-55.6--23.3)	0.9 (0.7-1.1)	0.4 (0.3-0.6)	-47.7 (-53.9--40.6)	0.1 (0.1-0.1)	0.6 (0.4-0.7)	0.1 (0-0.1)	0.4 (0.3-0.5)	61	70	25	32
Wyoming	4.8 (2.8-7.7)	2.6 (1.3-4.8)	-47.1 (-62.8--32.8)	2.7 (1.6-4.5)	1.5 (0.8-2.8)	-45.8 (-60--33.5)	0.1 (0.1-0.1)	0 (0-0.1)	-18.5 (-32.5--3)	0.1 (0-0.1)	0 (0-0.1)	-50.6 (-67.2--36.6)	0 (0-0.1)	0 (0-0)	-45.8 (-59.8--24.4)	0.1 (0.1-0.2)	0 (0-0.1)	-72.1 (-89.4--40.7)	0 (0-0)	0.1 (0-0.1)	0 (0-0)	0 (0-0.1)	80	84	67	78

Location	Birth Incidence rate (per 100,000 live births), 2000	Birth Incidence rate (per 100,000 live births), 2021	Birth Incidence rate (per 100,000 live births) percent change (2000-2021)	All age rate (per 100,000 population), 2000	All age rate (per 100,000 population), 2021	All age rate (per 100,000 population) percent change (2000-2021)	All age cause-specific SCD mortality rate (per 100,000 population), 2000	All age cause-specific SCD mortality rate (per 100,000 population), 2021	All age cause-specific SCD mortality rate (per 100,000 population) percent change (2000-2021)	All age total SCD mortality rate (per 100,000 population), 2000	All age total SCD mortality rate (per 100,000 population), 2021	All age total SCD mortality rate (per 100,000 population) percent change (2000-2021)	Under-5 cause-specific SCD mortality rate (per 100,000 population), 2000	Under-5 cause-specific SCD mortality rate (per 100,000 population), 2021	Under-5 cause-specific SCD mortality rate (per 100,000 population) percent change (2000-2021)	Under-5 total SCD mortality rate (per 100,000 population), 2000	Under-5 total SCD mortality rate (per 100,000 population), 2021	Under-5 total SCD mortality rate (per 100,000 population) percent change (2000-2021)	Under-5 cause-specific mortality cause fraction, 2000	Under-5 total SCD mortality cause fraction, 2000	Under-5 cause-specific mortality cause fraction, 2021	Under-5 total SCD mortality cause fraction, 2021	Under-5 cause-specific mortality rank 2000	Under-5 cause-specific mortality rank 2021	Under-5 total SCD mortality rank 2000	Under-5 total SCD mortality rank 2021
**Southern Latin America**	0.6 (0.5-0.8)	0.8 (0.6-1)	24.1 (13.8-43.9)	0.1 (0.1-0.2)	0.1 (0.1-0.2)	9.1 (-1-22.4)	0 (0-0)	0 (0-0)	-20.7 (-33.3--8.5)	0 (0-0)	0 (0-0)	3.6 (-7.1-25)	0 (0-0)	0 (0-0)	-62.4 (-76.3--45.2)	0.1 (0.1-0.1)	0.1 (0.1-0.1)	24.4 (9.5-54.1)	0 (0-0)	0 (0-0)	0 (0-0)	0.1 (0-0.1)	127	130	81	57
Argentina	0.4 (0.3-0.5)	0.4 (0.3-0.5)	0.6 (-8.9-34.6)	0.1 (0-0.1)	0.1 (0-0.1)	-10.2 (-21.1-8.4)	0 (0-0)	0 (0-0)	-29.8 (-43.2--14.5)	0 (0-0)	0 (0-0)	-17.1 (-27.8-10)	0 (0-0)	0 (0-0)	-68.9 (-80.7--50.3)	0.1 (0-0.1)	0.1 (0-0.1)	-0.6 (-13.8-34.2)	0 (0-0)	0 (0-0)	0 (0-0)	0 (0-0)	126	129	93	74
Chile	1.4 (0.9-1.8)	2 (1.6-2.5)	52.3 (32.1-84.5)	0.3 (0.2-0.3)	0.3 (0.3-0.4)	22.7 (7.6-45.4)	0 (0-0)	0 (0-0)	-52.6 (-62--41.3)	0 (0-0)	0 (0-0)	20.4 (3.5-59.6)	0 (0-0)	0 (0-0)	-70.6 (-80.8--54.8)	0.2 (0.1-0.2)	0.3 (0.2-0.3)	52.9 (29-107)	0 (0-0)	0.1 (0-0.1)	0 (0-0)	0.3 (0.2-0.4)	132	132	63	33
Uruguay	0.4 (0.2-0.6)	0.4 (0.3-0.6)	0 (-2.9-14.8)	0.1 (0-0.1)	0.1 (0-0.1)	-11 (-22.7-4.2)	0 (0-0)	0 (0-0)	45.5 (14.8-80.9)	0 (0-0)	0 (0-0)	-16 (-24.6--8.9)	0 (0-0)	0 (0-0)	-24.9 (-61.1-37.3)	0.1 (0-0.1)	0.1 (0-0.1)	-0.1 (-9-8.5)	0 (0-0)	0 (0-0)	0 (0-0)	0.1 (0-0.1)	108	102	88	67
**Western Europe**	31.2 (26.1-37.4)	29.5 (24.4-35)	-5.4 (-7.9--2.7)	6.2 (5.4-7.1)	5.5 (4.8-6.3)	-11.3 (-13.2--9.6)	0 (0-0)	0 (0-0)	1.3 (-3.1-5)	0.4 (0.3-0.4)	0.3 (0.3-0.4)	-10.3 (-12.4--7.8)	0 (0-0)	0 (0-0)	-20.1 (-29.1--8.3)	2 (1.6-2.6)	1.9 (1.5-2.5)	-6.1 (-9.9--1.9)	0 (0-0)	1.8 (1.4-2.2)	0 (0-0)	2.8 (2.1-3.6)	80	72	14	10
Andorra	19.2 (14.9-24.2)	19.3 (15.1-24.6)	0.7 (-6.3-8.7)	4.1 (3.3-5)	3.4 (2.8-4)	-17 (-22.4--12.3)	0 (0-0)	0 (0-0)	-45.3 (-66.7--13.7)	0.2 (0.2-0.3)	0.2 (0.1-0.2)	-18.8 (-24.4--12.6)	0.1 (0-0.2)	0 (0-0.1)	-70.1 (-90.8--26.8)	1.3 (0.9-1.7)	1.3 (0.9-1.7)	-0.5 (-10.1-10.1)	0.1 (0.1-0.3)	1.9 (1.2-2.7)	0.1 (0.1-0.3)	5.6 (3.3-8.4)	55	56	16	5
Austria	16.2 (14.5-17.9)	22.9 (20.2-26)	41.7 (31.8-52.5)	3.8 (3.4-4.2)	4.2 (3.7-4.6)	9.8 (5.5-15.6)	0 (0-0)	0 (0-0)	8.7 (-2.8-23.6)	0.2 (0.2-0.2)	0.2 (0.2-0.3)	28.1 (18.8-38)	0 (0-0)	0 (0-0)	-18.4 (-41.3-5)	0.8 (0.7-1)	1.2 (1-1.6)	48.8 (31.4-67.7)	0 (0-0)	0.8 (0.6-1)	0 (0-0)	2.4 (1.8-3)	85	80	21	11
Belgium	13.2 (10.3-16.6)	12.7 (9.6-16.1)	-4.1 (-11.9-4.1)	2.2 (1.8-2.6)	2 (1.7-2.4)	-7.5 (-13.6--0.9)	0 (0-0)	0 (0-0)	9.3 (-3.9-23.3)	0.2 (0.1-0.2)	0.1 (0.1-0.2)	-8.2 (-15-0.7)	0 (0-0)	0 (0-0)	-27.3 (-45.2--3.2)	1 (0.7-1.4)	0.9 (0.7-1.3)	-5.8 (-17.2-6.1)	0 (0-0)	0.8 (0.6-1.2)	0 (0-0)	1.4 (0.9-2.1)	74	72	23	17
Cyprus	55 (46-65.5)	55.4 (46.5-65.6)	0.8 (-6.3-9.1)	14.9 (13.1-17)	12.2 (10.6-13.9)	-17.9 (-22.3--12.7)	0.1 (0.1-0.1)	0 (0-0)	-63.6 (-73.7--51.7)	0.8 (0.6-0.9)	0.7 (0.6-0.8)	-13.5 (-19.8--6.3)	0 (0-0.1)	0 (0-0)	-67.6 (-81--36.2)	3.9 (2.9-5.2)	4 (2.9-5.3)	2.4 (-9.3-16.6)	0 (0-0)	3 (2.2-3.8)	0 (0-0)	8.2 (5.3-11.2)	77	77	10	4
Denmark	24.2 (19.5-29.3)	24.4 (19.8-29.2)	0.8 (-7.4-7.7)	5.8 (4.9-6.8)	5.3 (4.5-6.1)	-8.8 (-14.3--2.8)	0 (0-0)	0 (0-0)	5.1 (-9.3-25.2)	0.3 (0.2-0.4)	0.3 (0.2-0.3)	-7.9 (-14.3--1.9)	0 (0-0)	0 (0-0)	-8.6 (-39.7-25.3)	1.5 (1.1-2)	1.5 (1.1-2)	1.5 (-8.7-12)	0 (0-0)	1.4 (1-1.8)	0 (0-0)	2 (1.4-2.7)	104	93	14	9
Finland	22.5 (18.5-27.1)	22.1 (17.8-27.2)	-1.9 (-11.2-5.2)	5.1 (4.4-5.9)	4.4 (3.6-5.1)	-14.8 (-20.2--10.3)	0 (0-0)	0 (0-0)	17.6 (2.4-29.9)	0.3 (0.2-0.3)	0.2 (0.2-0.3)	-13.8 (-20--7.6)	0 (0-0)	0 (0-0)	-3.1 (-31.2-22.6)	1.3 (1-1.7)	1.2 (0.9-1.6)	-2.7 (-13.2-8.6)	0 (0-0)	1.6 (1.1-2)	0 (0-0)	3 (2.1-4.1)	104	95	14	10
France	57.8 (49.3-68.2)	53.6 (44.7-63.4)	-7.3 (-13.5-0)	11.1 (9.5-12.8)	9.8 (8.3-11.6)	-11.3 (-15.5--6.1)	0 (0-0)	0 (0-0)	7.6 (-3.3-19.6)	0.7 (0.6-0.9)	0.6 (0.5-0.7)	-15.3 (-20.5--9.8)	0.1 (0-0.1)	0.1 (0-0.1)	-1.5 (-22.3-23.4)	4.2 (3.2-5.4)	3.8 (2.9-4.8)	-11 (-18.9--2.5)	0.1 (0-0.1)	3.8 (3-4.9)	0.1 (0.1-0.1)	4.9 (3.6-6.3)	68	61	7	6
Germany	12.9 (11-15.2)	13 (10.9-14.9)	0.5 (-5.4-6.6)	2.9 (2.5-3.4)	2.6 (2.2-3)	-8.8 (-12.6--4)	0 (0-0)	0 (0-0)	29.8 (16.3-43.2)	0.1 (0.1-0.2)	0.1 (0.1-0.2)	-5.7 (-12.3-0.6)	0 (0-0)	0 (0-0)	-5.4 (-23.8-14.8)	0.8 (0.6-1)	0.8 (0.6-1)	0.6 (-8.8-11.4)	0 (0-0)	0.7 (0.6-0.9)	0 (0-0)	1.2 (0.9-1.5)	86	76	23	15
Greece	94.4 (77.6-117)	89.9 (74.7-107)	-4.8 (-16-8.7)	23 (19.8-26.6)	18.8 (16.5-21.1)	-18.3 (-22.7--11.9)	0 (0-0)	0.1 (0.1-0.1)	67.6 (45.5-95.5)	1 (0.9-1.3)	0.9 (0.7-1.1)	-13.9 (-23.7--3.7)	0 (0-0.1)	0.1 (0-0.1)	4 (-18.5-30.3)	5.9 (4.1-8)	5.7 (4.1-7.8)	-3.8 (-18.7-12.9)	0 (0-0)	4.8 (3.3-6.6)	0.1 (0-0.1)	6.8 (4.7-9.7)	63	57	4	4
Iceland	12.8 (9.3-17.1)	12.9 (9.4-17)	1 (-7.3-10.5)	3.3 (2.5-4.1)	2.9 (2.2-3.6)	-12.4 (-18.7--6.2)	0 (0-0)	0 (0-0)	38.1 (19.7-62.1)	0.2 (0.1-0.2)	0.2 (0.1-0.2)	-13.9 (-20.7--5.2)	0 (0-0)	0 (0-0)	-16.5 (-37.6-12.6)	0.7 (0.4-1.1)	0.7 (0.4-1.1)	-0.4 (-10.4-13.5)	0 (0-0)	1 (0.6-1.5)	0 (0-0)	1.2 (0.7-2)	82	78	21	16
Ireland	18 (14-22.7)	18.2 (13.7-23.1)	0.9 (-8.3-9.3)	4.6 (3.7-5.5)	4.1 (3.2-4.9)	-9.9 (-14.8--3.8)	0 (0-0)	0 (0-0)	-19 (-31.4--6.2)	0.3 (0.2-0.3)	0.2 (0.2-0.3)	-8.3 (-15.8--1.1)	0 (0-0)	0 (0-0)	-36.1 (-51.8--18.5)	1.1 (0.8-1.6)	1.1 (0.8-1.6)	0.8 (-10.4-13.2)	0 (0-0)	0.8 (0.6-1.1)	0 (0-0)	2 (1.4-2.9)	82	66	21	14
Israel	45.3 (36.1-57.4)	34.9 (27.4-43.8)	-22.8 (-32.7--15.4)	10.4 (8.7-12.5)	8.6 (7-10.4)	-17.3 (-24.5--12.2)	0 (0-0)	0 (0-0)	-14.7 (-25--0.2)	0.8 (0.6-1.1)	0.6 (0.5-0.8)	-26.9 (-36.1--16)	0.1 (0-0.1)	0 (0-0)	-56.7 (-68.9--44.1)	3.6 (2.4-5)	2.6 (1.8-3.6)	-28.3 (-39.6--14.1)	0 (0-0)	2.6 (1.8-3.5)	0.1 (0-0.1)	6.7 (4.5-9.7)	71	66	9	5
Italy	20 (17.1-23.1)	21.5 (18.4-24.5)	7.3 (4.8-10.9)	4.6 (4-5.2)	4.4 (3.8-5)	-5.5 (-8.7--3)	0 (0-0)	0 (0-0)	-43.1 (-49--37.1)	0.2 (0.2-0.2)	0.2 (0.2-0.2)	-0.1 (-2.9-3.2)	0 (0-0)	0 (0-0)	-52.7 (-63--43.3)	0.9 (0.7-1.1)	0.9 (0.7-1.2)	7.2 (2.8-11.9)	0 (0-0)	0.8 (0.6-1)	0 (0-0)	1.8 (1.4-2.2)	83	81	21	11
Abruzzo	7.8 (5.6-10.5)	8.6 (6.2-11.5)	10 (-3.7-22)	1.5 (1.1-2)	1.4 (1.1-1.9)	-7.2 (-17.8-2)	0 (0-0)	0 (0-0)	-44.5 (-52.2--34.8)	0.1 (0.1-0.1)	0.1 (0.1-0.1)	-3.7 (-13.1-7.2)	0 (0-0)	0 (0-0)	-49.6 (-64.6--33.4)	0.3 (0.2-0.5)	0.4 (0.2-0.5)	9.8 (-5.1-26.4)	0 (0-0)	0.3 (0.2-0.4)	0 (0-0)	0.6 (0.4-0.9)	89	85	36	26
Basilicata	5.7 (3.7-8.4)	7.5 (5-11.3)	32.3 (17.4-53.8)	1.5 (1-2.1)	1.6 (1.1-2.1)	6.5 (-5-18.9)	0 (0-0)	0 (0-0)	-42.1 (-51.8--32.9)	0.1 (0-0.1)	0.1 (0-0.1)	6.8 (-6.2-23.6)	0 (0-0)	0 (0-0)	-40.7 (-61.7--4.8)	0.2 (0.1-0.4)	0.3 (0.2-0.5)	35.8 (12.7-64.3)	0 (0-0)	0.2 (0.1-0.3)	0 (0-0)	0.5 (0.3-0.8)	86	85	47	29
Calabria	23.8 (20.3-27.5)	24.2 (19.9-28.5)	1.6 (-7-12.1)	7.1 (6.1-8.1)	6.2 (5.4-7.1)	-12.5 (-18.1--5.5)	0 (0-0)	0 (0-0)	-31.6 (-42.3--14.8)	0.3 (0.2-0.3)	0.2 (0.2-0.3)	-13.9 (-21.7--5.8)	0 (0-0)	0 (0-0)	-45.1 (-58.6--21.4)	0.9 (0.7-1.3)	1 (0.7-1.3)	1 (-11.9-14.9)	0 (0-0)	0.7 (0.5-1)	0 (0-0)	1.2 (0.9-1.5)	76	79	16	12
Campania	10.1 (8.5-11.9)	12.1 (10.5-13.9)	19.6 (10.2-28.7)	3 (2.5-3.4)	2.9 (2.5-3.3)	-1.4 (-8.4-4.5)	0 (0-0)	0 (0-0)	-41.3 (-50.4--25.1)	0.1 (0.1-0.2)	0.1 (0.1-0.1)	-0.7 (-7.5-7.1)	0 (0-0)	0 (0-0)	-49.9 (-61.7--33.3)	0.5 (0.4-0.6)	0.6 (0.5-0.8)	20.7 (9.8-34)	0 (0-0)	0.4 (0.3-0.5)	0 (0-0)	1 (0.7-1.4)	84	84	27	14
Emilia-Romagna	46.7 (39.2-53.8)	50.9 (43.4-57)	8.9 (-0.2-16.4)	8.1 (7.2-9.1)	8.3 (7.4-9.2)	2.5 (-2.9-8.3)	0 (0-0)	0 (0-0)	-47.3 (-58--38.4)	0.4 (0.4-0.5)	0.5 (0.4-0.5)	12.5 (5.1-20.6)	0 (0-0)	0 (0-0)	-56.4 (-68.8--41.2)	2.1 (1.7-2.7)	2.3 (1.8-2.9)	10.1 (-0.1-22.7)	0 (0-0)	2 (1.6-2.5)	0 (0-0)	4.8 (3.7-6.1)	84	81	11	7
Friuli-Venezia Giulia	34.5 (28-40.6)	35.8 (29.4-41.7)	3.8 (-4-18.7)	5.6 (4.7-6.5)	5.4 (4.6-6.2)	-2.3 (-9.1-6.6)	0 (0-0)	0 (0-0)	-49.9 (-57.9--40.5)	0.3 (0.3-0.4)	0.3 (0.3-0.4)	3.5 (-4.5-12.9)	0 (0-0)	0 (0-0)	-37.8 (-58.1--7.3)	1.4 (1.1-1.9)	1.5 (1.1-2)	2.7 (-8-14.4)	0 (0-0)	1.9 (1.4-2.7)	0 (0-0)	3.3 (2.4-4.5)	90	82	13	9
Lazio	13.6 (11.5-15.4)	15.9 (13.4-18.4)	16.7 (9.6-26.4)	3.3 (2.9-3.9)	3.4 (3-3.9)	1.9 (-2.9-9.1)	0 (0-0)	0 (0-0)	-46.3 (-55.6--35.3)	0.1 (0.1-0.2)	0.2 (0.1-0.2)	8 (1.2-16.1)	0 (0-0)	0 (0-0)	-62.1 (-71.7--51.3)	0.6 (0.4-0.7)	0.6 (0.5-0.8)	12.8 (1.9-24.9)	0 (0-0)	0.5 (0.4-0.6)	0 (0-0)	1.5 (1.1-2)	81	83	27	13
Liguria	34.1 (29-39.5)	36.7 (32-42.7)	7.9 (0.6-14.8)	6.5 (5.7-7.4)	6.5 (5.8-7.3)	-0.7 (-5.7-4.7)	0 (0-0)	0 (0-0)	-45 (-55.5--34.9)	0.3 (0.3-0.3)	0.3 (0.3-0.4)	5.3 (-1.8-12.3)	0 (0-0)	0 (0-0)	-49.7 (-63.9--26)	1.6 (1.2-2.1)	1.8 (1.4-2.4)	12.4 (-0.7-26.8)	0 (0-0)	1.5 (1.1-1.9)	0 (0-0)	3.5 (2.6-4.5)	80	79	11	9
Lombardia	13.3 (11-16)	14 (11.8-16.1)	5.3 (-3.2-13.1)	3.1 (2.6-3.7)	3 (2.6-3.5)	-3.6 (-10.6-3.1)	0 (0-0)	0 (0-0)	-49.1 (-58.4--37.3)	0.1 (0.1-0.2)	0.1 (0.1-0.2)	3.6 (-5.2-12.6)	0 (0-0)	0 (0-0)	-51.6 (-67.7--36)	0.5 (0.4-0.7)	0.5 (0.4-0.7)	7.2 (-3.6-19.7)	0 (0-0)	0.6 (0.4-0.7)	0 (0-0)	1.2 (0.9-1.5)	86	85	23	16
Marche	26.9 (22.2-32.6)	28.9 (23.8-34.4)	7.3 (-0.4-15.7)	5.3 (4.5-6.2)	5.1 (4.5-6.1)	-2.8 (-8-2.5)	0 (0-0)	0 (0-0)	-44.8 (-55.3--32.3)	0.3 (0.2-0.3)	0.3 (0.2-0.3)	2.1 (-5.4-10.2)	0 (0-0)	0 (0-0)	-56.5 (-67.8--45.8)	1.2 (0.9-1.6)	1.3 (1-1.7)	5.9 (-4.9-17.7)	0 (0-0)	1.2 (0.9-1.5)	0 (0-0)	2.9 (2.1-3.7)	85	80	19	9
Molise	14.1 (9.1-21)	13.5 (8.8-20.4)	-4.1 (-13.3-8.2)	2.9 (2-3.9)	2.3 (1.6-3.1)	-20.2 (-26.4--11.7)	0 (0-0)	0 (0-0)	-45 (-54.9--35.7)	0.1 (0.1-0.2)	0.1 (0.1-0.2)	-20.3 (-28.1--10.9)	0 (0-0)	0 (0-0)	-45.6 (-66.9--17.1)	0.6 (0.3-1)	0.6 (0.3-0.9)	-4.1 (-17.5-9.5)	0 (0-0)	0.5 (0.3-0.9)	0 (0-0)	1 (0.6-1.7)	97	94	25	17

Location	Birth Incidence rate (per 100,000 live births), 2000	Birth Incidence rate (per 100,000 live births), 2021	Birth Incidence rate (per 100,000 live births) percent change (2000-2021)	All age rate (per 100,000 population), 2000	All age rate (per 100,000 population), 2021	All age rate (per 100,000 population) percent change (2000-2021)	All age cause-specific SCD mortality rate (per 100,000 population), 2000	All age cause-specific SCD mortality rate (per 100,000 population), 2021	All age cause-specific SCD mortality rate (per 100,000 population) percent change (2000-2021)	All age total SCD mortality rate (per 100,000 population), 2000	All age total SCD mortality rate (per 100,000 population), 2021	All age total SCD mortality rate (per 100,000 population) percent change (2000-2021)	Under-5 cause-specific SCD mortality rate (per 100,000 population), 2000	Under-5 cause-specific SCD mortality rate (per 100,000 population), 2021	Under-5 cause-specific SCD mortality rate (per 100,000 population) percent change (2000-2021)	Under-5 total SCD mortality rate (per 100,000 population), 2000	Under-5 total SCD mortality rate (per 100,000 population), 2021	Under-5 total SCD mortality rate (per 100,000 population) percent change (2000-2021)	Under-5 cause-specific mortality cause fraction, 2000	Under-5 total SCD mortality cause fraction, 2000	Under-5 cause-specific mortality cause fraction, 2021	Under-5 total SCD mortality cause fraction, 2021	Under-5 cause-specific mortality rank 2000	Under-5 cause-specific mortality rank 2021	Under-5 total SCD mortality rank 2000	Under-5 total SCD mortality rank 2021
Piemonte	19.5 (15.9-23.7)	22 (18.4-25.6)	13.1 (5.5-20.1)	3.2 (2.7-3.7)	3.2 (2.7-3.7)	1.6 (-4.6-6.9)	0 (0-0)	0 (0-0)	-48.3 (-56.3--38.8)	0.2 (0.1-0.2)	0.2 (0.2-0.2)	11 (3.4-19.6)	0 (0-0)	0 (0-0)	-49.5 (-67.8--27.2)	0.8 (0.6-1.1)	0.9 (0.7-1.2)	16.1 (3.6-30.3)	0 (0-0)	0.8 (0.6-1.1)	0 (0-0)	2.1 (1.5-2.6)	92	89	22	11
Provincia autonoma di Bolzano	15.3 (10.9-21.2)	15.8 (11.5-21.6)	3.5 (-5.9-19.5)	3.5 (2.6-4.4)	3.2 (2.4-3.9)	-8.3 (-13.9--0.2)	0 (0-0)	0 (0-0)	-55 (-62.5--42.9)	0.2 (0.1-0.2)	0.2 (0.1-0.2)	-5.8 (-14.7-5)	0 (0-0)	0 (0-0)	-42.8 (-63.1--12.3)	0.6 (0.4-0.9)	0.6 (0.4-0.9)	2.5 (-9.3-18)	0 (0-0)	0.7 (0.4-1)	0 (0-0)	1.2 (0.7-1.7)	91	91	24	16
Provincia autonoma di Trento	13.6 (10.2-18.4)	14 (10.4-19.3)	2.8 (-8.8-13.3)	3 (2.3-3.7)	2.7 (2.1-3.4)	-7 (-15.1-1.2)	0 (0-0)	0 (0-0)	-48.3 (-56.7--34.5)	0.1 (0.1-0.2)	0.1 (0.1-0.2)	-2.5 (-12.2-8.3)	0 (0-0)	0 (0-0)	-43.8 (-63.6--20.9)	0.6 (0.4-0.8)	0.6 (0.4-0.9)	3.5 (-10.7-19.7)	0 (0-0)	0.7 (0.5-1)	0 (0-0)	1.2 (0.8-1.7)	86	84	24	19
Puglia	8.4 (6.6-10.7)	8.3 (6.5-10.2)	-1.8 (-12.3-8.2)	2.4 (1.9-2.9)	1.9 (1.6-2.2)	-18.9 (-26.2--11.5)	0 (0-0)	0 (0-0)	-39.6 (-49.3--26.4)	0.1 (0.1-0.1)	0.1 (0.1-0.1)	-18.7 (-26.9--9.6)	0 (0-0)	0 (0-0)	-55.6 (-67.1--38)	0.3 (0.2-0.5)	0.3 (0.2-0.4)	-3.2 (-16.3-11.1)	0 (0-0)	0.3 (0.2-0.3)	0 (0-0)	0.6 (0.4-0.7)	88	85	35	25
Sardegna	7.2 (5.1-9.8)	7.9 (5.6-10.7)	9 (-1.7-26.7)	1.6 (1.2-2.1)	1.4 (1-1.8)	-15.5 (-21.2--7.9)	0 (0-0)	0 (0-0)	-27.3 (-43.8--9.1)	0.1 (0.1-0.1)	0.1 (0-0.1)	-11.3 (-19.9--0.6)	0 (0-0)	0 (0-0)	-50.1 (-66.1--26.3)	0.3 (0.2-0.4)	0.3 (0.2-0.5)	9.6 (-6.7-28.2)	0 (0-0)	0.3 (0.2-0.4)	0 (0-0)	0.7 (0.4-1)	88	89	35	21
Sicilia	39.7 (34.6-45.3)	38 (33.2-43.1)	-4.4 (-10.2-1.5)	12.1 (10.7-14)	10.4 (9.3-11.7)	-13.9 (-18.3--9.4)	0 (0-0)	0 (0-0)	-34.7 (-45.6--20.5)	0.5 (0.4-0.5)	0.4 (0.3-0.4)	-16.8 (-22.1--11.2)	0.1 (0.1-0.1)	0 (0-0)	-46.2 (-61.3--27.3)	2 (1.6-2.6)	1.9 (1.5-2.3)	-8.7 (-18.4-1.5)	0 (0-0.1)	1.3 (1-1.7)	0 (0-0.1)	2.3 (1.8-3.2)	64	68	11	10
Toscana	14.4 (11.6-17.4)	15.2 (12.3-18.9)	5.2 (-3.4-16.9)	2.7 (2.2-3.2)	2.6 (2.2-3.1)	-3.9 (-11.1-3.7)	0 (0-0)	0 (0-0)	-44.8 (-55.2--34.4)	0.1 (0.1-0.2)	0.1 (0.1-0.2)	6.2 (-2-16.2)	0 (0-0)	0 (0-0)	-61.7 (-74.4--49)	0.5 (0.4-0.7)	0.6 (0.4-0.8)	7.1 (-4.7-21.3)	0 (0-0)	0.6 (0.4-0.8)	0 (0-0)	1.6 (1.1-2.1)	87	88	23	11
Umbria	20.9 (16.9-25.3)	22.3 (17.9-27.4)	7.1 (-2-17.5)	3.8 (3.1-4.5)	3.7 (3.2-4.4)	-0.8 (-7.6-5.2)	0 (0-0)	0 (0-0)	-49.4 (-57.9--37.1)	0.2 (0.2-0.2)	0.2 (0.2-0.2)	4.1 (-4-13.4)	0 (0-0.1)	0 (0-0)	-63.9 (-75.2--47.2)	1 (0.7-1.3)	1.1 (0.8-1.4)	8.5 (-4-23.2)	0 (0-0)	0.9 (0.7-1.3)	0 (0-0)	2.3 (1.5-3)	68	73	20	11
Valle d'Aosta	9.7 (5.4-14.8)	8.7 (4.9-14.3)	-10.2 (-26.1-6.6)	1.3 (0.8-1.9)	1 (0.7-1.6)	-16.8 (-27.2--3.8)	0 (0-0)	0 (0-0)	-60.2 (-67.5--53.3)	0.1 (0.1-0.1)	0.1 (0-0.1)	-10.1 (-22.4-1.5)	0 (0-0)	0 (0-0)	-52 (-66.9--28.7)	0.5 (0.3-0.8)	0.4 (0.2-0.7)	-10.5 (-24.1-4.7)	0 (0-0)	0.4 (0.2-0.7)	0 (0-0)	1.1 (0.5-1.8)	95	95	32	25
Veneto	24.7 (20.9-28.4)	27 (23.4-30.3)	9.4 (0.9-14.8)	5.5 (4.9-6.2)	5.4 (4.8-6)	-2.9 (-9.4-1.5)	0 (0-0)	0 (0-0)	-49.3 (-58.1--38.3)	0.3 (0.2-0.3)	0.3 (0.2-0.3)	3.2 (-2.9-9.7)	0 (0-0)	0 (0-0)	-46.3 (-62.5--29)	1.1 (0.9-1.4)	1.2 (0.9-1.5)	7.8 (-1.9-18.4)	0 (0-0)	1.3 (1.1-1.7)	0 (0-0)	2.6 (2-3.3)	86	85	13	10
Luxembourg	83.1 (71.5-97.4)	78 (65.7-93.1)	-6.1 (-13.7-2)	23.8 (20.7-27.2)	20.1 (17.5-23.4)	-15.6 (-20.5--10.2)	0 (0-0)	0 (0-0)	-14.2 (-24.4-0)	1.1 (0.9-1.2)	0.9 (0.8-1.1)	-12.1 (-18.2--4.4)	0 (0-0)	0 (0-0)	-6.9 (-31.1-37.2)	4.8 (3.5-6.5)	4.6 (3.3-6.2)	-4.4 (-15.6-9.2)	0 (0-0)	5.7 (4.1-7.3)	0 (0-0)	6.7 (4.3-9.8)	76	68	6	5
Malta	27.1 (22.1-32.8)	27.1 (21.7-32.6)	0 (-5.9-7.4)	6.9 (5.8-8.2)	5.4 (4.4-6.4)	-20.8 (-24.8--16.4)	0 (0-0)	0 (0-0)	41.9 (18.3-74.2)	0.3 (0.3-0.4)	0.3 (0.2-0.3)	-18.5 (-23.4--12.2)	0 (0-0)	0 (0-0)	25.2 (-13.2-96.2)	1.8 (1.3-2.3)	1.8 (1.3-2.3)	0.9 (-7.9-11.9)	0 (0-0)	1.3 (1-1.7)	0 (0-0)	1.7 (1.1-2.4)	84	72	12	12
Monaco	17.7 (13.8-22.9)	17.9 (13.7-22.6)	0.9 (-6.3-11.5)	3.1 (2.5-3.7)	3.1 (2.5-3.7)	-1.4 (-6.1-5.1)	0 (0-0)	0 (0-0)	-22.8 (-44.9-18.2)	0.2 (0.1-0.2)	0.2 (0.1-0.2)	2 (-5.3-10.5)	0 (0-0.1)	0 (0-0)	-73.9 (-90.5--26.1)	1.1 (0.8-1.5)	1.1 (0.8-1.5)	1.8 (-8.8-14.9)	0 (0-0.1)	1.2 (0.8-1.8)	0 (0-0.1)	3.5 (2.4-5)	67	70	16	7
Netherlands	2.4 (1.9-3)	2.5 (2-3)	2 (-5.6-11.7)	0.5 (0.4-0.6)	0.4 (0.3-0.5)	-12.6 (-17.5--6.3)	0 (0-0)	0 (0-0)	1.7 (-8.5-12.8)	0 (0-0)	0 (0-0)	-11.1 (-17.2--1.4)	0 (0-0.1)	0 (0-0)	-30.4 (-46.7--10.4)	0.2 (0.1-0.2)	0.2 (0.1-0.2)	2.2 (-7.5-16.7)	0 (0-0)	0.1 (0.1-0.2)	0 (0-0.1)	0.2 (0.2-0.3)	70	69	45	33
Norway	16.8 (11.2-24.7)	16.7 (10.9-25.1)	-0.5 (-4.7-4.2)	2.4 (1.7-3.4)	2.1 (1.5-3)	-12.4 (-15.5--9.6)	0 (0-0)	0 (0-0)	-53.4 (-58.5--48.3)	0.2 (0.1-0.3)	0.2 (0.1-0.3)	-13.9 (-17.7--9.2)	0 (0-0)	0 (0-0)	-57.6 (-67--45.2)	1.3 (0.7-2.2)	1.3 (0.7-2.1)	-1.2 (-7.8-6)	0 (0-0)	1.4 (0.7-2.3)	0 (0-0)	3.5 (1.8-5.8)	101	100	13	9
Agder	18.1 (11.2-27.5)	17.7 (11.1-26.2)	-2.3 (-17.5-10.9)	2.6 (1.8-3.7)	2.2 (1.5-3.1)	-14.9 (-22.3--7)	0 (0-0)	0 (0-0)	-59.1 (-66--47)	0.2 (0.1-0.4)	0.2 (0.1-0.3)	-15.8 (-26.7--3.4)	0 (0-0)	0 (0-0)	-61.2 (-72.3--47.7)	1.5 (0.7-2.5)	1.4 (0.7-2.3)	-2.8 (-21.2-16.8)	0 (0-0)	1.4 (0.7-2.3)	0 (0-0)	3.2 (1.5-5.5)	107	110	15	9
Innlandet	17.5 (11-26.1)	17.6 (11.3-26.7)	0.2 (-13.4-16.4)	2.2 (1.6-3.1)	1.9 (1.3-2.7)	-15.3 (-23.7--6.6)	0 (0-0)	0 (0-0)	-53.3 (-63.8--39.4)	0.2 (0.1-0.3)	0.2 (0.1-0.3)	-16.4 (-28.2--3.7)	0 (0-0)	0 (0-0)	-54.7 (-65--37.7)	1.4 (0.7-2.4)	1.4 (0.7-2.4)	-0.1 (-20.8-22.7)	0 (0-0)	1.5 (0.7-2.4)	0 (0-0)	3.6 (1.9-6.1)	106	109	14	8
Møre og Romsdal	17.3 (10.6-25.4)	17.3 (10.6-26.6)	0 (-12.5-16.8)	2.4 (1.7-3.4)	2.1 (1.4-2.9)	-15.2 (-23.8--7.7)	0 (0-0)	0 (0-0)	-49.7 (-59.8--34.6)	0.2 (0.1-0.3)	0.2 (0.1-0.3)	-16.4 (-26.2--5)	0 (0-0)	0 (0-0)	-49.9 (-63.9--30.7)	1.5 (0.7-2.4)	1.4 (0.7-2.5)	-0.3 (-19.4-22.7)	0 (0-0)	1.6 (0.8-2.7)	0 (0-0)	3.5 (1.6-5.7)	103	104	14	9
Nordland	17.8 (11.3-26.8)	17.5 (10.8-25.9)	-1.5 (-12.8-11.3)	2.5 (1.7-3.5)	2 (1.4-2.9)	-18.3 (-25.6--10.4)	0 (0-0)	0 (0-0)	-37.8 (-51.5--19.8)	0.2 (0.1-0.4)	0.2 (0.1-0.3)	-20.7 (-30.1--10.9)	0 (0-0)	0 (0-0)	-37.2 (-57--13.4)	1.5 (0.7-2.4)	1.4 (0.7-2.4)	-2.1 (-18.5-17.9)	0 (0-0)	1.6 (0.8-2.7)	0 (0-0)	3 (1.6-4.8)	103	102	12	9
Oslo	16.8 (10.9-25.2)	16.9 (10.7-25.2)	0.2 (-10.1-11.3)	2.3 (1.6-3.1)	2.2 (1.6-3.1)	-1 (-8.2-5)	0 (0-0)	0 (0-0)	-65.4 (-73.7--53.3)	0.2 (0.1-0.3)	0.2 (0.1-0.3)	-1.1 (-12.4-12.5)	0 (0-0)	0 (0-0)	-63 (-75--47.2)	1.3 (0.7-2.2)	1.4 (0.7-2.2)	1 (-16-21.3)	0 (0-0)	1.1 (0.6-1.7)	0 (0-0)	4.1 (2-7.1)	108	107	16	7
Rogaland	16.2 (9.9-23.2)	15.9 (10.3-23.7)	-1.7 (-13.2-11.4)	2.7 (1.9-3.8)	2.3 (1.6-3.2)	-14.5 (-21.5--5.9)	0 (0-0)	0 (0-0)	-51.6 (-61.6--40.5)	0.2 (0.1-0.4)	0.2 (0.1-0.3)	-15.6 (-26.6--2.5)	0 (0-0)	0 (0-0)	-59.5 (-70.8--45.3)	1.2 (0.6-2)	1.2 (0.6-2)	-2.5 (-20.2-21.1)	0 (0-0)	1.3 (0.7-2.2)	0 (0-0)	3.5 (1.7-6)	91	87	14	8
Troms og Finnmark	18.2 (11.6-27)	18.2 (11.6-27)	0.1 (-15-17.6)	2.6 (1.8-3.6)	2.1 (1.5-2.9)	-19.2 (-29--9.9)	0 (0-0)	0 (0-0)	-52.9 (-63.3--37.3)	0.3 (0.2-0.4)	0.2 (0.1-0.3)	-22.3 (-32.5--9.1)	0 (0-0)	0 (0-0)	-56.8 (-70.3--38.2)	1.5 (0.8-2.5)	1.5 (0.8-2.5)	-3.4 (-22.5-23.8)	0 (0-0)	1.5 (0.7-2.4)	0 (0-0)	3.4 (1.7-5.8)	101	99	15	9
Trøndelag	16.3 (9.9-24.1)	16.3 (10.1-24.1)	-0.3 (-14.9-17.8)	2.4 (1.7-3.4)	2.1 (1.5-3)	-12.1 (-20.9--3.2)	0 (0-0)	0 (0-0)	-48 (-60.9--29.7)	0.2 (0.1-0.3)	0.2 (0.1-0.3)	-14.2 (-25.9--0.8)	0 (0-0)	0 (0-0)	-52 (-64.3--31)	1.3 (0.6-2.2)	1.3 (0.6-2.1)	-0.9 (-20.1-23.6)	0 (0-0)	1.6 (0.8-2.7)	0 (0-0)	3.7 (1.8-6.5)	104	104	13	8
Vestfold og Telemark	17 (10.8-25.4)	17 (10.7-25)	-0.3 (-11.6-13.9)	2.4 (1.7-3.4)	2 (1.4-2.8)	-14.9 (-21.1--7.1)	0 (0-0)	0 (0-0)	-64.1 (-70.8--54.4)	0.2 (0.1-0.3)	0.2 (0.1-0.3)	-16 (-26.2--4.4)	0 (0-0)	0 (0-0)	-58.8 (-69.7--39.6)	1.4 (0.7-2.2)	1.3 (0.7-2.2)	-1.1 (-18.5-23.8)	0 (0-0)	1.4 (0.7-2.3)	0 (0-0)	3.5 (1.8-5.9)	89	90	14	9
Vestland	16.2 (10-23.7)	16.2 (10.4-24.2)	0.1 (-11.5-14.1)	2.5 (1.8-3.5)	2.2 (1.6-3.1)	-12.6 (-19.4--3.2)	0 (0-0)	0 (0-0)	-47.3 (-57--32.1)	0.2 (0.1-0.3)	0.2 (0.1-0.3)	-14.2 (-25.6--2.3)	0 (0-0)	0 (0-0)	-41.7 (-57.5--20.3)	1.2 (0.6-2)	1.2 (0.6-2.1)	-0.2 (-19.1-22.7)	0 (0-0)	1.5 (0.8-2.5)	0 (0-0)	3.2 (1.6-5)	107	100	14	9
Viken	16.1 (9.9-24.2)	16.2 (10.5-24.4)	0.4 (-16.5-14.5)	2.4 (1.7-3.3)	2.1 (1.5-2.9)	-12 (-19.9--3.2)	0 (0-0)	0 (0-0)	-55.7 (-63.7--45.1)	0.2 (0.1-0.3)	0.2 (0.1-0.3)	-12.4 (-25.6-0)	0 (0-0)	0 (0-0)	-46.7 (-61.3--21.9)	1.3 (0.6-2.2)	1.3 (0.6-2.2)	0.3 (-22.1-22.7)	0 (0-0)	1.4 (0.7-2.3)	0 (0-0)	3.5 (1.7-6.1)	106	105	13	9
Portugal	98.8 (76.9-126)	89.6 (70.3-115)	-9.3 (-20.2-4.5)	20.6 (17.6-23.7)	14.3 (11.9-17.1)	-30.4 (-39--25)	0 (0-0)	0 (0-0)	-17.8 (-24.3--9.7)	1.1 (0.9-1.4)	0.8 (0.6-1)	-27.1 (-35.4--17.1)	0.1 (0.1-0.1)	0 (0-0.1)	-36.8 (-55.4--19.4)	7.2 (4.8-10.2)	6.6 (4.4-9.4)	-8.2 (-24-10.4)	0 (0-0.1)	4.7 (3.2-6.6)	0.1 (0.1-0.1)	10.2 (6.4-14.6)	76	68	6	3
San Marino	16.8 (13-21.5)	16.9 (13-21.8)	0.3 (-6.9-9.3)	4 (3.2-4.8)	3.5 (2.9-4.3)	-10.9 (-16.3--5.1)	0 (0-0)	0 (0-0)	-42.8 (-63.7-1.4)	0.2 (0.2-0.3)	0.2 (0.1-0.2)	-11.8 (-19.1--4.4)	0.1 (0-0.2)	0 (0-0)	-77.3 (-94.2--39.8)	1.1 (0.8-1.5)	1.1 (0.8-1.5)	-0.2 (-10.4-12.2)	0.1 (0-0.2)	1.2 (0.8-1.8)	0.1 (0-0.2)	6.5 (4.1-9.9)	63	62	22	3
Spain	24.2 (19.9-28.9)	24.3 (19.6-29.2)	0.3 (-9.1-9.1)	4.5 (3.8-5.2)	4.1 (3.5-4.7)	-9.3 (-15.6--3)	0 (0-0)	0 (0-0)	4.7 (-7.9-17.2)	0.3 (0.2-0.3)	0.2 (0.2-0.3)	-5.7 (-13.6-3.9)	0 (0-0)	0 (0-0)	-16.1 (-33-5.2)	1.4 (1-1.9)	1.4 (1-1.9)	-0.5 (-13-14.6)	0 (0-0)	1.2 (0.9-1.6)	0 (0-0)	2.8 (2.1-3.9)	84	74	16	10

Location	Birth Incidence rate (per 100,000 live births), 2000	Birth Incidence rate (per 100,000 live births), 2021	Birth Incidence rate (per 100,000 live births) percent change (2000-2021)	All age rate (per 100,000 population), 2000	All age rate (per 100,000 population), 2021	All age rate (per 100,000 population) percent change (2000-2021)	All age cause-specific SCD mortality rate (per 100,000 population), 2000	All age cause-specific SCD mortality rate (per 100,000 population), 2021	All age cause-specific SCD mortality rate (per 100,000 population) percent change (2000-2021)	All age total SCD mortality rate (per 100,000 population), 2000	All age total SCD mortality rate (per 100,000 population), 2021	All age total SCD mortality rate (per 100,000 population) percent change (2000-2021)	Under-5 cause-specific SCD mortality rate (per 100,000 population), 2000	Under-5 cause-specific SCD mortality rate (per 100,000 population), 2021	Under-5 cause-specific SCD mortality rate (per 100,000 population) percent change (2000-2021)	Under-5 total SCD mortality rate (per 100,000 population), 2000	Under-5 total SCD mortality rate (per 100,000 population), 2021	Under-5 total SCD mortality rate (per 100,000 population) percent change (2000-2021)	Under-5 cause-specific mortality cause fraction, 2000	Under-5 total cause fraction, 2000	Under-5 cause-specific mortality cause fraction, 2021	Under-5 total SCD mortality cause fraction, 2021	Under-5 cause-specific mortality rank 2000	Under-5 cause-specific mortality rank 2021	Under-5 total SCD mortality rank 2000	Under-5 total SCD mortality rank 2021
Sweden	17.1 (12.9-22.5)	18.8 (13.9-24.5)	10 (0-20.9)	4.1 (3.3-5)	4.3 (3.4-5.4)	6.1 (-0.3-13.4)	0 (0-0)	0 (0-0)	105 (71.6-141)	0.2 (0.2-0.3)	0.2 (0.2-0.3)	8.1 (-1.6-18.1)	0 (0-0)	0 (0-0)	14.6 (-9-49.6)	0.8 (0.5-1.1)	0.9 (0.6-1.3)	10.4 (-0.8-22.5)	0 (0-0)	1 (0.7-1.5)	0 (0-0)	2.1 (1.4-3.1)	90	81	16	13
Stockholm	17.1 (13.1-22)	18.8 (14.3-24.5)	10.2 (1.1-21.3)	4.3 (3.4-5.3)	4.6 (3.5-5.6)	6.9 (0.2-14.2)	0 (0-0)	0 (0-0)	29.8 (4.2-61.4)	0.2 (0.2-0.3)	0.2 (0.2-0.3)	10.3 (-0.6-21.8)	0 (0-0)	0 (0-0)	-10.8 (-33.1-20.6)	0.8 (0.5-1.2)	0.9 (0.6-1.3)	11.1 (-3-27.3)	0 (0-0)	1 (0.7-1.5)	0 (0-0)	2.4 (1.4-3.5)	89	82	17	12
Sweden except Stockholm	17.1 (12.8-22.4)	18.8 (13.9-24.7)	10 (-1-25.4)	4.1 (3.3-5)	4.3 (3.3-5.3)	5.7 (-1.7-15.1)	0 (0-0)	0 (0-0)	136 (90.8-174)	0.2 (0.2-0.3)	0.2 (0.2-0.3)	7.4 (-4.2-20.5)	0 (0-0)	0 (0-0)	24.1 (-6.9-59.7)	0.8 (0.5-1.1)	0.9 (0.6-1.3)	10.1 (-3.8-24.6)	0 (0-0)	1.1 (0.7-1.5)	0 (0-0)	2.1 (1.4-3)	91	82	17	14
Switzerland	47.9 (43.3-55)	49 (44.5-54.9)	2.4 (-2.5-8.2)	12.6 (11.6-13.7)	11.5 (10.5-12.4)	-9.1 (-13.6--4.3)	0 (0-0)	0 (0-0)	-4.2 (-16.5-10.8)	0.6 (0.5-0.7)	0.6 (0.5-0.6)	-4.5 (-9.7-0.8)	0 (0-0)	0 (0-0)	-22.5 (-49.2-4.8)	2.6 (2.1-3.2)	2.7 (2.1-3.4)	3.6 (-8.1-16.5)	0 (0-0)	2.2 (1.7-2.7)	0 (0-0)	4 (3-5.1)	87	77	12	8
United Kingdom	26.9 (21.8-33.2)	27.8 (22.7-34.4)	3.5 (2.2-4.6)	5.1 (4-6.1)	5 (4-6)	-1.7 (-3--0.2)	0 (0-0)	0 (0-0)	-9.3 (-12.1--6.4)	0.3 (0.3-0.4)	0.3 (0.2-0.4)	-1.9 (-3.6-0)	0 (0-0)	0 (0-0)	-19 (-24.9--12.8)	1.8 (1.3-2.4)	1.9 (1.4-2.6)	7.2 (4.4-10)	0 (0-0)	1.5 (1.1-2)	0 (0-0)	2 (1.4-2.6)	76	70	13	11
England	28.4 (23-34.7)	29.3 (23.6-35.9)	3.2 (2-4.4)	5.3 (4.2-6.4)	5.2 (4.1-6.4)	-0.6 (-2-1)	0 (0-0)	0 (0-0)	-11.8 (-15.1--8.5)	0.3 (0.3-0.4)	0.3 (0.3-0.4)	-0.9 (-2.5-0.9)	0 (0-0)	0 (0-0)	-16 (-22.1--9.9)	1.9 (1.4-2.6)	2.1 (1.5-2.7)	7 (4.3-9.5)	0 (0-0)	1.6 (1.1-2.1)	0 (0-0)	2 (1.4-2.7)	77	69	13	11
East Midlands	20.2 (15.6-25.4)	21.2 (16.7-26.9)	5.2 (1.6-9.7)	3.6 (2.8-4.4)	3.5 (2.8-4.3)	-1.2 (-4.3-3.8)	0 (0-0)	0 (0-0)	-3.2 (-11.4-6.7)	0.2 (0.2-0.3)	0.2 (0.2-0.3)	-1.5 (-5.5-3.1)	0 (0-0)	0 (0-0)	3 (-8.9-18.4)	1.4 (1-1.9)	1.5 (1-2)	7.8 (2.5-14.4)	0 (0-0)	1.2 (0.8-1.6)	0 (0-0)	1.5 (1.1-2)	90	80	14	12
Derby	20.8 (15.3-28.6)	21.3 (16.3-28.7)	2.3 (-7.6-13.4)	3.9 (2.8-5.3)	4 (2.9-5.3)	0.6 (-6.9-8.4)	0 (0-0)	0 (0-0)	-6.7 (-17.2-5.9)	0.3 (0.2-0.4)	0.3 (0.2-0.4)	0.8 (-9-10.3)	0 (0-0)	0.1 (0-0.1)	61.2 (27.7-106)	1.5 (1-2.1)	1.6 (1-2.2)	1.9 (-11.6-15)	0 (0-0)	1 (0.7-1.5)	0 (0-0)	1 (0.7-1.5)	79	68	14	14
Derbyshire	10.9 (7.3-15.3)	11 (8-15.4)	1.4 (-9.8-12.7)	1.6 (1.2-2.2)	1.5 (1.1-1.9)	-11.3 (-18.4-1.8)	0 (0-0)	0 (0-0)	21.3 (1.8-45.1)	0.1 (0.1-0.2)	0.1 (0.1-0.2)	-13.3 (-23.1--2.4)	0 (0-0)	0 (0-0)	33.7 (5.4-71.6)	0.8 (0.5-1.2)	0.8 (0.5-1.2)	0.2 (-15-17.7)	0 (0-0)	0.8 (0.5-1.2)	0 (0-0)	0.8 (0.5-1.3)	95	85	17	19
Leicester	32 (24.2-40.8)	32.2 (25.4-41.2)	0.6 (-5.8-8.7)	7.1 (5.3-9.1)	6.9 (5.4-9)	-2.4 (-9.1-4.6)	0 (0-0)	0 (0-0)	-21.5 (-31.1--9.3)	0.4 (0.3-0.6)	0.4 (0.3-0.6)	-2.2 (-10.2-6.5)	0 (0-0)	0 (0-0)	0.3 (-26.1-37.8)	2.1 (1.5-2.8)	2.1 (1.5-2.9)	1.3 (-9-12.9)	0 (0-0)	1.2 (0.8-1.7)	0 (0-0)	1.4 (1-1.9)	77	72	13	14
Leicestershire	10.6 (7.2-15.5)	10.9 (7.6-15.2)	2.1 (-10-18.2)	1.6 (1.2-2.1)	1.5 (1.1-2)	-7.6 (-15.9-7.1)	0 (0-0)	0 (0-0)	24.2 (4.2-47.8)	0.1 (0.1-0.2)	0.1 (0.1-0.2)	-9.2 (-19.5-5.8)	0 (0-0)	0 (0-0)	35.9 (14.2-70.3)	0.8 (0.5-1.2)	0.8 (0.5-1.2)	0.7 (-14.5-20.9)	0 (0-0)	0.8 (0.5-1.2)	0 (0-0)	0.8 (0.4-1.2)	103	97	17	20
Lincolnshire	11.3 (7.7-16.3)	11.5 (8.3-15.8)	1.8 (-11.4-18.3)	1.6 (1.2-2.1)	1.5 (1.1-1.9)	-7 (-15.7-14)	0 (0-0)	0 (0-0)	23 (2-46.5)	0.1 (0.1-0.2)	0.1 (0.1-0.2)	-9.7 (-21-6.2)	0 (0-0)	0 (0-0)	7.1 (-16.6-30.6)	0.8 (0.5-1.3)	0.8 (0.5-1.3)	0.6 (-13.6-20.2)	0 (0-0)	0.7 (0.4-1.1)	0 (0-0)	1 (0.6-1.5)	104	98	18	17
Northamptonshire	34.2 (25.8-43.1)	34.7 (26.9-43.3)	1.5 (-6.2-9.3)	7.5 (5.7-9.8)	7 (5.3-8.9)	-6.8 (-14.2-0.1)	0 (0-0)	0 (0-0)	-15 (-27.6--1.2)	0.4 (0.3-0.6)	0.4 (0.3-0.5)	-5.4 (-12-1.4)	0 (0-0)	0 (0-0)	-54.2 (-64.4--43.1)	2.2 (1.5-3)	2.2 (1.6-3)	2.8 (-6.7-13.1)	0 (0-0)	1.9 (1.3-2.5)	0 (0-0)	5 (3.5-7.1)	79	71	9	5
Nottingham	51 (38.6-66.3)	51.7 (40.3-68.4)	1.4 (-4.7-8.6)	10.6 (8.1-13.7)	10.5 (8.1-13.5)	-0.9 (-7.4-5.3)	0 (0-0)	0 (0-0)	-14.1 (-25.1--0.6)	0.7 (0.5-0.9)	0.7 (0.5-0.9)	2.5 (-5.8-11.1)	0 (0-0)	0 (0-0)	-6.3 (-26.7-11.6)	3.9 (2.7-5.4)	4 (2.8-5.7)	3.5 (-9.3-16.5)	0 (0-0)	2.1 (1.4-2.8)	0 (0-0)	3 (2-4.2)	89	80	9	5
Nottinghamshire	11.2 (7.6-15.6)	11.3 (8.2-15.7)	0.6 (-8.3-15)	1.6 (1.2-2.2)	1.5 (1.1-2)	-7.3 (-13.2-1.3)	0 (0-0)	0 (0-0)	4 (-11.1-21.8)	0.1 (0.1-0.2)	0.1 (0.1-0.2)	-8.3 (-17.5-4.7)	0 (0-0)	0 (0-0)	22.5 (-5.2-55.2)	0.8 (0.5-1.3)	0.8 (0.5-1.3)	-0.8 (-13.6-17.6)	0 (0-0)	0.8 (0.5-1.3)	0 (0-0)	0.8 (0.5-1.3)	95	89	17	19
Rutland	10.8 (7.8-15.5)	11.2 (8-16.4)	3.4 (-10.4-22.6)	1.6 (1.2-2.3)	1.5 (1.1-2.4)	-8.1 (-19.2-17)	0 (0-0)	0 (0-0)	-13.3 (-30.5-6.1)	0.1 (0.1-0.2)	0.1 (0.1-0.2)	-11.2 (-22.2-6.6)	0 (0-0.1)	0 (0-0)	-54.8 (-70.2--31.8)	0.8 (0.5-1.3)	0.9 (0.5-1.4)	1.6 (-13.7-22.2)	0.1 (0-0.1)	1.1 (0.6-1.7)	0 (0-0.1)	2.4 (1.3-3.6)	68	65	19	11
East of England	22.6 (17.5-29.5)	21.6 (16.4-28.4)	-4.4 (-8--1.6)	4.5 (3.4-5.6)	4 (3-5)	-11.2 (-14.2--8.6)	0 (0-0)	0 (0-0)	-0.9 (-8.7-7.7)	0.3 (0.2-0.4)	0.3 (0.2-0.3)	-10.9 (-14--7.9)	0 (0-0)	0 (0-0)	13.1 (0.7-25.7)	1.5 (1-2.1)	1.4 (1-2)	-4.2 (-8.4-0.1)	0 (0-0)	1.5 (1-2.1)	0 (0-0)	1.6 (1.1-2.1)	84	74	13	12
Bedford	46.5 (32.7-61.8)	38.2 (27.3-50.2)	-17.8 (-25.3--10.3)	9.4 (6.6-12.7)	7.3 (5.1-9.9)	-22.4 (-29.7--14.8)	0 (0-0)	0 (0-0)	-1.7 (-17.1-13.7)	0.6 (0.4-0.8)	0.5 (0.3-0.6)	-22.8 (-29.2--15.6)	0 (0-0)	0 (0-0)	12.7 (-19-44)	3.6 (2.4-5.2)	2.9 (2-4.1)	-19.2 (-28.7--8.1)	0 (0-0)	2.9 (1.8-4.1)	0 (0-0)	2.3 (1.4-3.1)	78	75	7	11
Cambridgeshire	14.3 (10.7-19.5)	14.8 (10.8-20.1)	3.2 (-5.4-13.5)	2.6 (2-3.5)	2.4 (1.8-3.2)	-5.8 (-12.9-2.1)	0 (0-0)	0 (0-0)	2.9 (-12.6-18.9)	0.2 (0.1-0.2)	0.2 (0.1-0.2)	-6.6 (-15-2.2)	0 (0-0)	0 (0-0)	7.4 (-18.3-39.7)	1 (0.7-1.4)	1 (0.7-1.5)	3.1 (-9.2-17.2)	0 (0-0)	1.1 (0.7-1.6)	0 (0-0)	1.3 (0.8-1.9)	95	86	17	14
Central Bedfordshire	10.9 (8.2-14.9)	11.1 (7.9-15.4)	1.6 (-7.9-17.8)	1.8 (1.3-2.4)	1.6 (1.2-2.2)	-9.6 (-17-8)	0 (0-0)	0 (0-0)	-10.4 (-23.3-5.9)	0.1 (0.1-0.2)	0.1 (0.1-0.2)	-10.9 (-19-1.1)	0 (0-0)	0 (0-0)	55.2 (19.3-98.1)	0.8 (0.5-1.3)	0.8 (0.5-1.3)	0.2 (-13.6-17.2)	0 (0-0)	0.9 (0.5-1.3)	0 (0-0)	0.8 (0.5-1.2)	87	75	18	22
Essex	21.3 (16.2-28.6)	22 (16.4-28.7)	3.2 (-4.2-9.3)	4.3 (3.1-5.6)	4.1 (2.9-5.2)	-5.4 (-11.4--0.8)	0 (0-0)	0 (0-0)	11 (-4.7-26.4)	0.3 (0.2-0.4)	0.3 (0.2-0.3)	-5.2 (-12.1-1.2)	0 (0-0)	0 (0-0)	15.3 (-9.2-44)	1.3 (0.9-1.9)	1.4 (0.9-1.9)	4 (-7.8-15)	0 (0-0)	1.5 (1-2.1)	0 (0-0)	1.6 (1.1-2.4)	84	71	13	11
Hertfordshire	20.6 (16-26.6)	21 (16.3-27.5)	2 (-4.5-8.2)	4.4 (3.3-5.7)	4.3 (3.2-5.6)	-3.3 (-9.1-2)	0 (0-0)	0 (0-0)	-8.1 (-19.8-4.7)	0.3 (0.2-0.3)	0.3 (0.2-0.3)	-2.6 (-8.9-5.1)	0 (0-0)	0 (0-0)	8.8 (-13.8-44.9)	1.3 (0.9-1.8)	1.4 (0.9-1.9)	3 (-7-15)	0 (0-0)	1.5 (1-2)	0 (0-0)	1.6 (1.1-2.3)	83	70	14	12
Luton	30.2 (22.3-40.6)	30.8 (22.1-40.9)	1.9 (-3.9-7.8)	6.3 (4.5-8.4)	6.3 (4.5-8.4)	0.1 (-4.6-5.6)	0.1 (0.1-0.1)	0 (0-0.1)	-13.9 (-24.4--1.4)	0.4 (0.3-0.6)	0.4 (0.3-0.6)	1.1 (-6.2-8.3)	0.1 (0.1-0.1)	0.1 (0.1-0.1)	-4.5 (-24-18.5)	2.4 (1.6-3.4)	2.4 (1.6-3.4)	3.2 (-7-12.7)	0 (0-0.1)	1.3 (0.8-1.8)	0.1 (0-0.1)	1.6 (1-2.2)	65	64	14	12
Norfolk	15.9 (11.7-21.6)	16.2 (12.4-21.6)	2 (-5.5-11.2)	2.7 (2-3.3)	2.5 (2-3.2)	-4.3 (-10-1.7)	0 (0-0)	0 (0-0)	10.6 (-5.9-31.9)	0.2 (0.1-0.2)	0.2 (0.1-0.2)	-4.8 (-12.1-3.8)	0 (0-0)	0 (0-0)	3.9 (-24.1-33.3)	1 (0.7-1.5)	1 (0.7-1.5)	1.3 (-10.5-14.3)	0 (0-0)	1 (0.7-1.5)	0 (0-0)	1.2 (0.9-1.9)	104	97	17	14
Peterborough	16.2 (11.6-21.8)	16.4 (11.8-22.3)	1.8 (-6.3-12.3)	2.8 (2-3.7)	2.9 (2-3.9)	2.8 (-5.8-10.8)	0 (0-0)	0 (0-0)	18.7 (2.4-40)	0.2 (0.2-0.3)	0.2 (0.2-0.3)	2.1 (-6.9-12.3)	0 (0-0)	0 (0-0)	-3.5 (-27.8-26.6)	1.3 (0.8-1.9)	1.3 (0.8-1.9)	1.2 (-10.9-15.6)	0 (0-0)	0.9 (0.5-1.3)	0 (0-0)	1.2 (0.8-1.8)	102	92	18	13
Southend-on-Sea	11.1 (7.8-15.4)	11.3 (8.2-15.6)	1.4 (-7.7-14.7)	1.7 (1.2-2.2)	1.6 (1.2-2.2)	-3.4 (-12-9.1)	0 (0-0)	0 (0-0)	-12.4 (-26-1.7)	0.1 (0.1-0.2)	0.1 (0.1-0.2)	-4.9 (-14.2-7.2)	0 (0-0)	0 (0-0)	-4.2 (-27-39.9)	0.9 (0.5-1.3)	0.9 (0.5-1.3)	0.2 (-13.4-16.2)	0 (0-0)	0.9 (0.5-1.3)	0 (0-0)	1.1 (0.6-1.7)	84	77	18	18
Suffolk	38.2 (28.8-51)	28.9 (20.7-39.5)	-24.3 (-33.3--15.2)	7.8 (5.7-10.4)	5.4 (3.8-7)	-31 (-38.9--23.9)	0 (0-0)	0 (0-0)	7.6 (-8.4-24.1)	0.5 (0.3-0.6)	0.3 (0.2-0.4)	-31.9 (-39--24.6)	0 (0-0)	0 (0-0)	18 (-7-47.2)	2.5 (1.7-3.6)	1.9 (1.2-2.7)	-26.1 (-36.5--14.6)	0 (0-0)	2.9 (2-4.1)	0 (0-0)	2.3 (1.5-3.1)	84	76	7	9
Thurrock	41.9 (30-56.3)	42.3 (30.3-54.3)	1.1 (-6-6.9)	9.4 (6.7-12.6)	9.2 (6.5-12.2)	-2.7 (-10-5.4)	0.1 (0.1-0.1)	0 (0-0.1)	-9.1 (-22.1-5.2)	0.6 (0.4-0.8)	0.6 (0.4-0.8)	-2.2 (-9.5-4.5)	0.1 (0.1-0.1)	0.1 (0.1-0.1)	22.9 (-2.6-52.3)	2.9 (1.9-4.1)	3 (1.9-4.2)	3.2 (-6.7-12.5)	0.1 (0.1-0.1)	2.7 (1.8-3.8)	0.1 (0.1-0.1)	2.6 (1.7-3.5)	65	60	8	9
Greater London	67.3 (54.1-82.8)	63.8 (51.9-78.7)	-5.2 (-7.3--3.5)	15 (12.2-18.7)	14.3 (11.6-17.9)	-4.2 (-6.1--2.1)	0.1 (0.1-0.1)	0.1 (0-0.1)	-32.6 (-37.6--27.8)	0.9 (0.7-1.1)	0.9 (0.7-1.1)	-3.8 (-6.2--1.4)	0.1 (0.1-0.1)	0.1 (0.1-0.1)	-44.1 (-48.2--39.3)	4.7 (3.4-6.2)	4.6 (3.4-6)	-2.4 (-5.3-0.5)	0.1 (0.1-0.1)	3.1 (2.3-4.2)	0.1 (0.1-0.1)	5.2 (3.9-6.8)	58	58	8	5

Location	Birth Incidence rate (per 100,000 live births), 2000	Birth Incidence rate (per 100,000 live births), 2021	Birth Incidence rate (per 100,000 live births) percent change (2000-2021)	All age rate (per 100,000 population), 2000	All age rate (per 100,000 population), 2021	All age rate (per 100,000 population) percent change (2000-2021)	All age cause-specific SCD mortality rate (per 100,000 population), 2000	All age cause-specific SCD mortality rate (per 100,000 population), 2021	All age cause-specific SCD mortality rate (per 100,000 population) percent change (2000-2021)	All age total SCD mortality rate (per 100,000 population), 2000	All age total SCD mortality rate (per 100,000 population), 2021	All age total SCD mortality rate (per 100,000 population) percent change (2000-2021)
Barking and Dagenham	81.9 (66-98.6)	77.7 (62.1-94.9)	-5.1 (-12.3-2.3)	19.9 (16.4-24.1)	21.6 (17.6-26.2)	8.1 (0.1-15.2)	0.1 (0.1-0.1)	0.1 (0.1-0.1)	-23.3 (-30.1--14.2)	1.2 (0.9-1.4)	1.3 (1-1.6)	6.7 (-1.3-15.4)
Barnet	56.1 (44.1-69.6)	54.4 (43.8-67.4)	-3 (-10.6-3.6)	13.7 (11-17.2)	13.4 (10.5-16.7)	-2.8 (-8.5-5.2)	0 (0-0.1)	0 (0-0)	-27.2 (-35.6--19.4)	0.8 (0.6-0.9)	0.7 (0.6-0.9)	-2.4 (-8.9-5.1)
Bexley	38.5 (27.8-50.1)	33.8 (24.9-44.6)	-12.2 (-19.2--4)	8.9 (6.4-11.8)	7.7 (5.6-10.1)	-13.1 (-20.5--4.5)	0 (0-0)	0 (0-0)	-22 (-32.8--10.3)	0.5 (0.4-0.7)	0.4 (0.3-0.6)	-11.7 (-18.4--4.2)
Brent	56.2 (44.7-68.5)	56.5 (44.6-68.2)	0.5 (-7.4-9.5)	12.5 (9.9-15.8)	12.4 (10.1-15.8)	-0.3 (-6.8-9.5)	0.1 (0.1-0.1)	0.1 (0.1-0.1)	-25.7 (-34.5--18.4)	0.8 (0.6-0.9)	0.8 (0.6-1)	1.8 (-5.9-10.7)
Bromley	28.9 (20.1-39.4)	29.5 (20.7-42)	2.1 (-4.6-9.9)	6.6 (4.4-8.9)	6.5 (4.3-8.7)	-1.3 (-8.6-7.2)	0 (0-0)	0 (0-0)	-23.7 (-34.2--14.2)	0.4 (0.2-0.5)	0.4 (0.2-0.5)	0 (-6.5-7.5)
Camden	58.5 (45-76.6)	47.6 (37-61.4)	-18.6 (-24.1--11.2)	12.8 (9.9-17)	9.6 (7.4-12.4)	-25.2 (-30.9--19.1)	0.1 (0.1-0.1)	0 (0-0.1)	-17.9 (-49-4.3)	0.8 (0.6-1)	0.6 (0.4-0.8)	-26.4 (-32.3--20.3)
Croydon	63 (51.5-77.4)	64.5 (52.5-78.7)	2.5 (-4.9-9.7)	15 (12-18.8)	15.5 (12.4-19.2)	3 (-5-10.9)	0.1 (0.1-0.1)	0.1 (0.1-0.1)	-19 (-29.4--10.1)	0.9 (0.7-1.1)	0.9 (0.7-1.1)	5.3 (-1.8-14.2)
Ealing	40.7 (30.9-52.8)	36.8 (29-46.5)	-9.6 (-15.5--2)	9.1 (6.9-11.9)	8.1 (6.3-10.4)	-10.9 (-15.3--4)	0.1 (0.1-0.1)	0.1 (0-0.1)	-24.4 (-31.7--16.4)	0.6 (0.4-0.7)	0.5 (0.4-0.6)	-9.5 (-15.4--2.7)
Enfield	132 (107-163)	122 (97.2-151)	-7.4 (-15-0.2)	33.3 (27.7-40.4)	32.4 (26.3-39.6)	-2.9 (-10.2-4.3)	0.1 (0.1-0.1)	0.1 (0-0.1)	-25.8 (-37--14.9)	1.8 (1.4-2.2)	1.7 (1.4-2.1)	-3.3 (-10.8-3.9)
Greenwich	70.1 (55.2-86.2)	69.4 (55.7-85)	-1 (-8.5-5.7)	16.6 (13.2-20.5)	16.5 (13.5-20.6)	-0.5 (-6.8-7)	0.1 (0.1-0.1)	0.1 (0.1-0.1)	-30.8 (-39.3--23.5)	1 (0.8-1.2)	1 (0.8-1.2)	0 (-7.5-7.4)
Hackney	185 (152-228)	175 (140-214)	-5.3 (-13.7-3.4)	47.3 (39.1-57.6)	41.8 (33.9-51)	-11.7 (-18.2--3.3)	0.2 (0.2-0.2)	0.1 (0.1-0.1)	-48.7 (-56.6--40.6)	2.8 (2.3-3.4)	2.5 (2-3.1)	-11.7 (-18.8--3.6)
Hammersmith and Fulham	56.5 (40.2-73.6)	48 (35.8-62.7)	-15.1 (-21.8--7.2)	12.2 (8.9-16.9)	9.8 (7.1-13.3)	-19.7 (-26.7--12.8)	0.1 (0.1-0.1)	0.1 (0-0.1)	-41.7 (-50.5--35.4)	0.8 (0.5-1)	0.6 (0.4-0.8)	-19.9 (-26.1--12.3)
Haringey	187 (149-227)	178 (142-220)	-4.6 (-11.2-2.9)	45.6 (37.8-55.8)	40.7 (33.6-50.6)	-10.7 (-16.9--1.7)	0.1 (0.1-0.1)	0.1 (0.1-0.1)	-33.2 (-40.3--26.3)	2.7 (2.2-3.4)	2.4 (1.9-3)	-12.5 (-18.8--4.7)
Harrow	15.1 (10.9-20.5)	15.4 (11-21.7)	2.5 (-6-11.8)	2.8 (2.1-3.8)	3 (2.2-4.1)	5.6 (-2.4-15.3)	0 (0-0)	0 (0-0)	-13.4 (-24.5--0.9)	0.2 (0.1-0.3)	0.2 (0.1-0.3)	7.9 (-1.2-16.8)
Havering	20.5 (13.7-29.1)	21 (14-30.5)	2.6 (-5.8-11.9)	4.2 (2.7-6.2)	4.3 (2.8-6.5)	3.9 (-3.3-10.9)	0 (0-0)	0 (0-0)	7.9 (-7.6-25.3)	0.3 (0.2-0.4)	0.3 (0.2-0.4)	5.4 (-2.2-14.4)
Hillingdon	21.2 (14.8-29)	21.9 (15.1-31)	3.2 (-5.5-11.4)	4.4 (3-6.5)	4.7 (3.1-6.9)	5.6 (-4-14.2)	0 (0-0)	0 (0-0)	-9 (-21.8-4.9)	0.3 (0.2-0.4)	0.3 (0.2-0.4)	6.6 (-1-14.3)
Hounslow	39.3 (27.8-50.8)	38.6 (27.3-51.2)	-1.6 (-10.3-7.3)	9 (6.2-12)	8.7 (6.2-11.2)	-3.4 (-12.6-6.2)	0 (0-0)	0 (0-0)	-32 (-42.7--19.4)	0.5 (0.4-0.7)	0.5 (0.4-0.7)	-2.5 (-10.3-5.4)
Islington	32.6 (23.5-42.4)	33 (23.1-44.2)	1.2 (-5.7-9.5)	6.7 (4.7-9)	6.2 (4.4-8.4)	-7.9 (-15.5--2.3)	0.1 (0.1-0.1)	0 (0-0)	-47.4 (-54.1--40)	0.5 (0.3-0.6)	0.4 (0.3-0.6)	-5.5 (-12.7-1.9)
Kensington and Chelsea	11.2 (8-15.7)	11.4 (8.2-16)	1.7 (-10.3-14.5)	1.6 (1.2-2.2)	1.5 (1.1-2.1)	-4.9 (-12.6-13)	0 (0-0.1)	0 (0-0)	-32.9 (-42.4--25)	0.1 (0.1-0.2)	0.1 (0.1-0.2)	-6 (-16.7-9)
Kingston upon Thames	24.2 (18.5-31.4)	24.4 (18.5-31.8)	1 (-7.4-10.1)	5.3 (4-7)	5.2 (3.9-6.7)	-2 (-10.5-7.7)	0 (0-0)	0 (0-0)	4.8 (-8.5-21.5)	0.3 (0.2-0.4)	0.3 (0.2-0.4)	-0.6 (-8.3-8.6)
Lambeth	137 (110-169)	127 (100-156)	-7.4 (-14.6-0.3)	33.3 (27.6-40.2)	28.7 (23.2-35.2)	-13.9 (-19.5--7.3)	0.2 (0.2-0.2)	0.1 (0.1-0.1)	-44.1 (-52.7--36.3)	2 (1.6-2.5)	1.7 (1.4-2.1)	-14.3 (-21.1--7.2)
Lewisham	100 (83.4-122)	95.4 (76.7-116)	-4.7 (-10.6-2.1)	24.4 (20.8-29.7)	22.9 (19-27.9)	-6.2 (-11.9-2.2)	0.1 (0.1-0.1)	0.1 (0.1-0.1)	-36 (-44.9--27)	1.4 (1.2-1.8)	1.4 (1.1-1.7)	-5.7 (-13.1-1.3)
Merton	47.8 (34.5-61.9)	48.1 (35.2-62)	0.7 (-5.3-8.1)	11.1 (8.2-15.2)	11.2 (8.3-15.1)	0.6 (-6.2-9.2)	0 (0-0)	0 (0-0)	-16.9 (-28.4--2.8)	0.7 (0.5-0.9)	0.7 (0.5-0.9)	1.2 (-6.1-10.4)
Newham	81.1 (67.4-96.9)	82.5 (68.1-102)	1.7 (-5-8.5)	20.7 (17.3-25.5)	19 (15.4-23.1)	-8.4 (-14.1--1.7)	0.2 (0.2-0.2)	0.1 (0.1-0.1)	-37.4 (-44.7--30.6)	1.3 (1.1-1.6)	1.2 (1-1.5)	-7.1 (-14.1-0.6)
Redbridge	33.1 (24.2-44)	33.6 (23.8-43.1)	1.4 (-5.1-8.7)	7.5 (5.3-9.8)	8.1 (5.6-10.7)	8.6 (1.4-15.7)	0 (0-0)	0 (0-0)	-24.4 (-36.5--15.7)	0.4 (0.3-0.6)	0.5 (0.3-0.7)	9.4 (2.2-18.7)
Richmond upon Thames	23.7 (16.4-33.5)	24.3 (16.6-34.6)	2.7 (-6-7.8)	5.3 (3.5-7.5)	5.3 (3.5-7.4)	-0.1 (-8.1-7.2)	0 (0-0)	0 (0-0)	-12.4 (-27.2-1.4)	0.3 (0.2-0.4)	0.3 (0.2-0.4)	-0.2 (-7.6-6.6)
Southwark	124 (98.6-154)	125 (99.8-153)	0.8 (-6-8)	29.3 (23.5-36)	27.7 (23-33.6)	-5.4 (-11.1-0.8)	0.2 (0.2-0.2)	0.1 (0.1-0.1)	-41.9 (-48.2--34.9)	1.8 (1.4-2.2)	1.7 (1.4-2.1)	-3.4 (-10.1-4.7)
Sutton	18.9 (14-25.5)	19.1 (14.4-25.2)	1 (-6.2-9.5)	4 (2.9-5.2)	3.8 (2.8-4.9)	-4.2 (-11.6-4.9)	0 (0-0)	0 (0-0)	-24.1 (-34--10.3)	0.2 (0.2-0.3)	0.2 (0.2-0.3)	-2.4 (-9.8-6)
Tower Hamlets	36.4 (28.8-45.4)	31 (24.1-40)	-14.8 (-23.7--6.6)	8.3 (6.4-10.6)	6.5 (5-8.6)	-21.4 (-29.2--11.7)	0.1 (0.1-0.1)	0 (0-0)	-43.5 (-49.7--35.9)	0.6 (0.4-0.7)	0.5 (0.3-0.6)	-19.8 (-28.2--10.1)

Location	Under-5 cause-specific SCD mortality rate (per 100,000 population), 2000	Under-5 cause-specific SCD mortality rate (per 100,000 population), 2021	Under-5 cause-specific SCD mortality rate (per 100,000 population) percent change (2000-2021)	Under-5 total SCD mortality rate (per 100,000 population), 2000	Under-5 total SCD mortality rate (per 100,000 population), 2021	Under-5 total SCD mortality rate (per 100,000 population) percent change (2000-2021)	Under-5 cause-specific mortality cause fraction, 2000	Under-5 total SCD mortality cause fraction, 2000	Under-5 cause-specific mortality cause fraction, 2021	Under-5 total SCD mortality cause fraction, 2021	Under-5 cause-specific mortality rank 2000	Under-5 cause-specific mortality rank 2021	Under-5 total SCD mortality rank 2000	Under-5 total SCD mortality rank 2021
Barking and Dagenham	0.1 (0.1-0.1)	0.1 (0.1-0.1)	-27.7 (-39.5--16.5)	5.6 (4.1-7.4)	5.4 (3.9-7.3)	-4.1 (-14.3-7.4)	0.1 (0.1-0.1)	4.5 (3.2-6)	0.1 (0.1-0.1)	5.5 (3.9-7.3)	55	55	7	5
Barnet	0.1 (0-0.1)	0 (0-0)	-23.2 (-35.6--7)	3.5 (2.5-4.7)	3.5 (2.5-4.8)	0 (-10.2-12.2)	0.1 (0-0.1)	3.3 (2.4-4.4)	0.1 (0.1-0.1)	4.7 (3.3-6.4)	58	55	7	5
Bexley	0.1 (0-0.1)	0.1 (0-0.1)	0 (-18.8-22.6)	2.4 (1.6-3.4)	2.1 (1.4-2.9)	-13.3 (-22.1--2.7)	0.1 (0-0.1)	2.5 (1.7-3.5)	0.1 (0-0.1)	2.2 (1.4-3.1)	66	61	9	10
Brent	0.2 (0.1-0.2)	0.1 (0.1-0.1)	-40.8 (-50.6--32.4)	4.4 (3.2-5.8)	4.5 (3.2-6.1)	1.8 (-9.2-15.6)	0.1 (0.1-0.1)	2.1 (1.5-2.9)	0.1 (0.1-0.1)	4.2 (3-5.8)	55	51	10	6
Bromley	0 (0-0)	0 (0-0)	-6.6 (-22.5-10.3)	1.7 (1.1-2.5)	1.8 (1.2-2.6)	3.9 (-6.6-15.3)	0 (0-0)	1.9 (1.3-2.7)	0 (0-0)	2.5 (1.6-3.6)	72	68	11	8
Camden	0.1 (0.1-0.1)	0 (0-0.1)	-38.9 (-52--24.6)	4.2 (2.9-5.8)	3.4 (2.4-4.7)	-17.9 (-26.3--8)	0.1 (0-0.1)	2.9 (2.1-4)	0.1 (0.1-0.1)	4.7 (3.3-6.5)	68	62	8	5
Croydon	0.1 (0.1-0.1)	0.1 (0.1-0.1)	-32.2 (-43--21.6)	4.2 (3.1-5.7)	4.5 (3.2-6.1)	5.7 (-5.2-17.4)	0.1 (0.1-0.1)	2.9 (2.2-4)	0.1 (0.1-0.1)	5 (3.5-6.8)	59	55	8	5
Ealing	0.1 (0.1-0.1)	0.1 (0.1-0.1)	-30.7 (-40.8--14.2)	2.8 (2-3.9)	2.6 (1.9-3.5)	-7 (-15.8-2.9)	0.1 (0.1-0.1)	2 (1.5-2.8)	0.1 (0.1-0.1)	3.3 (2.3-4.6)	59	52	10	7
Enfield	0.1 (0.1-0.1)	0.1 (0.1-0.1)	-34.6 (-44--20.8)	8.3 (6-11.3)	7.9 (5.7-10.7)	-4.5 (-14.8-8.2)	0.1 (0.1-0.1)	5.9 (4.1-7.8)	0.1 (0.1-0.1)	8.7 (6.3-11.6)	53	52	5	2
Greenwich	0.1 (0.1-0.1)	0.1 (0.1-0.1)	-18.1 (-27.1--5.4)	4.8 (3.5-6.5)	4.9 (3.5-6.4)	1.2 (-8.7-10.8)	0.1 (0.1-0.1)	3.3 (2.4-4.5)	0.1 (0.1-0.1)	4.3 (3.1-5.7)	63	57	8	5
Hackney	0.3 (0.3-0.3)	0.1 (0.1-0.1)	-73.4 (-77.5--68.3)	13.3 (9.5-17.9)	12.8 (9.3-17.2)	-3.9 (-14.3-7.8)	0.1 (0.1-0.1)	6 (4.4-8)	0.1 (0.1-0.2)	21.7 (15.2-30.8)	45	48	5	2
Hammersmith and Fulham	0.1 (0.1-0.2)	0.1 (0.1-0.1)	-36 (-45.9--22.1)	4.1 (2.7-5.8)	3.6 (2.4-5.3)	-11.1 (-19.7--1)	0.1 (0.1-0.1)	2.6 (1.8-3.7)	0.1 (0.1-0.1)	4.2 (2.8-5.9)	55	51	9	6
Haringey	0.3 (0.2-0.3)	0.1 (0.1-0.1)	-58 (-64.4--50.2)	13.9 (10.2-19)	13.4 (9.9-18.2)	-3.5 (-13.5-7.9)	0.1 (0.1-0.1)	6.6 (4.5-9.3)	0.1 (0.1-0.1)	14.7 (10.3-20.5)	48	52	3	2
Harrow	0.1 (0-0.1)	0 (0-0.1)	-7.4 (-22.8-12.9)	1.1 (0.7-1.6)	1.1 (0.7-1.6)	1.9 (-9.7-14.8)	0 (0-0)	0.7 (0.5-1.1)	0 (0-0.1)	1 (0.6-1.5)	65	61	21	18
Havering	0 (0-0)	0 (0-0)	-14.9 (-37.6-16.1)	1.4 (0.9-2)	1.4 (0.9-2.1)	3.1 (-7.8-15.3)	0 (0-0)	1.4 (0.9-2.1)	0 (0-0)	2 (1.2-3)	82	75	12	11
Hillingdon	0 (0-0)	0 (0-0)	-3.5 (-26.8-18.5)	1.5 (1-2.2)	1.5 (1-2.3)	4.1 (-6.8-15.5)	0 (0-0)	1.3 (0.8-2)	0 (0-0)	1.8 (1.1-2.7)	94	83	13	11
Hounslow	0 (0-0)	0 (0-0)	-14 (-30.1-6.4)	2.8 (1.9-4)	2.8 (1.9-3.9)	-1.4 (-10.3-9.5)	0 (0-0)	1.9 (1.3-2.8)	0 (0-0)	2.5 (1.7-3.6)	76	66	10	8
Islington	0.1 (0.1-0.1)	0 (0-0.1)	-53 (-58.8--42.5)	2.5 (1.7-3.5)	2.6 (1.7-3.7)	3.6 (-6.3-14.7)	0.1 (0.1-0.1)	1.6 (1.1-2.2)	0.1 (0.1-0.1)	3.9 (2.5-5.3)	65	59	13	6
Kensington and Chelsea	0.1 (0.1-0.1)	0.1 (0.1-0.1)	-14.8 (-32-3.5)	1 (0.6-1.4)	1 (0.6-1.4)	0.5 (-14.2-19.4)	0.1 (0.1-0.1)	0.8 (0.5-1.3)	0.1 (0.1-0.1)	1.3 (0.8-2)	67	60	24	16
Kingston upon Thames	0 (0-0)	0 (0-0)	39.5 (-7.4-89.8)	1.6 (1.1-2.2)	1.6 (1.1-2.2)	1.3 (-9.4-13.8)	0 (0-0)	1.8 (1.2-2.8)	0 (0-0)	1.9 (1.4-2.6)	93	83	12	12
Lambeth	0.2 (0.2-0.3)	0.1 (0.1-0.1)	-55.5 (-62.4--46.9)	9.9 (7.3-13.2)	9.2 (6.8-12.4)	-6.7 (-16.7-4.2)	0.1 (0.1-0.1)	4.8 (3.5-6.5)	0.1 (0.1-0.1)	9.4 (6.7-12.4)	47	51	7	2
Lewisham	0.2 (0.1-0.2)	0.1 (0.1-0.1)	-39 (-48.8--26)	7 (5.2-9.3)	6.8 (4.9-9.2)	-3.3 (-13.9-7.8)	0.1 (0.1-0.1)	4 (2.9-5.4)	0.1 (0.1-0.1)	6.5 (4.8-8.7)	53	52	7	3
Merton	0 (0-0)	0 (0-0)	-9.5 (-28.1-18.6)	3.2 (2.2-4.5)	3.3 (2.2-4.6)	2.5 (-7.1-13)	0 (0-0)	2.9 (2-4)	0 (0-0)	4.3 (3-6)	74	66	8	6
Newham	0.3 (0.3-0.3)	0.2 (0.1-0.2)	-46.1 (-53.6--36.6)	6.4 (4.7-8.6)	6.7 (4.9-8.8)	3.8 (-7.2-15.8)	0.1 (0.1-0.1)	3.1 (2.3-4.1)	0.1 (0.1-0.2)	6.4 (4.8-8.4)	48	49	9	3
Redbridge	0.1 (0.1-0.1)	0 (0-0.1)	-28.5 (-40.2--10.9)	2.2 (1.5-3.2)	2.3 (1.6-3.2)	2.7 (-5.8-13.1)	0 (0-0.1)	1.8 (1.1-2.4)	0.1 (0-0.1)	2.9 (1.9-4)	66	62	12	7
Richmond upon Thames	0 (0-0)	0 (0-0)	-11 (-30.7-10.4)	1.5 (1-2.2)	1.6 (1-2.3)	3.4 (-6.5-13.6)	0 (0-0)	1.7 (1.1-2.5)	0 (0-0)	2.5 (1.6-3.8)	93	85	13	8
Southwark	0.3 (0.2-0.3)	0.1 (0.1-0.1)	-64.6 (-70--58.4)	9.1 (6.5-12.4)	9.4 (6.8-12.9)	3.3 (-7.2-15.4)	0.1 (0.1-0.1)	4.2 (3-5.7)	0.1 (0.1-0.1)	11.2 (7.9-15.5)	45	48	7	2
Sutton	0 (0-0)	0 (0-0)	11 (-13.4-35.9)	1.3 (0.8-1.8)	1.3 (0.9-1.8)	0.3 (-10.5-11.9)	0 (0-0)	1.3 (0.8-1.9)	0 (0-0)	1.4 (1-2)	81	73	15	14
Tower Hamlets	0.1 (0.1-0.1)	0 (0-0.1)	-43.4 (-54--31.4)	3.1 (2.2-4.2)	2.6 (1.9-3.6)	-15.9 (-26.3--4)	0 (0-0.1)	1.8 (1.2-2.4)	0.1 (0-0.1)	2.8 (2-3.9)	72	63	12	6

Location	Birth Incidence rate (per 100,000 live births), 2000	Birth Incidence rate (per 100,000 live births), 2021	Birth Incidence rate (per 100,000 live births) percent change (2000-2021)	All age rate (per 100,000 population), 2000	All age rate (per 100,000 population), 2021	All age rate (per 100,000 population) percent change (2000-2021)	All age cause-specific SCD mortality rate (per 100,000 population), 2000	All age cause-specific SCD mortality rate (per 100,000 population), 2021	All age cause-specific SCD mortality rate (per 100,000 population) percent change (2000-2021)	All age total SCD mortality rate (per 100,000 population), 2000	All age total SCD mortality rate (per 100,000 population), 2021	All age total SCD mortality rate (per 100,000 population) percent change (2000-2021)	Under-5 cause-specific SCD mortality rate (per 100,000 population), 2000	Under-5 cause-specific SCD mortality rate (per 100,000 population), 2021	Under-5 cause-specific SCD mortality rate (per 100,000 population) percent change (2000-2021)	Under-5 total SCD mortality rate (per 100,000 population), 2000	Under-5 total SCD mortality rate (per 100,000 population), 2021	Under-5 total SCD mortality rate (per 100,000 population) percent change (2000-2021)	Under-5 cause-specific mortality cause fraction, 2000	Under-5 total SCD mortality cause fraction, 2000	Under-5 cause-specific mortality cause fraction, 2021	Under-5 total SCD mortality cause fraction, 2021	Under-5 cause-specific mortality rank 2000	Under-5 cause-specific mortality rank 2021	Under-5 total SCD mortality rank 2000	Under-5 total SCD mortality rank 2021
Waltham Forest	77.9 (61.5-95.6)	75.9 (61.1-91.4)	-2.6 (-9-3.8)	18 (14-23)	17.5 (13.8-22.4)	-2.7 (-10-4.6)	0.1 (0.1-0.1)	0.1 (0.1-0.1)	-36.4 (-45.5--27.9)	1.1 (0.9-1.4)	1.1 (0.9-1.4)	-2.3 (-9.7-6.1)	0.2 (0.2-0.2)	0.1 (0.1-0.1)	-47.7 (-54.9--39.3)	5.9 (4.2-7.9)	5.8 (4.2-8.1)	-1 (-12.4-10.2)	0.1 (0.1-0.1)	3.2 (2.3-4.4)	0.1 (0.1-0.1)	6.1 (4.3-8.4)	46	51	8	5
Wandsworth	37.5 (28.9-48.5)	38.1 (28.7-49)	1.5 (-5.2-7.6)	8.2 (6-10.9)	8.2 (6.2-10.7)	1.2 (-6.6-9.6)	0.1 (0.1-0.1)	0 (0-0.1)	-45.3 (-54.9--36.3)	0.5 (0.4-0.7)	0.5 (0.4-0.7)	1.8 (-5.4-10.6)	0.1 (0.1-0.1)	0.1 (0-0.1)	-53.5 (-61.3--43.1)	2.7 (1.9-3.7)	2.8 (1.9-3.8)	2.7 (-5.9-12.5)	0.1 (0.1-0.1)	1.8 (1.3-2.4)	0.1 (0.1-0.1)	4.2 (2.9-5.7)	57	53	11	5
Westminster	27.1 (19.2-37.5)	22 (15.3-30.2)	-18.8 (-29.7--8.1)	4.8 (3.2-6.5)	3.7 (2.6-5.4)	-22.2 (-32.8--11.7)	0.1 (0.1-0.1)	0 (0-0)	-36.7 (-48.7--28.6)	0.3 (0.2-0.4)	0.3 (0.2-0.4)	-21.5 (-31.5--12)	0.1 (0.1-0.1)	0.1 (0-0.1)	-48.7 (-59.5--35.4)	2.2 (1.5-3.2)	1.8 (1.2-2.6)	-19.7 (-31.8--7.5)	0.1 (0.1-0.1)	1.3 (0.8-1.9)	0.1 (0.1-0.1)	2.7 (1.8-4)	65	57	15	9
North East England	16.7 (12.8-22)	17.6 (13.5-23.2)	5.2 (1.3-8.9)	2.8 (2.1-3.5)	2.7 (2-3.5)	-4 (-7.5-0)	0 (0-0)	0 (0-0)	31.8 (21.5-42.7)	0.2 (0.2-0.3)	0.2 (0.1-0.3)	-4.1 (-7.5--0.1)	0 (0-0)	0 (0-0)	12 (-11.7-36.4)	1.3 (0.9-1.8)	1.3 (0.9-1.8)	5.5 (0.3-10.6)	0 (0-0)	1.1 (0.7-1.5)	0 (0-0)	1.5 (1-2.1)	107	101	15	14
County Durham	11.3 (7.7-15.7)	11.6 (8.3-15.8)	2.2 (-7.8-19.3)	1.6 (1.2-2.2)	1.5 (1.1-2)	-7.1 (-15.2-6.9)	0 (0-0)	0 (0-0)	30.7 (9.4-53.4)	0.1 (0.1-0.2)	0.1 (0.1-0.2)	-8.5 (-17.4-8.2)	0 (0-0)	0 (0-0)	18.5 (-12-62.4)	0.8 (0.5-1.3)	0.8 (0.5-1.3)	0.6 (-13.9-20)	0 (0-0)	0.7 (0.4-1.1)	0 (0-0)	0.9 (0.5-1.4)	103	101	19	19
Darlington	11.9 (8.2-16.8)	12 (8.5-16.8)	0.8 (-11.6-15.7)	1.7 (1.3-2.4)	1.6 (1.2-2.2)	-6 (-14.8-10)	0 (0-0)	0 (0-0)	38.1 (17.7-63)	0.1 (0.1-0.2)	0.1 (0.1-0.2)	-8.1 (-19.3-6)	0 (0-0)	0 (0-0)	-14.1 (-38.7-28.3)	1 (0.6-1.5)	1 (0.6-1.5)	-0.1 (-15.9-18)	0 (0-0)	0.8 (0.4-1.3)	0 (0-0)	1 (0.6-1.5)	103	96	21	16
Gateshead	19.5 (14.5-26.7)	19.9 (14.5-27.6)	2 (-6.9-10.1)	3.5 (2.5-4.7)	3.3 (2.4-4.5)	-5.3 (-12.3-4)	0 (0-0)	0 (0-0)	25 (6.6-45.1)	0.2 (0.2-0.3)	0.2 (0.2-0.3)	-5.4 (-13.4-2.9)	0 (0-0)	0 (0-0)	27.7 (-7.6-86.3)	1.3 (0.9-1.9)	1.3 (0.9-1.9)	1.4 (-11.2-13.9)	0 (0-0)	1.1 (0.7-1.6)	0 (0-0)	1.4 (0.9-2.1)	104	96	14	13
Hartlepool	12.1 (8.8-17.1)	12.5 (8.9-18)	3.2 (-7.8-27.5)	1.8 (1.3-2.5)	1.7 (1.2-2.4)	-7.6 (-15.7-26.6)	0 (0-0)	0 (0-0)	51.5 (15.8-92.8)	0.2 (0.1-0.2)	0.1 (0.1-0.2)	-11.5 (-23.2-7.7)	0 (0-0)	0 (0-0)	28 (-3.5-73.7)	1 (0.6-1.6)	1 (0.6-1.6)	0.9 (-15.3-27.9)	0 (0-0)	0.8 (0.5-1.2)	0 (0-0)	1.3 (0.8-1.9)	104	98	18	17
Middlesbrough	29.3 (21.4-38.9)	30 (21.5-40)	2.1 (-6.3-11)	5.4 (4-7.3)	5.2 (3.7-7)	-3.7 (-12.7-4.4)	0 (0-0)	0 (0-0)	35.9 (9.1-58.5)	0.4 (0.3-0.5)	0.4 (0.3-0.5)	0.9 (-6.9-10.4)	0 (0-0)	0 (0-0)	5.8 (-25.9-44.2)	2.5 (1.7-3.6)	2.6 (1.8-3.8)	4.6 (-5.9-17.4)	0 (0-0)	1.7 (1.1-2.4)	0 (0-0)	2.4 (1.5-3.3)	99	95	12	11
Newcastle upon Tyne	31.3 (22-41.7)	31.9 (22.9-42.4)	2.1 (-4.5-9.1)	6.1 (4.3-8.2)	6.2 (4.3-8.4)	1.6 (-4.7-8)	0 (0-0)	0 (0-0)	12.7 (-3.6-32.2)	0.4 (0.3-0.5)	0.4 (0.3-0.6)	4.2 (-3-11.5)	0 (0-0)	0 (0-0)	17.9 (-9.5-55.7)	2.3 (1.5-3.2)	2.4 (1.5-3.3)	3.7 (-5.5-13.4)	0 (0-0)	1.9 (1.2-2.8)	0 (0-0)	2.5 (1.6-3.5)	103	101	12	10
North Tyneside	24.9 (18.6-33.4)	25.4 (18.2-33.7)	2.2 (-6.4-10.8)	4.4 (3.2-5.8)	4.1 (2.9-5.4)	-7.9 (-16.3--0.1)	0 (0-0)	0 (0-0)	17.9 (-1.3-40.6)	0.3 (0.2-0.4)	0.3 (0.2-0.4)	-5.9 (-12.9-2)	0 (0-0)	0 (0-0)	-9 (-40.7-21.4)	1.9 (1.3-2.8)	2 (1.3-2.9)	2.8 (-7.2-14.6)	0 (0-0)	1.8 (1.1-2.5)	0 (0-0)	2.6 (1.7-3.6)	98	99	10	9
Northumberland	10.8 (7.8-14.7)	11 (7.6-15.4)	1.9 (-9.1-18.2)	1.6 (1.2-2.2)	1.4 (1-1.9)	-11.3 (-19.8-4.8)	0 (0-0)	0 (0-0)	25.6 (8.1-47.4)	0.1 (0.1-0.2)	0.1 (0.1-0.2)	-12.5 (-21.9--0.1)	0 (0-0)	0 (0-0)	3.7 (-28.5-50.7)	0.8 (0.5-1.2)	0.8 (0.5-1.2)	0.6 (-14.8-18.2)	0 (0-0)	0.8 (0.4-1.2)	0 (0-0)	1 (0.6-1.6)	109	104	17	18
Redcar and Cleveland	11.6 (8.5-16)	11.7 (8.5-16.3)	1.1 (-11.3-15.6)	1.7 (1.2-2.4)	1.6 (1.2-2.1)	-8.7 (-17.2-4)	0 (0-0)	0 (0-0)	84.2 (52.1-132)	0.1 (0.1-0.2)	0.1 (0.1-0.2)	-10.9 (-21.3-3)	0 (0-0)	0 (0-0)	-3.2 (-32.3-24.3)	0.9 (0.6-1.4)	0.9 (0.5-1.4)	0.2 (-15.5-20.1)	0 (0-0)	0.9 (0.6-1.3)	0 (0-0)	1.2 (0.7-1.9)	101	94	18	16
South Tyneside	11.3 (8.3-15.9)	11.6 (8.3-15.9)	2.3 (-9.1-17.1)	1.7 (1.2-2.3)	1.6 (1.2-2.1)	-7.3 (-15.6-10)	0 (0-0)	0 (0-0)	19.6 (0-43.9)	0.1 (0.1-0.2)	0.1 (0.1-0.2)	-9.8 (-19.7-5.9)	0 (0-0)	0 (0-0)	27.5 (-9.3-86.9)	0.9 (0.5-1.3)	0.9 (0.5-1.4)	1 (-14.1-22.6)	0 (0-0)	0.9 (0.5-1.3)	0 (0-0)	1 (0.6-1.5)	101	100	18	17
Stockton-on-Tees	16.8 (12-22.8)	17.2 (12.4-23.7)	2.1 (-7.8-11.1)	3.1 (2.2-4.2)	2.9 (2.2-4)	-6.2 (-15.1-2.2)	0 (0-0)	0 (0-0)	42 (16-67.2)	0.2 (0.2-0.3)	0.2 (0.1-0.3)	-6.2 (-14.4-1.9)	0 (0-0)	0 (0-0)	9.1 (-23.4-44.6)	1.2 (0.8-1.8)	1.2 (0.8-1.7)	0.7 (-11.7-12.7)	0 (0-0)	1 (0.7-1.5)	0 (0-0)	1.2 (0.8-1.7)	104	100	15	14
Sunderland	11.5 (8.4-16)	11.7 (8.5-16.4)	2 (-8.4-15.8)	1.7 (1.2-2.3)	1.5 (1.1-2.1)	-10.6 (-19.2-3.3)	0 (0-0)	0 (0-0)	40.3 (22.2-62.3)	0.1 (0.1-0.2)	0.1 (0.1-0.2)	-11.6 (-20.7-0)	0 (0-0)	0 (0-0)	17.1 (-11.2-56.8)	0.9 (0.5-1.4)	0.9 (0.5-1.4)	0.7 (-13.3-15.6)	0 (0-0)	0.7 (0.4-1.1)	0 (0-0)	1.1 (0.6-1.6)	103	100	18	16
North West England	22.8 (18-29.2)	25.1 (19.9-31.4)	10.3 (7.1-13.8)	4.1 (3.2-5)	4.2 (3.3-5.1)	2.5 (-1-5.8)	0 (0-0)	0 (0-0)	1.4 (-5-8.1)	0.3 (0.2-0.4)	0.3 (0.2-0.4)	2.3 (-1.1-6.3)	0 (0-0)	0 (0-0)	24.2 (9.1-38.4)	1.7 (1.2-2.2)	1.9 (1.4-2.5)	12.6 (7.2-18.9)	0 (0-0)	1.3 (0.9-1.7)	0 (0-0)	1.5 (1.1-2)	88	73	13	12
Blackburn with Darwen	27.1 (20.2-36.3)	27.6 (20.3-36.5)	2 (-5.5-11.8)	5.5 (3.9-7.3)	5 (3.6-6.5)	-9.7 (-15.3--3.3)	0 (0-0)	0 (0-0)	-2.3 (-13.5-14.2)	0.4 (0.3-0.5)	0.4 (0.3-0.5)	-11 (-17.8--3.8)	0.1 (0.1-0.1)	0.1 (0.1-0.1)	3.6 (-18.6-34.6)	2.2 (1.5-3.1)	2.2 (1.5-3.2)	2.8 (-7.6-14.6)	0 (0-0)	1.1 (0.8-1.6)	0 (0-0.1)	1.4 (1-2)	73	67	16	13
Blackpool	22.7 (17.1-30.5)	23.1 (17.3-31.7)	2 (-5.5-8.6)	3.3 (2.4-4.3)	3.2 (2.4-4.3)	-0.9 (-8.1-4.9)	0 (0-0)	0 (0-0)	36.2 (17.9-64.1)	0.3 (0.2-0.4)	0.3 (0.2-0.4)	-0.3 (-8.4-7.7)	0 (0-0)	0 (0-0)	30.5 (5.3-76.6)	2 (1.4-2.8)	2.1 (1.4-3)	4 (-6.5-15.4)	0 (0-0)	1.3 (0.9-1.9)	0 (0-0)	1.3 (0.9-1.8)	88	77	14	14
Bolton	30.7 (22.6-39.9)	31.3 (22.7-40.9)	2 (-5.8-10.8)	5.8 (4.2-7.8)	5.5 (3.9-7.2)	-5.1 (-12.4-3.8)	0 (0-0)	0 (0-0)	-1.7 (-16-17.2)	0.4 (0.3-0.5)	0.4 (0.3-0.5)	-4.4 (-11.8-3.7)	0 (0-0)	0 (0-0)	30 (-4-67.2)	2.4 (1.6-3.4)	2.5 (1.7-3.6)	3.6 (-7-15.4)	0 (0-0)	1.7 (1.2-2.4)	0 (0-0)	1.8 (1.2-2.5)	100	91	12	11
Bury	15.7 (11.4-21.1)	16.2 (11.6-22.1)	3.3 (-8.1-15.6)	2.6 (1.9-3.5)	2.5 (1.8-3.5)	-3.5 (-12.9-4.3)	0 (0-0)	0 (0-0)	8.2 (-4.5-24.4)	0.2 (0.1-0.3)	0.2 (0.1-0.3)	-3.7 (-12.5-5.6)	0 (0-0)	0.1 (0-0.1)	58 (18.3-95.9)	1.3 (0.8-1.9)	1.3 (0.9-2)	3.2 (-9.6-18)	0 (0-0)	1.2 (0.8-1.7)	0 (0-0)	1 (0.7-1.5)	77	68	14	19
Cheshire East	13.5 (9.8-17.8)	13.7 (10-18.6)	1.4 (-7.2-13.1)	2.2 (1.7-2.9)	2 (1.5-2.6)	-9.4 (-14.5--2.5)	0 (0-0)	0 (0-0)	9.7 (-7.3-26)	0.2 (0.1-0.2)	0.1 (0.1-0.2)	-10.9 (-19.2--2.2)	0 (0-0)	0 (0-0)	55 (25.4-99.3)	0.9 (0.6-1.4)	0.9 (0.6-1.4)	-0.2 (-12.3-14)	0 (0-0)	1 (0.6-1.5)	0 (0-0)	1 (0.6-1.5)	96	86	16	18
Cheshire West and Chester	11.3 (8-16)	11.5 (8.2-16.5)	1.7 (-11.3-14)	1.6 (1.2-2.2)	1.5 (1.1-2.1)	-9.5 (-17.8-6.5)	0 (0-0)	0 (0-0)	15 (-2.2-35.8)	0.1 (0.1-0.2)	0.1 (0.1-0.2)	-12.8 (-23.8--0.4)	0 (0-0)	0 (0-0)	74.3 (31.6-141)	0.9 (0.5-1.3)	0.9 (0.5-1.4)	0.2 (-16.4-21.3)	0 (0-0)	0.9 (0.5-1.4)	0 (0-0)	0.9 (0.5-1.4)	102	95	18	20
Cumbria	13.6 (9.9-18.2)	14.1 (10.2-18.9)	3.4 (-4.9-15)	2.1 (1.6-2.8)	1.9 (1.4-2.5)	-10.5 (-18--2.5)	0 (0-0)	0 (0-0)	32.1 (14.9-50.4)	0.2 (0.1-0.2)	0.1 (0.1-0.2)	-11.9 (-19.4--2.6)	0 (0-0)	0 (0-0)	26.8 (-5.1-73.2)	1 (0.6-1.5)	1 (0.7-1.5)	3.6 (-7.7-20.1)	0 (0-0)	1 (0.7-1.5)	0 (0-0)	1.1 (0.8-1.7)	106	96	16	16
Halton	11.9 (8.4-16.7)	12.1 (8.9-16.8)	1.8 (-7.3-17.3)	1.8 (1.3-2.5)	1.6 (1.2-2.2)	-9.5 (-16.4-2.8)	0 (0-0)	0 (0-0)	9.6 (-8.4-28.1)	0.2 (0.1-0.2)	0.1 (0.1-0.2)	-10.3 (-20.5-2.8)	0 (0-0)	0 (0-0)	15 (-16.7-59.9)	1 (0.6-1.5)	1 (0.6-1.5)	0.7 (-13.9-19.6)	0 (0-0)	0.7 (0.4-1.1)	0 (0-0)	1 (0.6-1.5)	104	98	22	20
Knowsley	12 (8.6-16.3)	12.2 (8.9-16.8)	2.2 (-10.3-22.2)	1.9 (1.4-2.6)	1.8 (1.3-2.4)	-6.4 (-15-12.3)	0 (0-0)	0 (0-0)	13 (-7.2-28.3)	0.2 (0.1-0.2)	0.2 (0.1-0.2)	-8.2 (-18.3-6.3)	0 (0-0)	0 (0-0)	5.8 (-23.9-42)	1 (0.6-1.4)	1 (0.6-1.5)	0.7 (-14.6-18.9)	0 (0-0)	0.8 (0.5-1.1)	0 (0-0)	0.8 (0.5-1.3)	100	94	20	22
Lancashire	13.4 (9.8-17.7)	12.8 (9.3-17.4)	-4.2 (-13.2-8.2)	2.1 (1.6-2.8)	1.8 (1.4-2.4)	-16.3 (-24.2--8.8)	0 (0-0)	0 (0-0)	28.3 (14.4-45.9)	0.2 (0.1-0.2)	0.1 (0.1-0.2)	-14.9 (-23.2--6.2)	0 (0-0)	0 (0-0)	31.3 (-4.7-76.5)	1 (0.6-1.5)	1 (0.6-1.4)	-4.4 (-16.1-9.4)	0 (0-0)	0.8 (0.5-1.2)	0 (0-0)	0.8 (0.5-1.4)	96	83	18	20
Liverpool	34.3 (25.4-44)	34.9 (26.5-44.9)	1.7 (-5.6-9.8)	6.9 (5-9.1)	6.4 (4.8-8.6)	-7.3 (-12.9--0.1)	0 (0-0)	0 (0-0)	-17.8 (-27.2--6.9)	0.4 (0.3-0.6)	0.4 (0.3-0.6)	-4 (-10.7-3.2)	0 (0-0)	0 (0-0)	26.8 (-2.8-63.5)	2.5 (1.7-3.4)	2.6 (1.8-3.5)	3.3 (-5.9-13.5)	0 (0-0)	1.8 (1.2-2.4)	0 (0-0)	1.8 (1.2-2.5)	73	68	11	12
Manchester	68.9 (56.4-86.2)	70.1 (55.6-87.3)	1.7 (-5.2-8.9)	15.5 (12.3-19.3)	15.7 (12.5-19.5)	1.5 (-5.1-8.2)	0.1 (0.1-0.1)	0.1 (0-0.1)	-28.4 (-36.5--19)	1 (0.8-1.2)	1 (0.8-1.2)	4.3 (-3.1-11.4)	0.1 (0.1-0.1)	0.1 (0.1-0.1)	-24.3 (-40.9--5.6)	5.2 (3.8-7.1)	5.4 (4-7.3)	3.9 (-6.9-14.8)	0 (0-0.1)	2.6 (1.9-3.6)	0 (0-0.1)	3.6 (2.6-5.1)	66	62	8	6

Location	Birth Incidence rate (per 100,000 live births), 2000	Birth Incidence rate (per 100,000 live births), 2021	Birth Incidence rate (per 100,000 live births) percent change (2000-2021)	All age rate (per 100,000 population), 2000	All age rate (per 100,000 population), 2021	All age rate (per 100,000 population) percent change (2000-2021)	All age cause-specific SCD mortality rate (per 100,000 population), 2000	All age cause-specific SCD mortality rate (per 100,000 population), 2021	All age cause-specific SCD mortality rate (per 100,000 population) percent change (2000-2021)	All age total SCD mortality rate (per 100,000 population), 2000	All age total SCD mortality rate (per 100,000 population), 2021	All age total SCD mortality rate (per 100,000 population) percent change (2000-2021)	Under-5 cause-specific SCD mortality rate (per 100,000 population), 2000	Under-5 cause-specific SCD mortality rate (per 100,000 population), 2021	Under-5 cause-specific SCD mortality rate (per 100,000 population) percent change (2000-2021)	Under-5 total SCD mortality rate (per 100,000 population), 2000	Under-5 total SCD mortality rate (per 100,000 population), 2021	Under-5 total SCD mortality rate (per 100,000 population) percent change (2000-2021)	Under-5 cause-specific mortality cause fraction, 2000	Under-5 total cause fraction, 2000	Under-5 cause-specific mortality cause fraction, 2021	Under-5 total SCD mortality cause fraction, 2021	Under-5 cause-specific mortality rank 2000	Under-5 cause-specific mortality rank 2021	Under-5 total SCD mortality rank 2000	Under-5 total SCD mortality rank 2021
Oldham	15.1 (11.2-20.3)	15.6 (11.4-20.8)	3.2 (-4.5-10.5)	2.6 (1.9-3.4)	2.6 (1.9-3.5)	-0.3 (-6.3-7.2)	0 (0-0)	0 (0-0)	16 (3.2-29.7)	0.2 (0.2-0.3)	0.2 (0.1-0.3)	-0.7 (-10.5-8.5)	0 (0-0)	0.1 (0-0.1)	28.8 (4-63.1)	1.2 (0.8-1.8)	1.2 (0.8-1.8)	2.2 (-12.4-15.9)	0 (0-0)	0.7 (0.5-1.1)	0 (0-0)	0.7 (0.4-1)	74	70	19	22
Rochdale	12.4 (8.9-17.2)	12.6 (9.1-17.6)	1.7 (-5.8-12.2)	1.9 (1.4-2.5)	1.8 (1.3-2.4)	-4.9 (-11.7-4.1)	0 (0-0)	0 (0-0)	30.4 (10.4-47.9)	0.2 (0.1-0.2)	0.2 (0.1-0.2)	-6.2 (-15.6-4.3)	0 (0-0)	0 (0-0)	63.3 (25.9-111)	1 (0.6-1.6)	1 (0.6-1.6)	0.7 (-11.4-16.4)	0 (0-0)	0.7 (0.4-1)	0 (0-0)	0.7 (0.4-1.1)	101	93	20	23
Salford	60.1 (47.5-74.3)	60.6 (47.3-76.1)	0.9 (-9.7-8.2)	12.7 (10.2-16)	13 (10.3-16.3)	1.7 (-7.6-9.2)	0 (0-0)	0 (0-0)	-10.2 (-22.2-5.7)	0.8 (0.6-1)	0.8 (0.6-1)	3.9 (-4-13.8)	0 (0-0)	0 (0-0.1)	66.4 (32.1-111)	4.2 (3-5.6)	4.3 (3-5.9)	2.7 (-7.2-15)	0 (0-0)	2.9 (2-4)	0 (0-0)	3 (2.1-4.1)	85	69	8	7
Sefton	11.3 (8.2-15.3)	11.5 (8.2-15.7)	1.7 (-7.2-17.1)	1.6 (1.2-2.3)	1.5 (1.1-2.1)	-8.9 (-18.3-9.8)	0 (0-0)	0 (0-0)	18.5 (1.6-38.9)	0.1 (0.1-0.2)	0.1 (0.1-0.2)	-11.1 (-20.6-2.5)	0 (0-0)	0 (0-0)	20.3 (-11.5-55)	0.9 (0.5-1.3)	0.9 (0.5-1.4)	0.1 (-15.9-18.1)	0 (0-0)	0.8 (0.5-1.3)	0 (0-0)	0.8 (0.5-1.2)	85	77	18	22
St Helens	11.8 (8.5-16.2)	11.9 (8.5-16.7)	1.1 (-11.1-12.6)	1.7 (1.3-2.3)	1.6 (1.2-2.2)	-9.1 (-17.4-1.6)	0 (0-0)	0 (0-0)	38.2 (15.4-66.1)	0.1 (0.1-0.2)	0.1 (0.1-0.2)	-10.6 (-20.5-0.9)	0 (0-0)	0 (0-0)	11.9 (-22.5-57.6)	0.9 (0.6-1.4)	0.9 (0.6-1.4)	0 (-16-17.3)	0 (0-0)	0.7 (0.4-1.1)	0 (0-0)	1 (0.6-1.5)	99	93	20	19
Stockport	16.2 (11.7-22.8)	16.7 (11.7-23.1)	2.8 (-5.9-13.2)	2.8 (2.1-3.8)	2.7 (2-3.6)	-4.8 (-10.9-4)	0 (0-0)	0 (0-0)	3 (-13.9-20.7)	0.2 (0.1-0.3)	0.2 (0.1-0.3)	-4 (-11.1-4.7)	0 (0-0)	0 (0-0)	44.4 (16.9-80.4)	1.2 (0.8-1.8)	1.2 (0.8-1.8)	2.7 (-8.7-15.7)	0 (0-0)	1.1 (0.8-1.7)	0 (0-0)	1.1 (0.7-1.6)	88	76	13	17
Tameside	37.3 (26.9-48.2)	37.6 (27.3-48.6)	1 (-6.3-9.4)	7.7 (5.5-10.1)	7.2 (5.2-9.4)	-7 (-12.9--0.3)	0 (0-0)	0 (0-0)	32 (10.9-57.9)	0.5 (0.3-0.7)	0.5 (0.3-0.6)	-5 (-11.3-1.6)	0 (0-0)	0 (0-0)	16.4 (-11.8-57)	2.6 (1.8-3.7)	2.7 (1.9-3.9)	2.6 (-6.1-12.4)	0 (0-0)	2.2 (1.5-3.1)	0 (0-0)	2.7 (1.8-3.7)	99	95	8	9
Trafford	11.3 (8.4-15.8)	11.5 (8.3-16)	1.5 (-9.6-11.9)	1.7 (1.2-2.3)	1.6 (1.2-2.2)	-1.2 (-11.1-16.2)	0 (0-0)	0 (0-0)	-27.1 (-39.7--13.7)	0.1 (0.1-0.2)	0.1 (0.1-0.2)	-0.2 (-12.7-13.2)	0 (0-0)	0 (0-0)	-3.9 (-24.2-25.6)	0.9 (0.5-1.3)	0.9 (0.5-1.4)	0.5 (-15.3-17.9)	0 (0-0)	0.8 (0.5-1.2)	0 (0-0)	1.1 (0.7-1.7)	86	77	19	15
Warrington	11.9 (8.6-16.6)	11.9 (8.5-17.1)	0.5 (-8.7-12.9)	1.8 (1.3-2.4)	1.6 (1.2-2.2)	-9.6 (-16.2--0.8)	0 (0-0)	0 (0-0)	-7 (-20.2-8.8)	0.2 (0.1-0.2)	0.1 (0.1-0.2)	-11.8 (-20.7--0.7)	0 (0-0)	0 (0-0)	32.4 (-1.3-69)	0.9 (0.6-1.4)	0.9 (0.6-1.4)	-1.1 (-15-14)	0 (0-0)	0.8 (0.5-1.2)	0 (0-0)	1 (0.6-1.5)	86	75	17	18
Wigan	20.9 (15.4-28.1)	21.2 (15.7-29.1)	1.5 (-7.5-10.4)	4 (2.9-5.2)	3.7 (2.7-4.9)	-8.3 (-15.2-0.1)	0 (0-0)	0 (0-0)	27.1 (7.8-48.7)	0.3 (0.2-0.4)	0.2 (0.2-0.3)	-8 (-15-0)	0 (0-0)	0 (0-0)	29.1 (-6.8-71.1)	1.4 (0.9-1.9)	1.4 (0.9-2)	1.7 (-9.8-16.1)	0 (0-0)	1.1 (0.8-1.6)	0 (0-0)	1.3 (0.8-1.8)	99	91	15	16
Wirral	15.9 (11.8-21.5)	14.8 (10.5-19.8)	-6.8 (-14.9-5)	2.8 (2.1-3.5)	2.2 (1.7-3)	-18.1 (-25.3--8.6)	0 (0-0)	0 (0-0)	8.4 (-5.1-21.2)	0.2 (0.1-0.3)	0.2 (0.1-0.2)	-16 (-23.7--7.7)	0 (0-0)	0 (0-0)	22.7 (-6.7-58.1)	1.1 (0.7-1.6)	1.1 (0.7-1.6)	-5.5 (-17.5-7.3)	0 (0-0)	0.9 (0.6-1.4)	0 (0-0)	0.9 (0.6-1.4)	82	70	17	20
South East England	18.3 (14.2-24.1)	18.6 (14.6-24.4)	1.9 (-0.9-4.7)	3.5 (2.6-4.4)	3.3 (2.5-4.1)	-4.8 (-7--2)	0 (0-0)	0 (0-0)	1 (-8-9.6)	0.2 (0.2-0.3)	0.2 (0.2-0.3)	-4.5 (-7.5--1.7)	0 (0-0)	0 (0-0)	9.7 (0-21.5)	1.2 (0.9-1.7)	1.3 (0.9-1.8)	3.5 (-1-7.6)	0 (0-0)	1.3 (0.9-1.8)	0 (0-0)	1.5 (1-2)	86	79	14	12
Bracknell Forest	10.9 (7.9-15.7)	11.1 (8-15.3)	1.8 (-12.4-16.2)	1.9 (1.4-2.6)	1.8 (1.3-2.4)	-8.2 (-17.8-12.2)	0 (0-0)	0 (0-0)	-20 (-34.5--5.7)	0.2 (0.1-0.2)	0.1 (0.1-0.2)	-8.7 (-21-7.1)	0 (0-0)	0 (0-0)	-50 (-63.4--35.4)	0.8 (0.5-1.3)	0.8 (0.5-1.3)	0.1 (-16.4-20.2)	0 (0-0)	1 (0.6-1.5)	0 (0-0)	1.8 (1-2.8)	76	74	19	12
Brighton and Hove	16.6 (12-22.6)	16.9 (12.4-23)	2.1 (-5.3-12.2)	2.8 (2-3.9)	2.8 (2-4)	-0.6 (-6.4-5.5)	0 (0-0)	0 (0-0)	-17.8 (-31.5--2.9)	0.2 (0.1-0.3)	0.2 (0.1-0.3)	-2.7 (-10.5-5.9)	0 (0-0)	0 (0-0)	-29.7 (-47.9--5.3)	1.2 (0.8-1.7)	1.2 (0.8-1.8)	1.7 (-10.2-15.9)	0 (0-0)	0.9 (0.6-1.3)	0 (0-0)	1.7 (1.1-2.5)	90	79	18	11
Buckinghamshire	23 (16.5-30.8)	23.6 (17.3-31.3)	2.5 (-5.8-10.2)	4.8 (3.4-6.4)	4.5 (3.1-5.9)	-6.7 (-13.4--0.7)	0 (0-0)	0 (0-0)	-5.5 (-22.7-12)	0.3 (0.2-0.4)	0.3 (0.2-0.4)	-6 (-12.3-1.2)	0 (0-0)	0 (0-0)	32 (3.8-59.5)	1.5 (1-2.1)	1.5 (1-2.2)	3.2 (-7.2-14.3)	0 (0-0)	1.5 (1-2.2)	0 (0-0)	1.4 (0.9-2)	94	84	13	13
East Sussex	11.3 (8.1-15.9)	11.5 (8.3-15.9)	1.1 (-11.4-15.6)	1.6 (1.1-2.1)	1.4 (1.1-2)	-7.8 (-18.6-5.7)	0 (0-0)	0 (0-0)	4.4 (-11.5-20.8)	0.1 (0.1-0.2)	0.1 (0.1-0.2)	-11.8 (-22.5-0.9)	0 (0-0)	0 (0-0)	2.7 (-23.7-36.9)	0.8 (0.5-1.3)	0.8 (0.5-1.3)	-1.4 (-17.1-16.8)	0 (0-0)	0.9 (0.5-1.4)	0 (0-0)	1 (0.6-1.6)	91	78	18	17
Hampshire	17.3 (13.2-22.9)	17.5 (13.4-22.6)	1.5 (-5.5-10.6)	3.5 (2.7-4.4)	3.1 (2.4-4)	-9.5 (-15.2--2.4)	0 (0-0)	0 (0-0)	12.1 (-1.5-30.1)	0.2 (0.2-0.3)	0.2 (0.1-0.3)	-9.2 (-16.6--1.6)	0 (0-0)	0 (0-0)	38.5 (6.4-86.5)	1.1 (0.7-1.6)	1.1 (0.7-1.5)	0.5 (-12.5-12.9)	0 (0-0)	1.3 (0.9-1.9)	0 (0-0)	1.1 (0.8-1.7)	98	88	14	14
Isle of Wight	11.2 (8.3-15.6)	11.5 (8.4-15.8)	2.5 (-8.8-19.3)	1.5 (1.1-2.2)	1.4 (1.1-1.9)	-7.1 (-15.9-8.5)	0 (0-0)	0 (0-0)	31.2 (8.4-53.2)	0.1 (0.1-0.2)	0.1 (0.1-0.2)	-10.4 (-20-3)	0 (0-0)	0 (0-0)	-13.4 (-38-22.4)	0.9 (0.5-1.3)	0.9 (0.5-1.4)	0.3 (-15-20.3)	0 (0-0)	1.1 (0.6-1.6)	0 (0-0)	1.4 (0.9-2.3)	102	97	17	12
Kent	20.8 (15.8-27.7)	19.5 (14.7-26)	-6.1 (-14.1-3.5)	4.1 (3-5.3)	3.6 (2.7-4.6)	-13.3 (-20.6--3.6)	0 (0-0)	0 (0-0)	15.9 (-1.3-30.2)	0.3 (0.2-0.3)	0.2 (0.2-0.3)	-12.2 (-20.5--3.8)	0 (0-0)	0 (0-0)	25.3 (-3.5-65.5)	1.4 (1-2)	1.3 (0.9-1.9)	-5.6 (-17.3-6.3)	0 (0-0)	1.6 (1-2.2)	0 (0-0)	1.4 (1-2)	86	77	13	13
Medway	22 (16.4-29.9)	22.5 (17.2-29.8)	2 (-6.7-11.7)	4.7 (3.4-6.2)	4.4 (3.2-5.7)	-6.8 (-15.7--0.1)	0 (0-0)	0 (0-0)	0.7 (-13.9-15.9)	0.3 (0.2-0.4)	0.3 (0.2-0.4)	-5.5 (-12.9-2.1)	0 (0-0)	0 (0-0)	-2.2 (-28-28)	1.5 (1-2)	1.5 (1-2.1)	1.8 (-8.9-13.5)	0 (0-0)	1.3 (0.9-1.7)	0 (0-0)	1.7 (1.1-2.4)	91	77	15	14
Milton Keynes	52.3 (41.5-66.2)	52.7 (42.1-66.1)	0.8 (-5.9-8.8)	12.7 (9.8-16.1)	11.9 (9.2-15.5)	-6.6 (-13.1-1.7)	0 (0-0)	0 (0-0)	-13.1 (-24.8-0.6)	0.7 (0.6-0.9)	0.7 (0.5-0.9)	-6.1 (-12.8-0.1)	0 (0-0)	0 (0-0)	-21.8 (-43.9-1.4)	3.6 (2.6-4.8)	3.6 (2.6-4.9)	2.8 (-6.9-14.4)	0 (0-0)	2.5 (1.9-3.5)	0 (0-0)	3.6 (2.4-4.8)	80	75	8	6
Oxfordshire	19.9 (15-26.7)	20.3 (15-27.3)	2.5 (-3.6-10.1)	4 (2.9-5.1)	3.8 (2.7-4.9)	-5.7 (-11.4-0.5)	0 (0-0)	0 (0-0)	-10.5 (-22.9-5.7)	0.3 (0.2-0.3)	0.2 (0.2-0.3)	-5.8 (-11.8-0.5)	0 (0-0)	0 (0-0)	-4.4 (-22.2-26.6)	1.3 (0.9-1.9)	1.4 (0.9-1.9)	2.7 (-7.6-14.2)	0 (0-0)	1.4 (1-2.1)	0 (0-0)	1.9 (1.3-2.7)	80	76	14	12
Portsmouth	11.4 (8.2-15.8)	11.6 (8.6-16.1)	1.6 (-7.3-12.8)	1.8 (1.3-2.4)	1.8 (1.3-2.4)	1.8 (-6.7-14.8)	0 (0-0)	0 (0-0)	-9.7 (-24.1-5)	0.1 (0.1-0.2)	0.1 (0.1-0.2)	1.7 (-8.2-14.4)	0 (0-0)	0 (0-0)	-23.1 (-38.7-2.1)	0.9 (0.5-1.4)	0.9 (0.5-1.3)	0.1 (-13.2-17.2)	0 (0-0)	0.7 (0.4-1.1)	0 (0-0)	1.4 (0.8-2.1)	103	91	19	14
Reading	11.7 (8.2-16.3)	12 (8.5-16.8)	2.6 (-9-19.1)	1.8 (1.4-2.4)	2 (1.5-2.7)	9.7 (0-24.5)	0 (0-0)	0 (0-0)	-5.3 (-18.7-8.2)	0.1 (0.1-0.2)	0.2 (0.1-0.2)	14.2 (0.9-36.8)	0.1 (0-0.1)	0 (0-0.1)	-17.9 (-37.8-4.2)	0.9 (0.6-1.4)	1 (0.6-1.5)	2.2 (-13.4-27.1)	0 (0-0)	0.6 (0.4-0.9)	0 (0-0)	0.9 (0.5-1.4)	74	72	25	18
Slough	35.8 (24.5-50.4)	36.6 (25.1-50.1)	2.2 (-4.2-9.5)	7.6 (5.1-10.2)	8.2 (5.4-11.3)	8.4 (1-20.5)	0.1 (0.1-0.1)	0.1 (0-0.1)	-32 (-39.8--24.4)	0.5 (0.3-0.7)	0.6 (0.4-0.8)	8.8 (1.8-16.9)	0.1 (0.1-0.1)	0.1 (0.1-0.1)	-1.8 (-21.1-27.2)	2.8 (1.8-4)	2.9 (1.9-4.1)	3.3 (-4.8-13)	0.1 (0.1-0.1)	1.9 (1.2-2.8)	0.1 (0.1-0.1)	2 (1.4-2.9)	64	61	11	10
Southampton	20 (14.4-27.3)	20.6 (14.9-29.1)	2.9 (-5-11.5)	3.6 (2.5-4.9)	3.7 (2.6-5.2)	2.2 (-6.7-10.4)	0 (0-0)	0 (0-0)	-8.5 (-20.3-4.8)	0.3 (0.2-0.3)	0.3 (0.2-0.4)	6.7 (-1.5-16.3)	0 (0-0)	0 (0-0)	-15.9 (-38.4-22.3)	1.6 (1-2.3)	1.7 (1.1-2.4)	4.3 (-7.5-17.4)	0 (0-0)	1.3 (0.8-1.9)	0 (0-0)	1.8 (1.1-2.6)	88	78	14	12
Surrey	14.9 (11-20.3)	15.3 (11.5-20)	2.5 (-6.1-11.8)	2.8 (2.1-3.5)	2.7 (2.1-3.4)	-2.2 (-9.4-4.6)	0 (0-0)	0 (0-0)	-3.5 (-15.7-10.4)	0.2 (0.1-0.2)	0.2 (0.1-0.2)	-2.1 (-10.4-6.4)	0 (0-0)	0 (0-0)	16.4 (-9.8-41.6)	1 (0.7-1.4)	1 (0.7-1.5)	3.1 (-9.8-16)	0 (0-0)	1.3 (0.8-1.8)	0 (0-0)	1.4 (0.9-2)	91	78	14	14
West Berkshire	10.9 (7.8-15.3)	11.1 (7.9-15.8)	1.5 (-8.9-18.6)	1.8 (1.3-2.4)	1.6 (1.2-2.2)	-8.7 (-16.6-3.8)	0 (0-0)	0 (0-0)	5.1 (-10.8-23)	0.1 (0.1-0.2)	0.1 (0.1-0.2)	-7.8 (-17.5-4.5)	0 (0-0)	0 (0-0)	27.1 (-7.1-72.3)	0.8 (0.5-1.3)	0.8 (0.5-1.3)	1.1 (-14.9-18)	0 (0-0)	0.9 (0.5-1.4)	0 (0-0)	1.1 (0.6-1.6)	102	98	18	18
West Sussex	12.4 (9-16.6)	12.7 (9.3-17.2)	2.5 (-8.3-13.1)	2 (1.5-2.6)	1.9 (1.4-2.5)	-3.6 (-9.9-5.7)	0 (0-0)	0 (0-0)	13.6 (1-32)	0.1 (0.1-0.2)	0.1 (0.1-0.2)	-5.4 (-16-6.3)	0 (0-0)	0 (0-0)	9.4 (-13.9-33.6)	0.9 (0.5-1.3)	0.9 (0.5-1.3)	1.4 (-13.3-18.3)	0 (0-0)	1 (0.7-1.6)	0 (0-0)	1 (0.7-1.5)	77	71	17	17

Location	Birth Incidence rate (per 100,000 live births), 2000	Birth Incidence rate (per 100,000 live births), 2021	Birth Incidence rate (per 100,000 live births) percent change (2000-2021)	All age rate (per 100,000 population), 2000	All age rate (per 100,000 population), 2021	All age rate (per 100,000 population) percent change (2000-2021)	All age cause-specific SCD mortality rate (per 100,000 population), 2000	All age cause-specific SCD mortality rate (per 100,000 population), 2021	All age cause-specific SCD mortality rate (per 100,000 population) percent change (2000-2021)	All age total SCD mortality rate (per 100,000 population), 2000	All age total SCD mortality rate (per 100,000 population), 2021	All age total SCD mortality rate (per 100,000 population) percent change (2000-2021)	Under-5 cause-specific SCD mortality rate (per 100,000 population), 2000	Under-5 cause-specific SCD mortality rate (per 100,000 population), 2021	Under-5 cause-specific SCD mortality rate (per 100,000 population) percent change (2000-2021)	Under-5 total SCD mortality rate (per 100,000 population), 2000	Under-5 total SCD mortality rate (per 100,000 population), 2021	Under-5 total SCD mortality rate (per 100,000 population) percent change (2000-2021)	Under-5 cause-specific mortality cause fraction, 2000	Under-5 total SCD mortality cause fraction, 2000	Under-5 cause-specific mortality cause fraction, 2021	Under-5 total SCD mortality cause fraction, 2021	Under-5 cause-specific mortality rank 2000	Under-5 cause-specific mortality rank 2021	Under-5 total SCD mortality rank 2000	Under-5 total SCD mortality rank 2021
Windsor and Maidenhead	10.8 (7.6-15.1)	11 (7.8-15.3)	2.1 (-9.4-17.2)	1.7 (1.2-2.3)	1.6 (1.2-2.2)	-4.4 (-13.1-9.9)	0 (0-0)	0 (0-0)	-14.3 (-26.4--1.5)	0.1 (0.1-0.2)	0.1 (0.1-0.2)	-4.8 (-15.8-10.2)	0 (0-0)	0 (0-0)	-3.1 (-27.5-25.7)	0.8 (0.5-1.2)	0.8 (0.5-1.3)	0.9 (-14.6-17.8)	0 (0-0)	0.8 (0.5-1.3)	0 (0-0)	1.2 (0.7-1.8)	78	72	21	17
Wokingham	10.3 (7.3-13.9)	10.4 (7.3-14)	1 (-12-15.1)	1.8 (1.3-2.3)	1.6 (1.1-2.2)	-9.1 (-17.2-1.9)	0 (0-0)	0 (0-0)	27 (8-46.3)	0.1 (0.1-0.2)	0.1 (0.1-0.2)	-7.4 (-17.3-4.7)	0 (0-0)	0 (0-0)	31.4 (-0.7-77.6)	0.7 (0.4-1.2)	0.7 (0.4-1.1)	0.1 (-14.7-15)	0 (0-0)	1 (0.6-1.5)	0 (0-0)	1 (0.6-1.5)	81	73	16	17
South West England	15.6 (12.1-20.5)	15.8 (12.1-20.8)	1.5 (-1.9-7.1)	2.6 (2.1-3.3)	2.5 (2-3.1)	-5.8 (-9-1)	0 (0-0)	0 (0-0)	6.6 (-2.2-16)	0.2 (0.1-0.2)	0.2 (0.1-0.2)	-6.9 (-10.4--1.7)	0 (0-0)	0 (0-0)	-10 (-25.3-5.8)	1.1 (0.7-1.5)	1.1 (0.8-1.5)	1.7 (-3.4-7.5)	0 (0-0)	1.1 (0.7-1.5)	0 (0-0)	1.5 (1-2)	93	88	17	12
Bath and North East Somerset	20.5 (14.8-27.6)	21.1 (15.3-29.5)	2.9 (-7.1-9.1)	3.9 (2.7-5)	3.8 (2.6-5.2)	-2.8 (-10.3-4.7)	0 (0-0)	0 (0-0)	-19.4 (-32.1--5.3)	0.2 (0.2-0.3)	0.2 (0.2-0.3)	-2.8 (-11.4-4.6)	0 (0-0)	0 (0-0)	-11.3 (-33.4-8.7)	1.5 (1-2.2)	1.6 (1-2.3)	4.6 (-8.3-15.9)	0 (0-0)	1.8 (1.1-2.5)	0 (0-0)	2.4 (1.5-3.3)	84	77	12	12
Bournemouth	33.1 (24.1-43.5)	25.4 (18.8-33)	-23.4 (-32.4--13.4)	6.6 (4.8-8.8)	5.1 (3.8-6.9)	-22 (-30.9--13.4)	0 (0-0)	0 (0-0)	-16.8 (-31.7-3)	0.4 (0.3-0.5)	0.3 (0.2-0.4)	-22 (-32--10.9)	0 (0-0)	0 (0-0)	-69.2 (-78.7--55.1)	2 (1.4-2.8)	1.5 (1.1-2.2)	-22.2 (-34.2--8.7)	0 (0-0)	1.8 (1.2-2.5)	0 (0-0)	5.7 (3.5-8.3)	102	92	11	5
Bristol, City of	41.2 (32.9-51.4)	38.3 (30-48.9)	-6.9 (-13.5-0.8)	9.2 (7.1-11.6)	8.6 (6.7-11)	-6.2 (-13.2-2.6)	0 (0-0)	0 (0-0)	-3.6 (-15.5-8.5)	0.6 (0.4-0.7)	0.5 (0.4-0.7)	-5.6 (-12.7-1.4)	0 (0-0)	0 (0-0)	-20.4 (-38.5-3)	2.8 (2-3.9)	2.6 (1.9-3.6)	-6.8 (-16.8-3.4)	0 (0-0)	2.3 (1.6-3)	0 (0-0)	3.6 (2.4-4.7)	78	68	8	7
Cornwall	11.1 (7.6-15.9)	11.3 (8.1-15.7)	1.4 (-13.4-21.6)	1.6 (1.1-2.1)	1.4 (1.1-2)	-6.3 (-16.2-10.2)	0 (0-0)	0 (0-0)	33.3 (12.4-58.4)	0.1 (0.1-0.2)	0.1 (0.1-0.2)	-9.7 (-20.7-5.3)	0 (0-0)	0 (0-0)	14.3 (-12.4-44)	0.8 (0.5-1.3)	0.8 (0.5-1.2)	-0.8 (-17.6-20.2)	0 (0-0)	0.8 (0.5-1.4)	0 (0-0)	0.9 (0.6-1.4)	106	103	17	17
Devon	10.5 (7.2-14.8)	10.7 (8-14.8)	1.6 (-11.2-20.7)	1.5 (1.1-2)	1.4 (1.1-2)	-6.5 (-17-12)	0 (0-0)	0 (0-0)	22.1 (-0.1-40.8)	0.1 (0.1-0.2)	0.1 (0.1-0.2)	-8.7 (-18.9-7.3)	0 (0-0)	0 (0-0)	3.4 (-23.2-46.4)	0.8 (0.4-1.2)	0.7 (0.4-1.1)	-0.2 (-16-19)	0 (0-0)	0.8 (0.5-1.2)	0 (0-0)	1 (0.6-1.4)	93	88	17	16
Dorset	10.9 (7.7-14.8)	11 (8.1-15.7)	1.6 (-12.2-14.6)	1.5 (1.1-2)	1.4 (1-1.9)	-8.9 (-18.1-8.2)	0 (0-0)	0 (0-0)	7 (-10.6-31.7)	0.1 (0.1-0.2)	0.1 (0.1-0.1)	-11.3 (-21.9-2.7)	0 (0-0)	0 (0-0)	4.3 (-19.4-41.2)	0.8 (0.5-1.2)	0.8 (0.5-1.2)	0.4 (-14.4-21.5)	0 (0-0)	0.9 (0.5-1.4)	0 (0-0)	1.2 (0.8-1.9)	104	99	18	13
Gloucestershire	14.7 (10.9-20.4)	15.2 (11-20.6)	2.9 (-8.3-14.3)	2.6 (2-3.5)	2.5 (1.9-3.3)	-6.7 (-14.7-0.8)	0 (0-0)	0 (0-0)	16.7 (-1.7-36)	0.2 (0.1-0.2)	0.2 (0.1-0.2)	-7.4 (-16.3-1.8)	0 (0-0)	0 (0-0)	-2.8 (-24.8-29.6)	1 (0.6-1.4)	1 (0.7-1.5)	2 (-12.4-16.1)	0 (0-0)	1 (0.7-1.4)	0 (0-0)	1.2 (0.8-1.7)	81	75	17	15
North Somerset	10.7 (7.6-14.9)	10.9 (7.8-15.1)	2.1 (-8.5-12.8)	1.6 (1.2-2.2)	1.5 (1.1-2.1)	-4.7 (-11.7-5.8)	0 (0-0)	0 (0-0)	4.1 (-11.1-26.8)	0.1 (0.1-0.2)	0.1 (0.1-0.2)	-5.1 (-15.6-5.8)	0 (0-0)	0 (0-0)	-8.8 (-30.7-22.2)	0.8 (0.5-1.2)	0.8 (0.5-1.2)	0.8 (-13.8-16.7)	0 (0-0)	0.8 (0.5-1.2)	0 (0-0)	1.2 (0.7-1.9)	102	93	19	15
Plymouth	11.3 (8.3-15.8)	11.5 (8.3-15.5)	1.2 (-13.9-19.8)	1.7 (1.2-2.3)	1.6 (1.2-2.2)	-3.1 (-12.8-13.3)	0 (0-0)	0 (0-0)	17.9 (1.7-43.2)	0.1 (0.1-0.2)	0.1 (0.1-0.2)	-5.5 (-16-8.3)	0 (0-0)	0 (0-0)	-10.7 (-33-21.2)	0.9 (0.5-1.4)	0.9 (0.5-1.4)	0 (-14.3-18.1)	0 (0-0)	0.7 (0.4-1.1)	0 (0-0)	1.2 (0.7-1.7)	103	96	20	15
Poole	11.1 (8.1-15.7)	11.3 (8.5-15.8)	2.3 (-7.2-16.3)	1.6 (1.2-2.2)	1.5 (1.2-2.1)	-3.1 (-10.2-12.4)	0 (0-0)	0 (0-0)	-16.7 (-33.2-4.9)	0.1 (0.1-0.2)	0.1 (0.1-0.2)	-4.5 (-14-9.3)	0 (0-0)	0 (0-0)	-78.4 (-84.4--69.4)	0.9 (0.5-1.4)	0.9 (0.5-1.4)	1 (-13.5-20.7)	0 (0-0)	0.8 (0.5-1.3)	0 (0-0)	3.4 (1.8-5)	79	77	21	9
Somerset	10.8 (7.3-14.7)	11 (8.2-15.1)	1.6 (-12.2-20.4)	1.6 (1.2-2.1)	1.5 (1.1-1.9)	-6.9 (-16.1-11.5)	0 (0-0)	0 (0-0)	10.3 (-5.4-29.3)	0.1 (0.1-0.2)	0.1 (0.1-0.2)	-9.1 (-19.9-6.9)	0 (0-0)	0 (0-0)	4.1 (-31.6-42.6)	0.8 (0.4-1.2)	0.8 (0.5-1.2)	-0.2 (-16.1-21.8)	0 (0-0)	0.9 (0.5-1.3)	0 (0-0)	0.9 (0.6-1.4)	93	88	17	18
South Gloucestershire	17.4 (12.8-23.8)	18 (13.2-25.3)	3.4 (-5.6-12.6)	3.4 (2.4-4.6)	3.1 (2.3-4.3)	-6.3 (-13.3-1.4)	0 (0-0)	0 (0-0)	-10.6 (-23.1-4.2)	0.2 (0.2-0.3)	0.2 (0.1-0.3)	-7 (-15.2-0.7)	0 (0-0)	0 (0-0)	25.5 (-0.5-55.4)	1.2 (0.8-1.8)	1.3 (0.8-1.9)	5.2 (-7.6-17.9)	0 (0-0)	1.4 (0.9-1.9)	0 (0-0)	1.7 (1.1-2.4)	101	92	13	12
Swindon	11.2 (7.9-15.8)	11.3 (8.1-15.9)	1.1 (-7.1-15.5)	1.8 (1.3-2.5)	1.7 (1.2-2.3)	-5 (-13.2-4.1)	0 (0-0)	0 (0-0)	-7.3 (-20.2-11.8)	0.1 (0.1-0.2)	0.1 (0.1-0.2)	-6.2 (-15.1-6.3)	0 (0-0)	0 (0-0)	-12.8 (-35.3-19.3)	0.9 (0.5-1.4)	0.9 (0.5-1.4)	-0.5 (-13-18.6)	0 (0-0)	0.8 (0.5-1.1)	0 (0-0)	1.3 (0.7-1.9)	106	97	18	14
Torbay	11.5 (8.4-16.1)	11.7 (8.5-16.4)	2.3 (-9.3-16.1)	1.5 (1.1-2.1)	1.5 (1.2-2.1)	-1.3 (-11.7-15.8)	0 (0-0)	0 (0-0)	29.2 (10.5-50.4)	0.1 (0.1-0.2)	0.1 (0.1-0.2)	-1.9 (-13.3-13.7)	0 (0-0)	0 (0-0)	-4.2 (-28.8-41.4)	0.9 (0.5-1.4)	0.9 (0.5-1.4)	1 (-15.1-19.9)	0 (0-0)	0.9 (0.5-1.4)	0 (0-0)	1.4 (0.8-2)	103	99	20	15
Wiltshire	10.9 (7.4-15.4)	11.1 (8-15.2)	1.6 (-9.6-20.2)	1.7 (1.2-2.3)	1.6 (1.1-2.1)	-8.9 (-17.3-10.6)	0 (0-0)	0 (0-0)	8.9 (-9.1-24.3)	0.1 (0.1-0.2)	0.1 (0.1-0.2)	-11 (-21.3-4.3)	0 (0-0)	0 (0-0)	53.7 (13.2-104)	0.8 (0.5-1.2)	0.8 (0.5-1.2)	-0.3 (-14.2-18.5)	0 (0-0)	0.9 (0.5-1.4)	0 (0-0)	0.8 (0.5-1.3)	108	99	18	21
West Midlands	23.9 (19-29.8)	24.8 (19.6-31.1)	3.6 (0.2-7.9)	4.4 (3.5-5.4)	4.3 (3.5-5.3)	-1.3 (-4.3-3.3)	0 (0-0)	0 (0-0)	-1.2 (-7.7-6.7)	0.3 (0.2-0.4)	0.3 (0.2-0.4)	-1.4 (-4.9-2.4)	0 (0-0)	0 (0-0)	4.7 (-6-17.8)	1.7 (1.2-2.3)	1.8 (1.3-2.4)	5.5 (0.3-11)	0 (0-0)	1.2 (0.9-1.6)	0 (0-0)	1.2 (0.9-1.7)	75	68	14	14
Birmingham	42.2 (33.6-52.1)	44.8 (35.4-55.8)	6.3 (-0.6-16.1)	9.7 (7.7-12.1)	10.1 (8.1-12.8)	4.3 (-1.2-15.8)	0.1 (0.1-0.1)	0.1 (0.1-0.1)	-7.6 (-16.3-3.9)	0.6 (0.5-0.7)	0.6 (0.5-0.8)	4.7 (-2.1-12.9)	0.1 (0.1-0.1)	0.1 (0.1-0.1)	-9.2 (-24.7-8.3)	3.1 (2.3-4)	3.3 (2.4-4.4)	7.8 (-2.3-19.5)	0 (0-0.1)	1.5 (1.1-2)	0 (0-0)	1.8 (1.3-2.4)	66	57	13	10
Coventry	32.1 (23.3-42)	32.9 (23.6-43.2)	2.6 (-3.4-10.7)	6.5 (4.5-8.9)	6.6 (4.6-8.8)	1.1 (-7.2-7.7)	0 (0-0)	0 (0-0)	2.4 (-9.5-15.1)	0.4 (0.3-0.6)	0.4 (0.3-0.6)	3.7 (-3.3-10.7)	0 (0-0)	0 (0-0)	2.7 (-14.6-25)	2.4 (1.6-3.4)	2.5 (1.6-3.6)	4 (-5.3-14.3)	0 (0-0)	1.5 (1.1-2.2)	0 (0-0)	2.1 (1.3-3)	73	71	13	10
Dudley	26.9 (20.9-33.8)	22.9 (17.7-29.7)	-14.8 (-23.2--3.4)	5.3 (4-7)	4.2 (3.2-5.4)	-21.2 (-28.9--11.4)	0 (0-0)	0 (0-0)	8.9 (-6.8-27.5)	0.3 (0.2-0.4)	0.3 (0.2-0.3)	-18.3 (-26.2--10.1)	0 (0-0)	0 (0-0)	23.1 (-3.3-49.9)	1.8 (1.2-2.4)	1.5 (1.1-2.1)	-13.1 (-23.4--1)	0 (0-0)	1.4 (1-1.9)	0 (0-0)	1.4 (0.9-1.9)	82	72	14	14
Herefordshire, County of	11.5 (8.3-15.8)	11.6 (8.2-16)	1.4 (-7.8-15.8)	1.6 (1.2-2.2)	1.5 (1.1-2)	-9.5 (-17.6-4.1)	0 (0-0)	0 (0-0)	59.2 (36.7-97)	0.1 (0.1-0.2)	0.1 (0.1-0.2)	-13.3 (-23.6--0.1)	0 (0-0)	0 (0-0)	59 (11.3-103)	0.9 (0.5-1.4)	0.9 (0.5-1.4)	-0.8 (-15.8-18.7)	0 (0-0)	1.1 (0.6-1.8)	0 (0-0)	0.9 (0.5-1.4)	102	95	16	22
Sandwell	41 (30.9-53.7)	36 (27.2-46.9)	-12.2 (-17.8--4.4)	8.4 (6.1-11.1)	7.5 (5.5-9.7)	-11.3 (-16.5--4.8)	0.1 (0.1-0.1)	0.1 (0.1-0.1)	-12.7 (-21.7--3.6)	0.5 (0.4-0.7)	0.5 (0.4-0.6)	-11.2 (-17--4.3)	0.1 (0.1-0.1)	0.1 (0.1-0.1)	-10.6 (-23.1-5.9)	3.1 (2.1-4.2)	2.7 (1.8-3.7)	-13.3 (-20.5--4.7)	0 (0-0.1)	1.8 (1.2-2.4)	0 (0-0.1)	1.6 (1.1-2.3)	64	58	11	12
Shropshire	11.2 (8.1-15.3)	11.3 (7.9-15.8)	0.8 (-9.4-13)	1.6 (1.2-2.1)	1.4 (1-2)	-11.8 (-19.4-3.5)	0 (0-0)	0 (0-0)	17.4 (1.2-36.7)	0.1 (0.1-0.2)	0.1 (0.1-0.2)	-14.8 (-22.9--3.8)	0 (0-0)	0 (0-0)	34.4 (3.8-72.9)	0.9 (0.5-1.3)	0.9 (0.5-1.3)	-1.1 (-15.3-16.9)	0 (0-0)	0.9 (0.5-1.4)	0 (0-0)	0.9 (0.6-1.5)	104	96	20	19
Solihull	17.3 (12.5-24.3)	17.6 (12.8-24.4)	2 (-5.8-14.8)	3.3 (2.4-4.5)	3.1 (2.2-4.2)	-5.6 (-13.8-3.2)	0 (0-0)	0 (0-0)	3.6 (-10.4-20.9)	0.2 (0.2-0.3)	0.2 (0.1-0.3)	-5.6 (-14.1-4.1)	0 (0-0)	0 (0-0)	17.8 (-9.6-64)	1.2 (0.8-1.7)	1.2 (0.8-1.7)	0.8 (-11-15.5)	0 (0-0)	1.2 (0.8-1.8)	0 (0-0)	1 (0.6-1.5)	86	79	14	17
Staffordshire	12.8 (9.5-17.2)	12.2 (9.1-16.1)	-4 (-11.8-9.4)	2.1 (1.6-2.8)	1.8 (1.3-2.3)	-17.9 (-24.9--3.9)	0 (0-0)	0 (0-0)	3.9 (-11.4-21.4)	0.2 (0.1-0.2)	0.1 (0.1-0.2)	-16.7 (-25.7--4.3)	0 (0-0)	0 (0-0)	45.5 (20.8-70.6)	0.9 (0.6-1.3)	0.9 (0.5-1.3)	-3.1 (-18.3-15.9)	0 (0-0)	0.8 (0.5-1.2)	0 (0-0)	0.6 (0.4-1)	85	75	17	23
Stoke-on-Trent	12.2 (9-17.1)	12.5 (9.2-17.3)	2.1 (-10-18.5)	1.7 (1.3-2.4)	1.7 (1.3-2.4)	0.6 (-8.1-16)	0 (0-0)	0 (0-0)	41.7 (17.3-64.5)	0.2 (0.1-0.2)	0.2 (0.1-0.2)	2.2 (-9-17.9)	0 (0-0)	0 (0-0)	55.7 (13.6-111)	1 (0.6-1.5)	1 (0.6-1.6)	1.8 (-12.2-22.9)	0 (0-0)	0.5 (0.3-0.8)	0 (0-0)	0.5 (0.3-0.7)	104	102	21	26
Telford and Wrekin	35.5 (26.8-47)	36 (27.9-47.8)	1.3 (-9-10.8)	7.2 (5.6-9.5)	6.5 (4.9-8.7)	-9.7 (-19-0.1)	0 (0-0)	0 (0-0)	21.3 (2.9-41.9)	0.5 (0.4-0.7)	0.4 (0.3-0.6)	-9.3 (-17.1--0.8)	0 (0-0)	0 (0-0)	25.4 (-5.5-62.4)	2.9 (2-4)	3 (2-4.2)	3.9 (-8.3-18.2)	0 (0-0)	2.2 (1.5-3)	0 (0-0)	2.5 (1.7-3.6)	97	91	9	8

Location	Birth Incidence rate (per 100,000 live births), 2000	Birth Incidence rate (per 100,000 live births), 2021	Birth Incidence rate (per 100,000 live births) percent change (2000-2021)	All age rate (per 100,000 population), 2000	All age rate (per 100,000 population), 2021	All age rate (per 100,000 population) percent change (2000-2021)	All age cause-specific SCD mortality rate (per 100,000 population), 2000	All age cause-specific SCD mortality rate (per 100,000 population), 2021	All age cause-specific SCD mortality rate (per 100,000 population) percent change (2000-2021)	All age total SCD mortality rate (per 100,000 population), 2000	All age total SCD mortality rate (per 100,000 population), 2021	All age total SCD mortality rate (per 100,000 population) percent change (2000-2021)	Under-5 cause-specific SCD mortality rate (per 100,000 population), 2000	Under-5 cause-specific SCD mortality rate (per 100,000 population), 2021	Under-5 cause-specific SCD mortality rate (per 100,000 population) percent change (2000-2021)	Under-5 total SCD mortality rate (per 100,000 population), 2000	Under-5 total SCD mortality rate (per 100,000 population), 2021	Under-5 total SCD mortality rate (per 100,000 population) percent change (2000-2021)	Under-5 cause-specific mortality cause fraction, 2000	Under-5 total SCD mortality cause fraction, 2000	Under-5 cause-specific mortality cause fraction, 2021	Under-5 total SCD mortality cause fraction, 2021	Under-5 cause-specific mortality rank 2000	Under-5 cause-specific mortality rank 2021	Under-5 total SCD mortality rank 2000	Under-5 total SCD mortality rank 2021
Walsall	12.3 (9-17.2)	12.5 (9.4-17.2)	2 (-7.1-15.3)	1.8 (1.4-2.5)	1.8 (1.3-2.4)	-1.6 (-9.9-14.5)	0 (0-0)	0 (0-0)	-12.9 (-24.9-0)	0.2 (0.1-0.2)	0.2 (0.1-0.2)	-2.4 (-13.6-11.5)	0 (0-0)	0 (0-0)	15 (-6.5-34.9)	1 (0.6-1.6)	1 (0.6-1.6)	1.1 (-15-19.1)	0 (0-0)	0.7 (0.4-1)	0 (0-0)	0.6 (0.4-1)	81	74	20	25
Warwickshire	11 (7.4-15.6)	11.2 (8-15.5)	2.3 (-7.1-16.9)	1.6 (1.2-2.1)	1.5 (1.2-2.1)	-6.8 (-16-9.5)	0 (0-0)	0 (0-0)	25 (6-48)	0.1 (0.1-0.2)	0.1 (0.1-0.2)	-8 (-17.2-6.9)	0 (0-0)	0 (0-0)	55.8 (20.2-101)	0.8 (0.4-1.2)	0.8 (0.5-1.2)	1.4 (-11.9-21.3)	0 (0-0)	0.8 (0.4-1.3)	0 (0-0)	0.7 (0.4-1)	93	82	18	23
Wolverhampton	19.3 (14.5-25)	19.7 (14.8-25.2)	1.7 (-5.9-8.6)	3.5 (2.6-4.5)	3.5 (2.7-4.5)	-0.1 (-6.3-7.1)	0.1 (0.1-0.1)	0.1 (0.1-0.1)	-16.2 (-24.1--4.8)	0.2 (0.2-0.3)	0.3 (0.2-0.3)	0.7 (-7.4-9.6)	0.1 (0.1-0.1)	0.1 (0.1-0.1)	-23.3 (-37.9--8.1)	1.4 (1-2)	1.4 (1-2)	1.4 (-9.8-14.2)	0.1 (0-0.1)	0.8 (0.6-1.1)	0.1 (0-0.1)	1 (0.7-1.5)	63	58	16	17
Worcestershire	11 (7.8-15.4)	11.2 (7.9-15.2)	1.7 (-8-19.7)	1.6 (1.2-2.1)	1.5 (1.1-2)	-9.3 (-17.8-5.8)	0 (0-0)	0 (0-0)	38.8 (20.6-61.3)	0.1 (0.1-0.2)	0.1 (0.1-0.2)	-10.7 (-20.6-4.8)	0 (0-0)	0 (0-0)	27.7 (1.6-64.1)	0.8 (0.5-1.3)	0.8 (0.5-1.3)	0.4 (-14.8-23.4)	0 (0-0)	0.7 (0.5-1.2)	0 (0-0)	0.7 (0.5-1.2)	96	84	20	22
Yorkshire and the Humber	16 (12.1-21.3)	16.6 (12.4-21.9)	3.9 (-0.3-8.2)	2.7 (2.1-3.5)	2.6 (2-3.4)	-2.8 (-6-1.5)	0 (0-0)	0 (0-0)	10.5 (1.6-18.3)	0.2 (0.2-0.3)	0.2 (0.1-0.3)	-4 (-7.6--0.3)	0 (0-0)	0 (0-0)	-1.9 (-12.6-9.6)	1.2 (0.8-1.7)	1.2 (0.8-1.7)	3.6 (-1.5-8.7)	0 (0-0)	0.8 (0.5-1.2)	0 (0-0)	1.1 (0.8-1.6)	90	77	17	17
Barnsley	11.8 (8.1-16.6)	12 (8.6-16.3)	1.2 (-8.1-14.1)	1.7 (1.3-2.3)	1.6 (1.2-2.2)	-6.9 (-13.7-2.7)	0 (0-0)	0 (0-0)	37 (15.7-60.1)	0.2 (0.1-0.2)	0.1 (0.1-0.2)	-8.6 (-17.9-2.3)	0 (0-0)	0 (0-0)	18 (-11.3-52.7)	0.9 (0.6-1.4)	0.9 (0.6-1.4)	-0.6 (-15.3-14.9)	0 (0-0)	0.7 (0.5-1.1)	0 (0-0)	0.9 (0.5-1.3)	102	95	20	20
Bradford	20.1 (15-26.9)	20.5 (15.4-27.5)	2.3 (-7-13.8)	3.7 (2.8-4.7)	3.7 (2.8-4.8)	-0.5 (-9.9-10.4)	0 (0-0)	0 (0-0)	21 (4.1-39.7)	0.3 (0.2-0.4)	0.3 (0.2-0.4)	-2.2 (-10.8-7.1)	0 (0-0)	0 (0-0)	-4.8 (-26.9-26.5)	1.6 (1.1-2.2)	1.6 (1.1-2.3)	2.2 (-9.8-16.2)	0 (0-0)	0.7 (0.5-1)	0 (0-0)	1 (0.7-1.5)	84	71	20	18
Calderdale	12.1 (8.6-16.6)	12.4 (8.9-17.5)	2 (-10-13.9)	1.8 (1.3-2.4)	1.6 (1.2-2.2)	-8.6 (-17.1-3.8)	0 (0-0)	0 (0-0)	21.3 (2.8-46.5)	0.2 (0.1-0.2)	0.1 (0.1-0.2)	-10.7 (-21.5-0.5)	0 (0-0)	0 (0-0)	8.9 (-21.2-42.9)	1 (0.6-1.5)	1 (0.6-1.6)	0.6 (-14.8-17.3)	0 (0-0)	0.6 (0.4-1)	0 (0-0)	1 (0.6-1.5)	101	93	21	17
Doncaster	11.9 (8.7-16.5)	12.1 (9.1-17.1)	1.9 (-9.3-14.3)	1.7 (1.3-2.4)	1.6 (1.2-2.2)	-6.6 (-15.1-11.3)	0 (0-0)	0 (0-0)	34.7 (15.2-57.6)	0.2 (0.1-0.2)	0.1 (0.1-0.2)	-8.4 (-18-4.4)	0 (0-0)	0 (0-0)	30.3 (-2-71.8)	1 (0.6-1.4)	1 (0.6-1.5)	1.8 (-12.1-19.1)	0 (0-0)	0.7 (0.4-1.1)	0 (0-0)	0.8 (0.5-1.1)	103	98	20	21
East Riding of Yorkshire	11.2 (8.3-15.5)	11.3 (8.1-15.8)	1.6 (-12-14.1)	1.6 (1.2-2.2)	1.4 (1-1.9)	-13.4 (-21.4-1.8)	0 (0-0)	0 (0-0)	3.6 (-14.5-28.6)	0.1 (0.1-0.2)	0.1 (0.1-0.2)	-15.6 (-25.7--4.7)	0 (0-0)	0 (0-0)	1.8 (-24.2-34.6)	0.8 (0.5-1.3)	0.8 (0.5-1.3)	-0.2 (-16.3-15.5)	0 (0-0)	0.8 (0.5-1.3)	0 (0-0)	1.1 (0.6-1.7)	107	102	19	18
Kingston upon Hull, City of	24.4 (16.7-33.7)	25.1 (17.3-35.2)	2.8 (-5.1-9.7)	4.6 (3-6.5)	4.5 (3-6.5)	-1.8 (-10-4.6)	0 (0-0)	0 (0-0)	6 (-14.1-23.2)	0.3 (0.2-0.5)	0.3 (0.2-0.5)	-0.2 (-6.9-6.5)	0 (0-0)	0 (0-0)	-7 (-30.5-18.4)	1.9 (1.2-2.7)	2 (1.3-2.9)	4.1 (-5.1-13)	0 (0-0)	1.3 (0.9-1.9)	0 (0-0)	1.8 (1.1-2.6)	86	76	15	12
Kirklees	12.2 (8.4-16.7)	12.3 (8.9-16.7)	1.2 (-8.4-16.9)	1.8 (1.3-2.3)	1.6 (1.2-2.2)	-7.7 (-16.2-7.3)	0 (0-0)	0 (0-0)	5 (-10.1-22.9)	0.2 (0.1-0.2)	0.1 (0.1-0.2)	-10 (-20.6-4.3)	0 (0-0)	0 (0-0)	16.2 (-6.1-46.5)	1 (0.6-1.6)	1 (0.6-1.5)	-0.7 (-16-17.7)	0 (0-0)	0.6 (0.4-1)	0 (0-0)	0.8 (0.5-1.1)	84	71	20	20
Leeds	25.6 (19.1-33.2)	26 (19.7-33.3)	1.9 (-4.6-9.3)	5.2 (3.8-6.8)	5.2 (3.8-6.8)	-0.2 (-7.9-8.7)	0 (0-0)	0 (0-0)	8.6 (-5.8-21.5)	0.3 (0.2-0.4)	0.3 (0.2-0.4)	1.8 (-4.9-8.6)	0 (0-0)	0 (0-0)	-15.5 (-31.9-3.4)	1.8 (1.2-2.4)	1.8 (1.3-2.4)	2.7 (-6.3-13)	0 (0-0)	1.3 (0.9-1.7)	0 (0-0)	1.7 (1.2-2.5)	78	69	13	11
North East Lincolnshire	12 (8.6-16.8)	12.3 (8.7-16.9)	2.1 (-9.9-18.5)	1.8 (1.3-2.4)	1.6 (1.2-2.2)	-8.5 (-17.2-6.5)	0 (0-0)	0 (0-0)	16.5 (-0.3-34)	0.2 (0.1-0.2)	0.1 (0.1-0.2)	-11.3 (-21.5-2.8)	0 (0-0)	0 (0-0)	16.9 (-10-54.6)	1 (0.6-1.5)	1 (0.6-1.5)	0.8 (-15.1-19.3)	0 (0-0)	0.8 (0.5-1.2)	0 (0-0)	1 (0.6-1.5)	100	95	19	19
North Lincolnshire	11.6 (8.4-16.1)	11.8 (8.3-16.5)	1.1 (-8.2-12.9)	1.7 (1.2-2.4)	1.6 (1.2-2.2)	-7.4 (-13.8-3)	0 (0-0)	0 (0-0)	-10.5 (-26-3.8)	0.1 (0.1-0.2)	0.1 (0.1-0.2)	-8.5 (-17.3-2.1)	0 (0-0)	0 (0-0)	-7.4 (-31.3-23.8)	0.9 (0.5-1.4)	0.9 (0.5-1.4)	-0.3 (-13.3-15.5)	0 (0-0)	0.7 (0.4-1.1)	0 (0-0)	1.2 (0.7-1.9)	105	97	21	16
North Yorkshire	10.7 (7.4-15)	10.9 (8-15.4)	1.9 (-12.6-20.6)	1.6 (1.1-2.1)	1.4 (1-1.9)	-10.2 (-18.7-10.1)	0 (0-0)	0 (0-0)	7.9 (-10.4-29)	0.1 (0.1-0.2)	0.1 (0.1-0.2)	-11.9 (-22.9-1.8)	0 (0-0)	0 (0-0)	-4.8 (-26.5-34)	0.8 (0.5-1.2)	0.8 (0.5-1.2)	-0.1 (-17.2-18.8)	0 (0-0)	0.9 (0.5-1.4)	0 (0-0)	1.1 (0.7-1.8)	94	85	18	15
Rotherham	11.7 (8.6-16.1)	11.9 (8.3-16.4)	1.1 (-9.4-14.3)	1.8 (1.3-2.4)	1.6 (1.2-2.2)	-7.3 (-15.9-8.6)	0 (0-0)	0 (0-0)	15.5 (-3-36.4)	0.2 (0.1-0.2)	0.1 (0.1-0.2)	-9.4 (-18.8-2.6)	0 (0-0)	0 (0-0)	-24.1 (-41.9-0.7)	0.9 (0.5-1.4)	0.9 (0.6-1.4)	-1 (-16-16.6)	0 (0-0)	0.7 (0.4-1)	0 (0-0)	1 (0.6-1.4)	88	80	21	18
Sheffield	18.2 (13.2-24.9)	18.5 (13.7-25.1)	1.4 (-6.8-10.1)	3.5 (2.5-4.7)	3.6 (2.6-4.9)	2.1 (-6.6-13.1)	0 (0-0)	0 (0-0)	-2.3 (-14.2-11.8)	0.2 (0.2-0.3)	0.2 (0.2-0.3)	0.7 (-7.6-9.6)	0 (0-0)	0 (0-0)	-18.4 (-34.9-3.2)	1.2 (0.8-1.8)	1.2 (0.8-1.8)	0.6 (-11.7-13.8)	0 (0-0)	0.8 (0.5-1.2)	0 (0-0)	1.4 (0.9-2)	77	69	17	16
Wakefield	11.7 (8.4-15.9)	11.8 (8.6-16.6)	1 (-10.4-15)	1.7 (1.3-2.3)	1.6 (1.2-2.2)	-7.1 (-15-10.9)	0 (0-0)	0 (0-0)	22.1 (6.5-39.3)	0.1 (0.1-0.2)	0.1 (0.1-0.2)	-8.2 (-19.4-3.2)	0 (0-0)	0 (0-0)	26.2 (-5-56.7)	0.9 (0.5-1.4)	0.9 (0.5-1.4)	-0.6 (-15.6-17.2)	0 (0-0)	0.7 (0.4-1)	0 (0-0)	0.8 (0.5-1.3)	90	79	19	22
York	11.2 (8.1-15.5)	11.2 (8.2-15.7)	-0.1 (-11.8-10.4)	1.6 (1.2-2.1)	1.5 (1.1-2.1)	-3.8 (-13.8-10.2)	0 (0-0)	0 (0-0)	24.4 (6.7-49.2)	0.1 (0.1-0.2)	0.1 (0.1-0.2)	-6.9 (-18-3.7)	0 (0-0)	0 (0-0)	-22.8 (-42.4-4.1)	0.9 (0.5-1.3)	0.9 (0.5-1.4)	-2.2 (-16.8-13.6)	0 (0-0)	0.7 (0.4-1.1)	0 (0-0)	1.3 (0.8-2.1)	105	97	22	15
Northern Ireland	18.7 (14.4-23.6)	18.9 (14.7-24.5)	1.1 (-8-8.1)	4.4 (3.5-5.3)	4 (3.2-4.7)	-10.6 (-17.4--5.5)	0 (0-0)	0 (0-0)	-8.8 (-17.6-2)	0.3 (0.2-0.3)	0.2 (0.2-0.3)	-11.3 (-17.8--4)	0 (0-0)	0 (0-0)	-47.7 (-59.1--31.7)	1.2 (0.9-1.7)	1.2 (0.9-1.7)	1.1 (-8.6-13.2)	0 (0-0)	1 (0.7-1.5)	0 (0-0)	1.9 (1.3-2.6)	71	63	14	10
Scotland	19 (14.6-24)	19 (14.6-24.2)	-0.2 (-8.2-7.6)	4 (3.3-4.8)	3.5 (2.8-4.2)	-11.2 (-17--4.5)	0 (0-0)	0 (0-0)	5.8 (-8-17)	0.2 (0.2-0.3)	0.2 (0.2-0.3)	-12.1 (-18.5--5.5)	0 (0-0)	0 (0-0)	-31.1 (-50.2--10.5)	1.2 (0.9-1.6)	1.2 (0.9-1.6)	0.1 (-10.4-10.9)	0 (0-0)	1 (0.7-1.4)	0 (0-0)	1.7 (1.2-2.4)	70	64	15	12
Wales	19.2 (14.8-23.8)	19.1 (15-23.5)	-0.2 (-9.7-8.5)	4.2 (3.4-5)	3.8 (3.1-4.6)	-8.7 (-14.5--3.3)	0 (0-0)	0 (0-0)	-2.3 (-10.3-7.5)	0.2 (0.2-0.3)	0.2 (0.2-0.3)	-9.8 (-18.2--1)	0 (0-0)	0 (0-0)	-32.3 (-44--18.8)	1.2 (0.9-1.7)	1.2 (0.9-1.7)	-0.2 (-12.4-12.2)	0 (0-0)	1.1 (0.8-1.4)	0 (0-0)	1.5 (1.1-2.1)	71	64	13	10
Latin America and Caribbean	38.7 (33.6-43.5)	45.3 (39.6-51.7)	17.1 (13.3-22.6)	11.1 (9.6-12.6)	9.5 (8.1-10.9)	-14.8 (-17--12.6)	0.2 (0.2-0.3)	0.2 (0.2-0.3)	-1.3 (-11.1-12.6)	0.7 (0.6-0.9)	0.7 (0.6-0.8)	-8 (-11.2--4.4)	0.5 (0.4-0.7)	0.4 (0.2-0.6)	-18 (-41.1-7.4)	2.1 (1.7-2.5)	2.6 (2.1-3.3)	25.7 (18.2-34.8)	0.1 (0.1-0.1)	0.3 (0.2-0.4)	0.1 (0.1-0.2)	0.7 (0.5-0.9)	67	59	35	21
**Andean Latin America**	10.6 (8.4-13)	10.9 (8.5-13.5)	3 (-2.5-9.2)	4.5 (3.7-5.5)	3.9 (3.2-4.7)	-13.2 (-17.4--9.5)	0 (0-0.1)	0 (0-0)	-45.3 (-63.6--13.7)	0.2 (0.2-0.3)	0.2 (0.1-0.2)	-12.7 (-18.7--6.8)	0 (0-0)	0 (0-0)	-64 (-82.8--30.9)	0.1 (0.1-0.2)	0.1 (0.1-0.2)	4.6 (-3.4-14.3)	0 (0-0)	0 (0-0)	0 (0-0)	0 (0-0.1)	124	128	97	74
Bolivia (Plurinational State of)	11.6 (8.5-14.9)	11.5 (8.5-15.5)	-0.5 (-10.3-10.6)	5.1 (3.9-6.6)	4.5 (3.4-5.7)	-12.7 (-19.7--5.3)	0.1 (0.1-0.1)	0 (0-0.1)	-50.7 (-67.3--13.8)	0.2 (0.2-0.3)	0.2 (0.1-0.3)	-16.2 (-25.8--6.7)	0 (0-0.1)	0 (0-0)	-72.7 (-89--43.2)	0.2 (0.1-0.3)	0.2 (0.1-0.3)	-0.2 (-13.7-16.5)	0 (0-0)	0 (0-0)	0 (0-0)	0 (0-0.1)	124	123	99	77
Ecuador	10.3 (9.3-11.1)	11.4 (10.3-12.6)	10.7 (3.6-17.5)	4.5 (4.1-4.9)	4 (3.6-4.4)	-11.1 (-17.6--6)	0 (0-0)	0 (0-0)	258 (182-354)	0.2 (0.2-0.2)	0.2 (0.2-0.2)	-3.1 (-10-6)	0 (0-0)	0 (0-0)	141 (35-296)	0.1 (0.1-0.1)	0.1 (0.1-0.2)	12.6 (-0.1-27)	0 (0-0)	0 (0-0)	0 (0-0)	0 (0-0.1)	129	114	93	67
Peru	10.3 (7.6-13.7)	10.3 (7.6-13.7)	-0.1 (-10.7-7.7)	4.3 (3.4-5.6)	3.6 (2.8-4.7)	-16.1 (-23.5--10.4)	0.1 (0-0.1)	0 (0-0)	-60.3 (-77.2--33.2)	0.2 (0.1-0.3)	0.2 (0.1-0.2)	-17.4 (-26--8.6)	0 (0-0)	0 (0-0)	-79.3 (-93.1--57.8)	0.1 (0.1-0.2)	0.1 (0.1-0.2)	1.2 (-11.3-16.4)	0 (0-0)	0 (0-0)	0 (0-0)	0 (0-0.1)	122	129	95	74
**Caribbean**	339 (292-385)	396 (340-457)	17.1 (11.6-24.2)	74.2 (63-85.6)	65.9 (55-77.5)	-11.1 (-14.6--8)	1.4 (1.1-1.8)	1.2 (0.9-1.6)	-11.7 (-27.8-10.2)	6.1 (5.2-7.1)	6.1 (5.2-7)	-1.1 (-5.4-4)	3.6 (1.8-5.6)	3.1 (1.3-5.2)	-14.5 (-45-28.8)	22.5 (18-27.1)	28.3 (22-35)	25.4 (17.3-35.6)	0.3 (0.2-0.5)	2.1 (1.7-2.6)	0.4 (0.2-0.6)	3.2 (2.3-4)	33	31	12	9

Location	Birth Incidence rate (per 100,000 live births), 2000	Birth Incidence rate (per 100,000 live births), 2021	Birth Incidence rate (per 100,000 live births) percent change (2000-2021)	All age rate (per 100,000 population), 2000	All age rate (per 100,000 population), 2021	All age rate (per 100,000 population) percent change (2000-2021)	All age cause-specific SCD mortality rate (per 100,000 population), 2000	All age cause-specific SCD mortality rate (per 100,000 population), 2021	All age cause-specific SCD mortality rate (per 100,000 population) percent change (2000-2021)	All age total SCD mortality rate (per 100,000 population), 2000	All age total SCD mortality rate (per 100,000 population), 2021	All age total SCD mortality rate (per 100,000 population) percent change (2000-2021)	Under-5 cause-specific SCD mortality rate (per 100,000 population), 2000	Under-5 cause-specific SCD mortality rate (per 100,000 population), 2021	Under-5 cause-specific SCD mortality rate (per 100,000 population) percent change (2000-2021)	Under-5 total SCD mortality rate (per 100,000 population), 2000	Under-5 total SCD mortality rate (per 100,000 population), 2021	Under-5 total SCD mortality rate (per 100,000 population) percent change (2000-2021)	Under-5 cause-specific mortality cause fraction, 2000	Under-5 total SCD mortality cause fraction, 2000	Under-5 cause-specific mortality cause fraction, 2021	Under-5 total SCD mortality cause fraction, 2021	Under-5 cause-specific mortality rank 2000	Under-5 cause-specific mortality rank 2021	Under-5 total SCD mortality rank 2000	Under-5 total SCD mortality rank 2021
Antigua and Barbuda	161 (120-209)	181 (135-244)	12.8 (0.1-26.7)	48.3 (37.1-61.7)	34.8 (26-44)	-28.1 (-35.2--21.7)	2 (1.8-2.1)	2 (1.8-2.1)	2.4 (-9.1-13.1)	2.7 (2-3.7)	2.4 (1.8-3.2)	-12.1 (-22--0.7)	2.1 (1.6-2.7)	1.5 (1.1-2)	-28.3 (-51.3-4.2)	5.8 (3.8-8.3)	8.4 (5.1-12.6)	46.2 (18-78.4)	0.8 (0.7-0.8)	2.1 (1.3-3.1)	1 (0.9-1.1)	5.7 (3.1-8.4)	18	16	11	5
Bahamas	298 (223-398)	347 (268-449)	16.3 (2-33.2)	85.9 (63.3-109)	65.1 (49.8-79.9)	-24.2 (-33.3--14.8)	1.8 (1.6-2)	1.8 (1.4-2.2)	-2.5 (-23.8-26)	5.4 (3.9-7.5)	5 (3.7-6.6)	-8 (-20.5-5.6)	1 (0.8-1.2)	0.9 (0.6-1.4)	-7.7 (-35.9-52)	6.5 (4.6-8.8)	12 (8-17.2)	83.7 (55-123)	0.4 (0.4-0.5)	3 (2-3.9)	0.5 (0.4-0.5)	6.3 (3.6-8.9)	24	28	8	8
Barbados	172 (126-222)	196 (139-254)	13.7 (2-26)	41.5 (31.7-51.6)	30.8 (22.8-38.6)	-25.9 (-32.9--18.8)	1.2 (1.1-1.3)	0.7 (0.6-1)	-38.1 (-51.9--19.2)	2.5 (1.8-3.3)	2.2 (1.6-3)	-9.4 (-20.6-1.9)	0.9 (0.7-1.1)	0.5 (0.3-0.6)	-51.1 (-66--30.8)	6.4 (4.1-9.2)	9.3 (5.7-14.3)	44.2 (16.8-75.5)	0.3 (0.3-0.3)	2.1 (1.3-2.9)	0.2 (0.2-0.2)	4.4 (2.4-6.5)	32	38	10	7
Belize	262 (200-329)	301 (224-378)	14.9 (1.9-30.5)	79 (60.9-99.4)	61.8 (46.6-79.5)	-21.8 (-30.4--13.5)	0.6 (0.6-0.7)	0.5 (0.5-0.6)	-16.2 (-25.8--2.6)	6.2 (4.5-8)	5.7 (4.2-7.3)	-7.8 (-17.6-4.2)	1.1 (0.9-1.2)	0.5 (0.4-0.7)	-48.6 (-60.1--33.3)	17.9 (11.5-26.5)	23.6 (15.2-35.7)	32.3 (7.9-61.7)	0.2 (0.1-0.2)	2.8 (1.7-4)	0.2 (0.2-0.2)	8.1 (5-12.1)	47	43	8	4
Bermuda	121 (86-168)	135 (95.8-186)	11.6 (-3.3-30.2)	31.1 (23.6-38.5)	21.8 (16.5-27.9)	-30 (-35.7--21.6)	1.1 (1-1.2)	1.1 (1-1.3)	3.6 (-10.4-28.9)	1.5 (1.1-2.1)	1.3 (0.9-1.8)	-10.6 (-21.1-2)	0.7 (0.5-1)	0.6 (0.4-0.8)	-23.7 (-50-20.8)	2.2 (1.5-3)	3.3 (2-5)	48.6 (18.4-82.1)	0.6 (0.5-0.7)	2 (1.3-2.8)	0.8 (0.7-1.1)	5 (3-7.5)	24	21	12	5
Cuba	62.3 (52.1-73.2)	99.7 (80.1-123)	59.9 (41.7-82.2)	13.3 (11-16.3)	12.3 (9.9-15.3)	-7.8 (-16.6-4.3)	0.4 (0.4-0.4)	0.4 (0.3-0.4)	0.7 (-16.6-13.8)	0.9 (0.7-1.1)	1 (0.8-1.2)	16.1 (2.4-31.7)	0.3 (0.3-0.4)	0.2 (0.2-0.3)	-34.4 (-53--13.3)	3.6 (2.7-4.6)	6.3 (4.3-8.8)	75.6 (48.1-114)	0.2 (0.2-0.2)	2.1 (1.6-2.7)	0.2 (0.2-0.3)	6.8 (4.3-9.8)	42	40	10	5
Dominica	180 (136-234)	202 (153-264)	12 (-0.3-24)	49.7 (38.3-63.3)	36.2 (27.1-46)	-27.2 (-32.8--19.4)	1 (0.8-1.3)	1 (0.8-1.3)	-3.9 (-28.5-25.9)	3.3 (2.4-4.3)	2.8 (2.1-3.7)	-14.9 (-25.4--4.3)	1.4 (0.9-2)	1.8 (1.2-2.8)	28.8 (-25.4-111)	8 (5.2-11.1)	10.7 (6.9-15.9)	35.2 (10.2-62.3)	0.4 (0.3-0.6)	2.4 (1.5-3.3)	0.4 (0.3-0.6)	2.6 (1.4-3.8)	28	27	10	8
Dominican Republic	368 (302-429)	403 (335-480)	9.4 (-1-18.8)	92.3 (72.6-110)	77.2 (61.1-93.7)	-16.4 (-24.5--9.4)	1.1 (0.9-1.4)	0.7 (0.5-0.9)	-40.5 (-55.1--25.1)	8.2 (6.6-9.9)	7.3 (5.9-8.7)	-11.3 (-18.7--2.2)	2.9 (2.2-3.8)	1.2 (0.7-1.9)	-59.2 (-73.7--36.4)	26.3 (19.4-34.2)	30.6 (23.1-39.3)	16.5 (3.2-35.1)	0.4 (0.3-0.4)	3.3 (2.2-4.3)	0.2 (0.1-0.3)	6.2 (4.5-8.1)	30	29	7	5
Grenada	201 (154-251)	227 (170-299)	13.2 (-0.3-25.7)	55.3 (42.7-70)	41.4 (31.6-53.7)	-25.2 (-32.7--17.1)	2.5 (2.3-2.7)	2.9 (2.6-3.3)	18.1 (2.1-34)	3.8 (2.8-5)	3.3 (2.4-4.3)	-13.5 (-24.2--2.1)	2.6 (2.1-3.3)	1.9 (1.4-2.4)	-28.5 (-45.5--6.2)	10.3 (6.7-14.7)	13.9 (8.8-20.9)	35.4 (13.9-63.3)	0.6 (0.6-0.7)	2.4 (1.4-3.2)	0.7 (0.6-0.8)	5.1 (3-7.3)	19	19	7	7
Guyana	211 (157-271)	233 (174-300)	10.6 (-2.1-24.3)	66.7 (51.3-86.9)	52.2 (39.3-67.2)	-21.6 (-30.2--12.3)	2.2 (1.9-2.5)	1.8 (1.4-2.3)	-16.3 (-35.9-13.3)	4.4 (3.3-5.7)	4 (3-5.3)	-8.5 (-18.2-2.8)	2.4 (1.9-3)	1.6 (1.2-2.1)	-35.3 (-57.1--7.6)	9.9 (6.6-14.3)	12.8 (8-18.5)	29.9 (7.3-55.9)	0.3 (0.2-0.3)	1.2 (0.7-1.6)	0.3 (0.3-0.4)	2.9 (1.7-4.1)	26	27	12	8
Haiti	493 (427-558)	505 (428-567)	2.3 (-4.9-12.6)	140 (118-163)	117 (96.3-136)	-16.5 (-22.3--7.9)	3.5 (2-5.2)	2.7 (1.7-4)	-23.6 (-43.2-10.6)	12.6 (10.7-14.4)	11.1 (9.5-12.6)	-11.6 (-17.5--4.2)	8.3 (2.5-14.5)	6.4 (2-11.4)	-23.3 (-51.2-45)	35.6 (27.9-42.9)	36.9 (29.6-45.2)	3.6 (-5.9-16.1)	0.3 (0.1-0.6)	1.5 (1.1-1.8)	0.4 (0.1-0.7)	2.2 (1.6-2.8)	33	30	14	11
Jamaica	596 (566-627)	573 (498-654)	-3.9 (-15.1-10.6)	152 (137-167)	91.6 (78.5-109)	-39.7 (-46.6--32)	1.7 (1.6-1.8)	1.4 (1.1-1.8)	-15.6 (-34-5.7)	11.4 (10.5-12.3)	8.1 (7-9.3)	-28.7 (-37.4--20)	1.9 (1.5-2.4)	0.9 (0.6-1.2)	-53.9 (-65.8--33.3)	43.8 (37.9-49.2)	46.3 (35.6-58.2)	5.8 (-13.5-26.9)	0.4 (0.4-0.5)	10.2 (7.7-13.1)	0.3 (0.3-0.4)	17.1 (11.7-23.7)	20	25	3	2
Puerto Rico	132 (96.8-176)	147 (105-197)	11.1 (-1.4-23.9)	38.3 (28.5-48.3)	24.1 (18-31)	-37.1 (-44.3--31.1)	0.2 (0.1-0.2)	0.2 (0.1-0.2)	-0.5 (-19.8-18.2)	2 (1.4-2.8)	1.6 (1.1-2.2)	-20.3 (-29.4--9)	0.1 (0.1-0.1)	0.1 (0.1-0.1)	-41.5 (-60.3--17.7)	2.5 (1.7-3.5)	3.6 (2.4-5.3)	45.6 (20.2-70)	0.1 (0-0.1)	1.1 (0.8-1.6)	0.1 (0.1-0.1)	4.1 (2.5-5.7)	65	56	13	6
Saint Kitts and Nevis	178 (132-226)	198 (152-257)	11.5 (-3.9-24.6)	50.9 (39.7-64.8)	35.4 (27.6-45.6)	-30.5 (-38.1--23.1)	1.6 (1.4-1.8)	1.4 (1.2-1.7)	-13.4 (-29.3-4.3)	3.3 (2.4-4.3)	2.6 (1.9-3.4)	-20.6 (-29.6--10.7)	1.8 (1.5-2.1)	1 (0.7-1.4)	-43.1 (-60.6--16.3)	7.3 (4.9-10.7)	9.8 (6.3-15)	35.4 (14.1-64.2)	0.4 (0.4-0.5)	1.7 (1.1-2.4)	0.5 (0.4-0.5)	4.5 (2.5-6.6)	23	26	11	6
Saint Lucia	204 (154-262)	231 (167-297)	13.1 (1-26.1)	60.4 (47.8-77.4)	36.6 (28.2-46.4)	-39.4 (-45.6--33.7)	2 (1.9-2.2)	1.9 (1.6-2.3)	-6.1 (-21.5-12.7)	4 (2.9-5.3)	2.8 (2-3.6)	-29.3 (-36.6--19.5)	1.8 (1.4-2.4)	1.1 (0.7-1.6)	-38.3 (-57.5--4.6)	9.6 (6.2-14.1)	13.4 (8.7-19.7)	39.9 (17.3-69.1)	0.5 (0.5-0.6)	2.9 (1.7-4.4)	0.4 (0.3-0.4)	4.8 (2.7-7.5)	18	24	8	6
Saint Vincent and the Grenadines	203 (157-264)	227 (173-296)	11.9 (0.1-26.5)	59.2 (46.4-77.5)	42 (32.3-54.1)	-29.1 (-36--21.8)	2 (1.8-2.1)	1.9 (1.7-2.2)	-0.8 (-13.2-16.8)	4 (2.9-5.4)	3.2 (2.4-4.2)	-18.8 (-27.4--8.4)	1.8 (1.4-2.3)	1.1 (0.8-1.5)	-42.2 (-60--17)	9.5 (6.4-13.8)	12.7 (7.9-19.4)	33.9 (13.1-59.9)	0.4 (0.4-0.4)	2 (1.4-2.9)	0.4 (0.4-0.5)	5.4 (3.1-8.1)	24	26	11	6
Suriname	212 (160-265)	237 (184-302)	11.9 (0.7-23.4)	56.9 (43.6-72.2)	47.3 (36.4-61.3)	-16.9 (-24.4--8.9)	0.8 (0.5-0.9)	0.6 (0.4-0.8)	-20.9 (-38.1--0.5)	4.1 (3.1-5.3)	3.8 (2.9-5)	-6.9 (-16.4-3.9)	1.5 (1-1.9)	1.2 (0.8-1.7)	-23.9 (-51.2-28.5)	11.5 (7.6-16.3)	14.6 (9.6-21.1)	26.5 (6.5-47.1)	0.2 (0.1-0.2)	1.4 (0.9-2)	0.2 (0.1-0.3)	2.7 (1.7-3.9)	38	39	13	9
Trinidad and Tobago	196 (150-251)	220 (163-282)	12 (-6.2-28)	50.3 (38.4-63.9)	38 (29.3-48.7)	-24.5 (-32.7--14.9)	1.3 (1.2-1.4)	1 (0.8-1.3)	-19.5 (-37.4-0.6)	3.5 (2.5-4.6)	2.8 (2.1-3.7)	-19 (-30.4--8.7)	1.5 (1.3-1.8)	0.7 (0.5-0.9)	-52.5 (-66.3--31.9)	8.7 (5.8-12.4)	11.7 (7.5-17.3)	34.5 (12.2-59.4)	0.3 (0.2-0.3)	1.7 (1.1-2.4)	0.3 (0.2-0.3)	4.6 (2.6-6.5)	31	32	12	7
United States Virgin Islands	156 (114-202)	172 (126-233)	10.1 (-0.2-22.4)	44.5 (34.1-56.1)	32.7 (24.3-42.9)	-26.4 (-33.6--18.3)	0.4 (0.3-0.5)	0.3 (0.2-0.5)	-27.1 (-47.3-8)	2.5 (1.8-3.4)	2.1 (1.6-2.9)	-15 (-24.8--4.5)	0.4 (0.2-0.5)	0.1 (0.1-0.2)	-71.3 (-85.4--43.5)	3.8 (2.6-5.3)	5.2 (3.3-7.4)	37.1 (14.7-62.4)	0.1 (0.1-0.2)	1.6 (1-2.1)	0.2 (0.1-0.2)	7.7 (4.3-11)	46	53	14	4
**Central Latin America**	5.7 (4.7-6.8)	4.2 (3.4-5)	-26.9 (-31.7--21.3)	1.9 (1.5-2.2)	0.8 (0.6-1)	-58 (-62.1--53.7)	0.1 (0-0.1)	0.1 (0-0.1)	1.8 (-13-17.9)	0.1 (0.1-0.1)	0.1 (0.1-0.1)	-41.3 (-45.6--36.6)	0.1 (0.1-0.1)	0.1 (0-0.1)	-17.8 (-37.5-12.7)	0.4 (0.3-0.5)	0.4 (0.3-0.5)	-2.8 (-11.2-8.1)	0 (0-0)	0.1 (0-0.1)	0 (0-0)	0.1 (0.1-0.2)	102	95	71	52
Colombia	5.8 (4.3-7.3)	6 (4.6-8)	3.9 (-9.1-19.4)	0.5 (0.4-0.6)	0.4 (0.3-0.5)	-28.5 (-33.8--19.1)	0.1 (0.1-0.1)	0.1 (0.1-0.1)	-2.7 (-18-19)	0.1 (0.1-0.2)	0.1 (0.1-0.1)	-28.1 (-34.8--18.2)	0.1 (0.1-0.2)	0.1 (0.1-0.1)	-33.1 (-59-9.2)	1 (0.7-1.3)	1 (0.7-1.4)	5.6 (-6.3-21.8)	0 (0-0)	0.2 (0.1-0.2)	0 (0-0.1)	0.5 (0.3-0.8)	87	75	38	27
Costa Rica	0.2 (0.1-0.2)	0.2 (0.1-0.2)	3.4 (-4.1-26.6)	0 (0-0.1)	0 (0-0.1)	-21 (-28.9--8.1)	0.1 (0.1-0.1)	0.1 (0.1-0.1)	22.8 (5.3-38.2)	0 (0-0)	0 (0-0)	-21.2 (-27.9-3)	0.1 (0.1-0.1)	0 (0-0.1)	-42.5 (-55.8--25.4)	0 (0-0)	0 (0-0)	6.4 (-4.5-59.1)	0 (0-0)	0 (0-0)	0 (0-0)	0 (0-0)	77	75	105	90
El Salvador	0.6 (0.4-0.9)	0.7 (0.4-0.9)	6.7 (-1.8-42.5)	0.1 (0-0.1)	0 (0-0.1)	-22.4 (-33.8--1.9)	0 (0-0)	0 (0-0)	-67 (-81.6--35.7)	0 (0-0)	0 (0-0)	-24.4 (-34.6-12.1)	0 (0-0)	0 (0-0)	-69.6 (-87.2--21.2)	0.1 (0.1-0.2)	0.1 (0.1-0.2)	8.3 (-8-66.3)	0 (0-0)	0 (0-0)	0 (0-0)	0.1 (0-0.1)	121	121	84	67
Guatemala	0.7 (0.4-0.9)	0.7 (0.5-1.1)	8.4 (-4.5-66.2)	0.1 (0-0.1)	0 (0-0.1)	-29.5 (-42.6--4.5)	0 (0-0)	0 (0-0)	-16.4 (-31.5--0.1)	0 (0-0)	0 (0-0)	-34.9 (-43.8--4.6)	0 (0-0)	0 (0-0)	-31.6 (-58.4--1.9)	0.1 (0.1-0.2)	0.1 (0.1-0.2)	5.4 (-9.6-59.3)	0 (0-0)	0 (0-0)	0 (0-0)	0 (0-0)	125	123	88	75
Honduras	0.2 (0.1-0.3)	0.2 (0.1-0.3)	7.2 (-12.4-104)	0 (0-0)	0 (0-0)	-17.2 (-31-36.6)	0 (0-0)	0 (0-0)	-69.5 (-85.8--23.8)	0 (0-0)	0 (0-0)	-17.9 (-39.2-53.9)	0 (0-0)	0 (0-0)	-70 (-92.9--15.4)	0 (0-0)	0 (0-0.1)	8.8 (-20.8-118)	0 (0-0)	0 (0-0)	0 (0-0)	0 (0-0)	124	126	116	95
Mexico	2.6 (2-3.5)	2.6 (1.9-3.6)	1 (-3.4-6.3)	1.1 (0.8-1.5)	0.8 (0.6-1.2)	-21.8 (-24.9--18.5)	0 (0-0)	0 (0-0)	13.2 (2.1-25.1)	0 (0-0.1)	0 (0-0.1)	-16.3 (-20.2--11.3)	0 (0-0)	0 (0-0)	-35.3 (-49.7--14.4)	0 (0-0.1)	0 (0-0.1)	9.5 (-1.6-32.1)	0 (0-0)	0 (0-0)	0 (0-0)	0 (0-0)	112	103	109	91
Aguascalientes	0.3 (0.1-0.7)	0.3 (0.1-0.7)	18.3 (-3.7-239)	0.1 (0-0.3)	0.1 (0-0.2)	-10.9 (-29.3-305)	0 (0-0)	0 (0-0)	25.4 (1.5-50)	0 (0-0)	0 (0-0)	-6.5 (-27.9-126)	0 (0-0)	0 (0-0)	-21.7 (-46.8-15.2)	0 (0-0)	0 (0-0)	27.8 (-1.9-253)	0 (0-0)	0 (0-0)	0 (0-0)	0 (0-0)	120	113	116	102
Baja California	2.5 (1.3-4)	2.7 (1.5-4.3)	8.8 (-1.4-34.7)	1 (0.6-1.6)	0.8 (0.5-1.3)	-18 (-25.8-2)	0 (0-0)	0 (0-0)	33 (10.4-55.6)	0 (0-0.1)	0 (0-0.1)	-8.6 (-16-9.3)	0 (0-0)	0 (0-0)	-32.8 (-52.4--7)	0 (0-0)	0 (0-0.1)	18 (-0.4-75.2)	0 (0-0)	0 (0-0)	0 (0-0)	0 (0-0)	110	104	105	93
Baja California Sur	4.5 (2.6-7.1)	4.8 (2.8-7.4)	6.2 (-6.4-18.3)	2 (1.2-3)	1.6 (1-2.5)	-17.4 (-24.8--7.9)	0 (0-0)	0 (0-0)	8.3 (-7.2-26.7)	0.1 (0-0.1)	0.1 (0-0.1)	-8.4 (-18.5-2)	0 (0-0.1)	0 (0-0)	-34 (-56.2--5.3)	0 (0-0.1)	0 (0-0.1)	12.7 (-4.3-49.8)	0 (0-0)	0 (0-0)	0 (0-0)	0 (0-0)	97	94	97	85

Location	Birth Incidence rate (per 100,000 live births), 2000	Birth Incidence rate (per 100,000 live births), 2021	Birth Incidence rate (per 100,000 live births) percent change (2000-2021)	All age rate (per 100,000 population), 2000	All age rate (per 100,000 population), 2021	All age rate (per 100,000 population) percent change (2000-2021)	All age cause-specific SCD mortality rate (per 100,000 population), 2000	All age cause-specific SCD mortality rate (per 100,000 population), 2021	All age cause-specific SCD mortality rate (per 100,000 population) percent change (2000-2021)	All age total SCD mortality rate (per 100,000 population), 2000	All age total SCD mortality rate (per 100,000 population), 2021	All age total SCD mortality rate (per 100,000 population) percent change (2000-2021)	Under-5 cause-specific SCD mortality rate (per 100,000 population), 2000	Under-5 cause-specific SCD mortality rate (per 100,000 population), 2021	Under-5 cause-specific SCD mortality rate (per 100,000 population) percent change (2000-2021)	Under-5 total SCD mortality rate (per 100,000 population), 2000	Under-5 total SCD mortality rate (per 100,000 population), 2021	Under-5 total SCD mortality rate (per 100,000 population) percent change (2000-2021)	Under-5 cause-specific mortality cause fraction, 2000	Under-5 total SCD mortality cause fraction, 2000	Under-5 cause-specific mortality cause fraction, 2021	Under-5 total SCD mortality cause fraction, 2021	Under-5 cause-specific mortality rank 2000	Under-5 cause-specific mortality rank 2021	Under-5 total SCD mortality rank 2000	Under-5 total SCD mortality rank 2021
Campeche	5.4 (3.6-7.6)	5.8 (3.8-8)	5.6 (-2.5-19.1)	2.3 (1.6-3.2)	1.9 (1.2-2.6)	-18.9 (-26.1--10.8)	0 (0-0)	0 (0-0)	21.2 (-1.4-52.5)	0.1 (0.1-0.2)	0.1 (0.1-0.1)	-15.5 (-25.3--4.3)	0 (0-0.1)	0 (0-0)	-34.4 (-55.9--5.3)	0.1 (0-0.1)	0.1 (0-0.1)	11.6 (-6.1-48.2)	0 (0-0)	0 (0-0)	0 (0-0)	0 (0-0)	103	94	100	84
Chiapas	3.3 (2.3-4.6)	3.1 (2.1-4.4)	-6.7 (-17.1-4.7)	1.5 (1-2)	1.1 (0.8-1.6)	-22.4 (-30.4--16.3)	0 (0-0)	0 (0-0)	-11.9 (-29.7-6.3)	0.1 (0-0.1)	0.1 (0-0.1)	-21.1 (-30.4--11.5)	0.1 (0.1-0.1)	0 (0-0.1)	-35 (-53.6--3.8)	0.1 (0-0.1)	0.1 (0-0.1)	3.7 (-14.9-52.7)	0 (0-0)	0 (0-0)	0 (0-0)	0 (0-0)	109	100	112	95
Chihuahua	1.2 (0.5-2)	1.3 (0.7-2.5)	16.1 (-4.7-69.8)	0.5 (0.2-0.8)	0.4 (0.2-0.8)	-10.1 (-24.3-36)	0 (0-0)	0 (0-0)	42.4 (18.4-74.8)	0 (0-0)	0 (0-0)	-1.9 (-16.6-52)	0 (0-0)	0 (0-0)	-5.9 (-41.1-31)	0 (0-0)	0 (0-0.1)	24.8 (-2.7-144)	0 (0-0)	0 (0-0)	0 (0-0)	0 (0-0)	115	111	116	107
Coahuila	1.6 (1-2.2)	1.7 (1.1-2.5)	11.2 (1.5-37.2)	0.7 (0.4-1)	0.6 (0.4-0.9)	-10.1 (-17.1-2.8)	0 (0-0)	0 (0-0)	18.9 (3.7-37.1)	0 (0-0)	0 (0-0)	-3.3 (-15.1-22.5)	0 (0-0)	0 (0-0)	-27 (-53-12.8)	0 (0-0.1)	0 (0-0.1)	24.5 (-1.8-128)	0 (0-0)	0 (0-0)	0 (0-0)	0 (0-0)	114	108	106	91
Colima	1.9 (0.8-4)	2 (0.7-4.2)	1.7 (-31.7-19.9)	0.8 (0.3-1.6)	0.6 (0.2-1.3)	-22.9 (-51.8--11.8)	0 (0-0)	0 (0-0)	32.5 (7.6-60)	0 (0-0.1)	0 (0-0.1)	-18 (-42.9--3.8)	0.1 (0-0.1)	0 (0-0)	-49.1 (-65.5--23.8)	0 (0-0.1)	0 (0-0.1)	11.8 (-14-73.4)	0 (0-0)	0 (0-0)	0 (0-0)	0 (0-0)	104	101	116	94
Durango	1.5 (0.7-2.7)	1.7 (0.7-3.2)	17.5 (-3.9-49.9)	0.7 (0.3-1.2)	0.6 (0.2-1.2)	-5.9 (-23.3-27)	0 (0-0)	0 (0-0)	-0.9 (-15.8-21.3)	0 (0-0.1)	0 (0-0.1)	0.5 (-15.2-27.5)	0 (0-0)	0 (0-0)	-39.7 (-62.4--15.1)	0 (0-0.1)	0 (0-0.1)	22.2 (-0.8-96.6)	0 (0-0)	0 (0-0)	0 (0-0)	0 (0-0)	118	113	114	99
Guanajuato	1.2 (0.8-1.9)	1.3 (0.8-2)	5 (-30.4-57.6)	0.5 (0.3-0.8)	0.4 (0.2-0.6)	-24.5 (-50.5-9)	0 (0-0)	0 (0-0)	52 (23.4-88.5)	0 (0-0)	0 (0-0)	-15.5 (-40.5-22.8)	0 (0-0)	0 (0-0)	-29.1 (-49.6--5.9)	0 (0-0.1)	0 (0-0.1)	23.5 (-8.1-126)	0 (0-0)	0 (0-0)	0 (0-0)	0 (0-0)	115	110	104	91
Guerrero	6.8 (5.4-8.6)	6.4 (5-8.1)	-5.2 (-13.9-2.4)	3 (2.4-3.7)	2.3 (1.8-2.9)	-25 (-31.7--19.4)	0 (0-0)	0 (0-0)	-15.7 (-37.2-8.6)	0.1 (0.1-0.2)	0.1 (0.1-0.1)	-21.4 (-28.8--13)	0.1 (0.1-0.2)	0.1 (0-0.1)	-38.3 (-65.1-13.5)	0.1 (0.1-0.2)	0.1 (0.1-0.2)	1.4 (-15.5-34.4)	0 (0-0)	0 (0-0)	0 (0-0)	0 (0-0)	105	95	108	89
Hidalgo	1.1 (0.6-2)	1.3 (0.7-2.3)	11.5 (-3.8-45.5)	0.5 (0.2-0.9)	0.4 (0.2-0.8)	-15 (-26.1-4)	0 (0-0)	0 (0-0)	9.5 (-9.5-31.8)	0 (0-0)	0 (0-0)	-8.9 (-23.1-17.6)	0 (0-0)	0 (0-0)	-41.4 (-59.3--15.7)	0 (0-0)	0 (0-0.1)	19.8 (-5-146)	0 (0-0)	0 (0-0)	0 (0-0)	0 (0-0)	119	109	113	95
Jalisco	1.9 (1.2-2.9)	1.6 (0.8-2.6)	-14.5 (-42.9-2.8)	0.8 (0.5-1.2)	0.5 (0.3-0.8)	-36.4 (-55.9--23.7)	0 (0-0)	0 (0-0)	33 (11-62.9)	0 (0-0.1)	0 (0-0)	-27.5 (-47.4--15.6)	0 (0-0)	0 (0-0)	-24 (-47.1-3)	0 (0-0)	0 (0-0)	1.2 (-24.4-63.4)	0 (0-0)	0 (0-0)	0 (0-0)	0 (0-0)	110	106	109	98
México	0.5 (0.2-1)	0.6 (0.2-1.2)	16.4 (-9.8-171)	0.2 (0.1-0.4)	0.2 (0.1-0.4)	-8.2 (-28.1-136)	0 (0-0)	0 (0-0)	6.5 (-8.3-23.7)	0 (0-0)	0 (0-0)	-2.5 (-19.7-126)	0 (0-0)	0 (0-0)	-59.3 (-72.3--39.5)	0 (0-0)	0 (0-0)	22.4 (-2.9-164)	0 (0-0)	0 (0-0)	0 (0-0)	0 (0-0)	114	105	110	93
Mexico City	1.6 (1.1-2.1)	1.7 (1.3-2.2)	8.5 (-1.3-28.9)	0.6 (0.4-0.8)	0.5 (0.3-0.6)	-18.8 (-25.5--7.3)	0 (0-0)	0 (0-0)	52.6 (27-88.3)	0 (0-0)	0 (0-0)	-12.7 (-21.5-4.5)	0 (0-0)	0 (0-0)	1.4 (-32-49.8)	0 (0-0.1)	0 (0-0.1)	16.7 (-4.1-66.7)	0 (0-0)	0 (0-0)	0 (0-0)	0 (0-0)	116	106	94	87
Michoacán de Ocampo	1.7 (1-2.9)	1.8 (1-3.1)	6.9 (-16.4-31)	0.7 (0.4-1.2)	0.6 (0.3-1)	-17.7 (-35.7-4.2)	0 (0-0)	0 (0-0)	26 (6.8-52.9)	0 (0-0.1)	0 (0-0.1)	-14.7 (-31.7-5.4)	0 (0-0)	0 (0-0)	-15.5 (-43.8-17.7)	0 (0-0.1)	0 (0-0.1)	16.8 (-7.4-91.5)	0 (0-0)	0 (0-0)	0 (0-0)	0 (0-0)	117	112	114	99
Morelos	1.9 (0.8-3.9)	1.7 (0.7-3.7)	-10.4 (-38.9-6.6)	0.8 (0.3-1.6)	0.5 (0.2-1.2)	-32.8 (-58.2--14.8)	0 (0-0)	0 (0-0)	47.6 (14-87.8)	0 (0-0.1)	0 (0-0.1)	-25.5 (-49.6--12.5)	0 (0-0)	0 (0-0)	-40.9 (-57.9--15)	0 (0-0.1)	0 (0-0.1)	5.4 (-22.3-83.3)	0 (0-0)	0 (0-0)	0 (0-0)	0 (0-0)	111	106	106	91
Nayarit	2.2 (1.1-3.8)	2.5 (1.2-4.5)	11.9 (-4.1-30.6)	1 (0.5-1.6)	0.9 (0.4-1.6)	-9.7 (-23.3-3)	0 (0-0)	0 (0-0)	11.6 (-7.5-35.3)	0 (0-0.1)	0 (0-0.1)	-4.4 (-17.1-14.5)	0 (0-0)	0 (0-0)	-39.6 (-59.2--8.2)	0 (0-0.1)	0 (0-0.1)	19 (-4.5-92.8)	0 (0-0)	0 (0-0)	0 (0-0)	0 (0-0)	114	109	111	95
Nuevo León	3.3 (2.2-4.6)	2.7 (2-3.9)	-17.1 (-25.5--9)	1.4 (1-2)	1 (0.7-1.4)	-32.3 (-38.6--24.8)	0 (0-0)	0 (0-0)	40.3 (18.6-66.9)	0.1 (0-0.1)	0 (0-0.1)	-26.7 (-35.8--18.2)	0 (0-0)	0 (0-0)	-14.4 (-44.5-36.7)	0 (0-0.1)	0 (0-0.1)	-5.5 (-23.1-32.5)	0 (0-0)	0 (0-0)	0 (0-0)	0 (0-0)	114	107	109	95
Oaxaca	5.9 (4.4-7.7)	6 (4.6-7.8)	2.2 (-4.9-11.4)	2.5 (2-3.2)	1.9 (1.5-2.4)	-22.8 (-28.6--17.5)	0 (0-0)	0 (0-0)	-11.5 (-26.8-8.4)	0.1 (0.1-0.2)	0.1 (0.1-0.1)	-18.5 (-25.3--10.7)	0.1 (0-0.1)	0 (0-0.1)	-40.1 (-60.7--4.9)	0.1 (0.1-0.1)	0.1 (0.1-0.1)	8.4 (-7.9-43.3)	0 (0-0)	0 (0-0)	0 (0-0)	0 (0-0)	105	98	100	85
Puebla	1 (0.5-1.8)	1.1 (0.6-2)	14.8 (-4.8-113)	0.4 (0.2-0.7)	0.3 (0.2-0.6)	-12.8 (-27.6-55.5)	0 (0-0)	0 (0-0)	-1.5 (-17.2-19.2)	0 (0-0)	0 (0-0)	-7.5 (-23.5-57.5)	0 (0-0)	0 (0-0)	-37.9 (-57.7--4.9)	0 (0-0.1)	0 (0-0.1)	20.2 (-4.3-137)	0 (0-0)	0 (0-0)	0 (0-0)	0 (0-0)	116	109	117	98
Querétaro	0.3 (0.1-0.7)	0.3 (0.1-0.8)	18 (-3-150)	0.1 (0-0.3)	0.1 (0-0.2)	-14.2 (-31.5-184)	0 (0-0)	0 (0-0)	29 (6.6-55.1)	0 (0-0)	0 (0-0)	-9.9 (-29.5-121)	0 (0-0)	0 (0-0)	-11.2 (-36.6-34)	0 (0-0)	0 (0-0)	27 (-2.6-240)	0 (0-0)	0 (0-0)	0 (0-0)	0 (0-0)	114	105	112	102
Quintana Roo	5 (3.1-7.6)	4.7 (2.9-7.3)	-5.8 (-15.3-6.2)	2.1 (1.4-3.2)	1.6 (1-2.3)	-27.8 (-34--19.1)	0 (0-0)	0 (0-0)	17.6 (-9.4-52.6)	0.1 (0.1-0.2)	0.1 (0-0.1)	-22.1 (-28.5--13.2)	0 (0-0.1)	0 (0-0)	-33.3 (-54-4.7)	0.1 (0-0.1)	0.1 (0-0.1)	3.6 (-12.2-47)	0 (0-0)	0 (0-0)	0 (0-0)	0 (0-0)	106	99	100	90
San Luis Potosí	1.8 (0.8-3.1)	2 (0.9-3.6)	9.4 (-10.1-60.2)	0.7 (0.3-1.3)	0.6 (0.3-1.1)	-17.4 (-30.7-22.2)	0 (0-0)	0 (0-0)	18.7 (0.3-45.4)	0 (0-0.1)	0 (0-0.1)	-11.9 (-25.2-13.5)	0 (0-0)	0 (0-0)	-42.8 (-59.2--20.1)	0 (0-0.1)	0 (0-0.1)	17.8 (-2.8-107)	0 (0-0)	0 (0-0)	0 (0-0)	0 (0-0)	113	104	111	93
Sinaloa	3 (2-4.3)	3.2 (2.1-4.6)	7.5 (-2.6-18.4)	1.3 (0.8-1.8)	1 (0.7-1.5)	-19.4 (-29--12.2)	0 (0-0)	0 (0-0)	17.6 (0.6-38.7)	0.1 (0-0.1)	0 (0-0.1)	-13.6 (-22.9--1.9)	0 (0-0)	0 (0-0)	-36.4 (-55.5--9.5)	0 (0-0.1)	0 (0-0.1)	12.9 (-6.4-56.2)	0 (0-0)	0 (0-0)	0 (0-0)	0 (0-0)	119	113	109	91
Sonora	2.3 (1.4-3.6)	1.9 (1.2-3.1)	-16 (-34.3--1.1)	0.9 (0.6-1.4)	0.6 (0.4-1)	-35.4 (-45.2--25.4)	0 (0-0)	0 (0-0)	44 (22.5-75)	0 (0-0.1)	0 (0-0.1)	-29.5 (-47.5--15.5)	0 (0-0)	0 (0-0)	-41.6 (-59--20.6)	0 (0-0.1)	0 (0-0.1)	-1.2 (-24-54.1)	0 (0-0)	0 (0-0)	0 (0-0)	0 (0-0)	115	111	109	94
Tabasco	12.8 (10.7-15.8)	13.5 (11.2-16.5)	5.3 (-2.6-15.4)	5.4 (4.6-6.4)	4.3 (3.6-5.1)	-20.1 (-25.8--14.1)	0 (0-0)	0 (0-0.1)	36.4 (15.5-57.5)	0.3 (0.2-0.3)	0.2 (0.2-0.3)	-15.3 (-23.9--5.7)	0.1 (0.1-0.1)	0.1 (0.1-0.1)	-30.3 (-48.4--3.9)	0.2 (0.1-0.3)	0.2 (0.1-0.3)	12.2 (-4.8-37.5)	0 (0-0)	0 (0-0)	0 (0-0)	0.1 (0-0.1)	91	90	86	66
Tamaulipas	6.4 (5-8.2)	6.7 (5.1-8.6)	4.2 (-4.4-15.3)	2.7 (2.1-3.4)	2.2 (1.7-2.8)	-18.1 (-24.1--10.3)	0 (0-0)	0 (0-0)	27.7 (8-52.3)	0.1 (0.1-0.1)	0.1 (0.1-0.1)	-9.9 (-19.5--0.3)	0 (0-0)	0 (0-0)	-11.3 (-38.4-19.6)	0.1 (0-0.1)	0.1 (0.1-0.1)	9.2 (-9.2-40.9)	0 (0-0)	0 (0-0)	0 (0-0)	0 (0-0)	111	101	90	84
Tlaxcala	1 (0.6-1.9)	1.1 (0.6-2.2)	9.4 (-9.4-44.5)	0.5 (0.2-0.9)	0.4 (0.2-0.8)	-14.5 (-32.5-5.4)	0 (0-0)	0 (0-0)	19.1 (-4.1-43.4)	0 (0-0)	0 (0-0)	-7.4 (-21.9-23.9)	0 (0-0)	0 (0-0)	-27.8 (-53.1-5.2)	0 (0-0)	0 (0-0.1)	22.1 (-4.1-148)	0 (0-0)	0 (0-0)	0 (0-0)	0 (0-0)	117	105	109	97
Veracruz de Ignacio de la Llave	4.5 (3.4-5.9)	4.9 (3.8-6.3)	7.8 (-0.7-17.9)	1.9 (1.5-2.4)	1.5 (1.2-2)	-18.8 (-26.3--11.8)	0 (0-0)	0 (0-0)	7.8 (-8-25.1)	0.1 (0.1-0.1)	0.1 (0.1-0.1)	-13.4 (-21.7--3.6)	0.1 (0-0.1)	0 (0-0)	-50.3 (-63.3--33.6)	0.1 (0-0.1)	0.1 (0-0.1)	11.8 (-6.4-52)	0 (0-0)	0 (0-0)	0 (0-0)	0 (0-0)	101	91	103	83
Yucatán	5.2 (3.5-7.5)	5.5 (3.6-7.9)	4.6 (-6.1-14.2)	2.1 (1.4-3)	1.7 (1.1-2.3)	-20 (-28.6--12.8)	0 (0-0)	0 (0-0)	31.3 (11.4-52.3)	0.1 (0.1-0.1)	0.1 (0.1-0.1)	-15.8 (-25.4--5.6)	0 (0-0)	0 (0-0)	-8.2 (-35.8-27.3)	0.1 (0-0.1)	0.1 (0-0.1)	10.7 (-6.6-45.5)	0 (0-0)	0 (0-0)	0 (0-0)	0 (0-0)	105	100	97	84
Zacatecas	0.3 (0.1-0.7)	0.4 (0.1-0.9)	18 (-6.1-144)	0.1 (0-0.3)	0.1 (0-0.2)	-10.7 (-30.4-125)	0 (0-0)	0 (0-0)	64.1 (38.4-89.2)	0 (0-0)	0 (0-0)	-7.2 (-26-145)	0 (0-0)	0 (0-0)	-27.1 (-47--1.9)	0 (0-0)	0 (0-0)	26.1 (-2.1-253)	0 (0-0)	0 (0-0)	0 (0-0)	0 (0-0)	117	112	115	106

Location	Birth Incidence rate (per 100,000 live births), 2000	Birth Incidence rate (per 100,000 live births), 2021	Birth Incidence rate (per 100,000 live births) percent change (2000-2021)	All age rate (per 100,000 population), 2000	All age rate (per 100,000 population), 2021	All age rate (per 100,000 population) percent change (2000-2021)	All age cause-specific SCD mortality rate (per 100,000 population), 2000	All age cause-specific SCD mortality rate (per 100,000 population), 2021	All age cause-specific SCD mortality rate (per 100,000 population) percent change (2000-2021)	All age total SCD mortality rate (per 100,000 population), 2000	All age total SCD mortality rate (per 100,000 population), 2021	All age total SCD mortality rate (per 100,000 population) percent change (2000-2021)	Under-5 cause-specific SCD mortality rate (per 100,000 population), 2000	Under-5 cause-specific SCD mortality rate (per 100,000 population), 2021	Under-5 cause-specific SCD mortality rate (per 100,000 population) percent change (2000-2021)	Under-5 total SCD mortality rate (per 100,000 population), 2000	Under-5 total SCD mortality rate (per 100,000 population), 2021	Under-5 total SCD mortality rate (per 100,000 population) percent change (2000-2021)	Under-5 cause-specific mortality cause fraction, 2000	Under-5 total SCD mortality cause fraction, 2000	Under-5 cause-specific mortality cause fraction, 2021	Under-5 total SCD mortality cause fraction, 2021	Under-5 cause-specific mortality rank 2000	Under-5 cause-specific mortality rank 2021	Under-5 total SCD mortality rank 2000	Under-5 total SCD mortality rank 2021
Nicaragua	0.9 (0.6-1.3)	0.9 (0.7-1.3)	8 (-4.8-37)	0.1 (0.1-0.1)	0.1 (0-0.1)	-16.7 (-32.3-8.3)	0 (0-0)	0 (0-0)	-71.2 (-85--37.9)	0 (0-0)	0 (0-0)	-18.4 (-29.8-13.6)	0.1 (0-0.1)	0 (0-0)	-69.3 (-86.2--15.7)	0.2 (0.1-0.2)	0.2 (0.1-0.2)	8.8 (-7.9-53.2)	0 (0-0)	0 (0-0)	0 (0-0)	0.1 (0-0.1)	105	109	88	60
Panama	0.8 (0.6-1)	0.8 (0.6-1.1)	6.8 (-19.4-42.8)	0.1 (0.1-0.1)	0.1 (0-0.1)	-11.8 (-25.9-8.1)	0.4 (0.4-0.5)	0.4 (0.3-0.4)	-20.2 (-32.8--6.2)	0 (0-0)	0 (0-0)	-10.7 (-27.2-25)	0.6 (0.5-0.7)	0.3 (0.2-0.4)	-52.2 (-64.6--33.5)	0.1 (0.1-0.2)	0.1 (0.1-0.2)	6.9 (-14.4-55.1)	0.1 (0.1-0.1)	0 (0-0)	0.1 (0.1-0.1)	0.1 (0-0.1)	49	57	85	68
Venezuela (Bolivarian Republic of)	29.9 (24.6-35.2)	16.3 (13.3-19.8)	-45.2 (-52.7--38.2)	10.4 (8.4-12.3)	2.5 (2-3.1)	-75.9 (-78.3--72.8)	0.1 (0.1-0.1)	0.2 (0.1-0.2)	23.7 (-2-53.5)	0.6 (0.5-0.7)	0.3 (0.2-0.3)	-54.3 (-59.8--47.6)	0.1 (0.1-0.2)	0.2 (0.2-0.3)	65.4 (22.2-119)	1.6 (1.2-2)	1.5 (1.1-2)	-6.3 (-22-14.6)	0 (0-0)	0.3 (0.2-0.4)	0 (0-0)	0.2 (0.1-0.3)	85	76	22	34
**Tropical Latin America**	20.8 (18.3-23.2)	25.9 (21.8-29.5)	24.3 (18.5-29.4)	9 (8.1-9.8)	8.9 (7.8-9.9)	-0.8 (-4.3-2.8)	0.2 (0.2-0.2)	0.3 (0.2-0.3)	26.1 (17.7-36.6)	0.4 (0.3-0.4)	0.4 (0.3-0.4)	3.5 (-2-9.3)	0.6 (0.5-0.6)	0.4 (0.3-0.5)	-25.8 (-45.2--1.4)	0.3 (0.2-0.3)	0.3 (0.2-0.4)	19.7 (13-27)	0.1 (0.1-0.1)	0 (0-0)	0.1 (0.1-0.2)	0.1 (0.1-0.1)	60	54	77	60
Brazil	20.4 (18-22.7)	25.6 (21.6-29.2)	25.5 (19.2-31)	8.8 (7.9-9.6)	8.8 (7.8-9.7)	-0.2 (-4.1-3.7)	0.2 (0.2-0.2)	0.3 (0.3-0.3)	26.7 (18.3-37.1)	0.4 (0.3-0.4)	0.4 (0.3-0.4)	4.5 (-1.4-10.5)	0.6 (0.5-0.7)	0.4 (0.3-0.5)	-25.7 (-45.2--1.3)	0.2 (0.2-0.3)	0.3 (0.2-0.4)	21 (13.8-29.2)	0.1 (0.1-0.1)	0 (0-0)	0.1 (0.1-0.2)	0.1 (0.1-0.1)	59	52	79	59
Acre	18.6 (14.9-22.4)	24 (18.8-30)	29.1 (15-44)	7.7 (6.3-9)	7.8 (6.4-9.3)	1.3 (-5.3-9.8)	0.2 (0.2-0.2)	0.2 (0.2-0.2)	10.1 (-11.6-37.2)	0.4 (0.3-0.5)	0.4 (0.3-0.5)	6.2 (-4.9-18.4)	0.7 (0.6-1)	0.6 (0.4-0.9)	-16.5 (-46.6-38.2)	0.2 (0.1-0.3)	0.3 (0.2-0.4)	20.3 (5-38)	0.1 (0-0.1)	0 (0-0)	0.1 (0.1-0.2)	0.1 (0-0.1)	69	58	90	77
Alagoas	6.2 (5.1-7.5)	5.9 (4.7-7.2)	-4.8 (-17-11.7)	2.9 (2.5-3.4)	2.4 (2-3)	-16.8 (-28.3--0.8)	0.2 (0.2-0.2)	0.2 (0.2-0.2)	-1.1 (-18.4-21.4)	0.1 (0.1-0.1)	0.1 (0.1-0.1)	-18.6 (-29.9--5.2)	0.8 (0.6-1)	0.4 (0.3-0.6)	-50.5 (-68.7--25.7)	0.1 (0.1-0.1)	0.1 (0.1-0.1)	-1.4 (-19.8-25)	0.1 (0-0.1)	0 (0-0)	0.1 (0.1-0.2)	0 (0-0)	72	56	109	84
Amapá	8.1 (5.7-11.4)	9 (6-13.1)	11.3 (-5.3-26.8)	3 (2.2-4)	2.8 (2-3.9)	-7.7 (-17.8-3)	0.1 (0.1-0.2)	0.2 (0.2-0.3)	73.2 (41.2-128)	0.2 (0.1-0.3)	0.2 (0.1-0.2)	-14.5 (-25--1.4)	0.3 (0.2-0.4)	0.6 (0.4-0.7)	69.6 (5.7-148)	0.1 (0-0.1)	0.1 (0-0.1)	4.6 (-15.2-27)	0 (0-0.1)	0 (0-0)	0.1 (0.1-0.1)	0 (0-0)	69	53	96	90
Amazonas	8.9 (7.3-10.2)	11.9 (9.6-13.8)	33.1 (23.6-41.3)	5.6 (4.8-6.5)	6.4 (5.3-7.3)	13 (6.3-19.9)	0.1 (0.1-0.1)	0.1 (0.1-0.2)	106 (68.4-164)	0.1 (0.1-0.2)	0.2 (0.1-0.2)	27.5 (17.8-38)	0.2 (0.1-0.3)	0.3 (0.3-0.4)	51.7 (3.6-122)	0.1 (0.1-0.1)	0.1 (0.1-0.2)	36.5 (22.4-54.4)	0 (0-0)	0 (0-0)	0.1 (0.1-0.1)	0 (0-0)	80	64	93	85
Bahia	19.1 (16.6-21.6)	35.3 (29.2-41.4)	84.5 (65-104)	9.2 (8.1-10.4)	13 (11.3-14.6)	40.5 (29.9-49)	0.4 (0.4-0.5)	0.5 (0.5-0.6)	26.6 (8.7-48.6)	0.3 (0.3-0.4)	0.5 (0.4-0.6)	58.3 (41.4-76.6)	1.4 (1.1-1.7)	0.8 (0.6-1.1)	-39.1 (-58--10.9)	0.2 (0.2-0.3)	0.4 (0.3-0.5)	73.5 (50.9-103)	0.1 (0.1-0.2)	0 (0-0)	0.3 (0.2-0.3)	0.1 (0.1-0.2)	46	40	84	55
Ceará	5.1 (4.2-6)	6.8 (5.5-8.1)	33.4 (22.6-47.3)	2.3 (2-2.7)	2.5 (2.1-3)	10.4 (0.7-23.7)	0.1 (0.1-0.1)	0.1 (0.1-0.2)	18 (1.1-39.2)	0.1 (0.1-0.1)	0.1 (0.1-0.1)	10 (-0.5-22.1)	0.4 (0.3-0.4)	0.2 (0.1-0.3)	-42.8 (-62.7--10.7)	0.1 (0-0.1)	0.1 (0.1-0.1)	28.7 (12.5-48.7)	0 (0-0)	0 (0-0)	0.1 (0.1-0.1)	0 (0-0)	72	62	105	78
Distrito Federal	76.5 (66.8-85.8)	71.6 (62.7-81.2)	-6.5 (-17.6-2.3)	34 (30.7-37.4)	26.2 (22.8-28.9)	-23 (-27.8--17.9)	0.4 (0.3-0.4)	0.3 (0.3-0.3)	-17.3 (-25.2--7.3)	1.3 (1.1-1.5)	1 (0.8-1.1)	-22.3 (-30.3--14.6)	0.7 (0.6-0.8)	0.4 (0.4-0.6)	-31.3 (-44.6--12.7)	0.6 (0.5-0.8)	0.6 (0.4-0.8)	-6.1 (-17.6-7.8)	0.1 (0.1-0.2)	0.1 (0.1-0.2)	0.2 (0.2-0.2)	0.2 (0.2-0.3)	43	39	45	32
Espírito Santo	35.5 (29.9-41.1)	46.3 (38.1-55.8)	30.6 (16.2-45.7)	12.2 (10.7-13.5)	12.4 (10.6-14.1)	1.6 (-7.7-9.7)	0.2 (0.2-0.3)	0.3 (0.3-0.3)	24.2 (9.8-39.2)	0.6 (0.5-0.8)	0.6 (0.5-0.8)	0.7 (-11-13.6)	0.5 (0.4-0.6)	0.5 (0.4-0.7)	7.3 (-23.5-49.3)	0.3 (0.2-0.4)	0.4 (0.3-0.5)	24.6 (4.3-48.7)	0.1 (0.1-0.1)	0.1 (0-0.1)	0.2 (0.2-0.2)	0.1 (0.1-0.2)	54	45	64	53
Goiás	24.7 (21.4-28.2)	32.6 (26.7-38.1)	31.7 (19.4-46.3)	10.3 (9.2-11.5)	10.1 (8.8-11.3)	-1.8 (-9.2-5.6)	0.3 (0.2-0.3)	0.3 (0.3-0.4)	33.6 (15.8-56.5)	0.4 (0.4-0.5)	0.4 (0.4-0.5)	6.8 (-4.3-19.3)	0.6 (0.5-0.8)	0.5 (0.4-0.7)	-19 (-43.9-17.8)	0.2 (0.2-0.3)	0.3 (0.2-0.4)	26 (8.2-52.3)	0.1 (0.1-0.1)	0 (0-0.1)	0.2 (0.1-0.2)	0.1 (0.1-0.1)	54	45	76	58
Maranhão	13.3 (11.1-15.4)	15.8 (12.8-19.1)	18.7 (4.9-32.9)	5.6 (4.8-6.5)	6.1 (5.1-7.2)	9.1 (-1-18.4)	0.5 (0.4-0.7)	0.4 (0.3-0.4)	-27.6 (-47.4--10.6)	0.3 (0.2-0.3)	0.3 (0.2-0.4)	1.9 (-10.1-16.5)	1.6 (1-2.7)	0.6 (0.4-0.8)	-62.6 (-79.8--36.2)	0.2 (0.1-0.2)	0.2 (0.1-0.3)	13.8 (-2.5-31.9)	0.1 (0.1-0.2)	0 (0-0)	0.2 (0.2-0.3)	0.1 (0-0.1)	51	43	100	75
Mato Grosso	11.6 (9.8-13.4)	15.5 (12.9-18.3)	34.2 (22.7-44.4)	5.2 (4.4-5.9)	5.3 (4.4-6)	0 (-7-7.2)	0.2 (0.2-0.2)	0.3 (0.3-0.3)	42.3 (26.1-59.5)	0.2 (0.2-0.2)	0.2 (0.2-0.3)	9.9 (-0.2-20.9)	0.5 (0.4-0.6)	0.6 (0.5-0.8)	35 (0.4-77.3)	0.1 (0.1-0.2)	0.2 (0.1-0.2)	29.3 (15.2-48.1)	0.1 (0.1-0.1)	0 (0-0)	0.2 (0.1-0.2)	0 (0-0.1)	57	51	91	78
Mato Grosso do Sul	13 (11.1-15.3)	17.1 (13.8-20.2)	31.6 (20.7-42.5)	6.3 (5.3-7.2)	6.2 (5.2-7.1)	-1.1 (-7.8-4.7)	0.1 (0.1-0.1)	0.2 (0.1-0.2)	46.1 (26.3-68.8)	0.2 (0.2-0.3)	0.2 (0.2-0.3)	12.4 (1.6-24.9)	0.2 (0.2-0.3)	0.3 (0.2-0.3)	11.8 (-16.1-54.9)	0.1 (0.1-0.2)	0.2 (0.1-0.2)	30.4 (12.5-53.9)	0 (0-0)	0 (0-0)	0.1 (0.1-0.1)	0.1 (0-0.1)	74	64	86	75
Minas Gerais	28.5 (24.7-32.8)	35.4 (30.1-41.7)	24.2 (12.8-39.6)	10.3 (9.1-11.4)	9.7 (8.3-10.9)	-6.5 (-13.7-3.6)	0.3 (0.2-0.3)	0.4 (0.3-0.4)	36.4 (23.8-53)	0.5 (0.4-0.6)	0.5 (0.4-0.6)	-4.8 (-14-6.7)	0.6 (0.5-0.8)	0.5 (0.4-0.7)	-19.3 (-40-23.3)	0.3 (0.2-0.4)	0.4 (0.3-0.5)	13.9 (-2.5-36.5)	0.1 (0.1-0.1)	0.1 (0-0.1)	0.2 (0.2-0.2)	0.1 (0.1-0.2)	48	44	65	51
Pará	10.2 (8.7-11.6)	11.1 (9.3-13.2)	8.8 (-2.7-19.2)	4.7 (4-5.3)	4.6 (3.9-5.2)	-2.8 (-11.8-3.8)	0.2 (0.1-0.2)	0.2 (0.2-0.2)	31.3 (9.3-63.9)	0.2 (0.2-0.2)	0.2 (0.1-0.2)	-11.1 (-20.7-0)	0.5 (0.4-0.7)	0.4 (0.3-0.5)	-27.5 (-52.5-10.5)	0.1 (0.1-0.2)	0.1 (0.1-0.2)	8.2 (-6.4-25.1)	0.1 (0-0.1)	0 (0-0)	0.1 (0.1-0.2)	0 (0-0.1)	71	58	96	80
Paraíba	8.8 (7.3-10.2)	12.7 (10.5-15)	45 (36.6-52.9)	3.6 (3-4.2)	4.4 (3.7-5.2)	22.1 (14.7-29.6)	0.1 (0.1-0.2)	0.2 (0.1-0.2)	12.8 (-0.4-34)	0.2 (0.1-0.2)	0.2 (0.2-0.2)	22.4 (12.9-32.9)	0.5 (0.4-0.6)	0.2 (0.1-0.3)	-58.2 (-75.2--33.5)	0.2 (0.1-0.3)	0.3 (0.2-0.4)	49.5 (34-67.5)	0.1 (0-0.1)	0 (0-0)	0.1 (0.1-0.1)	0.1 (0.1-0.2)	69	61	80	52
Paraná	6.6 (5.7-7.4)	10.2 (8.5-12.1)	53.2 (40-66)	3.2 (2.9-3.6)	3.6 (3.1-4.1)	10.3 (3.2-17.9)	0.1 (0.1-0.1)	0.1 (0.1-0.1)	58.6 (40.4-79.8)	0.1 (0.1-0.1)	0.1 (0.1-0.2)	30.7 (19.4-44.4)	0.2 (0.1-0.2)	0.2 (0.1-0.2)	16.7 (-17.7-65.3)	0.1 (0-0.1)	0.1 (0.1-0.1)	47.4 (28.1-70.7)	0 (0-0)	0 (0-0)	0.1 (0.1-0.1)	0 (0-0)	72	64	90	76
Pernambuco	4.8 (3.9-5.8)	7.1 (5.9-8.4)	48.7 (34.1-64.5)	2.3 (1.9-2.6)	2.8 (2.4-3.4)	20.1 (8.4-32.5)	0.2 (0.2-0.2)	0.2 (0.2-0.2)	9.5 (-4.8-26.3)	0.1 (0.1-0.1)	0.1 (0.1-0.1)	32 (18.9-47)	0.6 (0.4-0.7)	0.3 (0.2-0.4)	-51 (-66.7--22.7)	0.1 (0-0.1)	0.1 (0.1-0.1)	54.6 (28.5-88.8)	0.1 (0-0.1)	0 (0-0)	0.1 (0.1-0.1)	0 (0-0)	66	58	107	78
Piaui	28.9 (24.6-33.3)	33.4 (28.5-39.7)	15.6 (4.9-28.9)	13 (11.3-14.5)	12.5 (10.7-14.1)	-3.9 (-9.6-3.1)	0.4 (0.3-0.5)	0.4 (0.4-0.5)	15.1 (-1.5-35)	0.5 (0.4-0.6)	0.5 (0.4-0.6)	-3.7 (-14.1-7.9)	1.2 (0.8-1.5)	0.6 (0.4-0.8)	-51.9 (-69.1--25.5)	0.3 (0.2-0.4)	0.4 (0.3-0.5)	16.6 (-1.2-38.2)	0.1 (0.1-0.2)	0 (0-0.1)	0.2 (0.2-0.3)	0.1 (0.1-0.2)	46	39	79	48
Rio de Janeiro	43.3 (38.2-48.2)	46.4 (39.3-52.9)	7.1 (-2.4-15.5)	15 (13.6-16.4)	12.5 (11-13.9)	-16.4 (-22.1--11.3)	0.3 (0.3-0.3)	0.4 (0.4-0.4)	40.8 (26.8-53.3)	0.7 (0.6-0.8)	0.6 (0.5-0.7)	-11.6 (-20--2.9)	0.5 (0.4-0.6)	0.6 (0.5-0.7)	12.1 (-13.6-45.4)	0.6 (0.5-0.8)	0.7 (0.5-0.9)	9.3 (-5.9-27.2)	0.1 (0.1-0.1)	0.1 (0.1-0.2)	0.2 (0.2-0.2)	0.2 (0.2-0.3)	52	47	46	40
Rio Grande do Norte	6.8 (5.4-8.1)	9.2 (6.9-11.6)	36.1 (23.3-58.3)	2.9 (2.4-3.5)	3.1 (2.5-3.8)	6.1 (-3.7-20.4)	0.1 (0.1-0.1)	0.2 (0.1-0.2)	24.7 (5.9-49.3)	0.1 (0.1-0.2)	0.1 (0.1-0.2)	12.1 (0.2-29.1)	0.5 (0.3-0.6)	0.2 (0.2-0.3)	-47.4 (-66.5--17.7)	0.1 (0.1-0.1)	0.1 (0.1-0.1)	35 (14.2-62.5)	0 (0-0.1)	0 (0-0)	0.1 (0.1-0.1)	0 (0-0.1)	72	61	107	77
Rio Grande do Sul	8.3 (7.2-9.4)	12.6 (10.8-14.5)	51.9 (38.8-65.6)	3.9 (3.3-4.3)	4.4 (3.8-5.1)	14.4 (6.2-21.7)	0.1 (0.1-0.1)	0.1 (0.1-0.1)	55.1 (38.4-71.8)	0.1 (0.1-0.1)	0.2 (0.1-0.2)	28.1 (17-40.3)	0.1 (0.1-0.2)	0.2 (0.2-0.2)	25.9 (-1.6-70)	0.1 (0.1-0.1)	0.1 (0.1-0.1)	50.6 (31.5-72.6)	0 (0-0)	0 (0-0)	0.1 (0.1-0.1)	0 (0-0.1)	71	60	84	73
Rondônia	20.4 (16.6-24.5)	26.3 (20.8-31.8)	28.5 (15.6-43.1)	7.8 (6.5-8.8)	7.3 (6.2-8.4)	-5.8 (-13.4-2.5)	0.1 (0.1-0.1)	0.1 (0.1-0.1)	4.5 (-9.5-23.3)	0.4 (0.3-0.5)	0.4 (0.3-0.5)	-5.8 (-16.6-5.3)	0.3 (0.3-0.5)	0.3 (0.2-0.4)	-6.7 (-30.5-40.3)	0.2 (0.1-0.3)	0.2 (0.2-0.3)	19.1 (0.2-39.6)	0 (0-0.1)	0 (0-0)	0.1 (0.1-0.1)	0.1 (0.1-0.1)	71	59	82	66
Roraima	30.1 (23.6-36.6)	28.4 (22.1-35.7)	-5.6 (-14.4-3.9)	12.4 (10.3-14.6)	10 (8.2-11.9)	-19.7 (-27.8--12.7)	0.1 (0.1-0.1)	0.1 (0.1-0.2)	54.1 (14.2-109)	0.6 (0.5-0.8)	0.5 (0.4-0.6)	-24.3 (-34.5--13.4)	0.1 (0.1-0.2)	0.4 (0.3-0.5)	176 (67.5-386)	0.3 (0.2-0.4)	0.2 (0.2-0.4)	-12.7 (-27.8-5.9)	0 (0-0)	0.1 (0-0.1)	0.1 (0.1-0.1)	0.1 (0-0.1)	76	66	64	74
Santa Catarina	4.5 (3.7-5.2)	6 (4.9-7)	32.7 (24.1-42.3)	2.3 (1.9-2.7)	2.4 (2-2.8)	2.6 (-4.2-11.7)	0.1 (0-0.1)	0.1 (0.1-0.1)	51.4 (31.3-73.7)	0.1 (0.1-0.1)	0.1 (0.1-0.1)	14.7 (4.5-26.6)	0.1 (0.1-0.1)	0.1 (0.1-0.2)	12.4 (-23.2-65.9)	0 (0-0.1)	0.1 (0-0.1)	40.3 (20-68.2)	0 (0-0)	0 (0-0)	0.1 (0-0.1)	0 (0-0)	78	68	93	82

Location	Birth Incidence rate (per 100,000 live births), 2000	Birth Incidence rate (per 100,000 live births), 2021	Birth Incidence rate (per 100,000 live births) percent change (2000-2021)	All age rate (per 100,000 population), 2000	All age rate (per 100,000 population), 2021	All age rate (per 100,000 population) percent change (2000-2021)	All age cause-specific SCD mortality rate (per 100,000 population), 2000	All age cause-specific SCD mortality rate (per 100,000 population), 2021	All age cause-specific SCD mortality rate (per 100,000 population) percent change (2000-2021)	All age total SCD mortality rate (per 100,000 population), 2000	All age total SCD mortality rate (per 100,000 population), 2021	All age total SCD mortality rate (per 100,000 population) percent change (2000-2021)	Under-5 cause-specific SCD mortality rate (per 100,000 population), 2000	Under-5 cause-specific SCD mortality rate (per 100,000 population), 2021	Under-5 cause-specific SCD mortality rate (per 100,000 population) percent change (2000-2021)	Under-5 total SCD mortality rate (per 100,000 population), 2000	Under-5 total SCD mortality rate (per 100,000 population), 2021	Under-5 total SCD mortality rate (per 100,000 population) percent change (2000-2021)	Under-5 cause-specific mortality cause fraction, 2000	Under-5 total SCD mortality cause fraction, 2000	Under-5 cause-specific mortality cause fraction, 2021	Under-5 total SCD mortality cause fraction, 2021	Under-5 cause-specific mortality rank 2000	Under-5 cause-specific mortality rank 2021	Under-5 total SCD mortality rank 2000	Under-5 total SCD mortality rank 2021
São Paulo	31.7 (28.2-35.4)	42.1 (35.7-48.6)	32.6 (20.5-43.8)	13.1 (11.7-14.1)	13.2 (11.6-14.7)	0.5 (-5.2-6.2)	0.2 (0.1-0.2)	0.2 (0.2-0.2)	43.5 (29.6-58.5)	0.5 (0.4-0.6)	0.6 (0.5-0.6)	8.5 (-0.7-18.8)	0.3 (0.2-0.3)	0.4 (0.3-0.4)	26.6 (-4.7-58.7)	0.4 (0.3-0.5)	0.5 (0.4-0.7)	28 (15.9-41.7)	0.1 (0.1-0.1)	0.1 (0.1-0.1)	0.1 (0.1-0.1)	0.2 (0.1-0.2)	65	54	55	44
Sergipe	17.3 (14.5-20.5)	21 (17.2-24.7)	21.4 (10.2-34.6)	7.6 (6.5-8.6)	7.3 (6.2-8.8)	-3.6 (-12.1-4.3)	0.3 (0.2-0.3)	0.3 (0.3-0.3)	11.4 (-2.6-33.5)	0.3 (0.2-0.4)	0.3 (0.2-0.4)	-1.6 (-13.9-10.6)	0.9 (0.6-1.1)	0.7 (0.5-0.9)	-25.7 (-50.1-9.9)	0.2 (0.1-0.3)	0.3 (0.2-0.3)	23.1 (5.3-44.2)	0.1 (0.1-0.1)	0 (0-0)	0.2 (0.1-0.2)	0.1 (0-0.1)	55	47	80	69
Tocantins	35.8 (30-41.3)	49.9 (40.9-58.6)	39.3 (29.4-54.9)	15.4 (13.3-17.3)	17 (14.5-19)	10.3 (2.3-19.8)	0.3 (0.2-0.4)	0.3 (0.3-0.4)	15.1 (-1.5-36.4)	0.7 (0.6-0.8)	0.8 (0.6-0.9)	11.7 (1.6-25)	0.9 (0.7-1.2)	0.7 (0.5-1.1)	-21.8 (-50.2-15.2)	0.4 (0.3-0.6)	0.6 (0.4-0.7)	31.8 (17.5-49.2)	0.1 (0.1-0.1)	0 (0-0.1)	0.2 (0.2-0.3)	0.2 (0.1-0.3)	51	42	76	49
Paraguay	32.4 (25.5-40.7)	32.5 (25.6-40.6)	0.3 (-7.4-7.9)	15 (12.1-17.8)	12.6 (10.3-15.2)	-15.7 (-19.9--11)	0.1 (0.1-0.1)	0.1 (0.1-0.1)	-4.8 (-30.5-34)	0.6 (0.5-0.9)	0.5 (0.4-0.7)	-16.7 (-24--8.4)	0.1 (0.1-0.1)	0.1 (0-0.1)	-16.7 (-55.9-66.1)	0.6 (0.4-0.7)	0.6 (0.4-0.7)	0.5 (-8.6-10.2)	0 (0-0)	0.1 (0.1-0.1)	0 (0-0)	0.1 (0.1-0.2)	90	93	51	48
North Africa and Middle East	242 (218-271)	164 (141-189)	-32.2 (-36.1--28)	168 (149-187)	98 (81.4-114)	-41.7 (-45.5--37.8)	0.3 (0.3-0.4)	0.2 (0.2-0.3)	-26.1 (-38.3--11.4)	3.1 (2.7-3.6)	2.2 (1.9-2.7)	-29.1 (-33.7--24.3)	0.4 (0.2-0.6)	0.2 (0.1-0.3)	-49.1 (-65.3--13)	5.5 (4.1-7.4)	5 (3.6-6.8)	-9.3 (-13.3--4.8)	0 (0-0)	0.5 (0.3-0.6)	0 (0-0.1)	1.1 (0.8-1.4)	82	86	29	16
**North Africa and Middle East**	242 (218-271)	164 (141-189)	-32.2 (-36.1--28)	168 (149-187)	98 (81.4-114)	-41.7 (-45.5--37.8)	0.3 (0.3-0.4)	0.2 (0.2-0.3)	-26.1 (-38.3--11.4)	3.1 (2.7-3.6)	2.2 (1.9-2.7)	-29.1 (-33.7--24.3)	0.4 (0.2-0.6)	0.2 (0.1-0.3)	-49.1 (-65.3--13)	5.5 (4.1-7.4)	5 (3.6-6.8)	-9.3 (-13.3--4.8)	0 (0-0)	0.5 (0.3-0.6)	0 (0-0.1)	1.1 (0.8-1.4)	82	86	29	16
Afghanistan	131 (105-159)	131 (105-159)	0.1 (-5.7-6)	84.5 (67-106)	82.2 (64.5-106)	-2.7 (-8.7-4)	0.8 (0.5-1.3)	0.6 (0.4-1)	-25.6 (-50.3-6.2)	2.3 (1.8-3)	2.2 (1.8-2.9)	-1.7 (-10-6.3)	0.8 (0.3-1.6)	0.4 (0.2-0.7)	-45.3 (-66.2-28.1)	5.1 (3.5-7.6)	5.1 (3.4-7.7)	0.5 (-11.1-12.3)	0 (0-0)	0.1 (0.1-0.2)	0 (0-0.1)	0.4 (0.3-0.6)	78	79	44	31
Algeria	22.7 (19.3-26.1)	22.7 (19.6-26.7)	0.3 (-4.7-5.1)	11.2 (9.3-12.9)	9.9 (8.3-11.5)	-12.3 (-16.8--6.3)	0.1 (0.1-0.1)	0.1 (0-0.1)	-42.5 (-62.2--16.1)	0.4 (0.3-0.4)	0.4 (0.3-0.4)	-1.2 (-7.4-5.9)	0.2 (0.1-0.3)	0.1 (0-0.1)	-59.1 (-77.2--26.4)	1.5 (1-2)	1.5 (1-2.1)	0.7 (-9.8-13.6)	0 (0-0)	0.2 (0.1-0.3)	0 (0-0)	0.4 (0.3-0.5)	83	85	39	28
Bahrain	1,260 (1,040-1,500)	1,040 (834-1,290)	-17.6 (-29.5--9)	949 (773-1,160)	618 (494-764)	-34.9 (-43.9--28.1)	3.2 (2.6-3.8)	1.9 (1.5-2.3)	-40.3 (-49.8--26.4)	15.9 (12.5-20)	15.5 (11.8-19.9)	-2.9 (-19.5-10.9)	1.4 (0.9-2)	1.6 (1.1-2.3)	11.6 (-18.2-55.6)	15.6 (11.2-21.7)	14.5 (10-20.8)	-6.8 (-18.1-5.5)	0.6 (0.4-0.9)	7.1 (5-10.2)	0.7 (0.5-0.9)	6.2 (4.3-8.7)	26	27	4	4
Egypt	151 (132-172)	151 (131-169)	-0.1 (-7.9-5.9)	110 (93.3-126)	104 (89.3-117)	-5.9 (-15.3--0.2)	0.1 (0.1-0.2)	0.2 (0.1-0.2)	4 (-22.3-46)	2 (1.7-2.3)	2 (1.7-2.3)	0 (-6.5-7)	0.1 (0.1-0.2)	0.1 (0.1-0.2)	-25.3 (-59.6-47.6)	3.2 (2.5-4.1)	3.2 (2.5-4.1)	-1 (-10.3-8.3)	0 (0-0)	0.3 (0.2-0.4)	0 (0-0)	0.9 (0.7-1.2)	80	79	33	14
Iran (Islamic Republic of)	227 (180-277)	154 (123-189)	-32.4 (-33.6--30.5)	170 (133-211)	94.7 (74-119)	-44.2 (-45.6--42.3)	0.3 (0.2-0.4)	0.2 (0.1-0.2)	-44.2 (-54.2--28.6)	2.6 (2-3.4)	2.1 (1.6-2.6)	-22.2 (-27--17.5)	0.2 (0.1-0.3)	0 (0-0)	-90.8 (-94.8--84.2)	2.8 (2-3.9)	2.4 (1.7-3.4)	-15.1 (-20.7--9.9)	0 (0-0)	0.4 (0.3-0.6)	0 (0-0)	3.8 (2.7-5.2)	80	85	27	7
Alborz	214 (166-264)	144 (114-183)	-32.7 (-37.2--27.5)	167 (130-208)	91.5 (71.2-115)	-45.2 (-48.8--40.5)	0.1 (0.1-0.2)	0.1 (0.1-0.1)	-40.6 (-54.1--20.2)	2.5 (1.8-3.2)	2 (1.5-2.6)	-19.8 (-28.6--10.9)	0 (0-0.1)	0 (0-0)	-98.2 (-99.6--94)	2 (1.5-2.8)	1.7 (1.2-2.4)	-16.1 (-26.2--3.5)	0 (0-0)	0.6 (0.4-0.9)	0 (0-0)	28.5 (16.2-46.4)	88	94	22	2
Ardebil	221 (174-274)	150 (117-187)	-32.2 (-36.2--27.3)	168 (130-209)	92.4 (71.2-115)	-45 (-49.4--40.8)	0.2 (0.1-0.2)	0.1 (0.1-0.1)	-38.8 (-55--15)	2.5 (1.9-3.2)	2 (1.5-2.6)	-17.3 (-26.2--7.5)	0.1 (0.1-0.3)	0 (0-0)	-92.8 (-96.6--85.3)	2.8 (2-3.8)	2.4 (1.7-3.3)	-15.4 (-25.5--5.2)	0 (0-0)	0.3 (0.2-0.4)	0 (0-0)	2.9 (2-3.9)	84	92	34	7
Bushehr	244 (193-297)	166 (133-206)	-31.7 (-37--26.8)	185 (142-232)	107 (82.7-134)	-41.8 (-45.9--37.8)	0.4 (0.2-0.5)	0.2 (0.1-0.3)	-45.8 (-60.4--27.5)	2.8 (2.2-3.6)	2.2 (1.7-2.9)	-21.7 (-28.2--15.1)	0.3 (0.1-0.5)	0 (0-0.1)	-90.5 (-95.3--83.1)	3.6 (2.5-5.2)	3.1 (2.1-4.6)	-12.9 (-23.3--0.5)	0 (0-0.1)	0.6 (0.4-0.9)	0 (0-0.1)	4.7 (3.1-7)	64	73	22	6
Chahar Mahaal and Bakhtiari	239 (186-294)	164 (131-200)	-31.4 (-36.3--26.5)	183 (141-227)	102 (78.6-130)	-44 (-47.6--40)	0.3 (0.2-0.4)	0.1 (0.1-0.2)	-52.4 (-67.2--33.6)	2.7 (2.1-3.4)	2.2 (1.7-2.8)	-18.2 (-25.7--10.4)	0.2 (0.1-0.4)	0 (0-0)	-92.7 (-95.8--86.2)	3.7 (2.6-5.4)	3.3 (2.3-4.8)	-10.6 (-21.9-1.5)	0 (0-0.1)	0.7 (0.5-1)	0 (0-0.1)	7.5 (4.8-11)	68	76	21	4
East Azarbayejan	226 (173-278)	152 (120-188)	-32.8 (-37.6--26.1)	164 (124-208)	90.3 (70-113)	-45 (-49.5--39.5)	0.2 (0.1-0.3)	0.1 (0.1-0.1)	-54.6 (-67.6--37.1)	2.7 (2-3.5)	2.1 (1.6-2.7)	-23.6 (-31.2--14.1)	0.1 (0.1-0.2)	0 (0-0)	-94.6 (-97.4--89.5)	2.8 (2-3.9)	2.4 (1.7-3.3)	-16.6 (-25.3--6.5)	0 (0-0)	0.3 (0.2-0.5)	0 (0-0)	5 (3.4-7.3)	86	95	30	6
Fars	248 (196-310)	168 (134-207)	-32.3 (-37.6--27.7)	181 (140-227)	97.9 (74.1-124)	-46.1 (-50.7--42.1)	0.6 (0.4-0.8)	0.3 (0.2-0.4)	-49 (-62.2--29.3)	3 (2.3-4)	2.3 (1.8-3)	-23.8 (-32.3--16.1)	0.3 (0.1-0.5)	0 (0-0.1)	-90.8 (-94.7--83.4)	3.6 (2.6-5.1)	3.1 (2.1-4.5)	-13.7 (-23.3--3.3)	0 (0-0.1)	0.6 (0.4-0.8)	0 (0-0.1)	4.3 (3-6.1)	64	78	21	5
Gilan	218 (170-273)	146 (114-185)	-32.9 (-37.9--28.4)	159 (126-202)	83.5 (63.1-106)	-47.6 (-51.2--43.7)	0.3 (0.2-0.4)	0.2 (0.1-0.2)	-37.8 (-52.3--17.7)	2.5 (1.9-3.3)	2 (1.5-2.6)	-21.3 (-29.3--12.7)	0.1 (0-0.2)	0 (0-0)	-97.1 (-98.8--93.8)	2 (1.4-2.7)	1.6 (1.2-2.3)	-16.7 (-26.4--5.7)	0 (0-0)	0.5 (0.4-0.7)	0 (0-0)	14.1 (7.9-24.1)	74	78	22	3
Golestan	233 (180-292)	158 (125-197)	-32.3 (-37.2--27.2)	177 (136-223)	101 (77.3-123)	-43.3 (-47--38)	0.4 (0.2-0.6)	0.3 (0.2-0.3)	-35.1 (-53.2--11.2)	2.8 (2.1-3.6)	2.2 (1.7-2.8)	-21.8 (-29.3--11.6)	0.2 (0.1-0.4)	0 (0-0)	-91.5 (-95.2--82.6)	2.8 (2-3.8)	2.3 (1.6-3.3)	-16.3 (-25.9--5.8)	0 (0-0.1)	0.4 (0.2-0.5)	0 (0-0.1)	3 (2.1-4.1)	73	83	31	7
Hamadan	231 (183-288)	158 (125-196)	-31.9 (-36.2--26.3)	169 (132-212)	91 (69-116)	-46 (-49.6--41.7)	0.2 (0.1-0.3)	0.1 (0.1-0.2)	-41.4 (-58--21.7)	2.8 (2.1-3.6)	2.1 (1.6-2.8)	-22.5 (-30.6--14.6)	0.1 (0.1-0.2)	0 (0-0)	-91.8 (-95.6--82.7)	3 (2.1-4.2)	2.6 (1.9-3.8)	-13.9 (-23.3--3.7)	0 (0-0)	0.4 (0.2-0.5)	0 (0-0)	3 (2.2-4.1)	81	90	26	8
Hormozgan	231 (181-290)	157 (123-193)	-32.1 (-36.5--27.3)	179 (138-229)	105 (80.9-131)	-41.4 (-45.8--37.4)	0.6 (0.2-0.9)	0.4 (0.1-0.5)	-37.9 (-55.2--9.5)	2.7 (2-3.5)	2.1 (1.6-2.7)	-21.5 (-28.8--13.7)	0.4 (0.1-0.7)	0 (0-0.1)	-90.7 (-95.1--81.6)	2.9 (2-4)	2.5 (1.8-3.5)	-13.2 (-23--2.4)	0 (0-0.1)	0.4 (0.3-0.5)	0 (0-0.1)	3.2 (2.4-4.6)	66	74	31	7
Ilam	232 (181-289)	157 (125-194)	-32.3 (-35.9--27)	177 (136-223)	96.5 (74-122)	-45.5 (-49--41.2)	0.3 (0.2-0.4)	0.2 (0.2-0.2)	-30.2 (-47.5--2.2)	2.7 (2.1-3.5)	2.2 (1.7-2.8)	-19.1 (-28--10.1)	0.2 (0.1-0.4)	0 (0-0.1)	-89.9 (-95.5--75.9)	3 (2.1-4.2)	2.6 (1.8-3.7)	-14.3 (-23.9--3.6)	0 (0-0.1)	0.4 (0.3-0.6)	0 (0-0)	3 (1.8-4.6)	72	81	25	7
Isfahan	229 (180-284)	156 (123-189)	-31.9 (-36.5--27.1)	167 (131-211)	92 (70-114)	-45 (-48.7--40.2)	0.2 (0.1-0.3)	0.1 (0.1-0.1)	-43.3 (-54.3--25.6)	2.7 (2-3.4)	2.1 (1.6-2.7)	-22 (-29.2--13.8)	0.1 (0.1-0.2)	0 (0-0)	-87.5 (-92.9--76.2)	3.1 (2.2-4.4)	2.7 (1.9-3.9)	-12.9 (-23.4-1)	0 (0-0)	0.6 (0.4-0.8)	0 (0-0)	3.6 (2.5-5.1)	76	87	23	7
Kerman	230 (180-289)	155 (122-194)	-32.8 (-37.2--27.7)	176 (136-222)	99.4 (76.9-126)	-43.4 (-46.9--38.8)	0.4 (0.3-0.6)	0.2 (0.1-0.3)	-48.8 (-63.8--28.2)	2.7 (2-3.5)	2.1 (1.6-2.7)	-23.1 (-30.7--14.6)	0.2 (0.1-0.3)	0 (0-0)	-86.4 (-92--76.7)	2.6 (1.9-3.6)	2.2 (1.6-3.2)	-15.5 (-25.3--5.3)	0 (0-0)	0.4 (0.3-0.6)	0 (0-0)	2.4 (1.7-3.4)	73	83	25	8
Kermanshah	238 (186-300)	161 (126-200)	-32.3 (-36.8--28.2)	176 (139-223)	94.2 (70.6-119)	-46.6 (-50--42.6)	0.3 (0.2-0.4)	0.2 (0.1-0.2)	-46.9 (-59.6--29.6)	2.9 (2.2-3.8)	2.2 (1.7-2.9)	-22.6 (-30.8--13.9)	0.1 (0.1-0.2)	0 (0-0)	-93.2 (-96.8--84.2)	2.9 (2.1-4.1)	2.5 (1.8-3.6)	-14.6 (-23.8--3.1)	0 (0-0)	0.3 (0.2-0.5)	0 (0-0)	3.4 (2.4-4.7)	82	89	28	6
Khorasan-e-Razavi	230 (176-284)	155 (123-192)	-32.8 (-37.8--27.1)	173 (132-219)	97.7 (75.5-123)	-43.4 (-48.2--38.3)	0.2 (0.2-0.3)	0.1 (0.1-0.1)	-52.2 (-63.8--34.3)	2.7 (2.1-3.6)	2.1 (1.6-2.7)	-23.1 (-30.6--14.7)	0.1 (0.1-0.2)	0 (0-0)	-93.1 (-96.8--85.7)	2.9 (2.1-4)	2.4 (1.7-3.4)	-16.3 (-25.1--3.1)	0 (0-0)	0.3 (0.2-0.5)	0 (0-0)	3.7 (2.4-5.3)	85	90	31	7
Khuzestan	252 (198-314)	172 (138-215)	-31.7 (-36.2--27.9)	191 (148-237)	108 (84.5-136)	-43.3 (-47.1--40)	0.5 (0.3-0.7)	0.3 (0.2-0.4)	-39.7 (-53.1--19.3)	3 (2.3-3.9)	2.3 (1.8-3)	-22.2 (-29.6--14.9)	0.3 (0.1-0.6)	0 (0-0.1)	-89.1 (-94.2--83.5)	3.8 (2.7-5.4)	3.4 (2.3-4.9)	-12.5 (-23.5--0.6)	0 (0-0.1)	0.6 (0.4-0.8)	0 (0-0.1)	4.3 (3-6)	64	72	23	5
Kohgiluyeh and Boyer-Ahmad	256 (203-318)	177 (140-219)	-30.9 (-35.8--24.6)	189 (147-237)	108 (83.4-138)	-43 (-47.4--37.3)	0.7 (0.4-0.9)	0.4 (0.3-0.5)	-43.2 (-58.8--19.8)	3.2 (2.5-4.1)	2.5 (1.9-3.2)	-23.2 (-31.3--13.9)	0.4 (0.2-0.7)	0 (0-0.1)	-93.4 (-96.8--84.8)	4.4 (3.1-6.3)	4 (2.7-6)	-9.8 (-22.1-2.9)	0.1 (0-0.1)	0.6 (0.4-0.9)	0 (0-0.1)	7.4 (4.8-11.1)	66	71	22	4
Kurdistan	227 (180-282)	154 (120-192)	-32.2 (-37--27.5)	171 (131-218)	94 (71.6-118)	-44.9 (-48.5--39.9)	0.1 (0.1-0.2)	0.1 (0-0.1)	-50.9 (-65.4--34)	2.6 (2-3.4)	2.1 (1.6-2.7)	-20.5 (-28.9--12.1)	0.1 (0.1-0.2)	0 (0-0)	-94.2 (-97.2--87.1)	3.2 (2.3-4.4)	2.7 (1.9-3.8)	-14.6 (-24.1--3.6)	0 (0-0)	0.3 (0.2-0.4)	0 (0-0)	3.3 (2.3-4.6)	85	94	30	6

Location	Birth Incidence rate (per 100,000 live births), 2000	Birth Incidence rate (per 100,000 live births), 2021	Birth Incidence rate (per 100,000 live births) percent change (2000-2021)	All age rate (per 100,000 population), 2000	All age rate (per 100,000 population), 2021	All age rate (per 100,000 population) percent change (2000-2021)	All age cause-specific SCD mortality rate (per 100,000 population), 2000	All age cause-specific SCD mortality rate (per 100,000 population), 2021	All age cause-specific SCD mortality rate (per 100,000 population) percent change (2000-2021)	All age total SCD mortality rate (per 100,000 population), 2000	All age total SCD mortality rate (per 100,000 population), 2021	All age total SCD mortality rate (per 100,000 population) percent change (2000-2021)	Under-5 cause-specific SCD mortality rate (per 100,000 population), 2000	Under-5 cause-specific SCD mortality rate (per 100,000 population), 2021	Under-5 cause-specific SCD mortality rate (per 100,000 population) percent change (2000-2021)	Under-5 total SCD mortality rate (per 100,000 population), 2000	Under-5 total SCD mortality rate (per 100,000 population), 2021	Under-5 total SCD mortality rate (per 100,000 population) percent change (2000-2021)	Under-5 cause-specific mortality cause fraction, 2000	Under-5 total SCD mortality cause fraction, 2000	Under-5 cause-specific mortality cause fraction, 2021	Under-5 total SCD mortality cause fraction, 2021	Under-5 cause-specific mortality rank 2000	Under-5 cause-specific mortality rank 2021	Under-5 total SCD mortality rank 2000	Under-5 total SCD mortality rank 2021
Lorestan	233 (182-293)	157 (125-195)	-32.3 (-37--26.8)	179 (137-226)	97.7 (74.6-125)	-45.4 (-50--41.2)	0.3 (0.2-0.4)	0.2 (0.1-0.2)	-38.6 (-53.6--8.9)	2.6 (2-3.5)	2.1 (1.7-2.8)	-18.3 (-26.3--10.2)	0.2 (0.1-0.3)	0 (0-0)	-94.6 (-97.1--88.4)	3 (2.1-4.3)	2.6 (1.8-3.7)	-14.2 (-23.7--3.6)	0 (0-0.1)	0.5 (0.3-0.7)	0 (0-0.1)	7.6 (5.3-10.3)	76	79	26	4
Markazi	227 (177-279)	154 (120-190)	-32.3 (-37.7--27.1)	166 (128-205)	89.8 (69.1-111)	-45.8 (-50.5--39.8)	0.2 (0.1-0.3)	0.1 (0.1-0.1)	-54 (-66.4--38.1)	2.7 (2-3.5)	2.1 (1.6-2.7)	-21.5 (-29.2--12.6)	0.1 (0-0.1)	0 (0-0)	-92.3 (-96.3--84.6)	2.6 (1.9-3.7)	2.2 (1.6-3.3)	-15 (-25--4.8)	0 (0-0)	0.4 (0.3-0.6)	0 (0-0)	3.8 (2.6-5.5)	81	92	23	8
Mazandaran	213 (167-267)	144 (114-179)	-32.7 (-37.1--28.8)	160 (123-202)	85.5 (66.2-107)	-46.5 (-49.6--43)	0.4 (0.2-0.5)	0.2 (0.1-0.3)	-34.7 (-52.3--9.3)	2.5 (1.9-3.2)	2 (1.5-2.5)	-21.3 (-28.9--13.6)	0.1 (0-0.2)	0 (0-0)	-93 (-97--86.1)	2 (1.4-2.8)	1.7 (1.2-2.4)	-16.5 (-26.5--6.2)	0 (0-0)	0.5 (0.3-0.7)	0 (0-0.1)	6.2 (4.1-9.4)	74	78	25	6
North Khorasan	231 (180-288)	157 (122-196)	-32.2 (-36.4--27.5)	173 (132-217)	97.7 (74.3-124)	-43.4 (-46.5--39.4)	0.1 (0.1-0.3)	0.1 (0.1-0.2)	-37.1 (-49.7--17.4)	2.8 (2.1-3.6)	2.1 (1.6-2.7)	-24.6 (-31.4--17.1)	0.1 (0-0.1)	0 (0-0)	-90.6 (-94.6--75.4)	3 (2.1-4.1)	2.5 (1.8-3.5)	-16.8 (-27.4--7)	0 (0-0)	0.4 (0.3-0.5)	0 (0-0)	3.3 (2.4-4.7)	89	97	32	8
Qazvin	222 (169-274)	149 (116-186)	-32.7 (-36.4--28.1)	169 (129-210)	93.1 (70.5-116)	-44.9 (-48.4--40.1)	0.1 (0.1-0.2)	0.1 (0.1-0.1)	-44.8 (-54.7--25.1)	2.5 (1.9-3.2)	2 (1.6-2.6)	-17.7 (-27.2--8.6)	0.1 (0.1-0.1)	0 (0-0)	-94.5 (-97.6--87.3)	2.6 (1.9-3.6)	2.2 (1.6-3.2)	-16 (-24.7--4.5)	0 (0-0)	0.4 (0.3-0.5)	0 (0-0)	5 (3.4-7.3)	82	92	29	6
Qom	219 (172-271)	148 (116-182)	-32.4 (-37.1--26.7)	169 (132-212)	96.8 (75.2-120)	-42.7 (-46.5--38.4)	0.2 (0.1-0.2)	0.1 (0.1-0.1)	-52.4 (-62.6--33.9)	2.5 (1.9-3.2)	2 (1.5-2.5)	-20.4 (-28.5--12.3)	0.1 (0-0.1)	0 (0-0)	-91.8 (-95.9--84)	2.5 (1.8-3.4)	2.1 (1.5-3)	-14.8 (-24.6--2.9)	0 (0-0)	0.5 (0.3-0.7)	0 (0-0)	5 (3.3-7.2)	87	95	24	6
Semnan	216 (170-269)	146 (116-183)	-32.4 (-39--26.3)	162 (130-206)	93.3 (71.7-117)	-42.2 (-47.8--36.8)	0.2 (0.2-0.3)	0.1 (0.1-0.1)	-51.3 (-66.9--31.4)	2.5 (1.9-3.2)	1.9 (1.5-2.5)	-21.3 (-30--12.5)	0.1 (0-0.2)	0 (0-0)	-92.6 (-96.9--82)	2.2 (1.6-3)	1.9 (1.3-2.6)	-16.1 (-26--3.9)	0 (0-0)	0.4 (0.3-0.6)	0 (0-0)	4 (2.4-6.4)	77	84	25	8
Sistan and Baluchistan	239 (187-303)	162 (128-204)	-32.2 (-37.8--26.3)	189 (146-241)	116 (89.3-146)	-38.6 (-43.5--34.1)	0.6 (0.3-0.9)	0.4 (0.2-0.6)	-27.6 (-46.5-2.1)	2.9 (2.2-3.8)	2.2 (1.7-2.8)	-24.6 (-31.5--17)	0.5 (0.2-0.8)	0.1 (0-0.1)	-88.3 (-93.6--79.6)	3.3 (2.3-4.5)	2.7 (2-3.9)	-15.9 (-25.2--5.6)	0 (0-0.1)	0.3 (0.2-0.4)	0 (0-0.1)	1.9 (1.4-2.6)	71	82	31	10
South Khorasan	217 (168-270)	146 (114-179)	-32.6 (-36.6--27.6)	162 (123-208)	94.9 (72.7-121)	-41.5 (-45.2--37.5)	0.1 (0.1-0.2)	0.1 (0-0.1)	-38.3 (-52.3--13.8)	2.4 (1.9-3.1)	1.9 (1.5-2.4)	-22.2 (-31.3--12.9)	0.1 (0-0.1)	0 (0-0)	-91.3 (-95.4--79.8)	2.9 (2.1-4)	2.4 (1.7-3.4)	-16.1 (-25.8--4.6)	0 (0-0)	0.3 (0.2-0.4)	0 (0-0)	2.8 (2-3.9)	90	96	34	8
Tehran	200 (159-248)	135 (104-166)	-32.8 (-37.6--28.3)	152 (119-188)	85.2 (65.8-108)	-44.1 (-48.3--40.2)	0.4 (0.2-0.5)	0.2 (0.1-0.2)	-52.8 (-65--33.8)	2.3 (1.7-2.9)	1.8 (1.4-2.3)	-21.6 (-29.4--13.2)	0.1 (0-0.2)	0 (0-0)	-95.1 (-98.5--87.6)	1.7 (1.2-2.4)	1.4 (1-2.1)	-15.9 (-25.9--5)	0 (0-0)	0.5 (0.3-0.7)	0 (0-0)	6.5 (3.1-13.1)	78	82	24	5
West Azarbayejan	227 (178-285)	153 (119-190)	-32.4 (-36--27.1)	169 (134-214)	95.9 (73.8-121)	-43.2 (-46.2--39)	0.2 (0.1-0.3)	0.1 (0.1-0.1)	-54.4 (-66.3--37.1)	2.7 (2-3.5)	2.1 (1.6-2.6)	-23.6 (-30.3--15.1)	0.1 (0.1-0.2)	0 (0-0)	-93.4 (-96.6--85.3)	2.9 (2.1-3.9)	2.4 (1.7-3.4)	-15.6 (-25.3--3.5)	0 (0-0)	0.3 (0.2-0.4)	0 (0-0)	3.4 (2.4-4.7)	83	92	31	6
Yazd	221 (174-271)	150 (119-188)	-31.9 (-39.2--26.3)	166 (129-207)	97.5 (75.6-122)	-41.2 (-47.6--36.3)	0.2 (0.1-0.2)	0.1 (0.1-0.1)	-50.9 (-64.4--32.3)	2.5 (1.9-3.2)	2 (1.5-2.5)	-21.6 (-29--13.9)	0.1 (0-0.1)	0 (0-0)	-88.9 (-93.3--79.4)	2.5 (1.8-3.5)	2.1 (1.5-3.1)	-13.5 (-24.2--0.9)	0 (0-0)	0.4 (0.3-0.6)	0 (0-0)	2.7 (1.9-3.8)	80	88	23	8
Zanjan	218 (172-270)	148 (115-181)	-32.3 (-36.7--26.8)	166 (128-210)	91.1 (70.2-115)	-45 (-48.6--41.9)	0.1 (0.1-0.2)	0.1 (0-0.1)	-54.7 (-67.5--37.8)	2.4 (1.8-3.1)	2 (1.5-2.5)	-17.4 (-25.7--9.6)	0.1 (0.1-0.2)	0 (0-0)	-93.7 (-97.1--86.4)	2.8 (2-3.9)	2.4 (1.7-3.4)	-15.2 (-24.6--4.3)	0 (0-0)	0.3 (0.2-0.4)	0 (0-0)	3.1 (2.2-4.6)	83	96	31	8
Iraq	215 (185-256)	214 (183-249)	-0.2 (-7.6-7)	111 (98.1-127)	97.6 (86.1-111)	-12.4 (-17.4--7.1)	0.5 (0.3-0.6)	0.2 (0.2-0.3)	-49.1 (-61--29.8)	4.4 (3.5-5.6)	3.6 (2.9-4.4)	-18.4 (-25.7--10.2)	0.4 (0.3-0.6)	0.2 (0.1-0.3)	-52.3 (-70.2--14.7)	13.1 (9.2-18.9)	13.1 (8.9-18.8)	-0.5 (-13.3-15.2)	0.1 (0-0.1)	1.8 (1.3-2.6)	0.1 (0-0.1)	5.1 (3.2-7.1)	64	62	9	7
Jordan	103 (82.8-124)	103 (83.3-123)	0.1 (-5.4-8.5)	72 (57.9-89.6)	65.4 (51.8-82.8)	-9.2 (-14.2--3.4)	0.3 (0.2-0.4)	0.1 (0.1-0.2)	-52.9 (-66.1--35.4)	1.4 (1.1-1.8)	1.4 (1.1-1.8)	0.3 (-9.2-10.2)	0.2 (0.1-0.3)	0.1 (0-0.1)	-59.6 (-75--37.4)	3.1 (2-5)	3.1 (2-5)	-0.4 (-13.3-15.6)	0 (0-0)	0.6 (0.3-0.9)	0 (0-0.1)	1.5 (0.9-2.4)	71	75	25	11
Kuwait	71.5 (57.2-85.2)	71.6 (58.1-87.1)	0.1 (-5.8-7.5)	39.2 (30.9-46.9)	33.7 (26.8-39.9)	-14.1 (-19.6--9.8)	0.2 (0.2-0.2)	0.1 (0.1-0.1)	-55.5 (-62.2--46.6)	1.1 (0.9-1.4)	1 (0.8-1.3)	-7.5 (-17.4-2.5)	0.1 (0.1-0.1)	0 (0-0.1)	-54.6 (-68.3--40.3)	3.3 (1.9-5.4)	3.4 (1.9-5.5)	0.9 (-15.6-18.4)	0 (0-0)	1.5 (0.9-2.4)	0 (0-0)	2.8 (1.6-4.7)	67	74	11	8
Lebanon	77.6 (65-92)	79.2 (65.4-93.3)	2.1 (-4.1-9)	49.5 (40-59.2)	45.7 (36.7-54.7)	-7.6 (-14.1--0.9)	0.2 (0.2-0.3)	0.2 (0.1-0.2)	-34 (-48.1--10.8)	1 (0.8-1.2)	1.1 (0.9-1.3)	5.1 (-2-12.9)	0.1 (0-0.1)	0 (0-0)	-80.5 (-89.9--55.4)	1.5 (1-2.2)	1.6 (1.1-2.3)	3.3 (-8.4-16.9)	0 (0-0)	0.4 (0.2-0.5)	0 (0-0)	1.7 (1-2.9)	86	89	31	13
Libya	867 (672-1,160)	873 (670-1,140)	0.7 (-8.9-8.4)	299 (252-352)	227 (193-264)	-24.2 (-30.5--18.8)	0.5 (0.4-0.7)	0.5 (0.4-0.8)	15.2 (-17.7-69.4)	17.4 (12.7-24.1)	11.6 (8.9-15)	-33.4 (-41--23.4)	0.8 (0.5-1.1)	1.3 (0.8-1.8)	62.3 (11.1-164)	82.7 (52.8-127)	83.6 (53.3-126)	1 (-12.7-18.2)	0.1 (0.1-0.2)	15.5 (10.2-23.2)	0.2 (0.1-0.2)	11.4 (7-17.6)	53	49	2	3
Morocco	29 (24.2-33.8)	29.1 (23.8-34)	0.5 (-4.9-6.8)	13.1 (10.7-15.2)	11.2 (9.2-13.1)	-14.5 (-19--9.2)	0.1 (0.1-0.2)	0.1 (0-0.1)	-39.1 (-62.4-6.6)	0.5 (0.4-0.6)	0.4 (0.3-0.5)	-17.3 (-23.9--10.3)	0.2 (0.1-0.3)	0.1 (0-0.2)	-62.4 (-81.3--26.9)	2.2 (1.5-3)	2.2 (1.5-3)	-0.4 (-11.9-12.7)	0 (0-0)	0.2 (0.1-0.3)	0 (0-0)	0.6 (0.5-0.9)	82	88	38	19
Oman	481 (410-544)	503 (418-596)	4.7 (-4.7-14.3)	289 (248-327)	273 (223-319)	-5.6 (-14.4-3.5)	0.7 (0.5-0.8)	0.4 (0.3-0.5)	-46.6 (-58.6--28.7)	7.5 (6.2-9)	7.6 (6.2-9.1)	0.5 (-10.3-12.4)	0.7 (0.5-1.1)	0.3 (0.2-0.4)	-56.3 (-69.4--40.5)	22.7 (15-32.7)	22.8 (15-32.1)	0.4 (-14-14.1)	0.2 (0.2-0.3)	7.4 (4.7-10.7)	0.2 (0.1-0.3)	15.7 (10.4-23.1)	34	40	5	2
Palestine	99.9 (79.7-122)	99.5 (81-121)	-0.4 (-6.2-5.9)	73 (57.6-93.1)	67.4 (52.9-85)	-7.7 (-14.7--1.3)	0.1 (0.1-0.2)	0.1 (0.1-0.1)	-34.8 (-51.4--11.6)	1.4 (1.1-1.8)	1.3 (1-1.7)	-6 (-15.6-4)	0.1 (0.1-0.2)	0.1 (0-0.1)	-52.7 (-73.4--6.6)	3.1 (2-4.8)	3.1 (2-4.8)	0.8 (-12.2-17.9)	0 (0-0)	0.5 (0.4-0.8)	0 (0-0)	1.5 (0.9-2.4)	76	84	22	11
Qatar	117 (95.6-147)	118 (98.8-143)	0.4 (-5.8-7.5)	72.6 (57.3-91.5)	66.7 (51.9-83.4)	-8.1 (-15.8--1.3)	0.1 (0.1-0.2)	0 (0-0.1)	-59.1 (-70.4--42.7)	1.7 (1.3-2.2)	1.6 (1.3-2.1)	-6.2 (-14.9-3.5)	0.1 (0-0.1)	0 (0-0)	-66.4 (-81.5--41.6)	3.5 (2.1-5.8)	3.6 (2.1-6)	1.9 (-12.7-21)	0 (0-0.1)	1.5 (0.9-2.5)	0 (0-0.1)	4.8 (2.8-8.3)	73	74	15	5
Saudi Arabia	1,510 (1,400-1,610)	379 (318-449)	-74.8 (-78.7--70.5)	1,260 (1,180-1,340)	289 (236-345)	-77 (-81.1--73.3)	0.8 (0.6-1)	0.7 (0.5-1)	-9.7 (-42.4-32.7)	18.9 (16.9-21.1)	5.5 (4.4-6.8)	-70.8 (-76.6--64.4)	0.4 (0.2-0.6)	0.1 (0-0.2)	-77.2 (-88.3--60)	7.6 (5.8-9.7)	4.2 (3-5.5)	-45 (-55.5--33.2)	0.1 (0.1-0.1)	1.7 (1.2-2.2)	0.1 (0.1-0.2)	4.4 (2.9-6.1)	59	61	9	6
Sudan	715 (601-845)	489 (398-590)	-31.7 (-38.4--23.1)	562 (469-668)	360 (288-439)	-36 (-43--27.7)	1 (0.6-1.3)	0.6 (0.4-0.9)	-37.5 (-56.8--6.2)	8.8 (7.3-10.8)	6.4 (5.2-7.8)	-27.9 (-35.7--19.8)	1.3 (0.5-2.2)	0.5 (0.3-0.8)	-58.5 (-75.1-8.8)	10.7 (8.4-13.9)	9.1 (7.2-11.8)	-14.9 (-23.3--5.6)	0.1 (0-0.1)	0.5 (0.4-0.6)	0.1 (0-0.1)	1.2 (0.9-1.6)	68	70	26	15
Syrian Arab Republic	32.3 (26.6-39.6)	32.4 (27.1-39.1)	0.2 (-7.4-8.2)	12.7 (10.6-14.6)	9.7 (8-11.2)	-24.2 (-30.2--19.6)	0.2 (0.1-0.2)	0.1 (0.1-0.2)	-30.7 (-56-8.4)	0.7 (0.6-0.9)	0.5 (0.4-0.6)	-35.5 (-42--27.8)	0.1 (0.1-0.2)	0.2 (0.1-0.3)	7 (-42.6-86.4)	2.9 (2-4)	2.9 (2-4)	0.6 (-11.1-14.8)	0 (0-0.1)	0.8 (0.6-1.2)	0 (0-0.1)	0.7 (0.4-0.9)	73	77	23	25
Tunisia	106 (84.6-128)	105 (87.4-126)	-0.4 (-6.8-6.2)	67.6 (53.4-85.3)	56.7 (44.4-70.5)	-16.2 (-23.3--9.2)	0.2 (0.1-0.2)	0.1 (0.1-0.2)	-23.8 (-47.9-9.8)	1.4 (1.1-1.7)	1.4 (1.1-1.7)	2.6 (-6-11.7)	0.1 (0.1-0.2)	0 (0-0.1)	-70.1 (-83.6--38.9)	3.2 (2.2-4.6)	3.2 (2.1-4.5)	-0.2 (-12.7-14.2)	0 (0-0)	0.6 (0.4-0.8)	0 (0-0)	1.5 (1-2.2)	78	82	26	13
Türkiye	37.8 (33.7-44)	31.3 (27.3-36.1)	-17.2 (-23.9--9.9)	21.1 (19-23.7)	13.2 (11.7-15)	-37.5 (-42.9--30.7)	0.2 (0.1-0.2)	0.1 (0.1-0.1)	-55.3 (-68.6--38.6)	0.5 (0.5-0.6)	0.4 (0.4-0.5)	-26.2 (-32.1--20.2)	0.2 (0.1-0.5)	0.1 (0-0.1)	-69.6 (-81.7--31.7)	1.8 (1.3-2.5)	1.8 (1.3-2.5)	-1.5 (-14.3-12.8)	0 (0-0.1)	0.2 (0.2-0.3)	0 (0-0)	0.7 (0.5-1)	79	90	40	23
United Arab Emirates	29.6 (24.7-34)	28.8 (24.3-32.9)	-2.6 (-6.8-3.1)	21.1 (17.5-25)	16.5 (13.4-19.2)	-21.8 (-26.2--15.6)	0.1 (0.1-0.1)	0.1 (0-0.1)	-48.3 (-62.7--26.2)	0.4 (0.3-0.5)	0.5 (0.4-0.6)	18.9 (5.3-32)	0 (0-0)	0 (0-0)	-76.7 (-85.9--61.4)	0.5 (0.3-0.7)	0.5 (0.3-0.7)	-0.8 (-11.2-12.1)	0 (0-0)	0.2 (0.1-0.3)	0 (0-0)	0.7 (0.5-1)	105	112	40	22
Yemen	29.3 (24.9-34.9)	29.4 (24.8-34.7)	0.4 (-4.1-5)	13.8 (11.5-16.2)	12.9 (10.7-14.8)	-6.6 (-13.1--1.4)	0.1 (0.1-0.2)	0.1 (0.1-0.1)	-32 (-58.2-20.8)	0.7 (0.5-0.9)	0.6 (0.5-0.7)	-17.4 (-23.5--10.5)	0.3 (0.1-0.5)	0.1 (0.1-0.3)	-47.2 (-70-25.5)	2.4 (1.7-3.2)	2.3 (1.7-3.2)	-1.3 (-11-9.7)	0 (0-0)	0.1 (0.1-0.1)	0 (0-0)	0.3 (0.2-0.4)	84	88	47	38

Location	Birth Incidence rate (per 100,000 live births), 2000	Birth Incidence rate (per 100,000 live births), 2021	Birth Incidence rate (per 100,000 live births) percent change (2000-2021)	All age rate (per 100,000 population), 2000	All age rate (per 100,000 population), 2021	All age rate (per 100,000 population) percent change (2000-2021)	All age cause-specific SCD mortality rate (per 100,000 population), 2000	All age cause-specific SCD mortality rate (per 100,000 population), 2021	All age cause-specific SCD mortality rate (per 100,000 population) percent change (2000-2021)	All age total SCD mortality rate (per 100,000 population), 2000	All age total SCD mortality rate (per 100,000 population), 2021	All age total SCD mortality rate (per 100,000 population) percent change (2000-2021)	Under-5 cause-specific SCD mortality rate (per 100,000 population), 2000	Under-5 cause-specific SCD mortality rate (per 100,000 population), 2021	Under-5 cause-specific SCD mortality rate (per 100,000 population) percent change (2000-2021)	Under-5 total SCD mortality rate (per 100,000 population), 2000	Under-5 total SCD mortality rate (per 100,000 population), 2021	Under-5 total SCD mortality rate (per 100,000 population) percent change (2000-2021)	Under-5 cause-specific mortality cause fraction, 2000	Under-5 total SCD mortality cause fraction, 2000	Under-5 cause-specific mortality cause fraction, 2021	Under-5 total SCD mortality cause fraction, 2021	Under-5 cause-specific mortality rank 2000	Under-5 cause-specific mortality rank 2021	Under-5 total SCD mortality rank 2000	Under-5 total SCD mortality rank 2021
South Asia	263 (187-351)	251 (179-335)	-4.6 (-6.9--1.9)	90 (66.4-118)	68.9 (51-90.2)	-23.4 (-25.4--21.4)	0.2 (0.1-0.2)	0.1 (0.1-0.1)	-61.7 (-70.3--51.6)	6.1 (4.3-8.5)	4.8 (3.5-6.7)	-20.9 (-23.9--17.4)	0.4 (0.2-0.5)	0.1 (0.1-0.2)	-62.8 (-80.8--27.1)	10.5 (6.8-15.5)	9.7 (6.3-14.1)	-8.5 (-12.7--4.1)	0 (0-0)	0.6 (0.4-0.9)	0 (0-0)	1.3 (0.8-2)	83	95	22	13
**South Asia**	263 (187-351)	251 (179-335)	-4.6 (-6.9--1.9)	90 (66.4-118)	68.9 (51-90.2)	-23.4 (-25.4--21.4)	0.2 (0.1-0.2)	0.1 (0.1-0.1)	-61.7 (-70.3--51.6)	6.1 (4.3-8.5)	4.8 (3.5-6.7)	-20.9 (-23.9--17.4)	0.4 (0.2-0.5)	0.1 (0.1-0.2)	-62.8 (-80.8--27.1)	10.5 (6.8-15.5)	9.7 (6.3-14.1)	-8.5 (-12.7--4.1)	0 (0-0)	0.6 (0.4-0.9)	0 (0-0)	1.3 (0.8-2)	83	95	22	13
Bangladesh	1.7 (1.3-2.2)	1.7 (1.2-2.2)	-0.6 (-13.2-8.9)	0.3 (0.3-0.5)	0.2 (0.2-0.3)	-32.9 (-44.8--26.4)	0 (0-0)	0 (0-0)	-66.1 (-81.8--26.2)	0 (0-0.1)	0 (0-0)	-33.9 (-42.6--27.9)	0 (0-0)	0 (0-0)	-64.4 (-85.4-27.7)	0.2 (0.1-0.3)	0.2 (0.1-0.3)	0 (-10.5-11.5)	0 (0-0)	0 (0-0)	0 (0-0)	0 (0-0)	130	130	87	67
Bhutan	1.6 (1.2-2.2)	1.6 (1.2-2.2)	-0.2 (-8.4-7.9)	0.3 (0.2-0.4)	0.2 (0.2-0.3)	-29.8 (-38.5--24.8)	0 (0-0)	0 (0-0)	-61.9 (-80.5--21.2)	0 (0-0.1)	0 (0-0)	-31.3 (-37.9--25.6)	0 (0-0.1)	0 (0-0)	-48.3 (-83.9-78.3)	0.2 (0.1-0.3)	0.2 (0.1-0.3)	-0.5 (-12.9-12.2)	0 (0-0)	0 (0-0)	0 (0-0)	0 (0-0)	120	119	100	85
India	364 (258-486)	360 (256-480)	-1.3 (-3.6-1.6)	116 (85.3-152)	89.6 (66.3-117)	-22.6 (-24.5--20.5)	0.2 (0.2-0.3)	0.1 (0.1-0.1)	-61.4 (-69.9--51.3)	7.9 (5.5-10.9)	6.3 (4.5-8.7)	-20 (-23--16.5)	0.5 (0.3-0.7)	0.2 (0.1-0.3)	-62 (-80.6--25.1)	14.4 (9.2-21.1)	13.6 (8.8-19.8)	-5.7 (-10.1--1.3)	0 (0-0)	0.9 (0.6-1.3)	0 (0-0)	2 (1.3-3.2)	75	80	19	10
Nepal	3.4 (2.8-4)	3.3 (2.8-4)	-0.3 (-8.5-7.1)	1 (0.8-1.2)	0.8 (0.6-1)	-24.4 (-30.1--16.6)	0 (0-0)	0 (0-0)	-41.7 (-71.2-16.9)	0.1 (0.1-0.1)	0.1 (0.1-0.1)	-23.9 (-30.5--16.6)	0 (0-0)	0 (0-0)	-60.7 (-86.3-50.2)	0.2 (0.2-0.3)	0.2 (0.2-0.3)	-0.9 (-11.7-10.9)	0 (0-0)	0 (0-0)	0 (0-0)	0 (0-0.1)	126	124	90	72
Pakistan	0.8 (0.5-1.2)	0.8 (0.5-1.2)	-2.1 (-9-4.7)	0.1 (0.1-0.2)	0.1 (0.1-0.1)	-21.5 (-27.5--15.5)	0 (0-0)	0 (0-0)	-50.3 (-79-18)	0 (0-0)	0 (0-0)	-23.5 (-31.2--14.3)	0 (0-0)	0 (0-0)	-28.5 (-66.1-96.5)	0.1 (0.1-0.2)	0.1 (0.1-0.2)	-3.4 (-12.9-9.5)	0 (0-0)	0 (0-0)	0 (0-0)	0 (0-0)	114	126	97	102
Azad Jammu & Kashmir	0.8 (0.5-1.1)	0.8 (0.5-1.1)	-2 (-21.8-8.6)	0.1 (0.1-0.2)	0.1 (0.1-0.1)	-30.3 (-37.8--24.6)	0 (0-0)	0 (0-0)	-62.5 (-84--0.7)	0 (0-0)	0 (0-0)	-32.6 (-44.7--25)	0 (0-0)	0 (0-0)	-47.3 (-79.6-34.2)	0.1 (0.1-0.2)	0.1 (0.1-0.2)	-3.2 (-24.1-8.6)	0 (0-0)	0 (0-0)	0 (0-0)	0 (0-0)	114	128	99	100
Balochistan	0.8 (0.5-1.2)	0.8 (0.5-1.2)	-0.3 (-13.3-16.7)	0.1 (0.1-0.2)	0.1 (0.1-0.2)	-6.4 (-16.3-7.8)	0 (0-0)	0 (0-0)	-40.1 (-70.3-33.5)	0 (0-0)	0 (0-0)	-7 (-21.9-11.6)	0 (0-0)	0 (0-0)	-25.9 (-65.1-105)	0.1 (0.1-0.2)	0.1 (0.1-0.2)	0 (-17.6-22.3)	0 (0-0)	0 (0-0)	0 (0-0)	0 (0-0)	116	127	97	107
Gilgit-Baltistan	0.8 (0.5-1.1)	0.8 (0.5-1.1)	-1.7 (-14.2-11.2)	0.1 (0.1-0.2)	0.1 (0.1-0.2)	-7.6 (-17.5-2.6)	0 (0-0)	0 (0-0)	-60.2 (-84.7-40.6)	0 (0-0)	0 (0-0)	-8.2 (-23.2-6.5)	0 (0-0)	0 (0-0)	-67.2 (-86.3--6.6)	0.1 (0.1-0.2)	0.1 (0.1-0.2)	-2.2 (-20.7-14.5)	0 (0-0)	0 (0-0)	0 (0-0)	0 (0-0)	117	128	100	97
Islamabad Capital Territory	0.7 (0.4-0.9)	0.6 (0.4-0.9)	-0.6 (-19.7-37.5)	0.1 (0.1-0.2)	0.1 (0.1-0.1)	-17.8 (-28.1--0.5)	0 (0-0)	0 (0-0)	-72.2 (-89.9--12.5)	0 (0-0)	0 (0-0)	-19 (-35.7-9.1)	0 (0-0)	0 (0-0)	-70.5 (-90.7--8.6)	0.1 (0-0.1)	0.1 (0-0.1)	0.9 (-24.8-45.2)	0 (0-0)	0 (0-0)	0 (0-0)	0 (0-0.1)	118	130	89	80
Khyber Pakhtunkhwa	0.7 (0.4-1.1)	0.7 (0.4-1)	-1.8 (-11.8-10.4)	0.1 (0.1-0.2)	0.1 (0.1-0.2)	-9.2 (-19.3--0.1)	0 (0-0)	0 (0-0)	-40.3 (-80.6-70.4)	0 (0-0)	0 (0-0)	-10.3 (-30.2-5.2)	0 (0-0)	0 (0-0)	-26.1 (-72.7-100)	0.1 (0.1-0.2)	0.1 (0.1-0.2)	-4.6 (-28.2-15.6)	0 (0-0)	0 (0-0)	0 (0-0)	0 (0-0)	118	128	95	98
Punjab	0.8 (0.5-1.2)	0.8 (0.6-1.2)	-0.7 (-11.2-13.1)	0.1 (0.1-0.2)	0.1 (0.1-0.1)	-27.6 (-35.4--20.3)	0 (0-0)	0 (0-0)	-48.9 (-79.3-38.8)	0 (0-0)	0 (0-0)	-29.5 (-39.9--16.2)	0 (0-0)	0 (0-0)	-12.9 (-61.4-200)	0.1 (0.1-0.2)	0.1 (0.1-0.2)	-1.5 (-17-20.5)	0 (0-0)	0 (0-0)	0 (0-0)	0 (0-0)	113	125	95	103
Sindh	0.8 (0.5-1.2)	0.8 (0.5-1.2)	-0.4 (-13.2-11)	0.1 (0.1-0.2)	0.1 (0.1-0.1)	-22.2 (-30.1--13.7)	0 (0-0)	0 (0-0)	-56.6 (-82.1-0.9)	0 (0-0)	0 (0-0)	-23.3 (-34.1--12.9)	0 (0-0)	0 (0-0)	-41.7 (-78.3-77)	0.1 (0.1-0.2)	0.1 (0.1-0.2)	-0.6 (-15.5-14.4)	0 (0-0)	0 (0-0)	0 (0-0)	0 (0-0)	111	126	98	97
Southeast Asia, East Asia, and Oceania	2.3 (1.8-2.9)	2.3 (1.8-2.9)	0.8 (-0.7-2.3)	1.4 (1-1.7)	1 (0.8-1.3)	-24.2 (-25.9--22.5)	0 (0-0)	0 (0-0)	-36.6 (-53.4--13.7)	0 (0-0.1)	0 (0-0)	-15.4 (-19.6--11.2)	0 (0-0)	0 (0-0)	-72.1 (-84.4--49.2)	0 (0-0.1)	0 (0-0.1)	-0.3 (-3.3-3.2)	0 (0-0)	0 (0-0)	0 (0-0)	0 (0-0)	137	137	126	111
**East Asia**	3.7 (2.9-4.7)	3.7 (2.9-4.7)	0.4 (-1.2-2)	1.9 (1.5-2.4)	1.5 (1.1-1.9)	-21.5 (-23.2--19.8)	0 (0-0)	0 (0-0)	-33.4 (-51.6--8.5)	0.1 (0-0.1)	0 (0-0.1)	-12.2 (-16.8--7.6)	0 (0-0)	0 (0-0)	-83.9 (-91--68.7)	0.1 (0-0.1)	0.1 (0-0.1)	-0.9 (-4.8-3)	0 (0-0)	0 (0-0)	0 (0-0)	0 (0-0.1)	134	136	121	87
China	3.7 (2.9-4.7)	3.8 (2.9-4.8)	0 (-1.6-1.5)	1.9 (1.5-2.4)	1.5 (1.1-1.9)	-22 (-23.8--20.3)	0 (0-0)	0 (0-0)	-34.3 (-52.4--9.3)	0.1 (0-0.1)	0 (0-0.1)	-12.6 (-17.1--8.1)	0 (0-0)	0 (0-0)	-84.3 (-91.2--68.9)	0.1 (0-0.1)	0.1 (0-0.1)	-1.8 (-5.6-2.1)	0 (0-0)	0 (0-0)	0 (0-0)	0 (0-0.1)	134	134	121	87
Democratic People's Republic of Korea	3.6 (2.7-4.7)	3.6 (2.8-4.6)	-0.3 (-7.9-7.1)	2.1 (1.6-2.7)	1.7 (1.2-2.1)	-20.3 (-26.3--14.8)	0 (0-0)	0 (0-0)	5.3 (-29.2-51.3)	0.1 (0-0.1)	0 (0-0.1)	-5.3 (-14.7-4)	0 (0-0)	0 (0-0)	-46.3 (-74.1-13.9)	0 (0-0.1)	0 (0-0.1)	0.2 (-12.3-18.4)	0 (0-0)	0 (0-0)	0 (0-0)	0 (0-0)	132	132	115	106
Taiwan (Province of China)	3.1 (2.4-4)	3.8 (3.1-4.7)	25.2 (11.6-42.9)	1.7 (1.3-2.2)	1.8 (1.5-2.2)	5.9 (-6-21.9)	0 (0-0)	0 (0-0)	-89.4 (-90.9--87.7)	0 (0-0.1)	0 (0-0.1)	9.1 (-5.7-27.4)	0 (0-0)	0 (0-0)	-95.8 (-97.1--94.1)	0 (0-0)	0 (0-0)	21.6 (4.3-42.5)	0 (0-0)	0 (0-0)	0 (0-0)	0 (0-0)	112	127	92	80
**Oceania**	0.1 (0-0.1)	0.1 (0-0.1)	0.7 (-4-6)	0 (0-0.1)	0 (0-0)	-7.6 (-12.8-1)	0 (0-0)	0 (0-0)	-23.5 (-48.6-8.2)	0 (0-0)	0 (0-0)	-4.8 (-7--0.7)	0 (0-0)	0 (0-0)	-43.2 (-66.2--7.8)	0 (0-0)	0 (0-0)	3 (-0.5-8)	0 (0-0)	0 (0-0)	0 (0-0)	0 (0-0)	139	141	127	124
American Samoa	0 (0-0.1)	0 (0-0.1)	-0.8 (-24.9-4.1)	0 (0-0.1)	0 (0-0)	-15.1 (-32.7--9.4)	0 (0-0)	0 (0-0)	49.7 (-20.4-131)	0 (0-0)	0 (0-0)	-14.4 (-30.6--7)	0 (0-0)	0 (0-0)	-37.3 (-73.7-49.5)	0 (0-0)	0 (0-0)	-2.1 (-28.9-4.4)	0 (0-0)	0 (0-0)	0 (0-0)	0 (0-0)	131	132	121	106
Cook Islands	0 (0-0.1)	0 (0-0.1)	-1.4 (-27.3-5.1)	0 (0-0)	0 (0-0)	-22.2 (-53.6--16.2)	0 (0-0)	0 (0-0)	-37 (-60.7--1.9)	0 (0-0)	0 (0-0)	-19.2 (-40.1--13.5)	0 (0-0)	0 (0-0)	-87.1 (-97.1--57.2)	0 (0-0)	0 (0-0)	-0.3 (-7.2-5.6)	0 (0-0)	0 (0-0)	0 (0-0)	0 (0-0)	132	131	110	93
Fiji	0.1 (0-0.1)	0.1 (0-0.1)	0 (0-0)	0 (0-0.1)	0 (0-0)	-11.3 (-14.9--4.6)	0 (0-0)	0 (0-0)	-10.2 (-37.3-29.8)	0 (0-0)	0 (0-0)	-9.8 (-11.1--8.4)	0 (0-0)	0 (0-0)	-6.9 (-60.9-137)	0 (0-0)	0 (0-0)	-0.2 (-5.4-4.8)	0 (0-0)	0 (0-0)	0 (0-0)	0 (0-0)	128	133	116	114
Guam	0 (0-0.1)	0 (0-0.1)	0 (-0.6-0.1)	0 (0-0.1)	0 (0-0)	-11.8 (-15.3--7.9)	0 (0-0)	0 (0-0)	-48.4 (-66.3-7.6)	0 (0-0)	0 (0-0)	-10.5 (-13.5--8.3)	0 (0-0)	0 (0-0)	-59.4 (-82.8--8.3)	0 (0-0)	0 (0-0)	-0.6 (-6.2-4.5)	0 (0-0)	0 (0-0)	0 (0-0)	0 (0-0)	133	137	114	111
Kiribati	0.1 (0-0.1)	0.1 (0-0.1)	1.6 (-7.1-95.2)	0 (0-0.1)	0 (0-0)	-6.2 (-17-181)	0 (0-0)	0 (0-0)	-17.9 (-53.6-34.9)	0 (0-0)	0 (0-0)	-6.7 (-11-22.9)	0 (0-0)	0 (0-0)	-47.4 (-76.2-9.1)	0 (0-0)	0 (0-0)	0.1 (-4.3-6.6)	0 (0-0)	0 (0-0)	0 (0-0)	0 (0-0)	131	131	121	119
Marshall Islands	0.1 (0-0.1)	0.1 (0-0.1)	-0.6 (-20.4-22.3)	0 (0-0.1)	0 (0-0)	-18.6 (-33.2-10.9)	0 (0-0)	0 (0-0)	-26.1 (-51.7-20.9)	0 (0-0)	0 (0-0)	-16.6 (-35.9--10.4)	0 (0-0)	0 (0-0)	-55.3 (-80.6-78.2)	0 (0-0)	0 (0-0)	-1.7 (-29.5-5.9)	0 (0-0)	0 (0-0)	0 (0-0)	0 (0-0)	134	135	117	112
Micronesia (Federated States of)	0.1 (0-0.1)	0.1 (0-0.1)	0 (-0.1-0)	0 (0-0.1)	0 (0-0)	-18.5 (-24.2--14.8)	0 (0-0)	0 (0-0)	-30.1 (-56.2-14)	0 (0-0)	0 (0-0)	-16.2 (-24.2--9.4)	0 (0-0)	0 (0-0)	-60.6 (-79.7-43)	0 (0-0)	0 (0-0)	-0.1 (-4.6-3.8)	0 (0-0)	0 (0-0)	0 (0-0)	0 (0-0)	135	135	113	107
Nauru	0.1 (0-0.1)	0.1 (0-0.1)	-1.5 (-47.2-6)	0 (0-0.1)	0 (0-0.1)	-8.5 (-68.4-3.1)	0 (0-0)	0 (0-0)	-38.6 (-60.9-13.8)	0 (0-0)	0 (0-0)	-9.3 (-33.4--2.9)	0 (0-0)	0 (0-0)	-51 (-77-40.1)	0 (0-0)	0 (0-0)	-1.4 (-9.7-4.3)	0 (0-0)	0 (0-0)	0 (0-0)	0 (0-0)	135	135	119	114
Niue	0.1 (0-0.1)	0 (0-0.1)	-2.2 (-19.8-8.3)	0 (0-0)	0 (0-0)	-13.9 (-21.3--8.3)	0 (0-0)	0 (0-0)	-35.8 (-56.7--0.6)	0 (0-0)	0 (0-0)	-12.8 (-38.8--6.5)	0 (0-0)	0 (0-0)	-38.6 (-78-51.7)	0 (0-0)	0 (0-0)	-3.4 (-39.5-5.1)	0 (0-0)	0 (0-0)	0 (0-0)	0 (0-0)	135	136	114	111

Location	Birth Incidence rate (per 100,000 live births), 2000	Birth Incidence rate (per 100,000 live births), 2021	Birth Incidence rate (per 100,000 live births) percent change (2000-2021)	All age rate (per 100,000 population), 2000	All age rate (per 100,000 population), 2021	All age rate (per 100,000 population) percent change (2000-2021)	All age cause-specific SCD mortality rate (per 100,000 population), 2000	All age cause-specific SCD mortality rate (per 100,000 population), 2021	All age cause-specific SCD mortality rate (per 100,000 population) percent change (2000-2021)	All age total SCD mortality rate (per 100,000 population), 2000	All age total SCD mortality rate (per 100,000 population), 2021	All age total SCD mortality rate (per 100,000 population) percent change (2000-2021)	Under-5 cause-specific SCD mortality rate (per 100,000 population), 2000	Under-5 cause-specific SCD mortality rate (per 100,000 population), 2021	Under-5 cause-specific SCD mortality rate (per 100,000 population) percent change (2000-2021)	Under-5 total SCD mortality rate (per 100,000 population), 2000	Under-5 total SCD mortality rate (per 100,000 population), 2021	Under-5 total SCD mortality rate (per 100,000 population) percent change (2000-2021)	Under-5 cause-specific mortality cause fraction, 2000	Under-5 total SCD mortality cause fraction, 2000	Under-5 cause-specific mortality cause fraction, 2021	Under-5 total SCD mortality cause fraction, 2021	Under-5 cause-specific mortality rank 2000	Under-5 cause-specific mortality rank 2021	Under-5 total SCD mortality rank 2000	Under-5 total SCD mortality rank 2021
Northern Mariana Islands	0 (0-0.1)	0 (0-0.1)	0.4 (-4.1-19.6)	0 (0-0.1)	0 (0-0)	-31 (-34.9--13)	0 (0-0)	0 (0-0)	-37.7 (-61.6-15.8)	0 (0-0)	0 (0-0)	-27 (-35.7--15.5)	0 (0-0)	0 (0-0)	-58.2 (-84.1-10.4)	0 (0-0)	0 (0-0)	0.5 (-6.6-13.3)	0 (0-0)	0 (0-0)	0 (0-0)	0 (0-0)	134	137	117	116
Palau	0.1 (0-0.1)	0.1 (0-0.1)	0.6 (-4.4-25.2)	0 (0-0)	0 (0-0)	-26.6 (-32--4.6)	0 (0-0)	0 (0-0)	-27.9 (-51.9-2.4)	0 (0-0)	0 (0-0)	-25.7 (-33.3--20.2)	0 (0-0)	0 (0-0)	-33.1 (-64.4-44.9)	0 (0-0)	0 (0-0)	-0.2 (-5.1-5.3)	0 (0-0)	0 (0-0)	0 (0-0)	0 (0-0)	129	131	110	109
Papua New Guinea	0.1 (0-0.1)	0.1 (0-0.1)	0 (-4.7-3.6)	0 (0-0.1)	0 (0-0)	-6.6 (-12.5-4.1)	0 (0-0)	0 (0-0)	-23.1 (-54.1-13)	0 (0-0)	0 (0-0)	-6.1 (-8.2--3.9)	0 (0-0)	0 (0-0)	-43.9 (-67.7--9.7)	0 (0-0)	0 (0-0)	-0.2 (-3.8-3.7)	0 (0-0)	0 (0-0)	0 (0-0)	0 (0-0)	137	139	124	121
Samoa	0.1 (0-0.1)	0.1 (0-0.1)	-1.4 (-27.6-1.6)	0 (0-0.1)	0 (0-0.1)	-6.3 (-27.3-2.2)	0 (0-0)	0 (0-0)	-20.7 (-58.1-31)	0 (0-0)	0 (0-0)	-6.2 (-28--2.4)	0 (0-0)	0 (0-0)	-56.6 (-82.9--2.7)	0 (0-0)	0 (0-0)	-1 (-28.2-5)	0 (0-0)	0 (0-0)	0 (0-0)	0 (0-0)	134	136	115	108
Solomon Islands	0.1 (0-0.1)	0.1 (0-0.1)	-1.1 (-7-1.5)	0 (0-0.1)	0 (0-0.1)	-9.5 (-15.6--4.4)	0 (0-0)	0 (0-0)	-21.6 (-52.9-30.6)	0 (0-0)	0 (0-0)	-10.8 (-29.5--5)	0 (0-0)	0 (0-0)	-57 (-78.5--10)	0 (0-0)	0 (0-0)	-2 (-23.5-3.2)	0 (0-0)	0 (0-0)	0 (0-0)	0 (0-0)	136	137	118	116
Tokelau	0 (0-0.1)	0 (0-0.1)	0 (-9.2-15.2)	0 (0-0.1)	0 (0-0)	-13.4 (-21.1--1.1)	0 (0-0)	0 (0-0)	-38.1 (-60.7-11.9)	0 (0-0)	0 (0-0)	-9.8 (-18.8--4.6)	0 (0-0)	0 (0-0)	-70.3 (-89.7--33)	0 (0-0)	0 (0-0)	0.1 (-12.7-8.3)	0 (0-0)	0 (0-0)	0 (0-0)	0 (0-0)	135	135	115	103
Tonga	0 (0-0.1)	0 (0-0.1)	-0.5 (-6.8-1.1)	0 (0-0.1)	0 (0-0.1)	-8.3 (-16--4.5)	0 (0-0)	0 (0-0)	-24.9 (-47.9-9.8)	0 (0-0)	0 (0-0)	-7.8 (-16.7--4.1)	0 (0-0)	0 (0-0)	-46.8 (-75.3-0.7)	0 (0-0)	0 (0-0)	-0.9 (-11.1-4.3)	0 (0-0)	0 (0-0)	0 (0-0)	0 (0-0)	134	135	118	115
Tuvalu	0.1 (0-0.1)	0.1 (0-0.1)	0.8 (-1.1-9.5)	0 (0-0)	0 (0-0)	-8.9 (-15.4-1)	0 (0-0)	0 (0-0)	-40 (-62.1--14.5)	0 (0-0)	0 (0-0)	-9.8 (-18.9--0.5)	0 (0-0)	0 (0-0)	-61.2 (-82.1--13.5)	0 (0-0)	0 (0-0)	1.1 (-4.4-8)	0 (0-0)	0 (0-0)	0 (0-0)	0 (0-0)	135	135	118	111
Vanuatu	0.1 (0-0.1)	0.1 (0-0.1)	-1.5 (-28.5-1.6)	0 (0-0.1)	0 (0-0.1)	-10.1 (-32.7--2.5)	0 (0-0)	0 (0-0)	-25.9 (-53.9-15.3)	0 (0-0)	0 (0-0)	-12.1 (-34--6.1)	0 (0-0)	0 (0-0)	-57.7 (-79.4--0.3)	0 (0-0)	0 (0-0)	-2.7 (-30.9-3.6)	0 (0-0)	0 (0-0)	0 (0-0)	0 (0-0)	134	137	117	113
**Southeast Asia**	0.3 (0.2-0.3)	0.3 (0.2-0.3)	1.8 (-0.7-7.2)	0 (0-0.1)	0 (0-0.1)	-22.1 (-25.4--18.2)	0 (0-0)	0 (0-0)	-56.9 (-70.9--41.8)	0 (0-0)	0 (0-0)	-23.1 (-26.4--17.5)	0 (0-0)	0 (0-0)	-50.6 (-72.1--17.3)	0 (0-0)	0 (0-0)	2.8 (-1.1-11.1)	0 (0-0)	0 (0-0)	0 (0-0)	0 (0-0)	132	136	123	113
Cambodia	0.3 (0.2-0.4)	0.3 (0.2-0.4)	2.7 (-4.2-25.4)	0 (0-0.1)	0 (0-0.1)	-21.3 (-29.7-3.1)	0 (0-0)	0 (0-0)	-59.4 (-77.8--22.9)	0 (0-0)	0 (0-0)	-22.4 (-30.4-19.3)	0 (0-0)	0 (0-0)	-62 (-81--2.4)	0 (0-0.1)	0 (0-0.1)	3.4 (-10.1-62.8)	0 (0-0)	0 (0-0)	0 (0-0)	0 (0-0)	138	138	123	107
Indonesia	0.3 (0.2-0.4)	0.3 (0.2-0.4)	3 (-0.7-15.5)	0 (0-0.1)	0 (0-0.1)	-23.6 (-27.8--18.8)	0 (0-0)	0 (0-0)	-55.2 (-73.6--31.3)	0 (0-0)	0 (0-0)	-24.5 (-28.9--14.3)	0 (0-0)	0 (0-0)	-42.6 (-66.2--1.8)	0 (0-0)	0 (0-0)	4 (-1.1-21.9)	0 (0-0)	0 (0-0)	0 (0-0)	0 (0-0)	129	130	122	109
Aceh	0.3 (0.2-0.4)	0.3 (0.2-0.4)	1.6 (-6-18.6)	0 (0-0.1)	0 (0-0.1)	-20.9 (-29.5--12.2)	0 (0-0)	0 (0-0)	-55.3 (-79.6--8.8)	0 (0-0)	0 (0-0)	-22.6 (-28--2.1)	0 (0-0)	0 (0-0)	-53.2 (-81.2-34.2)	0 (0-0.1)	0 (0-0.1)	1.5 (-6.7-34.8)	0 (0-0)	0 (0-0)	0 (0-0)	0 (0-0)	128	130	121	106
Bali	0.3 (0.2-0.3)	0.3 (0.2-0.4)	1.2 (-2.4-39.6)	0 (0-0.1)	0 (0-0.1)	-27 (-32.6--14.9)	0 (0-0)	0 (0-0)	-65 (-81.3--39.7)	0 (0-0)	0 (0-0)	-27.7 (-32.5--8.5)	0 (0-0)	0 (0-0)	-56.6 (-79.2--7.9)	0 (0-0)	0 (0-0)	1.9 (-5.8-37.4)	0 (0-0)	0 (0-0)	0 (0-0)	0 (0-0)	127	133	106	103
Bangka-Belitung Islands	0.3 (0.2-0.4)	0.3 (0.2-0.4)	1.9 (-17-24.4)	0 (0-0.1)	0 (0-0.1)	-26.4 (-32.7--16.4)	0 (0-0)	0 (0-0)	-49.6 (-72.9--19.3)	0 (0-0)	0 (0-0)	-27.8 (-37.8--6.8)	0 (0-0)	0 (0-0)	-20.3 (-56.6-68.7)	0 (0-0.1)	0 (0-0.1)	1.7 (-16.3-36.8)	0 (0-0)	0 (0-0)	0 (0-0)	0 (0-0)	128	129	112	110
Banten	0.3 (0.2-0.4)	0.3 (0.2-0.4)	3 (-3.6-40.7)	0 (0-0.1)	0 (0-0.1)	-21 (-27.7--6.7)	0 (0-0)	0 (0-0)	-63.6 (-81.1--37.3)	0 (0-0)	0 (0-0)	-21.7 (-26-7.2)	0 (0-0)	0 (0-0)	-58.1 (-80.3--5.8)	0 (0-0.1)	0 (0-0.1)	3.5 (-5.1-48.4)	0 (0-0)	0 (0-0)	0 (0-0)	0 (0-0)	126	129	118	106
Bengkulu	0.3 (0.2-0.4)	0.3 (0.2-0.4)	2.7 (-4.5-61.7)	0 (0-0.1)	0 (0-0.1)	-24.2 (-31.7--1.3)	0 (0-0)	0 (0-0)	-63.3 (-82.4--27.7)	0 (0-0)	0 (0-0)	-25.4 (-29.4-2.5)	0 (0-0)	0 (0-0)	-54.4 (-79.7-10)	0 (0-0.1)	0 (0-0.1)	2.8 (-4.6-48.2)	0 (0-0)	0 (0-0)	0 (0-0)	0 (0-0)	124	129	120	109
Gorontalo	0.3 (0.2-0.4)	0.3 (0.2-0.4)	2.1 (-2.5-20.6)	0 (0-0.1)	0 (0-0.1)	-22.1 (-29.2--12.3)	0 (0-0)	0 (0-0)	-39.4 (-70.8-13.9)	0 (0-0)	0 (0-0)	-27.4 (-32.5--3.9)	0 (0-0)	0 (0-0)	-36.7 (-70.4-46.4)	0 (0-0.1)	0 (0-0.1)	2.1 (-5.7-41.7)	0 (0-0)	0 (0-0)	0 (0-0)	0 (0-0)	123	125	117	109
Jakarta	0.3 (0.2-0.4)	0.3 (0.2-0.4)	1.9 (-10.6-29.4)	0 (0-0.1)	0 (0-0.1)	-22.5 (-29.6--15)	0 (0-0)	0 (0-0)	-62.2 (-77.3--37.2)	0 (0-0)	0 (0-0)	-22.9 (-33.5-6.6)	0 (0-0)	0 (0-0)	-54.6 (-74.7--11.6)	0 (0-0)	0 (0-0)	1.6 (-15.8-51.8)	0 (0-0)	0 (0-0)	0 (0-0)	0 (0-0)	127	129	117	108
Jambi	0.3 (0.2-0.4)	0.3 (0.2-0.4)	2.7 (-4.4-37.3)	0 (0-0.1)	0 (0-0.1)	-24.4 (-32.1--10)	0 (0-0)	0 (0-0)	-58.4 (-76.6--29.5)	0 (0-0)	0 (0-0)	-25.3 (-29.7-1.6)	0 (0-0)	0 (0-0)	-42.5 (-73.5-28.7)	0 (0-0)	0 (0-0)	3.7 (-3.6-51)	0 (0-0)	0 (0-0)	0 (0-0)	0 (0-0)	130	132	119	107
West Java	0.3 (0.2-0.4)	0.3 (0.2-0.4)	4 (-4.1-54.9)	0 (0-0.1)	0 (0-0.1)	-23.9 (-29.9--6.3)	0 (0-0)	0 (0-0)	-56.1 (-74.8--23.9)	0 (0-0)	0 (0-0)	-26.2 (-31.7-6.3)	0 (0-0)	0 (0-0)	-45.5 (-70.6-22.8)	0 (0-0.1)	0 (0-0.1)	4.7 (-5.2-62.9)	0 (0-0)	0 (0-0)	0 (0-0)	0 (0-0)	127	128	116	103
Central Java	0.3 (0.2-0.4)	0.3 (0.2-0.4)	1.4 (-5.8-36.6)	0 (0-0.1)	0 (0-0.1)	-26.9 (-34.3--15.2)	0 (0-0)	0 (0-0)	-59.5 (-78.6--30.6)	0 (0-0)	0 (0-0)	-28.9 (-38.5--3.1)	0 (0-0)	0 (0-0)	-46.2 (-73.5-32.5)	0 (0-0)	0 (0-0)	1.9 (-14.2-50.5)	0 (0-0)	0 (0-0)	0 (0-0)	0 (0-0)	127	129	115	102
East Java	0.3 (0.2-0.4)	0.3 (0.2-0.4)	1.3 (-3.9-11.9)	0 (0-0.1)	0 (0-0)	-25.7 (-31.9--17.9)	0 (0-0)	0 (0-0)	-54.5 (-77.5--32.8)	0 (0-0)	0 (0-0)	-27.3 (-32--7.1)	0 (0-0)	0 (0-0)	-47.3 (-75.3-6.1)	0 (0-0)	0 (0-0)	1.7 (-7-36.1)	0 (0-0)	0 (0-0)	0 (0-0)	0 (0-0)	125	129	113	102
West Kalimantan	0.3 (0.2-0.4)	0.3 (0.2-0.4)	3.9 (-1.2-43.7)	0 (0-0.1)	0 (0-0.1)	-22.9 (-29.5--5.8)	0 (0-0)	0 (0-0)	-59.5 (-77.4--12.3)	0 (0-0)	0 (0-0)	-24.1 (-29.3-17.2)	0 (0-0)	0 (0-0)	-47.4 (-75.8-44.5)	0 (0-0.1)	0 (0-0.1)	4.5 (-4.8-72.3)	0 (0-0)	0 (0-0)	0 (0-0)	0 (0-0)	128	131	122	114
South Kalimantan	0.3 (0.2-0.4)	0.3 (0.2-0.4)	2.6 (-2.8-36.1)	0 (0-0.1)	0 (0-0.1)	-26.5 (-33.2--13.4)	0 (0-0)	0 (0-0)	-60.4 (-77.8--33.1)	0 (0-0)	0 (0-0)	-28.5 (-33--4.6)	0 (0-0)	0 (0-0)	-51.1 (-73.2-31.6)	0 (0-0.1)	0 (0-0.1)	3.1 (-4.5-45.8)	0 (0-0)	0 (0-0)	0 (0-0)	0 (0-0)	127	131	120	107
Central Kalimantan	0.3 (0.2-0.4)	0.3 (0.2-0.4)	2.3 (-2.2-40)	0 (0-0.1)	0 (0-0.1)	-24.8 (-31--12.6)	0 (0-0)	0 (0-0)	-48.9 (-69.9--13)	0 (0-0)	0 (0-0)	-26.5 (-30.9--1.2)	0 (0-0)	0 (0-0)	-25.3 (-62.4-56.2)	0 (0-0.1)	0 (0-0.1)	2.4 (-4.9-44.8)	0 (0-0)	0 (0-0)	0 (0-0)	0 (0-0)	125	129	116	111
East Kalimantan	0.3 (0.2-0.4)	0.3 (0.2-0.4)	2.4 (-2.6-30.7)	0 (0-0.1)	0 (0-0.1)	-19.7 (-24.7--8.1)	0 (0-0)	0 (0-0)	-58.5 (-74.9--33.3)	0 (0-0)	0 (0-0)	-19.6 (-23.9-8.7)	0 (0-0)	0 (0-0)	-53 (-77.2--1.6)	0 (0-0)	0 (0-0)	3 (-5.4-48.3)	0 (0-0)	0 (0-0)	0 (0-0)	0 (0-0)	130	131	115	108
North Kalimantan	0.3 (0.2-0.4)	0.3 (0.2-0.4)	1.7 (-5.4-19.4)	0.1 (0-0.1)	0 (0-0.1)	-24.7 (-33--16.4)	0 (0-0)	0 (0-0)	-64.8 (-77.8--34.5)	0 (0-0)	0 (0-0)	-30.5 (-34.9--11)	0 (0-0)	0 (0-0)	-47.1 (-70.6-24.4)	0 (0-0.1)	0 (0-0.1)	1 (-6.7-37.1)	0 (0-0)	0 (0-0)	0 (0-0)	0 (0-0)	128	128	118	111
Riau Islands	0.3 (0.2-0.4)	0.3 (0.2-0.4)	2.9 (-4-32.9)	0 (0-0.1)	0 (0-0.1)	-15.9 (-22.8--2.6)	0 (0-0)	0 (0-0)	-64 (-79.2--23.4)	0 (0-0)	0 (0-0)	-12.5 (-17.2-17)	0 (0-0)	0 (0-0)	-60.6 (-78.5--1.8)	0 (0-0)	0 (0-0.1)	2.6 (-6.4-43.7)	0 (0-0)	0 (0-0)	0 (0-0)	0 (0-0)	128	128	116	108

Location	Birth Incidence rate (per 100,000 live births), 2000	Birth Incidence rate (per 100,000 live births), 2021	Birth Incidence rate (per 100,000 live births) percent change (2000-2021)	All age rate (per 100,000 population), 2000	All age rate (per 100,000 population), 2021	All age rate (per 100,000 population) percent change (2000-2021)	All age cause-specific SCD mortality rate (per 100,000 population), 2000	All age cause-specific SCD mortality rate (per 100,000 population), 2021	All age cause-specific SCD mortality rate (per 100,000 population) percent change (2000-2021)	All age total SCD mortality rate (per 100,000 population), 2000	All age total SCD mortality rate (per 100,000 population), 2021	All age total SCD mortality rate (per 100,000 population) percent change (2000-2021)	Under-5 cause-specific SCD mortality rate (per 100,000 population), 2000	Under-5 cause-specific SCD mortality rate (per 100,000 population), 2021	Under-5 cause-specific SCD mortality rate (per 100,000 population) percent change (2000-2021)	Under-5 total SCD mortality rate (per 100,000 population), 2000	Under-5 total SCD mortality rate (per 100,000 population), 2021	Under-5 total SCD mortality rate (per 100,000 population) percent change (2000-2021)	Under-5 cause-specific mortality cause fraction, 2000	Under-5 total SCD mortality cause fraction, 2000	Under-5 cause-specific mortality cause fraction, 2021	Under-5 total SCD mortality cause fraction, 2021	Under-5 cause-specific mortality rank 2000	Under-5 cause-specific mortality rank 2021	Under-5 total SCD mortality rank 2000	Under-5 total SCD mortality rank 2021
Lampung	0.3 (0.2-0.4)	0.3 (0.2-0.4)	3 (-8.3-32.3)	0 (0-0.1)	0 (0-0.1)	-28.2 (-35.5--16.5)	0 (0-0)	0 (0-0)	-47.8 (-68.1--5.8)	0 (0-0)	0 (0-0)	-29.2 (-34.1--2.1)	0 (0-0)	0 (0-0)	-18.7 (-55.3-102)	0 (0-0.1)	0 (0-0.1)	3.3 (-5.4-50.6)	0 (0-0)	0 (0-0)	0 (0-0)	0 (0-0)	128	129	113	110
Maluku	0.3 (0.2-0.4)	0.3 (0.2-0.4)	2.8 (-8.5-35.5)	0 (0-0.1)	0 (0-0.1)	-19.9 (-28.5--4.6)	0 (0-0)	0 (0-0)	-62.8 (-80.6--10.5)	0 (0-0)	0 (0-0)	-22.7 (-27.5-11.8)	0 (0-0)	0 (0-0)	-54 (-76.5-23.6)	0 (0-0.1)	0 (0-0.1)	2.4 (-5.8-55.5)	0 (0-0)	0 (0-0)	0 (0-0)	0 (0-0)	129	130	124	110
North Maluku	0.3 (0.2-0.4)	0.3 (0.2-0.4)	2.1 (-4.7-57.7)	0 (0-0.1)	0 (0-0.1)	-25.6 (-34--3.5)	0 (0-0)	0 (0-0)	-53 (-71.7--6.8)	0 (0-0)	0 (0-0)	-28.6 (-32.8--6.9)	0 (0-0)	0 (0-0)	-30.9 (-66.1-73.3)	0 (0-0.1)	0 (0-0.1)	1.7 (-4.9-39.1)	0 (0-0)	0 (0-0)	0 (0-0)	0 (0-0)	128	129	121	115
West Nusa Tenggara	0.3 (0.2-0.4)	0.3 (0.2-0.4)	3.2 (-1.8-38.1)	0 (0-0.1)	0 (0-0.1)	-22.4 (-29--3.8)	0 (0-0)	0 (0-0)	-58.7 (-85.7--23.3)	0 (0-0)	0 (0-0)	-23.3 (-27.7-7.7)	0 (0-0)	0 (0-0)	-55.6 (-81.3-29.1)	0 (0-0.1)	0 (0-0.1)	3.5 (-4.6-54.1)	0 (0-0)	0 (0-0)	0 (0-0)	0 (0-0)	131	131	117	101
East Nusa Tenggara	0.3 (0.2-0.4)	0.3 (0.2-0.4)	2.7 (-7.2-52.4)	0 (0-0.1)	0 (0-0.1)	-20.1 (-28.2-1.3)	0 (0-0)	0 (0-0)	-54.5 (-76.7--12.4)	0 (0-0)	0 (0-0)	-23.6 (-28.3-2.2)	0 (0-0)	0 (0-0)	-46.5 (-74.3-38.3)	0 (0-0.1)	0 (0-0.1)	2.6 (-4.4-46)	0 (0-0)	0 (0-0)	0 (0-0)	0 (0-0)	126	128	116	111
Papua	0.3 (0.2-0.4)	0.3 (0.2-0.4)	2.4 (-4.2-51.3)	0.1 (0-0.1)	0 (0-0.1)	-19.8 (-28.2-3.5)	0 (0-0)	0 (0-0)	-40.7 (-64.7-6.6)	0 (0-0)	0 (0-0)	-21.9 (-25.9-4.3)	0 (0-0)	0 (0-0)	-15.8 (-53.7-75.1)	0 (0-0.1)	0 (0-0.1)	1.8 (-5-40.8)	0 (0-0)	0 (0-0)	0 (0-0)	0 (0-0)	128	131	122	122
West Papua	0.3 (0.2-0.4)	0.3 (0.2-0.4)	2.9 (-6.4-83.9)	0.1 (0-0.1)	0 (0-0.1)	-20.9 (-30.3-14.5)	0 (0-0)	0 (0-0)	-51.5 (-74.1--2.2)	0 (0-0)	0 (0-0)	-21.1 (-25.5-11.6)	0 (0-0)	0 (0-0)	-26 (-65.5-74)	0 (0-0.1)	0 (0-0.1)	3.3 (-4.8-53.3)	0 (0-0)	0 (0-0)	0 (0-0)	0 (0-0)	129	131	117	115
Riau	0.3 (0.2-0.4)	0.3 (0.2-0.4)	2.6 (-1.4-30.1)	0.1 (0-0.1)	0 (0-0.1)	-19.5 (-25--6)	0 (0-0)	0 (0-0)	-68.1 (-81.7--48.2)	0 (0-0)	0 (0-0)	-20.5 (-24.8-8.1)	0 (0-0)	0 (0-0)	-63.8 (-81.4--34)	0 (0-0)	0 (0-0.1)	3 (-4.5-51.3)	0 (0-0)	0 (0-0)	0 (0-0)	0 (0-0)	129	132	122	106
West Sulawesi	0.3 (0.2-0.4)	0.3 (0.2-0.4)	1.9 (-0.9-22.9)	0 (0-0.1)	0 (0-0.1)	-24.2 (-31.8--13.5)	0 (0-0)	0 (0-0)	-54.4 (-72--14.2)	0 (0-0)	0 (0-0)	-28.3 (-32.3--12)	0 (0-0)	0 (0-0)	-33.9 (-66.4-96.6)	0 (0-0.1)	0 (0-0.1)	1.8 (-3.8-28.2)	0 (0-0)	0 (0-0)	0 (0-0)	0 (0-0)	128	129	119	114
South Sulawesi	0.3 (0.2-0.4)	0.3 (0.2-0.4)	2.8 (-4.8-50.7)	0 (0-0.1)	0 (0-0.1)	-25.2 (-33--7.7)	0 (0-0)	0 (0-0)	-51.8 (-73.3--11.6)	0 (0-0)	0 (0-0)	-27.5 (-32.5--4)	0 (0-0)	0 (0-0)	-40.3 (-69.4-48.1)	0 (0-0)	0 (0-0)	3.2 (-5.2-42.8)	0 (0-0)	0 (0-0)	0 (0-0)	0 (0-0)	131	133	114	101
Central Sulawesi	0.3 (0.2-0.4)	0.3 (0.2-0.4)	2.7 (-3.1-43.5)	0 (0-0.1)	0 (0-0.1)	-25.5 (-34.1--8.7)	0 (0-0)	0 (0-0)	-54.7 (-73.8--24.4)	0 (0-0)	0 (0-0)	-28.4 (-33.6--2.4)	0 (0-0)	0 (0-0)	-37 (-65.3-54.3)	0 (0-0.1)	0 (0-0.1)	2.7 (-5.9-46.5)	0 (0-0)	0 (0-0)	0 (0-0)	0 (0-0)	128	131	119	115
Southeast Sulawesi	0.3 (0.2-0.4)	0.3 (0.2-0.4)	2.2 (-2.6-34.3)	0 (0-0.1)	0 (0-0.1)	-22.7 (-29.1--10.4)	0 (0-0)	0 (0-0)	-44.4 (-61.1--14.1)	0 (0-0)	0 (0-0)	-24.7 (-28.8--1.8)	0 (0-0)	0 (0-0)	-15.5 (-52.1-58.5)	0 (0-0.1)	0 (0-0.1)	1.9 (-6.1-40)	0 (0-0)	0 (0-0)	0 (0-0)	0 (0-0)	126	128	117	116
North Sulawesi	0.3 (0.2-0.4)	0.3 (0.2-0.4)	1.3 (-7.1-15.9)	0 (0-0.1)	0 (0-0.1)	-29.4 (-36--23.6)	0 (0-0)	0 (0-0)	-47.4 (-67--14.1)	0 (0-0)	0 (0-0)	-32.5 (-37.4--17.4)	0 (0-0)	0 (0-0)	11.9 (-34.5-86.5)	0 (0-0)	0 (0-0)	1.1 (-7.2-29.6)	0 (0-0)	0 (0-0)	0 (0-0)	0 (0-0)	129	128	108	114
West Sumatra	0.3 (0.2-0.4)	0.3 (0.2-0.4)	0.8 (-4.7-22.9)	0 (0-0.1)	0 (0-0.1)	-23.9 (-32.5--15)	0 (0-0)	0 (0-0)	-65.6 (-83--36)	0 (0-0)	0 (0-0)	-27.4 (-41.9--4.5)	0 (0-0)	0 (0-0)	-59.4 (-82-4.5)	0 (0-0.1)	0 (0-0.1)	0.6 (-22.4-40.9)	0 (0-0)	0 (0-0)	0 (0-0)	0 (0-0)	123	128	117	105
South Sumatra	0.3 (0.2-0.4)	0.3 (0.2-0.4)	2.2 (-7.2-22.8)	0 (0-0.1)	0 (0-0.1)	-25.5 (-37.3--15.9)	0 (0-0)	0 (0-0)	-59.4 (-76.7--21.8)	0 (0-0)	0 (0-0)	-26.4 (-34.9--2.6)	0 (0-0)	0 (0-0)	-51.1 (-73-11.5)	0 (0-0.1)	0 (0-0.1)	2.3 (-10.9-45.5)	0 (0-0)	0 (0-0)	0 (0-0)	0 (0-0)	125	127	117	106
North Sumatra	0.3 (0.2-0.4)	0.3 (0.2-0.4)	2.3 (-2.9-24)	0 (0-0.1)	0 (0-0.1)	-22.9 (-30.4--11.5)	0 (0-0)	0 (0-0)	-42.6 (-65.9--12.9)	0 (0-0)	0 (0-0)	-24.6 (-29.4-0.3)	0 (0-0)	0 (0-0)	-16.9 (-51.2-52.2)	0 (0-0.1)	0 (0-0.1)	2.3 (-7.6-44)	0 (0-0)	0 (0-0)	0 (0-0)	0 (0-0)	123	126	113	112
Yogyakarta	0.2 (0.2-0.3)	0.3 (0.2-0.3)	1.9 (-3.6-19.6)	0 (0-0.1)	0 (0-0.1)	-22 (-27.7-1.2)	0 (0-0)	0 (0-0)	-41 (-64.2--6.1)	0 (0-0)	0 (0-0)	-22.1 (-26.4--3.1)	0 (0-0)	0 (0-0)	-35.2 (-65.3-31.3)	0 (0-0)	0 (0-0)	2.2 (-6.2-37.4)	0 (0-0)	0 (0-0)	0 (0-0)	0 (0-0)	128	130	103	99
Lao People's Democratic Republic	0.3 (0.2-0.4)	0.3 (0.2-0.4)	1.4 (-2.8-35.9)	0 (0-0.1)	0 (0-0.1)	-22.4 (-28.5--2.4)	0 (0-0)	0 (0-0)	-64.3 (-78.8--23.1)	0 (0-0)	0 (0-0)	-24.9 (-27.4-1.3)	0 (0-0)	0 (0-0)	-63.8 (-80.3--8.4)	0 (0-0.1)	0 (0-0.1)	1.8 (-5.4-41.9)	0 (0-0)	0 (0-0)	0 (0-0)	0 (0-0)	136	136	121	110
Malaysia	0.2 (0.2-0.3)	0.2 (0.2-0.3)	1.1 (-7.1-12.9)	0 (0-0.1)	0 (0-0.1)	-21.2 (-28--14.1)	0 (0-0)	0 (0-0)	-36.9 (-61.7-9.8)	0 (0-0)	0 (0-0)	-24 (-33.4-9)	0 (0-0)	0 (0-0)	-60.6 (-80.6--28.3)	0 (0-0)	0 (0-0)	1.8 (-14.5-56.3)	0 (0-0)	0 (0-0)	0 (0-0)	0 (0-0)	133	135	98	93
Maldives	0.2 (0.2-0.3)	0.3 (0.2-0.3)	1.5 (-1.6-22.7)	0 (0-0.1)	0 (0-0.1)	-33.7 (-42.2--21)	0 (0-0)	0 (0-0)	-61.1 (-79.6--40.9)	0 (0-0)	0 (0-0)	-38.4 (-43.8--19.5)	0 (0-0)	0 (0-0)	-66.1 (-81.4--18.5)	0 (0-0)	0 (0-0)	1.5 (-8.3-38.3)	0 (0-0)	0 (0-0)	0 (0-0)	0 (0-0)	132	132	102	84
Mauritius	0.2 (0.2-0.3)	0.2 (0.2-0.3)	1.4 (-5.7-34.9)	0 (0-0.1)	0 (0-0.1)	-31.3 (-37.9--17.7)	0.3 (0.3-0.3)	0.1 (0.1-0.1)	-69.5 (-73.6--65.2)	0 (0-0)	0 (0-0)	-33.6 (-40.7--15.3)	0.2 (0.2-0.3)	0.1 (0-0.1)	-78.1 (-84.7--71.1)	0 (0-0)	0 (0-0)	2.5 (-12.1-41.7)	0.1 (0.1-0.1)	0 (0-0)	0 (0-0)	0 (0-0)	49	73	89	87
Myanmar	0.3 (0.2-0.4)	0.3 (0.2-0.4)	1.5 (-1.1-14.1)	0 (0-0.1)	0 (0-0.1)	-19.7 (-26.7-4.5)	0 (0-0)	0 (0-0)	-67 (-79.4--35.8)	0 (0-0)	0 (0-0)	-22.1 (-26.1--0.4)	0 (0-0)	0 (0-0)	-64.5 (-79.6--9.3)	0 (0-0.1)	0 (0-0.1)	1.5 (-3.8-33.2)	0 (0-0)	0 (0-0)	0 (0-0)	0 (0-0)	133	137	123	115
Philippines	0.3 (0.2-0.3)	0.3 (0.2-0.3)	1.1 (-0.5-8.8)	0.1 (0-0.1)	0 (0-0.1)	-15.1 (-17.4--11)	0 (0-0)	0 (0-0)	-42.3 (-68.1--8.2)	0 (0-0)	0 (0-0)	-16 (-18.3--10.2)	0 (0-0)	0 (0-0)	-54.9 (-81.4--11.7)	0 (0-0)	0 (0-0)	1.2 (-1.2-9.1)	0 (0-0)	0 (0-0)	0 (0-0)	0 (0-0)	129	131	115	112
Abra	0.2 (0.2-0.3)	0.2 (0.2-0.3)	1.6 (-8.5-33.6)	0.1 (0-0.1)	0 (0-0.1)	-16.9 (-27.6--1.4)	0 (0-0)	0 (0-0)	-4.3 (-53.7-62.7)	0 (0-0)	0 (0-0)	-17.7 (-26.1-8.2)	0 (0-0)	0 (0-0)	-54.8 (-82.1-6.3)	0 (0-0)	0 (0-0)	1.8 (-9.8-43.4)	0 (0-0)	0 (0-0)	0 (0-0)	0 (0-0)	130	132	105	96
Agusan Del Norte	0.3 (0.2-0.4)	0.3 (0.2-0.4)	1.7 (-1.7-16.4)	0 (0-0.1)	0 (0-0.1)	-14.5 (-20.6--5.8)	0 (0-0)	0 (0-0)	-63 (-82.4--39.2)	0 (0-0)	0 (0-0)	-15 (-18-12.8)	0 (0-0)	0 (0-0)	-62.4 (-83.2--30)	0 (0-0)	0 (0-0.1)	1.8 (-6.3-43.4)	0 (0-0)	0 (0-0)	0 (0-0)	0 (0-0)	125	127	118	107
Agusan Del Sur	0.3 (0.2-0.4)	0.3 (0.2-0.4)	0.9 (-2.5-30.6)	0.1 (0-0.1)	0 (0-0.1)	-17 (-23.1--4.2)	0 (0-0)	0 (0-0)	-6.4 (-48.8-54.9)	0 (0-0)	0 (0-0)	-18.4 (-29.9-11.3)	0 (0-0)	0 (0-0)	-8.2 (-63.7-113)	0 (0-0.1)	0 (0-0.1)	0.4 (-16-41.7)	0 (0-0)	0 (0-0)	0 (0-0)	0 (0-0)	130	133	118	118
Aklan	0.3 (0.2-0.3)	0.3 (0.2-0.3)	1.7 (-5.1-35)	0 (0-0.1)	0 (0-0.1)	-12.2 (-17.7-6.5)	0 (0-0)	0 (0-0)	-21.9 (-61.8-29.8)	0 (0-0)	0 (0-0)	-11.3 (-14-16.8)	0 (0-0)	0 (0-0)	-37.2 (-74.8-35)	0 (0-0)	0 (0-0)	2.3 (-5.3-40.5)	0 (0-0)	0 (0-0)	0 (0-0)	0 (0-0)	125	128	111	111
Albay	0.3 (0.2-0.3)	0.3 (0.2-0.3)	0.5 (-0.6-10)	0.1 (0-0.1)	0 (0-0.1)	-16.1 (-20.4--10.1)	0 (0-0)	0 (0-0)	-50.1 (-72.8--20.2)	0 (0-0)	0 (0-0)	-17.3 (-19.3--5.3)	0 (0-0)	0 (0-0)	-59 (-83.7--14.1)	0 (0-0)	0 (0-0)	0.6 (-5.4-17.9)	0 (0-0)	0 (0-0)	0 (0-0)	0 (0-0)	126	128	108	100
Antique	0.3 (0.2-0.3)	0.3 (0.2-0.4)	1.5 (-2.1-30.6)	0.1 (0-0.1)	0 (0-0.1)	-13 (-19.4-3.6)	0 (0-0)	0 (0-0)	-38.3 (-70.1-7.4)	0 (0-0)	0 (0-0)	-13.6 (-16.7-15.7)	0 (0-0)	0 (0-0)	-60.6 (-86.7--9.8)	0 (0-0)	0 (0-0)	1.8 (-5.6-41.1)	0 (0-0)	0 (0-0)	0 (0-0)	0 (0-0)	124	128	114	103

Location	Birth Incidence rate (per 100,000 live births), 2000	Birth Incidence rate (per 100,000 live births), 2021	Birth Incidence rate (per 100,000 live births) percent change (2000-2021)	All age rate (per 100,000 population), 2000	All age rate (per 100,000 population), 2021	All age rate (per 100,000 population) percent change (2000-2021)	All age cause-specific SCD mortality rate (per 100,000 population), 2000	All age cause-specific SCD mortality rate (per 100,000 population), 2021	All age cause-specific SCD mortality rate (per 100,000 population) percent change (2000-2021)	All age total SCD mortality rate (per 100,000 population), 2000	All age total SCD mortality rate (per 100,000 population), 2021	All age total SCD mortality rate (per 100,000 population) percent change (2000-2021)	Under-5 cause-specific SCD mortality rate (per 100,000 population), 2000	Under-5 cause-specific SCD mortality rate (per 100,000 population), 2021	Under-5 cause-specific SCD mortality rate (per 100,000 population) percent change (2000-2021)	Under-5 total SCD mortality rate (per 100,000 population), 2000	Under-5 total SCD mortality rate (per 100,000 population), 2021	Under-5 total SCD mortality rate (per 100,000 population) percent change (2000-2021)	Under-5 cause-specific mortality cause fraction, 2000	Under-5 total SCD mortality cause fraction, 2000	Under-5 cause-specific mortality cause fraction, 2021	Under-5 total SCD mortality cause fraction, 2021	Under-5 cause-specific mortality rank 2000	Under-5 cause-specific mortality rank 2021	Under-5 total SCD mortality rank 2000	Under-5 total SCD mortality rank 2021
Apayao	0.3 (0.2-0.4)	0.3 (0.2-0.4)	0.8 (-7.7-16)	0.1 (0-0.1)	0 (0-0.1)	-22.1 (-28.6--13.9)	0 (0-0)	0 (0-0)	-49.3 (-76.4--9.7)	0 (0-0)	0 (0-0)	-24.4 (-34.1-2.5)	0 (0-0)	0 (0-0)	-63.2 (-85.9-19.4)	0 (0-0.1)	0 (0-0.1)	0.5 (-14.3-44.5)	0 (0-0)	0 (0-0)	0 (0-0)	0 (0-0)	124	127	117	109
Aurora	0.3 (0.2-0.3)	0.3 (0.2-0.4)	1.5 (-2.5-18.7)	0.1 (0-0.1)	0 (0-0.1)	-17.1 (-22.9--6.2)	0 (0-0)	0 (0-0)	-50.6 (-77.6--9.5)	0 (0-0)	0 (0-0)	-17.4 (-21.1-11.5)	0 (0-0)	0 (0-0)	-74.6 (-90.8--38.5)	0 (0-0)	0 (0-0)	1.5 (-4.9-44.8)	0 (0-0)	0 (0-0)	0 (0-0)	0 (0-0)	124	127	119	100
Basilan	0.3 (0.2-0.3)	0.3 (0.2-0.3)	1.1 (-2.9-28.9)	0.1 (0-0.1)	0.1 (0-0.1)	-11.5 (-16.5-2.1)	0 (0-0)	0 (0-0)	-50.9 (-78.8--5.2)	0 (0-0)	0 (0-0)	-12.5 (-15.2-6.2)	0 (0-0)	0 (0-0)	-62.3 (-86.4-9.3)	0 (0-0)	0 (0-0)	1.2 (-6.6-28)	0 (0-0)	0 (0-0)	0 (0-0)	0 (0-0)	127	130	105	93
Bataan	0.3 (0.2-0.3)	0.3 (0.2-0.3)	1.2 (-12.5-42)	0.1 (0-0.1)	0 (0-0.1)	-17.8 (-26.3--0.1)	0 (0-0)	0 (0-0)	-24.5 (-62.2-37.1)	0 (0-0)	0 (0-0)	-18 (-20.8-7.6)	0 (0-0)	0 (0-0)	-28.8 (-73.8-80.2)	0 (0-0)	0 (0-0)	1.4 (-5.2-40.2)	0 (0-0)	0 (0-0)	0 (0-0)	0 (0-0)	129	129	111	111
Batanes	0.3 (0.2-0.3)	0.3 (0.2-0.3)	0.7 (-3.6-22.5)	0 (0-0.1)	0 (0-0.1)	-21.7 (-26.8--11.8)	0 (0-0)	0 (0-0)	-44.7 (-73.4-2)	0 (0-0)	0 (0-0)	-23.1 (-27--16.6)	0 (0-0)	0 (0-0)	-58.9 (-83.6-4.9)	0 (0-0)	0 (0-0)	0.4 (-7.4-10.2)	0 (0-0)	0 (0-0)	0 (0-0)	0 (0-0)	125	127	106	97
Batangas	0.2 (0.2-0.3)	0.3 (0.2-0.3)	0.8 (-11.4-14.2)	0.1 (0-0.1)	0 (0-0.1)	-14 (-20.8--5.9)	0 (0-0)	0 (0-0)	-41 (-70.2--2.1)	0 (0-0)	0 (0-0)	-14 (-24.9-7.7)	0 (0-0)	0 (0-0)	-63.1 (-83.5--1.9)	0 (0-0)	0 (0-0)	1.1 (-14.7-31.1)	0 (0-0)	0 (0-0)	0 (0-0)	0 (0-0)	127	129	109	98
Benguet	0.2 (0.2-0.3)	0.2 (0.2-0.3)	0.7 (-8.4-24)	0.1 (0-0.1)	0 (0-0.1)	-13.8 (-21.3--1.5)	0 (0-0)	0 (0-0)	-67.9 (-83.2--43.2)	0 (0-0)	0 (0-0)	-13.6 (-22.4-9.7)	0 (0-0)	0 (0-0)	-80.6 (-92.1--55)	0 (0-0)	0 (0-0)	1.1 (-11-35.1)	0 (0-0)	0 (0-0)	0 (0-0)	0 (0-0)	127	130	113	94
Biliran	0.2 (0.2-0.3)	0.2 (0.2-0.3)	0.8 (-3.3-34.2)	0.1 (0-0.1)	0 (0-0.1)	-14.1 (-20.3-4.3)	0 (0-0)	0 (0-0)	-19.7 (-57.2-33.7)	0 (0-0)	0 (0-0)	-13.7 (-16.4--4.8)	0 (0-0)	0 (0-0)	-59.2 (-83.4--12.7)	0 (0-0)	0 (0-0)	1 (-7.1-19)	0 (0-0)	0 (0-0)	0 (0-0)	0 (0-0)	123	130	105	95
Bohol	0.3 (0.2-0.3)	0.3 (0.2-0.3)	1.6 (-3.4-40.3)	0.1 (0-0.1)	0 (0-0.1)	-19.7 (-28.7--2.1)	0 (0-0)	0 (0-0)	-29.7 (-64-24.9)	0 (0-0)	0 (0-0)	-22 (-25.7-5.2)	0 (0-0)	0 (0-0)	-42.8 (-78-16.4)	0 (0-0)	0 (0-0)	1.7 (-5.1-44.6)	0 (0-0)	0 (0-0)	0 (0-0)	0 (0-0)	130	131	111	111
Bukidnon	0.3 (0.2-0.4)	0.3 (0.2-0.4)	2.1 (-10.7-23.6)	0.1 (0-0.1)	0 (0-0.1)	-19.5 (-30.1--8.8)	0 (0-0)	0 (0-0)	-24.5 (-64.7-39.3)	0 (0-0)	0 (0-0)	-21 (-30-11)	0 (0-0)	0 (0-0)	-27.2 (-73.4-72.8)	0 (0-0.1)	0 (0-0.1)	2.1 (-12.8-49.9)	0 (0-0)	0 (0-0)	0 (0-0)	0 (0-0)	127	130	115	118
Bulacan	0.3 (0.2-0.3)	0.3 (0.2-0.3)	0.9 (-3.5-39.8)	0.1 (0-0.1)	0 (0-0.1)	-18.3 (-24--4)	0 (0-0)	0 (0-0)	-22.8 (-61.6-26)	0 (0-0)	0 (0-0)	-20 (-23--2.5)	0 (0-0)	0 (0-0)	-45.8 (-75.9-32.4)	0 (0-0)	0 (0-0)	1.2 (-5.5-30.9)	0 (0-0)	0 (0-0)	0 (0-0)	0 (0-0)	128	132	107	103
Cagayan	0.3 (0.2-0.4)	0.3 (0.2-0.4)	1.2 (-6.1-8.6)	0 (0-0.1)	0 (0-0.1)	-18.3 (-24.2--10)	0 (0-0)	0 (0-0)	-43.1 (-73.2--3.4)	0 (0-0)	0 (0-0)	-19.2 (-28.6-5.8)	0 (0-0)	0 (0-0)	-56.9 (-84.7--7.5)	0 (0-0)	0 (0-0)	1.3 (-13-37.1)	0 (0-0)	0 (0-0)	0 (0-0)	0 (0-0)	125	127	117	112
Camarines Norte	0.3 (0.2-0.3)	0.3 (0.2-0.4)	1.1 (-1.8-23.7)	0.1 (0-0.1)	0 (0-0.1)	-14.8 (-19.1--6.3)	0 (0-0)	0 (0-0)	-5.5 (-48.9-67.7)	0 (0-0)	0 (0-0)	-15.4 (-18-8.2)	0 (0-0)	0 (0-0)	-5.8 (-63.3-97.5)	0 (0-0)	0 (0-0)	1.1 (-6.8-36.3)	0 (0-0)	0 (0-0)	0 (0-0)	0 (0-0)	127	127	105	110
Camarines Sur	0.3 (0.2-0.4)	0.3 (0.2-0.4)	1.1 (-1.6-10.2)	0.1 (0-0.1)	0 (0-0.1)	-13.3 (-18.9--4.7)	0 (0-0)	0 (0-0)	-36.6 (-71.5-9.2)	0 (0-0)	0 (0-0)	-14.7 (-17.1-4.8)	0 (0-0)	0 (0-0)	-49.9 (-80.2-16)	0 (0-0)	0 (0-0)	1 (-4.7-29.4)	0 (0-0)	0 (0-0)	0 (0-0)	0 (0-0)	129	128	116	114
Camiguin	0.2 (0.2-0.3)	0.2 (0.2-0.3)	1.2 (-4.7-47.3)	0.1 (0-0.1)	0 (0-0.1)	-12.8 (-17.8-3.6)	0 (0-0)	0 (0-0)	-40.4 (-74.4-9.8)	0 (0-0)	0 (0-0)	-11.8 (-22.9-21.6)	0 (0-0)	0 (0-0)	-75.2 (-92.6--16.3)	0 (0-0)	0 (0-0)	2.1 (-11.1-47.5)	0 (0-0)	0 (0-0)	0 (0-0)	0 (0-0)	124	127	107	91
Capiz	0.3 (0.2-0.3)	0.3 (0.2-0.4)	2.4 (-0.6-24.6)	0 (0-0.1)	0 (0-0.1)	-16 (-20.3--4.8)	0 (0-0)	0 (0-0)	-40.4 (-68.1-3.3)	0 (0-0)	0 (0-0)	-16.1 (-19.9-12.1)	0 (0-0)	0 (0-0)	-46 (-81.2-12.6)	0 (0-0)	0 (0-0)	3.2 (-6.2-46.5)	0 (0-0)	0 (0-0)	0 (0-0)	0 (0-0)	126	130	113	112
Catanduanes	0.3 (0.2-0.4)	0.3 (0.2-0.4)	1.9 (-4.6-23.2)	0.1 (0-0.1)	0 (0-0.1)	-15.9 (-22.8--6)	0 (0-0)	0 (0-0)	-30.4 (-61.4-12.7)	0 (0-0)	0 (0-0)	-17 (-25.7-11.1)	0 (0-0)	0 (0-0)	-31 (-70-39.8)	0 (0-0.1)	0 (0-0.1)	1.7 (-8.7-40)	0 (0-0)	0 (0-0)	0 (0-0)	0 (0-0)	124	125	117	117
Cavite	0.2 (0.2-0.3)	0.3 (0.2-0.3)	2 (-4.1-43.6)	0.1 (0-0.1)	0 (0-0.1)	-15.9 (-21.6-4.9)	0 (0-0)	0 (0-0)	-20.3 (-60.3-40.4)	0 (0-0)	0 (0-0)	-16.5 (-20.3-16)	0 (0-0)	0 (0-0)	-44 (-79.5-27)	0 (0-0)	0 (0-0)	2.6 (-6.8-52.9)	0 (0-0)	0 (0-0)	0 (0-0)	0 (0-0)	127	130	107	99
Cebu	0.3 (0.2-0.3)	0.3 (0.2-0.3)	1.1 (-3.9-40.5)	0 (0-0.1)	0 (0-0.1)	-10.5 (-16.8-12.1)	0 (0-0)	0 (0-0)	-50.8 (-77.6--13.1)	0 (0-0)	0 (0-0)	-9.5 (-11.4-18.7)	0 (0-0)	0 (0-0)	-72.3 (-90.4--24.9)	0 (0-0)	0 (0-0)	1.5 (-5.8-44.2)	0 (0-0)	0 (0-0)	0 (0-0)	0 (0-0)	129	129	116	101
Cotabato (North Cotabato)	0.3 (0.2-0.3)	0.3 (0.2-0.4)	1.2 (-10.2-8.1)	0.1 (0-0.1)	0 (0-0.1)	-12.2 (-18.7--8)	0 (0-0)	0 (0-0)	-33.8 (-66.9-19.2)	0 (0-0)	0 (0-0)	-11.8 (-17.5-17.8)	0 (0-0)	0 (0-0)	-58.2 (-84.8-9.3)	0 (0-0)	0 (0-0)	1.6 (-9.9-42.5)	0 (0-0)	0 (0-0)	0 (0-0)	0 (0-0)	126	129	114	112
Davao de Oro	0.3 (0.2-0.3)	0.3 (0.2-0.3)	0.3 (-5.5-22.5)	0.1 (0-0.1)	0 (0-0.1)	-18.2 (-24.2--10.9)	0 (0-0)	0 (0-0)	-7.4 (-54.5-53.6)	0 (0-0)	0 (0-0)	-18.6 (-24--4.8)	0 (0-0)	0 (0-0)	-30.7 (-72.2-70.6)	0 (0-0)	0 (0-0)	0.9 (-7.2-23)	0 (0-0)	0 (0-0)	0 (0-0)	0 (0-0)	129	131	112	112
Davao Del Norte	0.3 (0.2-0.3)	0.3 (0.2-0.3)	1.6 (-2.4-31.9)	0.1 (0-0.1)	0 (0-0.1)	-20.6 (-25.6--6.9)	0 (0-0)	0 (0-0)	-21.4 (-60.5-28.3)	0 (0-0)	0 (0-0)	-20.7 (-24.7-3.7)	0 (0-0)	0 (0-0)	-29.7 (-73.9-72)	0 (0-0)	0 (0-0)	2.3 (-5.1-42.8)	0 (0-0)	0 (0-0)	0 (0-0)	0 (0-0)	128	132	112	108
Davao Del Sur	0.3 (0.2-0.3)	0.3 (0.2-0.3)	0.9 (-1.9-19.8)	0 (0-0.1)	0 (0-0.1)	-15.5 (-20.4--6.1)	0 (0-0)	0 (0-0)	-84.7 (-92.1--75)	0 (0-0)	0 (0-0)	-15.1 (-17.4-11.2)	0 (0-0)	0 (0-0)	-90.6 (-96.5--79.1)	0 (0-0)	0 (0-0)	1.1 (-5.6-38.6)	0 (0-0)	0 (0-0)	0 (0-0)	0 (0-0.1)	129	129	118	85
Davao Occidental	0.3 (0.2-0.3)	0.3 (0.2-0.4)	1.4 (-1-28.6)	0.1 (0-0.1)	0 (0-0.1)	-14.4 (-21-0.3)	0 (0-0)	0 (0-0)	-6.6 (-56.7-54.9)	0 (0-0)	0 (0-0)	-18.5 (-23.8-9)	0 (0-0)	0 (0-0)	-29.8 (-69-46)	0 (0-0)	0 (0-0)	0.9 (-7.3-40.3)	0 (0-0)	0 (0-0)	0 (0-0)	0 (0-0)	124	128	116	111
Davao Oriental	0.3 (0.2-0.4)	0.3 (0.2-0.4)	1.3 (-2.3-12.4)	0.1 (0-0.1)	0 (0-0.1)	-21.2 (-26.5--15.7)	0 (0-0)	0 (0-0)	-4.5 (-50.8-67.7)	0 (0-0)	0 (0-0)	-24.4 (-27.9--5.5)	0 (0-0)	0 (0-0)	-4.6 (-57.5-141)	0 (0-0)	0 (0-0)	1 (-6.5-32.5)	0 (0-0)	0 (0-0)	0 (0-0)	0 (0-0)	129	130	113	117
Dinagat Islands	0.3 (0.2-0.3)	0.3 (0.2-0.4)	1.4 (-6.7-18.4)	0.1 (0-0.1)	0 (0-0.1)	-19.8 (-25.6--8.6)	0 (0-0)	0 (0-0)	14.4 (-41.6-112)	0 (0-0)	0 (0-0)	-21.5 (-28.8-3.1)	0 (0-0)	0 (0-0)	-41.5 (-77.2-39.8)	0 (0-0)	0 (0-0)	1.7 (-8.2-40.2)	0 (0-0)	0 (0-0)	0 (0-0)	0 (0-0)	127	131	111	105
Eastern Samar	0.3 (0.2-0.4)	0.3 (0.2-0.4)	1.5 (-6.3-19.1)	0.1 (0-0.1)	0 (0-0.1)	-15.2 (-24--4.6)	0 (0-0)	0 (0-0)	-24.7 (-62.1-33.7)	0 (0-0)	0 (0-0)	-17.2 (-22.8-8.4)	0 (0-0)	0 (0-0)	-28.4 (-70.3-59.3)	0 (0-0.1)	0 (0-0.1)	1.6 (-5.3-37.6)	0 (0-0)	0 (0-0)	0 (0-0)	0 (0-0)	126	132	113	114
Guimaras	0.2 (0.2-0.3)	0.3 (0.2-0.3)	1.1 (-30.3-31.3)	0.1 (0-0.1)	0 (0-0.1)	-17.2 (-33--5)	0 (0-0)	0 (0-0)	-25.8 (-63.4-30.4)	0 (0-0)	0 (0-0)	-16.5 (-20-8.7)	0 (0-0)	0 (0-0)	-56.5 (-85.1-7.4)	0 (0-0)	0 (0-0)	1.3 (-6.1-39)	0 (0-0)	0 (0-0)	0 (0-0)	0 (0-0)	127	133	105	93
Ifugao	0.3 (0.2-0.4)	0.3 (0.2-0.3)	1.6 (-3.3-30.7)	0.1 (0-0.1)	0 (0-0.1)	-11.9 (-17.4-9.2)	0 (0-0)	0 (0-0)	-43.4 (-70.7-0.1)	0 (0-0)	0 (0-0)	-10.1 (-13.1-17.5)	0 (0-0)	0 (0-0)	-54.5 (-84.4-11.6)	0 (0-0)	0 (0-0)	2 (-5.2-40.1)	0 (0-0)	0 (0-0)	0 (0-0)	0 (0-0)	126	131	114	108

Location	Birth Incidence rate (per 100,000 live births), 2000	Birth Incidence rate (per 100,000 live births), 2021	Birth Incidence rate (per 100,000 live births) percent change (2000-2021)	All age rate (per 100,000 population), 2000	All age rate (per 100,000 population), 2021	All age rate (per 100,000 population) percent change (2000-2021)	All age cause-specific SCD mortality rate (per 100,000 population), 2000	All age cause-specific SCD mortality rate (per 100,000 population), 2021	All age cause-specific SCD mortality rate (per 100,000 population) percent change (2000-2021)	All age total SCD mortality rate (per 100,000 population), 2000	All age total SCD mortality rate (per 100,000 population), 2021	All age total SCD mortality rate (per 100,000 population) percent change (2000-2021)	Under-5 cause-specific SCD mortality rate (per 100,000 population), 2000	Under-5 cause-specific SCD mortality rate (per 100,000 population), 2021	Under-5 cause-specific SCD mortality rate (per 100,000 population) percent change (2000-2021)	Under-5 total SCD mortality rate (per 100,000 population), 2000	Under-5 total SCD mortality rate (per 100,000 population), 2021	Under-5 total SCD mortality rate (per 100,000 population) percent change (2000-2021)	Under-5 cause-specific mortality cause fraction, 2000	Under-5 total SCD mortality cause fraction, 2000	Under-5 cause-specific mortality cause fraction, 2021	Under-5 total SCD mortality cause fraction, 2021	Under-5 cause-specific mortality rank 2000	Under-5 cause-specific mortality rank 2021	Under-5 total SCD mortality rank 2000	Under-5 total SCD mortality rank 2021
Ilocos Norte	0.3 (0.2-0.3)	0.3 (0.2-0.3)	1.3 (-9.7-39.6)	0 (0-0.1)	0 (0-0.1)	-13.7 (-21.2-6)	0 (0-0)	0 (0-0)	-43.6 (-74.8--1.6)	0 (0-0)	0 (0-0)	-12.3 (-14.4-15.2)	0 (0-0)	0 (0-0)	-70.4 (-88.3--28)	0 (0-0)	0 (0-0)	1.7 (-6-41.5)	0 (0-0)	0 (0-0)	0 (0-0)	0 (0-0)	127	128	113	100
Ilocos Sur	0.3 (0.2-0.3)	0.3 (0.2-0.3)	1.1 (-28.8-31.1)	0 (0-0.1)	0 (0-0.1)	-14.8 (-30.4--0.4)	0 (0-0)	0 (0-0)	-40 (-71.8-6.4)	0 (0-0)	0 (0-0)	-13.9 (-16-10.3)	0 (0-0)	0 (0-0)	-64.1 (-85--3.1)	0 (0-0)	0 (0-0)	1.1 (-7.2-36.7)	0 (0-0)	0 (0-0)	0 (0-0)	0 (0-0)	127	129	109	101
Iloilo	0.3 (0.2-0.3)	0.3 (0.2-0.3)	1.7 (-3.8-35.5)	0 (0-0.1)	0 (0-0.1)	-11.3 (-16.7-14.6)	0 (0-0)	0 (0-0)	-41.2 (-69.4--4.3)	0 (0-0)	0 (0-0)	-10.1 (-12.9-16.8)	0 (0-0)	0 (0-0)	-61.6 (-82.8--13.2)	0 (0-0)	0 (0-0)	2.3 (-6.4-42.6)	0 (0-0)	0 (0-0)	0 (0-0)	0 (0-0)	127	131	109	99
Isabela	0.3 (0.2-0.3)	0.3 (0.2-0.3)	0.8 (-1.3-5.8)	0 (0-0.1)	0 (0-0.1)	-15 (-21.1--8.8)	0 (0-0)	0 (0-0)	-30.3 (-63-14.9)	0 (0-0)	0 (0-0)	-14.2 (-16.2--1.7)	0 (0-0)	0 (0-0)	-58.3 (-81.9--9.9)	0 (0-0)	0 (0-0)	1 (-5.2-21.2)	0 (0-0)	0 (0-0)	0 (0-0)	0 (0-0)	124	125	113	98
Kalinga	0.2 (0.2-0.3)	0.3 (0.2-0.3)	1.2 (-2.1-27)	0.1 (0-0.1)	0 (0-0.1)	-16.4 (-21.3--6.1)	0 (0-0)	0 (0-0)	5.3 (-53.2-84.3)	0 (0-0)	0 (0-0)	-16.1 (-19.1-7.4)	0 (0-0)	0 (0-0)	-56.5 (-86.7-12.8)	0 (0-0)	0 (0-0)	2.3 (-6.3-36.4)	0 (0-0)	0 (0-0)	0 (0-0)	0 (0-0)	122	123	104	94
La Union	0.3 (0.2-0.3)	0.3 (0.2-0.3)	1 (-2.4-18.3)	0 (0-0.1)	0 (0-0.1)	-16.2 (-20.7-0.8)	0 (0-0)	0 (0-0)	-43.3 (-74.8--4.1)	0 (0-0)	0 (0-0)	-15.8 (-17.7--3.1)	0 (0-0)	0 (0-0)	-62.6 (-87.4--5)	0 (0-0)	0 (0-0)	1.3 (-4.3-20.7)	0 (0-0)	0 (0-0)	0 (0-0)	0 (0-0)	122	126	114	103
Laguna	0.3 (0.2-0.3)	0.3 (0.2-0.3)	1.8 (-3.4-31.6)	0.1 (0-0.1)	0 (0-0.1)	-12.9 (-17.7-6.4)	0 (0-0)	0 (0-0)	-34 (-66.4-13.2)	0 (0-0)	0 (0-0)	-11.5 (-14.4-18.9)	0 (0-0)	0 (0-0)	-56.3 (-79.8--12)	0 (0-0)	0 (0-0)	2.4 (-6-44)	0 (0-0)	0 (0-0)	0 (0-0)	0 (0-0)	127	130	109	101
Lanao Del Norte	0.3 (0.2-0.3)	0.3 (0.2-0.3)	1.4 (-3.4-38.5)	0.1 (0-0.1)	0 (0-0.1)	-10.7 (-16.9-8.6)	0 (0-0)	0 (0-0)	-70 (-84--51.6)	0 (0-0)	0 (0-0)	-10.4 (-13.1-15.6)	0 (0-0)	0 (0-0)	-75.1 (-90.8--44.5)	0 (0-0)	0 (0-0)	2 (-5.8-36.5)	0 (0-0)	0 (0-0)	0 (0-0)	0 (0-0)	126	126	114	95
Lanao Del Sur	0.3 (0.2-0.4)	0.3 (0.2-0.4)	0.6 (-3.7-2.8)	0.1 (0-0.1)	0.1 (0-0.1)	-9.3 (-15.6--5.7)	0 (0-0)	0 (0-0)	-56.9 (-82--13.9)	0 (0-0)	0 (0-0)	-9.1 (-14.1-9.8)	0 (0-0)	0 (0-0)	-62.7 (-86.3-65.4)	0 (0-0)	0 (0-0)	1.2 (-5.9-25.3)	0 (0-0)	0 (0-0)	0 (0-0)	0 (0-0)	126	130	120	113
Leyte	0.3 (0.2-0.4)	0.3 (0.2-0.4)	1.1 (-4.2-5.9)	0.1 (0-0.1)	0 (0-0.1)	-15.3 (-21.9--10.8)	0 (0-0)	0 (0-0)	-45.3 (-72--1.1)	0 (0-0)	0 (0-0)	-16.5 (-19.2-10)	0 (0-0)	0 (0-0)	-56.5 (-83--9.8)	0 (0-0)	0 (0-0)	1.3 (-7-36.1)	0 (0-0)	0 (0-0)	0 (0-0)	0 (0-0)	126	128	113	107
Maguindanao	0.3 (0.2-0.4)	0.3 (0.2-0.4)	1.5 (-1-16.7)	0.1 (0-0.1)	0.1 (0-0.1)	-11.2 (-16.5-1.6)	0 (0-0)	0 (0-0)	-35.3 (-66.2-26.9)	0 (0-0)	0 (0-0)	-11.7 (-15-10.1)	0 (0-0)	0 (0-0)	-24.2 (-70.4-109)	0 (0-0)	0 (0-0)	1.4 (-4.8-30.3)	0 (0-0)	0 (0-0)	0 (0-0)	0 (0-0)	128	133	119	118
Marinduque	0.3 (0.2-0.3)	0.3 (0.2-0.3)	0.6 (-4-23.9)	0.1 (0-0.1)	0 (0-0.1)	-22.2 (-28.6--12.6)	0 (0-0)	0 (0-0)	-39.6 (-71.7-10.6)	0 (0-0)	0 (0-0)	-24.7 (-28.1--6.8)	0 (0-0)	0 (0-0)	-42.1 (-74.9-14.6)	0 (0-0)	0 (0-0)	0.3 (-6.9-26.5)	0 (0-0)	0 (0-0)	0 (0-0)	0 (0-0)	125	129	110	112
Masbate	0.3 (0.2-0.4)	0.3 (0.2-0.4)	1.9 (-0.1-5)	0.1 (0-0.1)	0 (0-0.1)	-10.9 (-15.7--6.9)	0 (0-0)	0 (0-0)	-10 (-51.9-55.6)	0 (0-0)	0 (0-0)	-11.9 (-17.2-18.4)	0 (0-0)	0 (0-0)	-12.7 (-64.9-85.6)	0 (0-0)	0 (0-0)	1.9 (-5.9-41.1)	0 (0-0)	0 (0-0)	0 (0-0)	0 (0-0)	125	127	114	117
Misamis Occidental	0.3 (0.2-0.4)	0.3 (0.2-0.4)	1.9 (-6.8-13)	0 (0-0.1)	0 (0-0.1)	-12.6 (-20.5--5.2)	0 (0-0)	0 (0-0)	-47.2 (-72.6--5)	0 (0-0)	0 (0-0)	-11.8 (-19.6-21)	0 (0-0)	0 (0-0)	-62.4 (-88--8.9)	0 (0-0)	0 (0-0)	2.6 (-6-48.6)	0 (0-0)	0 (0-0)	0 (0-0)	0 (0-0)	126	130	118	113
Misamis Oriental	0.3 (0.2-0.4)	0.3 (0.2-0.4)	1.9 (-1.4-24.4)	0 (0-0.1)	0 (0-0.1)	-11.1 (-15.9-2)	0 (0-0)	0 (0-0)	-64.8 (-83.5--40.7)	0 (0-0)	0 (0-0)	-10 (-14.9-19)	0 (0-0)	0 (0-0)	-74.7 (-90.2--41.4)	0 (0-0)	0 (0-0)	2.5 (-4.8-44)	0 (0-0)	0 (0-0)	0 (0-0)	0 (0-0)	129	129	119	98
Mountain Province	0.3 (0.2-0.3)	0.3 (0.2-0.4)	0.9 (-15.1-24.8)	0.1 (0-0.1)	0 (0-0.1)	-13.4 (-20.8-0.5)	0 (0-0)	0 (0-0)	-31.7 (-69.8-16)	0 (0-0)	0 (0-0)	-13 (-18.3-13.3)	0 (0-0)	0 (0-0)	-55.5 (-84.1--3.7)	0 (0-0)	0 (0-0)	0.9 (-9.1-32.9)	0 (0-0)	0 (0-0)	0 (0-0)	0 (0-0)	128	130	108	102
National Capital Region	0.3 (0.2-0.3)	0.3 (0.2-0.3)	1.6 (-20.2-54.1)	0 (0-0.1)	0 (0-0.1)	-18.7 (-31.5-1.8)	0 (0-0)	0 (0-0)	-52.8 (-77.3--18.9)	0 (0-0)	0 (0-0)	-20.2 (-24.1-5.7)	0 (0-0)	0 (0-0)	-63.9 (-83.9--25.3)	0 (0-0)	0 (0-0)	2.2 (-6.1-45.4)	0 (0-0)	0 (0-0)	0 (0-0)	0 (0-0)	129	132	112	99
Negros Occidental	0.3 (0.2-0.3)	0.3 (0.2-0.3)	1 (-2.4-28.6)	0.1 (0-0.1)	0 (0-0.1)	-15.8 (-21.4--4.2)	0 (0-0)	0 (0-0)	-30.2 (-60.9-20)	0 (0-0)	0 (0-0)	-16.6 (-18.7-3.6)	0 (0-0)	0 (0-0)	-26.5 (-73.3-58.6)	0 (0-0)	0 (0-0)	0.9 (-6-28.5)	0 (0-0)	0 (0-0)	0 (0-0)	0 (0-0)	124	125	111	111
Negros Oriental	0.3 (0.2-0.4)	0.3 (0.2-0.4)	1.4 (-0.6-7.5)	0 (0-0.1)	0 (0-0.1)	-16.7 (-20.8--11.3)	0 (0-0)	0 (0-0)	-23.8 (-63.1-26.3)	0 (0-0)	0 (0-0)	-17.7 (-20.6-10.6)	0 (0-0)	0 (0-0)	-27.6 (-72.3-61.4)	0 (0-0)	0 (0-0)	1.7 (-5.5-47.9)	0 (0-0)	0 (0-0)	0 (0-0)	0 (0-0)	124	128	113	116
Northern Samar	0.3 (0.2-0.4)	0.3 (0.2-0.4)	2.1 (-2.9-16.4)	0.1 (0-0.1)	0 (0-0.1)	-14.3 (-22.5--3.3)	0 (0-0)	0 (0-0)	-57.9 (-83.7--13.5)	0 (0-0)	0 (0-0)	-15.5 (-21.9-13.6)	0 (0-0)	0 (0-0)	-68.4 (-88.4--25.7)	0 (0-0)	0 (0-0.1)	2.5 (-6.7-43.7)	0 (0-0)	0 (0-0)	0 (0-0)	0 (0-0)	125	128	117	104
Nueva Ecija	0.3 (0.2-0.3)	0.3 (0.2-0.3)	1.9 (-3.8-39.6)	0 (0-0.1)	0 (0-0.1)	-14 (-18.7-3)	0 (0-0)	0 (0-0)	-29.9 (-65.4-22.9)	0 (0-0)	0 (0-0)	-13.5 (-16.3-14.3)	0 (0-0)	0 (0-0)	-49.6 (-76.5-11.9)	0 (0-0)	0 (0-0)	2 (-6.3-45)	0 (0-0)	0 (0-0)	0 (0-0)	0 (0-0)	127	130	107	99
Nueva Vizcaya	0.3 (0.2-0.3)	0.3 (0.2-0.3)	0.9 (-1.6-13.9)	0.1 (0-0.1)	0 (0-0.1)	-15.3 (-19.3--10.1)	0 (0-0)	0 (0-0)	-39.4 (-72.8-6.7)	0 (0-0)	0 (0-0)	-14.9 (-16.9--1.8)	0 (0-0)	0 (0-0)	-64.2 (-85.2--14.3)	0 (0-0)	0 (0-0)	0.8 (-6.4-22.7)	0 (0-0)	0 (0-0)	0 (0-0)	0 (0-0)	125	127	112	101
Occidental Mindoro	0.3 (0.2-0.4)	0.3 (0.2-0.4)	1.6 (-2.6-10.1)	0.1 (0-0.1)	0 (0-0.1)	-17 (-22--7.6)	0 (0-0)	0 (0-0)	-49.8 (-75.7-8.9)	0 (0-0)	0 (0-0)	-18.1 (-27.5-11.6)	0 (0-0)	0 (0-0)	-65.5 (-87.9--5.8)	0 (0-0)	0 (0-0)	1.2 (-13.2-47.4)	0 (0-0)	0 (0-0)	0 (0-0)	0 (0-0)	129	129	116	109
Oriental Mindoro	0.3 (0.2-0.4)	0.3 (0.2-0.4)	1.9 (-4.7-7.9)	0.1 (0-0.1)	0 (0-0.1)	-19.1 (-25.5--15.3)	0 (0-0)	0 (0-0)	-29.7 (-68.5-21.8)	0 (0-0)	0 (0-0)	-21.3 (-25.2-4.6)	0 (0-0)	0 (0-0)	-37.3 (-76.1-47.6)	0 (0-0)	0 (0-0)	2.1 (-5.2-43)	0 (0-0)	0 (0-0)	0 (0-0)	0 (0-0)	129	132	118	110
Palawan	0.3 (0.2-0.3)	0.3 (0.2-0.4)	2 (-0.7-16.1)	0.1 (0-0.1)	0 (0-0.1)	-14.9 (-19.4--2.3)	0 (0-0)	0 (0-0)	-52.6 (-75.4--22.2)	0 (0-0)	0 (0-0)	-16.4 (-19.9-10)	0 (0-0)	0 (0-0)	-61.7 (-85.4--12.2)	0 (0-0)	0 (0-0)	2 (-6.1-40.1)	0 (0-0)	0 (0-0)	0 (0-0)	0 (0-0)	127	128	116	109
Pampanga	0.3 (0.2-0.3)	0.3 (0.2-0.3)	1.9 (-4.2-41.1)	0.1 (0-0.1)	0 (0-0.1)	-15.1 (-20.7-1.8)	0 (0-0)	0 (0-0)	-29.8 (-63.6-28.9)	0 (0-0)	0 (0-0)	-15.6 (-18.5-16.4)	0 (0-0)	0 (0-0)	-29.2 (-69.5-28.1)	0 (0-0)	0 (0-0)	2.2 (-4.9-48)	0 (0-0)	0 (0-0)	0 (0-0)	0 (0-0)	127	129	105	105
Pangasinan	0.3 (0.2-0.3)	0.3 (0.2-0.3)	1.2 (-1.9-4.4)	0.1 (0-0.1)	0 (0-0.1)	-14.2 (-18.1--9.5)	0 (0-0)	0 (0-0)	-42.1 (-73.6-5.7)	0 (0-0)	0 (0-0)	-13.6 (-15.7-7.5)	0 (0-0)	0 (0-0)	-63 (-84.7--6.2)	0 (0-0)	0 (0-0)	1.4 (-6.4-34.1)	0 (0-0)	0 (0-0)	0 (0-0)	0 (0-0)	126	127	109	103
Quezon	0.3 (0.2-0.3)	0.3 (0.2-0.3)	1.2 (-2.4-30.8)	0.1 (0-0.1)	0 (0-0.1)	-15.4 (-20.2--1.8)	0 (0-0)	0 (0-0)	-52.6 (-76.7--12.9)	0 (0-0)	0 (0-0)	-16.3 (-19-7.9)	0 (0-0)	0 (0-0)	-73.1 (-89.6--33.3)	0 (0-0)	0 (0-0)	1.1 (-5.2-33.6)	0 (0-0)	0 (0-0)	0 (0-0)	0 (0-0)	127	127	116	99
Quirino	0.3 (0.2-0.4)	0.3 (0.2-0.4)	1.4 (-2.4-28.9)	0.1 (0-0.1)	0 (0-0.1)	-20.9 (-25.6--10.8)	0 (0-0)	0 (0-0)	-50.2 (-74.4--5.6)	0 (0-0)	0 (0-0)	-21.8 (-25.3-4.3)	0 (0-0)	0 (0-0)	-65.1 (-83--23.4)	0 (0-0)	0 (0-0)	1.2 (-5.5-42.3)	0 (0-0)	0 (0-0)	0 (0-0)	0 (0-0)	124	126	112	103

Location	Birth Incidence rate (per 100,000 live births), 2000	Birth Incidence rate (per 100,000 live births), 2021	Birth Incidence rate (per 100,000 live births) percent change (2000-2021)	All age rate (per 100,000 population), 2000	All age rate (per 100,000 population), 2021	All age rate (per 100,000 population) percent change (2000-2021)	All age cause-specific SCD mortality rate (per 100,000 population), 2000	All age cause-specific SCD mortality rate (per 100,000 population), 2021	All age cause-specific SCD mortality rate (per 100,000 population) percent change (2000-2021)	All age total SCD mortality rate (per 100,000 population), 2000	All age total SCD mortality rate (per 100,000 population), 2021	All age total SCD mortality rate (per 100,000 population) percent change (2000-2021)	Under-5 cause-specific SCD mortality rate (per 100,000 population), 2000	Under-5 cause-specific SCD mortality rate (per 100,000 population), 2021	Under-5 cause-specific SCD mortality rate (per 100,000 population) percent change (2000-2021)	Under-5 total SCD mortality rate (per 100,000 population), 2000	Under-5 total SCD mortality rate (per 100,000 population), 2021	Under-5 total SCD mortality rate (per 100,000 population) percent change (2000-2021)	Under-5 cause-specific mortality cause fraction, 2000	Under-5 total SCD mortality cause fraction, 2000	Under-5 cause-specific mortality cause fraction, 2021	Under-5 total SCD mortality cause fraction, 2021	Under-5 cause-specific mortality rank 2000	Under-5 cause-specific mortality rank 2021	Under-5 total SCD mortality rank 2000	Under-5 total SCD mortality rank 2021
Rizal	0.3 (0.2-0.3)	0.3 (0.2-0.3)	1.1 (-13-9.9)	0.1 (0-0.1)	0 (0-0.1)	-13.1 (-22.1--9)	0 (0-0)	0 (0-0)	-27.4 (-65.9-15.3)	0 (0-0)	0 (0-0)	-13.9 (-16.3-9.8)	0 (0-0)	0 (0-0)	-50.4 (-81.1--2.2)	0 (0-0)	0 (0-0)	1.3 (-6-34.3)	0 (0-0)	0 (0-0)	0 (0-0)	0 (0-0)	130	131	110	107
Romblon	0.3 (0.2-0.4)	0.3 (0.2-0.4)	1.5 (-9.6-8)	0.1 (0-0.1)	0 (0-0.1)	-17.8 (-24.1--13.2)	0 (0-0)	0 (0-0)	-43.1 (-72.9-11.5)	0 (0-0)	0 (0-0)	-18.6 (-24.9-9.1)	0 (0-0)	0 (0-0)	-64.6 (-85.9--15.7)	0 (0-0)	0 (0-0)	1.9 (-7.7-42.5)	0 (0-0)	0 (0-0)	0 (0-0)	0 (0-0)	125	127	116	107
Samar (Western Samar)	0.3 (0.2-0.4)	0.3 (0.2-0.4)	2.8 (-3.9-65)	0 (0-0.1)	0 (0-0.1)	-16.4 (-22.9-9.4)	0 (0-0)	0 (0-0)	-29.9 (-64.3-30.9)	0 (0-0)	0 (0-0)	-18 (-22-15.3)	0 (0-0)	0 (0-0)	-25.2 (-71.2-89.1)	0 (0-0.1)	0 (0-0.1)	3.5 (-3.8-51.8)	0 (0-0)	0 (0-0)	0 (0-0)	0 (0-0)	127	129	122	121
Sarangani	0.3 (0.2-0.3)	0.3 (0.2-0.3)	1.6 (-2.5-40.1)	0.1 (0-0.1)	0 (0-0.1)	-16.4 (-21.6--3.4)	0 (0-0)	0 (0-0)	-14 (-54.7-43.8)	0 (0-0)	0 (0-0)	-17.8 (-20.8-11.8)	0 (0-0)	0 (0-0)	-34.5 (-77.1-25.6)	0 (0-0)	0 (0-0)	1.8 (-5-43.8)	0 (0-0)	0 (0-0)	0 (0-0)	0 (0-0)	131	133	114	113
Siquijor	0.3 (0.2-0.3)	0.3 (0.2-0.3)	1 (-4.3-45.8)	0 (0-0.1)	0 (0-0.1)	-16.1 (-20.8--0.5)	0 (0-0)	0 (0-0)	-35 (-68.2-14.9)	0 (0-0)	0 (0-0)	-16.4 (-21.5-13.5)	0 (0-0)	0 (0-0)	-68.7 (-88--9.8)	0 (0-0)	0 (0-0)	1.8 (-7-46.9)	0 (0-0)	0 (0-0)	0 (0-0)	0 (0-0)	124	129	111	96
Sorsogon	0.3 (0.2-0.3)	0.3 (0.2-0.3)	1.3 (-2.5-31)	0.1 (0-0.1)	0 (0-0.1)	-15.5 (-21.7--2.3)	0 (0-0)	0 (0-0)	-39.7 (-73.4-3.8)	0 (0-0)	0 (0-0)	-16.7 (-19.5-6.5)	0 (0-0)	0 (0-0)	-62.6 (-86.9-3.8)	0 (0-0)	0 (0-0)	1.5 (-5.6-34.4)	0 (0-0)	0 (0-0)	0 (0-0)	0 (0-0)	127	129	111	99
South Cotabato	0.3 (0.2-0.4)	0.3 (0.2-0.4)	1.1 (-1.5-12.5)	0.1 (0-0.1)	0 (0-0.1)	-16.1 (-21.3--10.1)	0 (0-0)	0 (0-0)	-55.9 (-76.3--29.9)	0 (0-0)	0 (0-0)	-16.2 (-20.3-7.7)	0 (0-0)	0 (0-0)	-62.4 (-85.8--16.6)	0 (0-0)	0 (0-0)	1.6 (-5.2-34.8)	0 (0-0)	0 (0-0)	0 (0-0)	0 (0-0)	128	127	116	108
Southern Leyte	0.3 (0.2-0.4)	0.3 (0.2-0.4)	0.5 (-6.7-7)	0 (0-0.1)	0 (0-0.1)	-16.3 (-23.8--12)	0 (0-0)	0 (0-0)	-56 (-81.1--20.4)	0 (0-0)	0 (0-0)	-17.7 (-32.5-9.1)	0 (0-0)	0 (0-0)	-67.2 (-87--27.8)	0 (0-0)	0 (0-0)	0.5 (-20.3-38.9)	0 (0-0)	0 (0-0)	0 (0-0)	0 (0-0)	125	130	116	107
Sultan Kudarat	0.3 (0.2-0.3)	0.3 (0.2-0.3)	1.2 (-0.8-18.9)	0.1 (0-0.1)	0 (0-0.1)	-17.1 (-21.2--7.5)	0 (0-0)	0 (0-0)	-14.2 (-54.9-29.4)	0 (0-0)	0 (0-0)	-18.1 (-21.3-5.1)	0 (0-0)	0 (0-0)	-19.5 (-68-65.3)	0 (0-0)	0 (0-0)	1.6 (-5.3-33.9)	0 (0-0)	0 (0-0)	0 (0-0)	0 (0-0)	129	133	112	114
Sulu	0.3 (0.2-0.4)	0.3 (0.2-0.4)	1.3 (-1-13.1)	0.1 (0-0.1)	0 (0-0.1)	-11.9 (-19.1--3.9)	0 (0-0)	0 (0-0)	-41.5 (-66.9-16.5)	0 (0-0)	0 (0-0)	-16.4 (-20.6-6)	0 (0-0)	0 (0-0)	-40.7 (-71.4-86.4)	0 (0-0.1)	0 (0-0.1)	0.3 (-6.3-31.5)	0 (0-0)	0 (0-0)	0 (0-0)	0 (0-0)	127	130	118	116
Surigao Del Norte	0.3 (0.2-0.4)	0.3 (0.2-0.4)	1.1 (-4-27.1)	0 (0-0.1)	0 (0-0.1)	-14 (-18.9--4.8)	0 (0-0)	0 (0-0)	-45.3 (-74.7--6.2)	0 (0-0)	0 (0-0)	-14.1 (-20-14.4)	0 (0-0)	0 (0-0)	-64.4 (-85.1--24.8)	0 (0-0)	0 (0-0)	1.7 (-6.9-42.5)	0 (0-0)	0 (0-0)	0 (0-0)	0 (0-0)	127	128	117	107
Surigao Del Sur	0.3 (0.2-0.4)	0.3 (0.2-0.4)	1.8 (-7.8-23.6)	0.1 (0-0.1)	0 (0-0.1)	-16.7 (-23.8--8)	0 (0-0)	0 (0-0)	-34.3 (-69.5-17.2)	0 (0-0)	0 (0-0)	-17.1 (-26.3-15)	0 (0-0)	0 (0-0)	-46.6 (-78-47.9)	0 (0-0)	0 (0-0.1)	2.5 (-8.9-48.2)	0 (0-0)	0 (0-0)	0 (0-0)	0 (0-0)	128	130	119	114
Tarlac	0.3 (0.2-0.3)	0.3 (0.2-0.3)	1 (-5.4-40.3)	0.1 (0-0.1)	0 (0-0.1)	-17.7 (-23.8-0.6)	0 (0-0)	0 (0-0)	-18.7 (-59.9-40.5)	0 (0-0)	0 (0-0)	-18.2 (-20.7-1.6)	0 (0-0)	0 (0-0)	-31.1 (-70.5-61.1)	0 (0-0)	0 (0-0)	1.3 (-6.1-28.4)	0 (0-0)	0 (0-0)	0 (0-0)	0 (0-0)	127	128	109	111
Tawi-Tawi	0.2 (0.2-0.3)	0.3 (0.2-0.3)	1.8 (-4-34.2)	0.1 (0-0.1)	0.1 (0-0.1)	-10.8 (-16.2-6.2)	0 (0-0)	0 (0-0)	-30.5 (-64.6-26)	0 (0-0)	0 (0-0)	-10.9 (-15-16.5)	0 (0-0)	0 (0-0)	-43.1 (-81.6-51.3)	0 (0-0)	0 (0-0)	2.8 (-8.8-40.8)	0 (0-0)	0 (0-0)	0 (0-0)	0 (0-0)	128	130	107	103
Zambales	0.3 (0.2-0.3)	0.3 (0.2-0.3)	0.8 (-6.8-8)	0 (0-0.1)	0 (0-0.1)	-13.8 (-21.5--8)	0 (0-0)	0 (0-0)	-53.6 (-76--24)	0 (0-0)	0 (0-0)	-13.9 (-17.3-3.3)	0 (0-0)	0 (0-0)	-64.9 (-85.4--21.4)	0 (0-0)	0 (0-0)	0.6 (-7.8-28.1)	0 (0-0)	0 (0-0)	0 (0-0)	0 (0-0)	128	129	112	96
Zamboanga Del Norte	0.3 (0.2-0.4)	0.3 (0.2-0.3)	0.9 (-6.5-7.3)	0.1 (0-0.1)	0 (0-0.1)	-15.5 (-23.7--8.5)	0 (0-0)	0 (0-0)	-35.5 (-67.7-16.5)	0 (0-0)	0 (0-0)	-16.1 (-19.2-8.7)	0 (0-0)	0 (0-0)	-56.7 (-83.1-20.9)	0 (0-0)	0 (0-0)	1.2 (-6-37.2)	0 (0-0)	0 (0-0)	0 (0-0)	0 (0-0)	126	128	113	108
Zamboanga Del Sur	0.3 (0.2-0.3)	0.3 (0.2-0.3)	0.8 (-2.9-9.9)	0.1 (0-0.1)	0 (0-0.1)	-14.5 (-19.7--6.8)	0 (0-0)	0 (0-0)	-57.9 (-76--28.1)	0 (0-0)	0 (0-0)	-15.5 (-18.1--1.3)	0 (0-0)	0 (0-0)	-71.4 (-89.8--46.5)	0 (0-0)	0 (0-0)	1.1 (-5.3-21.4)	0 (0-0)	0 (0-0)	0 (0-0)	0 (0-0)	128	131	114	97
Zamboanga Sibugay	0.3 (0.2-0.3)	0.3 (0.2-0.3)	1.3 (-1.2-30.2)	0.1 (0-0.1)	0 (0-0.1)	-14.7 (-20.5-0.4)	0 (0-0)	0 (0-0)	-48 (-73.8-0.3)	0 (0-0)	0 (0-0)	-16.1 (-18.8-10.6)	0 (0-0)	0 (0-0)	-66.8 (-88.2--8)	0 (0-0)	0 (0-0)	1.3 (-5.5-36.5)	0 (0-0)	0 (0-0)	0 (0-0)	0 (0-0)	127	131	115	110
Seychelles	0.2 (0.2-0.3)	0.2 (0.2-0.3)	0.3 (-30.7-27.2)	0 (0-0.1)	0 (0-0.1)	-20.9 (-35.5--7.1)	0 (0-0)	0 (0-0)	-49.8 (-67.6--20.8)	0 (0-0)	0 (0-0)	-21.1 (-35.4--5.1)	0 (0-0)	0 (0-0)	-37.7 (-63.9-11.9)	0 (0-0)	0 (0-0)	0.3 (-21.2-26.3)	0 (0-0)	0 (0-0)	0 (0-0)	0 (0-0)	118	121	93	93
Sri Lanka	0.2 (0.2-0.3)	0.2 (0.2-0.3)	1.5 (-6.4-23.1)	0 (0-0.1)	0 (0-0.1)	-19.1 (-25--9.9)	0 (0-0)	0 (0-0)	-71.3 (-85.6--37.6)	0 (0-0)	0 (0-0)	-17.7 (-27.8-12.1)	0 (0-0)	0 (0-0)	-82.6 (-91.3--68.1)	0 (0-0)	0 (0-0)	2.9 (-13.4-54.9)	0 (0-0)	0 (0-0)	0 (0-0)	0 (0-0)	129	131	106	86
Thailand	0.2 (0.2-0.3)	0.2 (0.2-0.3)	1.8 (-4.5-48.4)	0 (0-0.1)	0 (0-0)	-36.6 (-43.3--24)	0 (0-0)	0 (0-0)	-72 (-82.8--51.7)	0 (0-0)	0 (0-0)	-38.3 (-42.5--18.4)	0 (0-0)	0 (0-0)	-87 (-93.9--61.4)	0 (0-0)	0 (0-0)	2.4 (-6.5-48.9)	0 (0-0)	0 (0-0)	0 (0-0)	0 (0-0)	136	141	106	93
Timor-Leste	0.3 (0.2-0.4)	0.3 (0.2-0.4)	1.2 (-18.3-50)	0.1 (0-0.1)	0 (0-0.1)	-15.7 (-31-12.4)	0 (0-0)	0 (0-0)	-45.8 (-69.1-7)	0 (0-0)	0 (0-0)	-21 (-30.8-6.3)	0 (0-0)	0 (0-0)	-45.4 (-70.1-35.8)	0 (0-0.1)	0 (0-0.1)	0.7 (-11.3-40.5)	0 (0-0)	0 (0-0)	0 (0-0)	0 (0-0)	133	133	120	109
Viet Nam	0.2 (0.2-0.3)	0.3 (0.2-0.3)	2 (-0.9-34.3)	0 (0-0.1)	0 (0-0.1)	-26.3 (-31.5--7.8)	0 (0-0)	0 (0-0)	-21.6 (-52.6-11.4)	0 (0-0)	0 (0-0)	-27.4 (-31.2--3)	0 (0-0)	0 (0-0)	-40.1 (-69-11.7)	0 (0-0)	0 (0-0)	2.6 (-5.5-46)	0 (0-0)	0 (0-0)	0 (0-0)	0 (0-0)	133	136	106	93
Sub-Saharan Africa	1,130 (959-1,340)	1,080 (911-1,270)	-5.1 (-7.9--2.8)	523 (448-610)	499 (420-582)	-4.6 (-7.1--2.3)	3.5 (2.6-4.5)	2.6 (1.8-3.5)	-25.9 (-44.3-1.2)	24.7 (20.5-29.9)	23.3 (19.3-28.3)	-5.9 (-8.4--3.6)	9.1 (5.8-13.4)	6 (3.7-8.2)	-34 (-53.9--2.2)	36.7 (26.8-48.6)	35.6 (25.8-47.5)	-2.9 (-6.7-0.4)	0.3 (0.2-0.4)	1 (0.8-1.4)	0.4 (0.3-0.5)	2.2 (1.5-3)	32	26	17	11
**Central Sub-Saharan Africa**	1,440 (1,250-1,650)	1,440 (1,250-1,660)	0.4 (-7-5.3)	680 (576-796)	640 (537-738)	-5.8 (-12.2--0.2)	3.3 (0.8-8.7)	2.6 (0.7-8.2)	-21.1 (-48.5-15.4)	33.9 (28.2-39.8)	32.9 (27.3-38.3)	-2.8 (-8.8-2.8)	6.1 (1.2-12.2)	3.5 (1-9.1)	-42.7 (-68-23.9)	49.5 (34.4-67.5)	51.4 (34.8-72)	3.8 (-6.5-14.2)	0.2 (0-0.3)	1.4 (1-1.9)	0.3 (0.1-0.6)	3.7 (2.3-5.1)	35	31	15	8
Angola	1,240 (1,040-1,490)	1,210 (1,010-1,440)	-2.8 (-10-4.5)	592 (496-709)	562 (460-675)	-5.1 (-11.9-3)	2.1 (0.6-3.9)	1.4 (0.5-2.6)	-32.6 (-53.1-6.4)	29.3 (23.7-36.3)	28.5 (22.7-35.6)	-2.7 (-12-6.9)	4.3 (0.9-10)	2.5 (0.6-4.5)	-42.8 (-62.5-63.1)	40.9 (27.5-59.3)	41.3 (26.9-61.2)	0.9 (-14-16.2)	0.1 (0-0.3)	1 (0.7-1.4)	0.2 (0-0.3)	2.9 (1.9-4.2)	44	39	19	10
Central African Republic	889 (747-1,060)	890 (759-1,050)	0 (-8-9.3)	405 (336-477)	383 (320-463)	-5.3 (-10.7-2.8)	2.6 (0.6-12)	2.5 (0.6-11.5)	-4.9 (-38.1-53.5)	20.9 (17.2-25.9)	20.1 (16.7-24.4)	-3.5 (-11.3-4.5)	4.2 (0.6-12.2)	4.2 (0.9-16.3)	-0.8 (-44.9-116)	29.9 (19.9-43)	30.8 (20.4-43.9)	2.9 (-11.8-19.2)	0.1 (0-0.3)	0.7 (0.5-1.1)	0.1 (0-0.6)	1.1 (0.7-1.5)	44	40	24	19
Congo	636 (545-731)	601 (512-696)	-5.4 (-11-1.7)	296 (250-341)	250 (212-299)	-15.4 (-21.9--9.1)	3 (1-5.6)	1.9 (0.6-3.6)	-39 (-66.3-6.4)	13.9 (11.5-16.5)	12.2 (10.2-14.4)	-12.1 (-19--5.4)	4.5 (1.1-10.4)	1.6 (0.6-3.1)	-64.2 (-83.9-0.8)	19.7 (14-26.3)	19.1 (13.6-25.6)	-3.1 (-13.4-8.5)	0.2 (0-0.4)	0.9 (0.6-1.2)	0.2 (0.1-0.4)	2.5 (1.6-3.3)	33	36	22	10
Democratic Republic of the Congo	1,570 (1,360-1,810)	1,610 (1,380-1,840)	2.3 (-8.1-9.4)	750 (628-874)	708 (594-814)	-5.6 (-13.7-2.3)	3.8 (0.8-11.2)	3.1 (0.7-11.1)	-17 (-48-26.8)	37.4 (31.1-43.9)	36.5 (30.1-42.1)	-2.4 (-10-4.8)	6.9 (1.1-14.8)	4 (0.9-12.2)	-42.6 (-72.5-30.9)	54.8 (37.8-75.4)	58 (39.5-80.9)	5.8 (-6.7-20.6)	0.2 (0-0.4)	1.6 (1.1-2.1)	0.3 (0.1-0.9)	4.5 (2.8-6.2)	35	27	14	7

Location	Birth Incidence rate (per 100,000 live births), 2000	Birth Incidence rate (per 100,000 live births), 2021	Birth Incidence rate (per 100,000 live births) percent change (2000-2021)	All age rate (per 100,000 population), 2000	All age rate (per 100,000 population), 2021	All age rate (per 100,000 population) percent change (2000-2021)	All age cause-specific SCD mortality rate (per 100,000 population), 2000	All age cause-specific SCD mortality rate (per 100,000 population), 2021	All age cause-specific SCD mortality rate (per 100,000 population) percent change (2000-2021)	All age total SCD mortality rate (per 100,000 population), 2000	All age total SCD mortality rate (per 100,000 population), 2021	All age total SCD mortality rate (per 100,000 population) percent change (2000-2021)	Under-5 cause-specific SCD mortality rate (per 100,000 population), 2000	Under-5 cause-specific SCD mortality rate (per 100,000 population), 2021	Under-5 cause-specific SCD mortality rate (per 100,000 population) percent change (2000-2021)	Under-5 total SCD mortality rate (per 100,000 population), 2000	Under-5 total SCD mortality rate (per 100,000 population), 2021	Under-5 total SCD mortality rate (per 100,000 population) percent change (2000-2021)	Under-5 cause-specific mortality cause fraction, 2000	Under-5 total SCD mortality cause fraction, 2000	Under-5 cause-specific mortality cause fraction, 2021	Under-5 total SCD mortality cause fraction, 2021	Under-5 cause-specific mortality rank 2000	Under-5 cause-specific mortality rank 2021	Under-5 total SCD mortality rank 2000	Under-5 total SCD mortality rank 2021
Equatorial Guinea	2,090 (1,760-2,510)	2,060 (1,710-2,480)	-1.5 (-10.4-6.8)	1,010 (850-1,190)	852 (703-1,000)	-16 (-22.8--10)	2.5 (0.3-4.8)	1.4 (0.1-3.2)	-45.3 (-71.9--2.9)	52.2 (42.1-64.4)	46.4 (37.6-55.9)	-11.1 (-17.9--3.5)	3.8 (0.3-7.9)	1.8 (0.1-3.1)	-53.1 (-72.8-24.7)	83.6 (61-112)	83.9 (60.5-113)	0.3 (-10.3-11.7)	0.2 (0-0.3)	3.4 (2.3-4.4)	0.2 (0-0.3)	8.4 (4.8-12)	34	35	6	5
Gabon	1,010 (809-1,220)	959 (784-1,150)	-5.2 (-11.7-1.5)	466 (373-561)	383 (318-465)	-17.9 (-23.7--12.5)	2.3 (0.1-4.4)	1.6 (0.1-3.9)	-28.9 (-61.9-163)	21.3 (17.1-26.3)	18.2 (14.8-22.4)	-14.3 (-20.6--6.9)	2.7 (0.1-5.3)	1.9 (0.2-4.4)	-30.8 (-62.5-84.6)	31.4 (22.4-42.1)	30.1 (21.8-40.1)	-4 (-14.2-7.7)	0.2 (0-0.3)	2.1 (1.5-2.9)	0.3 (0-0.5)	4.2 (2.5-6.3)	32	31	12	6
**Eastern Sub-Saharan Africa**	355 (301-421)	345 (289-408)	-2.6 (-5.6-1.2)	167 (141-198)	150 (125-177)	-9.7 (-12.4--6.9)	0.6 (0.4-1.2)	0.5 (0.3-0.9)	-27.4 (-50.7-20.9)	8.9 (7-11.1)	8.5 (6.8-10.6)	-4.6 (-7.4--1.2)	1.2 (0.4-2)	0.7 (0.3-1.1)	-40.9 (-61.8-18.3)	12.9 (8.8-17.8)	13.2 (8.9-18.2)	2.4 (-2.8-9.1)	0 (0-0.1)	0.4 (0.3-0.6)	0.1 (0-0.1)	1 (0.7-1.4)	77	71	25	18
Burundi	70.8 (58.1-88.2)	64.5 (52.5-79.8)	-8.8 (-22-3.7)	29.4 (24-36.2)	26.2 (21.5-32.1)	-11.2 (-20.8--1.4)	0.5 (0.3-0.8)	0.3 (0.2-0.5)	-34 (-65.9-25.6)	1.8 (1.4-2.3)	1.6 (1.3-2.1)	-11.7 (-24.4--1.1)	0.8 (0.2-1.7)	0.3 (0.2-0.6)	-62.4 (-81.6-32.6)	3.4 (2.4-4.7)	3.1 (2.2-4.3)	-7.8 (-21.5-7.4)	0 (0-0)	0.1 (0.1-0.1)	0 (0-0)	0.2 (0.2-0.3)	83	77	47	28
Comoros	6.5 (4.8-9)	6.4 (4.8-9)	-0.1 (-9.2-9.5)	2.1 (1.6-2.8)	1.6 (1.3-2.1)	-21.9 (-27.4--15)	0.1 (0-0.1)	0 (0-0.1)	-25.1 (-60.3-38.8)	0.2 (0.1-0.2)	0.1 (0.1-0.2)	-21.2 (-28.2--12.2)	0.2 (0-0.4)	0.1 (0-0.2)	-30.8 (-69-65.4)	0.5 (0.3-0.7)	0.5 (0.3-0.7)	-0.3 (-13.3-13.8)	0 (0-0)	0 (0-0)	0 (0-0)	0 (0-0.1)	104	103	83	74
Djibouti	21.9 (16.6-28)	21.6 (16.3-27.3)	-1.1 (-9.8-6)	9.3 (7.1-11.7)	7.7 (5.9-9.7)	-17 (-23.3--11.5)	0.1 (0.1-0.2)	0.1 (0-0.2)	-42.2 (-68.4-15)	0.5 (0.4-0.7)	0.4 (0.3-0.6)	-12.6 (-21.2--4.9)	0.2 (0.1-0.5)	0.1 (0.1-0.2)	-47 (-76.3-43.1)	0.9 (0.6-1.2)	0.9 (0.6-1.2)	1.4 (-12.6-15.5)	0 (0-0)	0 (0-0.1)	0 (0-0)	0.1 (0.1-0.2)	101	102	74	56
Eritrea	24.7 (18.3-31.5)	24.7 (18.4-31.6)	-0.2 (-8.1-9.5)	10.5 (7.9-13.6)	9.1 (7-11.7)	-13.4 (-19.6--7)	0.2 (0.1-0.3)	0.2 (0.1-0.3)	-14.6 (-52-48.2)	0.6 (0.5-0.9)	0.6 (0.4-0.7)	-11.3 (-18.8--1.2)	0.3 (0.1-0.8)	0.3 (0.1-0.6)	-16.7 (-57.7-175)	1.2 (0.8-1.7)	1.2 (0.8-1.6)	1 (-11.4-16.8)	0 (0-0)	0.1 (0-0.1)	0 (0-0)	0.1 (0.1-0.2)	100	98	66	60
Ethiopia	4.9 (3.7-6.9)	4.9 (3.7-6.7)	-0.6 (-4.3-3.2)	1.6 (1.2-2.1)	1.4 (1.1-1.9)	-11.2 (-15.5--6.9)	0 (0-0.1)	0 (0-0)	-43.7 (-73.1-15.9)	0.1 (0.1-0.2)	0.1 (0.1-0.2)	-10.2 (-15.2--5.6)	0.1 (0-0.2)	0 (0-0.1)	-57.9 (-80--3.9)	0.4 (0.2-0.6)	0.4 (0.2-0.6)	0 (-6.7-6.7)	0 (0-0)	0 (0-0)	0 (0-0)	0 (0-0.1)	122	123	99	80
Addis Ababa	4.1 (3.1-5.6)	4 (3.1-5.5)	-1.1 (-8.8-7.4)	1.3 (1-1.6)	0.9 (0.7-1.2)	-29.2 (-37.8--23.2)	0 (0-0.1)	0 (0-0)	-65.1 (-86.2--21.4)	0.1 (0.1-0.1)	0.1 (0.1-0.1)	-26.2 (-33--18.7)	0 (0-0)	0 (0-0)	-54.5 (-80.7--4.4)	0.2 (0.1-0.3)	0.2 (0.1-0.3)	-0.3 (-14.4-14)	0 (0-0)	0 (0-0)	0 (0-0)	0.1 (0.1-0.1)	121	122	85	68
Afar	5.4 (4-7.5)	5.3 (3.8-7.3)	-1.1 (-9.3-10.8)	1.6 (1.2-2.1)	1.5 (1.1-2)	-5.3 (-13.9-3.6)	0.1 (0-0.1)	0 (0-0.1)	-35 (-67-52.7)	0.2 (0.1-0.2)	0.1 (0.1-0.2)	-4.8 (-14.5-5.7)	0.1 (0-0.2)	0.1 (0-0.1)	-46.8 (-72.9-42.5)	0.4 (0.3-0.7)	0.4 (0.3-0.7)	-0.5 (-13.9-14.6)	0 (0-0)	0 (0-0)	0 (0-0)	0 (0-0)	120	122	98	85
Amhara	4.8 (3.5-6.8)	4.8 (3.5-6.7)	-0.7 (-8.7-6.8)	1.6 (1.2-2)	1.3 (1-1.7)	-15.8 (-22.7--9.5)	0 (0-0.1)	0 (0-0)	-51.1 (-78.5-20.3)	0.1 (0.1-0.2)	0.1 (0.1-0.2)	-14 (-22.3--4.7)	0.1 (0-0.2)	0 (0-0.1)	-66.2 (-87-12)	0.4 (0.2-0.6)	0.4 (0.2-0.6)	-1 (-14.1-13.5)	0 (0-0)	0 (0-0)	0 (0-0)	0 (0-0.1)	122	124	100	77
Benishangul-Gumuz	5.8 (4.2-7.9)	5.7 (4.2-7.8)	-1.2 (-11.2-7.4)	1.8 (1.3-2.3)	1.6 (1.2-2)	-13.4 (-20.9--6.2)	0.1 (0-0.1)	0 (0-0.1)	-36.4 (-69.3-40.6)	0.2 (0.1-0.2)	0.2 (0.1-0.2)	-12.6 (-20.9--4.2)	0.1 (0-0.3)	0.1 (0-0.1)	-47.6 (-74.7-64.1)	0.5 (0.3-0.7)	0.5 (0.3-0.7)	-0.8 (-13.3-11.2)	0 (0-0)	0 (0-0)	0 (0-0)	0 (0-0.1)	122	121	101	83
Dire Dawa	4.6 (3.5-6.5)	4.6 (3.4-6.5)	-0.9 (-8.3-7.4)	1.4 (1-1.8)	1.1 (0.9-1.5)	-18.1 (-24--11.1)	0 (0-0.1)	0 (0-0)	-61.2 (-82.8--17.5)	0.1 (0.1-0.2)	0.1 (0.1-0.1)	-17.8 (-25.1--10)	0.1 (0-0.2)	0 (0-0.1)	-69.6 (-87.6--9.1)	0.4 (0.2-0.6)	0.4 (0.2-0.6)	-0.7 (-12.9-14.9)	0 (0-0)	0 (0-0)	0 (0-0)	0.1 (0-0.1)	118	123	104	74
Gambella	5.3 (3.9-7.4)	5.2 (3.9-7.2)	-0.6 (-9.1-7.8)	1.6 (1.2-2.1)	1.3 (1-1.7)	-18.8 (-24.4--11.9)	0 (0-0.1)	0 (0-0)	-54.3 (-79.6--4.9)	0.2 (0.1-0.2)	0.1 (0.1-0.2)	-18.7 (-28.9--10.2)	0.1 (0-0.2)	0 (0-0.1)	-66.5 (-87.3-5.9)	0.4 (0.3-0.7)	0.4 (0.3-0.7)	0.1 (-15.6-14.1)	0 (0-0)	0 (0-0)	0 (0-0)	0.1 (0-0.1)	120	124	97	76
Harari	4.7 (3.4-6.6)	4.6 (3.4-6.3)	-0.9 (-8.2-7.7)	1.4 (1.1-1.9)	1.2 (0.9-1.5)	-18 (-24.4--10.4)	0 (0-0.1)	0 (0-0)	-54 (-80.2-7.6)	0.1 (0.1-0.2)	0.1 (0.1-0.1)	-17.7 (-24.4--9.1)	0.1 (0-0.1)	0 (0-0)	-59.1 (-84.5-31.1)	0.3 (0.2-0.5)	0.3 (0.2-0.5)	-0.3 (-12.7-13.3)	0 (0-0)	0 (0-0)	0 (0-0)	0 (0-0.1)	119	123	93	74
Oromia	4.9 (3.6-6.8)	4.8 (3.5-6.6)	-0.5 (-8.3-7.2)	1.6 (1.2-2.2)	1.5 (1.1-1.9)	-8.1 (-15.5-0.3)	0 (0-0.1)	0 (0-0)	-43.6 (-73.7-14.3)	0.1 (0.1-0.2)	0.1 (0.1-0.2)	-7.6 (-16.4-1.8)	0.1 (0-0.2)	0 (0-0.1)	-58 (-78.3-5.9)	0.4 (0.2-0.6)	0.4 (0.2-0.6)	0.5 (-12.3-14.5)	0 (0-0)	0 (0-0)	0 (0-0)	0 (0-0.1)	121	122	97	78
Somali	4.9 (3.6-6.8)	4.8 (3.4-6.7)	-0.9 (-9.5-7.9)	1.6 (1.2-2.1)	1.6 (1.1-2)	-5.1 (-13.1-2.2)	0 (0-0.1)	0 (0-0.1)	-21.9 (-62.3-79.3)	0.1 (0.1-0.2)	0.1 (0.1-0.2)	-3.7 (-13.2-6.1)	0.1 (0-0.2)	0.1 (0-0.1)	-32.8 (-68.8-99.2)	0.4 (0.2-0.6)	0.4 (0.2-0.6)	-0.3 (-12.5-14.2)	0 (0-0)	0 (0-0)	0 (0-0)	0 (0-0)	120	122	98	87
Southern Nations, Nationalities, and Peoples	5.2 (3.8-7.1)	5.1 (3.7-7)	-1.8 (-10.1-6.2)	1.7 (1.3-2.3)	1.5 (1.1-2)	-12.4 (-18.9--5.8)	0.1 (0-0.1)	0 (0-0.1)	-40.5 (-71.7-26)	0.2 (0.1-0.2)	0.1 (0.1-0.2)	-12.4 (-21.3--3.2)	0.1 (0-0.3)	0 (0-0.1)	-60.1 (-83-16.1)	0.4 (0.3-0.7)	0.4 (0.3-0.6)	-2 (-15.5-11.7)	0 (0-0)	0 (0-0)	0 (0-0)	0 (0-0.1)	122	122	103	79
Tigray	4.8 (3.6-6.6)	4.7 (3.5-6.6)	-0.7 (-7.6-7.2)	1.6 (1.2-2.1)	1.3 (1-1.7)	-17.4 (-24.7--9.4)	0 (0-0.1)	0 (0-0)	-50.7 (-81.4--1.2)	0.1 (0.1-0.2)	0.1 (0.1-0.2)	-15.3 (-23.2--6.6)	0.1 (0-0.2)	0 (0-0)	-64.7 (-85.9-19.3)	0.3 (0.2-0.5)	0.3 (0.2-0.5)	-0.1 (-12.3-13.6)	0 (0-0)	0 (0-0)	0 (0-0)	0 (0-0.1)	122	125	98	75
Kenya	1,300 (957-1,740)	1,330 (976-1,780)	2.4 (-0.1-4.8)	584 (447-762)	499 (386-645)	-14.5 (-16.1--12.4)	0.7 (0.4-1)	0.5 (0.3-0.8)	-26 (-44.7--1.4)	30.9 (22.2-43.2)	29.1 (21-40.2)	-5.9 (-8.7--3)	1.3 (0.6-2.1)	0.6 (0.3-1)	-48.3 (-63.1--12.1)	41.3 (26-61.3)	45.9 (29.6-67.3)	11.2 (6.2-16.9)	0.1 (0-0.1)	2.1 (1.3-3.3)	0.1 (0.1-0.1)	7.1 (4.4-10.7)	47	51	13	7
Baringo	1,130 (823-1,550)	1,170 (842-1,600)	3.2 (-8.7-15.8)	523 (389-695)	452 (332-609)	-13.7 (-22.1--4)	0.7 (0.6-1)	0.5 (0.3-0.7)	-35.2 (-57.8--0.6)	29.1 (20.3-41)	27.5 (19.1-39.1)	-5.5 (-16.8-7.7)	1.2 (0.6-2.2)	0.6 (0.4-0.9)	-52.7 (-70.2--6.8)	38.9 (24-59.6)	42.1 (25.8-66.1)	8.1 (-9-31.8)	0.1 (0-0.1)	1.8 (1.2-3)	0.1 (0-0.1)	5 (2.9-8.3)	56	59	14	7
Bomet	1,070 (754-1,430)	1,110 (795-1,500)	3.6 (-6-14.7)	565 (415-741)	475 (353-619)	-16 (-23.5--9)	0.2 (0.1-0.3)	0.2 (0.1-0.4)	45.3 (0.5-131)	25.1 (17.3-35)	24.1 (17.1-34.2)	-4 (-14.4-7.6)	0.2 (0.1-0.3)	0.1 (0.1-0.2)	-45.9 (-65.9--7.3)	24.9 (15.3-36.1)	26.9 (17.2-39.7)	8.2 (-9.9-28.2)	0 (0-0)	2.6 (1.5-4)	0 (0-0.1)	10.5 (6.3-16.3)	58	56	11	5
Bungoma	1,340 (996-1,810)	1,400 (1,020-1,920)	4.3 (-6-15.3)	647 (483-861)	581 (428-766)	-10.2 (-17.9--1.8)	0.7 (0.4-1.1)	0.5 (0.3-0.9)	-29.7 (-50.7-10.2)	33.8 (23.8-48)	33.4 (23.6-47.2)	-1.2 (-12.5-11.5)	2 (0.7-3.7)	1.4 (0.8-2.3)	-29.5 (-62.4-66.6)	46.4 (29.4-69.4)	53 (33.1-79.4)	14.2 (-3.1-35.3)	0.1 (0-0.2)	2 (1.3-3)	0.1 (0.1-0.2)	4.2 (2.5-6.7)	46	46	13	7
Busia	1,780 (1,290-2,360)	1,860 (1,340-2,490)	4.5 (-9.9-17.8)	842 (651-1,090)	756 (575-985)	-10.2 (-21.3--1.7)	1.5 (0.5-2.4)	1 (0.3-1.8)	-34 (-60.7-4.1)	45.6 (32.1-64.4)	44.7 (31-62.5)	-2 (-14.5-9.8)	3.3 (1.1-6.2)	1.2 (0.4-2.1)	-63.2 (-74.9--27.6)	65.3 (40.3-98.2)	74.2 (47-113)	13.8 (-4.4-36.3)	0.1 (0-0.2)	2.2 (1.4-3.3)	0.1 (0-0.2)	8.4 (5.1-13.2)	41	41	14	4
Elgeyo-Marakwet	1,080 (777-1,490)	1,120 (809-1,510)	3.7 (-6.5-14.4)	531 (396-699)	435 (333-571)	-18.1 (-24.4--9.4)	0.5 (0.3-0.7)	0.4 (0.2-0.6)	-12.6 (-43.6-37.9)	26.5 (17.9-38.4)	24.4 (16.8-35)	-7.8 (-18-4.7)	0.4 (0.3-0.6)	0.5 (0.3-0.9)	15.7 (-34.3-123)	29.1 (17.2-43.4)	31.9 (19.9-47.7)	9.6 (-7.9-32)	0 (0-0)	2.2 (1.3-3.5)	0.1 (0-0.1)	4.5 (2.6-7.3)	58	55	11	9
Embu	1,000 (719-1,370)	1,020 (724-1,410)	2 (-10.3-16.8)	441 (328-588)	333 (248-437)	-24.6 (-31.3--16.9)	0.3 (0.2-0.5)	0.3 (0.1-0.5)	-5.3 (-40.1-50.4)	22.4 (15.6-31.7)	18.8 (13.2-27)	-16.2 (-27.5--4)	0.4 (0.3-0.7)	0.5 (0.3-0.7)	7.6 (-30.8-74.5)	27.3 (16.6-42.2)	29 (18.5-44.4)	6.3 (-14.3-28.8)	0 (0-0.1)	2.2 (1.3-3.5)	0.1 (0-0.1)	4.2 (2.5-7)	59	57	11	8
Garissa	1,070 (781-1,450)	1,110 (808-1,530)	3.1 (-5.5-11.7)	526 (405-692)	511 (398-688)	-2.8 (-10.4-5.2)	0.5 (0.3-0.8)	0.3 (0.2-0.6)	-35.6 (-62.5-2)	27.4 (19.1-39.2)	27.4 (19.1-39.3)	-0.1 (-10.7-12.4)	0.7 (0.4-1.3)	0.2 (0.1-0.4)	-71.3 (-84.5--38.6)	32.8 (19.4-50.2)	35.1 (21.2-55)	7 (-10.5-25.1)	0 (0-0.1)	1.9 (1.1-3.3)	0 (0-0.1)	7.4 (4.4-11.7)	56	60	15	6
Homa Bay	2,300 (1,670-3,040)	2,400 (1,810-3,070)	4.1 (-8.3-15.2)	1,030 (778-1,320)	949 (725-1,210)	-7.6 (-17.8-1.4)	2 (0.5-3.7)	1.4 (0.4-2.8)	-27.8 (-55.2-39.2)	59.3 (41.8-82.5)	60.1 (43-81)	1.4 (-11.4-12.9)	4.3 (1-8.2)	2.3 (0.5-4.1)	-47.3 (-66.9-15)	85.6 (53.8-129)	99.9 (64.2-147)	16.7 (-3.1-37.1)	0.1 (0-0.2)	1.8 (1.1-2.7)	0.2 (0.1-0.4)	10.1 (6.2-15.2)	39	37	13	2
Isiolo	1,180 (843-1,550)	1,210 (868-1,630)	3 (-7.8-15.4)	510 (372-659)	442 (332-584)	-13.2 (-20.7--3.6)	0.7 (0.4-0.9)	0.5 (0.3-0.7)	-21.2 (-50.8-38.6)	29.9 (20.1-42.6)	27.9 (19.6-38.7)	-6.8 (-18.1-8.3)	1.6 (0.6-3.1)	1.2 (0.7-2)	-23.8 (-54.2-93.2)	44.7 (26.4-68.5)	48.5 (28.8-75.4)	8.4 (-11.5-31.4)	0 (0-0.1)	1.2 (0.7-1.9)	0.1 (0-0.1)	2 (1.2-3.4)	57	63	18	13

Location	Birth Incidence rate (per 100,000 live births), 2000	Birth Incidence rate (per 100,000 live births), 2021	Birth Incidence rate (per 100,000 live births) percent change (2000-2021)	All age rate (per 100,000 population), 2000	All age rate (per 100,000 population), 2021	All age rate (per 100,000 population) percent change (2000-2021)	All age cause-specific SCD mortality rate (per 100,000 population), 2000	All age cause-specific SCD mortality rate (per 100,000 population), 2021	All age cause-specific SCD mortality rate (per 100,000 population) percent change (2000-2021)	All age total SCD mortality rate (per 100,000 population), 2000	All age total SCD mortality rate (per 100,000 population), 2021	All age total SCD mortality rate (per 100,000 population) percent change (2000-2021)	Under-5 cause-specific SCD mortality rate (per 100,000 population), 2000	Under-5 cause-specific SCD mortality rate (per 100,000 population), 2021	Under-5 cause-specific SCD mortality rate (per 100,000 population) percent change (2000-2021)	Under-5 total SCD mortality rate (per 100,000 population), 2000	Under-5 total SCD mortality rate (per 100,000 population), 2021	Under-5 total SCD mortality rate (per 100,000 population) percent change (2000-2021)	Under-5 cause-specific mortality cause fraction, 2000	Under-5 total SCD mortality cause fraction, 2000	Under-5 cause-specific mortality cause fraction, 2021	Under-5 total SCD mortality cause fraction, 2021	Under-5 cause-specific mortality rank 2000	Under-5 cause-specific mortality rank 2021	Under-5 total SCD mortality rank 2000	Under-5 total SCD mortality rank 2021
Kajiado	1,050 (766-1,460)	1,080 (777-1,490)	2.4 (-7.5-15.2)	503 (384-656)	403 (305-529)	-19.9 (-27.4--11.5)	0.4 (0.2-0.7)	0.3 (0.1-0.5)	-30.7 (-67.2-26.3)	24.9 (17.1-35.6)	22.6 (16-31.9)	-9.6 (-19.9-2.9)	0.7 (0.4-1.1)	0.5 (0.2-1.2)	-23.1 (-71.4-63.7)	28.3 (17-43.7)	30.4 (18.7-46)	7.4 (-9.6-28.1)	0 (0-0.1)	2 (1.2-3.2)	0.1 (0-0.1)	4.1 (2.3-7)	52	52	12	8
Kakamega	1,430 (1,050-1,930)	1,490 (1,090-1,950)	3.9 (-8.7-15.3)	675 (511-887)	571 (440-740)	-15.4 (-23.9--7.2)	1 (0.6-1.4)	0.6 (0.4-0.9)	-41.1 (-64.2-5)	36.7 (25.4-52.2)	34.1 (24.6-46.5)	-7.1 (-20-6.2)	2.4 (0.9-4.7)	0.7 (0.3-1.2)	-69.3 (-80.2--34.5)	52.3 (31.2-80.4)	58.3 (37-84.9)	11.6 (-8.3-36.7)	0.1 (0-0.2)	2 (1.2-2.9)	0.1 (0.1-0.2)	10.8 (6.5-17.3)	45	46	14	2
Kericho	1,070 (764-1,470)	1,110 (814-1,450)	3.2 (-6.7-14.8)	531 (394-719)	425 (325-553)	-20 (-26.9--11.8)	0.3 (0.2-0.5)	0.3 (0.2-0.6)	4.8 (-27.3-58.8)	25.8 (17.8-37.2)	23.4 (16.6-32.7)	-9.2 (-19.4-2.2)	0.3 (0.2-0.5)	1.1 (0.7-1.9)	257 (128-444)	29.9 (18.4-44.4)	33.2 (20.5-50.4)	11.1 (-8.1-31.8)	0 (0-0)	2.5 (1.6-3.9)	0.1 (0-0.1)	2.1 (1.2-3.4)	56	54	10	11
Kiambu	1,040 (749-1,400)	1,060 (757-1,430)	2.2 (-8.7-15.9)	445 (333-580)	344 (259-449)	-22.7 (-29.5--15.1)	0.5 (0.3-0.7)	0.3 (0.2-0.6)	-26 (-48.7-4.4)	23.1 (16-32.1)	19.9 (14.1-27.8)	-13.9 (-23.6--1.5)	0.5 (0.3-0.7)	0.5 (0.1-1)	7.3 (-65.6-115)	27.1 (16.7-40.6)	29.2 (17.8-44.9)	7.8 (-11.5-28.7)	0 (0-0.1)	2.3 (1.5-3.5)	0.1 (0-0.1)	4.2 (2.4-6.9)	55	55	12	7
Kilifi	1,180 (922-1,470)	1,300 (1,030-1,650)	10.1 (3.1-18.3)	569 (460-702)	526 (422-653)	-7.6 (-14--1.2)	0.8 (0.3-1.6)	0.8 (0.3-1.5)	-9 (-40.8-60.7)	28.5 (21.8-36.7)	29.8 (22.7-39.6)	4.7 (-5.3-14.6)	1.7 (0.7-3)	1.2 (0.5-2.2)	-27.6 (-55.8-42.1)	40.8 (27.2-57.5)	50.2 (34.4-70.2)	23.1 (5.6-42.1)	0.1 (0-0.1)	1.5 (1-2.1)	0.1 (0.1-0.2)	5.6 (3.7-8.2)	46	44	14	7
Kirinyaga	1,010 (737-1,390)	1,050 (755-1,460)	3.7 (-6.1-14.1)	415 (314-553)	303 (228-409)	-26.8 (-33.8--20.1)	0.3 (0.2-0.6)	0.3 (0.2-0.5)	-12.6 (-49.3-32)	22.1 (15.5-30.9)	18.1 (12.8-25.9)	-18 (-26.5--6.4)	0.5 (0.3-0.9)	0.5 (0.1-0.9)	-13.2 (-79.3-63.3)	28.7 (17.2-43.7)	31.2 (18.8-48)	8.7 (-9.7-32.8)	0 (0-0.1)	2.3 (1.4-3.3)	0.1 (0-0.1)	4.3 (2.5-7)	56	60	12	8
Kisii	1,490 (1,100-2,000)	1,540 (1,140-2,010)	3.2 (-5.4-12.1)	695 (528-901)	532 (415-680)	-23.5 (-30.1--17.3)	1.4 (0.6-2.2)	1 (0.5-1.6)	-31.4 (-54.9-7.5)	37.9 (27-54.2)	32.9 (23.4-45.7)	-13.2 (-22.7--2.8)	2 (0.8-3.6)	1 (0.5-1.8)	-47.9 (-68.2-12.2)	49.3 (31.3-72.7)	55.6 (35.1-81.2)	12.9 (-5.4-33)	0.1 (0-0.2)	2.2 (1.5-3.3)	0.1 (0.1-0.2)	7.7 (4.5-11.7)	45	43	13	6
Kisumu	2,550 (2,030-3,160)	2,590 (2,110-3,270)	1.7 (-8.6-9.8)	1,010 (811-1,260)	825 (660-996)	-18.2 (-25.7--10.5)	1.2 (0.7-2)	0.9 (0.5-1.6)	-21.7 (-48.8-22.7)	65.9 (51-84.5)	58.7 (46.4-75.5)	-10.9 (-19.6--0.8)	2 (0.9-3.5)	1.4 (0.7-2.2)	-30.3 (-54.8-38.6)	116 (73.3-173)	124 (83.1-180)	6.7 (-10.1-27.9)	0.1 (0-0.1)	3.7 (2.4-5.8)	0.1 (0.1-0.2)	12.9 (8.2-19.4)	45	41	9	2
Kitui	1,060 (761-1,460)	1,090 (789-1,490)	2.6 (-8-13.4)	510 (384-669)	425 (321-559)	-16.8 (-24.2--7.2)	0.4 (0.2-0.6)	0.2 (0.1-0.4)	-33.5 (-59.7-1.4)	25.8 (17.7-36.9)	23.8 (16.7-34.4)	-7.6 (-18.3-4)	0.5 (0.3-0.8)	0.3 (0.2-0.5)	-40.9 (-66.9-22.6)	31.4 (19.4-47)	33.9 (20.9-50.8)	7.8 (-11.8-29.9)	0 (0-0.1)	2.1 (1.3-3.3)	0.1 (0-0.1)	6.5 (3.7-10.8)	58	59	12	6
Kwale	1,560 (1,130-2,070)	1,610 (1,180-2,130)	3.6 (-8.9-14.9)	724 (546-930)	648 (501-847)	-10.6 (-19.5--2)	1 (0.4-1.8)	0.8 (0.4-1.4)	-16.4 (-48.9-44.3)	38.5 (26.9-53.3)	37.2 (25.7-51.5)	-3.2 (-14.1-10.7)	1.7 (0.7-3.3)	1.2 (0.6-2.1)	-28.7 (-56-37.7)	53.7 (33.4-83.2)	60.1 (37-90.4)	12 (-6.2-35.3)	0.1 (0-0.1)	2.4 (1.6-3.5)	0.1 (0.1-0.2)	5.7 (3.4-8.9)	43	43	12	6
Laikipia	1,020 (733-1,350)	1,050 (745-1,400)	3.1 (-9-15.6)	500 (378-659)	394 (299-518)	-21.3 (-28.7--13.9)	0.3 (0.2-0.5)	0.2 (0.1-0.4)	-37.3 (-64.1-0.2)	23.2 (15.7-33.7)	20.8 (14.3-29)	-10.3 (-21.1-3)	0.3 (0.2-0.5)	0.2 (0.1-0.5)	-26.6 (-67.3-63.6)	23.8 (14.3-35.9)	25.7 (15.9-38.6)	8.1 (-11.8-31.9)	0 (0-0)	2.4 (1.4-3.7)	0.1 (0-0.1)	7.9 (4.4-12.7)	59	59	11	5
Lamu	1,090 (809-1,460)	1,120 (814-1,510)	2.6 (-9.3-13.3)	472 (361-607)	393 (303-515)	-16.6 (-23.9--9.9)	0.5 (0.3-0.8)	0.4 (0.2-0.7)	-23.2 (-58-39.8)	25.6 (18-35.9)	23.2 (16.5-32.6)	-9.5 (-20.1-1.8)	1.1 (0.4-1.8)	0.7 (0.4-1.1)	-38.8 (-66.3-35.5)	36.3 (21.6-57.3)	38.9 (23.1-60.3)	7.3 (-10.6-27.9)	0 (0-0.1)	1.5 (0.9-2.5)	0.1 (0-0.1)	3.7 (2.2-5.9)	55	61	14	10
Machakos	1,010 (726-1,360)	1,040 (734-1,390)	2.6 (-6.1-14)	475 (355-627)	350 (268-454)	-26.3 (-33.1--18.4)	0.2 (0.1-0.4)	0.2 (0.1-0.4)	-8 (-36.8-36.2)	23.3 (15.8-33.4)	19.5 (13.5-27.2)	-16.3 (-26.3--4.5)	0.4 (0.2-0.6)	0.4 (0.2-0.7)	15.9 (-45.6-116)	27.6 (16.4-42.5)	29.8 (18.2-45.5)	7.8 (-10.6-30.1)	0 (0-0)	2.2 (1.3-3.5)	0.1 (0-0.1)	4 (2.2-6.7)	58	56	10	8
Makueni	1,080 (790-1,510)	1,110 (787-1,510)	2.3 (-6.6-13.8)	516 (385-692)	418 (310-552)	-19.1 (-25--12.3)	0.2 (0.1-0.4)	0.2 (0.1-0.3)	-24 (-58.5-14.5)	25.2 (17.5-37)	23 (15.8-32.2)	-8.8 (-18.5-3.2)	0.2 (0.1-0.4)	0.1 (0-0.2)	-64.4 (-82.1--30)	29.2 (18.3-43.7)	31 (19.3-47)	6.3 (-9.1-26.5)	0 (0-0)	2.5 (1.6-3.8)	0 (0-0.1)	14.9 (8.8-23.8)	61	58	10	1
Mandera	1,130 (816-1,520)	1,160 (814-1,580)	2.7 (-6.8-16.6)	550 (409-715)	506 (383-658)	-7.9 (-17.7-5)	0.5 (0.3-0.7)	0.4 (0.2-0.6)	-26 (-51.2-16.7)	30.5 (20.8-44)	29.3 (20.3-41.5)	-3.8 (-14.9-9.5)	1 (0.4-1.7)	0.2 (0.1-0.4)	-75.7 (-85.4--55)	39.6 (23.7-61.8)	40.8 (24.7-61.6)	3.3 (-14.8-24.6)	0 (0-0.1)	1.9 (1.1-3.2)	0.1 (0-0.1)	10 (6-16)	58	59	15	4
Marsabit	1,090 (800-1,470)	1,120 (808-1,520)	2.3 (-9.8-12.5)	510 (379-685)	462 (343-591)	-9.5 (-17.6--1.5)	0.4 (0.2-0.6)	0.4 (0.2-0.6)	7.6 (-30.2-78)	26.9 (18.9-39.3)	26 (18.3-37.2)	-3.4 (-14-9.2)	0.6 (0.3-1)	0.5 (0.3-0.8)	-4.1 (-37.2-59.2)	34.6 (20.5-53.7)	36.2 (21.9-55.5)	4.6 (-12.9-26.8)	0 (0-0.1)	2 (1.2-3.1)	0 (0-0.1)	3.3 (2-5.4)	59	64	12	7
Meru	1,040 (756-1,420)	1,060 (750-1,420)	2.8 (-5.6-15.6)	459 (344-607)	351 (262-454)	-23.5 (-29.5--15.4)	0.2 (0.1-0.4)	0.3 (0.2-0.6)	39.8 (-16.3-131)	23.8 (16.3-33.1)	20.4 (14.1-28.3)	-14.2 (-23.4--2.6)	0.4 (0.2-0.6)	0.8 (0.4-1.2)	88.1 (-10.9-192)	30.8 (18.6-46.8)	33.2 (19.9-50.7)	7.8 (-7.8-30.6)	0 (0-0)	2.3 (1.5-3.5)	0.1 (0-0.1)	3.6 (2.1-5.7)	59	57	11	9
Migori	2,210 (1,660-2,920)	2,330 (1,680-3,040)	5.3 (-2.9-19.4)	994 (762-1,270)	955 (725-1,250)	-3.9 (-11.2-5.6)	2.2 (0.6-4.3)	1.8 (0.5-3.3)	-20.6 (-54.5-48.3)	58 (41-80.5)	61.1 (43.3-84.8)	5.5 (-5.7-18.2)	5.3 (1-10.6)	2.4 (0.5-4.4)	-54.8 (-72.8-24.4)	87.1 (55.2-131)	103 (66.5-154)	17.9 (0.3-38)	0.1 (0-0.2)	1.8 (1.1-2.7)	0.2 (0-0.4)	8.6 (5.7-13.6)	38	36	14	5
Mombasa	1,310 (931-1,760)	1,350 (980-1,790)	2.9 (-7.1-13)	556 (422-709)	485 (371-629)	-12.8 (-20.8--6.9)	0.5 (0.2-1)	0.4 (0.3-0.8)	-6.8 (-56.3-68.7)	28.6 (20.6-39.7)	27.2 (19.8-36.9)	-5 (-14.8-5.4)	0.5 (0.3-0.8)	0.3 (0.1-0.5)	-44.3 (-70.1-3.5)	38.2 (24.2-56.9)	42.6 (26.8-61.9)	11.4 (-6-33.6)	0 (0-0.1)	2.4 (1.5-3.5)	0.1 (0-0.1)	11.1 (6.8-17.4)	51	49	10	3
Murang'a	998 (699-1,350)	1,020 (736-1,360)	2.4 (-6.8-12)	445 (337-585)	308 (235-399)	-30.9 (-36.6--24.8)	0.3 (0.1-0.5)	0.2 (0.1-0.4)	-5.8 (-39.6-58.8)	22.1 (15.1-31.6)	17.5 (12.4-24.7)	-20.6 (-29.6--10.1)	0.3 (0.2-0.5)	0.3 (0.1-0.7)	-0.7 (-68.2-70.6)	25.8 (15.3-39.7)	27.2 (16.5-40.7)	5.5 (-11.9-24.3)	0 (0-0.1)	2.6 (1.6-4.2)	0.1 (0-0.1)	5.2 (3-8)	57	56	12	7
Nairobi	1,070 (771-1,450)	1,100 (786-1,500)	2.9 (-6.8-15)	425 (321-549)	364 (276-486)	-14.3 (-21.7--6.2)	0.5 (0.3-0.8)	0.4 (0.2-0.5)	-31.3 (-58-13.2)	23.1 (16.3-32.1)	21.5 (15.3-29.7)	-7.2 (-17.3-3.9)	0.6 (0.4-0.9)	0.1 (0-0.3)	-79.4 (-95.6--43.3)	28 (16.9-41.9)	30.7 (19-45.7)	9.6 (-7.7-31.2)	0 (0-0.1)	2.1 (1.2-3.2)	0.1 (0-0.1)	14.5 (7.6-24.7)	56	61	11	1
Nakuru	1,020 (737-1,400)	1,040 (748-1,400)	2.1 (-10.1-14.5)	491 (374-641)	385 (294-497)	-21.6 (-29.2--14.9)	0.3 (0.2-0.5)	0.3 (0.1-0.4)	-12.8 (-37.8-30.1)	23.8 (16.1-33.6)	21.2 (15-29.3)	-10.8 (-20.9--0.3)	0.3 (0.2-0.5)	0.4 (0.2-0.7)	30.7 (-23.1-115)	25.9 (15.9-39.1)	27.5 (16.9-40.5)	6.1 (-10.2-27.5)	0 (0-0.1)	2.7 (1.7-4.2)	0.1 (0-0.1)	4.8 (2.7-7.9)	55	56	10	8
Nandi	1,120 (800-1,520)	1,150 (838-1,520)	2.5 (-7.7-14.5)	575 (435-745)	470 (359-622)	-18.2 (-25.2--9.2)	0.3 (0.2-0.6)	0.3 (0.2-0.5)	-10.8 (-38.5-25.1)	26.4 (18.3-37.9)	24.4 (17.2-34.7)	-7.3 (-17.8-2.9)	0.3 (0.2-0.5)	0.4 (0.2-0.7)	21.1 (-21.7-97.9)	29.2 (17.7-44.6)	31.6 (19.5-46.7)	8.2 (-9-27.2)	0 (0-0)	2.8 (1.7-4.6)	0.1 (0-0.1)	6.1 (3.8-9.5)	54	54	10	7
Narok	1,120 (817-1,500)	1,160 (806-1,590)	2.8 (-9-14.1)	592 (437-768)	519 (382-690)	-12.3 (-21.1--2.8)	0.4 (0.2-0.6)	0.3 (0.2-0.6)	-10.8 (-46.6-34.4)	28.3 (19.6-40.5)	27.3 (19.1-38.5)	-3.7 (-14.3-9.6)	0.3 (0.1-0.5)	0.1 (0.1-0.2)	-48 (-68.9--9.6)	30.9 (19-47.4)	32.8 (20.1-49.8)	6.4 (-7.9-24.6)	0 (0-0)	3 (1.8-4.9)	0.1 (0-0.1)	12.1 (7.3-19.5)	60	59	12	3
Nyamira	1,310 (934-1,740)	1,340 (941-1,790)	2.1 (-6.7-13.3)	614 (460-798)	511 (372-666)	-16.8 (-22.4--9.3)	0.7 (0.4-1)	0.8 (0.5-1.2)	19.2 (-22.3-85)	30.8 (21.4-43.9)	29.1 (20-42.2)	-5.5 (-16.3-6.3)	0.4 (0.2-0.6)	0.3 (0.2-0.6)	-11.2 (-45.7-63)	32.5 (19.8-48.6)	35 (21.8-50.9)	7.7 (-8.1-30.5)	0 (0-0.1)	3 (1.9-4.5)	0.1 (0-0.1)	9.3 (5.7-13.5)	50	50	10	5
Nyandarua	1,060 (775-1,460)	1,090 (796-1,480)	2.9 (-10.7-12)	508 (386-674)	387 (291-503)	-23.8 (-30.9--18.3)	0.4 (0.2-0.6)	0.4 (0.2-0.7)	7 (-30-67.1)	25.1 (17.5-35.7)	21.8 (15.2-31.1)	-13.1 (-21.2--3.6)	0.3 (0.1-0.4)	0.2 (0.1-0.4)	-14.1 (-73-81.9)	24.7 (14.9-37.6)	26.4 (16.1-41.2)	6.9 (-10.6-26.5)	0 (0-0)	3 (1.8-4.9)	0.1 (0-0.1)	7.1 (4.1-11.6)	58	62	11	6
Nyeri	1,040 (760-1,390)	1,070 (755-1,460)	2.5 (-7.6-14)	442 (334-581)	311 (237-405)	-29.7 (-34.8--23.3)	0.4 (0.2-0.6)	0.3 (0.2-0.6)	-10 (-44-68.6)	22.5 (16.1-32.2)	18.1 (12.7-25.2)	-19.8 (-29--9.2)	0.2 (0.1-0.4)	0.3 (0.1-0.5)	23.4 (-59.8-123)	25.2 (15.4-38.2)	26.7 (16.1-41.1)	6 (-12-30.6)	0 (0-0)	2.9 (1.7-4.6)	0.1 (0-0.1)	6.4 (3.7-10.4)	61	59	10	7
Samburu	1,100 (781-1,490)	1,140 (826-1,580)	3.4 (-5.2-14.3)	557 (412-737)	502 (373-674)	-9.8 (-14.9--2.2)	0.3 (0.2-0.6)	0.3 (0.2-0.6)	7.9 (-29.1-84.9)	27.3 (18.6-39.4)	27 (18.5-39.5)	-1.1 (-13.2-12.1)	0.4 (0.2-0.6)	0.4 (0.2-0.6)	1.8 (-33.8-70.9)	29 (17.4-43.1)	31 (18.9-47)	6.9 (-9.7-28)	0 (0-0)	1.8 (1.1-2.7)	0 (0-0.1)	3.3 (1.9-5.4)	65	65	16	8
Siaya	2,100 (1,550-2,650)	2,180 (1,580-2,850)	3.9 (-5.5-19.5)	900 (695-1,150)	786 (597-1,020)	-12.6 (-20--2.3)	2.1 (0.5-4.1)	1.1 (0.3-2.3)	-46.3 (-65.2--6.5)	52.2 (37.8-70.8)	50.2 (35.7-68.5)	-3.8 (-14.4-9.2)	5.7 (1.3-11.6)	2.5 (0.5-4.9)	-55.5 (-69.8-4)	82.5 (52.5-124)	95.1 (61.8-140)	15.3 (-1.3-38.2)	0.1 (0-0.3)	1.8 (1.1-2.7)	0.2 (0-0.4)	7.5 (4.6-11.4)	37	40	14	6

Location	Birth Incidence rate (per 100,000 live births), 2000	Birth Incidence rate (per 100,000 live births), 2021	Birth Incidence rate (per 100,000 live births) percent change (2000-2021)	All age rate (per 100,000 population), 2000	All age rate (per 100,000 population), 2021	All age rate (per 100,000 population) percent change (2000-2021)	All age cause-specific SCD mortality rate (per 100,000 population), 2000	All age cause-specific SCD mortality rate (per 100,000 population), 2021	All age cause-specific SCD mortality rate (per 100,000 population) percent change (2000-2021)	All age total SCD mortality rate (per 100,000 population), 2000	All age total SCD mortality rate (per 100,000 population), 2021	All age total SCD mortality rate (per 100,000 population) percent change (2000-2021)	Under-5 cause-specific SCD mortality rate (per 100,000 population), 2000	Under-5 cause-specific SCD mortality rate (per 100,000 population), 2021	Under-5 cause-specific SCD mortality rate (per 100,000 population) percent change (2000-2021)	Under-5 total SCD mortality rate (per 100,000 population), 2000	Under-5 total SCD mortality rate (per 100,000 population), 2021	Under-5 total SCD mortality rate (per 100,000 population) percent change (2000-2021)	Under-5 cause-specific mortality cause fraction, 2000	Under-5 total SCD mortality cause fraction, 2000	Under-5 cause-specific mortality cause fraction, 2021	Under-5 total SCD mortality cause fraction, 2021	Under-5 cause-specific mortality rank 2000	Under-5 cause-specific mortality rank 2021	Under-5 total SCD mortality rank 2000	Under-5 total SCD mortality rank 2021
Taita Taveta	1,140 (834-1,530)	1,170 (848-1,580)	2.9 (-8.9-14.2)	476 (362-616)	363 (278-483)	-23.9 (-30.8--15.6)	0.4 (0.2-0.7)	0.4 (0.2-0.7)	-3.6 (-50.6-65)	25.7 (18.1-36.5)	21.8 (15.5-30.4)	-15 (-25.2--4)	0.7 (0.5-1.1)	0.6 (0.3-1.1)	-11.7 (-57.4-60.7)	34.7 (21.1-52.2)	37.5 (22.4-56.9)	7.8 (-11.8-30.4)	0 (0-0.1)	1.9 (1.3-3)	0.1 (0-0.1)	5.7 (3.4-9.7)	52	56	11	6
Tana River	1,230 (899-1,700)	1,280 (915-1,720)	3.4 (-8.4-15.4)	579 (442-770)	558 (411-730)	-3.5 (-12.1-4.5)	0.7 (0.5-1)	0.6 (0.4-0.9)	-16.5 (-49-40.3)	31.4 (22-44.2)	32 (22.4-45.1)	2 (-9.8-14.7)	1 (0.5-1.6)	0.7 (0.4-1.1)	-31.8 (-62.4-31.6)	42.3 (26.2-63.9)	45.8 (28.5-70.6)	8.3 (-10.4-27.7)	0 (0-0.1)	1.8 (1.1-2.8)	0.1 (0-0.1)	4.1 (2.3-6.3)	55	55	15	8
Tharaka Nithi	1,080 (758-1,480)	1,110 (818-1,500)	2.4 (-6.1-11.4)	464 (343-610)	394 (305-521)	-15.1 (-22--7.2)	0.6 (0.4-0.9)	0.4 (0.2-0.6)	-30.9 (-57.6-0)	24.9 (17.3-35.8)	23.4 (16.6-32.9)	-6 (-16.8-7.3)	0.9 (0.4-1.5)	1 (0.5-1.8)	22.6 (-46-190)	33.5 (20.4-51.3)	35.7 (21.4-54.3)	6.7 (-9.4-28.6)	0.1 (0-0.1)	2 (1.2-3.3)	0.1 (0-0.1)	2.5 (1.5-4.1)	56	57	15	11
Trans Nzoia	1,190 (872-1,610)	1,220 (881-1,660)	2.9 (-6.9-14.8)	583 (441-763)	476 (353-614)	-18.4 (-25.9--11.5)	0.4 (0.2-0.7)	0.3 (0.2-0.6)	-19.4 (-49-33.9)	29 (20-40.6)	26.7 (18.9-37.2)	-7.8 (-17.7-5)	0.4 (0.3-0.7)	0.4 (0.2-0.7)	-6.4 (-44.3-61.4)	34.5 (21.2-53.3)	37.7 (23.6-56.9)	9.3 (-7.7-33.2)	0 (0-0.1)	2.9 (1.7-4.3)	0.1 (0.1-0.1)	8.7 (5.2-13.2)	47	52	10	3
Turkana	1,210 (861-1,660)	1,240 (904-1,670)	3 (-6.4-14.9)	585 (444-773)	542 (422-703)	-7.2 (-14.7-2.1)	0.4 (0.2-0.6)	0.3 (0.1-0.6)	-21.5 (-57.8-48.8)	30.3 (21.1-43.3)	30.2 (21.4-41.6)	-0.4 (-13.4-13.2)	0.4 (0.2-0.6)	0.1 (0.1-0.2)	-68.6 (-82.2--39.2)	37 (22.7-56.6)	40.4 (25.6-60.3)	9.1 (-7.7-26.7)	0 (0-0)	2 (1.2-3.1)	0 (0-0.1)	9.4 (5.7-14.6)	61	62	13	4
Uasin Gishu	1,070 (776-1,480)	1,100 (798-1,490)	2.5 (-7.3-15.2)	525 (390-692)	423 (331-545)	-19.3 (-26.1--11.1)	0.4 (0.2-0.6)	0.3 (0.2-0.5)	-15.8 (-41.4-22.6)	25.3 (17.4-36.8)	23.1 (16.4-32.5)	-8.6 (-18.1-2.8)	0.4 (0.2-0.5)	0.6 (0.4-0.9)	65 (0.4-150)	29.3 (18.5-44.4)	31.8 (20-46.8)	8.4 (-9.1-28.3)	0 (0-0)	2.6 (1.7-4)	0.1 (0.1-0.1)	4.7 (2.8-7.3)	54	54	10	7
Vihiga	1,530 (1,120-2,020)	1,590 (1,160-2,140)	3.8 (-9.6-21.8)	669 (516-875)	571 (438-747)	-14.7 (-23.8--4)	0.9 (0.4-1.6)	0.8 (0.4-1.6)	-6.2 (-44.2-53.2)	35.7 (24.4-50.5)	33.9 (24.3-47.8)	-5.1 (-16.1-9.6)	1.6 (0.7-2.8)	0.6 (0.3-1)	-61.8 (-74.1--31.9)	51.2 (31.4-77.9)	56.9 (36.3-84.3)	11.1 (-7-35.7)	0.1 (0-0.1)	2.5 (1.5-3.7)	0.1 (0.1-0.2)	12.1 (7.7-19.6)	41	40	11	2
Wajir	1,120 (803-1,500)	1,160 (831-1,540)	3 (-10.6-15.3)	538 (406-706)	528 (394-682)	-2 (-11.8-9.1)	0.6 (0.4-0.8)	0.4 (0.2-0.6)	-37.1 (-58.8--2.6)	29.8 (20.9-41.9)	30.1 (21-42.7)	1 (-12.7-15.7)	1.1 (0.5-2.1)	0.4 (0.2-0.6)	-65.6 (-78.7--31.9)	38.4 (23.2-59.9)	40.5 (25.1-63.7)	5.5 (-13.9-25.1)	0 (0-0.1)	1.3 (0.8-2.3)	0 (0-0)	3.4 (2.1-5.7)	61	66	15	8
West Pokot	1,180 (851-1,600)	1,220 (890-1,640)	3.2 (-7.5-13)	574 (431-775)	531 (403-694)	-7.4 (-16-0.5)	0.6 (0.4-1)	0.5 (0.3-0.7)	-26.4 (-55.4-25.7)	30.7 (21.4-44.6)	30.8 (21.2-43.8)	0.2 (-12.6-12.3)	0.8 (0.4-1.6)	0.3 (0.2-0.5)	-59.9 (-74.1--20)	39.7 (23.8-62.1)	43.1 (26.2-66.1)	8.5 (-10.3-29.5)	0 (0-0.1)	2.2 (1.3-3.2)	0.1 (0-0.1)	7 (4.2-11.3)	53	56	14	6
Madagascar	373 (315-439)	369 (310-440)	-0.9 (-9.1-8.2)	162 (136-191)	144 (121-171)	-10.6 (-17.2--2.1)	0.9 (0.5-1.3)	0.7 (0.4-1.1)	-22 (-45-21.9)	9.2 (7.6-11.2)	8.4 (7-10.2)	-8.3 (-15.2--0.7)	1.6 (0.5-3.2)	0.9 (0.4-1.7)	-41.9 (-63.4-35.5)	15 (10.2-20.8)	15 (10-20.4)	0 (-12.3-14.4)	0.1 (0-0.1)	0.6 (0.4-0.8)	0.1 (0-0.1)	1.2 (0.7-1.6)	52	57	20	17
Malawi	76.3 (66.3-92.3)	76.4 (65.9-94.1)	0.1 (-8.7-8.3)	32.4 (27.3-39.5)	29.6 (24.6-35.5)	-8.5 (-16.4-0.2)	0.5 (0.3-0.8)	0.3 (0.2-0.5)	-39.9 (-68.2-17.7)	1.9 (1.6-2.4)	1.8 (1.5-2.2)	-5.8 (-13.6-1.6)	1 (0.3-2.1)	0.4 (0.2-0.7)	-58.4 (-78.1-24.6)	3.5 (2.5-4.7)	3.6 (2.5-4.8)	2.7 (-10.6-16.4)	0 (0-0.1)	0.1 (0.1-0.1)	0 (0-0.1)	0.3 (0.2-0.4)	81	80	50	32
Mozambique	18.7 (15.5-22.1)	18.9 (15.7-22.7)	1.2 (-4-6.6)	6.5 (5.4-7.4)	6.4 (5.4-7.4)	-1.2 (-5.9-3.3)	0.2 (0.1-0.3)	0.1 (0.1-0.2)	-34.9 (-64.7-35.5)	0.5 (0.4-0.6)	0.5 (0.4-0.6)	-1.2 (-7.8-5.4)	0.4 (0.1-0.8)	0.2 (0.1-0.3)	-56.2 (-78.2-38)	1.3 (1-1.8)	1.3 (1-1.8)	1.4 (-8.6-11.8)	0 (0-0)	0 (0-0)	0 (0-0)	0.1 (0.1-0.1)	96	96	75	54
Rwanda	23.5 (18.9-28.3)	23.6 (18.9-28.9)	0.4 (-6.7-8.3)	9.2 (7.5-11.2)	7.7 (6.2-9.2)	-16.3 (-21.9--7.5)	0.3 (0.2-0.5)	0.1 (0.1-0.2)	-56.2 (-76.6--5.2)	0.6 (0.5-0.8)	0.5 (0.4-0.7)	-13.7 (-21--6.4)	0.5 (0.1-1)	0.2 (0.1-0.4)	-60.6 (-81.1-22.6)	1.3 (0.9-1.8)	1.3 (0.9-1.8)	0.4 (-10.7-12.7)	0 (0-0)	0 (0-0.1)	0 (0-0)	0.1 (0.1-0.2)	100	92	77	47
Somalia	5.2 (3.8-7.3)	5.2 (3.7-7.1)	-0.6 (-10.3-9.9)	1.6 (1.2-2.1)	1.6 (1.1-2)	-3.1 (-10.9-5.3)	0.1 (0-0.1)	0 (0-0.1)	-22.6 (-57.1-41.3)	0.2 (0.1-0.2)	0.2 (0.1-0.2)	-4.6 (-15.1-6.1)	0.1 (0-0.3)	0.1 (0-0.2)	-33.2 (-67-65)	0.5 (0.3-0.7)	0.5 (0.3-0.7)	-0.8 (-14.4-13.2)	0 (0-0)	0 (0-0)	0 (0-0)	0 (0-0)	116	121	101	94
South Sudan	29.8 (22.3-37)	30 (23-37.4)	0.7 (-8.6-9.5)	11.5 (8.8-14.4)	10.9 (8.6-13.8)	-4.7 (-12.1-1.5)	0.2 (0.1-0.3)	0.2 (0.1-0.4)	37.1 (-13.2-128)	0.8 (0.6-1)	0.7 (0.6-1)	-4.6 (-13.3-4.5)	0.4 (0.1-0.8)	0.6 (0.2-1.2)	67.1 (-5-346)	1.7 (1.2-2.4)	1.7 (1.2-2.4)	1.1 (-10.8-14.8)	0 (0-0)	0 (0-0.1)	0 (0-0)	0 (0-0.1)	101	103	64	77
Uganda	579 (495-663)	577 (481-655)	-0.2 (-7.5-8.5)	271 (231-314)	248 (209-286)	-8.6 (-15.8-0.5)	1.1 (0.5-1.6)	0.7 (0.4-1.2)	-31 (-55.5-10.4)	15.3 (12.5-17.8)	14.4 (11.4-17)	-5.6 (-13.5-1.7)	1.8 (0.5-3.2)	1.1 (0.5-1.9)	-39.4 (-62.1-55.8)	23 (15.3-32.2)	23.4 (14.5-33.3)	2 (-11.9-17.9)	0.1 (0-0.1)	0.7 (0.5-1)	0.1 (0-0.1)	1.8 (1.1-2.7)	52	52	19	13
United Republic of Tanzania	611 (517-704)	617 (514-703)	1 (-7.6-12.5)	289 (241-332)	274 (227-318)	-4.9 (-12.9-4.7)	1.7 (0.7-5.7)	1.3 (0.5-4.7)	-24.9 (-56.9-24.1)	14.4 (11.7-16.9)	14.1 (11.2-16.9)	-2.2 (-11.3-9.4)	3.2 (0.8-7)	2.1 (0.7-5.4)	-36.1 (-65-28.1)	22 (14-30.1)	22.9 (15.7-31.8)	4.2 (-9.2-26)	0.1 (0-0.2)	0.8 (0.5-1)	0.2 (0.1-0.6)	2.2 (1.4-3.2)	46	45	19	12
Zambia	743 (641-910)	746 (648-904)	0.4 (-5.7-11.1)	339 (290-409)	309 (262-379)	-8.7 (-15.3-0.3)	1.8 (0.7-2.9)	1.1 (0.4-1.9)	-38.6 (-58.2-0.3)	18.8 (15.6-23.3)	17.5 (14.5-21.8)	-7 (-15-2.1)	3.6 (1-7)	1.8 (0.6-3.1)	-48.8 (-66.3-10.2)	28.8 (19.3-39.7)	29.5 (20.5-40.7)	2.4 (-11.3-16.1)	0.1 (0-0.2)	0.9 (0.6-1.2)	0.2 (0.1-0.3)	3 (1.8-4)	45	45	19	11
**Southern Sub-Saharan Africa**	6.8 (5.7-7.9)	6.8 (5.8-8.2)	-1.2 (-7.2-9.1)	2.1 (1.8-2.5)	1.9 (1.6-2.2)	-13.4 (-18.1--5.6)	0 (0-0.1)	0 (0-0.1)	5.9 (-31.3-51)	0.1 (0.1-0.2)	0.1 (0.1-0.1)	-12.2 (-17.7--3.5)	0 (0-0)	0.1 (0-0.1)	77.4 (-3.6-202)	0.4 (0.3-0.5)	0.4 (0.3-0.5)	-1 (-8.8-8.5)	0 (0-0)	0 (0-0)	0 (0-0)	0 (0-0)	109	109	63	73
Botswana	43.4 (36.1-51.4)	43.5 (36.3-52.3)	0.2 (-7-8)	21.2 (18.1-25.1)	17.4 (14.7-20.2)	-18.1 (-24.7--11.8)	0.2 (0.1-0.3)	0.1 (0.1-0.2)	-34.1 (-58.3-4.4)	0.9 (0.7-1.1)	0.8 (0.6-0.9)	-10.5 (-18.5--3.3)	0.1 (0.1-0.1)	0.1 (0-0.2)	29.3 (-45.1-146)	1.3 (0.9-1.7)	1.3 (1-1.7)	1.3 (-10-13.8)	0 (0-0)	0.1 (0.1-0.1)	0 (0-0)	0.2 (0.1-0.2)	93	98	38	42
Eswatini	3.3 (2.6-4.2)	3.3 (2.5-4.2)	0.3 (-6.3-6.9)	0.9 (0.7-1.2)	0.8 (0.6-1)	-18.3 (-24.6--10.3)	0 (0-0)	0 (0-0)	-21.1 (-54.6-22.4)	0.1 (0.1-0.1)	0.1 (0.1-0.1)	-16.5 (-22.3--10.5)	0 (0-0)	0 (0-0)	8 (-37.2-70.7)	0.3 (0.2-0.4)	0.3 (0.2-0.4)	0.3 (-9.2-11.5)	0 (0-0)	0 (0-0)	0 (0-0)	0 (0-0)	117	120	75	77
Lesotho	2.9 (2.2-3.9)	2.9 (2.2-3.8)	0.2 (-9.4-9.8)	0.7 (0.6-0.9)	0.6 (0.5-0.7)	-17.6 (-25.6--9.4)	0 (0-0)	0 (0-0)	15 (-32.8-99.9)	0.1 (0.1-0.1)	0.1 (0-0.1)	-16 (-23--8.5)	0 (0-0)	0 (0-0)	42.3 (-22.1-150)	0.2 (0.2-0.4)	0.3 (0.2-0.4)	0.5 (-10.6-12.5)	0 (0-0)	0 (0-0)	0 (0-0)	0 (0-0)	114	118	67	76
Namibia	3.2 (2.5-4.1)	3.1 (2.4-4)	-1.9 (-8.7-4.2)	0.9 (0.8-1.1)	0.8 (0.6-1)	-15.8 (-21.4--9.4)	0 (0-0)	0 (0-0)	-31.9 (-64.6-16)	0.1 (0.1-0.1)	0.1 (0.1-0.1)	-13.3 (-20.8--5.8)	0 (0-0)	0 (0-0)	11.5 (-40.2-105)	0.3 (0.2-0.4)	0.2 (0.2-0.4)	-2 (-14.1-10)	0 (0-0)	0 (0-0)	0 (0-0)	0 (0-0.1)	117	120	66	69
South Africa	1.8 (1.3-2.5)	1.7 (1.2-2.4)	-1.7 (-5.6-2.1)	0.3 (0.2-0.4)	0.3 (0.2-0.3)	-19.7 (-23.1--16.9)	0 (0-0)	0 (0-0)	-36.8 (-58.7--2.5)	0 (0-0.1)	0 (0-0)	-19.5 (-22.8--16.2)	0 (0-0)	0 (0-0)	15 (-30.3-82.3)	0.2 (0.1-0.3)	0.2 (0.1-0.3)	-3.8 (-9-1.8)	0 (0-0)	0 (0-0)	0 (0-0)	0 (0-0)	122	121	79	81
Eastern Cape	1.8 (1.3-2.6)	1.8 (1.3-2.6)	0 (-7.7-10.7)	0.4 (0.3-0.5)	0.3 (0.2-0.4)	-22 (-27.7--15.9)	0 (0-0)	0 (0-0)	-33.6 (-54.5-5)	0 (0-0.1)	0 (0-0)	-21 (-29--11.1)	0 (0-0)	0 (0-0)	-15.3 (-51.4-54.9)	0.2 (0.1-0.3)	0.2 (0.1-0.3)	-0.3 (-13.5-16.5)	0 (0-0)	0 (0-0)	0 (0-0)	0 (0-0)	120	128	85	80
Free State	1.8 (1.3-2.5)	1.8 (1.3-2.5)	0.3 (-7.4-12.2)	0.3 (0.2-0.4)	0.3 (0.2-0.3)	-17.4 (-23.7--9)	0 (0-0)	0 (0-0)	-34.2 (-59.5-9.5)	0 (0-0.1)	0 (0-0)	-16.6 (-25.7--4.4)	0 (0-0)	0 (0-0)	32.7 (-20.8-146)	0.2 (0.1-0.3)	0.2 (0.1-0.3)	-0.2 (-14.1-19.8)	0 (0-0)	0 (0-0)	0 (0-0)	0 (0-0)	113	117	77	83
Gauteng	1.6 (1.1-2.2)	1.6 (1.2-2.2)	0.8 (-8.1-18.3)	0.3 (0.2-0.3)	0.2 (0.2-0.3)	-7.8 (-15.7-0.5)	0 (0-0)	0 (0-0)	-43.3 (-64.7--7.5)	0 (0-0)	0 (0-0)	-7.8 (-17-7.9)	0 (0-0)	0 (0-0)	6.6 (-50.4-111)	0.1 (0.1-0.2)	0.1 (0.1-0.2)	0.3 (-13.5-24.3)	0 (0-0)	0 (0-0)	0 (0-0)	0 (0-0)	114	118	78	80
KwaZulu-Natal	1.9 (1.3-2.6)	1.9 (1.3-2.6)	-0.8 (-10-8)	0.4 (0.3-0.5)	0.3 (0.2-0.4)	-19.7 (-25.8--10.4)	0 (0-0)	0 (0-0)	-47.8 (-66.6--15.1)	0 (0-0.1)	0 (0-0)	-19.1 (-26.6--10)	0 (0-0)	0 (0-0)	43.4 (-25.8-164)	0.2 (0.1-0.3)	0.2 (0.1-0.3)	-1.5 (-13.7-13.3)	0 (0-0)	0 (0-0)	0 (0-0)	0 (0-0)	120	123	71	78
Limpopo	2 (1.4-2.8)	2 (1.4-2.8)	0.4 (-6.7-10.7)	0.5 (0.3-0.6)	0.4 (0.3-0.5)	-21.6 (-27.1--15.3)	0 (0-0)	0 (0-0)	24.5 (-24.6-97)	0 (0-0.1)	0 (0-0.1)	-19.6 (-26.3--9.5)	0 (0-0)	0 (0-0)	79.6 (-6.3-236)	0.2 (0.1-0.3)	0.2 (0.1-0.3)	0 (-12.3-17.1)	0 (0-0)	0 (0-0)	0 (0-0)	0 (0-0)	116	119	72	87

Location	Birth Incidence rate (per 100,000 live births), 2000	Birth Incidence rate (per 100,000 live births), 2021	Birth Incidence rate (per 100,000 live births) percent change (2000-2021)	All age rate (per 100,000 population), 2000	All age rate (per 100,000 population), 2021	All age rate (per 100,000 population) percent change (2000-2021)	All age cause-specific SCD mortality rate (per 100,000 population), 2000	All age cause-specific SCD mortality rate (per 100,000 population), 2021	All age cause-specific SCD mortality rate (per 100,000 population) percent change (2000-2021)	All age total SCD mortality rate (per 100,000 population), 2000	All age total SCD mortality rate (per 100,000 population), 2021	All age total SCD mortality rate (per 100,000 population) percent change (2000-2021)	Under-5 cause-specific SCD mortality rate (per 100,000 population), 2000	Under-5 cause-specific SCD mortality rate (per 100,000 population), 2021	Under-5 cause-specific SCD mortality rate (per 100,000 population) percent change (2000-2021)	Under-5 total SCD mortality rate (per 100,000 population), 2000	Under-5 total SCD mortality rate (per 100,000 population), 2021	Under-5 total SCD mortality rate (per 100,000 population) percent change (2000-2021)	Under-5 cause-specific mortality cause fraction, 2000	Under-5 total SCD mortality cause fraction, 2000	Under-5 cause-specific mortality cause fraction, 2021	Under-5 total SCD mortality cause fraction, 2021	Under-5 cause-specific mortality rank 2000	Under-5 cause-specific mortality rank 2021	Under-5 total SCD mortality rank 2000	Under-5 total SCD mortality rank 2021
Mpumalanga	2 (1.4-2.8)	1.9 (1.3-2.8)	-1.6 (-12.1-7.1)	0.4 (0.3-0.5)	0.3 (0.2-0.4)	-20.2 (-28.6--12)	0 (0-0)	0 (0-0)	-41.5 (-64.5-4.4)	0 (0-0.1)	0 (0-0.1)	-19.1 (-30.2--10)	0 (0-0)	0 (0-0)	53.3 (-14-165)	0.2 (0.1-0.3)	0.2 (0.1-0.3)	-2.1 (-20-14.1)	0 (0-0)	0 (0-0)	0 (0-0)	0 (0-0)	115	117	74	79
North-West	1.8 (1.3-2.6)	1.8 (1.2-2.5)	-0.4 (-9.7-9.5)	0.3 (0.2-0.4)	0.3 (0.2-0.4)	-12.6 (-20.5--3)	0 (0-0)	0 (0-0)	-21.8 (-50.8-28.2)	0 (0-0.1)	0 (0-0)	-12.5 (-22.2-0.1)	0 (0-0)	0 (0-0)	-1.7 (-44.7-61.8)	0.2 (0.1-0.3)	0.2 (0.1-0.3)	-1.4 (-15.6-19.6)	0 (0-0)	0 (0-0)	0 (0-0)	0 (0-0)	114	118	79	77
Northern Cape	1.7 (1.3-2.3)	1.7 (1.2-2.3)	0.2 (-14.2-14.8)	0.3 (0.2-0.4)	0.3 (0.2-0.3)	-15.7 (-23.9--5.8)	0 (0-0)	0 (0-0)	-47.2 (-66.4--12.6)	0 (0-0)	0 (0-0)	-14.8 (-25--0.6)	0 (0-0)	0 (0-0)	-15.7 (-56.4-45.7)	0.2 (0.1-0.2)	0.2 (0.1-0.2)	-0.4 (-16.6-22.2)	0 (0-0)	0 (0-0)	0 (0-0)	0 (0-0)	117	118	83	80
Western Cape	1.1 (0.8-1.6)	1.1 (0.8-1.7)	0 (-18.7-19.5)	0.2 (0.2-0.3)	0.2 (0.1-0.2)	-21.8 (-35.3--7.6)	0 (0-0)	0 (0-0)	-57.8 (-73.7--34.9)	0 (0-0)	0 (0-0)	-20.9 (-34.7--6.4)	0 (0-0)	0 (0-0)	-46.6 (-79.4--6.5)	0.1 (0.1-0.2)	0.1 (0.1-0.2)	-0.9 (-22.5-20.9)	0 (0-0)	0 (0-0)	0 (0-0)	0 (0-0)	120	122	87	78
Zimbabwe	17.2 (14-20.9)	15.4 (12.5-19.7)	-10.5 (-18.6-4)	6.8 (5.6-8.3)	5.6 (4.5-6.9)	-17.2 (-24.2--4.3)	0.2 (0.1-0.2)	0.2 (0.1-0.3)	11.2 (-34.3-71.2)	0.4 (0.3-0.5)	0.3 (0.3-0.4)	-15.8 (-24.6--0.9)	0.1 (0.1-0.1)	0.2 (0.1-0.3)	85.9 (-8-229)	0.8 (0.6-1)	0.7 (0.5-1)	-7.1 (-19.6-9.7)	0 (0-0)	0 (0-0.1)	0 (0-0)	0.1 (0-0.1)	87	90	48	62
**Western Sub-Saharan Africa**	1,940 (1,610-2,330)	1,710 (1,410-2,050)	-12 (-14.3--9.5)	948 (810-1,110)	852 (713-1,000)	-10.1 (-13--7.2)	7.1 (4.9-9.3)	4.9 (3.1-6.9)	-31.3 (-48--6.6)	43.3 (35.4-53.2)	37.5 (30.7-46.4)	-13.4 (-16.2--10.7)	19.3 (11.4-28.4)	12 (6.8-17.1)	-37.7 (-55.6--10.5)	62.1 (46-83.3)	54.6 (39.8-72.2)	-12.2 (-16.6--8.4)	0.5 (0.3-0.7)	1.5 (1.1-2)	0.6 (0.4-0.8)	2.7 (1.9-3.6)	22	21	14	8
Benin	3,450 (2,860-3,990)	2,560 (2,100-3,070)	-25.8 (-33.2--18.8)	1,930 (1,630-2,190)	1,340 (1,100-1,590)	-30.4 (-37.3--24)	17.4 (8.1-28.2)	11 (5.7-17.5)	-36.8 (-54.2--2.6)	73 (60.5-87.3)	55.1 (44.3-67.5)	-24.5 (-33.1--16.1)	51.1 (16.5-92.1)	29.4 (12.6-53.2)	-42.5 (-62.9-3.5)	93.5 (68.1-127)	74.2 (53.3-101)	-20.6 (-31.2--9.2)	1.6 (0.5-2.8)	2.9 (2.1-3.9)	1.7 (0.8-2.8)	4.3 (2.9-6.2)	13	11	9	7
Burkina Faso	3,170 (2,940-3,400)	2,230 (1,830-2,600)	-29.7 (-42--17.1)	1,790 (1,680-1,920)	1,160 (956-1,360)	-35.2 (-46.2--24.4)	17.6 (1.4-75.8)	10.9 (0.8-54.4)	-38 (-59.9--3.7)	65.4 (57.7-74.7)	47.1 (38.7-56.8)	-27.9 (-39.2--15)	55.5 (3.1-246)	32.1 (1.6-177)	-42.1 (-70.4--2.8)	87.2 (66.5-115)	68.2 (50.7-89.8)	-21.8 (-35.7--3.7)	1.3 (0.1-5.6)	2 (1.5-2.7)	1.5 (0.1-8.5)	3.3 (2.3-4.4)	14	13	12	9
Cabo Verde	118 (97.3-143)	118 (94.5-143)	0.4 (-6.1-7.7)	64.7 (53.5-79.6)	50.3 (41.1-62)	-22.2 (-27.2--16.6)	0.7 (0.5-0.9)	0.4 (0.2-0.5)	-47.8 (-64.3--24.6)	2.1 (1.7-2.6)	2 (1.6-2.4)	-7.9 (-14.4-0.1)	1.2 (0.5-2.4)	0.3 (0.1-0.6)	-76.8 (-91.4--29.4)	2.7 (2.1-3.6)	2.7 (2.1-3.5)	0.1 (-8.3-10.5)	0.1 (0.1-0.2)	0.3 (0.2-0.3)	0.1 (0.1-0.2)	1.2 (0.8-1.6)	46	52	35	16
Cameroon	794 (676-889)	805 (703-910)	1.3 (-5-8)	428 (358-488)	408 (346-464)	-4.8 (-10.8-2.9)	4.6 (1.2-7.3)	3.1 (1-5.8)	-31.2 (-50.6-7.6)	16.5 (13.5-19.1)	16.1 (13.5-18.8)	-2 (-10.3-6)	10 (1.8-19.6)	6.6 (1.6-12.2)	-34.1 (-54.9-9.7)	23.5 (15.8-32.2)	24.5 (16.8-33.9)	4.2 (-9.4-18.5)	0.3 (0.1-0.7)	0.8 (0.6-1.1)	0.4 (0.1-0.7)	1.4 (1-2.1)	28	26	21	12
Chad	216 (190-244)	213 (185-239)	-1.2 (-7.8-5.5)	110 (92.7-127)	110 (93.8-127)	0.1 (-7.8-7.3)	2.4 (1.1-4.1)	1.8 (0.9-3.3)	-23.9 (-45.8-13.8)	5.2 (4.3-6.1)	5.2 (4.2-6)	-0.6 (-7.2-6.6)	5.8 (1.9-11.5)	3.8 (1.4-6.7)	-34.7 (-54.9-28.2)	9.6 (7.3-12.3)	9.6 (7.1-12.3)	-0.6 (-9.5-8.5)	0.1 (0-0.3)	0.2 (0.2-0.3)	0.1 (0-0.2)	0.3 (0.2-0.5)	37	36	34	26
Côte d'Ivoire	677 (606-728)	673 (602-739)	-0.5 (-5.9-5.6)	300 (265-328)	274 (245-306)	-8.7 (-14.5--1.9)	2.9 (1.8-4.4)	1.9 (1.2-2.7)	-32.5 (-52-0.8)	16.3 (14.3-18.5)	14.9 (13.2-16.8)	-8 (-13.1--2.7)	6 (3.4-9.8)	3.8 (2.4-5.5)	-36.8 (-59.1-5)	36.1 (29-44.3)	36 (29.1-44.6)	-0.1 (-8.2-9.2)	0.2 (0.1-0.3)	1.2 (0.9-1.5)	0.2 (0.2-0.3)	2.3 (1.8-3.1)	34	34	15	10
Gambia	649 (572-737)	611 (524-707)	-5.9 (-13.1-1)	355 (310-402)	317 (264-362)	-10.9 (-18.6--4.4)	5.5 (1.3-23.6)	4 (1-16.6)	-26.9 (-53.4-21.4)	13.5 (11.4-15.7)	12.2 (10.3-14.3)	-9.5 (-15.9--1.9)	13.6 (2.3-59.6)	7.8 (1.7-38.8)	-43.1 (-79.6-37.9)	19.2 (14.3-25)	18.8 (13.8-24.3)	-2.5 (-12.5-9.4)	0.6 (0.1-2.6)	0.8 (0.6-1)	0.8 (0.2-4.1)	1.8 (1.2-2.4)	21	22	19	14
Ghana	1,730 (1,610-1,830)	1,580 (1,390-1,800)	-8.6 (-18.5-3.5)	893 (821-956)	722 (641-824)	-19.1 (-26.4--9)	11 (7.4-13.5)	7.2 (4.6-10.2)	-34.3 (-52--8)	34.7 (31.9-37.6)	30.7 (27.1-35.2)	-11.3 (-22.2-0.8)	24.2 (14.9-34.2)	15.9 (6.6-27.8)	-34.2 (-60.6-10.6)	43.7 (34.6-54.6)	44.1 (34.2-56.6)	0.9 (-12.8-16.4)	1.1 (0.7-1.6)	2 (1.6-2.5)	1.5 (0.7-2.5)	4.3 (3-6.4)	16	14	11	6
Guinea	1,870 (1,650-2,150)	1,870 (1,630-2,140)	0.1 (-7.1-7)	960 (826-1,090)	968 (837-1,140)	0.8 (-7.2-9.8)	8.2 (1.3-13.9)	6 (1.2-10.5)	-26.4 (-48.1-36)	39.9 (32.9-46.8)	40.2 (33.3-47.1)	0.7 (-6.4-9.7)	23.8 (2.7-45.3)	13.1 (2.6-23.6)	-45 (-64.8-30)	59.6 (40.5-81.8)	60.4 (42.1-82.7)	1.3 (-10.8-15.9)	0.5 (0.1-1)	1.4 (0.9-1.9)	0.7 (0.1-1.2)	3 (2-4.2)	20	21	13	9
Guinea-Bissau	69 (55.4-82.8)	65.5 (52.8-79.6)	-5.1 (-11.1-3.1)	30 (24.4-35.9)	26.3 (21.6-31.9)	-12.3 (-18.8--4.6)	3.2 (0.7-14.3)	1.9 (0.4-8.6)	-40.3 (-68.5-7.4)	1.7 (1.4-2.1)	1.5 (1.2-1.9)	-12.3 (-18.2--5)	7.2 (1-32.1)	2.8 (0.4-15.3)	-61.3 (-85.2--3.9)	4 (2.8-5.6)	3.8 (2.8-5.5)	-3.5 (-12.6-7.3)	0.2 (0-0.9)	0.1 (0.1-0.1)	0.2 (0-1)	0.2 (0.2-0.3)	37	41	48	38
Liberia	512 (443-581)	514 (441-584)	0.3 (-3.5-4.6)	198 (168-224)	174 (148-196)	-11.8 (-16--7.7)	3.5 (1.2-10)	2.2 (0.7-8)	-38.4 (-67.3-10.2)	13.2 (11.3-15.7)	11.2 (9.6-13)	-15.7 (-20.9--10.2)	9.4 (2.4-22)	3.9 (1.2-14.7)	-58.3 (-83--9.9)	36.8 (28.8-47)	36.8 (29.2-47.6)	0.2 (-8.3-9.3)	0.2 (0.1-0.5)	0.8 (0.6-1)	0.2 (0.1-0.9)	2.3 (1.6-3.1)	34	32	19	12
Mali	660 (584-737)	656 (584-729)	-0.6 (-5.2-6.1)	333 (294-375)	332 (291-375)	-0.2 (-5.3-6.9)	6.9 (3-12)	4.2 (2.1-7.9)	-39.1 (-58.5--10.6)	14.9 (12.8-17.1)	15 (12.9-17.2)	0.6 (-5.6-6.8)	18.6 (6.1-36.7)	8.9 (3.8-17.4)	-52 (-70.7--1.4)	23.9 (18.4-30.6)	24 (18.4-31.2)	0.4 (-7.6-9.4)	0.4 (0.1-0.7)	0.5 (0.4-0.6)	0.4 (0.2-0.7)	1 (0.7-1.2)	23	25	20	17
Mauritania	204 (171-238)	203 (168-231)	-0.4 (-8-5.7)	110 (91.8-131)	103 (85.8-122)	-6.5 (-14-0.2)	1.4 (0.8-2.2)	0.8 (0.4-1.2)	-44.7 (-61.1--16.7)	4.1 (3.3-4.9)	3.9 (3.1-4.6)	-5.9 (-13.3-1.9)	3.4 (1.5-5.8)	1.5 (0.8-2.5)	-54.2 (-70--17.3)	6.4 (4.7-8.5)	6.4 (4.7-8.4)	0 (-10.3-10.8)	0.2 (0.1-0.3)	0.4 (0.2-0.5)	0.2 (0.1-0.3)	0.8 (0.5-1.1)	36	40	28	21
Niger	1,510 (1,290-1,770)	1,180 (1,020-1,350)	-21.7 (-29.8--14.8)	793 (693-925)	623 (533-725)	-21.4 (-30.5--14)	5.3 (2-9)	2.9 (1.4-4.2)	-45.4 (-62.1--5.5)	34.9 (29.8-41.2)	28.4 (24.3-33.7)	-18.6 (-25.5--11.5)	14.7 (4.2-29.4)	6.6 (2.8-10.2)	-54.9 (-69.2--2.3)	55.9 (43.8-71.9)	48.2 (38.1-61.2)	-13.8 (-22.3--3.8)	0.3 (0.1-0.5)	1 (0.8-1.4)	0.3 (0.1-0.5)	2.2 (1.6-2.9)	27	25	15	13
Nigeria	2,530 (1,920-3,220)	2,290 (1,760-2,870)	-9.4 (-12.4--6.1)	1,190 (942-1,490)	1,130 (912-1,410)	-4.6 (-7.5--0.7)	6.5 (1.5-10.5)	4.8 (1.6-8.2)	-26 (-49.4-16.4)	58.6 (44.4-76.7)	50.7 (39.1-65.7)	-13.5 (-17.2--9.9)	17.7 (3.1-33.2)	12.2 (3-21.3)	-31.2 (-53.3-17.7)	82.8 (56.8-118)	70.6 (49.2-98)	-14.7 (-20.4--9.6)	0.4 (0.1-0.7)	1.9 (1.3-2.6)	0.5 (0.2-0.9)	3.1 (2-4.6)	23	21	11	8
Sao Tome and Principe	1,020 (812-1,250)	1,020 (819-1,230)	-0.1 (-7.4-8.6)	583 (468-722)	511 (409-628)	-12.3 (-20.5--6.3)	3.3 (0.8-5.4)	2 (0.6-3.6)	-40.4 (-60.3-3.7)	19.7 (15.1-25.2)	18.4 (14.3-23.2)	-6.3 (-15.3-3.6)	6.1 (1.1-10)	2.8 (0.7-6.1)	-55 (-76.4--6.5)	27.6 (19.1-38.7)	27.9 (19.2-38.5)	1 (-11.9-15.3)	0.3 (0.1-0.6)	1.6 (1-2.2)	0.7 (0.2-1.6)	7.3 (4.5-10.7)	28	24	11	5
Senegal	558 (483-632)	557 (488-625)	-0.2 (-7.3-5.5)	301 (262-349)	285 (247-332)	-5.2 (-12.4-2.6)	3.4 (1.3-7.9)	2.2 (0.9-5.4)	-34.3 (-57.5-3.6)	11.3 (9.5-13.1)	10.8 (9.1-12.5)	-4.6 (-11-2.4)	9 (3-18)	4.2 (1.6-11)	-53.8 (-74.1--15.6)	17 (12.5-21.9)	17.3 (12.8-22.9)	1.5 (-9.3-13)	0.3 (0.1-0.6)	0.6 (0.5-0.8)	0.5 (0.2-1.2)	1.9 (1.4-2.5)	28	29	19	11
Sierra Leone	2,570 (2,260-2,870)	2,530 (2,220-2,850)	-1.6 (-8.4-5)	1,300 (1,110-1,500)	1,230 (1,060-1,430)	-5.4 (-12.3-1)	7.8 (1-13.8)	4.8 (0.8-9.1)	-37.7 (-56.3-17.7)	54.6 (45.1-63.2)	52.2 (43-60.5)	-4.4 (-12-3.6)	27.8 (2.8-56.1)	12.3 (1.9-21.5)	-55.6 (-68.5-3.5)	82.4 (56.3-113)	83.5 (55.5-114)	1.3 (-10.6-16.2)	0.5 (0-0.9)	1.4 (0.9-1.8)	0.5 (0.1-0.9)	3.7 (2.3-5.5)	21	21	15	8
Togo	2,110 (1,870-2,370)	2,070 (1,820-2,340)	-2.2 (-8.8-4.3)	1,110 (983-1,270)	971 (857-1,120)	-12.3 (-19--5.7)	15.8 (8.5-25.2)	10.2 (4.8-18.9)	-35.3 (-56.1-2.5)	45.8 (40-53)	41.5 (36-47.8)	-9.5 (-14.8--3.4)	38.3 (16.5-63.3)	20.8 (10.1-39.6)	-45.8 (-67.4-5.3)	64.3 (49-82.4)	63.5 (48.1-82.5)	-1.2 (-8.8-8.4)	1.4 (0.7-2.3)	2.3 (1.7-3)	1.6 (0.8-3.1)	5.1 (3.5-6.8)	14	10	9	7

Table S14: Homozygous sickle cell and severe sickle cell/beta-thalassemia disease frequency: Birth rate, age-specific and all-age prevalence rates , 2000 - 2021

Location	Birth Incidence rate (per 100,000 live births), 2021	Birth Incidence rate (per 100,000 live births) percent change (2000-2021)	Under-5 rate (per 100,000 population), 2021	Under-5 rate (per 100,000 population) percent change (2000-2021)	5-14 rate (per 100,000 population), 2021	5-14 rate (per 100,000 population) percent change (2000-2021)	15-49 rate (per 100,000 population), 2021	15-49 rate (per 100,000 population) percent change (2000-2021)	50plus rate (per 100,000 population), 2021	50plus rate (per 100,000 population) percent change (2000-2021)	All age rate (per 100,000 population), 2021	All age rate (per 100,000 population) percent change (2000-2021)
Global	292 (232-369)	13.4 (10.8-16.3)	265 (208-335)	17.5 (15-20.5)	167 (132-216)	32 (28.9-35.2)	47.2 (37.1-59.1)	24.5 (19.6-29.1)	1.6 (1.3-2.1)	-14.8 (-19--10.6)	74.4 (59.8-92.1)	8.8 (5.9-11.8)
Central Europe, Eastern Europe, and Central Asia	0.1 (0-0.1)	-5.2 (-31.7-7.6)	0 (0-0.1)	-8 (-35.9-5.1)	0 (0-0)	-3.8 (-36.6-12.2)	0 (0-0)	-18.3 (-44.3--1.5)	0 (0-0)	-16.6 (-45.2-7.7)	0 (0-0)	-6.2 (-36.2-8.6)
**Central Asia**	0 (0-0.1)	-3.1 (-32.5-0.7)	0 (0-0.1)	-3.2 (-32.7-2)	0 (0-0)	1.1 (-29.7-10.6)	0 (0-0)	-14.9 (-46.2--3.1)	0 (0-0)	0.4 (-41.2-23.5)	0 (0-0)	-9.8 (-37.2--4)
Armenia	0 (0-0.1)	-2.4 (-32.3-0)	0 (0-0.1)	-2.7 (-33.2-4.4)	0 (0-0)	5.8 (-25.9-25.2)	0 (0-0)	-7.2 (-40.5-19.3)	0 (0-0)	-7 (-54.2-31.2)	0 (0-0)	-11.4 (-39.3-0.1)
Azerbaijan	0 (0-0.1)	-3 (-32.1-0.1)	0 (0-0.1)	-2.1 (-31.2-4.8)	0 (0-0)	-1.2 (-34.2-15.8)	0 (0-0)	-9.4 (-44.3-20.8)	0 (0-0)	23.3 (-41.8-94.7)	0 (0-0)	-22.8 (-47.6--14.7)
Georgia	0.1 (0-0.1)	-2.4 (-33-0)	0 (0-0.1)	-1.9 (-33.1-3.9)	0 (0-0)	5.1 (-26.5-25.2)	0 (0-0)	-8.3 (-38.6-22.5)	0 (0-0)	-3.6 (-45.6-46.3)	0 (0-0)	1.2 (-31.8-14)
Kazakhstan	0 (0-0.1)	-2.4 (-33.4-0.1)	0 (0-0.1)	-3.1 (-31.6-3.6)	0 (0-0)	6.1 (-22.1-25.5)	0 (0-0)	-11.9 (-41-16.3)	0 (0-0)	-1.6 (-54-63.8)	0 (0-0)	11 (-22-23.5)
Kyrgyzstan	0 (0-0.1)	-2.1 (-33.3-0.4)	0 (0-0.1)	-1.2 (-29.7-5.6)	0 (0-0)	2.8 (-23.3-21.8)	0 (0-0)	-5.8 (-33.4-26.8)	0 (0-0)	24.5 (-31.8-94.9)	0 (0-0)	2.2 (-26.5-13.3)
Mongolia	0 (0-0.1)	-3.6 (-33.6-0)	0 (0-0.1)	-4.6 (-34.9-2.9)	0 (0-0)	10.4 (-22.3-30.8)	0 (0-0)	-20.3 (-50.5-4.6)	0 (0-0)	16.7 (-48-88.1)	0 (0-0)	-3.2 (-33.1-6.5)
Tajikistan	0 (0-0.1)	-2.5 (-33.4-0)	0 (0-0.1)	-2.2 (-30.1-3.6)	0 (0-0)	2 (-25.1-19.5)	0 (0-0)	-9.2 (-39.1-20.4)	0 (0-0)	33.1 (-25.2-105)	0 (0-0)	-14.9 (-39.5--8)
Turkmenistan	0 (0-0.1)	-2 (-33.4-0.2)	0 (0-0.1)	-2 (-32.8-4)	0 (0-0)	2.2 (-29.6-20.3)	0 (0-0)	-11.3 (-39.7-18.6)	0 (0-0)	12.7 (-40.3-81.3)	0 (0-0)	-12.5 (-38.9--5.1)
Uzbekistan	0 (0-0.1)	-3.6 (-33.1-0.6)	0 (0-0.1)	-2 (-31.6-6.2)	0 (0-0)	-0.1 (-32.2-18.1)	0 (0-0)	-12.6 (-43.2-15.9)	0 (0-0)	22 (-40.6-91)	0 (0-0)	-12.6 (-40.2--3.1)
**Central Europe**	0.1 (0.1-0.2)	4.3 (-31.6-35.2)	0.1 (0.1-0.1)	1.9 (-33.5-31.9)	0 (0-0)	-4 (-38.3-27.8)	0 (0-0)	-15.7 (-46.1-14)	0 (0-0)	-19.8 (-47.9-12)	0 (0-0)	-14.3 (-44.8-12)
Albania	0.3 (0.1-0.4)	0.2 (0-0.2)	0.1 (0.1-0.2)	3.4 (0.2-6.2)	0 (0-0)	3.7 (-9.4-17.8)	0 (0-0)	-1.5 (-24.1-25.7)	0 (0-0)	-9.7 (-35.4-39.3)	0 (0-0)	-33.9 (-37.7--30)
Bosnia and Herzegovina	0 (0-0.1)	0.2 (0-0)	0 (0-0.1)	0.8 (-3.1-3.8)	0 (0-0)	3 (-11.3-19.4)	0 (0-0)	-5.2 (-23.6-17.8)	0 (0-0)	-12.2 (-37.1-18.5)	0 (0-0)	-24.8 (-31.8--17.1)
Bulgaria	0 (0-0.1)	0.1 (0-0)	0 (0-0.1)	-3 (-6.7-0.8)	0 (0-0)	5.9 (-7.9-21.8)	0 (0-0)	-10.5 (-31.4-14.4)	0 (0-0)	-15.2 (-45.2-23.3)	0 (0-0)	-2.1 (-9.6-6.1)
Croatia	0 (0-0.1)	-0.4 (0-0)	0 (0-0.1)	0.8 (-2-5.1)	0 (0-0)	0.7 (-12-14.9)	0 (0-0)	-3.6 (-21.4-19.1)	0 (0-0)	-9.8 (-32.2-19)	0 (0-0)	-19.1 (-25.9--11.5)
Czechia	0 (0-0.1)	0 (0-0)	0 (0-0.1)	-0.2 (-3.6-3.1)	0 (0-0)	0.3 (-11.2-12.8)	0 (0-0)	-11.3 (-28.9-9.1)	0 (0-0)	-24.8 (-43.9-2.4)	0 (0-0)	0.7 (-8.8-11.5)
Hungary	0 (0-0.1)	-1.6 (-46.3-0)	0 (0-0.1)	-2.2 (-47.2-2.9)	0 (0-0)	-1.8 (-49.6-14.4)	0 (0-0)	-8.3 (-50.7-14.5)	0 (0-0)	-14.2 (-44.2-16.9)	0 (0-0)	-14 (-54.1--4.4)
Montenegro	0 (0-0.1)	2.2 (-0.1-99.6)	0 (0-0.1)	2.8 (-3.1-106)	0 (0-0)	4.1 (-12-105)	0 (0-0)	-1.7 (-23.2-74.5)	0 (0-0)	-7.7 (-33.1-31.6)	0 (0-0)	-17.4 (-26.1-61.3)
North Macedonia	0 (0-0.1)	-2.6 (0-0)	0 (0-0.1)	-1.3 (-3.8-5.9)	0 (0-0)	-0.5 (-14.1-19.6)	0 (0-0)	-8.9 (-31.1-21.1)	0 (0-0)	-12.7 (-39.1-27.1)	0 (0-0)	-22.4 (-28.4--12.4)
Poland	0.3 (0.2-0.6)	-3 (-40.7-34.7)	0.2 (0.1-0.4)	-3.3 (-41.3-34.5)	0 (0-0.1)	-6.5 (-42.3-32.6)	0 (0-0)	-21.7 (-51.3-11.8)	0 (0-0)	-28.4 (-55.7-7.9)	0 (0-0)	-18.9 (-50.1-12.8)
Romania	0 (0-0.1)	0 (0-0)	0 (0-0.1)	-1.6 (-5.1-2)	0 (0-0)	5.1 (-8.2-20.1)	0 (0-0)	-11.5 (-29.2-13.2)	0 (0-0)	-8.7 (-36-37.2)	0 (0-0)	-12 (-18.9--5.5)
Serbia	0 (0-0.1)	0 (0-0)	0 (0-0.1)	-0.5 (-4.3-3.3)	0 (0-0)	-4.3 (-18.3-10.6)	0 (0-0)	-4.6 (-26.1-20)	0 (0-0)	-11.7 (-39.7-22)	0 (0-0)	-23.8 (-31--16.4)
Slovakia	0 (0-0.1)	-1.4 (-41.5-0)	0 (0-0.1)	0.2 (-40-5.4)	0 (0-0)	2.5 (-36.7-19.7)	0 (0-0)	-10.3 (-39.3-16.8)	0 (0-0)	-16.2 (-39.9-19.4)	0 (0-0)	-11.3 (-44.5--1.4)
Slovenia	0 (0-0.1)	0 (0-0.4)	0 (0-0.1)	-0.2 (-3.2-2.9)	0 (0-0)	6.6 (-6-20.1)	0 (0-0)	-5.4 (-23-17.2)	0 (0-0)	-8.1 (-32.6-26.6)	0 (0-0)	-7.2 (-16.1-2.1)
**Eastern Europe**	0.1 (0-0.1)	0.3 (-0.9-2)	0 (0-0.1)	-0.8 (-2.7-1.3)	0 (0-0)	14.7 (8.7-21.2)	0 (0-0)	-10.3 (-16.5--2.3)	0 (0-0)	-6.4 (-18-7.9)	0 (0-0)	13.8 (6.9-19.8)
Belarus	0 (0-0.1)	0 (0-0)	0 (0-0.1)	-2.1 (-5.8-2)	0 (0-0)	10.8 (-3.2-28.3)	0 (0-0)	-8.2 (-27.8-18.3)	0 (0-0)	2.9 (-35.1-60.3)	0 (0-0)	6.6 (-2.8-17.1)
Estonia	0 (0-0.1)	-0.1 (0-0)	0 (0-0.1)	-0.1 (-4.2-3.6)	0 (0-0)	5.6 (-7.8-23.8)	0 (0-0)	-7.2 (-25.9-16.4)	0 (0-0)	-15.3 (-41.7-33.2)	0 (0-0)	1.1 (-7.9-12.5)

Location	Birth Incidence rate (per 100,000 live births), 2021	Birth Incidence rate (per 100,000 live births) percent change (2000-2021)	Under-5 rate (per 100,000 population), 2021	Under-5 rate (per 100,000 population) percent change (2000-2021)	5-14 rate (per 100,000 population), 2021	5-14 rate (per 100,000 population) percent change (2000-2021)	15-49 rate (per 100,000 population), 2021	15-49 rate (per 100,000 population) percent change (2000-2021)	50plus rate (per 100,000 population), 2021	50plus rate (per 100,000 population) percent change (2000-2021)	All age rate (per 100,000 population), 2021	All age rate (per 100,000 population) percent change (2000-2021)
Latvia	0 (0-0.1)	0.6 (0-0)	0 (0-0.1)	-1.5 (-5.5-2.6)	0 (0-0)	6.8 (-9.2-24.4)	0 (0-0)	-7.5 (-27.3-19.3)	0 (0-0)	-8.4 (-43.9-50.5)	0 (0-0)	7.7 (-3.4-20.3)
Lithuania	0 (0-0.1)	-0.3 (0-0)	0 (0-0.1)	-0.4 (-4.2-3.9)	0 (0-0)	5.5 (-8.2-21.2)	0 (0-0)	-5.5 (-23.7-21.3)	0 (0-0)	-5.7 (-37-38.5)	0 (0-0)	-13.6 (-21.2--5)
Republic of Moldova	0 (0-0.1)	0 (0-0)	0 (0-0.1)	0.7 (-3.5-4.5)	0 (0-0)	5.9 (-9.3-22.7)	0 (0-0)	-10.8 (-30.6-11.2)	0 (0-0)	-4.1 (-40-41.7)	0 (0-0)	-22.9 (-29.2--16.8)
Russian Federation	0.1 (0-0.1)	0 (-0.6-0)	0 (0-0.1)	-1.2 (-2.3--0.5)	0 (0-0)	17.3 (11.5-23.2)	0 (0-0)	-9.5 (-15.2--4)	0 (0-0)	-6.3 (-16.1-4.3)	0 (0-0)	21.1 (13.6-28.3)
Ukraine	0.1 (0-0.1)	-0.1 (0-0)	0 (0-0.1)	-0.6 (-4.1-2.8)	0 (0-0)	8.1 (-6.4-23)	0 (0-0)	-12.7 (-31.4-12.2)	0 (0-0)	-5.1 (-36.5-48.7)	0 (0-0)	-2.7 (-10.4-6.2)
High-income	15.3 (13.3-17.8)	-3.8 (-5.6--1.9)	13.7 (12-16)	-1.8 (-3.5-0.1)	10.3 (9.2-11.7)	-0.2 (-1.8-1.3)	6.5 (5.9-7.1)	3.2 (1.6-4.7)	2 (1.7-2.3)	4.3 (2.2-6.4)	5.5 (5-6)	-7.4 (-8.6--6.1)
**Australasia**	1.9 (1.4-2.5)	3.5 (-3.6-12.5)	1.4 (1.1-1.8)	2.2 (-4.8-11.1)	0.8 (0.6-1.1)	-0.8 (-7.7-7.2)	0.5 (0.4-0.6)	0.2 (-6.5-8.6)	0.1 (0.1-0.1)	-10.7 (-18.4--1.3)	0.4 (0.3-0.6)	-9.2 (-14.9--2)
Australia	1.5 (1.1-2)	-0.7 (-9.5-10.8)	1.1 (0.8-1.5)	-0.1 (-8.7-11.2)	0.7 (0.5-0.9)	-0.7 (-10.3-10.7)	0.4 (0.3-0.5)	-0.7 (-9.8-11.3)	0.1 (0-0.1)	-9.6 (-19.9-4)	0.4 (0.3-0.5)	-9.5 (-17.6-1)
New Zealand	3.7 (2.9-4.7)	17.8 (4.2-34.4)	2.8 (2.2-3.5)	14.3 (2.4-28.8)	1.6 (1.3-2)	1.8 (-9.3-13.7)	1 (0.8-1.3)	3 (-7.5-14.5)	0.2 (0.1-0.2)	-13.6 (-24.2--2.3)	0.9 (0.7-1.1)	-7.3 (-16.7-2.8)
**High-income Asia Pacific**	0.2 (0.2-0.3)	1.9 (-7.7-8.4)	0.2 (0.1-0.2)	1.3 (-8.5-7.9)	0.1 (0.1-0.1)	3.1 (-6.2-11.5)	0.1 (0-0.1)	-0.8 (-9.7-7.7)	0 (0-0)	-13.2 (-23.5--0.8)	0 (0-0.1)	-18.1 (-25.4--11)
Brunei Darussalam	0.4 (0.2-0.5)	-0.4 (-11.9-0.6)	0.2 (0.1-0.3)	0.7 (-11.3-9.3)	0.1 (0.1-0.1)	3.7 (-14.3-23.2)	0.1 (0-0.1)	2 (-16.3-22.3)	0 (0-0)	-5.4 (-27.2-20.3)	0.1 (0-0.1)	-16.7 (-28.5--4.7)
Japan	0.2 (0.1-0.3)	5.4 (-2.4-15.9)	0.1 (0.1-0.2)	5.2 (-2-15.5)	0.1 (0.1-0.1)	8.1 (0.4-19.2)	0 (0-0.1)	0.4 (-6.3-9.9)	0 (0-0)	-18.1 (-25.4--9.8)	0 (0-0.1)	-13.5 (-19.4--5.6)
Aichi	1 (0.7-1.3)	-0.2 (-13.2-14.9)	0.7 (0.5-1)	-0.3 (-12-14.2)	0.5 (0.4-0.6)	0.8 (-10.2-16.5)	0.3 (0.2-0.4)	-4.6 (-15.8-9.3)	0 (0-0)	-21.5 (-34.9--6.2)	0.2 (0.1-0.3)	-16.1 (-25.7--3.7)
Akita	0.1 (0-0.2)	-0.5 (-0.2-0)	0.1 (0-0.2)	-1.4 (-4.4-2.6)	0 (0-0.1)	-0.5 (-7.8-8.1)	0 (0-0.1)	-2.9 (-13.8-8)	0 (0-0)	-22.8 (-35.4--8.7)	0 (0-0)	-26.4 (-32.7--19.3)
Aomori	0.1 (0-0.2)	0.6 (-0.2-0)	0.1 (0-0.2)	1 (-3.6-3.6)	0 (0-0.1)	2.3 (-7.1-10.1)	0 (0-0.1)	-2.3 (-12.8-8.2)	0 (0-0)	-23.8 (-36.1--8.9)	0 (0-0)	-25.5 (-32.4--18.7)
Chiba	0.4 (0.2-0.5)	0.4 (-0.2-27.2)	0.3 (0.1-0.4)	0.3 (-5.1-27.1)	0.2 (0.1-0.2)	0.6 (-10-31.2)	0.1 (0.1-0.1)	-7.4 (-20.4-15.1)	0 (0-0)	-26.2 (-40.9--3.6)	0.1 (0-0.1)	-21.5 (-30.2--1.8)
Ehime	0.1 (0-0.2)	0.1 (-0.2-0)	0.1 (0-0.2)	0.8 (-3.5-3.8)	0 (0-0.1)	1.4 (-6.9-8.4)	0 (0-0.1)	-1.9 (-11.3-7.6)	0 (0-0)	-22.2 (-33.9--9.6)	0 (0-0)	-18.4 (-24.7--12.1)
Fukui	0.1 (0-0.2)	-0.3 (-0.2-0)	0.1 (0-0.2)	0.3 (-4.5-4.9)	0 (0-0.1)	0.4 (-11-10)	0 (0-0.1)	-2.5 (-15.8-8.6)	0 (0-0)	-20.5 (-36.2--6.3)	0 (0-0)	-18.2 (-28--9.9)
Fukuoka	0.1 (0-0.2)	2 (-0.2-0)	0.1 (0-0.1)	1.6 (-4.6-3.7)	0 (0-0.1)	3.6 (-6.6-11.1)	0 (0-0)	-2.8 (-14.7-6.9)	0 (0-0)	-20.7 (-32.9--4.2)	0 (0-0)	-13.8 (-22.8--6.7)
Fukushima	0.1 (0-0.2)	-0.4 (-0.2-0)	0.1 (0-0.2)	-0.4 (-3.9-4.1)	0 (0-0.1)	0.9 (-7.3-9.4)	0 (0-0.1)	-0.9 (-12.6-10.5)	0 (0-0)	-19.2 (-35.7--2.8)	0 (0-0)	-24.3 (-31.5--17.7)
Gifu	0.1 (0-0.2)	-1.4 (-0.2-0)	0.1 (0-0.2)	-1.6 (-3.6-3.4)	0 (0-0.1)	0.2 (-5.6-9.7)	0 (0-0.1)	-3.2 (-12.2-8.5)	0 (0-0)	-23 (-35.7--9.1)	0 (0-0)	-18.7 (-24.5--10.4)
Gunma	0.1 (0-0.2)	-1.3 (-6.4-0)	0.1 (0-0.2)	-1.5 (-6.6-3.7)	0 (0-0.1)	-0.5 (-11.2-9.1)	0 (0-0.1)	-4 (-15.5-8)	0 (0-0)	-23.2 (-39.6--8.1)	0 (0-0)	-20.3 (-28.9--11.8)
Hiroshima	0.1 (0-0.2)	0.4 (-0.2-0)	0.1 (0-0.2)	-0.1 (-4.4-3)	0 (0-0.1)	1.6 (-7-8.6)	0 (0-0.1)	-2.8 (-13.9-8.5)	0 (0-0)	-22.1 (-33.7--9.2)	0 (0-0)	-14.8 (-22.9--7)
Hokkaidō	0.1 (0-0.2)	-5 (-55-0)	0.1 (0-0.1)	-5.4 (-56.1-4.4)	0 (0-0.1)	-4.7 (-59.7-9.6)	0 (0-0)	-9.3 (-59.8-6.6)	0 (0-0)	-27.1 (-59.4--11.1)	0 (0-0)	-26.3 (-66.7--14.4)
Hyōgo	0.2 (0.1-0.3)	-1.5 (-35.3-0.2)	0.1 (0.1-0.2)	-1.7 (-33.8-4.1)	0.1 (0-0.1)	-0.4 (-27.9-11.1)	0 (0-0.1)	-5.4 (-33.6-7.9)	0 (0-0)	-21.7 (-51.2--3.6)	0 (0-0.1)	-19.3 (-44.8--9.2)
Ibaraki	0.3 (0.2-0.5)	-2.4 (-34.6-0.2)	0.2 (0.1-0.4)	-2.2 (-32.7-6.8)	0.1 (0.1-0.2)	-0.4 (-27.8-17.6)	0.1 (0-0.1)	-5.9 (-32.3-12.9)	0 (0-0)	-22.8 (-46.5--1.6)	0.1 (0-0.1)	-21.8 (-44.3--6.5)
Ishikawa	0.1 (0-0.2)	-1 (-0.2-0)	0.1 (0-0.2)	-1 (-4.7-4.2)	0 (0-0.1)	-1.6 (-9.6-9.6)	0 (0-0.1)	-4.6 (-15.8-7.4)	0 (0-0)	-24 (-37.6--10.1)	0 (0-0)	-18.1 (-25.8--9.3)
Iwate	0.1 (0-0.2)	-0.4 (-0.2-0)	0.1 (0-0.2)	-1.4 (-6.3-2.7)	0 (0-0.1)	1.5 (-6.9-10.3)	0 (0-0.1)	-2.5 (-11.4-8.2)	0 (0-0)	-18.5 (-31.6--3.3)	0 (0-0)	-21.4 (-27.9--13.6)
Kagawa	0.1 (0-0.2)	-3 (-0.5-0)	0.1 (0-0.2)	-3.5 (-9.1-2.9)	0 (0-0.1)	-2.8 (-12.4-8.2)	0 (0-0.1)	-5.1 (-17.5-7.6)	0 (0-0)	-25.5 (-42.8--10.4)	0 (0-0)	-19.9 (-28.5--10.3)

Location	Birth Incidence rate (per 100,000 live births), 2021	Birth Incidence rate (per 100,000 live births) percent change (2000-2021)	Under-5 rate (per 100,000 population), 2021	Under-5 rate (per 100,000 population) percent change (2000-2021)	5-14 rate (per 100,000 population), 2021	5-14 rate (per 100,000 population) percent change (2000-2021)	15-49 rate (per 100,000 population), 2021	15-49 rate (per 100,000 population) percent change (2000-2021)	50plus rate (per 100,000 population), 2021	50plus rate (per 100,000 population) percent change (2000-2021)	All age rate (per 100,000 population), 2021	All age rate (per 100,000 population) percent change (2000-2021)
Kagoshima	0.1 (0-0.2)	-1.3 (-0.2-0)	0.1 (0-0.2)	-1.2 (-6.5-4.5)	0 (0-0.1)	0.4 (-12.4-12.7)	0 (0-0)	-3.3 (-16.6-11.6)	0 (0-0)	-18.8 (-37.3--1.8)	0 (0-0)	-18.6 (-27.5--8.8)
Kanagawa	0.1 (0-0.3)	-1.5 (-29.6-0)	0.1 (0-0.2)	-2.1 (-27.6-3.2)	0.1 (0-0.1)	-0.6 (-15.3-9.9)	0 (0-0.1)	-8.7 (-22-5.3)	0 (0-0)	-21.2 (-42.5--1.9)	0 (0-0.1)	-20.8 (-31.2--10.7)
Kōchi	0.1 (0-0.2)	0.9 (-0.2-0)	0.1 (0-0.2)	1.1 (-3.5-4.3)	0 (0-0.1)	0.5 (-7.6-9.1)	0 (0-0.1)	-3.4 (-13.6-9)	0 (0-0)	-24.3 (-37.2--7.3)	0 (0-0)	-19.1 (-26.2--11)
Kumamoto	0.1 (0-0.2)	-4.3 (-45-0)	0.1 (0-0.2)	-5 (-45.4-3.9)	0 (0-0.1)	-4 (-46-10.5)	0 (0-0.1)	-6.2 (-45.8-10.6)	0 (0-0)	-23.2 (-55.3--2.4)	0 (0-0)	-19.4 (-52.9--6.1)
Kyōto	0.1 (0-0.2)	0.5 (-23.7-10.7)	0.1 (0-0.2)	0.3 (-19-9.4)	0 (0-0.1)	1.4 (-15.8-15.3)	0 (0-0.1)	-5 (-19.7-10.2)	0 (0-0)	-23 (-38.6--3.6)	0 (0-0)	-17.2 (-30.3--5.3)
Mie	0.1 (0-0.2)	-0.2 (-0.2-0)	0.1 (0-0.2)	-1 (-6.2-4.1)	0 (0-0.1)	-0.8 (-9.9-9.8)	0 (0-0.1)	-3.3 (-13.8-11.6)	0 (0-0)	-20.8 (-33.9-5.2)	0 (0-0)	-18.7 (-26.1--7.1)
Miyagi	0.1 (0-0.2)	-1.7 (-0.2-0)	0.1 (0-0.2)	-2.6 (-4-2.5)	0 (0-0.1)	-1 (-6-7.7)	0 (0-0.1)	-5.3 (-13-5.8)	0 (0-0)	-21.2 (-33.4--5.8)	0 (0-0)	-21.5 (-26.8--13.1)
Miyazaki	0.1 (0-0.2)	0.1 (-0.2-0)	0.1 (0-0.2)	-0.7 (-6.6-4.5)	0 (0-0.1)	1.4 (-8.7-12)	0 (0-0.1)	-1.3 (-13.8-15)	0 (0-0)	-23.6 (-39.5--0.5)	0 (0-0)	-18.4 (-27--7.9)
Nagano	0.1 (0-0.2)	-0.5 (-9.7-0)	0.1 (0-0.2)	-1 (-6.8-3.8)	0 (0-0.1)	0.3 (-10.1-9.6)	0 (0-0.1)	-3.2 (-14.4-8.2)	0 (0-0)	-18.6 (-34--3.5)	0 (0-0)	-19 (-28--10.6)
Nagasaki	0.1 (0-0.2)	-1.8 (-11.9-0)	0.1 (0-0.2)	-2.4 (-12-3)	0 (0-0.1)	-1.5 (-12.5-8.7)	0 (0-0.1)	-2.1 (-22.9-11.5)	0 (0-0)	-22.3 (-39.9--6.8)	0 (0-0)	-21.1 (-33.8--11.9)
Nara	0.1 (0-0.2)	1 (-0.2-0)	0.1 (0-0.2)	0.1 (-4.3-3.1)	0 (0-0.1)	1.9 (-7.4-11.2)	0 (0-0.1)	-3.1 (-13.4-9.9)	0 (0-0)	-27 (-40.1--12.2)	0 (0-0)	-21.4 (-28.8--12.7)
Niigata	0.1 (0-0.2)	1.5 (-0.2-32.7)	0.1 (0-0.2)	1.2 (-4.4-27.9)	0 (0-0.1)	2.2 (-7.3-18)	0 (0-0.1)	-1.6 (-11.5-17.8)	0 (0-0)	-19.3 (-32-15.3)	0 (0-0)	-18 (-25.1--3.3)
Ōita	0.1 (0-0.2)	-2.4 (-16.2-0)	0.1 (0-0.2)	-1.6 (-13.1-4)	0 (0-0.1)	-1.4 (-10.4-9.2)	0 (0-0.1)	-4 (-19.4-9.6)	0 (0-0)	-24.2 (-41.6--8.8)	0 (0-0)	-18.5 (-29.3--8.9)
Okayama	0.1 (0-0.2)	-1.4 (-0.2-0)	0.1 (0-0.2)	-1.6 (-4.5-3.3)	0 (0-0.1)	0.3 (-7.7-11.8)	0 (0-0.1)	-4.3 (-15.8-7.7)	0 (0-0)	-23.1 (-36.7--8.2)	0 (0-0)	-16.1 (-24.4--7)
Okinawa	0.2 (0.1-0.4)	-1.8 (-16.3-0)	0.1 (0.1-0.3)	-3.3 (-13.6-3.6)	0.1 (0-0.2)	-2.7 (-13.8-10.4)	0 (0-0.1)	-5.3 (-22.2-10.2)	0 (0-0)	-16.8 (-39.9-9.4)	0 (0-0.1)	-18.8 (-30.7--8.1)
Ōsaka	0.1 (0-0.2)	-0.8 (-0.2-0)	0.1 (0-0.2)	-1.4 (-4.7-3.1)	0 (0-0.1)	0 (-7.8-8)	0 (0-0.1)	-7.4 (-16.8-3.9)	0 (0-0)	-24.7 (-35.9--7.7)	0 (0-0)	-19.8 (-26.9--11.4)
Saga	0.1 (0-0.2)	0 (-0.2-0)	0.1 (0-0.2)	-1.8 (-5.7-1.9)	0 (0-0.1)	-0.6 (-8.3-7.6)	0 (0-0.1)	-1.8 (-11.3-8.2)	0 (0-0)	-20.2 (-32.6--7.6)	0 (0-0)	-17.7 (-24.3--10.9)
Saitama	0.1 (0-0.2)	-1 (-0.2-0)	0.1 (0-0.1)	-1.4 (-5.8-3)	0 (0-0.1)	-0.8 (-9.4-7.8)	0 (0-0.1)	-7 (-17-4)	0 (0-0)	-26 (-38.5--13.5)	0 (0-0)	-21.5 (-28.5--13.2)
Shiga	0.1 (0-0.2)	-3.8 (-52.1-0)	0.1 (0-0.1)	-4.3 (-53.9-2.6)	0 (0-0.1)	-3 (-55.9-7.9)	0 (0-0.1)	-7.1 (-55.7-4.4)	0 (0-0)	-23.1 (-54.5--7.3)	0 (0-0)	-19.2 (-61--9.8)
Shimane	0.1 (0-0.2)	2.5 (-0.2-35.7)	0.1 (0-0.2)	3 (-3.6-26.9)	0 (0-0.1)	5.6 (-5.9-22.4)	0 (0-0.1)	2 (-11.1-25.6)	0 (0-0)	-19.5 (-31.7-2.4)	0 (0-0)	-12.2 (-21.9-4.5)
Shizuoka	0.1 (0-0.2)	-2.1 (-0.2-0)	0.1 (0-0.1)	-2.7 (-5.6-3.1)	0 (0-0.1)	-2.4 (-9.5-8.6)	0 (0-0.1)	-5.8 (-17.7-7.2)	0 (0-0)	-24.3 (-41.7--10.3)	0 (0-0)	-21.2 (-29.4--12)
Tochigi	0.1 (0-0.2)	-0.2 (-0.2-0)	0.1 (0-0.2)	-1 (-4.1-2.9)	0 (0-0.1)	0.6 (-6.9-8.8)	0 (0-0.1)	-3.4 (-13-6.2)	0 (0-0)	-22.2 (-35.4--9.1)	0 (0-0)	-19.1 (-26.2--12.4)
Tokushima	0.1 (0-0.2)	-1.9 (-0.2-0)	0.1 (0-0.2)	-1.6 (-4.4-3.9)	0 (0-0.1)	-1.5 (-8.9-8.7)	0 (0-0.1)	-4.4 (-15-6.8)	0 (0-0)	-23.3 (-39.7--8.4)	0 (0-0)	-20.7 (-27.8--11.9)
Tōkyō	0.3 (0.1-0.4)	-1.9 (-28.7-0)	0.2 (0.1-0.3)	-2.4 (-27.2-3.2)	0.1 (0.1-0.2)	-0.6 (-23.1-10)	0.1 (0-0.1)	-8.7 (-30.6-3.2)	0 (0-0)	-15.7 (-39.5--0.7)	0.1 (0-0.1)	-13.5 (-34.6--3.6)
Tottori	0.1 (0-0.2)	0.1 (-0.2-0)	0.1 (0-0.2)	0.4 (-3.5-4.2)	0 (0-0.1)	3 (-5-12.2)	0 (0-0.1)	-3 (-12.2-8.1)	0 (0-0)	-20.9 (-32.2--8.5)	0 (0-0)	-15.9 (-22.2--8.2)
Toyama	0.1 (0-0.2)	0.5 (-0.2-0)	0.1 (0-0.2)	-0.3 (-4.3-2.9)	0 (0-0.1)	1.8 (-5.8-10.1)	0 (0-0.1)	-2.7 (-13.1-7.6)	0 (0-0)	-23.2 (-36--8.1)	0 (0-0)	-17.4 (-25.3--10.3)
Wakayama	0.1 (0-0.2)	-1.2 (-0.2-0)	0.1 (0-0.2)	-1.1 (-4.3-3.6)	0 (0-0.1)	-0.2 (-8-8.7)	0 (0-0.1)	-3.9 (-14.3-6.4)	0 (0-0)	-22.6 (-34.9--8.4)	0 (0-0)	-20 (-26.7--12.2)
Yamagata	0.1 (0-0.2)	-0.9 (-27-48)	0.1 (0-0.2)	-2.3 (-27.9-43.8)	0 (0-0.1)	-0.6 (-32.6-28.7)	0 (0-0.1)	-2.9 (-34.3-29)	0 (0-0)	-21.1 (-46-26.3)	0 (0-0)	-21.1 (-46.1-7.6)
Yamaguchi	0.1 (0-0.2)	-0.4 (-0.2-0)	0.1 (0-0.2)	-0.4 (-4.8-4.7)	0 (0-0.1)	0.1 (-10.1-11.5)	0 (0-0.1)	-3.1 (-14.8-9.8)	0 (0-0)	-26 (-39.7--9)	0 (0-0)	-17.6 (-26.3--8.5)

Location	Birth Incidence rate (per 100,000 live births), 2021	Birth Incidence rate (per 100,000 live births) percent change (2000-2021)	Under-5 rate (per 100,000 population), 2021	Under-5 rate (per 100,000 population) percent change (2000-2021)	5-14 rate (per 100,000 population), 2021	5-14 rate (per 100,000 population) percent change (2000-2021)	15-49 rate (per 100,000 population), 2021	15-49 rate (per 100,000 population) percent change (2000-2021)	50plus rate (per 100,000 population), 2021	50plus rate (per 100,000 population) percent change (2000-2021)	All age rate (per 100,000 population), 2021	All age rate (per 100,000 population) percent change (2000-2021)
Yamanashi	0.1 (0-0.2)	0.4 (-0.2-0)	0.1 (0-0.2)	-1 (-5.1-3.8)	0 (0-0.1)	0.5 (-6.8-9.1)	0 (0-0.1)	-1.2 (-10.1-10.4)	0 (0-0)	-17.2 (-30--2.5)	0 (0-0)	-21.3 (-27.7--13.9)
Republic of Korea	0.3 (0.2-0.5)	-1.4 (-18.9--0.8)	0.2 (0.1-0.3)	-1.1 (-18.1-7.3)	0.1 (0.1-0.1)	-2.1 (-20.4-14.1)	0.1 (0-0.1)	-3.4 (-22.1-16.2)	0 (0-0)	-4 (-26.5-24.1)	0 (0-0.1)	-29 (-41.9--16.7)
Singapore	0.3 (0.2-0.4)	-0.2 (-11.5-11.2)	0.2 (0.1-0.3)	-0.9 (-12.2-10.8)	0.1 (0.1-0.2)	-5.5 (-18.4-9.5)	0.1 (0-0.1)	-9.1 (-23.1-8.1)	0 (0-0)	-8.6 (-27.8-13.8)	0.1 (0-0.1)	-22 (-33.4--9.9)
**High-income North America**	20.2 (18.5-22)	-3.8 (-5.7--2.3)	19.6 (18-21.4)	-2.7 (-4.5--1.1)	18.4 (16.8-20)	-2.8 (-4.6--1.4)	15.1 (13.9-16.4)	2.6 (0.7-4.3)	4.9 (4.2-5.7)	1.2 (-1-3.5)	12 (11.1-13)	-7.9 (-9.6--6.4)
Canada	0.5 (0.3-0.7)	10.5 (-35.9-60.2)	0.5 (0.3-0.7)	10.6 (-36-59.6)	0.4 (0.3-0.6)	10.5 (-36.3-58.8)	0.3 (0.2-0.5)	8.7 (-37.9-52.1)	0.1 (0.1-0.1)	-3.8 (-46.1-29.8)	0.3 (0.2-0.4)	-3 (-44.6-36.1)
Greenland	0.2 (0-0.6)	9.4 (0-323)	0.2 (0-0.6)	9.9 (-0.6-320)	0.2 (0-0.5)	9.4 (-2-316)	0.1 (0-0.3)	14.4 (0.9-309)	0 (0-0.1)	6.3 (-8.1-225)	0.1 (0-0.3)	-5.2 (-15.7-240)
United States of America	22.2 (20.4-24.2)	-2.1 (-4--0.6)	21.6 (19.8-23.6)	-1.6 (-3.5--0.1)	20.3 (18.6-22.1)	-2.5 (-4.2--1.1)	16.7 (15.4-18.1)	2.3 (0.5-4)	5.5 (4.7-6.4)	2.4 (0.1-4.7)	13.3 (12.3-14.4)	-7.5 (-9.1--6)
Alabama	45.2 (41.2-49.9)	-3.2 (-9.4-3.1)	43.9 (39.9-48.4)	-3 (-9-3.5)	41.4 (37.6-45.7)	-2.2 (-8.1-4.2)	32.7 (29.9-35.8)	2.4 (-3.3-8.4)	10 (8.5-11.6)	2.6 (-4.8-9.8)	26.1 (24-28.5)	-6.9 (-12.2--1.4)
Alaska	7.6 (5-11.1)	-5.2 (-14.3-3.3)	7.4 (4.8-10.8)	-4.8 (-13.8-3.8)	6.9 (4.5-10)	-4 (-13-4.4)	5.3 (3.5-7.7)	3.7 (-5.7-12.8)	1.4 (0.8-2.1)	-10.1 (-19.1-0.4)	4.4 (2.9-6.3)	-12.6 (-20.9--5.4)
Arizona	11.8 (10.5-13.1)	7.3 (-0.1-14.9)	11.5 (10.2-12.8)	7.3 (0-14.9)	10.9 (9.7-12.1)	7.7 (0.5-15.2)	8.8 (7.9-9.8)	10.6 (3.2-18.1)	2.7 (2.2-3.3)	8 (0.4-15.3)	7 (6.3-7.8)	-0.4 (-7-6.2)
Arkansas	29 (25.5-32.6)	-9.2 (-15.6--3)	28.1 (24.8-31.7)	-9 (-15.4--2.8)	26.5 (23.2-29.9)	-8.3 (-14.8--2.2)	21.1 (18.6-23.6)	-3.9 (-10.5-2.2)	6 (4.8-7.2)	-1.3 (-8.5-6.1)	16.7 (14.8-18.7)	-11.3 (-17.3--5.9)
California	9.8 (9-10.8)	-2.1 (-7.9-4.5)	9.6 (8.7-10.5)	-2 (-7.9-4.7)	9.2 (8.4-10.1)	-1.6 (-7.5-4.9)	7.6 (7-8.3)	1.5 (-4.3-7.6)	2.8 (2.5-3.3)	1.4 (-4.9-7.8)	6.3 (5.7-6.8)	-8.3 (-13.6--2.7)
Colorado	5.3 (4.3-6.3)	-9.9 (-17.8--3)	5.1 (4.2-6.1)	-9.8 (-17.6--2.8)	4.9 (4-5.8)	-9.3 (-16.9--2.2)	3.9 (3.3-4.7)	-5.3 (-13.2-2)	1.3 (0.9-1.7)	-9.1 (-17.4--0.6)	3.2 (2.6-3.8)	-15.9 (-22.7--9.1)
Connecticut	21.2 (19-23.7)	-3.1 (-8.8-4)	20.7 (18.6-23.1)	-3 (-8.5-4.1)	19.9 (17.8-22.1)	-2.5 (-8.1-4.5)	16.1 (14.6-17.8)	3.1 (-2.8-9.6)	5.1 (4.3-6.1)	3.3 (-2.8-9.9)	12.3 (11.1-13.6)	-9.2 (-14.3--3.4)
Delaware	46.7 (41.7-52)	0.2 (-6-7.7)	45.5 (40.6-50.5)	0.4 (-5.9-7.9)	43.2 (38.7-48.2)	0.9 (-5.5-8.4)	34.8 (31.3-38.8)	5.8 (-0.8-13.1)	10.7 (9-12.6)	4 (-2.8-11.7)	26.9 (24.2-29.7)	-6.7 (-12.5-0)
District of Columbia	69.1 (55.9-81.8)	-20 (-27.5--11.6)	66.5 (53.5-79)	-19.5 (-26.9--11)	62.1 (50.1-73.6)	-18.5 (-25.6--9.6)	44.9 (36.9-52.7)	-17.2 (-24--9.6)	11.8 (9.1-15)	-2.2 (-10-6.4)	38.4 (31.7-44.9)	-18.5 (-25.3--11)
Florida	43.6 (40-47.6)	-4.8 (-10.4-1.1)	42.3 (38.8-46.2)	-4.6 (-10.3-1.2)	40.1 (36.6-43.7)	-3.9 (-9.7-1.9)	32.2 (29.7-34.9)	0.8 (-4.4-6.4)	10 (8.7-11.5)	6.5 (1-12.4)	24.5 (22.7-26.6)	-7.5 (-12.3--2.5)
Georgia	46.8 (42.8-51.3)	0.4 (-5-6.2)	45.5 (41.5-49.8)	0.6 (-4.9-6.2)	43 (39.3-47)	1.3 (-4-6.9)	34.8 (32-38)	6.2 (0.3-11.9)	11.6 (10.1-13.4)	4.7 (-1.2-10.8)	28.5 (26.3-31)	-5 (-10.1-0.2)
Hawaii	1.9 (0.7-4.1)	-34.9 (-72.7--14.9)	1.9 (0.6-4)	-34.7 (-72.7--14.6)	1.8 (0.6-3.8)	-34.4 (-72.5--13.8)	1.4 (0.5-3.1)	-31.8 (-71.3--10.1)	0.4 (0.1-1)	-32.6 (-70.7--10.5)	1.1 (0.4-2.4)	-39 (-74.2--19.5)
Idaho	1.4 (1-2)	1.5 (-11-16.2)	1.4 (1-1.9)	1.8 (-10.8-16.2)	1.3 (0.9-1.8)	2.8 (-9.8-17.6)	1 (0.7-1.4)	6.4 (-7.2-22)	0.2 (0.1-0.3)	6.9 (-8.7-28.2)	0.8 (0.6-1.1)	-3.6 (-15.9-10.5)
Illinois	15.2 (13.7-16.7)	-11.3 (-16.7--5.5)	14.8 (13.4-16.2)	-11.1 (-16.5--5.4)	14.1 (12.7-15.5)	-10.3 (-15.7--4.5)	11.4 (10.4-12.5)	-5.5 (-10.8-0.2)	3.7 (3.1-4.4)	-1.1 (-6.6-5.8)	9.1 (8.3-10)	-15.1 (-19.7--9.8)
Indiana	14.9 (13.6-16.5)	-4.8 (-11.2-1.6)	14.5 (13.2-16)	-4.7 (-11.1-1.8)	13.8 (12.5-15.2)	-3.9 (-10.1-2.5)	11 (10-12.1)	1.6 (-4.5-7.7)	3.4 (2.8-4)	3.8 (-3.7-11.2)	8.8 (8-9.7)	-8.1 (-13.5--2.5)
Iowa	9.7 (8.3-11.3)	6.6 (-0.5-14.8)	9.5 (8.1-11)	6.6 (-0.5-15.1)	9 (7.6-10.5)	7.2 (-0.1-15.6)	7.4 (6.2-8.5)	11.4 (4.2-20.1)	2.2 (1.6-2.7)	11.7 (3.4-21.4)	5.8 (4.9-6.7)	2.5 (-4.1-10.1)
Kansas	9.5 (7.9-11.3)	-9.9 (-16.9--2.9)	9.2 (7.7-11)	-9.7 (-16.8--2.8)	8.8 (7.3-10.5)	-9 (-16--2)	7.2 (6-8.6)	-4.4 (-11.5-3.1)	2.3 (1.8-2.9)	-0.3 (-8.9-9)	5.8 (4.9-7)	-12.1 (-18.9--5.2)
Kentucky	8.3 (7.2-9.4)	-2.2 (-8.5-4.2)	8.1 (7-9.2)	-2 (-8.5-4.3)	7.7 (6.7-8.8)	-1.3 (-7.8-5.1)	6.1 (5.3-6.9)	2.9 (-3.9-9.8)	1.8 (1.4-2.2)	2.9 (-6.1-11.1)	4.8 (4.2-5.4)	-6.8 (-12.9--0.6)
Louisiana	32.6 (29.6-35.8)	-12.2 (-18.2--5.8)	31.7 (28.6-34.7)	-12 (-18.1--5.6)	29.8 (26.9-32.8)	-11 (-17.3--4.4)	23.7 (21.5-25.8)	-6.3 (-12.5-0.1)	7.8 (6.8-9.1)	-0.8 (-7.7-6.5)	19.5 (17.8-21.2)	-14.7 (-20.2--9.2)
Maine	0.8 (0.4-1.4)	-46.8 (-70.7--21.8)	0.7 (0.4-1.4)	-46.5 (-70.5--21.1)	0.7 (0.3-1.3)	-46.2 (-70.2--20.6)	0.5 (0.3-1)	-43.1 (-68.7--14.8)	0.1 (0.1-0.2)	-46.2 (-69.3--15.1)	0.4 (0.2-0.7)	-52.5 (-73.4--28.8)
Maryland	57.2 (52.7-62.7)	-1.4 (-7.5-4.9)	55.8 (51.2-61)	-1.2 (-7.4-5)	53.3 (48.8-58.2)	-0.7 (-6.7-5.4)	43.5 (40-47.1)	3.5 (-2.5-9.8)	14.5 (12.6-16.8)	-0.8 (-6.7-5)	34.8 (32.2-37.6)	-7.2 (-12.4--1.7)
Massachusetts	15.4 (13.8-17.2)	-8.2 (-14.4--2.1)	15 (13.5-16.8)	-8.1 (-14.3--1.8)	14.4 (12.9-16)	-7.5 (-13.5--1.2)	11.9 (10.7-13.2)	-2.3 (-8.4-3.7)	3.8 (3.1-4.5)	2.7 (-4.1-9.6)	9.2 (8.3-10.2)	-12.5 (-17.9--6.9)

Location	Birth Incidence rate (per 100,000 live births), 2021	Birth Incidence rate (per 100,000 live births) percent change (2000-2021)	Under-5 rate (per 100,000 population), 2021	Under-5 rate (per 100,000 population) percent change (2000-2021)	5-14 rate (per 100,000 population), 2021	5-14 rate (per 100,000 population) percent change (2000-2021)	15-49 rate (per 100,000 population), 2021	15-49 rate (per 100,000 population) percent change (2000-2021)	50plus rate (per 100,000 population), 2021	50plus rate (per 100,000 population) percent change (2000-2021)	All age rate (per 100,000 population), 2021	All age rate (per 100,000 population) percent change (2000-2021)
Michigan	22 (20.2-24.1)	-4.4 (-9.7-1.4)	21.5 (19.7-23.5)	-4.2 (-9.5-1.4)	20.5 (18.8-22.3)	-3.6 (-8.9-1.9)	16.6 (15.3-17.9)	1.8 (-3.6-7.7)	5.4 (4.7-6.2)	1.3 (-4.3-7)	12.9 (12-14)	-11.1 (-15.8--6.2)
Minnesota	8.2 (7-9.8)	-6.2 (-13.4-3)	8.1 (6.8-9.6)	-6.1 (-13.4-3.1)	7.7 (6.4-9.2)	-5.5 (-12.8-3.7)	6.3 (5.3-7.4)	-1 (-8.7-8.2)	2.1 (1.6-2.6)	1.2 (-7.9-11.7)	5 (4.2-5.9)	-11.1 (-18.1--3)
Mississippi	48.7 (44.2-53.6)	-6.7 (-12.7--0.1)	47.2 (42.6-52.1)	-6.5 (-12.4-0.1)	44.2 (39.9-48.7)	-5.5 (-11.1-1.3)	34.4 (31.4-37.5)	-0.6 (-6.1-5.7)	10 (8.5-11.7)	3.7 (-2.2-10.5)	27.8 (25.4-30.2)	-10.6 (-15.7--5)
Missouri	20.5 (18.6-22.7)	-6.3 (-12.6-0.4)	20 (18-22)	-6.1 (-12.3-0.5)	18.9 (17.1-20.9)	-5.3 (-11.3-1.2)	15 (13.6-16.6)	0 (-5.9-6.6)	4.6 (3.9-5.4)	2.8 (-3.9-8.9)	11.9 (10.8-13.1)	-9.7 (-14.8--3.9)
Montana	0.7 (0.3-1.3)	-3.5 (-33.8-34.7)	0.7 (0.3-1.3)	-3.4 (-33.6-34.9)	0.7 (0.3-1.2)	-2.5 (-33.3-35.9)	0.5 (0.2-0.9)	3.3 (-30-42.8)	0.1 (0.1-0.3)	-2.5 (-39.8-28.9)	0.4 (0.2-0.7)	-9.9 (-38.7-26)
Nebraska	5.7 (4.1-7.6)	-24.7 (-33.7--15.3)	5.5 (4-7.4)	-24.7 (-33.7--15.2)	5.3 (3.8-7)	-24.1 (-33--14.5)	4.4 (3.2-5.8)	-19.9 (-29--9.9)	1.4 (1-2)	-14.6 (-24.6--2.9)	3.6 (2.6-4.7)	-25.6 (-34.1--16.1)
Nevada	19.3 (17-21.9)	-0.5 (-8-6.7)	18.8 (16.6-21.4)	-0.4 (-8-6.8)	17.9 (15.8-20.3)	0.2 (-7.5-7.4)	14.6 (12.8-16.4)	4.8 (-3.5-12)	4.6 (3.7-5.5)	0.8 (-7.5-9.1)	11.7 (10.4-13.2)	-5.2 (-12.6-1.5)
New Hampshire	1.6 (1.1-2.4)	1 (-10.3-18)	1.6 (1-2.3)	0.7 (-10.4-17.7)	1.5 (1-2.2)	1.5 (-9.8-18.9)	1.2 (0.8-1.7)	9 (-2.4-28.1)	0.3 (0.2-0.5)	7.9 (-4-35.6)	0.9 (0.6-1.3)	-9.8 (-19.4-6.4)
New Jersey	29.8 (27.3-32.7)	-5.2 (-11.2-0.3)	29.1 (26.7-31.9)	-5 (-11.1-0.6)	27.8 (25.5-30.4)	-4.5 (-10.4-1.1)	22.6 (20.7-24.4)	0.2 (-5.3-5.4)	7.7 (6.7-8.9)	2.8 (-2.3-8.8)	17.9 (16.5-19.4)	-9.3 (-14.2--4.5)
New Mexico	3 (2.1-4)	-3.9 (-11.8-7.4)	2.9 (2.1-3.9)	-3.6 (-11.4-7.7)	2.7 (1.9-3.7)	-3 (-10.7-8.4)	2.1 (1.5-2.8)	3 (-5.1-14.3)	0.5 (0.3-0.7)	-1.2 (-10.9-10.3)	1.6 (1.1-2.2)	-11.4 (-18.4--2.2)
New York	37.3 (34.2-40.9)	-1.7 (-7.6-3.8)	36.7 (33.6-40.2)	-1.6 (-7.4-3.8)	35.5 (32.5-38.9)	-1.2 (-6.9-4.5)	30.2 (27.7-32.8)	1.9 (-3.9-7.3)	11.3 (9.7-13)	2.4 (-2.8-8.2)	24.1 (22.3-26.2)	-6.7 (-11.4--2.1)
North Carolina	36 (33.1-39.2)	-3.9 (-9.8-1.6)	35 (32.1-38)	-3.8 (-9.8-1.6)	33.2 (30.3-36.1)	-3.2 (-9.1-2)	26.7 (24.6-28.9)	1.1 (-5-6.7)	8.4 (7.3-9.7)	-0.2 (-5.9-6.1)	21.2 (19.7-22.9)	-8.9 (-14.2--4.1)
North Dakota	0.5 (0.1-1.2)	-9.8 (-56.7-0)	0.5 (0.1-1.2)	-9.6 (-56.5-0.5)	0.5 (0.1-1.1)	-8.9 (-56.2-1.8)	0.4 (0.1-0.9)	-4.8 (-53.4-7.4)	0.1 (0-0.3)	0.2 (-48.9-16.4)	0.3 (0.1-0.7)	-10.5 (-55.8-1)
Ohio	17.1 (15.5-18.7)	-4.7 (-11-0.7)	16.6 (15.1-18.2)	-4.6 (-11-0.8)	15.8 (14.3-17.3)	-3.9 (-10.4-1.7)	12.7 (11.6-13.9)	1.8 (-4.7-7.6)	4.1 (3.5-4.8)	4.4 (-2.5-11.3)	10 (9.1-10.9)	-8.9 (-14.7--4)
Oklahoma	8.8 (7.5-10.2)	-16 (-22.2--9.6)	8.5 (7.3-10)	-15.8 (-22.1--9.4)	8.1 (6.8-9.4)	-14.9 (-21--8.5)	6.4 (5.5-7.5)	-10 (-15.7--3.2)	1.8 (1.4-2.3)	-4.8 (-12.7-4)	5.2 (4.4-6)	-16.7 (-22.2--10.5)
Oregon	4.5 (3.6-5.5)	-3.3 (-10.9-5.9)	4.4 (3.5-5.4)	-3 (-10.7-6.3)	4.2 (3.3-5.1)	-2.4 (-10.1-6.9)	3.3 (2.6-4)	1.3 (-6.9-10.9)	0.9 (0.6-1.2)	-0.1 (-10.3-10.6)	2.5 (2-3.1)	-10.2 (-17--2.4)
Pennsylvania	14 (12.7-15.4)	-5.6 (-11.6-1.5)	13.6 (12.4-15)	-5.4 (-11.3-1.7)	13 (11.8-14.3)	-4.7 (-10.7-2.4)	10.4 (9.5-11.4)	1.4 (-4.5-8.8)	3.1 (2.6-3.7)	5.6 (-1.6-13.2)	8 (7.3-8.8)	-8.5 (-13.7--2.1)
Rhode Island	19 (15.5-22.9)	-9 (-16.7--1.6)	18.6 (15-22.4)	-8.9 (-16.7--1.5)	17.8 (14.4-21.4)	-8.5 (-16.2--1)	14.9 (12.1-17.9)	-4 (-12-3.8)	4.9 (3.8-6.2)	3.4 (-6.1-13.7)	11.4 (9.4-13.7)	-13.6 (-20.7--6.4)
South Carolina	39.5 (35.9-43.2)	-10.9 (-16.6--5.1)	38.4 (34.8-42)	-10.7 (-16.4--4.9)	36.3 (32.8-39.7)	-9.8 (-15.6--4.1)	29 (26.6-31.6)	-4.8 (-10.9-1)	9.4 (8.2-11)	-4 (-10-2.3)	23.1 (21.2-25.1)	-14.7 (-20.1--9.7)
South Dakota	1.8 (0.9-3.1)	-0.7 (-10.6-12.2)	1.8 (0.9-3.1)	-0.3 (-10.3-12.7)	1.7 (0.9-2.9)	0.7 (-8.9-13.5)	1.3 (0.7-2.2)	6.5 (-3.5-20.2)	0.3 (0.1-0.5)	14.7 (-2.3-33.2)	1 (0.5-1.7)	-3.4 (-12.3-8.7)
Tennessee	23 (21-25.2)	-3.5 (-9.5-3.5)	22.3 (20.3-24.5)	-3.6 (-9.5-3)	21.1 (19.1-23.2)	-4.1 (-9.6-2.3)	16.5 (15-18)	0.8 (-5-7.2)	4.8 (4-5.7)	0.1 (-6-6.6)	13.1 (12-14.3)	-8.9 (-14.1--3.5)
Texas	22.8 (20.7-25.1)	4.9 (-1.1-10.9)	22.2 (20.1-24.5)	4.7 (-1.2-10.7)	21.1 (19.1-23.3)	4.8 (-1.1-10.7)	17.2 (15.7-18.8)	8.3 (2.1-14.2)	5.7 (4.9-6.7)	5.6 (-1.1-11.9)	14.5 (13.4-15.8)	-0.6 (-6.1-4.4)
Utah	3 (2.3-3.9)	-7.2 (-15.1-0.9)	3 (2.3-3.8)	-7.1 (-15-0.9)	2.8 (2.2-3.7)	-6.5 (-14.2-1.4)	2.4 (1.8-3.1)	-4.5 (-12.4-4.1)	0.8 (0.6-1.1)	-1.4 (-11-8.2)	2.1 (1.6-2.7)	-10.3 (-17.9--2.1)
Vermont	4.9 (2.9-7.8)	6.2 (-8.4-21.2)	4.8 (2.8-7.7)	6.1 (-8.2-21.1)	4.6 (2.7-7.3)	6.6 (-7.5-21.5)	3.9 (2.3-6.1)	10.9 (-3-25.8)	1.4 (0.8-2.1)	6.7 (-3.6-20.8)	2.9 (1.7-4.5)	-4.1 (-14.8-8.2)
Virginia	29.7 (27.1-32.4)	-2.9 (-8.7-2.8)	29 (26.3-31.6)	-2.8 (-8.5-3)	27.5 (25-30.1)	-2.1 (-7.7-3.6)	22.4 (20.4-24.4)	2.5 (-3.5-7.9)	7.5 (6.5-8.8)	0.5 (-5.3-7.1)	18 (16.5-19.6)	-7.5 (-12.7--2.2)
Washington	6.5 (5.6-7.4)	2.7 (-4.5-10.7)	6.3 (5.5-7.2)	2.8 (-4.5-11)	6.1 (5.2-6.9)	3.3 (-3.8-11.6)	4.9 (4.3-5.6)	6.7 (-0.6-14.3)	1.5 (1.2-1.9)	2.6 (-5.4-10.9)	3.9 (3.4-4.4)	-4.5 (-10.9-2.4)
West Virginia	4.8 (3.6-6.2)	-9.2 (-15.8--3)	4.7 (3.5-6)	-9.2 (-16--2.9)	4.4 (3.3-5.7)	-8.4 (-15.2--1.9)	3.5 (2.7-4.5)	-4.2 (-11.4-3.5)	0.9 (0.6-1.3)	-4.8 (-13.3-6.1)	2.6 (2-3.3)	-14.2 (-20.8--7.1)
Wisconsin	11.4 (9.9-12.9)	-11.6 (-17.8--5.3)	11.2 (9.6-12.6)	-11.5 (-17.7--5.2)	10.6 (9.2-12.1)	-10.9 (-17--4.7)	8.7 (7.5-9.8)	-6.5 (-13--0.1)	2.6 (2.1-3.2)	-3.3 (-9.3-3.7)	6.7 (5.8-7.6)	-16.7 (-22--10.8)
Wyoming	2.4 (1.1-4.7)	-41.8 (-57.4--28.4)	2.4 (1-4.6)	-41.7 (-57.4--28.4)	2.2 (1-4.3)	-41.3 (-57.1--27.8)	1.8 (0.8-3.4)	-38.1 (-54.9--24)	0.5 (0.2-0.9)	-40.3 (-58.4--27.3)	1.4 (0.6-2.7)	-44.4 (-59.6--31.9)

Location	Birth Incidence rate (per 100,000 live births), 2021	Birth Incidence rate (per 100,000 live births) percent change (2000-2021)	Under-5 rate (per 100,000 population), 2021	Under-5 rate (per 100,000 population) percent change (2000-2021)	5-14 rate (per 100,000 population), 2021	5-14 rate (per 100,000 population) percent change (2000-2021)	15-49 rate (per 100,000 population), 2021	15-49 rate (per 100,000 population) percent change (2000-2021)	50plus rate (per 100,000 population), 2021	50plus rate (per 100,000 population) percent change (2000-2021)	All age rate (per 100,000 population), 2021	All age rate (per 100,000 population) percent change (2000-2021)
**Southern Latin America**	0.7 (0.6-0.9)	25.6 (12.4-56.2)	0.4 (0.3-0.5)	26.1 (14-52.1)	0.2 (0.1-0.2)	24.3 (11.3-47)	0.1 (0.1-0.1)	22.9 (9.2-45.2)	0 (0-0)	41.1 (20.4-73.2)	0.1 (0.1-0.2)	12 (0.8-32.7)
Argentina	0.3 (0.2-0.5)	0.8 (-7.9-39.5)	0.2 (0.1-0.3)	-0.8 (-10-37.7)	0.1 (0-0.1)	0.7 (-19.1-25.5)	0 (0-0.1)	-2.1 (-24.6-25)	0 (0-0)	-3 (-27.4-29.8)	0.1 (0-0.1)	-9.8 (-26.8-15.9)
Chile	2 (1.5-2.5)	52.6 (31-100)	1.2 (1-1.6)	51.4 (31.3-97.4)	0.5 (0.4-0.7)	49.3 (29.8-82.4)	0.3 (0.2-0.4)	46.9 (26.7-78.9)	0.1 (0-0.1)	41.5 (17.4-85.6)	0.3 (0.2-0.4)	25.2 (9.3-55.7)
Uruguay	0.3 (0.2-0.5)	-0.3 (-12.4--0.2)	0.2 (0.1-0.3)	-0.5 (-12.9-9.4)	0.1 (0-0.1)	-0.7 (-15.8-22.7)	0 (0-0.1)	-0.9 (-18.9-23.9)	0 (0-0)	-1.7 (-22.9-28.3)	0 (0-0.1)	-10.3 (-23.2-6.7)
**Western Europe**	19.7 (15.5-25)	-9.8 (-12.9--6.3)	16.6 (13.3-20.8)	-7.5 (-10.5--4.1)	9 (7.3-11.2)	-3.8 (-6.9--0.7)	3.2 (2.5-4)	-8.7 (-11.9--5.6)	1.1 (0.8-1.3)	-7.4 (-10.6--4.3)	3.6 (3-4.3)	-13.9 (-16.4--11.5)
Andorra	13 (9.4-17.9)	1 (-9-10.6)	10.9 (8-15)	0.9 (-9.2-10.4)	5.6 (4.2-7.5)	-2.2 (-10.3-7.4)	2.1 (1.6-2.8)	-0.9 (-8.7-7.7)	0.8 (0.6-1.1)	2.6 (-5.5-12.1)	2.2 (1.7-2.8)	-15.6 (-21.7--8.7)
Austria	18.6 (16-21.8)	46.7 (32.9-61.6)	16.2 (14.1-18.7)	45.9 (31.9-59.7)	9.7 (8.4-11.2)	33.7 (23.6-44.2)	2.7 (2.2-3.3)	8.7 (-0.1-17.7)	0.9 (0.7-1.1)	-2.8 (-12.1-6.5)	3.3 (2.9-3.8)	10.9 (3.4-18.2)
Belgium	10 (7.4-13.4)	-4.8 (-13.5-6.8)	8.2 (6.1-10.9)	-4.4 (-13.5-6)	3.6 (2.8-4.8)	-2.5 (-10.9-7.1)	1.1 (0.8-1.5)	-0.5 (-10.5-12.6)	0.3 (0.2-0.5)	-2.7 (-14.4-10.5)	1.5 (1.2-1.9)	-7.5 (-15-0.6)
Cyprus	14.1 (10-19.6)	0.8 (-11.3-11.5)	11.7 (8.4-16.5)	1.8 (-9.5-12.1)	6 (4.5-8.2)	2 (-8.1-11.3)	2.1 (1.5-2.7)	-4.4 (-12.4-5)	0.8 (0.6-1)	-5.3 (-14.6-4.5)	2.6 (2-3.4)	-13.4 (-20.5--5.8)
Denmark	12.7 (9.1-17.2)	1.2 (-9-11.2)	10.8 (7.9-14.8)	1.8 (-8.5-11.5)	5.9 (4.3-7.9)	-1.6 (-10-8.3)	2.3 (1.7-3)	3 (-5.1-12.2)	0.7 (0.5-1)	-6.2 (-14.8-3.3)	2.5 (1.9-3.2)	-7.9 (-14.9-0)
Finland	12.9 (9.1-17.8)	0.5 (-10.5-13.2)	11.2 (8-15.4)	0.5 (-9.5-12.7)	6.3 (4.7-8.3)	0 (-8.8-10.8)	2.2 (1.6-2.9)	1.3 (-7.4-10)	0.7 (0.5-0.9)	-13.3 (-21.7--4.5)	2.4 (1.9-3)	-11.4 (-18.3--3.3)
France	35 (27.5-44)	-9.5 (-17.2--0.9)	28.8 (23-36.2)	-9.2 (-17.2--1.1)	14.2 (11-18.2)	-2.9 (-11.6-6.1)	4.8 (3.6-6.3)	2.9 (-6.7-13.4)	1.5 (1.1-2)	-3 (-12.4-7.6)	5.9 (4.8-7.2)	-10.9 (-17.8--4)
Germany	10.1 (8.4-12.2)	0.1 (-8.4-8.3)	8.7 (7.2-10.4)	0.2 (-8.3-8.3)	5.3 (4.4-6.4)	1.9 (-5.8-10.6)	2 (1.6-2.5)	1.5 (-6.2-9.9)	0.6 (0.5-0.8)	-2.6 (-10.9-6.9)	2 (1.7-2.3)	-7.9 (-13.8--1.3)
Greece	81.7 (65.9-100)	-5.8 (-17.1-6.8)	69.4 (56.8-84.4)	-5.8 (-16.4-6.1)	41.7 (35.5-49.3)	-7.4 (-15.3-1.3)	19 (16.4-21.8)	-12 (-18.2--5.3)	5.9 (4.8-7.2)	-14.2 (-20.1--7.3)	17.6 (15.3-20)	-18.5 (-24.3--12.3)
Iceland	12.2 (8.8-16.5)	1.1 (-6.8-11.4)	10.6 (7.6-14.3)	1.4 (-6.2-12)	6 (4.4-8)	0.3 (-6.9-8.8)	2.2 (1.6-2.9)	-0.7 (-8.1-7.6)	0.8 (0.6-1.1)	-2 (-9.4-7)	2.7 (2.1-3.5)	-12.1 (-18.1--5.5)
Ireland	13.1 (9.4-17.5)	1.3 (-10.8-12.1)	11 (8-14.8)	1 (-10.6-11.3)	6 (4.5-8.2)	2.6 (-5.5-12.7)	2.2 (1.6-2.9)	-2.8 (-10.4-5.8)	0.8 (0.6-1)	-2.6 (-11.4-7.3)	2.8 (2.2-3.6)	-7.3 (-14.2-0.3)
Israel	26.9 (19.8-36.2)	-27.6 (-38--15.1)	22 (16.3-29.7)	-26.2 (-36.5--14.3)	10.6 (8-14.1)	-15.8 (-25.5--5.2)	3.6 (2.7-4.9)	-7.2 (-16.9-4.8)	1.3 (0.9-1.7)	-9.9 (-19.9-2.4)	6.1 (4.8-7.7)	-19.9 (-28.6--10.8)
Italy	14.2 (11.6-17.3)	7.4 (3.3-12.3)	13.1 (10.6-15.9)	8.4 (4.3-13.3)	7.9 (6.6-9.6)	5.5 (1.8-9.7)	2.8 (2.3-3.4)	-1.3 (-4.8-2.5)	1.2 (1-1.5)	-2.1 (-5.7-1.6)	2.9 (2.5-3.4)	-6.7 (-9.9--3.3)
Abruzzo	7.3 (5-10.4)	16 (4.6-30.2)	6.7 (4.5-9.5)	15.7 (4.6-30.3)	3.6 (2.6-5)	13 (3.7-26.3)	1 (0.7-1.5)	3.8 (-7-17.2)	0.4 (0.3-0.6)	8.9 (-4.2-24)	1.2 (0.8-1.6)	-1.7 (-9.9-10)
Basilicata	7.1 (4.6-11.2)	36.6 (18.6-58.8)	6.4 (4.1-10.2)	36.3 (18.6-59.1)	3.8 (2.5-5.7)	31.1 (16.1-51.6)	1.5 (1-2.1)	24 (8.9-43.7)	0.7 (0.5-0.9)	29.4 (13-52.7)	1.5 (1-2)	9.9 (-2.2-27.5)
Calabria	21.3 (17.4-25.5)	3.1 (-7.3-13.4)	19.6 (16.1-23.4)	3.1 (-7.2-13.1)	12.7 (10.9-15.1)	2.6 (-5-11)	5.7 (4.9-6.7)	-1.1 (-7-6.4)	2.6 (2.1-3.1)	2.9 (-3.9-10.8)	5.5 (4.8-6.3)	-10.7 (-16.3--4.1)
Campania	6 (4.8-7.3)	12.5 (1.8-25.6)	5.5 (4.4-6.7)	12.3 (1.9-25.2)	3.5 (2.9-4.3)	9.6 (-0.2-19.7)	1.6 (1.3-2)	5.3 (-3.5-14.7)	0.7 (0.6-0.9)	8.6 (-1.2-19.5)	1.6 (1.3-1.9)	-5.7 (-13.4-2.9)
Emilia-Romagna	34.3 (27.9-40.8)	9 (-2.3-21.5)	31.2 (25.5-37.1)	8.4 (-2.7-20.7)	17.4 (14.2-21.2)	3.9 (-6.3-14.4)	4.3 (3.5-5.1)	-5.9 (-14-2.1)	1.8 (1.5-2.2)	-5.3 (-13.2-3.4)	5.3 (4.6-6)	-0.2 (-6.8-7.2)
Friuli-Venezia Giulia	25.7 (19.7-32.5)	5.8 (-5.6-20.3)	23.6 (18.3-30)	5.3 (-6.5-19.9)	13.2 (10.4-16.6)	4.9 (-4.7-17.6)	3.2 (2.6-4)	4 (-5.4-15.4)	1.2 (1-1.6)	0.8 (-9-12.1)	3.8 (3.1-4.5)	-0.7 (-8.4-8.7)
Lazio	10.6 (8.6-13)	20.2 (9.3-33.3)	9.8 (8-11.9)	19.9 (9.7-32.4)	6.2 (5.2-7.6)	16.8 (6.6-28)	2.3 (1.8-2.8)	7.5 (-0.5-15.9)	1 (0.8-1.2)	6.4 (-2.2-16)	2.3 (2-2.7)	3.7 (-3.5-11.6)
Liguria	21.1 (17-26)	4.6 (-6.7-16.2)	19.5 (15.7-24)	4.1 (-7-15.1)	11.8 (9.8-14.2)	0.5 (-8.6-9.3)	4.6 (3.8-5.4)	0.7 (-6.6-8.8)	1.8 (1.5-2.2)	-0.7 (-7.7-7)	4.2 (3.5-4.8)	-3.5 (-10.1-3.5)
Lombardia	9.6 (7.7-11.9)	0.5 (-11.7-12.8)	8.9 (7.1-11)	0.2 (-11.6-12.8)	5.7 (4.6-7.1)	-1.6 (-12.9-9.3)	2 (1.6-2.5)	-5.6 (-14.2-4.9)	0.8 (0.7-1)	-9 (-17.5-1.9)	2.1 (1.8-2.5)	-8.8 (-17.5-0.2)
Marche	21.3 (16.6-26.8)	8.3 (-3.1-20.1)	19.4 (15-24.5)	8 (-3-19.9)	11 (8.7-14)	5.1 (-3.5-14.8)	3.2 (2.5-4.1)	-0.6 (-9.3-8.7)	1.4 (1.1-1.8)	0.7 (-9.2-10.7)	3.6 (3-4.4)	-3.4 (-10.7-4)
Molise	12.9 (8-20.1)	-3.4 (-12-8.4)	11.7 (7.3-18.5)	-3 (-11.7-9)	6.3 (4-9.4)	-3.4 (-11.4-7.2)	2.1 (1.4-3.1)	-9.1 (-17.4-1)	0.8 (0.5-1.2)	-5.3 (-14.6-5.9)	2.1 (1.4-3.1)	-19 (-25.7--10.9)

Location	Birth Incidence rate (per 100,000 live births), 2021	Birth Incidence rate (per 100,000 live births) percent change (2000-2021)	Under-5 rate (per 100,000 population), 2021	Under-5 rate (per 100,000 population) percent change (2000-2021)	5-14 rate (per 100,000 population), 2021	5-14 rate (per 100,000 population) percent change (2000-2021)	15-49 rate (per 100,000 population), 2021	15-49 rate (per 100,000 population) percent change (2000-2021)	50plus rate (per 100,000 population), 2021	50plus rate (per 100,000 population) percent change (2000-2021)	All age rate (per 100,000 population), 2021	All age rate (per 100,000 population) percent change (2000-2021)
Piemonte	15.4 (12.2-19)	8.1 (-2.1-21)	14.2 (11.2-17.4)	7.7 (-2.3-20.5)	7.1 (5.7-8.8)	2.4 (-6-12)	1.6 (1.2-2)	-2.9 (-12.8-9.8)	0.6 (0.5-0.8)	-5.1 (-15.7-7.5)	2 (1.7-2.4)	-3.8 (-11.6-4.4)
Provincia autonoma di Bolzano	13.2 (8.7-19.3)	4.1 (-7.9-19.8)	12.2 (8-17.7)	4.2 (-7.4-19.2)	7.1 (4.9-9.9)	3 (-7.2-15.9)	2.2 (1.5-3)	1.6 (-7.6-13.2)	0.9 (0.6-1.3)	0 (-10-11.5)	2.6 (1.9-3.5)	-8 (-15.6-2.8)
Provincia autonoma di Trento	12.5 (8.7-17.8)	6 (-5.2-19.4)	11.4 (7.9-16.3)	5.5 (-6.2-18.1)	6.3 (4.7-8.8)	2.9 (-6.4-13.1)	2.2 (1.6-2.9)	5 (-5.6-17.2)	1 (0.7-1.3)	4.6 (-7.1-18.5)	2.4 (1.8-3.2)	-3.9 (-12-5.6)
Puglia	6.8 (5.1-8.8)	-2.5 (-11.5-8.9)	6.2 (4.7-8.1)	-2.5 (-11.5-8.7)	3.9 (3.1-5)	-2.9 (-12.2-6.7)	1.6 (1.2-2)	-7.5 (-16.4-1.8)	0.7 (0.5-0.9)	-8.3 (-17.1-2.2)	1.6 (1.2-1.9)	-19 (-26.2--10.8)
Sardegna	6.9 (4.6-9.8)	14.3 (1.8-29.1)	6.3 (4.2-9)	13.7 (1.3-28.4)	3.6 (2.5-5)	10.3 (0.5-22.4)	1.2 (0.8-1.6)	1.2 (-8.1-12.2)	0.5 (0.3-0.7)	1.3 (-9.4-13.8)	1.2 (0.9-1.6)	-10.7 (-18.3--1.5)
Sicilia	21.6 (18.1-25.6)	-2.2 (-10.1-7.3)	19.9 (16.9-23.6)	-2.2 (-10-7.4)	14 (12-16.4)	-2 (-8.8-6.8)	7.4 (6.3-8.6)	-2.1 (-8.9-5.1)	3.4 (2.9-4)	0.3 (-6.8-8.3)	6.8 (5.9-7.7)	-11.7 (-17.5--4.8)
Toscana	12.1 (9.3-15.6)	7.4 (-3.5-20.5)	11.2 (8.7-14.4)	7.1 (-3.8-19.9)	6.3 (5-8)	3.5 (-4.9-12.5)	1.8 (1.4-2.3)	-2.4 (-10.9-7)	0.7 (0.6-1)	-2.3 (-11.9-7.6)	2 (1.6-2.5)	-1.8 (-8.6-6.8)
Umbria	15.9 (11.8-20.8)	11.9 (-1.2-24.7)	14.5 (10.8-18.8)	11.4 (-1.2-24.2)	7.9 (6.2-10.1)	8.9 (-1.2-19.7)	2.4 (1.9-3.1)	4.5 (-4.6-15)	1 (0.7-1.3)	4.5 (-5.4-15.3)	2.7 (2.1-3.2)	2.9 (-5.3-11.8)
Valle d'Aosta	7 (3.2-12.8)	-9.8 (-23.9-6.1)	6.4 (2.9-11.8)	-10.2 (-24.1-5.4)	3 (1.5-5.1)	-9.9 (-23.2-4.2)	0.6 (0.3-1)	-12.9 (-30.6-6.7)	0.2 (0.1-0.3)	-13.9 (-32-7.4)	0.8 (0.4-1.4)	-17.3 (-29.1--4)
Veneto	15.3 (12.3-18.9)	8.5 (-1.9-19.7)	14.1 (11.3-17.4)	8.2 (-2-18.5)	8.4 (7-10.3)	4.1 (-4.5-13.8)	2.7 (2.2-3.3)	-1.8 (-10-6.7)	1.2 (0.9-1.5)	-3.7 (-11.7-4.9)	3 (2.5-3.4)	-6.1 (-12.6-1.7)
Luxembourg	12.3 (8.9-16.7)	1 (-8.3-11.2)	10.5 (7.7-14.2)	0.9 (-7.9-11.3)	6 (4.5-8)	0.6 (-7.4-11.2)	2.3 (1.7-3)	1.4 (-6.6-10.5)	0.8 (0.6-1.1)	4.5 (-4.6-16)	2.6 (2-3.3)	-8.1 (-14.8--0.8)
Malta	13 (9.3-17.7)	1.4 (-7.3-15.4)	11 (7.8-15)	2 (-7-15.9)	6 (4.5-8)	3.2 (-5.1-13.6)	2.2 (1.7-3)	-2.2 (-10.6-6.8)	0.7 (0.5-1)	-16.2 (-23.9--7.8)	2.4 (1.8-3.1)	-16.1 (-22.5--8.6)
Monaco	13 (9.5-17.5)	1.3 (-9.3-13.3)	11.1 (8-15)	0.5 (-9.9-11.3)	6.1 (4.6-8.1)	0.6 (-8.1-9.7)	2.2 (1.6-2.9)	4.2 (-4.2-13)	0.7 (0.5-0.9)	3.6 (-5.2-14.6)	2.2 (1.7-2.8)	-0.6 (-7.7-6.7)
Netherlands	1.4 (1-2)	3 (-6.8-20.3)	1.2 (0.9-1.6)	3.1 (-7.1-19.6)	0.5 (0.4-0.7)	1.2 (-9.1-16.7)	0.2 (0.1-0.2)	3.4 (-9.9-19.4)	0.1 (0-0.1)	-5.4 (-18-10)	0.2 (0.1-0.3)	-11.1 (-19.3-2.5)
Norway	15.6 (8.9-23.5)	-0.5 (-4.9-5.1)	13 (7.6-19.7)	-0.6 (-4.6-4.5)	4.8 (3.1-7.2)	-1.3 (-5.2-2.9)	1.1 (0.7-1.6)	1.1 (-3.2-5.7)	0.3 (0.2-0.4)	0.4 (-5.1-5.7)	1.9 (1.2-2.6)	-12.6 (-15.7--9.2)
Agder	16.5 (9.2-25)	-2.4 (-16.7-12.2)	13.8 (7.9-21.1)	-1.6 (-14.5-13.5)	5.1 (3.2-7.8)	-2 (-14.8-11.2)	1.1 (0.7-1.7)	-1 (-14.3-12.8)	0.3 (0.2-0.4)	-1.2 (-17.4-18.7)	2 (1.3-2.8)	-14.9 (-24.5--4.9)
Innlandet	16.4 (9.4-25.4)	0.3 (-14.6-16.9)	13.6 (7.9-21.1)	-0.2 (-13.8-15.6)	4.9 (3.1-7.5)	-1.4 (-13.8-12.4)	1.1 (0.7-1.7)	3 (-10.3-18.5)	0.3 (0.2-0.4)	-0.9 (-15.8-17.4)	1.7 (1.1-2.4)	-15.4 (-25.2--4)
Møre og Romsdal	16.3 (8.9-25.5)	0.1 (-12.9-14.5)	13.4 (7.4-21)	-0.3 (-12.6-14.7)	4.7 (3-7.2)	-0.9 (-13.2-12.9)	1.1 (0.7-1.7)	1 (-11.9-17.4)	0.3 (0.2-0.4)	-0.2 (-15.5-20.2)	1.9 (1.2-2.7)	-15.1 (-24.5--5.2)
Nordland	16.4 (9.6-25.2)	-1.6 (-13.8-10.5)	13.5 (8.1-21.1)	-1.4 (-12.5-11)	5 (3.1-7.5)	-1.2 (-12.7-11.5)	1.1 (0.7-1.7)	2.8 (-9.6-20.9)	0.3 (0.2-0.4)	-1.8 (-17.5-18.4)	1.8 (1.2-2.6)	-18.6 (-26.8--8.9)
Oslo	15.7 (9-24.5)	0.3 (-11.8-14.6)	13.3 (7.8-20.7)	0.8 (-10.8-14.1)	4.8 (3.1-7.3)	-0.6 (-12-11.7)	1.1 (0.7-1.6)	0.1 (-12.2-13.2)	0.3 (0.2-0.4)	9.1 (-6.3-29)	2 (1.3-2.8)	-0.9 (-10.5-9.6)
Rogaland	14.8 (8.4-23)	-1.8 (-15.4-14.2)	12.4 (7.1-19.2)	-2.2 (-15.1-10.9)	4.9 (3.1-7.3)	-1.4 (-12.9-12.2)	1.1 (0.7-1.7)	-1.4 (-13.4-12.9)	0.3 (0.2-0.5)	1.2 (-13.7-17.2)	2 (1.3-2.9)	-14.5 (-23.4--4.3)
Troms og Finnmark	17 (9.6-26.1)	0.1 (-13.5-18.5)	14 (8.1-21.3)	-0.5 (-13.3-17.1)	4.9 (3.1-7.4)	-2.6 (-14.7-12.4)	1.1 (0.7-1.7)	3.9 (-10.7-21)	0.3 (0.1-0.4)	-4.9 (-19.1-15)	1.9 (1.2-2.6)	-19.2 (-28.8--8)
Trøndelag	15.2 (8.5-23.7)	-0.3 (-14.1-16.9)	12.7 (7.4-19.6)	0.1 (-12.6-15.7)	4.8 (3.1-7.2)	-0.7 (-12.6-12.7)	1.2 (0.7-1.7)	2.4 (-11.9-16.9)	0.3 (0.2-0.4)	-0.5 (-16.1-15.8)	1.9 (1.2-2.7)	-12.5 (-21.1--2.3)
Vestfold og Telemark	15.8 (9.4-23.9)	-0.2 (-12.5-15.6)	13.2 (8-19.8)	-0.4 (-11.9-14.2)	4.9 (3.2-7.4)	-0.6 (-10.4-13.1)	1.1 (0.7-1.7)	1.9 (-10.6-17.1)	0.3 (0.2-0.4)	-1.2 (-15.8-15.7)	1.8 (1.2-2.5)	-15.1 (-23.2--4.8)
Vestland	15 (8.7-23.2)	0.2 (-14.2-15.5)	12.7 (7.4-19.6)	0.6 (-12.9-15)	4.8 (3.1-7.1)	-0.8 (-13.2-12.4)	1.1 (0.7-1.7)	0.1 (-13.1-13.9)	0.3 (0.2-0.5)	0.9 (-15.2-17.9)	1.9 (1.3-2.7)	-12.8 (-22--3.3)
Viken	14.9 (8.6-23.6)	0.3 (-15.3-14.8)	12.5 (7.3-19.3)	-0.3 (-15.3-12.9)	4.7 (3.1-7)	-1.5 (-15-10.6)	1.1 (0.7-1.6)	2.4 (-10.9-16)	0.3 (0.2-0.4)	-1.5 (-15.8-12.4)	1.8 (1.2-2.6)	-12.3 (-22.6--2.4)
Portugal	82.2 (61.1-109)	-10.1 (-22.2-4.6)	66.8 (50.5-86.9)	-10.7 (-21.8-2.7)	34.2 (27.2-43)	-14.8 (-25.3--4)	12.8 (10-16.1)	-21.4 (-32.4--11.5)	4.1 (3.1-5.2)	-21.3 (-31.9--11.1)	12.9 (10.5-15.8)	-29.9 (-38.2--21.9)
San Marino	12 (8.5-16.4)	0.6 (-9.4-11.5)	10.2 (7.3-13.7)	0.8 (-9.6-11.3)	5.4 (4-7.2)	-0.4 (-9.4-8.2)	2.2 (1.7-2.9)	0.3 (-8.2-9.3)	0.8 (0.6-1.1)	2 (-7.7-13.5)	2.4 (1.9-3.1)	-9.7 (-17--2.6)
Spain	22.9 (18.6-28.2)	0.4 (-8.2-11.2)	19.6 (16.1-23.6)	-0.1 (-8.7-10.6)	10.7 (8.8-13.1)	0.8 (-7.3-9.6)	3.3 (2.7-4.1)	-5 (-12.7-4)	1.1 (0.8-1.4)	1.3 (-8.1-12)	3.8 (3.2-4.5)	-8.3 (-14.8--1)

Location	Birth Incidence rate (per 100,000 live births), 2021	Birth Incidence rate (per 100,000 live births) percent change (2000-2021)	Under-5 rate (per 100,000 population), 2021	Under-5 rate (per 100,000 population) percent change (2000-2021)	5-14 rate (per 100,000 population), 2021	5-14 rate (per 100,000 population) percent change (2000-2021)	15-49 rate (per 100,000 population), 2021	15-49 rate (per 100,000 population) percent change (2000-2021)	50plus rate (per 100,000 population), 2021	50plus rate (per 100,000 population) percent change (2000-2021)	All age rate (per 100,000 population), 2021	All age rate (per 100,000 population) percent change (2000-2021)
Sweden	16.7 (12.1-22.7)	10.5 (0.2-21)	15 (11-20.5)	10.5 (-0.2-21.2)	9.1 (6.9-12)	10.2 (0.7-19.9)	3.5 (2.6-4.6)	9 (1-18.1)	1 (0.7-1.3)	-0.8 (-8.3-7.7)	3.9 (3-5)	7 (-0.9-15.4)
Stockholm	16.8 (12.4-22.6)	10.7 (-1.5-23.2)	15 (11.2-20.2)	10.7 (-1.2-22.5)	9 (6.9-12.1)	9.4 (-1.3-20.3)	3.5 (2.6-4.5)	8.9 (-0.2-18.4)	1.1 (0.8-1.4)	2.3 (-6.9-12.6)	4 (3.1-5.2)	8.1 (-1.1-17.2)
Sweden except Stockholm	16.7 (12.1-22.8)	10.4 (-1.9-25.2)	15 (11-20.5)	10.5 (-1.7-25.3)	9.1 (6.8-12)	10.4 (-0.6-22.5)	3.5 (2.6-4.6)	9.1 (-0.1-20.1)	1 (0.7-1.3)	-1.7 (-11-8.2)	3.8 (3-4.8)	6.5 (-2.8-16.7)
Switzerland	12.7 (9.1-17.5)	0.6 (-9.5-11)	10.8 (7.8-15)	0.9 (-9-11.4)	5.9 (4.4-8)	1.1 (-8.1-10.9)	2.1 (1.6-2.8)	0.3 (-7.8-9.7)	0.8 (0.6-1)	-1.4 (-10.2-8.8)	2.4 (1.9-3.1)	-6.8 (-14.2-1)
United Kingdom	11.4 (8-15.7)	0.2 (-1.3-1.6)	9.4 (6.7-13)	0.7 (-0.8-2.3)	4 (3-5.5)	-1.2 (-2.9-0.3)	1.2 (0.9-1.6)	-1.5 (-3.3-0.3)	0.4 (0.3-0.5)	-1.8 (-3.9-0.4)	1.7 (1.3-2.2)	-6.7 (-8--5.3)
England	11.1 (7.7-15.4)	0.7 (-0.5-1.9)	9.1 (6.5-12.7)	1.4 (0.2-2.6)	3.6 (2.7-5)	0 (-1.2-1.1)	1 (0.7-1.4)	-0.6 (-1.7-0.6)	0.3 (0.2-0.4)	-1.9 (-3.4--0.4)	1.5 (1.2-2)	-5 (-6--4)
East Midlands	11.1 (7.8-15.5)	0.9 (-3.4-5.1)	9.2 (6.5-12.9)	1.7 (-2.6-6)	3.7 (2.7-5.1)	0 (-4.2-4.1)	1 (0.7-1.4)	0.1 (-3.4-4.1)	0.3 (0.2-0.4)	-3.2 (-7.7-1.6)	1.5 (1.1-2)	-6.9 (-10--3.8)
Derby	11.9 (8.2-16.7)	1.2 (-12-13.9)	9.6 (6.7-13.5)	1.8 (-10.8-14.3)	3.6 (2.6-4.9)	0.5 (-9.3-12.2)	1 (0.7-1.3)	-1.8 (-11.7-8.5)	0.3 (0.2-0.4)	5.1 (-7.5-19.4)	1.6 (1.2-2.2)	-1.3 (-10.4-7.5)
Derbyshire	10.7 (7.4-15.1)	0.7 (-10.4-10.9)	9 (6.2-12.7)	1.5 (-10-11.4)	3.7 (2.7-5.1)	-1 (-11.1-9.7)	1 (0.7-1.4)	1.1 (-9.8-11.9)	0.3 (0.2-0.4)	-4.8 (-15.8-6.9)	1.4 (1-1.8)	-12.2 (-19.6--3.9)
Leicester	11.7 (7.9-16.4)	0.4 (-9.7-12.7)	9.6 (6.5-13.4)	1 (-8.9-12.9)	3.6 (2.6-5)	-0.6 (-10.2-10.8)	1 (0.7-1.4)	-2.3 (-12.5-8.3)	0.3 (0.2-0.4)	7.8 (-5.5-21.3)	1.7 (1.3-2.2)	-3.1 (-11.2-6)
Leicestershire	10.5 (7.2-14.8)	1 (-9.8-14.6)	8.8 (6.1-12.4)	1.8 (-8.9-15.2)	3.6 (2.6-5)	0.1 (-9.1-12)	1 (0.7-1.4)	1.7 (-8-13.4)	0.3 (0.2-0.4)	-6.6 (-16.4-4.7)	1.4 (1.1-1.8)	-9.1 (-16.7-0.8)
Lincolnshire	11.2 (7.8-15.5)	0.8 (-11.4-13.4)	9.3 (6.5-13.1)	1.8 (-10.3-15.2)	3.8 (2.8-5.3)	1.1 (-9.5-12.8)	1 (0.7-1.4)	1.4 (-8.3-13.7)	0.3 (0.2-0.4)	-5.4 (-16.1-6.8)	1.4 (1.1-1.8)	-8.4 (-16.6-1.2)
Northamptonshire	10.9 (7.6-15.4)	0.9 (-8.8-11.5)	9 (6.3-12.9)	1.5 (-8.4-12.1)	3.7 (2.7-5.1)	0.6 (-8.7-10.5)	1 (0.7-1.4)	-0.4 (-10-10.1)	0.3 (0.2-0.4)	-3.9 (-14.1-8.1)	1.6 (1.2-2)	-5.8 (-13-2.4)
Nottingham	12.1 (8.5-16.7)	0.3 (-10-12.4)	9.9 (7-13.6)	1.1 (-8.6-12.4)	3.8 (2.7-5.2)	0.2 (-9.4-11)	1 (0.7-1.4)	-2.4 (-12.8-9)	0.3 (0.2-0.4)	12.2 (-0.5-27.4)	1.7 (1.3-2.2)	1.4 (-7.1-11.1)
Nottinghamshire	11 (7.4-15.4)	-0.1 (-9.4-12.7)	9.2 (6.4-12.8)	0.7 (-9.1-12.5)	3.7 (2.6-5.1)	-0.3 (-8.8-10.1)	1 (0.7-1.4)	0.1 (-9.4-10.3)	0.3 (0.2-0.4)	-4.4 (-14.9-6.8)	1.4 (1.1-1.9)	-8.2 (-15.1-0.1)
Rutland	10.3 (7-14.4)	0.4 (-11.1-13)	8.5 (5.9-12)	1.5 (-10.1-13.9)	3.3 (2.4-4.6)	0.4 (-9.3-11.4)	1 (0.8-1.4)	1.2 (-7.8-11)	0.3 (0.2-0.4)	-14 (-23.5--4.2)	1.3 (1-1.7)	-12.5 (-20--4.8)
East of England	10.7 (7.5-15)	0.5 (-3.6-4.2)	8.9 (6.3-12.5)	1.2 (-3.1-4.8)	3.6 (2.7-5)	-0.5 (-4.2-3.6)	1 (0.7-1.4)	0.2 (-3.5-3.9)	0.3 (0.2-0.4)	-3.6 (-7.7-0.3)	1.5 (1.1-2)	-6.4 (-9.3--3.6)
Bedford	11.6 (7.9-16.1)	0.8 (-10-13.6)	9.3 (6.5-13)	1.5 (-9.2-13.5)	3.5 (2.6-4.9)	0.1 (-10-10.6)	1 (0.7-1.4)	0.3 (-10.3-10.9)	0.3 (0.2-0.4)	-1.9 (-13.3-10.4)	1.6 (1.2-2)	-4.8 (-12.7-3.2)
Cambridgeshire	10.4 (7.1-14.7)	0.5 (-10.3-11.4)	8.7 (6-12.4)	1.2 (-9.3-11.9)	3.6 (2.6-5)	-0.5 (-9.9-10.6)	1 (0.7-1.4)	-1 (-9.7-9.2)	0.3 (0.2-0.4)	-5.5 (-14.3-5.5)	1.5 (1.1-1.9)	-8.1 (-15.5-0.2)
Central Bedfordshire	10.6 (7.4-14.8)	0.7 (-8.7-13)	8.8 (6.1-12.3)	1.4 (-7.6-13.8)	3.6 (2.6-5)	0.4 (-8.4-10.6)	1 (0.7-1.4)	0.8 (-8.6-11.4)	0.3 (0.2-0.4)	-6.7 (-16.4-4.5)	1.5 (1.1-2)	-10.8 (-17.7--2.7)
Essex	10.6 (7.3-15)	0.5 (-11.1-12.6)	8.9 (6.3-12.6)	1.3 (-9.7-12.9)	3.7 (2.7-5.2)	-0.1 (-9.4-9.4)	1 (0.7-1.4)	1.6 (-7.7-12.9)	0.3 (0.2-0.4)	-4.6 (-14.1-6.7)	1.5 (1.1-1.9)	-7.6 (-15.5--0.1)
Hertfordshire	10.2 (7-14.2)	0.5 (-10.7-14.2)	8.6 (6-12.1)	0.8 (-10.1-14.3)	3.6 (2.6-5)	-1 (-10.7-11.2)	1 (0.7-1.4)	0.7 (-9.2-13.9)	0.3 (0.2-0.5)	-0.8 (-11.6-14.6)	1.6 (1.2-2)	-4.7 (-12.6-5.2)
Luton	12.1 (8.4-16.7)	0.5 (-12.1-12.2)	9.7 (6.8-13.4)	0.9 (-11.4-13.4)	3.5 (2.6-4.9)	-0.2 (-11.9-11.9)	1 (0.7-1.3)	-1.4 (-12.5-10.3)	0.3 (0.2-0.4)	1.3 (-10.3-13.6)	1.8 (1.4-2.4)	0.3 (-10.2-10.2)
Norfolk	11 (7.6-15.5)	0.8 (-10-12.2)	9.2 (6.4-12.9)	1.6 (-8.5-13)	3.8 (2.8-5.2)	0.3 (-9.2-11.6)	1 (0.7-1.4)	0.4 (-9.6-10.3)	0.3 (0.2-0.4)	-6.4 (-17.3-4.4)	1.4 (1.1-1.8)	-6.3 (-14.1-2.6)
Peterborough	11.8 (8.1-16.5)	-0.4 (-11.1-11.6)	9.6 (6.7-13.3)	0.3 (-10.3-11.8)	3.6 (2.6-5.1)	0.1 (-10-10.4)	1 (0.7-1.3)	-2 (-11.7-8.8)	0.3 (0.2-0.4)	0.5 (-10.8-13.5)	1.7 (1.3-2.3)	-0.1 (-9-8.9)
Southend-on-Sea	10.8 (7.4-15.2)	0.7 (-8.8-11.9)	8.9 (6.1-12.6)	1.5 (-8.2-12.7)	3.6 (2.6-5.1)	-0.5 (-9.6-10.7)	1 (0.7-1.4)	-2.1 (-11.6-8)	0.3 (0.2-0.4)	4.2 (-7.4-16.7)	1.5 (1.1-2)	-4.3 (-12.1-4.2)
Suffolk	10.3 (7.1-14.7)	-0.6 (-10.6-11)	8.7 (6.1-12.4)	0.2 (-10-10.9)	3.6 (2.7-5)	-1.3 (-10.7-9)	1 (0.7-1.4)	0.1 (-9.7-10.9)	0.3 (0.2-0.4)	-5 (-15.3-6.7)	1.4 (1.1-1.8)	-10.5 (-18--2.3)
Thurrock	11.2 (7.8-15.7)	0.2 (-10.1-11.6)	9.2 (6.4-12.7)	0.8 (-9.3-11.5)	3.6 (2.6-4.9)	-0.5 (-10.7-10.1)	1 (0.7-1.4)	-1.3 (-11.5-10.5)	0.3 (0.2-0.4)	-1 (-11.4-13.2)	1.7 (1.3-2.2)	-2.7 (-10.6-5.8)
Greater London	11.2 (7.8-15.5)	0.5 (-1.7-2.5)	9.1 (6.5-12.7)	1 (-1.1-3.1)	3.6 (2.6-4.9)	0.2 (-1.9-2.4)	1 (0.7-1.4)	-1.8 (-3.7-0)	0.3 (0.2-0.5)	4.5 (2.2-7.1)	1.7 (1.3-2.2)	1.2 (-0.6-3.1)

Location	Birth Incidence rate (per 100,000 live births), 2021	Birth Incidence rate (per 100,000 live births) percent change (2000-2021)	Under-5 rate (per 100,000 population), 2021	Under-5 rate (per 100,000 population) percent change (2000-2021)	5-14 rate (per 100,000 population), 2021	5-14 rate (per 100,000 population) percent change (2000-2021)	15-49 rate (per 100,000 population), 2021	15-49 rate (per 100,000 population) percent change (2000-2021)	50plus rate (per 100,000 population), 2021	50plus rate (per 100,000 population) percent change (2000-2021)	All age rate (per 100,000 population), 2021	All age rate (per 100,000 population) percent change (2000-2021)
Barking and Dagenham	11.6 (7.8-16.3)	-0.1 (-11.4-11)	9.4 (6.5-13.1)	0.4 (-10.3-11.5)	3.7 (2.7-5.1)	-0.8 (-10.9-9.7)	1 (0.7-1.4)	-0.7 (-10.7-10.7)	0.3 (0.2-0.5)	19.3 (5.3-35)	2.1 (1.6-2.8)	15 (4.8-25.9)
Barnet	10.3 (7.2-14.3)	0.3 (-11-13.7)	8.6 (6-12)	0.7 (-10.5-13.5)	3.5 (2.6-4.8)	-1.1 (-10-9)	1 (0.7-1.4)	-0.6 (-10.8-10.4)	0.3 (0.2-0.5)	2.5 (-8.6-14.4)	1.7 (1.3-2.2)	2.6 (-5.3-12)
Bexley	10.4 (7.2-14.3)	0.3 (-9.4-13.6)	8.7 (6.1-12)	1 (-8.8-14.3)	3.5 (2.6-4.9)	0.1 (-9.9-10.6)	1.1 (0.8-1.5)	2.5 (-8.1-13.6)	0.3 (0.2-0.5)	-0.2 (-10.6-10.6)	1.6 (1.2-2.1)	0.1 (-8.7-9.2)
Brent	11.3 (7.8-15.8)	0 (-10.5-11.8)	9.1 (6.4-12.6)	0 (-10.1-10.8)	3.5 (2.5-4.8)	0.1 (-11.2-10.6)	1 (0.7-1.4)	-1.8 (-12.2-9.9)	0.3 (0.2-0.5)	2.8 (-9.4-16.2)	1.7 (1.3-2.2)	4.4 (-5.4-13.5)
Bromley	9.9 (6.7-14)	0.1 (-11.3-13.7)	8.4 (5.8-12)	0.8 (-10.8-14.9)	3.5 (2.6-4.8)	-0.2 (-10-10.7)	1 (0.8-1.4)	0.7 (-9-12.1)	0.3 (0.2-0.5)	0 (-10.8-13.7)	1.5 (1.2-2)	-2.4 (-10.3-6.8)
Camden	11.6 (7.9-16.1)	0.3 (-9.1-11.6)	9.6 (6.6-13.2)	0.9 (-9-12.8)	3.7 (2.7-5.1)	-0.7 (-10.3-10.6)	1 (0.7-1.3)	-2.4 (-12.6-8.8)	0.3 (0.2-0.4)	-5.6 (-16.2-6.2)	1.5 (1.1-2)	-6.4 (-14.2-2.6)
Croydon	10.9 (7.5-15)	0.4 (-10.3-11.2)	9 (6.3-12.7)	0.9 (-9.7-11.3)	3.7 (2.7-5.1)	1.2 (-8.2-12.2)	1 (0.7-1.4)	0.7 (-9.3-12.2)	0.3 (0.2-0.5)	4.6 (-5.7-16.9)	1.8 (1.4-2.3)	4.1 (-4.4-13.3)
Ealing	11.2 (7.8-15.6)	0.1 (-11.3-11.5)	9.2 (6.4-12.8)	0.2 (-10.5-11.6)	3.5 (2.6-4.9)	-0.4 (-10.1-10.7)	1 (0.7-1.3)	-1.4 (-11.7-10.4)	0.3 (0.2-0.4)	2.5 (-9.4-14.8)	1.7 (1.3-2.2)	2.6 (-6.2-12.2)
Enfield	10.6 (7.2-14.8)	1.3 (-10.4-15)	8.8 (6-12.4)	1.6 (-10.2-15.8)	3.5 (2.5-4.8)	-1.5 (-11.7-9.9)	1 (0.7-1.4)	1.1 (-8.7-12.2)	0.3 (0.2-0.5)	6.5 (-4.7-19.7)	1.8 (1.3-2.3)	5.5 (-3.7-16.6)
Greenwich	11.8 (8.1-16.2)	0.8 (-10.1-10.6)	9.6 (6.7-13.2)	1.2 (-9.4-11)	3.7 (2.6-5)	0.4 (-9.1-10.7)	1 (0.7-1.3)	-3.4 (-12.8-6.7)	0.3 (0.2-0.5)	10.4 (-1.8-25.1)	1.8 (1.4-2.4)	2.3 (-6.2-11.3)
Hackney	12 (8.4-16.8)	0.6 (-9.2-11.7)	9.7 (6.8-13.4)	1.2 (-8.4-12.5)	3.7 (2.6-5)	1.3 (-8.4-12.9)	1 (0.7-1.3)	-4.2 (-13.7-7.1)	0.3 (0.2-0.4)	12 (-0.3-26.4)	1.8 (1.3-2.3)	-7 (-14.7-1.2)
Hammersmith and Fulham	11.4 (8-15.9)	0.1 (-9.6-13.7)	9.3 (6.6-13)	0.5 (-8.6-13.7)	3.7 (2.7-5.1)	-0.5 (-9.7-10.1)	1 (0.7-1.3)	-3.5 (-13.2-7.2)	0.3 (0.2-0.5)	8.1 (-3.6-22.9)	1.6 (1.2-2.1)	-2 (-10-7.4)
Haringey	11.6 (7.8-16.2)	0.3 (-11.4-14.2)	9.3 (6.4-13.2)	0.3 (-10.6-14)	3.5 (2.5-4.8)	-1.6 (-11.7-9.7)	1 (0.7-1.3)	-3.3 (-13.4-6.7)	0.3 (0.2-0.5)	6.3 (-5.5-19.4)	1.6 (1.3-2.1)	-5.2 (-13.7-4.1)
Harrow	10.6 (7.3-15.3)	0.9 (-9.4-11.3)	8.7 (6-12.5)	1.4 (-9-10.6)	3.4 (2.5-4.7)	1.5 (-7.1-10.4)	1 (0.7-1.4)	-0.1 (-9.8-9.4)	0.3 (0.2-0.5)	-1.5 (-11.6-8.8)	1.6 (1.3-2.1)	5 (-3.5-13)
Havering	10.1 (7-14.2)	-0.7 (-11.1-11.1)	8.5 (5.9-11.9)	0.3 (-9.8-12.2)	3.6 (2.6-4.9)	0.6 (-8.2-9.7)	1.1 (0.8-1.4)	1.1 (-8.2-11.3)	0.3 (0.2-0.5)	-2.8 (-12.5-9)	1.6 (1.2-2.1)	2 (-6-10.9)
Hillingdon	10.9 (7.6-15.3)	0.1 (-9.8-11.6)	9.1 (6.5-12.7)	0.8 (-9-11.9)	3.7 (2.7-5.1)	0.7 (-8.2-11.3)	1 (0.7-1.4)	-0.4 (-9.7-10.3)	0.3 (0.2-0.5)	2.8 (-8.1-15.7)	1.8 (1.4-2.3)	3.2 (-4.7-12.5)
Hounslow	11.3 (7.7-16)	1.2 (-11.5-11.8)	9.2 (6.4-12.8)	1.5 (-10.8-12.8)	3.5 (2.5-4.9)	2 (-8.5-13.2)	1 (0.7-1.4)	-2.6 (-11.9-7.9)	0.3 (0.2-0.5)	1.3 (-10-13.9)	1.7 (1.3-2.3)	2.4 (-6.6-11.7)
Islington	11.4 (7.9-15.8)	0.8 (-9.1-13.8)	9.3 (6.5-13)	1.8 (-8.8-14.3)	3.7 (2.7-5.1)	1.4 (-9.3-12.4)	1 (0.7-1.4)	-3.4 (-14.5-6.8)	0.3 (0.2-0.4)	6 (-7.6-18.8)	1.6 (1.2-2)	-4.9 (-13.5-3.5)
Kensington and Chelsea	11 (7.4-15.4)	0.9 (-9.6-14.7)	8.9 (6.1-12.4)	1.5 (-9-15.3)	3.4 (2.5-4.8)	-1 (-10.4-10.4)	1 (0.7-1.3)	-2.3 (-12.9-8.3)	0.3 (0.2-0.5)	-2.6 (-14.2-9.1)	1.4 (1.1-1.8)	-6.2 (-14.4-3)
Kingston upon Thames	10.4 (7.2-14.6)	0.3 (-9.7-10.9)	8.6 (6-11.9)	0.9 (-9.2-11.5)	3.4 (2.5-4.6)	0.7 (-9.5-10.9)	1 (0.7-1.4)	0.3 (-9.9-12.4)	0.3 (0.2-0.5)	-1 (-12.1-12.3)	1.6 (1.2-2.1)	2.5 (-6.8-12.8)
Lambeth	11.9 (8.2-16.5)	1.4 (-10.3-13.4)	9.7 (6.8-13.3)	1.9 (-9.4-13.2)	3.7 (2.6-5)	0.3 (-10.5-10.9)	1 (0.7-1.3)	-2.1 (-11.8-9.7)	0.3 (0.2-0.5)	13 (-0.3-28.3)	1.7 (1.3-2.1)	-3.9 (-12.6-4.7)
Lewisham	11.6 (8.1-16.3)	1.1 (-10.1-13.2)	9.5 (6.7-13.2)	1.6 (-9.3-13.3)	3.8 (2.7-5.3)	0.8 (-9.1-11.5)	1 (0.7-1.4)	-1.4 (-11-9.9)	0.3 (0.2-0.5)	12.1 (-0.3-27.5)	1.8 (1.4-2.4)	2.5 (-6.3-12)
Merton	10.8 (7.4-15.3)	1.3 (-8.8-12)	8.9 (6.2-12.7)	1.7 (-8.2-12)	3.6 (2.6-4.9)	1.3 (-8.3-12.7)	1 (0.7-1.4)	-1.3 (-11.1-9.4)	0.3 (0.2-0.5)	5.1 (-6.1-17.7)	1.7 (1.3-2.2)	4.5 (-4-14)
Newham	12.3 (8.3-17)	0.8 (-8.7-12.5)	9.8 (6.7-13.5)	1.4 (-8.2-13.7)	3.6 (2.6-5)	2 (-7.9-12.6)	0.9 (0.7-1.3)	-6.2 (-15.8-4.5)	0.3 (0.2-0.5)	12.5 (-0.1-26.6)	1.8 (1.4-2.4)	-8.3 (-15.9-0.2)
Redbridge	10.9 (7.5-15.4)	0.7 (-10.4-12.1)	8.9 (6.3-12.5)	1 (-9.3-12.1)	3.5 (2.5-4.8)	-0.1 (-9.3-10.1)	1 (0.7-1.4)	-0.2 (-10.9-10.9)	0.3 (0.2-0.5)	3.3 (-8.7-17.4)	1.8 (1.4-2.3)	8.9 (-0.8-19)
Richmond upon Thames	9.9 (6.7-14.2)	0.7 (-10.3-11.4)	8.3 (5.7-11.9)	0.7 (-9.6-11)	3.5 (2.5-4.8)	-0.4 (-8.9-9.5)	1 (0.8-1.4)	0.8 (-8.2-10.8)	0.4 (0.2-0.5)	-0.7 (-11.3-10.1)	1.6 (1.2-2.1)	-1.3 (-9.2-6.8)
Southwark	11.9 (8.3-16.5)	0.7 (-9.7-14.5)	9.7 (6.8-13.3)	1.3 (-8.8-14.7)	3.7 (2.6-5.2)	-0.4 (-10.8-10.8)	1 (0.7-1.3)	-2.1 (-12-8.5)	0.3 (0.2-0.5)	17.3 (4.5-32.9)	1.7 (1.3-2.2)	-2.3 (-10.9-7)
Sutton	10.6 (7.2-15)	0.2 (-9.3-11.3)	8.7 (6-12.4)	1.1 (-8-11.6)	3.5 (2.5-4.7)	0.8 (-8.1-11.2)	1 (0.7-1.4)	0.1 (-9.4-11.3)	0.3 (0.2-0.5)	1.7 (-10-13.5)	1.6 (1.2-2.1)	-2.4 (-10.1-6.2)
Tower Hamlets	11.6 (8-16)	-0.6 (-14.3-13.2)	9.4 (6.5-12.9)	0 (-13-13)	3.7 (2.7-5.2)	2.2 (-8.8-13.2)	1 (0.7-1.3)	-5.5 (-15.2-6.5)	0.3 (0.2-0.4)	15.2 (0.8-31.1)	1.8 (1.4-2.4)	-3.9 (-12.8-5.3)

Location	Birth Incidence rate (per 100,000 live births), 2021	Birth Incidence rate (per 100,000 live births) percent change (2000-2021)	Under-5 rate (per 100,000 population), 2021	Under-5 rate (per 100,000 population) percent change (2000-2021)	5-14 rate (per 100,000 population), 2021	5-14 rate (per 100,000 population) percent change (2000-2021)	15-49 rate (per 100,000 population), 2021	15-49 rate (per 100,000 population) percent change (2000-2021)	50plus rate (per 100,000 population), 2021	50plus rate (per 100,000 population) percent change (2000-2021)	All age rate (per 100,000 population), 2021	All age rate (per 100,000 population) percent change (2000-2021)
Waltham Forest	11.4 (7.9-15.8)	0.2 (-10.1-13.9)	9.3 (6.5-12.8)	0.7 (-9.7-13.3)	3.6 (2.6-4.9)	0.5 (-9-11.7)	1 (0.7-1.4)	-1.5 (-12.7-10.8)	0.3 (0.2-0.5)	10.2 (-3.1-23.5)	1.8 (1.4-2.4)	2.8 (-6.7-13.2)
Wandsworth	11.2 (7.8-15.5)	0.8 (-9.9-11.8)	9.2 (6.4-12.8)	0.9 (-9.6-11.7)	3.7 (2.7-5)	-0.6 (-10.5-10.5)	1 (0.7-1.4)	-3.1 (-13.1-7.3)	0.3 (0.2-0.5)	9.7 (-4-23.8)	1.7 (1.3-2.2)	5.6 (-3.3-15.3)
Westminster	11.7 (7.8-16.3)	0.8 (-11.9-12.7)	9.4 (6.4-13.4)	0.9 (-10.8-12.7)	3.4 (2.5-4.7)	-1.8 (-11.8-9)	1 (0.7-1.3)	-3.3 (-13.6-7.6)	0.3 (0.2-0.4)	1.1 (-10.8-14.7)	1.5 (1.1-1.9)	2.2 (-6.5-12.1)
North East England	11.2 (7.8-15.5)	0.8 (-2.5-5)	9.2 (6.5-12.8)	1.7 (-1.7-5.8)	3.7 (2.7-5.1)	0.6 (-2.5-4)	1 (0.7-1.4)	-0.5 (-3.8-3.1)	0.3 (0.2-0.4)	-2.6 (-6.5-1.4)	1.5 (1.1-1.9)	-7.7 (-10.1--4.9)
County Durham	11.2 (7.7-15.4)	0.9 (-7.9-14.1)	9.3 (6.4-13.1)	1.8 (-7.2-14.6)	3.8 (2.8-5.4)	0.5 (-8.3-11.1)	1 (0.7-1.4)	0 (-9.7-10.1)	0.3 (0.2-0.4)	-4.6 (-14.8-7.2)	1.4 (1.1-1.9)	-8.8 (-15.9--0.9)
Darlington	11.3 (7.6-15.9)	-0.7 (-12.5-10.9)	9.2 (6.3-12.9)	0 (-11.4-11.1)	3.5 (2.6-4.9)	-1.6 (-10.8-8.1)	1 (0.7-1.4)	0.5 (-9.8-11.3)	0.3 (0.2-0.4)	-2.7 (-13.7-9.6)	1.5 (1.1-1.9)	-8 (-15.8-0.1)
Gateshead	11.2 (7.8-15.6)	0.2 (-10.7-11.4)	9.2 (6.4-12.8)	1 (-9.7-12.2)	3.7 (2.7-5.1)	0.6 (-8.6-11.1)	1 (0.7-1.3)	-1.5 (-11.3-9.5)	0.3 (0.2-0.4)	-1 (-12.2-11.2)	1.4 (1.1-1.9)	-6.8 (-14-1.7)
Hartlepool	11.7 (8.1-16.1)	0.3 (-11.9-12.2)	9.4 (6.6-12.9)	1.4 (-10.5-12.7)	3.6 (2.7-5.2)	0.4 (-9.2-11.4)	1 (0.7-1.4)	0.5 (-10.4-12.7)	0.3 (0.2-0.4)	-1.2 (-12.6-13.8)	1.5 (1.1-2)	-11.4 (-19--2.7)
Middlesbrough	12 (8.3-16.6)	0.7 (-9.8-12.3)	9.6 (6.9-13.5)	1.4 (-9.2-13)	3.7 (2.7-5.1)	2.2 (-8.2-13.4)	1 (0.7-1.4)	-0.7 (-11.1-11.1)	0.3 (0.2-0.4)	0.3 (-11.8-14.4)	1.7 (1.3-2.2)	-1.3 (-10-7.3)
Newcastle upon Tyne	11.6 (8-16.2)	1.1 (-9.7-12.5)	9.6 (6.6-13.4)	2.1 (-9.2-13.9)	3.7 (2.7-5.2)	1.4 (-9.1-12.4)	1 (0.7-1.4)	-1.8 (-11.7-9.6)	0.3 (0.2-0.4)	3.7 (-9.3-17.6)	1.6 (1.2-2.1)	1.7 (-7-10.9)
North Tyneside	11 (7.7-15.4)	0.9 (-10-12.7)	9.1 (6.3-12.7)	1.5 (-8.7-13.2)	3.6 (2.6-5)	0 (-9.3-11)	1 (0.7-1.4)	-0.9 (-11.6-10)	0.3 (0.2-0.4)	0.4 (-11.9-13.2)	1.4 (1.1-1.9)	-6.2 (-14-2.6)
Northumberland	10.6 (7.3-14.7)	1 (-9.7-12.6)	8.8 (6.2-12.6)	1.7 (-9.1-12.7)	3.6 (2.6-5.1)	0 (-9.3-9.7)	1 (0.8-1.4)	2.1 (-7.9-13.2)	0.3 (0.2-0.4)	-7.8 (-17.6-3)	1.3 (1-1.7)	-12.5 (-19.8--4.2)
Redcar and Cleveland	11.1 (7.6-15.3)	-0.3 (-12.4-12.7)	9.1 (6.3-12.5)	0.6 (-10.6-13.1)	3.6 (2.6-5.1)	-0.4 (-10.6-10.8)	1 (0.7-1.4)	-0.1 (-11.2-11.6)	0.3 (0.2-0.4)	-5.6 (-17.6-6.9)	1.4 (1.1-1.9)	-10.4 (-18.5--2.2)
South Tyneside	11 (7.6-15.2)	0.8 (-10-14)	9.1 (6.4-12.6)	1.8 (-9-14.7)	3.6 (2.7-5)	1.8 (-7.4-14.2)	1 (0.7-1.4)	0.3 (-10-12.9)	0.3 (0.2-0.4)	3.3 (-8.8-18.9)	1.4 (1.1-1.8)	-9.2 (-17.2-0.3)
Stockton-on-Tees	11.2 (7.7-15.6)	0.6 (-9-12.8)	9.2 (6.4-12.7)	1.4 (-8.1-13.5)	3.6 (2.7-5)	1.1 (-8.2-11.3)	1 (0.7-1.4)	-1 (-10.2-11.2)	0.3 (0.2-0.4)	-3.7 (-14.8-9.5)	1.5 (1.2-2)	-8 (-15.5-0.7)
Sunderland	11.3 (7.8-15.8)	1.1 (-8.9-12.5)	9.2 (6.5-13)	2 (-8.4-13.6)	3.6 (2.6-5.2)	0.3 (-9.5-10.8)	1 (0.7-1.4)	-1.7 (-12.1-8.4)	0.3 (0.2-0.4)	-3.7 (-15.6-8.5)	1.4 (1.1-1.8)	-11.6 (-18.8--3.4)
North West England	11.5 (8-16.1)	0.8 (-2.1-3.6)	9.4 (6.7-13.1)	1.6 (-1.1-4.5)	3.7 (2.7-5.1)	0.5 (-2.1-3)	1 (0.7-1.4)	-1.2 (-4-1.6)	0.3 (0.2-0.4)	-2.6 (-5.9-0.4)	1.5 (1.2-2)	-6.8 (-9--4.8)
Blackburn with Darwen	12.5 (8.5-17.3)	0.3 (-11.1-13.5)	9.8 (6.8-13.7)	1.2 (-11-13)	3.5 (2.5-4.9)	-0.8 (-11.8-10)	1 (0.7-1.3)	-1.5 (-11.8-9.9)	0.3 (0.2-0.4)	1.9 (-10.2-16.9)	1.7 (1.3-2.2)	-11.8 (-20.5--3.5)
Blackpool	12.6 (8.7-17.5)	0.7 (-9.4-13)	9.9 (6.9-13.7)	1.8 (-8.6-13.8)	3.7 (2.7-5.3)	0.4 (-8.9-11)	1 (0.7-1.3)	0.8 (-9.2-11.9)	0.2 (0.2-0.3)	4 (-8.3-17.3)	1.5 (1.1-2)	-2 (-10.1-7.1)
Bolton	11.7 (8-16.6)	-0.6 (-12.9-13.7)	9.5 (6.6-13.4)	0.3 (-11.6-14.2)	3.6 (2.6-5.1)	-0.8 (-11-9.8)	1 (0.7-1.4)	-0.8 (-12.2-9.9)	0.3 (0.2-0.4)	-1.8 (-13.2-10.6)	1.6 (1.2-2.1)	-6.2 (-15.4-2.5)
Bury	11.4 (7.9-15.8)	0.7 (-10.6-14.1)	9.2 (6.4-12.9)	1.5 (-9-14.5)	3.6 (2.6-4.9)	1.1 (-7.7-12.1)	1 (0.7-1.4)	1 (-8.8-12.4)	0.3 (0.2-0.4)	-3.8 (-14.4-10)	1.6 (1.2-2)	-5.4 (-13-4)
Cheshire East	10.8 (7.5-15.3)	0.1 (-9.8-11.9)	9 (6.2-12.6)	1 (-8.6-13.2)	3.6 (2.6-5)	-0.9 (-10.1-8.9)	1 (0.7-1.4)	0.8 (-8.8-11.8)	0.3 (0.2-0.4)	-7.3 (-18.3-4)	1.4 (1-1.8)	-11.7 (-18.8--3.6)
Cheshire West and Chester	11.1 (7.5-15.6)	0.5 (-11.6-11.9)	9.2 (6.3-12.8)	1.4 (-10.3-12.4)	3.6 (2.6-5)	0.1 (-9.9-10.6)	1 (0.7-1.4)	0.6 (-9-11.3)	0.3 (0.2-0.4)	-5.6 (-15.5-6)	1.4 (1-1.8)	-11.5 (-18.5--3.6)
Cumbria	11 (7.5-15.4)	1.2 (-8.1-13.9)	9.1 (6.3-12.9)	2.1 (-7.3-13.9)	3.7 (2.7-5.2)	-0.2 (-8.8-10.2)	1 (0.7-1.3)	0.9 (-8.3-11.8)	0.3 (0.2-0.4)	-5.9 (-17.1-6.6)	1.3 (1-1.7)	-12.9 (-19.3--5)
Halton	11.5 (8-16.5)	0.5 (-10-13.7)	9.3 (6.7-13.2)	1.1 (-9.1-14.1)	3.6 (2.6-5)	0.5 (-10.1-11.8)	1 (0.7-1.4)	-1.6 (-12.4-9.2)	0.3 (0.2-0.4)	-7.7 (-19.2-4.5)	1.5 (1.1-2)	-10.9 (-19.3--2)
Knowsley	11.6 (8-16.1)	0.6 (-9.2-12.5)	9.5 (6.6-13.3)	1.7 (-8.3-14.1)	3.8 (2.7-5.2)	1.8 (-8.2-12.9)	1 (0.7-1.4)	0.5 (-9.1-12.5)	0.3 (0.2-0.4)	1.3 (-9.9-14.3)	1.6 (1.2-2.1)	-8.1 (-15.2-1)
Lancashire	11.3 (7.9-15.9)	0.5 (-9.7-12.5)	9.3 (6.5-13)	1.7 (-8.4-12.9)	3.7 (2.7-5.1)	0.6 (-8.8-11.4)	1 (0.7-1.3)	-1.5 (-11.3-9.5)	0.3 (0.2-0.4)	-5.8 (-17-6)	1.4 (1.1-1.9)	-9.8 (-17--1.3)
Liverpool	11.9 (8.1-16.6)	0.4 (-11.3-11.7)	9.7 (6.7-13.5)	1.4 (-9.9-13.3)	3.8 (2.7-5.2)	3.9 (-6.9-15.7)	1 (0.7-1.4)	-4.2 (-14.2-8.4)	0.3 (0.2-0.4)	3.5 (-7.8-18.2)	1.6 (1.2-2)	-5.4 (-13.8-3.5)
Manchester	12.3 (8.4-17.3)	0.4 (-9.6-13.7)	9.8 (6.8-13.6)	1 (-9.1-13)	3.6 (2.6-5)	1.2 (-8.9-12.6)	1 (0.7-1.4)	-4.7 (-14.3-6.3)	0.3 (0.2-0.4)	15.6 (1.5-31)	1.8 (1.4-2.3)	3 (-6.4-12.9)

Location	Birth Incidence rate (per 100,000 live births), 2021	Birth Incidence rate (per 100,000 live births) percent change (2000-2021)	Under-5 rate (per 100,000 population), 2021	Under-5 rate (per 100,000 population) percent change (2000-2021)	5-14 rate (per 100,000 population), 2021	5-14 rate (per 100,000 population) percent change (2000-2021)	15-49 rate (per 100,000 population), 2021	15-49 rate (per 100,000 population) percent change (2000-2021)	50plus rate (per 100,000 population), 2021	50plus rate (per 100,000 population) percent change (2000-2021)	All age rate (per 100,000 population), 2021	All age rate (per 100,000 population) percent change (2000-2021)
Oldham	12.1 (8.3-16.8)	1.5 (-8.7-13.1)	9.7 (6.8-13.3)	2.4 (-7.9-13.6)	3.7 (2.7-5)	0.8 (-7.8-10.7)	1 (0.7-1.4)	0.5 (-9.1-10.5)	0.3 (0.2-0.4)	-2.5 (-12.9-10.6)	1.7 (1.3-2.2)	-2.7 (-10.7-5.3)
Rochdale	12.2 (8.5-16.7)	1.1 (-8.4-12.6)	9.8 (6.9-13.4)	2 (-8-13.3)	3.7 (2.7-5.2)	0.4 (-8.3-10.4)	1 (0.7-1.3)	0 (-10.2-10.9)	0.3 (0.2-0.4)	-2.6 (-13.2-9.3)	1.7 (1.2-2.2)	-5.8 (-13-1.9)
Salford	12.2 (8.4-17)	-0.6 (-12-11.2)	9.8 (6.7-13.7)	0.2 (-10.5-12.3)	3.7 (2.7-5.2)	1 (-9.1-11.6)	1 (0.7-1.3)	-2.6 (-12.9-7.8)	0.3 (0.2-0.4)	4.3 (-8.7-18.7)	1.7 (1.3-2.2)	4.7 (-4.8-14)
Sefton	11 (7.6-15.2)	0.8 (-9.8-14.3)	9.1 (6.3-12.6)	1.8 (-9-14.3)	3.7 (2.7-5.1)	1.1 (-8.1-12.1)	1 (0.7-1.4)	-0.7 (-10.7-9.7)	0.3 (0.2-0.4)	-4 (-15.5-7.8)	1.4 (1-1.8)	-10.2 (-17.7--1.7)
St Helens	11.4 (7.9-15.9)	0.5 (-10.7-12.8)	9.3 (6.5-13.1)	1.6 (-9.1-13.7)	3.7 (2.6-5)	0.2 (-9.3-11.8)	1 (0.7-1.4)	-1.4 (-10.9-10.2)	0.3 (0.2-0.4)	-7.9 (-18.1-5.1)	1.5 (1.1-1.9)	-9.8 (-17.4--1.2)
Stockport	10.6 (7.4-14.8)	0.3 (-9.4-12.2)	8.8 (6.1-12.2)	1 (-8.3-13.4)	3.7 (2.7-5.1)	1 (-8.4-11.3)	1 (0.7-1.4)	0 (-9.6-11.1)	0.3 (0.2-0.4)	-4.5 (-14.9-8)	1.5 (1.1-2)	-5.9 (-13.3-2.6)
Tameside	11.3 (7.8-15.6)	0 (-10.9-13.9)	9.2 (6.5-12.7)	0.7 (-9.8-13.4)	3.7 (2.7-5.2)	0.9 (-8.7-12.3)	1 (0.7-1.4)	-0.4 (-11.1-11.5)	0.3 (0.2-0.4)	-1.7 (-13.2-10.9)	1.6 (1.2-2.1)	-5.9 (-14.2-2.9)
Trafford	11.1 (7.7-15.2)	0.6 (-11.7-12.3)	9.1 (6.3-12.5)	1 (-11-12.4)	3.5 (2.6-4.8)	0 (-10.7-11)	1 (0.7-1.4)	0 (-10.1-10)	0.3 (0.2-0.4)	0.8 (-10.6-12.3)	1.5 (1.2-2)	-2.4 (-11.3-6)
Warrington	11.5 (7.9-15.9)	-0.3 (-10.1-12.2)	9.4 (6.4-13.2)	0.4 (-9.4-12.7)	3.7 (2.7-5.1)	-0.2 (-9.2-10.4)	1 (0.7-1.3)	0 (-9.8-10.5)	0.3 (0.2-0.4)	-4.1 (-15.6-6.8)	1.5 (1.1-1.9)	-10.8 (-18.2--2.5)
Wigan	11.3 (7.8-15.7)	0.7 (-10-14.3)	9.3 (6.5-13.1)	1.4 (-9.8-15)	3.7 (2.7-5.3)	-0.3 (-9.8-10.2)	1 (0.7-1.4)	-0.9 (-10.1-10)	0.3 (0.2-0.4)	-5.8 (-16.9-6.6)	1.5 (1.1-1.9)	-9.4 (-17.1--1.1)
Wirral	11.2 (7.8-15.6)	0 (-10.7-10.8)	9.2 (6.5-12.7)	0.9 (-9.5-11.8)	3.7 (2.6-5.1)	0.3 (-8.6-10.4)	1 (0.7-1.4)	-0.8 (-10.4-10.8)	0.3 (0.2-0.4)	-2.8 (-14-10.1)	1.4 (1.1-1.9)	-9.9 (-17--1.8)
South East England	10.7 (7.5-15)	0.8 (-2.8-3.8)	8.9 (6.3-12.4)	1.4 (-2.2-4.4)	3.6 (2.7-5)	-0.7 (-3.6-2.1)	1 (0.7-1.4)	0.1 (-3.2-3.3)	0.3 (0.2-0.4)	-2.8 (-6.7-1.2)	1.5 (1.1-1.9)	-6.1 (-8.6--3.7)
Bracknell Forest	10.5 (7.2-14.4)	0.2 (-13.2-12.5)	8.7 (6.1-12.1)	0.8 (-12-12.8)	3.5 (2.6-4.8)	0.3 (-9.6-11.7)	1 (0.7-1.4)	0 (-9.5-10.6)	0.3 (0.2-0.5)	-3.5 (-13.2-7.7)	1.6 (1.2-2)	-10.3 (-18.6--2)
Brighton and Hove	11.3 (7.8-15.6)	-0.3 (-11.3-13.3)	9.3 (6.5-12.9)	0.4 (-10-12.8)	3.7 (2.7-5.1)	-2.5 (-12.7-8.7)	1 (0.7-1.4)	-1.6 (-11.5-8.8)	0.3 (0.2-0.4)	14.3 (1.8-28.7)	1.5 (1.1-1.9)	-4.7 (-13-4.1)
Buckinghamshire	10.8 (7.5-15.1)	0.4 (-11.5-14.3)	9 (6.3-12.6)	1 (-10.2-12.9)	3.6 (2.6-4.9)	-1.3 (-11-8.7)	1 (0.7-1.4)	0.8 (-9.8-11.6)	0.3 (0.2-0.4)	-6.7 (-18.2-4.3)	1.5 (1.1-1.9)	-9 (-17.1--0.5)
East Sussex	11 (7.7-15.3)	0 (-12.6-13.6)	9.2 (6.5-12.8)	0.8 (-11-14.8)	3.8 (2.7-5.2)	-0.6 (-11.4-11)	1 (0.7-1.4)	1.6 (-9.2-14.3)	0.3 (0.2-0.4)	1.7 (-10.3-15.5)	1.4 (1-1.8)	-9.5 (-18-0.4)
Hampshire	10.3 (7.1-14.4)	0.1 (-12.8-11.3)	8.7 (6-12.1)	0.8 (-12-12)	3.6 (2.6-4.9)	-0.6 (-9.8-9.3)	1 (0.8-1.4)	0.8 (-9.2-11.7)	0.3 (0.2-0.4)	-7.2 (-17.7-4)	1.4 (1.1-1.8)	-10.6 (-18.1--3.2)
Isle of Wight	10.8 (7.5-15.1)	0.8 (-10-13.1)	9 (6.2-12.7)	2 (-8.6-13.1)	3.6 (2.6-5)	0.6 (-8.6-11.4)	1 (0.7-1.4)	0.7 (-8.3-12.1)	0.3 (0.2-0.4)	-4 (-13.9-7.9)	1.3 (1-1.6)	-9.3 (-16.8--0.9)
Kent	10.5 (7.2-14.8)	0.7 (-10.4-12.7)	8.8 (6.2-12.4)	1.4 (-9.4-13.5)	3.7 (2.7-5)	-1.1 (-9.7-8.9)	1 (0.8-1.4)	0.3 (-10.2-10.9)	0.3 (0.2-0.4)	-3.4 (-14.1-9.3)	1.5 (1.1-2)	-6.2 (-14.4-2.1)
Medway	11.6 (8-16.5)	1.6 (-10.4-14.4)	9.5 (6.6-13.4)	2.7 (-9-15.1)	3.7 (2.7-5.1)	1.6 (-8.5-11.6)	1 (0.7-1.4)	0.1 (-10.5-11)	0.3 (0.2-0.4)	-5 (-16.6-6.7)	1.6 (1.2-2.1)	-6.3 (-14.5-1.6)
Milton Keynes	11 (7.7-15.5)	1 (-9.8-13.8)	9.1 (6.3-12.7)	1.1 (-9.4-13.4)	3.6 (2.6-5)	0.1 (-9.4-11.2)	1 (0.7-1.4)	-1.2 (-11.2-8.8)	0.3 (0.2-0.4)	-5.3 (-16.1-6.4)	1.7 (1.3-2.2)	-3.5 (-11.7-5.5)
Oxfordshire	10.7 (7.3-14.9)	0.3 (-11-12.6)	9 (6.2-12.5)	0.9 (-9.8-13.2)	3.5 (2.5-4.9)	-1.1 (-9.2-9.8)	1 (0.7-1.4)	-0.6 (-10-10.1)	0.3 (0.2-0.4)	-4.6 (-14.5-7.8)	1.5 (1.1-1.9)	-7.5 (-14.7-1)
Portsmouth	11.2 (7.8-15.4)	1 (-9.7-13.9)	9.2 (6.4-12.7)	1.6 (-8.8-14.2)	3.7 (2.7-5.3)	1 (-7.4-12.2)	1 (0.7-1.4)	-1.7 (-11.3-8.7)	0.3 (0.2-0.4)	7.9 (-4.7-22.1)	1.6 (1.3-2.2)	1 (-6.8-10.1)
Reading	11.4 (7.7-15.9)	0.9 (-10.3-19.3)	9.3 (6.4-13.1)	1.1 (-9.9-18.7)	3.7 (2.7-5.1)	1.2 (-8.9-13.4)	1 (0.7-1.4)	-2 (-11.2-8.8)	0.3 (0.2-0.4)	5.7 (-6.1-18.6)	1.8 (1.3-2.4)	7.7 (-1.5-19.7)
Slough	12.1 (8.3-16.9)	1.2 (-9.3-13.9)	9.7 (6.7-13.5)	1 (-9.1-13.6)	3.6 (2.6-5)	0.4 (-9.8-12.3)	1 (0.7-1.3)	-1.5 (-11.3-10.1)	0.3 (0.2-0.4)	12.2 (-1-27.9)	2 (1.5-2.5)	9.8 (-0.5-21.5)
Southampton	11.6 (8-16.3)	0.5 (-9.9-11.5)	9.4 (6.6-13.2)	1.3 (-9.5-12.5)	3.7 (2.7-5.3)	0.1 (-8.9-10)	1 (0.7-1.4)	-3.7 (-12.5-6.5)	0.3 (0.2-0.4)	5.8 (-5.2-20.3)	1.7 (1.3-2.2)	3.6 (-4.6-12.5)
Surrey	10.2 (7.1-14.3)	1.2 (-9.7-13.9)	8.6 (6-12)	1.6 (-9.4-14.3)	3.6 (2.6-4.9)	-0.9 (-10.7-8.8)	1 (0.7-1.4)	0.8 (-9.3-12.3)	0.3 (0.2-0.5)	-3 (-14.3-8.7)	1.5 (1.1-1.9)	-4.3 (-13-3.8)
West Berkshire	10.6 (7.2-14.9)	1 (-10.4-14.5)	8.8 (6-12.2)	1.4 (-9.2-14.3)	3.5 (2.5-4.8)	-1.4 (-11.3-9.4)	1 (0.7-1.4)	1 (-8.8-12.3)	0.3 (0.2-0.4)	-6.5 (-16.8-5.6)	1.5 (1.1-1.9)	-9.6 (-16.7--0.8)
West Sussex	10.7 (7.4-15.1)	0.4 (-11.1-14.9)	8.9 (6.3-12.7)	1 (-10.2-15.6)	3.7 (2.7-5.1)	-0.8 (-10.7-9.1)	1 (0.7-1.4)	0.3 (-9.5-11.1)	0.3 (0.2-0.4)	-0.4 (-11.5-12)	1.4 (1.1-1.9)	-6.9 (-14.8-2.1)

Location	Birth Incidence rate (per 100,000 live births), 2021	Birth Incidence rate (per 100,000 live births) percent change (2000-2021)	Under-5 rate (per 100,000 population), 2021	Under-5 rate (per 100,000 population) percent change (2000-2021)	5-14 rate (per 100,000 population), 2021	5-14 rate (per 100,000 population) percent change (2000-2021)	15-49 rate (per 100,000 population), 2021	15-49 rate (per 100,000 population) percent change (2000-2021)	50plus rate (per 100,000 population), 2021	50plus rate (per 100,000 population) percent change (2000-2021)	All age rate (per 100,000 population), 2021	All age rate (per 100,000 population) percent change (2000-2021)
Windsor and Maidenhead	10.4 (7.2-14.6)	0.4 (-11-10.9)	8.7 (6-12)	0.7 (-9.6-10.8)	3.4 (2.5-4.6)	-1 (-9.7-8.9)	1 (0.7-1.4)	-0.4 (-10.6-11)	0.3 (0.2-0.5)	-5.9 (-16.4-5.2)	1.5 (1.1-1.9)	-7 (-14.2-1.1)
Wokingham	9.9 (6.8-13.8)	0.3 (-10.4-12.5)	8.3 (5.7-11.5)	0.5 (-10-11.9)	3.4 (2.5-4.7)	-0.8 (-11.2-9.7)	1 (0.7-1.4)	0.6 (-10.6-11.4)	0.3 (0.2-0.5)	-11.8 (-22.3--1.2)	1.5 (1.1-1.9)	-10.1 (-18.2--2)
South West England	10.7 (7.4-15)	0.5 (-2.8-4.3)	8.9 (6.3-12.5)	1.3 (-2-5)	3.6 (2.7-5)	0.2 (-2.9-3.2)	1 (0.7-1.4)	0.3 (-2.7-3.5)	0.3 (0.2-0.4)	-3.1 (-6.6-0.7)	1.4 (1.1-1.9)	-6.6 (-9.1--4.1)
Bath and North East Somerset	10 (6.9-14.1)	-0.2 (-11.5-11.7)	8.4 (5.8-11.7)	0.8 (-10.6-12.5)	3.5 (2.6-4.8)	-0.5 (-9.3-10.8)	1.1 (0.8-1.4)	0.7 (-8.7-11.7)	0.3 (0.2-0.4)	-1.9 (-12.3-8.8)	1.4 (1.1-1.8)	-5.8 (-13.7-2.3)
Bournemouth	10.8 (7.6-15)	-0.4 (-13.3-12.5)	9 (6.4-12.5)	0.7 (-11.5-13.7)	3.7 (2.8-5.2)	1.3 (-9.4-12.1)	1 (0.7-1.4)	-5.4 (-14.5-4.9)	0.3 (0.2-0.4)	8.4 (-3.9-22)	1.5 (1.2-2)	3.6 (-5.9-13.5)
Bristol, City of	10.8 (7.5-15.2)	0.5 (-10.1-14.4)	9 (6.3-12.5)	1.1 (-9.9-13.7)	3.7 (2.7-5.1)	2 (-8.4-13.1)	1 (0.7-1.4)	-3 (-12.5-7.5)	0.3 (0.2-0.4)	4.8 (-6.9-18.4)	1.7 (1.3-2.2)	3.5 (-5.4-12.9)
Cornwall	10.9 (7.5-15.3)	0.1 (-11.4-14.3)	9.1 (6.3-12.8)	1.2 (-10-15.7)	3.7 (2.7-5.1)	0.8 (-9.3-12)	1 (0.7-1.4)	1.1 (-9.3-12.5)	0.3 (0.2-0.4)	-6 (-16.7-6.7)	1.4 (1-1.8)	-8.4 (-16.6-0.8)
Devon	10.3 (7-14.5)	0.4 (-10.2-12.3)	8.7 (6-12.2)	1.2 (-8.9-13.1)	3.6 (2.7-5)	-0.3 (-10.6-10.2)	1 (0.8-1.4)	1 (-9.1-11.8)	0.3 (0.2-0.4)	-4.8 (-15.6-6.8)	1.3 (1-1.7)	-8.3 (-16.4--0.1)
Dorset	10.6 (7.3-15.1)	0.7 (-11.8-15.1)	8.9 (6.2-12.5)	1.5 (-10.3-16.1)	3.5 (2.6-4.9)	-0.8 (-11.1-9.9)	1 (0.7-1.4)	2 (-7.8-13)	0.3 (0.2-0.4)	-5 (-16.3-7.7)	1.2 (1-1.6)	-10.4 (-18.5--2.4)
Gloucestershire	10.7 (7.4-15.1)	0.9 (-10.9-12.3)	9 (6.2-12.7)	1.6 (-10-13.6)	3.7 (2.7-5.2)	0.5 (-8.5-10.4)	1 (0.7-1.4)	0.4 (-10.2-11.8)	0.3 (0.2-0.4)	-3 (-15.3-9.1)	1.4 (1.1-1.9)	-9 (-15.9--0.8)
North Somerset	10.4 (7.3-14.5)	1.4 (-9.4-13.4)	8.6 (6.1-12)	2.2 (-8.7-13.5)	3.6 (2.6-4.8)	0.4 (-8.7-10.7)	1 (0.7-1.4)	1.5 (-8.1-12)	0.3 (0.2-0.4)	-5.7 (-16-5.1)	1.4 (1.1-1.8)	-5.7 (-12.9-2.5)
Plymouth	11 (7.6-15.4)	0.3 (-10.3-13.2)	9.1 (6.3-12.5)	1.1 (-9.4-13.8)	3.6 (2.7-5)	1.2 (-8.7-12.6)	1 (0.7-1.4)	-1.4 (-11.8-10.6)	0.3 (0.2-0.4)	-2.8 (-13.4-9.5)	1.5 (1.1-2)	-4.2 (-12.3-5.4)
Poole	10.7 (7.4-15)	0.8 (-9.3-14.3)	8.8 (6.1-12.2)	1.1 (-8.4-13.2)	3.5 (2.5-4.7)	0 (-8.4-9.9)	1 (0.7-1.4)	-1.3 (-11.4-9.4)	0.3 (0.2-0.4)	-0.9 (-12.8-12.4)	1.4 (1.1-1.8)	-5.2 (-12.9-3)
Somerset	10.6 (7.4-15.2)	0.2 (-10.4-14.7)	8.9 (6.2-12.9)	1 (-9.8-15.2)	3.7 (2.7-5)	0.1 (-10.1-10.9)	1 (0.7-1.4)	1.9 (-8.1-13.5)	0.3 (0.2-0.4)	-4.8 (-15.3-7.7)	1.4 (1.1-1.8)	-8.9 (-16.4--0.2)
South Gloucestershire	10.5 (7.3-14.7)	0.9 (-10.8-12.9)	8.8 (6.2-12.3)	1.7 (-9.9-13.3)	3.6 (2.6-4.9)	-1 (-11.4-9.4)	1 (0.7-1.4)	0.7 (-8.9-11.2)	0.3 (0.2-0.4)	-6.9 (-16.4-5.1)	1.5 (1.1-1.9)	-9.3 (-16.9--1.4)
Swindon	10.8 (7.5-14.8)	0.3 (-9-13.1)	8.9 (6.2-12.2)	0.9 (-8.4-13.3)	3.5 (2.6-4.9)	0 (-8.8-9.5)	1 (0.7-1.4)	-0.6 (-10-9.8)	0.3 (0.2-0.5)	2.1 (-8.4-13.9)	1.6 (1.2-2)	-6.1 (-13.1-2.6)
Torbay	11.1 (7.7-15.8)	0.5 (-10.4-12.1)	9.1 (6.4-13)	1.5 (-9-12.4)	3.7 (2.6-5.1)	1.7 (-7.2-11.7)	1 (0.7-1.4)	0.9 (-8.9-11.8)	0.3 (0.2-0.4)	-2.2 (-14.7-10.8)	1.4 (1-1.8)	-3.3 (-11.1-5.1)
Wiltshire	10.7 (7.4-14.9)	0.1 (-9.5-11.6)	9 (6.3-12.4)	0.8 (-9.4-12.7)	3.7 (2.7-5.1)	-1.2 (-11-9.9)	1 (0.7-1.4)	2.1 (-7.1-12.5)	0.3 (0.2-0.4)	-3.7 (-13.7-7.2)	1.4 (1.1-1.9)	-11.1 (-19--2.9)
West Midlands	11.4 (7.9-15.8)	0.9 (-2.9-5.6)	9.3 (6.5-12.8)	1.7 (-2.2-6)	3.6 (2.6-5)	0.2 (-3.4-3.9)	1 (0.7-1.4)	-0.3 (-3.8-3.3)	0.3 (0.2-0.4)	-3.3 (-7-0.4)	1.5 (1.2-2)	-5.3 (-8.3--2.1)
Birmingham	11.8 (8-16.4)	0.7 (-8.8-15.8)	9.4 (6.5-13.3)	1.4 (-8.5-15.1)	3.5 (2.5-4.9)	-0.1 (-8.8-12.3)	1 (0.7-1.4)	-1.3 (-11-9.7)	0.3 (0.2-0.4)	6.7 (-5.8-20.8)	1.8 (1.3-2.3)	-1.5 (-10-9.6)
Coventry	11.9 (8.2-16.7)	1.1 (-12.6-13.3)	9.6 (6.7-13.3)	2 (-11.5-14.5)	3.7 (2.7-5)	1.9 (-8.3-13.4)	1 (0.7-1.4)	-1.3 (-11.9-9.2)	0.3 (0.2-0.4)	2 (-10.2-14.8)	1.7 (1.3-2.3)	2.4 (-7.1-11.9)
Dudley	11.4 (7.8-15.9)	0.8 (-11.9-14.1)	9.3 (6.4-12.8)	1.4 (-10.5-14.1)	3.6 (2.6-5)	0.7 (-10.4-12.1)	1 (0.7-1.4)	1.3 (-9-14)	0.3 (0.2-0.4)	-5.2 (-16-8)	1.5 (1.1-2)	-3.9 (-13.4-5.1)
Herefordshire, County of	11 (7.4-15.4)	-0.1 (-11.1-12.4)	9 (6.2-12.5)	1.1 (-9.9-13.2)	3.5 (2.5-4.9)	0.5 (-9.3-10.1)	1 (0.7-1.3)	0.7 (-9.3-12)	0.3 (0.2-0.4)	-6 (-16.4-5.5)	1.3 (1-1.7)	-11.6 (-19.1--3.9)
Sandwell	11.8 (8.1-16.5)	0.4 (-10.4-12)	9.6 (6.6-13.3)	1 (-10-12.2)	3.7 (2.7-5.1)	0.4 (-9.6-11.2)	1 (0.7-1.4)	-0.9 (-10.2-9.2)	0.3 (0.2-0.4)	5.5 (-5.6-19.3)	1.7 (1.3-2.3)	1.8 (-7.1-10.4)
Shropshire	10.9 (7.3-15.2)	-0.2 (-10.2-12.2)	9 (6.2-12.6)	0.6 (-9.8-12.9)	3.6 (2.6-4.9)	-0.4 (-9.7-9.9)	1 (0.7-1.4)	1.5 (-9.6-12.2)	0.3 (0.2-0.4)	-7.9 (-18.9-3.6)	1.3 (1-1.7)	-13.3 (-20.8--6)
Solihull	10.6 (7.2-14.7)	0.3 (-10-12.3)	8.8 (6.1-12.1)	1.3 (-9.7-12.5)	3.5 (2.5-4.8)	0.2 (-9.2-9.8)	1 (0.7-1.4)	1 (-9.7-11.2)	0.3 (0.2-0.4)	-7.4 (-18-3)	1.4 (1.1-1.9)	-7.6 (-14.9-0.6)
Staffordshire	10.6 (7.3-14.9)	0.7 (-11.1-13.9)	8.8 (6.2-12.5)	1.4 (-10.2-13.8)	3.6 (2.6-4.8)	-0.1 (-10.1-11.3)	1 (0.7-1.4)	0.2 (-10.4-12.5)	0.3 (0.2-0.4)	-8.4 (-18.9-3.1)	1.4 (1-1.8)	-12.1 (-20.3--4)
Stoke-on-Trent	12 (8.3-16.7)	1.3 (-9.4-16.6)	9.7 (6.8-13.5)	1.7 (-8.5-16.3)	3.7 (2.7-5.3)	1.2 (-9.2-12.1)	1 (0.7-1.3)	-2.1 (-12-9.3)	0.3 (0.2-0.4)	0.4 (-11.2-14.6)	1.6 (1.2-2.1)	-0.2 (-8.7-10.3)
Telford and Wrekin	11.4 (7.9-15.8)	1.3 (-10-14.2)	9.3 (6.5-12.8)	2 (-8.8-14)	3.6 (2.6-5)	0.2 (-8.8-10.7)	1 (0.7-1.4)	-0.2 (-10.1-10.7)	0.3 (0.2-0.4)	-6.3 (-17-6.2)	1.6 (1.2-2.1)	-9.1 (-16.3--0.6)

Location	Birth Incidence rate (per 100,000 live births), 2021	Birth Incidence rate (per 100,000 live births) percent change (2000-2021)	Under-5 rate (per 100,000 population), 2021	Under-5 rate (per 100,000 population) percent change (2000-2021)	5-14 rate (per 100,000 population), 2021	5-14 rate (per 100,000 population) percent change (2000-2021)	15-49 rate (per 100,000 population), 2021	15-49 rate (per 100,000 population) percent change (2000-2021)	50plus rate (per 100,000 population), 2021	50plus rate (per 100,000 population) percent change (2000-2021)	All age rate (per 100,000 population), 2021	All age rate (per 100,000 population) percent change (2000-2021)
Walsall	12.1 (8.3-16.7)	1.1 (-10-12.8)	9.7 (6.8-13.4)	1.9 (-9-14)	3.6 (2.6-5)	-0.1 (-10.2-11.3)	1 (0.7-1.3)	-0.2 (-11.1-11.6)	0.3 (0.2-0.4)	-2.4 (-14.2-11.2)	1.6 (1.2-2.1)	-2.7 (-10.8-6.3)
Warwickshire	10.8 (7.6-15.1)	1.1 (-8.1-13.2)	9.1 (6.4-12.8)	1.8 (-8.2-13.8)	3.8 (2.7-5.2)	0.4 (-9-10.2)	1 (0.7-1.4)	0.2 (-9.8-11.6)	0.3 (0.2-0.4)	-6.9 (-17.4-4.5)	1.4 (1.1-1.9)	-8.3 (-15.7-0.1)
Wolverhampton	11.8 (8.1-16.3)	0.4 (-10-12.4)	9.5 (6.7-13.1)	1 (-9.6-13.9)	3.7 (2.6-5.2)	0.7 (-8.7-11.6)	1 (0.7-1.4)	-1.8 (-11.8-8.7)	0.3 (0.2-0.4)	4.3 (-8.2-17.9)	1.6 (1.2-2.2)	-0.2 (-8.1-9.4)
Worcestershire	10.8 (7.4-15.1)	0.5 (-10.2-15.6)	9 (6.2-12.7)	1.3 (-8.8-15.9)	3.6 (2.6-5)	-0.5 (-9.5-10.4)	1 (0.7-1.4)	0.8 (-8.8-11.6)	0.3 (0.2-0.4)	-9.4 (-19.8-1.4)	1.4 (1-1.8)	-11.1 (-18.3--2.8)
Yorkshire and the Humber	11.4 (8-15.8)	0.3 (-3.6-3.8)	9.3 (6.6-12.9)	1.2 (-2.4-4.7)	3.6 (2.7-5)	0.2 (-2.7-2.9)	1 (0.7-1.4)	-1 (-3.9-2.2)	0.3 (0.2-0.4)	-2.4 (-5.7-1)	1.5 (1.2-2)	-6.1 (-8.7--3.6)
Barnsley	11.5 (8-15.7)	0.4 (-9.5-11)	9.4 (6.6-12.9)	1.4 (-8.4-12.1)	3.8 (2.7-5.3)	0.1 (-8.4-11)	1 (0.7-1.3)	0.6 (-8.7-12.9)	0.3 (0.2-0.4)	-2.4 (-13.3-11.3)	1.5 (1.1-2)	-8 (-14.9--0.2)
Bradford	12.3 (8.3-17.1)	0.2 (-11.1-13.5)	9.6 (6.6-13.3)	0.4 (-10.7-13.4)	3.5 (2.5-4.8)	-1.5 (-12-10.8)	1 (0.7-1.3)	-1.7 (-12.3-11.2)	0.3 (0.2-0.4)	2.5 (-9.7-16.5)	1.7 (1.3-2.2)	-4.5 (-13.7-4.7)
Calderdale	11.9 (8.2-16.5)	1.4 (-10.7-13.1)	9.6 (6.7-13.3)	2.6 (-8.7-14.4)	3.6 (2.6-5.1)	0.7 (-9.4-12.8)	1 (0.7-1.3)	-0.2 (-10.1-12.1)	0.3 (0.2-0.4)	-0.8 (-12.2-12.6)	1.5 (1.1-1.9)	-9.5 (-17.9-0.1)
Doncaster	11.7 (8.1-16.3)	1.1 (-9.2-13.2)	9.5 (6.7-13.3)	1.8 (-8.7-13.9)	3.7 (2.7-5.1)	-0.3 (-9.3-11.2)	1 (0.7-1.3)	-0.6 (-11.7-10.1)	0.3 (0.2-0.4)	-2.1 (-13.7-12.8)	1.5 (1.1-2)	-7.9 (-15.6-1.6)
East Riding of Yorkshire	10.9 (7.4-15.4)	0.7 (-11.1-11.8)	9.1 (6.2-12.7)	1.5 (-10.3-12.5)	3.6 (2.6-5)	-0.2 (-10.4-9.8)	1 (0.7-1.4)	0.9 (-8.7-12.1)	0.3 (0.2-0.4)	-9.6 (-19.9-1.3)	1.3 (1-1.6)	-14.8 (-22.6--7)
Kingston upon Hull, City of	12.1 (8.4-17.1)	0.2 (-9.8-12.1)	9.8 (6.8-13.7)	0.9 (-9.4-12.6)	3.8 (2.7-5.2)	1.3 (-8.4-12.4)	1 (0.7-1.3)	-3 (-13.4-8.1)	0.3 (0.2-0.4)	4.9 (-7.5-19.3)	1.7 (1.3-2.2)	-2.3 (-11.1-6.5)
Kirklees	11.9 (8.3-16.3)	0 (-10.8-12.3)	9.7 (6.7-13.5)	0.8 (-9.3-12.8)	3.6 (2.6-5)	0.1 (-9.9-9.8)	1 (0.7-1.3)	-1 (-11.4-9.5)	0.3 (0.2-0.4)	-3.2 (-15.7-10.2)	1.5 (1.2-2)	-9.4 (-17.1--1.6)
Leeds	11.2 (7.8-15.5)	1.2 (-10-12.9)	9.2 (6.5-12.7)	2.1 (-8.5-13.7)	3.8 (2.8-5.2)	2.9 (-7.4-13)	1 (0.7-1.4)	-3.2 (-13.3-6.8)	0.3 (0.2-0.4)	1 (-10.3-14.8)	1.6 (1.3-2.1)	1.6 (-7.1-10.9)
North East Lincolnshire	11.7 (8.1-16.1)	0.7 (-10-12.1)	9.5 (6.7-13.1)	1.8 (-8.8-12.8)	3.7 (2.7-5.2)	1.7 (-9.2-12.2)	1 (0.7-1.3)	-1.5 (-11.5-10.4)	0.3 (0.2-0.4)	-2.6 (-13.3-11.9)	1.5 (1.1-2)	-10.3 (-18.2--1.9)
North Lincolnshire	11.3 (7.7-15.8)	0.7 (-8.6-12)	9.2 (6.4-13.1)	1.6 (-7.8-13.1)	3.6 (2.6-5.1)	-0.3 (-9-11)	1 (0.7-1.4)	0.4 (-9.8-10.9)	0.3 (0.2-0.4)	-4.6 (-15.2-7.3)	1.5 (1.1-1.9)	-7.9 (-14.9-0.1)
North Yorkshire	10.6 (7.3-14.9)	0.8 (-11.5-12.9)	8.8 (6.2-12.6)	1.7 (-10.1-12.9)	3.6 (2.6-5)	0.4 (-9.2-11.7)	1 (0.7-1.4)	0.3 (-9.9-12.6)	0.3 (0.2-0.4)	-6.7 (-18.2-5.2)	1.3 (1-1.7)	-11.8 (-19.4--3.7)
Rotherham	11.4 (7.9-15.8)	0.3 (-9.7-11.6)	9.3 (6.5-12.9)	1.3 (-9.2-12.1)	3.7 (2.7-5.1)	0.1 (-9.1-9.4)	1 (0.7-1.4)	0.6 (-9.5-11.6)	0.3 (0.2-0.4)	-5.7 (-17.3-6.3)	1.5 (1.1-1.9)	-8.6 (-16--0.7)
Sheffield	10.9 (7.5-15.2)	-0.5 (-12-10.6)	9 (6.2-12.6)	0.4 (-10.9-11.6)	3.6 (2.6-4.9)	-1.3 (-10.8-8.6)	1 (0.8-1.4)	-0.6 (-10.4-10.5)	0.3 (0.2-0.4)	2.2 (-9.4-16.6)	1.6 (1.2-2)	-0.8 (-9.8-8.6)
Wakefield	11.3 (7.8-15.7)	0.1 (-11.6-11.8)	9.3 (6.5-12.9)	1 (-10.3-13)	3.7 (2.7-5.1)	0.8 (-9.7-10.9)	1 (0.7-1.4)	-0.4 (-11.5-10.3)	0.3 (0.2-0.4)	-4.1 (-15.8-8)	1.5 (1.1-1.9)	-8.2 (-16.7--0.1)
York	10.8 (7.4-15)	-0.9 (-13-9.1)	8.9 (6.2-12.2)	0.1 (-11-10.2)	3.5 (2.5-4.8)	-0.2 (-9.6-10.1)	1 (0.7-1.4)	-2 (-12.1-9.1)	0.3 (0.2-0.4)	-2.8 (-13.5-9.6)	1.4 (1.1-1.8)	-5.2 (-13.6-3)
Northern Ireland	13.6 (9.9-18.6)	1.4 (-7.1-11.8)	11.5 (8.4-15.7)	2.2 (-6.7-12.8)	6.1 (4.5-8.2)	1.1 (-7.3-10.1)	2.2 (1.6-2.9)	-0.9 (-9.3-8)	0.7 (0.5-1)	-0.5 (-9-9.9)	2.7 (2.1-3.5)	-9.8 (-17.1--2.5)
Scotland	13.6 (9.9-18.3)	-0.3 (-9.9-9.5)	11.5 (8.5-15.5)	0.3 (-9.3-10)	6.2 (4.7-8.3)	-1.6 (-9.6-7.9)	2.2 (1.7-2.9)	-0.2 (-8.2-9.3)	0.7 (0.5-0.9)	-2.4 (-12.1-8.9)	2.5 (1.9-3.1)	-11.1 (-17.6--3.4)
Wales	13.1 (9.5-17.7)	-0.5 (-12.1-12.3)	11.1 (8.1-15.1)	0.3 (-11.6-13.2)	6.1 (4.5-8.1)	-0.1 (-10.1-9.7)	2.2 (1.7-3)	0.3 (-8.4-9.2)	0.7 (0.5-1)	-3.1 (-12.2-6.4)	2.5 (1.9-3.2)	-8.7 (-16.5--1.1)
Latin America and Caribbean	31.9 (26-37.9)	14.8 (10.2-19.7)	30.6 (25.1-36.4)	15.8 (11.3-20.7)	21.1 (17.3-25.3)	12.2 (7.9-16.7)	3.9 (3.1-4.8)	-13.3 (-16.6--9.7)	0.7 (0.5-1)	-22.4 (-25.9--19)	8.1 (6.8-9.5)	-15 (-17.7--12.2)
**Andean Latin America**	10.8 (8.3-13.7)	3 (-2.9-8.8)	10.5 (8.2-13.3)	3 (-2.7-8.8)	8 (6.4-10)	3.6 (-1.8-8.9)	2.3 (1.8-2.9)	-4.2 (-9.4-1.1)	0.6 (0.5-0.9)	-4.3 (-9.6-1.5)	3.8 (3.1-4.6)	-12.4 (-16.7--8)
Bolivia (Plurinational State of)	11.4 (8.2-16)	-0.5 (-10.5-9.6)	11.1 (7.9-15.6)	-0.5 (-10.5-9.6)	8.2 (5.9-11.2)	0.9 (-7.8-9.9)	2.3 (1.7-3)	-5.4 (-13.3-4.2)	0.6 (0.4-0.8)	-2.7 (-11.4-6.9)	4.3 (3.2-5.7)	-12 (-18.8--4.9)
Ecuador	11.2 (10-12.4)	10.7 (3.5-19.5)	11 (9.8-12.1)	10.6 (3.5-19.5)	8.6 (7.7-9.5)	9 (2.3-16.7)	2.3 (1.9-2.7)	-2.5 (-10.3-5.6)	0.7 (0.6-0.9)	-7.1 (-14.9-0.8)	4 (3.6-4.4)	-9.6 (-14.8--3.9)
Peru	10.2 (7.3-13.7)	-0.1 (-10-9.8)	9.9 (7.2-13.4)	-0.2 (-10-9.7)	7.5 (5.5-10.1)	0.8 (-8.9-10)	2.2 (1.6-3)	-5.3 (-13.2-3)	0.6 (0.4-0.9)	-3.7 (-11.8-5.9)	3.5 (2.7-4.4)	-15.8 (-22.9--8.6)
**Caribbean**	265 (212-314)	14.1 (8.2-21.2)	254 (203-302)	14.6 (8.7-21.6)	173 (141-206)	12.5 (6.8-19.7)	20.6 (15.6-27)	-14.7 (-21.7--7.8)	3 (2.2-4)	-27.5 (-34--21.5)	59.8 (49.9-70.5)	-10 (-14.1--5)

Location	Birth Incidence rate (per 100,000 live births), 2021	Birth Incidence rate (per 100,000 live births) percent change (2000-2021)	Under-5 rate (per 100,000 population), 2021	Under-5 rate (per 100,000 population) percent change (2000-2021)	5-14 rate (per 100,000 population), 2021	5-14 rate (per 100,000 population) percent change (2000-2021)	15-49 rate (per 100,000 population), 2021	15-49 rate (per 100,000 population) percent change (2000-2021)	50plus rate (per 100,000 population), 2021	50plus rate (per 100,000 population) percent change (2000-2021)	All age rate (per 100,000 population), 2021	All age rate (per 100,000 population) percent change (2000-2021)
Antigua and Barbuda	141 (97.7-197)	6.8 (-6.6-22.6)	140 (96.6-195)	6.8 (-6.6-22.6)	107 (76-146)	4.6 (-7.9-19.8)	16.3 (12-21.5)	-20.3 (-30.2--9.7)	3.8 (2.7-5.2)	-21.8 (-31.4--11.2)	32.1 (24.3-42.2)	-26.8 (-33.9--18.6)
Bahamas	294 (210-398)	8.4 (-6.1-25.3)	289 (206-391)	8 (-6.5-24.8)	208 (149-275)	3.9 (-10.2-18.5)	26.9 (19.5-36.6)	-0.5 (-14-16.4)	4.4 (3.1-6.2)	0 (-11.7-15.1)	61.9 (47-79.6)	-24 (-32.9--13.5)
Barbados	151 (103-211)	7.8 (-6.1-25.8)	149 (102-209)	7.8 (-6-26)	115 (80.4-157)	5.4 (-7.7-20.2)	16.5 (12.1-22.1)	-17.8 (-28.9--6)	3.3 (2.4-4.5)	-22.9 (-32.8--12.1)	28.7 (21.5-37.2)	-24.3 (-32--16)
Belize	189 (132-261)	7.7 (-6.8-24.6)	185 (129-257)	7.7 (-6.8-24.5)	136 (95.5-183)	4.2 (-8.5-19.4)	21.4 (15.7-28.6)	-19 (-29.4--8)	4.1 (2.9-5.7)	-17.3 (-27.5--6.8)	55.8 (42-73.2)	-20.2 (-29.1--10.3)
Bermuda	120 (82-168)	8.6 (-5.3-25.9)	119 (81-166)	8.7 (-5.2-25.9)	93.5 (65-127)	5.9 (-7-21.5)	14.5 (10.7-19.1)	-18.9 (-29.1--7.5)	3.3 (2.4-4.5)	-33.8 (-43--24.9)	20.8 (15.9-26.7)	-28.5 (-35.5--20.2)
Cuba	67.2 (51.6-85.5)	52.9 (29.9-76.7)	66.4 (51-84.3)	52.8 (29.9-76.6)	50.8 (38.7-64.9)	49.2 (28.1-71.8)	5 (3.8-6.5)	-23.9 (-37.6--4.9)	1.2 (0.8-1.6)	-32.6 (-45.6--15)	11.2 (8.9-14.1)	-5.2 (-17.2-9.7)
Dominica	151 (105-209)	6.6 (-8.8-22.5)	149 (104-206)	6.6 (-8.9-22.5)	109 (77.3-151)	4.2 (-9-18.8)	17 (12.5-22.6)	-17.8 (-28.5--6.4)	3.5 (2.5-4.8)	-10.6 (-22.5-1.1)	33.8 (25.6-44.2)	-26.4 (-34--17.7)
Dominican Republic	259 (193-327)	5.7 (-6-19.7)	254 (189-320)	5.8 (-6-19.7)	183 (137-229)	6.2 (-5-19)	23.2 (17.4-30.8)	-11.4 (-22.5-1)	4.2 (3-5.7)	-13.2 (-24--1.3)	70.7 (55.1-86.3)	-15.3 (-23.8--6.3)
Grenada	161 (113-225)	7 (-8.6-22.5)	159 (111-221)	7.5 (-8.2-22.9)	119 (84.4-162)	6.4 (-7.6-22.2)	18.9 (13.9-25.2)	-20.7 (-29.7--10.9)	3.8 (2.7-5.3)	-23.7 (-32.8--13)	38.3 (28.8-50.3)	-24 (-32.3--14.1)
Guyana	175 (126-238)	6.7 (-5.7-20.7)	172 (123-234)	6.7 (-5.6-20.6)	126 (90.9-170)	3.7 (-8.1-16.5)	18.7 (13.7-25.2)	-15.5 (-25.5--5.5)	3 (2.1-4.1)	-24.7 (-33.9--14.7)	48 (35.7-63.2)	-20 (-28--11.8)
Haiti	343 (280-398)	0.9 (-6.7-12.1)	334 (274-390)	0.9 (-6.8-12)	224 (186-267)	-0.5 (-8.2-9.9)	28 (20.6-37.3)	-28.5 (-37.9--18.6)	4.3 (3.1-6)	-27.7 (-37.3--17.6)	105 (87.9-123)	-16.1 (-22.2--8.1)
Jamaica	339 (283-404)	-6.3 (-19.2-7.4)	337 (281-401)	-6.3 (-19.2-7.4)	257 (212-307)	-9.3 (-20.9-3.6)	38.1 (28.4-50)	-38.8 (-48.4--26.9)	8.1 (5.9-10.9)	-37.7 (-46.7--26.8)	82.2 (69.6-98.1)	-37.5 (-45--29.4)
Puerto Rico	130 (88.1-184)	8.1 (-4.3-24.4)	129 (87.2-182)	8.1 (-4.3-24.3)	102 (71.7-139)	4.8 (-7-19.5)	15.6 (11.5-20.9)	-21.3 (-31.1--10.1)	2.8 (2-3.7)	-37.7 (-46.4--28.5)	23 (17.4-29.8)	-35.9 (-42--28.6)
Saint Kitts and Nevis	152 (106-211)	6.2 (-7.3-20.2)	149 (105-208)	6.3 (-7.2-20.4)	111 (79.6-153)	4.5 (-7.9-18.2)	16.4 (11.9-21.8)	-21.4 (-31.2--12.1)	3.6 (2.6-5)	-5.4 (-17.6-8.8)	32.8 (25.2-42.6)	-29.4 (-36.6--21.9)
Saint Lucia	165 (111-229)	6.4 (-6.4-23.1)	163 (109-226)	6.6 (-6.2-23.5)	122 (84.5-168)	3.9 (-7.7-19.3)	18.2 (13.4-24.1)	-25.1 (-34.2--16.1)	3.8 (2.8-5.2)	-20.1 (-29--9.9)	33.6 (25.4-43.9)	-38.1 (-44.1--30.9)
Saint Vincent and the Grenadines	166 (117-228)	6.6 (-6.3-21.9)	164 (115-224)	6.6 (-6.2-22.1)	123 (86.8-166)	4.6 (-6.8-18.8)	17.9 (13-24)	-21.5 (-31.5--12)	3.4 (2.4-4.6)	-16.8 (-26.7--5.9)	38.6 (29.3-50.6)	-28 (-35.4--19.4)
Suriname	168 (120-232)	5.9 (-7-21.1)	165 (118-228)	6.1 (-6.6-21.8)	123 (87.9-169)	5.7 (-6.2-21)	17.6 (12.8-23.3)	-19.5 (-29.4--9.7)	3.3 (2.4-4.6)	-21.8 (-31.1--11.9)	43.4 (32.9-57.4)	-15.6 (-24.3--5.9)
Trinidad and Tobago	162 (113-222)	5.8 (-11.8-20.9)	160 (112-220)	5.8 (-11.9-21)	122 (85.7-166)	6.6 (-9.6-21)	17 (12.4-22.3)	-25.2 (-35.1--16.3)	3.2 (2.2-4.3)	-30.2 (-39.1--20.4)	35 (26.5-45.8)	-23 (-32.9--14.5)
United States Virgin Islands	148 (102-206)	7.2 (-6-22.1)	146 (101-204)	7.2 (-6-22.2)	115 (80.1-160)	6.3 (-6.3-20.8)	15.4 (11.3-20.8)	-19 (-29.2--7.1)	2.7 (1.9-3.7)	-40.5 (-48.5--31.6)	30.8 (22.8-41.1)	-25.2 (-32.7--16.5)
**Central Latin America**	2.2 (1.6-3)	-40.2 (-46.4--32.7)	2.2 (1.6-3)	-39.4 (-45.7--31.8)	1.7 (1.2-2.3)	-43.8 (-49.9--36.9)	0.4 (0.3-0.5)	-63.1 (-68.2--57.6)	0.1 (0-0.1)	-69 (-75.9--63.2)	0.7 (0.5-0.9)	-59.6 (-64--54.7)
Colombia	0.6 (0.5-0.8)	0 (-16.6-20.4)	0.6 (0.5-0.8)	-0.1 (-16.5-20.2)	0.4 (0.3-0.5)	-1.8 (-17.5-17.2)	0.1 (0-0.1)	-4.3 (-19.4-14.1)	0 (0-0)	-7.9 (-23.6-13.5)	0.1 (0.1-0.2)	-27 (-37.6--13.7)
Costa Rica	0.1 (0-0.1)	0.2 (-18-0.1)	0.1 (0-0.1)	0.2 (-18.3-0.3)	0 (0-0.1)	0.5 (-18.7-2.8)	0 (0-0)	-4.9 (-22.6-3.9)	0 (0-0)	-5.3 (-27.5-9.9)	0 (0-0)	-20.5 (-35.9--16.6)
El Salvador	0 (0-0.1)	0 (0-0)	0 (0-0.1)	0 (-0.2-0.3)	0 (0-0.1)	-0.6 (-2.9-1.8)	0 (0-0)	-4.3 (-14.2-5.1)	0 (0-0)	-3.1 (-18.5-12)	0 (0-0)	-21.8 (-26.1--18.3)
Guatemala	0 (0-0.1)	-0.9 (0-0)	0 (0-0.1)	-1 (-0.6-0.3)	0 (0-0.1)	-2.1 (-4.5-1.4)	0 (0-0)	-8 (-17.2-3.1)	0 (0-0)	-5.2 (-23.4-10.9)	0 (0-0)	-25 (-28.6--20.7)
Honduras	0 (0-0)	0.8 (0-0)	0 (0-0)	0.8 (-0.3-0.8)	0 (0-0)	0.3 (-3.7-3.5)	0 (0-0)	-4.6 (-14.9-9.2)	0 (0-0)	-3.7 (-19.4-16.9)	0 (0-0)	-16.3 (-20.7--12.6)
Mexico	2.5 (1.7-3.6)	0 (-4.2-5.2)	2.4 (1.7-3.5)	-0.7 (-4.8-4.5)	1.9 (1.3-2.7)	-2.2 (-6.1-2.9)	0.6 (0.4-0.8)	-8.8 (-12.3--5.2)	0.1 (0-0.1)	-11 (-15--6.8)	0.8 (0.6-1.1)	-21.9 (-24.9--18.3)
Aguascalientes	0.2 (0-0.6)	12.2 (0-631)	0.2 (0-0.6)	12.1 (-0.4-632)	0.2 (0-0.5)	10.6 (-3.2-625)	0 (0-0.2)	4.7 (-13.4-578)	0 (0-0)	5 (-18.3-559)	0.1 (0-0.2)	-11.3 (-24.1-472)
Baja California	2.6 (1.4-4.4)	8 (-0.7-30.1)	2.5 (1.3-4.3)	8 (-0.7-30.1)	2 (1.1-3.4)	6.1 (-2.3-28.8)	0.5 (0.3-0.8)	-2.9 (-13.5-26.4)	0.1 (0-0.1)	0.5 (-14.3-24.5)	0.8 (0.4-1.3)	-17.9 (-24.5-2.7)
Baja California Sur	4.7 (2.6-7.4)	5.8 (-6.5-16.3)	4.6 (2.5-7.3)	5.7 (-6.6-16.2)	3.7 (2-5.8)	4.2 (-7.2-14.4)	1.1 (0.7-1.8)	-4.9 (-14.7-6.3)	0.2 (0.1-0.3)	-2.1 (-15.9-11.5)	1.6 (0.9-2.5)	-16.5 (-24.5--8.1)

Location	Birth Incidence rate (per 100,000 live births), 2021	Birth Incidence rate (per 100,000 live births) percent change (2000-2021)	Under-5 rate (per 100,000 population), 2021	Under-5 rate (per 100,000 population) percent change (2000-2021)	5-14 rate (per 100,000 population), 2021	5-14 rate (per 100,000 population) percent change (2000-2021)	15-49 rate (per 100,000 population), 2021	15-49 rate (per 100,000 population) percent change (2000-2021)	50plus rate (per 100,000 population), 2021	50plus rate (per 100,000 population) percent change (2000-2021)	All age rate (per 100,000 population), 2021	All age rate (per 100,000 population) percent change (2000-2021)
Campeche	5.6 (3.5-8.4)	5.1 (-6.4-16.9)	5.5 (3.4-8.2)	5.1 (-6.4-16.9)	4.3 (2.7-6.3)	3.6 (-6.8-14.8)	1.1 (0.6-1.6)	-10.1 (-19.5-0.4)	0.2 (0.1-0.3)	-10.3 (-22.4-1.7)	1.8 (1.1-2.6)	-19.6 (-26.9--11.3)
Chiapas	3 (2-4.2)	-7.9 (-18.2-0.9)	2.9 (2-4.1)	-7.9 (-18.3-0.8)	2.2 (1.5-3.1)	-8.1 (-17.1-0.7)	0.7 (0.4-0.9)	-12.7 (-21.9--2.7)	0.1 (0-0.1)	-17 (-29.2--1.8)	1.1 (0.8-1.5)	-22.7 (-29.9--15.5)
Chihuahua	1.2 (0.5-2.4)	14.5 (0-75.8)	1.2 (0.5-2.4)	14.4 (0-75.6)	0.9 (0.4-1.8)	12.3 (-2.3-74.8)	0.3 (0.1-0.5)	2.9 (-12.6-60)	0 (0-0.1)	5.7 (-18.1-57.1)	0.4 (0.2-0.8)	-10.3 (-22.4-39.8)
Coahuila	1.4 (0.8-2.1)	7.3 (-4.7-23.8)	1.3 (0.8-2.1)	7.1 (-4.8-23.5)	1.1 (0.6-1.7)	6.2 (-5.4-22.5)	0.4 (0.2-0.7)	0.5 (-12.1-15.2)	0.1 (0-0.1)	-0.3 (-16.7-20.3)	0.5 (0.3-0.8)	-10.7 (-20.5-2.5)
Colima	1.8 (0.6-4.1)	0.4 (-33.4-17)	1.8 (0.6-4)	0.4 (-33.3-17)	1.4 (0.4-3.2)	-0.6 (-33.5-16.3)	0.4 (0.1-0.8)	-9.8 (-34-6.8)	0.1 (0-0.1)	-13.7 (-36.9-5.7)	0.6 (0.2-1.3)	-22.7 (-45.4--10.1)
Durango	1.6 (0.6-3.3)	16.7 (0.4-48)	1.6 (0.6-3.2)	16.5 (0.3-47.6)	1.2 (0.5-2.5)	15.9 (-0.2-46)	0.5 (0.2-0.9)	8.9 (-6.6-37.7)	0.1 (0-0.1)	6.3 (-14.4-33.3)	0.6 (0.2-1.2)	-5.9 (-19.2-16.9)
Guanajuato	1 (0.5-1.8)	-1.1 (-34.5-48.9)	1 (0.5-1.7)	-1.2 (-34.5-48.7)	0.8 (0.4-1.3)	-2.7 (-35.3-45.9)	0.2 (0.1-0.4)	-13.4 (-42.2-14.7)	0 (0-0.1)	-10 (-39-27.8)	0.3 (0.2-0.6)	-25.7 (-50.1-7.4)
Guerrero	6.3 (4.8-8.1)	-5.9 (-14.8-3)	6.1 (4.6-7.9)	-5.9 (-14.8-3)	4.7 (3.6-6.1)	-6.4 (-15-2.5)	1.4 (1.1-1.9)	-10.4 (-18.7--1.2)	0.2 (0.1-0.2)	-16.4 (-27.6--3.1)	2.2 (1.8-2.8)	-25.4 (-31.7--18.5)
Hidalgo	1.1 (0.5-2.2)	10 (-5.3-43.5)	1.1 (0.5-2.2)	9.9 (-5.2-43.3)	0.9 (0.4-1.7)	9.2 (-5.6-41.2)	0.3 (0.1-0.6)	-0.1 (-14.1-21.9)	0 (0-0.1)	-4.4 (-21.1-25.4)	0.4 (0.2-0.8)	-15.2 (-26.8-6.6)
Jalisco	1.5 (0.7-2.6)	-16.2 (-43.3--2.8)	1.5 (0.7-2.5)	-16.3 (-43.4--3)	1.2 (0.6-2)	-18 (-44.7--4.3)	0.3 (0.2-0.5)	-32.8 (-55.6--19.1)	0 (0-0.1)	-32.5 (-52.6--15.7)	0.5 (0.2-0.8)	-36.8 (-57.4--26.6)
México	0.5 (0.2-1.2)	15.9 (0-221)	0.5 (0.2-1.2)	15.8 (-0.3-221)	0.4 (0.1-0.9)	14.8 (-2.9-217)	0.1 (0-0.2)	2.9 (-16.8-194)	0 (0-0)	-0.1 (-25.4-161)	0.2 (0.1-0.4)	-7.9 (-23.2-144)
Mexico City	1.2 (0.8-1.8)	5.4 (-2.1-23.5)	1.2 (0.8-1.8)	5.3 (-2.3-23.4)	1 (0.7-1.5)	4.6 (-3.2-21.4)	0.3 (0.2-0.5)	-3.4 (-13.7-8.9)	0 (0-0.1)	-8 (-21.2-7.2)	0.4 (0.3-0.6)	-18.8 (-26.2--6.8)
Michoacán de Ocampo	1.7 (0.8-3.1)	5.7 (-16-26.5)	1.6 (0.8-3)	5.7 (-16.1-26.5)	1.3 (0.7-2.3)	5.2 (-16.6-24.9)	0.4 (0.2-0.6)	-2.1 (-19.9-15.7)	0 (0-0.1)	-5.4 (-25.8-17)	0.6 (0.3-1)	-17.9 (-33.8--4.1)
Morelos	1.6 (0.6-3.6)	-12.4 (-46.4-0)	1.6 (0.5-3.5)	-12.4 (-46.4-0)	1.2 (0.4-2.7)	-14 (-47.4--0.5)	0.3 (0.1-0.7)	-26 (-56.7--7)	0 (0-0.1)	-22.8 (-50--5)	0.5 (0.2-1.1)	-33.3 (-59.2--20.1)
Nayarit	2.4 (1.1-4.3)	11 (-1.2-30.6)	2.3 (1-4.2)	10.9 (-1.4-30.5)	1.9 (0.8-3.3)	10.3 (-2.5-28.9)	0.7 (0.3-1.1)	2 (-12.1-18.9)	0.1 (0-0.2)	-1.4 (-18.9-17.6)	0.9 (0.4-1.5)	-9.8 (-21.7-4.3)
Nuevo León	2.6 (1.8-3.8)	-18.2 (-28.7--9.6)	2.6 (1.7-3.7)	-18.3 (-28.7--9.7)	2.1 (1.4-3)	-19.1 (-29.2--10.4)	0.8 (0.5-1.1)	-23.1 (-32.4--13.6)	0.1 (0.1-0.2)	-20.7 (-32--8)	1 (0.6-1.4)	-32.6 (-40.3--25.1)
Oaxaca	5.9 (4.4-7.7)	1.8 (-5.2-9.7)	5.7 (4.3-7.5)	1.7 (-5.3-9.6)	4.4 (3.3-5.7)	0.6 (-6.6-8.2)	1.1 (0.8-1.5)	-9.3 (-17.7--0.5)	0.1 (0.1-0.2)	-13.6 (-25.1-0.3)	1.9 (1.4-2.4)	-22.9 (-28--17.2)
Puebla	1 (0.4-1.9)	13.7 (-4.1-103)	0.9 (0.4-1.9)	13.6 (-4.1-103)	0.7 (0.3-1.4)	11.9 (-6.2-95.1)	0.2 (0.1-0.3)	-1.4 (-21.7-64.9)	0 (0-0)	0.5 (-23.3-65.9)	0.3 (0.1-0.6)	-12.9 (-27.7-47.7)
Querétaro	0.2 (0-0.6)	11.4 (0-629)	0.2 (0-0.6)	11.4 (-0.3-628)	0.2 (0-0.5)	10.3 (-2.9-613)	0 (0-0.1)	-0.6 (-18.1-550)	0 (0-0)	2.2 (-18.8-560)	0.1 (0-0.2)	-15.1 (-27.2-444)
Quintana Roo	4.5 (2.6-7.3)	-6.8 (-15.3-2)	4.4 (2.6-7.2)	-6.8 (-15.3-2)	3.5 (2-5.5)	-8.1 (-16.4-0.4)	1 (0.6-1.4)	-20.2 (-29.3--9)	0.2 (0.1-0.3)	-17.7 (-29--4.1)	1.5 (0.9-2.3)	-28.4 (-35--20)
San Luis Potosí	1.8 (0.8-3.6)	8.4 (-11-41.3)	1.8 (0.8-3.5)	8.4 (-11-41)	1.4 (0.6-2.8)	6.9 (-11.6-40.4)	0.4 (0.2-0.7)	-1.5 (-23.2-32.5)	0.1 (0-0.1)	-2.9 (-23.7-30.9)	0.6 (0.3-1.1)	-17.5 (-32.6-9)
Sinaloa	3.1 (2-4.6)	6.9 (-4.7-20.3)	3 (1.9-4.4)	6.7 (-4.6-20)	2.4 (1.5-3.4)	5.6 (-5.1-18.8)	0.7 (0.4-1.1)	-3.3 (-15.6-10)	0.1 (0-0.2)	-8.6 (-23.6-8.5)	1 (0.6-1.5)	-19.2 (-27.7--9.6)
Sonora	1.8 (1-3.1)	-17.9 (-40--2.6)	1.8 (0.9-3)	-18 (-40.1--2.6)	1.4 (0.7-2.3)	-18.9 (-39.6--4.5)	0.4 (0.2-0.6)	-21.3 (-33.8--9.2)	0.1 (0-0.1)	-23.4 (-38.3--10.2)	0.6 (0.3-0.9)	-35.3 (-49--24.4)
Tabasco	13 (10.5-15.7)	4.5 (-5.3-16.1)	12.7 (10.2-15.4)	4.5 (-5.4-15.9)	9.8 (8-11.9)	3.4 (-5.9-13.6)	2.5 (2-3.1)	-10 (-18.8--0.8)	0.4 (0.2-0.5)	-9.1 (-19.5-2.2)	4.2 (3.5-5)	-20.5 (-26.6--13.6)
Tamaulipas	6.5 (4.9-8.6)	3.8 (-6.2-13.3)	6.4 (4.8-8.4)	3.7 (-6.3-13)	5 (3.8-6.5)	2.2 (-7.2-11.3)	1.6 (1.2-2.1)	-5.2 (-14.1-4.8)	0.2 (0.1-0.3)	-3.1 (-14.5-10.5)	2.2 (1.7-2.8)	-18.1 (-25.3--10.7)
Tlaxcala	1 (0.4-2)	7.2 (-8.4-44.5)	1 (0.4-2)	7.2 (-8.4-44.5)	0.8 (0.3-1.5)	6.2 (-9.1-44)	0.3 (0.1-0.6)	-3.8 (-19.7-26.6)	0 (0-0.1)	-0.7 (-22.6-34.6)	0.4 (0.2-0.7)	-14.7 (-28.1-13.2)
Veracruz de Ignacio de la Llave	4.8 (3.6-6.3)	7.4 (-2.8-17.9)	4.7 (3.5-6.2)	7.4 (-2.7-17.8)	3.6 (2.7-4.8)	6.5 (-2.9-16.5)	1.1 (0.8-1.4)	-0.9 (-10-9.5)	0.1 (0.1-0.2)	-7.2 (-19.2-8)	1.5 (1.2-2)	-18.7 (-25.5--11)
Yucatán	5.3 (3.4-7.9)	4.2 (-7.6-14.8)	5.2 (3.4-7.8)	4.2 (-7.6-14.8)	4 (2.6-5.9)	2.5 (-8.2-12.4)	1.1 (0.7-1.6)	-9 (-19-1)	0.2 (0.1-0.3)	-10.1 (-21.1-3.3)	1.6 (1.1-2.4)	-20.6 (-28--13.2)
Zacatecas	0.2 (0-0.7)	13.9 (0-411)	0.2 (0-0.7)	13.8 (-0.3-412)	0.2 (0-0.5)	12.9 (-3.2-423)	0 (0-0.2)	1.8 (-18.4-349)	0 (0-0)	6.6 (-18.8-347)	0.1 (0-0.2)	-10.6 (-24.9-309)

Location	Birth Incidence rate (per 100,000 live births), 2021	Birth Incidence rate (per 100,000 live births) percent change (2000-2021)	Under-5 rate (per 100,000 population), 2021	Under-5 rate (per 100,000 population) percent change (2000-2021)	5-14 rate (per 100,000 population), 2021	5-14 rate (per 100,000 population) percent change (2000-2021)	15-49 rate (per 100,000 population), 2021	15-49 rate (per 100,000 population) percent change (2000-2021)	50plus rate (per 100,000 population), 2021	50plus rate (per 100,000 population) percent change (2000-2021)	All age rate (per 100,000 population), 2021	All age rate (per 100,000 population) percent change (2000-2021)
Nicaragua	0 (0-0)	10.9 (0-1560)	0 (0-0)	10.9 (-0.4-1560)	0 (0-0)	10.5 (-3.4-1510)	0 (0-0)	2.1 (-17.1-1130)	0 (0-0)	8.8 (-15.8-1470)	0 (0-0)	-9.2 (-22.4-1130)
Panama	0 (0-0)	-3.3 (0-0)	0 (0-0)	-3.3 (-0.6-0.3)	0 (0-0)	-3 (-3.5-2.1)	0 (0-0)	-5 (-16.9-6)	0 (0-0)	-7.5 (-23.1-6.8)	0 (0-0)	-13.6 (-16.2--7.9)
Venezuela (Bolivarian Republic of)	9.1 (6.8-11.9)	-59 (-64.8--51.5)	8.8 (6.6-11.6)	-59 (-64.8--51.5)	6.1 (4.6-8.1)	-64.1 (-69--57.6)	1 (0.7-1.3)	-84.6 (-87.7--81.3)	0.1 (0.1-0.2)	-88.7 (-91.5--86)	2.2 (1.7-2.9)	-77.2 (-80.2--73.4)
**Tropical Latin America**	21.7 (17.7-26.1)	25.8 (17.9-33.2)	21.5 (17.5-25.8)	25.3 (17.6-32.6)	15.8 (13.2-19)	28.1 (21.3-35.3)	4.8 (4-5.9)	11.1 (4.9-17.6)	1 (0.7-1.3)	3.6 (-3.2-9.1)	6.6 (5.6-7.7)	-0.7 (-5.6-4.3)
Brazil	21.6 (17.6-25.9)	27 (18.8-35.3)	21.3 (17.5-25.6)	26.7 (18.5-35.1)	15.7 (13.2-18.9)	29.6 (22.5-37.4)	4.8 (3.9-5.8)	11.5 (5.1-17.9)	1 (0.7-1.2)	3.9 (-3.4-9.6)	6.5 (5.6-7.5)	0 (-5.2-5)
Acre	21.9 (16.7-28.4)	32.1 (18.8-46.8)	21.4 (16.3-27.8)	32.4 (19.1-47.1)	13.3 (10.4-17)	29.5 (18.5-43)	3 (2.2-4)	13 (1.3-27.1)	0.6 (0.4-0.9)	10.8 (-2.6-25.7)	6.2 (5-7.6)	3 (-5.5-13.5)
Alagoas	4.8 (3.7-6.2)	-13.1 (-24.7-0.1)	4.7 (3.6-6)	-12.8 (-24.4-0.5)	3.4 (2.6-4.3)	-11.8 (-23.3-1)	1.1 (0.8-1.5)	-23.4 (-33.8--12.3)	0.2 (0.1-0.3)	-23.2 (-35.9--10.2)	1.7 (1.3-2.1)	-28.4 (-37.6--17.7)
Amapá	8.9 (5.8-13.4)	11 (-4.1-28.7)	8.7 (5.7-13.1)	11.2 (-3.9-28.9)	6 (4-8.6)	14.3 (0-29.4)	1.4 (0.9-2.1)	16.1 (1.6-34.6)	0.3 (0.2-0.5)	15.3 (0.4-34.5)	2.7 (1.8-3.7)	-8.1 (-18-2.5)
Amazonas	5.8 (4.4-7.6)	40.7 (26.8-55.9)	5.7 (4.3-7.5)	40.9 (27.2-55.9)	4.2 (3.2-5.5)	40.2 (26.5-55.4)	1.5 (1.1-2)	26.2 (12.5-39.9)	0.3 (0.2-0.5)	22.5 (8-40.1)	2.2 (1.7-2.9)	15.6 (4.9-27.5)
Bahia	30.8 (24.7-37.3)	97.6 (75.2-121)	30.1 (24.3-36.8)	98 (75.8-122)	22 (18.1-26.8)	96.8 (77.9-120)	7.1 (5.8-8.8)	57.8 (43.4-72.7)	1.5 (1-2)	43.5 (26.8-58.3)	10.1 (8.5-11.9)	52.8 (39.1-67.9)
Ceará	5.5 (4.3-7)	34.7 (22.5-45.5)	5.4 (4.2-6.9)	34.9 (22.7-45.9)	3.8 (3-4.8)	38.6 (26.9-50.1)	1.1 (0.8-1.4)	19.9 (8.1-31.9)	0.2 (0.2-0.4)	21.8 (8.3-36.7)	1.7 (1.4-2.1)	10.2 (1-19.2)
Distrito Federal	61.2 (50.9-73.3)	-8.6 (-18.1-1.8)	60.3 (50.3-72.3)	-8.5 (-18-1.9)	45.6 (38.6-54.1)	-6.7 (-15.3-3)	15.4 (12.8-18.3)	-10.7 (-18.3--2.2)	4 (3-5)	-18.2 (-25.7--9.6)	19.6 (16.9-22.7)	-25.9 (-31.9--19.6)
Espírito Santo	44.6 (36.3-54.2)	31.1 (15.2-48.5)	43.8 (35.6-53.2)	31.2 (15.2-48.6)	30.5 (25.1-36.6)	36.8 (21.3-52.2)	6.8 (5.5-8.2)	20.3 (9.8-31.5)	1.4 (1-1.8)	8.8 (-2-20.2)	11.1 (9.5-12.8)	2.3 (-6.2-10.9)
Goiás	29 (23.6-35)	34.2 (19.2-50.1)	28.5 (23.2-34.3)	34.4 (19.6-50.2)	20 (16.8-23.9)	33.5 (19.7-46.8)	5.7 (4.6-6.9)	13.9 (3.3-25)	1.2 (0.9-1.5)	0.8 (-9.7-11.4)	7.9 (6.7-9.2)	-0.8 (-9.5-8.3)
Maranhão	14.2 (11-18.3)	18.1 (4.9-35.6)	13.8 (10.7-17.9)	18.2 (4.9-35.6)	9.4 (7.5-11.9)	25.7 (13.1-40.8)	2.6 (2-3.4)	20.5 (7.6-34.3)	0.5 (0.3-0.7)	22.3 (7-40.1)	4.9 (4-6)	8.7 (-1.8-20.4)
Mato Grosso	13.5 (10.6-16.9)	34.6 (22.7-47.3)	13.2 (10.4-16.5)	34.8 (23-47.6)	9.3 (7.5-11.6)	33.1 (22.2-45)	2.7 (2.1-3.5)	6.9 (-4-17.3)	0.6 (0.4-0.8)	-2.9 (-14.9-8.3)	3.9 (3.2-4.8)	-2 (-10.2-6.5)
Mato Grosso do Sul	13.9 (10.7-17.9)	36.8 (21.9-52.8)	13.7 (10.5-17.6)	37 (22.1-52.9)	10 (7.9-12.7)	35.4 (22.4-49)	3.2 (2.5-4)	14 (2.8-25.3)	0.7 (0.4-0.9)	4.1 (-7.2-16.5)	4.2 (3.4-5.2)	2.5 (-6.8-11.6)
Minas Gerais	32.2 (26.4-39)	27.2 (13.9-43.6)	31.5 (25.7-38)	27.5 (14.1-43.9)	21.4 (17.6-25.5)	29 (16.7-43.4)	5.3 (4.2-6.4)	14.7 (4.1-27.1)	0.8 (0.6-1.1)	-1.8 (-13.2-9.6)	7.7 (6.5-8.9)	-5.2 (-13-3.7)
Pará	9 (7-11.3)	5.7 (-7-19.8)	8.8 (6.9-11)	5.9 (-6.8-20)	6.3 (5-7.8)	11 (-1.4-24)	2.1 (1.6-2.6)	11.6 (-1.6-25.4)	0.5 (0.3-0.6)	9.1 (-5.8-23.2)	3.1 (2.5-3.8)	-6.8 (-17-2.9)
Paraíba	7 (5.2-9.2)	45.4 (28.8-60.7)	6.8 (5.1-9)	45.8 (29.3-61.5)	4.8 (3.7-6.3)	49 (32.9-64.4)	1.4 (1-1.9)	26.1 (12.2-39.9)	0.3 (0.2-0.4)	30.4 (13.6-47)	2.2 (1.7-2.8)	20.7 (8.1-32.1)
Paraná	8 (6.3-10)	62.9 (46.5-82)	7.9 (6.2-9.8)	63.2 (46.7-82.2)	5.8 (4.7-7.2)	59.7 (44.8-76.7)	1.8 (1.4-2.3)	29.2 (17.1-42.1)	0.4 (0.2-0.5)	14.9 (0.8-28.6)	2.3 (1.9-2.7)	15.5 (5.4-26.7)
Pernambuco	5.3 (4.1-6.8)	47 (31.5-65.7)	5.2 (4-6.6)	47.2 (31.7-66.2)	3.7 (3-4.7)	44.5 (32-59.9)	1.2 (0.9-1.6)	19 (8.1-30.3)	0.2 (0.1-0.3)	17.1 (3.3-31)	1.7 (1.4-2.1)	15.1 (6-25.7)
Piaui	29.6 (24.4-36.1)	13.8 (1-27.6)	29 (23.9-35.4)	14 (1.2-27.8)	21.2 (17.7-25.4)	17 (5.6-28.5)	7.1 (5.8-8.5)	-0.7 (-8.7-8.5)	1.5 (1.1-2)	-5.1 (-13.7-4.4)	10.2 (8.6-11.9)	-6.2 (-14.5-2)
Rio de Janeiro	39.8 (33.3-47.2)	6.6 (-5.4-19.4)	38.7 (32.3-46)	6.7 (-5.2-19.3)	27.6 (23.2-32.8)	6.9 (-3.3-17.6)	7.4 (6.1-8.9)	-5 (-13.4-3.3)	0.9 (0.6-1.3)	-19.3 (-28.9--10.2)	9.7 (8.4-11.2)	-17.7 (-24.8--10.9)
Rio Grande do Norte	7.7 (5.6-10.5)	35 (19.7-54.2)	7.6 (5.5-10.3)	35.3 (19.8-54.5)	5.2 (3.8-7)	34.9 (20-52.1)	1.3 (0.9-1.9)	3.6 (-11.9-18.9)	0.3 (0.2-0.5)	6.1 (-11.8-23.7)	2.2 (1.7-2.8)	1.8 (-9.1-14.6)
Rio Grande do Sul	9.1 (7.2-11.4)	56 (40.2-74.9)	9 (7.1-11.2)	56.3 (40.7-75.1)	6.6 (5.4-8.2)	54.7 (40.8-70.8)	2.1 (1.6-2.7)	36.4 (23.8-50)	0.4 (0.3-0.6)	15.2 (1.4-29.2)	2.5 (2-3)	14.7 (5-26.2)
Rondônia	24 (18.4-30.5)	30 (14.8-46.8)	23.5 (18.1-29.9)	30.3 (15.1-46.7)	15.2 (12-19.3)	32.6 (18.9-47.2)	3.2 (2.4-4.1)	11.5 (0.1-24.4)	0.7 (0.4-0.9)	2.4 (-9.3-15.4)	5.8 (4.7-7)	-6.2 (-14.4-3)
Roraima	28.1 (21.5-35.8)	-6 (-17.3-6)	27.6 (21-35.1)	-5.7 (-17.1-6.3)	19.5 (15.5-24.6)	-0.4 (-10.2-10.7)	5.4 (4.3-6.9)	-3.3 (-13.1-6.8)	1.1 (0.8-1.5)	-12.5 (-22.1--2.4)	9.6 (7.7-11.7)	-20.1 (-27.5--12.7)
Santa Catarina	3.8 (2.9-5)	27.7 (15.2-41.8)	3.8 (2.8-4.9)	27.8 (15.3-42.1)	2.8 (2.1-3.6)	26 (15-39.1)	0.9 (0.7-1.2)	7 (-4.9-18.2)	0.2 (0.1-0.3)	-5.4 (-17.2-7.1)	1.1 (0.9-1.4)	-6.8 (-15.6-2.6)

Location	Birth Incidence rate (per 100,000 live births), 2021	Birth Incidence rate (per 100,000 live births) percent change (2000-2021)	Under-5 rate (per 100,000 population), 2021	Under-5 rate (per 100,000 population) percent change (2000-2021)	5-14 rate (per 100,000 population), 2021	5-14 rate (per 100,000 population) percent change (2000-2021)	15-49 rate (per 100,000 population), 2021	15-49 rate (per 100,000 population) percent change (2000-2021)	50plus rate (per 100,000 population), 2021	50plus rate (per 100,000 population) percent change (2000-2021)	All age rate (per 100,000 population), 2021	All age rate (per 100,000 population) percent change (2000-2021)
São Paulo	33.7 (27.8-40.8)	36 (22.4-51.3)	33.2 (27.3-40.1)	36.1 (22.6-51.3)	25.3 (21-30)	34.6 (22.2-48.6)	7.6 (6.2-9.2)	14.5 (4.9-23.9)	1.6 (1.2-2.1)	1.6 (-8.6-10.7)	9.6 (8.2-11)	1.5 (-6.5-9.4)
Sergipe	17.7 (13.5-22.7)	18.6 (3.4-33.4)	17.3 (13.3-22.2)	18.8 (3.5-33.8)	12.2 (9.6-15.7)	20.3 (6.9-34.9)	3.6 (2.7-4.6)	0.4 (-11.1-11.2)	0.8 (0.5-1.1)	-2.8 (-14.8-9.3)	5.3 (4.3-6.6)	-8.5 (-18.2-1.1)
Tocantins	44.6 (36-54.1)	40.4 (26.9-56.3)	43.7 (35.1-52.9)	40.6 (27.1-56.5)	30.7 (25.5-36.8)	44.4 (32.2-58.6)	8.8 (7.2-10.7)	24.5 (14.7-36.1)	1.8 (1.4-2.4)	13.8 (3.9-25.4)	13.9 (11.8-16.2)	10.6 (2.7-20.3)
Paraguay	25.9 (18.7-35.7)	0.2 (-9-9.5)	25.4 (18.3-34.9)	0.2 (-9-9.3)	18.1 (13.4-24.1)	-0.2 (-8.4-7.8)	6.2 (4.6-8.2)	-3.3 (-11.5-5.2)	1.4 (1-1.9)	-2.2 (-10.6-6.8)	9.2 (7-12)	-16.7 (-23.6--9.8)
North Africa and Middle East	131 (107-157)	-36.9 (-41.7--32.4)	128 (105-153)	-36.9 (-41.9--32.4)	121 (99.4-145)	-36.9 (-41.7--32.6)	92.9 (76.6-111)	-39.8 (-44.7--35.2)	15.6 (11.6-20.3)	-32.9 (-38.3--27.1)	88.3 (72.6-105)	-42.7 (-47.2--38.5)
**North Africa and Middle East**	131 (107-157)	-36.9 (-41.7--32.4)	128 (105-153)	-36.9 (-41.9--32.4)	121 (99.4-145)	-36.9 (-41.7--32.6)	92.9 (76.6-111)	-39.8 (-44.7--35.2)	15.6 (11.6-20.3)	-32.9 (-38.3--27.1)	88.3 (72.6-105)	-42.7 (-47.2--38.5)
Afghanistan	102 (77.3-131)	0 (-7.6-7.6)	100 (75.7-129)	0 (-7.7-7.6)	93.7 (70.7-121)	-0.4 (-8-7.2)	66.9 (50.4-87.4)	-1.2 (-8.3-6.9)	10.1 (6.9-14)	45.7 (31.6-61.3)	75.3 (56.8-97.4)	-1.6 (-8.7-5.8)
Algeria	12 (9.5-14.7)	-0.1 (-6.5-8.1)	11.9 (9.4-14.4)	-0.2 (-6.6-8.1)	11.3 (9-13.8)	0 (-6.5-7.8)	8.4 (6.7-10.2)	-6.3 (-12.3-0.8)	1.4 (1-1.9)	4.3 (-4.7-13.8)	7.9 (6.4-9.6)	-11.4 (-17.1--4.9)
Bahrain	950 (743-1,200)	-18.9 (-31.4--9.3)	939 (733-1,190)	-18.9 (-31.4--9.3)	908 (711-1,150)	-18.9 (-31.1--9.3)	632 (496-794)	-25.4 (-36.3--16.7)	156 (112-210)	-11.4 (-21.4--0.5)	570 (450-716)	-34.6 (-44--27)
Egypt	135 (114-154)	-0.1 (-7.3-7.7)	133 (112-152)	-0.2 (-7.5-7.6)	126 (107-145)	-0.1 (-7.4-7.6)	95.4 (80.1-110)	-2.3 (-9.6-5.2)	14.8 (10.9-19.5)	-2.4 (-11-6.8)	93.6 (79.5-108)	-4.6 (-11.4-2.6)
Iran (Islamic Republic of)	141 (108-181)	-34.2 (-35.6--32.7)	139 (106-178)	-34.3 (-35.7--32.7)	133 (101-169)	-34.3 (-35.6--32.7)	95.3 (73.5-120)	-40.1 (-41.9--38.3)	20 (14.5-26.8)	-34.7 (-36.3--32.7)	89.2 (69.1-112)	-44.1 (-45.5--42.4)
Alborz	135 (103-177)	-34.2 (-39.2--27.8)	133 (101-174)	-34.2 (-39.2--27.9)	128 (96.9-167)	-34.2 (-39.3--27.9)	92.9 (70.6-120)	-40.3 (-45.4--34.8)	20.9 (15.1-28.4)	-38 (-43--32.6)	86 (65.8-111)	-45.3 (-49.9--40.4)
Ardebil	139 (104-180)	-33.9 (-39--28)	137 (102-177)	-33.9 (-38.9--28.1)	130 (97-168)	-33.8 (-38.8--27.9)	94.4 (71.6-120)	-40.7 (-45.1--35.3)	19.9 (14.1-26.8)	-31.1 (-37.3--24.7)	87.3 (65.9-111)	-45 (-49.2--40)
Bushehr	148 (112-190)	-34.2 (-39.3--28.6)	146 (111-187)	-34.2 (-39.3--28.6)	139 (105-180)	-34.2 (-39.3--28.6)	104 (79.3-133)	-39.1 (-43.7--33.8)	22.6 (16.2-30.2)	-35.8 (-41.5--30.3)	101 (77.1-129)	-41.5 (-45.9--36.5)
Chahar Mahaal and Bakhtiari	145 (110-186)	-34.2 (-39.1--28.4)	143 (108-183)	-34.2 (-39.1--28.6)	136 (103-175)	-34.2 (-39.1--28.5)	102 (77.3-129)	-40.2 (-44.7--35.2)	21.7 (15.6-28.8)	-33.6 (-39--28.1)	96 (73-122)	-43.9 (-48.2--39.3)
East Azarbayejan	141 (106-180)	-34.4 (-39.5--28.1)	139 (104-177)	-34.5 (-39.6--28.2)	131 (98.9-168)	-34.3 (-39.5--28)	92 (71.1-116)	-41.5 (-45.9--36.1)	19.3 (13.9-26.1)	-33.9 (-39.2--28.1)	85.4 (65.8-108)	-44.6 (-48.7--39.3)
Fars	150 (113-194)	-34.7 (-40.6--29.2)	148 (111-191)	-34.7 (-40.5--29.2)	141 (106-182)	-34.6 (-40--29)	97.7 (73.3-126)	-41.6 (-46.7--36.5)	19.4 (13.9-25.9)	-35.7 (-41.2--29.6)	91.4 (69.5-117)	-46.1 (-50.7--41.4)
Gilan	137 (103-178)	-34.3 (-39.2--28.9)	136 (102-175)	-34.3 (-39.3--28.9)	130 (98.1-169)	-34.3 (-39.2--28.7)	93.6 (70.8-121)	-40.1 (-44.8--35.1)	19.7 (14.2-26.3)	-33.1 (-38.3--27.1)	79 (60.1-102)	-47.4 (-51.4--43.3)
Golestan	147 (112-189)	-33.8 (-39.4--27.9)	145 (110-185)	-33.9 (-39.4--28)	138 (105-177)	-33.8 (-39.2--28)	97.4 (74.3-123)	-40.5 (-45.2--35)	19.3 (13.8-26.2)	-32.4 (-38.2--25.2)	94.6 (72.2-120)	-43.3 (-48.1--38.3)
Hamadan	144 (109-184)	-33.9 (-39.6--28.5)	142 (107-181)	-33.9 (-39.6--28.5)	135 (102-172)	-33.8 (-39.5--28.4)	93.6 (71.6-120)	-40.9 (-45.8--36.2)	18.3 (13.1-24.6)	-36 (-41.7--30.9)	85.5 (65.4-110)	-46 (-50.3--41.8)
Hormozgan	144 (110-184)	-34 (-39.3--28.1)	142 (108-181)	-34.1 (-39.4--28.1)	135 (102-172)	-34 (-39.3--27.9)	98.1 (74.7-124)	-39.4 (-44.5--33.9)	19.3 (13.6-26)	-28.9 (-35--22.6)	98.4 (75.3-124)	-41.3 (-46.1--35.9)
Ilam	143 (109-184)	-34.3 (-39.7--28.4)	141 (107-181)	-34.3 (-39.8--28.4)	135 (102-172)	-34.3 (-39.6--28.1)	96.2 (73.1-123)	-41 (-45.8--35.1)	19.9 (14.3-26.8)	-34.8 (-39.8--28.8)	90.8 (69-116)	-45.5 (-49.9--40.2)
Isfahan	140 (106-178)	-34.2 (-39.1--28.8)	138 (104-175)	-34.3 (-39.1--28.9)	132 (99-168)	-34.3 (-39.2--28.9)	95.7 (72.6-122)	-40.2 (-44.9--35)	21.3 (15.3-28.4)	-34.7 (-39.7--29)	86.2 (65.1-109)	-45.1 (-49.4--40.3)
Kerman	144 (108-185)	-34.4 (-39.9--29.6)	142 (107-182)	-34.4 (-39.9--29.6)	135 (102-173)	-34.3 (-39.8--29.5)	96 (73.9-121)	-40.7 (-45.6--35.8)	18.8 (13.5-25.2)	-33.4 (-39.6--27.8)	94 (72.2-119)	-43 (-47.8--38.6)
Kermanshah	149 (112-192)	-34.1 (-38.7--29)	146 (110-189)	-34.1 (-38.7--29.1)	139 (105-180)	-34 (-38.5--29)	96.1 (73.4-123)	-41 (-45.7--36.1)	18.4 (12.9-24.8)	-35.8 (-41.2--29.9)	88.4 (67.9-114)	-46.6 (-50.7--42.3)
Khorasan-e-Razavi	144 (109-185)	-34.4 (-39.1--28.1)	142 (107-181)	-34.4 (-39.2--28.2)	134 (101-171)	-34.4 (-39--28.2)	94.9 (72.8-120)	-40.8 (-45.3--35.6)	19.1 (13.9-25.8)	-32.1 (-37.9--25)	92.2 (71.4-116)	-43.2 (-47.3--38.1)
Khuzestan	153 (115-197)	-34.3 (-39.8--28.5)	150 (113-194)	-34.4 (-39.8--28.5)	143 (109-185)	-34.2 (-39.6--28.5)	103 (78.7-132)	-40.3 (-45.3--35.2)	20.6 (14.8-27.7)	-36.6 (-41.9--30.7)	101 (77.4-129)	-43.3 (-48.1--38.6)
Kohgiluyeh and Boyer-Ahmad	153 (116-199)	-34 (-39.4--27)	151 (114-196)	-34.1 (-39.5--27.1)	143 (108-185)	-34 (-39.4--27.2)	101 (77.1-129)	-40.4 (-45.1--34.2)	22.1 (16-29.5)	-31.3 (-36.9--25.1)	101 (77-129)	-43.1 (-47.6--37.3)
Kurdistan	142 (107-181)	-34.1 (-39.1--28.6)	139 (105-178)	-34.1 (-39.1--28.7)	132 (99.2-169)	-34 (-39--28.7)	94.5 (72.1-120)	-40.6 (-45--35.9)	19 (13.5-25.5)	-36.5 (-41.5--31.3)	88.7 (67.6-112)	-44.8 (-48.8--40.4)

Location	Birth Incidence rate (per 100,000 live births), 2021	Birth Incidence rate (per 100,000 live births) percent change (2000-2021)	Under-5 rate (per 100,000 population), 2021	Under-5 rate (per 100,000 population) percent change (2000-2021)	5-14 rate (per 100,000 population), 2021	5-14 rate (per 100,000 population) percent change (2000-2021)	15-49 rate (per 100,000 population), 2021	15-49 rate (per 100,000 population) percent change (2000-2021)	50plus rate (per 100,000 population), 2021	50plus rate (per 100,000 population) percent change (2000-2021)	All age rate (per 100,000 population), 2021	All age rate (per 100,000 population) percent change (2000-2021)
Lorestan	144 (110-185)	-34.3 (-38.6--29.4)	141 (108-182)	-34.4 (-38.6--29.5)	135 (103-174)	-34.3 (-38.6--29.3)	98.2 (75.8-125)	-40.9 (-45.3--36.2)	19.7 (14-26.7)	-34.4 (-39.5--28.8)	92 (71.7-117)	-45.3 (-49.2--41.1)
Markazi	141 (107-180)	-34.1 (-39.3--27.9)	139 (105-178)	-34.2 (-39.4--28)	133 (101-169)	-34.1 (-39.3--28)	93.8 (71.6-119)	-41 (-45.4--35.8)	18.7 (13.4-25.4)	-32.1 (-38.3--26.1)	84.5 (64.5-107)	-45.7 (-49.9--40.9)
Mazandaran	135 (102-172)	-34.1 (-39.3--29.4)	133 (101-169)	-34.1 (-39.3--29.4)	128 (96.4-163)	-34 (-39.2--29.3)	92.1 (70.3-116)	-40 (-45--35.9)	20.1 (14.4-26.7)	-36 (-40.9--31.2)	80.9 (61.7-102)	-46.4 (-50.8--42.7)
North Khorasan	146 (111-188)	-33.8 (-39.2--27.9)	143 (108-185)	-33.9 (-39.3--28)	135 (102-176)	-33.9 (-39.1--28.1)	96.2 (72.4-123)	-39.2 (-43.9--34)	18.8 (13.6-25.2)	-34.6 (-40.4--28.9)	92.3 (69.8-118)	-43.1 (-47.6--38)
Qazvin	139 (105-177)	-34.3 (-39.1--28.8)	137 (104-174)	-34.3 (-39.2--28.8)	130 (98.7-167)	-34.2 (-39--28.6)	94.5 (72-120)	-40.9 (-45.9--35.7)	20.3 (14.3-27.6)	-31.2 (-36.9--24.6)	87.8 (67-111)	-44.8 (-49.3--40.1)
Qom	137 (103-174)	-34.1 (-40.3--28.6)	135 (102-171)	-34.2 (-40.3--28.7)	129 (97.3-164)	-34 (-39.9--28.8)	95.8 (73.7-121)	-39.4 (-44.4--34.8)	20.9 (15.4-27.7)	-33.2 (-38.4--26.9)	91.2 (69.9-115)	-42.5 (-47.4--38.1)
Semnan	137 (105-176)	-33.8 (-40.4--27.7)	135 (103-174)	-33.9 (-40.4--27.7)	129 (98.7-167)	-33.8 (-39.8--27.4)	96.7 (74-125)	-38.4 (-44.4--32.5)	19.5 (13.9-26.2)	-32.6 (-39--25.8)	87.9 (67.5-113)	-42.2 (-47.5--36.6)
Sistan and Baluchistan	150 (114-196)	-34 (-39.1--27.7)	148 (111-192)	-34 (-39.2--27.8)	139 (105-181)	-34.1 (-39.2--27.9)	101 (76.6-130)	-38.8 (-43.7--33.1)	18.7 (13.5-25.2)	-32.9 (-39--26.3)	109 (82.4-141)	-38.5 (-43.2--32.7)
South Khorasan	135 (103-173)	-34.3 (-40.9--28.1)	133 (102-170)	-34.3 (-40.9--28.1)	126 (96.2-161)	-34.2 (-40.7--28.1)	93.8 (72.2-121)	-39.6 (-45.1--34.1)	19.3 (14-25.5)	-29.7 (-35.8--22.5)	89.7 (69.2-114)	-41.3 (-46.8--36)
Tehran	126 (95.4-161)	-34.2 (-39.9--28.8)	125 (94.1-159)	-34.3 (-39.9--28.9)	120 (91.1-154)	-34.1 (-39.7--28.7)	90.9 (70.3-116)	-38.8 (-43.9--33.6)	21 (15.3-28.1)	-38.6 (-43.2--32.8)	80.7 (62.1-102)	-43.9 (-48.5--39.2)
West Azarbayejan	142 (107-180)	-34.2 (-39.6--28.3)	140 (106-177)	-34.2 (-39.6--28.4)	132 (99.8-168)	-34.1 (-39.4--28.4)	94.2 (72-119)	-40 (-44.8--34.5)	20.2 (14.6-27.1)	-32.3 (-37.8--26.3)	90.8 (69-114)	-42.9 (-47.3--37.8)
Yazd	138 (105-177)	-33.8 (-39.2--27.4)	136 (103-175)	-33.9 (-39.2--27.4)	131 (99.2-168)	-33.7 (-39.1--27.2)	96 (73.5-123)	-39.3 (-44.2--33.2)	19.9 (14.3-26.5)	-32.1 (-38.3--25.9)	91.9 (70.4-117)	-41.1 (-45.6--35.2)
Zanjan	137 (104-174)	-34.1 (-38.9--29.3)	134 (102-171)	-34.1 (-38.9--29.3)	128 (97-164)	-34 (-38.4--28.9)	93.5 (70.9-119)	-40.7 (-44.6--36.4)	19.7 (14.3-26.3)	-33.6 (-38.6--28.1)	86.4 (65.8-109)	-44.7 (-48.4--40.5)
Iraq	102 (87.3-120)	-0.2 (-6.9-7.4)	101 (86.3-119)	-0.2 (-7-7.4)	96.2 (82-113)	-0.4 (-7.3-6.9)	72.7 (62.3-84.6)	-2.9 (-9.4-4.4)	11.2 (8.2-15)	2.6 (-6.5-12.1)	72.6 (62.2-84.1)	-8.7 (-14.6--1.8)
Jordan	79.4 (60.4-102)	0.3 (-7.1-8.7)	78.5 (59.7-101)	0.3 (-7.1-8.6)	75.5 (57.4-97.9)	0.2 (-7.3-8.4)	59.9 (46-77.8)	-3.6 (-11-4.4)	12 (8.4-16.4)	9.5 (0-19)	58 (44.6-74.9)	-7.8 (-14.8--0.3)
Kuwait	41.7 (32.4-52.4)	-0.1 (-8.7-8)	41.3 (32-51.7)	-0.2 (-8.7-7.9)	40 (31-50.3)	-0.2 (-8.7-8)	30.2 (23.5-37.6)	-8.8 (-16.5--1.1)	7.5 (5.2-10.1)	18.5 (6.7-31.7)	28.4 (22.3-35.4)	-12.6 (-20--5.5)
Lebanon	67.9 (53.6-83.5)	1.8 (-5.2-9.5)	66.8 (52.7-82.2)	1.8 (-5.2-9.6)	64.2 (50.7-79.4)	1.9 (-5-9.9)	46.7 (37.1-57.3)	-2.4 (-8.8-5.1)	6.3 (4.4-8.6)	2.2 (-6.5-12.4)	42.7 (34.3-52.2)	-5.4 (-11.7-1.9)
Libya	230 (178-291)	0.2 (-8.1-9.6)	226 (175-285)	0.2 (-8.2-9.2)	216 (167-273)	-0.1 (-8.3-9.1)	158 (124-198)	-8.2 (-15.9-0)	32.1 (23.1-43.2)	15.4 (5.5-25.3)	147 (115-183)	-15.7 (-22.8--8.4)
Morocco	14.1 (11-17.2)	0.3 (-6.3-7.9)	13.9 (10.8-17)	0.2 (-6.4-7.8)	13.3 (10.3-16.2)	0.1 (-6.3-7.6)	9.9 (7.7-12)	-3.6 (-9.8-3.8)	1.5 (1-2.1)	4.2 (-4-13.1)	9 (7.1-10.9)	-11.9 (-17.7--5.1)
Oman	336 (268-403)	7.5 (-6.2-20.6)	332 (264-398)	7.5 (-6.2-20.6)	319 (254-383)	7.6 (-5.9-21)	231 (182-279)	0.3 (-12.2-12.8)	45.9 (32.8-61.8)	9.6 (-3.9-24.6)	236 (186-284)	-2.2 (-14.3-9.8)
Palestine	78.4 (59.5-101)	-0.6 (-10.1-7.4)	77.3 (58.6-100)	-0.7 (-10-7.4)	74.3 (56.3-96.4)	-0.8 (-9.9-7.4)	60.5 (45.9-77.7)	-2.2 (-11.1-5.3)	11.9 (8.4-16)	12.8 (3.6-23)	60.1 (45.7-77.2)	-6.5 (-14.6-1)
Qatar	85.6 (64.7-109)	0.1 (-7.9-7.8)	84.7 (64-108)	0.1 (-7.9-7.8)	82.2 (62.3-104)	0.1 (-7.8-7.8)	62.5 (47.5-79.1)	2 (-6.3-11.1)	15.2 (10.8-20.7)	-7.5 (-16.5-2.4)	60.4 (46-76)	-4.5 (-11.7-4)
Saudi Arabia	355 (287-430)	-76.1 (-80.3--71.5)	353 (286-427)	-75.8 (-80.1--71.3)	349 (282-423)	-75.2 (-79.6--70.6)	296 (240-357)	-76.5 (-80.6--72.1)	92.5 (69.7-118)	-60.9 (-68.2--51.6)	280 (228-337)	-77.1 (-81.1--72.8)
Sudan	448 (361-551)	-33.6 (-41.7--25.8)	440 (355-540)	-33.5 (-41.6--25.6)	415 (334-512)	-33.8 (-42--26.1)	317 (256-393)	-34.9 (-42.5--26.9)	55 (40-72.9)	-23.6 (-33.2--13)	330 (266-406)	-36.4 (-44.1--28.7)
Syrian Arab Republic	10.7 (8.4-13.3)	1 (-5.4-8.3)	10.6 (8.3-13.1)	1 (-5.3-8.3)	10 (7.8-12.4)	0.4 (-5.7-7.7)	7.6 (6-9.3)	-4 (-9.9-3.1)	1.2 (0.8-1.7)	4 (-4.8-14.4)	6.8 (5.4-8.3)	-17.3 (-22.8--11.2)
Tunisia	83.7 (64.8-106)	-0.6 (-9.3-7.8)	82.6 (64-105)	-0.6 (-9.4-7.7)	79.4 (61.6-101)	-0.6 (-9.1-7.7)	60.6 (47.2-76)	-5.3 (-13.1-3)	11.5 (8.4-15.4)	1.9 (-6.6-10.4)	52.2 (40.6-65.2)	-14.8 (-21.8--7.3)
Türkiye	18.1 (15.4-21.2)	-26.6 (-32.8--19.9)	17.8 (15.2-20.8)	-26.6 (-32.8--19.9)	17.1 (14.5-20)	-26.8 (-33.2--20.2)	13 (11.2-15)	-30.1 (-36--23.7)	2.3 (1.7-2.9)	-22.8 (-29.8--14.9)	11 (9.6-12.8)	-37.6 (-42.9--32.1)
United Arab Emirates	25.2 (20.5-29.4)	-2.8 (-9-4)	25 (20.4-29)	-2.8 (-9-4)	24.2 (19.8-28.2)	-2.8 (-9-4)	15.7 (12.8-18.5)	-15.2 (-21.9--7.5)	4.6 (3.3-6.1)	-3.2 (-11.2-6.3)	15.2 (12.4-17.8)	-20.6 (-26.5--13.8)
Yemen	14.2 (11.5-17)	0.3 (-6.4-7.2)	13.9 (11.3-16.7)	0.2 (-6.4-7.2)	13.1 (10.7-15.8)	0.3 (-6.1-7.4)	9.7 (7.9-11.6)	-2.8 (-8.8-3.8)	1.2 (0.8-1.7)	2.9 (-6.7-13.5)	10.4 (8.5-12.4)	-5.1 (-10.9-0.7)

Location	Birth Incidence rate (per 100,000 live births), 2021	Birth Incidence rate (per 100,000 live births) percent change (2000-2021)	Under-5 rate (per 100,000 population), 2021	Under-5 rate (per 100,000 population) percent change (2000-2021)	5-14 rate (per 100,000 population), 2021	5-14 rate (per 100,000 population) percent change (2000-2021)	15-49 rate (per 100,000 population), 2021	15-49 rate (per 100,000 population) percent change (2000-2021)	50plus rate (per 100,000 population), 2021	50plus rate (per 100,000 population) percent change (2000-2021)	All age rate (per 100,000 population), 2021	All age rate (per 100,000 population) percent change (2000-2021)
South Asia	239 (166-332)	-4.8 (-7.3--1.8)	227 (159-315)	-4.2 (-6.6--1.4)	148 (107-203)	-3.4 (-5.9--0.9)	29.6 (21.6-40)	-8.7 (-11--6.4)	0.7 (0.4-1)	-1.4 (-4.6-1.9)	63.9 (47.5-85.4)	-23.9 (-26.2--21.5)
**South Asia**	239 (166-332)	-4.8 (-7.3--1.8)	227 (159-315)	-4.2 (-6.6--1.4)	148 (107-203)	-3.4 (-5.9--0.9)	29.6 (21.6-40)	-8.7 (-11--6.4)	0.7 (0.4-1)	-1.4 (-4.6-1.9)	63.9 (47.5-85.4)	-23.9 (-26.2--21.5)
Bangladesh	0.7 (0.5-1)	-1.6 (-26.9-10.1)	0.6 (0.4-0.9)	-1.8 (-26.9-8.9)	0.4 (0.3-0.6)	-4.5 (-28.5-8)	0.1 (0-0.1)	-12.7 (-32-5.4)	0 (0-0)	-13.2 (-40.7-22.2)	0.2 (0.1-0.2)	-31.2 (-48.1--22.7)
Bhutan	0.7 (0.5-0.9)	-0.6 (-19.7-6.2)	0.6 (0.4-0.9)	-0.7 (-19.2-5.7)	0.4 (0.3-0.5)	-1.1 (-21-7)	0.1 (0.1-0.1)	-20.2 (-36--6.9)	0 (0-0)	-7.3 (-33.2-25.5)	0.2 (0.1-0.2)	-28.6 (-41.6--22.5)
India	342 (238-475)	-1.4 (-4.1-1.6)	320 (225-444)	-1.2 (-3.8-1.6)	202 (146-276)	0 (-2.5-2.6)	38 (27.7-51.4)	-7.3 (-9.6--5)	0.8 (0.5-1.2)	-0.6 (-3.8-2.7)	83.1 (61.7-111)	-23 (-25.4--20.6)
Nepal	2.3 (1.9-2.9)	-0.1 (-10.9-12)	2.2 (1.8-2.7)	0 (-10.5-12.2)	1.5 (1.2-1.8)	-1 (-12.1-10.1)	0.3 (0.2-0.4)	-6.6 (-17.7-7.1)	0 (0-0)	-10.4 (-33.9-22.6)	0.7 (0.5-0.8)	-23.6 (-31.6--14.8)
Pakistan	0.1 (0-0.1)	-0.4 (-2.4-0)	0.1 (0-0.1)	-0.5 (-2.7-0.3)	0 (0-0.1)	-1.2 (-5-1.5)	0 (0-0)	-3.6 (-12.8-4)	0 (0-0)	3.6 (-15.6-24)	0 (0-0.1)	-13 (-16.5--10.1)
Azad Jammu & Kashmir	0.1 (0-0.1)	-0.2 (0-0)	0.1 (0-0.1)	-0.3 (-1.1-0.8)	0 (0-0.1)	-1 (-6.6-4.6)	0 (0-0)	-13.2 (-24.1--1.8)	0 (0-0)	-2.8 (-29.1-31.9)	0 (0-0)	-22.3 (-26.8--17.9)
Balochistan	0.1 (0-0.1)	-0.1 (0-0)	0.1 (0-0.1)	-0.1 (-1.3-0.9)	0 (0-0.1)	-1.3 (-7.9-3.7)	0 (0-0)	3.5 (-9.9-18.7)	0 (0-0)	5.5 (-24.6-47.9)	0 (0-0.1)	-2.2 (-7.7-1.8)
Gilgit-Baltistan	0.1 (0-0.1)	-0.1 (0-0)	0.1 (0-0.1)	-0.1 (-1.2-0.8)	0 (0-0.1)	-0.3 (-6-4.2)	0 (0-0)	-4.2 (-16.4-8.1)	0 (0-0)	-15.9 (-40.1-12.8)	0 (0-0.1)	-3.4 (-7.4-0.4)
Islamabad Capital Territory	0.1 (0-0.1)	-0.4 (0-0)	0.1 (0-0.1)	-0.4 (-0.7-1.1)	0 (0-0.1)	-0.5 (-3.4-3)	0 (0-0)	-11.7 (-20--3)	0 (0-0)	5.8 (-18.3-36.2)	0 (0-0.1)	-13.7 (-16.8--9.7)
Khyber Pakhtunkhwa	0.1 (0-0.1)	-0.2 (0-0)	0.1 (0-0.1)	-0.6 (-2.2-0.5)	0 (0-0.1)	-1.5 (-7.1-2.8)	0 (0-0)	-1.4 (-13.2-9.2)	0 (0-0)	1.6 (-29.8-38.8)	0 (0-0.1)	-3.1 (-7.4-0.4)
Punjab	0.1 (0-0.1)	-0.3 (0-0)	0.1 (0-0.1)	-0.4 (-1-0.6)	0 (0-0.1)	-1.5 (-6.7-3)	0 (0-0)	-5.6 (-18.7-6)	0 (0-0)	3.7 (-27.3-39.5)	0 (0-0)	-18.3 (-23.2--14.1)
Sindh	0.1 (0-0.1)	-0.2 (0-0)	0.1 (0-0.1)	-0.4 (-1.4-0.8)	0 (0-0.1)	-1.4 (-7.5-4.3)	0 (0-0)	-2.5 (-15.3-12.6)	0 (0-0)	0.7 (-33.6-40)	0 (0-0.1)	-13.7 (-18.9--9)
Southeast Asia, East Asia, and Oceania	2.1 (1.6-2.7)	0.9 (-0.9-2.7)	2 (1.5-2.6)	-0.4 (-2.2-1.6)	1.9 (1.4-2.5)	-5 (-6.6--3.2)	1.1 (0.8-1.4)	-14.9 (-16.9--13)	0.2 (0.2-0.3)	1.8 (-0.4-4.5)	1 (0.7-1.2)	-22.8 (-24.7--20.6)
**East Asia**	3.6 (2.7-4.7)	0.4 (-1.4-2.2)	3.5 (2.6-4.6)	-0.1 (-1.9-1.9)	3.1 (2.3-4.1)	0.9 (-0.9-2.7)	1.6 (1.2-2.1)	-6.3 (-8.4--4.2)	0.3 (0.2-0.4)	1.7 (-0.6-4.4)	1.4 (1.1-1.9)	-20 (-22--17.7)
China	3.6 (2.7-4.7)	0.1 (-1.8-2)	3.5 (2.6-4.6)	-0.5 (-2.4-1.5)	3.1 (2.3-4.1)	0.6 (-1.2-2.5)	1.6 (1.2-2.1)	-6.9 (-9--4.8)	0.3 (0.2-0.4)	-0.4 (-2.7-2.1)	1.4 (1.1-1.8)	-20.4 (-22.4--18.2)
Democratic People's Republic of Korea	3.6 (2.6-4.7)	-0.3 (-9.8-7.9)	3.5 (2.6-4.6)	-0.5 (-9.9-7.8)	3.2 (2.4-4.2)	-0.6 (-10-7.8)	1.6 (1.2-2.1)	-6.8 (-15.8-0.7)	0.3 (0.2-0.4)	-2.3 (-13.8-9.6)	1.5 (1.1-2)	-19.9 (-27.1--13.1)
Taiwan (Province of China)	3.8 (3-4.7)	25.4 (10.7-44)	3.8 (3-4.7)	25.4 (10.4-44)	3.5 (2.8-4.4)	25.8 (10.8-44.1)	2.1 (1.7-2.6)	28.5 (11.9-48.2)	0.7 (0.5-0.9)	101 (69.6-144)	1.8 (1.4-2.1)	6.2 (-8.1-23.5)
**Oceania**	0 (0-0.1)	0.3 (0-7.3)	0 (0-0.1)	0.3 (-0.6-8)	0 (0-0.1)	-0.1 (-2.9-9.5)	0 (0-0)	-5.3 (-12.1-4)	0 (0-0)	2.2 (-12.6-23.1)	0 (0-0)	-6.9 (-10.3-0.6)
American Samoa	0 (0-0.1)	0 (0-0.4)	0 (0-0.1)	0 (-0.2-0.5)	0 (0-0.1)	-0.5 (-1.7-0.5)	0 (0-0)	3.9 (-0.4-7.9)	0 (0-0)	1.3 (-21.4-17.7)	0 (0-0)	-13.7 (-15.9--12.1)
Cook Islands	0 (0-0.1)	-2.9 (-46.8-0.1)	0 (0-0.1)	-3 (-46.7-0.2)	0 (0-0.1)	-3.5 (-47.3-0.5)	0 (0-0)	-5.6 (-56.2-1.6)	0 (0-0)	-9.4 (-54.4-11.1)	0 (0-0)	-22.8 (-61.1--18.2)
Fiji	0 (0-0.1)	0 (0-0)	0 (0-0.1)	-0.1 (-0.5-0.3)	0 (0-0.1)	0.1 (-1.7-1.7)	0 (0-0)	-5.4 (-10.2--0.6)	0 (0-0)	-10.8 (-24.2-5.4)	0 (0-0)	-11 (-13.4--8.7)
Guam	0 (0-0.1)	0 (0-0.2)	0 (0-0.1)	0 (-0.2-0.3)	0 (0-0.1)	-0.1 (-1-0.8)	0 (0-0)	2.1 (-1.1-7.3)	0 (0-0)	-13.5 (-22.4-1.7)	0 (0-0)	-11.9 (-13.5--9.8)
Kiribati	0 (0-0.1)	3.4 (0-1250)	0 (0-0.1)	3.4 (-0.4-1250)	0 (0-0.1)	3.4 (-1.8-1200)	0 (0-0)	0.1 (-9.5-956)	0 (0-0)	1.3 (-23.7-273)	0 (0-0)	-4.9 (-10.8-1040)
Marshall Islands	0 (0-0.1)	1.3 (0-0)	0 (0-0.1)	1.3 (-0.3-0.6)	0 (0-0.1)	1.4 (-1.3-2.4)	0 (0-0)	-11.3 (-17.5--6.7)	0 (0-0)	-5.3 (-23.6-14.8)	0 (0-0)	-16.6 (-20--15)
Micronesia (Federated States of)	0 (0-0.1)	0 (-0.1-0)	0 (0-0.1)	0 (-0.4-0.3)	0 (0-0.1)	-0.3 (-2-1.2)	0 (0-0)	-5.1 (-9.9--0.2)	0 (0-0)	3.2 (-13.4-25)	0 (0-0)	-17.5 (-20--15.3)
Nauru	0 (0-0.1)	-2.3 (-92.6-0.1)	0 (0-0.1)	-2.4 (-92.6-0.3)	0 (0-0.1)	-2.5 (-92.4-2)	0 (0-0)	-0.3 (-91.3-8.5)	0 (0-0)	-2.5 (-79.5-24.9)	0 (0-0)	-7 (-92.5--2.1)
Niue	0 (0-0.1)	0.1 (0-0.1)	0 (0-0.1)	0.2 (-0.3-2)	0 (0-0.1)	0 (-1.6-1.8)	0 (0-0)	-0.9 (-5.9-3.5)	0 (0-0)	7.8 (-8.1-33.4)	0 (0-0)	-11.9 (-14.4--9.7)

Location	Birth Incidence rate (per 100,000 live births), 2021	Birth Incidence rate (per 100,000 live births) percent change (2000-2021)	Under-5 rate (per 100,000 population), 2021	Under-5 rate (per 100,000 population) percent change (2000-2021)	5-14 rate (per 100,000 population), 2021	5-14 rate (per 100,000 population) percent change (2000-2021)	15-49 rate (per 100,000 population), 2021	15-49 rate (per 100,000 population) percent change (2000-2021)	50plus rate (per 100,000 population), 2021	50plus rate (per 100,000 population) percent change (2000-2021)	All age rate (per 100,000 population), 2021	All age rate (per 100,000 population) percent change (2000-2021)
Northern Mariana Islands	0 (0-0.1)	-0.2 (-0.1-0)	0 (0-0.1)	-0.3 (-0.4-0.5)	0 (0-0.1)	-0.9 (-1.9-0.1)	0 (0-0)	7.6 (0.5-43.1)	0 (0-0)	-7.8 (-43.9-10.1)	0 (0-0)	-30.3 (-33--20.7)
Palau	0 (0-0.1)	1.3 (0-0.1)	0 (0-0.1)	1.3 (-0.3-0.7)	0 (0-0.1)	1 (-2-1.4)	0 (0-0)	-6.8 (-12.6--0.6)	0 (0-0)	3.3 (-16.9-24.8)	0 (0-0)	-25.4 (-28.6--23.1)
Papua New Guinea	0 (0-0.1)	0 (0-0.1)	0 (0-0.1)	-0.1 (-0.6-0.5)	0 (0-0.1)	0.1 (-3.1-2.8)	0 (0-0)	-4.4 (-13-3.4)	0 (0-0)	9.3 (-12.9-35)	0 (0-0)	-6.5 (-10.7--3.1)
Samoa	0 (0-0.1)	0 (0-0.1)	0 (0-0.1)	0.1 (-0.2-0.5)	0 (0-0.1)	-0.5 (-2.5-0.7)	0 (0-0)	0.5 (-5.1-5.1)	0 (0-0)	12.4 (-3.3-32.6)	0 (0-0)	-5.2 (-8.2--3)
Solomon Islands	0 (0-0.1)	0 (0-0)	0 (0-0.1)	-0.1 (-0.5-0.2)	0 (0-0.1)	-0.1 (-1.7-1.3)	0 (0-0)	-8 (-13.6--3)	0 (0-0)	18.8 (-6.9-46.4)	0 (0-0)	-7.7 (-10.3--5.6)
Tokelau	0 (0-0.1)	-0.3 (0-0.1)	0 (0-0.1)	0.2 (-0.3-4.7)	0 (0-0.1)	0.2 (-0.9-3)	0 (0-0)	-5.3 (-9--1.8)	0 (0-0)	6.1 (-10.5-22.7)	0 (0-0)	-12.8 (-14.5--10.3)
Tonga	0 (0-0.1)	0 (0-0.1)	0 (0-0.1)	0 (-0.3-0.2)	0 (0-0.1)	-0.1 (-1.8-1.2)	0 (0-0)	-4.5 (-9.9--1)	0 (0-0)	5.4 (-8.8-21.7)	0 (0-0)	-7.7 (-10.4--6.1)
Tuvalu	0 (0-0.1)	0 (0-0.1)	0 (0-0.1)	0 (-0.5-0.5)	0 (0-0.1)	-0.6 (-2.9-1.8)	0 (0-0)	5.3 (-1.8-15.6)	0 (0-0)	-5.5 (-23.3-29.8)	0 (0-0)	-7.5 (-11--3.8)
Vanuatu	0 (0-0.1)	0 (-0.2-0)	0 (0-0.1)	-0.1 (-0.5-0.2)	0 (0-0.1)	-0.1 (-1.6-1.4)	0 (0-0)	-3.4 (-9.2-1.6)	0 (0-0)	-6.3 (-22.7-17.2)	0 (0-0)	-8 (-10.5--5.9)
**Southeast Asia**	0 (0-0.1)	0.9 (-0.4-12.6)	0 (0-0.1)	1 (-0.1-13.2)	0 (0-0.1)	1.6 (-0.7-17.1)	0 (0-0)	-6.6 (-9.2-3.2)	0 (0-0)	3 (-4-8.7)	0 (0-0)	-16.7 (-19--7.4)
Cambodia	0 (0-0.1)	2 (0-88.4)	0 (0-0.1)	2 (-0.5-88.5)	0 (0-0.1)	2.3 (-2.3-88.2)	0 (0-0)	-7.1 (-16.3-85.5)	0 (0-0)	-2 (-25.1-222)	0 (0-0)	-17.3 (-22.5-56)
Indonesia	0 (0-0.1)	0.1 (0-1.5)	0 (0-0.1)	0.1 (-0.2-1.5)	0 (0-0.1)	-0.3 (-1-1.1)	0 (0-0)	-8.9 (-11.2--6.9)	0 (0-0)	2.4 (-3.5-8.8)	0 (0-0)	-17.2 (-18.7--15.7)
Aceh	0 (0-0.1)	0 (0-0)	0 (0-0.1)	-0.1 (-0.4-0.2)	0 (0-0.1)	0 (-1.6-2.1)	0 (0-0)	-9.2 (-13.8--4.1)	0 (0-0)	4 (-11-26)	0 (0-0)	-14.7 (-17.3--11.6)
Bali	0 (0-0.1)	0 (0-0)	0 (0-0.1)	0 (-0.3-0.3)	0 (0-0.1)	-0.5 (-2.1-1.3)	0 (0-0)	-8.3 (-13.3--2.8)	0 (0-0)	1.7 (-14.5-18.2)	0 (0-0)	-19.1 (-21.9--16)
Bangka-Belitung Islands	0 (0-0.1)	0 (0-0)	0 (0-0.1)	0 (-0.5-0.4)	0 (0-0.1)	0.1 (-2-2.5)	0 (0-0)	-14.9 (-20.9--8.3)	0 (0-0)	2.8 (-18.3-28.1)	0 (0-0)	-20.1 (-23.3--17.2)
Banten	0 (0-0.1)	0 (0-0)	0 (0-0.1)	0 (-0.6-0.4)	0 (0-0.1)	-0.2 (-2.8-2)	0 (0-0)	-9.5 (-16.1--3.9)	0 (0-0)	7.9 (-11.2-27.7)	0 (0-0)	-15.7 (-19.3--12.9)
Bengkulu	0 (0-0.1)	0.8 (0-0)	0 (0-0.1)	0.8 (-0.5-0.6)	0 (0-0.1)	0.7 (-2.2-4.2)	0 (0-0)	-9.8 (-16--0.9)	0 (0-0)	3.7 (-17.2-28.4)	0 (0-0)	-18.3 (-21.8--13.8)
Gorontalo	0 (0-0.1)	0 (0-0)	0 (0-0.1)	0 (-0.5-0.5)	0 (0-0.1)	-1 (-3.3-1.5)	0 (0-0)	1.8 (-4.7-9.1)	0 (0-0)	15.4 (-5.7-39.8)	0 (0-0)	-11.5 (-15.2--7.4)
Jakarta	0 (0-0.1)	0.3 (0-0)	0 (0-0.1)	0.2 (-0.5-0.2)	0 (0-0.1)	0.3 (-1.7-1.9)	0 (0-0)	-12.9 (-18--8.5)	0 (0-0)	-3.7 (-15.1-11.4)	0 (0-0)	-18.5 (-21.6--16.1)
Jambi	0 (0-0.1)	0 (0-0)	0 (0-0.1)	0 (-0.4-0.3)	0 (0-0.1)	-0.3 (-2.1-1.7)	0 (0-0)	-10.7 (-15.5--5.4)	0 (0-0)	-2.6 (-17.8-19.2)	0 (0-0)	-18.6 (-21.1--15.9)
West Java	0 (0-0.1)	0 (0-0)	0 (0-0.1)	0 (-0.5-0.3)	0 (0-0.1)	-0.3 (-2.2-1.8)	0 (0-0)	-8.7 (-14.2--4.2)	0 (0-0)	5.9 (-12.3-25.3)	0 (0-0)	-17.5 (-20.5--14.9)
Central Java	0 (0-0.1)	0 (0-0)	0 (0-0.1)	0 (-0.3-0.3)	0 (0-0.1)	-0.2 (-2.1-1.4)	0 (0-0)	-7.7 (-12--3.1)	0 (0-0)	4.2 (-8.2-21.7)	0 (0-0)	-19.9 (-22.5--17.3)
East Java	0 (0-0.1)	0 (0-0)	0 (0-0.1)	0 (-0.3-0.3)	0 (0-0.1)	-0.3 (-1.8-1.5)	0 (0-0)	-6.9 (-11--2.5)	0 (0-0)	2 (-13.3-24)	0 (0-0)	-19.2 (-21.6--16.8)
West Kalimantan	0 (0-0.1)	0 (0-0)	0 (0-0.1)	0 (-0.4-0.4)	0 (0-0.1)	-0.1 (-2.6-2.5)	0 (0-0)	-9.8 (-16.3--3.5)	0 (0-0)	-4.8 (-20.7-16.7)	0 (0-0)	-17.2 (-20.6--14.3)
South Kalimantan	0 (0-0.1)	-0.1 (0-0)	0 (0-0.1)	-0.1 (-0.6-0.3)	0 (0-0.1)	-0.4 (-2.9-1.9)	0 (0-0)	-10.5 (-17.3--4.2)	0 (0-0)	2.7 (-17.1-28.1)	0 (0-0)	-19.9 (-23.6--16.9)
Central Kalimantan	0 (0-0.1)	0 (0-0)	0 (0-0.1)	-0.1 (-0.4-0.4)	0 (0-0.1)	-0.3 (-2.2-1.8)	0 (0-0)	-11.9 (-17.6--6.2)	0 (0-0)	3.3 (-15.2-23.5)	0 (0-0)	-18.6 (-21.8--16)
East Kalimantan	0 (0-0.1)	0 (0-0)	0 (0-0.1)	-0.1 (-0.5-0.2)	0 (0-0.1)	-0.1 (-2.3-1.5)	0 (0-0)	-10.2 (-15.5--4.9)	0 (0-0)	-1.7 (-18.7-17.4)	0 (0-0)	-15 (-17.9--12.6)
North Kalimantan	0 (0-0.1)	1.9 (0-0)	0 (0-0.1)	1.8 (-0.4-0.5)	0 (0-0.1)	1.5 (-2.1-2.5)	0 (0-0)	-6.1 (-13.1--1.4)	0 (0-0)	2.6 (-13.7-20.6)	0 (0-0)	-14.4 (-19.1--12.3)
Riau Islands	0 (0-0.1)	0 (0-0)	0 (0-0.1)	-0.1 (-0.5-0.3)	0 (0-0.1)	0.6 (-1.7-3)	0 (0-0)	-20.7 (-27.2--14.5)	0 (0-0)	6.4 (-11.5-26.7)	0 (0-0)	-14.9 (-18.1--11.3)

Location	Birth Incidence rate (per 100,000 live births), 2021	Birth Incidence rate (per 100,000 live births) percent change (2000-2021)	Under-5 rate (per 100,000 population), 2021	Under-5 rate (per 100,000 population) percent change (2000-2021)	5-14 rate (per 100,000 population), 2021	5-14 rate (per 100,000 population) percent change (2000-2021)	15-49 rate (per 100,000 population), 2021	15-49 rate (per 100,000 population) percent change (2000-2021)	50plus rate (per 100,000 population), 2021	50plus rate (per 100,000 population) percent change (2000-2021)	All age rate (per 100,000 population), 2021	All age rate (per 100,000 population) percent change (2000-2021)
Lampung	0 (0-0.1)	0 (0-0)	0 (0-0.1)	0 (-0.4-0.3)	0 (0-0.1)	-0.4 (-2.1-1.3)	0 (0-0)	-10.8 (-16.4--6.3)	0 (0-0)	0.7 (-15.5-20)	0 (0-0)	-20.5 (-23.4--18)
Maluku	0 (0-0.1)	-0.8 (0-0)	0 (0-0.1)	-0.8 (-0.8-0.4)	0 (0-0.1)	-1.2 (-4-2.3)	0 (0-0)	-4.1 (-13.6-3.3)	0 (0-0)	5.4 (-19.4-31)	0 (0-0)	-12.4 (-17--8.7)
North Maluku	0 (0-0.1)	-0.7 (0-0)	0 (0-0.1)	-0.8 (-0.9-0.4)	0 (0-0.1)	-1.2 (-4.3-1.9)	0 (0-0)	-10.2 (-18--3.1)	0 (0-0)	0.1 (-24.1-26.7)	0 (0-0)	-17.8 (-21.4--14.3)
West Nusa Tenggara	0 (0-0.1)	0.8 (0-0)	0 (0-0.1)	0.7 (-0.5-0.8)	0 (0-0.1)	0.7 (-2.6-3.4)	0 (0-0)	-9.4 (-17.2--1)	0 (0-0)	0.5 (-21.3-25.8)	0 (0-0)	-16.7 (-20.8--12.8)
East Nusa Tenggara	0 (0-0.1)	-0.1 (0-0)	0 (0-0.1)	-0.1 (-0.6-0.4)	0 (0-0.1)	-0.8 (-3.2-1.4)	0 (0-0)	-1.8 (-9.5-4.8)	0 (0-0)	-0.1 (-21.4-23.8)	0 (0-0)	-11.6 (-15--8.9)
Papua	0 (0-0.1)	0 (0-0)	0 (0-0.1)	0 (-0.5-0.6)	0 (0-0.1)	-0.3 (-3.2-2.5)	0 (0-0)	-7.3 (-15-0.2)	0 (0-0)	3.9 (-17.8-31.9)	0 (0-0)	-13.1 (-16.9--9.7)
West Papua	0 (0-0.1)	0 (0-0)	0 (0-0.1)	0 (-0.5-0.4)	0 (0-0.1)	0.1 (-2.2-2.8)	0 (0-0)	-9.5 (-15.8--2.6)	0 (0-0)	-18 (-33.9-4.8)	0 (0-0)	-15.8 (-19--12.5)
Riau	0 (0-0.1)	0 (0-0)	0 (0-0.1)	0 (-0.4-0.2)	0 (0-0.1)	-0.1 (-2.2-2)	0 (0-0)	-9.7 (-15.2--4.4)	0 (0-0)	6.5 (-10.4-28.5)	0 (0-0)	-13.8 (-16.7--11)
West Sulawesi	0 (0-0.1)	0 (0-0)	0 (0-0.1)	-0.1 (-0.6-0.4)	0 (0-0.1)	-0.8 (-3.4-1.8)	0 (0-0)	-5.1 (-12.5-2)	0 (0-0)	0.5 (-19.8-30)	0 (0-0)	-14.8 (-18.5--11.2)
South Sulawesi	0 (0-0.1)	0 (0-0)	0 (0-0.1)	0 (-0.4-0.4)	0 (0-0.1)	-0.5 (-2.6-1.9)	0 (0-0)	-7 (-13.1--1.1)	0 (0-0)	-0.5 (-19.5-20.9)	0 (0-0)	-17.3 (-20.7--14)
Central Sulawesi	0 (0-0.1)	0.2 (0-0)	0 (0-0.1)	0.1 (-0.5-0.4)	0 (0-0.1)	-0.6 (-3.2-1.7)	0 (0-0)	-7.7 (-13.6--2)	0 (0-0)	-3.7 (-21.2-16.3)	0 (0-0)	-17.2 (-20.3--14.3)
Southeast Sulawesi	0 (0-0.1)	0 (0-0)	0 (0-0.1)	-0.1 (-0.5-0.4)	0 (0-0.1)	-0.3 (-2.9-1.8)	0 (0-0)	-8.7 (-14.7--3)	0 (0-0)	-1.1 (-20.3-24.8)	0 (0-0)	-15.6 (-18.7--13)
North Sulawesi	0 (0-0.1)	0 (0-0)	0 (0-0.1)	0 (-0.4-0.3)	0 (0-0.1)	-0.7 (-2.6-1.2)	0 (0-0)	-7.7 (-12.9--1.7)	0 (0-0)	-3.7 (-20.7-16.1)	0 (0-0)	-21.5 (-24.4--18.7)
West Sumatra	0 (0-0.1)	0 (0-0)	0 (0-0.1)	0 (-0.4-0.4)	0 (0-0.1)	-0.4 (-2.4-1.9)	0 (0-0)	-5.6 (-11.5-0.4)	0 (0-0)	3.8 (-14.6-29.4)	0 (0-0)	-16.3 (-19.2--13.1)
South Sumatra	0 (0-0.1)	0 (0-0)	0 (0-0.1)	0 (-0.4-0.3)	0 (0-0.1)	0 (-2-2.1)	0 (0-0)	-10.8 (-15.8--4.9)	0 (0-0)	-0.8 (-14.9-15.8)	0 (0-0)	-18.6 (-21.4--15.6)
North Sumatra	0 (0-0.1)	0 (0-0)	0 (0-0.1)	0 (-0.4-0.3)	0 (0-0.1)	-0.3 (-2.4-1.8)	0 (0-0)	-8.2 (-14.6--1.8)	0 (0-0)	2.7 (-15.3-27.6)	0 (0-0)	-16.8 (-19.7--13.8)
Yogyakarta	0 (0-0.1)	1.1 (0-0)	0 (0-0.1)	1.1 (-0.2-0.3)	0 (0-0.1)	1.1 (-1.2-1.6)	0 (0-0)	-7.4 (-12.8--2.5)	0 (0-0)	13.8 (-2.4-38.3)	0 (0-0)	-17.3 (-20.7--15.2)
Lao People's Democratic Republic	0 (0-0.1)	1.1 (0-0)	0 (0-0.1)	1.1 (-0.5-0.8)	0 (0-0.1)	0.9 (-2.8-4.1)	0 (0-0)	-7 (-15.5-1.9)	0 (0-0)	7.4 (-16.1-45.6)	0 (0-0)	-16.3 (-21--13)
Malaysia	0 (0-0.1)	0 (0-0.2)	0 (0-0.1)	0.1 (-0.1-0.2)	0 (0-0.1)	-0.1 (-1-0.8)	0 (0-0)	-3.3 (-7-1)	0 (0-0)	-13.1 (-22.3-0.5)	0 (0-0)	-15.3 (-17.5--13)
Maldives	0 (0-0.1)	3.3 (0-93.6)	0 (0-0.1)	3.3 (-0.2-92.9)	0 (0-0.1)	3.9 (-0.7-92.9)	0 (0-0)	-15.4 (-23.8-53.5)	0 (0-0)	24.9 (6.7-128)	0 (0-0)	-22.9 (-29.8-41.7)
Mauritius	0 (0-0.1)	1.1 (0-0.7)	0 (0-0.1)	1.2 (-0.1-2)	0 (0-0.1)	0.8 (-1.4-5.3)	0 (0-0)	-1.1 (-5.6-10)	0 (0-0)	-9.6 (-23-9.7)	0 (0-0)	-23.9 (-26.8--18.1)
Myanmar	0 (0-0.1)	1.5 (0-88)	0 (0-0.1)	1.5 (-0.6-89.5)	0 (0-0.1)	1.6 (-3.1-92.3)	0 (0-0)	-4.1 (-13.4-92.8)	0 (0-0)	10 (-16.2-231)	0 (0-0)	-14.7 (-19.8-65.1)
Philippines	0 (0-0.1)	0 (0-0.5)	0 (0-0.1)	0 (-0.1-0.5)	0 (0-0.1)	0.1 (-0.5-0.7)	0 (0-0)	-3.6 (-5.1--2.3)	0 (0-0)	-3.2 (-6.8--0.1)	0 (0-0)	-11.4 (-12.5--10.2)
Abra	0 (0-0.1)	0.7 (0-0)	0 (0-0.1)	0.7 (-0.5-0.3)	0 (0-0.1)	0.8 (-2-1.8)	0 (0-0)	-3.7 (-9.9-0.8)	0 (0-0)	-3.2 (-23-11.2)	0 (0-0)	-12 (-15.5--10.3)
Agusan Del Norte	0 (0-0.1)	0 (0-0)	0 (0-0.1)	0 (-0.5-0.4)	0 (0-0.1)	0 (-2.7-2.3)	0 (0-0)	-3.2 (-9.8-3.4)	0 (0-0)	-4.7 (-23.5-16.6)	0 (0-0)	-11.3 (-14.5--8.5)
Agusan Del Sur	0 (0-0.1)	0 (0-0)	0 (0-0.1)	0 (-0.4-0.4)	0 (0-0.1)	0.2 (-2.1-2.5)	0 (0-0)	-3.7 (-9.7-2.6)	0 (0-0)	-4 (-20.9-16.5)	0 (0-0)	-11.8 (-14.4--9.1)
Aklan	0 (0-0.1)	0 (0-0)	0 (0-0.1)	0 (-0.3-0.3)	0 (0-0.1)	0.4 (-1.1-1.8)	0 (0-0)	-4.6 (-9--0.6)	0 (0-0)	-3.2 (-18.2-14.4)	0 (0-0)	-10.3 (-12.5--8.3)
Albay	0 (0-0.1)	0 (0-0)	0 (0-0.1)	0 (-0.3-0.4)	0 (0-0.1)	-0.1 (-1.8-1.9)	0 (0-0)	-2.8 (-8.1-2.5)	0 (0-0)	-6.1 (-22.4-13.6)	0 (0-0)	-11.7 (-14.4--9.3)
Antique	0 (0-0.1)	0 (0-0)	0 (0-0.1)	0 (-0.5-0.3)	0 (0-0.1)	0.6 (-1.5-2.4)	0 (0-0)	-2.3 (-8.3-3.4)	0 (0-0)	-1.6 (-18.3-16.8)	0 (0-0)	-9 (-11.8--6.5)

Location	Birth Incidence rate (per 100,000 live births), 2021	Birth Incidence rate (per 100,000 live births) percent change (2000-2021)	Under-5 rate (per 100,000 population), 2021	Under-5 rate (per 100,000 population) percent change (2000-2021)	5-14 rate (per 100,000 population), 2021	5-14 rate (per 100,000 population) percent change (2000-2021)	15-49 rate (per 100,000 population), 2021	15-49 rate (per 100,000 population) percent change (2000-2021)	50plus rate (per 100,000 population), 2021	50plus rate (per 100,000 population) percent change (2000-2021)	All age rate (per 100,000 population), 2021	All age rate (per 100,000 population) percent change (2000-2021)
Apayao	0 (0-0.1)	-0.2 (0-0)	0 (0-0.1)	-0.3 (-0.6-0.3)	0 (0-0.1)	0 (-2.3-2.9)	0 (0-0)	-5.7 (-11.9-1.1)	0 (0-0)	-1.8 (-18-17.9)	0 (0-0)	-14.9 (-17.9--11.6)
Aurora	0 (0-0.1)	-0.7 (0-0)	0 (0-0.1)	-0.7 (-0.6-0.4)	0 (0-0.1)	-0.5 (-2.7-2.5)	0 (0-0)	-4.6 (-11.3-2.2)	0 (0-0)	-6.8 (-21.9-11.1)	0 (0-0)	-13.7 (-16.5--10.4)
Basilan	0 (0-0.1)	0 (0-0)	0 (0-0.1)	0 (-0.3-0.3)	0 (0-0.1)	-0.2 (-1.9-1.8)	0 (0-0)	-2 (-6.6-3.4)	0 (0-0)	0.9 (-18-18.4)	0 (0-0.1)	-7.2 (-9.4--4.7)
Bataan	0 (0-0.1)	0 (0-0)	0 (0-0.1)	0 (-0.3-0.3)	0 (0-0.1)	0 (-1.8-1.6)	0 (0-0)	-4.5 (-10.3--0.6)	0 (0-0)	-9.4 (-23.7-3.6)	0 (0-0)	-13.7 (-16.7--11.7)
Batanes	0 (0-0.1)	0.3 (0-0)	0 (0-0.1)	0.2 (-0.4-0.3)	0 (0-0.1)	0.6 (-1.4-2.2)	0 (0-0)	-10.3 (-15--6.2)	0 (0-0)	5.8 (-8.6-22)	0 (0-0)	-16 (-18.7--13.9)
Batangas	0 (0-0.1)	0 (0-0)	0 (0-0.1)	0 (-0.2-0.3)	0 (0-0.1)	0.1 (-1.1-1.7)	0 (0-0)	-4.9 (-9.3--0.8)	0 (0-0)	-2 (-14.8-13.4)	0 (0-0)	-11.4 (-13.6--9.1)
Benguet	0 (0-0.1)	0.3 (0-0)	0 (0-0.1)	0.3 (-0.3-0.3)	0 (0-0.1)	0.4 (-1.3-1.6)	0 (0-0)	-1.7 (-6.3-1.9)	0 (0-0)	-7.2 (-19.2-7.7)	0 (0-0)	-10.4 (-12.9--8.7)
Biliran	0 (0-0.1)	0 (0-0)	0 (0-0.1)	0 (-0.2-0.3)	0 (0-0.1)	0.3 (-0.9-1.7)	0 (0-0)	-2.7 (-6.7-1.8)	0 (0-0)	-8.2 (-23.5-9.3)	0 (0-0)	-10.5 (-12.2--8.8)
Bohol	0 (0-0.1)	1 (0-0)	0 (0-0.1)	0.9 (-0.4-0.4)	0 (0-0.1)	1 (-2-2.6)	0 (0-0)	-3.7 (-9.2-3)	0 (0-0)	3.2 (-15.8-23.1)	0 (0-0)	-12.4 (-15.8--9.8)
Bukidnon	0 (0-0.1)	-0.8 (0-0)	0 (0-0.1)	-0.9 (-0.8-0.3)	0 (0-0.1)	-0.7 (-3.2-2.6)	0 (0-0)	-5.4 (-12.8-1.1)	0 (0-0)	-6.7 (-23.3-12.7)	0 (0-0)	-13.7 (-17--10.4)
Bulacan	0 (0-0.1)	0 (0-0)	0 (0-0.1)	0 (-0.3-0.4)	0 (0-0.1)	-0.3 (-2.3-1.6)	0 (0-0)	-3.4 (-8.9-1.8)	0 (0-0)	-6.5 (-21.3-8.3)	0 (0-0)	-13.1 (-16.2--10.8)
Cagayan	0 (0-0.1)	0 (0-0)	0 (0-0.1)	0 (-0.4-0.3)	0 (0-0.1)	-0.2 (-2.5-1.9)	0 (0-0)	-5.3 (-10.7-0.3)	0 (0-0)	-3.2 (-21-14.1)	0 (0-0)	-14.4 (-17.2--11.6)
Camarines Norte	0 (0-0.1)	0 (0-0)	0 (0-0.1)	0 (-0.3-0.4)	0 (0-0.1)	0.1 (-1.8-1.8)	0 (0-0)	-1.9 (-7.2-3.5)	0 (0-0)	-4.8 (-21.9-12.4)	0 (0-0)	-10.5 (-12.9--8.4)
Camarines Sur	0 (0-0.1)	0.1 (0-0)	0 (0-0.1)	0.1 (-0.4-0.5)	0 (0-0.1)	0.2 (-2.1-2.6)	0 (0-0)	-0.9 (-6.8-5.5)	0 (0-0)	-2.6 (-22.6-18.6)	0 (0-0)	-8.8 (-12--5.9)
Camiguin	0 (0-0.1)	0 (0-0)	0 (0-0.1)	0 (-0.3-0.4)	0 (0-0.1)	0.2 (-1.4-1.8)	0 (0-0)	-3.8 (-8.4-0.4)	0 (0-0)	-5 (-17.6-9.7)	0 (0-0)	-10.4 (-12.7--8.3)
Capiz	0 (0-0.1)	0 (0-0)	0 (0-0.1)	0 (-0.3-0.4)	0 (0-0.1)	0.6 (-1-2.7)	0 (0-0)	-3.6 (-8.3-1.8)	0 (0-0)	-9.1 (-22.9-8)	0 (0-0)	-13.3 (-15.8--10.8)
Catanduanes	0 (0-0.1)	0 (0-0)	0 (0-0.1)	-0.1 (-0.5-0.4)	0 (0-0.1)	0.2 (-2-2.9)	0 (0-0)	-3.9 (-10.2-3)	0 (0-0)	1.9 (-16.6-25.1)	0 (0-0)	-10.3 (-13.2--6.9)
Cavite	0 (0-0.1)	0 (0-0)	0 (0-0.1)	0 (-0.2-0.3)	0 (0-0.1)	-0.2 (-1.8-1.1)	0 (0-0)	-3.8 (-8.3-0.5)	0 (0-0)	-5.7 (-20.1-9.8)	0 (0-0)	-12.5 (-14.9--10.1)
Cebu	0 (0-0.1)	0 (0-0)	0 (0-0.1)	0 (-0.3-0.4)	0 (0-0.1)	0.1 (-1.9-2)	0 (0-0)	-3.8 (-9.5-1.4)	0 (0-0)	-3.9 (-21.3-16.7)	0 (0-0)	-8.3 (-11.1--5.8)
Cotabato (North Cotabato)	0 (0-0.1)	0 (0-0)	0 (0-0.1)	0 (-0.4-0.4)	0 (0-0.1)	0.2 (-1.8-2.3)	0 (0-0)	-4.3 (-8.8-1.2)	0 (0-0)	-2.5 (-18-13.8)	0 (0-0)	-10.2 (-12.5--7.6)
Davao de Oro	0 (0-0.1)	0 (0-0)	0 (0-0.1)	-0.1 (-0.4-0.3)	0 (0-0.1)	0 (-1.8-1.6)	0 (0-0)	-3.8 (-8.3-0.1)	0 (0-0)	-3.5 (-16.1-13.1)	0 (0-0)	-12.2 (-14.4--10.3)
Davao Del Norte	0 (0-0.1)	-0.8 (0-0)	0 (0-0.1)	-0.9 (-0.5-0.3)	0 (0-0.1)	-0.6 (-1.8-1.8)	0 (0-0)	-5.9 (-11.5--0.8)	0 (0-0)	-8.2 (-23.3-10.8)	0 (0-0)	-14.6 (-17--11.8)
Davao Del Sur	0 (0-0.1)	0 (0-0)	0 (0-0.1)	0 (-0.4-0.4)	0 (0-0.1)	0.2 (-2-2.2)	0 (0-0)	-5.2 (-11.2--0.3)	0 (0-0)	-13.2 (-27.5-4.2)	0 (0-0)	-13.1 (-16--10.6)
Davao Occidental	0 (0-0.1)	0 (0-0)	0 (0-0.1)	-0.1 (-0.5-0.2)	0 (0-0.1)	-1.1 (-3-0.4)	0 (0-0)	2.6 (-3.2-8.1)	0 (0-0)	16 (-5.2-43.7)	0 (0-0)	-2.8 (-5.4--0.7)
Davao Oriental	0 (0-0.1)	0 (0-0)	0 (0-0.1)	-0.1 (-0.4-0.3)	0 (0-0.1)	-0.1 (-2.2-2)	0 (0-0)	-3.5 (-9.1-1.9)	0 (0-0)	0.5 (-13.8-19.2)	0 (0-0)	-13.7 (-16.3--11.1)
Dinagat Islands	0 (0-0.1)	1 (0-0)	0 (0-0.1)	0.9 (-0.5-0.3)	0 (0-0.1)	0.8 (-2.1-1.9)	0 (0-0)	-2.2 (-8.7-2.8)	0 (0-0)	-12.8 (-29.2-7.6)	0 (0-0)	-12.9 (-16.5--11.4)
Eastern Samar	0 (0-0.1)	0 (0-0)	0 (0-0.1)	0 (-0.5-0.4)	0 (0-0.1)	0 (-2.6-2.2)	0 (0-0)	-1.3 (-8.6-4.8)	0 (0-0)	3.1 (-15-25.1)	0 (0-0)	-10 (-13.5--7.2)
Guimaras	0 (0-0.1)	0 (0-0)	0 (0-0.1)	0 (-0.4-0.3)	0 (0-0.1)	0.4 (-0.9-1.8)	0 (0-0)	-7.5 (-11.2--3.2)	0 (0-0)	-4.3 (-17.7-10.5)	0 (0-0)	-14.1 (-16--12)
Ifugao	0 (0-0.1)	1 (0-0)	0 (0-0.1)	1 (-0.4-0.4)	0 (0-0.1)	1.2 (-1.6-2)	0 (0-0)	-5.6 (-12--1.4)	0 (0-0)	0.7 (-16.9-17.5)	0 (0-0)	-10.6 (-14.2--8.8)

Location	Birth Incidence rate (per 100,000 live births), 2021	Birth Incidence rate (per 100,000 live births) percent change (2000-2021)	Under-5 rate (per 100,000 population), 2021	Under-5 rate (per 100,000 population) percent change (2000-2021)	5-14 rate (per 100,000 population), 2021	5-14 rate (per 100,000 population) percent change (2000-2021)	15-49 rate (per 100,000 population), 2021	15-49 rate (per 100,000 population) percent change (2000-2021)	50plus rate (per 100,000 population), 2021	50plus rate (per 100,000 population) percent change (2000-2021)	All age rate (per 100,000 population), 2021	All age rate (per 100,000 population) percent change (2000-2021)
Ilocos Norte	0 (0-0.1)	0 (0-0)	0 (0-0.1)	0 (-0.4-0.3)	0 (0-0.1)	0.3 (-1.2-1.8)	0 (0-0)	-6.1 (-10.4--1.9)	0 (0-0)	-0.9 (-15.7-14.4)	0 (0-0)	-12.3 (-14.6--10.3)
Ilocos Sur	0 (0-0.1)	0 (0-0)	0 (0-0.1)	0 (-0.4-0.2)	0 (0-0.1)	0.1 (-1.9-1.7)	0 (0-0)	-6.2 (-11.6--2)	0 (0-0)	-1.2 (-14.9-13)	0 (0-0)	-12.4 (-15.3--10.1)
Iloilo	0 (0-0.1)	1 (0-0)	0 (0-0.1)	1 (-0.3-0.4)	0 (0-0.1)	1.3 (-1.5-2.4)	0 (0-0)	-4.3 (-9.9-0)	0 (0-0)	-2.5 (-18.2-16)	0 (0-0)	-10.1 (-13.5--8.5)
Isabela	0 (0-0.1)	0 (0-0)	0 (0-0.1)	0 (-0.3-0.4)	0 (0-0.1)	0.2 (-1.8-2.1)	0 (0-0)	-6.3 (-10.4--1)	0 (0-0)	-5 (-18.4-13.6)	0 (0-0)	-13.7 (-15.8--11)
Kalinga	0 (0-0.1)	0.3 (0-0)	0 (0-0.1)	0.2 (-0.4-0.3)	0 (0-0.1)	0.7 (-1.2-1.9)	0 (0-0)	-4.4 (-9.3--0.1)	0 (0-0)	1.7 (-12.6-16.3)	0 (0-0)	-11.1 (-13.8--9.1)
La Union	0 (0-0.1)	1 (0-0)	0 (0-0.1)	1 (-0.3-0.6)	0 (0-0.1)	1.3 (-1.4-3.3)	0 (0-0)	-5.2 (-11.3-0.5)	0 (0-0)	-2.7 (-20.5-26.8)	0 (0-0)	-13.7 (-17.2--11)
Laguna	0 (0-0.1)	0 (0-0)	0 (0-0.1)	0 (-0.3-0.3)	0 (0-0.1)	0.3 (-1.4-1.9)	0 (0-0)	-5 (-10.1--0.6)	0 (0-0)	-4.6 (-20.3-10.7)	0 (0-0)	-11.6 (-14.3--9.4)
Lanao Del Norte	0 (0-0.1)	0 (0-0)	0 (0-0.1)	0 (-0.4-0.3)	0 (0-0.1)	0.1 (-1.6-2)	0 (0-0)	-1.6 (-6.2-3.8)	0 (0-0)	-3.4 (-18.1-14.7)	0 (0-0)	-7.5 (-9.7--4.9)
Lanao Del Sur	0 (0-0.1)	0 (0-0)	0 (0-0.1)	0 (-0.5-0.4)	0 (0-0.1)	0 (-2.4-2.3)	0 (0-0)	-6.8 (-12.8--0.9)	0 (0-0)	8.5 (-11.3-31.6)	0 (0-0.1)	-6 (-8.8--3.6)
Leyte	0 (0-0.1)	0 (0-0)	0 (0-0.1)	0.1 (-0.4-0.5)	0 (0-0.1)	0.1 (-2-2.6)	0 (0-0)	-2.1 (-7.9-4.4)	0 (0-0)	-7.1 (-22.9-11)	0 (0-0)	-11.8 (-14.6--8.8)
Maguindanao	0 (0-0.1)	0 (0-0)	0 (0-0.1)	-0.1 (-0.5-0.3)	0 (0-0.1)	0 (-2.1-2.7)	0 (0-0)	-4.5 (-10.3-1.5)	0 (0-0)	0.4 (-14.6-19.5)	0 (0-0.1)	-6.5 (-9.2--3.6)
Marinduque	0 (0-0.1)	0 (0-0)	0 (0-0.1)	0 (-0.4-0.2)	0 (0-0.1)	-0.3 (-2-1.2)	0 (0-0)	-4.1 (-8.5-0.7)	0 (0-0)	-10.8 (-26.5-5.5)	0 (0-0)	-14.4 (-16.8--12.2)
Masbate	0 (0-0.1)	0.1 (0-0)	0 (0-0.1)	0 (-0.5-0.3)	0 (0-0.1)	0 (-2.5-1.8)	0 (0-0)	0.7 (-6.4-6.6)	0 (0-0)	-2.9 (-20.8-16.8)	0 (0-0)	-6.6 (-9.7--4.2)
Misamis Occidental	0 (0-0.1)	-0.1 (0-0)	0 (0-0.1)	-0.1 (-0.4-0.5)	0 (0-0.1)	0.1 (-2.1-2.7)	0 (0-0)	-2.7 (-8.8-3.9)	0 (0-0)	-3.2 (-20.4-18.5)	0 (0-0)	-10.7 (-13.5--7.6)
Misamis Oriental	0 (0-0.1)	0 (0-0)	0 (0-0.1)	0 (-0.4-0.4)	0 (0-0.1)	0.3 (-1.7-2.2)	0 (0-0)	-3.6 (-8.6-2)	0 (0-0)	-4.9 (-19.9-12.3)	0 (0-0)	-10 (-12.5--7.3)
Mountain Province	0 (0-0.1)	0 (0-0)	0 (0-0.1)	0 (-0.3-0.4)	0 (0-0.1)	0.7 (-1-2.8)	0 (0-0)	-3.4 (-8.5-1.7)	0 (0-0)	4.7 (-12.4-23.1)	0 (0-0)	-10.3 (-12.7--7.9)
National Capital Region	0 (0-0.1)	0 (0-0)	0 (0-0.1)	0 (-0.3-0.3)	0 (0-0.1)	-0.3 (-1.8-1.2)	0 (0-0)	-4.2 (-9.2-0.5)	0 (0-0)	-9.4 (-23.9-6)	0 (0-0)	-13.3 (-16.1--10.8)
Negros Occidental	0 (0-0.1)	0 (0-0)	0 (0-0.1)	0 (-0.4-0.3)	0 (0-0.1)	0 (-1.9-1.8)	0 (0-0)	-4.2 (-9.2-0.6)	0 (0-0)	-4.7 (-21-12.3)	0 (0-0)	-12.9 (-15.3--10.5)
Negros Oriental	0 (0-0.1)	0 (0-0)	0 (0-0.1)	0 (-0.4-0.4)	0 (0-0.1)	0.1 (-2.3-2.3)	0 (0-0)	-3.4 (-8.6-2.4)	0 (0-0)	-3 (-20.3-13.8)	0 (0-0)	-12.8 (-15.4--10.4)
Northern Samar	0 (0-0.1)	0.1 (0-0)	0 (0-0.1)	0.1 (-0.4-0.5)	0 (0-0.1)	-0.2 (-2.6-2.2)	0 (0-0)	-0.1 (-6.2-6.4)	0 (0-0)	-3.2 (-22-21.1)	0 (0-0)	-9.8 (-12.8--7)
Nueva Ecija	0 (0-0.1)	0 (0-0)	0 (0-0.1)	0 (-0.3-0.3)	0 (0-0.1)	-0.1 (-1.8-1.4)	0 (0-0)	-4.4 (-9.4-0)	0 (0-0)	-1.5 (-15.9-17.1)	0 (0-0)	-12 (-14.7--9.9)
Nueva Vizcaya	0 (0-0.1)	0 (0-0)	0 (0-0.1)	0 (-0.4-0.3)	0 (0-0.1)	0.1 (-1.6-1.9)	0 (0-0)	-5 (-11.3-0.5)	0 (0-0)	-7.6 (-23-8.3)	0 (0-0)	-13.1 (-15.9--10.4)
Occidental Mindoro	0 (0-0.1)	0.1 (0-0)	0 (0-0.1)	0 (-0.5-0.3)	0 (0-0.1)	0 (-2-1.8)	0 (0-0)	-5.4 (-10--0.2)	0 (0-0)	-8.5 (-24.1-8.8)	0 (0-0)	-12.1 (-14.3--9.8)
Oriental Mindoro	0 (0-0.1)	0.1 (0-0)	0 (0-0.1)	0 (-0.5-0.4)	0 (0-0.1)	0.2 (-2-2.5)	0 (0-0)	-4 (-9.2-1.9)	0 (0-0)	-8.9 (-24.3-8.7)	0 (0-0)	-13.2 (-15.7--10.7)
Palawan	0 (0-0.1)	1 (0-0)	0 (0-0.1)	1 (-0.4-0.4)	0 (0-0.1)	1.1 (-2.1-2.6)	0 (0-0)	-1.1 (-8.1-4.3)	0 (0-0)	-7.5 (-26-14)	0 (0-0)	-9.6 (-13.3--7.2)
Pampanga	0 (0-0.1)	0.5 (0-0)	0 (0-0.1)	0.6 (-0.3-0.4)	0 (0-0.1)	0.6 (-1.7-2.3)	0 (0-0)	-3.6 (-9-1.3)	0 (0-0)	-4 (-22.7-15.9)	0 (0-0)	-12 (-15.1--9.4)
Pangasinan	0 (0-0.1)	0 (0-0)	0 (0-0.1)	0 (-0.4-0.3)	0 (0-0.1)	0 (-1.9-2)	0 (0-0)	-5.6 (-10.6--0.3)	0 (0-0)	-7.5 (-25.6-12.1)	0 (0-0)	-12 (-14.7--9.6)
Quezon	0 (0-0.1)	0 (0-0)	0 (0-0.1)	0 (-0.3-0.3)	0 (0-0.1)	0 (-1.9-1.6)	0 (0-0)	-3.2 (-8.3-1.6)	0 (0-0)	-3.7 (-20.6-17.3)	0 (0-0)	-11.8 (-14.2--9.6)
Quirino	0 (0-0.1)	0 (0-0)	0 (0-0.1)	-0.1 (-0.4-0.4)	0 (0-0.1)	0.2 (-1.7-2.4)	0 (0-0)	-7.1 (-12.5--0.6)	0 (0-0)	-6.3 (-19.6-13.8)	0 (0-0)	-15.7 (-18.5--12.7)

Location	Birth Incidence rate (per 100,000 live births), 2021	Birth Incidence rate (per 100,000 live births) percent change (2000-2021)	Under-5 rate (per 100,000 population), 2021	Under-5 rate (per 100,000 population) percent change (2000-2021)	5-14 rate (per 100,000 population), 2021	5-14 rate (per 100,000 population) percent change (2000-2021)	15-49 rate (per 100,000 population), 2021	15-49 rate (per 100,000 population) percent change (2000-2021)	50plus rate (per 100,000 population), 2021	50plus rate (per 100,000 population) percent change (2000-2021)	All age rate (per 100,000 population), 2021	All age rate (per 100,000 population) percent change (2000-2021)
Rizal	0 (0-0.1)	0 (0-0)	0 (0-0.1)	0 (-0.3-0.4)	0 (0-0.1)	-0.3 (-2.3-1.8)	0 (0-0)	-1.2 (-6.7-3.8)	0 (0-0)	-10.8 (-26.1-5.2)	0 (0-0)	-9.6 (-12.5--7.2)
Romblon	0 (0-0.1)	0 (0-0)	0 (0-0.1)	0 (-0.5-0.3)	0 (0-0.1)	0.2 (-1.7-2.2)	0 (0-0)	-2.9 (-8.5-2)	0 (0-0)	-9.4 (-26.6-7.8)	0 (0-0)	-12.6 (-15.1--10.5)
Samar (Western Samar)	0 (0-0.1)	-0.7 (0-0)	0 (0-0.1)	-0.7 (-0.8-0.4)	0 (0-0.1)	-1.1 (-4.2-2.2)	0 (0-0)	-1.1 (-9.9-6.9)	0 (0-0)	0 (-22.7-23.6)	0 (0-0)	-11.8 (-15.8--8.2)
Sarangani	0 (0-0.1)	0 (0-0)	0 (0-0.1)	0 (-0.2-0.4)	0 (0-0.1)	-0.2 (-2.2-1.6)	0 (0-0)	-4.5 (-10-0.1)	0 (0-0)	-2.5 (-18.5-10.6)	0 (0-0)	-12.1 (-14.9--9.7)
Siquijor	0 (0-0.1)	0 (0-0)	0 (0-0.1)	0 (-0.3-0.3)	0 (0-0.1)	0.4 (-1-2.1)	0 (0-0)	-4.5 (-8.5-0.4)	0 (0-0)	-0.4 (-15.8-15.3)	0 (0-0)	-11.1 (-13.2--8.8)
Sorsogon	0 (0-0.1)	0 (0-0)	0 (0-0.1)	0 (-0.3-0.3)	0 (0-0.1)	-0.2 (-2-1.4)	0 (0-0)	-1.1 (-6.1-3)	0 (0-0)	-1.7 (-18.1-15.1)	0 (0-0)	-10.9 (-13.4--8.6)
South Cotabato	0 (0-0.1)	0 (0-0)	0 (0-0.1)	0 (-0.3-0.4)	0 (0-0.1)	0.2 (-1.7-2.1)	0 (0-0)	-5.2 (-10.2--0.4)	0 (0-0)	-2.1 (-20.9-14)	0 (0-0)	-12.6 (-15.1--10)
Southern Leyte	0 (0-0.1)	-0.8 (0-0)	0 (0-0.1)	-0.8 (-0.6-0.3)	0 (0-0.1)	-0.7 (-2.8-2)	0 (0-0)	-4 (-10.7-1.6)	0 (0-0)	0.3 (-17.2-20.2)	0 (0-0)	-12.3 (-15.6--9.2)
Sultan Kudarat	0 (0-0.1)	0.3 (0-0)	0 (0-0.1)	0.2 (-0.4-0.2)	0 (0-0.1)	0.5 (-1.4-1.7)	0 (0-0)	-3.9 (-8.6-0.2)	0 (0-0)	-5.2 (-21-12.1)	0 (0-0)	-10.9 (-13.3--9)
Sulu	0 (0-0.1)	0.8 (0-0)	0 (0-0.1)	0.7 (-0.6-0.8)	0 (0-0.1)	0.2 (-2.9-4)	0 (0-0)	-3.1 (-10-8)	0 (0-0)	-2.5 (-24.8-29.3)	0 (0-0)	-1.8 (-5.6-3.7)
Surigao Del Norte	0 (0-0.1)	-0.1 (0-0)	0 (0-0.1)	0 (-0.4-0.4)	0 (0-0.1)	0.2 (-2.1-2.4)	0 (0-0)	-4.2 (-11.6-1.6)	0 (0-0)	-6.4 (-23.4-13.9)	0 (0-0)	-11 (-14.4--8.2)
Surigao Del Sur	0 (0-0.1)	0 (0-0)	0 (0-0.1)	0 (-0.4-0.4)	0 (0-0.1)	0 (-2.3-2)	0 (0-0)	-4.4 (-9.5-0.6)	0 (0-0)	-6.6 (-23.1-11.7)	0 (0-0)	-13.4 (-15.7--11)
Tarlac	0 (0-0.1)	0 (0-0)	0 (0-0.1)	0 (-0.3-0.2)	0 (0-0.1)	-0.1 (-1.8-1.6)	0 (0-0)	-4.8 (-9.8--0.2)	0 (0-0)	-5.5 (-20.1-9)	0 (0-0)	-13.2 (-15.7--10.8)
Tawi-Tawi	0 (0-0.1)	0.3 (0-0)	0 (0-0.1)	0.2 (-0.4-0.3)	0 (0-0.1)	0.3 (-1.6-1.5)	0 (0-0)	-2 (-6.4-1.8)	0 (0-0)	-10.1 (-21.6-3.4)	0 (0-0.1)	-7.1 (-9.4--5.3)
Zambales	0 (0-0.1)	0 (0-0)	0 (0-0.1)	0 (-0.4-0.4)	0 (0-0.1)	0.1 (-1.8-2)	0 (0-0)	-3 (-8.5-1.8)	0 (0-0)	-14.1 (-27.3-1.6)	0 (0-0)	-10.6 (-13.3--8.3)
Zamboanga Del Norte	0 (0-0.1)	-1 (0-0)	0 (0-0.1)	-1 (-0.6-0.3)	0 (0-0.1)	-1.1 (-3-2)	0 (0-0)	-3.6 (-9.1-3)	0 (0-0)	-9.9 (-27-8.7)	0 (0-0)	-11.6 (-13.9--8.1)
Zamboanga Del Sur	0 (0-0.1)	0 (0-0)	0 (0-0.1)	0 (-0.3-0.3)	0 (0-0.1)	-0.1 (-1.6-1.4)	0 (0-0)	-2.6 (-6.4-1.4)	0 (0-0)	-4.7 (-20.6-14.1)	0 (0-0)	-11.4 (-13.4--9.4)
Zamboanga Sibugay	0 (0-0.1)	0 (0-0)	0 (0-0.1)	0 (-0.3-0.3)	0 (0-0.1)	0.1 (-1.6-1.9)	0 (0-0)	-2.6 (-7.4-2.4)	0 (0-0)	-5.8 (-21.5-12.7)	0 (0-0)	-10.1 (-12.3--7.8)
Seychelles	0 (0-0.1)	3.2 (0-85.5)	0 (0-0.1)	3.2 (-0.2-85.5)	0 (0-0.1)	3.4 (-0.8-85.8)	0 (0-0)	-7.4 (-14.7-80.6)	0 (0-0)	23.4 (1.6-381)	0 (0-0)	-16.8 (-21.8-56.1)
Sri Lanka	0 (0-0.1)	0.5 (0-0)	0 (0-0.1)	0.5 (-0.2-0.2)	0 (0-0.1)	0.4 (-1.3-1)	0 (0-0)	-3.9 (-8.4--0.5)	0 (0-0)	-12.6 (-24.3--1.4)	0 (0-0)	-14.8 (-17.3--13.2)
Thailand	0 (0-0.1)	0 (0-0)	0 (0-0.1)	0 (-0.3-0.2)	0 (0-0.1)	-0.2 (-1.5-1.3)	0 (0-0)	-9.4 (-13.5--4.8)	0 (0-0)	-7 (-19.9-6.6)	0 (0-0)	-30.7 (-33.1--28.2)
Timor-Leste	0 (0-0.1)	2.2 (0-1240)	0 (0-0.1)	2.1 (-0.5-1230)	0 (0-0.1)	1.7 (-2.8-1200)	0 (0-0)	8.1 (-0.9-1210)	0 (0-0)	-14.9 (-30.4-785)	0 (0-0)	-4.6 (-9.7-1100)
Viet Nam	0 (0-0.1)	0.9 (0-0)	0 (0-0.1)	0.9 (-0.3-0.5)	0 (0-0.1)	1.2 (-1-2.1)	0 (0-0)	-9.7 (-15.2--5.6)	0 (0-0)	19.1 (1.8-39.4)	0 (0-0)	-21.6 (-25.1--19)
Sub-Saharan Africa	783 (632-975)	-1.9 (-4.8-0.9)	736 (593-917)	0 (-2.9-2.7)	513 (412-652)	7.4 (4.5-10.3)	217 (168-278)	8.9 (5.7-12.4)	1.4 (0.9-2.3)	21.7 (15.1-28.2)	352 (287-436)	0.2 (-2.5-2.9)
**Central Sub-Saharan Africa**	1,360 (1,150-1,580)	0.6 (-4.8-6.1)	1,280 (1,080-1,500)	0.3 (-5-6)	835 (680-1,010)	0.2 (-5.8-6.8)	323 (252-415)	1.4 (-5.6-8.9)	2.2 (1.3-3.5)	11.4 (-4.7-31.7)	579 (486-689)	-5.6 (-11.2-0)
Angola	1,180 (971-1,440)	-2.7 (-10.7-6)	1,110 (917-1,350)	-3 (-11.1-5.6)	728 (582-912)	-3 (-11.3-5.2)	282 (220-368)	-2.2 (-11.8-7.5)	2 (1.1-3.1)	-0.2 (-16.2-20.9)	525 (430-649)	-5.8 (-13.5-2)
Central African Republic	880 (743-1,060)	0 (-7.4-7.2)	826 (699-997)	-0.2 (-7.3-7)	528 (427-644)	-1 (-7.4-6.6)	189 (145-242)	-0.4 (-9.2-9.5)	1 (0.6-1.7)	9 (-11.4-33.2)	359 (299-435)	-5.4 (-11.1-2.2)
Congo	525 (435-622)	-4.8 (-12.2-2.7)	497 (412-590)	-5 (-12.2-2.1)	345 (284-419)	-4.8 (-12.6-2.3)	124 (97.5-160)	-14 (-21.4--6.3)	0.8 (0.4-1.3)	11.4 (-8.2-36)	210 (175-254)	-14.8 (-21.3--8.2)
Democratic Republic of the Congo	1,520 (1,270-1,750)	2.1 (-5.5-9.4)	1,420 (1,190-1,650)	1.8 (-5.5-9)	926 (753-1,120)	1.6 (-6.4-10.3)	360 (278-462)	2.2 (-6.8-11.8)	2.4 (1.4-3.9)	14.6 (-6.5-43.3)	639 (536-761)	-5.3 (-12.1-2.2)

Location	Birth Incidence rate (per 100,000 live births), 2021	Birth Incidence rate (per 100,000 live births) percent change (2000-2021)	Under-5 rate (per 100,000 population), 2021	Under-5 rate (per 100,000 population) percent change (2000-2021)	5-14 rate (per 100,000 population), 2021	5-14 rate (per 100,000 population) percent change (2000-2021)	15-49 rate (per 100,000 population), 2021	15-49 rate (per 100,000 population) percent change (2000-2021)	50plus rate (per 100,000 population), 2021	50plus rate (per 100,000 population) percent change (2000-2021)	All age rate (per 100,000 population), 2021	All age rate (per 100,000 population) percent change (2000-2021)
Equatorial Guinea	1,290 (1,030-1,600)	-1.1 (-10.2-8.7)	1,210 (967-1,510)	-1.4 (-10.3-8.3)	792 (629-1,010)	-2.9 (-11-6.4)	324 (245-430)	-1.1 (-10.3-9.7)	2 (1.1-3.2)	16.5 (-6.7-44.7)	529 (427-663)	-15.4 (-23.1--7.2)
Gabon	703 (550-892)	-4.7 (-13-6.2)	668 (526-849)	-4.8 (-13-5.8)	477 (373-607)	-5.3 (-13.1-4)	187 (142-240)	-7.5 (-16.2-1.9)	1.2 (0.7-2)	22.9 (0.8-51.1)	275 (218-345)	-17.3 (-24.3--9.2)
**Eastern Sub-Saharan Africa**	329 (266-401)	-2.8 (-5.8-0.2)	311 (254-384)	-2.8 (-5.6-0.1)	207 (167-261)	-2.2 (-5.1-0.7)	70.7 (54.3-91.7)	-5 (-8.2--1.7)	0.2 (0.1-0.4)	16.2 (6.8-26.2)	138 (113-170)	-9.8 (-12.2--7.1)
Burundi	52.8 (41.7-69.8)	-10.8 (-24.6-1.5)	48.9 (38.6-64.4)	-10.9 (-24.7-0.8)	30.4 (23.3-40)	-9.4 (-22.8-2.9)	9.9 (7.2-13.2)	-8.8 (-22.6-3.3)	0 (0-0.1)	-2.7 (-26.6-26)	21.4 (16.9-27.6)	-12.6 (-25.5--1.8)
Comoros	0 (0-0.1)	1.3 (0-25)	0 (0-0.1)	1.2 (-1.2-25.3)	0 (0-0.1)	0.2 (-7.4-27.2)	0 (0-0)	-7.7 (-19.2-17.3)	0 (0-0)	9.1 (-24.7-52.8)	0 (0-0)	-18 (-24.7-3.9)
Djibouti	17.7 (12.5-24.6)	-1.3 (-12.2-8)	16.5 (11.7-23)	-1.6 (-12.2-7.8)	10.9 (8-15.1)	-0.7 (-10.5-8.3)	3.3 (2.4-4.3)	-16.3 (-25.2--6.8)	0 (0-0)	-6.5 (-29.6-22.3)	6.2 (4.6-8.4)	-16.5 (-24.6--8.9)
Eritrea	19.9 (14-27.5)	-0.1 (-9-11.8)	18.4 (13.1-25.6)	-0.4 (-9.2-10.9)	11.4 (8.4-15.7)	-1.6 (-10.3-8.6)	3.6 (2.6-5)	-4.3 (-14.8-7.5)	0 (0-0)	-1.5 (-25.8-34)	7.2 (5.3-9.7)	-13.6 (-20.9--4.9)
Ethiopia	0.1 (0.1-0.2)	-6.2 (-48.3-43.4)	0.1 (0.1-0.2)	-6.6 (-48.6-42.7)	0.1 (0-0.1)	-7.7 (-49.6-42.4)	0 (0-0)	-11.5 (-51.8-32.5)	0 (0-0)	-5 (-49.5-42.2)	0.1 (0-0.1)	-15.4 (-53.5-30.5)
Addis Ababa	0.1 (0.1-0.2)	-8.4 (-54.2-47)	0.1 (0.1-0.2)	-9.2 (-55.3-44.6)	0.1 (0-0.2)	-9.5 (-56.4-42.7)	0 (0-0)	-34.1 (-67.2-7.8)	0 (0-0)	-13.3 (-53.9-60.4)	0 (0-0.1)	-31.8 (-66.3-9.5)
Afar	0.2 (0.1-0.3)	-7.4 (-51.4-52.9)	0.1 (0.1-0.3)	-7.1 (-51-54)	0.1 (0-0.1)	-6.1 (-49.7-56.6)	0 (0-0)	-9.2 (-47.2-52.4)	0 (0-0)	5.2 (-40.4-138)	0.1 (0-0.1)	-10.8 (-52.4-49.5)
Amhara	0.1 (0.1-0.2)	-6.6 (-50.7-46.4)	0.1 (0.1-0.2)	-7 (-51.1-45.7)	0.1 (0-0.1)	-7.7 (-52.7-45.4)	0 (0-0)	-12.1 (-54.4-34.5)	0 (0-0)	-6.9 (-54.3-46.7)	0.1 (0-0.1)	-19.3 (-58.6-27.1)
Benishangul-Gumuz	0.2 (0.1-0.3)	-6.8 (-53.3-52.9)	0.1 (0.1-0.2)	-7.2 (-53.7-52.5)	0.1 (0-0.1)	-8.1 (-54-47.4)	0 (0-0)	-12.2 (-57.6-43.2)	0 (0-0)	5.2 (-39.6-125)	0.1 (0-0.1)	-17.2 (-58.4-34.8)
Dire Dawa	0.1 (0.1-0.2)	-6.6 (-53.1-47.9)	0.1 (0.1-0.2)	-7 (-53.9-46.4)	0.1 (0-0.1)	-7.6 (-54.7-46.6)	0 (0-0)	-17.7 (-59.1-27.1)	0 (0-0)	-4.9 (-55.8-59.1)	0 (0-0.1)	-21.3 (-60.8-24.4)
Gambella	0.1 (0.1-0.2)	-6.5 (-48.3-43.8)	0.1 (0.1-0.2)	-6.7 (-48.5-43.7)	0.1 (0-0.1)	-8 (-49.7-42.2)	0 (0-0)	-10.4 (-53.6-33.5)	0 (0-0)	17.2 (-48.2-87.4)	0 (0-0.1)	-21.9 (-57.5-19.6)
Harari	0.1 (0.1-0.2)	-6.6 (-53.1-46.5)	0.1 (0.1-0.2)	-7.1 (-53.2-45.5)	0.1 (0-0.1)	-8.4 (-52.9-41.8)	0 (0-0)	-15.9 (-58.6-27.3)	0 (0-0)	-5.7 (-56.8-52.2)	0 (0-0.1)	-21.5 (-59.7-22.1)
Oromia	0.1 (0.1-0.2)	-6.2 (-53.1-48)	0.1 (0.1-0.2)	-6.6 (-53.6-46.6)	0.1 (0-0.1)	-7.9 (-53-48.1)	0 (0-0)	-8.9 (-53.3-48.1)	0 (0-0)	-5.1 (-54.2-62.9)	0.1 (0-0.1)	-12.9 (-55.5-39.5)
Somali	0.1 (0.1-0.3)	-5.6 (-53.1-57.2)	0.1 (0.1-0.2)	-5.2 (-52.3-59.7)	0.1 (0-0.1)	-3.2 (-49.4-68.6)	0 (0-0)	-13.7 (-53.8-52.6)	0 (0-0)	8.8 (-40.3-126)	0.1 (0-0.1)	-8.8 (-52.6-57.2)
Southern Nations, Nationalities, and Peoples	0.1 (0.1-0.3)	-6.1 (-50.1-47.9)	0.1 (0.1-0.2)	-6.5 (-50.5-46.1)	0.1 (0-0.1)	-8.5 (-51.5-42.5)	0 (0-0)	-8.4 (-51.4-37.1)	0 (0-0)	-5.8 (-55.7-56.2)	0.1 (0-0.1)	-15.9 (-55-31.2)
Tigray	0.1 (0.1-0.2)	-6.5 (-50.6-48)	0.1 (0.1-0.2)	-6.8 (-50.9-46.5)	0.1 (0-0.1)	-8.2 (-52.3-42.3)	0 (0-0)	-13.1 (-55.2-32.4)	0 (0-0)	-5.4 (-55.5-55.2)	0.1 (0-0.1)	-20.1 (-58.2-24.3)
Kenya	1,250 (900-1,710)	1.6 (-0.6-3.9)	1,170 (844-1,610)	2 (-0.1-4.3)	779 (573-1,050)	0.3 (-1.6-2.4)	237 (174-322)	-9.8 (-11.8--7.8)	0.6 (0.3-1)	2.6 (-2-7)	461 (341-612)	-14 (-15.9--12.2)
Baringo	1,120 (783-1,580)	2.4 (-9.9-15.8)	1,050 (730-1,500)	2.3 (-9.9-15.7)	669 (481-925)	0.6 (-10-13)	216 (156-299)	-4.9 (-14.6-6.3)	0.6 (0.3-1)	-1.4 (-19.6-21.5)	431 (316-583)	-13 (-21.8--3.3)
Bomet	1,060 (754-1,490)	3 (-7.1-15.1)	1,010 (718-1,420)	3 (-6.7-14.5)	741 (534-1,020)	1.4 (-7.9-11.9)	249 (181-342)	-8.8 (-17.1-0.7)	0.6 (0.3-0.9)	10.2 (-12.3-39.9)	449 (326-605)	-15.6 (-23.3--7)
Bungoma	1,280 (913-1,810)	2.9 (-8.7-14.8)	1,200 (848-1,700)	2.6 (-8.4-14.2)	782 (570-1,070)	0.9 (-9.1-11.8)	269 (196-371)	-3 (-12.9-8)	0.7 (0.4-1.1)	-9.8 (-27.5-11.1)	523 (385-708)	-8.9 (-17.7-0.3)
Busia	1,660 (1,130-2,320)	2.6 (-10.4-13.8)	1,550 (1,070-2,190)	2.5 (-9.9-13.3)	994 (710-1,370)	1.2 (-10.9-11.7)	312 (227-427)	-5 (-15.5-6.5)	0.7 (0.4-1.2)	6.4 (-14.8-35)	650 (473-885)	-9.9 (-20.3--1)
Elgeyo-Marakwet	1,070 (744-1,510)	3.1 (-6.8-16.3)	1,020 (709-1,440)	2.9 (-6.9-16.2)	707 (499-998)	1 (-8.5-13.2)	232 (170-326)	-6.5 (-15.5-4.9)	0.5 (0.2-0.8)	-17.1 (-32.7-2.6)	417 (303-571)	-16.9 (-24.6--7.3)
Embu	987 (696-1,400)	1.5 (-10.4-14.7)	934 (661-1,330)	1.4 (-10.4-14.1)	652 (467-899)	0.3 (-10.2-11.8)	198 (145-273)	-15.8 (-24.4--5.6)	0.6 (0.3-1)	4.8 (-15.6-29.6)	321 (237-430)	-24.5 (-32.8--16)
Garissa	1,070 (754-1,500)	2.4 (-7.3-13.9)	1,010 (714-1,420)	2.6 (-7.5-13.2)	671 (491-932)	1.4 (-7.4-11.7)	230 (164-316)	-2.3 (-11.3-7.2)	0.7 (0.3-1.1)	-3.4 (-22.1-19.3)	476 (349-652)	-3.9 (-11.9-5.3)
Homa Bay	2,110 (1,500-2,890)	1.7 (-11.1-13.3)	1,970 (1,400-2,710)	1.5 (-11.2-12.4)	1,240 (900-1,720)	1.1 (-9.7-10.8)	365 (264-504)	-4.7 (-14.2-5.2)	0.6 (0.3-0.9)	6.7 (-14.8-32.6)	820 (604-1,110)	-7.6 (-17.2-1.4)
Isiolo	1,170 (831-1,620)	2.3 (-9.1-14.7)	1,090 (773-1,510)	2.1 (-9.2-13.7)	664 (483-907)	0.4 (-9.3-9.9)	192 (137-265)	-5.8 (-14.8-3.8)	0.5 (0.3-0.9)	-3.5 (-20.9-18.1)	413 (304-555)	-12.7 (-20.7--4.8)

Location	Birth Incidence rate (per 100,000 live births), 2021	Birth Incidence rate (per 100,000 live births) percent change (2000-2021)	Under-5 rate (per 100,000 population), 2021	Under-5 rate (per 100,000 population) percent change (2000-2021)	5-14 rate (per 100,000 population), 2021	5-14 rate (per 100,000 population) percent change (2000-2021)	15-49 rate (per 100,000 population), 2021	15-49 rate (per 100,000 population) percent change (2000-2021)	50plus rate (per 100,000 population), 2021	50plus rate (per 100,000 population) percent change (2000-2021)	All age rate (per 100,000 population), 2021	All age rate (per 100,000 population) percent change (2000-2021)
Kajiado	1,040 (739-1,460)	2 (-8.5-14.5)	987 (698-1,390)	1.9 (-8.5-14)	701 (504-978)	-0.1 (-10-11.9)	208 (150-284)	-14.8 (-22.8--6.2)	0.7 (0.3-1.1)	16.1 (-7.6-43.6)	388 (283-523)	-19.4 (-27--10.8)
Kakamega	1,370 (985-1,890)	2.4 (-10.8-15)	1,280 (917-1,760)	2.3 (-10.6-14.7)	817 (600-1,120)	1.5 (-9.2-13)	251 (183-346)	-9.1 (-18.2-1.4)	0.6 (0.3-1.1)	11.4 (-10.1-40.4)	512 (380-684)	-14.7 (-23.9--4.4)
Kericho	1,050 (754-1,450)	2.6 (-9.3-13.8)	996 (714-1,360)	2.3 (-9.2-13.1)	697 (508-955)	0.6 (-9.5-11)	224 (166-304)	-13.5 (-22.4--4.9)	0.6 (0.3-1)	-13.9 (-29.5-5.4)	406 (299-541)	-19.2 (-27.3--11)
Kiambu	1,030 (731-1,440)	1.7 (-9.3-15.1)	974 (690-1,360)	1.5 (-9.6-14.6)	688 (496-939)	0.3 (-9.8-12.3)	188 (137-254)	-19.1 (-27--9.8)	0.6 (0.3-1.1)	9.1 (-12.3-34.5)	335 (244-442)	-22.4 (-29.6--13.6)
Kilifi	1,150 (865-1,530)	8.4 (-1.5-17.9)	1,080 (812-1,450)	8.1 (-1.7-17.6)	718 (551-957)	4.9 (-4.7-14.2)	241 (179-325)	-2.4 (-12.3-7.5)	0.6 (0.3-1.1)	-3 (-21.2-20.1)	452 (352-586)	-6.2 (-14.5-2.2)
Kirinyaga	1,020 (720-1,450)	3.1 (-6.8-17.7)	958 (678-1,370)	3 (-7-17.2)	657 (473-921)	1.7 (-8.6-13.5)	180 (131-251)	-19.6 (-27.9--11.6)	0.7 (0.4-1.1)	12.1 (-9.7-40.9)	296 (217-402)	-26.6 (-33.5--18.3)
Kisii	1,410 (997-1,960)	1.7 (-9.3-12.4)	1,320 (939-1,840)	1.4 (-9.7-11.8)	862 (623-1,180)	-0.3 (-11.5-10.4)	274 (198-377)	-9.8 (-19.8-0.1)	0.6 (0.3-1.1)	-16.4 (-33-2.8)	484 (357-643)	-22.3 (-31.5--14.3)
Kisumu	2,440 (1,910-3,150)	0.5 (-8.6-9.7)	2,220 (1,750-2,870)	0.6 (-8.2-9.6)	1,280 (981-1,670)	-1.5 (-10.7-7.7)	329 (244-446)	-11.2 (-19.8--1.4)	0.5 (0.3-0.8)	-4.2 (-26-20.6)	741 (585-952)	-18.2 (-25.1--10.7)
Kitui	1,050 (755-1,490)	2 (-8.7-13.4)	990 (708-1,400)	1.9 (-8.8-13.1)	675 (491-927)	0.3 (-9.5-11.6)	226 (166-305)	-7 (-16.3-3.1)	0.5 (0.3-0.9)	7.9 (-11.1-35.7)	410 (300-553)	-15.7 (-23.5--6.5)
Kwale	1,460 (1,010-2,050)	1.8 (-10.4-15.2)	1,370 (950-1,920)	1.8 (-10.6-15.1)	894 (644-1,240)	0.2 (-10.5-11.9)	282 (203-389)	-4.4 (-15.5-5.8)	0.7 (0.4-1.1)	-7.9 (-25.4-16.5)	558 (405-753)	-10.5 (-19.8--0.6)
Laikipia	1,020 (706-1,400)	2.7 (-8.4-16.2)	969 (678-1,330)	2.5 (-8.1-15.9)	707 (511-961)	0.8 (-9.8-13.1)	221 (163-306)	-12.5 (-21.7--3.1)	0.6 (0.3-1)	6 (-15.1-31.1)	381 (278-510)	-20.6 (-28.5--11.3)
Lamu	1,080 (769-1,500)	2 (-10.1-13)	1,010 (715-1,420)	1.9 (-9.4-12.6)	655 (476-896)	0.5 (-10.6-11.2)	197 (143-270)	-9.3 (-18.5-0.2)	0.6 (0.3-1)	1.4 (-17.8-25)	366 (269-491)	-15.9 (-24.5--7.8)
Machakos	1,000 (703-1,390)	2.2 (-8.4-14.8)	948 (664-1,310)	2 (-8.3-14.4)	663 (480-913)	0.8 (-8.8-12)	200 (146-273)	-18.8 (-27.6--9.6)	0.6 (0.3-0.9)	-3.8 (-22.3-18.6)	334 (248-448)	-25.6 (-32.5--17.6)
Makueni	1,070 (744-1,480)	1.8 (-7.9-13.7)	1,010 (703-1,400)	1.8 (-7.8-13.4)	714 (510-974)	0.3 (-8.7-10.4)	228 (167-309)	-8.7 (-18-0.7)	0.5 (0.2-0.8)	16.4 (-6.9-42.9)	406 (295-545)	-18.5 (-25.3--10.5)
Mandera	1,120 (792-1,560)	2.1 (-8.9-15.9)	1,050 (749-1,470)	2.5 (-8.6-15.4)	667 (485-915)	0.2 (-9-12)	236 (171-323)	6.5 (-3.7-18.7)	0.7 (0.3-1.1)	3.9 (-18.9-28.4)	473 (348-634)	-7.9 (-16.4-2.9)
Marsabit	1,080 (765-1,490)	1.6 (-8.3-13.1)	1,010 (723-1,410)	1.7 (-7.7-13.5)	672 (491-922)	0.6 (-9.3-11.5)	223 (162-304)	-1.6 (-11.1-8.8)	0.6 (0.3-1)	10.2 (-12.6-40.8)	439 (323-586)	-9.1 (-17.6--0.1)
Meru	1,030 (715-1,410)	2.3 (-7.2-16.1)	970 (666-1,330)	2.1 (-7.1-15.7)	661 (478-903)	0.2 (-8.5-11.7)	202 (148-278)	-13.9 (-22.4--3.8)	0.6 (0.3-0.9)	1.3 (-18-25.9)	338 (249-452)	-23.3 (-30.3--14.5)
Migori	2,040 (1,430-2,860)	3.1 (-8-15.8)	1,900 (1,350-2,670)	2.9 (-7.9-14.9)	1,170 (851-1,630)	1.7 (-8.4-12.7)	356 (254-493)	-2.1 (-12.2-8.5)	0.7 (0.4-1.2)	13.4 (-9.7-40)	827 (606-1,120)	-3.2 (-12.4-6.9)
Mombasa	1,250 (892-1,720)	1.8 (-9.3-13.5)	1,180 (839-1,630)	1.5 (-9.3-12.8)	816 (598-1,120)	0.5 (-9-10.6)	226 (168-311)	-14.2 (-23.3--4.7)	0.8 (0.4-1.3)	-1.7 (-22.5-21.4)	424 (317-575)	-11.5 (-19.8--2.8)
Murang'a	989 (704-1,370)	1.9 (-7.8-13.5)	939 (665-1,310)	1.9 (-8-13.6)	666 (483-912)	0.8 (-8.3-11.6)	198 (145-269)	-17.9 (-26--9.3)	0.6 (0.3-1)	11.5 (-8.8-36.6)	300 (221-401)	-30.6 (-36.9--23.5)
Nairobi	1,070 (755-1,490)	2.4 (-9.1-14.7)	1,010 (718-1,420)	2.1 (-9.2-14.4)	712 (514-975)	0.9 (-9.8-11.1)	188 (136-259)	-17.3 (-25.6--8.7)	0.7 (0.4-1.2)	-5.8 (-23.7-16.1)	351 (256-474)	-13.9 (-22.6--5.6)
Nakuru	1,010 (717-1,390)	1.7 (-9.8-12.7)	958 (676-1,320)	1.6 (-9.9-12.4)	685 (497-931)	-0.1 (-10.2-9.5)	216 (158-296)	-13.2 (-21.7--3.8)	0.6 (0.3-1)	2.1 (-16.8-26.6)	371 (276-498)	-21 (-28.6--13.2)
Nandi	1,090 (769-1,510)	1.9 (-7.9-11.5)	1,030 (725-1,440)	1.8 (-8.1-11.4)	734 (531-1,010)	0.4 (-9.2-10.1)	242 (176-329)	-9 (-17.8-0.2)	0.6 (0.3-1)	4.7 (-16.3-32)	427 (314-578)	-17.2 (-25.2--9.5)
Narok	1,110 (779-1,540)	2.1 (-8.3-15.5)	1,050 (745-1,460)	2.2 (-8.1-15.2)	742 (533-1,020)	1 (-9.2-12.3)	235 (173-320)	-6.9 (-15.9-2.3)	0.6 (0.3-1)	29.7 (6.5-58.5)	491 (359-659)	-11.9 (-19.8--2.3)
Nyamira	1,250 (866-1,780)	1.3 (-9.3-13.1)	1,190 (821-1,690)	1.2 (-9.2-12.8)	854 (607-1,170)	0 (-9.8-10.4)	261 (192-359)	-8.7 (-16.8-0.7)	0.6 (0.3-1)	22.5 (-2-52.7)	483 (348-647)	-16.4 (-23.8--7.9)
Nyandarua	1,060 (741-1,500)	2.5 (-6.5-10.5)	1,010 (711-1,430)	2.4 (-6.2-10.5)	718 (517-978)	0.2 (-8.2-8.2)	216 (159-293)	-10.6 (-18.6--2.2)	0.6 (0.3-1)	12.1 (-9.7-43.2)	376 (276-502)	-23.9 (-29.8--17.1)
Nyeri	1,040 (727-1,440)	2.1 (-9.6-13.5)	986 (693-1,360)	2 (-9.6-13.1)	703 (507-958)	0.6 (-9.3-10.7)	194 (140-267)	-18.2 (-26.5--9.9)	0.5 (0.3-0.9)	11.4 (-10.5-39.4)	303 (221-406)	-29.4 (-36.3--22.5)
Samburu	1,100 (769-1,570)	2.9 (-8.1-16.3)	1,040 (732-1,490)	2.9 (-8-16)	728 (517-1,000)	1 (-9.2-12.7)	233 (169-325)	-4.5 (-13.4-5.6)	0.5 (0.3-0.9)	13.7 (-7.8-43.1)	485 (350-661)	-9.6 (-18.7-0.8)
Siaya	1,920 (1,350-2,650)	1.4 (-9.8-13.6)	1,780 (1,260-2,480)	1.2 (-9.9-13.6)	1,110 (812-1,530)	1.1 (-9.1-12.4)	338 (244-468)	-6.3 (-16.3-4.5)	0.5 (0.3-0.9)	-10.7 (-28.7-11.5)	688 (507-920)	-11.8 (-20.5--2.4)

Location	Birth Incidence rate (per 100,000 live births), 2021	Birth Incidence rate (per 100,000 live births) percent change (2000-2021)	Under-5 rate (per 100,000 population), 2021	Under-5 rate (per 100,000 population) percent change (2000-2021)	5-14 rate (per 100,000 population), 2021	5-14 rate (per 100,000 population) percent change (2000-2021)	15-49 rate (per 100,000 population), 2021	15-49 rate (per 100,000 population) percent change (2000-2021)	50plus rate (per 100,000 population), 2021	50plus rate (per 100,000 population) percent change (2000-2021)	All age rate (per 100,000 population), 2021	All age rate (per 100,000 population) percent change (2000-2021)
Taita Taveta	1,120 (786-1,560)	2.2 (-10.1-14.6)	1,050 (747-1,480)	2.1 (-9.8-14.5)	716 (523-991)	0.9 (-10.9-12.5)	201 (147-274)	-16.6 (-26--7.4)	0.5 (0.3-0.9)	3 (-17.9-32.4)	346 (258-467)	-23.2 (-31.9--15)
Tana River	1,220 (861-1,680)	2.6 (-8.4-14.3)	1,140 (803-1,580)	2.6 (-8.1-13.9)	738 (534-1,020)	1.4 (-8.6-11.9)	236 (171-319)	0.6 (-10.1-10.8)	0.5 (0.2-0.8)	3 (-18-29.2)	507 (369-687)	-3.1 (-12.4-6.2)
Tharaka Nithi	1,070 (764-1,490)	1.8 (-8.8-14.8)	1,000 (717-1,400)	1.7 (-8.8-14.1)	676 (487-927)	-0.2 (-9.3-11)	216 (156-298)	-6 (-15.3-4)	0.5 (0.3-0.9)	36.7 (8.5-68.8)	381 (282-507)	-14.6 (-22.2--5.1)
Trans Nzoia	1,150 (814-1,590)	2 (-8.2-13.6)	1,080 (770-1,500)	1.9 (-8.3-13.7)	750 (540-1,020)	0 (-9.4-11.1)	250 (180-343)	-7.9 (-16.7-2.6)	0.6 (0.3-1)	-2.1 (-22.4-23.1)	450 (327-601)	-17.6 (-25.2--8.3)
Turkana	1,190 (841-1,640)	2.2 (-10.3-14.2)	1,120 (796-1,550)	2.1 (-10-14.2)	745 (538-1,010)	1.2 (-10-12.7)	245 (180-339)	-1.4 (-11.5-8.9)	0.5 (0.2-0.8)	-14.2 (-31.5-6.8)	507 (374-672)	-6.6 (-16.4-3.7)
Uasin Gishu	1,050 (755-1,470)	1.8 (-8.3-15)	994 (710-1,380)	1.7 (-8.1-15.2)	705 (509-977)	0.5 (-9.4-12)	226 (164-308)	-12.4 (-21--3.4)	0.7 (0.4-1.2)	6.5 (-14.1-33)	398 (292-536)	-18.5 (-25.8--9.9)
Vihiga	1,450 (1,020-2,030)	2.3 (-8-17)	1,360 (951-1,920)	2.3 (-7.4-16.7)	890 (646-1,240)	0.6 (-9-12.8)	291 (210-406)	1.3 (-8.6-13.3)	0.6 (0.3-0.9)	10.6 (-11.6-37.8)	508 (373-694)	-14.3 (-22.5--4)
Wajir	1,120 (791-1,570)	2.5 (-10.8-15.3)	1,050 (743-1,480)	2.7 (-10.4-15.6)	663 (486-908)	1.5 (-10-14.6)	227 (164-314)	4 (-6.6-15.8)	0.7 (0.3-1.1)	-3.2 (-22.5-19.2)	488 (360-657)	-1.9 (-13.2-10)
West Pokot	1,150 (811-1,610)	2.2 (-10.2-13.7)	1,080 (761-1,510)	2.2 (-10.2-13.9)	703 (508-978)	0.6 (-9.5-10.1)	238 (172-328)	2.1 (-8.4-12.3)	0.7 (0.3-1.1)	3.3 (-16.5-27.6)	499 (362-671)	-6.6 (-16-2.4)
Madagascar	357 (300-435)	-0.9 (-8.2-6.6)	331 (281-404)	-1.1 (-8.1-6.2)	203 (164-256)	-1.6 (-9.5-6.9)	65.8 (50-86.2)	-3.1 (-12.7-7.6)	0.3 (0.1-0.5)	6.2 (-16.5-35.2)	133 (111-162)	-10.9 (-17.3--4.1)
Malawi	66.4 (55.2-83.1)	0.1 (-8.5-8)	61.3 (51.3-78.2)	-0.4 (-8.4-7.4)	37.3 (29.9-48.5)	-1.2 (-9.2-7.7)	12.6 (9.4-16.7)	-0.5 (-11.1-12.1)	0 (0-0.1)	0.2 (-24.1-31.7)	25 (20.6-31.8)	-9.2 (-16.2--1.6)
Mozambique	8.1 (6.2-10.2)	0.2 (-7.6-8.8)	7.4 (5.8-9.4)	0 (-7.6-8.7)	4.5 (3.4-5.7)	-0.6 (-9.5-8.1)	1.4 (1-1.9)	2.5 (-9.4-15.2)	0 (0-0)	0.4 (-27.5-42.2)	3.2 (2.5-4)	-1.3 (-9.2-7.2)
Rwanda	16.4 (12.3-21.8)	0.1 (-9.2-9.6)	15.2 (11.3-20.2)	0 (-9.3-10.1)	9.3 (7.1-12.4)	0.8 (-8.4-11)	3 (2.2-4)	-7.2 (-16.9-3.8)	0 (0-0)	-3.6 (-28.1-31.8)	5.7 (4.4-7.5)	-15 (-22.7--7.2)
Somalia	0.1 (0-0.4)	4.4 (-55.8-71.9)	0.1 (0-0.3)	4.1 (-55.9-70.8)	0.1 (0-0.2)	3.5 (-57.1-70.7)	0 (0-0.1)	-1.9 (-61.5-61.9)	0 (0-0)	-24 (-76.1-29.6)	0.1 (0-0.1)	1.7 (-57.3-65.6)
South Sudan	21.6 (15.4-29.9)	0 (-10.3-11.6)	19.9 (14.1-27.7)	0.1 (-9.8-11.3)	11.3 (8.3-15.9)	-1.9 (-11.2-9)	3.7 (2.7-5.1)	-1.3 (-12.9-10)	0 (0-0)	8.9 (-18.3-44.1)	8.1 (6-11)	-5.4 (-14.5-4.2)
Uganda	571 (467-663)	-0.2 (-7.8-7.1)	528 (433-615)	-0.5 (-7.9-7)	323 (260-393)	-1 (-9-7.9)	109 (83.9-141)	-2.8 (-11.9-8.4)	0.3 (0.1-0.5)	13.6 (-12.1-45.2)	232 (193-274)	-8.7 (-15.7--1.6)
United Republic of Tanzania	603 (489-715)	0.9 (-7.7-12)	563 (461-668)	0.6 (-7.7-11.1)	368 (299-448)	0.5 (-8.7-10.5)	131 (102-168)	-3.6 (-12.8-7.8)	0.5 (0.2-0.7)	13 (-10.3-40.4)	251 (207-300)	-5.1 (-13.2-4.2)
Zambia	721 (610-905)	0.3 (-8-9.4)	669 (572-844)	0.1 (-8-9.2)	424 (341-541)	-0.3 (-8.1-9.7)	135 (101-178)	-6.4 (-15.4-4.4)	0.4 (0.2-0.6)	10.1 (-11.9-38.6)	280 (234-354)	-10.7 (-17.7--2.9)
**Southern Sub-Saharan Africa**	4.2 (3.1-5.4)	-3.1 (-11.9-12.3)	3.8 (2.9-5)	-3.1 (-11.8-11.8)	2.6 (2-3.4)	4.1 (-4.7-17.9)	0.8 (0.6-1)	-8.7 (-15.9-0.2)	0 (0-0)	6 (-27.5-36.1)	1.3 (1-1.7)	-12.9 (-19.7--2)
Botswana	38 (30.8-46.6)	0.1 (-8.8-8.8)	36.1 (29.2-44.3)	0.1 (-8.7-8.8)	27.4 (22.2-33.6)	0.4 (-7.8-8.4)	10.7 (8.7-13)	-14.1 (-20.9--7.4)	0.1 (0-0.2)	-1.2 (-42.1-35.8)	15 (12.5-17.9)	-16.9 (-23.3--10.7)
Eswatini	0.2 (0.1-0.3)	-1.2 (-27.6-0.3)	0.2 (0.1-0.3)	0.2 (-25.7-3)	0.1 (0.1-0.2)	2.3 (-24.6-11.2)	0 (0-0.1)	-8.7 (-30.1-9.4)	0 (0-0)	-7.7 (-41.6-36.4)	0.1 (0-0.1)	-13.7 (-35.9--5.6)
Lesotho	0.2 (0.1-0.3)	-2 (-29.9-0)	0.2 (0.1-0.3)	-2 (-29.8-1.8)	0.1 (0.1-0.2)	-2.1 (-29-10.8)	0 (0-0.1)	-11.4 (-34.4-6)	0 (0-0)	-5.8 (-40.6-39.5)	0.1 (0-0.1)	-15.4 (-37.4--3.7)
Namibia	0.1 (0.1-0.2)	-1.6 (-28.6-0)	0.1 (0.1-0.1)	-1.7 (-29-0.8)	0.1 (0-0.1)	-1.7 (-26.5-5.3)	0 (0-0)	-10.3 (-32.2-2.7)	0 (0-0)	9.6 (-25.7-50.8)	0 (0-0.1)	-12.4 (-35.2--5.9)
South Africa	0 (0-0.1)	-0.8 (-3.5-0)	0 (0-0.1)	-1 (-4-0.3)	0 (0-0.1)	-0.1 (-3.8-3.1)	0 (0-0)	-11.4 (-18.4--6)	0 (0-0)	4.8 (-15.4-23.8)	0 (0-0)	-16.4 (-20.1--13.4)
Eastern Cape	0 (0-0.1)	-0.8 (0-0)	0 (0-0.1)	-0.8 (-0.9-0.7)	0 (0-0.1)	-0.4 (-3.7-4.5)	0 (0-0)	-12.3 (-21.4--2.8)	0 (0-0)	1.7 (-27-51.1)	0 (0-0)	-19.7 (-23.2--15.3)
Free State	0 (0-0.1)	-0.2 (0-0)	0 (0-0.1)	-0.3 (-0.9-0.5)	0 (0-0.1)	0 (-4-3.9)	0 (0-0)	-13 (-20.4--3.6)	0 (0-0)	-4.4 (-31.6-28.7)	0 (0-0)	-16.7 (-20.6--12.2)
Gauteng	0 (0-0.1)	1.1 (0-0)	0 (0-0.1)	0.9 (-0.8-0.7)	0 (0-0.1)	2 (-2.2-5.6)	0 (0-0)	-8.9 (-17.5-2)	0 (0-0)	-9 (-30.2-25.9)	0 (0-0)	-7.5 (-12.7--2.2)
KwaZulu-Natal	0 (0-0.1)	-0.4 (0-0)	0 (0-0.1)	-0.6 (-0.8-0.8)	0 (0-0.1)	-0.6 (-3.7-4.4)	0 (0-0)	-12.1 (-20.3--2.6)	0 (0-0)	-4.7 (-30.8-29.8)	0 (0-0)	-16.4 (-20.1--11.3)
Limpopo	0 (0-0.1)	-0.3 (0-0)	0 (0-0.1)	-0.4 (-0.8-0.7)	0 (0-0.1)	-0.1 (-4-4.6)	0 (0-0)	-14.3 (-21.6--5.9)	0 (0-0)	8 (-21.9-47.3)	0 (0-0)	-18.6 (-22.3--14.3)

Location	Birth Incidence rate (per 100,000 live births), 2021	Birth Incidence rate (per 100,000 live births) percent change (2000-2021)	Under-5 rate (per 100,000 population), 2021	Under-5 rate (per 100,000 population) percent change (2000-2021)	5-14 rate (per 100,000 population), 2021	5-14 rate (per 100,000 population) percent change (2000-2021)	15-49 rate (per 100,000 population), 2021	15-49 rate (per 100,000 population) percent change (2000-2021)	50plus rate (per 100,000 population), 2021	50plus rate (per 100,000 population) percent change (2000-2021)	All age rate (per 100,000 population), 2021	All age rate (per 100,000 population) percent change (2000-2021)
Mpumalanga	0 (0-0.1)	0 (0-0)	0 (0-0.1)	-0.1 (-0.8-0.6)	0 (0-0.1)	0.2 (-4-3.5)	0 (0-0)	-11.4 (-20.2--4)	0 (0-0)	0.7 (-25.1-32.3)	0 (0-0)	-16.4 (-20.7--12.9)
North-West	0 (0-0.1)	-2.7 (0-0)	0 (0-0.1)	-2.8 (-1.2-0.5)	0 (0-0.1)	-2.3 (-4.6-4.3)	0 (0-0)	-12.8 (-21.1--0.7)	0 (0-0)	-12.4 (-35.3-27.7)	0 (0-0)	-14.4 (-17.7--7.8)
Northern Cape	0 (0-0.1)	-0.1 (0-0)	0 (0-0.1)	-0.1 (-0.9-0.6)	0 (0-0.1)	-0.1 (-3.9-3.8)	0 (0-0)	-5.9 (-16-3)	0 (0-0)	-3.3 (-29.8-35.7)	0 (0-0)	-13 (-17.8--8.6)
Western Cape	0 (0-0)	0 (0-0)	0 (0-0)	-0.1 (-0.7-0.4)	0 (0-0)	0.3 (-2.2-3.1)	0 (0-0)	-10 (-17.6--1.5)	0 (0-0)	-4.4 (-26-19.8)	0 (0-0)	-18.1 (-21.9--14.2)
Zimbabwe	11.2 (8.2-15.3)	-14.1 (-24.8-5.7)	10.5 (7.6-14.3)	-14 (-24.4-5.4)	7.1 (5.2-9.5)	-12.2 (-22.9-5.5)	2.3 (1.6-3.1)	-20.1 (-31.2--6.5)	0 (0-0)	-2.9 (-30.4-38.9)	4.4 (3.3-5.9)	-19 (-28.4--3.3)
**Western Sub-Saharan Africa**	1,090 (846-1,410)	-7.6 (-11.3--3.7)	1,030 (804-1,330)	-6.3 (-9.9--2.5)	749 (585-977)	0.6 (-2.9-4.4)	362 (280-468)	6.6 (2.6-10.9)	2.7 (1.6-4.2)	22.2 (14.5-29.4)	538 (425-679)	-2.9 (-6.2-0.8)
Benin	917 (710-1,180)	-27.7 (-40--16.9)	870 (672-1,120)	-27.9 (-40.1--17.4)	648 (492-841)	-29.4 (-40.9--19.5)	347 (263-456)	-32.3 (-42.9--22.7)	2.8 (1.7-4.4)	-8.6 (-28.8-18.8)	491 (381-628)	-32.1 (-42.8--22.7)
Burkina Faso	1,120 (866-1,420)	-22.9 (-37.9--6.2)	1,060 (823-1,360)	-23.5 (-38.3--7)	798 (617-1,030)	-29 (-42.6--13.9)	423 (321-544)	-40.3 (-52.5--27.1)	3.7 (2.2-5.7)	-19.3 (-44.5-15.7)	602 (472-759)	-32.7 (-45.6--18.8)
Cabo Verde	61.6 (46.7-80.7)	2.4 (-6.6-11.8)	59.3 (44.9-77.9)	2.5 (-6.5-11.7)	49.6 (37.3-65.4)	2.6 (-6.3-11.5)	27.7 (20.5-35.8)	-4.9 (-13.7-4.6)	0.2 (0.1-0.4)	72.4 (39.8-112)	29.5 (22.4-38.1)	-15.9 (-23.5--8)
Cameroon	764 (648-878)	1.2 (-5.7-9)	723 (613-829)	1 (-6.1-8.5)	526 (438-623)	0.6 (-6.9-7.9)	263 (210-326)	-1.1 (-10.7-8.3)	1.5 (0.9-2.3)	6.2 (-13.7-32.1)	372 (313-437)	-5.1 (-11.8-2.1)
Chad	174 (140-203)	-0.8 (-7.5-6.9)	164 (133-193)	-1 (-7.8-6.7)	116 (94-141)	-1.5 (-8.8-7.3)	59 (46.2-74.7)	1.1 (-8.6-11.2)	0.4 (0.2-0.6)	12.4 (-8.8-40.8)	93 (76.9-113)	0 (-7-8.3)
Côte d'Ivoire	303 (249-351)	-0.1 (-8-7.9)	287 (237-335)	-0.3 (-7.5-7.6)	210 (174-255)	-0.2 (-7.8-8.1)	101 (79-126)	-6 (-14.9-2.3)	0.6 (0.3-1)	1 (-19.8-27.2)	147 (123-176)	-7 (-13.8-0.4)
Gambia	478 (390-567)	-2 (-9.8-7.4)	454 (372-542)	-2.4 (-10-6.8)	339 (275-408)	-2.7 (-10.5-5.8)	180 (141-222)	-3 (-12.4-6.1)	1 (0.6-1.7)	5.1 (-15.7-28.5)	243 (200-291)	-8 (-15.5--0.1)
Ghana	739 (656-918)	-14.8 (-25.6-1.9)	702 (623-877)	-15 (-25.9-1.7)	533 (455-672)	-15.7 (-25.8-0)	273 (222-343)	-15.7 (-25.6--1.3)	1.9 (1.1-2.9)	-1 (-21.5-22.1)	353 (301-442)	-21.8 (-31.1--7.6)
Guinea	1,550 (1,280-1,800)	-0.1 (-7.8-9.6)	1,460 (1,210-1,700)	-0.2 (-7.8-9.1)	1,020 (846-1,230)	-0.5 (-8.7-8.7)	510 (407-641)	1.2 (-8.2-11.2)	3 (1.9-4.7)	12.1 (-8.5-40.3)	766 (642-918)	-0.2 (-8.4-8.6)
Guinea-Bissau	33.3 (25.4-43.3)	-8.1 (-18-3.6)	31.4 (24-41)	-8.2 (-18.1-3.3)	22.7 (17.4-29.8)	-7.7 (-16.6-2)	11.1 (8.4-14.5)	-8.9 (-18.6-0.4)	0.1 (0-0.1)	1.9 (-18.8-28.2)	16.2 (12.5-20.9)	-13.1 (-21.9--4)
Liberia	210 (171-247)	0.1 (-6.5-8.3)	198 (162-234)	-0.3 (-6.8-7.7)	141 (114-170)	-1.4 (-8.3-6.3)	68.7 (53.6-86.3)	-6.6 (-14.3-1.8)	0.5 (0.3-0.8)	28 (3.3-58.6)	96.6 (78.8-117)	-10.2 (-16.2--3.5)
Mali	322 (268-383)	0.1 (-7.5-7.3)	303 (254-361)	0 (-7.4-7)	213 (173-262)	-0.5 (-8.3-6.2)	110 (86.6-137)	2.6 (-6.9-11.3)	0.7 (0.4-1.1)	7.2 (-12.7-35)	165 (138-201)	0.3 (-7-7)
Mauritania	161 (126-192)	-0.1 (-8.5-8.5)	153 (120-183)	-0.2 (-8.4-8.3)	117 (93.1-144)	-0.8 (-9.8-8)	64.2 (49.5-80.2)	0.8 (-8.4-10.8)	0.4 (0.2-0.7)	3.7 (-16.1-28.3)	82 (65.5-98.5)	-6.4 (-14.5-2.1)
Niger	521 (451-657)	-1.3 (-10.2-12.3)	492 (426-623)	-1.7 (-10.5-11.9)	350 (288-464)	-4.3 (-13.4-8.5)	188 (148-248)	-2.1 (-12.4-11.3)	1.3 (0.8-2)	-15.5 (-35.1-8.3)	286 (242-370)	-2.2 (-10.9-10.6)
Nigeria	1,570 (1,150-2,150)	-4 (-9.4-1.9)	1,490 (1,090-2,040)	-3.7 (-9.1-2)	1,040 (771-1,400)	6.1 (0.8-11.5)	490 (364-650)	23.1 (17.1-29.9)	3.8 (2.2-5.9)	42.3 (32.8-52.1)	748 (557-991)	4.6 (-0.3-9.6)
Sao Tome and Principe	906 (694-1,140)	0.1 (-9.1-10.5)	863 (661-1,080)	-0.1 (-9.4-10.2)	658 (516-835)	-0.2 (-9.2-9.2)	354 (273-448)	-6.8 (-14.9-1.7)	2.6 (1.5-3.9)	41.2 (14.3-73.6)	439 (341-550)	-12.1 (-19.7--4.3)
Senegal	457 (377-537)	-0.3 (-7.8-6.8)	433 (358-514)	-0.5 (-7.9-6.7)	321 (267-385)	-0.8 (-8.2-6.3)	172 (138-214)	-1.7 (-9.4-6.3)	1.1 (0.7-1.7)	-1.9 (-19.9-25.8)	227 (190-270)	-6.2 (-12.8-0.6)
Sierra Leone	2,150 (1,770-2,520)	-1.5 (-9.7-6.4)	2,030 (1,680-2,380)	-1.8 (-9.3-6.2)	1,400 (1,150-1,690)	-1.9 (-9.8-5.7)	692 (548-866)	0.8 (-8.2-10.3)	4.2 (2.6-6.6)	12.2 (-9.9-37.2)	995 (830-1,190)	-5.9 (-13.3-1.4)
Togo	1,130 (944-1,330)	0.4 (-7.1-7.6)	1,070 (896-1,260)	0.2 (-7.3-7.5)	784 (649-941)	-0.1 (-7.9-7.9)	382 (300-480)	-4.5 (-12.6-4.5)	2.6 (1.6-4)	2.8 (-16.7-26.4)	534 (451-639)	-9.4 (-16.1--2.2)

Table S15: Hemoglobin SC disease disease frequency: Birth rate, age-specific and all-age prevalence rates , 2000 - 2021

Location	Birth Incidence rate (per 100,000 live births), 2021	Birth Incidence rate (per 100,000 live births) percent change (2000-2021)	Under-5 rate (per 100,000 population), 2021	Under-5 rate (per 100,000 population) percent change (2000-2021)	5-14 rate (per 100,000 population), 2021	5-14 rate (per 100,000 population) percent change (2000-2021)	15-49 rate (per 100,000 population), 2021	15-49 rate (per 100,000 population) percent change (2000-2021)	50plus rate (per 100,000 population), 2021	50plus rate (per 100,000 population) percent change (2000-2021)	All age rate (per 100,000 population), 2021	All age rate (per 100,000 population) percent change (2000-2021)
Global	75.1 (56.6-98.1)	14.7 (9.4-19.5)	63.8 (48.1-82.7)	20.2 (14.3-25.5)	43.6 (32.9-56)	36.2 (28.7-42.7)	8 (6-10.5)	22.3 (16-27.8)	0.1 (0-0.1)	-11.7 (-14.6--8.9)	16.7 (12.8-21.4)	9.8 (4-14.7)
Central Europe, Eastern Europe, and Central Asia	0.1 (0.1-0.1)	-12.1 (-17.3--7.2)	0.1 (0.1-0.1)	-14.1 (-19.2--9.2)	0.1 (0.1-0.1)	-10.9 (-16--6.3)	0 (0-0)	-18.7 (-23.6--14.4)	0 (0-0)	-15.9 (-21.1--11.5)	0 (0-0)	-20.8 (-25.2--16.8)
**Central Asia**	0 (0-0)	-1.7 (-15.2-0.6)	0 (0-0)	-1.3 (-15.7-3.4)	0 (0-0)	0 (-12.3-7.7)	0 (0-0)	-8.7 (-19.4--1.5)	0 (0-0)	23.5 (9.4-37.6)	0 (0-0)	-11.1 (-22.2--4.9)
Armenia	0 (0-0)	1.4 (-0.5-44.5)	0 (0-0)	1.3 (-2.8-41)	0 (0-0)	2.8 (-3.7-41.7)	0 (0-0)	-5 (-12.7-22.4)	0 (0-0)	1.7 (-8.9-19.7)	0 (0-0)	-14.2 (-19.5-14.8)
Azerbaijan	0 (0-0)	-2.9 (0-0.1)	0 (0-0)	-1.9 (-3.5-4.3)	0 (0-0)	-1.1 (-6.6-8.5)	0 (0-0)	-7.6 (-15.9-2.1)	0 (0-0)	37.1 (21-55.6)	0 (0-0)	-19.2 (-24.4--12.1)
Georgia	0 (0-0)	-1.6 (-1.8-0)	0 (0-0)	-1.2 (-6.4-2.9)	0 (0-0)	0 (-9.9-5.6)	0 (0-0)	-7.4 (-21.1-0.1)	0 (0-0)	5.8 (-8.9-18.7)	0 (0-0)	-10.2 (-18.3--4.5)
Kazakhstan	0 (0-0)	3.7 (-22.1-381)	0 (0-0)	3.6 (-22.3-358)	0 (0-0)	5.7 (-20.9-341)	0 (0-0)	-5.3 (-32.5-235)	0 (0-0)	11.6 (-28.5-146)	0 (0-0)	0.9 (-25.8-304)
Kyrgyzstan	0 (0-0)	-3.1 (-45.5-0)	0 (0-0)	-2.2 (-41.3-4)	0 (0-0)	-1.4 (-38.8-8.1)	0 (0-0)	-6.4 (-35.8-3.2)	0 (0-0)	33.5 (15.7-51)	0 (0-0)	-6.2 (-40.1-2.3)
Mongolia	0 (0-0)	-2.2 (-0.7-0)	0 (0-0)	-2.6 (-8-3.2)	0 (0-0)	0.5 (-10.9-11.7)	0 (0-0)	-19.6 (-39.4--7.9)	0 (0-0)	20 (-30.7-48.9)	0 (0-0)	-14.8 (-23.6--5.6)
Tajikistan	0 (0-0)	-2.6 (0-0)	0 (0-0)	-1.8 (-4.6-4.7)	0 (0-0)	-0.6 (-9.6-9.7)	0 (0-0)	-7.4 (-17.7-3.1)	0 (0-0)	47.4 (22.2-69.8)	0 (0-0)	-13 (-19.9--5)
Turkmenistan	0 (0-0)	-4.5 (-31.9-0)	0 (0-0)	-3.9 (-30.9-4.7)	0 (0-0)	-2.2 (-31.1-11.3)	0 (0-0)	-11.7 (-39.3-1.6)	0 (0-0)	21.6 (-27.8-48.1)	0 (0-0)	-16.5 (-40.9--6)
Uzbekistan	0 (0-0)	-2.8 (-42.3-0.4)	0 (0-0)	-1.6 (-41.3-5.3)	0 (0-0)	-1.5 (-40.4-10.2)	0 (0-0)	-11.1 (-51.2-0.7)	0 (0-0)	29.3 (-42.5-53.1)	0 (0-0)	-15.7 (-51--6.4)
**Central Europe**	0.3 (0.3-0.4)	1.9 (-4.5-9.6)	0.3 (0.2-0.3)	1.1 (-5.5-8.6)	0.2 (0.2-0.2)	-0.1 (-6.7-7.4)	0.1 (0.1-0.1)	-13.4 (-19.5--7.4)	0 (0-0)	-17 (-23.1--10.7)	0.1 (0.1-0.1)	-19.3 (-24.5--13.6)
Albania	0.2 (0.2-0.3)	-1.6 (-22.3-0.1)	0.2 (0.1-0.2)	0.1 (-21-4.8)	0.1 (0.1-0.1)	0.2 (-20-8.1)	0 (0-0.1)	-3.4 (-22.4-5.1)	0 (0-0)	-12.6 (-31.4--1.6)	0 (0-0.1)	-28.6 (-43.2--23.6)
Bosnia and Herzegovina	0.1 (0.1-0.2)	-0.6 (-18-0)	0.1 (0.1-0.2)	-0.2 (-19-3.3)	0.1 (0.1-0.1)	0.6 (-17.3-6)	0 (0-0.1)	-4.1 (-21.5-2.1)	0 (0-0)	-10.4 (-28--0.5)	0 (0-0.1)	-21.4 (-36--17.1)
Bulgaria	0.2 (0.1-0.3)	-1.6 (-28.6-0.1)	0.1 (0.1-0.2)	-2.2 (-28.3-3.3)	0.1 (0.1-0.1)	0.5 (-25.5-9.5)	0 (0-0.1)	-10.9 (-33.4-2.9)	0 (0-0)	-18 (-41.2--2.6)	0 (0-0)	-13.4 (-36.1--2.4)
Croatia	1.3 (1-1.7)	-6.9 (-16.7-2.6)	1.1 (0.9-1.5)	-5.8 (-15.7-4)	0.8 (0.7-1.1)	-4.2 (-14.1-5.9)	0.4 (0.3-0.5)	-5.3 (-13.6-3.4)	0 (0-0.1)	-8.5 (-16.9-1.4)	0.3 (0.2-0.4)	-18.5 (-26.1--10.9)
Czechia	0 (0-0)	-0.7 (-18.3-0)	0 (0-0)	-0.5 (-17.7-2.4)	0 (0-0)	0 (-16.7-5)	0 (0-0)	-10.7 (-27.9--3.1)	0 (0-0)	-27.9 (-45.5--21.2)	0 (0-0)	-12.4 (-28.4--6.9)
Hungary	0.1 (0.1-0.2)	-2.1 (-27.4-0)	0.1 (0.1-0.2)	-2 (-26.7-1.9)	0.1 (0.1-0.1)	-1.6 (-26.6-4.1)	0 (0-0.1)	-9.6 (-33--2.8)	0 (0-0)	-19.5 (-45--10.6)	0 (0-0)	-16.9 (-38.2--11.9)
Montenegro	0.2 (0.1-0.2)	-1.4 (-25.1-0)	0.1 (0.1-0.2)	-1.2 (-24.7-2.7)	0.1 (0.1-0.1)	-0.4 (-23.4-5.8)	0 (0-0.1)	-4.2 (-28.9-4.7)	0 (0-0)	-7.7 (-37.9-4.1)	0 (0-0.1)	-16.4 (-37--10.4)
North Macedonia	0.2 (0.1-0.3)	-1.1 (-25.7-9.3)	0.2 (0.1-0.2)	-0.4 (-24.6-8.6)	0.1 (0.1-0.1)	0.3 (-22.3-9.1)	0 (0-0.1)	-5.7 (-26.7-5.4)	0 (0-0)	-7.7 (-29.1-3.4)	0 (0-0.1)	-18 (-36.7--10.2)
Poland	0.4 (0.3-0.5)	11.8 (-5.9-30.8)	0.4 (0.3-0.5)	10.8 (-6.9-29.2)	0.2 (0.2-0.3)	1.6 (-13.6-18.7)	0.1 (0.1-0.1)	-19.3 (-31.5--6.4)	0 (0-0)	-32.7 (-43.3--20.6)	0.1 (0.1-0.1)	-21.4 (-32.7--8.3)
Romania	0.5 (0.4-0.7)	2.4 (-3.4-27.9)	0.4 (0.3-0.5)	2.2 (-4.5-26.6)	0.3 (0.2-0.3)	4.2 (-6.2-26.7)	0.1 (0.1-0.1)	-6.8 (-17.6-11.2)	0 (0-0)	0.2 (-14.5-20.7)	0.1 (0.1-0.1)	-15.7 (-24.2-1.4)
Serbia	0 (0-0.1)	1.6 (0-33.2)	0 (0-0)	1.4 (-2.7-28.6)	0 (0-0)	1.8 (-4.3-27.9)	0 (0-0)	-1.5 (-8.5-25.2)	0 (0-0)	-11 (-19.5-8)	0 (0-0)	-13.9 (-19.1-8.6)
Slovakia	0.4 (0.3-0.5)	-5.3 (-25.8-21.6)	0.3 (0.3-0.4)	-4.7 (-25.6-22.8)	0.2 (0.2-0.3)	-4.3 (-24.9-22.7)	0.1 (0.1-0.1)	-12.6 (-29.2-11.3)	0 (0-0)	-18.6 (-31.2-6.4)	0.1 (0.1-0.1)	-21 (-36.8-1.5)
Slovenia	0.3 (0.2-0.3)	-3.3 (-32.7-0.4)	0.2 (0.2-0.3)	-3.3 (-32.7-2)	0.2 (0.1-0.2)	-1.7 (-31.2-5.8)	0.1 (0.1-0.1)	-8.5 (-33.9-0.5)	0 (0-0)	-12.9 (-36.2--1.9)	0.1 (0.1-0.1)	-17.3 (-40.7--10.5)
**Eastern Europe**	0.1 (0.1-0.1)	-2.2 (-9.4-0.1)	0.1 (0-0.1)	-2.3 (-9.5--0.1)	0 (0-0.1)	0.2 (-7.3-3.4)	0 (0-0)	-8.9 (-16.8--5)	0 (0-0)	-7.5 (-15.7--2.9)	0 (0-0)	-10 (-16.4--7)
Belarus	0.1 (0-0.1)	-2.9 (-37.8-0)	0.1 (0-0.1)	-2.9 (-35.6-1.8)	0 (0-0.1)	-0.6 (-32.9-6.2)	0 (0-0)	-8 (-39.7-0.1)	0 (0-0)	3.4 (-33.5-15.3)	0 (0-0)	-12.5 (-42--5.9)
Estonia	0.1 (0-0.1)	-2 (-42.2-0)	0.1 (0-0.1)	-1.7 (-42-2)	0 (0-0.1)	-0.1 (-41.7-5.6)	0 (0-0)	-8.6 (-49.4--1.1)	0 (0-0)	-18.7 (-55.7--8.8)	0 (0-0)	-13 (-50.7--7)

Location	Birth Incidence rate (per 100,000 live births), 2021	Birth Incidence rate (per 100,000 live births) percent change (2000-2021)	Under-5 rate (per 100,000 population), 2021	Under-5 rate (per 100,000 population) percent change (2000-2021)	5-14 rate (per 100,000 population), 2021	5-14 rate (per 100,000 population) percent change (2000-2021)	15-49 rate (per 100,000 population), 2021	15-49 rate (per 100,000 population) percent change (2000-2021)	50plus rate (per 100,000 population), 2021	50plus rate (per 100,000 population) percent change (2000-2021)	All age rate (per 100,000 population), 2021	All age rate (per 100,000 population) percent change (2000-2021)
Latvia	0.1 (0.1-0.1)	-4.8 (-41.8--0.6)	0.1 (0.1-0.1)	-4.8 (-41.3-1.2)	0.1 (0-0.1)	-3.1 (-40.4-5.1)	0 (0-0)	-9.6 (-45.3--0.1)	0 (0-0)	-14.6 (-47.4--1.9)	0 (0-0)	-16.6 (-49.1--8.9)
Lithuania	0.2 (0.1-0.2)	2.7 (0-36.2)	0.1 (0.1-0.2)	3.2 (-1.1-36.8)	0.1 (0.1-0.1)	4.4 (-1.8-40.3)	0.1 (0-0.1)	-1.3 (-8.2-38.2)	0 (0-0)	-2.7 (-12.2-44.5)	0 (0-0.1)	-16.1 (-21.7-15.1)
Republic of Moldova	0.1 (0.1-0.1)	-2.1 (-22.2-0.1)	0.1 (0-0.1)	-1.5 (-22.2-3.9)	0 (0-0.1)	-0.1 (-22.4-7.3)	0 (0-0)	-8.9 (-29.3-0.5)	0 (0-0)	-4 (-26.2-9.4)	0 (0-0)	-21.4 (-38.4--14.5)
Russian Federation	0.1 (0.1-0.1)	-1.9 (-11.5-0.1)	0.1 (0-0.1)	-1.8 (-11.3-0.3)	0 (0-0.1)	1.4 (-8.1-4.4)	0 (0-0)	-8.5 (-16.2--4.7)	0 (0-0)	-8.9 (-16.1--4.4)	0 (0-0)	-7.8 (-15.6--4.6)
Ukraine	0.1 (0.1-0.1)	-1.4 (-21.1-0)	0.1 (0-0.1)	-1.1 (-20.6-1.6)	0 (0-0.1)	0.8 (-19.3-5.8)	0 (0-0)	-8.8 (-27.1--1.7)	0 (0-0)	-2.7 (-24.5-9.2)	0 (0-0)	-11.8 (-29.5--6.7)
High-income	6.3 (5.3-7.6)	-6.1 (-9--3.5)	4.9 (4.1-5.8)	2.6 (0.2-5)	3.7 (3.1-4.4)	10.3 (7.7-13.3)	1.7 (1.5-2.1)	12 (9.4-14.6)	0.2 (0.2-0.3)	5.4 (1.5-9.4)	1.5 (1.3-1.8)	-3.6 (-5.6--1.5)
**Australasia**	0.2 (0.1-0.2)	-4 (-17.9-12.2)	0.1 (0.1-0.2)	-5.9 (-19.3-5.9)	0.1 (0.1-0.1)	-7.7 (-21.2-2.7)	0 (0-0.1)	-6.6 (-19.3-7)	0 (0-0)	-19.6 (-29.8--9.1)	0 (0-0.1)	-16.3 (-28.2--5.3)
Australia	0.1 (0.1-0.2)	0.6 (-11.4-25.6)	0.1 (0.1-0.1)	0.7 (-6.4-19)	0.1 (0-0.1)	-0.1 (-9-16.3)	0 (0-0)	-1.4 (-11.4-18.2)	0 (0-0)	-15.6 (-26.3-3.3)	0 (0-0)	-10.4 (-18.7-6.5)
New Zealand	0.4 (0.3-0.5)	-5.6 (-29.9-4.3)	0.3 (0.2-0.4)	-9.1 (-33.3-2.5)	0.2 (0.2-0.3)	-15.8 (-37.9--3.1)	0.1 (0.1-0.1)	-13 (-33.7-0.5)	0 (0-0)	-25.9 (-42.7--12.4)	0.1 (0.1-0.1)	-23.6 (-42.6--12.5)
**High-income Asia Pacific**	0.1 (0-0.1)	-0.3 (-14.8-5.8)	0 (0-0.1)	-3.7 (-16-2.3)	0 (0-0)	-6.5 (-17.6-1.9)	0 (0-0)	-3 (-16.2-6.4)	0 (0-0)	8.2 (-16.2-46.9)	0 (0-0)	-21.4 (-32.3--14.5)
Brunei Darussalam	0.2 (0.1-0.2)	0.5 (-0.3-33.5)	0.1 (0.1-0.1)	0.2 (-6.6-28.1)	0.1 (0-0.1)	-0.4 (-13.5-23.3)	0 (0-0)	-5.4 (-18.3-18.2)	0 (0-0)	-5.9 (-21.8-23)	0 (0-0)	-20.5 (-30.1--0.5)
Japan	0 (0-0.1)	-0.2 (-5.7-0.4)	0 (0-0)	-0.8 (-6.3-0.5)	0 (0-0)	-0.4 (-6.6-1.9)	0 (0-0)	-6.2 (-9.9--3.3)	0 (0-0)	-30.4 (-32.7--28.1)	0 (0-0)	-19.8 (-23.2--17.7)
Aichi	0 (0-0.1)	-1.2 (-23.3-0.2)	0 (0-0)	-1.9 (-24.8-1.9)	0 (0-0)	-1.6 (-24.6-4.5)	0 (0-0)	-7.5 (-26.8--1.4)	0 (0-0)	-30.7 (-41.8--24.5)	0 (0-0)	-18.6 (-36.2--13.9)
Akita	0 (0-0.1)	-2.9 (-45.1-0.1)	0 (0-0)	-3.5 (-45-2.2)	0 (0-0)	-3.8 (-45.3-4.8)	0 (0-0)	-8 (-41.8-2.3)	0 (0-0)	-35.9 (-50.5--26.4)	0 (0-0)	-30.9 (-57.4--24.4)
Aomori	0 (0-0.1)	0 (0-0.1)	0 (0-0)	-0.3 (-2.8-2.3)	0 (0-0)	-0.1 (-5-5.5)	0 (0-0)	-4.9 (-11.1-1.2)	0 (0-0)	-33.7 (-40.8--27.4)	0 (0-0)	-28.2 (-32.1--24.1)
Chiba	0 (0-0.1)	0 (0-0.2)	0 (0-0)	-0.6 (-3.6-2.5)	0 (0-0)	-0.4 (-6.2-4.9)	0 (0-0)	-7.1 (-14.4--0.5)	0 (0-0)	-36.3 (-42--29.4)	0 (0-0)	-22.3 (-27.5--17.6)
Ehime	0 (0-0.1)	-2.9 (-45.1-0.1)	0 (0-0)	-3.6 (-47.7-2.3)	0 (0-0)	-3.5 (-47.9-4.9)	0 (0-0)	-8.7 (-46.1-0.4)	0 (0-0)	-35.8 (-51.5--27.1)	0 (0-0)	-24.1 (-56.2--17.3)
Fukui	0 (0-0.1)	0 (0-0.2)	0 (0-0)	-0.4 (-2.7-2.4)	0 (0-0)	-0.9 (-5.2-4.1)	0 (0-0)	-4.7 (-10.1-1.2)	0 (0-0)	-31.7 (-37.7--25.4)	0 (0-0)	-20.7 (-24.4--16.4)
Fukuoka	0 (0-0.1)	0 (0-0.2)	0 (0-0)	-0.6 (-3.3-2.2)	0 (0-0)	0 (-4.9-5.2)	0 (0-0)	-7.3 (-13.3--1.6)	0 (0-0)	-33.2 (-39.4--27.2)	0 (0-0)	-17.8 (-22.3--13.9)
Fukushima	0 (0-0.1)	0.3 (0-0.1)	0 (0-0)	0 (-2.8-3)	0 (0-0)	0.1 (-4.9-5.8)	0 (0-0)	-5.8 (-12-1)	0 (0-0)	-31.1 (-37.9--24.1)	0 (0-0)	-28.2 (-32.3--23.9)
Gifu	0 (0-0.1)	-1.2 (-23.3-0.2)	0 (0-0)	-1.8 (-25.5-2.1)	0 (0-0)	-1.3 (-25.8-5.4)	0 (0-0)	-6.3 (-25.8-1.7)	0 (0-0)	-33.6 (-44.5--26.1)	0 (0-0)	-21.4 (-38.6--15.8)
Gunma	0 (0-0.1)	0.1 (0-0.2)	0 (0-0)	-0.5 (-3.2-2)	0 (0-0)	-0.3 (-5.3-4.5)	0 (0-0)	-5.3 (-11.7-0)	0 (0-0)	-33.2 (-38.8--26.9)	0 (0-0)	-21.7 (-26.3--17.9)
Hiroshima	0 (0-0.1)	-0.1 (0-0.2)	0 (0-0)	-0.8 (-3.6-1.7)	0 (0-0)	-0.3 (-5.8-4.2)	0 (0-0)	-6.6 (-13--0.2)	0 (0-0)	-33.6 (-40--27.5)	0 (0-0)	-17.9 (-22.8--13.5)
Hokkaidō	0 (0-0.1)	1.3 (0-30.5)	0 (0-0)	0.8 (-3.3-31)	0 (0-0)	1.3 (-5.1-31.8)	0 (0-0)	-5.8 (-12.7-17)	0 (0-0)	-34.8 (-41.4--24)	0 (0-0)	-23.8 (-28.7--3.8)
Hyōgo	0 (0-0.1)	-1.2 (-23.3-0.2)	0 (0-0)	-1.6 (-24.7-2.8)	0 (0-0)	-1.5 (-25.4-5.7)	0 (0-0)	-7.2 (-26.1--0.4)	0 (0-0)	-32.4 (-43.6--25.4)	0 (0-0)	-21.8 (-38.6--16.7)
Ibaraki	0 (0-0.1)	0 (0-0.2)	0 (0-0)	-0.7 (-3.6-1.7)	0 (0-0)	-0.2 (-5.5-5)	0 (0-0)	-6.2 (-13-0.7)	0 (0-0)	-35.9 (-42.7--30)	0 (0-0)	-23.9 (-28.4--19.5)
Ishikawa	0 (0-0.1)	1.3 (0-30.5)	0 (0-0)	0.9 (-2.9-31.3)	0 (0-0)	0.9 (-5.1-32)	0 (0-0)	-4.3 (-10.7-20.6)	0 (0-0)	-33.9 (-40.3--24.1)	0 (0-0)	-18.3 (-23.2-3.9)
Iwate	0 (0-0.1)	-0.2 (0-0.1)	0 (0-0)	-0.9 (-3.3-2)	0 (0-0)	-0.1 (-5-5)	0 (0-0)	-5.8 (-11.5-0.2)	0 (0-0)	-29.7 (-35.5--21.5)	0 (0-0)	-24.3 (-27.9--20.1)
Kagawa	0 (0-0.1)	0 (0-0.2)	0 (0-0)	-0.7 (-3.2-1.8)	0 (0-0)	-0.1 (-4.8-5.1)	0 (0-0)	-6.3 (-13--0.4)	0 (0-0)	-35.4 (-41.3--28.2)	0 (0-0)	-20.5 (-24.8--16.2)

Location	Birth Incidence rate (per 100,000 live births), 2021	Birth Incidence rate (per 100,000 live births) percent change (2000-2021)	Under-5 rate (per 100,000 population), 2021	Under-5 rate (per 100,000 population) percent change (2000-2021)	5-14 rate (per 100,000 population), 2021	5-14 rate (per 100,000 population) percent change (2000-2021)	15-49 rate (per 100,000 population), 2021	15-49 rate (per 100,000 population) percent change (2000-2021)	50plus rate (per 100,000 population), 2021	50plus rate (per 100,000 population) percent change (2000-2021)	All age rate (per 100,000 population), 2021	All age rate (per 100,000 population) percent change (2000-2021)
Kagoshima	0 (0-0.1)	0 (0-0.1)	0 (0-0)	-0.7 (-3.2-2.4)	0 (0-0)	-0.4 (-5.2-5.3)	0 (0-0)	-5.8 (-11.2-1.3)	0 (0-0)	-27.7 (-33.1--18.5)	0 (0-0)	-20.6 (-24.2--15.6)
Kanagawa	0 (0-0.1)	-1 (0-0.2)	0 (0-0)	-1.9 (-4.3-1.4)	0 (0-0)	-1.3 (-6.6-4.4)	0 (0-0)	-7.4 (-14.5--0.9)	0 (0-0)	-29.3 (-36.9--23)	0 (0-0)	-20.7 (-25.6--15.9)
Kōchi	0 (0-0.1)	0.3 (0-0.1)	0 (0-0)	-0.3 (-3.6-2.4)	0 (0-0)	-0.5 (-6-4.8)	0 (0-0)	-6.8 (-12.5--0.9)	0 (0-0)	-35.1 (-41.8--29)	0 (0-0)	-22 (-26.3--18)
Kumamoto	0 (0-0.1)	0 (0-0.2)	0 (0-0)	-0.6 (-3-2.9)	0 (0-0)	-0.2 (-4.7-6.9)	0 (0-0)	-4.9 (-10.3-3.1)	0 (0-0)	-30.3 (-36.7--22.2)	0 (0-0)	-18.1 (-22--12.1)
Kyōto	0 (0-0.1)	1 (0-0.2)	0 (0-0)	0.5 (-3-2.6)	0 (0-0)	0.6 (-5-5.8)	0 (0-0)	-6.2 (-12.8-0)	0 (0-0)	-33.2 (-39.4--25.8)	0 (0-0)	-18.9 (-23.9--14.3)
Mie	0 (0-0.1)	0 (0-0.2)	0 (0-0)	-0.7 (-3.4-1.9)	0 (0-0)	-0.4 (-5.4-4.7)	0 (0-0)	-5.8 (-11.9-0.1)	0 (0-0)	-30.4 (-35.8--22.9)	0 (0-0)	-20.7 (-25--16.5)
Miyagi	0 (0-0.1)	0 (0-0.2)	0 (0-0)	-0.8 (-3.3-1.8)	0 (0-0)	0 (-4.9-4.9)	0 (0-0)	-7 (-12.2--1.6)	0 (0-0)	-31.6 (-37.5--25)	0 (0-0)	-23 (-26.5--18.8)
Miyazaki	0 (0-0.1)	0 (0-0.1)	0 (0-0)	-0.7 (-3.4-1.7)	0 (0-0)	0.2 (-5.1-5.9)	0 (0-0)	-4.4 (-10.4-2)	0 (0-0)	-35.9 (-41.5--29.4)	0 (0-0)	-20.9 (-25.1--16.6)
Nagano	0 (0-0.1)	0 (0-0.2)	0 (0-0)	-0.6 (-3-1.6)	0 (0-0)	-0.4 (-4.9-3.5)	0 (0-0)	-5.1 (-11.3-0.2)	0 (0-0)	-27.8 (-34.2--20.6)	0 (0-0)	-21 (-25.2--17.6)
Nagasaki	0 (0-0.1)	-3 (-45.1-0.1)	0 (0-0)	-3.6 (-46-2.3)	0 (0-0)	-3.4 (-47.5-4.9)	0 (0-0)	-6.9 (-43.6-1.8)	0 (0-0)	-33.9 (-48.3--25.3)	0 (0-0)	-24.8 (-55--18.5)
Nara	0 (0-0.1)	0 (0-0.2)	0 (0-0)	-0.8 (-3.2-1.4)	0 (0-0)	-0.2 (-4.8-4)	0 (0-0)	-5.4 (-10.9-0)	0 (0-0)	-38.3 (-43.9--32.9)	0 (0-0)	-24 (-27.6--20.3)
Niigata	0 (0-0.1)	0 (0-0.2)	0 (0-0)	-0.5 (-3.1-1.9)	0 (0-0)	-0.2 (-4.4-4.5)	0 (0-0)	-5.2 (-10.3-0.4)	0 (0-0)	-30.8 (-36.2--24.9)	0 (0-0)	-21.5 (-25.1--17.4)
Ōita	0 (0-0.1)	-0.1 (0-0.2)	0 (0-0)	-0.4 (-3.2-2.2)	0 (0-0)	0 (-4.4-4.8)	0 (0-0)	-5.8 (-11.5--0.4)	0 (0-0)	-35.1 (-41--29.4)	0 (0-0)	-19.9 (-23.9--16.2)
Okayama	0 (0-0.1)	0.1 (0-0.1)	0 (0-0)	-0.4 (-3.7-2.6)	0 (0-0)	-0.1 (-5.7-6.1)	0 (0-0)	-5.7 (-12.2-2.2)	0 (0-0)	-33.7 (-39.8--26.2)	0 (0-0)	-17.6 (-22.4--11.7)
Okinawa	0 (0-0.1)	-3 (-45.1-0.1)	0 (0-0)	-3.8 (-46.3-2.4)	0 (0-0)	-3.3 (-46-5.7)	0 (0-0)	-7.2 (-42.1-1.6)	0 (0-0)	-22 (-38.8--12.9)	0 (0-0)	-19.9 (-52.2--12.8)
Ōsaka	0 (0-0.1)	0 (0-0.2)	0 (0-0)	-0.6 (-3.2-2.9)	0 (0-0)	-0.3 (-5.1-5.8)	0 (0-0)	-7 (-12.4-0.4)	0 (0-0)	-31.5 (-36.9--25.2)	0 (0-0)	-20 (-24.2--14.7)
Saga	0 (0-0.1)	-0.2 (0-0.1)	0 (0-0)	-1 (-3.8-2)	0 (0-0)	-0.4 (-5.7-5.6)	0 (0-0)	-4.5 (-10.6-2.3)	0 (0-0)	-31.6 (-38.6--25.1)	0 (0-0)	-19.7 (-24.2--15.1)
Saitama	0 (0-0.1)	0 (0-0.2)	0 (0-0)	-0.6 (-3.4-2.2)	0 (0-0)	-0.3 (-5.2-5)	0 (0-0)	-7.1 (-12.9--1.3)	0 (0-0)	-36.6 (-42.6--31.2)	0 (0-0)	-22.3 (-26.6--18.1)
Shiga	0 (0-0.1)	-1.2 (-23.3-0.2)	0 (0-0)	-2 (-25.4-2.4)	0 (0-0)	-1.7 (-25.7-5.6)	0 (0-0)	-7.5 (-27.5--0.1)	0 (0-0)	-31 (-43--23.9)	0 (0-0)	-19.6 (-37.4--13.7)
Shimane	0 (0-0.1)	-0.1 (0-0.2)	0 (0-0)	-0.5 (-2.7-1.8)	0 (0-0)	0.1 (-4.2-4.4)	0 (0-0)	-4.8 (-10.2-0.8)	0 (0-0)	-34.1 (-39.9--27.5)	0 (0-0)	-18.2 (-21.9--14.3)
Shizuoka	0 (0-0.1)	0 (0-0.2)	0 (0-0)	-0.7 (-3.1-2.5)	0 (0-0)	-0.5 (-5.3-5.8)	0 (0-0)	-5.7 (-11.2-1)	0 (0-0)	-33.8 (-39.2--28.5)	0 (0-0)	-21.4 (-25.3--16.7)
Tochigi	0 (0-0.1)	0.1 (0-0.1)	0 (0-0)	-0.8 (-3.4-1.9)	0 (0-0)	0 (-4.7-5.4)	0 (0-0)	-6.5 (-12.3--0.5)	0 (0-0)	-33.6 (-39.6--26.8)	0 (0-0)	-21.7 (-25.7--17.4)
Tokushima	0 (0-0.1)	-2.7 (-45.1-0.1)	0 (0-0)	-3.4 (-47.2-2.7)	0 (0-0)	-3.3 (-46.7-6.2)	0 (0-0)	-7.7 (-40.5-1.3)	0 (0-0)	-33.9 (-47.5--26)	0 (0-0)	-23.8 (-52.9--17.1)
Tōkyō	0 (0-0.1)	3 (0-0.2)	0 (0-0)	2.6 (-3.2-2.8)	0 (0-0)	3.2 (-5.1-7)	0 (0-0)	-5.1 (-13.6-1.8)	0 (0-0)	-16.5 (-24.6--8.8)	0 (0-0)	-10.1 (-17.4--4.9)
Tottori	0 (0-0.1)	0 (0-0.2)	0 (0-0)	-0.4 (-3.3-1.9)	0 (0-0)	0.3 (-5.1-4.9)	0 (0-0)	-4.5 (-9.9-1.3)	0 (0-0)	-32.3 (-38.2--26.5)	0 (0-0)	-18.2 (-22.1--14)
Toyama	0 (0-0.1)	0 (0-0.2)	0 (0-0)	-1.1 (-4.2-1.3)	0 (0-0)	-0.3 (-6.3-4.2)	0 (0-0)	-6.5 (-13.1--0.9)	0 (0-0)	-36 (-41.2--29.9)	0 (0-0)	-20.9 (-25.4--16.9)
Wakayama	0 (0-0.1)	0 (0-0.2)	0 (0-0)	-0.5 (-3.3-1.7)	0 (0-0)	-0.3 (-5.3-4.1)	0 (0-0)	-5.1 (-10.6-0.1)	0 (0-0)	-30.2 (-35.8--24)	0 (0-0)	-21.2 (-25--17.3)
Yamagata	0 (0-0.1)	0 (0-0.2)	0 (0-0)	-0.5 (-3.3-3.1)	0 (0-0)	-0.3 (-5.7-5.8)	0 (0-0)	-3.9 (-9.8-2.6)	0 (0-0)	-30.7 (-36.2--22.9)	0 (0-0)	-22.6 (-26.9--17.8)
Yamaguchi	0 (0-0.1)	-3 (-45.1-0.1)	0 (0-0)	-3.8 (-47-2.2)	0 (0-0)	-3.5 (-47.5-5.6)	0 (0-0)	-8.8 (-46.1-0.6)	0 (0-0)	-39 (-53.6--30.9)	0 (0-0)	-22.4 (-54.8--15.1)

Location	Birth Incidence rate (per 100,000 live births), 2021	Birth Incidence rate (per 100,000 live births) percent change (2000-2021)	Under-5 rate (per 100,000 population), 2021	Under-5 rate (per 100,000 population) percent change (2000-2021)	5-14 rate (per 100,000 population), 2021	5-14 rate (per 100,000 population) percent change (2000-2021)	15-49 rate (per 100,000 population), 2021	15-49 rate (per 100,000 population) percent change (2000-2021)	50plus rate (per 100,000 population), 2021	50plus rate (per 100,000 population) percent change (2000-2021)	All age rate (per 100,000 population), 2021	All age rate (per 100,000 population) percent change (2000-2021)
Yamanashi	0 (0-0.1)	-2.9 (-45.1-0.1)	0 (0-0)	-3.6 (-45.9-2.7)	0 (0-0)	-4 (-48.1-5.3)	0 (0-0)	-6.1 (-42.8-4.4)	0 (0-0)	-29.3 (-46.8--19)	0 (0-0)	-25.9 (-56.3--18.8)
Republic of Korea	0.1 (0.1-0.2)	-0.2 (-20.7-7.7)	0.1 (0.1-0.1)	-0.2 (-19.4-7.4)	0.1 (0-0.1)	-1 (-18.6-10.8)	0 (0-0)	-9.5 (-27.1-1.3)	0 (0-0)	-11 (-29.8-4.6)	0 (0-0)	-31.2 (-44.8--22.1)
Singapore	0.1 (0.1-0.2)	-0.4 (-20.8-0.5)	0.1 (0.1-0.1)	-2.5 (-22-3.1)	0.1 (0-0.1)	-0.3 (-21.2-11.6)	0 (0-0)	-10.5 (-27.6-0.1)	0 (0-0)	-15 (-28.2--2.9)	0 (0-0)	-23 (-37.6--14)
**High-income North America**	7.4 (6.4-8.4)	-23 (-25.8--20.6)	5.8 (5.2-6.5)	-8 (-10.2--5.9)	4.6 (4.1-5.1)	6.6 (2.8-10.8)	2.9 (2.6-3.2)	13.1 (9.1-17.1)	0.5 (0.4-0.6)	2 (-2.7-6.6)	2.4 (2.1-2.6)	-5.3 (-8.3--2.4)
Canada	0.3 (0.2-0.4)	-74.2 (-81.9--62)	0.2 (0.2-0.3)	-64.7 (-73.4--51.1)	0.2 (0.1-0.2)	-53.1 (-65.1--35.5)	0.1 (0.1-0.1)	-48.6 (-61.1--29.1)	0 (0-0)	-52.2 (-64.1--33.5)	0.1 (0.1-0.1)	-58.7 (-68.6--43.6)
Greenland	2.1 (1.5-2.8)	-1 (-11-11.6)	1.2 (0.9-1.6)	1.5 (-6.9-12)	0.7 (0.5-0.9)	0 (-11.3-11.2)	0.4 (0.3-0.5)	8.5 (-3.6-22.8)	0.1 (0-0.1)	1.6 (-10.1-16.3)	0.4 (0.3-0.5)	-14.3 (-22.7--4.4)
United States of America	8.1 (7-9.2)	-21.2 (-24--18.7)	6.4 (5.7-7.2)	-6.5 (-8.8--4.3)	5 (4.5-5.7)	7.4 (3.5-11.6)	3.1 (2.8-3.6)	13.3 (9.3-17.3)	0.5 (0.4-0.6)	4.2 (-0.6-8.8)	2.6 (2.4-2.9)	-4.4 (-7.5--1.5)
Alabama	17.7 (14.8-20.8)	-25 (-32.6--16.6)	13.3 (11.3-15.4)	-8.7 (-16.1--1)	10.1 (8.5-11.6)	10.4 (0.2-22.2)	5.9 (5.1-6.8)	15.6 (4.5-27)	0.9 (0.7-1.1)	6.2 (-5.5-17.8)	5 (4.3-5.7)	-2.2 (-10.8-6.8)
Alaska	0.6 (0.1-1.5)	-25.4 (-72-0)	0.4 (0.1-1.1)	-9.5 (-65.8-24.6)	0.3 (0.1-0.8)	8.9 (-53.3-55.9)	0.2 (0-0.4)	15.2 (-54.5-67.4)	0 (0-0.1)	-18.3 (-72.4-23.7)	0.1 (0-0.4)	-9.7 (-64.9-29.3)
Arizona	4.1 (3.3-5)	0.1 (-8.3-10.5)	3.2 (2.6-3.8)	12.5 (4-22.2)	2.5 (2-3)	25.3 (14.1-38.3)	1.5 (1.2-1.9)	27.3 (16.3-40.3)	0.2 (0.2-0.3)	11.5 (-1.1-25.5)	1.3 (1-1.5)	7.8 (-1.4-18)
Arkansas	7.3 (5.4-9.5)	-50 (-56.3--42.5)	5.3 (4-6.7)	-32.4 (-40.3--23.1)	3.9 (3-5)	-8.4 (-21.9-7.4)	2.1 (1.6-2.8)	-1.5 (-16.2-16.4)	0.3 (0.2-0.4)	-4 (-19.5-14.4)	1.9 (1.4-2.4)	-18.4 (-29.6--5.5)
California	3 (2.6-3.4)	-20.1 (-26.8--11.7)	2.4 (2.1-2.7)	-7.4 (-13.4--0.7)	1.9 (1.7-2.1)	5 (-2.7-13.6)	1.2 (1.1-1.4)	9.1 (1.2-17.8)	0.2 (0.2-0.3)	0.4 (-8.6-10.7)	1 (0.9-1.1)	-9 (-15.4--2.1)
Colorado	2.9 (2-3.9)	-23.8 (-32.4--15)	2.3 (1.6-3)	-13.7 (-24--3)	1.8 (1.3-2.4)	-2.3 (-15.4-10.5)	1.1 (0.8-1.6)	1.8 (-11.3-15.7)	0.2 (0.1-0.3)	-16.9 (-28.7--3.9)	1 (0.7-1.3)	-15.1 (-25.9--4)
Connecticut	9.2 (7.7-11)	-19.4 (-26.9--11.6)	7.5 (6.3-8.8)	-6.6 (-14.4-1.4)	6.2 (5.2-7.2)	5.1 (-4.4-15.2)	3.7 (3.1-4.4)	13.9 (3.9-24.6)	0.6 (0.5-0.8)	3.9 (-6.7-15.4)	2.9 (2.5-3.5)	-8 (-15.8-0)
Delaware	26.8 (22.3-31.9)	-4.2 (-13.9-4.4)	20.8 (17.4-24.2)	8.3 (-0.7-17.6)	16.2 (13.7-18.9)	20.6 (10.9-32.2)	9.7 (8.2-11.5)	25.3 (15-37.6)	1.4 (1-1.7)	7.8 (-2-18.8)	7.8 (6.6-9.1)	3.4 (-4.5-12.8)
District of Columbia	25.1 (18-32.9)	-47.8 (-56.2--39.2)	16.3 (12.2-21.2)	-25.1 (-36.2--14.1)	10.6 (7.9-13.7)	12.6 (-7.4-35.8)	6.3 (4.7-8.2)	14.1 (-5.8-39.2)	1 (0.7-1.3)	11.1 (-10.1-38.7)	5.8 (4.3-7.5)	2.1 (-14.4-21.9)
Florida	13 (11.4-14.7)	-19.9 (-26.2--12.5)	10.3 (9.2-11.4)	-5.9 (-12-1.2)	8.2 (7.4-9.1)	8.6 (0.6-17.4)	5 (4.6-5.5)	15.3 (7.6-24.2)	0.8 (0.7-0.9)	16 (6.6-26.9)	3.9 (3.6-4.3)	-1.4 (-7.8-5.8)
Georgia	19.1 (16.8-21.5)	-11.3 (-19.2--2.9)	15 (13.4-16.6)	0 (-6.4-7.3)	11.8 (10.6-13.1)	12.3 (5.2-20.5)	7.4 (6.7-8.2)	17.7 (10.3-25.7)	1.3 (1.1-1.6)	4.4 (-3.2-13.5)	6.3 (5.8-6.9)	-1.1 (-7.2-5.6)
Hawaii	1 (0.3-2.1)	-52.2 (-80.5--26.7)	0.7 (0.3-1.6)	-43.4 (-76.5--10.8)	0.6 (0.2-1.3)	-33.8 (-72.8-7.8)	0.4 (0.1-0.8)	-31.2 (-71.9-10.6)	0.1 (0-0.1)	-40.2 (-74.5--5)	0.3 (0.1-0.6)	-42.4 (-76.4--7.6)
Idaho	1 (0.6-1.4)	-4.5 (-17.6-11.6)	0.8 (0.5-1.2)	5.5 (-8.6-23.1)	0.7 (0.5-1)	15.2 (-0.6-33.8)	0.4 (0.2-0.6)	17.6 (1.7-37.3)	0 (0-0.1)	0 (-16.3-19.5)	0.3 (0.2-0.5)	2.4 (-11.5-18.8)
Illinois	4.9 (4.1-5.7)	-45.8 (-52.4--39)	3.7 (3.2-4.2)	-26.4 (-32.6--19.4)	2.9 (2.4-3.3)	0 (-10.1-11.6)	1.8 (1.5-2.1)	6.4 (-4.7-18.2)	0.3 (0.2-0.4)	1.3 (-10.8-14.6)	1.5 (1.3-1.7)	-14.4 (-22.4--5.9)
Indiana	6.5 (5.5-7.5)	-18.2 (-25.9--10.1)	5.1 (4.4-5.8)	-4.4 (-11.7-2.9)	4 (3.5-4.6)	10.1 (1-19.5)	2.4 (2.1-2.8)	14.4 (5-24.3)	0.4 (0.3-0.5)	2.2 (-7.9-12.8)	2.1 (1.8-2.4)	-2.1 (-10.2-5.5)
Iowa	2 (1.2-2.8)	-35.6 (-46.2--24.6)	1.6 (1-2.2)	-25.2 (-37.4--13)	1.3 (0.8-1.8)	-14.4 (-29.1-0.7)	0.8 (0.5-1.1)	-11.1 (-27-5.4)	0.1 (0.1-0.2)	-17 (-33--0.2)	0.7 (0.4-0.9)	-21.8 (-35.5--8.3)
Kansas	4.6 (3.5-6)	-25.5 (-33.7--16.3)	3.6 (2.7-4.5)	-10.4 (-20.2--0.6)	2.8 (2.1-3.5)	7.5 (-6-21.2)	1.8 (1.4-2.3)	12.1 (-2.1-26.6)	0.3 (0.2-0.4)	3.7 (-11.5-19.5)	1.5 (1.1-1.9)	-3.3 (-14.8-8.5)
Kentucky	3.6 (2.7-4.6)	-19.8 (-27.4--11.4)	2.7 (2.1-3.5)	-4.6 (-13.5-5)	2.1 (1.6-2.7)	12.6 (0.6-26.3)	1.2 (0.9-1.5)	18.2 (5.6-33.5)	0.1 (0.1-0.2)	6.8 (-6.5-24.2)	1 (0.8-1.3)	0.1 (-9.8-11.6)
Louisiana	15.5 (13.1-17.9)	-31.2 (-38--22.9)	11.5 (10.1-13)	-14.5 (-21.2--6.8)	8.6 (7.6-9.8)	7.3 (-2.5-17.8)	5.2 (4.6-5.9)	11 (0.9-21.9)	0.8 (0.7-1)	3.5 (-7.3-15)	4.5 (4-5.1)	-6.3 (-14.5-2)
Maine	0.3 (0.1-0.7)	-17.1 (-71.7-0)	0.2 (0.1-0.5)	-4.3 (-64.2-18.8)	0.2 (0.1-0.4)	8 (-58.2-37.8)	0.1 (0-0.2)	14 (-58.5-49.4)	0 (0-0)	-4.2 (-69.7-31.4)	0.1 (0-0.2)	-10.2 (-66.5-15.4)
Maryland	19.8 (17-22.5)	-20.5 (-28.3--13.4)	15.6 (13.6-17.5)	-5.3 (-12.1-1.4)	12.4 (10.9-13.9)	10.7 (2-19.7)	7.5 (6.7-8.5)	16.6 (8-26.3)	1.3 (1-1.6)	1.8 (-6.5-10.9)	6.3 (5.6-7)	-2.7 (-9.9-4.8)
Massachusetts	8.5 (7.2-9.8)	-18.5 (-25.3--11.4)	7.1 (6-8.1)	-7.2 (-13.8-0)	5.9 (5-6.8)	3.1 (-5.5-12.2)	3.8 (3.2-4.4)	8.2 (-0.7-17.4)	0.7 (0.5-0.8)	1.9 (-7.6-12.4)	3 (2.5-3.5)	-8.8 (-16.1--1.4)

Location	Birth Incidence rate (per 100,000 live births), 2021	Birth Incidence rate (per 100,000 live births) percent change (2000-2021)	Under-5 rate (per 100,000 population), 2021	Under-5 rate (per 100,000 population) percent change (2000-2021)	5-14 rate (per 100,000 population), 2021	5-14 rate (per 100,000 population) percent change (2000-2021)	15-49 rate (per 100,000 population), 2021	15-49 rate (per 100,000 population) percent change (2000-2021)	50plus rate (per 100,000 population), 2021	50plus rate (per 100,000 population) percent change (2000-2021)	All age rate (per 100,000 population), 2021	All age rate (per 100,000 population) percent change (2000-2021)
Michigan	9.4 (8.2-10.7)	-23.8 (-31.2--17.3)	7.5 (6.6-8.4)	-9.1 (-15.5--2.9)	6 (5.3-6.7)	6.4 (-1.8-15.7)	3.6 (3.2-4.1)	13.6 (4.9-22.9)	0.5 (0.4-0.7)	-1.4 (-10.3-9.1)	2.9 (2.6-3.2)	-9.4 (-15.8--2.5)
Minnesota	2.8 (1.9-3.7)	-24.3 (-35.2--14.1)	2.2 (1.5-2.9)	-11.3 (-23.4-1)	1.7 (1.2-2.3)	2.8 (-13.2-20.6)	1.1 (0.8-1.5)	8.7 (-7.8-28.5)	0.2 (0.1-0.3)	0.3 (-15.9-20.1)	0.9 (0.6-1.2)	-8.7 (-22.3-7)
Mississippi	23.4 (19.6-27.6)	-29.9 (-36.8--22.5)	17.1 (14.7-19.8)	-12.3 (-19.1--5.6)	12.5 (10.7-14.5)	10.4 (-0.1-21.1)	7.2 (6.2-8.3)	14.2 (4.1-25.3)	1 (0.8-1.2)	7.9 (-2.9-19.6)	6.2 (5.4-7.1)	-5.2 (-13-3)
Missouri	9.4 (7.9-10.9)	-24.4 (-31--17.1)	7.4 (6.3-8.5)	-10.8 (-18.1--3.7)	5.8 (5-6.7)	4.4 (-4.9-13.7)	3.4 (2.9-4)	11.5 (2-21.3)	0.5 (0.4-0.6)	3.9 (-6.3-15.3)	2.8 (2.5-3.3)	-6.5 (-13.9-1.3)
Montana	0.1 (0-0.3)	-18.9 (-88.2-0)	0.1 (0-0.2)	-5.1 (-84.7-20.6)	0 (0-0.2)	9.8 (-82.6-45.5)	0 (0-0.1)	14.2 (-82.2-54.7)	0 (0-0)	-8.7 (-87.9-28.8)	0 (0-0.1)	-5.6 (-84.7-25.7)
Nebraska	2.7 (1.7-3.9)	-32.3 (-43--22.6)	2 (1.3-3)	-18.9 (-31.8--7.5)	1.6 (1-2.2)	-2.5 (-19.9-14.8)	1 (0.6-1.5)	3.2 (-14.9-22.1)	0.2 (0.1-0.3)	-1.6 (-19.2-18.4)	0.9 (0.5-1.3)	-10.1 (-25.7-5.5)
Nevada	5.5 (4.3-6.9)	-24.3 (-34--15.5)	4.2 (3.4-5)	-7.5 (-16.9-2.7)	3.2 (2.5-3.9)	11.9 (-1.2-27.5)	2 (1.6-2.5)	17.2 (3-33.8)	0.4 (0.3-0.5)	0.3 (-12.5-15.6)	1.7 (1.4-2.1)	-1.7 (-12.6-10.9)
New Hampshire	1.1 (0.7-1.7)	-12.1 (-33.9-6.8)	1 (0.6-1.5)	-1.7 (-24.1-18.4)	0.8 (0.5-1.2)	7.4 (-16.9-31.2)	0.4 (0.3-0.7)	15.1 (-14.2-43)	0.1 (0-0.1)	-6 (-37.3-22.1)	0.3 (0.2-0.5)	-12.5 (-33.8-7.4)
New Jersey	10 (8.7-11.4)	-24.3 (-30.2--17.7)	8.2 (7.2-9.1)	-11.4 (-17.8--4.8)	6.7 (5.9-7.5)	1.3 (-6.4-9.7)	4.2 (3.7-4.7)	8.2 (-0.8-17.4)	0.7 (0.6-0.9)	2.8 (-6.6-13)	3.4 (3-3.8)	-9.1 (-15.9--2.3)
New Mexico	0.1 (0-0.2)	-8.7 (-71.9-0)	0.1 (0-0.2)	6.7 (-63.2-21.7)	0 (0-0.1)	23.8 (-54.2-48)	0 (0-0.1)	30.5 (-57.2-58)	0 (0-0)	9 (-71.4-36.9)	0 (0-0.1)	4.3 (-64.8-23.7)
New York	10.9 (9.8-12.1)	-17.4 (-24.4--10.6)	9.6 (8.6-10.6)	-6.2 (-12.4--0.3)	8.3 (7.4-9.2)	4.1 (-2.9-11.2)	5.3 (4.8-5.9)	10.4 (3.7-17.1)	1 (0.8-1.2)	4.4 (-3.5-13.3)	4.3 (3.9-4.7)	-5.9 (-11.4--0.3)
North Carolina	13 (11.4-14.9)	-17.9 (-25--10.2)	10.2 (9.1-11.4)	-4.8 (-10.9-2.1)	8.1 (7.2-9)	9.9 (2.9-17.9)	5.1 (4.6-5.7)	15.1 (7.7-23.5)	0.9 (0.7-1)	1.9 (-6.3-11.1)	4.2 (3.8-4.6)	-2.5 (-8.7-4.2)
North Dakota	0.1 (0-0.3)	-14.4 (-77-0)	0.1 (0-0.2)	0.8 (-71.1-21.8)	0.1 (0-0.2)	16.6 (-64.6-46.4)	0 (0-0.1)	18.6 (-65.7-51.5)	0 (0-0)	12.8 (-71.4-51.9)	0 (0-0.1)	8.5 (-68.8-36.7)
Ohio	6.8 (5.8-7.8)	-25.1 (-32.2--17.4)	5.4 (4.7-6)	-8.8 (-15.4--2.1)	4.2 (3.7-4.8)	9.2 (0-18.5)	2.6 (2.3-2.9)	16.4 (6.9-26.3)	0.4 (0.3-0.5)	5.2 (-5.3-16.9)	2.1 (1.9-2.4)	-4 (-11.6-3.4)
Oklahoma	4.2 (3-5.4)	-40.6 (-48.1--33)	3 (2.3-3.9)	-26.4 (-35.3--17.4)	2.3 (1.7-2.9)	-7.6 (-21.1-7.6)	1.3 (1-1.8)	-1.7 (-15.3-14.4)	0.2 (0.1-0.3)	-6.1 (-20.6-10.9)	1.2 (0.9-1.5)	-15.8 (-27.1--3.3)
Oregon	2.4 (1.6-3.3)	-7 (-14.7-5.1)	1.9 (1.4-2.6)	6.6 (-2.4-18.3)	1.6 (1.1-2.1)	20.2 (8.7-35.6)	0.9 (0.6-1.3)	23.5 (11.4-39)	0.1 (0.1-0.2)	2.6 (-10.6-17.7)	0.7 (0.5-1)	2.9 (-6.5-15)
Pennsylvania	6.6 (5.7-7.6)	-22.6 (-30.3--15.1)	5.2 (4.5-5.9)	-7 (-13.5--0.4)	4.1 (3.6-4.7)	9.2 (0.2-19.1)	2.5 (2.2-2.9)	15 (5.8-25)	0.4 (0.3-0.5)	7.5 (-2.2-18.4)	2 (1.8-2.2)	-2.6 (-10-4.9)
Rhode Island	2.4 (1.3-3.7)	-72 (-78.4--64.9)	1.8 (1-2.9)	-66 (-73.6--57.2)	1.5 (0.8-2.2)	-60 (-69.6--48.9)	1 (0.5-1.5)	-55.5 (-65.9--43.3)	0.2 (0.1-0.3)	-51.5 (-63.7--37.6)	0.7 (0.4-1.2)	-63.6 (-72.1--54)
South Carolina	12.7 (10.9-14.4)	-33.8 (-39.8--26.2)	9.7 (8.5-10.9)	-17.2 (-22.8--10.8)	7.5 (6.6-8.4)	3.5 (-4.8-12.7)	4.5 (4-5.1)	9.4 (0.3-18.6)	0.7 (0.6-0.9)	-4.7 (-14.3-5)	3.7 (3.4-4.2)	-10.1 (-16.9--3.2)
South Dakota	0.4 (0.1-1)	-33.9 (-77.5-0)	0.3 (0.1-0.7)	-21.5 (-71.2-21.6)	0.2 (0.1-0.5)	-6.9 (-64.5-48.3)	0.1 (0-0.3)	-2.8 (-65.5-56.5)	0 (0-0)	-6.2 (-70.4-55.4)	0.1 (0-0.3)	-15.6 (-68.9-34)
Tennessee	13.5 (11.6-15.3)	-17.3 (-24.6--9.6)	10.4 (9.1-11.7)	-3.2 (-9.8-3.7)	8 (7-9.1)	12.2 (3.4-21.6)	4.9 (4.3-5.6)	18.1 (9.1-27.5)	0.8 (0.6-0.9)	4.2 (-5.1-13.7)	4.1 (3.6-4.6)	0.4 (-6.9-8)
Texas	7.4 (6.4-8.3)	-10.9 (-17.9--1.9)	6.1 (5.4-6.8)	-0.5 (-7.1-7)	4.9 (4.4-5.5)	9.7 (2.3-18.2)	3.2 (2.9-3.5)	15.1 (7.8-23)	0.6 (0.5-0.7)	7.8 (-0.7-17.2)	2.8 (2.5-3.1)	0.1 (-6.1-6.6)
Utah	1.4 (0.9-1.9)	-17 (-30.7--3.8)	1.1 (0.8-1.5)	-6.4 (-20.7-7.4)	0.9 (0.6-1.2)	5.3 (-10.4-22.6)	0.5 (0.4-0.7)	4.1 (-13.2-23.7)	0.1 (0-0.1)	-1 (-22.7-23)	0.5 (0.4-0.7)	-6.8 (-21.7-9.4)
Vermont	0.1 (0-0.4)	-84.2 (-99-0)	0.1 (0-0.3)	-81.5 (-98.8-15.1)	0.1 (0-0.3)	-78.9 (-98.6-29.1)	0 (0-0.2)	-77.7 (-98.6-38.8)	0 (0-0)	-82.1 (-98.9-13.4)	0 (0-0.1)	-82.5 (-98.9-8.6)
Virginia	12 (10.3-13.8)	-24.3 (-31.8--15.7)	9.3 (8.2-10.5)	-7.4 (-13.9-0.8)	7.3 (6.4-8.3)	12.5 (2.5-23.2)	4.6 (4-5.3)	17.6 (7.4-28.9)	0.8 (0.7-1)	1.9 (-7.9-13.7)	3.8 (3.4-4.3)	-1 (-9.2-7.8)
Washington	2.5 (1.9-3.2)	-10.3 (-18.6--1.4)	2.1 (1.6-2.6)	0.3 (-9-10.5)	1.7 (1.3-2.2)	10.4 (-0.2-23.2)	1 (0.8-1.4)	13.1 (2.2-26.1)	0.2 (0.1-0.2)	-6 (-16.9-6.1)	0.9 (0.7-1.1)	-3.7 (-12.8-6.6)
West Virginia	1.1 (0.6-1.7)	-32.1 (-43--18.7)	0.8 (0.4-1.2)	-15.1 (-31.4-2.2)	0.6 (0.3-0.9)	8.3 (-17.5-35.4)	0.3 (0.2-0.6)	11 (-12.8-38.4)	0 (0-0.1)	-5 (-24-18.1)	0.3 (0.2-0.4)	-7.1 (-26.6-15)
Wisconsin	5.1 (4.1-6.2)	-27.5 (-35--20)	4.1 (3.3-5)	-15.8 (-23.9--7.6)	3.4 (2.7-4.1)	-4.1 (-14-6.7)	2 (1.6-2.5)	0.7 (-9.5-12)	0.3 (0.2-0.4)	-7.5 (-18.2-3.8)	1.6 (1.3-2)	-16 (-24.3--7)
Wyoming	0.1 (0-0.3)	-83.6 (-99-0)	0.1 (0-0.3)	-80.5 (-98.9-16.2)	0.1 (0-0.2)	-77 (-98.6-34.8)	0 (0-0.1)	-76.3 (-98.5-42.5)	0 (0-0)	-80.8 (-98.8-19.3)	0 (0-0.1)	-79.7 (-98.8-20.5)

Location	Birth Incidence rate (per 100,000 live births), 2021	Birth Incidence rate (per 100,000 live births) percent change (2000-2021)	Under-5 rate (per 100,000 population), 2021	Under-5 rate (per 100,000 population) percent change (2000-2021)	5-14 rate (per 100,000 population), 2021	5-14 rate (per 100,000 population) percent change (2000-2021)	15-49 rate (per 100,000 population), 2021	15-49 rate (per 100,000 population) percent change (2000-2021)	50plus rate (per 100,000 population), 2021	50plus rate (per 100,000 population) percent change (2000-2021)	All age rate (per 100,000 population), 2021	All age rate (per 100,000 population) percent change (2000-2021)
**Southern Latin America**	0 (0-0.1)	0.8 (-4.6-25.9)	0 (0-0)	-0.7 (-8.2-26.4)	0 (0-0)	-0.1 (-10.9-29.7)	0 (0-0)	-6 (-17.3-15.5)	0 (0-0)	-2.9 (-18.6-18.4)	0 (0-0)	-14.8 (-23.8-6.5)
Argentina	0 (0-0.1)	-0.4 (-4.8-32.3)	0 (0-0)	-1.9 (-9.9-33.3)	0 (0-0)	-1 (-15-36.4)	0 (0-0)	-7.7 (-21.6-20.5)	0 (0-0)	-6.4 (-23.3-14.8)	0 (0-0)	-14.1 (-24.9-14.3)
Chile	0 (0-0.1)	2.9 (0-88.7)	0 (0-0)	1.9 (-10.3-77.7)	0 (0-0)	2.1 (-19.6-70.3)	0 (0-0)	-1.9 (-23.7-72.7)	0 (0-0)	-4.4 (-27.2-85.6)	0 (0-0)	-17.4 (-33.2-43)
Uruguay	0 (0-0.1)	2.6 (-0.2-37.4)	0 (0-0)	2.6 (-5.7-45.2)	0 (0-0)	1.9 (-11.6-49.9)	0 (0-0)	-2.5 (-15.7-37.1)	0 (0-0)	-1.3 (-18.9-27.9)	0 (0-0)	-11.3 (-21.5-26.9)
**Western Europe**	9.2 (7.2-11.6)	6.5 (3.2-9.9)	7.1 (5.7-8.9)	5.2 (2.1-8.5)	5.1 (4-6.3)	6.5 (3-9.9)	1.9 (1.5-2.5)	8.1 (4.9-11.4)	0.1 (0.1-0.2)	-1.7 (-5.5-2.2)	1.8 (1.4-2.2)	-3.8 (-6.7--0.9)
Andorra	6.3 (4.6-8.4)	0.2 (-7.8-9.6)	5 (3.7-6.5)	-0.3 (-8.1-8.6)	3.7 (2.7-4.8)	-1 (-9.2-8.2)	1.4 (1-1.8)	-3.3 (-11.7-5)	0.2 (0.1-0.2)	0.2 (-9.1-10.2)	1.2 (0.9-1.6)	-18.8 (-25.8--11.6)
Austria	3.8 (2.9-4.7)	25.3 (13-38.2)	3 (2.3-3.6)	24.9 (13-36.2)	2.2 (1.7-2.7)	23.1 (12.6-34.9)	0.8 (0.6-1.1)	21.9 (10.8-33.1)	0.1 (0-0.1)	22.7 (8.6-37.4)	0.8 (0.6-0.9)	9.2 (-0.1-18.4)
Belgium	2.6 (1.9-3.4)	-1.4 (-8.8-7.8)	2 (1.5-2.7)	-1.4 (-9.3-6.9)	1.5 (1.1-1.9)	-1.7 (-9.7-6.5)	0.5 (0.4-0.7)	-0.4 (-7.6-7.4)	0 (0-0.1)	-6 (-14.3-3.7)	0.5 (0.4-0.7)	-7.9 (-14.8--1.2)
Cyprus	41.4 (33.3-50.9)	0.9 (-7.5-11.2)	32.4 (26.4-39.1)	1.3 (-6.6-10.8)	23.4 (19.4-27.9)	0.4 (-7.2-9.3)	9.4 (7.8-11.3)	-10.7 (-16.7--3.7)	1 (0.7-1.3)	-14.1 (-21.4--5.7)	9.5 (8-11.1)	-18.2 (-23.9--11.7)
Denmark	11.8 (9.4-14.6)	0.2 (-7.8-8.9)	9.5 (7.6-11.6)	0.6 (-6.5-8.1)	7.1 (5.8-8.6)	-0.6 (-7.9-7)	3.1 (2.5-3.8)	4.9 (-2.6-12.7)	0.3 (0.2-0.3)	-15.1 (-22.4--7.6)	2.7 (2.3-3.3)	-8.2 (-14.5--1.4)
Finland	9.2 (7.5-11.3)	-5 (-13.8-4.8)	7.6 (6.2-9.2)	-5.4 (-13.8-3.3)	5.8 (4.8-7)	-5.2 (-13.3-3.7)	2.2 (1.8-2.7)	-5.8 (-13.6-2.4)	0.1 (0.1-0.2)	-35.2 (-43.1--26.2)	1.9 (1.6-2.3)	-18.3 (-25.2--11.4)
France	18.1 (14.6-22.3)	-3.1 (-10.1-5.1)	14.2 (11.5-17.5)	-3.1 (-10.3-5.2)	10.5 (8.3-13)	-2.1 (-10-6.1)	4.2 (3.3-5.3)	-0.2 (-8.1-8.3)	0.3 (0.2-0.4)	-14.8 (-23.3--6.4)	3.9 (3.1-4.9)	-11.7 (-18.5--4.7)
Germany	2.8 (2.1-3.7)	1.9 (-6.4-11.3)	2.2 (1.6-2.9)	1.7 (-6.3-11.7)	1.6 (1.2-2.1)	2.3 (-5.6-12.3)	0.6 (0.4-0.8)	6.2 (-1.1-15.4)	0 (0-0.1)	6.7 (-4.4-19)	0.5 (0.4-0.7)	-8 (-14.6-0)
Greece	1.4 (1-1.9)	-2.1 (-10.6-6.2)	1.1 (0.8-1.5)	-2.5 (-10.6-6)	0.8 (0.6-1.1)	-3 (-11.3-5.8)	0.3 (0.2-0.4)	-11.8 (-19.4--3.6)	0 (0-0)	-6.9 (-16.1-4.1)	0.3 (0.2-0.3)	-19.1 (-25.8--12)
Iceland	0.7 (0.5-0.9)	-0.4 (-19.2-17.9)	0.5 (0.4-0.8)	-0.3 (-18-17.8)	0.4 (0.3-0.6)	-0.6 (-16-16.4)	0.2 (0.1-0.2)	-2.7 (-16.8-14.1)	0 (0-0)	-12.2 (-24.7-1.4)	0.2 (0.1-0.2)	-15 (-27.9-0)
Ireland	5.1 (3.7-6.8)	-0.1 (-8.9-9.1)	4 (2.9-5.4)	-0.8 (-8.9-7.8)	3 (2.2-4)	-0.3 (-8.2-8.3)	1.1 (0.9-1.5)	-9.5 (-16.3--2.1)	0.1 (0.1-0.2)	-10.5 (-17.9--1.3)	1.2 (0.9-1.6)	-13 (-19.3--6)
Israel	8 (5.8-10.7)	-1.4 (-9.8-7.2)	6.2 (4.6-8.2)	-1.4 (-8.9-6.6)	4.5 (3.3-5.9)	-1.4 (-8.9-6.8)	2 (1.5-2.6)	-5 (-12.4-2.5)	0.2 (0.1-0.2)	-17.8 (-24.8--9.7)	2.4 (1.8-3.1)	-7.2 (-14.2-0.3)
Italy	3.8 (2.8-4.9)	17.1 (12.5-22.2)	3.3 (2.5-4.2)	19.6 (14.8-25.4)	2.5 (1.9-3.2)	24.6 (19-31.1)	0.9 (0.7-1.2)	16.8 (11.6-22.7)	0.1 (0.1-0.1)	24.3 (18.5-31.2)	0.8 (0.6-1)	5.2 (0.5-10.2)
Abruzzo	1 (0.5-1.8)	-14 (-54.5-3.4)	0.9 (0.4-1.6)	-14 (-54.5-3.3)	0.7 (0.3-1.2)	-13.7 (-54.1-3.9)	0.2 (0.1-0.4)	-17.1 (-54.5-1.9)	0 (0-0)	0.3 (-44.7-27.5)	0.2 (0.1-0.3)	-26.7 (-60.5--11)
Basilicata	0.1 (0-0.3)	-13.1 (-82.5-0)	0.1 (0-0.2)	-13.1 (-82.4-1.5)	0 (0-0.2)	-12.9 (-82.2-3.5)	0 (0-0.1)	-17.1 (-83-0.7)	0 (0-0)	-0.1 (-80.1-28.7)	0 (0-0)	-32.5 (-86.1--18.9)
Calabria	0.8 (0.4-1.2)	-14.7 (-44.2-5.2)	0.6 (0.4-1)	-14.8 (-43-5.5)	0.5 (0.3-0.7)	-14.2 (-41.5-5.5)	0.2 (0.1-0.3)	-23.1 (-47.1--5.7)	0 (0-0)	-2.5 (-30.5-24.5)	0.2 (0.1-0.2)	-32.8 (-54.4--17.3)
Campania	2.9 (1.9-4.1)	20.3 (6.6-36.4)	2.5 (1.7-3.5)	20.7 (7-36.7)	1.9 (1.3-2.7)	21.6 (8.7-37.5)	0.7 (0.5-1)	17 (4.9-32.1)	0.1 (0-0.1)	31.2 (15.8-52.5)	0.6 (0.4-0.9)	-3.2 (-13.4-9)
Emilia-Romagna	11.7 (9.4-14.1)	12.4 (3.3-21.2)	9.9 (8.1-11.9)	11.2 (2.5-20.6)	7.6 (6.2-9.2)	10.9 (1.9-19.5)	2.5 (2-3)	14.2 (5.4-24.2)	0.2 (0.1-0.3)	28.5 (16.9-42.8)	2.1 (1.8-2.6)	10.9 (2.7-19.6)
Friuli-Venezia Giulia	6.3 (4.5-8.4)	2.4 (-6.5-13.8)	5.4 (4-7.3)	1.8 (-7-13.6)	4.3 (3.1-5.7)	2.6 (-6.4-13.5)	1.2 (0.9-1.7)	8.4 (-1-20.6)	0.1 (0-0.1)	9.9 (-1.6-24.9)	1 (0.8-1.4)	-2.6 (-10.5-7.6)
Lazio	3.3 (2.3-4.5)	31.9 (18.1-47.1)	2.9 (2-3.9)	32.1 (17.9-47.3)	2.2 (1.6-3)	32.5 (18.6-47.2)	0.8 (0.6-1.1)	23.4 (10.6-37.6)	0.1 (0-0.1)	35.1 (18.3-54.6)	0.7 (0.5-0.9)	14.2 (2.9-26.3)
Liguria	5.2 (3.5-7.2)	8.9 (-1.3-17.9)	4.5 (3.1-6.3)	8.3 (-1.8-17.4)	3.5 (2.4-4.8)	8.1 (-1.8-16.8)	1.1 (0.8-1.6)	10.7 (1.1-20.4)	0.1 (0-0.1)	18.6 (6.1-33)	0.9 (0.6-1.2)	2 (-7-10.7)
Lombardia	2.7 (2-3.4)	16.4 (6.2-27.2)	2.3 (1.7-3)	16.3 (5.7-27.3)	1.8 (1.4-2.4)	16.9 (6.8-27.9)	0.7 (0.5-0.9)	22.4 (11.5-36)	0.1 (0-0.1)	33.9 (18.3-53.6)	0.6 (0.4-0.7)	10.7 (1.1-21.9)
Marche	4.9 (3.4-6.8)	18.3 (7-31.5)	4.1 (2.9-5.7)	18.5 (7.7-31.4)	3.2 (2.2-4.3)	19.7 (8.8-32.3)	1.2 (0.9-1.7)	24.3 (13.3-37.2)	0.1 (0.1-0.2)	50.6 (34-69.9)	1 (0.7-1.3)	12 (1.7-23.7)
Molise	0.6 (0.3-1.2)	-16.3 (-61.9-0)	0.5 (0.2-1)	-15.8 (-61.6-3.3)	0.4 (0.2-0.8)	-14.6 (-61.6-6.4)	0.1 (0.1-0.3)	-16.5 (-62.1-5.5)	0 (0-0)	-4.3 (-54.9-26.9)	0.1 (0-0.2)	-31 (-68.9--14.4)

Location	Birth Incidence rate (per 100,000 live births), 2021	Birth Incidence rate (per 100,000 live births) percent change (2000-2021)	Under-5 rate (per 100,000 population), 2021	Under-5 rate (per 100,000 population) percent change (2000-2021)	5-14 rate (per 100,000 population), 2021	5-14 rate (per 100,000 population) percent change (2000-2021)	15-49 rate (per 100,000 population), 2021	15-49 rate (per 100,000 population) percent change (2000-2021)	50plus rate (per 100,000 population), 2021	50plus rate (per 100,000 population) percent change (2000-2021)	All age rate (per 100,000 population), 2021	All age rate (per 100,000 population) percent change (2000-2021)
Piemonte	4.9 (3.6-6.5)	26 (13.3-37.9)	4.2 (3.1-5.6)	25.9 (13.9-37.2)	3.2 (2.4-4.3)	26 (14.7-38.3)	1 (0.8-1.4)	21 (10.4-32.6)	0.1 (0.1-0.1)	19.8 (6.4-35.9)	0.9 (0.7-1.2)	13.4 (4.1-24.2)
Provincia autonoma di Bolzano	1.4 (0.8-2.1)	14 (-2.5-31.6)	1.2 (0.7-1.8)	13.8 (-2.7-30.6)	0.9 (0.5-1.3)	12.9 (-4.1-29.6)	0.4 (0.2-0.5)	21.7 (4.3-40.1)	0 (0-0.1)	45.9 (23.1-70.8)	0.3 (0.2-0.5)	2.7 (-12.2-17.6)
Provincia autonoma di Trento	0.8 (0.3-1.6)	-14 (-53.4-0)	0.7 (0.2-1.4)	-14.1 (-53.7-1.2)	0.5 (0.2-1)	-14.5 (-54.7-2.3)	0.2 (0.1-0.4)	-8.6 (-49.2-11.8)	0 (0-0)	-5.4 (-45-19.6)	0.2 (0.1-0.3)	-21.6 (-56.6--6.2)
Puglia	1.3 (0.8-1.8)	9.3 (-17-35.5)	1.1 (0.7-1.6)	9.6 (-15.9-35.3)	0.8 (0.5-1.2)	10.5 (-14.9-36.7)	0.3 (0.2-0.5)	15.1 (-12.9-42)	0 (0-0)	28 (-6.5-65.3)	0.3 (0.2-0.4)	-9.2 (-30.9-11.1)
Sardegna	0.6 (0.3-1)	-14.5 (-47.4-6.3)	0.5 (0.3-0.8)	-14.1 (-46.6-4.8)	0.4 (0.2-0.6)	-14.2 (-46.4-5.6)	0.1 (0.1-0.2)	-23.5 (-50.5--5)	0 (0-0)	-15.5 (-45.8-7.2)	0.1 (0.1-0.2)	-36.4 (-59.5--21.4)
Sicilia	4 (2.9-5.3)	11.7 (2.3-22.5)	3.4 (2.5-4.5)	11.5 (2.1-22.2)	2.6 (2-3.4)	11.5 (1.8-22.3)	1.1 (0.8-1.4)	7.6 (-0.7-17.9)	0.1 (0.1-0.2)	18.6 (7.3-32.4)	0.9 (0.7-1.1)	-8.3 (-15.5-0.7)
Toscana	1.9 (1.1-2.8)	-5.3 (-13.6-4.3)	1.6 (1-2.4)	-5.6 (-13.9-3.7)	1.3 (0.8-1.9)	-5.2 (-13.4-3.6)	0.5 (0.3-0.7)	-5.8 (-13.4-3.5)	0 (0-0.1)	3.6 (-7.4-16.4)	0.4 (0.2-0.5)	-10.6 (-17.5--2)
Umbria	3.1 (2-4.7)	-8 (-20.2-1.7)	2.7 (1.8-4)	-8.7 (-20.8-0.8)	2.1 (1.4-3)	-8 (-20.7-1.6)	0.6 (0.4-0.9)	-7.6 (-21.5-2.7)	0 (0-0.1)	3 (-17.4-17.4)	0.5 (0.4-0.8)	-12.7 (-25.5--4)
Valle d'Aosta	0.1 (0-0.4)	-10.9 (-77.8-0)	0.1 (0-0.3)	-11.4 (-77.9-1.1)	0.1 (0-0.3)	-11.3 (-77.7-3.5)	0 (0-0.1)	-5.5 (-76.4-12.5)	0 (0-0)	-0.4 (-74.3-27.4)	0 (0-0.1)	-17.3 (-79.3--2.9)
Veneto	6 (4.6-7.6)	16.6 (7.5-27.4)	5.2 (4-6.6)	16.5 (7.4-27.4)	4.1 (3.2-5.2)	17.1 (8.1-27.3)	1.5 (1.1-1.9)	17.8 (9-28.3)	0.1 (0.1-0.2)	23 (10.6-37.1)	1.2 (1-1.5)	4.7 (-2.9-13.3)
Luxembourg	65.7 (53.7-79.7)	-7.3 (-15.2-3.2)	54 (44.8-63.9)	-7.9 (-15.7-0.9)	42.6 (35.7-50.5)	-7.6 (-15.2-1.1)	19.1 (16.1-22.5)	-8 (-14.3--1.1)	2.1 (1.6-2.8)	2.3 (-7.1-12.2)	17.4 (14.8-20.3)	-16.3 (-22--9.6)
Malta	14.1 (10.4-18.4)	-1.3 (-9-7.2)	11.2 (8.4-14.2)	-0.9 (-8.4-7.8)	8.4 (6.4-10.7)	-1.2 (-8.6-7)	3.5 (2.7-4.4)	-9.1 (-16.3--2.5)	0.3 (0.2-0.4)	-39.9 (-45.6--34)	3 (2.3-3.8)	-23.5 (-29.6--17.9)
Monaco	4.8 (3.5-6.5)	0 (-8.8-10.6)	3.9 (2.8-5.2)	-0.9 (-9.7-7.9)	2.9 (2.1-3.9)	-0.9 (-9.8-7.3)	1.1 (0.8-1.4)	7.7 (-1.1-17.5)	0.1 (0.1-0.1)	1.2 (-8.4-12.9)	0.9 (0.6-1.1)	-1.9 (-10.2-6.4)
Netherlands	1 (0.8-1.4)	0.8 (-9.2-15.6)	0.8 (0.6-1.1)	0.4 (-9-13.9)	0.6 (0.5-0.8)	-0.1 (-8.4-13)	0.3 (0.2-0.3)	5.1 (-3.2-16.5)	0 (0-0)	-16.7 (-25.4--6.5)	0.2 (0.2-0.3)	-12.9 (-19.2--2.4)
Norway	1 (0.7-1.5)	-0.1 (-6.8-5.5)	0.9 (0.6-1.2)	-0.2 (-7.1-4.9)	0.7 (0.4-1)	-0.2 (-7.3-4.3)	0.2 (0.1-0.3)	2.4 (-3.8-7.2)	0 (0-0)	-6.2 (-12--1.1)	0.2 (0.1-0.3)	-9.3 (-15.1--4.8)
Agder	1 (0.6-1.5)	-1.3 (-13.5-12.4)	0.9 (0.5-1.2)	-0.5 (-13.3-13)	0.6 (0.4-1)	-1.5 (-13.8-12)	0.2 (0.1-0.3)	-0.2 (-12-12.9)	0 (0-0)	-7.7 (-20.6-9.2)	0.2 (0.1-0.3)	-11.8 (-22--0.7)
Innlandet	1 (0.6-1.5)	-0.9 (-29.2-15.1)	0.8 (0.5-1.3)	-1.5 (-28.8-14.4)	0.7 (0.4-1)	-0.9 (-28.2-15.4)	0.2 (0.1-0.3)	4.2 (-22.9-21.4)	0 (0-0)	-10.5 (-33.7-8.2)	0.2 (0.1-0.3)	-12.7 (-35.6-1.8)
Møre og Romsdal	0.9 (0.6-1.4)	-1.7 (-27.8-7)	0.8 (0.5-1.2)	-2.2 (-28.6-6.4)	0.6 (0.4-0.9)	-2.6 (-29.2-6.6)	0.2 (0.1-0.3)	0.2 (-25.5-11.5)	0 (0-0)	-11.8 (-31.7-1.3)	0.2 (0.1-0.3)	-13.8 (-36.3--5.3)
Nordland	1 (0.6-1.5)	-0.4 (-12.9-9.5)	0.8 (0.5-1.2)	-0.3 (-13-10)	0.6 (0.4-0.9)	-0.5 (-13.4-10.9)	0.2 (0.1-0.3)	4.5 (-7.8-17.9)	0 (0-0)	-10.8 (-24.1-4.2)	0.2 (0.1-0.3)	-14.9 (-25.2--4.8)
Oslo	1 (0.6-1.5)	-1.1 (-14.5-16.2)	0.9 (0.5-1.3)	-0.5 (-13.2-15.5)	0.7 (0.4-1)	-1.7 (-14.7-13.3)	0.2 (0.1-0.3)	-0.7 (-14.1-15.2)	0 (0-0)	4.3 (-12.6-22.9)	0.2 (0.1-0.4)	-0.2 (-13.6-14.6)
Rogaland	1 (0.6-1.4)	-0.3 (-13.4-12.1)	0.8 (0.5-1.2)	-1.1 (-13.9-10.8)	0.7 (0.4-1)	-1.2 (-14.1-10.9)	0.2 (0.1-0.3)	-1 (-14.6-9.8)	0 (0-0)	-4.5 (-23.1-10.3)	0.2 (0.2-0.4)	-11.7 (-23.2--2.1)
Troms og Finnmark	1 (0.6-1.5)	-0.1 (-11.9-11.4)	0.8 (0.5-1.2)	-1.4 (-13-9.2)	0.6 (0.4-0.9)	-1.5 (-13.4-9.7)	0.2 (0.1-0.3)	6.4 (-7-18.8)	0 (0-0)	-14.5 (-27.1--0.3)	0.2 (0.1-0.3)	-14.7 (-25.2--5.3)
Trøndelag	1 (0.6-1.5)	0 (-18.5-13.8)	0.8 (0.5-1.2)	0.2 (-18.1-14.8)	0.6 (0.4-0.9)	0.5 (-16.5-14.7)	0.2 (0.1-0.3)	4.7 (-15.5-19.6)	0 (0-0)	-8.3 (-23.9-8.4)	0.2 (0.1-0.3)	-8.6 (-25.3-6.2)
Vestfold og Telemark	1 (0.6-1.5)	-0.4 (-14.3-16)	0.8 (0.5-1.3)	-0.8 (-13.6-14.9)	0.7 (0.4-1)	0.5 (-12.7-16.5)	0.2 (0.1-0.3)	3.9 (-9.4-19.4)	0 (0-0)	-9.4 (-22.3-8.8)	0.2 (0.1-0.3)	-12 (-23.5-1.7)
Vestland	1 (0.6-1.5)	0.3 (-10.3-13.9)	0.9 (0.5-1.2)	0.6 (-9.9-14.2)	0.7 (0.4-1)	-0.3 (-10.8-13.1)	0.2 (0.1-0.3)	0.9 (-9.6-14.1)	0 (0-0)	-5.2 (-16.5-9.3)	0.2 (0.2-0.4)	-10.1 (-19.4-2.6)
Viken	1 (0.7-1.6)	1 (-12.6-23.5)	0.9 (0.6-1.3)	0.3 (-13.2-23.3)	0.7 (0.4-1)	0.7 (-11.4-22.4)	0.2 (0.1-0.3)	4.6 (-9.3-24.3)	0 (0-0)	-6.7 (-19.5-8.7)	0.2 (0.1-0.3)	-8.3 (-19.6-10.2)
Portugal	7.5 (5.3-9.9)	-0.5 (-7.7-10.2)	5.6 (4.1-7.4)	-1.3 (-8.4-8.5)	3.9 (2.8-5.1)	-1.3 (-9.5-8.5)	1.4 (1-1.8)	-9.4 (-16.4--1.1)	0.1 (0.1-0.1)	-7.8 (-16.9-2.5)	1.2 (0.9-1.5)	-22.6 (-28.5--15.7)
San Marino	4.8 (3.5-6.4)	-0.3 (-7.5-7.9)	3.9 (2.8-5.1)	-0.6 (-7.8-7.9)	2.9 (2.1-3.8)	-1 (-8.6-7.7)	1.2 (0.9-1.6)	-2.1 (-9.8-5.9)	0.1 (0.1-0.2)	6.3 (-2.7-16.9)	1.1 (0.8-1.4)	-12.7 (-19.3--5.7)
Spain	0.8 (0.6-1.1)	-0.6 (-16.4-10)	0.7 (0.4-0.9)	-1.7 (-16-7.6)	0.5 (0.3-0.7)	-0.9 (-15.5-8.4)	0.2 (0.1-0.2)	-11.7 (-23.1--3.3)	0 (0-0)	6 (-7.2-18.5)	0.2 (0.1-0.2)	-15.2 (-26.7--7.7)

Location	Birth Incidence rate (per 100,000 live births), 2021	Birth Incidence rate (per 100,000 live births) percent change (2000-2021)	Under-5 rate (per 100,000 population), 2021	Under-5 rate (per 100,000 population) percent change (2000-2021)	5-14 rate (per 100,000 population), 2021	5-14 rate (per 100,000 population) percent change (2000-2021)	15-49 rate (per 100,000 population), 2021	15-49 rate (per 100,000 population) percent change (2000-2021)	50plus rate (per 100,000 population), 2021	50plus rate (per 100,000 population) percent change (2000-2021)	All age rate (per 100,000 population), 2021	All age rate (per 100,000 population) percent change (2000-2021)
Sweden	1.8 (1.4-2.5)	6.6 (-1-14.3)	1.5 (1.1-2)	6.3 (-1-14.1)	1.1 (0.8-1.5)	5.7 (-1.6-13.4)	0.4 (0.3-0.6)	6.1 (-0.4-13.8)	0 (0-0.1)	-8.8 (-15.6--0.6)	0.4 (0.3-0.6)	2.6 (-3.8-9.8)
Stockholm	1.8 (1.3-2.5)	7 (-1.9-17.8)	1.5 (1.1-2)	6.1 (-2.5-17.1)	1.1 (0.8-1.5)	5.6 (-2.8-16.5)	0.4 (0.3-0.6)	5.1 (-2.9-15.7)	0 (0-0.1)	-4.2 (-13.6-7.7)	0.5 (0.3-0.6)	3.9 (-3.9-14.1)
Sweden except Stockholm	1.8 (1.4-2.5)	6.5 (-2.2-15.9)	1.5 (1.1-2)	6.4 (-2.1-15.7)	1.1 (0.9-1.5)	5.8 (-2.5-14.7)	0.5 (0.3-0.6)	6.6 (-1.2-15.7)	0 (0-0)	-10.3 (-18.2--0.1)	0.4 (0.3-0.6)	2.1 (-4.9-10.3)
Switzerland	36.4 (31.5-40.9)	3.1 (-3.8-10.9)	30.6 (26.9-34.1)	2.7 (-4.1-9.7)	24.6 (21.9-27.6)	2.5 (-4.1-9.5)	9.6 (8.5-10.9)	2 (-3.8-8.3)	0.9 (0.7-1.2)	0.1 (-8.6-10.1)	8.6 (7.8-9.6)	-7.9 (-13.3--2.2)
United Kingdom	16.4 (11.8-22)	5.8 (3.9-7.7)	12.8 (9.3-17.1)	10.6 (8.8-12.5)	8.4 (6-11.3)	11.1 (9.2-13)	3.3 (2.3-4.6)	6.4 (4.5-8.4)	0.2 (0.1-0.3)	5 (2.6-7.8)	3.3 (2.4-4.4)	1 (-0.6-2.8)
England	18.2 (13.1-24.4)	4.8 (2.9-6.8)	14.1 (10.2-18.9)	9.2 (7.4-11.1)	9.3 (6.6-12.6)	10.5 (8.5-12.5)	3.6 (2.6-5.1)	6 (4.1-8)	0.2 (0.1-0.3)	6.4 (3.8-9.5)	3.6 (2.6-4.9)	1.4 (-0.3-3.1)
East Midlands	10.1 (6.9-14.3)	10.5 (4.7-17.5)	8.1 (5.6-11.4)	15 (8.8-22.7)	5.6 (3.8-7.9)	14.1 (7.6-23.1)	2 (1.4-3)	12.8 (6.6-22.5)	0.1 (0.1-0.2)	2.9 (-4.5-16.3)	2 (1.4-2.9)	3.6 (-1.4-11.7)
Derby	9.4 (5-15.6)	3.6 (-9.7-17.2)	7.9 (4.2-13)	3.2 (-8.4-17.6)	5.8 (3-9.5)	3.2 (-9.4-18.1)	2 (1-3.5)	4.7 (-8.4-20.7)	0.1 (0.1-0.3)	15.5 (-2.5-37.6)	2.3 (1.2-3.8)	2.1 (-9.9-17)
Derbyshire	0.3 (0-1.3)	36.9 (0-2460)	0.2 (0-1.1)	36.8 (-1.7-2440)	0.2 (0-0.8)	33.8 (-4.6-2390)	0.1 (0-0.3)	43.7 (-1.4-2610)	0 (0-0)	23.1 (-18.2-2420)	0.1 (0-0.3)	19.5 (-15.8-2120)
Leicester	20.5 (14.2-28.6)	0.8 (-11.1-12.4)	17.1 (12-24.1)	0 (-11.5-10.6)	12.3 (8.5-17)	0.1 (-10.9-11.9)	4.4 (3-6.3)	0.5 (-11.7-13.8)	0.3 (0.2-0.5)	8.7 (-7.5-28.3)	5.2 (3.7-7.2)	-2 (-13.2-9.3)
Leicestershire	0.3 (0-1.8)	53.4 (0-2590)	0.3 (0-1.5)	53.6 (-1.8-2610)	0.2 (0-1.1)	51.2 (-4.4-2530)	0.1 (0-0.5)	59.9 (-2.5-2650)	0 (0-0)	30.4 (-21.8-2280)	0.1 (0-0.4)	37 (-14.6-2260)
Lincolnshire	0.3 (0-1.8)	52.6 (0-2590)	0.3 (0-1.5)	53.1 (-1.5-2560)	0.2 (0-1.1)	50.8 (-3.9-2430)	0.1 (0-0.4)	58 (-3.4-2470)	0 (0-0)	35.8 (-18.7-2170)	0.1 (0-0.4)	37.2 (-13.7-2190)
Northamptonshire	23.8 (16.8-32.3)	1.8 (-7.9-11.7)	19.9 (14-26.8)	1.6 (-8.1-11.9)	14.7 (10.2-20.1)	-0.3 (-10.9-10.1)	4.9 (3.3-7.2)	1.4 (-6.9-11.4)	0.4 (0.2-0.6)	-9.2 (-18.7-2.7)	5.3 (3.8-7.4)	-7.2 (-15.8-2.1)
Nottingham	39.6 (26.5-56)	1.7 (-8.6-12.9)	30.9 (21.2-43.7)	1.4 (-8.6-11.3)	20.5 (13.7-28.9)	-0.2 (-9.5-9.2)	7.5 (5.2-10.9)	-4.2 (-13.5-4.3)	0.5 (0.3-0.8)	24.3 (10.1-41.4)	8.7 (6-12.5)	-0.9 (-10.3-7.4)
Nottinghamshire	0.3 (0-1.3)	36.6 (0-2150)	0.2 (0-1.1)	36.2 (-1.8-2080)	0.2 (0-0.8)	34.1 (-4.2-2040)	0.1 (0-0.3)	41 (-2.3-2220)	0 (0-0)	22.8 (-16.7-1850)	0.1 (0-0.3)	23.3 (-12.7-1870)
Rutland	1 (0-4.7)	53.2 (0-957)	0.8 (0-3.9)	55.4 (-1.6-949)	0.6 (0-2.8)	53.9 (-4.3-918)	0.3 (0-1.2)	63.5 (-1.2-889)	0 (0-0.1)	21.8 (-27.9-638)	0.2 (0-1)	34.8 (-16.9-752)
East of England	10.9 (6.4-16.6)	-8.8 (-14.6--3.9)	9.1 (5.3-13.7)	-8.3 (-14--3.6)	6.6 (3.9-10.1)	-8 (-13.7--3.4)	2.5 (1.4-3.9)	-5.6 (-11.5--0.9)	0.2 (0.1-0.3)	-10.9 (-17.8--5.2)	2.4 (1.4-3.7)	-14 (-19.4--9.8)
Bedford	26.6 (16.2-40.4)	-23.9 (-32.4--15.4)	21.1 (12.9-31.7)	-22.7 (-31.2--13.7)	14.8 (8.9-22.1)	-21.1 (-29.1--12.3)	5.4 (3.3-8.3)	-19.9 (-28.7--10.2)	0.4 (0.2-0.7)	-19 (-29.5--6.2)	5.7 (3.5-8.4)	-26.3 (-33.9--17.9)
Cambridgeshire	4.4 (1.9-8.1)	9.8 (-2.7-26.5)	3.6 (1.6-6.8)	9.5 (-3.3-26)	2.5 (1.1-4.7)	7.4 (-4.7-23.7)	1 (0.4-1.9)	5.1 (-8.8-22.2)	0.1 (0-0.2)	-2.2 (-17.9-17.6)	1 (0.4-1.8)	-2.4 (-14.2-12.5)
Central Bedfordshire	0.4 (0-2)	29.2 (0-1490)	0.4 (0-1.7)	28.8 (-1.8-1490)	0.3 (0-1.2)	27.3 (-4.6-1510)	0.1 (0-0.5)	33.1 (-2.8-1540)	0 (0-0)	10.7 (-22.4-1310)	0.1 (0-0.5)	12.9 (-15.9-1290)
Essex	11.4 (6.4-17.7)	5.9 (-2.2-14.2)	9.7 (5.4-14.9)	5.7 (-2.4-13.6)	7.2 (4-11)	4.2 (-3.3-11.8)	2.7 (1.5-4.4)	8.1 (-0.9-17.9)	0.2 (0.1-0.4)	-5.3 (-15.8-7.3)	2.6 (1.4-4)	-4.3 (-11.4-2.8)
Hertfordshire	10.9 (6.8-15.8)	3.6 (-2.6-10.6)	9.1 (5.7-13.4)	3.3 (-3-10.3)	6.8 (4.2-10)	2.1 (-5.4-9.6)	2.7 (1.6-4.2)	5 (-3.2-14.1)	0.2 (0.1-0.4)	0.2 (-10.4-12.2)	2.7 (1.7-4.1)	-2.5 (-9.8-4.8)
Luton	18.6 (10.7-28.6)	2.9 (-4.2-10.7)	14.7 (8.4-22)	2.1 (-4.9-9.6)	9.9 (5.6-15)	0.9 (-6.5-9.9)	3.5 (2-5.8)	-2.3 (-10.4-6.2)	0.3 (0.1-0.5)	7 (-5.8-20.1)	4.5 (2.6-6.8)	0.1 (-7-8.2)
Norfolk	5.3 (3-8.3)	4.6 (-6.1-18.1)	4.6 (2.6-7.1)	5.2 (-5.5-18.5)	3.6 (2-5.6)	4.8 (-6-18.7)	1.2 (0.7-2)	8.4 (-5.6-22.8)	0.1 (0-0.1)	-8.9 (-23.9-8.3)	1.1 (0.6-1.8)	-2.1 (-12.9-10.3)
Peterborough	4.6 (1.9-10)	7.9 (-4.2-28.1)	3.8 (1.5-8.3)	9.5 (-2.9-28.7)	2.8 (1.1-5.8)	8.3 (-4.7-26.5)	0.9 (0.4-1.9)	7.3 (-6.4-25.9)	0.1 (0-0.2)	8.5 (-9.2-30.8)	1.1 (0.4-2.3)	7.7 (-4.7-25.9)
Southend-on-Sea	0.5 (0-2.1)	21.2 (0-1030)	0.4 (0-1.7)	21.4 (-2-1010)	0.3 (0-1.2)	20.4 (-4.9-985)	0.1 (0-0.5)	18.1 (-8.5-943)	0 (0-0)	31.6 (-2.8-1040)	0.1 (0-0.5)	15.3 (-9.2-932)
Suffolk	18.6 (10.9-28.2)	-33.1 (-43.2--22.9)	15.4 (9.1-23.4)	-32.1 (-42.2--22.7)	11.2 (6.8-16.6)	-30.6 (-41.1--21)	4.3 (2.6-6.7)	-26 (-37--16)	0.3 (0.2-0.6)	-27.9 (-38.6--17.2)	4 (2.4-6)	-36.4 (-45.8--27.8)
Thurrock	31.1 (19.6-44.4)	1.5 (-7.4-9.6)	25.2 (15.8-35.9)	0.7 (-8.5-9.2)	18.1 (11.1-26.2)	-0.5 (-9.2-8.1)	6.4 (3.9-9.8)	0.2 (-8.9-9)	0.6 (0.3-1)	-0.6 (-11.8-13.2)	7.4 (4.7-10.6)	-2.8 (-11.7-5.2)
Greater London	52.6 (40.5-66.8)	-6.4 (-9--4)	41.6 (32.3-52.4)	-5.3 (-7.9--3.1)	29 (21.8-36.8)	-5.2 (-7.5--3.1)	10.7 (8.1-14.2)	-5.7 (-8.1--3.5)	1 (0.6-1.4)	14.2 (11-17.6)	12.5 (9.7-16)	-4.8 (-7.1--2.8)

Location	Birth Incidence rate (per 100,000 live births), 2021	Birth Incidence rate (per 100,000 live births) percent change (2000-2021)	Under-5 rate (per 100,000 population), 2021	Under-5 rate (per 100,000 population) percent change (2000-2021)	5-14 rate (per 100,000 population), 2021	5-14 rate (per 100,000 population) percent change (2000-2021)	15-49 rate (per 100,000 population), 2021	15-49 rate (per 100,000 population) percent change (2000-2021)	50plus rate (per 100,000 population), 2021	50plus rate (per 100,000 population) percent change (2000-2021)	All age rate (per 100,000 population), 2021	All age rate (per 100,000 population) percent change (2000-2021)
Barking and Dagenham	66.1 (51.8-83.9)	-6 (-13.7-3.6)	53.4 (42-66.6)	-6 (-13.1-3.3)	38.1 (29.5-48.3)	-6.4 (-13.8-1.9)	14.1 (10.9-18.3)	-5.1 (-12.4-3.2)	1.4 (0.9-2)	31.9 (20-45.2)	19.2 (15.2-24.1)	7.4 (-0.6-16.6)
Barnet	44.1 (32.8-57.2)	-3.8 (-11.2-3.9)	36.4 (27.3-46.8)	-4.3 (-12.1-3.5)	26.7 (20-34.7)	-5.7 (-12.9-2.3)	10.4 (7.8-13.8)	-5.2 (-12.4-2.4)	1 (0.7-1.4)	0.1 (-8.9-10.3)	11.6 (8.8-14.9)	-3.6 (-10.8-3.9)
Bexley	23.4 (14.9-33.4)	-16.8 (-25.2--8.8)	19.8 (12.8-28.3)	-15.7 (-24.6--7.6)	14.8 (9.6-21.3)	-15.5 (-24.3--7)	5.8 (3.6-8.7)	-11.2 (-19.7--2.5)	0.5 (0.3-0.8)	-11.6 (-21.2-0.3)	6 (3.9-8.6)	-16.1 (-24.3--8.4)
Brent	45.3 (34.5-58.8)	0.6 (-7.8-9.7)	35.2 (26.9-44.6)	-1.2 (-9.4-7.3)	23.6 (17.9-30.7)	-2.2 (-10.3-6.5)	8.9 (6.7-11.9)	-4.8 (-12.2-3.6)	0.8 (0.5-1.2)	7.5 (-2.7-20.5)	10.6 (8.3-13.7)	-0.9 (-8.8-7.1)
Bromley	19.5 (11-29.5)	3.2 (-5.5-11.8)	16.5 (9.4-25.1)	2.7 (-5.9-12.6)	12.6 (7.1-19)	1.7 (-6.2-12.5)	4.9 (2.7-7.9)	4.6 (-3.2-14.1)	0.5 (0.2-0.8)	3.8 (-5.9-14.8)	4.9 (2.7-7.5)	-1.2 (-8.5-8.2)
Camden	36 (25.1-49.7)	-23.2 (-30.7--15.9)	29.2 (20.4-40.6)	-23.3 (-30.5--16.4)	20.5 (14.2-28.1)	-23.4 (-30.8--15.4)	7.5 (5.3-10.6)	-24 (-30.8--16.3)	0.6 (0.4-0.9)	-22.8 (-31.3--12.9)	7.9 (5.7-11)	-27.8 (-34.2--21)
Croydon	53.6 (40.8-68.5)	2.9 (-5-13.3)	43.3 (33.1-55)	2.3 (-5.4-12.5)	30.9 (23.4-40)	1.3 (-6.3-11.2)	11.5 (8.5-15.3)	2.6 (-6.2-11.3)	1 (0.7-1.6)	6 (-4.9-16.3)	13.5 (10.4-17.2)	2.7 (-4.7-12)
Ealing	25.6 (18-34.5)	-13.2 (-20.2--6.1)	20.7 (14.7-28)	-14 (-21.2--6.8)	14.5 (10.1-19.7)	-15.2 (-22--7.9)	5.4 (3.7-7.8)	-15.4 (-22.6--7.3)	0.5 (0.3-0.8)	-5.3 (-15.5-6.4)	6.3 (4.5-8.8)	-13.9 (-20.8--6.8)
Enfield	111 (88.1-138)	-8.2 (-15.5--0.7)	91.3 (72.8-112)	-8.7 (-16.3--1.4)	66.1 (52.4-81.6)	-9.5 (-17--2.3)	26.9 (21.1-34.6)	-3.9 (-11.5-3.7)	2.6 (1.8-3.7)	7.6 (-2.5-17.8)	30.3 (24.4-37.6)	-3.4 (-10.8-3.7)
Greenwich	57.6 (43.9-73.7)	-1.4 (-9.4-6.8)	46.7 (35.6-59.9)	-1.7 (-9.9-6.7)	32.8 (24.9-42.2)	-3.1 (-11.2-5.5)	11.7 (8.7-15.6)	-4.6 (-12.9-3.9)	1.1 (0.7-1.7)	19.5 (7.3-32.6)	14.6 (11.3-18.7)	-0.9 (-8.6-7.4)
Hackney	164 (129-203)	-5.7 (-13.6-3.6)	131 (103-162)	-6.1 (-13.8-3)	90.7 (70.3-114)	-6.1 (-13.9-3.1)	31.2 (23.9-40.2)	-10.2 (-17.4--2)	3.2 (2.2-4.7)	16.5 (5.1-29.3)	39.6 (31.5-49.3)	-11.9 (-18.4--3.7)
Hammersmith and Fulham	36.6 (23.7-52.8)	-18.9 (-26.5--9.7)	29.2 (19-41.6)	-20 (-27.7--11)	20.4 (13.4-28.9)	-20.6 (-28.6--11.9)	7 (4.6-10.4)	-23.2 (-30.8--13.7)	0.6 (0.4-1.1)	-1 (-12.8-12.8)	8.1 (5.4-11.5)	-22.4 (-29.5--14.1)
Haringey	167 (131-209)	-4.9 (-12.2-3.6)	132 (105-163)	-5.5 (-12.2-3.3)	90.7 (70.5-115)	-5.8 (-12.9-2.8)	32.8 (25.6-42.6)	-9.1 (-16.1--1.4)	3.4 (2.3-4.9)	11.9 (1.5-23.3)	38.7 (31-48.5)	-10.9 (-17.2--3.7)
Harrow	4.8 (2-9.1)	6.1 (-6.3-21.9)	4.1 (1.7-7.8)	4 (-7.7-18.5)	3.1 (1.3-5.9)	2.8 (-9.1-17)	1.2 (0.5-2.5)	7.4 (-6.2-24.7)	0.1 (0-0.2)	2.4 (-13.7-24.1)	1.3 (0.6-2.6)	6.4 (-7-21.8)
Havering	10.9 (4.7-19.8)	5.9 (-2.8-19.9)	9 (3.8-16.6)	6.1 (-2.6-20.3)	6.8 (2.8-12.3)	4.9 (-4-19)	2.7 (1.1-5.1)	8.4 (-0.8-21.6)	0.2 (0.1-0.5)	0.1 (-12.2-15.1)	2.7 (1.1-5)	4.9 (-3.9-18.9)
Hillingdon	11 (4.7-19.7)	6.6 (-4.6-17.4)	8.9 (3.8-16.2)	6.4 (-4.4-16.1)	6.4 (2.7-11.6)	5.1 (-5.2-14.1)	2.5 (1-4.6)	6.1 (-4.2-16.1)	0.2 (0.1-0.5)	13.4 (-0.7-31.4)	2.9 (1.2-5.1)	7.5 (-3.1-16.2)
Hounslow	27.4 (17.3-39.4)	-2.7 (-12-6.9)	22.3 (14-31.6)	-2.9 (-12.8-5.9)	15.9 (9.8-22.7)	-2.9 (-12.9-6.5)	5.8 (3.5-8.8)	-8 (-16.9-0.4)	0.5 (0.3-0.9)	-0.6 (-12.2-12.2)	6.9 (4.3-10)	-4.6 (-13.7-4)
Islington	21.6 (13.2-32)	1.4 (-8.5-11.6)	16.9 (10.2-25)	1.2 (-8.8-10.2)	11.6 (6.9-17.1)	-0.4 (-10.1-8.8)	3.9 (2.3-6.1)	-8.9 (-18.5--0.4)	0.3 (0.2-0.6)	13.7 (0.2-30.3)	4.6 (2.7-6.9)	-8.5 (-17.5--0.3)
Kensington and Chelsea	0.4 (0-2)	28.4 (0-1470)	0.4 (0-1.7)	28.1 (-2.1-1460)	0.3 (0-1.2)	26.8 (-5.3-1420)	0.1 (0-0.4)	21.2 (-11-1280)	0 (0-0)	18.5 (-15.8-1160)	0.1 (0-0.5)	18.5 (-11.7-1290)
Kingston upon Thames	14 (9.1-19.9)	1.5 (-9.6-18.4)	11.8 (7.7-16.7)	1.2 (-9.4-17.5)	8.8 (5.8-12.5)	-0.6 (-10.8-15.5)	3.2 (2-4.8)	-8.2 (-18.6-5.5)	0.3 (0.2-0.5)	-2.3 (-15.2-13.7)	3.6 (2.3-5.2)	-4 (-13.5-11.8)
Lambeth	115 (91-144)	-8.2 (-15.3--0.8)	92.5 (73.8-115)	-8.2 (-15.3--0.7)	64.3 (49.9-80.4)	-8.4 (-15.5--1.2)	22.5 (17.3-29.3)	-12.7 (-19.1--5.1)	2.4 (1.6-3.4)	23.5 (11.5-35.7)	26.7 (21.2-33.4)	-14.3 (-20.3--7.5)
Lewisham	83.8 (65.8-105)	-5.4 (-13.5-2.2)	67.3 (53.7-83.3)	-5.7 (-13.3-2.3)	47.3 (37.2-59.3)	-6.1 (-13.3-1.3)	16.9 (13.1-22)	-7.9 (-15.4--0.7)	1.7 (1.1-2.5)	19.3 (7.4-31.3)	20.8 (16.8-26)	-6.8 (-13.6-0.4)
Merton	37.3 (25.8-52.4)	0.5 (-7.9-8.9)	30.5 (20.9-43.2)	0.2 (-8.1-8.4)	22.1 (14.8-30.4)	-0.5 (-8.6-8.9)	7.8 (5.2-11.5)	-4.6 (-12.7-4.4)	0.8 (0.5-1.3)	8.9 (-1.9-22.3)	9.3 (6.5-13)	-0.2 (-8.2-8.8)
Newham	70.2 (55.2-88.9)	1.8 (-6.5-11.1)	54.7 (43.2-67.8)	1.3 (-6.3-10)	36.3 (27.5-46.5)	0.5 (-7.5-8.8)	12.8 (9.7-16.8)	-9.4 (-16.6--1)	1.3 (0.9-1.9)	24.7 (12.9-38.3)	16.9 (13.2-21.1)	-8.4 (-14.9--0.3)
Redbridge	22.7 (14-33.2)	1.6 (-7-11.2)	18.6 (11.4-27.3)	1.3 (-6.9-11.3)	13.5 (8.2-19.9)	1 (-7.7-11.4)	5.2 (3.1-8.1)	1.4 (-6.4-12.3)	0.5 (0.2-0.8)	3 (-7.9-15.4)	6.2 (3.8-9.2)	8.6 (-0.1-19.8)
Richmond upon Thames	14.4 (7.2-24.2)	4.1 (-4.9-11.9)	12.1 (6-20)	3.4 (-6-11)	9.1 (4.5-15)	2.8 (-7.4-11.4)	3.5 (1.7-6)	3 (-7.3-11.8)	0.4 (0.2-0.7)	-1.8 (-13.8-10.4)	3.7 (1.8-6.2)	0.3 (-8.9-8.4)
Southwark	113 (87.4-142)	0.9 (-7-10)	89.8 (69.8-113)	0.8 (-6.6-9.5)	61.5 (46.9-77.3)	-0.4 (-8.2-8)	20.5 (15.8-26.9)	-7.2 (-14--0.2)	2.2 (1.4-3.1)	37.8 (24.6-51)	25.7 (20.2-31.9)	-5.4 (-11.8-2.1)
Sutton	8.5 (4.9-13.3)	1.9 (-9.1-15.1)	7.3 (4.2-11.4)	1.8 (-9.8-14.9)	5.6 (3.2-8.8)	-0.9 (-12.2-11.5)	2 (1.1-3.3)	-1.5 (-12.5-11.2)	0.2 (0.1-0.4)	4.8 (-8.5-21.4)	2.2 (1.3-3.5)	-5.5 (-15.6-6.4)
Tower Hamlets	19.4 (13.2-27.7)	-21.5 (-32.8--9.6)	14.7 (10-20.6)	-22.4 (-32.6--10.3)	9.7 (6.6-13.8)	-21.1 (-31.3--9)	3.5 (2.4-5.1)	-30.6 (-40.5--20)	0.3 (0.2-0.5)	7 (-10.1-26.6)	4.6 (3.2-6.4)	-26.5 (-35.6--15.4)

Location	Birth Incidence rate (per 100,000 live births), 2021	Birth Incidence rate (per 100,000 live births) percent change (2000-2021)	Under-5 rate (per 100,000 population), 2021	Under-5 rate (per 100,000 population) percent change (2000-2021)	5-14 rate (per 100,000 population), 2021	5-14 rate (per 100,000 population) percent change (2000-2021)	15-49 rate (per 100,000 population), 2021	15-49 rate (per 100,000 population) percent change (2000-2021)	50plus rate (per 100,000 population), 2021	50plus rate (per 100,000 population) percent change (2000-2021)	All age rate (per 100,000 population), 2021	All age rate (per 100,000 population) percent change (2000-2021)
Waltham Forest	64.5 (48.9-84.3)	-3.1 (-11.6-5.7)	50.7 (38.6-65.5)	-3.3 (-11.2-4.7)	34.3 (25.6-44.7)	-3.5 (-11.3-4.4)	12.6 (9.4-16.8)	-6.4 (-13.8-1.3)	1.2 (0.8-1.9)	17.9 (7-29.9)	15.5 (11.9-20.1)	-3 (-10.7-4.4)
Wandsworth	26.8 (18.4-37.5)	1.8 (-4.6-10.7)	21.7 (14.9-30.1)	1 (-5.3-9.6)	15.4 (10.4-21.6)	0.2 (-7.2-8.6)	5.4 (3.5-8.1)	-7.9 (-16-1.3)	0.5 (0.3-0.9)	21.4 (7-37.1)	6.4 (4.5-9.2)	0.3 (-7.8-9.7)
Westminster	10.4 (4.8-18.5)	-33.4 (-48.9--20)	8.2 (3.8-14.7)	-32.8 (-48.3--19.4)	5.6 (2.5-9.9)	-32 (-47.8--18.7)	2.1 (1-3.8)	-36.2 (-51.3--24)	0.2 (0.1-0.4)	-25.6 (-43.6--7.2)	2.2 (1-4)	-32.5 (-48.3--19.7)
North East England	6.4 (3.5-10.6)	14.1 (8-20.8)	5 (2.7-8.2)	14.8 (8.8-22)	3.3 (1.7-5.4)	10.9 (5.1-18.9)	1.2 (0.7-2.2)	12.4 (5.2-21.4)	0.1 (0-0.1)	-0.6 (-9.3-11.1)	1.2 (0.6-2)	1.3 (-4.1-8.8)
County Durham	0.4 (0-1.8)	62.8 (0-4160)	0.3 (0-1.5)	62.9 (-1.3-4120)	0.2 (0-1.1)	60.8 (-4-4080)	0.1 (0-0.4)	68.5 (-3.7-4170)	0 (0-0)	49.9 (-17.1-3560)	0.1 (0-0.4)	46.3 (-14.1-3610)
Darlington	0.6 (0-2.5)	41.1 (0-3590)	0.5 (0-2)	39.9 (-2-3440)	0.4 (0-1.4)	35.9 (-4.9-3090)	0.1 (0-0.5)	43.3 (-2.7-3560)	0 (0-0)	33 (-14-3340)	0.1 (0-0.5)	29 (-10.8-3120)
Gateshead	8.7 (4.3-14.7)	4.5 (-10.5-17.4)	7.4 (3.7-12.4)	4.5 (-10-16.8)	5.6 (2.7-9.3)	2.6 (-10.7-15.3)	1.8 (0.8-3.1)	4 (-9.1-19.1)	0.1 (0-0.2)	2.5 (-13.2-21.8)	1.9 (0.9-3.1)	-3.9 (-16.3-9.1)
Hartlepool	0.8 (0-3.6)	77.7 (0-5710)	0.6 (0-2.8)	77.7 (-1.7-5750)	0.5 (0-2)	74.3 (-5.6-5800)	0.2 (0-0.8)	82 (-3.9-5650)	0 (0-0)	77.3 (-13.3-5330)	0.2 (0-0.7)	55.1 (-16.6-4980)
Middlesbrough	18 (10.3-27.8)	3.1 (-7.1-18.1)	13.4 (7.6-20.6)	2.6 (-8.1-17.5)	8.7 (5-13.3)	1.3 (-11.4-15.3)	3.1 (1.8-4.9)	-1.8 (-13.6-12)	0.1 (0.1-0.3)	2.2 (-15.8-23.4)	3.5 (2-5.5)	-4.6 (-15.3-8.2)
Newcastle upon Tyne	20.3 (11.5-31)	2.6 (-5.2-11.1)	16.3 (9.3-24.9)	2.6 (-4.7-10.1)	11.3 (6.3-17.3)	1.4 (-5.7-8.6)	4.3 (2.4-7)	0.1 (-8.9-8.4)	0.2 (0.1-0.4)	6.4 (-6.1-19.9)	4.6 (2.5-7.2)	1.7 (-6-9.1)
North Tyneside	14.4 (8.1-22.2)	3.2 (-8.2-16.6)	10.9 (6.2-16.8)	1.1 (-10.5-14.4)	7.3 (4.1-11.4)	-2 (-13.7-11.5)	2.7 (1.5-4.4)	-1.3 (-13.9-13.3)	0.2 (0.1-0.3)	5 (-13.2-29.3)	2.6 (1.5-4.2)	-8.7 (-18.4-3.7)
Northumberland	0.4 (0-2.1)	30.7 (0-1490)	0.4 (0-1.7)	30.6 (-1.6-1500)	0.3 (0-1.3)	29.1 (-4-1490)	0.1 (0-0.5)	37.4 (-0.8-1550)	0 (0-0)	5.5 (-28.4-1180)	0.1 (0-0.4)	12.3 (-17.2-1270)
Redcar and Cleveland	0.6 (0-2.4)	38.2 (0-3620)	0.5 (0-1.9)	37.4 (-1.5-3530)	0.3 (0-1.4)	34.2 (-4.3-3260)	0.1 (0-0.5)	39.4 (-3.4-3440)	0 (0-0)	20.7 (-21.5-2500)	0.1 (0-0.5)	20 (-15.1-2970)
South Tyneside	0.6 (0-2.4)	39.1 (0-3610)	0.5 (0-2)	38.2 (-1.5-3570)	0.4 (0-1.4)	35.4 (-4.3-3450)	0.1 (0-0.5)	38.7 (-4.1-3530)	0 (0-0)	39.3 (-8.7-3600)	0.1 (0-0.5)	20 (-15.7-3060)
Stockton-on-Tees	6 (2.6-11.1)	5.2 (-7.8-20.8)	5 (2.1-9.4)	3 (-9.6-19.1)	3.7 (1.6-6.9)	2.6 (-9.7-19.9)	1.3 (0.6-2.5)	12.6 (-1.8-30.1)	0.1 (0-0.2)	-4.9 (-22.2-16.6)	1.4 (0.6-2.5)	-4.1 (-16-12.9)
Sunderland	0.4 (0-2.1)	31.1 (0-1520)	0.4 (0-1.7)	30.5 (-1.8-1520)	0.3 (0-1.2)	29.1 (-4.5-1500)	0.1 (0-0.5)	28.9 (-7.1-1530)	0 (0-0)	24.4 (-15.3-1480)	0.1 (0-0.4)	11 (-18.1-1290)
North West England	13.6 (9.6-18.9)	19.9 (14.6-26)	10.5 (7.4-14.6)	22.8 (17.1-29.4)	6.9 (4.7-9.5)	17.2 (11.9-23.2)	2.7 (1.9-3.8)	17.2 (11.4-24.1)	0.1 (0.1-0.2)	5.1 (-0.6-11.4)	2.6 (1.9-3.7)	8.6 (3.8-14.3)
Blackburn with Darwen	15.1 (8.5-23.6)	3.5 (-6.3-14)	11.9 (6.8-19)	2.8 (-6.7-14)	8.2 (4.8-12.8)	0.2 (-9.6-12.3)	2.8 (1.5-4.6)	2 (-7.6-14.8)	0.2 (0.1-0.3)	6.2 (-7.5-26.8)	3.3 (1.9-5.1)	-8.9 (-16.9-0.9)
Blackpool	10.6 (5.9-16.8)	3.5 (-7.6-16)	7.7 (4.3-12)	4.3 (-6.6-16.1)	4.8 (2.6-7.5)	2.7 (-9.2-15.4)	1.6 (0.9-2.6)	5.2 (-8.4-20.5)	0.1 (0-0.2)	12.9 (-9.1-41.5)	1.7 (0.9-2.7)	0.3 (-11-13.6)
Bolton	19.5 (11.9-29.3)	3.7 (-4.9-14.3)	15.2 (9.2-22.9)	3.9 (-5.4-14.2)	10.1 (5.9-15.4)	1.2 (-8.3-11.9)	3.5 (2-5.6)	1.4 (-8.9-11.8)	0.2 (0.1-0.4)	-0.2 (-13.1-13.9)	3.9 (2.3-6)	-4.7 (-13.4-5)
Bury	4.9 (2-10)	9.8 (-1-23.6)	3.7 (1.5-7.7)	8 (-4.3-23.7)	2.4 (1-5)	6.8 (-8.5-25.7)	0.9 (0.4-1.9)	9.2 (-5.5-28.4)	0.1 (0-0.1)	2.7 (-14-24.9)	0.9 (0.4-2)	-0.4 (-13.8-16.3)
Cheshire East	2.9 (1.2-5.8)	7.1 (-6.6-26.5)	2.5 (1-5)	8.8 (-4.2-28.3)	1.9 (0.8-3.8)	7 (-4.7-25.3)	0.7 (0.3-1.4)	14.4 (0.7-40)	0 (0-0.1)	-8.2 (-23.2-15.2)	0.6 (0.3-1.2)	-4.4 (-14.9-13.4)
Cheshire West and Chester	0.4 (0-2)	47.8 (0-1470)	0.4 (0-1.7)	48.6 (-1.4-1500)	0.3 (0-1.2)	47.8 (-4.6-1480)	0.1 (0-0.5)	52.5 (-2.7-1540)	0 (0-0)	32.3 (-19.3-1320)	0.1 (0-0.4)	29.6 (-16.2-1290)
Cumbria	3.1 (1.2-6.6)	11.7 (-4.1-30.4)	2.5 (1-5.4)	12.3 (-2.7-33.2)	1.7 (0.7-3.7)	10.6 (-3.6-34.4)	0.6 (0.2-1.4)	13.3 (-1.8-35.6)	0 (0-0.1)	1.5 (-18.3-26.8)	0.5 (0.2-1.2)	-5 (-17.3-14.2)
Halton	0.6 (0-2.4)	36.8 (0-3620)	0.5 (0-1.9)	36 (-2.6-3550)	0.3 (0-1.4)	33 (-5.8-3450)	0.1 (0-0.5)	33.2 (-7.2-3550)	0 (0-0)	9.4 (-28.6-2760)	0.1 (0-0.5)	17.5 (-16.8-3090)
Knowsley	0.6 (0-2.4)	46.8 (0-3950)	0.5 (0-1.9)	46.3 (-1.1-3750)	0.3 (0-1.4)	43.9 (-4.3-3540)	0.1 (0-0.5)	49.4 (-3.8-3660)	0 (0-0)	50.6 (-8.4-3400)	0.1 (0-0.5)	28 (-15.5-3150)
Lancashire	1.5 (0.6-3)	-30.2 (-50.9--10.7)	1.2 (0.5-2.5)	-30.5 (-51.6--10.1)	0.9 (0.4-1.8)	-30.6 (-51--10.1)	0.3 (0.1-0.7)	-27.5 (-49.3--6.2)	0 (0-0)	-36.5 (-56.6--14.9)	0.3 (0.1-0.6)	-37.2 (-55.7--18.9)
Liverpool	23 (15.2-32.9)	2.3 (-6.4-11.8)	18.5 (12.2-26.3)	2.5 (-6.7-11.6)	12.7 (8.2-18)	1.6 (-8.2-10.8)	4.5 (2.9-6.8)	-6 (-14.9-2.2)	0.3 (0.1-0.5)	5.2 (-8-19.1)	4.8 (3.1-7)	-8.1 (-16.7-0.1)
Manchester	57.8 (44.6-74.4)	2 (-5.6-9.8)	45.6 (35.1-59)	1.7 (-6.5-9.1)	30.7 (22.9-39.5)	0.6 (-7.6-8.4)	11.2 (8.5-15.1)	-7.7 (-15.2-0.4)	0.9 (0.6-1.3)	32.7 (18.2-48.7)	13.8 (10.6-17.7)	1.2 (-6.4-9.2)

Location	Birth Incidence rate (per 100,000 live births), 2021	Birth Incidence rate (per 100,000 live births) percent change (2000-2021)	Under-5 rate (per 100,000 population), 2021	Under-5 rate (per 100,000 population) percent change (2000-2021)	5-14 rate (per 100,000 population), 2021	5-14 rate (per 100,000 population) percent change (2000-2021)	15-49 rate (per 100,000 population), 2021	15-49 rate (per 100,000 population) percent change (2000-2021)	50plus rate (per 100,000 population), 2021	50plus rate (per 100,000 population) percent change (2000-2021)	All age rate (per 100,000 population), 2021	All age rate (per 100,000 population) percent change (2000-2021)
Oldham	3.5 (1.4-7.5)	9.6 (-0.2-37.9)	2.9 (1.1-6.1)	7.9 (-2.5-34.2)	2.1 (0.8-4.4)	5.4 (-6.1-29.6)	0.7 (0.3-1.6)	16.7 (3.7-43.5)	0 (0-0.1)	8.4 (-9-32.2)	0.8 (0.3-1.8)	5.5 (-5.2-30.3)
Rochdale	0.5 (0-2.1)	22.3 (0-1110)	0.4 (0-1.6)	22.7 (-2.2-1120)	0.3 (0-1.1)	21.4 (-5.7-1070)	0.1 (0-0.4)	24.3 (-4.8-1130)	0 (0-0)	16.5 (-15.5-1000)	0.1 (0-0.5)	13.9 (-11-1030)
Salford	48.4 (35.6-64)	1.3 (-7.5-12.9)	39.5 (29.6-51.7)	0.2 (-9.3-11.7)	27.8 (20.7-36.2)	-0.5 (-9.6-9.9)	9.3 (6.8-12.5)	-5.2 (-13.9-4.4)	0.7 (0.4-1)	12.9 (0-27.9)	11.2 (8.5-14.5)	1.3 (-7-11.5)
Sefton	0.4 (0-2.1)	28.8 (0-1490)	0.4 (0-1.7)	28.7 (-1.8-1480)	0.3 (0-1.2)	27.6 (-4.3-1430)	0.1 (0-0.5)	32.2 (-3-1490)	0 (0-0)	21.7 (-15.4-1310)	0.1 (0-0.4)	12.1 (-16.2-1230)
St Helens	0.5 (0-2.1)	19.4 (0-762)	0.4 (0-1.7)	19.8 (-1.3-756)	0.3 (0-1.2)	18.5 (-3.7-751)	0.1 (0-0.5)	18.7 (-5.4-733)	0 (0-0)	6.2 (-20.1-633)	0.1 (0-0.4)	4.8 (-15.2-643)
Stockport	6 (2.7-10.6)	7.5 (-2.3-24)	4.7 (2.1-8.5)	6.4 (-3-23.2)	3.2 (1.4-5.9)	4.4 (-6.1-20.7)	1.2 (0.5-2.3)	6 (-6.9-23)	0.1 (0-0.2)	0.3 (-16.7-22.4)	1.2 (0.5-2.2)	-3.7 (-13.6-11.8)
Tameside	26.4 (16.6-38.1)	1.5 (-5.9-10.9)	21.2 (13.2-30.5)	1.3 (-6.1-9.8)	15.1 (9.3-21.7)	0.3 (-7.3-9.3)	5.2 (3.1-7.9)	1 (-7.1-9.6)	0.3 (0.2-0.6)	-2.1 (-15.1-11.2)	5.5 (3.4-8)	-7.4 (-14.5--0.4)
Trafford	0.4 (0-2)	27.1 (0-1470)	0.4 (0-1.7)	26 (-2.7-1440)	0.3 (0-1.2)	24.9 (-6-1420)	0.1 (0-0.5)	28.9 (-4.8-1460)	0 (0-0)	32.3 (-6.8-1430)	0.1 (0-0.5)	21.8 (-8.8-1410)
Warrington	0.5 (0-2.1)	21.3 (0-1050)	0.4 (0-1.7)	21.3 (-2-1050)	0.3 (0-1.2)	20.6 (-4.9-1030)	0.1 (0-0.4)	24.8 (-3.7-1070)	0 (0-0)	15.1 (-16.8-960)	0.1 (0-0.4)	8.8 (-14.4-927)
Wigan	9.9 (5.2-16)	2.5 (-5-13.2)	8.5 (4.5-13.6)	3.2 (-5.1-14.4)	6.4 (3.4-10.2)	2.7 (-7.4-14.4)	2.1 (1.1-3.5)	3.2 (-7.5-16.7)	0.1 (0.1-0.3)	-3 (-18.7-16.4)	2.2 (1.2-3.5)	-7.6 (-16.3-3)
Wirral	3.6 (1.5-7.1)	-22.9 (-38--7.2)	3.1 (1.3-6.2)	-22.7 (-37.5--7.4)	2.4 (1-4.7)	-23.6 (-37.8--6.9)	0.8 (0.3-1.7)	-17.6 (-34.5-1)	0 (0-0.1)	-28.1 (-46.3--9)	0.8 (0.3-1.6)	-29.9 (-43.4--14.9)
South East England	7.9 (4.7-12.1)	3.5 (-0.9-7.7)	6.6 (3.9-10)	5 (0.5-9.4)	4.8 (2.9-7.4)	4.6 (0.1-9.1)	1.8 (1-2.8)	3.2 (-1.5-7.6)	0.1 (0.1-0.2)	-4.1 (-9.6-1.9)	1.8 (1-2.7)	-3.7 (-7.7-0.2)
Bracknell Forest	0.6 (0-2.4)	39.8 (0-3960)	0.5 (0-2)	39.1 (-1.2-3830)	0.4 (0-1.5)	36.9 (-3.6-3660)	0.1 (0-0.6)	39.1 (-4.5-3870)	0 (0-0.1)	25.7 (-18.7-3630)	0.1 (0-0.6)	21.5 (-15-3350)
Brighton and Hove	5.7 (2.3-11.9)	7.4 (-3.2-17.7)	4.7 (1.9-9.8)	7 (-4-19.1)	3.4 (1.4-7.1)	6 (-4.3-18.1)	1.2 (0.5-2.7)	7.4 (-4.7-19.8)	0.1 (0-0.2)	41.1 (19.5-63.2)	1.3 (0.5-2.6)	5 (-5.7-17.1)
Buckinghamshire	12.8 (7-19.9)	4.3 (-2.5-13.4)	10.7 (5.8-17)	3.6 (-3.6-12.7)	8 (4.3-12.2)	2.4 (-5.7-11.1)	3 (1.6-4.9)	8.4 (-1.3-18.6)	0.3 (0.1-0.5)	-9 (-19.6-3.2)	3 (1.6-4.7)	-5.7 (-13.2-2.1)
East Sussex	0.4 (0-2)	44.8 (0-874)	0.3 (0-1.6)	45.5 (-1.3-882)	0.2 (0-1.2)	45.5 (-4.2-868)	0.1 (0-0.5)	53.7 (-1.5-920)	0 (0-0)	44.1 (-12.5-825)	0.1 (0-0.4)	33.6 (-12.2-792)
Hampshire	7.2 (4.4-10.8)	3.6 (-5.6-12.3)	6.3 (3.8-9.3)	3.5 (-6.1-12.7)	4.9 (2.9-7.2)	2.6 (-7.2-12.2)	1.8 (1.1-2.9)	5.8 (-4.2-16.3)	0.1 (0.1-0.3)	-11 (-22.1-2.1)	1.7 (1-2.6)	-8.7 (-16.6--0.1)
Isle of Wight	0.6 (0-2.5)	42.8 (0-3610)	0.5 (0-2)	41.7 (-1.1-3470)	0.4 (0-1.5)	38 (-3.7-3140)	0.1 (0-0.6)	46.4 (0-3530)	0 (0-0)	32.5 (-15.6-3270)	0.1 (0-0.5)	26.1 (-12.9-2980)
Kent	9 (5-14)	-13 (-26.9-0.3)	7.3 (4-11.5)	-12.7 (-26.6-0.2)	5.3 (2.8-8.4)	-13.5 (-27.2--0.3)	2.1 (1.1-3.5)	-10 (-24.3-3.8)	0.1 (0.1-0.3)	-15.4 (-31.4-2.9)	2 (1-3.2)	-18 (-31.3--5.8)
Medway	10.9 (6.2-16.8)	2.5 (-8.7-13.4)	9.2 (5.3-14.1)	0.1 (-11.7-10.9)	6.8 (3.9-10.5)	-1.3 (-12.4-9.6)	2.6 (1.4-4.2)	6.1 (-6.4-18.6)	0.2 (0.1-0.4)	-8.5 (-22.2-7.7)	2.7 (1.5-4.2)	-7 (-17.7-2.7)
Milton Keynes	41.7 (30.7-55.3)	0.7 (-8.2-9)	33.6 (25.1-45)	-0.1 (-8.1-7.7)	24.1 (17.5-31.9)	-0.9 (-8.9-8.2)	8.5 (6.2-11.8)	-3.5 (-11.2-5.3)	0.7 (0.5-1.1)	-16.3 (-24.5--6)	10.1 (7.6-13.3)	-7 (-14.6-0.7)
Oxfordshire	9.6 (5-15.6)	5 (-2.2-11.6)	8 (4.2-12.8)	4.7 (-2.7-11.7)	5.8 (3-9.2)	3.5 (-5.3-11.4)	2.3 (1.2-3.9)	3.4 (-5.4-10.9)	0.2 (0.1-0.4)	-7.1 (-17.2-3.5)	2.2 (1.1-3.7)	-4.9 (-12.6-1.5)
Portsmouth	0.5 (0-2.1)	20 (0-1030)	0.4 (0-1.7)	20.2 (-1.9-1030)	0.3 (0-1.2)	20 (-3.9-1020)	0.1 (0-0.5)	19.9 (-5.6-1050)	0 (0-0)	38.4 (4.2-1250)	0.1 (0-0.5)	20.2 (-3.9-1040)
Reading	0.6 (0-2.4)	50.4 (0-3610)	0.5 (0-2)	48.8 (-2.4-3470)	0.3 (0-1.4)	46.9 (-5.1-3330)	0.1 (0-0.5)	37.9 (-14.2-3300)	0 (0-0)	65.8 (-0.6-3700)	0.1 (0-0.6)	49.3 (-4.1-3510)
Slough	24.5 (13.5-38.1)	2.7 (-4.7-14.7)	19.3 (10.6-30)	1.5 (-5.9-12.7)	13.3 (7.2-20.8)	0.6 (-7.2-12.2)	4.4 (2.3-7.2)	-2.4 (-10.4-8.2)	0.4 (0.2-0.7)	18 (5.6-35.3)	6.2 (3.4-9.8)	8.1 (0.4-20)
Southampton	9 (4.2-15.7)	6.2 (-6.8-23.3)	6.8 (3.1-12)	5.4 (-7.3-20.7)	4.5 (2.1-7.8)	1.8 (-11.3-17.9)	1.7 (0.8-3.1)	-5.2 (-18.6-11.3)	0.1 (0-0.2)	13.9 (-4.8-41.7)	1.9 (0.9-3.4)	1.2 (-12-16.9)
Surrey	5.1 (2.9-8.1)	5.4 (-7-16.2)	4.2 (2.3-6.6)	5.5 (-6.9-17.6)	3.1 (1.7-4.8)	5.5 (-6.3-17)	1.2 (0.7-2.1)	7.5 (-4.5-20.5)	0.1 (0-0.2)	-0.2 (-15-17.2)	1.2 (0.6-1.9)	0.2 (-10.5-10.9)
West Berkshire	0.5 (0-2.1)	16.7 (0-1030)	0.4 (0-1.7)	16.6 (-2.2-1040)	0.3 (0-1.3)	15.7 (-5.2-1020)	0.1 (0-0.5)	20.5 (-3-1050)	0 (0-0)	1.8 (-20.7-842)	0.1 (0-0.5)	4.4 (-14.6-904)
West Sussex	2.1 (0.8-4.5)	14.7 (-1-41.8)	1.8 (0.7-4)	15.5 (-0.9-41.2)	1.4 (0.6-3.1)	14.8 (-1.6-38.6)	0.5 (0.2-1.2)	18.5 (2.5-45.5)	0 (0-0.1)	12.2 (-3.8-37.5)	0.5 (0.2-1.1)	7.1 (-7.7-30.4)

Location	Birth Incidence rate (per 100,000 live births), 2021	Birth Incidence rate (per 100,000 live births) percent change (2000-2021)	Under-5 rate (per 100,000 population), 2021	Under-5 rate (per 100,000 population) percent change (2000-2021)	5-14 rate (per 100,000 population), 2021	5-14 rate (per 100,000 population) percent change (2000-2021)	15-49 rate (per 100,000 population), 2021	15-49 rate (per 100,000 population) percent change (2000-2021)	50plus rate (per 100,000 population), 2021	50plus rate (per 100,000 population) percent change (2000-2021)	All age rate (per 100,000 population), 2021	All age rate (per 100,000 population) percent change (2000-2021)
Windsor and Maidenhead	0.6 (0-2.4)	47.3 (0-3610)	0.5 (0-2)	46.1 (-1.9-3550)	0.4 (0-1.4)	43.6 (-4.2-3310)	0.1 (0-0.6)	47.3 (-3.4-3470)	0 (0-0.1)	35.3 (-14.5-2890)	0.1 (0-0.6)	36 (-9.8-3200)
Wokingham	0.5 (0-2.1)	19.4 (0-747)	0.4 (0-1.7)	19.3 (-2.2-745)	0.3 (0-1.3)	18.7 (-4.5-734)	0.1 (0-0.6)	21 (-4.8-731)	0 (0-0.1)	-3 (-26.2-546)	0.1 (0-0.5)	5.1 (-15.9-631)
South West England	5.2 (3.3-8.2)	3.5 (-4.2-15.5)	4.1 (2.6-6.6)	5.8 (-2.1-18.4)	2.7 (1.7-4.4)	5.2 (-2.7-18.7)	1.2 (0.7-1.9)	1.4 (-7.3-14.2)	0.1 (0-0.1)	-4.4 (-13.5-11.9)	1 (0.7-1.6)	-5.1 (-12.6-7)
Bath and North East Somerset	11.1 (5.6-18.3)	5.7 (-5.5-17.3)	8.7 (4.4-14.2)	6.1 (-5.7-19.3)	6 (3-9.8)	2.1 (-10.5-15)	2.6 (1.3-4.4)	7.2 (-5.6-20.9)	0.2 (0.1-0.4)	0.9 (-12.4-18.9)	2.3 (1.1-3.8)	-1.6 (-13.3-10.4)
Bournemouth	14.6 (9.1-22.2)	-34.6 (-45.4--24)	12.5 (7.8-19)	-36.3 (-46.7--25.9)	9.7 (6.1-14.5)	-34.7 (-45.9--23.5)	3.5 (2.2-5.3)	-30.9 (-40.6--19.3)	0.3 (0.2-0.5)	0.5 (-15-18.1)	3.6 (2.3-5.3)	-29.5 (-40.3--17.7)
Bristol, City of	27.5 (19.2-37.9)	-9.5 (-18.7--1.2)	22.3 (15.7-30.8)	-9.5 (-18.4--1.7)	16.2 (11.3-22.2)	-8.2 (-16.7--0.5)	6.1 (4.2-8.7)	-14 (-22.6--5.7)	0.5 (0.3-0.7)	1.4 (-10.9-16.3)	6.8 (4.8-9.5)	-8.8 (-17.5--1.6)
Cornwall	0.4 (0-1.9)	62.3 (0-4100)	0.3 (0-1.6)	62.4 (-1.7-3990)	0.2 (0-1.2)	59.8 (-4.4-3750)	0.1 (0-0.5)	69.4 (-1.9-4050)	0 (0-0)	40.1 (-22.7-3220)	0.1 (0-0.4)	47.9 (-12.3-3540)
Devon	0.3 (0-1.8)	53.7 (0-2660)	0.3 (0-1.5)	54.3 (-1.4-2740)	0.2 (0-1.2)	52.2 (-4-2710)	0.1 (0-0.5)	62 (-1.1-2890)	0 (0-0)	36.6 (-18.3-2580)	0.1 (0-0.4)	40 (-12.3-2490)
Dorset	0.4 (0-2)	30.5 (0-1490)	0.4 (0-1.7)	30.4 (-1.3-1510)	0.3 (0-1.3)	28.9 (-4-1470)	0.1 (0-0.5)	39.6 (1.9-1580)	0 (0-0)	18.7 (-18.1-1330)	0.1 (0-0.4)	16.8 (-13.5-1320)
Gloucestershire	4.4 (1.9-8.7)	7.9 (-7.5-22.6)	3.8 (1.6-7.4)	7.7 (-7.2-22)	3 (1.2-5.6)	5.4 (-9.2-19.5)	1 (0.4-2.1)	12.4 (-3.1-29.6)	0.1 (0-0.2)	-2.1 (-19.9-14.6)	1 (0.4-1.9)	-3.3 (-16.5-10)
North Somerset	0.5 (0-2.1)	20.5 (0-1040)	0.4 (0-1.7)	20.7 (-1.8-1050)	0.3 (0-1.3)	19.6 (-4.5-1020)	0.1 (0-0.5)	24.9 (-2.6-1080)	0 (0-0)	8.1 (-18.3-895)	0.1 (0-0.5)	12.5 (-10.6-966)
Plymouth	0.4 (0-2.1)	28.9 (0-1470)	0.4 (0-1.7)	28.6 (-2-1460)	0.3 (0-1.2)	27 (-5.1-1410)	0.1 (0-0.5)	26.3 (-8-1290)	0 (0-0)	22.5 (-14.7-1080)	0.1 (0-0.5)	20 (-10.6-1280)
Poole	0.6 (0-2.4)	38.5 (0-3620)	0.5 (0-1.9)	37.1 (-1.8-3560)	0.4 (0-1.4)	34.3 (-4.1-3240)	0.1 (0-0.5)	36.4 (-4.1-3500)	0 (0-0)	37.8 (-7.8-3700)	0.1 (0-0.5)	28.2 (-9.2-3250)
Somerset	0.4 (0-1.8)	65 (0-4160)	0.3 (0-1.6)	64.6 (-1.5-4090)	0.2 (0-1.2)	61.6 (-4.1-4040)	0.1 (0-0.5)	75.1 (-0.3-4300)	0 (0-0)	46.9 (-18.9-3310)	0.1 (0-0.4)	48.5 (-13.1-3620)
South Gloucestershire	7.6 (3.4-13.3)	7.1 (-6.8-23.3)	6.1 (2.7-10.9)	8.2 (-5.8-24.7)	4.3 (1.9-7.6)	7.3 (-6.3-21.8)	1.7 (0.7-3)	9.2 (-4.8-25.8)	0.1 (0-0.3)	-2.4 (-19.7-18.7)	1.6 (0.7-2.9)	-3.7 (-15.7-10.1)
Swindon	0.5 (0-2.1)	24.7 (0-1470)	0.4 (0-1.7)	24.5 (-1.5-1480)	0.3 (0-1.2)	23.5 (-4.2-1440)	0.1 (0-0.5)	24 (-6.1-1360)	0 (0-0)	31.1 (-4.7-1350)	0.1 (0-0.5)	15.1 (-11.2-1310)
Torbay	0.6 (0-2.5)	42.9 (0-3620)	0.5 (0-2)	41.9 (-1.7-3580)	0.4 (0-1.5)	38.8 (-4-3340)	0.1 (0-0.6)	45.7 (-2.4-3440)	0 (0-0)	37.4 (-14.3-3260)	0.1 (0-0.5)	32.1 (-9.6-3160)
Wiltshire	0.4 (0-1.9)	68.1 (0-4160)	0.3 (0-1.6)	67.9 (-1.2-4090)	0.2 (0-1.2)	65.9 (-3.7-4060)	0.1 (0-0.5)	81.3 (1.5-4350)	0 (0-0)	54.2 (-15.5-3600)	0.1 (0-0.4)	51.9 (-12.9-3660)
West Midlands	13.4 (9.3-18.7)	6 (0.7-12)	10.5 (7.4-14.6)	7.7 (2.6-13.8)	7.3 (5-10.1)	8.4 (3.6-14.8)	2.7 (1.8-3.9)	8.7 (3.1-15.6)	0.1 (0.1-0.2)	0.3 (-6.3-8.6)	2.8 (2-3.9)	1.2 (-3.5-7.1)
Birmingham	33 (24.1-43.9)	8.4 (0.2-18.9)	26.5 (19.4-35.2)	8.2 (-0.3-19.3)	18.7 (13.5-24.8)	7.4 (-0.5-19)	7.1 (5.1-9.9)	4.7 (-3.8-15)	0.5 (0.3-0.8)	22 (9-39.1)	8.3 (6.1-11)	5.7 (-1.8-15.6)
Coventry	21 (12.1-32)	3.5 (-4.7-12.1)	16.7 (9.7-25.6)	3 (-4.6-11.3)	11.4 (6.6-17.7)	1.8 (-7-11)	4.2 (2.4-6.8)	-1.9 (-11.8-6.6)	0.3 (0.1-0.5)	7.7 (-7.5-22.1)	4.8 (2.8-7.5)	0.6 (-8-8.5)
Dudley	11.5 (7.2-17.3)	-26.1 (-36.3--14.9)	9.7 (6-14.5)	-27.1 (-38--15.5)	7.1 (4.5-10.6)	-28 (-38.9--17)	2.7 (1.7-4.2)	-19.2 (-31.1--7.3)	0.2 (0.1-0.4)	-24.3 (-35.4--10.6)	2.6 (1.7-4)	-28.7 (-38.8--18.5)
Herefordshire, County of	0.6 (0-2.4)	40.2 (0-3950)	0.5 (0-1.9)	39.5 (-1.2-3710)	0.3 (0-1.4)	35.9 (-4.5-3420)	0.1 (0-0.5)	43.5 (-1.7-3730)	0 (0-0)	21.3 (-20-3130)	0.1 (0-0.5)	21.7 (-15.2-3130)
Sandwell	24.2 (15.8-34.6)	-17.3 (-24.9--9.1)	19.4 (12.8-27.6)	-16.6 (-24.2--8.2)	13.6 (8.8-19.4)	-15.8 (-23.4--7.9)	4.9 (3.1-7.4)	-15.8 (-23.5--7.6)	0.3 (0.2-0.6)	1.9 (-9.4-15.8)	5.7 (3.7-8.1)	-14.6 (-22--6.7)
Shropshire	0.4 (0-2.1)	30.9 (0-1490)	0.4 (0-1.7)	30.8 (-1.8-1490)	0.3 (0-1.2)	29.2 (-4.9-1460)	0.1 (0-0.5)	36.3 (-1.6-1520)	0 (0-0)	15.3 (-20.8-1220)	0.1 (0-0.4)	13.2 (-17.2-1250)
Solihull	7 (3.2-12.6)	4.7 (-5.1-20.2)	6 (2.7-10.5)	3.4 (-6.6-19.2)	4.5 (2.1-7.9)	4.2 (-6.2-20.4)	1.7 (0.8-3)	11.1 (-0.3-31.2)	0.1 (0-0.3)	-8.6 (-22.8-9.6)	1.6 (0.7-2.8)	-4.1 (-13.5-10.7)
Staffordshire	1.7 (0.7-3.9)	-25.6 (-45.5-10.7)	1.4 (0.6-3.4)	-25.3 (-45.1-10.6)	1.1 (0.5-2.7)	-25.6 (-45.4-11)	0.4 (0.2-1)	-21.5 (-43.4-18.3)	0 (0-0.1)	-36.2 (-55.6--4.3)	0.4 (0.1-0.9)	-34.2 (-51.9--1.3)
Stoke-on-Trent	0.4 (0-2.1)	28.9 (0-1470)	0.4 (0-1.6)	27.9 (-2.4-1440)	0.2 (0-1.1)	26.8 (-5.5-1410)	0.1 (0-0.4)	24.9 (-8.4-1370)	0 (0-0)	31.8 (-8.1-1330)	0.1 (0-0.5)	24.1 (-7.3-1370)
Telford and Wrekin	24.5 (16.5-36)	1.3 (-9.6-14.3)	18.6 (12.5-26.8)	0.6 (-10.1-13.9)	12.6 (8.3-17.7)	1.4 (-10.7-14.6)	4.6 (3-6.5)	0.8 (-11.2-15.1)	0.3 (0.2-0.5)	-11.1 (-23.2-5)	4.9 (3.3-6.9)	-10 (-19.7-1.9)

Location	Birth Incidence rate (per 100,000 live births), 2021	Birth Incidence rate (per 100,000 live births) percent change (2000-2021)	Under-5 rate (per 100,000 population), 2021	Under-5 rate (per 100,000 population) percent change (2000-2021)	5-14 rate (per 100,000 population), 2021	5-14 rate (per 100,000 population) percent change (2000-2021)	15-49 rate (per 100,000 population), 2021	15-49 rate (per 100,000 population) percent change (2000-2021)	50plus rate (per 100,000 population), 2021	50plus rate (per 100,000 population) percent change (2000-2021)	All age rate (per 100,000 population), 2021	All age rate (per 100,000 population) percent change (2000-2021)
Walsall	0.4 (0-2.1)	31.2 (0-1490)	0.4 (0-1.6)	30.7 (-2.9-1500)	0.2 (0-1.1)	29.2 (-6.6-1480)	0.1 (0-0.4)	32.8 (-7-1530)	0 (0-0)	34.5 (-10.6-1620)	0.1 (0-0.5)	26.4 (-8.9-1440)
Warwickshire	0.3 (0-1.8)	54.2 (0-2450)	0.3 (0-1.6)	54.3 (-1.5-2440)	0.2 (0-1.2)	52 (-4.4-2400)	0.1 (0-0.5)	59.6 (-2.6-2630)	0 (0-0)	36.4 (-19.1-2460)	0.1 (0-0.4)	40.4 (-12.3-2250)
Wolverhampton	7.8 (4.5-12.6)	3.6 (-6.2-17.1)	6.5 (3.7-10.3)	1.6 (-8-14.6)	4.8 (2.8-7.5)	0.1 (-9.7-13.1)	1.6 (0.9-2.7)	2.6 (-9.3-16)	0.1 (0-0.2)	14 (-3.4-34.3)	1.8 (1.1-2.9)	0.1 (-9.9-12.4)
Worcestershire	0.4 (0-1.8)	62 (0-2980)	0.3 (0-1.5)	61.7 (-1.8-2810)	0.2 (0-1.2)	59.2 (-4.2-2610)	0.1 (0-0.5)	69.9 (-1.4-2950)	0 (0-0)	34.4 (-23.9-2490)	0.1 (0-0.4)	42.8 (-15-2420)
Yorkshire and the Humber	5.2 (2.9-8.5)	12.7 (6.3-20.9)	4.2 (2.4-6.8)	15 (8.5-23.5)	2.9 (1.6-4.7)	10.1 (3.5-18.4)	1.1 (0.6-1.9)	7.2 (0.1-15.4)	0.1 (0-0.1)	-0.3 (-8.7-9.9)	1.1 (0.6-1.8)	1.9 (-4.3-9.4)
Barnsley	0.5 (0-2.1)	26 (0-1470)	0.4 (0-1.7)	26.7 (-1.5-1470)	0.3 (0-1.2)	25 (-4.6-1420)	0.1 (0-0.4)	28.4 (-2.9-1480)	0 (0-0)	25 (-10.3-1400)	0.1 (0-0.4)	14.1 (-12.7-1290)
Bradford	8.2 (4.2-13.7)	5.6 (-9.1-21.2)	6.7 (3.5-10.9)	6.4 (-7.7-23.2)	4.8 (2.4-7.8)	5 (-9.2-23.3)	1.7 (0.8-2.9)	7 (-8.2-25.5)	0.1 (0-0.2)	5.5 (-12.6-24.9)	2 (1-3.3)	3.2 (-11.3-19.4)
Calderdale	0.5 (0-2.1)	22.6 (0-1040)	0.4 (0-1.7)	23 (-2.2-1050)	0.3 (0-1.1)	21.4 (-5.8-1040)	0.1 (0-0.4)	25.5 (-3.5-1070)	0 (0-0)	19 (-12.6-994)	0.1 (0-0.4)	10.2 (-14-928)
Doncaster	0.4 (0-2.1)	31.3 (0-1490)	0.4 (0-1.7)	31.1 (-2.4-1500)	0.3 (0-1.2)	29.4 (-5.4-1500)	0.1 (0-0.4)	32.5 (-4.9-1500)	0 (0-0)	26.2 (-14.9-1390)	0.1 (0-0.4)	20.1 (-12.2-1380)
East Riding of Yorkshire	0.4 (0-2.1)	30.8 (0-1490)	0.4 (0-1.7)	30.7 (-1.4-1510)	0.3 (0-1.2)	29.2 (-4.2-1490)	0.1 (0-0.5)	35.1 (-2.3-1540)	0 (0-0)	8.1 (-24.7-1210)	0.1 (0-0.4)	9.2 (-19.8-1240)
Kingston upon Hull, City of	12.9 (5.8-22.4)	5.3 (-3.2-13.7)	10.3 (4.6-17.7)	5 (-2.9-13.4)	7.1 (3.2-12.2)	4.1 (-4.5-13.7)	2.4 (1-4.3)	-1.8 (-12-8.5)	0.1 (0.1-0.3)	11.2 (-5-28.4)	2.8 (1.2-4.8)	-2 (-10.4-7.2)
Kirklees	0.4 (0-1.8)	61.3 (0-4100)	0.3 (0-1.5)	60.9 (-2.4-4030)	0.2 (0-1.1)	57.9 (-5.5-3700)	0.1 (0-0.4)	62.1 (-6.1-3670)	0 (0-0)	48.7 (-14.6-3060)	0.1 (0-0.4)	45.9 (-13.2-3400)
Leeds	14.9 (9.5-21.3)	2.4 (-3.6-10.4)	12.2 (7.7-17.5)	2.4 (-4.3-10)	8.9 (5.5-12.7)	1.4 (-5.7-10)	3.2 (1.9-4.9)	-3 (-11.1-5.5)	0.2 (0.1-0.4)	5.2 (-6.2-17.6)	3.5 (2.2-5.1)	-1.1 (-7.7-7.2)
North East Lincolnshire	0.6 (0-2.4)	40.1 (0-3950)	0.5 (0-1.9)	39.2 (-1.6-3670)	0.3 (0-1.4)	36.2 (-4.1-3520)	0.1 (0-0.5)	38.1 (-6.2-3630)	0 (0-0)	32.3 (-15.4-3510)	0.1 (0-0.5)	21 (-15.9-3170)
North Lincolnshire	0.5 (0-2.1)	12.6 (0-1030)	0.4 (0-1.7)	12.8 (-1.7-1030)	0.3 (0-1.2)	11.4 (-4.6-1010)	0.1 (0-0.5)	15.5 (-2.3-1060)	0 (0-0)	2 (-18.6-926)	0.1 (0-0.4)	2.6 (-12-927)
North Yorkshire	0.3 (0-1.8)	52.2 (0-2660)	0.3 (0-1.5)	52.6 (-1.5-2730)	0.2 (0-1.2)	51.4 (-3.8-2720)	0.1 (0-0.5)	62.1 (-0.6-2860)	0 (0-0)	30.3 (-22.4-2310)	0.1 (0-0.4)	33.8 (-15.8-2370)
Rotherham	0.4 (0-2.1)	30.7 (0-1470)	0.4 (0-1.7)	30.7 (-1.7-1470)	0.3 (0-1.2)	29 (-4.7-1440)	0.1 (0-0.5)	33.8 (-2.6-1460)	0 (0-0)	20.8 (-16-1240)	0.1 (0-0.4)	17.6 (-13-1290)
Sheffield	7.6 (3.7-12.8)	4.2 (-7.2-17.8)	6.5 (3.2-10.7)	3.8 (-7.9-17.3)	5 (2.4-8.2)	3.3 (-8.6-17)	1.9 (0.9-3.4)	5.2 (-8.5-21)	0.1 (0.1-0.3)	13.5 (-4.9-36.9)	2 (0.9-3.3)	4.2 (-7.9-18.9)
Wakefield	0.4 (0-2)	30.6 (0-1490)	0.4 (0-1.7)	30.3 (-1.7-1500)	0.3 (0-1.2)	28.8 (-4.6-1490)	0.1 (0-0.4)	31.1 (-5.1-1460)	0 (0-0)	21.4 (-16-1310)	0.1 (0-0.4)	16.2 (-14.3-1300)
York	0.5 (0-2.1)	22 (0-1040)	0.4 (0-1.7)	22.3 (-1.8-1040)	0.3 (0-1.2)	21.3 (-4.5-1020)	0.1 (0-0.5)	20.6 (-5.8-1010)	0 (0-0)	18.2 (-12.8-976)	0.1 (0-0.5)	15.6 (-8.5-971)
Northern Ireland	5.2 (3.8-7.2)	0.3 (-7.5-8.9)	4.2 (3-5.6)	0.9 (-6.4-9.4)	3 (2.2-4)	-0.4 (-7.8-8)	1.1 (0.8-1.5)	-4.5 (-11.2-2.8)	0.1 (0.1-0.1)	-3.1 (-11.8-6.4)	1.2 (0.9-1.6)	-13.6 (-19.5--7.2)
Scotland	5.3 (3.8-7.1)	0.1 (-8.6-9.8)	4.2 (3-5.6)	0.6 (-8.4-9.9)	3.1 (2.3-4.1)	-0.7 (-9.4-8.3)	1.1 (0.8-1.5)	-0.6 (-8.4-8.2)	0.1 (0.1-0.1)	-7.8 (-17.3-2.4)	1.1 (0.8-1.4)	-12.7 (-19.4--5.3)
Wales	6 (4.3-7.9)	0.4 (-7.3-8.6)	4.7 (3.5-6.2)	0.8 (-6.9-8.6)	3.5 (2.6-4.6)	-0.3 (-8.3-8.1)	1.4 (1-1.8)	0 (-7-7.7)	0.1 (0.1-0.1)	-9.4 (-17.9-0.7)	1.3 (0.9-1.6)	-10.4 (-16.6--3.7)
Latin America and Caribbean	11.1 (8.4-14.3)	24.5 (16-34.4)	2.8 (2.2-3.5)	3 (-2.6-9.2)	0.6 (0.4-0.7)	-15.1 (-20.8--9.3)	0.2 (0.2-0.3)	-20 (-25.1--13.9)	0 (0-0)	-22.8 (-29.7--15.6)	0.4 (0.3-0.5)	-26.6 (-30.4--22.3)
**Andean Latin America**	0.1 (0.1-0.2)	6.8 (-0.1-42.4)	0.1 (0-0.1)	0.2 (-9.1-27.6)	0 (0-0)	-2.9 (-16.1-22.5)	0 (0-0)	-12.3 (-24.9-10.4)	0 (0-0)	-16.8 (-31-12.4)	0 (0-0)	-18.4 (-28.1-3)
Bolivia (Plurinational State of)	0.1 (0.1-0.2)	5.1 (0-78.7)	0 (0-0.1)	-0.6 (-11.5-58.8)	0 (0-0)	-6 (-24.4-39.5)	0 (0-0)	-16.5 (-33.6-23.7)	0 (0-0)	-21.7 (-39.8-17.7)	0 (0-0)	-15.4 (-28.1-26.7)
Ecuador	0.1 (0.1-0.2)	5.5 (0-48.1)	0.1 (0-0.1)	0.4 (-9-36.8)	0 (0-0)	-3.3 (-17.9-34.2)	0 (0-0)	-11.3 (-26.2-24.6)	0 (0-0)	-18 (-36.5-30.9)	0 (0-0)	-20.4 (-31.3-11.7)
Peru	0.1 (0.1-0.2)	7.5 (0-79.3)	0 (0-0.1)	1.3 (-11.1-68.3)	0 (0-0)	-2.6 (-21.9-53.2)	0 (0-0)	-13.1 (-31.3-37.6)	0 (0-0)	-16.2 (-35-37.5)	0 (0-0)	-19.8 (-33.2-30.2)
**Caribbean**	116 (86.7-148)	25.3 (16.3-36.8)	25.1 (19.3-31.5)	1.1 (-5.6-9.1)	1.3 (0.9-1.8)	-54.7 (-61.4--47.7)	0.4 (0.3-0.5)	-61 (-65.9--55.8)	0 (0-0)	-75.1 (-79.3--71)	2.5 (1.9-3)	-31.8 (-37.5--25.4)

Location	Birth Incidence rate (per 100,000 live births), 2021	Birth Incidence rate (per 100,000 live births) percent change (2000-2021)	Under-5 rate (per 100,000 population), 2021	Under-5 rate (per 100,000 population) percent change (2000-2021)	5-14 rate (per 100,000 population), 2021	5-14 rate (per 100,000 population) percent change (2000-2021)	15-49 rate (per 100,000 population), 2021	15-49 rate (per 100,000 population) percent change (2000-2021)	50plus rate (per 100,000 population), 2021	50plus rate (per 100,000 population) percent change (2000-2021)	All age rate (per 100,000 population), 2021	All age rate (per 100,000 population) percent change (2000-2021)
Antigua and Barbuda	38.2 (21.9-58.3)	43.2 (13.5-76.5)	9.9 (6.4-14.2)	7.5 (-11.4-27.2)	1.2 (0.8-1.8)	-45.9 (-58.4--30.7)	0.4 (0.3-0.5)	-54.9 (-63.7--44)	0 (0-0.1)	-68.4 (-74.9--60.7)	0.9 (0.7-1.3)	-45.9 (-54.8--36.6)
Bahamas	53 (32.8-79.3)	93.8 (62.3-141)	12.3 (8.3-17.2)	60.5 (37.1-88.7)	1 (0.6-1.4)	-23.9 (-43.6-4.5)	0.3 (0.2-0.4)	-33.9 (-48.4--12.6)	0 (0-0)	-58.7 (-70--45.6)	1 (0.7-1.3)	-21.6 (-33.7--6)
Barbados	42.9 (25.3-67.6)	42.7 (14.6-77.9)	11.4 (7.5-16.4)	4.6 (-12.6-25.8)	1.5 (1-2.1)	-46.1 (-58.8--28.7)	0.4 (0.3-0.6)	-54.3 (-63.7--42.5)	0 (0-0)	-71 (-76.9--62.8)	0.9 (0.7-1.2)	-47.1 (-56--37.9)
Belize	108 (65.9-166)	30.8 (6.1-61.7)	26 (17.4-37.2)	5.1 (-10.2-23.1)	2 (1.4-2.8)	-48 (-60.7--33.8)	0.6 (0.4-0.9)	-56.4 (-65.6--46.1)	0 (0-0.1)	-69.5 (-75.7--62.8)	3 (2.2-4.1)	-38.3 (-47.7--29.5)
Bermuda	13.8 (7.9-22.3)	47.9 (16.2-83.7)	4 (2.6-5.8)	7.2 (-9.9-26.4)	0.7 (0.5-1)	-42.1 (-54.3--26.6)	0.2 (0.1-0.3)	-52.9 (-61.9--41.3)	0 (0-0)	-72.4 (-78.3--65.9)	0.3 (0.2-0.4)	-51.4 (-58.4--43.1)
Cuba	28.9 (18.6-41.6)	94.7 (60.2-143)	5.9 (4.1-8)	33 (9.7-59.4)	0.3 (0.2-0.5)	-51.2 (-64.1--35.5)	0.1 (0.1-0.2)	-58.4 (-68.4--45.7)	0 (0-0)	-68.3 (-76--59)	0.4 (0.3-0.5)	-32.6 (-46--17.7)
Dominica	48.4 (28.9-74.4)	33.9 (6-63.9)	11.8 (7.7-16.8)	5.3 (-12.8-25)	1 (0.7-1.5)	-48.4 (-61.2--33.8)	0.3 (0.2-0.5)	-56.1 (-66.2--44.8)	0 (0-0)	-62.3 (-71--52.9)	1 (0.7-1.4)	-45.7 (-54.5--35.8)
Dominican Republic	123 (86.2-165)	20.4 (4.9-43.5)	27.4 (20-35.5)	-0.9 (-12.7-14.8)	1.5 (1-2.1)	-48.1 (-62.6--29.4)	0.4 (0.3-0.6)	-57 (-67.3--44.2)	0 (0-0)	-71.9 (-78.3--64.7)	3.2 (2.4-4.1)	-31.5 (-40.1--21.8)
Grenada	63.4 (38.4-97.9)	33.4 (10.7-62.3)	15.6 (10.3-22.4)	4.7 (-10.2-22.2)	1.4 (1-2)	-46.5 (-59.1--31)	0.5 (0.3-0.6)	-57.8 (-67.5--47.3)	0 (0-0.1)	-71.8 (-78.3--64.6)	1.5 (1.1-2)	-42.3 (-51.7--31.4)
Guyana	55.7 (32.9-84.1)	25.8 (2-54.3)	13.6 (8.6-19.1)	3.2 (-13.7-21.4)	1.1 (0.7-1.6)	-50.5 (-62.9--35.8)	0.3 (0.2-0.4)	-55.7 (-66.1--44.8)	0 (0-0)	-77.3 (-84.1--70.5)	1.6 (1.1-2.2)	-35.5 (-46.2--23.8)
Haiti	141 (109-180)	6.4 (-5.2-22.4)	30.8 (24-38.4)	-12.4 (-20.6--2)	1.2 (0.8-1.7)	-52.8 (-63.3--40.9)	0.3 (0.2-0.5)	-62.6 (-70.2--54.5)	0 (0-0)	-76.9 (-83.5--71)	4.1 (3.2-5.1)	-32.5 (-39.2--24.6)
Jamaica	222 (168-281)	-0.2 (-19.3-20)	50.7 (39.2-62.7)	-22.3 (-36.5--8.2)	3.7 (2.6-5.2)	-67.9 (-76.3--56.9)	1.3 (0.9-1.8)	-73 (-79.5--65.4)	0.1 (0.1-0.2)	-80 (-84.6--74.4)	4.5 (3.6-5.5)	-60.8 (-68.5--52.8)
Puerto Rico	16.1 (9.8-24.8)	44.6 (17.8-69.6)	4.6 (3.2-6.5)	4.1 (-10.4-20.6)	0.8 (0.5-1.1)	-44.7 (-56.4--30.3)	0.2 (0.2-0.3)	-55.5 (-64.2--45.6)	0 (0-0)	-76 (-81.8--69.6)	0.3 (0.3-0.5)	-59.5 (-65.6--52.4)
Saint Kitts and Nevis	44.4 (26.8-69.1)	35.3 (12.8-68.2)	11 (7.2-15.8)	4.2 (-11.4-22.5)	1.1 (0.7-1.5)	-47.2 (-59.4--33.8)	0.3 (0.2-0.4)	-59.2 (-67.4--50.4)	0 (0-0)	-61.7 (-70--53.1)	0.9 (0.7-1.2)	-48 (-56.2--39.2)
Saint Lucia	63.1 (39.1-94.8)	36.7 (12.9-67.3)	16.1 (10.9-22.5)	4.5 (-9.3-22.8)	1.8 (1.2-2.5)	-46.9 (-58.5--33.6)	0.5 (0.4-0.7)	-60.5 (-67.9--51.5)	0 (0-0.1)	-68.5 (-75.1--61)	1.3 (1-1.7)	-56.2 (-62.8--48)
Saint Vincent and the Grenadines	58.1 (34.1-91.7)	31.7 (8.6-60.6)	14.5 (9.6-21.1)	2.7 (-12.8-22.1)	1.4 (1-2)	-48.5 (-60.6--34.2)	0.4 (0.3-0.6)	-59.6 (-68.2--49)	0 (0-0)	-69.6 (-76.6--61.7)	1.4 (1-1.8)	-47 (-56.2--37.3)
Suriname	66.5 (41.4-100)	31.3 (9-53.7)	15.3 (10.3-21.4)	-0.6 (-14.9-13.2)	1.2 (0.8-1.7)	-48.1 (-61.5--32.2)	0.3 (0.2-0.5)	-59.1 (-69.2--49.1)	0 (0-0)	-71.6 (-78.7--64.7)	1.6 (1.1-2.1)	-36.5 (-45.7--26.3)
Trinidad and Tobago	55.1 (32.9-85.8)	36 (11.5-63.1)	13.5 (8.9-18.9)	0.7 (-14.7-16.9)	1.5 (1-2.1)	-46.8 (-60.2--29.8)	0.4 (0.3-0.5)	-62.9 (-71.2--51.9)	0 (0-0)	-77.4 (-82.7--71.4)	1.2 (0.8-1.5)	-43.4 (-53.2--32.4)
United States Virgin Islands	22.7 (13.4-33.8)	34.7 (10.7-61.6)	6.4 (4.2-8.9)	-0.3 (-14.2-16.1)	1 (0.7-1.5)	-44.9 (-58.5--30.5)	0.2 (0.2-0.3)	-54.1 (-64.3--43.5)	0 (0-0)	-81 (-85.6--75.8)	0.6 (0.4-0.8)	-46.5 (-55.1--37.6)
**Central Latin America**	1.8 (1.3-2.4)	-2.4 (-10.9-8.4)	0.5 (0.4-0.7)	-11.6 (-18.1--3.7)	0.1 (0.1-0.1)	-35.8 (-45.8--25.7)	0 (0-0)	-37.7 (-48.1--28.7)	0 (0-0)	-33.6 (-46.8--20.9)	0.1 (0.1-0.1)	-41.6 (-47.5--36.1)
Colombia	5 (3.5-6.8)	4.8 (-6.2-20.9)	1.5 (1.1-2)	3.4 (-5.7-15.6)	0.2 (0.1-0.3)	-3 (-20.9-16.8)	0.1 (0-0.1)	-8 (-25.4-8.6)	0 (0-0)	-18.8 (-40.1-3.3)	0.2 (0.1-0.2)	-27.9 (-36--19.5)
Costa Rica	0.1 (0.1-0.1)	5.5 (0-63.3)	0 (0-0.1)	4.6 (-7.3-69.1)	0 (0-0)	3.3 (-14-57.3)	0 (0-0)	-6.5 (-23.3-46)	0 (0-0)	-10.8 (-29.4-37.6)	0 (0-0)	-20.6 (-33.3-25.9)
El Salvador	0.7 (0.4-0.9)	7 (-8.2-59.7)	0.2 (0.2-0.3)	6.4 (-9.8-50.5)	0.1 (0-0.1)	0.8 (-21.4-42.1)	0 (0-0)	-8.1 (-30.8-26.5)	0 (0-0)	-8.7 (-39.7-33.5)	0 (0-0.1)	-22.5 (-37.1-5.4)
Guatemala	0.7 (0.5-1)	8.9 (-6.9-62.2)	0.2 (0.1-0.3)	2.5 (-14.5-46.4)	0 (0-0.1)	0.8 (-27.3-50.6)	0 (0-0)	-9.6 (-35.4-35.1)	0 (0-0)	-10.5 (-41-32.7)	0 (0-0.1)	-30.5 (-45--2.3)
Honduras	0.2 (0.1-0.3)	8.1 (-22-110)	0.1 (0-0.1)	6.4 (-25.3-82.9)	0 (0-0)	1.9 (-30.7-73.7)	0 (0-0)	-8 (-37.9-55.7)	0 (0-0)	-13.3 (-43.8-50.9)	0 (0-0)	-16.9 (-44-39.7)
Mexico	0.1 (0-0.2)	25.5 (0-119)	0 (0-0.1)	21.6 (-3.6-104)	0 (0-0)	18.7 (-6.5-105)	0 (0-0)	4.6 (-19.1-92.6)	0 (0-0)	2.1 (-22.7-103)	0 (0-0)	-9 (-28.9-55.4)
Aguascalientes	0.1 (0-0.2)	23.9 (-0.8-319)	0 (0-0.1)	20 (-8.3-263)	0 (0-0)	15.2 (-19.6-210)	0 (0-0)	4.3 (-28.4-185)	0 (0-0)	1.3 (-33.5-243)	0 (0-0)	-10.9 (-36.2-155)
Baja California	0.1 (0-0.2)	33 (-0.6-484)	0 (0-0.1)	29.8 (-6.5-430)	0 (0-0)	25.7 (-13.7-359)	0 (0-0)	12.1 (-23.6-335)	0 (0-0)	9.3 (-28.5-486)	0 (0-0)	-5 (-33.9-263)
Baja California Sur	0.1 (0-0.2)	28.9 (-0.8-484)	0 (0-0.1)	24.3 (-6.6-390)	0 (0-0)	19.8 (-14-293)	0 (0-0)	6.4 (-24.7-273)	0 (0-0)	10.1 (-26.9-405)	0 (0-0)	-6.6 (-32.4-243)

Location	Birth Incidence rate (per 100,000 live births), 2021	Birth Incidence rate (per 100,000 live births) percent change (2000-2021)	Under-5 rate (per 100,000 population), 2021	Under-5 rate (per 100,000 population) percent change (2000-2021)	5-14 rate (per 100,000 population), 2021	5-14 rate (per 100,000 population) percent change (2000-2021)	15-49 rate (per 100,000 population), 2021	15-49 rate (per 100,000 population) percent change (2000-2021)	50plus rate (per 100,000 population), 2021	50plus rate (per 100,000 population) percent change (2000-2021)	All age rate (per 100,000 population), 2021	All age rate (per 100,000 population) percent change (2000-2021)
Campeche	0.1 (0-0.3)	23.9 (-0.8-317)	0 (0-0.1)	21.5 (-8.1-265)	0 (0-0)	18.4 (-20.1-222)	0 (0-0)	2.7 (-31.7-192)	0 (0-0)	2.5 (-33.3-241)	0 (0-0)	-8.4 (-35.2-162)
Chiapas	0.1 (0-0.2)	24.7 (-0.8-464)	0 (0-0.1)	21.4 (-7.8-365)	0 (0-0)	16.4 (-22.8-248)	0 (0-0)	4.6 (-32.5-213)	0 (0-0)	-5.5 (-42.1-269)	0 (0-0)	-3.8 (-31.8-227)
Chihuahua	0.1 (0-0.2)	27.4 (-0.8-464)	0 (0-0.1)	23.3 (-8.1-350)	0 (0-0)	19.3 (-18-298)	0 (0-0)	9.4 (-26.9-267)	0 (0-0)	8 (-35.7-442)	0 (0-0)	-8.9 (-35.5-217)
Coahuila	0.1 (0-0.2)	25.6 (-0.7-446)	0 (0-0.1)	22.5 (-7.8-352)	0 (0-0)	17.7 (-17.8-231)	0 (0-0)	7.2 (-25.7-219)	0 (0-0)	2.7 (-31.3-300)	0 (0-0)	-4.2 (-30.8-222)
Colima	0.1 (0-0.2)	23.4 (-0.6-489)	0 (0-0.1)	20.1 (-8-404)	0 (0-0)	16.8 (-17-383)	0 (0-0)	0.1 (-28.2-297)	0 (0-0)	0.6 (-31.2-453)	0 (0-0)	-12 (-35.2-259)
Durango	0.1 (0-0.2)	24.4 (-0.7-321)	0 (0-0.1)	21.4 (-8.2-271)	0 (0-0)	19.4 (-18.4-218)	0 (0-0)	5.7 (-27.4-214)	0 (0-0)	3.8 (-31-303)	0 (0-0)	-8.6 (-34.8-166)
Guanajuato	0.1 (0-0.2)	28.2 (-0.6-496)	0 (0-0.1)	23.4 (-7.3-428)	0 (0-0)	19.4 (-14.5-386)	0 (0-0)	5.2 (-28-359)	0 (0-0)	10.3 (-31.2-590)	0 (0-0)	-11.5 (-36.4-286)
Guerrero	0.1 (0-0.3)	23.5 (-0.7-246)	0 (0-0.1)	20.3 (-9-203)	0 (0-0)	17.8 (-21.3-208)	0 (0-0)	4.3 (-29.6-166)	0 (0-0)	-6.8 (-40.1-203)	0 (0-0)	-12.1 (-36.9-117)
Hidalgo	0.1 (0-0.2)	22.5 (-0.8-360)	0 (0-0.1)	18.5 (-7.8-334)	0 (0-0)	15.4 (-16.1-250)	0 (0-0)	2 (-28.6-225)	0 (0-0)	1 (-35.4-266)	0 (0-0)	-14.2 (-37-180)
Jalisco	0.1 (0-0.2)	28.5 (-0.8-496)	0 (0-0.1)	25.3 (-7.2-426)	0 (0-0)	21.1 (-14.7-337)	0 (0-0)	5.9 (-27.8-308)	0 (0-0)	2.8 (-35.3-508)	0 (0-0)	-6 (-34-260)
México	0.1 (0-0.2)	27.9 (-0.8-454)	0 (0-0.1)	24 (-7.7-378)	0 (0-0)	22.2 (-13.7-283)	0 (0-0)	7.6 (-24.6-258)	0 (0-0)	0.3 (-33.3-390)	0 (0-0)	-4 (-30.5-232)
Mexico City	0 (0-0.1)	20.7 (-0.6-887)	0 (0-0.1)	17.4 (-5.7-773)	0 (0-0)	16.5 (-9.4-663)	0 (0-0)	3.5 (-21.2-657)	0 (0-0)	-4.3 (-32.2-868)	0 (0-0)	-12.6 (-32-533)
Michoacán de Ocampo	0.1 (0-0.2)	26.2 (-0.6-490)	0 (0-0.1)	22.5 (-7.5-409)	0 (0-0)	18.8 (-17.4-378)	0 (0-0)	0.9 (-29.9-324)	0 (0-0)	0.8 (-34.8-544)	0 (0-0)	-10.6 (-35.6-279)
Morelos	0.1 (0-0.2)	22.8 (-0.8-281)	0 (0-0.1)	20.3 (-6.5-215)	0 (0-0)	17.7 (-15.4-192)	0 (0-0)	3.8 (-26.2-153)	0 (0-0)	-1.6 (-34.6-270)	0 (0-0)	-9.1 (-32.7-117)
Nayarit	0.1 (0-0.2)	27.1 (-0.8-351)	0 (0-0.1)	24.3 (-7.2-300)	0 (0-0)	21.4 (-17-271)	0 (0-0)	5.7 (-28.9-245)	0 (0-0)	4.5 (-32.7-366)	0 (0-0)	-5 (-33-199)
Nuevo León	0.1 (0-0.2)	25.2 (-0.7-489)	0 (0-0.1)	22.4 (-6.4-421)	0 (0-0)	20.9 (-11.3-344)	0 (0-0)	9.2 (-21.9-345)	0 (0-0)	5.2 (-26.8-473)	0 (0-0)	-4 (-29.4-288)
Oaxaca	0.1 (0-0.2)	22.6 (-0.8-464)	0 (0-0.1)	19.3 (-7.7-374)	0 (0-0)	15.8 (-16.5-239)	0 (0-0)	2.1 (-28.9-222)	0 (0-0)	-4.3 (-39.6-357)	0 (0-0)	-13.8 (-36.4-200)
Puebla	0.1 (0-0.3)	19.6 (-0.8-337)	0 (0-0.1)	16.1 (-10.5-261)	0 (0-0)	13.7 (-22.6-212)	0 (0-0)	0.7 (-32.3-169)	0 (0-0)	1.3 (-40.7-262)	0 (0-0)	-13.9 (-38-152)
Querétaro	0.1 (0-0.2)	22.6 (-0.8-260)	0 (0-0.1)	20 (-8.2-195)	0 (0-0)	17 (-16.6-215)	0 (0-0)	0.8 (-31.3-150)	0 (0-0)	3.8 (-32.2-189)	0 (0-0)	-12.9 (-36.8-109)
Quintana Roo	0.1 (0-0.3)	24.2 (-0.7-317)	0 (0-0.1)	21.4 (-6.9-262)	0 (0-0)	18.1 (-16.7-213)	0 (0-0)	1.1 (-29.4-188)	0 (0-0)	5.3 (-31.7-272)	0 (0-0)	-9.8 (-34.6-158)
San Luis Potosí	0.1 (0-0.2)	21.3 (-0.8-317)	0 (0-0.1)	17.5 (-7.7-244)	0 (0-0)	13.4 (-16.9-190)	0 (0-0)	1.4 (-27-163)	0 (0-0)	0.8 (-30.5-210)	0 (0-0)	-14.8 (-36.7-129)
Sinaloa	0.1 (0-0.2)	24.6 (-0.7-447)	0 (0-0.1)	21.9 (-7.2-367)	0 (0-0)	17.7 (-16.5-332)	0 (0-0)	3.4 (-28.1-288)	0 (0-0)	-1.9 (-35.2-527)	0 (0-0)	-13.2 (-37.2-239)
Sonora	0.1 (0-0.2)	27.2 (-0.6-484)	0 (0-0.1)	23.8 (-6.9-422)	0 (0-0)	19.4 (-14.9-344)	0 (0-0)	9.1 (-24-342)	0 (0-0)	1.9 (-32.7-500)	0 (0-0)	-8.4 (-33.5-271)
Tabasco	0.1 (0-0.3)	23.3 (-0.8-246)	0 (0-0.1)	21.2 (-9.3-212)	0 (0-0)	18.3 (-22.8-184)	0 (0-0)	0.6 (-34.5-156)	0 (0-0)	-1.7 (-38.6-202)	0 (0-0)	-11.4 (-38.4-125)
Tamaulipas	0.1 (0-0.2)	25.9 (-0.6-376)	0 (0-0.1)	21.2 (-7.7-336)	0 (0-0)	18.4 (-14.8-289)	0 (0-0)	6.6 (-24.1-276)	0 (0-0)	6.6 (-27.3-428)	0 (0-0)	-9 (-33.3-211)
Tlaxcala	0.1 (0-0.3)	25.5 (-0.8-446)	0 (0-0.1)	22.7 (-7.8-321)	0 (0-0)	20 (-18.1-215)	0 (0-0)	6.1 (-29.2-183)	0 (0-0)	11.5 (-28.7-273)	0 (0-0)	-8.2 (-35.3-158)
Veracruz de Ignacio de la Llave	0.1 (0-0.2)	24.2 (-0.7-319)	0 (0-0.1)	20.7 (-7.7-255)	0 (0-0)	16.5 (-17.2-206)	0 (0-0)	4.3 (-27.9-184)	0 (0-0)	-3.9 (-36.9-245)	0 (0-0)	-14.7 (-38-134)
Yucatán	0.1 (0-0.2)	21.3 (-0.8-322)	0 (0-0.1)	17.8 (-7.8-270)	0 (0-0)	12.9 (-17.8-205)	0 (0-0)	-1.2 (-30.1-182)	0 (0-0)	-2.5 (-33.2-223)	0 (0-0)	-13.1 (-35.3-155)
Zacatecas	0.1 (0-0.2)	23.3 (-0.8-446)	0 (0-0.1)	18.7 (-9.1-349)	0 (0-0)	13.8 (-19.5-277)	0 (0-0)	-1.1 (-32.2-222)	0 (0-0)	6.9 (-31.1-327)	0 (0-0)	-11.4 (-35.5-200)

Location	Birth Incidence rate (per 100,000 live births), 2021	Birth Incidence rate (per 100,000 live births) percent change (2000-2021)	Under-5 rate (per 100,000 population), 2021	Under-5 rate (per 100,000 population) percent change (2000-2021)	5-14 rate (per 100,000 population), 2021	5-14 rate (per 100,000 population) percent change (2000-2021)	15-49 rate (per 100,000 population), 2021	15-49 rate (per 100,000 population) percent change (2000-2021)	50plus rate (per 100,000 population), 2021	50plus rate (per 100,000 population) percent change (2000-2021)	All age rate (per 100,000 population), 2021	All age rate (per 100,000 population) percent change (2000-2021)
Nicaragua	0.9 (0.6-1.3)	8.1 (-7.7-49.9)	0.3 (0.2-0.4)	6.9 (-9.6-43.1)	0.1 (0-0.1)	3.7 (-19.8-40.7)	0 (0-0)	-9.5 (-31.2-23)	0 (0-0)	-5.8 (-32.9-29)	0.1 (0-0.1)	-17.2 (-32.1-8.3)
Panama	0.8 (0.6-1.1)	7.2 (-12.7-50.5)	0.3 (0.2-0.4)	4.8 (-11.8-39.1)	0.1 (0.1-0.1)	2.8 (-15.6-37.2)	0 (0-0)	-3.6 (-21.8-29.2)	0 (0-0)	-12.3 (-31.6-18.6)	0.1 (0-0.1)	-10.7 (-24.9-16.5)
Venezuela (Bolivarian Republic of)	7.2 (5-9.6)	-5.6 (-21.5-15.4)	2.1 (1.6-2.7)	-23 (-33.6--9.5)	0.3 (0.2-0.4)	-53.9 (-64--41.1)	0.1 (0.1-0.1)	-55.5 (-65.6--42.5)	0 (0-0)	-52.9 (-65.5--35.7)	0.3 (0.2-0.3)	-53.7 (-60.2--45.7)
**Tropical Latin America**	1.7 (1.4-2.1)	12.8 (6.3-19.7)	1.5 (1.2-1.8)	9.9 (3.6-16.3)	1.2 (1-1.5)	15.3 (8.9-21.5)	0.5 (0.4-0.6)	5.9 (-1.1-12.5)	0.1 (0-0.1)	7.6 (-2.1-17)	0.5 (0.4-0.7)	-8.4 (-13.7--2.9)
Brazil	1.6 (1.3-2)	13.2 (6.4-20.5)	1.5 (1.2-1.8)	10.4 (3.8-17.2)	1.2 (1-1.5)	16 (9.3-22.6)	0.5 (0.4-0.6)	6.3 (-0.9-13.1)	0.1 (0-0.1)	8.1 (-1.9-17.7)	0.5 (0.4-0.7)	-8.1 (-13.6--2.3)
Acre	0.1 (0-0.2)	66.4 (0-2570)	0.1 (0-0.2)	64 (-3.2-2520)	0 (0-0.1)	68.9 (-3.5-2510)	0 (0-0)	64.1 (-10.1-2480)	0 (0-0)	97.2 (6-3150)	0 (0-0.1)	39 (-21-2080)
Alagoas	0.1 (0-0.2)	76.8 (0-2570)	0 (0-0.1)	72.9 (-4.3-2460)	0 (0-0.1)	79.4 (-3.6-2500)	0 (0-0)	68.1 (-12.7-2330)	0 (0-0)	112 (4.6-2970)	0 (0-0)	46.2 (-21.5-2040)
Amapá	0.1 (0-0.2)	57.3 (0-2570)	0.1 (0-0.2)	55.2 (-2.4-2530)	0 (0-0.1)	57.8 (-2.6-2540)	0 (0-0)	54.4 (-8.6-2490)	0 (0-0)	86.1 (4.4-3290)	0 (0-0.1)	28.7 (-21.4-2070)
Amazonas	0.2 (0.1-0.4)	79.5 (0-515)	0.1 (0.1-0.3)	76.9 (-2.2-500)	0.1 (0-0.2)	79.8 (-1.6-491)	0 (0-0.1)	73.8 (-8.8-467)	0 (0-0)	109 (2.6-756)	0.1 (0-0.1)	49.5 (-19.5-398)
Bahia	1 (0.6-1.4)	48.1 (0-146)	0.8 (0.5-1.2)	45.3 (-2.2-140)	0.6 (0.4-0.8)	49.6 (0.7-146)	0.2 (0.1-0.3)	31.4 (-12.2-118)	0 (0-0)	73 (12.4-199)	0.3 (0.2-0.4)	18 (-20.7-94.3)
Ceará	0.1 (0-0.1)	83.2 (0-1080)	0 (0-0.1)	80.1 (-2.5-1040)	0 (0-0.1)	85.7 (-1-1080)	0 (0-0)	74.6 (-12.1-1070)	0 (0-0)	135 (9.1-1950)	0 (0-0)	51.3 (-21-895)
Distrito Federal	1.4 (0.9-2.1)	60 (6.3-171)	1.2 (0.8-1.8)	58.2 (5.2-165)	1 (0.6-1.4)	60.9 (7.4-169)	0.3 (0.2-0.5)	46.3 (-1.5-143)	0 (0-0.1)	51.5 (3.9-151)	0.4 (0.3-0.6)	23.2 (-17.3-105)
Espírito Santo	0.5 (0.3-0.8)	93.2 (0-332)	0.4 (0.2-0.7)	90.1 (-1.8-327)	0.3 (0.2-0.6)	91.5 (-1.5-334)	0.1 (0-0.2)	67.7 (-13.9-270)	0 (0-0)	102 (-2.6-561)	0.1 (0.1-0.2)	43.9 (-25.9-221)
Goiás	0.5 (0.3-0.8)	40.6 (0-166)	0.4 (0.2-0.7)	38.7 (-2.4-160)	0.3 (0.2-0.5)	41.8 (-1.7-166)	0.1 (0-0.2)	37.9 (-7.5-158)	0 (0-0)	46.7 (-5.1-194)	0.1 (0.1-0.2)	10.2 (-24.8-104)
Maranhão	0 (0-0.1)	68.2 (0-1410)	0 (0-0.1)	64.8 (-4-1380)	0 (0-0.1)	72.5 (-2-1440)	0 (0-0)	53.9 (-15.6-1300)	0 (0-0)	118 (9.4-2190)	0 (0-0)	47.3 (-16.3-1230)
Mato Grosso	0.1 (0-0.1)	72.2 (0-1810)	0 (0-0.1)	70.1 (-2.9-1770)	0 (0-0.1)	74.5 (-2.8-1810)	0 (0-0)	64.1 (-12.1-1690)	0 (0-0)	99.4 (1.7-2120)	0 (0-0)	33.1 (-26.5-1370)
Mato Grosso do Sul	0.4 (0.2-0.7)	88.5 (-0.1-359)	0.4 (0.2-0.6)	86.4 (-1.4-354)	0.3 (0.1-0.5)	92.5 (0.8-365)	0.1 (0-0.2)	94.6 (0.9-360)	0 (0-0)	106 (4-416)	0.1 (0.1-0.2)	54 (-19.6-270)
Minas Gerais	0.5 (0.3-0.8)	-41.1 (-59.1--16.6)	0.4 (0.3-0.7)	-42.8 (-60.3--19.5)	0.3 (0.2-0.5)	-42.9 (-59.9--19.3)	0.1 (0.1-0.2)	-39.7 (-57.2--17)	0 (0-0)	-27.2 (-54-11)	0.1 (0.1-0.2)	-54 (-67.6--36.1)
Pará	0.1 (0-0.2)	85.2 (0-742)	0.1 (0-0.2)	82.5 (-2.6-701)	0.1 (0-0.1)	88 (-1.8-659)	0 (0-0)	80.9 (-9.9-667)	0 (0-0)	122 (7.3-1240)	0 (0-0.1)	54.9 (-20-555)
Paraíba	4 (2.9-5.4)	44.9 (32.3-59.1)	3.4 (2.4-4.6)	42.9 (30.4-57)	2.5 (1.7-3.3)	48 (33.4-62.4)	0.7 (0.5-1)	31.9 (16.5-49.7)	0 (0-0.1)	74.6 (48.9-107)	1 (0.8-1.4)	20.4 (8.6-32.3)
Paraná	0.1 (0.1-0.3)	88.4 (-0.1-375)	0.1 (0.1-0.2)	86.5 (-1.8-363)	0.1 (0-0.2)	92.3 (0-380)	0 (0-0.1)	95.5 (-1.9-389)	0 (0-0)	104 (-0.9-559)	0 (0-0.1)	49 (-23.9-275)
Pernambuco	0.3 (0.1-0.5)	94.4 (0-410)	0.2 (0.1-0.4)	91.3 (-2.7-404)	0.2 (0.1-0.3)	94.3 (-3-412)	0 (0-0.1)	66.5 (-18.9-326)	0 (0-0)	107 (0.1-498)	0.1 (0-0.1)	53.3 (-23.4-300)
Piaui	0.5 (0.3-0.9)	90.7 (0-331)	0.4 (0.2-0.7)	87.4 (-2-321)	0.3 (0.2-0.5)	95.2 (1.1-320)	0.1 (0-0.2)	74.5 (-12.6-281)	0 (0-0)	111 (6.3-437)	0.1 (0.1-0.2)	57 (-19.8-245)
Rio de Janeiro	4.2 (3.4-5.2)	9.2 (0.5-18.3)	3.8 (3-4.6)	8.3 (-0.4-17.3)	3 (2.4-3.7)	9.9 (1.7-18.6)	1 (0.8-1.2)	4.6 (-2.4-12.7)	0.1 (0-0.1)	-4.8 (-16.3-6.7)	1.1 (0.9-1.4)	-12.4 (-18.8--5.9)
Rio Grande do Norte	0.2 (0.1-0.3)	93.4 (-0.1-683)	0.1 (0-0.3)	90 (-3.1-668)	0.1 (0-0.2)	96.6 (-0.8-658)	0 (0-0.1)	90.8 (-8.3-648)	0 (0-0)	139 (8-1040)	0 (0-0.1)	60.2 (-20.8-530)
Rio Grande do Sul	0.3 (0.2-0.5)	70.3 (0-348)	0.3 (0.1-0.5)	68.9 (-1.5-344)	0.2 (0.1-0.4)	72.2 (-0.4-349)	0.1 (0-0.1)	71.8 (-1.2-332)	0 (0-0)	55.8 (-10.3-311)	0.1 (0-0.1)	31.5 (-24-238)
Rondônia	0.2 (0.1-0.4)	-11.7 (-75.3-39.1)	0.2 (0.1-0.3)	-12.4 (-74.7-36.9)	0.1 (0-0.2)	-8 (-73.2-44)	0 (0-0.1)	-4.8 (-71.3-49.9)	0 (0-0)	7.9 (-68.5-61)	0 (0-0.1)	-29.6 (-79.5-8.6)
Roraima	0.1 (0-0.2)	62.9 (0-1920)	0.1 (0-0.2)	60.7 (-2.7-1790)	0 (0-0.1)	65.8 (-2.4-1830)	0 (0-0)	60.7 (-9.6-1750)	0 (0-0)	89.8 (-4-3890)	0 (0-0.1)	32.5 (-22.1-1450)
Santa Catarina	0.3 (0.1-0.5)	89.6 (0-398)	0.2 (0.1-0.4)	87.8 (-1.5-395)	0.2 (0.1-0.3)	89.5 (-1.7-403)	0.1 (0-0.1)	75.2 (-11.4-359)	0 (0-0)	84.7 (-10.4-423)	0.1 (0-0.1)	41 (-27.6-272)

Location	Birth Incidence rate (per 100,000 live births), 2021	Birth Incidence rate (per 100,000 live births) percent change (2000-2021)	Under-5 rate (per 100,000 population), 2021	Under-5 rate (per 100,000 population) percent change (2000-2021)	5-14 rate (per 100,000 population), 2021	5-14 rate (per 100,000 population) percent change (2000-2021)	15-49 rate (per 100,000 population), 2021	15-49 rate (per 100,000 population) percent change (2000-2021)	50plus rate (per 100,000 population), 2021	50plus rate (per 100,000 population) percent change (2000-2021)	All age rate (per 100,000 population), 2021	All age rate (per 100,000 population) percent change (2000-2021)
São Paulo	6.1 (4.8-7.6)	21.3 (11.7-30.8)	5.3 (4.3-6.6)	19.9 (10.6-29.2)	4.1 (3.3-5.1)	22.3 (13.1-31.7)	1.5 (1.1-1.9)	14.9 (5.6-24.3)	0.2 (0.1-0.2)	10.1 (-2.2-23.5)	1.6 (1.3-2)	-4.3 (-11-3.3)
Sergipe	0.7 (0.4-1.1)	58.1 (0-197)	0.6 (0.3-0.9)	54.6 (-2.5-193)	0.4 (0.2-0.7)	54.7 (-2.4-194)	0.1 (0.1-0.2)	35.1 (-17.1-149)	0 (0-0)	103 (10.2-406)	0.2 (0.1-0.3)	18.9 (-25.8-125)
Tocantins	2.2 (1.4-3.1)	37.1 (10-66.1)	1.8 (1.2-2.6)	35.7 (9.1-64.8)	1.3 (0.9-1.9)	41.4 (12.9-75.5)	0.4 (0.3-0.6)	30.7 (5-61.2)	0 (0-0.1)	42.3 (19.4-67.9)	0.6 (0.4-0.9)	10.7 (-10.5-35.6)
Paraguay	2.3 (1.7-3.2)	0.8 (-8.2-12.2)	1.8 (1.3-2.4)	0 (-8.6-9.9)	1.2 (0.9-1.6)	-0.2 (-8.5-9.6)	0.5 (0.4-0.7)	-8.5 (-16.6--0.4)	0 (0-0.1)	-9.8 (-19.3-0.7)	0.7 (0.5-0.9)	-18.1 (-25--10.6)
North Africa and Middle East	0.3 (0.3-0.4)	-5.2 (-11.7-0.3)	0.2 (0.2-0.3)	-8.2 (-13.7--2.9)	0.1 (0.1-0.2)	-15 (-19.6--9.5)	0.1 (0-0.1)	-22 (-26.7--17)	0 (0-0)	1.2 (-6.5-9.3)	0.1 (0.1-0.1)	-26.9 (-31.3--22.4)
**North Africa and Middle East**	0.3 (0.3-0.4)	-5.2 (-11.7-0.3)	0.2 (0.2-0.3)	-8.2 (-13.7--2.9)	0.1 (0.1-0.2)	-15 (-19.6--9.5)	0.1 (0-0.1)	-22 (-26.7--17)	0 (0-0)	1.2 (-6.5-9.3)	0.1 (0.1-0.1)	-26.9 (-31.3--22.4)
Afghanistan	0.2 (0.1-0.3)	-2.3 (-32.5-0)	0.1 (0.1-0.1)	-2.9 (-32.6-6.8)	0 (0-0.1)	-4.8 (-31.9-11.9)	0 (0-0)	0.3 (-27.3-20.4)	0 (0-0)	77.8 (26.6-135)	0 (0-0)	-11.9 (-38.1-0.6)
Algeria	1.1 (0.8-1.4)	0.4 (-9.4-13.1)	0.7 (0.5-1)	-0.1 (-10.3-11.5)	0.4 (0.3-0.5)	1.6 (-9.6-14)	0.1 (0.1-0.2)	-18.3 (-27.4--7.4)	0 (0-0)	7.2 (-6.2-23.9)	0.2 (0.2-0.3)	-16.3 (-24.8--6.9)
Bahrain	0.3 (0.2-0.4)	-3.6 (-29.5-0)	0.2 (0.2-0.3)	-4.3 (-29.5-2.8)	0.1 (0.1-0.2)	-3.4 (-26.3-7)	0.1 (0-0.1)	-19 (-38.4--8.7)	0 (0-0)	-0.7 (-24.8-13.4)	0.1 (0-0.1)	-34.3 (-50.9--26.6)
Egypt	0.2 (0.1-0.3)	-1.6 (-31.4-21.2)	0.1 (0.1-0.2)	-3.6 (-32.7-20)	0.1 (0-0.1)	-0.7 (-29.4-27.6)	0 (0-0)	-7.3 (-31.8-18.5)	0 (0-0)	-3.7 (-27.2-25.6)	0 (0-0.1)	-10.3 (-36.1-15.4)
Iran (Islamic Republic of)	0.5 (0.4-0.6)	-1.8 (-7.9-5.1)	0.3 (0.3-0.4)	-3.3 (-8.9-3)	0.2 (0.2-0.2)	-0.6 (-6-5.3)	0.1 (0-0.1)	-23.4 (-29.8--17.7)	0 (0-0)	8 (0.8-19)	0.1 (0.1-0.1)	-25.5 (-30--20.6)
Alborz	0.4 (0.3-0.5)	-3 (-31.6-24.3)	0.3 (0.2-0.4)	-3.9 (-31.8-22.9)	0.2 (0.1-0.2)	-0.6 (-27.6-28.8)	0.1 (0-0.1)	-24.6 (-43.2-1.6)	0 (0-0)	-2.1 (-24.1-34.8)	0.1 (0.1-0.1)	-29 (-47.3--6.1)
Ardebil	0.4 (0.3-0.5)	-1.3 (-30.4-28.5)	0.3 (0.2-0.4)	-2.8 (-30.7-26)	0.1 (0.1-0.2)	0.3 (-27.4-30)	0 (0-0.1)	-24.6 (-42.7-0.4)	0 (0-0)	18 (-7.3-64.9)	0.1 (0.1-0.1)	-29.7 (-47.7--7.2)
Bushehr	0.7 (0.5-0.9)	-4.4 (-27.9-17.2)	0.5 (0.4-0.7)	-5.6 (-28-16)	0.3 (0.2-0.4)	-3.7 (-25.7-17.2)	0.1 (0.1-0.2)	-20.1 (-36.9--1.6)	0 (0-0)	1.9 (-17.7-28.6)	0.2 (0.1-0.2)	-21.8 (-38.8--4.4)
Chahar Mahaal and Bakhtiari	0.7 (0.5-1)	-0.5 (-23.2-20.3)	0.5 (0.4-0.7)	-1.6 (-23.4-18.3)	0.3 (0.2-0.4)	1.2 (-19.3-22.1)	0.1 (0.1-0.1)	-22.3 (-36--3.4)	0 (0-0)	9.2 (-8.3-44)	0.2 (0.1-0.2)	-24.9 (-38.9--8.3)
East Azarbayejan	0.4 (0.3-0.5)	-1.5 (-33-29.7)	0.3 (0.2-0.4)	-3.8 (-32.3-28.2)	0.1 (0.1-0.2)	0.8 (-26.5-32.1)	0 (0-0.1)	-26.7 (-45.9--1.2)	0 (0-0)	11.3 (-16-55)	0.1 (0.1-0.1)	-24.5 (-44.7-0.9)
Fars	0.7 (0.5-0.9)	-2.2 (-27.2-18.7)	0.5 (0.4-0.7)	-3.5 (-27.3-17.1)	0.3 (0.2-0.4)	-0.4 (-22.8-19.2)	0.1 (0.1-0.1)	-28.5 (-42--12)	0 (0-0)	2.3 (-16.1-31.2)	0.1 (0.1-0.2)	-30.7 (-45--16.7)
Gilan	0.3 (0.3-0.5)	-1 (-28.2-28.1)	0.3 (0.2-0.4)	-1.6 (-28.3-27.4)	0.2 (0.1-0.2)	0.6 (-24.7-31.1)	0.1 (0-0.1)	-21.2 (-38.8-6.7)	0 (0-0)	12.4 (-10.2-59.5)	0.1 (0.1-0.1)	-31.5 (-47.3--9)
Golestan	0.4 (0.3-0.5)	-0.6 (-30.9-27.1)	0.3 (0.2-0.4)	-2.5 (-31.1-25.4)	0.2 (0.1-0.2)	1.3 (-25.6-30.7)	0 (0-0.1)	-24.6 (-43.2-2.4)	0 (0-0)	11.9 (-12.7-57.9)	0.1 (0.1-0.1)	-23.1 (-43.4-0.9)
Hamadan	0.5 (0.4-0.7)	-2 (-34-26.7)	0.3 (0.3-0.5)	-3.3 (-34.3-23.4)	0.2 (0.1-0.3)	0.6 (-29-28.9)	0.1 (0-0.1)	-25.9 (-45--1.4)	0 (0-0)	4.7 (-17-48.5)	0.1 (0.1-0.1)	-30.2 (-49.6--10)
Hormozgan	0.5 (0.3-0.6)	3.8 (-17.5-33.6)	0.3 (0.2-0.4)	2.6 (-16.8-31.5)	0.2 (0.1-0.2)	5 (-14.8-35.5)	0.1 (0-0.1)	-18.5 (-32.8-8.4)	0 (0-0)	24.8 (-0.3-84.1)	0.1 (0.1-0.1)	-17.7 (-33.3-7.4)
Ilam	0.5 (0.4-0.7)	-1.5 (-29.5-25)	0.4 (0.3-0.5)	-2.6 (-28.7-23.3)	0.2 (0.2-0.3)	0.7 (-24-26.8)	0.1 (0-0.1)	-24.6 (-42.4--1.2)	0 (0-0)	6.3 (-14.3-44.7)	0.1 (0.1-0.1)	-31.4 (-48.6--12.2)
Isfahan	0.6 (0.5-0.8)	-1.4 (-28.7-22.6)	0.5 (0.3-0.6)	-2.4 (-28.5-21.7)	0.3 (0.2-0.4)	0.2 (-24.3-26.2)	0.1 (0.1-0.1)	-23.6 (-39.2--1.1)	0 (0-0)	6.9 (-13.9-43.9)	0.1 (0.1-0.2)	-27.7 (-44.2--7.5)
Kerman	0.4 (0.3-0.6)	-0.5 (-30.4-25)	0.3 (0.2-0.4)	-1.8 (-29.6-23.8)	0.2 (0.1-0.2)	1.1 (-26.1-28.6)	0.1 (0-0.1)	-25.7 (-45.1--3.1)	0 (0-0)	9.9 (-13.8-48.2)	0.1 (0.1-0.1)	-22.7 (-43.9--0.4)
Kermanshah	0.5 (0.4-0.7)	-1.5 (-33.2-29.4)	0.4 (0.3-0.5)	-2.4 (-33-27.9)	0.2 (0.1-0.3)	1.2 (-28.1-31.5)	0.1 (0-0.1)	-27.5 (-48.1--2.2)	0 (0-0)	3.2 (-19.9-46.7)	0.1 (0.1-0.1)	-31.3 (-51.5--8.9)
Khorasan-e-Razavi	0.4 (0.3-0.5)	-1.4 (-30.9-29.6)	0.3 (0.2-0.4)	-3.2 (-30.9-26.2)	0.1 (0.1-0.2)	0.9 (-24.1-31.3)	0 (0-0.1)	-24.4 (-41.6-2.2)	0 (0-0)	16.7 (-6.5-64)	0.1 (0.1-0.1)	-22.9 (-42.3-1.7)
Khuzestan	0.8 (0.6-1)	-1.3 (-24.9-21.5)	0.6 (0.4-0.7)	-2.6 (-25.1-19.4)	0.3 (0.2-0.4)	0.2 (-21.8-22.7)	0.1 (0.1-0.1)	-25.5 (-40.9--7.6)	0 (0-0)	0.5 (-18.4-29.8)	0.2 (0.1-0.2)	-23.6 (-39.4--6.6)
Kohgiluyeh and Boyer-Ahmad	0.9 (0.7-1.2)	-1.8 (-25.4-23.9)	0.7 (0.5-0.9)	-2.9 (-25.8-20.4)	0.4 (0.3-0.5)	0.3 (-19.7-24.4)	0.1 (0.1-0.1)	-26.1 (-40.2--6.3)	0 (0-0)	15.4 (-4.7-52.5)	0.2 (0.1-0.2)	-27.2 (-42.2--8.9)
Kurdistan	0.5 (0.3-0.6)	-1.9 (-33.2-25.9)	0.3 (0.2-0.4)	-2.9 (-32.1-25.3)	0.2 (0.1-0.2)	0.3 (-28.8-26.9)	0.1 (0-0.1)	-24.3 (-44--0.6)	0 (0-0)	1.9 (-21.5-44.4)	0.1 (0.1-0.1)	-29.1 (-48.7--8.5)

Location	Birth Incidence rate (per 100,000 live births), 2021	Birth Incidence rate (per 100,000 live births) percent change (2000-2021)	Under-5 rate (per 100,000 population), 2021	Under-5 rate (per 100,000 population) percent change (2000-2021)	5-14 rate (per 100,000 population), 2021	5-14 rate (per 100,000 population) percent change (2000-2021)	15-49 rate (per 100,000 population), 2021	15-49 rate (per 100,000 population) percent change (2000-2021)	50plus rate (per 100,000 population), 2021	50plus rate (per 100,000 population) percent change (2000-2021)	All age rate (per 100,000 population), 2021	All age rate (per 100,000 population) percent change (2000-2021)
Lorestan	0.5 (0.4-0.7)	-3 (-32.5-24.2)	0.4 (0.3-0.5)	-3.9 (-31.6-23.5)	0.2 (0.2-0.3)	-0.7 (-26.8-31.2)	0.1 (0.1-0.1)	-24.8 (-43.3-3.3)	0 (0-0)	7.5 (-14-55)	0.1 (0.1-0.1)	-29.3 (-47.6--5.5)
Markazi	0.5 (0.4-0.6)	-1.5 (-32.5-29.6)	0.4 (0.3-0.5)	-2.4 (-32.8-27.4)	0.2 (0.2-0.3)	1.2 (-27.7-30.9)	0.1 (0-0.1)	-24.3 (-43.5-1.1)	0 (0-0)	18.8 (-5.7-65.9)	0.1 (0.1-0.1)	-28.9 (-48.5--6.4)
Mazandaran	0.4 (0.3-0.5)	-1.4 (-31.9-26.7)	0.3 (0.2-0.4)	-2.2 (-31.7-26.4)	0.2 (0.1-0.2)	-0.1 (-27.7-31.4)	0.1 (0-0.1)	-22.8 (-42.6-3.3)	0 (0-0)	3.4 (-20.1-47.3)	0.1 (0.1-0.1)	-30.7 (-49--8.8)
North Khorasan	0.4 (0.3-0.6)	-1.2 (-30.9-35.3)	0.3 (0.2-0.4)	-3.3 (-31.6-30.5)	0.1 (0.1-0.2)	0.3 (-26.3-33.4)	0 (0-0.1)	-19.1 (-39.5-11.9)	0 (0-0)	8.8 (-16.2-62.5)	0.1 (0.1-0.1)	-25.9 (-45.6-0.5)
Qazvin	0.4 (0.3-0.5)	-0.6 (-27.1-32.4)	0.3 (0.2-0.4)	-1.6 (-26.6-30.9)	0.2 (0.1-0.2)	1.4 (-22.9-32.5)	0.1 (0-0.1)	-23.7 (-40.2-2.7)	0 (0-0)	19.5 (-2.7-69.6)	0.1 (0.1-0.1)	-26.3 (-43.3--2.8)
Qom	0.4 (0.3-0.6)	0.1 (-25.2-29.4)	0.3 (0.3-0.4)	-0.9 (-25.2-27.2)	0.2 (0.1-0.3)	1.9 (-22.1-30.4)	0.1 (0-0.1)	-19.4 (-37.1-5.9)	0 (0-0)	12.2 (-9.3-59)	0.1 (0.1-0.1)	-20.7 (-38.8-1.9)
Semnan	0.4 (0.3-0.5)	-0.6 (-29.7-28.6)	0.3 (0.2-0.4)	-1 (-29-26.5)	0.2 (0.1-0.2)	1 (-24.4-29.6)	0.1 (0-0.1)	-19.1 (-39.4-6.8)	0 (0-0)	13.1 (-11.9-54.8)	0.1 (0.1-0.1)	-22.5 (-41.9-2)
Sistan and Baluchistan	0.4 (0.3-0.6)	0.2 (-30.9-33.8)	0.3 (0.2-0.4)	-1.6 (-31.1-31.5)	0.1 (0.1-0.2)	0.3 (-27.5-34.4)	0 (0-0.1)	-17.5 (-37.6-13.6)	0 (0-0)	13.9 (-12.2-70.1)	0.1 (0.1-0.1)	-12.4 (-37.5-18.1)
South Khorasan	0.4 (0.3-0.5)	-0.5 (-29.8-29.7)	0.3 (0.2-0.4)	-2.1 (-28.2-29)	0.1 (0.1-0.2)	-0.3 (-24-29.3)	0 (0-0.1)	-19.9 (-37.7-6.4)	0 (0-0)	21.8 (-2.4-72.2)	0.1 (0.1-0.1)	-20.9 (-39.5-4.2)
Tehran	0.3 (0.3-0.5)	-0.8 (-25.3-23.8)	0.3 (0.2-0.4)	-1.9 (-25.9-22.8)	0.2 (0.1-0.2)	1.1 (-22.5-28.2)	0.1 (0-0.1)	-19.5 (-36-3.2)	0 (0-0)	-1.9 (-20.8-32)	0.1 (0.1-0.1)	-23.5 (-40.3--3.6)
West Azarbayejan	0.4 (0.3-0.6)	-0.3 (-27.2-29.7)	0.3 (0.2-0.4)	-2.1 (-28.2-29.3)	0.1 (0.1-0.2)	1.9 (-23.5-34.3)	0 (0-0.1)	-22.6 (-40-3.5)	0 (0-0)	16.1 (-6.4-69.4)	0.1 (0.1-0.1)	-19.9 (-39.6-5.8)
Yazd	0.5 (0.3-0.6)	0.1 (-28.2-25.3)	0.4 (0.3-0.5)	-1.4 (-28.8-21.7)	0.2 (0.2-0.3)	1.3 (-25.3-27.2)	0.1 (0.1-0.1)	-21.5 (-40.1-1.7)	0 (0-0)	15.9 (-10.3-59)	0.1 (0.1-0.1)	-18.4 (-39.4-3.3)
Zanjan	0.4 (0.3-0.6)	-0.7 (-28.2-32)	0.3 (0.2-0.4)	-2.1 (-28.2-31)	0.2 (0.1-0.2)	2 (-22.5-34.3)	0.1 (0-0.1)	-23.6 (-41.6-3.2)	0 (0-0)	12.9 (-9.6-63.4)	0.1 (0.1-0.1)	-27 (-44.2--2.5)
Iraq	0.2 (0.1-0.3)	-1.8 (-28.1-0)	0.1 (0.1-0.2)	-1.9 (-27.7-4.2)	0.1 (0.1-0.1)	-2.2 (-25-8.3)	0 (0-0)	-7.7 (-27.2-3.2)	0 (0-0)	1.7 (-19.2-16.8)	0 (0-0.1)	-21.3 (-40.6--14)
Jordan	0 (0-0.1)	-0.8 (-13.2-0)	0 (0-0.1)	-1.1 (-13.1-2.6)	0 (0-0)	-1.1 (-11.7-5.9)	0 (0-0)	-7.1 (-15.7-1.5)	0 (0-0)	14.1 (1.5-28.8)	0 (0-0)	-15 (-23--9.3)
Kuwait	0.2 (0.2-0.3)	-1.2 (-22.9-0.4)	0.2 (0.1-0.3)	-1.3 (-23.7-3.5)	0.1 (0.1-0.2)	-0.9 (-21.8-6.5)	0.1 (0-0.1)	-12.2 (-29.4--2.3)	0 (0-0)	24.9 (-0.3-41.6)	0.1 (0.1-0.1)	-20.1 (-35.6--13.2)
Lebanon	0.3 (0.2-0.4)	0.2 (-17.6-23.6)	0.2 (0.2-0.3)	-0.3 (-19.2-22.5)	0.2 (0.1-0.2)	1.1 (-18-23.5)	0.1 (0-0.1)	-9.9 (-23.3-8.3)	0 (0-0)	3 (-11.7-27.2)	0.1 (0.1-0.1)	-12 (-27.4-5.9)
Libya	0.2 (0.2-0.3)	-2.4 (-33.8-0)	0.2 (0.1-0.2)	-2.8 (-33.5-9)	0.1 (0.1-0.1)	-1.9 (-30-18.2)	0 (0-0)	-20.3 (-40.5--7)	0 (0-0)	18.9 (-8.6-49.4)	0 (0-0.1)	-33 (-51.3--23.5)
Morocco	0.2 (0.1-0.2)	-2.2 (-30.6-0.2)	0.1 (0.1-0.2)	-3.7 (-29.5-2.8)	0.1 (0-0.1)	-1.6 (-28.7-10.2)	0 (0-0)	-10.1 (-33.9-1.8)	0 (0-0)	1.6 (-24.5-17.8)	0 (0-0)	-22.5 (-43.5--13.8)
Oman	0.1 (0.1-0.2)	-1.5 (-27.6-0)	0.1 (0.1-0.1)	-1.9 (-27.3-4)	0.1 (0-0.1)	0 (-24.2-10.2)	0 (0-0)	-18.8 (-38.2--7.1)	0 (0-0)	4.4 (-19.2-21)	0 (0-0)	-21.3 (-40.3--13.6)
Palestine	0.2 (0.1-0.2)	-2.4 (-28.5-16.1)	0.1 (0.1-0.2)	-3.5 (-28.3-16.8)	0.1 (0.1-0.1)	-2.8 (-28.8-18.6)	0 (0-0)	-5.9 (-29.4-14.8)	0 (0-0)	16.5 (-12.4-42.2)	0.1 (0-0.1)	-16.7 (-38.8-2.2)
Qatar	0.3 (0.2-0.3)	-2 (-24.3-0)	0.2 (0.2-0.3)	-2.3 (-24.4-2.5)	0.2 (0.1-0.2)	-1.3 (-22.5-6)	0.1 (0-0.1)	1.4 (-22.5-15)	0 (0-0)	-11.4 (-31.9-2.4)	0.1 (0.1-0.1)	-16.9 (-35.3--7.8)
Saudi Arabia	0.2 (0.1-0.3)	-1 (-24.2-0)	0.1 (0.1-0.2)	-0.5 (-22.3-5.5)	0.1 (0.1-0.1)	0.4 (-19.7-11.6)	0 (0-0)	-18.3 (-34.3--7.2)	0 (0-0)	8.6 (-13.3-28.3)	0 (0-0.1)	-30.8 (-45.2--23.7)
Sudan	0.3 (0.2-0.4)	1.9 (-21.4-47.5)	0.2 (0.1-0.3)	-0.8 (-22.2-42.3)	0.1 (0-0.1)	1.4 (-21.9-40.8)	0 (0-0)	-1.9 (-23.9-35.2)	0 (0-0)	16.2 (-13.1-62.9)	0.1 (0-0.1)	-14.1 (-33.5-19.9)
Syrian Arab Republic	0.3 (0.2-0.3)	-1.8 (-26.5-11.4)	0.2 (0.1-0.3)	-1.6 (-24.6-12.7)	0.1 (0.1-0.1)	-4.2 (-26-11.3)	0 (0-0.1)	-6.8 (-27.1-9.9)	0 (0-0)	2.8 (-22.3-22.7)	0.1 (0-0.1)	-30.3 (-45.6--19.5)
Tunisia	0.1 (0.1-0.2)	-6.3 (-38.7-0)	0.1 (0.1-0.1)	-7.1 (-38.1-1.8)	0.1 (0-0.1)	-5 (-35.4-7.4)	0 (0-0)	-17.4 (-41.3--6)	0 (0-0)	-3.5 (-32.9-11.5)	0 (0-0)	-26.1 (-49.5--16.9)
Türkiye	0.8 (0.6-1)	0.2 (-16.8-18.9)	0.5 (0.4-0.7)	0.4 (-15-19)	0.3 (0.2-0.4)	0.6 (-13.5-18.6)	0.1 (0.1-0.2)	-7.2 (-18.6-9)	0 (0-0)	-2.9 (-15.8-13)	0.1 (0.1-0.2)	-25 (-35.1--12)
United Arab Emirates	0 (0-0)	-2.1 (-33.7-0)	0 (0-0)	-2.8 (-33.2-1)	0 (0-0)	-2.2 (-31.9-3.7)	0 (0-0)	-27 (-53.8--17.5)	0 (0-0)	-3.4 (-39.5-9.2)	0 (0-0)	-31.8 (-54.9--25)
Yemen	0.2 (0.1-0.3)	-2.2 (-27.9-0)	0.1 (0.1-0.1)	-4.7 (-29.8-3.1)	0 (0-0.1)	-1.9 (-26.4-12.2)	0 (0-0)	-12.1 (-34.7-2.7)	0 (0-0)	-2.8 (-29.3-17.8)	0 (0-0.1)	-19 (-39.1--9.4)

Location	Birth Incidence rate (per 100,000 live births), 2021	Birth Incidence rate (per 100,000 live births) percent change (2000-2021)	Under-5 rate (per 100,000 population), 2021	Under-5 rate (per 100,000 population) percent change (2000-2021)	5-14 rate (per 100,000 population), 2021	5-14 rate (per 100,000 population) percent change (2000-2021)	15-49 rate (per 100,000 population), 2021	15-49 rate (per 100,000 population) percent change (2000-2021)	50plus rate (per 100,000 population), 2021	50plus rate (per 100,000 population) percent change (2000-2021)	All age rate (per 100,000 population), 2021	All age rate (per 100,000 population) percent change (2000-2021)
South Asia	0.1 (0.1-0.1)	-5.6 (-12.1--1.3)	0.1 (0-0.1)	-5.3 (-11.8--0.9)	0 (0-0)	-6.9 (-12.5--2)	0 (0-0)	-10.7 (-16.4--5.4)	0 (0-0)	-1.5 (-8.7-5.8)	0 (0-0)	-23.9 (-29.3--19.6)
**South Asia**	0.1 (0.1-0.1)	-5.6 (-12.1--1.3)	0.1 (0-0.1)	-5.3 (-11.8--0.9)	0 (0-0)	-6.9 (-12.5--2)	0 (0-0)	-10.7 (-16.4--5.4)	0 (0-0)	-1.5 (-8.7-5.8)	0 (0-0)	-23.9 (-29.3--19.6)
Bangladesh	0 (0-0)	-2.9 (-45.1-0)	0 (0-0)	-3.2 (-45-3.2)	0 (0-0)	-3.3 (-43.4-8.3)	0 (0-0)	-9.2 (-41.9-3.4)	0 (0-0)	-11.7 (-46.2-5.5)	0 (0-0)	-28 (-56.8--20)
Bhutan	0 (0-0)	2.6 (0-82.4)	0 (0-0)	2 (-4.9-77)	0 (0-0)	3.4 (-8.4-77.6)	0 (0-0)	-9.4 (-21.5-59)	0 (0-0)	-2.5 (-16.9-52.6)	0 (0-0)	-21.5 (-29.8-34.9)
India	0.1 (0.1-0.1)	-3.3 (-10.5-1.4)	0.1 (0.1-0.1)	-3.4 (-10.1-1.5)	0 (0-0.1)	-4.8 (-10.4-0.3)	0 (0-0)	-9.9 (-15.7--4.3)	0 (0-0)	-0.8 (-8.3-7.4)	0 (0-0)	-23.6 (-28.9--19)
Nepal	0 (0-0)	-0.1 (0-0)	0 (0-0)	-0.6 (-4.2-3.8)	0 (0-0)	0.1 (-7.8-10)	0 (0-0)	-2.3 (-10.9-8.8)	0 (0-0)	-7.3 (-18.3-6.6)	0 (0-0)	-21.1 (-26.9--14.3)
Pakistan	0 (0-0.1)	-5.2 (-50.7-0)	0 (0-0)	-5.8 (-50.9-1.3)	0 (0-0)	-4.2 (-48.2-6.3)	0 (0-0)	-5.5 (-46.8-6)	0 (0-0)	0.9 (-39.4-17.3)	0 (0-0)	-16.5 (-55.3--8.3)
Azad Jammu & Kashmir	0 (0-0.1)	-2.6 (-52-0)	0 (0-0)	-3.2 (-50.7-3)	0 (0-0)	-2.5 (-47.9-9.3)	0 (0-0)	-10.8 (-51.6-3.3)	0 (0-0)	-6.2 (-49.8-17.5)	0 (0-0)	-22.1 (-58.6--13.8)
Balochistan	0 (0-0.1)	-5.9 (-78.1-0)	0 (0-0)	-6.2 (-78.1-3)	0 (0-0)	-5.9 (-77-8)	0 (0-0)	-1.2 (-75.2-15.1)	0 (0-0)	-0.1 (-75.1-21.1)	0 (0-0)	-7.8 (-77.9-3.7)
Gilgit-Baltistan	0 (0-0.1)	-2.2 (-19.6-0)	0 (0-0)	-2.7 (-20.5-2.4)	0 (0-0)	-1.6 (-20.1-9.6)	0 (0-0)	-3.5 (-21.3-11.1)	0 (0-0)	-17.7 (-39.1-1.9)	0 (0-0)	-5.4 (-22.8-2.8)
Islamabad Capital Territory	0 (0-0)	-2.7 (-28.2-0)	0 (0-0)	-3.1 (-28.4-1.8)	0 (0-0)	-1.9 (-25.6-6)	0 (0-0)	-8.8 (-32.6-0.5)	0 (0-0)	2.6 (-23.8-18.7)	0 (0-0)	-12.6 (-34.9--6)
Khyber Pakhtunkhwa	0 (0-0.1)	-1.9 (-23.9-0)	0 (0-0)	-2.4 (-25.7-2.5)	0 (0-0)	-1 (-24.3-7.2)	0 (0-0)	-0.9 (-24.6-9.1)	0 (0-0)	5 (-23.5-22.3)	0 (0-0)	-3.9 (-26-2.7)
Punjab	0 (0-0.1)	-4.8 (-45.4-0)	0 (0-0)	-6 (-45.6-2.8)	0 (0-0)	-5.6 (-45.1-10.1)	0 (0-0)	-7.7 (-44.7-9.9)	0 (0-0)	1.3 (-39-28.1)	0 (0-0)	-21.9 (-54.5--11.1)
Sindh	0 (0-0.1)	-4.6 (-45.4-0)	0 (0-0)	-5.8 (-46.9-2.9)	0 (0-0)	-5 (-47.2-8.5)	0 (0-0)	-5.6 (-48.5-11.3)	0 (0-0)	-4.6 (-46.1-18.7)	0 (0-0)	-17.9 (-54.7--7.6)
Southeast Asia, East Asia, and Oceania	0.1 (0.1-0.1)	1.3 (-4.5-6.1)	0.1 (0.1-0.1)	-5.2 (-10.6--0.4)	0 (0-0.1)	-3.8 (-9-0.7)	0 (0-0)	-14.1 (-19.1--9.5)	0 (0-0)	5.1 (-0.5-11.5)	0 (0-0)	-24.3 (-28.6--20.6)
**East Asia**	0.1 (0.1-0.2)	0.9 (-5.8-5.9)	0.1 (0.1-0.1)	-5.5 (-11.7--0.2)	0.1 (0.1-0.1)	1.1 (-4.7-6.1)	0 (0-0)	-6.8 (-12.4--1.6)	0 (0-0)	5 (-0.7-11.7)	0 (0-0)	-22.4 (-26.9--18.2)
China	0.2 (0.1-0.2)	-0.7 (-7.3-4.2)	0.1 (0.1-0.1)	-6.9 (-13--1.6)	0.1 (0.1-0.1)	0.6 (-5.2-5.6)	0 (0-0)	-6.6 (-12.2--1.3)	0 (0-0)	4.7 (-1.1-11.4)	0 (0-0)	-22.5 (-27--18.3)
Democratic People's Republic of Korea	0 (0-0.1)	-1.2 (0-0)	0 (0-0)	-1.5 (-2.6-1.7)	0 (0-0)	-1.6 (-5.4-2.9)	0 (0-0)	-5.8 (-12.7-0.6)	0 (0-0)	7.6 (-8.1-19.7)	0 (0-0)	-24.6 (-28.9--20.1)
Taiwan (Province of China)	0 (0-0)	-0.3 (0-0)	0 (0-0)	-0.7 (-2-1.1)	0 (0-0)	0 (-2.8-2.8)	0 (0-0)	-7.4 (-12--3.4)	0 (0-0)	-9 (-16.3--2.5)	0 (0-0)	-28.5 (-31.5--25.3)
**Oceania**	0 (0-0.1)	1.2 (-0.4-8.2)	0 (0-0)	-0.4 (-4.2-7.2)	0 (0-0)	-2.6 (-9.5-4.8)	0 (0-0)	-9.5 (-17.2-1.2)	0 (0-0)	-1.6 (-10.6-10.8)	0 (0-0)	-9.2 (-14.5--1.4)
American Samoa	0 (0-0)	-2.7 (-57.8-0)	0 (0-0)	-2.5 (-57.5-1.8)	0 (0-0)	-3.1 (-57.1-2.7)	0 (0-0)	3.6 (-49-12)	0 (0-0)	-1.8 (-43.2-11)	0 (0-0)	-16.8 (-62.5--11.7)
Cook Islands	0 (0-0)	-0.2 (0-0)	0 (0-0)	-0.2 (-1.3-1.6)	0 (0-0)	-0.6 (-3.3-2.6)	0 (0-0)	-0.2 (-4.6-4.8)	0 (0-0)	-6 (-14.3-4.8)	0 (0-0)	-19.7 (-22.2--17)
Fiji	0 (0-0)	0 (0-0)	0 (0-0)	-0.4 (-3.2-2.2)	0 (0-0)	0.3 (-4.7-5.9)	0 (0-0)	-5.9 (-12.4-3)	0 (0-0)	-12.1 (-22.8-1.6)	0 (0-0)	-11 (-15.2--6.1)
Guam	0 (0-0)	-0.1 (0-0)	0 (0-0)	0.1 (-1.4-1.9)	0 (0-0)	-0.2 (-3.2-3.4)	0 (0-0)	3 (-2.1-8.9)	0 (0-0)	-13.8 (-21.7--5.8)	0 (0-0)	-11.7 (-14.5--7.9)
Kiribati	0 (0-0.1)	0 (0-0)	0 (0-0)	-0.4 (-4.4-3.5)	0 (0-0)	0 (-8-8.4)	0 (0-0)	-3.8 (-13.4-6.6)	0 (0-0)	-1.1 (-18-19.4)	0 (0-0)	-9.3 (-15.5--3)
Marshall Islands	0 (0-0)	-3.6 (-45.6-0)	0 (0-0)	-4 (-44.9-2.2)	0 (0-0)	-3.9 (-42.8-5.3)	0 (0-0)	-17.3 (-47.5--7.4)	0 (0-0)	-12.1 (-35.4-8.8)	0 (0-0)	-22.2 (-53--15.3)
Micronesia (Federated States of)	0 (0-0)	0 (0-0)	0 (0-0)	0 (-2.3-2.6)	0 (0-0)	-0.2 (-4.9-5.1)	0 (0-0)	-5.9 (-12.8-1.5)	0 (0-0)	4.1 (-12.3-22.5)	0 (0-0)	-19.5 (-23.6--15.2)
Nauru	0 (0-0)	-1 (0-0)	0 (0-0)	-1.8 (-4.5-1.8)	0 (0-0)	-1.3 (-7.7-6)	0 (0-0)	0.6 (-8.7-10.9)	0 (0-0)	-6.2 (-21.4-12.7)	0 (0-0)	-8.5 (-13.5--2.4)
Niue	0 (0-0)	-3.4 (-79.3-0)	0 (0-0)	-3.3 (-78-2.4)	0 (0-0)	-3.6 (-77.3-3.8)	0 (0-0)	-4 (-74-6.5)	0 (0-0)	6 (-58.5-24.5)	0 (0-0)	-15.7 (-79.1--9)

Location	Birth Incidence rate (per 100,000 live births), 2021	Birth Incidence rate (per 100,000 live births) percent change (2000-2021)	Under-5 rate (per 100,000 population), 2021	Under-5 rate (per 100,000 population) percent change (2000-2021)	5-14 rate (per 100,000 population), 2021	5-14 rate (per 100,000 population) percent change (2000-2021)	15-49 rate (per 100,000 population), 2021	15-49 rate (per 100,000 population) percent change (2000-2021)	50plus rate (per 100,000 population), 2021	50plus rate (per 100,000 population) percent change (2000-2021)	All age rate (per 100,000 population), 2021	All age rate (per 100,000 population) percent change (2000-2021)
Northern Mariana Islands	0 (0-0)	1.6 (0-2.4)	0 (0-0)	0 (-3.1-3.5)	0 (0-0)	0.3 (-4-9)	0 (0-0)	11.7 (3.7-25.5)	0 (0-0)	-9.7 (-18.8-5.2)	0 (0-0)	-28.5 (-32.4--22.2)
Palau	0 (0-0)	-0.2 (0-0)	0 (0-0)	-0.9 (-3.1-1.9)	0 (0-0)	-0.8 (-4.9-4.5)	0 (0-0)	-5.6 (-12.9-1.3)	0 (0-0)	-1.5 (-14.1-11.5)	0 (0-0)	-26.9 (-30.9--23.2)
Papua New Guinea	0 (0-0.1)	0 (0-0)	0 (0-0)	0 (-3.5-3.5)	0 (0-0)	0.3 (-8.2-8.4)	0 (0-0)	-4.9 (-14.8-4.3)	0 (0-0)	8.8 (-6.4-26.9)	0 (0-0)	-7 (-12.9--1)
Samoa	0 (0-0)	-4 (-42.1-0)	0 (0-0)	-3 (-42.3-4)	0 (0-0)	-4.8 (-44.1-5.2)	0 (0-0)	-2.9 (-44-9.1)	0 (0-0)	7.6 (-44-26.3)	0 (0-0)	-9 (-47.4-0.4)
Solomon Islands	0 (0-0)	-2.4 (-42.4-0)	0 (0-0)	-3.1 (-41.1-2.1)	0 (0-0)	-2.7 (-40.5-5.3)	0 (0-0)	-10.6 (-49.6--0.6)	0 (0-0)	13.3 (-44.8-38.3)	0 (0-0)	-11.9 (-47.7--5)
Tokelau	0 (0-0)	0.3 (-23.4-0)	0 (0-0)	0.3 (-24.3-1.9)	0 (0-0)	0.8 (-22.4-5.8)	0 (0-0)	-5.1 (-28.3-3.6)	0 (0-0)	7 (-21.5-32.7)	0 (0-0)	-12.9 (-33.8--8.8)
Tonga	0 (0-0)	-1.3 (-22-0)	0 (0-0)	-1.4 (-21.1-2.2)	0 (0-0)	-1.1 (-20.4-5.1)	0 (0-0)	-4.8 (-26.7-2.2)	0 (0-0)	5.7 (-22.6-19.8)	0 (0-0)	-8.9 (-28.3--3.9)
Tuvalu	0 (0-0)	2 (0-0)	0 (0-0)	2.4 (-3-5.1)	0 (0-0)	1 (-7.8-7.3)	0 (0-0)	6.1 (-4.4-14.6)	0 (0-0)	-5.6 (-19.5-10)	0 (0-0)	-9.3 (-16.6--4.7)
Vanuatu	0 (0-0)	-4.4 (-42.6-0)	0 (0-0)	-4.9 (-43-2.2)	0 (0-0)	-5.1 (-44.2-5.5)	0 (0-0)	-9.3 (-52-2.8)	0 (0-0)	-15.5 (-66-4.9)	0 (0-0)	-14.5 (-51--6.1)
**Southeast Asia**	0 (0-0)	1.4 (-0.3-33)	0 (0-0)	1.1 (-1.1-30.6)	0 (0-0)	0.5 (-3.5-27.9)	0 (0-0)	-7.3 (-11.4-13.1)	0 (0-0)	3.8 (-1.7-14)	0 (0-0)	-18.5 (-21.4-2)
Cambodia	0 (0-0.1)	1 (0-0)	0 (0-0)	0.7 (-3.3-4.8)	0 (0-0)	1.2 (-7.4-11.6)	0 (0-0)	-10.7 (-20.2-2.6)	0 (0-0)	-3 (-17.3-20.5)	0 (0-0)	-20.6 (-26.7--13.6)
Indonesia	0 (0-0.1)	2.4 (0-70)	0 (0-0)	1.1 (-1.9-68.7)	0 (0-0)	0.2 (-3.9-58.5)	0 (0-0)	-8.4 (-13.6-41.8)	0 (0-0)	5.1 (-2.7-49.7)	0 (0-0)	-18.7 (-22.5-29.1)
Aceh	0 (0-0.1)	-1.5 (-51.7-19.6)	0 (0-0)	-2.4 (-51.6-16.8)	0 (0-0)	-1.5 (-51.2-16.2)	0 (0-0)	-10.7 (-54.6-7)	0 (0-0)	5.2 (-43.2-29.5)	0 (0-0)	-18.4 (-58.7--3.1)
Bali	0 (0-0.1)	0.2 (0-0)	0 (0-0)	-0.6 (-3.2-1.6)	0 (0-0)	-0.8 (-5.7-5)	0 (0-0)	-7.1 (-13.8--0.6)	0 (0-0)	1.9 (-12-18.1)	0 (0-0)	-20.7 (-24.9--16.6)
Bangka-Belitung Islands	0 (0-0.1)	3.6 (0-208)	0 (0-0)	2.4 (-4.6-198)	0 (0-0)	2.7 (-7.6-192)	0 (0-0)	-14.3 (-26-136)	0 (0-0)	7 (-12.6-197)	0 (0-0)	-20.9 (-28.9-125)
Banten	0 (0-0.1)	4.5 (0-73.8)	0 (0-0)	3.5 (-4.5-69.2)	0 (0-0)	3.7 (-8.8-70)	0 (0-0)	-6 (-18.5-63.6)	0 (0-0)	13.9 (-4.3-104)	0 (0-0)	-14.7 (-24.3-45.7)
Bengkulu	0 (0-0.1)	3.1 (0-49)	0 (0-0)	2 (-4.2-50.3)	0 (0-0)	2.5 (-7.1-52.9)	0 (0-0)	-8.7 (-18.6-34.2)	0 (0-0)	6.6 (-10.3-53)	0 (0-0)	-19.5 (-26.5-18)
Gorontalo	0 (0-0.1)	1.6 (0-81.8)	0 (0-0)	1 (-3.7-78)	0 (0-0)	-1 (-10.6-68.7)	0 (0-0)	4.4 (-7.4-66.6)	0 (0-0)	17.2 (-4.2-99.5)	0 (0-0)	-15.8 (-24.1-45.2)
Jakarta	0 (0-0.1)	1.6 (0-31)	0 (0-0)	0.2 (-4.1-29.9)	0 (0-0)	1.6 (-6.9-32.3)	0 (0-0)	-11.8 (-20.8-14)	0 (0-0)	-2.3 (-18.3-32.5)	0 (0-0)	-18.5 (-25.3-6)
Jambi	0 (0-0.1)	3.3 (0-60.4)	0 (0-0)	2.5 (-3.1-58.3)	0 (0-0)	2.5 (-6.7-59.6)	0 (0-0)	-8.5 (-18.6-37.9)	0 (0-0)	2.2 (-15.2-57.7)	0 (0-0)	-18.8 (-25.7-25)
West Java	0 (0-0.1)	2.2 (0-81.8)	0 (0-0)	1.4 (-3.7-85.4)	0 (0-0)	1 (-7.7-91.3)	0 (0-0)	-7.2 (-16.4-85.8)	0 (0-0)	9.3 (-5.6-144)	0 (0-0)	-18.8 (-25.4-57.2)
Central Java	0 (0-0.1)	0.7 (0-0.1)	0 (0-0)	0.2 (-3.1-3.3)	0 (0-0)	-0.3 (-7.4-8.6)	0 (0-0)	-7.1 (-16.6-2.5)	0 (0-0)	7.1 (-8.1-39)	0 (0-0)	-21.4 (-26.8--15.5)
East Java	0 (0-0.1)	0.7 (0-0)	0 (0-0)	-0.1 (-3.6-4.7)	0 (0-0)	-0.5 (-7-9)	0 (0-0)	-6.5 (-14.7-8)	0 (0-0)	4.1 (-13.1-29.4)	0 (0-0)	-20.7 (-26--12)
West Kalimantan	0 (0-0.1)	3.6 (0-49.6)	0 (0-0)	2.5 (-4.4-50.2)	0 (0-0)	2.9 (-9.2-51.2)	0 (0-0)	-7.4 (-20.3-35.5)	0 (0-0)	-1.9 (-20.4-45.1)	0 (0-0)	-17.8 (-26.7-20.8)
South Kalimantan	0 (0-0.1)	3.5 (0-49.6)	0 (0-0)	2.3 (-4.1-48.2)	0 (0-0)	2.1 (-9.3-53.5)	0 (0-0)	-7.2 (-20.4-45.7)	0 (0-0)	8 (-13.1-88.6)	0 (0-0)	-20.6 (-29.2-20.2)
Central Kalimantan	0 (0-0.1)	3.4 (0-107)	0 (0-0)	2.6 (-4.1-104)	0 (0-0)	2.5 (-8.2-100)	0 (0-0)	-9.5 (-20.3-72.6)	0 (0-0)	6.8 (-9.2-105)	0 (0-0)	-18.8 (-26.7-57.7)
East Kalimantan	0 (0-0.1)	0.7 (0-0.1)	0 (0-0)	-0.4 (-3.1-3.4)	0 (0-0)	0.3 (-5.3-11.4)	0 (0-0)	-9.6 (-16.9-1.6)	0 (0-0)	-0.7 (-14.5-19.9)	0 (0-0)	-16.2 (-20.9--8.8)
North Kalimantan	0 (0-0.1)	2.4 (0-71.5)	0 (0-0)	1.3 (-4.1-67.1)	0 (0-0)	1.3 (-6.9-64.4)	0 (0-0)	-5.8 (-15.7-66)	0 (0-0)	1.4 (-10.6-69.7)	0 (0-0)	-17.7 (-24.7-37.6)
Riau Islands	0 (0-0.1)	1.7 (0-31)	0 (0-0)	-0.3 (-5.4-28.3)	0 (0-0)	2.5 (-6.1-30.9)	0 (0-0)	-21.1 (-29.5--1.2)	0 (0-0)	9.6 (-3.4-43.3)	0 (0-0)	-14.3 (-21-8.9)

Location	Birth Incidence rate (per 100,000 live births), 2021	Birth Incidence rate (per 100,000 live births) percent change (2000-2021)	Under-5 rate (per 100,000 population), 2021	Under-5 rate (per 100,000 population) percent change (2000-2021)	5-14 rate (per 100,000 population), 2021	5-14 rate (per 100,000 population) percent change (2000-2021)	15-49 rate (per 100,000 population), 2021	15-49 rate (per 100,000 population) percent change (2000-2021)	50plus rate (per 100,000 population), 2021	50plus rate (per 100,000 population) percent change (2000-2021)	All age rate (per 100,000 population), 2021	All age rate (per 100,000 population) percent change (2000-2021)
Lampung	0 (0-0.1)	2.6 (0-22.6)	0 (0-0)	1.8 (-3.6-24.7)	0 (0-0)	1.5 (-7.6-28.6)	0 (0-0)	-8.6 (-20.2-17.6)	0 (0-0)	6 (-10.3-46.7)	0 (0-0)	-21.1 (-28.9--0.1)
Maluku	0 (0-0.1)	2.7 (0-73.8)	0 (0-0)	1.2 (-5.1-70.7)	0 (0-0)	2 (-8.8-73)	0 (0-0)	-0.6 (-14-66.7)	0 (0-0)	9 (-12.3-55)	0 (0-0)	-14 (-22.1-45.3)
North Maluku	0 (0-0.1)	2.6 (0-73.8)	0 (0-0)	1.3 (-4.2-67.7)	0 (0-0)	1.1 (-8.9-67.1)	0 (0-0)	-8.7 (-19.6-47.8)	0 (0-0)	4.3 (-14.3-28)	0 (0-0)	-20 (-27.5-32.8)
West Nusa Tenggara	0 (0-0.1)	4.5 (0-81.8)	0 (0-0)	3.6 (-4.6-76.2)	0 (0-0)	4.3 (-8.9-72.7)	0 (0-0)	-6.9 (-21.5-57.6)	0 (0-0)	5.4 (-16.8-67.5)	0 (0-0)	-16.8 (-26.7-41.5)
East Nusa Tenggara	0 (0-0.1)	4.3 (0-81.8)	0 (0-0)	3.1 (-4.4-81.6)	0 (0-0)	2 (-10.6-85.3)	0 (0-0)	2.5 (-12.2-96.5)	0 (0-0)	6.4 (-14-110)	0 (0-0)	-12.9 (-22.3-61.8)
Papua	0 (0-0.1)	2.2 (0-73.8)	0 (0-0)	0.7 (-4.6-57.3)	0 (0-0)	0.8 (-9.4-49)	0 (0-0)	-6 (-17.3-40.6)	0 (0-0)	6.5 (-13.1-56.1)	0 (0-0)	-15.6 (-23.1-28.2)
West Papua	0 (0-0.1)	3 (0-101)	0 (0-0)	2.2 (-3.9-97)	0 (0-0)	3.2 (-7.3-96.7)	0 (0-0)	-7.9 (-19.7-64.7)	0 (0-0)	-17.8 (-31.9-16.2)	0 (0-0)	-16 (-23.8-58.1)
Riau	0 (0-0.1)	2.6 (0-107)	0 (0-0)	1.6 (-3.6-101)	0 (0-0)	1.9 (-6-91.9)	0 (0-0)	-8.4 (-17.7-68.2)	0 (0-0)	10.1 (-5.7-95.1)	0 (0-0)	-14.3 (-21.3-62.6)
West Sulawesi	0 (0-0.1)	2.6 (0-73.8)	0 (0-0)	1.7 (-4-71.9)	0 (0-0)	1.1 (-9.1-66.5)	0 (0-0)	-2.6 (-14.7-65.5)	0 (0-0)	2.4 (-17.3-57.2)	0 (0-0)	-18.3 (-26.1-34.5)
South Sulawesi	0 (0-0.1)	2.2 (0-60.4)	0 (0-0)	1.2 (-4.1-56.1)	0 (0-0)	0.8 (-8-53.2)	0 (0-0)	-5.1 (-15.2-40.7)	0 (0-0)	1.8 (-12.3-50.1)	0 (0-0)	-19.1 (-26.1-23)
Central Sulawesi	0 (0-0.1)	3.9 (0-178)	0 (0-0)	2.7 (-4.5-184)	0 (0-0)	2.7 (-8.8-193)	0 (0-0)	-3.9 (-16.1-171)	0 (0-0)	1 (-17.1-173)	0 (0-0)	-18.3 (-26.7-129)
Southeast Sulawesi	0 (0-0.1)	1.8 (0-22.4)	0 (0-0)	0.7 (-4-25.3)	0 (0-0)	0.9 (-7.8-33.1)	0 (0-0)	-7.4 (-18.8-25.6)	0 (0-0)	2.8 (-14.4-39.9)	0 (0-0)	-17.9 (-25-8)
North Sulawesi	0 (0-0.1)	0.5 (0-0)	0 (0-0)	-0.2 (-3.5-2.8)	0 (0-0)	-0.7 (-7.3-5.9)	0 (0-0)	-5.9 (-13.2-2)	0 (0-0)	-0.8 (-13.8-14.6)	0 (0-0)	-23.4 (-28.9--18.8)
West Sumatra	0 (0-0.1)	1.3 (-1.3-30.8)	0 (0-0)	0.5 (-9-29.3)	0 (0-0)	0.5 (-14.4-35.8)	0 (0-0)	-5.1 (-24-34.8)	0 (0-0)	5.7 (-26.8-53.7)	0 (0-0)	-18.6 (-30.1-8.9)
South Sumatra	0 (0-0.1)	0.3 (0-49)	0 (0-0)	-0.6 (-5.5-47)	0 (0-0)	0 (-8.7-49.7)	0 (0-0)	-10.9 (-23.4-35.8)	0 (0-0)	2.1 (-18.1-68.3)	0 (0-0)	-20.9 (-28.6-17.5)
North Sumatra	0 (0-0.1)	2.1 (0-35.8)	0 (0-0)	1.3 (-3.1-34.5)	0 (0-0)	1.4 (-7-39.3)	0 (0-0)	-6.6 (-16-33.5)	0 (0-0)	7.2 (-8-61.5)	0 (0-0)	-18 (-24.3-11.1)
Yogyakarta	0 (0-0)	3.9 (0-69.2)	0 (0-0)	3.3 (-2.1-68.5)	0 (0-0)	3.8 (-4-70.9)	0 (0-0)	-4.6 (-13.5-65.2)	0 (0-0)	21.1 (3.3-126)	0 (0-0)	-15.7 (-22.4-40.6)
Lao People's Democratic Republic	0 (0-0.1)	0.4 (0-0)	0 (0-0)	0.1 (-4.4-3.6)	0 (0-0)	0.1 (-9.2-9.2)	0 (0-0)	-9.5 (-19.3-0.7)	0 (0-0)	6.3 (-10.2-28.6)	0 (0-0)	-20.5 (-26.6--15.2)
Malaysia	0 (0-0)	0.3 (0-0.1)	0 (0-0)	0.9 (-1-3)	0 (0-0)	0.1 (-3.7-4.5)	0 (0-0)	-3.7 (-9.1-2.8)	0 (0-0)	-14.2 (-22.3--3.8)	0 (0-0)	-16.5 (-20.3--12.6)
Maldives	0 (0-0)	3.5 (0-381)	0 (0-0)	3.7 (-2.9-367)	0 (0-0)	4 (-6.3-354)	0 (0-0)	-18.3 (-30.1-304)	0 (0-0)	30.2 (9-452)	0 (0-0)	-26.7 (-35.4-236)
Mauritius	0 (0-0)	0.1 (0-0.1)	0 (0-0)	0.4 (-1.8-1.9)	0 (0-0)	-0.7 (-4.6-3.1)	0 (0-0)	-2.7 (-8.1-3.1)	0 (0-0)	-14.3 (-22.1--4.1)	0 (0-0)	-25.6 (-29.1--22.4)
Myanmar	0 (0-0.1)	0 (0-0)	0 (0-0)	-0.5 (-4.3-4.1)	0 (0-0)	-0.5 (-9-10.5)	0 (0-0)	-6.7 (-16.2-5.2)	0 (0-0)	6.1 (-12.3-27.2)	0 (0-0)	-18.7 (-24.9--11.8)
Philippines	0 (0-0.1)	0 (-4.6-2.9)	0 (0-0)	0.2 (-3.7-3.3)	0 (0-0)	0.2 (-2.1-4.2)	0 (0-0)	-3.2 (-6.1-1.1)	0 (0-0)	-0.8 (-4-2.3)	0 (0-0)	-12.5 (-15.7--10.2)
Abra	0 (0-0)	3.1 (0-62.8)	0 (0-0)	2.5 (-2.9-62.3)	0 (0-0)	2.7 (-4.9-61.7)	0 (0-0)	-2.4 (-10.9-42.1)	0 (0-0)	-1.8 (-14.7-30.8)	0 (0-0)	-11.8 (-18.3-33.8)
Agusan Del Norte	0 (0-0.1)	-0.1 (0-0)	0 (0-0)	0 (-3.9-3.5)	0 (0-0)	0.6 (-7.7-8.8)	0 (0-0)	-2.7 (-13.1-7.7)	0 (0-0)	-2.4 (-17.3-15.1)	0 (0-0)	-12.6 (-18.8--6.9)
Agusan Del Sur	0 (0-0.1)	0.2 (0-14.3)	0 (0-0)	-0.6 (-5.5-11.4)	0 (0-0)	0.8 (-9.2-16.2)	0 (0-0)	-3.5 (-14.2-13.8)	0 (0-0)	-1.9 (-19.3-36)	0 (0-0)	-14 (-21.1--2.6)
Aklan	0 (0-0.1)	0 (0-0)	0 (0-0)	0 (-2.1-2.2)	0 (0-0)	0.4 (-4.2-6.1)	0 (0-0)	-5.4 (-12.3-1.8)	0 (0-0)	-2.6 (-14.4-8.6)	0 (0-0)	-11.3 (-15.2--6.8)
Albay	0 (0-0.1)	-0.3 (0-0)	0 (0-0)	0 (-2-2.8)	0 (0-0)	-0.1 (-6.9-6)	0 (0-0)	-2.4 (-10.6-5.4)	0 (0-0)	-4.1 (-18.2-8.8)	0 (0-0)	-13.2 (-17.8--8.8)
Antique	0 (0-0.1)	0 (0-0)	0 (0-0)	0 (-2.4-2.5)	0 (0-0)	0.9 (-4.8-7.1)	0 (0-0)	-2.7 (-9.8-6)	0 (0-0)	-0.5 (-13.9-15.1)	0 (0-0)	-10.9 (-15.4--6.2)

Location	Birth Incidence rate (per 100,000 live births), 2021	Birth Incidence rate (per 100,000 live births) percent change (2000-2021)	Under-5 rate (per 100,000 population), 2021	Under-5 rate (per 100,000 population) percent change (2000-2021)	5-14 rate (per 100,000 population), 2021	5-14 rate (per 100,000 population) percent change (2000-2021)	15-49 rate (per 100,000 population), 2021	15-49 rate (per 100,000 population) percent change (2000-2021)	50plus rate (per 100,000 population), 2021	50plus rate (per 100,000 population) percent change (2000-2021)	All age rate (per 100,000 population), 2021	All age rate (per 100,000 population) percent change (2000-2021)
Apayao	0 (0-0.1)	0.1 (0-0)	0 (0-0)	-1.1 (-5.3-4.2)	0 (0-0)	0.1 (-9.2-17.7)	0 (0-0)	-5.9 (-16.6-12)	0 (0-0)	-1.2 (-18.7-49.1)	0 (0-0)	-18.4 (-24.8--10.1)
Aurora	0 (0-0.1)	1.5 (-20.4-2.9)	0 (0-0)	1.3 (-19.6-8.9)	0 (0-0)	2 (-17.1-21.4)	0 (0-0)	-1.9 (-15.5-47.9)	0 (0-0)	-3.5 (-18.5-66.8)	0 (0-0)	-13.4 (-27.8-1.7)
Basilan	0 (0-0.1)	0 (0-0)	0 (0-0)	-0.1 (-2.6-2.1)	0 (0-0)	-0.2 (-5.7-5.1)	0 (0-0)	-1.5 (-8.3-5.3)	0 (0-0)	2.5 (-10-16.3)	0 (0-0)	-8.6 (-13--4.1)
Bataan	0 (0-0.1)	0 (0-0)	0 (0-0)	0 (-1.6-2)	0 (0-0)	0.1 (-5.5-4.6)	0 (0-0)	-4.9 (-11.4-2.2)	0 (0-0)	-9.4 (-22.7-2.2)	0 (0-0)	-15.1 (-18.9--11.5)
Batanes	0 (0-0.1)	-0.6 (0-0)	0 (0-0)	-1 (-3.1-1.8)	0 (0-0)	-0.3 (-5.7-6.2)	0 (0-0)	-10.3 (-16.7--2.9)	0 (0-0)	9 (-2.4-23.4)	0 (0-0)	-17.9 (-22.2--13.1)
Batangas	0 (0-0)	0.3 (0-0)	0 (0-0)	0.7 (-1.3-2.7)	0 (0-0)	0.7 (-4.1-5.9)	0 (0-0)	-4.4 (-11.6-3.1)	0 (0-0)	0.4 (-12.5-14.7)	0 (0-0)	-11.5 (-15.6--7)
Benguet	0 (0-0)	-2.4 (0-0)	0 (0-0)	-2.2 (-1.7-1.9)	0 (0-0)	-2.1 (-5.6-5.1)	0 (0-0)	-4.9 (-15.3-5)	0 (0-0)	-8.8 (-25.8-5.6)	0 (0-0)	-13.4 (-17.5--7)
Biliran	0 (0-0.1)	-0.6 (0-0)	0 (0-0)	-0.6 (-2.1-1.6)	0 (0-0)	0.1 (-5.1-5.5)	0 (0-0)	-3.4 (-11.6-4.3)	0 (0-0)	-5.7 (-19-8.7)	0 (0-0)	-12.1 (-16.4--7.8)
Bohol	0 (0-0.1)	0.6 (0-0)	0 (0-0)	-0.2 (-3-2.2)	0 (0-0)	0.2 (-5.6-5.9)	0 (0-0)	-4.1 (-11.5-3.3)	0 (0-0)	3.9 (-8.1-18.4)	0 (0-0)	-15.5 (-20.2--11.3)
Bukidnon	0 (0-0.1)	-1.3 (-13.3-0)	0 (0-0)	-2.4 (-14.5-2.5)	0 (0-0)	-1.7 (-15.5-7.6)	0 (0-0)	-6.2 (-21.5-6.3)	0 (0-0)	-6.8 (-32.9-12.1)	0 (0-0)	-17.6 (-29.1--10.5)
Bulacan	0 (0-0.1)	0 (0-0)	0 (0-0)	0.2 (-2.4-2.3)	0 (0-0)	-0.3 (-5.8-4.7)	0 (0-0)	-2.8 (-9.3-4.3)	0 (0-0)	-5.7 (-17.1-5.7)	0 (0-0)	-14.5 (-18.6--10.5)
Cagayan	0 (0-0.1)	2.4 (0-36.1)	0 (0-0)	2.4 (-2.6-36.1)	0 (0-0)	1.9 (-6.8-37.3)	0 (0-0)	-3.8 (-13-26.4)	0 (0-0)	1 (-11.9-36.7)	0 (0-0)	-14.3 (-20.8-13.4)
Camarines Norte	0 (0-0.1)	-0.1 (0-0)	0 (0-0)	-0.4 (-2.7-2.2)	0 (0-0)	-0.5 (-6.6-5.3)	0 (0-0)	-2.1 (-10.4-5.8)	0 (0-0)	-3.2 (-15.5-9.3)	0 (0-0)	-12.5 (-16.9--8.3)
Camarines Sur	0 (0-0.1)	2.4 (0-32.5)	0 (0-0)	2.3 (-3.6-35.4)	0 (0-0)	2.1 (-8.4-35)	0 (0-0)	1.1 (-11.3-35.1)	0 (0-0)	-0.5 (-17.7-53)	0 (0-0)	-9 (-17.4-19.7)
Camiguin	0 (0-0)	-0.7 (0-0)	0 (0-0)	-0.7 (-2.7-1.9)	0 (0-0)	-0.5 (-6.2-4.5)	0 (0-0)	-3.7 (-10.2-3.7)	0 (0-0)	-2.6 (-14.4-11)	0 (0-0)	-11.3 (-15.1--7.4)
Capiz	0 (0-0.1)	0.7 (0-0)	0 (0-0)	0.9 (-2.3-3.9)	0 (0-0)	1.9 (-4.6-9.2)	0 (0-0)	-3.4 (-11.5-6.7)	0 (0-0)	-7 (-19.6-13.2)	0 (0-0)	-14.2 (-19.5--9)
Catanduanes	0 (0-0.1)	0 (0-0)	0 (0-0)	-1 (-4.4-1.8)	0 (0-0)	0 (-8.9-7.8)	0 (0-0)	-4.3 (-14.2-5.6)	0 (0-0)	6.6 (-7.5-21.3)	0 (0-0)	-13.4 (-19.2--8.5)
Cavite	0 (0-0)	2.4 (0-0)	0 (0-0)	2.6 (-1.4-2.7)	0 (0-0)	2.2 (-4.8-7)	0 (0-0)	-0.7 (-10.4-6.3)	0 (0-0)	-0.4 (-15.2-11.4)	0 (0-0)	-11.2 (-17.4--7.8)
Cebu	0 (0-0.1)	0.1 (0-0)	0 (0-0)	0.1 (-2.1-2.9)	0 (0-0)	0.2 (-5.7-7.3)	0 (0-0)	-3.6 (-11.2-7)	0 (0-0)	0.1 (-13.7-21)	0 (0-0)	-8.7 (-13.4--3.1)
Cotabato (North Cotabato)	0 (0-0.1)	-1.1 (0-0)	0 (0-0)	-1.2 (-3.9-2.5)	0 (0-0)	-1.3 (-10.6-5.4)	0 (0-0)	-6.2 (-21-2.2)	0 (0-0)	-2.9 (-28.7-10.6)	0 (0-0)	-12.3 (-19.9--7.2)
Davao de Oro	0 (0-0.1)	-2.1 (-32.7-0)	0 (0-0)	-3 (-32.1-1.6)	0 (0-0)	-2.4 (-29.7-5.6)	0 (0-0)	-5.7 (-35.1-4.7)	0 (0-0)	-3.5 (-36.9-12.5)	0 (0-0)	-15.9 (-40.9--9.7)
Davao Del Norte	0 (0-0.1)	0 (0-0)	0 (0-0)	-0.7 (-2.5-1.5)	0 (0-0)	0.1 (-5.1-5.5)	0 (0-0)	-5.9 (-12.4-0.7)	0 (0-0)	-7.1 (-18.8-4.8)	0 (0-0)	-16.6 (-20.7--12.7)
Davao Del Sur	0 (0-0.1)	-1 (0-0)	0 (0-0)	-1 (-2.6-2.1)	0 (0-0)	-0.3 (-6.9-7.6)	0 (0-0)	-5.9 (-14-4.2)	0 (0-0)	-12.8 (-26.3-4)	0 (0-0)	-14.6 (-19.4--9.2)
Davao Occidental	0 (0-0.1)	0 (0-0)	0 (0-0)	-1 (-3.7-1.9)	0 (0-0)	-1.6 (-8.1-4.8)	0 (0-0)	4.2 (-5.2-13.5)	0 (0-0)	23 (6.3-42.9)	0 (0-0)	-7.6 (-13.6--2.1)
Davao Oriental	0 (0-0.1)	0 (0-0)	0 (0-0)	-0.3 (-3.3-2.6)	0 (0-0)	0.2 (-7.3-8)	0 (0-0)	-2.7 (-11.3-6.4)	0 (0-0)	3.7 (-9-19.2)	0 (0-0)	-17 (-22.2--12)
Dinagat Islands	0 (0-0.1)	-0.3 (0-0)	0 (0-0)	-1.4 (-4.9-2.9)	0 (0-0)	-0.9 (-7.9-8.8)	0 (0-0)	-4 (-14.3-7.4)	0 (0-0)	-14.3 (-29.2--1.1)	0 (0-0)	-16.9 (-22.5--10.3)
Eastern Samar	0 (0-0.1)	-1.1 (0-0)	0 (0-0)	-1.3 (-4.6-3.7)	0 (0-0)	-1.7 (-10-8.3)	0 (0-0)	-2.6 (-15.2-8.3)	0 (0-0)	5.3 (-19.1-22.7)	0 (0-0)	-14.1 (-19.6--7.2)
Guimaras	0 (0-0)	0.8 (0-0)	0 (0-0)	0.6 (-2.4-2.5)	0 (0-0)	1.3 (-4.5-7.9)	0 (0-0)	-7 (-14.6-1.9)	0 (0-0)	-1.6 (-12.2-10.8)	0 (0-0)	-14.1 (-18.6--9.2)
Ifugao	0 (0-0.1)	0.3 (0-0)	0 (0-0)	0.1 (-2.7-2.7)	0 (0-0)	0.3 (-6.3-6.1)	0 (0-0)	-7.4 (-15.4--0.3)	0 (0-0)	1 (-11-12.5)	0 (0-0)	-11.8 (-16.7--7.6)

Location	Birth Incidence rate (per 100,000 live births), 2021	Birth Incidence rate (per 100,000 live births) percent change (2000-2021)	Under-5 rate (per 100,000 population), 2021	Under-5 rate (per 100,000 population) percent change (2000-2021)	5-14 rate (per 100,000 population), 2021	5-14 rate (per 100,000 population) percent change (2000-2021)	15-49 rate (per 100,000 population), 2021	15-49 rate (per 100,000 population) percent change (2000-2021)	50plus rate (per 100,000 population), 2021	50plus rate (per 100,000 population) percent change (2000-2021)	All age rate (per 100,000 population), 2021	All age rate (per 100,000 population) percent change (2000-2021)
Ilocos Norte	0 (0-0.1)	0 (0-0)	0 (0-0)	0 (-2.2-2.1)	0 (0-0)	0.4 (-4.9-6)	0 (0-0)	-6.1 (-13-0.4)	0 (0-0)	1.1 (-9.5-13.9)	0 (0-0)	-12.5 (-16.5--8.3)
Ilocos Sur	0 (0-0.1)	0 (0-0)	0 (0-0)	-0.1 (-2.3-2.1)	0 (0-0)	0 (-5.2-6.3)	0 (0-0)	-6.2 (-12.7-2.5)	0 (0-0)	1.6 (-10.7-16.7)	0 (0-0)	-13 (-16.8--8.3)
Iloilo	0 (0-0.1)	0 (0-0)	0 (0-0)	0.1 (-2.4-2.1)	0 (0-0)	0.7 (-4.1-6.4)	0 (0-0)	-5.4 (-12-1.3)	0 (0-0)	-1.7 (-16.7-10.3)	0 (0-0)	-11.1 (-15--7)
Isabela	0 (0-0.1)	-0.5 (0-0)	0 (0-0)	-0.4 (-2.7-2.5)	0 (0-0)	0 (-8.2-6.7)	0 (0-0)	-6.6 (-16.3-2)	0 (0-0)	-4.6 (-25.7-10.5)	0 (0-0)	-14.2 (-20--9.1)
Kalinga	0 (0-0.1)	0 (0-0)	0 (0-0)	-0.7 (-2.9-0.9)	0 (0-0)	0.6 (-4.8-5.6)	0 (0-0)	-5 (-10.9-1.9)	0 (0-0)	2 (-7.5-12.5)	0 (0-0)	-13.2 (-17.1--9.7)
La Union	0 (0-0.1)	0 (0-0)	0 (0-0)	0 (-2.1-2.4)	0 (0-0)	0.1 (-5-6.7)	0 (0-0)	-6.6 (-13.2-0.4)	0 (0-0)	-3.6 (-15.2-7.4)	0 (0-0)	-15.3 (-19.2--10.8)
Laguna	0 (0-0.1)	0.3 (0-0)	0 (0-0)	0.3 (-1.5-1.8)	0 (0-0)	0.5 (-3.9-4.6)	0 (0-0)	-4.9 (-11-2.4)	0 (0-0)	-3.9 (-15-11.4)	0 (0-0)	-11.8 (-15.3--8.3)
Lanao Del Norte	0 (0-0.1)	0.6 (0-0)	0 (0-0)	0.1 (-2.8-2.7)	0 (0-0)	0.6 (-4.8-7)	0 (0-0)	-1.5 (-8.8-7)	0 (0-0)	-2.5 (-13.3-13.2)	0 (0-0)	-8.3 (-12.8--3.1)
Lanao Del Sur	0 (0-0.1)	-1 (-32.6-0)	0 (0-0)	-1.7 (-30.8-2.6)	0 (0-0)	-1.3 (-29.2-8.9)	0 (0-0)	-8.8 (-38.6-2.9)	0 (0-0)	11.4 (-31.8-32)	0 (0-0)	-9.1 (-36.5--2.1)
Leyte	0 (0-0.1)	0 (0-0)	0 (0-0)	0.2 (-2.5-3.2)	0 (0-0)	0.2 (-6.2-7.5)	0 (0-0)	-2.4 (-10.4-6.5)	0 (0-0)	-4.3 (-17.2-12.1)	0 (0-0)	-13.5 (-18.1--8.4)
Maguindanao	0 (0-0.1)	2.3 (0-48.3)	0 (0-0)	1.3 (-4-47.8)	0 (0-0)	2.6 (-7.4-56.3)	0 (0-0)	-2.3 (-12.4-61.1)	0 (0-0)	2.2 (-11.9-95.9)	0 (0-0)	-6.8 (-14.2-41.7)
Marinduque	0 (0-0.1)	-1 (0-0)	0 (0-0)	-1.4 (-3.5-2.4)	0 (0-0)	-1.3 (-7.1-5.7)	0 (0-0)	-4.5 (-13.7-4.2)	0 (0-0)	-10.1 (-24.6-2.8)	0 (0-0)	-18.1 (-22.9--13)
Masbate	0 (0-0.1)	-1.4 (-32.6-0)	0 (0-0)	-1.7 (-32-3.9)	0 (0-0)	-1.4 (-31.4-8.6)	0 (0-0)	0.6 (-31.9-13.2)	0 (0-0)	-0.8 (-43.7-15.9)	0 (0-0)	-9.8 (-38.2--1.9)
Misamis Occidental	0 (0-0.1)	-2 (-47.9-0)	0 (0-0)	-1.9 (-47.5-3)	0 (0-0)	-1.4 (-46.8-8.1)	0 (0-0)	-3.8 (-45.8-6.5)	0 (0-0)	-1.5 (-36.1-18.2)	0 (0-0)	-13 (-52.8--6.4)
Misamis Oriental	0 (0-0.1)	-0.1 (0-22.2)	0 (0-0)	0.2 (-4-20.1)	0 (0-0)	0.8 (-11-17.6)	0 (0-0)	-3.5 (-22-19.7)	0 (0-0)	-2.6 (-30-26.9)	0 (0-0)	-10.4 (-20.3-6.4)
Mountain Province	0 (0-0.1)	0.6 (0-31.3)	0 (0-0)	0.1 (-3.5-29.3)	0 (0-0)	1.8 (-5.6-31.5)	0 (0-0)	-3.3 (-11.7-19.5)	0 (0-0)	8.4 (-8-29.4)	0 (0-0)	-10.9 (-16.5-13.8)
National Capital Region	0 (0-0.1)	0.6 (0-0)	0 (0-0)	0.5 (-2.1-2.7)	0 (0-0)	-0.5 (-5.9-7.2)	0 (0-0)	-4.3 (-11-5.1)	0 (0-0)	-7.4 (-18.8-8.1)	0 (0-0)	-15.1 (-19.8--9.7)
Negros Occidental	0 (0-0.1)	0.3 (0-0)	0 (0-0)	0.4 (-2-3)	0 (0-0)	0.5 (-4.8-7.2)	0 (0-0)	-3.5 (-10.3-4.7)	0 (0-0)	-3.3 (-14.7-11.3)	0 (0-0)	-13.7 (-17.7--9.1)
Negros Oriental	0 (0-0.1)	1.1 (0-22.2)	0 (0-0)	0.9 (-3.2-22.9)	0 (0-0)	1 (-6-27.1)	0 (0-0)	-2.5 (-11.2-26.9)	0 (0-0)	-0.9 (-16.1-40.6)	0 (0-0)	-13.7 (-19.4-9.6)
Northern Samar	0 (0-0.1)	-0.1 (-32.6-0.4)	0 (0-0)	-0.1 (-32.9-6.7)	0 (0-0)	-0.6 (-33.8-12.9)	0 (0-0)	0.3 (-36.7-17.9)	0 (0-0)	-0.9 (-50.8-25)	0 (0-0)	-12.5 (-42.4--2.5)
Nueva Ecija	0 (0-0.1)	0.6 (0-0)	0 (0-0)	0.7 (-2.1-3.8)	0 (0-0)	0.6 (-5.3-7.6)	0 (0-0)	-3.2 (-10.4-11.2)	0 (0-0)	2.5 (-9.3-36.8)	0 (0-0)	-11.7 (-16.4--5.5)
Nueva Vizcaya	0 (0-0.1)	0 (0-0)	0 (0-0)	-0.2 (-2.5-2.2)	0 (0-0)	0.2 (-4.6-6.2)	0 (0-0)	-5.2 (-11.3-2.5)	0 (0-0)	-8.3 (-17.9-2.8)	0 (0-0)	-13.7 (-17.4--9.2)
Occidental Mindoro	0 (0-0.1)	2.4 (0-36.1)	0 (0-0)	1.3 (-3.6-35.4)	0 (0-0)	1.8 (-7.4-34.2)	0 (0-0)	-3.7 (-14.2-22.6)	0 (0-0)	-4.8 (-19.3-17.7)	0 (0-0)	-12.8 (-19.8-15.3)
Oriental Mindoro	0 (0-0.1)	0.3 (0-0)	0 (0-0)	0.1 (-3.9-3.9)	0 (0-0)	0.7 (-7.8-10.5)	0 (0-0)	-3.8 (-14.6-5.9)	0 (0-0)	-5.7 (-20-14.4)	0 (0-0)	-15.9 (-22.3--9.9)
Palawan	0 (0-0.1)	0.4 (0-0)	0 (0-0)	0.2 (-3.2-3)	0 (0-0)	0.5 (-6.7-7.6)	0 (0-0)	-1.8 (-10.2-6.4)	0 (0-0)	-8.5 (-18.4-4.1)	0 (0-0)	-12.4 (-17.4--7.8)
Pampanga	0 (0-0.1)	2 (0-1)	0 (0-0)	2.3 (-1.8-5.6)	0 (0-0)	2.1 (-5.4-11.4)	0 (0-0)	-1.6 (-10.4-9.4)	0 (0-0)	0.4 (-13.6-20.1)	0 (0-0)	-11.3 (-17.4--4.6)
Pangasinan	0 (0-0.1)	0 (0-0)	0 (0-0)	0.1 (-1.7-2.2)	0 (0-0)	0.4 (-4.4-5.8)	0 (0-0)	-5.2 (-12.1-2)	0 (0-0)	-5.5 (-16.5-6.3)	0 (0-0)	-12.4 (-16--8.5)
Quezon	0 (0-0.1)	-0.4 (0-0)	0 (0-0)	-0.4 (-2.8-2.4)	0 (0-0)	-0.6 (-6.9-5.7)	0 (0-0)	-3.3 (-11.1-4.8)	0 (0-0)	-1.9 (-14.1-13.8)	0 (0-0)	-13.6 (-18--8.9)
Quirino	0 (0-0.1)	-1 (-13.3-0)	0 (0-0)	-1.9 (-13-2.5)	0 (0-0)	-1.1 (-12.9-7.6)	0 (0-0)	-8.4 (-20.9-1.9)	0 (0-0)	-6.9 (-22-5.1)	0 (0-0)	-19 (-29.1--12.2)

Location	Birth Incidence rate (per 100,000 live births), 2021	Birth Incidence rate (per 100,000 live births) percent change (2000-2021)	Under-5 rate (per 100,000 population), 2021	Under-5 rate (per 100,000 population) percent change (2000-2021)	5-14 rate (per 100,000 population), 2021	5-14 rate (per 100,000 population) percent change (2000-2021)	15-49 rate (per 100,000 population), 2021	15-49 rate (per 100,000 population) percent change (2000-2021)	50plus rate (per 100,000 population), 2021	50plus rate (per 100,000 population) percent change (2000-2021)	All age rate (per 100,000 population), 2021	All age rate (per 100,000 population) percent change (2000-2021)
Rizal	0 (0-0.1)	-0.3 (0-0)	0 (0-0)	0.1 (-1.8-2.3)	0 (0-0)	-0.6 (-5.2-5.7)	0 (0-0)	-0.9 (-7-6.4)	0 (0-0)	-10.6 (-20.2--0.2)	0 (0-0)	-10.7 (-14.4--6.8)
Romblon	0 (0-0.1)	-0.3 (0-0)	0 (0-0)	-0.8 (-4.1-3.1)	0 (0-0)	0.1 (-8.1-9.3)	0 (0-0)	-3 (-14.3-7.9)	0 (0-0)	-5.8 (-22.5-14)	0 (0-0)	-15.1 (-20.9--8.9)
Samar (Western Samar)	0 (0-0.1)	0 (0-0)	0 (0-0)	0.6 (-3.5-4.5)	0 (0-0)	-0.2 (-9.8-9.8)	0 (0-0)	0.9 (-9.8-13.7)	0 (0-0)	3.2 (-11.8-23.4)	0 (0-0)	-14.2 (-20.3--7.7)
Sarangani	0 (0-0.1)	-0.6 (0-0)	0 (0-0)	-0.4 (-2.7-2)	0 (0-0)	-1.1 (-6.2-4.5)	0 (0-0)	-5 (-13.1-2.4)	0 (0-0)	-2.5 (-13.8-10.5)	0 (0-0)	-14.1 (-18.8--9.8)
Siquijor	0 (0-0.1)	0 (0-0)	0 (0-0)	-0.5 (-2.7-1.6)	0 (0-0)	0.8 (-4.4-6.7)	0 (0-0)	-4.5 (-11-4.6)	0 (0-0)	3.1 (-7.5-20)	0 (0-0)	-12.7 (-17--7.9)
Sorsogon	0 (0-0.1)	0.9 (0-31.3)	0 (0-0)	1 (-2-27.5)	0 (0-0)	0.2 (-6.5-23.9)	0 (0-0)	0.3 (-7.5-21.1)	0 (0-0)	2.2 (-12.1-15.9)	0 (0-0)	-11.9 (-17.1-8.9)
South Cotabato	0 (0-0.1)	-1.1 (0-0)	0 (0-0)	-1 (-4.9-3.2)	0 (0-0)	-1 (-10.9-8.5)	0 (0-0)	-6.4 (-22.6-3.5)	0 (0-0)	-1.6 (-26.4-15.1)	0 (0-0)	-14.8 (-21.2--8.2)
Southern Leyte	0 (0-0.1)	-1.7 (0-0)	0 (0-0)	-1.8 (-4.3-2.8)	0 (0-0)	-1.9 (-7.4-6.1)	0 (0-0)	-5 (-14.8-4.8)	0 (0-0)	1.7 (-19.7-17.3)	0 (0-0)	-15 (-19.3--9.2)
Sultan Kudarat	0 (0-0.1)	0 (0-0)	0 (0-0)	-1 (-3.4-0.9)	0 (0-0)	-0.3 (-5.9-5.2)	0 (0-0)	-4.7 (-11.1-2.9)	0 (0-0)	-3.7 (-13.9-8.1)	0 (0-0)	-13.6 (-18.1--9.5)
Sulu	0 (0-0.1)	0 (0-0)	0 (0-0)	-2.2 (-6.5-0.9)	0 (0-0)	-1.9 (-12.1-7.1)	0 (0-0)	-4.7 (-15.6-6.4)	0 (0-0)	-2.5 (-18.6-18.6)	0 (0-0)	-8.8 (-16.6--2.5)
Surigao Del Norte	0 (0-0.1)	-1.2 (-51.9-0)	0 (0-0)	-0.8 (-50.9-3.5)	0 (0-0)	-0.5 (-47.8-12.1)	0 (0-0)	-5.7 (-48.4-7.3)	0 (0-0)	-5.6 (-36.6-21.6)	0 (0-0)	-13.3 (-55--4.8)
Surigao Del Sur	0 (0-0.1)	1.7 (0-48.3)	0 (0-0)	2.1 (-3-49)	0 (0-0)	1.7 (-8-50.6)	0 (0-0)	-3 (-14.4-48.7)	0 (0-0)	-3.1 (-18.5-75.5)	0 (0-0)	-13.5 (-20.8-27.7)
Tarlac	0 (0-0.1)	0 (0-0)	0 (0-0)	0 (-2.4-2.3)	0 (0-0)	0.1 (-5.1-5.1)	0 (0-0)	-4.5 (-11-2.2)	0 (0-0)	-4.5 (-17.1-7.1)	0 (0-0)	-14.4 (-18.7--10.3)
Tawi-Tawi	0 (0-0)	2.4 (0-0)	0 (0-0)	1.9 (-2.7-2.2)	0 (0-0)	2.2 (-5.7-8.7)	0 (0-0)	-0.2 (-9.1-7.8)	0 (0-0)	-7.2 (-21-16.1)	0 (0-0)	-6.6 (-13.3--1.5)
Zambales	0 (0-0.1)	-0.8 (-23.8-0)	0 (0-0)	-0.6 (-22.6-3.2)	0 (0-0)	-0.9 (-20.7-7.3)	0 (0-0)	-4.2 (-19.7-5.3)	0 (0-0)	-13.2 (-25.8--1.2)	0 (0-0)	-12.4 (-29.8--6.1)
Zamboanga Del Norte	0 (0-0.1)	1 (0-0)	0 (0-0)	0.6 (-2.8-3.5)	0 (0-0)	0.7 (-5.3-8.7)	0 (0-0)	-1.8 (-9.8-7.9)	0 (0-0)	-7 (-19.1-7.9)	0 (0-0)	-11.7 (-17.1--6.6)
Zamboanga Del Sur	0 (0-0.1)	-0.6 (0-0)	0 (0-0)	-0.5 (-2.8-2.1)	0 (0-0)	-0.7 (-6.6-5.1)	0 (0-0)	-2.9 (-11.4-5.8)	0 (0-0)	-5.1 (-23.8-8.2)	0 (0-0)	-13 (-17.7--8.2)
Zamboanga Sibugay	0 (0-0.1)	0.5 (0-0.5)	0 (0-0)	0.2 (-3-4)	0 (0-0)	0.4 (-6.7-12.2)	0 (0-0)	-2.3 (-10.1-13)	0 (0-0)	-3.8 (-13.8-12.3)	0 (0-0)	-11.6 (-16.9--3.8)
Seychelles	0 (0-0)	-0.1 (0-0)	0 (0-0)	-0.3 (-1.9-1.3)	0 (0-0)	-0.1 (-3.5-3)	0 (0-0)	-10.8 (-16.3--6.1)	0 (0-0)	20.9 (9.8-32.1)	0 (0-0)	-19.4 (-22.8--16.4)
Sri Lanka	0 (0-0)	0.4 (0-0.3)	0 (0-0)	0.2 (-2-1.5)	0 (0-0)	0.2 (-4.4-4)	0 (0-0)	-4.1 (-10.5-2.4)	0 (0-0)	-16.8 (-24.7--7.9)	0 (0-0)	-15.3 (-19.7--11.7)
Thailand	0 (0-0)	-0.1 (-0.1-0)	0 (0-0)	-0.2 (-2.1-1.4)	0 (0-0)	-0.6 (-5.2-3)	0 (0-0)	-8.9 (-15.3--3.3)	0 (0-0)	-7 (-17.9-5.4)	0 (0-0)	-31.6 (-35.5--28.2)
Timor-Leste	0 (0-0.1)	0.4 (0-0)	0 (0-0)	-0.4 (-4.7-3.6)	0 (0-0)	-0.7 (-8.6-9.3)	0 (0-0)	7.1 (-3.4-19.1)	0 (0-0)	-17.4 (-28.1--4.6)	0 (0-0)	-11.1 (-17.1--4.4)
Viet Nam	0 (0-0)	0 (0-0.7)	0 (0-0)	-0.3 (-2.8-2.5)	0 (0-0)	0.5 (-4.7-5.8)	0 (0-0)	-11.6 (-17.6--5.4)	0 (0-0)	21.8 (10.5-36.2)	0 (0-0)	-23.3 (-27--19)
Sub-Saharan Africa	264 (199-345)	-14.1 (-18--10.5)	241 (182-313)	-14.3 (-18.5--10.5)	192 (145-247)	-13.6 (-18.3--9.4)	55.9 (41.2-73.1)	-16.6 (-20.8--12.8)	0.1 (0.1-0.2)	15.6 (3.6-26.8)	114 (87.4-147)	-18.7 (-23.1--15.1)
**Central Sub-Saharan Africa**	72 (50.9-100)	-2.4 (-10.5-6.2)	61 (43.2-84.1)	-3.8 (-11.5-5)	43.5 (30.3-58.2)	-3 (-10.3-5.8)	12.2 (8.5-16.5)	0.7 (-6.6-9.8)	0 (0-0)	11.9 (-7.9-37.3)	27.1 (19.4-36.2)	-8.9 (-15.8--1)
Angola	26.1 (18.4-35.5)	-6.6 (-14.4-1.6)	21.8 (15.5-29.4)	-7.7 (-15.2-0.4)	15 (10.5-19.9)	-7.2 (-14.7-1.7)	4 (2.7-5.4)	-7.1 (-14.9-2.1)	0 (0-0)	-3.1 (-31.7-38.9)	9.8 (7-13)	-10.2 (-17.4--2.1)
Central African Republic	8 (5.6-11.2)	-0.2 (-8.2-7.6)	6.7 (4.7-9.4)	-0.9 (-8.6-6.3)	4.4 (3.1-6)	-1.5 (-9.8-6.6)	1.1 (0.7-1.5)	-0.3 (-8.5-8.2)	0 (0-0)	7.2 (-34.4-69.4)	2.7 (2-3.7)	-6.4 (-13.7-0.8)
Congo	72.6 (50.1-101)	-9.9 (-18.6--1.4)	63 (43.9-86.3)	-10.7 (-18.9--1.9)	46.3 (31.9-62.8)	-10.1 (-18.4--0.9)	10.5 (7.2-14.4)	-23.9 (-31.7--15.8)	0 (0-0)	9.6 (-18-43)	24.6 (17.2-33)	-20.9 (-28.1--12.9)
Democratic Republic of the Congo	82.2 (57.4-115)	4.4 (-6-16.3)	69 (48.5-96.1)	3.4 (-6.7-15.6)	47.9 (33.2-64.7)	3.5 (-6.8-16.2)	12.8 (8.7-17.5)	1.6 (-8.7-13.6)	0 (0-0)	15.5 (-11.7-51.8)	29.6 (20.8-39.6)	-5 (-14.1-6.1)

Location	Birth Incidence rate (per 100,000 live births), 2021	Birth Incidence rate (per 100,000 live births) percent change (2000-2021)	Under-5 rate (per 100,000 population), 2021	Under-5 rate (per 100,000 population) percent change (2000-2021)	5-14 rate (per 100,000 population), 2021	5-14 rate (per 100,000 population) percent change (2000-2021)	15-49 rate (per 100,000 population), 2021	15-49 rate (per 100,000 population) percent change (2000-2021)	50plus rate (per 100,000 population), 2021	50plus rate (per 100,000 population) percent change (2000-2021)	All age rate (per 100,000 population), 2021	All age rate (per 100,000 population) percent change (2000-2021)
Equatorial Guinea	768 (546-1,050)	-2.3 (-13-10.4)	658 (475-887)	-3.2 (-13.6-9.6)	470 (331-633)	-3 (-14.3-9.9)	136 (98.5-183)	-4.3 (-15-7.8)	0.1 (0.1-0.2)	14.8 (-7.7-45.8)	276 (198-369)	-18.6 (-27.7--7.7)
Gabon	250 (178-348)	-6.8 (-16.3-4.2)	220 (156-301)	-7 (-16.5-3.5)	167 (119-225)	-7.4 (-16.6-3.3)	40.7 (29.1-55.6)	-14.9 (-23.5--5.2)	0.1 (0-0.1)	17 (-10.5-51)	81.7 (59.3-110)	-22.3 (-29.8--13.4)
**Eastern Sub-Saharan Africa**	2.1 (1.5-2.9)	-0.1 (-3-3.2)	1.8 (1.3-2.4)	-0.1 (-3-3)	1.2 (0.9-1.6)	1 (-1.9-3.9)	0.3 (0.2-0.4)	-3.7 (-6.5--0.9)	0 (0-0)	8.5 (-6.5-25.3)	0.7 (0.5-1)	-8.2 (-10.8--5.3)
Burundi	2 (1.4-2.8)	-0.5 (-10.5-7.8)	1.7 (1.2-2.4)	-0.8 (-10.9-6.8)	1.1 (0.7-1.6)	-0.3 (-9.5-7.7)	0.3 (0.2-0.4)	-6.1 (-16-1.7)	0 (0-0)	-0.9 (-39.6-61.4)	0.7 (0.5-1)	-5 (-13.8-2.1)
Comoros	1.2 (0.8-1.6)	-0.9 (-13.2-6.5)	1 (0.7-1.4)	-1.2 (-13.1-6.3)	0.7 (0.5-1)	-1.5 (-12.9-8.2)	0.2 (0.1-0.2)	-13 (-23.7--4.4)	0 (0-0)	6.8 (-29.8-47.5)	0.4 (0.3-0.5)	-22 (-31.4--14.9)
Djibouti	0.7 (0.5-1.1)	-0.9 (-19.3-0)	0.6 (0.4-0.9)	-1.8 (-20.2-0.7)	0.5 (0.3-0.7)	-0.6 (-19.2-4.2)	0.1 (0.1-0.1)	-25.9 (-38.5--20)	0 (0-0)	-4.3 (-40.4-30.3)	0.2 (0.1-0.3)	-18.7 (-33.7--15.2)
Eritrea	0.9 (0.6-1.2)	-0.9 (-16.7-5.6)	0.7 (0.5-1.1)	-1.5 (-16.9-4.1)	0.5 (0.3-0.7)	-1.6 (-16.3-5.3)	0.1 (0.1-0.2)	-6.9 (-19.6-0.5)	0 (0-0)	-3 (-46.5-55.9)	0.3 (0.2-0.4)	-15.2 (-27.8--10)
Ethiopia	0.9 (0.6-1.2)	-1.3 (-10.6-0.9)	0.8 (0.5-1.1)	-1.8 (-10.2-0.5)	0.5 (0.3-0.7)	-1.5 (-9-2.2)	0.1 (0.1-0.2)	-7.6 (-14.3--3.4)	0 (0-0)	3.1 (-17.8-29.7)	0.3 (0.2-0.4)	-11.1 (-18.1--8.1)
Addis Ababa	0.8 (0.5-1.1)	-1.9 (-23.3-0)	0.7 (0.5-1)	-2.6 (-23-0.3)	0.5 (0.3-0.8)	-0.9 (-21.8-4.5)	0.1 (0.1-0.1)	-41.5 (-53.2--36.9)	0 (0-0)	-6.2 (-40.9-51.4)	0.2 (0.1-0.3)	-30.3 (-45.1--26.7)
Afar	0.9 (0.6-1.3)	-1.9 (-21.9-2.7)	0.8 (0.5-1.1)	-2.5 (-22.2-2.8)	0.5 (0.3-0.7)	-1.2 (-21.3-7.5)	0.1 (0.1-0.1)	-8.8 (-26.5-1.8)	0 (0-0)	8.5 (-47-133)	0.3 (0.2-0.4)	-6.9 (-25.1--0.7)
Amhara	0.8 (0.6-1.2)	-1.2 (-16.6-2.8)	0.7 (0.5-1)	-1.6 (-16.7-2.7)	0.5 (0.3-0.7)	-1.1 (-15.4-5)	0.1 (0.1-0.2)	-9.1 (-22.2--0.7)	0 (0-0)	3 (-27.6-66.2)	0.3 (0.2-0.4)	-15.3 (-27.7--11.2)
Benishangul-Gumuz	1 (0.6-1.4)	-1.8 (-20.8-3.4)	0.8 (0.5-1.2)	-2.5 (-21.7-3)	0.5 (0.3-0.7)	-1.9 (-20.3-6.7)	0.1 (0.1-0.1)	-10.4 (-25.9-0.1)	0 (0-0)	6.5 (-42.6-119)	0.3 (0.2-0.5)	-13 (-29.2--6.5)
Dire Dawa	0.8 (0.5-1.2)	-0.7 (-16.5-0.4)	0.7 (0.5-1)	-1.5 (-17.1-0.7)	0.5 (0.3-0.7)	-0.8 (-15.2-4.8)	0.1 (0.1-0.1)	-16.6 (-29.1--9.6)	0 (0-0)	2.4 (-39.8-71.8)	0.2 (0.2-0.3)	-17.4 (-29.5--13.5)
Gambella	0.9 (0.6-1.3)	-1.2 (-16.9-2.6)	0.8 (0.5-1.1)	-2 (-17.7-2.2)	0.5 (0.3-0.7)	-2.1 (-17.5-4.2)	0.1 (0.1-0.2)	-3.8 (-18.7-5.3)	0 (0-0)	33.9 (-21.7-127)	0.3 (0.2-0.4)	-18 (-30.9--13.2)
Harari	0.8 (0.5-1.2)	-0.8 (-18.8-0.4)	0.7 (0.5-1)	-1.7 (-19.3-1.4)	0.5 (0.3-0.7)	-1.4 (-19.1-7.2)	0.1 (0.1-0.1)	-12.7 (-26.8--5.5)	0 (0-0)	6.7 (-33-75.4)	0.2 (0.2-0.3)	-17.2 (-32--12)
Oromia	0.8 (0.6-1.2)	-1.9 (-20.6-0)	0.7 (0.5-1.1)	-2.4 (-20.6-0.9)	0.5 (0.3-0.7)	-2.1 (-19.2-4.4)	0.1 (0.1-0.2)	-3.6 (-20.4-4.5)	0 (0-0)	2.5 (-34.8-46.2)	0.3 (0.2-0.4)	-8.5 (-24.2--3.4)
Somali	0.8 (0.6-1.2)	-1.2 (-16.4-5.4)	0.7 (0.5-1)	-1.5 (-16.9-4.5)	0.5 (0.3-0.7)	-0.1 (-15.6-7.8)	0.1 (0.1-0.2)	-17.5 (-30.5--9.6)	0 (0-0)	5.9 (-38.4-69.4)	0.3 (0.2-0.5)	-6.9 (-21.4--0.4)
Southern Nations, Nationalities, and Peoples	0.9 (0.6-1.3)	-1.4 (-17.1-2.2)	0.8 (0.5-1.1)	-1.9 (-17.4-1.8)	0.5 (0.3-0.7)	-2 (-16.3-4.7)	0.1 (0.1-0.2)	-1.3 (-16.2-7.2)	0 (0-0)	3.2 (-35.7-57.7)	0.3 (0.2-0.5)	-11.2 (-25--6.3)
Tigray	0.8 (0.6-1.2)	-0.9 (-14.6-3)	0.7 (0.5-1)	-1.4 (-14.6-2.6)	0.5 (0.3-0.7)	-1.5 (-13.7-4.9)	0.1 (0.1-0.2)	-9.9 (-21.8--2.1)	0 (0-0)	5.3 (-36-65.8)	0.3 (0.2-0.4)	-16.2 (-26.6--12)
Kenya	1.9 (1.4-2.6)	-5.8 (-8--3.7)	1.7 (1.3-2.3)	-4.5 (-6.6--2.5)	1.2 (0.9-1.6)	-4.5 (-6.4--2.7)	0.3 (0.2-0.4)	-15.5 (-17.3--13.9)	0 (0-0)	-7.8 (-19.1-0.3)	0.6 (0.5-0.9)	-19.5 (-21.4--17.8)
Baringo	1.1 (0.8-1.6)	-0.3 (-10-9.4)	1 (0.7-1.4)	-0.7 (-10-9)	0.7 (0.5-1)	-1 (-11.1-8.8)	0.2 (0.1-0.3)	-7.7 (-17.5-1.6)	0 (0-0)	-2.1 (-35.1-35.2)	0.4 (0.3-0.6)	-15.5 (-24.1--7.4)
Bomet	1.2 (0.9-1.7)	-0.5 (-12-14)	1.1 (0.8-1.6)	-0.6 (-12.2-13.9)	0.9 (0.6-1.2)	-0.8 (-12.3-13.6)	0.2 (0.2-0.3)	-14.2 (-24.2--2.3)	0 (0-0)	8.6 (-31.6-68.8)	0.5 (0.4-0.7)	-19.2 (-28.2--7.4)
Bungoma	2.7 (1.8-3.8)	-0.9 (-7.9-10)	2.4 (1.6-3.4)	-1.3 (-7.8-9.6)	1.7 (1.2-2.3)	-1.1 (-7.7-8.3)	0.5 (0.3-0.7)	-5.1 (-12.4-4.6)	0 (0-0)	-7.4 (-42.1-41.4)	1 (0.7-1.5)	-11.4 (-17.1--3.1)
Busia	4.4 (3-6)	-1.1 (-9-8.2)	3.9 (2.7-5.4)	-1.4 (-9.3-7.9)	2.7 (1.8-3.7)	-1.4 (-8.2-6.9)	0.7 (0.4-1)	-9.2 (-16.3--1.2)	0 (0-0)	8.1 (-30.3-91.8)	1.6 (1.1-2.2)	-13.3 (-19.4--5.4)
Elgeyo-Marakwet	1.2 (0.8-1.6)	-0.6 (-8.6-8.9)	1.1 (0.8-1.5)	-1 (-9.2-8.4)	0.8 (0.6-1.1)	-1.6 (-10.1-6.9)	0.2 (0.1-0.3)	-11.6 (-19.3--3.8)	0 (0-0)	-19.6 (-44.8-25.1)	0.4 (0.3-0.6)	-20.5 (-27.1--13.3)
Embu	0.8 (0.6-1.2)	-1.1 (-18.2-8.3)	0.8 (0.5-1.1)	-1.4 (-18.7-7.9)	0.6 (0.4-0.8)	-1.3 (-18.5-7.6)	0.1 (0.1-0.2)	-20.9 (-33.5--13.3)	0 (0-0)	7.9 (-31-49.5)	0.3 (0.2-0.4)	-27.5 (-39.5--20.8)
Garissa	0.9 (0.6-1.3)	-1.2 (-18.5-1.7)	0.8 (0.5-1.2)	-1.3 (-19.6-2)	0.6 (0.4-0.8)	-1.3 (-19.7-4.5)	0.2 (0.1-0.3)	-4 (-19.7-2.9)	0 (0-0)	-4.7 (-40.1-37.9)	0.4 (0.3-0.6)	-6.9 (-23.7--2)
Homa Bay	6 (4.1-8.3)	-0.4 (-7.3-7.1)	5.3 (3.7-7.4)	-1 (-8.1-5.8)	3.5 (2.4-4.8)	-0.7 (-8.3-6.2)	0.8 (0.5-1.1)	-8.1 (-16--0.4)	0 (0-0)	7.4 (-41.1-118)	2.2 (1.5-3)	-10.2 (-16.9--4)
Isiolo	1 (0.6-1.4)	-1 (-19.4-2.1)	0.8 (0.5-1.2)	-1.4 (-19.1-1.6)	0.5 (0.4-0.8)	-1.2 (-18.9-5.1)	0.1 (0.1-0.2)	-11.5 (-22.8--4.1)	0 (0-0)	-2.4 (-35.7-48.1)	0.3 (0.2-0.4)	-16.3 (-30.6--12.1)

Location	Birth Incidence rate (per 100,000 live births), 2021	Birth Incidence rate (per 100,000 live births) percent change (2000-2021)	Under-5 rate (per 100,000 population), 2021	Under-5 rate (per 100,000 population) percent change (2000-2021)	5-14 rate (per 100,000 population), 2021	5-14 rate (per 100,000 population) percent change (2000-2021)	15-49 rate (per 100,000 population), 2021	15-49 rate (per 100,000 population) percent change (2000-2021)	50plus rate (per 100,000 population), 2021	50plus rate (per 100,000 population) percent change (2000-2021)	All age rate (per 100,000 population), 2021	All age rate (per 100,000 population) percent change (2000-2021)
Kajiado	0.8 (0.6-1.2)	-0.6 (-18.2-10)	0.8 (0.5-1.1)	-0.9 (-17.8-9.7)	0.6 (0.4-0.8)	-1.1 (-17.7-9)	0.1 (0.1-0.2)	-20.5 (-32.8--13.2)	0 (0-0)	17.9 (-25.7-84)	0.3 (0.2-0.4)	-22.5 (-35.4--15.2)
Kakamega	2.6 (1.8-3.7)	-0.6 (-8.1-8.9)	2.4 (1.6-3.3)	-0.7 (-8-8.7)	1.6 (1.1-2.2)	-0.7 (-8.7-8.6)	0.4 (0.3-0.6)	-14.5 (-22.4--6.7)	0 (0-0)	11.9 (-28.2-76)	0.9 (0.6-1.3)	-17.9 (-24.4--11)
Kericho	1.3 (0.9-1.8)	-0.9 (-10.7-7.5)	1.2 (0.8-1.7)	-1.3 (-11.1-7.3)	0.9 (0.6-1.3)	-1.1 (-10.1-7.6)	0.2 (0.2-0.3)	-19.5 (-26.9--10.3)	0 (0-0)	-11.8 (-38.7-28.1)	0.5 (0.3-0.7)	-22.4 (-29.8--15.8)
Kiambu	0.8 (0.6-1.2)	-0.9 (-17.6-9.5)	0.8 (0.5-1.1)	-1.3 (-17.7-9.2)	0.6 (0.4-0.8)	-1.1 (-17.5-7.8)	0.1 (0.1-0.2)	-26.1 (-37.3--18.6)	0 (0-0)	8.7 (-29.5-71.9)	0.3 (0.2-0.4)	-25.8 (-37.7--19.3)
Kilifi	3.3 (2.2-4.7)	-0.8 (-9.9-7.6)	3 (2-4.1)	-1.1 (-9.9-6.6)	2.1 (1.4-3)	-1.5 (-8.8-6.7)	0.6 (0.4-0.8)	-4.6 (-11.7-3.5)	0 (0-0)	0.2 (-30.5-57.8)	1.2 (0.9-1.7)	-12.6 (-19.3--5)
Kirinyaga	0.8 (0.6-1.2)	-1.3 (-18.2-5.7)	0.8 (0.5-1.1)	-1.7 (-18.4-4.8)	0.6 (0.4-0.8)	-1.4 (-18.3-4)	0.1 (0.1-0.2)	-25.6 (-37.4--20.1)	0 (0-0)	12.9 (-27.6-78.5)	0.2 (0.2-0.3)	-30.3 (-42--26.5)
Kisii	3 (2.1-4.2)	-0.7 (-8.2-6.7)	2.7 (1.9-3.8)	-1.2 (-8.8-5.9)	1.9 (1.3-2.7)	-1.2 (-9.7-6.4)	0.5 (0.3-0.7)	-13.1 (-21.4--5.7)	0 (0-0)	-18.4 (-47.6-34.4)	1 (0.7-1.4)	-24.5 (-30.8--18.5)
Kisumu	3.4 (2.3-4.7)	-0.9 (-8.5-6.3)	3 (2-4.1)	-1.4 (-8.9-5.5)	2 (1.4-2.8)	-1.4 (-8.8-6.6)	0.4 (0.3-0.6)	-14.2 (-21.6--6.2)	0 (0-0)	0.1 (-45.2-101)	1.1 (0.7-1.5)	-18.9 (-24.9--12.3)
Kitui	1 (0.6-1.4)	-1 (-18.8-10.8)	0.9 (0.6-1.2)	-1.3 (-19.1-10)	0.6 (0.4-0.9)	-1.5 (-18.7-8.8)	0.2 (0.1-0.3)	-9.9 (-24.6-0.2)	0 (0-0)	8.9 (-36.7-77.9)	0.4 (0.2-0.5)	-18.4 (-32.7--9.4)
Kwale	3.3 (2.3-4.6)	-0.8 (-8.2-6.7)	3 (2-4.1)	-1 (-8-6.4)	2.1 (1.4-2.9)	-1.3 (-9.2-7.7)	0.5 (0.3-0.8)	-5.6 (-13.3-3.2)	0 (0-0)	-6.5 (-36.8-41.9)	1.2 (0.8-1.7)	-12.7 (-19.3--5.5)
Laikipia	0.8 (0.6-1.2)	-1 (-17.6-0.1)	0.8 (0.5-1.1)	-1.3 (-17.4-0.7)	0.6 (0.4-0.9)	-1.6 (-17.2-2.6)	0.1 (0.1-0.2)	-17.1 (-28.6--12.2)	0 (0-0)	5.7 (-30.6-64.4)	0.3 (0.2-0.4)	-24 (-35.8--21.3)
Lamu	1 (0.7-1.4)	-0.9 (-13.7-3)	0.9 (0.6-1.3)	-1.2 (-13.6-3.1)	0.6 (0.4-0.9)	-1.4 (-14.3-4)	0.2 (0.1-0.2)	-11.1 (-21.7--4.9)	0 (0-0)	4.5 (-33.1-54.6)	0.3 (0.2-0.5)	-18.4 (-29.2--14.3)
Machakos	0.9 (0.6-1.2)	-1 (-17.1-7)	0.8 (0.5-1.1)	-1.4 (-17.1-6.6)	0.6 (0.4-0.8)	-1.3 (-16.7-5.6)	0.1 (0.1-0.2)	-26.6 (-38.1--19.9)	0 (0-0)	-1.4 (-33.9-53.4)	0.3 (0.2-0.4)	-29.6 (-40.6--24.4)
Makueni	0.9 (0.6-1.3)	-0.5 (-9.6-0.2)	0.8 (0.5-1.2)	-0.7 (-9.6-1.2)	0.6 (0.4-0.9)	-0.8 (-9.7-2.7)	0.2 (0.1-0.2)	-13 (-21.2--8.1)	0 (0-0)	20.3 (-26.5-106)	0.3 (0.2-0.5)	-21.3 (-28.6--18.6)
Mandera	0.9 (0.6-1.2)	-0.5 (-14.1-9.7)	0.8 (0.5-1.1)	-0.1 (-13.7-10.3)	0.5 (0.4-0.7)	-1.1 (-15.2-7.6)	0.2 (0.1-0.2)	10.2 (-5.8-21)	0 (0-0)	5.3 (-34.4-66.1)	0.4 (0.2-0.5)	-9.5 (-22.5--2)
Marsabit	0.9 (0.6-1.3)	-0.8 (-14.3-0.7)	0.8 (0.5-1.2)	-0.8 (-14.3-1.3)	0.6 (0.4-0.8)	-1.1 (-14.3-3.2)	0.2 (0.1-0.2)	-3.8 (-16.2-3.1)	0 (0-0)	13.5 (-29.4-63.4)	0.4 (0.2-0.5)	-11.8 (-23.5--8.3)
Meru	0.9 (0.6-1.2)	-0.5 (-15-10.3)	0.8 (0.5-1.1)	-0.9 (-15.1-10.9)	0.6 (0.4-0.8)	-1.1 (-15.3-10.8)	0.1 (0.1-0.2)	-19 (-29.7--11.5)	0 (0-0)	-0.3 (-39-47.4)	0.3 (0.2-0.4)	-26.4 (-36.2--18.9)
Migori	5.9 (4-8.2)	-0.6 (-8-8.2)	5.2 (3.5-7.2)	-1 (-8.3-7.3)	3.3 (2.3-4.5)	-0.8 (-7.6-6.6)	0.8 (0.5-1.1)	-3.7 (-10.9-5.2)	0 (0-0)	16.8 (-34-110)	2.2 (1.5-3)	-6.2 (-12.9-1.3)
Mombasa	2.1 (1.5-3)	-0.5 (-7.9-9)	2 (1.4-2.7)	-0.9 (-8.7-8.6)	1.4 (1-2)	-0.7 (-8.3-8)	0.3 (0.2-0.4)	-18.4 (-25.4--11.5)	0 (0-0)	-5.1 (-38.9-57.2)	0.7 (0.5-0.9)	-13.3 (-20--6)
Murang'a	0.8 (0.6-1.2)	-0.5 (-16.5-5.4)	0.8 (0.5-1.1)	-0.7 (-16.5-4.8)	0.6 (0.4-0.8)	-0.8 (-15-3.9)	0.1 (0.1-0.2)	-24.3 (-34.3--18.7)	0 (0-0)	11.9 (-29.5-73)	0.2 (0.2-0.3)	-34 (-43.5--30.9)
Nairobi	0.9 (0.6-1.3)	-0.6 (-18.9-10.7)	0.8 (0.5-1.2)	-1.1 (-19.1-10)	0.6 (0.4-0.9)	-0.7 (-17.6-10.6)	0.1 (0.1-0.2)	-23.7 (-35.9--15.4)	0 (0-0)	-9.3 (-42.3-40.8)	0.3 (0.2-0.4)	-16.5 (-30.8--6.8)
Nakuru	0.9 (0.6-1.2)	-0.7 (-18.9-7.7)	0.8 (0.5-1.1)	-1 (-19.1-7.5)	0.6 (0.4-0.9)	-1.2 (-17.9-8.5)	0.2 (0.1-0.2)	-17.2 (-31.6--5.6)	0 (0-0)	3 (-33.7-62.3)	0.3 (0.2-0.4)	-23.6 (-36.3--16.2)
Nandi	1.5 (1-2)	-1.3 (-8.4-6.6)	1.4 (0.9-1.9)	-1.5 (-8.7-6.7)	1 (0.7-1.4)	-1.6 (-10.1-6.6)	0.3 (0.2-0.4)	-13.5 (-21.4--5.7)	0 (0-0)	10.4 (-29.3-81.6)	0.6 (0.4-0.8)	-20.4 (-27--14)
Narok	1.2 (0.8-1.6)	-0.9 (-12.1-8.1)	1.1 (0.8-1.5)	-0.9 (-12.1-8.3)	0.8 (0.6-1.1)	-1.2 (-11.3-7.3)	0.2 (0.1-0.3)	-10 (-19.8--2)	0 (0-0)	30 (-17.2-127)	0.5 (0.4-0.7)	-14.9 (-23.7--7.2)
Nyamira	2.1 (1.4-2.9)	-1.2 (-8.2-5.4)	1.9 (1.3-2.7)	-1.4 (-8.6-5.1)	1.5 (1-2.1)	-1.3 (-9-5.6)	0.4 (0.2-0.5)	-12.7 (-19.6--5.8)	0 (0-0)	27.1 (-26.8-117)	0.8 (0.5-1.1)	-19.1 (-25.4--13.3)
Nyandarua	0.8 (0.6-1.2)	-1.4 (-18.6-2)	0.8 (0.5-1.1)	-1.5 (-18.7-1.9)	0.6 (0.4-0.8)	-2.2 (-19.4-2.1)	0.1 (0.1-0.2)	-13.6 (-27.4--7.7)	0 (0-0)	12 (-34.2-82.5)	0.3 (0.2-0.4)	-27.2 (-39.6--23.9)
Nyeri	0.8 (0.5-1.1)	-0.5 (-13.5-0)	0.7 (0.5-1.1)	-0.7 (-13.4-0.7)	0.6 (0.4-0.8)	-1 (-12.7-3)	0.1 (0.1-0.2)	-23.4 (-31.8--18.2)	0 (0-0)	17.5 (-21.2-83.7)	0.2 (0.1-0.3)	-32.5 (-40.5--29.9)
Samburu	0.9 (0.6-1.3)	-1 (-18.8-7.5)	0.9 (0.6-1.2)	-1.1 (-18.2-7.5)	0.7 (0.4-0.9)	-1.4 (-17.8-6.6)	0.2 (0.1-0.2)	-6.6 (-18.2-2.1)	0 (0-0)	13.3 (-26.4-89.6)	0.4 (0.3-0.6)	-13 (-26.4--5.7)
Siaya	5.5 (3.8-7.7)	-1.1 (-12.2-6.2)	4.8 (3.4-6.7)	-1.6 (-11.7-5.3)	3.2 (2.2-4.4)	-0.9 (-9.9-6.8)	0.8 (0.5-1.1)	-11.9 (-20.2--4.2)	0 (0-0)	-4.9 (-44.2-72.1)	1.8 (1.3-2.5)	-15 (-22.6--8.3)

Location	Birth Incidence rate (per 100,000 live births), 2021	Birth Incidence rate (per 100,000 live births) percent change (2000-2021)	Under-5 rate (per 100,000 population), 2021	Under-5 rate (per 100,000 population) percent change (2000-2021)	5-14 rate (per 100,000 population), 2021	5-14 rate (per 100,000 population) percent change (2000-2021)	15-49 rate (per 100,000 population), 2021	15-49 rate (per 100,000 population) percent change (2000-2021)	50plus rate (per 100,000 population), 2021	50plus rate (per 100,000 population) percent change (2000-2021)	All age rate (per 100,000 population), 2021	All age rate (per 100,000 population) percent change (2000-2021)
Taita Taveta	1.2 (0.8-1.6)	-1.3 (-14.3-8.8)	1.1 (0.8-1.5)	-1.6 (-14.4-8.5)	0.8 (0.6-1.1)	-1.7 (-14.9-8.8)	0.2 (0.1-0.2)	-23.3 (-33.5--14.2)	0 (0-0)	6.8 (-36.2-78.1)	0.3 (0.2-0.5)	-27.3 (-37--19.2)
Tana River	1.3 (0.9-1.8)	-2.2 (-17.4-5.9)	1.1 (0.8-1.6)	-2.4 (-17.6-6)	0.8 (0.5-1.1)	-2.5 (-17.8-5.5)	0.2 (0.1-0.3)	-0.1 (-14.5-9.2)	0 (0-0)	3.2 (-41.3-70.9)	0.5 (0.4-0.7)	-6.8 (-20.6-0.7)
Tharaka Nithi	0.9 (0.6-1.3)	-0.4 (-15.2-13)	0.8 (0.5-1.1)	-0.8 (-15.4-11.7)	0.6 (0.4-0.8)	-1 (-14.7-8.8)	0.1 (0.1-0.2)	-6.8 (-18.3-5.1)	0 (0-0)	42.1 (-23.2-125)	0.3 (0.2-0.4)	-16.5 (-28.1--7.5)
Trans Nzoia	1.7 (1.2-2.4)	-0.7 (-7.5-8.5)	1.6 (1.1-2.2)	-1 (-7.9-8.2)	1.2 (0.8-1.6)	-1.4 (-8.8-7.9)	0.3 (0.2-0.4)	-11.1 (-17.8--2.2)	0 (0-0)	-0.5 (-38.4-62)	0.6 (0.4-0.9)	-20.3 (-26.3--12.7)
Turkana	1.3 (0.9-1.8)	-0.4 (-7.4-8.8)	1.1 (0.8-1.6)	-0.6 (-7.6-8.3)	0.8 (0.6-1.1)	-0.1 (-7.8-9.1)	0.2 (0.1-0.3)	-2.9 (-10.8-7.3)	0 (0-0)	-8.6 (-49.1-54.1)	0.5 (0.4-0.7)	-8.8 (-15.5--0.2)
Uasin Gishu	1.3 (0.9-1.8)	-0.5 (-8.6-7)	1.2 (0.8-1.6)	-0.9 (-9-6.6)	0.9 (0.6-1.2)	-1.1 (-8.7-6)	0.2 (0.1-0.3)	-18.5 (-24.8--11.9)	0 (0-0)	7.5 (-24.2-59.8)	0.5 (0.3-0.6)	-21.8 (-27.8--16.2)
Vihiga	3.2 (2.2-4.5)	-1.2 (-8.7-7.9)	2.9 (2-4)	-1.3 (-9.4-7.9)	2 (1.4-2.8)	-1.4 (-10.3-7.2)	0.6 (0.4-0.8)	1 (-7.2-9.4)	0 (0-0)	12.4 (-29-63.3)	1.1 (0.8-1.5)	-16.9 (-24--9.4)
Wajir	0.9 (0.6-1.3)	-0.8 (-16.5-0)	0.8 (0.5-1.2)	-0.9 (-16.3-1.2)	0.6 (0.4-0.8)	-1.5 (-17.7-2.8)	0.2 (0.1-0.2)	5.1 (-12-12.2)	0 (0-0)	-4.4 (-36.6-43.3)	0.4 (0.3-0.5)	-4.7 (-19.9--1.1)
West Pokot	1.5 (1-2.1)	-0.8 (-8.8-7.4)	1.4 (0.9-1.9)	-1 (-9.2-7.3)	1 (0.6-1.4)	-1.5 (-9.7-6.8)	0.3 (0.2-0.4)	2.2 (-6.8-11.8)	0 (0-0)	2.8 (-29.2-52.3)	0.6 (0.4-0.9)	-9 (-16.2--1)
Madagascar	2.2 (1.5-3.1)	-2.8 (-12.2-7.3)	1.9 (1.3-2.7)	-3.2 (-12-6.2)	1.3 (0.9-1.8)	-3 (-10.5-5.7)	0.3 (0.2-0.4)	-4.9 (-13.3-3.8)	0 (0-0)	5.8 (-33.3-56.1)	0.8 (0.5-1.1)	-12.9 (-19.9--4.8)
Malawi	1.7 (1.1-2.5)	0.1 (-9.1-8.1)	1.4 (1-2.1)	-1 (-10-7.1)	0.9 (0.6-1.3)	-0.7 (-8.9-7.2)	0.2 (0.1-0.3)	3.9 (-4.6-12.8)	0 (0-0)	2.1 (-41-73)	0.6 (0.4-0.8)	-8.8 (-16.2--1.5)
Mozambique	1.4 (1-2)	1.4 (-6.5-13.5)	1.2 (0.8-1.7)	0.5 (-7-12.2)	0.8 (0.5-1.1)	0.5 (-8.1-11.8)	0.2 (0.1-0.3)	7.9 (-1.7-19.6)	0 (0-0)	1.9 (-41.1-72.2)	0.5 (0.4-0.7)	0.5 (-7.7-11.6)
Rwanda	0.6 (0.4-0.9)	0.4 (-16.3-25.1)	0.5 (0.3-0.8)	-0.1 (-16.5-25.6)	0.4 (0.2-0.5)	0.4 (-15.4-24.3)	0.1 (0.1-0.1)	-14.4 (-28.9-5.2)	0 (0-0)	-6 (-36.7-39.3)	0.2 (0.1-0.3)	-17.2 (-31.2-3.5)
Somalia	0.9 (0.6-1.2)	-0.8 (-20.3-14.4)	0.7 (0.5-1)	-1.5 (-20.5-13.1)	0.5 (0.3-0.7)	-1.6 (-19.7-10.8)	0.1 (0.1-0.2)	-8.1 (-23.5-3)	0 (0-0)	-28.5 (-60.5-20.8)	0.3 (0.2-0.4)	-3.4 (-21.5-9)
South Sudan	1.4 (1-2)	1.6 (-7.2-10.5)	1.2 (0.9-1.7)	1.4 (-6.9-10)	0.8 (0.5-1.1)	0.5 (-7.5-9.5)	0.2 (0.1-0.3)	10.8 (1.2-21.8)	0 (0-0)	13.1 (-30.1-81.8)	0.5 (0.3-0.7)	-1.7 (-9.8-6.7)
Uganda	4.6 (3.1-6.5)	-0.6 (-8.1-9.8)	3.9 (2.7-5.5)	-1.3 (-7.9-8.9)	2.7 (1.8-3.7)	-1.1 (-8.2-8)	0.7 (0.5-1)	-2.5 (-9.4-6.2)	0 (0-0)	13 (-33.6-85.2)	1.8 (1.2-2.5)	-9.2 (-15.4--1)
United Republic of Tanzania	2.6 (1.8-3.6)	1.7 (-6.5-12.2)	2.2 (1.5-3.1)	1.2 (-6.6-10.8)	1.5 (1-2.1)	1.7 (-5.7-11.3)	0.4 (0.3-0.5)	-2.9 (-11.8-5.8)	0 (0-0)	14.5 (-29.6-91.5)	0.9 (0.7-1.3)	-4.5 (-11.5-4.2)
Zambia	6.1 (4.2-8.7)	-0.3 (-7.6-8.8)	5.2 (3.6-7.4)	-0.9 (-8.4-7.4)	3.6 (2.4-4.9)	-0.8 (-8.6-7.9)	0.8 (0.5-1.2)	-9.6 (-17.3--1.8)	0 (0-0)	9.4 (-37.2-87.1)	2.1 (1.5-3)	-12.4 (-19.2--5.8)
**Southern Sub-Saharan Africa**	0.9 (0.7-1.2)	5.3 (0.3-11)	0.8 (0.6-1)	4.3 (-0.6-9.5)	0.6 (0.4-0.7)	8.5 (3.3-14)	0.1 (0.1-0.1)	-15.1 (-19.3--10.8)	0 (0-0)	11.3 (-8.1-51.9)	0.2 (0.2-0.3)	-11.3 (-15.4--6.8)
Botswana	4.1 (2.8-5.7)	1.3 (-5.3-12)	3.6 (2.5-5)	1 (-5.7-11.6)	2.7 (1.9-3.7)	1.6 (-5.6-11.5)	0.5 (0.3-0.7)	-27.8 (-33.6--21.2)	0 (0-0)	5.6 (-45-90.9)	1.2 (0.8-1.6)	-22 (-27.2--15)
Eswatini	1.4 (1-1.9)	0.7 (-8.9-9.1)	1.2 (0.8-1.6)	0.2 (-8.6-8.9)	0.8 (0.6-1.2)	0.7 (-8.9-9.4)	0.2 (0.1-0.3)	-19.4 (-27.5--11.5)	0 (0-0)	-5.8 (-50.4-101)	0.4 (0.3-0.6)	-17.7 (-25.5--10.6)
Lesotho	1.2 (0.9-1.7)	0.4 (-11.8-13.9)	1 (0.8-1.4)	0 (-12.9-13)	0.7 (0.5-1)	0.4 (-11.9-13.5)	0.1 (0.1-0.2)	-20.9 (-32.1--9.8)	0 (0-0)	5.2 (-43.7-93.4)	0.3 (0.2-0.5)	-17.3 (-27.8--6.2)
Namibia	1.4 (1-1.9)	-1.2 (-11.1-5.8)	1.2 (0.8-1.7)	-1.5 (-12.2-5.9)	0.9 (0.6-1.2)	-1.4 (-12.8-6.2)	0.2 (0.1-0.3)	-16.7 (-26.1--9.6)	0 (0-0)	12.2 (-25.3-87.5)	0.5 (0.3-0.6)	-14.7 (-24.4--8.2)
South Africa	0.3 (0.2-0.4)	-0.9 (-8.6-5.8)	0.3 (0.2-0.4)	-1.4 (-9.4-5.6)	0.2 (0.1-0.3)	-0.4 (-8.9-5.9)	0 (0-0)	-23.4 (-28.6--18.4)	0 (0-0)	14.3 (-12.7-74)	0.1 (0.1-0.1)	-20.6 (-26.9--15.4)
Eastern Cape	0.3 (0.2-0.5)	1.2 (-14.6-35.9)	0.3 (0.2-0.4)	0.9 (-14.8-34.8)	0.2 (0.1-0.3)	1.5 (-15.1-35.1)	0 (0-0.1)	-19.9 (-33.1-5.1)	0 (0-0)	10.4 (-48-166)	0.1 (0.1-0.1)	-21.3 (-33.9-4.3)
Free State	0.3 (0.2-0.5)	0.6 (-17.2-31.5)	0.3 (0.2-0.4)	0.2 (-18-30.9)	0.2 (0.1-0.3)	1 (-17.7-29.6)	0 (0-0.1)	-22.2 (-36.7-0)	0 (0-0)	-0.7 (-52.9-113)	0.1 (0.1-0.1)	-18.7 (-33.9-4.6)
Gauteng	0.3 (0.2-0.5)	0.8 (-12-30.6)	0.3 (0.2-0.4)	0.2 (-12.1-30)	0.2 (0.1-0.3)	1.4 (-10.9-32)	0 (0-0)	-20.4 (-31-4.7)	0 (0-0)	-6.1 (-44-94.9)	0.1 (0-0.1)	-9.7 (-20.7-17.8)
KwaZulu-Natal	0.3 (0.2-0.5)	1.2 (-11-32.6)	0.3 (0.2-0.4)	0.8 (-11.1-32.5)	0.2 (0.1-0.3)	1.3 (-9.8-32.7)	0 (0-0.1)	-21.5 (-30.9-2.2)	0 (0-0)	-2.5 (-50.7-93.3)	0.1 (0.1-0.1)	-18.8 (-27.8-6.4)
Limpopo	0.4 (0.2-0.5)	0.5 (-15.6-22.9)	0.3 (0.2-0.5)	0.2 (-15.6-22.6)	0.2 (0.2-0.3)	0.4 (-13.4-21.7)	0.1 (0-0.1)	-24.9 (-36--9.1)	0 (0-0)	12 (-31.3-93.3)	0.1 (0.1-0.2)	-22 (-33.6--4.9)

Location	Birth Incidence rate (per 100,000 live births), 2021	Birth Incidence rate (per 100,000 live births) percent change (2000-2021)	Under-5 rate (per 100,000 population), 2021	Under-5 rate (per 100,000 population) percent change (2000-2021)	5-14 rate (per 100,000 population), 2021	5-14 rate (per 100,000 population) percent change (2000-2021)	15-49 rate (per 100,000 population), 2021	15-49 rate (per 100,000 population) percent change (2000-2021)	50plus rate (per 100,000 population), 2021	50plus rate (per 100,000 population) percent change (2000-2021)	All age rate (per 100,000 population), 2021	All age rate (per 100,000 population) percent change (2000-2021)
Mpumalanga	0.3 (0.2-0.5)	-0.7 (-21.1-16.5)	0.3 (0.2-0.4)	-0.9 (-21.3-16.8)	0.2 (0.1-0.3)	-0.2 (-20.8-15.8)	0 (0-0.1)	-22.8 (-38--8.4)	0 (0-0)	3.2 (-44.1-89.2)	0.1 (0.1-0.1)	-20.7 (-37--7.1)
North-West	0.3 (0.2-0.5)	1.1 (-17.2-38)	0.3 (0.2-0.4)	0.6 (-17.5-36.9)	0.2 (0.1-0.3)	1.4 (-16.4-37.3)	0 (0-0.1)	-18.6 (-33.5-7.1)	0 (0-0)	-7.3 (-47.1-80)	0.1 (0.1-0.1)	-13.2 (-29-17.7)
Northern Cape	0.3 (0.2-0.5)	0.9 (-15.3-30.4)	0.3 (0.2-0.4)	0.7 (-16-30.3)	0.2 (0.1-0.3)	1.1 (-16.4-30.9)	0 (0-0.1)	-11.8 (-27-14.1)	0 (0-0)	2.3 (-40.7-84.3)	0.1 (0.1-0.1)	-15.3 (-29.5-9.4)
Western Cape	0.1 (0-0.2)	0 (0-0)	0 (0-0.2)	-0.2 (-1.1-0.5)	0 (0-0.1)	0.5 (-1.5-2.6)	0 (0-0)	-22.1 (-25.6--18.3)	0 (0-0)	-4.2 (-33-53.6)	0 (0-0)	-22.7 (-24.2--20.9)
Zimbabwe	1.8 (1.2-2.6)	2 (-5.6-10.8)	1.6 (1.1-2.2)	1.4 (-6.1-10.5)	1.1 (0.8-1.6)	2.2 (-5.5-11.6)	0.3 (0.2-0.4)	-13.1 (-19.9--5.1)	0 (0-0)	6.6 (-40.5-80.7)	0.6 (0.4-0.9)	-6.7 (-13.6-1.8)
**Western Sub-Saharan Africa**	564 (425-735)	-20.1 (-23.9--16.7)	527 (396-682)	-20.9 (-24.9--17.3)	425 (321-546)	-22.3 (-26.7--18.5)	130 (96.2-170)	-20.8 (-24.9--17.2)	0.2 (0.1-0.4)	15.8 (3.6-27.1)	260 (199-332)	-23.9 (-28--20.3)
Benin	1,630 (1,230-2,070)	-24.8 (-35.4--13.2)	1,510 (1,130-1,930)	-25.2 (-35.6--13.6)	1,240 (923-1,580)	-26.8 (-37.1--15.6)	385 (281-498)	-32.9 (-43.9--22.2)	1 (0.6-1.5)	-17.4 (-39.8-12.1)	784 (587-996)	-30.8 (-40.7--20.3)
Burkina Faso	1,070 (819-1,380)	-36.4 (-49.6--20.4)	988 (757-1,270)	-36.7 (-49.8--20.4)	794 (594-1,030)	-36.4 (-50.5--20.1)	234 (174-307)	-33.4 (-48.7--16.1)	0.4 (0.2-0.7)	15.6 (-24.5-68.4)	501 (382-643)	-38.4 (-51.9--22.7)
Cabo Verde	48.7 (34.1-66.6)	-1.7 (-11-8.1)	46.1 (32.2-63)	-1.7 (-11.5-7.8)	40.9 (28.6-55.5)	-1.7 (-11.4-7.7)	10.1 (7.1-13.7)	-28.9 (-36--21.5)	0 (0-0)	62.6 (19.7-135)	17.1 (12-23.2)	-30.7 (-37.3--23.3)
Cameroon	25.4 (20.2-33.1)	2.8 (-4.8-10.9)	23.5 (18.6-30.6)	2.4 (-4.9-10.5)	19.1 (14.7-24.8)	2.6 (-4.9-10.7)	5.5 (4.1-7.3)	-3.3 (-10.2-4.4)	0 (0-0)	10.1 (-20.8-67)	11.1 (8.7-14.3)	-5.8 (-12.7-1.7)
Chad	11.8 (8.6-15.7)	-3 (-10-6.5)	10.8 (8-14.6)	-3.1 (-10.2-6.5)	8.6 (6.3-11.5)	-3.2 (-10.3-5.8)	2.7 (1.9-3.6)	3.2 (-4.3-12.5)	0 (0-0)	8 (-24.8-68.9)	5.9 (4.4-7.9)	-1.2 (-8.4-8.1)
Côte d'Ivoire	204 (160-248)	-1.4 (-10.6-7.2)	189 (146-226)	-1.7 (-10.4-6.9)	154 (116-186)	-1.4 (-10.3-7.4)	39.6 (29.7-49.6)	-12.4 (-20.6--4.4)	0 (0-0.1)	-1.1 (-22.9-28.5)	87.1 (67-105)	-9.7 (-18--2.2)
Gambia	108 (81.3-141)	-20.4 (-29.9--11.7)	100 (75.7-132)	-20.8 (-30.1--11.7)	83.2 (61.8-109)	-20.9 (-30.2--11.6)	25.4 (18.7-33.7)	-20.2 (-29.4--10.9)	0 (0-0.1)	6.2 (-20.4-40.6)	48.8 (36.7-63.5)	-26.3 (-34.7--17.8)
Ghana	818 (666-975)	-2.3 (-19.6-15.3)	760 (613-911)	-3.3 (-20.6-14.5)	629 (504-756)	-5.4 (-22.1-11.7)	165 (130-205)	-14.8 (-29-0)	0.2 (0.1-0.4)	-1.5 (-27.8-32.9)	324 (263-388)	-17 (-31.5--2.3)
Guinea	319 (220-436)	1 (-8.5-12.5)	292 (203-397)	0.7 (-8.3-11.9)	231 (161-317)	0.7 (-8.8-12.6)	65.7 (45.6-91.4)	1.1 (-9.2-13.3)	0.1 (0-0.1)	13.5 (-10.7-46)	144 (102-197)	-0.4 (-9.8-11.3)
Guinea-Bissau	14 (9.7-19.6)	-1.5 (-9.5-6.9)	12.9 (9-18)	-1.8 (-9.9-6.4)	10.3 (7.2-14.1)	-1.7 (-9.7-6.4)	2.7 (1.9-3.9)	-11.8 (-18.9--4.3)	0 (0-0)	2.7 (-39.1-60.7)	6 (4.2-8.3)	-11 (-18.1--3.8)
Liberia	110 (77.3-152)	0.8 (-7.8-10.4)	101 (71.1-139)	0.4 (-8-10.1)	80.5 (56.9-111)	0 (-8.6-9.9)	21.8 (15.3-30.3)	-11.1 (-19.3--2.3)	0 (0-0)	27.6 (-4.2-69.2)	44.6 (31.7-61)	-12 (-19.5--3.3)
Mali	311 (242-375)	-1.5 (-9.8-8.5)	285 (219-343)	-1.6 (-9.8-8.2)	223 (169-273)	-1.6 (-10-8.3)	68.7 (51.5-86.9)	4.8 (-4.5-16)	0.1 (0-0.1)	5.1 (-20.7-37.9)	146 (112-177)	-1.1 (-9.1-8.9)
Mauritania	26.7 (20-35)	-1 (-9.5-8.9)	24.9 (18.6-32.4)	-1.3 (-9.8-8.4)	20.9 (15.7-27.4)	-1.6 (-10.2-7.9)	6.4 (4.8-8.3)	1 (-8.2-10.2)	0 (0-0)	5.3 (-23.3-41)	11.8 (8.9-15.4)	-8.8 (-16.9--0.1)
Niger	556 (415-715)	-36.2 (-44.4--27.4)	513 (386-657)	-36.8 (-44.8--28.2)	409 (307-525)	-38 (-46--29.7)	139 (103-181)	-32.4 (-41.5--23.3)	0.3 (0.1-0.4)	-30.3 (-48.4--7.4)	289 (219-370)	-35.5 (-44--26.8)
Nigeria	665 (474-916)	-20.6 (-23.3--17.6)	629 (452-861)	-21.6 (-24.4--18.8)	507 (369-683)	-23.9 (-26.4--21.3)	163 (116-219)	-19.6 (-22.7--16.7)	0.3 (0.2-0.5)	30.8 (20.9-41.8)	314 (229-419)	-23.4 (-25.9--20.7)
Sao Tome and Principe	80.1 (57.8-106)	-1.8 (-10.8-8.1)	74.7 (54.2-99.2)	-2.1 (-10.9-7.1)	62.9 (45.4-82.8)	-2.2 (-11.2-7.2)	18 (12.8-24)	-18.8 (-26.4--11)	0 (0-0)	35.4 (3.8-75)	32.7 (23.6-43)	-19.6 (-26.8--11.8)
Senegal	79.6 (60.4-98.8)	-0.1 (-7.9-7.8)	73.7 (55.5-91.4)	-0.2 (-8.1-7.7)	60.7 (46.2-75.6)	0.1 (-7.9-8.2)	18 (13.6-22.8)	-3.5 (-11.4-4.3)	0 (0-0)	0.4 (-26.1-35.6)	34.7 (26.6-42.9)	-7.5 (-15.1--0.3)
Sierra Leone	360 (280-443)	-1.9 (-10.9-7)	330 (257-403)	-2.3 (-11.3-6.7)	259 (197-320)	-2.3 (-11.1-6.8)	72.2 (54.3-92)	0.7 (-8.4-10.5)	0.1 (0-0.1)	9.6 (-15.8-39.6)	149 (116-184)	-8.6 (-16.6--0.2)
Togo	898 (710-1,140)	-5.4 (-13.3-3)	832 (659-1,050)	-5.5 (-13.7-2.9)	679 (529-861)	-5.4 (-13.2-3.3)	179 (136-233)	-13.4 (-21.1--4.7)	0.2 (0.1-0.4)	1.1 (-21.7-27.2)	374 (294-470)	-16.5 (-23.6--8.9)

Table S16: Mild sickle cell/beta-thalassemia disease frequency: Birth rate, age-specific and all-age prevalence rates , 2000 - 2021

Location	Birth Incidence rate (per 100,000 live births), 2021	Birth Incidence rate (per 100,000 live births) percent change (2000-2021)	Under-5 rate (per 100,000 population), 2021	Under-5 rate (per 100,000 population) percent change (2000-2021)	5-14 rate (per 100,000 population), 2021	5-14 rate (per 100,000 population) percent change (2000-2021)	15-49 rate (per 100,000 population), 2021	15-49 rate (per 100,000 population) percent change (2000-2021)	50plus rate (per 100,000 population), 2021	50plus rate (per 100,000 population) percent change (2000-2021)	All age rate (per 100,000 population), 2021	All age rate (per 100,000 population) percent change (2000-2021)
Global	14.7 (10.6-21)	12.6 (10.6-14.9)	9.3 (6.6-12.9)	13.9 (11.9-16)	2.6 (1.8-3.5)	23.1 (19.4-26.3)	0.5 (0.4-0.7)	11.7 (8.1-15.2)	0 (0-0)	-8.9 (-11.4--6.5)	1.5 (1.1-2)	-1.5 (-3.5-0.8)
Central Europe, Eastern Europe, and Central Asia	0.4 (0.3-0.6)	-42.6 (-50.1--34.8)	0.3 (0.2-0.4)	-46.4 (-53.2--39.7)	0.1 (0-0.1)	-41.2 (-48.2--33)	0 (0-0)	-47.5 (-54--39.4)	0 (0-0)	-15.6 (-38.6-11.4)	0 (0-0)	-44.2 (-50.2--38.2)
**Central Asia**	0 (0-0)	-0.2 (-0.4-0)	0 (0-0)	-0.3 (-3-1.7)	0 (0-0)	3.6 (-5-10.6)	0 (0-0)	-22.1 (-32.6--12.7)	0 (0-0)	-5.8 (-32.7-25.4)	0 (0-0)	-7.8 (-12.7--4.3)
Armenia	0 (0-0)	-1.4 (0-0)	0 (0-0)	-1.9 (-5.2-3.6)	0 (0-0)	4.9 (-12-22.6)	0 (0-0)	-22 (-46.2-3.9)	0 (0-0)	-10.7 (-62.7-75.7)	0 (0-0)	-13 (-21.7--3.4)
Azerbaijan	0 (0-0)	0 (0-0)	0 (0-0)	0.9 (-3.3-5)	0 (0-0)	1.7 (-12-17.4)	0 (0-0)	-18.5 (-39.8-9.5)	0 (0-0)	33.9 (-33.6-162)	0 (0-0)	-22.1 (-27.9--16.4)
Georgia	0 (0-0)	-0.1 (0-0)	0 (0-0)	0.4 (-2.9-3.7)	0 (0-0)	4.6 (-7.9-19.9)	0 (0-0)	-16.4 (-35.9-11.4)	0 (0-0)	1.7 (-49.8-92.2)	0 (0-0)	3 (-5.3-13.2)
Kazakhstan	0 (0-0)	-0.3 (0-0)	0 (0-0)	-1 (-4.6-3.2)	0 (0-0)	7.9 (-5.1-26)	0 (0-0)	-19.4 (-39.2-4.8)	0 (0-0)	4.1 (-54.4-126)	0 (0-0)	13.5 (5.3-23.3)
Kyrgyzstan	0 (0-0)	0 (0-0)	0 (0-0)	0.6 (-3.3-4.7)	0 (0-0)	2.9 (-14.3-21.4)	0 (0-0)	-13.9 (-36.3-15.7)	0 (0-0)	29 (-37.9-164)	0 (0-0)	3 (-5.2-10.9)
Mongolia	0 (0-0)	-0.5 (0-0)	0 (0-0)	-1.7 (-6-3.1)	0 (0-0)	11.4 (-8.8-29.1)	0 (0-0)	-35.5 (-53.9--14)	0 (0-0)	18.7 (-46.5-182)	0 (0-0)	-1 (-9.2-6.7)
Tajikistan	0 (0-0)	0 (0-0)	0 (0-0)	0.5 (-3.2-4.7)	0 (0-0)	3.9 (-11.6-20.8)	0 (0-0)	-15.8 (-37.5-12.7)	0 (0-0)	38.6 (-26.4-166)	0 (0-0)	-13.5 (-18.7--8.2)
Turkmenistan	0 (0-0)	0 (0-0)	0 (0-0)	0.1 (-3.2-4.3)	0 (0-0)	3.9 (-10.5-23.3)	0 (0-0)	-21.1 (-41.6-10.4)	0 (0-0)	12.1 (-43.2-167)	0 (0-0)	-11.4 (-17.1--4.9)
Uzbekistan	0 (0-0)	-0.3 (0-0)	0 (0-0)	1.3 (-5-5.6)	0 (0-0)	3.3 (-14.5-19)	0 (0-0)	-21.2 (-44.2-3.1)	0 (0-0)	27.3 (-39.5-159)	0 (0-0)	-10.7 (-19--4.3)
**Central Europe**	2.1 (1.4-3)	-29.3 (-38.6--19.6)	1.4 (0.9-2)	-30.9 (-39.7--22.1)	0.3 (0.2-0.5)	-30.3 (-38.6--20.4)	0 (0-0)	-42.7 (-49.9--33.8)	0 (0-0)	-14.5 (-37.8-13)	0.1 (0.1-0.2)	-40.8 (-47.1--34.3)
Albania	0.3 (0.2-0.3)	-0.9 (-22.5-0)	0.1 (0.1-0.2)	2.5 (-18.3-7.9)	0 (0-0)	3.2 (-16.8-19.3)	0 (0-0)	-7.5 (-31.2-21.6)	0 (0-0)	-8.8 (-51.7-64.2)	0 (0-0)	-35.9 (-48.1--30.6)
Bosnia and Herzegovina	0 (0-0)	-2.2 (-96.7-0)	0 (0-0)	-2.1 (-96.5-2.9)	0 (0-0)	0.3 (-95.7-18.5)	0 (0-0)	-11.8 (-95.2-15.1)	0 (0-0)	-12.7 (-88.7-50.8)	0 (0-0)	-28.6 (-97.2--20.3)
Bulgaria	0 (0-0)	0 (-0.1-0)	0 (0-0)	-2.5 (-6.5-1.2)	0 (0-0)	6.2 (-8.4-24.3)	0 (0-0)	-21.2 (-40.3-7.5)	0 (0-0)	-17.8 (-55.6-65.7)	0 (0-0)	-1.4 (-10.1-6.6)
Croatia	0 (0-0)	-5 (-45.2-0)	0 (0-0)	-4.3 (-45.8-3.7)	0 (0-0)	-3.9 (-47.7-16.1)	0 (0-0)	-13.3 (-63.9-21.5)	0 (0-0)	-21.1 (-82.4-72.5)	0 (0-0)	-24.3 (-59.2--12.9)
Czechia	0 (0-0)	-1.6 (-78.3-70.8)	0 (0-0)	-1.8 (-78.7-70.8)	0 (0-0)	-0.7 (-79.3-71.8)	0 (0-0)	-25 (-85.6-46.7)	0 (0-0)	-28.5 (-89-140)	0 (0-0)	0 (-79.4-79.8)
Hungary	0 (0-0)	-4.8 (-45.2-0)	0 (0-0)	-5.9 (-47.3-2.7)	0 (0-0)	-7 (-54.3-15.1)	0 (0-0)	-23.3 (-70.2-8.3)	0 (0-0)	-27.7 (-87.6-60.5)	0 (0-0)	-19.5 (-58.5--6.6)
Montenegro	0 (0-0)	-0.2 (0-0.1)	0 (0-0)	-0.4 (-3.5-2.6)	0 (0-0)	1.1 (-13.3-16.8)	0 (0-0)	-8 (-31.1-19.7)	0 (0-0)	-7.4 (-51-81.2)	0 (0-0)	-21.1 (-27--14.6)
North Macedonia	0 (0-0)	0 (0-0)	0 (0-0)	0.7 (-3.5-4.6)	0 (0-0)	2.4 (-12.4-17.8)	0 (0-0)	-13.1 (-36.4-12.6)	0 (0-0)	-5.8 (-48.8-61.5)	0 (0-0)	-22.7 (-29.1--16.5)
Poland	6 (3.9-8.8)	-37.6 (-45.9--29.1)	4.1 (2.7-5.9)	-36.9 (-45.1--28.9)	0.9 (0.6-1.4)	-32.4 (-40.5--22.8)	0.1 (0.1-0.1)	-46.1 (-52.9--37.7)	0 (0-0)	-22.1 (-43.4-3.1)	0.3 (0.2-0.5)	-45 (-50.9--39)
Romania	0 (0-0)	0 (-0.1-0)	0 (0-0)	-1.4 (-5.1-3.2)	0 (0-0)	5.9 (-8.1-26.9)	0 (0-0)	-17.5 (-37.3-10.1)	0 (0-0)	-5.8 (-51.3-83.2)	0 (0-0)	-11.7 (-18.3--4.1)
Serbia	0 (0-0)	0 (0-0)	0 (0-0)	-0.7 (-4.3-5.2)	0 (0-0)	-3.5 (-18.9-13.5)	0 (0-0)	-8.5 (-32.4-17.9)	0 (0-0)	-13.8 (-55.3-69.2)	0 (0-0)	-25.5 (-32.5--17.9)
Slovakia	0 (0-0)	0 (0-0)	0 (0-0)	1.1 (-1.9-4.4)	0 (0-0)	4.7 (-8.2-20.4)	0 (0-0)	-22.1 (-40.1--0.1)	0 (0-0)	-17.9 (-59.3-63.1)	0 (0-0)	-11.9 (-19.1--4.3)
Slovenia	0 (0-0)	1.7 (-29.3-107)	0 (0-0)	1 (-30.3-97.6)	0 (0-0)	7.8 (-24.5-94.7)	0 (0-0)	-13.3 (-46.3-35.7)	0 (0-0)	-9.9 (-59.7-71.9)	0 (0-0)	-5.3 (-34.2-70.3)
**Eastern Europe**	0 (0-0)	-0.3 (-7.2-1.8)	0 (0-0)	-1.4 (-8-0.9)	0 (0-0)	11.6 (4.9-18.6)	0 (0-0)	-25.6 (-31.8--19.1)	0 (0-0)	-6.7 (-25.3-18.5)	0 (0-0)	12.5 (5-19.5)
Belarus	0 (0-0)	0 (0-0)	0 (0-0)	-1.6 (-4.8-1.7)	0 (0-0)	9.8 (-4.2-26.8)	0 (0-0)	-23 (-41.1--1.6)	0 (0-0)	0.3 (-49-101)	0 (0-0)	5.3 (-4.1-16.1)
Estonia	0 (0-0)	0 (0-0)	0 (0-0)	-0.2 (-2.8-3)	0 (0-0)	4.4 (-8.9-20.1)	0 (0-0)	-18.9 (-37.9-3)	0 (0-0)	-15.6 (-58.2-53.3)	0 (0-0)	-0.2 (-9.3-8.8)

Location	Birth Incidence rate (per 100,000 live births), 2021	Birth Incidence rate (per 100,000 live births) percent change (2000-2021)	Under-5 rate (per 100,000 population), 2021	Under-5 rate (per 100,000 population) percent change (2000-2021)	5-14 rate (per 100,000 population), 2021	5-14 rate (per 100,000 population) percent change (2000-2021)	15-49 rate (per 100,000 population), 2021	15-49 rate (per 100,000 population) percent change (2000-2021)	50plus rate (per 100,000 population), 2021	50plus rate (per 100,000 population) percent change (2000-2021)	All age rate (per 100,000 population), 2021	All age rate (per 100,000 population) percent change (2000-2021)
Latvia	0 (0-0)	0 (0-0)	0 (0-0)	-1.7 (-4.8-1.1)	0 (0-0)	4.5 (-8-20.9)	0 (0-0)	-22.2 (-40.7-3.1)	0 (0-0)	-18 (-56.5-82.9)	0 (0-0)	4.8 (-4.8-17.1)
Lithuania	0 (0-0)	0 (0-0)	0 (0-0)	0.2 (-2.7-3.5)	0 (0-0)	5.6 (-8-21.7)	0 (0-0)	-12.9 (-32.9-13.6)	0 (0-0)	-10 (-49.5-88.2)	0 (0-0)	-15.2 (-22.4--7.1)
Republic of Moldova	0 (0-0)	-0.6 (0-0)	0 (0-0)	-0.3 (-3.9-3.7)	0 (0-0)	4.4 (-11.9-21.2)	0 (0-0)	-26.7 (-48.4--3.2)	0 (0-0)	-6 (-56.1-97.2)	0 (0-0)	-26.2 (-33--19.9)
Russian Federation	0 (0-0)	-0.9 (-11.3-0)	0 (0-0)	-2.1 (-12.3--0.8)	0 (0-0)	13.5 (3.4-20.7)	0 (0-0)	-25.2 (-31.3--20.3)	0 (0-0)	-4.1 (-18.2-16.9)	0 (0-0)	19.8 (8.9-27.9)
Ukraine	0 (0-0)	0 (-0.4-0)	0 (0-0)	-0.7 (-3.3-2.3)	0 (0-0)	6.6 (-5.5-20.5)	0 (0-0)	-27.6 (-44.6--4.8)	0 (0-0)	-7.4 (-55.4-93.2)	0 (0-0)	-3.8 (-11.2-4.4)
High-income	1.4 (1.1-1.6)	-8.4 (-10--6.7)	1.2 (1-1.4)	-6.9 (-8.6--5.2)	1 (0.9-1.2)	-4.6 (-6.6--2.8)	0.7 (0.6-0.8)	1.3 (-0.8-3.4)	0.1 (0.1-0.1)	1.8 (-1.6-5.4)	0.5 (0.5-0.6)	-11.8 (-13.4--10.1)
**Australasia**	0 (0-0)	0.8 (0-47.6)	0 (0-0)	1.7 (-2.3-49.6)	0 (0-0)	0.1 (-12.5-46.5)	0 (0-0)	-4 (-23.5-25.7)	0 (0-0)	-14.9 (-55.8-50.7)	0 (0-0)	-11.5 (-17.1-28.3)
Australia	0 (0-0)	0 (0-0)	0 (0-0)	1 (-2.8-4.6)	0 (0-0)	-0.1 (-13.7-13.4)	0 (0-0)	-4.7 (-25.9-22.5)	0 (0-0)	-15.3 (-57.7-59.6)	0 (0-0)	-10.8 (-16.9--4.8)
New Zealand	0 (0-0)	4.7 (0-274)	0 (0-0)	5.2 (-2.3-296)	0 (0-0)	0.7 (-15.6-250)	0 (0-0)	0.3 (-26.4-150)	0 (0-0)	-10.6 (-55.4-108)	0 (0-0)	-14.9 (-23.5-207)
**High-income Asia Pacific**	0 (0-0)	0 (-0.1-0)	0 (0-0)	0 (-1.4-1.9)	0 (0-0)	3 (-1.7-8.3)	0 (0-0)	-14.9 (-20.6--8.8)	0 (0-0)	-33.3 (-42.4--22.2)	0 (0-0)	-27.5 (-29.4--25.2)
Brunei Darussalam	0 (0-0)	0 (0-0)	0 (0-0)	0 (-3-3.3)	0 (0-0)	-1.3 (-15.7-14.8)	0 (0-0)	-10.9 (-33.8-19.1)	0 (0-0)	-6.4 (-53.6-103)	0 (0-0)	-34.7 (-38.6--30.2)
Japan	0 (0-0)	0 (0-0)	0 (0-0)	-0.9 (-1.7-0)	0 (0-0)	1.5 (-1.5-4.5)	0 (0-0)	-11.3 (-16.8--5.8)	0 (0-0)	-28.7 (-38.4--17.6)	0 (0-0)	-21.8 (-23.3--20.4)
Aichi	0 (0-0)	0 (0-0)	0 (0-0)	-1 (-4.4-2.6)	0 (0-0)	0.5 (-12-15.4)	0 (0-0)	-11.1 (-29.6-11.2)	0 (0-0)	-29.7 (-58.2-30.2)	0 (0-0)	-21.1 (-26.8--14.7)
Akita	0 (0-0)	0 (0-0)	0 (0-0)	-0.5 (-4.2-3.4)	0 (0-0)	-1.1 (-14.6-14.4)	0 (0-0)	-12.1 (-32.6-12.9)	0 (0-0)	-36.8 (-68.4-21.6)	0 (0-0)	-34.2 (-39.2--28.6)
Aomori	0 (0-0)	0 (0-0)	0 (0-0)	-0.5 (-4.1-3.3)	0 (0-0)	0.8 (-11.3-18.9)	0 (0-0)	-8.8 (-28-16.9)	0 (0-0)	-32.5 (-65.6-30.6)	0 (0-0)	-32.1 (-36.8--26.2)
Chiba	0 (0-0)	0 (0-0)	0 (0-0)	-0.9 (-4.3-2.6)	0 (0-0)	-0.6 (-14.1-14.5)	0 (0-0)	-11.7 (-32.7-15.6)	0 (0-0)	-34 (-64.3-24.3)	0 (0-0)	-25.5 (-31.2--19)
Ehime	0 (0-0)	0 (0-0)	0 (0-0)	-1 (-4.8-2.1)	0 (0-0)	1.7 (-14.1-17.2)	0 (0-0)	-10.2 (-31.1-16)	0 (0-0)	-30.3 (-61.8-27.4)	0 (0-0)	-24.5 (-30.7--18.3)
Fukui	0 (0-0)	0 (0-0)	0 (0-0)	-0.2 (-4-3.5)	0 (0-0)	-0.7 (-14.9-14.7)	0 (0-0)	-8 (-28.8-22)	0 (0-0)	-30.2 (-62.3-28.2)	0 (0-0)	-26.3 (-32.2--20.1)
Fukuoka	0 (0-0)	0 (0-0)	0 (0-0)	-1 (-5-3.2)	0 (0-0)	2.5 (-11.7-18.6)	0 (0-0)	-14.4 (-34.8-10.1)	0 (0-0)	-28.8 (-64.8-32.1)	0 (0-0)	-15.2 (-22.3--8.3)
Fukushima	0 (0-0)	-0.1 (0-0)	0 (0-0)	-0.7 (-4.1-3.3)	0 (0-0)	-1 (-14.6-13)	0 (0-0)	-14.2 (-32.2-11)	0 (0-0)	-32 (-62.7-27.1)	0 (0-0)	-33.4 (-38--28.3)
Gifu	0 (0-0)	0 (0-0)	0 (0-0)	-1 (-4.9-3.1)	0 (0-0)	0.4 (-14.9-16.9)	0 (0-0)	-8.8 (-33.6-19.5)	0 (0-0)	-30.5 (-64.7-25.3)	0 (0-0)	-24.9 (-31.4--18.2)
Gunma	0 (0-0)	0 (0-0)	0 (0-0)	-1 (-5-2.9)	0 (0-0)	-1.1 (-16.7-13.3)	0 (0-0)	-9.5 (-30.2-17.4)	0 (0-0)	-33.7 (-63.9-33.9)	0 (0-0)	-28.6 (-35.1--22.7)
Hiroshima	0 (0-0)	0 (0-0)	0 (0-0)	-1.2 (-5.1-2.5)	0 (0-0)	1.7 (-13.4-19)	0 (0-0)	-11.7 (-32.7-15.1)	0 (0-0)	-32.2 (-64.1-30.3)	0 (0-0)	-19.7 (-26.5--12.9)
Hokkaidō	0 (0-0)	0 (0-0)	0 (0-0)	-1.1 (-5-2.9)	0 (0-0)	1.4 (-12.7-17.1)	0 (0-0)	-13.2 (-35.3-18)	0 (0-0)	-34.5 (-67.8-32)	0 (0-0)	-26.9 (-33--20.5)
Hyōgo	0 (0-0)	0 (0-0)	0 (0-0)	-0.7 (-4.2-3.4)	0 (0-0)	1.3 (-12-16.6)	0 (0-0)	-9.4 (-28.3-17.1)	0 (0-0)	-29.6 (-62.6-31.3)	0 (0-0)	-23.4 (-29.3--16.7)
Ibaraki	0 (0-0)	0 (0-0)	0 (0-0)	-1 (-4.7-3)	0 (0-0)	0.3 (-13.4-17.9)	0 (0-0)	-11.7 (-34.5-17.5)	0 (0-0)	-35.7 (-66.7-22)	0 (0-0)	-28.2 (-33.5--21.8)
Ishikawa	0 (0-0)	0 (0-0)	0 (0-0)	-0.6 (-4-3.5)	0 (0-0)	0 (-14.2-16.8)	0 (0-0)	-9.1 (-29.7-19.7)	0 (0-0)	-31.1 (-62.5-29.8)	0 (0-0)	-23.3 (-29.5--16.2)
Iwate	0 (0-0)	0.1 (0-0)	0 (0-0)	-0.8 (-4.9-2.8)	0 (0-0)	1.6 (-14.1-17.9)	0 (0-0)	-9.7 (-33.7-16.1)	0 (0-0)	-29.3 (-65.5-37.7)	0 (0-0)	-28.3 (-34.9--22.1)
Kagawa	0 (0-0)	0 (0-0)	0 (0-0)	-0.9 (-5-3.1)	0 (0-0)	1.7 (-12.9-16.7)	0 (0-0)	-8.8 (-29.6-17.6)	0 (0-0)	-33.3 (-65.2-31.5)	0 (0-0)	-24.7 (-31--18.3)

Location	Birth Incidence rate (per 100,000 live births), 2021	Birth Incidence rate (per 100,000 live births) percent change (2000-2021)	Under-5 rate (per 100,000 population), 2021	Under-5 rate (per 100,000 population) percent change (2000-2021)	5-14 rate (per 100,000 population), 2021	5-14 rate (per 100,000 population) percent change (2000-2021)	15-49 rate (per 100,000 population), 2021	15-49 rate (per 100,000 population) percent change (2000-2021)	50plus rate (per 100,000 population), 2021	50plus rate (per 100,000 population) percent change (2000-2021)	All age rate (per 100,000 population), 2021	All age rate (per 100,000 population) percent change (2000-2021)
Kagoshima	0 (0-0)	0 (0-0)	0 (0-0)	-0.8 (-4.7-3.1)	0 (0-0)	3.2 (-11.8-18.9)	0 (0-0)	-12.4 (-32.7-12.2)	0 (0-0)	-27.2 (-62.5-37.2)	0 (0-0)	-17.7 (-24.3--11.5)
Kanagawa	0 (0-0)	0 (0-0)	0 (0-0)	-1.5 (-5.6-2.3)	0 (0-0)	-0.1 (-15.9-17.4)	0 (0-0)	-10.6 (-32.1-15.4)	0 (0-0)	-25.9 (-58.7-27.4)	0 (0-0)	-22.5 (-29.7--15.1)
Kōchi	0 (0-0)	0.1 (0-0)	0 (0-0)	-0.7 (-4.5-2.6)	0 (0-0)	0.4 (-13-16.6)	0 (0-0)	-11.3 (-31.5-16.6)	0 (0-0)	-33.3 (-66.2-24.7)	0 (0-0)	-24.2 (-30.1--18.3)
Kumamoto	0 (0-0)	0 (0-0)	0 (0-0)	-1.1 (-4.9-2.9)	0 (0-0)	2.5 (-12-16.8)	0 (0-0)	-10.9 (-30.3-12.2)	0 (0-0)	-29.5 (-64.4-24)	0 (0-0)	-15.6 (-22.2--9.5)
Kyōto	0 (0-0)	0 (0-0)	0 (0-0)	-0.5 (-4.2-2.9)	0 (0-0)	0.5 (-15.9-16.6)	0 (0-0)	-12.3 (-32.5-14.8)	0 (0-0)	-29.7 (-64-29.8)	0 (0-0)	-21.8 (-28.9--14.9)
Mie	0 (0-0)	0.3 (0-0)	0 (0-0)	-0.7 (-4.3-2.7)	0 (0-0)	1.3 (-13-15.4)	0 (0-0)	-8.9 (-29.3-16.3)	0 (0-0)	-28.3 (-61-33.6)	0 (0-0)	-25.3 (-31.6--19.1)
Miyagi	0 (0-0)	0 (0-0)	0 (0-0)	-1.3 (-5.1-2.3)	0 (0-0)	1.9 (-12-18.3)	0 (0-0)	-15.7 (-35.8-8.8)	0 (0-0)	-32.6 (-64.2-28.7)	0 (0-0)	-25.7 (-31.8--19.3)
Miyazaki	0 (0-0)	0 (0-0)	0 (0-0)	-1 (-5-2.9)	0 (0-0)	2.6 (-10.9-18.6)	0 (0-0)	-8.4 (-30.9-20.8)	0 (0-0)	-34 (-65.5-44.4)	0 (0-0)	-21.4 (-27.7--15)
Nagano	0 (0-0)	0 (0-0)	0 (0-0)	-0.9 (-4.8-2.7)	0 (0-0)	-1.6 (-15.4-14.1)	0 (0-0)	-8.1 (-29.3-15.9)	0 (0-0)	-28.5 (-64.7-23.6)	0 (0-0)	-26.6 (-32.5--20.6)
Nagasaki	0 (0-0)	0 (0-0)	0 (0-0)	-0.4 (-4.4-3.7)	0 (0-0)	2.6 (-12.9-18.6)	0 (0-0)	-8.2 (-29.7-19.1)	0 (0-0)	-29.5 (-64.3-34.9)	0 (0-0)	-23.1 (-29.2--16.5)
Nara	0 (0-0)	0 (0-0)	0 (0-0)	-1.4 (-5.4-2.7)	0 (0-0)	1.4 (-13.7-18.6)	0 (0-0)	-8 (-27.5-19.7)	0 (0-0)	-35.4 (-64.3-13)	0 (0-0)	-26.3 (-32.8--19.9)
Niigata	0 (0-0)	0 (0-0)	0 (0-0)	-0.6 (-4-2.8)	0 (0-0)	1.3 (-12.8-18.4)	0 (0-0)	-10.4 (-30.7-16.7)	0 (0-0)	-29 (-63-44.1)	0 (0-0)	-25.7 (-31.6--19)
Ōita	0 (0-0)	0 (0-0)	0 (0-0)	-0.9 (-4.7-2.9)	0 (0-0)	0.8 (-13.8-19.2)	0 (0-0)	-13.1 (-33.8-16.7)	0 (0-0)	-35.4 (-66-33.2)	0 (0-0)	-20.9 (-27.3--13.6)
Okayama	0 (0-0)	0.1 (0-0)	0 (0-0)	-0.8 (-4.8-3.2)	0 (0-0)	1 (-13-16.4)	0 (0-0)	-10.6 (-30.3-18.5)	0 (0-0)	-32.2 (-64.7-32.7)	0 (0-0)	-20.6 (-26.7--13.7)
Okinawa	0 (0-0)	0 (0-0)	0 (0-0)	-1.3 (-4.4-2.3)	0 (0-0)	2.1 (-10.2-18.8)	0 (0-0)	-9.9 (-28.4-18.2)	0 (0-0)	-20 (-57-62.2)	0 (0-0)	-16.9 (-22.4--10)
Ōsaka	0 (0-0)	0 (0-0)	0 (0-0)	-0.8 (-4.9-3.1)	0 (0-0)	0.2 (-14.1-17.8)	0 (0-0)	-11.5 (-32.3-17.4)	0 (0-0)	-29.6 (-62.8-29.1)	0 (0-0)	-23.8 (-30.5--16.4)
Saga	0 (0-0)	0 (0-0)	0 (0-0)	-0.9 (-4.8-3.1)	0 (0-0)	2.8 (-11.2-17.4)	0 (0-0)	-10 (-30.8-13.8)	0 (0-0)	-33.5 (-64-29.1)	0 (0-0)	-21.3 (-27.4--15)
Saitama	0 (0-0)	0 (0-0)	0 (0-0)	-1.1 (-4.9-2.8)	0 (0-0)	-0.4 (-13.2-13.7)	0 (0-0)	-13.2 (-31.7-11.2)	0 (0-0)	-37.2 (-62.5-11.4)	0 (0-0)	-25.1 (-30.8--18.5)
Shiga	0 (0-0)	0 (0-0)	0 (0-0)	-1.1 (-4.8-3.3)	0 (0-0)	0.5 (-12.8-16.3)	0 (0-0)	-11.7 (-31.3-13.2)	0 (0-0)	-29.4 (-60.6-26.1)	0 (0-0)	-22.8 (-28.9--15.8)
Shimane	0 (0-0)	0 (0-0)	0 (0-0)	-0.7 (-4.2-3)	0 (0-0)	2.2 (-10.8-17.7)	0 (0-0)	-9.9 (-28.4-18.8)	0 (0-0)	-36 (-66.9-34.8)	0 (0-0)	-18 (-23.7--10.8)
Shizuoka	0 (0-0)	0 (0-0)	0 (0-0)	-1.1 (-4.6-2.6)	0 (0-0)	0.7 (-12.2-17.2)	0 (0-0)	-10.2 (-30.4-18.7)	0 (0-0)	-33.9 (-64.3-25)	0 (0-0)	-24.6 (-30.2--18.2)
Tochigi	0 (0-0)	0 (0-0)	0 (0-0)	-1.5 (-5.2-2.7)	0 (0-0)	1 (-12-16.2)	0 (0-0)	-12.4 (-31.5-11.4)	0 (0-0)	-32.9 (-66.3-18.1)	0 (0-0)	-24.8 (-30.6--18.9)
Tokushima	0 (0-0)	-0.1 (0-0)	0 (0-0)	-1.4 (-5.1-2.7)	0 (0-0)	0.8 (-14.1-16.8)	0 (0-0)	-11.9 (-32-12.7)	0 (0-0)	-33.4 (-62.1-17.7)	0 (0-0)	-23.7 (-29.7--17.8)
Tōkyō	0 (0-0)	0 (0-0)	0 (0-0)	-1 (-4.8-2.6)	0 (0-0)	1.2 (-12.6-18.1)	0 (0-0)	-17.8 (-37.5-6.8)	0 (0-0)	-21.3 (-56.2-39.6)	0 (0-0)	-7.2 (-15.2-1.5)
Tottori	0 (0-0)	0 (0-0)	0 (0-0)	-0.9 (-4-3.1)	0 (0-0)	3 (-9-21.2)	0 (0-0)	-8.1 (-27.1-15)	0 (0-0)	-31.1 (-62.2-24.7)	0 (0-0)	-18.2 (-23.7--11.3)
Toyama	0 (0-0)	0 (0-0)	0 (0-0)	-1.7 (-5.5-2.6)	0 (0-0)	0.7 (-13.9-15.5)	0 (0-0)	-9.9 (-30.8-16.1)	0 (0-0)	-34.2 (-65.2-21.6)	0 (0-0)	-24.5 (-30.4--18.4)
Wakayama	0 (0-0)	0 (0-0)	0 (0-0)	-0.8 (-4.5-3.3)	0 (0-0)	2.5 (-11-19)	0 (0-0)	-7.2 (-27.4-18.3)	0 (0-0)	-27 (-61.5-35)	0 (0-0)	-24.6 (-30--18.3)
Yamagata	0 (0-0)	0 (0-0)	0 (0-0)	-0.8 (-4.3-3.4)	0 (0-0)	0.6 (-12.8-16.7)	0 (0-0)	-7.2 (-28.3-20.2)	0 (0-0)	-30.4 (-63.6-33.4)	0 (0-0)	-27.3 (-32.7--20.5)
Yamaguchi	0 (0-0)	0 (0-0)	0 (0-0)	-1.2 (-5.3-2.8)	0 (0-0)	2.3 (-13.4-21.6)	0 (0-0)	-11.4 (-31.7-15.6)	0 (0-0)	-37 (-64.5-17.8)	0 (0-0)	-21.8 (-28.7--14.8)

Location	Birth Incidence rate (per 100,000 live births), 2021	Birth Incidence rate (per 100,000 live births) percent change (2000-2021)	Under-5 rate (per 100,000 population), 2021	Under-5 rate (per 100,000 population) percent change (2000-2021)	5-14 rate (per 100,000 population), 2021	5-14 rate (per 100,000 population) percent change (2000-2021)	15-49 rate (per 100,000 population), 2021	15-49 rate (per 100,000 population) percent change (2000-2021)	50plus rate (per 100,000 population), 2021	50plus rate (per 100,000 population) percent change (2000-2021)	All age rate (per 100,000 population), 2021	All age rate (per 100,000 population) percent change (2000-2021)
Yamanashi	0 (0-0)	0 (0-0)	0 (0-0)	-0.2 (-4.2-3.5)	0 (0-0)	-1 (-15.5-15.1)	0 (0-0)	-3.8 (-25.8-26.5)	0 (0-0)	-26.1 (-61.3-46.8)	0 (0-0)	-30.4 (-36.6--24.5)
Republic of Korea	0 (0-0)	0 (0-0)	0 (0-0)	0.7 (-2.9-5)	0 (0-0)	-0.7 (-14.7-17.2)	0 (0-0)	-19.3 (-39-11)	0 (0-0)	-17.1 (-60.4-72.7)	0 (0-0)	-41.7 (-45.6--37)
Singapore	0 (0-0)	0 (0-0)	0 (0-0)	-3.4 (-7.2-0.8)	0 (0-0)	2.3 (-12.4-17.2)	0 (0-0)	-25.5 (-44--1.4)	0 (0-0)	-16.7 (-55.5-45.9)	0 (0-0)	-23.9 (-30.1--17.9)
**High-income North America**	3 (2.5-3.5)	-8.9 (-11--7.1)	2.8 (2.4-3.3)	-7.5 (-9.5--5.6)	2.5 (2.1-2.9)	-7.3 (-9.3--5.3)	2 (1.7-2.3)	-2.9 (-5--0.7)	0.3 (0.3-0.4)	-8.1 (-11.3--4.7)	1.5 (1.3-1.7)	-15.2 (-17--13.5)
Canada	0 (0-0)	-35.4 (-63.2-0)	0 (0-0)	-35.3 (-63.7-0.9)	0 (0-0)	-35.5 (-64.1-1.7)	0 (0-0)	-35 (-64-4)	0 (0-0)	-48.3 (-82.1--11.9)	0 (0-0)	-44.7 (-68.5--12.2)
Greenland	0 (0-0)	0 (0-0)	0 (0-0)	0.9 (-1.4-3.4)	0 (0-0)	-0.3 (-5.8-5.4)	0 (0-0)	10.7 (-1.2-24)	0 (0-0)	1.8 (-23-30)	0 (0-0)	-16.4 (-22.5--10.8)
United States of America	3.3 (2.8-3.8)	-7.3 (-9.3--5.4)	3.1 (2.6-3.6)	-6.4 (-8.5--4.6)	2.7 (2.3-3.2)	-7 (-8.9--5)	2.2 (1.9-2.5)	-3.1 (-5.3--0.9)	0.4 (0.3-0.5)	-6.7 (-10--3.4)	1.6 (1.4-1.9)	-14.8 (-16.6--13.1)
Alabama	6.8 (5.6-8.2)	-7 (-14.1-0.3)	6.4 (5.2-7.7)	-6.2 (-13.6-1.4)	5.6 (4.7-6.8)	-4.9 (-12.1-3.3)	4.2 (3.5-5.1)	-1.8 (-9.4-6.4)	0.6 (0.5-0.8)	-5.1 (-14-4.4)	3.2 (2.7-3.8)	-12.7 (-19.2--5.9)
Alaska	2 (1.4-2.7)	-8.5 (-20.5-5)	1.8 (1.3-2.5)	-6.5 (-18.1-7.4)	1.6 (1.2-2.1)	-4.4 (-16.9-10)	1.2 (0.9-1.6)	2.9 (-10.6-17.5)	0.1 (0.1-0.2)	-33.5 (-43--20.8)	0.9 (0.7-1.3)	-16.8 (-27.5--5)
Arizona	2.8 (2.2-3.4)	3.1 (-4.9-11.7)	2.6 (2-3.2)	3.6 (-4.5-12.2)	2.3 (1.8-2.9)	4.4 (-3.9-13.4)	1.8 (1.4-2.2)	6.5 (-2-15.7)	0.3 (0.2-0.4)	-1.5 (-11-9)	1.3 (1.1-1.7)	-6.8 (-14.1-1.2)
Arkansas	4.4 (3.4-5.5)	0.7 (-8.3-10.9)	4.1 (3.2-5.2)	1.4 (-7.5-11.8)	3.6 (2.9-4.6)	2.5 (-6.4-12.9)	2.7 (2.1-3.5)	5.1 (-3.5-15.4)	0.4 (0.3-0.5)	-1.3 (-9.5-9)	2.1 (1.6-2.6)	-4.7 (-12.4-4.9)
California	2 (1.6-2.4)	-6.8 (-14.9-1)	1.8 (1.5-2.2)	-6.2 (-14.5-1.4)	1.7 (1.4-2)	-5.5 (-13.4-1.7)	1.3 (1.1-1.6)	-4.1 (-12.5-3.2)	0.2 (0.2-0.3)	-11.5 (-20.5--3.8)	1 (0.8-1.2)	-16.5 (-23.6--10.2)
Colorado	1.3 (0.9-2)	-23.1 (-38.9--11.3)	1.3 (0.9-1.9)	-22.8 (-38.7--11)	1.1 (0.8-1.7)	-22 (-38.1--10.4)	0.9 (0.6-1.3)	-19.9 (-36--7.9)	0.1 (0.1-0.2)	-30.8 (-44.7--20)	0.7 (0.5-1)	-31 (-45--20.7)
Connecticut	3.1 (2.4-4)	-16.4 (-24.6--8.5)	2.9 (2.3-3.8)	-16.1 (-24.4--7.9)	2.7 (2.1-3.5)	-15.6 (-23.9--7.4)	2.1 (1.6-2.7)	-12.1 (-20.5--3.4)	0.4 (0.2-0.5)	-14.7 (-23.2--5.1)	1.5 (1.2-1.9)	-24.9 (-31.9--18.2)
Delaware	10.3 (8.2-12.9)	-5.7 (-13.5-1.5)	9.7 (7.8-12.1)	-5 (-12.8-2.3)	8.7 (7-10.9)	-4 (-11.6-3)	6.6 (5.3-8.2)	-0.4 (-8.5-7.4)	0.9 (0.7-1.2)	-8.2 (-16.1-0.7)	4.8 (3.9-5.9)	-15.3 (-22--8.9)
District of Columbia	16 (12.3-20.8)	0.4 (-12.6-13.8)	14.5 (11.1-18.9)	2.7 (-10.3-16.5)	12.4 (9.5-16.1)	5 (-8.2-18.6)	8 (6.1-10.3)	-0.3 (-13-12.4)	0.7 (0.5-1)	7 (-8.7-22.4)	6.7 (5.2-8.6)	-1.6 (-13.7-10.8)
Florida	6.9 (6.1-7.7)	-10 (-16.6--3.4)	6.5 (5.8-7.3)	-9.2 (-16.1--2.6)	5.8 (5.2-6.6)	-7.9 (-14.7--1.6)	4.5 (4-5.1)	-4.7 (-11.7-2.1)	0.8 (0.6-1)	1.4 (-6.3-10.1)	3.2 (2.9-3.6)	-14.4 (-20.3--8.4)
Georgia	4.8 (4.2-5.6)	-6.1 (-13.1-1.1)	4.5 (3.9-5.2)	-5.5 (-12.5-1.8)	4 (3.5-4.7)	-4.3 (-11.6-2.9)	3.1 (2.7-3.6)	-1.3 (-9-5.7)	0.6 (0.4-0.7)	-7 (-15.1-2.2)	2.4 (2.1-2.8)	-13.9 (-20.7--7.9)
Hawaii	0.1 (0-0.2)	-2.1 (0-0)	0 (0-0.1)	-1.4 (-0.2-1.3)	0 (0-0.1)	-0.5 (-0.3-2.8)	0 (0-0.1)	1.7 (-0.2-6)	0 (0-0)	-11.2 (-20.7--2.8)	0 (0-0.1)	-11.8 (-13.2--8.2)
Idaho	0.6 (0.4-1)	-2.7 (-20.5-40.3)	0.6 (0.3-0.9)	-1.6 (-20-41.5)	0.5 (0.3-0.8)	0.4 (-18.3-46.1)	0.4 (0.2-0.6)	2.4 (-16.4-53.6)	0 (0-0.1)	-15.3 (-34.4-20)	0.3 (0.2-0.5)	-9.3 (-25.8-36.3)
Illinois	1.9 (1.5-2.4)	-29.3 (-36--22.1)	1.8 (1.4-2.2)	-28.8 (-35.6--21.6)	1.6 (1.3-2)	-27.8 (-34.8--20.8)	1.3 (1-1.6)	-25.4 (-32.5--18)	0.2 (0.2-0.3)	-22.9 (-30.5--13.7)	1 (0.8-1.2)	-34.6 (-40.8--28.6)
Indiana	2.4 (1.9-3)	-11.4 (-19.5--4.2)	2.3 (1.8-2.9)	-11 (-19.2--3.6)	2 (1.6-2.5)	-9.9 (-18.1--2.3)	1.6 (1.2-2)	-6.9 (-15.2-1.2)	0.2 (0.2-0.3)	-11.6 (-21.2--1.3)	1.2 (1-1.5)	-17.6 (-25--10.8)
Iowa	2.1 (1.5-3)	-16.3 (-25.4--7)	2 (1.4-2.9)	-15.8 (-24.9--6)	1.8 (1.3-2.6)	-14.8 (-23.7--5.1)	1.4 (1-2)	-12.2 (-21.7--2.2)	0.2 (0.1-0.3)	-18.2 (-29.1--7.8)	1 (0.7-1.5)	-21.2 (-29.7--12.3)
Kansas	1.3 (0.8-2.1)	-56.5 (-65.3--47.9)	1.2 (0.8-2)	-56.1 (-65--47.5)	1.1 (0.7-1.8)	-55.4 (-64.3--46.5)	0.9 (0.6-1.4)	-53.6 (-62.9--44.7)	0.1 (0.1-0.2)	-49.7 (-59.2--39.8)	0.7 (0.4-1)	-58.3 (-66.5--50.1)
Kentucky	0.7 (0.5-0.9)	1.9 (-15.3-40.7)	0.6 (0.4-0.9)	2.5 (-14.5-42.2)	0.6 (0.4-0.8)	3.6 (-13.4-44.3)	0.4 (0.3-0.6)	6.6 (-10.9-51)	0 (0-0.1)	-4.6 (-18.9-55.4)	0.3 (0.2-0.4)	-6.3 (-21.5-32.7)
Louisiana	4.7 (3.9-5.6)	-16.7 (-23.2--10.6)	4.4 (3.6-5.2)	-15.9 (-21.9--9.7)	3.9 (3.2-4.6)	-13.9 (-19.8--7.3)	2.9 (2.4-3.4)	-10.9 (-16.9--4.1)	0.4 (0.3-0.5)	-11.7 (-21--1.3)	2.2 (1.9-2.7)	-21.4 (-26.5--15.4)
Maine	0 (0-0.1)	-8.9 (-80-0)	0 (0-0.1)	-8.4 (-79.6-0.9)	0 (0-0.1)	-7.6 (-79-2.4)	0 (0-0.1)	-4.4 (-77.1-7.4)	0 (0-0)	-17.2 (-69.2-0)	0 (0-0.1)	-22.1 (-81.5--12.6)
Maryland	7.4 (6.3-8.7)	-6.3 (-13.9-1.6)	7 (5.9-8.2)	-5.7 (-13.6-2.4)	6.3 (5.4-7.5)	-4.7 (-12.5-2.9)	4.9 (4.2-5.8)	-1 (-8.5-6.5)	0.9 (0.7-1.1)	-8.2 (-15--0.3)	3.8 (3.2-4.4)	-13.6 (-19.8--7.1)
Massachusetts	3.2 (2.5-4.1)	10.4 (1.7-18.9)	3.1 (2.4-3.9)	10.6 (1.9-19.2)	2.8 (2.2-3.6)	11 (2.7-19.9)	2.2 (1.8-2.9)	13.5 (5.2-23.1)	0.4 (0.3-0.5)	4.6 (-5.3-15.4)	1.6 (1.3-2.1)	-1.3 (-8.4-6.5)

Location	Birth Incidence rate (per 100,000 live births), 2021	Birth Incidence rate (per 100,000 live births) percent change (2000-2021)	Under-5 rate (per 100,000 population), 2021	Under-5 rate (per 100,000 population) percent change (2000-2021)	5-14 rate (per 100,000 population), 2021	5-14 rate (per 100,000 population) percent change (2000-2021)	15-49 rate (per 100,000 population), 2021	15-49 rate (per 100,000 population) percent change (2000-2021)	50plus rate (per 100,000 population), 2021	50plus rate (per 100,000 population) percent change (2000-2021)	All age rate (per 100,000 population), 2021	All age rate (per 100,000 population) percent change (2000-2021)
Michigan	3.4 (2.8-4)	-11.2 (-17.9--3)	3.2 (2.7-3.8)	-10.6 (-17.1--2.6)	2.9 (2.4-3.4)	-9.4 (-16--1.6)	2.2 (1.9-2.6)	-5.9 (-12.6-2)	0.3 (0.2-0.4)	-13.5 (-20.7--5.8)	1.6 (1.3-1.9)	-21.1 (-26.6--14.8)
Minnesota	0.9 (0.6-1.3)	2.5 (-22.4-41.3)	0.8 (0.6-1.3)	2.7 (-22.6-42.2)	0.8 (0.5-1.2)	3.3 (-21.8-43.8)	0.6 (0.4-0.9)	5.6 (-19.7-48.4)	0.1 (0.1-0.2)	-1.9 (-21.8-43.2)	0.5 (0.3-0.7)	-7.4 (-29.8-28.9)
Mississippi	6.9 (5.5-8.7)	-24.3 (-31.7--16.5)	6.4 (5.1-8)	-23.3 (-31--14.9)	5.6 (4.4-7)	-21.1 (-29.3--12.1)	4 (3.2-5.1)	-18.3 (-26.4--9.4)	0.5 (0.4-0.7)	-16.3 (-25.2--6)	3.1 (2.5-3.9)	-28.6 (-35.6--21)
Missouri	2.6 (2-3.3)	-11.4 (-20.6--0.8)	2.5 (1.9-3.1)	-10.7 (-19.9--0.6)	2.2 (1.7-2.8)	-9.5 (-18.7--0.1)	1.7 (1.3-2.1)	-6.2 (-15.6-3.9)	0.2 (0.2-0.3)	-8.9 (-19.1-2.4)	1.2 (1-1.6)	-17.5 (-25.6--9.3)
Montana	0 (0-0.1)	-10.7 (-84.1-0)	0 (0-0.1)	-10.3 (-83.9-1.2)	0 (0-0.1)	-9.2 (-83.5-3.3)	0 (0-0.1)	-5.8 (-82.6-9.1)	0 (0-0)	-22.3 (-80.2--4.9)	0 (0-0.1)	-19.7 (-85--7.7)
Nebraska	1 (0.6-1.7)	2.9 (-24.2-49.5)	1 (0.6-1.6)	3 (-24.2-50.2)	0.9 (0.5-1.5)	3.7 (-24-51.4)	0.7 (0.4-1.1)	6.3 (-21.5-54.8)	0.1 (0.1-0.2)	0.2 (-21-54)	0.5 (0.3-0.9)	-3.3 (-28.6-41.5)
Nevada	5 (4-6.3)	0.9 (-6.1-9.4)	4.7 (3.8-5.9)	1.3 (-5.7-9.8)	4.2 (3.4-5.3)	2 (-5.5-10.3)	3.2 (2.6-4.1)	4.6 (-3.7-12.8)	0.5 (0.4-0.7)	-7.1 (-15-2.4)	2.5 (2-3.1)	-7.4 (-14.4-0.1)
New Hampshire	0.3 (0.2-0.6)	-4.1 (-42.7--0.1)	0.3 (0.2-0.6)	-3.9 (-42.7-1.1)	0.3 (0.1-0.5)	-2.4 (-41.9-5)	0.2 (0.1-0.4)	3.3 (-39.4-12.9)	0 (0-0)	-17 (-48.9-1.1)	0.1 (0.1-0.3)	-19.1 (-52.5--12.6)
New Jersey	4.2 (3.5-5)	-3.7 (-11.1-4.3)	4 (3.4-4.7)	-3.2 (-10.4-4.8)	3.6 (3-4.3)	-2.4 (-9.4-5.6)	2.8 (2.4-3.4)	0.7 (-6.5-9)	0.5 (0.4-0.6)	-1.8 (-10.7-7.6)	2.1 (1.8-2.5)	-11.5 (-17.8--4.7)
New Mexico	0.3 (0.1-0.5)	9.1 (0-208)	0.3 (0.1-0.5)	10.8 (0.3-210)	0.2 (0.1-0.4)	12.3 (0.5-211)	0.2 (0.1-0.3)	18.3 (3.9-215)	0 (0-0)	-7.5 (-26.3-122)	0.1 (0.1-0.2)	-2.2 (-13.1-161)
New York	5.1 (4.6-5.8)	2.7 (-3.4-9.7)	4.9 (4.4-5.5)	3.1 (-3-9.7)	4.5 (4-5)	3.6 (-2.7-10.3)	3.6 (3.2-4)	5.5 (-0.9-12)	0.8 (0.6-0.9)	-0.7 (-7.2-7.5)	2.7 (2.5-3)	-6.2 (-11.7--0.5)
North Carolina	4.7 (4-5.5)	-8.5 (-14.7--1.4)	4.4 (3.8-5.2)	-8 (-14.1--1.1)	4 (3.4-4.6)	-7 (-13.1-0)	3.1 (2.6-3.6)	-4.4 (-10.4-2.2)	0.5 (0.4-0.7)	-9.5 (-16.2--1.2)	2.3 (2-2.7)	-15.7 (-21--9.8)
North Dakota	0 (0-0.1)	-17.4 (-87-0)	0 (0-0.1)	-16.8 (-86.7-1.2)	0 (0-0.1)	-15.5 (-86.2-3.4)	0 (0-0.1)	-13.4 (-85.3-7.6)	0 (0-0)	-16.1 (-83.1-8.1)	0 (0-0.1)	-19.8 (-86.6--1)
Ohio	2.2 (1.8-2.7)	6.8 (-0.2-16)	2 (1.7-2.5)	7.2 (0.1-16.3)	1.9 (1.5-2.3)	8 (0.6-17.6)	1.4 (1.2-1.8)	11.3 (3.5-21.1)	0.2 (0.2-0.3)	1.7 (-7.6-14.1)	1 (0.9-1.3)	-3.4 (-10.1-5.1)
Oklahoma	1.1 (0.6-1.8)	-59.8 (-75.3--48.2)	1 (0.6-1.7)	-59.5 (-75.1--47.8)	0.9 (0.5-1.5)	-58.9 (-74.6--47.1)	0.6 (0.4-1.1)	-57.8 (-73.9--45.2)	0.1 (0-0.1)	-61.2 (-74.7--46)	0.5 (0.3-0.8)	-61.7 (-76.4--50.2)
Oregon	0.3 (0.2-0.5)	14.6 (-6.4-194)	0.3 (0.2-0.5)	16 (-5.8-195)	0.3 (0.1-0.5)	17.9 (-4.7-200)	0.2 (0.1-0.3)	19.3 (-4.6-205)	0 (0-0)	-3.7 (-28.1-142)	0.1 (0.1-0.2)	2.1 (-18.5-160)
Pennsylvania	2.8 (2.2-3.3)	-3.3 (-11.3-4.4)	2.6 (2.1-3.2)	-2.8 (-10.8-5.1)	2.4 (1.9-2.9)	-1.9 (-9.8-6.1)	1.8 (1.5-2.2)	1.9 (-6.2-10.8)	0.3 (0.2-0.4)	-0.7 (-11.5-10)	1.3 (1.1-1.6)	-10.3 (-17.3--2.5)
Rhode Island	2.4 (1.9-3.1)	-10.1 (-22.4-1.3)	2.3 (1.8-3)	-10.5 (-22.6-0.8)	2.1 (1.6-2.8)	-10.4 (-22.6-0.9)	1.8 (1.4-2.3)	-8.3 (-20.5-2.6)	0.4 (0.3-0.5)	-4 (-15.1-6.2)	1.3 (1-1.6)	-19 (-29.7--8.9)
South Carolina	5.3 (4.5-6.3)	-24.2 (-30.1--17.3)	5 (4.2-5.8)	-23.5 (-29.6--16.7)	4.4 (3.8-5.2)	-21.6 (-28--15.1)	3.3 (2.9-3.9)	-18 (-24.2--11.5)	0.5 (0.4-0.6)	-19.3 (-27.6--10.2)	2.5 (2.1-2.9)	-29 (-34.3--23.4)
South Dakota	0 (0-0.1)	-20.6 (-87-0)	0 (0-0.1)	-20 (-86.8-1.2)	0 (0-0.1)	-18.8 (-86.5-3.3)	0 (0-0.1)	-16.5 (-85.7-7.5)	0 (0-0)	-19.7 (-86.1-8.8)	0 (0-0.1)	-25 (-87.3--3.9)
Tennessee	4.7 (3.9-5.5)	-16.5 (-23.4--10)	4.3 (3.6-5.2)	-15.7 (-22.5--9.2)	3.9 (3.2-4.6)	-14.1 (-20.5--7.8)	2.9 (2.4-3.5)	-10.4 (-17.5--3.6)	0.4 (0.3-0.6)	-12.2 (-20.4--4.1)	2.2 (1.8-2.6)	-21 (-27--15.1)
Texas	2.7 (2.2-3.1)	2.3 (-4.5-8.7)	2.5 (2.1-2.9)	2.5 (-4.2-9)	2.3 (2-2.7)	3 (-3.8-9.6)	1.8 (1.6-2.1)	5.1 (-2-11.9)	0.3 (0.3-0.5)	0.8 (-9.1-10.5)	1.5 (1.3-1.7)	-5.1 (-11.4-0.6)
Utah	0.1 (0-0.3)	-17.7 (-69.5-0)	0.1 (0-0.2)	-17.4 (-69.2-0.9)	0.1 (0-0.2)	-16.5 (-68.5-2.8)	0.1 (0-0.2)	-16.4 (-68-4.2)	0 (0-0)	-20.3 (-67.9-4.3)	0.1 (0-0.1)	-22.9 (-70.7--4.5)
Vermont	0 (0-0.1)	-8.1 (-74.3-0)	0 (0-0.1)	-7.7 (-73.6-1)	0 (0-0.1)	-6.8 (-72.7-2.6)	0 (0-0.1)	-3.3 (-70.8-7.6)	0 (0-0)	-18.9 (-69.3--4)	0 (0-0.1)	-21.3 (-76.1--12.7)
Virginia	4.1 (3.4-5.1)	-8.2 (-16.1--0.6)	3.9 (3.2-4.8)	-7.7 (-15.5-0.3)	3.5 (2.9-4.3)	-6.5 (-14.5-1.8)	2.8 (2.3-3.4)	-3.5 (-12.2-5.1)	0.5 (0.4-0.6)	-9.3 (-18.2--0.1)	2.1 (1.7-2.6)	-15 (-22.2--7.5)
Washington	2 (1.4-2.7)	-24.3 (-32.8--14.2)	1.9 (1.4-2.5)	-23.9 (-32.4--13.5)	1.7 (1.2-2.3)	-23.1 (-31.7--12.8)	1.3 (1-1.8)	-20.9 (-29.7--10.2)	0.2 (0.1-0.3)	-29.9 (-38.5--19.9)	1 (0.7-1.4)	-31.2 (-38.7--22.5)
West Virginia	0.3 (0.1-0.5)	6.8 (-11.6-202)	0.3 (0.1-0.5)	6.5 (-11.8-204)	0.2 (0.1-0.5)	7.3 (-10.7-210)	0.2 (0.1-0.3)	7.5 (-9.9-207)	0 (0-0.1)	-5 (-23.9-183)	0.1 (0.1-0.2)	-5.3 (-20.9-174)
Wisconsin	1.7 (1.2-2.3)	-30.5 (-41.7--16.9)	1.6 (1.1-2.2)	-30.1 (-41.2--16.2)	1.4 (1-2)	-29.1 (-40.5--14.9)	1.1 (0.8-1.5)	-26.6 (-38.4--12.4)	0.2 (0.1-0.2)	-28.3 (-38.9--15.7)	0.8 (0.6-1.1)	-36.7 (-46.8--24.5)
Wyoming	0 (0-0.1)	-2 (-0.1-0)	0 (0-0.1)	-1.7 (-0.6-1)	0 (0-0.1)	-0.6 (-1.1-3.1)	0 (0-0.1)	2.4 (-0.6-8)	0 (0-0)	-16.1 (-28--6.6)	0 (0-0.1)	-10.1 (-12.2--5.7)

Location	Birth Incidence rate (per 100,000 live births), 2021	Birth Incidence rate (per 100,000 live births) percent change (2000-2021)	Under-5 rate (per 100,000 population), 2021	Under-5 rate (per 100,000 population) percent change (2000-2021)	5-14 rate (per 100,000 population), 2021	5-14 rate (per 100,000 population) percent change (2000-2021)	15-49 rate (per 100,000 population), 2021	15-49 rate (per 100,000 population) percent change (2000-2021)	50plus rate (per 100,000 population), 2021	50plus rate (per 100,000 population) percent change (2000-2021)	All age rate (per 100,000 population), 2021	All age rate (per 100,000 population) percent change (2000-2021)
**Southern Latin America**	0 (0-0)	23.5 (0.1-80.6)	0 (0-0)	23.8 (-1.9-85.9)	0 (0-0)	32.7 (-2.5-112)	0 (0-0)	29.7 (-14.8-138)	0 (0-0)	102 (-10.3-480)	0 (0-0)	0.3 (-22.8-53)
Argentina	0 (0-0)	0.2 (0-0.2)	0 (0-0)	-1.1 (-5.8-3.1)	0 (0-0)	1.3 (-14.6-19.3)	0 (0-0)	-11.7 (-39.2-20.7)	0 (0-0)	-2.8 (-61.6-123)	0 (0-0)	-20.1 (-26.2--14)
Chile	0 (0-0.1)	111 (0.2-499)	0 (0-0)	112 (-0.7-488)	0 (0-0)	127 (0.6-516)	0 (0-0)	127 (-7.9-568)	0 (0-0)	149 (-17.3-1020)	0 (0-0)	62.3 (-25.6-346)
Uruguay	0 (0-0)	-0.2 (-0.3-0)	0 (0-0)	-0.1 (-5.1-4.9)	0 (0-0)	-1.1 (-17.3-17.6)	0 (0-0)	-7 (-32.9-28.9)	0 (0-0)	-3.8 (-56.4-110)	0 (0-0)	-18.9 (-25.2--11.9)
**Western Europe**	0.6 (0.5-0.8)	-16.1 (-19.9--12.2)	0.5 (0.4-0.6)	-11.8 (-15.4--8.1)	0.3 (0.2-0.3)	-2 (-6.1-1.9)	0.1 (0.1-0.1)	-1.3 (-5.2-2.8)	0 (0-0)	29.2 (21.6-37.3)	0.1 (0.1-0.1)	-11.8 (-15--8.6)
Andorra	0 (0-0)	0 (-0.1-0)	0 (0-0)	-0.4 (-4.4-3.6)	0 (0-0)	-5.8 (-20.1-8)	0 (0-0)	1.7 (-24.8-34)	0 (0-0)	12.7 (-45.2-129)	0 (0-0)	-35.5 (-41.5--29)
Austria	0.6 (0.4-0.8)	14.5 (-4.7-67.8)	0.4 (0.3-0.5)	15.1 (-3.8-65.5)	0.1 (0.1-0.2)	11.5 (-10.9-54.5)	0 (0-0)	14.1 (-22.9-71)	0 (0-0)	24.9 (-47.6-185)	0 (0-0)	2.4 (-16.2-42.8)
Belgium	0 (0-0.1)	0.7 (-16.2-32.2)	0 (0-0)	0.7 (-15.6-32.7)	0 (0-0)	0.6 (-17.3-33.9)	0 (0-0)	5.3 (-23.2-49.5)	0 (0-0)	-3.9 (-53.9-106)	0 (0-0)	-4.9 (-21.3-26)
Cyprus	0 (0-0)	0 (0-0.1)	0 (0-0)	2.1 (-2-6.6)	0 (0-0)	1.9 (-13-20.3)	0 (0-0)	-29.5 (-48.7--6.4)	0 (0-0)	-7.2 (-55.9-93.4)	0 (0-0)	-16.7 (-22.8--10.4)
Denmark	0 (0-0)	-3.2 (-51.6-0.1)	0 (0-0)	-1.4 (-49.8-6.2)	0 (0-0)	-8.7 (-51.4-11.2)	0 (0-0)	10.8 (-33.4-52.8)	0 (0-0)	-11.8 (-54.1-68.6)	0 (0-0)	-16.9 (-57.3--6.9)
Finland	0 (0-0)	0 (-0.1-0)	0 (0-0)	-0.8 (-4.5-3.1)	0 (0-0)	-2.3 (-16.7-13.8)	0 (0-0)	-1 (-24.9-31.7)	0 (0-0)	-28.3 (-66.7-62)	0 (0-0)	-18.6 (-25--11.8)
France	0.5 (0.4-0.7)	0.4 (-3.1-18.4)	0.3 (0.2-0.3)	-1.2 (-7.1-14.4)	0 (0-0)	0.2 (-16.5-22)	0 (0-0)	4.5 (-26.5-42)	0 (0-0)	-13.8 (-53-66.3)	0 (0-0)	-13 (-20--2.1)
Germany	0 (0-0.1)	-1.1 (-26.7-0.1)	0 (0-0)	-1.4 (-28.1-4.1)	0 (0-0)	1.3 (-29.3-19.7)	0 (0-0)	2.4 (-28.5-34.7)	0 (0-0)	1.4 (-51.6-93.5)	0 (0-0)	-7.1 (-32.7-1.8)
Greece	6.9 (5.1-9.2)	7.8 (-5.4-22)	4.4 (3.4-5.8)	8.3 (-4.5-21.6)	1.9 (1.4-2.4)	13.8 (-1-28)	0.6 (0.4-0.7)	4.6 (-7.8-16.5)	0 (0-0)	-2 (-18.3-15.8)	0.6 (0.5-0.8)	-5.1 (-15.1-5)
Iceland	0 (0-0)	0 (-0.1-0)	0 (0-0)	0.7 (-3.3-4.8)	0 (0-0)	-3.9 (-17.6-12.9)	0 (0-0)	-8.8 (-31.1-20.4)	0 (0-0)	-10.4 (-57.9-83.5)	0 (0-0)	-21.9 (-27.7--15.3)
Ireland	0 (0-0)	-7.2 (-45.4-0)	0 (0-0)	-8.4 (-46.2-3)	0 (0-0)	-4.6 (-46.9-18)	0 (0-0)	-23.8 (-64-8.7)	0 (0-0)	-25.8 (-83.4-86.5)	0 (0-0)	-18.7 (-53.2--4.6)
Israel	0 (0-0)	0 (0-0.1)	0 (0-0)	0.2 (-3.5-4)	0 (0-0)	1.4 (-14.2-17.2)	0 (0-0)	-7.2 (-33.8-21.9)	0 (0-0)	-17.1 (-59.1-63.7)	0 (0-0)	-2.2 (-9.1-4.1)
Italy	3.5 (2.6-4.8)	-1.8 (-6.3-3.2)	2.8 (2.1-3.8)	-1.5 (-5.8-3.1)	1.7 (1.3-2.3)	-1.4 (-5.6-2.9)	0.8 (0.6-1)	9 (4.4-14.4)	0.1 (0.1-0.1)	34.1 (26.3-42.7)	0.6 (0.5-0.8)	-7.1 (-10.8--3.1)
Abruzzo	0.2 (0.1-0.5)	-21.1 (-74.1-0.1)	0.2 (0.1-0.4)	-20.7 (-74-2.8)	0.1 (0-0.2)	-17.4 (-73.1-10.7)	0 (0-0.1)	-16.7 (-72.6-19.7)	0 (0-0)	12.3 (-65.1-84.9)	0 (0-0.1)	-27.5 (-76.1-0.4)
Basilicata	0.3 (0.1-0.8)	-13.8 (-70.1-47.9)	0.3 (0.1-0.7)	-12.4 (-69.4-50.6)	0.2 (0.1-0.4)	-10.6 (-67.1-50.9)	0.1 (0-0.2)	-11.6 (-67.3-54.2)	0 (0-0)	20.4 (-56.2-112)	0.1 (0-0.1)	-28.2 (-73.7-24.6)
Calabria	2.1 (1.2-3.5)	-6.6 (-16.3-6)	1.7 (0.9-2.8)	-5.8 (-15.8-7.4)	1 (0.6-1.7)	-3.1 (-13-10.8)	0.5 (0.3-0.7)	-0.6 (-13.5-15.7)	0.1 (0-0.1)	26.9 (4.2-55.3)	0.4 (0.2-0.6)	-18 (-26.9--5.9)
Campania	3.3 (2.3-4.8)	34.4 (17.5-58.7)	2.6 (1.8-3.8)	35.5 (19.1-57.3)	1.6 (1.1-2.2)	39.2 (22.5-62.2)	0.8 (0.5-1)	44.8 (25.5-69.3)	0.1 (0.1-0.1)	80.4 (51.4-120)	0.6 (0.5-0.9)	17.4 (2.8-36.2)
Emilia-Romagna	4.9 (3.6-7)	0.8 (-9.2-11.3)	3.9 (2.8-5.3)	0.5 (-9.5-12)	2.3 (1.7-3)	4 (-5.6-14.9)	0.9 (0.7-1.2)	13.3 (0.3-27.9)	0.1 (0.1-0.1)	47.6 (21.8-83.8)	0.8 (0.6-1)	6.4 (-3.8-16.9)
Friuli-Venezia Giulia	3.9 (2.4-6)	-5.6 (-16.7-8.2)	3.1 (1.9-4.7)	-6 (-16.8-7.4)	1.9 (1.2-2.8)	-4 (-14.6-9.2)	0.7 (0.4-1.1)	3.9 (-9.5-19.8)	0.1 (0-0.1)	29.6 (4.3-62.1)	0.6 (0.4-0.8)	-7.7 (-17.4-5.2)
Lazio	2 (1.3-3)	-13.8 (-24.8--3)	1.6 (1-2.4)	-13.7 (-24.1--3.3)	1 (0.7-1.5)	-11.9 (-22.3--2.7)	0.4 (0.3-0.6)	-8.1 (-21.6-4.8)	0.1 (0-0.1)	18.4 (-6.6-47.1)	0.3 (0.2-0.5)	-18.8 (-29.3--9.5)
Liguria	10.5 (7.9-14.5)	14.7 (1.7-30.4)	8.3 (6.3-11.1)	14.5 (2.5-28.9)	4.8 (3.7-6.1)	12.6 (1.7-24.9)	1.8 (1.4-2.3)	21.4 (9.6-34.3)	0.2 (0.1-0.3)	47.7 (27.4-72.7)	1.4 (1.1-1.8)	10.3 (0.4-21.8)
Lombardia	1.8 (1.2-2.6)	19 (5-52)	1.4 (1-2.1)	18.8 (5-50.9)	0.9 (0.6-1.3)	23.2 (8.7-52.2)	0.4 (0.3-0.5)	33.7 (15.9-63.4)	0 (0-0.1)	62.5 (27.5-109)	0.3 (0.2-0.5)	18.7 (4.9-45.7)
Marche	2.7 (1.6-4.5)	-13.4 (-23.7--4.8)	2.1 (1.2-3.5)	-13.4 (-22.8--3.5)	1.3 (0.8-2.1)	-11 (-19.7--0.7)	0.6 (0.3-0.9)	-6.5 (-18.5-6.6)	0.1 (0-0.1)	22.7 (-3.3-55.8)	0.4 (0.3-0.7)	-16.1 (-25.4--6.1)
Molise	0.1 (0-0.2)	-12.8 (-90.1-0)	0.1 (0-0.2)	-12.1 (-90.1-3.4)	0 (0-0.1)	-9 (-89.7-13.9)	0 (0-0)	-5.1 (-89.1-24.6)	0 (0-0)	29.2 (-85.3-92.5)	0 (0-0)	-23 (-91.2--3.7)

Location	Birth Incidence rate (per 100,000 live births), 2021	Birth Incidence rate (per 100,000 live births) percent change (2000-2021)	Under-5 rate (per 100,000 population), 2021	Under-5 rate (per 100,000 population) percent change (2000-2021)	5-14 rate (per 100,000 population), 2021	5-14 rate (per 100,000 population) percent change (2000-2021)	15-49 rate (per 100,000 population), 2021	15-49 rate (per 100,000 population) percent change (2000-2021)	50plus rate (per 100,000 population), 2021	50plus rate (per 100,000 population) percent change (2000-2021)	All age rate (per 100,000 population), 2021	All age rate (per 100,000 population) percent change (2000-2021)
Piemonte	1.7 (1.1-2.6)	29.5 (10-69.8)	1.4 (0.9-2.1)	29.4 (8.6-67)	0.8 (0.5-1.2)	31.6 (12.7-67.5)	0.3 (0.2-0.5)	38.8 (15.8-77.5)	0 (0-0.1)	66.9 (26.9-129)	0.3 (0.2-0.4)	25 (6.7-58.6)
Provincia autonoma di Bolzano	1.2 (0.6-2.4)	-10.7 (-26.6-7.4)	1 (0.5-2)	-10.1 (-26-7.3)	0.6 (0.3-1.1)	-9 (-24.8-9.3)	0.2 (0.1-0.4)	-1.1 (-24.2-27)	0 (0-0.1)	35.5 (-12.7-101)	0.2 (0.1-0.4)	-17.3 (-32.3-1.4)
Provincia autonoma di Trento	0.7 (0.3-1.4)	-21.3 (-56.7-0)	0.6 (0.3-1.1)	-21.1 (-56.8-2.1)	0.3 (0.2-0.6)	-19.7 (-55.3-6.9)	0.1 (0.1-0.2)	-8.8 (-48.2-25.8)	0 (0-0)	11 (-37.7-70.8)	0.1 (0.1-0.2)	-24 (-57.5-1.8)
Puglia	0.3 (0.1-0.5)	-25.2 (-72.7-0.1)	0.2 (0.1-0.4)	-24.9 (-72.9-3)	0.1 (0.1-0.3)	-22.5 (-72.5-9.4)	0.1 (0-0.1)	-24.2 (-73.3-13)	0 (0-0)	-9 (-67.4-49.5)	0 (0-0.1)	-37.7 (-77.8--9.5)
Sardegna	0.4 (0.1-0.8)	-21.3 (-64.9-0)	0.3 (0.1-0.6)	-21.1 (-65.7-2.6)	0.2 (0.1-0.4)	-18.7 (-64.1-9.7)	0.1 (0-0.1)	-17.9 (-59.6-20.2)	0 (0-0)	1.9 (-49.9-66.3)	0.1 (0-0.1)	-35.4 (-69.7--9.4)
Sicilia	12.4 (10-15.6)	-11.9 (-21.9--1.3)	9.7 (7.9-12.2)	-11 (-20.5--1.2)	6.3 (5.2-7.6)	-7.6 (-15.9-1.8)	3.2 (2.6-3.8)	-2.8 (-10.7-5.7)	0.5 (0.4-0.6)	20.3 (9-33.1)	2.6 (2.1-3.1)	-18.8 (-25.3--11.8)
Toscana	1.2 (0.7-2)	2.6 (-21.2-44)	1 (0.6-1.6)	1.8 (-21.9-42.3)	0.6 (0.4-1)	1.4 (-22.2-41.1)	0.2 (0.1-0.4)	4.7 (-21.4-42.8)	0 (0-0.1)	32.4 (-4.1-86.9)	0.2 (0.1-0.3)	-1.6 (-24.6-32.3)
Umbria	3.3 (2.2-4.9)	1.9 (-14.8-20.6)	2.6 (1.7-3.8)	1.3 (-15-18.7)	1.5 (1-2.1)	0.8 (-14.9-16.9)	0.6 (0.4-0.8)	3.6 (-14.1-22.3)	0.1 (0-0.1)	29.5 (2.8-67.6)	0.5 (0.3-0.7)	-2.6 (-18.8-13.8)
Valle d'Aosta	1.6 (0.8-3)	-12 (-25.4-3.4)	1.2 (0.6-2.3)	-12.4 (-26.4-2.5)	0.7 (0.3-1.2)	-11 (-25.7-8.8)	0.2 (0.1-0.4)	1.2 (-21.8-31.6)	0 (0-0)	29.3 (-12.1-107)	0.2 (0.1-0.4)	-14.6 (-29.7-4.9)
Veneto	5.8 (4.4-7.5)	4.8 (-4.6-15.2)	4.7 (3.6-6)	5.1 (-3.4-15.1)	3.1 (2.4-3.8)	7.8 (-0.7-17.6)	1.4 (1.1-1.7)	17.5 (7.5-27.5)	0.2 (0.1-0.3)	44.9 (26.6-64.7)	1.1 (0.9-1.4)	1.3 (-6.8-9.9)
Luxembourg	0 (0-0)	0 (-0.2-0)	0 (0-0)	-0.1 (-4.1-3.5)	0 (0-0)	-1.4 (-15.8-14)	0 (0-0)	0.4 (-24.5-31)	0 (0-0)	9.3 (-43.9-123)	0 (0-0)	-19.3 (-25.6--12.9)
Malta	0 (0-0)	0 (0-0.5)	0 (0-0)	1.8 (-2.4-6)	0 (0-0)	4.6 (-10.7-23.9)	0 (0-0)	-20.7 (-40.4-7)	0 (0-0)	-40.5 (-67.8-26)	0 (0-0)	-20 (-26.5--12.9)
Monaco	0 (0-0)	-7.2 (-45.3-0)	0 (0-0)	-8.8 (-45.2-2.2)	0 (0-0)	-6.2 (-37.1-15.1)	0 (0-0)	18.7 (-23.8-65)	0 (0-0)	-8.5 (-73.8-118)	0 (0-0)	-6.2 (-40.9-8.3)
Netherlands	0 (0-0)	0 (0-0)	0 (0-0)	-0.1 (-3.8-3.3)	0 (0-0)	-1.6 (-16.4-14.3)	0 (0-0)	15.6 (-15.1-51.9)	0 (0-0)	-13.4 (-58.6-70.4)	0 (0-0)	-17.5 (-23.9--11.5)
Norway	0.2 (0-0.5)	-0.2 (-14.9-12.9)	0.1 (0-0.3)	-0.1 (-14.4-15.3)	0 (0-0.1)	-1.4 (-15.3-15.5)	0 (0-0)	2.4 (-15.9-24.4)	0 (0-0)	-2.3 (-31.4-35.2)	0 (0-0)	-18.1 (-29.3--5.9)
Agder	0.2 (0-0.6)	0.5 (0-0)	0.1 (0-0.3)	-0.3 (-6.7-6.9)	0 (0-0.1)	-1.2 (-22.5-25.7)	0 (0-0)	-0.9 (-34.9-50.8)	0 (0-0)	-4 (-61.7-142)	0 (0-0)	-20 (-28.7--10.1)
Innlandet	0.2 (0-0.5)	-0.2 (0-0)	0.1 (0-0.3)	0.2 (-5.6-7.2)	0 (0-0.1)	-2.5 (-23.4-22.4)	0 (0-0)	10.8 (-32-64.2)	0 (0-0)	-4.1 (-63.9-141)	0 (0-0)	-21.8 (-30.3--10.8)
Møre og Romsdal	0.2 (0-0.5)	-0.3 (0-0)	0.1 (0-0.3)	-0.4 (-8-6.7)	0 (0-0.1)	-1.4 (-22.9-20.1)	0 (0-0)	-0.1 (-37.4-56.7)	0 (0-0)	-8.9 (-64.4-150)	0 (0-0)	-21.6 (-31--11.1)
Nordland	0.2 (0-0.6)	1.7 (0-0)	0.1 (0-0.3)	1.9 (-5.7-7)	0 (0-0.1)	-0.1 (-19.7-24.7)	0 (0-0)	4.5 (-28.8-57.7)	0 (0-0)	-6.8 (-60.8-150)	0 (0-0)	-23.8 (-31.7--15)
Oslo	0.2 (0-0.5)	0.1 (0-0)	0.1 (0-0.3)	-1.3 (-7-4.8)	0 (0-0.1)	-0.4 (-21.8-27.3)	0 (0-0)	4 (-36.9-60.2)	0 (0-0)	5.8 (-60.1-153)	0 (0-0)	-5.2 (-14.6-6)
Rogaland	0.2 (0-0.5)	-1.5 (0-0)	0.1 (0-0.3)	-1.7 (-7.1-5.7)	0 (0-0.1)	-0.7 (-21.3-25.2)	0 (0-0)	-3.1 (-41.2-43.5)	0 (0-0)	7.8 (-57.2-142)	0 (0-0)	-20 (-28.2--10.1)
Troms og Finnmark	0.2 (0-0.6)	0.1 (0-0)	0.1 (0-0.3)	3.3 (-2.6-8.9)	0 (0-0.1)	-2.2 (-22.9-20.3)	0 (0-0)	5.1 (-31.6-59.1)	0 (0-0)	-6.2 (-61.7-140)	0 (0-0)	-24.3 (-31.7--16.5)
Trøndelag	0.2 (0-0.5)	-1.2 (-50.5-0)	0.1 (0-0.3)	-1.5 (-51.8-8.9)	0 (0-0.1)	-3.7 (-47.3-26.4)	0 (0-0)	1.4 (-37.3-64.6)	0 (0-0)	-2.1 (-61.1-137)	0 (0-0)	-20.1 (-58.2--7.3)
Vestfold og Telemark	0.2 (0-0.5)	-1.4 (0-0)	0.1 (0-0.3)	-1.3 (-7.3-7.5)	0 (0-0.1)	-6 (-26.8-19.6)	0 (0-0)	1.3 (-36-55.5)	0 (0-0)	-5.6 (-64.9-145)	0 (0-0)	-23.2 (-31.7--12.1)
Vestland	0.2 (0-0.5)	-1.5 (0-0)	0.1 (0-0.3)	-1.8 (-7.4-6.6)	0 (0-0.1)	-1.9 (-24.8-23.9)	0 (0-0)	1.2 (-35.3-59.3)	0 (0-0)	-2.8 (-60.8-117)	0 (0-0)	-19.4 (-29.1--7)
Viken	0.2 (0-0.5)	-0.8 (0-0)	0.1 (0-0.3)	-0.8 (-8.1-6.3)	0 (0-0.1)	-3.7 (-26.4-19.9)	0 (0-0)	2.4 (-38.7-54.4)	0 (0-0)	-7.2 (-64.6-123)	0 (0-0)	-19.2 (-29.5--8.2)
Portugal	0 (0-0)	0 (-0.4-0)	0 (0-0)	-1.2 (-5.1-2.4)	0 (0-0)	-2.1 (-17.7-12.2)	0 (0-0)	-12 (-35.3-21.2)	0 (0-0)	-3.6 (-57.5-121)	0 (0-0)	-27.2 (-32.1--22.3)
San Marino	0 (0-0)	0 (0-0)	0 (0-0)	0.1 (-3.8-4.5)	0 (0-0)	-2.9 (-17.3-14.8)	0 (0-0)	0 (-22.3-33.4)	0 (0-0)	14.9 (-40.9-142)	0 (0-0)	-19.8 (-25.5--13.5)
Spain	0.5 (0.4-0.7)	0 (-19.7-33.4)	0.4 (0.3-0.5)	-1.6 (-19.6-27.3)	0.1 (0.1-0.2)	-0.6 (-18.8-23.4)	0 (0-0)	-18.5 (-37.5-6)	0 (0-0)	9.2 (-41.2-95.9)	0 (0-0)	-11 (-26.1-12.6)

Location	Birth Incidence rate (per 100,000 live births), 2021	Birth Incidence rate (per 100,000 live births) percent change (2000-2021)	Under-5 rate (per 100,000 population), 2021	Under-5 rate (per 100,000 population) percent change (2000-2021)	5-14 rate (per 100,000 population), 2021	5-14 rate (per 100,000 population) percent change (2000-2021)	15-49 rate (per 100,000 population), 2021	15-49 rate (per 100,000 population) percent change (2000-2021)	50plus rate (per 100,000 population), 2021	50plus rate (per 100,000 population) percent change (2000-2021)	All age rate (per 100,000 population), 2021	All age rate (per 100,000 population) percent change (2000-2021)
Sweden	0.2 (0.2-0.3)	3.9 (-5.4-71.4)	0.2 (0.1-0.2)	4 (-4.9-65.5)	0.1 (0-0.1)	4.1 (-12.9-53.5)	0 (0-0)	7.8 (-16.2-55.3)	0 (0-0)	-10.2 (-47.2-54.6)	0 (0-0)	4.7 (-11.4-55.4)
Stockholm	0.2 (0.2-0.3)	4.1 (0.1-77)	0.2 (0.1-0.2)	4 (-5.3-75.7)	0.1 (0-0.1)	3.4 (-17.2-74.3)	0 (0-0)	7.7 (-24.1-83.3)	0 (0-0)	-4 (-54.7-99.8)	0 (0-0)	4.7 (-14.7-77.1)
Sweden except Stockholm	0.2 (0.2-0.4)	3.9 (0.1-71.4)	0.2 (0.1-0.3)	4.1 (-5.2-69.3)	0.1 (0-0.1)	4.3 (-14-65.8)	0 (0-0)	8.3 (-22.8-66)	0 (0-0)	-12.1 (-55.7-75.9)	0 (0-0)	4.6 (-13.3-67)
Switzerland	0 (0-0)	-1.6 (-29.3-0)	0 (0-0)	-1.3 (-29.2-4.2)	0 (0-0)	0 (-29.8-16.7)	0 (0-0)	-3.8 (-37-28.9)	0 (0-0)	-6.3 (-60.7-83.9)	0 (0-0)	-8.8 (-34.9--0.6)
United Kingdom	0 (0-0)	0.7 (0-11.8)	0 (0-0)	2.3 (0.9-12.7)	0 (0-0)	-0.5 (-3.3-7.5)	0 (0-0)	-1.4 (-6-5.7)	0 (0-0)	-4.6 (-13.3-6.1)	0 (0-0)	-6.1 (-8-2.8)
England	0 (0-0)	0.3 (-0.7-13.3)	0 (0-0)	1.5 (0.6-13.7)	0 (0-0)	-1 (-2.9-7.9)	0 (0-0)	-1.4 (-4.8-6.5)	0 (0-0)	-4.3 (-12.6-5.3)	0 (0-0)	-5.3 (-6.6-5.3)
East Midlands	0 (0-0)	0.1 (0-1.4)	0 (0-0)	1.6 (0-3.7)	0 (0-0)	-1.5 (-7-4.6)	0 (0-0)	0.4 (-9.8-13.6)	0 (0-0)	-8.3 (-32.8-25.3)	0 (0-0)	-7.7 (-10.4--4.7)
Derby	0 (0-0)	-0.1 (0-0)	0 (0-0)	0.6 (-3.4-4.4)	0 (0-0)	-1 (-15.2-14.8)	0 (0-0)	-4.8 (-30-30.3)	0 (0-0)	12.1 (-41.7-157)	0 (0-0)	-2.5 (-9.4-4.4)
Derbyshire	0 (0-0)	0.3 (0-0)	0 (0-0)	2.1 (-1.9-6)	0 (0-0)	-1.9 (-14.4-13.8)	0 (0-0)	7.7 (-19-46.3)	0 (0-0)	-9.8 (-54.6-80.5)	0 (0-0)	-14.7 (-20.6--8.3)
Leicester	0 (0-0)	0.6 (0-0)	0 (0-0)	1.9 (-2.4-5.9)	0 (0-0)	-1.3 (-16.8-18.3)	0 (0-0)	-9.3 (-32.2-24.2)	0 (0-0)	15.7 (-51.1-111)	0 (0-0)	-4 (-11.2-3.6)
Leicestershire	0 (0-0)	0 (0-0)	0 (0-0)	1.8 (-2-5.7)	0 (0-0)	-0.8 (-14.9-13.5)	0 (0-0)	6.4 (-19.8-42.6)	0 (0-0)	-10.4 (-57.1-82.8)	0 (0-0)	-10.2 (-16.5--3.7)
Lincolnshire	0 (0-0)	0.2 (0-0)	0 (0-0)	2.8 (-1.3-6.9)	0 (0-0)	1.6 (-13.7-17.7)	0 (0-0)	3.5 (-24.2-37)	0 (0-0)	-7.9 (-53.4-76)	0 (0-0)	-8 (-15--1.2)
Northamptonshire	0 (0-0)	0 (0-0)	0 (0-0)	1.3 (-2.7-5.8)	0 (0-0)	-0.6 (-15.6-21.2)	0 (0-0)	0.1 (-25.3-34)	0 (0-0)	-12.6 (-57.4-70.6)	0 (0-0)	-5.7 (-12.9-2.5)
Nottingham	0 (0-0)	0 (0-0)	0 (0-0)	1.5 (-2.6-5.8)	0 (0-0)	-0.4 (-16.9-15.7)	0 (0-0)	-14.7 (-37.8-15.8)	0 (0-0)	21.3 (-44.1-155)	0 (0-0)	3.3 (-4.6-11.1)
Nottinghamshire	0 (0-0)	-0.3 (0-0)	0 (0-0)	1.4 (-2-5.5)	0 (0-0)	-0.9 (-12.4-18.6)	0 (0-0)	3.4 (-23-42.3)	0 (0-0)	-12.3 (-55.8-99.8)	0 (0-0)	-7.8 (-13.7-0)
Rutland	0 (0-0)	0.5 (0-0)	0 (0-0)	2.5 (-1.6-7.1)	0 (0-0)	0.3 (-13.9-18.8)	0 (0-0)	5.1 (-23.1-38.4)	0 (0-0)	-22.4 (-62.2-50)	0 (0-0)	-13.3 (-20.6--4.9)
East of England	0 (0-0)	0 (-3.6-2.5)	0 (0-0)	1.1 (-2.6-3.5)	0 (0-0)	-1.6 (-8.7-4)	0 (0-0)	1.8 (-9.8-13.5)	0 (0-0)	-6.6 (-29.1-23.6)	0 (0-0)	-7.6 (-11.7--4.5)
Bedford	0 (0-0)	0.4 (0-0)	0 (0-0)	2 (-2.3-6.9)	0 (0-0)	-0.6 (-15.1-19.9)	0 (0-0)	0.9 (-24-34.3)	0 (0-0)	-0.4 (-52.8-102)	0 (0-0)	-4.2 (-10.6-4.8)
Cambridgeshire	0 (0-0)	-0.3 (0-0)	0 (0-0)	1 (-3.2-5.4)	0 (0-0)	-2.4 (-19.8-12.6)	0 (0-0)	-4.4 (-29.1-29.8)	0 (0-0)	-10.3 (-56.6-87.5)	0 (0-0)	-10.6 (-19--3.5)
Central Bedfordshire	0 (0-0)	0 (0-0)	0 (0-0)	1.3 (-2.4-5.5)	0 (0-0)	-0.1 (-14.6-16.3)	0 (0-0)	3.9 (-22.6-42.3)	0 (0-0)	-16.2 (-56.1-68.6)	0 (0-0)	-12.4 (-18.8--5.5)
Essex	0 (0-0)	-0.4 (0-0)	0 (0-0)	1.3 (-2.9-5.4)	0 (0-0)	-1.3 (-18.3-16.1)	0 (0-0)	5.3 (-22.3-41.5)	0 (0-0)	-10 (-53.9-71.4)	0 (0-0)	-8.9 (-16.9--1)
Hertfordshire	0 (0-0)	0 (0-0)	0 (0-0)	1 (-2.5-4.5)	0 (0-0)	-1 (-14.7-15.2)	0 (0-0)	5.6 (-19-42.6)	0 (0-0)	-2.4 (-48.7-100)	0 (0-0)	-5.2 (-12.2-2.4)
Luton	0 (0-0)	1.3 (0-0)	0 (0-0)	1.8 (-3.4-6.3)	0 (0-0)	0.2 (-14.8-23.2)	0 (0-0)	-7.5 (-34.1-43.5)	0 (0-0)	2.8 (-46.1-121)	0 (0-0)	1.5 (-6.1-9.4)
Norfolk	0 (0-0)	0 (0-0)	0 (0-0)	1.6 (-2.4-5.8)	0 (0-0)	-0.8 (-15.3-13.7)	0 (0-0)	0.9 (-24.7-32.6)	0 (0-0)	-11 (-53.9-83.6)	0 (0-0)	-6.8 (-13.5-0.2)
Peterborough	0 (0-0)	0 (0-0)	0 (0-0)	1.4 (-4.1-6.7)	0 (0-0)	-0.5 (-16.4-20.6)	0 (0-0)	-8.4 (-32.3-28.4)	0 (0-0)	4.8 (-52.7-135)	0 (0-0)	2.1 (-6.2-11.5)
Southend-on-Sea	0 (0-0)	0 (0-0)	0 (0-0)	1.4 (-2-5.7)	0 (0-0)	-1.8 (-14.9-14.7)	0 (0-0)	-5.6 (-29.3-28.2)	0 (0-0)	7.1 (-43.8-120)	0 (0-0)	-6.7 (-12.6-0.9)
Suffolk	0 (0-0)	0.6 (0-23.3)	0 (0-0)	2.2 (-2.6-23.8)	0 (0-0)	-0.5 (-16.7-20)	0 (0-0)	4.5 (-24.3-37.6)	0 (0-0)	-8.1 (-52.2-73.5)	0 (0-0)	-12 (-19.7-5.3)
Thurrock	0 (0-0)	-1.5 (0-0)	0 (0-0)	-0.8 (-4.8-4.6)	0 (0-0)	-2.2 (-21.6-16.7)	0 (0-0)	-1.4 (-30.2-31.8)	0 (0-0)	-0.1 (-52.9-89.5)	0 (0-0)	-4.3 (-11.6-4.6)
Greater London	0 (0-0)	0.4 (-1.5-74.4)	0 (0-0)	1 (-1.5-78.2)	0 (0-0)	-0.6 (-4.1-67.9)	0 (0-0)	-6.7 (-12.2-31.6)	0 (0-0)	1.8 (-13.4-21.8)	0 (0-0)	2.7 (-0.4-79.3)

Location	Birth Incidence rate (per 100,000 live births), 2021	Birth Incidence rate (per 100,000 live births) percent change (2000-2021)	Under-5 rate (per 100,000 population), 2021	Under-5 rate (per 100,000 population) percent change (2000-2021)	5-14 rate (per 100,000 population), 2021	5-14 rate (per 100,000 population) percent change (2000-2021)	15-49 rate (per 100,000 population), 2021	15-49 rate (per 100,000 population) percent change (2000-2021)	50plus rate (per 100,000 population), 2021	50plus rate (per 100,000 population) percent change (2000-2021)	All age rate (per 100,000 population), 2021	All age rate (per 100,000 population) percent change (2000-2021)
Barking and Dagenham	0 (0-0)	0.9 (0-0)	0 (0-0)	1.7 (-5.3-7.1)	0 (0-0)	0.1 (-21-24.3)	0 (0-0)	-0.2 (-30.3-36)	0 (0-0)	41.2 (-35.9-209)	0 (0-0)	21.5 (9.1-34.7)
Barnet	0 (0-0)	-0.4 (0-0)	0 (0-0)	0.5 (-3.2-4.6)	0 (0-0)	-2.5 (-17-12.6)	0 (0-0)	-1.4 (-24.9-32)	0 (0-0)	4.6 (-47.5-83)	0 (0-0)	4.6 (-3-13.4)
Bexley	0 (0-0)	0.3 (0-7.8)	0 (0-0)	2 (-2.1-7.3)	0 (0-0)	-1.1 (-14.9-16.1)	0 (0-0)	9.7 (-19.4-44)	0 (0-0)	-3 (-53.2-84.7)	0 (0-0)	2.7 (-4.5-11.9)
Brent	0 (0-0)	0 (0-0)	0 (0-0)	-0.5 (-4.2-3.3)	0 (0-0)	0.2 (-13.2-17.3)	0 (0-0)	-6.2 (-29.7-28.1)	0 (0-0)	-3.6 (-52.1-118)	0 (0-0)	9.5 (2.4-17.6)
Bromley	0 (0-0)	-0.4 (0-1.2)	0 (0-0)	0.9 (-3.3-5.7)	0 (0-0)	-0.3 (-14.6-15.5)	0 (0-0)	7.3 (-17.4-44.8)	0 (0-0)	-1.1 (-47.4-83.2)	0 (0-0)	-1.4 (-9.3-6.7)
Camden	0 (0-0)	-1 (0-7.8)	0 (0-0)	0 (-4.6-8.5)	0 (0-0)	-4 (-20.1-13.7)	0 (0-0)	-11.8 (-34.8-23.2)	0 (0-0)	-12.6 (-56.5-90.9)	0 (0-0)	-9.8 (-17.1--1.6)
Croydon	0 (0-0)	5.7 (-6.4-1570)	0 (0-0)	6.3 (-6.1-1490)	0 (0-0)	4.6 (-15-1220)	0 (0-0)	7.7 (-23.7-988)	0 (0-0)	5.5 (-50.5-318)	0 (0-0)	13.4 (-1.4-1490)
Ealing	0 (0-0)	0.5 (0-0)	0 (0-0)	0.2 (-5.8-6.8)	0 (0-0)	0.6 (-19.4-24.6)	0 (0-0)	-2.7 (-32.3-45)	0 (0-0)	5.7 (-54.6-129)	0 (0-0)	6 (-4-17.8)
Enfield	0 (0-0)	0.1 (0-0)	0 (0-0)	0.4 (-3.5-4.5)	0 (0-0)	-3.2 (-17.1-12.1)	0 (0-0)	7.2 (-20.5-44.2)	0 (0-0)	14.8 (-40.4-119)	0 (0-0)	7.6 (-0.2-15.5)
Greenwich	0 (0-0)	0 (-7.2-0)	0 (0-0)	0.8 (-8.1-5.6)	0 (0-0)	-0.1 (-18.3-26.1)	0 (0-0)	-10.9 (-32.9-33.4)	0 (0-0)	21 (-36.8-150)	0 (0-0)	3 (-7.8-12.5)
Hackney	0 (0-0)	0 (0-0)	0 (0-0)	0.6 (-3.5-4.5)	0 (0-0)	0.4 (-15.6-17.6)	0 (0-0)	-19.4 (-42.6-13.3)	0 (0-0)	12.9 (-46.2-156)	0 (0-0)	-12 (-17.6--6.2)
Hammersmith and Fulham	0 (0-0)	0 (0-0)	0 (0-0)	0.2 (-3.5-4.4)	0 (0-0)	-1.6 (-17.2-14.6)	0 (0-0)	-14.3 (-38.4-19.3)	0 (0-0)	15.7 (-43.8-163)	0 (0-0)	-3.1 (-9.4-3.6)
Haringey	0 (0-0)	0 (0-0)	0 (0-0)	-0.3 (-3.6-3.2)	0 (0-0)	-2.8 (-15.2-13.7)	0 (0-0)	-9.5 (-29.1-19.8)	0 (0-0)	3.8 (-45.6-139)	0 (0-0)	-7.7 (-13.4--1.3)
Harrow	0 (0-0)	0 (0-0)	0 (0-0)	0.5 (-3.6-5)	0 (0-0)	1.4 (-13.7-20.7)	0 (0-0)	0.1 (-24-36.2)	0 (0-0)	-1.2 (-53.4-96.2)	0 (0-0)	10.8 (2.2-20.8)
Havering	0 (0-0)	0.4 (0-0)	0 (0-0)	2.6 (-1.8-7.5)	0 (0-0)	1 (-12-19.8)	0 (0-0)	3.9 (-19.4-37.7)	0 (0-0)	-6.7 (-52.3-87.8)	0 (0-0)	9.6 (1.7-20)
Hillingdon	0 (0-0)	-0.3 (-7.2-0)	0 (0-0)	0.8 (-5.7-5.2)	0 (0-0)	-0.2 (-14.8-18.2)	0 (0-0)	0.6 (-24.1-39.2)	0 (0-0)	5.8 (-50.4-109)	0 (0-0)	6.1 (-2.2-14.8)
Hounslow	0 (0-0)	0 (0-0)	0 (0-0)	-0.2 (-3.9-3.7)	0 (0-0)	-0.4 (-14.5-15.4)	0 (0-0)	-11.8 (-33.5-13.1)	0 (0-0)	0.6 (-51.4-108)	0 (0-0)	3.6 (-3.1-10.6)
Islington	0 (0-0)	0 (0-0)	0 (0-0)	1.8 (-1.8-5.9)	0 (0-0)	0.3 (-12.2-17.8)	0 (0-0)	-17.9 (-38.8-12.2)	0 (0-0)	9 (-51.3-151)	0 (0-0)	-6.8 (-12.5--0.5)
Kensington and Chelsea	0 (0-0)	0 (0-0)	0 (0-0)	1.4 (-2.9-5.7)	0 (0-0)	-2.2 (-15.1-15.2)	0 (0-0)	-7.9 (-30.5-24.9)	0 (0-0)	-7.3 (-54.2-98.6)	0 (0-0)	-10.5 (-16.3--3.5)
Kingston upon Thames	0 (0-0)	-0.4 (0-0)	0 (0-0)	0.9 (-3.4-5.6)	0 (0-0)	-0.6 (-15.8-17.4)	0 (0-0)	-5 (-28.5-29.6)	0 (0-0)	-13 (-54.7-78.5)	0 (0-0)	5.7 (-2.5-14.9)
Lambeth	0 (0-0)	0 (0-0)	0 (0-0)	0.7 (-3.2-5.1)	0 (0-0)	-0.2 (-14.8-15.8)	0 (0-0)	-9.5 (-33.4-22.9)	0 (0-0)	26.1 (-40.7-176)	0 (0-0)	-5.8 (-11.7-0.5)
Lewisham	0 (0-0)	0 (0-0)	0 (0-0)	0.8 (-3.3-4.9)	0 (0-0)	-0.5 (-14.8-15.7)	0 (0-0)	-5.4 (-29.2-30.6)	0 (0-0)	23.6 (-40.1-166)	0 (0-0)	4.7 (-2.4-11.9)
Merton	0 (0-0)	0.4 (0-231)	0 (0-0)	0.9 (-5.2-231)	0 (0-0)	0 (-17.3-226)	0 (0-0)	-6.8 (-32-153)	0 (0-0)	2.5 (-53-139)	0 (0-0)	7.6 (-2.1-251)
Newham	0 (0-0)	0 (0-0)	0 (0-0)	1.1 (-2.8-5.2)	0 (0-0)	1.2 (-12.9-18.9)	0 (0-0)	-23.5 (-43.7-5)	0 (0-0)	15.3 (-40.4-138)	0 (0-0)	-12.8 (-18.4--6.8)
Redbridge	0 (0-0)	-1.1 (0-0.4)	0 (0-0)	-0.5 (-4.7-5.6)	0 (0-0)	-1.4 (-18.5-20.3)	0 (0-0)	-1.4 (-27.8-34.3)	0 (0-0)	0 (-52.2-109)	0 (0-0)	12.2 (2.8-23.7)
Richmond upon Thames	0 (0-0)	-0.8 (-20.4-0)	0 (0-0)	-1.3 (-20.6-3.2)	0 (0-0)	-1.6 (-19.6-13.4)	0 (0-0)	4.7 (-22-38.7)	0 (0-0)	-6.5 (-51.3-80.9)	0 (0-0)	-3 (-22.9-5)
Southwark	0 (0-0)	0.3 (0-0)	0 (0-0)	1.6 (-4.4-9.8)	0 (0-0)	-0.6 (-18.7-32.3)	0 (0-0)	-14.7 (-38.6-35.9)	0 (0-0)	28.3 (-40.2-163)	0 (0-0)	-3.2 (-11.2-9.5)
Sutton	0 (0-0)	0 (0-0)	0 (0-0)	1.5 (-2.4-5.8)	0 (0-0)	-1.2 (-16.1-14)	0 (0-0)	0 (-25-32.1)	0 (0-0)	-3.9 (-52.9-87.8)	0 (0-0)	-1.2 (-7.9-6.4)
Tower Hamlets	0 (0-0)	0.1 (0-0)	0 (0-0)	0.8 (-3.3-4.4)	0 (0-0)	3.2 (-11.2-21.1)	0 (0-0)	-26.6 (-46.6-2.7)	0 (0-0)	31.2 (-35.4-189)	0 (0-0)	-6 (-11.9-0.6)

Location	Birth Incidence rate (per 100,000 live births), 2021	Birth Incidence rate (per 100,000 live births) percent change (2000-2021)	Under-5 rate (per 100,000 population), 2021	Under-5 rate (per 100,000 population) percent change (2000-2021)	5-14 rate (per 100,000 population), 2021	5-14 rate (per 100,000 population) percent change (2000-2021)	15-49 rate (per 100,000 population), 2021	15-49 rate (per 100,000 population) percent change (2000-2021)	50plus rate (per 100,000 population), 2021	50plus rate (per 100,000 population) percent change (2000-2021)	All age rate (per 100,000 population), 2021	All age rate (per 100,000 population) percent change (2000-2021)
Waltham Forest	0 (0-0)	-0.4 (0-0)	0 (0-0)	0.2 (-3.9-4.2)	0 (0-0)	0 (-14-16.6)	0 (0-0)	-4.9 (-29.5-26.4)	0 (0-0)	20.9 (-41.7-128)	0 (0-0)	4 (-3.1-11.2)
Wandsworth	0 (0-0)	0.2 (0-6.9)	0 (0-0)	0.1 (-3.8-6.2)	0 (0-0)	-1.3 (-14.6-13.4)	0 (0-0)	-12.3 (-36-16.2)	0 (0-0)	15.4 (-41.8-136)	0 (0-0)	10.2 (3.3-18.1)
Westminster	0 (0-0)	0 (0-0)	0 (0-0)	-0.6 (-4-3.5)	0 (0-0)	-3.2 (-17-14.5)	0 (0-0)	-9.3 (-34.6-26.8)	0 (0-0)	-1.6 (-53.6-115)	0 (0-0)	3.1 (-3.3-11.5)
North East England	0 (0-0)	0 (0-1.8)	0 (0-0)	1.8 (0.1-5.7)	0 (0-0)	-0.3 (-6.1-8.3)	0 (0-0)	-2.9 (-13.2-7.3)	0 (0-0)	-5.6 (-27.4-21.9)	0 (0-0)	-8.2 (-11.6--3.6)
County Durham	0 (0-0)	0 (0-0)	0 (0-0)	1.9 (-2-5.8)	0 (0-0)	-0.7 (-14.1-13.2)	0 (0-0)	-2.3 (-25.6-27.2)	0 (0-0)	-10.7 (-52.2-82.6)	0 (0-0)	-9.5 (-16.2--3.2)
Darlington	0 (0-0)	-1.1 (0-0)	0 (0-0)	0.2 (-4.3-5.5)	0 (0-0)	-3 (-19.6-14.1)	0 (0-0)	3.8 (-26.3-37.6)	0 (0-0)	-5.2 (-51.6-95.8)	0 (0-0)	-10.7 (-18.1--2.6)
Gateshead	0 (0-0)	0 (0-0)	0 (0-0)	1.4 (-2.8-5.6)	0 (0-0)	0.2 (-14.4-16.7)	0 (0-0)	-8.3 (-31.3-21.4)	0 (0-0)	-1.7 (-52.9-93.9)	0 (0-0)	-8.6 (-15--1.8)
Hartlepool	0 (0-0)	-1.1 (0-0)	0 (0-0)	1.2 (-3.5-6.8)	0 (0-0)	-1.7 (-20.2-15.9)	0 (0-0)	-0.5 (-29.8-34.3)	0 (0-0)	-1.7 (-49.1-121)	0 (0-0)	-14.5 (-21.2--7.3)
Middlesbrough	0 (0-0)	1 (0-0)	0 (0-0)	2.4 (-2.4-7.3)	0 (0-0)	3.3 (-11.6-26.3)	0 (0-0)	-4.8 (-28.4-37.4)	0 (0-0)	1.3 (-50.5-125)	0 (0-0)	3.6 (-4.1-13)
Newcastle upon Tyne	0 (0-0)	-1.1 (0-0)	0 (0-0)	0.8 (-3-6)	0 (0-0)	0.5 (-17.6-17.9)	0 (0-0)	-7.7 (-36.7-22.8)	0 (0-0)	2.4 (-51-119)	0 (0-0)	3 (-6-11.7)
North Tyneside	0 (0-0)	0 (0-0)	0 (0-0)	1.4 (-2.6-6.2)	0 (0-0)	0.3 (-14.8-16.8)	0 (0-0)	-3.3 (-27.5-28.2)	0 (0-0)	-3.2 (-53.5-110)	0 (0-0)	-6.2 (-12.8-1.2)
Northumberland	0 (0-0)	0.2 (0-0)	0 (0-0)	1.7 (-2.6-6.6)	0 (0-0)	-2.6 (-16.7-15.7)	0 (0-0)	6.6 (-19.6-47.8)	0 (0-0)	-15.1 (-59.3-80.5)	0 (0-0)	-15.3 (-21.6--6.2)
Redcar and Cleveland	0 (0-0)	1.6 (0-0.1)	0 (0-0)	3.8 (-2.1-8.6)	0 (0-0)	3.1 (-13.6-25.9)	0 (0-0)	1.6 (-29.5-51)	0 (0-0)	-3.5 (-57.2-96.9)	0 (0-0)	-7.8 (-16-1.1)
South Tyneside	0 (0-0)	0 (0-0)	0 (0-0)	2.1 (-1.6-6.2)	0 (0-0)	0.4 (-11.6-15.6)	0 (0-0)	-4.1 (-28.4-25.5)	0 (0-0)	4.6 (-52.1-130)	0 (0-0)	-10.1 (-15.8--3.6)
Stockton-on-Tees	0 (0-0)	0.8 (0-3.9)	0 (0-0)	2.6 (-3.8-12.6)	0 (0-0)	2.2 (-19.8-38.6)	0 (0-0)	-0.2 (-32-64)	0 (0-0)	-4.2 (-51.8-100)	0 (0-0)	-7.1 (-17.5-11.8)
Sunderland	0 (0-0)	0 (0-0)	0 (0-0)	1.8 (-2.1-6.1)	0 (0-0)	-1.4 (-14-13.7)	0 (0-0)	-7.1 (-32.1-20.1)	0 (0-0)	-2.6 (-54.2-85)	0 (0-0)	-12.2 (-18.2--5.7)
North West England	0 (0-0)	0.1 (0-1.1)	0 (0-0)	1.7 (0.4-3.3)	0 (0-0)	-1 (-4.8-3.3)	0 (0-0)	-4.7 (-11.7-3.6)	0 (0-0)	-5.4 (-23.6-16.9)	0 (0-0)	-7.4 (-9.5--5)
Blackburn with Darwen	0 (0-0)	1.1 (0-0)	0 (0-0)	2.4 (-2.5-6.1)	0 (0-0)	-2.4 (-15.5-15.4)	0 (0-0)	-7.5 (-29-35.3)	0 (0-0)	-4.2 (-53-121)	0 (0-0)	-16.7 (-22.6--10.7)
Blackpool	0 (0-0)	0 (0-0)	0 (0-0)	2.3 (-1.4-6)	0 (0-0)	0.4 (-13.4-16.4)	0 (0-0)	3.4 (-21-39.1)	0 (0-0)	5.4 (-47.3-129)	0 (0-0)	-1.5 (-7.9-4.9)
Bolton	0 (0-0)	-1.5 (0-0)	0 (0-0)	0.4 (-4.7-6.4)	0 (0-0)	-3.2 (-19.9-13.9)	0 (0-0)	-3.6 (-34.5-33.7)	0 (0-0)	-8.1 (-60-90.4)	0 (0-0)	-7.7 (-14.1-0.4)
Bury	0 (0-0)	0 (0-0)	0 (0-0)	1.4 (-2.3-5.6)	0 (0-0)	1 (-13.2-18.5)	0 (0-0)	3.6 (-21.6-37.6)	0 (0-0)	-6.9 (-53.5-111)	0 (0-0)	-5.4 (-12.1-1.9)
Cheshire East	0 (0-0)	0 (0-0)	0 (0-0)	2.1 (-1.9-6)	0 (0-0)	-1.5 (-16.3-15.4)	0 (0-0)	6.5 (-22.5-43)	0 (0-0)	-10.8 (-54.5-82.6)	0 (0-0)	-13 (-19.7--6.4)
Cheshire West and Chester	0 (0-0)	-1.9 (-1.5-0)	0 (0-0)	0.4 (-4.8-6.8)	0 (0-0)	-4.8 (-26.5-13.6)	0 (0-0)	-0.8 (-40.4-36.1)	0 (0-0)	-10.8 (-56.5-70.5)	0 (0-0)	-15 (-24.8--6.6)
Cumbria	0 (0-0)	-0.2 (0-0)	0 (0-0)	1.6 (-3.2-6)	0 (0-0)	-1.8 (-16.8-15.1)	0 (0-0)	3.3 (-23.5-44.2)	0 (0-0)	-13.9 (-60-79)	0 (0-0)	-15.8 (-22.8--8.8)
Halton	0 (0-0)	0.8 (0-1.6)	0 (0-0)	1.7 (-4.6-10.2)	0 (0-0)	1.7 (-18.9-35.3)	0 (0-0)	-1 (-35.7-37.4)	0 (0-0)	-14.7 (-61-87.5)	0 (0-0)	-11.6 (-20.4-1.2)
Knowsley	0 (0-0)	-0.8 (0-0)	0 (0-0)	1.5 (-3.7-6.8)	0 (0-0)	1.6 (-17.2-19.8)	0 (0-0)	-1.5 (-33.8-31.9)	0 (0-0)	1.6 (-51.2-95.4)	0 (0-0)	-5.7 (-13.5-2.6)
Lancashire	0 (0-0)	0 (0-0)	0 (0-0)	2 (-1.8-6.6)	0 (0-0)	-0.3 (-14-16.1)	0 (0-0)	-2.8 (-27.4-30.6)	0 (0-0)	-12.1 (-55.6-92.1)	0 (0-0)	-10.4 (-16.5--3.6)
Liverpool	0 (0-0)	0 (0-0)	0 (0-0)	2.2 (-1.6-6.3)	0 (0-0)	4.4 (-9.9-22.3)	0 (0-0)	-18.4 (-39.4-7.9)	0 (0-0)	5.9 (-49.6-115)	0 (0-0)	-4.1 (-11.3-3.2)
Manchester	0 (0-0)	0 (0-0)	0 (0-0)	1.3 (-2.6-5.4)	0 (0-0)	2.1 (-13-19.7)	0 (0-0)	-20.8 (-41.5-10.4)	0 (0-0)	33 (-39.7-190)	0 (0-0)	4.2 (-2.9-11.8)

Location	Birth Incidence rate (per 100,000 live births), 2021	Birth Incidence rate (per 100,000 live births) percent change (2000-2021)	Under-5 rate (per 100,000 population), 2021	Under-5 rate (per 100,000 population) percent change (2000-2021)	5-14 rate (per 100,000 population), 2021	5-14 rate (per 100,000 population) percent change (2000-2021)	15-49 rate (per 100,000 population), 2021	15-49 rate (per 100,000 population) percent change (2000-2021)	50plus rate (per 100,000 population), 2021	50plus rate (per 100,000 population) percent change (2000-2021)	All age rate (per 100,000 population), 2021	All age rate (per 100,000 population) percent change (2000-2021)
Oldham	0 (0-0)	0 (0-0)	0 (0-0)	1.4 (-2.3-5.4)	0 (0-0)	-1.8 (-15.6-16.1)	0 (0-0)	3.5 (-22.3-42.4)	0 (0-0)	-3.4 (-51.4-100)	0 (0-0)	-3.8 (-10-2.9)
Rochdale	0 (0-0)	0 (0-0)	0 (0-0)	2.1 (-2-6)	0 (0-0)	-0.1 (-14.3-18.8)	0 (0-0)	1.3 (-25.9-33.1)	0 (0-0)	-1.6 (-52.5-103)	0 (0-0)	-6 (-12.1-0.7)
Salford	0 (0-0)	0.8 (0-1.6)	0 (0-0)	2.4 (-3.8-9.3)	0 (0-0)	3.3 (-18.9-36.7)	0 (0-0)	-16.2 (-45.2-17.7)	0 (0-0)	4.7 (-49-111)	0 (0-0)	11.1 (-0.8-23.1)
Sefton	0 (0-0)	0 (0-0)	0 (0-0)	2.4 (-2.2-7.1)	0 (0-0)	1.2 (-12.8-18.3)	0 (0-0)	-2.4 (-28.1-32)	0 (0-0)	-7.4 (-57.4-78.5)	0 (0-0)	-8.7 (-15.7--1.2)
St Helens	0 (0-0)	1.6 (0-0.1)	0 (0-0)	3.7 (-1.5-8)	0 (0-0)	1.5 (-14.1-24.2)	0 (0-0)	-2.8 (-30.2-61.8)	0 (0-0)	-7.6 (-59.9-93.3)	0 (0-0)	-7.3 (-14.9-0.6)
Stockport	0 (0-0)	0 (0-0)	0 (0-0)	1.3 (-2.8-5.6)	0 (0-0)	0.2 (-15.5-16.6)	0 (0-0)	3.7 (-26.6-35.6)	0 (0-0)	-7.7 (-57.4-75.8)	0 (0-0)	-4.3 (-12.2-3.4)
Tameside	0 (0-0)	-0.8 (-1.5-0)	0 (0-0)	1 (-6.3-7.2)	0 (0-0)	-1.4 (-27.6-28.5)	0 (0-0)	-4.5 (-42.2-42.4)	0 (0-0)	-4.7 (-56.3-113)	0 (0-0)	-5.6 (-16.4-5.2)
Trafford	0 (0-0)	0 (0-0)	0 (0-0)	0.1 (-3.7-4)	0 (0-0)	-2.6 (-15.3-14.2)	0 (0-0)	4.8 (-22.9-43.8)	0 (0-0)	4.1 (-53-112)	0 (0-0)	-3.2 (-10.3-4.5)
Warrington	0 (0-0)	0 (0-0)	0 (0-0)	1.2 (-2.8-5.7)	0 (0-0)	-1.4 (-17.1-15)	0 (0-0)	4.1 (-24.3-43.3)	0 (0-0)	-3.2 (-56.1-109)	0 (0-0)	-13.6 (-19.7--7.7)
Wigan	0 (0-0)	1.1 (0-0)	0 (0-0)	2.6 (-2.6-8.2)	0 (0-0)	-1.1 (-17.5-26.7)	0 (0-0)	-1 (-27.8-50.5)	0 (0-0)	-8.3 (-53.1-114)	0 (0-0)	-10.3 (-18.2--0.7)
Wirral	0 (0-0)	0 (0-0)	0 (0-0)	1.8 (-2.2-6.2)	0 (0-0)	0.2 (-14.9-17.6)	0 (0-0)	-0.1 (-25.1-34.8)	0 (0-0)	-4.3 (-54.5-99.7)	0 (0-0)	-10.8 (-17.4--4)
South East England	0 (0-0)	0.6 (-0.2-2.5)	0 (0-0)	1.6 (-0.4-4.2)	0 (0-0)	-2.1 (-7.2-4.2)	0 (0-0)	2.2 (-7.8-15)	0 (0-0)	-6.4 (-27.1-18.2)	0 (0-0)	-7.2 (-9.8--4.3)
Bracknell Forest	0 (0-0)	2 (0-7.8)	0 (0-0)	2.5 (-3-10.1)	0 (0-0)	0.2 (-14.3-25.3)	0 (0-0)	3.1 (-21-53.9)	0 (0-0)	-9.6 (-53.7-82.1)	0 (0-0)	-11.5 (-18.9-0.7)
Brighton and Hove	0 (0-0)	0 (0-0)	0 (0-0)	1.3 (-2.6-5.2)	0 (0-0)	-4 (-18.7-11)	0 (0-0)	-4.9 (-29.4-26.9)	0 (0-0)	23.9 (-41.7-141)	0 (0-0)	-10.1 (-16.6--3.6)
Buckinghamshire	0 (0-0)	-0.5 (-1.2-0)	0 (0-0)	0.7 (-3.6-5.1)	0 (0-0)	-3.2 (-18.7-15.6)	0 (0-0)	8.3 (-17.7-44.8)	0 (0-0)	-11 (-53.5-70.9)	0 (0-0)	-11.1 (-18.9--3.5)
East Sussex	0 (0-0)	1.2 (0-0.1)	0 (0-0)	3.5 (-1.7-9)	0 (0-0)	-0.6 (-15.3-20.6)	0 (0-0)	6.7 (-21.1-47.7)	0 (0-0)	-2.1 (-49.5-96)	0 (0-0)	-12 (-19.4--0.6)
Hampshire	0 (0-0)	-0.1 (0-0)	0 (0-0)	1.3 (-2.6-5.1)	0 (0-0)	-2.1 (-16.2-12.9)	0 (0-0)	5.4 (-19.5-35.9)	0 (0-0)	-13.1 (-59.2-76.9)	0 (0-0)	-12.8 (-19--6.1)
Isle of Wight	0 (0-0)	0 (0-0)	0 (0-0)	2.9 (-1-6.9)	0 (0-0)	-0.9 (-14.7-15.4)	0 (0-0)	4.7 (-19.4-39.8)	0 (0-0)	-6.1 (-50.2-85.2)	0 (0-0)	-10.6 (-17--4.1)
Kent	0 (0-0)	1.5 (0-0.1)	0 (0-0)	3.2 (-2.3-8.8)	0 (0-0)	-1 (-16.9-20.3)	0 (0-0)	4 (-22.5-55.9)	0 (0-0)	-7.4 (-53.5-79.1)	0 (0-0)	-5.8 (-14.2-3.2)
Medway	0 (0-0)	0 (0-0)	0 (0-0)	2.1 (-2.2-6.2)	0 (0-0)	-0.5 (-13.9-13.9)	0 (0-0)	1.1 (-22.5-39)	0 (0-0)	-7.9 (-48.9-97.3)	0 (0-0)	-6.7 (-13.1--0.5)
Milton Keynes	0 (0-0)	0 (0-0)	0 (0-0)	-0.1 (-4.1-4.2)	0 (0-0)	-1.8 (-16.3-13.7)	0 (0-0)	-5.4 (-30.4-24.5)	0 (0-0)	-13.3 (-59-66.1)	0 (0-0)	-3.7 (-11-3.6)
Oxfordshire	0 (0-0)	0.2 (0-0)	0 (0-0)	1.5 (-2.2-5.4)	0 (0-0)	-1.6 (-14.3-13.8)	0 (0-0)	-2.2 (-27-31.7)	0 (0-0)	-8.6 (-57.4-76.7)	0 (0-0)	-8.6 (-14.9--2.3)
Portsmouth	0 (0-0)	0.8 (0-25.6)	0 (0-0)	1.8 (-2.7-26.8)	0 (0-0)	1 (-14.5-32.1)	0 (0-0)	-2.8 (-27.1-43.2)	0 (0-0)	15.3 (-42.9-155)	0 (0-0)	2.5 (-5.6-28.3)
Reading	0 (0-0)	0.4 (0-0)	0 (0-0)	-0.1 (-4.3-5)	0 (0-0)	0.5 (-12.9-19.5)	0 (0-0)	-14 (-36.8-16.5)	0 (0-0)	4.9 (-47.7-127)	0 (0-0)	13 (4.6-23.9)
Slough	0 (0-0)	0.2 (0-0)	0 (0-0)	-0.8 (-4.7-2.8)	0 (0-0)	-1.6 (-16.9-15.2)	0 (0-0)	-5 (-30.5-33.3)	0 (0-0)	19.9 (-50.8-158)	0 (0-0)	11.9 (4.4-19.5)
Southampton	0 (0-0)	0.2 (0-6.9)	0 (0-0)	1.6 (-3-7.2)	0 (0-0)	0 (-13.8-17.4)	0 (0-0)	-18.4 (-38.7-13.7)	0 (0-0)	10.3 (-43.4-123)	0 (0-0)	8.8 (0.9-19)
Surrey	0 (0-0)	0 (0-0)	0 (0-0)	0.8 (-2.9-4.9)	0 (0-0)	-2.9 (-16-12)	0 (0-0)	8.1 (-21.9-44.5)	0 (0-0)	-3.5 (-47.9-80.9)	0 (0-0)	-5.6 (-12.4-1.9)
West Berkshire	0 (0-0)	0 (0-0)	0 (0-0)	0.8 (-2.8-5.2)	0 (0-0)	-3.4 (-15.9-12.7)	0 (0-0)	6.7 (-16.3-39.8)	0 (0-0)	-16.3 (-57.4-60)	0 (0-0)	-12 (-17.7--5.2)
West Sussex	0 (0-0)	1.8 (0-6.9)	0 (0-0)	3.1 (-2.1-9.8)	0 (0-0)	0.2 (-14-23.8)	0 (0-0)	1.8 (-23.3-42.8)	0 (0-0)	-0.8 (-45.8-92.1)	0 (0-0)	-6.9 (-13.9-3.2)

Location	Birth Incidence rate (per 100,000 live births), 2021	Birth Incidence rate (per 100,000 live births) percent change (2000-2021)	Under-5 rate (per 100,000 population), 2021	Under-5 rate (per 100,000 population) percent change (2000-2021)	5-14 rate (per 100,000 population), 2021	5-14 rate (per 100,000 population) percent change (2000-2021)	15-49 rate (per 100,000 population), 2021	15-49 rate (per 100,000 population) percent change (2000-2021)	50plus rate (per 100,000 population), 2021	50plus rate (per 100,000 population) percent change (2000-2021)	All age rate (per 100,000 population), 2021	All age rate (per 100,000 population) percent change (2000-2021)
Windsor and Maidenhead	0 (0-0)	-0.3 (0-0)	0 (0-0)	0.2 (-4.5-4.6)	0 (0-0)	-3.5 (-18.1-14.3)	0 (0-0)	2.8 (-25.1-39.6)	0 (0-0)	-8.7 (-51.4-82.3)	0 (0-0)	-10.3 (-17.9--3.1)
Wokingham	0 (0-0)	-0.1 (0-0)	0 (0-0)	0 (-3.2-4.1)	0 (0-0)	-2.9 (-14.4-13.8)	0 (0-0)	5.1 (-18.6-41.4)	0 (0-0)	-19.5 (-56.2-45.8)	0 (0-0)	-11.9 (-17.9--3.8)
South West England	0 (0-0)	0 (-1.5-3.8)	0 (0-0)	1.6 (0.1-5.8)	0 (0-0)	-0.9 (-5.4-4.2)	0 (0-0)	0.9 (-7.6-9.6)	0 (0-0)	-7 (-24.1-15.4)	0 (0-0)	-7.4 (-9.7--4)
Bath and North East Somerset	0 (0-0)	0.2 (0-0)	0 (0-0)	2.6 (-1.7-6.5)	0 (0-0)	-0.5 (-15.7-15.7)	0 (0-0)	2.2 (-24.1-31.9)	0 (0-0)	-2.1 (-53.9-86.8)	0 (0-0)	-6.6 (-14-0.9)
Bournemouth	0 (0-0)	-0.2 (-6.9-0)	0 (0-0)	1.8 (-4.9-6.1)	0 (0-0)	0.7 (-15.8-19.7)	0 (0-0)	-20.7 (-41.4-6.7)	0 (0-0)	8.4 (-41.1-124)	0 (0-0)	6.5 (-3.2-16.5)
Bristol, City of	0 (0-0)	-0.5 (0-0)	0 (0-0)	0.9 (-3.9-5.5)	0 (0-0)	2 (-14.2-18)	0 (0-0)	-15.1 (-36.3-16.6)	0 (0-0)	2.4 (-44.7-131)	0 (0-0)	5.8 (-3-14)
Cornwall	0 (0-0)	0 (0-0)	0 (0-0)	2.1 (-1.9-6.2)	0 (0-0)	-0.6 (-13.5-16.5)	0 (0-0)	2.3 (-21-36.9)	0 (0-0)	-14.5 (-58-78)	0 (0-0)	-10 (-16.5--2.9)
Devon	0 (0-0)	-0.2 (0-0)	0 (0-0)	1.7 (-2.6-6)	0 (0-0)	-0.5 (-14-15.8)	0 (0-0)	4.7 (-21.3-37.7)	0 (0-0)	-8.4 (-55.3-84)	0 (0-0)	-8.6 (-15.1--1.4)
Dorset	0 (0-0)	-0.5 (-18.9-0)	0 (0-0)	1.3 (-15.5-6.5)	0 (0-0)	-1.9 (-15.7-15.6)	0 (0-0)	11.2 (-14.9-49.2)	0 (0-0)	-8.8 (-53.6-79.9)	0 (0-0)	-12.4 (-24.8--4.4)
Gloucestershire	0 (0-0)	-0.1 (0-0)	0 (0-0)	1.8 (-2-6.3)	0 (0-0)	-1.2 (-15.8-13.9)	0 (0-0)	3.2 (-21.6-37.4)	0 (0-0)	-6.3 (-51.8-99.7)	0 (0-0)	-10.9 (-17.6--4.1)
North Somerset	0 (0-0)	-0.1 (0-0)	0 (0-0)	1.6 (-1.8-5.8)	0 (0-0)	-1.1 (-14-15)	0 (0-0)	5.7 (-18.5-41.6)	0 (0-0)	-9.8 (-53.1-88.1)	0 (0-0)	-6.8 (-12.8-0.2)
Plymouth	0 (0-0)	0.5 (0-0)	0 (0-0)	2.3 (-2.2-7.1)	0 (0-0)	0.9 (-13.9-20.2)	0 (0-0)	-9.7 (-34-21.6)	0 (0-0)	-0.9 (-50.9-99.5)	0 (0-0)	-3 (-10.3-5.9)
Poole	0 (0-0)	-0.4 (0-0)	0 (0-0)	0.5 (-4.4-5.8)	0 (0-0)	-2.1 (-18.8-14.5)	0 (0-0)	-4.2 (-28.7-26.4)	0 (0-0)	-7.9 (-55.5-94.6)	0 (0-0)	-7.2 (-16.2-1.2)
Somerset	0 (0-0)	-0.4 (0-0)	0 (0-0)	1.5 (-1.9-5.7)	0 (0-0)	-0.6 (-16.5-13.7)	0 (0-0)	7 (-22.1-39.7)	0 (0-0)	-13.7 (-59.3-82.9)	0 (0-0)	-9.7 (-16.8--3.2)
South Gloucestershire	0 (0-0)	-0.1 (0-0)	0 (0-0)	1.3 (-2.2-5.4)	0 (0-0)	-2.8 (-15.3-11.6)	0 (0-0)	7.5 (-18-39.4)	0 (0-0)	-8.8 (-53.5-66.7)	0 (0-0)	-10.7 (-16.6--4.1)
Swindon	0 (0-0)	0 (0-0)	0 (0-0)	1.1 (-2.9-5.7)	0 (0-0)	-0.7 (-15.3-14.9)	0 (0-0)	0.4 (-24.7-33.2)	0 (0-0)	2.5 (-48.9-104)	0 (0-0)	-7.2 (-13.4-0)
Torbay	0 (0-0)	0.8 (0-0)	0 (0-0)	2.8 (-2.4-7.1)	0 (0-0)	1.5 (-13.3-20)	0 (0-0)	3.4 (-22.6-39.8)	0 (0-0)	-8.2 (-57.2-91.6)	0 (0-0)	0.7 (-7.9-8.7)
Wiltshire	0 (0-0)	1.4 (0-50.3)	0 (0-0)	2.8 (-2.4-52.3)	0 (0-0)	-2.6 (-16.6-41.6)	0 (0-0)	11.4 (-17.4-61.3)	0 (0-0)	-9.7 (-57.4-91.1)	0 (0-0)	-14.1 (-21.1-26.1)
West Midlands	0 (0-0)	0 (0-0)	0 (0-0)	1.3 (0.1-2.7)	0 (0-0)	-1.5 (-6.3-3.5)	0 (0-0)	-1 (-10.3-9.8)	0 (0-0)	-5.1 (-25.3-20.2)	0 (0-0)	-6.5 (-8.6--4.2)
Birmingham	0 (0-0)	0 (0-0)	0 (0-0)	1.3 (-2.6-5)	0 (0-0)	0 (-14-16.4)	0 (0-0)	-5.5 (-28.6-26.3)	0 (0-0)	16.3 (-45-159)	0 (0-0)	-3.1 (-9.2-3.2)
Coventry	0 (0-0)	0.1 (0-0)	0 (0-0)	1.6 (-2.4-5.4)	0 (0-0)	0.2 (-14.3-16.8)	0 (0-0)	-12.2 (-34.2-18.9)	0 (0-0)	4.9 (-47.1-94.2)	0 (0-0)	4.3 (-2.9-12)
Dudley	0 (0-0)	0.1 (0-0)	0 (0-0)	1.6 (-2.3-5)	0 (0-0)	0.2 (-12.3-15.6)	0 (0-0)	7.4 (-18.7-40.7)	0 (0-0)	-1.2 (-48.9-84.6)	0 (0-0)	-3.1 (-9.8-3.3)
Herefordshire, County of	0 (0-0)	0.1 (0-0)	0 (0-0)	2.7 (-1.1-6.7)	0 (0-0)	0.7 (-12.9-18.3)	0 (0-0)	5.9 (-21.3-42.9)	0 (0-0)	-11.3 (-58-71.6)	0 (0-0)	-13.5 (-19.5--6.2)
Sandwell	0 (0-0)	-0.1 (0-0)	0 (0-0)	1.2 (-2.4-5.1)	0 (0-0)	-0.2 (-14.7-16.2)	0 (0-0)	-3.2 (-28-29.2)	0 (0-0)	16.7 (-41.7-169)	0 (0-0)	2.2 (-4.3-9.4)
Shropshire	0 (0-0)	0 (0-0)	0 (0-0)	2.1 (-1.8-6.1)	0 (0-0)	-0.8 (-15.2-15.6)	0 (0-0)	5.3 (-21-41.8)	0 (0-0)	-16.6 (-55.7-83.3)	0 (0-0)	-16.1 (-21.5--9.9)
Solihull	0 (0-0)	-0.3 (0-0)	0 (0-0)	1.9 (-2.1-6.6)	0 (0-0)	-0.7 (-15.3-13.5)	0 (0-0)	5.6 (-20.9-38.6)	0 (0-0)	-11.5 (-55.9-70.8)	0 (0-0)	-6.8 (-13.4-0.3)
Staffordshire	0 (0-0)	0 (0-0)	0 (0-0)	1.9 (-1.9-5.8)	0 (0-0)	-0.6 (-14.6-16.3)	0 (0-0)	2.6 (-24.5-36.6)	0 (0-0)	-11.4 (-59.1-70.9)	0 (0-0)	-14.3 (-20.6--7.4)
Stoke-on-Trent	0 (0-0)	0 (0-0)	0 (0-0)	0.7 (-3.1-4.7)	0 (0-0)	0.2 (-12.9-17.1)	0 (0-0)	-6 (-29.2-28.9)	0 (0-0)	2.2 (-56.1-116)	0 (0-0)	1.1 (-5.6-8.9)
Telford and Wrekin	0 (0-0)	0 (0-0)	0 (0-0)	2 (-2-6)	0 (0-0)	0.4 (-15.3-16.2)	0 (0-0)	2.2 (-25.9-41.1)	0 (0-0)	-11.3 (-61-99.2)	0 (0-0)	-10.4 (-17--3.9)

Location	Birth Incidence rate (per 100,000 live births), 2021	Birth Incidence rate (per 100,000 live births) percent change (2000-2021)	Under-5 rate (per 100,000 population), 2021	Under-5 rate (per 100,000 population) percent change (2000-2021)	5-14 rate (per 100,000 population), 2021	5-14 rate (per 100,000 population) percent change (2000-2021)	15-49 rate (per 100,000 population), 2021	15-49 rate (per 100,000 population) percent change (2000-2021)	50plus rate (per 100,000 population), 2021	50plus rate (per 100,000 population) percent change (2000-2021)	All age rate (per 100,000 population), 2021	All age rate (per 100,000 population) percent change (2000-2021)
Walsall	0 (0-0)	0 (0-0)	0 (0-0)	1.6 (-2.2-5.2)	0 (0-0)	-1.2 (-16.6-14.9)	0 (0-0)	0.8 (-26.5-34)	0 (0-0)	2.2 (-53.1-99.5)	0 (0-0)	-2.8 (-9-3.2)
Warwickshire	0 (0-0)	0 (0-0)	0 (0-0)	1.4 (-2.4-5.3)	0 (0-0)	-1 (-14.7-13.9)	0 (0-0)	2.6 (-21.4-36.2)	0 (0-0)	-11 (-55.1-78.9)	0 (0-0)	-10 (-16.2--3.6)
Wolverhampton	0 (0-0)	-0.1 (0-0)	0 (0-0)	0.6 (-2.8-4.6)	0 (0-0)	-1.3 (-14.4-13.3)	0 (0-0)	-6.8 (-29.9-25.1)	0 (0-0)	13.8 (-42.8-161)	0 (0-0)	0.9 (-5.6-7.9)
Worcestershire	0 (0-0)	0 (0-0)	0 (0-0)	1.7 (-2.3-5.8)	0 (0-0)	-1.7 (-16.8-13.3)	0 (0-0)	4.3 (-22.7-36.9)	0 (0-0)	-17 (-58.7-56.8)	0 (0-0)	-12.6 (-19.1--6.3)
Yorkshire and the Humber	0 (0-0)	-0.2 (0-2.1)	0 (0-0)	1.4 (0-3.7)	0 (0-0)	-1.2 (-7.8-4.3)	0 (0-0)	-4.1 (-14.1-4.4)	0 (0-0)	-6.8 (-27.5-16.2)	0 (0-0)	-6.9 (-9.9--3.9)
Barnsley	0 (0-0)	0 (0-0)	0 (0-0)	2.3 (-1.4-6.6)	0 (0-0)	-1.5 (-14.1-13.8)	0 (0-0)	0.8 (-27.3-35.9)	0 (0-0)	-4.4 (-56.4-105)	0 (0-0)	-8.4 (-14.2--1.3)
Bradford	0 (0-0)	-0.8 (0-0)	0 (0-0)	-0.2 (-4.3-4.2)	0 (0-0)	-2.6 (-18.3-12.9)	0 (0-0)	-2.1 (-34-32)	0 (0-0)	3 (-54.2-110)	0 (0-0)	-7.8 (-14--0.5)
Calderdale	0 (0-0)	1.8 (0-1.4)	0 (0-0)	4.2 (-2-10.9)	0 (0-0)	1.5 (-17.2-31.3)	0 (0-0)	6.2 (-22.3-60.8)	0 (0-0)	-3.5 (-52.9-133)	0 (0-0)	-11 (-19--3.3)
Doncaster	0 (0-0)	0 (0-0)	0 (0-0)	1.8 (-2.6-6.2)	0 (0-0)	-1.9 (-16.4-14.7)	0 (0-0)	-4 (-29-29.9)	0 (0-0)	-5.8 (-54.3-112)	0 (0-0)	-10.3 (-17--3.1)
East Riding of Yorkshire	0 (0-0)	0 (0-0)	0 (0-0)	1.8 (-2.1-6.7)	0 (0-0)	-1.4 (-15.7-14.5)	0 (0-0)	3.7 (-20.5-36.8)	0 (0-0)	-17.9 (-58.4-64)	0 (0-0)	-17.5 (-23.4--10.8)
Kingston upon Hull, City of	0 (0-0)	0 (0-0)	0 (0-0)	1 (-3-5.3)	0 (0-0)	-0.1 (-14.3-16.5)	0 (0-0)	-17.9 (-40.5-12.6)	0 (0-0)	4.1 (-52.6-115)	0 (0-0)	-1 (-8-6.4)
Kirklees	0 (0-0)	-2.8 (-36-0)	0 (0-0)	-1.8 (-35.7-5.3)	0 (0-0)	-5.5 (-37.2-14.5)	0 (0-0)	-8.4 (-40.6-29.6)	0 (0-0)	-15.3 (-65.4-80.6)	0 (0-0)	-15.7 (-44.6--7.1)
Leeds	0 (0-0)	-0.3 (0-0.1)	0 (0-0)	1.5 (-2.3-5.5)	0 (0-0)	1.4 (-13.4-19.2)	0 (0-0)	-14.7 (-35.9-13.1)	0 (0-0)	-1.6 (-50.6-96.1)	0 (0-0)	5 (-2.8-13.6)
North East Lincolnshire	0 (0-0)	0.1 (0-0)	0 (0-0)	2.4 (-1.7-7.4)	0 (0-0)	2.6 (-12.5-20.6)	0 (0-0)	-5.9 (-31.6-31.2)	0 (0-0)	-0.7 (-55.4-115)	0 (0-0)	-11 (-17.3--3.9)
North Lincolnshire	0 (0-0)	0.4 (0-0)	0 (0-0)	2 (-2.7-6.2)	0 (0-0)	-0.6 (-15.4-23.2)	0 (0-0)	1.4 (-24.2-43.7)	0 (0-0)	-12 (-58.1-92)	0 (0-0)	-8.6 (-15.9-0.3)
North Yorkshire	0 (0-0)	0.5 (0-0)	0 (0-0)	2.4 (-3.6-8.6)	0 (0-0)	1.7 (-18.1-24.9)	0 (0-0)	8.2 (-26.6-57.8)	0 (0-0)	-13.2 (-58.9-92.9)	0 (0-0)	-12.4 (-22.7--2.7)
Rotherham	0 (0-0)	0 (0-0)	0 (0-0)	2.3 (-2.2-6.4)	0 (0-0)	-0.8 (-15.5-14.9)	0 (0-0)	2 (-25-34.5)	0 (0-0)	-12.6 (-57.1-89.4)	0 (0-0)	-8.9 (-15.6--2.7)
Sheffield	0 (0-0)	0.3 (0-0)	0 (0-0)	2 (-2.2-6.1)	0 (0-0)	-2.6 (-17-13)	0 (0-0)	-4.8 (-30.6-27.2)	0 (0-0)	5.8 (-51.3-113)	0 (0-0)	-0.4 (-8.2-6.7)
Wakefield	0 (0-0)	0.3 (0-0)	0 (0-0)	2.6 (-1.5-6.7)	0 (0-0)	1.9 (-15.1-19.5)	0 (0-0)	1.5 (-26.4-37.6)	0 (0-0)	-1.5 (-54.7-112)	0 (0-0)	-6.2 (-13-0.5)
York	0 (0-0)	0 (0-0)	0 (0-0)	2.1 (-1.7-6.2)	0 (0-0)	-0.5 (-13.6-15.9)	0 (0-0)	-8.4 (-31.9-21.9)	0 (0-0)	-6.9 (-54-88.4)	0 (0-0)	-5.4 (-12-2.2)
Northern Ireland	0 (0-0)	0 (0-0.1)	0 (0-0)	2.8 (-1.9-7.5)	0 (0-0)	-0.6 (-15.3-18.8)	0 (0-0)	-10 (-32.8-21.2)	0 (0-0)	1.6 (-53-104)	0 (0-0)	-14.2 (-20.6--7.2)
Scotland	0 (0-0)	0 (0-0.1)	0 (0-0)	1.8 (-2-6.8)	0 (0-0)	-4.1 (-21.2-16.8)	0 (0-0)	-4.2 (-34-36.2)	0 (0-0)	-6.3 (-57.5-118)	0 (0-0)	-17.1 (-24.3--8.7)
Wales	0 (0-0)	0 (0-0.1)	0 (0-0)	2.1 (-2-5.9)	0 (0-0)	-1 (-16.9-16.4)	0 (0-0)	-2.9 (-27.7-33.4)	0 (0-0)	-6.9 (-56.8-114)	0 (0-0)	-12.1 (-18.8--5.4)
Latin America and Caribbean	2.2 (1.7-3)	15.2 (10.3-19.7)	1.6 (1.2-2)	15.8 (10.9-20)	0.8 (0.7-1.1)	14.1 (8.2-19.5)	0.7 (0.5-0.9)	11.5 (5.2-17.6)	0.1 (0.1-0.1)	6.2 (-0.7-12.8)	0.6 (0.5-0.8)	-0.9 (-6-3.5)
**Andean Latin America**	0 (0-0)	15.4 (0.1-56.6)	0 (0-0)	13.9 (-3.7-59.1)	0 (0-0)	28.8 (-1.7-94.9)	0 (0-0)	22.3 (-16.7-116)	0 (0-0)	53.4 (-26.8-239)	0 (0-0)	-3.1 (-21.9-42)
Bolivia (Plurinational State of)	0 (0-0)	1.5 (0-6.1)	0 (0-0)	1 (-4.2-6.2)	0 (0-0)	5.2 (-10.2-25)	0 (0-0)	-15.8 (-38-19.1)	0 (0-0)	-3.5 (-50.7-114)	0 (0-0)	-12 (-18.7--5)
Ecuador	0.1 (0-0.1)	35.2 (-0.2-131)	0 (0-0.1)	36.2 (-2.2-140)	0 (0-0)	48.8 (-3.5-181)	0 (0-0)	51.2 (-13.5-261)	0 (0-0)	96.3 (-30-582)	0 (0-0)	11.5 (-24.8-105)
Peru	0 (0-0)	0.6 (-0.1-23.4)	0 (0-0)	-0.2 (-4-22)	0 (0-0)	0.1 (-13.4-20.6)	0 (0-0)	-18.2 (-39.4-7.7)	0 (0-0)	-4.1 (-49.8-83.4)	0 (0-0)	-22.6 (-28.7--7.5)
**Caribbean**	16.1 (11.5-22.2)	12.5 (6.1-19.1)	8.6 (6.2-11.9)	12 (5.8-18.5)	0.9 (0.6-1.2)	11.4 (3.4-20.3)	0 (0-0)	-3.2 (-14.2-10.6)	0 (0-0)	-3.5 (-30.5-37)	0.9 (0.6-1.2)	-10 (-14.8--4.6)

Location	Birth Incidence rate (per 100,000 live births), 2021	Birth Incidence rate (per 100,000 live births) percent change (2000-2021)	Under-5 rate (per 100,000 population), 2021	Under-5 rate (per 100,000 population) percent change (2000-2021)	5-14 rate (per 100,000 population), 2021	5-14 rate (per 100,000 population) percent change (2000-2021)	15-49 rate (per 100,000 population), 2021	15-49 rate (per 100,000 population) percent change (2000-2021)	50plus rate (per 100,000 population), 2021	50plus rate (per 100,000 population) percent change (2000-2021)	All age rate (per 100,000 population), 2021	All age rate (per 100,000 population) percent change (2000-2021)
Antigua and Barbuda	1.8 (1.1-2.8)	0.7 (-9.8-13.1)	1.2 (0.8-1.9)	0.8 (-8.8-13.2)	0.2 (0.1-0.3)	-3.1 (-15.7-12.3)	0 (0-0)	-7.4 (-28-16.8)	0 (0-0)	3.4 (-47.2-84)	0.1 (0.1-0.2)	-34.6 (-40.7--26.6)
Bahamas	0.1 (0.1-0.2)	-0.5 (-28.2-34.9)	0.1 (0.1-0.1)	0.6 (-24.7-32.7)	0 (0-0)	-8.1 (-25.9-18)	0 (0-0)	-3.8 (-28.9-30)	0 (0-0)	2.3 (-50.8-97)	0 (0-0)	-38.4 (-52--20.8)
Barbados	2.1 (1.3-3.1)	0.1 (-10-11.7)	1.4 (0.9-2.2)	-0.6 (-10.5-10.7)	0.3 (0.1-0.4)	-3.4 (-16.1-12.2)	0 (0-0)	0 (-20.6-25.4)	0 (0-0)	-2.3 (-46.1-80.1)	0.1 (0.1-0.1)	-32.5 (-39--25.6)
Belize	4.4 (2.8-6.8)	2.9 (-7.3-15)	2.6 (1.6-3.9)	2.9 (-6.9-13.9)	0.3 (0.2-0.5)	-2.4 (-17.7-13.8)	0 (0-0)	-6.3 (-26.8-18.9)	0 (0-0)	16.5 (-40.7-116)	0.3 (0.2-0.5)	-27.7 (-35--19.8)
Bermuda	0.8 (0.5-1.2)	-0.8 (-21.7-15.8)	0.6 (0.4-0.9)	-0.9 (-21.3-14.3)	0.1 (0.1-0.2)	-4 (-21.1-12.3)	0 (0-0)	-1.1 (-21.3-27.7)	0 (0-0)	-20 (-52.9-41.5)	0 (0-0.1)	-36.1 (-47.5--27.7)
Cuba	3.6 (2.4-5.3)	0.7 (-11.5-11.1)	2.4 (1.6-3.4)	-0.9 (-10.3-8.9)	0.4 (0.2-0.5)	10.6 (-3.1-26.3)	0 (0-0)	-1.9 (-22.7-24.5)	0 (0-0)	3 (-44.9-81.4)	0.2 (0.1-0.2)	-26.4 (-33.2--19.2)
Dominica	2.2 (1.4-3.4)	0.2 (-11.4-10.9)	1.4 (0.8-2.1)	0.7 (-11.6-10.7)	0.2 (0.1-0.3)	-3.2 (-17.6-13.4)	0 (0-0)	-4.3 (-25.3-26.9)	0 (0-0)	22.4 (-31.8-134)	0.1 (0.1-0.2)	-35.5 (-43--28.6)
Dominican Republic	20.8 (14.7-28.3)	-1.1 (-11.5-8.1)	11.4 (8.1-15.5)	-1.7 (-11.5-8)	1.3 (0.9-1.8)	-0.2 (-12.3-14)	0.1 (0-0.1)	-12.8 (-30.7-6.7)	0 (0-0)	-2.1 (-44.3-82.4)	1.4 (1-1.9)	-23 (-30.6--15.7)
Grenada	2.8 (1.8-4.4)	0.2 (-11.8-10.2)	1.7 (1.1-2.6)	0.4 (-10.8-10.5)	0.3 (0.1-0.4)	-1.2 (-14.9-14.2)	0 (0-0)	-14.7 (-33.2-9.6)	0 (0-0)	-3.7 (-44-81.2)	0.2 (0.1-0.2)	-30.2 (-37.3--23.2)
Guyana	2.3 (1.4-3.5)	-0.4 (-9.1-10.3)	1.3 (0.8-2)	1 (-7.5-11.2)	0.2 (0.1-0.3)	-4.7 (-17.9-9.3)	0 (0-0)	-1.6 (-24.7-28.8)	0 (0-0)	-6.4 (-54.7-100)	0.2 (0.1-0.2)	-24.4 (-31.1--17.1)
Haiti	20.4 (14.7-28.4)	-0.5 (-8.5-8.2)	10.9 (7.8-14.9)	-2.2 (-10.5-6.3)	1 (0.7-1.4)	0.5 (-10.5-11.6)	0 (0-0.1)	-17.6 (-34.2-7.1)	0 (0-0)	4.8 (-35.9-82)	1.5 (1.1-2.1)	-17.6 (-24.8--10.6)
Jamaica	11.9 (8.3-17)	0 (-10-12.2)	7.4 (5.2-10.5)	1 (-8.9-13.2)	0.9 (0.6-1.4)	-3.9 (-16.2-11.8)	0 (0-0.1)	-9.1 (-27.7-14.5)	0 (0-0)	9 (-35.6-79.2)	0.6 (0.5-0.9)	-33.7 (-39.4--26.7)
Puerto Rico	0.8 (0.6-1.3)	-1 (-19.9-21.1)	0.6 (0.4-0.9)	-1.2 (-19.6-20.5)	0.1 (0.1-0.2)	-5.3 (-20.6-13.4)	0 (0-0)	-11.1 (-28.9-11.4)	0 (0-0)	-23.7 (-56.4-44.5)	0 (0-0.1)	-48.9 (-57.4--38.9)
Saint Kitts and Nevis	2 (1.3-3.1)	0.8 (-9.6-12.8)	1.3 (0.8-1.9)	0.6 (-9.4-11.3)	0.2 (0.1-0.3)	-0.1 (-13-16.5)	0 (0-0)	-13.6 (-32.5-12.5)	0 (0-0)	23.4 (-33.6-141)	0.1 (0.1-0.2)	-36 (-42.4--29.2)
Saint Lucia	2.9 (1.8-4.3)	-0.5 (-9.8-9.4)	1.9 (1.2-2.9)	-0.3 (-9.4-9.6)	0.3 (0.2-0.5)	-4.6 (-16.9-9.1)	0 (0-0)	-21.9 (-37.9--1.8)	0 (0-0)	8.3 (-41.9-106)	0.1 (0.1-0.2)	-48.1 (-53.1--42.7)
Saint Vincent and the Grenadines	2.6 (1.6-4.1)	-0.5 (-9.2-11)	1.6 (1-2.5)	-0.4 (-9.8-11.5)	0.2 (0.1-0.4)	-2.2 (-15.8-12.2)	0 (0-0)	-16.6 (-35.5-6.1)	0 (0-0)	5.8 (-41.5-85.2)	0.1 (0.1-0.2)	-34.9 (-41--27.2)
Suriname	2.7 (1.7-4.1)	0.8 (-9.2-12.2)	1.5 (1-2.3)	-1.2 (-11.5-10.3)	0.2 (0.1-0.3)	2.4 (-12.7-20.6)	0 (0-0)	-9.5 (-30.7-14.8)	0 (0-0)	3.1 (-50.9-98.7)	0.2 (0.1-0.2)	-20.9 (-29.1--11.2)
Trinidad and Tobago	2.5 (1.6-3.7)	-0.3 (-9.3-8.7)	1.6 (1-2.3)	-0.9 (-8.9-7.9)	0.3 (0.2-0.4)	7.2 (-7-23.3)	0 (0-0)	-26.7 (-41.7--8.1)	0 (0-0)	-18.7 (-55.9-64.4)	0.1 (0.1-0.2)	-21.3 (-27.7--14.2)
United States Virgin Islands	1.1 (0.7-1.8)	-0.5 (-19.4-19.9)	0.8 (0.5-1.2)	-0.9 (-19.2-19.3)	0.2 (0.1-0.2)	-1.7 (-17.6-17.4)	0 (0-0)	-7 (-29.3-20.8)	0 (0-0)	-28.4 (-66.2-43.1)	0.1 (0-0.1)	-30.5 (-42.1--17.6)
**Central Latin America**	0.1 (0.1-0.2)	-1 (-15.3-24.1)	0.1 (0.1-0.2)	1.4 (-12.1-28.2)	0 (0-0.1)	0.7 (-15.5-38.4)	0 (0-0)	-16.6 (-32.4-13.9)	0 (0-0)	-6.4 (-36.3-39.1)	0 (0-0)	-27.2 (-37.7--5.5)
Colombia	0.4 (0.3-0.6)	-0.7 (-24.8-15.6)	0.3 (0.2-0.4)	-0.6 (-22.5-14.3)	0.1 (0-0.1)	-3.8 (-20.3-14.6)	0 (0-0)	-9.9 (-29.3-19.2)	0 (0-0)	-3.2 (-46.8-101)	0 (0-0)	-32.6 (-46.5--24.2)
Costa Rica	0 (0-0)	3.8 (-79.1-77.4)	0 (0-0)	3.7 (-78.9-75.2)	0 (0-0)	4.3 (-77.8-66.4)	0 (0-0)	-10.1 (-75.9-21.6)	0 (0-0)	-1.2 (-49.3-62.6)	0 (0-0)	-26.1 (-84.1-17.1)
El Salvador	0 (0-0)	5.9 (0-92.3)	0 (0-0)	6.2 (-3-91.6)	0 (0-0)	3.6 (-15.5-79.2)	0 (0-0)	-8 (-33.9-28)	0 (0-0)	8.6 (-48.5-132)	0 (0-0)	-25.4 (-34.5-29.8)
Guatemala	0 (0-0)	-2.8 (-50.6-0)	0 (0-0)	-4.2 (-50.6-2.2)	0 (0-0)	-6 (-47.9-13.3)	0 (0-0)	-18.2 (-59.4-13.4)	0 (0-0)	-5 (-64-123)	0 (0-0)	-37.8 (-67.4--30.8)
Honduras	0 (0-0)	0 (0-0)	0 (0-0)	0.1 (-3-3.2)	0 (0-0)	-0.8 (-14.2-14.6)	0 (0-0)	-10.2 (-32.1-18.3)	0 (0-0)	7.2 (-44.7-83.2)	0 (0-0)	-23.6 (-28.5--18.4)
Mexico	0.1 (0-0.2)	15.3 (-14.1-96)	0.1 (0-0.2)	14.8 (-13.5-93.6)	0 (0-0.1)	11.8 (-14.7-88.2)	0 (0-0)	-15.5 (-38.4-30.4)	0 (0-0)	-9 (-47.6-60.1)	0 (0-0)	-18.8 (-38.5-37.2)
Aguascalientes	0.1 (0-0.2)	34.6 (0-810)	0 (0-0.2)	33.6 (-3.4-806)	0 (0-0.1)	26.4 (-17.2-816)	0 (0-0)	4.8 (-35.4-707)	0 (0-0)	19.5 (-44.7-481)	0 (0-0)	-7.3 (-36.4-558)
Baja California	0.1 (0-0.2)	29.1 (0-663)	0 (0-0.1)	28.1 (-2.7-688)	0 (0-0.1)	21.6 (-13-737)	0 (0-0)	3.7 (-31.2-479)	0 (0-0)	21.6 (-51.1-600)	0 (0-0)	-12.3 (-36.1-464)
Baja California Sur	0.1 (0-0.2)	27.5 (0-810)	0 (0-0.1)	26.5 (-2.6-786)	0 (0-0.1)	21 (-13.1-663)	0 (0-0)	-1.4 (-35.4-465)	0 (0-0)	23.2 (-43.7-466)	0 (0-0)	-11.5 (-35.1-475)

Location	Birth Incidence rate (per 100,000 live births), 2021	Birth Incidence rate (per 100,000 live births) percent change (2000-2021)	Under-5 rate (per 100,000 population), 2021	Under-5 rate (per 100,000 population) percent change (2000-2021)	5-14 rate (per 100,000 population), 2021	5-14 rate (per 100,000 population) percent change (2000-2021)	15-49 rate (per 100,000 population), 2021	15-49 rate (per 100,000 population) percent change (2000-2021)	50plus rate (per 100,000 population), 2021	50plus rate (per 100,000 population) percent change (2000-2021)	All age rate (per 100,000 population), 2021	All age rate (per 100,000 population) percent change (2000-2021)
Campeche	0.1 (0-0.3)	29.3 (0-1210)	0.1 (0-0.2)	28.1 (-3.4-1230)	0 (0-0.1)	22.7 (-13.4-1370)	0 (0-0)	-5.5 (-38.3-792)	0 (0-0)	29.5 (-47.1-541)	0 (0-0)	-9.3 (-34.1-889)
Chiapas	0.1 (0-0.2)	32.8 (0-663)	0 (0-0.2)	31.4 (-3.2-665)	0 (0-0.1)	25.6 (-16.3-640)	0 (0-0)	0.3 (-39.4-462)	0 (0-0)	30.9 (-52.4-545)	0 (0-0)	0.9 (-28.8-489)
Chihuahua	0.1 (0-0.2)	32.9 (0-663)	0 (0-0.2)	31.3 (-3.5-637)	0 (0-0.1)	23.3 (-17.1-581)	0 (0-0)	8 (-34.2-530)	0 (0-0)	38.9 (-55-639)	0 (0-0)	-8.2 (-35.8-409)
Coahuila	0.3 (0.1-0.6)	30 (0.3-283)	0.2 (0.1-0.5)	28.7 (-2.7-284)	0.1 (0-0.3)	22.4 (-14.6-272)	0 (0-0)	4.4 (-35.3-201)	0 (0-0)	14.6 (-44.3-258)	0 (0-0.1)	-3.7 (-30.9-190)
Colima	0.1 (0-0.2)	26.9 (0-1210)	0.1 (0-0.2)	25.4 (-2.8-1230)	0 (0-0.1)	18 (-15-1260)	0 (0-0)	-8 (-40.6-821)	0 (0-0)	32.3 (-47.8-535)	0 (0-0)	-14.5 (-36.5-834)
Durango	0.1 (0-0.2)	27.8 (0-556)	0 (0-0.2)	26.9 (-3.4-562)	0 (0-0.1)	22.6 (-14.4-608)	0 (0-0)	-1.6 (-35.6-334)	0 (0-0)	22.6 (-43.9-508)	0 (0-0)	-10.2 (-34.7-381)
Guanajuato	0.3 (0.1-0.6)	30.9 (0-363)	0.2 (0.1-0.4)	29.3 (-3.4-364)	0.1 (0-0.2)	24.3 (-16-351)	0 (0-0)	0.3 (-42.4-226)	0 (0-0)	12.6 (-49.6-301)	0 (0-0.1)	-13.5 (-39-206)
Guerrero	0.1 (0-0.3)	36.2 (0-1440)	0.1 (0-0.3)	34.5 (-4.3-1460)	0 (0-0.1)	27.8 (-21.2-1560)	0 (0-0)	1.9 (-40.9-1010)	0 (0-0)	13.3 (-51.6-1390)	0 (0-0)	-6.8 (-37.3-1020)
Hidalgo	0.1 (0-0.2)	29 (0-1210)	0.1 (0-0.2)	27.6 (-2.9-1240)	0 (0-0.1)	20 (-14-1280)	0 (0-0)	-3.8 (-38-822)	0 (0-0)	32.8 (-49.3-693)	0 (0-0)	-14.9 (-37.6-803)
Jalisco	0 (0-0.1)	13.6 (0-413)	0 (0-0.1)	12.4 (-3.5-414)	0 (0-0.1)	8.8 (-14-476)	0 (0-0)	-11.1 (-37.6-307)	0 (0-0)	6.4 (-49.9-277)	0 (0-0)	-19.3 (-34.3-292)
México	0 (0-0.1)	12.5 (0-396)	0 (0-0.1)	11.5 (-3-401)	0 (0-0.1)	8.7 (-12.3-409)	0 (0-0)	-10.2 (-35.8-268)	0 (0-0)	3.4 (-50.8-255)	0 (0-0)	-17 (-31.4-270)
Mexico City	0.5 (0.3-0.8)	16.9 (-0.5-101)	0.4 (0.2-0.7)	16.3 (-2.7-104)	0.2 (0.1-0.4)	14.6 (-10.9-107)	0 (0-0)	-8.1 (-33.3-53.1)	0 (0-0)	-8.5 (-50.8-74.2)	0.1 (0-0.1)	-19.1 (-36-42.2)
Michoacán de Ocampo	0.1 (0-0.2)	31.5 (0-810)	0 (0-0.1)	30.3 (-3.2-792)	0 (0-0.1)	27.4 (-13.4-742)	0 (0-0)	-4 (-38.1-503)	0 (0-0)	14.2 (-47.9-680)	0 (0-0)	-9.2 (-35.6-504)
Morelos	0.1 (0-0.2)	36.1 (0-810)	0.1 (0-0.2)	35.1 (-3-806)	0 (0-0.1)	29 (-12.6-921)	0 (0-0)	3.6 (-35.1-648)	0 (0-0)	37.1 (-47.7-702)	0 (0-0)	-3.8 (-33.3-573)
Nayarit	0.1 (0-0.2)	30.6 (0-988)	0.1 (0-0.2)	29.2 (-3.1-990)	0 (0-0.1)	22.4 (-14.2-1100)	0 (0-0)	-4.6 (-38.9-678)	0 (0-0)	29.4 (-48.2-418)	0 (0-0)	-7.2 (-32.8-732)
Nuevo León	0.1 (0-0.2)	24.2 (0-499)	0 (0-0.1)	23 (-2.8-512)	0 (0-0.1)	17.5 (-14.3-585)	0 (0-0)	-0.9 (-35.3-410)	0 (0-0)	15.8 (-42.6-435)	0 (0-0)	-10.3 (-32.9-380)
Oaxaca	0.1 (0-0.2)	33.9 (0-963)	0 (0-0.2)	32.5 (-3.4-957)	0 (0-0.1)	24.7 (-14.8-879)	0 (0-0)	-3.6 (-40.3-745)	0 (0-0)	30.2 (-53.1-745)	0 (0-0)	-10.3 (-36.6-609)
Puebla	0.1 (0-0.2)	28.6 (0-1150)	0 (0-0.2)	26.6 (-3.7-1170)	0 (0-0.1)	18.1 (-16.5-1180)	0 (0-0)	-6 (-40.2-786)	0 (0-0)	30.4 (-44.7-503)	0 (0-0)	-12.2 (-35.7-810)
Querétaro	0.1 (0-0.2)	37.5 (0-870)	0 (0-0.2)	36.4 (-3.5-900)	0 (0-0.1)	30.1 (-13.7-882)	0 (0-0)	1.2 (-39-652)	0 (0-0)	43.8 (-44.1-667)	0 (0-0)	-8.1 (-37.4-592)
Quintana Roo	0.1 (0-0.3)	33.2 (0-1230)	0.1 (0-0.2)	31.8 (-3.3-1240)	0 (0-0.1)	25.3 (-15.2-1420)	0 (0-0)	-3.4 (-40-808)	0 (0-0)	30.9 (-48.4-458)	0 (0-0)	-8.4 (-35.8-892)
San Luis Potosí	0.1 (0-0.2)	32 (0-810)	0.1 (0-0.2)	30.6 (-3.4-812)	0 (0-0.1)	22.7 (-15.4-814)	0 (0-0)	-1 (-38.4-631)	0 (0-0)	37.8 (-49.5-551)	0 (0-0)	-12.6 (-38.2-519)
Sinaloa	0.1 (0-0.2)	28.8 (0-491)	0 (0-0.2)	27.8 (-3.3-507)	0 (0-0.1)	22.2 (-15-535)	0 (0-0)	-2.2 (-37.9-291)	0 (0-0)	13.3 (-49.2-526)	0 (0-0)	-14.7 (-38.7-316)
Sonora	0.1 (0-0.2)	29.7 (0-810)	0 (0-0.1)	28.6 (-2.8-790)	0 (0-0.1)	22.7 (-13.6-716)	0 (0-0)	6 (-30.6-547)	0 (0-0)	17.2 (-46.8-639)	0 (0-0)	-10.7 (-35.7-492)
Tabasco	0.4 (0.2-0.9)	34 (0.5-280)	0.3 (0.1-0.8)	32.4 (-3-275)	0.2 (0.1-0.3)	27 (-15-262)	0 (0-0)	-2.8 (-41.3-170)	0 (0-0)	21.4 (-49.3-291)	0.1 (0-0.1)	-7.9 (-35.9-159)
Tamaulipas	0.1 (0-0.2)	30.4 (0-556)	0 (0-0.2)	28.8 (-3.1-553)	0 (0-0.1)	22.4 (-14.7-562)	0 (0-0)	2.6 (-35.2-319)	0 (0-0)	24.6 (-48.9-454)	0 (0-0)	-10.1 (-35.7-362)
Tlaxcala	0.1 (0-0.3)	30.9 (0-1210)	0.1 (0-0.2)	29.1 (-3.4-1260)	0 (0-0.1)	21.9 (-13-1450)	0 (0-0)	-1 (-34.8-1040)	0 (0-0)	47.5 (-41.7-794)	0 (0-0)	-9.9 (-34.8-949)
Veracruz de Ignacio de la Llave	0.1 (0-0.2)	28.1 (0-665)	0 (0-0.1)	27 (-3.8-673)	0 (0-0.1)	21.8 (-15.7-625)	0 (0-0)	-1.1 (-37.6-459)	0 (0-0)	18 (-55.7-558)	0 (0-0)	-16.1 (-39.3-411)
Yucatán	0.1 (0-0.2)	26.5 (0-810)	0 (0-0.1)	25.8 (-3.5-782)	0 (0-0.1)	20.1 (-13.6-728)	0 (0-0)	-7.1 (-38.6-464)	0 (0-0)	16 (-44.7-362)	0 (0-0)	-13.4 (-36.3-482)
Zacatecas	0.1 (0-0.2)	28.4 (0-560)	0.1 (0-0.2)	27 (-3.2-593)	0 (0-0.1)	20.7 (-14.8-670)	0 (0-0)	-6 (-42.4-438)	0 (0-0)	39.9 (-51.6-611)	0 (0-0)	-10 (-34.5-442)

Location	Birth Incidence rate (per 100,000 live births), 2021	Birth Incidence rate (per 100,000 live births) percent change (2000-2021)	Under-5 rate (per 100,000 population), 2021	Under-5 rate (per 100,000 population) percent change (2000-2021)	5-14 rate (per 100,000 population), 2021	5-14 rate (per 100,000 population) percent change (2000-2021)	15-49 rate (per 100,000 population), 2021	15-49 rate (per 100,000 population) percent change (2000-2021)	50plus rate (per 100,000 population), 2021	50plus rate (per 100,000 population) percent change (2000-2021)	All age rate (per 100,000 population), 2021	All age rate (per 100,000 population) percent change (2000-2021)
Nicaragua	0 (0-0)	0.1 (0-0.6)	0 (0-0)	0.1 (-2.8-3.3)	0 (0-0)	-0.3 (-13.2-14.1)	0 (0-0)	-18.5 (-39-4.1)	0 (0-0)	5.5 (-44.1-91.1)	0 (0-0)	-24.8 (-30.3--19)
Panama	0 (0-0)	0 (0-0)	0 (0-0)	-0.3 (-2.7-2.4)	0 (0-0)	1.5 (-8.6-12.5)	0 (0-0)	-4.3 (-21.8-19.4)	0 (0-0)	-2.4 (-41.9-79.3)	0 (0-0)	-14.6 (-19.7--9.5)
Venezuela (Bolivarian Republic of)	0.1 (0.1-0.2)	-1.6 (-27.1-23.2)	0.1 (0.1-0.1)	-1.4 (-26.1-23.1)	0 (0-0)	-3.9 (-24.5-25.7)	0 (0-0)	-19.4 (-41.3-7.2)	0 (0-0)	-13.4 (-56.7-65.8)	0 (0-0)	-30.2 (-46.2--12.3)
**Tropical Latin America**	2.5 (2-3.2)	19.8 (12.9-26.3)	2.4 (1.9-3.1)	18.8 (12.1-25.3)	2.2 (1.7-2.8)	18.8 (12.4-25.3)	1.7 (1.4-2.2)	15.6 (9-21.9)	0.2 (0.1-0.3)	5.7 (-1.2-12.2)	1.4 (1.1-1.9)	2.8 (-3-8.4)
Brazil	2.4 (1.9-3.1)	20.9 (13.6-27.9)	2.3 (1.9-3)	20 (12.8-27)	2.1 (1.7-2.7)	19.8 (13-26.7)	1.7 (1.4-2.2)	16 (9.2-22.5)	0.2 (0.1-0.3)	5.9 (-1.2-12.6)	1.4 (1.1-1.8)	3.4 (-2.9-9.4)
Acre	2.1 (1.3-3.2)	3.5 (-13.2-18.3)	2 (1.3-3.1)	3.3 (-13.3-18)	1.8 (1.1-2.8)	2.8 (-13.7-16.8)	1.4 (0.9-2.2)	1.3 (-14.6-14.5)	0.2 (0.1-0.3)	4.2 (-10.8-19.8)	1.3 (0.9-2.1)	-5.8 (-20.7-6.8)
Alagoas	1.1 (0.7-1.7)	58.2 (0-180)	1 (0.6-1.6)	57.4 (-0.6-178)	0.9 (0.6-1.5)	56.1 (-1.7-174)	0.7 (0.5-1.2)	51.8 (-5.3-163)	0.1 (0-0.2)	52.7 (-7.6-164)	0.7 (0.4-1)	40 (-12.4-144)
Amapá	0 (0-0.1)	40.2 (0-1930)	0 (0-0.1)	39.8 (-0.7-1920)	0 (0-0.1)	38.4 (-2.4-1860)	0 (0-0.1)	33.8 (-6.6-1660)	0 (0-0)	32.7 (-12-1530)	0 (0-0.1)	24.2 (-13-1580)
Amazonas	5.9 (4.7-7.3)	25.5 (14.7-38.9)	5.7 (4.6-7.1)	25.1 (14.4-38.3)	5.2 (4.1-6.4)	24.1 (13.6-36.5)	4.2 (3.3-5.2)	20.1 (10.5-31.8)	0.5 (0.3-0.7)	10.8 (-0.4-24)	3.9 (3.1-4.9)	11.5 (2.5-22.7)
Bahia	3.5 (2.9-4.3)	22.3 (12.2-34.4)	3.4 (2.8-4.2)	21.8 (11.8-34.1)	3.1 (2.5-3.7)	20.8 (11.2-33)	2.4 (2-3)	15.4 (5.7-26.4)	0.3 (0.2-0.4)	13.1 (0.2-27.4)	2.1 (1.7-2.6)	5.3 (-3.3-15.5)
Ceará	1.2 (0.9-1.7)	26.6 (0-72)	1.2 (0.8-1.6)	26.3 (-0.2-71.3)	1 (0.7-1.4)	25.5 (-0.7-70.7)	0.8 (0.6-1.1)	21.3 (-4.2-64.4)	0.1 (0.1-0.2)	24.8 (-2.1-68)	0.7 (0.5-1)	11.8 (-11.6-51.2)
Distrito Federal	8.9 (7.4-10.7)	3.2 (-5.4-11)	8.7 (7.2-10.3)	2.9 (-5.6-10.8)	7.9 (6.6-9.4)	2.3 (-6.1-10.1)	6.3 (5.2-7.5)	-1.3 (-8.9-6.5)	0.8 (0.6-1.1)	-14.1 (-22.4--5.4)	5.4 (4.5-6.4)	-12.9 (-19.9--6)
Espírito Santo	1.3 (0.8-1.9)	5.8 (-18.6-29.3)	1.2 (0.8-1.8)	5.5 (-18.5-29)	1.1 (0.7-1.7)	5 (-18.9-28.2)	0.9 (0.6-1.3)	2 (-20.7-25.3)	0.1 (0.1-0.2)	-7.7 (-28.2-14.3)	0.7 (0.5-1.1)	-11 (-31-8.8)
Goiás	3.1 (2.5-3.9)	12 (2.1-22.8)	3 (2.4-3.8)	11.7 (2-22.2)	2.7 (2.2-3.4)	10.7 (0.8-21)	2.1 (1.7-2.6)	7 (-2.4-16.4)	0.2 (0.2-0.3)	-1.8 (-12.9-10.7)	1.8 (1.4-2.2)	-6 (-14.3-2.4)
Maranhão	1.5 (1.1-2.1)	23.3 (6.4-46.5)	1.5 (1.1-2)	22.6 (6-45.7)	1.3 (0.9-1.7)	21.6 (5.2-44.2)	1 (0.7-1.4)	17 (1.7-36.4)	0.1 (0.1-0.2)	19.6 (0.7-40.7)	1 (0.7-1.3)	12.4 (-2.4-32.6)
Mato Grosso	2 (1.4-2.8)	30.9 (15.8-50)	1.9 (1.4-2.7)	30.6 (15.3-49.6)	1.7 (1.2-2.4)	29.3 (14.2-48.2)	1.3 (1-1.9)	24.4 (10.1-43)	0.2 (0.1-0.3)	11.5 (-4.2-28.9)	1.1 (0.8-1.6)	9.6 (-3-26)
Mato Grosso do Sul	2.8 (2-3.8)	6.8 (-6.4-17.7)	2.7 (1.9-3.7)	6.5 (-6.5-17.2)	2.5 (1.8-3.4)	5.6 (-7.3-16.1)	1.9 (1.4-2.7)	2.1 (-10.1-12)	0.2 (0.1-0.3)	-7.4 (-20.5-4.7)	1.6 (1.2-2.2)	-10 (-20.9--1.2)
Minas Gerais	2.7 (2.1-3.4)	16.5 (3.5-29.3)	2.6 (2.1-3.3)	16.3 (3.4-29.2)	2.4 (1.9-3)	15.4 (2.5-27.5)	2 (1.6-2.4)	12.1 (0-23.4)	0.2 (0.1-0.3)	-1.8 (-14.4-11.1)	1.6 (1.3-1.9)	-3.2 (-13.5-6.6)
Pará	2 (1.5-2.5)	22.4 (13-32.7)	1.9 (1.4-2.5)	22 (12.6-32.1)	1.7 (1.3-2.2)	20.8 (11.1-30.8)	1.3 (1-1.7)	16.8 (6.6-26.8)	0.2 (0.1-0.2)	13.1 (1.1-26.2)	1.2 (0.9-1.6)	8.7 (-0.5-18.4)
Paraíba	1.8 (1.2-2.5)	43.7 (17.5-76.2)	1.7 (1.2-2.5)	43.3 (16.8-76.2)	1.6 (1.1-2.3)	42.6 (16.4-74.9)	1.2 (0.8-1.8)	37.2 (11.8-69.6)	0.1 (0.1-0.2)	44 (14.9-85.1)	1.1 (0.7-1.5)	27.4 (3.9-57)
Paraná	2 (1.6-2.6)	22.6 (12.3-31.9)	1.9 (1.5-2.5)	22.3 (12.3-31.8)	1.8 (1.4-2.3)	21.5 (11.5-30.7)	1.4 (1.1-1.8)	18.6 (9.4-28.1)	0.2 (0.1-0.2)	5.5 (-4.7-17.2)	1.1 (0.9-1.5)	1.8 (-6-9.8)
Pernambuco	1.5 (1.1-2.2)	48.2 (16.5-103)	1.4 (1-2.1)	47.7 (15.9-102)	1.3 (0.9-1.9)	46.6 (14.5-101)	1 (0.7-1.5)	40.8 (10.3-91.8)	0.1 (0.1-0.2)	40.4 (6.8-90.6)	0.9 (0.6-1.3)	29.7 (1.5-77.8)
Piaui	3.3 (2.5-4.3)	25.1 (13.5-34.9)	3.1 (2.4-4.1)	24.7 (13-34.5)	2.8 (2.1-3.6)	23.9 (12.5-33.6)	2.2 (1.7-2.9)	18.8 (7.3-29.2)	0.2 (0.2-0.4)	12.3 (-1.1-25.4)	2 (1.5-2.5)	8.9 (-1.9-17.7)
Rio de Janeiro	2.4 (1.9-3)	13.1 (2.7-25.9)	2.3 (1.8-3)	12.9 (2.7-25.6)	2.1 (1.7-2.7)	12.2 (2-24.2)	1.6 (1.3-2.1)	9.6 (-0.2-20.1)	0.2 (0.1-0.2)	-6.8 (-18.4-5.8)	1.3 (1-1.6)	-4.1 (-12.5-5.5)
Rio Grande do Norte	1.3 (0.9-2)	37.5 (0-112)	1.3 (0.9-1.9)	37 (-0.3-111)	1.1 (0.8-1.7)	36 (-0.9-108)	0.9 (0.6-1.4)	31.1 (-4.4-101)	0.1 (0.1-0.2)	34.5 (-2.8-106)	0.8 (0.5-1.2)	19.4 (-13.1-82.8)
Rio Grande do Sul	3.2 (2.4-4.3)	39.9 (24.9-57.7)	3.1 (2.3-4.2)	39.6 (24.7-57.3)	2.9 (2.1-3.8)	38.6 (23.8-55.8)	2.3 (1.7-3)	35.7 (21.6-53.4)	0.2 (0.1-0.3)	6.1 (-8.6-22.1)	1.7 (1.3-2.3)	14.2 (2.3-28.7)
Rondônia	2.1 (1.4-3.1)	18.8 (6.9-33)	2 (1.4-3)	18.5 (6.4-32.7)	1.8 (1.2-2.7)	17.2 (5.3-31.7)	1.4 (1-2)	12.5 (1.1-27.2)	0.2 (0.1-0.3)	3.9 (-10.7-19)	1.2 (0.8-1.8)	-1.1 (-11.1-11.8)
Roraima	0.2 (0.1-0.5)	25.4 (-47-368)	0.2 (0.1-0.4)	24.7 (-47.4-366)	0.2 (0.1-0.4)	21.7 (-48.5-357)	0.1 (0.1-0.3)	14.4 (-51.5-330)	0 (0-0)	-2.7 (-60.1-246)	0.1 (0.1-0.3)	6.2 (-55.2-297)
Santa Catarina	1.9 (1.4-2.6)	38 (21.5-55.3)	1.8 (1.3-2.5)	37.7 (21.2-55.4)	1.7 (1.2-2.3)	36.8 (20.7-54.6)	1.4 (1-1.8)	33.3 (18.4-51.2)	0.2 (0.1-0.3)	14.4 (-0.6-31.7)	1.1 (0.8-1.5)	14.3 (1.3-29.5)

Location	Birth Incidence rate (per 100,000 live births), 2021	Birth Incidence rate (per 100,000 live births) percent change (2000-2021)	Under-5 rate (per 100,000 population), 2021	Under-5 rate (per 100,000 population) percent change (2000-2021)	5-14 rate (per 100,000 population), 2021	5-14 rate (per 100,000 population) percent change (2000-2021)	15-49 rate (per 100,000 population), 2021	15-49 rate (per 100,000 population) percent change (2000-2021)	50plus rate (per 100,000 population), 2021	50plus rate (per 100,000 population) percent change (2000-2021)	All age rate (per 100,000 population), 2021	All age rate (per 100,000 population) percent change (2000-2021)
São Paulo	2.3 (1.9-2.8)	18.2 (6.7-30.7)	2.2 (1.8-2.7)	18 (6.5-30.4)	2 (1.6-2.5)	17.4 (5.8-29.3)	1.6 (1.3-2)	14.2 (2.9-25.5)	0.2 (0.1-0.2)	-1.9 (-13.3-10.7)	1.3 (1-1.6)	-0.9 (-10.6-8.6)
Sergipe	2.7 (1.9-3.8)	34.4 (21.2-49.8)	2.6 (1.8-3.6)	33.9 (21-48.8)	2.3 (1.6-3.2)	32.7 (19.5-46.6)	1.8 (1.2-2.5)	27.5 (14.5-40.2)	0.2 (0.1-0.3)	28.2 (11.6-45.3)	1.6 (1.1-2.2)	15.4 (4.1-27.3)
Tocantins	3.1 (2.2-4.2)	27 (14.1-39.3)	3 (2.1-4.1)	26.5 (13.8-38.8)	2.7 (1.9-3.6)	25.3 (13.1-37.9)	2.1 (1.5-2.9)	20.6 (8.9-33.1)	0.2 (0.2-0.4)	17.6 (3.7-33)	1.9 (1.4-2.5)	10.1 (-0.5-21.5)
Paraguay	4.3 (3.2-5.8)	0.3 (-8.7-9.2)	4 (2.9-5.4)	0.3 (-8.6-9.1)	3.3 (2.5-4.5)	0.2 (-8-8.9)	2.4 (1.7-3.1)	-1.6 (-10-7.8)	0.2 (0.1-0.3)	-3.5 (-16.6-10.1)	2.3 (1.7-3.1)	-10.4 (-17.6--2.5)
North Africa and Middle East	32.3 (23.9-43.7)	-3.4 (-6.9-0.8)	20.3 (15-26.8)	-7.5 (-11--3.6)	6.8 (4.9-9)	-3.5 (-8.2-1.6)	2 (1.4-2.6)	-5.2 (-9.8-0)	0.1 (0-0.1)	18.1 (10.1-26.8)	4.3 (3.3-5.5)	-23.4 (-26.8--19.8)
**North Africa and Middle East**	32.3 (23.9-43.7)	-3.4 (-6.9-0.8)	20.3 (15-26.8)	-7.5 (-11--3.6)	6.8 (4.9-9)	-3.5 (-8.2-1.6)	2 (1.4-2.6)	-5.2 (-9.8-0)	0.1 (0-0.1)	18.1 (10.1-26.8)	4.3 (3.3-5.5)	-23.4 (-26.8--19.8)
Afghanistan	28.7 (18.3-42.9)	0.3 (-11.3-12.3)	16.3 (10.6-23.9)	0 (-11.2-11.4)	3.8 (2.6-5.4)	-3.1 (-15.9-10.7)	0.9 (0.6-1.3)	3.2 (-10.8-20.1)	0 (0-0)	82.6 (31.7-157)	4.2 (3-5.9)	-11.7 (-20.3--2.2)
Algeria	9.6 (6.9-13.2)	0.7 (-8.1-10.5)	5.9 (4.3-7.9)	-0.2 (-8.8-9.7)	1.7 (1.1-2.4)	4.2 (-9.7-19.7)	0.4 (0.3-0.5)	-19.4 (-31.1--6.4)	0 (0-0)	6.6 (-17.4-38.9)	1.1 (0.8-1.4)	-13.9 (-22.2--5.5)
Bahrain	85.9 (59.1-124)	0 (-12.1-12.4)	57.4 (40.3-79.7)	-1.6 (-13.2-9.3)	19.1 (12.9-26.4)	-1.7 (-14.9-10.7)	3.8 (2.7-5.2)	-23.4 (-33--11.5)	0.2 (0.1-0.4)	2.2 (-16.8-22.8)	7.1 (5.2-9.2)	-44.7 (-50.2--38.8)
Egypt	16.3 (12.6-20.8)	-0.2 (-9.8-10.2)	9.5 (7.3-12)	-2.9 (-13-7.1)	2.7 (1.9-3.6)	1.6 (-11-16.1)	0.7 (0.5-1)	-7 (-18.5-7.4)	0 (0-0)	-2.8 (-23.5-25.8)	2 (1.5-2.4)	-12.3 (-20.3--3.5)
Iran (Islamic Republic of)	11.8 (7.8-17.9)	-1 (-3.9-2.3)	7.6 (5.1-11.3)	-3.2 (-5.9--0.4)	2.3 (1.6-3.2)	2.5 (-1.1-6)	0.4 (0.3-0.6)	-28.9 (-33.3--24.5)	0 (0-0)	5.1 (-2.3-12.9)	1.2 (0.9-1.7)	-26.2 (-28.2--24.2)
Alborz	8.8 (5.8-13.7)	-0.3 (-10.7-13.2)	6.3 (4.1-9.6)	-2.1 (-12.4-10.6)	2.1 (1.4-3)	2.7 (-10.8-17.1)	0.4 (0.3-0.5)	-30.7 (-39.3--20.7)	0 (0-0)	-4 (-23.5-24.8)	1 (0.7-1.4)	-30.6 (-37.4--22.5)
Ardebil	10.2 (6.6-15.4)	0.8 (-12.5-15.3)	6.1 (4-8.8)	-1 (-13.5-12.5)	1.6 (1.1-2.3)	4.5 (-10.6-18.8)	0.3 (0.2-0.4)	-29.9 (-40.7--17.8)	0 (0-0)	15.4 (-10.3-51)	0.9 (0.6-1.2)	-31 (-38.9--23)
Bushehr	18.1 (11.9-27.8)	-3.2 (-14.8-11.1)	11.9 (7.8-17.5)	-5 (-16.9-7.8)	3.6 (2.5-5.2)	-1.7 (-13-10.6)	0.8 (0.6-1.1)	-24 (-32.9--13.6)	0 (0-0)	1.3 (-17.2-24.4)	2.2 (1.6-3)	-23.6 (-31.5--14.9)
Chahar Mahaal and Bakhtiari	18.6 (12.1-27.4)	1.1 (-12.1-16.2)	11.9 (7.7-17.2)	-0.7 (-12.6-12.6)	3.4 (2.3-4.8)	4.2 (-9.8-19.6)	0.7 (0.5-1)	-28.1 (-38.4--16.4)	0 (0-0)	6.9 (-14.6-33.7)	2 (1.4-2.8)	-26.2 (-34.1--17.2)
East Azarbayejan	10.4 (6.8-15.9)	-0.5 (-10.3-13.6)	6.4 (4.2-9.4)	-3.9 (-13.9-8.6)	1.7 (1.1-2.4)	4 (-10.5-20.4)	0.3 (0.2-0.4)	-32.3 (-43.4--19.9)	0 (0-0)	8 (-19.6-42.7)	0.9 (0.6-1.3)	-24.2 (-32--15.2)
Fars	17.2 (11.2-25.8)	-2.5 (-13-9.1)	11.3 (7.5-16.9)	-4.1 (-14.4-7)	3.4 (2.3-4.9)	2.5 (-10.4-17.4)	0.6 (0.4-0.8)	-33.8 (-42.7--22.8)	0 (0-0)	1 (-21.5-34.1)	1.7 (1.2-2.3)	-31.3 (-38.6--24.1)
Gilan	8.3 (5.4-12.7)	0.2 (-11.3-13.3)	5.8 (3.8-8.5)	-0.9 (-11.8-11.3)	1.9 (1.3-2.8)	2.5 (-10.4-17.1)	0.4 (0.2-0.5)	-28.1 (-39.2--16.6)	0 (0-0)	8.1 (-19.6-37.6)	0.8 (0.5-1)	-35.3 (-42.3--28.3)
Golestan	10.1 (6.6-15.7)	-0.1 (-13.7-14)	6.3 (4.1-9.5)	-2.7 (-16.2-10.5)	1.8 (1.2-2.6)	4 (-9.4-18.7)	0.3 (0.2-0.5)	-29.7 (-39.7--18)	0 (0-0)	7.2 (-19.9-43.4)	1.1 (0.8-1.5)	-23.3 (-31.7--13.9)
Hamadan	12.7 (8.4-19.4)	0.4 (-10.8-12.7)	7.9 (5.2-11.5)	-1.5 (-12.8-11)	2.3 (1.6-3.3)	5.5 (-8.6-19.7)	0.4 (0.3-0.6)	-30.7 (-40.6--20.6)	0 (0-0)	1.7 (-23.2-31.6)	1.1 (0.8-1.6)	-30.1 (-37.5--22.7)
Hormozgan	11.7 (7.7-17.6)	3.5 (-9.6-18.2)	7.3 (4.8-10.7)	1.4 (-10.2-16.3)	2.1 (1.4-3)	6.5 (-6.9-22.2)	0.4 (0.3-0.6)	-23.3 (-33.6--11.6)	0 (0-0)	19.1 (-12.6-55.5)	1.4 (1-1.9)	-19.4 (-27.3--10.3)
Ilam	13.1 (8.6-19.7)	-0.9 (-11.2-12.1)	8.3 (5.6-12.2)	-2.8 (-12.6-9.4)	2.5 (1.7-3.5)	3 (-9.9-18.4)	0.5 (0.3-0.7)	-30.7 (-41--18.8)	0 (0-0)	4.1 (-19.6-33.4)	1.2 (0.9-1.7)	-35.4 (-41.7--27.9)
Isfahan	15.2 (10-23.1)	-1 (-13.8-14.8)	10 (6.6-14.9)	-2.5 (-13.9-11.2)	3.1 (2.1-4.4)	2.8 (-9.8-17)	0.6 (0.4-0.8)	-29.5 (-39.8--19.5)	0 (0-0)	4.7 (-18.5-30.6)	1.4 (1-1.9)	-30.5 (-37.5--22.6)
Kerman	10.6 (7-16.3)	0.1 (-11.7-12.9)	6.7 (4.5-10)	-1.9 (-12.6-11.5)	2.1 (1.4-3)	3.2 (-8.9-17.1)	0.4 (0.3-0.5)	-30.4 (-39.3--19.6)	0 (0-0)	6.6 (-16.6-43.5)	1.2 (0.9-1.7)	-22.3 (-30--13.3)
Kermanshah	12.5 (8.3-19.4)	-0.7 (-12.6-12.1)	7.9 (5.2-11.9)	-2.1 (-13.9-8.8)	2.5 (1.7-3.6)	5.6 (-8-21)	0.4 (0.3-0.6)	-31.8 (-41.3--20.6)	0 (0-0)	0.8 (-23.8-35.1)	1.2 (0.8-1.6)	-30.7 (-38.3--22.8)
Khorasan-e-Razavi	10.5 (6.8-16.2)	-0.5 (-12.1-15)	6.4 (4.2-9.5)	-2.9 (-13.3-10.8)	1.7 (1.1-2.4)	4.2 (-8.5-21.2)	0.3 (0.2-0.4)	-28.9 (-39.7--16.4)	0 (0-0)	13.1 (-15.6-48.9)	1.1 (0.8-1.5)	-22.5 (-30.1--13.4)
Khuzestan	18.9 (12-28.2)	-0.8 (-13.8-14.7)	12.2 (8.1-17.8)	-3 (-15.7-10.8)	3.7 (2.5-5.3)	4 (-10.3-18.6)	0.7 (0.5-1)	-29.9 (-39.5--19.1)	0 (0-0)	0.3 (-20.4-27.7)	2.2 (1.6-3.1)	-22.7 (-31.5--13.5)
Kohgiluyeh and Boyer-Ahmad	23 (14.7-34.3)	0.4 (-13.6-14.2)	14.8 (9.4-21.6)	-1.4 (-14.6-11.4)	4.3 (2.9-6.2)	3.9 (-10.5-18.4)	0.7 (0.5-1)	-30.4 (-40.4--19.3)	0 (0-0)	11.1 (-10.6-40.1)	2.5 (1.8-3.6)	-27.6 (-36.2--18.9)
Kurdistan	12.1 (8-18.4)	-1 (-12.2-13.8)	7.2 (4.7-10.6)	-2.2 (-13.1-11.5)	1.9 (1.2-2.6)	3.8 (-9.4-19)	0.4 (0.2-0.5)	-29.6 (-39.3--18.4)	0 (0-0)	-0.7 (-23-31.9)	1 (0.8-1.4)	-30.1 (-37--21.4)

Location	Birth Incidence rate (per 100,000 live births), 2021	Birth Incidence rate (per 100,000 live births) percent change (2000-2021)	Under-5 rate (per 100,000 population), 2021	Under-5 rate (per 100,000 population) percent change (2000-2021)	5-14 rate (per 100,000 population), 2021	5-14 rate (per 100,000 population) percent change (2000-2021)	15-49 rate (per 100,000 population), 2021	15-49 rate (per 100,000 population) percent change (2000-2021)	50plus rate (per 100,000 population), 2021	50plus rate (per 100,000 population) percent change (2000-2021)	All age rate (per 100,000 population), 2021	All age rate (per 100,000 population) percent change (2000-2021)
Lorestan	13.4 (8.7-20.2)	-0.7 (-11.9-11.5)	8.6 (5.6-12.8)	-2.5 (-13.5-8.3)	2.6 (1.7-3.7)	4.4 (-10-19.7)	0.5 (0.3-0.7)	-29.5 (-40.3--18)	0 (0-0)	6.3 (-18.8-37.4)	1.4 (1-2)	-30.3 (-37.9--22.5)
Markazi	11.9 (7.9-18.1)	-1.4 (-14.3-13.5)	7.8 (5.1-11.7)	-2.9 (-14.7-10.9)	2.4 (1.6-3.4)	3.8 (-9.6-18.2)	0.4 (0.3-0.6)	-30.8 (-39.4--20.8)	0 (0-0)	13.9 (-9.8-45.4)	1.1 (0.8-1.5)	-30.9 (-38--23.2)
Mazandaran	8.6 (5.7-12.9)	-0.4 (-11.5-13.5)	6 (3.9-8.6)	-1.7 (-11.8-10.8)	1.9 (1.3-2.8)	2.4 (-8.6-16.1)	0.4 (0.2-0.5)	-29 (-38.1--17.8)	0 (0-0)	0.9 (-20.5-35.7)	0.8 (0.6-1.1)	-33.9 (-40.1--26.7)
North Khorasan	10.6 (6.7-16.7)	-1.3 (-15.3-13.2)	6.4 (4.2-9.5)	-4.2 (-16-9.9)	1.7 (1.1-2.4)	1.7 (-12.5-16.5)	0.3 (0.2-0.4)	-22.7 (-33.4--9)	0 (0-0)	6.1 (-18.7-43.5)	1 (0.7-1.5)	-30 (-38.1--20.7)
Qazvin	10.2 (6.5-15.4)	-0.6 (-12.6-13.3)	6.4 (4.1-9.4)	-2.2 (-14.4-12.1)	1.9 (1.2-2.6)	4.5 (-7.8-19.5)	0.4 (0.2-0.5)	-30.1 (-39.9--18)	0 (0-0)	14.9 (-13.4-51.2)	1 (0.7-1.3)	-27.6 (-35.1--18.6)
Qom	10.8 (6.7-16.8)	-0.2 (-12.7-14.4)	7.2 (4.5-10.8)	-1.5 (-13.6-11.7)	2.2 (1.5-3.2)	3.7 (-8.5-17.5)	0.4 (0.3-0.6)	-26.1 (-36.3--14.9)	0 (0-0)	7.1 (-18.6-36.6)	1.2 (0.9-1.7)	-22.2 (-30.2--13.8)
Semnan	9.1 (5.9-13.7)	-0.9 (-13.4-13.4)	6.1 (3.9-9.2)	-1.7 (-13.8-11.6)	1.9 (1.3-2.8)	3.5 (-9.8-18.4)	0.4 (0.3-0.6)	-24.2 (-34.1--12)	0 (0-0)	10.3 (-14.5-40.8)	1 (0.7-1.3)	-23.9 (-32.6--14.7)
Sistan and Baluchistan	11.6 (7.6-17.7)	-0.1 (-11.1-10.7)	6.9 (4.5-10.1)	-2.6 (-13.2-8.8)	1.7 (1.2-2.5)	-0.1 (-13.6-14.9)	0.4 (0.2-0.5)	-20.8 (-32.2--7.1)	0 (0-0)	6.4 (-25.4-45.2)	1.6 (1.1-2.3)	-13.4 (-22.4--3.8)
South Khorasan	10.4 (6.9-16.2)	-0.7 (-11.4-14)	6.2 (4.1-9.2)	-2.6 (-13-10.3)	1.6 (1.1-2.3)	2.2 (-10.5-18.2)	0.3 (0.2-0.5)	-24.1 (-34.4--11.7)	0 (0-0)	16.5 (-9.1-48.6)	1.1 (0.7-1.5)	-23.1 (-30.8--14.2)
Tehran	8 (5.1-12.3)	-0.5 (-11.8-11.4)	5.8 (3.8-8.8)	-2.2 (-12.8-9.1)	2 (1.4-2.9)	4.6 (-7.7-18.5)	0.4 (0.3-0.6)	-26.6 (-37.1--15.5)	0 (0-0)	-5.4 (-26-18)	0.9 (0.6-1.2)	-25 (-32.4--17.5)
West Azarbayejan	10.8 (7-16.6)	1.1 (-10.1-16)	6.6 (4.3-9.9)	-1.1 (-12.6-11.6)	1.8 (1.2-2.5)	6.2 (-6.8-21.4)	0.3 (0.2-0.5)	-26.8 (-37--13.8)	0 (0-0)	12.7 (-14.6-47.3)	1.1 (0.8-1.5)	-17.3 (-25.7--7.8)
Yazd	11.6 (7.6-17.7)	1 (-12.7-14.3)	7.8 (5.2-11.3)	-1.2 (-14-11)	2.5 (1.6-3.5)	5.4 (-7.8-21.1)	0.5 (0.3-0.7)	-26.6 (-36.4--15.3)	0 (0-0)	13.9 (-10.4-45)	1.4 (1-1.9)	-17.7 (-26.4--7.9)
Zanjan	10.7 (6.9-16.7)	0.1 (-14.6-14.2)	6.4 (4.2-9.6)	-1.9 (-14.5-12.5)	1.7 (1.2-2.4)	5.3 (-9.6-21.6)	0.3 (0.2-0.5)	-29.6 (-39.4--17.8)	0 (0-0)	11.1 (-15.1-44.5)	1 (0.7-1.3)	-28.3 (-36.6--19.3)
Iraq	112 (81.9-149)	-0.2 (-11.2-13)	77.3 (58.1-102)	-0.8 (-10.8-11.1)	31.6 (23.1-41.8)	-1.5 (-11.7-9.9)	9.9 (7.3-12.9)	-6.9 (-15.9-2.3)	0.3 (0.2-0.5)	3.2 (-12.1-18.8)	20.3 (15.6-26)	-24.3 (-31.6--16.1)
Jordan	23.4 (15.1-35.5)	-0.3 (-12.2-14.9)	16.2 (10.7-23.6)	-1 (-12.4-14.2)	5.4 (3.6-7.4)	-0.8 (-12.7-12.7)	1.6 (1.1-2.2)	-8.8 (-20.2-2.8)	0.1 (0-0.1)	15.2 (-7.3-37.1)	3.6 (2.6-4.9)	-21.7 (-29.3--12.8)
Kuwait	29.6 (19.3-43.9)	0.5 (-13.1-14.6)	21.6 (14.1-31.4)	0 (-12.7-13.9)	8 (5.5-11.1)	0.4 (-11.8-14.9)	1.9 (1.4-2.7)	-14.9 (-24.9--3)	0.1 (0.1-0.2)	24.3 (3.3-49.1)	3.6 (2.6-4.9)	-28 (-35.8--20)
Lebanon	11 (7.5-15.9)	3.7 (-7.1-17.6)	8.1 (5.5-11.5)	2.5 (-8.3-15)	3.1 (2.1-4.3)	7.2 (-6.4-21.5)	0.8 (0.5-1.1)	-9.1 (-19.6-4.2)	0 (0-0)	6.1 (-14.3-31.4)	1.7 (1.2-2.2)	-12.3 (-21--3.3)
Libya	643 (442-910)	0.9 (-9.9-14.9)	410 (288-566)	-0.5 (-11.9-12.3)	138 (97.7-191)	-2.3 (-14.2-11.5)	36.9 (26.5-49.8)	-22.1 (-31--11.3)	1.7 (1.1-2.5)	20.5 (3.4-41.1)	66.8 (49.3-88.6)	-38.8 (-45.2--30.9)
Morocco	14.8 (10.7-19.8)	0.7 (-9.9-11.8)	8.8 (6.3-11.7)	-1.9 (-11.8-8.9)	2.4 (1.6-3.3)	-0.5 (-12.8-13.8)	0.6 (0.4-0.8)	-10.2 (-21.3-2.5)	0 (0-0)	4.3 (-17.9-35.1)	1.4 (1.1-1.8)	-27.8 (-35.2--20.5)
Oman	167 (117-227)	-0.4 (-13-11.1)	110 (77.9-146)	-1.3 (-12.7-10.5)	34.4 (23.5-47.4)	3 (-10.1-19.5)	7.2 (5-10.1)	-21.2 (-31.8--8.1)	0.4 (0.2-0.5)	7.4 (-11.9-31.9)	20.4 (15.4-26)	-24.2 (-31.4--15.6)
Palestine	21 (13.6-31.9)	0.7 (-11.8-15.3)	13.7 (9-20)	-1.3 (-12.5-14.5)	4.1 (2.8-5.8)	-2.2 (-14.1-13.3)	1.4 (0.9-1.9)	-5.8 (-17.2-7.7)	0.1 (0-0.1)	15.7 (-6.2-38.9)	3.3 (2.4-4.5)	-23.9 (-32.3--12.5)
Qatar	32 (20.9-49.3)	1.3 (-11-16.6)	23.8 (15.7-36.1)	-0.2 (-12-13.3)	9.8 (6.8-13.6)	0.9 (-10.4-13.3)	2.3 (1.6-3.2)	3.3 (-8.1-16.3)	0.1 (0.1-0.2)	-9.9 (-26.8-10.3)	4 (2.9-5.3)	-27.4 (-35.1--19.4)
Saudi Arabia	24.6 (18-32.2)	1.2 (-8.2-11)	16.1 (11.8-20.8)	1 (-8.5-11.2)	5.6 (3.9-7.6)	0.2 (-11.8-12.8)	1.2 (0.8-1.7)	-20.6 (-31.6--6.9)	0 (0-0.1)	11.2 (-14.2-40.2)	2.5 (1.9-3.2)	-39.6 (-45.9--33.2)
Sudan	40.7 (31.7-53.3)	-1.5 (-10.8-8.7)	23.4 (18.4-30.7)	-4.1 (-13.1-6.4)	5.9 (4.1-8.1)	-2.2 (-15.2-12.7)	1.6 (1.1-2.2)	-4.4 (-16.6-9.5)	0 (0-0.1)	12.7 (-12.8-43.8)	5.3 (4.2-6.7)	-21.4 (-29.8--13.3)
Syrian Arab Republic	21.4 (15.9-28.3)	-0.1 (-9.2-13)	13.9 (10.3-18)	-0.6 (-10.3-11.8)	3.8 (2.7-5.2)	-7.4 (-18.8-6.6)	1.1 (0.8-1.6)	-8.2 (-19.8-5)	0 (0-0.1)	2.2 (-18-32.1)	2.3 (1.7-2.9)	-40.4 (-46.2--33.9)
Tunisia	21.3 (15.2-29.3)	0.1 (-12-12.8)	13.7 (9.6-18.7)	-2 (-12.6-9.5)	4 (2.8-5.7)	1.6 (-11.6-18.1)	1 (0.7-1.4)	-17.5 (-27.8--5.6)	0 (0-0.1)	1.5 (-17.2-24.9)	2.1 (1.6-2.7)	-26.1 (-33.3--18.2)
Türkiye	12.5 (9.4-17)	0.5 (-10.6-12.1)	7.8 (6-10.5)	0.2 (-9.9-10.8)	2.8 (2.2-3.6)	0.2 (-10.1-13)	0.9 (0.7-1.2)	-8.5 (-17.4-2.3)	0 (0-0.1)	-3.2 (-20.6-14.1)	1.3 (1.1-1.6)	-31.3 (-37.1--24.6)
United Arab Emirates	3.5 (2.5-5)	-1.7 (-11-10.7)	2.6 (1.9-3.7)	-3 (-12.5-8.7)	1 (0.7-1.4)	-2 (-12.2-10.4)	0.2 (0.1-0.2)	-36.2 (-46.5--23.5)	0 (0-0)	-2.3 (-30.5-32.5)	0.3 (0.2-0.4)	-39.8 (-45.4--33.6)
Yemen	15 (11.2-20.2)	0.6 (-7.7-11.2)	8.6 (6.4-11.5)	-2.6 (-11.5-6.8)	2.2 (1.5-3.1)	-0.4 (-12.3-14.3)	0.5 (0.4-0.8)	-10.6 (-22.5-4.7)	0 (0-0)	0.9 (-24.7-38.3)	2 (1.5-2.6)	-22.1 (-29.8--14.5)

Location	Birth Incidence rate (per 100,000 live births), 2021	Birth Incidence rate (per 100,000 live births) percent change (2000-2021)	Under-5 rate (per 100,000 population), 2021	Under-5 rate (per 100,000 population) percent change (2000-2021)	5-14 rate (per 100,000 population), 2021	5-14 rate (per 100,000 population) percent change (2000-2021)	15-49 rate (per 100,000 population), 2021	15-49 rate (per 100,000 population) percent change (2000-2021)	50plus rate (per 100,000 population), 2021	50plus rate (per 100,000 population) percent change (2000-2021)	All age rate (per 100,000 population), 2021	All age rate (per 100,000 population) percent change (2000-2021)
South Asia	12.7 (8.2-19)	-1.2 (-4.1-1.4)	7.8 (5-11.6)	-1.2 (-4-1.3)	1.1 (0.7-1.7)	-5.4 (-8.8--2)	0 (0-0.1)	-14.5 (-19.3--9.7)	0 (0-0)	6.7 (-7.8-23.6)	0.9 (0.6-1.3)	-31.3 (-33.4--29.4)
**South Asia**	12.7 (8.2-19)	-1.2 (-4.1-1.4)	7.8 (5-11.6)	-1.2 (-4-1.3)	1.1 (0.7-1.7)	-5.4 (-8.8--2)	0 (0-0.1)	-14.5 (-19.3--9.7)	0 (0-0)	6.7 (-7.8-23.6)	0.9 (0.6-1.3)	-31.3 (-33.4--29.4)
Bangladesh	1 (0.6-1.5)	0.1 (-11-11.7)	0.5 (0.3-0.8)	-0.3 (-11.1-11.7)	0.1 (0-0.1)	-1.3 (-16.4-20.1)	0 (0-0)	-13.2 (-41.5-28.7)	0 (0-0)	9.5 (-54.6-140)	0.1 (0-0.1)	-37.9 (-45.2--29.7)
Bhutan	1 (0.6-1.5)	0.1 (-12.6-12.4)	0.5 (0.3-0.8)	-1.4 (-14.1-11.5)	0.1 (0-0.1)	-0.4 (-19.2-23)	0 (0-0)	-24.5 (-47.1-11.4)	0 (0-0)	-4.2 (-59-127)	0.1 (0-0.1)	-35.6 (-44.1--27.1)
India	17.8 (11.4-26.7)	2.1 (-0.9-4.9)	10.8 (7-16.1)	1.7 (-1.1-4.4)	1.5 (1-2.3)	-2.2 (-5.7-1.4)	0.1 (0-0.1)	-13.3 (-18.3--8.3)	0 (0-0)	7.4 (-7.2-24.7)	1.2 (0.8-1.7)	-30.6 (-32.7--28.6)
Nepal	1 (0.6-1.5)	-0.7 (-12.7-12.8)	0.6 (0.3-0.8)	-2.1 (-13.7-12.9)	0.1 (0-0.1)	-4.2 (-21-15.8)	0 (0-0)	-7.7 (-36.1-32.3)	0 (0-0)	-2.6 (-58.4-119)	0.1 (0-0.1)	-36.1 (-43.6--26.1)
Pakistan	0.7 (0.4-1.1)	-2.1 (-13.5-10)	0.4 (0.3-0.6)	-3.4 (-12.8-8.2)	0.1 (0-0.1)	-3.4 (-17.1-12.4)	0 (0-0)	-2 (-22.7-26.2)	0 (0-0)	4.6 (-35.4-80.4)	0.1 (0-0.1)	-25 (-33.1--15.7)
Azad Jammu & Kashmir	0.7 (0.4-1.1)	-2.3 (-23.2-8.9)	0.4 (0.2-0.6)	-4.1 (-23.1-8.3)	0 (0-0.1)	-5.7 (-24.7-19.7)	0 (0-0)	-21.2 (-47-17)	0 (0-0)	-3.4 (-63.8-166)	0 (0-0.1)	-34.6 (-46.4--25)
Balochistan	0.7 (0.4-1.1)	-0.1 (-16.4-21.9)	0.4 (0.2-0.6)	-0.3 (-17.7-18.7)	0 (0-0.1)	-2.6 (-24.9-28.2)	0 (0-0)	2 (-29.7-52.4)	0 (0-0)	7.4 (-51.4-168)	0.1 (0-0.1)	-8.1 (-24.7-11.2)
Gilgit-Baltistan	0.7 (0.4-1)	-1.9 (-20-16.2)	0.4 (0.2-0.6)	-2.4 (-19.6-15.4)	0 (0-0.1)	-1 (-22.6-23.4)	0 (0-0)	-4.1 (-34.8-44.1)	0 (0-0)	-5.9 (-61.6-123)	0.1 (0-0.1)	-9.1 (-24.7-6.7)
Islamabad Capital Territory	0.6 (0.3-0.9)	-0.5 (-24.2-39.1)	0.4 (0.2-0.5)	0.5 (-21.1-37)	0.1 (0-0.1)	1.2 (-23.8-30.1)	0 (0-0)	-18.7 (-43.6-23.5)	0 (0-0)	9.2 (-45.9-156)	0.1 (0-0.1)	-21 (-37.6-4)
Khyber Pakhtunkhwa	0.6 (0.4-1)	-2 (-25.9-17.2)	0.4 (0.2-0.6)	-5.1 (-27-14.2)	0.1 (0-0.1)	-7 (-27.7-21.1)	0 (0-0)	0.6 (-32.3-51)	0 (0-0)	-5.8 (-59-123)	0.1 (0-0.1)	-11.7 (-31.8-7.4)
Punjab	0.7 (0.5-1.1)	-0.5 (-15.3-19.4)	0.4 (0.3-0.7)	-2.2 (-16.5-18.7)	0.1 (0-0.1)	-3.5 (-26.6-25.3)	0 (0-0)	-4.1 (-35-46.8)	0 (0-0)	4.3 (-58.3-178)	0.1 (0-0.1)	-31.6 (-42.1--16.7)
Sindh	0.7 (0.4-1.1)	-0.2 (-14.8-15.2)	0.4 (0.2-0.6)	-2 (-16.9-14.2)	0.1 (0-0.1)	-2.7 (-22.5-21)	0 (0-0)	0.2 (-32.5-48)	0 (0-0)	0.4 (-57.1-143)	0.1 (0-0.1)	-25.6 (-37.3--13)
Southeast Asia, East Asia, and Oceania	0.1 (0.1-0.1)	-0.2 (-3.9-6.9)	0.1 (0-0.1)	0 (-3.8-7.1)	0 (0-0)	8.6 (2.9-17.3)	0 (0-0)	-1.7 (-10.3-8.6)	0 (0-0)	3.2 (-12.8-25.8)	0 (0-0)	-20.7 (-23.7--14.6)
**East Asia**	0 (0-0)	0.2 (-4-1.8)	0 (0-0)	-3.6 (-9--1.3)	0 (0-0)	11.1 (5.8-16.4)	0 (0-0)	-15.5 (-21--9.7)	0 (0-0)	1.5 (-13.2-21.2)	0 (0-0)	-21.9 (-25.7--19.7)
China	0 (0-0)	-0.3 (-5-0.1)	0 (0-0)	-4.1 (-10--2.5)	0 (0-0)	11.6 (6.2-17.4)	0 (0-0)	-15.4 (-21.1--9.4)	0 (0-0)	1.5 (-13.7-22.9)	0 (0-0)	-21.2 (-25.3--19.2)
Democratic People's Republic of Korea	0 (0-0)	0 (0-0)	0 (0-0)	-0.6 (-3.7-2.5)	0 (0-0)	-3.9 (-16.2-9.3)	0 (0-0)	-8.8 (-30.8-17.3)	0 (0-0)	7 (-42.5-143)	0 (0-0)	-40.9 (-45--36.6)
Taiwan (Province of China)	0 (0-0)	-0.1 (-0.6-0)	0 (0-0)	-0.9 (-3.4-1.8)	0 (0-0)	2.4 (-8.3-15.2)	0 (0-0)	-17.9 (-35.9-7.2)	0 (0-0)	-5.1 (-46.9-69.4)	0 (0-0)	-39.1 (-44.4--33.4)
**Oceania**	0 (0-0)	0.3 (-10.1-1.7)	0 (0-0)	-1.6 (-12.9-1.8)	0 (0-0)	-5.5 (-21-5.2)	0 (0-0)	-21.3 (-37.7--6.2)	0 (0-0)	-14.3 (-35.6-12.1)	0 (0-0)	-10.6 (-22.6--6.2)
American Samoa	0 (0-0)	0 (-0.3-0.3)	0 (0-0)	0.2 (-2.9-2.9)	0 (0-0)	-4 (-15.3-8.5)	0 (0-0)	13.1 (-13.1-47.7)	0 (0-0)	-1.7 (-47.5-101)	0 (0-0)	-27.2 (-32.6--21.5)
Cook Islands	0 (0-0)	0 (0-0)	0 (0-0)	0.1 (-2.1-2.6)	0 (0-0)	-2.9 (-14-9.2)	0 (0-0)	-0.2 (-20.9-27.7)	0 (0-0)	-2.4 (-51.2-82.4)	0 (0-0)	-28.6 (-34.1--22.8)
Fiji	0 (0-0)	0 (0-0)	0 (0-0)	-0.8 (-4.3-2.7)	0 (0-0)	1.5 (-12.1-16.1)	0 (0-0)	-13.5 (-35.2-12.7)	0 (0-0)	-17.3 (-53.4-61.5)	0 (0-0)	-11.6 (-17.8--6)
Guam	0 (0-0)	0 (0-0)	0 (0-0)	0.2 (-2.5-2.8)	0 (0-0)	-0.6 (-11.2-10.5)	0 (0-0)	8 (-12.7-36.3)	0 (0-0)	-12.4 (-54.6-60)	0 (0-0)	-15.5 (-20.8--10)
Kiribati	0 (0-0)	0 (0-0)	0 (0-0)	-0.5 (-4.4-3.9)	0 (0-0)	0.2 (-14.5-15.7)	0 (0-0)	-10.7 (-34.3-19.9)	0 (0-0)	2.5 (-46.4-92)	0 (0-0)	-12.6 (-18--7.2)
Marshall Islands	0 (0-0)	0.1 (0-0)	0 (0-0)	-0.8 (-4.9-3.2)	0 (0-0)	0.6 (-13.5-18.1)	0 (0-0)	-21.5 (-43.5-5.5)	0 (0-0)	-6.5 (-52.9-85.5)	0 (0-0)	-23.5 (-29--17.7)
Micronesia (Federated States of)	0 (0-0)	0 (0-0)	0 (0-0)	-0.1 (-3.5-4.3)	0 (0-0)	-1.9 (-13.9-14.9)	0 (0-0)	-10.7 (-34.5-20.8)	0 (0-0)	14 (-49.4-129)	0 (0-0)	-29 (-33.7--23.3)
Nauru	0 (0-0)	0 (0-0)	0 (0-0)	-2 (-5.7-1.8)	0 (0-0)	-2.4 (-16-13.3)	0 (0-0)	1.1 (-26.4-35.3)	0 (0-0)	-3.2 (-54.2-89.2)	0 (0-0)	-17.4 (-22.4--12.2)
Niue	0 (0-0)	-5.3 (-44.9-0)	0 (0-0)	-6 (-47.1-2.7)	0 (0-0)	-7.5 (-55.4-13.7)	0 (0-0)	-12.3 (-61.3-24.5)	0 (0-0)	-9.5 (-84-122)	0 (0-0)	-25.7 (-60.6--15)

Location	Birth Incidence rate (per 100,000 live births), 2021	Birth Incidence rate (per 100,000 live births) percent change (2000-2021)	Under-5 rate (per 100,000 population), 2021	Under-5 rate (per 100,000 population) percent change (2000-2021)	5-14 rate (per 100,000 population), 2021	5-14 rate (per 100,000 population) percent change (2000-2021)	15-49 rate (per 100,000 population), 2021	15-49 rate (per 100,000 population) percent change (2000-2021)	50plus rate (per 100,000 population), 2021	50plus rate (per 100,000 population) percent change (2000-2021)	All age rate (per 100,000 population), 2021	All age rate (per 100,000 population) percent change (2000-2021)
Northern Mariana Islands	0 (0-0)	0 (0-0)	0 (0-0)	-3 (-5.5--0.7)	0 (0-0)	-6.6 (-17.8-5)	0 (0-0)	37.6 (4.6-90.9)	0 (0-0)	2.2 (-46-100)	0 (0-0)	-43.6 (-49.4--36.9)
Palau	0 (0-0)	0 (0-0)	0 (0-0)	-1.5 (-5.3-1.8)	0 (0-0)	-5 (-16.5-8.9)	0 (0-0)	-4.4 (-27.9-23.8)	0 (0-0)	-3 (-48.1-84.6)	0 (0-0)	-38.4 (-43.1--33.2)
Papua New Guinea	0 (0-0)	0 (0-0)	0 (0-0)	-0.5 (-4.3-3.1)	0 (0-0)	1.4 (-12.6-19.2)	0 (0-0)	-8.8 (-34.4-28)	0 (0-0)	11.3 (-47.7-109)	0 (0-0)	-8.4 (-13.8--3.1)
Samoa	0 (0-0)	0 (0-0)	0 (0-0)	1.4 (-2.3-5.9)	0 (0-0)	-4.4 (-17.1-9.5)	0 (0-0)	3.2 (-21.4-35.4)	0 (0-0)	10.7 (-41.4-111)	0 (0-0)	-8.7 (-15.1--1.4)
Solomon Islands	0 (0-0)	-0.1 (0-0)	0 (0-0)	-1.5 (-5.1-2.4)	0 (0-0)	-0.9 (-16.1-16.8)	0 (0-0)	-11.6 (-34.4-23.8)	0 (0-0)	12.8 (-43.8-172)	0 (0-0)	-16.8 (-21.8--10)
Tokelau	0 (0-0)	0 (0-0)	0 (0-0)	-0.2 (-2.6-2.2)	0 (0-0)	2.3 (-7.9-17.7)	0 (0-0)	-11.1 (-30-15.4)	0 (0-0)	4.7 (-41.7-84.3)	0 (0-0)	-16.4 (-21.1--10.6)
Tonga	0 (0-0)	0 (0-0)	0 (0-0)	-0.8 (-4-2.5)	0 (0-0)	-0.9 (-13.2-11.9)	0 (0-0)	-8 (-29.2-15.9)	0 (0-0)	1.8 (-43.4-77)	0 (0-0)	-12.1 (-18.2--6.5)
Tuvalu	0 (0-0)	0 (0-0)	0 (0-0)	0.5 (-3.6-4.6)	0 (0-0)	-5 (-17.9-11.3)	0 (0-0)	6.5 (-19.6-50.9)	0 (0-0)	-10.8 (-53.2-83.2)	0 (0-0)	-23 (-28--17.4)
Vanuatu	0 (0-0)	0 (0-0)	0 (0-0)	-1 (-5-2.9)	0 (0-0)	-0.9 (-15.6-14.3)	0 (0-0)	-8.1 (-36.1-28)	0 (0-0)	-10.4 (-65-122)	0 (0-0)	-17.9 (-23.9--11.7)
**Southeast Asia**	0.2 (0.2-0.3)	2 (-2-9.8)	0.1 (0.1-0.2)	1.3 (-2.8-9.2)	0 (0-0)	-0.8 (-6.5-7.9)	0 (0-0)	-15.6 (-23.4--6.8)	0 (0-0)	5.4 (-21.2-44.8)	0 (0-0)	-26.1 (-29.2--19.8)
Cambodia	0.2 (0.2-0.4)	3 (-8.9-63)	0.1 (0.1-0.2)	2.7 (-10.1-65.1)	0 (0-0)	3.9 (-16.7-60)	0 (0-0)	-22.1 (-45.2-17.2)	0 (0-0)	-2.4 (-49.2-89.7)	0 (0-0)	-24.1 (-34.2-23.4)
Indonesia	0.2 (0.2-0.3)	3.5 (-1.2-21.3)	0.1 (0.1-0.2)	1.5 (-3.3-17.3)	0 (0-0)	-1.6 (-8.5-14)	0 (0-0)	-13.8 (-24-2)	0 (0-0)	4.8 (-18.5-41)	0 (0-0)	-28.3 (-32.4--17.6)
Aceh	0.2 (0.1-0.3)	2.2 (0-35.8)	0.1 (0.1-0.2)	0.7 (-7-30.4)	0 (0-0)	0.9 (-17.9-31.1)	0 (0-0)	-14.5 (-39.1-24.1)	0 (0-0)	8.9 (-45-115)	0 (0-0)	-25.2 (-33.1--4.4)
Bali	0.2 (0.1-0.3)	1.5 (0-36.2)	0.1 (0.1-0.2)	0.1 (-5.4-32.2)	0 (0-0)	-2.9 (-20.6-29.5)	0 (0-0)	-5.4 (-33.6-37.2)	0 (0-0)	6.3 (-50.4-114)	0 (0-0)	-32.2 (-39.4--9.7)
Bangka-Belitung Islands	0.2 (0.2-0.3)	2 (-16.1-38.9)	0.1 (0.1-0.2)	0.2 (-16.4-37.4)	0 (0-0)	0 (-18.7-35.5)	0 (0-0)	-27.2 (-48.9-5)	0 (0-0)	1.4 (-53.9-111)	0 (0-0)	-31.3 (-41.8--9.4)
Banten	0.2 (0.2-0.4)	3.3 (0-53.2)	0.1 (0.1-0.2)	1.6 (-6.5-48.1)	0 (0-0)	-0.1 (-19.5-35.4)	0 (0-0)	-17.1 (-43-21.8)	0 (0-0)	4.3 (-51.4-139)	0 (0-0)	-24.7 (-32.8-6.8)
Bengkulu	0.2 (0.2-0.4)	3 (0-54.5)	0.1 (0.1-0.2)	1.5 (-6.6-50)	0 (0-0)	1.5 (-17.8-45.6)	0 (0-0)	-17.3 (-40.9-25.3)	0 (0-0)	-2.1 (-56.5-150)	0 (0-0)	-28.2 (-35.7-5.2)
Gorontalo	0.2 (0.2-0.4)	2.5 (0-44.6)	0.1 (0.1-0.2)	2 (-6.9-42)	0 (0-0)	-5.1 (-25.6-30.9)	0 (0-0)	10.5 (-25.2-82.4)	0 (0-0)	15.8 (-50.1-176)	0 (0-0)	-30.3 (-38.9--4.2)
Jakarta	0.2 (0.1-0.3)	2.1 (-14.8-53)	0.1 (0.1-0.2)	0.1 (-17.3-49.4)	0 (0-0)	2.8 (-16.9-48.2)	0 (0-0)	-24.8 (-46.6-12.2)	0 (0-0)	-5.5 (-51.4-71.2)	0 (0-0)	-26.1 (-37.6-7.7)
Jambi	0.2 (0.1-0.3)	3.1 (0-51.4)	0.1 (0.1-0.2)	1.6 (-4.9-46.3)	0 (0-0)	-1.6 (-16.7-39.6)	0 (0-0)	-15.7 (-38.2-26.7)	0 (0-0)	-5.5 (-54.9-125)	0 (0-0)	-29.2 (-35.6-1.9)
West Java	0.2 (0.2-0.4)	4.7 (0-63.8)	0.1 (0.1-0.2)	3.1 (-6.1-56.5)	0 (0-0)	0.5 (-19.3-54.6)	0 (0-0)	-10.7 (-37.4-47.2)	0 (0-0)	13.7 (-39.2-156)	0 (0-0)	-29.2 (-37.6-6.8)
Central Java	0.2 (0.1-0.3)	1.7 (-14.5-53.5)	0.1 (0.1-0.2)	0.7 (-15.3-48.6)	0 (0-0)	-2 (-21.1-35.4)	0 (0-0)	-11.2 (-37.1-28)	0 (0-0)	6.9 (-43.8-122)	0 (0-0)	-32.6 (-43.7--4.2)
East Java	0.2 (0.1-0.3)	1.7 (0-40)	0.1 (0.1-0.2)	0.6 (-6.6-35)	0 (0-0)	-1.9 (-20.1-25.9)	0 (0-0)	-7.1 (-33.5-34.6)	0 (0-0)	9.5 (-44.1-116)	0 (0-0)	-31.1 (-38.9--9.5)
West Kalimantan	0.2 (0.2-0.4)	4.5 (0-74.2)	0.1 (0.1-0.2)	2.7 (-6.5-71.1)	0 (0-0)	1.5 (-18.4-55.7)	0 (0-0)	-13.9 (-41.6-31.7)	0 (0-0)	-7.4 (-62.8-107)	0 (0-0)	-27.1 (-35.6-18.6)
South Kalimantan	0.2 (0.2-0.4)	3 (0-47.2)	0.1 (0.1-0.2)	1.1 (-6.4-41.6)	0 (0-0)	-2.2 (-22.2-36.6)	0 (0-0)	-14.5 (-40.8-30.3)	0 (0-0)	2.4 (-48-101)	0 (0-0)	-32.1 (-39.4--7.3)
Central Kalimantan	0.2 (0.1-0.3)	2.5 (0-47.8)	0.1 (0.1-0.2)	0.7 (-6.3-47.1)	0 (0-0)	-0.2 (-18.6-34.4)	0 (0-0)	-17.2 (-40.3-20.5)	0 (0-0)	2.3 (-48.6-130)	0 (0-0)	-29.8 (-37.3-0.3)
East Kalimantan	0.2 (0.1-0.3)	2.9 (0-45.7)	0.1 (0.1-0.2)	0.8 (-5.9-43.1)	0 (0-0)	0.9 (-17.4-37.3)	0 (0-0)	-14.1 (-38.9-23.8)	0 (0-0)	-3.9 (-54.4-97.9)	0 (0-0)	-23 (-30.3-7.9)
North Kalimantan	0.2 (0.1-0.3)	1.6 (0-37.4)	0.1 (0.1-0.2)	-0.2 (-6.8-31)	0 (0-0)	-2.3 (-21.1-28.7)	0 (0-0)	-13.3 (-38.2-25)	0 (0-0)	1 (-42.9-88.8)	0 (0-0)	-33.7 (-40.9--15.2)
Riau Islands	0.2 (0.1-0.3)	3.3 (0-46.3)	0.1 (0.1-0.2)	-0.5 (-9-40.1)	0 (0-0)	7.1 (-11.8-50.1)	0 (0-0)	-38.3 (-56.4--6.9)	0 (0-0)	9.5 (-52.5-141)	0 (0-0)	-15.1 (-25-15.5)

Location	Birth Incidence rate (per 100,000 live births), 2021	Birth Incidence rate (per 100,000 live births) percent change (2000-2021)	Under-5 rate (per 100,000 population), 2021	Under-5 rate (per 100,000 population) percent change (2000-2021)	5-14 rate (per 100,000 population), 2021	5-14 rate (per 100,000 population) percent change (2000-2021)	15-49 rate (per 100,000 population), 2021	15-49 rate (per 100,000 population) percent change (2000-2021)	50plus rate (per 100,000 population), 2021	50plus rate (per 100,000 population) percent change (2000-2021)	All age rate (per 100,000 population), 2021	All age rate (per 100,000 population) percent change (2000-2021)
Lampung	0.2 (0.1-0.3)	3.5 (0-55.6)	0.1 (0.1-0.2)	2.1 (-6.2-48.8)	0 (0-0)	0 (-17.3-38.6)	0 (0-0)	-14.3 (-38.7-28.4)	0 (0-0)	7.3 (-49.1-111)	0 (0-0)	-32.3 (-39.6--1.7)
Maluku	0.3 (0.2-0.4)	3.2 (0-61.1)	0.1 (0.1-0.2)	1.1 (-7.7-56.9)	0 (0-0)	-0.3 (-22.1-49.3)	0 (0-0)	0.8 (-33.3-60.1)	0 (0-0)	8.7 (-56.9-152)	0 (0-0)	-25.2 (-33.9-14.6)
North Maluku	0.3 (0.2-0.4)	2.4 (0-41.9)	0.1 (0.1-0.2)	0.4 (-8.6-38.6)	0 (0-0)	-4.4 (-22.9-33.1)	0 (0-0)	-13 (-42.5-33)	0 (0-0)	1.1 (-60.5-140)	0 (0-0)	-31.6 (-39.5--5.5)
West Nusa Tenggara	0.2 (0.2-0.4)	3.5 (0-59.1)	0.1 (0.1-0.2)	2.1 (-6.2-58)	0 (0-0)	2.2 (-17.5-46.2)	0 (0-0)	-14.6 (-40.1-31.9)	0 (0-0)	-3.5 (-59-120)	0 (0-0)	-26 (-34.1-13.6)
East Nusa Tenggara	0.2 (0.2-0.4)	2.9 (0-46.2)	0.1 (0.1-0.2)	1.4 (-6.4-39.5)	0 (0-0)	-2.8 (-21.6-35.3)	0 (0-0)	5.1 (-27.7-61.1)	0 (0-0)	0 (-52.2-125)	0 (0-0)	-26.3 (-34.4-1.7)
Papua	0.3 (0.2-0.4)	2.7 (0-45.2)	0.1 (0.1-0.2)	0.5 (-7.7-38.2)	0 (0-0)	-0.3 (-19.3-34)	0 (0-0)	-3.5 (-32.5-41.3)	0 (0-0)	-1.2 (-55.9-131)	0 (0-0)	-24.3 (-32-3.6)
West Papua	0.2 (0.2-0.3)	3.3 (0-55.6)	0.1 (0.1-0.2)	1.6 (-6.4-53.6)	0 (0-0)	0.2 (-18.2-47.4)	0 (0-0)	-23.1 (-47.9-23.9)	0 (0-0)	-18.3 (-67.9-93.7)	0 (0-0)	-24.1 (-32.3-13.1)
Riau	0.2 (0.1-0.3)	3 (0-49.7)	0.1 (0.1-0.2)	1.2 (-5.8-42.2)	0 (0-0)	1.4 (-14.6-39.5)	0 (0-0)	-13.6 (-35.4-26.4)	0 (0-0)	10.8 (-42.9-143)	0 (0-0)	-23.6 (-30.6-5)
West Sulawesi	0.3 (0.2-0.4)	2.2 (0-31.4)	0.1 (0.1-0.2)	0.6 (-5.8-30.3)	0 (0-0)	-3.1 (-19.7-28.1)	0 (0-0)	-2.7 (-31-46)	0 (0-0)	1.3 (-51.9-139)	0 (0-0)	-31.5 (-37.5--11.6)
South Sulawesi	0.2 (0.1-0.3)	3.3 (0-47.5)	0.1 (0.1-0.2)	2 (-6-42.3)	0 (0-0)	-1.8 (-21.5-33.8)	0 (0-0)	-8.5 (-38.1-38.3)	0 (0-0)	0.2 (-52.5-110)	0 (0-0)	-30.9 (-38.7--5.2)
Central Sulawesi	0.2 (0.2-0.4)	2.9 (0-47.8)	0.1 (0.1-0.2)	1 (-7.6-45.7)	0 (0-0)	-3.3 (-22.3-34.7)	0 (0-0)	-7 (-36.5-39.7)	0 (0-0)	-5.5 (-57.9-109)	0 (0-0)	-32 (-39.6--2.2)
Southeast Sulawesi	0.2 (0.2-0.4)	2.6 (0-41.9)	0.1 (0.1-0.2)	0.9 (-7.1-38.7)	0 (0-0)	-0.1 (-19.2-36.3)	0 (0-0)	-10 (-37.7-34)	0 (0-0)	-1.4 (-54-122)	0 (0-0)	-27.3 (-35.5--1.8)
North Sulawesi	0.2 (0.1-0.3)	1.6 (-7.5-31.5)	0.1 (0.1-0.2)	0.5 (-7-26.9)	0 (0-0)	-3.7 (-22.8-23.2)	0 (0-0)	-6.8 (-35.8-33.9)	0 (0-0)	-3 (-56.1-93)	0 (0-0)	-36.2 (-43.5--19.4)
West Sumatra	0.2 (0.1-0.3)	0.9 (-22.4-41.1)	0.1 (0.1-0.2)	-0.2 (-24.7-37.4)	0 (0-0)	-1.9 (-25.7-36)	0 (0-0)	-8.1 (-38.7-37.4)	0 (0-0)	0.2 (-48.3-101)	0 (0-0)	-30.2 (-47.3--4.8)
South Sumatra	0.2 (0.2-0.4)	2.8 (-10.2-46.3)	0.1 (0.1-0.2)	1.2 (-10.9-40.9)	0 (0-0)	1.9 (-19.1-40.3)	0 (0-0)	-19.2 (-43.5-17.6)	0 (0-0)	2.1 (-46.7-103)	0 (0-0)	-29.2 (-37.9--3.7)
North Sumatra	0.2 (0.2-0.3)	2.8 (-5.7-46.5)	0.1 (0.1-0.2)	1.4 (-8.8-43.2)	0 (0-0)	0.7 (-18.6-44.1)	0 (0-0)	-11.8 (-39.3-30.4)	0 (0-0)	8 (-48.1-120)	0 (0-0)	-27.1 (-36.7-1.8)
Yogyakarta	0.2 (0.1-0.3)	1.8 (0-35.1)	0.1 (0.1-0.2)	1.2 (-4-34.9)	0 (0-0.1)	1.2 (-14.8-25.7)	0 (0-0)	-15.2 (-37.5-16.9)	0 (0-0)	10.2 (-42.4-127)	0 (0-0)	-26.3 (-33.8--6.9)
Lao People's Democratic Republic	0.2 (0.2-0.4)	1.6 (-0.1-44.7)	0.1 (0.1-0.2)	1.3 (-6-41.5)	0 (0-0)	0.4 (-18.8-36.5)	0 (0-0)	-18.9 (-42.3-23.7)	0 (0-0)	5.6 (-46.5-119)	0 (0-0)	-26.6 (-33.3-1.9)
Malaysia	0.2 (0.1-0.3)	1.3 (-15.3-55.7)	0.1 (0.1-0.2)	2.2 (-14.1-54.3)	0 (0-0.1)	-0.6 (-15.2-40.6)	0 (0-0)	-10.9 (-32.2-23.8)	0 (0-0)	-15 (-51.1-44.2)	0 (0-0)	-26.5 (-36.8-7.8)
Maldives	0.2 (0.1-0.3)	1.1 (-7.6-39.3)	0.1 (0.1-0.2)	1.6 (-5.2-35.6)	0 (0-0)	7.8 (-11.5-36.9)	0 (0-0)	-41.1 (-59.5--18.3)	0 (0-0)	28.9 (-34.7-141)	0 (0-0)	-42 (-48.2--24)
Mauritius	0.2 (0.1-0.3)	1.5 (-14.2-39.6)	0.1 (0.1-0.2)	2.1 (-11.8-39.2)	0 (0-0.1)	-4.4 (-19.9-24.5)	0 (0-0)	-6.4 (-30.3-27.1)	0 (0-0)	-11.9 (-51.5-61.5)	0 (0-0)	-37.7 (-46.4--18.4)
Myanmar	0.2 (0.2-0.4)	1.6 (0-40.2)	0.1 (0.1-0.2)	1 (-5-37.3)	0 (0-0)	1 (-16.2-32.9)	0 (0-0)	-10.8 (-36.6-27.1)	0 (0-0)	1.2 (-48.1-100)	0 (0-0)	-23.7 (-30-2)
Philippines	0.2 (0.1-0.3)	1.5 (-0.5-10)	0.1 (0.1-0.2)	1.4 (-0.8-10)	0 (0-0)	1.4 (-2.8-10.2)	0 (0-0)	-7.3 (-13.7-1.1)	0 (0-0)	-1.5 (-13.9-11.5)	0 (0-0)	-17.5 (-19.7--10.5)
Abra	0.2 (0.1-0.3)	1.5 (-4.2-42)	0.1 (0.1-0.2)	0.2 (-7.1-37.5)	0 (0-0.1)	2.4 (-14.5-35.1)	0 (0-0)	-8.8 (-32.7-29)	0 (0-0)	-7 (-49.6-76.1)	0 (0-0)	-20.7 (-28.9-6.2)
Agusan Del Norte	0.2 (0.1-0.3)	2.2 (0-45.7)	0.1 (0.1-0.2)	1.7 (-6.2-42.9)	0 (0-0)	2.3 (-17.1-37)	0 (0-0)	-8 (-36.7-35.5)	0 (0-0)	-3.6 (-51.6-88.2)	0 (0-0)	-16.5 (-25.2-14.4)
Agusan Del Sur	0.2 (0.1-0.3)	1.2 (-15.1-45.7)	0.1 (0.1-0.2)	-0.4 (-15.3-42.9)	0 (0-0)	2.9 (-16.5-45.6)	0 (0-0)	-8.3 (-35.7-36.2)	0 (0-0)	-4.8 (-51.7-103)	0 (0-0)	-20.4 (-30.3-13.6)
Aklan	0.2 (0.1-0.3)	2.2 (0-40.8)	0.1 (0.1-0.2)	2.1 (-4.4-41.1)	0 (0-0)	5.3 (-13.6-40.2)	0 (0-0)	-11.9 (-36.7-25.4)	0 (0-0)	-1.2 (-42.4-95.8)	0 (0-0)	-12.7 (-21.6-19.3)
Albay	0.2 (0.1-0.3)	0.8 (0-18.4)	0.1 (0.1-0.2)	0.8 (-4.2-18.5)	0 (0-0)	-0.1 (-16.4-21.8)	0 (0-0)	-6.5 (-35.8-28.6)	0 (0-0)	-10.1 (-53.3-76.6)	0 (0-0)	-18.8 (-25.2--6.1)
Antique	0.2 (0.1-0.3)	1.9 (0-45.7)	0.1 (0.1-0.2)	1.4 (-5.2-44.5)	0 (0-0)	5 (-14.3-45.6)	0 (0-0)	-6.9 (-34.9-30.9)	0 (0-0)	-5 (-54.9-86.2)	0 (0-0)	-15.6 (-23.8-19.9)

Location	Birth Incidence rate (per 100,000 live births), 2021	Birth Incidence rate (per 100,000 live births) percent change (2000-2021)	Under-5 rate (per 100,000 population), 2021	Under-5 rate (per 100,000 population) percent change (2000-2021)	5-14 rate (per 100,000 population), 2021	5-14 rate (per 100,000 population) percent change (2000-2021)	15-49 rate (per 100,000 population), 2021	15-49 rate (per 100,000 population) percent change (2000-2021)	50plus rate (per 100,000 population), 2021	50plus rate (per 100,000 population) percent change (2000-2021)	All age rate (per 100,000 population), 2021	All age rate (per 100,000 population) percent change (2000-2021)
Apayao	0.2 (0.1-0.3)	1 (-14.1-45.7)	0.1 (0.1-0.2)	-1 (-16.4-40)	0 (0-0)	1.4 (-18.6-35.5)	0 (0-0)	-12.1 (-38.2-21.4)	0 (0-0)	-7.2 (-54.5-82.8)	0 (0-0)	-27.4 (-38.5-1.9)
Aurora	0.2 (0.1-0.3)	1.9 (0-46.4)	0.1 (0.1-0.2)	1.1 (-5.3-40.5)	0 (0-0)	2.8 (-15.6-41)	0 (0-0)	-7.2 (-35-35.8)	0 (0-0)	-5.8 (-49.4-93)	0 (0-0)	-19.2 (-26.3-12.4)
Basilan	0.2 (0.1-0.3)	1.3 (0-26.4)	0.1 (0.1-0.2)	1 (-5.2-25.9)	0 (0-0)	-0.6 (-17.5-25.4)	0 (0-0)	-6 (-30.3-26.6)	0 (0-0)	2.5 (-47.6-111)	0 (0-0)	-14 (-22.1-6.9)
Bataan	0.2 (0.1-0.3)	1.5 (0-43.9)	0.1 (0.1-0.2)	1 (-5.7-40.2)	0 (0-0)	2 (-16-37.1)	0 (0-0)	-6.8 (-31.9-36.4)	0 (0-0)	-7.4 (-51.5-84.3)	0 (0-0)	-20 (-27.6-10)
Batanes	0.2 (0.1-0.3)	0.9 (0-9.3)	0.1 (0.1-0.2)	-0.3 (-6.4-8.2)	0 (0-0)	0.9 (-14.6-22.5)	0 (0-0)	-15.1 (-35.7-14.3)	0 (0-0)	4.6 (-47.7-117)	0 (0-0)	-25.9 (-31.8--17.4)
Batangas	0.2 (0.1-0.3)	1 (-13.5-35.2)	0.1 (0.1-0.2)	1.4 (-12.3-32.1)	0 (0-0.1)	2 (-15.7-30.9)	0 (0-0)	-13.7 (-37.1-23.4)	0 (0-0)	-3.1 (-51.1-83.4)	0 (0-0)	-15.1 (-27-9.7)
Benguet	0.2 (0.1-0.3)	1.1 (-9.6-37.4)	0.1 (0.1-0.2)	0.8 (-10.4-34.2)	0 (0-0)	0.7 (-19-28.3)	0 (0-0)	-9.6 (-35-25.9)	0 (0-0)	-11.1 (-56-71.4)	0 (0-0)	-14.8 (-24.9-10.2)
Biliran	0.2 (0.1-0.3)	1.2 (0-13.9)	0.1 (0.1-0.2)	0.9 (-3.7-14.3)	0 (0-0.1)	3.7 (-12.1-26.2)	0 (0-0)	-5.7 (-29.1-29.5)	0 (0-0)	-4.3 (-48.6-65.5)	0 (0-0)	-15.6 (-22.2--5.3)
Bohol	0.2 (0.1-0.3)	1.8 (0-44.8)	0.1 (0.1-0.2)	0.7 (-5-41.2)	0 (0-0)	1.2 (-15.9-33)	0 (0-0)	-7.7 (-34-29.8)	0 (0-0)	-1.8 (-48.2-85.6)	0 (0-0)	-25.1 (-31.9-3.3)
Bukidnon	0.2 (0.1-0.3)	3 (-12-52.3)	0.1 (0.1-0.2)	1.1 (-10.9-45.6)	0 (0-0)	2.7 (-16.2-46.1)	0 (0-0)	-10 (-36-31.4)	0 (0-0)	-9.7 (-52.7-62.9)	0 (0-0)	-23.3 (-32.9-9.8)
Bulacan	0.2 (0.1-0.3)	1.3 (0-30.6)	0.1 (0.1-0.2)	1.5 (-3.3-26.9)	0 (0-0)	-0.9 (-17.1-23)	0 (0-0)	-3.2 (-27.7-34.3)	0 (0-0)	-8.5 (-49.7-66)	0 (0-0)	-21.9 (-28.3--4.1)
Cagayan	0.2 (0.1-0.3)	1.2 (-14.6-41.6)	0.1 (0.1-0.2)	0.8 (-13.3-41.5)	0 (0-0)	-0.2 (-19.8-37.7)	0 (0-0)	-14.7 (-39.5-21.5)	0 (0-0)	-8 (-54.2-77)	0 (0-0)	-21.4 (-31.5-5.6)
Camarines Norte	0.2 (0.1-0.3)	1.4 (0-35.2)	0.1 (0.1-0.2)	0.8 (-6.3-33)	0 (0-0)	0.7 (-17.9-28.2)	0 (0-0)	-5.3 (-33-39.5)	0 (0-0)	-8.6 (-53.5-76.9)	0 (0-0)	-17.2 (-25.7-6.5)
Camarines Sur	0.2 (0.1-0.3)	1.1 (0-31.1)	0.1 (0.1-0.2)	0.7 (-5.8-29.1)	0 (0-0)	1.2 (-15.9-25.5)	0 (0-0)	-1.7 (-28.8-36.1)	0 (0-0)	-3 (-46.5-103)	0 (0-0)	-16.3 (-23.2-5.2)
Camiguin	0.2 (0.1-0.3)	1.6 (-12.4-49.9)	0.1 (0.1-0.2)	1.6 (-12.2-49.4)	0 (0-0)	1.7 (-16.1-39.1)	0 (0-0)	-10 (-35-25.4)	0 (0-0)	-5.6 (-50.7-87.6)	0 (0-0)	-13.4 (-25.9-24.4)
Capiz	0.2 (0.1-0.3)	3 (-0.1-45.7)	0.1 (0.1-0.2)	3.3 (-7.2-44.2)	0 (0-0)	7.9 (-15-47)	0 (0-0)	-11.4 (-37.1-31.6)	0 (0-0)	-9.3 (-55.9-67)	0 (0-0)	-17.6 (-28.2-12.8)
Catanduanes	0.2 (0.1-0.3)	2.5 (-10.3-44)	0.1 (0.1-0.2)	0.4 (-10.9-42.7)	0 (0-0)	3 (-15.5-44.7)	0 (0-0)	-7.3 (-36.1-40.2)	0 (0-0)	7.7 (-43.8-110)	0 (0-0)	-19.3 (-28.9-13.2)
Cavite	0.2 (0.1-0.3)	2.3 (-3.4-51.7)	0.1 (0.1-0.2)	2.6 (-4.4-49.5)	0 (0-0)	-0.2 (-16.9-37.2)	0 (0-0)	-4.5 (-29.4-37.3)	0 (0-0)	-6.5 (-49.5-74.5)	0 (0-0)	-18.4 (-26-17.4)
Cebu	0.2 (0.1-0.3)	1.5 (0-45)	0.1 (0.1-0.2)	1.3 (-4.5-42.5)	0 (0-0)	0.7 (-19.1-35.3)	0 (0-0)	-8.5 (-34.1-34.1)	0 (0-0)	-1 (-45.4-82.9)	0 (0-0)	-10.6 (-19.6-22.7)
Cotabato (North Cotabato)	0.2 (0.1-0.3)	1.6 (-0.9-46.4)	0.1 (0.1-0.2)	1.3 (-7-44.6)	0 (0-0)	3.8 (-15.2-38.7)	0 (0-0)	-10 (-33-26.3)	0 (0-0)	-3.5 (-48-81.1)	0 (0-0)	-12.5 (-22.6-20.1)
Davao de Oro	0.2 (0.1-0.3)	0.7 (-4.9-21)	0.1 (0.1-0.2)	-1.1 (-7.6-18.1)	0 (0-0)	0.7 (-17.3-22.8)	0 (0-0)	-10.1 (-35-23.3)	0 (0-0)	-3.7 (-48.8-75)	0 (0-0)	-21.4 (-28.9--7.4)
Davao Del Norte	0.2 (0.1-0.3)	2.2 (0-40.8)	0.1 (0.1-0.2)	0.5 (-4.8-36.9)	0 (0-0)	2.6 (-13.5-31.4)	0 (0-0)	-12.8 (-38.2-24.7)	0 (0-0)	-9.1 (-50.5-73)	0 (0-0)	-25 (-30.9--0.5)
Davao Del Sur	0.2 (0.1-0.3)	1.2 (0-41.1)	0.1 (0.1-0.2)	1.2 (-4.1-40.6)	0 (0-0)	3.1 (-15.6-34.6)	0 (0-0)	-12.8 (-40.7-29.4)	0 (0-0)	-18.8 (-61.8-52.4)	0 (0-0)	-16.3 (-23.3-15)
Davao Occidental	0.2 (0.1-0.3)	1.9 (0-42.1)	0.1 (0.1-0.2)	-0.4 (-7-36.1)	0 (0-0)	-7.2 (-24.8-26.7)	0 (0-0)	10.6 (-24.2-56.2)	0 (0-0)	16.9 (-37-130)	0 (0-0)	-22.5 (-30-8)
Davao Oriental	0.2 (0.1-0.3)	1.7 (0-32.7)	0.1 (0.1-0.2)	0.6 (-6.4-29.8)	0 (0-0)	1.1 (-17.7-33.4)	0 (0-0)	-6.3 (-32.4-37.1)	0 (0-0)	0.8 (-47.3-97.6)	0 (0-0)	-26.7 (-33.6--4.8)
Dinagat Islands	0.2 (0.1-0.3)	1.6 (-0.2-41.3)	0.1 (0.1-0.2)	-0.4 (-7.9-36.7)	0 (0-0)	0.2 (-17.9-30.6)	0 (0-0)	-7.4 (-33-28.2)	0 (0-0)	-12.8 (-57-79.1)	0 (0-0)	-24.7 (-32.2-1.1)
Eastern Samar	0.2 (0.1-0.3)	2.1 (0-42)	0.1 (0.1-0.2)	1.8 (-5.9-41)	0 (0-0)	2.2 (-16.2-44.4)	0 (0-0)	-2.8 (-31.4-46.9)	0 (0-0)	5.4 (-45.3-108)	0 (0-0)	-18.3 (-26-13.9)
Guimaras	0.2 (0.1-0.3)	1.3 (-3.8-39.1)	0.1 (0.1-0.2)	0.2 (-6.1-36.6)	0 (0-0)	4.1 (-16.7-31.1)	0 (0-0)	-19.4 (-42.3-10)	0 (0-0)	-3.5 (-54.2-74.7)	0 (0-0)	-18.6 (-26.9-5.7)
Ifugao	0.2 (0.1-0.3)	1.9 (0-42)	0.1 (0.1-0.2)	1.4 (-5-39.7)	0 (0-0)	4.3 (-17.3-41.4)	0 (0-0)	-15.7 (-41.3-20.4)	0 (0-0)	-3.1 (-51.8-88.9)	0 (0-0)	-11.2 (-19.5-21.4)

Location	Birth Incidence rate (per 100,000 live births), 2021	Birth Incidence rate (per 100,000 live births) percent change (2000-2021)	Under-5 rate (per 100,000 population), 2021	Under-5 rate (per 100,000 population) percent change (2000-2021)	5-14 rate (per 100,000 population), 2021	5-14 rate (per 100,000 population) percent change (2000-2021)	15-49 rate (per 100,000 population), 2021	15-49 rate (per 100,000 population) percent change (2000-2021)	50plus rate (per 100,000 population), 2021	50plus rate (per 100,000 population) percent change (2000-2021)	All age rate (per 100,000 population), 2021	All age rate (per 100,000 population) percent change (2000-2021)
Ilocos Norte	0.2 (0.1-0.3)	1.7 (0-40.7)	0.1 (0.1-0.2)	1.4 (-5.1-39.5)	0 (0-0)	3.7 (-13.4-38.3)	0 (0-0)	-11.5 (-34.4-23.6)	0 (0-0)	0.2 (-45.7-90.9)	0 (0-0)	-13.3 (-21.9-16.1)
Ilocos Sur	0.2 (0.1-0.3)	1.4 (0-36.2)	0.1 (0.1-0.2)	0.8 (-4.7-34.1)	0 (0-0)	3.4 (-13.4-28.6)	0 (0-0)	-12.1 (-36.3-22.7)	0 (0-0)	0.6 (-47.1-92)	0 (0-0)	-15.5 (-22.9-9.3)
Iloilo	0.2 (0.1-0.3)	2.1 (0-39.5)	0.1 (0.1-0.2)	1.9 (-4.6-39.7)	0 (0-0)	4.5 (-12-38.4)	0 (0-0)	-11.4 (-36-30.1)	0 (0-0)	-1.5 (-52.9-91.5)	0 (0-0)	-10.9 (-18.6-18.8)
Isabela	0.2 (0.1-0.3)	1 (0-18.5)	0.1 (0.1-0.2)	0.8 (-4.4-15.7)	0 (0-0)	3.4 (-13.3-23.6)	0 (0-0)	-13.5 (-37.9-19.4)	0 (0-0)	-8.8 (-52.2-62.6)	0 (0-0)	-14.8 (-22--4)
Kalinga	0.2 (0.1-0.3)	1.5 (0-37.4)	0.1 (0.1-0.2)	0 (-4.8-34.2)	0 (0-0.1)	4 (-11.9-29.7)	0 (0-0)	-10.1 (-32.3-23.2)	0 (0-0)	-0.4 (-47.4-90.4)	0 (0-0)	-19.6 (-25.8-8.2)
La Union	0.2 (0.1-0.3)	1.2 (0-19.7)	0.1 (0.1-0.2)	0.9 (-4.2-19.5)	0 (0-0)	2.2 (-14-21.8)	0 (0-0)	-14.3 (-37-16.1)	0 (0-0)	-3.8 (-48.9-79.2)	0 (0-0)	-17.1 (-23.4--2.2)
Laguna	0.2 (0.1-0.3)	2.3 (0-47.9)	0.1 (0.1-0.2)	2.1 (-3.7-46.4)	0 (0-0)	4 (-14.1-45.5)	0 (0-0)	-9.5 (-36-33.8)	0 (0-0)	-4.6 (-52.3-82.8)	0 (0-0)	-12.4 (-19.8-27.2)
Lanao Del Norte	0.2 (0.1-0.3)	1.6 (0-35.7)	0.1 (0.1-0.2)	0.7 (-5.8-33.9)	0 (0-0)	0.9 (-16.2-29.4)	0 (0-0)	-4.3 (-30-37.6)	0 (0-0)	-3 (-50.2-84.9)	0 (0-0)	-12.4 (-20.5-14.1)
Lanao Del Sur	0.2 (0.1-0.3)	0.9 (-6.2-26)	0.1 (0.1-0.2)	0.1 (-7.2-26.1)	0 (0-0)	1.6 (-13.6-30.8)	0 (0-0)	-14 (-36.8-17.3)	0 (0-0)	9.9 (-43-113)	0 (0-0)	-10.9 (-18.5-12.8)
Leyte	0.2 (0.1-0.3)	1.4 (0-40.1)	0.1 (0.1-0.2)	1.7 (-6.2-41.8)	0 (0-0)	1.7 (-15.7-35)	0 (0-0)	-8 (-35.2-35)	0 (0-0)	-4.3 (-49.2-87.7)	0 (0-0)	-17.6 (-25-11.7)
Maguindanao	0.2 (0.1-0.3)	1.6 (0-32.7)	0.1 (0.1-0.2)	-0.5 (-6.4-28.6)	0 (0-0)	2.2 (-15.2-30.5)	0 (0-0)	-8.2 (-32.2-30.8)	0 (0-0)	1.2 (-49.9-97)	0 (0-0)	-14.3 (-21.1-9.8)
Marinduque	0.2 (0.1-0.3)	0.9 (0-31.2)	0.1 (0.1-0.2)	-0.1 (-5.5-28.5)	0 (0-0)	-0.7 (-18-25.5)	0 (0-0)	-7.8 (-36-32.9)	0 (0-0)	-13.5 (-54.3-80.9)	0 (0-0)	-27.5 (-34.1--7)
Masbate	0.2 (0.1-0.3)	2.6 (0-51.5)	0.1 (0.1-0.2)	1.8 (-5.8-46.1)	0 (0-0)	1.6 (-17-42.1)	0 (0-0)	4.1 (-25.3-57.2)	0 (0-0)	0.6 (-48-111)	0 (0-0)	-13.1 (-21.5-23.1)
Misamis Occidental	0.2 (0.1-0.3)	2.8 (0-47.7)	0.1 (0.1-0.2)	2.5 (-5.3-48.1)	0 (0-0)	5.1 (-13.4-49.2)	0 (0-0)	-2.2 (-28.1-42.3)	0 (0-0)	2 (-47.2-103)	0 (0-0)	-12.4 (-20.3-26.5)
Misamis Oriental	0.2 (0.1-0.3)	2.5 (0-46.4)	0.1 (0.1-0.2)	2.4 (-4-43.8)	0 (0-0)	4.8 (-12.5-44)	0 (0-0)	-6.9 (-30.6-32.1)	0 (0-0)	-3.4 (-48.6-95.7)	0 (0-0)	-10.6 (-18.1-23.7)
Mountain Province	0.2 (0.1-0.3)	1 (0-35.6)	0.1 (0.1-0.2)	-0.1 (-7.6-31.4)	0 (0-0)	4.7 (-14.5-33.7)	0 (0-0)	-10.7 (-37.6-37.7)	0 (0-0)	6.7 (-49.5-134)	0 (0-0)	-14.8 (-23.5-10.4)
National Capital Region	0.2 (0.1-0.3)	2 (0-47.8)	0.1 (0.1-0.2)	1.7 (-4.4-42.2)	0 (0-0)	-1.6 (-19.2-40.3)	0 (0-0)	-6.8 (-33.4-37.8)	0 (0-0)	-11.6 (-55.8-73.5)	0 (0-0)	-23.4 (-30.5-3.9)
Negros Occidental	0.2 (0.1-0.3)	1.2 (0-28.8)	0.1 (0.1-0.2)	1.5 (-5-31.9)	0 (0-0)	2.8 (-16.3-29.8)	0 (0-0)	-9.8 (-35.5-27.8)	0 (0-0)	-7.8 (-48.7-74.5)	0 (0-0)	-17.6 (-25-6.4)
Negros Oriental	0.2 (0.1-0.3)	1.8 (0-42.3)	0.1 (0.1-0.2)	1.3 (-6.2-41.5)	0 (0-0)	1.9 (-17.1-34.6)	0 (0-0)	-8.4 (-34.3-28.4)	0 (0-0)	-6.7 (-52.1-84)	0 (0-0)	-19.4 (-27.5-11)
Northern Samar	0.2 (0.1-0.3)	2.7 (-5.5-46.4)	0.1 (0.1-0.2)	2.4 (-6.9-45.5)	0 (0-0)	1.2 (-20.5-47)	0 (0-0)	2.1 (-30.6-56.7)	0 (0-0)	-1 (-50.3-93.3)	0 (0-0)	-16.9 (-26.2-19.1)
Nueva Ecija	0.2 (0.1-0.3)	2.4 (0-44.6)	0.1 (0.1-0.2)	2 (-4.1-44)	0 (0-0)	0.3 (-15.1-32)	0 (0-0)	-8.9 (-34.5-30.6)	0 (0-0)	-0.2 (-47.2-96.8)	0 (0-0)	-14.6 (-21.7-16.4)
Nueva Vizcaya	0.2 (0.1-0.3)	1.1 (0-19.9)	0.1 (0.1-0.2)	0.8 (-5.3-18.1)	0 (0-0)	3.2 (-11.3-24.2)	0 (0-0)	-10.7 (-33.6-20)	0 (0-0)	-8.8 (-52.1-67.1)	0 (0-0)	-16 (-22.2--2.7)
Occidental Mindoro	0.2 (0.1-0.3)	1.7 (-12.3-46.4)	0.1 (0.1-0.2)	0.1 (-13.7-42.2)	0 (0-0)	1.1 (-17.6-38.1)	0 (0-0)	-9.5 (-36.7-24.5)	0 (0-0)	-9.4 (-53.2-77.3)	0 (0-0)	-20.5 (-30.7-12.7)
Oriental Mindoro	0.2 (0.1-0.3)	2.4 (0-44.9)	0.1 (0.1-0.2)	1.5 (-7.2-42.6)	0 (0-0)	1.6 (-17.9-37.8)	0 (0-0)	-9.3 (-38.8-28.9)	0 (0-0)	-7.7 (-54.3-79.6)	0 (0-0)	-23.7 (-32.4-6.2)
Palawan	0.2 (0.1-0.3)	2.2 (-0.6-42)	0.1 (0.1-0.2)	1.5 (-6.5-40.1)	0 (0-0)	2.6 (-15.5-41.7)	0 (0-0)	-5.7 (-29.4-34.6)	0 (0-0)	-12.3 (-54-69.9)	0 (0-0)	-18.4 (-26.4-10.7)
Pampanga	0.2 (0.1-0.3)	2.1 (0-49.9)	0.1 (0.1-0.2)	2.4 (-3.5-45.8)	0 (0-0)	2.3 (-14.6-40.1)	0 (0-0)	-8.1 (-31.1-30.9)	0 (0-0)	-6.8 (-48.9-69.4)	0 (0-0)	-17.1 (-24.4-16.2)
Pangasinan	0.2 (0.1-0.3)	1.4 (0-35.6)	0.1 (0.1-0.2)	1.1 (-4.8-27.6)	0 (0-0)	3 (-14.4-29.2)	0 (0-0)	-10.3 (-36-25.9)	0 (0-0)	-7.1 (-51.8-70.6)	0 (0-0)	-14.7 (-22.1-8)
Quezon	0.2 (0.1-0.3)	1.6 (0-39.1)	0.1 (0.1-0.2)	1.5 (-4.4-37.2)	0 (0-0)	1.6 (-16.8-29.3)	0 (0-0)	-4 (-34.3-35.7)	0 (0-0)	-1.5 (-58.5-95.7)	0 (0-0)	-17.4 (-24.6-9.6)
Quirino	0.2 (0.1-0.3)	1.9 (0-46.4)	0.1 (0.1-0.2)	0.3 (-6.6-41.8)	0 (0-0)	2.9 (-14.6-35.4)	0 (0-0)	-13.3 (-39.1-21.2)	0 (0-0)	-9.7 (-49.8-74)	0 (0-0)	-24.1 (-31.3-4.8)

Location	Birth Incidence rate (per 100,000 live births), 2021	Birth Incidence rate (per 100,000 live births) percent change (2000-2021)	Under-5 rate (per 100,000 population), 2021	Under-5 rate (per 100,000 population) percent change (2000-2021)	5-14 rate (per 100,000 population), 2021	5-14 rate (per 100,000 population) percent change (2000-2021)	15-49 rate (per 100,000 population), 2021	15-49 rate (per 100,000 population) percent change (2000-2021)	50plus rate (per 100,000 population), 2021	50plus rate (per 100,000 population) percent change (2000-2021)	All age rate (per 100,000 population), 2021	All age rate (per 100,000 population) percent change (2000-2021)
Rizal	0.2 (0.1-0.3)	1.4 (0-35.2)	0.1 (0.1-0.2)	1.3 (-5-30.9)	0 (0-0)	-1.4 (-18.8-23.5)	0 (0-0)	-1.7 (-30.5-39.9)	0 (0-0)	-14.9 (-55.3-67.8)	0 (0-0)	-14.9 (-22.9-8.7)
Romblon	0.2 (0.1-0.3)	2 (-8.9-46.4)	0.1 (0.1-0.2)	0.7 (-10.4-42)	0 (0-0)	1.9 (-20.3-42.3)	0 (0-0)	-7.3 (-36.4-40.2)	0 (0-0)	-7.1 (-55.4-90.8)	0 (0-0)	-20.9 (-31.1-10.4)
Samar (Western Samar)	0.2 (0.1-0.3)	3.5 (0-55.5)	0.1 (0.1-0.2)	3.5 (-4.8-54.5)	0 (0-0)	-0.9 (-17.9-44.1)	0 (0-0)	-1.2 (-29.3-54.7)	0 (0-0)	5.3 (-46.9-115)	0 (0-0)	-19.3 (-27.1-20.6)
Sarangani	0.2 (0.1-0.3)	2.1 (0-47.7)	0.1 (0.1-0.2)	2.4 (-4-45.8)	0 (0-0)	0.8 (-16.8-40.8)	0 (0-0)	-9.8 (-33.7-30.2)	0 (0-0)	-5.8 (-46.2-82.2)	0 (0-0)	-19.1 (-26.6-13.6)
Siquijor	0.2 (0.1-0.3)	1.3 (-6.9-43.7)	0.1 (0.1-0.2)	0.9 (-5.9-42.1)	0 (0-0)	4.8 (-13.7-37.8)	0 (0-0)	-11.9 (-37.4-24.6)	0 (0-0)	-3.1 (-55.1-83.8)	0 (0-0)	-19.4 (-27.2-12.5)
Sorsogon	0.2 (0.1-0.3)	1.6 (0-35.1)	0.1 (0.1-0.2)	1.9 (-4.1-32.6)	0 (0-0)	-0.2 (-18.6-28.3)	0 (0-0)	-3 (-29.5-45.4)	0 (0-0)	0.2 (-50.5-100)	0 (0-0)	-18.3 (-25.4-4.1)
South Cotabato	0.2 (0.1-0.3)	1.4 (0-37.7)	0.1 (0.1-0.2)	1.1 (-4.9-36.9)	0 (0-0)	0.9 (-17.8-30.8)	0 (0-0)	-13.1 (-36.8-18.6)	0 (0-0)	-6.8 (-49.6-88.3)	0 (0-0)	-17.8 (-25.4-7.8)
Southern Leyte	0.2 (0.1-0.3)	1 (-20-43.7)	0.1 (0.1-0.2)	1.1 (-18.8-40.1)	0 (0-0)	2.2 (-21.6-34.1)	0 (0-0)	-7.9 (-33.1-35.9)	0 (0-0)	0.3 (-47.1-94.2)	0 (0-0)	-18.7 (-34.4-11.1)
Sultan Kudarat	0.2 (0.1-0.3)	1.5 (0-37.3)	0.1 (0.1-0.2)	-0.4 (-6.1-32.8)	0 (0-0)	2.2 (-14.5-32.3)	0 (0-0)	-9.9 (-32.8-24.9)	0 (0-0)	-4.4 (-50.1-80.6)	0 (0-0)	-21.3 (-28.1-5)
Sulu	0.2 (0.1-0.3)	1.6 (0-38.4)	0.1 (0.1-0.2)	-0.8 (-7.7-35.9)	0 (0-0)	-2.7 (-19.7-33.2)	0 (0-0)	-3.7 (-32.9-41)	0 (0-0)	-1 (-53-100)	0 (0-0)	-19.3 (-26.6-8.8)
Surigao Del Norte	0.2 (0.1-0.3)	1.7 (0-42)	0.1 (0.1-0.2)	2 (-5.6-40.4)	0 (0-0)	3.6 (-14.9-35.5)	0 (0-0)	-10.5 (-36.2-25.9)	0 (0-0)	-8.8 (-52.8-78.9)	0 (0-0)	-15.2 (-23.7-16)
Surigao Del Sur	0.2 (0.1-0.3)	2.2 (-10.3-53.2)	0.1 (0.1-0.2)	2.2 (-11-49.3)	0 (0-0)	1.8 (-16.6-37.9)	0 (0-0)	-11.8 (-37.7-26.2)	0 (0-0)	-14.4 (-56.6-67.8)	0 (0-0)	-18.7 (-29-17.7)
Tarlac	0.2 (0.1-0.3)	1.3 (0-30.8)	0.1 (0.1-0.2)	0.8 (-4.7-30.4)	0 (0-0)	0.6 (-16.1-30.1)	0 (0-0)	-10 (-34-32.6)	0 (0-0)	-7 (-49.7-76.8)	0 (0-0)	-20.4 (-27.1-2.4)
Tawi-Tawi	0.2 (0.1-0.3)	2.1 (-4.2-40.8)	0.1 (0.1-0.2)	1.4 (-5.7-37.3)	0 (0-0)	1.4 (-17-34.1)	0 (0-0)	-9.1 (-34.3-35.4)	0 (0-0)	-17.2 (-55.6-60.6)	0 (0-0)	-13.5 (-22.5-13.8)
Zambales	0.2 (0.1-0.3)	1 (0-26.6)	0.1 (0.1-0.2)	1 (-5-22.9)	0 (0-0)	1.6 (-15.1-26.4)	0 (0-0)	-5.1 (-30.7-32.7)	0 (0-0)	-14.9 (-56.6-69)	0 (0-0)	-15 (-22.3-2.1)
Zamboanga Del Norte	0.2 (0.1-0.3)	1.2 (0-37.6)	0.1 (0.1-0.2)	0.4 (-5.7-34.7)	0 (0-0)	0.4 (-19.1-35.5)	0 (0-0)	-5.2 (-31.8-40.3)	0 (0-0)	-12.9 (-52.9-72.9)	0 (0-0)	-18 (-25.4-10)
Zamboanga Del Sur	0.2 (0.1-0.3)	1.1 (0-21.2)	0.1 (0.1-0.2)	1.2 (-4.3-20.3)	0 (0-0)	0.9 (-16.8-25.7)	0 (0-0)	-6 (-32.7-32.9)	0 (0-0)	-6.4 (-48.8-72.5)	0 (0-0)	-16.9 (-24.1--0.5)
Zamboanga Sibugay	0.2 (0.1-0.3)	1.6 (0-41.5)	0.1 (0.1-0.2)	0.9 (-5.6-40.5)	0 (0-0)	1.9 (-17.8-36.3)	0 (0-0)	-7.2 (-34.5-31.5)	0 (0-0)	-9.7 (-52.1-70.1)	0 (0-0)	-17.9 (-25.5-12.9)
Seychelles	0.2 (0.1-0.3)	-0.1 (-22.1-24.4)	0.1 (0.1-0.2)	-0.6 (-23.4-21.6)	0 (0-0.1)	1.4 (-22-21.7)	0 (0-0)	-19.2 (-41.6-11.5)	0 (0-0)	20.1 (-34.6-140)	0 (0-0)	-23.4 (-39.9--8.8)
Sri Lanka	0.2 (0.1-0.3)	1.8 (-14.3-47.8)	0.1 (0.1-0.2)	1 (-13.9-46.5)	0 (0-0.1)	-0.7 (-19.2-31.5)	0 (0-0)	-9 (-33.3-27.8)	0 (0-0)	-13.5 (-54-68.4)	0 (0-0)	-20.4 (-33.5-10.4)
Thailand	0.2 (0.1-0.3)	2.2 (-0.1-45.5)	0.1 (0.1-0.2)	1.9 (-4.4-41.1)	0 (0-0.1)	-1.1 (-17.4-34.8)	0 (0-0)	-18.2 (-38.6-17.3)	0 (0-0)	-9.8 (-54.7-84.4)	0 (0-0)	-42.3 (-48.3--21.7)
Timor-Leste	0.2 (0.2-0.4)	1.1 (-13.3-41.8)	0.1 (0.1-0.2)	0 (-16.6-38.7)	0 (0-0)	-2.9 (-25.7-29.4)	0 (0-0)	15.4 (-19.1-80.3)	0 (0-0)	-14.2 (-60.3-84.1)	0 (0-0)	-22.5 (-35.7-5.6)
Viet Nam	0.2 (0.1-0.3)	2.3 (0-45.3)	0.1 (0.1-0.2)	1.7 (-4.8-40.5)	0 (0-0)	3.4 (-14.9-36.1)	0 (0-0)	-27.1 (-45.3-0.2)	0 (0-0)	18.9 (-36.8-139)	0 (0-0)	-30.3 (-37.5--7.3)
Sub-Saharan Africa	30.3 (21.9-42.7)	2.3 (0-5)	20.1 (14.4-27.8)	0.5 (-1.7-3)	6.8 (4.7-9.4)	-4.9 (-8.6--1.5)	1.4 (1-2)	-14.6 (-19.6--10.1)	0 (0-0)	1.4 (-7.6-8.4)	5.5 (4-7.3)	-11.8 (-14.2--9.3)
**Central Sub-Saharan Africa**	7.8 (5.6-10.5)	0.9 (-7.4-9.9)	4.6 (3.3-6.1)	-1.9 (-9.8-6.5)	0.9 (0.6-1.3)	-0.9 (-12.7-12.9)	0.1 (0.1-0.2)	0.8 (-13.6-18.7)	0 (0-0)	13.3 (-25.3-80.6)	1 (0.7-1.3)	-15.3 (-22.7--7.7)
Angola	3.7 (2.5-5.2)	-6.7 (-15.5-2.9)	2.1 (1.5-3)	-9.2 (-17.9-0.6)	0.4 (0.2-0.6)	-6.2 (-20-11.5)	0 (0-0.1)	-4.4 (-22.9-19.8)	0 (0-0)	1.4 (-43.9-76)	0.5 (0.3-0.7)	-17.6 (-25.6--8.8)
Central African Republic	1.7 (1.1-2.5)	0.6 (-9-14.5)	1 (0.6-1.4)	-0.4 (-9.1-13.4)	0.2 (0.1-0.3)	0.6 (-14.1-18.3)	0 (0-0)	4.4 (-18.2-34.4)	0 (0-0)	10.1 (-36.2-102)	0.2 (0.1-0.3)	-9.4 (-17.7-3)
Congo	3.4 (2.2-5.3)	-8.3 (-18.1-0.6)	2.2 (1.4-3.3)	-10.5 (-19.8--2.1)	0.5 (0.3-0.8)	-7.6 (-20-6.9)	0.1 (0-0.1)	-20.5 (-36.1--2.9)	0 (0-0)	11.8 (-34.1-92.2)	0.4 (0.3-0.6)	-24.2 (-31.8--16)
Democratic Republic of the Congo	10.1 (7.2-13.6)	5.2 (-4.9-17.1)	5.9 (4.3-7.8)	2.4 (-7.2-13)	1.1 (0.7-1.6)	3.8 (-10.7-22.1)	0.1 (0.1-0.2)	2.9 (-14.1-25)	0 (0-0)	17.1 (-27.6-100)	1.2 (0.9-1.6)	-13.1 (-21.7--3.8)

Location	Birth Incidence rate (per 100,000 live births), 2021	Birth Incidence rate (per 100,000 live births) percent change (2000-2021)	Under-5 rate (per 100,000 population), 2021	Under-5 rate (per 100,000 population) percent change (2000-2021)	5-14 rate (per 100,000 population), 2021	5-14 rate (per 100,000 population) percent change (2000-2021)	15-49 rate (per 100,000 population), 2021	15-49 rate (per 100,000 population) percent change (2000-2021)	50plus rate (per 100,000 population), 2021	50plus rate (per 100,000 population) percent change (2000-2021)	All age rate (per 100,000 population), 2021	All age rate (per 100,000 population) percent change (2000-2021)
Equatorial Guinea	5.4 (3.4-8.4)	-1.8 (-12.7-10.6)	3.3 (2.1-4.9)	-4.1 (-14.1-7.2)	0.7 (0.4-1)	-4 (-17.4-11.5)	0.1 (0.1-0.2)	-5.2 (-22.3-13.8)	0 (0-0)	19.3 (-31.4-102)	0.6 (0.4-0.9)	-32.3 (-40.1--24.1)
Gabon	5.9 (3.8-9)	-6.6 (-17-4.5)	3.9 (2.5-5.8)	-7.2 (-17-3.3)	1 (0.6-1.5)	-7.9 (-20.4-6.5)	0.1 (0.1-0.2)	-11.4 (-28.3-8.7)	0 (0-0)	23.7 (-25.8-106)	0.7 (0.5-1)	-27.8 (-35.1--19.8)
**Eastern Sub-Saharan Africa**	14.3 (9.7-20.6)	3 (-0.7-6.8)	11.6 (7.9-16.3)	-1.3 (-5.3-2.4)	6.6 (4.5-9.2)	-12.5 (-17.9--7.6)	1.7 (1.2-2.5)	-26.4 (-31.9--21)	0 (0-0)	-5.5 (-19-6.9)	4.4 (3.1-6.1)	-18.2 (-21.9--14.4)
Burundi	9.8 (6.6-14.3)	2.1 (-8.5-14.4)	7.3 (5-10.8)	1.9 (-9-12.6)	3.7 (2.5-5.2)	2.4 (-8.6-14.6)	0.9 (0.6-1.3)	-1.9 (-15.4-13)	0 (0-0)	3 (-29.6-51.7)	2.7 (1.9-3.8)	-2 (-11.8-8.3)
Comoros	5.2 (3.5-7.6)	0 (-9.8-12.3)	4.1 (2.7-6)	-0.4 (-10.3-11.6)	2.3 (1.6-3.2)	-1.3 (-11.6-11.3)	0.6 (0.4-0.8)	-11.7 (-22.7-0.8)	0 (0-0)	9.6 (-27.3-61.5)	1.2 (0.8-1.7)	-22.3 (-29.8--13.2)
Djibouti	3.2 (2.1-4.7)	0 (-11.1-10.3)	2.5 (1.7-3.7)	-1.1 (-12.4-8.6)	1.5 (1-2.1)	0.8 (-11.6-11.5)	0.3 (0.2-0.4)	-21.8 (-32.8--9.6)	0 (0-0)	-5.3 (-36.9-47.3)	0.8 (0.5-1.1)	-17.8 (-27--9.2)
Eritrea	3.9 (2.6-5.8)	-0.4 (-9.9-10.2)	3 (2-4.3)	-0.9 (-9.5-8.8)	1.6 (1-2.3)	-1.3 (-10.7-9.2)	0.4 (0.3-0.6)	-5.6 (-18.4-7.5)	0 (0-0)	0.1 (-40.9-55.3)	1 (0.7-1.4)	-15.7 (-22.8--7.6)
Ethiopia	3.9 (2.6-5.7)	-0.2 (-5.5-4.7)	3.1 (2-4.4)	-0.9 (-5.9-4.1)	1.5 (1-2.2)	-0.8 (-6.3-4.6)	0.4 (0.2-0.5)	-5.8 (-12.5-1)	0 (0-0)	3.3 (-17.3-28.7)	1 (0.7-1.5)	-11 (-15.4--6.5)
Addis Ababa	3.1 (2.1-4.5)	-0.5 (-10.6-12.1)	2.7 (1.8-3.9)	-1.5 (-11.2-10.4)	1.7 (1.1-2.4)	0.2 (-10.5-11.6)	0.3 (0.2-0.4)	-36.5 (-45.2--25.2)	0 (0-0)	-6.4 (-39.8-45.7)	0.7 (0.5-1)	-28.4 (-35.9--19.7)
Afar	4.3 (2.9-6.3)	-0.6 (-11.3-12.5)	3.3 (2.2-4.8)	-1 (-11.2-11.8)	1.5 (1-2.2)	0.4 (-10-14)	0.3 (0.2-0.5)	-5.2 (-19-11.4)	0 (0-0)	4.3 (-36.5-81.3)	1.1 (0.8-1.6)	-5.2 (-14.8-6.4)
Amhara	3.8 (2.6-5.6)	-0.3 (-11-11.1)	3 (2-4.4)	-0.6 (-10.7-10.4)	1.5 (1-2.1)	-0.3 (-11.7-12.4)	0.4 (0.2-0.5)	-7 (-20.9-9)	0 (0-0)	0.5 (-36-54.4)	1 (0.7-1.4)	-15.2 (-24--5.4)
Benishangul-Gumuz	4.6 (3-6.7)	-0.9 (-10.6-10.6)	3.5 (2.4-5.1)	-1.7 (-11.1-9.2)	1.6 (1.1-2.4)	-0.8 (-12-12)	0.3 (0.2-0.5)	-7.9 (-21.3-9.8)	0 (0-0)	5.9 (-37.1-78.8)	1.2 (0.8-1.7)	-13 (-21.5--2.9)
Dire Dawa	3.6 (2.4-5.5)	-0.8 (-11.3-11.1)	2.9 (1.9-4.2)	-1.8 (-11.6-9)	1.4 (1-2.1)	-1.1 (-11.4-9.7)	0.3 (0.2-0.5)	-14.8 (-26.8--2.4)	0 (0-0)	1.6 (-34.7-56.5)	0.8 (0.6-1.2)	-18.5 (-26.2--10.6)
Gambella	4.2 (2.8-6.2)	-0.2 (-13-10.2)	3.3 (2.1-4.8)	-1.3 (-13.6-9.5)	1.6 (1-2.2)	-1.4 (-12.4-10.4)	0.4 (0.2-0.6)	-1.8 (-15.4-14.7)	0 (0-0)	32.5 (-18-114)	1 (0.7-1.4)	-19.2 (-28.1--10.3)
Harari	3.6 (2.5-5.4)	-0.6 (-10.4-11.4)	2.9 (2-4.2)	-1.8 (-11.2-9.4)	1.5 (1-2.1)	-1.4 (-11.4-11.2)	0.3 (0.2-0.5)	-11.3 (-22.8-3)	0 (0-0)	8 (-33-66.8)	0.9 (0.6-1.2)	-18.7 (-26--9.8)
Oromia	3.9 (2.6-5.6)	0.1 (-9.6-10.3)	3 (2-4.4)	-0.7 (-10.3-10.5)	1.5 (1-2.1)	-0.9 (-12.1-11.5)	0.4 (0.3-0.6)	-2.2 (-16.6-13.9)	0 (0-0)	3.5 (-33.8-64.7)	1.1 (0.8-1.6)	-8.2 (-17.1-1.9)
Somali	3.9 (2.5-5.8)	-0.6 (-10.4-10.7)	3 (2-4.6)	-0.8 (-10.2-9.8)	1.5 (1-2.1)	1.4 (-8.9-13.8)	0.4 (0.2-0.5)	-13.8 (-25.8-0.2)	0 (0-0)	3.9 (-34.2-67.7)	1.1 (0.8-1.6)	-4.6 (-13.7-5.3)
Southern Nations, Nationalities, and Peoples	4 (2.7-5.9)	-1.7 (-13-9.4)	3.1 (2.1-4.5)	-2 (-12.6-8.7)	1.5 (1-2.2)	-1.8 (-13.8-9.5)	0.4 (0.2-0.6)	0.2 (-13.3-16.3)	0 (0-0)	7.1 (-30.8-63)	1.1 (0.8-1.6)	-12.2 (-22.1--3.2)
Tigray	3.7 (2.5-5.4)	-0.4 (-9.9-10.5)	3 (2-4.4)	-1.1 (-10.4-9.5)	1.5 (1-2.2)	-1.9 (-11.7-8.6)	0.4 (0.2-0.5)	-8.2 (-20.9-6)	0 (0-0)	3.7 (-36.3-60.6)	1 (0.7-1.4)	-17.2 (-25.1--8.7)
Kenya	83.1 (56.1-121)	17.3 (10.3-24.5)	67.3 (45.9-96)	10.7 (4-17.2)	36.6 (25.2-51.6)	-11.3 (-18.5--4.3)	8.6 (5.9-12.1)	-36.1 (-42.4--29.7)	0 (0-0)	-24 (-38.1--8.4)	21.8 (15.4-30.3)	-22.6 (-27.4--17.8)
Baringo	47.4 (31.1-69.7)	24.4 (10.2-39.3)	38.9 (25.9-56.5)	16 (3.1-29.9)	20.3 (13.9-29.3)	-9.3 (-21.7-3.8)	5.4 (3.6-7.8)	-30.9 (-42.2--19.3)	0 (0-0)	-21.3 (-49.7-16.2)	13.4 (9.4-19.1)	-18.8 (-28.2--8.9)
Bomet	47.1 (32.2-67.2)	17.1 (3.5-32)	41.3 (27.8-58.7)	11.9 (-0.8-26.3)	27.4 (18.4-38.6)	-4.5 (-16.3-7.3)	7.5 (5.1-10.6)	-31.6 (-41.1--20.7)	0 (0-0)	-12.3 (-41.7-29)	16.2 (11-22.6)	-21.6 (-30.3--12)
Bungoma	115 (77.8-169)	24 (10.8-42.4)	92.5 (61.5-131)	14.5 (2.9-30.5)	49.7 (33.5-70.8)	-9.6 (-21.3-3.6)	14.1 (9.6-20.2)	-29.4 (-39.3--17.3)	0 (0-0)	-24.9 (-48.4-7.1)	34.1 (23.8-48)	-15.7 (-25--4.4)
Busia	194 (129-284)	24.2 (9.3-42.7)	156 (104-225)	15 (1.7-31.4)	81.2 (54.3-116)	-8.9 (-20-4.7)	20.9 (14.2-29.8)	-31.5 (-42.4--20.2)	0 (0-0)	-16.8 (-42.5-19.5)	55 (37.6-76.3)	-15.8 (-25.2--4.5)
Elgeyo-Marakwet	47.7 (31.9-70)	19.3 (5.7-36)	40.7 (27.4-58.8)	12.5 (-0.1-27.5)	24.8 (16.9-35.2)	-7.3 (-18.7-5.2)	6.6 (4.5-9.3)	-32 (-41.6--20.2)	0 (0-0)	-33.2 (-56.2-0.1)	14.4 (10-20)	-24.1 (-32.8--14.2)
Embu	32.9 (22.3-47.8)	18.3 (4.6-32.2)	27.7 (18.9-39.5)	11.1 (-2.3-23.9)	16.9 (11.6-23.8)	-7.9 (-19.4-3.6)	4.1 (2.8-5.8)	-38.8 (-48.3--29.5)	0 (0-0)	-11.2 (-41.8-31.9)	8.1 (5.6-11.1)	-32.1 (-40.4--24.2)
Garissa	39 (25.7-57.1)	22.7 (6.8-41.3)	32.9 (21.8-47.4)	15.5 (1.1-34.3)	18.8 (12.9-27.4)	-7 (-19.6-6)	5.4 (3.7-7.8)	-26.3 (-38.3--13.6)	0 (0-0)	-21.3 (-48.8-20.1)	13.6 (9.5-19)	-8.5 (-19.4-4.1)
Homa Bay	287 (193-425)	25.7 (10.3-41.8)	225 (151-328)	14.6 (1-28.8)	112 (76.5-158)	-9.7 (-21-3.2)	26.8 (18.2-38.4)	-32.1 (-42.7--20.4)	0 (0-0)	-21.5 (-47.9-14)	78.3 (54.8-110)	-13.2 (-22.9--2.5)
Isiolo	43.4 (29.3-63.3)	27.3 (11.8-46.2)	34 (22.7-49.1)	16.4 (2-33.5)	16.5 (11.2-23.4)	-10 (-23.3-4.5)	3.8 (2.5-5.4)	-34 (-45.5--21.8)	0 (0-0)	-25.4 (-52.3-13.1)	10.6 (7.4-15)	-18.5 (-29--7.1)

Location	Birth Incidence rate (per 100,000 live births), 2021	Birth Incidence rate (per 100,000 live births) percent change (2000-2021)	Under-5 rate (per 100,000 population), 2021	Under-5 rate (per 100,000 population) percent change (2000-2021)	5-14 rate (per 100,000 population), 2021	5-14 rate (per 100,000 population) percent change (2000-2021)	15-49 rate (per 100,000 population), 2021	15-49 rate (per 100,000 population) percent change (2000-2021)	50plus rate (per 100,000 population), 2021	50plus rate (per 100,000 population) percent change (2000-2021)	All age rate (per 100,000 population), 2021	All age rate (per 100,000 population) percent change (2000-2021)
Kajiado	34.1 (23.6-48.8)	19.1 (4.6-33.5)	28.7 (19.7-41.1)	12.1 (-0.3-25)	17.8 (12.3-25.2)	-6.7 (-17.5-5.4)	4.1 (2.8-5.8)	-38.5 (-48.2--28.1)	0 (0-0)	-5.8 (-41.1-39.1)	9.6 (6.7-13.4)	-26.3 (-33.7--17.4)
Kakamega	117 (78.8-170)	24.5 (10.1-40.8)	93.7 (64-135)	15.4 (2.8-31.2)	48.7 (33.1-68.8)	-9.5 (-21.5-4.9)	12.1 (8.2-17.1)	-36.1 (-46.8--24.7)	0 (0-0)	-9.3 (-41.5-38)	31.5 (22.3-43.9)	-21.1 (-30.2--9.9)
Kericho	53.2 (35.7-78.1)	19.2 (2.4-36.4)	44.8 (30.4-65.3)	11.9 (-3.3-27.7)	27.3 (18.8-38.8)	-7.2 (-20.4-6.6)	7 (4.8-10)	-37.1 (-46.7--26.2)	0 (0-0)	-29 (-52.9-2.8)	15.6 (10.9-21.8)	-26.1 (-35.5--16)
Kiambu	34.4 (23.7-49.7)	19.3 (5.4-33.9)	29.6 (20.2-42.2)	12.8 (-0.3-27.8)	18.3 (12.5-26.1)	-6.1 (-17.1-5.9)	3.7 (2.5-5.2)	-43 (-51.4--34.3)	0 (0-0)	-11.4 (-42.6-37.2)	8.5 (5.8-11.6)	-29.5 (-37.4--21)
Kilifi	152 (104-225)	24.8 (9.6-43.7)	122 (84.5-177)	15.4 (1.4-31.6)	65.3 (44.3-92.7)	-9 (-22.2-5)	17.3 (11.7-24.9)	-29.4 (-40.6--16.6)	0 (0-0)	-17.8 (-47.7-21.7)	41.9 (29.3-58.6)	-16.5 (-27.2--5)
Kirinyaga	33.4 (22.2-48)	20.2 (7-35.6)	28.3 (18.8-40.7)	13.2 (1-27.4)	16.8 (11.5-23.9)	-7.3 (-18.6-4.8)	3.5 (2.4-5)	-43.3 (-52--33.4)	0 (0-0)	-8.1 (-39.3-35.1)	7.2 (5.1-10.3)	-34.6 (-41.5--26.2)
Kisii	131 (88.8-194)	22.2 (7.1-41.3)	107 (73.3-156)	13.6 (-1.3-29.8)	58.8 (40.3-83.4)	-9.5 (-21.3-4.3)	15.1 (10.4-21.4)	-35.2 (-45.1--24)	0 (0-0)	-30.4 (-55.5-6)	32.8 (23.3-46)	-29.5 (-38.2--19.5)
Kisumu	156 (103-226)	24.5 (8.2-47.5)	124 (81.8-178)	14.4 (1.1-33.1)	64.9 (43.8-91.9)	-9.3 (-20.7-3.6)	15 (10.2-21.6)	-35.2 (-45.2--24.7)	0 (0-0)	-23 (-49.2-10.4)	37.8 (26.1-52.1)	-22.6 (-31.4--12.5)
Kitui	39.9 (27.1-57.4)	21.9 (5.3-40.2)	33.5 (22.9-47.5)	14.5 (-0.5-30.5)	19.5 (13.4-27.3)	-7 (-19.2-5.4)	5.3 (3.6-7.5)	-30.1 (-40.9--18)	0 (0-0)	-12.3 (-43.6-37.7)	11.9 (8.4-16.2)	-20.8 (-30.2--10.8)
Kwale	148 (98.9-218)	23.5 (9-41.6)	120 (79.8-175)	14.6 (1-30.8)	65.3 (44.2-92.5)	-9.1 (-21.5-3.7)	16.7 (11.4-23.9)	-30 (-41.9--18)	0 (0-0)	-23 (-49.9-10.4)	41.3 (28.8-57.7)	-16.8 (-27--6)
Laikipia	32.4 (22.2-47)	17.2 (2.3-30.4)	28.4 (19.6-41.1)	11.8 (-1.9-24)	18.6 (12.9-26.2)	-4.9 (-15.8-6.7)	4.7 (3.3-6.5)	-33.8 (-44.1--21.8)	0 (0-0)	-17.4 (-44.2-23.4)	9.7 (6.9-13.5)	-26.4 (-34.9--18)
Lamu	42.6 (28.6-61.4)	23.5 (9.3-41.3)	34.8 (23.6-50.2)	14.7 (1-31.3)	18.7 (12.8-27)	-9.7 (-22-4)	4.6 (3.1-6.7)	-34.8 (-45.5--23.2)	0 (0-0)	-15.1 (-45.2-25.3)	10.5 (7.3-14.8)	-23.3 (-32.2--12.9)
Machakos	34.1 (23.2-49.6)	18 (4.3-33.7)	29.2 (19.9-41.8)	11.5 (-2.1-27.2)	17.8 (12.3-25.1)	-8.1 (-19.4-5.1)	4.2 (3-6.1)	-42.3 (-51.2--32.4)	0 (0-0)	-20 (-49.2-18.3)	8.7 (6.1-12)	-33.6 (-41.1--24.7)
Makueni	37.5 (25.1-54.4)	18.5 (4.6-34.6)	32.2 (21.9-47.3)	12.3 (-0.5-26.7)	19.8 (13.6-27.9)	-7.1 (-18.9-5.8)	5.1 (3.5-7.2)	-31.6 (-41.8--21.4)	0 (0-0)	-9.6 (-42.5-38.9)	11.1 (7.8-15.5)	-24.4 (-32.6--14.9)
Mandera	37.6 (25.1-55)	23.8 (8.3-38.1)	30.9 (20.7-44.7)	16.3 (1.7-29.8)	16.3 (11.1-23.2)	-9.4 (-21.1-3.5)	5 (3.4-7.3)	-18.5 (-31.9--4.4)	0 (0-0)	-13.6 (-46.1-30.6)	12 (8.4-16.9)	-12.6 (-22.3--1.7)
Marsabit	38.8 (25.8-57.2)	23.3 (8.5-39.3)	32.3 (21.3-47.7)	16.1 (3.2-31.2)	18 (12.2-25.4)	-7.2 (-20-6.3)	4.9 (3.3-7.1)	-26.3 (-38.9--13)	0 (0-0)	-8.4 (-43.2-37.5)	12 (8.3-17.1)	-13.3 (-24.1--2)
Meru	34.9 (23.4-52.3)	20.7 (6.8-38.3)	29.1 (19.4-43.4)	13 (0.6-28.3)	17 (11.6-24.6)	-8.2 (-19.7-4.7)	4.1 (2.8-6)	-38.1 (-48.1--27.6)	0 (0-0)	-18.3 (-47.8-21.6)	8.5 (5.9-12)	-31 (-38.9--22.1)
Migori	278 (190-410)	25.8 (11.8-45.8)	215 (145-303)	14.4 (2-31.9)	104 (71.5-148)	-10.8 (-22.7-4.4)	26 (17.6-37.3)	-30.3 (-41.4--18.9)	0 (0-0)	-13.6 (-43-25.1)	78.5 (54.7-109)	-9.7 (-19.1-3.6)
Mombasa	92.7 (63-135)	20.4 (4.8-37.7)	77.9 (52.6-112)	13 (-2.3-28.7)	45.9 (31.3-65.3)	-7.1 (-20.9-7.2)	9.6 (6.6-13.7)	-40.2 (-50--29.7)	0 (0-0)	-21.8 (-49.6-16.1)	23.1 (16.2-32.7)	-20.2 (-31.2--8.6)
Murang'a	32.3 (21.9-47.3)	18.4 (5.7-33)	27.8 (18.8-39.8)	12.4 (-0.2-26.2)	17.5 (11.9-25.1)	-6.1 (-18.1-6.2)	4.1 (2.8-5.8)	-40.1 (-48.6--29)	0 (0-0)	-9.6 (-41.6-31.5)	7.5 (5.3-10.4)	-37.3 (-44.3--29)
Nairobi	35.9 (24.7-50.6)	19.6 (5.9-39.5)	30.9 (21-43.6)	13.1 (0.5-29)	18.9 (12.9-27)	-5.2 (-16.6-7.9)	3.6 (2.5-5.2)	-43 (-51.7--33.2)	0 (0-0)	-26.8 (-55.4-14.4)	8.9 (6.2-12.4)	-22.4 (-31.2--12.5)
Nakuru	34.3 (23.1-49.8)	17.2 (3.9-34.3)	29.6 (19.9-42.9)	11.1 (-0.8-26.5)	18.8 (12.8-27.1)	-6.4 (-17.8-6.5)	4.7 (3.2-6.7)	-35.6 (-45.9--25)	0 (0-0)	-17.7 (-46.4-24.3)	9.8 (6.9-13.9)	-27.8 (-36.2--18.6)
Nandi	59.8 (41.1-86.1)	17.9 (4.1-34.8)	51.6 (35.4-73.5)	12 (-0.5-27.2)	32.4 (22.3-46.1)	-6.5 (-17.2-6.3)	8.5 (5.9-12)	-32.5 (-41.9--22.9)	0 (0-0)	-15.2 (-44.5-22.4)	18.4 (13-25.7)	-23.8 (-32.2--14.1)
Narok	48.3 (33.2-73)	18.9 (5.3-36)	41.7 (28.4-62.2)	13.1 (-1.2-28.5)	25.7 (17.7-36.9)	-6.4 (-18.4-6.8)	6.5 (4.5-9.4)	-31.1 (-41.4--18.3)	0 (0-0)	3.1 (-33.6-55.9)	17.1 (12-24.8)	-16.7 (-26.9--5.2)
Nyamira	86 (58-125)	17.4 (1.5-33.7)	74.9 (50.5-109)	11.6 (-3.4-26.8)	47.9 (33.1-69.5)	-5.4 (-17.5-7.4)	11.7 (8-16.8)	-31.3 (-43.2--20.4)	0 (0-0)	-6.5 (-39.6-30.2)	26.4 (18.4-37.5)	-22 (-31.4--11.7)
Nyandarua	33.3 (23.1-48.2)	17.2 (3.7-33.7)	29.7 (20.4-43.1)	12.7 (-0.2-28.8)	19 (13.2-26.9)	-5.4 (-16.2-6.7)	4.5 (3.1-6.5)	-32.5 (-41.8--21.9)	0 (0-0)	-12.4 (-43.9-30.1)	9.6 (6.8-13.3)	-29.3 (-37.1--20.6)
Nyeri	31.1 (20.8-44.8)	17.6 (3.6-31.7)	27.2 (18-38.9)	12.1 (-0.7-25.5)	17.3 (11.8-24.3)	-5.7 (-16.7-7.3)	3.7 (2.6-5.3)	-39.9 (-49--29.7)	0 (0-0)	-8.8 (-43.3-38)	7.1 (4.9-9.8)	-36 (-43--27.8)
Samburu	39.1 (26.3-56.1)	20.2 (3.1-37.9)	33.5 (22.7-48.1)	13.8 (-1.9-29.5)	20.1 (13.8-28.3)	-6.2 (-18.1-6.6)	5.3 (3.6-7.5)	-26.8 (-38.6--14)	0 (0-0)	-9.4 (-42.9-38.8)	13.6 (9.7-19)	-14 (-25.1--2.8)
Siaya	257 (170-382)	26.7 (11.1-49.3)	199 (134-290)	15.3 (1.8-34.3)	98 (67.3-139)	-10.4 (-22-3.5)	24 (16.2-34.6)	-34.2 (-44.2--24.1)	0 (0-0)	-29.5 (-52-3.4)	63.4 (44.9-88.3)	-18.2 (-27.4--6.6)

Location	Birth Incidence rate (per 100,000 live births), 2021	Birth Incidence rate (per 100,000 live births) percent change (2000-2021)	Under-5 rate (per 100,000 population), 2021	Under-5 rate (per 100,000 population) percent change (2000-2021)	5-14 rate (per 100,000 population), 2021	5-14 rate (per 100,000 population) percent change (2000-2021)	15-49 rate (per 100,000 population), 2021	15-49 rate (per 100,000 population) percent change (2000-2021)	50plus rate (per 100,000 population), 2021	50plus rate (per 100,000 population) percent change (2000-2021)	All age rate (per 100,000 population), 2021	All age rate (per 100,000 population) percent change (2000-2021)
Taita Taveta	51.5 (35.1-77)	21.3 (7.2-36.6)	42.9 (28.9-62.5)	13.6 (0.7-27.3)	24.9 (16.9-35.1)	-7.5 (-19.7-4.1)	5.5 (3.8-8)	-40.1 (-50.3--30)	0 (0-0)	-17 (-46.9-28.6)	11.7 (8.2-16.4)	-30.8 (-39.3--22.9)
Tana River	58.4 (38.9-87.2)	22.8 (9-39.6)	48 (31.3-71.1)	14.7 (1.4-31)	25.4 (17.1-36)	-9.9 (-22.6-4.2)	6.6 (4.4-9.6)	-27.1 (-39.7--14.1)	0 (0-0)	-21.4 (-49.6-16.8)	18.1 (12.4-25.5)	-9.6 (-20.4-3.3)
Tharaka Nithi	37.5 (25.3-54.4)	22 (6.2-39.6)	30.7 (20.7-45)	13.6 (-0.4-29.6)	17.6 (11.8-25.2)	-8.4 (-20.2-5.5)	4.5 (3.1-6.6)	-30.5 (-42--18.8)	0 (0-0)	9 (-31.6-73.4)	9.8 (6.7-13.8)	-21.6 (-31.3--11.1)
Trans Nzoia	70.7 (48.2-99.9)	19.3 (7.7-35.3)	59.7 (40.4-85.8)	12.4 (1.2-27.6)	35.6 (24.3-51.4)	-7.7 (-19.7-5.5)	9.7 (6.5-13.8)	-31.5 (-42.3--20.3)	0 (0-0)	-21 (-46.6-16.6)	21.1 (14.8-29.7)	-24.1 (-33.2--14)
Turkana	55 (36.8-81)	22.6 (7.1-40.3)	45.6 (30.7-66)	14.7 (0.4-30.5)	25.7 (18-36.2)	-6.4 (-18.7-7.5)	7 (4.7-10.1)	-26 (-38.6--12.4)	0 (0-0)	-31 (-55-3.5)	17.9 (12.5-25.1)	-10.9 (-21.5-0.4)
Uasin Gishu	50.3 (34.9-72)	19.5 (5.5-36.9)	42.9 (29.6-62)	12.6 (-1-28.4)	26.7 (18.6-37.7)	-6.4 (-18.1-7.5)	6.8 (4.6-9.6)	-36.5 (-46.1--24.8)	0 (0-0)	-12.7 (-42.2-31.2)	14.6 (10.3-20.4)	-25.7 (-34--15.1)
Vihiga	141 (94.5-206)	23.3 (8.4-40.7)	116 (77-169)	15.5 (1.9-31.6)	63.1 (43.7-90.2)	-8.6 (-20.5-4.5)	17.2 (11.7-24.8)	-24.3 (-35.8--11.4)	0 (0-0)	-13.4 (-43.1-26.8)	36.4 (25.6-51.4)	-20.5 (-29.4--10.3)
Wajir	39.4 (26.6-57)	24.5 (6.5-43.9)	32.2 (21.4-46.5)	15.9 (-0.4-34.6)	16.9 (11.4-23.8)	-9.3 (-23-4.6)	5 (3.4-7.1)	-21.9 (-35.1--7.6)	0 (0-0)	-22.7 (-52.2-15.4)	13.1 (9.1-18.1)	-7 (-19.6-6)
West Pokot	65.4 (44.3-96)	23.8 (8.8-42.6)	53.8 (36.8-78.1)	15.7 (1.2-32.3)	29.1 (20.1-40.7)	-8.7 (-21-5.5)	8.1 (5.6-11.7)	-24.9 (-37.1--12.9)	0 (0-0)	-18 (-45.4-21.2)	21.4 (15.2-29.7)	-11.3 (-21.4-0.9)
Madagascar	10.3 (7-15.2)	-2.2 (-11.9-8.8)	7.9 (5.2-11.4)	-2.8 (-13.1-8)	4.1 (2.7-5.9)	-3.1 (-13.4-9.2)	1 (0.7-1.5)	-4.7 (-16.9-10.9)	0 (0-0)	5.3 (-28.5-56.6)	2.7 (1.9-3.8)	-13.9 (-22.3--4.3)
Malawi	8.4 (5.6-12.2)	0.6 (-10.6-11.8)	6.2 (4.2-9.1)	-1 (-12-10)	3 (2-4.4)	-0.5 (-11-11.1)	0.8 (0.5-1.2)	2.1 (-12.9-17.9)	0 (0-0)	0 (-35-56.6)	2.1 (1.4-3)	-11.7 (-21.8--2.5)
Mozambique	9.4 (6.7-12.7)	2 (-7.6-12.9)	6.9 (5-9.3)	1 (-8-11.3)	3.3 (2.2-4.6)	1 (-9.4-12.5)	0.8 (0.5-1.2)	6.2 (-10.1-25.3)	0 (0-0)	5.3 (-35.8-73.5)	2.5 (1.8-3.4)	-1 (-10.1-9.1)
Rwanda	6.6 (4.3-9.7)	1.2 (-8.6-13.4)	4.9 (3.3-7.1)	0.7 (-8.4-11.6)	2.5 (1.7-3.6)	1.8 (-7.9-14.2)	0.6 (0.4-0.9)	-10.6 (-22.6-3.1)	0 (0-0)	-1.2 (-38.9-56.6)	1.6 (1.1-2.2)	-16.8 (-24.3--7.3)
Somalia	4.2 (2.8-6.1)	-0.7 (-11.9-11)	3.1 (2-4.4)	-1.5 (-11.3-9.8)	1.5 (1-2.1)	-0.8 (-11.3-11.3)	0.4 (0.2-0.5)	-6.3 (-20.1-8.9)	0 (0-0)	-23.1 (-54.3-22.8)	1.1 (0.8-1.6)	-3.5 (-13-6.8)
South Sudan	7 (4.7-10.2)	2.4 (-7.9-12.7)	5.1 (3.5-7.4)	2.6 (-7-13.7)	2.4 (1.6-3.4)	1.1 (-9.2-13.1)	0.6 (0.4-0.9)	6.2 (-7.8-23.1)	0 (0-0)	14.2 (-23.3-72.1)	1.8 (1.2-2.5)	-2.9 (-11.6-7.7)
Uganda	2 (1.4-2.8)	-0.1 (-11.1-10.4)	1.4 (1-2)	-1 (-10.9-8.5)	0.7 (0.5-1)	-1 (-12.3-9.2)	0.2 (0.1-0.3)	-2.2 (-16.2-14.2)	0 (0-0)	14.3 (-29.3-91.9)	0.5 (0.4-0.7)	-10.5 (-19.6--2.6)
United Republic of Tanzania	12.1 (8.1-17.5)	2.9 (-9.2-14.5)	9.2 (6.2-13.1)	2 (-9.5-13.6)	5 (3.3-7)	2.5 (-7.8-14.3)	1.3 (0.9-1.9)	-1.6 (-14.4-13.2)	0 (0-0)	14.5 (-28.6-69.3)	3.4 (2.4-4.8)	-4.4 (-13.7-5.2)
Zambia	18.9 (12.7-27.8)	2 (-9.3-16.5)	14.2 (9.7-20.7)	1.2 (-8.9-15.6)	7.4 (4.9-10.5)	0.7 (-9.6-13.9)	1.8 (1.2-2.6)	-7.7 (-19.8-7.6)	0 (0-0)	11.7 (-28.8-78.2)	5 (3.5-7)	-11.8 (-20.1--1.1)
**Southern Sub-Saharan Africa**	1.7 (1.2-2.5)	0.2 (-4.4-5.4)	1.2 (0.8-1.7)	-0.2 (-4.7-4.9)	0.4 (0.3-0.6)	2.7 (-2.7-8.5)	0.1 (0-0.1)	-18.8 (-24.4--11.6)	0 (0-0)	13.3 (-9.4-45.4)	0.2 (0.2-0.3)	-15.6 (-19.4--11.7)
Botswana	1.5 (1-2.2)	0.4 (-11.7-20.1)	1 (0.7-1.5)	0.1 (-11.6-21.4)	0.4 (0.2-0.6)	0.8 (-13.1-16.8)	0.1 (0-0.1)	-26.8 (-39.8--10.8)	0 (0-0)	6 (-40.8-79.4)	0.2 (0.1-0.3)	-22.5 (-31.9--10)
Eswatini	1.7 (1.1-2.6)	0.2 (-8.4-11.4)	1.2 (0.8-1.7)	-0.6 (-10-11.3)	0.4 (0.2-0.6)	-1.4 (-14.3-13.7)	0.1 (0-0.1)	-20.1 (-34.3--2.8)	0 (0-0)	-3.6 (-46.4-68.2)	0.3 (0.2-0.4)	-20.4 (-28.4--10.7)
Lesotho	1.5 (1-2.3)	0.3 (-12.2-12.5)	1 (0.7-1.6)	-0.5 (-13-11.4)	0.3 (0.2-0.5)	-0.1 (-14.3-13.7)	0 (0-0.1)	-21 (-36.2--5.1)	0 (0-0)	2.1 (-43.5-79.3)	0.2 (0.1-0.3)	-19.4 (-29.5--10.3)
Namibia	1.6 (1.1-2.5)	-2.4 (-16.5-10)	1.2 (0.7-1.7)	-2.1 (-16.2-10.5)	0.4 (0.3-0.6)	-2.9 (-14.9-10.3)	0.1 (0-0.1)	-17.5 (-30.8-1.3)	0 (0-0)	12.4 (-33.6-87.3)	0.3 (0.2-0.4)	-15.7 (-25.3--5.5)
South Africa	1.4 (1-2.1)	-1.9 (-6.5-2.7)	1 (0.7-1.5)	-1.9 (-6.3-2.6)	0.4 (0.2-0.6)	1.4 (-3.5-6.6)	0 (0-0.1)	-21.2 (-27.4--14.5)	0 (0-0)	11.7 (-13.3-47.8)	0.2 (0.1-0.3)	-19.4 (-22.7--15.6)
Eastern Cape	1.5 (1-2.2)	-0.2 (-12.2-14.4)	1 (0.7-1.5)	-0.3 (-11.8-13.8)	0.3 (0.2-0.5)	0.3 (-12.5-16.2)	0 (0-0.1)	-20.7 (-35.3-0.4)	0 (0-0)	4.8 (-42.1-99.2)	0.2 (0.1-0.3)	-22.5 (-30.7--11.3)
Free State	1.5 (1-2.2)	0.3 (-12.2-16.1)	1 (0.7-1.5)	0 (-11.5-14.7)	0.4 (0.2-0.6)	1.4 (-11.6-19.1)	0 (0-0.1)	-21.3 (-36.7--3.5)	0 (0-0)	-2 (-43.6-64.3)	0.2 (0.1-0.2)	-17.3 (-26.4--4)
Gauteng	1.3 (0.9-1.9)	0.7 (-11.9-24.7)	0.9 (0.7-1.4)	-0.3 (-12-20.1)	0.4 (0.2-0.6)	2.9 (-9.7-22.1)	0 (0-0.1)	-18.1 (-32.3-1.9)	0 (0-0)	-7.2 (-46.1-67.6)	0.2 (0.1-0.2)	-6.7 (-17.2-11.4)
KwaZulu-Natal	1.5 (1-2.3)	-1.2 (-11.8-11.1)	1.1 (0.7-1.6)	-1.2 (-11.6-12.1)	0.4 (0.2-0.6)	0.2 (-12.3-16.9)	0 (0-0.1)	-21.4 (-37.3--3)	0 (0-0)	-5.2 (-47.6-62.5)	0.2 (0.1-0.3)	-20.4 (-28.7--8.4)
Limpopo	1.6 (1.1-2.4)	0.4 (-9.2-15.5)	1.2 (0.8-1.7)	0.6 (-8.3-12.7)	0.4 (0.3-0.6)	1 (-10.2-16.2)	0.1 (0-0.1)	-23.5 (-35.3--7.5)	0 (0-0)	11.6 (-34-83.2)	0.2 (0.2-0.3)	-21.1 (-28.4--11.7)

Location	Birth Incidence rate (per 100,000 live births), 2021	Birth Incidence rate (per 100,000 live births) percent change (2000-2021)	Under-5 rate (per 100,000 population), 2021	Under-5 rate (per 100,000 population) percent change (2000-2021)	5-14 rate (per 100,000 population), 2021	5-14 rate (per 100,000 population) percent change (2000-2021)	15-49 rate (per 100,000 population), 2021	15-49 rate (per 100,000 population) percent change (2000-2021)	50plus rate (per 100,000 population), 2021	50plus rate (per 100,000 population) percent change (2000-2021)	All age rate (per 100,000 population), 2021	All age rate (per 100,000 population) percent change (2000-2021)
Mpumalanga	1.6 (1-2.5)	-1.9 (-17.5-11.7)	1.1 (0.7-1.7)	-1.7 (-16.9-11.4)	0.4 (0.3-0.6)	-0.8 (-14.4-13.5)	0.1 (0-0.1)	-22.2 (-35.9--7)	0 (0-0)	-0.2 (-36.7-73.1)	0.2 (0.1-0.3)	-20.4 (-30.9--11.2)
North-West	1.4 (0.9-2.2)	-0.7 (-13.3-18.8)	1 (0.7-1.5)	-1 (-14.1-17.7)	0.4 (0.2-0.6)	0.9 (-13.7-15.7)	0 (0-0.1)	-19.1 (-35.7--0.8)	0 (0-0)	-7.9 (-47.7-53.4)	0.2 (0.1-0.3)	-12.6 (-24.3-0.7)
Northern Cape	1.3 (0.9-2)	0 (-15.6-20.3)	1 (0.7-1.4)	0.4 (-14.1-20.8)	0.4 (0.2-0.6)	0.8 (-14.1-18)	0 (0-0.1)	-12.1 (-28.6-6.7)	0 (0-0)	-7.2 (-44.8-59)	0.2 (0.1-0.3)	-16.3 (-28.2--0.8)
Western Cape	1.1 (0.7-1.6)	0 (-18.4-19.6)	0.8 (0.6-1.2)	-0.2 (-18.2-18.4)	0.4 (0.3-0.6)	1.2 (-17.1-21.4)	0.1 (0-0.1)	-20.6 (-37-0.7)	0 (0-0)	-3.1 (-44.6-59.3)	0.1 (0.1-0.2)	-21.8 (-35.8--7)
Zimbabwe	2.4 (1.5-3.6)	0.4 (-11.4-13)	1.6 (1-2.4)	-0.4 (-11.2-10.5)	0.6 (0.4-0.8)	0.9 (-12.3-14.6)	0.1 (0-0.1)	-13.9 (-27.8-3.6)	0 (0-0)	5.4 (-37.5-67.9)	0.4 (0.3-0.6)	-8.6 (-18.6-1.5)
**Western Sub-Saharan Africa**	52.6 (38.2-73.3)	-3.7 (-6.3--0.9)	33.9 (24.6-46.4)	-5.1 (-7.5--2.3)	9.4 (6.4-13.2)	-6.5 (-9.8--3.1)	1.7 (1.1-2.5)	-6.1 (-9.8--2)	0 (0-0)	4.7 (-3.3-13.9)	8.7 (6.4-11.4)	-13.3 (-15.6--10.7)
Benin	11.4 (8.2-16)	0.1 (-9.7-10.1)	7.2 (5.2-9.8)	-0.8 (-10.7-8.7)	1.9 (1.2-2.8)	-2.9 (-16.2-11.9)	0.3 (0.2-0.5)	-0.6 (-16.7-19.9)	0 (0-0)	12.5 (-27-81.5)	1.9 (1.4-2.6)	-8.8 (-17.1-0.4)
Burkina Faso	40.2 (31.2-52.3)	0.8 (-8.3-11.6)	24.8 (19.2-32.9)	0 (-9.4-10.2)	6.3 (4.2-9.1)	-1.3 (-13.9-13.9)	1 (0.7-1.6)	-5.6 (-19.3-13)	0 (0-0)	11.6 (-29.4-77.2)	6.6 (5.1-8.5)	-7.1 (-16.1-3.2)
Cabo Verde	7.7 (5-11.5)	-1.8 (-12.7-11.9)	5.7 (3.7-8.3)	-2.2 (-12.7-10.5)	2.5 (1.6-3.6)	-2.4 (-13.8-9.3)	0.4 (0.3-0.6)	-25.1 (-36.6--13.9)	0 (0-0)	46.1 (-12.8-125)	1.2 (0.8-1.7)	-32 (-39.5--24.2)
Cameroon	15 (11.3-19.9)	2.9 (-5.9-13.2)	9.5 (7.1-12.5)	0.5 (-8.5-9.6)	2.7 (1.8-3.9)	-0.1 (-11.6-13.3)	0.5 (0.3-0.7)	-3.4 (-18.1-15.1)	0 (0-0)	8.5 (-31.4-72)	2.2 (1.7-3)	-14 (-22.4--6.2)
Chad	27.2 (20.6-35.8)	-3.4 (-12.2-6)	16.9 (12.9-22.3)	-4.3 (-13.1-4.7)	4.3 (3-6.2)	-5.4 (-17.3-8.7)	0.8 (0.5-1.2)	1.6 (-14.5-19.9)	0 (0-0)	8.1 (-32.9-72.6)	5 (3.9-6.5)	-5.6 (-14.1-3.4)
Côte d'Ivoire	166 (131-215)	-0.2 (-9.5-9.6)	108 (84.4-138)	-1.6 (-10.2-8.2)	31.8 (21.7-44.4)	-1.8 (-13.2-14.5)	4.9 (3.2-7.1)	-11.1 (-24-5.4)	0 (0-0)	2.4 (-30.3-55.8)	26.6 (21-33.9)	-12.5 (-20--3.2)
Gambia	25.6 (16.4-39.6)	-1.9 (-12.5-13)	17.2 (11.2-25.9)	-3.9 (-14.5-8.8)	5.2 (3.3-7.6)	-3 (-15.6-10)	1 (0.6-1.4)	-2.1 (-16.4-14.6)	0 (0-0)	8 (-33-70.5)	4.3 (3-6)	-17.6 (-25.9--8)
Ghana	21.5 (16-28.2)	0.1 (-8.7-10.1)	14.4 (10.7-18.7)	-0.3 (-8.8-9.3)	4.3 (2.9-6)	-1.5 (-13.2-11.6)	0.7 (0.4-1)	-5.8 (-21.2-11.7)	0 (0-0)	6.1 (-36-69.3)	3.1 (2.4-4)	-17.9 (-25--10.3)
Guinea	6.7 (4.7-9.6)	-0.5 (-11.4-11.1)	4.1 (2.8-5.9)	-2 (-11.9-8.9)	1 (0.7-1.5)	-3 (-15.5-12.4)	0.2 (0.1-0.3)	-0.7 (-17.4-20.8)	0 (0-0)	9.8 (-31.9-86.8)	1 (0.8-1.4)	-7 (-16.4-3.7)
Guinea-Bissau	18.2 (11.5-28)	-2.1 (-12.3-9.6)	11.6 (7.5-17.2)	-3.6 (-13.7-8)	3.1 (2-4.6)	-3.2 (-16-11.6)	0.5 (0.3-0.7)	-11.1 (-24.9-6.1)	0 (0-0)	3.9 (-36.6-78.6)	2.8 (1.9-4)	-16.1 (-24--6.6)
Liberia	193 (148-249)	0.4 (-9-10.3)	117 (91.1-151)	-2.1 (-11.1-7.8)	30.7 (20.7-43.7)	-3.9 (-15.7-9.6)	5.1 (3.4-7.4)	-11 (-23.4-3.7)	0 (0-0)	27.9 (-14.2-87.4)	25 (19.3-32)	-23 (-30.3--15.4)
Mali	23.9 (18-32.2)	1.9 (-9.1-11.7)	14.8 (11.3-19.8)	0.9 (-9.6-10.8)	3.7 (2.5-5.2)	-0.2 (-13.8-15.2)	0.7 (0.4-1)	7.7 (-8.6-28.8)	0 (0-0)	10.7 (-29.8-79.1)	4.1 (3.1-5.3)	-2.9 (-12.2-6.6)
Mauritania	15.4 (9.8-23.4)	-2.4 (-13.1-10.3)	10.6 (6.8-15.8)	-3.7 (-13.7-7.8)	3.5 (2.2-5.2)	-4.9 (-16.3-8.4)	0.7 (0.4-1)	-0.4 (-14.6-15.7)	0 (0-0)	2.6 (-30-66.1)	2.6 (1.8-3.7)	-18.7 (-26.7--9.4)
Niger	102 (81-134)	-4.4 (-12.7-5.2)	62.8 (49.6-81.8)	-5.8 (-14.4-3.5)	15.4 (10.4-22.2)	-6.9 (-18.9-8.2)	3 (1.9-4.3)	5.6 (-10.6-25.2)	0 (0-0)	-11.7 (-41.2-37)	18.9 (14.9-24.1)	-7 (-15-2.6)
Nigeria	55.5 (35.6-85.4)	-1.8 (-4.4-0.8)	36.8 (24.2-54.7)	-4.7 (-6.8--2.2)	10.4 (6.9-15)	-8.5 (-11.5--5.7)	2 (1.3-3)	-0.4 (-3.7-3.1)	0 (0-0)	4.8 (-4.3-14.9)	9.4 (6.5-13.2)	-12.6 (-15.1--9.9)
Sao Tome and Principe	32 (20.3-49.3)	-0.6 (-12.7-13.3)	21.8 (13.7-32.3)	-2.1 (-13.8-10.2)	7 (4.6-10.2)	-3.4 (-14.7-10.2)	1.3 (0.8-1.9)	-16.9 (-28.7--3.3)	0 (0-0)	37.2 (-9.2-105)	4.7 (3.2-6.7)	-26.7 (-35.1--18.2)
Senegal	20.3 (15.2-27.4)	1 (-6.4-10.4)	13.4 (9.9-17.9)	-0.2 (-8.2-9.4)	3.8 (2.5-5.4)	-1.9 (-14.5-13.3)	0.7 (0.4-1)	-3.8 (-19.6-12.8)	0 (0-0)	2.4 (-31.1-59.2)	3.1 (2.4-4.1)	-12.8 (-20.3--4.5)
Sierra Leone	15 (10.5-21.6)	-2 (-12.3-9.4)	9.1 (6.4-12.8)	-4.2 (-14-5.9)	2.3 (1.5-3.2)	-4.5 (-16.5-9.8)	0.4 (0.2-0.6)	0.1 (-16.1-20.5)	0 (0-0)	10.5 (-36.6-71.2)	2.1 (1.5-2.8)	-16.4 (-24.7--8.5)
Togo	43.8 (32.5-58.3)	0.6 (-7.8-10.5)	28.4 (20.8-37.8)	-0.8 (-9.3-8.8)	8.2 (5.5-11.7)	-1.3 (-14.4-13.5)	1.3 (0.8-1.9)	-7.9 (-21.8-7.4)	0 (0-0)	5 (-32.4-63.8)	6.6 (4.9-8.5)	-17.3 (-24.7--8.8)

Table S17: Under-5 sickle cell disease mortality metrics in 2000, 2010, and 2021

Location	Under-5 cause-specific SCD mortality rate (per 100,000 population), 2000	Under-5 total SCD mortality rate (per 100,000 population), 2000	Under-5 cause-specific SCD mortality rate (per 100,000 population), 2010	Under-5 total SCD mortality rate (per 100,000 population), 2010	Under-5 cause-specific SCD mortality rate (per 100,000 population), 2021	Under-5 total SCD mortality rate (per 100,000 population), 2021	Under-5 cause-specific mortality cause fraction, 2000	Under-5 total SCD mortality cause fraction, 2000	Under-5 cause-specific mortality cause fraction, 2010	Under-5 total SCD mortality cause fraction, 2010	Under-5 cause-specific mortality cause fraction, 2021	Under-5 total SCD mortality cause fraction, 2021	Under-5 cause-specific mortality rank 2000	Under-5 total SCD mortality rank 2000	Under-5 cause-specific mortality rank 2010	Under-5 total SCD mortality rank 2010	Under-5 cause-specific mortality rank 2021	Under-5 total SCD mortality rank 2021
Global	1.9 (1.2-2.7)	10.6 (7.7-14.2)	1.8 (1.2-2.6)	11.3 (8.2-15.1)	1.7 (1-2.2)	12.5 (9-16.6)	0.1 (0.1-0.2)	0.7 (0.5-0.9)	0.2 (0.1-0.2)	1 (0.7-1.4)	0.2 (0.2-0.3)	1.7 (1.1-2.3)	51	22	44	16	40	12
Central Europe, Eastern Europe, and Central Asia	0 (0-0)	0.1 (0.1-0.2)	0 (0-0)	0.1 (0.1-0.1)	0 (0-0)	0.1 (0-0.1)	0 (0-0)	0 (0-0)	0 (0-0)	0 (0-0)	0 (0-0)	0 (0-0)	136	81	134	79	136	72
**Central Asia**	0 (0-0)	0 (0-0)	0 (0-0)	0 (0-0)	0 (0-0)	0 (0-0)	0 (0-0)	0 (0-0)	0 (0-0)	0 (0-0)	0 (0-0)	0 (0-0)	136	126	135	120	135	118
Armenia	0 (0-0)	0 (0-0)	0 (0-0)	0 (0-0)	0 (0-0)	0 (0-0)	0 (0-0)	0 (0-0)	0 (0-0)	0 (0-0)	0 (0-0)	0 (0-0)	132	106	128	109	132	100
Azerbaijan	0 (0-0)	0 (0-0)	0 (0-0)	0 (0-0)	0 (0-0)	0 (0-0)	0 (0-0)	0 (0-0)	0 (0-0)	0 (0-0)	0 (0-0)	0 (0-0)	128	119	126	113	127	107
Georgia	0 (0-0)	0 (0-0)	0 (0-0)	0 (0-0)	0 (0-0)	0 (0-0)	0 (0-0)	0 (0-0)	0 (0-0)	0 (0-0)	0 (0-0)	0 (0-0)	136	108	131	108	135	100
Kazakhstan	0 (0-0)	0 (0-0)	0 (0-0)	0 (0-0)	0 (0-0)	0 (0-0)	0 (0-0)	0 (0-0)	0 (0-0)	0 (0-0)	0 (0-0)	0 (0-0)	134	115	134	114	132	104
Kyrgyzstan	0 (0-0)	0 (0-0)	0 (0-0)	0 (0-0)	0 (0-0)	0 (0-0)	0 (0-0)	0 (0-0)	0 (0-0)	0 (0-0)	0 (0-0)	0 (0-0)	134	114	132	115	134	111
Mongolia	0 (0-0)	0 (0-0)	0 (0-0)	0 (0-0)	0 (0-0)	0 (0-0)	0 (0-0)	0 (0-0)	0 (0-0)	0 (0-0)	0 (0-0)	0 (0-0)	126	124	127	117	128	110
Tajikistan	0 (0-0)	0 (0-0)	0 (0-0)	0 (0-0)	0 (0-0)	0 (0-0)	0 (0-0)	0 (0-0)	0 (0-0)	0 (0-0)	0 (0-0)	0 (0-0)	127	116	125	114	129	114
Turkmenistan	0 (0-0)	0 (0-0)	0 (0-0)	0 (0-0)	0 (0-0)	0 (0-0)	0 (0-0)	0 (0-0)	0 (0-0)	0 (0-0)	0 (0-0)	0 (0-0)	135	121	130	117	129	113
Uzbekistan	0 (0-0)	0 (0-0)	0 (0-0)	0 (0-0)	0 (0-0)	0 (0-0)	0 (0-0)	0 (0-0)	0 (0-0)	0 (0-0)	0 (0-0)	0 (0-0)	132	113	132	113	125	111
**Central Europe**	0 (0-0)	0.4 (0.3-0.7)	0 (0-0)	0.3 (0.2-0.4)	0 (0-0)	0.3 (0.2-0.5)	0 (0-0)	0.2 (0.1-0.3)	0 (0-0)	0.2 (0.1-0.3)	0 (0-0)	0.3 (0.2-0.5)	122	45	123	42	126	32
Albania	0 (0-0.1)	0.1 (0.1-0.1)	0 (0-0.1)	0.1 (0.1-0.1)	0 (0-0)	0.1 (0.1-0.1)	0 (0-0)	0 (0-0)	0 (0-0)	0 (0-0)	0 (0-0)	0 (0-0.1)	102	88	103	75	110	71
Bosnia and Herzegovina	0 (0-0)	0 (0-0)	0 (0-0)	0 (0-0)	0 (0-0)	0 (0-0)	0 (0-0)	0 (0-0)	0 (0-0)	0 (0-0)	0 (0-0)	0 (0-0)	123	97	125	89	127	89
Bulgaria	0 (0-0)	0 (0-0)	0 (0-0)	0 (0-0)	0 (0-0)	0 (0-0)	0 (0-0)	0 (0-0)	0 (0-0)	0 (0-0)	0 (0-0)	0 (0-0)	109	101	113	92	108	80
Croatia	0 (0-0)	0.1 (0.1-0.1)	0 (0-0)	0.1 (0.1-0.1)	0 (0-0)	0.1 (0-0.1)	0 (0-0)	0.1 (0-0.1)	0 (0-0)	0.1 (0.1-0.1)	0 (0-0)	0.1 (0-0.1)	118	56	117	52	113	50
Czechia	0 (0-0)	0 (0-0)	0 (0-0)	0 (0-0)	0 (0-0)	0 (0-0)	0 (0-0)	0 (0-0)	0 (0-0)	0 (0-0)	0 (0-0)	0 (0-0)	122	105	122	94	119	83
Hungary	0 (0-0)	0 (0-0)	0 (0-0)	0 (0-0)	0 (0-0)	0 (0-0)	0 (0-0)	0 (0-0)	0 (0-0)	0 (0-0)	0 (0-0)	0 (0-0)	124	101	120	92	115	85
Montenegro	0 (0-0)	0 (0-0)	0 (0-0)	0 (0-0)	0 (0-0)	0 (0-0)	0 (0-0)	0 (0-0)	0 (0-0)	0 (0-0)	0 (0-0)	0 (0-0)	119	95	118	86	117	71
North Macedonia	0 (0-0)	0 (0-0)	0 (0-0)	0 (0-0)	0 (0-0)	0 (0-0)	0 (0-0)	0 (0-0)	0 (0-0)	0 (0-0)	0 (0-0)	0 (0-0)	121	99	123	77	123	64
Poland	0 (0-0)	1.3 (0.8-2)	0 (0-0)	0.8 (0.5-1.2)	0 (0-0)	0.8 (0.5-1.3)	0 (0-0)	0.7 (0.5-1.1)	0 (0-0)	0.7 (0.4-1)	0 (0-0)	1 (0.6-1.6)	117	19	119	18	115	13
Romania	0 (0-0)	0 (0-0.1)	0 (0-0)	0 (0-0.1)	0 (0-0)	0 (0-0.1)	0 (0-0)	0 (0-0)	0 (0-0)	0 (0-0)	0 (0-0)	0 (0-0.1)	123	92	124	80	124	70
Serbia	0 (0-0)	0 (0-0)	0 (0-0)	0 (0-0)	0 (0-0)	0 (0-0)	0 (0-0)	0 (0-0)	0 (0-0)	0 (0-0)	0 (0-0)	0 (0-0)	130	115	131	96	131	92
Slovakia	0 (0-0)	0 (0-0)	0 (0-0)	0 (0-0)	0 (0-0)	0 (0-0)	0 (0-0)	0 (0-0)	0 (0-0)	0 (0-0)	0 (0-0)	0 (0-0)	120	82	121	75	124	70
Slovenia	0 (0-0)	0 (0-0)	0 (0-0)	0 (0-0)	0 (0-0)	0 (0-0)	0 (0-0)	0 (0-0)	0 (0-0)	0 (0-0)	0 (0-0)	0 (0-0)	125	82	129	72	125	63
**Eastern Europe**	0 (0-0)	0 (0-0)	0 (0-0)	0 (0-0)	0 (0-0)	0 (0-0)	0 (0-0)	0 (0-0)	0 (0-0)	0 (0-0)	0 (0-0)	0 (0-0)	132	115	131	107	130	91
Belarus	0 (0-0)	0 (0-0)	0 (0-0)	0 (0-0)	0 (0-0)	0 (0-0)	0 (0-0)	0 (0-0)	0 (0-0)	0 (0-0)	0 (0-0)	0 (0-0)	134	106	136	101	135	84
Estonia	0 (0-0)	0 (0-0)	0 (0-0)	0 (0-0)	0 (0-0)	0 (0-0)	0 (0-0)	0 (0-0)	0 (0-0)	0 (0-0)	0 (0-0)	0 (0-0)	135	104	132	95	132	82

Location	Under-5 cause-specific SCD mortality rate (per 100,000 population), 2000	Under-5 total SCD mortality rate (per 100,000 population), 2000	Under-5 cause-specific SCD mortality rate (per 100,000 population), 2010	Under-5 total SCD mortality rate (per 100,000 population), 2010	Under-5 cause-specific SCD mortality rate (per 100,000 population), 2021	Under-5 total SCD mortality rate (per 100,000 population), 2021	Under-5 cause-specific mortality cause fraction, 2000	Under-5 total SCD mortality cause fraction, 2000	Under-5 cause-specific mortality cause fraction, 2010	Under-5 total SCD mortality cause fraction, 2010	Under-5 cause-specific mortality cause fraction, 2021	Under-5 total SCD mortality cause fraction, 2021	Under-5 cause-specific mortality rank 2000	Under-5 total SCD mortality rank 2000	Under-5 cause-specific mortality rank 2010	Under-5 total SCD mortality rank 2010	Under-5 cause-specific mortality rank 2021	Under-5 total SCD mortality rank 2021
Latvia	0 (0-0)	0 (0-0)	0 (0-0)	0 (0-0)	0 (0-0)	0 (0-0)	0 (0-0)	0 (0-0)	0 (0-0)	0 (0-0)	0 (0-0)	0 (0-0)	134	109	133	96	135	81
Lithuania	0 (0-0)	0 (0-0)	0 (0-0)	0 (0-0)	0 (0-0)	0 (0-0)	0 (0-0)	0 (0-0)	0 (0-0)	0 (0-0)	0 (0-0)	0 (0-0)	133	101	134	94	129	75
Republic of Moldova	0 (0-0)	0 (0-0)	0 (0-0)	0 (0-0)	0 (0-0)	0 (0-0)	0 (0-0)	0 (0-0)	0 (0-0)	0 (0-0)	0 (0-0)	0 (0-0)	134	110	133	108	135	101
Russian Federation	0 (0-0)	0 (0-0)	0 (0-0)	0 (0-0)	0 (0-0)	0 (0-0)	0 (0-0)	0 (0-0)	0 (0-0)	0 (0-0)	0 (0-0)	0 (0-0)	133	117	134	105	133	90
Ukraine	0 (0-0)	0 (0-0)	0 (0-0)	0 (0-0)	0 (0-0)	0 (0-0)	0 (0-0)	0 (0-0)	0 (0-0)	0 (0-0)	0 (0-0)	0 (0-0)	122	98	121	100	119	89
High-income	0.1 (0.1-0.1)	1.2 (1-1.5)	0.1 (0.1-0.1)	1.2 (1-1.5)	0 (0-0)	1 (0.8-1.3)	0 (0-0)	0.8 (0.7-1)	0 (0-0)	1 (0.8-1.2)	0 (0-0)	1.2 (0.9-1.5)	77	21	73	17	76	17
[truncated: 1,264,652 more chars]
